# Supplementary figures and images for: The Elephant in the room: What can we learn from California regarding the use of sport hunting of pumas (Puma concolor) as a management tool?
Source: PLoS One. 2020 Feb 13;15(2):e0224638. doi: 10.1371/journal.pone.0224638 (PMC7018503; doi:10.1371/journal.pone.0224638)

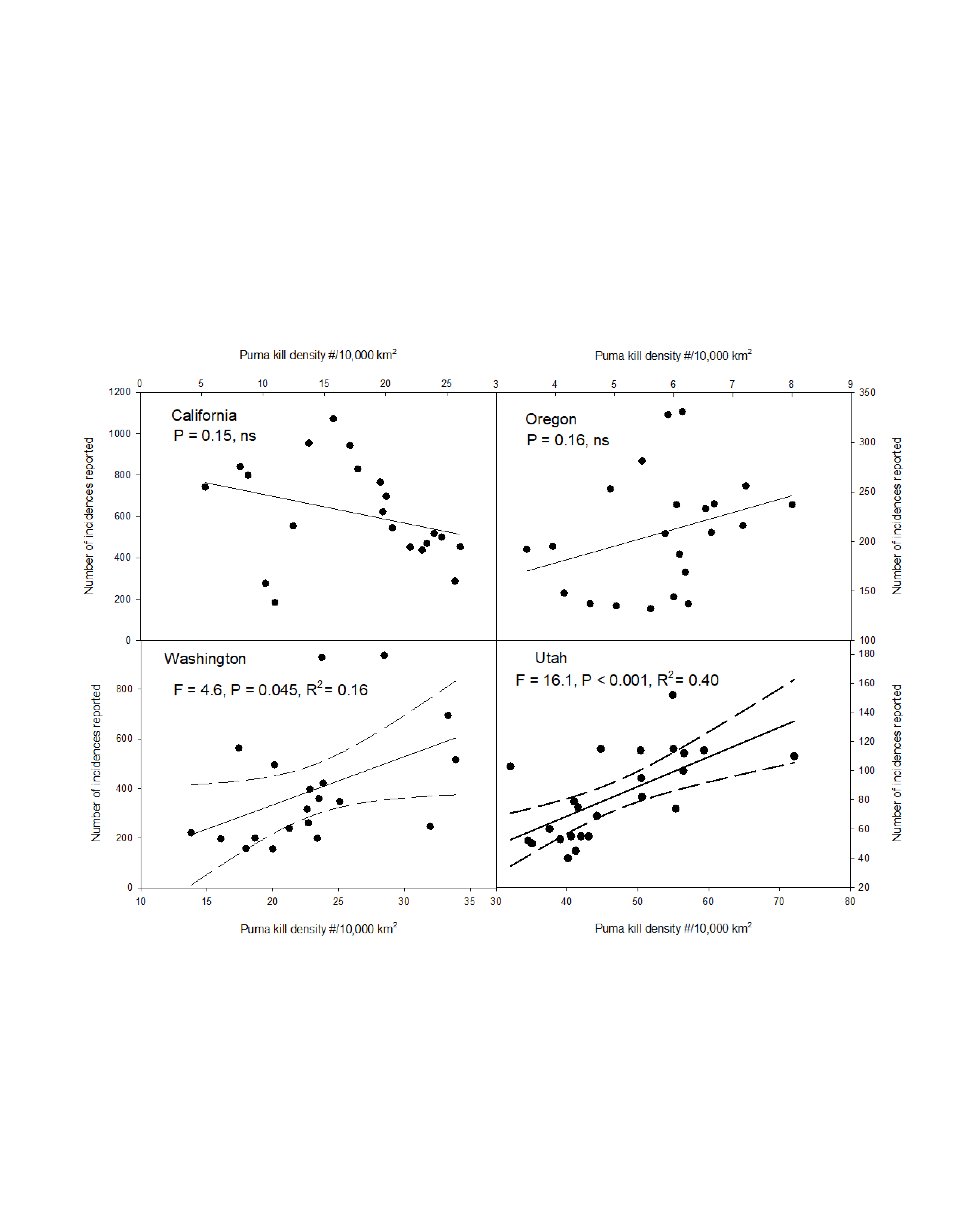

Supplement: S1 Fig — Data are for California and the three states (Oregon, Washington, and Utah) for which these data were available. (TIF) [file pone.0224638.s001.tif]
