# Supplementary material for: The Elephant in the room: What can we learn from California regarding the use of sport hunting of pumas (Puma concolor) as a management tool?
Source: PLoS One. 2020 Feb 13;15(2):e0224638. doi: 10.1371/journal.pone.0224638 (PMC7018503; doi:10.1371/journal.pone.0224638)
Supplement: S2 File — This is a zip file that contains sources of the data used in the analyses that are no longer available on the internet. (ZIP) [file pone.0224638.s003.zip › 1995 California agricultural statistics.pdf]

CALIFORNIA AGRICULTURAL STATISTICS

1995

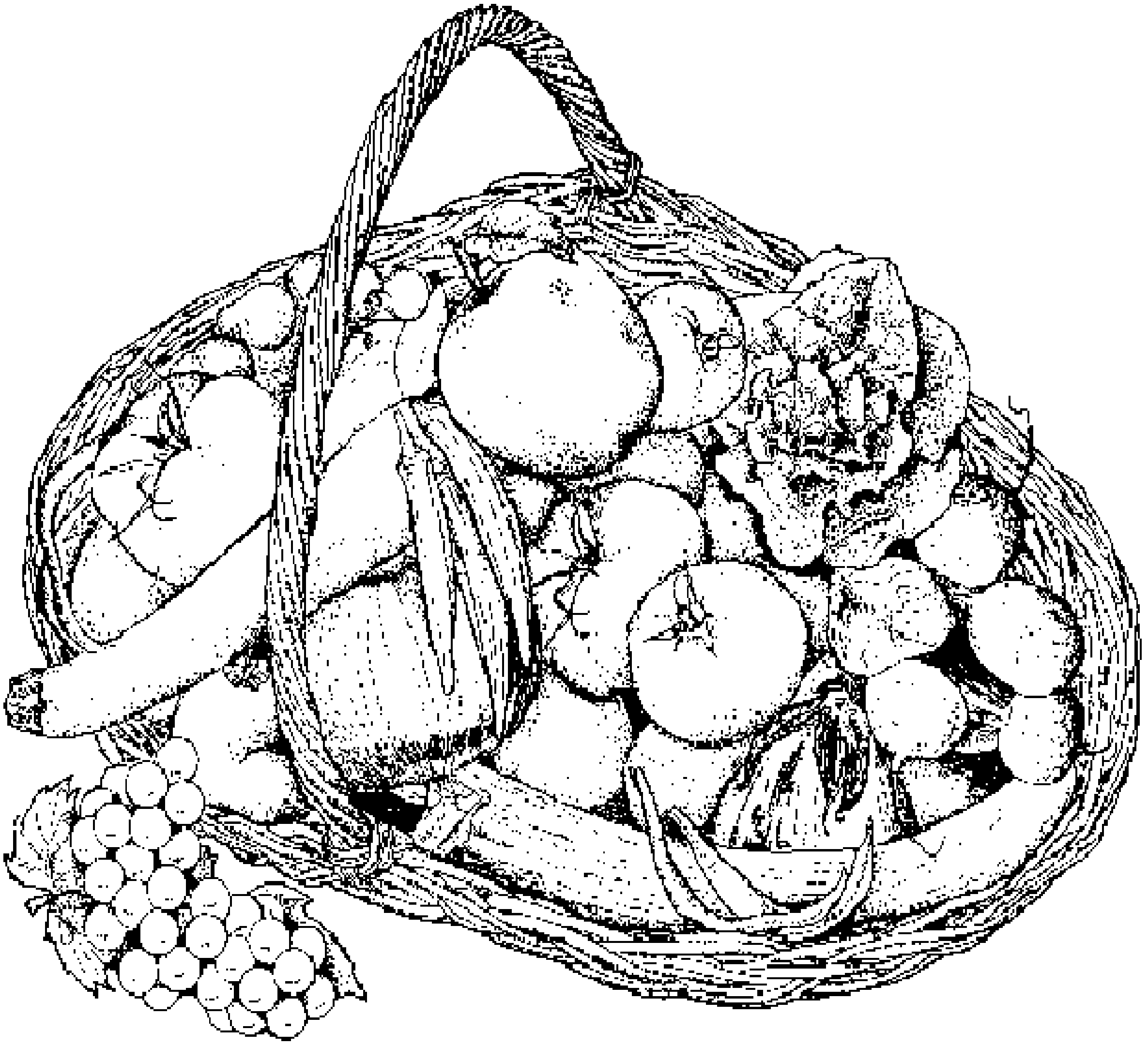

CALIFORNIA
AGRICULTURAL STATISTICS

Issued Cooperatively By

California Dept. of Food and Agriculture
Ann M. Veneman
Secretary

And

U.S. Department of Agriculture
National Agricultural Statistics Service
Donald M. Bay, Administrator

Compiled By

CALIFORNIA AGRICULTURAL STATISTICS SERVICE

1220 N Street, Room 243
P.O. Box 1258
Sacramento, California 95812-1258
Phone: (916) 498-5161
FAX: (916) 498-5186

Jim Tippett, State Statistician
Dwaine Nelson, Deputy State Statistician

September 1996

TABLE OF CONTENTS

CHAPTER **PAGE**

Agricultural Overview 1

California Fruit and Nut Crops 15

California Vegetables and Melons 43

California Field Crops 51

California Floriculture 73

California Livestock and Dairy 75

California Department of Food and Agriculture 91

California County Agricultural Commissioners 92

California Cooperative Extension County Offices 94

California Agricultural Statistics Service Publication Order Form 95

CALIFORNIA AGRICULTURE OVERVIEW

CALIFORNIA'S CASH RECEIPTS JUMP 4% TO A NEW RECORD HIGH

California's ranchers and farmers sold an estimated $22.1 billion in agricultural products in 1995, up 4% from 1994. Fruits and nuts, vegetables and melons, and livestock and poultry, each comprised 25% of the total farm marketings. Field crops accounted for 15%, while nursery and greenhouse products, and Christmas trees accounted for the remaining 10%. Fruit and nut cash receipts decreased 1% to $5.53 billion, while field crops, and livestock and poultry each increased 1% to $3.28 billion and $5.55 billion, respectively. Vegetable crops jumped 12% to $5.56 billion, and nursery and greenhouse products increased 9% to $2.17 billion. Notable increases in marketings: cotton, +17%; sweet potatoes, +24%; Freestone peaches, +43%; plums, +49%; pistachios, +18%; lettuce, +34%; celery, +27%; carrots, +26%; onions, +28%; milk and cream, +4%; and melons (cantaloupes, honeydew, watermelon), +38%. Significant decreases in marketings: rice, -29%; sugar beets, -18%; grapes, -5%; cattle and calves, -10%; and fresh market tomatoes, -23%.

RAIN BRINGS MUCH NEEDED RELIEF TO CALIFORNIA

The Golden State experienced a very wet year (July 1994-June 1995) with precipitation much above normal for most of the State. Heavy rainfall across the State in January and March caused severe flooding and many crops were damaged. Because of the saturated soils and strong winds many trees were blown over, but especially hard hit were the almonds. The cool, rainy weather during the critical pollination reduced the production of many fruit and nut crops. Crops affected and reductions in production from the preceding year were: almonds, -50%; apricots, -64%; cherries, -62%; plums, -50%; and strawberries, -10%. March storms wrecked havoc in Monterey, California's leading vegetable county. Many vegetable crops actually showed an increase for the year. However, the storms reduced the production of the following: artichokes, -57%; asparagus, -21%; and head lettuce, -16%. Late plantings of field crops, caused by the wet, cool spring weather, and combined with heavy pest infestations in cotton, substantially reduced production of the following: Upland cotton, -15%; potatoes, -11%; rice, -14%; sugar beets, -18%; and wheat, -26%. Significant increases in production were; dry beans, +7%; Pima cotton, +21%; all hay, +10%; and sweet potatoes, +14%. Red meat production was up a modest 1% (beef, 1%; lambs and mutton, 17%; and pork, -7%). Poultry production was up 4%. California continues to lead the Nation in milk production; milk is the Golden State's leading agricultural commodity.

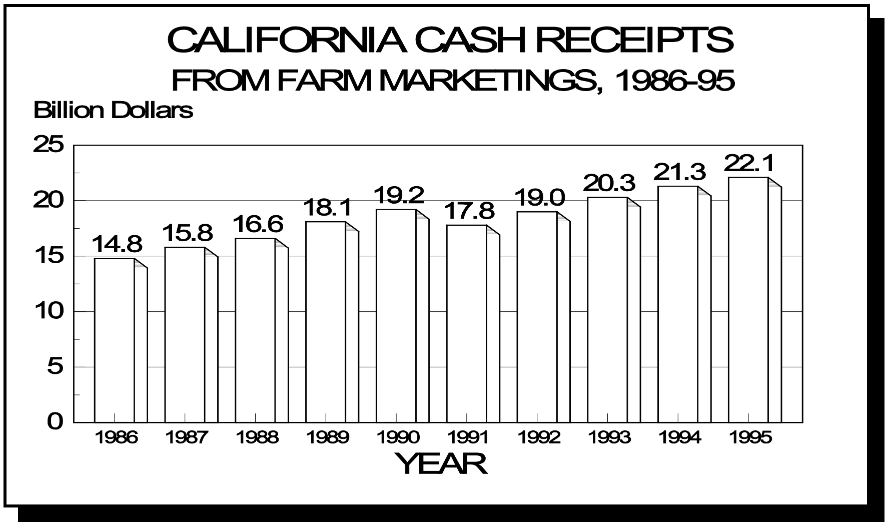

CALIFORNIA FIRSTS

Some 250 crops are recognized in California, including seeds, flowers and ornamentals.

This report contains information about 65 major crops grown on a large commercial scale in California:

13 Field Crops
25 Fruits and Nuts
25 Vegetables and Melons
Nursery Products and Cut Flowers

In addition, data are shown for 10 livestock, poultry, and other products.

California leads the Nation in the crops shown in the table below. A large number are specialty crops in which the Golden State accounts for most of the U.S. production.

CROP AND LIVESTOCK COMMODITIES IN WHICH CALIFORNIA LEADS THE NATION

Alfalfa Hay	Dates	Oriental Vegetables
Alfalfa Seed	Dry Baby Lima Beans	Parsley
Almonds	Dry Large Lima Beans	Peaches
Apricots	Eggs	Pears, Bartlett
Artichokes	Figs	Persian Melons
Asparagus	Garbanzo Beans	Persimmons
Avocados	Garlic	Pigeons & Squabs
Bedding Plants	Goat's Milk	Pistachios
Bell Peppers	Green Lima Beans	Plums
Bermuda Grass Seed	Green Onions	Pomegranates
Blackeye Beans	Greenhouse Vegetables	Prunes
Broccoli	Herbs	Rabbits
Brussels Sprouts	Honey	Raisins
Bulbs	Honeydew Melons	Safflower
Cantaloupes	Indoor Plants	Sod
Carrots	Dry Kidney Beans	Spinach
Casaba Melons	Kiwifruit	Strawberries
Cauliflower	Ladino Clover Seed	Sudan Grass
Celery	Lemons	Sweet Rice
Chicary	Romaine Lettuce	Table Grapes
Chinchillas	Leaf Lettuce	Processing Tomatoes
Chinese Cabbage	Head Lettuce	Vegetable & Flower Seed
Chinese Peas	Milk	Walnuts
Crenshaw Melons	Nectarines	Wild Rice
Currants	Olives	Wine Grapes
Cut Flowers	Onions	

CALIFORNIA FARM INCOME INDICATORS, 1991-95

Item	1991	1992	1993	1994	1995
	Million Dollars				
Gross Farm Income	19,363.5	20,309.9	22,185.4	22,733.6	23,309.7
Gross Cash Income	18,601.4	19,982.1	21,469.5	22,096.0	22,895.1
Farm Marketings	17,774.2	19,036.3	20,337.9	21,274.5	22,108.4
Crops	12,527.1	13,936.3	14,956.4	15,785.8	16,559.9
Livestock and Products	5,247.1	5,100.0	5,381.5	5,488.7	5,548.5
Government Payments	260.8	430.4	522.1	272.7	237.8
Farm-Related Income	566.4	515.5	609.5	548.8	548.9
Noncash Income	533.8	531.0	578.7	636.9	634.1
Value of Home Consumption	27.3	23.4	23.4	15.6	17.9
Rental Value of Dwellings	506.4	507.5	555.2	621.4	616.3
Operator and Other Dwellings 1/	440.5	423.6	499.1	556.8	558.3
Hired Laborer Dwellings	65.9	84.0	56.2	64.5	57.9
Value of Inventory Adjustment	228.4	-203.2	137.2	0.7	-219.5
Total Production Expenses	15,198.8	14,972.7	16,297.0	17,176.3	19,365.1
Intermediate Product Expenses	8,670.4	8,415.1	9,517.1	10,104.0	11,780.9
Farm Origin	2,650.4	2,604.5	2,933.3	2,886.4	3,374.5
Feed Purchased	1,610.5	1,691.1	2,003.4	1,960.8	2,380.5
Livestock and Poultry Purchased	616.1	523.7	512.6	526.7	521.5
Seed Purchased	423.7	389.8	417.4	398.9	472.5
Manufactured Inputs	1,667.6	1,686.3	1,744.1	1,821.2	2,326.9
Fertilizer and Lime	602.3	572.3	593.6	665.7	773.3
Pesticides	654.0	724.5	754.6	779.4	881.2
Fuel and Oil	411.3	389.5	395.9	376.1	872.4
Other	4,352.5	4,124.3	4,839.7	5,396.4	6,079.5
Repair and Maintenance	603.5	614.7	704.6	648.4	704.3
Other Miscellaneous	3,749.0	3,509.6	4,135.1	4,748.1	5,375.2
Interest	978.8	937.1	990.4	1,103.5	1,202.0
Real Estate	438.0	460.0	520.6	563.9	617.6
Non-Real Estate	540.8	477.0	469.9	539.5	584.4
Contract and Hired Labor Expenses	3,699.9	3,718.9	3,958.8	4,006.9	4,530.5
Net Rent to Non-Operator Landlords 2/	534.3	554.9	465.8	532.0	378.2
Capital Consumption	927.9	937.2	925.1	951.7	948.5
Property Taxes	387.5	409.4	439.8	478.3	525.0
NET FARM INCOME 3/	4,164.7	5,337.2	5,888.4	5,557.2	3,944.7
Gross Receipts of Farms	18,923.0	19,886.3	21,686.3	22,176.7	22,751.4
Farm Production Expenses	14,898.0	14,689.8	16,022.8	16,884.2	19,048.6
Non-Factor Payments	9,724.2	9,521.1	10,646.7	11,282.6	12,980.2
Intermediate Product Expenses	8,566.1	8,361.4	9,470.0	10,052.0	11,719.9
Capital Consumption	797.7	781.6	772.0	793.8	780.2
Property Taxes	360.4	378.1	404.7	436.8	480.1
Factor Payments	5,173.8	5,168.7	5,376.1	5,601.6	6,068.4
Interest	939.6	894.9	951.5	1,062.7	1,159.7
Contract and Hired Labor Expenses	3,699.9	3,718.9	3,958.8	4,006.9	4,530.5
Net Rent to Non-Operator Landlords 2/	534.3	554.9	465.8	532.0	378.2
RETURNS TO OPERATORS 4/	4,025.0	5,196.5	5,663.5	5,292.6	3,702.8
Gross Cash Income	18,601.4	19,982.1	21,469.5	22,096.0	22,895.1
Cash Expenses	14,074.4	13,864.2	15,233.5	16,065.6	18,250.1
Cash Expenses, Excluding Net Rent	13,500.1	13,269.3	14,728.8	15,493.8	17,832.3
Intermediate Product Expenses	8,566.1	8,361.4	9,470.0	10,052.0	11,719.9
Interest	939.6	894.9	951.5	1,062.7	1,159.7
Cash Labor Expenses	3,634.0	3,634.9	3,902.6	3,942.4	4,472.5
Property Taxes	360.4	378.1	404.7	436.8	480.1
Net Rent to Non-Operator Landlords 5/	574.2	594.9	504.7	571.8	417.8
NET CASH INCOME	4,527.0	6,117.9	6,236.0	6,030.3	4,645.0
Number Of Farms	83,000	82,000	79,000	79,000	80,000

1/ Value added to gross income. Value added to net farm income equals difference in net farm income and returns to operators. 2/ Includes landlord capital consumption. 3/ Statistics in and above the Net Farm Income line represent the farm sector, defined as including farm operators' dwellings located on farms. Statistics below the Net Farm Income line represent only the farm businesses to the exclusion of the operators' dwellings. 4/ Returns to operators is equivalent to net farm income excluding the income and expenses associated with farm operators' dwellings. 5/ Excludes landlord capital consumption.

CASH RECEIPTS DEFINED

Cash receipt data as prepared by the Economic Research Service (ERS) reflect income derived from the sale of agricultural commodities during a calendar year for only that portion of the commodity that is sold. Whereas, value of production data for crops and livestock products as prepared by the National Agricultural Statistics Service (NASS) reflect the total value of the commodity produced based on a marketing year average price. For certain commodities such as some fruits and vegetables which are normally sold in the same calendar year in which they were produced, cash receipt data and value of production data will be in close agreement. However, for most field crops, the marketing year will span portions of two calendar years, making the two data series non comparable. Data users should be aware of the differences between the ERS and NASS data series in their use of the data.

CALIFORNIA CASH INCOME BY COMMODITIES AND COMMODITY GROUPS, 1993-95

Source of Income	1993	1994	1995
		$1,000	
FIELD CROPS			
Alfalfa Seed	24,116	40,381	40,400
Barley	23,956	29,242	35,093
Beans, Dry	67,301	81,665	98,764
Corn for Grain	58,429	65,425	67,581
Cotton Lint	933,908	1,119,392	1,307,977
Cottonseed	116,120	100,575	84,922
Hay, All	528,708	643,660	633,059
Oats	3,312	3,111	3,109
Oil Crops	62,844	63,052	56,748
Potatoes	180,695	171,643	177,328
Rice	147,207	394,808	279,042
Sugar Beets	125,528	132,258	107,870
Sweet potatoes	40,221	45,682	56,784
Wheat	120,202	156,334	136,347
Other Field Crops	174,236	191,827	193,687
TOTAL	2,606,783	3,239,055	3,278,711
Percent of Gross Cash Income	12.1	14.7	14.3
FRUIT & NUT CROPS			
Apples	138,131	138,478	140,467
Apricots	32,288	43,700	20,405
Cherries	43,203	63,512	41,540
Figs	24,341	23,713	18,357
Nectarines	102,421	68,168	93,990
Peaches, Clingstone	113,033	97,470	90,310
Peaches, Freestone	90,072	67,918	97,406
Pears	77,806	70,269	62,261
Plums	93,954	79,358	117,849
Prunes	162,845	172,496	202,294
Almonds	930,618	900,375	888,000
Pecans	2,880	1,740	3,584
Pistachios	162,640	118,809	139,722
Walnuts	361,400	232,000	234,000
Grapes	1,823,455	1,719,464	1,638,418
Kiwifruit	16,502	18,413	15,859
Avocados	135,678	248,527	232,440
Dates	22,756	18,609	22,701
Olives	56,974	38,893	49,755
Grapefruit	53,058	51,708	51,741
Lemons	230,044	193,720	178,218
Oranges, Navel & Miscellaneous	303,114	277,759	241,600
Oranges, Valencia	171,360	188,700	188,700
Tangerines	27,249	25,804	19,144
Bushberries	43,336	34,047	27,535
Strawberries	457,808	634,126	634,133
Other Fruits & Nuts	70,950	80,642	79,150
TOTAL	5,747,916	5,608,418	5,529,579
Percent of Gross Cash Income	26.8	25.4	24.2
CHRISTMAS TREES	21,000	18,807	19,000
Percent of Gross Cash Income	0.1	0.1	0.1
NURSERY & GREENHOUSE PRODUCTS	1,902,610	1,986,450	2,171,904
Percent of Gross Cash Income	8.9	9.0	9.5

Source of Income	1993	1994	1995
		$1,000	
VEGETABLE CROPS			
Artichokes	45,762	61,577	34,299
Asparagus	88,177	101,694	92,028
Beans, Snap 1/	32,706	33,518	43,928
Broccoli	247,863	304,070	317,604
Brussels Sprouts	24,761	15,471	16,452
Cabbage	59,184	51,590	75,517
Carrots	180,640	227,753	287,070
Cauliflower	159,245	154,594	171,559
Celery	230,545	194,181	246,229
Corn, Sweet	50,401	56,260	62,916
Cucumbers 1/	46,198	39,428	59,211
Garlic	132,470	181,038	179,834
Lettuce	1,135,240	1,033,085	1,385,397
Mushrooms	134,476	140,235	146,090
Onions	161,668	102,128	130,656
Peppers, Green	149,057	152,287	150,020
Spinach	46,075	42,636	47,383
Tomatoes, Fresh	266,288	248,942	192,888
Tomatoes, Proc.	529,038	655,638	672,472
Cantaloupe	188,955	179,145	237,319
Honeydew	44,088	45,177	78,914
Watermelon	59,094	71,164	91,005
Other Veg. Crops	666,130	841,438	841,919
TOTAL	4,678,061	4,933,049	5,560,710
Percent of Gross Cash Income	21.8	22.3	24.3
LIVESTOCK, POULTRY & PRODUCTS			
Aquaculture	13,258	9,767	12,885
Broilers	352,512	373,230	383,175
Cattle & Calves	1,590,174	1,428,205	1,289,765
Eggs	265,988	255,277	288,369
Farm Chickens	1,651	1,058	1,033
Hogs	50,537	45,186	35,887
Honey	22,500	12,240	23,436
Milk & Cream	2,663,209	2,967,085	3,078,480
Sheep & Lambs	73,486	54,698	68,920
Turkeys	208,980	193,242	212,520
Wool	2,780	3,457	5,355
Other Livestock & Poultry	136,421	145,265	148,702
TOTAL	5,381,496	5,488,710	5,548,527
Percent of Gross Cash Income	25.1	24.8	24.2
TOTAL CASH INCOME FROM MARKETINGS	20,337,866	21,274,489	22,108,431
GOVERNMENT PAYMENTS	522,100	272,700	237,800
Percent of Gross Cash Income	2.4	1.2	1.0
FARM RELATED INCOME	593,500	548,800	548,900
Percent of Gross Cash Income	2.8	2.5	2.4
GROSS CASH INCOME FROM FARMING	21,453,500	22,095,900	22,895,100

1/ Income for 1993 includes fresh market.

SOURCE: USDA Economic Research Service

COMMODITY RANKING, ACREAGE, PRODUCTION, VALUE, AND LEADING PRODUCING COUNTIES, 1995

Commodity	National Ranking 1/	California Share of U.S. Production	Harvested Acreage	Short Tons (2,000 Lbs.)	Value 2/	Rank Among All California Commodities		Harvest Season	Leading Counties 3/
						1994	1995		
	Number	*Percent*	*1,000 Acres*	*1,000 Tons*	*$1,000*	*Number*			
**VEGETABLES -- TOTAL VALUE -- $ 5,546,499,000**									
Artichokes	1	100	8.3	23.6	34,299	49	61	Continuous	Monterey, Riverside, Santa Cruz, Orange, San Mateo
Asparagus	2	38	28.0	39.2	91,728	40	44	Feb. 15-June 30	San Joaquin, Imperial, Monterey, Santa Barbara
Beans, Snap	2	18	8.8	39.6	40,392	62	59	Apr. 20-Dec. 15	Tulare, Riverside, Orange
Broccoli	1	88	89.5	537.0	317,604	14	15	Continuous	Monterey, Santa Barbara, San Luis Obispo, Imperial
Brussels Sprouts 4/	1	93	3.8	32.3	16,452	68	68	Aug. 1-Mar. 15	Santa Cruz, San Mateo, Monterey
Cabbage	2	20	13.0	240.5	75,517	52	47	Continuous	Ventura, San Benito, Monterey, Santa Barbara
Carrots	1	55	63.5	702.2	287,070	20	18	Continuous	Imperial, Kern, Monterey
Cauliflower	1	78	40.7	254.4	171,559	31	31	Continuous	Monterey, Santa Barbara, Imperial
Celery	1	90	22.7	749.1	246,229	24	19	Continuous	Ventura, Monterey, Santa Barbara, San Luis Obispo
Corn, Sweet, Fresh Market	2	16	22.3	167.2	62,552	50	51	May 1-Dec. 10	Riverside, Imperial, Fresno, San Joaquin, Contra Costa
Cucumbers, Fresh Market	3	17	5.2	81.9	43,079	61	56	Mar. 20-Nov. 30	San Joaquin, San Diego, Riverside, Orange
Processing	5	8	5.2	42.1	16,132	71	69	July 1-Sept. 30	San Joaquin , San Diego, Riverside, Orange
Garlic 4/	1	88	31.0	255.8	179,834	26	29	Apr. 1-Sept. 15	Fresno, Kern, Monterey
Lettuce, Head	1	69	136.0	2,006.0	986,952	9	6	Continuous	Monterey, Fresno, Imperial, Santa Barbara
Leaf	1	82	35.0	367.5	225,645	22	23	Continuous	Monterey, Imperial, Ventura, Riverside
Romaine	1	80	24.0	360.0	172,800	37	30	Continuous	Monterey, Riverside, Ventura
Melons, Cantaloupe	1	65	59.3	682.0	237,319	27	20	May 15-Nov. 30	Fresno, Imperial, Kern, Riverside, Merced
Honeydew	1	75	21.1	211.0	78,914	56	46	June 1-Nov. 30	Fresno, Sutter, Riverside, Imperial, Stanislaus
Watermelon	4	16	17.2	318.2	91,005	49	45	May 25-Oct. 15	Kern, Riverside, San Joaquin, Imperial, Fresno
Mushrooms, Agaricus	2	17	0.5	67.4	140,796	36	35	Continuous	Monterey, Santa Clara, San Mateo, San Diego
Onions	1	25	38.0	797.9	130,666	39	37	Apr. 1-Oct. 31	Imperial, Fresno, Kern, San Joaquin
Peppers, Bell	1	44	20.0	290.0	150,020	32	33	July 1-Dec. 10	Riverside, San Benito, Ventura, Fresno, San Joaquin
Spinach, Fresh Market	1	64	6.7	62.0	42,656	60	57	Continuous	Monterey, Stanislaus, Santa Barbara
Tomatoes, Fresh Market	2	31	10.3	513.0	192,888	18	25	May 15-Dec. 31	Fresno, San Joaquin, Merced, San Diego
Processing	1	93	317.0	10,606.8	672,472	10	9	June 20-Nov. 10	Fresno, Yolo, Colusa, San Joaquin, Sutter
Other Vegetables & Melons, Etc.	---	---	---	---	841,919	---	---	---	---

See Footnotes on Page 8.

COMMODITY RANKING, ACREAGE, PRODUCTION, VALUE, AND LEADING PRODUCING COUNTIES, 1995

Commodity	National Ranking 1/	California Share of U.S. Production	Harvested Acreage	Short Tons (2,000 Lbs.)	Value 2/	Rank Among All California Commodities 1994	Rank Among All California Commodities 1995	Harvest Season	Leading Counties 3/
	Number	*Percent*	*1,000 Acres*	*1,000 Tons*	*$1,000*	*Number*			
**FRUITS AND NUTS -- TOTAL VALUE -- $ 5,775,304,000**									
Almonds (Shelled)	1	99.9	400.0	185.0	857,771	6	7	Aug. 1-Oct. 31	Kern, Stanislaus, Merced, Fresno, Madera
Apples	4	8.1	35	425.0	155,750	34	32	July 15-Oct. 30	Kern, San Joaquin, Fresno, Tulare, Madera
Apricots	1	88.9	19.8	52.0	20,405	57	65	May 1-July 15	Stanislaus, Fresno, Kern, San Joaquin, Tulare
Avocados	1	88.5	67.6	156.0	232,440	17	21	Continuous	San Diego, Ventura, Santa Barbara, Riverside
Boysenberries	2	27.4	0.3	0.9	1,710	75	75	June 1-July 15	---
Cherries, Sweet	4	12.0	12.5	19.8	41,540	47	58	May 20-June 25	San Joaquin, Stanislaus, Tulare, Santa Clara
Dates 4/	1	99.5	5.5	22.0	16,456	67	67	Oct. 1-Dec. 15	Riverside, Imperial
Figs 4/	1	99.9	15.0	50.0	18,357	65	66	June 10-Sep. 15	Madera, Merced, Fresno
Grapefruit, All	2	13.1	18.4	311.6	52,827	53	53	---	Riverside, Tulare, San Diego, Kern
Desert	---	---	8.0	110.6	14,423	---	---	Nov. 10-June 10	---
Other	---	---	10.4	201.0	38,404	---	---	Mar. 15-Oct. 25	---
Grapes, All	1	88.4	645.2	5,248.0	1,838,900	2	2	---	Fresno, Tulare, Kern, San Joaquin, Madera
Raisin Type	---	---	268.0	2,255.0	496,860	---	---	May 15-Nov. 15	---
Table Type	---	---	76.2	718.0	376,890	---	---	May 25-Dec. 15	---
Wine Type	--	---	301.0	2,275.0	965,150	---	---	Aug. 5-Dec. 15	---
Kiwifruit 4/	1	99.5	6.6	38.0	15,089	66	70	Oct. 1-May 31	Tulare, Butte, Fresno, Yuba, Kern
Lemons	1	85.1	45.7	779.0	228,308	23	22	Continuous	Ventura, San Diego, Kern, Riverside
Nectarines 4/	1	93.0	32.4	176.0	93,990	46	42	June 10-Sep. 5	Fresno, Tulare, Kern, Kings
Olives	1	100.0	33.7	77.5	47,528	58	54	Sep. 25-Mar. 15	Tulare, Glenn, Tehama, Kern, Madera
Oranges, All	2	19.0	191.0	2,287.5	458,130	12	12	---	Tulare, Kern, Fresno, Ventura, San Diego
Navel & Misc.	---	---	118.0	1,312.5	273,750	---	---	Nov. 25-May 15	---
Valencia	---	---	73.0	975.0	184,380	---	---	May 15-Oct. 15	---
Peaches, All	1	62.4	60.6	683.5	183,485	29	28	---	Fresno, Tulare, Stanislaus, Sutter, Kern
Clingstone	---	100.0	28.1	432.5	90,310	---	---	July 15-Sep. 15	---
Freestone	---	36.7	32.5	251.0	93,175	---	---	May 10-Sep. 15	---
Pears, All	2	28.2	23.9	267.0	63,187	45	50	Aug. 5-Oct. 5	Sacramento, Lake, Mendocino, Tulare
Pecans	9	1.1	2.6	1.4	3,712	74	74	Sep. 1-Nov. 30	Tulare
Pistachios	1	100	60.3	74.0	141,636	38	34	Sep. 15-Dec. 10	Kern, Madera, Tulare, Kings, Fresno
Plums	1	84.4	42.0	124.0	117,849	43	39	May 25-Aug. 20	Tulare, Fresno, Kern
Prunes, Dried Basis	1	100.0	78.8	181.0	188,240	21	27	Aug. 15-Oct. 10	Sutter, Butte, Yuba, Tulare, Tehama
Raspberries	3	17.7	1.9	7.6	23,325	63	64	June 1-Oct. 31	Santa Cruz, Monterey

See Footnotes on Page 8.

COMMODITY RANKING, ACREAGE, PRODUCTION, VALUE, AND LEADING PRODUCING COUNTIES, 1995

Commodity	National Ranking 1/	California Share of U.S. Production	Harvested Acreage	Short Tons (2,000 Lbs.)	Value 2/	Rank Among All California Commodities		Harvest Season	Leading Counties 3/
						1994	1995		
	Number	*Percent*	*1,000 Acres*	*1,000 Tons*	*$1,000*	*Number*			
Strawberries	1	79.6	23.6	595.9	551,698	11	11	Feb. 20-Nov. 15	Monterey, Ventura, Santa Barbara, Santa Cruz, Orange
Fresh Market	1	76.7	---	400.0	459,257	---	---	---	---
Processing	1	86.1	---	195.9	92,441	---	---	---	---
Tangerines, Mandarins, Tangelos, & Tangors	2	19.8	8.5	82.5	30,261	64	62	Nov. 1-Apr. 30	Tulare, Riverside, Imperial
Walnuts 4/	1	99.5	169.0	234.0	313,560	19	16	Sep. 5-Nov. 10	San Joaquin, Stanislaus, Butte, Tulare, Sutter
Other Fruit and Nuts	---	---	---	---	79,150	---	---	---	---
**FIELD AND SEED CROPS -- TOTAL VALUE -- $ 3,511,814,000**									
Barley	6	3.9	200.0	336.0	45,500	59	55	May 15-Oct. 1	Tulare, Siskiyou, Kings, Modoc, Kern
Beans, Dry	4	9.5	137.0	148.0	101,824	41	41	Aug. 20-Nov. 15	Stanislaus, Fresno, San Joaquin, Tulare, Kern
Corn for Grain	25	0.3	150.0	672.0	92,400	42	43	Sept. 1-Dec. 1	San Joaquin, Sacramento, Yolo, Solano, Glenn
All Cotton, Lint	2	14.2	1,280.0	608.8	1,063,505	5	5	Oct. 1-Jan. 1	Fresno, Kern, Kings, Tulare, Merced
All Cotton, Seed	2	13.7	---	940.0	124,080	33	38	---	Fresno, Kings, Kern, Tulare, Merced
Hay, Alfalfa & Other	2	5.8	1,600.0	9,000.0	841,200	7	8	Mar. 1-Nov. 5	Imperial, Kern, Fresno, Merced, Tulare
Oats	17	1.6	30.0	40.8	5,228	72	73	June 15-Oct. 1	San Joaquin, Siskiyou, Riverside, Merced, Solano
Potatoes	10	3.3	41.3	731.0	190,894	28	26	Continuous	Kern, Siskiyou, Riverside, Modoc, San Joaquin
Rice 5/ 6/	2	20.3	465.0	1,767.6	318,168	15	14	Sept. 1-Nov. 30	Colusa, Sutter, Glenn, Butte, Yuba
Sugar Beets 5/ 6/	3	11.5	115.0	3,220.0	107,870	35	40	Apr. 1-Dec. 10	Imperial, Fresno, San Joaquin, Solano, Kern
Sweet Potatoes	3	12.8	8.3	83.0	63,360	54	49	July 15-Nov. 15	Merced, Stanislaus
Wheat, All	19	1.5	493.0	981.8	139,953	30	36	May 20-Sept. 1	Imperial, Tulare, Kings, Kern, Solano
Oil Crops 7/	N/A	37.1	179.2	183.3	56,748	48	52	---	---
Other Field Crops	---	---	---	---	361,084	---	---	---	---
**NURSERY AND FLOWER CROPS -- TOTAL VALUE -- $ 2,171,904,000**									
Nursery Products 4/ 6/ 8/	1	24.6	---	---	1,500,000	4	3	Continuous	San Diego, Monterey, San Mateo, Kern, Ventura
Flowers & Foliage 9/	1	20.5	---	---	671,904	8	10	Continuous	San Diego, San Mateo, Santa Barbara, Monterey, Santa Cruz

See Footnotes on Page 8.

COMMODITY RANKING, VALUE, AND LEADING PRODUCING COUNTIES, 1995

Commodity	National Ranking 1/	California Share of U.S. Production	Marketings	Cash Receipts 2/	Rank Among All California Commodities		Leading Counties 3/
					1994	1995	
	Number	Percent	1,000 Pounds	$1,000	Number		
**LIVESTOCK, DAIRY, POULTRY, AND APIARY -- TOTAL VALUE -- $ 5,548,527,000**							
Aquaculture	---	---	---	12,885	70	71	Riverside, Imperial, Sacramento
Cattle and Calves	7	4.8	2,703,670	1,289,765	3	4	Tulare, Imperial, Fresno, Kern, San Bernardino
Chickens, All	---	3.5	1,233,360	384,208	13	13	Fresno, Merced, Stanislaus, San Bernardino
Broilers & Fryers	10	3.4	1,179,000	383,175	---	---	---
Other	8	5.1	54,360	1,033	---	---	---
Hogs & Pigs	26	0.4	89,366	35,887	55	60	Tulare, Merced, Fresno
Honey	1	18.6	39,060	23,436	69	63	Tulare, Kern, Riverside, Los Angeles, Kings
Milk & Cream	1	16.2	24,742,000	3,078,480	1	1	Tulare, San Bernardino, Merced, Stanislaus, Riverside
Sheep & Lambs	2	12.3	98,218	68,920	51	48	Merced, Tulare, Kern, Solano, San Joaquin
Turkeys	5	6.8	462,000	212,520	25	24	Fresno, Stanislaus, Merced, Kings, Madera
Wool	3	8.3	5,250	5,355	73	72	---
Eggs, Chicken	1	8.7	6,444	288,369	16	17	Riverside, San Diego, Stanislaus, San Bernardino, Merced
Other Livestock & Poultry	---	---	---	148,702	---	---	---

1/ Based on quantity produced for crops and on quantity marketed for livestock and poultry products.
2/ Based on value of quantity harvested for crops, value of quantity marketed for livestock and value of quantity produced for poultry products.
3/ Based on values published in the Annual County Agricultural Commissioners' Reports.
4/ Share of U.S. production based on 1987 Census of Agriculture.
5/ The 1995 price and value are based on the 1994 California average price.
6/ Extracted from Agricultural Commissioners' Annual Crop Reports.
7/ Includes Sunflower, Safflower, and Canola.
8/ Includes trees, shrubs, vines, bulbs, turf, etc., not included in flowers and foliage category.
9/ Includes cut flowers, potted plants, foliage plants, bedding plants and indoor decoratives.

CALIFORNIA'S LEADING FARM PRODUCTS

- California's "Top Twenty" crop and livestock commodities account for 72% of the State's gross farm income.

California's Diversity

- California agriculture is considered one of the most diversified in the world, with no one crop dominating the State's farm economy.

- This is illustrated by the fact that only one product exceeds 10%, and the five leading commodities account for about two-fifths, of the State's total gross farm income.

- California continues to lead the nation with many commodities, primarily fruit and nut crops and vegetables. Fruit, nut and vegetable crops produced in California account for over half of the nation's production.

CALIFORNIA'S TOP TWENTY

Farm Product	Commodity Ranking		Value		Percent of Total	
	1994	1995	1994	1995	1994	1995
	Number		*$1,000*		*Percent*	
Milk and Cream	1	1	2,967,085	3,078,480	13.8	13.7
Grapes	2	2	1,712,939	1,838,900	7.9	8.2
Nursery Products	4	3	1,285,002	1,500,000	6.0	6.7
Cattle and Calves	3	4	1,428,205	1,289,765	6.6	5.7
Cotton Lint	5	5	1,137,718	1,063,505	5.3	4.7
Lettuce, Head	9	6	698,610	986,952	3.2	4.4
Almonds	6	7	965,202	857,771	4.5	3.8
Hay	7	8	853,220	847,050	3.6	3.8
Tomatoes, Processing	10	9	655,638	672,472	3.0	3.0
Flowers and Foliage	8	10	701,448	671,904	3.3	3.0
Strawberries	11	11	647,575	551,698	3.0	2.5
Oranges	12	12	462,567	458,130	2.1	2.0
Chickens	13	13	374,288	384,208	1.7	1.7
Rice	15	14	287,331	318,168	1.3	1.4
Broccoli	14	15	304,070	317,604	1.4	1.4
Walnuts	19	16	232,000	313,560	1.1	1.4
Eggs, Chicken	16	17	255,277	288,369	1.2	1.3
Carrots	20	18	227,753	287,070	1.1	1.3
Celery	22	19	194,181	246,229	0.9	1.1
Cantaloupe	27	20	179,145	237,319	0.8	1.1

CALIFORNIA'S LEADING AGRICULTURAL COUNTIES
BY TOTAL VALUE OF PRODUCTION -- 1995 1/

Rank		County	Value of Production	Leading Crops 2/
1994	1995		$1,000	
1	1	Fresno	3,167,157	Cotton, Grapes, Poultry, Tomatoes, Milk
2	2	Tulare	2,610,290	Milk, Oranges, Grapes, Cattle & Calves, Cotton Lint & Cottonseed
4	3	Monterey	2,028,492	Head Lettuce, Broccoli, Strawberries, Leaf Lettuce, Cauliflower
3	4	Kern	1,978,319	Grapes, Cotton & Cottonseed, Citrus, Almonds & By-Products, Milk
7	5	San Joaquin	1,222,600	Grapes, Milk, Almonds, Tomatoes, Walnuts
5	6	Merced	1,220,175	Milk, Almonds, Chickens, Cotton, Tomatoes
8	7	Riverside	1,163,430	Milk, Table Grapes, Eggs, Nursery, Cattle & Calves
6	8	Stanislaus	1,115,489	Milk, Almonds, Chickens, Chicken Eggs, Cattle & Calves
9	9	San Diego	1,048,721	Indoor Decoratives, Ornamental Trees & Shrubs, Bedding Plants & Turf, Avocados, Eggs
10	10	Imperial	1,008,653	Cattle, Alfalfa, Head Lettuce, Carrots, Leaf Lettuce
11	11	Ventura	921,744	Lemons, Strawberries, Celery, Avocados, Nursery Stock
12	12	Kings	837,899	Cotton & Cottonseed, Milk, Cattle & Calves, Turkeys, Grapes
13	13	San Bernardino	607,607	Milk, Cattle & Calves, Eggs, Nursery Products, Alfalfa Hay & Greenchop
14	14	Madera	598,565	Almonds, Raisin Grapes, Wine Grapes, Market Milk, Cotton Lint
15	15	Santa Barbara	533,803	Broccoli, Strawberries, Avocados, Grapes, Head Lettuce
17	16	Sonoma	342,550	Wine Grapes, Market Milk, Misc. Livestock & Poultry, Vegetables, Cattle & Calves
16	17	Sutter	328,925	Rice & Seed, Prunes, Tomatoes & Seed, Walnuts, Peaches
18	18	San Luis Obispo	321,598	Grapes, Broccoli, Head Letuce, Cattle & Calves, Vegetable Transplants
20	19	Colusa	304,900	Rice, Processing Tomatoes, Almonds, Wheat, Rice Seed
19	20	Yolo	297,200	Tomatoes, Wine Grapes, Rice, Almonds, Alfalfa Hay
21	21	Butte	260,273	Rice, Almonds, Walnuts, Prunes, Nursery Stock
22	22	Glenn	237,048	Rice, Dairy Products, Almonds, Prunes, Alfalfa Hay
23	23	Santa Cruz	235,047	Strawberries, Head Lettuce, Raspberries, Hybrid Tea Roses, Apples
25	24	Orange	234,015	Nursery & Cut Flowers, Strawberries, Avocados, Celery, Bell Peppers
24	25	Sacramento	228,761	Milk, Wine Grapes, Bartlett Pears, Field Corn, Processing Tomatoes
26	26	Los Angeles	222,725	Ornamental Trees & Shrubs, Bedding Plants, Dry Onions, Peaches, Indoor Plants & Foliage
27	27	San Mateo	194,752	Ornamental Nursery Stock, Potted Foliage Plants, Mushrooms, Snapdragons, Potted Orchids
28	28	Solano	180,387	Processing Tomatoes, Nursery Stock, Alfalfa Hay, Sugar Beets, Field Corn
31	29	San Benito	160,474	Head Lettuce, Bell Peppers, Nursery Stock, Baby Lettuce, Leaf Lettuce
29	30	Santa Clara	159,686	Mushrooms, Nursery Crops, Cut Flowers, Bell Peppers, Leaf Lettuce
30	31	Napa	150,405	Grapes, Floral & Nursery Products, Cattle & Calves, Milk, Pasture & Range
33	32	Siskiyou	120,784	Cattle & Calves, Alfalfa Hay, Potatoes & Seed, Pasture & Range, Nursery Products
32	33	Yuba	120,776	Rice, Prunes, Walnuts, Peaches, Cattle & Calves
34	34	Tehama	96,738	Walnuts, Prunes, Almonds, Cattle & Calves, Pasture & Range
36	35	Mendocino	89,220	Wine Grapes, Bartlett Pears, Cattle & Calves, Pasture, Milk
35	36	Contra Costa	75,818	Bedding Plants, Apples, Tomatoes, Milk, Sweet Corn

1/ Timber not included.
2/ Extracted from County Commissioner Reports where shown.

SOURCE: California Agricultural Commissioners' Reports

CALIFORNIA'S 1994-95 AND 1995-96 SEASON RAINFALL WITH COMPARISONS TO NORMAL

Stations	1994-95		1995-96		Normal Annual Rainfall
	Precipitation July 1 to June 30	Percent of Normal	Precipitation July 1 to June 30	Percent of Normal	
	Inches		*Inches*		*Inches*
**NORTH COAST**					
Eureka	57.78	154	43.62	116	37.53
Ukiah	Missing	---	23.29	61	37.96
Santa Rosa	47.82	158	36.72	121	30.30
Lakeport	56.92	182	35.96	115	31.25
St. Helena	58.35	166	46.49	132	35.19
**CENTRAL COAST**					
San Francisco AP	29.61	150	24.67	125	19.70
San Jose	23.59	164	17.20	119	14.42
Livermore	21.02	148	19.76	139	14.21
Salinas AP/Monterey 1/	28.50	152	13.61	109	12.44
Monterey	---	---	20.94	112	18.72
King City	19.24	168	10.41	91	11.44
Paso Robles AP	25.89	186	11.21	80	13.95
**SACRAMENTO VALLEY**					
Redding	57.22	172	39.29	118	33.30
Willows	34.04	195	20.39	117	17.49
Chico	56.56	215	26.40	100	26.32
Marysville	38.29	182	24.29	115	21.04
Woodland	32.05	165	25.40	131	19.43
Sacramento AP	27.63	158	21.05	120	17.52
**SAN JOAQUIN VALLEY**					
Stockton AP	17.50	125	17.03	122	13.95
Newman	18.86	178	14.59	138	10.58
Merced	17.70	147	12.96	108	12.01
Fresno AP	19.03	179	12.10	114	10.60
Five Points	13.22	198	5.13	77	6.67
Kettleman Station	13.50	204	5.34	81	6.61
Porterville	15.90	144	9.65	87	11.03
Bakersfield	10.29	180	6.26	109	5.72
**SOUTH COAST**					
Santa Maria AP	26.50	214	12.88	104	12.36
Santa Barbara AP	36.21	223	Missing	---	16.25
Oxnard	28.66	199	11.44	80	14.38
Los Angeles	24.35	165	9.95	67	14.77
Riverside	16.89	176	2.52	26	9.58
San Diego AP	17.12	173	4.75	48	9.90
**SOUTHEASTERN INTERIOR**					
Bishop	Missing	---	0.91	17	5.37
Daggett AP	Missing	---	1.05	27	3.93
Thermal AP	2.93	93	0.10	3	3.16
Blythe AP	Missing	---	1.87	52	3.60
Imperial	3.14	114	0.28	10	2.75
**CASCADE-SIERRA**					
Tulelake	14.13	128	14.49	131	11.05
Alturas	17.09	142	16.03	133	12.01
Mt. Shasta City	65.50	177	35.14	95	37.02
Blue Canyon AP	95.71	143	87.18	130	67.04
Yosemite Valley	63.66	172	41.89	113	37.05
Huntington Lake	80.41	218	43.79	119	36.93

1/ Weather Station has moved from Salinas (1993-94) to Monterey (1994/95).

SOURCE: National Weather Service Office, Sacramento, CA

CALIFORNIA FRUIT AND VEGETABLE CROP SEASONS

Commodity	Jan.	Feb.	Mar.	Apr.	May	June	July	Aug.	Sept.	Oct.	Nov.	Dec.
**FRUIT**												
Apples							◗	●	●	●		
Apricots					●	●	◖					
Boysenberries						●	◖					
Olallieberries						●	●	●				
Cherries					◗	◖						
Dates										●	●	◖
Figs						◗	●	●	◖			
Grapefruit	●	●	●	●	●	●	●	●	●	◖	◗	●
Grapes					◗	●	●	●	●	●	◖	
Kiwifruit	●	●	●	●	●					●	●	●
Lemons	●	●	●	●	●	●	●	●	●	●	●	●
Cantaloupe					◗	●	●	●	●	●	●	
Melons, Honeydew						◗	●	●	●	●	●	
Melons, Persian						◗	●	●	●	●		
Oranges, Navel	●	●	●	●	◖						◗	●
Oranges, Valencia					◗	●	●	●	●	◖		
Peaches					◗	●	●	●	◖			
Pears								●	●	◖		
Plums					◗	●	●	●				
Pomegranates									◗	●	◖	
Prunes								◗	●	◖		
Strawberries		◗	●	●	●	●	●	●	●	●	◖	
Tangerines	●	●	●								●	●
Tangelos	●	●	●								●	●
Watermelons					◗	●	●	●	●	◖		
**VEGETABLES**												
Artichokes	●	●	●	●	●	●	●	●	●	●	●	●
Asparagus		◗	●	●	●	●						
Avocados	●	●	●	●	●	●	●	●	●	●	●	●
Beans, Dry								◗	●	●	◖	
Beans, Snap				●	●	●	●	●	●	●	●	◖
Broccoli	●	●	●	●	●	●	●	●	●	●	●	●
Brussels Sprouts	●	●	◖					●	●	●	●	●
Cabbage	●	●	●	●	●	●	●	●	●	●	●	●
Carrots	●	●	●	●	●	●	●	●	●	●	●	●
Cauliflower	●	●	●	●	●	●	●	●	●	●	●	●
Celery	●	●	●	●	●	●	●	●	●	●	●	●
Corn					●	●	●	●	●	●	●	◖
Cucumbers			◗	●	●	●	●	●	●	●	●	
Garlic				●	●	●	●	●	◖			
Lettuce	●	●	●	●	●	●	●	●	●	●	●	●
Mushrooms	●	●	●	●	●	●	●	●	●	●	●	●
Onions				●	●	●	●	●	●	●		
Peppers, Green							●	●	●	●	●	◖
Peppers, Chili									●	●	●	◖
Potatoes	●	●	●	●	●	●	●	●	●	◖	◗	●
Potatoes, Sweet							◗	●	●	●	◖	
Spinach	●	●	●	●	●	●	●	●	●	●	●	●
Squash	●	●	●	●	●	●	●	●	●	●	●	●
Tomatoes					◗	●	●	●	●	●	●	●

● = Full Month ◖ = 1st Half of Month ◗ = 2nd Half of Month

CALIFORNIA 1995 FARM FACTS

- About 11.6% of the national gross cash receipts for farming are produced in California on 80,000 farms.
- This constitutes 3.9% of the Nation's total farms (based on the Census of Agriculture definition of farms as places with annual sales of agricultural products of $1,000 or more).

CALIFORNIA NUMBER OF FARMS, LAND IN FARMS, AND SIZE OF FARM, 1950-95

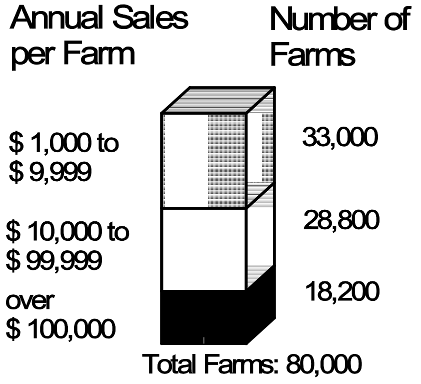

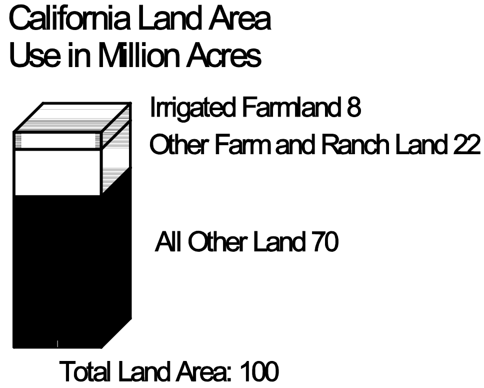

CALIFORNIA NUMBER OF FARMS, LAND IN FARMS, AND SIZE OF FARM -- 1950-1995

Year	Number of Farms	Land in Farms	Average Size of Farm
		1,000 Acres	Acres
1950 a/	144,000	37,500	260
1955	124,000	39,000	316
1960	108,000	38,800	359
1965	82,000	37,800	461
1970	64,000	36,600	572
1975 b/	73,000	34,300	470
1980	81,000	33,800	417
1985	79,000	32,900	416
1986	79,000	32,800	415
1987	83,000	31,900	384
1988	84,000	31,600	376
1989	84,000	31,300	373
1990	85,000	30,800	362
1991	83,000	30,500	367
1992	82,000	30,200	368
1993	79,000	30,000	380
1994	79,000	29,900	378
1995	80,000	30,000	375

a/ Old definition of farm: Places of 10 or more acres that had annual sales of agricultural products of $50 or more and places of less than 10 acres that had annual sales of $250 or more.
b/ New definition of farm: Places with annual sales of agricultural products of $1,000 or more.

PROFILE OF CALIFORNIA'S AGRICULTURE

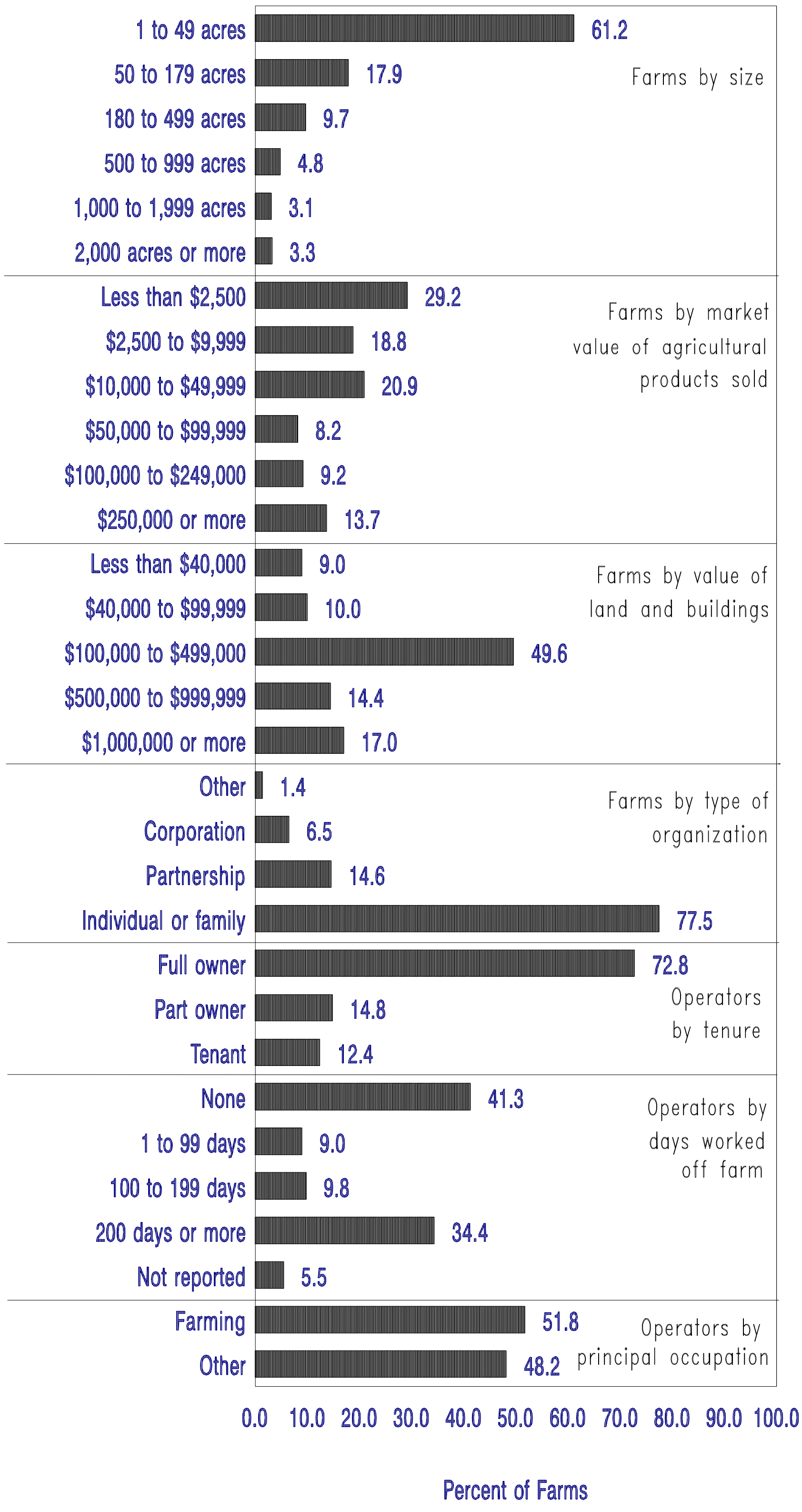

SOURCE: 1992 Census of Agriculture

CALIFORNIA FIELD CROPS

SUMMARY OF FIELD CROPS

California's 1995 field crop production was worth $3.51 billion at the farm-gate level. This value was 2% below the previous year.

Alfalfa, wheat, Upland cotton, cottonseed and oats recorded significantly lower farm returns. Although wheat prices were up, production was down considerably. The declining production was primarily due to widespread flooding in the Sacramento Valley, and persistent wet conditions which led to disease problems. Upland cotton prices were up slightly, but production was off dramatically. This was caused by the cool, wet spring and heavy insect damage throughout the growing season.

Rice, barley, potatoes, sweet potatoes, other hay, and American Pima cotton showed significantly higher farm values, which nearly offset the declines in wheat and Upland cotton. Rice, barley and potato production were down, but sharply higher prices helped push their total values above 1994 levels. For sweet potatoes and American Pima cotton, production and prices were considerably higher than the previous year.

The average value per harvested acre in 1995 dropped to $670 from the $700 per acre value in 1994.

Production of the major field crops totaled 26.8 million tons, up fractionally from the 26.7 million tons produced on 1994. Harvested acreage was higher than the previous year for cotton, dry beans, corn for silage, hay and sweet potatoes. Wheat, rice, barley, corn for grain, sugarbeets and potatoes showed declines in harvested acreage.

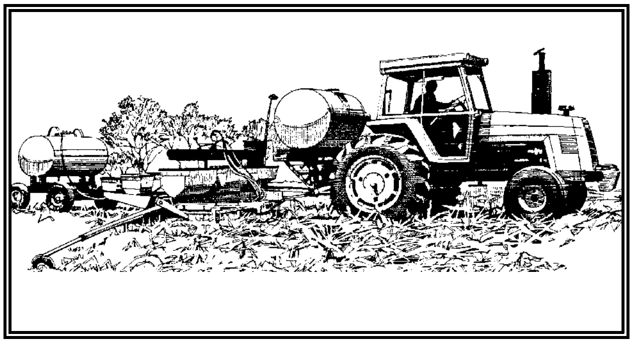

Upland cotton was again the state's highest valued field crop with a total value of $927 million. Nationally, California ranked second in Upland cotton production with 2.31 million bales produced, at an average price of 83.5 cents per pound.

All hay was California's second leading field crop, with a total value of $841 million. At the U.S. level, the state ranked second in the production of all hay with a total of 9.00 million tons.

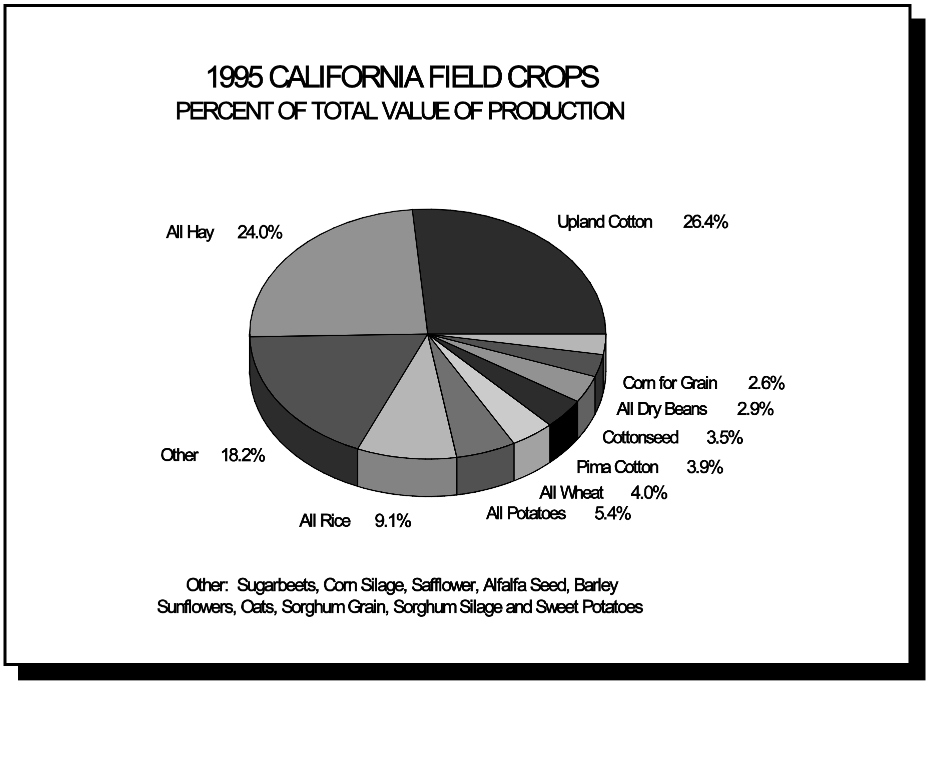

CALIFORNIA FIELD CROPS: Acreage, Production, Price And Value, 1986-95

Year	Acreage		Yield Per Acre	Production	Average Value Per Unit	Value of Production
	Planted	Harvested				
**Alfalfa Seed a/**						
	Acres d/	*Acres*	*Pounds*	*Pounds*	*Dollars Per Cwt.*	*$1,000*
1986	---	93,000	500	46,500,000	92.00	42,780
1987	---	67,000	605	40,541,000	108.00	43,784
1988	---	67,000	529	35,466,000	120.00	42,559
1989	---	67,000	489	32,755,000	130.00	42,582
1990	---	71,000	494	35,065,000	144.00	50,494
1991	---	68,000	603	41,012,000	123.00	50,445
1992	---	46,000	664	30,548,000	112.00	34,214
1993 b/	---	---	---	---	---	---
1994 b/	---	---	---	---	---	---
1995 b/	---	---	---	---	---	---
**Barley**						
	Acres	*Acres*	*Tons*	*Tons*	*Dollars Per Ton*	*$1,000*
1986	470,000	400,000	1.42	566,400	85.83	48,616
1987	400,000	330,000	1.30	427,700	89.58	38,313
1988	360,000	280,000	1.46	409,900	114.58	46,970
1989	320,000	250,000	1.39	348,000	110.83	38,570
1990	300,000	230,000	1.39	320,200	109.17	34,951
1991	240,000	160,000	1.42	226,600	105.82	23,978
1992	230,000	190,000	1.49	282,700	107.09	30,275
1993	250,000	200,000	1.56	312,000	105.00	32,760
1994	290,000	220,000	1.56	343,200	111.25	38,181
1995	260,000	200,000	1.68	336,000	135.42	45,500
**Beans, Dry Edible c/**						
	Acres	*Acres*	*Cwt.*	*Cwt.*	*Dollars Per Cwt.*	*$1,000*
1986	157,000	155,000	18.46	2,862,000	28.50	81,567
1987	172,000	168,000	18.68	3,138,000	27.50	86,295
1988	151,000	147,000	19.63	2,885,000	36.20	104,437
1989	185,000	180,000	18.65	3,357,000	34.80	116,824
1990	175,000	166,000	18.42	3,058,000	30.90	94,492
1991	153,000	150,000	21.57	3,235,000	26.20	84,757
1992	121,000	117,000	20.90	2,445,000	24.40	59,658
1993	109,000	106,000	20.85	2,210,000	35.30	78,013
1994	136,000	131,000	21.15	2,771,000	34.80	96,431
1995	145,000	137,000	21.60	2,960,000	34.40	101,824
**Corn For Grain**						
	Acres	*Acres*	*Tons*	*Tons*	*Dollars Per Ton*	*$1,000*
1986	500,000	250,000	4.26	1,064,000	76.79	81,700
1987	423,000	221,000	4.48	990,100	86.07	85,218
1988	375,000	187,000	4.06	759,200	112.86	85,683
1989	380,000	185,000	4.48	828,800	107.14	88,800
1990	375,000	160,000	4.48	716,800	108.21	77,568
1991	330,000	115,000	4.48	515,200	110.71	57,040
1992	375,000	150,000	4.62	693,000	101.79	70,538
1993	400,000	170,000	4.62	785,400	113.93	89,480
1994	420,000	180,000	4.76	856,800	106.43	91,188
1995	435,000	150,000	4.48	672,000	137.50	92,400

See Footnotes on Page 58.

CALIFORNIA FIELD CROPS: Acreage, Production, Price And Value, 1986-95

Year	Acreage		Yield Per Acre	Production	Average Value Per Unit	Value of Production
	Planted	Harvested				
**Corn For Silage**						
	Acres d/	*Acres*	*Tons*	*Tons*	*Dollars Per Ton d/*	*$1,000 d/*
1986	---	243,000	24	5,832,000	---	---
1987	---	196,000	26	5,096,000	---	---
1988	---	182,000	23	4,186,000	---	---
1989	---	189,000	24	4,536,000	---	---
1990	---	210,000	25	5,250,000	---	---
1991	---	210,000	25	5,250,000	---	---
1992	---	220,000	25	5,500,000	---	---
1993	---	225,000	25	5,625,000	---	---
1994	---	235,000	25	5,875,000	---	---
1995	---	280,000	25	7,000,000	---	---
**Cotton, All**						
	Acres	*Acres*	*Pounds*	*Bales e/*	*Cents Per Lb.*	*$1,000*
1986	1,000,000	990,000	1,088	2,245,000	59.1	636,862
1987	1,150,900	1,140,900	1,258	2,991,200	68.9	989,663
1988	1,351,800	1,336,800	1,015	2,827,200	64.8	878,833
1989	1,068,000	1,057,900	1,226	2,701,200	72.2	935,534
1990	1,125,700	1,115,500	1,201	2,791,400	77.5	1,038,655
1991	1,044,000	1,041,000	1,242	2,694,200	68.2	882,054
1992	1,110,000	1,105,000	1,351	3,110,700	62.3	930,357
1993	1,141,000	1,136,000	1,324	3,132,600	67.0	1,006,747
1994	1,181,000	1,175,800	1,185	2,901,800	81.5	1,137,718
1995	1,285,000	1,280,000	951	2,536,500	87.4	1,063,505
**Cotton, American-Pima**						
	Acres	*Acres*	*Pounds*	*Bales e/*	*Cents Per Lb.*	*$1,000*
1986 d/	---	---	---	---	---	---
1987	900	900	1,173	2,200	104.0	1,098
1988	1,800	1,800	853	3,200	118.0	1,812
1989	18,000	17,900	1,078	40,200	95.6	18,447
1990	25,700	25,500	1,080	57,400	107.0	29,481
1991	64,000	64,000	1,097	146,200	96.2	67,509
1992	110,000	110,000	1,282	293,700	78.7	110,948
1993	91,000	91,000	1,132	214,600	84.0	86,527
1994	81,000	80,800	1,098	184,800	102.0	90,478
1995	115,000	115,000	937	224,500	127.0	136,855
**Cotton, Upland**						
	Acres	*Acres*	*Pounds*	*Bales e/*	*Cents Per Lb.*	*$1,000*
1986	1,000,000	990,000	1,088	2,245,000	59.1	636,862
1987	1,150,000	1,140,000	1,259	2,989,000	69.6	988,565
1988	1,350,000	1,335,000	1,015	2,824,000	64.7	877,021
1989	1,050,000	1,040,000	1,228	2,661,000	71.8	917,087
1990	1,100,000	1,090,000	1,204	2,734,000	76.9	1,009,174
1991	980,000	977,000	1,252	2,548,000	66.6	814,545
1992	1,000,000	995,000	1,359	2,817,000	60.5	819,409
1993	1,050,000	1,045,000	1,340	2,918,000	65.7	920,220
1994	1,100,000	1,095,000	1,191	2,717,000	80.3	1,047,240
1995	1,170,000	1,165,000	953	2,312,000	83.5	926,650

See Footnotes on Page 58.

CALIFORNIA FIELD CROPS: Acreage, Production, Price And Value, 1986-95

Year	Acreage: Planted	Acreage: Harvested	Yield Per Acre	Production	Average Value Per Unit	Value of Production
**Cottonseed**						
	Acres d/	*Acres d/*	*Tons d/*	*Tons*	*Dollars Per Ton*	*$1,000*
1986	---	---	---	875,000	102.00	89,250
1987	---	---	---	1,151,800	89.50	103,086
1988	---	---	---	1,116,300	147.00	164,096
1989	---	---	---	1,040,000	93.50	97,240
1990	---	---	---	1,079,000	148.00	159,692
1991	---	---	---	1,073,000	104.00	111,592
1992	---	---	---	1,148,000	120.00	137,760
1993	---	---	---	1,142,000	132.00	150,744
1994	---	---	---	1,063,000	143.00	152,009
1995	---	---	---	940,000	132.00	124,080
**Hay, All**						
	Acres d/	*Acres*	*Tons*	*Tons*	*Dollars Per Ton*	*$1,000*
1986	---	1,680,000	5.14	8,628,000	72.50	625,530
1987	---	1,620,000	5.30	8,586,000	77.50	665,415
1988	---	1,680,000	5.15	8,652,000	94.00	813,288
1989 f/	---	1,670,000	5.10	8,524,000	100.00	828,888
1990 f/	---	1,630,000	5.10	8,307,000	101.00	828,531
1991 f/	---	1,680,000	5.13	8,610,000	80.50	678,458
1992 f/	---	1,450,000	5.35	7,755,000	79.50	600,006
1993 f/	---	1,380,000	5.50	7,590,000	102.00	758,448
1994 f/	---	1,470,000	5.59	8,210,000	107.00	853,220
1995 f/	---	1,600,000	5.63	9,000,000	98.50	841,200
**Hay, Alfalfa**						
	Acres d/	*Acres*	*Tons*	*Tons*	*Dollars Per Ton*	*$1,000*
1986	---	1,080,000	6.60	7,128,000	d/	d/
1987	---	1,080,000	6.70	7,236,000	d/	d/
1988	---	1,100,000	6.60	7,260,000	d/	d/
1989	---	1,020,000	6.70	6,834,000	102.00	697,068
1990	---	1,060,000	6.60	6,996,000	104.00	727,584
1991	---	1,050,000	6.70	7,035,000	82.00	576,870
1992	---	960,000	6.70	6,432,000	83.00	533,856
1993	---	920,000	6.90	6,348,000	105.00	666,540
1994	---	950,000	7.00	6,650,000	112.00	744,800
1995	---	1,000,000	6.90	6,900,000	100.00	690,000
**Hay, Other**						
	Acres d/	*Acres*	*Tons*	*Tons*	*Dollars Per Ton*	*$1,000*
1986	---	600,000	2.50	1,500,000	d/	d/
1987	---	540,000	2.50	1,350,000	d/	d/
1988	---	580,000	2.40	1,392,000	d/	d/
1989	---	650,000	2.60	1,690,000	78.00	131,820
1990	---	570,000	2.30	1,311,000	77.00	100,947
1991	---	630,000	2.50	1,575,000	64.50	101,588
1992	---	490,000	2.70	1,323,000	50.00	66,150
1993	---	460,000	2.70	1,242,000	74.00	91,908
1994	---	520,000	3.00	1,560,000	69.50	108,420
1995	---	600,000	3.50	2,100,000	72.00	151,200

See Footnotes on Page 58.

CALIFORNIA FIELD CROPS: Acreage, Production, Price And Value, 1986-95

Year	Acreage		Yield Per Acre	Production	Average Value Per Unit	Value of Production
	Planted	Harvested				
**Oats**						
	Acres	*Acres*	*Tons*	*Tons*	*Dollars Per Ton*	*$1,000*
1986	360,000	45,000	1.12	50,400	95.63	4,820
1987	380,000	40,000	1.12	44,800	112.50	5,040
1988	365,000	35,000	1.25	43,700	153.13	6,689
1989	400,000	45,000	1.20	54,000	103.75	5,603
1990	380,000	45,000	1.20	54,000	101.25	5,468
1991	380,000	45,000	1.20	54,000	96.26	5,198
1992	380,000	35,000	1.28	44,800	103.13	4,620
1993	300,000	30,000	1.28	38,400	115.63	4,440
1994	310,000	30,000	1.28	38,400	183.13	7,032
1995	350,000	30,000	1.36	40,800	128.13	5,228
**Potatoes, All**						
	Acres	*Acres*	*Cwt.*	*Cwt.*	*Dollars Per Cwt.*	*$1,000*
1986	48,400	48,400	381	18,451,000	7.85	144,136
1987	50,800	50,600	376	19,039,000	8.45	158,908
1988	47,200	47,200	355	16,765,000	9.15	153,136
1989	48,700	48,700	366	17,831,000	11.40	203,038
1990	50,300	50,300	359	18,082,000	10.00	178,769
1991	46,200	45,200	362	16,361,000	9.90	160,293
1992	43,000	42,900	363	15,592,000	9.85	153,097
1993	41,900	41,500	360	14,932,000	12.40	184,559
1994	44,700	44,700	368	16,467,000	10.60	172,086
1995	41,500	41,300	354	14,620,000	13.10	190,894
**Potatoes, Winter**						
	Acres	*Acres*	*Cwt.*	*Cwt.*	*Dollars Per Cwt.*	*$1,000*
1986	5,100	5,100	290	1,479,000	7.00	10,353
1987	4,600	4,600	235	1,081,000	11.30	12,215
1988	5,200	5,200	230	1,196,000	10.40	12,438
1989	5,500	5,500	240	1,320,000	16.10	21,252
1990	5,500	5,500	230	1,265,000	24.40	30,866
1991	5,600	4,600	245	1,127,000	17.10	19,272
1992	5,300	5,300	260	1,378,000	14.10	19,430
1993	5,600	5,200	200	1,040,000	20.20	21,008
1994	4,500	4,500	215	968,000	21.10	20,425
1995	5,000	5,000	260	1,300,000	18.20	23,660
**Potatoes, Spring**						
	Acres	*Acres*	*Cwt.*	*Cwt.*	*Dollars Per Cwt.*	*$1,000*
1986	19,500	19,500	390	7,605,000	8.55	65,023
1987	21,300	21,300	370	7,881,000	11.50	90,632
1988	19,600	19,600	385	7,546,000	8.90	67,159
1989	21,000	21,000	375	7,875,000	11.00	86,625
1990	22,500	22,500	375	8,438,000	10.50	88,599
1991	21,800	21,800	380	8,284,000	12.60	104,378
1992	19,300	19,300	375	7,238,000	9.20	66,590
1993	19,500	19,500	385	7,508,000	13.00	97,604
1994	20,500	20,500	380	7,790,000	11.60	90,364
1995	18,000	17,800	350	6,230,000	13.60	84,728

See Footnotes on Page 58.

CALIFORNIA FIELD CROPS: Acreage, Production, Price And Value, 1986-95

Year	Acreage		Yield Per Acre	Production	Average Value Per Unit	Value of Production
	Planted	Harvested				
**Potatoes, Summer**						
	Acres	*Acres*	*Cwt.*	*Cwt.*	*Dollars Per Cwt.*	*$1,000*
1986	6,800	6,800	365	2,482,000	9.95	24,696
1987	6,600	6,400	345	2,208,000	10.60	23,405
1988	5,900	5,900	325	1,918,000	12.40	23,783
1989	6,100	6,100	360	2,196,000	18.70	41,065
1990	5,300	5,300	330	1,749,000	12.30	21,513
1991	4,800	4,800	325	1,560,000	8.85	14,274
1992	4,400	4,300	320	1,376,000	14.60	23,117
1993	4,800	4,800	330	1,584,000	13.30	21,067
1994	5,700	5,700	370	2,109,000	13.00	27,417
1995	5,500	5,500	320	1,760,000	17.20	30,272
**Potatoes, Fall**						
	Acres	*Acres*	*Cwt.*	*Cwt.*	*Dollars Per Cwt.*	*$1,000*
1986	17,000	17,000	405	6,885,000	6.40	44,064
1987	18,300	18,300	430	7,869,000	4.15	32,656
1988	16,500	16,500	370	6,105,000	8.15	49,756
1989	16,100	16,100	400	6,440,000	8.40	54,096
1990	17,000	17,000	390	6,630,000	5.70	37,791
1991	14,000	14,000	385	5,390,000	4.15	22,369
1992	14,000	14,000	400	5,600,000	7.85	43,960
1993	12,000	12,000	400	4,800,000	9.35	44,880
1994	14,000	14,000	400	5,600,000	6.05	33,880
1995	13,000	13,000	410	5,330,000	9.80	52,234
**Rice, All g/**						
	Acres	*Acres*	*Cwt.*	*Cwt.*	*Dollars Per Cwt.*	*$1,000*
1986	363,000	360,000	77.00	27,727,000	3.18	88,172
1987	374,000	370,000	75.50	27,935,000	6.72	187,723
1988	430,000	425,000	70.20	29,840,000	6.15	183,516
1989	415,000	410,000	79.00	32,390,000	6.27	203,085
1990	400,000	395,000	77.00	30,429,000	5.93	180,444
1991	351,000	350,000	81.00	28,350,000	6.65	188,528
1992	396,000	394,000	85.00	33,490,000	5.64	188,884
1993	440,000	437,000	83.00	36,271,000	8.27	299,961
1994	487,000	485,000	85.00	41,224,000	6.97	287,331
1995	467,000	465,000	76.00	35,352,000	9.00	318,168
**Rice, Long**						
	Acres	*Acres*	*Cwt.*	*Cwt.*	*Dollars/Cwt. d/*	*$1,000 d/*
1986	20,000	20,000	76.00	1,520,000	---	---
1987	36,000	36,000	72.00	2,592,000	---	---
1988	60,000	60,000	70.00	4,200,000	---	---
1989	30,000	30,000	75.00	2,250,000	---	---
1990	18,000	18,000	73.00	1,314,000	---	---
1991	16,000	16,000	73.00	1,168,000	---	---
1992	15,000	15,000	80.00	1,200,000	---	---
1993	14,000	14,000	81.80	1,145,000	---	---
1994	7,000	7,000	81.00	567,000	---	---
1995	8,000	8,000	75.00	600,000	---	---

See Footnotes on Page 58.

CALIFORNIA FIELD CROPS: Acreage, Production, Price And Value, 1986-95

Year	Acreage		Yield Per Acre	Production	Average Value Per Unit	Value of Production
	Planted	Harvested				
**Rice, Medium**						
	Acres	*Acres*	*Cwt.*	*Cwt.*	*Dollars/Cwt. d/*	*$1,000 d/*
1986	288,000	285,000	76.90	21,917,000	---	---
1987	299,000	295,000	76.00	22,496,000	---	---
1988	320,000	315,000	70.00	22,050,000	---	---
1989	335,000	330,000	79.75	26,315,000	---	---
1990	370,000	365,000	77.30	28,215,000	---	---
1991	326,000	325,000	81.50	26,489,000	---	---
1992	369,000	367,000	85.40	31,342,000	---	---
1993	413,000	410,000	83.20	34,112,000	---	---
1994	470,000	468,000	85.10	39,827,000	---	---
1995	449,000	447,000	76.00	33,972,000	---	---
**Rice, Short**						
	Acres	*Acres*	*Cwt.*	*Cwt.*	*Dollars/Cwt. d/*	*$1,000 d/*
1986	55,000	55,000	78.00	4,290,000	---	---
1987	39,000	39,000	73.00	2,847,000	---	---
1988	50,000	50,000	71.80	3,590,000	---	---
1989	50,000	50,000	76.50	3,825,000	---	---
1990	12,000	12,000	75.00	900,000	---	---
1991	9,000	9,000	77.00	693,000	---	---
1992	12,000	12,000	79.00	948,000	---	---
1993	13,000	13,000	78.00	1,014,000	---	---
1994	10,000	10,000	83.00	830,000	---	---
1995	10,000	10,000	78.00	780,000	---	---
**Sugar Beets h/**						
	Acres	*Acres*	*Tons*	*Tons*	*Dollars Per Ton*	*$1,000*
1986	193,000	189,000	25.50	4,820,000	35.70	172,502
1987	219,000	216,000	28.20	6,091,000	33.60	204,658
1988	215,000	212,000	25.00	5,300,000	36.70	194,510
1989	176,000	169,000	27.30	4,614,000	42.30	195,172
1990	173,000	168,000	25.80	4,334,000	41.60	180,294
1991	165,000	158,000	25.50	4,029,000	37.10	149,476
1992	154,000	150,000	28.20	4,230,000	35.90	151,857
1993	138,000	136,000	26.00	3,536,000	35.50	125,528
1994	143,000	141,000	28.00	3,948,000	33.50	132,258
1995	117,000	115,000	28.00	3,220,000	i/	i/
**Sweet Potatoes**						
	Acres	*Acres*	*Cwt.*	*Cwt.*	*Dollars Per Cwt.*	*$1,000*
1986	6,800	6,800	200	1,360,000	11.70	15,912
1987	6,600	6,600	205	1,353,000	20.40	27,601
1988	7,100	7,100	170	1,207,000	12.70	15,329
1989	8,300	8,300	175	1,453,000	20.70	30,077
1990	8,300	8,300	175	1,453,000	16.70	24,265
1991	8,200	8,200	185	1,517,000	27.10	41,111
1992	9,000	9,000	205	1,845,000	20.30	37,454
1993	8,300	8,300	210	1,743,000	24.30	42,355
1994	8,200	8,200	205	1,681,000	28.70	48,245
1995	9,600	9,600	200	1,920,000	33.00	63,360

See Footnotes on Page 58.

CALIFORNIA FIELD CROPS: Acreage, Production, Price And Value, 1986-95

Year	Acreage		Yield Per Acre	Production	Average Value Per Unit	Value of Production
	Planted	Harvested				
**Wheat, All**						
	Acres	*Acres*	*Tons*	*Tons*	*Dollars Per Ton*	*$1,000*
1986	730,000	675,000	2.29	1,545,800	97.33	150,318
1987	620,000	567,000	2.32	1,316,700	96.67	127,133
1988	590,000	549,000	2.53	1,388,300	127.33	176,669
1989	731,000	675,000	2.34	1,578,200	127.33	201,132
1990	680,000	619,000	2.33	1,445,000	110.33	157,871
1991	483,000	442,000	2.45	1,084,800	110.87	120,269
1992	642,000	590,000	2.22	1,309,100	114.88	150,385
1993	591,000	540,000	2.35	1,269,000	107.78	136,779
1994	650,000	569,000	2.34	1,331,000	121.92	162,274
1995	650,000	493,000	1.99	981,800	142.55	139,953
**Wheat, Excluding Durum**						
	Acres	*Acres*	*Tons*	*Tons*	*Dollars Per Ton*	*$1,000*
1986	650,000	600,000	2.25	1,350,000	94.33	127,350
1987	560,000	510,000	2.28	1,162,800	94.33	109,691
1988	530,000	490,000	2.49	1,220,100	123.67	150,886
1989	625,000	570,000	2.31	1,316,700	128.00	168,538
1990	620,000	560,000	2.28	1,276,800	107.67	137,469
1991	450,000	410,000	2.40	984,000	110.00	108,240
1992	585,000	535,000	2.16	1,155,600	114.33	132,124
1993	550,000	500,000	2.31	1,155,000	105.00	121,275
1994	590,000	510,000	2.28	1,162,800	117.33	136,435
1995	580,000	425,000	1.83	777,800	136.67	106,293
**Wheat, Durum**						
	Acres	*Acres*	*Tons*	*Tons*	*Dollars Per Ton*	*$1,000*
1986	80,000	75,000	2.61	195,800	117.33	22,968
1987	60,000	57,000	2.70	153,900	113.33	17,442
1988	60,000	59,000	2.85	168,200	153.33	25,783
1989	106,000	105,000	2.49	261,500	124.67	32,594
1990	60,000	59,000	2.85	168,200	121.33	20,402
1991	33,000	32,000	3.15	100,800	119.34	12,029
1992	57,000	55,000	2.79	153,500	118.96	18,261
1993	41,000	40,000	2.85	114,000	136.00	15,504
1994	60,000	59,000	2.85	168,200	153.67	25,839
1995	70,000	68,000	3.00	204,000	165.00	33,660

a/ Production is on a clean basis
b/ Estimate discontinued.
c/ Excludes beans grown for garden seed.
d/ Not estimated.
e/ Cotton: 480 lbs. net-weight per bale.
f/ Estimated marketings of alfalfa and other hay used as weights to calculate all hay price for 1989-95.
g/ Excludes sweet and wild rice.
h/ Includes some acreage carried over and harvested the following spring.
i/ Not available.

CALIFORNIA DRY EDIBLE BEANS: Acreage And Production, 1986-95

Year	Acreage		Yield Per Acre	Production (Clean)
	Planted	Harvested		
**Large Lima**				
	Acres	*Acres*	*Pounds*	*Cwt.*
1986	19,000	18,500	2,080	385,000
1987	22,000	21,000	2,070	435,000
1988	29,000	28,000	2,050	574,000
1989	33,000	32,000	1,930	618,000
1990	24,000	23,000	2,000	460,000
1991	31,000	30,000	2,270	681,000
1992	26,000	25,000	2,460	615,000
1993	15,000	15,000	2,330	350,000
1994	24,000	23,500	1,830	429,000
1995	21,000	20,000	2,250	450,000
**Baby Lima**				
	Acres	*Acres*	*Pounds*	*Cwt.*
1986	30,000	29,500	2,160	637,000
1987	22,000	21,000	2,310	485,000
1988	30,000	29,000	2,320	673,000
1989	36,000	35,000	2,080	728,000
1990	25,000	25,000	2,280	570,000
1991	40,000	39,000	2,500	975,000
1992	21,000	20,000	2,490	498,000
1993	14,000	14,000	2,510	351,000
1994	26,000	24,500	2,490	609,000
1995	23,000	22,000	2,430	535,000
**Blackeye**				
	Acres	*Acres*	*Pounds*	*Cwt.*
1986	43,000	42,000	1,730	727,000
1987	63,500	62,500	1,780	1,110,000
1988	44,000	43,000	1,960	843,000
1989	40,000	39,000	2,040	797,000
1990	49,000	46,000	1,970	906,000
1991	42,000	41,000	2,250	923,000
1992	23,000	22,000	2,320	510,000
1993	24,000	23,000	2,380	548,000
1994	32,000	30,000	2,570	771,000
1995	44,500	43,500	2,300	1,000,000
**Light Red Kidney**				
	Acres	*Acres*	*Pounds*	*Cwt.*
1986	27,000	27,000	1,820	491,000
1987	34,500	33,500	1,800	603,000
1988	23,000	23,000	1,580	363,000
1989	38,000	37,000	1,540	571,000
1990	33,000	31,000	1,620	502,000
1991	14,000	14,000	1,640	230,000
1992	20,000	19,000	1,820	346,000
1993	20,000	19,000	2,120	402,000
1994	14,000	13,500	1,950	263,000
1995	13,000	12,000	1,830	220,000

See Footnotes on Page 60.

CALIFORNIA DRY EDIBLE BEANS: Acreage And Production, 1986-95

Year	Acreage		Yield Per Acre	Production (Clean)
	Planted	Harvested		
**Dark Red Kidney**				
	Acres	*Acres*	*Pounds*	*Cwt.*
1986	7,500	7,500	1,790	134,000
1987	11,500	11,500	1,830	210,000
1988	9,000	8,000	1,950	156,000
1989	10,000	10,000	1,740	177,000
1990	15,000	14,000	1,800	252,000
1991	10,000	10,000	1,670	167,000
1992	7,500	7,500	1,640	123,000
1993	7,000	7,000	1,730	121,000
1994	7,000	7,000	1,790	125,000
1995	4,500	4,500	1,670	75,000
**Pink**				
	Acres	*Acres*	*Pounds*	*Cwt.*
1986	18,000	18,000	1,800	324,000
1987	5,500	5,500	1,710	94,000
1988	5,000	5,000	1,800	90,000
1989	16,000	16,000	1,610	258,000
1990	17,000	17,000	1,260	214,000
1991 a/	---	---	---	---
1992	4,500	4,500	1,930	87,000
1993	4,500	4,500	2,000	90,000
1994	7,000	7,000	2,160	151,000
1995	7,000	6,000	1,830	110,000
**Garbanzo**				
	Acres	*Acres*	*Pounds*	*Cwt.*
1986	6,000	6,000	800	48,000
1987	4,000	4,000	880	35,000
1988	1,000	1,000	1,100	11,000
1989 a/	---	---	---	---
1990 a/	---	---	---	---
1991 a/	---	---	---	---
1992	15,000	15,000	1,260	189,000
1993	17,500	16,500	1,350	222,000
1994	14,000	14,000	1,870	262,000
1995	19,000	17,000	1,760	300,000
**Other b/**				
	Acres	*Acres*	*Pounds*	*Cwt.*
1986	6,500	6,500	1,780	116,000
1987	9,000	9,000	1,844	166,000
1988	10,000	10,000	1,750	175,000
1989	12,000	11,000	1,890	208,000
1990	12,000	10,000	1,540	154,000
1991	16,000	16,000	1,620	259,000
1992	4,000	4,000	1,930	77,000
1993	7,000	7,000	1,800	126,000
1994	12,000	11,500	1,400	161,000
1995	13,000	12,000	2,250	270,000

a/ Included in "Other".
b/ Includes varieties not listed separately. Garden seed beans have been excluded. Estimates for the "small white" variety were discontinued in 1989 and will no longer be published separately.

CALIFORNIA PRICES RECEIVED: MONTHLY AVERAGE, SPECIFIED COMMODITIES, 1986-95

Year	Jan.	Feb.	Mar.	Apr.	May	June	July	Aug.	Sept.	Oct.	Nov.	Dec.
**Barley**												
Dollars Per Cwt.												
1986	5.50	5.00	5.21	4.65	4.40	4.29	3.60	3.67	4.48	4.19	4.54	4.00
1987	4.04	4.52	4.38	4.35	4.77	4.42	4.42	4.08	4.00	4.10	4.50	4.35
1988	4.71	4.38	4.54	4.96	4.69	5.15	6.13	6.02	6.02	5.94	5.17	5.77
1989	5.83	5.58	5.67	5.29	5.94	5.60	5.88	5.27	5.13	5.00	5.19	5.56
1990	5.27	5.69	5.40	5.63	5.67	5.75	5.67	5.15	4.81	4.69	5.04	4.85
1991	5.06	5.90	5.44	5.27	5.29	5.31	4.94	5.15	5.04	5.25	5.00	5.69
1992	4.92	6.17	5.92	5.67	5.21	5.65	5.10	5.38	5.44	5.50	5.50	5.48
1993	5.60	5.52	5.75	5.46	5.02	5.33	5.04	5.04	5.17	4.67	5.17	5.04
1994	5.62	a/	5.29	5.31	5.38	5.40	5.21	5.42	a/	a/	a/	5.65
1995	a/	5.67	5.60	a/	6.10	a/	a/	5.77	6.17	a/	6.96	7.10
**Beans, Dry Edible b/**												
Dollars Per Cwt.												
1986	22.00	21.20	21.00	20.90	21.20	23.00	23.50	23.20	23.00	25.00	28.00	28.00
1987	31.00	30.00	30.00	30.00	29.00	30.00	31.00	29.00	25.00	25.00	24.00	24.00
1988	25.00	28.00	30.00	30.00	30.00	29.00	34.00	34.00	31.00	35.00	36.00	36.00
1989	35.00	36.00	38.00	38.00	38.00	38.00	38.00	36.00	33.00	35.00	36.00	36.00
1990	35.00	35.00	35.00	34.00	34.00	35.00	34.00	33.50	31.00	30.50	30.00	30.00
1991	30.40	30.00	32.20	32.60	32.40	31.20	31.60	30.80	31.40	28.30	27.70	27.70
1992	26.70	26.50	26.00	25.00	24.00	23.50	22.50	22.50	22.00	23.50	23.80	25.20
1993	25.00	24.80	24.70	24.20	23.60	24.20	24.80	27.40	29.40	31.20	35.50	34.80
1994	36.90	36.70	36.30	37.00	36.00	36.00	37.80	36.90	36.20	33.00	35.10	34.10
1995	35.40	35.10	35.40	34.60	35.00	35.70	35.20	34.40	34.40	34.00	34.60	33.80
**Cotton Lint, Upland c/**												
Cents Per Pound												
1986	57.00	61.00	62.40	63.90	67.10	67.50	70.70	68.00	59.50	59.30	56.50	57.40
1987	66.90	56.30	55.10	57.90	67.50	73.30	70.10	70.10	72.80	68.90	70.20	71.30
1988	67.20	65.50	65.80	67.20	67.50	75.90	64.20	63.90	61.60	63.70	64.80	64.50
1989	60.70	64.40	65.70	69.90	71.00	70.10	69.40	78.90	72.70	73.10	71.90	69.70
1990	66.80	70.00	73.80	75.70	81.20	73.50	73.90	78.70	76.70	74.90	75.30	78.50
1991	79.30	81.90	79.70	82.00	83.70	77.80	76.10	68.20	68.50	69.40	70.60	66.60
1992	60.10	60.90	60.10	64.40	a/	66.10	a/	54.50	60.30	57.80	59.20	61.90
1993	62.50	63.30	62.90	62.40	59.50	59.70	a/	57.60	54.50	56.10	60.20	62.30
1994	67.10	70.40	72.20	72.40	72.40	76.40	70.80	65.40	64.80	68.80	75.40	79.30
1995	94.50	94.80	108.00	113.20	107.90	97.90	99.40	80.60	85.90	81.00	81.70	84.50
**Cottonseed c/**												
Dollars Per Ton												
1986	a/	a/	---	---	---	---	---	---	---	100.00	107.00	90.00
1987	a/	a/	---	---	---	---	---	---	75.00	75.00	102.00	97.00
1988	110.00	a/	---	---	---	---	---	---	---	147.00	148.00	147.00
1989	147.00	a/	---	---	---	---	---	---	---	76.00	105.00	124.00
1990	a/	a/	---	---	---	---	---	---	---	145.00	146.00	158.00
1991	178.00	178.00	---	---	---	---	---	---	---	104.00	103.00	104.00
1992	a/	a/	---	---	---	---	---	---	---	118.00	124.00	123.00
1993	a/	a/	---	---	---	---	---	---	---	125.00	139.00	136.00
1994	140.00	---	---	---	---	---	---	---	---	---	141.00	147.00
1995	157.00	---	---	---	---	---	---	---	---	---	132.00	131.00

See Footnotes on Page 63.

CALIFORNIA PRICES RECEIVED: MONTHLY AVERAGE, SPECIFIED COMMODITIES, 1986-95

Year	Jan.	Feb.	Mar.	Apr.	May	June	July	Aug.	Sept.	Oct.	Nov.	Dec.
**Hay, All (Baled)**												
Dollars Per Ton												
1986	85.00	79.00	80.00	71.00	71.00	74.00	71.00	66.00	66.00	70.00	73.00	77.00
1987	79.00	81.00	82.00	84.00	84.00	76.00	72.00	72.00	74.00	76.00	72.00	73.00
1988	76.00	78.00	89.00	87.00	92.00	89.00	86.00	84.00	87.00	92.00	96.00	96.00
1989	105.00	105.00	109.00	111.00	106.00	94.00	92.00	90.00	96.00	91.00	96.00	101.00
1990	107.00	107.00	108.00	121.00	111.00	103.00	97.00	94.00	96.00	100.00	101.00	95.00
1991	102.00	102.00	101.00	107.00	94.00	89.00	81.00	76.00	77.00	77.00	79.00	78.00
1992	75.00	75.00	75.00	74.00	88.00	84.00	67.00	76.00	71.00	77.00	76.00	75.00
1993	81.00	85.00	89.00	96.00	104.00	97.00	89.00	92.00	101.00	104.00	108.00	110.00
1994	116.00	123.00	120.00	122.00	107.00	111.00	104.00	99.00	109.00	112.00	116.00	124.00
1995	114.00	110.00	109.00	99.00	95.00	100.00	96.00	96.00	96.00	104.00	98.00	116.00
**Hay, Alfalfa (Baled)**												
Dollars Per Ton												
1986	86.00	79.00	79.00	73.00	72.00	75.00	71.00	66.00	66.00	71.00	74.00	79.00
1987	80.00	82.00	83.00	86.00	87.00	78.00	73.00	73.00	75.00	78.00	73.00	76.00
1988	78.00	79.00	90.00	88.00	95.00	90.00	87.00	84.00	88.00	93.00	97.00	96.00
1989	105.00	106.00	109.00	113.00	107.00	96.00	93.00	91.00	97.00	93.00	97.00	103.00
1990	110.00	109.00	109.00	123.00	115.00	105.00	100.00	97.00	99.00	103.00	103.00	96.00
1991	104.00	104.00	101.00	108.00	96.00	92.00	82.00	78.00	78.00	79.00	80.00	78.00
1992	76.00	76.00	76.00	76.00	93.00	88.00	69.00	78.00	74.00	80.00	79.00	80.00
1993	86.00	91.00	92.00	97.00	107.00	100.00	91.00	94.00	103.00	107.00	112.00	116.00
1994	119.00	126.00	123.00	124.00	111.00	116.00	108.00	106.00	114.00	116.00	120.00	131.00
1995	124.00	113.00	110.00	100.00	96.00	104.00	98.00	97.00	98.00	105.00	98.00	120.00
**Oats**												
Dollars Per Cwt.												
1986	5.25	4.75	4.19	a/	4.59	3.69	4.75	4.06	5.34	6.00	a/	5.59
1987	5.81	5.50	5.38	5.25	5.00	5.03	4.75	5.00	5.00	5.44	5.75	5.81
1988	5.81	6.00	6.09	6.00	a/	a/	a/	7.50	7.84	8.00	9.00	7.63
1989	a/	7.50	a/	6.50	a/	6.00	a/	4.91	5.16	5.59	5.56	5.28
1990	5.09	5.59	5.06	5.03	4.88	5.03	5.00	5.00	5.06	5.09	5.13	5.06
1991	5.09	a/	5.03	5.06	5.84	4.56	4.50	5.00	4.50	5.03	4.50	a/
1992	5.22	6.31	a/	a/	a/	5.25	4.63	5.16	a/	a/	a/	5.22
1993	a/	a/	a/	a/	a/	a/	a/	a/	a/	a/	a/	a/
1994	a/	a/	a/	a/	a/	a/	a/	a/	a/	a/	a/	a/
1995	a/	a/	a/	a/	a/	a/	a/	a/	a/	a/	a/	a/
**Potatoes**												
Dollars Per Cwt.												
1986	6.35	6.30	5.85	6.30	7.85	8.10	9.15	9.55	8.35	7.00	6.90	6.90
1987	7.30	7.45	7.55	6.20	12.50	10.70	10.10	9.80	6.75	7.00	4.00	4.65
1988	6.00	6.35	5.45	5.50	10.10	7.15	8.30	11.30	10.10	7.55	7.75	8.70
1989	10.70	11.80	9.85	10.60	10.90	11.10	13.30	18.80	10.70	10.10	8.60	8.10
1990	12.80	12.30	15.60	11.00	12.70	9.75	10.00	12.70	6.70	6.50	8.80	11.60
1991	10.40	8.80	6.75	5.35	11.30	12.10	11.20	9.40	6.95	4.10	5.80	6.75
1992	6.45	7.00	6.10	7.90	7.45	8.65	11.20	15.30	11.00	8.75	10.70	11.20
1993	10.90	10.90	9.20	13.60	11.80	11.10	14.70	15.10	11.00	8.50	13.30	13.90
1994	13.70	11.60	11.60	10.70	11.70	11.00	12.10	11.50	6.75	6.55	8.45	8.40
1995	8.50	9.80	9.15	8.55	12.10	12.10	14.30	16.80	19.30	13.40	13.50	12.50

See Footnotes on Page 63.

CALIFORNIA PRICES RECEIVED: MONTHLY AVERAGE, SPECIFIED COMMODITIES, 1986-95

Year	Jan.	Feb.	Mar.	Apr.	May	June	July	Aug.	Sept.	Oct.	Nov.	Dec.
**Wheat, All**												
Dollars Per Cwt.												
1986	6.12	5.88	5.93	5.30	4.93	5.02	4.60	5.02	5.20	4.25	4.53	4.62
1987	5.20	5.02	4.65	4.38	5.02	4.67	4.53	4.32	5.15	5.50	5.02	4.77
1988	5.25	4.88	5.72	a/	5.67	6.23	6.70	6.25	6.03	6.52	6.33	7.33
1989	7.55	7.18	7.28	7.43	7.47	6.40	6.40	6.25	6.23	6.67	6.35	6.15
1990	6.18	5.58	6.23	6.42	6.08	5.80	5.83	5.22	4.83	4.82	4.55	4.58
1991	5.55	4.52	5.08	4.92	4.73	5.33	5.17	5.17	5.18	5.52	5.40	5.25
1992	6.27	6.63	6.35	6.37	5.73	5.87	5.82	5.43	5.63	5.83	5.75	5.57
1993	5.88	6.18	5.53	5.33	5.30	5.30	5.00	5.25	5.02	4.97	5.67	5.93
1994	6.25	5.77	5.73	5.77	6.22	6.30	5.63	5.73	6.27	6.77	6.77	6.63
1995	7.21	6.40	7.00	6.23	7.12	7.53	6.73	a/	7.07	7.50	8.83	7.72

a/ Not published -- insufficient sales or disclosure problem.
b/ The marketing season for dry beans is September 1 to August 31.
c/ The marketing season for cotton and cottonseed is August 1 to July 31.

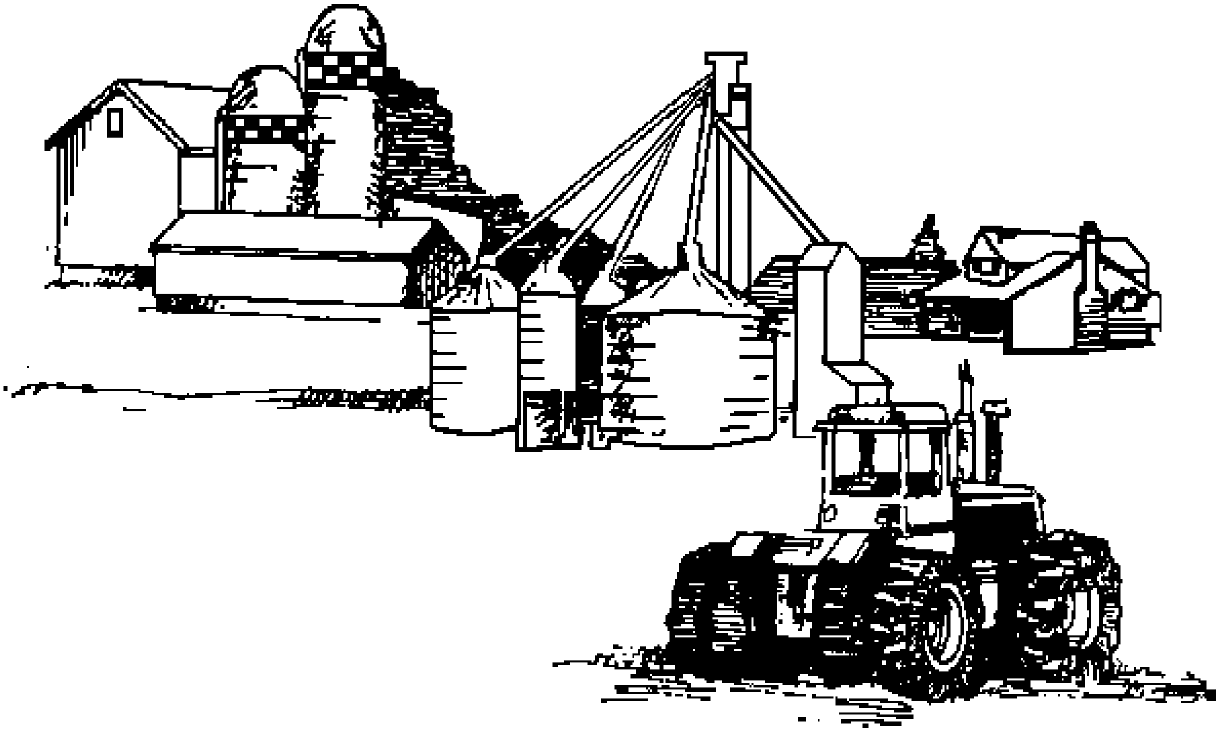

CALIFORNIA BARLEY: Acreage, Yield And Production By Counties, 1994-95

County	1994				1995			
	Planted	Harvested	Yield/Acre	Production	Planted	Harvested	Yield/Acre	Production
	Acres		Bushels		Acres		Bushels	
Del Norte	---	---	---	---	---	---	---	---
Humboldt	---	---	---	---	---	---	---	---
Mendocino	---	---	---	---	---	---	---	---
	---	---	---	---	---	---	---	---
Shasta	1,100	700	64.3	45,000	800	500	80.0	40,000
Siskiyou	17,900	16,300	92.3	1,505,000	14,700	13,000	106.2	1,380,000
Trinity	---	---	---	---	---	---	---	---
	19,000	17,000	91.2	1,550,000	15,500	13,500	105.2	1,420,000
Lassen	1,400	1,300	76.9	100,000	1,200	1,000	78.0	78,000
Modoc	18,600	15,700	100.0	1,570,000	16,300	13,500	115.7	1,562,000
Plumas	---	---	---	---	---	---	---	---
	20,000	17,000	98.2	1,670,000	17,500	14,500	113.1	1,640,000
Alameda	500	200	55.0	11,000	400	200	60.0	12,000
Contra Costa	600	500	50.0	25,000	600	500	52.0	26,000
Lake	---	---	---	---	---	---	---	---
Marin	---	---	---	---	---	---	---	---
Monterey	23,200	15,200	38.9	592,000	19,500	13,700	42.3	580,000
Napa	---	---	---	---	---	---	---	---
San Benito	7,900	5,400	54.1	292,000	7,500	5,300	54.2	287,000
San Francisco	---	---	---	---	---	---	---	---
San Luis Obispo	36,500	32,600	49.0	1,597,000	28,300	22,800	50.4	1,150,000
San Mateo	400	300	46.7	14,000	300	200	50.0	10,000
Santa Clara	800	300	46.7	14,000	700	300	50.0	15,000
Santa Cruz	---	---	---	---	---	---	---	---
Sonoma	---	---	---	---	---	---	---	---
Combined 1/	100	---	---	---	200	---	---	---
	70,000	54,500	46.7	2,545,000	57,500	43,000	48.4	2,080,000
Butte	3,700	2,300	45.2	104,000	3,100	1,600	48.1	77,000
Colusa	1,600	1,500	46.7	70,000	1,400	1,100	49.1	54,000
Glenn	2,500	2,400	46.3	111,000	2,200	1,900	48.9	93,000
Sacramento	1,500	1,300	70.8	92,000	1,300	1,000	72.0	72,000
Solano	19,000	17,800	53.4	951,000	16,500	14,400	54.5	785,000
Sutter	1,700	900	62.2	56,000	1,400	700	62.9	44,000
Tehama	1,900	1,900	39.5	75,000	1,600	1,200	43.3	52,000
Yolo	4,100	1,900	61.1	116,000	3,500	1,600	59.4	95,000
Yuba	---	---	---	---	---	---	---	---
	36,000	30,000	52.5	1,575,000	31,000	23,500	54.1	1,272,000
Fresno	17,300	15,000	85.0	1,275,000	16,000	13,800	86.4	1,193,000
Kern	30,000	24,000	66.7	1,600,000	28,800	27,400	70.2	1,923,000
Kings	33,800	27,000	70.0	1,890,000	34,800	31,800	73.9	2,349,000
Madera	3,900	3,400	88.2	300,000	3,500	2,700	94.8	256,000
Merced	3,500	2,500	94.0	235,000	3,100	2,000	95.5	191,000
San Joaquin	3,300	2,500	76.0	190,000	3,100	1,500	88.7	133,000
Stanislaus	2,500	1,700	61.8	105,000	2,000	1,000	69.0	69,000
Tulare	25,200	11,900	73.9	880,000	23,200	12,800	77.5	992,000
	119,500	88,000	73.6	6,475,000	114,500	93,000	76.4	7,106,000
Alpine	---	---	---	---	---	---	---	---
Amador	400	400	72.5	29,000	300	300	83.3	25,000
Calaveras	---	---	---	---	---	---	---	---
El Dorado	---	---	---	---	---	---	---	---
Inyo	---	---	---	---	---	---	---	---
Mariposa	---	---	---	---	---	---	---	---
Mono	---	---	---	---	---	---	---	---
Nevada	---	---	---	---	---	---	---	---
Placer	---	---	---	---	---	---	---	---
Sierra	---	---	---	---	---	---	---	---
Tuolumne	---	---	---	---	---	---	---	---
Combined 1/	100	100	60.0	6,000	200	200	60.0	12,000
	500	500	70.0	35,000	500	500	74.0	37,000
Imperial	---	---	---	---	---	---	---	---
Los Angeles	4,000	2,000	58.0	116,000	3,800	2,900	51.4	149,000
Orange	---	---	---	---	---	---	---	---
Riverside	11,300	5,800	31.0	180,000	10,600	4,400	33.9	149,000
San Bernardino	3,100	2,000	22.5	45,000	3,000	2,000	27.0	54,000
San Diego	2,000	1,500	30.0	45,000	1,900	1,400	31.4	44,000
Santa Barbara	3,200	800	50.0	40,000	3,000	600	48.3	29,000
Ventura	1,000	600	25.0	15,000	900	500	28.0	14,000
Combined 1/	400	300	30.0	9,000	300	200	30.0	6,000
	25,000	13,000	34.6	450,000	23,500	12,000	37.1	445,000
STATE	290,000	220,000	65.0	14,300,000	260,000	200,000	70.0	14,000,000

1/ These figures include estimates for one or more counties which normally have less than 200 acres planted.

CALIFORNIA DRY EDIBLE BEANS: Acreage, Yield And Production By Counties, 1994-95

County	1994				1995			
	Planted	Harvested	Yield/Acre	Production	Planted	Harvested	Yield/Acre	Production
	Acres		*Pounds*	*Cwt.*	*Acres*		*Pounds*	*Cwt.*
Del Norte	---	---	---	---	---	---	---	---
Humboldt	---	---	---	---	---	---	---	---
Mendocino	200	200	2,000	4,000	200	200	2,000	4,000
	200	200	2,000	4,000	200	200	2,000	4,000
Shasta	---	---	---	---	---	---	---	---
Siskiyou	---	---	---	---	---	---	---	---
Trinity	---	---	---	---	---	---	---	---
	---	---	---	---	---	---	---	---
Lassen	---	---	---	---	---	---	---	---
Modoc	---	---	---	---	---	---	---	---
Plumas	---	---	---	---	---	---	---	---
	---	---	---	---	---	---	---	---
Alameda	---	---	---	---	---	---	---	---
Contra Costa	---	---	---	---	---	---	---	---
Lake	---	---	---	---	---	---	---	---
Marin	---	---	---	---	---	---	---	---
Monterey	3,000	2,900	2,310	67,000	3,100	3,000	2,300	69,000
Napa	---	---	---	---	---	---	---	---
San Benito	---	---	---	---	---	---	---	---
San Francisco	---	---	---	---	---	---	---	---
San Luis Obispo	1,600	1,600	1,500	24,000	1,800	1,700	1,471	25,000
San Mateo	200	200	2,000	4,000	200	200	2,000	4,000
Santa Clara	500	500	2,400	12,000	500	500	2,400	12,000
Santa Cruz	---	---	---	---	---	---	---	---
Sonoma	---	---	---	---	---	---	---	---
	5,300	5,200	2,058	107,000	5,600	5,400	2,037	110,000
Butte	2,000	2,000	2,200	44,000	2,300	2,100	2,143	45,000
Colusa	10,400	9,400	2,160	203,000	10,600	10,000	2,000	200,000
Glenn	3,500	3,300	2,000	66,000	3,700	3,400	2,088	71,000
Sacramento	1,600	1,600	2,063	33,000	1,600	1,600	2,188	35,000
Solano	5,000	4,800	1,979	95,000	5,700	5,400	2,037	110,000
Sutter	9,600	9,300	1,968	183,000	9,800	9,100	1,934	176,000
Tehama	1,400	1,400	1,500	21,000	1,400	1,400	1,500	21,000
Yolo	3,000	3,000	1,667	50,000	3,500	3,300	1,758	58,000
Yuba	200	200	2,000	4,000	200	200	2,000	4,000
	36,700	35,000	1,997	699,000	38,800	36,500	1,973	720,000
Fresno	15,900	15,600	2,135	333,000	19,000	17,400	2,011	350,000
Kern	7,000	6,700	2,269	152,000	7,200	6,800	2,500	170,000
Kings	3,000	3,000	1,900	57,000	3,300	3,100	2,290	71,000
Madera	1,600	1,600	2,188	35,000	1,700	1,700	2,000	34,000
Merced	4,500	4,300	2,279	98,000	4,900	4,500	2,533	114,000
San Joaquin	18,100	17,500	2,131	373,000	18,600	17,800	2,135	380,000
Stanislaus	24,000	23,200	2,349	545,000	25,000	24,000	2,479	595,000
Tulare	9,000	8,600	2,395	206,000	9,700	9,100	2,637	240,000
	83,100	80,500	2,235	1,799,000	89,400	84,400	2,315	1,954,000
Alpine	---	---	---	---	---	---	---	---
Amador	---	---	---	---	---	---	---	---
Calaveras	---	---	---	---	---	---	---	---
El Dorado	---	---	---	---	---	---	---	---
Inyo	---	---	---	---	---	---	---	---
Mariposa	---	---	---	---	---	---	---	---
Mono	---	---	---	---	---	---	---	---
Nevada	---	---	---	---	---	---	---	---
Placer	---	---	---	---	---	---	---	---
Sierra	---	---	---	---	---	---	---	---
Tuolumne	---	---	---	---	---	---	---	---
	---	---	---	---	---	---	---	---
Imperial	---	---	---	---	---	---	---	---
Los Angeles	---	---	---	---	---	---	---	---
Orange	1,000	900	2,222	20,000	1,100	1,000	2,200	22,000
Riverside	1,000	1,000	1,600	16,000	1,000	1,000	1,600	16,000
San Bernardino	---	---	---	---	---	---	---	---
San Diego	200	200	1,500	3,000	200	200	1,500	3,000
Santa Barbara	8,300	7,800	1,526	119,000	8,400	8,000	1,563	125,000
Ventura	200	200	2,000	4,000	300	300	2,000	6,000
	10,700	10,100	1,604	162,000	11,000	10,500	1,638	172,000
Other Counties 1/	---	---	---	---	---	---	---	---
STATE	136,000	131,000	2,115	2,771,000	145,000	137,000	2,160	2,960,000

1/ Includes those counties which normally have less than 200 acres planted

CALIFORNIA CORN: Acreage, Yield And Production By Counties, 1994-95

County	1994				1995			
	Planted	Harvested	Yield/Acre	Production	Planted	Harvested	Yield/Acre	Production
	Acres		*Bushels*		*Acres*		*Bushels*	
Del Norte	---	---	---	---	---	---	---	---
Humboldt	---	---	---	---	---	---	---	---
Mendocino	---	---	---	---	---	---	---	---
	---	---	---	---	---	---	---	---
Shasta	300	---	---	---	200	---	---	---
Siskiyou	700	---	---	---	300	---	---	---
Trinity	---	---	---	---	---	---	---	---
	1,000	---	---	---	500	---	---	---
Lassen	---	---	---	---	---	---	---	---
Modoc	---	---	---	---	---	---	---	---
Plumas	---	---	---	---	---	---	---	---
	---	---	---	---	---	---	---	---
Alameda	500	500	160.0	80,000	200	200	145.0	29,000
Contra Costa	6,800	6,500	145.4	945,000	5,500	5,200	150.6	783,000
Lake	---	---	---	---	---	---	---	---
Marin	---	---	---	---	---	---	---	---
Monterey	200	200	175.0	35,000	200	200	160.0	32,000
Napa	---	---	---	---	---	---	---	---
San Benito	300	---	---	---	400	---	---	---
San Francisco	---	---	---	---	---	---	---	---
San Luis Obispo	---	---	---	---	---	---	---	---
San Mateo	---	---	---	---	---	---	---	---
Santa Clara	200	200	150.0	30,000	200	200	140.0	28,000
Santa Cruz	---	---	---	---	---	---	---	---
Sonoma	500	200	150.0	30,000	500	200	140.0	28,000
	8,500	7,600	147.4	1,120,000	7,000	6,000	150.0	900,000
Butte	1,000	800	156.3	125,000	1,100	600	166.7	100,000
Colusa	4,100	3,600	150.0	540,000	3,800	2,800	160.7	450,000
Glenn	12,000	9,900	177.8	1,760,000	11,900	8,500	155.3	1,320,000
Sacramento	28,900	24,000	144.0	3,455,000	32,000	28,000	154.8	4,335,000
Solano	24,900	16,900	180.5	3,050,000	24,500	16,300	160.1	2,610,000
Sutter	3,600	2,900	184.5	535,000	3,000	2,400	160.4	385,000
Tehama	1,500	1,400	175.0	245,000	1,400	700	150.0	105,000
Yolo	21,400	18,700	177.5	3,320,000	20,600	17,300	158.4	2,740,000
Yuba	1,600	1,000	170.0	170,000	1,700	700	150.0	105,000
	99,000	79,200	166.7	13,200,000	100,000	77,300	157.2	12,150,000
Fresno	21,600	3,400	155.9	530,000	23,500	1,400	142.9	200,000
Kern	5,400	2,000	177.5	355,000	6,000	500	150.0	75,000
Kings	22,000	3,300	162.1	535,000	25,400	1,500	150.0	225,000
Madera	12,200	5,900	165.3	975,000	14,700	5,500	140.0	770,000
Merced	55,800	3,800	146.1	555,000	56,600	4,000	140.0	560,000
San Joaquin	80,600	60,000	179.8	10,790,000	76,300	43,000	170.9	7,350,000
Stanislaus	36,000	900	138.9	125,000	36,500	600	158.3	95,000
Tulare	72,900	12,200	175.0	2,135,000	82,500	8,000	171.3	1,370,000
	306,500	91,500	174.9	16,000,000	321,500	64,500	165.0	10,645,000
Alpine	---	---	---	---	---	---	---	---
Amador	500	500	200.0	100,000	500	500	150.0	75,000
Calaveras	---	---	---	---	---	---	---	---
El Dorado	---	---	---	---	---	---	---	---
Inyo	---	---	---	---	---	---	---	---
Mariposa	---	---	---	---	---	---	---	---
Mono	---	---	---	---	---	---	---	---
Nevada	---	---	---	---	---	---	---	---
Placer	500	500	200.0	100,000	1,000	1,000	150.0	150,000
Sierra	---	---	---	---	---	---	---	---
Tuolumne	---	---	---	---	---	---	---	---
	1,000	1,000	200.0	200,000	1,500	1,500	150.0	225,000
Imperial	500	300	100.0	30,000	500	300	100.0	30,000
Los Angeles	---	---	---	---	---	---	---	---
Orange	---	---	---	---	---	---	---	---
Riverside	1,200	200	100.0	20,000	1,500	200	100.0	20,000
San Bernardino	1,300	---	---	---	1,500	---	---	---
San Diego	---	---	---	---	---	---	---	---
Santa Barbara	500	---	---	---	500	---	---	---
Ventura	---	---	---	---	---	---	---	---
	3,500	500	100.0	50,000	4,000	500	100.0	50,000
Combined 1/	500	200	150.0	30,000	500	200	150.0	30,000
STATE	420,000	180,000	170.0	30,600,000	435,000	150,000	160.0	24,000,000

1/ Includes those counties which normally have less than 200 acres planted.

CALIFORNIA COTTON, UPLAND: Acreage, Yield And Production By Counties, 1994-95

County	1994				1995			
	Planted	Harvested	Yield/Acre	Production	Planted	Harvested	Yield/Acre	Production
	Acres		*Pounds*	*Bales*	*Acres*		*Pounds*	*Bales*
DEL NORTE	---	---	---	---	---	---	---	---
HUMBOLT	---	---	---	---	---	---	---	---
MENDOCINO	---	---	---	---	---	---	---	---
	---	---	---	---	---	---	---	---
SHASTA	---	---	---	---	---	---	---	---
SISKIYOU	---	---	---	---	---	---	---	---
TRINITY	---	---	---	---	---	---	---	---
	---	---	---	---	---	---	---	---
LASSEN	---	---	---	---	---	---	---	---
MODOC	---	---	---	---	---	---	---	---
PLUMAS	---	---	---	---	---	---	---	---
	---	---	---	---	---	---	---	---
ALAMEDA	---	---	---	---	---	---	---	---
CONTRA COSTA	---	---	---	---	---	---	---	---
LAKE	---	---	---	---	---	---	---	---
MARIN	---	---	---	---	---	---	---	---
MONTEREY	---	---	---	---	---	---	---	---
NAPA	---	---	---	---	---	---	---	---
SAN BENITO	---	---	---	---	---	---	---	---
SAN FRANCISCO	---	---	---	---	---	---	---	---
SAN LUIS OBISP	---	---	---	---	---	---	---	---
SAN MATEO	---	---	---	---	---	---	---	---
SANTA CLARA	---	---	---	---	---	---	---	---
SANTA CRUZ	---	---	---	---	---	---	---	---
SONOMA	---	---	---	---	---	---	---	---
	---	---	---	---	---	---	---	---
BUTTE	---	---	---	---	---	---	---	---
COLUSA	---	---	---	---	4,800	4,100	468	4,000
GLEN	---	---	---	---	800	700	480	700
SACRAMENTO	---	---	---	---	---	---	---	---
SOLANO	---	---	---	---	---	---	---	---
SUTTER	---	---	---	---	---	---	---	---
TEHAMA	---	---	---	---	---	---	---	---
YOLO	---	---	---	---	500	500	768	800
YUBA	---	---	---	---	---	---	---	---
COMBINED 1/	1,100	1,100	1,309	3,000	1,200	1,000	624	1,300
	1,100	1,100	1,309	3,000	7,300	6,300	518	6,800
FRESNO	347,000	345,000	1,295	931,000	364,000	363,000	1,091	825,000
KERN	266,000	265,000	1,186	655,000	270,000	268,000	856	478,000
KINGS	226,000	225,000	1,082	507,000	245,000	245,000	876	447,000
MADERA	39,000	39,000	1,255	102,000	45,000	45,000	875	82,000
MERCED	76,000	76,000	1,301	206,000	83,000	83,000	1,105	191,000
SAN JOAQUIN	---	---	---	---	---	---	---	---
STANISLAUS	---	---	---	---	---	---	---	---
TULARE	126,000	125,000	1,010	263,000	134,000	133,000	830	230,000
	1,080,000	1,075,000	1,190	2,664,000	1,141,000	1,137,000	951	2,253,000
ALPINE	---	---	---	---	---	---	---	---
AMADOR	---	---	---	---	---	---	---	---
CALAVERAS	---	---	---	---	---	---	---	---
EL DORADO	---	---	---	---	---	---	---	---
INYO	---	---	---	---	---	---	---	---
MARIPOSA	---	---	---	---	---	---	---	---
MONO	---	---	---	---	---	---	---	---
NEVADA	---	---	---	---	---	---	---	---
PLACER	---	---	---	---	---	---	---	---
SIERRA	---	---	---	---	---	---	---	---
TUOLUMNE	---	---	---	---	---	---	---	---
	---	---	---	---	---	---	---	---
IMPERIAL	8,600	8,600	1,284	23,000	8,800	8,800	1,184	21,700
LOS ANGELES	---	---	---	---	---	---	---	---
ORANGE	---	---	---	---	---	---	---	---
RIVERSIDE	9,800	9,800	1,273	26,000	12,200	12,200	1,133	28,800
SAN	---	---	---	---	---	---	---	---
SAN DIEGO	---	---	---	---	---	---	---	---
SANTA BARBARA	---	---	---	---	---	---	---	---
VENTURA	---	---	---	---	---	---	---	---
	18,400	18,400	1,278	49,000	21,000	21,000	1,154	50,500
COMBINED 2/	500	500	960	1,000	700	700	1,166	1,700
STATE - TOTAL	1,100,000	1,095,000	1,191	2,717,000	1,170,000	1,165,000	953	2,312,000

1/ Some or all individual county totals not published due to possible disclosure of individual operations.
2/ Includes those counties which normally have less than 500 acres planted.

CALIFORNIA COTTON, AMERICAN PIMA: Acreage, Yield And Production By Counties, 1994-95

County	1994				1995			
	Planted	Harvested	Yield/Acre	Production	Planted	Harvested	Yield/Acre	Production
	Acres		*Pounds*	*Bales*	*Acres*		*Pounds*	*Bales*
DEL NORTE	---	---	---	---	---	---	---	---
HUMBOLT	---	---	---	---	---	---	---	---
MENDOCINO	---	---	---	---	---	---	---	---
	---	---	---	---	---	---	---	---
SHASTA	---	---	---	---	---	---	---	---
SISKIYOU	---	---	---	---	---	---	---	---
TRINITY	---	---	---	---	---	---	---	---
	---	---	---	---	---	---	---	---
LASSEN	---	---	---	---	---	---	---	---
MODOC	---	---	---	---	---	---	---	---
PLUMAS	---	---	---	---	---	---	---	---
	---	---	---	---	---	---	---	---
ALAMEDA	---	---	---	---	---	---	---	---
CONTRA COSTA	---	---	---	---	---	---	---	---
LAKE	---	---	---	---	---	---	---	---
MARIN	---	---	---	---	---	---	---	---
MONTEREY	---	---	---	---	---	---	---	---
NAPA	---	---	---	---	---	---	---	---
SAN BENITO	---	---	---	---	---	---	---	---
SAN FRANCISCO	---	---	---	---	---	---	---	---
SAN LUIS OBISP	---	---	---	---	---	---	---	---
SAN MATEO	---	---	---	---	---	---	---	---
SANTA CLARA	---	---	---	---	---	---	---	---
SANTA CRUZ	---	---	---	---	---	---	---	---
SONOMA	---	---	---	---	---	---	---	---
	---	---	---	---	---	---	---	---
BUTTE	---	---	---	---	---	---	---	---
COLUSA	---	---	---	---	---	---	---	---
GLEN	---	---	---	---	---	---	---	---
SACRAMENTO	---	---	---	---	---	---	---	---
SOLANO	---	---	---	---	---	---	---	---
SUTTER	---	---	---	---	---	---	---	---
TEHAMA	---	---	---	---	---	---	---	---
YOLO	---	---	---	---	---	---	---	---
YUBA	---	---	---	---	---	---	---	---
	---	---	---	---	---	---	---	---
FRESNO	30,700	30,500	1,201	76,300	48,500	48,500	1,044	105,500
KERN	25,000	25,000	1,089	56,700	34,800	34,800	848	61,500
KINGS	24,000	24,000	978	48,900	30,300	30,300	870	54,900
MADERA	---	---	---	---	---	---	---	---
MERCED	500	500	1,152	1,200	400	400	1,080	900
SAN JOAQUIN	---	---	---	---	---	---	---	---
STANISLAUS	---	---	---	---	---	---	---	---
TULARE	500	500	1,056	1,100	700	700	823	1,200
	80,700	80,500	1,098	184,200	114,700	114,700	937	224,000
ALPINE	---	---	---	---	---	---	---	---
AMADOR	---	---	---	---	---	---	---	---
CALAVERAS	---	---	---	---	---	---	---	---
EL DORADO	---	---	---	---	---	---	---	---
INYO	---	---	---	---	---	---	---	---
MARIPOSA	---	---	---	---	---	---	---	---
MONO	---	---	---	---	---	---	---	---
NEVADA	---	---	---	---	---	---	---	---
PLACER	---	---	---	---	---	---	---	---
SIERRA	---	---	---	---	---	---	---	---
TUOLUMNE	---	---	---	---	---	---	---	---
	---	---	---	---	---	---	---	---
IMPERIAL	---	---	---	---	---	---	---	---
LOS ANGELES	---	---	---	---	---	---	---	---
ORANGE	---	---	---	---	---	---	---	---
RIVERSIDE	---	---	---	---	---	---	---	---
SAN	---	---	---	---	---	---	---	---
SAN DIEGO	---	---	---	---	---	---	---	---
SANTA BARBARA	---	---	---	---	---	---	---	---
VENTURA	---	---	---	---	---	---	---	---
	---	---	---	---	---	---	---	---
COMBINED 1/	300	300	960	600	300	300	800	500
STATE - TOTAL	81,000	80,800	1,098	184,800	115,000	115,000	937	224,500

1/ Includes those counties which normally have less than 200 acres planted.

CALIFORNIA OATS: Acreage, Yield And Production By Counties, 1994-95

County	1994				1995			
	Planted	Harvested	Yield/Acre	Production	Planted	Harvested	Yield/Acre	Production
	Acres		*Bushels*		*Acres*		*Bushels*	
Del Norte	---	---	---	---	---	---	---	---
Humboldt	300	200	50.0	10,000	---	---	---	---
Mendocino	2,600	200	50.0	10,000	2,500	200	80.0	16,000
Combined 1/	---	---	---	---	400	100	80.0	8,000
	2,900	400	50.0	20,000	2,900	300	80.0	24,000
Shasta	1,200	300	100.0	30,000	1,100	300	116.7	35,000
Siskiyou	6,000	3,800	113.9	433,000	5,800	3,400	111.5	379,000
Trinity	---	---	---	---	---	---	---	---
	7,200	4,100	112.9	463,000	6,900	3,700	111.9	414,000
Lassen	6,400	400	75.0	30,000	6,600	300	100.0	30,000
Modoc	8,300	1,000	100.0	100,000	9,100	800	128.8	103,000
Plumas	900	---	---	---	1,000	---	---	---
	15,600	1,400	92.9	130,000	16,700	1,100	120.9	133,000
Alameda	3,000	200	75.0	15,000	3,000	200	70.0	14,000
Contra Costa	2,100	400	80.0	32,000	2,300	300	76.7	23,000
Lake	3,300	200	70.0	14,000	3,200	400	67.5	27,000
Marin	6,000	600	70.0	42,000	6,200	900	66.7	60,000
Monterey	3,200	600	80.0	48,000	3,400	400	82.5	33,000
Napa	1,200	---	---	---	1,400	---	---	---
San Benito	3,100	200	65.0	13,000	3,300	400	62.5	25,000
San Francisco	---	---	---	---	---	---	---	---
San Luis Obispo	15,100	1,500	54.7	82,000	17,400	1,400	58.6	82,000
San Mateo	1,500	800	71.3	57,000	1,600	900	67.8	61,000
Santa Clara	2,800	600	80.0	48,000	3,000	600	83.3	50,000
Santa Cruz	900	---	---	---	1,000	---	---	---
Sonoma	24,300	1,500	46.0	69,000	25,200	1,600	48.8	78,000
	66,500	6,600	63.6	420,000	71,000	7,100	63.8	453,000
Butte	5,700	300	63.3	19,000	6,100	300	80.0	24,000
Colusa	700	200	75.0	15,000	700	400	82.5	33,000
Glenn	4,700	400	67.5	27,000	5,300	300	80.0	24,000
Sacramento	5,200	400	65.0	26,000	5,900	500	90.0	45,000
Solano	3,600	1,500	80.0	120,000	4,300	1,200	83.3	100,000
Sutter	2,900	500	92.0	46,000	3,300	400	90.0	36,000
Tehama	3,700	300	80.0	24,000	4,200	200	75.0	15,000
Yolo	11,400	200	75.0	15,000	12,500	200	95.0	19,000
Yuba	1,100	400	75.0	30,000	1,100	300	80.0	24,000
	39,000	4,200	76.7	322,000	43,400	3,800	84.2	320,000
Fresno	10,900	1,700	94.1	160,000	12,600	1,900	100.5	191,000
Kern	4,200	500	106.0	53,000	4,600	600	118.3	71,000
Kings	3,600	200	90.0	18,000	4,000	200	95.0	19,000
Madera	8,000	1,000	100.0	100,000	8,300	1,300	105.4	137,000
Merced	42,500	3,700	84.3	312,000	48,100	3,600	96.1	346,000
San Joaquin	23,100	900	91.1	82,000	24,200	1,000	102.0	102,000
Stanislaus	29,000	800	93.8	75,000	35,900	900	102.2	92,000
Tulare	31,700	2,000	75.0	150,000	33,900	1,900	71.6	136,000
	153,000	10,800	88.0	950,000	171,600	11,400	96.0	1,094,000
Alpine	---	---	---	---	---	---	---	---
Amador	600	---	---	---	800	---	---	---
Calaveras	800	200	55.0	11,000	1,000	200	65.0	13,000
El Dorado	200	---	---	---	300	---	---	---
Inyo	200	---	---	---	300	---	---	---
Mariposa	200	---	---	---	200	---	---	---
Mono	500	---	---	---	600	---	---	---
Nevada	---	---	---	---	---	---	---	---
Placer	3,100	500	48.0	24,000	3,700	400	65.0	26,000
Sierra	600	---	---	---	700	---	---	---
Tuolumne	200	---	---	---	300	---	---	---
	6,400	700	50.0	35,000	7,900	600	65.0	39,000
Imperial	400	---	---	---	500	---	---	---
Los Angeles	800	200	30.0	6,000	1,200	200	35.0	7,000
Orange	200	---	---	---	300	---	---	---
Riverside	7,400	900	31.1	28,000	11,500	1,100	35.5	39,000
San Bernardino	2,000	---	---	---	2,900	---	---	---
San Diego	3,800	400	37.5	15,000	6,400	400	40.0	16,000
Santa Barbara	3,900	300	36.7	11,000	5,500	300	36.7	11,000
Ventura	900	---	---	---	1,300	---	---	---
	19,400	1,800	33.3	60,000	29,600	2,000	36.5	73,000
STATE	310,000	30,000	80.0	2,400,000	350,000	30,000	85.0	2,550,000

1/ These figures include estimates for one or more counties which normally have less than 200 acres planted.

CALIFORNIA RICE: Acreage, Yield And Production By Counties, 1994-95

County	1994				1995			
	Planted	Harvested	Yield/Acre	Production	Planted	Harvested	Yield/Acre	Production
	Acres		*Pounds*	*Cwt.*	*Acres*		*Pounds*	*Cwt.*
Del Norte	---	---	---	---	---	---	---	---
Humboldt	---	---	---	---	---	---	---	---
Mendocino	---	---	---	---	---	---	---	---
	---	---	---	---	---	---	---	---
Shasta	---	---	---	---	---	---	---	---
Siskiyou	---	---	---	---	---	---	---	---
Trinity	---	---	---	---	---	---	---	---
	---	---	---	---	---	---	---	---
Lassen	---	---	---	---	---	---	---	---
Modoc	---	---	---	---	---	---	---	---
Plumas	---	---	---	---	---	---	---	---
	---	---	---	---	---	---	---	---
Alameda	---	---	---	---	---	---	---	---
Contra Costa	---	---	---	---	---	---	---	---
Lake	---	---	---	---	---	---	---	---
Marin	---	---	---	---	---	---	---	---
Monterey	---	---	---	---	---	---	---	---
Napa	---	---	---	---	---	---	---	---
San Benito	---	---	---	---	---	---	---	---
San Francisco	---	---	---	---	---	---	---	---
San Luis Obispo	---	---	---	---	---	---	---	---
San Mateo	---	---	---	---	---	---	---	---
Santa Clara	---	---	---	---	---	---	---	---
Santa Cruz	---	---	---	---	---	---	---	---
Sonoma	---	---	---	---	---	---	---	---
	---	---	---	---	---	---	---	---
Butte	88,000	87,700	8,438	7,400,000	83,000	82,700	7,573	6,263,000
Colusa	123,000	122,700	8,341	10,234,000	122,000	121,400	7,790	9,457,000
Glenn	81,000	80,700	8,400	6,779,000	79,000	78,700	7,535	5,930,000
Sacramento	11,500	11,500	8,435	970,000	10,300	10,300	6,505	670,000
Solano	---	---	---	---	---	---	---	---
Sutter	90,000	89,700	8,740	7,840,000	82,000	81,700	7,408	6,052,000
Tehama	1,800	1,800	8,333	150,000	1,700	1,700	7,412	126,000
Yolo	26,700	26,600	8,876	2,361,000	27,000	26,900	7,509	2,020,000
Yuba	34,000	33,800	8,896	3,007,000	32,000	31,900	8,091	2,581,000
	456,000	454,500	8,524	38,741,000	437,000	435,300	7,604	33,099,000
Fresno	3,000	3,000	7,933	238,000	4,000	4,000	7,375	295,000
Kern	---	---	---	---	---	---	---	---
Kings	---	---	---	---	---	---	---	---
Madera	---	---	---	---	---	---	---	---
Merced	5,000	4,900	8,102	397,000	5,000	4,900	8,265	405,000
San Joaquin	6,000	5,900	7,966	470,000	5,000	4,900	7,653	375,000
Stanislaus	3,000	3,000	7,500	225,000	3,000	3,000	7,400	222,000
Tulare	---	---	---	---	---	---	---	---
	17,000	16,800	7,917	1,330,000	17,000	16,800	7,720	1,297,000
Alpine	---	---	---	---	---	---	---	---
Amador	---	---	---	---	---	---	---	---
Calaveras	---	---	---	---	---	---	---	---
El Dorado	---	---	---	---	---	---	---	---
Inyo	---	---	---	---	---	---	---	---
Mariposa	---	---	---	---	---	---	---	---
Mono	---	---	---	---	---	---	---	---
Nevada	---	---	---	---	---	---	---	---
Placer	14,000	13,700	8,416	1,153,000	13,000	12,900	7,411	956,000
Sierra	---	---	---	---	---	---	---	---
Tuolumne	---	---	---	---	---	---	---	---
	14,000	13,700	8,416	1,153,000	13,000	12,900	7,411	956,000
Imperial	---	---	---	---	---	---	---	---
Los Angeles	---	---	---	---	---	---	---	---
Orange	---	---	---	---	---	---	---	---
Riverside	---	---	---	---	---	---	---	---
San Bernardino	---	---	---	---	---	---	---	---
San Diego	---	---	---	---	---	---	---	---
Santa Barbara	---	---	---	---	---	---	---	---
Ventura	---	---	---	---	---	---	---	---
	---	---	---	---	---	---	---	---
STATE	487,000	485,000	8,500	41,224,000	467,000	465,000	7,600	35,352,000

CALIFORNIA SUGAR BEETS: Acreage, Yield And Production By Counties, 1994-95

County	1994					1995				
	Planted	Harvested	Yield/Acre	Production	Percent	Planted	Harvested	Yield/Acre	Production	Percent
	Acres		*Tons*		*Percent*	*Acres*		*Tons*		*Percent*
Del Norte	---	---	---	---	---	---	---	---	---	---
Humboldt	---	---	---	---	---	---	---	---	---	---
Mendocino	---	---	---	---	---	---	---	---	---	---
	---	---	---	---	---	---	---	---	---	---
Shasta	500	500	16.0	8,000	17.92	500	500	18.0	9,000	17.80
Siskiyou	1,700	1,600	19.4	31,000	18.91	1,800	1,800	18.9	34,000	18.81
Trinity	---	---	---	---	---	---	---	---	---	---
	2,200	2,100	18.6	39,000	18.71	2,300	2,300	18.7	43,000	18.60
Lassen	1,000	900	14.4	13,000	18.60	900	900	13.3	12,000	18.58
Modoc	3,600	3,600	24.4	88,000	18.75	3,500	3,500	19.7	69,000	18.67
Plumas	---	---	---	---	---	---	---	---	---	---
	4,600	4,500	22.4	101,000	18.73	4,400	4,400	18.4	81,000	18.66
Alameda	---	---	---	---	---	---	---	---	---	---
Contra Costa	---	---	---	---	---	---	---	---	---	---
Lake	---	---	---	---	---	---	---	---	---	---
Marin	---	---	---	---	---	---	---	---	---	---
Monterey	---	---	---	---	---	---	---	---	---	---
Napa	---	---	---	---	---	---	---	---	---	---
San Benito	---	---	---	---	---	---	---	---	---	---
San Francisco	---	---	---	---	---	---	---	---	---	---
San Luis Obispo	---	---	---	---	---	---	---	---	---	---
San Mateo	---	---	---	---	---	---	---	---	---	---
Santa Clara	---	---	---	---	---	---	---	---	---	---
Santa Cruz	---	---	---	---	---	---	---	---	---	---
Sonoma	---	---	---	---	---	---	---	---	---	---
	---	---	---	---	---	---	---	---	---	---
Butte	1,800	1,800	32.2	58,000	13.01	1,200	1,200	26.7	32,000	13.18
Colusa	5,000	4,700	27.2	128,000	14.54	3,300	3,200	27.5	88,000	13.51
Glenn	10,000	9,700	34.5	335,000	13.34	5,000	4,800	29.2	140,000	13.49
Sacramento	5,100	5,100	23.7	121,000	15.21	4,100	4,000	27.0	108,000	14.61
Solano	9,500	9,200	24.0	221,000	15.44	8,300	8,000	26.9	215,000	15.00
Sutter	1,900	1,900	24.2	46,000	14.75	1,500	1,500	26.7	40,000	14.10
Tehama	---	---	---	---	---	---	---	---	---	---
Yolo	7,100	7,100	26.6	189,000	14.74	5,900	5,700	26.0	148,000	14.92
Yuba	---	---	---	---	---	---	---	---	---	---
	40,400	39,500	27.8	1,098,000	14.39	29,300	28,400	27.1	771,000	14.37
Fresno	20,600	20,400	28.1	574,000	14.96	15,600	15,300	26.2	401,000	14.08
Kern	8,800	8,800	29.0	255,000	14.43	7,600	7,500	31.5	236,000	14.80
Kings	4,100	4,000	25.0	100,000	13.72	3,000	2,900	30.3	88,000	13.62
Madera	700	600	30.0	18,000	14.30	500	500	28.0	14,000	14.26
Merced	8,400	8,400	23.9	201,000	15.49	6,600	6,500	26.0	169,000	15.03
San Joaquin	16,000	16,000	25.1	402,000	15.36	12,100	11,800	25.2	297,000	15.32
Stanislaus	---	---	---	---	---	---	---	---	---	---
Tulare	3,200	3,200	26.9	86,000	13.29	2,600	2,600	30.8	80,000	13.60
	61,800	61,400	26.6	1,636,000	14.87	48,000	47,100	27.3	1,285,000	14.56
Alpine	---	---	---	---	---	---	---	---	---	---
Amador	---	---	---	---	---	---	---	---	---	---
Calaveras	---	---	---	---	---	---	---	---	---	---
El Dorado	---	---	---	---	---	---	---	---	---	---
Inyo	---	---	---	---	---	---	---	---	---	---
Mariposa	---	---	---	---	---	---	---	---	---	---
Mono	---	---	---	---	---	---	---	---	---	---
Nevada	---	---	---	---	---	---	---	---	---	---
Placer	---	---	---	---	---	---	---	---	---	---
Sierra	---	---	---	---	---	---	---	---	---	---
Tuolumne	---	---	---	---	---	---	---	---	---	---
	---	---	---	---	---	---	---	---	---	---
Imperial	31,800	31,600	32.1	1,013,000	16.36	31,000	30,900	32.0	989,000	16.88
Los Angeles	---	---	---	---	---	---	---	---	---	---
Orange	---	---	---	---	---	---	---	---	---	---
Riverside	---	---	---	---	---	---	---	---	---	---
San Bernardino	---	---	---	---	---	---	---	---	---	---
San Diego	---	---	---	---	---	---	---	---	---	---
Santa Barbara	---	---	---	---	---	---	---	---	---	---
Ventura	---	---	---	---	---	---	---	---	---	---
	31,800	31,600	32.1	1,013,000	16.36	31,000	30,900	32.0	989,000	16.88
Other Counties 1/	2,200	1,900	32.1	61,000	14.73	2,000	1,900	26.8	51,000	14.40
STATE	143,000	141,000	28.0	3,948,000	15.25	117,000	115,000	28.0	3,220,000	15.38

1/ Includes those counties which normally have less than 500 acres planted.

CALIFORNIA WHEAT, ALL: Acreage, Yield And Production By Counties, 1994-95

County	1994				1995			
	Planted	Harvested	Yield/Acre	Production	Planted	Harvested	Yield/Acre	Production
	Acres		*Bushels*		*Acres*		*Bushels*	
Del Norte	---	---	---	---	---	---	---	---
Humboldt	---	---	---	---	---	---	---	---
Mendocino	---	---	---	---	---	---	---	---
	---	---	---	---	---	---	---	---
Shasta	2,000	2,000	67.0	134,000	2,000	1,000	72.0	72,000
Siskiyou	8,000	6,000	73.7	442,000	7,500	6,500	71.7	466,000
Trinity	---	---	---	---	---	---	---	---
	10,000	8,000	72.0	576,000	9,500	7,500	71.7	538,000
Lassen	2,000	2,000	50.5	101,000	2,000	1,500	41.3	62,000
Modoc	4,000	3,000	76.3	229,000	3,500	3,500	80.0	280,000
Plumas	---	---	---	---	---	---	---	---
	6,000	5,000	66.0	330,000	5,500	5,000	68.4	342,000
Alameda	1,000	1,000	75.0	75,000	2,000	2,000	47.0	94,000
Contra Costa	3,500	3,000	77.3	232,000	4,500	4,000	69.8	279,000
Lake	---	---	---	---	---	---	---	---
Marin	---	---	---	---	---	---	---	---
Monterey	4,500	3,500	41.1	144,000	5,500	4,500	42.2	190,000
Napa	---	---	---	---	---	---	---	---
San Benito	4,500	3,500	62.6	219,000	4,500	2,000	51.0	102,000
San Francisco	---	---	---	---	---	---	---	---
San Luis Obispo	8,000	7,500	33.6	252,000	7,500	6,000	24.2	145,000
San Mateo	---	---	---	---	---	---	---	---
Santa Clara	2,000	1,500	83.3	125,000	2,000	1,500	78.0	117,000
Santa Cruz	---	---	---	---	---	---	---	---
Sonoma	1,500	1,000	45.0	45,000	1,500	1,000	44.0	44,000
	25,000	21,000	52.0	1,092,000	27,500	21,000	46.2	971,000
Butte	10,000	9,000	77.2	695,000	10,000	7,000	45.7	320,000
Colusa	45,000	43,000	85.8	3,690,000	40,000	30,000	57.1	1,714,000
Glenn	29,000	27,000	79.0	2,134,000	26,500	18,000	57.9	1,043,000
Sacramento	24,000	22,000	76.5	1,682,000	23,000	15,000	59.9	898,000
Solano	40,000	37,000	76.1	2,814,000	40,500	33,000	53.0	1,749,000
Sutter	23,000	22,000	85.4	1,878,000	23,500	17,000	60.9	1,036,000
Tehama	5,000	5,000	53.0	265,000	4,500	3,500	27.7	97,000
Yolo	74,000	69,000	80.4	5,551,000	66,000	51,000	63.1	3,217,000
Yuba	1,000	1,000	87.0	87,000	1,000	500	64.0	32,000
	251,000	235,000	80.0	18,796,000	235,000	175,000	57.7	10,106,000
Fresno	48,000	38,000	88.8	3,373,000	50,000	32,500	65.5	2,130,000
Kern	52,000	42,000	65.7	2,758,000	50,000	36,000	61.5	2,215,000
Kings	42,000	36,000	84.0	3,024,000	43,000	36,000	76.2	2,743,000
Madera	21,000	18,000	78.2	1,407,000	20,500	15,500	74.1	1,149,000
Merced	12,000	11,000	80.5	885,000	12,000	10,000	64.4	644,000
San Joaquin	40,000	34,000	79.0	2,686,000	40,000	32,000	62.5	2,000,000
Stanislaus	7,000	3,000	70.0	210,000	7,500	2,000	64.0	128,000
Tulare	44,000	31,000	62.7	1,943,000	45,000	24,000	62.6	1,503,000
	266,000	213,000	76.5	16,286,000	268,000	188,000	66.6	12,512,000
Alpine	---	---	---	---	---	---	---	---
Amador	---	---	---	---	---	---	---	---
Calaveras	---	---	---	---	---	---	---	---
El Dorado	---	---	---	---	---	---	---	---
Inyo	---	---	---	---	---	---	---	---
Mariposa	---	---	---	---	---	---	---	---
Mono	---	---	---	---	---	---	---	---
Nevada	---	---	---	---	---	---	---	---
Placer	2,000	2,000	58.0	116,000	3,000	2,500	46.4	116,000
Sierra	---	---	---	---	---	---	---	---
Tuolumne	---	---	---	---	---	---	---	---
	2,000	2,000	58.0	116,000	3,000	2,500	46.4	116,000
Imperial	62,000	61,000	94.2	5,745,000	67,000	64,500	101.2	6,527,000
Los Angeles	1,500	1,000	48.0	48,000	1,500	1,000	48.0	48,000
Orange	---	---	---	---	---	---	---	---
Riverside	22,000	19,000	62.5	1,187,000	28,000	24,000	56.1	1,347,000
San Bernardino	---	---	---	---	---	---	---	---
San Diego	3,000	3,000	46.3	139,000	3,000	3,000	46.7	140,000
Santa Barbara	1,500	1,000	50.0	50,000	2,000	1,500	52.0	78,000
Ventura	---	---	---	---	---	---	---	---
	90,000	85,000	84.3	7,169,000	101,500	94,000	86.6	8,140,000
STATE	650,000	569,000	78.0	44,365,000	650,000	493,000	66.4	32,725,000

CALIFORNIA FLORICULTURE: Production, Sales, and Value, 1994 1/

Kind	Number of Producers	Plants Grown	Production Area	Sales: Unit	Sales: Number Sold	Sales: Percent of Sales at Wholesale	Wholesale Price 2/	Value of Sales at Wholesale
	Number	*1,000*	*1,000 Sq. Ft.*	*1,000*	*1,000*	*Percent*	*Dollars*	*$1,000*
Cut Flowers 3/	---	---	---	---	---	---	---	263,238
Carnations	---	17,301	12,669	---	---	---	---	22,392
Standard	63	10,029	7,882	Blooms	96,598	100	0.14	13,939
Miniature	67	7,272	4,787	Bunches	6,308	98	1.34	8,453
Chrysanthemums	---	41,008	11,310	---	---	---	---	19,194
Standard	15	10,424	3,104	Blooms	10,192	98	0.46	4,634
Pompon	32	30,584	8,206	Bunches	11,287	96	1.29	14,560
Roses	---	15,383	24,899	---	303,433	---	---	75,931
Hybrid Tea	93	13,904	22,827	Blooms	264,444	99	0.26	69,311
Sweetheart	47	1,479	2,072	Blooms	38,989	99	0.17	6,620
Others	184	---	197,272	---	---	99	---	137,892
Potted Flowering Plants								137,055
African Violets	22	---	1,220	Pots	2,990	100	1.00	2,976
Chrysanthemums	33	---	4,432	Pots	8,380	100	2.67	22,339
Cyclamens	61	---	1,369	Pots	1,682	97	2.33	3,912
Finished Florist Azaleas	35	---	906	Pots	1,442	89	4.32	6,231
Kalanchoes	20	---	746	Pots	1,304	100	2.18	2,839
Easter Lilies	27	---	773	Pots	995	100	3.81	3,791
Other Lilies	21	---	131	Pots	130	98	4.04	525
Poinsettias	108	---	9,994	Pots	8,185	99	3.45	28,217
Others	139	---	15,016	Pots	25,077	97	2.64	66,225
Foliage Plants								86,634
Hanging Baskets	67	---	---	Baskets	4,637	94	3.35	15,534
Potted Foliage	144	---	17,767	---	---	95	---	71,100
Bedding/Garden Plants 4/								182,480
Flats								121,756
Geraniums	24	---	298	Flats	416	100	9.70	4,035
Impatiens	60	---	5,583	Flats	2,302	98	5.59	12,868
Petunias	62	---	4,752	Flats	1,962	98	6.35	12,459
Other (Include Foliar)	92	---	28,579	Flats	10,585	98	7.84	85,338
Vegetable Type	49	---	2,576	Flats	920	97	7.67	7,056
Potted								53,898
Hardy/Garden Chrysanthemums	44	---	3,043	Pots	4,205	100	0.89	3,737
Geraniums (Cutting)	44	---	891	Pots	1,837	100	1.38	2,538
Geraniums (Seed)	29	---	394	Pots	941	99	1.29	1,218
Impatiens	51	---	2,526	Pots	4,802	95	0.77	3,675
New Guinea Impatiens	32	---	443	Pots	517	99	1.64	849
Petunias	30	---	2,303	Pots	4,187	97	0.65	2,737
Other (Include Foliar)	73	---	20,477	Pots	38,885	97	0.93	36,215
Vegetable Type	32	---	1,182	Pots	3,839	100	0.76	2,929
Hanging Baskets								6,043
Geraniums	32	---	---	Baskets	147	97	6.10	897
Impatiens	32	---	---	Baskets	150	98	4.77	716
New Guinea Impatiens	29	---	---	Baskets	155	98	6.27	972
Petunias	18	---	---	Baskets	39	99	5.59	218
Flowering	49	---	---	Baskets	664	88	4.88	3,240
Total Cut Cultivated	35	---	876	---	---	99	---	16,231
TOTAL ALL PLANTS 5/	953							685,638

1/ The total covered growing area of 123,844,000 square feet consisted of the following: 17,359,000 square feet of shade and temporary cover; 16,456,000 square feet of glass; 44,008,000 square feet of fiberglass and other rigid greenhouses; and 46,021,000 square feet of film plastic (single/multi) greenhouses. In addition, plants were produced on 7,547 acres of open ground.
2/ For potted plants, price represents a weighted average for plants sold in pots less than 5 inches and in pots 5 inches or more.
3/ Total includes Gladioli, which are not published to avoid disclosure of individual operations.
4/ Total includes New Guinea Impatiens, which are not published to avoid disclosure of individual operations.
5/ Based on equivalent wholesale value of all sales for all crops, except potted foliage plants which are based on net value of sales.

CALIFORNIA FLORICULTURE: Production, Sales, and Value, 1995 1/

Kind	Number of Producers	Plants Grown	Production Area	Sales			Wholesale Price 2/	Value of Sales at Wholesale
				Unit	Number Sold	Percent of Sales at Wholesale		
	Number	1,000	1,000 Sq. Ft.	1,000	1,000	Percent	Dollars	$1,000
Cut Flowers 3/	---	---	---	---	---	---	---	252,985
Carnations	---	17,406	12,875	---	---	---	---	23,480
Standard	62	10,384	8,211	Blooms	97,070	100	0.14	13,687
Miniature	72	7,022	4,664	Bunches	8,590	86	1.14	9,793
Chrysanthemums	---	40,002	11,983	---	---	---	---	19,829
Standard	18	10,571	3,303	Blooms	9,813	96	0.50	4,871
Pompon	35	29,431	8,680	Bunches	12,465	97	1.20	14,958
Roses	---	13,171	21,962	---	269,328	---	---	68,869
Hybrid Tea	82	12,017	20,224	Blooms	239,930	98	0.27	63,989
Sweetheart	43	1,154	1,738	Blooms	29,398	100	0.17	4,880
Others	171	---	185,828	---	---	98	---	131,963
Potted Flowering Plants								126,440
African Violets	20	---	460	Pots	1,807	100	1.04	1,982
Chrysanthemums	32	---	3,896	Pots	8,031	99	2.06	20,265
Cyclamens	57	---	1,571	Pots	1,928	99	2.39	4,610
Finished Florist Azaleas	26	---	1,013	Pots	1,527	89	4.46	6,809
Kalanchoes	22	---	640	Pots	1,150	100	2.17	2,492
Easter Lilies	29	---	708	Pots	957	100	3.78	3,617
Other Lilies	21	---	203	Pots	227	99	3.72	844
Poinsettias	101	---	10,318	---	6,971	99	3.79	26,409
Others	131	---	14,163	Pots	20,487	98	2.90	59,412
Foliage Plants								85,356
Hanging Baskets	61	---	---	Baskets	6,076	90	3.09	18,775
Potted Foliage	130	---	17,152	---	---	98	---	66,581
Bedding/Garden Plants 4/								173,154
Flats	---	---	---	---	---	---	---	110,835
Geraniums	28	---	533	Flats	238	92	7.68	1,828
Impatiens	69	---	4,794	Flats	2,018	98	6.70	13,521
Petunias	68	---	5,042	Flats	2,387	99	7.32	17,473
Other (Include Foliar)	79	---	24,145	Flats	9,165	98	7.08	64,888
Vegetable Type	47	---	4,195	Flats	1,793	98	7.32	13,125
Potted								54,274
Hardy/Garden Chrysanthemums	36	---	2,386	Pots	3,358	99	0.82	2,752
Geraniums (Cutting)	43	---	1,282	Pots	3,300	100	1.25	4,137
Geraniums (Seed)	26	---	431	Pots	991	98	1.28	1,264
Impatiens	46	---	2,706	Pots	4,863	95	0.71	3,472
New Guinea Impatiens	28	---	636	Pots	649	99	1.67	1,086
Petunias	34	---	1,884	Pots	3,698	97	0.61	2,247
Other (Include Foliar)	82	---	20,594	Pots	32,679	96	1.12	36,600
Vegetable Type	32	---	1,152	Pots	3,537	99	0.77	2,716
Hanging Baskets								7,084
Geraniums	43	---	---	Baskets	256	98	5.48	1,403
Impatiens	38	---	---	Baskets	198	96	4.82	954
New Guinea Impatiens	31	---	---	Baskets	153	97	6.45	987
Petunias	25	---	---	Baskets	41	97	5.48	225
Flowering	43	---	---	Baskets	630	94	5.58	3,515
Total Cut Cultivated	46	---	912	---	---	100	---	16,344
TOTAL ALL PLANTS 5/	952	---	---	---	---	---	---	654,279

1/ The total covered growing area of 120,849,000 square feet consisted of the following: 15,162,000 square feet of shade and temporary cover; 13,764,000 square feet of glass; 42,305,000 square feet of fiberglass and other rigid greenhouses; and 49,618,000 square feet of film plastic (single/multi) greenhouses. In addition, plants were produced on 6,814 acres of open ground.
2/ For potted plants, price represents a weighted average for plants sold in pots less than 5 inches and in pots 5 inches or more.
3/ Total includes Gladioli, which are not published to avoid disclosure of individual operations.
4/ Total includes New Guinea Impatiens, which are not published to avoid disclosure of individual operations.
5/ Based on equivalent wholesale value of all sales for all crops, except potted foliage plants which are based on net value of sales.

FRUIT AND NUT CROPS

SUMMARY OF FRUIT AND NUT CROPS

California produced 13.1 million tons of fruit and nut crops in 1995, 8% less than 1994. The Golden State grew 39% of U.S. fruit and nut production in 1995, down from 42% last year. The total value of fruit and nut production was $5.78 billion in 1995, up 1% from 1994. This was 55% of the U.S. total value. California accounts for virtually all of the U.S. production of dates, figs, kiwifruit, nectarines, olives, almonds, pistachios, and English walnuts.

California dominates U.S. production of apricots, avocados, grapes, lemons, plums, prunes, and strawberries. Several crops had major changes in production from 1994 to 1995. They include the following changes: apricots, down 64%; sweet cherries, down 62%; nectarines, down 27%; Cling peaches, down 23%; Freestone peaches, down 21%; pears, down 26%; plums, down 50%; almonds, down 50%; pecans up 93%, pistachios, up 15%; and avocodos, up 12%.

Precipitation during the winter was well above normal. Several storms hit the State in March with strong winds, heavy rains, and flooding. Particularly hard hit were almonds where many trees were uprooted and the poor weather during the critical pollination period reduced the crop to the smallest since 1986. The strawberry crop also suffered extreme damage, especially in Monterey and Santa Cruz counties. Many other fruit crops were also affected by the storms and production of some crops was down substantially. Because of the cool rainy weather, crop development was generally behind normal. However, mild weather during the summer and fall months permitted the crops to be harvested without any major difficulties.

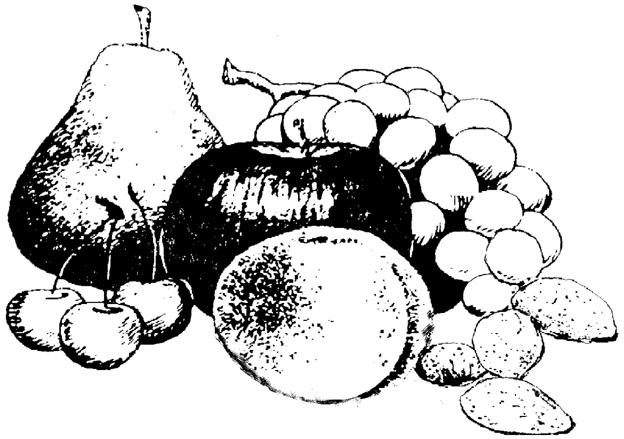

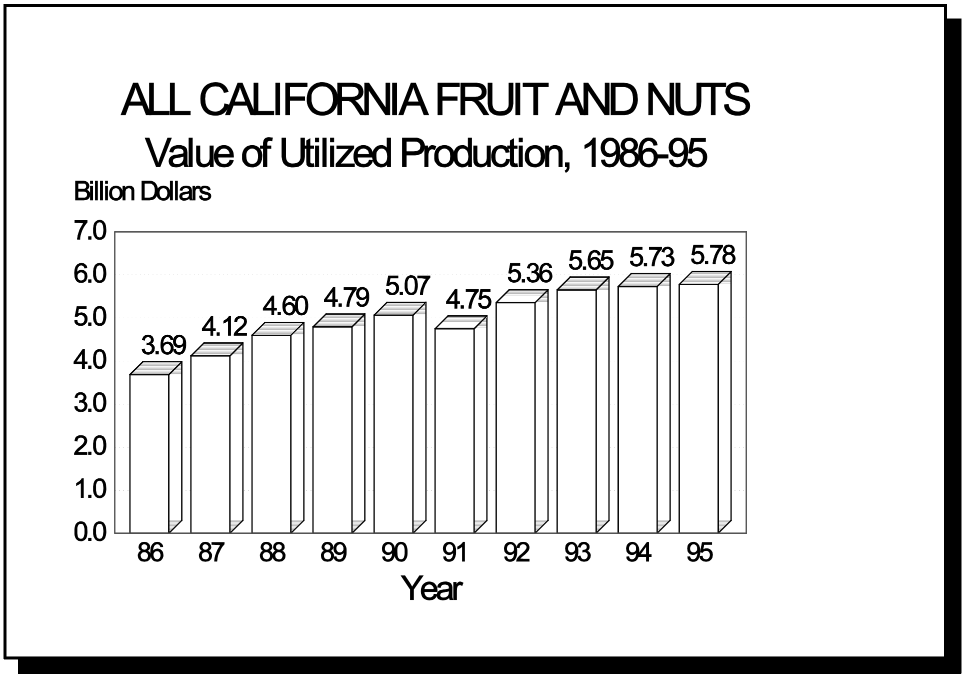

NOTES FOR USERS

Total production is the quantity actually harvested plus quantities which would have been acceptable for fresh market or processing but were not harvested. It also includes fruit that was harvested but not sold due to marketing conditions. **Utilized production** is the amount sold, plus the quantities used at home or held in storage. **Crop year production** for citrus and avocados are from the bloom of the first year shown. For example, 1995-96 would represent the 1996 production from the 1995 bloom.

Utilization refers to first, but not necessarily final, utilization. For example, frozen fruit used for preserves is included as frozen. **Canned** includes all canned products -- jam, jelly, baby food, nectar, juice, puree, and all fruit packs combined.

The price quoted is at the packinghouse door, which includes the transportation costs. Drying costs are excluded from prices on all fruit crops except grapes. **Crop value** estimates cover the marketing season or crop year and should not be confused with **cash receipts** from these crops which are calculated on a single calendar year. The season average price is applied to utilized production to compute **value of production**.

Price and value for citrus fruits are based on equivalent packinghouse door returns for the entire data series. The processing price for berries is based on equivalent returns at the processing plant door. The fresh market price is on a packed and loaded basis, F.O.B. shipping point.

CALIFORNIA NON-CITRUS FRUITS: Acreage, Production, Price And Value, 1986-95

Crop	Crop Year	Bearing	Non-Bearing	Yield Per Acre	Total Production	Price 1/	Total Value
		Acres		*Tons*		*$/Ton*	*$1,000*
Apples	1986	21,200	8,400	12.10	257,500	326.00	83,903
	1987	22,700	8,100	14.30	325,000	222.00	72,070
	1988	27,600	4,400	11.40	315,000	374.00	117,750
	1989	29,900	3,000	11.30	337,500	300.00	101,023
	1990	31,400	2,900	12.40	390,000	312.00	121,700
	1991	31,800	2,200	12.60	400,000	420.00	167,950
	1992	33,800	2,000	12.50	420,000	406.00	170,160
	1993	34,700	4,200	12.70	440,000	314.00	138,320
	1994	34,900	4,700	15.00	525,000	266.00	139,300
	1995	35,000	4,200	12.20	425,000	366.00	155,750
Apricots	1986	18,500	1,400	2.70	50,000	371.00	18,564
	1987	18,000	2,500	6.11	110,000	333.00	33,970
	1988	17,900	2,800	5.31	95,000	340.00	29,613
	1989	17,400	3,000	6.78	118,000	323.00	37,821
	1990	16,900	3,200	6.80	115,000	323.00	36,536
	1991	17,300	2,600	5.20	90,000	374.00	32,183
	1992	18,100	2,700	5.47	99,000	328.00	32,458
	1993	19,300	1,500	4.61	89,000	363.00	32,288
	1994	19,800	N/A	7.32	145,000	331.00	43,700
	1995	19,800	N/A	2.63	52,000	392.00	20,405
Avocados	85-86	74,500	1,300	2.15	160,000	1,020.00	163,200
	86-87	75,000	1,000	3.71	278,000	338.00	93,964
	87-88	74,800	700	2.41	180,000	1,140.00	205,200
	88-89	75,200	700	2.19	165,000	1,260.00	207,900
	89-90	75,000	600	1.40	105,000	2,280.00	239,400
	90-91	74,200	400	1.83	136,000	1,410.00	191,760
	91-92	73,500	400	2.12	156,000	1,170.00	182,520
	92-93	72,900	300	3.90	284,000	400.00	113,600
	93-94	72,500	N/A	1.92	139,000	1,810.00	251,590
	94-95	67,600	N/A	2.31	156,000	1,490.00	232,440
Cherries, Sweet	1986	10,400	1,500	0.81	8,400	1,220.00	10,211
	1987	10,300	1,500	4.37	45,000	636.00	28,615
	1988	10,000	2,000	2.60	26,000	771.00	20,040
	1989	10,300	2,400	2.52	26,000	939.00	24,418
	1990	10,600	2,700	2.08	22,000	891.00	19,610
	1991	10,800	2,600	3.33	36,000	957.00	34,434
	1992	11,200	3,000	2.77	31,000	1,240.00	38,342
	1993	11,700	2,700	1.62	19,000	2,270.00	43,203
	1994	12,100	N/A	4.30	52,000	1,220.00	63,512
	1995	12,500	N/A	1.58	19,800	2,100.00	41,540
Dates	1986	4,500	1,300	3.96	17,800	828.00	14,738
	1987	4,900	1,100	3.96	19,400	872.00	16,917
	1988	5,100	1,200	4.31	22,000	896.00	19,712
	1989	5,000	1,000	4.40	22,000	1,040.00	22,880
	1990	5,000	900	4.80	24,000	820.00	19,680
	1991	5,200	600	4.23	22,000	970.00	21,340
	1992	5,300	400	3.96	21,000	1,060.00	22,260
	1993	5,500	200	5.27	29,000	790.00	22,910
	1994	5,500	N/A	4.18	23,000	750.00	17,250
	1995	5,500	N/A	4.00	22,000	748.00	16,456

See Footnotes on Page 21.

CALIFORNIA NON-CITRUS FRUITS: Acreage, Production, Price And Value, 1986-95

Crop	Crop Year	Bearing	Non-Bearing	Yield Per Acre	Total Production	Price 1/	Total Value
		Acres		Tons		$/Ton	$1,000
Figs	1986	18,000	500	2.78	50,000	283.00	14,173
	1987	18,100	100	2.89	52,300	331.00	17,336
	1988	17,700	60	3.14	55,500	352.00	19,530
	1989	16,900	50	2.84	48,000	379.00	18,198
	1990	16,700	300	2.97	49,600	350.00	17,370
	1991	16,000	400	2.82	45,100	369.00	16,626
	1992	14,400	500	3.26	46,900	405.00	18,985
	1993	12,200	500	4.98	60,700	401.00	24,341
	1994	14,600	N/A	3.88	56,700	419.00	23,768
	1995	15,000	N/A	3.33	50,000	367.00	18,357
Grapes, All 2/ 6/	1986	670,700	36,900	7.68	4,770,000	222.00	1,060,245
	1987	662,800	38,700	7.19	4,660,000	258.00	1,202,840
	1988	656,400	46,500	8.74	5,520,000	263.00	1,451,883
	1989	641,700	58,100	8.40	5,390,000	314.00	1,692,085
	1990	639,000	61,500	8.11	5,185,000	291.00	1,508,045
	1991	636,500	58,500	8.08	4,980,000	314.00	1,562,627
	1992	644,300	48,600	8.64	5,460,000	309.00	1,687,225
	1993	655,800	40,400	8.47	5,383,000	340.00	1,828,330
	1994	654,800	51,900	8.03	5,256,000	326.00	1,712,939
	1995	645,200	71,600	8.46	5,248,000	350.00	1,838,900
Grapes, Raisin 2/ 3/	1986	277,000	3,900	9.01	2,045,000	213.00	434,665
	1987	275,000	2,900	8.35	2,170,000	223.00	483,870
	1988	274,000	4,400	10.30	2,570,000	205.00	525,830
	1989	271,000	7,000	9.48	2,570,000	258.00	663,880
	1990	270,000	8,000	8.69	2,345,000	237.00	554,901
	1991	266,000	10,100	8.80	2,165,000	247.00	535,127
	1992	267,000	10,100	10.50	2,670,000	230.00	614,595
	1993	266,000	10,200	9.57	2,354,000	255.00	599,404
	1994	270,000	8,500	8.85	2,389,000	229.00	547,002
	1995	268,000	8,700	9.28	2,255,000	220.00	496,860
Grapes, Table	1986	81,700	12,400	7.59	620,000	307.00	190,140
	1987	83,800	10,200	6.44	540,000	435.00	235,040
	1988	85,400	7,400	9.02	770,000	363.00	279,363
	1989	80,700	8,700	7.81	630,000	449.00	283,005
	1990	78,000	9,300	8.27	645,000	429.00	276,874
	1991	75,500	9,900	8.21	620,000	438.00	271,700
	1992	77,300	7,000	8.34	645,000	356.00	229,630
	1993	77,800	5,000	8.12	632,000	574.00	362,544
	1994	77,800	6,200	7.74	602,000	515.00	310,022
	1995	76,200	9,300	9.42	718,000	525.00	376,890
Grapes, Wine	1986	312,000	20,600	6.75	2,105,000	207.00	435,440
	1987	304,000	25,600	6.41	1,950,000	248.00	483,930
	1988	297,000	34,700	7.34	2,180,000	297.00	646,690
	1989	290,000	42,400	7.55	2,190,000	340.00	745,200
	1990	291,000	44,200	7.54	2,195,000	308.00	676,270
	1991	295,000	38,500	7.44	2,195,000	344.00	755,800
	1992	300,000	31,500	7.15	2,145,000	393.00	843,000
	1993	312,000	25,200	7.68	2,397,000	361.00	866,382
	1994	307,000	37,200	7.38	2,265,000	378.00	855,915
	1995	301,000	53,500	7.56	2,275,000	424.00	965,150

See Footnotes on Page 21.

CALIFORNIA NON-CITRUS FRUITS: Acreage, Production, Price And Value, 1986-95

Crop	Crop Year	Bearing	Non-Bearing	Yield Per Acre	Total Production	Price 1/	Total Value
		Acres		Tons		$/Ton	$1,000
Kiwifruit	1986	5,600	1,300	4.34	24,300	1,030.00	24,102
	1987	6,800	800	4.26	29,000	710.00	18,886
	1988	7,100	700	4.61	32,700	760.00	22,420
	1989	7,200	500	5.56	40,000	400.00	14,800
	1990	7,300	300	5.34	39,000	415.00	14,110
	1991	7,300	50	4.05	29,600	820.00	21,976
	1992	7,300	40	7.16	52,300	290.00	13,833
	1993	7,200	10	6.83	49,200	370.00	16,502
	1994	6,900	N/A	5.71	39,400	491.00	18,413
	1995	6,600	N/A	5.76	38,000	473.00	15,089
Nectarines	1986	22,800	3,200	7.54	172,000	440.00	75,720
	1987	23,600	3,300	8.09	191,000	343.00	65,545
	1988	25,300	4,300	8.58	217,000	394.00	85,594
	1989	24,400	4,200	9.02	220,000	398.00	87,645
	1990	25,500	4,300	9.10	232,000	474.00	109,999
	1991	26,400	4,700	8.14	215,000	402.00	86,457
	1992	26,500	9,300	8.91	236,000	312.00	73,710
	1993	27,100	8,300	7.56	205,000	500.00	102,421
	1994	28,800	N/A	8.40	242,000	282.00	68,168
	1995	32,400	N/A	5.43	176,000	534.00	93,990
Olives 4/	1986	32,300	1,600	3.45	111,500	587.00	65,407
	1987	31,600	1,900	2.14	67,500	608.00	41,053
	1988	31,500	2,700	2.78	87,500	518.00	45,316
	1989	29,800	3,400	4.13	123,000	467.00	57,458
	1990	30,400	4,000	4.33	131,500	423.00	55,663
	1991	29,700	4,400	2.19	65,000	559.00	36,306
	1992	30,100	4,600	5.48	165,000	549.00	90,561
	1993	30,100	4,400	4.05	122,000	467.00	56,991
	1994	32,000	N/A	2.63	84,000	464.00	38,994
	1995	33,700	N/A	2.30	77,500	613.00	47,528
Peaches, All	1986	54,300	9,800	13.10	714,000	207.00	141,882
	1987	54,800	10,400	13.10	715,500	219.00	149,002
	1988	54,700	12,000	14.10	770,000	242.00	177,672
	1989	54,600	14,100	14.10	770,500	249.00	183,672
	1990	54,600	15,500	14.80	806,000	263.00	204,305
	1991	54,400	16,100	15.20	828,500	236.00	188,644
	1992	59,500	14,900	15.30	912,500	228.00	200,146
	1993	59,700	14,400	14.20	850,000	248.00	203,105
	1994	60,300	N/A	14.60	882,000	193.00	165,388
	1995	60,600	N/A	11.30	683,500	277.00	183,485
Peaches, Clingstone 4/	1986	29,300	6,600	15.90	466,500	168.00	73,374
	1987	28,900	6,700	15.90	460,000	189.00	80,750
	1988	27,600	7,900	18.40	508,500	207.00	98,072
	1989	27,600	10,200	18.00	496,000	214.00	98,868
	1990	27,000	11,900	18.70	506,000	214.00	102,185
	1991	26,200	12,600	19.70	515,000	218.00	105,730
	1992	30,700	8,500	19.30	591,500	215.00	120,636
	1993	30,200	9,000	18.20	548,500	218.00	113,033
	1994	30,200	N/A	18.70	565,000	180.00	97,470
	1995	28,100	N/A	15.40	432,500	220.00	90,310

See Footnotes on Page 21.

CALIFORNIA NON-CITRUS FRUITS: Acreage, Production, Price And Value, 1986-95

Crop	Crop Year	Bearing	Non-Bearing	Yield Per Acre	Total Production	Price 1/	Total Value
		Acres		*Tons*		*$/Ton*	*$1,000*
Peaches, Freestone	1986	25,000	3,200	9.90	247,500	276.00	68,508
	1987	25,900	3,700	9.86	255,500	268.00	68,252
	1988	27,100	4,100	9.65	261,500	304.00	79,600
	1989	27,000	3,900	10.20	274,500	308.00	84,804
	1990	27,600	3,600	10.90	300,000	340.00	102,120
	1991	28,200	3,500	11.10	313,500	264.00	82,914
	1992	28,800	6,400	11.20	321,000	248.00	79,510
	1993	29,500	5,400	10.20	301,500	298.00	90,072
	1994	30,100	N/A	10.60	317,000	214.00	67,918
	1995	32,500	N/A	7.70	251,000	372.00	93,175
Pears, All	1986	23,100	2,100	12.70	294,000	232.00	68,140
	1987	23,100	1,900	14.60	337,000	192.00	64,794
	1988	23,300	2,100	13.00	302,000	247.00	74,540
	1989	23,500	2,300	13.40	315,000	271.00	85,250
	1990	23,300	2,300	14.20	332,000	251.00	83,248
	1991	23,400	1,900	13.50	317,000	263.00	83,403
	1992	23,900	1,500	14.10	338,000	246.00	83,156
	1993	24,100	1,000	12.80	308,000	256.00	78,848
	1994	24,300	N/A	14.90	363,000	191.00	69,327
	1995	23,900	N/A	11.20	267,000	237.00	63,187
Pears, Bartlett	1986	21,900	400	13.00	285,000	223.00	63,445
	1987	21,700	300	15.00	325,000	176.00	57,058
	1988	21,400	300	13.60	291,000	238.00	69,290
	1989	20,900	200	14.30	298,000	265.00	79,040
	1990	20,200	200	15.50	314,000	242.00	75,867
	1991	20,200	200	14.90	300,000	252.00	75,624
	1992	20,200	100	15.60	315,000	238.00	74,970
	1993	19,900	100	14.50	288,000	237.00	68,287
	1994	19,500	N/A	17.10	333,000	183.00	60,904
	1995	19,100	N/A	12.90	247,000	212.00	52,453
Pears, Other	1986	1,200	1,700	7.50	9,000	522.00	4,695
	1987	1,400	1,600	8.57	12,000	645.00	7,736
	1988	1,900	1,800	5.79	11,000	477.00	5,250
	1989	2,600	2,100	6.54	17,000	365.00	6,210
	1990	3,100	2,100	5.81	18,000	410.00	7,381
	1991	3,200	1,700	5.31	17,000	458.00	7,779
	1992	3,700	1,400	6.22	23,000	356.00	8,186
	1993	4,200	900	4.76	20,000	528.00	10,561
	1994	4,800	N/A	6.25	30,000	281.00	8,423
	1995	4,800	N/A	4.17	20,000	537.00	10,734
Plums	1986	38,200	4,700	3.98	152,000	657.00	99,857
	1987	39,900	4,600	6.14	245,000	308.00	75,361
	1988	42,800	4,300	5.05	216,000	475.00	102,661
	1989	41,100	4,000	5.26	216,000	445.00	96,146
	1990	41,800	3,300	5.33	223,000	603.00	134,412
	1991	42,400	2,600	5.14	218,000	449.00	97,894
	1992	42,400	1,700	5.90	250,000	252.00	63,033
	1993	41,200	3,500	4.49	185,000	508.00	93,954
	1994	41,600	N/A	5.94	247,000	321.00	79,358
	1995	42,000	N/A	2.95	124,000	950.00	117,849

See Footnotes on Page 21.

CALIFORNIA NON-CITRUS FRUITS: Acreage, Production, Price And Value, 1986-95

Crop	Crop Year	Bearing	Non-Bearing	Yield Per Acre	Total Production	Price 1/	Total Value
		Acres		Tons		$/Ton	$1,000
Pomegranates 5/	1986	3,500	300	6.29	22,000	395.00	7,666
	1987	3,400	300	5.44	18,500	340.00	5,617
	1988	3,500	100	5.71	20,000	359.00	6,454
	1989	3,100	300	6.13	19,000	273.00	4,906
	1990	---	---	---	---	---	---
	1991	---	---	---	---	---	---
	1992	---	---	---	---	---	---
	1993	---	---	---	---	---	---
	1994	---	---	---	---	---	---
	1995	---	---	---	---	---	---
Prunes (Dried Basis) 6/	1986	72,600	10,400	1.36	99,000	819.00	81,081
	1987	75,600	7,700	3.03	229,000	734.00	168,086
	1988	77,200	6,900	1.96	151,000	782.00	118,082
	1989	77,400	6,500	2.92	226,000	779.00	176,054
	1990	80,100	5,800	1.84	147,000	873.00	128,331
	1991	80,200	6,500	2.33	187,000	940.00	175,780
	1992	80,400	8,000	2.29	184,000	1,030.00	189,520
	1993	78,200	8,400	1.55	121,000	1,120.00	135,520
	1994	78,900	12,720	2.45	193,000	1,090.00	210,370
	1995	78,800	20,000	2.30	181,000	1,040.00	188,240
Miscellaneous Fruits & Nuts 7/	1986	---	---	---	---	---	---
	1987	13,389	---	---	676,810	---	37,840
	1988	13,750	---	---	587,680	---	50,322
	1989	13,750	---	---	597,670	---	50,670
	1990	13,750	---	---	597,680	---	60,070
	1991	8,859	---	---	609,345	---	66,800
	1992	8,700	---	---	600,000	---	62,750
	1993	6,600	---	---	600,000	---	70,950
	1994	6,600	---	---	600,000	---	80,642
	1995	6,600	---	---	600,000	---	79,150

1/ Return at processing plant door or packinghouse door.
2/ Acres of raisin type grapes enrolled in the California Raisin Industry Diversion (RID) program were; in 1986, 50,000; in 1987, 15,000; in 1988, 25,000; in 1991, 20,000; in 1992, 12,500; in 1993, 20,000; in 1994, zero; in 1995, 25,000. These acres were deducted from bearing acres before calculating yield.
3/ Raisin type production is the fresh equivalent of dried and not dried.
4/ Total value based on grower return.
5/ Reported as miscellaneous after 1989.
6/ Acreage by county, variety, and year of planting for 1995 are available upon request.
7/ These crops are almond hulls, black walnuts, jojobas (until 1991), limes, persimmons, pomegranates (starting in 1990), and in 1991, bushberries. Acres exclude almond hulls.

DRYING RATIO -- TONS OF GREEN FRUIT REQUIRED TO PRODUCE ONE DRIED TON

Year	Apples	Apricots	Figs	Raisins	Freestone Peaches	Bartlett Pears	Prunes
1986	8.50	6.80	3.00	4.26	9.03	6.40	2.94
1987	8.30	7.90	3.00	4.02	9.00	7.10	2.98
1988	8.80	6.70	3.00	4.99	8.20	6.50	3.11
1989	9.10	6.40	3.00	4.30	8.40	5.90	3.33
1990	8.40	6.90	3.00	4.40	9.00	6.00	3.15
1991	N/A	6.50	3.00	4.53	9.30	6.00	3.15
1992	N/A	6.40	3.00	4.00	9.60	6.60	2.90
1993	N/A	6.60	3.00	4.30	8.10	6.50	3.10
1994	N/A	6.40	3.00	4.50	9.30	6.20	3.08
1995	N/A	6.50	3.00	5.00	9.03	6.10	3.30

CALIFORNIA NON-CITRUS FRUITS: Utilized Production And Average Grower Return Per Ton, By Utilization Category, 1986-95

Crop	Crop Year	Total Utilized Production	For Fresh Market		For Processing			
					Total		Canned	
			Quantity	Price	Quantity	Price	Quantity	Price
		Short Tons	*Tons*	*$/Ton*	*Tons*	*$/Ton*	*Tons*	*$/Ton*
Apples 1/	1986	257,500	108,500	614.00	149,000	115.00	24,500	142.00
	1987	325,000	115,000	466.00	210,000	87.00	40,000	123.00
	1988	315,000	152,500	640.00	162,500	123.00	22,000	156.00
	1989	337,500	139,000	524.00	198,500	141.00	30,500	150.00
	1990	390,000	155,000	582.00	235,000	134.00	40,000	159.00
	1991	400,000	175,000	754.00	225,000	159.00	15,000	147.00
	1992	420,000	185,000	714.00	235,000	162.00	15,000	187.00
	1993	440,000	185,000	574.00	255,000	126.00	20,500	152.00
	1994	525,000	175,000	568.00	350,000	113.00	45,000	145.00
	1995	425,000	150,000	734.00	275,000	165.00	37,500	150.00
Apricots	1986	50,000	6,300	1,060.00	43,700	272.00	27,000	263.00
	1987	102,000	12,300	625.00	89,700	293.00	53,000	286.00
	1988	87,000	12,600	626.00	74,400	292.00	44,500	282.00
	1989	117,000	14,000	590.00	103,000	287.00	67,000	280.00
	1990	113,000	17,000	568.00	96,000	280.00	64,000	274.00
	1991	86,000	15,000	735.00	71,000	298.00	43,000	287.00
	1992	99,000	17,000	496.00	82,000	293.00	53,000	282.00
	1993	89,000	14,500	711.00	74,500	295.00	45,000	266.00
	1994	132,000	20,000	477.00	112,000	305.00	71,000	303.00
	1995	52,000	11,000	748.00	41,000	297.00	28,000	287.00
Avocados 2/	85-86	160,000	160,000	1,020.00	---	---	---	---
	86-87	278,000	278,000	338.00	---	---	---	---
	87-88	180,000	180,000	1,140.00	---	---	---	---
	88-89	165,000	153,000	1,260.00	12,000	---	---	---
	89-90	105,000	93,000	2,280.00	12,000	---	---	---
	90-91	136,000	124,000	1,410.00	12,000	---	---	---
	91-92	156,000	143,000	1,170.00	13,000	---	---	---
	92-93	284,000	265,000	400.00	19,000	---	---	---
	93-94	139,000	132,000	1,810.00	7,000	---	---	---
	94-95	156,000	152,700	1,490.00	3,300	---	---	---
Cherries, Sweet 3/	1986	8,400	6,600	1,390.00	1,800	576.00	---	---
	1987	45,000	34,000	686.00	11,000	481.00	---	---
	1988	26,000	19,500	874.00	6,500	461.00	---	---
	1989	26,000	18,500	1,120.00	7,500	493.00	---	---
	1990	22,000	16,300	1,080.00	5,700	352.00	---	---
	1991	36,000	27,000	1,050.00	9,000	667.00	---	---
	1992	31,000	25,500	1,380.00	5,500	573.00	---	---
	1993	19,000	14,500	2,820.00	4,500	514.00	---	---
	1994	52,000	36,000	1,550.00	16,000	482.00	---	---
	1995	19,800	11,000	3,410.00	8,800	458.00	---	---
Dates	1986	17,800	17,800	828.00	---	---	---	---
	1987	19,400	19,400	872.00	---	---	---	---
	1988	22,000	22,000	896.00	---	---	---	---
	1989	22,000	22,000	1,040.00	---	---	---	---
	1990	24,000	24,000	820.00	---	---	---	---
	1991	22,000	22,000	970.00	---	---	---	---
	1992	21,000	21,000	1,060.00	---	---	---	---
	1993	29,000	29,000	790.00	---	---	---	---
	1994	23,000	23,000	750.00	---	---	---	---
	1995	22,000	22,000	748.00	---	---	---	---

See Footnotes on Page 31.

CALIFORNIA NON-CITRUS FRUITS: Utilized Production And Average Grower Return Per Ton, By Utilization Category, 1986-95

Crop	Crop Year	For Processing							
		Juice or Crushed		Frozen		Dried (Fresh Basis)		Dried Basis	
		Quantity	Price	Quantity	Price	Quantity	Price	Quantity	Price
		Tons	*$/Ton*	*Tons*	*$/Ton*	*Tons*	*$/Ton*	*Tons*	*$/Ton*
Apples 1/	1986	108,000	105.00	---	---	---	---	---	---
	1987	146,000	72.00	---	---	---	---	---	---
	1988	122,500	117.00	---	---	---	---	---	---
	1989	142,000	142.00	---	---	---	---	---	---
	1990	159,000	127.00	---	---	---	---	---	---
	1991	157,500	157.00	---	---	---	---	---	---
	1992	162,500	155.00	---	---	---	---	---	---
	1993	175,000	122.00	---	---	---	---	---	---
	1994	250,000	110.00	---	---	---	---	---	---
	1995	200,000	165.00	---	---	---	---	---	---
Apricots	1986	---	---	7,200	307.00	9,500	271.00	1,400	1,840.00
	1987	---	---	13,100	291.00	23,600	310.00	2,980	2,450.00
	1988	---	---	12,200	294.00	17,700	314.00	2,640	2,100.00
	1989	---	---	11,000	310.00	25,000	296.00	3,900	1,890.00
	1990	---	---	11,000	305.00	21,000	287.00	3,050	1,950.00
	1991	---	---	11,000	308.00	17,000	319.00	2,600	2,110.00
	1992	---	---	13,000	319.00	16,000	306.00	2,500	1,960.00
	1993	---	---	11,500	315.00	18,000	353.00	2,730	2,320.00
	1994	---	---	10,500	350.00	30,500	295.00	4,800	1,890.00
	1995	---	---	5,200	317.00	7,800	321.00	1,200	2,090.00
Avocados 2/	85-86	---	---	---	---	---	---	---	---
	86-87	---	---	---	---	---	---	---	---
	87-88	---	---	---	---	---	---	---	---
	88-89	---	---	---	---	---	---	---	---
	89-90	---	---	---	---	---	---	---	---
	90-91	---	---	---	---	---	---	---	---
	91-92	---	---	---	---	---	---	---	---
	92-93	---	---	---	---	---	---	---	---
	93-94	---	---	---	---	---	---	---	---
	94-95	---	---	---	---	---	---	---	---
Cherries, Sweet 3/	1986	---	---	---	---	---	---	---	---
	1987	---	---	---	---	---	---	---	---
	1988	---	---	---	---	---	---	---	---
	1989	---	---	---	---	---	---	---	---
	1990	---	---	---	---	---	---	---	---
	1991	---	---	---	---	---	---	---	---
	1992	---	---	---	---	---	---	---	---
	1993	---	---	---	---	---	---	---	---
	1994	---	---	---	---	---	---	---	---
	1995	---	---	---	---	---	---	---	---
Dates	1986	---	---	---	---	---	---	---	---
	1987	---	---	---	---	---	---	---	---
	1988	---	---	---	---	---	---	---	---
	1989	---	---	---	---	---	---	---	---
	1990	---	---	---	---	---	---	---	---
	1991	---	---	---	---	---	---	---	---
	1992	---	---	---	---	---	---	---	---
	1993	---	---	---	---	---	---	---	---
	1994	---	---	---	---	---	---	---	---
	1995	---	---	---	---	---	---	---	---

See Footnotes on Page 31.

CALIFORNIA NON-CITRUS FRUITS: Utilized Production And Average Grower Return Per Ton, By Utilization Category, 1986-95

Crop	Crop Year	Total Utilized Production	For Fresh Market		For Processing			
					Total		Canned	
			Quantity	Price	Quantity	Price	Quantity	Price
		Short Tons	*Tons*	*$/Ton*	*Tons*	*$/Ton*	*Tons*	*$/Ton*
Figs 4/	1986	50,000	1,400	---	---	---	---	---
	1987	52,300	1,750	---	---	---	---	---
	1988	55,500	1,500	---	---	---	---	---
	1989	48,000	1,500	---	---	---	---	---
	1990	49,600	1,600	---	---	---	---	---
	1991	45,100	1,300	---	---	---	---	---
	1992	46,900	1,300	---	---	---	---	---
	1993	60,700	2,800	---	---	---	---	---
	1994	56,700	2,100	---	---	---	---	---
	1995	50,000	2,000	---	---	---	---	---
Grapes, All 5/	1986	4,770,000	746,500	442.00	4,023,500	182.00	40,000	210.00
	1987	4,660,000	685,000	519.00	3,975,000	213.00	40,000	220.00
	1988	5,520,000	801,000	493.00	4,719,000	224.00	40,000	225.00
	1989	5,390,000	760,000	592.00	4,630,000	268.00	40,000	235.00
	1990	5,185,000	821,000	525.00	4,364,000	247.00	40,000	235.00
	1991	4,980,000	773,000	537.00	4,207,000	273.00	41,000	249.00
	1992	5,460,000	745,000	416.00	4,715,000	292.00	46,000	260.00
	1993	5,383,000	774,000	670.00	4,609,000	284.00	45,000	256.00
	1994	5,256,000	779,000	565.00	4,477,000	284.00	38,000	256.00
	1995	5,248,000	837,000	607.00	4,411,000	302.00	35,000	257.00
Grapes, Raisin 5/	1986	2,045,000	320,000	512.00	1,725,000	157.00	40,000	210.00
	1987	2,170,000	260,000	502.00	1,910,000	185.00	40,000	220.00
	1988	2,570,000	305,000	469.00	2,265,000	169.00	40,000	225.00
	1989	2,570,000	310,000	596.00	2,260,000	212.00	40,000	235.00
	1990	2,345,000	307,000	513.00	2,038,000	195.00	40,000	235.00
	1991	2,165,000	278,000	547.00	1,887,000	203.00	41,000	249.00
	1992	2,670,000	285,000	374.00	2,385,000	213.00	46,000	260.00
	1993	2,354,000	270,000	630.00	2,084,000	206.00	45,000	256.00
	1994	2,389,000	270,000	472.00	2,119,000	198.00	38,000	256.00
	1995	2,255,000	258,000	579.00	1,997,000	174.00	35,000	257.00
Grapes, Table	1986	620,000	376,500	419.00	243,500	133.00	---	---
	1987	540,000	370,000	570.00	170,000	142.00	---	---
	1988	770,000	441,000	535.00	329,000	132.00	---	---
	1989	630,000	405,000	626.00	225,000	131.00	---	---
	1990	645,000	464,000	546.00	181,000	130.00	---	---
	1991	620,000	450,000	549.00	170,000	145.00	---	---
	1992	645,000	415,000	458.00	230,000	172.00	---	---
	1993	632,000	468,000	720.00	164,000	156.00	---	---
	1994	602,000	464,000	628.00	138,000	135.00	---	---
	1995	718,000	534,000	651.00	184,000	159.00	---	---
Grapes, Wine	1986	2,105,000	50,000	160.00	2,055,000	208.00	---	---
	1987	1,950,000	55,000	254.00	1,895,000	248.00	---	---
	1988	2,180,000	55,000	283.00	2,125,000	297.00	---	---
	1989	2,190,000	45,000	258.00	2,145,000	342.00	---	---
	1990	2,195,000	50,000	398.00	2,145,000	306.00	---	---
	1991	2,195,000	45,000	360.00	2,150,000	344.00	---	---
	1992	2,145,000	45,000	300.00	2,100,000	395.00	---	---
	1993	2,397,000	36,000	325.00	2,361,000	362.00	---	---
	1994	2,265,000	45,000	471.00	2,220,000	376.00	---	---
	1995	2,275,000	45,000	238.00	2,230,000	428.00	---	---

See Footnotes on Page 31.

CALIFORNIA NON-CITRUS FRUITS: Utilized Production
And Average Grower Return Per Ton, By Utilization Category, 1986-95

Crop	Crop Year	For Processing							
		Juice or Crushed		Frozen		Dried (Fresh Basis)		Dried Basis	
		Quantity	Price	Quantity	Price	Quantity	Price	Quantity	Price
		Tons	*$/Ton*	*Tons*	*$/Ton*	*Tons*	*$/Ton*	*Tons*	*$/Ton*
Figs 4/	1986	---	---	---	---	48,600	267.00	16,200	801.00
	1987	---	---	---	---	50,550	317.00	16,850	951.00
	1988	---	---	---	---	54,000	337.00	18,000	1,010.00
	1989	---	---	---	---	46,500	373.00	15,500	1,120.00
	1990	---	---	---	---	48,000	320.00	16,000	960.00
	1991	---	---	---	---	43,800	347.00	14,600	1,040.00
	1992	---	---	---	---	45,600	393.00	15,200	1,180.00
	1993	---	---	---	---	57,900	367.00	19,300	1,100.00
	1994	---	---	---	---	54,600	400.00	18,200	1,200.00
	1995	---	---	---	---	48,000	353.00	16,000	1,060.00
Grapes, All 5/	1986	2,795,000	183.00	---	---	1,188,500	177.00	278,900	756.00
	1987	2,496,000	219.00	---	---	1,439,000	203.00	357,950	817.00
	1988	2,852,000	253.00	---	---	1,827,000	180.00	366,500	897.00
	1989	2,726,000	297.00	---	---	1,864,000	227.00	433,200	975.00
	1990	2,583,000	276.00	---	---	1,741,000	205.00	395,500	902.00
	1991	2,591,000	310.00	---	---	1,575,000	212.00	348,000	962.00
	1992	3,102,000	325.00	---	---	1,567,000	228.00	391,500	911.00
	1993	2,895,000	323.00	---	---	1,669,000	218.00	388,300	936.00
	1994	2,540,000	344.00	---	---	1,899,000	204.00	422,000	920.00
	1995	2,832,000	372.00	---	---	1,544,000	175.00	308,800	873.00
Grapes, Raisin 5/	1986	505,000	105.00	---	---	1,180,000	177.00	277,000	757.00
	1987	440,000	123.00	---	---	1,430,000	203.00	356,000	817.00
	1988	415,000	116.00	---	---	1,810,000	180.00	363,000	898.00
	1989	370,000	132.00	---	---	1,850,000	227.00	430,000	977.00
	1990	268,000	126.00	---	---	1,730,000	205.00	393,000	903.00
	1991	284,000	150.00	---	---	1,562,000	212.00	345,000	963.00
	1992	785,000	182.00	---	---	1,554,000	228.00	388,300	911.00
	1993	397,000	152.00	---	---	1,642,000	218.00	382,000	937.00
	1994	197,000	118.00	---	---	1,884,000	205.00	418,600	923.00
	1995	432,000	165.00	---	---	1,530,000	175.00	306,000	875.00
Grapes, Table	1986	235,000	133.00	---	---	8,500	130.00	1,900	582.00
	1987	161,000	140.00	---	---	9,000	174.00	1,950	783.00
	1988	312,000	131.00	---	---	17,000	158.00	3,500	767.00
	1989	211,000	128.00	---	---	14,000	176.00	3,200	770.00
	1990	170,000	127.00	---	---	11,000	183.00	2,500	805.00
	1991	157,000	141.00	---	---	13,000	194.00	3,000	842.00
	1992	217,000	170.00	---	---	13,000	213.00	3,200	866.00
	1993	137,000	147.00	---	---	27,000	204.00	6,300	874.00
	1994	123,000	136.00	---	---	15,000	125.00	3,400	550.00
	1995	170,000	162.00	---	---	14,000	120.00	2,800	600.00
Grapes, Wine	1986	2,055,000	208.00	---	---	---	---	---	---
	1987	1,895,000	248.00	---	---	---	---	---	---
	1988	2,125,000	297.00	---	---	---	---	---	---
	1989	2,145,000	342.00	---	---	---	---	---	---
	1990	2,145,000	306.00	---	---	---	---	---	---
	1991	2,150,000	344.00	---	---	---	---	---	---
	1992	2,100,000	395.00	---	---	---	---	---	---
	1993	2,361,000	362.00	---	---	---	---	---	---
	1994	2,220,000	376.00	---	---	---	---	---	---
	1995	2,230,000	428.00	---	---	---	---	---	---

See Footnotes on Page 31.

CALIFORNIA NON-CITRUS FRUITS: Utilized Production And Average Grower Return Per Ton, By Utilization Category, 1986-95

Crop	Crop Year	Total Utilized Production	For Fresh Market		For Processing			
					Total		Canned	
			Quantity	Price	Quantity	Price	Quantity	Price
		Short Tons	*Tons*	*$/Ton*	*Tons*	*$/Ton*	*Tons*	*$/Ton*
Kiwifruit	1986	23,400	23,400	1,030.00	---	---	---	---
	1987	26,600	26,600	710.00	---	---	---	---
	1988	29,500	29,500	760.00	---	---	---	---
	1989	37,000	37,000	400.00	---	---	---	---
	1990	34,000	34,000	415.00	---	---	---	---
	1991	26,800	26,800	820.00	---	---	---	---
	1992	47,700	47,700	290.00	---	---	---	---
	1993	44,600	44,600	370.00	---	---	---	---
	1994	37,500	37,500	491.00	---	---	---	---
	1995	31,900	31,900	473.00	---	---	---	---
Nectarines 6/	1986	172,000	170,000	---	2,000	---	---	---
	1987	191,000	190,500	---	500	---	---	---
	1988	217,000	216,000	---	1,000	---	---	---
	1989	220,000	219,000	---	1,000	---	---	---
	1990	232,000	229,500	---	2,500	---	---	---
	1991	215,000	211,000	---	4,000	---	---	---
	1992	236,000	233,000	---	3,000	---	---	---
	1993	205,000	201,000	---	4,000	---	---	---
	1994	242,000	238,000	---	4,000	---	---	---
	1995	176,000	170,000	---	6,000	---	---	---
Olives 7/								
	1986	111,500	500	500.00	111,000	587.00	85,000	709.00
	1987	67,500	500	500.00	67,000	609.00	55,000	702.00
	1988	87,500	500	500.00	87,000	518.00	70,000	603.00
	1989	123,000	500	500.00	122,500	467.00	94,000	563.00
	1990	131,500	500	500.00	131,000	423.00	88,000	553.00
	1991	65,000	500	500.00	64,500	559.00	53,700	631.00
	1992	165,000	500	500.00	164,500	549.00	121,000	676.00
	1993	122,000	500	500.00	121,500	467.00	93,000	558.00
	1994	84,000	500	500.00	83,500	464.00	66,500	551.00
	1995	77,500	500	500.00	77,000	614.00	58,500	742.00
Peaches, All 8/	1986	684,250	151,000	354.00	533,250	168.00	460,750	168.00
	1987	680,500	155,000	334.00	525,500	184.00	449,000	188.00
	1988	741,500	160,000	396.00	573,000	199.00	495,150	206.00
	1989	736,500	174,500	392.00	562,000	205.00	484,200	213.00
	1990	777,500	192,000	434.00	585,500	207.00	497,100	214.00
	1991	798,500	201,000	314.00	597,500	210.00	503,700	218.00
	1992	879,500	215,000	286.00	664,500	208.00	573,900	215.00
	1993	820,000	193,000	370.00	627,000	210.00	533,200	217.00
	1994	857,500	220,000	232.00	638,500	179.00	556,600	181.00
	1995	661,500	161,300	482.00	500,200	211.00	422,500	219.00
Peaches, Clingstone 8/	1986	436,750	---	---	436,750	168.00	436,750	168.00
	1987	425,000	---	---	425,000	189.00	425,000	189.00
	1988	471,500	---	---	471,500	207.00	471,500	207.00
	1989	462,000	---	---	462,000	214.00	462,000	214.00
	1990	477,500	---	---	477,500	214.00	477,500	214.00
	1991	485,000	---	---	485,000	218.00	485,000	218.00
	1992	558,500	---	---	558,500	215.00	558,500	215.00
	1993	518,500	---	---	518,500	218.00	518,500	218.00
	1994	541,500	---	---	541,500	180.00	541,500	180.00
	1995	410,500	---	---	410,500	220.00	410,500	220.00

See Footnotes on Page 31.

CALIFORNIA NON-CITRUS FRUITS: Utilized Production And Average Grower Return Per Ton, By Utilization Category, 1986-95

Crop	Crop Year	For Processing							
		Juice or Crushed		Frozen		Dried (Fresh Basis)		Dried Basis	
		Quantity	Price	Quantity	Price	Quantity	Price	Quantity	Price
		Tons	*$/Ton*	*Tons*	*$/Ton*	*Tons*	*$/Ton*	*Tons*	*$/Ton*
Kiwifruit	1986	---	---	---	---	---	---	---	---
	1987	---	---	---	---	---	---	---	---
	1988	---	---	---	---	---	---	---	---
	1989	---	---	---	---	---	---	---	---
	1990	---	---	---	---	---	---	---	---
	1991	---	---	---	---	---	---	---	---
	1992	---	---	---	---	---	---	---	---
	1993	---	---	---	---	---	---	---	---
	1994	---	---	---	---	---	---	---	---
	1995	---	---	---	---	---	---	---	---
Nectarines 6/	1986	---	---	---	---	---	---	---	---
	1987	---	---	---	---	---	---	---	---
	1988	---	---	---	---	---	---	---	---
	1989	---	---	---	---	---	---	---	---
	1990	---	---	---	---	---	---	---	---
	1991	---	---	---	---	---	---	---	---
	1992	---	---	---	---	---	---	---	---
	1993	---	---	---	---	---	---	---	---
	1994	---	---	---	---	---	---	---	---
	1995	---	---	---	---	---	---	---	---
Olives 7/				- Limited -		- Undersize -			
	1986	6,000	17.00	20,000	240.00	---	---	---	---
	1987	3,000	15.00	9,000	236.00	---	---	---	---
	1988	3,000	13.00	8,500	310.00	5,500	28.00	---	---
	1989	5,500	12.00	14,000	289.00	9,000	22.00	---	---
	1990	5,000	10.90	22,000	295.00	16,000	13.90	---	---
	1991	1,800	10.30	7,300	291.00	1,700	23.90	---	---
	1992	5,700	10.50	31,500	266.00	6,300	10.50	---	---
	1993	5,300	10.50	19,700	235.00	3,500	37.20	---	---
	1994	4,400	11.00	8,400	234.00	4,200	14.00	---	---
	1995	4,000	11.00	9,300	403.00	5,200	16.00	---	---
Peaches, All 8/	1986	---	---	56,250	165.00	16,250	96.00	1,800	864.00
	1987	---	---	59,000	178.00	17,500	101.00	1,950	909.00
	1988	---	---	57,250	165.00	20,600	117.00	2,500	960.00
	1989	---	---	63,500	167.00	14,300	110.00	1,700	924.00
	1990	---	---	74,850	178.00	13,550	108.00	1,500	975.00
	1991	---	---	71,600	183.00	22,200	116.00	2,400	1080.00
	1992	---	---	70,400	182.00	20,200	93.00	2,100	893.00
	1993	---	---	78,800	185.00	15,000	82.00	1,850	713.00
	1994	---	---	68,400	183.00	13,500	93.00	1,450	856.00
	1995	---	---	63,700	186.00	14,000	86.00	1,550	894.00
Peaches, Clingstone 8/	1986	---	---	---	---	---	---	---	---
	1987	---	---	---	---	---	---	---	---
	1988	---	---	---	---	---	---	---	---
	1989	---	---	---	---	---	---	---	---
	1990	---	---	---	---	---	---	---	---
	1991	---	---	---	---	---	---	---	---
	1992	---	---	---	---	---	---	---	---
	1993	---	---	---	---	---	---	---	---
	1994	---	---	---	---	---	---	---	---
	1995	---	---	---	---	---	---	---	---

See Footnotes on Page 31.

CALIFORNIA NON-CITRUS FRUITS: Utilized Production And Average Grower Return Per Ton, By Utilization Category, 1986-95

Crop	Crop Year	Total Utilized Production	For Fresh Market		For Processing			
					Total		Canned	
			Quantity	Price	Quantity	Price	Quantity	Price
		Short Tons	Tons	$/Ton	Tons	$/Ton	Tons	$/Ton
Peaches, Freestone 8/	1986	247,500	151,000	354.00	96,500	156.00	24,000	177.00
	1987	255,500	155,000	334.00	100,500	164.00	24,000	176.00
	1988	261,500	160,000	396.00	101,500	160.00	23,650	181.00
	1989	274,500	174,500	392.00	100,000	164.00	22,200	192.00
	1990	300,000	192,000	434.00	108,000	174.00	19,600	204.00
	1991	313,500	201,000	314.00	112,500	176.00	18,700	217.00
	1992	321,000	215,000	286.00	106,000	170.00	15,400	213.00
	1993	301,500	193,000	370.00	108,500	172.00	14,700	196.00
	1994	317,000	220,000	232.00	97,000	174.00	15,100	205.00
	1995	251,000	161,300	482.00	89,700	172.00	12,000	190.00
Pears, All 6/	1986	294,000	---	---	---	---	---	---
	1987	337,000	---	---	---	---	---	---
	1988	302,000	---	---	---	---	---	---
	1989	315,000	---	---	---	---	---	---
	1990	332,000	---	---	---	---	---	---
	1991	317,000	---	---	---	---	---	---
	1992	338,000	---	---	---	---	---	---
	1993	308,000	---	---	---	---	---	---
	1994	363,000	---	---	---	---	---	---
	1995	267,000	---	---	---	---	---	---
Pears, Bartlett 8/	1986	285,000	70,000	332.00	215,000	187.00	---	---
	1987	325,000	74,500	164.00	250,500	179.00	---	---
	1988	291,000	63,700	317.00	227,300	216.00	---	---
	1989	298,000	64,000	299.00	234,000	256.00	---	---
	1990	314,000	75,000	285.00	239,000	228.00	---	---
	1991	300,000	72,000	284.00	228,000	242.00	---	---
	1992	315,000	63,000	238.00	252,000	238.00	---	---
	1993	288,000	61,000	271.00	227,000	228.00	---	---
	1994	333,000	88,000	113.00	245,000	208.00	---	---
	1995	247,000	60,000	254.00	187,000	199.00	---	---
Pears, Other 6/	1986	9,000	---	---	---	---	---	---
	1987	12,000	---	---	---	---	---	---
	1988	11,000	---	---	---	---	---	---
	1989	17,000	---	---	---	---	---	---
	1990	18,000	---	---	---	---	---	---
	1991	17,000	---	---	---	---	---	---
	1992	23,000	---	---	---	---	---	---
	1993	20,000	---	---	---	---	---	---
	1994	30,000	---	---	---	---	---	---
	1995	20,000	---	---	---	---	---	---
Plums 6/	1986	152,000	---	---	---	---	---	---
	1987	245,000	---	---	---	---	---	---
	1988	216,000	---	---	---	---	---	---
	1989	216,000	---	---	---	---	---	---
	1990	223,000	---	---	---	---	---	---
	1991	218,000	---	---	---	---	---	---
	1992	250,000	---	---	---	---	---	---
	1993	185,000	---	---	---	---	---	---
	1994	247,000	---	---	---	---	---	---
	1995	124,000	---	---	---	---	---	---

See Footnotes on Page 31.

CALIFORNIA NON-CITRUS FRUITS: Utilized Production And Average Grower Return Per Ton, By Utilization Category, 1986-95

Crop	Crop Year	For Processing							
		Juice or Crushed		Frozen		Dried (Fresh Basis)		Dried Basis	
		Quantity	Price	Quantity	Price	Quantity	Price	Quantity	Price
		Tons	*$/Ton*	*Tons*	*$/Ton*	*Tons*	*$/Ton*	*Tons*	*$/Ton*
Peaches, Freestone 8/	1986	---	---	56,250	165.00	16,250	96.00	1,800	864.00
	1987	---	---	59,000	178.00	17,500	101.00	1,950	909.00
	1988	---	---	57,250	165.00	20,600	117.00	2,500	960.00
	1989	---	---	63,500	167.00	14,300	110.00	1,700	924.00
	1990	---	---	74,850	178.00	13,550	108.00	1,500	975.00
	1991	---	---	71,600	183.00	22,200	116.00	2,400	1,080.00
	1992	---	---	70,400	182.00	20,200	93.00	2,100	893.00
	1993	---	---	78,800	185.00	15,000	82.00	1,850	713.00
	1994	---	---	68,400	183.00	13,500	93.00	1,450	856.00
	1995	---	---	63,700	186.00	14,000	86.00	1,550	894.00
Pears, All 6/	1986	---	---	---	---	---	---	---	---
	1987	---	---	---	---	---	---	---	---
	1988	---	---	---	---	---	---	---	---
	1989	---	---	---	---	---	---	---	---
	1990	---	---	---	---	---	---	---	---
	1991	---	---	---	---	---	---	---	---
	1992	---	---	---	---	---	---	---	---
	1993	---	---	---	---	---	---	---	---
	1994	---	---	---	---	---	---	---	---
	1995	---	---	---	---	---	---	---	---
Pears, Bartlett 8/	1986	---	---	---	---	9,000	105.00	1,410	672.00
	1987	---	---	---	---	8,000	106.00	1,130	753.00
	1988	---	---	---	---	7,900	147.00	1,220	956.00
	1989	---	---	---	---	6,700	146.00	1,140	861.00
	1990	---	---	---	---	7,600	126.00	1,270	756.00
	1991	---	---	---	---	9,300	150.00	1,550	900.00
	1992	---	---	---	---	9,900	171.00	1,500	1,130.00
	1993	---	---	---	---	9,700	149.00	1,500	970.00
	1994	---	---	---	---	8,000	152.00	1,300	942.00
	1995	---	---	---	---	4,900	150.00	800	919.00
Pears, Other 6/	1986	---	---	---	---	---	---	---	---
	1987	---	---	---	---	---	---	---	---
	1988	---	---	---	---	---	---	---	---
	1989	---	---	---	---	---	---	---	---
	1990	---	---	---	---	---	---	---	---
	1991	---	---	---	---	---	---	---	---
	1992	---	---	---	---	---	---	---	---
	1993	---	---	---	---	---	---	---	---
	1994	---	---	---	---	---	---	---	---
	1995	---	---	---	---	---	---	---	---
Plums 6/	1986	---	---	---	---	---	---	---	---
	1987	---	---	---	---	---	---	---	---
	1988	---	---	---	---	---	---	---	---
	1989	---	---	---	---	---	---	---	---
	1990	---	---	---	---	---	---	---	---
	1991	---	---	---	---	---	---	---	---
	1992	---	---	---	---	---	---	---	---
	1993	---	---	---	---	---	---	---	---
	1994	---	---	---	---	---	---	---	---
	1995	---	---	---	---	---	---	---	---

See Footnotes on Page 31.

CALIFORNIA NON-CITRUS FRUITS: Utilized Production And Average Grower Return Per Ton, By Utilization Category, 1986-95

Crop	Crop Year	Total Utilized Production	For Fresh Market		For Processing			
					Total		Canned	
			Quantity	Price	Quantity	Price	Quantity	Price
		Short Tons	*Tons*	*$/Ton*	*Tons*	*$/Ton*	*Tons*	*$/Ton*
Pomegranates 9/	1986	19,400	---	---	---	---	---	---
	1987	16,500	---	---	---	---	---	---
	1988	18,000	---	---	---	---	---	---
	1989	18,000	---	---	---	---	---	---
	1990	---	---	---	---	---	---	---
	1991	---	---	---	---	---	---	---
	1992	---	---	---	---	---	---	---
	1993	---	---	---	---	---	---	---
	1994	---	---	---	---	---	---	---
	1995	---	---	---	---	---	---	---
Prunes, Dried Basis	1986	99,000	---	---	99,000	819.00	---	---
	1987	229,000	---	---	229,000	734.00	---	---
	1988	151,000	---	---	151,000	782.00	---	---
	1989	226,000	---	---	226,000	779.00	---	---
	1990	147,000	---	---	147,000	873.00	---	---
	1991	187,000	---	---	187,000	940.00	---	---
	1992	184,000	---	---	184,000	1,030.00	---	---
	1993	121,000	---	---	121,000	1,120.00	---	---
	1994	193,000	---	---	193,000	1,090.00	---	---
	1995	181,000	---	---	181,000	1,040.00	---	---

See Footnotes on Page 31.

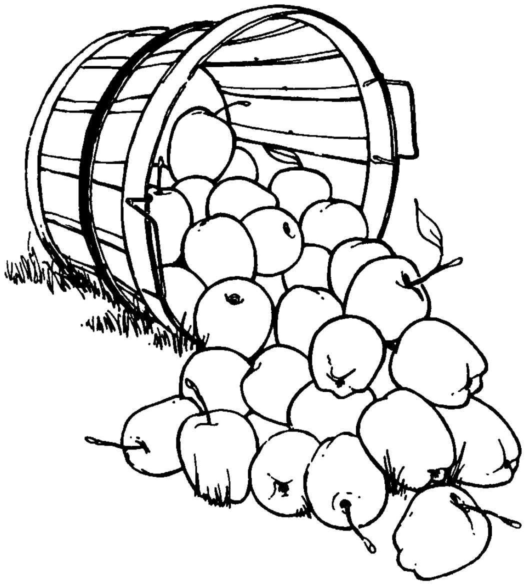

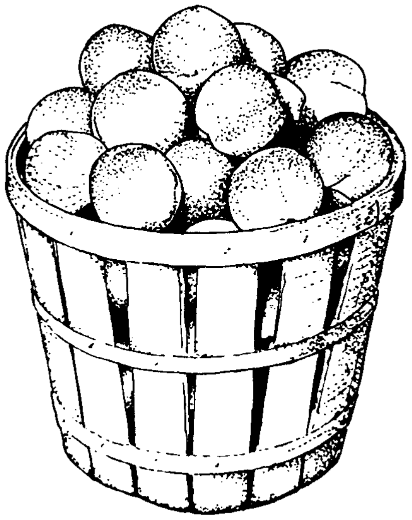

CALIFORNIA NON-CITRUS FRUITS: Utilized Production And Average Grower Return Per Ton, By Utilization Category, 1986-95

Crop	Crop Year	For Processing							
		Juice or Crushed		Frozen		Dried (Fresh Basis)		Dried Basis	
		Quantity	Price	Quantity	Price	Quantity	Price	Quantity	Price
		Tons	*$/Ton*	*Tons*	*$/Ton*	*Tons*	*$/Ton*	*Tons*	*$/Ton*
Pomegranates 9/	1986	---	---	---	---	---	---	---	---
	1987	---	---	---	---	---	---	---	---
	1988	---	---	---	---	---	---	---	---
	1989	---	---	---	---	---	---	---	---
	1990	---	---	---	---	---	---	---	---
	1991	---	---	---	---	---	---	---	---
	1992	---	---	---	---	---	---	---	---
	1993	---	---	---	---	---	---	---	---
	1994	---	---	---	---	---	---	---	---
	1995	---	---	---	---	---	---	---	---
Prunes, Dried Basis	1986	---	---	---	---	291,100	279.00	99,000	819.00
	1987	---	---	---	---	682,400	246.00	229,000	734.00
	1988	---	---	---	---	469,600	251.00	151,000	782.00
	1989	---	---	---	---	754,800	233.00	226,000	779.00
	1990	---	---	---	---	463,100	266.00	147,000	873.00
	1991	---	---	---	---	589,000	298.00	187,000	940.00
	1992	---	---	---	---	534,000	355.00	184,000	1,030.00
	1993	---	---	---	---	375,000	374.00	121,000	1,120.00
	1994	---	---	---	---	594,000	354.00	193,000	1,090.00
	1995	---	---	---	---	597,300	320.00	181,000	1,040.00

1/ Dried and frozen fruit included in total processed to avoid disclosure of individual operations.
2/ Processed fruit included in fresh market total to avoid disclosure of individual operations.
3/ Total processed includes canned, crushed, frozen, and brined.
4/ For 1986-95, prices for processing and fresh totals were not published to avoid disclosure of individual operations. Small quantities processed included in fresh to avoid disclosure of individual operations.
5/ Includes substandard raisins diverted to other uses.
6/ Processed and fresh fruit not published to avoid disclosure of individual operations but included in total utilized.
7/ Limited and undersized combined prior to 1988.
8/ Processed mostly canned, but includes other small quantities not published separately to avoid disclosure of individual operations.
9/ Pomegranates reported as miscellaneous after 1989 crop.

CALIFORNIA CITRUS FRUITS: Acreage, Production, Price And Value, 1985-95

Crop	Crop Year	Bearing	Non-Bearing	Yield Per Acre	Total Production	Price 1/	Total Value
		Acres		*Cartons*	*1,000 Cartons*	*Dollars*	*$1,000*
Grapefruit, All 2/	85-86	20,900	1,300	775	16,200	3.67	59,280
	86-87	20,800	1,500	894	18,600	3.03	56,251
	87-88	20,700	1,100	879	18,200	3.12	56,600
	88-89	19,900	1,800	804	16,000	3.11	49,459
	89-90	19,200	2,100	979	18,800	4.01	75,457
	90-91	18,300	2,400	874	16,000	3.20	51,128
	91-92	18,500	2,400	1,082	20,000	3.06	61,120
	92-93	17,800	2,600	1,034	18,400	2.46	45,089
	93-94	18,000	N/A	1,034	18,600	2.72	50,579
	94-95	18,400	N/A	1,010	18,600	2.84	52,827
Grapefruit, Desert Valley 3/	85-86	9,400	500	766	7,200	3.41	24,504
	86-87	9,200	600	935	8,600	2.74	23,578
(Net weight per carton:	87-88	9,000	600	933	8,400	2.54	21,304
33.5 pounds)	88-89	8,200	900	854	7,000	2.33	16,314
	89-90	7,600	1,100	921	7,000	4.55	31,799
	90-91	7,200	1,200	972	7,000	3.13	21,905
	91-92	7,300	1,300	958	7,000	3.09	21,613
	92-93	7,300	1,600	958	7,000	2.09	14,639
	93-94	7,700	N/A	884	6,800	1.85	12,541
	94-95	8,000	N/A	826	6,600	2.19	14,423
Grapefruit, Other Areas	85-86	11,500	800	783	9,000	3.87	34,776
	86-87	11,600	900	862	10,000	3.27	32,673
(Net weight per carton:	87-88	11,700	500	838	9,800	3.60	35,296
33.5 pounds)	88-89	11,700	900	769	9,000	3.69	33,145
	89-90	11,600	1,000	1,017	11,800	3.70	43,658
	90-91	11,100	1,200	810	9,000	3.25	29,223
	91-92	11,200	1,100	1,160	13,000	3.04	39,507
	92-93	10,500	1,000	1,086	11,400	2.67	30,450
	93-94	10,300	N/A	1,146	11,800	3.23	38,038
	94-95	10,400	N/A	1,154	12,000	3.20	38,404
Oranges, All	85-86	174,600	7,600	617	107,800	3.39	364,952
	86-87	172,900	12,700	670	115,800	3.75	434,124
(All varieties --	87-88	172,600	13,900	684	118,000	3.89	458,180
net weight per carton:	88-89	177,600	13,800	663	117,800	3.93	462,264
37.5 pounds)	89-90	175,100	15,000	816	142,800	3.89	555,055
	90-91	178,400	15,500	286	51,200	7.27	372,284
	91-92	181,800	16,200	742	134,800	3.22	433,994
	92-93	184,000	16,600	726	133,600	3.54	472,010
	93-94	185,000	N/A	688	127,200	3.64	462,567
	94-95	191,000	N/A	638	122,000	3.76	458,130

See Footnotes on Page 33.

CALIFORNIA CITRUS FRUITS: Acreage, Production, Price And Value, 1985-95

Crop	Crop Year	Bearing	Non-Bearing	Yield Per Acre	Total Production	Price 1/	Total Value
		Acres		*Cartons*	*1,000 Cartons*	*Dollars*	*$1,000*
Oranges, Navel & Misc.	85-86	107,000	4,200	617	66,000	3.58	235,980
	86-87	106,000	7,400	651	69,000	3.52	242,840
	87-88	106,000	8,100	594	63,000	3.91	246,462
	88-89	108,000	9,200	630	68,000	3.79	257,328
	89-90	106,000	10,200	836	88,600	4.02	355,661
	90-91	109,000	10,000	290	31,600	5.04	159,330
	91-92	112,000	9,800	626	70,200	4.36	305,811
	92-93	114,000	10,600	768	87,600	3.16	277,008
	93-94	114,000	N/A	642	73,200	3.99	291,807
	94-95	118,000	N/A	594	70,000	3.91	273,750
Oranges, Valencia	85-86	67,600	3,400	618	41,800	3.09	128,972
	86-87	66,900	5,300	700	46,800	4.09	191,284
	87-88	66,600	5,800	826	55,000	3.85	211,718
	88-89	69,600	4,600	716	49,800	4.12	204,936
	89-90	69,100	4,800	784	54,200	3.68	199,394
	90-91	69,400	5,500	282	19,600	10.87	212,954
	91-92	69,800	6,400	926	64,600	1.99	128,183
	92-93	70,000	6,000	658	46,000	4.24	195,002
	93-94	71,000	N/A	760	54,000	3.16	170,760
	94-95	73,000	N/A	712	52,000	3.55	184,380
Lemons	85-86	48,800	1,500	619	30,200	5.65	170,426
	86-87	48,400	1,100	888	43,000	3.56	152,890
(Net weight per carton:	87-88	49,000	700	694	34,000	5.02	170,628
38 pounds)	88-89	48,400	800	669	32,400	6.19	200,524
	89-90	47,800	1,400	661	31,600	7.60	239,979
	90-91	46,700	1,600	634	29,600	8.19	242,269
	91-92	46,400	1,900	650	30,200	6.32	190,894
	92-93	46,400	2,100	880	40,800	4.97	202,827
	93-94	45,500	N/A	910	41,400	5.00	207,020
	94-95	45,700	N/A	898	41,000	5.57	228,308
Tangerines	85-86	8,500	600	424	3,600	6.10	21,940
	86-87	8,600	600	519	4,460	5.76	25,697
(Including Mandarins & Mandarin Hybrids,	87-88	8,400	900	498	4,180	7.73	32,297
Tangelos and Tangors.)	88-89	8,000	1,200	510	4,080	7.04	28,733
	89-90	7,300	1,500	452	3,300	9.45	31,193
(Net weight per carton: 37.5 pounds)	90-91	7,600	1,400	356	2,700	9.09	24,528
	91-92	7,800	1,400	626	4,880	7.25	35,357
	92-93	7,700	1,300	546	4,200	6.00	25,186
	93-94	8,000	N/A	576	4,600	5.58	25,651
	94-95	8,500	N/A	518	4,400	6.88	30,261

1/ Equivalent packinghouse door returns.
2/ Season average prices for all grapefruit are derived by weighting the season average prices for each area by their carton weights.
3/ Prior to 1993-94 net weight per carton was 32 pounds.

CALIFORNIA NUTS: Acreage, Production, Price And Value, 1986-95

Crop	Crop Year	Bearing	Non-Bearing	Yield Per Acre	Production: Marketable In-Shell	Production: Shelling Stock	Production: Total	Price	Total Value
		Acres		*Pounds*	*1,000 Pounds*			*$/Lb.*	*$1,000*
Almonds 1/ 2/	1986	416,000	17,200	601	---	---	250,000	1.92	461,568
	1987	417,000	17,500	1,580	---	---	660,000	1.00	648,000
(Shelled	1988	419,000	16,700	1,410	---	---	590,000	1.05	600,075
Basis)	1989	411,000	21,700	1,190	---	---	490,000	1.02	480,930
	1990	411,000	32,400	1,610	---	---	660,000	0.93	597,990
	1991	405,000	33,000	1,210	---	---	490,000	1.19	564,179
	1992	401,000	33,600	1,370	---	---	548,000	1.30	691,340
	1993	402,000	26,600	1,220	---	---	490,000	1.94	930,618
	1994	409,000	36,900	1,800	---	---	735,000	1.34	965,202
	1995	400,000	56,900	925	---	---	370,000	2.39	857,771
Pecans 3/	1986	1,400	1,300	---	---	---	---	---	---
	1987	1,800	1,200	---	---	---	---	---	---
(In-Shell	1988	2,500	500	880	---	---	2,200	0.70	1,540
Basis)	1989	2,500	700	800	---	---	2,000	0.90	1,804
	1990	2,600	600	1,080	---	---	2,800	1.25	3,500
	1991	2,500	600	920	---	---	2,300	1.32	3,036
	1992	2,600	600	1,000	---	---	2,600	1.60	4,160
	1993	2,500	500	1,200	---	---	3,000	0.96	2,880
	1994	2,600	N/A	577	---	---	1,500	1.16	1,740
	1995	2,600	N/A	1,120	---	---	2,900	1.28	3,712
Pistachios 2/	1986	34,200	20,400	2,190	57,500	17,400	74,900	1.12	83,888
	1987	41,000	16,400	807	27,200	5,900	33,100	1.37	45,347
(In-Shell	1988	47,200	10,300	1,990	76,100	17,900	94,000	1.22	114,680
Basis)	1989	50,900	12,000	766	33,000	6,000	39,000	1.63	63,570
	1990	53,700	12,300	2,230	94,600	25,400	120,000	1.02	122,400
	1991	55,700	13,300	1,380	59,000	18,000	77,000	1.25	96,250
	1992	56,500	13,900	2,600	114,500	32,500	147,000	1.03	151,410
	1993	57,000	15,700	2,670	113,000	39,000	152,000	1.07	162,640
	1994	57,500	16,600	2,240	94,600	34,400	129,000	0.92	118,809
	1995	60,300	13,400	2,450	107,500	40,500	148,000	0.96	141,636
		Acres		*Tons*	*Tons*			*$/Ton*	*$1,000*
Walnuts 2/	1986	180,000	20,800	1.00	---	---	180,000	1,080	194,400
	1987	176,000	25,300	1.40	---	---	247,000	984	243,048
(In-Shell	1988	177,000	22,900	1.18	---	---	209,000	922	192,698
Basis)	1989	179,000	20,800	1.28	---	---	229,000	1,070	245,030
	1990	181,000	17,700	1.25	---	---	227,000	1,040	236,080
	1991	181,000	16,600	1.43	---	---	259,000	1,060	274,540
	1992	178,000	17,200	1.14	---	---	203,000	1,410	286,230
	1993	176,000	20,000	1.48	---	---	260,000	1,390	361,400
	1994	171,000	19,800	1.36	---	---	232,000	1,000	232,000
	1995	169,000	26,600	1.38	---	---	234,000	1,340	313,560

1/ Price and value are based on the edible portion of the crop only. Included in production are inedible quantities of no value as follows: 1986 -- 9.8 million pounds, 1987 -- 12.0 million pounds, 1988 -- 18.5 million pounds, 1989 -- 18.5 million pounds, 1990 -- 17.0 million pounds, 1991 -- 15.9 million pounds, 1992 -- 16.2 million pounds, 1993 -- 10.3 million pounds, 1994 -- 14.7 million pounds, 1995 -- 11.1 milliion pounds.

2/ Data for 1995 acreage by county, variety, and year of planting available upon request.

3/ Pecan production data begins with the 1988 crop.

CALIFORNIA BERRIES: Acreage, Production, Price And Value, 1986-95

Crop	Crop Year	Harvested Acreage	Average Yield	Production	Price Per Cwt. 1/	Total Value
		Acres	*Cwt.*	*1,000 Cwt.*	*Dollars*	*$1,000*
Boysenberries	1986	510	65	33	72.20	2,383
	1987	450	73	33	71.60	2,364
	1988	400	53	21	181.60	3,814
	1989	460	93	43	121.70	5,232
	1990	450	80	36	88.00	3,168
	1991	N/A	N/A	N/A	N/A	N/A
	1992	200	85	17	91.10	1,548
	1993	250	90	23	88.00	1,980
	1994	250	80	20	75.50	1,510
	1995	270	67	18	95.00	1,710
Olallieberries	1986	200	90	18	79.50	1,431
	1987	150	107	16	79.70	1,275
	1988	200	110	22	93.40	2,054
	1989	200	80	16	78.50	1,256
	1990	200	110	22	80.00	1,760
	1991	N/A	N/A	N/A	N/A	N/A
	1992	N/A	N/A	N/A	N/A	N/A
	1993	N/A	N/A	N/A	N/A	N/A
	1994	N/A	N/A	N/A	N/A	N/A
	1995	N/A	N/A	N/A	N/A	N/A
Raspberries	1986	600	100	60	177.60	10,653
	1987	1,000	82	82	154.60	12,677
	1988	1,500	111	167	158.10	26,407
	1989	1,400	104	145	165.40	23,978
	1990	1,700	104	177	165.00	29,228
	1991	N/A	N/A	N/A	N/A	N/A
	1992	1,600	165	264	183.00	48,276
	1993	1,850	142	263	148.00	39,000
	1994	1,900	110	210	143.00	30,000
	1995	1,900	80	153	152.00	23,325
Strawberries, All	1986	15,600	505	7,878	48.90	385,245
	1987	17,500	470	8,225	49.50	407,251
	1988	19,200	450	8,640	45.10	389,830
	1989	20,400	425	8,670	42.90	372,201
	1990	21,000	470	9,870	43.30	417,728
	1991	21,100	520	10,972	42.40	465,515
	1992	24,000	430	10,320	49.70	513,028
	1993	25,100	455	11,421	40.10	457,855
	1994	23,300	570	13,281	48.80	647,575
	1995	23,600	505	11,918	46.30	551,698
Strawberries, Fresh Market	1986	---	---	5,804	58.30	338,373
	1987	---	---	5,995	58.00	347,710
	1988	---	---	6,590	52.00	342,680
	1989	---	---	6,570	49.30	323,901
	1990	---	---	6,705	52.00	338,603
	1991	---	---	7,652	50.90	389,487
	1992	---	---	7,630	59.20	451,696
	1993	---	---	7,736	46.80	362,045
	1994	---	---	9,016	59.10	532,846
	1995	---	---	8,001	57.40	459,257

See Footnote on Page 36.

CALIFORNIA BERRIES: Acreage, Production, Price And Value, 1986-95

Crop	Crop Year	Harvested Acreage	Average Yield	Production	Price Per Cwt. 1/	Total Value
		Acres	*Cwt.*	*1,000 Cwt.*	*Dollars*	*$1,000*
Strawberries, Processing	1986	---	---	2,074	22.60	46,872
	1987	---	---	2,230	26.70	59,541
	1988	---	---	2,050	23.00	47,150
	1989	---	---	2,100	23.00	48,300
	1990	---	---	3,165	25.00	79,125
	1991	---	---	3,320	22.90	76,028
	1992	---	---	2,690	22.80	61,332
	1993	---	---	3,685	26.00	95,810
	1994	---	---	4,265	26.90	114,729
	1995	---	---	3,917	23.60	92,441

1/ The processed price is based on equivalent returns at the processing plant door. The fresh market price is on a packed and loaded basis, F.O. B. shipping point.

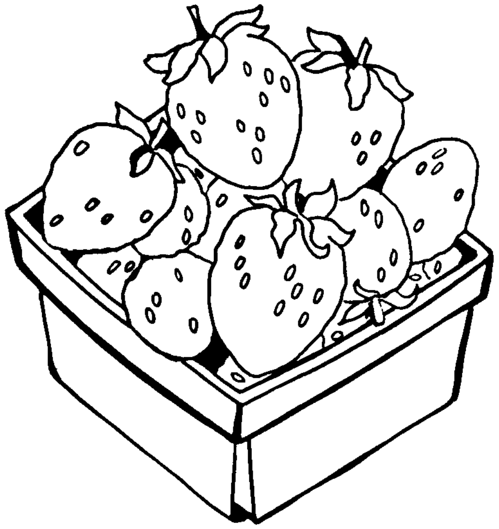

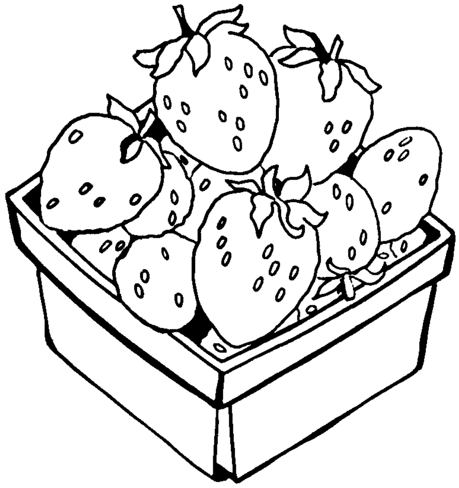

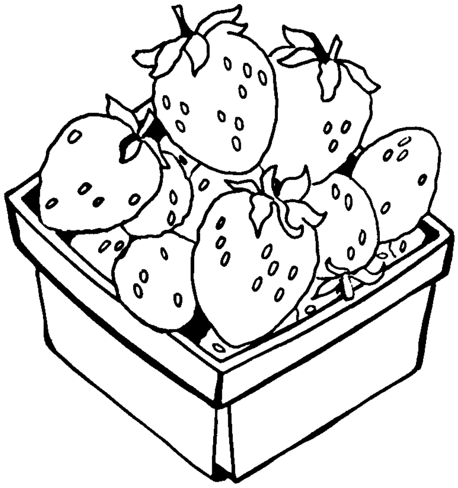

CALIFORNIA CITRUS FRUITS: Sales By Utilization And Price Per Carton, 1985-95

Crop	Crop Year	All Sales Quantity	All Sales Price 1/	Fresh Market Quantity	Fresh Market Price 1/	Processing Quantity	Processing Price 1/
		1,000 Cartons	*$/Ctn.*	*1,000 Cartons*	*$/Ctn.*	*1,000 Cartons*	*$/Ctn.*
Grapefruit, All 2/	85-86	16,200	3.67	11,400	5.07	4,800	0.33
	86-87	18,600	3.03	11,700	4.58	6,900	0.41
	87-88	18,200	3.12	12,000	4.45	6,200	0.54
	88-89	16,000	3.11	11,000	4.50	5,000	0.06
	89-90	18,800	4.01	12,000	6.11	6,800	0.30
	90-91	16,000	3.20	10,400	4.97	5,600	-0.08
	91-92	20,000	3.06	12,800	4.56	7,200	0.40
	92-93	18,400	2.46	11,400	4.08	7,000	-0.17
	93-94	18,600	2.72	12,160	4.16	6,440	0.00
	94-95	18,600	2.84	11,820	4.53	6,780	-0.10
Grapefruit, Desert Valley 3/	85-86	7,200	3.41	4,800	4.93	2,400	0.35
	86-87	8,600	2.74	5,200	4.24	3,400	0.45
(Net weight per carton:	87-88	8,400	2.54	5,200	3.74	3,200	0.58
33.5 pounds)	88-89	7,000	2.33	5,100	3.21	1,900	-0.03
	89-90	7,000	4.55	4,800	6.54	2,200	0.19
	90-91	7,000	3.13	5,000	4.42	2,000	-0.09
	91-92	7,000	3.09	5,200	4.08	1,800	0.24
	92-93	7,000	2.09	4,800	3.13	2,200	-0.18
	93-94	6,800	1.85	4,560	2.76	2,240	-0.01
	94-95	6,600	2.19	4,420	3.31	2,180	-0.09
Grapefruit, Other Areas	85-86	9,000	3.87	6,600	5.16	2,400	0.30
	86-87	10,000	3.27	6,500	4.83	3,500	0.37
(Net weight per carton:	87-88	9,800	3.60	6,800	4.97	3,000	0.50
33.5 pounds)	88-89	9,000	3.69	5,900	5.56	3,100	0.11
	89-90	11,800	3.70	7,200	5.84	4,600	0.35
	90-91	9,000	3.25	5,400	5.47	3,600	-0.08
	91-92	13,000	3.04	7,600	4.88	5,400	0.46
	92-93	11,400	2.67	6,600	4.73	4,800	-0.16
	93-94	11,800	3.23	7,600	5.01	4,200	0.00
	94-95	12,000	3.20	7,400	5.26	4,600	-0.11
Oranges, All	85-86	107,800	3.39	87,200	4.16	20,600	0.13
	86-87	115,800	3.75	83,400	5.08	32,400	0.32
(All varieties --	87-88	118,000	3.89	82,600	5.15	35,400	0.93
net weight per carton:	88-89	117,800	3.93	81,200	5.04	36,600	1.45
37.5 pounds)	89-90	142,800	3.89	94,200	5.00	48,600	1.73
	90-91	51,200	7.27	32,400	11.21	18,800	0.49
	91-92	134,800	3.22	82,700	4.92	52,100	0.52
	92-93	133,600	3.54	98,200	4.84	35,400	-0.08
	93-94	127,200	3.64	92,200	5.02	35,000	-0.01
	94-95	122,000	3.76	87,000	5.35	35,000	-0.19

See Foonotes on Page 38.

CALIFORNIA CITRUS FRUITS: Sales By Utilization And Price Per Carton, 1985-95

Crop	Crop Year	All Sales		Fresh Market		Processing	
		Quantity	Price 1/	Quantity	Price 1/	Quantity	Price 1/
		1,000 Cartons	*$/Ctn.*	*1,000 Cartons*	*$/Ctn.*	*1,000 Cartons*	*$/Ctn.*
Oranges, Navel & Misc.	85-86	66,000	3.58	53,000	4.45	13,000	0.01
	86-87	69,000	3.52	53,000	4.60	16,000	-0.06
	87-88	63,000	3.91	49,800	4.79	13,200	0.60
	88-89	68,000	3.79	51,800	4.68	16,200	0.92
	89-90	88,600	4.02	64,600	4.84	24,000	1.81
	90-91	31,600	5.04	18,400	8.34	13,200	0.45
	91-92	70,200	4.36	53,000	5.53	17,200	0.76
	92-93	87,600	3.16	65,400	4.30	22,200	-0.18
	93-94	73,200	3.99	58,200	5.01	15,000	0.02
	94-95	70,000	3.91	53,000	5.21	17,000	-0.14
Oranges, Valencia	85-86	41,800	3.09	34,200	3.70	7,600	0.32
	86-87	46,800	4.09	30,400	5.92	16,400	0.69
	87-88	55,000	3.85	32,800	5.69	22,200	1.13
	88-89	49,800	4.12	29,400	5.68	20,400	1.86
	89-90	54,200	3.68	29,600	5.37	24,600	1.65
	90-91	19,600	10.87	14,000	14.98	5,600	0.59
	91-92	64,600	1.99	29,700	3.84	34,900	0.41
	92-93	46,000	4.24	32,800	5.92	13,200	0.08
	93-94	54,000	3.16	34,000	5.04	20,000	-0.03
	94-95	52,000	3.55	34,000	5.55	18,000	-0.24
Lemons	85-86	30,200	5.65	19,000	8.51	11,200	0.78
	86-87	43,000	3.56	20,000	6.69	23,000	0.83
(Net weight per carton:	87-88	34,000	5.02	20,200	8.16	13,800	0.42
38 pounds)	88-89	32,400	6.19	20,600	9.19	11,800	0.95
	89-90	31,600	7.60	21,000	10.63	10,600	1.59
	90-91	29,600	8.19	18,780	12.16	10,820	1.29
	91-92	30,200	6.32	18,180	9.93	12,020	0.87
	92-93	40,800	4.97	19,800	9.22	21,000	0.97
	93-94	41,400	5.00	19,000	10.10	22,400	0.68
	94-95	41,000	5.57	21,960	9.95	10,040	0.52
Tangerines	85-86	3,600	6.10	2,540	8.55	1,060	0.21
	86-87	4,460	5.76	3,264	7.62	1,196	0.69
(Including Mandarins &	87-88	4,180	7.73	3,048	10.34	1,132	0.69
Mandarin Hybrids,	88-89	4,080	7.04	2,950	9.33	1,130	1.07
Tangelos and Tangors)	89-90	3,300	9.45	2,480	12.12	820	1.40
	90-91	2,700	9.09	1,850	13.13	850	0.29
	91-92	4,880	7.25	3,360	10.29	1,520	0.52
(Net weight per carton:	92-93	4,200	6.00	2,930	8.72	1,270	-0.28
37.5 pounds)	93-94	4,600	5.58	3,200	8.04	1,400	-0.06
	94-95	4,400	6.88	3,000	10.15	1,400	-0.14

1/ Equivalent packinghouse door returns.
2/ Season average prices for all grapefruit are derived by weighting the season average prices for each area by their carton weights.
3/ Prior to 1993-94 net weight per carton was 32 pounds.

CALIFORNIA CITRUS FRUITS: Grower Returns, Month And Season Average Prices (Dollars Per Carton), 1985-95

Grapefruit, Desert	Crop Year	Oct.	Nov.	Dec.	Jan.	Feb.	Mar.	Apr.	May	June	July	Aug.	Sept.	Season Avg. Price
Fresh	85-86	---	6.33	5.78	5.68	5.08	4.73	3.64	4.64	4.34	---	---	---	4.93
	86-87	---	4.24	4.14	3.84	4.04	3.54	3.69	4.29	4.89	4.89	---	---	4.24
	87-88	---	5.39	4.29	3.79	3.69	3.54	3.39	3.64	3.74	3.49	3.29	---	3.74
	88-89	---	4.16	3.11	2.86	2.56	3.11	3.51	3.56	2.16	2.06	---	---	3.21
	89-90	---	4.94	3.69	6.09	6.34	6.84	6.99	7.04	7.34	8.19	---	---	6.54
	90-91	---	3.64	3.74	3.94	3.69	4.49	4.59	4.29	3.84	3.64	---	---	4.42
	91-92	---	4.23	3.88	4.08	3.88	3.93	4.38	3.88	4.33	3.93	---	---	4.08
	92-93	---	5.03	3.78	3.78	3.23	2.93	2.73	2.43	2.78	2.78	---	---	3.13
	93-94	---	3.97	3.06	2.70	2.63	2.51	2.16	2.43	3.00	3.52	---	---	2.76
	94-95	---	5.37	2.47	2.55	2.97	2.98	2.91	3.21	3.69	3.91	-- -	-- -	3.31
Processing	85-86	---	0.33	0.33	0.33	0.33	0.33	0.33	0.33	0.36	0.36	---	---	0.35
	86-87	---	0.46	0.46	0.46	0.46	0.46	0.46	0.46	0.44	0.44	---	---	0.45
	87-88	---	0.58	0.58	0.58	0.58	0.58	0.58	0.58	0.58	0.58	0.58	---	0.58
	88-89	---	-0.01	-0.09	-0.09	-0.06	-0.12	-0.07	-0.03	0.13	0.13	---	---	-0.03
	89-90	---	0.22	0.21	0.22	0.11	0.17	0.14	0.28	0.11	---	---	---	0.19
	90-91	---	-0.15	-0.14	-0.15	-0.15	-0.15	-0.15	-0.13	-0.11	-0.12	---	---	-0.09
	91-92	---	-0.07	-0.12	---	0.03	0.23	0.28	0.31	0.34	0.34	---	---	0.24
	92-93	---	0.25	-0.14	-0.17	-0.18	-0.18	-0.18	-0.18	-0.18	-0.18	---	---	-0.18
	93-94	---	0.18	-0.03	-0.03	-0.03	-0.03	-0.03	-0.03	-0.03	-0.03	---	---	-0.01
	94-95	---	-0.09	-0.11	-0.11	-0.11	-0.11	-0.09	-0.03	-0.11	-0.11	---	---	-0.09
All Sales	85-86	---	5.55	4.77	4.76	3.73	3.40	2.62	2.92	2.62	0.36	---	---	3.41
	86-87	---	3.85	3.45	3.58	3.02	2.69	2.48	2.71	2.52	1.18	---	---	2.74
	87-88	---	5.09	3.92	3.10	3.23	2.95	2.53	2.63	2.40	1.09	1.03	---	2.54
	88-89	---	3.64	2.69	2.24	2.02	2.33	2.41	2.40	1.67	1.10	---	---	2.33
	89-90	---	4.42	3.26	5.51	4.98	5.51	4.20	3.81	3.00	8.19	---	---	4.55
	90-91	---	3.35	3.19	3.51	2.84	3.60	2.79	2.93	2.12	1.64	---	---	3.13
	91-92	---	3.85	3.43	3.59	2.97	2.99	3.08	2.69	3.41	2.73	---	---	3.09
	92-93	---	4.80	3.50	3.37	2.50	1.88	1.59	1.55	1.43	2.45	---	---	2.09
	93-94	---	2.63	2.81	2.44	1.92	1.75	1.49	1.48	1.32	1.16	---	---	1.85
	94-95	---	4.01	2.19	2.31	2.01	1.78	2.09	2.10	2.00	0.57	---	---	2.19

Grapefruit, Other Areas	Crop Year	Nov.	Dec.	Jan.	Feb.	Mar.	Apr.	May	June	July	Aug.	Sept.	Oct.	Season Avg. Price
Fresh	85-86	---	---	---	---	5.98	3.38	4.65	5.40	5.10	5.05	5.45	---	5.16
	86-87	---	---	---	---	5.95	3.60	4.85	5.47	5.17	4.07	4.67	4.52	4.83
	87-88	---	---	11.87	6.62	2.47	2.37	4.62	4.47	5.07	4.97	5.22	4.72	4.97
	88-89	---	13.41	9.71	3.71	2.18	2.26	6.11	4.76	5.31	5.86	6.91	4.66	5.56
	89-90	9.69	11.64	7.64	6.04	7.19	7.89	8.44	7.74	5.84	4.44	2.07	1.79	5.84
	90-91	14.14	11.29	11.94	6.69	7.14	7.79	6.94	6.24	5.59	4.64	2.67	2.07	5.47
	91-92	18.08	17.43	7.33	3.93	3.88	5.13	6.18	4.98	4.58	4.68	4.68	3.83	4.88
	92-93	16.18	15.43	11.08	3.53	3.03	5.48	4.98	4.78	4.83	4.68	5.03	3.38	4.73
	93-94	15.48	10.77	11.61	9.15	3.95	4.83	5.44	5.58	4.59	4.70	4.80	4.69	5.01
	94-95	16.82	14.05	12.47	7.86	4.68	4.40	5.69	5.64	4.41	4.66	---	---	5.26
Processing	85-86	---	---	---	---	---	0.31	0.30	0.30	0.30	0.30	0.30	---	0.30
	86-87	---	---	---	---	0.40	0.40	0.40	0.36	0.36	0.36	0.36	0.36	0.37
	87-88	---	---	---	---	0.50	0.50	0.50	0.50	0.50	0.50	0.50	0.50	0.50
	88-89	---	---	-0.12	0.21	0.28	0.16	0.10	0.17	0.10	0.06	0.09	0.18	0.11
	89-90	0.46	0.63	0.38	0.32	0.33	0.54	0.80	0.44	0.61	-0.01	-0.11	-0.05	0.35
	90-91	-0.14	-0.13	-0.12	-0.14	-0.15	-0.12	-0.12	-0.13	-0.14	-0.14	-0.10	-0.10	-0.08
	91-92	-0.02	-0.02	-0.02	-0.02	0.35	0.46	0.45	0.55	0.49	0.43	0.36	0.32	0.46
	92-93	-0.18	-0.18	-0.18	-0.18	-0.18	-0.18	-0.18	-0.16	-0.15	-0.15	-0.17	-0.17	-0.16
	93-94	---	---	---	0.16	0.12	-0.07	-0.04	0.10	0.00	-0.04	-0.04	0.07	0.00
	94-95	-0.11	-0.11	-0.11	-0.11	-0.11	-0.11	-0.11	-0.11	-0.11	-0.11	---	---	-0.11
All Sales	85-86	---	---	---	---	5.98	2.12	2.33	4.51	3.73	3.73	4.69	---	3.87
	86-87	---	---	---	---	4.10	2.89	3.15	3.56	3.58	2.98	2.93	3.35	3.27
	87-88	---	---	11.87	6.62	2.08	1.75	2.97	3.72	3.76	3.64	3.32	3.35	3.60
	88-89	---	13.41	7.75	2.31	0.91	0.58	1.61	3.06	3.75	3.96	4.55	4.21	3.69
	89-90	5.08	6.14	6.73	4.61	3.76	6.53	5.35	4.97	3.65	2.45	1.16	1.40	3.70
	90-91	7.01	7.49	9.53	5.72	3.83	5.91	2.85	3.86	3.45	2.58	1.56	1.62	3.25
	91-92	9.03	8.71	3.66	2.94	3.29	2.87	3.18	3.17	2.91	2.82	3.06	2.92	3.04
	92-93	8.00	7.63	5.45	2.79	2.63	1.31	0.71	2.45	3.19	2.65	3.00	2.65	2.67
	93-94	15.48	10.77	11.61	4.15	1.78	3.95	3.54	3.45	3.19	2.75	3.59	1.80	3.23
	94-95	15.69	10.78	11.54	5.20	3.31	2.64	2.47	3.50	2.19	3.28	---	---	3.20

CALIFORNIA CITRUS FRUITS: Grower Returns, Month And Season Average Prices (Dollars Per Carton), 1985-95

Oranges, Navel/Misc.	Crop Year	Oct.	Nov.	Dec.	Jan.	Feb.	Mar.	Apr.	May	June	July	Aug.	Sept.	Season Avg. Price
Fresh	85-86	7.05	5.45	5.30	4.75	4.05	4.15	3.80	3.70	3.25	---	---	---	4.45
	86-87	6.00	5.60	4.75	4.65	4.35	4.20	3.95	4.80	5.20	---	---	---	4.60
	87-88	12.84	6.94	4.44	4.79	4.19	4.04	4.24	6.24	3.54	---	---	---	4.79
	88-89	7.08	7.23	5.68	4.38	3.93	4.08	4.13	5.13	4.83	---	---	---	4.68
	89-90	7.59	5.99	4.94	4.79	4.74	4.44	4.24	4.99	4.99	---	---	---	4.84
	90-91	6.09	6.84	6.49	12.79	14.49	13.19	13.54	---	---	---	---	---	8.34
	91-92	---	9.28	7.38	6.68	4.88	4.33	4.43	4.73	3.38	---	---	---	5.53
	92-93	6.55	5.20	4.65	4.20	3.95	3.80	4.25	4.40	2.95	---	---	---	4.30
	93-94	6.86	6.86	5.11	4.06	4.06	5.06	5.31	6.01	---	---	---	---	5.01
	94-95	6.06	6.26	5.46	5.26	4.81	4.46	5.66	5.11	---	---	---	---	5.21
Processing	85-86	0.01	0.01	0.01	0.01	0.01	0.01	0.01	0.01	0.01	---	---	---	0.01
	86-87	-0.06	-0.06	-0.06	-0.06	-0.06	-0.06	-0.06	-0.06	-0.06	---	---	---	-0.06
	87-88	0.60	0.60	0.60	0.60	0.60	0.60	0.60	0.60	0.60	---	---	---	0.60
	88-89	0.71	0.71	0.36	0.49	0.67	1.03	1.24	1.61	1.74	---	---	---	0.92
	89-90	0.54	1.11	1.39	1.44	1.80	1.93	1.93	2.04	2.04	---	---	---	1.81
	90-91	0.45	0.45	0.45	0.45	0.45	0.45	0.45	---	---	---	---	---	0.45
	91-92	---	0.65	0.74	0.93	0.93	0.84	0.65	0.65	0.65	---	---	---	0.76
	92-93	-0.19	-0.19	-0.19	-0.19	-0.19	-0.19	-0.19	-0.10	0.18	---	---	---	-0.18
	93-94	-0.09	-0.18	-0.18	-0.14	0.09	0.18	-0.01	-0.01	---	---	---	---	0.02
	94-95	-0.12	-0.12	-0.03	-0.03	-0.12	-0.18	-0.22	-0.22	---	---	---	---	-0.14
All Sales	85-86	4.71	4.86	4.76	4.00	3.37	3.27	2.63	2.67	2.04	---	---	---	3.58
	86-87	4.88	4.80	4.05	3.71	3.20	3.11	2.79	3.29	2.95	---	---	---	3.52
	87-88	11.79	6.50	3.96	3.96	3.39	3.17	3.26	4.45	2.56	---	---	---	3.91
	88-89	5.41	6.09	4.89	3.54	3.08	3.25	3.40	4.18	3.60	---	---	---	3.79
	89-90	6.41	5.20	4.38	4.20	3.99	3.62	3.41	3.96	3.82	---	---	---	4.02
	90-91	4.87	5.94	5.45	3.94	4.67	6.82	10.27	---	---	---	---	---	5.04
	91-92	---	8.06	6.42	5.75	4.09	3.44	3.20	3.25	2.25	---	---	---	4.36
	92-93	4.64	4.14	3.85	3.31	2.91	2.65	2.92	2.88	1.57	---	---	---	3.16
	93-94	5.99	6.07	4.43	3.34	3.25	3.84	3.88	4.29	---	---	---	---	3.99
	94-95	4.52	4.88	4.36	4.40	3.52	3.12	3.98	4.27	---	---	---	---	3.91

Oranges, Valencia	Crop Year	Feb.	Mar.	Apr.	May	June	July	Aug.	Sept.	Oct.	Nov.	Dec.	Jan.	Season Avg. Price
Fresh	85-86	3.30	2.80	3.20	3.95	3.45	3.45	3.60	3.95	4.15	4.00	---	---	3.70
	86-87	---	4.92	5.42	5.67	5.87	6.07	5.07	6.37	6.97	6.07	---	---	5.92
	87-88	---	6.24	6.89	7.19	6.84	5.69	5.29	5.49	3.59	2.84	---	---	5.69
	88-89	---	6.43	5.68	5.48	5.73	6.08	5.78	5.88	5.08	4.53	---	---	5.68
	89-90	---	5.66	5.61	6.76	6.01	5.61	5.01	5.12	4.27	3.97	---	---	5.37
	90-91	15.24	14.99	16.59	14.99	14.69	14.09	14.64	15.53	14.63	---	---	---	14.98
	91-92	---	4.89	4.84	4.74	4.04	3.59	3.44	3.44	3.54	3.54	---	---	3.84
	92-93	3.52	4.62	5.02	4.37	4.47	5.02	6.62	9.67	9.52	4.17	---	---	5.92
	93-94	6.09	5.89	5.34	5.59	5.24	4.74	5.09	4.84	4.09	4.79	---	---	5.04
	94-95	5.20	5.15	5.50	6.05	5.85	5.50	5.55	---	---	---	---	---	5.55
Processing	85-86	0.32	0.32	0.32	0.32	0.32	0.32	0.32	0.32	0.32	0.32	---	---	0.32
	86-87	---	0.69	0.69	0.69	0.69	0.69	0.69	0.69	0.69	0.69	---	---	0.69
	87-88	---	1.13	1.13	1.13	1.13	1.13	1.13	1.13	1.13	1.13	---	---	1.13
	88-89	---	0.97	1.22	1.44	2.08	2.17	1.96	1.75	1.75	2.17	---	---	1.86
	89-90	---	2.08	2.11	2.24	2.24	2.01	1.41	1.27	1.27	0.65	---	---	1.65
	90-91	0.51	0.51	0.41	0.61	0.61	0.61	0.51	0.54	0.64	---	---	---	0.59
	91-92	---	0.87	0.65	0.65	0.65	0.55	0.34	0.24	0.24	0.34	---	---	0.41
	92-93	-0.27	-0.27	-0.27	-0.16	-0.06	0.16	0.26	0.36	0.36	-0.16	---	---	0.08
	93-94	0.07	0.18	-0.03	-0.03	-0.03	-0.03	-0.03	-0.03	-0.03	-0.03	---	---	-0.03
	94-95	-0.12	-0.19	-0.24	-0.24	-0.24	-0.24	-0.24	---	---	---	---	---	-0.24
All Sales	85-86	2.71	2.54	2.85	3.41	2.97	2.70	2.86	3.20	3.65	3.85	---	---	3.09
	86-87	---	3.46	3.49	4.09	3.97	3.88	3.56	4.57	5.13	3.92	---	---	4.09
	87-88	---	4.97	5.31	5.64	4.96	4.13	3.60	3.66	2.30	1.75	---	---	3.85
	88-89	---	4.79	3.87	4.09	4.28	4.54	4.16	4.17	3.54	3.29	---	---	4.12
	89-90	---	4.47	4.19	5.04	4.53	3.92	3.24	3.22	2.82	2.56	---	---	3.68
	90-91	11.56	10.17	10.92	11.19	10.91	9.85	10.38	11.81	11.46	---	---	---	10.87
	91-92	---	3.79	3.24	3.08	2.45	1.94	1.51	1.53	1.67	1.59	---	---	1.99
	92-93	2.89	3.19	3.25	3.24	3.25	3.56	4.65	6.95	6.90	1.70	---	---	4.24
	93-94	4.59	4.65	4.14	4.35	3.82	3.06	3.14	2.74	1.78	2.42	---	---	3.16
	94-95	3.43	4.39	4.29	4.48	4.03	4.07	3.17	---	---	---	---	---	3.55

CALIFORNIA CITRUS FRUITS: Grower Returns, Month And Season Average Prices (Dollars Per Carton), 1985-95

Lemons, All	Crop Year	Aug.	Sept.	Oct.	Nov.	Dec.	Jan.	Feb.	Mar.	Apr.	May	June	July	Season Avg. Price
Fresh	85-86	18.41	19.41	15.01	9.76	7.91	7.41	5.71	5.26	5.71	5.51	7.86	8.51	8.51
	86-87	5.44	3.29	5.14	5.74	5.54	5.54	6.09	6.89	6.69	6.64	8.19	9.99	6.69
	87-88	11.21	10.66	8.01	6.31	5.31	4.76	5.41	6.86	7.21	7.96	9.96	11.06	8.16
	88-89	12.54	11.14	8.64	7.24	6.19	6.14	6.74	7.64	8.24	9.59	10.89	11.59	9.19
	89-90	12.38	12.63	12.33	9.38	7.58	8.03	9.23	10.03	10.28	10.58	11.18	11.53	10.63
	90-91	10.26	10.46	10.36	5.81	5.91	15.41	11.56	10.91	12.71	14.36	13.81	16.96	12.16
	91-92	16.38	16.78	14.13	11.58	4.78	6.23	7.18	7.68	8.63	8.73	9.38	9.63	9.93
	92-93	9.82	10.82	8.37	4.47	5.57	6.12	6.17	6.87	7.82	9.32	14.12	14.62	9.22
	93-94	17.25	16.90	13.15	9.15	6.10	4.95	4.25	5.35	7.15	8.55	12.30	16.00	10.10
	94-95	18.45	16.25	10.40	7.85	6.85	6.65	6.10	6.10	7.70	9.95	14.55	16.95	9.95
Processing	85-86	0.78	0.78	0.78	0.78	0.78	0.78	0.78	0.78	0.78	0.78	0.78	0.78	0.78
	86-87	0.83	0.83	0.83	0.83	0.83	0.83	0.83	0.83	0.83	0.83	0.83	0.83	0.83
	87-88	0.42	0.42	0.42	0.42	0.42	0.42	0.42	0.42	0.42	0.42	0.42	0.42	0.42
	88-89	0.42	0.42	0.42	1.11	1.13	1.03	1.03	1.05	1.06	1.07	1.07	1.07	0.95
	89-90	1.15	1.15	1.15	1.28	1.49	1.63	1.70	1.73	1.73	1.73	1.73	1.77	1.59
	90-91	1.78	1.81	1.81	1.64	1.19	1.02	1.02	1.02	1.02	1.02	1.02	1.02	1.29
	91-92	0.98	0.98	0.98	0.84	0.88	0.91	0.88	0.84	0.84	0.84	0.88	0.88	0.87
	92-93	0.87	0.90	0.88	1.01	1.01	1.01	1.01	1.01	1.01	0.94	0.94	0.94	0.97
	93-94	0.93	0.93	0.93	0.72	0.72	0.65	0.65	0.65	0.65	0.65	0.65	0.65	0.68
	94-95	0.65	0.65	0.65	0.51	0.51	0.51	0.51	0.51	0.51	0.51	0.51	0.51	0.52
All Sales	85-86	12.42	12.50	10.57	6.92	5.45	4.95	3.88	3.55	3.63	3.65	5.31	5.83	5.65
	86-87	3.45	2.00	2.94	2.71	2.40	2.60	2.91	3.11	3.14	3.93	5.71	6.80	3.56
	87-88	7.57	5.91	5.31	3.44	2.81	2.26	2.58	4.40	4.73	5.46	7.49	8.15	5.02
	88-89	9.56	7.60	4.85	4.37	3.83	3.91	4.06	4.45	5.52	7.49	8.98	9.29	6.19
	89-90	9.86	9.87	8.56	6.14	5.33	6.20	6.59	6.63	7.11	7.61	8.13	8.21	7.60
	90-91	7.11	7.58	6.89	3.90	3.12	4.64	6.98	8.68	10.51	12.00	10.89	12.68	8.19
	91-92	11.72	11.97	10.74	7.49	2.98	3.57	4.24	4.91	5.24	5.37	6.13	6.25	6.32
	92-93	6.29	7.22	4.72	2.59	2.78	3.23	3.14	3.11	3.56	5.17	9.54	10.59	4.97
	93-94	12.94	12.45	8.51	5.44	3.04	2.12	2.00	2.41	3.25	4.12	6.80	7.11	5.00
	94-95	15.69	11.91	6.37	4.84	3.14	3.78	2.80	3.12	3.61	6.43	11.15	11.35	5.57

Tangerines	Crop Year	Oct.	Nov.	Dec.	Jan.	Feb.	Mar.	Apr.	May	June	July	Aug.	Sept.	Season Avg. Price
Fresh	85-86	---	13.90	11.90	8.85	9.85	5.95	6.40	7.35	2.40	---	---	---	8.55
	86-87	---	13.47	8.67	7.17	7.92	6.72	5.92	7.32	---	---	---	---	7.62
	87-88	---	20.89	13.99	7.34	10.34	10.09	9.89	5.79	5.79	5.79	---	---	10.34
	88-89	---	14.98	11.43	9.38	9.63	8.43	6.43	6.63	6.63	---	---	---	9.33
	89-90	---	16.66	14.36	13.21	14.26	9.86	9.11	9.56	---	---	---	---	12.12
	90-91	---	13.79	11.59	12.34	12.99	15.84	14.44	14.39	---	---	---	---	13.13
	91-92	19.99	16.59	9.99	9.09	7.69	7.59	7.79	7.44	7.44	---	---	---	10.29
	92-93	13.92	8.52	8.07	8.17	8.27	9.52	9.22	---	---	---	---	---	8.72
	93-94	13.69	10.24	7.29	6.09	7.09	7.34	7.34	5.79	---	---	---	---	8.04
	94-95	13.95	10.40	7.85	9.30	10.75	11.25	13.25	13.25	---	---	---	---	10.15
Processing	85-86	---	0.21	0.21	0.21	0.21	0.21	0.21	0.21	0.21	---	---	---	0.21
	86-87	---	0.69	0.69	0.69	0.69	0.69	0.69	0.69	---	---	---	---	0.69
	87-88	---	---	0.69	0.69	0.69	0.69	0.69	0.69	0.69	0.69	---	---	0.69
	88-89	---	---	1.07	1.07	1.07	1.07	1.07	1.07	1.07	---	---	---	1.07
	89-90	---	0.67	0.93	1.35	1.53	1.55	1.67	1.67	1.67	---	---	---	1.40
	90-91	---	0.26	0.26	0.26	0.26	0.26	0.26	0.26	---	---	---	---	0.29
	91-92	---	0.41	0.46	0.62	0.62	0.53	0.38	0.38	0.46	---	---	---	0.52
	92-93	-0.28	-0.28	-0.28	-0.28	-0.28	-0.28	-0.28	-0.20	---	---	---	---	-0.28
	93-94	-0.16	-0.24	-0.24	-0.20		0.08	-0.09	-0.09	---	---	---	---	-0.06
	94-95	-0.16	-0.16	-0.08	-0.08	-0.16	-0.21	-0.25	-0.25	---	---	---	---	-0.14
All Sales	85-86	---	11.51	7.61	7.28	6.90	4.08	4.32	4.40	1.67	---	---	---	6.10
	86-87	---	10.59	6.61	6.75	6.17	4.38	3.89	5.52	---	---	---	---	5.76
	87-88	---	20.89	12.96	7.23	8.73	6.25	4.30	2.25	1.97	1.74	---	---	7.73
	88-89	---	14.98	9.71	8.76	7.40	5.53	4.56	2.79	2.59	---	---	---	7.04
	89-90	---	11.86	12.02	12.22	10.68	7.54	6.63	7.58	1.67	---	---	---	9.45
	90-91	---	10.23	10.01	10.89	2.30	10.55	9.72	7.33	---	---	---	---	9.09
	91-92	19.99	15.92	9.69	7.15	4.76	4.02	4.32	4.41	1.85	---	---	---	7.25
	92-93	11.68	7.30	6.11	5.04	5.12	6.31	1.63	-0.20	---	---	---	---	6.00
	93-94	12.06	9.59	6.43	3.51	4.39	3.71	4.37	3.83	---	---	---	---	5.58
	94-95	10.15	7.55	5.33	5.52	7.17	7.89	10.08	5.15	---	---	---	---	6.88

CALIFORNIA BERRIES: Monthly And Season Average Prices (Dollars Per Cwt.), 1986-95

Straw-berries 1/	Crop Year	Jan.	Feb.	Mar.	Apr.	May	June	July	Aug.	Sept.	Oct.	Nov.	Dec.	Season Avg. Price
Fresh	1986	99.60	85.60	69.00	52.90	43.00	53.20	55.00	83.70	82.50	144.00	122.00	---	58.30
Sales	1987	101.00	125.00	92.70	46.10	50.40	54.90	55.90	54.00	68.10	116.00	146.00	---	58.00
	1988	---	74.30	64.30	42.70	50.00	43.90	50.00	55.00	60.00	70.00	160.00	---	52.00
	1989	---	110.00	95.00	45.00	35.00	55.00	30.00	35.00	65.00	95.00	175.00	---	49.30
	1990	150.00	115.00	115.00	50.00	35.00	45.00	40.00	50.00	55.00	75.00	100.00	125.00	52.00
	1991	---	110.00	100.00	60.00	50.00	35.00	45.00	35.00	35.00	55.00	110.00	---	50.90
	1992	135.00	95.00	85.00	50.00	41.00	64.20	49.30	89.20	61.10	70.70	115.00	---	59.20
	1993	102.00	80.20	57.70	42.40	38.30	42.50	31.60	46.90	48.90	64.20	93.70	---	46.80
	1994	77.90	99.00	94.40	57.50	56.40	50.40	49.30	49.00	46.60	82.40	89.90	---	59.10
	1995	---	67.00	65.50	57.60	66.90	53.10	50.70	63.30	41.50	49.70	115.00	---	57.40

1/ Prices are F.O.B. shipping point.

CALIFORNIA RAISINS: Production And Average Grower Returns, By Variety, 1986-95

Raisins 1/	Crop Year	All Varieties	Natural Dried				Dehydrated, All Types
			Thompson Seedless	Muscats	Sultanas	Zante Currant	
		Tons					
Production	1986	277,000	247,750	600	350	3,300	25,000
	1987	356,000	325,900	400	300	3,000	26,400
	1988	363,000	329,600	300	300	4,100	28,700
	1989	430,000	396,000	100	300	3,900	29,700
	1990	393,000	357,400	600	250	4,450	30,300
	1991	345,000	313,500	230	270	4,200	26,800
	1992	388,300	346,850	50	280	4,720	36,400
	1993	382,000	338,000	170	330	5,700	37,800
	1994	418,600	378,400	230	270	5,800	33,900
	1995	306,000	1/97	1/97	1/97	1/97	1/97
		Dollars/Ton					
Average Grower Returns	1986	757	744	495	650	770	900
	1987	817	809	600	640	910	910
	1988	898	890	473	750	983	985
	1989	977	969	599	807	1,080	1,060
	1990	903	884	730	799	1,290	1,070
	1991	963	943	749	813	1,400	1,130
	1992	911	873	800	825	1,560	1,190
	1993	937	908	1,080	817	1,260	1,150
	1994	923	908	1,000	773	1,000	1,080
	1995	875	1/97	1/97	1/97	1/97	1/97

1/ Excludes raisins produced from table type grapes.

CALIFORNIA NON-CITRUS FRUITS: Unutilized Production, 1986-95

Crop	1986	1987	1988	1989	1990	1991	1992	1993	1994	1995
	Unharvested Tons									
Apricots	---	8,000	8,000	1,000	2,000	4,000	---	---	13,000	---
	Harvested Not Sold Tons									
Kiwifruit	900	2,400	3,200	3,000	5,000	2,800	4,600	4,600	1,900	6,100
Peaches, Clingstone	29,750	35,000	37,000	34,000	28,500	30,000	33,000	30,000	23,500	22,000
Pomegranate	2,600	2,000	2,000	1,000	---	---	---	---	---	---

CALIFORNIA LIVESTOCK AND DAIRY

SUMMARY OF LIVESTOCK INVENTORY AND CASH RECEIPTS

The increase in California's milk and cream cash receipts offset declines in most other livestock. Livestock cash receipts totaled $5.55 billion, 1% above 1994.

Red meat production, at 2.12 billion pounds, was 1% above last year's production. Beef production, at 1.97 billion pounds, was up 1% from a year ago. Lamb and mutton production of 60.1 million pounds was up 17%. Pork production of 88.6 million pounds was 7% below a year ago.

Poultry production, at 1.70 billion pounds, increased 4% from 1994. Broiler production of 1.18 billion pounds increased 4% from the previous year. Turkey production was 3% higher at 462 million pounds. The 54.4 million pounds of other chickens produced was up 3% from a year ago.

Milk production, at 25.3 billion pounds, was fractionally above a year ago. Egg production, at 6.44 billion eggs, was down 2%. Honey production of 39.1 million pounds increased by 63% from the 1994 production year. Wool production of 5.25 million pounds was 1% less than 1994.

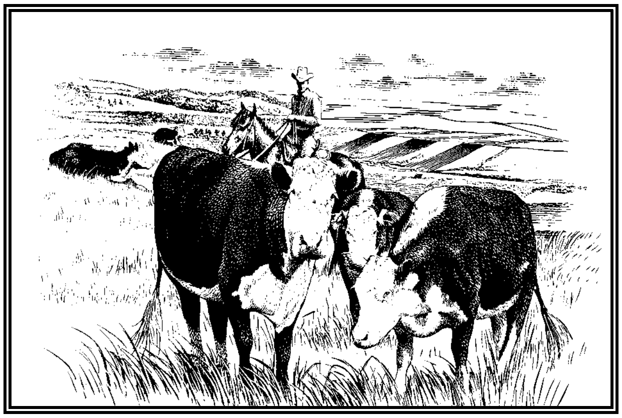

CALIFORNIA LIVESTOCK CASH RECEIPTS

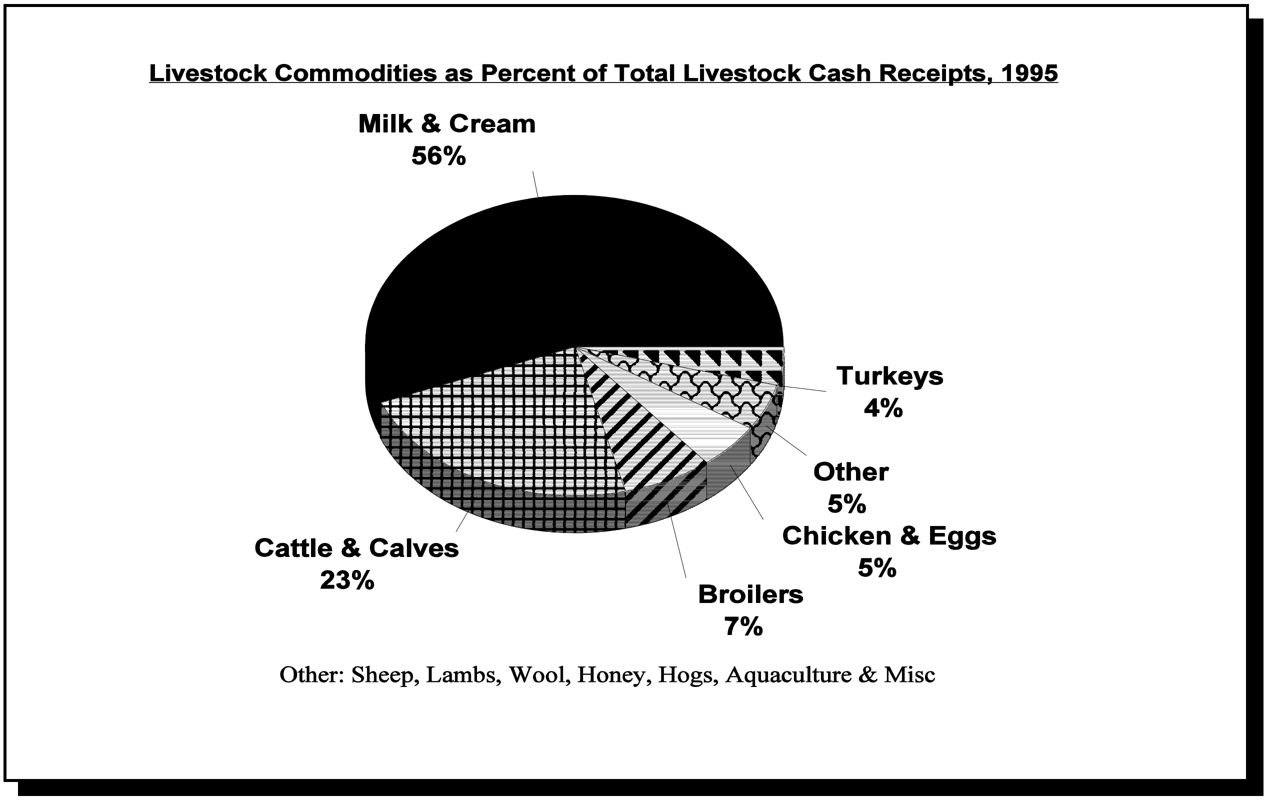

CALIFORNIA CATTLE BY CLASS, JANUARY 1, 1987-96

Year	Cows That Have Calved			Heifers 500 Lbs. & Over			Other Cattle			
	Beef	Dairy	All	Replacements		Other Heifers	Calves Under 500 Lbs.	Bulls 500 Lbs. & Over	Steers 500 Lbs. & Over	All Cattle & Calves
				Beef Cow	Milk Cow					
	1,000 Head									
1987	970	1,062	2,032	155	470	175	1,068	65	785	4,750
1988	920	1,071	1,991	155	475	164	1,020	65	780	4,650
1989	925	1,095	2,020	150	485	190	985	65	805	4,700
1990	955	1,115	2,070	155	525	185	945	70	750	4,700
1991	900	1,150	2,050	155	505	185	1,015	70	620	4,600
1992	880	1,160	2,040	145	555	190	920	70	630	4,550
1993	840	1,200	2,040	140	560	190	930	70	670	4,600
1994	810	1,220	2,030	140	590	160	980	70	580	4,550
1995	840	1,250	2,090	160	585	180	970	75	590	4,650
1996	840	1,260	2,100	160	600	170	950	70	550	4,600

CALIFORNIA CATTLE INVENTORY, SUPPLY AND DISPOSITION, 1986-95

Year	Beginning Inventory January 1	Calf Crop	Inshipments	Marketings 1/		Farm Slaughter Cattle & Calves 2/	Deaths		Ending Inventory January 1
				Cattle	Calves		Cattle	Calves	
	1,000 Head								
1986	5,000	1,780	1,220	2,554	380	26	100	190	4,750
1987	4,750	1,740	846	2,123	286	25	90	162	4,650
1988	4,650	1,770	867	2,100	247	25	75	140	4,700
1989	4,700	1,770	850	2,170	228	17	70	135	4,700
1990	4,700	1,750	737	2,185	178	19	75	130	4,600
1991	4,600	1,700	728	2,069	180	19	70	140	4,550
1992	4,550	1,720	630	1,930	150	20	75	125	4,600
1993	4,600	1,700	600	1,927	180	18	70	155	4,550
1994	4,550	1,750	700	1,961	162	17	75	135	4,650
1995	4,650	1,780	800	2,135	252	18	90	135	4,600

1/ Includes custom slaughter for use on farms where produced, but excludes interfarm sales.
2/ Excludes custom slaughter for farmers at commercial establishments.

CALIFORNIA CATTLE OPERATIONS AND INVENTORY, PERCENTAGE BY SIZE GROUPS, 1986-95

Year	1 - 99 Head		100 - 499 Head		500-999 Head 1/		1,000+ Head	
	Operation	Inventory	Operation	Inventory	Operation	Inventory	Operation	Inventory
	Percent							
1986	80.6	8.4	13.2	23.2	6.2	68.4	N/A	N/A
1987	80.0	8.5	13.6	24.4	6.4	67.1	N/A	N/A
1988	79.4	8.4	13.6	23.4	7.0	68.2	N/A	N/A
1989	77.7	7.7	14.7	22.3	7.6	70.0	N/A	N/A
1990	76.9	8.0	15.2	21.0	7.9	71.0	N/A	N/A
1991	77.3	7.5	14.5	20.5	8.2	72.0	N/A	N/A
1992	76.5	6.7	14.6	19.3	8.9	74.0	N/A	N/A
1993	76.8	6.5	14.0	18.5	4.8	18.5	4.4	56.5
1994	75.8	6.0	14.6	18.0	5.0	19.0	4.6	57.0
1995	75.6	6.0	14.8	18.0	5.2	20.0	4.4	56.0

1/ Starting in 1993, class 500+ was broken out into classes 500-999 and 1,000+.

CALIFORNIA BEEF COW OPERATIONS AND INVENTORY, PERCENTAGE BY SIZE GROUPS, 1989-95

Year	1 - 99 Head		100-499 Head 1/		500+ Head	
	Operations	Inventory	Operations	Inventory	Operations	Inventory
	Percent					
1989	86.7	25.0	13.3	75.0	N/A	N/A
1990	87.2	25.0	12.8	75.0	N/A	N/A
1991	87.6	28.0	12.4	72.0	N/A	N/A
1992	86.7	25.0	13.3	75.0	N/A	N/A
1993	86.0	24.0	12.0	43.0	2.0	33.0
1994	86.0	24.0	12.0	44.0	2.0	32.0
1995	86.3	24.0	11.9	44.0	1.9	32.0

1/ Classed as 100+ head for 1989-92.

CALIFORNIA CATTLE AND CALVES MARKETED FROM FEEDLOTS, 1986-95

Year	January-March	April-June	July-September	October-December	Total
	1,000 Head				
1986	203	190	220	226	839
1987	185	160	200	220	765
1988	205	200	210	235	850
1989	240	240	200	250	930
1990	220	185	190	220	815
1991	200	180	180	170	730
1992	180	165	140	155	640
1993	165	150	135	135	585
1994	130	150	145	155	580
1995	150	160	150	135	595

CALIFORNIA CATTLE AND CALVES SLAUGHTERED UNDER FEDERAL AND STATE INSPECTION, 1986-95

Year	Jan.	Feb.	Mar.	Apr.	May	June	July	Aug.	Sept.	Oct.	Nov.	Dec.	Annual Total 1/
	1,000 Head												
CATTLE													
1986	131	104	112	127	136	134	145	133	128	135	109	126	1,521
1987	120	97	107	114	107	122	124	114	106	109	98	110	1,328
1988	105	100	110	100	102	108	103	111	94	95	93	95	1,215
1989	99	89	98	90	106	101	93	99	94	102	97	92	1,158
1990	105	90	101	97	109	104	100	105	86	97	91	87	1,172
1991	101	88	92	95	95	91	95	93	83	91	77	81	1,082
1992	87	71	77	72	71	80	75	70	74	77	68	77	899
1993	73	67	72	72	70	75	74	72	70	71	71	71	857
1994	70	62	73	69	74	80	74	80	75	79	83	82	901
1995	82	72	82	72	79	89	80	89	82	89	88	81	985
CALVES													
1986	29	27	28	30	31	30	30	24	25	24	21	26	325
1987	26	24	28	23	22	24	24	22	21	18	19	21	272
1988	19	18	19	16	14	13	14	15	13	11	10	12	174
1989	13	11	11	8	9	9	9	10	9	8	7	8	112
1990	10	9	9	7	7	7	8	7	6	7	7	8	92
1991	11	8	9	6	5	6	7	7	6	6	6	6	84
1992	8	7	7	6	6	7	7	8	7	7	7	8	83
1993	8	7	7	6	5	5	6	6	5	5	5	6	70
1994	6	5	6	6	7	8	9	9	8	13	15	14	106
1995	16	12	14	14	21	21	19	19	16	13	15	18	196

1/ Totals may not equal sum of parts due to rounding.

CALIFORNIA AVERAGE LIVE WEIGHTS OF CATTLE AND CALVES SLAUGHTERED UNDER FEDERAL AND STATE INSPECTIONS, 1986-95

Year	Jan.	Feb.	Mar.	Apr.	May	June	July	Aug.	Sept.	Oct.	Nov.	Dec.	Annual Avg.
	Pounds												
CATTLE													
1986	1,125	1,129	1,129	1,112	1,102	1,103	1,130	1,113	1,134	1,140	1,145	1,135	1,124
1987	1,149	1,146	1,131	1,114	1,107	1,111	1,115	1,114	1,132	1,144	1,152	1,148	1,130
1988	1,156	1,168	1,166	1,161	1,159	1,148	1,145	1,160	1,147	1,164	1,169	1,171	1,159
1989	1,169	1,172	1,166	1,166	1,151	1,155	1,170	1,179	1,191	1,204	1,198	1,189	1,176
1990	1,202	1,207	1,216	1,188	1,195	1,180	1,174	1,187	1,204	1,216	1,222	1,229	1,201
1991	1,235	1,244	1,238	1,223	1,227	1,227	1,224	1,234	1,249	1,258	1,242	1,244	1,237
1992	1,260	1,253	1,244	1,236	1,234	1,232	1,228	1,224	1,234	1,243	1,251	1,251	1,241
1993	1,222	1,206	1,211	1,210	1,223	1,210	1,213	1,219	1,238	1,253	1,247	1,245	1,225
1994	1,264	1,261	1,232	1,245	1,235	1,213	1,215	1,213	1,206	1,224	1,223	1,236	1,230
1995	1,231	1,222	1,207	1,228	1,240	1,237	1,226	1,223	1,232	1,243	1,233	1,229	1,229
CALVES													
1986	167	168	167	185	172	178	190	177	164	167	177	157	173
1987	169	161	166	175	182	183	174	171	189	185	191	197	177
1988	183	201	190	212	237	242	216	211	219	232	226	196	211
1989	197	203	199	204	197	218	213	225	235	245	229	213	213
1990	220	277	246	223	221	221	245	237	241	229	232	260	239
1991	252	273	287	285	291	311	326	322	301	280	305	292	291
1992	282	274	285	280	289	271	295	297	298	317	326	334	296
1993	314	334	345	345	342	351	369	351	335	362	377	360	349
1994	366	373	370	368	377	360	352	344	367	347	314	352	352
1995	400	320	377	332	296	290	322	326	338	338	370	364	337

CALIFORNIA AVERAGE PRICES RECEIVED FOR BEEF CATTLE, STEERS AND HEIFERS, COWS AND CALVES, 1986-95

Year	Jan.	Feb.	Mar.	Apr.	May	June	July	Aug.	Sept.	Oct.	Nov.	Dec.	Annual Average
	Dollars Per Cwt.												
**ALL BEEF CATTLE**													
1986	49.40	47.80	46.30	42.40	43.40	42.40	45.60	47.20	49.70	49.90	48.90	49.40	46.70
1987	50.50	52.80	53.80	55.50	57.10	56.60	56.50	57.50	60.00	58.20	56.00	59.30	56.30
1988	59.10	62.60	63.00	63.70	64.70	61.80	59.50	62.50	62.30	60.10	59.50	60.60	61.80
1989	59.60	62.10	63.50	60.30	63.40	61.70	62.70	63.40	62.00	62.30	62.70	62.10	62.20
1990	61.70	64.20	64.80	63.90	66.90	69.10	67.00	66.50	64.20	64.40	64.50	62.80	65.00
1991	60.90	63.50	69.20	62.30	68.60	68.20	61.80	63.70	62.40	62.20	63.40	60.60	64.10
1992	57.70	60.90	58.70	56.90	58.50	59.80	58.10	61.00	61.30	59.40	59.70	56.10	59.10
1993	66.50	66.10	65.50	66.00	66.00	67.70	65.80	65.10	63.60	61.60	61.80	62.10	64.80
1994	64.00	65.90	64.50	59.40	58.80	56.80	55.30	56.00	52.20	50.50	51.30	51.40	57.40
1995	54.10	55.00	52.00	48.40	47.90	48.40	45.80	46.30	43.50	42.20	41.00	42.20	47.00
**BEEF STEERS AND HEIFERS**													
1986	58.30	56.10	55.30	51.50	50.20	48.30	52.50	54.30	56.00	56.80	56.80	57.80	54.30
1987	56.90	60.50	61.20	64.20	66.40	65.80	63.90	65.50	67.70	67.50	64.90	67.30	64.50
1988	66.70	69.00	70.10	72.50	73.90	70.10	67.50	70.60	71.20	70.60	72.00	70.80	70.40
1989	73.20	74.00	75.30	72.80	74.00	73.20	74.20	75.00	71.50	73.00	74.80	73.80	73.70
1990	75.00	77.40	76.50	78.20	78.40	80.50	75.40	77.00	74.00	74.20	74.30	76.70	76.40
1991	75.70	76.20	81.70	77.40	80.50	76.50	73.40	75.00	73.90	77.40	74.10	71.40	76.30
1992	75.60	77.90	73.50	70.80	71.30	72.90	74.20	75.20	75.10	75.70	77.10	77.70	74.50
1993	81.00	79.00	79.20	78.70	79.40	83.00	81.00	79.50	79.00	78.50	76.50	78.50	79.40
1994	80.00	79.80	78.30	71.50	69.70	68.00	66.70	66.20	62.60	61.50	63.20	64.30	69.20
1995	68.80	66.20	62.30	57.90	57.20	57.30	55.00	54.60	51.80	51.00	51.50	53.00	57.00
**BEEF COWS 1/**													
1986	34.60	36.40	36.70	31.60	33.00	33.60	34.80	36.60	37.50	36.80	36.10	36.90	35.30
1987	40.00	42.50	44.30	44.20	43.40	43.40	44.00	44.80	46.20	45.00	44.60	45.70	44.00
1988	46.40	48.10	48.20	47.90	48.10	45.50	45.70	47.00	47.60	45.50	44.80	44.50	46.70
1989	44.70	47.60	48.40	46.60	46.20	46.10	45.80	47.80	49.20	47.50	45.90	47.20	46.90
1990	48.90	51.70	52.80	51.80	51.90	51.90	54.60	52.90	50.80	47.50	45.60	47.30	50.60
1991	47.20	50.40	49.10	48.70	52.00	49.80	47.30	48.60	48.60	46.50	44.00	44.80	48.20
1992	46.70	47.10	46.30	46.40	45.10	46.70	45.80	46.60	44.30	43.70	44.70	44.50	45.70
1993	46.20	46.90	46.50	46.70	46.00	46.50	46.40	45.20	44.30	43.30	43.50	42.50	45.40
1994	43.70	46.00	45.40	44.60	43.10	40.00	40.30	41.30	38.50	36.40	34.80	35.00	41.10
1995	35.50	38.80	37.80	35.80	33.90	35.50	33.70	34.40	32.40	31.00	27.00	29.00	33.50
**CALVES**													
1986	53.60	53.20	53.80	50.40	49.60	50.10	51.30	52.70	56.20	58.20	55.90	56.40	53.70
1987	61.00	66.00	68.30	68.40	72.40	73.70	72.40	75.10	77.60	74.40	71.10	71.10	71.60
1988	79.80	85.40	86.80	84.70	85.20	78.10	76.30	82.60	84.90	82.20	84.30	83.10	82.70
1989	84.90	86.50	82.80	83.90	82.10	82.50	83.00	81.10	82.90	87.90	81.40	78.10	83.20
1990	88.00	87.50	92.20	93.00	91.90	94.70	84.50	87.60	83.30	85.80	86.00	91.40	89.40
1991	89.10	94.20	98.30	93.20	97.50	101.00	90.70	87.50	85.70	85.30	93.60	85.60	93.10
1992	87.10	89.70	95.70	91.00	90.40	85.30	87.10	83.80	81.80	89.00	92.60	92.10	89.30
1993	93.60	92.70	98.30	97.30	95.50	95.80	92.70	91.50	91.00	93.00	94.00	93.00	94.30
1994	94.70	95.50	97.10	89.10	86.90	80.00	79.00	80.00	75.80	73.90	76.20	78.70	85.60
1995	81.50	82.20	78.50	72.20	71.50	71.00	66.90	64.30	62.60	62.50	61.00	63.00	69.40

1/ Beef cows sold for slaughter and herd replacement and cull dairy cows sold for slaughter.

CALIFORNIA MILK COWS, PRODUCTION AND PRICE, 1986-95

Year	Milk Cows & Heifers Calved		Annual Production Per Milk Cow		Total Annual Production On Farms		Wholesale Milk Price
	January 1	Average During Year	Milk	Milk Fat	Milk	Milk Fat	
	1,000 Head		*Pounds*		*Million Pounds*		*$/Cwt.*
1986	1,076	1,070	16,095	584	17,222	625	11.77
1987	1,062	1,061	16,897	613	17,928	651	11.43
1988	1,071	1,083	17,181	629	18,607	681	10.94
1989	1,095	1,104	17,591	644	19,420	711	12.37
1990	1,115	1,135	18,456	668	20,947	758	12.02
1991	1,150	1,155	18,534	678	21,407	784	11.22
1992	1,160	1,180	18,722	685	22,092	809	11.61
1993	1,200	1,210	18,948	693	22,927	839	11.45
1994	1,220	1,235	20,439	746	25,242	921	11.52
1995	1,250	1,254	20,197	735	25,327	922	11.68

CALIFORNIA MILK COW OPERATIONS AND INVENTORY, PERCENTAGE BY SIZE GROUPS, 1986-95

Year	1 - 49 Head		50 - 99 Head		100-199 Head 1/		200+ Head	
	Operation	Inventory	Operation	Inventory	Operation	Inventory	Operation	Inventory
	Percent							
1986	58.7	1.0	6.7	2.7	34.6	96.3	N/A	N/A
1987	55.0	0.9	7.0	2.8	38.0	96.3	N/A	N/A
1988	49.8	0.8	7.7	2.6	42.5	96.6	N/A	N/A
1989	47.7	0.8	7.6	2.4	44.7	96.8	N/A	N/A
1990	45.3	0.6	8.0	2.0	46.7	97.4	N/A	N/A
1991	40.9	0.6	6.7	1.6	52.4	97.8	N/A	N/A
1992	40.9	0.6	6.7	1.7	52.4	97.7	N/A	N/A
1993	43.0	0.6	4.5	1.2	7.5	3.7	45.0	94.5
1994	39.5	0.6	4.4	1.1	8.9	3.3	47.2	95.0
1995	31.6	0.4	4.5	0.9	9.4	3.7	54.5	95.0

1/ Starting in 1993, class 100+ was broken out into classes 100-199 and 200+.

CALIFORNIA AVERAGE PRICES RECEIVED FOR MILK COWS, 1986-95 1/

Year	Jan.	Apr.	July	Oct.	Annual Average
	Dollars Per Head				
1986	1,000	965	1,020	1,010	1,000
1987	1,050	1,090	1,120	1,160	1,110
1988	1,170	1,210	1,180	1,170	1,180
1989	1,210	1,200	1,190	1,210	1,200
1990	1,280	1,260	1,320	1,360	1,310
1991	1,240	1,200	1,200	1,250	1,220
1992	1,260	1,280	1,320	1,310	1,290
1993	1,270	1,320	1,320	1,330	1,310
1994	1,340	1,320	1,230	1,290	1,300
1995	1,270	1,230	1,230	1,190	1,230

1/ For dairy herd replacement.

CALIFORNIA MANUFACTURED DAIRY PRODUCTS

Commodity	1993	1994	1995
	1,000 Pounds		
Butter	333,468	344,657	342,765
All Cheese Total	848,436	922,887	921,461
American Cheese Total	334,445	351,149	345,952
Cheddar	240,810	259,626	250,058
Monterey, Jack and Other	93,635	91,523	95,894
Italian Cheese Total	448,552	499,087	503,318
Mozzarella	404,679	450,837	443,648
Other Italian	43,873	48,250	59,670
All Other Cheese	65,439	72,651	72,191
Cream Cheese	20,157	N/A	N/A
Creamed and Low Fat Cottage Cheese Total	90,426	92,290	74,475
Creamed	34,688	32,746	26,474
Low Fat	55,738	59,544	48,001
Curd Cottage Cheese Total	59,977	61,072	47,031
Yogurt, Plain and Flavored	284,944	286,766	251,651
Milk, Nonfat Dry	415,496	429,761	442,466
Milk, Unsweetened Condensed Skim	484,182	525,493	516,518
	1,000 Gallons		
Ice Cream	109,453	111,194	110,069
Lowfat Ice Cream Total 1/	36,159	33,328	32,926
Hard	13,875	16,344	18,172
Soft	22,284	16,984	14,754
Milk Sherbet	5,610	5,076	6,665
Water Ices	4,093	5,516	4,318

1/ Previously referred to as Ice Milk.

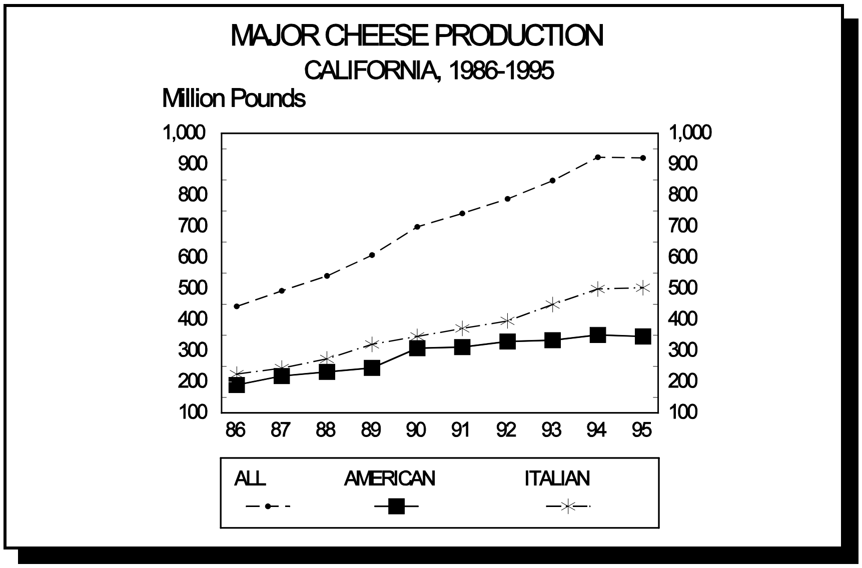

CALIFORNIA SHEEP AND LAMBS BY CLASS, JANUARY 1, 1987-96

Year	All Sheep 1/	Sheep & Lambs On Feed For Market 1/	Stock Sheep			
			Total	Ewes 1 Yr. +	Replacement Lambs	Wethers & Rams 1 Yr. +
	1,000 Head					
1987	980	185	795	685	85	25
1988	970	170	800	685	90	25
1989	940	160	780	632	124	24
1990	1,000	225	775	620	129	26
1991	1,015	280	735	576	132	27
1992	995	285	710	562	125	23
1993	895	305	590	450	117	23
1994	1,120	600	520	400	100	20
1995	1,060	520	540	430	90	20
1996	1,000	520	480	390	75	15

1/ Beginning in 1994, totals include new crop lambs.

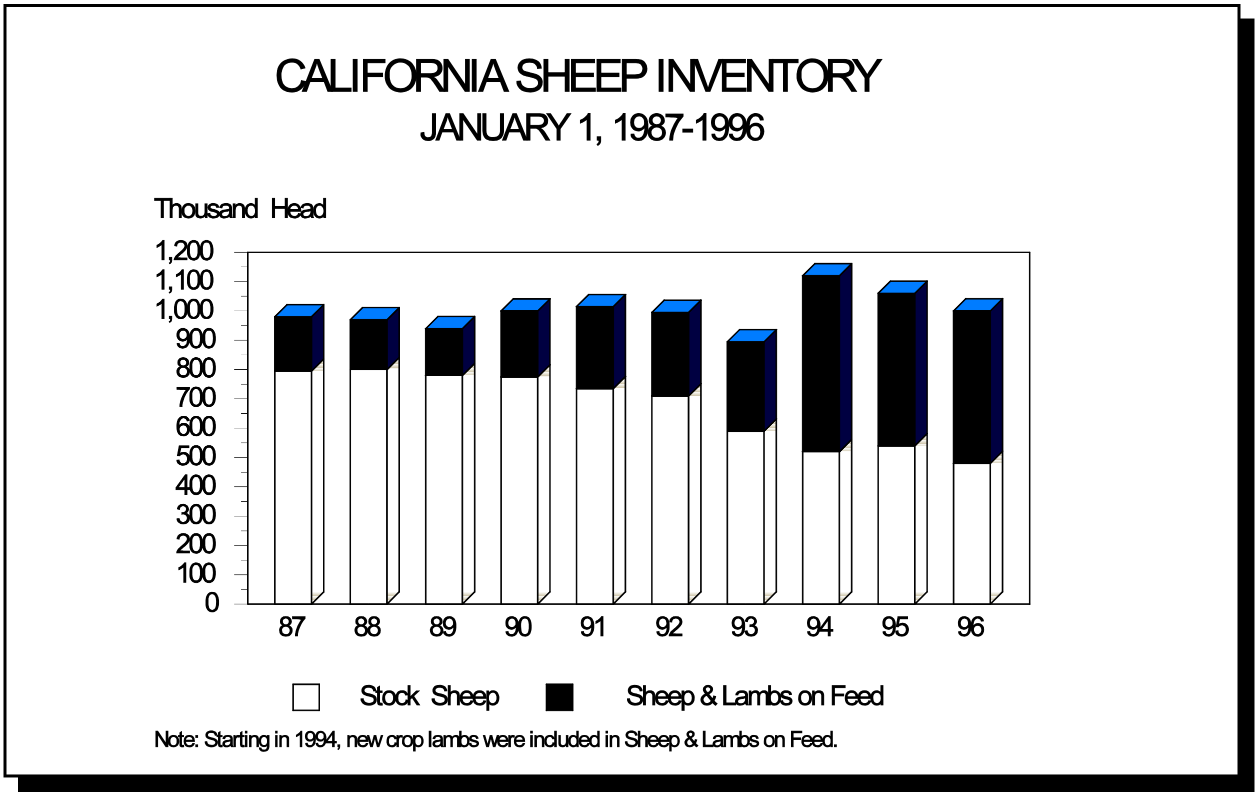

CALIFORNIA SHEEP AND LAMBS INVENTORY, SUPPLY AND DISPOSITION, 1986-95

Year	Beginning Inventory January 1 2/	Lamb Crop	Inshipments	Marketings 1/		Farm Slaughter Sheep & Lambs	Deaths		Ending Inventory January 1 2/
				Sheep	Lambs		Sheep	Lambs	
	1,000 Head								
1986	1,065	630	198	111	685	24	43	50	980
1987	980	625	298	43	797	13	30	50	970
1988	970	590	213	75	673	10	37	38	940
1989	940	550	268	97	580	8	37	36	1,000
1990	1,000	535	260	144	566	8	32	30	1,015
1991	1,015	520	248	130	583	7	34	34	995
1992	995	530	285	217	624	8	34	32	895
1993	895	390	469	139	718	5	25	27	840
1994	1,120	365	370	85	656	6	24	24	1,060
1995	1,060	380	380	120	649	5	29	17	1,000

1/ Includes custom slaughter for use on farms where produced, but excludes interfarm sales within the State.
2/ Beginning in 1994, totals include new crop lambs.

CALIFORNIA SHEEP AND LAMBS SHORN, WOOL PRODUCTION AND VALUE, 1986-95

Year	Sheep and Lambs Shorn	Wool Produced		Wool Value Per Pound	Total Wool Value
		Per Animal	Total		
	1,000 Head	*Pounds*	*1,000 Pounds*	*Dollars*	*$1,000*
1986	1,088	7.6	8,229	0.660	5,431
1987	1,020	7.1	7,241	0.870	6,300
1988	955	7.4	7,103	1.430	10,157
1989	990	7.7	7,624	1.370	10,445
1990	1,015	7.5	7,646	0.820	6,270
1991	930	7.7	7,160	0.560	4,010
1992	880	7.7	6,780	0.670	4,543
1993	800	7.6	6,044	0.460	2,780
1994	695	7.7	5,318	0.650	3,457
1995	690	7.6	5,250	1.020	5,355

CALIFORNIA SHEEP AND LAMBS AVERAGE PRICES RECEIVED, 1986-95

Year	Jan.	Feb.	Mar.	Apr.	May	June	July	Aug.	Sept.	Oct.	Nov.	Dec.	Annual Avg. 1/
	Dollars Per Cwt.												
SHEEP													
1986	30.00	24.70	23.60	22.20	19.20	23.60	24.90	23.30	22.90	22.30	21.10	23.40	23.20
1987	24.30	25.40	25.30	24.40	22.40	21.90	24.10	26.10	24.70	25.80	29.60	32.70	25.10
1988	34.40	27.70	30.60	21.50	19.20	20.40	27.60	21.40	19.70	19.30	19.00	20.90	23.60
1989	30.50	27.20	30.50	22.20	17.80	18.90	19.00	20.10	20.20	17.50	19.90	26.10	21.10
1990	26.20	27.30	27.10	21.50	16.90	14.40	19.30	18.50	17.70	15.70	25.20	22.60	18.90
1991	20.50	17.30	18.90	22.00	18.00	15.20	20.40	17.10	20.70	20.20	20.40	22.60	17.90
1992	27.00	26.60	29.20	27.30	20.40	19.70	20.70	20.90	21.60	22.80	28.00	30.60	22.50
1993	30.00	31.10	32.00	29.00	26.00	24.50	24.00	26.00	25.00	23.00	24.00	27.50	25.80
1994	32.20	32.70	31.50	29.10	27.10	29.10	24.30	30.80	29.00	27.70	26.70	35.80	29.10
1995	29.90	34.70	28.60	26.10	22.40	24.00	25.10	24.00	24.00	23.70	22.00	27.00	24.80
LAMBS													
1986	64.30	65.00	64.30	65.10	72.80	74.00	71.60	70.30	66.80	64.50	75.80	72.50	68.60
1987	73.50	73.80	76.00	80.00	85.60	84.10	78.20	76.50	71.90	63.30	59.90	67.00	74.90
1988	75.70	82.50	74.60	81.00	77.30	51.30	57.30	58.10	61.80	63.80	65.50	67.10	70.30
1989	67.10	67.40	67.40	75.80	74.40	72.70	70.40	67.30	68.20	64.80	67.30	67.80	69.30
1990	68.00	63.80	68.40	63.30	61.10	58.80	58.90	56.30	57.30	54.90	53.30	51.10	60.10
1991	51.90	47.10	51.70	55.30	59.70	53.50	58.10	54.10	55.20	52.10	53.30	53.80	53.80
1992	56.40	58.40	64.10	71.80	72.00	66.20	65.70	57.20	59.40	57.20	59.50	65.40	62.90
1993	65.10	71.70	74.50	71.00	66.00	62.00	60.00	59.00	63.00	62.50	64.50	65.50	65.70
1994	60.90	58.80	58.30	55.30	54.40	61.90	71.50	76.00	74.80	73.80	72.70	76.70	65.60
1995	69.60	71.00	76.70	77.00	78.70	84.00	86.00	86.50	83.00	80.00	78.00	78.50	79.20

1/ Average weighted by amount sold each month.

CALIFORNIA SHEEP AND LAMBS SLAUGHTERED UNDER FEDERAL AND STATE INSPECTION, 1986-95

Year	Jan.	Feb.	Mar.	Apr.	May	June	July	Aug.	Sept.	Oct.	Nov.	Dec.	Annual Total
	1,000 Head												
1986	75	77	95	100	84	66	72	74	74	60	46	55	877
1987	64	75	69	80	70	73	83	72	81	76	66	74	883
1988	66	75	86	69	69	71	66	70	59	54	55	46	785
1989	52	61	70	50	57	49	51	64	55	53	55	53	670
1990	55	61	69	55	68	63	58	a/	---	---	---	---	---
1991	---	---	---	---	---	---	---	---	---	---	---	---	---
1992	---	---	---	---	---	---	---	---	---	---	---	---	---
1993	---	---	---	---	---	---	---	---	---	---	---	---	---
1994	---	---	---	---	---	---	---	---	---	---	---	---	---
1995	---	---	---	---	---	---	---	---	---	---	---	---	---

a/ Information not published after July 1990 to avoid disclosing individual operations.

CALIFORNIA AVERAGE LIVE WEIGHTS OF SHEEP AND LAMBS SLAUGHTERED UNDER FEDERAL AND STATE INSPECTIONS, 1986-95

Year	Jan.	Feb.	Mar.	Apr.	May	June	July	Aug.	Sept.	Oct.	Nov.	Dec.	Annual Avg.
	Pounds												
1986	119	123	122	117	116	112	112	116	117	119	116	120	117
1987	118	128	129	122	119	118	122	124	126	126	125	127	124
1988	127	131	133	129	127	124	128	120	121	121	118	124	126
1989	124	123	153	125	123	118	123	118	122	122	122	123	125
1990	123	131	128	126	126	124	119	a/	---	---	---	---	---
1991	---	---	---	---	---	---	---	---	---	---	---	---	---
1992	---	---	---	---	---	---	---	---	---	---	---	---	---
1993	---	---	---	---	---	---	---	---	---	---	---	---	---
1994	---	---	---	---	---	---	---	---	---	---	---	---	---
1995	---	---	---	---	---	---	---	---	---	---	---	---	---

a/ Information not published after July 1990 to avoid disclosing individual operations.

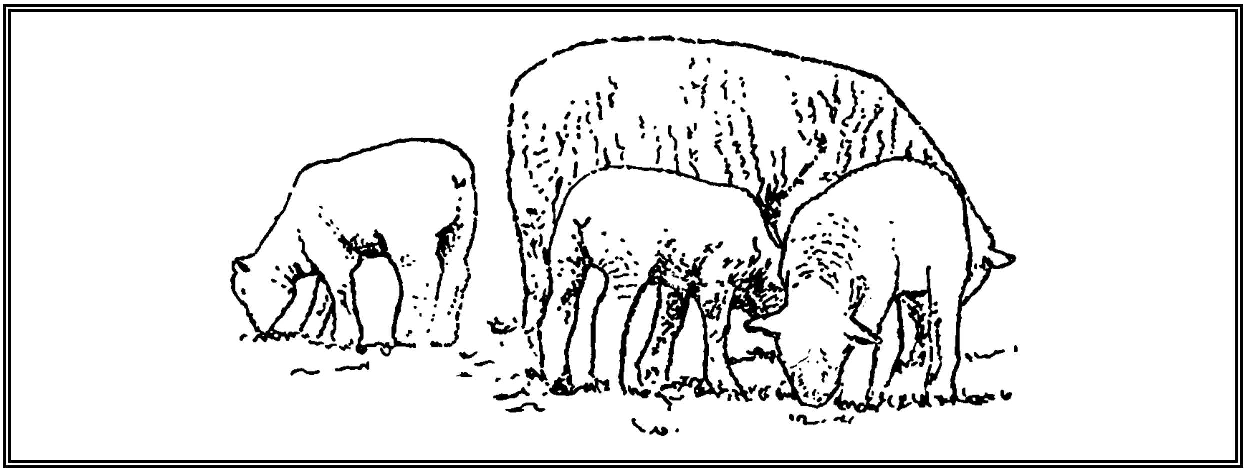

CALIFORNIA HOGS AND PIGS BY CLASS, DECEMBER 1, 1986-95

Year	Total Hogs & Pigs	Breeding Hogs & Pigs	Market Hogs: Under 60 Pounds	Market Hogs: 60-119 Pounds	Market Hogs: 120-179 Pounds	Market Hogs: 180 Pounds & Over	Market Hogs: Total
	1,000 Head						
1986	150	27	53	31	21	18	123
1987	140	25	49	28	21	17	115
1988	130	23	45	28	19	15	107
1989	140	23	42	40	19	16	117
1990	195	28	60	49	31	27	167
1991	215	29	64	51	40	31	186
1992	265	37	82	58	45	43	228
1993	260	33	74	60	49	44	227
1994	255	32	70	65	45	43	223
1995	240	31	65	58	44	42	209

CALIFORNIA SOWS FARROWED AND PIGS SAVED, 1986-95

Year	December - May: Sows Farrowed	December - May: Pigs Saved	December - May: Pigs Per Litter	June - November: Sows Farrowed	June - November: Pigs Saved	June - November: Pigs Per Litter
	1,000 Head		*Number*	*1,000 Head*		*Number*
1986	18	133	7.4	17	131	7.7
1987	17	131	7.7	17	131	7.7
1988	18	133	7.4	16	119	7.4
1989	15	112	7.5	16	120	7.5
1990	22	167	7.6	24	191	8.0
1991	23	188	8.2	25	199	8.0
1992	24	202	8.4	28	221	7.9
1993	29	238	8.2	29	246	8.5
1994	28	242	8.6	25	221	8.8
1995	25	223	8.9	23	199	8.7

CALIFORNIA HOGS AND PIGS INVENTORY, SUPPLY AND DISPOSITION, 1986-95

Year	Beginning Inventory Dec. 1 (Prev. Yr.)	Pig Crop: Dec.-May	Pig Crop: June-Nov.	Inshipments 1/	Marketings 2/	Farm Slaughter 3/	Deaths	Ending Inventory December 1
	1,000 Head							
1986	145	133	131	21	250	14	16	150
1987	150	131	131	20	262	14	16	140
1988	140	133	119	32	258	18	18	130
1989	130	112	120	32	224	15	15	140
1990	140	167	191	44	310	20	17	195
1991	195	188	199	31	357	22	19	215
1992	215	202	221	55	398	15	30	265
1993	265	238	246	40	462	25	42	260
1994	260	242	221	40	448	21	39	255
1995	255	223	199	40	413	24	40	240

1/ For feeding or breeding, excludes stock brought in for immediate slaughter.
2/ Includes custom slaughter for use on farms where produced and State outshipments, but excludes interfarm sales.
3/ Excludes custom slaughter for farmers at commercial establishments.

CALIFORNIA HOGS AND PIGS SLAUGHTERED UNDER FEDERAL AND STATE INSPECTION, 1986-95

Year	Jan.	Feb.	Mar.	Apr.	May	June	July	Aug.	Sept.	Oct.	Nov.	Dec.	Annual Total 1/
	1,000 Head												
1986	158	135	154	162	152	148	153	106	127	161	127	152	1,734
1987	150	134	149	148	142	147	146	139	158	163	157	183	1,816
1988	170	173	187	164	164	173	161	199	190	185	184	188	2,137
1989	195	165	200	170	189	184	165	194	179	179	169	171	2,159
1990	179	151	167	157	169	133	137	164	138	159	150	153	1,859
1991	159	144	148	151	155	142	155	164	152	164	155	162	1,850
1992	169	145	166	157	151	160	158	152	160	167	141	161	1,888
1993	145	141	151	150	145	163	155	162	160	165	155	163	1,856
1994	163	160	159	156	151	164	146	166	168	158	156	177	1,924
1995	169	141	176	152	177	164	155	175	157	164	163	171	1,964

1/ Totals may not equal sum of parts due to rounding.

CALIFORNIA AVERAGE LIVE WEIGHTS OF HOGS AND PIGS SLAUGHTERED UNDER FEDERAL AND STATE INSPECTIONS, 1986-95

Year	Jan.	Feb.	Mar.	Apr.	May	June	July	Aug.	Sept.	Oct.	Nov.	Dec.	Annual Avg.
	Pounds												
1986	228	224	227	227	227	220	219	218	218	221	218	225	223
1987	226	226	226	225	226	225	222	222	223	228	229	227	225
1988	228	227	226	226	228	226	227	223	224	231	230	229	227
1989	225	223	226	227	227	224	221	222	219	224	224	222	224
1990	219	221	223	222	224	220	218	219	221	222	224	223	221
1991	223	219	224	223	222	220	220	219	222	224	226	224	222
1992	229	221	227	232	223	226	220	221	221	225	228	220	224
1993	222	229	223	222	228	224	226	221	221	224	225	223	224
1994	223	222	219	227	221	227	217	217	216	224	224	229	222
1995	225	224	235	238	241	231	224	223	215	219	224	218	227

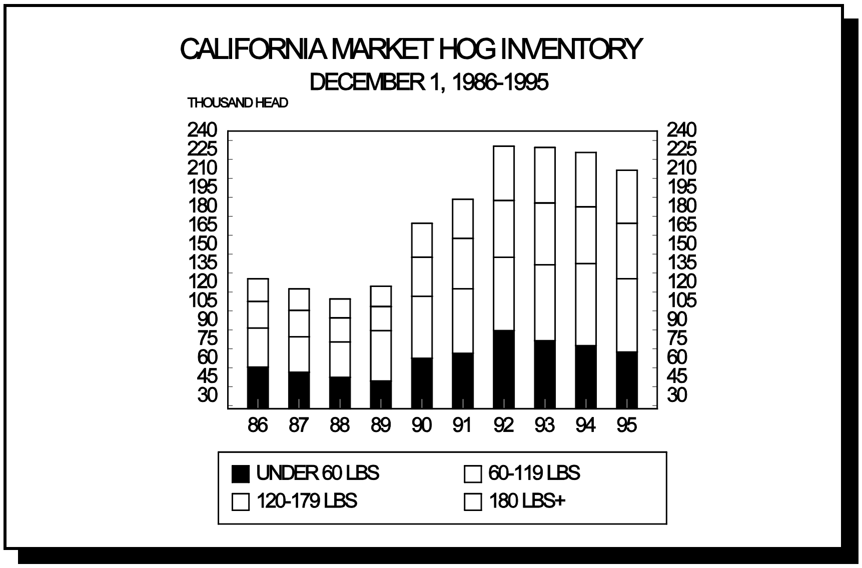

CALIFORNIA HENS AND PULLETS OF LAYING AGE (HPLA), AND EGG PRODUCTION, 1994-95

Month	Average Number of HPLA			Eggs Per 100 Layers			Total Egg Production		
	1994	1995	1995/1994	1994	1995	1995/1994	1994	1995	1995/1994
	Thousands		*Percent*	*Number*		*Percent*	*Millions*		*Percent*
December 1/	27,196	27,859	101	2,173	2,164	100	591	592	100
January	26,363	26,565	101	2,090	2,108	101	551	560	102
February	25,619	26,100	102	1,913	1,893	99	490	494	101
March	25,751	26,073	101	2,225	2,178	98	573	568	99
April	25,851	25,536	99	2,097	2,091	100	542	534	99
May	25,900	24,893	96	2,127	2,141	101	551	533	97
June	25,600	24,145	94	2,094	2,096	100	536	506	94
July	25,222	23,581	93	2,165	2,192	101	546	517	95
August	25,477	23,900	94	2,178	2,184	100	555	522	94
September	25,757	24,638	96	2,069	2,102	102	533	518	97
October	26,211	25,348	97	2,156	2,174	101	565	551	98
November	26,967	25,636	95	2,110	2,142	102	569	549	96
Avg./Total	25,995	25,312	97	2,116	2,122	100	6,602	6,444	98

1/ December of previous year.

CALIFORNIA EGG PRODUCTION AND VALUE, 1986-95

Year	Eggs Produced	Price Per Dozen	Value of Production
	Millions	*Cents*	*$1,000*
1986	7,850	53.1	347,362
1987	8,023	46.0	307,548
1988	7,917	46.2	304,805
1989	7,579	65.9	416,213
1990	7,711	63.3	406,755
1991	7,721	58.4	375,755
1992	7,224	47.6	286,552
1993	7,029	49.1	287,603
1994	6,602	46.4	255,277
1995	6,444	53.7	288,869

CALIFORNIA BROILER PRODUCTION AND VALUE, 1986-95

Year	Number Produced	Average Live Weight	Pounds Produced	Price Per Pound 1/	Value of Production 2/
	1,000 Head	*Pounds*	*1,000 Pounds*	*Cents*	*$1,000*
1986	184,832	4.7	868,710	36.0	312,736
1987	196,120	4.7	921,764	37.0	341,053
1988	212,199	4.7	997,335	34.0	339,094
1989	223,130	4.7	1,048,711	34.5	361,805
1990	231,100	4.8	1,109,280	30.5	338,330
1991	240,200	4.8	1,152,960	29.0	334,358
1992	233,000	5.1	1,188,300	29.5	350,549
1993	216,000	5.1	1,101,600	32.0	352,512
1994	226,200	5.0	1,131,000	33.0	373,230
1995	235,800	5.0	1,179,000	32.5	383,175

1/ Live weight equivalent price.
2/ Gross income including home consumption.

CALIFORNIA TURKEY PRODUCTION AND VALUE, 1986-95

Year	Number Raised	Pounds Produced 1/	Price Per Pound 2/	Value of Production
	1,000 Head	*1,000 Pounds*	*Cents*	*$1,000*
1986	21,900	464,280	45.0	208,926
1987	25,500	545,700	33.0	180,081
1988	26,500	572,400	35.0	200,340
1989	30,200	664,400	38.0	252,472
1990	31,500	711,900	38.0	270,522
1991	29,000	652,500	37.0	241,425
1992	24,000	508,800	39.0	198,432
1993	22,500	486,000	43.0	208,980
1994	21,000	449,400	43.0	193,242
1995	22,000	462,000	46.0	212,520

1/ Includes home consumption.
2/ Live weight equivalent price.

CALIFORNIA HONEY PRODUCTION, NUMBER OF COLONIES, AND VALUE, 1988-95

Year	Honey Producing Colonies	Yield Per Colony	Production	Average Price Per Pound	Value of Production
	1,000	Pounds	1,000 Pounds	Cents	$1,000
1988	600	40	24,000	49	11,760
1989	620	34	21,080	49	10,329
1990	480	42	20,160	54	10,886
1991	550	63	34,650	54	18,711
1992	490	67	32,830	52	17,072
1993	500	90	45,000	50	22,500
1994	400	60	24,000	51	12,240
1995	420	93	39,060	60	23,436

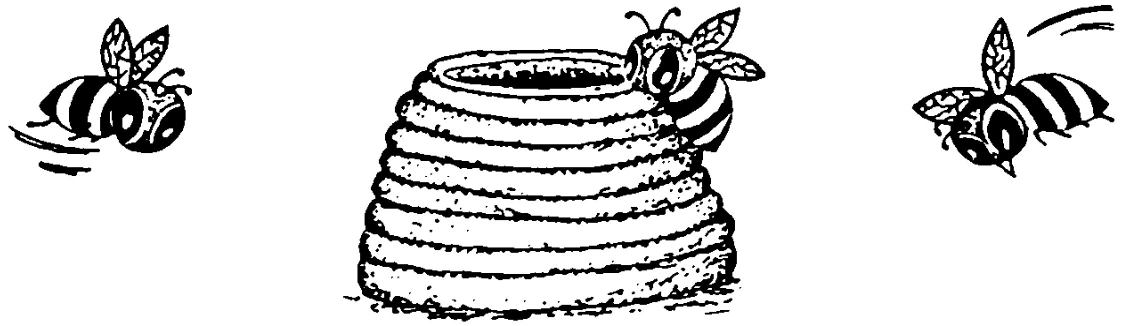

CALIFORNIA LIVESTOCK PRODUCTION AND INCOME, 1986-95

Year	Production 1/	Marketings 2/	Average Price Per 100 Pounds		Value of Production	Cash Receipts 3/ 4/	Value of Home Consumptio n	Gross Income
	1,000 Pounds		Dollars		$1,000			
TOTAL MEAT ANIMALS								
1986	1,983,133	3,020,041	---	---	945,784	1,433,008	22,937	1,455,945
1987	1,958,050	2,542,454	---	---	1,131,449	1,457,460	22,365	1,479,825
1988	2,196,792	2,707,013	---	---	1,362,906	1,678,329	26,352	1,704,681
1989	2,170,550	2,748,174	---	---	1,348,649	1,711,412	20,339	1,731,751
1990	2,029,815	2,809,866	---	---	1,322,423	1,820,131	21,950	1,842,081
1991	2,116,743	2,754,502	---	---	1,344,967	1,762,881	22,451	1,782,332
1992	2,071,286	2,601,468	---	---	1,224,197	1,532,069	19,387	1,551,456
1993	2,008,139	2,636,088	---	---	1,306,167	1,705,789	20,902	1,726,691
1994	2,089,703	2,646,374	---	---	1,173,763	1,528,090	12,863	1,540,951
1995	2,117,427	2,891,254	---	---	981,043	1,394,572	11,891	1,406,462
CATTLE AND CALVES								
			(Cattle)	(Calves)				
1986	1,851,090	2,873,100	46.70	53.70	868,352	1,347,044	18,302	1,365,346
1987	1,817,500	2,382,400	56.30	71.60	1,035,305	1,350,012	18,819	1,368,831
1988	2,070,710	2,557,200	61.80	82.70	1,287,672	1,590,632	23,240	1,613,872
1989	2,056,490	2,619,260	62.20	83.20	1,286,428	1,639,190	17,158	1,656,348
1990	1,890,920	2,654,880	65.00	89.40	1,245,792	1,736,037	18,447	1,754,484
1991	1,965,580	2,586,300	64.10	93.10	1,265,374	1,672,249	18,104	1,690,353
1992	1,908,900	2,409,800	59.10	89.30	1,142,883	1,437,117	16,461	1,453,578
1993	1,838,210	2,425,610	64.80	94.30	1,215,321	1,590,174	16,884	1,607,058
1994	1,943,243	2,460,650	57.40	85.60	1,101,603	1,428,205	10,168	1,438,373
1995	1,968,665	2,703,670	47.00	69.40	907,035	1,289,765	9,218	1,298,983
SHEEP AND LAMBS								
			(Sheep)	(Lambs)				
1986	73,668	93,115	23.20	68.60	46,988	57,830	2,114	59,944
1987	81,478	102,502	25.10	74.90	61,907	74,034	1,305	75,339
1988	72,224	93,609	23.60	70.30	50,091	61,250	990	62,240
1989	66,478	81,868	21.10	69.30	40,542	50,605	1,022	51,627
1990	67,224	87,706	18.90	60.10	34,833	44,583	840	45,423
1991	65,796	88,063	17.90	53.80	30,438	40,984	571	41,555
1992	68,420	104,322	22.50	62.90	37,471	53,520	832	54,352
1993	68,096	110,702	25.80	65.70	39,644	65,078	571	65,649
1994	51,215	89,832	29.10	65.60	27,842	54,699	745	55,443
1995	60,148	98,218	24.80	79.20	41,379	68,920	793	69,712
HOGS AND PIGS								
1986	58,375	53,826	52.20	---	30,444	28,134	2,521	30,655
1987	59,072	57,552	58.00	---	34,237	33,414	2,241	35,655
1988	53,585	56,204	47.00	---	25,143	26,447	2,122	28,569
1989	47,582	47,046	45.70	---	21,679	21,617	2,159	23,776
1990	71,671	67,280	58.40	---	41,798	39,511	2,663	42,174
1991	85,367	80,139	57.90	---	49,155	49,648	3,776	50,424
1992	93,966	87,346	47.10	---	43,843	41,432	2,094	43,526
1993	101,833	99,776	50.40	---	51,202	50,537	3,447	53,984
1994	95,245	95,892	46.70	---	44,318	45,186	1,950	47,135
1995	88,614	89,366	39.70	---	32,629	35,887	1,880	37,767

1/ Live weight; adjustments made for changes in inventory and for inshipments.
2/ Live weight; excludes custom slaughter for use on farms where produced and interfarm sales within the State.
3/ Receipts from marketing and sale of farm slaughter.
4/ Hogs and pigs receipts for 1994 and 1995 include allowance for higher average price of State inshipments and outshipments of feeder pigs.

CALIFORNIA VEGETABLES AND MELONS

SUMMARY OF VEGETABLE AND MELON CROPS

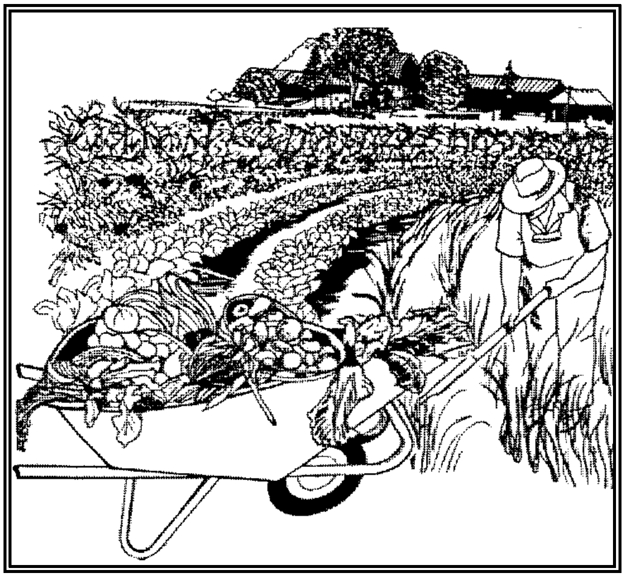

California produced 19.8 million tons of vegetables and melons in 1995, 2% less than in 1994. Total value of vegetable production was $5.55 billion, up 13% from 1994. Spring onions had the greatest percentage increase in value, which was 95% above 1994. Only fresh market artichokes, asparagus, garlic, bell peppers, and tomatoes, as well as processing broccoli and carrots, had lower total values than a year ago.

The Golden State continues to lead the Nation in vegetables and melons, accounting for 36% of the harvested acres, 54% of the production, and 63% of the total value.

The vegetable and melon estimates include commercial production, excluding garden production and vegetables grown for seed. Prices for processing crops and processed segments of dual utilization crops are based upon equivalent returns at the processing plant door.

For fresh market sales, the price is on a packed and loaded basis, F.O.B. shipping point. Not all data are published for California to avoid disclosure of individual plants.

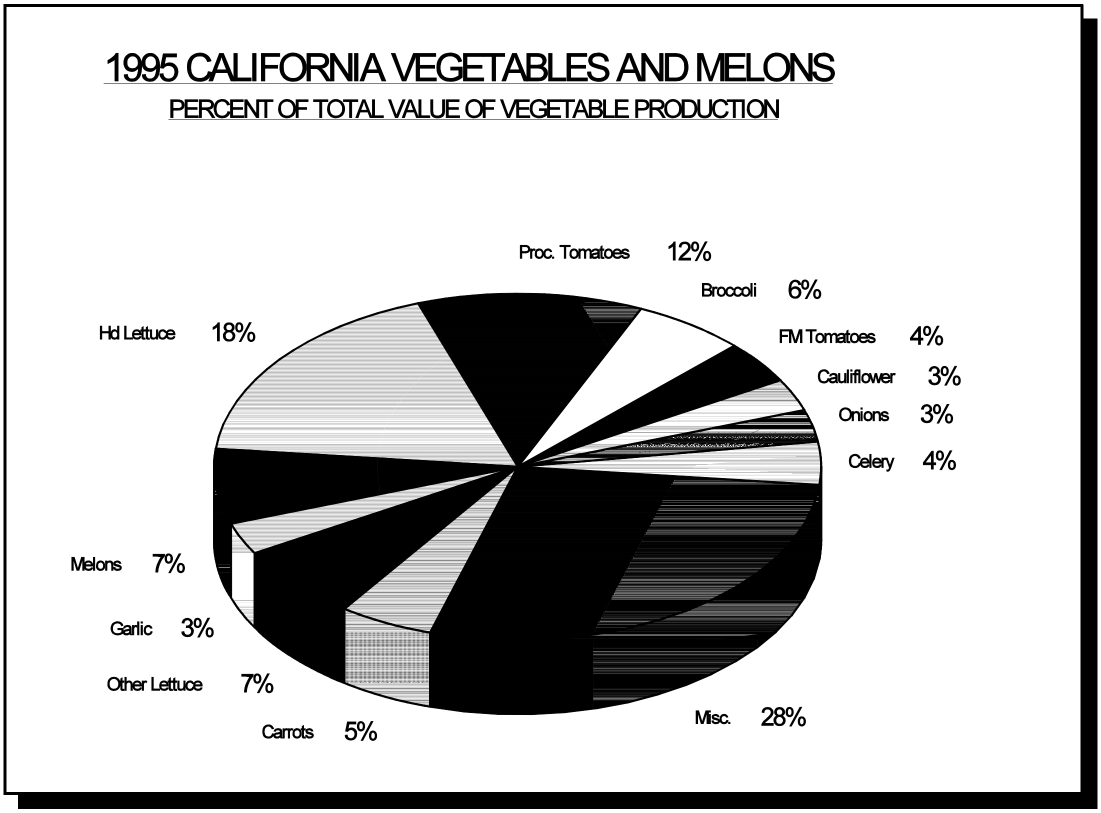

CALIFORNIA VEGETABLES AND MELONS: Processing Crops, 1986-95

Crop	Year	Harvested Acreage	Average Yield	Production	Value Per Ton		Total Value
					First Delivery Point	Processing Plant Door	
		Acres	*Tons*		*Dollars*		*$1,000*
**TOMATOES**	1986	210,400	30.80	6,480,320	51.00	62.20	403,076
For	1987	214,000	31.32	6,702,480	46.40	57.20	383,382
	1988	226,100	28.96	6,547,860	48.20	58.90	385,669
	1989	276,500	31.05	8,585,330	55.50	68.30	586,378
	1990	310,000	30.02	9,306,200	55.20	66.30	617,001
	1991	312,000	31.71	9,893,520	52.90	64.70	640,111
	1992	240,000	33.05	7,932,000	46.10	56.40	447,365
	1993	274,000	32.67	8,951,580	48.30	59.10	529,038
	1994	311,000	34.56	10,748,160	50.90	61.00	655,638
	1995	317,000	33.46	10,606,820	54.00	63.40	672,472
**CUCUMBERS**	1986	4,000	16.17	64,680	N/A	200.00	12,936
For Pickles	1987	4,100	15.00	61,500	N/A	198.00	12,177
	1988	3,800	18.16	69,010	N/A	213.00	14,699
	1989	4,300	12.20	52,460	N/A	247.00	12,958
	1990	4,700	13.30	62,510	N/A	214.00	13,377
	1991	4,200	13.01	54,640	N/A	230.00	12,567
	1992	2,500	16.10	40,250	N/A	209.00	8,412
	1993	4,200	10.24	43,010	N/A	192.00	8,258
	1994	4,900	9.26	45,370	N/A	183.00	8,303
	1995	5,200	8.10	42,120	N/A	383.00	16,132

CALIFORNIA VEGETABLES AND MELONS: Fresh Market And Dual Usage Crops, 1986-95

Crop	Year	Harvested Acreage	Average Yield	Production	Value Per Cwt. a/	Total Value
		Acres	*Cwt.*	*1,000 Cwt.*	*Dollars*	*$1,000*
**ARTICHOKES b/**	1992	9,600	115	1,104	39.40	43,447
	1993	8,300	110	913	50.10	45,762
	1994	8,500	130	1,105	55.70	61,577
	1995	8,300	57	473	72.50	34,299
**ASPARAGUS**	1986	37,800	29	1,096	68.90	75,513
	1987	39,700	30	1,191	62.80	74,746
	1988	40,100	29	1,163	71.70	83,431
	1989	37,500	29	1,088	66.20	71,978
	1990	35,900	29	1,041	68.00	70,833
	1991	33,500	28	938	78.10	73,258
	1992	34,000	29	986	92.70	91,386
	1993	32,100	29	931	94.70	88,177
	1994	30,200	33	997	102.00	101,694
	1995	28,000	28	784	117.00	91,728
**BEANS, SNAP b/**	1992	6,900	90	621	27.40	17,015
Fresh Market	1993	7,300	80	584	49.90	29,142
	1994	7,500	70	525	57.20	30,030
	1995	8,800	90	792	51.00	40,392
**BROCCOLI**	1986	---	---	7,667	21.40	164,074
Fresh Market	1987	---	---	7,830	21.40	167,562
	1988	---	---	9,167	23.90	219,091
	1989	---	---	9,792	20.40	199,757
	1990	---	---	9,113	22.30	203,220
	1991	---	---	8,560	21.60	184,896
	1992	---	---	9,625	23.50	226,188
	1993	---	---	8,625	26.20	225,975
	1994	---	---	10,119	28.10	284,344
	1995	---	---	9,780	30.60	299,268

See Footnotes on Page 48.

CALIFORNIA VEGETABLES AND MELONS: Fresh Market And Dual Usage Crops, 1986-95

Crop	Year	Harvested Acreage	Average Yield	Production	Value Per Cwt. a/	Total Value
		Acres	Cwt.	1,000 Cwt.	Dollars	$1,000
BROCCOLI Processing	1986	---	---	2,760	18.10	49,956
	1987	---	---	2,500	18.00	45,000
	1988	---	---	2,460	19.10	46,863
	1989	---	---	2,400	18.40	44,160
	1990	---	---	2,100	19.80	41,475
	1991	---	---	1,560	19.70	30,654
	1992	---	---	1,530	19.30	29,529
	1993	---	---	1,140	19.20	21,888
	1994	---	---	1,221	16.10	19,726
	1995	---	---	960	14.10	18,336
BROCCOLI Total	1986	106,400	98	10,427	20.50	214,030
	1987	107,600	96	10,330	20.60	212,562
	1988	101,100	115	11,627	22.90	265,954
	1989	101,600	120	12,192	20.00	243,917
	1990	97,500	115	11,213	21.80	244,695
	1991	88,000	115	10,120	21.30	215,550
	1992	97,000	115	11,155	22.90	255,717
	1993	93,000	105	9,765	25.40	247,863
	1994	94,500	120	11,340	26.80	304,070
	1995	89,500	120	10,740	29.60	317,604
BRUSSELS SPROUTS b/	1992	3,500	160	560	30.50	17,104
	1993	5,000	160	800	31.00	24,761
	1994	3,500	180	630	24.60	15,471
	1995	3,800	170	646	25.50	16,452
CABBAGE b/ Fresh Market	1992	11,500	335	3,853	9.64	37,143
	1993	12,000	360	4,320	13.70	59,184
	1994	10,000	385	3,850	13.40	51,590
	1995	13,000	370	4,810	15.70	75,517
CARROTS c/ Fresh Market	1986	---	---	10,960	13.00	142,480
	1987	---	---	14,600	10.60	154,760
	1988	---	---	12,022	13.00	156,286
	1989	---	---	14,675	13.70	201,048
	1990	---	---	15,852	11.20	177,542
	1991	---	---	14,240	14.00	199,360
	1992	---	---	14,940	13.90	207,666
	1993	51,200	300	15,400	11.10	170,940
	1994	58,300	325	18,948	11.50	218,118
	1995	63,500	290	18,415	15.10	278,067
CARROTS c/ Processing	1986	---	---	1,798	4.05	7,282
	1987	---	---	1,400	4.33	6,055
	1988	---	---	1,520	4.18	6,346
	1989	---	---	2,515	3.23	8,136
	1990	---	---	2,100	4.25	8,925
	1991	---	---	2,000	4.78	9,550
	1992	---	---	2,160	4.35	9,396
	1993	6,800	294	2,000	4.85	9,700
	1994	4,100	618	2,532	3.81	9,635
	1995	5,100	478	2,440	3.69	9,003
CARROTS Total	1986	40,500	315	12,758	11.70	149,762
	1987	50,000	320	16,000	10.10	160,815
	1988	51,100	265	13,542	12.00	162,632
	1989	57,300	300	17,190	12.20	209,184
	1990	56,100	320	17,952	10.40	186,467
	1991	56,000	290	16,240	12.90	208,910
	1992	60,000	285	17,100	12.70	217,062
	1993	58,000	300	17,400	10.40	180,640
	1994	62,400	344	21,480	10.60	227,753
	1995	68,600	305	20,855	13.80	287,070

See Footnotes on Page 48.

CALIFORNIA VEGETABLES AND MELONS: Fresh Market And Dual Usage Crops, 1986-95

Crop	Year	Harvested Acreage	Average Yield	Production	Value Per Cwt. a/	Total Value
		Acres	Cwt.	1,000 Cwt.	Dollars	$1,000
CAULIFLOWER Fresh Market	1986	---	---	4,670	28.50	133,095
	1987	---	---	4,661	28.40	132,372
	1988	---	---	5,340	27.60	147,384
	1989	---	---	5,378	27.80	149,508
	1990	---	---	5,506	24.80	136,549
	1991	---	---	5,110	26.40	134,904
	1992	---	---	d/	d/	d/
	1993	---	---	d/	d/	d/
	1994	---	---	d/	d/	d/
	1995	---	---	d/	d/	d/
CAULIFLOWER Processing	1986	---	---	1,160	14.80	17,110
	1987	---	---	960	15.40	14,784
	1988	---	---	900	15.70	14,130
	1989	---	---	660	17.50	11,550
	1990	---	---	650	25.20	16,380
	1991	---	---	350	29.00	10,150
	1992	---	---	d/	d/	d/
	1993	---	---	d/	d/	d/
	1994	---	---	d/	d/	d/
	1995	---	---	d/	d/	d/
CAULIFLOWER Total	1986	53,000	110	5,830	25.80	150,205
	1987	51,100	110	5,621	26.20	147,156
	1988	48,000	130	6,240	25.90	161,514
	1989	52,500	115	6,038	26.70	161,058
	1990	51,300	120	6,156	24.80	152,929
	1991	42,000	130	5,460	26.60	145,054
	1992	42,000	130	5,460	28.40	155,001
	1993	43,000	125	5,375	29.60	159,245
	1994	42,000	130	5,460	28.30	154,594
	1995	40,700	125	5,088	33.70	171,559
CELERY	1986	21,400	593	12,683	11.70	147,764
	1987	21,300	595	12,671	10.80	136,348
	1988	20,300	654	13,277	11.10	147,740
	1989	21,800	670	14,612	12.70	184,842
	1990	24,800	597	14,812	10.80	159,999
	1991	20,900	670	14,003	10.30	143,739
	1992	23,500	670	15,750	12.10	190,445
	1993	24,500	640	15,680	14.70	230,545
	1994	24,500	630	15,435	12.60	194,181
	1995	22,700	660	14,982	16.40	246,229
CORN, SWEET Fresh Market	1986	15,000	100	1,500	16.10	24,150
	1987	16,000	95	1,520	13.30	20,216
	1988	18,000	90	1,620	15.10	24,462
	1989	17,500	115	2,013	16.10	32,409
	1990	20,000	95	1,900	16.70	31,730
	1991	16,300	120	1,956	16.30	31,883
	1992	18,500	130	2,405	16.00	38,480
	1993	20,000	140	2,800	17.80	49,840
	1994	21,600	140	3,024	18.30	55,339
	1995	22,300	150	3,345	18.70	62,552
CUCUMBERS b/ Fresh Market	1992	4,500	300	1,350	28.30	38,205
	1993	5,600	250	1,400	27.10	37,940
	1994	5,000	250	1,250	24.90	31,125
	1995	5,200	315	1,638	26.30	43,079
GARLIC b/	1992	23,000	165	3,795	27.10	102,854
	1993	26,000	160	4,160	31.80	132,470
	1994	27,000	170	4,590	39.40	181,038
	1995	31,000	165	5,115	35.20	179,834

See Footnotes on Page 48.

CALIFORNIA VEGETABLES AND MELONS: Fresh Market And Dual Usage Crops, 1986-95

Crop	Year	Harvested Acreage	Average Yield	Production	Value Per Cwt. a/	Total Value
		Acres	Cwt.	1,000 Cwt.	Dollars	$1,000
LETTUCE Head	1986	145,500	290	42,195	11.50	485,243
	1987	157,100	315	49,487	15.10	747,254
	1988	166,700	310	51,677	12.20	630,459
	1989	168,400	340	57,256	11.70	669,895
	1990	162,200	345	55,959	12.20	682,700
	1991	152,000	335	50,920	11.80	600,856
	1992	147,000	360	52,920	13.00	687,960
	1993	141,000	360	50,760	16.50	837,540
	1994	145,000	330	47,850	14.60	698,610
	1995	136,000	295	40,120	24.60	986,952
LETTUCE Leaf b/	1992	32,000	215	6,880	22.40	154,112
	1993	35,000	205	7,175	27.70	198,748
	1994	33,000	225	7,425	28.10	208,643
	1995	35,000	210	7,350	30.70	225,645
LETTUCE Romaine b/	1992	15,500	300	4,650	18.10	84,165
	1993	19,000	280	5,320	18.60	98,952
	1994	21,000	280	5,880	21.40	125,832
	1995	24,000	300	7,200	24.00	172,800
MELONS Cantaloupe	1986	79,100	160	12,656	12.40	156,934
	1987	85,100	150	12,765	11.50	146,798
	1988	84,000	140	11,760	15.45	181,692
	1989	80,800	190	15,352	12.60	193,435
	1990	90,200	150	13,530	9.20	124,476
	1991	N/A	N/A	N/A	N/A	N/A
	1992	64,000	180	11,520	14.00	161,280
	1993	65,000	190	12,350	15.30	188,955
	1994	59,300	190	11,267	15.90	179,145
	1995	59,300	230	13,639	17.40	237,319
Honeydew	1986	19,000	200	3,800	11.00	41,800
	1987	20,600	180	3,708	11.90	44,125
	1988	21,300	170	3,621	13.10	47,435
	1989	21,300	190	4,047	11.80	47,755
	1990	19,000	180	3,420	15.80	54,036
	1991	18,200	140	2,548	15.60	39,749
	1992	17,500	180	3,150	13.00	40,950
	1993	16,500	160	2,640	16.70	44,088
	1994	18,100	155	2,806	16.10	45,177
	1995	21,100	200	4,220	18.70	78,914
Watermelon b/	1992	15,000	400	6,000	8.72	52,320
	1993	17,500	420	7,350	8.04	59,094
	1994	16,700	430	7,181	9.91	71,164
	1995	17,200	370	6,364	14.30	91,005
MUSHROOMS (Agaricus)	1986	518	2,064	1,069	97.70	104,444
	1987	495	2,242	1,110	110.00	122,064
	1988	501	2,248	1,126	97.90	110,189
	1989	529	2,440	1,291	100.00	129,457
	1990	545	2,440	1,330	96.00	127,701
	1991	532	2,453	1,305	97.60	127,446
	1992	488	2,498	1,219	99.40	121,208
	1993	528	2,434	1,285	101.00	129,866
	1994	501	2,475	1,240	105.57	130,911
	1995	503	2,682	1,349	104.37	140,796
ONIONS Spring	1986	7,800	370	2,886	10.20	29,437
	1987	8,200	390	3,198	17.50	55,965
	1988	8,500	390	3,315	9.71	32,189
	1989	7,700	400	3,080	11.30	34,804
	1990	8,500	420	3,570	11.10	39,627
	1991	8,000	395	3,160	18.00	56,880
	1992	9,000	400	3,600	11.80	42,480
	1993	7,500	440	3,300	18.00	59,400
	1994	6,700	440	2,948	8.02	23,643
	1995	7,500	440	3,300	14.00	46,200

See Footnotes on Page 48.

CALIFORNIA VEGETABLES AND MELONS: Fresh Market And Dual Usage Crops, 1986-95

Crop	Year	Harvested Acreage	Average Yield	Production	Value Per Cwt. a/	Total Value
		Acres	*Cwt.*	*1,000 Cwt.*	*Dollars*	*$1,000*
ONIONS Summer e/	1986	26,900	370	9,953	7.87	76,765
	1987	29,000	340	9,860	7.64	72,840
	1988	28,800	365	10,512	5.69	58,193
	1989	27,000	375	10,125	7.54	74,867
	1990	30,500	380	11,590	7.49	85,067
	1991	28,600	370	10,582	7.06	73,510
	1992	27,500	375	10,313	8.26	82,946
	1993	33,000	395	13,035	8.04	102,268
	1994	31,000	410	12,710	6.18	78,485
	1995	30,500	415	12,658	6.67	84,466
ONIONS Total e/	1986	34,700	370	12,839	8.40	106,202
	1987	37,200	351	13,058	10.10	128,805
	1988	37,300	371	13,827	6.67	90,382
	1989	34,700	381	13,205	8.43	109,671
	1990	39,000	389	15,160	8.35	124,694
	1991	36,600	375	13,742	9.61	130,390
	1992	36,500	381	13,913	9.20	125,426
	1993	40,500	403	16,335	10.10	161,668
	1994	37,700	415	15,658	6.52	102,128
	1995	38,000	420	15,958	8.19	130,666
PEPPERS, BELL b/	1992	20,000	285	5,700	18.30	104,470
	1993	18,500	300	5,550	26.90	149,057
	1994	20,500	290	5,945	25.60	152,287
	1995	20,000	290	5,800	25.90	150,020
SPINACH b/ Fresh Market	1992	7,000	205	1,435	18.20	26,117
	1993	6,000	215	1,290	31.00	39,990
	1994	5,800	205	1,189	30.80	36,621
	1995	6,700	185	1,240	34.40	42,656
TOMATOES Fresh Market	1986	28,600	278	7,938	22.80	180,956
	1987	29,500	300	8,850	21.50	190,275
	1988	37,500	245	9,188	29.90	274,721
	1989	38,400	260	9,984	24.00	239,616
	1990	38,000	255	9,690	28.20	273,258
	1991	40,000	235	9,400	25.00	235,000
	1992	36,000	280	10,080	34.10	343,728
	1993	37,500	270	10,125	26.30	266,288
	1994	36,500	275	10,038	24.80	248,942
	1995	38,000	270	10,260	18.80	192,888
MISCELLANEOUS VEGETABLES AND MELONS f/ Fresh Market & Processing	1986	182,300	---	---	---	629,256
	1987	241,690	---	---	---	702,180
	1988	249,098	---	---	---	692,478
	1989	201,132	---	---	---	669,884
	1990	219,350	---	---	---	711,961
	1991	303,700	---	---	---	884,344
	1992	131,118	---	---	---	356,077
	1993	132,470	---	---	---	666,130
	1994	122,084	---	---	---	841,438
	1995	151,572	---	---	---	841,919

a/ *The processed price is based on equivalent returns at the processing plant door. The fresh market price is on a packed and loaded basis, f.o.b. shipping point.*

b/ *Program initiated for 1992.*

c/ *Fresh market and processed acreage separated starting in 1993.*

d/ *Information unavailable due to disclosure.*

e/ *Onion production includes quantity harvested, but not marketed because of loss during storage (shrinkage): 1986 -- 200,000 cwt., 1987 -- 320,000 cwt., 1988 -- 280,000 cwt., 1989 -- 200,000 cwt., 1990 -- 234,000 cwt., 1991 -- 174,000 cwt., 1992 -- 275,000 cwt., 1993 -- 313,000 cwt., 1994 -- 248,000 cwt.; 1995 -- 253,000 cwt. Storage loss amounts are excluded when computing total value.*

f/ *Miscellaneous vegetables and melons include: Green lima beans for processing, oriental vegetables, green peas, chili peppers, squash, etc. The new crops added in 1992 are excluded from the 1992 to present miscellaneous totals. Miscellaneous figures come from County Agricultural Commissioners.*

CALIFORNIA TOMATOES FOR PROCESSING BY COUNTY, 1995

County	Contracted and Open Market			
	Planted Acres	Harvested Acres	Yield	Production 1/
	Acres		*Tons/Acre*	*Tons*
Contra Costa	4,200	4,000	34.58	138,300
San Benito	2,200	2,100	34.14	71,700
Santa Clara	1,200	1,100	36.45	40,100
Colusa	30,200	28,500	32.95	939,000
Sacramento	7,000	6,400	28.36	181,500
Solano	20,500	19,200	30.25	580,800
Sutter	22,900	21,500	31.52	677,700
Yolo	71,000	68,200	32.58	2,221,800
Fresno	101,000	98,000	34.46	3,377,400
Merced	10,000	9,600	41.94	402,600
San Joaquin	24,200	23,500	38.49	904,400
Stanislaus	18,200	17,400	28.73	499,900
Other Counties 2/	18,400	17,500	32.66	571,620
State Total	331,000	317,000	33.46	10,606,820

1/ On the basis of PAID FOR TONNAGE purchased from growers as reported by processors, dockage not included.
2/ To avoid disclosure of individual operations, OTHER COUNTIES include: Madera, Monterey, Kern, Kings, Imperial, Santa Barbara, Tulare, and Ventura.

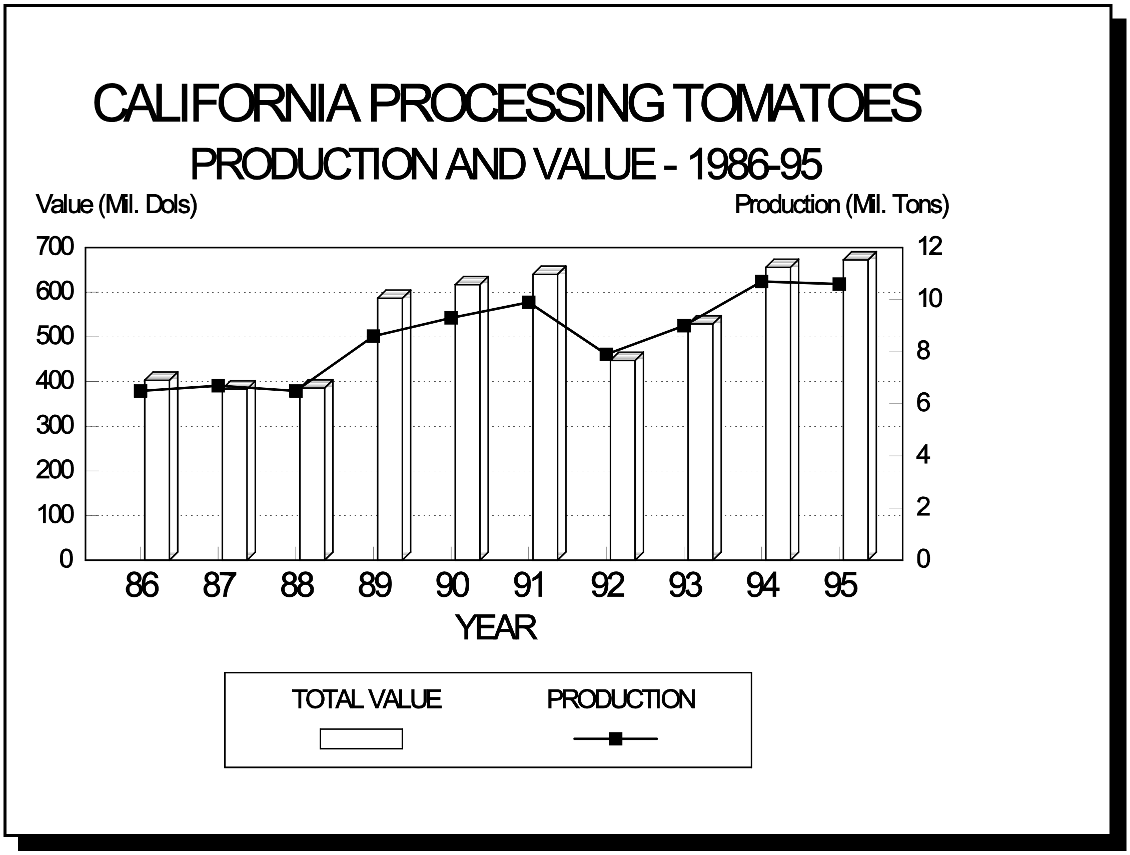

CALIFORNIA VEGETABLE CROPS: 1993-95 Monthly Prices, Selected Fresh Market Vegetables 1/

Crop	Jan.	Feb.	Mar.	Apr.	May	June	July	Aug.	Sep.	Oct.	Nov.	Dec.
	Dollars Per Hundredweight											
ASPARAGUS												
1993	121.00	113.00	80.70	107.00	72.40	111.00	164.00	171.00	176.00	146.00	---	---
1994	135.00	141.00	97.60	88.60	93.50	101.00	207.00	144.00	196.00	151.00	---	---
1995	150.00	118.00	93.60	161.00	90.00	107.00	---	---	---	---	---	---
BROCCOLI												
1993	32.60	28.10	28.60	23.70	22.30	26.80	24.50	20.00	36.60	22.40	24.20	30.00
1994	23.50	21.40	19.50	21.80	27.10	21.10	21.60	18.50	38.60	37.00	57.70	46.00
1995	23.70	33.30	53.40	33.00	25.50	26.30	18.50	30.30	26.70	22.60	22.00	26.90
CARROTS												
1993	18.20	13.00	11.00	12.40	10.90	10.10	8.74	8.33	8.33	9.38	10.30	10.80
1994	10.60	10.40	11.50	10.20	12.20	12.10	13.40	14.60	15.20	16.20	15.30	15.60
1995	17.00	14.50	16.50	17.50	17.50	14.00	13.00	13.00	13.00	14.00	14.00	14.00
CAULIFLOWER												
1993	34.30	29.10	24.70	44.90	26.90	37.00	27.70	27.20	38.40	25.90	21.60	30.90
1994	24.80	24.90	23.10	20.80	32.20	29.10	30.20	22.50	33.20	30.50	42.50	29.80
1995	23.70	33.30	53.60	33.00	25.50	26.30	18.50	30.30	26.70	22.60	22.00	55.00
CELERY												
1993	24.60	36.60	27.40	16.50	12.60	8.30	8.71	10.40	14.00	13.20	11.40	10.90
1994	11.20	9.01	7.65	8.40	13.80	8.69	12.10	14.70	11.90	11.50	13.90	25.50
1995	24.10	25.80	20.40	33.10	24.30	14.20	11.10	10.20	16.60	12.90	12.70	11.40
SWEET CORN												
1993	---	---	---	---	19.70	19.90	19.60	13.90	14.50	16.90	15.10	29.70
1994	---	---	---	22.10	20.50	18.10	20.60	11.90	15.30	22.50	22.10	30.60
1995	---	---	---	21.20	19.90	19.00	19.70	15.50	16.10	22.30	20.90	26.00
HONEYDEW MELON												
1993	---	---	---	---	26.60	19.40	15.20	14.60	16.40	21.30	22.10	---
1994	---	---	---	---	27.30	19.30	14.20	13.00	15.70	22.50	21.30	---
1995	---	---	---	---	23.50	27.60	22.00	16.90	13.70	21.20	18.40	---
HEAD LETTUCE												
1993	10.00	18.70	13.10	39.10	12.50	11.40	19.00	15.60	16.90	12.20	11.20	9.37
1994	7.70	11.50	9.99	11.80	11.40	13.70	10.30	10.90	17.40	21.90	21.20	39.10
1995	14.20	9.87	41.20	48.50	48.50	15.60	12.50	16.50	25.90	15.00	12.30	12.00
SPRING ONIONS												
1993	---	---	---	34.00	24.60	9.47	10.90	---	---	---	---	---
1994	---	---	---	8.03	7.15	7.86	10.20	---	---	---	---	---
1995	---	---	---	18.60	15.60	9.30	11.40	---	---	---	---	---
SUMMER ONIONS												
1993	26.10	28.40	27.20	---	---	---	10.90	12.40	11.50	11.00	18.00	22.40
1994	26.70	29.40	22.60	---	---	---	10.20	7.23	8.06	11.00	15.40	13.10
1995	25.00	25.00	25.00	---	---	---	11.40	9.50	12.10	14.10	15.00	---
TOMATOES												
1993	---	---	---	---	73.10	23.10	17.00	32.20	28.00	18.20	37.30	---
1994	---	---	---	---	24.10	28.40	23.30	27.40	19.10	25.70	28.80	---
1995	---	---	---	---	---	32.80	17.10	12.70	17.20	20.20	22.70	---

1/ Prices are F.O.B. shipping point.
