# Supplementary material for: The Elephant in the room: What can we learn from California regarding the use of sport hunting of pumas (Puma concolor) as a management tool?
Source: PLoS One. 2020 Feb 13;15(2):e0224638. doi: 10.1371/journal.pone.0224638 (PMC7018503; doi:10.1371/journal.pone.0224638)
Supplement: S2 File — This is a zip file that contains sources of the data used in the analyses that are no longer available on the internet. (ZIP) [file pone.0224638.s003.zip › 2004 California Agricultural statistics.pdf]

California Agricultural Statistics

2002

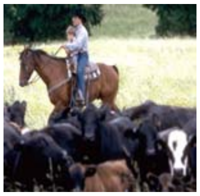
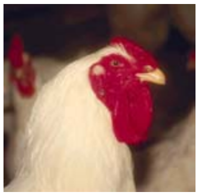
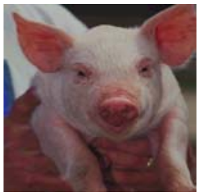
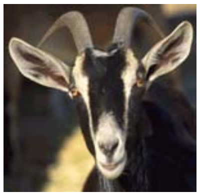
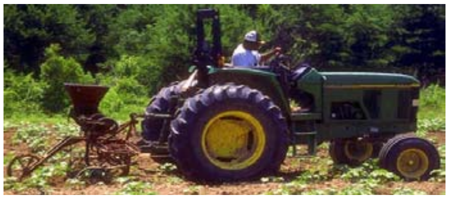
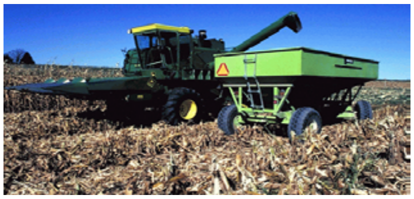
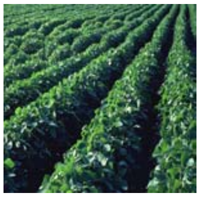
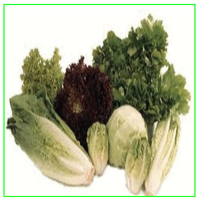
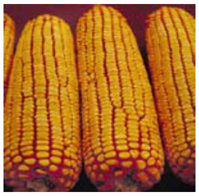
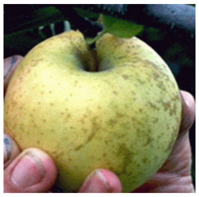

October 2003

TABLE OF CONTENTS

CHAPTER	PAGE
California Agricultural Overview	1
California Field Crops	11
California Floriculture	33
California Fruit and Nut Crops	37
California Livestock and Dairy	61
California Vegetables and Melons	75
California County Agricultural Commissioners	86
California Agricultural Statistics Service Publication Order Form	99

Compiled by:

CALIFORNIA AGRICULTURAL STATISTICS SERVICE

650 Capitol Mall, Suite 6-100
P.O. Box 1258
Sacramento, California 95812-1258
Phone: (916) 498-5161
Fax: (916) 498-5186

Vic Tolomeo, Director
Jack Rutz, Deputy Director

October 2003

California Agricultural Statistics Service publications are available on the Internet at:

http://www.nass.usda.gov/ca

California Agricultural Overview

The Golden State is, by far, the number-one agricultural producer and exporter in the United States. No other state in the union even comes close. In fact, last year the agricultural marketings of California's farmers and ranchers reached $27.5 billion.

Top 5 Agricultural States in Cash Receipts, 2002

State	Rank	Total Value (Billion Dollars)
California	1	26.1
Texas	2	12.7
Iowa	3	10.8
Nebraska	4	9.6
Kansas	5	7.9

California produces one out of every five glasses of milk consumed in the nation. The dairy industry continues to grow at an astonishing rate with milk production jumping 5 percent from 2001 to 2002. It follows then, that California also has some of the largest dairies in the country with more than 700 cows on average, while the United States average is less than 100. In 2002, the average milk production per cow in California was 21,180 pounds, which is 14 percent higher than the national average.

California produces most of the grapes grown in the United States. Warm days and cool nights are very favorable to grapes, so it comes as no surprise that California now produces many of the best wines in the world. In 2002, 91 percent of all grapes (raisin, table and wine) came from right here in the Golden State.

California's agricultural abundance includes 350 different crops. Among those, the state grows more than half of the nation's total of fruits, nuts and vegetables. Many of these commodities are specialty crops and almost solely produced in California. It will be hard to find these crops growing in any other state—artichokes, Brussels sprouts, almonds, dates, figs, kiwifruit, nectarines, olives, pistachios, dried plums (prunes) and walnuts.

California's Gross Cash Income for 2002

Agricultural production and gross cash income in 2002 increased to $27.5 billion, this is 2 percent lower than the previous year. Cash receipts from farm marketings totaled $26.1 billion, down 1 percent from 2001, while other farm related income totaled $1.37 billion.

Fruit and nut cash receipts, at $7.86 billion, were 10 percent above last year and comprised 29 percent of the total. Vegetable receipts increased 1 percent from $6.15 billion in 2001 to $6.21 billion in 2002 and comprised 23 percent of the total. Livestock and poultry receipts fell 15 percent and comprised 23 percent of the total. Nursery and flower crops, with 11 percent of the total, were down 4 percent from last year. Field crops accounted for 10 percent of the total, and cash receipts jumped 6 percent from the previous year.

The Golden State's two leading commodities in cash receipts are milk with $3.81 billion and grapes with $2.58 billion. Nationally, California has about 21 percent of the total milk receipts and 91 percent of the grape receipts.

Notable Increases in Marketings:

Alfalfa Seed 47%
Almonds 61%
Dates 37%
Figs 20%
Honey 58%
Lemons 21%
Oats 149%
Peaches, Clingstone 21%
Pecans 61%
Pistachios 107%
Plums 21%
Potatoes (Except Sweet) 71%
Spinach 29%
Strawberries 20%
Tomatoes, Processing 27%
Wheat 28%
Wool 25%

Significant Decreases in Marketings:

Asparagus 29%
Barley 28%
Olives 33%
Plums, Dried (Prunes) 30%
Rice 29%
Turkeys 25%
Walnuts 40%

California Farm Facts

California has 84,000 farming operations. This number represents less than 4 percent of the nation's total; however, these farms account for 14 percent of the national gross cash receipts from farming. In 2002, land in farms totaled 27.7 million acres. Average farm size in California is 330 acres. Some 350 crops are recognized in the state, including seeds, flowers, and ornamentals.

Crop and Livestock Commodities in which California Leads the Nation

**Almonds**	**Figs**	Melons, Honeydew	Plums
Apricots	Flowers, Bulbs	Melons, Persian	**Plums, Dried**
**Artichokes**	Flowers, Cut	Milk	Pomegranates
Asparagus	Flowers, Potted Plants	Milk, Goat	Rabbits
Avocados	Garlic	**Nectarines**	Rice, Sweet
Beans, Black-eyed	**Grapes, Raisins**	Nursery, Bedding Plants	Safflower
Beans, Dry Baby Lima	Grapes, Table	Nursery Crops	Seed, Alfalfa
Beans, Dry Large Lima	Grapes, Wine	**Olives**	Seed, Bermuda Grass
Beans, Garbanzo	Hay, Alfalfa	Onions	Seed, Ladino Clover
Broccoli	Herbs	Onions, Green	Seed, Sudan Grass
Brussels Sprouts	Kale	Parsley	Seed, Vegetable and Flower
Cabbage, Chinese	**Kiwifruit**	**Peaches, Clingstone**	Spinach
Carrots	Lemons	Peaches, Freestone	Strawberries
Cauliflower	Lettuce, Head	Pears, Bartlett	Tomatoes, Processing
Celery	Lettuce, Leaf	Peas, Chinese	Vegetables, Greenhouse
Chicory	Lettuce, Romaine	Peppers, Bell	Vegetables, Oriental
Currants	Melons, Cantaloupe	**Persimmons**	**Walnuts**
**Dates**	Melons, Casaba	Pigeons and Squabs	
Escarole/Endive	Melons, Crenshaw	**Pistachios**	

California is the sole producer (99% or more) of the commodities in bold.

California's Top 20 Commodities

California's top 20 crop and livestock commodities account for 74 percent of the state's gross farm income. Milk and cream production remained number one in 2002, followed by grapes and nursery products.

Leading Counties

California has some of the most agriculturally productive counties in the nation. According to the 1997 Census of Agriculture's ranking of market value of agricultural products sold, eight of the nation's top 10 producing counties are in California.

Fresno regained the number one spot from Tulare as the leading county in the nation, and in 2002 reached $3.42 billion in agricultural value. A large share of Tulare's agricultural productivity comes from milk and cream, which showed a big decrease in 2002. Fresno's number one commodity is grapes and the value jumped 17 percent from 2001. If ranked separately, the value of agricultural commodities in Fresno County would rank it ahead of more than half the other states in the nation.

California's Top 20 Commodities for 2000-2002

Commodity	Value and Rank					
	2000		2001		2002	
	$1,000					
Milk and Cream	3,707,255	(1)	4,625,431	(1)	3,812,371	(1)
Grapes, All	2,828,545	(2)	2,658,587	(2)	2,578,854	(2)
Nursery	2,247,256	(3)	2,107,711	(3)	2,347,697	(3)
Lettuce, All	1,494,114	(4)	1,344,551	(5)	1,277,895	(4)
Cattle and Calves	1,218,220	(5)	1,353,837	(4)	1,228,722	(5)
Almonds	666,487	(11)	740,012	(10)	1,189,870	(6)
Strawberries	839,926	(8)	826,478	(8)	990,583	(7)
Flowers	931,267	(7)	985,795	(7)	948,629	(8)
Tomatoes, All	948,469	(6)	752,282	(9)	925,775	(9)
Hay, All	768,978	(9)	1,018,989	(6)	913,509	(10)
Cotton, All	719,502	(10)	615,986	(11)	623,466	(11)
Oranges, All	345,654	(14)	514,460	(13)	558,843	(12)
Broccoli	573,562	(12)	439,387	(14)	488,442	(13)
Carrots, All	322,455	(17)	433,919	(15)	459,838	(14)
Chickens, All	471,081	(13)	532,452	(12)	452,353	(15)
Avocados	339,594	(15)	315,842	(17)	358,000	(16)
Pistachios	245,430	(22)	162,610	(31)	336,330	(17)
Potatoes, All	209,455	(28)	184,411	(26)	307,024	(18)
Walnuts	296,360	(18)	341,600	(16)	304,560	(19)
Lemons	253,876	(20)	212,725	(22)	287,026	(20)

California's Top 10 Agricultural Counties

County	Total Value				Leading Commodities
	2001		2002		
	$1,000				
Fresno	3,215,185	(2)	3,415,591	(1)	Grapes, Cotton, Poultry, Tomatoes, Milk
Tulare	3,474,470	(1)	3,200,552	(2)	Milk, Oranges, Grapes, Cattle and Calves, Peaches
Monterey	2,746,244	(3)	2,812,069	(3)	Lettuce, Broccoli, Strawberries, Nursery Products, Grapes
Kern	2,221,659	(4)	2,586,247	(4)	Grapes, Citrus, Carrots, Almonds, Milk
Merced	1,703,039	(5)	1,730,720	(5)	Milk, Chickens, Almonds, Cattle and Calves, Tomatoes
San Joaquin	1,353,300	(7)	1,367,971	(6)	Milk, Grapes, Tomatoes, Almonds, Walnuts
Stanislaus	1,389,876	(6)	1,343,808	(7)	Milk, Almonds, Chickens, Cattle and Calves, Fruit and Nut Crops
San Diego	1,289,699	(8)	1,297,258	(8)	Flowers, Nursery Products, Avocados, Eggs, Tomatoes
Riverside	1,010,321	(11)	1,224,109	(9)	Milk, Nursery Products, Table Grapes, Eggs, Hay
Ventura	1,053,636	(10)	1,161,070	(10)	Strawberries, Lemons, Nursery Stock, Celery, Avocados

Weather Highlights for 2002

In California, the 2002 season started with cooler than normal temperatures and above normal rainfall in January in the Sacramento and San Joaquin valleys, but below normal precipitation in the coastal and southern California areas. Growers performed the needed cultural practices as weather permitted. Cold temperatures late in January helped ease growers' concerns over lack of chilling hours required for most tree fruit, but also slowed the growth of some vegetables and there were reports of some lettuce damaged by frost.

Warmer temperatures in late February encouraged plant growth of the winter vegetables and harvesting resumed. Seasonal storms brought rain to the valleys, but precipitation remained below normal in the central and south coast, as well as the desert areas. Almonds began blooming in late February and were in full bloom by early March. A freeze in mid-March damaged the almonds and the crop was reduced significantly in the Arbuckle area of the Sacramento Valley. Harvesting of winter vegetables continued in the desert areas and then moved into the San Joaquin Valley. As March began, a strong high-pressure ridge aloft and an offshore flow at the lower levels of the atmosphere dominated weather conditions. This condition resulted in very dry, warm daytime conditions and cool nights. Warmer weather in March and April accelerated plant growth and pollination in orchards and vineyards. Weather conditions during the summer months were relatively mild with no extreme temperatures. Fall temperatures were also mild, which allowed harvesting of most crops to remain on schedule with few problems.

Cotton planting began in late April and early May and progressed very well. Cool spells during the spring initially slowed growth in some areas, but warm weather during the summer helped the crop progress well with good plant growth and boll retention. In general, insect pressure was light during the year. Harvesting of Upland cotton began in the Imperial Valley in mid-September, with picking following in the San Joaquin Valley by late September and early October. Harvest of American Pima cotton began in early October and progressed well with most of the crop harvested by early December.

Weather conditions for rice were also very favorable and provided for excellent yields. Dry conditions in southern California prevented or delayed planting of the wheat crop and production from that area was down. Rains in the Sacramento Valley during late May caused some quality problems for wheat, but overall quality of the crop was good.

Global Marketplace

California is the nation's leader in agricultural exports, annually shipping more than $6.50 billion in both food and agricultural commodities around the world. To highlight the significance of this figure, it is estimated that 14 percent of California's agricultural production is exported.

In terms of foreign markets, Canada remained the largest destination for California agriculture. Japan was surpassed by the European Union in 2002 and is now the third largest export destination for California agriculture. Collectively, Canada, the European Union, and Japan represent nearly 50 percent of the agricultural exports from the Golden State.

With key international markets in Asia, Europe, and North America, as shown in these two tables, California agriculture is clearly enjoyed around the world.

Cash Income by Commodity Groups, 2000-2002

Source of Income	2000	2001	2002
	$1,000		
**FIELD CROPS**			
Alfalfa Seed	43,096	20,435	30,000
Barley	18,177	16,579	11,897
Beans, Dry	61,509	53,086	51,421
Corn for Grain	59,427	56,508	57,740
Cotton Lint	480,036	582,371	623,462
Cottonseed	98,926	94,411	81,972
Grain, Sorghum	659	1,459	2,343
Hay, All	473,619	592,918	575,483
Oats	1,732	1,383	3,440
Oil Crops	38,258	28,209	26,371
Potatoes (Excl. Sweet)	210,101	177,316	302,520
Potatoes, Sweet	63,263	61,870	72,342
Rice	152,812	265,041	187,218
Sugar Beets	95,608	53,306	62,191
Wheat	79,978	87,528	112,249
Other Seed Crops	149,920	132,073	137,934
Other Field Crops	311,725	345,306	383,725
TOTAL	2,338,847	2,569,799	2,722,309
**Percent of Gross Cash Income**	8.6	9.1	9.9
**FRUIT AND NUT CROPS**			
Almonds	666,487	740,012	1,189,870
Apples	120,260	76,953	91,723
Apricots	26,679	22,330	22,964
Avocados	337,890	318,793	355,324
Berries, Bushberries	61,563	56,521	54,723
Berries, Strawberries	800,334	826,478	990,583
Cherries	71,564	79,814	93,315
Dates	22,872	25,498	34,938
Figs	15,226	15,012	18,012
Grapefruit	57,608	55,333	58,128
Grapes	2,832,692	2,659,343	2,578,753
Kiwifruit	13,888	15,340	18,097
Lemons	243,311	244,106	295,760
Nectarines	106,256	127,642	114,517
Olives	34,768	90,048	60,475
Oranges, Navel and Miscellaneous	383,862	391,886	371,558
Oranges, Valencia	160,086	165,886	165,886
Peaches, Clingstone	127,125	110,044	132,639
Peaches, Freestone	124,484	136,699	138,369
Pears	71,587	72,282	68,265
Pecans	5,168	2,997	4,826
Pistachios	245,430	162,610	336,330
Plums	87,115	64,362	77,586
Plums, Dried (Prunes)	95,100	146,195	102,647
Tangerines	21,731	33,145	39,274
Walnuts	296,360	341,600	204,560
Other Fruits and Nuts	143,446	144,110	140,700
TOTAL	7,172,892	7,125,039	7,859,822
**Percent of Gross Cash Income**	26.3	25.3	28.6
**CHRISTMAS TREES**			
TOTAL	10,669	10,345	10,500
**Percent of Gross Cash Income**	0.01	0.01	0.01
**NURSERY, GREENHOUSE AND FLORICULTURE**			
TOTAL	3,115,620	3,176,410	3,061,859
**Percent of Gross Cash Income**	11.5	11.3	11.1

Source of Income	2000	2001	2002
	$1,000		
**VEGETABLE CROPS**			
Artichokes	61,021	58,559	66,764
Asparagus	144,448	163,800	116,280
Beans, Green Lima	13,014	13,214	13,760
Beans, Snap, All	33,311	30,656	36,633
Broccoli, All	575,349	439,387	488,442
Brussels Sprouts	16,115	14,471	N/A
Cabbage	63,011	62,928	66,500
Carrots, All	322,455	433,919	459,838
Cauliflower	211,087	167,504	167,491
Celery	324,850	255,841	227,130
Corn, Sweet	66,132	83,402	97,920
Cucumbers, All	49,514	48,252	40,007
Garlic	140,447	160,420	138,146
Lettuce	1,494,114	1,344,551	1,277,895
Melons, Cantaloupe	226,148	252,372	223,287
Melons, Honeydew	68,820	72,890	63,403
Melons, Watermelon	76,998	68,169	76,429
Mushrooms	162,811	166,313	170,666
Onions	223,234	246,072	219,222
Peppers, Chili	28,165	17,697	17,914
Peppers, Green	171,788	182,343	197,987
Pumpkins	19,080	16,397	16,350
Spinach, All	115,524	94,126	121,501
Squash	29,951	28,060	33,049
Tomatoes, Fresh	345,680	255,474	293,370
Tomatoes, Processing	602,789	496,808	632,403
Other Vegetable Crops	948,534	979,292	948,130
TOTAL	6,534,390	6,152,917	6,210,517
**Percent of Gross Cash Income**	24.0	21.9	22.6
**LIVESTOCK, POULTRY AND PRODUCTS**			
Aquaculture	13,738	14,385	12,966
Cattle and Calves	1,218,220	1,353,837	1,228,722
Chickens, All	471,081	532,452	452,353
Eggs	237,561	235,921	200,051
Hogs	35,563	26,853	22,188
Honey	18,172	19,061	30,083
Milk and Cream	3,707,255	4,625,431	3,812,371
Sheep and Lambs	58,156	52,975	50,388
Turkeys	210,816	211,815	158,026
Wool	1,160	1,313	1,643
Other Livestock/Poultry	280,346	269,012	272,841
TOTAL	6,252,068	7,343,055	6,241,632
**Percent of Gross Cash Income**	23.0	26.1	22.7
**TOTAL CASH INCOME FROM MARKETINGS**			
TOTAL	25,424,486	26,377,565	26,106,639
**FARM RELATED INCOME**			
TOTAL	1,758,075	1,742,305	1,371,347
**Percent of Gross Cash Income**	6.5	6.2	5.0
**GROSS CASH INCOME FROM FARMING 1/**			
TOTAL	27,182,561	28,119,870	27,477,986

1/ Includes value of home consumption, value of inventory adjustments, machine hire and custom work, forest products sold, rental value of farm dwelling, government payments, and other miscellaneous farm income.

Source: USDA Economic Research Service

Farm Income Indicators, 1998-2002 1/

Item	1998	1999	2000	2001	2002
			$1,000		
**Value of Crop Production**	17,458,369	18,507,449	19,443,171	19,062,870	19,686,544
Food grains	453,770	387,309	232,790	352,569	299,467
Feed crops	535,584	515,038	553,613	668,847	650,904
Cotton	950,142	667,703	578,961	676,782	705,434
Oil crops	51,605	44,735	38,261	28,209	26,371
Tobacco	---	---	---	---	---
Fruits and tree nuts	6,536,089	6,640,438	7,172,890	7,121,820	7,748,106
Vegetables	5,802,199	6,272,068	6,698,937	6,270,933	6,458,121
All other crops	3,405,813	3,731,261	3,896,964	3,912,132	3,864,889
Home consumption	5,935	5,802	5,704	5,835	5,734
Value of inventory adjustment 2/	(282,768)	243,095	265,051	25,743	(72,482)
**Value of Livestock Production**	6,697,288	6,680,857	6,304,198	7,392,857	6,299,978
Meat animals	1,127,393	1,308,778	1,311,939	1,433,665	1,301,298
Dairy products	4,140,659	4,090,952	3,707,255	4,625,431	3,812,371
Poultry and eggs	1,048,318	1,027,944	978,700	1,021,942	864,217
Miscellaneous livestock	214,643	241,409	254,174	262,017	263,746
Home consumption	11,266	11,798	10,341	10,861	10,582
Value of inventory adjustment 2/	155,009	(24)	41,789	38,941	47,764
**Revenues from Services and Forestry**	1,487,751	1,570,263	1,435,192	1,664,143	1,491,464
Machine hire and custom work	282,648	292,721	374,381	277,185	288,566
Forest products sold	3,300	5,593	4,738	7,801	6,500
Other farm income	533,317	633,778	413,498	686,476	511,478
Gross imputed rental value of farm dwellings	668,486	638,171	642,575	692,681	684,920
**VALUE OF AGRICULTURAL SECTOR PRODUCTION**	**25,643,408**	**26,758,569**	**27,182,561**	**28,119,870**	**27,477,986**
less: **Purchased Inputs**	12,838,986	13,680,281	13,912,488	15,314,628	14,461,294
**Farm Origin**	3,368,593	3,712,901	4,045,816	4,059,961	3,919,111
Feed purchased	2,244,843	2,421,837	2,688,975	2,547,856	2,520,981
Livestock and poultry purchased	420,504	565,608	601,774	623,793	539,424
Seed purchased	703,246	725,456	755,067	888,312	858,706
**Manufactured Inputs**	2,778,867	2,857,558	2,941,395	3,259,384	2,772,825
Fertilizers and lime	763,324	750,646	804,669	812,009	719,322
Pesticides	1,068,372	1,016,436	964,504	1,038,689	934,821
Petroleum fuel and oils	434,110	488,116	573,710	540,610	506,513
Electricity	513,061	602,360	598,512	868,076	612,169
**Other Purchased Inputs**	6,691,526	7,109,822	6,925,277	7,995,283	7,769,358
Repair and maintenance of capital items	761,997	844,442	861,274	769,754	769,965
Machine hire and custom work	1,109,142	1,048,453	788,486	747,945	1,211,950
Marketing, storage, and transportation expenses	1,422,240	1,635,112	1,900,769	2,134,752	1,832,223
Contract labor	1,071,584	1,166,784	1,149,140	1,643,763	1,552,647
Miscellaneous expenses	2,326,563	2,415,031	2,225,608	2,699,069	2,402,573
plus: **Net Government Transactions**	(225,965)	65,918	66,921	(27,500)	(137,402)
+ Direct Government payments	357,340	668,501	667,466	586,761	451,495
- Motor vehicle registration and licensing fees	41,172	43,067	37,743	41,581	29,379
- Property taxes	542,133	559,516	562,802	572,680	559,518
**Gross Value Added**	12,578,457	13,144,206	13,336,993	12,777,742	12,879,290
less: **Capital Consumption**	1,034,134	1,015,830	1,003,457	1,017,925	1,044,326
**Net Value Added**	11,544,324	12,128,376	12,333,536	11,759,817	11,834,964
less: **Payments to Stakeholders**	5,988,411	6,640,534	6,845,820	6,667,935	6,749,972
Employee compensation (total hired labor)	4,237,847	4,865,338	4,956,621	4,948,075	4,996,603
Net rent received by non-operator landlords	470,083	462,610	463,311	395,150	444,957
Real estate and non-real estate interest	1,280,481	1,312,586	1,425,888	1,324,710	1,308,412
**NET FARM INCOME**	**5,555,913**	**5,487,842**	**5,487,716**	**5,091,882**	**5,084,992**

1/ Value of agricultural sector production is the gross value of commodities and services produced within a year. Net value-added is the sector's contribution to the national economy and is the sum of the income from production earned by all factors-of-production, regardless of ownership. Net farm income is the farm operators' share of income from the sector's production activities. The concept presented is consistent with that employed by the Organization for Economic Cooperation and Development.

2/ A positive value of inventory change represents current-year production not sold by December 31. A negative value is an offset to production from prior years included in current-year sales.

Source: USDA Economic Research Service

Commodity Rank, Acreage, Production, Value and Leading Producing Counties, 2002

Commodity	U.S. Rank 1/	CA Share of U.S. Prod.	Area Harvested	Short Tons (2,000 Lbs.)	Total Value 2/	California Rank		Harvest Season	Leading Counties 3/
						2001	2002		
	Number	*Percent*	*1,000 Acres*	*1,000 Tons*	*$1,000*	*Number*			
**VEGETABLES**									
**TOTAL VALUE -- $6,209,017,000**									
Artichokes 4/	1	99	8.2	47.2	66,764	49	45	Continuous	Monterey, Riverside, San Mateo, Orange
Asparagus	1	55	34.0	51.0	116,280	29	33	Feb. 15-June 30	San Joaquin, Monterey, Imperial, Fresno
Beans, Fresh Market Snap	4	9	6.5	26.0	32,396	59	58	Apr. 20-Dec. 15	Orange, San Diego, Santa Clara, San Luis Obispo
Broccoli	1	92	115.0	805.0	488,442	14	13	Continuous	Monterey, Santa Barbara, San Luis Obispo, Imperial, Fresno
Cabbage, Fresh Market	1	19	12.5	237.5	66,500	47	46	Continuous	Ventura, Santa Barbara, Monterey, San Luis Obispo, Imperial
Carrots	1	76	79.1	1,176.8	459,838	15	14	Continuous	Kern, Imperial, Monterey, Riverside
Cauliflower	1	86	37.0	277.5	167,491	27	29	Continuous	Monterey, Santa Barbara, Imperial, San Luis Obispo
Celery	1	95	25.0	887.5	227,130	18	22	Continuous	Ventura, Monterey, Santa Barbara, San Luis Obispo
Corn, Fresh Market Sweet	2	15	24.0	204.0	97,920	39	38	May 1-Dec. 10	Fresno, Imperial, Contra Costa, San Joaquin, Riverside
Cucumbers, Fresh Market	4	9	4.3	51.6	29,515	55	60	Mar. 20-Nov. 30	San Joaquin, San Diego, Tulare, Santa Clara, Orange
Garlic 4/	1	84	27.0	236.2	138,146	32	31	Apr. 1-Sept. 15	Fresno, Kern
Lettuce, Head	1	71	129.0	2,418.8	735,300	---	---	Continuous	Monterey, Imperial, Fresno, Santa Barbara
Lettuce, Leaf	1	85	49.0	526.7	296,034	---	---	Continuous	Imperial, Monterey, Fresno, Riverside, San Benito
Lettuce, Romaine	1	78	42.0	619.5	246,561	---	---	Continuous	Monterey, Riverside, Ventura, Santa Clara
Lettuce, All	1	74	220.0	3,565.0	1,277,895	5	4	---	Monterey, Imperial, Fresno, Santa Barbara, San Benito
Melons, Cantaloupe	1	58	54.9	672.6	223,287	19	23	May 15-Nov. 30	Fresno, Imperial, Merced, Riverside, Stanislaus
Melons, Honeydew	1	75	20.4	188.7	63,403	42	48	June 1-Nov. 30	Fresno, Sutter, Yolo, Imperial, Riverside
Melons, Watermelon	3	15	11.8	300.9	76,429	44	42	May 25-Oct. 15	Fresno, Riverside, Kern, San Joaquin, Imperial
Mushrooms, Agaricus	2	15	0.5	64.2	170,159	30	28	Continuous	Monterey, Santa Clara, San Mateo, San Diego
Onions, All	1	32	43.8	1,382.4	218,229	28	24	Apr. 1-Oct. 31	Fresno, Imperial, Kern, San Joaquin, Los Angeles
Peppers, Bell	1	43	21.0	346.5	197,987	---	---	July 1-Dec. 10	Kern, Ventura, Riverside, Fresno, San Joaquin
Peppers, Chili	2	22	2.8	32.2	17,914	---	---	---	Santa Clara, Riverside, San Diego
Peppers, All	---	---	23.8	378.7	215,901	25	25	---	Kern, Ventura, Riverside, Santa Clara
Pumpkins	2	19	6.0	75.0	16,350	64	66	---	San Joaquin, Sacramento, San Mateo, Ventura
Spinach, Fresh Market	1	67	20.0	160.0	112,640	38	36	Continuous	Monterey, Riverside, Imperial, San Benito, Satna Barbara
Squash	1	18	8.3	78.8	33,049	58	57	---	Santa Barbara, Fresno, Riverside, San Luis Obispo
Tomatoes, Fresh Market	2	31	38.5	577.5	293,370	---	---	May 15-Dec. 31	Merced, San Joaquin, Fresno, San Diego, Stanislaus
Tomatoes, Processing	1	95	291.0	11,056.0	632,403	---	---	June 20-Nov. 10	Fresno, Yolo, San Joaquin, Colusa, Merced
Tomatoes, All	1	86	329.5	11,633.5	925,773	9	9	---	Fresno, San Joaquin, Merced, Yolo, Colusa
Other Vegetables and Melons, Etc.	---	---	237.2	4,210.8	985,480	---	---	---	---
**FRUITS AND NUTS**									
**TOTAL VALUE -- $7,885,185,000**									
Almonds (Shelled) 4/	1	99	530.0	545.0	1,189,870	10	6	Aug. 1-Oct. 31	Kern, Stanislaus, Fresno, Merced, Madera
Apples	4	5	26.0	235.0	93,840	41	39	July 15-Oct. 30	San Joaquin, Kern, Fresno, Santa Cruz, Stanislaus
Apricots	1	93	17.0	85.0	22,964	62	61	May 1-July 15	Stanislaus, Fresno, San Joaquin Merced, Kern
Avocados	1	90	58.0	200.0	358,000	17	16	Continuous	San Diego, Ventura, Santa Barbara, Riverside
Berries, Boysenberries	2	32	0.2	1.0	1,372	73	74	June 1-July 15	---
Berries, Raspberries	2	26	2.4	14.6	40,851	54	54	June 1-Oct. 31	Santa Cruz, Monterey, Ventura
Berries, F.M. Strawberries	1	85	---	632.1	852,071	---	---	---	---
Berries, Proc. Strawberries	1	90	---	215.8	138,512	---	---	---	---
Berries, All Strawberries	1	86	28.5	847.9	990,583	8	7	Feb. 20-Nov. 15	Ventura, Monterey, Santa Barbara, Santa Cruz, Orange
Cherries, Sweet	2	31	26.0	55.5	93,315	40	40	May 20-June 25	San Joaquin, Fresno, Kern, Tulare, Stanislaus
Dates 4/	1	99	4.5	24.2	37,510	61	56	Oct. 1-Dec. 15	Riverside, Imperial
Figs 4/	1	99	12.9	54.4	18,012	66	65	June 10-Sept. 15	Madera, Merced
Grapefruit, All	3	10	14.0	197.7	47,485	50	53	Continuous	Riverside, Tulare, Kern, Imperial, San Diego
Grapes, Raisin Type	---	---	246.0	2,833.0	438,458	---	---	May 15-Nov. 15	---
Grapes, Table Type	---	---	88.0	739.0	456,944	---	---	May 25-Dec. 15	---
Grapes, Wine Type	---	---	486.0	3,149.0	1,683,452	---	---	Aug. 5-Dec. 15	---
Grapes, All	1	91	820.0	6,721.0	2,578,854	2	2	---	Kern, Fresno, Tulare, Napa, Sonoma

Commodity Rank, Acreage, Production, Value and Leading Producing Counties, 2002

Commodity	U.S. Rank 1/	CA Share of U.S. Prod.	Area Harvested	Short Tons (2,000 Lbs.)	Total Value 2/	California Rank		Harvest Season	Leading Counties 3/
						2001	2002		
	Number	*Percent*	*1,000 Acres*	*1,000 Tons*	*$1,000*	*Number*			
Kiwifruit 4/	1	99	4.5	26.1	18,097	65	64	Oct. 1-May 31	Tulare, Butte, Yuba, Fresno, Kern
Lemons	1	87	49.5	695.4	287,026	22	20	Continuous	Ventura, Kern, Riverside, Tulare, Imperial
Nectarines 4/	1	99	36.5	299.0	114,517	33	34	June 10-Sept. 5	Fresno, Tulare, Kings, Kern, Stanislaus
Olives 4/	1	99	36.0	103.0	60,725	37	49	Sept. 25-Mar. 15	Tulare, Glenn, Tehama, Madera, Butte
Oranges, Navel and Misc.	---	21	128.0	1,200.0	412,305	---	---	Nov. 25-May 15	---
Oranges, Valencia	---	18	65.0	731.3	146,538	---	---	May 15-Oct. 15	---
Oranges, All	2	19	193.0	1,931.3	558,843	13	12	---	Tulare, Kern, Fresno, Ventura, Riverside
Peaches, Clingstone	1	100	31.0	562.0	132,639	---	---	July 15-Sept. 15	---
Peaches, Freestone	1	55	39.0	398.0	138,369	---	---	May 10-Sept. 15	---
Peaches, All	1	75	70.0	960.0	271,008	20	21	---	Fresno, Tulare, Sutter, Stanislaus, Kings
Pears, All	2	30	17.3	262.0	68,270	43	44	Aug. 5-Oct. 5	Sacramento, Lake, Fresno, Mendocino, Tulare
Pecans	8	1	2.6	1.9	4,826	70	71	Sept. 1-Nov. 30	Tulare
Pistachios 4/	1	99	83.0	151.5	336,330	31	17	Sept. 15-Dec. 10	Kern, Madera, Tulare, Kings, Fresno
Plums	1	91	36.0	201.0	77,586	46	41	May 25-Aug. 20	Fresno, Tulare, Kings, Kern, Madera
Plums, Dried 4/ (Prunes)	1	99	74.0	171.0	131,220	36	32	Aug. 15-Oct. 10	Tehama, Yuba, Sutter, Butte, Glenn
Tangerines, Mandarins, Tangelos, and Tangors	2	23	9.0	82.5	38,821	56	55	Nov. 1-Apr. 30	Tulare, Riverside, San Diego, Placer, Imperial
Walnuts 4/	1	99	200.0	282.0	304,560	16	19	Sept. 5-Nov. 10	San Joaquin, Stanislaus, Butte, Tulare, Sutter
Other Fruits and Nuts	---	---	43.0	259.0	140,700	---	---	---	---
**FIELD AND SEED CROPS TOTAL VALUE -- $3,124,499,000**									
Barley	7	2	75.0	122.4	12,495	68	68	May 15-Oct. 1	Modoc, Siskiyou, Fresno, Kern, San Luis Obispo
Beans, Dry	6	6	89.0	88.1	56,384	53	51	Aug. 20-Nov. 15	Stanislaus, Fresno, San Joaquin, Colusa, Sutter
Cotton Lint, All	2	12	686.0	495.2	633,466	11	11	Oct. 1-Jan. 1	Fresno, Kings, Kern, Merced, Tulare
Cottonseed	2	12	---	731.0	101,609	34	37	---	Fresno, Kings, Kern, Tulare, Merced
Grain, Corn	24	1	150.0	714.0	71,400	45	43	Sept. 1-Dec. 1	San Joaquin, Glenn, Sacramento, Solano, Yolo
Grain, Sorghum	15	1	11.0	29.3	3,219	72	72		Solano, Tulare, Sutter, Sacramento
Hay, Alfalfa and Other	2	6	1,640.0	9,594.0	913,509	6	10	Mar. 1-Nov. 5	Imperial, Kern, Tulare, Merced, Fresno
Oats	17	1	27.0	34.6	5,832	71	70	June 15-Oct. 1	Merced, Siskiyou, Sonoma, Modoc, Solano
Potatoes (Excl. Sweet)	8	4	44.9	884.8	307,024	26	18	Continuous	Kern, Imperial, San Joaquin, Modoc, Siskiyou
Potatoes, Sweet	2	20	10.4	122.2	56,456	48	50	July 15-Nov. 15	Merced, Stanislaus
Rice 5/ 6/	2	20	528.0	2,149.4	214,945	24	26	Sept. 1-Nov. 30	Colusa, Sutter, Butte, Glenn, Yuba
Sugar Beets 5/ 6/	5	7	49.9	1,971.0	65,831	51	47	Apr. 1-Dec. 10	Imperial, Fresno, Merced, Kern, Kings
Wheat, All	18	2	390.0	945.0	114,300	35	35	May 20-Sept. 1	Kings, Kern, Fresno, Imperial, Tulare
Oil Crops 7/	---	---	109.0	23.7	26,370	57	63	---	---
Other Field Crops	---	---	4,712.9	9,241.4	551,659	---	---	---	---
**NURSERY, GREENHOUSE AND FLORICULTURE CROPS TOTAL VALUE -- $3,306,826,000**									
Nursery Products 4/ 6/ 8/	1	20	---	---	2,347,697	3	3	Continuous	San Diego, Orange, Riverside, Monterey, Ventura, Los Angeles
Flowers and Foliage 9/	1	---	---	---	948,629	7	8	Continuous	San Diego, Santa Barbara, San Luis Obispo, Ventura, San Mateo
Christmas Trees	—	—	---	---	10,500	69	69	Nov. 1-Dec. 24	El Dorado, San Diego, Siskiyou, Santa Clara

1/ Based on quantity produced for crops and on quantity marketed for livestock and poultry products.
2/ Based on value of quantity harvested for crops, value of quantity marketed for livestock, and value of quantity produced for poultry products.
3/ Based on values published in the county agricultural commissioners' annual crop reports.
4/ Share of U.S. production based on 1997 Census of Agriculture.
5/ The 2002 price and value are based on the 2001 California average price.
6/ Extracted from county agricultural commissioners' annual crop reports.
7/ Includes sunflower, safflower, and canola.
8/ Includes trees, shrubs, vines, bulbs, turf, etc., not included in flowers and foliage category.
9/ Includes cut flowers, potted plants, foliage plants, bedding plants, and indoor decoratives.

Commodity Rank, Value and Leading Producing Counties, 2002

Commodity	U.S. Rank 1/	CA Share of U.S. Prod.	Marketings	Cash Receipts 2/	California Rank		Leading Counties 3/
					2001	2002	
	Number	*Percent*	*1,000 Pounds*	*$1,000*	*Number*		
**LIVESTOCK, DAIRY, POULTRY, AND APIARY**							
**TOTAL VALUE -- $6,241,632,000**							
Aquaculture	---	---	---	12,966	67	67	Riverside, Imperial, Sacramento
Cattle and Calves	7	4	2,330,200	1,228,722	4	5	Tulare, Imperial, Fresno, Merced, San Bernardino
Chickens, All	---	---	---	452,353	12	15	Fresno, Merced, Stanislaus, San Bernardino
Hogs and Pigs 4/	29	---	63,950	22,188	60	62	Tulare, Fresno, Stanislaus, Merced
Honey	2	14	23,320	30,083	63	59	Tulare, Kern, Merced, Imperial, Fresno
Milk and Cream	1	21	34,884,000	3,812,371	1	1	Tulare, Merced, Stanislaus, San Bernardino, Kings
Sheep and Lambs	2	12	77,835	50,388	52	52	Kern, Solano, Riverside, Fresno, Imperial
Turkeys	6	6	438,960	158,026	23	30	Fresno, Merced, Stanislaus, Kings, Tulare
Wool	3	9	3,650	1,643	74	73	---
			Millions of Eggs				
Eggs, Chicken	4	7	6,124	200,051	21	27	Riverside, Merced, San Diego, San Bernardino, Fresno
Other Livestock and Poultry	---	---	---	272,841	---		---

1/ Based on quantity produced for crops and on quantity marketed for livestock and poultry products.
2/ Based on value of quantity harvested for crops, value of quantity marketed for livestock, and value of quantity produced for poultry products.
3/ Based on values published in the county agricultural commissioners' annual crop reports.
4/ California share of U.S. production is less than one-half percent.

California's Farm Numbers and Size, 1950-2002

Year 1/	Number of Farms	Land in Farms	Average Size of Farm
		1,000 Acres	*Acres*
1950	144,000	37,500	260
1955	124,000	39,000	316
1960	108,000	38,800	359
1965	82,000	37,800	461
1970	64,000	36,600	572
1975	73,000	34,300	470
1980	81,000	33,800	417
1985	79,000	32,900	416
1990	85,000	30,800	362
1991	83,000	30,500	367
1992	82,000	30,200	368
1993	85,000	29,900	352
1994	85,000	29,600	348
1995	86,000	29,300	341
1996	86,000	29,000	337
1997	87,000	28,700	330
1998	89,000	28,500	320
1999	89,000	27,800	312
2000	87,500	27,800	318
2001	85,000	27,700	326
2002	84,000	27,700	330

1/ Until 1950 the definition of a farm was: Places of 10 or more acres that had annual sales of agricultural products of $50 or more and places of less than 10 acres that had annual sales of $250 or more. Starting in 1975 the new definition of a farm is: Places with annual sales of agricultural products of $1,000 or more.

County Rank, Total Value of Production and Leading Commodities, 2002 1/

Rank 2001	Rank 2002	County	Total Value $1,000	Leading Commodities
2	1	Fresno	3,415,591	Grapes, Cotton, Poultry, Tomatoes, Milk
1	2	Tulare	3,200,552	Milk, Navel and Valencia Oranges, Grapes, Cattle and Calves, Clingstone and Freestone Peaches
3	3	Monterey	2,812,069	Leaf and Head Lettuce, Broccoli, Strawberries, Nursery Products, Grapes
4	4	Kern	2,586,247	Grapes, All Citrus, All Carrots, Almonds and By-Products, All Milk
5	5	Merced	1,730,720	Milk, Chickens, Almonds, Cattle and Calves, All Tomatoes
7	6	Stanislaus	1,367,971	All Milk, Almonds, All Chickens, All Cattle and Calves, Deciduous Fruit and Nuts
6	7	San Joaquin	1,343,808	All Milk, All Grapes, All Tomatoes, Almond Meats, English Walnuts
8	8	San Diego	1,297,258	Cut Flowers and Foliage Plants, Nursery Woody Ornamentals and Bedding Plants, Avocados, Eggs, Tomatoes
11	9	Imperial	1,224,109	Cattle, Leaf and Head Lettuce, Alfalfa, Carrots, Livestock
10	10	Ventura	1,161,070	Strawberries, Lemons, Nursery Stock, Celery, Avocados
9	11	Riverside	1,063,478	Milk, Nursery Products, Table Grapes, Eggs, All Hay
12	12	Kings	1,023,807	Milk, Cotton, Cattle and Calves, Alfalfa Hay, Turkeys
15	13	Madera	778,385	Grapes, Almonds, Milk, Pistachios, Replacement Heifers
13	14	Santa Barbara	775,051	Strawberries, Broccoli, Wine Grapes, Head Lettuce, Cauliflower
14	15	San Bernardino	631,550	Milk, Cattle and Calves, Replacement Heifers, Eggs, Nursery Trees and Shrubs
16	16	Sonoma	564,767	Wine Grapes, Market Milk, Livestock and Poultry, Nursery Ornamentals, Cattle and Calves
17	17	San Luis Obispo	478,872	Wine Grapes, Broccoli, Cattle and Calves, Head Lettuce, Indoor Decorative Flowers
18	18	Napa	387,863	Wine Grapes, Nursery Products, Cattle and Calves, Livestock Products, Range Pasture
20	19	Orange	344,289	Nursery Stock and Cut Flowers, Strawberries, Tomatoes, Bell and Misc. Peppers, Avocados
24	20	Glenn	303,892	Paddy Rice, Almonds, Dairy Products, Cattle and Calves, Alfalfa Hay
22	21	Yolo	299,780	Processing Tomatoes, Wine Grapes, Alfalfa Hay, Milling Rice, Vegetable Crops
26	22	Sutter	291,061	Rice, Peaches, Walnuts, Dried Plums, Tomatoes
25	23	Colusa	290,266	Rice, Processing Tomatoes, Almond Meats, Rice Seed, Cotton Lint
28	24	Butte	287,497	Rice, Almonds, English Walnuts, Dried Plums, Nursery Stock
19	25	Santa Cruz	284,808	Strawberries and Raspberries, Nursery Plants, Head and Leaf Lettuce, Vegetable Crops, Cut Flowers
27	26	Los Angeles	275,969	Nursery Plants, Root Vegetables, Dry Onions, Peaches, Indoor Foliage and Flowering Plants
21	27	Sacramento	275,937	Wine Grapes, Market Milk, Bartlett Pears, Nursery Stock, Cattle and Calves
23	28	Santa Clara	255,374	Nursery Crops, Mushrooms, Bell Peppers, Cut Flowers, Wine Grapes
29	29	San Benito	214,841	Nursery Stock, Baby, Leaf and Head Lettuce, Vegetable Crops, Wine Grapes, Bell Peppers
30	30	Solano	199,486	Nursery Stock, Processing Tomatoes, Cattle and Calves, Alfalfa, Wine Grapes
31	31	San Mateo	181,972	Ornamental and Potted Nursery Stock, Mushrooms, Cut and Potted Flowers, Brussels Sprouts
33	32	Yuba	133,484	Rice, Peaches, Dried Plums, Cattle and Calves, Walnuts
32	33	Mendocino	126,953	Wine Grapes, Bartlett Pears, Cattle and Calves, Milk, Nursery
34	34	Tehama	126,372	English Walnuts, Dried Plums, Market Milk, Olives, Almonds
36	35	Siskiyou	119,021	Alfalfa Hay, Strawberry Nursery Plants, Irish Potatoes, Fed Heifers and Steers, Irrigated Pasture
35	36	Humboldt	104,695	Nursery Products, All Milk, Cattle and Calves, Range Pasture, Biomass
37	37	Contra Costa	100,154	Nursery Bedding Plants, Cattle and Calves, Sweet Corn, Livestock Products, Grapes
41	38	Modoc	69,988	Alfalfa Hay, Irish Potatoes, Cattle and Calves, Vegetable Crops, Onions
38	39	Placer	66,556	Milling Rice, Nursery Products, Cattle and Calves, Chickens, Fruit and Nut Crops
39	40	Lake	63,907	Wine Grapes, Bartlett Pears, Nursery Products, Cattle and Calves, English Walnuts
40	41	Shasta	52,198	Cattle, Strawberry Nursery Plants, Alfalfa and Other Hay, Irrigated and Range Pasture, Wild Rice
42	42	Marin	44,477	Market Milk, Cattle and Calves, Range Pasture, Poultry, Fish
43	43	Lassen	43,349	All Hay, Livestock, Strawberry and Field Crop Nursery Plants, Pasture, Mint
45	44	Del Norte	31,092	All Milk, Nursery Bulbs, Woody Ornamentals and Misc. Products, Cattle and Calves, Irrigated Pasture
44	45	Alameda	30,013	Woody Ornamentals and Nursery Products, Wine Grapes, Cattle and Calves, Range Pasture, Vegetable Crops
46	46	El Dorado	26,544	Apples, Wine Grapes, Cattle and Calves, Christmas Trees and Cut Greens, Range Pasture
48	47	Amador	23,737	Wine Grapes, Cattle and Calves, Range and Irrigated Pasture, Livestock, English Walnuts
47	48	Mono	21,835	Carrots, Alfalfa and Other Hay, Cattle, Irrigated Pasture, Sheep and Lambs
50	49	Tuolumne	20,270	Livestock, Cattle and Calves, Irrigated and Range Pasture, Firewood, Bees
49	50	Mariposa	19,600	Cattle and Calves, Range Pasture, Livestock Products, Poultry, Fruit and Nut Crops
51	51	Calaveras	17,716	Cattle and Calves, Poultry, Wine Grapes, Livestock and Poultry Products, Walnuts
52	52	Plumas	14,617	Cattle, All Pasture, All Hay, Livestock, Fruit and Nut Crops
53	53	Inyo	14,240	Nursery Turf, Cattle, Alfalfa and Other Hay, Carrots, Irrigated and Range Pasture
54	54	Nevada	8,190	Cattle and Calves, Pasture and Range, Wine Grapes, Fruit and Vegetable Crops, Nursery Stock
55	55	Sierra	4,400	Cattle, Irrigated, Forage, and Range Pasture, Wild, Alfalfa and Grain Hay, Fruit and Nuts Crops, Livestock
57	56	Trinity 2/	2,139	Cattle and Calves, Irrigated and Range Pasture, Wine Grapes, Fruit and Nut Crops, Nursery Products
56	57	San Francisco	1,645	Vegetable Crops, Cut Flowers
58	58	Alpine 2/	0	—

1/ Totals vary slightly from those published by counties due to classification differences between county and state reports.

2/ Reports for Alpine and Trinity counties were not available at publication so 2001 data were used for this report.

Seasonal Rainfall with Comparisons to Normal, 2002-2003

Stations	2001-2002		2002-2003		Normal Annual Rainfall
	Precipitation July 1 to June 24	Percent of Normal	Precipitation July 1 to June 29	Percent of Normal	
	Inches		*Inches*		*Inches*
**North Coast**					
Eureka	39.77	106	52.40	140	37.53
Ukiah	29.21	77	41.94	110	38.01
Santa Rosa	32.20	108	34.76	116	29.92
**Central Coast**					
San Francisco Airport	19.37	98	23.82	120	19.79
San Jose	10.94	77	15.36	108	14.20
Livermore Tele	11.92	85	16.69	118	14.09
Salinas Airport	3.62	30	7.02	57	12.22
Monterey FAA	11.36	78	14.38	99	14.57
King City	6.44	58	11.16	100	11.12
Paso Robles Airport	5.59	44	10.48	82	12.71
**Sacramento Valley**					
Redding	28.62	101	34.99	123	28.36
Red Bluff FSS	18.68	83	27.06	120	22.51
Chico AFS	27.64	108	33.10	129	25.57
Marysville	21.91	104	20.52	97	21.05
Sacramento Airport	17.95	102	16.78	95	17.59
**San Joaquin Valley**					
Stockton WSO	12.12	87	12.13	87	13.97
Fresno	7.02	66	9.82	92	10.64
Bakersfield	3.59	63	5.94	104	5.73
**South Coast**					
Santa Maria Airport	6.46	52	12.59	101	12.47
Santa Barbara	9.18	56	22.32	137	16.29
Oxnard	6.21	44	17.85	126	14.20
Los Angeles	4.37	30	17.01	115	14.73
Riverside	3.30	33	11.40	114	9.98
San Diego Airport	3.01	30	10.65	107	9.91
**Southeastern Interior**					
Bishop	2.04	38	3.89	73	5.36
Lancaster	2.05	28	7.06	97	7.26
Daggett Airport	1.64	42	4.17	106	3.94
Thermal Airport	0.42	13	1.80	56	3.21
Blythe	0.66	18	2.40	66	3.63
Imperial	0.02	1	2.19	80	2.75
**Cascade-Sierra**					
Alturas	10.58	87	10.00	82	12.13
Mt. Shasta	37.44	103	54.70	151	36.22
Blue Canyon	61.71	91	54.10	80	67.85
Yosemite Valley	25.59	68	23.56	63	37.39

Source: Western Regional Climate Center, Reno, Nevada

California Field Crops

California's 2002 field crop production was worth $3.12 billion at the farm-gate level. The average value per harvested acre increased from $642 in 2001 to $663 in 2002. Production of the major field crops in California was 29.0 million tons, up 12 percent from the 26.0 million tons in 2001.

Notable Increases in Value:

Beans, Dry	21%
Oats	188%
Potatoes, All	66%
Sugar Beets	23%

Significant Decreases in Value:

Barley	9%
Cotton, Seed	16%
Hay, All	10%
Potatoes, Sweet	9%

Hay was the state's highest-valued field crop at $913 million, followed by cotton with a value of $623 million. Of the total hay value, alfalfa accounted for $797 million and other hay was $116 million. Nationally, the Golden State is the leading alfalfa-producing state and ranked second for all cotton and all hay production. The value of all hay decreased 10 percent. This was due to a 17 percent fall in unit price per ton—from $115.00 in 2001 to $95.50 in 2002. However, production of all hay increased by 8 percent in 2002. Hay was in strong demand to provide for the growing dairy industry.

All cotton production and harvested acreage dropped 14 and 21 percent, respectively, from last year. American Pima production fell 6 percent and Upland production declined 18 percent. The unit price of American Pima dropped fractionally, while Upland cotton increased by 29 percent. Planting began in mid-March, but cool, wet weather during the month and into early April caused a large amount of the acreage to be replanted. Due to poor Upland prices, a number of growers switched to American Pima. Warm weather during the growing season was beneficial and harvest began by mid-September in the Imperial Valley and late September in the San Joaquin Valley. Harvest progressed well and was nearly complete by mid-December.

Rice harvested acreage and production both increased 12 percent, while the value jumped by 6 percent. Ideal conditions throughout the growing season provided for excellent yields. The weather during harvest also helped to boost yields as very little rainfall occurred during the harvest season.

The large increase in dry bean value was attributed to a 5 percent increase in harvested acreage and an 18 percent increase in production. Harvest of the crop went well with good quality reported.

Corn for grain harvested acreage and production both dropped 6 percent from the previous year. Planting of the crop began in March and continued into April and May. Growth was normal during the growing season and harvest began the last week of July and continued through the fall.

Sugar beet acreage was up 12 percent, while the production and value both jumped 23 percent from 2001.

Field Crop Acreage, Production and Value, 1993-2002

Crop	Crop Year	Planted	Harvested	Yield Per Acre	Production	Value Per Unit	Total Value
Barley		*Acres*	*Acres*	*Tons*	*Tons*	*Dollars Per Ton*	*$1,000*
	1993	250,000	200,000	1.56	312,000	105.00	32,760
	1994	290,000	220,000	1.56	343,200	111.25	38,181
	1995	260,000	200,000	1.68	336,000	136.67	45,920
	1996	250,000	190,000	1.44	273,600	153.75	42,066
	1997	230,000	150,000	1.37	205,200	114.59	23,513
	1998	170,000	125,000	1.44	180,000	97.08	17,475
	1999	140,000	100,000	1.54	153,600	97.50	14,976
	2000	130,000	95,000	1.63	155,000	98.75	15,310
	2001	160,000	110,000	1.27	139,900	98.33	13,759
	2002	130,000	75,000	1.63	122,400	102.08	12,495
Beans, Black-Eyed Dry 1/ 2/		*Acres*	*Acres*	*Pounds*	*Cwt.*	*Dollars Per Cwt.*	*$1,000*
	1993	24,000	23,000	2,380	548,000	---	---
	1994	32,000	30,000	2,570	771,000	---	---
	1995	44,500	43,500	2,230	970,000	---	---
	1996	24,000	23,000	2,220	511,000	---	---
	1997	30,000	29,000	2,400	695,000	---	---
	1998	33,000	31,000	1,840	570,000	---	---
	1999	39,500	38,500	2,010	775,000	---	---
	2000	15,300	15,300	2,160	330,000	---	---
	2001	12,000	12,000	2,420	290,000	---	---
	2002	12,600	12,400	2,520	313,000	---	---
Beans, Garbanzo Dry 1/ 2/		*Acres*	*Acres*	*Pounds*	*Cwt.*	*Dollars Per Cwt.*	*$1,000*
	1993	17,500	16,500	1,350	222,000	---	---
	1994	14,000	14,000	1,870	262,000	---	---
	1995	19,000	17,000	1,690	288,000	---	---
	1996	25,000	23,000	1,530	352,000	---	---
	1997	9,000	9,000	1,670	150,000	---	---
	1998	5,000	5,000	1,600	80,000	---	---
	1999	16,500	16,500	1,730	285,000	---	---
	2000	24,500	23,500	1,460	343,000	---	---
	2001	29,000	27,000	1,270	342,000	---	---
	2002	18,500	18,000	1,600	288,000	---	---
Beans, Dark Red Kidney Dry 1/ 2/		*Acres*	*Acres*	*Pounds*	*Cwt.*	*Dollars Per Cwt.*	*$1,000*
	1993	7,000	7,000	1,730	121,000	---	---
	1994	7,000	7,000	1,790	125,000	---	---
	1995	4,500	4,500	1,600	72,000	---	---
	1996	5,000	5,000	1,640	82,000	---	---
	1997	5,000	5,000	1,800	90,000	---	---
	1998	5,500	5,500	850	47,000	---	---
	1999	3,500	3,500	1,310	46,000	---	---
	2000	6,000	6,000	1,370	82,000	---	---
	2001	2,500	2,500	1,600	40,000	---	---
	2002	2,500	2,500	1,640	41,000	---	---
Beans, Light Red Kidney Dry 1/ 2/		*Acres*	*Acres*	*Pounds*	*Cwt.*	*Dollars Per Cwt.*	*$1,000*
	1993	20,000	19,000	2,120	402,000	---	---
	1994	14,000	13,500	1,950	263,000	---	---
	1995	13,000	12,000	1,740	209,000	---	---
	1996	10,000	10,000	1,870	187,000	---	---
	1997	10,000	10,000	1,980	198,000	---	---
	1998	9,500	8,500	1,380	117,000	---	---
	1999	8,000	8,000	1,510	121,000	---	---
	2000	11,000	11,000	1,480	163,000	---	---
	2001	6,200	6,200	1,450	90,000	---	---
	2002	6,000	6,000	1,270	76,000	---	---

Field Crop Acreage, Production and Value, 1993-2002

Crop	Crop Year	Planted	Harvested	Yield Per Acre	Production	Value Per Unit	Total Value
Beans, Baby Lima Dry 1/ 2/		*Acres*	*Acres*	*Pounds*	*Cwt.*	*Dollars Per Cwt.*	*$1,000*
	1993	14,000	14,000	2,510	351,000	---	---
	1994	26,000	24,500	2,490	609,000	---	---
	1995	23,000	22,000	2,320	510,000	---	---
	1996	24,000	23,000	2,250	517,000	---	---
	1997	37,000	36,000	2,510	902,000	---	---
	1998	13,000	12,000	1,700	204,000	---	---
	1999	27,000	26,000	2,380	620,000	---	---
	2000	24,500	23,500	2,310	542,000	---	---
	2001	12,200	11,500	2,040	235,000	---	---
	2002	21,500	21,000	2,390	501,000	---	---
Beans, Large Lima Dry 1/ 2/		*Acres*	*Acres*	*Pounds*	*Cwt.*	*Dollars Per Cwt.*	*$1,000*
	1993	15,000	15,000	2,330	350,000	---	---
	1994	24,000	23,500	1,830	429,000	---	---
	1995	21,000	20,000	2,080	415,000	---	---
	1996	21,000	20,000	1,970	394,000	---	---
	1997	30,000	29,000	2,480	718,000	---	---
	1998	26,000	25,000	1,250	312,000	---	---
	1999	25,000	24,000	1,800	433,000	---	---
	2000	20,500	19,500	2,240	437,000	---	---
	2001	14,800	14,500	2,250	326,000	---	---
	2002	19,000	18,200	1,840	334,000	---	---
Beans, Pink Dry 1/ 2/		*Acres*	*Acres*	*Pounds*	*Cwt.*	*Dollars Per Cwt.*	*$1,000*
	1993	4,500	4,500	2,000	90,000	---	---
	1994	7,000	7,000	2,160	151,000	---	---
	1995	7,000	6,000	1,630	98,000	---	---
	1996	8,000	8,000	1,560	125,000	---	---
	1997	4,000	4,000	1,550	62,000	---	---
	1998	5,500	5,500	1,070	59,000	---	---
	1999	2,000	2,000	1,150	23,000	---	---
	2000	700	700	860	6,000	---	---
	2001	---	---	---	---	---	---
	2002	---	---	---	---	---	---
Beans, Other Dry 1/ 2/ 3/		*Acres*	*Acres*	*Pounds*	*Cwt.*	*Dollars Per Cwt.*	*$1,000*
	1993	7,000	7,000	1,800	126,000	---	---
	1994	12,000	11,500	1,400	161,000	---	---
	1995	13,000	12,000	1,480	178,000	---	---
	1996	11,000	11,000	1,430	157,000	---	---
	1997	10,000	10,000	1,850	185,000	---	---
	1998	12,500	12,500	1,320	165,000	---	---
	1999	13,500	13,500	1,130	152,000	---	---
	2000	12,500	12,500	1,250	156,000	---	---
	2001	11,300	11,300	1,530	173,000	---	---
	2002	11,900	10,900	1,920	209,000	---	---
Beans, All Dry Edible 3/		*Acres*	*Acres*	*Pounds*	*Cwt.*	*Dollars Per Cwt.*	*$1,000*
	1993	109,000	106,000	2,085	2,210,000	35.30	78,013
	1994	136,000	131,000	2,120	2,771,000	34.80	96,431
	1995	145,000	137,000	2,000	2,740,000	34.80	95,352
	1996	128,000	123,000	1,890	2,325,000	38.50	89,513
	1997	135,000	132,000	2,270	3,000,000	29.40	88,200
	1998	110,000	105,000	1,480	1,554,000	36.10	56,099
	1999	135,000	132,000	1,860	2,455,000	27.00	66,285
	2000	115,000	112,000	1,840	2,059,000	26.80	55,181
	2001	88,000	85,000	1,760	1,496,000	31.20	46,675
	2002	92,000	89,000	1,980	1,762,000	32.00	56,384

Field Crop Acreage, Production and Value, 1993-2002

Crop	Crop Year	Planted	Harvested	Yield Per Acre	Production	Value Per Unit	Total Value
Corn For Grain 4/		*Acres*	*Acres*	*Tons*	*Tons*	*Dollars Per Ton*	*$1,000*
	1993	400,000	170,000	4.62	785,400	113.93	89,480
	1994	420,000	180,000	4.76	856,800	106.43	91,188
	1995	435,000	150,000	4.48	672,000	141.07	94,800
	1996	500,000	220,000	4.48	985,600	120.36	118,624
	1997	585,000	265,000	4.76	1,261,400	108.93	137,403
	1998	600,000	245,000	4.48	1,097,600	95.00	104,272
	1999	525,000	185,000	4.76	880,600	89.29	78,625
	2000	540,000	205,000	4.76	975,800	87.14	85,034
	2001	480,000	160,000	4.76	761,600	87.50	66,640
	2002	545,000	150,000	4.76	714,000	100.00	71,400
Corn For Silage 2/		*Acres*	*Acres*	*Tons*	*Tons*	*Dollars Per Ton*	*$1,000*
	1993	---	225,000	25.00	5,625,000	---	---
	1994	---	235,000	25.00	5,875,000	---	---
	1995	---	280,000	25.00	7,000,000	---	---
	1996	---	275,000	25.00	6,875,000	---	---
	1997	---	315,000	26.00	8,190,000	---	---
	1998	---	350,000	25.00	8,750,000	---	---
	1999	---	335,000	26.00	8,710,000	---	---
	2000	---	330,000	26.00	8,580,000	---	---
	2001	---	315,000	26.00	8,190,000	---	---
	2002	---	390,000	26.00	10,140,000	---	---
Cotton, American-Pima 5/		*Acres*	*Acres*	*Pounds*	*Bales*	*Cents Per Lb.*	*$1,000*
	1993	91,000	91,000	1,132	214,600	84.0	86,527
	1994	81,000	80,800	1,098	184,800	102.0	90,478
	1995	115,000	115,000	937	224,500	126.0	135,778
	1996	165,000	164,000	1,098	375,000	107.0	192,600
	1997	185,000	184,000	1,141	437,200	102.0	214,053
	1998	200,000	180,000	941	352,800	92.3	156,305
	1999	240,000	239,000	1,210	602,700	85.1	246,191
	2000	145,000	144,000	1,154	346,300	101.0	167,886
	2001	240,000	239,000	1,283	639,000	85.6	262,552
	2002	210,000	209,000	1,386	603,300	85.1	246,436
Cotton, Upland 5/		*Acres*	*Acres*	*Pounds*	*Bales*	*Cents Per Lb.*	*$1,000*
	1993	1,050,000	1,045,000	1,340	2,918,000	65.7	920,220
	1994	1,100,000	1,095,000	1,191	2,717,000	80.3	1,047,240
	1995	1,170,000	1,165,000	953	2,312,000	82.1	911,113
	1996	1,000,000	995,000	1,153	2,390,000	76.5	877,608
	1997	880,000	875,000	1,202	2,191,000	73.2	769,830
	1998	650,000	620,000	887	1,146,000	67.8	372,954
	1999	610,000	605,000	1,254	1,580,000	56.2	426,221
	2000	775,000	770,000	1,378	2,210,000	52.0	551,616
	2001	630,000	625,000	1,359	1,770,000	41.6	353,434
	2002	480,000	477,000	1,469	1,460,000	53.8	377,030
Cotton, All 5/		*Acres*	*Acres*	*Pounds*	*Bales*	*Cents Per Lb.*	*$1,000*
	1993	1,141,000	1,136,000	1,324	3,132,600	67.0	1,006,747
	1994	1,181,000	1,175,800	1,185	2,901,800	81.7	1,137,718
	1995	1,285,000	1,280,000	951	2,536,500	86.0	1,046,891
	1996	1,165,000	1,159,000	1,145	2,765,000	80.6	1,070,208
	1997	1,065,000	1,059,000	1,191	2,628,200	78.0	983,883
	1998	850,000	800,000	899	1,498,800	73.6	529,259
	1999	850,000	844,000	1,241	2,182,700	64.2	672,412
	2000	920,000	914,000	1,342	2,556,300	58.6	719,502
	2001	870,000	864,000	1,338	2,409,000	53.3	615,986
	2002	690,000	686,000	1,406	2,063,300	63.0	623,466

Field Crop Acreage, Production and Value, 1993-2002

Crop	Crop Year	Planted	Harvested	Yield Per Acre	Production	Value Per Unit	Total Value
Cottonseed 2/		*Acres*	*Acres*	*Tons*	*Tons*	*Dollars Per Ton*	*$1,000*
	1993	---	---	---	1,142,000	132.00	150,744
	1994	---	---	---	1,063,000	143.00	152,009
	1995	---	---	---	940,000	132.00	124,080
	1996	---	---	---	1,020,000	176.00	179,520
	1997	---	---	---	942,000	174.00	163,908
	1998	---	---	---	544,000	177.00	96,288
	1999	---	---	---	799,000	137.00	109,463
	2000	---	---	---	909,000	139.00	126,351
	2001	---	---	---	849,000	142.00	120,558
	2002	---	---	---	731,000	139.00	101,609
Hay, Alfalfa 2/		*Acres*	*Acres*	*Tons*	*Tons*	*Dollars Per Ton*	*$1,000*
	1993	---	920,000	6.90	6,348,000	105.00	666,540
	1994	---	950,000	7.00	6,650,000	112.00	744,800
	1995	---	940,000	6.90	6,486,000	101.00	655,086
	1996	---	940,000	7.00	6,580,000	110.00	723,800
	1997	---	950,000	7.20	6,840,000	119.00	813,960
	1998	---	1,050,000	6.60	6,930,000	98.00	679,140
	1999	---	1,050,000	6.90	7,245,000	90.00	652,050
	2000	---	1,020,000	7.00	7,140,000	92.00	656,880
	2001	---	1,010,000	7.20	7,272,000	119.00	865,368
	2002	---	1,140,000	7.10	8,094,000	98.50	797,259
Hay, Other 2/		*Acres*	*Acres*	*Tons*	*Tons*	*Dollars Per Ton*	*$1,000*
	1993	---	460,000	2.70	1,242,000	74.00	91,908
	1994	---	520,000	3.00	1,560,000	69.50	108,420
	1995	---	530,000	3.50	1,855,000	67.50	125,213
	1996	---	510,000	2.80	1,428,000	82.00	117,096
	1997	---	560,000	2.80	1,568,000	98.00	153,664
	1998	---	580,000	2.80	1,624,000	68.00	110,432
	1999	---	530,000	2.90	1,537,000	75.00	115,275
	2000	---	510,000	2.80	1,428,000	78.50	112,098
	2001	---	530,000	3.10	1,643,000	93.50	153,621
	2002	---	500,000	3.00	1,500,000	77.50	116,250
Hay, All 2/ 6/		*Acres*	*Acres*	*Tons*	*Tons*	*Dollars Per Ton*	*$1,000*
	1993	---	1,380,000	5.50	7,590,000	102.00	758,448
	1994	---	1,470,000	5.59	8,210,000	107.00	853,220
	1995	---	1,470,000	5.67	8,341,000	98.50	780,299
	1996	---	1,450,000	5.52	8,008,000	108.00	840,896
	1997	---	1,510,000	5.57	8,408,000	117.00	967,624
	1998	---	1,630,000	5.25	8,554,000	96.00	789,572
	1999	---	1,580,000	5.56	8,782,000	89.00	767,325
	2000	---	1,530,000	5.60	8,568,000	90.50	768,978
	2001	---	1,540,000	5.79	8,915,000	115.00	1,018,989
	2002	---	1,640,000	5.85	9,594,000	95.50	913,509
Oats		*Acres*	*Acres*	*Tons*	*Tons*	*Dollars Per Ton*	*$1,000*
	1993	300,000	30,000	1.28	38,400	115.63	4,440
	1994	310,000	30,000	1.28	38,400	183.13	7,032
	1995	350,000	30,000	1.36	40,800	130.63	5,330
	1996	300,000	30,000	1.20	36,000	138.75	4,995
	1997	350,000	30,000	1.28	38,400	123.75	4,752
	1998	320,000	30,000	1.20	36,000	85.00	3,060
	1999	275,000	25,000	1.36	34,000	93.75	3,188
	2000	220,000	25,000	1.20	30,000	96.88	2,906
	2001	260,000	15,000	0.96	14,400	140.63	2,025
	2002	260,000	27,000	1.28	34,600	168.75	5,832

Field Crop Acreage, Production and Value, 1993-2002

Crop	Crop Year	Planted	Harvested	Yield Per Acre	Production	Value Per Unit	Total Value
Potatoes, Winter		*Acres*	*Acres*	*Cwt.*	*Cwt.*	*Dollars Per Cwt.*	*$1,000*
	1993	5,600	5,200	200	1,040,000	20.20	21,008
	1994	4,500	4,500	215	968,000	21.10	20,425
	1995	5,000	5,000	260	1,300,000	18.20	23,660
	1996	5,700	5,700	250	1,425,000	21.10	30,068
	1997	6,600	6,600	235	1,551,000	18.10	28,073
	1998	7,000	7,000	220	1,540,000	22.40	34,496
	1999	8,500	8,500	260	2,210,000	22.60	49,946
	2000	9,000	9,000	320	2,880,000	17.00	48,960
	2001	9,000	9,000	310	2,790,000	19.70	54,963
	2002	9,000	9,000	270	2,430,000	24.00	58,320
Potatoes, Spring		*Acres*	*Acres*	*Cwt.*	*Cwt.*	*Dollars Per Cwt.*	*$1,000*
	1993	19,500	19,500	385	7,508,000	13.00	97,604
	1994	20,500	20,500	380	7,790,000	11.60	90,364
	1995	18,000	17,800	350	6,230,000	13.60	84,728
	1996	20,100	20,100	375	7,538,000	13.00	97,994
	1997	20,700	20,700	390	8,073,000	9.85	79,519
	1998	18,500	18,500	335	6,198,000	10.50	65,079
	1999	19,000	19,000	400	7,600,000	10.80	82,080
	2000	18,800	18,800	395	7,426,000	12.20	90,597
	2001	15,500	15,500	390	6,045,000	12.40	74,958
	2002	19,000	19,000	405	7,695,000	19.80	152,361
Potatoes, Summer		*Acres*	*Acres*	*Cwt.*	*Cwt.*	*Dollars Per Cwt.*	*$1,000*
	1993	4,800	4,800	330	1,584,000	13.30	21,067
	1994	5,700	5,700	370	2,109,000	13.00	27,417
	1995	5,500	5,500	320	1,760,000	17.50	30,800
	1996	5,800	5,800	360	2,088,000	14.60	30,485
	1997	5,900	5,900	360	2,124,000	14.50	30,798
	1998	6,200	6,100	355	2,166,000	11.90	25,775
	1999	6,700	6,700	360	2,412,000	12.80	30,874
	2000	7,500	7,500	355	2,663,000	18.10	48,200
	2001	8,000	8,000	355	2,840,000	15.10	42,884
	2002	7,300	7,300	360	2,628,000	19.10	50,195
Potatoes, Fall		*Acres*	*Acres*	*Cwt.*	*Cwt.*	*Dollars Per Cwt.*	*$1,000*
	1993	12,000	12,000	400	4,800,000	9.35	44,880
	1994	14,000	14,000	400	5,600,000	6.05	33,880
	1995	13,000	13,000	410	5,330,000	9.65	51,435
	1996	11,500	11,500	400	4,600,000	4.80	22,080
	1997	10,500	10,500	400	4,200,000	8.10	34,020
	1998	10,300	10,300	360	3,708,000	8.40	31,147
	1999	9,000	9,000	445	4,005,000	7.35	29,437
	2000	8,700	8,700	430	3,741,000	5.80	21,698
	2001	3,200	3,200	445	1,424,000	8.15	11,606
	2002	8,000	8,200	520	4,264,000	8.45	36,031
Potatoes, All		*Acres*	*Acres*	*Cwt.*	*Cwt.*	*Dollars Per Cwt.*	*$1,000*
(Excluding Sweet)	1993	41,900	41,500	360	14,932,000	12.40	184,559
	1994	44,700	44,700	368	16,467,000	10.60	172,086
	1995	41,500	41,300	354	14,620,000	13.10	190,623
	1996	43,100	43,100	363	15,651,000	11.70	180,627
	1997	43,700	43,700	365	15,948,000	10.90	172,410
	1998	42,000	41,900	325	13,612,000	11.60	156,497
	1999	43,200	43,200	376	16,227,000	11.90	192,337
	2000	44,000	44,000	380	16,710,000	12.50	209,455
	2001	35,700	35,700	367	13,099,000	14.20	184,411
	2002	43,500	43,500	391	17,017,000	17.50	296,907

Field Crop Acreage, Production and Value, 1993-2002

Crop	Crop Year	Planted	Harvested	Yield Per Acre	Production	Value Per Unit	Total Value
Potatoes, Sweet		*Acres*	*Acres*	*Cwt.*	*Cwt.*	*Dollars Per Cwt.*	*$1,000*
	1993	8,300	8,300	210	1,743,000	24.30	42,355
	1994	8,200	8,200	205	1,681,000	28.70	48,245
	1995	9,600	9,600	200	1,920,000	33.00	63,360
	1996	9,600	9,600	225	2,160,000	26.10	56,376
	1997	9,700	9,700	205	1,989,000	28.00	55,692
	1998	9,700	9,700	220	2,134,000	25.80	55,057
	1999	10,000	10,000	240	2,400,000	28.80	69,120
	2000	10,500	10,500	250	2,625,000	24.10	63,263
	2001	10,000	10,000	230	2,300,000	26.90	61,870
	2002	10,400	10,400	235	2,444,000	23.10	56,456
Rice, Long 2/		*Acres*	*Acres*	*Cwt.*	*Cwt.*	*Dollars Per Cwt.*	*$1,000*
	1993	14,000	14,000	81.80	1,145,000	---	---
	1994	7,000	7,000	81.00	567,000	---	---
	1995	8,000	8,000	75.00	600,000	---	---
	1996	5,000	5,000	72.00	360,000	---	---
	1997	9,000	9,000	77.00	693,000	---	---
	1998	9,000	9,000	59.70	537,000	---	---
	1999	5,000	5,000	68.00	340,000	---	---
	2000	9,000	9,000	71.00	639,000	---	---
	2001	13,000	13,000	77.00	1,001,000	---	---
	2002	7,000	7,000	64.00	448,000	---	---
Rice, Medium 2/		*Acres*	*Acres*	*Cwt.*	*Cwt.*	*Dollars Per Cwt.*	*$1,000*
	1993	413,000	410,000	83.20	34,112,000	---	---
	1994	470,000	468,000	85.10	39,827,000	---	---
	1995	449,000	447,000	76.00	33,972,000	---	---
	1996	484,000	482,000	75.00	36,150,000	---	---
	1997	493,000	491,000	82.60	40,557,000	---	---
	1998	420,000	418,000	69.90	29,218,000	---	---
	1999	455,000	450,000	73.00	32,850,000	---	---
	2000	507,000	505,000	80.00	40,400,000	---	---
	2001	435,000	433,000	83.00	35,939,000	---	---
	2002	500,000	495,000	83.00	41,085,000	---	---
Rice, Short 2/		*Acres*	*Acres*	*Cwt.*	*Cwt.*	*Dollars Per Cwt.*	*$1,000*
	1993	13,000	13,000	78.00	1,014,000	---	---
	1994	10,000	10,000	83.00	830,000	---	---
	1995	10,000	10,000	78.00	780,000	---	---
	1996	13,000	13,000	73.00	949,000	---	---
	1997	16,000	16,000	81.00	1,296,000	---	---
	1998	31,000	31,000	52.60	1,631,000	---	---
	1999	50,000	50,000	70.00	3,500,000	---	---
	2000	34,000	34,000	73.00	2,482,000	---	---
	2001	25,000	25,000	62.00	1,550,000	---	---
	2002	26,000	26,000	56.00	1,456,000	---	---
Rice, All 7/		*Acres*	*Acres*	*Cwt.*	*Cwt.*	*Dollars Per Cwt.*	*$1,000*
	1993	440,000	437,000	83.00	36,271,000	8.27	299,961
	1994	487,000	485,000	85.00	41,224,000	6.97	287,331
	1995	467,000	465,000	76.00	35,352,000	8.79	310,744
	1996	502,000	500,000	74.90	37,459,000	7.91	296,301
	1997	518,000	516,000	82.50	42,546,000	7.95	338,241
	1998	460,000	458,000	68.50	31,386,000	9.19	288,437
	1999	510,000	505,000	72.70	36,690,000	6.97	255,729
	2000	550,000	548,000	79.40	43,521,000	4.99	217,170
	2001	473,000	471,000	81.70	38,490,000	5.28	203,227
	2002	533,000	528,000	81.40	42,989,000	5.00	214,945

Field Crop Acreage, Production and Value, 1993-2002

Crop	Crop Year	Planted	Harvested	Yield Per Acre	Production	Value Per Unit	Total Value
Sorghum For Grain 4/ 8/		*Acres*	*Acres*	*Tons*	*Tons*	*Dollars Per Ton*	*$1,000*
	2000	12,000	8,000	2.10	16,800	82.20	1,380
	2001	10,000	7,000	2.52	17,600	87.60	1,545
	2002	15,000	11,000	2.66	29,300	110.00	3,219
Sorghum For Silage 2/ 8/		*Acres*	*Acres*	*Tons*	*Tons*	*Dollars Per Ton*	*$1,000*
	2000	---	4,000	15.00	60,000	---	---
	2001	---	3,000	20.00	60,000	---	---
	2002	---	4,000	17.00	68,000	---	---
Sugar Beets 2/ 9/		*Acres*	*Acres*	*Tons*	*Tons*	*Dollars Per Ton*	*$1,000*
	1993	138,000	136,000	26.00	3,536,000	35.50	125,528
	1994	143,000	141,000	28.00	3,948,000	33.50	132,258
	1995	116,000	114,000	28.00	3,192,000	39.40	125,765
	1996	84,000	82,000	29.50	2,419,000	43.00	104,017
	1997	101,000	99,000	30.00	2,970,000	40.60	120,582
	1998	101,000	99,000	27.50	2,723,000	39.80	108,375
	1999	110,000	108,000	32.00	3,456,000	36.80	127,181
	2000	98,000	92,500	34.00	3,145,000	30.40	95,608
	2001	46,600	44,700	35.70	1,596,000	33.40	53,306
	2002	50,200	49,900	39.50	1,971,000	---	---
Wheat, Winter		*Acres*	*Acres*	*Tons*	*Tons*	*Dollars Per Ton*	*$1,000*
(Excluding Durum)	1993	550,000	500,000	2.31	1,155,000	105.00	121,275
	1994	590,000	510,000	2.28	1,162,800	117.33	136,435
	1995	580,000	425,000	1.83	777,800	138.33	107,589
	1996	640,000	550,000	2.07	1,138,500	163.33	185,955
	1997	510,000	400,000	2.10	840,000	123.67	103,880
	1998	500,000	380,000	1.80	684,000	98.67	67,488
	1999	500,000	370,000	2.34	865,800	91.67	79,365
	2000	535,000	390,000	2.10	819,000	89.00	72,891
	2001	530,000	380,000	2.10	798,000	97.67	77,938
	2002	530,000	300,000	2.25	675,000	116.67	78,750
Wheat, Durum		*Acres*	*Acres*	*Tons*	*Tons*	*Dollars Per Ton*	*$1,000*
	1993	41,000	40,000	2.85	114,000	136.00	15,504
	1994	60,000	59,000	2.85	168,200	153.67	25,839
	1995	70,000	68,000	3.00	204,000	165.00	33,660
	1996	140,000	138,000	3.00	414,000	171.33	70,932
	1997	145,000	144,000	2.85	410,400	142.67	58,550
	1998	180,000	175,000	2.70	472,500	127.67	60,323
	1999	90,000	85,000	3.15	267,800	123.33	33,023
	2000	100,000	97,000	3.00	291,000	106.33	30,943
	2001	85,000	81,000	3.15	255,200	129.00	32,914
	2002	95,000	90,000	3.00	270,000	131.67	35,550

Field Crop Acreage, Production and Value, 1993-2002

Crop	Crop Year	Planted	Harvested	Yield Per Acre	Production	Value Per Unit	Total Value
Wheat, All		*Acres*	*Acres*	*Tons*	*Tons*	*Dollars Per Ton*	*$1,000*
	1993	591,000	540,000	2.35	1,269,000	107.78	136,779
	1994	650,000	569,000	2.34	1,331,000	121.92	162,274
	1995	650,000	493,000	1.99	981,800	143.87	141,249
	1996	780,000	688,000	2.26	1,552,500	165.47	256,887
	1997	655,000	544,000	2.30	1,250,400	129.90	162,430
	1998	680,000	555,000	2.09	1,156,500	100.67	127,811
	1999	590,000	455,000	2.49	1,133,600	94.67	112,388
	2000	635,000	487,000	2.28	1,110,000	94.00	103,834
	2001	615,000	461,000	2.28	1,053,200	103.67	110,852
	2002	625,000	390,000	2.42	945,000	121.67	114,300

1/ Production is on a clean basis.
2/ Dashes represent data not published due to the estimate being discontinued, not estimated, or not available.
3/ Includes varieties not listed separately. Excludes beans grown for garden seed.
4/ Planted acreage is for all purposes.
5/ Cotton bales are 480 lbs. net-weight.
6/ Estimated marketings of alfalfa and other hay used as weights to calculate all hay price for 1993-02.
7/ Excludes sweet and wild rice.
8/ Initiated in 2000.
9/ Relates to year of intended harvest for fall planted beets in central California and to year of planting for overwintered beets in central and southern California.

Field Crop Monthly Average Prices Received, 1993-2002

Crop	Crop Year	Jan.	Feb.	Mar.	Apr.	May	June	July	Aug.	Sept.	Oct.	Nov.	Dec.
Barley							*Dollars Per Ton*						
	1993	112.00	110.00	115.00	109.00	100.00	107.00	101.00	101.00	103.00	93.40	103.00	101.00
	1994	113.00	1/	106.00	106.00	108.00	108.00	104.00	108.00	1/	1/	1/	113.00
	1995	1/	113.00	112.00	1/	122.00	1/	1/	115.00	123.00	1/	139.00	142.00
	1996	137.00	164.00	173.00	168.00	167.00	172.00	160.00	157.00	143.00	1/	127.00	1/
	1997	128.00	1/	126.00	1/	128.00	121.00	1/	121.00	1/	1/	1/	1/
	1998	118.00	117.00	115.00	1/	1/	1/	101.00	1/	1/	1/	1/	1/
	1999	1/	1/	1/	1/	89.20	88.30	94.60	93.30	97.50	90.40	105.00	1/
	2000	93.80	102.00	103.00	99.20	101.00	103.00	101.00	96.30	1/	1/	1/	1/
	2001	1/	1/	1/	1/	1/	99.60	93.80	90.80	91.30	100.80	1/	95.00
	2002	1/	1/	1/	1/	1/	102.90	1/	1/	1/	1/	1/	1/
Beans, Dry Edible							*Dollars Per Cwt.*						
	1993	25.00	24.80	24.70	24.20	23.60	24.20	24.80	27.40	29.40	31.20	35.50	34.80
	1994	36.90	36.70	36.30	37.00	36.00	36.00	37.80	36.90	36.20	33.00	35.10	34.10
	1995	35.40	35.10	35.40	34.60	35.00	35.70	35.20	34.40	34.40	34.00	34.60	33.80
	1996	34.10	34.60	34.30	34.70	35.30	36.20	36.30	36.90	37.20	36.10	37.70	38.70
	1997	38.90	39.60	39.50	39.40	39.60	38.70	39.10	37.80	31.10	30.00	29.70	29.60
	1998	29.10	28.60	28.10	28.60	27.80	28.60	30.40	31.20	31.20	31.30	31.90	37.80
	1999	39.40	39.80	39.40	38.90	37.60	36.20	36.20	34.50	29.30	27.60	27.20	27.60
	2000	27.10	26.80	26.70	27.00	26.40	26.60	26.00	25.80	27.20	27.10	27.20	26.60
	2001	26.90	26.90	26.60	26.80	26.80	26.30	26.30	27.00	27.20	28.60	31.50	31.20
	2002	31.80	31.60	32.70	32.20	32.30	31.90	32.40	33.40	32.50	31.70	31.60	32.60
Cotton Lint, Upland							*Cents Per Pound*						
	1993	62.50	63.30	62.90	62.40	59.50	59.70	1/	57.60	54.50	56.10	60.20	62.30
	1994	67.10	70.40	72.20	72.40	72.40	76.40	70.80	65.40	64.80	68.80	75.40	79.30
	1995	94.50	94.80	108.00	113.20	107.90	97.90	99.40	78.40	83.30	78.90	80.30	84.10
	1996	84.70	83.80	85.20	86.30	80.60	82.10	80.00	77.00	77.60	75.60	77.00	77.50
	1997	76.60	77.60	77.40	73.70	75.50	74.50	74.30	68.40	1/	76.90	74.70	69.70
	1998	73.40	71.30	71.30	68.60	71.50	77.50	1/	71.80	68.80	69.70	73.70	73.30
	1999	68.40	58.50	56.50	56.40	1/	55.20	49.70	51.40	1/	55.20	52.90	58.20
	2000	1/	57.90	1/	1/	58.70	1/	1/	1/	66.10	64.90	69.00	68.20
	2001	57.80	57.40	1/	39.60	39.70	36.50	37.10	1/	47.90	1/	35.10	53.30
	2002	39.40	1/	1/	1/	1/	1/	1/	1/	1/	51.90	54.50	55.50
Cottonseed							*Dollars Per Ton*						
	1993	---	---	---	---	---	---	---	---	---	125.00	139.00	136.00
	1994	140.00	---	---	---	---	---	---	---	---	---	141.00	147.00
	1995	157.00	---	---	---	---	---	---	---	---	---	132.00	131.00
	1996	---	---	---	---	---	---	---	---	---	175.00	---	174.00
	1997	189.00	---	---	---	---	---	---	---	---	171.00	175.00	177.00
	1998	---	---	---	---	---	---	---	---	---	135.00	175.00	179.00
	1999	180.00	199.00	---	---	---	---	---	---	---	149.00	141.00	135.00
	2000	116.00	137.00	---	---	---	---	---	---	---	145.00	140.00	134.00
	2001	136.00	143.00	---	---	---	---	---	---	---	146.00	141.00	145.00
	2002	132.00	137.00	---	---	---	---	---	---	---	150.00	142.00	132.00
Hay, Alfalfa (Baled)							*Dollars Per Ton*						
	1993	86.00	91.00	92.00	97.00	107.00	100.00	91.00	94.00	103.00	107.00	112.00	116.00
	1994	119.00	126.00	123.00	124.00	111.00	116.00	108.00	106.00	114.00	116.00	120.00	131.00
	1995	124.00	113.00	110.00	100.00	100.00	103.00	99.00	97.00	99.00	105.00	98.00	115.00
	1996	100.00	104.00	98.00	108.00	104.00	104.00	100.00	101.00	106.00	111.00	112.00	118.00
	1997	126.00	133.00	127.00	132.00	131.00	125.00	117.00	113.00	113.00	121.00	125.00	119.00
	1998	120.00	121.00	115.00	130.00	107.00	102.00	99.00	99.00	93.00	96.00	89.00	79.00
	1999	79.00	102.00	109.00	101.00	104.00	97.00	87.00	81.00	78.00	85.00	85.00	86.00
	2000	85.00	94.00	99.00	96.00	91.00	81.00	76.00	81.00	89.00	97.00	97.00	97.00
	2001	102.00	103.00	111.00	130.00	121.00	114.00	111.00	113.00	125.00	123.00	128.00	124.00
	2002	113.00	122.00	126.00	121.00	116.00	101.00	95.00	90.00	92.00	98.00	101.00	95.00

Field Crop Monthly Average Prices Received, 1993-2002

Crop	Crop Year	Jan.	Feb.	Mar.	Apr.	May	June	July	Aug.	Sept.	Oct.	Nov.	Dec.
Hay, All (Baled)							*Dollars Per Ton*						
	1993	81.00	85.00	89.00	96.00	104.00	97.00	89.00	92.00	101.00	104.00	108.00	110.00
	1994	116.00	123.00	120.00	122.00	107.00	111.00	104.00	99.00	109.00	112.00	116.00	124.00
	1995	114.00	110.00	109.00	100.00	97.00	99.00	96.00	94.00	98.00	104.00	96.00	106.00
	1996	103.00	96.00	105.00	99.00	102.00	103.00	98.00	100.00	104.00	110.00	110.00	117.00
	1997	124.00	132.00	126.00	128.00	128.00	122.00	115.00	111.00	113.00	110.00	123.00	118.00
	1998	118.00	120.00	113.00	129.00	103.00	100.00	99.00	97.00	91.00	92.00	86.00	79.00
	1999	76.00	100.00	103.00	98.00	103.00	96.00	85.00	82.00	78.00	84.00	82.00	83.00
	2000	83.00	88.00	98.00	96.00	90.00	81.00	77.00	81.00	87.00	93.00	95.00	94.00
	2001	100.00	100.00	110.00	128.00	116.00	112.00	108.00	110.00	121.00	117.00	117.00	115.00
	2002	111.00	119.00	123.00	119.00	115.00	98.00	92.00	88.00	89.00	94.00	95.00	94.00
Potatoes							*Dollars Per Cwt.*						
	1993	10.90	10.90	9.20	13.60	11.80	11.10	14.70	15.10	11.00	8.50	13.30	13.90
	1994	13.70	11.60	11.60	10.70	11.70	11.00	12.10	11.50	6.75	6.55	8.45	8.40
	1995	8.50	9.80	9.15	8.55	12.10	12.10	14.30	16.80	19.30	13.40	13.50	12.50
	1996	12.20	12.00	14.00	13.90	11.20	13.10	13.10	11.00	14.90	11.20	9.80	7.95
	1997	8.50	8.70	9.95	11.50	8.15	9.95	10.20	13.50	14.70	13.80	8.20	11.20
	1998	11.00	12.30	17.10	15.30	11.00	10.20	9.90	10.90	12.00	10.70	11.50	14.20
	1999	16.70	16.40	12.90	20.40	8.65	11.50	11.30	12.30	16.50	7.95	12.20	10.90
	2000	13.00	12.50	13.20	14.20	10.80	11.20	14.60	17.40	16.80	8.00	8.05	11.50
	2001	15.80	16.00	7.35	6.85	12.10	11.10	12.60	15.70	14.80	11.00	13.20	17.10
	2002	17.70	19.00	15.70	25.60	24.10	18.40	18.60	17.70	19.20	9.40	15.20	20.10
Wheat, All							*Dollars Per Ton*						
	1993	118.00	124.00	111.00	107.00	106.00	106.00	100.00	105.00	100.00	99.40	113.40	119.00
	1994	125.00	115.00	115.00	115.00	124.00	126.00	113.00	115.00	125.00	135.00	135.00	133.00
	1995	144.00	128.00	140.00	125.00	142.00	151.00	135.00	1/	1/	150.00	177.00	154.00
	1996	167.00	175.00	188.00	188.00	185.00	179.00	160.00	160.00	159.00	147.00	144.00	140.00
	1997	161.00	146.00	151.00	145.00	140.00	134.00	1/	1/	124.00	131.00	1/	1/
	1998	1/	1/	104.00	104.00	124.00	99.60	1/	1/	1/	1/	1/	1/
	1999	96.70	100.00	98.70	104.00	93.00	1/	1/	1/	106.00	1/	1/	1/
	2000	1/	85.00	86.70	1/	1/	1/	1/	1/	1/	1/	1/	1/
	2001	1/	1/	1/	1/	111.00	114.00	1/	1/	1/	1/	1/	1/
	2002	1/	1/	1/	1/	1/	118.00	1/	1/	1/	1/	1/	1/

1/ Not published due to insufficient sales or disclosure.

CALIFORNIA BARLEY: Acreage, Yield and Production by County, 2001-2002

County	2001				2002			
	Planted	Harvested	Yield/Acre	Production	Planted	Harvested	Yield/Acre	Production
	Acres		*Tons*		*Acres*		*Tons*	
Del Norte	---	---	---	---	---	---	---	---
Humboldt	---	---	---	---	---	---	---	---
Mendocino	---	---	---	---	---	---	---	---
Other Counties 1/	1,100	900	1.04	936	---	---	---	---
**District 10 Total**	**1,100**	**900**	**1.04**	**936**	**---**	**---**	**---**	**---**
Shasta	---	---	---	---	---	---	---	---
Siskiyou	13,300	10,200	1.54	15,720	12,100	7,000	2.07	14,520
Trinity	---	---	---	---	---	---	---	---
Other Counties 1/	700	300	1.28	384	400	200	1.92	384
**District 20 Total**	**14,000**	**10,500**	**1.53**	**16,104**	**12,500**	**7,200**	**2.07**	**14,904**
Lassen	---	---	---	---	---	---	---	---
Modoc	16,200	13,600	1.91	25,992	12,500	9,600	2.16	20,760
Plumas	---	---	---	---	---	---	---	---
Other Counties 1/	600	200	1.80	360	500	---	---	---
**District 30 Total**	**16,800**	**13,800**	**1.91**	**26,352**	**13,000**	**9,600**	**2.16**	**20,760**
Alameda	1,700	700	0.99	696	---	---	---	---
Contra Costa	---	---	---	---	---	---	---	---
Lake	---	---	---	---	---	---	---	---
Marin	---	---	---	---	---	---	---	---
Monterey	11,600	7,200	0.84	6,048	11,000	4,000	1.01	4,056
Napa	---	---	---	---	---	---	---	---
San Benito	1,900	800	0.90	720	1,900	900	0.96	864
San Francisco	---	---	---	---	---	---	---	---
San Luis Obispo	36,100	25,000	0.74	18,480	24,000	15,600	1.11	17,256
San Mateo	---	---	---	---	---	---	---	---
Santa Clara	---	---	---	---	---	---	---	---
Santa Cruz	---	---	---	---	---	---	---	---
Sonoma	---	---	---	---	---	---	---	---
Other Counties 1/	200	100	0.96	96	1,600	200	1.08	216
**District 40 Total**	**51,500**	**33,800**	**0.77**	**26,040**	**38,500**	**20,700**	**1.08**	**22,392**
Butte	---	---	---	---	500	400	1.38	552
Colusa	---	---	---	---	---	---	---	---
Glenn	---	---	---	---	3,400	2,800	1.67	4,680
Sacramento	1,000	700	1.03	720	500	200	1.92	384
Solano	5,600	4,200	1.63	6,864	6,000	4,700	1.47	6,888
Sutter	---	---	---	---	400	200	1.44	288
Tehama	1,500	1,100	0.85	936	600	400	1.56	624
Yolo	2,000	1,400	1.44	2,016	2,300	1,700	1.77	3,000
Yuba	---	---	---	---	---	---	---	---
Other Counties 1/	1,800	1,300	1.16	1,512	300	200	1.56	312
**District 50 Total**	**11,900**	**8,700**	**1.39**	**12,048**	**14,000**	**10,600**	**1.58**	**16,728**
Fresno	10,200	7,300	1.65	12,072	14,800	8,600	1.75	15,048
Kern	24,100	18,000	1.31	23,616	17,300	9,100	1.80	16,416
Kings	5,600	3,200	1.43	4,560	4,100	1,900	1.60	3,048
Madera	2,000	1,400	1.44	2,016	---	---	---	---
Merced	6,100	3,200	1.49	4,752	3,600	1,700	2.29	3,888
San Joaquin	---	---	---	---	---	---	---	---
Stanislaus	---	---	---	---	---	---	---	---
Tulare	4,100	3,100	1.45	4,488	2,000	600	1.68	1,008
Other Counties 1/	3,200	1,800	1.43	2,568	4,200	2,100	1.98	4,152
**District 51 Total**	**55,300**	**38,000**	**1.42**	**54,072**	**46,000**	**24,000**	**1.82**	**43,560**
Alpine	---	---	---	---	---	---	---	---
Amador	---	---	---	---	---	---	---	---
Calaveras	---	---	---	---	---	---	---	---
El Dorado	---	---	---	---	---	---	---	---
Inyo	---	---	---	---	---	---	---	---
Mariposa	---	---	---	---	---	---	---	---
Mono	---	---	---	---	---	---	---	---
Nevada	---	---	---	---	---	---	---	---
Placer	---	---	---	---	---	---	---	---
Sierra	---	---	---	---	---	---	---	---
Tuolumne	---	---	---	---	---	---	---	---
Other Counties 1/	200	100	0.96	96	---	---	---	---
**District 60 Total**	**200**	**100**	**0.96**	**96**	**---**	**---**	**---**	**---**
Imperial	---	---	---	---	300	200	1.44	288
Los Angeles	4,200	1,800	0.84	1,512	1,300	500	1.25	624
Orange	---	---	---	---	---	---	---	---
Riverside	4,400	2,200	1.15	2,520	3,000	1,800	1.41	2,544
San Bernardino	---	---	---	---	---	---	---	---
San Diego	---	---	---	---	---	---	---	---
Santa Barbara	---	---	---	---	400	200	1.44	288
Ventura	---	---	---	---	---	---	---	---
Other Counties 1/	600	200	1.20	240	1,000	200	1.56	312
**District 80 Total**	**9,200**	**4,200**	**1.02**	**4,272**	**6,000**	**2,900**	**1.40**	**4,056**
**STATE**	**160,000**	**110,000**	**1.27**	**139,920**	**130,000**	**75,000**	**1.63**	**122,400**

1/ Includes one or more counties within a district that normally have less than 200 acres planted.

CALIFORNIA DRY EDIBLE BEANS: Acreage, Yield And Production By County, 2001-2002

County	2001				2002			
	Planted	Harvested	Yield/Acre	Production	Planted	Harvested	Yield/Acre	Production
	Acres		*Pounds*	*Cwt.*	*Acres*		*Pounds*	*Cwt.*
Del Norte	---	---	---	---	---	---	---	---
Humboldt	---	---	---	---	---	---	---	---
Mendocino	200	200	1,500	3,000	---	---	---	---
**District 10 Total**	**200**	**200**	**1,500**	**3,000**	**---**	**---**	**---**	**---**
Shasta	200	200	1,500	3,000	---	---	---	---
Siskiyou	---	---	---	---	---	---	---	---
Trinity	---	---	---	---	---	---	---	---
**District 20 Total**	**200**	**200**	**1,500**	**3,000**	**---**	**---**	**---**	**---**
Lassen	---	---	---	---	---	---	---	---
Modoc	---	---	---	---	---	---	---	---
Plumas	---	---	---	---	---	---	---	---
**District 30 Total**	**---**	**---**	**---**	**---**	**---**	**---**	**---**	**---**
Alameda	200	200	1,000	2,000	---	---	---	---
Contra Costa	---	---	---	---	---	---	---	---
Lake	---	---	---	---	---	---	---	---
Marin	---	---	---	---	---	---	---	---
Monterey	1,300	1,300	2,231	29,000	1,200	1,100	2,909	32,000
Napa	---	---	---	---	---	---	---	---
San Benito	---	---	---	---	---	---	---	---
San Francisco	---	---	---	---	---	---	---	---
San Luis Obispo	300	300	1,000	3,000	400	400	1,250	5,000
San Mateo	200	200	1,500	3,000	200	200	1,500	3,000
Santa Clara	---	---	---	---	---	---	---	---
Santa Cruz	---	---	---	---	---	---	---	---
Sonoma	---	---	---	---	---	---	---	---
Other Counties 1/	---	---	---	---	100	100	1,000	1,000
**District 40 Total**	**2,000**	**2,000**	**1,850**	**37,000**	**1,900**	**1,800**	**2,278**	**41,000**
Butte	800	800	1,875	15,000	700	700	2,286	16,000
Colusa	7,500	7,400	1,689	125,000	8,500	8,100	1,790	145,000
Glenn	2,900	2,900	1,759	51,000	4,800	4,600	1,826	84,000
Sacramento	1,300	1,300	1,538	20,000	600	600	1,833	11,000
Solano	3,400	3,300	1,788	59,000	4,600	4,500	1,822	82,000
Sutter	7,600	7,400	1,743	129,000	10,200	9,800	1,755	172,000
Tehama	800	800	1,750	14,000	700	700	1,857	13,000
Yolo	900	900	1,778	16,000	3,100	3,000	1,833	55,000
Yuba	---	---	---	---	---	---	---	---
**District 50 Total**	**25,200**	**24,800**	**1,730**	**429,000**	**33,200**	**32,000**	**1,806**	**578,000**
Fresno	14,100	13,500	1,793	242,000	10,800	10,400	2,125	221,000
Kern	1,800	1,700	1,471	25,000	2,900	2,800	2,357	66,000
Kings	15,800	14,900	1,302	194,000	10,400	10,100	1,436	145,000
Madera	300	300	2,333	7,000	900	900	2,667	24,000
Merced	4,200	4,000	1,725	69,000	4,300	4,200	1,738	73,000
San Joaquin	11,000	10,600	2,208	234,000	14,900	14,500	2,290	332,000
Stanislaus	8,400	8,100	2,432	197,000	6,100	5,900	2,627	155,000
Tulare	2,900	2,900	2,655	77,000	2,000	1,900	2,947	56,000
Other Counties 1/	---	---	---	---	400	400	1,250	5,000
**District 51 Total**	**58,500**	**56,000**	**1,866**	**1,045,000**	**52,700**	**51,100**	**2,108**	**1,077,000**
Alpine	---	---	---	---	---	---	---	---
Amador	---	---	---	---	---	---	---	---
Calaveras	---	---	---	---	---	---	---	---
El Dorado	---	---	---	---	---	---	---	---
Inyo	---	---	---	---	---	---	---	---
Mariposa	---	---	---	---	---	---	---	---
Mono	---	---	---	---	---	---	---	---
Nevada	---	---	---	---	---	---	---	---
Placer	---	---	---	---	---	---	---	---
Sierra	---	---	---	---	---	---	---	---
Tuolumne	---	---	---	---	---	---	---	---
**District 60 Total**	**---**	**---**	**---**	**---**	**---**	**---**	**---**	**---**
Imperial	---	---	---	---	---	---	---	---
Los Angeles	---	---	---	---	---	---	---	---
Orange	1,500	1,400	1,500	21,000	1,000	1,000	1,700	17,000
Riverside	900	900	1,444	13,000	400	400	1,500	6,000
San Bernardino	---	---	---	---	---	---	---	---
San Diego	---	---	---	---	---	---	---	---
Santa Barbara	3,100	3,100	1,484	46,000	2,400	2,300	1,565	36,000
Ventura	---	---	---	---	---	---	---	---
Other Counties 1/	400	400	1,250	5,000	200	200	1,500	3,000
**District 80 Total**	**5,900**	**5,800**	**1,466**	**85,000**	**4,000**	**3,900**	**1,590**	**62,000**
Other Districts 2/	---	---	---	---	200	200	2,000	4,000
**STATE**	92,000	89,000	1,800	1,602,000	92,000	89,000	1,980	1,762,000

1/ Includes one or more counties within a district that normally have less than 200 acres planted.
2/ Includes counties from one or more districts that normally have less than 200 acres planted.

CALIFORNIA CORN FOR GRAIN: Acreage, Yield And Production By County, 2001-2002

County	2001				2002			
	Planted 1/	Harvested	Yield/Acre	Production	Planted 1/	Harvested	Yield/Acre	Production
	Acres		*Tons*		*Acres*		*Tons*	
Del Norte	---	---	---	---	---	---	---	---
Humboldt	---	---	---	---	700	200	4.48	896
Mendocino	---	---	---	---	---	---	---	---
**District 10 Total**	**---**	**---**	**---**	**---**	**700**	**200**	**4.48**	**896**
Shasta	200	---	---	---	---	---	---	---
Siskiyou	300	---	---	---	---	---	---	---
Trinity	---	---	---	---	---	---	---	---
Other Counties 2/	---	---	---	---	200	---	---	---
**District 20 Total**	**500**	**---**	**---**	**---**	**200**	**---**	**---**	**---**
Lassen	---	---	---	---	---	---	---	---
Modoc	---	---	---	---	---	---	---	---
Plumas	---	---	---	---	---	---	---	---
**District 30 Total**	**---**	**---**	**---**	**---**	**---**	**---**	**---**	**---**
Alameda	400	400	4.34	1,736	600	500	4.48	2,240
Contra Costa	5,800	5,600	3.33	18,620	6,700	5,300	3.64	19,292
Lake	---	---	---	---	---	---	---	---
Marin	---	---	---	---	---	---	---	---
Monterey	---	---	---	---	---	---	---	---
Napa	---	---	---	---	---	---	---	---
San Benito	---	---	---	---	---	---	---	---
San Francisco	---	---	---	---	---	---	---	---
San Luis Obispo	200	200	3.22	644	200	---	---	---
San Mateo	---	---	---	---	---	---	---	---
Santa Clara	---	---	---	---	---	---	---	---
Santa Cruz	---	---	---	---	---	---	---	---
Sonoma	300	---	---	---	400	---	---	---
Other Counties 2/	---	---	---	---	100	100	4.20	420
**District 40 Total**	**6,700**	**6,200**	**3.39**	**21,000**	**8,000**	**5,900**	**3.72**	**21,952**
Butte	500	300	3.73	1,120	600	400	5.11	2,044
Colusa	3,500	3,200	3.76	12,040	3,500	3,300	4.41	14,560
Glenn	16,500	13,000	5.82	75,600	16,000	11,000	5.81	63,924
Sacramento	29,000	23,600	4.15	98,000	35,000	25,000	3.92	98,000
Solano	15,000	14,000	5.30	74,200	14,400	10,900	4.88	53,200
Sutter	6,000	5,500	5.09	28,000	6,500	4,300	5.05	21,728
Tehama	1,000	600	4.67	2,800	1,500	800	4.62	3,696
Yolo	17,500	17,100	5.32	91,000	17,000	16,000	5.08	81,200
Yuba	1,000	700	5.20	3,640	1,500	800	5.50	4,396
**District 50 Total**	**90,000**	**78,000**	**4.95**	**386,400**	**96,000**	**72,500**	**4.73**	**342,748**
Fresno	20,000	2,500	4.48	11,200	25,000	3,000	4.90	14,700
Kern	20,000	500	4.48	2,240	34,000	2,500	5.04	12,600
Kings	42,500	5,500	4.99	27,440	46,000	4,400	5.85	25,760
Madera	17,000	3,000	4.90	14,700	19,500	3,000	4.81	14,420
Merced	53,500	3,500	5.40	18,900	68,000	3,800	5.08	19,320
San Joaquin	79,500	49,500	4.52	224,000	81,000	50,500	4.72	238,140
Stanislaus	51,000	3,000	3.73	11,200	51,500	300	4.67	1,400
Tulare	94,500	7,500	5.41	40,600	110,000	2,800	5.60	15,680
**District 51 Total**	**378,000**	**75,000**	**4.67**	**350,280**	**435,000**	**70,300**	**4.87**	**342,020**
Alpine	---	---	---	---	---	---	---	---
Amador	300	300	4.67	1,400	---	---	---	---
Calaveras	---	---	---	---	---	---	---	---
El Dorado	---	---	---	---	---	---	---	---
Inyo	---	---	---	---	---	---	---	---
Mariposa	---	---	---	---	---	---	---	---
Mono	---	---	---	---	---	---	---	---
Nevada	---	---	---	---	---	---	---	---
Placer	---	---	---	---	---	---	---	---
Sierra	---	---	---	---	---	---	---	---
Tuolumne	---	---	---	---	---	---	---	---
**District 60 Total**	**300**	**300**	**4.67**	**1,400**	**---**	**---**	**---**	**---**
Imperial	1,300	500	5.04	2,520	800	600	5.97	3,584
Los Angeles	---	---	---	---	---	---	---	---
Orange	---	---	---	---	---	---	---	---
Riverside	1,800	---	---	---	1,900	---	---	---
San Bernardino	600	---	---	---	1,300	---	---	---
San Diego	---	---	---	---	---	---	---	---
Santa Barbara	300	---	---	---	500	---	---	---
Ventura	---	---	---	---	---	---	---	---
Other Counties 2/	---	---	---	---	100	100	5.60	560
**District 80 Total**	**4,000**	**500**	**5.04**	**2,520**	**4,600**	**700**	**5.92**	**4,144**
Other Districts 3/	500	---	---	---	500	400	5.60	2,240
**STATE**	**480,000**	**160,000**	**4.76**	**761,600**	**545,000**	**150,000**	**4.76**	**714,000**

1/ Includes corn planted for all purposes.
2/ Includes one or more counties within a district that normally have less than 400 acres planted.
3/ Includes counties from one or more districts that normally have less than 400 acres planted .

CALIFORNIA CORN FOR SILAGE: Acreage, Yield And Production By County, 2001-2002

County	2001				2002			
	Planted 1/	Harvested	Yield/Acre	Production	Planted 1/	Harvested	Yield/Acre	Production
	Acres		*Tons*		*Acres*		*Tons*	
Del Norte	---	---	---	---	---	---	---	---
Humboldt	---	---	---	---	700	500	24.40	12,200
Mendocino	---	---	---	---	---	---	---	---
**District 10 Total**	**---**	**---**	**---**	**---**	**700**	**500**	**24.40**	**12,200**
Shasta	200	200	15.00	3,000	---	---	---	---
Siskiyou	300	300	20.00	6,000	---	---	---	---
Trinity	---	---	---	---	---	---	---	---
Other Counties 2/	---	---	---	---	200	200	19.00	3,800
**District 20 Total**	**500**	**500**	**18.00**	**9,000**	**200**	**200**	**19.00**	**3,800**
Lassen	---	---	---	---	---	---	---	---
Modoc	---	---	---	---	---	---	---	---
Plumas	---	---	---	---	---	---	---	---
**District 30 Total**	**---**	**---**	**---**	**---**	**---**	**---**	**---**	**---**
Alameda	400	---	---	---	600	---	---	---
Contra Costa	5,800	200	20.00	4,000	6,700	1,000	27.00	27,000
Lake	---	---	---	---	---	---	---	---
Marin	---	---	---	---	---	---	---	---
Monterey	---	---	---	---	---	---	---	---
Napa	---	---	---	---	---	---	---	---
San Benito	---	---	---	---	---	---	---	---
San Francisco	---	---	---	---	---	---	---	---
San Luis Obispo	200	---	---	---	200	---	---	---
San Mateo	---	---	---	---	---	---	---	---
Santa Clara	---	---	---	---	---	---	---	---
Santa Cruz	---	---	---	---	---	---	---	---
Sonoma	300	300	23.30	7,000	400	400	26.50	10,600
Other Counties 2/	---	---	---	---	100	300	16.30	4,900
**District 40 Total**	**6,700**	**500**	**22.00**	**11,000**	**8,000**	**1,700**	**25.00**	**42,500**
Butte	500	200	25.00	5,000	600	200	30.00	6,000
Colusa	3,500	300	21.70	6,500	3,500	---	---	---
Glenn	16,500	3,500	20.90	73,000	16,000	4,500	23.50	105,900
Sacramento	29,000	5,400	29.60	160,000	35,000	9,000	25.40	228,300
Solano	15,000	1,000	26.00	26,000	14,400	3,200	26.90	86,000
Sutter	6,000	500	28.00	14,000	6,500	1,800	28.00	50,400
Tehama	1,000	400	27.50	11,000	1,500	700	25.30	17,700
Yolo	17,500	400	27.50	11,000	17,000	600	31.70	19,000
Yuba	1,000	300	28.30	8,500	1,500	600	30.70	18,400
Other Counties 2/	---	---	---	---	---	100	22.00	2,200
**District 50 Total**	**90,000**	**12,000**	**26.30**	**315,000**	**96,000**	**20,700**	**25.80**	**533,900**
Fresno	20,000	17,500	27.70	485,000	25,000	21,900	23.60	517,500
Kern	20,000	19,500	23.10	450,000	34,000	31,000	24.00	744,700
Kings	42,500	37,000	23.50	870,000	46,000	41,500	25.40	1,055,000
Madera	17,000	14,000	28.60	400,000	19,500	16,500	25.40	418,900
Merced	53,500	49,000	28.60	1,400,000	68,000	64,000	25.70	1,645,000
San Joaquin	79,500	28,000	28.90	810,000	81,000	30,000	28.50	855,300
Stanislaus	51,000	47,000	26.40	1,243,000	51,500	51,000	27.60	1,410,000
Tulare	94,500	86,000	24.40	2,100,000	110,000	107,000	26.20	2,800,000
**District 51 Total**	**378,000**	**298,000**	**26.00**	**7,758,000**	**435,000**	**362,900**	**26.00**	**9,446,400**
Alpine	---	---	---	---	---	---	---	---
Amador	300	---	---	---	---	---	---	---
Calaveras	---	---	---	---	---	---	---	---
El Dorado	---	---	---	---	---	---	---	---
Inyo	---	---	---	---	---	---	---	---
Mariposa	---	---	---	---	---	---	---	---
Mono	---	---	---	---	---	---	---	---
Nevada	---	---	---	---	---	---	---	---
Placer	---	---	---	---	---	---	---	---
Sierra	---	---	---	---	---	---	---	---
Tuolumne	---	---	---	---	---	---	---	---
**District 60 Total**	**300**	**---**	**---**	**---**	**---**	**---**	**---**	**---**
Imperial	1,300	800	18.80	15,000	800	200	19.00	3,800
Los Angeles	---	---	---	---	---	---	---	---
Orange	---	---	---	---	---	---	---	---
Riverside	1,800	1,800	26.70	48,000	1,900	1,900	26.50	50,300
San Bernardino	600	600	25.00	15,000	1,300	1,300	24.50	31,900
San Diego	---	---	---	---	---	---	---	---
Santa Barbara	300	300	23.30	7,000	500	500	24.80	12,400
Ventura	---	---	---	---	---	---	---	---
Other Counties 2/	---	---	---	---	100	---	---	---
**District 80 Total**	**4,000**	**3,500**	**24.30**	**85,000**	**4,600**	**3,900**	**25.20**	**98,400**
Other Districts 3/	500	500	24.00	12,000	500	100	28.00	2,800
**STATE**	**480,000**	**315,000**	**26.00**	**8,190,000**	**545,000**	**390,000**	**26.00**	**10,140,000**

1/ Includes corn planted for all purposes.
2/ Includes one or more counties within a district that normally have less than 200 acres planted.
3/ Includes counties from one or more districts that normally have less than 200 acres planted.

CALIFORNIA COTTON, AMERICAN PIMA: Acreage, Yield And Production By County, 2001-2002

County	2001				2002			
	Planted	Harvested	Yield/Acre	Production	Planted	Harvested	Yield/Acre	Production
	Acres		*Pounds*	*Bales*	*Acres*		*Pounds*	*Bales*
Del Norte	---	---	---	---	---	---	---	---
Humboldt	---	---	---	---	---	---	---	---
Mendocino	---	---	---	---	---	---	---	---
**District 10 Total**	**---**	**---**	**---**	**---**	**---**	**---**	**---**	**---**
Shasta	---	---	---	---	---	---	---	---
Siskiyou	---	---	---	---	---	---	---	---
Trinity	---	---	---	---	---	---	---	---
**District 20 Total**	**---**	**---**	**---**	**---**	**---**	**---**	**---**	**---**
Lassen	---	---	---	---	---	---	---	---
Modoc	---	---	---	---	---	---	---	---
Plumas	---	---	---	---	---	---	---	---
**District 30 Total**	**---**	**---**	**---**	**---**	**---**	**---**	**---**	**---**
Alameda	---	---	---	---	---	---	---	---
Contra Costa	---	---	---	---	---	---	---	---
Lake	---	---	---	---	---	---	---	---
Marin	---	---	---	---	---	---	---	---
Monterey	---	---	---	---	---	---	---	---
Napa	---	---	---	---	---	---	---	---
San Benito	---	---	---	---	---	---	---	---
San Francisco	---	---	---	---	---	---	---	---
San Luis Obispo	---	---	---	---	---	---	---	---
San Mateo	---	---	---	---	---	---	---	---
Santa Clara	---	---	---	---	---	---	---	---
Santa Cruz	---	---	---	---	---	---	---	---
Sonoma	---	---	---	---	---	---	---	---
**District 40 Total**	**---**	**---**	**---**	**---**	**---**	**---**	**---**	**---**
Butte	---	---	---	---	---	---	---	---
Colusa	---	---	---	---	---	---	---	---
Glenn	---	---	---	---	---	---	---	---
Sacramento	---	---	---	---	---	---	---	---
Solano	---	---	---	---	---	---	---	---
Sutter	---	---	---	---	---	---	---	---
Tehama	---	---	---	---	---	---	---	---
Yolo	---	---	---	---	---	---	---	---
Yuba	---	---	---	---	---	---	---	---
**District 50 Total**	**---**	**---**	**---**	**---**	**---**	**---**	**---**	**---**
Fresno	105,500	105,100	1,388	303,900	75,200	74,800	1,509	235,200
Kern	31,200	31,000	1,394	90,000	41,800	41,500	1,400	121,000
Kings	95,300	94,900	1,128	223,000	87,300	87,000	1,277	231,400
Madera	400	400	1,320	1,100	---	---	---	---
Merced	3,000	3,000	1,296	8,100	2,300	2,300	1,273	6,100
San Joaquin	---	---	---	---	---	---	---	---
Stanislaus	---	---	---	---	---	---	---	---
Tulare	4,500	4,500	1,355	12,700	3,200	3,200	1,380	9,200
Other Counties 1/	---	---	---	---	100	100	960	200
**District 51 Total**	**239,900**	**238,900**	**1,283**	**638,800**	**209,900**	**208,900**	**1,386**	**603,100**
Alpine	---	---	---	---	---	---	---	---
Amador	---	---	---	---	---	---	---	---
Calaveras	---	---	---	---	---	---	---	---
El Dorado	---	---	---	---	---	---	---	---
Inyo	---	---	---	---	---	---	---	---
Mariposa	---	---	---	---	---	---	---	---
Mono	---	---	---	---	---	---	---	---
Nevada	---	---	---	---	---	---	---	---
Placer	---	---	---	---	---	---	---	---
Sierra	---	---	---	---	---	---	---	---
Tuolumne	---	---	---	---	---	---	---	---
**District 60 Total**	**---**	**---**	**---**	**---**	**---**	**---**	**---**	**---**
Imperial	---	---	---	---	---	---	---	---
Los Angeles	---	---	---	---	---	---	---	---
Orange	---	---	---	---	---	---	---	---
Riverside	---	---	---	---	---	---	---	---
San Bernardino	---	---	---	---	---	---	---	---
San Diego	---	---	---	---	---	---	---	---
Santa Barbara	---	---	---	---	---	---	---	---
Ventura	---	---	---	---	---	---	---	---
**District 80 Total**	**---**	**---**	**---**	**---**	**---**	**---**	**---**	**---**
Other Districts 2/	100	100	960	200	100	100	960	200
**STATE**	**240,000**	**239,000**	**1,283**	**639,000**	**210,000**	**209,000**	**1,386**	**603,300**

1/ Includes one or more counties within a district that normally have less than 200 acres planted.
2/ Includes counties from one or more districts that normally have less than 200 acres planted.

CALIFORNIA COTTON, UPLAND: Acreage, Yield And Production By County, 2001-2002

County	2001				2002			
	Planted	Harvested	Yield/Acre	Production	Planted	Harvested	Yield/Acre	Production
	Acres		*Pounds*	*Bales*	*Acres*		*Pounds*	*Bales*
Del Norte	---	---	---	---	---	---	---	---
Humboldt	---	---	---	---	---	---	---	---
Mendocino	---	---	---	---	---	---	---	---
**District 10 Total**	**---**	**---**	**---**	**---**	**---**	**---**	**---**	**---**
Shasta	---	---	---	---	---	---	---	---
Siskiyou	---	---	---	---	---	---	---	---
Trinity	---	---	---	---	---	---	---	---
**District 20 Total**	**---**	**---**	**---**	**---**	**---**	**---**	**---**	**---**
Lassen	---	---	---	---	---	---	---	---
Modoc	---	---	---	---	---	---	---	---
Plumas	---	---	---	---	---	---	---	---
**District 30 Total**	**---**	**---**	**---**	**---**	**---**	**---**	**---**	**---**
Alameda	---	---	---	---	---	---	---	---
Contra Costa	---	---	---	---	---	---	---	---
Lake	---	---	---	---	---	---	---	---
Marin	---	---	---	---	---	---	---	---
Monterey	---	---	---	---	---	---	---	---
Napa	---	---	---	---	---	---	---	---
San Benito	---	---	---	---	---	---	---	---
San Francisco	---	---	---	---	---	---	---	---
San Luis Obispo	---	---	---	---	---	---	---	---
San Mateo	---	---	---	---	---	---	---	---
Santa Clara	---	---	---	---	---	---	---	---
Santa Cruz	---	---	---	---	---	---	---	---
Sonoma	---	---	---	---	---	---	---	---
**District 40 Total**	**---**	**---**	**---**	**---**	**---**	**---**	**---**	**---**
Butte	---	---	---	---	---	---	---	---
Colusa	12,000	12,000	1,360	34,000	8,700	8,600	1,518	27,200
Glenn	2,400	2,400	1,220	6,100	2,100	2,000	1,200	5,000
Sacramento	---	---	---	---	---	---	---	---
Solano	---	---	---	---	---	---	---	---
Sutter	2,400	2,400	1,260	6,300	2,000	2,000	1,200	5,000
Tehama	---	---	---	---	---	---	---	---
Yolo	5,500	5,500	1,248	14,300	3,400	3,400	1,073	7,600
Yuba	---	---	---	---	---	---	---	---
**District 50 Total**	**22,300**	**22,300**	**1,307**	**60,700**	**16,200**	**16,000**	**1,344**	**44,800**
Fresno	165,500	164,500	1,415	485,000	150,000	149,300	1,491	463,800
Kern	125,600	124,900	1,335	347,300	84,800	84,500	1,395	245,500
Kings	107,500	106,500	1,318	292,500	88,200	87,500	1,494	272,300
Madera	27,000	26,900	1,310	73,400	19,500	19,400	1,475	59,600
Merced	66,000	65,100	1,425	193,200	50,400	50,000	1,465	152,600
San Joaquin	---	---	---	---	---	---	---	---
Stanislaus	---	---	---	---	---	---	---	---
Tulare	80,800	80,000	1,297	216,200	48,300	48,000	1,490	149,000
Other Counties 1/	---	---	---	---	100	100	960	200
**District 51 Total**	**572,400**	**567,900**	**1,359**	**1,607,600**	**441,300**	**438,800**	**1,469**	**1,343,000**
Alpine	---	---	---	---	---	---	---	---
Amador	---	---	---	---	---	---	---	---
Calaveras	---	---	---	---	---	---	---	---
El Dorado	---	---	---	---	---	---	---	---
Inyo	---	---	---	---	---	---	---	---
Mariposa	---	---	---	---	---	---	---	---
Mono	---	---	---	---	---	---	---	---
Nevada	---	---	---	---	---	---	---	---
Placer	---	---	---	---	---	---	---	---
Sierra	---	---	---	---	---	---	---	---
Tuolumne	---	---	---	---	---	---	---	---
**District 60 Total**	**---**	**---**	**---**	**---**	**---**	**---**	**---**	**---**
Imperial	12,800	12,500	1,440	37,500	10,200	10,000	1,555	32,400
Los Angeles	---	---	---	---	---	---	---	---
Orange	---	---	---	---	---	---	---	---
Riverside	20,600	20,400	1,395	59,300	11,400	11,300	1,572	37,000
San Bernardino	1,600	1,600	1,320	4,400	900	900	1,493	2,800
San Diego	---	---	---	---	---	---	---	---
Santa Barbara	---	---	---	---	---	---	---	---
Ventura	---	---	---	---	---	---	---	---
**District 80 Total**	**35,000**	**34,500**	**1,408**	**101,200**	**22,500**	**22,200**	**1,561**	**72,200**
Other Districts 2/	300	300	800	500	---	---	---	---
**STATE**	**630,000**	**625,000**	**1,359**	**1,770,000**	**480,000**	**477,000**	**1,469**	**1,460,000**

1/ Includes one or more counties within a district that normally have less than 500 acres planted.
2/ Includes counties from one or more districts that normally have less than 500 acres planted.

CALIFORNIA OATS: Acreage, Yield And Production By County, 2001-2002

County	2001				2002			
	Planted	Harvested	Yield/Acre	Production	Planted	Harvested	Yield/Acre	Production
	Acres		*Tons*		*Acres*		*Tons*	
Del Norte	---	---	---	---	---	---	---	---
Humboldt	400	---	---	---	---	---	---	---
Mendocino	1,600	200	1.20	240	---	---	---	---
Other Counties 1/	---	---	---	---	3,000	200	1.12	224
**District 10 Total**	**2,000**	**200**	**1.20**	**240**	**3,000**	**200**	**1.12**	**224**
Shasta	2,400	200	1.04	208	2,100	200	0.96	192
Siskiyou	5,600	500	1.09	544	4,900	800	1.44	1,152
Trinity	---	---	---	---	---	---	---	---
**District 20 Total**	**8,000**	**700**	**1.07**	**752**	**7,000**	**1,000**	**1.34**	**1,344**
Lassen	3,200	200	1.44	288	2,600	400	1.44	576
Modoc	5,100	300	1.17	352	3,200	600	1.63	976
Plumas	700	---	---	---	1,200	---	---	---
**District 30 Total**	**9,000**	**500**	**1.28**	**640**	**7,000**	**1,000**	**1.55**	**1,552**
Alameda	1,700	200	0.80	160	---	---	---	---
Contra Costa	1,200	200	0.80	160	---	---	---	---
Lake	1,200	---	---	---	1,400	300	1.39	416
Marin	4,000	---	---	---	3,200	---	---	---
Monterey	2,500	600	1.01	608	2,400	300	1.97	592
Napa	700	---	---	---	700	---	---	---
San Benito	2,500	500	0.77	384	2,100	500	1.09	544
San Francisco	---	---	---	---	---	---	---	---
San Luis Obispo	12,000	400	0.72	288	12,600	600	0.75	448
San Mateo	1,000	200	0.56	112	1,100	200	0.80	160
Santa Clara	1,400	---	---	---	2,200	500	0.64	320
Santa Cruz	800	---	---	---	700	---	---	---
Sonoma	22,000	1,200	0.89	1,072	19,000	1,400	0.93	1,296
Other Counties 1/	---	---	---	---	2,600	200	0.96	192
**District 40 Total**	**51,000**	**3,300**	**0.84**	**2,784**	**48,000**	**4,000**	**0.99**	**3,968**
Butte	2,400	200	0.72	144	1,800	300	0.91	272
Colusa	900	---	---	---	700	400	1.00	400
Glenn	5,100	200	0.96	192	5,100	300	1.12	336
Sacramento	5,200	---	---	---	---	---	---	---
Solano	3,500	200	0.96	192	3,400	800	1.42	1,136
Sutter	2,700	300	1.39	416	3,000	900	1.16	1,040
Tehama	5,300	200	1.04	208	---	---	---	---
Yolo	9,200	300	1.33	400	9,600	400	1.36	544
Yuba	700	200	1.04	208	2,000	600	1.07	640
Other Counties 1/	---	---	---	---	11,400	300	1.17	352
**District 50 Total**	**35,000**	**1,600**	**1.10**	**1,760**	**37,000**	**4,000**	**1.18**	**4,720**
Fresno	10,500	400	1.16	464	9,600	2,400	1.33	3,200
Kern	3,200	300	1.17	352	2,700	600	1.55	928
Kings	3,500	---	---	---	4,700	200	1.28	256
Madera	5,900	800	1.04	832	7,500	1,300	1.28	1,664
Merced	30,400	1,200	0.89	1,072	35,000	1,600	1.24	1,984
San Joaquin	18,000	700	1.21	848	16,500	2,500	1.59	3,968
Stanislaus	29,000	1,900	0.96	1,824	27,000	1,400	1.36	1,904
Tulare	19,500	2,000	0.91	1,824	18,000	4,500	1.43	6,448
**District 51 Total**	**120,000**	**7,300**	**0.99**	**7,216**	**121,000**	**14,500**	**1.40**	**20,352**
Alpine	---	---	---	---	---	---	---	---
Amador	2,000	---	---	---	1,300	---	---	---
Calaveras	2,200	300	1.17	352	---	---	---	---
El Dorado	---	---	---	---	---	---	---	---
Inyo	300	---	---	---	---	---	---	---
Mariposa	1,200	---	---	---	900	---	---	---
Mono	400	---	---	---	---	---	---	---
Nevada	300	---	---	---	---	---	---	---
Placer	4,100	400	0.72	288	4,400	200	0.96	192
Sierra	1,000	---	---	---	1,200	---	---	---
Tuolumne	300	---	---	---	---	---	---	---
Other Counties 1/	200	---	---	---	4,200	100	1.28	128
**District 60 Total**	**12,000**	**700**	**0.91**	**640**	**12,000**	**300**	**1.07**	**320**
Imperial	2,600	200	0.48	96	4,900	800	1.02	816
Los Angeles	700	---	---	---	700	---	---	---
Orange	---	---	---	---	---	---	---	---
Riverside	6,500	300	0.37	112	5,500	400	1.04	416
San Bernardino	1,500	---	---	---	1,700	---	---	---
San Diego	5,500	---	---	---	5,100	500	1.06	528
Santa Barbara	5,100	200	0.80	160	5,600	300	1.07	320
Ventura	1,100	---	---	---	1,500	---	---	---
**District 80 Total**	**23,000**	**700**	**0.53**	**368**	**25,000**	**2,000**	**1.04**	**2,080**
**STATE**	**260,000**	**15,000**	**0.96**	**14,400**	**260,000**	**27,000**	**1.28**	**34,560**

1/ Includes one or more counties within a district that normally have less than 200 acres planted.

CALIFORNIA RICE: Acreage, Yield And Production By County, 2001-2002

County	2001				2002			
	Planted	Harvested	Yield/Acre	Production	Planted	Harvested	Yield/Acre	Production
	Acres		*Pounds*	*Cwt.*	*Acres*		*Pounds*	*Cwt.*
Del Norte	---	---	---	---	---	---	---	---
Humboldt	---	---	---	---	---	---	---	---
Mendocino	---	---	---	---	---	---	---	---
**District 10 Total**	**---**	**---**	**---**	**---**	**---**	**---**	**---**	**---**
Shasta	---	---	---	---	---	---	---	---
Siskiyou	---	---	---	---	---	---	---	---
Trinity	---	---	---	---	---	---	---	---
**District 20 Total**	**---**	**---**	**---**	**---**	**---**	**---**	**---**	**---**
Lassen	---	---	---	---	---	---	---	---
Modoc	---	---	---	---	---	---	---	---
Plumas	---	---	---	---	---	---	---	---
**District 30 Total**	**---**	**---**	**---**	**---**	**---**	**---**	**---**	**---**
Alameda	---	---	---	---	---	---	---	---
Contra Costa	---	---	---	---	---	---	---	---
Lake	---	---	---	---	---	---	---	---
Marin	---	---	---	---	---	---	---	---
Monterey	---	---	---	---	---	---	---	---
Napa	---	---	---	---	---	---	---	---
San Benito	---	---	---	---	---	---	---	---
San Francisco	---	---	---	---	---	---	---	---
San Luis Obispo	---	---	---	---	---	---	---	---
San Mateo	---	---	---	---	---	---	---	---
Santa Clara	---	---	---	---	---	---	---	---
Santa Cruz	---	---	---	---	---	---	---	---
Sonoma	---	---	---	---	---	---	---	---
**District 40 Total**	**---**	**---**	**---**	**---**	**---**	**---**	**---**	**---**
Butte	86,800	86,400	8,397	7,255,000	100,000	99,000	8,373	8,289,000
Colusa	126,300	125,600	8,232	10,339,000	138,500	137,000	8,139	11,150,000
Glenn	78,300	78,000	8,272	6,452,000	87,500	86,500	8,254	7,140,000
Sacramento	7,800	7,800	8,064	629,000	8,200	8,200	7,683	630,000
Solano	---	---	---	---	700	700	9,000	63,000
Sutter	87,700	87,300	8,258	7,209,000	101,700	100,700	8,300	8,358,000
Tehama	800	800	6,750	54,000	900	900	6,667	60,000
Yolo	26,000	25,900	7,722	2,000,000	31,500	31,200	7,769	2,424,000
Yuba	37,100	37,000	7,954	2,943,000	36,000	35,800	7,821	2,800,000
**District 50 Total**	**450,800**	**448,800**	**8,218**	**36,881,000**	**505,000**	**500,000**	**8,183**	**40,914,000**
Fresno	2,000	2,000	7,150	143,000	1,300	1,300	6,308	82,000
Kern	---	---	---	---	---	---	---	---
Kings	---	---	---	---	---	---	---	---
Madera	---	---	---	---	---	---	---	---
Merced	4,300	4,300	7,744	333,000	6,600	6,600	8,136	537,000
San Joaquin	4,500	4,500	7,556	340,000	5,800	5,800	7,690	446,000
Stanislaus	2,200	2,200	6,727	148,000	2,300	2,300	7,174	165,000
Tulare	---	---	---	---	---	---	---	---
**District 51 Total**	**13,000**	**13,000**	**7,415**	**964,000**	**16,000**	**16,000**	**7,688**	**1,230,000**
Alpine	---	---	---	---	---	---	---	---
Amador	---	---	---	---	---	---	---	---
Calaveras	---	---	---	---	---	---	---	---
El Dorado	---	---	---	---	---	---	---	---
Inyo	---	---	---	---	---	---	---	---
Mariposa	---	---	---	---	---	---	---	---
Mono	---	---	---	---	---	---	---	---
Nevada	---	---	---	---	---	---	---	---
Placer	9,200	9,200	7,011	645,000	12,000	12,000	7,042	845,000
Sierra	---	---	---	---	---	---	---	---
Tuolumne	---	---	---	---	---	---	---	---
**District 60 Total**	**9,200**	**9,200**	**7,011**	**645,000**	**12,000**	**12,000**	**7,042**	**845,000**
Imperial	---	---	---	---	---	---	---	---
Los Angeles	---	---	---	---	---	---	---	---
Orange	---	---	---	---	---	---	---	---
Riverside	---	---	---	---	---	---	---	---
San Bernardino	---	---	---	---	---	---	---	---
San Diego	---	---	---	---	---	---	---	---
Santa Barbara	---	---	---	---	---	---	---	---
Ventura	---	---	---	---	---	---	---	---
**District 80 Total**	**---**	**---**	**---**	**---**	**---**	**---**	**---**	**---**
**STATE**	**473,000**	**471,000**	**8,172**	**38,490,000**	**533,000**	**528,000**	**8,142**	**42,989,000**

CALIFORNIA SORGHUM: Acreage, Yield And Production By County, 2001-2002 1/

County	2001				2002			
	Planted	Harvested	Yield/Acre	Production	Planted	Harvested	Yield/Acre	Production
	Acres		*Tons*		*Acres*		*Tons*	
Del Norte	---	---	---	---	---	---	---	---
Humboldt	---	---	---	---	---	---	---	---
Mendocino	---	---	---	---	---	---	---	---
**District 10 Total**	**---**	**---**	**---**	**---**	**---**	**---**	**---**	**---**
Shasta	---	---	---	---	---	---	---	---
Siskiyou	---	---	---	---	---	---	---	---
Trinity	---	---	---	---	---	---	---	---
**District 20 Total**	**---**	**---**	**---**	**---**	**---**	**---**	**---**	**---**
Lassen	---	---	---	---	---	---	---	---
Modoc	---	---	---	---	---	---	---	---
Plumas	---	---	---	---	---	---	---	---
**District 30 Total**	**---**	**---**	**---**	**---**	**---**	**---**	**---**	**---**
Alameda	---	---	---	---	---	---	---	---
Contra Costa	---	---	---	---	---	---	---	---
Lake	---	---	---	---	---	---	---	---
Marin	---	---	---	---	---	---	---	---
Monterey	---	---	---	---	---	---	---	---
Napa	---	---	---	---	---	---	---	---
San Benito	---	---	---	---	---	---	---	---
San Francisco	---	---	---	---	---	---	---	---
San Luis Obispo	---	---	---	---	---	---	---	---
San Mateo	---	---	---	---	---	---	---	---
Santa Clara	---	---	---	---	---	---	---	---
Santa Cruz	---	---	---	---	---	---	---	---
Sonoma	---	---	---	---	---	---	---	---
**District 40 Total**	**---**	**---**	**---**	**---**	**---**	**---**	**---**	**---**
Butte	---	---	---	---	---	---	---	---
Colusa	---	---	---	---	---	---	---	---
Glenn	---	---	---	---	---	---	---	---
Sacramento	---	---	---	---	1,100	900	2.58	2,324
Solano	---	---	---	---	1,000	900	2.77	2,492
Sutter	---	---	---	---	700	600	2.71	1,624
Tehama	---	---	---	---	---	---	---	---
Yolo	---	---	---	---	1,200	1,200	2.73	3,276
Yuba	---	---	---	---	---	---	---	---
Other Counties 2/	---	---	---	---	500	400	2.45	980
**District 50 Total**	**---**	**---**	**---**	**---**	**4,500**	**4,000**	**2.67**	**10,696**
Fresno	---	---	---	---	---	---	---	---
Kern	---	---	---	---	1,900	1,600	2.68	4,284
Kings	---	---	---	---	700	---	---	---
Madera	---	---	---	---	---	---	---	---
Merced	---	---	---	---	---	---	---	---
San Joaquin	---	---	---	---	---	---	---	---
Stanislaus	---	---	---	---	---	---	---	---
Tulare	---	---	---	---	3,300	3,000	2.66	7,980
Other Counties 2/	---	---	---	---	1,100	700	2.56	1,792
**District 51 Total**	**---**	**---**	**---**	**---**	**7,000**	**5,300**	**2.65**	**14,056**
Alpine	---	---	---	---	---	---	---	---
Amador	---	---	---	---	---	---	---	---
Calaveras	---	---	---	---	---	---	---	---
El Dorado	---	---	---	---	---	---	---	---
Inyo	---	---	---	---	---	---	---	---
Mariposa	---	---	---	---	---	---	---	---
Mono	---	---	---	---	---	---	---	---
Nevada	---	---	---	---	---	---	---	---
Placer	---	---	---	---	---	---	---	---
Sierra	---	---	---	---	---	---	---	---
Tuolumne	---	---	---	---	---	---	---	---
**District 60 Total**	**---**	**---**	**---**	**---**	**---**	**---**	**---**	**---**
Imperial	---	---	---	---	---	---	---	---
Los Angeles	---	---	---	---	---	---	---	---
Orange	---	---	---	---	---	---	---	---
Riverside	---	---	---	---	2,300	1,400	2.66	3,724
San Bernardino	---	---	---	---	500	---	---	---
San Diego	---	---	---	---	---	---	---	---
Santa Barbara	---	---	---	---	---	---	---	---
Ventura	---	---	---	---	---	---	---	---
Other Counties 2/	---	---	---	---	200	100	2.52	252
**District 80 Total**	**---**	**---**	**---**	**---**	**3,000**	**1,500**	**2.65**	**3,976**
Other Districts 3/	---	---	---	---	500	200	2.66	532
**STATE**	**---**	**---**	**---**	**---**	**15,000**	**11,000**	**2.66**	**29,260**

1/ Publication of sorghum county estimates data reinstated in 2002.
2/ Includes one or more counties within a district that normally have less than 400 acres planted.
3/ Includes counties from one or more districts that normally have less than 400 acres planted.

CALIFORNIA SUGAR BEETS: Acreage, Yield And Production By County, 2001-2002

County	2001					2002				
	Planted	Harvested	Yield/Acre	Production	Sucrose	Planted	Harvested	Yield/Acre	Production	Sucrose
	Acres		Tons		Percent	Acres		Tons		Percent
Del Norte	---	---	---	---	---	---	---	---	---	---
Humboldt	---	---	---	---	---	---	---	---	---	---
Mendocino	---	---	---	---	---	---	---	---	---	---
**District 10 Total**	**---**	**---**	**---**	**---**	**---**	**---**	**---**	**---**	**---**	**---**
Shasta	---	---	---	---	---	---	---	---	---	---
Siskiyou	---	---	---	---	---	---	---	---	---	---
Trinity	---	---	---	---	---	---	---	---	---	---
**District 20 Total**	**---**	**---**	**---**	**---**	**---**	**---**	**---**	**---**	**---**	**---**
Lassen	---	---	---	---	---	---	---	---	---	---
Modoc	---	---	---	---	---	---	---	---	---	---
Plumas	---	---	---	---	---	---	---	---	---	---
**District 30 Total**	**---**	**---**	**---**	**---**	**---**	**---**	**---**	**---**	**---**	**---**
Alameda	---	---	---	---	---	---	---	---	---	---
Contra Costa	---	---	---	---	---	---	---	---	---	---
Lake	---	---	---	---	---	---	---	---	---	---
Marin	---	---	---	---	---	---	---	---	---	---
Monterey	---	---	---	---	---	---	---	---	---	---
Napa	---	---	---	---	---	---	---	---	---	---
San Benito	---	---	---	---	---	---	---	---	---	---
San Francisco	---	---	---	---	---	---	---	---	---	---
San Luis Obispo	---	---	---	---	---	---	---	---	---	---
San Mateo	---	---	---	---	---	---	---	---	---	---
Santa Clara	---	---	---	---	---	---	---	---	---	---
Santa Cruz	---	---	---	---	---	---	---	---	---	---
Sonoma	---	---	---	---	---	---	---	---	---	---
**District 40 Total**	**---**	**---**	**---**	**---**	**---**	**---**	**---**	**---**	**---**	**---**
Butte	---	---	---	---	---	---	---	---	---	---
Colusa	---	---	---	---	---	---	---	---	---	---
Glenn	---	---	---	---	---	---	---	---	---	---
Sacramento	---	---	---	---	---	---	---	---	---	---
Solano	---	---	---	---	---	---	---	---	---	---
Sutter	---	---	---	---	---	---	---	---	---	---
Tehama	---	---	---	---	---	---	---	---	---	---
Yolo	---	---	---	---	---	---	---	---	---	---
Yuba	---	---	---	---	---	---	---	---	---	---
**District 50 Total**	**---**	**---**	**---**	**---**	**---**	**---**	**---**	**---**	**---**	**---**
Fresno	11,800	11,300	30.5	345,000	15.22	12,200	12,100	38.2	462,000	14.94
Kern	2,000	1,900	33.2	63,000	15.68	3,300	3,200	38.8	124,000	15.11
Kings	2,000	2,000	27.5	55,000	14.88	2,800	2,700	31.5	85,000	14.05
Madera	600	400	25.0	10,000	15.52	---	---	---	---	---
Merced	4,000	3,300	23.3	77,000	16.41	4,400	4,400	33.0	145,000	16.00
San Joaquin	---	---	---	---	---	---	---	---	---	---
Stanislaus	---	---	---	---	---	---	---	---	---	---
Tulare	900	800	32.5	26,000	12.20	1,000	1,000	48.0	48,000	12.41
Other Counties 1/	200	200	35.0	7,000	16.10	700	700	34.3	24,000	15.00
**District 51 Total**	**21,500**	**19,900**	**29.3**	**583,000**	**15.28**	**24,400**	**24,100**	**36.8**	**888,000**	**14.92**
Alpine	---	---	---	---	---	---	---	---	---	---
Amador	---	---	---	---	---	---	---	---	---	---
Calaveras	---	---	---	---	---	---	---	---	---	---
El Dorado	---	---	---	---	---	---	---	---	---	---
Inyo	---	---	---	---	---	---	---	---	---	---
Mariposa	---	---	---	---	---	---	---	---	---	---
Mono	---	---	---	---	---	---	---	---	---	---
Nevada	---	---	---	---	---	---	---	---	---	---
Placer	---	---	---	---	---	---	---	---	---	---
Sierra	---	---	---	---	---	---	---	---	---	---
Tuolumne	---	---	---	---	---	---	---	---	---	---
**District 60 Total**	---	---	---	---	---	---	---	---	---	---
Imperial	24,900	24,700	40.9	1,009,000	16.15	25,600	25,600	42.0	1,076,000	16.50
Los Angeles	---	---	---	---	---	---	---	---	---	---
Orange	---	---	---	---	---	---	---	---	---	---
Riverside	---	---	---	---	---	---	---	---	---	---
San Bernardino	---	---	---	---	---	---	---	---	---	---
San Diego	---	---	---	---	---	---	---	---	---	---
Santa Barbara	---	---	---	---	---	---	---	---	---	---
Ventura	---	---	---	---	---	---	---	---	---	---
**District 80 Total**	**24,900**	**24,700**	**40.9**	**1,009,000**	**16.15**	**25,600**	**25,600**	**42.0**	**1,076,000**	**16.50**
Other Districts 2/	200	100	40.0	4,000	14.95	200	200	35.0	7,000	15.20
**STATE**	**46,600**	**44,700**	**35.7**	**1,596,000**	**15.83**	**50,200**	**49,900**	**39.5**	**1,971,000**	**15.78**

1/ Includes one or more counties within a district that normally have less than 300 acres planted.
2/ Includes counties from one or more districts that normally have less than 300 acres planted.

CALIFORNIA WHEAT, ALL: Acreage, Yield And Production By County, 2001-2002

County	2001				2002			
	Planted	Harvested	Yield/Acre	Production	Planted	Harvested	Yield/Acre	Production
	Acres		*Tons*		*Acres*		*Tons*	
Del Norte	---	---	---	---	---	---	---	---
Humboldt	---	---	---	---	---	---	---	---
Mendocino	---	---	---	---	---	---	---	---
**District 10 Total**	**---**	**---**	**---**	**---**	**---**	**---**	**---**	**---**
Shasta	1,500	1,000	1.71	1,710	1,500	1,000	1.80	1,800
Siskiyou	4,500	3,000	2.14	6,420	6,500	5,000	1.60	8,010
Trinity	---	---	---	---	---	---	---	---
**District 20 Total**	**6,000**	**4,000**	**2.03**	**8,130**	**8,000**	**6,000**	**1.64**	**9,810**
Lassen	4,500	1,500	2.02	3,030	5,000	2,000	1.95	3,900
Modoc	5,500	1,500	1.90	2,850	7,000	3,000	2.05	6,150
Plumas	---	---	---	---	---	---	---	---
**District 30 Total**	**10,000**	**3,000**	**1.96**	**5,880**	**12,000**	**5,000**	**2.01**	**10,050**
Alameda	2,000	500	2.22	1,110	3,000	500	1.44	720
Contra Costa	4,000	2,500	1.90	4,740	3,500	1,500	1.60	2,400
Lake	---	---	---	---	---	---	---	---
Marin	---	---	---	---	---	---	---	---
Monterey	3,500	1,500	1.10	1,650	1,500	500	1.50	750
Napa	---	---	---	---	---	---	---	---
San Benito	3,000	1,500	1.52	2,280	2,500	1,000	1.50	1,500
San Francisco	---	---	---	---	---	---	---	---
San Luis Obispo	3,500	1,500	1.18	1,770	3,000	1,000	1.38	1,380
San Mateo	---	---	---	---	---	---	---	---
Santa Clara	---	---	---	---	---	---	---	---
Santa Cruz	---	---	---	---	---	---	---	---
Sonoma	1,000	500	1.80	900	1,500	500	1.50	750
**District 40 Total**	**17,000**	**8,000**	**1.56**	**12,450**	**15,000**	**5,000**	**1.50**	**7,500**
Butte	4,000	4,000	1.96	7,830	2,000	1,000	2.40	2,400
Colusa	24,500	23,500	2.21	51,990	24,000	17,000	2.12	36,000
Glenn	11,500	9,500	2.32	22,050	11,000	6,000	2.13	12,750
Sacramento	17,000	13,500	2.23	30,150	13,000	7,000	2.25	15,750
Solano	24,000	24,000	2.18	52,200	20,000	15,000	2.44	36,600
Sutter	13,000	11,000	2.10	23,100	11,000	7,000	2.46	17,250
Tehama	3,000	1,000	1.62	1,620	2,000	500	1.98	990
Yolo	42,500	40,000	2.51	100,440	31,000	23,000	2.41	55,500
Yuba	500	500	1.68	840	1,000	500	1.92	960
**District 50 Total**	**140,000**	**127,000**	**2.29**	**290,220**	**115,000**	**77,000**	**2.31**	**178,200**
Fresno	62,500	59,000	2.40	141,360	45,000	37,000	2.76	102,300
Kern	65,000	37,000	2.14	79,110	55,000	35,000	2.44	85,500
Kings	79,000	70,500	2.09	147,060	56,000	49,000	2.50	122,700
Madera	29,000	13,500	2.16	29,130	34,000	16,000	2.32	37,050
Merced	21,500	8,000	2.28	18,210	54,000	21,000	2.39	50,250
San Joaquin	38,000	32,000	2.24	71,700	22,000	18,000	2.49	44,850
Stanislaus	4,000	1,500	2.46	3,690	12,000	4,000	2.06	8,250
Tulare	61,000	29,500	2.37	69,840	115,000	56,000	2.18	122,100
**District 51 Total**	**360,000**	**251,000**	**2.23**	**560,100**	**393,000**	**236,000**	**2.43**	**573,000**
Alpine	---	---	---	---	---	---	---	---
Amador	---	---	---	---	---	---	---	---
Calaveras	---	---	---	---	---	---	---	---
El Dorado	---	---	---	---	---	---	---	---
Inyo	---	---	---	---	---	---	---	---
Mariposa	---	---	---	---	---	---	---	---
Mono	---	---	---	---	---	---	---	---
Nevada	---	---	---	---	---	---	---	---
Placer	4,500	2,000	2.24	4,470	5,000	2,000	2.34	4,680
Sierra	---	---	---	---	---	---	---	---
Tuolumne	---	---	---	---	---	---	---	---
**District 60 Total**	**4,500**	**2,000**	**2.24**	**4,470**	**5,000**	**2,000**	**2.34**	**4,680**
Imperial	41,500	38,500	3.16	121,830	43,000	40,500	3.11	126,000
Los Angeles	---	---	---	---	---	---	---	---
Orange	---	---	---	---	---	---	---	---
Riverside	34,000	25,500	1.82	46,500	32,500	17,500	1.96	34,260
San Bernardino	---	---	---	---	---	---	---	---
San Diego	1,500	1,500	2.00	3,000	1,000	500	1.50	750
Santa Barbara	500	500	1.14	570	500	500	1.50	750
Ventura	---	---	---	---	---	---	---	---
**District 80 Total**	**77,500**	**66,000**	**2.60**	**171,900**	**77,000**	**59,000**	**2.74**	**161,760**
**STATE**	**615,000**	**461,000**	**2.28**	**1,053,150**	**625,000**	**390,000**	**2.42**	**945,000**

California Floriculture

Floriculture halted its previously rapid growth in 2002. The industry enjoyed record wholesale values due to the strength of its leading producing states—California, Florida, Michigan, Texas and Ohio. These five states account for over half of the national value in floriculture.

California maintained its position as the leading floriculture state in the nation, with a total wholesale value of $949 million. This total represents 21 percent of the national value and a 4 percent decrease from the previous year. Aided by its favorable coastal climate, California now accounts for 68 percent of the national value for all fresh, cut flowers. At $279 million in sales, this represents a 3 percent decrease from the $287 million of 2001. The rose is the most popular cut flower in the nation, and California accounts for roughly 64 percent of the value for all fresh, cut roses grown in the United States. While cut flower demand has continued to rise, domestic production has been declining with over two-thirds of the major cut flowers now imported. Most imports are coming from Colombia, Costa Rica, Ecuador, Mexico and the Netherlands.

California's total sales of potted flowering plants fell 7 percent from $213 million in 2001 to $198 million in 2002. California is still the leading state with 24 percent of the national value. Sales of foliage plants showed a 6 percent decrease from $96.6 million in 2001 to $90.4 million in 2002.

The wholesale value of bedding and garden plants fell 1 percent from $303 million in 2001 to $301 million in 2002. The value of cut, cultivated greens was down fractionally, falling from $17.4 million in 2001 to $17.1 million in 2002.

The number of floriculture producers in the state has been in decline in recent years. Of those producers with sales greater than $10,000, California now has 802 growers compared to 917 in 2001.

Floriculture Production and Value, 2002 1/

Kind	Number of Producers	Sales: Unit	Sales: Number Sold	Sales: Wholesale	Wholesale Price 2/	Value of Sales at Wholesale
			1,000	*Percent*	*Dollars*	*$1,000*
**Cut Flowers**						**278,581**
Alstroemeria	37	Stems	14,490	99	0.16	2,304
Carnations, Standard	21	Stems	17,902	97	0.15	2,596
Chrysanthemums, Pompon	30	Bunches	11,532	99	1.30	14,992
Delphinium and Larkspur	58	Stems	37,773	100	0.22	8,348
Gerbera Daisy	27	Stems	73,557	100	0.29	21,626
Gladioli	17	Spikes	17,726	100	0.30	5,282
Iris	42	Stems	61,529	100	0.20	12,183
Lilies, All	51	Stems	78,819	99	0.59	46,503
Lisianthus	41	Stems	13,056	99	0.45	5,862
Orchids, All	13	Blooms	8,100	99	0.72	5,824
Roses, All	41	Stems	105,706	99	0.34	36,363
Snapdragons	53	Spikes	45,736	99	0.23	10,656
Tulips	30	Stems	47,860	99	0.31	14,837
Other Cut Flowers	158	---	---	91	---	91,205
**Potted Flowering Plants**						**197,940**
African Violets	14	Pots	1,622	100	1.74	2,817
Finished Florist Azaleas	24	Pots	1,515	97	5.17	7,835
Florist Chrysanthemums	19	Pots	6,693	100	2.81	18,805
Easter Lilies	30	Pots	1,415	100	4.30	6,088
Orchids	41	Pots	4,298	95	9.52	40,903
Poinsettias	89	Pots	9,360	95	4.07	38,077
Spring Flowering Bulbs	29	Pots	2,705	100	2.75	7,429
Other Potted Flowering Plants	80	Pots	19,870	99	3.37	75,986
**Foliage Plants for Indoor or Patio Use**						**90,412**
Hanging Baskets	60	Baskets	3,977	100	3.50	13,920
Potted Foliage	106	---	---	98	---	76,492
**Annual Bedding/Garden Plants**						**301,383**
Flats						
Begonias	51	Flats	461	96	8.46	3,900
Geraniums (Cutting)	19	Flats	181	98	11.96	2,165
Geraniums (Seed)	6	Flats	13	100	9.93	129
New Guinea Impatiens	13	Flats	111	97	10.85	1,204
Impatiens	62	Flats	1,844	99	9.95	18,348
Marigolds	57	Flats	1,055	99	10.43	11,004
Pansy/Viola	59	Flats	1,837	99	10.23	18,793
Petunias	62	Flats	1,662	97	10.09	16,770
Other Flowering and Foliar	64	Flats	6,857	99	9.70	66,526
Vegetable Type	46	Flats	1,590	99	10.29	16,361
Potted						
Begonias	50	Pots	2,127	78	1.10	2,341
Geraniums (Cutting)	48	Pots	7,303	100	1.52	11,077
Geraniums (Seed)	22	Pots	2,059	97	1.27	2,605
New Guinea Impatiens	47	Pots	2,285	100	1.48	3,378
Impatiens	50	Pots	5,324	89	0.82	4,363
Marigolds	38	Pots	1,954	92	0.95	1,864
Pansy/Viola	40	Pots	4,348	91	0.84	3,648
Petunias	40	Pots	4,081	96	1.03	4,184
Other Flowering and Foliar	64	Pots	33,671	97	1.38	46,484
Vegetable Type	40	Pots	6,319	99	0.92	5,883
Hanging Baskets						
Begonias	19	Baskets	25	94	7.08	177
Geraniums (Cutting)	30	Baskets	175	99	6.83	1,195
Geraniums (Seed)	6	Baskets	18	75	7.74	139
New Guinea Impatiens	26	Baskets	176	99	6.72	1,183
Impatiens	25	Baskets	140	99	5.62	787
Petunias	20	Baskets	53	97	6.06	321
Other Flowering	33	Baskets	795	99	7.77	6,177
Herbaceous Perennials 3/						
Hardy/Garden Chrysanthemums	39	Pots	5,745	100	1.29	7,383
Hosta	18	Pots	76	95	3.55	270
Other	82	Pots	19,898	97	2.15	42,724
**Total Cut Cultivated Greens** 4/	42	---	---	99	---	**17,052**
**Propagative (Unfinished)**						**63,261**
Cut Flowers	---	---	---	---	---	6,565
Potted Flowering Plants	---	---	---	---	---	18,011
Bedding/Garden Plants	---	---	---	---	---	30,297
Herbaceous Perennial Plants	---	---	---	---	---	4,423
Foliage Plants	---	---	---	---	---	3,965
**TOTAL ALL PLANTS**	---	---	---	---	---	**948,629**

1/ The total covered growing area of 119,788,000 square feet consisted of the following: 21,363,000 square feet of shade and temporary cover; 15,807,000 square feet of glass; 27,987,000 square feet of fiberglass and other rigid greenhouses; 54,631,000 square feet of film plastic (single/multi) greenhouses; and 7,544,000 open ground.
2/ For potted plants, price represents a weighted average for plants sold in pots less than 5 inches and in pots 5 inches or more.
3/ Pot price is a weighted average of all pots to avoid disclosure of individual operations.
4/ Total includes Leatherleaf Ferns, which are not published to avoid disclosure of individual operations.

Floriculture Production and Value, 2001 1/

Kind	Number of Producers	Sales			Wholesale Price 2/	Value of Sales at Wholesale
		Unit	Number Sold	Wholesale		
			1,000	*Percent*	*Dollars*	*$1,000*
**Cut Flowers**						**286,755**
Alstroemeria	48	Stems	16,179	98	0.16	2,572
Carnations, Standard	32	Stems	21,961	98	0.15	3,228
Chrysanthemums, Pompon	33	Bunches	12,169	99	1.28	15,576
Delphinium and Larkspur	63	Stems	40,613	100	0.23	9,138
Gerbera Daisy	29	Stems	70,310	100	0.30	21,093
Gladioli	26	Spikes	11,897	100	0.36	4,307
Iris	48	Stems	67,575	100	0.23	15,407
Lilies, All	55	Stems	76,927	99	0.64	49,233
Lisianthus	42	Stems	17,678	99	0.44	7,814
Orchids, All	13	Blooms	7,132	100	0.73	5,192
Roses, All	51	Stems	114,730	99	0.32	36,943
Snapdragons	61	Spikes	45,604	98	0.23	10,626
Tulips	34	Stems	34,346	99	0.39	13,223
Other Cut Flowers	177	---	---	92	---	92,403
**Potted Flowering Plants**						**213,163**
African Violets	13	Pots	1,621	100	1.70	2,756
Finished Florist Azaleas	24	Pots	2,425	98	5.22	12,649
Florist Chrysanthemums	21	Pots	7,175	100	2.79	19,990
Easter Lilies	32	Pots	1,527	99	4.06	6,198
Orchids	44	Pots	4,722	91	9.29	43,870
Poinsettias	92	Pots	9,982	93	4.10	40,933
Spring Flowering Bulbs	37	Pots	3,274	100	2.85	9,342
Other Potted Flowering Plants	81	Pots	20,509	100	3.29	77,425
**Foliage Plants for Indoor or Patio Use**						**96,613**
Hanging Baskets	63	Baskets	5008	100	3.24	16,226
Potted Foliage	109	---	---	97	---	80,387
**Annual Bedding/Garden Plants**						**303,108**
Flats						
Begonias	49	Flats	491	98	9.22	4,527
Geraniums (Cutting)	21	Flats	197	98	11.38	2,242
Geraniums (Seed)	8	Flats	18	100	9.35	168
New Guinea Impatiens	13	Flats	78	96	12.42	969
Impatiens	61	Flats	2,360	99	9.25	21,830
Marigolds	55	Flats	1,747	99	8.81	15,391
Pansy/Viola	56	Flats	2,185	99	9.01	19,687
Petunias	65	Flats	2,095	98	9.18	19,232
Other Flowering and Foliar	67	Flats	7,854	99	8.83	69,351
Vegetable Type	47	Flats	1,733	100	9.59	16,626
Potted						
Begonias	52	Pots	1,960	77	1.11	2,182
Geraniums (Cutting)	45	Pots	7,394	99	1.23	9,096
Geraniums (Seed)	25	Pots	2,178	100	1.15	2,513
New Guinea Impatiens	48	Pots	2,331	99	1.53	3,559
Impatiens	57	Pots	5,913	90	0.83	4,897
Marigolds	39	Pots	1,667	91	0.85	1,419
Pansy/Viola	42	Pots	3,585	90	0.79	2,841
Petunias	43	Pots	3,495	96	0.99	3,476
Other Flowering and Foliar	68	Pots	31,801	97	1.26	39,938
Vegetable Type	42	Pots	6,606	98	0.96	6,363
Hanging Baskets						
Begonias	18	Baskets	20	90	7.00	140
Geraniums (Cutting)	27	Baskets	142	98	6.99	993
Geraniums (Seed)	6	Baskets	16	94	7.29	117
New Guinea Impatiens	29	Baskets	177	99	6.22	1,101
Impatiens	25	Baskets	100	98	6.03	603
Petunias	21	Baskets	36	93	5.79	208
Other Flowering	35	Baskets	588	99	7.88	4,633
Herbaceous Perennials 3/						
Hardy/Garden Chrysanthemums	39	Pots	5,041	100	1.27	6,385
Hosta	22	Pots	190	73	3.46	657
Other	77	Pots	20,144	97	2.08	41,964
**Total Cut Cultivated Greens** 4/	42	---	---	100	---	**17,375**
**Propagative (Unfinished)**						**68,781**
Cut Flowers	---	---	---	---	---	6,688
Potted Flowering Plants	---	---	---	---	---	18,694
Bedding/Garden Plants	---	---	---	---	---	35,628
Herbaceous Perennial Plants	---	---	---	---	---	4,397
Foliage Plants	---	---	---	---	---	3,374
**TOTAL ALL PLANTS**	---	---	---	---	---	**985,795**

1/ The total covered growing area of 125,174,000 square feet consisted of the following: 22,860,000 square feet of shade and temporary cover; 17,329,000 square feet of glass; 30,288,000 square feet of fiberglass and other rigid greenhouses; 54,697,000 square feet of film plastic (single/multi) greenhouses; and 7,610,000 open ground.

2/ For potted plants, price represents a weighted average for plants sold in pots less than 5 inches and in pots 5 inches or more.

3/ Pot price is a weighted average of all pots to avoid disclosure of individual operations.

4/ Total includes Leatherleaf Ferns, which are not published to avoid disclosure of individual operations.

California Fruit and Nut Crops

California accounts for 41 percent of the national fruit and nut production and 62 percent of the national value of fruit and nut crops. In 2002, the state produced 14.4 million tons of fruit and nuts, representing a 6 percent increase over 2001. The state's total production value in 2002 was $7.89 billion, up 13 percent from the previous year.

Grapes continue to be the top-valued fruit and nut crop with $2.58 billion, despite a decline of 3 percent from 2001. Crops with record production in 2002 were almonds, cherries, pistachios and strawberries. California accounts for nearly all the national production of almonds, dates, figs, grapes for raisins, kiwifruit, nectarines, olives, Clingstone peaches, pistachios, dried plums (prunes) and walnuts.

Significant Increases in Production:

Almonds 31%
Dates 23%
Figs 33%
Grapes, Raisin 28%
Peaches, Clingstone 18%
Pistachios 88%
Plums, Dried (Prunes) 14%
Strawberries 24%

Notable Decreases in Production:
Apples 10%
Avocados 6%
Boysenberries 12%
Lemons 19%
Olives 23%
Oranges, Navels 10%
Pears, Bartlett 16%
Walnuts 8%

In January, the season started with cooler temperatures and above normal rainfall in the Sacramento and San Joaquin valleys, but below normal precipitation in the coastal and southern California areas. Growers performed the needed orchard and vineyard activities such as pruning, spraying, fertilizing, brush shredding, and preparing for new tree plantings when conditions permitted. The cold weather late in January helped ease growers' concerns over the lack of chilling hours required for most tree fruit. Almonds began blooming in late February and were in full bloom by early March. A freeze in mid-March damaged the almonds in the Sacramento Valley and the crop was reduced significantly in the Arbuckle area. Warmer weather in March and April also accelerated growth and pollination in orchards and vineyards. Thinning was active in the stone fruit during April and May.

Temperatures during the summer months were relatively mild with no extreme temperatures. The fruit and nut crops developed normally. Even with the frost that damaged the almond crop in the Arbuckle area of the Sacramento Valley, the production hit a record 1.09 billion pounds, 31 percent higher than the previous record set in 1999.

The weather during the fall months was also mild and allowed the harvest of fruit and nut crops with few problems. Most fruit and nuts had good quality.

The 2001-2002 Navel orange crop experienced a very low set per tree, but the fruit sizes were large and quality was good. The Valencia crop had a higher set than the previous year and production was up 3 percent. California's grapefruit crop had generally good quality with smooth texture. The lemon season progressed normally with good quality reported and normal shaped fruit. However, heat in the major producing areas during bloom resulted in a low set and production dropped 19 percent below the previous year. The tangerine crop experienced good color and quality.

Berry Acreage, Production and Value, 1993-2002

Crop	Crop Year	Harvested	Yield Per Acre	Production	Value Per Cwt. 1/	Total Value
		Acres	*Cwt.*	*1,000 Cwt.*	*Dollars*	*$1,000*
Boysenberries						
	1993	250	90	23	88.00	1,980
	1994	250	80	20	75.50	1,510
	1995	270	67	18	95.00	1,710
	1996	270	89	24	93.70	2,248
	1997	270	91	25	66.70	1,642
	1998	250	70	18	79.20	1,386
	1999	300	93	28	82.40	2,308
	2000	260	96	25	82.40	2,061
	2001	230	83	19	81.60	1,550
	2002	210	80	17	81.70	1,372
Raspberries						
	1993	1,850	142	263	148.00	39,000
	1994	1,900	111	210	143.00	30,000
	1995	1,900	81	153	152.00	23,325
	1996	1,800	83	149	171.00	25,520
	1997	1,700	85	145	139.00	20,225
	1998	1,700	87	148	192.00	28,470
	1999	1,800	116	208	147.00	30,604
	2000	2,000	113	226	190.00	42,940
	2001	2,300	109	250	166.00	41,500
	2002	2,400	122	292	140.00	40,851
Strawberries, Fresh Market						
	1993	---	---	7,736	46.80	362,045
	1994	---	---	9,016	59.10	532,846
	1995	---	---	9,048	57.40	519,355
	1996	---	---	9,989	52.50	524,423
	1997	---	---	9,618	61.40	590,545
	1998	---	---	9,086	68.70	624,208
	1999	---	---	10,603	71.30	755,994
	2000	---	---	11,518	61.40	707,205
	2001	---	---	10,338	70.60	729,863
	2002	---	---	12,642	67.40	852,071
Strawberries, Processing						
	1993	---	---	3,685	26.00	95,810
	1994	---	---	4,265	26.90	114,729
	1995	---	---	3,932	23.40	92,003
	1996	---	---	3,619	16.70	60,437
	1997	---	---	3,716	25.80	95,873
	1998	---	---	4,466	29.70	132,640
	1999	---	---	4,748	31.20	148,138
	2000	---	---	4,214	22.10	93,129
	2001	---	---	3,390	28.50	96,615
	2002	---	---	4,315	32.10	138,512
Strawberries, All						
	1993	25,100	455	11,421	40.10	457,855
	1994	23,300	570	13,281	48.80	647,575
	1995	23,600	550	12,980	47.10	611,358
	1996	25,200	540	13,608	43.00	584,860
	1997	22,600	590	13,334	51.50	686,418
	1998	24,200	560	13,552	55.80	756,848
	1999	25,800	595	15,351	58.90	904,132
	2000	27,600	570	15,732	50.90	800,334
	2001	26,400	520	13,728	60.20	826,478
	2002	28,500	595	16,957	58.40	990,583

1/ The processed value is based on equivalent returns at the processing plant door. The fresh market value is on a packed and loaded basis, F.O.B. shipping point.

Strawberry Monthly and Season Average Prices, 1993-2002 1/

Fresh Sales	Crop Year	Jan.	Feb.	Mar.	Apr.	May	June	July	Aug.	Sept.	Oct.	Nov.	Dec.	Season Average
		Dollars Per Cwt.												
	1993	102.00	80.20	57.70	42.40	38.30	42.50	31.60	46.90	48.90	64.20	93.70	---	46.80
	1994	77.90	99.00	94.40	57.50	56.40	50.40	49.30	49.00	46.60	82.40	89.90	---	59.10
	1995	65.50	67.00	65.50	57.60	66.90	53.10	50.70	63.30	41.50	49.70	115.00	---	57.40
	1996	---	80.30	76.70	38.50	45.60	65.00	44.10	54.80	61.50	40.70	---	---	52.50
	1997	116.00	98.30	85.80	42.70	54.30	74.80	56.20	53.10	67.50	69.80	---	---	61.40
	1998	120.00	147.00	86.50	67.50	67.40	54.60	56.30	65.00	59.00	80.20	116.00	---	68.70
	1999	167.00	148.00	125.00	88.20	61.00	62.60	57.90	66.20	58.30	45.60	79.00	---	71.30
	2000	95.80	107.00	75.70	68.90	46.10	58.20	56.80	49.60	62.20	95.40	---	---	61.40
	2001	153.00	125.00	96.10	75.70	51.60	61.50	68.70	87.40	78.10	80.70	96.40	---	70.60
	2002	185.00	133.00	96.70	60.00	63.40	57.30	53.10	82.60	58.40	68.40	108.00	---	67.40

1/ Prices are F.O.B. shipping point.

Citrus Acreage, Production and Value, 1993-2002

Crop	Crop Year	Bearing	Non-Bearing 1/	Yield Per Acre	Production	Value Per Unit 2/	Total Value
		Acres	*Acres*	*Cartons*	*1,000 Cartons*	*Dollars*	*$1,000*
Grapefruit, All							
(Net weight	92-93	17,800	2,600	1,034	18,400	2.46	45,089
per carton: 33.5 pounds)	93-94	18,000	---	1,034	18,600	2.72	50,579
	94-95	18,400	---	1,010	18,600	2.82	52,420
	95-96	18,800	---	862	16,200	3.29	53,277
	96-97	18,000	---	912	16,400	3.21	52,679
	97-98	16,800	1,800	952	16,000	4.21	67,330
	98-99	16,600	---	880	14,600	5.37	78,309
	99-00	16,600	---	868	14,400	4.09	58,798
	00-01	15,400	---	818	12,600	4.39	55,242
	01-02	14,000	---	842	11,800	4.03	47,485
Lemons							
(Net weight	92-93	46,400	2,100	880	40,800	4.97	202,827
per carton: 38 pounds)	93-94	45,500	---	910	41,400	5.00	207,020
	94-95	45,700	---	876	40,000	5.66	226,120
	95-96	46,400	---	906	42,000	5.20	218,544
	96-97	47,400	---	954	45,200	6.06	274,125
	97-98	48,700	6,500	862	42,000	5.16	216,659
	98-99	48,500	---	668	32,400	6.39	207,098
	99-00	48,500	---	784	38,000	6.68	253,876
	00-01	49,500	---	914	45,200	4.71	212,725
	01-02	49,500	---	740	36,600	7.84	287,026
Oranges, Navel and Misc.							
(Net weight	92-93	114,000	10,600	768	87,600	3.16	277,008
per carton: 37.5 pounds)	93-94	114,000	---	642	73,200	3.99	291,807
	94-95	118,000	---	594	70,000	3.91	273,750
	95-96	121,000	---	628	76,000	3.68	279,300
	96-97	124,000	---	646	80,000	4.68	373,890
	97-98	127,000	8,300	692	88,000	4.42	388,870
	98-99	128,000	---	328	42,000	5.23	219,640
	99-00	126,000	---	634	80,000	3.28	262,160
	00-01	128,000	---	554	71,000	4.99	354,320
	01-02	128,000	---	500	64,000	6.44	412,305
Oranges, Valencia							
(Net weight	92-93	70,000	6,000	658	46,000	4.24	195,002
per carton: 37.5 pounds)	93-94	71,000	---	760	54,000	3.16	170,760
	94-95	73,000	---	576	42,000	4.74	199,042
	95-96	75,000	---	533	40,000	5.26	210,190
	96-97	76,000	---	632	48,000	4.39	210,370
	97-98	73,200	4,800	684	50,000	4.52	225,740
	98-99	73,500	---	408	30,000	6.14	184,210
	99-00	69,500	---	690	48,000	1.74	83,494
	00-01	66,500	---	572	38,000	4.22	160,140
	01-02	65,000	---	600	39,000	3.76	146,538
Oranges, All							
(Net weight	92-93	184,000	16,600	726	133,600	3.54	472,010
per carton: 37.5 pounds)	93-94	185,000	---	688	127,200	3.64	462,567
	94-95	191,000	---	586	112,000	4.22	472,792
	95-96	196,000	---	592	116,000	4.22	489,490
	96-97	200,000	---	640	128,000	4.57	584,260
	97-98	200,200	13,100	690	138,000	4.46	614,610
	98-99	201,500	---	358	72,000	5.61	403,850
	99-00	195,500	---	654	128,000	2.70	345,654
	00-01	194,500	---	560	109,000	4.72	514,460
	01-02	193,000	---	534	103,000	5.43	558,843
Tangerines							
(Including Mandarins and	92-93	7,700	1,300	546	4,200	6.00	25,186
Hybrids, Tangelos and	93-94	8,000	---	576	4,600	5.58	25,651
Tangors)	94-95	8,600	---	582	5,000	7.09	35,452
(Net weight	95-96	8,800	---	590	5,200	5.42	28,184
per carton: 37.5 pounds)	96-97	8,700	---	598	5,200	7.56	39,298
	97-98	8,800	1,900	546	4,800	5.74	27,530
	98-99	8,600	---	348	3,000	7.34	22,023
	99-00	8,600	---	582	5,000	5.55	27,759
	00-01	8,800	---	500	4,400	7.08	31,137
	01-02	9,000	---	488	4,400	8.83	38,821

1/ Non-bearing shown only in years acreage surveys were conducted.
2/ Equivalent packinghouse door returns.

Citrus Sales by Utilization and Value Per Carton, 1993-2002

Crop	Crop Year	All Sales		Fresh Market		Processing	
		Quantity	Value 1/	Quantity	Value 1/	Quantity	Value 1/
		1,000 Cartons	*$/Carton*	*1,000 Cartons*	*$/Carton*	*1,000 Cartons*	*$/Carton*
Grapefruit, All 2/							
(Net weight	92-93	18,400	2.46	11,400	4.08	7,000	(0.17)
per carton: 33.5 pounds)	93-94	18,600	2.72	12,160	4.16	6,440	(0.01)
	94-95	18,600	2.82	11,820	4.56	6,780	(0.22)
	95-96	16,200	3.29	12,000	4.48	4,200	(0.12)
	96-97	16,400	3.21	12,120	4.38	4,280	(0.10)
	97-98	16,000	4.21	11,088	6.13	4,912	(0.13)
	98-99	14,600	5.37	13,276	5.91	1,324	(0.07)
	99-00	14,400	4.09	12,400	4.80	2,000	(0.33)
	00-01	12,600	4.39	10,800	5.14	1,800	(0.12)
	01-02	11,800	4.03	10,200	4.68	1,600	(0.13)
Lemons							
(Net weight	92-93	40,800	4.97	19,800	9.22	21,000	0.97
per carton: 38 pounds)	93-94	41,400	5.00	19,000	10.10	22,400	0.68
	94-95	40,000	5.66	21,800	9.93	18,200	0.53
	95-96	42,000	5.20	21,600	9.23	20,400	0.94
	96-97	45,200	6.06	23,080	10.43	22,120	1.51
	97-98	42,000	5.16	19,840	9.58	22,160	1.20
	98-99	32,400	6.39	19,570	10.33	12,830	0.39
	99-00	38,000	6.68	23,200	10.49	14,800	0.71
	00-01	45,200	4.71	23,020	9.00	22,180	0.25
	01-02	36,600	7.84	25,040	11.41	11,560	0.13
Oranges, Navel and Misc.							
(Net weight	92-93	87,600	3.16	65,400	4.30	22,200	(0.18)
per carton: 37.5 pounds)	93-94	73,200	3.99	58,200	5.01	15,000	0.02
	94-95	70,000	3.91	53,000	5.21	17,000	(0.14)
	95-96	76,000	3.68	58,000	4.89	18,000	(0.24)
	96-97	80,000	4.68	69,000	5.39	11,000	0.18
	97-98	88,000	4.42	73,000	5.29	15,000	0.18
	98-99	42,000	5.23	23,000	9.41	19,000	0.18
	99-00	80,000	3.28	62,000	4.24	18,000	(0.04)
	00-01	71,000	4.99	61,000	5.82	10,000	(0.07)
	01-02	64,000	6.44	55,800	7.40	8,200	(0.08)
Oranges, Valencia							
(Net weight	92-93	46,000	4.24	32,800	5.92	13,200	0.08
per carton: 37.5 pounds)	93-94	54,000	3.16	34,000	5.04	20,000	(0.03)
	94-95	42,000	4.74	35,200	5.70	6,800	(0.24)
	95-96	40,000	5.26	31,000	6.85	9,000	(0.24)
	96-97	48,000	4.39	34,000	5.65	14,000	1.31
	97-98	50,000	4.52	38,000	5.50	12,000	1.40
	98-99	30,000	6.14	16,000	10.21	14,000	1.50
	99-00	48,000	1.74	25,400	3.35	22,600	(0.07)
	00-01	38,000	4.22	31,000	5.08	7,000	0.38
	01-02	39,000	3.76	32,700	4.41	6,300	0.37
Oranges, All							
(Net weight	92-93	133,600	3.54	98,200	4.84	35,400	(0.08)
per carton: 37.5 pounds)	93-94	127,200	3.64	92,200	5.02	35,000	(0.01)
	94-95	112,000	4.22	88,200	5.41	23,800	(0.17)
	95-96	116,000	4.22	89,000	5.58	27,000	(0.24)
	96-97	128,000	4.57	103,000	5.48	25,000	0.81
	97-98	138,000	4.46	111,000	5.36	27,000	0.72
	98-99	72,000	5.61	39,000	9.74	33,000	0.74
	99-00	128,000	2.70	87,400	3.98	40,600	(0.06)
	00-01	109,000	4.72	92,000	5.57	17,000	0.12
	01-02	103,000	5.43	88,500	6.30	14,500	0.12
Tangerines							
(Including Mandarins and	92-93	4,200	6.00	2,930	8.72	1,270	(0.28)
Hybrids, Tangelos and	93-94	4,600	5.58	3,200	8.04	1,400	(0.06)
Tangors)	94-95	5,000	7.09	3,400	10.50	1,600	(0.16)
(Net weight	95-96	5,200	5.42	3,600	7.90	1,600	(0.16)
per carton: 37.5 pounds)	96-97	5,200	7.56	4,240	9.30	960	(0.14)
	97-98	4,800	5.74	3,296	8.35	1,504	0.01
	98-99	3,000	7.34	2,126	10.16	874	0.50
	99-00	5,000	5.55	3,400	8.20	1,600	(0.07)
	00-01	4,400	7.08	3,420	9.13	980	(0.09)
	01-02	4,400	8.83	3,940	9.81	460	0.37

1/ Equivalent packinghouse door returns.

2/ Estimates for "Grapefruit, Desert" and "Grapefruit, Other" were combined to "Grapefruit, All" starting in 1995-96. Season average values for all grapefruit are derived by weighting the season average value for each area by their carton weights. Prior to 1993-94, net weight per carton for Desert Valley was 32 pounds.

Citrus Monthly and Season Average Prices, 1993-2002

Crop	Crop Year	Nov.	Dec.	Jan.	Feb.	Mar.	Apr.	May	June	July	Aug.	Sept.	Oct.	Season Average
		Dollars Per Carton												
Grapefruit, All														
Fresh Sales	92-93	5.59	4.65	4.21	3.27	2.95	3.05	2.80	4.07	4.69	4.68	5.74	4.70	4.08
	93-94	6.70	3.55	3.39	2.73	2.58	2.50	3.56	4.86	4.56	4.70	4.81	4.71	4.16
	94-95	5.08	3.60	3.79	2.83	2.92	3.05	3.62	4.88	5.45	5.48	4.78	5.98	4.56
	95-96	7.28	4.93	4.18	3.63	3.23	3.38	4.88	3.98	3.33	5.63	7.08	7.08	4.48
	96-97	6.83	6.38	5.28	4.13	3.48	3.43	3.73	4.58	4.63	4.23	4.23	3.33	4.38
	97-98	3.48	4.73	4.63	3.58	3.88	4.38	5.13	5.58	6.23	7.23	9.22	9.22	6.13
	98-99	7.91	7.36	7.56	5.66	4.51	4.16	6.21	7.36	6.46	4.66	3.16	4.81	5.91
	99-00	6.90	6.50	6.25	5.25	4.35	3.45	4.05	5.10	4.65	4.50	4.70	5.40	4.80
	00-01	5.99	5.79	5.69	4.74	4.89	4.54	5.79	5.44	4.94	4.69	5.09	4.84	5.14
	01-02	5.38	5.53	5.38	5.08	2.98	3.53	4.68	4.98	5.03	4.88	4.18	4.43	4.68
Processing	92-93	0.03	(0.16)	(0.18)	(0.18)	(0.18)	(0.18)	(0.18)	(0.17)	(0.15)	(0.15)	---	---	(0.17)
	93-94	0.18	(0.03)	(0.03)	(0.02)	(0.01)	(0.03)	(0.03)	0.05	(0.01)	(0.04)	(0.04)	0.07	(0.01)
	94-95	(0.20)	(0.22)	(0.22)	(0.22)	(0.22)	(0.21)	(0.19)	(0.22)	(0.22)	(0.22)	(0.22)	(0.22)	(0.22)
	95-96	(0.11)	(0.11)	(0.11)	(0.11)	(0.11)	(0.11)	(0.11)	(0.11)	(0.11)	(0.11)	(0.14)	(0.14)	(0.12)
	96-97	(0.13)	(0.13)	0.09	0.10	(0.10)	(0.18)	(0.15)	(0.09)	(0.07)	0.01	(0.12)	(0.20)	(0.10)
	97-98	(0.13)	(0.13)	(0.13)	(0.13)	(0.13)	(0.13)	(0.13)	(0.13)	(0.13)	(0.13)	(0.15)	(0.15)	(0.13)
	98-99	(0.08)	(0.16)	(0.05)	(0.08)	(0.16)	(0.16)	(0.10)	(0.03)	(0.03)	(0.03)	(0.03)	(0.03)	(0.07)
	99-00	(0.33)	(0.33)	(0.33)	(0.33)	(0.33)	(0.33)	(0.33)	(0.33)	(0.33)	(0.33)	(0.33)	(0.33)	(0.33)
	00-01	(0.12)	(0.12)	(0.12)	(0.12)	(0.12)	(0.12)	(0.12)	(0.12)	(0.12)	(0.12)	(0.12)	(0.12)	(0.12)
	01-02	(0.13)	(0.13)	(0.13)	(0.13)	(0.13)	(0.13)	(0.13)	(0.13)	(0.13)	(0.13)	(0.13)	(0.13)	(0.13)
All Sales	92-93	5.09	4.04	3.57	2.53	1.98	1.52	1.21	2.09	3.15	2.65	5.74	4.70	2.46
	93-94	4.78	3.28	8.08	1.98	1.75	1.76	2.23	2.71	3.10	2.75	3.60	1.81	2.72
	94-95	3.95	3.15	3.44	1.88	1.72	2.05	1.87	2.85	2.59	3.83	3.25	5.08	2.82
	95-96	6.07	4.65	3.23	2.96	2.29	2.21	3.66	3.24	2.52	3.16	4.98	6.00	3.29
	96-97	6.40	5.70	4.36	3.18	2.28	2.11	1.98	3.37	4.37	2.88	2.75	1.85	3.21
	97-98	2.96	4.12	4.05	2.79	2.66	2.58	2.62	3.31	4.34	5.43	7.78	7.96	4.21
	98-99	6.90	6.52	6.64	4.63	3.79	2.97	6.06	7.04	5.97	4.14	2.90	3.62	5.37
	99-00	5.93	6.01	5.62	4.85	4.19	3.31	3.57	4.37	3.87	3.40	4.17	4.53	4.09
	00-01	5.05	5.30	5.06	4.33	4.69	4.35	5.09	4.68	4.14	3.58	4.53	4.07	4.39
	01-02	4.54	5.17	4.83	4.83	2.89	3.41	4.12	4.29	4.25	3.75	3.73	3.74	4.03

Crop	CropYear	Oct.	Nov.	Dec.	Jan.	Feb.	Mar.	Apr.	May	June	July	Aug.	Sept.	Season Average
		Dollars Per Carton												
Oranges,Navel														
and Misc.	92-93	6.55	5.20	4.65	4.20	3.95	3.80	4.25	4.40	2.95	---	---	---	4.30
Fresh Sales	93-94	6.86	6.86	5.11	4.06	4.06	5.06	5.31	6.01	---	---	---	---	5.01
	94-95	6.06	6.26	5.46	5.26	4.81	4.46	5.66	5.11	---	---	---	---	5.21
	95-96	---	6.99	4.54	4.24	3.79	5.04	5.84	7.79	---	---	---	---	4.89
	96-97	10.29	6.24	5.19	5.09	4.69	4.79	5.69	7.94	---	---	---	---	5.39
	97-98	6.24	5.34	4.99	4.44	4.44	4.99	6.69	7.14	8.14	---	---	---	5.29
	98-99	---	7.71	7.31	11.41	10.56	10.31	13.81	13.36	---	---	---	---	9.41
	99-00	---	6.94	4.74	4.29	3.69	3.64	3.49	4.24	6.39	---	---	---	4.24
	00-01	6.97	5.52	4.97	4.77	5.02	6.32	7.62	8.67	9.42	---	---	---	5.82
	01-02	---	9.70	8.10	7.55	6.90	6.45	7.25	8.55	---	---	---	---	7.40
Processing	92-93	(0.19)	(0.19)	(0.19)	(0.19)	(0.19)	(0.19)	(0.19)	(0.10)	0.18	---	---	---	(0.18)
	93-94	(0.09)	(0.18)	(0.18)	(0.14)	0.09	0.18	(0.01)	(0.01)	---	---	---	---	0.02
	94-95	(0.12)	(0.12)	(0.03)	(0.03)	(0.12)	(0.18)	(0.22)	(0.22)	---	---	---	---	(0.14)
	95-96	---	(0.24)	(0.24)	(0.24)	(0.24)	(0.24)	(0.24)	(0.24)	---	---	---	---	(0.24)
	96-97	(0.14)	(0.14)	(0.14)	(0.04)	0.30	0.32	0.32	0.33	---	---	---	---	0.18
	97-98	(0.14)	(0.14)	(0.14)	(0.02)	0.10	0.33	0.33	0.33	0.33	---	---	---	0.18
	98-99	---	0.23	0.31	0.42	0.12	(0.10)	(0.12)	0.03	---	---	---	---	0.18
	99-00	---	(0.04)	(0.04)	(0.04)	(0.04)	(0.04)	(0.04)	(0.04)	(0.04)	---	---	---	(0.04)
	00-01	(0.07)	(0.07)	(0.07)	(0.07)	(0.07)	(0.07)	(0.07)	(0.07)	(0.07)	---	---	---	(0.07)
	01-02	---	(0.08)	(0.08)	(0.08)	(0.08)	(0.08)	(0.08)	(0.08)	---	---	---	---	(0.08)
All Sales	92-93	4.64	4.14	3.85	3.31	2.91	2.65	2.92	2.88	1.57	---	---	---	3.16
	93-94	5.99	6.07	4.43	3.34	3.25	3.84	3.88	4.29	---	---	---	---	3.99
	94-95	4.52	4.88	4.36	4.40	3.52	3.12	3.98	4.27	---	---	---	---	3.91
	95-96	---	6.16	4.02	3.46	2.80	3.43	3.82	5.50	---	---	---	---	3.68
	96-97	7.97	5.43	4.66	4.58	4.06	4.13	4.72	6.80	---	---	---	---	4.68
	97-98	5.24	4.79	4.42	3.83	3.77	3.98	5.39	5.37	6.69	---	---	---	4.42
	98-99	---	6.56	6.10	3.65	5.49	4.93	6.61	6.69	---	---	---	---	5.23
	99-00	---	5.76	4.12	3.73	3.03	2.78	2.50	2.73	3.78	---	---	---	3.28
	00-01	4.63	4.66	4.41	4.31	4.44	5.30	6.12	7.13	9.42	---	---	---	4.99
	01-02	---	8.73	7.17	7.03	6.17	5.41	5.84	7.34	---	---	---	---	6.44

Citrus Monthly and Season Average Prices, 1993-2002

Crop	Crop Year	Feb.	Mar.	Apr.	May	June	July	Aug.	Sept.	Oct.	Nov.	Dec.	Jan.	Season Average
		Dollars Per Carton												
Oranges,														
Valencia	92-93	3.52	4.62	5.02	4.37	4.47	5.02	6.62	9.67	9.52	4.17	---	---	5.92
Fresh Sales	93-94	6.09	5.89	5.34	5.59	5.24	4.74	5.09	4.84	4.09	4.79	---	---	5.04
	94-95	5.20	5.15	5.50	6.05	5.85	5.50	5.60	5.80	5.50	---	---	---	5.70
	95-96	---	6.65	6.00	5.65	5.55	6.55	7.70	10.60	8.75	---	---	---	6.85
	96-97	6.35	6.35	5.40	5.20	5.25	5.45	6.30	6.40	5.75	---	---	---	5.65
	97-98	4.60	5.85	6.60	6.45	5.95	5.20	4.60	5.05	5.50	6.55	---	---	5.50
	98-99	12.56	11.76	11.76	12.01	10.76	9.21	8.61	10.36	10.01	7.61	---	---	10.21
	99-00	---	4.65	4.55	4.00	3.75	3.20	2.95	3.10	2.50	---	---	---	3.35
	00-01	5.53	5.73	6.08	5.63	4.88	4.33	4.48	5.38	4.83	---	---	---	5.08
	01-02	7.61	5.81	4.96	4.36	3.91	3.76	4.31	4.36	4.51	---	---	---	4.41
Processing	92-93	(0.27)	(0.27)	(0.27)	(0.16)	(0.06)	0.16	0.26	0.36	0.36	(0.16)	---	---	0.08
	93-94	0.07	0.18	(0.03)	(0.03)	(0.03)	(0.03)	(0.03)	(0.03)	(0.03)	(0.03)	---	---	(0.03)
	94-95	(0.12)	(0.19)	(0.24)	(0.24)	(0.24)	(0.24)	(0.24)	(0.24)	(0.24)	---	---	---	(0.24)
	95-96	(0.24)	(0.24)	(0.24)	(0.24)	(0.24)	(0.24)	(0.24)	(0.24)	(0.24)	---	---	---	(0.24)
	96-97	1.02	0.67	0.54	0.63	0.76	1.44	1.69	1.70	1.63	---	---	---	1.31
	97-98	0.98	0.98	0.68	1.27	1.55	1.55	1.55	1.78	0.92	0.76	---	---	1.40
	98-99	1.53	2.32	0.96	1.16	1.56	1.56	1.56	1.56	1.56	1.56	---	---	1.50
	99-00	---	(0.07)	(0.07)	(0.07)	(0.07)	(0.07)	(0.07)	(0.07)	(0.07)	---	---	---	(0.07)
	00-01	---	0.38	0.38	0.38	0.38	0.38	0.38	0.38	0.38	---	---	---	0.38
	01-02	0.37	0.37	0.37	0.37	0.37	0.37	0.37	0.37	0.37	---	---	---	0.37
All Sales	92-93	2.89	3.19	3.25	3.24	3.25	3.56	4.65	6.95	6.90	1.70	---	---	4.24
	93-94	4.59	4.65	4.14	4.35	3.82	3.06	3.14	2.74	1.78	2.42	---	---	3.16
	94-95	4.09	4.62	4.35	4.96	4.77	4.77	4.69	4.67	4.83	---	---	---	4.74
	95-96	---	5.93	4.79	4.77	4.11	4.63	5.50	7.89	6.24	---	---	---	5.26
	96-97	5.02	5.04	4.56	4.42	4.01	4.28	4.77	4.62	4.04	---	---	---	4.39
	97-98	3.21	4.23	5.19	5.60	5.07	4.43	3.82	4.06	4.26	5.69	---	---	4.52
	98-99	9.55	10.18	8.81	7.47	6.97	5.42	4.46	6.11	5.84	5.59	---	---	6.14
	99-00	---	3.86	3.93	3.19	2.63	1.66	1.33	1.30	0.87	---	---	---	1.74
	00-01	5.53	5.42	5.49	4.99	4.04	3.49	3.68	4.27	3.62	---	---	---	4.22
	01-02	6.41	4.82	3.94	3.66	3.27	3.26	3.84	3.86	3.89	---	---	---	3.76

Crop	Crop Year	Aug.	Sept.	Oct.	Nov.	Dec.	Jan.	Feb.	Mar.	Apr.	May	June	July	Season Average
		Dollars Per Carton												
Lemons,All														
Fresh Sales	92-93	9.82	10.82	8.37	4.47	5.57	6.12	6.17	6.87	7.82	9.32	14.12	14.62	9.22
	93-94	17.25	16.90	13.15	9.15	6.10	4.95	4.25	5.35	7.15	8.55	12.30	16.00	10.10
	94-95	18.48	16.28	10.43	7.88	6.88	6.68	6.13	6.13	7.73	9.98	14.48	17.63	9.93
	95-96	17.43	15.33	11.53	7.78	6.08	5.48	4.88	5.58	7.48	9.83	13.33	15.83	9.23
	96-97	14.58	13.48	11.03	9.98	8.68	7.08	5.58	5.28	6.93	11.68	16.73	19.88	10.43
	97-98	20.73	18.33	11.88	6.78	5.48	4.83	4.53	5.63	7.08	9.68	14.73	18.08	9.58
	98-99	16.68	13.43	14.18	11.03	8.58	10.28	7.38	7.13	8.68	10.33	13.08	15.28	10.33
	99-00	14.69	15.24	13.14	10.59	11.69	11.44	8.89	8.94	7.59	7.14	10.89	13.94	10.49
	00-01	14.30	10.80	7.80	5.85	5.70	5.25	5.20	6.35	8.75	9.65	10.55	13.40	9.00
	01-02	15.26	13.56	13.96	12.36	10.61	9.26	8.46	8.01	9.21	10.66	13.11	13.66	11.41
Processing	92-93	0.87	0.90	0.88	1.01	1.01	1.01	1.01	1.01	1.01	0.94	0.94	0.94	0.97
	93-94	0.93	0.93	0.93	0.72	0.72	0.65	0.65	0.65	0.65	0.65	0.65	0.65	0.68
	94-95	0.64	0.64	0.64	0.50	0.50	0.50	0.50	0.50	0.54	0.54	0.54	0.54	0.53
	95-96	0.94	0.94	0.94	0.94	0.94	0.94	0.94	0.94	0.94	0.94	0.94	0.94	0.94
	96-97	1.51	1.51	1.51	1.51	1.51	1.51	1.51	1.51	1.51	1.51	1.51	1.51	1.51
	97-98	1.51	1.51	1.51	1.23	1.23	1.23	1.23	1.13	1.13	1.13	1.13	1.13	1.20
	98-99	1.10	1.10	1.10	0.34	0.34	0.34	0.34	0.34	0.34	0.34	0.34	0.34	0.39
	99-00	0.71	0.71	0.71	0.71	0.71	0.71	0.71	0.71	0.71	0.71	0.71	0.71	0.71
	00-01	0.70	0.70	0.70	0.16	0.16	0.16	0.16	0.16	0.16	0.16	0.16	0.16	0.25
	01-02	0.15	0.15	0.15	0.12	0.12	0.12	0.12	0.12	0.12	0.12	0.12	0.12	0.13
All Sales	92-93	6.29	7.22	4.72	2.59	2.78	3.23	3.14	3.11	3.56	5.17	9.54	10.59	4.97
	93-94	12.94	12.45	8.51	5.44	3.04	2.12	2.00	2.41	3.25	4.12	6.80	7.11	5.00
	94-95	15.83	11.95	6.31	4.80	3.18	3.94	2.85	3.15	3.62	6.44	11.27	11.94	5.66
	95-96	11.38	9.64	6.83	4.80	3.60	2.87	2.74	3.16	4.26	5.36	7.52	8.58	5.20
	96-97	9.44	8.90	6.72	5.91	5.19	3.99	2.74	2.81	4.46	9.49	14.39	16.54	6.06
	97-98	13.65	11.12	7.11	4.17	3.30	2.84	2.71	2.69	3.24	5.26	10.05	13.49	5.16
	98-99	13.49	10.70	11.68	8.03	5.35	5.62	4.39	4.58	4.99	5.91	7.01	8.85	6.39
	99-00	10.50	11.95	9.70	7.51	7.85	7.74	6.86	6.34	4.77	3.84	5.44	7.97	6.68
	00-01	8.77	6.51	4.17	3.05	2.91	2.42	2.45	2.76	3.90	4.77	6.58	9.98	4.71
	01-02	11.32	9.69	11.04	9.85	6.86	5.32	4.29	5.08	6.33	7.38	9.84	10.95	7.84

Citrus Monthly and Season Average Prices, 1993-2002

Crop	Crop Year	Oct.	Nov.	Dec.	Jan.	Feb.	Mar.	Apr.	May	June	July	Aug.	Sept.	Season Average
		Dollars Per Carton												
Tangerines														
Fresh Sales	92-93	13.92	8.52	8.07	8.17	8.27	9.52	9.22	---	---	---	---	---	8.72
	93-94	13.69	10.24	7.29	6.09	7.09	7.34	7.34	5.79	---	---	---	---	8.04
	94-95	13.95	10.40	7.85	9.30	10.75	11.25	13.25	13.25	13.25	---	---	---	10.50
	95-96	14.45	9.80	7.10	5.40	6.10	6.95	6.85	6.15	---	---	---	---	7.90
	96-97	15.00	10.65	8.45	9.35	8.25	7.05	8.05	6.90	---	---	---	---	9.30
	97-98	15.45	10.25	8.85	8.00	7.00	7.10	6.35	(0.25)	(0.40)	---	---	---	8.35
	98-99	13.51	11.71	10.16	9.76	8.56	8.91	1.96	---	---	---	---	---	10.16
	99-00	18.10	12.50	8.30	7.20	6.25	7.95	7.75	6.55	---	---	---	---	8.20
	00-01	15.53	10.58	8.28	7.63	9.48	11.03	3.98	---	---	---	---	---	9.13
	01-02	16.91	10.76	8.26	10.41	10.01	9.26	9.86	10.46	---	---	---	---	9.81
Processing	92-93	(0.28)	(0.28)	(0.28)	(0.28)	(0.28)	(0.28)	(0.28)	(0.20)	---	---	---	---	(0.28)
	93-94	(0.16)	(0.24)	(0.24)	(0.20)	---	0.08	(0.09)	(0.09)	---	---	---	---	(0.06)
	94-95	(0.16)	(0.16)	(0.08)	(0.08)	(0.16)	(0.21)	(0.25)	(0.25)	(0.25)	---	---	---	(0.16)
	95-96	---	(0.16)	(0.16)	(0.16)	(0.16)	(0.16)	(0.16)	(0.16)	---	---	---	---	(0.16)
	96-97	(0.14)	(0.14)	(0.14)	(0.14)	(0.14)	(0.14)	(0.14)	---	---	---	---	---	(0.14)
	97-98	(0.14)	(0.14)	(0.14)	(0.04)	0.10	0.33	0.33	0.33	0.33	---	---	---	0.01
	98-99	0.21	0.21	0.69	0.73	0.69	0.35	(0.17)	---	---	---	---	---	0.50
	99-00	(0.07)	(0.07)	(0.07)	(0.07)	(0.07)	(0.07)	(0.07)	(0.07)	---	---	---	---	(0.07)
	00-01	(0.09)	(0.09)	(0.09)	(0.09)	(0.09)	(0.09)	(0.09)	---	---	---	---	---	(0.09)
	01-02	0.37	0.37	0.37	0.37	0.37	0.37	0.37	---	---	---	---	---	0.37
All Sales	92-93	11.68	7.30	6.11	5.04	5.12	6.31	1.63	(0.20)	---	---	---	---	6.00
	93-94	12.06	9.59	6.43	3.51	4.39	3.71	4.37	3.83	---	---	---	---	5.58
	94-95	9.25	7.76	5.69	5.28	7.12	7.43	6.50	6.50	10.55	---	---	---	7.09
	95-96	14.45	8.66	6.19	3.72	3.19	3.11	3.34	4.04	---	---	---	---	5.42
	96-97	12.47	8.56	6.69	7.85	6.50	5.71	7.30	6.90	---	---	---	---	7.56
	97-98	10.00	7.01	5.26	5.32	5.43	5.41	4.63	(0.09)	(0.17)	---	---	---	5.74
	98-99	9.88	8.54	7.57	6.86	6.24	5.89	1.59	---	---	---	---	---	7.34
	99-00	13.56	8.03	6.24	5.38	4.45	4.94	4.92	3.52	---	---	---	---	5.55
	00-01	10.23	8.20	6.12	5.71	6.96	9.05	3.59	---	---	---	---	---	7.08
	01-02	15.26	8.94	7.99	9.51	8.82	7.90	9.04	10.46	---	---	---	---	8.83

Drying Ratio -- Tons of Green Fruit Required to Produce One Dried Ton

Year	Apricots	Figs	Peaches, Freestone	Pears, Bartlett	Plums, Dried (Prunes)	Raisins
1993	6.60	3.00	8.10	6.50	3.10	4.30
1994	6.40	3.00	9.30	6.15	3.10	4.50
1995	6.50	3.00	9.00	6.10	3.30	5.00
1996	6.90	3.00	9.60	7.00	3.20	4.20
1997	6.90	3.00	9.00	6.75	3.06	4.15
1998	7.20	3.00	8.06	6.91	3.20	4.70
1999	6.03	3.00	8.49	6.93	2.90	4.17
2000	7.14	3.00	9.33	6.67	3.11	4.43
2001	7.32	3.00	10.07	6.00	2.80	4.15
2002	7.14	3.00	9.31	6.52	2.98	4.50

Non-Citrus Fruit Acreage, Production and Value, 1993-2002

Crop	Crop Year	Bearing	Non-Bearing 1/	Yield Per Acre	Production	Value Per Unit 2/	Total Value
		Acres	*Acres*	*Tons*	*Tons*	*$/Ton*	*$1,000*
Apples							
	1993	34,700	4,200	12.70	440,000	314.00	138,320
	1994	34,900	4,700	15.05	525,000	266.00	139,300
	1995	35,000	4,300	12.15	425,000	366.00	155,750
	1996	38,000	---	12.50	475,000	332.00	158,050
	1997	38,500	---	12.50	481,000	338.00	162,655
	1998	37,000	---	11.60	430,000	306.00	131,340
	1999	35,000	---	12.80	448,000	316.00	141,764
	2000	31,000	---	9.20	285,000	328.00	83,430
	2001	29,000	---	8.95	260,000	314.00	77,110
	2002	26,000	---	9.05	235,000	408.00	93,840
Apricots							
	1993	19,300	1,500	4.61	89,000	363.00	32,288
	1994	19,800	---	7.32	145,000	331.00	43,700
	1995	19,800	---	2.73	54,000	387.00	20,913
	1996	20,200	---	3.76	76,000	403.00	30,663
	1997	20,000	---	6.60	132,000	308.00	37,673
	1998	20,000	---	5.65	113,000	311.00	31,895
	1999	19,000	---	4.47	85,000	361.00	30,703
	2000	19,000	---	4.74	90,000	329.00	26,679
	2001	18,000	---	4.28	77,000	319.00	22,330
	2002	17,000	---	5.00	85,000	306.00	22,964
Avocados							
	92-93	72,900	300	3.90	284,000	400.00	113,600
	93-94	66,900	---	2.08	139,000	1,810.00	251,590
	94-95	61,300	---	2.53	155,000	1,480.00	229,894
	95-96	61,100	---	2.80	171,000	1,370.00	234,831
	96-97	59,900	---	2.79	167,000	1,560.00	260,162
	97-98	59,900	---	2.57	154,000	1,710.00	263,473
	98-99	59,400	---	2.29	136,000	2,400.00	327,002
	99-00	59,000	---	2.73	161,000	2,110.00	339,594
	00-01	59,000	---	3.61	213,000	1,480.00	315,842
	01-02	58,000	---	3.45	200,000	1,790.00	358,000
Cherries, Sweet							
	1993	11,700	2,700	1.62	19,000	2,270.00	43,203
	1994	13,500	---	3.85	52,000	1,220.00	63,512
	1995	15,000	---	1.32	19,800	2,100.00	41,540
	1996	16,500	---	1.49	24,600	1,850.00	45,430
	1997	17,000	---	2.89	49,200	1,290.00	63,375
	1998	17,500	---	0.87	15,200	1,550.00	23,512
	1999	18,500	---	4.38	81,000	866.00	68,866
	2000	22,000	---	2.14	47,000	1,570.00	71,564
	2001	25,000	---	2.21	55,300	1,560.00	79,814
	2002	26,000	---	2.13	55,500	1,740.00	93,315
Dates							
	1993	5,500	200	5.27	29,000	790.00	22,910
	1994	5,500	---	4.18	23,000	750.00	17,250
	1995	5,200	---	4.37	22,700	780.00	17,706
	1996	4,680	---	4.91	23,000	1,090.00	25,070
	1997	4,800	---	4.38	21,000	1,100.00	23,100
	1998	4,900	---	5.08	24,900	1,220.00	30,378
	1999	4,900	---	4.53	22,200	1,240.00	27,528
	2000	4,800	---	3.63	17,400	1,230.00	21,402
	2001	4,600	---	4.28	19,700	1,360.00	26,792
	2002	4,500	---	5.38	24,200	1,550.00	37,510

Non-Citrus Fruit Acreage, Production and Value, 1993-2002

Crop	Crop Year	Bearing	Non-Bearing 1/	Yield Per Acre	Production	Value Per Unit 2/	Total Value
		Acres	*Acres*	*Tons*	*Tons*	*$/Ton*	*$1,000*
Figs							
	1993	14,200	500	4.27	60,700	401.00	24,341
	1994	14,600	---	3.88	56,700	419.00	23,768
	1995	14,700	---	3.56	52,400	314.00	16,429
	1996	15,400	---	2.95	45,500	283.00	12,894
	1997	16,400	---	3.51	57,500	265.00	15,209
	1998	16,300	---	3.15	51,300	226.00	11,611
	1999	14,700	---	3.22	47,300	268.00	12,685
	2000	15,400	---	3.63	55,900	272.00	15,226
	2001	14,000	---	2.93	41,000	366.00	15,012
	2002	12,900	---	4.22	54,400	331.00	18,012
Grapes, Raisin 3/ 4/							
	1993	266,000	10,700	9.57	2,354,000	255.00	599,404
	1994	270,000	10,200	8.85	2,389,000	229.00	547,002
	1995	268,000	9,500	9.27	2,252,000	234.00	526,297
	1996	270,000	7,800	8.12	2,192,000	281.00	614,940
	1997	270,000	5,300	10.68	2,883,000	262.00	756,405
	1998	275,000	6,000	7.55	2,077,000	291.00	604,036
	1999	279,000	7,000	7.61	2,122,000	321.00	680,864
	2000	280,000	7,000	10.40	2,921,000	166.00	485,139
	2001	235,000	7,000	9.43	2,215,000	182.00	403,304
	2002	246,000	6,000	11.50	2,833,000	155.00	438,458
Grapes, Table							
	1993	77,800	6,400	8.12	632,000	574.00	362,544
	1994	78,000	8,100	7.72	602,000	515.00	310,022
	1995	79,000	11,600	8.95	707,000	523.00	370,097
	1996	80,000	14,800	7.40	592,000	650.00	384,505
	1997	81,000	13,100	10.19	825,000	448.00	369,420
	1998	83,000	15,000	7.75	643,000	499.00	320,693
	1999	87,000	13,000	8.71	758,000	552.00	418,497
	2000	89,000	11,000	8.70	774,000	565.00	437,448
	2001	88,000	10,000	8.10	713,000	610.00	435,177
	2002	88,000	9,000	8.40	739,000	618.00	456,944
Grapes, Wine							
	1993	312,000	33,400	7.68	2,397,000	361.00	866,382
	1994	322,000	47,100	7.03	2,265,000	378.00	855,915
	1995	328,000	64,400	6.94	2,275,000	424.00	965,150
	1996	350,000	76,200	6.36	2,225,000	536.00	1,192,185
	1997	374,000	78,300	7.86	2,940,000	598.00	1,756,935
	1998	385,000	122,000	6.68	2,570,000	581.00	1,491,908
	1999	424,000	130,000	6.28	2,662,000	585.00	1,556,405
	2000	458,000	110,000	7.34	3,364,000	567.00	1,908,649
	2001	480,000	90,000	6.36	3,051,000	597.00	1,820,106
	2002	486,000	70,000	6.48	3,149,000	535.00	1,683,452
Grapes, All 3/ 6/							
	1993	655,800	50,500	8.47	5,383,000	340.00	1,828,330
	1994	670,000	65,400	7.84	5,256,000	326.00	1,712,939
	1995	675,000	85,500	8.05	5,234,000	356.00	1,861,544
	1996	700,000	98,800	7.16	5,009,000	438.00	2,191,630
	1997	725,000	96,700	9.17	6,648,000	434.00	2,882,760
	1998	743,000	143,000	7.12	5,290,000	457.00	2,416,637
	1999	790,000	150,000	7.02	5,542,000	479.00	2,655,766
	2000	827,000	128,000	8.54	7,059,000	401.00	2,831,236
	2001	803,000	107,000	7.45	5,979,000	445.00	2,658,587
	2002	820,000	85,000	8.20	6,721,000	384.00	2,578,854

Non-Citrus Fruit Acreage, Production and Value, 1993-2002

Crop	Crop Year	Bearing	Non-Bearing 1/	Yield Per Acre	Production	Value Per Unit 2/	Total Value
		Acres	*Acres*	*Tons*	*Tons*	*$/Ton*	*$1,000*
Kiwifruit							
	1993	6,900	10	7.13	49,200	370.00	16,502
	1994	6,500	---	6.06	39,400	491.00	18,413
	1995	6,100	---	6.20	37,800	459.00	15,434
	1996	5,700	---	5.53	31,500	470.00	13,157
	1997	5,300	---	6.60	35,000	518.00	16,483
	1998	5,300	---	6.91	36,600	744.00	24,544
	1999	5,300	---	5.09	27,000	634.00	15,215
	2000	5,300	---	6.42	34,000	455.00	13,888
	2001	4,900	---	5.27	25,800	667.00	15,340
	2002	4,500	---	5.80	26,100	783.00	18,097
Nectarines							
	1993	28,000	8,300	7.32	205,000	500.00	102,421
	1994	31,000	---	7.81	242,000	282.00	68,168
	1995	34,000	---	5.18	176,000	534.00	93,990
	1996	35,000	---	7.06	247,000	474.00	116,977
	1997	36,000	---	7.33	264,000	375.00	98,895
	1998	35,500	---	6.31	224,000	471.00	105,466
	1999	35,500	---	7.72	274,000	411.00	112,497
	2000	35,500	---	7.52	267,000	398.00	106,256
	2001	36,500	---	7.53	275,000	464.00	127,642
	2002	36,500	---	8.19	299,000	383.00	114,517
Olives 5/							
	1993	30,100	4,400	4.05	122,000	467.00	56,991
	1994	32,000	---	2.63	84,000	464.00	38,994
	1995	33,700	---	2.30	77,500	646.00	50,069
	1996	33,700	---	4.93	166,000	617.00	102,364
	1997	35,300	---	2.95	104,000	642.00	66,801
	1998	35,300	---	2.55	90,000	459.00	41,331
	1999	35,300	---	4.02	142,000	387.00	55,011
	2000	36,000	---	1.47	53,000	656.00	34,743
	2001	36,000	---	3.72	134,000	672.00	90,096
	2002	36,000	---	2.86	103,000	590.00	60,725
Peaches, Clingstone 5/							
	1993	30,200	9,000	18.20	548,500	218.00	113,033
	1994	30,200	---	18.70	565,000	180.00	97,470
	1995	28,100	---	15.40	432,500	214.00	87,847
	1996	31,000	---	17.70	546,500	220.00	114,510
	1997	31,000	---	18.50	574,000	260.00	143,000
	1998	30,400	---	17.20	522,500	220.00	112,862
	1999	29,800	---	17.80	529,500	226.00	115,740
	2000	28,200	---	18.85	532,000	250.00	127,125
	2001	28,800	---	16.55	476,000	244.00	110,044
	2002	31,000	---	18.15	562,000	248.00	132,639
Peaches, Freestone							
	1993	29,500	5,400	10.20	301,500	298.00	90,072
	1994	30,100	---	10.60	317,000	214.00	67,918
	1995	32,500	---	7.70	251,000	372.00	93,175
	1996	34,300	---	9.90	337,000	434.00	146,306
	1997	35,200	---	10.50	369,500	244.00	89,937
	1998	37,000	---	9.20	340,500	314.00	107,155
	1999	38,000	---	10.00	381,500	320.00	122,420
	2000	39,000	---	10.15	395,500	314.00	124,484
	2001	39,000	---	9.95	387,500	352.00	136,699
	2002	39,000	---	10.20	398,000	348.00	138,369

Non-Citrus Fruit Acreage, Production and Value, 1993-2002

Crop	Crop Year	Bearing	Non-Bearing 1/	Yield Per Acre	Production	Value Per Unit 2/	Total Value
		Acres	*Acres*	*Tons*	*Tons*	*$/Ton*	*$1,000*
Peaches, All							
	1993	59,700	14,400	14.30	850,000	248.00	203,105
	1994	60,300	---	14.70	882,000	193.00	165,388
	1995	60,600	---	11.30	683,500	274.00	181,022
	1996	65,300	---	13.60	883,500	304.00	260,816
	1997	66,200	---	14.30	943,500	254.00	232,937
	1998	67,400	---	12.80	863,000	258.00	220,017
	1999	67,800	---	13.50	911,000	266.00	238,160
	2000	67,200	---	13.80	927,500	278.00	251,609
	2001	67,800	---	12.75	863,500	294.00	246,743
	2002	70,000	---	13.70	960,000	290.00	271,008
Pears, Bartlett							
	1993	19,900	100	14.50	288,000	237.00	68,287
	1994	19,500	---	17.10	333,000	183.00	60,904
	1995	18,400	---	13.40	247,000	212.00	52,453
	1996	17,500	---	16.40	287,000	267.00	76,594
	1997	15,600	---	18.10	282,000	263.00	74,054
	1998	15,000	---	18.50	277,000	246.00	68,121
	1999	15,000	---	20.70	311,000	208.00	64,676
	2000	15,000	---	18.80	282,000	205.00	54,666
	2001	14,000	---	19.60	275,000	255.00	61,202
	2002	13,000	---	19.60	232,000	242.00	56,120
Pears, Other							
	1993	4,200	900	4.76	20,000	528.00	10,561
	1994	4,800	---	6.25	30,000	281.00	8,423
	1995	4,600	---	4.35	20,000	537.00	10,734
	1996	4,500	---	6.67	30,000	500.00	15,000
	1997	4,400	---	6.82	30,000	371.00	11,130
	1998	4,300	---	6.98	30,000	436.00	13,080
	1999	4,300	---	6.98	30,000	295.00	8,850
	2000	4,300	---	6.98	30,000	437.00	13,110
	2001	4,300	---	6.98	30,000	369.00	11,070
	2002	4,300	---	6.98	30,000	405.00	12,150
Pears, All							
	1993	24,100	1,000	12.80	308,000	256.00	78,848
	1994	24,300	---	14.90	363,000	191.00	69,327
	1995	23,000	---	11.60	267,000	237.00	63,187
	1996	22,000	---	14.40	317,000	289.00	91,594
	1997	20,000	---	15.60	312,000	273.00	85,184
	1998	19,300	---	15.90	307,000	264.00	81,201
	1999	19,300	---	17.70	341,000	216.00	73,526
	2000	19,300	---	16.20	312,000	228.00	67,776
	2001	18,300	---	16.70	305,000	289.00	72,272
	2002	17,300	---	15.10	262,000	261.00	68,270
Plums							
	1993	41,200	3,500	4.49	185,000	508.00	93,954
	1994	41,600	---	5.94	247,000	321.00	79,358
	1995	42,000	---	2.95	124,000	950.00	117,849
	1996	42,600	---	5.35	228,000	420.00	95,831
	1997	42,000	---	5.86	246,000	312.00	76,825
	1998	42,000	---	4.48	188,000	529.00	99,388
	1999	40,000	---	4.90	196,000	419.00	82,041
	2000	38,000	---	5.18	197,000	442.00	87,115
	2001	37,000	---	5.68	210,000	306.00	64,362
	2002	36,000	---	5.58	201,000	386.00	77,586

Non-Citrus Fruit Acreage, Production and Value, 1993-2002

Crop	Crop Year	Bearing	Non-Bearing 1/	Yield Per Acre	Production	Value Per Unit 2/	Total Value
		Acres	*Acres*	*Tons*	*Tons*	*$/Ton*	*$1,000*
Plums, Dried (Prunes) 6/							
	1993	83,000	11,000	1.46	121,000	1,120.00	135,520
	1994	84,000	18,800	2.30	193,000	1,090.00	210,370
	1995	83,500	23,200	2.17	181,000	1,040.00	188,240
	1996	85,000	23,700	2.62	223,000	839.00	187,097
	1997	82,000	21,300	2.61	214,000	883.00	181,015
	1998	83,000	26,000	1.30	108,000	764.00	78,692
	1999	83,000	22,000	2.14	178,000	861.00	142,065
	2000	86,000	15,000	2.55	219,000	770.00	154,770
	2001	86,000	11,000	1.74	150,000	723.00	97,605
	2002	74,000	9,000	2.31	171,000	810.00	131,220
Miscellaneous							
Fruits and Nuts 7/	1993	25,000	---	---	165,000	---	70,950
	1994	29,000	---	---	193,000	---	80,642
	1995	22,000	---	---	132,000	---	62,750
	1996	27,000	---	---	165,000	---	81,872
	1997	41,000	---	---	288,000	---	144,664
	1998	39,000	---	---	232,000	---	113,024
	1999	46,000	---	---	253,000	---	135,393
	2000	49,000	---	---	309,000	---	143,446
	2001	48,000	---	---	273,000	---	144,110
	2002	43,000	---	---	262,000	---	140,700

1/ Non-bearing shown only in years acreage surveys were conducted.
2/ Return at processing plant door or packinghouse door.
3/ Acres of raisin type grapes enrolled in the California Raisin Industry Diversion (RID) program were; 1993 -- 20,000; 1994 -- zero; 1995 -- 25,000; 1996,1997, 1998, 1999 and 2000 -- zero; 2001 -- 41,000; 2002 – 27,000. These acres were deducted from bearing acres before calculating yield.
4/ Raisin-type production is the fresh equivalent of dried and not dried.
5/ Total value based on grower return.
6/ Acreage by county, variety and year of planting for 2002 are available upon request.
7/ These crops are almond hulls, apricot-plum crosses (aprium, plumcot, pluots), black walnuts, chayotes, cherimoyas, chestnuts, feijoas, guavas, jojobas, jujubes, kumquats, limes, loquats, Macadamia nuts, persimmons, pomegranates, prickly pears, quince, citrus by-products, and orchard Bio mass.

Non-Citrus Fruit Unutilized Production, 1993-2002

Crop	1993	1994	1995	1996	1997	1998	1999	2000	2001	2002
Unharvested Tons										
Apples	---	---	---	---	---	---	---	30,000	15,000	---
Apricots	---	13,000	---	---	9,600	10,400	---	9,000	7,000	10,000
Pears, Bartlett	---	---	---	---	---	---	---	15,000	35,000	---
Harvested Tons Not Sold										
Apples	---	---	---	---	---	---	---	---	---	5,000
Kiwifruit	4,600	1,900	4,200	3,500	3,200	3,600	3,000	3,500	2,800	3,000
Peaches, Clingstone	30,000	23,500	22,000	26,000	24,000	7,150	15,100	23,500	25,000	25,000
Plums, Dried (Prunes)	---	---	---	---	9,000	5,000	13,000	18,000	15,000	9,000

Non-Citrus Fruit Utilized Production and Average Grower Return, 1993-2002

Crop	Crop Year	Utilized Production	Fresh Market		Processing			
					Total		Canned	
			Quantity	Value	Quantity	Value	Quantity	Value
		Short Tons	*Tons*	*$/Ton*	*Tons*	*$/Ton*	*Tons*	*$/Ton*
Apples 1/								
	1993	440,000	185,000	574.00	255,000	126.00	20,500	152.00
	1994	525,000	175,000	568.00	350,000	113.00	45,000	145.00
	1995	425,000	150,000	734.00	275,000	165.00	37,500	150.00
	1996	475,000	175,000	646.00	300,000	150.00	48,500	184.00
	1997	481,000	187,500	664.00	293,500	130.00	37,500	144.00
	1998	430,000	200,000	544.00	230,000	98.00	10,000	144.00
	1999	448,000	210,000	496.00	238,000	158.00	20,000	148.00
	2000	255,000	130,000	486.00	125,000	162.00	3,000	152.00
	2001	245,000	110,000	458.00	135,000	198.00	8,500	146.00
	2002	230,000	115,000	606.00	115,000	210.00	11,000	208.00
Apricots								
	1993	89,000	14,500	711.00	74,500	295.00	45,000	266.00
	1994	132,000	20,000	477.00	112,000	305.00	71,000	303.00
	1995	54,000	11,000	748.00	43,000	295.00	19,200	310.00
	1996	76,000	10,500	1,080.00	65,500	295.00	20,000	320.00
	1997	122,400	20,900	467.00	101,500	275.00	46,700	320.00
	1998	102,600	18,500	533.00	84,100	262.00	40,700	330.00
	1999	85,000	21,000	563.00	64,000	295.00	33,500	330.00
	2000	81,000	21,000	459.00	60,000	288.00	32,000	322.00
	2001	70,000	14,000	551.00	56,000	260.00	31,000	292.00
	2002	75,000	14,000	490.00	61,000	271.00	30,500	286.00
Avocados 2/								
	92-93	284,000	265,000	400.00	19,000	---	---	---
	93-94	139,000	132,000	1,810.00	7,000	---	---	---
	94-95	155,000	153,500	1,490.00	1,500	---	---	---
	95-96	171,000	169,500	1,380.00	1,500	---	---	---
	96-97	167,000	165,000	1,570.00	2,000	---	---	---
	97-98	154,000	152,500	1,720.00	1,500	---	---	---
	98-99	136,000	136,000	2,400.00	---	---	---	---
	99-00	161,000	161,000	2,110.00	---	---	---	---
	00-01	213,000	213,000	1,480.00	---	---	---	---
	01-02	200,000	200,000	1,790.00	---	---	---	---
Cherries, Sweet 3/								
	1993	19,000	14,500	2,820.00	4,500	514.00	---	---
	1994	52,000	36,000	1,550.00	16,000	482.00	---	---
	1995	19,800	11,000	3,410.00	8,800	458.00	---	---
	1996	24,600	16,000	2,540.00	8,600	557.00	---	---
	1997	49,200	31,600	1,570.00	17,600	782.00	---	---
	1998	15,200	7,500	2,330.00	7,700	784.00	---	---
	1999	79,500	54,200	1,040.00	25,300	494.00	---	---
	2000	45,700	29,600	2,180.00	16,100	437.00	---	---
	2001	51,100	41,300	1,840.00	9,800	390.00	---	---
	2002	53,700	43,300	2,090.00	10,400	271.00	---	---
Dates								
	1993	29,000	29,000	790.00	---	---	---	---
	1994	23,000	23,000	750.00	---	---	---	---
	1995	22,700	22,700	780.00	---	---	---	---
	1996	23,000	23,000	1,090.00	---	---	---	---
	1997	21,000	21,000	1,100.00	---	---	---	---
	1998	24,900	24,900	1,220.00	---	---	---	---
	1999	22,200	22,200	1,240.00	---	---	---	---
	2000	17,400	17,400	1,230.00	---	---	---	---
	2001	19,700	19,700	1,360.00	---	---	---	---
	2002	24,200	24,200	1,550.00	---	---	---	---

Non-Citrus Fruit Utilized Production and Average Grower Return, 1993-2002

Crop	Crop Year	Utilized Production	Fresh Market		Processing			
					Total		Canned	
		Short Tons	Quantity	Value	Quantity	Value	Quantity	Value
			Tons	*$/Ton*	*Tons*	*$/Ton*	*Tons*	*$/Ton*
Figs 4/								
	1993	60,700	2,800	---	57,900	---	---	---
	1994	56,700	2,100	---	54,600	---	---	---
	1995	52,400	2,000	---	50,400	---	---	---
	1996	45,500	2,000	---	43,500	---	---	---
	1997	57,500	2,000	---	55,500	---	---	---
	1998	51,300	1,800	---	49,500	---	---	---
	1999	47,300	2,000	---	45,300	---	---	---
	2000	55,900	4,000	---	51,900	---	---	---
	2001	41,000	2,000	---	39,000	---	---	---
	2002	54,400	2,500	---	51,900	---	---	---
Grapes, Raisin 5/								
	1993	2,354,000	270,000	630.00	2,084,000	206.00	45,000	256.00
	1994	2,389,000	270,000	472.00	2,119,000	198.00	38,000	256.00
	1995	2,252,000	253,000	579.00	1,999,000	190.00	35,000	257.00
	1996	2,192,000	230,000	669.00	1,962,000	235.00	36,000	267.00
	1997	2,883,000	275,000	759.00	2,608,000	210.00	44,000	268.00
	1998	2,077,000	230,000	715.00	1,847,000	238.00	36,000	270.00
	1999	2,122,000	240,000	704.00	1,882,000	272.00	35,000	270.00
	2000	2,921,000	230,000	600.00	2,691,000	129.00	32,000	270.00
	2001	2,215,000	217,000	680.00	1,998,000	128.00	29,000	270.00
	2002	2,833,000	264,000	750.00	2,569,000	93.60	31,000	270.00
Grapes, Table								
	1993	632,000	468,000	720.00	164,000	156.00	---	---
	1994	602,000	464,000	628.00	138,000	135.00	---	---
	1995	707,000	523,000	651.00	184,000	161.00	---	---
	1996	592,000	465,000	775.00	127,000	190.00	---	---
	1997	825,000	595,000	554.00	230,000	173.00	---	---
	1998	643,000	489,000	607.00	154,000	155.00	---	---
	1999	758,000	583,000	659.00	175,000	196.00	---	---
	2000	774,000	613,000	680.00	161,000	128.00	---	---
	2001	713,000	587,000	720.00	126,000	99.50	---	---
	2002	739,000	662,000	680.00	77,000	88.10	---	---
Grapes, Wine								
	1993	2,397,000	36,000	325.00	2,361,000	362.00	---	---
	1994	2,265,000	45,000	471.00	2,220,000	376.00	---	---
	1995	2,275,000	45,000	238.00	2,230,000	428.00	---	---
	1996	2,225,000	45,000	333.00	2,180,000	540.00	---	---
	1997	2,940,000	45,000	250.00	2,895,000	603.00	---	---
	1998	2,570,000	42,000	250.00	2,528,000	586.00	---	---
	1999	2,662,000	45,000	275.00	2,617,000	590.00	---	---
	2000	3,364,000	45,000	300.00	3,319,000	571.00	---	---
	2001	3,051,000	45,000	300.00	3,006,000	601.00	---	---
	2002	3,149,000	45,000	300.00	3,104,000	538.00	---	---
Grapes, All 5/								
	1993	5,383,000	774,000	670.00	4,609,000	284.00	45,000	256.00
	1994	5,256,000	779,000	565.00	4,477,000	284.00	38,000	256.00
	1995	5,234,000	821,000	606.00	4,413,000	309.00	35,000	257.00
	1996	5,009,000	740,000	715.00	4,269,000	389.00	36,000	267.00
	1997	6,648,000	915,000	601.00	5,733,000	407.00	44,000	268.00
	1998	5,290,000	761,000	620.00	4,529,000	429.00	36,000	270.00
	1999	5,542,000	868,000	652.00	4,674,000	447.00	35,000	270.00
	2000	7,059,000	888,000	640.00	6,171,000	367.00	32,000	270.00
	2001	5,979,000	849,000	688.00	5,130,000	404.00	29,000	270.00
	2002	6,721,000	971,000	681.00	5,750,000	333.00	31,000	270.00

Non-Citrus Fruit Utilized Production and Average Grower Return, 1993-2002

Crop	Crop Year	Utilized Production	Fresh Market		Processing			
					Total		Canned	
		Short Tons	Quantity	Value	Quantity	Value	Quantity	Value
			Tons	*$/Ton*	*Tons*	*$/Ton*	*Tons*	*$/Ton*
Kiwifruit 6/								
	1993	44,600	44,600	---	---	---	---	---
	1994	37,500	37,500	---	---	---	---	---
	1995	33,600	32,500	---	1,100	---	---	---
	1996	28,000	26,100	---	1,900	---	---	---
	1997	31,800	31,300	---	500	---	---	---
	1998	33,000	32,000	---	1,000	---	---	---
	1999	24,000	23,100	---	900	---	---	---
	2000	30,500	28,500	---	2,000	---	---	---
	2001	23,000	22,200	---	800	---	---	---
	2002	23,100	22,300	---	800	---	---	---
Nectarines 6/								
	1993	205,000	201,000	---	4,000	---	---	---
	1994	242,000	238,000	---	4,000	---	---	---
	1995	176,000	170,000	---	6,000	---	---	---
	1996	247,000	239,800	---	7,200	---	---	---
	1997	264,000	258,500	---	5,500	---	---	---
	1998	224,000	207,600	---	16,400	---	---	---
	1999	274,000	256,300	---	17,700	---	---	---
	2000	267,000	260,700	---	6,300	---	---	---
	2001	275,000	265,400	---	9,600	---	---	---
	2002	299,000	299,000	---	---	---	---	---
Olives								
	1993	122,000	500	500.00	121,500	467.00	93,000	558.00
	1994	84,000	500	500.00	83,500	464.00	66,500	551.00
	1995	77,500	500	500.00	77,000	647.00	58,500	779.00
	1996	166,000	500	500.00	165,500	617.00	123,000	745.00
	1997	104,000	500	500.00	103,500	643.00	82,200	760.00
	1998	90,000	500	500.00	89,500	459.00	64,200	590.00
	1999	142,000	500	500.00	141,500	387.00	86,000	513.00
	2000	53,000	500	500.00	52,500	657.00	41,400	769.00
	2001	134,000	500	500.00	133,500	673.00	109,700	770.00
	2002	103,000	500	500.00	102,500	590.00	82,800	675.00
Peaches, Clingstone 7/								
	1993	518,500	---	---	518,500	218.00	485,500	224.00
	1994	541,500	---	---	541,500	180.00	493,500	189.00
	1995	410,500	---	---	410,500	213.00	381,500	220.00
	1996	520,500	---	---	520,500	219.00	480,500	220.00
	1997	550,000	---	---	550,000	260.00	529,000	264.00
	1998	515,350	---	---	515,350	219.00	469,500	230.00
	1999	514,400	---	---	514,400	225.00	476,200	232.00
	2000	508,500	---	---	508,500	250.00	486,950	251.00
	2001	451,000	---	---	451,000	244.00	435,100	244.00
	2002	537,000	---	---	537,000	247.00	511,500	244.00
Peaches, Freestone 8/ 9/								
	1993	301,500	193,000	370.00	108,500	172.00	14,700	196.00
	1994	317,000	220,000	232.00	97,000	174.00	15,100	205.00
	1995	251,000	161,300	482.00	89,700	172.00	11,000	190.00
	1996	337,000	229,350	560.00	107,650	166.00	9,100	204.00
	1997	369,500	249,050	276.00	120,450	194.00	10,650	246.00
	1998	340,500	215,800	396.00	124,700	186.00	12,750	215.00
	1999	381,500	254,150	396.00	127,350	171.00	10,800	216.00
	2000	395,500	269,000	380.00	126,500	176.00	---	---
	2001	387,500	269,000	428.00	118,500	182.00	---	---
	2002	398,000	277,850	418.00	120,150	185.00	---	---

Non-Citrus Fruit Utilized Production and Average Grower Return, 1993-2002

Crop	Crop Year	Utilized Production	Fresh Market		Processing			
					Total		Canned	
			Quantity	Value	Quantity	Value	Quantity	Value
		Short Tons	*Tons*	*$/Ton*	*Tons*	*$/Ton*	*Tons*	*$/Ton*
Peaches, All 7/ 10/								
	1993	820,000	193,000	370.00	627,000	210.00	500,200	223.00
	1994	858,500	220,000	232.00	638,500	179.00	508,600	189.00
	1995	661,500	161,300	482.00	500,200	206.00	392,500	219.00
	1996	857,500	229,350	560.00	628,150	210.00	489,600	220.00
	1997	919,500	249,050	276.00	670,450	248.00	539,650	264.00
	1998	855,850	215,800	396.00	640,050	213.00	482,250	230.00
	1999	895,900	254,150	396.00	641,750	214.00	487,000	232.00
	2000	904,000	269,000	380.00	635,000	235.00	486,950	251.00
	2001	838,500	269,000	428.00	569,500	231.00	435,100	244.00
	2002	935,000	277,850	418.00	657,150	236.00	511,500	244.00
Pears, Bartlett 7/ 10/								
	1993	288,000	61,000	271.00	227,000	228.00	---	---
	1994	333,000	88,000	113.00	245,000	208.00	---	---
	1995	247,000	60,000	254.00	187,000	199.00	---	---
	1996	287,000	77,000	362.00	210,000	232.00	---	---
	1997	282,000	68,000	318.00	214,000	247.00	---	---
	1998	277,000	53,000	309.00	224,000	231.00	---	---
	1999	311,000	84,000	143.00	227,000	235.00	---	---
	2000	267,000	81,000	159.00	186,000	220.00	---	---
	2001	240,000	67,000	317.00	173,000	232.00	---	---
	2002	232,000	69,000	270.00	163,000	231.00	---	---
Pears, Other 6/								
	1993	20,000	---	---	---	---	---	---
	1994	30,000	---	---	---	---	---	---
	1995	20,000	---	---	---	---	---	---
	1996	30,000	---	---	---	---	---	---
	1997	30,000	---	---	---	---	---	---
	1998	30,000	---	---	---	---	---	---
	1999	30,000	---	---	---	---	---	---
	2000	30,000	---	---	---	---	---	---
	2001	30,000	---	---	---	---	---	---
	2002	30,000	---	---	---	---	---	---
Pears, All 6/								
	1993	308,000	---	---	---	---	---	---
	1994	363,000	---	---	---	---	---	---
	1995	267,000	---	---	---	---	---	---
	1996	317,000	---	---	---	---	---	---
	1997	312,000	---	---	---	---	---	---
	1998	307,000	---	---	---	---	---	---
	1999	341,000	---	---	---	---	---	---
	2000	297,000	---	---	---	---	---	---
	2001	270,000	---	---	---	---	---	---
	2002	262,000	---	---	---	---	---	---
Plums 6/								
	1993	185,000	---	---	---	---	---	---
	1994	247,000	---	---	---	---	---	---
	1995	124,000	---	---	---	---	---	---
	1996	228,000	---	---	---	---	---	---
	1997	246,000	---	---	---	---	---	---
	1998	188,000	---	---	---	---	---	---
	1999	196,000	---	---	---	---	---	---
	2000	197,000	---	---	---	---	---	---
	2001	210,000	---	---	---	---	---	---
	2002	201,000	---	---	---	---	---	---

Non-Citrus Fruit Utilized Production and Average Grower Return, 1993-2002

Crop	Crop Year	Utilized Production	Fresh Market		Processing			
					Total		Canned	
		Short Tons	Quantity	Value	Quantity	Value	Quantity	Value
			Tons	$/Ton	Tons	$/Ton	Tons	$/Ton
Plums, Dried (Prunes)								
	1993	121,000	---	---	121,000	1,120.00	---	---
	1994	193,000	---	---	193,000	1,090.00	---	---
	1995	181,000	---	---	181,000	1,040.00	---	---
	1996	223,000	---	---	223,000	839.00	---	---
	1997	205,000	---	---	205,000	883.00	---	---
	1998	103,000	---	---	103,000	764.00	---	---
	1999	165,000	---	---	165,000	861.00	---	---
	2000	201,000	---	---	201,000	770.00	---	---
	2001	135,000	---	---	135,000	723.00	---	---
	2002	162,000	---	---	162,000	810.00	---	---

1/ Dried and frozen fruit included in total processed to avoid disclosure of individual operations.
2/ Processed fruit included in fresh market total to avoid disclosure of individual operations.
3/ Total processed includes canned, crushed, frozen and brined.
4/ Prices for processing and fresh totals were not published to avoid disclosure of individual operations. Small quantities processed included in fresh to avoid disclosure of individual operations.
5/ Includes substandard raisins diverted to other uses.
6/ Processed and fresh fruit not published to avoid disclosure of individual operations, but included in total utilized.
7/ Processed mostly canned, but includes other small quantities not published separately to avoid disclosure of individual operations.
8/ Quantity and value include dried, but price excludes dried.
9/ Not published separately to avoid disclosure of individual operations.
10/ Total processing value per ton excludes dried processing.

Non-Citrus Fruit Utilized Production and Average Grower Return, 1993-2002 (Continued)

Crop	Crop Year	Processing							
		Juice or Crushed		Frozen		Dried (Fresh Basis)		Dried Basis	
		Quantity	Value	Quantity	Value	Quantity	Value	Quantity	Value
		Tons	*$/Ton*	*Tons*	*$/Ton*	*Tons*	*$/Ton*	*Tons*	*$/Ton*
Apples 1/									
	1993	175,000	122.00	---	---	---	---	---	---
	1994	250,000	110.00	---	---	---	---	---	---
	1995	200,000	165.00	---	---	---	---	---	---
	1996	209,000	137.00	---	---	---	---	---	---
	1997	181,000	106.00	---	---	---	---	---	---
	1998	160,000	88.00	---	---	---	---	---	---
	1999	200,000	154.00	---	---	---	---	---	---
	2000	112,500	156.00	---	---	---	---	---	---
	2001	109,000	209.00	---	---	---	---	---	---
	2002	99,000	213.00	---	---	---	---	---	---
Apricots									
	1993	---	---	11,500	315.00	18,000	353.00	2,730	2,320.00
	1994	---	---	10,500	350.00	30,500	295.00	4,800	1,890.00
	1995	8,500	230.00	5,700	300.00	9,600	321.00	1,480	2,090.00
	1996	21,500	245.00	9,000	310.00	15,000	325.00	2,170	2,240.00
	1997	27,700	190.00	15,100	300.00	12,000	262.00	1,740	1,810.00
	1998	24,000	125.00	10,400	315.00	9,000	258.00	1,250	1,860.00
	1999	13,000	200.00	10,500	315.00	7,000	271.00	1,160	1,630.00
	2000	10,000	169.00	10,000	298.00	8,000	260.00	1,120	1,860.00
	2001	9,000	102.00	9,000	308.00	6,000	268.00	820	1,960.00
	2002	7,000	158.00	10,500	299.00	8,000	223.00	1,120	1,590.00
Avocados 2/									
	92-93	---	---	---	---	---	---	---	---
	93-94	---	---	---	---	---	---	---	---
	94-95	---	---	---	---	---	---	---	---
	95-96	---	---	---	---	---	---	---	---
	96-97	---	---	---	---	---	---	---	---
	97-98	---	---	---	---	---	---	---	---
	98-99	---	---	---	---	---	---	---	---
	99-00	---	---	---	---	---	---	---	---
	00-01	---	---	---	---	---	---	---	---
	01-02	---	---	---	---	---	---	---	---
Cherries, Sweet 3/									
	1993	---	---	---	---	---	---	---	---
	1994	---	---	---	---	---	---	---	---
	1995	---	---	---	---	---	---	---	---
	1996	---	---	---	---	---	---	---	---
	1997	---	---	---	---	---	---	---	---
	1998	---	---	---	---	---	---	---	---
	1999	---	---	---	---	---	---	---	---
	2000	---	---	---	---	---	---	---	---
	2001	---	---	---	---	---	---	---	---
	2002	---	---	---	---	---	---	---	---
Dates									
	1993	---	---	---	---	---	---	---	---
	1994	---	---	---	---	---	---	---	---
	1995	---	---	---	---	---	---	---	---
	1996	---	---	---	---	---	---	---	---
	1997	---	---	---	---	---	---	---	---
	1998	---	---	---	---	---	---	---	---
	1999	---	---	---	---	---	---	---	---
	2000	---	---	---	---	---	---	---	---
	2001	---	---	---	---	---	---	---	---
	2002	---	---	---	---	---	---	---	---

Non-Citrus Fruit Utilized Production and Average Grower Return, 1993-2002 (Continued)

Crop	Crop Year	Processing							
		Juice or Crushed		Frozen		Dried (Fresh Basis)		Dried Basis	
		Quantity	Value	Quantity	Value	Quantity	Value	Quantity	Value
		Tons	*$/Ton*	*Tons*	*$/Ton*	*Tons*	*$/Ton*	*Tons*	*$/Ton*
Figs 4/									
	1993	---	---	---	---	57,900	367.00	19,300	1,100.00
	1994	---	---	---	---	54,600	400.00	18,200	1,200.00
	1995	---	---	---	---	50,400	298.00	16,800	895.00
	1996	---	---	---	---	43,500	258.00	14,500	774.00
	1997	---	---	---	---	55,500	233.00	18,500	699.00
	1998	---	---	---	---	49,500	198.00	16,500	594.00
	1999	---	---	---	---	45,300	227.00	15,100	681.00
	2000	---	---	---	---	51,900	224.00	17,300	672.00
	2001	---	---	---	---	39,000	308.00	13,000	924.00
	2002	---	---	---	---	51,900	290.00	17,300	871.00
Grapes, Raisin 5/									
	1993	397,000	152.00	---	---	1,642,000	218.00	382,000	937.00
	1994	197,000	118.00	---	---	1,884,000	205.00	418,600	923.00
	1995	432,000	164.00	---	---	1,532,000	196.00	306,400	980.00
	1996	618,000	191.00	---	---	1,308,000	255.00	311,500	1,070.00
	1997	786,000	185.00	---	---	1,778,000	219.00	428,500	908.00
	1998	507,000	164.00	---	---	1,304,000	266.00	277,400	1,250.00
	1999	420,000	200.00	---	---	1,427,000	293.00	342,300	1,220.00
	2000	513,000	125.00	---	---	2,146,000	128.00	484,500	569.00
	2001	262,000	85.70	---	---	1,707,000	132.00	411,300	548.00
	2002	622,000	75.50	---	---	1,916,000	96.70	425,800	435.00
Grapes, Table									
	1993	137,000	147.00	---	---	27,000	204.00	6,300	874.00
	1994	123,000	136.00	---	---	15,000	125.00	3,400	550.00
	1995	170,000	162.00	---	---	14,000	150.00	2,800	750.00
	1996	117,000	188.00	---	---	10,000	210.00	2,400	880.00
	1997	212,000	169.00	---	---	18,000	220.00	4,300	914.00
	1998	136,000	150.00	---	---	18,000	197.00	3,800	933.00
	1999	150,000	187.00	---	---	25,000	247.00	6,000	1,030.00
	2000	120,000	118.00	---	---	41,000	157.00	9,200	700.00
	2001	101,000	87.20	---	---	25,000	149.00	5,800	644.00
	2002	61,000	80.50	---	---	16,000	117.00	3,600	520.00
Grapes, Wine									
	1993	2,361,000	362.00	---	---	---	---	---	---
	1994	2,220,000	376.00	---	---	---	---	---	---
	1995	2,230,000	428.00	---	---	---	---	---	---
	1996	2,180,000	540.00	---	---	---	---	---	---
	1997	2,895,000	603.00	---	---	---	---	---	---
	1998	2,528,000	586.00	---	---	---	---	---	---
	1999	2,617,000	590.00	---	---	---	---	---	---
	2000	3,319,000	571.00	---	---	---	---	---	---
	2001	3,006,000	601.00	---	---	---	---	---	---
	2002	3,104,000	538.00	---	---	---	---	---	---
Grapes, All 5/									
	1993	2,895,000	323.00	---	---	1,669,000	218.00	388,300	936.00
	1994	2,540,000	344.00	---	---	1,899,000	204.00	422,000	920.00
	1995	2,832,000	372.00	---	---	1,546,000	196.00	309,200	978.00
	1996	2,915,000	452.00	---	---	1,318,000	255.00	313,900	1,070.00
	1997	3,893,000	495.00	---	---	1,796,000	219.00	432,800	908.00
	1998	3,171,000	500.00	---	---	1,322,000	265.00	281,200	1,250.00
	1999	3,187,000	520.00	---	---	1,452,000	292.00	348,300	1,220.00
	2000	3,952,000	499.00	---	---	2,187,000	129.00	493,700	571.00
	2001	3,369,000	546.00	---	---	1,732,000	132.00	417,100	549.00
	2002	3,787,000	455.00	---	---	1,932,000	96.90	429,400	436.00

Non-Citrus Fruit Utilized Production and Average Grower Return, 1993-2002 (Continued)

Crop	Crop Year	Processing							
		Juice or Crushed		Frozen		Dried (Fresh Basis)		Dried Basis	
		Quantity	Value	Quantity	Value	Quantity	Value	Quantity	Value
		Tons	*$/Ton*	*Tons*	*$/Ton*	*Tons*	*$/Ton*	*Tons*	*$/Ton*
Kiwifruit									
	1993	---	---	---	---	---	---	---	---
	1994	---	---	---	---	---	---	---	---
	1995	---	---	---	---	---	---	---	---
	1996	---	---	---	---	---	---	---	---
	1997	---	---	---	---	---	---	---	---
	1998	---	---	---	---	---	---	---	---
	1999	---	---	---	---	---	---	---	---
	2000	---	---	---	---	---	---	---	---
	2001	---	---	---	---	---	---	---	---
	2002	---	---	---	---	---	---	---	---
Nectarines 6/									
	1993	---	---	---	---	---	---	---	---
	1994	---	---	---	---	---	---	---	---
	1995	---	---	---	---	---	---	---	---
	1996	---	---	---	---	---	---	---	---
	1997	---	---	---	---	---	---	---	---
	1998	---	---	---	---	---	---	---	---
	1999	---	---	---	---	---	---	---	---
	2000	---	---	---	---	---	---	---	---
	2001	---	---	---	---	---	---	---	---
	2002	---	---	---	---	---	---	---	---
Olives				*Limited*		*Undersize*			
	1993	5,300	10.50	19,700	235.00	3,500	37.20	---	---
	1994	4,400	11.00	8,400	234.00	4,200	14.00	---	---
	1995	4,000	11.00	9,300	443.00	5,200	16.00	---	---
	1996	7,000	11.00	29,000	355.00	6,500	11.00	---	---
	1997	3,600	11.00	10,200	386.00	7,500	11.00	---	---
	1998	4,100	11.00	12,800	240.00	8,400	11.00	---	---
	1999	5,000	200.00	36,500	259.00	14,000	11.00	---	---
	2000	3,000	300.00	5,100	333.00	3,000	11.00	---	---
	2001	3,000	300.00	15,300	287.00	5,500	6.00	---	---
	2002	6,000	240.00	9,900	309.00	3,800	17.00	---	---
Peaches, Clingstone 7/									
	1993	---	---	---	---	---	---	---	---
	1994	---	---	---	---	---	---	---	---
	1995	---	---	---	---	---	---	---	---
	1996	---	---	---	---	---	---	---	---
	1997	---	---	---	---	---	---	---	---
	1998	---	---	---	---	---	---	---	---
	1999	---	---	---	---	---	---	---	---
	2000	---	---	---	---	---	---	---	---
	2001	---	---	---	---	---	---	---	---
	2002	---	---	---	---	---	---	---	---
Peaches, Freestone 8/ 9/									
	1993	---	---	78,800	185.00	15,000	82.00	1,850	713.00
	1994	---	---	68,400	183.00	13,500	93.00	1,450	856.00
	1995	---	---	63,700	186.00	14,000	86.00	1,550	894.00
	1996	---	---	77,850	186.00	16,350	78.00	1,700	726.00
	1997	---	---	89,450	190.00	17,050	68.00	1,894	612.00
	1998	---	---	81,900	200.00	12,500	67.50	1,551	544.50
	1999	---	---	87,100	201.00	15,700	73.00	1,849	619.50
	2000	---	---	92,650	200.00	12,600	78.00	1,350	728.00
	2001	---	---	86,000	214.00	14,600	51.00	1,450	514.00
	2002	---	---	91,100	205.00	14,200	49.00	1,525	456.00

Non-Citrus Fruit Utilized Production and Average Grower Return, 1993-2002 (Continued)

Crop	Crop Year	Processing							
		Juice or Crushed		Frozen		Dried (Fresh Basis)		Dried Basis	
		Quantity	Value	Quantity	Value	Quantity	Value	Quantity	Value
		Tons	*$/Ton*	*Tons*	*$/Ton*	*Tons*	*$/Ton*	*Tons*	*$/Ton*
Peaches, All 7/ 8/ 9/ 10/									
	1993	---	---	78,800	185.00	15,000	82.00	1,850	713.00
	1994	---	---	68,400	183.00	13,500	93.00	1,450	856.00
	1995	---	---	63,700	186.00	14,000	86.00	1,550	894.00
	1996	---	---	77,850	186.00	16,350	78.00	1,700	726.00
	1997	---	---	89,450	190.00	17,050	68.00	1,894	612.00
	1998	---	---	81,900	200.00	12,500	67.50	1,551	544.50
	1999	---	---	87,100	201.00	15,700	73.00	1,849	619.50
	2000	---	---	92,650	200.00	12,600	78.00	1,350	728.00
	2001	---	---	86,000	214.00	14,600	51.00	1,450	514.00
	2002	---	---	91,100	205.00	14,200	49.00	1,525	456.00
Pears, Bartlett 7/ 10/									
	1993	---	---	---	---	9,700	149.00	1,500	970.00
	1994	---	---	---	---	8,000	152.00	1,300	942.00
	1995	---	---	---	---	4,900	150.00	800	919.00
	1996	---	---	---	---	4,900	184.00	700	1,290.00
	1997	---	---	---	---	5,400	151.00	800	1,020.00
	1998	---	---	---	---	7,600	217.00	1,100	1,500.00
	1999	---	---	---	---	7,000	150.00	1,010	1,040.00
	2000	---	---	---	---	4,000	136.00	600	909.00
	2001	---	---	---	---	3,000	202.00	500	1,210.00
	2002	---	---	---	---	3,000	202.00	460	1,315.00
Pears, Other 6/									
	1993	---	---	---	---	---	---	---	---
	1994	---	---	---	---	---	---	---	---
	1995	---	---	---	---	---	---	---	---
	1996	---	---	---	---	---	---	---	---
	1997	---	---	---	---	---	---	---	---
	1998	---	---	---	---	---	---	---	---
	1999	---	---	---	---	---	---	---	---
	2000	---	---	---	---	---	---	---	---
	2001	---	---	---	---	---	---	---	---
	2002	---	---	---	---	---	---	---	---
Pears, All 6/									
	1993	---	---	---	---	---	---	---	---
	1994	---	---	---	---	---	---	---	---
	1995	---	---	---	---	---	---	---	---
	1996	---	---	---	---	---	---	---	---
	1997	---	---	---	---	---	---	---	---
	1998	---	---	---	---	---	---	---	---
	1999	---	---	---	---	---	---	---	---
	2000	---	---	---	---	---	---	---	---
	2001	---	---	---	---	---	---	---	---
	2002	---	---	---	---	---	---	---	---
Plums 6/									
	1993	---	---	---	---	---	---	---	---
	1994	---	---	---	---	---	---	---	---
	1995	---	---	---	---	---	---	---	---
	1996	---	---	---	---	---	---	---	---
	1997	---	---	---	---	---	---	---	---
	1998	---	---	---	---	---	---	---	---
	1999	---	---	---	---	---	---	---	---
	2000	---	---	---	---	---	---	---	---
	2001	---	---	---	---	---	---	---	---
	2002	---	---	---	---	---	---	---	---

Non-Citrus Fruit Utilized Production and Average Grower Return, 1993-2002 (Continued)

Crop	Crop Year	Processing: Juice or Crushed Quantity	Juice or Crushed Value	Frozen Quantity	Frozen Value	Dried (Fresh Basis) Quantity	Dried (Fresh Basis) Value	Dried Basis Quantity	Dried Basis Value
		Tons	*$/Ton*	*Tons*	*$/Ton*	*Tons*	*$/Ton*	*Tons*	*$/Ton*
Plums, Dried (Prunes)									
	1993	---	---	---	---	375,000	374.00	121,000	1,120.00
	1994	---	---	---	---	594,000	354.00	193,000	1,090.00
	1995	---	---	---	---	597,300	320.00	181,000	1,040.00
	1996	---	---	---	---	704,000	262.00	223,000	839.00
	1997	---	---	---	---	627,300	289.00	205,000	883.00
	1998	---	---	---	---	329,600	239.00	103,000	764.00
	1999	---	---	---	---	478,500	297.00	165,000	861.00
	2000	---	---	---	---	625,100	248.00	201,000	770.00
	2001	---	---	---	---	378,000	258.00	135,000	723.00
	2002	---	---	---	---	483,000	272.00	162,000	810.00

Raisin Production and Average Grower Return, 1993-2002

Raisins 1/	Crop Year	All Varieties	Naturally Dried: Thompson Seedless	Muscats	Oleate 2/	Sultanas	Zante Currant	Dehydrated, All Types
Production 3/					*Tons*			
	1993	382,000	338,000	170	---	330	5,700	37,800
	1994	418,600	378,400	230	400	270	5,400	33,900
	1995	306,400	264,800	150	300	130	2,820	38,200
	1996	311,500	272,100	130	810	140	4,520	33,800
	1997	428,500	382,500	80	1,100	200	4,820	39,800
	1998	277,400	240,500	30	580	120	3,770	32,400
	1999	342,300	300,000	10	270	120	3,700	38,200
	2000	484,500	437,000	60	3,700	140	4,800	38,800
	2001	411,300	378,000	50	6,500	150	4,200	22,400
	2002	425,800	---	---	---	---	---	---
Average Grower Return 3/					*Dollars Per Ton*			
	1993	937	908	1,080	---	817	1,260	1,150
	1994	923	908	1,000	970	773	1,000	1,080
	1995	980	947	935	978	733	871	1,220
	1996	1,070	1,050	1,000	943	823	1,180	1,250
	1997	908	881	1,000	1,100	842	658	1,190
	1998	1,250	1,240	700	1,280	818	994	1,360
	1999	1,220	1,210	702	1,240	919	596	1,400
	2000	569	564	533	855	590	831	852
	2001	548	538	536	814	531	976	964
	2002	435	---	---	---	---	---	---

1/ Excludes raisins produced from table-type grapes.
2/ Miscellaneous seedless grapes with Oleate applied.
3/ Missing 2002 data will be available by the end of January 2004.

Nut Acreage, Production and Value, 1993-2002

Crop	Crop Year	Bearing	Non-Bearing	Yield Per Acre	Production: Marketable In-Shell	Production: Shelling Stock	Production: Total	Value Per Unit	Total Value
		Acres	*Acres*	*Pounds*	*1,000 Pounds*	*1,000 Pounds*	*1,000 Pounds*	*$/Pound*	*$1,000*
Almonds 1/ 2/									
(Shelled Basis)	1993	413,000	33,400	1,190	---	---	490,000	1.94	930,618
	1994	433,000	46,500	1,700	---	---	735,000	1.34	965,202
	1995	418,000	65,700	885	---	---	370,000	2.48	880,896
	1996	428,000	72,400	1,190	---	---	510,000	2.08	1,018,368
	1997	442,000	63,000	1,720	---	---	759,000	1.56	1,160,640
	1998	460,000	113,000	1,130	---	---	520,000	1.41	703,590
	1999	480,000	105,000	1,740	---	---	833,000	0.86	687,742
	2000	500,000	95,000	1,410	---	---	703,000	0.97	666,487
	2001	525,000	70,000	1,580	---	---	830,000	0.91	740,012
	2002	530,000	60,000	2,060	---	---	1,090,000	1.10	1,189,870
Pecans									
(In-Shell Basis)	1993	2,500	500	1,200	---	---	3,000	0.96	2,880
	1994	2,600	N/A	577	---	---	1,500	1.16	1,740
	1995	2,600	N/A	1,120	---	---	2,900	1.28	3,712
	1996	2,500	N/A	520	---	---	1,300	0.83	1,079
	1997	2,600	N/A	962	---	---	3,000	1.32	3,960
	1998	2,600	N/A	654	---	---	1,700	1.66	2,822
	1999	2,600	N/A	731	---	---	1,900	1.27	2,413
	2000	2,600	N/A	1,310	---	---	3,400	1.52	5,168
	2001	2,600	N/A	1,420	---	---	3,700	0.81	2,997
	2002	2,600	N/A	1,460	---	---	3,800	1.27	4,826
Pistachios									
	1993	57,000	15,700	2,670	113,000	39,000	152,000	1.07	162,640
	1994	57,500	16,600	2,240	94,600	34,400	129,000	0.92	118,809
	1995	60,300	13,400	2,450	107,500	40,500	148,000	1.09	161,320
	1996	64,300	17,100	1,630	85,000	20,000	105,000	1.16	121,800
	1997	65,400	17,000	2,750	137,000	43,000	180,000	1.13	203,400
	1998	68,000	19,300	2,760	138,000	50,000	188,000	1.03	193,640
	1999	71,000	21,000	1,730	105,000	18,000	123,000	1.33	163,590
	2000	74,600	21,700	3,260	190,000	53,000	243,000	1.01	245,430
	2001	78,000	23,500	2,060	126,500	34,500	161,000	1.01	162,610
	2002	83,000	N/A	3,650	242,000	61,000	303,000	1.11	336,330
Walnuts 2/		*Acres*	*Acres*	*Tons*	*Tons*	*Tons*	*Tons*	*$/Ton*	*$1,000*
(In-Shell Basis)									
	1993	185,000	20,000	1.41	---	---	260,000	1,390.00	361,400
	1994	189,000	25,600	1.23	---	---	232,000	1,030.00	238,960
	1995	193,000	23,300	1.21	---	---	234,000	1,400.00	327,600
	1996	192,000	23,200	1.08	---	---	208,000	1,580.00	328,640
	1997	193,000	20,900	1.39	---	---	269,000	1,430.00	384,670
	1998	193,000	28,000	1.18	---	---	227,000	1,050.00	238,350
	1999	191,000	29,000	1.48	---	---	283,000	886.00	250,738
	2000	193,000	30,000	1.24	---	---	239,000	1,240.00	296,360
	2001	196,000	28,000	1.56	---	---	305,000	1,120.00	341,600
	2002	200,000	26,000	1.41	---	---	282,000	1,080.00	304,560

1/ Values are based on the edible portion of the crop only. Included in production are inedible quantities of no value as follows: 1993 -- 10.3 million pounds, 1994 -- 14.7 million pounds, 1995 -- 14.8 million pounds, 1996 -- 20.4 million pounds, 1997 -- 15.0 million pounds, 1998 -- 21.0 million pounds, 1999 -- 33.3 million pounds, 2000 – 15.9 million pounds, 2001 – 16.8 million pounds, 2002 -- 8.30 million pounds.

2/ Data for 2002 acreage by county, variety and year of planting available upon request.

California Livestock and Dairy

The Golden State's dairy industry continued its phenomenal growth as new record highs were set for the number of milk cows and milk production. In 2002, California led the nation in total milk production with a record 34.9 billion pounds, representing a 5 percent increase from the previous year. The average number of cows in 2002 was 1.65 million, up 4 percent from 2001. Total milk value was $3.82 billion, down 18 percent. This decrease is due to a 22 percent decline in the unit value, which went from $13.94 to $10.94 per hundredweight.

The five leading counties in milk production and number of milk cows were Tulare, Merced, Stanislaus, San Bernardino and Kings. Collectively, these counties accounted for approximately two-thirds of the state's milk production and milk cows.

Nationally, California ranked first in nonfat dry milk production, first in butter production, and second in cheese production. California produced 48 percent of the nation's nonfat dry milk, 28 percent of the butter, and 20 percent of the cheese. Cheddar and mozzarella cheeses constituted approximately three-fourths of the cheese production in California.

Livestock cash receipts (excluding broilers) during 2002 totaled $6.24 billion, down 15 percent from 2001. The decrease was due mainly to an 18 percent decline in milk and cream, as well as a 9 percent fall in the value of cattle and calves.

Total production of the three red meat species (cattle, sheep and hogs) was 1.99 billion pounds, down 5 percent from the 2.10 billion pounds of a year ago. Total marketings of the three red meat species also declined by 4 percent, from 2.58 to 2.47 billion pounds. Cattle and calf marketings were 2.33 billion pounds, representing a 4 percent reduction. Sheep and lamb marketings of 77.8 million pounds were down 5 percent from 2001. Hog and pig marketings of 64.0 million pounds were up 8 percent from 2001.

Cattle and calves marketed from California feedlots totaled 671,000, which is an 11 percent increase over 2001. Turkey production decreased 3 percent to 439 million pounds in 2002. Egg production of 6.12 billion eggs was up 2 percent. Honey production was 23.3 million pounds, down 16 percent from 2001. Wool production was 3.65 million pounds, down 3 percent from the previous year.

Bee Colonies, Honey Production and Value, 1993-2002

Year	Bee Colonies	Yield Per Colony	Production	Average Price Per Pound	Total Value
	1,000	*Pounds*	*1,000 Pounds*	*Cents*	*$1,000*
1993	500	90	45,000	50	22,500
1994	400	60	24,000	51	12,240
1995	420	93	39,060	63	24,608
1996	390	70	27,300	84	22,932
1997	420	75	31,500	70	22,050
1998	450	83	37,350	62	23,157
1999	505	60	30,300	55	16,665
2000	440	70	30,800	59	18,172
2001	425	65	27,625	69	19,061
2002	440	53	23,320	129	30,083

Beef Cow Operations and Inventory, 1993-2002

Year	1 - 99 Head		100-499 Head 1/		500+ Head	
	Operations	Inventory	Operations	Inventory	Operations	Inventory
	Percent of Total					
1993	86.0	24.0	12.0	43.0	2.0	33.0
1994	86.0	24.0	12.0	44.0	2.0	32.0
1995	86.3	24.0	11.9	44.0	1.9	32.0
1996	86.0	24.0	12.0	43.0	2.0	33.0
1997	85.8	24.0	12.2	42.0	2.0	34.0
1998	85.5	23.0	12.4	43.0	2.1	34.0
1999	85.3	22.0	12.6	43.0	2.1	35.0
2000	85.2	21.0	12.7	44.0	2.1	35.0
2001	85.1	21.0	12.6	45.0	2.3	34.0
2002	84.1	19.0	13.6	45.0	2.3	36.0

1/ Starting in 1993, the 100+ Head class was broken out into 100-499 Head and 500+ Head classes.

Cattle Operations and Inventory, 1993-2002

Year	1 - 99 Head		100 - 499 Head		500-999 Head 1/		1,000+ Head	
	Operation	Inventory	Operation	Inventory	Operation	Inventory	Operation	Inventory
	Percent of Total							
1993	76.8	6.5	14.0	18.5	4.8	19.0	4.4	56.0
1994	75.8	6.0	14.6	18.0	5.0	19.0	4.6	57.0
1995	75.6	6.0	14.8	18.0	5.2	20.0	4.4	56.0
1996	75.0	6.0	15.4	19.0	5.0	19.0	4.6	56.0
1997	75.8	6.5	14.6	17.5	4.6	16.0	5.0	60.0
1998	75.2	6.0	14.8	16.0	4.8	17.0	5.2	61.0
1999	75.2	6.0	14.8	16.0	4.8	17.0	5.2	61.0
2000	75.0	6.0	14.5	15.0	5.0	16.0	5.5	63.0
2001	74.3	6.0	14.8	15.0	5.2	15.0	5.7	64.0
2002	73.5	5.5	15.0	14.5	5.5	15.0	6.0	65.0

1/ Starting in 1993, the 500+ Head class was broken out into 500-999 Head and 1,000+ Head classes.

Cattle and Calves Average Prices Received, 1993-2002

Year	Jan.	Feb.	Mar.	Apr.	May	June	July	Aug.	Sept.	Oct.	Nov.	Dec.	Annual Average
	Dollars Per Cwt.												
**All Beef Cattle 1/**													
1993	66.50	66.10	65.50	66.00	66.00	67.70	65.80	65.10	63.60	61.60	61.80	62.10	64.80
1994	64.00	65.90	64.50	59.40	58.80	56.80	55.30	56.00	52.20	50.50	51.30	51.40	57.40
1995	54.10	55.00	52.00	48.40	47.90	48.40	45.80	46.30	43.50	42.20	41.00	42.20	47.00
1996	42.20	41.20	40.90	38.20	38.20	41.80	43.60	45.10	44.50	43.80	43.60	44.10	42.40
1997	43.30	46.30	48.90	49.00	51.90	50.30	49.50	51.30	49.10	48.90	49.30	47.70	48.80
1998	48.40	48.50	49.80	50.30	51.20	49.90	47.00	47.20	45.80	46.00	46.00	44.50	47.80
1999	45.80	47.20	48.60	48.50	48.80	49.70	49.20	49.70	48.60	48.30	48.40	48.50	49.00
2000	48.10	48.90	49.50	52.50	52.00	51.80	51.10	50.80	48.10	48.10	49.40	50.80	50.00
2001	54.10	55.90	58.10	58.20	56.90	57.10	55.50	55.20	51.20	49.80	47.90	47.40	53.90
2002	48.20	51.90	55.50	52.70	51.70	51.50	50.50	49.40	49.50	47.80	49.10	52.10	50.80
**Beef Steers and Heifers 2/**													
1993	81.00	79.00	79.20	78.70	79.40	83.00	81.00	79.50	79.00	78.50	76.50	78.50	79.40
1994	80.00	79.80	78.30	71.50	69.70	68.00	66.70	66.20	62.60	61.50	63.20	64.30	69.20
1995	68.80	66.20	62.30	57.90	57.20	57.30	55.00	54.60	51.80	51.00	51.50	53.00	57.00
1996	52.00	49.50	49.50	46.00	44.50	51.00	53.00	55.00	56.00	56.00	57.00	58.00	52.50
1997	58.50	61.00	63.00	62.00	64.00	62.50	61.50	64.00	64.00	64.50	66.00	64.50	63.10
1998	65.00	63.50	64.00	64.00	64.00	62.00	60.00	59.00	58.00	60.00	61.00	60.00	61.50
1999	61.50	62.50	64.00	63.00	62.50	64.50	63.00	64.00	64.50	67.00	68.00	67.00	64.30
2000	67.00	67.50	69.00	72.00	71.00	70.00	68.50	67.00	67.00	70.00	73.00	76.00	69.80
2001	78.00	78.00	79.50	79.50	77.00	76.00	73.00	72.00	71.00	69.00	66.50	66.00	74.10
2002	69.00	72.00	73.00	68.00	67.00	65.50	65.00	65.00	67.00	66.50	71.00	74.50	68.40
**Beef Cows 3/**													
1993	46.20	46.90	46.50	46.70	46.00	46.50	46.40	45.20	44.30	43.30	43.50	42.50	45.40
1994	43.70	46.00	45.40	44.60	43.10	40.00	40.30	41.30	38.50	36.40	34.80	35.00	41.10
1995	35.50	38.80	37.80	35.80	33.90	35.50	33.70	34.40	32.40	31.00	27.00	29.00	33.50
1996	31.50	31.50	30.00	29.00	29.50	29.50	32.00	33.00	31.00	30.00	29.00	29.00	30.40
1997	28.00	31.00	33.00	35.00	36.50	34.00	36.00	37.00	33.00	32.00	32.00	31.50	33.10
1998	32.50	33.50	34.50	35.50	35.50	34.50	33.00	34.50	33.50	30.00	29.50	30.30	32.90
1999	33.50	34.60	35.40	35.70	36.20	35.50	36.50	36.00	34.50	33.00	33.00	34.00	34.80
2000	35.50	36.50	36.50	36.50	37.00	37.50	37.50	37.50	35.00	33.50	35.00	34.00	35.90
2001	37.50	40.50	42.00	41.50	40.50	41.00	40.00	42.00	38.50	37.00	35.50	35.00	38.90
2002	36.00	39.00	40.50	38.00	37.00	37.00	36.50	35.00	34.00	32.50	32.50	34.50	35.90
**Calves 4/**													
1993	93.60	92.70	98.30	97.30	95.50	95.80	92.70	91.50	91.00	93.00	94.00	93.00	94.30
1994	94.70	95.50	97.10	89.10	86.90	80.00	79.00	80.00	75.80	73.90	76.20	78.70	85.60
1995	81.50	82.20	78.50	72.20	71.50	71.00	66.90	64.30	62.60	62.50	61.00	63.00	69.40
1996	62.50	59.00	61.00	54.00	51.00	51.00	53.00	56.00	55.00	54.00	55.00	56.00	55.70
1997	59.00	65.00	68.00	67.00	71.00	69.00	71.00	71.00	71.00	70.00	67.00	69.00	68.10
1998	72.00	74.00	73.00	75.00	73.50	70.00	68.00	68.00	63.50	70.00	72.50	73.30	70.90
1999	77.50	81.00	79.00	76.00	74.00	75.50	76.00	76.50	81.50	80.00	83.50	87.50	79.10
2000	91.00	96.00	97.50	94.50	92.00	90.50	93.00	90.50	91.50	93.00	95.00	93.00	93.00
2001	98.00	95.50	98.00	99.50	95.00	91.00	94.00	94.00	90.00	89.00	90.00	94.00	94.10
2002	91.00	93.50	93.50	85.00	84.50	80.00	77.50	79.00	79.00	79.50	88.00	95.00	85.40

1/ Steers and heifers over 500 pounds (including fed cattle) and slaughter cows.
2/ Steers and heifers (all qualities) over 500 pounds (including fed cattle).
3/ Beef cows and cull dairy cows sold for slaughter.
4/ Steers and heifers (all qualities) under 500 pounds

Cattle and Calves Slaughtered Under Federal and State Inspections, 1993-2002

Year	Jan.	Feb.	Mar.	Apr.	May	June	July	Aug.	Sept.	Oct.	Nov.	Dec.	Annual Total 1/
	1,000 Head												
**Cattle**													
1993	73	67	72	72	70	75	74	72	70	71	71	71	857
1994	70	62	73	69	74	80	74	80	75	79	83	82	901
1995	82	72	82	72	79	89	80	89	82	89	88	81	985
1996	88	77	78	81	87	84	85	87	81	94	87	88	1,017
1997	95	75	82	86	84	83	88	84	84	93	81	95	1,030
1998	90	79	83	84	80	86	86	87	88	88	83	87	1,019
1999	81	74	86	82	79	86	81	85	83	86	86	87	997
2000	83	81	92	75	86	89	81	92	87	94	93	86	1,038
2001	92	77	89	82	89	91	89	96	85	99	90	83	1,062
2002	102	90	97	107	111	108	114	116	110	120	109	111	1,294
**Calves**													
1993	8	7	7	6	5	5	6	6	5	5	5	6	70
1994	6	5	6	6	7	8	9	9	8	13	15	14	106
1995	16	12	14	14	21	21	19	19	16	13	15	18	196
1996	21	24	24	23	25	24	27	25	21	20	17	18	269
1997	22	17	16	15	12	15	17	17	17	15	15	20	200
1998	19	17	16	14	12	15	22	19	16	14	13	16	191
1999	15	14	16	11	10	13	16	17	16	13	11	12	164
2000	12	11	12	8	10	11	10	11	10	10	9	8	121
2001	9	8	8	8	8	8	9	10	9	10	10	9	105
2002	10	9	9	10	10	9	12	11	10	10	10	12	121

1/ Totals may not equal sum of parts due to rounding.

Average Live Weights of Cattle and Calves Slaughtered Under Federal and State Inspections, 1993-2002

Year	Jan.	Feb.	Mar.	Apr.	May	June	July	Aug.	Sept.	Oct.	Nov.	Dec.	Annual Average
	Pounds												
**Cattle**													
1993	1,222	1,206	1,211	1,210	1,223	1,210	1,213	1,219	1,238	1,253	1,247	1,245	1,225
1994	1,264	1,261	1,232	1,245	1,235	1,213	1,215	1,213	1,206	1,224	1,223	1,236	1,230
1995	1,231	1,222	1,207	1,228	1,240	1,237	1,226	1,223	1,232	1,243	1,233	1,229	1,229
1996	1,244	1,240	1,233	1,224	1,225	1,224	1,229	1,200	1,219	1,219	1,222	1,224	1,225
1997	1,224	1,203	1,244	1,238	1,240	1,235	1,245	1,244	1,253	1,263	1,264	1,243	1,242
1998	1,245	1,243	1,217	1,224	1,240	1,248	1,237	1,237	1,245	1,250	1,275	1,267	1,244
1999	1,292	1,271	1,275	1,259	1,246	1,241	1,231	1,248	1,250	1,251	1,261	1,251	1,256
2000	1,257	1,237	1,230	1,238	1,244	1,240	1,233	1,233	1,247	1,244	1,231	1,264	1,241
2001	1,269	1,259	1,249	1,269	1,266	1,238	1,257	1,262	1,270	1,279	1,313	1,303	1,269
2002	1,305	1,295	1,308	1,290	1,312	1,294	1,273	1,269	1,280	1,288	1,275	1,286	1,289
**Calves**													
1993	314	334	345	345	342	351	369	351	335	362	377	360	349
1994	366	373	370	368	377	360	352	344	367	347	314	352	352
1995	400	320	377	332	296	290	322	326	338	338	370	364	337
1996	363	348	359	367	357	362	357	350	321	330	328	321	348
1997	324	334	351	349	352	374	356	350	346	352	336	256	338
1998	248	240	148	113	115	119	115	119	121	122	131	128	146
1999	123	126	135	149	149	162	134	130	136	146	163	155	141
2000	166	154	153	160	192	179	164	149	152	156	160	153	162
2001	153	157	157	157	149	156	160	159	157	185	173	180	162
2002	166	171	163	152	143	133	130	123	138	152	141	140	145

Cattle and Calves Marketed from Feedlots, 1993-2002

Year	January-March	April-June	July-September	October-December	Annual Total
	1,000 Head				
1993	165	150	135	135	585
1994	130	150	145	155	580
1995	150	160	150	135	595
1996	145	140	135	145	565
1997	135	150	140	145	570
1998	140	125	145	160	570
1999	145	150	153	142	590
2000	153	154	151	150	608
2001	161	151	149	145	606
2002	162	186	160	163	671

Cattle by Class as of January 1, 1994-2003

Year	Cows That Have Calved			Heifers 500+ Lbs.			Other Cattle			All Cattle and Calves
	Beef	Dairy	All	Cow Replacements		Other Heifers	Calves Under 500 Lbs.	Bulls 500+ Lbs.	Steers 500+ Lbs.	
				Beef	Milk					
	1,000 Head									
1994	860	1,230	2,090	155	610	170	980	75	670	4,750
1995	850	1,270	2,120	155	630	170	970	75	680	4,800
1996	840	1,320	2,160	150	650	180	980	70	660	4,850
1997	820	1,380	2,200	150	660	170	980	70	670	4,900
1998	820	1,400	2,220	145	670	165	990	65	645	4,900
1999	810	1,440	2,250	145	720	190	1,060	70	665	5,100
2000	790	1,490	2,280	145	720	175	1,050	70	660	5,100
2001	780	1,560	2,340	140	750	170	1,050	70	630	5,150
2002	760	1,620	2,380	135	770	170	1,040	65	640	5,200
2003	740	1,680	2,420	130	790	170	1,060	65	615	5,250

Cattle Inventory, Supply and Disposition, 1993-2002

Year	Beginning Inventory January 1	Calf Crop	Inshipments	Marketings 1/		Farm Slaughter Cattle and Calves 2/	Deaths		Ending Inventory January 1
				Cattle	Calves		Cattle	Calves	
	1,000 Head								
1993	4,600	1,750	700	1,889	162	19	75	155	4,750
1994	4,750	1,800	700	2,040	172	19	80	139	4,800
1995	4,800	1,820	800	2,106	212	20	95	137	4,850
1996	4,850	1,860	820	2,064	312	20	90	144	4,900
1997	4,900	1,880	785	2,167	261	22	90	125	4,900
1998	4,900	1,920	600	1,855	201	19	95	150	5,100
1999	5,100	1,950	700	2,119	282	19	85	145	5,100
2000	5,100	1,980	700	2,013	362	15	90	150	5,150
2001	5,150	1,990	750	2,054	387	14	90	145	5,200
2002	5,200	2,040	700	1,934	502	14	90	150	5,250

1/ Includes custom slaughter for use on farms where produced, but excludes inter-farm sales.
2/ Excludes custom slaughter for farmers at commercial establishments.

Manufactured Dairy Products, 1998-2002

Commodity	1998	1999	2000	2001	2002 1/
	1,000 Pounds				
Butter	312,749	343,389	360,013	341,103	379,462
All Cheese Total 2/	1,243,062	1,387,582	1,496,653	1,643,334	1,722,190
American Cheese Total	505,935	597,048	641,662	745,391	814,170
Cheddar	368,327	427,517	461,691	539,221	590,194
Monterey, Jack and Other	137,608	169,531	179,971	206,170	223,976
Italian Cheese Total	656,163	698,563	725,572	772,827	791,186
Mozzarella	555,126	619,470	634,006	675,623	697,751
Other Italian	101,037	79,093	91,566	97,204	93,435
Hispanic Cheese	50,313	58,388	65,252	70,636	78,669
All Other Cheese	8,977	11,314	37,781	24,423	12,775
Creamed and Low Fat Cottage Cheese Total	98,938	101,647	100,769	98,410	96,416
Creamed	33,247	34,065	33,496	31,690	31,086
Low Fat 3/	65,691	67,582	67,273	66,720	65,330
Curd Cottage Cheese Total	54,206	53,520	48,684	40,258	41,759
Yogurt, Plain and Flavored	200,363	238,110	257,211	264,304	266,804
Milk, Nonfat Dry for Human Consumption	507,135	652,040	692,954	694,459	758,985
Milk, Unsweetened Condensed Skim	547,715	648,213	522,391	468,728	504,448
	1,000 Gallons				
Ice Cream 4/	116,192	139,692	137,534	143,525	131,526
Low Fat Ice Cream Total 5/	42,979	35,684	31,355	26,859	31,645
Hard	10,297	6,603	8,038	4,965	6,922
Soft	32,682	29,081	23,317	21,894	24,723
Milk Sherbet	7,264	8,323	7,299	7,000	7,943
Water Ices	4,392	3,993	5,011	3,001	3,557

1/ Preliminary 2002 data.
2/ Excludes processed cheese, Hispanic cheese, cottage cheese and other cheeses not broken out separately.
3/ Includes low fat and nonfat cottage cheese.
4/ Includes nonfat ice cream.
5/ Previously referred to as Ice Milk.

Milk Cow Average Prices Received, 1993-2002 1/

Year	Jan.	Apr.	July	Oct.	Annual Average
	Dollars Per Head				
1993	1,270	1,320	1,320	1,330	1,310
1994	1,340	1,320	1,230	1,290	1,300
1995	1,270	1,230	1,230	1,190	1,230
1996	1,190	1,190	1,230	1,280	1,220
1997	1,230	1,250	1,240	1,240	1,240
1998	1,220	1,260	1,290	1,310	1,270
1999	1,340	1,340	1,360	1,470	1,380
2000	1,480	1,500	1,500	1,480	1,490
2001	1,480	1,480	1,650	1,850	1,620
2002	1,800	1,900	1,800	1,450	1,740

1/ For dairy herd replacement.

Milk Cows Calved, Milk Production and Value, 1993-2002

Year	Milk Cows and Heifers Calved		Production Per Milk Cow		Production on Farms		Value Per Cwt.	Total Value 1/
	January 1	Annual Average	Milk	Milk Fat	Milk	Milk Fat		
	1,000 Head	*1,000 Head*	*Pounds*	*Pounds*	*Million Pounds*	*Million Pounds*	*Dollars*	*$1,000*
1993	1,200	1,216	18,852	690	22,924	839	11.63	2,663,516
1994	1,230	1,249	20,203	737	25,234	921	11.97	3,016,105
1995	1,270	1,294	19,573	712	25,327	922	12.18	3,080,084
1996	1,320	1,349	19,161	701	25,848	946	14.39	3,714,624
1997	1,380	1,391	19,829	722	27,582	1,004	13.17	3,629,762
1998	1,400	1,420	19,475	715	27,654	1,015	15.01	4,140,659
1999	1,440	1,466	20,777	760	30,459	1,115	13.45	4,090,952
2000	1,490	1,526	21,149	778	32,273	1,188	11.50	3,707,255
2001	1,563	1,589	20,904	771	33,217	1,226	13.94	4,630,450
2002	1,624	1,647	21,180	782	34,884	1,287	10.94	3,816,310

1/ Milk valued at averaged returns per 100 pounds in combined marketings of milk and cream. Includes value of milk fed to calves.

Milk Cow Operations and Inventory, 1993-2002

Year	1 - 49 Head		50 - 99 Head		100-199 Head 1/		200+ Head 2/		200-499 Head		500+ Head	
	Operation	Inventory	Operation	Inventory	Operation	Inventory	Operation	Inventory	Operation	Inventory	Operation	Inventory
	Percent of Total											
1993	43.0	0.6	4.5	1.1	7.5	3.3	45.0	95.0	---	---	---	---
1994	36.7	0.6	4.4	1.0	8.9	3.4	50.0	95.0	---	---	---	---
1995	31.5	0.4	4.5	0.9	9.4	3.7	54.6	95.0	---	---	---	---
1996	23.1	0.4	4.5	0.8	10.3	3.8	62.1	95.0	---	---	---	---
1997	21.1	0.4	4.3	0.7	10.3	3.4	64.0	95.5	26.8	16.5	37.5	79.0
1998	18.9	0.4	4.1	0.7	10.0	3.4	67.0	95.5	27.8	17.5	39.3	78.0
1999	17.3	0.3	2.7	0.4	9.6	3.3	70.4	96.0	28.1	17.0	42.3	79.0
2000	14.8	0.3	2.8	0.3	9.2	2.9	73.2	96.5	29.2	16.5	44.0	80.0
2001	14.8	0.3	3.2	0.3	10.0	2.4	72.0	97.0	28.0	15.0	44.0	82.0
2002	14.0	0.3	3.6	0.4	10.4	2.3	72.0	97.0	28.0	14.0	44.0	83.0

1/ Starting in 1993, the 100+ Head class was broken out into 100-199 Head and 200+ Head classes.
2/ Starting in 1997, the 200+ Head class was broken out into 200+ Head, 200-499 Head, and 500+ Head classes.

Grazing Fee Annual Average Rates, 1993-2002 1/

Year	Per Animal Unit 2/	Cow-Calf	Per Head
	Dollars Per Month		
1993	10.40	13.30	11.20
1994	11.00	13.10	11.80
1995	10.50	12.80	11.10
1996	10.10	12.20	11.90
1997	10.60	13.00	12.00
1998	11.50	14.50	12.90
1999	12.10	15.00	12.50
2000	12.30	15.50	12.70
2001	13.00	16.00	13.50
2002	12.80	16.50	14.00

1/ The average rates are estimates (rates over $10.00 are rounded to the nearest dime) based on survey indications of monthly lease rates for private, non-irrigated grazing land from the January Agricultural Survey.
2/ Includes animal unit plus cow-calf rates. Cow-calf rate converted to animal unit (AUM) using (1 aum=cow-calf *0.833).

Hens and Pullets of Laying Age (HPLA) and Egg Production, 2001-2002

Month	Average Number of HPLA			Average Number of Eggs Per 100 Layers			Total Egg Production		
	2001	2002	2002/2001	2001	2002	2002/2001	2001	2002	2002/2001
	1,000	*1,000*	*Percent*	*Number*	*Number*	*Percent*	*Millions*	*Millions*	*Percent*
December 1/	23,828	23,932	100	2,187	2,215	101	521	530	102
January	23,536	23,914	102	2,129	2,162	102	501	517	103
February	23,683	24,037	101	1,896	1,956	103	449	470	105
March	23,303	24,082	103	2,159	2,201	102	503	530	105
April	23,407	23,723	101	2,097	2,104	100	491	499	102
May	23,641	23,479	99	2,141	2,173	101	506	510	101
June	23,270	23,593	101	2,114	2,137	101	492	504	102
July	23,118	23,425	101	2,189	2,208	101	506	517	102
August	22,910	23,269	102	2,196	2,205	100	503	513	102
September	23,139	23,649	102	2,122	2,136	101	491	505	103
October	23,660	23,664	100	2,198	2,215	101	520	524	101
November	23,749	23,059	97	2,169	2,190	101	515	505	98
**Average/Total**	**23,437**	**23,652**	**101**	**2,133**	**2,159**	**101**	**5,998**	**6,124**	**102**

1/ December of previous year.

Broiler Production and Value, 1993-2002 1/

Year	Broilers	Average Live Weight	Meat Produced	Value Per Pound 2/	Total Value 3/
	1,000 Head	*Pounds*	*1,000 Pounds*	*Cents*	*$1,000*
1993	216,000	5.1	1,101,600	32.0	352,512
1994	226,200	5.0	1,131,000	33.0	373,230
1995	235,800	5.0	1,179,000	32.5	383,175
1996	234,200	5.0	1,171,000	39.0	456,690
1997	237,300	5.0	1,210,200	39.0	471,978
1998	---	---	---	---	---
1999	---	---	---	---	---
2000	---	---	---	---	---
2001	---	---	---	---	---
2002	---	---	---	---	---

1/ Data not published in 1998, 1999, 2000, 2001 and 2002 to avoid disclosure of individual operations.
2/ Live weight equivalent price.
3/ Gross income including home consumption.

Egg Production and Value, 1993-2002 1/

Year	Eggs	Value Per Dozen 2/	Total Value
	Millions	*Cents*	*$1,000*
1993	7,029	49.1	287,603
1994	6,602	46.4	255,277
1995	6,444	53.7	288,869
1996	6,569	67.1	367,317
1997	6,663	62.1	344,810
1998	6,608	56.1	308,924
1999	6,606	47.9	263,690
2000	6,293	45.3	237,561
2001	5,998	47.2	235,921
2002	6,124	39.2	200,051

1/ Data covers the 12-month period of December 1 (of the previous year) through November 30.
2/ Average of all eggs, including hatching eggs.

Turkey Production and Value, 1993-2002

Year	Turkeys	Meat Produced	Value Per Pound 1/	Total Value
	1,000 Head	*1,000 Pounds*	*Cents*	*$1,000*
1993	22,500	486,000	43.0	208,980
1994	21,000	449,400	43.0	193,242
1995	22,000	462,000	46.0	212,520
1996	22,500	492,750	45.0	221,738
1997	21,000	480,900	42.0	201,978
1998	19,000	442,700	41.0	181,507
1999	17,500	428,750	45.0	192,938
2000	18,000	439,200	48.0	210,816
2001	18,700	450,670	47.0	211,815
2002	17,700	438,960	36.0	158,026

1/ Live weight equivalent price.

Hogs and Pigs by Class as of December 1, 1993-2002

Year	Hogs and Pigs		Market Hogs				
	All	Breeding	Under 60 Pounds	60-119 Pounds	120-179 Pounds	180 Pounds and Over	Total
	1,000 Head						
1993	260	33	74	60	49	44	227
1994	255	32	70	65	45	43	223
1995	240	31	65	58	44	42	209
1996	210	27	57	51	40	35	183
1997	210	27	60	45	45	33	183
1998	210	27	70	50	40	23	183
1999	190	25	55	50	35	25	165
2000	150	20	32	43	45	10	130
2001	110	20	35	25	11	19	90
2002	135	21	34	29	26	25	114

Sows Farrowed and Pig Crop, 1993-2002

Year	December - November		
	Sows Farrowed	Pig Crop	Pigs Per Litter
	1,000 Head	*1,000 Head*	*Number*
1993	58	484	8.3
1994	53	463	8.7
1995	48	422	8.8
1996	42	390	9.3
1997	45	416	9.2
1998	44	404	9.2
1999	43	399	9.3
2000	40	371	9.3
2001	27	239	8.9
2002	33	286	8.7

Hogs and Pigs Inventory, Supply and Disposition, 1993-2002

Year	Beginning Inventory December 1 (Previous Year)	Pig Crop (Dec.-Nov.)	Inshipments 1/	Marketings 2/	Farm Slaughter 3/	Deaths	Ending Inventory December 1
	1,000 Head						
1993	265	484	40	472	25	32	260
1994	260	463	40	448	21	39	255
1995	255	422	40	413	24	40	240
1996	240	390	30	383	22	45	210
1997	210	416	51	400	15	52	210
1998	210	404	40	377	12	55	210
1999	210	399	40	391	8	60	190
2000	190	371	45	374	7	75	150
2001	150	239	30	278	6	25	110
2002	110	295	45	290	5	20	135

1/ For feeding or breeding, excludes stock brought in for immediate slaughter.
2/ Includes custom slaughter for use on farms where produced and State outshipments, but excludes interfarm sales.
3/ Excludes custom slaughter for farmers at commercial establishments.

Hogs and Pigs Slaughtered Under Federal and State Inspections, 1993-2002

Year	Jan.	Feb.	Mar.	Apr.	May	June	July	Aug.	Sept.	Oct.	Nov.	Dec.	Annual Total 1/
	1,000 Head												
1993	145	141	151	150	145	163	155	162	160	165	155	163	1,856
1994	163	160	159	156	151	164	146	166	168	158	156	177	1,924
1995	169	141	176	152	177	164	155	175	157	164	163	171	1,964
1996	176	168	165	171	165	151	164	173	162	182	158	183	2,017
1997	172	152	160	159	155	154	160	156	161	171	149	190	1,937
1998	183	155	169	173	170	180	188	190	207	204	189	205	2,212
1999	208	188	203	204	193	190	191	192	199	193	188	202	2,350
2000	193	180	202	175	195	189	173	201	183	205	186	195	2,278
2001	205	172	196	186	191	179	182	205	184	219	218	231	2,367
2002	208	176	186	196	204	185	208	214	206	232	187	228	2,429

1/ Totals may not equal sum of parts due to rounding.

Average Live Weights of Hogs and Pigs Slaughtered Under Federal and State Inspections, 1993-2002

Year	Jan.	Feb.	Mar.	Apr.	May	June	July	Aug.	Sept.	Oct.	Nov.	Dec.	Annual Average
	Pounds												
1993	222	229	223	222	228	224	226	221	221	224	225	223	224
1994	223	222	219	227	221	227	217	217	216	224	224	229	222
1995	225	224	235	238	241	231	224	223	215	219	224	218	227
1996	219	216	217	216	217	214	215	213	212	216	216	214	215
1997	212	212	214	214	216	211	208	206	209	212	216	212	212
1998	210	215	215	210	213	208	208	207	211	211	213	210	211
1999	216	212	214	217	215	212	213	210	208	214	216	218	214
2000	219	213	219	217	218	215	213	216	218	223	222	220	218
2001	217	220	223	224	221	221	222	222	223	228	229	230	224
2002	229	225	231	234	239	236	229	226	230	234	236	238	232

Sheep and Lambs by Class as of January 1, 1994-2003

Year	All Sheep 1/	Sheep and Lambs On Feed for Market 1/	Stock Sheep			
			Ewes 1 Yr. +	Replacement Lambs	Wethers and Rams 1 Yr. +	Total
	1,000 Head					
1994	1,080	600	400	65	15	480
1995	1,020	520	430	55	15	500
1996	920	495	355	55	15	425
1997	880	455	360	50	15	425
1998	800	410	345	35	10	390
1999	810	420	330	45	15	390
2000	800	420	320	45	15	380
2001	840	465	320	40	15	375
2002	800	430	310	45	15	370
2003	790	435	295	50	10	355

1/ Beginning in 1994, totals include new crop lambs.

Sheep and Lambs Shorn, Wool Production and Value, 1993-2002

Year	Sheep and Lambs Shorn	Production		Value Per Pound	Total Value
		Per Animal	Total		
	1,000 Head	*Pounds*	*1,000 Pounds*	*Dollars*	*$1,000*
1993	800	7.6	6,044	0.46	2,780
1994	695	7.7	5,318	0.65	3,457
1995	690	7.6	5,250	1.02	5,355
1996	580	7.6	4,380	0.67	2,935
1997	470	8.1	3,800	0.79	3,002
1998	480	7.5	3,600	0.61	2,196
1999	480	7.6	3,630	0.35	1,271
2000	530	7.5	4,000	0.29	1,160
2001	510	7.4	3,750	0.35	1,313
2002	520	7.0	3,650	0.45	1,643

Sheep and Lambs Inventory, Supply and Disposition, 1993-2002

Year	Beginning Inventory January 1 1/	Lamb Crop	Inshipments	Marketings 2/		Farm Slaughter Sheep and Lambs	Deaths		Ending Inventory January 1 1/
				Sheep	Lambs		Sheep	Lambs	
	1,000 Head								
1993	895	370	469	139	718	5	25	27	1,080
1994	1,080	400	320	50	676	6	24	24	1,020
1995	1,020	330	375	120	634	5	29	17	920
1996	920	330	435	40	722	5	24	14	880
1997	880	320	365	57	667	5	22	14	800
1998	800	310	313	51	521	5	23	13	810
1999	810	290	360	56	564	5	23	12	800
2000	800	310	355	45	542	5	19	14	840
2001	840	290	305	47	553	5	17	13	800
2002	800	285	320	61	517	5	18	14	790

1/ Beginning in 1994, totals include new crop lambs.
2/ Includes custom slaughter for use on farms where produced, but excludes interfarm sales within the State.

Sheep and Lambs Average Prices Received, 1993-2002

Year	Jan.	Feb.	Mar.	Apr.	May	June	July	Aug.	Sept.	Oct.	Nov.	Dec.	Annual Averge 1/
	Dollars Per Cwt.												
**Sheep**													
1993	30.00	31.10	32.00	29.00	26.00	24.50	24.00	26.00	25.00	23.00	24.00	27.50	25.80
1994	32.20	32.70	31.50	29.10	27.10	29.10	24.30	30.80	29.00	27.70	26.70	35.80	29.10
1995	29.90	34.70	28.60	26.10	22.40	24.00	25.10	24.00	24.00	23.70	22.00	27.00	24.80
1996	30.00	31.00	29.00	27.00	25.00	24.50	26.50	27.50	24.50	28.50	28.50	34.00	26.70
1997	35.00	37.00	37.00	37.00	30.00	33.00	32.00	36.00	30.00	34.00	35.00	37.00	33.80
1998	35.50	33.00	36.50	35.50	30.00	32.00	29.00	29.00	27.50	26.50	25.50	28.50	30.50
1999	28.50	30.00	33.00	31.50	29.00	24.50	26.00	29.50	28.00	27.00	27.00	33.50	28.70
2000	30.00	31.50	37.50	36.00	31.50	28.00	31.00	31.50	28.50	30.50	31.50	34.00	31.50
2001	40.50	38.00	39.50	31.50	31.00	26.50	29.50	31.50	28.50	26.50	24.00	31.50	30.20
2002	30.00	30.00	31.00	27.50	27.00	22.00	26.00	29.50	30.50	25.50	34.00	36.50	27.10
**Lambs**													
1993	65.10	71.70	74.50	71.00	66.00	62.00	60.00	59.00	63.00	62.50	64.50	65.50	65.70
1994	60.90	58.80	58.30	55.30	54.40	61.90	71.50	76.00	74.80	73.80	72.70	76.70	65.60
1995	69.60	71.00	76.70	77.00	78.70	84.00	86.00	86.50	83.00	80.00	78.00	78.50	79.20
1996	79.50	84.00	85.50	86.00	89.00	98.00	100.00	94.00	90.00	90.00	84.00	88.00	89.40
1997	92.00	98.00	99.00	97.00	94.00	91.00	87.00	90.00	89.00	86.50	84.50	85.00	91.60
1998	81.50	78.00	71.00	69.00	63.00	90.00	84.00	79.00	78.00	71.00	64.00	66.00	74.80
1999	68.00	68.50	66.00	68.00	83.00	81.00	75.50	80.00	77.50	73.00	74.00	77.00	74.80
2000	72.00	72.00	80.00	81.00	91.00	90.00	87.00	83.00	79.00	75.00	70.00	68.00	79.10
2001	69.00	78.00	80.00	78.00	76.50	72.00	68.00	60.00	56.00	51.50	50.00	57.00	67.30
2002	60.50	63.50	63.00	61.00	62.00	72.50	75.00	73.50	74.50	74.50	77.00	79.00	69.40

1/ Average weighted by amount sold each month.

Livestock Production and Income, 1993-2002

Year	Production 1/	Marketings 2/	Average Price Per 100 Pounds		Value of Production	Cash Receipts 3/ 4/	Value of Home Consumption	Gross Income
	1,000 Pounds	*1,000 Pounds*	*Dollars*	*Dollars*	*$1,000*	*$1,000*	*$1,000*	*$1,000*
**All Livestock**								
1993	2,024,599	2,586,056	---	---	1,244,511	1,671,059	15,642	1,686,701
1994	1,943,849	2,539,514	---	---	1,061,164	1,468,824	13,820	1,482,644
1995	1,918,670	2,622,058	---	---	851,750	1,262,481	12,723	1,275,204
1996	1,872,910	2,588,462	---	---	762,188	1,158,997	11,067	1,170,064
1997	2,075,249	2,728,265	---	---	988,026	1,385,084	12,803	1,397,887
1998	1,974,094	2,323,708	---	---	912,615	1,127,393	11,266	1,138,659
1999	2,097,648	2,617,115	---	---	922,975	1,308,778	11,798	1,320,576
2000	2,045,587	2,516,273	---	---	900,474	1,311,939	10,341	1,322,280
2001	2,096,705	2,578,540	---	---	1,002,408	1,433,664	10,876	1,444,540
2002	1,993,138	2,471,985	---	---	924,271	1,301,298	10,294	1,311,592
**Cattle and Calves**			***(Cattle)***	***(Calves)***				
1993	1,829,597	2,372,090	64.80	94.30	1,138,047	1,553,634	12,755	1,566,389
1994	1,795,879	2,356,220	57.40	85.60	986,137	1,368,770	11,125	1,379,895
1995	1,775,864	2,436,000	47.00	69.40	777,753	1,159,032	10,050	1,169,082
1996	1,736,019	2,410,790	42.40	55.70	673,737	1,033,513	7,856	1,041,369
1997	1,936,801	2,550,150	48.80	68.10	895,678	1,257,018	10,205	1,267,223
1998	1,849,594	2,171,350	47.80	70.90	851,681	1,048,300	9,509	1,057,809
1999	1,972,129	2,454,890	49.00	79.10	861,952	1,223,123	10,240	1,233,363
2000	1,923,165	2,359,040	50.00	93.00	831,338	1,218,220	8,614	1,226,834
2001	1,992,326	2,437,100	53.90	94.10	945,486	1,353,837	9,239	1,363,076
2002	1,880,884	2,330,200	50.80	85.40	871,445	1,228,722	8,947	1,237,669
**Sheep and Lambs**			***(Sheep)***	***(Lambs)***				
1993	91,777	110,702	25.80	65.70	54,507	65,078	575	65,653
1994	52,725	87,402	29.10	65.60	30,709	54,868	745	55,613
1995	53,813	96,313	24.80	79.20	38,797	67,411	793	68,204
1996	59,076	97,247	26.70	89.40	48,011	83,589	975	84,564
1997	56,292	93,750	33.80	91.60	47,840	81,505	954	82,459
1998	49,167	73,493	30.50	74.80	35,458	51,960	798	52,758
1999	47,254	80,280	28.70	74.80	33,669	56,601	783	57,384
2000	49,807	77,308	31.50	79.10	37,338	58,156	813	58,969
2001	51,640	82,340	30.20	67.30	33,349	52,974	712	53,686
2002	47,849	77,835	27.10	69.40	31,434	50,388	710	51,098
**Hogs and Pigs**			***(Hogs and Pigs)***					
1993	103,225	103,264	50.40	---	51,957	52,347	2,312	54,659
1994	95,245	95,892	46.70	---	44,318	45,186	1,950	47,136
1995	88,993	89,745	39.70	---	35,200	36,038	1,880	37,918
1996	77,815	80,425	52.00	---	40,440	41,895	2,236	44,131
1997	82,156	84,365	54.70	---	44,508	46,561	1,644	48,205
1998	75,333	78,865	34.00	---	25,476	27,133	959	28,092
1999	78,265	81,945	35.10	---	27,354	29,054	775	29,829
2000	72,615	79,925	44.10	---	31,798	35,563	914	36,477
2001	52,739	59,100	44.90	---	23,573	26,853	925	27,778
2002	64,405	63,950	34.20	---	21,392	22,188	637	22,825

1/ Live weight; adjustments made for changes in inventory and for inshipments.
2/ Live weight; excludes custom slaughter for use on farms where produced and interfarm sales within the State.
3/ Receipts from marketing and sale of farm slaughter.
4/ Hogs and pigs receipts for 1995 and 1996 include allowance for higher average price of State inshipments and outshipments of feeder pigs.

California Livestock Inventory by Class and County, January 1, 2002-2003

County	2002			2003		
	All Cattle	Beef Cows	Milk Cows	All Cattle	Beef Cows	Milk Cows
Del Norte	6,000	1,000	---	9,000	1,000	---
Humboldt	70,000	22,000	16,700	63,000	19,000	18,000
Mendocino	27,000	12,000	---	22,000	10,000	---
**District 10 Total**	**103,000**	**35,000**	**16,700**	**94,000**	**30,000**	**18,000**
Shasta	40,000	22,000	---	41,000	24,000	---
Siskiyou	63,000	31,000	1,900	64,000	32,000	2,400
Trinity	4,000	3,000	---	5,000	2,000	---
**District 20 Total**	**107,000**	**56,000**	**1,900**	**110,000**	**58,000**	**2,400**
Lassen	50,000	25,000	---	50,000	28,000	---
Modoc	86,000	42,000	---	83,000	41,000	---
Plumas	14,000	6,000	---	14,000	6,000	---
**District 30 Total**	**150,000**	**73,000**	**---**	**147,000**	**75,000**	**---**
Alameda	28,000	12,000	---	31,000	12,000	---
Contra Costa	25,000	8,000	3,000	24,000	7,000	---
Lake	6,000	3,000	---	8,000	5,000	---
Marin	39,000	9,000	10,000	37,000	8,000	9,700
Monterey	102,000	32,000	2,200	96,000	26,000	1,300
Napa	8,000	4,000	---	8,000	3,000	---
San Benito	39,000	16,000	800	36,000	12,000	600
San Francisco	---	---	---	---	---	---
San Luis Obispo	90,000	39,000	---	94,000	36,000	800
San Mateo	4,000	2,000	---	3,000	1,000	---
Santa Clara	20,000	10,000	---	20,000	9,000	---
Santa Cruz	3,000	2,000	---	3,000	1,000	---
Sonoma	86,000	15,000	31,500	88,000	13,000	33,000
**District 40 Total**	**450,000**	**152,000**	**47,500**	**448,000**	**133,000**	**45,400**
Butte	17,000	7,000	800	19,000	8,000	900
Colusa	22,000	8,000	---	24,000	10,000	---
Glenn	68,000	18,000	15,700	70,000	18,000	17,000
Sacramento	63,000	14,000	17,000	73,000	15,000	16,700
Solano	25,000	8,000	1,500	40,000	14,000	3,900
Sutter	9,000	3,000	---	10,000	5,000	---
Tehama	76,000	32,000	5,500	71,000	30,000	5,300
Yolo	19,000	8,000	2,000	20,000	12,000	2,000
Yuba	36,000	6,000	3,500	33,000	6,000	3,200
**District 50 Total**	**335,000**	**104,000**	**46,000**	**360,000**	**118,000**	**49,000**
Fresno	413,000	20,000	88,000	400,000	20,000	85,000
Kern	220,000	41,000	78,000	235,000	40,000	85,000
Kings	200,000	4,000	132,000	250,000	5,000	149,000
Madera	128,000	16,000	48,000	125,000	18,000	48,000
Merced	428,000	32,000	221,000	440,000	30,000	225,000
San Joaquin	237,000	21,000	105,000	235,000	21,000	103,000
Stanislaus	435,000	46,000	164,000	450,000	45,000	164,000
Tulare	706,000	31,000	394,000	765,000	30,000	426,000
**District 51 Total**	**2,767,000**	**211,000**	**1,230,000**	**2,900,000**	**209,000**	**1,285,000**
Alpine	3,000	2,000	---	2,000	2,000	---
Amador	28,000	12,000	---	23,000	11,000	---
Calaveras	23,000	13,000	---	26,000	13,000	---
El Dorado	11,000	5,000	---	10,000	4,000	---
Inyo	14,000	8,000	---	16,000	8,000	---
Mariposa	19,000	8,000	---	17,000	6,000	---
Mono	7,000	4,000	---	6,000	3,000	---
Nevada	8,000	5,000	---	6,000	3,000	---
Placer	26,000	10,000	---	23,000	8,000	---
Sierra	7,000	3,000	---	9,000	4,000	---
Tuolumne	17,000	7,000	---	13,000	6,000	---
**District 60 Total**	**163,000**	**77,000**	**---**	**151,000**	**68,000**	**---**
Imperial	530,000	1,000	---	485,000	3,000	---
Los Angeles	13,000	5,000	---	7,000	3,000	---
Orange	3,000	1,000	---	2,000	1,000	---
Riverside	180,000	6,000	100,000	170,000	7,000	98,000
San Bernardino	277,000	3,000	153,000	277,000	3,000	159,000
San Diego	27,000	7,000	6,000	24,000	6,000	5,700
Santa Barbara	62,000	24,000	3,000	50,000	22,000	2,500
Ventura	13,000	5,000	---	10,000	4,000	---
**District 80 Total**	**1,105,000**	**52,000**	**262,000**	**1,025,000**	**49,000**	**265,200**
Other Districts 1/	20,000	---	15,900	15,000	---	15,000
**STATE**	**5,200,000**	**760,000**	**1,620,000**	**5,250,000**	**740,000**	**1,680,000**

1/ Includes counties from one or more districts to avoid disclosure of individual operations.

California Vegetable and Melon Crops

California's diverse climate allows for year-round production of many vegetables, beginning in the desert during winter months and then heading into the Central Valley and coastal areas as the season progresses.

California continues to lead the nation in vegetable and melon production, accounting for 41 percent of the harvested acreage, 67 percent of production, and 58 percent of total value.

In real terms, the Golden State produced 26.7 million tons of vegetables and melons in 2002. Total value of vegetable and melon production was $6.21 billion. These figures represent a 12 percent increase in production and a 2 percent increase in value of production from 2001.

Artichokes, broccoli, snap beans, sweet corn, leaf and romaine lettuce, spinach, fresh and processing tomatoes, and watermelon showed large increases in value. There were notable decreases in value for asparagus, cantaloupes, honeydews, celery, garlic, head lettuce, and fresh and processing cucumbers.

In 2002, California produced 95 percent of processing tomatoes in the country, with most coming from rich farmland in the Sacramento and San Joaquin valleys. The Golden State also accounts for more than 90 percent of the nation's production of artichokes, broccoli, and celery. Additionally, California is home to more than 75 percent of the national production of cauliflower, fresh market carrots, garlic, honeydews, and leaf and romaine lettuce. Monterey County's fertile Salinas Valley is known worldwide for its abundant, high-quality vegetable crops, particularly artichokes, broccoli, cauliflower, celery, and lettuce.

The month of January delivered the expected rainfall and cooler temperatures that slowed winter-vegetable crop development and delayed harvest. Precipitation was near normal in the Sacramento and San Joaquin valleys, but below normal in the coastal areas. In late January and early February, cold, frosty conditions slowed growth of some vegetables and there were reports of some lettuce damaged by frost.

Warmer temperatures in late February encouraged plant growth and harvesting resumed. Seasonal storms brought rain to the valleys, but precipitation remained below normal in the vegetable growing areas in the central and south coast, as well as the desert areas. Harvesting of the winter vegetables continued in the desert areas and then moved into the San Joaquin Valley. As March began, a strong high-pressure ridge aloft off the west coast and by the offshore flow at the lower levels of the atmosphere dominated weather conditions. This combination resulted in very dry, warm daytime conditions and cool nights. Temperatures remained below normal for much of March and precipitation was near normal in the valleys, but remained below in the coastal areas. By early April, warm weather aided crop development and the vegetable crops flourished. Temperatures averaged a few degrees below normal for most of May, but the vegetable crops developed normally.

The remainder of the vegetable growing season was uneventful and planting and harvesting proceeded on schedule. The summer months were generally mild with no temperature extremes.

Notable Changes in Vegetable Production from 2001 to 2002:

Beans, Snap	+ 28%
Carrots, Processing	– 38%
Cucumbers, Processing	+ 14%
Spinach, Fresh	+ 21%
Tomatoes, Fresh	+ 13%
Tomatoes, Processing	+ 28%

Note to the Reader

Vegetable and melon estimates include commercial production, but exclude garden production and vegetables grown for seed. Prices for processing crops and processed segments of dual utilization crops are based on equivalent returns at the processing plant door.

For fresh market sales, the price is on a packed and loaded basis, free-on-board (f.o.b.) shipping point. To avoid disclosure of individual plants, not all of California's data were published.

Vegetable and Melon Acreage, Production and Value, 1993-2002

Crop	Crop Year	Harvested	Yield Per Acre	Production	Value Per Cwt. 1/	Total Value
		Acres	*Cwt.*	*1,000 Cwt.*	*Dollars*	*$1,000*
Artichokes						
	1993	9,200	110	1,012	50.80	51,435
	1994	9,200	130	1,196	56.40	67,474
	1995	8,900	92	819	75.70	61,965
	1996	8,900	100	890	73.50	65,416
	1997	9,300	100	930	79.50	73,910
	1998	9,700	90	873	70.60	61,633
	1999	9,800	115	1,125	67.00	75,395
	2000	8,800	115	1,012	60.30	61,021
	2001	8,000	125	1,000	58.60	58,559
	2002	8,200	115	943	70.80	66,764
Asparagus						
	1993	32,100	29	931	94.70	88,177
	1994	30,200	33	997	102.00	101,694
	1995	28,000	28	784	117.00	91,728
	1996	29,000	26	754	85.80	64,691
	1997	30,100	27	813	115.00	93,224
	1998	32,200	26	837	138.00	115,270
	1999	33,800	33	1,115	142.00	158,330
	2000	37,000	32	1,184	122.00	144,448
	2001	35,000	30	1,050	156.00	163,800
	2002	34,000	30	1,020	114.00	116,280
Beans, Fresh Market Snap						
	1993	7,300	80	584	49.90	29,142
	1994	7,500	70	525	57.20	30,030
	1995	7,000	100	700	50.20	35,140
	1996	7,800	90	702	47.80	33,566
	1997	4,500	100	450	52.00	23,400
	1998	4,700	95	447	67.80	30,307
	1999	5,100	95	485	58.90	28,567
	2000	5,500	100	550	56.00	30,800
	2001	5,400	80	432	64.40	27,821
	2002	6,500	80	520	62.30	32,396
Broccoli						
	1993	105,000	105	11,025	25.50	280,875
	1994	118,000	120	14,160	27.30	387,160
	1995	115,000	125	14,375	27.40	394,272
	1996	122,000	120	14,640	26.60	388,753
	1997	119,000	130	15,470	27.90	431,301
	1998	121,000	130	15,730	28.90	454,383
	1999	130,000	140	18,200	23.80	432,973
	2000	133,000	140	18,620	30.90	575,349
	2001	122,000	140	17,080	25.70	439,387
	2002	115,000	140	16,100	30.30	488,442
Brussels Sprouts 2/						
	1993	5,000	160	800	30.90	24,756
	1994	3,500	180	630	24.60	15,471
	1995	3,400	165	561	25.70	14,390
	1996	3,800	180	684	29.40	20,120
	1997	3,200	160	512	41.40	21,180
	1998	3,200	160	512	41.00	20,985
	1999	3,200	180	576	37.20	21,419
	2000	2,600	180	468	34.40	16,115
	2001	2,200	180	396	36.50	14,471
	2002	---	---	---	---	---

Vegetable and Melon Acreage, Production and Value, 1993-2002

Crop	Crop Year	Harvested	Yield Per Acre	Production	Value Per Cwt. 1/	Total Value
		Acres	*Cwt.*	*1,000 Cwt.*	*Dollars*	*$1,000*
Cabbage, Fresh Market						
	1993	12,000	360	4,320	13.70	59,184
	1994	10,000	385	3,850	13.40	51,590
	1995	10,000	370	3,700	15.50	57,350
	1996	10,000	350	3,500	12.60	44,100
	1997	13,800	340	4,692	14.90	69,911
	1998	14,000	330	4,620	16.50	76,230
	1999	12,500	320	4,000	13.40	53,600
	2000	13,000	370	4,810	13.10	63,011
	2001	12,000	380	4,560	13.80	62,928
	2002	12,500	380	4,750	14.00	66,500
Carrots, Fresh Market						
	1993	75,200	300	22,560	11.10	250,416
	1994	75,900	360	27,324	12.70	347,015
	1995	72,500	300	21,750	16.60	361,050
	1996	85,700	300	25,710	13.60	349,656
	1997	83,200	360	29,998	13.00	389,974
	1998	86,500	330	28,545	11.40	325,413
	1999	83,500	295	24,633	17.20	423,688
	2000	80,500	290	23,345	13.30	310,489
	2001	81,000	290	23,490	18.10	425,169
	2002	77,000	290	22,330	20.30	453,299
Carrots, Processing						
	1993	6,800	294	2,000	4.85	9,700
	1994	4,100	618	2,532	3.81	9,635
	1995	5,500	462	2,542	3.64	9,254
	1996	4,300	670	2,880	3.02	8,698
	1997	2,700	641	1,730	3.28	5,666
	1998	4,500	628	2,826	3.35	9,453
	1999	4,400	623	2,739	3.61	9,877
	2000	4,900	600	2,940	4.07	11,966
	2001	3,300	586	1,934	4.52	8,750
	2002	2,100	574	1,205	5.43	6,539
Carrots, All						
	1993	82,000	300	24,560	10.59	260,116
	1994	80,000	373	29,856	11.95	356,650
	1995	78,000	311	24,292	15.24	370,304
	1996	90,000	318	28,590	12.53	358,354
	1997	85,900	369	31,728	12.47	395,640
	1998	91,000	345	31,371	10.67	334,866
	1999	87,900	311	27,372	15.84	433,565
	2000	85,400	308	26,285	12.27	322,455
	2001	84,300	302	25,424	17.07	433,919
	2002	79,100	298	23,535	19.54	459,838
Cauliflower						
	1993	48,000	136	6,540	29.70	194,545
	1994	47,000	140	6,611	28.40	187,654
	1995	43,000	137	5,875	33.70	198,275
	1996	41,500	152	6,310	32.30	203,499
	1997	37,500	154	5,790	31.40	181,829
	1998	39,000	150	5,850	31.20	182,650
	1999	41,000	155	6,355	27.30	173,725
	2000	38,500	180	6,930	30.50	211,087
	2001	38,500	155	5,968	28.10	167,504
	2002	37,000	150	5,550	30.20	167,491

Vegetable and Melon Acreage, Production and Value, 1993-2002

Crop	Crop Year	Harvested *Acres*	Yield Per Acre *Cwt.*	Production *1,000 Cwt.*	Value Per Cwt. 1/ *Dollars*	Total Value *$1,000*
Celery						
	1993	24,000	680	16,320	14.70	240,081
	1994	24,500	690	16,905	11.80	198,695
	1995	24,000	685	16,440	16.50	272,037
	1996	24,500	700	17,150	10.60	181,606
	1997	24,000	695	16,680	14.80	247,371
	1998	24,500	680	16,666	12.20	203,126
	1999	25,000	700	17,500	12.00	209,940
	2000	24,000	720	17,280	18.80	324,850
	2001	26,500	695	17,723	14.40	255,841
	2002	25,500	710	17,750	12.80	227,130
Corn, Fresh Market Sweet						
	1993	20,000	140	2,800	17.80	49,840
	1994	21,600	140	3,024	18.30	55,339
	1995	22,300	150	3,345	18.70	62,552
	1996	24,100	155	3,736	17.60	65,754
	1997	27,000	145	3,915	17.20	67,338
	1998	32,000	175	5,600	15.80	88,480
	1999	26,000	165	4,290	17.60	75,504
	2000	24,000	165	3,960	16.70	66,132
	2001	22,000	170	3,740	22.30	83,402
	2002	24,000	170	4,080	24.00	97,920
Cucumbers, Fresh Market						
	1993	5,600	250	1,400	27.10	37,940
	1994	5,000	250	1,250	24.90	31,125
	1995	5,200	315	1,638	25.90	42,424
	1996	6,000	330	1,980	28.20	55,836
	1997	6,300	315	1,985	24.70	49,030
	1998	6,000	320	1,920	30.90	59,328
	1999	6,500	310	2,015	26.80	54,002
	2000	4,500	285	1,283	28.50	36,566
	2001	4,700	240	1,128	30.00	33,840
	2002	4,300	240	1,032	28.60	29,515
Escarole and Endive 2/						
	2000	2,400	160	384	36.70	14,093
	2001	2,400	150	360	35.50	12,780
	2002	---	---	---	---	---
Eggplant 2/						
	2000	1,600	205	328	35.00	9,020
	2001	1,400	235	329	24.50	8,061
	2002	---	---	---	---	---
Garlic						
	1993	26,000	160	4,160	31.80	132,470
	1994	27,000	170	4,590	40.10	183,852
	1995	28,500	165	4,703	31.40	147,714
	1996	35,000	175	6,125	32.10	196,333
	1997	33,000	170	5,610	47.90	268,776
	1998	38,000	145	5,510	37.70	207,511
	1999	40,000	185	7,400	29.60	219,076
	2000	29,000	165	4,785	29.40	140,447
	2001	30,000	170	5,100	31.50	160,420
	2002	27,000	175	4,725	29.20	138,146
Greens, Collard 2/						
	2000	700	190	133	36.00	4,788
	2001	700	170	119	38.80	4,617
	2002	---	---	---	---	---

Vegetable and Melon Acreage, Production and Value, 1993-2002

Crop	Crop Year	Harvested *Acres*	Yield Per Acre *Cwt.*	Production *1,000 Cwt.*	Value Per Cwt. 1/ *Dollars*	Total Value *$1,000*
Greens, Kale 2/						
	2000	3,000	215	645	33.20	21,414
	2001	2,800	190	532	28.70	15,268
	2002	---	---	---	---	---
Greens, Mustard 2/						
	2000	3,200	170	544	36.80	20,019
	2001	3,400	140	476	36.90	17,564
	2002	---	---	---	---	---
Lettuce, Head						
	1993	141,000	360	50,760	16.50	837,540
	1994	153,000	330	50,490	14.60	737,154
	1995	144,000	295	42,480	25.00	1,062,000
	1996	150,500	290	43,645	15.40	672,133
	1997	141,000	350	49,350	19.40	957,390
	1998	135,000	315	42,525	16.30	693,158
	1999	140,000	380	53,200	13.70	728,840
	2000	130,000	390	50,700	18.80	953,160
	2001	128,000	380	48,640	18.50	899,840
	2002	129,000	375	48,375	15.20	735,300
Lettuce, Leaf						
	1993	37,000	205	7,585	27.70	210,105
	1994	35,000	225	7,875	28.10	221,288
	1995	37,000	210	7,770	31.90	247,863
	1996	36,000	210	7,560	25.10	189,756
	1997	42,000	205	8,660	28.30	245,385
	1998	38,000	215	8,170	30.50	249,185
	1999	43,000	220	9,460	23.80	225,148
	2000	45,000	235	10,575	29.60	313,020
	2001	46,000	215	9,890	24.90	246,261
	2002	49,000	215	10,535	28.10	296,034
Lettuce, Romaine						
	1993	19,000	280	5,320	18.60	98,952
	1994	24,000	280	6,720	21.40	143,808
	1995	23,000	280	6,440	24.40	157,136
	1996	23,500	275	6,463	18.60	120,212
	1997	25,000	280	7,000	19.20	134,400
	1998	26,000	280	7,280	21.60	157,248
	1999	31,000	315	9,765	18.00	175,770
	2000	36,000	315	11,340	20.10	227,934
	2001	40,500	280	11,340	17.50	198,450
	2002	42,000	295	12,390	19.90	246,561
Lettuce, All						
	1993	197,000	323	63,665	18.00	1,146,597
	1994	212,000	307	65,085	16.90	1,102,250
	1995	204,000	278	56,690	25.90	1,466,999
	1996	210,000	275	57,668	17.00	982,101
	1997	208,000	313	65,010	20.60	1,337,175
	1998	199,000	291	57,975	19.00	1,099,591
	1999	214,000	338	72,425	15.60	1,129,758
	2000	211,000	344	72,615	20.60	1,494,114
	2001	214,500	326	69,870	19.20	1,344,551
	2002	220,000	324	71,300	17.90	1,277,895

Vegetable and Melon Acreage, Production and Value, 1993-2002

Crop	Crop Year	Harvested *Acres*	Yield Per Acre *Cwt.*	Production *1,000 Cwt.*	Value Per Cwt. 1/ *Dollars*	Total Value *$1,000*
Melons, Cantaloupe						
	1993	65,000	190	12,350	15.30	188,955
	1994	59,300	190	11,267	15.90	179,145
	1995	59,300	200	11,860	17.30	205,178
	1996	59,000	220	12,980	17.90	232,342
	1997	62,300	210	13,083	17.70	231,569
	1998	58,000	220	12,760	17.10	218,196
	1999	61,000	210	12,810	17.30	221,613
	2000	57,500	230	13,225	17.10	226,148
	2001	54,500	245	13,353	18.90	252,372
	2002	54,900	245	13,451	16.60	223,287
Melons, Honeydew						
	1993	16,500	160	2,640	16.70	44,088
	1994	20,500	165	3,383	16.10	54,466
	1995	18,100	160	2,896	18.70	54,155
	1996	20,300	170	3,451	16.50	56,942
	1997	20,500	180	3,690	18.50	68,265
	1998	19,000	190	3,610	19.30	69,673
	1999	20,500	180	3,690	21.10	77,859
	2000	20,000	185	3,700	18.60	68,820
	2001	20,000	185	3,700	19.70	72,890
	2002	20,400	185	3,774	16.80	63,403
Melons, Watermelon						
	1993	16,000	420	6,720	8.00	54,029
	1994	16,700	430	7,181	9.90	71,164
	1995	16,800	380	6,364	14.30	91,291
	1996	17,100	440	7,524	10.50	79,002
	1997	17,000	460	7,820	10.90	85,283
	1998	15,000	450	6,750	12.30	83,025
	1999	14,700	430	6,321	9.80	61,946
	2000	12,300	535	6,581	11.70	76,998
	2001	11,800	530	6,254	10.90	68,169
	2002	11,800	510	6,018	12.70	76,429
Mushrooms, Agaricus						
	1993	528	2,434	1,285	101.00	129,866
	1994	501	2,475	1,240	106.00	130,911
	1995	503	2,682	1,349	104.00	140,796
	1996	514	2,549	1,310	109.00	142,642
	1997	529	2,461	1,302	120.00	155,554
	1998	563	2,378	1,338	119.00	158,638
	1999	548	2,412	1,322	122.00	161,612
	2000	537	2,408	1,293	126.00	162,279
	2001	526	2,456	1,292	126.00	162,988
	2002	523	2,453	1,283	133.00	170,159
Onions, Spring						
	1993	9,500	430	4,085	18.00	73,530
	1994	8,800	435	3,828	8.00	30,701
	1995	8,700	440	3,828	14.00	53,592
	1996	9,600	385	3,696	9.80	36,221
	1997	9,600	450	4,320	14.30	61,776
	1998	9,100	445	4,050	14.10	57,105
	1999	7,300	440	3,212	11.90	38,223
	2000	7,100	435	3,089	10.60	32,743
	2001	6,200	430	2,666	13.50	35,991
	2002	6,200	460	2,852	14.20	40,498

Vegetable and Melon Acreage, Production and Value, 1993-2002

Crop	Crop Year	Harvested *Acres*	Yield Per Acre *Cwt.*	Production *1,000 Cwt.*	Value Per Cwt. 1/ *Dollars*	Total Value *$1,000*
Onions, Summer Storage 3/ 4/						
	1993	36,500	395	14,418	8.00	113,115
	1994	33,200	410	13,612	6.30	83,915
	1995	32,100	415	13,322	7.20	93,649
	1996	33,500	430	14,405	7.70	108,860
	1997	31,300	440	13,772	7.84	105,793
	1998	37,700	415	15,646	9.71	149,439
	1999	39,000	435	16,965	7.53	125,936
	2000	36,300	445	16,154	7.98	126,875
	2001	29,800	405	12,069	7.26	85,748
	2002	30,400	405	12,312	7.12	85,847
Onions, Summer Non-Storage 5/						
	2000	6,200	395	2,449	12.00	29,388
	2001	7,000	450	3,150	13.50	42,525
	2002	7,200	470	3,384	12.90	43,654
Onions, Processed 6/						
	1998	---	---	9,327	6.55	61,092
	1999	---	---	12,550	6.55	82,203
	2000	---	---	11,304	6.30	71,215
	2001	---	---	9,125	5.50	50,188
	2002	---	---	9,100	5.30	48,230
Onions, All 3/						
	1993	46,000	402	18,503	10.30	186,645
	1994	42,000	415	17,440	6.67	114,616
	1995	40,800	420	17,150	8.72	147,241
	1996	43,100	420	18,101	8.15	145,081
	1997	40,900	442	18,092	9.41	167,569
	1998	46,800	421	19,696	10.60	206,544
	1999	46,300	436	20,177	8.24	164,159
	2000	49,600	437	21,692	8.81	189,006
	2001	43,000	416	17,885	9.31	164,264
	2002	43,800	423	18,548	9.29	169,999
Peppers, Bell						
	1993	21,100	300	6,330	27.50	174,251
	1994	21,500	290	6,235	25.90	161,190
	1995	24,000	290	6,960	27.10	188,523
	1996	25,500	300	7,650	22.70	173,506
	1997	21,000	300	6,300	24.60	155,040
	1998	22,000	285	6,270	27.20	170,374
	1999	22,500	330	7,425	29.80	220,997
	2000	21,000	300	6,300	27.30	171,788
	2001	22,000	325	7,150	25.50	182,343
	2002	21,000	330	6,930	28.60	197,987
Peppers, Chili 5/						
	2000	3,000	230	690	40.80	28,165
	2001	2,600	225	585	30.30	17,697
	2002	2,800	230	644	27.80	17,914
Pumpkins 5/						
	2000	5,900	305	1,800	10.60	19,080
	2001	6,100	240	1,464	11.20	16,397
	2002	6,000	250	1,500	10.90	16,350
Radishes 2/						
	2000	1,200	250	300	26.00	7,800
	2001	1,100	230	253	24.50	6,199
	2002	---	---	---	---	---

Vegetable and Melon Acreage, Production and Value, 1993-2002

Crop	Crop Year	Harvested	Yield Per Acre	Production	Value Per Cwt. 1/	Total Value
		Acres	*Cwt.*	*1,000 Cwt.*	*Dollars*	*$1,000*
Spinach, Fresh Market						
	1993	5,000	215	1,075	31.00	33,325
	1994	7,100	205	1,456	30.80	44,845
	1995	8,400	160	1,340	34.50	46,230
	1996	9,000	150	1,350	30.90	41,715
	1997	15,200	175	2,660	31.20	82,992
	1998	15,000	160	2,400	35.40	84,960
	1999	17,000	150	2,550	31.40	80,070
	2000	19,000	180	3,420	30.70	104,994
	2001	17,000	155	2,635	32.60	85,901
	2002	20,000	160	3,200	35.20	112,640
Squash 5/						
	2000	8,300	180	1,494	20.00	29,951
	2001	8,000	190	1,520	18.50	28,060
	2002	8,300	190	1,577	21.00	33,049
Tomatoes, Fresh Market						
	1993	37,500	300	11,250	26.30	295,875
	1994	40,000	275	11,000	24.80	272,800
	1995	38,000	270	10,260	18.70	191,862
	1996	37,400	280	10,472	24.80	259,706
	1997	34,000	285	9,860	26.50	261,630
	1998	41,000	240	9,840	33.10	325,704
	1999	44,000	260	11,440	22.30	255,112
	2000	40,000	290	11,600	29.80	345,680
	2001	38,000	270	10,260	24.90	255,474
	2002	38,500	300	11,550	25.40	293,370
Miscellaneous Vegetables						
and Melons, Fresh Market	1993	164,000	---	58,420	---	656,547
and Processing 7/	1994	208,000	---	78,500	---	827,313
	1995	184,000	---	67,200	---	825,877
	1996	238,000	---	86,960	---	953,535
	1997	166,000	---	62,400	---	796,332
	1998	210,000	---	88,220	---	938,160
	1999	211,000	---	101,180	---	927,630
	2000	180,000	---	102,900	---	878,201
	2001	199,000	---	97,880	---	918,918
	2002	204,000	---	111,440	---	985,480

1/ The processed price is based on equivalent returns at the processing plant door. The fresh market price is on a packed and loaded basis, F.O.B. shipping point.
2/ Program discontinued in 2002.
3/ Onion production includes quantity harvested, but not marketed because of loss during storage (shrinkage): 1993 -- 346,000 cwt., 1994 and 1995 -- 266,000 cwt., 1996 -- 292,000 cwt., 1997 -- 278,000 cwt., 1998 to 2002 -- 250,000 cwt. Storage loss amounts are excluded when computing total value.
4/ Includes fresh and processed.
5/ Program initiated in 2000.
6/ Program initiated in 1998.
7/ Miscellaneous vegetables and melons include: Green lima beans for processing, oriental vegetables, green peas, etc. The crops added in 2000 are excluded from the 2000 to present miscellaneous totals.

Processing Vegetable Acreage, Production and Value, 1993-2002

Crop	Crop Year	Harvested	Yield Per Acre	Production	Value Per Ton		Total Value
					First Delivery Point	Processing Plant Door	
		Acres	*Tons*	*Tons*	*Dollars*	*Dollars*	*$1,000*
Tomatoes, Processing							
	1993	274,000	32.67	8,951,580	48.30	59.10	529,038
	1994	311,000	34.55	10,745,560	50.90	61.00	655,479
	1995	317,000	33.46	10,605,787	54.00	62.30	660,740
	1996	313,000	34.05	10,658,741	53.50	61.10	651,249
	1997	260,000	35.93	9,342,309	50.85	59.50	555,867
	1998	280,000	31.76	8,892,800	54.65	64.20	570,918
	1999	329,000	37.20	12,239,300	59.85	70.50	862,871
	2000	271,000	37.96	10,286,500	50.75	58.60	602,789
	2001	254,000	34.02	8,640,140	49.40	57.50	496,808
	2002	291,000	37.99	11,056,000	50.30	57.20	632,403
Cucumbers, Pickles 1/							
	1993	4,200	10.24	43,010	---	192.00	8,258
	1994	4,900	9.26	45,370	---	183.00	8,303
	1995	5,200	8.10	42,120	---	383.00	16,132
	1996	6,200	7.00	43,400	---	370.00	16,058
	1997	4,200	7.19	30,200	---	296.00	8,939
	1998	4,500	6.56	29,520	---	235.00	6,937
	1999	---	---	---	---	---	---
	2000	---	---	---	---	---	---
	2001	---	---	---	---	---	---
	2002	---	---	---	---	---	---

1/ Data for 1999-02 not published to avoid disclosure of individual operations.

California Processing Tomatoes by County, 2002 Crop

County	Contracted and Open Market			
	Planted	Harvested	Yield Per Acre	Production 1/
	Acres	*Acres*	*Tons*	*Tons*
Colusa	19,000	18,700	40.84	763,800
Fresno	107,800	106,900	39.29	4,200,000
Glenn	1,000	1,000	34.30	34,300
Kings	14,000	13,400	37.87	507,400
Merced	19,200	18,200	35.91	653,500
Sacramento	3,900	3,900	44.62	174,000
San Joaquin	32,700	32,600	36.99	1,206,000
Solano	12,600	12,600	37.45	471,900
Stanislaus	16,700	16,600	36.45	605,000
Sutter	9,400	9,100	36.89	335,700
Yolo	41,800	41,600	36.18	1,505,000
Other Counties 2/	17,900	16,400	36.55	599,400
Total	296,000	291,000	37.99	11,056,000

1/ On the basis of PAID FOR TONNAGE purchased from growers as reported by processors, dockage not included.
2/ To avoid disclosure of individual operations, OTHER COUNTIES includes: Contra Costa, Imperial, Kern, Madera, San Benito, Santa Clara, and Tulare.

Vegetable and Melon Monthly Average Prices Received, 1997-2002 1/

Crop	Crop Year	Jan.	Feb.	Mar.	Apr.	May	June	July	Aug.	Sept.	Oct.	Nov.	Dec.
		Dollars Per Cwt.											
Asparagus													
	1997	161.00	140.00	116.00	109.00	92.40	154.00	---	---	---	---	---	---
	1998	179.00	158.00	144.00	134.00	123.00	164.00	234.00	211.00	199.00	152.00	148.00	---
	1999	141.00	119.00	178.00	128.00	123.00	158.00	214.00	---	---	---	---	---
	2000	147.00	99.70	98.60	139.00	134.00	172.00	206.00	205.00	---	---	---	---
	2001	219.00	256.00	147.00	150.00	133.00	177.00	176.00	145.00	---	137.00	129.00	---
	2002	218.00	162.00	119.00	99.80	119.00	129.00	146.00	---	---	---	---	---
Broccoli													
	1997	36.80	27.80	25.90	24.20	23.10	30.30	27.50	23.30	31.20	40.70	27.00	30.20
	1998	33.80	26.80	30.70	40.70	27.10	29.60	23.30	27.60	29.20	32.80	25.90	24.00
	1999	27.70	20.10	21.70	20.30	18.60	23.10	18.70	27.40	29.30	23.00	21.70	39.50
	2000	22.50	20.30	25.90	23.30	44.30	30.00	31.50	25.20	27.70	34.10	56.00	34.40
	2001	22.80	32.30	23.80	27.00	25.50	27.00	23.60	27.10	22.90	24.20	21.60	55.10
	2002	53.40	44.90	33.80	24.00	20.80	28.40	27.00	29.60	40.60	24.00	37.10	35.00
Carrots													
	1997	14.90	14.60	13.30	12.50	12.50	12.60	12.50	12.50	12.50	12.50	12.30	16.90
	1998	12.50	12.70	12.70	12.00	11.40	11.50	10.60	10.40	10.20	10.60	11.30	11.00
	1999	16.10	19.60	21.20	26.30	24.80	21.80	15.50	12.40	10.40	11.10	11.20	11.40
	2000	9.49	11.60	11.80	12.30	13.40	14.60	15.40	14.20	14.30	14.80	15.30	15.60
	2001	15.90	16.70	17.30	17.30	17.30	18.60	20.70	19.10	16.70	19.30	19.30	19.30
	2002	19.30	19.70	21.10	21.20	21.20	21.50	20.50	20.20	20.20	19.90	19.60	19.50
Cauliflower													
	1997	29.60	33.80	32.60	27.70	20.70	31.20	38.90	22.90	34.50	49.30	27.60	28.90
	1998	35.10	44.00	49.50	43.80	35.50	26.40	23.20	25.30	31.30	23.60	33.70	35.80
	1999	29.40	29.40	37.60	45.80	23.40	25.50	19.30	24.80	20.30	20.00	34.50	54.30
	2000	22.90	30.10	31.10	34.60	46.00	31.20	37.20	24.00	24.80	21.60	65.30	28.10
	2001	25.70	35.20	22.80	47.30	26.30	37.40	24.80	24.70	23.50	21.60	21.50	56.10
	2002	59.50	29.30	44.80	25.50	26.40	32.70	27.40	22.70	23.00	22.40	37.60	50.00
Celery													
	1997	16.20	16.20	12.30	10.50	15.40	9.89	19.00	16.40	14.00	13.40	18.40	19.10
	1998	11.20	11.40	16.40	13.80	15.40	12.40	9.99	9.39	9.80	9.91	11.90	14.00
	1999	9.51	8.47	8.35	10.20	12.80	18.30	13.80	9.81	9.77	8.48	12.80	17.20
	2000	19.20	16.00	12.90	21.20	25.60	29.10	18.60	20.70	15.20	12.80	19.40	21.50
	2001	14.60	15.00	15.80	19.10	24.00	33.70	13.10	8.15	8.04	7.46	8.64	9.62
	2002	10.10	19.50	23.50	18.60	12.30	9.37	10.10	9.94	10.60	9.34	15.30	9.50
Corn, Sweet													
	1997	---	---	---	20.30	18.80	14.90	15.80	14.90	16.50	18.20	19.80	19.70
	1998	---	---	---	33.60	20.00	14.80	12.60	11.00	15.20	26.20	27.30	17.30
	1999	---	---	---	---	19.30	14.60	13.90	15.80	12.10	17.60	25.10	---
	2000	---	---	---	21.90	14.30	18.80	15.50	19.20	26.90	16.30	20.80	---
	2001	---	---	---	28.60	29.40	18.90	18.00	18.50	24.00	26.50	23.20	20.50
	2002	---	---	---	29.60	25.00	19.40	19.00	15.70	19.50	17.40	19.40	---
Melons, Cantaloupe													
	1997	---	---	---	---	20.20	16.50	14.70	15.20	21.50	24.90	21.80	18.50
	1998	---	---	---	---	27.10	15.90	18.60	11.80	15.50	19.00	16.00	---
	1999	---	---	---	---	23.90	15.90	12.80	13.50	15.90	17.70	19.40	---
	2000	---	---	---	---	16.70	16.80	14.30	12.30	19.00	24.00	23.20	35.10
	2001	---	---	---	---	27.30	13.20	14.50	17.20	13.50	16.50	20.00	23.70
	2002	---	---	---	---	21.80	12.60	14.50	10.80	14.80	21.30	16.10	---
Melons, Honeydew													
	1997	---	---	---	---	28.50	21.00	14.40	12.60	22.70	31.90	21.20	18.00
	1998	---	---	---	---	25.20	23.00	22.30	15.00	13.00	15.00	17.80	16.00
	1999	---	---	---	---	---	22.60	16.60	16.00	20.20	28.50	20.70	---
	2000	---	---	---	---	18.70	17.30	17.00	13.80	21.60	24.80	22.20	---
	2001	---	---	---	---	30.70	19.00	18.30	17.90	16.10	14.80	20.40	---
	2002	---	---	---	---	22.40	17.30	19.50	12.30	11.60	17.10	14.10	---

Vegetable and Melon Monthly Average Prices Received, 1997-2002 1/

Crop	Crop Year	Jan.	Feb.	Mar.	Apr.	May	June	July	Aug.	Sept.	Oct.	Nov.	Dec.
		Dollars Per Cwt.											
Lettuce, Head													
	1997	15.20	10.00	13.80	15.80	10.50	14.70	17.00	22.80	22.30	35.10	26.90	32.50
	1998	19.40	11.30	16.60	28.10	14.70	11.40	15.40	16.20	14.00	21.30	10.60	12.50
	1999	11.10	15.50	37.60	20.80	14.00	11.40	12.50	11.90	13.00	13.00	10.80	17.50
	2000	15.00	10.00	12.40	23.30	23.50	13.40	15.00	19.20	29.40	16.10	18.00	12.20
	2001	14.50	24.10	15.00	21.90	18.50	12.00	16.40	26.90	26.20	11.30	10.90	29.60
	2002	29.00	43.10	80.60	13.60	9.97	10.50	11.30	14.60	14.30	13.30	11.80	30.00
Onions, Spring													
	1997	---	---	---	16.60	12.60	15.50	14.10	---	---	---	---	---
	1998	---	---	---	15.90	14.40	13.40	20.20	---	---	---	---	---
	1999	---	---	---	10.80	11.30	11.80	13.10	---	---	---	---	---
	2000	---	---	---	12.50	9.12	10.00	13.10	---	---	---	---	---
	2001	---	---	---	16.30	13.00	12.70	14.50	---	---	---	---	---
	2002	---	---	---	13.70	13.70	15.00	14.20	---	---	---	---	---
Onions, Summer													
	1997	19.60	14.60	---	---	---	---	14.10	14.20	11.20	9.90	12.40	16.20
	1998	17.00	21.00	23.00	---	---	---	20.20	13.50	13.40	12.20	13.80	16.50
	1999	17.50	16.70	12.80	---	---	---	11.20	10.70	10.10	9.22	9.28	---
	2000	---	---	---	---	---	---	13.00	11.10	12.20	12.90	14.10	16.30
	2001	---	---	---	---	---	---	14.50	12.60	12.80	12.60	11.90	11.90
	2002	11.60	11.20	11.20	---	---	---	14.20	12.50	11.00	11.30	11.30	11.30
Tomatoes													
	1997	---	---	---	---	36.90	27.30	25.40	25.40	23.20	23.30	41.10	---
	1998	---	---	---	---	29.20	25.70	43.10	20.40	26.60	43.10	37.60	---
	1999	---	---	---	---	26.10	27.80	20.30	22.50	25.30	18.90	23.40	30.70
	2000	---	---	---	---	25.10	24.50	21.80	31.30	27.80	40.40	38.40	38.40
	2001	---	---	---	---	35.30	24.00	24.70	26.20	19.60	26.60	28.40	---
	2002	---	---	---	---	40.70	27.30	24.80	22.50	21.30	25.90	38.10	---

1/ Prices are F.O.B. shipping point.

County Agricultural Commissioners

County	Commissioner	Address	Telephone	Fax
Alameda	Dennis F. Bray	224 W. Winton Avenue, Room 184, Hayward, CA 94544-1215	(510) 670-5232	783-3928
Alpine	Bill Stephans	311 Fair Lane, Placerville, CA 95667-4195	(530) 621-5520	626-4756
Amador	Michael E. Boitano	12200 Airport Road, Jackson, CA 95642-9527	(209) 223-6487	223-3312
Butte	Richard B. Price	316 Nelson Avenue, Oroville, CA 95965-3318	(530) 538-7381	538-7594
Calaveras	Jearl D. Howard	891 Mountain Ranch Road, San Andreas, CA 95249-9709<br>Government Center	(209) 754-6504	754-6521
Colusa	Harry A. Krug	100 Sunrise Blvd., Suite F, Colusa, CA 95932-1029	(530) 458-0580	458-5000
Contra Costa	Edward P. Meyer	2366 'A' Stanwell Circle, Concord, CA 94520-4804	(925) 646-5250	646-5732
Del Norte	Glenn E. Anderson	2650 Washington Blvd., Crescent City, CA 95531-8627	(707) 464-7231	464-6044
El Dorado	Bill Stephans	311 Fair Lane, Placerville, CA 95667-4195	(530) 621-5520	626-4756
Fresno	Jerry Prieto, Jr.	1730 S. Maple Avenue, Fresno, CA 93702-4596	(559) 456-7510	456-7379
Glenn	Bill Duckworth	P.O. Box 351, Willows, CA 95988<br>720 N. Colusa Street	(530) 934-6501	934-6503
Humboldt	John E. Falkenstrom	5630 S. Broadway, Eureka, CA 95503-6998	(707) 445-7223	445-7220
Imperial	Stephen L. Birdsall	150 South Ninth Street, El Centro, CA 92243-2850	(760) 482-4314	353-9420
Inyo	George L. Milovich	207 W. South Street, Bishop, CA 93514-3492<br>County Services Bldg.	(760) 873-7860	872-1610
Kern	Theodore K. Davis	1001 South Mt. Vernon Avenue, Bakersfield, CA 93307-2857	(661) 868-6300	868-6301
Kings	Tim Niswander	680 N. Campus Drive, Suite B, Hanford, CA 93230-5923	(559) 582-3211<br>Ext. 2830	582-5251
Lake	Steve Hajik	883 Lakeport Blvd., Lakeport, CA 95453	(707) 263-0217	263-1052
Lassen	Kenneth R. Smith	175 Russell Avenue, Susanville, CA 96130-4299	(530) 251-8110	257-6515
Los Angeles	Cato Fiksdal	12300 Lower Azusa Road, Arcadia, CA 91006-5872	(626) 575-5451	350-3243
Madera	Robert J. Rolan	332 Madera Avenue, Madera, CA 93637-5499	(559) 675-7876	674-4071
Marin	Stacy K. Carlsen	1682 Novato Blvd., Suite150-A, Novato, CA 94947-7021	(415) 499-6700	499-7543
Mariposa	Cathi Boze	P.O. Box 905, Mariposa, CA 95338-0905<br>5009 Fairgrounds Road, Mariposa, CA 95338-9435	(209) 966-2075	966-2056
Mendocino	David A. Bengston	579 Low Gap Road, Ukiah, CA 95482-3745	(707) 463-4208	463-0240
Merced	David A. Robinson	2139 Wardrobe Avenue, Merced, CA 95340-6495	(209) 385-7431	725-3536
Modoc	Joe A. Moreo	202 West Fourth Street, Alturas, CA 96101-3915	(530) 233-6401	233-5542
Mono	George L. Milovich	207 W. South Street, Bishop, CA 93514-3492<br>County Services Bldg.	(760) 873-7860	872-1610
Monterey	Eric Lauritzen	1428 Abbott Street, Salinas, CA 93901	(831) 759-7325	422-5003
Napa	David R. Whitmer	1710 Soscol Avenue, Suite 3, Napa, CA 94559-1315	(707) 253-4357	253-4881
Nevada	Paul Boch	255 S. Auburn Street, Grass Valley, CA 95945-7289	(530) 273-2648	273-1713
Orange	Richard M. Le Feuvre	10852 Douglass Road, Bldg. D, Anaheim, CA 92806-6050	(714) 447-7100	774-2741
Placer	Christine Turner	11477 'E' Avenue, Auburn, CA 95603-2799	(530) 889-7372	823-1698
Plumas	Karl Bishop	208 Fairgrounds Road, Quincy, CA 95971-9462	(530) 283-6365	283-4210
Riverside	John R. Snyder	P.O. Box 1089, Riverside, CA 92502-1089<br>4080 Lemon Street, Room 19	(909) 955-3000	955-3012
Sacramento	Frank E. Carl	4137 Branch Center Road, Sacramento, CA 95827-3897	(916) 875-6603	875-6150
San Benito	Paul Matulich	3224 Southside Road, Hollister, CA 95023-0699	(831) 637-5344	637-9015
San Bernardino	Edouard P. Layaye	777 E. Rialto Avenue, San Bernardino, CA 92415-0720	(909) 387-2115	387-2449
San Diego	Kathleen A. Thuner	5555 Overland Avenue, Bldg. 3, San Diego, CA 92123-1292	(858) 694-2741	565-7046
San Francisco	David C. Frieders	501 Cesar Chavez Street, Suite 109-A, San Francisco, CA 94124	(415) 285-5010	285-8776
San Joaquin	Scott T. Hudson	P.O. Box 1809, Stockton, CA 95201-1809<br>1868 E. Hazelton Avenue	(209) 468-3300	468-3330
San Luis Obispo	Robert Lilley	2156 Sierra Way, Suite A, San Luis Obispo, CA 93401-4556	(805) 781-5910	781-1035
San Mateo	Gail M. Raabe	P.O. Box 999, Redwood City, CA 94064-0999<br>728 Heller Street	(650) 363-4700	367-0130

County Agricultural Commissioners

County	Commissioner	Address	Telephone	Fax
Santa Barbara	William D. Gillette	263 Camino Del Remedio, Santa Barbara, CA 93110-1335	(805) 681-5600	681-5603
Santa Clara	Greg Van Wassenhove	1553 Berger Drive, Bldg. 1, San Jose, CA 95112-2795	(408) 918-4600	286-2460
Santa Cruz	David W. Moeller	175 Westridge Drive, Watsonville, CA 95076-2797	(831) 763-8080	763-8234
Shasta	Mary Pfeiffer	3179 Bechelli Lane, Suite 210, Redding, CA 96002-2041	(530) 224-4949	224-4951
Sierra	Karl Bishop	208 Fairgrounds Road, Quincy, CA 95971-9462	(530) 283-6365	283-4210
Siskiyou	Ag. Commissioner	525 S. Foothill Drive, Yreka, CA 96097-3090	(530) 841-4025	842-6690
Solano	Susan E. Cohen	501 Texas Street, Fairfield, CA 94533	(707) 421-7465	429-0827
Sonoma	John G. Westoby	133 Aviation Blvd., Suite 110, Santa Rosa, CA 95403	(707) 565-2371	565-3850
Stanislaus	Dennis Gudgel	3800 Cornucopia Way, Suite B, Modesto, CA 95358	(209) 525-4730	525-4789
Sutter	Mark P. Quisenberry	142 Garden Highway, Yuba City, CA 95991-5512	(530) 822-7500	822-7510
Tehama	Mark D. Black	P.O. Box 38, Red Bluff, CA 96080-0038<br>1760 Walnut Street	(530) 527-4504	529-1049
Trinity	Mark T. Lockhart	P.O. Box 1466, Weaverville, CA 96093-1466<br>173 Tombell Road	(530) 623-1356	623-1391
Tulare	Gary W. Kunkel	4437 South Laspina Street, Tulare, CA 93291-4584	(559) 685-3323	685-3336
Tuolumne	Gary Caseri	2 South Green Street, Sonora, CA 95370-4617<br>22365 S. Airport Road, Colombia, CA 95310	(209) 533-5691	533-5520
Ventura	W. Earl McPhail	P.O. Box 889, Santa Paula, CA 93061-0889<br>815 E. Santa Barbara Street	(805) 933-8415	525-8922
Yolo	Rick Landon	70 Cottonwood Street, Woodland, CA 95695-2593	(530) 666-8140	662-6094
Yuba	Dennis S. Pooler	938 - 14th Street, Marysville, CA 95901	(530) 749-5400	743-4442

California Agricultural Statistics Service publications are available on the Internet at:

http://www.nass.usda.gov/ca

CALIFORNIA AGRICULTURAL STATISTICS SERVICE

UNITED STATES
DEPARTMENT OF AGRICULTURE
NATIONAL AGRICULTURAL STATISTICS SERVICE

STATE OF CALIFORNIA
DEPT. OF FOOD AND AGRICULTURE
MARKETING SERVICES DIVISION

P.O. Box 1258
Sacramento, CA 95812-1258
Phone (916) 498-5161 --- Fax (916) 498-5186

PUBLICATION AND SUBSCRIPTION REQUEST FORM

Complete the "publication request" if you would like to purchase one or more publications on a one-time basis only. We will mail you the latest release unless you indicate otherwise. When ordering diskettes, please be sure to check the appropriate file format.

Complete the "subscription request" if you would like to be placed on our regular mailing list for upcoming releases.

For all requests, check the publications you wish to receive, enter the amount in the space provided and total the cost of all fees. Please be sure to complete the "requested by" information.

Publications on this order form can be viewed free of charge at ***www.nass.usda.gov/ca***. Please forward any inquires to our e-mail address at ***nass-ca@nass.usda.gov***.

PUBLICATION REQUEST				
Check Here	Publication Title and Description	List the Years You Are Buying	Domestic Charge	Enter Amount
	**HARD COPY**			
	**California Agricultural Resource Directory:** 2002 Annual Bulletin (2001 Crop)<br>Year-end summary for field crops, fruit and nut crops, vegetable and melon crops, livestock, dairy and poultry. Also includes cash receipts, exports, floriculture, top counties, and U.S. and state rankings.<br>**The 2003 Annual Bulletin (2002 Crop) is not available.**		$ 15.00	
	**County Agricultural Commissioners' Data:** Annual Bulletin<br>County acreage, production and value by commodity. Crop years available: 1980-2002		$ 20.00 per year	
	**Dot Maps:** 1990 Bulletin<br>Acreage, yield, production and value for 50 major commodities.		$ 15.00	
	**Grape Acreage:** Annual Bulletin<br>Grape acreage by year planted, variety and county.<br>Crop years available: 1961, 1964, 1969 and 1972-2002		$ 10.00 per year	
	**Grape Crush Report (Final):** Annual Bulletin<br>Tons purchased, crushed, Brix factors and price per ton by variety and district.<br>Crop years available: 1976-2002		$ 10.00 per year	
	**Summary of County Agricultural Commissioners' Reports:** Annual Bulletin<br>Gross values by commodity groups. Crop years available: 1981-2002		$ 5.00 per year	
	**DISKETTE**<br>All data files are IBM-PC compatible on 3.5 disks. Operating System: Windows 95/98<br>*Please indicate file format:* ☐ WordPerfect ☐ Spreadsheet ☐ Xbase			
	**County Agricultural Commissioners' Data:** Annual Bulletin<br>County acreage, production and value by commodity. Crop years available: 1986-2002		$ 20.00 per year	
	**Grape Acreage:** Annual Bulletin<br>Grape acreage by year planted, variety and county. Crop years available: 1991-2002		$ 20.00 per year	
	**Grape Crush Report (Final):** Annual Bulletin<br>Tons purchased, crushed, Brix factors and price per ton by variety and district.<br>Crop years available: 1991-2002		$ 20.00 per year	
			**TOTAL ONE-TIME CHARGE**	

Continued on back of page

CALIFORNIA AGRICULTURAL STATISTICS SERVICE

PUBLICATION AND SUBSCRIPTION REQUEST FORM — Continued

SUBSCRIPTION REQUEST				
Check Here	Publication Title and Description	Subscription Rate (First-Class Mail)		Enter Amount
		Foreign 1/	Domestic 2/	
907	**Crop Weather Report:** Weekly<br>Weather's effect on crops; planting and harvesting information; weekly and accumulated precipitation data, and temperatures by station.	$ 60.00	$ 30.00	
910	**Field Crop Review:** Monthly<br>Grain, cotton, hay, potatoes, sugar beets, etc.; acreage, production, value; price and /warehouse data.	$ 30.00	$ 15.00	
920	**Fruit and Nut Review:** Monthly except November<br>Berry, grape, citrus, deciduous fruit and nut acreage, production, price and utilization.	$ 30.00	$ 15.00	
922	**Grape Crush Report (Preliminary and Final):** February & March Bulletins<br>Tons purchased, crushed, Brix factors and price per ton by variety and district.	$ 40.00	$ 20.00	
923	**Grape Acreage:** Annual Bulletin<br>Grape acreage by year planted, variety and county.	$ 20.00	$ 10.00	
931	**Vegetable Review:** January, April, July, September and October<br>Processing and fresh market vegetable and potato acreage, production and value.	$ 14.00	$ 7.00	
940	**Livestock Review:** Monthly<br>Livestock inventories, intentions and values; pasture, slaughter and on-feed data for cattle and sheep.	$ 30.00	$ 15.00	
960	**Poultry Review:** Monthly<br>Chicken and turkey settings, hatchings, eggs produced, inventory, value and cold storage.	$ 30.00	$ 15.00	
	**TOTAL SUBSCRIPTION FEES**			

1/ Foreign mailing is via air mail. All checks must be drawn on a U.S. bank.
2/ Mexico's rate is the same as domestic rate.

PAYMENT	
Make check or money order payable to:	**CASS/CDFA**
Send this request form with remittance to:	California Agricultural Statistics Service<br>P.O. Box 1258<br>Sacramento, CA 95812-1258

REQUESTED BY
Company or Firm Name:
Name:
Mailing Address:
City, State & Zip:
E-mail Address:
