# Supplementary material for: The Elephant in the room: What can we learn from California regarding the use of sport hunting of pumas (Puma concolor) as a management tool?
Source: PLoS One. 2020 Feb 13;15(2):e0224638. doi: 10.1371/journal.pone.0224638 (PMC7018503; doi:10.1371/journal.pone.0224638)
Supplement: S2 File — This is a zip file that contains sources of the data used in the analyses that are no longer available on the internet. (ZIP) [file pone.0224638.s003.zip › Califonina Mountain Lion Depredation 1972-2013 .pdf]

CALIFORNIA DEPARTMENT OF FISH and WILDLIFE

Mountain Lion Depredation Statistics Summary

Pursuant to California Fish and Game Code Section 4802 (et. Seq.), the Department of Fish and Wildlife shall, upon request, issue (depredation) permits to individuals reporting livestock loss or damage caused by mountain lions, if the loss or damage is confirmed by CDFW staff to have been caused by mountain lions. The permittee is required to report the fate of the permit to the CDFW upon expiration or fulfillment of the permit. Below is a summary of the annual number of depredation permits issued by the CDFW and the resultant take as reported to CDFW Headquarters. Please note that some permits and reports may be outstanding at the time of this compilation.

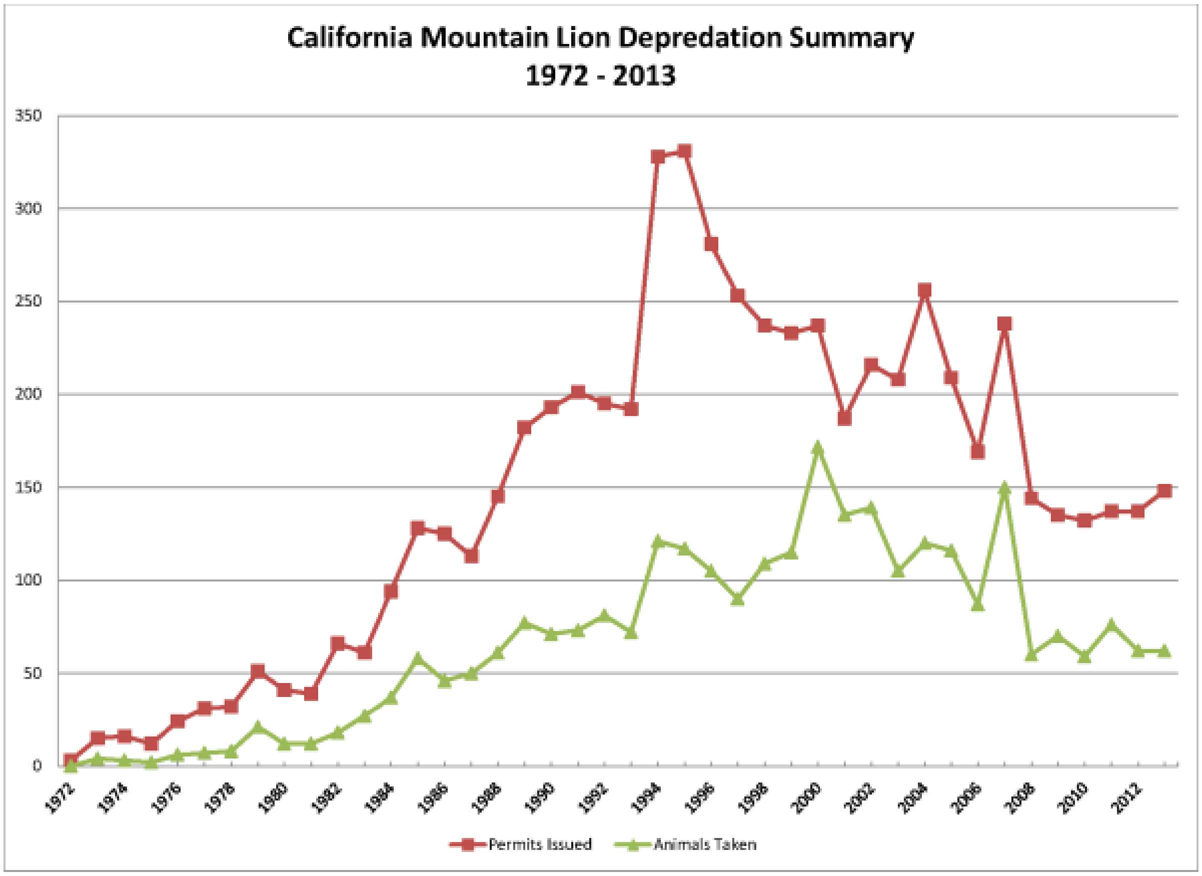

Click Graph to Enlarge

Year	Depredation Permits Issued	Animals Taken
1972	3	0
1973	15	4
1974	16	3

1975	12	2
1976	24	6
1977	31	7
1978	32	8
1979	51	21
1980	41	12
1981	39	12
1982	66	18
1983	61	27
1984	94	37
1985	128	58
1986	125	46
1987	113	50
1988	145	61
1989	182	77
1990	193	71
1991	201	73
1992	195	81
1993	192	72
1994	328	121
1995	331	117
1996	281	105
1997	253	90
1998	237	109
1999	233	115
2000	237	172
2001	187	135
2002	216	139
2003	208	105
2004	256	120
2005	209	116
2006	169	87
2007	238	150

2008	144	60
2009	135	70
2010	132	59
2011	137	76
2012	137	62
2013	148	62
**Total**	**6175**	**2816**

Updated 01/29/2014

Conditions of Use | Privacy Policy
