# Supplementary material for: The Elephant in the room: What can we learn from California regarding the use of sport hunting of pumas (Puma concolor) as a management tool?
Source: PLoS One. 2020 Feb 13;15(2):e0224638. doi: 10.1371/journal.pone.0224638 (PMC7018503; doi:10.1371/journal.pone.0224638)
Supplement: S2 File — This is a zip file that contains sources of the data used in the analyses that are no longer available on the internet. (ZIP) [file pone.0224638.s003.zip › Cattle predator death losses 1995.pdf]

HDR101200013003051796150OCATTLE
HDR201200013003051796150OCATTLE PREDATOR LOSS
Released May 17, 1996, by the National Agricultural Statistics Service (NASS), Agricultural Statistics Board, U.S. Department of Agriculture. For information on "Cattle Predator Loss" call Livestock, Dairy, and Poultry Branch at (202) 720-6146, office hours 8:00 a.m. to 4:30 p.m. ET. For assistance with general agricultural statistics, information about NASS, its products or services, contact the NASS Information Hotline at 1-800-727-9540 or E-mail: NASS@NASS.USDA.GOV.

1995 Cattle and Calf Death Losses Valued at $1.8 Billion

A total of 4.38 million head of cattle and calves died from all causes during 1995 in the United States. This estimate excludes Alaska. Predators accounted for 117,400 head or 2.7 percent, while other causes made up the remainder. These total losses cost ranchers and farmers a total of $1.80 billion with predator losses at $39.6 million and other losses at $1.76 billion.

Coyotes were the largest cause of cattle and calf predator losses at 69,350 head or 59 percent of the total loss to predators. The value of coyote losses was $21.8 million. Dogs were the second leading cause of predator losses, accounting for 19 percent of predator losses. The western and mountain region accounted for 67 percent of all predator losses. Respiratory problems led the other causes with 28 percent of the total deaths. The value of these losses was $478 million. The second leading other cause of deaths in cattle and calves was digestive problems with 20 percent of the total. The value of these losses was $316 million.

This report is released as a cooperative effort between the National Agricultural Statistics Service, and APHIS - Animal Damage Control and APHIS - National Animal Health Monitoring System.

Mt An 2 (5-96)

Losses of Cattle and Calves from All Causes, Number of Head and Total Value, United States, 1995 1/

Cause	Number of Head	Percent Total Deaths	Total Value
	Head	Percent	1,000 Dollars
Coyotes	69,350	1.6	21,798
Dogs	21,800	.5	6,952
Mountain Lions & Bobcats	8,050	.2	2,780
Bears	1,800	.0	813
Wolves	2,500	.1	891
Other Predators	13,900	.3	6,328
Total Predators	117,400	2.7	39,562
Digestive Problems	863,200	19.7	316,457
Respiratory Problems	1,203,350	27.5	478,158
Calving Problems	646,925	14.8	270,148
Weather	417,000	9.5	169,073
Poison	49,300	1.1	24,566
Theft	19,700	.4	8,285
Other Causes	399,150	9.1	204,055

Unknown Causes	664,375	15.2	291,097
Total Non-Predator Causes	4,263,000	97.3	1,761,839
Total Losses	4,380,400	100.0	1,801,402

1/ Totals may not add due to rounding.

Losses of Cattle by Predators, Other Causes and Total,
by State and United States, 1995 1/

State	Total	Predators		Other Causes	
	Cattle	Cattle	Percent of Total Deaths	Cattle	Percent of Total Deaths
	-------- Head -------		Percent	Head	Percent
AL	27,000	400	1.5	26,600	98.5
AZ	30,000	900	3.0	29,100	97.0
AR	30,000	400	1.3	29,600	98.7
CA	90,000	1,500	1.7	88,500	98.3
CO	45,000	400	.9	44,600	99.1
CT	1,000			1,000	100.0
DE	1,000			1,000	100.0
FL	30,000	600	2.0	29,400	98.0
GA	25,000	300	1.2	24,700	98.8
HI	4,000	100	2.5	3,900	97.5
ID	26,000	300	1.2	25,700	98.8
IL	25,000	100	.4	24,900	99.6
IN	15,000	200	1.3	14,800	98.7
IA	58,000	400	.7	57,600	99.3
KS	92,000	500	.5	91,500	99.5
KY	45,000	500	1.1	44,500	98.9
LA	15,000	200	1.3	14,800	98.7
ME	2,000			2,000	100.0
MD	5,000	75	1.5	4,925	98.5
MA	1,000	75	7.5	925	92.5
MI	25,000	200	.8	24,800	99.2
MN	60,000	600	1.0	59,400	99.0
MS	35,000	600	1.7	34,400	98.3

MO	65,000	700	1.1	64,300	98.9
MT	23,000	500	2.2	22,500	97.8
NE	80,000	500	.6	79,500	99.4
NV	6,000	200	3.3	5,800	96.7
NH	1,000			1,000	100.0
NJ	1,000			1,000	100.0
NM	22,000	600	2.7	21,400	97.3
NY	30,000	200	.7	29,800	99.3
NC	19,000	400	2.1	18,600	97.9
ND	19,000	200	1.1	18,800	98.9
OH	24,000	200	.8	23,800	99.2
OK	95,000	2,100	2.2	92,900	97.8
OR	28,000	300	1.1	27,700	98.9
PA	29,000	200	.7	28,800	99.3
RI	100			100	100.0
SC	10,000	100	1.0	9,900	99.0
SD	60,000	400	.7	59,600	99.3
TN	40,000	500	1.3	39,500	98.8
TX	240,000	4,000	1.7	236,000	98.3
UT	14,000	200	1.4	13,800	98.6
VT	5,000	75	1.5	4,925	98.5
VA	24,000	400	1.7	23,600	98.3
WA	22,000	200	.9	21,800	99.1
WV	6,000	75	1.3	5,925	98.8
WI	70,000	600	.9	69,400	99.1
WY	15,000	200	1.3	14,800	98.7
US	1,635,100	21,200	1.3	1,613,900	98.7

1/ Missing predator loss data are included with other causes to avoid disclosure of individual operations.

Losses of Calves by Predators, Other Causes and Total, by State and United States, 1995 1/

State	Total	Predators		Other Causes	
	Calves	Calves	Percent of Total Deaths	Calves	Percent of Total Deaths
	-------- Head -------		Percent	Head	Percent

	:					
AL	:	47,000	2,700	5.7	44,300	94.3
AZ	:	30,000	3,300	11.0	26,700	89.0
AR	:	45,000	1,700	3.8	43,300	96.2
CA	:	135,000	4,100	3.0	130,900	97.0
CO	:	60,000	3,500	5.8	56,500	94.2
CT	:	1,000			1,000	100.0
DE	:	1,000			1,000	100.0
FL	:	44,000	1,100	2.5	42,900	97.5
GA	:	34,000	1,400	4.1	32,600	95.9
HI	:	4,000	200	5.0	3,800	95.0
ID	:	52,000	1,300	2.5	50,700	97.5
IL	:	45,000	1,700	3.8	43,300	96.2
IN	:	31,000	700	2.3	30,300	97.7
IA	:	115,000	1,000	.9	114,000	99.1
KS	:	70,000	1,400	2.0	68,600	98.0
KY	:	85,000	4,000	4.7	81,000	95.3
LA	:	21,000	1,100	5.2	19,900	94.8
ME	:	4,000	75	1.9	3,925	98.1
MD	:	7,000	75	1.1	6,925	98.9
MA	:	1,000	75	7.5	925	92.5
MI	:	60,000	200	.3	59,800	99.7
MN	:	115,000	1,500	1.3	113,500	98.7
MS	:	42,000	1,800	4.3	40,200	95.7
MO	:	140,000	1,900	1.4	138,100	98.6
MT	:	57,000	1,800	3.2	55,200	96.8
NE	:	100,000	1,500	1.5	98,500	98.5
NV	:	12,000	1,300	10.8	10,700	89.2
NH	:	1,000			1,000	100.0
NJ	:	2,000			2,000	100.0
NM	:	36,000	2,000	5.6	34,000	94.4
NY	:	65,000	400	.6	64,600	99.4
NC	:	30,000	800	2.7	29,200	97.3
ND	:	58,000	1,300	2.2	56,700	97.8
OH	:	52,000	500	1.0	51,500	99.0
OK	:	150,000	8,500	5.7	141,500	94.3
OR	:	50,000	4,400	8.8	45,600	91.2
PA	:	52,000	500	1.0	51,500	99.0
RI	:	300			300	100.0
SC	:	15,000	700	4.7	14,300	95.3
SD	:	165,000	2,400	1.5	162,600	98.5

TN	80,000	3,800	4.8	76,200	95.3
TX	270,000	25,000	9.3	245,000	90.7
UT	26,000	1,000	3.8	25,000	96.2
VT	10,000	75	.8	9,925	99.3
VA	65,000	1,600	2.5	63,400	97.5
WA	29,000	1,000	3.4	28,000	96.6
WV	11,000	200	1.8	10,800	98.2
WI	190,000	1,000	.5	189,000	99.5
WY	30,000	1,600	5.3	28,400	94.7
US	2,745,300	96,200	3.5	2,649,100	96.5

1/ Missing predator loss data are included with other causes to avoid disclosure of individual operations.

Losses of Cattle and Calves by Coyotes and Dogs, by State and United States, 1995 1/

State	Coyotes				Dogs			
	Cattle	% of Total Pred.	Calves	% of Total Pred.	Cattle	% of Total Pred.	Calves	% of Total Pred.
	Head	Percent	Head	Percent	Head	Percent	Head	Percent
AL	200	50.0	2,000	74.1	100	25.0	600	22.2
AZ	400	44.4	1,500	45.5	100	11.1	200	6.1
AR	100	25.0	500	29.4	100	25.0	1,000	58.8
CA	500	33.3	1,900	46.3	200	13.3	700	17.1
CO	100	25.0	3,000	85.7			100	2.9
CT								
DE								
FL	200	33.3	400	36.4	100	16.7	400	36.4
GA	100	33.3	800	57.1	100	33.3	500	35.7
HI					100	100.0	200	100.0
ID	100	33.3	800	61.5			100	7.7
IL			900	52.9			400	23.5
IN	100	50.0	600	85.7				
IA	100	25.0	800	80.0				
KS	200	40.0	600	42.9	100	20.0	400	28.6
KY	300	60.0	2,800	70.0	100	20.0	800	20.0

LA			900	81.8			100	9.1
ME								
MD								
MA								
MI								
MN	100	16.7	700	46.7	100	16.7	100	6.7
MS	300	50.0	1,300	72.2	100	16.7	300	16.7
MO	200	28.6	1,100	57.9	300	42.9	600	31.6
MT			1,100	61.1			200	11.1
NE	200	40.0	1,200	80.0	100	20.0	100	6.7
NV			1,100	84.6			100	7.7
NH								
NJ								
NM	200	33.3	1,200	60.0			100	5.0
NY	100	50.0	200	50.0				
NC							700	87.5
ND	100	50.0	1,100	84.6			100	7.7
OH			100	20.0			200	40.0
OK	1,300	61.9	5,000	58.8	200	9.5	2,500	29.4
OR	100	33.3	3,300	75.0			500	11.4
PA			200	40.0			100	20.0
RI								
SC							600	85.7
SD	200	50.0	2,000	83.3	100	25.0	200	8.3
TN	200	40.0	1,600	42.1	200	40.0	1,600	42.1
TX	1,500	37.5	19,000	76.0	500	12.5	3,500	14.0
UT	100	50.0	600	60.0				
VT								
VA	100	25.0	800	50.0	100	25.0	700	43.8
WA			800	80.0			100	10.0
WV							100	50.0
WI			500	50.0			200	20.0
WY			1,100	68.8				
US	7,650	36.1	61,700	64.1	3,450	16.3	18,350	19.1

1/ Missing data are less than 100 head for the State or not published to avoid disclosure of individual operations. Data not published are included in U.S. total. Totals may not add due to rounding.

Losses of Cattle and Calves by Mountain Lions and Bobcats
by Selected States and United States, 1995 1/

State	Mountain Lions and Bobcats			
	Cattle	Percent of Total Predators	Calves	Percent of Total Predators
	Head	Percent	Head	Percent
AZ	300	33.3	1,500	45.5
CA	300	20.0	1,200	29.3
CO			200	5.7
ID			100	7.7
KS			100	7.1
MT	200	40.0	100	5.6
NV				
NM	100	16.7	300	15.0
OK			200	2.4
OR	100	33.3	300	6.8
TX	500	12.5	2,000	8.0
UT			100	10.0
VA				
WY				
US	1,750	8.3	6,300	6.5

1/ Missing data are less than 100 head for the State or not published to avoid disclosure of individual operations. Data not published are included in U.S. total. Totals may not add due to rounding.

Losses of Cattle and Calves by Other Predators
by State and United States, 1995 1/

State	All Other Predators			
	Cattle	Percent of Total Predators	Calves	Percent of Total Predators
	Head	Percent	Head	Percent
AL	100	25.0	100	3.7

AZ	:	100	11.1	100	3.0
AR	:	200	50.0	200	11.8
CA	:	500	33.3	300	7.3
CO	:	200	50.0	200	5.7
CT	:				
DE	:				
FL	:	300	50.0	300	27.3
GA	:	100	33.3	100	7.1
HI	:				
ID	:	100	33.3	300	23.1
IL	:	100	100.0	400	23.5
IN	:				
IA	:	300	75.0	200	20.0
KS	:	200	40.0	300	21.4
KY	:	100	20.0	400	10.0
LA	:	100	50.0	100	9.1
ME	:				
MD	:				
MA	:				
MI	:	100	50.0	100	50.0
MN	:	400	66.7	700	46.7
MS	:	200	33.3	200	11.1
MO	:	200	28.6	200	10.5
MT	:	200	40.0	400	22.2
NE	:	200	40.0	200	13.3
NV	:				
NH	:				
NJ	:				
NM	:	300	50.0	400	20.0
NY	:	100	50.0	200	50.0
NC	:	400	100.0	100	12.5
ND	:	100	50.0	100	7.7
OH	:	100	50.0	200	40.0
OK	:	600	28.6	800	9.4
OR	:	100	33.3	300	6.8
PA	:	100	50.0	200	40.0
RI	:				
SC	:			100	14.3
SD	:	100	25.0	200	8.3
TN	:	100	20.0	600	15.8
TX	:	1,500	37.5	500	2.0

UT			300	30.0
VT				
VA	200	50.0		
WA	100	50.0	100	10.0
WV				
WI	500	83.3	300	30.0
WY			350	21.9
US	8,350	39.4	9,850	10.2

1/ Missing data are less than 100 head for the State or not published to avoid disclosure of individual operations. Data not published are included in U.S. total. Totals may not add due to rounding.

Losses of Cattle and Calves by Digestive and Respiratory Problems, by State and United States, 1995 1/

State	Digestive Problems				Respiratory Problems			
	Cattle	% of Total Deaths	Calves	% of Total Deaths	Cattle	% of Total Deaths	Calves	% of Total Deaths
	Head	Percent	Head	Percent	Head	Percent	Head	Percent
AL	1,100	4.1	6,600	14.0	5,400	20.0	9,900	21.1
AZ	3,300	11.0	7,000	23.3	4,500	15.0	9,000	30.0
AR	2,500	8.3	6,700	14.9	3,200	10.7	13,500	30.0
CA	18,700	20.8	41,000	30.4	23,600	26.2	55,600	41.2
CO	5,900	13.1	10,800	18.0	12,800	28.4	14,900	24.8
CT	100	10.0	300	30.0	200	20.0	300	30.0
DE	100	10.0	400	40.0			200	20.0
FL	1,300	4.3	9,100	20.7	2,000	6.7	11,500	26.1
GA	3,200	12.8	7,100	20.9	2,800	11.2	6,500	19.1
HI	300	7.5	400	10.0	300	7.5	400	10.0
ID	4,600	17.7	17,600	33.8	6,600	25.4	12,700	24.4
IL	2,400	9.6	11,000	24.4	7,000	28.0	11,700	26.0
IN	1,400	9.3	7,800	25.2	3,300	22.0	10,300	33.2
IA	8,700	15.0	26,800	23.3	21,500	37.1	37,400	32.5
KS	12,500	13.6	13,700	19.6	48,800	53.0	28,100	40.1
KY	3,400	7.6	16,100	18.9	4,600	10.2	19,800	23.3
LA	1,300	8.7	4,200	20.0	2,800	18.7	5,700	27.1

ME	500	25.0	1,000	25.0	400	20.0	1,000	25.0
MD	400	8.0	2,400	34.3	700	14.0	2,000	28.6
MA	200	20.0	300	30.0	200	20.0	300	30.0
MI	3,500	14.0	23,600	39.3	7,100	28.4	17,800	29.7
MN	7,800	13.0	46,300	40.3	16,900	28.2	37,900	33.0
MS	3,500	10.0	7,200	17.1	3,700	10.6	12,900	30.7
MO	3,100	4.8	27,500	19.6	8,300	12.8	37,400	26.7
MT	2,600	11.3	10,000	17.5	3,600	15.7	8,600	15.1
NE	16,700	20.9	25,600	25.6	33,900	42.4	25,200	25.2
NV	900	15.0	3,100	25.8	1,500	25.0	3,400	28.3
NH			300	30.0	200	20.0	300	30.0
NJ	100	10.0	500	25.0	200	20.0	500	25.0
NM	3,200	14.5	8,500	23.6	3,900	17.7	10,900	30.3
NY	4,300	14.3	25,200	38.8	5,600	18.7	21,500	33.1
NC	1,400	7.4	6,300	21.0	3,400	17.9	5,800	19.3
ND	2,900	15.3	15,500	26.7	4,000	21.1	12,100	20.9
OH	5,100	21.3	14,700	28.3	4,600	19.2	14,000	26.9
OK	12,000	12.6	19,200	12.8	29,000	30.5	41,600	27.7
OR	3,600	12.9	12,000	24.0	5,000	17.9	16,600	33.2
PA	4,400	15.2	17,000	32.7	4,800	16.6	15,900	30.6
RI			100	33.3			100	33.3
SC	1,700	17.0	2,300	15.3	1,500	15.0	3,000	20.0
SD	6,800	11.3	37,000	22.4	14,300	23.8	30,000	18.2
TN	2,900	7.3	19,800	24.8	5,200	13.0	20,300	25.4
TX	19,400	8.1	31,600	11.7	80,900	33.7	85,500	31.7
UT	1,800	12.9	6,700	25.8	2,700	19.3	6,900	26.5
VT	500	10.0	2,600	26.0	1,100	22.0	3,000	30.0
VA	1,500	6.3	15,500	23.8	3,900	16.3	14,400	22.2
WA	5,500	25.0	6,800	23.4	4,500	20.5	10,000	34.5
WV	500	8.3	2,400	21.8	1,600	26.7	2,300	20.9
WI	7,600	10.9	82,300	43.3	13,800	19.7	69,600	36.6
WY	1,700	11.3	6,300	21.0	2,800	18.7	6,100	20.3
US	197,000	12.0	666,200	24.3	418,950	25.6	784,400	28.6

1/ Missing data are less than 100 head for the State or not published to avoid disclosure of individual operations. Data not published are included in U.S. total. Totals may not add due to rounding.

Losses of Cattle and Calves by Calving Problems and Weather, by State and United States, 1995 1/

State	Calving Problems				Weather			
	Cattle	% of Total Deaths	Calves	% of Total Deaths	Cattle	% of Total Deaths	Calves	% of Total Deaths
	Head	Percent	Head	Percent	Head	Percent	Head	Percent
AL	7,600	28.1	9,200	19.6	1,600	5.9	2,200	4.7
AZ	1,700	5.7	1,700	5.7	2,600	8.7	1,100	3.7
AR	7,500	25.0	9,900	22.0	2,600	8.7	4,000	8.9
CA	12,900	14.3	8,100	6.0	7,900	8.8	10,300	7.6
CO	3,100	6.9	7,400	12.3	2,700	6.0	7,100	11.8
CT	200	20.0	100	10.0				
DE					100	10.0		
FL	6,400	21.3	5,600	12.7	8,200	27.3	3,000	6.8
GA	7,600	30.4	8,300	24.4	1,400	5.6	900	2.6
HI	400	10.0	300	7.5	800	20.0	600	15.0
ID	2,900	11.2	5,900	11.3	1,000	3.8	4,100	7.9
IL	3,700	14.8	7,900	17.6	4,200	16.8	3,300	7.3
IN	2,100	14.0	4,000	12.9	1,600	10.7	2,500	8.1
IA	5,400	9.3	19,700	17.1	6,700	11.6	15,100	13.1
KS	4,000	4.3	10,200	14.6	6,300	6.8	7,500	10.7
KY	9,700	21.6	19,200	22.6	3,900	8.7	8,500	10.0
LA	3,400	22.7	3,300	15.7	1,400	9.3	2,100	10.0
ME	400	20.0	400	10.0			100	2.5
MD	1,000	20.0	1,200	17.1	700	14.0	100	1.4
MA	200	20.0	100	10.0				
MI	4,500	18.0	8,300	13.8	1,700	6.8	2,000	3.3
MN	9,600	16.0	9,300	8.1	3,900	6.5	5,100	4.4
MS	6,700	19.1	7,500	17.9	2,200	6.3	1,600	3.8
MO	17,900	27.5	26,600	19.0	7,600	11.7	17,800	12.7
MT	2,000	8.7	13,900	24.4	2,700	11.7	13,100	23.0
NE	5,700	7.1	18,800	18.8	9,400	11.8	25,200	25.2
NV	800	13.3	1,900	15.8	600	10.0	400	3.3
NH	400	40.0	200	20.0				
NJ	200	20.0	200	10.0	200	20.0	200	10.0
NM	4,700	21.4	3,700	10.3	1,600	7.3	3,700	10.3
NY	6,800	22.7	6,000	9.2	1,300	4.3	1,700	2.6
NC	6,200	32.6	6,200	20.7	1,000	5.3	1,000	3.3
ND	2,200	11.6	15,800	27.2	2,300	12.1	7,300	12.6

State	Cattle	% of Total Deaths	Calves	% of Total Deaths	Cattle	% of Total Deaths	Calves	% of Total Deaths
OH	3,700	15.4	13,000	25.0	1,200	5.0	1,800	3.5
OK	11,100	11.7	26,900	17.9	7,600	8.0	14,100	9.4
OR	5,500	19.6	7,400	14.8	400	1.4	3,000	6.0
PA	5,000	17.2	6,100	11.7	1,500	5.2	1,000	1.9
RI								
SC	1,600	16.0	4,200	28.0	600	6.0	700	4.7
SD	5,500	9.2	16,700	10.1	13,000	21.7	72,000	43.6
TN	10,600	26.5	14,800	18.5	3,200	8.0	3,400	4.3
TX	28,900	12.0	27,700	10.3	8,200	3.4	13,800	5.1
UT	1,800	12.9	5,400	20.8	100	.7	1,500	5.8
VT	800	16.0	1,200	12.0	200	4.0	700	7.0
VA	4,500	18.8	12,500	19.2	1,700	7.1	6,500	10.0
WA	3,400	15.5	4,700	16.2	200	.9	1,000	3.4
WV	1,000	16.7	2,600	23.6	100	1.7	1,300	11.8
WI	13,500	19.3	10,300	5.4	5,700	8.1	6,300	3.3
WY	1,600	10.7	5,800	19.3	2,000	13.3	4,200	14.0
US	246,625	15.1	400,300	14.6	134,000	8.2	283,000	10.3

1/ Missing data are less than 100 head for the State or not published to avoid disclosure of individual operations. Data not published are included in U.S. total. Totals may not add due to rounding.

Losses of Cattle and Calves by Poison and Theft, by State and United States, 1995 1/

State	Poison: Cattle	Poison: % of Total Deaths	Poison: Calves	Poison: % of Total Deaths	Theft: Cattle	Theft: % of Total Deaths	Theft: Calves	Theft: % of Total Deaths
	Head	Percent	Head	Percent	Head	Percent	Head	Percent
AL	500	1.9	600	1.3	100	.4	200	.4
AZ	700	2.3	200	.7	100	.3	100	.3
AR	1,100	3.7	300	.7	100	.3	100	.2
CA	500	.6	100	.1	900	1.0	1,100	.8
CO	900	2.0	700	1.2	200	.4	300	.5
CT	100	10.0						
DE								
FL	300	1.0	200	.5	400	1.3	100	.2

GA	:	1,800	7.2	600	1.8	100	.4	200	.6
HI	:								
ID	:	700	2.7	200	.4	300	1.2	200	.4
IL	:	300	1.2	100	.2	100	.4	300	.7
IN	:	300	2.0	400	1.3	100	.7	400	1.3
IA	:	600	1.0	100	.1	300	.5	1,200	1.0
KS	:	1,000	1.1	100	.1	600	.7	400	.6
KY	:	2,500	5.6	600	.7	200	.4	100	.1
LA	:	300	2.0	200	1.0	100	.7	200	1.0
ME	:								
MD	:								
MA	:								
MI	:	200	.8	100	.2			100	.2
MN	:	600	1.0	100	.1			100	.1
MS	:	800	2.3	400	1.0				
MO	:	1,100	1.7	900	.6	500	.8	800	.6
MT	:	800	3.5	900	1.6			200	.4
NE	:	600	.8	500	.5	200	.3	100	.1
NV	:	200	3.3						
NH	:								
NJ	:								
NM	:	1,300	5.9	600	1.7	600	2.7	300	.8
NY	:	500	1.7	100	.2	200	.7	100	.2
NC	:	400	2.1	100	.3	100	.5		
ND	:	200	1.1	100	.2	100	.5	100	.2
OH	:	600	2.5	100	.2			100	.2
OK	:	3,600	3.8	1,400	.9	800	.8	600	.4
OR	:	1,500	5.4	300	.6	300	1.1	400	.8
PA	:	400	1.4	400	.8	100	.3	100	.2
RI	:								
SC	:	200	2.0	700	4.7				
SD	:	1,000	1.7	200	.1	200	.3	200	.1
TN	:	600	1.5	900	1.1	100	.3	400	.5
TX	:	2,400	1.0	2,700	1.0	700	.3	2,700	1.0
UT	:	700	5.0	400	1.5	100	.7		
VT	:	200	4.0	300	3.0				
VA	:	600	2.5	100	.2				
WA	:	400	1.8	100	.3			100	.3
WV	:	300	5.0						
WI	:	800	1.1	400	.2	100	.1	100	.1
WY	:	800	5.3	500	1.7	200	1.3	200	.7

US	32,500	2.0	16,800	.6	8,000	.5	11,700	.4

1/ Missing data are less than 100 head for the State or not published to avoid disclosure of individual operations. Data not published are included in U.S. total. Totals may not add due to rounding.

Losses of Cattle and Calves by Other and Unknown Causes, by State and United States, 1995 1/

State	Other				Unknown			
	Cattle	% of Total Deaths	Calves	% of Total Deaths	Cattle	% of Total Deaths	Calves	% of Total Deaths
	Head	Percent	Head	Percent	Head	Percent	Head	Percent
AL	4,800	17.8	3,300	7.0	5,500	20.4	12,300	26.2
AZ	2,400	8.0	700	2.3	13,800	46.0	6,900	23.0
AR	6,300	21.0	4,000	8.9	6,300	21.0	4,800	10.7
CA	11,800	13.1	3,600	2.7	12,200	13.6	11,100	8.2
CO	4,900	10.9	2,500	4.2	14,100	31.3	12,800	21.3
CT	200	20.0			200	20.0	200	20.0
DE	100	10.0			300	30.0	200	20.0
FL	6,200	20.7	2,300	5.2	4,600	15.3	11,100	25.2
GA	4,400	17.6	2,100	6.2	3,400	13.6	6,900	20.3
HI	700	17.5	600	15.0	1,400	35.0	1,400	35.0
ID	4,400	16.9	3,300	6.3	5,200	20.0	6,700	12.9
IL	3,900	15.6	1,700	3.8	3,300	13.2	7,300	16.2
IN	2,100	14.0	1,600	5.2	3,900	26.0	3,300	10.6
IA	8,000	13.8	4,900	4.3	6,400	11.0	8,800	7.7
KS	5,500	6.0	1,500	2.1	12,800	13.9	7,100	10.1
KY	9,200	20.4	5,100	6.0	11,000	24.4	11,600	13.6
LA	2,900	19.3	1,700	8.1	2,600	17.3	2,500	11.9
ME	300	15.0	500	12.5	400	20.0	925	23.1
MD	1,100	22.0	500	7.1	1,025	20.5	725	10.4
MA	200	20.0	100	10.0	125	12.5		
MI	4,500	18.0	2,200	3.7	3,300	13.2	5,700	9.5
MN	11,300	18.8	3,900	3.4	9,300	15.5	10,800	9.4
MS	6,100	17.4	4,500	10.7	11,400	32.6	6,100	14.5
MO	14,200	21.8	12,600	9.0	11,600	17.8	14,500	10.4

MT	5,000	21.7	3,600	6.3	5,800	25.2	4,900	8.6
NE	7,400	9.3	1,200	1.2	5,600	7.0	1,900	1.9
NV	900	15.0	800	6.7	800	13.3	1,100	9.2
NH	100	10.0			100	10.0	200	20.0
NJ			100	5.0			500	25.0
NM	2,600	11.8	1,600	4.4	3,500	15.9	4,700	13.1
NY	6,100	20.3	2,600	4.0	5,000	16.7	7,400	11.4
NC	3,400	17.9	2,700	9.0	2,700	14.2	7,100	23.7
ND	3,300	17.4	1,700	2.9	3,800	20.0	4,100	7.1
OH	5,400	22.5	2,600	5.0	3,200	13.3	5,200	10.0
OK	10,100	10.6	5,400	3.6	18,700	19.7	32,300	21.5
OR	5,900	21.1	1,400	2.8	5,500	19.6	4,500	9.0
PA	7,500	25.9	5,100	9.8	5,100	17.6	5,900	11.3
RI			100	33.3				
SC	1,900	19.0	2,100	14.0	2,400	24.0	1,300	8.7
SD	8,500	14.2	1,500	.9	10,300	17.2	5,000	3.0
TN	8,700	21.8	5,800	7.3	8,200	20.5	10,800	13.5
TX	43,100	18.0	18,400	6.8	52,400	21.8	62,600	23.2
UT	2,500	17.9	1,100	4.2	4,100	29.3	3,000	11.5
VT	1,200	24.0	1,200	12.0	925	18.5	925	9.3
VA	4,900	20.4	3,300	5.1	6,500	27.1	11,100	17.1
WA	3,500	15.9	2,400	8.3	4,300	19.5	2,900	10.0
WV	1,425	23.8	1,200	10.9	1,000	16.7	900	8.2
WI	11,600	16.6	5,300	2.8	16,300	23.3	14,700	7.7
WY	2,700	18.0	1,300	4.3	3,000	20.0	4,000	13.3
US	263,350	16.1	135,800	4.9	313,475	19.2	350,900	12.8

1/ Missing data are less than 100 head for the State or not published to avoid disclosure of individual operations. Data not published are included in U.S. total. Totals may not add due to rounding.

Losses and Value of Losses of Cattle by Predators, Other Causes, and Total, by State and United States, 1995 1/

State	Total			Predators		Other Causes	
	All Losses	Value Per Head	Total Value	Losses	Total Value	Losses	Total Value

	Head	Dollars	1,000 Dol	Head	1,000 Dol	Head	1,000 Dol
AL	27,000	500	13,500	400	200	26,600	13,300
AZ	30,000	643	19,290	900	579	29,100	18,711
AR	30,000	548	16,440	400	219	29,600	16,221
CA	90,000	823	74,070	1,500	1,235	88,500	72,836
CO	45,000	605	27,225	400	242	44,600	26,983
CT	1,000	915	915			1,000	915
DE	1,000	873	873			1,000	873
FL	30,000	595	17,850	600	357	29,400	17,493
GA	25,000	583	14,575	300	175	24,700	14,400
HI	4,000	495	1,980	100	50	3,900	1,931
ID	26,000	655	17,030	300	197	25,700	16,834
IL	25,000	630	15,750	100	63	24,900	15,687
IN	15,000	673	10,095	200	135	14,800	9,960
IA	58,000	630	36,540	400	252	57,600	36,288
KS	92,000	520	47,840	500	260	91,500	47,580
KY	45,000	578	26,010	500	289	44,500	25,721
LA	15,000	593	8,895	200	119	14,800	8,776
ME	2,000	813	1,626			2,000	1,626
MD	5,000	808	4,040	75	61	4,925	3,979
MA	1,000	890	890	75	67	925	823
MI	25,000	818	20,450	200	164	24,800	20,286
MN	60,000	720	43,200	600	432	59,400	42,768
MS	35,000	513	17,955	600	308	34,400	17,647
MO	65,000	580	37,700	700	406	64,300	37,294
MT	23,000	630	14,490	500	315	22,500	14,175
NE	80,000	583	46,640	500	292	79,500	46,349
NV	6,000	585	3,510	200	117	5,800	3,393
NH	1,000	893	893			1,000	893
NJ	1,000	905	905			1,000	905
NM	22,000	658	14,476	600	395	21,400	14,081
NY	30,000	893	26,790	200	179	29,800	26,611
NC	19,000	548	10,412	400	219	18,600	10,193
ND	19,000	643	12,217	200	129	18,800	12,088
OH	24,000	713	17,112	200	143	23,800	16,969
OK	95,000	518	49,210	2,100	1,088	92,900	48,122
OR	28,000	620	17,360	300	186	27,700	17,174
PA	29,000	873	25,317	200	175	28,800	25,142
RI	100	810	81			100	81
SC	10,000	563	5,630	100	56	9,900	5,574

SD	60,000	610	36,600	400	244	59,600	36,356
TN	40,000	550	22,000	500	275	39,500	21,725
TX	240,000	543	130,320	4,000	2,172	236,000	128,148
UT	14,000	635	8,890	200	127	13,800	8,763
VT	5,000	968	4,840	75	73	4,925	4,767
VA	24,000	595	14,280	400	238	23,600	14,042
WA	22,000	745	16,390	200	149	21,800	16,241
WV	6,000	508	3,048	75	38	5,925	3,010
WI	70,000	855	59,850	600	513	69,400	59,337
WY	15,000	628	9,420	200	126	14,800	9,294
US	1,635,100		1,025,420	21,200	13,053	1,613,900	1,012,367

1/ Missing predator loss data are included with other causes to avoid disclosure of individual operations.

Losses and Value of Losses of Calves by Predators, Other Causes, and Total, by State and United States, 1995 1/

State	Total			Predators		Other Causes	
	All Losses	Value Per Head	Total Value	Losses	Total Value	Losses	Total Value
	Head	Dollars	1,000 Dol	Head	1,000 Dol	Head	1,000 Dol
AL	47,000	245	11,515	2,700	662	44,300	10,854
AZ	30,000	230	6,900	3,300	759	26,700	6,141
AR	45,000	278	12,510	1,700	473	43,300	12,037
CA	135,000	250	33,750	4,100	1,025	130,900	32,725
CO	60,000	345	20,700	3,500	1,208	56,500	19,493
CT	1,000	295	295			1,000	295
DE	1,000	385	385			1,000	385
FL	44,000	235	10,340	1,100	259	42,900	10,082
GA	34,000	248	8,432	1,400	347	32,600	8,085
HI	4,000	220	880	200	44	3,800	836
ID	52,000	273	14,196	1,300	355	50,700	13,841
IL	45,000	293	13,185	1,700	498	43,300	12,687
IN	31,000	285	8,835	700	200	30,300	8,636
IA	115,000	318	36,570	1,000	318	114,000	36,252

KS	: 70,000	295	20,650	1,400	413	68,600	20,237
KY	: 85,000	263	22,355	4,000	1,052	81,000	21,303
LA	: 21,000	260	5,460	1,100	286	19,900	5,174
ME	: 4,000	273	1,092	75	20	3,925	1,072
MD	: 7,000	325	2,275	75	24	6,925	2,251
MA	: 1,000	288	288	75	22	925	266
MI	: 60,000	273	16,380	200	55	59,800	16,325
MN	: 115,000	275	31,625	1,500	413	113,500	31,213
MS	: 42,000	223	9,366	1,800	401	40,200	8,965
MO	: 140,000	268	37,520	1,900	509	138,100	37,011
MT	: 57,000	350	19,950	1,800	630	55,200	19,320
NE	: 100,000	330	33,000	1,500	495	98,500	32,505
NV	: 12,000	310	3,720	1,300	403	10,700	3,317
NH	: 1,000	315	315			1,000	315
NJ	: 2,000	295	590			2,000	590
NM	: 36,000	285	10,260	2,000	570	34,000	9,690
NY	: 65,000	308	20,020	400	123	64,600	19,897
NC	: 30,000	223	6,690	800	178	29,200	6,512
ND	: 58,000	335	19,430	1,300	436	56,700	18,995
OH	: 52,000	298	15,496	500	149	51,500	15,347
OK	: 150,000	275	41,250	8,500	2,338	141,500	38,913
OR	: 50,000	305	15,250	4,400	1,342	45,600	13,908
PA	: 52,000	275	14,300	500	138	51,500	14,163
RI	: 300	295	89			300	89
SC	: 15,000	260	3,900	700	182	14,300	3,718
SD	: 165,000	330	54,450	2,400	792	162,600	53,658
TN	: 80,000	240	19,200	3,800	912	76,200	18,288
TX	: 270,000	265	71,550	25,000	6,625	245,000	64,925
UT	: 26,000	290	7,540	1,000	290	25,000	7,250
VT	: 10,000	310	3,100	75	23	9,925	3,077
VA	: 65,000	228	14,820	1,600	365	63,400	14,455
WA	: 29,000	275	7,975	1,000	275	28,000	7,700
WV	: 11,000	253	2,783	200	51	10,800	2,732
WI	: 190,000	285	54,150	1,000	285	189,000	53,865
WY	: 30,000	355	10,650	1,600	568	28,400	10,082
	:						
US	:2,745,300		775,982	96,200	26,510	2,649,100	749,472

1/ Missing predator loss data are included with other causes to avoid disclosure of individual operations.

Losses of Cattle and Calves by Cause and Total Value,
by Region and United States, 1995 1/

Region	Total Predators Losses: Cattle	Total Predators Losses: Calves	Total Predators Losses: Total Value	Total Non-Predator Losses: Cattle	Total Non-Predator Losses: Calves	Total Non-Predator Losses: Total Value
	------ Head -----		1,000 Dol	------- Head -------		1,000 Dol
Mountain and West	13,100	65,600	26,466	897,900	1,268,400	912,593
Midwest	3,400	10,200	5,225	368,600	782,800	478,184
South and East	4,700	20,400	7,872	347,400	597,900	371,063
US	21,200	96,200	39,562	1,613,900	2,649,100	1,761,839

Region	Total Losses: Cattle	Total Losses: Calves	Total Losses: Total Value
	------------- Head	-------------	1,000 Dol
Mountain and West	911,000	1,334,000	939,059
Midwest	372,000	793,000	483,408
South and East	352,100	618,300	378,935
US	1,635,100	2,745,300	1,801,402

See footnotes at end of table. --continued

Losses of Cattle and Calves by Cause and Total Value,
by Regions and United States, 1995 2/ (Continued)

	Coyotes	Dogs

Region	Cattle	Calves	Total Value	Cattle	Calves	Total Value
	------ Head -----		1,000 Dol	------ Head -----		1,000 Dol
Mountain and West	5,200	45,300	16,020	1,700	9,150	3,537
Midwest	750	5,250	1,994	700	2,600	1,182
South and East	1,700	11,150	3,784	1,050	6,600	2,234
US	7,650	61,700	21,798	3,450	18,350	6,952
	Mountain Lions and Bobcats			Bears		
	Cattle	Calves	Total Value	Cattle	Calves	Total Value
	----- Head -----		1,000 Dol	----- Head ----		1,000 Dol
Mountain and West	1,750	6,250	2,768	400	1,200	655
Midwest				200		158
South and East						
US	1,750	6,300	2,780	600	1,200	813
	Wolves			Other Predators		
	Cattle	Calves	Total Value	Cattle	Calves	Total Value
	----- Head ----		1,000 Dol	----- Head -----		1,000 Dol
Mountain and West	400	1,100	531	3,650	2,600	2,955
Midwest	200	600	310	1,550	1,750	1,581

South and East		200	50	1,950	2,400	1,792
US	600	1,900	891	7,150	6,750	6,328

See footnotes at end of table. --continued

Losses of Cattle and Calves by Cause and Total Value,
by Regions and United States, 1995 1/ (Continued)

Region	Digestive Problems			Respiratory Problems		
	Cattle	Calves	Total Value	Cattle	Calves	Total Value
	------ Head ------		1,000 Dol	------ Head ------		1,000 Dol
Mountain and West	122,400	272,800	156,670	282,700	377,600	274,026
Midwest	42,100	246,700	99,929	85,700	249,600	131,121
South and East	32,500	146,700	59,858	50,550	157,200	73,011
US	197,000	666,200	316,457	418,950	784,400	478,158

Region	Calving Problems			Weather		
	Cattle	Calves	Total Value	Cattle	Calves	Total Value
	------ Head ------		1,000 Dol	------ Head ------		1,000 Dol
Mountain and West	98,200	182,300	115,293	69,400	190,000	101,911
Midwest	67,900	109,000	77,645	35,200	57,900	40,348
South and East	80,525	109,000	77,210	29,400	35,100	26,814

US	246,625	400,300	270,148	134,000	283,000	169,073

See footnotes at end of table. --continued

Losses of Cattle and Calves by Cause and Total Value,
by Regions and United States, 1995 1/ (Continued)

Region	Poison			Theft		
	Cattle	Calves	Total Value	Cattle	Calves	Total Value
	------ Head -----		1,000 Dol	------ Head -----		1,000 Dol
Mountain and West	17,300	9,000	12,952	5,400	7,100	5,345
Midwest	5,600	2,500	4,415	1,200	3,200	1,688
South and East	9,600	5,300	7,198	1,400	1,400	1,252
US	32,500	16,800	24,566	8,000	11,700	8,285
	**Other Causes**			**Unknown Causes**		
	Cattle	Calves	Total Value	Cattle	Calves	Total Value
	------ Head ------		1,000 Dol	------ Head ------		1,000 Dol
Mountain and West	125,200	52,600	90,552	177,300	177,000	155,844
Midwest	67,300	38,800	57,143	63,600	75,100	65,893
South and East	70,850	44,400	56,360	72,575	98,800	69,361
US	263,350	135,800	204,055	313,475	350,900	291,097

1/ Totals may not add due to rounding.
2/ Missing data are less than 100 head for the Region or not published to avoid

disclosure of individual operations. Data not published are included in U.S. total. Totals may not add due to rounding.

Reliability of Cattle and Calf Death Loss Estimates

Survey Procedures: A random sample of U.S. producers was surveyed to provide data for these estimates. Survey procedures ensured that all cattle producers, regardless of size, had a chance to be included in the survey to collect death loss by cause. Large producers were sampled more heavily than small operations. Data were collected from about 50,000 operators during the first half of January by mail, telephone, and face-to-face personal interviews. Operators were asked to report death losses for cattle and calves for the 1995 calendar year.

Estimating Procedures: These estimates of total death losses and death loss by cause were prepared by the Livestock, Dairy and Poultry Branch, NASS, and reviewed by the 49 States involved. Total cattle and calf death losses from all causes were published in the 1995 "Meat Animals Production, Disposition, and Income" Report, released April 11, 1996. To estimate death loss by cause, cattle and calf losses from total predators were first determined. Next, the total predator estimate was broken down by specific predators, and a percent of total predator losses was calculated. This procedure was repeated to determine death losses from all other causes. All published loss estimates were rounded to the nearest 100 head. Value estimates were rounded to the nearest $1,000. State estimates of less than 100 head are not published, but are included in U.S. totals. States were combined into regions when sampling variability did not permit individual state data to be released.

Reliability: Since all cattle operators are not included in the sample, survey estimates are subject to sampling variability. Survey results are also subject to non-sampling errors such as omissions, duplications, and mistakes in reporting, recording, and processing the data. The effects of these non-sampling errors cannot be measured directly. They are minimized through rigid quality controls in the data collection process and through a careful review of all reported data for consistency and reasonableness.

Terms and Definitions:

Cattle: Includes all cows, bulls, steers, and heifers weighing over 500 pounds. This includes beef and milk breeds as well as cattle on feed.

Calves: Beef and milk breed steers, heifers, and bulls weighing less than 500 pounds.

Regions: Mountain and Western:

Arizona	Kansas	North Dakota	Texas
California	Montana	Oklahoma	Utah
Colorado	Nebraska	Oregon	Washington
Hawaii	Nevada	South Dakota	Wyoming
Idaho	New Mexico		

Midwest:

Arkansas	Iowa	Minnesota	Ohio
Illinois	Michigan	Missouri	Wisconsin
Indiana			

Southern and Eastern:

Alabama	Louisiana	New Jersey	Tennessee
Connecticut	Maine	New York	Vermont
Delaware	Maryland	North Carolina	Virginia
Florida	Massachusetts	Pennsylvania	West Virginia
Georgia	Mississippi	Rhode Island	
Kentucky	New Hampshire	South Carolina	

Value: Cattle - An Average of the inventory value per head of cattle over 500 pounds. These data were collected nationally during the January 1, 1995 and 1996 Cattle Surveys.

Calves - An average of the inventory value per head of calves less than

500 pounds. These data were collected nationally during the January 1. 1995 and 1996 Cattle Surveys.

Digestive Problems: Includes bloat, scours, parasites, etc.

Respiratory Problems: Includes shipping fever, pneumonia, etc.

Poison: Includes nitrate poisoning and noxious feeds or weeds.

Mountain Lions and Bobcats: Includes cougars, pumas and lynx.

Index

	Page
Losses and Value of Losses of Cattle and Calves from all causes, U.S.	2
Losses of Cattle by Predators, Other Causes and Total, by State and U.S.	4
Losses of Calves by Predators, Other Causes and Total, by State and U.S.	5
Losses of Cattle and Calves by Coyotes and Dogs, by State and U.S.	6
Losses of Cattle and Calves from Mountain Lions and Bobcats, by Selected States and U.S.	7
Losses of Cattle and Calves by Other Predators, by State and U.S.	8
Losses of Cattle and Calves by Digestive Problems and Respiratory Problems, by State and U.S.	9

Losses of Cattle and Calves by Calving Problems and Weather,
by State and U.S. 10

Losses of Cattle and Calves by Poison and Theft,
by State and U.S. 11

Losses of Cattle and Calves by Other and Unknown Causes,
by State and U.S. 12

Losses and Value of Losses of Cattle by Predators,
Other Causes, and Total, by State and U.S. 13

Losses and Value of Losses of Calves by Predators,
Other Causes, and Total, by State and U.S. 14

Losses of Cattle and Calves by Cause and Total Value,
by Region and U.S. 15

Survey Procedures and Reliability 20

The United States Department of Agriculture (USDA) prohibits discrimination in its programs on the basis of race, color, national origin, sex, religion, age, disability, political beliefs, and marital or familial status. Persons with disabilities who require alternative means for communication of program information (braille, large print, audiotape, etc.) should contact the USDA Office of Communications at (202) 720-5881 (voice) or (202) 720-7808 (TDD).

To file a complaint, write the Secretary of Agriculture, USDA, Washington, D.C., 20250, or call (202) 720-7327 (voice) or (202) 720-1127 (TDD). USDA is an equal employment opportunity employer.

SUBSCRIBE TODAY!!

For your convenience, there are several ways to obtain NASS reports, data products, and services:

INTERNET ACCESS

All NASS reports are now available free of charge on the worldwide Internet.
For access, connect to the Internet and select:

1. Worldwide Web:
   http://www.usda.gov/nass/ OR

2. For Gopher/Telenet/FTP access:
   HOST=usda.mannlib.cornell.edu OR

3. For a subscription direct to your e-mail address,
   send an e-mail message to:
   usda-reports@usda.mannlib.cornell.edu
   and in the body of the message type the word: list

- -

PRINTED REPORTS OR DATA PRODUCTS

CALL OUR TOLL-FREE ORDER DESK: 1-800-999-6779 (U.S. and Canada)
Other areas, please call l-703-834-0125 FAX: 1-703-834-0110
(Visa, MasterCard, check, or money order acceptable for payment.)

- -

ASSISTANCE

For assistance with general agricultural statistics or further information about NASS or its products or services, contact the NASS INFORMATION HOTLINE at 1-800-727-9540, 8:00 a.m. to 4:30 p.m., or e-mail: NASS@NASS.USDA.GOV.
