# Supplementary material for: The Elephant in the room: What can we learn from California regarding the use of sport hunting of pumas (Puma concolor) as a management tool?
Source: PLoS One. 2020 Feb 13;15(2):e0224638. doi: 10.1371/journal.pone.0224638 (PMC7018503; doi:10.1371/journal.pone.0224638)
Supplement: S2 File — This is a zip file that contains sources of the data used in the analyses that are no longer available on the internet. (ZIP) [file pone.0224638.s003.zip › Cattle death loss 1991.pdf]

Historic, archived document

Do not assume content reflects current scientific knowledge, policies, or practices.

United States
Department of
Agriculture

National
Agricultural
Statistics
Service

in cooperation with

Animal and
Plant
Health
Inspection
Service

NASS Staff Report
LDP Number 92-02

October 1992

CATTLE AND CALVES DEATH LOSS 1991

Cattle and Calves Death Loss, by Glenda L. Shepler, Livestock Section, Livestock, Dairy and Poultry Branch, Estimates Division, National Agricultural Statistics Service, U.S. Department of Agriculture, Washington, D.C. 20250. October 1992.

Abstract

Survey data was collected in January 1992 to pick up data on death losses of cattle and calves from all causes. This report provides a detailed breakdown of the death losses by class and size group.

Acknowledgements

The author would like to thank the Commodity Section, Statistical Methods Branch, for providing the class and size group breakdown data used to prepare this report.

National Agricultural Statistics Service

Animal and Plant Health Inspection Service

1991 Cattle and Calves Death Losses Total 4.37 Million Head

A total of 4.37 million head of cattle and calves were lost from all causes during 1991 in the United States. This report provides class and size group breakdowns of these losses. Beef cattle operations accounted for 37.2 percent of the total cattle death losses during 1991. This includes death losses from all causes except predators. Other operations, which include primarily feedlots and/or yearling operations, lost 477,800 head, or 30.5 percent of the total. Twenty eight percent of the cattle losses, excluding predators, were attributed to dairy cattle operations.

Beef calves also accounted for the largest share of the total losses to calves less than 500 pounds with 42.4 percent of the total death losses, excluding predators. Dairy calves accounted for 35.7 percent while other calves were 15.8 percent of the total.

Digestive, respiratory and calving complications accounted for most of the causes of losses to cattle across all classes. The most predominant losses to calves across all classes came from digestive and respiratory problems.

This report is released as a cooperative effort between the National Agricultural Statistics Service and the National Animal Health Monitoring System of the Animal and Plant Health Inspection Service. Both agencies are part of the U.S. Department of Agriculture. This is the second release of 1991 death loss data and includes a further breakdown of data from the first release by class and size group.

Losses of Cattle by Class

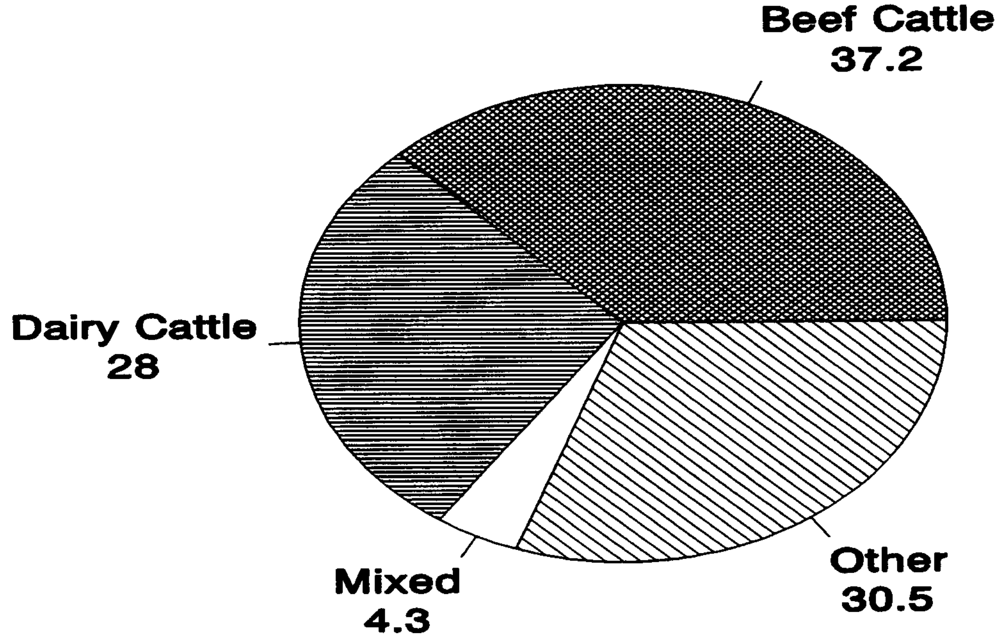

Losses of Calves by Class

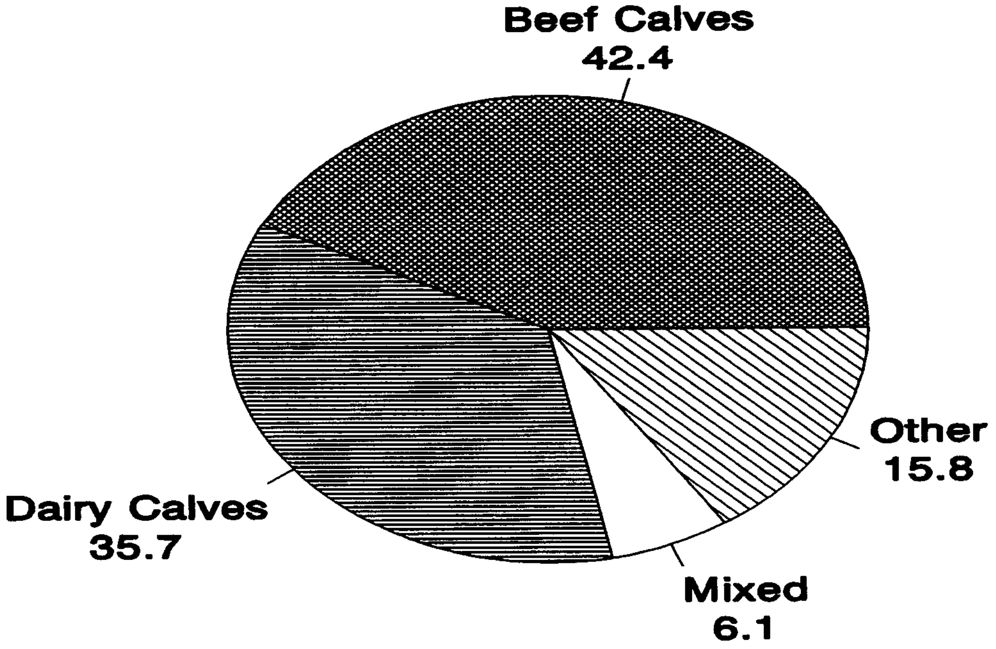

Losses of Cattle from All Causes, by Class and Total, by State, Region and United States, 1991

State	Total	Other					Predators
	Total Cattle	Total Cattle	Beef Cattle	Dairy Cattle	Mixed	Other	Total Cattle
	Head						
AL	23,000	22,700	12,400	5,200	900	4,200	300
AZ	25,000	23,400	11,900	7,400	200	3,900	1,600
AR	30,000	29,700	17,200	7,800	2,300	2,400	300
CA	75,000	73,900	18,200	33,700	1,900	20,100	1,100
CO	55,000	54,800	16,700	2,800	700	34,600	200
CT	2,000	2,000		2,000			0
DE	1,000	1,000		800		100	1/
FL	22,000	21,600	11,900	7,700	1,900	100	400
GA	32,000	31,600	14,500	14,300	1,800	1,000	400
HI	3,000	3,000	2,200	600	0	100	1/
ID	22,000	21,900	8,200	5,600	300	7,800	100
IL	25,000	24,800	10,200	2,800	500	11,300	200
IN	15,000	14,900	5,700	6,200	300	2,700	100
IA	65,000	64,700	22,300	7,200	1,600	33,600	300
KS	80,000	79,800	10,600	1,600	600	67,000	200
KY	45,000	44,700	22,400	12,900	4,200	5,200	300
LA	20,000	19,800	9,800	8,300	1,700		200
ME	2,000	2,000	100	1,300	100	400	1/
MD	5,000	4,900	1,000	3,500	400		100
MA	1,000	1,000		900			1/
MI	25,000	24,900	3,800	12,800	100	8,200	100
MN	45,000	44,900	8,700	16,700	1,800	17,700	100
MS	25,000	24,700	14,600	6,500	1,000	2,600	300
MO	70,000	69,500	35,200	15,700	8,100	10,500	500
MT	26,000	25,700	22,500	800	1,300	1,100	300
NE	80,000	79,800	19,000	1,300	300	59,200	200
NV	6,000	6,000	4,100	1,100	100	600	1/
NH	1,000	1,000		900			0
NJ	1,000	1,000		900			1/
NM	19,000	18,000	7,400	6,300	400	3,900	1,000
NY	33,000	33,000	600	29,500	1,500	1,300	1/
NC	17,000	16,700	7,000	7,500	1,100	1,100	300
ND	19,000	18,800	10,400	3,800	2,600	2,000	200
OH	25,000	25,000	4,100	13,300	600	7,000	0
OK	85,000	83,200	34,300	9,400	2,900	36,600	1,800
OR	25,000	24,800	15,700	7,300	1,100	700	200
PA	28,000	27,900	2,200	23,100	1,200	1,400	100
RI	100	100				0	0
SC	12,000	12,000	3,600	6,200	1,100	1,100	0
SD	55,000	54,700	30,000	4,000	1,900	18,800	300
TN	35,000	34,600	18,500	11,500	3,000	1,600	400
TX	240,000	237,000	104,500	37,400	7,000	88,100	3,000
UT	11,000	10,900	5,300	3,600	1,100	900	100
VT	11,000	10,900	200	10,200	500		100
VA	27,000	26,200	11,400	8,500	4,700	1,600	800
WA	27,000	26,800	5,600	11,900	600	8,700	200
WV	8,000	8,000	3,600	3,000	900	400	1/
WI	65,000	65,000	3,100	52,900	3,500	5,500	0
WY	15,000	14,900	12,400	100	100	2,300	100
Mt & West	868,100	857,500	339,000	138,900	23,200	356,400	10,600
Midwest	365,000	363,400	110,300	135,400	18,800	98,900	1,600
So & East	351,000	347,300	133,800	164,900	26,100	22,500	3,700
US	1,584,100	1,568,200	583,100	439,200	68,100	477,800	15,900

1/ Less than 100 head for the State, but number is included in U.S. total.
Totals may not add due to rounding.

Losses of Beef Cattle from All Causes, by Size Group, State, Region and United States, 1991

State	All Causes 1/			
	Beef			
	Total	1-49 Head	50-99 Head	100+ Head
	Head			
AL	12,400	2,700	2,500	7,200
AZ	11,900		100	11,800
AR	17,200	4,700	4,700	7,800
CA	18,200	1,400	800	16,000
CO	16,700	1,200	800	14,700
CT			0	0
DE				
FL	11,900	300	600	11,000
GA	14,500	4,400	3,300	6,800
HI	2,200			2,200
ID	8,200	400	400	7,400
IL	10,200	4,800	3,000	2,400
IN	5,700	1,600	300	3,800
IA	22,300	8,300	6,000	8,000
KS	10,600	2,600	1,800	6,200
KY	22,400	10,500	6,000	5,900
LA	9,800	1,800	2,300	5,700
ME	100		100	
MD	1,000	400	200	400
MA				
MI	3,800	1,200	500	2,100
MN	8,700	4,100	2,700	1,900
MS	14,600	2,900	2,700	9,000
MO	35,200	9,400	8,600	17,200
MT	22,500	700	1,400	20,400
NE	19,000	2,200	1,300	15,500
NV	4,100		100	4,000
NH			0	0
NJ				0
NM	7,400	500	600	6,300
NY	600	500	100	
NC	7,000	2,600	1,200	3,200
ND	10,400	1,300	2,600	6,500
OH	4,100	2,700	300	1,100
OK	34,300	9,800	6,600	17,900
OR	15,700	800	500	14,400
PA	2,200	1,500	300	400
RI			0	0
SC	3,600	1,000	800	1,800
SD	30,000	3,700	4,800	21,500
TN	18,500	6,500	5,100	6,900
TX	104,500	6,500	8,100	89,900
UT	5,300	600	700	4,000
VT	200	200		
VA	11,400	2,000	2,400	7,000
WA	5,600	500	300	4,800
WV	3,600	1,400	1,000	1,200
WI	3,100	1,600	400	1,100
WY	12,400	400	700	11,300
Mt & West	339,000	32,600	31,600	274,800
Midwest	110,300	38,400	26,500	45,400
So & East	133,800	38,700	28,600	66,500
US	583,100	109,700	86,700	386,700

1/ Losses from all causes excludes predators. Totals may not add due to rounding.

Losses of Dairy Cattle from All Causes, by Size Group, State, Region and United States, 1991

State	All Causes 1/				
	Dairy				
	Total	1-29 Head	30-49 Head	50-99 Head	100+ Head
	Head				
AL	5,200			500	4,700
AZ	7,400	0	0	0	7,400
AR	7,800		100	3,100	4,600
CA	33,700			100	33,600
CO	2,800			200	2,600
CT	2,000	100		100	1,800
DE	800		0		800
FL	7,700	0		100	7,600
GA	14,300	0		1,000	13,300
HI	600	0	0	0	600
ID	5,600		200	500	4,900
IL	2,800	200	400	1,000	1,200
IN	6,200	100	600	1,900	3,600
IA	7,200	500	1,100	2,200	3,400
KS	1,600	100		400	1,100
KY	12,900	700	1,900	3,600	6,700
LA	8,300			1,900	6,400
ME	1,300		100	300	900
MD	3,500		200	600	2,700
MA	900			200	700
MI	12,800	500	900	3,300	8,100
MN	16,700	1,200	3,900	6,000	5,600
MS	6,500	200		1,500	4,800
MO	15,700	900	1,000	4,600	9,200
MT	800		0	100	700
NE	1,300		100	400	800
NV	1,100		0		1,100
NH	900		100	200	600
NJ	900			200	700
NM	6,300		0		6,300
NY	29,500	200	1,500	7,800	20,000
NC	7,500		200	1,400	5,900
ND	3,800		500	1,100	2,200
OH	13,300	700	1,200	4,100	7,300
OK	9,400	200	200	1,000	8,000
OR	7,300			400	6,900
PA	23,100	900	3,000	7,000	12,200
RI					
SC	6,200	0	0	500	5,700
SD	4,000	200	400	700	2,700
TN	11,500	200	500	2,300	8,500
TX	37,400	100	500	1,200	35,600
UT	3,600		100	700	2,800
VT	10,200		1,400	2,200	6,600
VA	8,500		300	1,400	6,800
WA	11,900			500	11,400
WV	3,000	0		700	2,300
WI	52,900	2,700	7,600	17,600	25,000
WY	100				100
Mt & West	138,900	600	2,000	7,300	129,000
Midwest	135,400	6,800	16,800	43,800	68,000
So & East	164,900	2,300	9,200	33,500	119,900
US	439,200	9,700	28,000	84,600	316,900

1/ Losses from all causes excludes predators. Totals may not add due to rounding.

Losses of Calves from All Causes, by Class and Total, by State, Region and United States, 1991

State	Total	Other					Predators
	Total Calves	Total Calves	Beef Calves	Dairy Calves	Mixed	Other	Total Calves
				Head			
AL	30,000	27,900	15,900	7,300	1,700	3,000	2,100
AZ	40,000	36,700	2,400	4,500		29,800	3,300
AR	45,000	43,600	16,900	8,900	1,800	16,000	1,400
CA	140,000	135,700	23,100	82,000	2,700	27,900	4,300
CO	60,000	57,100	37,900	9,900	2,500	6,800	2,900
CT	2,000	2,000		1,900			1/
DE	1,000	1,000		1,000			1/
FL	35,000	34,100	16,100	13,500	4,400	100	900
GA	40,000	38,700	17,000	17,500	2,700	1,500	1,300
HI	4,000	3,800	2,100	1,100	500		200
ID	50,000	48,800	27,200	17,600	1,400	2,600	1,200
IL	55,000	53,200	29,100	13,900	2,600	7,600	1,800
IN	35,000	34,500	9,900	12,600	300	11,700	500
IA	130,000	128,400	69,800	29,300	9,200	20,100	1,600
KS	70,000	68,900	29,900	5,500	2,400	31,100	1,100
KY	85,000	81,700	44,400	23,300	9,900	4,100	3,300
LA	30,000	28,600	11,600	14,800	2,100	100	1,400
ME	4,000	4,000	400	3,100	300	100	1/
MD	8,000	8,000	1,700	5,500	800		0
MA	2,000	2,000	100	1,700	100		1/
MI	55,000	54,900	5,400	43,400	800	5,300	100
MN	120,000	118,600	29,700	61,300	7,200	20,400	1,400
MS	39,000	37,400	21,900	7,300	1,500	6,700	1,600
MO	135,000	133,200	77,500	32,300	18,300	5,100	1,800
MT	60,000	58,200	52,600	1,600	2,200	1,800	1,800
NE	90,000	88,200	72,000	6,500	3,400	6,300	1,800
NV	15,000	13,400	10,800	2,400	100	100	1,600
NH	2,000	2,000		1,800	200		1/
NJ	2,000	2,000	200	1,600	100		0
NM	33,000	31,200	8,700	13,900	900	7,700	1,800
NY	85,000	84,800	3,500	74,500	3,900	2,900	200
NC	31,000	30,300	10,900	16,000	2,600	800	700
ND	53,000	51,600	35,000	7,800	7,100	1,700	1,400
OH	50,000	49,900	8,300	25,400	1,200	15,000	100
OK	145,000	138,000	86,200	21,800	6,000	24,000	7,000
OR	55,000	50,500	35,000	10,900	4,200	400	4,500
PA	65,000	64,800	4,900	46,200	2,700	11,000	200
RI	300	300		100		0	0
SC	19,000	18,400	5,500	10,700	1,900	300	600
SD	110,000	106,300	82,800	13,200	6,200	4,100	3,700
TN	80,000	75,900	36,500	27,800	7,700	3,900	4,100
TX	300,000	276,600	111,000	78,100	9,700	77,800	23,400
UT	28,000	27,000	13,500	9,300	3,900	300	1,000
VT	20,000	19,800	700	17,900	1,100	100	200
VA	65,000	63,400	26,700	20,800	13,900	2,000	1,600
WA	30,000	29,200	7,500	19,300	800	1,600	800
WV	18,000	17,800	9,600	6,000	2,000	200	200
WI	185,000	184,600	6,200	107,500	7,100	63,800	400
WY	30,000	28,800	26,300	500	1,000	1,000	1,200
Mt & West	1,313,100	1,250,100	664,000	306,000	55,100	225,000	63,000
Midwest	810,000	800,900	252,800	334,600	48,500	165,000	9,100
So & East	663,200	644,800	227,600	320,600	59,700	36,900	18,400
US	2,786,300	2,695,800	1,144,400	961,200	163,300	426,900	90,500

1/ Less than 100 head for the State, but number is included in U.S. total.
Totals may not add due to rounding.

Losses of Beef Calves from All Causes, by Size Group, State, Region and United States, 1991

State	All Causes 1/			
	Beef			
	Total	1-49 Head	50-99 Head	100+ Head
	Head			
AL	15,900	3,800	2,800	9,300
AZ	2,400			2,400
AR	16,900	5,000	3,900	8,000
CA	23,100	1,300	1,900	19,900
CO	37,900	2,600	2,300	33,000
CT			0	0
DE				0
FL	16,100	700	700	14,700
GA	17,000	4,300	2,600	10,100
HI	2,100		100	2,000
ID	27,200	1,900	2,200	23,100
IL	29,100	13,300	7,600	8,200
IN	9,900	3,600	1,100	5,200
IA	69,800	24,600	18,900	26,300
KS	29,900	7,200	6,000	16,700
KY	44,400	20,100	12,100	12,200
LA	11,600	2,100	2,300	7,200
ME	400	300	100	
MD	1,700	800	300	600
MA	100		100	
MI	5,400	3,000	1,800	600
MN	29,700	14,800	7,300	7,600
MS	21,900	2,300	2,600	17,000
MO	77,500	18,200	18,200	41,100
MT	52,600	1,300	2,700	48,600
NE	72,000	7,800	6,200	58,000
NV	10,800	200		10,600
NH				0
NJ	200	100	100	
NM	8,700	900	800	7,000
NY	3,500	1,500	1,600	400
NC	10,900	4,900	2,000	4,000
ND	35,000	3,800	6,800	24,400
OH	8,300	4,100	1,000	3,200
OK	86,200	21,100	16,000	49,100
OR	35,000	2,000	1,500	31,500
PA	4,900	3,200	500	1,200
RI			0	0
SC	5,500	1,800	1,200	2,500
SD	82,800	8,500	11,500	62,800
TN	36,500	15,000	8,600	12,900
TX	111,000	10,100	10,700	90,200
UT	13,500	1,700	1,400	10,400
VT	700	400	100	200
VA	26,700	5,900	6,100	14,700
WA	7,500	600	700	6,200
WV	9,600	3,800	2,100	3,700
WI	6,200	3,100	1,000	2,100
WY	26,300	800	1,200	24,300
Mt & West	664,000	71,800	72,000	520,200
Midwest	252,800	89,700	60,800	102,300
So & East	227,600	71,000	45,900	110,700
US	1,144,400	232,500	178,700	733,200

1/ Losses from all causes excludes predators. Totals may not add due to rounding.

Losses of Dairy Calves from All Causes, by Size Group, State, Region and United States, 1991

State	All Causes 1/				
	Dairy				
	Total	1-29 Head	30-49 Head	50-99 Head	100+ Head
	Head				
AL	7,300	200		900	6,200
AZ	4,500	0	0	0	4,500
AR	8,900		200	3,500	5,200
CA	82,000			200	81,800
CO	9,900	100		500	9,300
CT	1,900			100	1,800
DE	1,000	0	0		1,000
FL	13,500	0		100	13,400
GA	17,500	0		1,300	16,200
HI	1,100	0	0	0	1,100
ID	17,600	300	600	1,400	15,300
IL	13,900	300	1,700	4,400	7,500
IN	12,600	600	1,500	5,100	5,400
IA	29,300	2,300	6,000	10,000	11,000
KS	5,500	400	200	1,200	3,700
KY	23,300	1,300	2,700	7,400	11,900
LA	14,800		200	2,300	12,300
ME	3,100		300	800	2,000
MD	5,500		200	1,400	3,900
MA	1,700			600	1,100
MI	43,400	900	2,700	11,500	28,300
MN	61,300	3,900	14,600	21,000	21,800
MS	7,300		200	1,900	5,200
MO	32,300	2,600	1,500	12,600	15,600
MT	1,600	100	0	500	1,000
NE	6,500	200	500	1,500	4,300
NV	2,400		0		2,400
NH	1,800		100	400	1,300
NJ	1,600			300	1,300
NM	13,900	500	0	100	13,300
NY	74,500	1,200	3,700	16,800	52,800
NC	16,000	0	200	2,300	13,500
ND	7,800	100	800	2,300	4,600
OH	25,400	1,300	2,300	7,100	14,700
OK	21,800	200	500	2,900	18,200
OR	10,900		200	900	9,800
PA	46,200	2,200	8,100	13,000	22,900
RI	100				100
SC	10,700		0	700	10,000
SD	13,200	900	2,300	1,900	8,100
TN	27,800	700	1,000	5,800	20,300
TX	78,100	200	200	1,700	76,000
UT	9,300		200	1,900	7,200
VT	17,900	300	2,100	4,900	10,600
VA	20,800	100	800	4,600	15,300
WA	19,300	1,000		900	17,400
WV	6,000		0	1,200	4,800
WI	107,500	4,600	16,900	37,700	48,300
WY	500	200		200	100
Mt & West	306,000	4,200	5,500	18,100	278,200
Midwest	334,600	16,500	47,400	112,900	157,800
So & East	320,600	6,000	19,600	66,800	228,200
US	961,200	26,700	72,500	197,800	664,200

1/ Losses from all causes excludes predators. Totals may not add due to rounding.

Losses of Cattle from Digestive Problems, by Class and Total, by State, Region and United States, 1991

State	Digestive Problems				
	Total Cattle	Beef Cattle	Dairy Cattle	Mixed	Other
	Head				
AL	1,600	800	800		
AZ	5,400	1,900	1,900	0	1,600
AR	2,200	1,500	700		
CA	12,000	1,200	6,000	200	4,600
CO	6,600	1,100	200	100	5,200
CT	100	0	100		0
DE	100	0	100		0
FL	1,000	300	600	100	
GA	3,700	1,700	1,700	200	100
HI	200	100	100	0	0
ID	3,000	900	900	100	1,100
IL	3,700	1,200	500	100	1,900
IN	1,800	400	1,100		300
IA	10,000	3,200	1,500	300	5,000
KS	19,600	500	100		19,000
KY	5,600	2,500	2,100	700	300
LA	3,500	2,000	1,300	200	0
ME	300		200	0	100
MD	800	100	600	100	
MA	100	0	100		
MI	4,600	1,200	2,500	100	800
MN	5,900	1,200	1,800	200	2,700
MS	2,600	1,500	700	300	100
MO	4,400	1,400	1,000	600	1,400
MT	4,000	3,300	400	200	100
NE	9,400	1,200	100		8,100
NV	400	200	100		100
NH	1/	0			0
NJ	100	0	100		0
NM	2,000	500	1,100	0	400
NY	5,200	100	4,600	300	200
NC	600	300	200	100	
ND	3,100	1,500	500	500	600
OH	3,300	400	1,700	100	1,100
OK	9,100	4,800	1,800	600	1,900
OR	4,600	2,600	2,000		
PA	3,300	200	2,800	100	200
RI	0	0	0	0	0
SC	2,500	800	1,100	500	100
SD	8,100	3,000	500	200	4,400
TN	3,200	1,400	1,700	100	0
TX	18,600	4,700	4,000	1,100	8,800
UT	2,100	700	800	300	300
VT	800		700	100	0
VA	2,600	1,200	1,100	200	100
WA	5,100	600	2,000	100	2,400
WV	1,100	500	400	200	
WI	10,100	300	8,500	700	600
WY	2,200	1,400	100		700
Mt & West	115,500	30,200	22,600	3,400	59,300
Midwest	46,000	10,800	19,300	2,100	13,800
So & East	38,900	13,400	21,000	3,200	1,300
US	200,400	54,400	62,900	8,700	74,400

1/ Less than 100 head for the State, but number is included in U.S. total.
Totals may not add due to rounding.

Losses of Beef Cattle from Digestive Problems, by Size Group,
State, Region and United States, 1991 1/

State	Digestive Problems			
	Beef			
	Total	1-49 Head	50-99 Head	100+ Head
	Head			
AL	800	100	300	400
AZ	1,900	0	0	1,900
AR	1,500	700	300	500
CA	1,200	100	100	1,000
CO	1,100	100		1,000
CT	0	0	0	0
DE	0	0	0	0
FL	300		100	200
GA	1,700	400	300	1,000
HI	100	0		100
ID	900	100		800
IL	1,200	700	300	200
IN	400	200		200
IA	3,200	1,400	1,000	800
KS	500	100	100	300
KY	2,500	1,700	300	500
LA	2,000	400	300	1,300
ME			0	0
MD	100	100		
MA	0	0	0	0
MI	1,200	200		1,000
MN	1,200	700	400	100
MS	1,500	200	200	1,100
MO	1,400	500	300	600
MT	3,300	100	200	3,000
NE	1,200	200	100	900
NV	200		0	200
NH	0	0	0	0
NJ	0	0	0	0
NM	500			500
NY	100	100		
NC	300	200	0	100
ND	1,500	100	500	900
OH	400	400		0
OK	4,800	1,400	800	2,600
OR	2,600		100	2,500
PA	200	100	100	
RI	0	0	0	0
SC	800	300	100	400
SD	3,000	400	700	1,900
TN	1,400	400	600	400
TX	4,700	300	300	4,100
UT	700	100	100	500
VT			0	0
VA	1,200	300	600	300
WA	600	100	100	400
WV	500	200	100	200
WI	300	200	0	100
WY	1,400		100	1,300
Mt & West	30,200	3,100	3,200	23,900
Midwest	10,800	5,000	2,300	3,500
So & East	13,400	4,500	3,000	5,900
US	54,400	12,600	8,500	33,300

1/ Totals may not add due to rounding.

Losses of Dairy Cattle from Digestive Problems, by Size Group, State, Region and United States, 1991 1/

State	Digestive Problems				
	Dairy				
	Total	1-29 Head	30-49 Head	50-99 Head	100+ Head
	Head				
AL	800	0	0	100	700
AZ	1,900	0	0	0	1,900
AR	700	0	0	300	400
CA	6,000		0		6,000
CO	200	0	0		200
CT	100	0	0	0	100
DE	100	0	0		100
FL	600	0	0		600
GA	1,700	0	0	100	1,600
HI	100	0	0	0	100
ID	900			100	800
IL	500	100	100	100	200
IN	1,100		200	400	500
IA	1,500	100	200	300	900
KS	100				100
KY	2,100		400	500	1,200
LA	1,300	0	0	400	900
ME	200	0	0		200
MD	600	0		200	400
MA	100	0			100
MI	2,500		100	600	1,800
MN	1,800	200	400	600	600
MS	700	0	0	200	500
MO	1,000	0	0	300	700
MT	400		0	100	300
NE	100		0		100
NV	100	0	0	0	100
NH		0	0		
NJ	100	0	0		100
NM	1,100	0	0		1,100
NY	4,600		200	1,200	3,200
NC	200		0	0	200
ND	500		100	100	300
OH	1,700	100	200	600	800
OK	1,800	0	0	100	1,700
OR	2,000	0			2,000
PA	2,800	100	400	700	1,600
RI	0	0	0	0	0
SC	1,100	0	0	100	1,000
SD	500		100		400
TN	1,700	0	0	400	1,300
TX	4,000	0	100	100	3,800
UT	800		100	200	500
VT	700	0		300	400
VA	1,100	0	0	200	900
WA	2,000	0			2,000
WV	400	0	0	200	200
WI	8,500	300	900	1,900	5,400
WY	100				100
Mt & West	22,600	0	400	700	21,500
Midwest	19,300	800	2,100	5,100	11,300
So & East	21,000	100	1,000	4,600	15,300
US	62,900	900	3,500	10,400	48,100

1/ Totals may not add due to rounding.

Losses of Calves from Digestive Problems, by Class and Total,
by State, Region and United States, 1991

State	Digestive Problems				
	Total Calves	Beef Calves	Dairy Calves	Mixed	Other
	Head				
AL	5,500	2,200	2,300	1,000	
AZ	12,000	400	1,700		9,900
AR	6,600	2,800	2,700	900	200
CA	28,900	3,800	22,600	600	1,900
CO	11,200	6,800	2,700	800	900
CT	900		900		
DE	300	0	300		
FL	7,800	1,100	4,700	2,000	0
GA	9,500	2,700	6,400	300	100
HI	200		200		0
ID	13,000	6,500	5,300	600	600
IL	12,500	5,000	5,300	1,000	1,200
IN	9,200	1,600	5,100	100	2,400
IA	28,300	13,600	8,800	2,600	3,300
KS	10,000	5,000	1,800	600	2,600
KY	21,700	8,200	6,700	5,200	1,600
LA	9,300	2,100	6,500	700	0
ME	1,400		1,200	200	
MD	3,400	500	2,700	200	
MA	900		800	100	0
MI	20,600	1,000	17,600	300	1,700
MN	43,200	7,200	27,800	2,100	6,100
MS	4,600	1,500	2,100	700	300
MO	39,000	15,700	15,000	7,400	900
MT	16,200	14,700	700	700	100
NE	27,200	22,600	2,700	1,000	900
NV	3,400	2,300	1,100		
NH	600		600		
NJ	1,000		900	100	
NM	2,500	600	1,600	0	300
NY	32,800	700	29,600	1,900	600
NC	8,100	1,300	5,300	1,100	400
ND	18,500	10,700	3,500	3,800	500
OH	19,800	2,300	10,100	400	7,000
OK	20,300	9,900	7,800	1,800	800
OR	10,800	6,200	3,200	1,400	
PA	23,100	1,300	19,600	900	1,300
RI	1/				0
SC	4,700	400	3,300	900	100
SD	33,400	25,500	4,400	2,500	1,000
TN	24,200	6,800	13,100	2,800	1,500
TX	31,900	7,400	20,500	3,100	900
UT	8,100	3,300	3,300	1,500	
VT	5,500	100	5,100	200	100
VA	16,300	4,700	6,200	5,200	200
WA	11,100	2,200	8,000	100	800
WV	5,100	1,500	2,800	800	0
WI	69,200	1,600	47,000	3,300	17,300
WY	7,300	6,200	200	400	500
Mt & West	266,000	134,100	91,300	18,900	21,700
Midwest	248,400	50,800	139,400	18,100	40,100
So & East	186,800	35,100	121,100	24,300	6,300
US	701,200	220,000	351,800	61,300	68,100

1/ Totals may not add due to rounding.

Losses of Beef Calves from Digestive Problems, by Size Group, State, Region and United States, 1991 1/

State	Digestive Problems			
	Beef			
	Total	1-49 Head	50-99 Head	100+ Head
	Head			
AL	2,200	200	200	1,800
AZ	400			400
AR	2,800	500	800	1,500
CA	3,800	300	100	3,400
CO	6,800	400	200	6,200
CT			0	0
DE	0	0	0	0
FL	1,100	100	100	900
GA	2,700	500	300	1,900
HI		0	0	
ID	6,500	500	600	5,400
IL	5,000	1,800	1,400	1,800
IN	1,600	600	300	700
IA	13,600	5,700	3,700	4,200
KS	5,000	500	1,300	3,200
KY	8,200	3,000	2,600	2,600
LA	2,100	400	400	1,300
ME		0	0	
MD	500	200		300
MA				0
MI	1,000	700	100	200
MN	7,200	3,400	1,900	1,900
MS	1,500	200	200	1,100
MO	15,700	2,700	2,700	10,300
MT	14,700	200	600	13,900
NE	22,600	1,600	2,100	18,900
NV	2,300			2,300
NH			0	0
NJ			0	0
NM	600	100		500
NY	700	200	200	300
NC	1,300	700	100	500
ND	10,700	900	1,800	8,000
OH	2,300	800	200	1,300
OK	9,900	2,100	1,000	6,800
OR	6,200	400	200	5,600
PA	1,300	800	300	200
RI			0	0
SC	400	100		300
SD	25,500	1,800	2,700	21,000
TN	6,800	2,100	1,900	2,800
TX	7,400	900	700	5,800
UT	3,300	300	400	2,600
VT	100	100	0	0
VA	4,700	400	1,000	3,300
WA	2,200	100	200	1,900
WV	1,500	300	300	900
WI	1,600	600	400	600
WY	6,200	100	300	5,800
Mt & West	134,100	10,200	12,200	111,700
Midwest	50,800	16,800	11,500	22,500
So & East	35,100	9,300	7,600	18,200
US	220,000	36,300	31,300	152,400

1/ Totals may not add due to rounding.

Losses of Dairy Calves from Digestive Problems, by Size Group, State, Region and United States, 1991 1/

State	Digestive Problems				
	Dairy				
	Total	1-29 Head	30-49 Head	50-99 Head	100+ Head
	Head				
AL	2,300	0		300	2,000
AZ	1,700	0	0	0	1,700
AR	2,700	0		900	1,800
CA	22,600			100	22,500
CO	2,700	0	0	200	2,500
CT	900	0			900
DE	300	0	0		300
FL	4,700	0	0		4,700
GA	6,400	0		600	5,800
HI	200	0	0	0	200
ID	5,300	100	200	600	4,400
IL	5,300	100	700	1,800	2,700
IN	5,100	100	900	2,200	1,900
IA	8,800	600	1,500	3,600	3,100
KS	1,800	200		400	1,200
KY	6,700	500	500	2,400	3,300
LA	6,500		100	900	5,500
ME	1,200		100	300	800
MD	2,700		100	700	1,900
MA	800	0		200	600
MI	17,600	200	1,600	5,000	10,800
MN	27,800	1,200	6,400	9,500	10,700
MS	2,100		100	300	1,700
MO	15,000	1,400	700	5,400	7,500
MT	700		0	200	500
NE	2,700	0	100	1,000	1,600
NV	1,100	0	0		1,100
NH	600			100	500
NJ	900			200	700
NM	1,600		0		1,600
NY	29,600	400	1,100	5,800	22,300
NC	5,300	0	100	800	4,400
ND	3,500		300	1,100	2,100
OH	10,100	500	700	2,500	6,400
OK	7,800		100	800	6,900
OR	3,200	0	100	100	3,000
PA	19,600	800	3,300	6,100	9,400
RI		0			
SC	3,300	0	0	200	3,100
SD	4,400	200	1,000	700	2,500
TN	13,100	200	200	2,100	10,600
TX	20,500	0	0	600	19,900
UT	3,300		100	600	2,600
VT	5,100	100	200	2,000	2,800
VA	6,200		200	700	5,300
WA	8,000			400	7,600
WV	2,800		0	500	2,300
WI	47,000	1,900	7,900	18,200	19,000
WY	200	100		100	
Mt & West	91,300	600	1,900	6,900	81,900
Midwest	139,400	6,000	20,400	49,100	63,900
So & East	121,100	2,000	6,000	24,200	88,900
US	351,800	8,600	28,300	80,200	234,700

1/ Totals may not add due to rounding.

Losses of Cattle from Respiratory Problems, by Class and Total, by State, Region and United States, 1991

State	Respiratory Problems				
	Total Cattle	Beef Cattle	Dairy Cattle	Mixed	Other
	Head				
AL	5,500	1,000	400	100	4,000
AZ	5,500	1,500	2,100	0	1,900
AR	6,700	3,100	1,300	100	2,200
CA	17,900	3,500	6,100	100	8,200
CO	27,100	6,200	400	300	20,200
CT	300		300	0	
DE	1/	0		0	
FL	700	200	400	100	0
GA	3,800	1,400	1,600	100	700
HI	200	100	100	0	
ID	3,800	900	600	100	2,200
IL	11,300	3,900	400	100	6,900
IN	2,100	200	400		1,500
IA	25,500	5,400	1,000	300	18,800
KS	34,700	2,200	100		32,400
KY	6,500	2,500	700	200	3,100
LA	2,700	900	1,300	500	
ME	500		200	0	300
MD	800	200	500	100	0
MA	100		100		
MI	8,200	1,600	1,800		4,800
MN	15,000	2,400	2,100	500	10,000
MS	4,600	2,100	600	100	1,800
MO	8,300	1,600	400	300	6,000
MT	4,000	3,200	100	200	500
NE	43,600	6,100	200		37,300
NV	700	200			500
NH	100	0	100	0	0
NJ	100		100	0	
NM	4,700	800	800	0	3,100
NY	6,000	100	5,200	200	500
NC	2,000	800	1,000		200
ND	4,100	1,600	1,300	200	1,000
OH	4,900	600	1,200	100	3,000
OK	16,300	1,900	200	100	14,100
OR	3,500	2,000	1,100		400
PA	2,500	300	1,400	100	700
RI	1/	0		0	0
SC	2,000	300	700	100	900
SD	16,500	4,600	600	200	11,100
TN	4,400	1,900	900	300	1,300
TX	70,000	2,000	600	200	67,200
UT	1,800	700	400	200	500
VT	1,200	100	1,000	100	0
VA	5,800	3,200	900	700	1,000
WA	6,300	800	300		5,200
WV	800	300	200	100	200
WI	13,500	400	8,700	600	3,800
WY	3,100	1,800			1,300
Mt & West	263,800	40,100	15,000	1,600	207,100
Midwest	95,500	19,200	17,300	2,000	57,000
So & East	50,500	15,300	17,600	2,800	14,800
US	409,800	74,600	49,900	6,400	278,900

1/ Less than 100 head for the State, but number is included in U.S. total.
Totals may not add due to rounding.

Losses of Beef Cattle from Respiratory Problems, by Size Group,
State, Region and United States, 1991 1/

State	Respiratory Problems			
	Beef			
	Total	1-49 Head	50-99 Head	100+ Head
	Head			
AL	1,000	100	100	800
AZ	1,500	0		1,500
AR	3,100	700	600	1,800
CA	3,500	800	300	2,400
CO	6,200	500	100	5,600
CT			0	0
DE	0	0	0	0
FL	200			200
GA	1,400	300	400	700
HI	100			100
ID	900	100		800
IL	3,900	1,400	1,700	800
IN	200	100		100
IA	5,400	1,700	1,800	1,900
KS	2,200	700	300	1,200
KY	2,500	900	300	1,300
LA	900	200	300	400
ME			0	
MD	200	100	0	100
MA			0	0
MI	1,600	400	200	1,000
MN	2,400	1,000	600	800
MS	2,100	500	300	1,300
MO	1,600	400	700	500
MT	3,200	100	200	2,900
NE	6,100	400	300	5,400
NV	200			200
NH	0	0	0	0
NJ			0	0
NM	800	100		700
NY	100	100	0	
NC	800	500	100	200
ND	1,600	200	400	1,000
OH	600	300	100	200
OK	1,900	400	300	1,200
OR	2,000	100	100	1,800
PA	300	300		0
RI	0	0	0	0
SC	300		100	200
SD	4,600	500	700	3,400
TN	1,900	300	500	1,100
TX	2,000	100	400	1,500
UT	700	100	100	500
VT	100	100	0	0
VA	3,200	300		2,900
WA	800			800
WV	300	100	100	100
WI	400	300	0	100
WY	1,800	100	100	1,600
Mt & West	40,100	4,200	3,300	32,600
Midwest	19,200	6,300	5,700	7,200
So & East	15,300	3,800	2,200	9,300
US	74,600	14,300	11,200	49,100

1/ Totals may not add due to rounding.

Losses of Dairy Cattle from Respiratory Problems, by Size Group, State, Region and United States, 1991 1/

State	Respiratory Problems				
	Dairy				
	Total	1-29 Head	30-49 Head	50-99 Head	100+ Head
	Head				
AL	400		0	100	300
AZ	2,100	0	0	0	2,100
AR	1,300	0	0	300	1,000
CA	6,100		0		6,100
CO	400	0			400
CT	300	0			300
DE		0	0	0	
FL	400	0	0		400
GA	1,600	0	0		1,600
HI	100	0	0	0	100
ID	600	0		0	600
IL	400		100	100	200
IN	400	0	0	100	300
IA	1,000	100	200	300	400
KS	100				100
KY	700		100	100	500
LA	1,300	0		300	1,000
ME	200	0			200
MD	500	0	0	100	400
MA	100	0	0		100
MI	1,800	100		400	1,300
MN	2,100	100	600	800	600
MS	600	0	0	200	400
MO	400	0		200	200
MT	100		0		100
NE	200			100	100
NV		0	0	0	
NH	100				100
NJ	100				100
NM	800		0	0	800
NY	5,200		300	600	4,300
NC	1,000	0		400	600
ND	1,300	0	100	100	1,100
OH	1,200	100	100	300	700
OK	200		0		200
OR	1,100		0		1,100
PA	1,400	200	200	400	600
RI		0	0		
SC	700	0	0	100	600
SD	600			100	500
TN	900		0		900
TX	600	0			600
UT	400	0		100	300
VT	1,000	0	300	200	500
VA	900	0	100	100	700
WA	300		0		300
WV	200	0	0		200
WI	8,700	200	700	2,700	5,100
WY		0			
Mt & West	15,000	0	100	400	14,500
Midwest	17,300	600	1,700	5,200	9,800
So & East	17,600	200	1,000	2,600	13,800
US	49,900	800	2,800	8,200	38,100

1/ Totals may not add due to rounding.

Losses of Calves from Respiratory Problems, by Class and Total, by State, Region and United States, 1991

State	Respiratory Problems				
	Total Calves	Beef Calves	Dairy Calves	Mixed	Other
	Head				
AL	9,000	3,200	2,400	500	2,900
AZ	16,000	800	2,100		13,100
AR	24,100	4,500	3,700	300	15,600
CA	50,400	8,200	30,700	800	10,700
CO	20,400	11,200	3,300	900	5,000
CT	400		400		
DE	100	0	100		0
FL	5,200	1,800	3,200	200	0
GA	10,700	4,000	4,300	1,200	1,200
HI	500	100	400		
ID	12,700	5,900	4,900	500	1,400
IL	18,800	8,200	4,100	800	5,700
IN	13,500	1,600	4,100	200	7,600
IA	49,900	19,400	12,400	4,000	14,100
KS	40,600	12,000	2,300	1,200	25,100
KY	19,000	8,100	7,000	2,300	1,600
LA	8,800	2,800	5,400	500	100
ME	1,000	100	800		100
MD	1,900	400	1,300	200	
MA	400		400	0	0
MI	19,500	1,800	14,000	300	3,400
MN	45,700	8,500	22,800	2,700	11,700
MS	21,500	12,800	2,400	300	6,000
MO	38,800	17,100	10,500	7,900	3,300
MT	9,500	8,600	500	400	
NE	30,900	22,100	2,800	1,700	4,300
NV	1,600	1,200	200	100	100
NH	700		600	100	
NJ	600	100	500	0	0
NM	11,300	1,200	2,800	0	7,300
NY	26,000	1,300	21,700	1,200	1,800
NC	9,700	2,400	5,600	1,300	400
ND	12,600	6,800	3,100	1,800	900
OH	17,400	2,300	8,500	300	6,300
OK	56,800	28,300	6,300	2,000	20,200
OR	14,900	8,000	4,500	2,200	200
PA	18,800	500	14,000	800	3,500
RI	1/			0	0
SC	5,600	1,400	3,700	400	100
SD	31,300	20,900	5,400	2,400	2,600
TN	24,800	9,900	8,900	3,800	2,200
TX	127,000	26,700	23,900	3,400	73,000
UT	6,100	2,200	2,800	1,000	100
VT	6,800	400	6,100	300	0
VA	21,900	6,600	8,500	5,400	1,400
WA	8,900	1,800	6,200	200	700
WV	4,100	1,700	1,800	600	0
WI	67,200	1,300	35,900	2,500	27,500
WY	5,000	4,400	200	200	200
Mt & West	456,500	170,400	102,400	18,800	164,900
Midwest	294,900	64,700	116,000	19,000	95,200
So & East	197,000	57,500	99,100	19,100	21,300
US	948,400	292,600	317,500	56,900	281,400

1/ Less than 100 head for the State, but number is included in U.S. total.
Totals may not add due to rounding.

Losses of Beef Calves from Respiratory Problems, by Size Group, State, Region and United States, 1991 1/

State	Respiratory Problems			
	Beef			
	Total	1-49 Head	50-99 Head	100+ Head
	Head			
AL	3,200	500	400	2,300
AZ	800			800
AR	4,500	700	800	3,000
CA	8,200	500	1,200	6,500
CO	11,200	1,000	600	9,600
CT			0	0
DE	0	0	0	0
FL	1,800	100		1,700
GA	4,000	900	400	2,700
HI	100	0		100
ID	5,900	600	300	5,000
IL	8,200	3,900	2,300	2,000
IN	1,600	700	100	800
IA	19,400	6,200	5,700	7,500
KS	12,000	3,800	2,000	6,200
KY	8,100	3,400	1,900	2,800
LA	2,800	300	200	2,300
ME	100	100	0	
MD	400	100	100	200
MA				0
MI	1,800	800	700	300
MN	8,500	4,100	2,400	2,000
MS	12,800	300	1,000	11,500
MO	17,100	2,900	5,200	9,000
MT	8,600	200	500	7,900
NE	22,100	2,200	1,600	18,300
NV	1,200			1,200
NH			0	0
NJ	100	100	0	
NM	1,200	100	100	1,000
NY	1,300	800	400	100
NC	2,400	1,200	400	800
ND	6,800	700	1,000	5,100
OH	2,300	900	400	1,000
OK	28,300	7,000	5,400	15,900
OR	8,000	500	500	7,000
PA	500	400	100	
RI			0	0
SC	1,400	200	200	1,000
SD	20,900	2,600	3,000	15,300
TN	9,900	2,800	2,300	4,800
TX	26,700	1,700	2,700	22,300
UT	2,200	300	200	1,700
VT	400	300	100	0
VA	6,600	1,000	1,300	4,300
WA	1,800	100	100	1,600
WV	1,700	200	300	1,200
WI	1,300	1,000	100	200
WY	4,400	100	200	4,100
Mt & West	170,400	21,400	19,400	129,600
Midwest	64,700	21,200	17,700	25,800
So & East	57,500	12,700	9,100	35,700
US	292,600	55,300	46,200	191,100

1/ Totals may not add due to rounding.

Losses of Dairy Calves from Respiratory Problems, by Size Group, State, Region and United States, 1991 1/

State	Respiratory Problems				
	Dairy				
	Total	1-29 Head	30-49 Head	50-99 Head	100+ Head
	Head				
AL	2,400	200		300	1,900
AZ	2,100	0	0	0	2,100
AR	3,700	0		1,600	2,100
CA	30,700			100	30,600
CO	3,300	0	0	100	3,200
CT	400				400
DE	100	0	0		100
FL	3,200	0	0		3,200
GA	4,300	0		300	4,000
HI	400	0	0	0	400
ID	4,900	100	100	400	4,300
IL	4,100	100	600	1,100	2,300
IN	4,100	300	300	1,500	2,000
IA	12,400	1,300	2,500	4,400	4,200
KS	2,300	100	200	500	1,500
KY	7,000	400	800	1,700	4,100
LA	5,400		100	900	4,400
ME	800		100	200	500
MD	1,300		100	400	800
MA	400			100	300
MI	14,000	400	500	4,100	9,000
MN	22,800	1,100	5,600	7,800	8,300
MS	2,400			700	1,700
MO	10,500	300	300	4,400	5,500
MT	500		0	200	300
NE	2,800		300	400	2,100
NV	200		0		200
NH	600			100	500
NJ	500			100	400
NM	2,800	500	0		2,300
NY	21,700	500	900	4,700	15,600
NC	5,600	0	100	800	4,700
ND	3,100	100	400	800	1,800
OH	8,500	300	1,000	2,200	5,000
OK	6,300	100	100	800	5,300
OR	4,500		100	400	4,000
PA	14,000	700	2,400	3,500	7,400
RI					
SC	3,700		0	400	3,300
SD	5,400	600	600	800	3,400
TN	8,900		500	2,500	5,900
TX	23,900	0	100	600	23,200
UT	2,800		100	500	2,200
VT	6,100	100	700	1,300	4,000
VA	8,500	100	300	2,100	6,000
WA	6,200			300	5,900
WV	1,800		0	300	1,500
WI	35,900	1,600	4,000	12,000	18,300
WY	200	100			100
Mt & West	102,400	1,600	2,000	5,900	92,900
Midwest	116,000	5,400	14,800	39,100	56,700
So & East	99,100	2,000	6,000	20,400	70,700
US	317,500	9,000	22,800	65,400	220,300

1/ Totals may not add due to rounding.

Losses of Cattle from Calving Problems, by Class and Total, by State, Region and United States, 1991

State	Calving Problems				
	Total Cattle	Beef Cattle	Dairy Cattle	Mixed	Other
			Head		
AL	6,000	4,300	1,400	300	
AZ	2,800	1,700	1,100	0	
AR	8,400	4,200	3,400	800	
CA	11,600	3,800	7,400	300	100
CO	3,100	1,500	900	100	600
CT	600		600		0
DE	200		200	0	0
FL	5,700	2,000	2,300	1,400	0
GA	7,900	4,100	3,100	700	0
HI	400	300	100		
ID	1,700	700	900		100
IL	2,400	1,500	600	100	200
IN	2,600	1,100	1,100	0	400
IA	5,600	2,500	2,300	300	500
KS	3,900	1,900	700	200	1,100
KY	10,500	6,000	3,200	1,200	100
LA	4,900	1,700	2,700	500	
ME	400		300	100	0
MD	1,300	400	800	100	
MA	200		200		
MI	4,900	400	4,400		100
MN	7,400	1,200	5,700	400	100
MS	6,500	4,000	1,900	300	300
MO	18,400	9,600	5,400	2,900	500
MT	3,000	2,600	100	200	100
NE	7,500	3,900	500	100	3,000
NV	400	300	100	0	0
NH	300		300	0	
NJ	400		400		0
NM	1,800	1,000	700	100	
NY	8,800	100	7,900	600	200
NC	4,600	2,100	2,200	300	
ND	1,500	800	400	300	0
OH	5,700	1,000	4,500	200	0
OK	13,900	8,800	3,800	1,200	100
OR	2,900	1,800	900	200	
PA	5,900	400	5,100	400	
RI	1/				0
SC	3,700	1,400	1,900	400	
SD	5,200	3,700	900	400	200
TN	11,100	5,500	4,500	1,000	100
TX	29,400	16,900	10,100	2,200	200
UT	1,400	700	600	100	
VT	2,500		2,400	100	0
VA	5,100	2,000	2,100	1,000	0
WA	4,700	1,200	3,300	100	100
WV	2,000	900	900	200	
WI	12,600	600	11,300	500	200
WY	1,500	1,500			0
Mt & West	96,700	53,100	32,500	5,500	5,600
Midwest	68,000	22,100	38,700	5,200	2,000
So & East	88,600	34,900	44,400	8,600	700
US	253,300	110,100	115,600	19,300	8,300

1/ Less than 100 head for the State, but number is included in U.S. total.
Totals may not add due to rounding.

Losses of Beef Cattle from Calving Problems, by Size Group, State, Region and United States, 1991 1/

State	Calving Problems			
	Beef			
	Total	1-49 Head	50-99 Head	100+ Head
	Head			
AL	4,300	800	900	2,600
AZ	1,700			1,700
AR	4,200	1,500	1,300	1,400
CA	3,800	200	100	3,500
CO	1,500	100	200	1,200
CT			0	0
DE				0
FL	2,000	100	100	1,800
GA	4,100	1,700	800	1,600
HI	300			300
ID	700		100	600
IL	1,500	700	300	500
IN	1,100	400	100	600
IA	2,500	1,200	500	800
KS	1,900	500	300	1,100
KY	6,000	2,500	1,700	1,800
LA	1,700	400	300	1,000
ME			0	0
MD	400	100	100	200
MA				0
MI	400	300	100	
MN	1,200	600	400	200
MS	4,000	700	600	2,700
MO	9,600	3,100	1,700	4,800
MT	2,600	100	100	2,400
NE	3,900	400	200	3,300
NV	300			300
NH			0	0
NJ				0
NM	1,000	100	100	800
NY	100	100		0
NC	2,100	800	500	800
ND	800	200	300	300
OH	1,000	500	100	400
OK	8,800	2,300	1,700	4,800
OR	1,800	200	100	1,500
PA	400	300	100	0
RI			0	0
SC	1,400	300	400	700
SD	3,700	400	400	2,900
TN	5,500	2,000	1,400	2,100
TX	16,900	1,500	1,900	13,500
UT	700	100	100	500
VT			0	0
VA	2,000	500	300	1,200
WA	1,200	100	100	1,000
WV	900	400	400	100
WI	600	400	100	100
WY	1,500	100	100	1,300
Mt & West	53,100	6,300	5,800	41,000
Midwest	22,100	8,700	4,600	8,800
So & East	34,900	10,700	7,600	16,600
US	110,100	25,700	18,000	66,400

1/ Totals may not add due to rounding.

Losses of Dairy Cattle from Calving Problems, by Size Group, State, Region and United States, 1991 1/

State	Calving Problems				
	Dairy				
	Total	1-29 Head	30-49 Head	50-99 Head	100+ Head
	Head				
AL	1,400	0		100	1,300
AZ	1,100	0	0	0	1,100
AR	3,400		100	1,300	2,000
CA	7,400	0		100	7,300
CO	900	0		100	800
CT	600	100			500
DE	200	0	0	0	200
FL	2,300	0	0		2,300
GA	3,100	0	0	400	2,700
HI	100	0	0	0	100
ID	900			100	800
IL	600	100		200	300
IN	1,100	100	100	400	500
IA	2,300	100	300	700	1,200
KS	700	100		200	400
KY	3,200	200	400	1,300	1,300
LA	2,700			600	2,100
ME	300		100	100	100
MD	800		100	100	600
MA	200	0			200
MI	4,400	200	300	1,000	2,900
MN	5,700	400	1,400	2,100	1,800
MS	1,900	100		400	1,400
MO	5,400	600	200	1,400	3,200
MT	100	0	0		100
NE	500	0	100	100	300
NV	100	0	0	0	100
NH	300		100	100	100
NJ	400	0		100	300
NM	700	0	0	0	700
NY	7,900	100	400	2,400	5,000
NC	2,200	0	100	200	1,900
ND	400		100	100	200
OH	4,500	200	600	1,600	2,100
OK	3,800	200		600	3,000
OR	900	0	0	100	800
PA	5,100	200	800	1,700	2,400
RI					
SC	1,900	0	0	200	1,700
SD	900	100	100	200	500
TN	4,500	100	200	1,100	3,100
TX	10,100	100		600	9,400
UT	600	0		200	400
VT	2,400	0	200	500	1,700
VA	2,100		100	300	1,700
WA	3,300		0	200	3,100
WV	900	0		300	600
WI	11,300	700	2,100	3,400	5,100
WY		0	0		
Mt & West	32,500	500	300	2,600	29,100
Midwest	38,700	2,400	5,100	12,100	19,100
So & East	44,400	800	2,500	9,900	31,200
US	115,600	3,700	7,900	24,600	79,400

1/ Totals may not add due to rounding.

Losses of Calves from Calving Problems, by Class and Total, by State, Region and United States, 1991

State	Calving Problems				
	Total Calves	Beef Calves	Dairy Calves	Mixed	Other
	Head				
AL	5,600	4,600	800	200	
AZ	500	200	300	0	
AR	5,800	4,100	1,400	300	
CA	11,600	4,500	6,900	200	
CO	8,200	5,800	2,300		100
CT	200		200	0	
DE	200		200	0	0
FL	5,100	3,700	1,300	100	0
GA	6,800	3,600	2,400	800	0
HI	600	500	100		0
ID	6,700	4,800	1,600	200	100
IL	9,500	7,800	1,400	200	100
IN	4,200	2,500	1,600		100
IA	22,500	16,900	4,200	1,100	300
KS	7,000	5,600	800	400	200
KY	13,000	8,700	3,200	1,000	100
LA	3,600	2,000	1,000	600	0
ME	600	100	500		0
MD	1,200	400	600	200	
MA	200		200	0	0
MI	6,300	1,200	5,000	100	
MN	10,000	5,100	3,700	1,100	100
MS	5,100	3,400	1,400	100	200
MO	20,500	16,100	2,900	1,300	200
MT	13,400	12,300	200	700	200
NE	17,000	16,300	300	200	200
NV	700	600	100		0
NH	400		300	100	0
NJ	200	100	100		
NM	4,000	1,200	2,500	300	
NY	8,800	300	8,300	200	
NC	3,800	2,500	1,200	100	
ND	11,200	9,300	800	900	200
OH	6,800	2,000	4,300	400	100
OK	18,100	14,100	2,600	1,300	100
OR	10,600	9,000	1,300	200	100
PA	6,000	700	4,700	600	
RI	1/			0	0
SC	4,800	2,200	2,100	400	100
SD	16,500	14,300	1,500	600	100
TN	8,600	6,800	1,700	100	0
TX	26,100	18,600	6,600	700	200
UT	4,900	2,600	1,700	600	
VT	3,200		2,900	300	0
VA	12,400	7,100	3,200	1,900	200
WA	2,400	1,000	1,300	100	
WV	5,400	3,900	900	500	100
WI	10,500	700	9,500	200	100
WY	6,000	5,900		100	
Mt & West	165,500	126,600	30,900	6,500	1,500
Midwest	96,100	56,400	34,000	4,700	1,000
So & East	95,200	50,100	37,200	7,200	700
US	356,800	233,100	102,100	18,400	3,200

1/ Less than 100 head for the State, but number is included in U.S. total. Totals may not add due to rounding.

Losses of Beef Calves from Calving Problems, by Size Group, State, Region and United States, 1991 1/

State	Calving Problems			
	Beef			
	Total	1-49 Head	50-99 Head	100+ Head
	Head			
AL	4,600	1,300	900	2,400
AZ	200			200
AR	4,100	1,600	800	1,700
CA	4,500	200	100	4,200
CO	5,800	500	600	4,700
CT			0	0
DE				0
FL	3,700	200	200	3,300
GA	3,600	1,100	700	1,800
HI	500		100	400
ID	4,800	300	600	3,900
IL	7,800	3,700	1,800	2,300
IN	2,500	1,100	400	1,000
IA	16,900	6,000	4,200	6,700
KS	5,600	1,200	1,200	3,200
KY	8,700	4,200	3,200	1,300
LA	2,000	400	700	900
ME	100	100	0	0
MD	400	200	100	100
MA			0	
MI	1,200	700	500	0
MN	5,100	2,300	1,400	1,400
MS	3,400	800	600	2,000
MO	16,100	5,400	3,700	7,000
MT	12,300	400	700	11,200
NE	16,300	1,900	1,600	12,800
NV	600	100		500
NH			0	0
NJ	100		100	0
NM	1,200	200	200	800
NY	300	200	100	
NC	2,500	1,100	700	700
ND	9,300	900	2,400	6,000
OH	2,000	1,100	300	600
OK	14,100	3,900	2,700	7,500
OR	9,000	500	400	8,100
PA	700	700	0	0
RI			0	0
SC	2,200	900	700	600
SD	14,300	1,900	2,400	10,000
TN	6,800	3,400	1,400	2,000
TX	18,600	1,700	1,900	15,000
UT	2,600	200	300	2,100
VT			0	0
VA	7,100	2,100	2,100	2,900
WA	1,000	100	200	700
WV	3,900	2,100	900	900
WI	700	400	200	100
WY	5,900	200	200	5,500
Mt & West	126,600	14,200	15,600	96,800
Midwest	56,400	22,300	13,300	20,800
So & East	50,100	18,800	12,400	18,900
US	233,100	55,300	41,300	136,500

1/ Totals may not add due to rounding.

Losses of Dairy Calves from Calving Problems, by Size Group, State, Region and United States, 1991 1/

State	Calving Problems				
	Dairy				
	Total	1-29 Head	30-49 Head	50-99 Head	100+ Head
	Head				
AL	800	0		200	600
AZ	300	0	0	0	300
AR	1,400		100	900	400
CA	6,900				6,900
CO	2,300			200	2,100
CT	200		0	0	200
DE	200	0	0	0	200
FL	1,300	0			1,300
GA	2,400	0	0		2,400
HI	100	0	0	0	100
ID	1,600		100	100	1,400
IL	1,400	100	300	500	500
IN	1,600	100	200	800	500
IA	4,200	200	900	800	2,300
KS	800	100		200	500
KY	3,200	100	200	1,500	1,400
LA	1,000		0	200	800
ME	500		100	200	200
MD	600			300	300
MA	200	0		100	100
MI	5,000	200	300	1,200	3,300
MN	3,700	700	1,100	1,600	300
MS	1,400			700	700
MO	2,900	200	100	1,400	1,200
MT	200		0	100	100
NE	300	100		100	100
NV	100	0	0	0	100
NH	300	0	0	100	200
NJ	100	0			100
NM	2,500	0	0	0	2,500
NY	8,300	100	600	1,700	5,900
NC	1,200	0	0	400	800
ND	800		100	400	300
OH	4,300	300	200	1,400	2,400
OK	2,600	100	200	900	1,400
OR	1,300	0		100	1,200
PA	4,700	400	1,000	1,000	2,300
RI					0
SC	2,100	0	0	100	2,000
SD	1,500		400	200	900
TN	1,700	300	100	600	700
TX	6,600	100		200	6,300
UT	1,700	0		300	1,400
VT	2,900	0	600	900	1,400
VA	3,200		100	800	2,300
WA	1,300	0	0	100	1,200
WV	900	0	0	200	700
WI	9,500	300	2,100	2,700	4,400
WY		0	0	0	
Mt & West	30,900	400	800	2,900	26,800
Midwest	34,000	2,100	5,300	11,300	15,300
So & East	37,200	900	2,700	9,000	24,600
US	102,100	3,400	8,800	23,200	66,700

1/ Totals may not add due to rounding.

Losses of Cattle from Weather, by Class and Total, by State, Region and United States, 1991

State	Weather				
	Total Cattle	Beef Cattle	Dairy Cattle	Mixed	Other
	Head				
AL	1,200	1,000	100	0	100
AZ	3,200	2,900	200	100	
AR	2,600	1,200	600	700	100
CA	2,400	600	1,700	0	100
CO	2,500	1,400	100	100	900
CT	0	0	0	0	0
DE	0	0	0	0	0
FL	3,600	2,000	1,600		0
GA	1,200	800	100	200	100
HI	400	400		0	0
ID	500	200	100		200
IL	1,100	700	100		300
IN	2,000	500	1,400		100
IA	8,700	5,000	600	300	2,800
KS	9,700	1,200	100	100	8,300
KY	3,000	1,500	800	500	200
LA	1,400	1,200	100	100	0
ME	1/	0		0	0
MD	300		300		
MA	1/			0	0
MI	700	100	400	0	200
MN	2,100	900	600	100	500
MS	1,500	1,200	200		100
MO	11,200	6,300	2,800	1,400	700
MT	3,600	3,400		100	100
NE	8,500	2,400	100		6,000
NV	300	300		0	0
NH	1/			0	0
NJ	1/	0		0	0
NM	1,400	500	600	100	200
NY	800	100	400	200	100
NC	600	500	100	0	
ND	3,200	2,500	100	400	200
OH	500	200	200		100
OK	8,800	2,800	700	100	5,200
OR	600	400	0	200	0
PA	800	100	600	100	0
RI	0	0	0	0	0
SC	600	100	400		100
SD	8,700	7,100	300	300	1,000
TN	1,000	900	100		
TX	9,400	3,700	1,200	100	4,400
UT	500	300	100		100
VT	300		300	0	0
VA	1,400	900	300	100	100
WA	1,200	200	500	100	400
WV	200	100	100		
WI	1,700	100	1,300	300	
WY	2,600	2,500			100
Mt & West	67,600	32,800	5,800	1,800	27,200
Midwest	30,600	15,000	8,000	2,800	4,800
So & East	18,100	10,400	5,500	1,300	900
US	116,300	58,200	19,300	5,900	32,900

1/ Less than 100 head for the State, but number is included in U.S. total. Totals may not add due to rounding.

Losses of Beef Cattle from Weather, by Size Group, State, Region and United States, 1991 1/

State	Weather			
	Beef			
	Total	1-49 Head	50-99 Head	100+ Head
	Head			
AL	1,000	300	200	500
AZ	2,900			2,900
AR	1,200	200	400	600
CA	600		100	500
CO	1,400	100	300	1,000
CT	0	0	0	0
DE	0	0	0	0
FL	2,000		100	1,900
GA	800	300	100	400
HI	400		0	400
ID	200	0		200
IL	700	500	100	100
IN	500	400		100
IA	5,000	1,600	900	2,500
KS	1,200	200	300	700
KY	1,500	500	800	200
LA	1,200	300	100	800
ME	0	0	0	0
MD			0	
MA			0	0
MI	100	100	0	
MN	900	500	300	100
MS	1,200	100	200	900
MO	6,300	1,400	2,400	2,500
MT	3,400	100	200	3,100
NE	2,400	200	200	2,000
NV	300	0	0	300
NH			0	0
NJ	0	0	0	0
NM	500		100	400
NY	100		100	
NC	500	100	100	300
ND	2,500	300	400	1,800
OH	200	200		0
OK	2,800	1,100	600	1,100
OR	400	100		300
PA	100	100	0	0
RI	0	0	0	0
SC	100			100
SD	7,100	700	1,000	5,400
TN	900	400	200	300
TX	3,700	400	300	3,000
UT	300	100		200
VT			0	0
VA	900	100	300	500
WA	200			200
WV	100			100
WI	100	100		0
WY	2,500	100	200	2,200
Mt & West	32,800	3,400	3,700	25,700
Midwest	15,000	5,000	4,100	5,900
So & East	10,400	2,200	2,200	6,000
US	58,200	10,600	10,000	37,600

1/ Totals may not add due to rounding.

Losses of Dairy Cattle from Weather, by Size Group, State, Region and United States, 1991 1/

State	Weather				
	Dairy				
	Total	1-29 Head	30-49 Head	50-99 Head	100+ Head
	Head				
AL	100	0	0	0	100
AZ	200	0	0	0	200
AR	600			300	300
CA	1,700	0	0		1,700
CO	100	0	0		100
CT	0	0	0	0	0
DE	0	0	0	0	0
FL	1,600	0	0		1,600
GA	100	0	0		100
HI		0	0	0	
ID	100	0	0		100
IL	100			100	
IN	1,400	0	0	100	1,300
IA	600		100	200	300
KS	100	0			100
KY	800	0	100	100	600
LA	100	0	0		100
ME		0	0	0	
MD	300	0			300
MA		0	0	0	
MI	400		100	100	200
MN	600	200	100	200	100
MS	200	0	0		200
MO	2,800	100	400	700	1,600
MT		0	0		0
NE	100	0			100
NV		0	0	0	
NH		0	0		
NJ		0	0		
NM	600	0	0	0	600
NY	400	0		200	200
NC	100	0	0	100	
ND	100		0	0	100
OH	200			100	100
OK	700		100	100	500
OR	0	0	0	0	0
PA	600	0	100		500
RI	0	0	0	0	0
SC	400	0	0		400
SD	300	0		100	200
TN	100	0	0		100
TX	1,200	0	0		1,200
UT	100	0		0	100
VT	300			100	200
VA	300	0	0	100	200
WA	500	0		0	500
WV	100	0	0		100
WI	1,300	100	300	500	400
WY		0	0	0	
Mt & West	5,800	0	100	200	5,500
Midwest	8,000	400	1,000	2,300	4,300
So & East	5,500	0	200	600	4,700
US	19,300	400	1,300	3,100	14,500

1/ Totals may not add due to rounding.

Losses of Calves from Weather, by Class and Total, by State, Region and United States, 1991

State	Weather: Total Calves	Weather: Beef Calves	Weather: Dairy Calves	Weather: Mixed	Weather: Other
	Head				
AL	700	600	100	0	0
AZ	5,000	500	200		4,300
AR	1,600	1,200	400		
CA	5,400	1,700	3,500	100	100
CO	3,600	2,800	400		400
CT	0	0	0	0	0
DE	1/	0		0	0
FL	1,500	1,300	100	100	
GA	1,300	900	200	100	100
HI	600	600		0	0
ID	4,500	3,900	500		100
IL	3,500	3,300	100		100
IN	1,200	700	100	0	400
IA	14,200	12,300	400	500	1,000
KS	5,100	2,300	100	100	2,600
KY	3,600	3,300	300	0	0
LA	1,400	1,300	100		
ME	1/			0	0
MD	500	100	400		0
MA	100	100		0	0
MI	1,100	600	400	0	100
MN	4,300	3,500	300	300	200
MS	1,300	900	100	300	
MO	12,800	11,200	1,000	500	100
MT	9,000	8,700	200	100	
NE	8,800	7,500	500	200	600
NV	900	900		0	0
NH	0	0	0	0	0
NJ	1/	0		0	0
NM	4,900	1,200	3,100	500	100
NY	1,300	200	900	100	100
NC	1,200	1,200		0	0
ND	3,700	3,400	100	200	
OH	900	600	100		200
OK	12,100	8,900	2,300	300	600
OR	2,100	1,700	100	300	0
PA	500	200	200		100
RI	1/			0	0
SC	200	200			0
SD	13,700	13,100	300	200	100
TN	2,400	1,900	400	100	
TX	14,100	7,600	4,200	800	1,500
UT	2,200	1,400	300	400	100
VT	400	0	300	100	0
VA	3,400	2,200	600	500	100
WA	1,500	500	800	200	0
WV	900	500	400	0	
WI	1,200	400	300	400	100
WY	8,700	8,300		200	200
Mt & West	105,900	75,000	16,600	3,600	10,700
Midwest	40,800	33,800	3,100	1,700	2,200
So & East	20,900	14,900	4,200	1,400	400
US	167,600	123,700	23,900	6,700	13,300

1/ Less than 100 head for the State, but number is included in U.S. total.
Totals may not add due to rounding.

Losses of Beef Calves from Weather, by Size Group, State, Region and United States, 1991 1/

State	Weather			
	Beef			
	Total	1-49 Head	50-99 Head	100+ Head
	Head			
AL	600	200	100	300
AZ	500			500
AR	1,200	400	400	400
CA	1,700	100	200	1,400
CO	2,800	300	200	2,300
CT	0	0	0	0
DE	0	0	0	0
FL	1,300	100		1,200
GA	900	200	100	600
HI	600		0	600
ID	3,900	100	200	3,600
IL	3,300	1,400	1,100	800
IN	700	400	100	200
IA	12,300	3,400	2,900	6,000
KS	2,300	600	700	1,000
KY	3,300	1,900	800	600
LA	1,300	300	300	700
ME			0	0
MD	100	100		
MA	100		100	0
MI	600	200	300	100
MN	3,500	2,100	600	800
MS	900	200	100	600
MO	11,200	2,200	2,400	6,600
MT	8,700	300	400	8,000
NE	7,500	1,000	500	6,000
NV	900		0	900
NH	0	0	0	0
NJ	0	0	0	0
NM	1,200	100	100	1,000
NY	200	100	100	0
NC	1,200	300	200	700
ND	3,400	600	600	2,200
OH	600	500	100	
OK	8,900	2,100	1,800	5,000
OR	1,700		100	1,600
PA	200	200		0
RI			0	0
SC	200	100		100
SD	13,100	1,100	2,000	10,000
TN	1,900	600	200	1,100
TX	7,600	800	800	6,000
UT	1,400	300		1,100
VT	0	0	0	0
VA	2,200	500	500	1,200
WA	500	100	100	300
WV	500	200	200	100
WI	400	200	100	100
WY	8,300	200	400	7,700
Mt & West	75,000	7,700	8,100	59,200
Midwest	33,800	10,800	8,000	15,000
So & East	14,900	5,000	2,700	7,200
US	123,700	23,500	18,800	81,400

1/ Totals may not add due to rounding.

Losses of Dairy Calves from Weather, by Size Group, State, Region and United States, 1991 1/

State	Weather				
	Dairy				
	Total	1-29 Head	30-49 Head	50-99 Head	100+ Head
	Head				
AL	100	0	0		100
AZ	200	0	0	0	200
AR	400	0		100	300
CA	3,500	0		0	3,500
CO	400	0			400
CT	0	0	0	0	0
DE		0	0	0	
FL	100	0	0	0	100
GA	200	0	0	0	200
HI		0	0	0	
ID	500		100		400
IL	100				100
IN	100	0	0	0	100
IA	400		100	100	200
KS	100	0	0	0	100
KY	300		100	100	100
LA	100	0	0	0	100
ME		0	0		
MD	400	0	0		400
MA		0			
MI	400	0	100	100	200
MN	300	100	100	100	
MS	100	0	0	0	100
MO	1,000	300	100	100	500
MT	200	100	0	0	100
NE	500	100	100		300
NV		0	0	0	
NH	0	0	0	0	0
NJ		0	0	0	
NM	3,100	0	0	0	3,100
NY	900		200	200	500
NC		0	0	0	
ND	100	0	0		100
OH	100	100		0	0
OK	2,300	0	0	200	2,100
OR	100	0		100	
PA	200	0		0	200
RI		0		0	0
SC		0	0	0	
SD	300	0	0		300
TN	400	0	0	200	200
TX	4,200	0	0	0	4,200
UT	300	0		100	200
VT	300	0		100	200
VA	600	0		0	600
WA	800	0	0		800
WV	400	0	0	100	300
WI	300	0		200	100
WY		0	0		
Mt & West	16,600	200	200	400	15,800
Midwest	3,100	500	400	700	1,500
So & East	4,200		300	700	3,200
US	23,900	700	900	1,800	20,500

1/ Totals may not add due to rounding.

Losses of Cattle from Poison, by Class and Total,
by State, Region and United States, 1991

State	Poison				
	Total Cattle	Beef Cattle	Dairy Cattle	Mixed	Other
	Head				
AL	700	600	100		0
AZ	1,200	1,200		0	
AR	1,400	1,200	100	100	
CA	1,100	1,000	100		0
CO	5,100	4,100		0	1,000
CT	0	0	0	0	0
DE	0	0	0	0	0
FL	200	200		0	0
GA	1,900	1,500	200	100	100
HI	1/	0	0	0	
ID	1,800	1,700	100	0	
IL	600	400	100	0	100
IN	600	100	400	100	
IA	500	300	100	0	100
KS	1,900	1,000	100		800
KY	2,300	1,500	400		400
LA	300	100	100	100	
ME	1/	0		0	0
MD	100	0	100	0	0
MA	1/			0	0
MI	200	100	100	0	
MN	500	100	300	100	0
MS	1,200	800	300	0	100
MO	2,400	1,600	500	300	0
MT	1,400	1,200	0	100	100
NE	1,200	900	100	100	100
NV	500	500	0		0
NH	0	0	0	0	0
NJ	1/	0		0	
NM	700	700		0	
NY	200		200	0	0
NC	200	200		0	0
ND	300	300	0	0	
OH	600	300	200	100	
OK	3,700	2,300	1,400	0	
OR	1,400	1,300	100	0	
PA	400	100	300		0
RI	0	0	0	0	0
SC	200	100	100		0
SD	2,000	2,000			0
TN	2,000	1,700	200	100	0
TX	4,100	2,600	800	500	200
UT	700	600	100	0	0
VT	400	0	400		0
VA	1,100	500	300	300	
WA	300	300			0
WV	300	100	0	100	100
WI	1,000		1,000	0	
WY	2,600	2,500			100
Mt & West	30,100	24,200	2,900	700	2,300
Midwest	7,800	4,100	2,800	700	200
So & East	11,600	7,400	2,700	700	800
US	49,500	35,700	8,400	2,100	3,300

1/ Less than 100 head for the State, but number is included in U.S. total.
Totals may not add due to rounding.

Losses of Beef Cattle from Poison, by Size Group, State, Region and United States, 1991 1/

State	Poison			
	Beef			
	Total	1-49 Head	50-99 Head	100+ Head
	Head			
AL	600	200	200	200
AZ	1,200	0		1,200
AR	1,200	200	300	700
CA	1,000			1,000
CO	4,100	100	100	3,900
CT	0	0	0	0
DE	0	0	0	0
FL	200		200	
GA	1,500	300	600	600
HI	0	0	0	0
ID	1,700			1,700
IL	400	300		100
IN	100	100		
IA	300	200		100
KS	1,000	200	100	700
KY	1,500	700	700	100
LA	100			100
ME	0	0	0	0
MD	0	0	0	0
MA		0		0
MI	100		100	0
MN	100	100		
MS	800	100	200	500
MO	1,600	200	600	800
MT	1,200			1,200
NE	900	100	200	600
NV	500		0	500
NH	0	0	0	0
NJ	0	0	0	0
NM	700		100	600
NY			0	0
NC	200	100	100	0
ND	300			300
OH	300	200	0	100
OK	2,300	500	600	1,200
OR	1,300			1,300
PA	100	100		0
RI	0	0	0	0
SC	100		100	0
SD	2,000	100	100	1,800
TN	1,700	400	300	1,000
TX	2,600	500	200	1,900
UT	600		100	500
VT	0	0	0	0
VA	500	100	100	300
WA	300	0	0	300
WV	100	100		
WI			0	0
WY	2,500	100		2,400
Mt & West	24,200	1,600	1,500	21,100
Midwest	4,100	1,300	1,000	1,800
So & East	7,400	2,100	2,500	2,800
US	35,700	5,000	5,000	25,700

1/ Totals may not add due to rounding.

Losses of Dairy Cattle from Poison, by Size Group, State, Region and United States, 1991 1/

State	Poison				
	Dairy				
	Total	1-29 Head	30-49 Head	50-99 Head	100+ Head
	Head				
AL	100	0	0		100
AZ		0	0	0	
AR	100	0	0	0	100
CA	100	0	0	0	100
CO		0	0	0	
CT	0	0	0	0	0
DE	0	0	0	0	0
FL		0	0	0	
GA	200	0	0	100	100
HI	0	0	0	0	0
ID	100	0	100	0	
IL	100	0	0	100	
IN	400	0		200	200
IA	100	0	0	100	0
KS	100	0	0		100
KY	400	200	100	100	0
LA	100	0	0	100	
ME		0			0
MD	100	0	0		100
MA		0			
MI	100	0	0		100
MN	300		0	300	0
MS	300	0		100	200
MO	500	0	0	100	400
MT	0	0	0	0	0
NE	100	0	0	0	100
NV	0	0	0	0	0
NH	0	0	0	0	0
NJ		0	0		
NM		0	0		0
NY	200		0	100	100
NC		0	0	0	
ND	0	0	0	0	0
OH	200		0	200	
OK	1,400	0	0	0	1,400
OR	100	0	0	0	100
PA	300			200	100
RI	0	0	0	0	0
SC	100	0	0	0	100
SD				0	0
TN	200	0	100		100
TX	800	0	0	0	800
UT	100	0		0	100
VT	400	0		0	400
VA	300		0	100	200
WA		0	0		
WV	0	0	0	0	0
WI	1,000	100			900
WY		0	0	0	
Mt & West	2,900	0	100	0	2,800
Midwest	2,800	100	0	1,000	1,700
So & East	2,700	200	200	800	1,500
US	8,400	300	300	1,800	6,000

1/ Totals may not add due to rounding.

Losses of Calves from Poison, by Class and Total, by State, Region and United States, 1991

State	Poison				
	Total Calves	Beef Calves	Dairy Calves	Mixed	Other
	Head				
AL	300	300		0	0
AZ	100	100	0	0	0
AR	200	200	0	0	
CA	200		200		0
CO	900	700	100	0	100
CT	1/		0	0	0
DE	0	0	0	0	0
FL	200	200	0	0	0
GA	600	400	200		
HI	1/	0	0	0	
ID	500	400	100	0	0
IL	600	400	100	0	100
IN	200	100	0	0	100
IA	1,100	300	200	0	600
KS	300	300	0		
KY	700	400	200		100
LA	200	200	0	0	0
ME	0	0	0	0	0
MD	0	0	0	0	0
MA	1/	0		0	0
MI	200		200	0	0
MN	500	100			400
MS	200	200		0	0
MO	1,000	1,000	0	0	0
MT	400	400		0	0
NE	200	100	100	0	
NV	200	200	0	0	0
NH	0	0	0	0	0
NJ	1/	0		0	0
NM	400	400	0	0	0
NY	300		300	0	0
NC	200	100	100	0	0
ND	500	500	0		0
OH	200		200	0	0
OK	2,300	2,200	100	0	0
OR	400	400	0	0	
PA	500	0	400	100	0
RI	0	0	0	0	0
SC	600	100	500	0	0
SD	300	300	0	0	0
TN	1,100	500	600	0	0
TX	2,100	1,400	600	100	
UT	300	300		0	0
VT	200	0		200	0
VA	400	400	0		0
WA	100	100		0	0
WV	200	200		0	0
WI	300	100	100	0	100
WY	700	500	100	0	100
Mt & West	10,000	8,300	1,400	100	200
Midwest	4,300	2,200	800	0	1,300
So & East	5,800	3,000	2,400	300	100
US	20,100	13,500	4,600	400	1,600

1/ Less than 100 head for the State, but number is included in U.S. total.
Totals may not add due to rounding.

Losses of Beef Calves from Poison, by Size Group, State, Region and United States, 1991 1/

State	Poison			
	Beef			
	Total	1-49 Head	50-99 Head	100+ Head
	Head			
AL	300	100	100	100
AZ	100	0		100
AR	200	100	100	0
CA		0	0	
CO	700		100	600
CT			0	0
DE	0	0	0	0
FL	200		100	100
GA	400		200	200
HI	0	0	0	0
ID	400	100		300
IL	400	200	200	
IN	100	100	0	0
IA	300	200	100	
KS	300	100		200
KY	400	400	0	
LA	200	100	100	0
ME	0	0	0	0
MD	0	0	0	0
MA	0	0	0	0
MI				0
MN	100		100	0
MS	200		100	100
MO	1,000	100	300	600
MT	400			400
NE	100			100
NV	200		0	200
NH	0	0	0	0
NJ	0	0	0	0
NM	400		100	300
NY			0	0
NC	100	100	0	0
ND	500		100	400
OH			0	0
OK	2,200	100	100	2,000
OR	400			400
PA	0	0	0	0
RI	0	0	0	0
SC	100	100	0	0
SD	300	0	0	300
TN	500	200	200	100
TX	1,400	300	200	900
UT	300			300
VT	0	0	0	0
VA	400	100	100	200
WA	100		0	100
WV	200		200	0
WI	100	100	0	0
WY	500	100		400
Mt & West	8,300	700	600	7,000
Midwest	2,200	800	800	600
So & East	3,000	1,100	1,100	800
US	13,500	2,600	2,500	8,400

1/ Totals may not add due to rounding.

Losses of Dairy Calves from Poison, by Size Group, State, Region and United States, 1991 1/

State	Poison				
	Dairy				
	Total	1-29 Head	30-49 Head	50-99 Head	100+ Head
	Head				
AL		0	0	0	
AZ	0	0	0	0	0
AR	0	0	0	0	0
CA	200	0	0	0	200
CO	100	0	0		100
CT	0	0	0	0	0
DE	0	0	0	0	0
FL	0	0	0	0	0
GA	200	0	0	200	
HI	0	0	0	0	0
ID	100	0	0	0	100
IL	100	0			100
IN	0	0	0	0	0
IA	200	0	100	100	0
KS	0	0	0	0	0
KY	200	0	200	0	0
LA	0	0	0	0	0
ME	0	0	0	0	0
MD	0	0	0	0	0
MA		0	0	0	
MI	200	0	0	200	0
MN		0	0		0
MS		0	0	0	
MO	0	0	0	0	0
MT		0	0		0
NE	100	0	0	0	100
NV	0	0	0	0	0
NH	0	0	0	0	0
NJ		0	0		
NM	0	0	0	0	0
NY	300	100	0		200
NC	100	0	0	100	0
ND	0	0	0	0	0
OH	200	0	200	0	0
OK	100	0	100	0	0
OR	0	0	0	0	0
PA	400	0	0	400	0
RI	0	0	0	0	0
SC	500	0	0	0	500
SD	0	0	0	0	0
TN	600	0	0	0	600
TX	600	0	0	0	600
UT		0	0	0	
VT		0	0	0	
VA	0	0	0	0	0
WA			0	0	0
WV		0	0		
WI	100	0	0	100	0
WY	100	0	0	100	0
Mt & West	1,400	0	100	100	1,200
Midwest	800	0	300	400	100
So & East	2,400	100	200	700	1,400
US	4,600	100	600	1,200	2,700

1/ Totals may not add due to rounding.

Losses of Cattle from Theft, by Class and Total, by State, Region and United States, 1991

State	Theft				
	Total Cattle	Beef Cattle	Dairy Cattle	Mixed	Other
	Head				
AL	100		100	0	0
AZ	100	100	0	0	0
AR	500	500		0	0
CA	500	200	100	100	100
CO	400	400	0	0	0
CT	0	0	0	0	0
DE	0	0	0	0	0
FL	200	100	100	0	0
GA	0	0	0	0	0
HI	100	100	0		0
ID	300	300	0		
IL	100		0	0	100
IN	100	0	100	0	0
IA	400	200	100	0	100
KS	800	500	0		300
KY	300	200	100	0	0
LA	700	700	0	0	0
ME	0	0	0	0	0
MD	100	100		0	0
MA	1/	0		0	0
MI	0	0	0	0	0
MN	0	0	0	0	0
MS	200	200	0	0	0
MO	1,300	1,100	200	0	0
MT	300	300	0		0
NE	200	100			100
NV	1/		0	0	0
NH	0	0	0	0	0
NJ	0	0	0	0	0
NM	300	200	100	0	0
NY	1/	0		0	0
NC	0	0	0	0	0
ND	300	300	0	0	0
OH	300	300		0	0
OK	4,600	4,200		0	400
OR	0	0	0	0	0
PA	0	0	0	0	0
RI	0	0	0	0	0
SC	0	0	0	0	0
SD	700	600	0	0	100
TN	600	300	100	200	
TX	900	800		0	100
UT	100	100	0		0
VT	0	0	0	0	0
VA	200	200		0	0
WA	600	600			
WV	1/			0	0
WI	100		100	0	
WY	500	500	0		
Mt & West	10,800	9,300	300	100	1,100
Midwest	2,800	2,100	500	0	200
So & East	2,600	1,800	600	200	0
US	16,200	13,200	1,400	300	1,300

1/ Less than 100 head for the State, but number is included in U.S. total.
Totals may not add due to rounding.

Losses of Beef Cattle from Theft, by Size Group, State, Region and United States, 1991 1/

State	Theft			
	Beef			
	Total	1-49 Head	50-99 Head	100+ Head
	Head			
AL				0
AZ	100	0	0	100
AR	500	0	100	400
CA	200			200
CO	400	0		400
CT	0	0	0	0
DE	0	0	0	0
FL	100			100
GA	0	0	0	0
HI	100		0	100
ID	300			300
IL				
IN	0	0	0	0
IA	200		100	100
KS	500	100	100	300
KY	200	0	100	100
LA	700	0	600	100
ME	0	0	0	0
MD	100	0	100	0
MA	0	0	0	0
MI	0	0	0	0
MN	0	0	0	0
MS	200	100		100
MO	1,100	100	100	900
MT	300	0		300
NE	100			100
NV		0	0	
NH	0	0	0	0
NJ	0	0	0	0
NM	200			200
NY	0	0	0	0
NC	0	0	0	0
ND	300	0		300
OH	300	300	0	0
OK	4,200	1,900	200	2,100
OR	0	0	0	0
PA	0	0	0	0
RI	0	0	0	0
SC	0	0	0	0
SD	600	0	100	500
TN	300	100	200	0
TX	800	100	200	500
UT	100	0	0	100
VT	0	0	0	0
VA	200	0	0	200
WA	600			600
WV			0	0
WI		0		0
WY	500		0	500
Mt & West	9,300	2,100	600	6,600
Midwest	2,100	400	300	1,400
So & East	1,800	200	1,000	600
US	13,200	2,700	1,900	8,600

1/ Totals may not add due to rounding.

Losses of Dairy Cattle from Theft, by Size Group, State, Region and United States, 1991 1/

State	Theft				
	Dairy				
	Total	1-29 Head	30-49 Head	50-99 Head	100+ Head
	Head				
AL	100	0	0	0	100
AZ	0	0	0	0	0
AR		0	0	0	
CA	100	0	0	0	100
CO	0	0	0	0	0
CT	0	0	0	0	0
DE	0	0	0	0	0
FL	100	0	0	0	100
GA	0	0	0	0	0
HI	0	0	0	0	0
ID	0	0	0	0	0
IL	0	0	0	0	0
IN	100	0	0	0	100
IA	100	0	0	100	0
KS	0	0	0	0	0
KY	100	0	100		0
LA	0	0	0	0	0
ME	0	0	0	0	0
MD		0		0	
MA		0	0	0	
MI	0	0	0	0	0
MN	0	0	0	0	0
MS	0	0	0	0	0
MO	200	0	0	0	200
MT	0	0	0	0	0
NE		0	0		0
NV	0	0	0	0	0
NH	0	0	0	0	0
NJ	0	0	0	0	0
NM	100	0	0	0	100
NY		0	0		
NC	0	0	0	0	0
ND	0	0	0	0	0
OH		0	0	0	
OK		0	0	0	
OR	0	0	0	0	0
PA	0	0	0	0	0
RI	0	0	0	0	0
SC	0	0	0	0	0
SD	0	0	0	0	0
TN	100	0	0	0	100
TX		0	0	0	
UT	0	0	0	0	0
VT	0	0	0	0	0
VA		0	0	0	
WA		0	0	0	
WV		0	0	0	
WI	100	0	0		100
WY	0	0	0	0	0
Mt & West	300	0	0	0	300
Midwest	500	0	0	100	400
So & East	600	0	100	0	500
US	1,400	0	100	100	1,200

1/ Totals may not add due to rounding.

Losses of Calves from Theft, by Class and Total, by State, Region and United States, 1991

State	Theft				
	Total Calves	Beef Calves	Dairy Calves	Mixed	Other
	Head				
AL	100	100		0	0
AZ	100	100		0	0
AR	200	200		0	0
CA	1,200	300	800	100	0
CO	400	400		0	0
CT	1/	0		0	0
DE	0	0	0	0	0
FL	300	200	100	0	0
GA	200	100	100	0	0
HI	1/		0	0	0
ID	500	500		0	
IL	100	100	0	0	0
IN	0	0	0	0	0
IA	400	200	200	0	
KS	400	200	0	100	100
KY	400	400	0	0	0
LA	200	200	0	0	0
ME	0	0	0	0	0
MD	100	100		0	0
MA	1/	0		0	0
MI	0	0	0	0	0
MN	200	100		0	100
MS	200	100	100	0	0
MO	200	200	0	0	0
MT	400	400	0		0
NE	200	200	0		
NV	100	100	0	0	0
NH	0	0	0	0	0
NJ	0	0	0	0	0
NM	300	200	100	0	0
NY	200	0	200	0	0
NC	100	100	0		0
ND	500	500	0	0	0
OH	100	100		0	0
OK	3,700	2,400	0	200	1,100
OR	200	200	0	0	0
PA	0	0	0	0	0
RI	0	0	0	0	0
SC	100	0	100	0	0
SD	900	800		0	100
TN	900	800	0	100	0
TX	1,500	1,300	100		100
UT	200	200			0
VT	0	0	0	0	0
VA	100	100	0	0	0
WA	300	300	0		0
WV	100	100	0	0	0
WI	800		100	0	700
WY	300	300	0	0	0
Mt & West	11,300	8,400	1,000	500	1,400
Midwest	2,000	900	300	0	800
So & East	3,100	2,300	700	100	0
US	16,400	11,600	2,000	600	2,200

1/ Less than 100 head for the State, but number is included in U.S. total. Totals may not add due to rounding.

Losses of Beef Calves from Theft, by Size Group, State, Region and United States, 1991 1/

State	Theft			
	Beef			
	Total	1-49 Head	50-99 Head	100+ Head
	Head			
AL	100	100	0	0
AZ	100		0	100
AR	200	0		200
CA	300		0	300
CO	400	100		300
CT	0	0	0	0
DE	0	0	0	0
FL	200	0		200
GA	100	0		100
HI		0	0	
ID	500			500
IL	100	100		0
IN	0	0	0	0
IA	200		100	100
KS	200	100		100
KY	400	100	0	300
LA	200	0	100	100
ME	0	0	0	0
MD	100		100	0
MA	0	0	0	0
MI	0	0	0	0
MN	100	0		100
MS	100	0	100	0
MO	200	200	0	0
MT	400		0	400
NE	200	100		100
NV	100	0	0	100
NH	0	0	0	0
NJ	0	0	0	0
NM	200	100		100
NY	0	0	0	0
NC	100	100		
ND	500		100	400
OH	100	100		0
OK	2,400	300	500	1,600
OR	200		100	100
PA	0	0	0	0
RI	0	0	0	0
SC	0	0	0	0
SD	800	0		800
TN	800	400	200	200
TX	1,300	800	100	400
UT	200			200
VT	0	0	0	0
VA	100		0	100
WA	300	0	0	300
WV	100		0	100
WI			0	0
WY	300		0	300
Mt & West	8,400	1,500	800	6,100
Midwest	900	400	100	400
So & East	2,300	700	500	1,100
US	11,600	2,600	1,400	7,600

1/ Totals may not add due to rounding.

Losses of Dairy Calves from Theft, by Size Group, State, Region and United States, 1991 1/

State	Theft				
	Dairy				
	Total	1-29 Head	30-49 Head	50-99 Head	100+ Head
	Head				
AL		0	0	0	
AZ		0	0	0	
AR		0	0	0	
CA	800	0	0	0	800
CO		0	0	0	
CT		0	0	0	
DE	0	0	0	0	0
FL	100	0	0	0	100
GA	100	0	0	0	100
HI	0	0	0	0	0
ID		0	0	0	
IL	0	0	0	0	0
IN	0	0	0	0	0
IA	200	0	0	200	
KS	0	0	0	0	0
KY	0	0	0	0	0
LA	0	0	0	0	0
ME	0	0	0	0	0
MD		0	0		0
MA		0	0	0	
MI	0	0	0	0	0
MN		0		0	0
MS	100	0	0		100
MO	0	0	0	0	0
MT	0	0	0	0	0
NE	0	0	0	0	0
NV	0	0	0	0	0
NH	0	0	0	0	0
NJ	0	0	0	0	0
NM	100	0	0	0	100
NY	200	0	0		200
NC	0	0	0	0	0
ND	0	0	0	0	0
OH			0	0	0
OK	0	0	0	0	0
OR	0	0	0	0	0
PA	0	0	0	0	0
RI	0	0	0	0	0
SC	100	0	0	0	100
SD		0	0	0	
TN	0	0	0	0	0
TX	100	0	0	0	100
UT		0	0	0	
VT	0	0	0		0
VA	0	0	0	0	0
WA	0	0	0	0	0
WV	0	0	0	0	0
WI	100	0	0	0	100
WY	0	0	0	0	0
Mt & West	1,000	0	0	0	1,000
Midwest	300	0	0	200	100
So & East	700	0	0	0	700
US	2,000	0	0	200	1,800

1/ Totals may not add due to rounding.

Losses of Cattle from Other and Unknown Causes, by Class and Total, by State, Region and United States, 1991

State	Other and Unknown				
	Total Cattle	Beef Cattle	Dairy Cattle	Mixed	Other
	Head				
AL	7,600	4,700	2,300	500	100
AZ	5,200	2,600	2,100	100	400
AR	7,900	5,500	1,700	600	100
CA	28,400	7,900	12,300	1,200	7,000
CO	10,000	2,000	1,200	100	6,700
CT	1,000		1,000		
DE	600		500		100
FL	10,200	7,100	2,700	300	100
GA	13,100	5,000	7,600	500	
HI	1,600	1,200	300		100
ID	10,800	3,500	3,000	100	4,200
IL	5,600	2,500	1,100	200	1,800
IN	5,700	3,400	1,700	200	400
IA	14,000	5,700	1,600	400	6,300
KS	9,200	3,300	500	300	5,100
KY	16,500	8,200	5,600	1,600	1,100
LA	6,300	3,200	2,800	300	0
ME	700	100	600		0
MD	1,500	200	1,200	100	
MA	500		500		0
MI	6,300	400	3,600		2,300
MN	14,000	2,900	6,200	500	4,400
MS	8,100	4,800	2,800	300	200
MO	23,500	13,600	5,400	2,600	1,900
MT	9,400	8,500	200	500	200
NE	9,400	4,400	300	100	4,600
NV	3,600	2,600	900	100	
NH	500		500		
NJ	300		300		0
NM	7,100	3,700	3,000	200	200
NY	11,900	200	11,200	200	300
NC	8,700	3,100	4,000	700	900
ND	6,300	3,400	1,500	1,200	200
OH	9,700	1,300	5,500	100	2,800
OK	26,800	9,500	1,500	900	14,900
OR	11,800	7,600	3,200	700	300
PA	15,000	1,100	12,900	500	500
RI	1/	0		0	0
SC	3,000	900	2,000	100	
SD	13,500	9,000	1,700	800	2,000
TN	12,300	6,800	4,000	1,300	200
TX	104,600	73,800	20,700	2,900	7,200
UT	4,300	2,200	1,600	500	
VT	5,700	100	5,400	200	
VA	10,000	3,400	3,800	2,400	400
WA	8,600	1,900	5,800	300	600
WV	3,500	1,700	1,400	300	100
WI	26,000	1,700	22,000	1,400	900
WY	2,400	2,200		100	100
Mt & West	273,000	149,300	59,800	10,100	53,800
Midwest	112,700	37,000	48,800	6,000	20,900
So & East	137,000	50,600	73,100	9,300	4,000
US	522,700	236,900	181,700	25,400	78,700

1/ Less than 100 head for the State, but number is included in U.S. total.
Totals may not add due to rounding.

Losses of Beef Cattle from Other and Unknown Causes, by Size Group, State, Region and United States, 1991 1/

State	Other and Unknown			
	Beef			
	Total	1-49 Head	50-99 Head	100+ Head
	Head			
AL	4,700	1,200	800	2,700
AZ	2,600		100	2,500
AR	5,500	1,400	1,700	2,400
CA	7,900	300	200	7,400
CO	2,000	300	100	1,600
CT			0	0
DE		0		
FL	7,100	200	100	6,800
GA	5,000	1,400	1,100	2,500
HI	1,200			1,200
ID	3,500	200	300	3,000
IL	2,500	1,200	600	700
IN	3,400	400	200	2,800
IA	5,700	2,200	1,700	1,800
KS	3,300	800	600	1,900
KY	8,200	4,200	2,100	1,900
LA	3,200	500	700	2,000
ME	100		100	
MD	200	100		100
MA				
MI	400	200	100	100
MN	2,900	1,200	1,000	700
MS	4,800	1,200	1,200	2,400
MO	13,600	3,700	2,800	7,100
MT	8,500	300	700	7,500
NE	4,400	900	300	3,200
NV	2,600		100	2,500
NH			0	0
NJ				0
NM	3,700	300	300	3,100
NY	200	200		
NC	3,100	900	400	1,800
ND	3,400	500	1,000	1,900
OH	1,300	800	100	400
OK	9,500	2,200	2,400	4,900
OR	7,600	400	200	7,000
PA	1,100	600	100	400
RI	0	0	0	0
SC	900	400	100	400
SD	9,000	1,600	1,800	5,600
TN	6,800	2,900	1,900	2,000
TX	73,800	3,600	4,800	65,400
UT	2,200	200	300	1,700
VT	100	100		
VA	3,400	700	1,100	1,600
WA	1,900	300	100	1,500
WV	1,700	600	400	700
WI	1,700	600	300	800
WY	2,200	100	200	1,900
Mt & West	149,300	12,000	13,500	123,800
Midwest	37,000	11,700	8,500	16,800
So & East	50,600	15,200	10,100	25,300
US	236,900	38,900	32,100	165,900

1/ Totals may not add due to rounding.

Losses of Dairy Cattle from Other and Unknown Causes, by Size Group, State, Region and United States, 1991 1/

State	Other and Unknown				
	Dairy				
	Total	1-29 Head	30-49 Head	50-99 Head	100+ Head
	Head				
AL	2,300		0	200	2,100
AZ	2,100	0	0	0	2,100
AR	1,700			900	800
CA	12,300	0			12,300
CO	1,200			100	1,100
CT	1,000		0	100	900
DE	500		0		500
FL	2,700	0		100	2,600
GA	7,600	0		400	7,200
HI	300	0	0	0	300
ID	3,000		100	300	2,600
IL	1,100		200	400	500
IN	1,700		300	700	700
IA	1,600	200	300	500	600
KS	500			200	300
KY	5,600	300	700	1,500	3,100
LA	2,800			500	2,300
ME	600			200	400
MD	1,200	0	100	200	900
MA	500			200	300
MI	3,600	200	400	1,200	1,800
MN	6,200	300	1,400	2,000	2,500
MS	2,800	100		600	2,100
MO	5,400	200	400	1,900	2,900
MT	200		0		200
NE	300			200	100
NV	900		0		900
NH	500			100	400
NJ	300			100	200
NM	3,000	0	0		3,000
NY	11,200	100	600	3,300	7,200
NC	4,000	0	100	700	3,200
ND	1,500	0	200	800	500
OH	5,500	300	300	1,300	3,600
OK	1,500		100	200	1,200
OR	3,200			300	2,900
PA	12,900	400	1,500	4,000	7,000
RI		0			0
SC	2,000	0	0	100	1,900
SD	1,700	100	200	300	1,100
TN	4,000	100	200	800	2,900
TX	20,700		400	500	19,800
UT	1,600	0		200	1,400
VT	5,400		900	1,100	3,400
VA	3,800		100	600	3,100
WA	5,800		0	300	5,500
WV	1,400	0		200	1,200
WI	22,000	1,300	3,600	9,100	8,000
WY			0		
Mt & West	59,800	100	1,000	3,400	55,300
Midwest	48,800	2,500	6,900	18,000	21,400
So & East	73,100	1,000	4,200	15,000	52,900
US	181,700	3,600	12,100	36,400	129,600

1/ Totals may not add due to rounding.

Losses of Calves from Other and Unknown Causes, by Class and Total, by State, Region and United States, 1991 1/

State	Other and Unknown				
	Total Calves	Beef Calves	Dairy Calves	Mixed	Other
			Head		
AL	6,700	4,900	1,700		100
AZ	3,000	300	200	0	2,500
AR	5,100	3,900	700	300	200
CA	38,000	4,600	17,300	900	15,200
CO	12,400	10,200	1,100	800	300
CT	400		400		
DE	400		400		
FL	14,000	7,800	4,100	2,000	100
GA	9,600	5,300	3,900	300	100
HI	1,800	900	400	500	
ID	10,900	5,200	5,200	100	400
IL	8,200	4,300	2,900	600	400
IN	6,200	3,400	1,700	0	1,100
IA	12,000	7,100	3,100	1,000	800
KS	5,500	4,500	500		500
KY	23,300	15,300	5,900	1,400	700
LA	5,100	3,000	1,800	300	
ME	900	200	600	100	
MD	900	200	500	200	
MA	300		300		
MI	7,200	800	6,200	100	100
MN	14,700	5,200	6,700	1,000	1,800
MS	4,500	3,000	1,200	100	200
MO	20,900	16,200	2,900	1,200	600
MT	9,300	7,500		300	1,500
NE	3,900	3,200	100	300	300
NV	6,500	5,500	1,000	0	
NH	300		300		
NJ	100		100	0	0
NM	7,800	3,900	3,800	100	
NY	15,400	1,000	13,500	500	400
NC	7,200	3,300	3,800	100	
ND	4,600	3,800	300	400	100
OH	4,700	1,000	2,200	100	1,400
OK	24,700	20,400	2,700	400	1,200
OR	11,500	9,500	1,800	100	100
PA	15,900	2,200	7,300	300	6,100
RI	100		100	0	0
SC	2,400	1,200	1,000	200	0
SD	10,200	7,900	1,600	500	200
TN	13,900	9,800	3,100	800	200
TX	73,900	48,000	22,200	1,600	2,100
UT	5,200	3,500	1,200	400	100
VT	3,700	200	3,500	0	0
VA	8,900	5,600	2,300	900	100
WA	4,900	1,600	3,000	200	100
WV	2,000	1,700	100	100	100
WI	35,400	2,100	14,600	700	18,000
WY	800	700		100	0
Mt & West	234,900	141,200	62,400	6,700	24,600
Midwest	114,400	44,000	41,000	5,000	24,400
So & East	136,000	64,700	55,900	7,300	8,100
US	485,300	249,900	159,300	19,000	57,100

1/ Totals may not add due to rounding.

Losses of Beef Calves from Other and Unknown Causes, by Size Group, State, Region and United States, 1991 1/

State	Other and Unknown			
	Beef			
	Total	1-49 Head	50-99 Head	100+ Head
	Head			
AL	4,900	1,400	1,100	2,400
AZ	300			300
AR	3,900	1,700	1,000	1,200
CA	4,600	200	300	4,100
CO	10,200	300	600	9,300
CT			0	0
DE			0	0
FL	7,800	200	300	7,300
GA	5,300	1,600	900	2,800
HI	900			900
ID	5,200	300	500	4,400
IL	4,300	2,200	800	1,300
IN	3,400	700	200	2,500
IA	7,100	3,100	2,200	1,800
KS	4,500	900	800	2,800
KY	15,300	7,100	3,600	4,600
LA	3,000	600	500	1,900
ME	200	100	100	
MD	200	200		
MA			0	0
MI	800	600	200	0
MN	5,200	2,900	900	1,400
MS	3,000	800	500	1,700
MO	16,200	4,700	3,900	7,600
MT	7,500	200	500	6,800
NE	3,200	1,000	400	1,800
NV	5,500	100		5,400
NH				0
NJ			0	0
NM	3,900	300	300	3,300
NY	1,000	200	800	
NC	3,300	1,400	600	1,300
ND	3,800	700	800	2,300
OH	1,000	700	0	300
OK	20,400	5,600	4,500	10,300
OR	9,500	600	200	8,700
PA	2,200	1,100	100	1,000
RI			0	0
SC	1,200	400	300	500
SD	7,900	1,100	1,400	5,400
TN	9,800	5,500	2,400	1,900
TX	48,000	3,900	4,300	39,800
UT	3,500	600	500	2,400
VT	200			200
VA	5,600	1,800	1,100	2,700
WA	1,600	200	100	1,300
WV	1,700	1,000	200	500
WI	2,100	800	200	1,100
WY	700	100	100	500
Mt & West	141,200	16,100	15,300	109,800
Midwest	44,000	17,400	9,400	17,200
So & East	64,700	23,400	12,500	28,800
US	249,900	56,900	37,200	155,800

1/ Totals may not add due to rounding.

Losses of Dairy Calves from Other and Unknown Causes, by Size Group, State, Region and United States, 1991 1/

State	Other and Unknown				
	Dairy				
	Total	1-29 Head	30-49 Head	50-99 Head	100+ Head
	Head				
AL	1,700		0	100	1,600
AZ	200	0	0	0	200
AR	700	0	100		600
CA	17,300	0	0		17,300
CO	1,100	100	0		1,000
CT	400		0	100	300
DE	400	0	0		400
FL	4,100	0	0	100	4,000
GA	3,900	0	0	200	3,700
HI	400	0	0	0	400
ID	5,200	100	100	300	4,700
IL	2,900		100	1,000	1,800
IN	1,700	100	100	600	900
IA	3,100	200	900	800	1,200
KS	500			100	400
KY	5,900	300	900	1,700	3,000
LA	1,800	0		300	1,500
ME	600			100	500
MD	500				500
MA	300			200	100
MI	6,200	100	200	900	5,000
MN	6,700	800	1,400	2,000	2,500
MS	1,200		100	200	900
MO	2,900	400	300	1,300	900
MT		0	0	0	
NE	100	0	0		100
NV	1,000	0	0	0	1,000
NH	300		100	100	100
NJ	100	0			100
NM	3,800		0	100	3,700
NY	13,500	100	900	4,400	8,100
NC	3,800	0	0	200	3,600
ND	300				300
OH	2,200	100	200	1,000	900
OK	2,700	0	0	200	2,500
OR	1,800			200	1,600
PA	7,300	300	1,400	2,000	3,600
RI	100				100
SC	1,000	0	0	0	1,000
SD	1,600	100	300	200	1,000
TN	3,100	200	200	400	2,300
TX	22,200	100	100	300	21,700
UT	1,200	0		400	800
VT	3,500	100	600	600	2,200
VA	2,300	0	200	1,000	1,100
WA	3,000	1,000		100	1,900
WV	100	0	0	100	
WI	14,600	800	2,900	4,500	6,400
WY			0	0	
Mt & West	62,400	1,400	500	1,900	58,600
Midwest	41,000	2,500	6,200	12,100	20,200
So & East	55,900	1,000	4,400	11,800	38,700
US	159,300	4,900	11,100	25,800	117,500

1/ Totals may not add due to rounding.

Losses of Cattle and Calves from Coyotes and Dogs, By State, Region and United States, 1991

State	Coyotes		Dogs	
	Cattle	Calves	Cattle	Calves
	Head			
AL	100	1,600	100	500
AZ	1,300	1,900	1/	100
AR	1/	300	1/	900
CA	200	2,600	200	1,000
CO	100	2,500	0	200
CT	0	1/	0	0
DE	0	0	0	0
FL	1/	200	200	500
GA	100	600	200	600
HI	0	0	0	200
ID	1/	900	0	1/
IL	0	1,400	0	200
IN	1/	400	1/	1/
IA	200	1,200	1/	100
KS	100	800	1/	100
KY	100	1,800	100	1,400
LA	100	1,200	0	100
ME	0	1/	0	0
MD	0	0	0	0
MA	1/	1/	0	0
MI	0	0	1/	100
MN	0	400	1/	100
MS	1/	1,300	1/	200
MO	200	1,000	200	600
MT	1/	1,200	1/	100
NE	100	1,700	1/	1/
NV	1/	1,500	0	100
NH	0	1/	0	0
NJ	0	0	0	0
NM	200	1,000	0	100
NY	1/	200	0	0
NC	0	0	200	600
ND	100	1,300	0	0
OH	0	1/	0	1/
OK	1,300	4,000	200	2,300
OR	1/	4,000	1/	100
PA	0	1/	0	100
RI	0	0	0	0
SC	0	0	0	600
SD	100	3,500	1/	200
TN	0	1,400	300	2,100
TX	1,000	16,200	300	2,300
UT	1/	800	0	1/
VT	1/	200	1/	0
VA	0	700	200	800
WA	100	700	1/	100
WV	0	1/	1/	100
WI	0	300	0	100
WY	1/	1,000	1/	1/
Mt & West	4,900	45,600	900	7,000
Midwest	400	5,000	500	2,200
So & East	600	9,400	1,400	7,600
US	5,900	60,000	2,800	16,800

1/ Less than 100 head for the region but number is included in U.S. total. Totals may not add due to rounding.

Losses of Cattle and Calves from Mountain Lions and Bobcats, by Selected States, Region, and United States, 1991

State	Mountain Lions and Bobcats	
	Cattle	Calves
AZ	200	1,200
CA	400	600
CO	1/	100
ID	1/	100
KS	1/	100
MO	0	1/
MT	100	200
NM	0	200
OK	0	100
OR	0	300
TX	800	1,700
UT	1/	1/
WA	1/	0
WY	100	100
Mt & West	1,600	4,700
Midwest	0	1/
So & East	0	0
US	1,600	4,700

1/ Less than 100 head for the State, but number is included in U.S. total. Totals may not add due to rounding.

Losses of Cattle and Calves from Bears and Wolves, By Region and United States, 1991

State	Bears		Wolves
	Cattle	Calves	Cattle & Calves
Mt & West	900	900	1,400
Midwest	0	100	300
So & East	0	0	1/
US	900	1,000	1,800

1/ Less than 100 head for the region but number is included in U.S. total. Totals may not add due to rounding.

Losses of Cattle and Calves from Other Predators, By State, Region and United States, 1991

State	All Other Predators	
	Cattle	Calves
AL	100	0
AZ	1/	1/
AR	200	200
CA	300	1/
CO	1/	1/
CT	0	0
DE	1/	1/
FL	100	200
GA	100	100
HI	1/	1/
ID	1/	100
IL	200	200
IN	1/	1/
IA	1/	300
KS	1/	100
KY	100	100
LA	1/	100
ME	1/	0
MD	100	0
MA	0	1/
MI	1/	0
MN	1/	500
MS	200	100
MO	100	100
MT	1/	100
NE	1/	1/
NV	1/	0
NH	0	0
NJ	1/	0
NM	0	1/
NY	0	0
NC	100	100
ND	100	100
OH	0	0
OK	300	500
OR	100	1/
PA	100	1/
RI	0	0
SC	0	0
SD	100	0
TN	100	600
TX	900	2,100
UT	0	1/
VT	1/	0
VA	600	100
WA	1/	0
WV	1/	1/
WI	0	0
WY	0	1/
Mt & West	2,200	3,500
Midwest	600	1,300
So & East	1,800	1,500
US	4,600	6,300

1/ Less than 100 head for the State, but number is included in U.S. total. Totals may not add due to rounding.

Reliability of Cattle and Calf Death Loss Estimates

Survey Procedures: A random sample of U.S. producers was surveyed to provide data for these estimates. Survey procedures ensured that all cattle producers, regardless of size, had a chance to be included in the survey to collect death loss by cause. Large producers were sampled more heavily than small operations. Data were collected from about 77,000 operators during the first half of January by mail, telephone, and face-to-face personal interviews. Regardless of when operators responded, they were asked to report death losses for cattle and calves for the 1991 calendar year.

Based on the original data collected during January, a report titled Cattle and Calves Death Loss was issued in May 1992. This report contained data for death loss by cause including predator losses and losses from other causes. These estimates were on a State and Regional level.

Data in the above mentioned report as well as an additional summary from the original data provided data for this report. The original data was resummarized to break the losses out by class and size group. The classes included beef cattle, dairy cattle, mixed and other cattle operations. These classes are further defined in the following terms and definitions section. Size groups included 1-49 head, 50-99 head and 100+ head for beef cattle and 1-29 head, 30-49 head, 50-99 head and 100+ head for dairy cattle. No size group breakdowns are included for other or mixed operations.

Estimating Procedures: These estimates of total death losses and death loss by cause were prepared by the Livestock, Dairy and Poultry Branch, NASS. Total cattle and calf death losses from all causes were published in the 1991 Meat Animals Production, Disposition, and Income report, released April 13, 1992. Breakdowns by cause of loss and value of loss were published in the Cattle and Calves Death Loss report issued in May 1992. Class estimates for this report were compiled by using the unexpanded percent each class was of the total loss by cause. This percent was then applied to the total death loss published in the May 1992 report. Once the beef and dairy totals were derived, the same procedure was used to estimate size groups within each of these two classes. All published loss estimates were rounded to the nearest 100 head. State estimates of less than 100 head are not published, but are included in U.S. totals. Throughout the report, a blank entry indicates that some deaths were reported for that category but the number is less than 50 head. A zero indicates no deaths were reported for that category. This does not imply that there were no deaths, simply that none were reported for this survey. The sample used for this report was not specifically selected to derive the breakdowns within. However, the estimates in this report should be a good indication of where the death losses occur and which classes and/or size groups are most impacted from the various death loss causes. States were combined into regions when sampling variability did not permit individual State data to be released.

Reliability: Since all cattle operators are not included in the sample, survey estimates are subject to sampling variability. Survey results are also subject to non-sampling errors such as omissions, duplications, and mistakes in reporting, recording, and processing the data. The effects of these non-sampling errors cannot be measured directly. They are minimized through rigid quality controls in the data collection process and through a careful review of all reported data for consistency and reasonableness.

Terms and Definitions:

Beef Cattle: On individual questionnaires, if beef cows were greater than milk cows and milk cows were less than 5 head, the operation was classified in this report as a beef cattle operation.

Cattle: Includes all cows, bulls, steers, and heifers weighing over 500 pounds. This includes beef and milk breeds as well as cattle on feed.

Calves: Beef and milk breed steers, heifers, and bulls weighing less than 500 pounds.

Dairy Cattle: On individual questionnaires, if milk cows were greater than beef cows and beef cows were less than 5 head, the operation was classified in this report as a dairy cattle operation.

Mixed: On individual questionnaires, if beef cows were greater than 5 head and milk cows were greater than 5 head, the operation was classified in this report as a mixed operation.

Other: On individual questionnaires, if an operation did not meet the criteria for a beef cattle, dairy cattle, or mixed operation, the operation was classified in this report as other.

Regions: Mountain and Western:

Arizona	Kansas	North Dakota	Texas
California	Montana	Oklahoma	Utah
Colorado	Nebraska	Oregon	Washington
Hawaii	Nevada	South Dakota	Wyoming
Idaho	New Mexico		

Midwest:

Arkansas	Iowa	Minnesota	Ohio
Illinois	Michigan	Missouri	Wisconsin
Indiana			

Southern and Eastern:

Alabama	Louisiana	New Jersey	Tennessee
Connecticut	Maine	New York	Vermont
Delaware	Maryland	North Carolina	Virginia
Florida	Massachusetts	Pennsylvania	West Virginia
Georgia	Mississippi	Rhode Island	
Kentucky	New Hampshire	South Carolina	

Digestive Problems: Includes bloat, scours, parasites, etc.

Respiratory Problems: Includes shipping fever, pneumonia, etc.

Poison: Includes nitrate poisoning and noxious feeds or weeds.

Mountain Lions and Bobcats: Includes cougars, pumas and lynx.

Index

	Page
Losses of Beef Calves from All Causes, by Size Group, State, Region and U.S.	7
Losses of Beef Calves from Calving Problems, by Size Group, State, Region and U.S.	25
Losses of Beef Calves from Digestive Problems, by Size Group, State, Region and U.S.	13
Losses of Beef Calves from Other and Unknown Causes, by Size Group, State, Region and U.S.	49
Losses of Beef Calves from Poison, by Size Group, State, Region and U.S.	37
Losses of Beef Calves from Respiratory Problems, by Size Group, State, Region and U.S.	19
Losses of Beef Calves from Theft, by Size Group, State, Region and U.S.	43
Losses of Beef Calves from Weather, by Size Group, State, Region and U.S.	31
Losses of Beef Cattle from All Causes, by Size Group, State, Region and U.S.	4
Losses of Beef Cattle from Calving Problems, by Size Group, State, Region and U.S.	22
Losses of Beef Cattle from Digestive Problems, by Size Group, State, Region and U.S.	10
Losses of Beef Cattle from Other and Unknown Causes, by Size Group, State, Region and U.S.	46
Losses of Beef Cattle from Poison, by Size Group, State, Region and U.S.	34
Losses of Beef Cattle from Respiratory Problems, by Size Group, State, Region and U.S.	16
Losses of Beef Cattle from Theft, by Size Group, State, Region and U.S.	40
Losses of Beef Cattle from Weather, by Size Group, State, Region and U.S.	28
Losses of Calves from All Causes, by Size Group, State, Region and U.S.	6

-- continued

Index (continued)

	Page
Losses of Calves from Calving Problems, by Size Group, State, Region and U.S.	24
Losses of Calves from Digestive Problems, by Size Group, State, Region and U.S.	12
Losses of Calves from Other and Unknown Causes, by Size Group, State, Region and U.S.	48
Losses of Calves from Poison, by Size Group, State, Region and U.S.	36
Losses of Calves from Respiratory Problems, by Size Group, State, Region and U.S.	18
Losses of Calves from Theft, by Size Group, State, Region and U.S.	42
Losses of Calves from Weather, by Size Group, State, Region and U.S.	30
Losses of Cattle and Calves from Coyotes and Dogs, by State, Region and U.S.	51
Losses of Cattle and Calves from Mountain Lions, Bobcats, Bears, and Wolves, by State, Region, and U.S.	52
Losses of Cattle and Calves from Other Predators, by State, Region and U.S.	53
Losses of Cattle from All Causes, by Size Group, State, Region and U.S.	3
Losses of Cattle from Calving Problems, by Size Group, State, Region and U.S.	21
Losses of Cattle from Digestive Problems, by Size Group, State, Region and U.S.	9
Losses of Cattle from Other and Unknown Causes, by Size Group, State, Region and U.S.	45
Losses of Cattle from Poison, by Size Group, State, Region and U.S.	33
Losses of Cattle from Respiratory Problems, by Size Group, State, Region and U.S.	15
Losses of Cattle from Theft, by Size Group, State, Region and U.S.	39

-- continued

Index (continued)

Page

Losses of Cattle from Weather, by Size Group, State, Region and U.S. 27

Losses of Dairy Calves from All Causes, by Size Group, State, Region and U.S. 8

Losses of Dairy Calves from Calving Problems, by Size Group, State, Region and U.S. 26

Losses of Dairy Calves from Digestive Problems, by Size Group, State, Region and U.S. 14

Losses of Dairy Calves from Other and Unknown Causes, by Size Group, State, Region and U.S. 50

Losses of Dairy Calves from Poison, by Size Group, State, Region and U.S. 38

Losses of Dairy Calves from Respiratory Problems, by Size Group, State, Region and U.S. 20

Losses of Dairy Calves from Theft, by Size Group, State, Region and U.S. 44

Losses of Dairy Calves from Weather, by Size Group, State, Region and U.S. 32

Losses of Dairy Cattle from All Causes, by Size Group, State, Region and U.S. 5

Losses of Dairy Cattle from Calving Problems, by Size Group, State, Region and U.S. 23

Losses of Dairy Cattle from Digestive Problems, by Size Group, State, Region and U.S. 11

Losses of Dairy Cattle from Other and Unknown Causes, by Size Group, State, Region and U.S. 47

Losses of Dairy Cattle from Poison, by Size Group, State, Region and U.S. 35

Losses of Dairy Cattle from Respiratory Problems, by Size Group, State, Region and U.S. 17

Losses of Dairy Cattle from Theft, by Size Group, State, Region and U.S. 41

Losses of Dairy Cattle from Weather, by Size Group, State, Region and U.S. 29

Survey Procedures and Reliability 54
