# Supplementary material for: The Elephant in the room: What can we learn from California regarding the use of sport hunting of pumas (Puma concolor) as a management tool?
Source: PLoS One. 2020 Feb 13;15(2):e0224638. doi: 10.1371/journal.pone.0224638 (PMC7018503; doi:10.1371/journal.pone.0224638)
Supplement: S2 File — This is a zip file that contains sources of the data used in the analyses that are no longer available on the internet. (ZIP) [file pone.0224638.s003.zip › Cattle predator loss 2000.pdf]

Cattle Predator Loss

NASS
FACT FINDERS FOR AGRICULTURE
UNITED STATES DEPARTMENT OF AGRICULTURE
Washington, D.C.

Released May 4, 2001, by the National Agricultural Statistics Service (NASS), Agricultural Statistics Board, U.S. Department of Agriculture. For information on "Cattle Predator Loss" call Steve Anderson at 202-720-3040, office hours 7:00 a.m. to 4:00 p.m. ET.

U.S. Cattle and Calves Predator Loss

Cattle and calf losses from animal predators in the United States totaled 147,000 head during 2000. This estimate excludes Alaska. This resulted in a loss to farmers and ranchers of $51.6 million due to predators.

Coyotes caused the majority of cattle and calf losses to predators. They accounted for 64.6 percent of the total head lost. Dogs were the second leading cause of predator losses, accounting for 17.7 percent.

Use of non-lethal predator controls is also highlighted in this publication. Farmers and ranchers throughout the United States spent $184.9 million on non-lethal methods to prevent predator loss of cattle and calves.

This report is released as a cooperative effort between the National Agricultural Statistics Service and Animal and Plant Health Inspection Service - Wildlife Services.

Losses of Cattle and Calves: Number of Head and Total Value, United States, 2000 [1]

Predator	Number of Head	% of Total Predators	Total Value
	Number	*Percent*	*1,000 Dollars*
Coyotes	95,000	64.6	31,754
Dogs	26,000	17.7	9,547
Mountain Lions & Bobcats [2]	11,000	7.5	4,334
Bears	2,800	1.9	1,804
Wolves	1,600	1.1	1,030
All Other Animals	10,600	7.2	3,168
US	147,000	100.0	51,637

[1] Totals may not add due to rounding. Cattle includes all cows, bulls, steers, and heifers weighing over 500 pounds. This includes beef and milk breeds as well as cattle on feed. Calves include beef and milk breed steers, heifers, and bulls weighing less than 500 pounds.
[2] Includes cougars, pumas, and lynx.

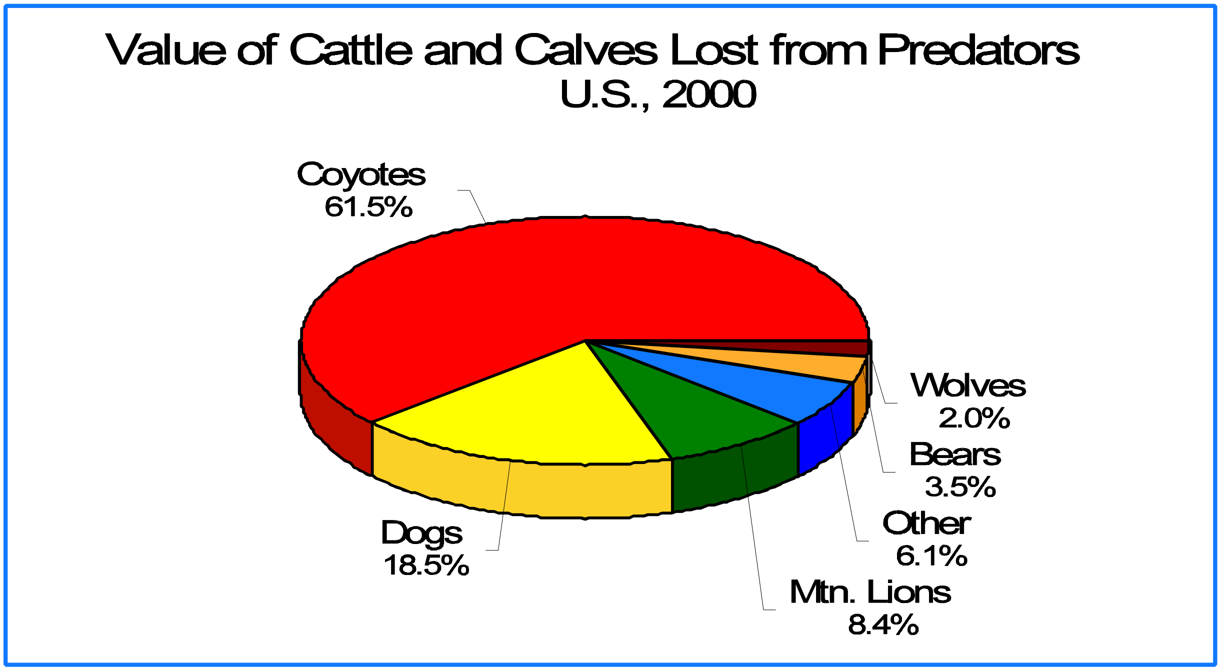
Value of Cattle and Calves Lost from Predators
U.S., 2000
Coyotes
61.5%
Wolves
2.0%
Bears
3.5%
Other
6.1%
Mtn. Lions
8.4%
Dogs
18.5%

Losses of Cattle and Calves by Predators, Total by State and United States, 2000 [1]

State	Cattle	Percent of U.S.	Calves	Percent of U.S.
	Head	*Percent*	*Head*	*Percent*
AL	800	3.8	3,700	2.9
AZ	500	2.4	3,200	2.5
AR	500	2.4	2,800	2.2
CA	2,400	11.4	12,500	9.9
CO	500	2.4	3,000	2.4
CT				
DE				
FL	600	2.9	2,500	2.0
GA	500	2.4	5,400	4.3
HI	1,500	7.1	700	0.6
ID	300	1.4	2,300	1.8
IL	800	3.8	2,100	1.7
IN	200	1.0	1,300	1.0
IA	100	0.5	1,100	0.9
KS	300	1.4	3,700	2.9
KY	800	3.8	2,200	1.7
LA	300	1.4	1,500	1.2
ME				
MD				
MA				
MI			300	0.2
MN	100	0.5	1,100	0.9
MS	200	1.0	3,800	3.0
MO	1,000	4.8	3,100	2.5
MT	600	2.9	3,200	2.5
NE			2,200	1.7
NV	300	1.4	1,500	1.2
NH				
NJ				
NM	700	3.3	5,000	4.0
NY	100	0.5	1,200	1.0
NC	500	2.4	2,100	1.7
ND	200	1.0	1,300	1.0
OH			600	0.5
OK	700	3.3	6,800	5.4
OR	300	1.4	4,100	3.3
PA				
RI				
SC	200	1.0	900	0.7
SD	400	1.9	2,600	2.1
TN	400	1.9	4,100	3.3
TX	2,800	13.3	22,000	17.5
UT	400	1.9	2,100	1.7
VT				
VA	600	2.9	2,300	1.8
WA	600	2.9	1,600	1.3
WV			900	0.7
WI	200	1.0	1,200	1.0
WY	300	1.4	3,600	2.9
Unpub	300	1.4	400	0.3
US	21,000	100.0	126,000	100.0

[1] Missing data are less than 100 head for the State or not published to avoid disclosure of individual operations. This data is included in Unpublished States and in United States. Totals may not add due to rounding.

Losses of Cattle and Calves by Coyotes and Dogs, by State and United States, 2000 [1]

State	Coyotes				Dogs			
	Cattle	% of Total Pred.	Calves	% of Total Pred.	Cattle	% of Total Pred.	Calves	% of Total Pred.
	Head	*Percent*	*Head*	*Percent*	*Head*	*Percent*	*Head*	*Percent*
AL	500	62.5	2,500	67.6	100	12.5	1,100	29.7
AZ			1,600	50.0	200	40.0	300	9.4
AR	200	40.0	1,600	57.1	200	40.0	500	17.9
CA	900	37.5	8,100	64.8	400	16.7	1,500	12.0
CO	200	40.0	2,600	86.7			100	3.3
CT								
DE								
FL	100	16.7	1,300	52.0	200	33.3	900	36.0
GA	200	40.0	3,500	64.8	200	40.0	1,700	31.5
HI					100	6.7	100	14.3
ID	100	33.3	1,600	69.6			100	4.3
IL	100	12.5	1,100	52.4	400	50.0	900	42.9
IN	100	50.0	1,000	76.9	100	50.0	200	15.4
IA			900	81.8			200	18.2
KS	200	66.7	2,900	78.4			500	13.5
KY	500	62.5	1,400	63.6	200	25.0	600	27.3
LA	200	66.7	1,100	73.3			300	20.0
ME								
MD								
MA								
MI			200	66.7				
MN			400	36.4			100	9.1
MS			2,700	71.1	100	50.0	1,100	28.9
MO			2,100	67.7	800	80.0	900	29.0
MT	200	33.3	2,300	71.9			100	3.1
NE			2,100	95.5				
NV	200	66.7	1,400	93.3				
NH								
NJ								
NM	200	28.6	4,000	80.0	100	14.3	200	4.0
NY			900	75.0				
NC	200	40.0	500	23.8	300	60.0	800	38.1
ND	200	100.0	1,300	100.0				
OH			500	83.3				
OK	200	28.6	3,800	55.9	400	57.1	2,200	32.4
OR	100	33.3	3,200	78.0			200	4.9
PA								
RI								
SC	100	50.0	300	33.3			600	66.7
SD	400	100.0	2,500	96.2				
TN	300	75.0	2,900	70.7			1,200	29.3
TX	1,800	64.3	17,000	77.3	300	10.7	3,100	14.1
UT	200	50.0	1,800	85.7				
VT								
VA	100	16.7	1,000	43.5	400	66.7	1,100	47.8
WA			700	43.8	100	16.7	200	12.5
WV			700	77.8				
WI	100	50.0	400	33.3	100	50.0		
WY	100	33.3	2,800	77.8				
Unpub	300		300		300		200	
US	8,000	38.1	87,000	69.0	5,000	23.8	21,000	16.7

[1] Missing data are less than 100 head for the State or not published to avoid disclosure of individual operations. This data is included in Unpublished States and in United States. Totals may not add due to rounding.

Losses of Cattle and Calves by Mountain Lions and Bobcats by Selected States and United States, 2000 [1] [2]

State	Mountain Lions and Bobcats			
	Cattle	Percent of Total Predators	Calves	Percent of Total Predators
	Head	*Percent*	*Head*	*Percent*
AZ	200	40.0	1,100	34.4
AR			100	3.6
CA	900	37.5	2,400	19.2
CO	200	40.0	100	3.3
ID	100	33.3	300	13.0
KS			200	5.4
MO	100	10.0		
MT			100	3.1
NV	100	33.3		
NM	300	42.9	700	14.0
NC			100	4.8
OK	100	14.3	300	4.4
OR	100	33.3	500	12.2
TX	100	3.6	300	1.4
UT	100	25.0	200	9.5
VA			100	4.3
WA	500	83.3	500	31.3
WV			100	11.1
WY	100	33.3	300	8.3
Unpub [3]	100		600	
US	3,000	14.3	8,000	6.3

[1] Missing data are less than 100 head for the State or not published to avoid disclosure of individual operations. This data is included in Unpublished States and in United States. Totals may not add due to rounding.
[2] Includes cougars, pumas, and lynx.
[3] Unpublished States may not be included in table.

Losses of Cattle and Calves by Other Predators by State and United States, 2000 [1]

State	All Other Predators			
	Cattle	Percent of Total Predators	Calves	Percent of Total Predators
	Head	*Percent*	*Head*	*Percent*
AL	200	25.0	100	2.7
AZ			200	6.3
AR	100	20.0	600	21.4
CA	200	8.3	500	4.0
CO	100	20.0	200	6.7
CT				
DE				
FL	300	50.0	300	12.0
GA	100	20.0	200	3.7
HI	1,400	93.3	600	85.7
ID	100	33.3	300	13.0
IL	300	37.5	100	4.8
IN			100	7.7
IA				
KS			100	2.7
KY	100	12.5	200	9.1
LA	100	33.3	100	6.7
ME				
MD				
MA				
MI			100	33.3
MN			600	54.5
MS				
MO	100	10.0		
MT	300	50.0	700	21.9
NE				
NV				
NH				
NJ				
NM	100	14.3	100	2.0
NY				
NC			700	33.3
ND				
OH				
OK			500	7.4
OR	100	33.3	200	4.9
PA				
RI				
SC				
SD				
TN				
TX	600	21.4	1,600	7.3
UT	100	25.0		
VT				
VA	100	16.7	100	4.3
WA			200	12.5
WV			100	11.1
WI			600	50.0
WY	100	33.3	500	13.9
Unpub	500		400	
US	5,000	23.8	10,000	7.9

[1] Missing data are less than 100 head for the State or not published to avoid disclosure of individual operations. This data is included in Unpublished States and in United States. Totals may not add due to rounding.

Losses of Cattle and Calves from Predators: Total Value per Head and Total Value, by State and United States, 2000 [1]

State	Value per Head		Total Value	
	Cattle [2]	Calves [3]	Cattle	Calves
	Dollars	*Dollars*	*1,000 Dollars*	*1,000 Dollars*
AL	613.00	306.00	490	1,132
AZ	577.00	306.00	289	979
AR	621.00	288.00	311	806
CA	649.00	279.00	1,558	3,488
CO	752.00	315.00	376	945
CT				
DE				
FL	586.00	303.00	352	758
GA	576.00	294.60	288	1,591
HI	401.00	183.00	602	128
ID	706.00	274.80	212	632
IL	697.00	345.00	558	725
IN	704.00	285.00	141	371
IA	783.00	306.00	78	337
KS	732.00	321.00	220	1,188
KY	648.00	288.30	518	634
LA	590.00	295.50	177	443
ME				
MD				
MA				
MI		306.00		92
MN	720.00	284.40	72	313
MS	613.00	282.60	123	1,074
MO	685.00	306.00	685	949
MT	795.00	309.00	477	989
NE		318.00		700
NV	720.00	336.00	216	504
NH				
NJ				
NM	629.00	303.00	440	1,515
NY	714.00	312.00	71	374
NC	613.00	288.60	307	606
ND	809.00	295.80	162	385
OH		284.10		170
OK	665.00	306.00	466	2,081
OR	668.00	279.00	200	1,144
PA				
RI				
SC	613.00	303.00	123	273
SD	817.00	324.00	327	842
TN	620.00	284.40	248	1,166
TX	623.00	321.00	1,744	7,062
UT	720.00	296.70	288	623
VT				
VA	629.00	300.00	377	690
WA	720.00	290.40	432	465
WV		262.50		236
WI	701.00	381.00	140	457
WY	779.00	321.00	234	1,156
Unpub	740.00	225.00	222	90
US [4]	644.00	302.00	13,524	38,113

[1] Missing data are less than 100 head for the State or not published to avoid disclosure of individual operations. This data is included in Unpublished States and in United States. [2] Cattle value per head is based on a two-year straight average of the value of beef cows reported in the January 1 Cattle survey from 2000 and 2001. [3] Calf value per head is based on the market year average calf price. An average weight of 300 pounds was used in all States. [4] U.S. value per head rounded to the nearest dollar. U.S. and unpublished value per head are derived. Totals may not add due to rounding.

Non-Lethal Methods Used to Prevent Losses of Cattle and Calves to Predators, by State and United States, 2000 [1] [2]

State	Guard Animals	Exclusion Fencing	Herding	Night Penning
	Percent	*Percent*	*Percent*	*Percent*
AL	40.3	32.8	6.0	4.5
AZ	28.6	28.6		
AR	55.3	21.1		
CA	19.6	48.0	10.8	13.7
CO	25.0	17.9	7.1	14.3
CT	33.3	33.3		16.7
DE	20.0	50.0		20.0
FL	24.4	16.7	1.3	2.6
GA	21.7	19.6		
HI	14.3	57.1	7.1	
ID	22.2	44.4	8.9	4.4
IL	20.0	57.1	2.9	11.4
IN	35.1	32.4	21.9	14.3
IA	13.0	40.7		11.1
KS	19.6	8.9	5.4	7.1
KY	38.5	26.9	1.9	3.8
LA	24.0	57.3	20.0	20.0
ME	26.7	60.0	6.7	20.0
MD			50.0	
MA	24.1	55.2	10.3	27.6
MI	18.2	68.2	4.5	13.6
MN	39.4	40.9	1.5	12.1
MS	40.0	14.0	2.0	
MO	50.0	21.4	3.6	7.1
MT	22.4	11.9	14.9	16.4
NE	20.7	12.1	5.2	20.7
NV	16.7	66.7		16.7
NH	30.8	38.5		23.1
NJ	13.3	26.7	6.7	33.3
NM	26.7	40.0	6.7	
NY	17.4	21.7	17.4	17.4
NC	7.1	42.9		7.1
ND	47.6	23.8		9.5
OH	46.4	35.7	3.6	14.3
OK	54.1	8.1		5.4
OR	19.7	45.1	12.7	18.3
PA	23.5	41.2	5.9	29.4
RI	20.0	60.0		40.0
SC		75.0	8.3	
SD	25.0	7.1	3.6	10.7
TN	50.0	19.6	8.9	1.8
TX	39.8	30.5	6.3	8.6
UT	20.0	30.0	30.0	
VT	10.0	50.0		30.0
VA	22.0	29.3	2.4	4.9
WA	26.1	34.8		8.7
WV	26.7	33.3	6.7	6.7
WI	9.6	7.7	1.3	5.1
WY	35.4	20.8	12.5	18.8
US	27.7	29.9	7.4	10.8

[1] Missing data are not published to avoid disclosure of individual operations.
[2] Use of multiple non-lethal methods may result in percentages summing to greater than 100.

Non-Lethal Methods Used to Prevent Losses of Cattle and Calves to Predators, by State and United States, 2000 [1 2]

State	Fright Tactics	Livestock Carcass Removal	Other Non-lethal
	Percent	*Percent*	*Percent*
AL	4.5	35.8	16.4
AZ	14.3	28.6	
AR	2.6	10.5	26.3
CA	7.8	46.1	11.8
CO	17.9	64.3	5.4
CT	16.7		
DE			
FL	6.4	56.4	10.3
GA	6.5	47.8	21.7
HI	7.1	14.3	35.7
ID	4.4	42.2	8.9
IL	11.4	20.0	14.3
IN	6.8	41.6	6.8
IA		42.6	11.1
KS	12.5	51.8	17.9
KY		25.0	11.5
LA	1.3	49.3	2.7
ME	6.7	6.7	6.7
MD	80.0	80.0	50.0
MA	13.8	24.1	20.7
MI	9.1	18.2	4.5
MN	6.1	27.3	3.0
MS	10.0	36.0	10.0
MO	14.3	21.4	10.7
MT	10.4	37.3	7.5
NE	6.9	44.8	13.8
NV	16.7	33.3	
NH		7.7	30.8
NJ	20.0	6.7	26.7
NM		20.0	13.3
NY	8.7	60.9	8.7
NC		50.0	7.1
ND	14.3	19.0	4.8
OH		3.6	3.6
OK	8.1	27.0	10.8
OR	9.9	40.8	11.3
PA		29.4	5.9
RI			
SC		8.3	8.3
SD	10.7	57.1	7.1
TN	7.1	41.1	8.9
TX	3.1	21.9	18.8
UT	30.0	20.0	10.0
VT		10.0	20.0
VA	12.2	34.1	12.2
WA		17.4	17.4
WV	6.7	13.3	13.3
WI	2.6	85.9	6.4
WY	10.4	29.2	14.6
US	6.8	40.4	11.3

[1] Missing data are not published to avoid disclosure of individual operations.
[2] Use of multiple non-lethal methods may result in percentages summing to greater than 100.

Reliability of Cattle and Calves Predator Loss Estimates

Survey Procedures: A random sample of U.S. producers was surveyed to provide data for these estimates. Survey procedures ensured that all cattle producers, regardless of size, had a chance to be included in the survey. Large producers were sampled more heavily than small operations. About 50,000 operators were contacted during the first half of January by mail, telephone and face-to-face personal interview.

Estimation Procedures: These estimates of death loss by cause were prepared by the Livestock Branch, NASS, and reviewed by the 49 States involved. Total cattle and calf death losses from all causes were published in the 2001 "*Meat Animals-Production, Disposition, and Income*" (released April 27, 2001). To estimate death loss by cause, cattle and calf losses from total predators were first determined. Next, the total predator losses was calculated. All published loss estimates were rounded to the nearest 100 head. Value estimates were rounded to the nearest $1,000. State estimates of less than 100 head are not published, but are included in U.S. totals. States were combined into regions when sampling variability did not permit individual state data to be released.

Revision Policy: Revisions to previous estimates are made to improve the current estimate. Previous year estimates are subject to revision when current estimates are made. Estimates for losses from all causes are subject to revision in next year's "*Meat Animals-Production, Disposition, and Income*". No revision to predator losses is planned.

Reliability: Since all cattle operators are not included in the sample, survey estimates are subject to sampling variability. Survey results are also subject to non-sampling errors such as omissions, duplications, and mistakes in reporting, recording, and processing the data. The effects of these non-sampling errors cannot be measured directly. They are minimized through rigid quality controls in the data collection process and through a careful review of all reported data for consistency and reasonableness.

The United States Department of Agriculture (USDA) prohibits discrimination in all its programs on the basis of race, color, national origin, gender, religion, age, disability, political beliefs, sexual orientation, and marital or family status. (Not all prohibited bases apply to all programs.) Persons with disabilities who require alternative means for communication of program information (braille, large print, audiotape, etc.) should contact the USDA's TARGET Center at 202-720-2600 (voice and TDD).

To file a complaint of discrimination, write USDA, Director, Office of Civil Rights, Room 326-W, Whitten Building, 14th and Independence Avenue, SW, Washington, D.C., 20250-9410, or call 202-720-5964 (voice or TDD). USDA is an equal opportunity provider and employer.

ACCESS TO REPORTS!!

For your convenience, there are several ways to obtain NASS reports, data products, and services:

INTERNET ACCESS

All NASS reports are available free of charge on the worldwide Internet. For access, connect to the Internet and go to the NASS Home Page at: **http:/www.usda.gov/nass/**. Select "Today's Reports" or Publications and then Reports by Calendar or Publications and then Search, by Title or Subject.

E-MAIL SUBSCRIPTION

There are two options for subscribing via e-mail. All NASS reports are available by subscription free of charge direct to your e-mail address. 1) Starting with the NASS Home Page at **http:/www.usda.gov/nass/**, click on **Publications**, then click on the **Subscribe by E-mail** button which takes you to the page describing e-mail delivery of reports. Finally, click on **Go to the Subscription Page** and follow the instructions. 2) If you do NOT have Internet access, send an e-mail message to: **usda-reports@usda.mannlib.cornell.edu**. In the body of the message type the word: **list**.

AUTOFAX ACCESS

NASSFax service is available for some reports from your fax machine. Please call 202-720-2000, using the handset attached to your fax. Respond to the voice prompts. Document 0411 is a list of available reports.

- -

PRINTED REPORTS OR DATA PRODUCTS

CALL OUR TOLL-FREE ORDER DESK: 800-999-6779 (U.S. and Canada)
Other areas, please call 703-834-0125 FAX: 703-834-0110
(Visa, MasterCard, check, or money order acceptable for payment.)

- -

ASSISTANCE

For **assistance** with general agricultural statistics or further information about NASS or its products or services, contact the **Agricultural Statistics Hotline** at **800-727-9540**, 7:30 a.m. to 4:00 p.m. ET, or e-mail: **nass@nass.usda.gov.**
