# Supplementary material for: The Elephant in the room: What can we learn from California regarding the use of sport hunting of pumas (Puma concolor) as a management tool?
Source: PLoS One. 2020 Feb 13;15(2):e0224638. doi: 10.1371/journal.pone.0224638 (PMC7018503; doi:10.1371/journal.pone.0224638)
Supplement: S2 File — This is a zip file that contains sources of the data used in the analyses that are no longer available on the internet. (ZIP) [file pone.0224638.s003.zip › Cattle predator loss 2005.pdf]

NASS
FACT FINDERS FOR AGRICULTURE
UNITED STATES DEPARTMENT OF AGRICULTURE

Washington, D.C.

Cattle Death Loss

Released May 5, 2006, by the National Agricultural Statistics Service (NASS), Agricultural Statistics Board, U.S. Department of Agriculture. For information on *Cattle Death Loss* call Mike Miller at 202-720-3040, office hours 7:30 a.m. to 4:00 p.m. ET.

Cattle and Calf Death Losses

Cattle and calf losses from animal predators and non-predator causes in the United States totaled 4.05 million head (excluding Alaska). This report provides detailed breakouts of cattle and calf losses by predators and non-predator causes, as well as data on non-lethal predator control measures.

Cattle and calf losses from animal predators totaled 190,000 head. This represented 4.7 percent of the total losses from all causes and resulted in a loss of $92.7 million to farmers and ranchers. Coyotes and dogs caused the majority of cattle and calf losses accounting for 51.1 percent and 11.5 percent respectively.

Cattle and calf losses from non-predator causes totaled 3.86 million head or 95.3 percent of the total losses. Respiratory problems was the leading cause of non-predator deaths accounting for 28.7 percent, followed by digestive problems at 16.8 percent.

Farmers and ranchers throughout the United States spent 199.1 million dollars on non-lethal methods to control predators. Use of guard animals was the most common method at 38.0 percent. Exclusion fencing, frequent checking, and culling were the next most commonly used methods of preventing cattle and calf losses at 34.0 percent, 21.8 percent, and 19.6 percent respectively.

This report is released as a cooperative effort between the National Agricultural Statistics Service and Animal and Plant Health Inspection Service - Wildlife Services and Veterinary Services.

Contents

Page

Number and Value of Losses of Cattle and Calves 1

Losses of Cattle and Calves by Predators and Non-Predator Causes 2

Losses of Cattle and Calves Value per Head and Total Value 3

Losses of Cattle and Calves by Predators 4

Losses of Cattle and Calves from Non-Predator Causes 7

Non-Lethal Prevention Methods 13

Reliability of Cattle and Calves Predator Loss Estimates 15

Information Contacts 15

Losses of Cattle and Calves: Predator and Non-Predator
Number of Head and Total Value, United States, 2005 [1]

Cause	Number of Head	% of Total	Total Value
	Number	*Percent*	*1,000 Dollars*
Predator			
Coyotes	97,000	51.1	43,911
Dogs	21,900	11.5	10,786
Mountain Lions & Bobcats [2]	14,700	7.7	7,590
Vultures [3]	8,600	4.5	3,807
Wolves	4,400	2.3	2,087
Bears	2,800	1.5	1,453
Other Predators	9,300	4.9	5,735
Unknown Predators [4]	31,300	16.5	17,305
Total Predator	190,000	100.0	92,674
Non-Predator			
Digestive Problems	648,000	16.8	367,441
Respiratory Problems	1,110,000	28.7	692,430
Metabolic Problems	78,000	2.0	64,991
Mastitis	67,000	1.7	64,695
Other Diseases	174,000	4.5	124,833
Lameness/Injury	132,000	3.4	104,427
Weather Related	275,000	7.1	172,491
Calving Problems	572,000	14.8	328,193
Poisoning	39,000	1.0	28,736
Theft	21,000	0.5	13,920
Other Non-Predator	271,000	7.0	225,066
Unknown Non-Predator	474,000	12.3	311,352
Total Non-Predator	3,861,000	100.0	2,498,575
US Total	4,051,000	100.0	2,591,249

[1] Totals may not add due to rounding. Cattle includes all cows, bulls, steers, and heifers weighing over 500 pounds. This includes beef and milk breeds as well as cattle on feed. Calves include beef and milk breed steers, heifers, and bulls weighing less than 500 pounds.
[2] Includes cougars, pumas, and lynx.
[3] Reports of death loss to cattle/calves by vultures is primarily due to black vultures and will occur only in states where this species ranges. See range map of black vultures at: http:/www.birds.cornell.edu/AllAboutBirds/BirdGuide/Black_Vulture_dtl.html#map.
[4] The category of unknown predators was not included in the "Cattle Predator Loss" report released May 2001.

Losses of Cattle and Calves: By All Causes, Predators and Non-Predators, Number by State and United States, 2005 [1]

State	All Causes		Predators		Non-Predators	
	Cattle	Calves	Cattle	Calves	Cattle	Calves
	Head	*Head*	*Head*	*Head*	*Head*	*Head*
AL	21,000	28,000	1,500	5,500	19,500	22,500
AZ	24,000	22,000	700	2,600	23,300	19,400
AR	30,000	43,000	1,800	4,500	28,200	38,500
CA	100,000	165,000	1,400	5,100	98,600	159,900
CO	50,000	55,000	700	4,000	49,300	51,000
CT	900	1,000			900	1,000
DE	600	700			600	700
FL	25,000	35,000	1,700	6,100	23,300	28,900
GA	21,000	31,000	1,700	5,900	19,300	25,100
HI	3,000	2,000	200	300	2,800	1,700
ID	42,000	63,000	500	2,000	41,500	61,000
IL	21,000	35,000	200	2,200	20,800	32,800
IN	15,000	29,000	300	1,200	14,700	27,800
IA	65,000	110,000		1,400	65,000	108,600
KS	135,000	70,000	700	3,900	134,300	66,100
KY	35,000	76,000	1,400	5,800	33,600	70,200
LA	26,000	20,000	900	3,700	25,100	16,300
ME	1,600	3,100			1,600	3,100
MD	3,000	5,000		100	3,000	4,900
MA	1,000	1,000		100	1,000	900
MI	25,000	47,000		400	25,000	46,600
MN	42,000	82,000	100	1,700	41,900	80,300
MS	22,000	29,000	1,200	2,600	20,800	26,400
MO	70,000	120,000	1,400	4,000	68,600	116,000
MT	20,000	46,000	600	2,400	19,400	43,600
NE	95,000	75,000	500	2,200	94,500	72,800
NV	6,000	13,000	200	1,100	5,800	11,900
NH	800	1,000			800	1,000
NJ	1,000	1,000			1,000	1,000
NM	26,000	35,000	2,200	4,800	23,800	30,200
NY	35,000	50,000	100	800	34,900	49,200
NC	15,000	22,000	500	1,800	14,500	20,200
ND	12,000	40,000	500	2,300	11,500	37,700
OH	22,000	33,000	400	1,900	21,600	31,100
OK	100,000	135,000	2,000	9,600	98,000	125,400
OR	23,000	42,000	400	4,100	22,600	37,900
PA	33,000	45,000	1,000	2,000	32,000	43,000
RI	100	200			100	200
SC	6,000	10,000	200	1,400	5,800	8,600
SD	53,000	85,000	300	2,300	52,700	82,700
TN	38,000	63,000	1,200	5,400	36,800	57,600
TX	300,000	270,000	4,100	35,000	295,900	235,000
UT	15,000	26,000	100	2,500	14,900	23,500
VT	6,000	8,000	100	200	5,900	7,800
VA	27,000	56,000	1,400	3,800	25,600	52,200
WA	21,000	23,000	900	1,600	20,100	21,400
WV	7,000	11,000	100	1,500	6,900	9,500
WI	65,000	140,000	300	2,700	64,700	137,300
WY	11,000	31,000	500	3,500	10,500	27,500
US	1,717,000	2,334,000	34,000	156,000	1,683,000	2,178,000

[1] Totals may not add due to rounding.

Losses of Cattle and Calves from Predators and Non-Predator Causes:
Total Value per Head and Total Value, by State and United States, 2005 [1]

State	Value per Head		Total Value Predator Losses		Total Value Non-Predator Losses	
	Cattle [2]	Calves [3]	Cattle	Calves	Cattle	Calves
	Dollars	*Dollars*	*1,000 Dollars*	*1,000 Dollars*	*1,000 Dollars*	*1,000 Dollars*
AL	864	381	1,296	2,096	16,848	8,573
AZ	873	378	611	983	20,341	7,333
AR	905	387	1,629	1,742	25,521	14,900
CA	962	363	1,347	1,851	94,853	58,044
CO	1,045	408	732	1,632	51,519	20,808
CT	996	300			896	300
DE	996	330			598	231
FL	836	390	1,421	2,379	19,479	11,271
GA	791	375	1,345	2,213	15,266	9,413
HI	483	288	97	86	1,352	490
ID	985	378	493	756	40,878	23,058
IL	989	363	198	799	20,571	11,906
IN	1,007	351	302	421	14,803	9,758
IA	1,080	393		550	70,200	42,680
KS	1,042	420	729	1,638	139,941	27,762
KY	917	369	1,284	2,140	30,811	25,904
LA	869	369	782	1,365	21,812	6,015
ME	996	315			1,594	977
MD	996	330		33	2,988	1,617
MA	996	300		30	996	270
MI	978	396		158	24,450	18,454
MN	970	399	97	678	40,643	32,040
MS	864	369	1,037	959	17,971	9,742
MO	991	399	1,387	1,596	67,983	46,284
MT	1,129	414	677	994	21,903	18,050
NE	1,119	426	560	937	105,746	31,013
NV	1,030	411	206	452	5,974	4,891
NH	996	315			797	315
NJ	996	390			996	390
NM	991	393	2,180	1,886	23,586	11,869
NY	996	420	100	336	34,760	20,664
NC	864	357	432	643	12,528	7,211
ND	1,153	405	577	932	13,260	15,269
OH	866	354	346	673	18,706	11,009
OK	918	396	1,836	3,802	89,964	49,658
OR	966	357	386	1,464	21,832	13,530
PA	895	396	895	792	28,640	17,028
RI	996	300			100	60
SC	864	369	173	517	5,011	3,173
SD	1,163	411	349	945	61,290	33,990
TN	891	366	1,069	1,976	32,789	21,082
TX	913	408	3,743	14,280	270,157	95,880
UT	1,030	402	103	1,005	15,347	9,447
VT	996	315	100	63	5,876	2,457
VA	854	366	1,196	1,391	21,862	19,105
WA	1,030	375	927	600	20,703	8,025
WV	996	336	100	504	6,872	3,192
WI	959	489	288	1,320	62,047	67,140
WY	1,113	420	557	1,470	11,687	11,550
US [4]	970	396	31,587	61,087	1,634,747	863,828

[1] Missing data are less than 100 head for the State or not published to avoid disclosure of individual operations. This data is included in the Unpublished States and in the United States.
[2] Cattle value per head is based on a two-year straight average of the value of beef cows reported in the January 1 Cattle survey from 2005 and 2006.
[3] Calf value per head is based on the market year average calf price. An average weight of 300 pounds was used in all States.
[4] U.S. value per head rounded to the nearest dollar. U.S. value per head derived. Totals may not add due to rounding.

Losses of Cattle and Calves: Number and Percent by Coyotes and Dogs, by State and United States, 2005 [1]

State	Coyotes				Dogs			
	Cattle	% Total Predator	Calves	% Total Predator	Cattle	% Total Predator	Calves	% Total Predator
	Head	*Percent*	*Head*	*Percent*	*Head*	*Percent*	*Head*	*Percent*
AL	500	33.3	3,200	58.2	300	20.0	1,000	18.2
AZ			600	23.1			200	7.7
AR	400	22.2	1,800	40.0	100	5.6	600	13.3
CA	100	7.1	3,000	58.8	200	14.3	600	11.8
CO	500	71.4	2,300	57.5			200	5.0
CT								
DE								
FL	700	41.2	3,600	59.0			500	8.2
GA	1,200	70.6	3,400	57.6	100	5.9	700	11.9
HI					100	50.0	200	66.7
ID			600	30.0			100	5.0
IL	100	50.0	1,100	50.0	100	50.0	300	13.6
IN	100	33.3	1,000	83.3			100	8.3
IA			1,100	78.6			100	7.1
KS	400	57.1	2,000	51.3	100	14.3	700	17.9
KY	400	28.6	3,700	63.8	300	21.4	400	6.9
LA			2,400	64.9	600	66.7	400	10.8
ME								
MD								
MA			100	100.0				
MI			200	50.0				
MN			700	41.2				
MS			600	23.1	100	8.3	700	26.9
MO			1,100	27.5	600	42.9	1,500	37.5
MT			1,300	54.2			100	4.2
NE	200	40.0	1,700	77.3			100	4.5
NV	100	50.0	1,000	90.9				
NH								
NJ								
NM	900	40.9	1,900	39.6	100	4.5	200	4.2
NY	100	100.0	700	87.5				
NC	400	80.0	900	50.0			600	33.3
ND	100	20.0	1,700	73.9				
OH	100	25.0	1,100	57.9			300	15.8
OK	800	40.0	4,300	44.8	200	10.0	2,300	24.0
OR	100	25.0	2,200	53.7				
PA			300	15.0				
RI								
SC	200	100.0	800	57.1			100	7.1
SD	200	66.7	1,700	73.9			200	8.7
TN	600	50.0	3,500	64.8			1,100	20.4
TX	1,700	41.5	21,000	60.0	600	14.6	2,900	8.3
UT			1,600	64.0				
VT			100	50.0				
VA	600	42.9	1,700	44.7	200	14.3	1,000	26.3
WA			600	37.5				
WV			1,300	86.7				
WI			2,000	74.1				
WY	100	20.0	2,100	60.0			100	2.9
Unpub	400				700		200	
US	11,000	32.4	86,000	55.1	4,400	12.9	17,500	11.2

[1] Missing data are less than 100 head for the State or not published to avoid disclosure of individual operations. This data is included in Unpublished States and in United States. Totals may not add due to rounding.

Losses of Cattle and Calves: Number and Percent by Mountain Lions, Bobcats and Other Predators, by State and United States, 2005 [1]

State<br>State	Mountain Lions and Bobcats				Other Predators			
	Cattle	% Total Predator	Calves	% Total Predator	Cattle	% Total Predator	Calves	% Total Predator
	Head	*Percent*	*Head*	*Percent*	*Head*	*Percent*	*Head*	*Percent*
AL					100	6.7	400	7.3
AZ	100	14.3	1,200	46.2			100	3.8
AR	100	5.6	300	6.7	100	5.6	300	6.7
CA	1,000	71.4	1,300	25.5				
CO			600	15.0	100	14.3	200	5.0
CT								
DE								
FL					700	41.2	1,100	18.0
GA					100	5.9	400	6.8
HI								
ID			200	10.0	400	80.0	600	30.0
IL								
IN					100	33.3		
IA								
KS			500	12.8			100	2.6
KY					200	14.3	200	3.4
LA							300	8.1
ME								
MD								
MA								
MI							200	50.0
MN							800	47.1
MS							200	7.7
MO			200	5.0	700	50.0	1,000	25.0
MT			200	8.3	200	33.3	500	20.8
NE	100	20.0	200	9.1				
NV			100	9.1				
NH								
NJ								
NM	700	31.8	1,700	35.4	200	9.1	200	4.2
NY								
NC							200	11.1
ND								
OH							300	15.8
OK	100	5.0	700	7.3	400	20.0	1,500	15.6
OR	200	50.0	1,300	31.7				
PA							200	10.0
RI								
SC								
SD			200	8.7				
TN							300	5.6
TX	200	4.9	1,000	2.9	700	17.1	6,900	19.7
UT			300	12.0			500	20.0
VT								
VA					400	28.6	600	15.8
WA	200	22.2	600	37.5	300	33.3	300	18.8
WV							200	13.3
WI					200	66.7	600	22.2
WY	100	20.0	400	11.4	300	60.0	900	25.7
Unpub	400		500		400		400	
US	3,200	9.4	11,500	7.4	5,600	16.5	19,500	12.5

[1] Missing data are less than 100 head for the State or not published to avoid disclosure of individual operations. Data not published are included in U.S. total. Totals may not add due to rounding.
[2] Includes cougars, pumas, and lynx.

Losses of Cattle and Calves: Number and Percent by Unknown Predators, by State and United States, 2005 [1]

State	Unknown Predators			
	Cattle	% Total Predators	Calves	% Total Predators
	Head	*Percent*	*Head*	*Percent*
AL	600	40.0	900	16.4
AZ	500	71.4	500	19.2
AR	1,100	61.1	1,500	33.3
CA	100	7.1	200	3.9
CO			700	17.5
CT				
DE				
FL	200	11.8	900	14.8
GA	300	17.6	1,400	23.7
HI			100	33.3
ID	100	20.0	500	25.0
IL			600	27.3
IN				
IA				
KS			600	15.4
KY	500	35.7	1,500	25.9
LA	100	11.1	600	16.2
ME				
MD				
MA				
MI				
MN			200	11.8
MS	1,000	83.3	1,100	42.3
MO			200	5.0
MT	200	33.3	300	12.5
NE				
NV				
NH				
NJ				
NM	300	13.6	800	16.7
NY				
NC	100	20.0	100	5.6
ND	400	80.0	500	21.7
OH	300	75.0	200	10.5
OK	500	25.0	800	8.3
OR			500	12.2
PA	1,000	100.0	1,500	75.0
RI				
SC			400	28.6
SD			200	8.7
TN	400	33.3	500	9.3
TX	900	22.0	3,200	9.1
UT			100	4.0
VT				
VA	200	14.3	500	13.2
WA	400	44.4	100	6.3
WV				
WI			100	3.7
WY				
Unpub	600		200	
US	9,800	28.8	21,500	13.8

[1] Missing data are less than 100 head for the State or not published to avoid disclosure of individual operations. This data is included in Unpublished States and in United States. Totals may not add due to rounding.

Losses of Cattle and Calves: Number and Percent from Digestive and Respiratory Problems, by State and United States, 2005 [1]

State	Digestive Problems				Respiratory Problems			
	Cattle	% Total Non-Pred	Calves	% Total Non Pred	Cattle	% Total Non-Pred	Calves	% Total Non-Pred
	Head	*Percent*	*Head*	*Percent*	*Head*	*Percent*	*Head*	*Percent*
AL	1,700	8.7	1,300	5.8	1,600	8.2	4,600	20.4
AZ	1,900	8.2	4,400	22.7	1,300	5.6	4,900	25.3
AR	1,700	6.0	3,800	9.9	4,500	16.0	11,300	29.4
CA	15,000	15.2	62,000	38.8	20,500	20.8	61,000	38.1
CO	7,800	15.8	10,500	20.6	16,600	33.7	15,200	29.8
CT			300	30.0			300	30.0
DE								
FL	1,900	8.2	5,100	17.6	2,100	9.0	6,100	21.1
GA	1,200	6.2	4,200	16.7	2,600	13.5	6,200	24.7
HI								
ID	3,200	7.7	20,000	32.8	6,900	16.6	19,600	32.1
IL	2,300	11.1	8,600	26.2	5,500	26.4	10,600	32.3
IN	1,700	11.6	6,800	24.5	3,100	21.1	11,200	40.3
IA	9,500	14.6	25,000	23.0	27,000	41.5	32,000	29.5
KS	28,500	21.2	6,600	10.0	77,200	57.5	28,000	42.4
KY	1,600	4.8	13,300	18.9	5,800	17.3	18,800	26.8
LA	800	3.2	900	5.5	1,600	6.4	3,900	23.9
ME	200	12.5	900	29.0	200	12.5	1,100	35.5
MD	200	6.7	500	10.2	500	16.7	1,800	36.7
MA			200	22.2			300	33.3
MI	3,400	13.6	17,200	36.9	5,300	21.2	14,100	30.3
MN	4,300	10.3	28,700	35.7	9,600	22.9	22,200	27.6
MS	800	3.8	1,100	4.2	2,300	11.1	5,900	22.3
MO	3,000	4.4	18,200	15.7	8,600	12.5	34,000	29.3
MT	1,500	7.7	5,500	12.6	3,100	16.0	7,800	17.9
NE	9,800	10.4	11,400	15.7	43,000	45.5	24,100	33.1
NV	500	8.6	1,800	15.1	1,000	17.2	5,800	48.7
NH	100	12.5	200	20.0			300	30.0
NJ	100	10.0	200	20.0			200	20.0
NM	1,300	5.5	1,600	5.3	3,300	13.9	6,100	20.2
NY	5,000	14.3	11,800	24.0	4,800	13.8	22,900	46.5
NC	1,600	11.0	3,400	16.8	3,500	24.1	3,300	16.3
ND	900	7.8	7,800	20.7	2,000	17.4	8,300	22.0
OH	4,900	22.7	11,400	36.7	7,300	33.8	10,400	33.4
OK	5,600	5.7	9,500	7.6	39,500	40.3	50,000	39.9
OR	1,600	7.1	9,700	25.6	7,500	33.2	12,600	33.2
PA	3,200	10.0	12,600	29.3	4,400	13.8	14,000	32.6
RI								
SC	400	6.9	1,000	11.6	300	5.2	1,300	15.1
SD	6,200	11.8	17,200	20.8	13,800	26.2	30,000	36.3
TN	4,600	12.5	10,100	17.5	5,000	13.6	14,900	25.9
TX	31,500	10.6	26,000	11.1	54,500	18.4	88,000	37.4
UT	1,700	11.4	6,300	26.8	2,900	19.5	6,900	29.4
VT	400	6.8	2,800	35.9	400	6.8	2,100	26.9
VA	1,500	5.9	5,300	10.2	3,200	12.5	8,700	16.7
WA	4,000	19.9	5,200	24.3	3,000	14.9	8,500	39.7
WV	800	11.6	1,000	10.5	600	8.7	1,500	15.8
WI	6,700	10.4	55,000	40.1	9,200	14.2	45,000	32.8
WY	1,200	11.4	5,500	20.0	2,700	25.7	6,000	21.8
Unpub	200		100		200		200	
US	186,000	11.1	462,000	21.2	418,000	24.8	692,000	31.8

[1] Missing data are less than 100 head for the State or not published to avoid disclosure of individual operations. Data not published are included in U.S. total. Totals may not add due to rounding.

Losses of Cattle and Calves: Number and Percent from Metabolic Problems and Mastitis, by State and United States, 2005 [1]

State	Metabolic Problems				Mastitis			
	Cattle	% Total Non-Pred	Calves	% Total Non-Pred	Cattle	% Total Non-Pred	Calves	% Total Non-Pred
	Head	*Percent*	*Head*	*Percent*	*Head*	*Percent*	*Head*	*Percent*
AL	300	1.5	600	2.7	200	1.0		
AZ	400	1.7			900	3.9		
AR	1,300	4.6	600	1.6	200	0.7		
CA	8,400	8.5	2,200	1.4	14,000	14.2		
CO	600	1.2	200	0.4	800	1.6		
CT					200	22.2		
DE								
FL	800	3.4	500	1.7	800	3.4		
GA	900	4.7	400	1.6	800	4.1		
HI								
ID	1,800	4.3			1,500	3.6		
IL	800	3.8	200	0.6	1,000	4.8		
IN	700	4.8	1,100	4.0	800	5.4		
IA	800	1.2	800	0.7	1,300	2.0		
KS	1,700	1.3	200	0.3	900	0.7		
KY	1,000	3.0	1,000	1.4	500	1.5		
LA	200	0.8			300	1.2		
ME					200	12.5		
MD	200	6.7			300	10.0		
MA					100	10.0		
MI	2,100	8.4	100	0.2	2,400	9.6		
MN	2,300	5.5	400	0.5	2,800	6.7		
MS					600	2.9		
MO	1,200	1.7	700	0.6	800	1.2		
MT	200	1.0	300	0.7				
NE	800	0.8	100	0.1				
NV	200	3.4			500	8.6		
NH								
NJ								
NM	3,000	12.6			5,400	22.7		
NY	3,900	11.2	200	0.4	5,600	16.0		
NC	300	2.1			300	2.1		
ND	300	2.6	300	0.8	100	0.9		
OH	1,300	6.0	800	2.6	1,400	6.5		
OK	900	0.9	600	0.5	400	0.4		
OR	1,700	7.5	300	0.8	1,700	7.5		
PA	3,000	9.4	1,000	2.3	3,800	11.9		
RI								
SC								
SD	1,400	2.7	200	0.2	1,900	3.6		
TN	1,700	4.6	600	1.0	900	2.4		
TX	2,700	0.9	3,100	1.3	3,500	1.2		
UT	600	4.0	200	0.9	700	4.7		
VT	500	8.5			1,000	16.9		
VA	700	2.7	100	0.2	800	3.1		
WA	2,600	12.9	300	1.4	1,400	7.0		
WV	400	5.8						
WI	7,600	11.7	500	0.4	5,400	8.3		
WY	200	1.9						
Unpub	500		400		800			
US	60,000	3.6	18,000	0.8	67,000	4.0		

[1] Missing data are less than 100 head for the State or not published to avoid disclosure of individual operations. Data not published are included in U.S. total. Totals may not add due to rounding.

Losses of Cattle and Calves: Number and Percent from Other Diseases and Lameness/Injury, by State and United States, 2005 [1]

State	Other Diseases				Lameness/Injury			
	Cattle	% Total Non-Pred	Calves	% Total Non-Pred	Cattle	% Total Non-Pred	Calves	% Total Non-Pred
	Head	*Percent*	*Head*	*Percent*	*Head*	*Percent*	*Head*	*Percent*
AL	300	1.5	1,000	4.4	900	4.6	800	3.6
AZ	300	1.3	2,800	14.4	600	2.6	300	1.5
AR	1,300	4.6	1,300	3.4	1,000	3.5	900	2.3
CA	7,500	7.6	3,500	2.2	7,300	7.4	7,300	4.6
CO	5,800	11.8	1,800	3.5	4,300	8.7	200	0.4
CT								
DE								
FL	1,000	4.3	700	2.4	700	3.0	400	1.4
GA	600	3.1	700	2.8	1,400	7.3	600	2.4
HI								
ID	2,100	5.1	2,500	4.1	2,600	6.3	500	0.8
IL	600	2.9	900	2.7	2,100	10.1	300	0.9
IN	600	4.1	800	2.9	900	6.1	500	1.8
IA	2,500	3.8	4,300	4.0	3,100	4.8	1,000	0.9
KS	1,900	1.4	2,100	3.2	1,900	1.4	500	0.8
KY	2,200	6.5	2,800	4.0	1,600	4.8	1,100	1.6
LA	1,000	4.0	800	4.9	600	2.4	300	1.8
ME					200	12.5		
MD	200	6.7	200	4.1	300	10.0		
MA								
MI	2,800	11.2	4,200	9.0	2,000	8.0	900	1.9
MN	2,400	5.7	2,900	3.6	4,100	9.8	1,000	1.2
MS	1,100	5.3	1,100	4.2	700	3.4	500	1.9
MO	4,500	6.6	4,000	3.4	2,300	3.4	1,600	1.4
MT	2,200	11.3	1,100	2.5	1,000	5.2	600	1.4
NE	3,200	3.4	1,000	1.4	3,900	4.1	800	1.1
NV			300	2.5	200	3.4		
NH								
NJ								
NM	2,200	9.2			300	1.3	1,700	5.6
NY	2,800	8.0	3,400	6.9	4,300	12.3	700	1.4
NC	700	4.8	400	2.0	800	5.5	500	2.5
ND	800	7.0	1,000	2.7	800	7.0	400	1.1
OH	300	1.4	1,000	3.2	1,000	4.6	300	1.0
OK	6,800	6.9	6,000	4.8	2,800	2.9	1,500	1.2
OR	1,600	7.1	800	2.1	1,600	7.1	1,200	3.2
PA	3,500	10.9	1,900	4.4	3,700	11.6	1,400	3.3
RI								
SC	200	3.4	500	5.8	300	5.2		
SD	4,900	9.3	1,900	2.3	3,200	6.1	700	0.8
TN	1,000	2.7	4,000	6.9	2,700	7.3	1,300	2.3
TX	20,000	6.8	6,700	2.9	8,900	3.0	4,800	2.0
UT	500	3.4	1,200	5.1	500	3.4		
VT	400	6.8	300	3.8	1,200	20.3	300	3.8
VA	1,400	5.5	1,700	3.3	1,200	4.7	500	1.0
WA	1,200	6.0	400	1.9	2,400	11.9	300	1.4
WV	300	4.3	100	1.1			200	2.1
WI	3,300	5.1	3,600	2.6	10,000	15.5	5,800	4.2
WY	600	5.7	800	2.9				
Unpub	400		500		600		300	
US	97,000	5.8	77,000	3.5	90,000	5.3	42,000	1.9

[1] Missing data are less than 100 head for the State or not published to avoid disclosure of individual operations. Data not published are included in U.S. total. Totals may not add due to rounding.

Losses of Cattle and Calves: Number and Percent from Weather Related and Calving Problems, by State and United States, 2005 [1]

State	Weather Related				Calving Problems			
	Cattle	% Total Non-Pred	Calves	% Total Non-Pred	Cattle	% Total Non-Pred	Calves	% Total Non-Pred
	Head	*Percent*	*Head*	*Percent*	*Head*	*Percent*	*Head*	*Percent*
AL	2,100	10.8	1,100	4.9	4,000	20.5	5,900	26.2
AZ	700	3.0	3,000	15.5	1,100	4.7	700	3.6
AR	1,200	4.3	2,400	6.2	5,700	20.2	9,900	25.7
CA	2,200	2.2	2,500	1.6	10,300	10.4	6,400	4.0
CO	1,700	3.4	3,400	6.7	2,700	5.5	14,100	27.6
CT					200	22.2		
DE							100	14.3
FL	2,700	11.6	3,100	10.7	3,200	13.7	3,300	11.4
GA	1,500	7.8	1,800	7.2	4,100	21.2	7,400	29.5
HI			200	11.8	200	7.1	100	5.9
ID	200	0.5	1,100	1.8	3,300	8.0	5,900	9.7
IL	900	4.3	3,600	11.0	3,000	14.4	5,100	15.5
IN	500	3.4	1,400	5.0	1,800	12.2	2,900	10.4
IA	3,500	5.4	9,400	8.7	6,500	10.0	25,000	23.0
KS	7,200	5.4	7,500	11.3	2,800	2.1	13,200	20.0
KY	2,500	7.4	4,800	6.8	5,400	16.1	12,500	17.8
LA	12,700	50.6	6,600	40.5	2,600	10.4	2,100	12.9
ME			300	9.7	200	12.5		
MD	200	6.7	100	2.0	200	6.7	700	14.3
MA					200	20.0		
MI	300	1.2	1,200	2.6	3,900	15.6	6,200	13.3
MN	1,500	3.6	4,200	5.2	4,800	11.5	14,000	17.4
MS	2,100	10.1	4,100	15.5	4,200	20.2	8,400	31.8
MO	5,700	8.3	13,300	11.5	13,000	19.0	25,100	21.6
MT	1,200	6.2	10,200	23.4	1,500	7.7	11,300	25.9
NE	13,300	14.1	12,000	16.5	2,400	2.5	11,900	16.3
NV	500	8.6	600	5.0	300	5.2	600	5.0
NH							100	10.0
NJ					300	30.0	400	40.0
NM	400	1.7	7,100	23.5	1,900	8.0	7,100	23.5
NY	500	1.4	1,300	2.6	3,700	10.6	2,600	5.3
NC	700	4.8	200	1.0	2,000	13.8	5,300	26.2
ND	1,800	15.7	3,200	8.5	800	7.0	12,500	33.2
OH	1,100	5.1	1,400	4.5	1,700	7.9	3,800	12.2
OK	4,400	4.5	3,700	3.0	9,800	10.0	34,000	27.1
OR	200	0.9	1,400	3.7	1,400	6.2	6,900	18.2
PA	400	1.3	900	2.1	5,100	15.9	6,500	15.1
RI								
SC	300	5.2	600	7.0	1,300	22.4	3,400	39.5
SD	5,200	9.9	12,200	14.8	2,800	5.3	15,500	18.7
TN	1,700	4.6	3,400	5.9	7,700	20.9	12,800	22.2
TX	23,600	8.0	10,500	4.5	44,400	15.0	44,000	18.7
UT	700	4.7	2,900	12.3	1,000	6.7	3,100	13.2
VT			100	1.3	1,000	16.9	600	7.7
VA	1,100	4.3	4,400	8.4	3,900	15.2	15,300	29.3
WA	300	1.5	800	3.7	1,300	6.5	3,200	15.0
WV	400	5.8	2,600	27.4	1,200	17.4	1,800	18.9
WI	1,500	2.3	4,200	3.1	7,200	11.1	6,200	4.5
WY	1,000	9.5	6,000	21.8	800	7.6	7,000	25.5
Unpub	300		200		100		100	
US	110,000	6.5	165,000	7.6	187,000	11.1	385,000	17.7

[1] Missing data are less than 100 head for the State or not published to avoid disclosure of individual operations. Data not published are included in U.S. total. Totals may not add due to rounding.

Losses of Cattle and Calves: Number and Percent from Poisoning and Theft, by State and United States, 2005 [1]

State	Poisoning				Theft			
	Cattle	% Total Non-Pred	Calves	% Total Non Pred	Cattle	% Total Non-Pred	Calves	% Total Non-Pred
	Head	*Percent*	*Head*	*Percent*	*Head*	*Percent*	*Head*	*Percent*
AL	400	2.1					200	0.9
AZ	900	3.9	200	1.0			200	1.0
AR	500	1.8	700	1.8	400	1.4	500	1.3
CA	200	0.2			800	0.8	1,900	1.2
CO	1,900	3.9	200	0.4			700	1.4
CT								
DE								
FL	1,300	5.6			100	0.4		
GA	400	2.1	100	0.4				
HI								
ID	500	1.2	300	0.5	100	0.2	100	0.2
IL			400	1.2				
IN								
IA	200	0.3						
KS	800	0.6	400	0.6	500	0.4	100	0.2
KY	600	1.8	300	0.4	100	0.3		
LA								
ME								
MD								
MA	100	10.0						
MI								
MN	200	0.5			100	0.2		
MS	200	1.0	200	0.8				
MO	1,900	2.8	2,000	1.7	700	1.0	200	0.2
MT	800	4.1	300	0.7			300	0.7
NE	800	0.8	500	0.7			300	0.4
NV	200	3.4			600	10.3	200	1.7
NH								
NJ								
NM	700	2.9	600	2.0				
NY	100	0.3						
NC								
ND	100	0.9			200	1.7		
OH	500	2.3	800	2.6				
OK	200	0.2	1,100	0.9	900	0.9	1,100	0.9
OR	400	1.8	600	1.6			100	0.3
PA	300	0.9	300	0.7				
RI								
SC					300	5.2		
SD	200	0.4	300	0.4	400	0.8	400	0.5
TN	400	1.1	800	1.4			600	1.0
TX	4,500	1.5	1,500	0.6	3,300	1.1	1,800	0.8
UT	1,200	8.1	300	1.3	500	3.4		
VT	100	1.7						
VA	500	2.0	500	1.0	100	0.4	1,200	2.3
WA	100	0.5			300	1.5		
WV	400	5.8					100	1.1
WI	400	0.6	2,600	1.9				
WY	800	7.6	700	2.5	100	1.0	500	1.8
Unpub	200		300		500		500	
US	23,000	1.4	16,000	0.7	10,000	0.6	11,000	0.5

[1] Missing data are less than 100 head for the State or not published to avoid disclosure of individual operations. Data not published are included in U.S. total. Totals may not add due to rounding.

Losses of Cattle and Calves: Number from Other and Unknown Causes, by State and United States, 2005 [1]

State	Other Non-Predator				Unknown Non-Predator			
	Cattle	% Total Non-Pred	Calves	% Total Non-Pred	Cattle	% Total Non-Pred	Calves	% Total Non-Pred
	Head	*Percent*	*Head*	*Percent*	*Head*	*Percent*	*Head*	*Percent*
AL	4,900	25.1	1,700	7.6	3,100	15.9	5,300	23.6
AZ	500	2.1			14,700	63.1	2,700	13.9
AR	5,200	18.4	1,000	2.6	5,200	18.4	6,100	15.8
CA	6,600	6.7	3,800	2.4	5,800	5.9	9,300	5.8
CO	3,200	6.5	700	1.4	3,900	7.9	4,000	7.8
CT	100	11.1						
DE					400	66.7	500	71.4
FL	4,200	18.0	900	3.1	4,500	19.3	8,600	29.8
GA	4,600	23.8	500	2.0	1,200	6.2	3,200	12.7
HI	400	14.3	200	11.8	1,900	67.9	1,100	64.7
ID	2,000	4.8	1,000	1.6	17,300	41.7	10,000	16.4
IL	2,800	13.5	200	0.6	1,800	8.7	2,900	8.8
IN	1,600	10.9	800	2.9	3,000	20.4	2,200	7.9
IA	6,300	9.7	5,000	4.6	4,300	6.6	6,100	5.6
KS	7,900	5.9	2,500	3.8	3,000	2.2	5,000	7.6
KY	4,700	14.0	1,200	1.7	7,600	22.6	14,400	20.5
LA	2,600	10.4	300	1.8	2,600	10.4	1,400	8.6
ME	200	12.5	100	3.2	200	12.5	600	19.4
MD	100	3.3			800	26.7	1,600	32.7
MA			100	11.1				
MI	1,200	4.8	400	0.9	1,500	6.0	2,300	4.9
MN	5,100	12.2	3,400	4.2	4,700	11.2	3,500	4.4
MS	5,900	28.4	1,100	4.2	2,700	13.0	4,000	15.2
MO	16,200	23.6	6,800	5.9	10,700	15.6	10,100	8.7
MT	3,500	18.0	900	2.1	4,200	21.6	5,300	12.2
NE	6,300	6.7	1,900	2.6	10,600	11.2	8,800	12.1
NV	600	10.3	200	1.7	1,200	20.7	2,400	20.2
NH	200	25.0	200	20.0			200	20.0
NJ	100	10.0			200	20.0		
NM	3,700	15.5	300	1.0	1,600	6.7	5,300	17.5
NY	2,300	6.6	2,600	5.3	1,900	5.4	3,700	7.5
NC	1,600	11.0	500	2.5	3,000	20.7	6,400	31.7
ND	2,200	19.1	600	1.6	1,500	13.0	3,400	9.0
OH	900	4.2			1,200	5.6	1,100	3.5
OK	15,200	15.5	3,200	2.6	11,500	11.7	14,700	11.7
OR	2,200	9.7	800	2.1	2,700	11.9	3,500	9.2
PA	1,500	4.7	400	0.9	3,100	9.7	4,000	9.3
RI								
SC	1,300	22.4	200	2.3	1,200	20.7	1,600	18.6
SD	6,400	12.1	800	1.0	6,300	12.0	3,500	4.2
TN	5,800	15.8	2,100	3.6	5,300	14.4	7,000	12.2
TX	55,300	18.7	7,600	3.2	43,700	14.8	41,000	17.4
UT	1,900	12.8	500	2.1	2,700	18.1	1,900	8.1
VT	300	5.1	400	5.1	600	10.2	1,100	14.1
VA	4,000	15.6	1,100	2.1	7,200	28.1	13,400	25.7
WA	1,400	7.0	700	3.3	2,100	10.4	2,000	9.3
WV	1,600	23.2	500	5.3	900	13.0	1,600	16.8
WI	3,200	4.9	1,500	1.1	10,200	15.8	12,900	9.4
WY	3,100	29.5	1,000	3.6				
Unpub	100		300		200		300	
US	211,000	12.5	60,000	2.8	224,000	13.3	250,000	11.5

[1] Missing data are less than 100 head for the State or not published to avoid disclosure of individual operations. Data not published are included in U.S. total. Totals may not add due to rounding.

Non-Lethal Methods Used to Prevent Losses of Cattle and Calves to Predators, by State and United States, 2005 [1] [2]

State	Guard Animals	Exclusion Fencing	Herding	Night Penning	Frequent Checks
	Percent	*Percent*	*Percent*	*Percent*	*Percent*
AL	48.8	55.5	4.4	0.2	9.6
AZ	35.5	53.2	14.3	22.9	38.2
AR	37.7	37.8	2.2		24.0
CA	20.1	66.3	0.9	5.1	8.2
CO	16.5	27.0	7.6	9.6	38.9
CT	12.7	27.0	4.8	69.8	52.3
DE					
FL	51.1	23.1	5.2	0.1	26.0
GA	42.3	57.7	2.1	3.6	12.0
HI	1.7	84.1	4.5		10.7
ID	16.9	14.8	1.7	1.4	60.9
IL	40.1	55.2	5.0	11.9	26.1
IN	23.7	17.1	2.3	19.6	47.2
IA	15.4	15.9		3.2	64.0
KS	7.2	45.9	2.7		13.8
KY	33.1	49.1	2.8	3.9	7.8
LA	25.2	52.3	10.8	1.8	13.5
ME		77.8		20.7	0.8
MD					
MA	25.2	63.3		30.3	
MI	70.8	29.4	6.4	0.8	12.6
MN	61.9	40.2	2.5	6.3	20.2
MS	30.0	25.0		0.5	3.1
MO	57.4	21.9	5.4	1.1	15.5
MT	21.8	21.1	9.6	9.9	31.4
NE	8.5	42.7	7.5	2.4	47.9
NV		1.6		26.1	56.9
NH	0.3	2.4		97.1	
NJ	40.3	70.2	15.6	43.6	23.5
NM	8.6	29.5	5.3	13.9	37.0
NY		12.2	77.0	8.2	70.5
NC	22.3	46.7	8.7	8.1	36.3
ND	34.5	19.7	0.3	6.0	29.0
OH	22.9	45.9	0.3	7.6	12.3
OK	49.2	32.9	6.8	0.2	23.3
OR	26.2	32.0	1.4	5.9	25.5
PA	46.0	40.5	9.0	17.9	28.5
RI					
SC	70.0	24.2	0.4		8.1
SD	21.9	4.6	2.7	33.6	14.7
TN	32.9	19.2	6.5	3.6	28.8
TX	62.9	17.3	0.9	10.2	15.2
UT	32.3	49.3	47.9	13.0	21.8
VT	64.8	33.0			
VA	29.6	48.9	0.1	1.1	18.7
WA	43.8	48.1		0.2	43.1
WV	38.0	30.4	1.9	3.5	52.5
WI	54.3	28.1		9.5	10.1
WY	20.9	20.5	10.0	21.6	48.0
US	38.0	34.0	3.8	9.7	21.8

[1] Missing data are not published to avoid disclosure of individual operations.
[2] Use of multiple non-lethal methods may result in percentages summing to greater than 100.

Non-Lethal Methods Used to Prevent Losses of Cattle and Calves to Predators, by State and United States, 2005 [1] [2]

State	Fright Tactics	Livestock Carcass Removal	Culling	Other Non-lethal
	Percent	*Percent*	*Percent*	*Percent*
AL	2.6	11.5	8.2	2.0
AZ	11.8	13.8	40.7	1.2
AR	13.4	13.5	14.6	5.7
CA		10.6	35.5	6.3
CO	7.0	15.5	14.3	0.1
CT		17.5	17.5	12.7
DE				
FL	0.5	20.4	29.3	0.1
GA	0.4	6.6	10.4	5.4
HI	1.7	9.1	7.9	8.0
ID	20.6	15.0	16.8	6.0
IL		17.0	26.7	
IN	5.7	26.7	36.4	0.1
IA		2.2	3.3	7.1
KS		22.1	30.2	0.2
KY		1.1	4.3	12.5
LA	2.2	14.5	3.4	13.8
ME		2.2	2.2	
MD				
MA				6.5
MI		15.6	9.1	1.6
MN	3.2	13.0	19.0	5.5
MS	3.1	13.1	25.6	4.3
MO	1.5	11.0	12.5	4.6
MT	7.8	18.5	31.7	11.7
NE	2.5	50.4	41.1	4.0
NV	15.4	30.9	30.9	
NH				0.2
NJ	3.4	18.9	3.4	6.3
NM	25.5	44.4	31.5	2.0
NY	9.2	74.4	89.2	17.2
NC	3.1	27.4	39.7	1.0
ND	2.8	16.4	19.4	11.8
OH	4.1	6.8	12.2	15.4
OK	2.5	29.4	5.9	11.2
OR	1.6	41.4	47.6	4.3
PA	2.9	18.2	22.4	4.7
RI				
SC	0.7	5.5	5.2	
SD	4.4	22.7	28.6	12.0
TN	0.8	32.3	30.1	5.9
TX	0.1	5.2	15.3	8.4
UT		4.5	38.0	
VT			1.5	0.7
VA		28.2	42.1	13.9
WA	4.2	13.6	14.1	13.7
WV		4.7	19.9	6.5
WI	2.6	14.7	9.4	6.8
WY	0.5	32.7	42.0	12.2
US	3.0	16.5	19.6	6.2

[1] Missing data are not published to avoid disclosure of individual operations.
[2] Use of multiple non-lethal methods may result in percentages summing to greater than 100.

Reliability of Cattle and Calves Predator Loss Estimates

Survey Procedures: A random sample of U.S. producers was surveyed to provide data for these estimates. Survey procedures ensured that all cattle producers, regardless of size, had a chance to be included in the survey. Large producers were sampled more heavily than small operations. About 50,000 operators were contacted during the first half of January by mail, telephone and face-to-face personal interview.

Estimation Procedures: These estimates of death loss by cause were prepared by the Livestock Branch, NASS, and reviewed by the 49 States involved. Total cattle and calf death losses from all causes were published in the 2006 "*Meat Animals-Production, Disposition, and Income*" (released April 27, 2006). In setting the predator and non-predator loss estimates, first total predator and non-predator losses were estimated as a percent of total losses, then specific predator and non-predator losses were estimated as a percent of total predator and non-predator losses. All published loss estimates were rounded to the nearest 100 head. Value estimates were rounded to the nearest $1,000. State estimates of less than 100 head are not published, but are included in U.S. totals.

Revision Policy: Revisions to previous estimates are made to improve the current estimate. Previous year estimates are subject to revision when current estimates are made. Estimates for losses from all causes are subject to revision in next year's "*Meat Animals-Production, Disposition, and Income*". No revision to predator losses is planned.

Reliability: Since all cattle operators are not included in the sample, survey estimates are subject to sampling variability. Survey results are also subject to non-sampling errors such as omissions, duplications, and mistakes in reporting, recording, and processing the data. The effects of these non-sampling errors cannot be measured directly. They are minimized through rigid quality controls in the data collection process and through a careful review of all reported data for consistency and reasonableness.

Information Contacts

Listed below are the commodity specialists in the Livestock Branch of the National Agricultural Statistics Service to contact for additional information.

Dan Kerestes, Chief, Livestock Branch . . . . (202) 720-3570

William Weaver, Head, Livestock Section . . . . (202) 720-3570
Bruce Boess - Hogs and Pigs . . . . (202) 720-3106
Shawn Clark - Dairy Products . . . . (202) 690-3236
Scott Hollis - Sheep, Lambs, Wool, Goats and Mohair . . . . (202) 720-4751
Mike Miller - Cattle . . . . (202) 720-3040
Katy Paulsen - Livestock Slaughter . . . . (515) 284-4340
Ann Summa - Milk and Milk Cows . . . . (202) 720-3278
Charmaine Wilson - Dairy Products Prices . . . . (202) 690-2168

ACCESS TO REPORTS!!

For your convenience, there are several ways to obtain NASS reports, data products, and services:

INTERNET ACCESS

All NASS reports are available free of charge on the worldwide Internet. For access, connect to the Internet and go to the NASS Home Page at: **www.nass.usda.gov**.

E-MAIL SUBSCRIPTION

All NASS reports are available by subscription free of charge direct to your e-mail address. Starting with the NASS Home Page at **www.nass.usda.gov**, under the right navigation, *Receive reports by Email*, click on **National** or **State**. Follow the instructions on the screen.

- -

PRINTED REPORTS OR DATA PRODUCTS

CALL OUR TOLL-FREE ORDER DESK: 800-999-6779 (U.S. and Canada)
Other areas, please call 703-605-6220 FAX: 703-605-6900
(Visa, MasterCard, check, or money order acceptable for payment.)

- -

ASSISTANCE

For **assistance** with general agricultural statistics or further information about NASS or its products or services, contact the **Agricultural Statistics Hotline** at **800-727-9540**, 7:30 a.m. to 4:00 p.m. ET, or e-mail: **nass@nass.usda.gov.**

The U.S. Department of Agriculture (USDA) prohibits discrimination in all its programs and activities on the basis of race, color, national origin, age, disability, and where applicable, sex, marital status, familial status, parental status, religion, sexual orientation, genetic information, political beliefs, reprisal, or because all or part of an individual's income is derived from any public assistance program. (Not all prohibited bases apply to all programs.) Persons with disabilities who require alternative means for communication of program information (Braille, large print, audiotape, etc.) should contact USDA's TARGET Center at (202) 720-2600 (voice and TDD).

To file a complaint of discrimination, write to USDA, Director, Office of Civil Rights, 1400 Independence Avenue, S.W., Washington, D.C. 20250-9410, or call (800) 795-3272 (voice) or (202) 720-6382 (TDD). USDA is an equal opportunity provider and employer.
