# Supplementary material for: The Elephant in the room: What can we learn from California regarding the use of sport hunting of pumas (Puma concolor) as a management tool?
Source: PLoS One. 2020 Feb 13;15(2):e0224638. doi: 10.1371/journal.pone.0224638 (PMC7018503; doi:10.1371/journal.pone.0224638)

REPORT TO THE FISH AND GAME COMMISSION

An Assessment of Mule and Black-tailed Deer Habitats and Populations in California

With Special Emphasis on Public Lands Administered by the Bureau of Land Management and the United States Forest Service
February 1998

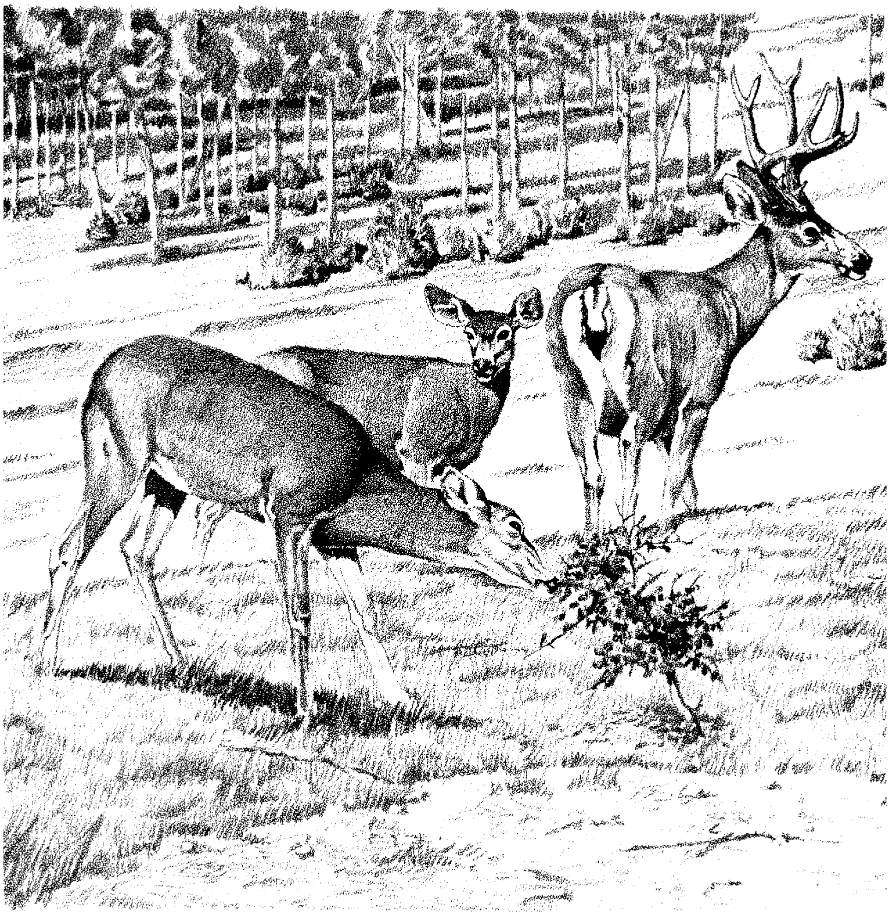

CONTENTS

I. EXECUTIVE SUMMARY 4

II. BACKGROUND 6
Objectives of the Workshop 6
Participation 7
Geographic Area of Analysis 7

III. INTRODUCTION 9
Using Deer as an "Umbrella" or Flagship Species for Habitat Conditions in California 10
Role of the Department of Fish and Game in managing deer and wildlife habitat 10
Role of the United States Forest Service in managing deer and wildlife habitat 11
Role of the Bureau of Land Management in managing deer and wildlife habitat 12
Who Owns and Administers Wildlife Habitat in California? 13

IV. DEER POPULATION TRENDS 13
California Deer Populations in Relation to Habitat Quality (How Did We Get Here?) 15

V. COMMON FACTORS AFFECTING DEER HABITATS 20
Habitat Components 20
Factors Affecting Deer 21

VI. COMMON FACTORS AFFECTING DEER HABITATS ON PUBLIC LANDS 21
Forests moving away from early successional habitats through the lack of fire and through fire suppression 22
Habitat changes on rangelands as influenced by fire 23
Forests moving away from early successional habitats through timber management practices and reforestation efforts 24
Forest Thinning and Whole-tree Removal (Biomass Thinning) 24
Herbicide Treatments 25
Livestock grazing impacts on important habitats and natural communities 25
Aspen 26
Mountain meadows and montane riparian zones 27
Great Basin and Desert Ranges- riparian, springs, seeps, and meadows 27
Great Basin shrub/grass communities (sagebrush, bitterbrush, mountain mahogany) 28
Hardwoods and associated shrub communities 29
Recognizing the role that private lands have in affecting deer range 29

Collaborative Effort & Document preparation, editing by:

Eric R. Loft, California Department of Fish & Game
Don Armentrout, Bureau of Land Management
Gary Smith, U.S. Forest Service
Diana Craig, U.S. Forest Service
Michael Chapel, U.S. Forest Service
John Willoughby, Bureau of Land Management
Carl Rountree, Bureau of Land Management
Terry Mansfield, California Department of Fish & Game
Sonke Mastrup, California Department of Fish and Game
Frank Hall, California Department of Fish & Game

We thank the participants listed on page 46 for their involvement in this effort.

Cover art: *Paul B. Johnson*

Compilation of a workshop held April 29, 1997 at the Feather River Inn, Portola, California. Prepared by: California Department of Fish & Game, Wildlife Management Division; United States Department of Interior, Bureau of Land Management; & United States Department of Agriculture, Forest Service. February 1998.

Copies of this report are available from:
California Department of Fish and Game
Wildlife Management Division
1416 Ninth St., Sacramento, CA 95814; or e-mail to: eloft@dfg.ca.gov. Internet users will be able to download a copy at: www.dfg.ca.gov/wmd/deer.html

VII. UNIT EVALUATIONS (DEER ASSESSMENT UNITS) 30
DAU 1- North Coast 30
DAU 2- Northeastern California 31
DAU 3- Northeast Sierra Nevada 33
DAU 4- Cascade-North Sierra Nevada 34
DAU 5- Central Sierra Nevada 35
DAU 6- Eastern Sierra Nevada 36
DAU 7- Southern Sierra Nevada 37
DAU 8- Central Coast (north) 38
DAU 9- Central Coast (south) 39
DAU 10- South Coast 40
DAU 11- Desert 41

VIII. DEVELOPMENT OF FOCUS AREAS FOR COLLABORATIVE EFFORTS TO ENHANCE CONDITIONS FOR DEER **42**
Identification of Priority Areas 42

IX. PILOT DAU MANAGEMENT STRATEGY (for focus DAUs #2 & #3) **43**

X. REFERENCES **44**

Appendix 1. Participants at Workshop **46**
Appendix 2. Additional Fish and Game Code sections applying to deer management **47**
Appendix 3. Tabular data and maps of 11 Deer Assessment Units (DAUs) **48**
Color graphics and photos referred to in text **50-56**

"Hopefully, we all recognized that the habitats on which deer depend in California, are most of the same habitats on which much of the more recent focus--on ecosystem management and biodiversity--also depend."

"We're talking about a process called "succession"-- the seemingly orderly replacement of plant communities, and the animals which depend on them, through a progression to a stable state. Deer and possibly many other unmonitored species are being orderly "replaced out" by the decline of significant, large-scale disturbances to California's forestlands. Reduced biological diversity cannot help but be a consequence of this progression away from a diversity of successional communities."

"More sunlight hitting the ground is in order, as Smokey himself would likely find today's forests less habitable than when he was born."

"Livestock can be fed on valley ranches, but deer and elk must find food in the foothills or starve. The habitable wintering belt is narrow, ... these scattering foothill clumps of bitterbrush, sage, and oak, now fast shrinking under the onslaught of cheat fires, are the key to wildlife survival in the whole region. Besides, these scattered bushed often harbor, under their mechanical protection, remnants of native perennial grasses.... While the sportsmen and stockmen wrangle over who should move first in easing the burden on the winter range, cheat grass is leaving less and less winter range to wrangle about." --A. Leopold, A Sand County Almanac. 1949.

"You in America have a great faith in research, statistics, surveys, and conferences. Words, theories and oratory will never raise one head of game. The chance for improvement lies in action and work." from: Rambling thoughts from a perverted Britisher--Capt. Percy R. Creed, reprinted in California Fish and Game (1930, p314).

I. EXECUTIVE SUMMARY

California's mule and black-tailed deer are among our most visible and widespread wildlife species, inhabiting much of the wildlands in the state. Consequently, their value as representatives of California's wildlife resources is high. Deer are enjoyed for viewing as in the mountain meadows of Yosemite National Park, along 17-mile drive on the Monterey Peninsula, or concentrated on winter range on the east side of the Sierra Nevada and Cascade ranges. Deer are an integral component in the food chain, from their role as grazers/browsers of wildland plants to their role as prey species to California's top carnivores particularly the mountain lion, black bear, coyote, and golden eagle.

Deer inhabit about 75 percent of California's wildlands in a wide variety of habitats. Most of that habitat is administered as public land by the federal government (e.g., U.S. Forest Service, Bureau of Land Management, National Park Service, Military) or is privately owned (e.g., timber holdings, ranches). Because of the diverse ownership and objectives of each, improving deer habitat condition may not be a high priority in many areas.

Deer are also California's most popular game mammal, attracting between 165,000-200,000 hunters to the field annually. The opportunity to go deer hunting provides for thousands of Californian's and their families, the chance to get out of the office, away from the cities and suburbs, to enjoy the wildlands of the state. Most of that opportunity is realized on public lands.

In recent years, California deer populations have experienced declines in several areas of the state, while in others, populations have remained fairly stable. The greatest declines have occurred in northeastern California and the Northern/Central Sierra Nevada. Conversely, deer populations along the western "half" of the state have remained stable to slightly increasing.

In response to a request from the Fish and Game Commission, the Department of Fish and Game, Bureau of Land Management, and U.S. Forest Service conducted a technical workshop in April 1997 to assess California's deer populations and habitat conditions on public lands. Essentially, the Commission asked whether the three agencies could initiate new work together to improve habitat conditions for deer on public lands.

With the state divided into 11 Deer Assessment Units (DAUs), workshop participants discussed deer population trends, habitat status, habitat issues, and opportunities for changes in habitat condition. This report is a product of that workshop and expands on the topics that were discussed to improve the understanding of concerns and issues related to deer habitat and management of public lands.

Based on the assessment of deer population trends, two DAUs- Northeastern California and Northeast Sierra Nevada were identified as the top priority to initiate efforts to improve conditions for deer. The second highest priority was determined to be either the Central Sierra Nevada where deer declines have been substantial, or a combination of the Eastern and Southern Sierra Nevada where there is opportunity to capitalize on several years of intensive research on deer and factors affecting them. The analysis in this report reflects the importance of priority placed on the various DAUs. Consequently, there is substantially more discussion that applies to Northeastern California and Sierra Nevada units than to the Coastal or Desert units.

On forest-dominated areas such as the west slope of the Sierra Nevada, or in chaparral communities of the Coast Range, deer thrive on early successional habitats that are a product of disturbances that open up the forest and shrub canopy to allow grass/forb/shrub growth to occur. Fire and logging have been the primary mechanisms enabling "sunlight to hit the ground" in forests. Management changes over the decades have decreased the disturbance to these habitats, resulting in declining habitat condition for deer. Because it occurs at a scale of millions of acres, this is likely the single greatest factor contributing to the lowered deer populations we experience today compared to the past several decades. Practices related to timber management such as stand thinning, biomass, underburning/fuel reduction, and herbicide spraying were identified as concerns affecting early successional vegetation important to deer.

Within forested summer ranges, mountain meadow, riparian, aspen, and montane shrub communities can provide valuable high quality habitats depending on the condition of forage and cover. However, livestock grazing can negatively affect the quality of those habitats when grazing/browsing is excessive. Concerns relating to conditions of specific habitat types or vegetation communities exist in Great Basin ranges in northeastern California and east of the Sierra Nevada. Here, livestock and wild horse/burro impacts on bitterbrush stands, aspen stands, and the small riparian-wetland areas are of concern because these communities are important in the life history of mule deer. More effective monitoring of livestock use and enforcement of existing standards would likely result in improvements to many habitats.

Impacts of juniper encroachment and expansion of cheatgrass range further degrade the condition of the range and exacerbate potential competition between wild and domestic herbivores. Development of winter range on private lands (e.g., near Susanville, Reno, and Bishop) removes acreage of deer winter range and increases deer-human conflict. Public lands occurring at this urban-wildland interface can become more important than in the past as the remaining habitats for wildlife. Incidence of summer wildfire on the east side has also contributed substantially to the problems faced by deer. Fire in these habitats frequently kills desirable browse species which are often replaced by undesirable, exotic annuals such as cheatgrass. Harsh and unreliable precipitation patterns causing either drought or winter die-off have both occurred in the past decade, further compounding factors that can be managed.

At lower elevation winter ranges of the Sierra Nevada, urbanization on private land increases the relative value to wildlife of remaining public lands. Increasing age (decadence) of shrub stands and decline in habitat quality is a concern as in higher elevation forested ranges. In some areas, competition can be a factor when livestock graze/browse vegetation during spring and summer that deer must rely on in winter.

Identification of factors negatively affecting deer habitat, how they relate to deer, and potential opportunities to alleviate the negative impacts were discussed. It is important to recognize that land management activities that could benefit deer, could also benefit many other game and nongame wildlife species. Development and implementation of a Pilot Management Strategy encompassing entire DAUs is proposed. A process to assess, more specifically, the factors affecting habitats is a component of this strategy, as is the need to involve other stakeholders interested in public lands management. As we proceed beyond this general technical assessment to specific management strategies, and ultimately implementation, all three agencies would need to make commitments to support projects and activities to improve habitat conditions.

II. BACKGROUND

At the request of the Fish and Game Commission, Wildlife Management Division staff in the Department of Fish and Game (hereafter Department or DFG) met with representatives of the U.S. Forest Service (hereafter USFS) and Bureau of Land Management (hereafter BLM) in late January 1997 to begin investigating the potential to increase efforts to improve conditions for deer on public lands administered by the USFS and BLM. The initial meeting concluded with a collective recommendation that an overall assessment of deer populations and deer habitat conditions on public land throughout the state was needed to help identify key problems and opportunities on an area by area basis.

This report is the product of that overall assessment and focuses specifically on public lands managed for multiple use. There is, however, an important link to private wildlands that is discussed because of the habitat implications to public land management as a consequence of management strategies occurring on private lands. Greater support from private landowners to maintain and enhance wildland habitats is highly desirable in the context of a landscape approach to habitat management. Gaining that support will require all the agencies to work together with private interests and local governments to develop programs, incentives, or other opportunities.

In assessing habitat status, we evaluated management activities contributing to the conditions that currently exist. With some activities, the DFG has historically been concerned about their impacts to deer and other wildlife. Some concerns may be easy to agree to, resolve, or take action. The easy ones are not the focus of this document. Rather, the intent here is to discuss some of the more difficult long-term issues that have affected the operations and relationships of all three of the agencies, and more importantly affected habitats, for several years, if not decades.

The agency representatives agreed that after the information contained herein has been reviewed and presented, we could begin a Pilot Management Strategy to incorporate possible alternatives to remedy deer habitat issues. This later stage will step beyond the technical recommendations and possible solutions to habitat issues in California, and involve the diverse perspectives and input from other interested parties (e.g., user groups representing hunting, fishing, livestock, and timber interests; other agencies and local governments, conservation organizations, or individuals).

Objectives of the Workshop

The recommendation for an assessment of deer populations and habitat conditions translated into a larger, one day workshop held April 29, 1997, at the Feather River Inn, Portola, California. The workshop objectives were straightforward:

1) On an area-by-area analysis, summarize:
   - -Area Description
   - -Location
   - -Ownership patterns
   - -Habitats of primary importance to deer

2) Discuss and capture local expert thoughts, ideas, and recommendations on:
   - -Assessment of deer population trends--an indicator of habitat quality

-Assessment of deer habitat trends
-Summarize factors affecting deer habitat: Positive Negative Neutral
-Opportunities to further enhance forest and rangeland habitats on public lands
-Constraints to further enhance forest and rangeland habitats on public lands

3) Summarize common and important themes and discuss future steps. Specifically, how can we be more effective at increasing the accommodation of early successional forested habitats and of preferred rangeland habitats on public lands to benefit deer?

Participation

The workshop provided an opportunity to bring together employees from the three agencies to improve the understanding of each agency's perspective on deer and wildlife habitat. This report is an expanded product of the one-day workshop, which in turn, is based on the decades of collective experience and professional opinions of the participants.

Workshop participants included: 17 from DFG, 1 from the Commission, 13 from USFS, and 7 from BLM (Appendix 1). Several local forest service personnel whose names were not captured on the attendance list were also present. DFG staff were present from throughout the state, USFS staff represented 6 of 18 national forests, and BLM staff were present from northeastern California and the Bakersfield District. Statewide representatives for each organization were also in attendance.

Collectively, we took a step back in time in discussing a game species and game management, a major focus of the two land management agencies during most of their existence, and always an important facet of the DFG. Hopefully, we all recognized that the habitats on which deer depend in California, are most of the same habitats on which much of the more recent focus--on ecosystem management and biodiversity--also depend.

Geographic Areas of Analysis

To help guide the assessment, the DFG provided a recently developed strategy for analyzing deer populations and habitat status. The intent of the new strategy is to have deer population/habitat analyses and deer harvest recommendations eventually based more on environmental and ecological factors than on the somewhat ecologically artificial boundaries of existing hunt zones. These preliminary Deer Assessment Units (or DAU's) were developed by DFG regional and staff biologists by combining existing deer hunt zones into DAU's based on similarities (Figure 1).

This new strategy would also reduce the number of geographic areas for data analysis from 45 to 11 (45 existing hunt zones versus 11 proposed DAU's) thereby providing more power to the analysis and reducing the amount of variability in DFG estimates (Appendix 3).

DEER ASSESSMENT UNITS (DAUs)

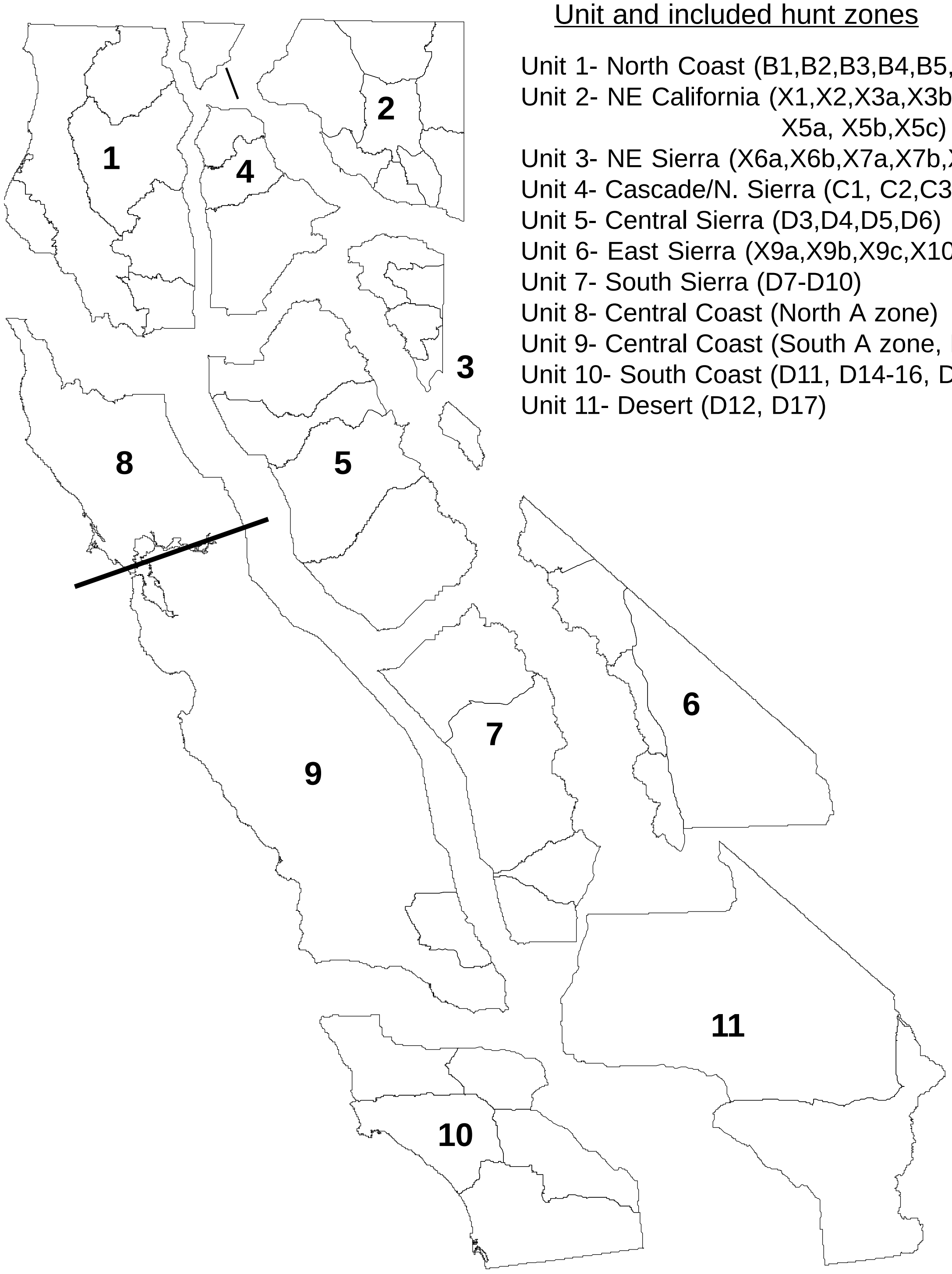

Unit and included hunt zones

Unit 1- North Coast (B1,B2,B3,B4,B5,B6)
Unit 2- NE California (X1,X2,X3a,X3b,X4, X5a, X5b,X5c)
Unit 3- NE Sierra (X6a,X6b,X7a,X7b,X8)
Unit 4- Cascade/N. Sierra (C1, C2,C3,C4)
Unit 5- Central Sierra (D3,D4,D5,D6)
Unit 6- East Sierra (X9a,X9b,X9c,X10,X12)
Unit 7- South Sierra (D7-D10)
Unit 8- Central Coast (North A zone)
Unit 9- Central Coast (South A zone, D13)
Unit 10- South Coast (D11, D14-16, D19)
Unit 11- Desert (D12, D17)

FIGURE 1. Deer population/hunting data in California is currently compiled on a zone basis. There are 45 primary hunt zones in the state. This graphic illustrates one possible grouping of these zones into 11 geographic areas (or deer assessment units) based primarily on environmental similarities.

III. INTRODUCTION

California's mule and black-tailed deer are among our most visible and widespread wildlife species, inhabiting much of the wildlands in the state (Figure 2). Consequently, their value as representatives of California's wildlife resources is high. Deer are enjoyed for viewing, as in the mountain meadows of Yosemite National Park, along 17-mile drive on the Monterey Peninsula, or concentrated on winter range on the east side of the Sierra Nevada and Cascade ranges. Deer are an integral component in the food chain, from their role as grazers/browsers of wildland plants to their role as prey species to California's top carnivores, particularly the mountain lion, black bear, coyote, and golden eagle.

Deer are also California's most popular game mammal, attracting between 165,000-200,000 hunters to the field annually. The opportunity to go deer hunting provides for thousands of Californians and their families the chance to get out of the office, away from the cities and suburbs, to enjoy the wildlands of the state. The economic value that the deer resource contributes to California through recreational activities was estimated in a detailed study in 1987 (Loomis et al. 1989) at over $450 million annually (at 1987 dollars and conditions).

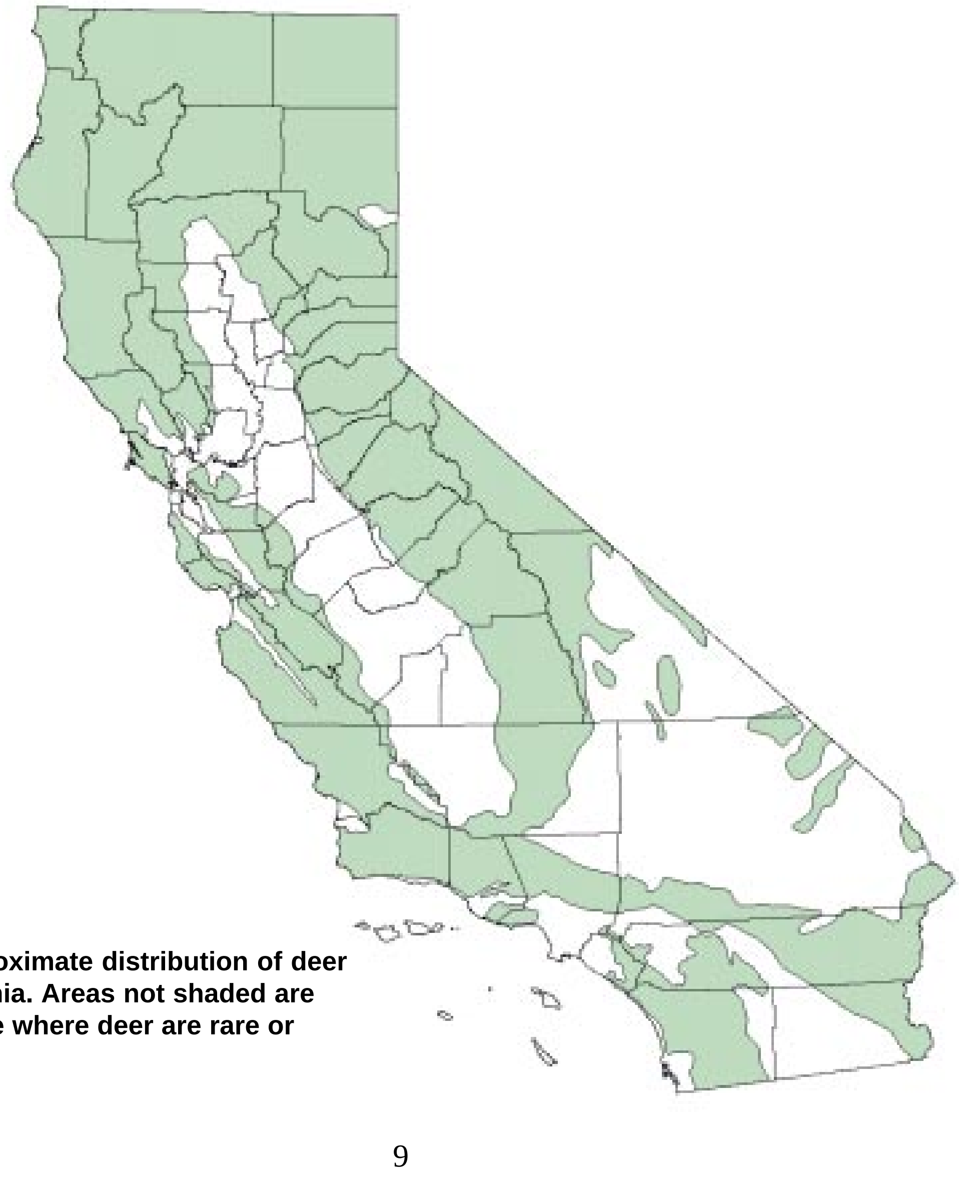

FIGURE 2. Approximate distribution of deer range in California. Areas not shaded are considered to be where deer are rare or absent.

Because of their role in nature, their widespread occurrence, and their long-standing popularity with hunting as well as non-hunting Californians, the conservation of deer and the habitats they occupy continues to be one of the fundamentally important aspects of wildlife conservation in California.

Using Deer as an "Umbrella" or Flagship Species for Habitat Conditions in California

Deer are among the most studied wildlife species in California thanks to decades of interest in them as a principal game animal. For some herds, data exist as far back as the early 1900s. From this long history of study, we have learned that deer often respond predictably to California's changing wildland environment, particularly to changes in forestland habitats that are dominated by a mix of herbaceous and shrub vegetation; and to changes in Great Basin shrub/grass ranges.

Because of the existence of long-term data on deer abundance and seasonal ranges and their well established popularity and economic value, deer are an important flagship species in the DFG's environmental review process. They are often the focus of attention in the DFG's review of proposed projects that are subject to the California Environmental Quality Act (CEQA) and in similar federal environmental review processes conducted by the USFS and BLM. Long-term trends in deer populations reflect the conditions of their habitat. Early successional habitat quantity/quality on forested lands, and quantity/quality of important habitat types on Great Basin and desert ranges influence these trends.

Role of the Department of Fish and Game in Managing Deer and Wildlife Habitat

The Department collects, compiles, and analyzes deer population data throughout the state and develops proposed hunting regulations for deer. DFG has monitored deer habitat conditions on public lands at varying levels of intensity over the years. Intensive efforts in the past were often collaborative ones, with all three agencies involved at some level. Currently, DFG is renewing an investigative effort in habitat assessment and mapping, particularly in northern California.

The Department is California's lead agency for fish, wildlife, and native plants- collectively called "wildlife." Fish and Game has trustee responsibility for the conservation and management of deer and other wildlife for the benefit and enjoyment of the public. As DFG administers a small amount of the state's wildland, it relies on collaborative efforts, cooperation, and mutual goals/objectives of public land managers (e.g., National Park Service, Fish & Wildlife Service, Bureau of Land Management, U.S. Forest Service); other state agencies (e.g, Parks & Recreation, Forestry & Fire Protection, Water Resources); city/county governments; private landowners; and environmental organizations to achieve consideration for wildlife values. DFG represents the interests of wildlife to these and other entities proposing to implement management strategies, land use plans, specific projects, and other resource use activities on California's wildlands.

The Department is guided by State policies and laws relating to deer and other wildlife. The Fish and Game Code (Section 450) states: "It is hereby declared to be the policy of the Legislature to encourage the conservation, restoration, maintenance, and utilization of California's wild deer populations. Such conservation shall be in accordance with the principles of conservation of wildlife resources set forth in Section 1801 and in accordance with the objectives and elements stated in "A Plan for California Deer, 1976."

Section 1801 of the Code establishes the overall Wildlife Conservation Policy for the Department: "It is hereby declared to be the policy of the state to encourage the preservation, conservation, and maintenance of wildlife resources under the jurisdiction and influence of the state. This policy shall include the following objectives:

(a) To maintain sufficient populations of all species of wildlife and the habitat necessary to achieve the objectives stated in subdivisions (b), (c), and (d).

(b) To provide for the beneficial use and enjoyment of wildlife by all citizens of the state.

(c) To perpetuate all species of wildlife for their intrinsic and ecological values, as well as for their direct benefits to all persons.

(d) To provide for aesthetic, educational, and nonappropriative uses of the various wildlife species.

(e) To maintain diversified recreational uses of wildlife, including the sport of hunting, as proper uses of certain designated species of wildlife, subject to regulations consistent with the maintenance of healthy, viable wildlife resources, the public safety, and a quality outdoor experience.

(f) To provide for economic contributions to the citizens of the state, through the recognition that wildlife is a renewable resource of the land by which economic return can accrue to the citizens of the state, individually and collectively, through regulated management. Such management shall be consistent with the maintenance of healthy and thriving wildlife resources and the public ownership status of the wildlife resources."

Section 1802 of the Code further establishes the department's role as it relates to lead agencies such as the USFS and BLM: "The department has jurisdiction over the conservation, protection, and management of fish, wildlife, native plants, and habitat necessary for biologically sustainable populations of those species. The department, as trustee for fish and wildlife resources, shall consult with lead and responsible agencies and shall provide, as available, the requisite biological expertise to review and comment upon environmental documents and impacts arising from project activities, as those terms are used in the California Environmental Protection Act."

Appendix 2 provides additional Fish and Game Code sections specifically related to the management of deer herds in the state.

Role of the United States Forest Service in Managing Deer and Wildlife Habitat

The USFS is authorized by Acts of Congress and by regulations issued by the Secretary of Agriculture to administer, manage, and protect National Forest System (NFS) lands for multiple uses, including the fish, wildlife, and plant resources. Therefore, the USFS is responsible for managing habitats (eg, food, water, and cover) for species, and coordinates with the Department, who is responsible for managing the animal populations.

Each National Forest is required to developed a Land and Resource Management Plan (LRMP), which sets the framework for multiple use management of the Forest. As directed by the Code of Federal Regulations (CFR) 36, Part 219.19, National Forests must identify management indicator species (MIS) in their LRMP. In developing LRMPs, National Forests are required to: (1) establish objectives for the maintenance and improvement of the habitat for MIS species, (2) evaluate the quantity and quality of habitat and of animal population trends of MIS species within planning alternatives, (3) consult biologists from State fish and wildlife agencies and other Federal

agencies to coordinate planning for fish and wildlife, and (4) monitor the trends of MIS species and determine the relationships to habitat changes determined. Most National Forests (16 of 18) in California have identified deer as a MIS species.

In addition, 36 CFR 219.19 directs the Forest Service to manage fish and wildlife habitat to maintain viable populations of existing native and desired non-native vertebrate species. Habitat within each planning area must be provided to support, at least, a minimum number of reproductive individuals and that habitat must be well distributed so that those individuals can interact with others in the planning area. This regulation defines a "viable population" as "one which has the estimated numbers and distribution of reproductive individuals to insure its continued existence is well distributed in the planning area." 37 CFR 219.27 further requires that habitat for MIS species is "maintained and improved to the degree consistent with multiple-use objectives established in the plan."

Role of the Bureau of Land Management in Managing Deer and Wildlife Habitat

The Taylor Grazing Act of 1934 stipulated that forage for wildlife would be taken into account when allocating forage in Grazing Districts established by The Grazing Service. On July 16, 1946 The Grazing Service and The Land Office were merged to form the Bureau of Land Management. The Taylor Grazing Act and its stipulations for wildlife forage stayed in effect until 1976. With passage of the Federal Land Policy and Management Act (FLPMA) on October 21, 1976 (Public Law 94-579), Congress expanded the Bureau of Land Management's (BLM) role in managing wildlife and wildlife habitat from strictly discussing forage to all habitat components found on Public Lands. Two paragraphs within Section 102. (a) of FLPMA, Declaration of Policy, have the most direct bearing on wildlife and wildlife habitat management on Public Lands. Paragraph 7 states: "it is the policy of the United States...that management be on the basis of multiple use and sustained yield...." Paragraph 8 states: "the public lands be managed in a manner that will protect the quality of scientific, scenic, historical, ecological, environmental, air and atmospheric, water resource, and archeological values; that where appropriate, will preserve and protect certain public lands in their natural condition; that will provide food and habitat for fish and wildlife and domestic animals; and that will provide for outdoor recreation and human occupancy and use...." The BLM, therefore, is charged with maintaining the health of the land while providing for several appropriate uses. The major uses for public lands defined in FLPMA "includes and is limited to, domestic livestock grazing, fish and wildlife development and utilization, mineral exploration and production, rights-of-way, outdoor recreation and timber production."

The Secretary of the Interior within the regulatory authority granted by 43 United States Code 1201 further establishes the BLM's role in wildlife and wildlife habitat management in Part 24 of the 43 Code of Federal Regulations (CFR) entitled: *Department of the Interior Fish and Wildlife Policy; State-Federal Relationships*. While the several states are recognized to "possess primary authority and responsibility for management of fish and resident wildlife on BLM lands, the Secretary, through the Bureau of Land Management, has custody of the land itself and the habitat upon fish and resident wildlife are dependent (Subpart 24.4 Resource management and public activities on Federal Lands, Paragraph (d))." While management of the habitat is a responsibility of the Federal Government, the Secretary of the Interior is directed to cooperate with the states in developing programs for the conservation and rehabilitation of fish and wildlife including specific habitat improvement projects. Federal agencies of the Department of the Interior are directed to

prepare fish and wildlife management plans in cooperation with state fish and wildlife agencies, and institute fish and wildlife habitat management practices within their statutory authority and subject to agency management priorities and strategies. Agency management priorities and strategies include the Rangeland Health Initiative. The Rangeland Health Initiative sets standards for rangeland health and establishes guidelines for grazing administration based on meeting rangeland health standards. One standard which guides management of wildlife habitat is that healthy, productive, and diverse populations of native species exist and are maintained.

The BLM in California manages approximately 4,886,000 acres of deer habitat which provide an estimated 2,795,000 hunter days for deer hunters with an estimated net economic value of more than $170 million. California BLM's policy is to: "maintain close cooperation and coordination with the California Department of Fish and Game (CDFG) on matters of wildlife, wildlife habitat, and other areas of common interest on BLM administered lands (BLM Manual Supplement, Rel. 6-18, Dated 5/10/84, entitled; *BLM-State Memorandum of Understanding)*." In developing and implementing the Master Memorandum of Understanding and its 5 Addenda the BLM and CDFG have agreed, within the context of their respective statutory authority, policies, and management strategies, to work cooperatively in the management of the State's wildlife and their habitats.

Who Owns and Administers Wildlife Habitat In California?

Deer inhabit about 64 million acres of California's approximately 85 million acres of forest, rangeland, and desert. About 50 percent of the deer range is public land administered by the federal government, primarily the USFS and the BLM (Figure 3). About 45 percent of the state's deer range is privately owned by individuals or businesses; among these, timber companies own a substantial amount of land that is deer range, particularly in the northern part of the state. A limited amount of deer range is owned and administered by the state as state parks, forests, and wildlife areas. The DFG has responsibility for the management of less than one percent of the state's deer habitat; about nine percent is managed largely to preserve natural conditions (e.g., national and state parks); and about 90 percent of the habitat is managed for specific single uses (e.g., private lands) or multiple uses (e.g., USFS and BLM lands) that may or may not be beneficial to deer or other wildlife. The majority of deer hunting by the public occurs on lands administered by the USFS and BLM.

IV. DEER POPULATION TRENDS

The DFG estimated deer populations by DAU, but also provided a general representation of deer population trends in California since 1800 (Figure 4). Figure 4 illustrates that deer populations in California peaked in the late 1950s to 1960s (see also Figure 5) and are now at a lower level of statewide population. The deer decline appears due largely to long-term declines in habitat quality throughout the state, brought about by various factors.

Deer population estimates were made for the period 1990-1996. Annual variation in specific deer population estimates may be quite high due to localized changes in environmental conditions, so it is more appropriate to have at least a several-year period upon which to evaluate trend (stable, upward, or downward). The DAU system fits reasonably well with the late 1940s assessment conducted by Longhurst et al. (1952), and their estimate of population is included for each of the

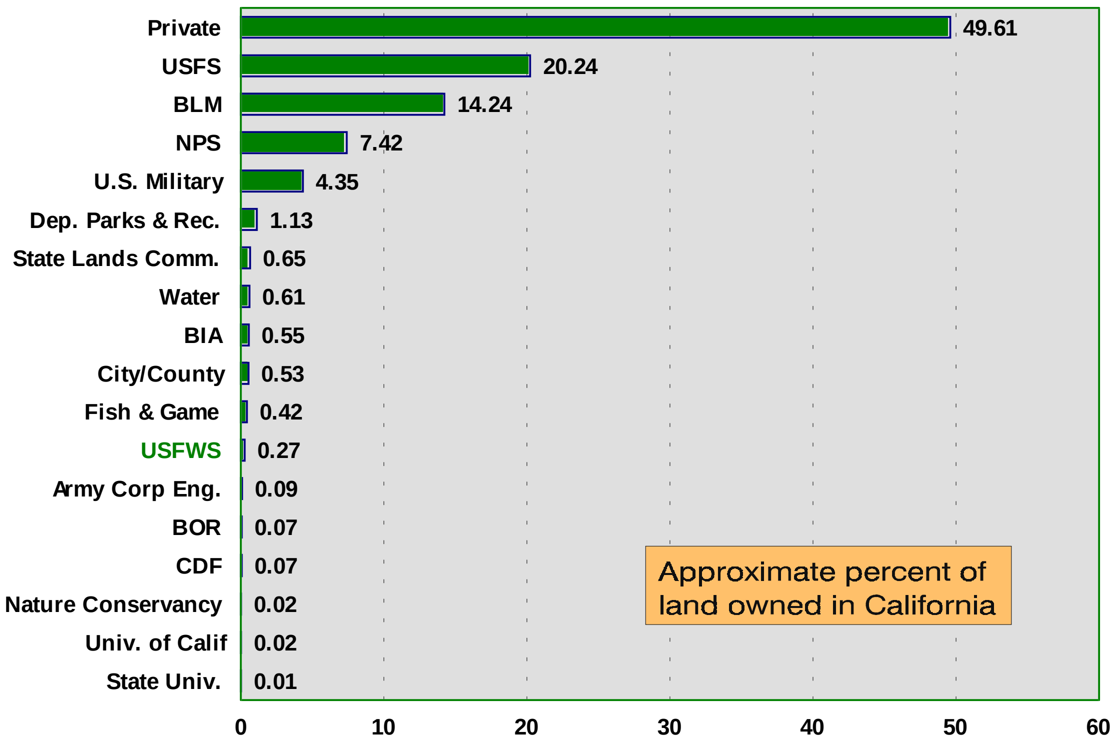

FIGURE 3. Major ownership and administration of deer habitat occurs on private lands and on USFS and BLM lands.

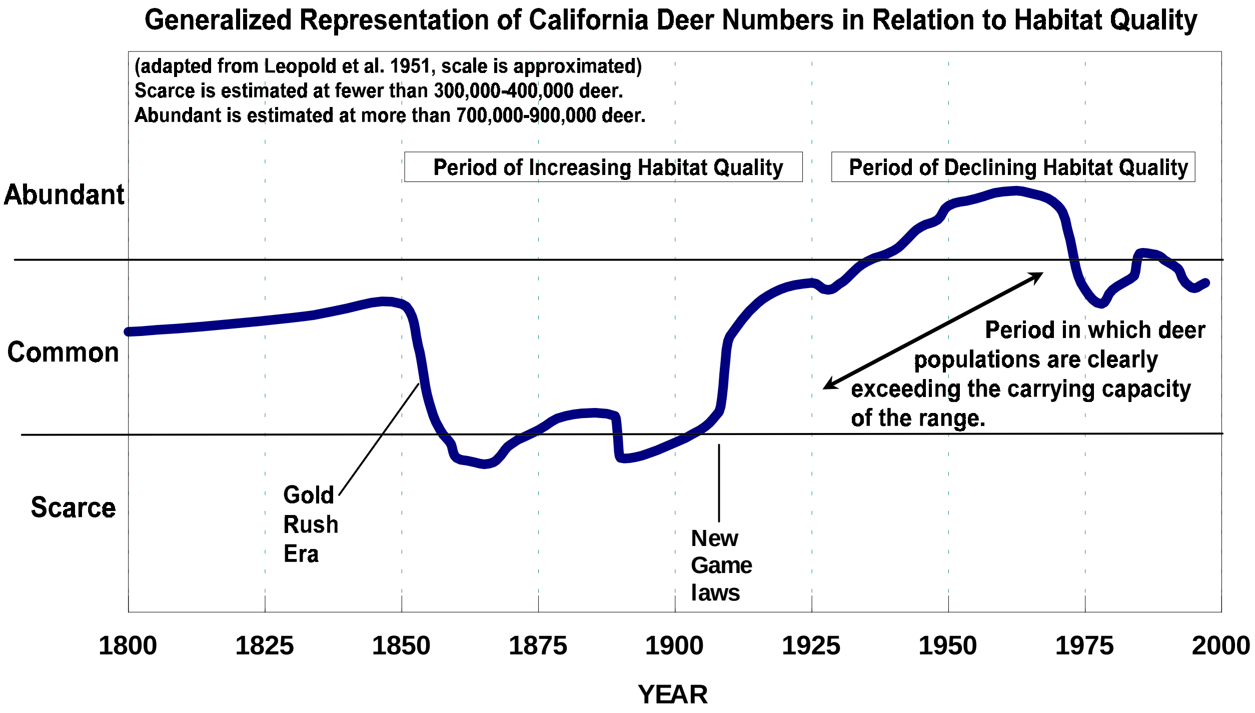

FIGURE 4. Generalized deer population trends as they relate to key periods of increasing habitat quality due to disturbances (e.g., fire and logging) and decreasing habitat quality due to declining disturbance (fewer fires and more regulated logging). Opening of forests as a result of post World War II logging activities likely contributed to the final peak in deer numbers in the 1960s, but also signaled the start of the decline as those forests began to "close" again.

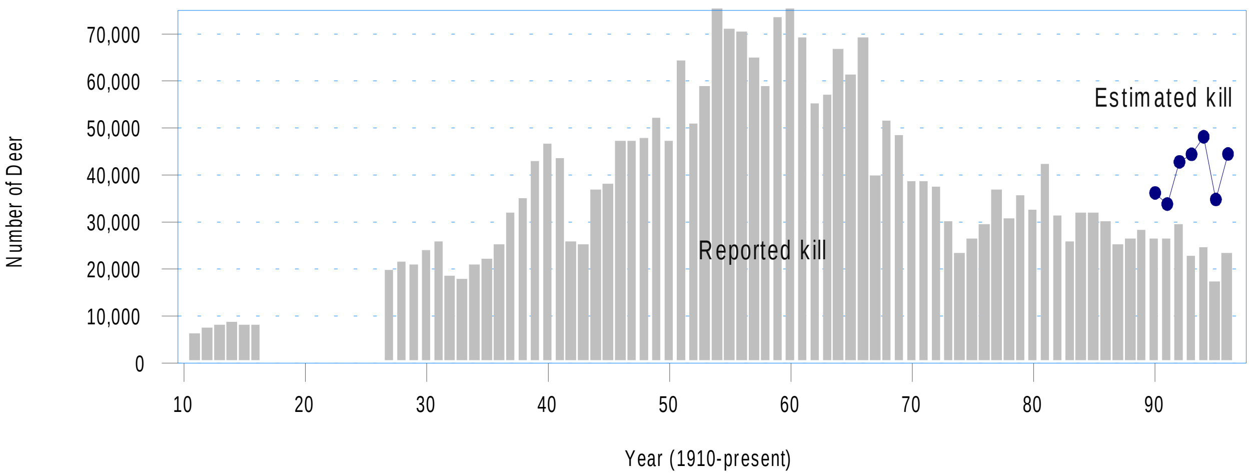

(Reported kill numbers are based on tag returns only and represent the minimum # harvested. Estimated kill reporting began in 1990 and accounts for those tags not returned by successful hunters).

FIGURE 5. Deer harvest in California reflects the general changes in deer populations, and is affected by long-term changes in habitat quality. (Beginning in 1967, a change to self-validation of deer tags was imposed on hunters and there was a marked drop in the deer tag return. Self-validation was dropped in 1970, however the tag return rate remained low, with a 30-40 percent estimated non-return rate.)

---

specific DAU sections (Longhurst's numbers do not reflect the ultimate high point in deer numbers that continued to increase into the 1960s, then began trending downward to present levels).

Deer population trend is considered increasing in DAU 9, the south central Coast (Figure 6). Populations were considered fairly stable in DAUs 1, 7, 8, and 10 (Figure 6) and populations were declining in DAUs 2, 3, 4, 5, 6, and 11 (Figure 7). Northeastern California has experienced the sharpest percentage decline in deer, followed by the northeastern Sierra. Deer populations on the eastside have also declined substantially.

California Deer Populations in Relation to Habitat Quality (How Did we Get Here?)

The most notable fact about deer populations in California is they have decreased from the record highs of the 1950s-60s, and it is of concern to hunters (and others interested in deer) from one end of the state to the other because of the declining hunting opportunity (Figure 5). This benchmark period is important because it is the basis upon which the DFG deer management program's success is, and has been, compared.

Not coincidentally, much of the poor public relations that deer management in California has experienced for the past 40-50 years was specifically a result of the active programs begun in 1946 to address the growing "deer problem... that occurs when deer populations become out of balance with their habitats" (Dasmann et al. 1958). Generally, there were two possible approaches to address the deer problem:

a) increase the quality (carrying capacity) of deer habitats; and/or
b) reduce the deer populations by instituting doe harvests, thereby maintaining or increasing buck numbers and potential harvest while keeping the deer population in balance with existing habitat conditions.

FIGURE 6. Stable or upward-trending DAUs. Note scales vary among DAUs.

Unit 1- North Coast (B1,B2,B3,B4,B5,B6)

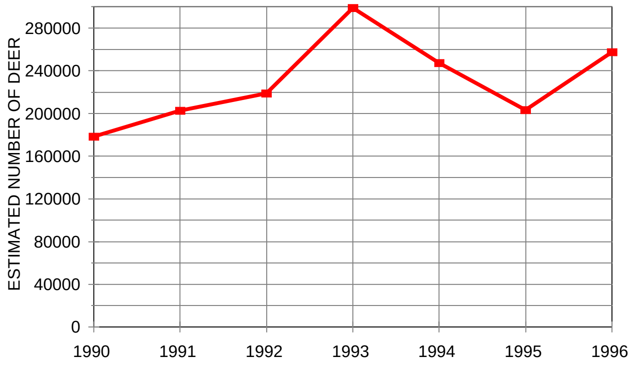

Unit 7- South Sierra (D7-D10)

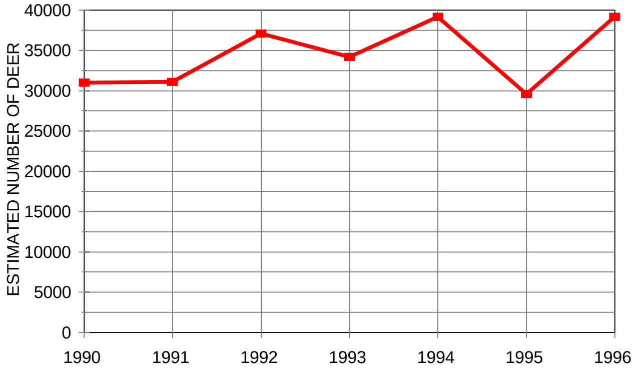

Unit 8- Central Coast (North A zone)

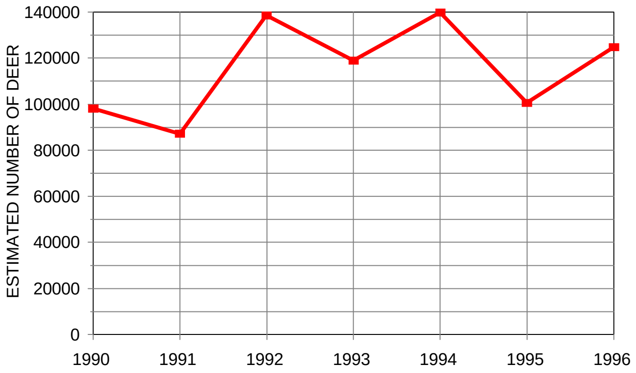

Unit 9- Central Coast (South A zone, D13)

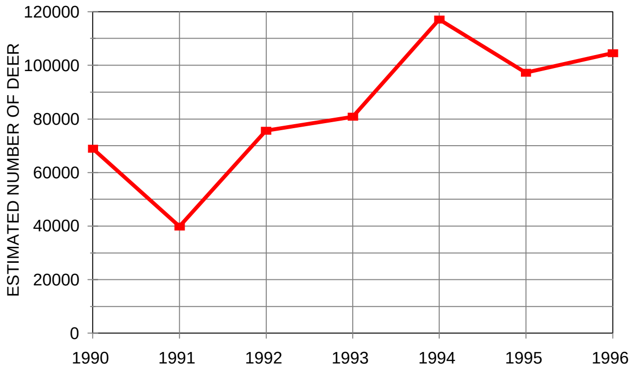

Unit 10- South Coast (D11, D14-16, D19)

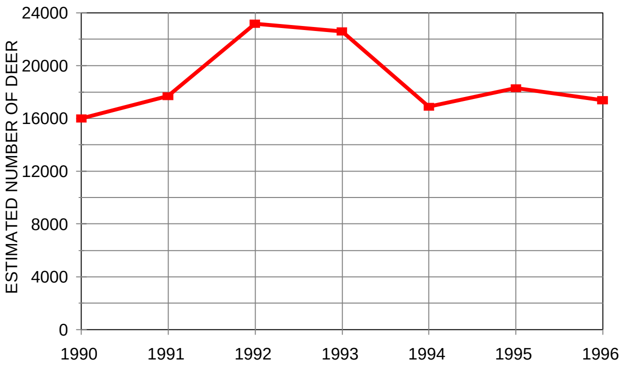

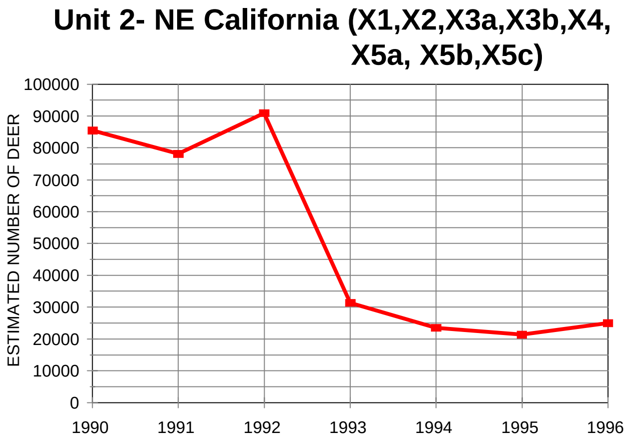

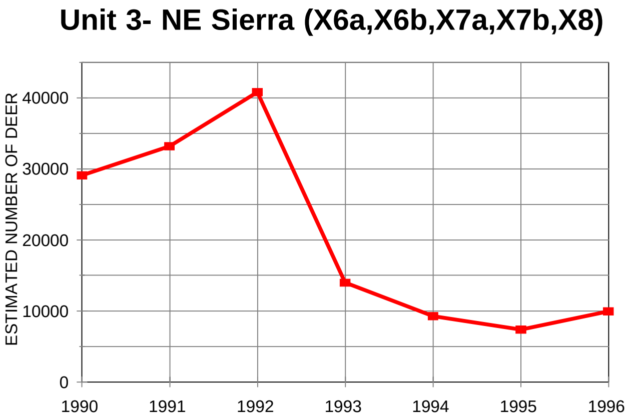

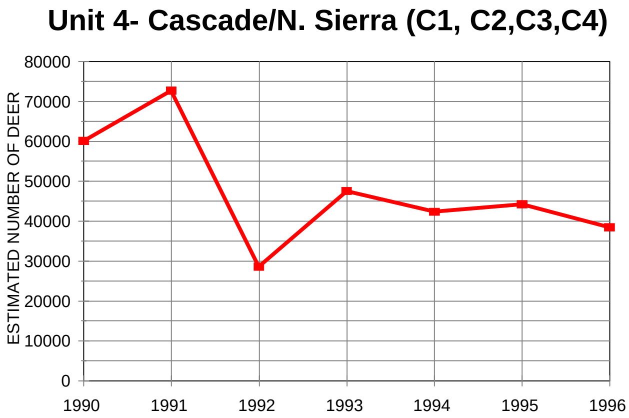

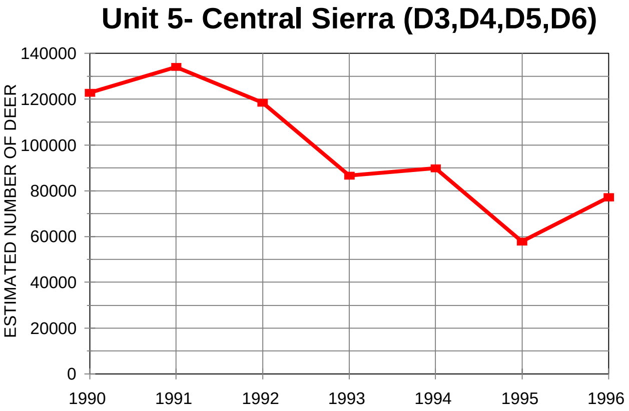

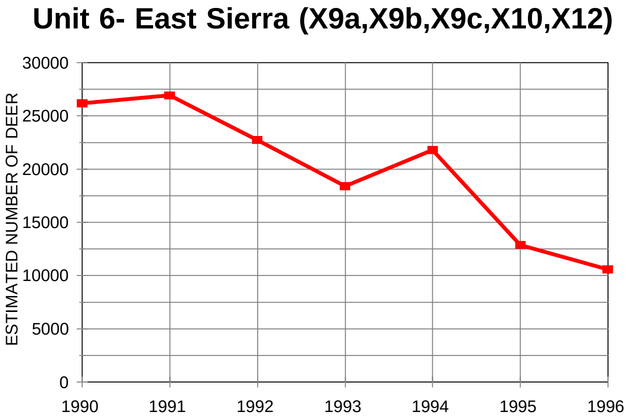

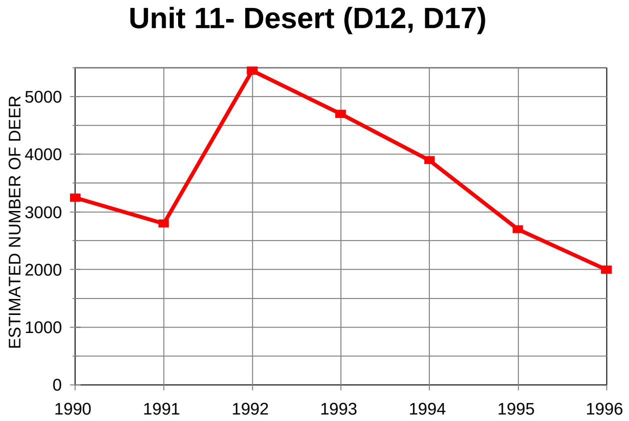

FIGURE 7. Downward-trending DAUs. Note scales vary among DAUs.

DFG had no direct ability to implement projects designed to increase the quality of habitats on public or private land, although several interagency efforts were implemented during this period. The agency did have authority to implement approach “b” in 1950 with California’s first special antlerless deer hunts. There were several successful antlerless hunts conducted and these were followed by the first and still controversial “either-sex” deer hunt in 1956 (Dasmann et al. 1958).

Ideally, both approaches “a” and “b” would be used (e.g., Longhurst and Connolly 1970), but in reality the problems identified (declining deer populations were a symptom of the problem) were largely attributable to declining quality of deer habitats rather than an increase in quality. The decline began as a consequence of long-term change in management of wildlands that began in the early 1900s, particularly, the move to more regulated and intensive forest management and improved fire suppression. The institutionalization of these management changes by the federal government (USFS and BLM), state government (California Department of Forestry and Fire Protection), and by large private landholdings meant a decline in disturbance that perpetuates early successional habitats and the beginning of a reversal as far as deer habitat quality was concerned.

An indirect, long-term consequence of these changes includes increased competition with livestock as herbivores had to now share a resource that was declining rather than increasing. Significant reductions in grazing over much of the state occurred as range management evolved. Much of the early successional vegetation created since the start of the gold rush in 1849 was being replaced by forage-limited, second-growth forest (Leopold 1950) and by decadent shrubfields dominated by unavailable or low quality browse and having little herbaceous vegetation (Salwasser et al. 1978, Storer 1932).

Opening of forests as a result of post-World War II logging activities (Laudenslayer and Darr 1990) likely contributed to the final peak in deer numbers in the 1960s. Deer numbers then began to decline as those forests began to “close” again. The relationship between understory forage (herbaceous and shrub) and overstory canopy (Figure 8) is typical of much of California’s forested ranges--as canopy increases, forage decreases. The expansion of urbanization and residential development on private lands into the Sierra Nevada on both the West and East Slope further reduces available deer habitat, virtually eliminating the potential to purposely restore large-scale disturbances, such as fire, into the system in many areas.

It’s well-documented that deer thrive on early successional vegetation in forested communities (Leopold 1950, Wallmo and Schoen 1981), and there is a period encompassing about 2-30 years following major disturbances such as fire or logging when herbaceous and shrub species are abundant, available, and in highest quality (Figure 9). Livestock and perhaps hundreds of largely unstudied species of wildlife such as blue grouse or mountain quail, also rely on the vegetation produced in forest openings where sunlight is allowed to “hit the ground” and enable plants to grow and be available for consumption or as cover.

Habitat quality constantly changes for better or worse as a result of fire, logging, grazing, succession, and other processes (Wallmo and others 1976), while habitat quantity is continually declining because of urbanization and development. To sustain deer populations, we would need to counter the loss of habitat with more efficient use of remaining habitats.

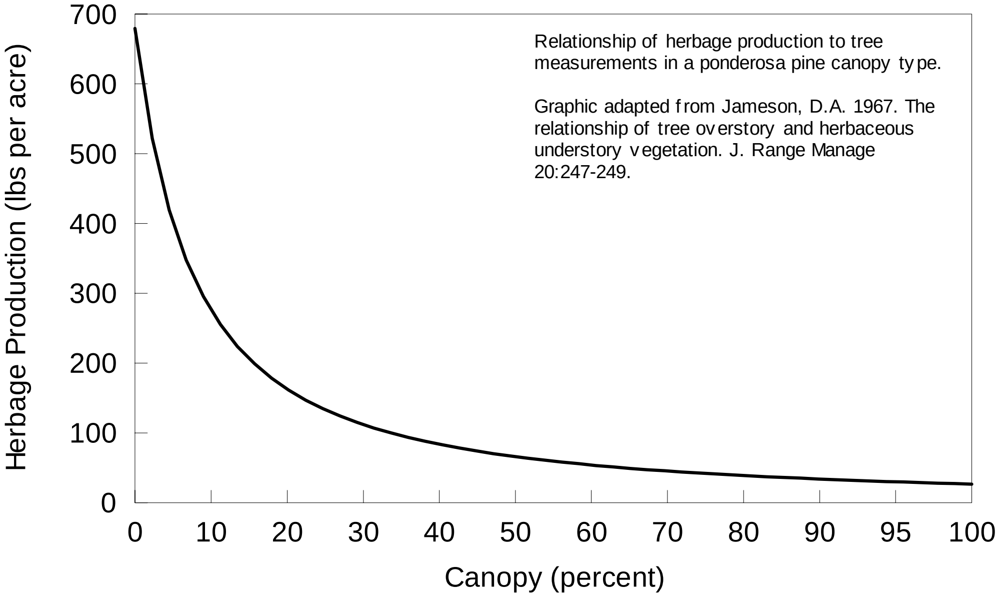

FIGURE 8. Generalized representation of the relationship between grass/forb herbage production per acre and the overstory canopy on a pine system. Increasing tree canopy cover even a small amount, such as from 10 to 30 percent, causes significant declines in the amount of herbage produced as a result of competition for sunlight, moisture, and nutrients.

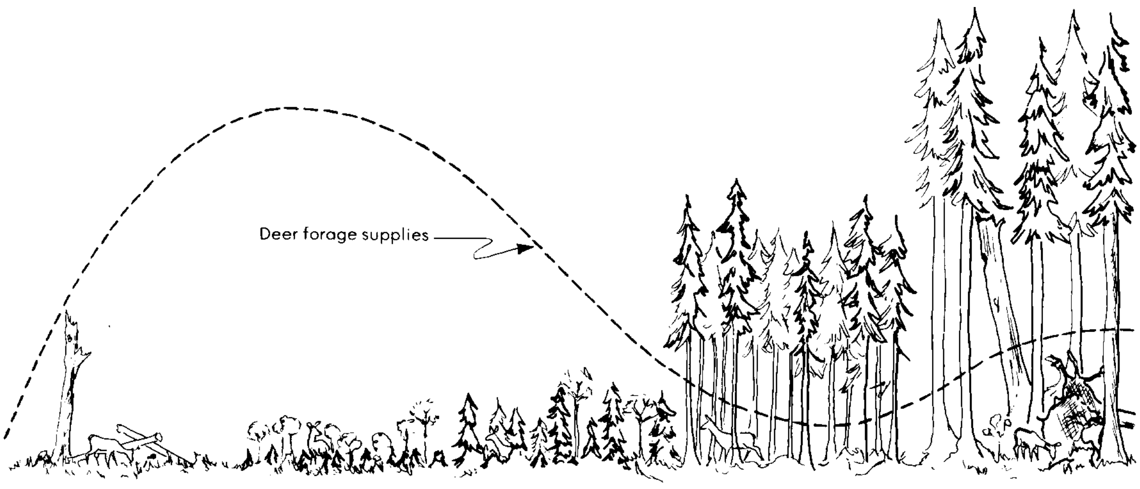

New clearcut	Seedling conifer	Advanced regeneration	Second growth timber	Old growth forest
Forb - grass - sedge	Shrub - herb	Young tree - shrub	Minimum undergrowth	Moderate undergrowth
1-5 years	6-10	10-20	20-200	200 +

FIGURE 9. Generalized representation of the relationship between deer forage supplies and the successional process as influenced by timber harvest and plant succession. Graphic from Wallmo and Schoen (1981). Forest management for deer. Pages 434-457 *in* O.C. Wallmo, Ed. Mule and black-tailed deer of North America. Wildlife Management Institute, Univ. of Nebraska Press.

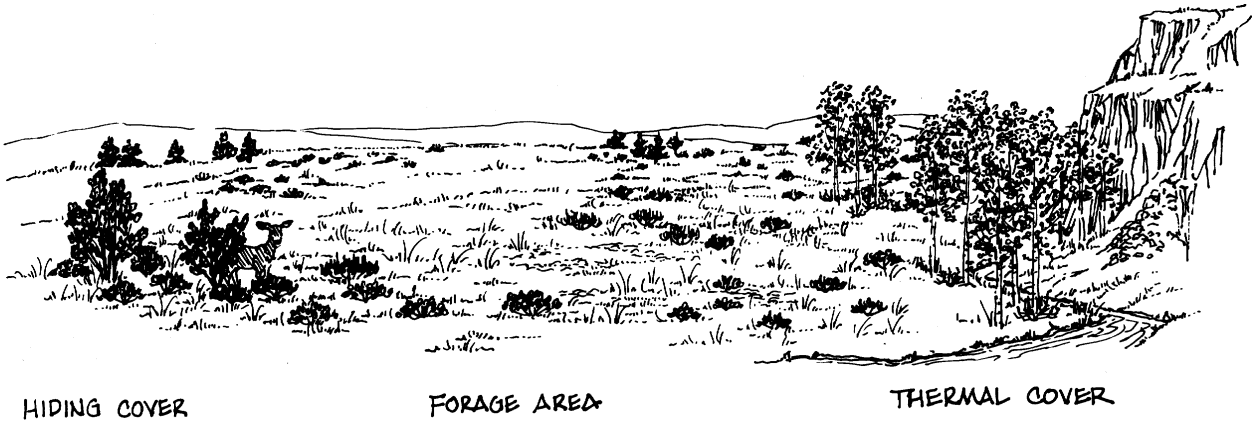

Figure 10. Example of habitat components on Great Basin range illustrating the importance of forage and cover areas in proximity. Source: Leckenby, et al. 1986. Mule deer. Wildlife habitats in managed rangelands- the Great Basin of southeastern Oregon. Gen. Tech. Rep. PNW-139.

V. COMMON FACTORS AFFECTING DEER HABITATS

Habitat Components

Historically, the phrase "quantity and quality" has been used in discussions of deer habitat and factors limiting deer populations. Quantity refers to the amount of habitat available, accounting for those areas that may have become geographically isolated (fragmented) or modified by human activity and are no longer viable deer habitat. Quality describes our understanding of the habitat's value in meeting life history requirements of deer. The variety of habitats available do not have the same value to deer. The value, or quality of deer habitat is determined either subjectively or is actually measured. Habitat quality is based primarily on how the following three components are distributed (Figure 10) and made available for use by deer:

Forage- Deer food must contain sufficient amounts of protein, carbohydrate, minerals, and trace elements. Nutritious vegetation must be available (normally five feet or less in height) and digestible. Many plant species that are available do not provide forage because deer cannot easily digest them. Typically, young tender shoots and leaves of plants are higher in nutrients and more digestible than old plants. Hence, valuable forages are primarily young shrubs, new shoots of shrubs, and succulent grasses and forbs. Other food items, such as acorns, are seasonally important.

Cover- The quality of deer habitat is influenced by the availability of cover and its proximity to food. Deer require cover for hiding, escape, and for regulating their body temperature. Hiding cover is particularly important from early to midsummer for young fawns; and for all deer in areas subject to predation and/or human disturbance. Escape cover is generally the same as hiding cover, but refers specifically to cover which is near open foraging habitats. Having suitable escape cover in proximity to desirable foraging areas increases the quality of the area as habitat and its likelihood of being used by deer. Thermal cover is used by deer to minimize their energy expenditure and is typically provided by overstory trees and large shrubs. Thermal cover is important to deer inhabiting areas that experience hot summers, cold winters, or both. High quality thermal cover is generally that which provides cool, moist environments during summer

days, and mild temperatures during winter nights. Protection from wind, snow, rain, and sun are also features of desirable thermal cover. Overall, habitat quality is increased when all of these cover elements are in proximity to good feeding areas.

Water- In arid areas of the state, water can be limiting and preclude deer from inhabiting otherwise suitable habitat. Drinking water is particularly important during summer when adult females are nursing fawns and seasonal water sources dry up. Generally, the closer a permanent water source, the greater the likelihood that deer would use a habitat.

Factors Affecting Deer

Numerous factors continually affect deer abundance, condition, and health in California (CDFG, *A Strategic Plan for California Deer*, draft, in prep.). Many of these factors are at work simultaneously, confounding our ability to point to any single reason for a decline or increase in deer numbers. Factors potentially having significant impacts on deer include:

- ✓ Habitat loss and conversion.
- ✓ Habitat condition- acreage of habitat and quality of forage and cover.
- ✓ Timber harvest and reforestation practices- e.g., biomass thinning, hardwood removal, and clear-cutting.
- ✓ Livestock grazing.
- ✓ Wildfire, prescribed fire, and fire suppression.
- ✓ Developments- residential, reservoirs, ski areas, golf courses, and agriculture.
- ✓ Predation by mountain lion, coyote, black bear, and domestic dog.
- ✓ Regulated hunting, including antlerless hunting.
- ✓ Illegal kill.
- ✓ Diseases.
- ✓ Annual or short-term weather patterns, such as severe winters and drought, and long-term climatic change.
- ✓ The size, sex and age structure of deer populations relative to their habitat.
- ✓ Competition with non-native wildlife species.
- ✓ Highway mortality.

The significance of any of the above factors will vary among deer populations and each factor can change in its importance or influence. We do not expect to find solutions to deal with all factors, although each is considered in deer management planning efforts. Priority, or importance, of factors should be based on the potential impacts to deer and the likelihood of a successful solution to any factor that may be causing problems. Our ability to determine cause and effect relationships from any individual, or combination of these factors affecting deer populations can enhance our understanding of each factors importance.

VI. COMMON FACTORS AFFECTING DEER HABITATS ON PUBLIC LANDS

Of those factors manageable on public lands, timber management practices, fire, and livestock grazing were identified as having the most widespread impact on deer habitats. Some of the issues related to these activities were common to several DAUs and should be addressed throughout the state as well as regionally. The following sections describe some of the factors common in much of the state and introduce why the issue is relevant to deer habitat management and ultimately, deer populations. Where applicable, these factors are mentioned in the individual DAU evaluations.

Forests Moving Away from Early Successional Habitats Through the Lack of Fire and Through Fire Suppression

Declining abundance of early successional vegetation communities in forestland was considered to have the greatest effect on long-term deer populations. The primary mechanism to establish those communities is fire, either wildfire or prescribed. In California, society has passively contributed to the aging and declining quality of deer habitats by not instititutionalizing prescribed fire or natural fire (let-burn) on a significant scale. For example, of the approximately 32 million wildland acres that the California Department of Forestry and Fire Protection has jurisdiction over, an average 250,000 acres has burned annually since 1953-- only about 1/10 of one percent. A corollary is that we actively move away from diverse ecosystems having adequate representation of the varied successional communities, in part because of fire suppression efforts (Martin and Sapsis 1992). State and Federal programs to integrate fire into wildlands should be increased dramatically.

Evidence there is a positive response by deer to fires includes increased fat reserves, body weight, and productivity several years following fire (Taber and Dasmann 1958). On many forested ranges, enhancement of grass/forb growth and shrubs such as deerbrush (*Ceanothus integerrimus*) and hardwood species is a common and desirable benefit for deer after fire (Grifantini 1991). The 1987 fires on the Klamath (Gallagher and McCullough 1992) and Stanislaus forests are examples of large fires that benefit deer and other early successional wildlife on a landscape perspective. These benefits translate into more deer available for hunters (Figure 11), although the benefit could be short-lived in the absence of either-sex hunting (Longhurst and Connolly 1970), because of the maturing vegetation (as in Figure 9), or because other factors become limiting.

Prescribed fire to benefit deer should occur at times of the year when the greatest likelihood of achieving the desired plant response will be achieved. For example, dry season burns tend to result in better regeneration of shrub species from seed than moist season burns. Fire-adapted shrub species are typically favored when burning occurs at the time of year that plants have adapted to-- usually in the late summer or early fall.

A common use of prescribed fire is as prevention against large wildland fires that endanger structures and valuable timber stands (Biswell 1989, CDF 1995). However, such fires do not necessarily benefit deer or other wildlife because of the timing, as well as the location. Timber stand enhancements that use prescribed fire are another example in which the understory forage and cover may be lost because of a dense overstory canopy.

DFG, USFS, BLM, and the CDFFP have been involved in prescribed fire programs to reduce fuels, enhance forage, and enhance wildlife habitat since the 1950s. DFG’s deer herd restoration program provided additional funding to enhance deer range on about 50,000 acres since 1985 in cooperation with the USFS and BLM. Over the years we have learned that the most effective fires are those greater than 400 acres in size; are a component of a larger watershed approach that establishes mosaics of varying successional stages; and are conducted where wildlife value is the priority (compared to fuel reduction or timber stand improvement as priority).

Encroachment by development into privately owned wildlands necessitates greater vigilance and fire suppression on these and nearby lands (public and private) that otherwise could benefit from

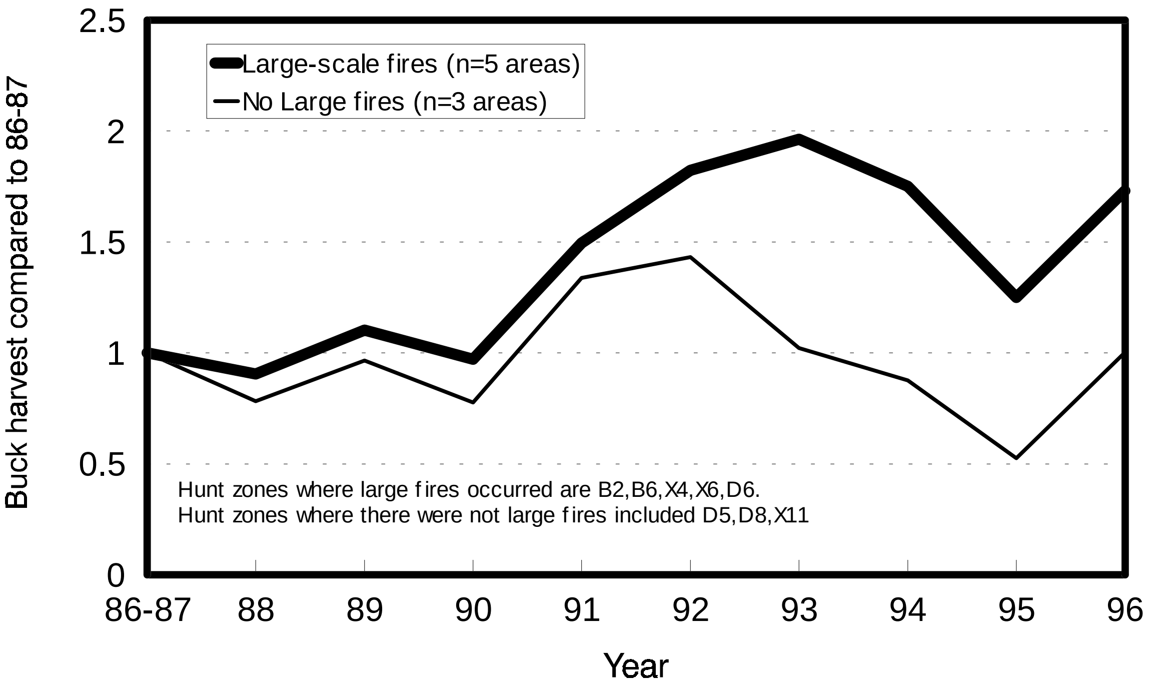

FIGURE 11. Buck deer harvest in years following 1987 fire year on forested deer ranges. Numbers reflect proportional change in deer harvest compared to 1987 values in five areas with, and three areas without, large fires. These zones comprise portions of the DAUs. Fires were each greater than 30,000 acres in size. (Recall the generalized model presented in Figure 9.)

fire. Because of fire suppression efforts on private lands, the ability to interject some diversity in chaparral and forest on public lands by fire is a desirable long-term objective.

Habitat Changes on Rangelands as Influenced by Fire

On shrub winter ranges east of the Sierra Nevada, fire is a different story, with largely negative effects on deer habitat. For a time, recently burned areas provide little or no browse, thermal cover, or hiding cover for wintering deer (Loft and Menke 1990; and see Figure 10). Contributing to the habitat concerns on deer ranges between Susanville and Reno are the large acreages burned in the mid-1980s (Figure 12). Faster and more effective response to suppress summer wildfires in east side shrub winter ranges is desirable. Summer prescribed burning in these communities to reduce juniper or sagebrush and enhance herbaceous production may result in fire so hot that some desirable shrub species may be killed.

Post-fire rehabilitation of burned sites can also affect deer. Establishing diverse stands of native forbs, grasses, and shrubs provide more beneficial habitat than use of one or few exotic plant species. Annual ryegrass on chaparral burned sites, or planting of crested wheatgrass in the Great Basin are examples of non-native rehabilitation efforts that do little to enhance deer habitat. Quick action to rehabilite burned ranges before invasive species dominate appears to be the most likely means of recovery for burned ranges (Evans and Young 1978).

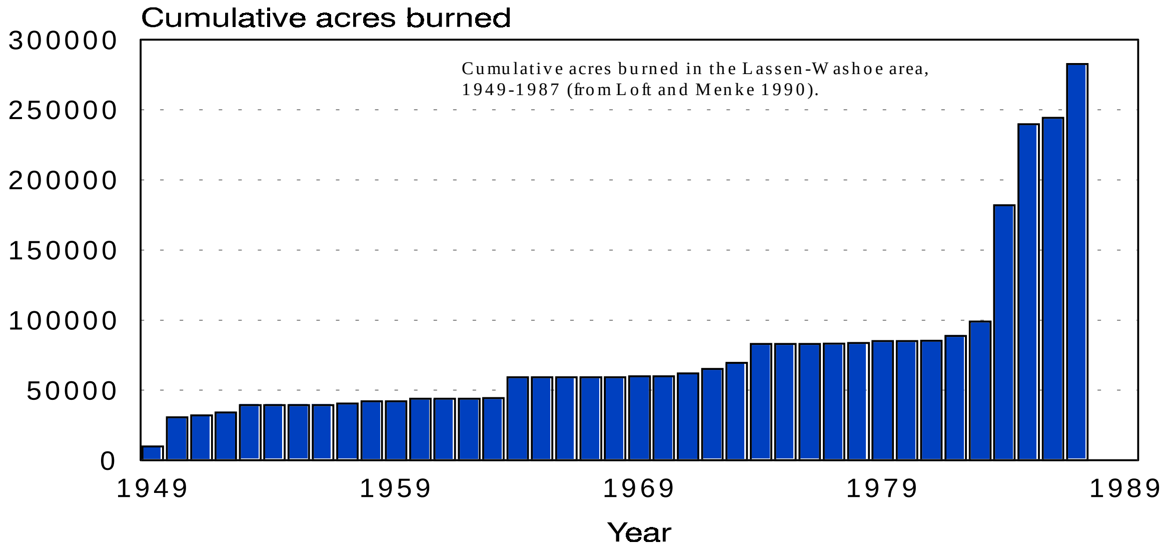

FIGURE 12. Cumulative acreage burned on the Lassen-Washoe deer winter ranges between Reno and Susanville areas (up to 1987). The summer wildfire years of 1984, 1985, and 1987 resulted in portions of the Lassen-Washoe shrub range to be recolonized by plants such as cheatgrass which are of low value to deer as food/cover; and are ephemeral (unreliable) in occurrence.

Forests Moving Away From Early Successional Habitats Through Timber Management Practices and Reforestation Efforts

Historically, logging and post-fire rehabilitation typically involved clear-cutting an area, piling/ burning slash debris, and reforesting the cleared area with seedling trees. There are both positive and negative consequences for wildlife, depending on species, to clear-cutting, as well as consequences to soils and watercourses (Tuchmann et al. 1996). Over the years, clear-cutting, except in small patches, has gone out of favor on public lands because the potential negative impacts often exceed the positive. However, early successional vegetation in clear-cut areas was a positive response that benefitted deer in many cases.

Current timber management practices appear to suppress the diversity of habitats and early successional stages (Photo 1a, 1b). For example, renewed and intensive efforts to improve tree growth and health through whole-tree (biomass) thinning and herbicide spraying results in a meager understory of forage and cover for deer and other early successional dependent wildlife. Salvage-logging following fire can alter the successional process and reduce the abundance of desirable browse species (Grifantini 1991).

Forest thinning and Whole-tree removal (biomass thinning)

Thinning is used to help restore tree stands to a more healthy condition by removing undesirable trees. It “increases merchantable yields on trees by distributing growth to a lesser number of larger stems, similar to thinning a row of carrots” (USDA Forest Service 1996a). While this can improve health of the tree stand, it decreases some of the components (and successional stages) of a diverse forest system. Manipulated second-growth forest stands typically have minimal understory vegetation in them (see Figure 9 and Photo 1a).

Kucera and Barrett (1995) assessed the effects of thinning on wildlife habitat in Northern California. This activity is conducted on several forests in Northern California (Plumas, Lassen, Shasta-Trinity, and Modoc for example). The Lassen National Forest reportedly has thinned more than 7,000 acres per year; approximately 60,000 acres are thinned annually statewide. However, the majority of thinning occurs on private lands between Redding and Susanville.

Thinning results in an immediate decrease in thermal and hiding cover, and may result in a decline in forage. Wildlife that benefit from post-fire shrubfields or dense understory, such as deer, may not benefit in the short term. The authors concluded that the structural consequences of biomass harvest as currently practiced are not consistent with good deer habitat, and livestock grazing tends to compound the problem.

The long-term effects of whole-tree thinning on wildlife and wildlife habitat are not well-understood, but research indicates they appear to be negative for at least 10 years; that development of a shrub layer following treatments is rare; and that the use of this practice on private timberlands increases the concern about conducting the practice on public lands where there are multiple use mandates.

Herbicide treatments

Herbicide sprays are frequently used following fire in conifer forests to kill the reestablishing herbaceous and woody shrub vegetation prior to transplanting conifer seedlings on such sites (Photo 2a). This practice typically modifies normal successional trends and has been common on private timberlands (examples can be seen west of Burney on Hwy 299 and east of Placerville on Hwy 50) and has been reinstituted in the past few years on public lands administered by the Forest Service (e.g., Stanislaus and El Dorado forests). Aerial application of herbicides can eliminate large tracts of herbaceous and shrub vegetation as suitable deer habitat.

A recent study of post-fire herbicide spraying by DiTomaso et al. (1997) indicated very low shrub cover (1, 7, and 11 percent cover) in three sprayed areas after 2, 8, and 12 years following fire, respectively, compared to 75, 44, and 103 percent cover in the same respective areas not treated with herbicide. Herbicides clearly reduced shrubs, and the potential application of such treatments over large tracts of land is a concern from a forage perspective. Conversely, allowing post-fire areas to become dominated by shrub species (typically deerbrush) in excess of 60 percent cover is not desirable from a deer habitat perspective either (Photo 2b).

A management strategy that would maintain a diverse mix of herbaceous, shrub, and conifer species for forage and cover consistent with typical successional processes (e.g., grass/forb to shrub to tree) would be more desirable to support the diverse wildlife species that occur in forest communities (Thomas et al. 1979) than a mix that rapidly is dominated by herbaceous and conifer cover. This would be especially true in systems such as the west slope of the Sierra Nevada where shrub species are such an integral component.

Livestock Grazing Impacts on Important Habitats and Natural Communities

Discussion of livestock impacts on deer (or other large native herbivores) in California frequently recognizes that deer populations were at their highest at about the same time that livestock

numbers were at their highest. However, this was during the period that we were still "flying high" with an abundance of early successional habitats throughout forested ranges in the state and we now have fewer of both species on forests/rangelands, with overuse still occurring on some ranges.

Historically, the competitive effects of livestock on deer were likely overshadowed by the tremendous level of habitat disturbance that took place between 1849 and the early 1900's. There was likely enough early successional habitat available on forested ranges that livestock and deer did not significantly compete. Since that time, the acreage and quality of deer habitat has declined to the extent that cattle and deer may now be competing for resources on summer ranges in mutually preferred meadow-riparian and aspen habitats (Loft and Menke 1988); on winter and spring-fall ranges characterized by declining hardwood resources and shrinking forest openings (west side of Sierra and Coast Ranges; Bronson 1992); and on winter range shrub communities (east and west side of Sierra Crest, Coast Ranges; e.g., Longhurst et al. 1977).

The decreasing role of fire and logging as mechanisms for creating early successional habitat in forested ranges indirectly results in greater potential for competition between deer and cattle on remaining ranges. Grazing by cattle in the spring and summer on west slope deer winter ranges may have a negative impact on browse availability the following winter. Cattle may also directly compete with deer for mast crops during fall and winter (Leach and Hiehle 1957). Barrett (1982) reported that cattle excluded deer from preferred oak-woodland habitat and suggested negative social interactions were detrimental to deer.

On east side shrub/grass ranges, the continuous growing season-long grazing that had been the usual practice was largely replaced in the 1970s by grazing systems that allow for periods of rest from livestock. While these systems have benefitted upland perennial grass species in some areas, they largely ignored effects on riparian-wetland areas and on browse species. More attention is being paid to reducing livestock use on key browse species such as bitterbrush and to implementing grazing practices that will enhance riparian-wetland vegetation. These efforts need to be continued and expanded. Grazing can have positive impacts for deer habitat in some cases. Consumption of grass that competes with desirable forbs and shrubs is an example. Urness (1990) indicates that these positive impacts have largely been fortuitious rather than prescriptive. Greater effort to establish grazing prescriptions to benefit wildlife habitat would likely enhance public lands and help meet the goals of the three agencies.

The following sections summarize some of the key habitats important to deer around the state and how livestock grazing can affect them:

Aspen- In natural settings, aspen habitat supports a wide variety of wildlife species because of the presence of a productive overstory and understory providing food and cover. Livestock also highly prefer these summer habitats in California, congregate in them, and can degrade the stand. Hanley and Page (1982) illustrate this impact: "lush vegetation of perennial forbs... was not present in the livestock-grazed aspen stands..." and that there was "absence of successful aspen reproduction due to consumption of the root sprouts by livestock." In the absence of livestock, aspen was the most highly preferred habitat by mule deer in the Sierra Nevada (Loft and Menke 1988). Many aspen stands are declining in California (Photo 3a), however no comprehensive assessment of this habitat has been conducted in the state. One forest, the Stanislaus, has

identified aspen as a species/ecosystem component of interest to "conserve biological diversity" in the Research Natural Area program (USDA 1996b).

The decline in aspen is largely attributable to season-long livestock grazing and preference for these desirable stands (Pillsbury 1994, Dale 1996). Even after understory forage is consumed, livestock congregate in aspen stands for shade as this habitat is usually associated with water sources and meadow-riparian habitat (Loft and Menke 1988). The result is repeated browsing and trampling of the new shoots (suckers) that would form new trees in openings in the aspen clone. This impact precludes survival of the suckers to establish trees and is why many aspen stands in managed forests are characterized by few, large trees of similar age with many dead and down logs scattered about to delineate the original stand (Photo 3b). Lack of fire can also plays an important role as it can stimulate vigorous sprouting. Aspen habitat has a short-term forage value for livestock in summer, but summer-long value as resting cover. In the absence of livestock, this habitat provides abundant understory and ground cover for wildlife summer-long (Photo series 3c). In addition to livestock, wild horses/burros can also impact aspen stands on east side ranges.

Mountain meadows and montane riparian zones- Livestock highly prefer these summer habitats in California and congregate in them summer-long because of the availability of high quality forage in proximity to water. Distributing livestock to prevent excessive utilization is one of the most difficult challenges to grazing management (Photo 4a, 4b). These habitats are highly preferred by deer and other wildlife. These and associated aspen habitats are often regarded as key fawning areas and population centers, critical for female deer trying to nurture young fawns at this most nutritionally demanding time of the year (Hanley and McKendrick 1985). Overuse of herbaceous and shrub vegetation through the summer reduces hiding and escape cover, and leaves little in terms of quality forage in September-October as deer attempt to build reserves for the winter period. Negative effects of livestock on mountain meadow vegetation cover and diversity have been documented from the coastal mountains of Southern California (Bowyer and Bleich 1984), to the Sierra Nevada (Loft and Menke 1988, Kie et al. 1991), and to the Klamath Mountains near the Oregon border (Van Sickle 1994).

Inadequate monitoring and lack of implementation of management change are important issues in management of meadow-riparian habitats. The habitats comprise a relatively small amount of geographic area while serving a critical role in providing areas of high quality forage, cover, and water in proximity. Case studies indicate livestock exclusion for a period of time can result in recovery. Placing recovery as a first priority, then bringing in modified and well-managed (monitored) grazing systems would benefit these habitats.

Great Basin and Desert ranges- riparian, springs, seeps, and meadows: These small "oases" in the desert make surrounding uplands inhabitable by deer and other wildlife for up to several miles distance. Water, succulent herbaceous forage, and cover are typically available at these small isolated spots if they have not been degraded (Photo 5a). These areas are often heavily used by livestock and wild horses/burros resulting in utilization levels being exceeded (Photo 5b). Many of the more productive and larger riparian-wetland areas in this category are privately owned, thereby increasing the importance of managing sites on public lands for multiple uses. Incentive programs for private holdings for maintaining habitat quality would also be beneficial.

FIGURE 13. Relationship between bitterbrush use (by livestock and deer) and deer fawns produced in the following year (from Dasmann and Blaisdell 1954).

The authors concluded that fawn survival would decline moderately if utilization exceeded 25 percent, and steeply if it exceeded 34 percent. Alleviating browsing pressure from livestock is one opportunity to help retain browse for deer.

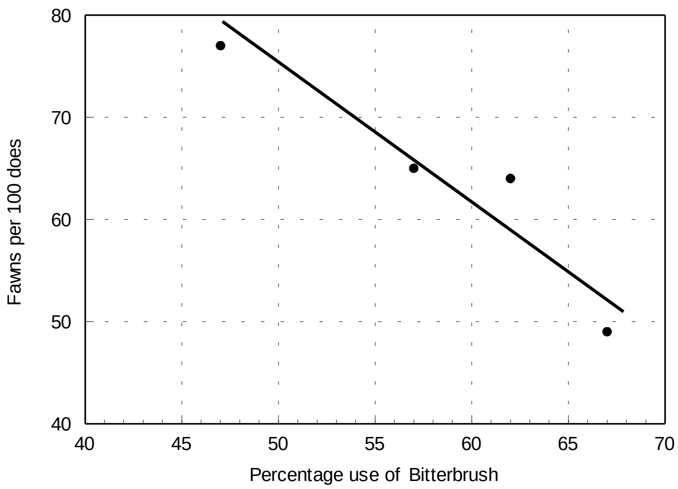

Grazing impacts at these typically small sites can be a determining factor for when livestock should be moved on many ranges. In productive (e.g., wet) years, an abundance of upland vegetation nearby may be lightly grazed because of livestock behavior, while nearby riparian-wetlands may be severely grazed. In dry years, the riparian-wetlands become even more heavily used by all herbivores. However, whether in drought or wet year, inadequate monitoring and management of livestock use on riparian-wetland areas can contribute to degradation of the habitat for deer populations. Setting allowable use levels conservatively enough to allow for the needed improvement of these sites can have long-term benefits.

An increasing, and year-round, wild horse population in northeastern California further impacts available resources. As feral animals are present year-round, the potential for competition and displacement can occur at stressful times for deer populations such as summer (when adult female deer are lactating) and winter (maintenance survival conditions).

Great Basin shrub/grass communities (big sagebrush, antelope bitterbrush, curlleaf mountain mahogany): These habitats are considered the traditional deer habitats on the east side. Deer historically have relied on bitterbrush for feed during the fall and early winter, and on sagebrush for the bulk of the winter (Leach 1956). Mahogany stands when available also provide valuable forage and cover.

Livestock browsing of these key species, particularly bitterbrush, during the growing season can reduce potential flowering and consequent seed production, as well as reduce leader availability for deer. Also, previous high deer populations substantially contributed to declining shrub range conditions on the east side. A strong correlation between percent utilization of bitterbrush and the following season’s fawn survival has been demonstrated (Figure 13; Dasmann and Blaisdell 1954). For some areas, establishing more conservative browse limits for livestock use could be an important step in attempting to maintain valuable shrub stands. Early intensive grazing to reduce grass competition with bitterbrush is one possibility for using livestock to benefit deer habitat. This strategy should be evaluated and increased if it can be demonstrated to be effective.
The lush growth of herbaceous grasses and forbs are important to deer in spring and summer (Figure 14, [color figure on page 50]). Lactating does have their highest energy demands in summer and need high quality forage to nourish their growing fawn(s). The effects of livestock and wild horse/burro grazing of lush vegetation and on plant species composition can be negative.

Conversely, these larger, more general diet herbivores can remove some of the older, rank plant material and stimulate new growth desired by deer.

Other activities of concern related to livestock grazing, or more specifically range management activities, are grass seedings and prescribed fire to increase grass production for livestock; and in some cases, fencing. These activities can be at the expense of deer habitat (Longhurst et al. 1977).

Hardwoods and associated west slope shrub communities: Oak mast (acorns) and oak browse are staple food item for deer on west slope Sierra Nevada and in the Coast Range areas. Long grazing seasons (fall to spring) on winter range reduces forage available to deer (Bronson 1992). The effects are increased in years when herbaceous forage is scarce and cattle reduce oak mast biomass that could otherwise be available for deer. Kie and Boroski (1995) reported that competition with cattle on west slope winter ranges resulted in larger home range sizes for deer and recommended grazing not be permitted before mid-January. Spring grazing can also have an impact on browse species that deer would rely on in the following fall and winter (e.g., buckbrush, *Ceanothus cuneatus*).

Recognizing the Role That Private Lands Have in Affecting Deer Range

Many private lands are, and will remain, wildland. Additional efforts by the three agencies should be encouraged to provide assistance and incentive to private landowners to maintain high quality deer habitat, or enhance habitats through manipulation. The Enhancement and Management of Fish and Wildlife and their Habitat on Private Lands (PLM) program is an example of providing an economic incentive to maintaining deer habitat. For many ranches there is an important link to public land management because they have grazing permits on federal land that help keep the entire ranching operation viable. Greater incentive to maintain private holdings as wildland should be encouraged by all three agencies.

Susanville, Bishop, Reno, Santa Rosa, Auburn, Paso Robles, or the San Diego area all are examples of California’s intrusion with houses, subdivisions, or other permanent development on California’s wildlands. As it affects deer, much of the development occurs on winter range areas on either side of the Sierra Nevada or year-round range in the coastal mountains. As California proceeds with development on private lands, the remaining public lands administered by the BLM and USFS are becoming increasingly important as sustaining habitats for deer and other wildlife. It is becoming more important that these public lands be able to support the diverse habitats and successional processes that wildlife depend on.

The necessity to suppress fire on public lands at the urban-wildland interface, as well as attempts to reduce fuels through prescribed fire, indicates that these lands may not be managed for their potential value as deer habitat. The same applies to private forestlands managed for timber production. Maintaining or enhancing deer habitat is not usually a high priority because it can increase the cost of doing business. Greater effort to develop and prescribe cost-effective methods to accomplish timber objectives while deriving an enhanced benefit to habitat conditions are needed.

VII. UNIT EVALUATIONS

The following are brief discussions of each of the 11 DAUs deer populations, habitat conditions, and some opportunities discussed at the April 1997 workshop. More specific, detailed evaluations will be developed as needed for each Pilot Management Strategy to be implemented.

DAU 1- North Coast

Description

The North Coast unit comprises about 16,500 square miles south from the Oregon border and west of Interstate 5. Deer populations, of the Columbian black-tailed (*Odocoileus hemionus columbianus*) variety, occur at comparatively higher densities in this unit than elsewhere in the state. Primary ownership in the DAU is private (48%) and USFS (44%). Deer are migratory in some areas where topographic variation is high such as the Trinity Alps and Marble Mountains area. Elsewhere they seasonally move about within a year-round home range and are considered resident deer.

The DAU is typified by coastal redwood forest, Douglas fir, ponderosa pine, and other conifer species forests, montane and coastal shrubfields and chaparral, riparian-wetland, aspen, and oak woodland-grassland.

Deer population trend

The deer population in DAU 1 is considered fairly stable in recent years from about 170,000-250,000 (Figure 5). This DAU is the most productive (based on a per unit area evaluation) in terms of deer/square mile. Longhurst et al. (1952) estimated 190,000 deer in this geographic area within the present range estimated.

Habitat issues and opportunities

Longhurst et al. (1952) rated (possible ratings were very poor, to poor, fair, good, or excellent) all the deer range that comprises this DAU as very poor, poor, or fair. However, workshop participants were not as negative in their assessment although potential for improving was discussed.

Greater emphasis to improve declining summer and fall habitat conditions so that deer populations can be sent to winter ranges in as good a condition as possible was recommended. Early successional habitats are considered to be declining in quality and abundance over time, but little actual monitoring data is collected other than long-term photo-documentation. Recent Landsat image data from the Trinity River Basin indicate low amounts of feeding range and high amounts of cover for deer and other wildlife (B. Boroski, pers. comm.). Deer winter ranges in some areas were identified as lacking forage. Declining habitat conditions in some areas may be a consequence of increased competition between native herbivores and livestock. There are some riparian concerns related to overgrazing/browsing by native and domestic herbivores in the Mendocino portion of the DAU.

Historically, clear-cutting and wild/prescribed fire have been the disturbance factors contributing the most to increasing early successional habitats by opening tree-dominated stands or dense chaparral. Recommendations to improve habitat conditions in this unit focused on the need for large-scale disturbances such as fire. Fire was discussed as being easier to implement in this unit compared to others because of fewer air quality constraints. However, the Northwest Forest Plan and accomodations for late successional reserves were considered to be constraints to implementing a program designed to favor early successional habitat. The USFS Forest Health Protection Plan was identified as a potential means to advocate and interject more prescribed fire into the system.

DAU 2 Northeastern California

Description

The Northeastern California unit comprises about 10,200 square miles in the extreme corner of the state. Deer populations are comprised of both black-tailed and Rocky Mountain mule deer (*Odocoileus hemionus hemionus*). Primary ownership in the DAU is USFS (42%), private (34%), and BLM (21%).

The DAU is comprised of Great Basin vegetation dominated by big sagebrush, antelope bitterbrush, perennial grasses, juniper, and annual grasses. Montane forest communities consisting of mixed conifer, pine/grass, and pine/bitterbrush types are common at higher elevations. Riparian-wetland, mountain meadow, aspen, mountain mahogany, and oak woodland are some other key habitats or vegetation types of importance to deer. Private lands in agriculture (e.g., alfalfa) have replaced some of the wintering habitat of deer, but deer use these fields heavily in some areas.

Deer population trend

In recent years, the deer population in DAU 2 has declined more than any other in the state (est. from about 90,000 in 1992 to 25,000 in 1996). Longhurst et al. (1952) estimated 100,000 deer in this unit's area. This area of the state historically has been considered the destination for hunting big mule deer, and most of the "X" hunt zones comprising the area are highly sought after by hunters. The decline in deer populations in this DAU, and in DAU 3, as reflected in decreased hunter opportunity, is considered an important cause of dissatisfaction among California deer hunters.

Habitat issues and opportunities

Habitat concerns in DAU 2 focus on fire, grazing, and encroachment of less desirable plant species as having the greatest negative impacts. Thinning and/or underburning of timber stands to reduce the risk of wildfire is also of concern where elimination of understory forage and cover is reduced. The relatively low and variable precipitation in this DAU makes opportunities and success for habitat improvements less predictable than desired.

Great Basin shrub communities dominated by sagebrush/bitterbrush are typically reduced by fire that occurs in the dry season. Resprouting and seed germination of bitterbrush can occur in some of the moister environments, such as the pine-bitterbrush communities, or higher elevation shrub ranges, but in the treeless winter range areas of the DAU, burned sagebrush/bitterbrush sites are often recolonized by exotic annual grasses (primarily cheatgrass) to the detriment of native plant species (Evans and Young 1978). To date, rehabilitation efforts in these areas have met with limited success, although an agressive program may be a desirable opportunity. The inability to ensure rejuvenation of shrub stands in Great Basin communities has led to opposition to the use of prescribed fire. Fire-proofing valuable stands is one consideration--greenstripping is a method that can be used adjacent to highways. Using livestock in the early season to eat herbaceous forage in shrub stands is commonly advocated although monitoring to assure minimal use of bitterbrush would be needed.

Bitterbrush is unproductive over much of it's range in the DAU because of natural maturation of the stands and repeated heavy browsing by livestock and wildlife. For example, little regeneration of bitterbrush has been documented on East Lassen-Washoe deer ranges since 1951 (DFG unpubl. data 1996). Decreasing allowable use levels on bitterbrush by livestock so that potential seed production is increased, and use reserved for deer, is an opportunity to attempt recovery of stands. Some bitterbrush improvement was identified as a consequence of depressed deer populations and good water supplies in recent years. Taking advantage of such circumstances by planting/protecting bitterbrush seedlings in the future is desirable.

Mountain mahogany stands, of limited area but a highly valued habitat that provides food and cover, are also impacted by fire. Seeds frequently germinate following fire, but because of herbivores, appear to rarely get established. Barton (1995) recently observed the apparent successful regeneration of mountain mahogany seeds around and under pruned limbs.

Aspen provides a multi-tiered habitat structure that is valuable for deer, other wildlife, and livestock. Aspen stands are declining in many areas, primarily due to a long history of livestock use. Because of the limited distribution of aspen stands and typically small patch size, efforts to maintain and enhance them could be a focused opportunity that would benefit all. Similarly, small meadows, springs, seeps, and riparian areas are important habitats to deer. Improved grazing management to avoid exceeding prescribed utilization levels are needed to ensure resotration and maintenance of these patch habitats. More timely monitoring and herding of livestock would help alleviate potential problems on key habitats. Fencing is also an option, although costly.

Encroachment of juniper into shrub/grass communities has been ongoing for decades. For deer, juniper provides valuable cover and a small amount of winter browse, however it is not as desirable as the shrub/grass communities it replaces. A desirable treatment for deer habitat enhancement would be to eliminate juniper in patches so that a more diverse mix of cover stands and openings is created. Mechanisms to reduce juniper include mechanical treatments and prescribed fire. One concern with juniper control efforts is the possibility of killing desirable grass/shrub species with a hot fire in summer. More work on site-adapted seed sources is needed to effectively accomplish revegetation efforts. Several organizations would be interested in addressing this unmet need.

In forest stands, use of prescribed burns to fireproof timber stands in the DAU are being used as a preventive tool against summer wildfire. However, the resultant effects on deer and other wildlife are not well documented.

Development issues on deer winter range, particularly in the vicinity of Susanville and Alturas, place greater impact and importance on winter ranges administered by the USFS and BLM.

DAU 3- Northeast Sierra Nevada

Description

The Northeast Sierra Nevada unit comprises about 3,600 square miles from Susanville to south of Lake Tahoe along the California-Nevada border. The DAU runs east of Highway 89 north of Lake Tahoe and east of the Sierra Crest in the south. Deer populations are comprised of some black-tailed in the northwest, but are primarily Rocky Mountain mule deer (*Odocoileus hemionus hemionus*) that summer in the Sierra Nevada and winter on the east side including parts of Nevada. Primary ownership in the DAU is USFS (63%) and private land (33%) (California only). BLM manages about 3 percent in California side, but quite a bit of additional winter range in Nevada.

The DAU is comprised of Great Basin vegetation dominated by big sagebrush, antelope bitterbrush, perennial grasses, pinyon-juniper, and annual grasses. The summer range is dominated by montane forest communities consisting of Jeffrey pine/grass, fir, and pine/Ceanothus types are common on FS lands. Riparian-wetland, aspen, shrub communities of Ceanothus species, and oak woodland are some other key habitats or vegetation types of importance to deer. Private lands in agriculture (e.g., alfalfa) have replaced some of the wintering habitat of deer, but deer use heavily use these fields in some areas.

Deer population trend

In recent years, the deer population in DAU 3 has declined nearly as dramatically as DAU 2 (est. from about 40,000 in 1992 to 10,000 in 1996). Like DAU 2, this area of the state has also been a desirable place to go hunting for big mule deer. The decline in deer populations in this DAU, and in DAU 2, as reflected in decreased hunter opportunity, are considered the leading cause of dissatisfaction among California deer hunters.

Habitat issues and opportunities

The habitat issues identified in this DAU are declining winter range conditions as affected by summer fires and grazing; development on winter ranges from the Carson City area to Susanville, especially around Reno and on summer ranges around Lake Tahoe/Truckee; grazing impacts on summer range habitats (mt. meadow, riparian, aspen); lack of fire in forested ranges; and forest understory thinning. Thinning and burning on summer range could be modified to enhance rather than degrade deer habitat. Forest plans could incorporate modifications to benefit habitat; however, this opportunity is not there in areas where fire suppression must occur.

Competition with livestock on summer ranges was identified as a problem, with aspen and riparian habitats declining in area. A compounding factor is the decline in habitats that provide the herbaceous and shrub forage used by both deer and livestock. Understory vegetation is considered to be in poor condition on most of the summer range. Modifying timber harvest practices to encourage more aspen regeneration is an opportunity. Keeping livestock out of aspen stands and reducing white fir encroachment are others.

The winter range has suffered dramatic change recently due to extended drought and fire (over 200,000 acres burned in the 1980s [Figure 11]; Loft and Menke 1990). Fires and grazing have impacted bitterbrush, and restoration efforts have had little success.

DAU 4- Cascade-North Sierra Nevada unit

Description

The Cascade-North Sierra Nevada unit comprises about 7,000 square miles from the Oregon border south to the Lake Almanor area and Feather River drainage. Deer populations are comprised of black-tailed and Rocky Mountain mule deer. Primary ownership in the DAU is private (61%) and USFS (33%).

The DAU is comprised of conifer forest dominated summer ranges, with pine/bitterbrush associations in the north and Sierra mixed conifer to the south. Common species included within the forest community are oak, aspen, riparian-wetland areas, willow, Ceanothus, and manzanita. Winter ranges are composed largely of oak-woodland, oak-annual grass savanna, chaparral shrub stands, agricultural fields, and sagebrush/bitterbrush/grass communities. Because of the large private forest ownership, a significant portion of the DAU is commercial forest dominated.

Deer population trend

The deer population has moved from 60,000-70,000 animals down to 35,000-45,000 in the past several years. Longhurst et al. (1952) estimated there were 69,000 in these areas. Deer productivity in the winter ranges of Shasta-Tehama counties has been linked to fall rains and the germination of annual vegetation. Recent deer declines may be partially attributable to a hard freeze several years ago that killed desirable browse species in some parts of the summer range.

Habitat issues and opportunities

The main habitat issues affecting deer in the DAU are associated with forestry practices, lack of habitat disturbance that favors early successional communities, and localized overuse by livestock on summer range habitats. Obvious overbrowsing by deer is apparent in the Almanor Basin, and includes severe hedging of desirable browse (e.g., mountain whitethorn) as well as seemingly less desirable browse (e.g., greenleaf manzanita and fir seedlings). This suggests a lack of disturbance and consequent lack of high quality early successional habitat.

Biomass harvest and forest thinning practices are regarded as activities detrimental to early successional habitat in the DAU. Shrub response and abundance is reduced by these activities, thereby exacerbating forage limitations. Adequate prescriptions are needed for biomass and thinning practices to accomodate early successsional conditions.

Decadent shrubfields dominate much of the range, and in some areas shrub stands may serve as the climax community. There is a need to develop prescriptions for mixes of shrub age stands. The USFS's "Forest Health" is developing as a policy. There is a need to help define forest health from a wildlife or vegetation succession standpoint. It was suggested there may be some potential to swap land units so that the timber base is maintained and there is reduced resistance to accomodating deer.

It was recommended that more effective monitoring of use and removal of livestock is needed when target utilization levels are reached on riparian-wetland, willow, bitterbrush, and aspen ranges.

DAU 5- Central Sierra Nevada

Description

The Central Sierra Nevada unit comprises about 10,500 square miles from the Feather River drainage (and Highway 70) south to Yosemite National Park, and generally west of Highway 89 and the Sierra Crest. Deer populations are comprised of black-tailed and California mule deer, although Rocky Mountain mule deer share some of the summer range. Primary ownership in the DAU is private (45%), USFS (39%), National Park Service (11%), and BLM (3%). Checkerboard ownership of private/USFS land occurs in the northern half of the DAU; much of the winter range is on private lands in the north, and on public lands in the south.

The DAU is typified by mixed conifer forest summer ranges. Common species/habitats important to deer that are included within the forest community are oak, aspen, riparian-wetland areas, willow, Ceanothus, and manzanita. Winter ranges are composed largely of oak-woodland, oak-annual grass savanna, chaparral shrub stands, and agricultural fields.

Deer population trend

The deer population has moved from 120,000-130,000 animals down to 50,000-90,000 in the past several years. Longhurst et al. (1952) reported of "repeated die-offs" and attributed them to malnutrition caused by "too many deer on too little winter range." They forecast a continued downward trend for the estimated 150,000 deer on the west slope because of habitat issues.

Habitat issues and opportunities

The main habitat issues affecting deer in the DAU are associated with forestry practices, lack of habitat disturbance that favors early successional communities, and overuse by livestock on key summer range habitats. Human development and encroachment onto private wildlands has been significant in many areas. Presence of this urban interface, adjacent to public lands, restricts options for use of fire to manipulate habitat, thereby resulting in declining early successional habitat.

Wildfire burned areas on forest land have been intensively reworked with herbicides and tree planting to the detriment of deer. Use of herbicides following fire on private forest land is increasing, thereby exacerbating public land impacts and importance for meeting wildlife objectives. Planned spring burning is being sold as ecosystem process, but does not mimic natural events and could be detrimental to deer habitat. Also, some burn projects have been labeled wildlife enhancement work when they were not. However, there is a need to integrate fuel reduction projects with habitat work when possible. "Open and park-like" goals for forests (favored for tree production) may not be desirable from an ecosystem perspective except at localized scale.

Livestock grazing is considered an important issue on the summer range. Competition for forage and negative impacts to habitats such as meadow-riparian, aspen, and willow are of concern on most forests. Where other than continuous season-long grazing systems are in place (Kennedy Meadows cited as an example), rotation grazing appears to have benefitted deer populations. Other opportunities include improved grazing management to reduce impacts to key habitats and application of fire to enhance early successional deer habitat in forest communities.

DAU 6- Eastern Sierra Nevada

Description

The eastern Sierra Nevada unit comprises about 7,500 square miles on the east side of the Sierra Nevada from Mono County south through Inyo County. Deer populations are comprised of Rocky Mountain and Inyo mule deer. These deer may share summer ranges with California mule deer. Primary ownership in the DAU is USFS (48%), BLM (28%), National Park Service (13%), and private (4%). Much of the summer range is FS land and much of the winter range is BLM land.

The DAU is typified by mixed conifer forest summer ranges. Common species/habitats important to deer that are included within the forest community are aspen, riparian-wetland areas, willow, ceanothus, and manzanita. Winter ranges are typified by bitterbrush, sagebrush, pinon pine, mahogany, blackbush, and some agricultural/pasture on private land.

Deer population trend

The deer population has moved from about 26,000-28,000 animals down to 10,000-13,000 in the past several years. Longhurst et al. (1952) estimated about 65,000 deer on the east slope from the Walker River south in this DAU. They attributed deer range problems to livestock on winter ranges and to overuse by deer. Currently, studies indicate fawn mortality is high on summer ranges, while adult mortality primarily occurs on winter ranges. Causitive factors on summer range are not well-documented.

Habitat issues and opportunities

Public land habitat issues were livestock grazing impacts on key summer range habitats, increasing pinon pine on some winter range areas to the detriment of more desirable browse, and loss of key bitterbrush winter range to fire. On private lands, development in Nevada and California is becoming a greater problem for deer as it continually reduces the amount of winter range.

There is high concern about loss of winter range bitterbrush to fire (Round Valley near Bishop is an example). Winter range shrub range manipulation opportunities are considered few because of the unreliable chance for success, but experimental approaches are being initiated by BLM and the DFG. Additionally, successional changes are slow in this DAU because of harsh environment on both winter and summer range, hence recovery from mistakes (e.g., overgrazing or inappropriate use of fire) are long-lasting. It was mentioned that deer population trends may be affected by other factors more than habitat quantity and quality. Grazing is a serious issue on summer ranges, particularly in mountain meadow and riparian habitats. Poor fawn survival could be a consequence of livestock impacts as shown in recent Sierra Nevada studies, although more specific knowledge is still needed.

It was recommended that we evaluate purchase of important wildlife habitats to enhance recovery opportunity for deer on winter range. Pinon pine invasion is increasing and possibly becoming an issue on winter ranges. Other opportunities include improved grazing management to reduce impacts to key habitats and application of fire to enhance early successional deer habitat in forest communities.

DAU 7- South Sierra

Description

The south Sierra Nevada unit comprises about 8,800 square miles on the west side of the Sierra Nevada from Yosemite National Park south to the Kern River drainage area, then across the Tehachapi's to Interstate 5. Deer populations are comprised of California mule deer, although these deer may share summer ranges with Rocky Mountain and Inyo mule deer. Primary ownership in the DAU is USFS (40%), private (39%), National Park Service (15%), and BLM (4%). Much of the summer range is FS and NPS land and much of the winter range is private or FS land.

The DAU is typified by mixed conifer forest summer ranges. Common species/habitats important to deer that are included within the forest community are aspen, riparian-wetland areas, willow, ceanothus, and manzanita. Winter ranges are composed largely of oak-woodland, oak-annual grass savanna, chaparral shrub stands, and agricultural fields/pasture. The southeastern portion of the DAU gets into Mojave Desert influenced plant communities.

Deer population trend

The deer population has been fairly stable at 30,000-40,000 animals in the past several years. Longhurst et al. (1952) estimated about 95,000 deer in this DAU, illustrating how far we have declined since the late 1940s. They attributed deer range problems to livestock on winter ranges and to overuse by deer. Currently, studies indicate fawn mortality is high on summer ranges, while adult mortality primarily occurs on winter ranges. Causitive factors on summer range are not well-documented. Resident deer were considered to be responding positively to wildfires, while migratory deer populations were not.

Habitat issues and opportunities

The primary habitat issues are livestock grazing impacts on key summer range habitats (e.g., aspen, mountain meadow, riparian), and on winter range (e.g., Rodgers Ridge); lack of fire on some of the summer range forested areas, and too much fire in some winter range shrub communities. There is some concern about the implications of prescribed burning on summer range brushfields (huckleberry oak stands).

Opportunities include improved grazing management to reduce impacts to key habitats and application of fire to enhance early successional deer habitat in forest communities.

DAU 8 Central Coast (North)

Description

The Central Coast unit comprises about 6,300 square miles from Mendocino County east to Interstate 5 and south to the Delta and San Francisco Bay. Deer populations are comprised of black-tailed deer. Primary ownership in the DAU is private (83%), USFS (5%), and BLM (7%). Deer in the unit are largely resident animals that exhibit some upslope/downslope movement with seasonal changes in weather and forage conditions. However, some winter range areas have been delineated in the Mendocino National Forest.

The DAU is typified by mixed conifer forest summer ranges. Common species/habitats important to deer that are included within the forest community are several oak species, western mountain mahogany, chamise, riparian-wetland areas, willow/birch, ceanothus, and manzanita. Oak-woodland, oak-annual grass savanna, chaparral shrub stands, are common at lower elevations. Agricultural fields, pastures, and vineyards occur throughout the area in valley bottoms.

Deer population trend

The deer population has varied from about 90,000-140,000 in the past several years, but appears fairly stable over the seven year period 1990-96. Longhurst et al. (1952) estimated 119,000 deer in this unit, within the range of current estimates, but fewer than the peak in the late 1950-60s. Long-term studies (e.g., Taber and Dasmann 1958) have nicely described the factors affecting deer populations in this unit and how they respond to land management activities, especially fire.

Habitat issues and opportunities

Deer use in some areas is concentrated on riparian habitat and stream corridors to the detriment of the habitat. Part of the problem is believed to be a shortage of early successional habitat and forage during summer. Public land habitats are considered to be decadent shrubfields in many areas. Concern that spring burning tends to cause a switch from a diverse mix of browse to chamise dominated stands

Past opportunity has been missed with USFS burn programs, and more planning efforts to promote mosaic patterns of habitats are needed. There is a desire to move to fall burning and/or mechanical manipulation and away from spring burning. Private lands are very important and there is a need to support private landowner efforts to maintain/enhance wildlands through programs such as the Department’s PLM program or other incentives.

DAU 9- Central Coast (South)

Description

The Central Coast south unit comprises about 15,600 square miles from the Delta and San Francisco Bay west of Interstate 5 and south through Ventura County. Deer populations are comprised of black-tailed in the north and California mule deer in the south. Primary ownership in the DAU is private (71%), USFS (18%), and BLM (4%). Deer in the unit are resident animals that exhibit some upslope/downslope movement with seasonal changes in weather and forage conditions.

The DAU is typified by pine forest summer ranges. Common species/habitats important to deer that are included within the forest community are several oak species, pinon-juniper, chamise, riparian-wetland areas, willow/birch, ceanothus, and manzanita. Oak-woodland, oak-annual grass savanna, chaparral shrub stands, are common at lower elevations. Agricultural fields, pastures, and vineyards occur throughout the area in valley bottoms.

Deer population trend

The deer population has varied from about 70,000-120,000 in the past several years, but appears stable to increasing over the seven year period 1990-96. Longhurst et al. (1952) estimated 202,000 deer in this unit, within the range of current estimates, but fewer than the peak in the late 1950-60s.

Habitat issues and opportunities

Because of the dominance of private land, there is need to focus on providing incentive and support for deer management on these lands. Developing vineyards are converting much habitat, and result in increases in depredation problems. On public lands, summer forage is considered laking because of decadent shrub dominated stands. Recent large fires in the San Luis Obispo area occurred and should soon provide benefits in the area. Lack of blue oak regeneration is considered a problem throughout the unit.

Past opportunity has been missed with USFS burn programs, and more planning efforts to promote mosaic patterns of habitats are needed. There is a desire to move to fall burning and/or mechanical manipulation and away from spring burning. Fall burns to date have been generally successful. In some areas, north slope burning should be increased, as long as potential loss of hardwoods is considered to not be significant.

Post-wildfire prescriptions need to better develop the long-term habitat objectives for an area so as to maintain mosaic patterns of habitat. Private lands are very important and there is a need to support private landowner efforts to maintain/enhance wildlands through programs such as the Department’s PLM program or other incentives.

DAU 10- South Coast

Description

The South Coast south unit comprises about 7,800 square miles from Los Angeles County south to the border with Mexico and east to Interstate 10. It includes the San Bernardino Mountains. Deer populations are comprised of California and Southern mule deer, with some burro deer in the Santa Rosa Mountains. Primary ownership in the DAU is private (45%), USFS (34%), other public lands (10%), and BLM (8%). Deer inhabiting the higher mountains are migratory (Nicholson 1995), while deer in the coastal areas and lower elevations are resident animals that exhibit some upslope/downslope movement with seasonal changes in weather and forage conditions.

The DAU is typified by chamise chaparral, oak woodland, grassland, coastal scrub, sagebrush, and pine communities. Other habitats important to deer include montane riparian and meadow.

Deer population trend

The deer population has varied from about 16,000-24,000 in the past several years, but appears fairly stable over the seven year period 1990-96. Longhurst et al. (1952) estimated 79,000 deer in this unit, far more than current estimates, and somewhat understandable given the changes to Southern California's landscape.

Habitat issues and opportunities

Encroachment by development on private lands adjacent to the public land interface is a primary habitat issue in the unit. On public lands, people management was considered the biggest issue. Human disturbance is regarded as a problem for deer populations, and may be impacting deer use of key habitats.

Focused programs to implement prescribed burning programs have been effective at maintaining early successional habitats in some watersheds. Restrictions on burning can make it difficult to implement habitat improvement efforts. Further, ongoing conservation planning efforts in part of the unit may dictate habitat manipulation potential. Representative habitat guidelines (e.g., Laguna Morena) are often not implemented. Livestock grazing is of concern on key meadow habitats in the southern mountains (Bowyer and Bleich 1984).

DAU 11- Desert

Description

The Desert unit comprises about 7,500 square miles of the Mojave and Colorado/Sonoran Desert areas and extends east to the California border. It includes the Colorado River area and isolated mountain ranges in the eastern counties.

Deer populations are comprised of burro deer. Primary ownership in the DAU is BLM (51%), NPS (22%), private (14%), and military (9%). Deer movements occur seasonally, and in response to need for reliable water and forage sources. Movement down to near permanent water sources such as the Colorado River area or permanent canals typically occurs in late spring.

The DAU is typified by desert scrub, with habitats important to deer occurring in the riparian drainages and desert washes. In some of the ranges, waxy bitterbrush, sagebrush, and juniper occur.

Deer population trend

The extreme environment results in low densities of deer. Annual fluctuation in forage and water conditions away from the permanent sources of water, likely play an important role in annual variations in population. The overall deer population has varied from about 2,000-5,000 in the past several years, and appears to be stable at around 2,000 because Longhurst et al. (1952) estimated 2,100 deer in this unit, similar to current estimates.

Habitat issues and opportunities

Competition for water, forage, space with burros is a long-term problem, particularly in preferred habitats. Other public land uses that have some effect, mostly localized, are mining operations and OHV use. Development and agriculture in riparian bottomlands along the Colorado River removes native habitat, decreases carrying capacity, and increases potential problems relating to depredation.

The primary opportunities in the unit are continued development and maintenance of effective water sources and implementation of some control over burro populations. Habitat conservation/protection measures would also contribute to maintaining deer and other wildlife populations.

VIII. DEVELOPMENT OF FOCUS AREAS FOR COLLABORATIVE EFFORTS TO ENHANCE CONDITIONS FOR DEER

Identification of priority areas

Workshop participants were aware that resources of each agency are limited in their ability to accomplish positive activities to enhance early successional habitats and other habitats important to deer. Instead of attempting a statewide effort, focus areas based on the results of the assessment were identified for more intensive collaboration, but with the realization that deer throughout the state were important.

Northeastern California and Northeastern Sierra (DAUs 2 and 3) were the top priority areas to attempt to reverse the decline in deer populations through habitat-based efforts. The primary habitat targets are shrub-dominated winter ranges, riparian-wetland areas, and forested understory communities.

The number two priority area identified would include parts of the Southern and Eastern Sierra Nevada (DAUs 6 and 7), where mountain meadow and aspen summer ranges and west slope forest/chaparral ranges that have become closed timber or shrub stands with little forage available are the target communities.

A requirement for developing and implementing any positive actions for deer is a core group of personnel in a focus area with the interest and motivation to follow-through with an effort. There are staff from each agency in the area comprising DAUs 2 and 3 who participated in the workshop and believe an effort is desirable. For DAUs 6 and 7, there were BLM and DFG staff at the workshop, but no FS staff; hence a strong commitment from FS in that area is still tentative. As well as local support, commitment from the leadership of all three agencies is also needed as we move from identifying concerns, issues, or problems to implementing actions on-the-ground.

IX. PILOT DAU MANAGEMENT STRATEGY

The next step in working toward achieving improved habitat conditions for deer on public lands will involve a regional effort, or what we have identified as a "Pilot DAU Management Strategy." Specific management strategies (changes), or habitat improvement/manipulation efforts that can be implemented on public lands to address deer habitat issues must be crafted at the DAU level and involve stakeholders interested in habitat management on public lands. These strategies for northeastern California and the Sierra Nevada will be developed among, and by, regional participants. Some of the primary components of this strategy will include:

1. DAU Location Maps to illustrate scale and area of emphasis for pilot strategy.
2. Definition of the issues including those resulting from agency statutory authorities and management priorities.
3. Stakeholder involvement (e.g. counties, municipalities, county F&G comm., public groups, etc.).
4. Deer population trend and habitat assessment (expanded for that DAU using data gathered in that DAU).
5. Visual identification and display of key/essential habitats such as winter ranges.
6. Consolidation of available data and identification of data gaps. A prioritization for filling data gaps.
7. Definition of the role mule deer play in the broader issues of land management agencies.
8. Habitat objectives and recommended strategies for reaching the objectives.
9. An interagency, stakeholder monitoring effort to ensure objectives are being met and management actions are accomplishing the intended task.
10. Projects implemented to meet the objectives.

X. REFERENCES

Barrett, R.H. 1982. Habitat preferences of feral hogs, deer, and cattle on a Sierra foothill range. J. Range Manage. 35:342-346.

Biswell, H.H. 1989. Prescribed burning in California wildlands vegetation management. Univ. of Calif. Press, Berkeley. 255 p.

Bronson, M. 1992. Effects of longer versus shorter short-duration cattle grazing on winter forage available to mule deer in the northern Sierra Nevada foothills. M.S. thesis, Univ. of Calif., Davis. Dept. of Agronomy and Range Sci. 41 p.

Bowyer, R.T., and V.C. Bleich. 1984. Effects of cattle grazing on selected habitats of southern mule deer. Calif. Fish Game 70:240-247.

California Dept. of Forestry & Fire Protection (CDFFP). 1995. California Fire Plan. (On-line at http://frap.cdf.ca.gov/fire_plan/)

Dale, J.W. 1996. Tech. Coord. California Forest Health in 1994 and 1995. Rep. R5-FPM-PR-002. U.S. Dep. Agric., Forest Service, San Francisco. 63 p.

Dasmann, W.P., and J.A. Blaisdell. 1954. Deer and forage relationship on the Lassen-Washoe Interstate winter deer range. Calif. Fish Game 40:215-234.

Dasmann, W.P., H.A. Hjersman, and D. Gilsenan. 1958. California's first general either-sex deer hunting season. Calif. Fish Game 44:231-251.

DiTomaso, J.M., D. Marcum, M. Rasmussen, E. Healy, and G.B. Kyser. 1997. Post-fire herbicide sprays enhance native plant diversity. Calif. Agric. 51:6-11.

Evans, R. A., and J.A. Young. 1978. Effectiveness of rehabilitation practices following wildfire in a degraded big sagebrush-downy brome community. J. Range Manage. 31:185-188.

Gallagher, J.F., and D.R. McCullough. 1992. Deer response to the 1987 wildfires and subsequent timber harvest practices, Klamath National Forest. Final Rep. to Calif. Fish and Game. Univ. of Calif., Berkeley. 58 p.

Grifantini, M.C. 1991. Early-seral changes following wildfire, salvage-logging, and reforestation, Klamath Mountains, CA. Final Rep. Humboldt State Univ., Arcata. 47 p.

Hanley, T.A. and J.L. Page. 1982. Differential effects of livestoc use on habitat structure and rodent populations in great basin communities. Calif. Fish and Game 68:160-174.

Hanley, T.A., and J.D. McKendrick. 1985. Potential nutritional limitations for black-tailed deer in a spruce-hemlock forest, southeastern Alaska. J. Wildl. Manage 49:103-114.

Kie, J.G., C.J. Evans, E.R. Loft, and J.W. Menke. 1991. Foraging behavior by mule deer: the influence of cattle grazing. J. Wildl. Manage 55:665-674.

Kie, J.G., and B.B. Boroski. 1995. The effects of cattle grazing on black-tailed deer during winter on the Tehama Wildlife Management Area. Final Rep. USDA Forest Service, Fresno, CA. 36 p.

Kucera, T.E., and R.H. Barrett. 1995. Effects of whole-tree removal on wildlife habitat in forests of Northern California. Final Rep. To Dep. Fish and Game. 32 p.

Laudenslayer, W.F., and H.H. Darr. 1990. Historical effects of logging on the forests of the Cascade and Sierra Nevada ranges of California. Trans. West. Sec. Wildl. Soc. 26:12-23.

Leach, H.R., and J.L. Hiehle. 1957. Food habits of the Tehama deer herd. Calif. Fish and Game 43:161-178.

Leckenby, D. et al. 1982. Mule deer. Wildlife habitats in managed rangelands — the Great Basin of southeastern Oregon. U. S. For. Serv. Gen. Tech. Rep. PNW-l39.

Leopold, A.S. 1950. Deer in relation to plant succession. Trans. N. Amer. Wildl. Conf., 15:571-579.

Loft, E. R., and J.W. Menke. 1988. Habitat and spatial relationships between mule deer and cattle in a Sierra Nevada Forest zone. Dep. Agron. and Range Sci., Univ. of Calif., Davis. Final Rep. to U.S. For. Serv. 144 p.

Loft, E.R., and J.W. Menke. 1990. Evaluation of fire effects on mule deer habitat in Lassen County. Final. Rep. Univ. of Calif., Dept. of Agronomy and Range Sci. 30 p.

Longhurst, W. M., Leopold, A. S., & Dasmann, R. F. 1952. A survey of California deer herds: Their ranges and management problems. California Department of Fish & Game Bulletin No. 6.

Longhurst, W.M., and G.E. Connolly. 1970. The effects of brush burning on deer. Cal-Neva Wildl. Trans. 1970:134-155.

Longhurst, W. M., Garton, E. 0., Heady, N. F., & Connolly, G. E. 1976. The California deer decline and possibilities for restoration. Transactions of the Western Section of the Wildlife Society, 16, 74-101.

Longhurst, W.M., and others. 1977. Livestock and wild ungulates. Pages 42-64 in J.W. Menke, ed. Proc. of workshop on livestock and wildlife-fisheries relationships in the Great Basin. Sparks, NV. Univ. of Calif., Special Publ. 3301, Berkeley, CA.

Loomis, J., M. Creel, and J. Cooper. 1989. Economic benefits of deer in California: hunting and viewing values. Univ. of Calif., Davis. Inst. of Ecology Rep. 32.

Martin, R.E., and D.B. Sapsis. 1992. Fires as agents of biodiversity: pyrodiversity promotes biodiversity. Pages 150-157 in R.R. Harris et al., eds. Proc. Symp. on Biodiversity of Northwestern California.

Nicholson, M. 1995. Habitat selection by mule deer: effects of migration and population density. Ph.D. diss. Univ. of Alaska, Fairbanks. 108 p.

Pillsbury, N.H. 1994. Aspen inventory, assessment, and management on the Kern Plateau. Final Rep. Cal. Poly State Univ. San Luis Obispo. 68 p +appendices.

Salwasser, H., Holl, S., & Ashcraft, G. 1978. Fawn production and survival in the North Kings deer herd. California Fish and Game, 64, 38-52.

Storer, T. I. 1932. Factors influencing wildlife in California, past and present. Ecology 13:315-327.

Taber, R.D., and R.F. Dasmann. 1958. The black-tailed deer of the chaparral. Game Bull. 8, Calif. Dep. Fish and Game. 163 p.

Thomas, J.W., H. Black, Jr., R. Scherzinger, and R.J. Pedersen. 1979. Deer and elk. Pages 104-127 in J.W. Thomas, ed. Wildlife habitats in managed forests. USDA Forest Service, Agric. Handbook 553.

Thomas, J.W., C. Maser, and J.E. Rodiek. 1979. Riparian zones. *In* Wildlife habitats in managed rangelands- the Great Basin of southeastern Oregon. USDA/USDI Gen. Tech. Rep. PNW-80. 18 p.

Tuchmann, E.T., K. Connaughton, L. Freedman, and C. Moriwaki. The Northwest forest plan. USDA, For. Serv., Pacific NW Res. Sta. 253 p.

Urness, P.J. 1990. Livestock as manipulators of mule deer winter habitats in northern Utah. Pages 25-40 *in* K.E. Severson, Ed., Can livestock be used as a tool to enhance wildlife habitat? USDA For. Serv. Rocky Mt. For. and Range Exp. Sta. Gen. Tech. Rep. RM-194.

USDA, Forest Service. 1996a. Thinning in Support of Forest Health. Paper available at website http:// 162.79.41.7/fh/fh_issue_papers/thinning.html. 2 p.

USDA. 1996b. Strategic Management Report, Winter/Spring 1996. The USDA Forest Service, Pacific Southwest Region. 24 p.

Van Sickle, V. 1995. Comparison of vegetation patch composition, biodiversity and wildlife, human and livestock use of Marble Mountain Wilderness Area meadow basins. M.S. thesis, Dep. Agron. and Range Sci., Univ. of Calif., Davis. 95 p.

Wallmo, O.C., D.F. Reed, and L.H. Carpenter. 1976. Alteration of mule deer habitat by wildfire, logging, highways, agriculture, and housing developments. Pages 37-47 *in* Workman and Low, eds., Mule deer decline in the West: A symposium. Utah State Univ.

Wallmo, O.C., and J.W. Schoen. 1981. Forest management for deer. Pages 434-448 in O.C. Wallmo, ed. Mule and black-tailed deer of North America. Univ. of Nebraska Press.

Wright, H.A., and A.W. Bailey. 1982. Fire ecology: United States and southern Canada. J. Wiley and Sons. 501 p.

Appendix 1. Participants at the Feather River Inn Workshop on Deer (April 29, 1997).

California Department of Fish and Game

Headquarters-	Terry Mansfield	Sonke Mastrup	Dr. Eric Loft	
	Ken Mayer	Russ Mohr		
Region 1-	Don Koch	Tim Burton	Dave Smith	
Region 2-	Ron Bertram	Syd Kahre	Jeff Finn	
Region 3-	Jim Lidberg	Jack Booth		
Region 4-	Ed Smith	Jim Maddox		
Region 5-	Bob Schaefer	Dr. Vern Bleich		
Fish and Game Commission Staff-		Ron Pelzman		

Forest Service

Forest Service Regional Office-	Mike Chapel	Barry Davis	
Lassen National Forest-	Barbara Dutman	Todd Johnson	
	Tom Rickman	Gary Smith	
Mendocino National Forest-	Linda Tatum		
Modoc National Forest-	Tom Ratcliff		
Plumas National Forest-	Debbie Bliss	Tina Hopkins	Gary Rotta
Tahoe National Forest-	Quentin Youngblood		
Toiyabe National Forest-	Pat Shanley		

Pacific Southwest Research Station, Fresno- Brian Boroski

Bureau of Land Management

State Office-	Carl Rountree	John Willoughby
NE California-	Roger Farschon	Don Armentrout
Eastern Sierra-	Steve Nelson	Jim Ramakka (Carson City)
Central California-	Larry Saslaw	

Acknowledgment: We thank the participants above for their interest and contributions; and thank Tom Lupo and Ann Mahaney from DFG's Wildlife Management GIS lab for their assistance.

Appendix 2. Additional Fish and Game Code sections applying specifically to deer herd management in California.

450. (in text of report)

451. As used in this chapter "general deer hunting season" means the annual season for the area in question as is set by the commission under its general regulatory powers, or set by statute, for the taking of male deer.

452. The department shall designate deer herd management units and designate the manager for the units. Such units may encompass a single deer herd or a group of deer herds having similar management and habitat requirements and characteristics. Boundaries of such units, unless appropriate, need not follow county boundary lines.

453. The department shall develop plans for such deer herd management units. The objectives of such plans shall be the restoration and maintenance of healthy deer herds in the wild state and to provide for high quality and diversified use of deer in California.

454. Such management plans shall contain the following program elements:

(a) Document existing information on deer herd management units and programs to obtain information that may be needed.

(b) Develop programs to maintain and increase the quality of deer habitat statewide. Such programs will emphasize cooperative action between the department and the appropriate land management entities, both public and private. Emphasis shall be directed towards identifying critical deer habitat areas and the maintenance and management of such areas.

(c) Develop programs to reduce natural mortalities where such reduction may be critical to meeting deer herd plan objectives.

(d) Develop programs to decrease the illegal taking of deer through modern law enforcement methods supported by public and private cooperative efforts.

(e) Develop diversified recreational use programs, including both hunting and nonhunting uses, consistent with the basic individual deer herd management unit capabilities.

455. Deer herd management unit plans shall be reviewed annually and shall be the basis for department recommendations to the commission pursuant to this chapter.

456. (a) The department shall biennially report to the Legislature and to the Fish and Game Commission on the progress that is being made toward the restoration and maintenance of California's deer herds. The first report shall be submitted on or before October 1, 1989. The report shall include program activities regarding deer habitat, particularly addressing problems dealing with identification and preservation of critical deer habitat areas; the amount of revenue derived from the sale of deer tags during the two previous fiscal years; a list of expenditures during the two previous fiscal years and proposed expenditures during the current fiscal year; and a report of general benefits accrued to the deer resources as a result of the program.

Appendix 3. Tabular data of 11 DAUs in California.

Square miles of deer range by DAU and by ownership:

DAU	BLM	MILITARY	NPS	OTHER PUBLIC	PRIVATE	USFS	
1	558	3	181	495	7927	7296	
2	2075	6	72	213	3353	4097	
3	94	3	0	28	1069	2042	
4	59	0	165	108	3068	1663	
5	334	97	1156	91	4711	4125	
6	2058	75	954	524	306	3544	
7	384	15	1343	108	3422	3532	
8	442	28	119	217	5159	288	
9	701	546	55	483	11028	2783	
10	623	199	0	788	3487	2672	
11	3819	686	1617	345	1055	0	
TOTAL	11147	1658	5662	3400	44585	32042	98494 square miles

Percentage ownership of each DAU by major landowners/agencies.

DAU	Sq. miles BLM	Percentage
1	558	5.0%
2	2075	18.6%
3	94	0.8%
4	59	0.5%
5	334	3.0%
6	2058	18.5%
7	384	3.4%
8	442	4.0%
9	701	6.3%
10	623	5.6%
11	3819	34.3%
TOTAL	11147	100.0%

DAU	Sq. miles OTHER PUBLIC	Percentage
1	495	14.6%
2	213	6.3%
3	28	0.8%
4	108	3.2%
5	91	2.7%
6	524	15.4%
7	108	3.2%
8	217	6.4%
9	483	14.2%
10	788	23.2%
11	345	10.1%
TOTAL	3400	100.0%

DAU	Sq. miles MILITARY	Percentage
1	3	0.2%
2	6	0.4%
3	3	0.2%
4	0	0.0%
5	97	5.9%
6	75	4.5%
7	15	0.9%
8	28	1.7%
9	546	32.9%
10	199	12.0%
11	686	41.4%
TOTAL	1658	100.0%

DAU	Sq. miles PRIVATE	Percentage
1	7927	17.8%
2	3353	7.5%
3	1069	2.4%
4	3068	6.9%
5	4711	10.6%
6	306	0.7%
7	3422	7.7%
8	5159	11.6%
9	11028	24.7%
10	3487	7.8%
11	1055	2.4%
TOTAL	44585	100.0%

DAU	Sq. miles NPS	Percentage
1	181	3.2%
2	72	1.3%
3	0	0.0%
4	165	2.9%
5	1156	20.4%
6	954	16.8%
7	1343	23.7%
8	119	2.1%
9	55	1.0%
10	0	0.0%
11	1617	28.6%
TOTAL	5662	100.0%

DAU	Sq. miles USFS	Percentage
1	7296	22.8%
2	4097	12.8%
3	2042	6.4%
4	1663	5.2%
5	4125	12.9%
6	3544	11.1%
7	3532	11.0%
8	288	0.9%
9	2783	8.7%
10	2672	8.3%
11	0	0.0%
TOTAL	32042	100.0%
Sq. mi of deer range	98,494	

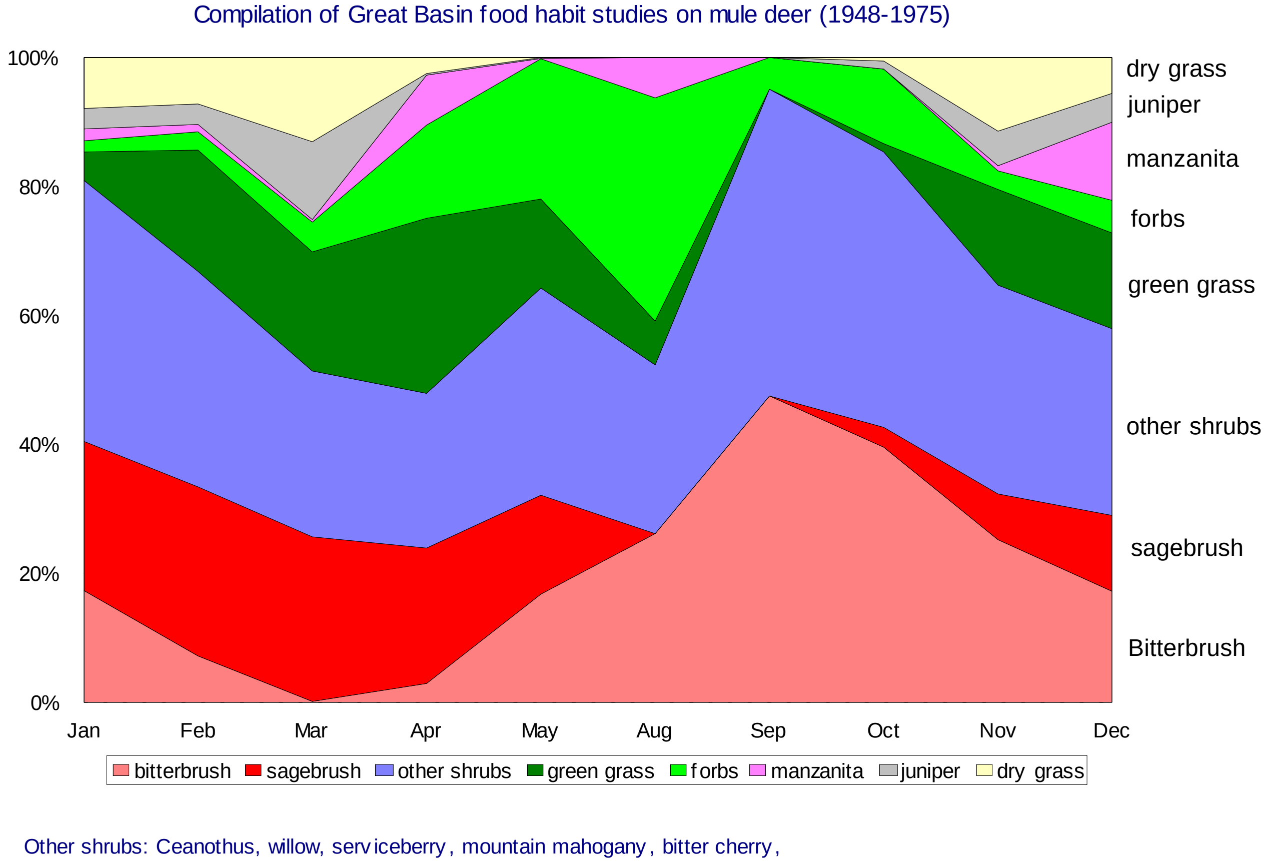

Figure 14. Compilation of all deer food habit studies (does not include studies of deer die-offs) in northeastern California by month. Bitterbrush, sagebrush, and other shrubs dominate the deer diet, with lush green grass and forb being important during spring to mid-summer. (original studies by Howard Leach, unpubl. data; compiled by DFG 1997)

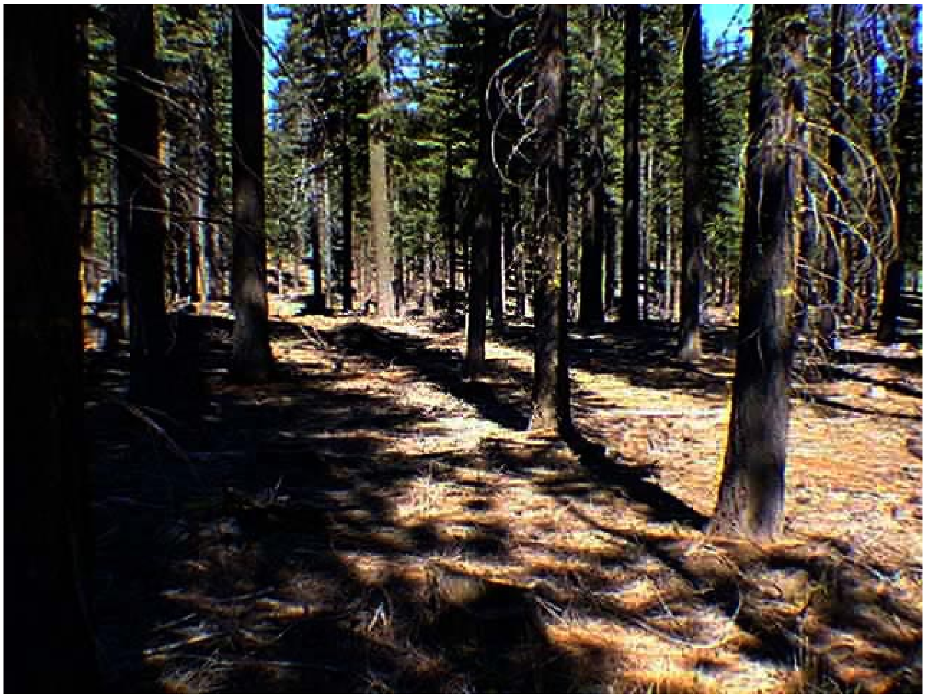

PHOTO 1a. The use of prescribed fire, stand thinning, or biomass thinning can result in the lack of understory vegetation as shown here. There is little food or hiding cover available for herbivores. As the stand continues to mature and the canopy further closes, habitat quality disappears for early successional favored species like deer, elk, and cattle.

These two digital photos from the Almanor Basin, September 1997.

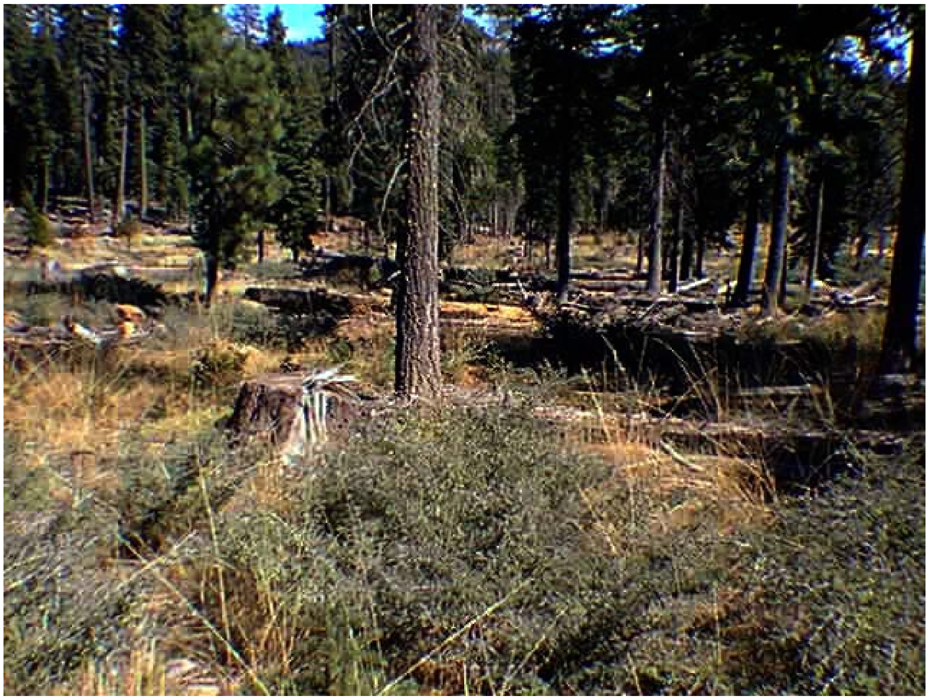

PHOTO 1b. Nearby, a more open stand illustrates the abundance of grass and shrub vegetation that is associated with disturbance, as long as the disturbance is not followed by intensive site preparation for reforestation and timber stand enhancement.

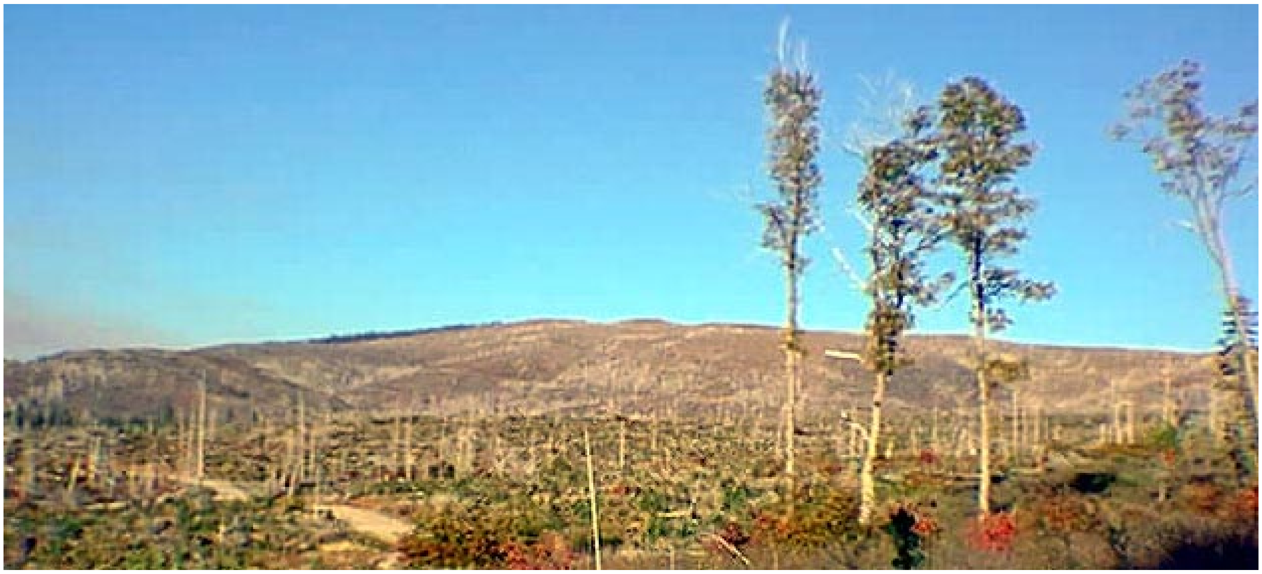

PHOTO 2a. November 1997 digital photo of summer 1992 Fountain fire area along Hwy 299 in northern California. Herbicide spraying illustrates the impact on early successional vegetation and lack of shrub cover on hillsides in background. Note standing oak trees have resprouted. Smoke on left is a prescribed fire.

PHOTO 2b. Wildfire burned slope (1987 fire) on the Stanislaus National Forest. Use of herbicides as proposed here would be beneficial to reforestation effort. However, opportunities to develop a mix of reforested sites with shrub stands for wildlife habitat exist in such areas. Deer and other wildlife would benefit if spraying were not broadcast over the entire area, but rather, in a mosaic pattern to reserve some cover and browse. Untouched, areas such as this frequently come back in very dense cover of deerbrush (light green in background), more so than desired from a habitat perspective.

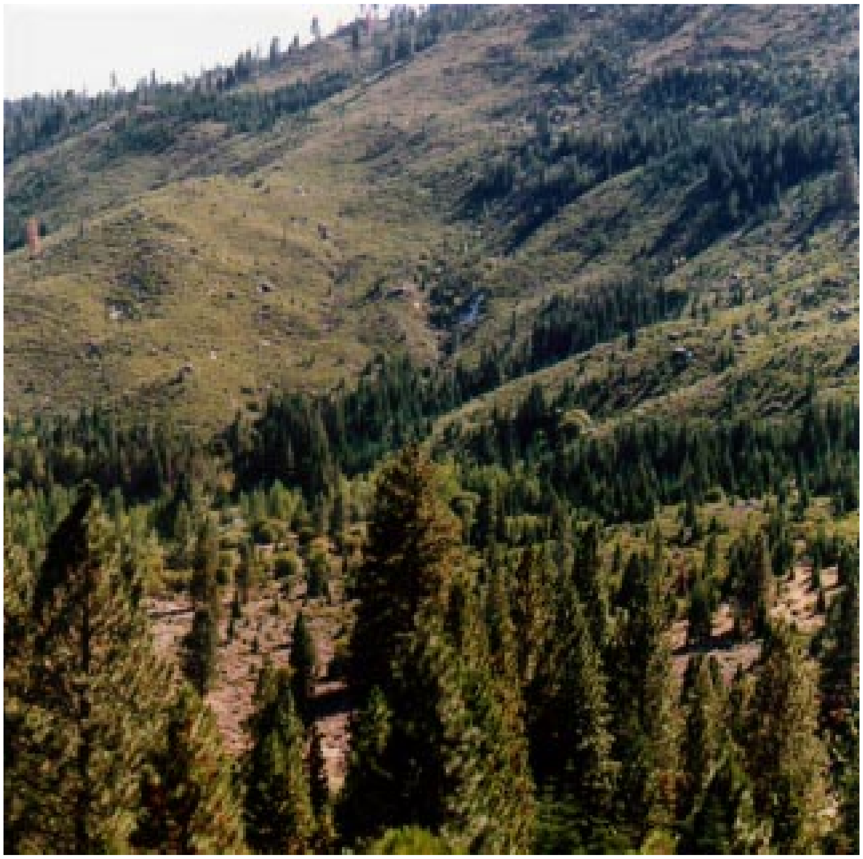

PHOTO 3a. Aspen stand in Modoc NF illustrating lack of understory typical of a grazed system. Over time, these stands may decline to the extent that aspen dies out.

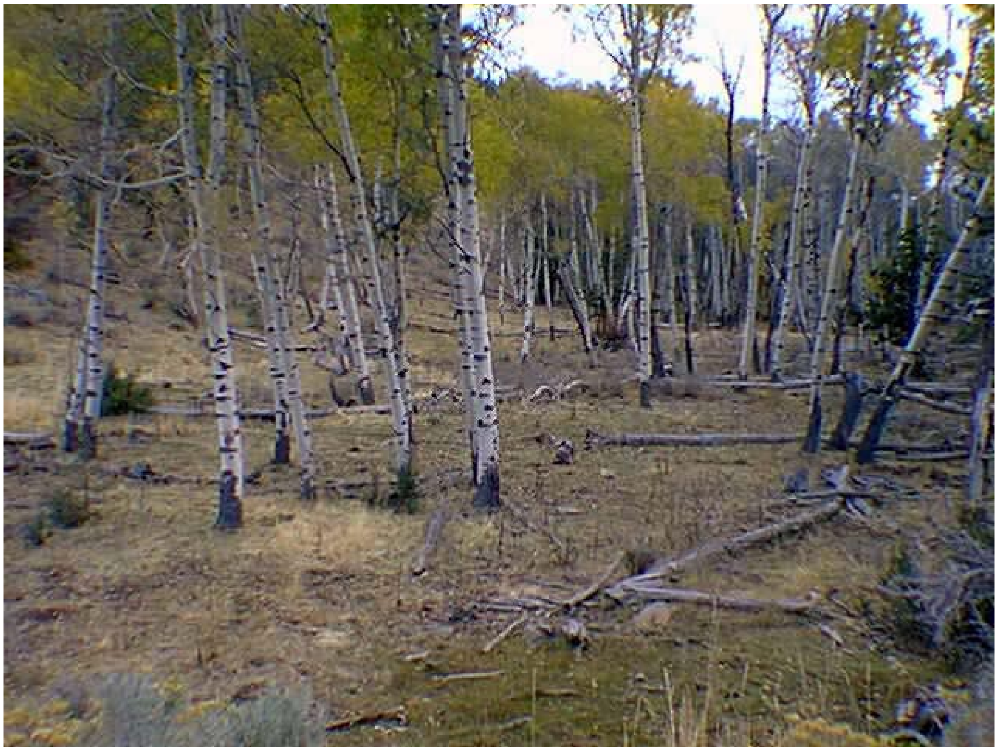

Digital photos- 1997

PHOTO 3b. Aspen stand in Modoc NF illustrating down and dead logs indicative of a former stand. The presence of these logs on summer ranges is of concern in grazed systems in California.

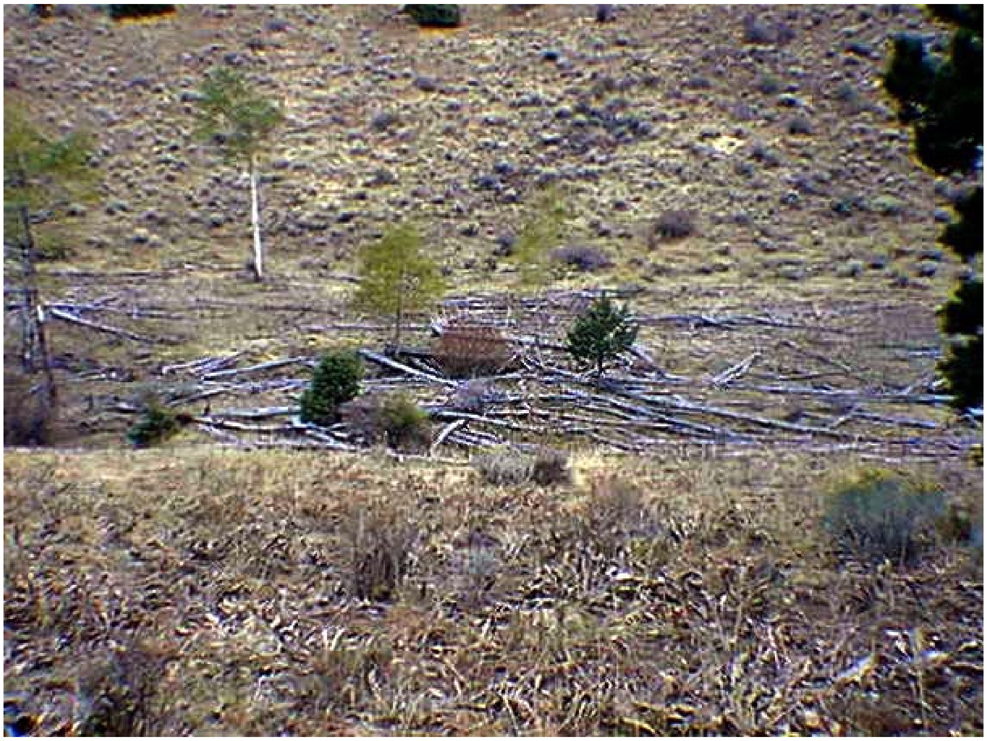

PHOTO series 3c. Photo plots of aspen understory near end of grazing season under different grazing treatments (from Loft and Menke 1988) during 1983-84, Stanislaus NF; and again on October 1, 1997. Heavy grazing has contributed to a decline in aspen cover. Tree in right background is the same, although dead (left of center) in 1997 photo.

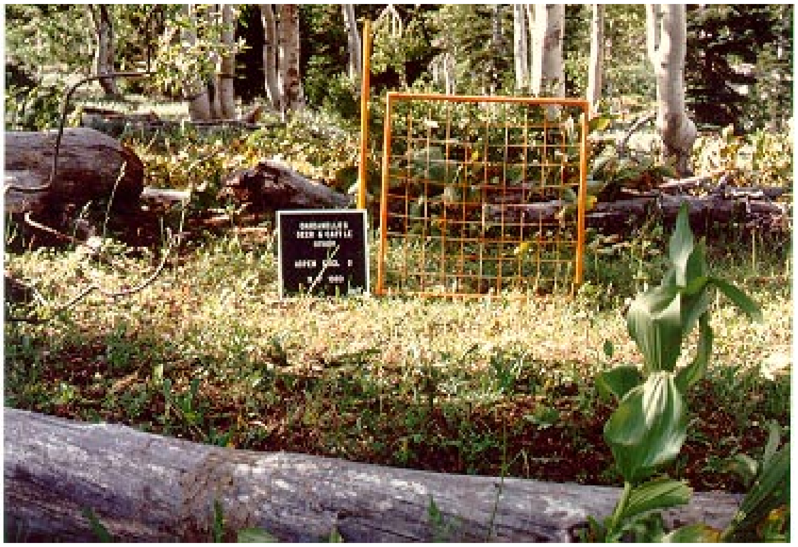

Moderate grazing in 1983; this approximated the USFS recommended stocking rate

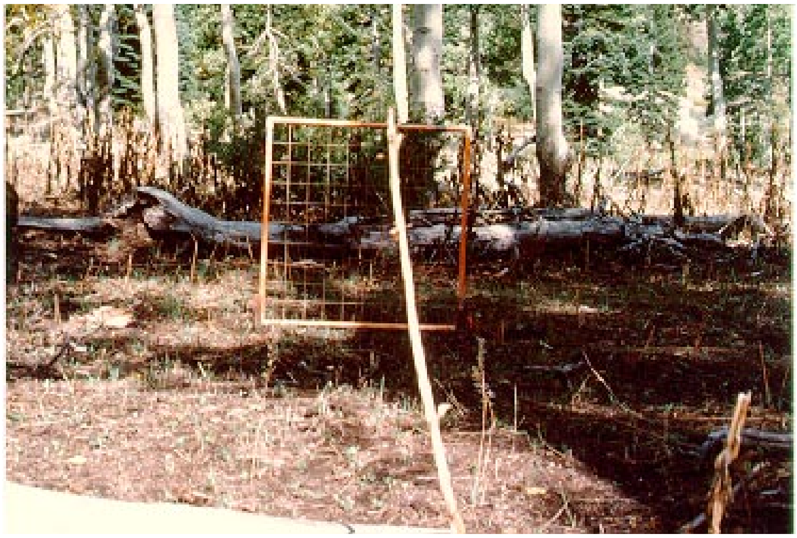

Heavy grazing in 1984; this was approximately 1.5x the recommended stocking rate

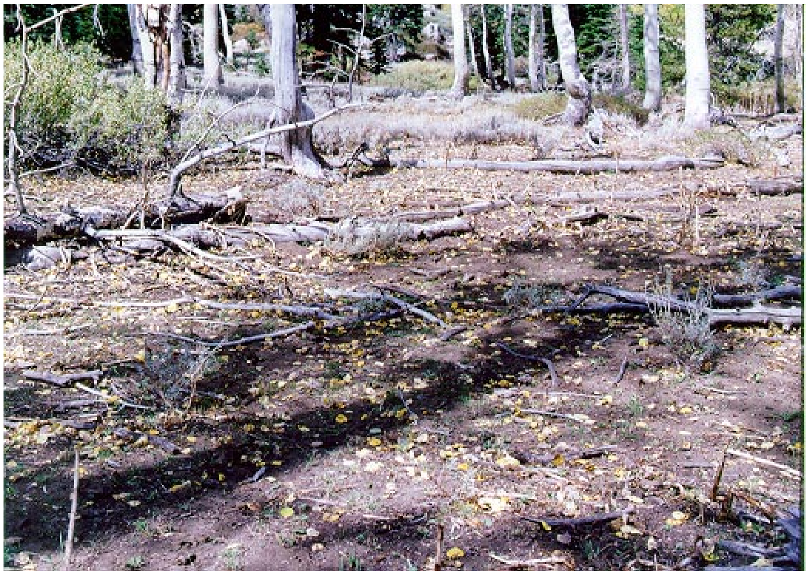

1997 grazing season and influence of intervening 13 years indicate aspen is on the decline at this site

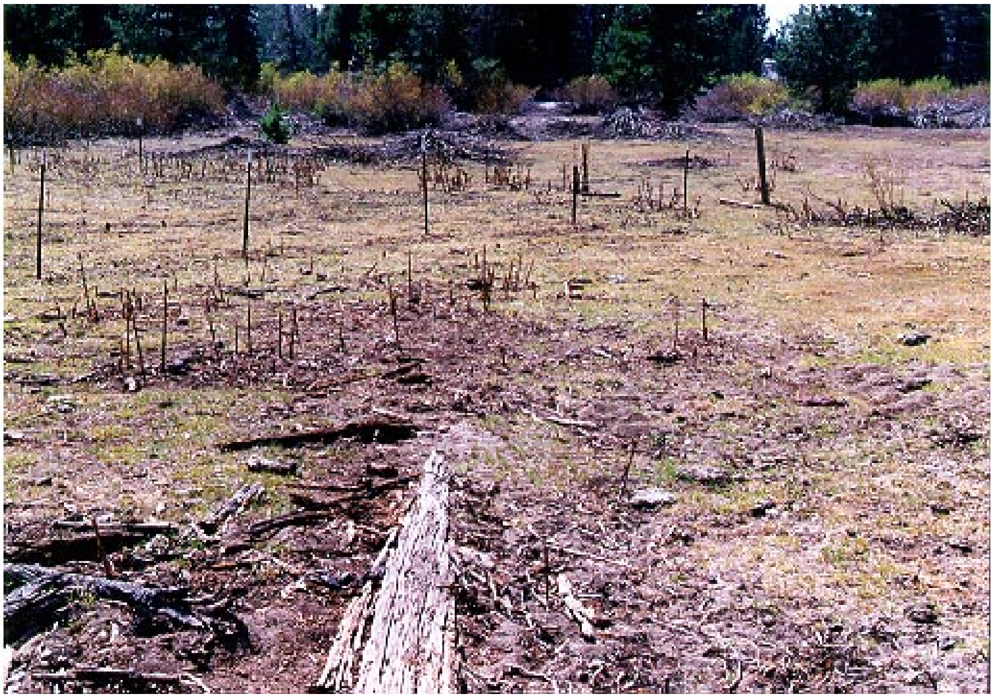

PHOTO 4a. Mountain meadow and willow-riparian site (October 1, 1997). Occurrence of bare ground and killed willows in background is indicative of severe grazing conditions over time. (the exclosure is no longer maintained). McCormick Creek, Stanislaus NF.

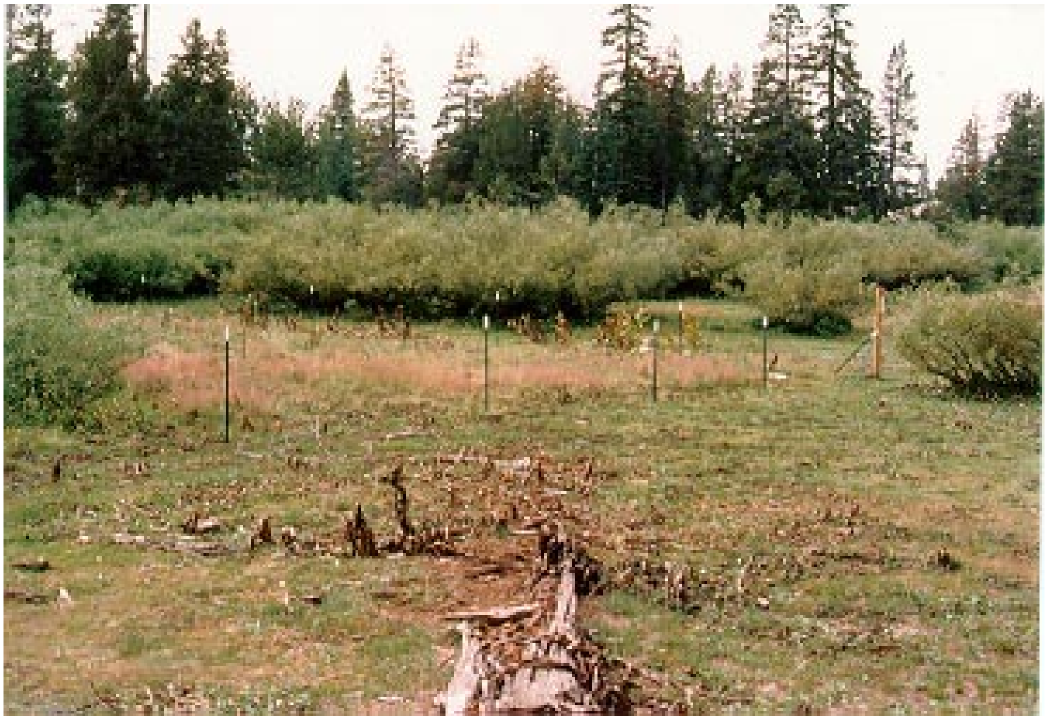

PHOTO 4b. Same mountain meadow and willow-riparian habitat under experimentally applied heavy grazing in 1983 (Loft and Menke 1988). Fenced area in middle of photograph is a livestock exclosure. Note herbaceous growth in area, and willows in background (beyond exclosure) in this late September photograph.

PHOTO 5a. Riparian drainage that provides vertical structure and cover as habitat in Great Basin communities. Drainages with adequate water can develop woody riparian cover as shown here. Willow and members of the rose family are common.

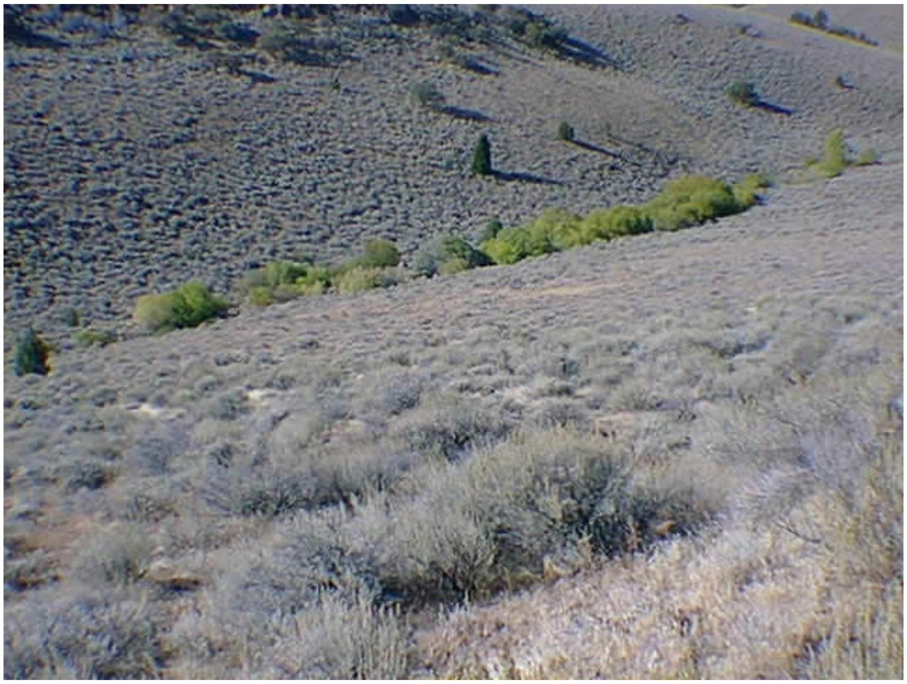

PHOTO 5b. Example of small Great Basin meadow/spring site receiving excessive use.

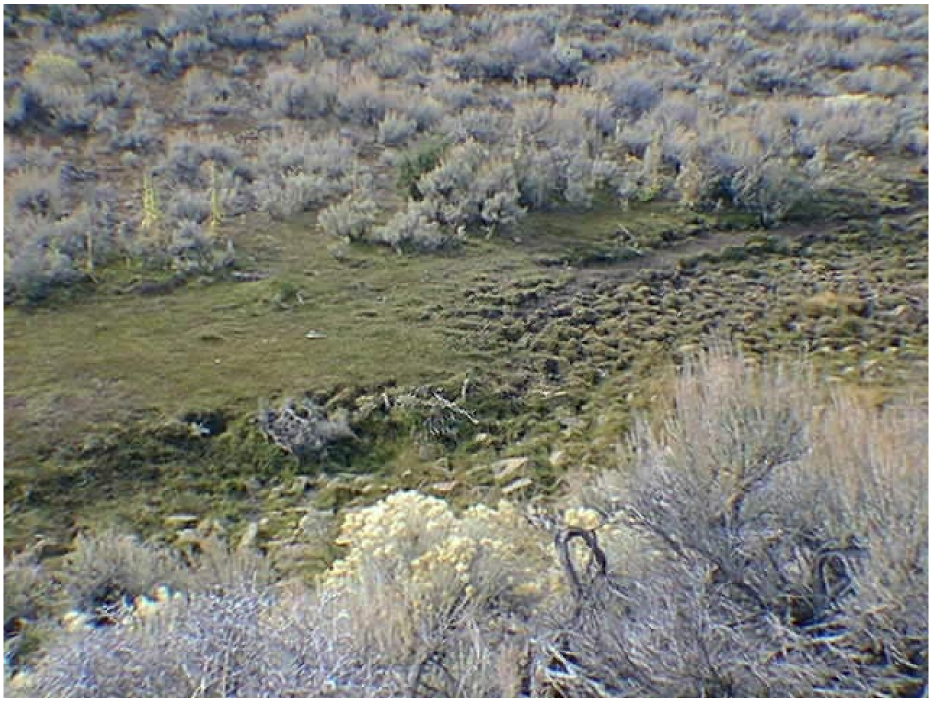

Supplement: S2 File — This is a zip file that contains sources of the data used in the analyses that are no longer available on the internet. (ZIP) [file pone.0224638.s003.zip › CDFW_1998_DeerAssessment.pdf]
