# Supplementary material for: The Elephant in the room: What can we learn from California regarding the use of sport hunting of pumas (Puma concolor) as a management tool?
Source: PLoS One. 2020 Feb 13;15(2):e0224638. doi: 10.1371/journal.pone.0224638 (PMC7018503; doi:10.1371/journal.pone.0224638)
Supplement: S2 File — This is a zip file that contains sources of the data used in the analyses that are no longer available on the internet. (ZIP) [file pone.0224638.s003.zip › cougar_mortality_stats to 2018.pdf]

Table. Trends in cougar complaints, damage, harvest, and other mortality in Oregon during 1992–2018. Complaint and mortality data current through 11/29/18. Numbers may change as late data are added.

Year	Number of Complaints[a]	Tags Sold[b]	Hunter-Harvest	Damage[c]	Human Safety[d]	Administrative Removals[e]	Other[f]	Total
1992	184	517	184	17	3	0	22	226
1993	276	560	162	20	7	0	21	210
1994	554	588	199	29	11	0	20	259
1995	742	385	22	41	22	0	12	97
1996	840	779	43	64	34	0	25	166
1997	798	935	61	82	20	0	18	181
1998	954	11,761	111	93	20	0	17	241
1999	1,072	14,564	169	91	39	0	25	324
2000[g]	942	22,386	188	120	27	0	17	352
2001	829	28,447	221	97	27	0	21	366
2002	765	32,126	229	111	25	0	35	400
2003	697	34,135	248	111	28	0	25	412
2004	545	34,071	265	95	28	0	35	423
2005	622	38,079	224	125	28	0	30	407
2006	451	38,719	289	106	26	0	32	453
2007	453	41,813	309	114	21	52	41	537
2008	515	43,211	272	109	23	34	54	492
2009	432	45,375	274	110	31	21	37	473
2010	465	48,776	240	99	25	79	39	482
2011	500	50,889	241	139	23	71	32	506
2012	419	53,698	253	130	46	56	45	530
2013	359	55,072	292	148	24	36	31	531
2014	404	56,114	209	124	27	0	25	385
2015	444	57,387	233	134	22	0	44	433
2016	421	58,091	267	151	18	71	37	544
2017	462	57,950	292	151	27	60	25	571
2018*	618	61,022	225	156	36	56	30	503

[a]Number of complaints received during the calendar year. Sightings not associated with damage or public safety concerns are not included.

[b]Includes general and additional tags (including Sports Pac licenses).

[c]Number of animals killed as a result of damage during a calendar year.

[d]Animals killed as a result of real or perceived threat to humans or pets.

[e]Adminstrative removals on cougar target areas (2007–present only).

[f]Includes roadkill, accidental, found dead, and illegal kill.

[g]Hunting season changed to calendar year.

*as of 11/29/18
