# Supplementary material for: The Elephant in the room: What can we learn from California regarding the use of sport hunting of pumas (Puma concolor) as a management tool?
Source: PLoS One. 2020 Feb 13;15(2):e0224638. doi: 10.1371/journal.pone.0224638 (PMC7018503; doi:10.1371/journal.pone.0224638)
Supplement: S2 File — This is a zip file that contains sources of the data used in the analyses that are no longer available on the internet. (ZIP) [file pone.0224638.s003.zip › HuntAZ2009.pdf]

Hunt Arizona

2009 Edition

Survey, Harvest and Hunt Data for Big and Small Game

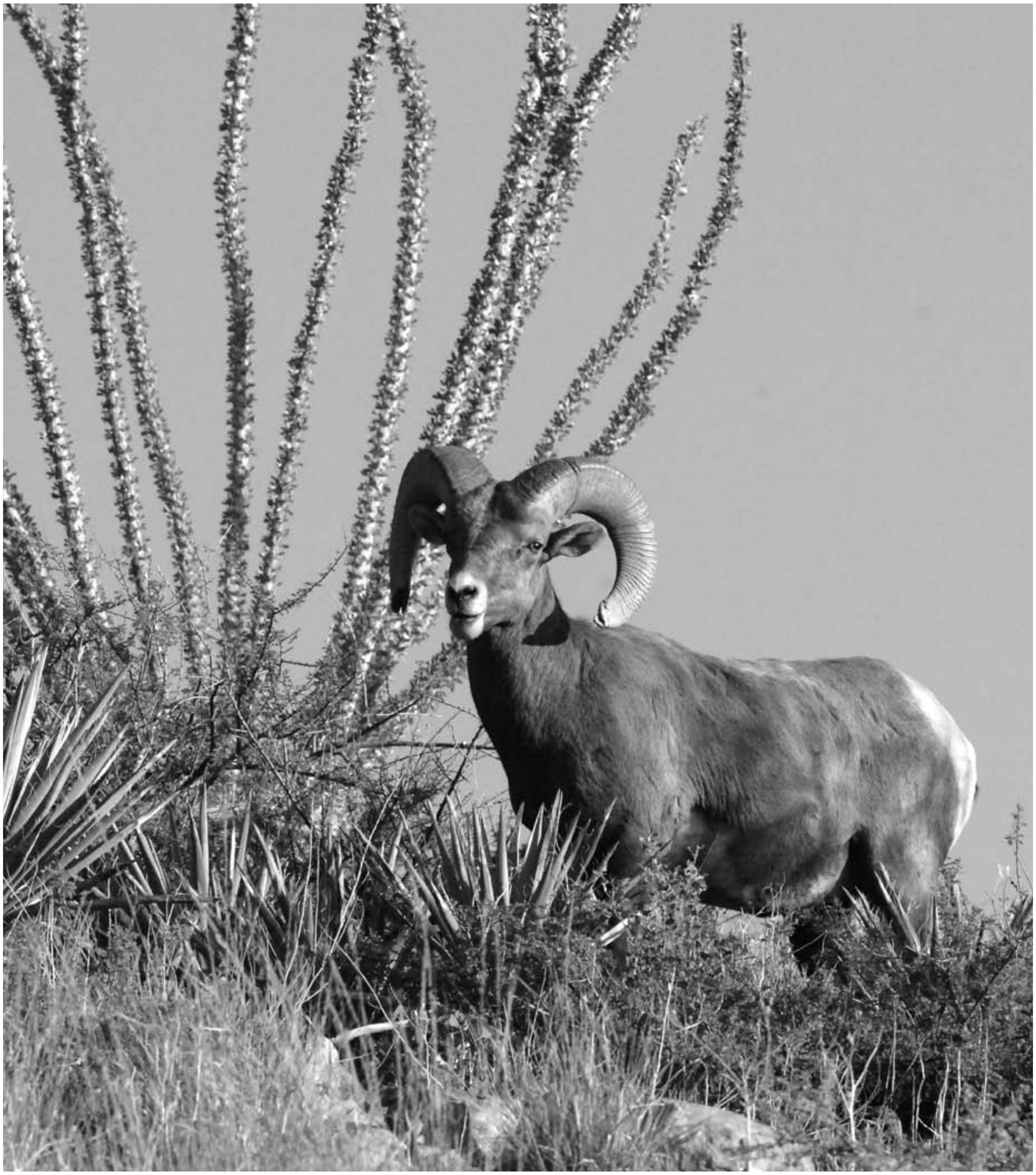

ARIZONA GAME AND FISH DEPARTMENT

ARIZONA GAME AND FISH COMMISSION

Bob Hernbrode, Chair .... Tucson
Jennifer L. Martin .... Phoenix
Robert R. Woodhouse .... Roll
Norman W. Freeman .... Chino Valley
Jack F. Husted .... Springerville

ARIZONA GAME AND FISH DEPARTMENT

5000 W. Carefree Highway
Phoenix, AZ 85086
(602) 942-3000
www.azgfd.gov

Larry D. Voyles .... Director
Gary R. Hovatter .... Deputy Director
Robert D. Broscheid .... Deputy Director

ASSISTANT DIRECTORS

Mike Senn .... Wildlife Management
Harry Seck .... Special Services
Ty Gray .... Information and Education

REGIONAL OFFICES

Region I — Jon Cooley, Supervisor
2878 E. White Mountain Blvd., Pinetop, AZ 85935, (928) 367-4281

Region II — Ron Sieg, Supervisor
3500 S. Lake Mary Road, Flagstaff, AZ 86001, (928) 774-5045

Region III — Bob Posey, Supervisor
5325 N. Stockton Hill Rd., Kingman, AZ 86409, (928) 692-7700

Region IV — Pat Barber, Supervisor
9140 E. 28th St., Yuma, AZ 85365, (928) 342-0091

Region V — Leonard Ordway, Supervisor
555 N. Greasewood Rd., Tucson, AZ 85745, (520) 628-5376

Region VI — Rod Lucas, Supervisor
7200 E. University, Mesa, AZ 85207, (480) 981-9400

Published by the Arizona Game and Fish Department
Information and Education Division, Information Branch, Publications Section

©May 2009

Cover photograph: George Andrejko

The Arizona Game and Fish Department prohibits discrimination on the basis of race, color, sex, national origin, age, or disability in its programs and activities. If anyone believes that they have been discriminated against in any of the AGFD's programs or activities, including employment practices, they may file a complaint with the Director's Office, 5000 W. Carefree Highway Phoenix, AZ 85086, (602) 942-3000, or with the Fish and Wildlife Service, 4040 N. Fairfax Dr. Ste. 130, Arlington, VA 22203. Persons with a disability may request a reasonable accommodation or this document in an alternative format by contacting the Director's Office as listed above.

Table of Contents

How to Use Survey and Harvest Data.....1-2
Bonus Points by Species............................ 2-5

Deer
Mule Deer Natural History 6
Mule Deer Hunt History.................................7
White-tailed Deer Natural History 7
White-tailed Deer Hunt History.....................8
Survey Data 9
Harvest Data 18
Hunt Data 22

Pronghorn Antelope
Natural History...40
Hunt History 41
Survey Data 42
Harvest Data 46
Hunt Data 49

Elk
Natural History...54
Hunt History 55
Survey Data 56
Harvest Data 59
Hunt Data 62

Turkey
Natural History...78
Hunt History 79
Survey Data 80
Harvest Data 82
Hunt Data 85

Javelina
Natural History...92
Hunt History 93
Survey Data 94
Harvest Data 99
Hunt Data 102

Bighorn Sheep
Natural History... 111
Hunt History 112
Survey Data 113
Harvest Data 117
Hunt Data 118
Successful Hunter Scores 122
Measurement Data....................................124
Hunt Application Data 125

Buffalo
Natural History...126
Hunt History 127
Survey Data 128
Harvest Data 130
Hunt Data 131

Black Bear
Natural History...133
Hunt History 134
Harvest Data 135
Hunt Data 136

Mountain Lion
Natural History...139
Hunt History 140
Harvest Data 141
Hunt Data 142

Small Game
Quail 147
Natural History..147
Hunt History 148
White-winged Dove 149
Natural History..149
Hunt History 150
Mourning Dove 150
Natural History..150
Hunt History 151
Cottontail Rabbit..151
Natural History..151
Hunt History 152
Tree Squirrels...152
Natural History..152
Hunt History 153
Band-tailed Pigeon.....................................153
Natural History..153
Hunt History 153
Blue Grouse 153
Natural History..153
Hunt History 154
Pheasant...154
Natural History..154
Hunt History 154
Small Game Harvest Data 155

Predators
Coyotes...159
Natural History..159
Hunting and Trapping History.................159
Bobcats 160
Natural History..160
Hunting and Trapping History.................160
Foxes 160
Natural History..161
Hunting and Trapping History.................161
Skunks 161
Natural History..162
Trapping History......................................162

Furbearers
Beaver 163
Natural History..163
Trapping History......................................164
Muskrat 164
Natural History..164
Trapping History......................................164
Raccoon..164
Natural History..165
Trapping and Hunt History......................165
Ringtail...165
Natural History..165
Trapping History......................................165
Otter...166
Natural History..166
Trapping and Hunt History......................166
Weasel..166
Natural History..166
Trapping History......................................166
Badger..167
Natural History..167
Trapping History......................................167
Trapping..167

Predator and Furbearer
Harvest Data 168

Waterfowl
Natural History...170
Hunt History 171
Survey and Harvest Data............................173

Sandhill Crane
Natural History...175
Hunt History 176
Harvest Data 177

Other Birds and Mammals
Pigeon (Rock Dove) 178
Natural History and Status......................178
House (English) Sparrow 178
Natural History and Status......................178
European Starling.......................................179
Natural History and Status......................179
Peach-faced Lovebird.................................179
Natural History and Status......................179
American Crow 179
Natural History and Status......................180
Coati 180
Natural History and Status......................180
Gunnison's Prairie Dog...............................181
Natural History and Status......................181
Black-tailed Prairie Dog..............................181
Natural History and Status......................181
Specially Protected Mammals....................181
Bats 181
Natural History and Status......................181
Black-footed Ferret.....................................182
Natural History and Status......................182
Hualapai Mexican Vole 182
Natural History and Status......................182
Jaguar...183
Natural History and Status......................183
Jaguarundi..183
Natural History and Status......................183
Ocelot...183
Natural History and Status......................184
Otter (see Furbearers) 184
Porcupine 184
Natural History and Status......................184
Gray Wolf 185
Natural History and Status......................185

Game Management Unit Map................. 191

Arizona Game and Fish Web Site

www.azgfd.gov

Arizona Small and Big Game Hunt Information Now Available on the World Wide Web

Written by Arizona Game and Fish Department Wildlife Managers

Hunting information is available for deer, elk, antelope, bighorn sheep, javelina, turkey, quail, dove, tree squirrel, waterfowl and other small and big game species. The more than 470 written accounts provide hunt forecasts, areas to hunt, access information, and tips to improve hunt success for 80 game management units statewide. Best of all, the new, up-to-date information was written by Arizona Game and Fish Department Wildlife Managers. These professionals are on the ground everyday and are now sharing their knowledge with you in a way that's easy to access. Go to www.azgfd.gov and click on the Wildlife & Fish link then click on Statewide, Unit by Unit Hunting Information. It's that easy!

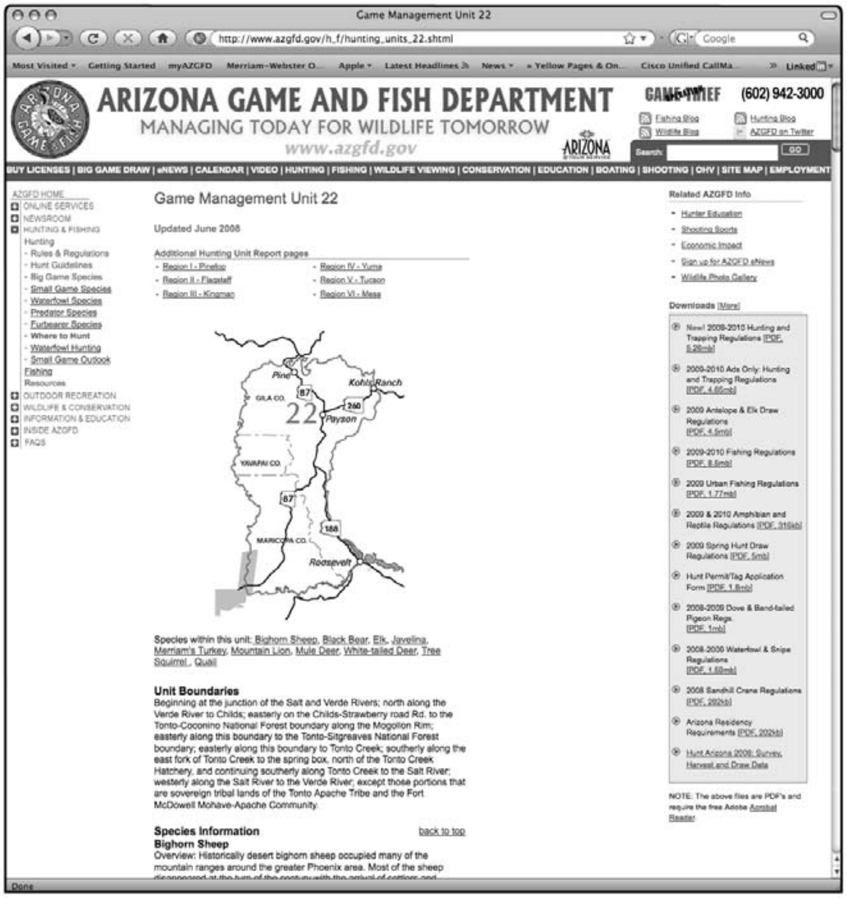

(Actual web page for Unit 22 - Bighorn Sheep showing just part of the information available.)

Your purchase of hunting equipment supports Wildlife Restoration

How to Use Survey and Harvest Data

Both novice and experienced hunters will find this book a valuable resource to help in making informed decisions regarding hunt selections. The book is a compendium of facts about hunting in Arizona, including up-to-date information on:

- Which game management units have the most big-game permits,
- The units with the narrowest male to female ratios,
- Units and hunts with the highest hunt success,
- Hunts that have the best drawing odds, and
- Historical survey and hunt information the reader can use to compare trends for the major game species in each management unit.

The information is relatively simple to use. Looking through the section on deer, for example, you will find a summary of the survey data for both mule deer and white-tailed deer in each game management unit having these species. This information will help you determine whether a unit has a high proportion of bucks and whether it is experiencing good fawn production. Bear in mind, however, that due to differences in survey methods the male to female and female to young ratios are only estimates.

By checking the unit hunt information summary, you can determine the hunter success rate, how many permits were available in the past, and the drawing odds for previous hunts. Be aware that some units have several authorized hunts, each limited to a specific kind (or kinds) of weapon. Your selection of a hunt for which to apply will depend on your own preference of hunt area, weapon type, season dates, and the kind of animal you wish to harvest.

Beating the Odds

Permits for big-game hunts in Arizona are issued through a drawing system. Since the best predictor of the future is the past, the best estimate of your draw odds for an upcoming hunt is the draw rate for that hunt in the previous draw. Draw odds for each hunt are calculated by dividing successful first choice applicants by the total first choice applicants. Even though some permits may have been issued to second choice applicants, this method accurately reflects the applicant's chances of receiving their first choice.

The odds of receiving a permit for a second choice hunt instead of a first choice hunt are calculated by subtracting the draw rate for the first choice hunt from the draw rate for the second choice hunt. The odds for receiving your first or second choice would therefore be the same as your highest odds choice. For example, if your first choice selection had a 40 percent draw rate last year, and your second choice selection had a 60 percent draw rate, your odds this year are 40 percent for getting your first choice, 20 percent for getting your second choice, and 60 percent overall (assuming that the results of this year's draw will be similar to those of the previous draw). It therefore makes little sense to apply for a second choice hunt with a lower draw rate than your first choice hunt. Only those hunts that did not fill with first or second choice applications are considered for third, fourth, or fifth choices. Therefore, only hunts with draw odds of 100 percent are good candidates for these choices.

While draw rates are relatively favorable for most deer, turkey and javelina hunts, they are much more competitive for elk, antelope, buffalo, and bighorn sheep. Beginning in 1991, the Arizona Game and Fish Department began issuing bonus points to unsuccessful applicants for these species. In 1999, unsuccessful applicants for deer began to receive bonus points. In 2005, turkey, javelina, and spring bear began receiving bonus points. Each point accumulated gives the applicant an extra entry in the hunt drawing for that species. For more information about the bonus point system, please refer to R12-4-107 in the 2009-2010 Hunting Regulations.

A summary of 2008 draw odds seems to indicate little advantage to having many bonus points. Further analysis, however, reveals that applicants with the largest number of bonus points are applying for hunts with the poorest draw odds, which obscures the benefits of having multiple bonus points. For example, elk applicants without any bonus points applied for hunts with draw odds that averaged 25 percent, while those with 19 bonus points applied for hunts with draw odds averaging less than 1.0 percent. This tendency held true for their second choices as well.

Comparing applicants on the basis of their first choice hunts reveals a truer picture of the advantages of bonus points, particularly for those applying for hunts with high draw odds. General antelope applicants with 19 bonus points, for example, were drawn for their first choice hunt at over 83 times the rate of applicants with no bonus points (83.3 percent versus 0.2 percent). General elk applicants with 19 bonus points were drawn for their first choice hunt at over 88 times the rate of applicants with no bonus points (100 percent versus 11.9 percent).

Draw odds in the tables contained in this report are computed without regard to numbers of bonus points and therefore represent your odds if you have an average number of bonus points. In the 2009 draw, the average applicant for elk tags will have about 2.9 bonus points while those applying for antelope, bighorn sheep, buffalo, and deer tags will have 6.0, 6.8, 6.5, and 1.9 respectively.

Another point to consider when choosing hunts is the number of people on your application. This can be an important factor when applying for hunts with low numbers of permits, since no permits will be issued if there are not enough for everyone on the application. Applying with three other people for a 10-permit hunt, for example, cuts your odds by 30 percent. Applying with people who have fewer bonus points than you have will also decrease your odds, since the number of bonus points assigned to an application is the average accumulated by the group.

One last tip to keep in mind is that new hunts, or hunts in which permits have been recently increased, generally have slightly better draw odds than other hunts. Conversely, hunts with reduced numbers of permits generally have poorer odds.

Bonus Points By Species

Bonus points listed below include the permanent hunter education point and the loyalty point (earn by submitting a valid application for 5 consecutive years).

For all species except antelope and elk, the tables below are a summary of group bonus points resulting from the 2008 Fall Draw (Section A) and individual bonus points going into the 2009 Fall Draw (Section B). For antelope and elk, the tables are a summary of group bonus points resulting from the 2009 Antelope and Elk Draw (Section A) and individual bonus points going into the 2010 Antelope and Elk Draw (Section B). Group bonus points are the average number of bonus points per hunt application. A hunt application can be submitted with 1 to 4 applicants. The bonus points, which may differ for each individual on an application, are averaged to come up with "group bonus points." Individual bonus points in Section B are the count of all hunters in each bonus point level. Both group and individual bonus points include the permanent hunter education point and the loyalty point. All potential hunters may not be represented in Section A if an individual with bonus points did not apply during the recent Draw. Also, keep in mind that applicants with the greatest number of bonus points often apply for hunts with poorest draw odds, which obscures the benefits of having multiple bonus points. Refer to the narrative on the previous pages about "Beating the Odds."

Remember, all potential hunters may not apply in a given year. Also, Section A does NOT reflect individuals who may have purchased a bonus point for a given species.

DEER

A			B			
RESULTS OF THE 2008 FALL DRAW			GOING INTO 2009 FALL DRAW			
Group Bonus Points going into the 2008 Fall Draw	No. Hunters per Bonus Point going into the 2008 Fall Draw	Percent Drawn during the 2008 Fall Draw within a Bonus Point grouping	Individual Bonus Points going into the 2009 Fall Draw	No. Hunters per Bonus Point going into the 2009 Fall Draw		
				Resident	Nonresident	Total
0	12,252	56.4%	1	106,140	7,768	113,908
1	24,977	66.8%	2	29,281	2,844	32,125
2	17,063	64.8%	3	9,979	1,941	11,920
3	6,150	59.1%	4	4,102	983	5,085
4	2,848	54.6%	5	1,850	1,218	3,068
5	1,467	41.7%	6	1,012	858	1,870
6	888	33.3%	7	578	668	1,246
7	629	25.9%	8	394	602	996
8	494	15.4%	9	267	459	726
9	352	11.7%	10	150	375	525
10	511	11.2%	11	118	573	691
11	200	32.0%	12	61	171	232

Bonus Points by Species

ANTELOPE (Section A does NOT reflect individuals who purchased a bonus point)

A			B			
RESULTS OF THE 2009 FALL DRAW			GOING INTO 2010 FALL DRAW			
Group Bonus Points going into the 2009 Fall Draw	No. Hunters per Bonus Point going into the 2009 Fall Draw	Percent Drawn during the 2009 Fall Draw within a Bonus Point grouping	Individual Bonus Points going into the 2010 Fall Draw	No. Hunters per Bonus Point going into the 2010 Fall Draw		
				Resident	Nonresident	Total
0	1,458	1.1%	1	86,360	5,143	91,503
1	1,746	1.7%	2	9,414	1,526	10,940
2	2,226	2.2%	3	5,894	1,172	7,066
3	1,950	2.3%	4	3,824	490	4,314
4	1,597	3.5%	5	2,854	657	3,511
5	1,427	5.1%	6	2,904	479	3,383
6	1,458	5.4%	7	2,333	426	2,759
7	1,382	5.1%	8	2,130	326	2,456
8	1,285	4.0%	9	1,819	333	2,152
9	1,167	7.4%	10	1,597	229	1,826
10	959	7.0%	11	1,340	149	1,489
11	894	5.6%	12	1,104	141	1,245
12	747	6.0%	13	939	106	1,045
13	638	7.8%	14	833	88	921
14	546	6.4%	15	730	46	776
15	477	8.2%	16	594	25	619
16	373	7.0%	17	433	23	456
17	310	11.3%	18	339	7	346
18	154	27.3%	19	175	2	177
19	88	65.9%	20	59	1	60
20	4	100%	21	8	0	8

ELK (Section A does NOT reflect individuals who purchased a bonus point)

A			B			
RESULTS OF THE 2009 FALL DRAW			GOING INTO 2010 FALL DRAW			
Group Bonus Points going into the 2009 Fall Draw	No. Hunters per Bonus Point going into the 2009 Fall Draw	Percent Drawn during the 2009 Fall Draw within a Bonus Point grouping	Individual Bonus Points going into the 2010 Fall Draw	No. Hunters per Bonus Point going into the 2010 Fall Draw		
				Resident	Nonresident	Total
0	10,445	20.4%	1	99,290	11,552	110,842
1	18,505	32.6%	2	34,506	4,763	39,269
2	18,160	36.4%	3	18,062	3,316	21,378
3	10,853	36.5%	4	9,905	1,582	11,487
4	6,599	37.7%	5	5,619	2,371	8,530
5	4,658	32.0%	6	4,027	1,954	5,981
6	3,048	34.7%	7	2,491	1,320	3,811
7	2,173	37.4%	8	1,604	934	2,538
8	1,358	36.2%	9	1,008	772	1,780
9	1,000	28.0%	10	651	546	1,197
10	681	22.0%	11	442	489	931
11	505	17.4%	12	317	348	665
12	368	20.1%	13	241	191	432
13	225	12.9%	14	193	130	323
14	189	13.8%	15	133	104	237
15	154	18.2%	16	96	67	163
16	83	27.7%	17	58	40	98
17	37	83.8%	18	5	7	12
18	4	100%	19	5	2	7
19	2	100%	20	1	1	2
20	0	–	21	0	1	1

Bonus Points by Species

TURKEY (Accrue bonus points through both the spring and fall draws; Section A does NOT reflect individuals who purchased a bonus point)

A			B			
RESULTS OF THE 2008 FALL DRAW			GOING INTO 2009 FALL DRAW			
Group Bonus Points going into the 2008 Fall Draw	No. Hunters per Bonus Point going into the 2008 Fall Draw	Percent Drawn during the 2008 Fall Draw within a Bonus Point grouping	Individual Bonus Points going into the 2009 Fall Draw	No. Hunters per Bonus Point going into the 2009 Fall Draw		
				Resident	Nonresident	Total
0	1,699	49.6%	1	99,493	3,857	103,350
1	3,104	71.4%	2	10,214	199	10,413
2	2,117	79.2%	3	2,462	91	2,553
3	791	97.9%	4	600	25	625
4	98	100%	5	125	13	138
5	12	100%	6	42	7	49
			7	28	5	33
			8	30	7	37

JAVELINA (Accrue bonus points through both the spring and fall draws; Section A does NOT reflect individuals who purchased a bonus point)

A			B			
RESULTS OF THE 2008 FALL DRAW			GOING INTO 2009 FALL DRAW			
Group Bonus Points going into the 2008 Fall Draw	No. Hunters per Bonus Point going into the 2008 Fall Draw	Percent Drawn during the 2008 Fall Draw within a Bonus Point grouping	Individual Bonus Points going into the 2009 Fall Draw	No. Hunters per Bonus Point going into the 2009 Fall Draw		
	(Juniors-only hunts)			Resident	Nonresident	Total
0	159	92.5%	1	99,564	4,514	104,078
1	378	96.8%	2	6,480	321	6,801
2	69	92.8%	3	192	89	281
3	5	100%	4	34	31	65
			5	5	9	14
			6	7	3	10
			7	8	3	11
			8	4	1	5

BIGHORN (Section A does NOT reflect individuals who purchased a bonus point)

A			B			
RESULTS OF THE 2008 FALL DRAW			GOING INTO 2009 FALL DRAW			
Group Bonus Points going into the 2008 Fall Draw	No. Hunters per Bonus Point going into the 2008 Fall Draw	Percent Drawn during the 2008 Fall Draw within a Bonus Point grouping	Individual Bonus Points going into the 2009 Fall Draw	No. Hunters per Bonus Point going into the 2009 Fall Draw		
				Resident	Nonresident	Total
0	537	5.96%	1	91,417	5,140	96,557
1	594	6.59%	2	3,461	1,630	5,091
2	637	7.06%	3	2,179	1,330	3,509
3	588	6.52%	4	1,600	600	2,200
4	374	4.15%	5	1,035	784	1,819
5	510	5.66%	6	1,002	688	1,690
6	606	6.72%	7	944	568	1,512
7	657	7.29%	8	808	507	1,315
8	622	6.90%	9	782	349	1,131
9	488	5.41%	10	569	293	862
10	405	4.49%	11	449	240	689
11	410	4.55%	12	426	240	666
12	366	4.06%	13	391	187	578
13	363	4.03%	14	387	170	557
14	357	3.96%	15	351	139	490
15	334	3.70%	16	314	127	441
16	286	3.17%	17	271	96	367
17	259	2.87%	18	267	68	335
18	308	3.42%	19	294	39	333
19	316	3.50%	20	304	13	317

Bonus Points by Species

BUFFALO (Accrue bonus points through both the spring and fall draws; Section A does NOT reflect individuals who purchased a bonus point)

A			B			
RESULTS OF THE 2008 FALL DRAW			GOING INTO 2009 FALL DRAW			
Group Bonus Points going into the 2008 Fall Draw	No. Hunters per Bonus Point going into the 2008 Fall Draw	Percent Drawn during the 2008 Fall Draw within a Bonus Point grouping	Individual Bonus Points going into the 2009 Fall Draw	No. Hunters per Bonus Point going into the 2009 Fall Draw		
				Resident	Nonresident	Total
0	16	0%	1	95,971	3,851	99,822
1	28	0%	2	2,074	321	2,395
2	23	0%	3	1,245	199	1,444
3	32	0%	4	845	113	958
4	20	5.0%	5	549	76	625
5	21	0%	6	415	42	457
6	25	0%	7	339	23	362
7	23	0%	8	268	24	292
8	32	0%	9	259	9	268
9	19	5.3%	10	153	5	158
10	28	3.6%	11	149	2	151
11	24	0%	12	150	1	151
12	22	0%	13	103	4	107
13	20	5.0%	14	88	2	90
14	22	4.6%	15	80	3	83
15	13	0%	16	58	0	58
16	12	0%	17	50	0	50
17	4	0%	18	33	0	33
18	4	0%	19	21	0	21
19	3	0%	20	16	0	16
20	4	0%	21	12	1	13
21	1	0%	22	16	0	16
22	1	0%	23	6	0	6
23	0	–	24	10	0	10
24	2	50.0%	25	7	0	7
25	0	–	26	1	1	2
26	0	–	27	2	0	2
			28	2	0	2
			29	2	0	2
			30	0	0	0
			31	1	0	1

BEAR (Spring draw only)

A			B			
RESULTS OF THE 2009 FALL DRAW			GOING INTO 2010 FALL DRAW			
Group Bonus Points going into the 2009 Spring Draw	No. Hunters per Bonus Point going into the 2009 Spring Draw	Percent Drawn during the 2009 Spring Draw within a Bonus Point grouping	Individual Bonus Points going into the 2010 Spring Draw	No. Hunters per Bonus Point going into the 2010 Spring Draw		
	(Spring)			Resident	Nonresident	Total
0	47	61.7%	1	99,607	3,707	103,314
1	145	68.3%	2	772	8	780
2	83	62.6%	3	129	6	135
3	28	50.0%	4	46	1	47
4	5	100%	5	11	0	11

Deer

Two species of deer occur in Arizona, the mule deer (Odocoileus hemionus) and the white-tailed deer (Odocoileus virginianus).

Mule Deer

Natural History

Mule deer are the most abundant big-game animal in Arizona, with the statewide population estimated at 120,000 post-hunt adults. They can be found in most areas of the state, from sparsely vegetated deserts upward into high, forested mountains. Rocky Mountain mule deer occur primarily in northern Arizona above the Mogollon Rim in game management units 1 through 13, while the so-called desert mule deer is found in all of the more southern units (15 through 46).

The mule deer gets its name from its large ears. Its coat is reddish-brown in summer, turning to a blue-gray or a chocolate brown in winter. The forehead is much darker than the face, while the animal's throat, belly, and inner leg surfaces are white. One of the mule deer's most distinguishing characteristics is a white rump patch and a narrow, black-tipped white tail.

The mule deer is the larger of Arizona's deer species. Adult bucks may weigh more than 200 pounds and stand up to 42 inches tall at the shoulder. Does average about 125 pounds.

Mule deer antlers typically branch into two main beams, each of which may fork into two or more tines. The size and number of points is dependent on a combination of the buck's age, nutrition, and genetic background. The antlers develop under a layer of soft skin, called velvet, which supplies them with nutrients. When fully grown, the antlers harden and the now dry velvet is rubbed off. The bony antlers are retained until spring, after the breeding season has passed. Buck deer are polygamous and use their antlers to intimidate other males and drive them away from the does during the winter breeding season.

After a gestation period of about 190 days, the does give birth to one or two spotted fawns. Fawns in northern Ari-

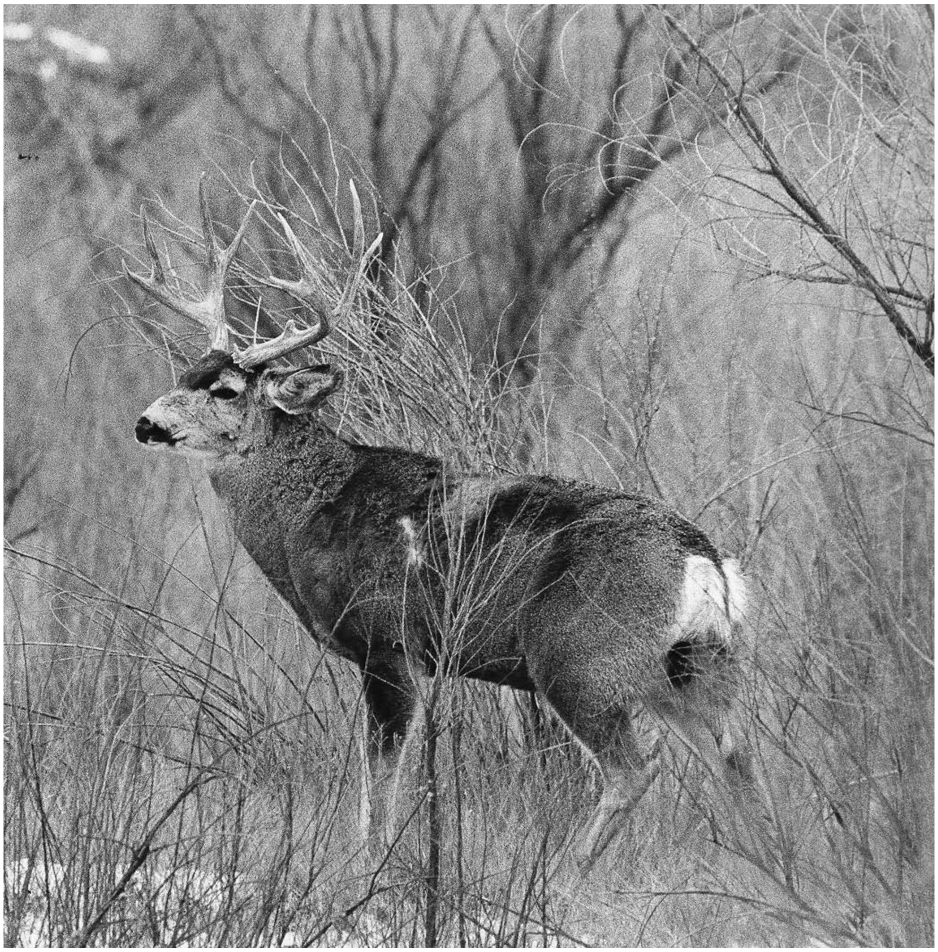

BOB MILES

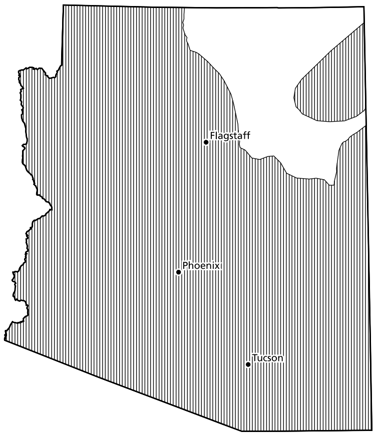

Mule deer distribution

zona are born in late spring, while those in southern Arizona usually arrive in midsummer. A fawn's spots disappear in about two months. The young remain with their mother until the following spring. Both sexes attain maturity in about one year and have a life span of about 10 years.

Research has shown that mule deer population levels are largely determined by the number of fawns that survive to be yearlings. Fawn survival, in turn, is largely determined by climatic events, with wet, mild winters contributing to high fawn survival rates. Dry winters and springs usually result in poor fawn survival, and heavy snows and freezing temperatures occasionally reduce the population levels of both fawn and adult Rocky Mountain mule deer. Another limiting factor for mule deer is predation. In Arizona, the mountain lion is the principal mule deer predator.

Mule deer are primarily browsers, although they feed largely on forbs and new grass growth in the spring and summer. Other major diet items are twigs, bark, buds, leaves, and nuts. Important browse plants include mountain mahogany, cliff rose, sagebrush, and oak in northern Arizona, with jojoba, buck brush, and mountain mahogany being favored in southern Arizona. Most feeding is done at dawn and dusk, although human activity and a full moon may cause a shift to more feeding at night.

Hunt History

As befits Arizona's principal game animal, deer received some protection as early as 1887 when a four-month season of October 1 through January 31 was established by the territorial legislature. Buck-only hunting was instituted in 1893, and the season was gradually reduced until 1913 when the new state legislature authorized a two-month season and a two-buck bag limit. Even this was deemed excessive by the state's sportsmen, and a public initiative in 1916 reduced the limit to one buck deer to be taken during the month of October.

Despite a serious overpopulation of deer on the North Kaibab in the 1920s, deer numbers appeared to decline in the rest of the state. In 1929, the mule deer season was closed south of the Gila River, and even as recently as 1946, fewer than 5,000 mule deer (more than 80 percent of all deer killed) were harvested in Arizona. Then, for reasons that are still unclear, deer populations began to increase. As the populations rose, doe and "any-deer" hunts were authorized. In 1961, an all-time high of 91,120 deer hunters took 35,897 deer. More than 86 percent of these were mule deer and nearly 10,000 were antlerless animals. Archery deer hunting was also now beginning to provide a significant hunting opportunity.

A series of years of poor fawn survival followed. By 1970 fewer than 16,000 deer were taken, and hunt success had fallen to 16 percent. With the institution of permit-only deer hunting the following year, hunter numbers dropped from more than 97,000 to fewer than 68,000. Only about 9,500 mule deer were reported harvested.

Deer permit numbers gradually increased after 1972, leveling off at around 70,000 per year between 1976 and 1982, when hunters took more than 12,000 mule deer, approximately 75 percent of the total deer harvest. Then, a series of wet winters resulted in an increase in fawn survival rates, and hunter numbers and the numbers of deer bagged increased accordingly until 1986, when nearly 86,000 hunters took 25,566 deer, of which 77 percent were mule deer.

Since then, another series of droughts has occurred, and deer hunting opportunity is again being curtailed. In 1998, 44,524 hunters reported taking fewer than 10,500 deer. Of the total deer harvested that year only 60 percent were mule deer. Prospects in the near future are even more discouraging, but mule deer are "boom and bust" animals. With the advent of better than average winter rains, mule deer populations will once again improve. The only question is when.

White-tailed Deer

Natural History

Arizona's other deer is a small subspecies of the white-tailed deer. These Coues (pronounced Cows) deer are most common in the state's southeastern mountains, but range northward to the edge of the Mogollon Rim, up into the White Mountains, and as far west as Sycamore Canyon in Unit 8. The statewide population is estimated at 82,000 post-hunt adults. Coues whitetails require areas of predictable summer precipitation and are most common in oak woodlands and on chaparral covered hillsides with oaks and pines. This species, while more resilient than mule deer to hunt pressure, is less tolerant of droughts and appears to be more affected by livestock grazing.

In contrast to the mule deer's branching antlers, the tines or points of a whitetail's antlers originate from a forward-curving main beam. Mature bucks generally have three to four tines per side. The coat color is grayish-brown salt-and-pepper with white underparts; the face is marked with white halos around the eyes and a

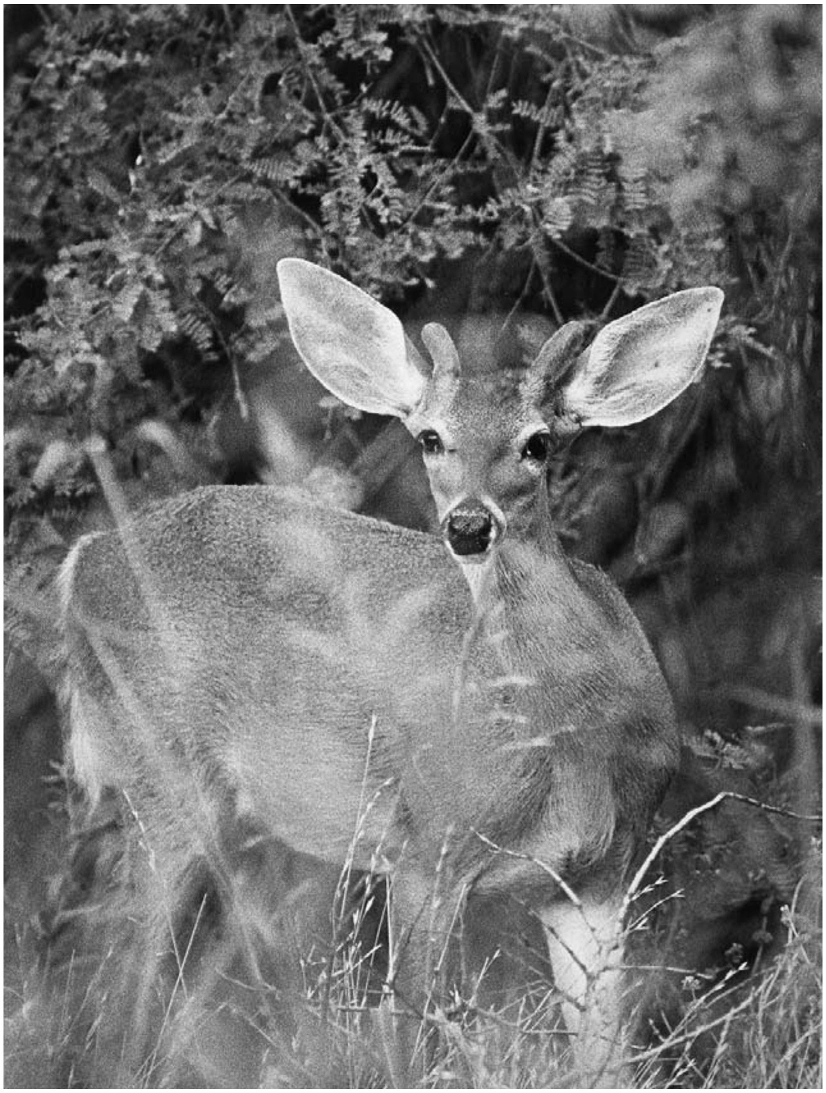
BOB MILES

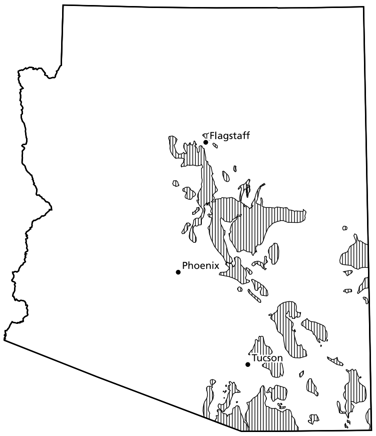

White-tailed deer distribution

and seeing two generations running together is not uncommon. Unlike mule deer, white-tailed deer rarely form herds, and most observations are of fewer than six animals.

When seen at a distance, white-tailed deer can often be distinguished from mule deer by their cautious, running gait and flagging white tail. Whitetails never use the stiff legged, bounding gait sometimes employed by mule deer. Habitat preferences also differ. In Arizona's southern mountain ranges, whitetails are generally found at higher elevations and in rougher country than are mule deer.

white band across the muzzle. The most distinguishing characteristic of the whitetail, however, is a long, fluffy tail that is all white on the underside, gray to reddish-black on top, and often lifted upward as an alarm signal.

The Coues deer is much smaller than most of its eastern cousins. Bucks stand just over 30 inches at the shoulder and rarely weigh more than 100 pounds. Does average 65 pounds. The rutting season usually runs from December through February, and the fawn drop coincides with the new growth of forbs that results from the summer rains.

A doe's first pregnancy usually results in a single fawn; thereafter she may bear twins. White-tailed deer fawns may stay with their mothers for more than a year,

Hunt History

The Coues white-tailed deer is perhaps Arizona's finest game animal. Wary, and expert at using cover, whitetails rarely offer the hunter a standing shot once jumped. Perhaps for this reason, the species has become increasingly important in the harvest. Although the statewide take has varied from 1,500 to more than 7,000 whitetails a year, depending on the vagaries of drought and fawn survival, the recent trend has been for this species to constitute an ever greater proportion of the statewide harvest. For example, whitetails comprised less than 15 percent of Arizona's deer harvest in 1961 but nearly 40 percent in 1998.

Historic Summary of Mule Deer Survey Data

Year	Bucks	Does	Fawns	Unclassified	Total	Bucks/100 Does	Fawns/100 Does
1948	95	293	143	77	608	32	49
1949	149	387	189	73	798	39	49
1950	373	1326	690	103	2492	28	52
1951	432	1553	887	424	3296	28	57
1952	804	2398	1551	747	5500	34	65
1953	636	2101	1512	559	4808	30	72
1954	616	2373	1020	553	4562	26	43
1955	1052	2276	841	594	4763	46	37
1956	352	1184	539	279	2354	30	46
1957	735	2079	886	540	4240	35	43
1958	552	1810	1057	350	3769	31	58
1959	1049	2748	1388	698	5883	38	51
1960	1125	2890	1179	409	5603	39	41
1961	1162	2806	1212	522	5702	41	43
1962	1213	3072	1205	478	5968	39	39
1963	1185	3186	1189	350	5910	37	37
1964	1118	3269	1467	353	6207	34	45
1965	1260	3460	1775	377	6872	36	51
1966	1299	4370	2240	486	8395	30	51
1967	1341	4715	2462	320	8838	28	52
1968	1029	3708	1620	324	6681	28	44
1969	1173	4494	2324	392	8383	26	52
1970	1306	5218	2669	383	9576	25	51
1971	1551	6018	2649	597	10815	26	44
1972	1262	4385	2093	346	8086	29	48
1973	1089	4363	2514	286	8252	25	58
1974	1009	4184	1999	319	7511	24	48
1975	1126	4275	1911	439	7751	26	45
1976	1029	4320	1820	263	7432	24	42
1977	1022	4402	1696	467	7587	23	39
1978	1329	5719	2573	472	10093	23	45
1979	1119	4824	2249	288	8480	23	47
1980	1255	5815	2428	311	9809	22	42
1981	1367	6315	2694	254	10630	22	43
1982	1299	5992	3033	249	10573	22	51
1983	1360	6540	3361	361	11622	21	51
1984	1401	6259	3411	407	11478	22	55
1985	2102	9093	4312	345	15852	23	47
1986	2148	10521	4989	210	17868	20	47
1987	2227	10193	4139	175	16734	22	41
1988	2157	11383	4577	145	18262	19	40
1989	1976	10272	3465	214	15927	19	34
1990	1778	10361	4024	203	16366	17	39
1991	1798	10532	4444	220	16994	17	42
1992	1689	9500	4332	100	15621	18	46
1993	1910	10177	4190	167	16444	19	41
1994	2103	11504	3833	159	17599	18	33
1995	1820	11082	3668	265	16835	16	33
1996	1590	9954	3001	124	14669	16	30
1997	1351	8756	3168	100	13375	15	36
1998	1404	8041	3919	53	13417	17	49
1999	1705	8559	3786	44	14094	20	44
2000	1732	8416	2794	80	13022	21	33
2001	1502	7408	3051	45	12006	20	41
2002	1321	7069	1838	142	10370	19	26
2003	1268	6190	2524	36	10018	20	41
2004	1134	5148	2309	59	8650	22	45
2005	1054	4738	2333	69	8194	22	49
2006	1146	5143	2150	89	8528	22	42
2007	1180	4931	2071	59	8241	24	42
2008	1132	4164	1965	106	7367	27	47

Historic Summary of White-tailed Deer Survey Data

Year	Bucks	Does	Fawns	Unclassified	Total	Bucks/100 Does	Fawns/100 Does
1946	35	53	28	18	134	66	53
1947	58	72	11	26	167	81	15
1948	32	96	61	54	243	33	64
1949	49	95	37	67	248	52	39
1950	136	223	109	108	576	61	49
1951	25	68	22	23	138	37	32
1952	145	272	139	146	702	53	51
1953	151	336	387	181	1055	45	115
1954	367	828	309	204	1708	44	37
1955	227	497	217	120	1061	46	44
1956	247	533	167	219	1166	46	31
1957	266	455	147	170	1038	58	32
1958	221	420	148	88	877	53	35
1959	177	453	137	93	860	39	30
1960	159	440	164	124	887	36	37
1961	266	484	174	113	1037	55	36
1962	263	586	193	135	1177	45	33
1963	291	630	212	152	1285	46	34
1964	291	581	243	143	1258	50	42
1965	211	502	224	124	1061	42	45
1966	222	484	222	100	1028	46	46
1967	164	391	164	80	799	42	42
1968	152	382	144	105	783	40	38
1969	131	350	152	71	704	37	43
1970	149	373	138	49	709	40	37
1971	170	398	150	94	812	43	38
1972	145	312	133	70	660	46	43
1973	113	316	149	54	632	36	47
1974	101	244	95	54	494	41	39
1975	147	448	195	65	855	33	44
1976	171	577	183	73	1004	30	32
1977	165	577	178	76	996	29	31
1978	202	644	336	84	1266	31	52
1979	226	752	312	54	1344	30	41
1980	306	766	267	62	1401	40	35
1981	329	1069	404	48	1850	31	38
1982	315	1020	471	59	1865	31	46
1983	296	978	528	50	1852	30	54
1984	283	1016	538	56	1893	28	53
1985	424	1388	690	42	2544	31	50
1986	439	1403	544	112	2498	31	39
1987	444	1648	493	34	2619	27	30
1988	425	1584	551	29	2589	27	35
1989	461	1749	567	87	2864	26	32
1990	568	1970	742	53	3333	29	38
1991	483	1814	671	107	3075	27	37
1992	466	1859	634	58	3017	25	34
1993	479	1764	528	62	2833	27	30
1994	541	2000	518	192	3251	27	26
1995	538	2227	588	102	3455	24	26
1996	620	2697	729	91	4137	23	27
1997	484	2380	569	45	3478	20	24
1998	475	1967	679	52	3173	24	35
1999	422	1885	679	32	3018	22	36
2000	405	1734	499	57	2695	23	29
2001	451	1925	711	132	3219	23	37
2002	475	2023	558	44	3100	23	28
2003	550	2165	761	45	3521	25	35
2004	636	2423	777	45	3881	26	32
2005	544	2031	738	41	3354	27	36
2006	581	2055	738	38	3412	28	36
2007	684	2319	800	56	3859	30	35
2008	658	2164	799	33	3654	30	37

5-Year: 2004-2008 Deer Survey Data

Unit	Year	Bucks	Does	Fawns	Unclassified	Total	Bucks/100 Does	Fawns/100 Does
**MULE DEER**								
1	2004	11	66	36	0	113	17	55
1	2005	11	91	32	1	135	12	35
1	2006	7	39	16	0	62	18	41
1	2007	8	35	20	1	64	23	57
1	2008	9	41	18	3	71	22	44
2	2005	7	43	25	0	75	16	58
2	2006	6	41	15	0	62	15	37
2	2007	5	44	15	0	64	11	34
2	2008	12	75	9	0	96	16	12
3A/3C	2004	5	38	15	0	58	13	39
3A/3C	2005	14	75	35	0	124	19	47
3A/3C	2006	14	38	18	0	70	37	47
3A/3C	2007	9	41	31	0	81	22	76
3A/3C	2008	16	44	18	0	78	36	41
3B	2004	16	56	1	0	73	29	2
3B	2005	8	10	6	0	24	80	60
3B	2006	12	38	6	0	56	32	16
3B	2007	15	43	18	0	76	35	42
3B	2008	17	37	16	0	70	46	43
4	2005	8	27	16	0	51	30	59
4	2006	15	34	10	0	59	44	29
4	2007	10	20	10	0	40	50	50
4	2008	8	19	8	0	35	42	42
5	2004	41	177	61	0	279	23	34
5	2005	26	74	39	0	139	35	53
5	2006	15	112	26	0	153	13	23
5	2007	14	103	37	0	154	14	36
5	2008	18	59	20	0	97	31	34
6A	2004	28	125	50	0	203	22	40
6A	2005	23	140	52	0	215	16	37
6A	2006	26	106	46	0	178	25	43
6A	2007	28	122	50	0	200	23	41
6A	2008	25	109	43	0	177	23	39
6B	2004	6	40	25	4	75	15	63
6B	2005	18	66	37	1	122	27	56
6B	2006	24	79	37	1	141	30	47
6B	2007	15	60	28	4	107	25	47
6B	2008	8	40	17	0	65	20	43
7	2004	17	102	53	1	173	17	52
7	2005	23	106	60	0	189	22	57
7	2006	17	77	34	1	129	22	44
7	2007	18	101	28	0	147	18	28
7	2008	30	105	47	0	182	29	45
8	2004	15	82	40	0	137	18	49
8	2005	16	117	53	1	187	14	45
8	2006	21	109	50	0	180	19	46
8	2007	16	99	29	0	144	16	29
8	2008	30	78	36	1	145	38	46
9	2004	18	130	51	0	199	14	39
9	2005	7	103	39	0	149	7	38
9	2006	34	206	72	0	312	17	35
9	2007	38	217	89	0	344	18	41
9	2008	8	45	26	0	79	18	58
10	2004	27	129	50	0	206	21	39
10	2005	22	107	72	0	201	21	67
10	2006	7	22	2	0	31	32	9
10	2007	13	41	12	0	66	32	29
10	2008	11	42	19	27	99	26	45
12AE	2004	4	36	28	0	68	11	78
12AE	2005	8	30	25	0	63	27	83
12AE	2006	46	172	118	2	338	27	69
12AE	2007	5	23	17	0	45	22	74
12AE	2008	44	197	142	0	383	22	72
12AW	2004	71	237	244	5	557	30	103

5-Year: 2004-2008 Deer Survey Data

Unit	Year	Bucks	Does	Fawns	Unclassified	Total	Bucks/100 Does	Fawns/100 Does
**MULE DEER (continued)**								
12AW	2005	84	362	313	14	773	23	86
12AW	2006	92	336	265	0	693	27	79
12AW	2007	99	314	304	5	722	32	97
12AW	2008	67	210	161	15	453	32	77
12B	2004	78	253	173	0	504	31	68
12B	2005	19	108	82	14	223	18	76
12B	2006	62	183	133	5	383	34	73
12B	2008	2	1	0	0	3	200	0
12BW	2007	86	277	140	6	509	31	51
12BW	2008	57	161	114	6	338	35	71
13A	2004	25	63	47	5	140	40	75
13A	2005	21	56	47	12	136	38	84
13A	2006	46	107	65	12	230	43	61
13A	2007	40	74	28	3	145	54	38
13A	2008	25	50	21	1	97	50	42
13B	2004	18	53	30	1	102	34	57
13B	2005	35	54	39	0	128	65	72
13B	2006	40	59	38	6	143	68	64
13B	2007	48	61	25	18	152	79	41
13B	2008	34	76	48	0	158	45	63
15A/15B	2006	11	22	1	1	35	50	5
15A/15B	2008	5	18	7	0	30	28	39
16A	2004	17	41	21	0	79	41	51
16A	2005	19	41	10	0	70	46	24
16A	2006	9	27	7	0	43	33	26
16A	2007	14	34	8	0	56	41	24
16A	2008	12	37	15	0	64	32	41
17	2004	30	86	44	3	163	35	51
17A	2005	2	9	2	0	13	22	22
17A	2006	8	30	9	0	47	27	30
17A	2007	5	27	4	0	36	19	15
17A	2008	15	51	14	0	80	29	27
17B	2005	16	41	26	0	83	39	63
17B	2006	6	69	26	0	101	9	38
17B	2007	18	80	15	0	113	23	19
17B	2008	17	51	19	0	87	33	37
18A	2004	30	124	58	0	212	24	47
18A	2005	32	71	55	0	158	45	77
18A	2006	6	24	2	0	32	25	8
18A	2007	12	62	16	0	90	19	26
18A	2008	20	71	36	0	127	28	51
18B	2004	19	86	43	0	148	22	50
18B	2005	11	60	31	0	102	18	52
18B	2006	28	161	42	13	244	17	26
18B	2007	25	76	22	0	123	33	29
18B	2008	22	41	23	9	95	54	56
19A	2004	4	35	12	2	53	11	34
19A	2005	18	65	43	0	126	28	66
19A	2006	19	87	21	0	127	22	24
19A	2007	9	49	17	1	76	18	35
19A	2008	16	56	29	0	101	29	52
19B	2004	30	88	24	0	142	34	27
19B	2005	22	90	26	0	138	24	29
19B	2006	19	54	9	13	95	35	17
19B	2007	20	44	20	0	84	45	45
19B	2008	18	43	19	2	82	42	44
20A	2004	9	70	20	4	103	13	29
20A	2005	9	39	14	0	62	23	36
20A	2006	8	34	6	0	48	24	18
20A	2007	16	47	14	0	77	34	30
20A	2008	2	18	10	0	30	11	56
20B	2004	17	43	15	0	75	40	35
20B	2005	31	66	24	0	121	47	36
20B	2006	34	85	28	0	147	40	33

5-Year: 2004-2008 Deer Survey Data

Unit	Year	Bucks	Does	Fawns	Unclassified	Total	Bucks/100 Does	Fawns/100 Does
**MULE DEER (continued)**								
20B	2007	25	71	19	0	115	35	27
20B	2008	19	36	15	1	71	53	42
20C	2004	23	109	35	0	167	21	32
20C	2005	30	63	36	1	130	48	57
20C	2006	43	142	52	0	237	30	37
20C	2007	23	75	28	0	126	31	37
20C	2008	29	66	28	0	123	44	42
21	2004	9	34	14	0	57	26	41
21	2005	15	51	23	0	89	29	45
21	2006	14	48	25	0	87	29	52
21	2007	18	68	19	0	105	26	28
21	2008	21	77	37	0	135	27	48
22	2004	52	138	61	1	252	38	44
22	2005	32	81	39	0	152	40	48
22	2006	42	119	69	0	230	35	58
22	2007	31	113	56	0	200	27	50
22	2008	27	138	60	0	225	20	43
23	2004	22	107	45	0	174	21	42
23	2005	40	110	70	0	220	36	64
23	2006	30	122	62	0	214	25	51
23	2007	30	123	52	0	205	24	42
23	2008	30	101	46	0	177	30	46
24A	2004	25	121	48	2	196	21	40
24A	2005	17	92	44	0	153	18	48
24A	2006	7	53	17	5	82	13	32
24A	2007	6	60	33	2	101	10	55
24A	2008	22	60	42	0	124	37	70
24B	2004	20	87	62	0	169	23	71
24B	2005	17	113	37	0	167	15	33
24B	2006	5	43	42	0	90	12	98
24B	2007	24	90	47	0	161	27	52
24B	2008	22	82	43	0	147	27	52
25M	2006	1	8	2	0	11	13	25
26M	2006	4	5	2	0	11	80	40
27	2004	52	231	68	0	351	23	29
27	2005	38	222	35	0	295	17	16
27	2006	51	239	57	0	347	21	24
27	2007	77	294	108	0	479	26	37
27	2008	42	190	72	0	304	22	38
28	2004	32	170	70	1	273	19	41
28	2005	22	161	56	0	239	14	35
28	2006	19	157	59	1	236	12	38
28	2007	26	184	72	0	282	14	39
28	2008	25	123	50	0	198	20	41
29	2004	16	148	28	0	192	11	19
29	2005	21	175	43	0	239	12	25
29	2006	19	161	37	0	217	12	23
29	2007	27	171	24	0	222	16	14
29	2008	30	150	28	0	208	20	19
30A	2004	20	240	63	0	323	8	26
30A	2005	42	205	66	1	314	20	32
30A	2006	21	217	67	0	305	10	31
30A	2007	32	186	66	0	284	17	35
30A	2008	31	155	68	1	255	20	44
30B	2004	31	172	93	0	296	18	54
30B	2005	15	154	80	2	251	10	52
30B	2006	10	100	37	0	147	10	37
30B	2007	25	123	79	1	228	20	64
30B	2008	8	49	29	3	89	16	59
31	2004	35	144	45	0	224	24	31
31	2005	10	91	27	0	128	11	30
31	2006	20	127	35	3	185	16	28
31	2007	24	143	42	1	210	17	29
31	2008	23	135	42	0	200	17	31

5-Year: 2004–2008 Deer Survey Data

Unit	Year	Bucks	Does	Fawns	Unclassified	Total	Bucks/100 Does	Fawns/100 Does
**MULE DEER (continued)**								
32	2004	23	298	105	17	443	8	35
32	2005	25	289	116	0	430	9	40
32	2006	28	231	59	0	318	12	26
32	2007	34	340	112	6	492	10	33
32	2008	33	272	94	19	418	12	35
33	2004	10	42	26	0	78	24	62
33	2005	5	25	13	0	43	20	52
33	2006	7	52	15	0	74	13	29
33	2007	5	22	10	0	37	23	45
33	2008	8	47	17	3	75	17	36
34A	2004	3	16	3	0	22	19	19
34A	2005	1	20	10	0	31	5	50
34A	2006	0	4	1	0	5	0	25
34A	2007	10	35	17	0	62	29	49
34A	2008	1	5	5	0	11	20	100
34B	2004	14	20	9	10	53	70	45
34B	2005	4	14	9	0	27	29	64
34B	2006	1	20	10	0	31	5	50
34B	2007	5	16	9	0	30	31	56
34B	2008	1	8	5	0	14	13	63
35A	2004	9	85	48	0	142	11	56
35A	2005	8	52	23	1	84	15	44
35A	2006	8	27	17	0	52	30	63
35A	2007	12	46	18	0	76	26	39
35A	2008	3	27	11	0	41	11	41
35B	2004	17	56	24	0	97	30	43
35B	2005	9	44	18	0	71	20	41
35B	2006	1	12	6	0	19	8	50
35B	2007	6	16	11	0	33	38	69
35B	2008	5	22	13	0	40	23	59
36A	2004	29	152	73	0	254	19	48
36A	2005	29	160	82	3	274	18	51
36A	2006	22	183	56	17	278	12	31
36A	2007	13	182	76	2	273	7	42
36A	2008	7	71	46	3	127	10	65
36B	2004	18	137	85	0	240	13	62
36B	2005	13	105	60	0	178	12	57
36B	2006	12	105	51	3	171	11	49
36B	2007	11	77	33	1	122	14	43
36B	2008	11	137	80	0	228	8	58
36C	2004	17	109	54	0	180	16	50
36C	2005	5	43	16	8	72	12	37
36C	2006	10	56	11	0	77	18	20
36C	2007	2	40	19	0	61	5	48
36C	2008	5	30	20	4	59	17	67
37A	2004	7	15	1	0	23	47	7
37A	2005	4	10	6	0	20	40	60
37A	2006	5	6	3	0	14	83	50
37A	2007	17	21	10	0	48	81	48
37A	2008	16	15	6	0	37	107	40
37B	2004	5	20	6	0	31	25	30
37B	2005	9	12	6	4	31	75	50
37B	2006	8	24	11	1	44	33	46
37B	2007	5	25	18	2	50	20	72
37B	2008	17	36	28	0	81	47	78
39	2004	6	37	14	0	57	16	38
39	2005	8	29	15	0	52	28	52
39	2006	8	46	26	0	80	17	57
39	2007	11	50	9	0	70	22	18
39	2008	11	25	10	4	50	44	40
39M	2005	1	0	0	0	1	–	–
40	2004	8	10	5	1	24	80	50
40	2005	4	7	1	0	12	57	14
40	2006	9	20	10	0	39	45	50

5-Year: 2004–2008 Deer Survey Data

Unit	Year	Bucks	Does	Fawns	Unclassified	Total	Bucks/100 Does	Fawns/100 Does
**MULE DEER (continued)**								
40	2007	3	7	3	0	13	43	43
40	2008	2	8	3	0	13	25	38
41	2004	20	70	24	0	114	29	34
41	2005	21	53	27	2	103	40	51
41	2006	23	152	76	4	255	15	50
41	2007	20	52	24	0	96	38	46
41	2008	26	55	21	3	105	47	38
42	2004	29	62	19	1	111	47	31
42	2005	28	114	72	0	214	25	63
42	2006	32	137	64	0	233	23	47
42	2007	16	43	17	0	76	37	40
42	2008	47	104	43	1	195	45	41
43A	2004	2	7	1	0	10	29	14
43A	2005	2	10	6	0	18	20	60
43A	2006	2	8	3	0	13	25	38
43A	2007	3	17	4	5	29	18	24
43A	2008	6	12	3	0	21	50	25
43B	2004	3	13	5	0	21	23	38
43B	2005	4	17	10	0	31	24	59
43B	2006	1	12	5	0	18	8	42
43B	2007	3	11	3	0	17	27	27
43B	2008	8	24	13	0	45	33	54
44A	2004	22	69	20	1	112	32	29
44A	2005	39	88	41	4	172	44	47
44A	2006	24	102	41	0	167	24	40
44A	2007	24	76	18	0	118	32	24
44A	2008	29	73	37	0	139	40	51
45	2004	21	76	16	0	113	28	21
45	2005	26	77	43	0	146	34	56
45	2006	27	54	20	1	102	50	37
45	2007	26	60	18	1	105	43	30
45	2008	19	56	15	0	90	34	27
**WHITE-TAILED DEER**								
1	2004	1	4	1	0	6	25	25
1	2005	0	1	0	0	1	0	0
1	2007	0	10	6	0	16	0	60
1	2008	2	1	0	0	3	200	0
4	2005	1	1	0	0	2	100	0
4	2007	0	2	0	0	2	0	0
4	2008	4	5	3	0	12	80	60
5	2004	1	0	0	0	1	–	–
5	2005	1	3	2	0	6	33	67
6A	2004	26	58	25	3	112	45	43
6A	2005	21	66	30	0	117	32	45
6A	2006	17	47	19	0	83	36	40
6A	2007	26	53	10	1	90	49	19
6A	2008	22	51	19	0	92	43	37
6B	2004	1	13	3	2	19	8	23
6B	2005	2	6	3	0	11	33	50
6B	2006	4	10	1	0	15	40	10
6B	2007	4	13	1	0	18	31	8
6B	2008	2	9	4	0	15	22	44
8	2004	0	0	0	1	1	–	–
8	2005	1	7	1	0	9	14	14
8	2006	2	0	0	0	2	–	–
8	2007	2	6	0	0	8	33	0
8	2008	13	24	6	3	46	54	25
19A	2005	0	2	2	0	4	0	100
19A	2006	2	6	0	0	8	33	0
21	2004	9	39	18	0	66	23	46
21	2005	16	52	20	0	88	31	38
21	2006	25	51	15	4	95	49	29
21	2007	17	63	12	0	92	27	19

5-Year: 2004–2008 Deer Survey Data

Unit	Year	Bucks	Does	Fawns	Unclassified	Total	Bucks/100 Does	Fawns/100 Does
**WHITE-TAILED DEER (continued)**								
21	2008	20	66	17	0	103	30	26
22	2004	29	93	11	1	134	31	12
22	2005	38	92	23	0	153	41	25
22	2006	29	112	17	0	158	26	15
22	2007	78	173	62	0	313	45	36
22	2008	38	108	43	0	189	35	40
23	2004	36	66	31	1	134	55	47
23	2005	17	54	22	1	94	31	41
23	2006	29	73	34	0	136	40	47
23	2007	19	54	20	1	94	35	37
23	2008	28	95	52	0	175	29	55
24A	2004	19	103	43	3	168	18	42
24A	2005	30	64	18	0	112	47	28
24A	2006	25	77	28	0	130	32	36
24A	2007	29	73	26	1	129	40	36
24A	2008	37	75	36	0	148	49	48
24B	2004	28	78	28	0	134	36	36
24B	2005	24	88	28	0	140	27	32
24B	2006	45	110	46	2	203	41	42
24B	2007	46	107	29	0	182	43	27
24B	2008	34	69	39	0	142	49	57
27	2004	10	38	6	0	54	26	16
27	2005	16	67	8	0	91	24	12
27	2006	15	49	15	0	79	31	31
27	2007	19	55	21	0	95	35	38
27	2008	18	43	20	0	81	42	47
28	2004	1	2	1	0	4	50	50
28	2006	0	1	2	0	3	0	200
28	2008	1	2	0	0	3	50	0
29	2004	35	162	13	1	211	22	8
29	2005	22	106	30	5	163	21	28
29	2006	20	121	39	1	181	17	32
29	2007	23	120	17	3	163	19	14
29	2008	22	104	14	0	140	21	13
30A	2004	28	112	19	0	159	25	17
30A	2005	20	83	30	1	134	24	36
30A	2006	17	106	29	0	152	16	27
30A	2007	17	70	17	1	105	24	24
30A	2008	31	68	19	2	120	46	28
30B	2004	30	93	50	2	175	32	54
30B	2005	18	90	62	5	175	20	69
30B	2006	20	93	30	2	145	22	32
30B	2007	43	112	77	4	236	38	69
30B	2008	36	116	39	3	194	31	34
31	2004	28	104	37	4	173	27	36
31	2005	23	73	21	0	117	32	29
31	2006	33	82	22	1	138	40	27
31	2007	20	79	17	1	117	25	22
31	2008	18	81	15	1	115	22	19
32	2004	47	181	52	5	285	26	29
32	2005	33	123	38	3	197	27	31
32	2006	25	89	16	1	131	28	18
32	2007	49	189	43	8	289	26	23
32	2008	32	133	30	5	200	24	23
33	2004	59	270	107	3	439	22	40
33	2005	48	154	37	0	239	31	24
33	2006	53	208	64	8	333	25	31
33	2007	96	368	155	18	637	26	42
33	2008	83	408	140	2	633	20	34
34A	2004	29	189	35	3	256	15	19
34A	2005	41	211	80	1	333	19	38
34A	2006	30	170	81	8	289	18	48
34A	2007	39	211	71	5	326	18	34
34A	2008	38	138	50	7	233	28	36

5-Year: 2004–2008 Deer Survey Data

Unit	Year	Bucks	Does	Fawns	Unclassified	Total	Bucks/100 Does	Fawns/100 Does
**WHITE-TAILED DEER (continued)**								
34B	2004	16	50	15	5	86	32	30
34B	2005	19	113	25	4	161	17	22
34B	2006	30	101	47	2	180	30	47
34B	2007	25	102	41	5	173	25	40
34B	2008	20	80	16	0	116	25	20
35A	2004	31	102	31	2	166	30	30
35A	2005	16	58	23	0	97	28	40
35A	2006	29	52	28	0	109	56	54
35A	2007	22	72	28	2	124	31	39
35A	2008	31	76	38	0	145	41	50
35B	2004	27	101	39	3	170	27	39
35B	2005	28	82	29	1	140	34	35
35B	2006	34	102	57	5	198	33	56
35B	2007	33	88	38	2	161	38	43
35B	2008	32	75	28	5	140	43	37
36A	2004	42	171	61	2	276	25	36
36A	2005	37	182	87	2	308	20	48
36A	2006	25	106	44	1	176	24	42
36A	2007	31	133	54	4	222	23	41
36A	2008	24	103	54	1	182	23	52
36B	2004	62	278	115	1	456	22	41
36B	2005	47	173	86	8	314	27	50
36B	2006	28	143	57	1	229	20	40
36B	2007	24	77	31	0	132	31	40
36B	2008	37	154	78	3	272	24	51
36C	2004	41	116	35	3	195	35	30
36C	2005	23	80	33	10	146	29	41
36C	2006	42	143	46	2	233	29	32
36C	2007	20	86	24	0	130	23	28
36C	2008	32	78	39	1	150	41	50
37A	2005	2	0	0	0	2	–	–
37A	2006	2	1	1	0	4	200	100
37A	2007	1	1	0	0	2	100	0
37A	2008	3	2	0	0	5	150	0
37B	2004	0	0	1	0	1	–	–
37B	2006	0	2	0	0	2	0	0
37B	2007	1	2	0	0	3	50	0

Historic Summary of General Deer Hunts[1]

Year[2]	1st Choice Applicants	Permits Issued	Hunters	Hunter Days	Deer Harvest					Percent Success
					Mule Deer		Whitetail		Total	
					Bucks	A-less	Bucks	A-less		
1946	–	–	–	–	4733	–	991	–	5724	–
1947	–	–	–	–	6420	–	1152	–	7572	–
1948	–	–	–	–	7358	–	1347	–	8705	–
1949	–	–	–	–	7465	386	1203	–	9054	–
1950	–	–	–	–	9009	798	1175	–	10982	–
1951	–	–	–	–	9618	658	1234	–	11510	–
1952	–	–	–	–	10575	2707	1490	–	14772	–
1953	–	–	–	–	12590	3948	1791	–	18329	–
1954	–	–	–	–	11662	6425	1500	–	19587	–
1955	–	–	53791	–	15220	5483	1489	300	22492	42
1956	–	–	64123	–	16175	8943	2066	357	27541	43
1957	–	–	56499	–	15307	4859	1808	593	22567	40
1958	–	–	76358	259876	17994	9840	3394	1702	32930	43
1959	–	–	78102	290686	16329	7769	3105	1687	28890	37
1960	–	–	87986	318806	19291	8380	3871	1111	32653	37
1961	–	–	91120	350200	22459	8307	3891	843	35500	39
1962	–	–	93337	373035	16658	7579	3211	983	28431	31
1963	–	–	92594	371619	14082	6262	2859	1463	24666	27
1964	–	–	86867	335508	12613	2362	3207	1116	19298	22
1965	–	–	87548	316911	11357	2002	2871	741	16971	19
1966	–	–	88230	354586	12158	2040	2390	597	17185	20
1967	–	–	90361	365358	12350	1388	2404	258	16400	18
1968	–	–	88253	359684	12298	741	2722	205	15966	18
1969	–	–	91575	358833	12203	567	2124	78	14972	16
1970	–	–	97113	395038	13167	420	2197	35	15819	16
1971	–	77437	67263	256100	9129	334	1517	18	10998	16
1972	66905	74096	63269	241882	9137	338	1653	17	11145	18
1973	83334	75200	64120	243322	11114	402	2080	22	13618	21
1974	79664	82650	72352	255592	11715	533	3221	0	15469	21
1975	80929	79750	69262	253721	12576	408	2870	0	15854	23
1976	86829	83125	72049	228763	10578	261	2656	0	13495	19
1977	83593	84265	72472	255850	9871	6	2295	24	12196	17
1978	84017	81675	69709	264624	9075	38	2247	40	11400	16
1979	85072	78215	66451	270068	10347	0	3207	54	13608	21
1980	94285	79409	66909	278520	11111	0	3480	46	14637	22
1981	92679	77755	66308	274028	10825	0	3466	38	14329	22
1982	91673	83045	71123	296368	12187	0	3965	34	16186	23
1983	71826	94285	77106	309699	12767	0	4173	51	16991	22
1984	72989	92545	82618	328231	17102	0	7030	75	24207	29
1985	80014	92345	84079	333156	16292	273	6782	110	23457	28
1986	82982	94871	84687	331015	16493	2961	5829	86	25369	30
1987	84145	87340	79557	304440	15081	2191	4777	92	22141	28
1988	85084	79135	72796	290084	13744	1781	4505	75	20105	28
1989	84485	75925	69974	277264	13516	694	4293	84	18587	27
1990	82911	76620	70901	284643	11278	2809	4368	62	18517	26
1991	79466	68304	63109	256780	12101	0	5268	76	17445	28
1992	85343	68910	64143	256592	11997	0	5639	75	17711	28
1993	87558	70348	65151	260399	11879	0	5489	58	17426	27
1994	92904	68849	63330	256856	10867	0	5336	0	16203	26
1995	92139	63708	58649	242281	8824	0	4876	0	13700	23
1996	88529	57570	52679	212116	7229	0	4091	0	11320	22
1997	89627	51222	47210	195719	6065	0	4154	33	10252	22
1998	88329	46694	42753	173577	5877	0	4095	7	9979	24
1999	92104	47065	42970	175908	5924	310	3264	8	9506	22

[1] Muzzleloader hunt data included up until 1984. Juniors-0nly hunt data not included in this table.

[2] 1994 and 1995 data does not include results of hunts at Ft. Huachuca. Beginning with 1996, Ft. Huachuca data is based on questionnaire returns, not data gathered by the Fort.

Historic Summary of General Deer Hunts[1]

Year[2]	1st Choice Applicants	Permits Issued	Hunters	Hunter Days	Deer Harvest					Percent Success
					Mule Deer		Whitetail		Total	
					Bucks	A-less	Bucks	A-less		
2000	85091	46072	41677	166780	5025	188	4121	0	9334	22
2001	83808	44978	41110	170820	5226	623	3369	0	9218	22
2002	84384	42020	38368	163098	4540	0	3595	0	8135	21
2003	86546	37260	33905	144027	3753	0	3937	0	7690	23
2004	90057	36665	33395	136377	4037	0	4515	0	8552	26
2005	83264	37918	34883	144949	4357	0	4214	0	8571	25
2006	85534	38138	35016	147433	4811	0	4158	0	8969	26
2007	68625	39834	37002	158215	5388	0	4362	0	9750	26
2008	62236	41958	38770	157646	5215	0	5094	0	10309	27

[1] Muzzleloader hunt data included up until 1984. Juniors-0nly hunt data not included in this table.
[2] 1994 and 1995 data does not include results of hunts at Ft. Huachuca. Beginning with 1996, Ft. Huachuca data is based on questionnaire returns, not data gathered by the Fort.

Historic Summary of Juniors-Only Deer Hunts

Year	1st Choice Applicants	Permits Issued	Hunters	Hunter Days	Deer Harvest					Percent Success
					Mule Deer		Whitetail		Total	
					Bucks	A-less	Bucks	A-less		
1992	299	350	336	1386	147	0	2	0	149	44
1993	403	150	148	569	88	0	7	0	95	64
1994	608	275	264	1073	158	0	14	0	172	65
1995	837	339	331	1450	177	0	18	0	195	59
1996	1076	706	649	2262	178	0	21	0	199	31
1997	1155	603	543	2083	159	0	35	0	194	36
1998	1497	808	763	2502	263	0	42	0	305	40
1999	1897	1224	1100	2719	280	360	41	0	681	61
2000	2427	1250	1134	2959	167	395	57	0	619	55
2001	2571	1625	1449	3858	166	591	47	0	804	55
2002	2863	1510	1394	4117	141	462	37	0	640	46
2003	2855	980	904	2704	114	301	44	0	459	51
2004	2815	1030	923	2711	149	225	54	0	428	46
2005	2634	1280	1143	3258	140	269	34	0	443	39
2006	2581	1332	1219	4469	291	64	148	0	503	41
2007	2520	1769	1633	5601	342	269	223	0	834	51
2008	2668	2109	1941	6032	367	364	195	0	926	48

Historic Summary of Muzzleloader Deer Hunts

Year	1st Choice Applicants	Permits Issued	Hunters	Hunter Days	Deer Harvest					Percent Success
					Mule Deer		Whitetail		Total	
					Bucks	A-less	Bucks	A-less		
1984	424	950	664	3035	200	0	11	0	211	32
1985	263	950	739	3154	201	0	10	0	211	29
1986	337	950	840	3947	178	0	19	0	197	23
1987	402	750	664	2651	134	0	26	0	160	24
1988	556	1000	821	3545	171	0	20	0	191	23
1989	877	1250	1110	5660	259	0	10	0	269	24
1990	713	1139	996	4822	130	0	19	0	149	15
1991	772	1181	1074	5424	205	0	31	0	236	22
1992	964	1300	1189	5808	216	0	21	0	237	20
1993	970	1625	1437	6950	285	0	2	0	287	20
1994	1070	1821	1667	7875	303	0	13	0	316	19
1995	1213	1626	1456	7135	278	0	5	0	283	19
1996	1267	1479	1309	6323	189	0	14	0	203	16
1997	1540	1335	1179	5605	184	0	7	0	191	16
1998	1621	1120	1008	4372	164	0	16	0	180	18
1999	1541	1055	949	4063	157	0	26	0	183	19
2000	1489	915	822	3812	111	0	26	0	137	17
2001	1456	869	782	3775	170	0	24	0	194	25
2002	1775	995	874	4020	143	0	18	0	161	18
2003	1585	745	675	3189	150	0	32	0	182	27
2004	1896	783	679	2988	119	0	33	0	152	22
2005	1498	859	768	3189	193	0	23	0	216	28
2006	1724	924	807	3726	190	0	29	0	219	27
2007	1506	940	873	4350	233	0	13	0	246	28
2008	1756	1015	940	4456	217	0	40	0	257	27

Summary Of Archery Deer Hunts (Draw Hunts)

Year	1st Choice Applicants	Permits Issued	Hunters	Hunter Days	Deer Harvest					Percent Success
					Mule Deer		Whitetail		Total	
					Bucks	A-less	Bucks	A-less		
2008	1167	1912	1607	10373	225	0	0	0	225	14

Summary of Archery Deer Hunts (OTC Hunts)

Year	Tags Sold	Hunters	Hunter Days	DEER HARVEST					Percent Success
				Mule Deer		Whitetail		Total	
				Buck	A-less	Buck	A-less		
1952	–	104	–	21	0	0	0	21	20
1954	–	156	–	5	0	0	0	5	3
1955	–	98	–	12	0	0	0	12	12
1956	–	670	–	49	0	0	0	49	7
1957	–	–	–	33	0	0	0	33	–
1958	2736	2181	11736	175	226	1	1	403	19
1959	3451	3165	16292	143	224	0	8	375	12
1960	2349	2245	9517	82	93	2	6	183	8
1961	1695	1384	5518	15	20	5	2	42	3
1962	4625	4319	19768	141	172	47	44	404	9
1963	4567	4225	16922	88	91	18	17	214	5
1964	3596	3246	12809	50	63	13	15	141	4
1965	3835	3798	–	–	–	–	–	122	3
1966	3596	3387	–	–	–	–	–	150	4
1967	4679	4390	–	–	–	–	–	206	5
1968	4510	4216	–	–	–	–	–	176	4
1969	5107	4664	–	–	–	–	–	208	5
1970	5855	5275	–	–	–	–	–	228	4
1971	7261	6412	–	–	–	–	–	285	4
1972	–	6832	–	–	–	–	–	315	5
1973	–	7000	–	–	–	–	–	310	4
1974	–	7420	–	–	–	–	–	419	6
1975	–	7163	–	–	–	–	–	346	4
1976	–	7517	–	–	–	–	–	373	5
1977	–	9038	–	–	–	–	–	416	5
1978	–	7313	–	–	–	–	–	381	5
1979	–	8425	–	–	–	–	–	620	7
1980	–	7157	–	–	–	–	–	237	3
1981	19814	12862	77011	327	40	88	16	471	4
1982	15109	10212	63099	287	51	60	0	398	4
1983	11934	9689	63071	248	61	71	0	380	4
1984	12628	10619	70553	417	35	65	0	517	5
1985	14249	12302	85328	534	71	138	0	743	6
1986	16554	14397	104288	742	130	94	0	966	7
1987	18666	16163	111826	748	58	115	0	921	6
1988	20883	17909	119793	704	223	108	0	1035	6
1989	22399	19423	125848	598	201	189	0	988	5
1990	22398	19325	125940	674	35	100	0	809	4
1991	20324	18051	126545	773	0	129	0	902	5
1992	18883	17505	120123	691	0	100	0	791	5
1993	21580	19559	133174	1084	63	136	27	1310	7
1994	23445	20646	144881	942	105	212	39	1298	6
1995	23329	20383	149129	916	97	166	64	1243	6
1996	23568	20698	143789	996	83	145	36	1260	6
1997	23166	19939	148077	691	30	138	18	877	4
1998	23022	19375	146101	1050	63	166	38	1317	7
1999	24293	20738	158187	1032	27	227	28	1314	6
2000	25338	21148	155575	1209	55	177	37	1478	7
2001	23783	20408	148346	827	12	194	25	1058	5
2002	23082	19595	151816	929	7	247	37	1220	6
2003	22447	18512	139107	621	5	291	20	937	5
2004	22675	18305	138856	779	0	262	0	1041	6
2005	22949	18824	14847	803	0	377	0	1180	6
2006	24538	20352	153887	939	0	410	0	1349	7
2007	23907	19265	157895	870	0	393	0	1263	7
2008[1]	19653	15737	116631	549	0	421	0	970	6

[1] 2008 data is preliminary.

5-Year: 2004-2008 Harvest

Unit	Year	Hunt Type	Dates	Permits Authorized	1st Choice Applicants	Permits Issued	Draw Odds	Hunters	Hunter Days	Harvest					Hunt Success
										MD-Buck	MD-Aless	WT- Buck	WT-Aless	Total	
**GENERAL**															
1	2004	AA	10/29/-11/07	175	1428	175	11.4	161	695	34	0	2	0	36	22
1	2005	AA	10/28/-11/06	200	1317	200	13.7	171	826	38	0	0	0	38	22
1	2006	AA	11/10/-11/19	200	1435	200	12.2	185	830	33	0	5	0	38	21
1	2007	AA	11/09/-11/18	200	817	200	21.8	181	831	17	0	0	0	17	9
1	2008	AA	10/24/-11/02	260	628	260	33.3	227	964	59	0	2	0	61	27
2	2004	AA	10/29/-11/07	80	531	80	13.6	80	331	49	0	0	0	49	61
2	2005	AA	10/28/-11/06	90	587	90	12.6	84	523	17	0	0	0	17	20
2	2006	AA	11/10/-11/19	90	551	90	14.0	86	464	14	0	0	0	14	16
2	2007	AA	11/09/-11/18	80	355	80	19.4	77	387	37	0	0	0	37	48
2	2008	AA	10/31/-11/09	80	354	80	17.8	67	341	27	0	0	0	27	40
3A/3C	2004	AA	10/29/-11/07	125	584	125	19.0	109	550	18	0	0	0	18	17
3A/3C	2005	AA	10/28/-11/06	125	704	125	15.8	112	487	34	0	0	0	34	30
3A/3C	2006	AA	11/10/-11/19	150	971	148	13.4	138	743	46	0	0	0	46	33
3A/3C	2007	AA	11/09/-11/18	200	964	200	17.3	191	883	68	0	0	0	68	36
3A/3C	2008	AA	10/31/-11/09	300	1001	300	25.5	296	1331	123	0	0	0	123	42
4	2004	AA	10/29/-11/07	150	946	150	13.5	143	739	17	0	2	0	19	13
4	2005	AA	10/28/-11/06	150	760	150	15.5	140	695	14	0	0	0	14	10
4	2006	AA	11/10/-11/19	150	775	150	16.6	134	756	16	0	0	0	16	12
4	2007	AA	11/09/-11/18	150	414	150	20.8	139	739	18	0	2	0	20	14
4	2008	AA	10/31/-11/09	150	386	150	29.5	139	823	24	0	0	0	24	17
5	2004	AA	10/29/-11/07	350	2619	350	12.1	322	1549	70	0	0	0	70	22
5	2005	AA	10/28/-11/06	350	2282	350	14.1	342	1521	72	0	6	0	78	23
5	2006	AA	10/27/-11/05	450	2816	450	13.0	419	1885	93	0	12	0	105	25
5	2007	AA	10/26/-11/04	450	1866	450	20.8	432	2080	69	0	10	0	79	18
5	2008	AA	10/31/-11/09	400	1553	400	23.2	376	1912	59	0	0	0	59	16
6A	2004	MD	10/29/-11/01	275	2556	275	10.1	260	1096	65	0	0	0	65	25
6A	2005	MD	10/28/-11/06	275	2270	275	10.8	259	1165	71	0	0	0	71	27
6A	2006	MD	10/27/-11/05	275	2419	275	10.7	259	1162	88	0	0	0	88	34
6A	2007	MD	10/26/-11/04	325	1992	325	14.8	311	1506	74	0	0	0	74	24
6A	2008	MD	10/31/-11/06	400	1846	400	20.7	366	1495	76	0	0	0	76	21
6A	2005	WT	10/28/-10/31	175	200	175	48.0	145	498	0	0	9	0	9	6
6A E	2006	WT	10/27/-11/05	400	317	400	56.5	362	1612	0	0	60	0	60	17
6A E	2007	WT	10/26/-10/31	250	170	250	74.7	233	907	0	0	31	0	31	13
6A E	2008	WT	10/24/-10/30	200	135	200	59.3	187	789	0	0	49	0	49	26
6A M	2007	WT	11/02/-11/11	200	76	200	82.9	181	821	0	0	36	0	36	20
6A M	2008	WT	11/07/-11/13	200	168	200	56.0	198	819	0	0	34	0	34	17
6A L	2004	WT	12/17/-12/31	250	783	250	23.6	233	1174	0	0	66	0	66	28
6A L	2005	WT	12/16/-12/31	150	565	150	15.9	145	876	0	0	37	0	37	26
6A L	2006	WT	12/15/-12/31	50	465	50	8.6	44	286	0	0	16	0	16	36
6A L	2007	WT	12/14/-12/31	50	344	50	10.2	44	262	0	0	21	0	21	48
6A L	2008	WT	12/12/-12/31	75	482	75	12.9	69	444	0	0	42	0	42	61
6B	2004	AA	11/05/-11/14	150	395	150	22.5	132	506	29	0	7	0	36	27
6B	2005	AA	11/04/-11/13	150	379	150	25.6	136	652	8	0	0	0	8	6
6B	2006	MD	11/10/-11/19	190	274	190	33.2	180	823	48	0	0	0	48	27
6B	2007	MD	11/09/-11/18	250	387	250	40.3	240	1185	36	0	0	0	36	15
6B	2008	MD	11/07/-11/13	275	332	275	48.5	250	1091	25	0	0	0	25	10
6B E	2006	WT	10/27/-11/05	20	12	20	75.0	20	90	0	0	0	0	0	0
6B E	2007	WT	10/26/-11/04	20	22	20	54.5	20	87	0	0	3	0	3	15
6B E	2008	WT	10/24/-10/30	55	21	55	81.0	49	206	0	0	8	0	8	16
6B/8	2004	WT	12/17/-12/31	50	86	50	34.9	48	302	0	0	13	0	13	27
6B/8	2005	WT	12/16/-12/31	50	78	51	35.9	51	299	0	0	15	0	15	29
6B/8 L	2006	WT	12/15/-12/31	50	112	50	21.4	46	254	0	0	21	0	21	46
6B/8 L	2007	WT	12/14/-12/31	50	166	50	15.7	50	352	0	0	21	0	21	42
6B/8 L	2008	WT	12/12/-12/31	25	125	25	10.4	23	102	0	0	12	0	12	52
7	2004	AA	10/29/-11/07	700	2380	700	22.1	646	2771	70	0	0	0	70	11
7	2005	AA	10/28/-11/06	700	1792	700	27.1	652	2593	155	0	2	0	157	24
7	2006	AA	10/27/-11/05	800	2562	798	23.0	723	3093	131	0	2	0	133	18
7	2007	AA	10/26/-11/04	800	1644	800	35.2	759	3338	187	0	11	0	198	26
7	2008	AA	10/31/-11/09	800	1492	800	40.8	743	3571	138	0	0	0	138	19
8	2004	AA	10/29/-11/07	500	2231	500	19.4	453	2018	91	0	2	0	93	21
8	2005	AA	10/28/-11/06	500	2165	500	20.2	464	1996	96	0	9	0	105	23
8	2006	MD	10/27/-11/05	500	2114	500	20.3	466	2034	169	0	0	0	169	36

AA = Any Antlered Deer **MD** = Mule Deer **WT** = Whitetail Deer **ALS** = Antlerless **CN** = Camp Navajo **FTHU** = Fort Huachuca,
C = CHAMP Hunt; in the unit column **E** = early or 1st season **M** = 2nd season **T** = 3rd season **L** = late or 4th season.

5-Year: 2004-2008 Harvest

Unit	Year	Hunt Type	Dates	Permits Authorized	1st Choice Applicants	Permits Issued	Draw Odds	Hunters	Hunter Days	Harvest					Hunt Success
										MD-Buck	MD-Aless	WT- Buck	WT-Aless	Total	
**GENERAL**															
8	2007	MD	10/26/-11/04	600	1861	600	29.5	588	2943	135	0	0	0	135	23
8	2008	MD	10/31/-11/06	600	1421	599	36.4	552	2450	78	0	0	0	78	14
8 E	2006	WT	10/27/-11/05	50	34	50	70.6	46	209	0	0	2	0	2	4
8 E	2007	WT	10/26/-11/04	50	46	50	60.9	44	179	0	0	4	0	4	9
8 E	2008	WT	10/24/-11/02	75	35	75	91.4	68	345	0	0	15	0	15	22
9 E	2004	AA	10/29/-11/07	650	843	650	43.7	598	2774	137	0	2	0	139	23
9 E	2005	AA	10/28/-11/06	650	1148	650	37.2	615	2986	132	0	4	0	136	22
9 E	2006	AA	10/27/-11/05	400	979	400	29.0	372	1751	144	0	2	0	146	39
9 E	2007	AA	10/26/-11/04	400	986	400	30.3	378	1844	119	0	0	0	119	31
9 E	2008	AA	10/31/-11/09	400	737	400	42.3	367	1669	109	0	0	0	109	30
10	2004	AA	10/29/-11/14	700	2286	700	22.3	647	3691	53	0	0	0	53	8
10	2005	AA	10/28/-11/13	700	1563	700	29.9	651	3644	92	0	2	0	94	14
10	2006	AA	10/27/-11/05	725	1756	721	29.3	678	3315	134	0	4	0	138	20
10	2007	AA	10/26/-11/04	725	1128	725	42.6	691	3694	105	0	0	0	105	15
10	2008	AA	10/24/-11/02	725	927	725	56.4	683	3472	91	0	0	0	91	13
12A Champ	2008	AA	10/10/-10/16	10	31	10	29.0	7	24	7	0	0	0	7	100
12AE E	2004	AA	10/29/-11/01	200	1539	204	10.1	190	958	83	0	0	0	83	44
12AE E	2005	AA	10/28/-11/06	200	1207	200	10.4	194	1017	79	0	0	0	79	41
12AE E	2006	AA	10/27/-11/05	150	1139	150	8.8	146	785	85	0	0	0	85	58
12AE E	2007	AA	10/26/-11/04	175	882	175	13.3	171	870	76	0	0	0	76	44
12AE E	2008	AA	10/31/-11/09	150	630	150	15.4	134	650	76	0	0	0	76	57
12AE L	2004	AA	11/19/-11/28	50	2545	62	2.2	57	347	30	0	0	0	30	53
12AE L	2005	AA	11/18/-11/27	50	1845	50	2.2	49	304	28	0	0	0	28	57
12AE L	2006	AA	11/24/-12/03	50	2068	50	2.3	50	292	40	0	0	0	40	80
12AE L	2007	AA	11/23/-12/02	50	1764	50	2.8	50	224	42	0	0	0	42	84
12AE L	2008	AA	11/21/-11/30	50	1319	50	3.3	46	217	37	0	0	0	37	80
12AW E	2004	AA	10/29/-11/07	500	4803	500	8.6	449	1981	214	0	0	0	214	48
12AW E	2005	AA	10/28/-11/06	1000	5898	1000	13.2	948	4685	428	0	0	0	428	45
12AW E	2006	AA	10/27/-11/05	500	4716	500	7.9	470	2254	241	0	0	0	241	51
12AW E	2007	AA	10/26/-11/04	650	3707	646	12.8	614	2930	339	0	0	0	339	55
12AW E	2008	AA	10/31/-11/09	850	3258	850	18.9	796	3846	427	0	0	0	427	54
12AW L	2004	AA	11/19/-11/28	125	3552	154	3.3	144	723	89	0	0	0	89	62
12AW L	2005	AA	11/18/-11/27	175	2852	175	4.0	170	1073	105	0	0	0	105	62
12AW L	2006	AA	11/24/-12/03	175	4125	175	3.4	169	1003	118	0	0	0	118	70
12AW L	2007	AA	11/23/-12/02	175	3924	175	3.5	173	919	125	0	0	0	125	72
12AW L	2008	AA	11/21/-11/30	175	2980	177	4.6	170	1094	98	0	0	0	98	58
12B E	2004	AA	10/29/-11/07	25	357	25	4.5	25	87	20	0	0	0	20	80
12B E	2005	AA	10/28/-11/06	25	332	25	4.2	24	106	16	0	0	0	16	67
12B E	2006	AA	10/27/-11/05	25	384	25	3.9	22	118	12	0	0	0	12	55
12B E	2007	AA	10/26/-11/04	25	184	25	10.9	24	119	18	0	0	0	18	75
12B E	2008	AA	10/31/-11/09	25	212	25	8.5	24	143	14	0	0	0	14	58
12B L	2004	AA	11/19/-11/28	10	1037	12	1.1	12	66	9	0	0	0	9	75
12B L	2005	AA	11/18/-11/27	65	1961	65	2.8	62	314	46	0	0	0	46	74
12B L	2006	AA	11/24/-12/03	75	1991	75	2.7	75	375	51	0	0	0	51	68
12B L	2007	AA	11/23/-12/02	75	1584	75	4.2	75	380	62	0	0	0	62	83
12B L	2008	AA	11/21/-11/30	75	1390	75	4.7	73	389	51	0	0	0	51	70
12BW	2004	AA	11/19/-11/28	65	1493	87	4.2	84	460	55	0	0	0	55	65
12BW E	2004	AA	10/29/-11/07	100	395	114	16.5	106	476	58	0	0	0	58	55
12BW E	2005	AA	10/28/-11/06	125	300	125	14.3	120	658	81	0	0	0	81	68
12BW E	2006	AA	10/27/-11/05	185	468	185	16.5	179	815	120	0	0	0	120	67
12BW E	2007	AA	10/26/-11/04	175	350	175	24.9	169	750	120	0	0	0	120	71
12BW E	2008	AA	10/31/-11/09	175	466	175	17.6	166	811	122	0	0	0	122	73
13A	2004	AA	11/12/-11/21	40	1217	53	3.0	53	322	27	0	0	0	27	51
13A	2005	AA	11/11/-11/20	30	953	30	1.0	29	170	21	0	0	0	21	72
13A	2006	AA	11/17/-11/26	40	1263	40	2.7	40	189	36	0	0	0	36	90
13A	2007	AA	11/16/-11/25	55	1253	55	3.4	52	387	37	0	0	0	37	71
13A	2008	AA	11/14/-11/23	45	977	45	3.0	43	223	36	0	0	0	36	84
13B	2004	AA	11/05/-11/14	55	6997	72	1.0	68	432	41	0	0	0	41	60
13B	2005	AA	11/04/-11/13	60	5068	60	1.2	60	434	39	0	0	0	39	65
13B	2006	AA	11/10/-11/19	75	5591	75	1.3	70	454	50	0	0	0	50	71
13B	2007	AA	11/09/-11/18	85	3280	85	2.6	85	649	40	0	0	0	40	47
13B	2008	AA	11/07/-11/16	45	2987	45	1.5	45	244	32	0	0	0	32	71

AA = Any Antlered Deer **MD** = Mule Deer **WT** = Whitetail Deer **ALS** = Antlerless **CN** = Camp Navajo **FTHU** = Fort Huachuca,
C = CHAMP Hunt; in the unit column **E** = early or 1st season **M** = 2nd season **T** = 3rd season **L** = late or 4th season.

5-Year: 2004–2008 Harvest

Unit	Year	Hunt Type	Dates	Permits Authorized	1st Choice Applicants	Permits Issued	Draw Odds	Hunters	Hunter Days	Harvest					Hunt Success
										MD-Buck	MD-Aless	WT- Buck	WT-Aless	Total	
**GENERAL**															
16A	2004	AA	10/29/-11/07	600	712	600	58.1	540	2474	89	0	0	0	89	16
16A	2005	AA	10/28/-11/06	600	789	600	57.0	535	2488	98	0	0	0	98	18
16A	2006	AA	10/27/-11/05	600	757	598	59.6	561	2674	127	0	0	0	127	23
16A	2007	AA	10/26/-11/04	650	558	650	88.0	597	2845	127	0	0	0	127	21
16A	2008	AA	10/24/-11/02	650	485	650	100.0	576	2699	105	0	0	0	105	18
17A	2004	AA	10/29/-11/07	375	687	375	42.4	339	1484	44	0	0	0	44	13
17A	2005	AA	10/28/-11/06	375	687	375	44.0	337	1628	42	0	0	0	42	12
17A	2006	AA	10/27/-11/05	375	675	375	40.0	343	1635	80	0	0	0	80	23
17A	2007	AA	10/26/-11/04	375	634	371	42.9	341	1697	61	0	0	0	61	18
17A	2008	AA	10/24/-11/02	350	585	350	49.4	326	1424	74	0	0	0	74	23
17B	2004	AA	10/29/-11/07	400	803	400	35.1	364	1526	60	0	0	0	60	16
17B	2005	AA	10/28/-11/06	400	793	400	39.1	385	1574	71	0	0	0	71	18
17B	2006	AA	10/27/-11/05	400	847	400	35.1	357	1443	78	0	0	0	78	22
17B	2007	AA	10/26/-11/04	400	550	400	53.8	366	1520	67	0	0	0	67	18
17B	2008	AA	10/24/-11/02	400	534	400	58.1	389	1709	77	0	0	0	77	20
18A	2004	AA	10/29/-11/14	500	539	500	56.6	424	2367	55	0	0	0	55	13
18A	2005	AA	10/28/-11/13	500	407	500	66.6	466	2933	101	0	0	0	101	22
18A	2006	AA	10/27/-11/05	600	592	600	63.3	541	2658	96	0	0	0	96	18
18A	2007	AA	10/26/-11/04	600	421	600	90.7	554	2859	95	0	0	0	95	17
18A	2008	AA	10/24/-11/02	600	439	600	98.6	547	2921	117	0	0	0	117	21
18B	2004	AA	10/29/-11/14	500	797	500	43.5	438	2080	73	0	0	0	73	17
18B	2005	AA	10/28/-11/13	500	651	500	54.4	443	2206	97	0	0	0	97	22
18B	2006	AA	10/27/-11/05	550	886	549	46.4	507	2109	153	0	0	0	153	30
18B	2007	AA	10/26/-11/04	600	712	600	62.9	574	2541	123	0	0	0	123	21
18B	2008	AA	10/24/-11/02	650	593	650	87.5	603	2569	168	0	0	0	168	28
19A	2004	AA	10/29/-11/07	425	880	425	31.8	402	1779	81	0	0	0	81	20
19A	2005	AA	10/28/-11/06	350	848	350	29.1	327	1482	68	0	5	0	73	22
19A	2006	AA	10/27/-11/05	400	893	400	33.6	387	1604	139	0	4	0	143	37
19A	2007	AA	10/26/-11/04	450	705	450	45.4	431	1744	143	0	4	0	147	34
19A	2008	AA	10/24/-11/02	450	789	450	47.3	437	1770	148	0	7	0	155	35
19B	2004	AA	10/29/-11/14	250	366	250	40.2	224	1171	31	0	0	0	31	14
19B	2005	AA	10/28/-11/13	250	285	250	44.9	241	1382	26	0	0	0	26	11
19B	2006	AA	10/27/-11/05	200	224	199	52.7	183	872	49	0	0	0	49	27
19B	2007	AA	10/26/-11/04	250	269	250	59.9	230	1126	46	0	0	0	46	20
19B	2008	AA	10/24/-11/02	300	223	300	81.6	283	1384	53	0	0	0	53	19
20A	2004	AA	10/29/-11/14	550	1836	550	25.5	519	2519	126	0	0	0	126	24
20A	2005	AA	10/28/-11/13	550	1729	550	28.1	502	2475	91	0	0	0	91	18
20A	2006	AA	10/27/-11/05	550	1472	550	31.3	501	2040	159	0	0	0	159	32
20A	2007	AA	10/26/-11/04	600	1130	596	45.4	564	2330	167	0	0	0	167	30
20A	2008	AA	10/24/-11/02	700	1031	700	55.3	650	3040	113	0	0	0	113	17
20B	2004	AA	11/12/-11/21	200	454	200	32.8	181	726	29	0	0	0	29	16
20B	2005	AA	11/11/-11/20	225	356	225	46.1	213	793	45	0	0	0	45	21
20B	2006	AA	11/10/-11/19	300	473	300	43.1	272	983	48	0	0	0	48	18
20B	2007	AA	11/09/-11/18	350	312	350	74.0	332	1204	61	0	0	0	61	18
20B	2008	AA	11/14/-11/23	350	283	350	77.0	322	1273	35	0	0	0	35	11
20C	2004	AA	10/29/-11/01	250	293	250	53.9	216	612	29	0	0	0	29	13
20C	2004	AA	11/05/-11/14	250	334	250	51.2	229	982	44	0	0	0	44	19
20C	2005	AA	10/28/-10/31	225	229	225	68.1	210	627	35	0	0	0	35	17
20C	2005	AA	11/04/-11/13	225	293	225	49.8	208	856	44	0	0	0	44	21
20C E	2006	AA	10/27/-11/01	300	337	300	60.8	266	823	68	0	0	0	68	26
20C E	2007	AA	10/26/-10/31	350	275	350	86.2	318	1105	102	0	0	0	102	32
20C E	2008	AA	10/31/-11/06	300	412	300	56.8	276	959	69	0	0	0	69	25
20C L	2006	AA	11/03/-11/12	150	217	150	47.0	136	577	18	0	0	0	18	13
20C L	2007	AA	11/02/-11/11	200	121	200	87.6	182	749	42	0	0	0	42	23
20C L	2008	AA	11/07/-11/13	250	98	250	99.0	227	869	62	0	0	0	62	27
21	2004	MD	11/05/-11/14	400	949	400	31.9	367	1449	52	0	0	0	52	14
21	2005	MD	11/04/-11/13	400	1003	400	29.3	366	1380	54	0	0	0	54	15
21	2006	MD	11/10/-11/19	400	823	400	36.3	368	1495	71	0	0	0	71	19
21	2007	MD	11/09/-11/18	450	581	450	55.2	426	1842	104	0	0	0	104	24
21	2008	MD	11/07/-11/13	450	668	450	46.7	419	1553	82	0	0	0	82	20
21	2004	WT	12/17/-12/31	300	484	300	37.2	279	1256	0	0	88	0	88	32
21	2005	WT	12/16/-12/31	300	461	300	30.2	279	1529	0	0	86	0	86	31

AA = Any Antlered Deer **MD** = Mule Deer **WT** = Whitetail Deer **ALS** = Antlerless **CN** = Camp Navajo **FTHU** = Fort Huachuca,
C = CHAMP Hunt; in the unit column **E** = early or 1st season **M** = 2nd season **T** = 3rd season **L** = late or 4th season.

5-Year: 2004-2008 Harvest

Unit	Year	Hunt Type	Dates	Permits Authorized	1st Choice Applicants	Permits Issued	Draw Odds	Hunters	Hunter Days	Harvest					Hunt Success
										MD-Buck	MD-Aless	WT- Buck	WT-Aless	Total	
**GENERAL**															
21 E	2006	WT	10/27/-11/05	350	114	350	100.0	317	1300	0	0	69	0	69	22
21 E	2007	WT	10/26/-11/04	400	197	400	93.4	380	1640	0	0	62	0	62	16
21 E	2008	WT	10/24/-10/30	460	167	460	99.4	429	1597	0	0	93	0	93	22
21 L	2006	WT	12/15/-12/31	50	318	50	12.6	41	239	0	0	17	0	17	41
21 L	2007	WT	12/14/-12/31	50	281	50	13.5	47	228	0	0	19	0	19	40
21 L	2008	WT	12/12/-12/31	20	217	20	6.5	20	114	0	0	10	0	10	50
22	2004	MD	10/29/-11/07	500	1571	500	25.3	443	1958	61	0	0	0	61	14
22	2005	MD	10/28/-11/06	500	1482	500	26.9	454	1918	82	0	0	0	82	18
22	2006	MD	10/27/-11/05	500	1293	498	29.3	473	2073	79	0	0	0	79	17
22	2007	MD	10/26/-11/04	600	952	600	42.9	566	2445	99	0	0	0	99	17
22	2008	MD	10/24/-11/02	625	924	625	47.4	583	2529	88	0	0	0	88	15
22	2004	WT	12/17/-12/31	450	1498	450	23.1	415	1923	0	0	164	0	164	40
22	2005	WT	12/16/-12/31	450	1434	450	23.2	416	1997	0	0	117	0	117	28
22 E	2006	WT	10/27/-11/05	500	434	500	59.4	458	2123	0	0	78	0	78	17
22 E	2007	WT	10/26/-11/04	500	365	500	69.6	441	1806	0	0	107	0	107	24
22 E	2008	WT	10/24/-10/30	660	379	660	91.8	622	2384	0	0	121	0	121	19
22 L	2006	WT	12/15/-12/31	50	749	50	5.5	50	289	0	0	18	0	18	36
22 L	2007	WT	12/14/-12/31	50	522	50	6.1	48	271	0	0	33	0	33	69
22 L	2008	WT	12/12/-12/31	25	464	25	4.5	25	154	0	0	17	0	17	68
23	2004	MD	10/29/-11/07	450	1745	450	21.7	437	1818	99	0	0	0	99	23
23	2005	MD	10/28/-11/06	450	1849	450	20.6	405	1755	69	0	0	0	69	17
23	2006	MD	10/27/-11/05	525	1772	524	24.2	506	2316	97	0	0	0	97	19
23	2007	MD	10/26/-11/04	575	1494	575	32.7	555	2488	121	0	0	0	121	22
23	2008	MD	10/31/-11/06	610	1432	610	36.2	555	2354	98	0	0	0	98	18
23	2004	WT	12/17/-12/31	300	1164	300	19.0	260	1209	0	0	123	0	123	47
23 E	2005	WT	10/28/-10/31	300	257	300	44.4	259	806	0	0	50	0	50	19
23 E	2006	WT	10/27/-11/05	425	402	425	47.8	397	1698	0	0	82	0	82	21
23 E	2007	WT	10/26/-11/04	600	457	600	61.3	551	2458	0	0	120	0	120	22
23 E	2008	WT	10/24/-10/30	575	446	575	64.6	526	2009	0	0	104	0	104	20
23 L	2005	WT	12/16/-12/31	150	1144	150	12.0	140	756	0	0	48	0	48	34
23 L	2006	WT	12/15/-12/31	50	872	50	4.7	43	209	0	0	11	0	11	26
23 L	2007	WT	12/14/-12/31	50	747	50	5.8	44	244	0	0	27	0	27	61
23 L	2008	WT	12/12/-12/31	85	905	85	7.1	76	321	0	0	51	0	51	67
24A	2004	MD	11/12/-11/21	125	527	125	18.6	111	447	43	0	0	0	43	39
24A	2005	MD	11/11/-11/20	175	551	175	26.9	164	716	61	0	0	0	61	37
24A	2006	MD	11/10/-11/19	175	547	175	24.5	162	664	51	0	0	0	51	31
24A	2007	MD	10/26/-11/04	140	435	140	25.5	138	660	63	0	0	0	63	46
24A	2008	MD	11/14/-11/20	165	440	165	33.0	156	591	52	0	0	0	52	33
24A	2004	WT	12/17/-12/31	375	995	375	27.5	356	1634	0	0	180	0	180	51
24A	2005	WT	12/16/-12/31	375	1019	375	28.9	354	1824	0	0	152	0	152	43
24A E	2006	WT	11/10/-11/19	550	359	550	73.3	526	2315	0	0	175	0	175	33
24A E	2007	WT	11/09/-11/18	600	381	600	79.8	566	2354	0	0	159	0	159	28
24A E	2008	WT	10/24/-10/30	355	188	355	97.3	331	1217	0	0	127	0	127	38
24A M	2008	WT	11/07/-11/13	355	219	355	91.8	323	1323	0	0	58	0	58	18
24A L	2006	WT	12/15/-12/31	50	533	50	8.1	50	266	0	0	25	0	25	50
24A L	2007	WT	12/14/-12/31	50	451	50	9.3	47	232	0	0	24	0	24	51
24A L	2008	WT	12/12/-12/31	30	309	30	7.1	28	92	0	0	21	0	21	75
24B	2004	MD	11/12/-11/21	350	601	350	38.4	324	1473	39	0	0	0	39	12
24B	2005	MD	11/11/-11/20	550	669	550	56.2	513	2242	90	0	0	0	90	18
24B	2006	MD	11/03/-11/12	450	642	449	42.7	390	1639	53	0	0	0	53	14
24B	2007	MD	11/02/-11/11	450	491	448	64.2	435	1883	59	0	0	0	59	14
24B	2008	MD	11/14/-11/20	450	452	450	68.1	427	1450	95	0	0	0	95	22
24B E	2006	WT	10/27/-11/01	250	100	250	100.0	231	806	0	0	54	0	54	23
24B E	2007	WT	10/26/-10/31	250	137	250	97.8	230	743	0	0	67	0	67	29
24B E	2008	WT	10/24/-10/30	305	140	305	98.6	287	918	0	0	62	0	62	22
24B M	2006	WT	11/10/-11/19	200	113	200	69.0	177	738	0	0	40	0	40	23
24B M	2007	WT	11/09/-11/18	250	110	250	97.3	224	943	0	0	54	0	54	24
24B M	2008	WT	11/07/-11/13	305	152	305	96.1	295	1109	0	0	91	0	91	31
24B L	2004	WT	12/17/-12/31	350	678	350	33.2	307	1260	0	0	151	0	151	49
24B L	2005	WT	12/16/-12/31	350	707	350	34.8	328	1482	0	0	137	0	137	42
24B L	2006	WT	12/15/-12/31	50	356	50	10.4	45	205	0	0	23	0	23	51
24B L	2007	WT	12/14/-12/31	50	379	50	9.2	50	269	0	0	27	0	27	54

AA = Any Antlered Deer **MD** = Mule Deer **WT** = Whitetail Deer **ALS** = Antlerless **CN** = Camp Navajo **FTHU** = Fort Huachuca,
C = CHAMP Hunt; in the unit column **E** = early or 1st season **M** = 2nd season **T** = 3rd season **L** = late or 4th season.

5-Year: 2004-2008 Harvest

Unit	Year	Hunt Type	Dates	Permits Authorized	1st Choice Applicants	Permits Issued	Draw Odds	Hunters	Hunter Days	Harvest					Hunt Success
										MD-Buck	MD-Aless	WT- Buck	WT-Aless	Total	
GENERAL															
24B L	2008	WT	12/12/-12/31	25	225	25	8.0	20	130	0	0	7	0	7	35
27 E	2004	MD	10/29/-11/07	800	2699	800	22.8	724	3486	157	0	0	0	157	22
27 E	2005	MD	10/28/-11/06	800	2345	800	25.4	743	3286	168	0	0	0	168	23
27 E	2006	MD	11/03/-11/12	725	2454	725	22.0	676	3107	132	0	0	0	132	20
27 E	2007	MD	11/02/-11/11	725	1914	725	30.4	653	2900	186	0	0	0	186	28
27 E	2008	MD	11/07/-11/13	900	1696	900	43.2	839	3509	203	0	0	0	203	24
27/28	2004	WT	12/17/-12/31	250	402	250	34.6	215	1095	0	0	80	0	80	37
27/28	2005	WT	12/16/-12/31	250	375	250	39.7	230	1235	0	0	41	0	41	18
27/28 E	2006	WT	11/10/-11/19	150	100	150	59.0	139	694	0	0	24	0	24	17
27/28 E	2007	WT	11/09/-11/18	150	103	150	64.1	131	681	0	0	36	0	36	27
27/28 E	2008	WT	10/24/-10/30	350	142	350	95.8	311	1309	0	0	72	0	72	23
27/28 L	2006	WT	12/15/-12/31	50	265	50	15.5	42	215	0	0	19	0	19	45
27/28 L	2007	WT	12/14/-12/31	50	192	50	17.7	46	246	0	0	15	0	15	33
27/28 L	2008	WT	12/12/-12/31	15	140	15	9.3	13	72	0	0	8	0	8	62
28 E	2004	AA	10/29/-11/01	400	450	400	50.4	385	1188	82	0	3	0	85	22
28 E	2005	AA	10/28/-10/31	400	432	400	55.1	367	1098	86	0	5	0	91	25
28 L	2004	AA	11/05/-11/14	400	513	400	54.4	376	1666	72	0	5	0	77	20
28 L	2005	AA	11/04/-11/13	400	408	400	61.8	371	1822	68	0	0	0	68	18
28 E	2006	MD	10/27/-11/01	400	439	399	54.7	381	1355	76	0	0	0	76	20
28 E	2007	MD	10/26/-10/31	400	355	400	73.2	385	1455	86	0	0	0	86	22
28 E	2008	MD	10/31/-11/06	400	451	400	63.0	385	1540	96	0	0	0	96	25
28 L	2006	MD	11/03/-11/12	400	336	400	62.2	380	2138	82	0	0	0	82	22
28 L	2007	MD	11/02/-11/11	400	282	400	78.7	371	1743	117	0	0	0	117	32
28 L	2008	MD	11/14/-11/20	400	228	400	95.2	373	1349	139	0	0	0	139	37
29 E	2004	MD	10/29/-11/01	200	176	200	86.9	180	580	27	0	0	0	27	15
29 E	2005	MD	10/28/-10/31	150	119	150	97.5	145	440	8	0	0	0	8	6
29 E	2006	MD	10/27/-11/01	125	83	125	96.4	100	382	7	0	0	0	7	7
29 E	2008	MD	10/31/-11/06	75	84	75	67.9	66	259	13	0	0	0	13	20
29 L	2004	MD	11/05/-11/14	200	177	200	78.5	161	772	28	0	0	0	28	17
29 L	2005	MD	11/04/-11/13	150	67	150	100.0	136	524	20	0	0	0	20	15
29 L	2006	MD	11/03/-11/12	125	62	125	98.4	106	465	14	0	0	0	14	13
29 L	2007	MD	11/02/-11/11	150	123	150	82.9	145	543	48	0	0	0	48	33
29 L	2008	MD	11/14/-11/20	75	43	75	100.0	68	265	18	0	0	0	18	26
29 T	2008	WT	11/28/-12/04	275	39	275	100.0	233	829	0	0	50	0	50	21
29 E	2004	WT	10/29/-11/01	350	191	350	95.8	322	946	0	0	104	0	104	32
29 E	2005	WT	10/28/-10/31	400	243	400	98.8	356	1069	0	0	62	0	62	17
29 E	2006	WT	10/27/-11/01	400	185	400	97.8	337	1142	0	0	49	0	49	15
29 E	2007	WT	10/26/-10/31	400	154	400	100.0	320	1054	0	0	66	0	66	21
29 E	2008	WT	10/24/-10/30	285	162	285	99.4	252	924	0	0	45	0	45	18
29 M	2004	WT	11/12/-11/21	550	284	550	100.0	509	2097	0	0	148	0	148	29
29 M	2005	WT	11/11/-11/20	500	297	500	95.6	452	2068	0	0	88	0	88	19
29 M	2006	WT	11/10/-11/19	450	229	449	95.2	387	1683	0	0	72	0	72	19
29 M	2007	WT	11/09/-11/18	400	186	400	96.2	374	1598	0	0	58	0	58	16
29 M	2008	WT	11/07/-11/13	275	89	275	100.0	247	848	0	0	75	0	75	30
29 L	2004	WT	12/17/-12/31	50	368	50	11.1	45	248	0	0	13	0	13	29
29 L	2005	WT	12/16/-12/31	50	323	50	12.7	50	284	0	0	12	0	12	24
29 L	2006	WT	12/15/-12/31	75	297	74	18.2	69	412	0	0	7	0	7	10
29 L	2007	WT	12/14/-12/31	75	284	75	21.8	67	292	0	0	30	0	30	45
29 L	2008	WT	12/12/-12/31	40	180	40	13.9	36	215	0	0	19	0	19	53
30A E	2004	MD	10/29/-11/01	300	368	300	70.1	272	730	61	0	0	0	61	22
30A E	2005	MD	10/28/-10/31	300	259	300	92.3	270	804	45	0	0	0	45	17
30A E	2006	MD	10/27/-11/01	300	277	300	80.1	287	882	55	0	0	0	55	19
30A E	2007	MD	10/26/-10/31	300	247	300	96.0	288	964	112	0	0	0	112	39
30A E	2008	MD	10/31/-11/06	325	389	325	71.0	305	1021	124	0	0	0	124	41
30A L	2004	MD	11/05/-11/14	350	387	350	76.2	318	1522	93	0	0	0	93	29
30A L	2005	MD	11/04/-11/13	350	292	350	94.9	334	1543	85	0	0	0	85	25
30A L	2006	MD	11/03/-11/12	350	298	349	96.3	320	1621	61	0	0	0	61	19
30A L	2007	MD	11/02/-11/11	300	211	300	95.3	287	1215	102	0	0	0	102	36
30A L	2008	MD	11/14/-11/20	325	150	325	92.7	301	1109	102	0	0	0	102	34
30A T	2008	WT	11/28/-12/04	175	16	168	100.0	135	496	0	0	57	0	57	42
30A E	2004	WT	10/29/-11/01	250	45	250	100.0	208	593	0	0	63	0	63	30

AA = Any Antlered Deer **MD** = Mule Deer **WT** = Whitetail Deer **ALS** = Antlerless **CN** = Camp Navajo **FTHU** = Fort Huachuca,
C = CHAMP Hunt; in the unit column **E** = early or 1st season **M** = 2nd season **T** = 3rd season **L** = late or 4th season.

5-Year: 2004-2008 Harvest

Unit	Year	Hunt Type	Dates	Permits Authorized	1st Choice Applicants	Permits Issued	Draw Odds	Hunters	Hunter Days	Harvest MD-Buck	MD-Aless	WT- Buck	WT-Aless	Total	Hunt Success
GENERAL															
30A E	2005	WT	10/28/-10/31	250	36	250	88.9	213	593	0	0	35	0	35	16
30A E	2006	WT	10/27/-11/01	250	28	250	100.0	203	618	0	0	54	0	54	27
30A E	2007	WT	10/26/-10/31	250	69	250	100.0	212	631	0	0	52	0	52	25
30A E	2008	WT	10/24/-10/30	200	70	200	78.6	167	538	0	0	38	0	38	23
30A M	2004	WT	11/12/-11/21	300	66	300	97.0	251	902	0	0	35	0	35	14
30A M	2005	WT	11/11/-11/20	300	51	300	100.0	254	994	0	0	80	0	80	31
30A M	2006	WT	11/10/-11/19	275	93	275	90.3	247	1000	0	0	54	0	54	22
30A M	2007	WT	11/09/-11/18	275	49	275	100.0	240	1002	0	0	52	0	52	22
30A M	2008	WT	11/07/-11/13	200	24	194	100.0	163	543	0	0	47	0	47	29
30A L	2004	WT	12/17/-12/31	50	216	50	13.9	46	217	0	0	13	0	13	28
30A L	2005	WT	12/16/-12/31	50	154	50	22.1	41	195	0	0	11	0	11	27
30A L	2006	WT	12/15/-12/31	75	192	75	24.5	71	357	0	0	16	0	16	23
30A L	2007	WT	12/14/-12/31	75	128	75	32.8	73	336	0	0	19	0	19	26
30A L	2008	WT	12/12/-12/31	40	149	40	20.8	38	181	0	0	17	0	17	45
30B E	2004	MD	10/29/-11/01	400	288	400	97.6	375	1106	53	0	0	0	53	14
30B E	2005	MD	10/28/-10/31	400	221	400	97.3	360	1133	88	0	0	0	88	24
30B E	2006	MD	10/27/-11/01	400	265	399	98.9	344	1160	65	0	0	0	65	19
30B E	2007	MD	10/26/-10/31	350	272	350	98.5	334	1226	68	0	0	0	68	20
30B E	2008	MD	10/31/-11/06	400	374	400	93.0	393	1577	93	0	0	0	93	24
30B L	2004	MD	11/05/-11/14	400	356	400	85.4	371	1802	95	0	0	0	95	26
30B L	2005	MD	11/04/-11/13	400	396	400	82.6	358	1784	61	0	0	0	61	17
30B L	2006	MD	11/03/-11/12	400	312	400	94.2	369	1985	61	0	0	0	61	17
30B L	2007	MD	11/02/-11/11	350	223	350	99.6	327	1725	101	0	0	0	101	31
30B L	2008	MD	11/14/-11/20	400	145	400	100.0	369	1469	92	0	0	0	92	25
30B T	2008	WT	11/28/-12/04	150	16	131	100.0	116	413	0	0	30	0	30	26
30B E	2004	WT	10/29/-11/01	250	39	250	100.0	211	645	0	0	42	0	42	20
30B E	2005	WT	10/28/-10/31	300	43	300	100.0	269	788	0	0	26	0	26	10
30B E	2006	WT	10/27/-11/01	250	33	250	100.0	211	583	0	0	51	0	51	24
30B E	2007	WT	10/26/-10/31	250	35	250	100.0	220	725	0	0	30	0	30	14
30B E	2008	WT	10/24/-10/30	150	36	150	94.4	117	384	0	0	23	0	23	20
30B M	2004	WT	11/12/-11/21	250	53	252	100.0	217	886	0	0	45	0	45	21
30B M	2005	WT	11/11/-11/20	300	91	300	90.1	257	1014	0	0	58	0	58	23
30B M	2006	WT	11/10/-11/19	250	38	249	89.5	226	941	0	0	50	0	50	22
30B M	2007	WT	11/09/-11/18	250	26	250	92.3	204	880	0	0	43	0	43	21
30B M	2008	WT	11/07/-11/13	150	25	118	96.0	111	475	0	0	30	0	30	27
30B L	2006	WT	12/15/-12/31	50	77	50	33.8	47	216	0	0	16	0	16	34
30B L	2007	WT	12/14/-12/31	50	115	50	27.0	48	179	0	0	21	0	21	44
30B L	2008	WT	12/12/-12/31	75	145	75	32.4	73	357	0	0	34	0	34	47
31 E	2004	MD	10/29/-11/01	100	221	100	37.6	90	223	40	0	0	0	40	44
31 E	2005	MD	10/28/-10/31	100	285	100	26.7	95	286	26	0	0	0	26	27
31 E	2006	MD	10/27/-11/01	100	302	100	27.5	83	248	13	0	0	0	13	16
31 E	2008	MD	10/31/-11/06	150	222	150	50.0	146	552	35	0	0	0	35	24
31 L	2004	MD	11/05/-11/14	200	258	200	52.3	179	839	37	0	0	0	37	21
31 L	2005	MD	11/04/-11/13	200	267	200	49.8	188	872	53	0	0	0	53	28
31 L	2006	MD	11/03/-11/12	200	278	200	47.5	174	818	76	0	0	0	76	44
31 L	2007	MD	11/02/-11/11	300	493	300	46.5	274	1282	64	0	0	0	64	23
31 L	2008	MD	11/14/-11/20	150	99	150	82.8	146	581	31	0	0	0	31	21
31 T	2008	WT	11/28/-12/04	300	90	300	96.7	256	1024	0	0	85	0	85	33
31 E	2004	WT	10/29/-11/01	450	338	450	84.0	392	1124	0	0	113	0	113	29
31 E	2005	WT	10/28/-10/31	450	291	451	75.9	394	1064	0	0	135	0	135	34
31 E	2006	WT	10/27/-11/01	450	319	450	80.3	395	1285	0	0	104	0	104	26
31 E	2007	WT	10/26/-10/31	550	319	550	97.8	497	1763	0	0	127	0	127	26
31 E	2008	WT	10/24/-10/30	325	298	325	74.5	290	1048	0	0	93	0	93	32
31 M	2004	WT	11/12/-11/21	550	416	550	65.9	493	2028	0	0	161	0	161	33
31 M	2005	WT	11/11/-11/20	550	344	550	78.2	506	2152	0	0	134	0	134	26
31 M	2006	WT	11/10/-11/19	550	380	550	69.2	509	2236	0	0	145	0	145	28
31 M	2007	WT	11/09/-11/18	550	290	546	87.9	506	2390	0	0	97	0	97	19
31 M	2008	WT	11/07/-11/13	325	112	326	97.3	295	1192	0	0	64	0	64	22
31 L	2004	WT	12/17/-12/31	50	563	50	8.5	45	168	0	0	32	0	32	71
31 L	2005	WT	12/16/-12/31	50	586	51	7.8	49	235	0	0	29	0	29	59
31 L	2006	WT	12/15/-12/31	100	696	100	12.4	91	511	0	0	43	0	43	47
31 L	2007	WT	12/14/-12/31	100	618	100	11.2	96	465	0	0	55	0	55	57

AA = Any Antlered Deer **MD** = Mule Deer **WT** = Whitetail Deer **ALS** = Antlerless **CN** = Camp Navajo **FTHU** = Fort Huachuca,
C = CHAMP Hunt; in the unit column **E** = early or 1st season **M** = 2nd season **T** = 3rd season **L** = late or 4th season.

5-Year: 2004–2008 Harvest

Unit	Year	Hunt Type	Dates	Permits Authorized	1st Choice Applicants	Permits Issued	Draw Odds	Hunters	Hunter Days	Harvest					Hunt Success
										MD-Buck	MD-Aless	WT- Buck	WT-Aless	Total	
**GENERAL**															
31 L	2008	WT	12/12/-12/31	125	644	125	15.1	120	688	0	0	75	0	75	63
32 E	2004	MD	10/29/-11/01	375	1120	375	28.1	350	1020	112	0	0	0	112	32
32 E	2005	MD	10/28/-10/31	350	1063	350	29.5	329	911	116	0	0	0	116	35
32 E	2006	MD	10/27/-11/01	450	1077	450	36.4	421	1304	108	0	0	0	108	26
32 E	2007	MD	10/26/-10/31	200	667	199	27.3	187	592	68	0	0	0	68	36
32 E	2008	MD	10/31/-11/06	450	956	450	44.2	422	1432	142	0	0	0	142	34
32 L	2004	MD	11/05/-11/14	575	985	575	44.5	535	2236	147	0	0	0	147	27
32 L	2005	MD	11/04/-11/13	600	838	600	47.0	565	2452	149	0	0	0	149	26
32 L	2006	MD	11/03/-11/12	450	767	449	42.5	428	1872	94	0	0	0	94	22
32 L	2007	MD	11/02/-11/11	600	692	596	56.1	553	2251	165	0	0	0	165	30
32 L	2008	MD	11/14/-11/20	450	355	450	67.9	431	1708	100	0	0	0	100	23
32 T	2008	WT	11/28/-12/04	460	161	460	95.0	410	1513	0	0	117	0	117	29
32 E	2004	WT	10/29/-11/01	650	383	650	82.8	614	1851	0	0	251	0	251	41
32 E	2005	WT	10/28/-10/31	650	450	650	80.4	590	1790	0	0	198	0	198	34
32 E	2006	WT	10/27/-11/01	650	501	649	71.5	597	1999	0	0	202	0	202	34
32 E	2007	WT	10/26/-10/31	650	428	649	92.5	597	1999	0	0	186	0	186	31
32 E	2008	WT	10/24/-10/30	500	387	500	81.9	449	1508	0	0	156	0	156	35
32 M	2004	WT	11/12/-11/21	800	576	800	66.8	732	3059	0	0	280	0	280	38
32 M	2005	WT	11/11/-11/20	800	615	800	68.5	717	2757	0	0	228	0	228	32
32 M	2006	WT	11/10/-11/19	650	463	650	64.1	560	2341	0	0	169	0	169	30
32 M	2007	WT	11/09/-11/18	650	406	648	77.6	593	2631	0	0	156	0	156	26
32 M	2008	WT	11/07/-11/13	500	260	500	98.1	432	1626	0	0	123	0	123	28
32 L	2004	WT	12/17/-12/31	100	1053	100	7.2	91	366	0	0	64	0	64	70
32 L	2005	WT	12/16/-12/31	100	830	100	9.2	98	468	0	0	49	0	49	50
32 L	2006	WT	12/15/-12/31	200	1144	200	12.6	178	901	0	0	81	0	81	46
32 L	2007	WT	12/14/-12/31	175	880	175	14.0	162	768	0	0	90	0	90	56
32 L	2008	WT	12/12/-12/31	40	541	40	5.7	40	169	0	0	29	0	29	73
33 E	2004	MD	10/29/-11/01	350	395	350	64.3	331	1013	77	0	0	0	77	23
33 E	2005	MD	10/28/-10/31	400	445	400	65.6	364	1169	56	0	0	0	56	15
33 E	2006	MD	10/27/-11/01	350	418	349	62.9	323	1084	57	0	0	0	57	18
33 E	2007	MD	10/26/-10/31	350	319	350	72.7	325	1077	46	0	0	0	46	14
33 E	2008	MD	10/31/-11/06	350	341	350	70.1	319	1120	39	0	0	0	39	12
33 L	2004	MD	11/05/-11/14	250	220	250	72.3	226	1002	55	0	0	0	55	24
33 L	2005	MD	11/04/-11/13	300	296	300	68.2	276	1116	46	0	0	0	46	17
33 L	2006	MD	11/03/-11/12	250	230	249	63.9	226	1001	33	0	0	0	33	15
33 L	2007	MD	11/02/-11/11	250	161	250	77.6	224	937	26	0	0	0	26	12
33 L	2008	MD	11/14/-11/20	250	101	250	100.0	241	945	23	0	0	0	23	10
33 T	2008	WT	11/28/-12/04	650	400	650	77.5	621	2204	0	0	208	0	208	33
33 E	2004	WT	10/29/-11/01	700	442	700	82.8	623	1693	0	0	275	0	275	44
33 E	2005	WT	10/28/-10/31	750	599	750	68.1	699	1898	0	0	281	0	281	40
33 E	2006	WT	10/27/-11/01	725	837	725	55.1	678	2112	0	0	269	0	269	40
33 E	2007	WT	10/26/-10/31	750	786	743	61.1	692	2244	0	0	265	0	265	38
33 E	2008	WT	10/24/-10/30	650	956	650	49.4	614	2113	0	0	272	0	272	44
33 M	2004	WT	11/12/-11/21	700	552	700	56.0	654	2568	0	0	276	0	276	42
33 M	2005	WT	11/11/-11/20	750	677	750	49.6	707	2719	0	0	316	0	316	45
33 M	2006	WT	11/10/-11/19	725	837	723	47.0	667	2847	0	0	244	0	244	37
33 M	2007	WT	11/09/-11/18	750	799	750	49.7	718	2955	0	0	260	0	260	36
33 M	2008	WT	11/07/-11/13	650	452	650	73.5	612	2231	0	0	223	0	223	36
33 L	2004	WT	12/17/-12/31	100	1385	100	6.4	91	336	0	0	58	0	58	64
33 L	2005	WT	12/16/-12/31	100	1375	100	6.1	96	416	0	0	47	0	47	49
33 L	2006	WT	12/15/-12/31	150	1499	150	8.5	142	717	0	0	80	0	80	56
33 L	2007	WT	12/14/-12/31	150	1464	149	9.2	143	637	0	0	95	0	95	66
33 L	2008	WT	12/12/-12/31	40	1217	40	3.3	40	243	0	0	30	0	30	75
34A E	2004	MD	10/29/-11/01	75	315	75	21.6	64	208	19	0	0	0	19	30
34A E	2005	MD	10/28/-10/31	75	320	75	20.9	75	233	5	0	0	0	5	7
34A E	2006	MD	10/27/-11/01	75	284	75	24.3	75	280	16	0	0	0	16	21
34A E	2007	MD	10/26/-10/31	25	168	25	14.3	20	83	3	0	0	0	3	15
34A E	2008	MD	10/31/-11/06	25	153	25	15.0	20	102	2	0	0	0	2	10
34A T	2008	WT	11/28/-12/04	550	161	550	100.0	515	2047	0	0	125	0	125	24
34A E	2004	WT	10/29/-11/01	950	530	950	98.1	872	2511	0	0	209	0	209	24
34A E	2005	WT	10/28/-10/31	900	630	900	81.1	851	2447	0	0	163	0	163	19
34A E	2006	WT	10/27/-11/01	750	459	750	97.8	689	2276	0	0	147	0	147	21

AA = Any Antlered Deer **MD** = Mule Deer **WT** = Whitetail Deer **ALS** = Antlerless **CN** = Camp Navajo **FTHU** = Fort Huachuca,
C = CHAMP Hunt; in the unit column **E** = early or 1st season **M** = 2nd season **T** = 3rd season **L** = late or 4th season.

5-Year: 2004-2008 Harvest

Unit	Year	Hunt Type	Dates	Permits Authorized	1st Choice Applicants	Permits Issued	Draw Odds	Hunters	Hunter Days	Harvest					Hunt Success
										MD-Buck	MD-Aless	WT- Buck	WT-Aless	Total	
**GENERAL**															
34A E	2007	WT	10/26/-10/31	800	438	800	97.7	713	2287	0	0	147	0	147	21
34A E	2008	WT	10/24/-10/30	600	428	600	93.2	537	1834	0	0	159	0	159	30
34A M	2004	WT	11/12/-11/21	950	917	950	67.6	887	3756	0	0	148	0	148	17
34A M	2005	WT	11/11/-11/20	900	853	900	73.0	841	3647	0	0	156	0	156	19
34A M	2006	WT	11/10/-11/19	750	661	749	71.7	703	2991	0	0	126	0	126	18
34A M	2007	WT	11/09/-11/18	750	514	750	88.1	705	3040	0	0	117	0	117	17
34A M	2008	WT	11/07/-11/13	600	299	600	98.0	550	2022	0	0	145	0	145	26
34A L	2004	WT	12/17/-12/31	25	635	25	3.5	25	108	0	0	10	0	10	40
34A L	2005	WT	12/16/-12/31	25	533	25	3.9	23	154	0	0	10	0	10	43
34A L	2006	WT	12/15/-12/31	150	700	150	16.7	132	676	0	0	47	0	47	36
34A L	2007	WT	12/14/-12/31	150	590	150	20.3	146	847	0	0	72	0	72	49
34A L	2008	WT	12/12/-12/31	40	513	40	6.6	35	170	0	0	14	0	14	40
34B E	2004	MD	10/29/-11/01	100	124	100	50.8	94	287	19	0	0	0	19	20
34B E	2005	MD	10/28/-10/31	100	181	100	45.3	96	291	13	0	0	0	13	14
34B E	2006	MD	10/27/-11/01	100	138	98	55.1	87	288	19	0	0	0	19	22
34B E	2007	MD	10/26/-10/31	100	139	100	57.6	100	339	16	0	0	0	16	16
34B E	2008	MD	10/31/-11/06	100	162	100	51.9	96	327	23	0	0	0	23	24
34B L	2004	MD	11/05/-11/14	100	233	100	34.8	94	383	17	0	0	0	17	18
34B L	2005	MD	11/04/-11/13	100	131	100	59.5	90	388	14	0	0	0	14	16
34B L	2006	MD	11/03/-11/12	100	139	100	44.6	89	369	31	0	0	0	31	35
34B L	2007	MD	11/02/-11/11	100	136	100	58.1	93	347	40	0	0	0	40	43
34B L	2008	MD	11/14/-11/20	100	108	100	76.9	96	369	20	0	0	0	20	21
34B T	2008	WT	11/28/-12/04	100	34	100	100.0	92	355	0	0	12	0	12	13
34B E	2004	WT	10/29/-11/01	275	92	275	100.0	244	695	0	0	58	0	58	24
34B E	2005	WT	10/28/-10/31	275	64	275	100.0	228	689	0	0	32	0	32	14
34B E	2006	WT	10/27/-11/01	175	47	175	100.0	158	512	0	0	24	0	24	15
34B E	2007	WT	10/26/-10/31	175	43	175	100.0	155	509	0	0	36	0	36	23
34B E	2008	WT	10/24/-10/30	150	57	150	100.0	138	514	0	0	34	0	34	25
34B M	2004	WT	11/12/-11/21	275	96	275	97.9	251	965	0	0	28	0	28	11
34B M	2005	WT	11/11/-11/20	275	104	275	95.2	245	1036	0	0	21	0	21	9
34B M	2006	WT	11/10/-11/19	200	67	200	100.0	190	824	0	0	15	0	15	8
34B M	2007	WT	11/09/-11/18	200	56	200	100.0	188	871	0	0	32	0	32	17
34B M	2008	WT	11/07/-11/13	150	53	150	92.5	119	440	0	0	17	0	17	14
34B L	2004	WT	12/17/-12/31	25	198	25	8.1	25	143	0	0	8	0	8	32
34B L	2005	WT	12/16/-12/31	25	147	25	12.2	21	102	0	0	6	0	6	29
34B L	2006	WT	12/15/-12/31	50	162	50	18.5	45	223	0	0	23	0	23	51
34B L	2007	WT	12/14/-12/31	50	156	50	19.9	48	226	0	0	28	0	28	58
34B L	2008	WT	12/12/-12/31	40	137	40	19.0	34	198	0	0	16	0	16	47
35A T	2008	WT	11/28/-12/04	200	48	200	95.8	187	778	0	0	52	0	52	28
35A E	2004	WT	10/29/-11/01	300	111	300	100.0	245	721	0	0	53	0	53	22
35A E	2005	WT	10/28/-10/31	300	125	300	100.0	261	762	0	0	35	0	35	13
35A E	2006	WT	10/27/-11/01	300	106	300	100.0	255	864	0	0	36	0	36	14
35A E	2007	WT	10/26/-10/31	400	90	400	100.0	358	1274	0	0	80	0	80	22
35A E	2008	WT	10/24/-10/30	300	119	300	89.1	275	1064	0	0	94	0	94	34
35A M	2004	WT	11/12/-11/21	250	159	250	81.1	222	953	0	0	36	0	36	16
35A M	2005	WT	11/11/-11/20	250	143	250	91.6	220	950	0	0	34	0	34	15
35A M	2006	WT	11/10/-11/19	250	101	250	95.0	230	1018	0	0	47	0	47	20
35A M	2007	WT	11/09/-11/18	300	141	300	95.7	278	1277	0	0	53	0	53	19
35A M	2008	WT	11/07/-11/13	300	81	300	100.0	281	1067	0	0	33	0	33	12
35A L	2004	WT	12/17/-12/31	50	273	50	13.9	48	231	0	0	23	0	23	48
35A L	2005	WT	12/16/-12/31	50	284	50	14.1	40	194	0	0	13	0	13	33
35A L	2006	WT	12/15/-12/31	50	214	50	14.0	50	256	0	0	15	0	15	30
35A L	2007	WT	12/14/-12/31	50	203	50	23.2	48	253	0	0	28	0	28	58
35A L	2008	WT	12/12/-12/31	40	271	40	12.9	40	184	0	0	18	0	18	45
35B T	2008	WT	11/28/-12/04	250	53	251	98.1	218	774	0	0	69	0	69	32
35B E	2004	WT	10/29/-11/01	400	151	400	100.0	372	1129	0	0	69	0	69	19
35B E	2005	WT	10/28/-10/31	400	168	400	100.0	358	1056	0	0	47	0	47	13
35B E	2006	WT	10/27/-11/01	400	178	400	100.0	362	1136	0	0	67	0	67	19
35B E	2007	WT	10/26/-10/31	500	165	500	100.0	447	1407	0	0	104	0	104	23
35B E	2008	WT	10/24/-10/30	400	203	400	100.0	351	1259	0	0	110	0	110	31
35B M	2004	WT	11/12/-11/21	400	284	400	83.8	365	1423	0	0	58	0	58	16
35B M	2005	WT	11/11/-11/20	400	281	400	85.1	378	1525	0	0	63	0	63	17

AA = Any Antlered Deer **MD** = Mule Deer **WT** = Whitetail Deer **ALS** = Antlerless **CN** = Camp Navajo **FTHU** = Fort Huachuca,
C = CHAMP Hunt; in the unit column **E** = early or 1st season **M** = 2nd season **T** = 3rd season **L** = late or 4th season.

Deer Hunt Data

5-Year: 2004-2008 Harvest

Unit	Year	Hunt Type	Dates	Permits Authorized	1st Choice Applicants	Permits Issued	Draw Odds	Hunters	Hunter Days	Harvest					Hunt Success
										MD-Buck	MD-Aless	WT- Buck	WT-Aless	Total	
**GENERAL**															
35B M	2006	WT	11/10/-11/19	400	312	398	85.9	356	1526	0	0	59	0	59	17
35B M	2007	WT	11/09/-11/18	450	237	450	99.6	418	1694	0	0	82	0	82	20
35B M	2008	WT	11/07/-11/13	400	185	400	97.8	367	1427	0	0	83	0	83	23
35B L	2004	WT	12/17/-12/31	50	272	50	16.2	42	197	0	0	13	0	13	31
35B L	2005	WT	12/16/-12/31	50	236	50	17.4	48	188	0	0	25	0	25	52
35B L	2006	WT	12/15/-12/31	50	261	46	13.0	46	260	0	0	14	0	14	30
35B L	2007	WT	12/14/-12/31	50	239	50	18.0	43	198	0	0	15	0	15	35
35B L	2008	WT	12/12/-12/31	40	206	40	15.5	40	170	0	0	15	0	15	38
36A E	2004	MD	10/29/-11/01	300	541	300	45.7	279	819	92	0	0	0	92	33
36A E	2005	MD	10/28/-10/31	350	628	350	47.0	332	999	69	0	0	0	69	21
36A E	2006	MD	10/27/-11/01	350	579	350	52.2	324	1101	82	0	0	0	82	25
36A E	2007	MD	10/26/-10/31	350	466	350	68.0	325	1124	58	0	0	0	58	18
36A E	2008	MD	10/31/-11/06	350	513	350	57.5	331	1267	71	0	0	0	71	21
36A L	2004	MD	11/05/-11/14	400	683	400	43.2	379	1643	118	0	0	0	118	31
36A L	2005	MD	11/04/-11/13	450	633	450	54.3	418	1872	109	0	0	0	109	26
36A L	2006	MD	11/03/-11/12	450	620	450	58.9	432	1842	105	0	0	0	105	24
36A L	2007	MD	11/02/-11/11	450	459	450	74.1	406	1963	69	0	0	0	69	17
36A L	2008	MD	11/14/-11/20	450	324	450	93.2	429	1614	83	0	0	0	83	19
36A T	2008	WT	11/28/-12/04	350	41	336	92.7	307	1066	0	0	101	0	101	33
36A E	2004	WT	10/29/-11/01	400	191	400	99.5	345	1032	0	0	78	0	78	23
36A E	2005	WT	10/28/-10/31	400	207	400	99.5	367	1021	0	0	77	0	77	21
36A E	2006	WT	10/27/-11/01	400	180	400	100.0	370	1150	0	0	82	0	82	22
36A E	2007	WT	10/26/-10/31	450	140	446	100.0	404	1291	0	0	120	0	120	30
36A E	2008	WT	10/24/-10/30	350	156	350	94.9	333	1071	0	0	88	0	88	26
36A M	2004	WT	11/12/-11/21	550	237	550	100.0	511	2107	0	0	123	0	123	24
36A M	2005	WT	11/11/-11/20	550	246	550	99.6	520	2022	0	0	141	0	141	27
36A M	2006	WT	11/10/-11/19	550	268	550	100.0	500	1953	0	0	137	0	137	27
36A M	2007	WT	11/09/-11/18	550	215	550	100.0	499	2268	0	0	72	0	72	14
36A M	2008	WT	11/07/-11/13	450	86	436	100.0	409	1348	0	0	80	0	80	20
36A L	2004	WT	12/17/-12/31	50	380	50	10.5	40	228	0	0	21	0	21	53
36A L	2005	WT	12/16/-12/31	50	297	50	11.4	50	245	0	0	15	0	15	30
36A L	2006	WT	12/15/-12/31	100	238	100	26.1	96	617	0	0	38	0	38	40
36A L	2007	WT	12/14/-12/31	100	242	100	27.3	91	441	0	0	35	0	35	38
36A L	2008	WT	12/12/-12/31	40	212	40	10.4	37	265	0	0	15	0	15	41
36B E	2004	MD	10/29/-11/01	100	307	100	27.7	93	265	48	0	0	0	48	52
36B E	2005	MD	10/28/-10/31	125	310	125	31.3	115	341	55	0	0	0	55	48
36B E	2006	MD	10/27/-11/01	150	323	147	33.7	138	405	43	0	0	0	43	31
36B E	2007	MD	10/26/-10/31	175	242	175	62.8	167	574	51	0	0	0	51	31
36B E	2008	MD	10/31/-11/06	200	304	200	56.9	188	658	77	0	0	0	77	41
36B L	2004	MD	11/05/-11/14	100	280	100	30.4	98	404	42	0	0	0	42	43
36B L	2005	MD	11/04/-11/13	125	238	125	45.0	118	484	31	0	0	0	31	26
36B L	2006	MD	11/03/-11/12	150	167	150	55.1	147	631	58	0	0	0	58	39
36B L	2007	MD	11/02/-11/11	225	214	225	75.7	223	910	40	0	0	0	40	18
36B L	2008	MD	11/14/-11/20	200	102	200	94.1	179	710	41	0	0	0	41	23
36B T	2008	WT	11/28/-12/04	700	129	700	96.9	663	2481	0	0	153	0	153	23
36B E	2004	WT	10/29/-11/01	1000	483	1000	100.0	895	2559	0	0	210	0	210	23
36B E	2005	WT	10/28/-10/31	1000	567	1000	99.3	926	2713	0	0	229	0	229	25
36B E	2006	WT	10/27/-11/01	1000	598	1000	99.8	895	2946	0	0	202	0	202	23
36B E	2007	WT	10/26/-10/31	1025	477	1025	98.1	934	3236	0	0	211	0	211	23
36B E	2008	WT	10/24/-10/30	800	449	800	87.3	743	2745	0	0	201	0	201	27
36B M	2004	WT	11/12/-11/21	1000	783	1000	83.0	923	3988	0	0	190	0	190	21
36B M	2005	WT	11/11/-11/20	1000	674	1000	93.0	906	3858	0	0	268	0	268	30
36B M	2006	WT	11/10/-11/19	1000	644	998	93.6	925	3829	0	0	208	0	208	22
36B M	2007	WT	11/09/-11/18	1025	571	1025	98.2	946	4259	0	0	159	0	159	17
36B M	2008	WT	11/07/-11/13	800	314	800	97.1	735	2733	0	0	171	0	171	23
36B L	2004	WT	12/17/-12/31	150	995	150	12.3	138	581	0	0	50	0	50	36
36B L	2005	WT	12/16/-12/31	150	828	150	14.6	141	735	0	0	63	0	63	45
36B L	2006	WT	12/15/-12/31	200	796	200	17.3	198	1080	0	0	99	0	99	50
36B L	2007	WT	12/14/-12/31	200	634	200	24.4	184	930	0	0	98	0	98	53
36B L	2008	WT	12/12/-12/31	40	473	40	6.1	34	201	0	0	25	0	25	74
36C E	2004	MD	10/29/-11/01	100	143	100	52.4	98	276	28	0	0	0	28	29
36C E	2005	MD	10/28/-10/31	125	216	125	47.7	120	340	35	0	0	0	35	29

AA = Any Antlered Deer **MD** = Mule Deer **WT** = Whitetail Deer **ALS** = Antlerless **CN** = Camp Navajo **FTHU** = Fort Huachuca, **C** = CHAMP Hunt; in the unit column **E** = early or 1st season **M** = 2nd season **T** = 3rd season **L** = late or 4th season.

5-Year: 2004-2008 Harvest

Unit	Year	Hunt Type	Dates	Permits Authorized	1st Choice Applicants	Permits Issued	Draw Odds	Hunters	Hunter Days	Harvest					Hunt Success
										MD-Buck	MD-Aless	WT- Buck	WT-Aless	Total	
GENERAL															
36C E	2006	MD	10/27/-11/01	100	224	100	39.3	96	372	28	0	0	0	28	29
36C E	2007	MD	10/26/-10/31	100	180	100	48.3	100	356	44	0	0	0	44	44
36C E	2008	MD	10/31/-11/06	125	213	125	50.2	121	489	24	0	0	0	24	20
36C L	2004	MD	11/05/-11/14	100	179	100	40.8	100	420	50	0	0	0	50	50
36C L	2005	MD	11/04/-11/13	125	235	125	40.0	118	588	46	0	0	0	46	39
36C L	2006	MD	11/03/-11/12	100	198	100	40.9	98	444	26	0	0	0	26	27
36C L	2007	MD	11/02/-11/11	100	120	100	58.3	90	431	35	0	0	0	35	39
36C L	2008	MD	11/14/-11/20	125	109	125	81.7	115	461	25	0	0	0	25	22
36C T	2008	WT	11/28/-12/04	150	42	150	100.0	136	608	0	0	34	0	34	25
36C E	2004	WT	10/29/-11/01	300	102	300	100.0	261	718	0	0	83	0	83	32
36C E	2005	WT	10/28/-10/31	325	178	325	96.6	276	769	0	0	121	0	121	44
36C E	2006	WT	10/27/-11/01	325	247	321	82.6	297	906	0	0	90	0	90	30
36C E	2007	WT	10/26/-10/31	350	154	350	100.0	328	1109	0	0	91	0	91	28
36C E	2008	WT	10/24/-10/30	200	102	200	91.2	188	613	0	0	53	0	53	28
36C M	2004	WT	11/12/-11/21	300	170	300	78.2	264	1121	0	0	74	0	74	28
36C M	2005	WT	11/11/-11/20	325	208	325	82.2	289	1114	0	0	89	0	89	31
36C M	2006	WT	11/10/-11/19	325	181	325	86.7	300	1105	0	0	110	0	110	37
36C M	2007	WT	11/09/-11/18	350	180	350	93.3	318	1291	0	0	47	0	47	15
36C M	2008	WT	11/07/-11/13	200	59	202	100.0	155	542	0	0	47	0	47	30
36C L	2004	WT	12/17/-12/31	75	517	75	10.8	62	298	0	0	41	0	41	66
36C L	2005	WT	12/16/-12/31	75	462	75	11.3	73	434	0	0	41	0	41	56
36C L	2006	WT	12/15/-12/31	75	462	75	13.0	72	387	0	0	26	0	26	36
36C L	2007	WT	12/14/-12/31	75	330	75	18.8	68	307	0	0	36	0	36	53
36C L	2008	WT	12/12/-12/31	100	415	100	17.1	100	609	0	0	62	0	62	62
37A E	2008	MD	10/31/-11/06	75	120	75	52.5	65	272	12	0	0	0	12	18
37A L	2004	MD	11/05/-11/14	200	247	200	57.5	190	905	28	0	0	0	28	15
37A L	2005	MD	11/04/-11/13	200	247	200	53.4	181	734	11	0	0	0	11	6
37A L	2006	MD	11/10/-11/19	200	283	197	51.2	179	866	21	0	0	0	21	12
37A L	2007	MD	11/09/-11/18	150	212	150	57.1	143	707	7	0	0	0	7	5
37A L	2008	MD	11/14/-11/20	75	103	75	64.1	63	247	6	0	0	0	6	10
37B E	2008	AA	10/31/-11/06	250	466	250	46.4	243	924	52	0	2	0	54	22
37B L	2004	AA	11/05/-11/14	500	1004	500	41.9	471	2029	75	0	0	0	75	16
37B L	2005	AA	11/04/-11/13	500	967	500	45.0	467	2090	83	0	0	0	83	18
37B L	2006	AA	11/10/-11/19	500	1113	500	36.7	463	2019	44	0	2	0	46	10
37B L	2007	AA	11/09/-11/18	500	876	500	49.7	476	2213	87	0	2	0	89	19
37B L	2008	AA	11/14/-11/20	250	310	250	58.4	243	920	27	0	3	0	30	12
39/40	2004	AA	11/05/-11/14	175	351	175	44.4	159	656	18	0	0	0	18	11
39/40	2005	AA	11/04/-11/13	250	450	250	48.9	220	916	37	0	0	0	37	17
39/40	2006	AA	11/10/-11/19	300	493	300	50.1	280	1196	50	0	0	0	50	18
39/40	2007	AA	11/09/-11/18	300	406	300	60.3	270	1099	95	0	0	0	95	35
39/40 E	2008	AA	10/31/-11/06	150	320	150	39.7	138	499	23	0	0	0	23	17
39/40 L	2008	AA	11/07/-11/13	150	134	150	55.2	135	506	44	0	0	0	44	33
41	2004	AA	11/05/-11/14	400	864	400	41.6	357	1607	25	0	0	0	25	7
41	2005	AA	11/04/-11/13	400	767	400	46.2	374	1631	40	0	0	0	40	11
41	2006	AA	11/10/-11/19	400	690	400	49.9	383	1510	105	0	0	0	105	27
41	2007	AA	11/09/-11/18	475	648	475	58.2	429	2002	138	0	0	0	138	32
41	2008	AA	11/07/-11/16	475	630	475	62.5	445	1993	60	0	0	0	60	13
42	2004	AA	11/05/-11/14	200	368	200	41.3	189	831	27	0	0	0	27	14
42	2005	AA	11/04/-11/13	225	368	225	44.0	201	931	14	0	0	0	14	7
42	2006	AA	11/10/-11/19	300	333	300	63.4	278	1383	56	0	0	0	56	20
42	2007	AA	11/02/-11/11	300	394	300	58.9	280	1231	90	0	0	0	90	32
42	2008	AA	11/07/-11/16	300	318	300	65.7	271	1150	48	0	0	0	48	18
43/44	2004	AA	11/05/-11/14	500	834	500	47.1	451	2082	38	0	0	0	38	8
43/44	2005	AA	11/04/-11/13	500	797	500	52.8	458	2176	46	0	0	0	46	10
43/44	2006	AA	11/10/-11/19	650	676	650	74.3	579	2667	105	0	0	0	105	18
43/44	2007	AA	11/09/-11/18	650	600	650	80.7	606	2773	158	0	0	0	158	26
43/44	2008	AA	11/07/-11/16	650	606	650	84.2	606	3096	147	0	0	0	147	24
45A/45B/45C	2004	AA	11/05/-11/14	275	339	275	61.4	228	996	25	0	0	0	25	11
45A/45B/45C	2005	AA	11/04/-11/13	225	353	225	56.4	206	872	21	0	0	0	21	10
45A/45B/45C	2006	AA	11/10/-11/19	275	287	274	75.3	230	969	46	0	0	0	46	20
45A/45B/45C	2007	AA	11/09/-11/18	275	253	275	89.7	249	1126	54	0	0	0	54	22
45A/45B/45C	2008	AA	11/07/-11/16	275	257	275	82.9	249	1062	32	0	0	0	32	13

AA = Any Antlered Deer **MD** = Mule Deer **WT** = Whitetail Deer **ALS** = Antlerless **CN** = Camp Navajo **FTHU** = Fort Huachuca,
C = CHAMP Hunt; in the unit column **E** = early or 1st season **M** = 2nd season **T** = 3rd season **L** = late or 4th season.

5-Year: 2004-2008 Harvest

Unit	Year	Hunt Type	Dates	Permits Authorized	1st Choice Applicants	Permits Issued	Draw Odds	Hunters	Hunter Days	Harvest					Hunt Success
										MD-Buck	MD-Aless	WT- Buck	WT-Aless	Total	
**GENERAL**															
FTHU	2004	AM	11/05/-11/16	10	29	10	34.5	9	29	6	0	0	0	6	67
FTHU	2005	AM	11/04/-11/15	10	38	10	26.3	10	41	5	0	0	0	5	50
FTHU	2006	AM	11/17/-11/25	10	22	10	45.5	10	38	7	0	0	0	7	70
FTHU	2007	AM	11/16/-11/25	10	30	10	30.0	8	20	8	0	0	0	8	100
FTHU	2008	AM	11/01/-12/31	10	35	10	25.7	8	47	3	0	0	0	3	38
FTHU	2004	AW	11/05/-11/16	85	36	85	100.0	76	437	0	0	16	0	16	21
FTHU	2004	AW	12/17/-12/30	120	92	120	97.8	98	509	0	0	54	0	54	55
FTHU	2005	AW	11/04/-11/15	75	47	75	100.0	64	430	0	0	13	0	13	20
FTHU	2005	AW	12/16/-12/30	124	102	124	98.0	102	564	0	0	36	0	36	35
FTHU	2006	AW	11/17/-11/25	75	27	68	100.0	65	283	0	0	22	0	22	34
FTHU	2006	AW	12/15/-12/30	125	97	125	90.7	106	603	0	0	48	0	48	45
FTHU	2007	AW	11/16/-11/25	70	28	67	100.0	60	255	0	0	23	0	23	38
FTHU	2007	AW	12/14/-12/31	130	105	130	99.0	127	741	0	0	72	0	72	57
FTHU	2008	AW	11/01/-12/31	70	34	65	100.0	63	241	0	0	27	0	27	43
FTHU	2008	AW	11/01/-12/31	130	112	140	98.2	130	601	0	0	85	0	85	65
FTHU CHAMP	2004	DD	11/07/-11/18	10	11	10	90.9	9	17	3	3	3	0	9	100
FTHU CHAMP	2005	DD	11/04/-11/15	10	19	10	52.6	9	17	1	1	1	1	4	44
FTHU CHAMP	2006	DD	11/17/-11/25	10	11	10	90.9	7	15	0	0	0	5	5	71
FTHU CHAMP	2007	DD	11/16/-11/25	10	11	10	90.9	10	28	2	0	7	0	9	90
**JUNIORS ONLY**															
10	2004	AA	10/22/-10/25	20	167	20	10.8	17	57	3	0	0	0	3	18
10	2005	AA	10/21/-10/24	20	112	20	17.9	20	60	3	0	0	0	3	15
10	2006	AA	10/06/-10/15	100	252	98	32.5	98	394	32	0	0	0	32	33
10	2007	AA	10/05/-10/14	100	191	100	39.8	95	376	43	0	0	0	43	45
10	2008	AA	10/10/-10/19	100	186	100	48.4	96	398	28	0	0	0	28	29
12AW	2004	ALS	10/22/-10/25	500	931	500	45.0	437	1059	0	225	0	0	225	51
12AW	2005	ALS	11/11/-11/14	750	755	751	80.9	653	1655	0	269	0	0	269	41
12AW	2006	ALS	11/10/-11/16	100	424	99	19.8	79	196	0	64	0	0	64	81
12AW	2007	ALS	11/09/-11/12	400	422	400	83.6	363	828	0	269	0	0	269	74
12AW	2008	ALS	11/14/-11/17	600	438	599	95.9	520	1240	0	364	0	0	364	70
16A	2006	AA	10/06/-10/15	20	28	20	53.6	18	85	7	0	0	0	7	39
16A	2007	AA	10/05/-10/14	20	33	20	45.5	18	58	4	0	0	0	4	22
16A	2008	AA	10/10/-10/19	20	24	20	70.8	20	76	13	0	0	0	13	65
17B	2004	AA	10/08/-10/11	10	39	10	15.4	10	33	3	0	0	0	3	30
17B	2005	AA	10/07/-10/10	10	36	10	22.2	10	23	7	0	0	0	7	70
17B	2006	AA	10/06/-10/15	60	104	60	38.5	52	170	16	0	0	0	16	31
17B	2007	AA	10/05/-10/14	60	63	60	61.9	57	242	17	0	0	0	17	30
17B	2008	AA	10/10/-10/19	60	49	60	79.6	55	231	5	0	0	0	5	9
18B	2004	AA	12/17/-12/31	10	218	10	3.7	10	33	8	0	0	0	8	80
18B	2005	AA	12/16/-12/31	10	196	10	4.6	10	27	7	0	0	0	7	70
18B	2006	AA	11/17/-11/26	50	95	50	36.8	43	155	17	0	0	0	17	40
18B	2007	AA	11/16/-11/25	50	69	50	53.6	50	220	11	0	0	0	11	22
18B	2008	AA	11/21/-11/30	75	62	75	77.4	66	239	23	0	0	0	23	35
20A	2004	AA	10/08/-10/11	10	69	10	11.6	10	27	5	0	0	0	5	50
20A	2005	AA	10/07/-10/10	10	75	10	10.7	10	24	4	0	0	0	4	40
20A	2006	AA	10/06/-10/15	30	86	30	30.2	30	79	17	0	0	0	17	57
20A	2007	AA	10/05/-10/14	50	93	50	37.6	48	126	22	0	0	0	22	46
20A	2008	AA	10/10/-10/19	100	111	100	70.3	93	323	33	0	0	0	33	35
20C	2004	AA	11/19/-11/28	50	81	50	48.1	39	135	9	0	0	0	9	23
20C	2005	AA	11/18/-11/27	50	64	50	42.2	37	135	11	0	0	0	11	30
20C	2006	AA	11/17/-11/26	100	105	99	58.1	84	294	28	0	0	0	28	33
20C	2007	AA	11/16/-11/25	100	75	100	81.3	93	361	24	0	0	0	24	26
20C	2008	AA	11/21/-11/30	100	66	100	100.0	94	363	29	0	0	0	29	31
23	2005	AA	10/21/-10/27	100	385	100	24.2	91	277	13	0	6	0	19	21
23	2006	AA	10/06/-10/15	150	425	146	29.6	139	575	47	0	24	0	71	51
23	2007	AA	10/05/-10/14	150	432	150	30.1	142	494	52	0	33	0	85	60
23	2008	AA	10/10/-10/16	150	409	150	35.7	144	490	45	0	12	0	57	40
24B	2004	AA	10/22/-10/28	100	197	100	40.1	96	318	13	0	11	0	24	25
27	2004	AA	10/15/-10/18	50	316	50	15.8	46	133	19	0	3	0	22	48
27	2005	AA	10/14/-10/17	50	271	50	18.5	50	136	34	0	2	0	36	72
27	2006	AA	10/06/-10/15	100	343	100	26.5	91	331	49	0	3	0	52	57
27	2007	AA	10/05/-10/14	100	325	100	29.2	95	392	58	0	8	0	66	69

AA = Any Antlered Deer **MD** = Mule Deer **WT** = Whitetail Deer **ALS** = Antlerless **CN** = Camp Navajo **FTHU** = Fort Huachuca, **C** = CHAMP Hunt; in the unit column **E** = early or 1st season **M** = 2nd season **T** = 3rd season **L** = late or 4th season.

5-Year: 2004-2008 Harvest

Unit	Year	Hunt Type	Dates	Permits Authorized	1st Choice Applicants	Permits Issued	Draw Odds	Hunters	Hunter Days	Harvest					Hunt Success
										MD-Buck	MD-Aless	WT- Buck	WT-Aless	Total	
**JUNIORS ONLY**															
27	2008	AA	10/10/-10/16	140	269	140	43.9	128	370	63	0	15	0	78	61
28/29/30A 31/32	2008	AA	10/10/-10/16	100	352	100	25.3	97	292	48	0	19	0	67	69
32	2004	AA	11/26/-12/05	100	345	100	25.2	89	326	20	0	26	0	46	52
32	2005	AA	11/25/-12/04	100	356	100	22.5	95	300	21	0	13	0	34	36
32	2006	AA	11/17/-11/26	150	269	150	46.1	142	525	22	0	25	0	47	33
32	2007	AA	11/16/-11/25	150	195	150	60.5	144	551	36	0	27	0	63	44
33	2006	AA	11/17/-11/26	175	148	175	76.4	168	565	12	0	58	0	70	42
33	2007	AA	10/05/-10/14	250	295	249	67.8	234	766	12	0	132	0	144	62
33	2008	AA	10/10/-10/16	150	297	150	43.4	142	346	6	0	79	0	85	60
33	2008	AA	11/21/-11/27	150	120	150	73.3	144	468	2	0	28	0	30	21
36A	2005	AA	11/18/-12/04	100	270	100	32.6	98	421	17	0	13	0	30	31
36A	2006	AA	11/17/-11/26	200	176	200	79.0	186	742	18	0	38	0	56	30
36A	2007	AA	11/16/-11/25	200	111	200	94.6	171	699	32	0	17	0	49	29
36A	2008	AA	10/10/-10/16	50	39	50	71.8	48	150	10	0	15	0	25	52
36A	2008	AA	11/21/-11/27	150	61	150	96.7	142	488	15	0	14	0	29	20
36A/36C	2004	AA	11/19/-12/05	100	364	100	22.5	94	302	33	0	14	0	47	50
36B	2008	AA	11/21/-11/27	50	29	50	89.7	42	139	10	0	10	0	20	48
36C	2007	AA	11/16/-11/25	25	47	25	42.6	16	52	3	0	2	0	5	31
42	2004	AA	11/19/-11/28	50	37	50	56.8	50	181	19	0	0	0	19	38
42	2005	AA	11/18/-11/27	50	47	50	57.4	48	145	14	0	0	0	14	29
42	2006	AA	11/17/-11/26	75	43	75	93.0	64	238	16	0	0	0	16	25
42	2007	AA	11/16/-11/25	75	48	75	89.6	67	272	18	0	0	0	18	27
42	2008	AA	11/21/-11/30	75	55	75	96.4	72	267	24	0	0	0	24	33
FTHU	2007	AA	11/10/-11/25	10	8	10	100.0	10	44	0	0	4	0	4	40
FTHU	2008	AA	11/08/-11/23	10	15	10	53.3	10	17	3	0	3	0	6	60
**MUZZLELOADER**															
03B	2004	AA	10/29/-11/07	60	188	60	26.6	56	240	15	0	0	0	15	27
03B	2005	AA	10/28/-11/06	60	171	60	30.4	56	247	18	0	2	0	20	36
03B	2006	AA	11/10/-11/19	70	166	70	28.3	53	248	15	0	0	0	15	28
03B	2007	AA	11/09/-11/18	70	124	70	44.4	68	356	15	0	0	0	15	22
03B	2008	AA	10/31/-11/09	80	119	80	48.7	78	397	19	0	0	0	19	24
06B	2004	AA	10/29/-11/01	150	197	150	46.7	123	385	8	0	0	0	8	7
06B	2005	AA	10/28/-10/31	150	183	150	51.4	134	431	19	0	3	0	22	16
06B	2006	AA	10/27/-11/05	190	243	190	55.6	170	733	45	0	7	0	52	31
06B	2007	AA	10/26/-11/04	250	224	250	76.3	242	1193	55	0	2	0	57	24
06B	2008	AA	10/24/-10/30	275	184	275	84.8	253	1106	35	0	11	0	46	18
12AE	2004	AA	11/12/-11/18	50	428	63	8.4	61	312	19	0	0	0	19	31
12AE	2006	AA	11/10/-11/16	50	333	50	11.4	44	212	22	0	0	0	22	50
12AE	2008	AA	11/14/-11/20	50	518	50	6.9	48	239	37	0	0	0	37	77
12B	2005	AA	11/11/-11/17	35	251	35	5.6	33	143	22	0	0	0	22	67
12BW	2007	AA	11/09/-11/18	35	305	35	6.9	35	188	32	0	0	0	32	91
15	2004	AA	10/29/-11/07	150	156	150	64.1	124	583	17	0	0	0	17	14
15	2005	AA	10/28/-11/06	150	171	150	68.4	138	668	35	0	0	0	35	25
15	2006	AA	10/27/-11/05	175	162	175	77.2	154	702	55	0	0	0	55	36
15	2007	AA	10/26/-11/04	175	158	175	84.8	158	762	47	0	0	0	47	30
15	2008	AA	10/24/-11/02	200	145	200	100.0	187	948	43	0	0	0	43	23
20B	2005	AA	10/28/-11/06	175	90	175	100.0	145	509	23	0	0	0	23	16
20B	2006	AA	10/27/-11/05	150	65	150	100.0	130	515	16	0	0	0	16	12
20B	2007	AA	10/26/-11/04	150	38	150	100.0	132	569	18	0	0	0	18	14
20B	2008	AA	10/31/-11/09	150	26	150	100.0	134	532	23	0	0	0	23	17
24A	2004	AA	10/29/-11/07	75	132	75	40.9	72	279	8	0	15	0	23	32
34A	2004	AA	11/05/-11/14	100	114	100	59.6	80	376	16	0	2	0	18	23
34A	2005	AA	11/04/-11/13	100	94	100	54.3	84	343	10	0	8	0	18	21
34A	2006	AA	11/03/-11/12	100	106	99	53.8	86	427	3	0	0	0	3	3
34A	2007	AA	11/02/-11/11	100	87	100	78.2	85	412	12	0	0	0	12	14
34A	2008	AA	11/14/-11/20	100	65	100	83.1	90	408	18	0	4	0	22	24
35 E	2004	AA	11/05/-11/14	75	64	75	43.8	71	375	11	0	7	0	18	25
35 E	2005	AA	11/04/-11/13	75	54	75	44.4	68	296	9	0	6	0	15	22
35 E	2006	AA	11/03/-11/12	75	29	75	58.6	64	313	0	0	11	0	11	17
35 E	2007	AA	11/02/-11/11	50	29	50	69.0	50	239	14	0	4	0	18	36
35 E	2008	AA	10/31/-11/06	50	39	50	41.0	48	227	9	0	4	0	13	27

AA = Any Antlered Deer **MD** = Mule Deer **WT** = Whitetail Deer **ALS** = Antlerless **CN** = Camp Navajo **FTHU** = Fort Huachuca,
C = CHAMP Hunt; in the unit column **E =** early or 1st season **M =** 2nd season **T =** 3rd season **L =** late or 4th season.

5-Year: 2004–2008 Harvest

Unit	Year	Hunt Type	Dates	Permits Authorized	1st Choice Applicants	Permits Issued	Draw Odds	Hunters	Hunter Days	Harvest					Hunt Success
										MD-Buck	MD-Aless	WT- Buck	WT-Aless	Total	
**MUZZLELOADER**															
35 L	2004	AA	12/17/-12/31	50	303	50	12.2	40	177	10	0	4	0	14	35
35 L	2005	AA	12/16/-12/31	50	215	52	18.6	48	242	24	0	4	0	28	58
35 L	2006	AA	12/15/-12/31	50	209	50	15.8	45	252	11	0	7	0	18	40
35 L	2007	AA	12/14/-12/31	50	173	50	22.5	47	279	18	0	7	0	25	53
35 L	2008	AA	12/12/-12/31	50	260	50	16.5	46	233	15	0	19	0	34	74
39/40/41/42	2004	AA	12/17/-12/31	50	295	50	12.5	42	224	11	0	0	0	11	26
39/40/41/42	2005	AA	12/16/-12/31	50	250	50	14.8	50	228	31	0	0	0	31	62
39/40/41/42	2006	AA	12/15/-12/31	50	394	50	10.2	47	239	18	0	0	0	18	38
39/40/41/42	2007	AA	12/14/-12/31	50	347	50	11.0	46	294	19	0	0	0	19	41
39/40/41/42	2008	AA	12/12/-12/31	50	385	50	10.9	46	322	14	0	0	0	14	30
FTHU	2004	AA	12/17/-12/30	10	19	10	52.6	10	36	4	0	5	0	9	90
FTHU	2005	AA	12/16/-12/30	12	19	12	57.9	12	82	2	0	0	0	2	17
FTHU	2006	AA	12/15/-12/31	15	17	15	76.5	14	85	5	0	4	0	9	64
FTHU	2007	AA	12/14/-12/30	10	21	10	38.1	10	58	3	0	0	0	3	30
FTHU	2008	AA	10/24/-12/31	10	17	10	35.3	10	44	4	0	2	0	6	60
**MUZZLELOADER - JUNIORS ONLY**															
16A	2004	AA	12/10/-12/31	30	51	30	29.4	25	107	17	0	0	0	17	68
16A	2005	AA	12/10/-12/31	30	67	30	28.4	21	55	9	0	0	0	9	43
16A	2006	AA	12/15/-12/31	30	83	30	30.1	25	120	10	0	0	0	10	40
16A	2007	AA	12/14/-12/31	30	113	30	23.9	30	120	10	0	0	0	10	33
16A	2008	AA	12/19/-12/31	30	86	30	29.1	28	135	10	0	0	0	10	36
**ARCHERY DEER – DRAW UNITS**															
1	2008	AA	8/22-9/11	190	74	190		145	890	10	0	0	0	10	7
3A/3C	2008	AA	8/22-9/11	150	79	150		140	1099	16	0	0	0	16	11
7	2008	AA	8/22-9/11	800	147	417		355	1972	43	0	0	0	43	12
12A	2008	AA	8/22-9/11	1000	580	1000		862	5664	130	0	0	0	130	15
12B	2008	AA	8/22-9/11	15	13	15		12	66	-	0	0	0	–	–
12BW	2008	AA	8/22-9/11	75	2	65		29	152	-	0	0	0	–	–
13A	2008	AA	8/22-9/11	45	40	45		36	251	15	0	0	0	15	42
13B	2008	AA	8/22-9/11	30	232	30		28	279	11	0	0	0	11	39

AA = Any Antlered Deer **MD** = Mule Deer **WT** = Whitetail Deer **ALS** = Antlerless **CN** = Camp Navajo **FTHU** = Fort Huachuca,
C = CHAMP Hunt; in the unit column **E** = early or 1st season **M** = 2nd season **T** = 3rd season **L** = late or 4th season.

5-Year: 2004-2008 Archery Deer Harvest (Over-the-Counter hunts only)

Unit	Year	Hunters	Hunter Days	Deer Harvest					Percent Success
				Mule Deer		Whitetail		Total	
				Buck	A-less	Buck	A-less		
1	2004	843	3627	36	0	0	0	36	4
1	2005	731	3100	13	0	0	0	13	2
1	2006	673	2953	18	0	0	0	18	3
1	2007	657	3136	19	0	0	0	19	3
2	2004	59	394	5	0	5	0	10	17
2	2005	47	234	0	0	4	0	4	9
2	2006	48	136	0	0	0	0	0	0
2	2007	38	278	0	0	0	0	0	0
2	2008	50	249	5	0	0	0	5	10
3A/3C	2004	317	1406	14	0	0	0	14	4
3A/3C	2005	293	1382	9	0	0	0	9	3
3A/3C	2006	502	2139	13	0	0	0	13	3
3A/3C	2007	527	2628	19	0	0	0	19	4
3B	2004	249	993	5	0	5	0	10	4
3B	2005	174	646	9	0	0	0	9	5
3B	2006	180	766	0	0	0	0	0	0
3B	2007	149	782	0	0	0	0	0	0
3B	2008	181	960	9	0	0	0	9	5
4	2004	512	1832	0	0	0	0	0	0
4	2005	302	1250	0	0	0	0	0	0
4	2006	414	1466	4	0	0	0	4	1
4	2007	288	1050	0	0	0	0	0	0
4	2008	448	1947	9	0	5	0	14	3
5	2004	1084	4969	32	0	0	0	32	3
5	2005	872	3980	13	0	0	0	13	1
5	2006	990	4397	13	0	0	0	13	1
5	2007	873	3807	0	0	5	0	5	1
5	2008	779	3540	0	0	0	0	0	0
6A	2004	1533	6379	23	0	5	0	28	2
6A	2005	1191	5328	21	0	4	0	25	2
6A	2006	1259	5761	4	0	4	0	8	1
6A	2007	1357	5620	23	0	6	0	29	2
6A	2008	1413	6383	18	0	18	0	36	3
6B	2004	336	1492	9	0	0	0	9	3
6B	2005	310	1182	9	0	0	0	9	3
6B	2006	343	1391	4	0	0	0	4	1
6B	2007	503	2297	5	0	5	0	10	2
6B	2008	430	2182	5	0	0	0	5	1
7	2004	1025	4783	36	0	0	0	36	4
7	2005	1038	4622	30	0	0	0	30	3
7	2006	1268	5977	57	0	0	0	57	4
7	2007	1367	6948	38	0	5	0	43	3
7M	2004	181	839	18	0	0	0	18	10
7M	2005	234	1233	4	0	0	0	4	2
8	2004	585	2707	0	0	0	0	0	0
8	2005	685	2960	11	0	5	0	16	2
8	2006	651	2764	4	0	0	0	4	1
8	2007	623	2752	10	0	5	0	15	2
8	2008	679	3060	0	0	5	0	5	1
9	2004	141	612	9	0	0	0	9	6
9	2005	145	727	0	0	0	0	0	0
9	2006	167	678	4	0	0	0	4	2
9	2007	163	609	0	0	0	0	0	0
9	2008	172	629	0	0	0	0	0	0
10	2004	880	3899	9	0	0	0	9	1
10	2005	957	4669	30	0	0	0	30	3
10	2006	1021	5018	9	0	0	0	9	1
10	2007	844	4277	14	0	0	0	14	2
10	2008	706	3133	14	0	0	0	14	2
11M	2006	277	1004	9	0	0	0	9	3
11M	2007	479	2297	19	0	0	0	19	4
11M	2008	326	1888	5	0	0	0	5	2

Note: 2008 data is preliminary

5-Year: 2004-2008 Archery Deer Harvest (Over-the-Counter hunts only)

Unit	Year	Hunters	Hunter Days	Deer Harvest					Percent Success
				Mule Deer		Whitetail		Total	
				Buck	A-less	Buck	A-less		
12A	2004	2208	11503	172	0	5	0	177	8
12A	2005	1705	8998	106	0	0	0	106	6
12A	2006	1391	7949	158	0	0	0	158	11
12A	2007	1362	7710	110	0	0	0	110	8
12B	2004	131	653	0	0	0	0	0	0
12B	2005	81	251	0	0	0	0	0	0
12B	2006	62	238	4	0	0	0	4	6
12B	2007	58	225	5	0	0	0	5	9
13A	2004	231	1297	14	0	0	0	14	6
13A	2005	145	578	4	0	0	0	4	3
13A	2006	97	546	13	0	0	0	13	13
13A	2007	105	633	14	0	0	0	14	13
15A/15B	2004	172	1034	0	0	0	0	0	0
15A/15B	2005	234	1327	21	0	0	0	21	9
15A/15B	2006	286	1695	48	0	0	0	48	17
15A/15B	2007	245	1486	19	0	0	0	19	8
15A/15B	2008	163	874	5	0	0	0	5	3
15C/15D	2004	14	63	0	0	0	0	0	0
15C/15D	2005	26	132	4	0	0	0	4	15
15C/15D	2006	26	84	0	0	0	0	0	0
15C/15D	2007	10	34	0	0	0	0	0	0
15C/15D	2008	9	23	5	0	0	0	5	56
16A	2004	240	1056	5	0	0	0	5	2
16A	2005	187	1021	4	0	0	0	4	2
16A	2006	207	849	9	0	4	0	13	6
16A	2007	264	1400	10	0	0	0	10	4
16A	2008	181	711	9	0	0	0	9	5
17A	2004	417	1818	5	0	0	0	5	1
17A	2005	404	2139	21	0	0	0	21	5
17A	2006	484	1937	35	0	0	0	35	7
17A	2007	393	2134	5	0	0	0	5	1
17A	2008	272	1177	14	0	0	0	14	5
17B	2004	394	1877	9	0	0	0	9	2
17B	2005	446	2330	4	0	4	0	8	2
17B	2006	603	2905	26	0	0	0	26	4
17B	2007	412	1726	24	0	0	0	24	6
17B	2008	462	2150	18	0	0	0	18	4
18A	2004	286	1111	9	0	0	0	9	3
18A	2005	327	1399	9	0	0	0	9	3
18A	2006	335	1549	4	0	0	0	4	1
18A	2007	393	1889	5	0	0	0	5	1
18A	2008	217	1041	5	0	0	0	5	2
18B	2004	218	907	0	0	0	0	0	0
18B	2005	332	1522	0	0	0	0	0	0
18B	2006	365	1725	4	0	0	0	4	1
18B	2007	355	1649	5	0	0	0	5	1
18B	2008	281	1471	14	0	0	0	14	5
19A	2004	263	1161	0	0	0	0	0	0
19A	2005	213	1046	4	0	0	0	4	2
19A	2006	251	1039	4	0	0	0	4	2
19A	2007	235	1012	10	0	0	0	10	4
19A	2008	317	1499	9	0	0	0	9	3
19B	2004	240	975	5	0	0	0	5	2
19B	2005	213	940	0	0	0	0	0	0
19B	2006	255	1272	4	0	0	0	4	2
19B	2007	230	1156	5	0	0	0	5	2
19B	2008	231	996	9	0	0	0	9	4
20A	2004	1047	5717	50	0	0	0	50	5
20A	2005	906	5451	55	0	0	0	55	6
20A	2006	1017	5687	62	0	0	0	62	6
20A	2007	906	5433	53	0	0	0	53	6

Note: 2008 data is preliminary

5-Year: 2004-2008 Archery Deer Harvest (Over-the-Counter hunts only)

Unit	Year	Hunters	Hunter Days	Deer Harvest					Percent Success
				Mule Deer		Whitetail		Total	
				Buck	A-less	Buck	A-less		
20A	2008	593	3088	5	0	0	0	5	1
20B	2004	367	1347	9	0	0	0	9	2
20B	2005	421	1947	9	0	0	0	9	2
20B	2006	374	1567	4	0	0	0	4	1
20B	2007	451	2100	0	0	0	0	0	0
20B	2008	353	1254	0	0	0	0	0	0
20C	2004	286	1433	14	0	0	0	14	5
20C	2005	319	1752	26	0	0	0	26	8
20C	2006	352	1796	0	0	0	0	0	0
20C	2007	340	1928	10	0	0	0	10	3
20C	2008	258	1236	23	0	0	0	23	9
21	2004	671	3160	5	0	0	0	5	1
21	2005	685	2972	21	0	0	0	21	3
21	2006	766	2808	9	0	4	0	13	2
21	2007	921	4090	24	0	0	0	24	3
21	2008	982	4699	9	0	14	0	23	2
22	2004	1256	6085	9	0	27	0	36	3
22	2005	1399	7463	18	0	67	0	85	6
22	2006	1690	8688	18	0	97	0	115	7
22	2007	1601	8137	0	0	53	0	53	3
22	2008	1023	4360	0	0	32	0	32	3
23	2004	1247	5808	32	0	54	0	86	7
23	2005	1373	7008	34	0	30	0	64	5
23	2006	1774	8332	64	0	37	0	101	6
23	2007	1525	7748	34	0	34	0	68	4
23	2008	1250	6175	9	0	36	0	45	4
24A	2004	476	2743	0	0	14	0	14	3
24A	2005	532	2832	13	0	13	0	26	5
24A	2006	700	3534	4	0	35	0	39	6
24A	2007	561	3121	10	0	19	0	29	5
24A	2008	697	3789	18	0	18	0	36	5
24B	2004	494	2802	0	0	0	0	0	0
24B	2005	604	3210	0	0	9	0	9	1
24B	2006	612	2975	9	0	9	0	18	3
24B	2007	623	3414	5	0	5	0	10	2
24B	2008	697	3672	18	0	5	0	23	3
25M	2007	14	43	0	0	0	0	0	0
25M	2008	5	23	0	0	0	0	0	0
26M	2006	44	110	0	0	0	0	0	0
26M	2007	139	671	10	0	0	0	10	7
26M	2008	91	611	9	0	0	0	9	10
27	2004	766	3138	41	0	14	0	55	7
27	2005	761	3355	17	0	13	0	30	4
27	2006	775	3719	31	0	13	0	44	6
27	2007	595	3016	29	0	19	0	48	8
27	2008	688	3165	36	0	27	0	63	9
28	2004	227	857	0	0	0	0	0	0
28	2005	247	1072	9	0	0	0	9	4
28	2006	277	1351	4	0	0	0	4	1
28	2007	302	1654	19	0	0	0	19	6
28	2008	303	1494	9	0	5	0	14	5
29	2004	186	934	5	0	9	0	14	8
29	2005	327	1646	0	0	26	0	26	8
29	2006	326	1743	4	0	18	0	22	7
29	2007	302	1884	10	0	24	0	34	11
29	2008	244	1286	14	0	23	0	37	15
30A	2004	272	1333	23	0	9	0	32	12
30A	2005	323	1684	13	0	4	0	17	5
30A	2006	335	1739	4	0	18	0	22	7
30A	2007	273	1621	5	0	5	0	10	4
30A	2008	222	1222	14	0	14	0	28	13

Note: 2008 data is preliminary

5-Year: 2004-2008 Archery Deer Harvest (Over-the-Counter hunts only)

Unit	Year	Hunters	Hunter Days	Deer Harvest					Percent Success
				Mule Deer		Whitetail		Total	
				Buck	A-less	Buck	A-less		
30B	2004	290	1886	9	0	9	0	18	6
30B	2005	323	2062	13	0	9	0	22	7
30B	2006	357	2157	4	0	0	0	4	1
30B	2007	360	2445	10	0	5	0	15	4
30B	2008	299	1566	14	0	9	0	23	8
31	2004	535	2779	14	0	9	0	23	4
31	2005	472	2186	0	0	13	0	13	3
31	2006	528	2482	13	0	18	0	31	6
31	2007	432	2143	24	0	5	0	29	7
31	2008	453	2286	14	0	18	0	32	7
32	2004	757	3537	14	0	36	0	50	7
32	2005	812	3784	10	0	15	0	25	3
32	2006	845	3741	13	0	13	0	26	3
32	2007	782	3730	19	0	10	0	29	4
32	2008	566	2712	12	0	6	0	18	3
33	2004	952	5250	18	0	0	0	18	2
33	2005	1310	7458	9	0	37	0	46	4
33	2006	1413	7350	19	0	34	0	53	4
33	2007	1429	7542	38	0	53	0	91	6
33	2008	1204	6221	23	0	81	0	104	9
34A	2004	975	5835	32	0	23	0	55	6
34A	2005	1131	6544	39	0	33	0	72	6
34A	2006	1144	6083	31	0	18	0	49	4
34A	2007	1103	6478	24	0	48	0	72	7
34A	2008	933	5632	18	0	14	0	32	3
34B	2004	326	1505	5	0	0	0	5	2
34B	2005	366	1799	9	0	0	0	9	2
34B	2006	493	2152	18	0	0	0	18	4
34B	2007	350	1971	5	0	0	0	5	1
34B	2008	326	1639	5	0	5	0	10	3
35A	2004	367	2204	5	0	9	0	14	4
35A	2005	446	2981	4	0	30	0	34	8
35A	2006	431	2760	0	0	26	0	26	6
35A	2007	417	3328	5	0	19	0	24	6
35A	2008	403	2531	9	0	45	0	54	13
35B	2004	190	812	9	0	5	0	14	7
35B	2005	217	1046	4	0	13	0	17	8
35B	2006	233	924	0	0	13	0	13	6
35B	2007	235	1501	0	0	38	0	38	16
35B	2008	199	1499	14	0	18	0	32	16
36A	2004	943	5604	14	0	14	0	28	3
36A	2005	1191	7144	31	0	20	0	51	4
36A	2006	1034	5563	31	0	18	0	49	5
36A	2007	964	5226	34	0	14	0	48	5
36A	2008	878	4505	45	0	9	0	54	6
36B	2004	721	3709	5	0	0	0	5	1
36B	2005	833	4410	36	0	15	0	51	6
36B	2006	889	4195	40	0	18	0	58	7
36B	2007	786	4028	37	0	16	0	53	7
36B	2008	738	3486	9	0	14	0	23	3
36C	2004	490	2457	27	0	5	0	32	7
36C	2005	515	2636	13	0	13	0	26	5
36C	2006	585	2685	0	0	9	0	9	2
36C	2007	441	2316	10	0	0	0	10	2
36C	2008	521	2639	0	0	0	0	0	0
37A	2004	295	1619	5	0	0	0	5	2
37A	2005	336	1841	9	0	0	0	9	3
37A	2006	383	1756	13	0	0	0	13	3
37A	2007	408	2095	0	0	0	0	0	0
37A	2008	244	1141	9	0	0	0	9	4
37B	2004	390	2371	0	0	5	0	5	1

Note: 2008 data is preliminary

5-Year: 2004-2008 Archery Deer Harvest (Over-the-Counter hunts only)

Unit	Year	Hunters	Hunter Days	Deer Harvest					Percent Success
				Mule Deer		Whitetail		Total	
				Buck	A-less	Buck	A-less		
37B	2005	583	2602	9	0	0	0	9	2
37B	2006	621	3006	0	0	0	0	0	0
37B	2007	537	2412	0	0	0	0	0	0
37B	2008	512	2639	0	0	0	0	0	0
38M	2004	145	889	14	0	0	0	14	10
38M	2005	327	2020	17	0	0	0	17	5
38M	2006	281	1770	17	0	4	0	21	7
38M	2007	288	2115	24	0	0	0	24	8
38M	2008	236	1675	23	0	0	0	23	10
39/40	2004	113	576	0	0	0	0	0	0
39/40	2005	132	1033	13	0	0	0	13	10
39/40	2006	185	1021	9	0	0	0	9	5
39/40	2007	206	1146	14	0	0	0	14	7
39/40	2008	177	905	14	0	0	0	14	8
39M	2005	21	149	0	0	0	0	0	0
41	2004	131	748	0	0	0	0	0	0
41	2005	170	893	4	0	0	0	4	2
41	2006	273	1329	4	0	0	0	4	1
41	2007	283	1530	14	0	0	0	14	5
41	2008	294	1947	9	0	0	0	9	3
42	2004	394	2562	0	0	0	0	0	0
42	2005	395	2475	34	0	0	0	34	9
42	2006	445	2267	22	0	0	0	22	5
42	2007	551	3658	29	0	0	0	29	5
42	2008	330	2178	5	0	0	0	5	2
42M	2004	86	458	0	0	0	0	0	0
42M	2005	128	714	0	0	0	0	0	0
42M	2006	40	216	0	0	0	0	0	0
42M	2008	9	18	0	0	0	0	0	0
43/44	2004	163	667	0	0	0	0	0	0
43/44	2005	157	748	4	0	0	0	4	3
43/44	2006	224	1268	40	0	0	0	40	18
43/44	2007	192	1189	0	0	0	0	0	0
43/44	2008	131	706	0	0	0	0	0	0
45	2004	41	277	0	0	0	0	0	0
45	2005	43	315	0	0	0	0	0	0
45	2006	35	154	4	0	0	0	4	11
45	2007	62	187	10	0	0	0	10	16
45	2008	27	149	9	0	0	0	9	33
46A	2006	4	13	0	0	0	0	0	0
46B	2006	9	35	0	0	0	0	0	0
47M	2006	44	158	0	0	0	0	0	0
47M	2007	72	374	5	0	0	0	5	7
47M	2008	45	199	0	0	0	0	0	0
Unknown	2004	104	367	5	0	0	0	5	5
Unknown	2005	115	519	4	0	0	0	4	3
Unknown	2006	88	515	0	0	0	0	0	0
Unknown	2007	38	86	0	0	0	0	0	0
Unknown	2008	54	371	0	0	0	0	0	0

Note: 2008 data is preliminary

Pronghorn Antelope *(Antilocapra americana)*

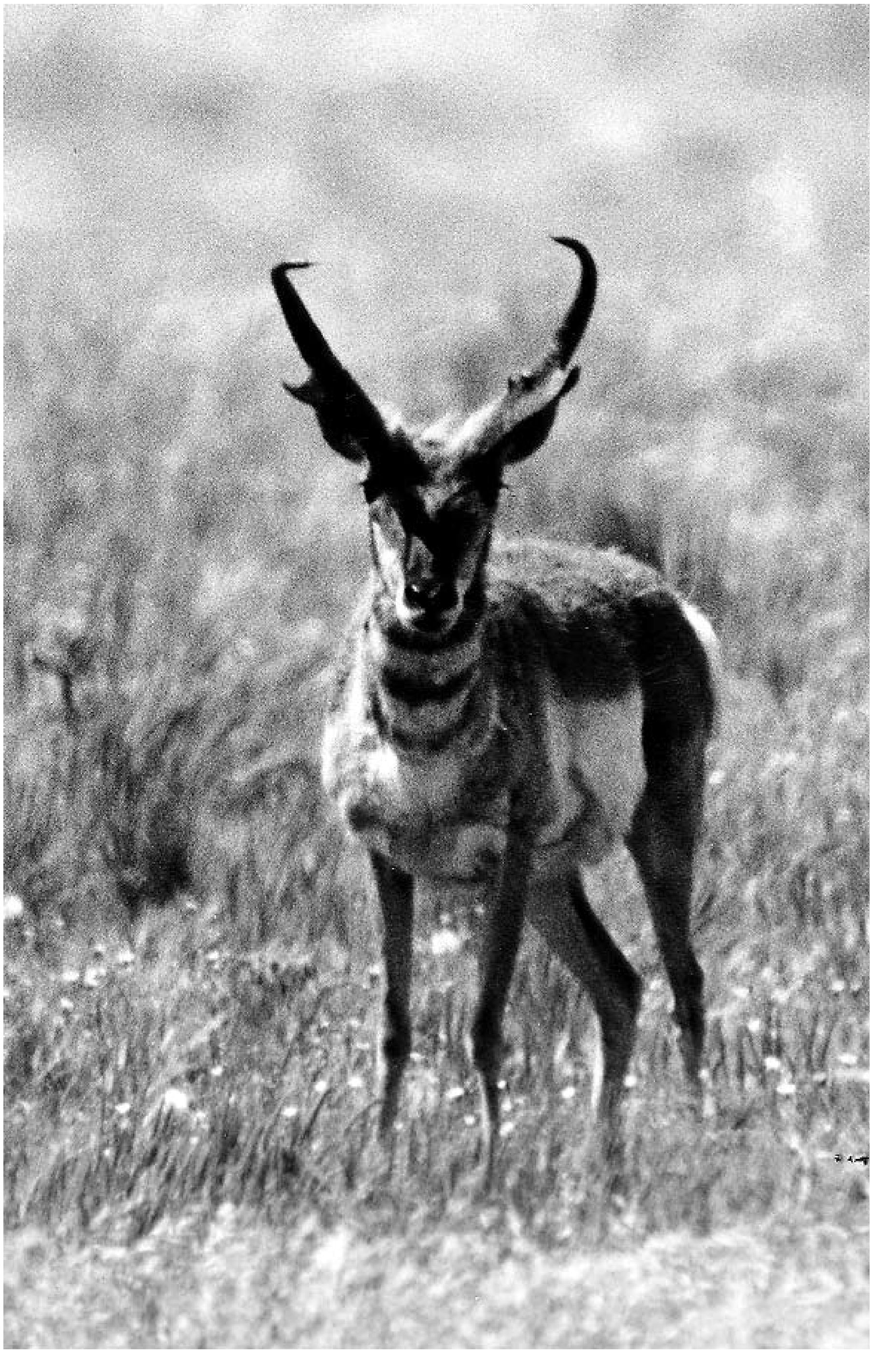

GEORGE ANDREJKO

Natural History

Pronghorn antelope are native to the prairies of North America. At one time they numbered in the millions and were found from the Mississippi River to the Pacific Ocean, and from central Canada to Mexico. With the European settlement of the plains, the population was reduced nearly to extinction. In Arizona, antelope persisted primarily in the northern plains. They also inhabit high elevation meadows between forested areas, and scattered herds are again found in the grasslands of southeastern Arizona. The endangered Sonoran pronghorn is restricted to the extreme desert lands of southwestern Arizona and northern Sonora, Mexico. The statewide population of pronghorn is estimated at 7,800 post-hunt adults.

The name pronghorn comes from the sharply pointed prong on the horn of the buck antelope. The doe's horns, if present at all, are smaller and more slender. Antelope have true horns in that the horny tissue is composed of fused hairs, which form over a bony core. Horn length reaches maximum size during the summer before the outer sheaths are shed, usually sometime in the fall.

Antelope have exceptional eyesight, which is often compared to high-powered binoculars. These "prairie goats" are also one of the fastest mammals, being able to run in excess of 60 mph. Despite their speed, antelope are reluctant to jump over objects, preferring to crawl under or through fences rather than leap over them.

A conspicuous characteristic of the antelope is the white rump patch. When an animal is alarmed, its rump hairs stand erect and appear as a white flash that can be seen for miles. The dominant body color is an apricot tan, with sharply contrasting white markings on the belly, head, and neck. The top of the buck's muzzle is brown or black, and below the ear he will usually have a triangular black cheek patch, which is lacking on the doe. A short mane is present along the top of the neck. Shedding is continuous, with the individual hairs being loosely attached to the skin, making the hide nearly worthless. Since the hairs are hollow and can be erected at will, prong-horns are able to adjust to great extremes in temperature.

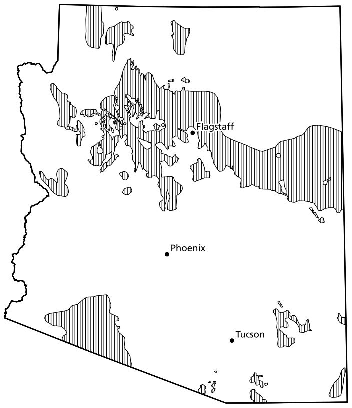

Antelope distribution

Adult bucks usually weigh between 90 and 120 pounds The does are about 20 pounds lighter. Antelope are primarily browsers, feeding mostly on weeds and short browse plants, with grass being only a minor food source. Because of Arizona's mild winters, antelope tend to live longer than the six- to eight-year average life span of their northern cousins, one reason that a disproportionate number of Arizona bucks are trophy animals with horns in excess of 15 inches in length.

Antelope are gregarious and usually seen in mixed herds, except in the spring when the bucks are alone or in small bachelor groups. Later, in the summer and early fall, these same bucks will collect harems of does, which may number up to 15 animals, which they then defend from other bucks. Antelope breed in August and September, and the young are born in May and June. A doe will typically produce one or two fawns. The young are not spotted like the fawns of the deer family, but instead have markings similar to those of adults. The fawns remain hidden, with the doe feeding them several times a day, until they are about two to three weeks old and strong enough to travel with the adults. During this time, pronghorn fawns, or "kids," are the most vulnerable to coyotes, which may take 75 percent or more of the year's production. Adult antelope are taken by mountain lions, as well as by coyotes.

Hunt History

Once second only to deer as a game animal, Arizona's antelope were first given a closed season in 1893. The response must have been less than satisfactory, however, as the season was completely closed in 1905. By 1922, the state's antelope population was estimated to be less than 1,000 animals.

Then, for reasons that still are not fully understood, pronghorn antelope began to make a comeback. Aided by a closed season, government predator control programs, and the abandonment of numerous homesteads, pronghorn numbers steadily increased until fears were expressed that some northern Arizona populations were in danger of exceeding their food supply. Accordingly, a limited hunt of 400 buck permits was authorized for northern Arizona in 1941.

After a closed season from 1944 to 1948, antelope hunting in Arizona recommenced in 1949. Hunts were liberalized gradually, until 1954 when 1,600 permits were issued and 1,146 bucks were taken. Despite the issuance of a number of antlerless antelope permits between 1961 and 1975, this level of harvest has never again been equaled. Annual harvests since 1990 have varied between 500 and 700 bucks, with archers taking a proportionally larger percent of the harvest in recent years. Plagued by encroaching subdivisions, increasing highway construction, and other land-use changes, maintaining even the present number of antelope is dependent on citizen involvement and an aggressive translocation program. Approximately 10 percent of the 1998 antelope harvest was in areas having reintroduced herds.

Pronghorn Antelope Survey Data

Historic Summary of Antelope Survey Data

Year	Bucks	Does	Fawns	Unclassified	Total	Bucks/100 Does	Fawns/100 Does
1948	943	1944	1536	0	4423	49	79
1949	1302	2153	1397	0	4852	60	65
1950	1208	2280	1550	0	5038	53	68
1951	1134	2007	1089	0	4230	57	54
1952	1130	2312	1840	0	5282	49	80
1953	1119	2230	1040	0	4389	50	47
1954	1098	2086	1344	0	4528	53	64
1955	1148	2283	969	0	4400	50	42
1956	862	2056	891	0	3809	42	43
1957	782	2169	806	0	3757	36	37
1958	819	2396	1096	0	4311	34	46
1959	994	2545	1631	0	5170	39	64
1960	1006	2745	1689	0	5440	37	62
1961	835	2180	1067	123	4205	38	49
1962	817	2711	1158	37	4723	30	43
1963	893	2699	1386	30	5008	33	51
1964	874	2905	1410	12	5201	30	49
1965	1014	2948	1040	0	5002	34	35
1966	969	2851	1181	44	5045	34	41
1967	1060	3086	1329	22	5497	34	43
1968	590	2249	938	0	3777	26	42
1969	799	2472	1053	2	4326	32	43
1970	866	2730	1728	1	5325	32	63
1971	993	2559	636	2	4190	39	25
1972	749	2028	841	23	3641	37	41
1973	1211	3005	1275	14	5505	40	42
1974	1006	2878	941	6	4831	35	33
1975	910	2926	1086	0	4922	31	37
1976	950	3347	932	1	5230	28	28
1977	936	3177	727	0	4840	29	23
1978	937	3473	1352	0	5762	27	39
1979	1071	3706	1204	1	5982	29	32
1980	1190	3750	1173	0	6113	32	31
1981	1292	3833	899	0	6024	34	23
1982	1029	3388	1300	5	5722	30	38
1983	1157	3753	1471	3	6384	31	39
1984	1264	3611	1190	12	6077	35	33
1985	1563	4881	1477	1	7922	32	30
1986	1800	5327	1610	0	8737	34	30
1987	1685	5249	1632	2	8568	32	31
1988	1915	6013	1413	0	9341	32	24
1989	1572	4967	1131	4	7674	32	23
1990	1731	5738	1323	3	8795	30	23
1991	1581	5326	1825	9	8741	30	34
1992	1916	5663	1831	1	9411	34	32
1993	2133	6187	2294	34	10648	34	37
1994	2019	5809	1427	0	9255	35	25
1995	2236	6638	1787	14	10675	34	27
1996	2036	5498	435	7	7976	37	8
1997	1998	6426	2037	28	10489	31	32
1998	1997	6152	1651	11	9811	32	27
1999	1814	5420	1076	8	8318	33	20
2000	1455	4453	1002	7	6917	33	23
2001	1739	5702	1773	15	9229	31	31
2002	1503	4305	353	8	6169	35	8
2003	1313	4484	1459	1	7257	29	33
2004	1353	4502	1494	1	7350	30	33
2005	1292	3626	1485	16	6419	36	41
2006	1205	3006	596	16	4823	40	40
2007	952	2778	620	16	4366	34	22
2008	1014	2816	538	12	4380	36	19

5-year: 2004-2008 Antelope Survey Data

Unit	Year	Bucks	Does	Fawns	Unclassified	Total	Bucks/100 Does	Fawns/100 Does
1	2004	69	274	63	0	406	25	23
1	2005	80	263	52	0	395	30	20
1	2006	68	205	21	0	294	33	10
1	2007	55	218	37	0	310	25	17
1	2008	48	160	29	1	238	30	18
2A	2004	33	115	21	1	170	29	18
2A	2006	49	162	20	10	241	30	12
2A	2007	60	149	26	2	237	40	17
2A	2008	33	121	7	0	161	27	6
2B	2004	46	140	27	0	213	33	19
2B	2005	42	119	14	0	175	35	12
2B	2006	21	91	9	0	121	23	10
2B	2007	16	105	29	2	152	15	28
2B	2008	35	125	17	0	177	28	14
2C	2004	49	237	13	0	299	21	5
2C	2005	48	269	58	0	375	18	22
2C	2006	32	241	46	0	319	13	19
2C	2007	15	129	29	0	173	12	22
2C	2008	14	79	12	0	105	18	15
3A	2004	61	183	29	0	273	33	16
3A	2005	47	141	63	0	251	33	45
3A	2006	66	111	14	0	191	59	13
3A	2007	29	84	15	0	128	35	18
3A	2008	34	150	26	0	210	23	17
3B	2004	23	121	20	0	164	19	17
3B	2005	19	110	27	0	156	17	25
3B	2006	18	78	17	0	113	23	22
3B	2007	21	73	9	0	103	29	12
3B	2008	10	46	9	0	65	22	20
3C	2004	13	68	16	0	97	19	24
3C	2005	19	65	20	0	104	29	31
3C	2006	26	72	27	0	125	36	38
3C	2007	15	65	7	0	87	23	11
3C	2008	5	53	7	0	65	9	13
4A	2004	56	245	54	0	355	23	22
4A	2005	21	137	49	0	207	15	36
4A	2006	45	166	11	0	222	27	7
4A	2007	56	210	55	0	321	27	26
4A	2008	61	173	14	0	248	35	8
4B	2004	52	200	42	0	294	26	21
4B	2005	23	137	66	0	226	17	48
4B	2006	32	60	14	0	106	53	23
4B	2007	24	95	27	0	146	25	28
4B	2008	18	81	12	1	112	22	15
5A	2004	36	79	27	0	142	46	34
5A	2005	15	38	15	0	68	39	39
5A	2006	9	22	1	0	32	41	5
5A	2007	23	61	22	0	106	38	36
5A	2008	37	57	15	0	109	65	26
5B	2004	61	164	74	0	299	37	45
5B	2005	36	99	36	0	171	36	36
5B	2006	33	109	20	2	164	30	18
5B	2007	77	142	47	0	266	54	33
5B	2008	83	152	23	0	258	55	15
6A	2004	11	33	13	0	57	33	39
6A	2005	4	11	7	0	22	36	64
6A	2006	6	14	11	0	31	43	79
6A	2007	2	5	4	0	11	40	80
6A	2008	6	6	1	0	13	100	17
6B	2004	4	23	10	0	37	17	43
6B	2005	10	15	4	0	29	67	27
6B	2006	4	20	4	0	28	20	20
6B	2007	7	19	3	0	29	37	16
6B	2008	5	25	3	0	33	20	12

5-year: 2004-2008 Antelope Survey Data

Unit	Year	Bucks	Does	Fawns	Unclassified	Total	Bucks/100 Does	Fawns/100 Does
7	2004	43	168	74	0	285	26	44
7	2005	33	108	60	0	201	31	56
7	2006	73	93	22	0	188	78	24
7	2007	32	98	21	2	153	33	21
7	2008	70	177	58	0	305	40	33
8	2004	37	103	58	0	198	36	56
8	2005	22	101	55	2	180	22	54
8	2006	26	55	30	0	111	47	55
8	2007	24	95	30	0	149	25	32
8	2008	18	115	19	0	152	16	17
9	2004	31	95	31	0	157	33	33
9	2005	33	67	30	0	130	49	45
9	2006	12	62	8	0	82	19	13
9	2007	42	61	27	2	132	69	44
9	2008	30	85	23	0	138	35	27
10	2004	134	412	134	0	680	33	33
10	2005	128	285	131	5	549	45	46
10	2006	126	324	56	0	506	39	17
10	2007	44	89	10	0	143	49	11
10	2008	35	117	25	0	177	30	21
12	2004	7	37	4	0	48	19	11
12	2005	5	32	19	0	56	16	59
12A/12B	2006	12	37	4	0	53	32	11
12A/12B	2007	9	36	11	0	56	25	31
12A/12B	2008	3	14	3	0	20	21	21
13A	2004	49	192	117	0	358	26	61
13A	2005	36	179	115	0	330	20	64
13A	2006	44	148	29	0	221	30	20
13A	2007	40	137	7	0	184	29	5
13A	2008	15	79	6	1	101	19	8
13B	2004	18	57	29	0	104	32	51
13B	2005	19	35	21	9	84	54	60
13B	2006	21	34	13	0	68	62	38
13B	2007	21	52	2	0	75	40	4
13B	2008	18	47	17	0	82	38	36
15A/15B	2004	14	19	11	0	44	74	58
15A/15B	2005	19	3	1	0	23	633	33
15A/15B	2006	2	5	4	1	12	40	80
15A/15B	2007	5	25	4	1	35	20	16
15A/15B	2008	3	12	2	0	17	25	17
17A	2004	4	11	9	0	24	36	82
17A	2005	32	47	22	0	101	68	47
17A	2006	11	2	1	0	14	550	50
17A	2007	2	18	1	0	21	11	6
17A	2008	10	50	3	0	63	20	6
17B	2004	44	92	41	0	177	48	45
17B	2005	79	64	27	0	170	123	42
17B	2006	78	74	12	0	164	105	16
17B	2007	30	55	2	0	87	55	4
17B	2008	33	33	2	0	68	100	6
18A	2004	69	200	71	0	340	35	36
18A	2005	59	209	71	0	339	28	34
18A	2006	42	89	20	0	151	47	22
18A	2007	7	72	4	3	86	10	6
18A	2008	10	68	5	0	83	15	7
18B	2004	47	174	70	0	291	27	40
18B	2005	50	148	74	0	272	34	50
18B	2006	40	157	39	0	236	25	25
18B	2007	25	49	13	0	87	51	27
18B	2008	44	146	58	0	248	30	40
19A	2004	144	393	193	0	730	37	49
19A	2005	166	337	215	0	718	49	64
19A	2006	86	155	52	0	293	55	34
19A	2007	75	156	51	0	282	48	33

5-year: 2004-2008 Antelope Survey Data

Unit	Year	Bucks	Does	Fawns	Unclassified	Total	Bucks/100 Does	Fawns/100 Does
19A	2008	117	156	66	0	339	75	42
19B	2004	90	342	140	0	572	26	41
19B	2005	135	280	92	0	507	48	33
19B	2006	105	143	26	3	277	73	18
19B	2007	108	245	17	0	370	44	7
19B	2008	98	221	22	0	341	44	10
21	2004	22	54	29	0	105	41	54
21	2005	19	55	21	0	95	35	38
21	2006	27	82	36	0	145	33	44
21	2007	15	47	30	0	92	32	64
21	2008	15	38	13	9	75	39	34
27	2004	12	15	11	0	38	80	73
27	2005	8	21	6	0	35	38	29
27	2006	5	11	1	0	17	45	9
27	2007	10	16	6	0	32	63	38
27	2008	5	11	7	0	23	45	64
28	2004	2	7	1	0	10	29	14
30A	2004	42	135	33	0	210	31	24
30A	2005	20	105	57	0	182	19	54
30A	2006	41	67	5	0	113	61	7
30A	2007	14	41	17	1	73	34	41
30A	2008	34	89	12	0	135	38	13
31/32	2004	8	29	6	0	43	28	21
31/32	2005	22	43	12	0	77	51	28
31/32	2006	16	38	11	0	65	42	29
31/32	2007	19	49	33	0	101	39	67
31/32	2008	33	47	16	0	96	70	34
32	2004	3	20	2	0	25	15	10
34B	2004	4	12	7	0	23	33	58
34B	2005	7	26	15	0	48	27	58
34B	2006	5	14	2	0	21	36	14
34B	2007	13	25	6	3	47	52	24
34B	2008	11	14	2	0	27	79	14
35	2004	7	32	5	0	44	22	16
35	2005	29	65	28	0	122	45	43
35	2006	14	56	10	0	80	25	18
35	2007	12	43	18	0	73	28	42
35	2008	18	65	3	0	86	28	5
36A	2004	3	4	3	0	10	75	75
36A	2006	2	1	0	0	3	200	0
36B	2004	5	17	6	0	28	29	35
36B	2005	7	12	2	0	21	58	17
36B	2006	8	8	0	0	16	100	0
36B	2007	5	14	0	0	19	36	0
36B	2008	5	4	1	0	10	125	25

Pronghorn Antelope Harvest Data

Historic Summary of General Antelope Hunts (Juniors-Only Hunts listed separately)

Year	1st Choice Applicants	Permits Issued	Hunters	Hunter Days	Harvest			Percent Success
					Bucks	Does/Fawns[1]	Total	
1941	–	400	387	–	286	0	286	74
1942	–	750	721	–	487	0	487	68
1943	–	1072	991	–	522	0	522	53
1949	–	606	575	–	437	0	437	76
1950	–	520	502	–	382	0	382	76
1951	–	835	794	–	548	0	548	69
1952	–	1233	1201	–	739	0	739	62
1953	–	1340	1283	–	828	0	828	65
1954	–	1600	1561	–	1146	0	1146	73
1955	–	955	914	–	578	0	578	63
1956	–	445	430	–	297	0	297	69
1957	–	305	296	–	205	0	205	69
1958	–	490	476	–	317	0	317	67
1959	–	990	974	–	589	0	589	61
1960	–	1200	1174	–	722	0	722	62
1961	–	1411	1373	–	687	68	755	55
1962	–	1215	1173	–	559	53	612	52
1963	–	1281	1257	–	690	39	729	58
1964	–	1413	1377	–	724	125	849	62
1965	–	1278	1248	–	652	25	677	54
1966	6781	1180	1150	–	542	20	562	49
1967	5895	1336	1297	–	667	27	694	54
1968	4291	800	782	–	352	2	354	45
1969	5178	810	791	–	406	0	406	51
1970	6769	1124	1103	–	589	28	617	56
1971	6493	909	896	–	559	0	559	62
1972	5594	997	972	–	480	20	500	51
1973	6161	1219	1205	–	642	21	663	55
1974	6435	1213	1181	2445	685	31	716	61
1975	6340	1196	1163	2293	652	18	670	58
1976	7680	974	937	1983	522	0	522	56
1977	9138	970	796	1713	425	0	425	53
1978	9751	880	849	1955	415	0	415	49
1979	9557	844	810	1816	427	0	427	53
1980	9493	713	683	1513	444	0	444	65
1981	9888	730	713	1502	456	0	456	64
1982	9571	835	814	1904	506	0	506	62
1983	7978	834	795	1816	521	0	521	66
1984	7357	841	810	1701	558	0	558	69
1985	7965	780	768	1621	584	0	584	76
1986	8354	740	728	1526	533	0	533	73
1987	8682	591	571	1177	426	0	426	75
1988	9035	647	640	1374	489	0	489	76
1989	8988	647	633	1341	488	0	488	77
1990	8812	601	587	1366	424	0	424	72
1991	9047	574	565	1225	442	0	442	78
1992	10095	528	507	1105	417	0	417	82
1993	11204	645	633	1496	484	0	484	76
1994	11888	652	640	1411	521	0	521	81
1995	12933	656	650	1427	534	0	534	82
1996	14116	651	630	1308	540	0	540	86
1997	15138	556	545	1214	435	0	435	80
1998	16728	543	534	1248	427	0	427	80
1999	17168	497	484	1088	407	0	407	84
2000	16989	459	454	943	402	0	402	89
2001	16450	450	442	898	356	0	356	81
2002	20082	437	428	929	357	0	357	83
2003	22727	360	350	807	295	0	295	84
2004	25822	353	345	825	283	0	283	82
2005	18627	422	413	976	356	0	356	86
2006	23632	455	440	1083	389	0	389	88
2007	28042	473	466	1257	414	0	414	89
2008	18931	503	485	1226	432	0	432	89

[1] Harvest classifications prior to 1968 are unavailable for some hunts. In these cases, all harvest has been listed as bucks.

Pronghorn Antelope Harvest Data

Historic Summary of Juniors-Only Antelope Hunts

Year	1st Choice Applicants	Permits Issued	Hunters	Hunter Days	Harvest			Percent Success
					Bucks	Does/Fawns	Total	
1999	443	15	15	29	13	0	13	87
2000	485	15	15	41	12	0	12	80
2001	509	15	15	22	13	0	13	87
2002	664	15	15	26	14	0	14	93
2003	761	12	12	39	8	0	8	67
2004	776	12	12	39	5	0	5	42
2005	No juniors hunts offered							
2006	No juniors hunts offered							
2007	No juniors hunts offered							
2008	No juniors hunts offered							

Historic Summary of Muzzleloader Antelope Hunts

Year	1st Choice Applicants	Permits Issued	Hunters	Hunter Days	Harvest			Percent Success
					Bucks	Does/Fawns	Total	
1982	89	40	40	154	13	0	13	33
1983	87	45	44	135	13	0	13	30
1984	132	75	68	181	23	0	23	34
1985	181	65	60	166	19	0	19	32
1986	246	78	78	206	32	0	32	41
1987	358	123	117	361	40	0	40	34
1988	365	122	119	316	58	0	58	49
1989	454	147	144	378	64	0	64	44
1990	528	145	135	370	68	0	68	50
1991	608	143	138	441	55	0	55	40
1992	587	143	141	481	61	0	61	43
1993	628	153	149	486	80	0	80	54
1994	729	148	146	495	67	0	67	46
1995	821	142	136	460	53	0	53	39
1996	824	106	103	302	62	0	62	60
1997	831	91	91	261	57	0	57	63
1998	865	96	95	254	56	0	56	59
1999	988	91	89	245	57	0	57	64
2000	1027	99	97	289	59	0	59	61
2001	1017	93	92	212	62	0	62	67
2002	1319	94	94	199	72	0	72	77
2003	1561	87	83	240	55	0	55	66
2004	1746	92	89	292	50	0	50	56
2005	1446	97	91	297	56	0	56	62
2006	1618	103	103	336	68	0	68	66
2007	2154	103	94	320	67	0	67	71
2008	1691	113	108	413	76	0	76	70

Historic Summary of Archery Antelope Hunts

Year	1st Choice Applicants	Permits Issued	Hunters	Hunter Days	Harvest			Percent Success
					Bucks	Does/Fawns	Total	
1974	16	50	37	168	2	0	2	5.4
1975	17	50	25	62	0	0	0	.0
1976	36	100	57	209	3	0	3	5.3
1977	84	119	93	405	5	1	6	6.5
1978	106	160	142	498	11	2	13	9.2
1979	116	210	170	683	7	6	13	7.6
1980	203	225	214	1133	21	0	21	9.8
1981	364	225	203	1203	13	0	13	6.4
1982	338	236	218	1370	15	0	15	6.9
1983	249	289	268	1357	20	2	22	8.2
1984	298	339	315	1543	33	3	36	11.4
1985	332	364	345	1791	32	1	33	9.6
1986	385	423	401	2175	31	3	34	8.5
1987	483	473	451	2315	32	0	32	7.1
1988	468	497	475	2596	52	1	53	11.2
1989	564	508	475	2565	54	0	54	11.4
1990	625	484	456	2490	53	0	53	11.6
1991	678	549	521	2999	46	0	46	8.8
1992	831	657	631	3646	75	0	75	11.9
1993	1046	666	615	3391	111	0	111	18.0
1994	1183	683	621	3474	116	0	116	18.7
1995	1233	671	617	3580	106	0	106	17.2
1996	1373	611	568	3160	101	0	101	17.8
1997	1497	585	549	3065	106	0	106	19.3
1998	1582	587	560	3155	110	0	110	19.6
1999	1812	588	562	3417	97	0	97	17.3
2000	1933	558	516	3102	70	0	70	13.6
2001	1943	536	503	3156	82	0	82	16.3
2002	2319	514	493	2667	143	0	143	29.0
2003	2482	433	408	2557	57	0	57	14.0
2004	2502	416	388	2622	73	0	73	18.8
2005	2069	415	392	2452	59	0	59	15.0
2006	2376	400	361	2383	71	0	71	20.0
2007	2697	399	370	2420	89	0	89	24.0
2008	2074	394	371	2432	111	0	111	30.0

5-Year: 2004-2008 Harvest

Unit	Year	Dates	Permits Authorized	1st Choice Applicants	Permits Issued	Draw Odds	Hunters	Hunter Days	Harvest	Hunt Success
**FIREARMS**										
1	2004	9/24-9/29	30	4729	30	0.6	29	53	27	93
1	2005	9/23-9/28	30	3097	30	0.9	30	80	29	97
1	2006	9/08-9/17	30	3805	30	0.7	30	90	25	83
1	2007	9/07-9/16	30	3988	31	0.8	31	82	26	84
1	2008	9/05-9/14	30	2691	30	1.1	30	86	28	93
2A	2004	9/24-9/29	30	795	30	1.3	28	45	25	89
2A	2005	9/23-9/28	30	640	30	2.0	29	68	24	83
2A	2006	9/08-9/17	30	779	30	2.2	30	67	27	90
2A	2007	9/07-9/16	30	950	30	2.3	30	64	28	93
2A	2008	9/05-9/14	35	702	35	2.0	35	75	33	94
2C	2004	9/24-9/29	16	603	16	1.5	16	32	15	94
2C	2005	9/23-9/28	20	479	20	1.9	20	40	20	100
2C	2006	9/08-9/17	15	508	15	2.4	15	47	14	93
2C	2007	9/07-9/16	8	390	8	0.8	7	23	5	71
2C	2008	9/05-9/14	5	217	5	1.4	4	11	3	75
3A	2004	9/24-9/29	10	549	10	1.3	10	25	10	100
3A	2005	9/23-9/28	15	558	15	1.6	15	39	14	93
3A	2006	9/08-9/17	15	606	15	1.8	15	25	12	80
3A	2007	9/07-9/16	20	835	20	1.8	19	45	18	95
3A	2008	9/05-9/14	25	644	25	2.6	25	77	23	92
3B	2004	9/24-9/29	5	281	5	1.4	4	8	4	100
3B	2005	9/23-9/28	5	226	5	1.3	5	6	5	100
3B	2006	9/08-9/17	5	273	5	1.5	5	9	5	100
3B North	2004	9/24-9/29	10	334	10	2.1	10	23	8	80
3B North	2005	9/23-9/28	10	132	10	3.8	10	22	8	80
3B North	2006	9/08-9/17	5	94	5	3.2	5	8	5	100
3B North	2007	9/07-9/16	5	331	5	1.2	5	16	3	60
3C	2004	9/24-9/29	5	132	5	2.3	5	14	4	80
3C	2005	9/23-9/28	5	113	5	1.8	5	13	5	100
3C	2006	9/08-9/17	5	166	5	2.4	5	13	3	60
3C	2007	9/07-9/16	7	228	7	3.1	7	19	7	100
3C	2008	9/05-9/14	7	175	7	2.3	5	16	5	100
4A	2004	9/24-9/29	15	1170	15	1.3	15	48	12	80
4A	2005	9/23-9/28	15	871	15	1.5	15	39	10	67
4A	2006	9/08-9/17	15	964	15	1.1	14	28	13	93
4A	2007	9/07-9/16	10	889	10	0.8	10	37	8	80
4A	2008	9/05-9/14	10	628	10	1.4	10	24	9	90
4B	2004	9/24-9/29	10	524	10	1.3	10	18	6	60
4B	2005	9/23-9/28	10	384	10	2.1	9	15	9	100
4B	2006	9/08-9/17	5	314	5	1.3	5	11	5	100
4B	2007	9/07-9/16	10	555	10	1.1	8	46	6	75
4B	2008	9/05-9/14	15	406	15	3.2	14	36	11	79
5A	2004	10/01-10/06	3	399	3	0.8	3	6	3	100
5A	2005	9/30-10/05	6	353	6	1.1	6	21	3	50
5A	2006	9/08-9/17	6	357	6	1.7	6	28	6	100
5A	2007	9/07-9/16	6	422	6	1.2	6	26	6	100
5A	2008	9/05-9/14	10	353	10	2.3	10	22	10	100
5B	2004	10/01-10/06	5	550	5	0.5	5	13	5	100
5B	2005	9/30-10/05	7	519	7	1.3	7	28	6	86
5B	2006	9/08-9/17	7	651	7	1.1	6	18	5	83
5B	2007	9/07-9/16	5	609	5	0.8	5	6	5	100
5B	2008	9/05-9/14	20	733	20	2.6	20	83	15	75
6A	2004	10/01-10/06	3	319	3	0.6	3	8	2	67
6A	2005	9/30-10/05	3	178	3	1.1	3	7	2	67
6A	2006	9/08-9/17	3	224	3	1.3	3	8	3	100
6A	2007	9/07-9/16	3	355	3	0.8	3	6	3	100
6A	2008	9/05-9/14	3	172	3	1.2	3	16	3	100
7	2004	10/01-10/06	46	3824	46	1.0	46	142	29	63
7	2005	9/30-10/05	55	2689	55	1.6	54	114	48	89
7	2006	9/08-9/17	55	3225	55	1.6	55	148	49	89
7	2007	9/07-9/16	65	4339	65	1.2	65	198	51	78
7	2008	9/05-9/14	50	2085	50	1.9	48	103	44	92

CN = Camp Navajo **E** = Early Season **L** = Late Season

5-Year: 2004–2008 Harvest

Unit	Year	Dates	Permits Authorized	1st Choice Applicants	Permits Issued	Draw Odds	Hunters	Hunter Days	Harvest	Hunt Success
**FIREARMS (continued)**										
9	2004	10/01-10/06	15	860	15	1.2	15	57	14	93
9	2005	9/30-10/05	15	579	15	1.7	15	28	14	93
9	2006	9/08-9/17	20	828	20	2.1	20	53	19	95
9	2007	9/07-9/16	20	1011	20	1.4	20	48	19	95
9	2008	9/05-9/14	25	753	25	2.4	25	69	24	96
10	2004	10/01-10/06	20	4203	20	0.5	18	57	15	83
10	2005	9/30-10/05	40	3177	40	1.2	40	111	29	73
10	2006	9/08-9/17	50	4450	50	1.0	48	132	41	85
10	2007	9/07-9/16	50	5795	50	0.9	49	144	45	92
10	2008	9/05-9/14	60	4571	60	1.3	60	146	53	88
12	2006	9/08-9/17	2	91	2	2.2	2	4	2	100
12	2007	9/07-9/16	2	132	2	1.5	2	14	2	100
12	2008	9/05-9/14	2	64	2	3.1	2	20	2	100
13A	2004	10/01-10/06	15	318	15	3.8	14	36	10	71
13A	2005	9/30-10/05	20	307	20	4.9	20	44	19	95
13A	2006	9/08-9/17	35	485	35	3.5	34	86	30	88
13A	2007	9/07-9/16	40	805	40	3.2	40	94	40	100
13A	2008	9/05-9/14	40	491	40	5.9	36	80	31	86
13B	2004	10/01-10/06	8	172	8	2.3	8	15	5	63
13B	2005	9/30-10/05	10	98	10	9.2	7	17	7	100
13B	2006	9/08-9/17	20	364	20	4.1	17	34	14	82
13B	2007	9/07-9/16	25	185	25	4.3	24	94	17	71
13B	2008	9/05-9/14	20	183	20	7.1	20	55	18	90
17A	2004	9/24-9/27	3	255	3	1.2	3	5	3	100
17A	2005	9/23-9/28	3	175	3	1.7	3	4	3	100
17A	2006	9/08-9/17	4	217	4	1.8	4	5	4	100
17A	2007	9/07-9/16	4	292	4	1.4	4	4	4	100
17A	2008	9/05-9/14	4	163	4	2.5	4	4	4	100
18A	2004	10/01-10/06	10	536	10	1.3	10	26	10	100
18A	2005	9/30-10/05	15	419	16	2.4	15	32	13	87
18A	2006	9/15-9/20	15	579	15	1.7	15	29	14	93
18A	2007	9/14-9/19	15	662	14	1.7	14	33	12	86
18A	2008	9/12-9/18	15	453	15	1.5	15	35	8	53
18B	2004	9/24-9/29	25	525	25	1.9	24	64	14	58
18B	2005	9/23-9/28	25	316	25	2.5	24	60	18	75
18B	2006	9/08-9/17	30	500	30	5.0	27	55	21	78
18B	2007	9/07-9/16	30	796	31	2.1	30	79	28	93
18B	2008	9/05-9/14	30	442	30	4.5	29	86	27	93
19A	2004	10/01-10/06	15	803	15	1.5	15	19	14	93
19A	2004	9/24-9/29	15	827	15	1.3	15	39	13	87
19A	2005	9/30-10/05	15	667	15	1.5	14	25	14	100
19A	2005	9/23-9/28	20	643	20	2.3	20	53	16	80
19A	2006	9/15-9/20	15	926	15	1.6	15	33	12	80
19A	2006	9/08-9/17	10	520	10	1.7	9	12	9	100
19A North	2007	9/14-9/19	15	927	15	1.3	15	23	15	100
19A South	2007	9/14-9/19	10	463	10	1.1	10	16	10	100
19A North	2008	9/12-9/18	15	534	15	2.4	13	15	13	100
19A South	2008	9/05-9/14	10	292	10	2.4	10	25	10	100
19B	2004	9/24-9/27	26	1750	26	1.3	26	48	22	85
19B	2005	9/23-9/26	30	1188	30	1.8	30	78	24	80
19B	2006	9/08-9/17	40	1655	40	2.0	39	97	37	95
19B	2007	9/07-9/16	40	2006	40	1.5	40	84	38	95
19B	2008	9/05-9/14	50	1385	50	2.9	48	105	43	90
21	2004	10/01-10/06	2	234	2	0.9	2	6	2	100
21	2005	9/30-10/05	4	173	4	2.3	4	12	3	75
21	2006	9/08-9/17	5	253	5	2.0	5	17	4	80
21	2007	9/07-9/16	7	280	7	2.5	7	20	6	86
21	2008	9/05-9/14	10	329	10	2.4	8	21	5	63
30A	2004	9/24-9/27	6	237	6	2.5	6	10	6	100
30A	2005	9/23-9/26	8	177	8	4.0	8	13	8	100
30A	2006	9/08-9/17	8	214	8	2.8	6	11	6	100
30A	2007	9/07-9/16	10	290	10	2.1	10	18	8	80

CN = Camp Navajo **E** = Early Season **L** = Late Season

5-Year: 2004-2008 Harvest

Unit	Year	Dates	Permits Authorized	1st Choice Applicants	Permits Issued	Draw Odds	Hunters	Hunter Days	Harvest	Hunt Success
**FIREARMS (continued)**										
30A	2008	9/05-9/14	8	166	8	3.0	8	8	7	88
31/32	2004	10/01-10/06	4	481	4	0.8	4	7	4	100
31/32	2005	9/30-10/05	4	268	4	1.5	4	6	4	100
31/32	2006	9/08-9/17	4	330	4	1.2	4	14	3	75
31/32	2007	9/07-9/16	3	271	4	0.4	4	17	3	75
31/32	2008	9/05-9/14	3	183	3	1.1	2	7	2	100
34B	2004	10/01-10/06	1	412	1	0.2	1	1	1	100
34B	2005	9/30-10/05	1	201	1	0.5	1	1	1	100
34B	2006	9/15-9/20	1	254	1	0.4	1	1	1	100
34B	2007	9/14-9/19	1	236	1	0.4	1	1	1	100
34B	2008	9/12-9/18	1	116	1	0.9	1	1	1	100
**JUNIORS ONLY**										
2C	2004	9/24-9/29	4	270	4	0.7	4	19	1	25
7	2004	10/01-10/06	4	294	4	1.4	4	4	4	100
19B	2004	10/01-10/06	4	202	4	1.5	4	16	0	0
**MUZZLELOADER**										
2B	2004	9/24-9/29	35	522	35	3.1	33	145	14	42
2B	2005	9/23-9/28	35	409	35	6.1	30	104	18	60
2B	2006	9/08-9/17	35	446	35	5.2	35	132	21	60
2B	2007	9/07-9/16	30	534	30	3.6	29	108	16	55
2B	2008	9/05-9/14	30	342	30	4.7	28	128	14	50
3B North	2008	9/05-9/14	8	74	8	8.1	8	45	5	63
3B South	2008	9/05-9/14	2	23	2	4.3	2	5	1	50
8	2004	10/01-10/06	25	551	25	3.4	24	64	14	58
8	2005	9/30-10/05	30	494	30	4.0	29	100	14	48
8	2006	9/08-9/17	35	516	35	4.1	35	126	19	54
8	2007	9/07-9/16	35	681	35	2.8	33	122	23	70
8	2008	9/05-9/14	35	526	35	4.4	33	148	23	70
15A/15B	2004	9/24-9/27	2	35	2	5.7	2	5	1	50
15A/15B	2005	9/23-9/26	2	48	2	4.2	2	8	0	0
15A/15B	2006	9/08-9/17	2	35	2	5.7	2	1	1	50
15A/15B	2007	9/07-9/16	2	44	2	0.0	2	4	2	100
15A/15B	2008	9/05-9/14	2	23	2	4.3	2	10	0	0
17B	2004	9/24-9/29	3	55	3	5.5	3	11	2	67
17B	2005	9/23-9/28	3	40	3	5.0	3	9	2	67
17B	2006	9/08-9/17	3	60	3	5.0	3	4	3	100
17B	2007	9/07-9/16	3	104	3	1.9	3	18	3	100
17B	2008	9/05-9/14	3	82	3	3.7	3	3	3	100
18A	2004	9/24-9/29	10	143	10	4.9	10	21	7	70
18A	2005	9/23-9/28	10	202	10	3.5	10	35	8	80
18A	2006	9/08-9/13	10	185	10	4.3	10	46	8	80
18A	2007	9/07-9/12	10	307	10	1.6	10	33	9	90
18A	2008	9/05-9/11	10	218	10	4.1	9	25	8	89
19A	2004	9/24-9/29	15	321	15	2.8	15	38	10	67
19A	2005	9/23-9/28	15	178	15	6.2	15	35	13	87
19A	2006	9/08-9/13	15	289	15	3.8	15	20	14	93
19A North	2007	9/07-9/12	15	289	15	3.8	10	12	10	100
19A North	2008	9/05-9/11	20	320	20	3.4	20	45	19	95
19A South	2007	9/07-9/12	5	79	5	2.5	5	17	2	40
34B	2004	9/24-9/29	1	41	1	0.0	1	3	1	100
34B	2005	9/23-9/28	1	25	1	4.0	1	4	0	0
34B	2006	9/08-9/13	1	28	1	0.0	1	1	1	100
34B	2007	9/07-9/12	1	37	1	2.7	0	0	0	-
34B	2008	9/05-9/11	1	18	1	0.0	1	1	1	100
35	2004	9/24-9/29	1	78	1	1.3	1	5	1	100
35	2005	9/23-9/28	1	50	1	2.0	1	2	1	100
35	2006	9/08-9/17	2	59	2	3.4	2	6	1	50
35	2007	9/07-9/16	2	79	2	2.5	2	6	2	100
35	2008	9/05-9/14	2	65	2	1.5	2	3	2	100
**ARCHERY**										
1	2004	8/27-9/09	30	315	30	7.3	28	219	2	7
1	2005	8/26-9/08	30	245	30	10.2	29	152	3	10

CN = Camp Navajo **E** = Early Season **L** = Late Season

5-Year: 2004-2008 Harvest

Unit	Year	Dates	Permits Authorized	1st Choice Applicants	Permits Issued	Draw Odds	Hunters	Hunter Days	Harvest	Hunt Success
**ARCHERY (continued)**										
1	2006	8/25-9/07	30	283	30	7.8	27	176	6	22
1	2007	8/24-9/06	30	289	30	7.3	26	212	2	8
1	2008	8/22-9/04	30	208	30	10.6	30	227	2	7
3A/3C	2004	8/27-9/09	20	125	20	11.2	20	133	3	15
3A/3C	2005	8/26-9/08	20	91	20	12.1	19	141	4	21
3A/3C	2006	8/25-9/07	25	128	25	13.3	21	144	1	5
3A/3C	2007	8/24-9/06	25	133	25	6.8	21	129	3	14
3A/3C	2008	8/22-9/04	25	108	25	12.0	25	190	4	16
3B	2004	8/27-9/09	10	38	10	18.4	5	28	0	0
3B	2005	8/26-9/08	10	42	10	11.9	10	62	0	0
3B	2006	8/25-9/07	10	49	10	12.2	10	60	1	10
3B	2007	8/24-9/06	10	36	10	16.7	10	59	0	0
3B North	2004	8/27-9/09	20	39	20	20.5	20	156	4	20
3B North	2005	8/26-9/08	20	43	20	7.0	19	101	1	5
3B North	2006	8/25-9/07	20	34	20	38.2	18	114	2	11
3B North	2007	8/24-9/06	20	83	20	6.0	19	136	5	26
3B North	2008	8/22-9/04	20	37	20	29.7	20	115	5	25
3B South	2008	8/22-9/04	5	18	5	11.1	5	45	0	0
4B	2004	8/27-9/09	15	102	15	9.8	15	92	0	0
4B	2005	8/26-9/08	15	65	15	16.9	15	107	3	20
4B	2006	8/25-9/07	20	117	19	9.4	16	114	1	6
4B	2007	8/24-9/06	20	91	20	13.2	20	125	5	25
4B	2008	8/22-9/04	20	96	20	13.5	17	144	0	0
5A	2004	8/27-9/09	4	37	4	10.8	4	48	0	0
5A	2005	8/26-9/08	5	35	5	11.4	4	43	1	25
5A	2006	8/25-9/07	5	26	5	7.7	5	36	0	0
5A	2007	8/24-9/06	5	42	5	11.9	5	22	1	20
5A	2008	8/22-9/04	5	42	5	9.5	5	10	3	60
5B	2004	8/27-9/09	10	117	10	6.0	10	71	2	20
5B	2005	8/26-9/08	10	92	10	9.8	8	36	1	13
5B	2006	8/25-9/07	10	114	10	7.0	10	73	0	0
5B	2007	8/24-9/06	10	100	10	6.0	10	83	2	20
5B	2008	8/22-9/04	10	105	10	7.6	10	96	0	0
6B	2004	8/27-9/09	25	88	25	11.4	25	208	2	8
6B	2005	8/26-9/08	25	58	25	10.3	25	162	4	16
6B	2006	8/25-9/07	25	87	25	14.9	23	133	0	0
6B	2007	8/24-9/06	25	69	25	14.5	21	167	3	14
6B	2008	8/22-9/04	25	55	25	21.8	25	200	5	20
10/18	2004	8/27-9/09	100	560	100	11.1	97	630	20	21
10/18	2005	8/26-9/08	100	461	100	15.0	94	619	13	14
10/18	2006	8/25-9/07	100	610	100	10.7	88	539	25	28
10/18	2007	8/24-9/06	100	852	100	8.5	94	575	28	30
10/18	2008	8/22-9/04	100	598	100	10.4	83	535	17	20
11M	2004	9/03-9/16	5	72	5	4.2	5	41	1	20
11M	2005	9/02-9/15	5	71	5	7.0	5	55	0	0
11M	2006	8/25-9/07	5	56	5	7.1	5	51	0	0
11M	2007	8/24-9/06	5	50	5	6.0	3	37	0	0
11M	2008	8/22-9/04	5	31	5	12.9	5	34	1	20
12	2004	8/27-9/09	5	18	5	5.6	0	0	0	–
12	2005	8/26-9/08	5	19	5	21.1	4	21	0	0
12	2006	8/25-9/07	5	18	5	11.1	5	63	3	60
12	2007	8/24-9/06	5	20	5	20.0	5	45	1	20
12	2008	8/22-9/04	5	21	5	4.8	4	31	3	75
15A/15B	2004	8/27-9/09	2	34	2	5.9	2	14	0	0
15A/15B	2005	8/26-9/08	2	13	2	15.4	2	24	0	0
15A/15B	2006	8/25-9/07	4	15	4	26.7	4	23	2	50
15A/15B	2007	8/24-9/06	4	35	4	5.7	4	32	1	25
15A/15B	2008	8/22-9/04	4	10	4	20.0	4	13	4	100
17B	2004	8/27-9/09	5	44	5	6.8	3	13	3	100
17B	2005	8/26-9/08	5	52	5	7.7	5	33	0	0
17B	2006	8/25-9/07	5	52	5	9.6	5	25	4	80
17B	2007	8/24-9/06	5	84	5	4.8	5	22	2	40

CN = Camp Navajo **E** = Early Season **L** = Late Season

5-Year: 2004-2008 Harvest

Unit	Year	Dates	Permits Authorized	1st Choice Applicants	Permits Issued	Draw Odds	Hunters	Hunter Days	Harvest	Hunt Success
**ARCHERY (continued)**										
17B	2008	8/22-9/04	5	61	5	4.9	5	30	2	40
19A	2004	8/20-9/02	60	480	60	9.6	58	348	12	21
19A	2004	9/03-9/16	60	69	60	21.7	51	346	12	24
19A	2005	8/19-9/01	60	328	60	14.9	56	327	7	13
19A	2005	9/02-9/15	60	103	60	23.3	57	345	10	18
19A	2006	8/11-8/24	40	281	40	8.5	36	234	4	11
19A	2006	8/25-9/07	40	106	40	14.2	40	224	11	28
19A	2007	8/10-8/23	40	286	40	9.1	32	225	7	22
19A	2007	8/24-9/06	40	134	40	14.9	40	236	7	18
19A E	2008	8/08-8/21	40	235	40	14.0	40	215	22	55
19A L	2008	8/22-9/04	40	94	40	20.2	40	220	20	50
19B North	2004	8/27-9/09	10	132	10	4.5	10	58	7	70
19B North	2005	8/26-9/08	10	143	10	4.2	9	46	6	67
19B North	2006	8/25-9/07	20	180	20	9.4	19	95	8	42
19B North	2007	8/24-9/06	20	207	20	8.7	20	86	14	70
19B North	2008	8/22-9/04	20	155	20	11.0	20	145	11	55
21	2004	8/27-9/09	10	31	10	19.4	10	56	0	0
21	2005	8/26-9/08	10	36	10	19.4	10	63	0	0
21	2006	8/25-9/07	10	37	10	8.1	8	38	0	0
21	2007	8/24-9/06	10	28	10	17.9	10	62	0	0
21	2008	8/22-9/04	10	47	10	21.3	8	58	2	25
31/32	2004	8/27-9/09	10	65	10	7.7	10	73	0	0
31/32	2005	8/26-9/08	10	54	10	9.3	8	57	3	38
31/32	2006	8/25-9/07	10	67	10	7.5	7	48	2	29
31/32	2007	8/24-9/06	10	44	10	15.9	10	88	3	30
31/32	2008	8/22-9/04	10	50	10	12.0	10	57	2	20
34B	2004	8/27-9/09	1	46	1	2.2	1	5	0	0
34B	2005	8/26-9/08	1	35	1	2.9	1	1	1	100
34B	2006	8/25-9/07	1	39	1	0.0	1	6	0	0
34B	2007	8/24-9/06	1	37	1	2.7	1	12	0	0
34B	2008	8/22-9/04	1	35	1	2.9	1	1	1	100
35	2004	8/27-9/09	8	85	8	5.9	8	53	5	63
35	2005	8/26-9/08	6	72	6	6.9	6	32	2	33
35	2006	8/25-9/07	10	71	10	7.0	9	55	1	11
35	2007	8/24-9/06	10	71	10	7.0	10	37	3	30
35	2008	8/22-9/04	10	64	10	9.4	10	60	7	70
CN	2004	8/27-9/10	4	3	4	100.0	4	20	0	0
CN	2004	8/27-9/10	2	2	2	50.0	2	10	0	0
CN	2005	8/19-8/28	4	5	4	80.0	4	16	0	0
CN	2005	8/19-8/28	2	6	2	16.7	2	9	0	0
CN	2006	8/25-9/07	4	4	4	100.0	2	20	0	0
CN	2006	8/25-9/07	2	2	2	100.0	2	12	0	0
CN	2007	8/24-9/06	4	6	4	66.7	4	30	2	50
CN	2008	8/22-9/04	4	4	4	100.0	4	6	0	0

CN = Camp Navajo **E** = Early Season **L** = Late Season

Elk *(Cervus elaphus)*

Natural History

Elk were at one time thinly distributed in Arizona from the White and Blue mountains westward along the Mogollon Rim to near the San Francisco Peaks. These native elk were eliminated sometime prior to 1900. In February 1913, private conservationists released 83 elk from Yellowstone National Park into Cabin Draw near Chevelon Creek. These, and two other transplants of Yellowstone elk in the 1920s—one south of Alpine, and another north of Williams—were great successes, and Arizona's elk population has now grown to approximately 24,000 post-hunt adults as of 2003.

Mountain meadows, ponderosa pine woodlands, spruce-fir forests, and other high elevation habitats between 7,000 and 10,500 feet elevation constitute the elk's principal summer range. Elk are rarely found more than one-half mile from water and tend to stay on the summer range as long as possible, arriving early in the year and remaining until forced down by deep snow. Their winter range, which is usually between 5,500 and 6,500 feet elevation, is more limited in extent and may only comprise about 10 percent of the animal's total habitat. Here, in the pinyon-juniper zone, elk remain until melting snows allow them to migrate upward.

Elk have distinct summer and winter coats, which they shed in late summer and spring, respectively. In winter, the head, belly, neck, and legs are dark brown, and the sides and back are a grayish-brown; the rump patch is a yellowish color bordered by a dark brownish stripe. While females are usually somewhat lighter in color than bulls, both sexes have heavy dark manes. In summer, the coat becomes a deep reddish brown. Elk

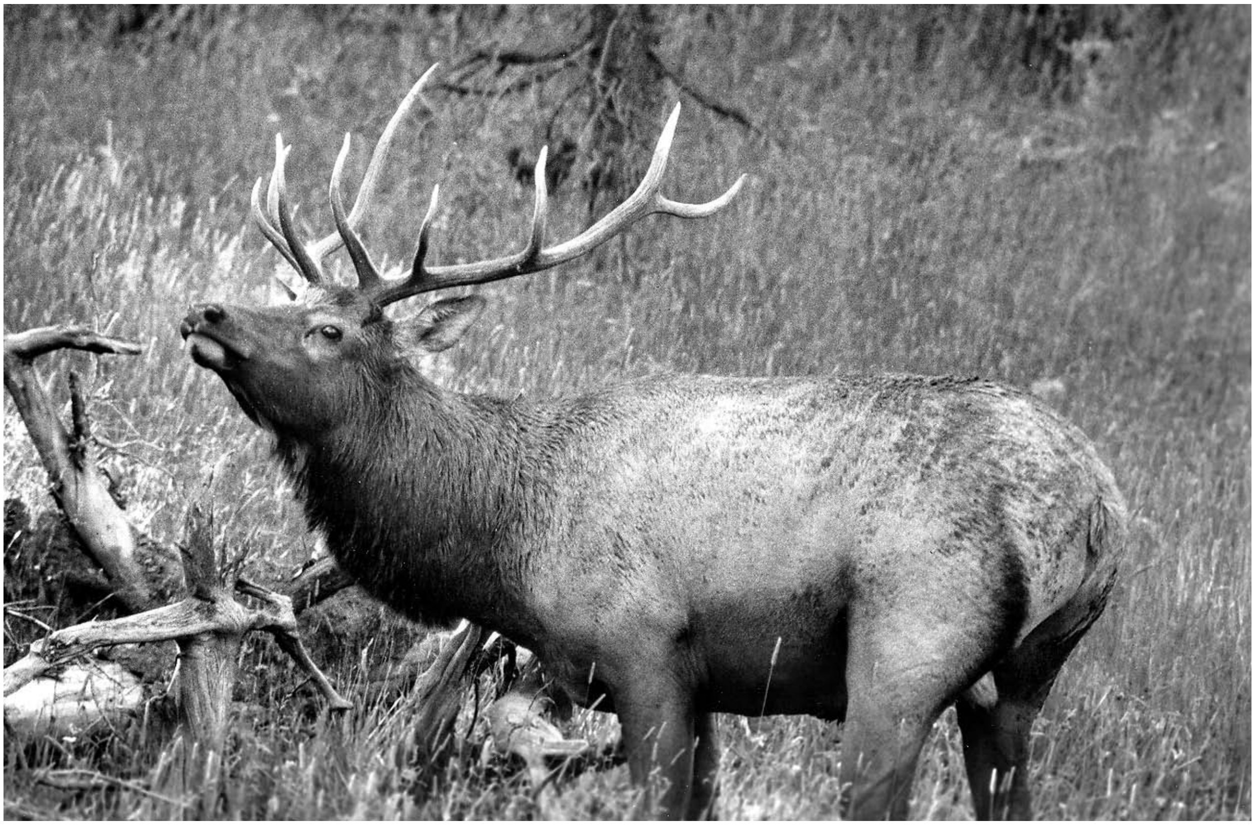

DAVE DAUGHTRY

have little to no undercoat, giving them a sleek, muscular appearance.

Calves are born between late May and early June after an 8-month gestation period. They are dark russet in color with white spots on the back and sides. Newly born calves weigh an average of nearly 30 pounds, with males averaging 4 pounds more than females. Twins are extremely rare.

When the time comes to give birth, a cow will drive off her previous year's calf and separate from the herd to seek out an area of dense cover for a nursery. Within hours after birth, the newborn is able to move and is led from the birthing spot to a safer place. After a week, the mother will band with other cow elk, and after two to three weeks, the calves, now able to run, will join the herd. Some of these matriarchal bands may number in the hundreds. By September, the calves will have shed their spotted coats and will be behaving much like their mothers.

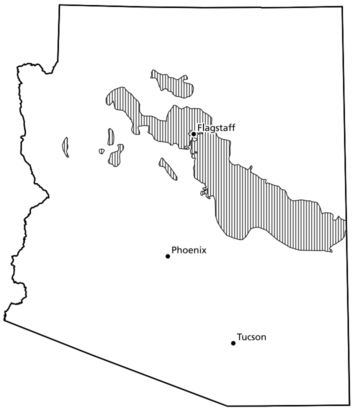

Elk distribution

An elk's natural life span is about 14 to 16 years for males and 15 to 17 for females, even though tagged animals of more than 25 years old have been documented.

Antler development and size is a function of age, the older, larger bulls having the most developed antlers. Old bulls shed their antlers between January and March, and yearling males sometime between March and June. As soon as antlers are shed, new ones begin growing, so it is possible to see yearlings with old spikes and bulls in velvet at the same time. The antlers continue to grow for a period ranging from 90 days for yearlings to 150 days for adult bulls.

By early August, antler growth is complete. The now dry velvet is stripped off the hardened antlers in a matter of hours as the bull polishes them against trees. By early September, the bull is in the rut, and bugling and harem formation occurs. Harems may number up to 30, depending on the size and vigor of the bull, but usually average 15 to 20.

A large bull may weigh up to 1,200 pounds, but most range between 600 to 800 pounds. The live weight of mature cows ranges from 450 to 600 pounds. Elk evolved as distance runners and can approach speeds of 40 mph for short periods, and maintain speeds of nearly 30 mph for longer periods. They are also strong swimmers—even calves can swim more than a mile—and high jumpers, a 10-foot fence may not stop an adult.

Elk are grass-eating animals, and one of the requirements of feeding in open country is to always be on the alert for danger. As herd animals, some elk can always be watching for predators while the others feed.

Hunt History

As with many game species in Arizona, elk hunting has had its ups and downs. With native elk having been extirpated, the closed season imposed by the territorial legislature in 1893 was too little too late. The releases of Yellowstone elk between 1913 and 1929 were successful, however, and in 1935 the population was deemed sufficient to support a limited, 266-permit bull hunt. One hundred and forty-five elk were harvested, and hunts were continued every year through 1943.

Because of World War II, no season was conducted in 1944 or 1945, but a limited hunt, which included the issuance of the first cow elk permits, was again authorized in 1946. Elk hunting opportunities expanded almost annually as biologists and ranchers feared that Arizona's elk population might now "rise out of control." These concerns culminated in 1953 when 6,288 permits were issued and 1,558 elk were taken—more than 1,000 of which were cows. Because of concerns about the "slaughter," elk permits were greatly curtailed in 1954 and remained below 5,000 until 1965, when more than 6,000 permits were again authorized. By 1967, elk permit numbers were exceeding 7,000, and the annual harvest exceeded 1,500 elk. Once again, elk permits were gradually lowered, although new hunts, including archery hunts, were being initiated.

By the mid-1980s, elk, and elk permit numbers, were again headed upward. This trend culminated in 1994, when nearly 11,000 elk were harvested—a number unimaginable just 20 years earlier. Since then, elk numbers and harvests have remained at a high level with more than 9,800 elk taken in 1999. This situation is expected to continue for the foreseeable future as wildlife managers and land managers continue to be concerned about habitat quality and elk-livestock competition.

Historic Summary of Elk Survey Data

Year	Spike	Bull	Cow	Calf	Unclassified	Total	Bulls[1] /100 Cows	Calves/100 Cows
1947	17	89	332	129	0	567	32	39
1948	44	138	357	182	0	721	51	51
1949	45	101	309	129	0	584	47	42
1950	30	91	290	141	0	552	42	49
1951	27	121	293	116	4	561	51	40
1952	11	93	241	93	0	438	43	39
1953	35	92	206	78	0	411	62	38
1954	14	77	202	79	35	407	45	39
1955	21	88	221	73	37	440	49	33
1956	14	48	122	54	15	253	51	44
1957	13	70	111	48	34	276	75	43
1958	10	62	74	40	16	202	97	54
1959	22	87	152	79	49	389	72	52
1960	23	43	127	70	37	300	52	55
1961	33	83	172	80	23	391	67	47
1962	18	51	164	86	16	335	42	52
1963	53	111	288	138	54	644	57	48
1964	25	94	228	124	51	522	52	54
1965	41	86	284	167	57	635	45	59
1966	54	121	387	233	41	836	45	60
1967	100	124	446	267	24	961	50	60
1968	39	132	486	271	21	949	35	56
1969	61	147	526	296	40	1070	40	56
1970	53	96	469	256	96	970	32	55
1971	86	148	495	267	270	1266	47	54
1972	67	126	471	274	150	1088	41	58
1973	56	88	438	280	230	1092	33	64
1974	60	126	597	353	244	1380	31	59
1975	68	139	598	393	192	1390	35	66
1976	85	148	546	330	158	1267	43	60
1977	93	185	678	404	117	1477	41	60
1978	122	158	775	473	68	1596	36	61
1979	156	196	1142	602	66	2162	31	53
1980	53	109	601	338	82	1183	27	56
1981	125	276	1121	618	199	2339	36	55
1982	163	154	1264	707	86	2374	25	56
1983	175	199	1186	691	43	2294	32	58
1984	365	281	2032	1172	131	3981	32	58
1985	286	250	1693	978	285	3492	32	58
1986	274	245	1827	903	204	3453	28	49
1987	384	405	2671	1504	203	5167	30	56
1988	447	434	2810	1537	263	5491	31	55
1989	752	599	4306	2142	461	8260	31	50
1990	647	678	4405	1813	198	7741	30	41
1991	639	869	5354	2860	931	10653	28	53
1992	947	895	5647	2671	399	10559	33	47
1993	926	889	7698	3892	324	13729	24	51
1994	934	1080	6530	2807	591	11942	31	43
1995	837	1111	6793	2809	105	11655	29	41
1996	869	1348	7493	2559	255	12524	30	34
1997	727	1383	6461	2423	178	11172	33	38
1998	670	1535	7052	3440	131	12828	31	49
1999	986	1330	6397	2901	432	12046	36	45
2000	965	1300	7684	3013	161	13123	29	39
2001	400	1224	4540	1251	29	7444	36	28
2002	344	1217	5409	1842	53	8865	29	34
2003	489	1460	4732	1589	117	8387	41	34
2004	493	1347	4585	2289	195	8909	40	50
2005	378	1082	4136	1894	62	7552	35	46
2006	592	1261	4984	1847	170	8854	37	37
2007	473	1077	4328	1641	2	7521	36	38
2008	336	1186	3764	1448	29	6763	40	38

[1] Includes spikes

5-Year: 2004-2008 Elk Survey Data

Unit	Year	Spike	Bull	Cow	Calf	Unclassified	Total	Bulls[1]/100 Cows	Calves/100 Cows
1	2004	36	122	311	141	6	616	51	45
1	2005	39	105	398	165	0	707	36	41
1	2006	81	164	544	174	62	1025	45	32
1	2007	82	113	501	217	0	913	39	43
1	2008	66	189	673	257	0	1185	38	38
2B	2004	5	37	53	20	0	115	79	38
3A/3C	2004	18	76	242	148	0	484	39	61
3A/3C	2005	3	114	377	197	0	691	31	52
3A/3C	2006	17	65	311	134	0	527	26	43
3A/3C	2007	54	122	395	193	0	764	45	49
3A/3C	2008	34	34	173	85	0	326	39	49
3B	2004	1	14	48	20	0	83	31	42
3B	2005	6	20	56	27	0	109	46	48
3B	2006	8	13	38	15	0	74	55	39
3B	2007	5	10	46	19	0	80	33	41
3B	2008	8	12	51	10	0	81	39	20
4A	2004	17	50	185	74	1	327	36	40
4A	2005	17	53	167	68	5	310	42	41
4A	2006	40	42	255	75	5	417	32	29
4A	2007	23	56	140	63	0	282	56	45
4A	2008	20	55	133	58	0	266	56	44
4B	2004	10	31	84	32	0	157	49	38
4B	2005	2	51	93	33	0	179	57	35
4B	2006	7	25	81	30	0	143	40	37
4B	2007	9	45	100	43	0	197	54	43
4B	2008	5	19	27	5	0	56	89	19
5A	2004	18	46	201	113	2	380	32	56
5A	2005	17	38	220	112	14	401	25	51
5A	2006	30	82	263	98	0	473	43	37
5A	2007	11	57	154	66	1	289	44	43
5A	2008	19	89	156	72	0	336	69	46
5BN	2004	49	91	254	101	0	495	55	40
5BN	2005	36	86	238	62	0	422	51	26
5BN	2006	15	34	194	73	0	316	25	38
5BN	2007	16	86	241	82	0	425	42	34
5BN	2008	25	85	240	59	0	409	46	25
5BS	2004	59	105	532	285	0	981	31	54
5BS	2005	32	72	358	155	0	617	29	43
5BS	2006	31	118	574	182	3	908	26	32
5BS	2007	21	39	202	54	0	316	30	27
5BS	2008	18	60	348	91	0	517	22	26
6A	2004	67	93	407	252	0	819	39	62
6A	2005	38	121	309	190	4	662	51	61
6A	2006	99	111	657	209	0	1076	32	32
6A	2007	38	61	344	121	0	564	29	35
6A	2008	6	59	193	58	3	319	34	30
6B	2004	13	39	90	54	0	196	58	60
6B	2005	12	17	108	52	0	189	27	48
6B	2006	48	38	117	60	51	314	74	51
6B	2007	14	18	144	62	0	238	22	43
6B	2008	8	18	73	37	1	137	36	51
7	2004	17	25	242	129	14	427	17	53
7	2005	25	47	181	62	0	315	40	34
7E	2006	16	40	159	71	1	287	35	45
7E	2007	11	24	66	34	0	135	53	52
7E	2008	15	43	77	50	0	185	75	65
7W	2006	30	41	205	91	0	367	35	44
7W	2007	41	38	328	103	0	510	24	31
7W	2008	6	26	165	67	0	264	19	41
8	2004	39	36	294	128	0	497	26	44
8	2005	40	13	155	78	34	320	34	50
8	2006	35	38	223	78	0	374	33	35
8	2007	14	23	146	51	0	234	25	35

[1] Includes spikes

5-Year: 2004–2008 Elk Survey Data

Unit	Year	Spike	Bull	Cow	Calf	Unclassified	Total	Bulls[1]/100 Cows	Calves/100 Cows
8	2008	6	29	98	43	12	188	36	44
9	2004	27	169	388	207	15	806	51	53
9	2005	18	72	213	122	0	425	42	57
9	2006	23	111	320	137	36	627	42	43
9	2007	23	53	315	81	0	472	24	26
9	2008	23	85	178	74	8	368	61	42
10	2004	33	108	398	189	148	876	35	47
10	2005	56	90	558	267	0	971	26	48
10	2006	13	40	109	52	2	216	49	48
10	2007	17	47	175	48	0	287	37	27
10	2008	0	57	165	67	0	289	35	41
18A	2004	11	27	51	26	0	115	75	51
18B	2006	0	0	0	2	2	4	–	–
19B	2006	0	7	0	0	2	9	–	–
21	2005	0	8	20	8	0	36	40	40
21	2006	0	9	15	6	0	30	60	40
21	2007	2	7	14	2	0	25	64	14
21	2008	0	11	23	8	0	42	48	35
22	2004	25	118	197	89	2	431	73	45
22	2005	7	53	123	62	0	245	49	50
22	2006	47	128	329	145	0	649	53	44
22	2007	40	115	278	113	0	546	56	41
22	2008	25	100	274	92	0	491	46	34
22N	2007	0	3	6	2	0	11	50	33
23	2004	16	91	202	80	5	394	53	40
23	2005	16	94	237	102	3	452	46	43
23	2006	23	101	196	65	0	385	63	33
23	2007	11	88	204	69	1	373	49	34
23	2008	30	124	342	154	0	650	45	45
23N	2007	0	9	28	11	0	48	32	39
27	2004	15	32	143	52	2	244	33	36
27	2005	9	18	117	42	2	188	23	36
27	2006	18	45	214	63	6	346	29	29
27	2007	6	38	116	32	0	192	38	28
27	2008	10	28	143	58	5	244	27	41
CN	2004	17	37	263	149	0	466	21	57
CN	2005	5	10	208	90	0	313	7	43
CN	2006	11	9	180	87	0	287	11	48
CN	2007	35	25	385	175	0	620	16	45
CN	2008	12	63	232	103	0	410	32	44

[1] Includes spikes
CN = Camp Navajo

Elk Harvest Data

Historic Summary of General Elk Hunts

Year	1st Choice Applicants	Permits Issued	Hunters	Hunter Days	Harvest[1]					Percent Success
					Bulls	Spikes	Cows	Calves	Total	
1935	–	–	266	–	137	8	0	0	145	55
1936	–	–	249	–	76	9	0	0	85	34
1937	–	–	230	–	47	18	0	0	65	28
1938	–	–	169	–	68	17	0	0	85	50
1939	–	–	238	–	77	27	6	0	110	46
1940	–	–	229	–	76	19	0	0	95	41
1941	–	–	581	–	114	19	0	0	133	23
1942	–	–	1167	–	223	96	0	0	319	27
1943	–	–	2047	–	152	98	0	0	250	12
1946	–	–	498	–	103	0	13	0	116	23
1947	–	–	1616	–	246	0	255	0	501	31
1948	–	–	2200	–	453	0	467	0	920	42
1949	–	2850	2675	–	290	0	566	0	856	32
1950	–	4250	3685	–	413	1	1070	0	1484	40
1951	–	6023	5788	–	467	41	1185	0	1693	29
1952	–	5476	5192	–	302	42	845	0	1189	23
1953	–	6288	6015	–	380	124	1054	0	1558	26
1954	–	2985	2846	–	176	58	395	0	629	22
1955	–	2225	2096	–	207	58	347	0	612	29
1956	–	1750	1581	–	115	29	119	39	302	19
1957	–	1275	1074	–	123	0	0	0	123	11
1958	–	1483	1321	–	181	0	0	0	181	14
1959	–	–	1136	–	282	0	0	0	282	25
1960	–	–	1661	–	312	93	131	54	590	36
1961	–	–	1492	–	343	104	107	34	588	39
1962	–	–	2266	–	402	110	172	86	770	34
1963	–	–	3184	–	528	180	339	107	1154	36
1964	–	–	4060	–	566	163	338	126	1193	29
1965	–	–	4941	–	590	185	426	168	1369	28
1966	7811	–	5687	–	709	241	500	188	1638	29
1967	7730	–	6526	–	745	304	442	191	1682	26
1968	8379	–	5845	–	613	279	376	135	1403	24
1969	9843	–	5771	–	551	266	355	87	1259	22
1970	11888	–	5208	–	500	239	202	77	1018	20
1971	10812	–	4866	–	742	407	330	105	1584	33
1972	12644	5561	5177	–	423	279	267	84	1053	20
1973	16078	5675	5321	–	460	296	295	91	1142	21
1974	18623	5972	5685	27227	437	368	309	72	1186	21
1975	19504	5758	5088	21248	443	317	172	44	976	19
1976	20511	5915	5528	23808	478	438	343	89	1348	24
1977	23198	6145	5792	26294	556	376	406	71	1409	24
1978	26745	5935	5502	22409	571	510	425	95	1601	29
1979	27041	5800	5456	24344	534	485	390	65	1474	27
1980	28198	5850	5479	26554	584	499	422	68	1573	29
1981	28286	5385	5093	22952	796	606	390	81	1873	37
1982	26507	5720	5522	24529	816	735	400	96	2047	37
1983	29572	6060	5757	24741	732	776	405	96	2009	35
1984	28780	6005	5791	24496	995	1031	442	74	2542	44
1985	31121	6730	6450	25782	1159	1169	867	220	3415	53
1986	33437	6385	6202	27613	1155	1115	592	126	2988	48
1987	34995	6300	6164	26477	1209	1010	693	91	3003	49
1988	37289	6955	6785	25600	1376	1165	1162	224	3927	58
1989	38965	7975	7796	28980	1473	1144	1069	184	3870	50
1990	41616	8585	8389	29148	1790	1233	1510	188	4721	56
1991	41415	9718	9349	30811	2047	1207	1784	271	5309	57
1992	49054	10491	10207	34757	2028	1351	2067	262	5708	56
1993	51919	11579	11309	38157	2011	962	3106	445	6524	58
1994	60849	14683	14382	46962	2201	1121	4867	630	8819	61
1995	63582	14891	14613	50862	2368	794	4132	522	7816	53
1996	63003	14229	13897	46444	2553	936	4262	512	8263	59
1997	66013	11683	11398	41591	2590	583	2490	306	6269	55
1998	66823	12110	11832	43552	2423	664	2744	385	6216	53

[1] In some years prior to 1960, spikes and calves were not differentiated from bulls and cows.

Historic Summary of General Elk Hunts

Year	1st Choice Applicants	Permits Issued	Hunters	Hunter Days	Harvest[1]					Percent Success
					Bulls	Spikes	Cows	Calves	Total	
1999	71839	15538	15158	55291	2082	724	4037	556	7399	49
2000	66652	15460	14940	54195	2260	724	3956	475	7415	50
2001	70809	18285	17628	66564	2214	393	4348	375	7330	42
2002	69798	16265	15767	62497	2276	282	3482	309	6349	40
2003	71514	13402	12983	52398	1949	313	2690	288	5240	40
2004	76542	14967	14399	56288	2159	357	3191	405	6112	42
2005	64684	15856	15254	63702	2077	367	3034	376	5585	38
2006	66873	16321	15773	68255	2303	532	3369	340	6544	41
2007	65190	16848	16189	72481	2412	496	3221	373	6502	40
2008	52044	17756	16968	77827	2712	444	3188	371	6715	40

[1] In some years prior to 1960, spikes and calves were not differentiated from bulls and cows.

Historic Summary of Juniors-Only Elk Hunts

Year	1st Choice Applicants	Permits Issued	Hunters	Hunter Days	Harvest					Percent Success
					Bulls	Spikes	Cows	Calves	Total	
1994	269	75	75	233	0	0	23	8	31	41
1995	291	100	100	233	0	0	59	6	65	65
1996	409	175	173	466	0	0	94	9	103	60
1997	654	200	195	526	0	0	101	14	115	59
1998	927	400	391	1061	0	0	208	31	239	61
1999	1372	1185	1162	3017	0	0	574	88	662	57
2000	2022	1200	1173	2959	0	0	638	68	706	60
2001	2416	1370	1352	3744	0	0	543	50	593	43
2002	2705	1088	1066	2923	0	0	498	51	549	52
2003	2744	1076	1054	2891	0	0	470	62	532	50
2003	2744	1076	1054	2891	0	0	470	62	532	50
2004	2668	1025	996	2555	0	0	532	76	608	61
2005	2462	1161	1123	3139	0	0	459	68	527	47
2006	2580	1142	1100	3286	0	0	532	53	585	53
2007	3017	1181	1100	3286	0	0	618	59	677	60
2008	2817	1335	1288	3786	0	0	573	92	665	52

Historic Summary of Muzzleloader Elk Hunts

Year	1st Choice Applicants	Permits Issued	Hunters	Hunter Days	Harvest					Percent Success
					Bulls	Spikes	Cows	Calves	Total	
1980	138	80	77	429	1	1	6	0	8	10
1981	98	50	49	200	6	0	0	0	6	12
1982	381	200	194	805	43	7	0	0	50	26
1983	420	130	124	518	37	2	0	0	39	31
1984	854	150	149	535	36	9	21	10	76	51
1985	880	200	197	811	37	8	11	3	59	30
1986	1030	200	200	753	57	12	25	1	95	48
1987	1307	200	194	805	51	16	17	3	87	45
1988	1215	225	222	809	56	12	69	4	141	64
1989	1089	225	225	766	42	12	74	10	138	61
1990	1389	225	223	886	47	3	37	10	97	43
1991	1876	265	263	1066	116	11	19	4	150	57
1992	1313	410	405	1472	46	8	150	18	222	55
1993	2244	451	450	1766	145	16	89	14	264	59
1994	2953	752	729	2796	133	12	167	36	348	48
1995	2707	766	753	2788	128	6	238	38	410	54
1996	4227	723	703	2802	96	5	156	22	279	40
1997	4486	937	919	3588	172	26	125	23	346	38
1998	3819	1120	1076	3947	163	24	217	20	424	39
1999	4118	1183	1148	4438	159	38	198	28	423	37
2000	5115	1168	1118	4033	225	27	199	47	498	45
2001	3591	1495	1437	5580	209	13	235	21	478	33
2002	5287	1015	977	3874	186	21	101	4	312	32
2003	5457	1087	1054	4332	180	17	147	24	368	35
2004	4814	1325	1279	5082	255	20	245	28	548	43

Historic Summary of Muzzleloader Elk Hunts (continued)

Year	1st Choice Applicants	Permits Issued	Hunters	Hunter Days	Harvest					Percent Success
					Bulls	Spikes	Cows	Calves	Total	
2005	4672	1276	1217	5116	203	31	161	28	423	35
2006	5238	1161	1101	4743	229	33	164	5	431	39
2007	4858	1206	1179	4963	208	45	200	36	489	41
2008	4723	1386	1327	6139	253	59	236	35	583	41

Historic Summary of Archery Elk Hunts

Year	1st Choice Applicants	Permits Issued	Hunters	Hunter Days	Harvest					Percent Success
					Bulls	Spikes	Cows	Calves	Total	
1978	3756	2865	2552	16941	62	38	46	1	147	6
1979	3854	2990	2802	19069	110	74	68	2	254	9
1980	4265	3450	3268	22590	164	57	91	9	321	10
1981	5037	2925	2805	18562	136	41	48	9	234	8
1982	5092	3600	3469	23906	154	75	61	12	302	9
1983	4454	3935	3775	25370	216	93	93	10	412	11
1984	4738	3760	3627	24543	208	105	80	12	405	11
1985	4954	3810	3696	24602	198	127	136	24	485	13
1986	5574	3699	3613	24471	281	135	125	26	567	16
1987	6236	3680	3599	25528	301	152	161	29	643	18
1988	6807	3615	3538	24391	308	123	188	17	636	18
1989	7776	3925	3837	27019	418	161	254	15	848	22
1990	8357	4230	4152	28730	545	126	191	19	881	21
1991	8900	4806	4729	33141	549	137	381	39	1106	23
1992	9831	5315	5184	35902	675	178	459	46	1358	26
1993	10201	5318	5225	38027	587	151	479	56	1273	24
1994	11256	6880	6731	46661	775	192	754	67	1788	27
1995	12167	6780	6654	47049	874	160	750	50	1834	28
1996	12898	5756	5638	41417	518	121	514	38	1191	21
1997	13807	6151	6033	43221	887	84	547	44	1562	26
1998	15301	5386	5288	35826	1074	65	631	55	1825	35
1999	17506	5440	5303	38333	743	74	475	42	1334	25
2000	18268	7168	6978	49801	675	129	998	79	1881	27
2001	17907	8507	8271	54328	1169	79	922	57	2227	27
2002	18581	5827	5662	42505	460	14	541	29	1044	18
2003	18833	6708	6537	47439	1042	57	737	59	1895	29
2004	20597	5577	5435	39360	962	35	584	56	1637	30
2005	20869	6676	6491	46313	1143	82	890	78	2193	34
2006	22653	6510	6367	45887	919	55	776	43	1793	28
2007	24684	5132	4963	38251	910	62	458	33	1463	29
2008	21625	5883	5675	44982	1241	73	357	23	1694	30

Elk Hunt Data

5-Year: 2004–2008 Harvest

Unit		Year	Hunt Type	Dates	Permits Authorized	1st Choice Applicants	Permits Issued	Draw Odds	Hunters	Hunter Days	Harvest					Hunt Success
											Bull	Spike	Cow	Calf	Total	
**GENERAL**																
1		2004	BE	10/01-10/06	40	6948	40	0.6	40	108	33	0	0	0	33	83
1		2005	BE	9/30-10/05	40	6016	40	0.6	40	125	38	0	0	0	38	95
1	CH	2008	ALS	10/10-10/16	10	38	10	26.3	10	37	0	0	10	0	10	100
1	ES	2004	ALS	12/17-12/23	75	68	75	26.5	69	247	0	0	34	4	38	55
1	ES	2005	ALS	12/16-12/22	75	61	75	55.7	73	266	0	0	21	2	23	32
1	ES	2006	ALS	12/15-12/21	65	50	65	36.0	63	233	0	0	25	0	25	40
1	ES	2007	ALS	12/14-12/20	50	86	50	39.5	46	137	0	0	20	0	20	43
1	ES	2008	ALS	12/12-12/18	50	49	50	61.2	46	189	0	0	13	0	13	28
1	RV	2004	ALS	12/10-12/14	30	5	30	100.0	26	81	0	0	0	0	0	0
1	RV	2004	ALS	12/24-12/28	30	6	30	100.0	27	87	0	0	3	0	3	11
1	RV	2005	ALS	12/09-12/13	30	15	30	86.7	30	118	0	0	3	0	3	10
1	RV	2005	ALS	12/23-12/27	30	4	30	100.0	28	100	0	0	3	0	3	11
1	RV	2006	ALS	12/08-12/12	30	6	28	100.0	25	89	0	0	8	0	8	32
1	RV	2006	ALS	12/22-12/26	30	5	30	100.0	24	75	0	0	0	0	0	0
1	RV	2007	ALS	12/07-12/11	30	21	30	66.7	30	98	0	0	11	0	11	37
1	RV	2008	ALS	12/19-12/25	40	11	40	100.0	34	74	0	0	19	0	19	56
1/2B/2C		2007	BE	9/28-10/04	40	5484	41	0.7	41	118	36	0	0	0	36	88
1/2B/2C		2004	B	11/19-11/25	360	3615	366	6.8	366	1482	174	52	0	0	226	62
1/2B/2C		2005	B	11/18-11/24	425	3318	425	7.6	412	1702	173	39	0	0	212	51
1/2B/2C		2006	B	11/24-11/30	425	4952	425	5.5	412	1809	147	59	0	0	206	50
1/2B/2C		2007	B	11/23-11/29	425	3395	425	7.7	415	1807	179	62	0	0	241	58
1/2B/2C		2008	B	11/28-12/04	425	3010	425	9.5	414	1853	167	58	0	0	225	54
1/2B/2C		2004	ALS	12/17-12/23	450	1757	450	16.7	434	1455	0	0	246	21	267	62
1/2B/2C		2005	ALS	12/16-12/22	420	1249	420	21.3	395	1446	0	0	204	19	223	56
1/2B/2C		2006	ALS	12/15-12/21	105	820	105	7.3	101	271	0	0	64	4	68	67
1/2B/2C		2007	ALS	12/14-12/20	70	357	70	9.8	67	127	0	0	49	7	56	84
1/2B/2C		2008	ALS	12/12-12/18	150	480	150	19.8	148	405	0	0	79	10	89	60
1E		2005	ALS	12/02-12/08	225	126	225	61.9	216	770	0	0	77	9	86	40
1E		2006	ALS	12/01-12/07	65	158	65	11.4	63	217	0	0	30	2	32	51
1E		2007	ALS	11/30-12/06	50	85	50	36.5	49	199	0	0	17	0	17	35
1E		2008	ALS	12/05-12/11	50	75	50	36.0	48	176	0	0	21	3	24	50
2A/2B		2008	ALSS	12/05-12/14	30	0	30	–	22	70	0	0	3	0	3	14
2A/2B		2008	ALS	9/12-9/21	40	12	40	100.0	35	190	0	0	5	0	5	14
2A/2B		2008	ALS	10/03-10/12	40	1	40	100.0	40	218	0	0	8	0	8	20
2A/2B		2008	ALS	10/17-10/26	30	3	30	100.0	30	135	0	0	2	2	4	13
2A/2B		2008	AE	9/12-9/21	20	69	20	24.6	20	82	5	2	0	0	7	35
2A/2B		2008	AE	10/03-10/12	20	37	20	16.2	20	104	10	2	4	0	16	80
2A/2B		2008	AE	10/17-10/26	15	5	15	40.0	15	75	0	0	4	0	4	27
2A/2B		2008	AE	12/05-12/14	15	4	15	50.0	15	69	2	2	2	0	6	40
2B		2004	ALS	9/10-9/19	25	14	25	78.6	25	128	0	0	13	0	13	52
2B		2004	ALS	10/01-10/10	25	8	25	100.0	21	81	0	0	6	0	6	29
2B		2004	ALS	10/15-10/24	25	9	25	100.0	23	130	0	0	3	0	3	13
2B		2005	ALS	9/09-9/18	40	18	40	94.4	32	144	0	0	5	0	5	16
2B		2005	ALS	9/30-10/09	35	2	35	100.0	32	172	0	0	3	0	3	9
2B		2005	ALS	10/14-10/23	25	2	25	100.0	23	111	0	0	2	0	2	9
2B		2005	ALS	12/02-12/11	20	5	20	40.0	18	80	0	0	3	5	8	44
2B		2006	ALS	9/15-9/24	40	9	40	77.8	38	205	0	0	5	2	7	18
2B		2006	ALS	10/06-10/12	35	2	34	100.0	32	138	0	0	2	0	2	6
2B		2006	ALS	10/20-10/29	25	7	25	100.0	25	148	0	0	0	0	0	0
2B		2006	ALS	12/08-12/17	20	0	20	–	18	104	0	0	0	0	0	0
2B		2007	ALS	8/24-9/06	25	4	25	100.0	21	135	0	0	2	0	2	10
2B		2007	ALS	10/05-10/14	25	6	25	100.0	23	94	0	0	6	0	6	26
2B		2007	ALS	10/19-10/28	20	3	20	100.0	19	73	0	0	7	3	10	53
2B		2007	ALS	12/07-12/16	20	6	19	100.0	18	99	0	0	3	0	3	17
2B		2004	AE	9/10-9/19	10	126	10	5.6	10	26	6	0	0	0	6	60
2B		2004	AE	10/01-10/10	10	23	10	4.3	10	37	7	0	0	0	7	70
2B		2004	AE	10/15-10/24	10	14	10	42.9	9	37	3	0	0	0	3	33
2B		2005	AE	9/09-9/18	20	78	20	15.4	20	97	4	0	0	0	4	20
2B		2005	AE	9/30-10/09	15	19	15	26.3	15	33	8	0	0	0	8	53
2B		2005	AE	10/14-10/23	15	28	15	35.7	11	38	5	0	0	0	5	45
2B		2005	AE	12/02-12/11	10	13	10	7.7	9	36	1	0	0	0	1	11

BE = Early Bull **B** = Bull **ALS** = Antlerless **AE** = Any Elk **CN** = Camp Navajo **CH** = CHAMP Hunt **DV** = Disabled Veteran

Herd-Units:

CC = Canyon Creek
ES (Unit 1) = Escudilla
GV = Grapevine
HM = Hutch Mtn.
M = Martinez
ML = Marshall Lake
MM = Melatone Mesa
MZ = Mazatzal
RV = Round Valley
SM = East Sunset/West Sunset/Meteor Crater
TT = Twin Arrows/Two Guns/Grapevine
VV = Verde Valley
WI = Winslow
WS (17/20A/20C) = Williamson Valley/Skull Valley/Kirkland Junction

5-Year: 2004-2008 Harvest

Unit		Year	Hunt Type	Dates	Permits Authorized	1st Choice Applicants	Permits Issued	Draw Odds	Hunters	Hunter Days	Harvest					Hunt Success
											Bull	Spike	Cow	Calf	Total	
**GENERAL (continued)**																
2B		2006	AE	-9/24	20	147	20	10.2	20	108	10	0	0	0	10	50
2B		2006	AE	10/06-10/15	15	31	15	12.9	15	78	4	0	0	1	5	33
2B		2006	AE	10/20-10/29	15	15	15	33.3	14	87	2	0	0	0	2	14
2B		2006	AE	12/08-12/17	10	12	10	50.0	10	25	0	0	0	0	0	0
2B		2007	AE	8/24-9/06	10	41	9	17.1	9	41	4	4	0	0	8	89
2B		2007	AE	10/05-10/14	10	37	10	18.9	10	45	8	0	0	0	8	80
2B		2007	AE	10/19-10/28	10	11	10	18.2	8	46	4	0	0	0	4	50
2B		2007	AE	12/07-12/16	10	26	9	15.4	9	45	0	0	2	0	2	22
3A/3C		2004	BE	10/01-10/06	20	2184	22	0.9	22	58	19	0	0	0	19	86
3A/3C		2005	BE	9/30-10/05	25	2272	25	1.1	25	96	19	0	0	0	19	76
3A/3C		2006	BE	10/06-10/12	25	2634	25	0.8	25	108	13	0	0	0	13	52
3A/3C		2008	BE	9/26-10/02	30	2552	30	1.1	30	103	29	0	0	0	29	97
3A/3C		2004	B	11/19-11/25	150	634	154	10.9	150	620	52	11	0	0	63	42
3A/3C		2005	B	11/18-11/24	175	572	175	14.2	171	811	51	14	0	0	65	38
3A/3C		2006	B	11/24-11/30	190	582	187	12.9	185	817	65	15	0	0	80	43
3A/3C		2007	B	11/23-11/29	190	907	190	13.0	175	698	55	19	0	0	74	42
3A/3C		2008	B	11/28-12/04	250	463	250	24.8	243	1187	72	11	0	0	83	34
3A/3C		2005	ALS	10/14-10/19	380	712	380	32.0	373	1438	0	0	135	11	146	39
3A/3C		2006	ALS	10/13-10/18	265	651	265	24.4	250	883	0	0	106	17	123	49
3A/3C		2006	ALS	12/08-12/14	250	127	250	72.4	233	894	0	0	109	2	111	48
3A/3C		2007	ALS	10/12-10/17	265	791	264	22.9	258	866	0	0	128	18	146	57
3A/3C		2007	ALS	12/07-12/13	250	258	250	55.4	250	958	0	0	90	7	97	39
3A/3C		2008	ALS	12/12-12/18	250	271	250	54.6	234	901	0	0	95	14	109	47
3A/3CE		2004	ALS	10/22-10/27	250	477	250	30.8	238	1013	0	0	93	13	106	45
3A/3CE		2005	ALS	10/21-10/26	300	85	300	80.0	288	1144	0	0	84	14	98	34
3A/3CE		2006	ALS	10/20-10/26	300	304	300	55.3	289	1180	0	0	102	17	119	41
3A/3CE		2007	ALS	10/19-10/25	300	196	300	57.1	280	1047	0	0	129	20	149	53
3A/3CE		2008	ALS	10/24-10/30	300	547	300	40.6	288	1202	0	0	107	12	119	41
3AE		2004	ALS	10/01-12/31	5	12	5	41.7	5	25	0	0	0	0	0	0
3AE		2005	ALS	8/12-8/25	5	4	5	25.0	0	0	0	0	0	0	0	–
3AE		2004	AE	10/01-12/31	5	56	5	7.1	5	33	2	2	0	0	4	80
3AE		2005	AE	8/12-8/25	5	43	5	11.6	5	30	0	0	0	0	0	0
3AW/4BN		2004	ALS	10/08-10/21	75	69	75	59.4	75	371	0	0	24	5	29	39
3AW/4BN		2005	ALS	10/07-10/20	75	63	75	58.7	72	345	0	0	24	0	24	33
3AW/4BN		2006	ALS	10/13-10/26	75	35	75	82.9	73	395	0	0	11	4	15	21
3AW/4BN		2007	ALS	10/12-10/25	75	37	75	81.1	68	283	0	0	38	13	51	75
3AW/4BN		2008	ALS	10/03-10/16	75	41	75	100.0	70	342	0	0	18	7	25	36
3B		2005	BE	9/30-10/05	30	618	30	1.5	27	90	23	0	0	0	23	85
3B		2006	BE	10/06-10/12	20	827	20	1.6	18	80	17	2	0	0	19	106
3B		2004	B	11/19-11/25	100	511	100	11.2	100	443	23	5	0	0	28	28
3B		2005	B	11/18-11/24	100	290	100	16.2	88	385	28	3	0	0	31	35
3B		2006	B	11/24-11/30	150	391	150	16.6	146	744	21	15	0	0	36	25
3B		2007	B	11/23-11/29	150	430	150	17.0	145	694	28	16	0	0	44	30
3B		2004	ALS	12/03-12/09	70	158	70	22.8	64	250	0	0	23	9	32	50
3B		2004	ALS	12/17-12/23	225	279	225	37.3	217	821	0	0	63	6	69	32
3B		2005	ALS	12/02-12/08	70	117	70	21.4	66	249	0	0	16	0	16	24
3B		2005	ALS	12/16-12/22	225	221	225	47.5	214	931	0	0	43	13	56	26
3B		2006	ALS	12/08-12/14	50	89	50	41.6	47	162	0	0	19	2	21	45
3B		2006	ALS	12/15-12/31	150	227	150	39.2	147	735	0	0	66	7	73	50
3B		2007	ALS	12/07-12/13	50	99	50	25.3	46	161	0	0	13	2	15	33
3B		2007	ALS	12/14-12/30	150	203	150	48.8	142	793	0	0	43	4	47	33
3B	CH	2004	ALS	10/22-10/27	10	24	10	37.5	9	34	0	0	6	0	6	67
3B	CH	2005	ALS	10/07-10/13	10	29	10	31.0	8	29	0	0	4	0	4	50
3B	CH	2006	ALS	10/13-10/19	10	29	10	31.0	10	38	0	0	8	0	8	80
3B	CH	2007	ALS	10/12-10/18	10	30	9	26.7	8	39	0	0	5	0	5	63
3CW		2004	ALS	10/22-10/27	150	352	150	26.7	145	482	0	0	63	2	65	45
3CW		2005	ALS	10/21-10/26	150	135	150	65.9	146	606	0	0	43	6	49	34
3CW		2006	ALS	10/20-10/26	150	158	150	60.1	148	533	0	0	57	10	67	45
3CW		2007	ALS	10/19-10/25	150	180	150	43.9	140	550	0	0	60	6	66	47
3CW		2008	ALS	10/24-10/30	150	301	150	36.5	141	524	0	0	68	4	72	51
4A		2004	BE	10/01-10/06	10	1482	13	0.7	12	40	10	0	0	0	10	83
4A		2006	BE	10/06-10/12	15	896	15	1.0	15	45	13	0	0	0	13	87
4A		2007	BE	9/28-10/04	15	658	15	1.5	14	32	13	0	0	0	13	93
4A		2004	B	11/19-11/25	205	1096	205	11.8	199	918	35	6	0	0	41	21
4A		2005	B	11/18-11/24	250	948	250	14.5	242	1061	25	15	0	0	40	17
4A		2006	B	11/24-11/30	250	820	250	18.4	248	1174	39	26	0	0	65	26
4A		2007	B	11/23-11/29	250	765	250	22.0	246	1145	54	2	0	0	56	23

BE = Early Bull **B** = Bull **ALS** = Antlerless **AE** = Any Elk **CN** = Camp Navajo **CH** = CHAMP Hunt **DV** = Disabled Veteran

5-Year: 2004–2008 Harvest

Unit		Year	Hunt Type	Dates	Permits Authorized	1st Choice Applicants	Permits Issued	Draw Odds	Hunters	Hunter Days	Harvest					Hunt Success
											Bull	Spike	Cow	Calf	Total	
**GENERAL (continued)**																
4A		2008	B	11/28-12/04	395	785	395	30.3	373	1755	77	14	0	0	91	24
4A		2004	ALS	11/12-11/15	300	861	300	22.1	284	809	0	0	107	12	119	42
4A		2005	ALS	11/11-11/14	250	631	250	30.1	241	714	0	0	72	9	81	34
4A		2006	ALS	10/27-11/02	170	484	170	22.1	158	556	0	0	69	4	73	46
4A		2007	ALS	10/26-11/01	100	347	100	22.2	98	372	0	0	33	5	38	39
4A		2008	ALS	10/24-10/30	100	298	100	28.9	96	320	0	0	39	6	45	47
4B		2005	BE	9/30-10/05	40	1222	40	2.0	40	182	22	2	0	0	24	60
4B		2007	BE	9/28-10/04	25	585	25	2.4	25	107	16	0	0	0	16	64
4B		2008	BE	9/26-10/02	50	1184	50	2.2	48	214	28	2	0	0	30	63
4B		2004	B	11/19-11/25	250	958	250	15.2	241	1076	56	7	0	0	63	26
4B		2005	B	11/18-11/24	275	632	275	25.6	257	1275	41	6	0	0	47	18
4B		2006	B	11/24-11/30	300	836	300	20.0	290	1325	48	15	0	0	63	22
4B		2007	B	11/23-11/29	300	750	300	23.6	296	1433	49	14	0	0	63	21
4B		2008	B	11/28-12/04	400	588	400	37.6	379	1808	71	18	0	0	89	23
4B		2008	ALS	12/05-12/11	200	63	200	100.0	189	840	0	0	29	6	35	19
4BN		2007	ALS	8/01-9/13	15	9	15	77.8	13	90	0	0	0	0	0	0
4BN		2007	ALS	10/12-11/01	15	1	15	100.0	15	95	0	0	5	0	5	33
4BN		2007	ALS	11/02-11/22	10	3	10	100.0	10	37	0	0	3	0	3	30
4BN		2007	ALS	11/30-12/31	5	0	5	-	5	13	0	0	1	0	1	20
4BN		2007	AE	8/01-9/13	5	76	5	3.9	5	67	5	0	0	0	5	100
4BN		2007	AE	11/30-12/31	5	23	5	0.0	3	3	0	0	3	0	3	100
5A		2006	BE	10/06-10/12	25	1253	25	1.7	25	100	23	0	0	0	23	92
5A		2004	B	11/19-11/25	150	1309	150	9.8	142	588	59	2	0	0	61	43
5A		2005	B	11/18-11/24	115	990	115	10.3	111	532	37	7	0	0	44	40
5A		2006	B	11/24-11/30	145	771	145	11.8	143	644	58	13	0	0	71	50
5A		2007	B	11/23-11/29	165	861	165	13.9	161	707	54	19	0	0	73	45
5A		2008	B	11/28-12/04	240	761	240	23.0	234	1136	73	6	0	0	79	34
5A		2004	ALS	10/15-10/21	300	1235	300	18.4	285	1016	0	0	133	29	162	57
5A		2004	ALS	12/03-12/09	300	363	300	41.3	280	1077	0	0	57	10	67	24
5A		2005	ALS	10/14-10/20	250	890	250	20.8	247	911	0	0	133	13	146	59
5A		2005	ALS	12/02-12/08	200	163	200	48.5	195	746	0	0	87	4	91	47
5A		2006	ALS	10/20-10/26	350	1041	350	26.5	338	1332	0	0	166	11	177	52
5A		2006	ALS	12/01-12/07	200	319	200	30.7	189	714	0	0	72	6	78	41
5A		2007	ALS	10/19-10/25	300	763	299	27.9	292	1196	0	0	122	14	136	47
5A		2007	ALS	11/30-12/06	200	154	200	59.7	190	812	0	0	65	10	75	39
5A		2008	ALS	10/17-10/23	325	772	325	32.6	315	1226	0	0	146	15	161	51
5A		2008	ALS	12/05-12/11	220	180	220	61.7	211	940	0	0	52	4	56	27
5A/5BN	SM	2004	ALS	10/15-10/21	50	7	50	100.0	50	192	0	0	5	3	8	16
5A/5BN	SM	2006	ALS	10/20-10/26	75	11	75	63.6	73	284	0	0	5	2	7	10
5A/5BN	SM	2007	ALS	10/19-10/25	75	12	75	100.0	68	284	0	0	19	0	19	28
5A/5BN	SM	2008	ALS	10/17-10/23	75	10	75	100.0	70	230	0	0	21	3	24	34
5A/5BN	SM	2004	AE	10/15-10/21	75	285	75	17.9	73	277	20	0	13	0	33	45
5A/5BN	SM	2005	AE	10/14-10/20	75	249	75	22.1	75	371	30	2	4	0	36	48
5A/5BN	SM	2006	AE	10/20-10/26	75	153	75	34.0	70	305	27	0	0	0	27	39
5A/5BN	SM	2007	AE	10/19-10/25	75	303	75	18.8	75	318	35	0	0	0	35	47
5A/5BN	SM	2008	AE	10/17-10/23	75	114	75	38.6	67	310	22	2	4	0	28	42
5BN		2008	BE	9/26-10/02	25	1193	25	1.8	25	76	19	0	0	0	19	76
5BN		2004	B	11/19-11/25	450	1994	450	13.8	442	1818	104	14	0	0	118	27
5BN		2005	B	11/18-11/24	450	1896	450	13.5	435	1830	138	23	0	0	161	37
5BN		2006	B	11/24-11/30	500	2205	500	13.9	484	1962	175	31	0	0	206	43
5BN		2007	B	11/23-11/29	425	1565	425	15.9	394	1835	116	25	0	0	141	36
5BN		2008	B	11/28-12/04	500	1375	500	19.9	476	2028	206	20	0	0	226	47
5BN		2004	ALS	12/03-12/09	125	225	125	24.0	117	367	0	0	55	2	57	49
5BN		2005	ALS	12/02-12/08	100	144	100	33.3	98	369	0	0	27	4	31	32
5BN		2006	ALS	12/01-12/07	125	200	125	42.0	123	462	0	0	45	11	56	46
5BN		2007	ALS	11/30-12/06	150	177	150	42.9	150	626	0	0	34	2	36	24
5BN		2008	ALS	10/17-10/23	300	518	300	31.5	281	1033	0	0	104	14	118	42
5BN		2008	ALS	12/05-12/11	150	75	150	82.7	143	540	0	0	39	5	44	31
5BN	ML	2004	ALS	10/15-10/21	200	352	200	38.9	196	709	0	0	83	11	94	48
5BN	ML	2005	ALS	10/14-10/20	300	406	300	43.1	290	980	0	0	113	28	141	49
5BN	ML	2006	ALS	10/20-10/26	300	380	298	46.3	288	1044	0	0	104	8	112	39

BE = Early Bull **B** = Bull **ALS** = Antlerless **AE** = Any Elk **CN** = Camp Navajo **CH** = CHAMP Hunt **DV** = Disabled Veteran

Herd-Units:

CC = Canyon Creek
ES (Unit 1) = Escudilla
GV = Grapevine
HM = Hutch Mtn.
M = Martinez
ML = Marshall Lake
MM = Melatone Mesa
MZ = Mazatzal
RV = Round Valley
SM = East Sunset/West Sunset/Meteor Crater
TT = Twin Arrows/Two Guns/Grapevine
VV = Verde Valley
WI = Winslow
WS (17/20A/20C) = Williamson Valley/Skull Valley/Kirkland Junction

5-Year: 2004-2008 Harvest

Unit		Year	Hunt Type	Dates	Permits Authorized	1st Choice Applicants	Permits Issued	Draw Odds	Hunters	Hunter Days	Harvest					Hunt Success
											Bull	Spike	Cow	Calf	Total	
**GENERAL (continued)**																
5BN	ML	2007	ALS	10/19-10/25	300	401	299	39.9	287	1163	0	0	124	9	133	46
5BN	TT	2004	ALS	10/15-10/21	140	81	140	70.4	138	556	0	0	35	0	35	25
5BN	TT	2005	ALS	10/14-10/20	140	67	140	100.0	135	541	0	0	16	14	30	22
5BN	TT	2006	ALS	10/20-10/26	140	59	140	84.7	136	524	0	0	28	6	34	25
5BN	TT	2007	ALS	10/19-10/25	100	54	100	92.6	95	363	0	0	30	2	32	34
5BN	TT	2008	ALS	10/17-10/23	125	43	125	100.0	113	435	0	0	31	0	31	27
5BN	GV	2004	AE	10/15-10/21	15	90	15	8.9	15	42	9	0	0	0	9	60
5BN	GV	2005	AE	10/14-10/20	20	83	20	18.1	18	88	9	2	0	0	11	61
5BN	TT	2004	AE	10/15-10/21	25	62	25	21.0	23	86	9	0	5	0	14	61
5BN	TT	2005	AE	10/14-10/20	20	66	20	21.2	20	74	10	0	2	0	12	60
5BN	TT	2006	AE	10/20-10/26	70	244	70	16.8	70	298	20	0	6	2	28	40
5BS		2004	B	11/19-11/25	200	2107	200	8.5	194	684	92	16	0	0	108	56
5BS		2005	B	11/18-11/24	175	1638	175	9.0	171	660	65	27	0	0	92	54
5BS		2006	B	11/24-11/30	255	1883	252	10.4	244	935	102	31	0	0	133	55
5BS		2007	B	11/23-11/29	350	2033	347	12.5	339	1366	157	33	0	0	190	56
5BS		2008	B	11/28-12/04	350	1627	350	16.8	343	1447	143	37	0	0	180	52
5BS		2004	ALS	12/03-12/09	400	766	400	28.7	384	1176	0	0	165	20	185	48
5BS		2005	ALS	12/02-12/08	475	785	475	33.2	450	1549	0	0	234	26	260	58
5BS		2006	ALS	12/01-12/07	520	933	520	33.1	504	1745	0	0	226	12	238	47
5BS		2007	ALS	10/19-10/25	625	2100	625	21.3	609	2092	0	0	316	29	345	57
5BS		2007	ALS	11/30-12/06	550	384	549	47.4	521	1859	0	0	154	20	174	33
5BS		2008	ALS	10/17-10/23	625	1986	625	25.2	612	2198	0	0	274	46	320	52
5BS		2008	ALS	12/12-12/18	420	312	420	60.3	396	1162	0	0	170	21	191	48
5BS	HM	2004	ALS	10/15-10/21	325	844	325	23.9	313	838	0	0	214	22	236	75
5BS	HM	2005	ALS	10/14-10/20	400	1077	400	29.2	400	1210	0	0	212	24	236	59
5BS	HM	2006	ALS	10/20-10/26	430	1178	429	27.2	414	1224	0	0	262	14	276	67
5BS	MM	2004	ALS	10/15-10/21	180	275	180	41.1	170	505	0	0	67	16	83	49
5BS	MM	2005	ALS	10/14-10/20	180	266	180	39.1	169	567	0	0	73	7	80	47
5BS	MM	2006	ALS	10/20-10/26	190	206	190	47.1	185	752	0	0	38	7	45	24
6A		2004	B	11/19-11/25	815	10448	815	6.9	795	3176	291	48	0	0	339	43
6A		2005	B	11/18-11/24	750	8226	750	8.2	724	2895	250	65	0	0	315	44
6A		2006	B	11/24-11/30	860	8369	858	9.0	838	3268	317	134	0	0	451	54
6A		2007	B	11/23-11/29	860	7773	860	9.3	834	3805	277	104	0	0	381	46
6A		2008	B	11/28-12/04	950	6144	950	13.1	921	4084	340	59	0	0	399	43
6A		2005	ALS	12/02-12/08	485	1444	485	19.8	474	1671	0	0	217	17	234	49
6A		2006	ALS	12/01-12/07	535	1498	533	18.5	522	1975	0	0	226	23	249	48
6A		2007	ALS	11/30-12/06	700	1502	699	23.8	672	2700	0	0	247	10	257	38
6A		2008	ALS	12/05-12/11	845	1245	845	35.1	817	3127	0	0	299	30	329	40
6A/19A/21	VV	2004	ALS	10/15-10/21	30	7	30	100.0	30	93	0	0	3	0	3	10
6A/19A/21	VV	2005	ALS	10/14-10/20	25	10	25	100.0	19	63	0	0	10	0	10	53
6A/19A/21	VV	2006	ALS	10/20-10/26	25	20	25	55.0	21	73	0	0	6	0	6	29
6A/19A/21	VV	2007	ALS	10/19-10/25	25	17	25	76.5	21	88	0	0	8	0	8	38
6A/19A/21	VV	2004	AE	11/19-11/25	15	28	15	28.6	15	83	0	0	0	0	0	0
6A/19A/21	VV	2005	AE	11/18-11/24	5	24	5	8.3	5	15	3	0	0	0	3	60
6A/19A/21	VV	2006	AE	11/24-11/30	10	64	10	9.4	10	40	5	0	0	0	5	50
6A/19A/21	VV	2007	AE	11/23-11/29	10	50	10	14.0	10	48	3	0	3	0	6	60
6AN/6AS		2004	ALS	12/03-12/09	505	1520	505	18.9	479	1626	0	0	199	17	216	45
6AN/6AW		2007	BE	9/28-10/04	25	2044	25	1.0	25	98	20	1	0	0	21	84
6AW		2004	ALS	10/15-10/21	35	180	35	13.9	35	113	0	0	20	2	22	63
6AW		2004	ALS	12/03-12/09	75	70	75	44.3	60	215	0	0	20	3	23	38
6AW		2005	ALS	10/14-10/20	50	144	50	21.5	50	154	0	0	22	0	22	44
6AW		2006	ALS	10/20-10/26	55	176	55	18.8	55	187	0	0	22	0	22	40
6AW		2007	ALS	10/19-10/25	75	137	75	32.8	75	277	0	0	32	4	36	48
6AW		2008	ALS	10/17-10/23	75	171	75	26.9	75	260	0	0	28	7	35	47
6B		2005	BE	9/30-10/05	25	949	25	2.2	25	74	24	0	0	0	24	96
6B		2004	B	11/19-11/25	170	598	174	18.6	168	653	57	17	0	0	74	44
6B		2005	B	11/18-11/24	175	552	175	15.0	169	658	63	12	0	0	75	44
6B		2006	B	11/24-11/30	190	592	190	19.8	186	697	76	23	0	0	99	53
6B		2007	B	11/23-11/29	230	715	230	21.3	224	924	87	11	0	0	98	44
6B		2008	B	11/28-12/04	200	531	200	24.9	195	865	62	19	0	0	81	42
6B		2004	ALS	12/03-12/09	340	392	340	48.7	319	1100	0	0	112	17	129	40
6B		2005	ALS	12/02-12/08	400	357	400	61.1	381	1609	0	0	76	11	87	23
6B		2006	ALS	12/01-12/07	400	357	399	51.0	388	1530	0	0	101	12	113	29
6B		2007	ALS	11/30-12/06	450	261	448	76.2	417	1809	0	0	97	14	111	27
6B		2008	ALS	12/05-12/11	450	308	450	80.8	423	1922	0	0	80	8	88	21
7E		2004	B	11/19-11/25	380	1025	380	23.7	373	1622	121	36	0	0	157	42
7E		2005	B	11/18-11/24	460	1307	460	20.8	450	2093	81	29	0	0	110	24

BE = Early Bull **B** = Bull **ALS** = Antlerless **AE** = Any Elk **CN** = Camp Navajo **CH** = CHAMP Hunt **DV** = Disabled Veteran

5-Year: 2004–2008 Harvest

Unit		Year	Hunt Type	Dates	Permits Authorized	1st Choice Applicants	Permits Issued	Draw Odds	Hunters	Hunter Days	Harvest					Hunt Success
											Bull	Spike	Cow	Calf	Total	
**GENERAL (continued)**																
7E		2006	B	11/24-11/30	375	777	373	26.9	371	1620	113	27	0	0	140	38
7E		2007	B	11/23-11/29	400	1037	400	27.2	398	1789	100	37	0	0	137	34
7E		2008	B	11/28-12/04	425	764	425	36.6	418	1810	136	28	0	0	164	39
7E		2004	ALS	10/15-10/21	340	386	340	45.1	334	1358	0	0	75	17	92	28
7E		2005	ALS	10/14-10/20	325	381	325	51.2	319	1331	0	0	75	10	85	27
7E		2006	ALS	10/20-10/26	220	225	219	52.9	214	752	0	0	107	8	115	54
7E		2007	ALS	10/19-10/25	325	373	325	52.5	313	1224	0	0	104	14	118	38
7E		2008	ALS	10/17-10/23	325	304	325	60.2	305	1263	0	0	109	6	115	38
7W		2004	B	11/19-11/25	475	2241	475	13.0	459	2008	110	48	0	0	158	34
7W		2005	B	11/18-11/24	400	1539	400	19.0	390	1862	86	50	0	0	136	35
7W		2006	B	11/24-11/30	400	1780	400	15.5	389	1845	126	46	0	0	172	44
7W		2007	B	11/23-11/29	450	1757	450	18.5	437	2034	79	37	0	0	116	27
7W		2008	B	11/28-12/04	450	1367	450	25.2	442	2169	106	23	0	0	129	29
7W		2004	ALS	10/15-10/21	350	762	350	31.2	347	1153	0	0	174	21	195	56
7W		2005	ALS	10/14-10/20	400	783	400	34.6	379	1455	0	0	147	15	162	43
7W		2006	ALS	10/20-10/26	475	1015	475	32.7	459	1637	0	0	230	33	263	57
7W		2007	ALS	10/19-10/25	500	1019	499	35.3	486	1791	0	0	225	36	261	54
7W		2008	ALS	10/17-10/23	520	989	520	37.7	507	2059	0	0	206	30	236	47
8		2004	BE	10/01-10/06	20	2031	22	0.8	22	105	18	0	0	0	18	82
8		2004	B	11/19-11/25	485	2069	565	14.5	544	2354	139	28	0	0	167	31
8		2005	B	11/18-11/24	425	1825	425	15.3	409	2009	78	22	0	0	100	24
8		2006	B	11/24-11/30	425	1706	424	16.1	418	1961	105	21	0	0	126	30
8		2007	B	11/23-11/29	500	1741	500	17.5	481	2205	114	43	0	0	157	33
8		2008	B	11/28-12/04	500	1298	500	27.0	491	2379	172	27	0	0	199	41
8		2004	ALS	12/03-12/09	400	630	400	38.7	388	1594	0	0	97	15	112	29
8		2005	ALS	12/02-12/08	200	368	200	28.8	188	798	0	0	48	4	52	28
8		2006	ALS	12/01-12/07	200	383	200	30.8	186	784	0	0	67	4	71	38
8		2007	ALS	11/30-12/06	200	288	200	40.6	187	809	0	0	52	0	52	28
8		2008	ALS	12/05-12/11	200	244	200	46.3	196	890	0	0	52	6	58	30
9		2006	BE	10/06-10/12	25	4053	25	0.5	25	85	25	0	0	0	25	100
9		2007	BE	9/28-10/04	25	4224	25	0.6	25	84	22	0	0	0	22	88
9		2008	BE	9/26-10/02	25	3955	25	0.6	25	102	23	0	0	0	23	92
9		2004	B	11/19-11/25	255	1532	282	14.5	278	1336	76	9	0	0	85	31
9		2005	B	11/18-11/24	280	1255	280	14.4	273	1343	109	12	0	0	121	44
9		2006	B	11/24-11/30	265	1086	265	16.1	254	1172	106	11	0	0	117	46
9		2007	B	11/23-11/29	300	1194	300	16.2	295	1380	143	14	0	0	157	53
9		2008	B	11/28-12/04	275	900	275	18.9	268	1365	96	10	0	0	106	40
9		2004	ALS	10/15-10/21	350	508	350	41.9	334	1233	0	0	160	24	184	55
9		2004	ALS	12/03-12/09	350	270	350	70.7	340	1297	0	0	164	18	182	54
9		2005	ALS	10/14-10/20	400	601	400	45.8	386	1574	0	0	149	13	162	42
9		2005	ALS	12/02-12/08	400	396	400	57.6	388	1443	0	0	167	26	193	50
9		2006	ALS	10/20-10/26	400	638	400	42.0	397	1535	0	0	175	27	202	51
9		2006	ALS	12/01-12/07	400	409	400	54.8	385	1658	0	0	135	10	145	38
9		2007	ALS	10/19-10/25	400	665	400	37.1	374	1499	0	0	152	20	172	46
9		2007	ALS	11/30-12/06	400	245	398	72.7	378	1463	0	0	149	13	162	43
9		2008	ALS	10/17-10/23	400	584	400	41.6	385	1546	0	0	212	17	229	59
9		2008	ALS	12/05-12/11	400	214	400	73.4	393	1688	0	0	146	13	159	40
10		2004	BE	10/01-10/06	50	6083	61	1.0	61	208	52	0	0	0	52	85
10		2005	BE	9/30-10/05	50	4855	50	1.0	48	144	42	0	0	0	42	88
10		2006	BE	10/06-10/12	25	3228	25	0.7	25	108	22	0	0	0	22	88
10		2007	BE	9/28-10/04	25	3448	27	0.6	25	113	19	0	0	0	19	76
10		2008	BE	9/26-10/02	25	1784	25	0.8	25	86	25	0	0	0	25	100
10		2004	B	11/19-11/25	350	2018	454	13.6	437	1791	166	25	0	0	191	44
10		2005	B	11/18-11/24	450	1854	450	13.6	425	2059	126	11	0	0	137	32
10		2006	B	11/24-11/30	400	1645	399	12.9	386	1861	108	15	0	0	123	32
10		2007	B	11/23-11/29	450	1602	450	15.9	430	2056	131	11	0	0	142	33
10		2008	B	11/28-12/04	450	1181	450	22.3	441	2089	150	17	0	0	167	38
10		2004	ALS	10/15-10/21	700	1138	700	41.2	653	2746	0	0	140	25	165	25
10		2004	ALS	12/03-12/12	700	581	700	64.5	652	2754	0	0	242	40	282	43
10		2005	ALS	10/14-10/20	800	904	800	51.1	764	3206	0	0	118	21	139	18
10		2005	ALS	12/02-12/11	800	746	800	60.6	761	3877	0	0	133	31	164	22

BE = Early Bull **B** = Bull **ALS** = Antlerless **AE** = Any Elk **CN** = Camp Navajo **CH** = CHAMP Hunt **DV** = Disabled Veteran

Herd-Units:

CC = Canyon Creek
ES (Unit 1) = Escudilla
GV = Grapevine
HM = Hutch Mtn.
M = Martinez
ML = Marshall Lake
MM = Melatone Mesa
MZ = Mazatzal
RV = Round Valley
SM = East Sunset/West Sunset/Meteor Crater
TT = Twin Arrows/Two Guns/Grapevine
VV = Verde Valley
WI = Winslow
WS (17/20A/20C) = Williamson Valley/Skull Valley/Kirkland Junction

5-Year: 2004-2008 Harvest

Unit		Year	Hunt Type	Dates	Permits Authorized	1st Choice Applicants	Permits Issued	Draw Odds	Hunters	Hunter Days	Harvest					Hunt Success
											Bull	Spike	Cow	Calf	Total	
**GENERAL (continued)**																
10		2006	ALS	10/20-10/26	800	841	800	54.3	770	3376	0	0	147	22	169	22
10		2006	ALS	12/01-12/07	800	559	800	68.7	757	3839	0	0	163	15	178	24
10		2007	ALS	10/19-10/25	600	646	600	51.7	574	2500	0	0	136	24	160	28
10		2007	ALS	11/30-12/06	600	289	599	85.8	568	2956	0	0	156	18	174	31
10		2008	ALS	10/17-10/23	600	555	600	55.5	576	2595	0	0	120	13	133	23
10		2008	ALS	12/05-12/14	600	286	600	93.7	567	2938	0	0	170	12	182	32
12		2004	AE	9/24-10/07	20	46	20	39.1	16	109	0	0	0	0	0	0
15A/15B/ 17A/18/19B		2004	AE	10/01-10/17	20	287	20	5.9	20	91	13	0	0	0	13	65
15A/15B/ 17A/18/19B		2004	AE	11/05-11/21	20	71	20	16.9	20	146	3	0	3	0	6	30
15A/15B/ 17A/18/19B		2004	AE	12/03-12/19	30	116	30	14.7	30	192	12	3	9	0	24	80
15A/15B/ 17A/18/19B		2005	AE	9/30-10/16	30	338	30	5.6	30	134	20	0	1	0	21	70
15A/15B/ 17A/18/19B		2005	AE	11/04-11/20	40	44	40	20.5	38	290	13	0	8	0	21	55
15A/15B/ 17A/18/19B		2005	AE	12/02-12/18	60	140	60	25.7	58	396	28	0	4	0	32	55
15A/15B/17/18/ 19B/20A/20C		2008	B	10/03-10/16	60	192	60	19.3	60	330	45	0	0	0	45	75
15A/15B/17/18/ 19B/20A/20C		2004	ALS	10/01-10/17	65	45	65	82.2	63	332	0	0	23	2	25	40
15A/15B/17/18/ 19B/20A/20C		2005	ALS	9/30-10/16	80	67	80	65.7	76	473	0	0	20	4	24	32
15A/15B/17/18/ 19B/20A/20C		2006	ALS	10/20-12/17	400	133	398	98.5	379	3578	0	0	77	14	91	24
15A/15B/17/18/ 19B/20A/20C		2007	ALS	10/19-12/16	400	127	398	100.0	392	4243	0	0	70	11	81	21
15A/15B/17/18/ 19B/20A/20C		2008	ALS	10/03-10/16	100	8	100	100.0	89	489	0	0	9	2	11	12
15A/15B/17/18/ 19B/20A/20C		2008	ALS	10/17-12/14	500	103	500	99.0	462	5351	0	0	70	15	85	18
15A/15B/17/18/ 19B/20A/20C		2006	AE	10/06-10/19	60	358	60	9.2	56	235	44	0	2	2	48	86
15A/15B/17/18/ 19B/20A/20C		2006	AE	10/20-12/17	200	781	200	15.7	193	1945	44	7	22	0	73	38
15A/15B/17/18/ 19B/20A/20C		2007	AE	10/05-10/18	60	255	60	12.5	60	375	32	0	3	0	35	58
15A/15B/17/18/ 19B/20A/20C		2007	AE	10/19-12/16	200	648	195	16.8	191	1959	57	2	7	4	70	37
15A/15B/17/18/ 19B/20A/20C		2008	AE	10/17-12/14	250	518	250	30.1	233	2476	60	6	19	0	85	36
17/20A/20C	WS	2004	ALS	10/01-10/17	15	6	15	83.3	11	66	0	0	2	0	2	18
17/20A/20C	WS	2004	ALS	11/05-11/21	15	0	15	–	15	108	0	0	6	0	6	40
17/20A/20C	WS	2004	ALS	12/03-12/19	15	2	15	100.0	15	83	0	0	0	3	3	20
17/20A/20C	WS	2005	ALS	9/30-10/16	20	6	20	100.0	18	88	0	0	5	0	5	28
17/20A/20C	WS	2005	ALS	11/04-11/20	20	0	20	–	20	142	0	0	2	0	2	10
17/20A/20C	WS	2005	ALS	12/02-12/18	20	6	20	100.0	20	147	0	0	3	0	3	15
17/20A/20C	WS	2004	AE	10/01-10/17	5	15	5	33.3	5	47	3	0	0	0	3	60
17/20A/20C	WS	2004	AE	11/05-11/21	5	10	5	30.0	5	37	3	0	0	0	3	60
17/20A/20C	WS	2004	AE	12/03-12/19	5	9	5	11.1	3	17	0	0	0	0	0	0
17/20A/20C	WS	2005	AE	9/30-10/16	10	26	10	7.7	10	80	4	0	0	0	4	40
17/20A/20C	WS	2005	AE	11/04-11/20	10	14	10	28.6	10	76	1	0	0	0	1	10
17/20A/20C	WS	2005	AE	12/02-12/18	10	2	10	100.0	8	42	0	2	0	0	2	25
19A		2004	B	11/19-11/25	10	111	10	6.3	10	37	7	0	0	0	7	70
19A		2005	B	11/18-11/24	15	136	15	7.4	15	64	9	0	0	0	9	60
19A		2006	B	11/24-11/30	20	74	20	13.5	19	64	13	3	0	0	16	84
19A		2007	B	11/23-11/29	20	76	20	11.8	20	67	9	2	0	0	11	55
19A		2008	B	11/28-12/04	40	110	40	25.5	40	174	18	2	0	0	20	50
19A		2004	ALS	10/15-10/24	25	31	25	64.5	25	86	0	0	9	0	9	36
19A		2004	ALS	12/03-12/12	25	9	25	100.0	25	88	0	0	5	0	5	20
19A		2005	ALS	10/14-10/23	40	33	40	72.7	40	207	0	0	9	0	9	23
19A		2005	ALS	12/02-12/11	20	11	20	72.7	20	106	0	0	0	0	0	0
19A		2006	ALS	10/20-10/26	50	22	50	100.0	50	217	0	0	5	0	5	10
19A		2006	ALS	12/01-12/07	20	10	20	100.0	13	37	0	0	0	0	0	0
19A		2007	ALS	10/19-10/25	50	39	50	79.5	45	218	0	0	5	0	5	11
19A		2007	ALS	11/30-12/06	20	14	20	78.6	16	49	0	0	3	0	3	19
19A		2008	ALS	10/17-10/23	50	20	50	100.0	50	252	0	0	4	0	4	8
19A		2008	ALS	12/05-12/14	40	7	40	100.0	0	0	0	0	0	0	0	–
21		2004	BE	10/01-10/06	5	332	6	1.5	5	11	5	0	0	0	5	100

BE = Early Bull **B** = Bull **ALS** = Antlerless **AE** = Any Elk **CN** = Camp Navajo **CH** = CHAMP Hunt **DV** = Disabled Veteran

5-Year: 2004–2008 Harvest

Unit		Year	Hunt Type	Dates	Permits Authorized	1st Choice Applicants	Permits Issued	Draw Odds	Hunters	Hunter Days	Harvest					Hunt Success
											Bull	Spike	Cow	Calf	Total	
**GENERAL (continued)**																
21		2006	BE	10/06-10/12	5	221	5	2.3	5	9	5	0	0	0	5	100
21		2008	BE	9/26-10/02	5	146	5	2.7	5	8	5	0	0	0	5	100
21		2007	B	11/30-12/06	5	80	5	6.3	0	0	0	0	0	0	0	–
21		2008	B	11/28-12/04	10	24	10	12.5	10	50	6	0	0	0	6	60
22		2004	B	12/10-12/16	175	1211	204	9.7	200	825	110	9	0	0	119	60
22	MZ	2004	B	10/22-10/28	5	50	6	8.0	5	20	2	0	0	0	2	40
22	MZ	2005	B	10/21-10/27	10	38	10	18.4	10	53	3	0	0	0	3	30
22N		2004	BE	10/01-10/06	30	1378	31	1.7	31	80	28	0	0	0	28	90
22N		2005	BE	9/30-10/05	30	1106	30	1.8	30	86	26	0	0	0	26	87
22N		2006	BE	10/06-10/12	30	1251	30	1.8	30	87	27	0	0	0	27	90
22N		2007	BE	9/28-10/04	30	1213	30	2.0	30	81	29	0	0	0	29	97
22N		2008	BE	9/26-10/02	30	959	30	2.4	30	90	26	0	0	0	26	87
22N		2005	B	12/02-12/08	100	696	100	10.2	97	347	64	3	0	0	67	69
22N		2005	B	12/09-12/15	130	306	130	13.7	121	490	61	4	0	0	65	54
22N		2006	B	12/01-12/07	150	716	150	11.7	150	630	73	6	0	0	79	53
22N		2006	B	12/08-12/14	220	495	220	21.0	214	949	92	15	0	0	107	50
22N		2007	B	11/30-12/06	300	807	300	23.7	288	1358	102	12	0	0	114	40
22N		2007	B	12/08-12/14	320	393	320	36.1	307	1388	104	13	0	0	117	38
22N		2008	B	11/28-12/04	350	629	350	31.3	332	1451	95	13	0	0	108	33
22N		2008	B	12/05-12/11	400	546	400	43.0	385	1819	87	35	0	0	122	32
22N		2004	ALS	10/22-10/28	85	219	85	23.3	83	272	0	0	50	2	52	63
22N		2005	ALS	10/21-10/27	85	269	85	22.7	83	273	0	0	47	6	53	64
22N		2006	ALS	10/20-10/26	130	272	130	29.4	126	390	0	0	70	8	78	62
22N		2007	ALS	10/19-10/25	225	319	225	47.3	220	820	0	0	71	9	80	36
22N		2008	ALS	10/17-10/23	225	355	225	42.3	221	771	0	0	121	14	135	61
22S		2005	BE	9/30-10/05	30	90	30	10.0	30	112	26	2	0	0	28	93
22S		2005	B	12/02-12/08	50	34	50	32.4	48	186	18	0	0	0	18	38
22S		2005	B	12/09-12/15	30	41	30	29.3	30	117	23	0	0	0	23	77
22S		2006	B	12/01-12/07	30	168	30	12.5	28	106	14	2	0	0	16	57
22S		2007	B	11/30-12/06	30	67	30	20.9	28	124	9	0	0	0	9	32
22S		2008	B	11/28-12/04	30	56	30	32.1	28	99	15	2	0	0	17	61
22S		2004	ALS	10/22-10/28	15	17	15	52.9	15	55	0	0	0	0	0	0
22S		2005	ALS	10/21-10/27	15	12	15	41.7	15	54	0	0	4	0	4	27
22S		2006	ALS	10/20-10/26	15	18	15	27.8	14	30	0	0	7	1	8	57
22S		2007	ALS	10/19-10/25	15	9	15	77.8	15	53	0	0	2	2	4	27
22S		2008	ALS	10/17-10/23	30	29	30	48.3	30	116	0	0	10	3	13	43
23		2004	B	12/03-12/09	100	847	117	9.0	113	458	59	4	0	0	63	56
23		2005	B	12/02-12/08	120	866	120	8.4	116	512	58	2	0	0	60	52
23		2006	B	12/01-12/07	150	728	150	10.7	144	671	49	2	0	0	51	35
23		2007	B	11/30-12/06	200	756	200	12.6	194	822	84	4	0	0	88	45
23		2008	B	11/28-12/04	150	570	150	16.5	142	628	73	3	0	0	76	54
23		2008	B	12/05-12/11	150	224	150	25.0	143	665	54	0	0	0	54	38
23	CC	2004	ALS	8/06-8/09	8	13	8	46.2	8	16	0	0	4	0	4	50
23	CC	2004	ALS	8/13-8/16	8	6	8	83.3	8	24	0	0	2	0	2	25
23	CC	2004	ALS	9/03-9/06	8	7	8	100.0	8	18	0	0	3	0	3	38
23	CC	2004	ALS	9/10-9/13	8	4	8	100.0	8	13	0	0	5	0	5	63
23	CC	2005	ALS	8/05-8/08	8	23	8	30.4	8	12	0	0	4	2	6	75
23	CC	2005	ALS	8/12-8/15	8	1	8	100.0	8	19	0	0	0	0	0	0
23	CC	2005	ALS	9/02-9/05	8	8	8	75.0	5	5	0	0	5	0	5	100
23	CC	2005	ALS	9/09-9/12	8	33	8	21.2	8	19	0	0	5	1	6	75
23	CC	2006	ALS	8/11-8/14	8	22	8	36.4	8	11	0	0	8	0	8	100
23	CC	2006	ALS	8/18-8/21	8	4	8	50.0	8	17	0	0	5	0	5	63
23	CC	2006	ALS	9/08-9/11	8	12	8	25.0	8	14	0	0	7	1	8	100
23	CC	2006	ALS	9/15-9/18	8	7	8	42.9	8	8	0	0	8	0	8	100
23	CC	2007	ALS	8/10-8/13	8	22	8	36.4	8	17	0	0	4	0	4	50
23	CC	2007	ALS	8/17-8/20	8	2	8	50.0	7	19	0	0	3	1	4	57
23	CC	2007	ALS	9/07-9/10	8	8	8	62.5	8	23	0	0	3	0	3	38
23	CC	2007	ALS	9/14-9/17	8	15	8	40.0	8	24	0	0	6	0	6	75
23	CC	2008	ALS	8/08-8/11	8	9	8	33.3	6	16	0	0	3	0	3	50
23	CC	2008	ALS	8/15-8/18	8	2	8	50.0	6	21	0	0	0	0	0	0
23	CC	2008	ALS	9/05-9/08	8	6	8	50.0	5	5	0	0	5	0	5	100

BE = Early Bull **B** = Bull **ALS** = Antlerless **AE** = Any Elk **CN** = Camp Navajo **CH** = CHAMP Hunt **DV** = Disabled Veteran

Herd-Units:

CC = Canyon Creek
ES (Unit 1) = Escudilla
GV = Grapevine
HM = Hutch Mtn.
M = Martinez
ML = Marshall Lake
MM = Melatone Mesa
MZ = Mazatzal
RV = Round Valley
SM = East Sunset/West Sunset/Meteor Crater
TT = Twin Arrows/Two Guns/Grapevine
VV = Verde Valley
WI = Winslow
WS (17/20A/20C) = Williamson Valley/Skull Valley/Kirkland Junction

5-Year: 2004–2008 Harvest

Unit		Year	Hunt Type	Dates	Permits Authorized	1st Choice Applicants	Permits Issued	Draw Odds	Hunters	Hunter Days	Harvest					Hunt Success
											Bull	Spike	Cow	Calf	Total	
**GENERAL (continued)**																
23	CC	2008	ALS	9/12-9/15	8	9	8	44.4	8	20	0	0	4	2	6	75
23N		2004	BE	10/01-10/06	20	1173	20	1.4	20	48	20	0	0	0	20	100
23N		2005	BE	9/30-10/05	20	1173	20	1.7	20	64	17	0	0	0	17	85
23N		2006	BE	10/06-10/12	20	1157	20	1.2	20	83	16	0	0	0	16	80
23N		2007	BE	9/28-10/04	20	1511	20	1.3	20	51	19	0	0	0	19	95
23N		2008	BE	9/26-10/02	15	1090	15	1.3	15	47	15	0	0	0	15	100
23N		2004	ALS	10/22-10/28	30	92	30	19.6	30	88	0	0	19	0	19	63
23N		2004	ALS	12/10-12/16	90	141	90	36.2	87	332	0	0	38	3	41	47
23N		2005	ALS	10/21-10/27	30	114	30	21.1	30	127	0	0	7	0	7	23
23N		2005	ALS	12/09-12/15	90	89	90	38.2	83	341	0	0	25	2	27	33
23N		2006	ALS	10/27-11/02	30	119	30	18.5	25	80	0	0	10	0	10	40
23N		2006	ALS	12/15-12/21	90	100	90	44.0	88	305	0	0	39	0	39	44
23N		2007	ALS	12/07-12/13	100	126	100	38.1	89	283	0	0	35	5	40	45
23N		2008	ALS	10/17-10/23	120	152	120	50.0	113	459	0	0	46	5	51	45
23S		2006	BE	10/06-10/12	15	385	15	1.6	14	52	11	0	0	0	11	79
23S		2004	ALS	10/22-10/28	20	33	20	27.3	17	63	0	0	3	0	3	18
23S		2004	ALS	12/10-12/16	20	6	20	100.0	16	80	0	0	4	0	4	25
23S		2005	ALS	10/21-10/27	20	25	20	12.0	20	77	0	0	7	0	7	35
23S		2005	ALS	12/09-12/15	20	38	20	28.9	16	60	0	0	4	0	4	25
23S		2006	ALS	10/27-11/02	20	7	20	14.3	20	120	0	0	4	0	4	20
23S		2006	ALS	12/15-12/21	20	14	20	42.9	19	76	0	0	4	0	4	21
23S		2007	ALS	10/26-11/01	20	21	20	33.3	17	77	0	0	3	0	3	18
23S		2007	ALS	12/07-12/13	20	6	20	100.0	16	64	0	0	0	0	0	0
24A		2005	ALS	12/01-12/12	7	2	7	50.0	7	41	0	0	1	0	1	14
24A		2006	ALS	12/01-12/12	10	4	10	100.0	10	63	0	0	3	0	3	30
24A		2007	ALS	11/30-12/11	10	1	10	100.0	7	33	0	0	0	0	0	0
24A		2008	ALS	11/28-12/09	10	1	10	100.0	10	53	0	0	0	0	0	0
24A		2004	AE	12/01-12/12	5	62	5	8.1	5	43	0	0	0	0	0	0
24A		2005	AE	12/01-12/12	3	21	3	14.3	3	23	0	0	0	0	0	0
24A		2006	AE	12/01-12/12	5	26	5	11.5	5	28	0	0	3	0	3	60
24A		2007	AE	12/01-12/12	5	17	5	17.6	5	48	0	0	0	0	0	0
24A		2008	AE	12/01-12/12	5	9	5	33.3	5	38	1	0	0	0	1	20
27		2004	BE	10/01-10/06	25	1485	32	1.1	31	109	18	0	0	0	18	58
27		2006	BE	10/06-10/12	25	2465	25	1.0	24	105	15	0	0	0	15	63
27		2007	BE	9/28-10/04	25	1328	25	1.1	25	87	22	0	0	0	22	88
27		2008	BE	9/26-10/02	25	1925	25	1.3	22	84	21	0	0	0	21	95
27		2004	B	11/19-11/25	350	1582	397	12.9	392	1746	117	15	0	0	132	34
27		2005	B	11/18-11/24	350	1377	350	14.2	348	1736	108	12	0	0	120	34
27		2006	B	11/24-11/30	350	1211	349	12.6	336	1528	99	6	0	0	105	31
27		2007	B	11/23-11/29	350	947	348	17.3	340	1625	113	10	0	0	123	36
27		2008	B	11/28-12/04	400	1045	400	17.1	385	1751	142	19	0	0	161	42
27		2004	ALS	10/22-10/25	175	656	175	20.7	167	528	0	0	51	3	54	32
27		2005	ALS	10/21-10/24	140	433	140	19.2	134	435	0	0	25	0	25	19
27		2006	ALS	10/27-11/02	70	298	70	17.8	68	273	0	0	34	3	37	54
27		2007	ALS	10/26-11/01	150	349	150	25.8	143	580	0	0	59	7	66	46
27		2008	ALS	10/17-10/23	170	375	170	27.7	160	647	0	0	71	6	77	48
27	M	2007	ALS	8/17-8/23	30	1	28	100.0	25	101	0	0	3	0	3	12
27	M	2008	ALS	8/15-8/21	30	2	30	100.0	12	66	0	0	6	0	6	50
27	M	2007	AE	8/17-8/23	20	28	16	32.1	16	59	8	0	0	0	8	50
27	M	2008	AE	8/15-8/21	20	14	20	92.9	17	60	3	0	0	0	3	18
27S		2005	ALS	8/19-8/25	50	16	50	100.0	45	160	0	0	12	0	12	27
27S		2006	ALS	8/18-8/24	75	39	75	100.0	69	257	0	0	2	0	2	3
27S		2007	ALS	8/17-8/23	50	12	50	100.0	48	191	0	0	4	6	10	21
27S		2008	ALS	8/15-8/21	50	9	50	100.0	41	174	0	0	0	3	3	7
28/31		2004	ALS	10/01-11/14	10	13	10	46.2	10	83	0	0	2	0	2	20
28/31		2004	ALS	11/19-12/31	10	7	10	57.1	10	140	0	0	0	0	0	0
28/31		2005	ALS	9/30-11/13	10	10	10	70.0	10	160	0	0	0	0	0	0
28/31		2005	ALS	11/18-12/31	10	10	10	50.0	5	70	0	0	0	0	0	0
28/31/32		2006	ALS	10/06-11/02	15	7	15	71.4	12	30	0	0	12	0	12	100
28/31/32		2006	ALS	11/03-11/30	15	1	15	100.0	13	145	0	0	3	0	3	23
28/31/32		2006	ALS	12/01-12/31	15	19	15	63.2	15	173	0	0	0	0	0	0
28/31/32		2007	ALS	10/05-11/01	15	1	12	100.0	12	129	0	0	3	0	3	25
28/31/32		2007	ALS	11/02-11/29	25	2	25	100.0	23	127	0	0	2	2	4	17
28/31/32		2007	ALS	11/30-12/31	25	2	25	100.0	21	152	0	0	2	0	2	10
28/31/32		2008	ALS	10/03-10/30	15	1	15	0.0	15	99	0	0	0	0	0	0
28/31/32		2008	ALS	10/31-11/29	25	1	25	100.0	25	167	0	0	0	0	0	0
28/31/32		2008	ALS	11/28-12/29	25	0	25	–	13	46	0	0	0	0	0	0

BE = Early Bull **B** = Bull **ALS** = Antlerless **AE** = Any Elk **CN** = Camp Navajo **CH** = CHAMP Hunt **DV** = Disabled Veteran

5-Year: 2004–2008 Harvest

Unit		Year	Hunt Type	Dates	Permits Authorized	1st Choice Applicants	Permits Issued	Draw Odds	Hunters	Hunter Days	Harvest					Hunt Success
											Bull	Spike	Cow	Calf	Total	
**GENERAL (continued)**																
28/31/32		2007	AE	10/05-11/01	10	43	10	9.3	10	64	6	0	0	0	6	60
28/31/32		2008	AE	10/03-10/30	10	34	10	14.7	10	40	0	5	0	0	5	50
CN		2004	ALS	11/08-11/28	35	19	35	100.0	33	142	0	0	10	0	10	30
CN		2004	ALS	11/08-11/28	5	21	5	19.0	5	10	0	0	2	0	2	40
CN		2004	ALS	10/08-10/14	5	0	5	–	5	7	0	0	3	0	3	60
CN		2004	ALS	10/08-10/14	2	3	2	66.7	2	6	0	0	2	0	2	100
CN		2005	ALS	11/07-11/27	35	18	35	100.0	35	216	0	0	8	0	8	23
CN		2005	ALS	11/07-11/27	5	17	5	23.5	5	18	0	0	3	0	3	60
CN		2005	ALS	10/07-10/13	5	0	5	–	4	13	0	0	2	0	2	50
CN		2005	ALS	10/07-10/13	2	5	2	0.0	2	4	0	0	0	1	1	50
CN		2006	ALS	11/24-12/07	35	15	35	100.0	32	105	0	0	15	2	17	53
CN		2006	ALS	11/24-12/07	5	7	5	42.9	5	23	0	0	3	0	3	60
CN		2006	ALS	9/29-10/05	5	1	5	100.0	5	18	0	0	5	0	5	100
CN		2006	ALS	9/29-10/05	2	1	2	100.0	2	8	0	0	0	2	2	100
CN		2007	ALS	11/23-12/06	35	14	35	100.0	35	120	0	0	20	2	22	63
CN		2007	ALS	11/23-12/06	5	12	5	41.7	4	8	0	0	3	0	3	75
CN		2007	ALS	9/28-10/04	5	3	5	66.7	5	15	0	0	3	0	3	60
CN		2007	ALS	9/28-10/04	2	1	2	0.0	1	5	0	0	1	0	1	100
CN		2008	ALS	9/26-10/02	5	0	5	-	5	23	0	0	0	3	3	60
CN		2008	ALS	9/26-10/02	2	4	2	0.0	2	9	0	0	2	0	2	100
CN		2008	ALS	11/21-12/11	35	9	35	100.0	31	116	0	0	20	2	22	71
CN		2008	ALS	11/21-12/11	5	12	5	41.7	5	22	0	0	2	2	4	80
CN	DV	2004	ALS	10/15-10/21	14	5	14	60.0	14	82	0	0	3	2	5	36
CN	DV	2005	ALS	10/14-10/20	14	3	14	100.0	14	56	0	0	3	2	5	36
CN	DV	2006	ALS	10/20-10/26	14	5	14	40.0	12	40	0	0	6	2	8	67
CN	DV	2007	ALS	10/19-10/25	14	7	14	57.1	13	46	0	0	7	0	7	54
CN	DV	2008	ALS	10/17-10/23	14	4	14	50.0	14	61	0	0	4	0	4	29
CN		2004	AE	10/08-10/14	5	36	5	13.9	5	20	2	0	3	0	5	100
CN		2004	AE	10/08-10/14	2	32	2	6.3	2	5	1	0	1	0	2	100
CN		2004	AE	10/22-10/28	25	22	25	63.6	24	61	7	0	7	6	20	83
CN		2004	AE	10/22-10/28	4	17	4	11.8	2	12	2	0	0	0	2	100
CN		2005	AE	10/07-10/13	5	45	5	8.9	5	18	1	1	1	0	3	60
CN		2005	AE	10/07-10/13	2	48	2	4.2	2	6	0	0	0	0	0	0
CN		2005	AE	10/21-10/27	25	45	25	31.1	25	106	6	0	8	2	16	64
CN		2005	AE	10/21-10/27	4	27	4	0.0	4	22	2	0	0	0	2	50
CN		2006	AE	9/29-10/05	5	51	5	9.8	5	15	5	0	0	0	5	100
CN		2006	AE	9/29-10/05	2	16	2	6.3	2	4	2	0	0	0	2	100
CN		2006	AE	10/27-11/02	25	29	25	44.8	25	105	2	5	2	0	9	36
CN		2006	AE	10/27-11/02	4	10	4	10.0	4	12	3	1	0	0	4	100
CN		2007	AE	9/28-10/04	5	57	5	8.8	4	12	2	1	0	0	3	75
CN		2007	AE	9/28-10/04	2	33	2	6.1	2	4	2	0	0	0	2	100
CN		2007	AE	10/26-11/01	25	36	25	30.6	23	100	0	0	5	0	5	22
CN		2007	AE	10/26-11/01	4	8	4	12.5	4	15	0	0	1	0	1	25
CN		2008	AE	9/26-10/02	5	48	5	8.3	5	13	5	0	0	0	5	100
CN		2008	AE	9/26-10/02	2	42	2	4.8	2	4	2	0	0	0	2	100
CN		2008	AE	10/24-10/30	25	27	25	55.6	21	98	2	0	2	0	4	19
CN		2008	AE	10/24-10/30	4	7	4	14.3	4	15	3	0	1	0	4	100
CN	DV	2004	AE	10/15-10/21	7	37	7	18.9	4	11	4	0	0	0	4	100
CN	DV	2005	AE	10/14-10/20	7	34	7	20.6	7	34	0	0	6	0	6	86
CN	DV	2006	AE	10/20-10/26	7	33	7	18.2	6	25	2	1	1	0	4	67
CN	DV	2007	AE	10/19-10/25	7	30	7	20.0	7	25	6	0	0	0	6	86
CN	DV	2008	AE	10/17-10/23	7	34	7	20.6	7	33	0	2	0	0	2	29
**JUNIORS-ONLY**																
1/2C		2004	ALS	10/15-10/18	150	479	150	26.1	146	342	0	0	94	9	103	71
1/2C		2005	ALS	10/14-10/17	150	444	150	25.9	144	366	0	0	71	15	86	60
1/2C		2006	ALS	10/20-10/26	65	414	65	10.6	56	154	0	0	48	0	48	86
1/2C		2007	ALS	10/19-10/25	160	592	160	21.3	158	419	0	0	116	14	130	82
1/2C		2008	ALS	10/17-10/23	160	492	160	27.6	156	352	0	0	103	6	109	70
3A/3C		2008	ALS	10/17-10/23	250	270	250	46.3	246	793	0	0	111	9	120	49
4A		2004	ALS	10/22-10/27	200	437	200	33.6	194	546	0	0	112	10	122	63
4A		2005	ALS	10/21-10/26	275	419	275	41.3	256	762	0	0	83	31	114	45

BE = Early Bull **B** = Bull **ALS** = Antlerless **AE** = Any Elk **CN** = Camp Navajo **CH** = CHAMP Hunt **DV** = Disabled Veteran

Herd-Units:

CC = Canyon Creek
ES (Unit 1) = Escudilla
GV = Grapevine
HM = Hutch Mtn.
M = Martinez
ML = Marshall Lake
MM = Melatone Mesa
MZ = Mazatzal
RV = Round Valley
SM = East Sunset/West Sunset/Meteor Crater
TT = Twin Arrows/Two Guns/Grapevine
VV = Verde Valley
WI = Winslow
WS (17/20A/20C) = Williamson Valley/Skull Valley/Kirkland Junction

5-Year: 2004-2008 Harvest

Unit		Year	Hunt Type	Dates	Permits Authorized	1st Choice Applicants	Permits Issued	Draw Odds	Hunters	Hunter Days	Harvest					Hunt Success
											Bull	Spike	Cow	Calf	Total	
**JUNIORS-ONLY (continued)**																
4A		2006	ALS	10/20-10/26	220	425	220	37.2	214	679	0	0	80	7	87	41
4A		2007	ALS	10/19-10/25	220	429	220	36.4	207	635	0	0	93	0	93	45
4A		2008	ALS	10/17-10/23	100	227	100	39.2	96	264	0	0	49	7	56	58
6AN/6AS		2004	ALS	10/15-10/18	575	1559	575	33.5	567	1424	0	0	291	48	339	60
6AN/6AS		2005	ALS	10/14-10/17	635	1466	636	37.8	625	1713	0	0	276	20	296	47
6AN/6AS		2006	ALS	10/20-10/26	765	1581	762	40.5	739	2170	0	0	355	40	395	53
6AN/6AS		2007	ALS	10/19-10/25	765	1868	761	35.0	720	2046	0	0	391	45	436	61
6AN/6AS		2008	ALS	10/17-10/23	765	1708	765	39.4	739	2200	0	0	285	68	353	48
22N		2007	ALS	10/12-10/18	20	67	20	16.4	18	76	0	0	7	0	7	39
22N		2008	ALS	10/10-10/16	30	96	30	28.1	30	83	0	0	19	2	21	70
23N		2007	ALS	10/19-10/25	20	61	20	27.9	18	63	0	0	11	0	11	61
23S		2008	ALS	10/17-10/23	30	24	30	70.8	21	94	0	0	6	0	6	29
27		2004	ALS	10/15-10/18	100	193	100	32.1	89	243	0	0	35	9	44	49
27		2005	ALS	10/14-10/17	100	133	100	51.9	98	298	0	0	29	2	31	32
27		2006	ALS	10/20-10/26	95	160	95	29.4	91	283	0	0	49	6	55	60
**MUZZLELOADER**																
1/2B/2C		2006	BE	10/06-10/12	40	1561	40	2.0	40	185	24	0	0	0	24	60
1/2B/2C		2008	BE	9/26-10/02	40	1515	40	2.0	40	139	39	0	0	0	39	98
3A/3C		2007	BE	9/28-10/04	30	1295	30	2.1	30	113	26	0	0	0	26	87
3B		2004	BE	10/01-10/06	30	546	34	4.6	34	150	27	0	0	0	27	79
3B		2007	BE	9/28-10/04	20	251	20	4.4	20	86	13	0	0	0	13	65
3B		2008	BE	9/26-10/02	20	310	20	1.9	20	87	13	0	0	0	13	65
3B		2008	BE	11/28-12/04	150	95	150	63.2	139	676	21	17	0	0	38	27
3B		2008	ALS	12/12-12/31	250	51	250	100.0	231	1267	0	0	95	10	105	45
4A		2005	BE	9/30-10/05	10	333	10	2.7	10	37	5	2	0	0	7	70
4B		2004	BE	10/01-10/06	40	631	42	4.3	42	176	20	0	0	0	20	48
4B		2006	BE	10/06-10/12	40	281	40	10.3	39	168	18	0	0	0	18	46
4B		2004	ALS	10/22-10/27	250	207	250	61.8	240	983	0	0	51	4	55	23
4B		2005	ALS	10/21-10/26	225	248	225	59.7	213	876	0	0	24	2	26	12
4B		2006	ALS	10/20-10/26	175	149	175	62.4	171	711	0	0	40	0	40	23
4B		2007	ALS	10/19-10/25	175	177	175	63.8	170	690	0	0	44	4	48	28
5BN		2005	BE	9/30-10/05	40	747	40	2.8	39	150	31	0	0	0	31	79
5BS		2004	BE	10/01-10/06	30	838	32	3.1	32	86	32	0	0	0	32	100
6A		2004	BE	11/12-11/17	300	1558	335	14.1	329	1297	152	19	0	0	171	52
6A		2005	BE	11/11-11/16	425	1705	425	17.0	412	1740	151	29	0	0	180	44
6A		2006	BE	10/06-10/12	50	1358	50	3.2	50	229	34	0	0	0	34	68
6A		2006	BE	11/17-11/23	390	1175	390	16.3	375	1571	139	30	0	0	169	45
6A		2007	BE	11/16-11/22	390	1566	390	15.7	385	1674	125	44	0	0	169	44
6A		2008	BE	11/14-11/20	450	1496	450	20.3	430	1952	131	38	0	0	169	39
6A		2004	ALS	11/12-11/17	360	346	360	38.7	340	1362	0	0	112	9	121	36
6A		2005	ALS	11/11-11/16	300	288	300	41.7	281	1121	0	0	86	17	103	37
6A		2006	ALS	11/17-11/23	250	328	250	37.8	231	997	0	0	68	2	70	30
6A		2007	ALS	11/16-11/22	325	294	325	37.8	320	1302	0	0	89	15	104	33
6A		2008	ALS	11/14-11/20	200	242	200	32.6	195	843	0	0	66	7	73	37
6AS		2007	BE	9/28-10/04	25	688	25	2.2	24	93	21	1	0	0	22	92
6B		2008	BE	9/26-10/02	25	277	25	5.8	25	125	14	1	0	0	15	60
8		2004	ALS	10/15-10/21	200	250	200	52.4	192	729	0	0	69	15	84	44
8		2005	ALS	10/14-10/20	200	290	200	42.4	190	852	0	0	41	9	50	26
8		2006	ALS	10/06-10/12	150	181	150	43.1	139	617	0	0	51	3	54	39
8		2007	ALS	9/28-10/04	150	263	150	34.2	144	619	0	0	55	16	71	49
8		2008	ALS	9/26-10/02	150	237	150	38.8	146	593	0	0	70	18	88	60
16A		2004	BE	10/01-10/06	3	39	3	5.1	3	3	3	0	0	0	3	100
16A		2005	BE	9/30-10/05	3	31	3	9.7	3	18	2	0	0	0	2	67
16A		2006	BE	10/06-10/12	3	14	3	7.1	0	0	0	0	0	0	0	–
16A		2007	BE	9/28-10/04	3	24	3	4.2	3	14	0	0	0	0	0	0
16A		2008	BE	9/26-10/02	3	13	3	7.7	3	12	2	0	0	0	2	67
21		2005	BE	9/30-10/05	5	125	5	2.4	5	18	5	0	0	0	5	100
21		2007	BE	9/28-10/04	5	89	5	3.4	5	16	5	0	0	0	5	100
22S		2006	BE	10/06-10/12	20	144	20	5.6	20	92	11	0	0	0	11	55
22S		2007	BE	9/28-10/04	40	172	40	12.2	37	181	15	0	0	0	15	41
22S		2008	BE	9/26-10/02	40	161	40	15.5	40	166	21	3	0	0	24	60
23S		2004	BE	10/01-10/06	20	331	26	4.8	24	118	14	0	0	0	14	58
23S		2008	BE	9/26-10/02	15	272	15	3.7	15	68	10	0	0	0	10	67
27		2005	BE	9/30-10/05	25	791	25	2.3	25	110	9	0	0	0	9	36
CN		2004	BE	9/24-10/01	10	31	10	29.0	10	40	4	1	0	0	5	50
CN		2004	BE	9/24-10/01	3	28	3	10.7	3	6	3	0	0	0	3	100
CN		2007	ALSS	10/05-10/11	25	0	25	–	23	85	0	0	10	0	10	43

BE = Early Bull **B** = Bull **ALS** = Antlerless **AE** = Any Elk **CN** = Camp Navajo **CH** = CHAMP Hunt **DV** = Disabled Veteran

5-Year: 2004–2008 Harvest

Unit		Year	Hunt Type	Dates	Permits Authorized	1st Choice Applicants	Permits Issued	Draw Odds	Hunters	Hunter Days	Harvest					Hunt Success
											Bull	Spike	Cow	Calf	Total	
**MUZZLELOADER (continued)**																
CN		2004	ALS	9/24-10/01	25	5	25	100.0	25	120	0	0	10	0	10	40
CN		2004	ALS	9/24-10/01	5	4	5	75.0	5	12	0	0	3	0	3	60
CN		2005	ALS	9/23-9/30	25	1	25	100.0	22	108	0	0	6	0	6	27
CN		2005	ALS	9/23-9/30	5	4	5	0.0	5	20	0	0	1	0	1	20
CN		2006	ALS	10/06-10/12	25	0	25	–	20	95	0	0	2	0	2	10
CN		2006	ALS	10/06-10/12	5	4	5	100.0	3	13	0	0	0	0	0	0
CN		2007	ALS	10/05-10/11	5	3	5	66.7	5	30	0	0	1	0	1	20
CN		2008	ALS	10/10-10/16	25	5	25	100.0	25	120	0	0	5	0	5	20
CN		2008	ALS	10/10-10/16	5	8	5	37.5	5	33	0	0	0	0	0	0
CN		2005	AE	9/23-9/30	10	49	10	16.3	10	53	0	0	3	0	3	30
CN		2005	AE	9/23-9/30	3	60	3	5.0	2	13	0	0	0	0	0	0
CN		2006	AE	10/06-10/12	10	31	10	32.3	10	47	3	3	3	0	9	90
CN		2006	AE	10/06-10/12	3	12	3	25.0	3	18	0	0	0	0	0	0
CN		2007	AE	10/05-10/11	10	24	10	25.0	10	39	3	0	1	1	5	50
CN		2007	AE	10/05-10/11	3	12	3	25.0	3	21	0	0	0	0	0	0
CN		2008	AE	10/10-10/16	10	27	10	25.9	10	50	0	0	0	0	0	0
CN		2008	AE	10/10-10/16	3	14	3	7.1	3	8	2	0	0	0	2	67
**ARCHERY**																
1		2004	B	9/17-9/30	150	2647	170	5.2	170	1420	76	0	0	0	76	45
1		2005	B	9/16-9/29	150	2344	150	5.1	150	1083	93	2	0	0	95	63
1		2004	ALS	9/17-9/30	250	213	250	42.3	236	1519	0	0	63	2	65	28
1		2005	ALS	9/16-9/29	325	202	325	48.0	317	2022	0	0	77	4	81	26
1/2B/2C		2006	B	9/22-10/05	150	2614	150	4.6	150	1214	70	0	0	0	70	47
1/2B/2C		2007	B	9/14-9/27	150	2847	150	3.9	144	1285	80	2	0	0	82	57
1/2B/2C		2008	B	9/12-9/25	150	2241	150	5.3	148	1218	73	4	0	0	77	52
1/2B/2C		2008	B	11/14-11/27	25	24	25	37.5	25	168	5	5	0	0	10	40
1/2B/2C		2006	ALS	9/22-10/05	170	137	170	27.0	168	1026	0	0	41	6	47	28
1/2B/2C		2007	ALS	9/14-9/27	50	51	50	9.8	50	308	0	0	10	2	12	24
1/2B/2C		2008	ALS	9/12-9/25	75	62	75	30.6	73	520	0	0	10	0	10	14
3A/3C		2004	B	9/17-9/30	50	960	54	4.8	54	406	38	0	0	0	38	70
3A/3C		2005	B	9/16-9/29	90	1392	90	5.5	90	667	67	0	0	0	67	74
3A/3C		2006	B	9/22-10/05	100	1463	100	5.5	100	770	63	0	0	0	63	63
3A/3C		2007	B	9/14-9/27	100	1722	100	3.8	97	838	55	5	0	0	60	62
3A/3C		2008	B	9/12-9/25	150	1647	150	7.0	150	1271	86	0	0	0	86	57
3A/3C		2008	B	11/14-11/27	25	10	25	100.0	25	194	6	0	0	0	6	24
3A/3C		2004	ALS	9/17-9/30	50	64	50	37.5	47	260	0	0	10	3	13	28
3A/3C		2005	ALS	9/16-9/29	50	34	50	61.8	50	326	0	0	6	3	9	18
3A/3C		2006	ALS	9/22-10/05	70	40	70	55.0	65	406	0	0	19	2	21	32
3A/3C		2007	ALS	9/14-9/27	70	42	70	42.9	65	427	0	0	14	2	16	25
3A/3C		2008	ALS	9/12-9/25	70	32	70	50.0	68	399	0	0	7	2	9	13
3B		2004	B	9/17-9/30	25	178	28	9.6	26	215	22	2	0	0	24	92
3B		2005	B	9/16-9/29	25	239	25	5.9	25	250	8	0	0	0	8	32
3B		2006	B	9/22-10/05	25	171	25	8.8	25	175	13	0	0	0	13	52
3B		2007	B	9/14-9/27	25	231	25	7.4	25	203	15	0	0	0	15	60
3B		2008	B	9/12-9/25	75	251	75	18.3	74	642	38	0	0	0	38	51
3B		2008	B	11/14-11/27	25	11	25	63.6	25	196	6	0	0	0	6	24
3B		2004	ALS	9/17-9/30	15	17	15	47.1	15	128	0	0	0	0	0	0
3B		2005	ALS	9/16-9/29	15	3	15	33.3	15	98	0	0	2	2	4	27
3B		2006	ALS	9/22-10/05	15	4	15	100.0	15	101	0	0	3	0	3	20
3B		2007	ALS	9/14-9/27	15	22	15	31.8	15	94	0	0	2	0	2	13
3B		2008	ALS	9/12-9/25	25	8	25	87.5	25	221	0	0	2	0	2	8
4A		2004	B	9/17-9/30	125	1065	129	9.1	127	925	46	0	0	0	46	36
4A		2005	B	9/16-9/29	150	1111	150	11.3	150	994	76	5	0	0	81	54
4A		2006	B	9/22-10/05	175	1269	175	11.3	171	1434	56	2	0	0	58	34
4A		2007	B	9/14-9/27	175	1242	175	11.0	171	1411	58	6	0	0	64	37
4A		2008	B	9/12-9/25	225	1179	225	13.5	219	1783	94	2	0	0	96	44
4A		2008	B	11/14-11/27	25	19	25	100.0	19	106	0	6	0	0	6	32
4A		2004	ALS	9/17-9/30	200	210	200	32.9	198	1142	0	0	78	8	86	43
4A		2005	ALS	9/16-9/29	300	202	300	57.9	294	1969	0	0	76	6	82	28
4A		2006	ALS	9/22-10/05	295	152	295	55.9	289	1972	0	0	64	2	66	23
4A		2007	ALS	9/14-9/27	80	39	80	17.9	74	392	0	0	36	2	38	51

BE = Early Bull **B** = Bull **ALS** = Antlerless **AE** = Any Elk **CN** = Camp Navajo **CH** = CHAMP Hunt **DV** = Disabled Veteran

Herd-Units:

CC = Canyon Creek
ES (Unit 1) = Escudilla
GV = Grapevine
HM = Hutch Mtn.
M = Martinez
ML = Marshall Lake
MM = Melatone Mesa
MZ = Mazatzal
RV = Round Valley
SM = East Sunset/West Sunset/Meteor Crater
TT = Twin Arrows/Two Guns/Grapevine
VV = Verde Valley
WI = Winslow
WS (17/20A/20C) = Williamson Valley/Skull Valley/Kirkland Junction

5-Year: 2004-2008 Harvest

Unit		Year	Hunt Type	Dates	Permits Authorized	1st Choice Applicants	Permits Issued	Draw Odds	Hunters	Hunter Days	Harvest					Hunt Success
											Bull	Spike	Cow	Calf	Total	
**ARCHERY (continued)**																
4A		2008	ALS	9/12-9/25	80	36	80	25.0	74	554	0	0	19	0	19	26
4B		2004	B	9/17-9/30	125	458	133	14.8	131	1106	37	0	0	0	37	28
4B		2005	B	9/16-9/29	150	417	150	14.9	150	1220	52	0	0	0	52	35
4B		2006	B	9/22-10/05	150	390	150	15.1	144	1270	22	0	0	0	22	15
4B		2007	B	9/14-9/27	150	416	151	14.7	146	1314	44	0	0	0	44	30
4B		2008	B	9/12-9/25	200	372	200	26.6	196	1747	57	4	0	0	61	31
4B		2008	B	11/14-11/27	25	11	25	100.0	23	153	0	0	0	0	0	0
4B		2004	ALS	9/17-9/30	100	34	100	58.8	96	724	0	0	2	0	2	2
4B		2005	ALS	9/16-9/29	75	17	75	100.0	65	438	0	0	9	0	9	14
4B		2006	ALS	9/22-10/05	50	24	50	70.8	50	324	0	0	7	0	7	14
4B		2007	ALS	9/14-9/27	50	24	50	37.5	50	336	0	0	6	2	8	16
4B		2008	ALS	9/12-9/25	50	15	50	73.3	50	333	0	0	6	0	6	12
4B/5A	WI	2004	ALS	9/01-9/30	5	1	5	0.0	0	0	0	0	0	0	0	–
4B/5A	WI	2004	ALS	9/17-10/14	10	2	10	100.0	10	20	0	0	0	0	0	0
4B/5A	WI	2004	ALS	10/01-10/31	5	0	7	–	7	47	0	0	2	0	2	29
4B/5A	WI	2004	ALS	10/14-11/14	10	0	10	–	10	60	0	0	0	0	0	0
4B/5A	WI	2005	ALS	9/01-9/29	5	3	5	100.0	5	43	0	0	0	0	0	0
4B/5A	WI	2005	ALS	9/16-10/13	10	0	10	–	10	58	0	0	0	0	0	0
4B/5A	WI	2005	ALS	9/30-10/31	5	0	5	–	5	15	0	0	0	0	0	0
4B/5A	WI	2005	ALS	10/14-11/13	10	0	10	–	6	26	0	0	0	0	0	0
4B/5A	WI	2004	AE	9/01-9/30	5	7	5	42.9	3	16	0	0	0	0	0	0
4B/5A	WI	2004	AE	10/01-10/31	5	7	5	42.9	0	0	0	0	0	0	0	–
4B/5A	WI	2005	AE	9/01-9/30	5	4	5	75.0	5	58	0	0	3	0	3	60
4B/5A	WI	2005	AE	9/30-10/31	5	0	5	–	5	8	0	0	0	0	0	0
5A		2004	B	9/17-9/30	80	1003	94	8.8	88	572	44	0	0	0	44	50
5A		2005	B	9/16-9/29	145	1023	145	9.9	145	1100	78	3	0	0	81	56
5A		2006	B	9/22-10/05	90	949	90	7.3	90	734	35	0	0	0	35	39
5A		2007	B	9/14-9/27	140	1164	140	9.5	138	1126	28	2	0	0	30	22
5A		2008	B	9/12-9/25	110	911	110	10.3	107	868	49	0	0	0	49	46
5A		2008	B	11/14-11/27	25	2	25	100.0	22	150	3	0	0	0	3	14
5A		2004	ALS	9/17-9/30	250	176	250	36.9	241	1591	0	0	82	7	89	37
5A		2005	ALS	9/16-9/29	300	160	300	65.6	283	1842	0	0	94	11	105	37
5A		2006	ALS	9/22-10/05	220	166	220	48.2	216	1354	0	0	72	2	74	34
5A		2007	ALS	9/14-9/27	200	158	200	35.4	195	1332	0	0	60	2	62	32
5A		2008	ALS	9/12-9/25	135	121	135	37.2	128	919	0	0	17	0	17	13
5BN		2004	B	9/17-9/30	200	944	215	15.0	209	1707	48	0	0	0	48	23
5BN		2005	B	9/16-9/29	200	858	200	14.8	195	1674	57	7	0	0	64	33
5BN		2006	B	9/22-10/05	200	1030	200	11.3	198	1490	64	4	0	0	68	34
5BN		2007	B	9/14-9/27	125	797	125	11.9	123	1039	31	2	0	0	33	27
5BN		2008	B	9/12-9/25	175	797	174	13.9	172	1484	69	2	0	0	71	41
5BN		2008	B	11/14-11/27	25	7	25	100.0	25	180	2	2	0	0	4	16
5BN		2004	ALS	9/17-9/30	175	56	175	69.6	163	1126	0	0	25	2	27	17
5BN		2005	ALS	9/16-9/29	250	99	250	69.7	240	1697	0	0	47	4	51	21
5BN		2006	ALS	9/22-10/05	200	71	200	74.6	196	1301	0	0	46	2	48	24
5BN		2007	ALS	9/14-9/27	100	55	100	38.2	98	774	0	0	21	0	21	21
5BN		2008	ALS	9/12-9/25	100	33	100	42.4	100	815	0	0	15	0	15	15
5BS		2004	B	9/17-9/30	125	1389	133	7.7	133	956	51	2	0	0	53	40
5BS		2005	B	9/16-9/29	150	1450	150	8.4	150	1104	84	12	0	0	96	64
5BS		2006	B	9/22-10/05	195	1742	195	8.6	193	1581	59	4	0	0	63	33
5BS		2007	B	9/14-9/27	200	2111	200	7.8	197	1381	87	3	0	0	90	46
5BS		2008	B	9/12-9/25	200	1796	200	8.7	197	1781	74	2	0	0	76	39
5BS		2008	B	11/14-11/27	25	20	25	30.0	25	205	5	0	0	0	5	20
5BS		2004	ALS	9/17-9/30	250	246	250	29.3	250	1622	0	0	92	2	94	38
5BS		2005	ALS	9/16-9/29	350	217	350	44.7	343	2161	0	0	137	7	144	42
5BS		2006	ALS	9/22-10/05	360	291	359	33.7	348	2290	0	0	102	9	111	32
5BS		2007	ALS	9/14-9/27	200	192	200	27.6	198	1337	0	0	48	4	52	26
5BS		2008	ALS	9/12-9/25	200	178	200	32.6	193	1424	0	0	43	0	43	22
6A		2008	B	11/21-11/27	25	17	25	35.3	25	118	3	0	0	0	3	12
6A/19A/21	VV	2007	ALSS	9/14-9/27	30	0	30	–	28	173	0	0	2	2	4	14
6A/19A/21	VV	2004	ALS	9/17-9/30	10	0	10	–	7	17	0	0	0	0	0	0
6A/19A/21	VV	2005	ALS	9/16-9/29	20	0	20	–	16	78	0	0	0	0	0	0
6A/19A/21	VV	2006	ALS	9/22-10/05	20	0	20	–	17	94	0	0	0	3	3	18
6A/19A/21	VV	2004	AE	9/17-9/30	5	11	5	18.2	5	40	1	0	0	0	1	20
6A/19A/21	VV	2005	AE	9/16-9/29	10	3	10	100.0	10	82	3	0	0	0	3	30
6A/19A/21	VV	2006	AE	9/22-10/05	15	27	15	29.6	15	95	1	0	0	0	1	7
6A/19A/21	VV	2007	AE	9/14-9/27	15	16	15	43.8	15	113	4	0	4	0	8	53
6AN		2004	B	9/17-9/30	80	791	91	8.5	91	728	33	4	0	0	37	41

BE = Early Bull **B** = Bull **ALS** = Antlerless **AE** = Any Elk **CN** = Camp Navajo **CH** = CHAMP Hunt **DV** = Disabled Veteran

Elk Hunt Data

5-Year: 2004-2008 Harvest

Unit		Year	Hunt Type	Dates	Permits Authorized	1st Choice Applicants	Permits Issued	Draw Odds	Hunters	Hunter Days	Harvest					Hunt Success
											Bull	Spike	Cow	Calf	Total	
**ARCHERY (continued)**																
6AN		2005	B	9/16-9/29	100	995	100	8.1	100	718	46	0	0	0	46	46
6AN		2006	B	9/22-10/05	115	1062	115	8.9	115	920	40	2	0	0	42	37
6AN		2007	B	9/14-9/27	115	1233	115	7.9	115	846	49	5	0	0	54	47
6AN		2008	B	9/12-9/25	150	1074	150	11.3	146	1311	65	2	0	0	67	46
6AN		2004	ALS	9/17-9/30	240	187	240	49.7	236	1484	0	0	38	4	42	18
6AN		2005	ALS	9/16-9/29	390	183	390	62.8	371	2359	0	0	107	4	111	30
6AN		2006	ALS	9/22-10/05	450	227	450	61.2	438	2880	0	0	99	2	101	23
6AN		2007	ALS	9/14-9/27	200	186	199	31.2	193	1291	0	0	58	0	58	30
6AN		2008	ALS	9/12-9/25	200	188	200	44.7	194	1301	0	0	39	2	41	21
6AS		2004	B	9/17-9/30	120	1036	133	10.6	131	1079	33	4	0	0	37	28
6AS		2005	B	9/16-9/29	100	723	100	11.5	100	752	30	6	0	0	36	36
6AS		2006	B	9/22-10/05	115	824	115	6.8	113	816	32	6	0	0	38	34
6AS		2007	B	9/14-9/27	115	745	115	12.1	115	944	37	2	0	0	39	34
6AS		2008	B	9/12-9/25	150	945	150	12.1	148	1344	35	2	0	0	37	25
6AS		2004	ALS	9/17-9/30	250	156	250	49.4	245	1540	0	0	54	5	59	24
6AS		2005	ALS	9/16-9/29	390	186	390	83.9	384	2719	0	0	66	6	72	19
6AS		2006	ALS	9/22-10/05	450	192	450	77.1	438	2924	0	0	65	0	65	15
6AS		2007	ALS	9/14-9/27	200	134	197	50.0	186	1322	0	0	42	2	44	24
6AS		2008	ALS	9/12-9/25	175	140	175	50.7	173	1176	0	0	29	2	31	18
6AW		2004	B	9/17-9/30	55	303	56	15.8	54	420	20	4	0	0	24	44
6AW		2005	B	9/16-9/29	50	241	50	11.6	50	424	14	4	0	0	18	36
6AW		2006	B	9/22-10/05	55	217	55	14.7	55	414	20	0	0	0	20	36
6AW		2007	B	9/14-9/27	55	281	55	14.6	54	412	11	0	0	0	11	20
6AW		2008	B	9/12-9/25	75	243	75	17.7	73	680	23	2	0	0	25	34
6AW		2004	ALS	9/17-9/30	50	29	50	72.4	50	335	0	0	25	5	30	60
6AW		2005	ALS	9/16-9/29	125	51	125	60.8	122	934	0	0	30	0	30	25
6AW		2006	ALS	9/22-10/05	150	26	150	100.0	147	1091	0	0	20	3	23	16
6AW		2007	ALS	9/14-9/27	50	24	50	54.2	50	403	0	0	9	0	9	18
6AW		2008	ALS	9/12-9/25	50	16	50	68.8	47	420	0	0	16	0	16	34
6B		2004	B	9/17-9/30	50	286	50	12.9	50	341	19	2	0	0	21	42
6B		2005	B	9/16-9/29	65	328	65	12.8	63	506	16	2	0	0	18	29
6B		2006	B	9/22-10/05	80	358	80	15.1	80	676	24	2	0	0	26	33
6B		2007	B	9/14-9/27	100	417	100	12.7	98	1000	17	0	0	0	17	17
6B		2008	B	9/12-9/25	100	299	100	20.4	98	806	24	2	0	0	26	27
6B		2008	B	11/14-11/27	25	5	25	100.0	23	212	2	0	0	0	2	9
6B		2004	ALS	9/17-9/30	50	42	50	50.0	48	405	0	0	10	0	10	21
6B		2005	ALS	9/16-9/29	100	46	100	71.7	98	781	0	0	28	2	30	31
6B		2006	ALS	9/22-10/05	100	42	100	54.8	95	639	0	0	24	0	24	25
6B		2007	ALS	9/14-9/27	100	32	95	75.0	91	656	0	0	13	2	15	16
6B		2008	ALS	9/12-9/25	100	78	100	46.2	96	758	0	0	10	0	10	10
7E		2004	B	9/17-9/30	115	317	138	23.0	136	1219	41	0	0	0	41	30
7E		2005	B	9/16-9/29	115	507	115	18.9	115	995	38	2	0	0	40	35
7E		2006	B	9/22-10/05	100	435	100	12.9	96	814	29	4	0	0	33	34
7E		2007	B	9/14-9/27	120	486	120	11.9	120	1093	36	0	0	0	36	30
7E		2008	B	9/12-9/25	130	357	130	20.4	130	1226	38	0	0	0	38	29
7E		2008	B	11/14-11/27	25	12	25	91.7	20	148	2	0	0	0	2	10
7E		2004	ALS	9/17-9/30	175	53	175	100.0	171	1138	0	0	19	0	19	11
7E		2005	ALS	9/16-9/29	235	50	235	100.0	228	1598	0	0	36	2	38	17
7E		2006	ALS	9/22-10/05	150	58	150	100.0	146	848	0	0	35	2	37	25
7E		2007	ALS	9/14-9/27	75	35	75	42.9	70	498	0	0	13	2	15	21
7E		2008	ALS	9/12-9/25	75	36	75	66.7	75	489	0	0	11	2	13	17
7M		2004	B	9/17-9/30	80	341	97	19.4	92	748	25	9	0	0	34	37
7M		2004	B	10/01-10/14	80	235	95	25.1	95	896	22	0	0	0	22	23
7M		2005	B	9/16-9/29	80	320	80	17.2	75	650	13	2	0	0	15	20
7M		2005	B	9/30-10/13	80	144	80	27.1	80	702	12	6	0	0	18	23
7M		2004	ALS	9/17-9/30	80	30	80	100.0	78	525	0	0	14	2	16	21
7M		2004	ALS	10/01-10/14	80	12	80	100.0	80	628	0	0	12	2	14	18
7M		2005	ALS	9/16-9/29	80	38	80	65.8	80	595	0	0	28	0	28	35
7M		2005	ALS	9/30-10/13	80	21	80	100.0	70	480	0	0	14	0	14	20
7W		2004	B	9/17-9/30	155	831	171	12.0	169	1520	58	0	0	0	58	34
7W		2005	B	9/16-9/29	150	717	150	14.4	150	1138	84	2	0	0	86	57

BE = Early Bull **B** = Bull **ALS** = Antlerless **AE** = Any Elk **CN** = Camp Navajo **CH** = CHAMP Hunt **DV** = Disabled Veteran

Herd-Units:

CC = Canyon Creek
ES (Unit 1) = Escudilla
GV = Grapevine
HM = Hutch Mtn.
M = Martinez
ML = Marshall Lake
MM = Melatone Mesa
MZ = Mazatzal
RV = Round Valley
SM = East Sunset/West Sunset/Meteor Crater
TT = Twin Arrows/Two Guns/Grapevine
VV = Verde Valley
WI = Winslow
WS (17/20A/20C) = Williamson Valley/Skull Valley/Kirkland Junction

5-Year: 2004-2008 Harvest

Unit		Year	Hunt Type	Dates	Permits Authorized	1st Choice Applicants	Permits Issued	Draw Odds	Hunters	Hunter Days	Harvest					Hunt Success
											Bull	Spike	Cow	Calf	Total	
**ARCHERY (continued)**																
7W		2006	B	9/22-10/05	150	1097	150	9.3	150	1278	51	0	0	0	51	34
7W		2007	B	9/14-9/27	150	1086	150	9.0	148	1317	38	5	0	0	43	29
7W		2008	B	9/12-9/25	150	844	150	12.1	150	1352	72	0	0	0	72	48
7W		2008	B	11/14-11/27	25	14	25	78.6	25	163	3	0	0	0	3	12
7W		2004	ALS	9/17-9/30	100	53	100	50.9	98	628	0	0	18	5	23	23
7W		2005	ALS	9/16-9/29	250	74	250	97.3	241	1635	0	0	41	11	52	22
7W		2006	ALS	9/22-10/05	225	85	225	52.9	221	1417	0	0	61	0	61	28
7W		2007	ALS	9/14-9/27	100	60	100	41.7	96	683	0	0	9	4	13	14
7W		2008	ALS	9/12-9/25	75	40	75	35.0	70	503	0	0	12	0	12	17
8		2004	B	9/17-9/30	100	573	127	12.0	127	1030	53	0	0	0	53	42
8		2005	B	9/16-9/29	100	734	100	7.9	100	931	43	0	0	0	43	43
8		2006	B	9/22-10/05	125	739	125	9.9	125	1201	45	4	0	0	49	39
8		2007	B	9/14-9/27	125	878	125	8.2	125	1330	23	2	0	0	25	20
8		2008	B	9/12-9/25	125	677	125	12.0	122	1130	42	9	0	0	51	42
8		2008	B	11/14-11/27	25	10	25	70.0	23	139	5	2	0	0	7	30
8		2006	ALS	9/22-10/05	50	14	50	85.7	50	410	0	0	12	2	14	28
8		2007	ALS	9/14-9/27	50	32	50	18.8	50	396	0	0	13	0	13	26
8		2008	ALS	9/12-9/25	50	9	50	100.0	50	330	0	0	16	0	16	32
9		2004	B	9/17-9/30	90	2613	123	4.4	120	943	72	0	0	0	72	60
9		2005	B	9/16-9/29	100	2835	100	3.2	100	674	82	0	0	0	82	82
9		2006	B	9/22-10/05	75	2885	75	2.4	75	646	53	1	0	0	54	72
9		2007	B	9/14-9/27	75	3314	75	1.7	75	572	51	0	0	0	51	68
9		2008	B	9/12-9/25	75	2651	74	2.5	74	584	54	0	0	0	54	73
9		2008	B	11/14-11/27	25	47	25	34.0	23	203	5	0	0	0	5	22
10		2004	B	9/17-9/30	150	1274	177	9.2	171	1338	89	0	0	0	89	52
10		2005	B	9/16-9/29	150	1060	151	7.1	149	1023	99	0	0	0	99	66
10		2006	B	9/22-10/05	125	1664	125	4.7	122	959	56	0	0	0	56	46
10		2007	B	9/14-9/27	150	1845	150	3.8	150	1403	36	0	0	0	36	24
10		2008	B	9/12-9/25	150	1052	150	8.3	148	1359	50	0	0	0	50	34
10		2008	B	11/14-11/27	25	16	25	75.0	23	183	4	0	0	0	4	17
10		2004	ALS	9/17-9/30	100	33	100	75.8	94	704	0	0	4	0	4	4
10		2005	ALS	9/16-9/29	100	17	100	100.0	90	523	0	0	21	2	23	26
10		2006	ALS	9/22-10/05	100	42	94	54.8	90	651	0	0	4	0	4	4
10		2007	ALS	9/14-9/27	75	18	72	88.9	62	497	0	0	10	0	10	16
10		2008	ALS	9/12-9/25	75	56	75	60.7	72	431	0	0	3	0	3	4
11M		2006	B	9/22-10/05	80	317	80	17.0	80	744	27	0	0	0	27	34
11M		2006	B	10/06-10/19	80	107	80	22.4	80	752	9	2	0	0	11	14
11M		2007	B	9/14-9/27	80	399	80	13.8	80	697	18	2	0	0	20	25
11M		2007	B	9/28-10/11	80	139	80	20.1	80	640	24	2	0	0	26	33
11M		2008	B	9/12-9/25	80	253	80	20.6	80	796	22	6	0	0	28	35
11M		2008	B	9/26-10/09	80	158	80	20.9	80	790	16	0	0	0	16	20
11M		2006	ALS	9/22-10/05	80	41	80	70.7	80	535	0	0	25	0	25	31
11M		2006	ALS	10/06-10/19	80	9	80	100.0	77	562	0	0	10	0	10	13
11M		2007	ALS	9/14-9/27	80	43	79	72.1	77	511	0	0	15	5	20	26
11M		2007	ALS	9/28-10/11	80	13	79	100.0	77	656	0	0	16	0	16	21
11M		2008	ALS	9/12-9/25	80	55	80	74.5	71	631	0	0	22	2	24	34
11M		2008	ALS	9/26-10/09	80	31	80	61.3	80	517	0	0	22	6	28	35
15A/15B/ 17A/18/19B		2004	ALS	9/17-9/30	25	3	25	100.0	25	211	0	0	2	0	2	8
15A/15B/ 17A/18/19B		2005	ALS	9/16-9/30	35	5	35	100.0	33	208	0	0	2	0	2	6
15A/15B/ 17A/18/19B		2004	AE	9/17-9/30	25	78	25	20.5	23	148	8	0	0	0	8	35
15A/15B/ 17A/18/19B		2005	AE	9/16-9/29	35	104	35	18.3	35	228	15	3	0	0	18	51
15A/15B/17/18/ 19B/20A/20C		2008	B	9/12-9/25	75	90	75	24.4	70	532	35	0	0	0	35	50
15A/15B/17/18/ 19B/20A/20C		2006	ALS	9/22-10/05	75	8	75	100.0	70	458	0	0	0	0	0	0
15A/15B/17/18/ 19B/20A/20C		2007	ALS	9/14-9/27	75	7	76	100.0	61	398	0	0	3	0	3	5
15A/15B/17/18/ 19B/20A/20C		2008	ALS	9/12-9/25	75	2	75	100.0	60	363	0	0	2	0	2	3
15A/15B/17/18/ 19B/20A/20C		2006	AE	9/22-10/05	75	163	75	27.6	66	398	23	0	0	0	23	35
15A/15B/17/18/ 19B/20A/20C		2007	AE	9/14-9/27	75	141	75	19.1	75	618	26	0	0	0	26	35
16A		2004	B	9/17-9/30	3	20	6	30.0	6	33	3	3	0	0	6	100
16A		2005	B	9/16-9/29	4	26	4	11.5	4	4	0	4	0	0	4	100

BE = Early Bull **B** = Bull **ALS** = Antlerless **AE** = Any Elk **CN** = Camp Navajo **CH** = CHAMP Hunt **DV** = Disabled Veteran

5-Year: 2004–2008 Harvest

Unit		Year	Hunt Type	Dates	Permits Authorized	1st Choice Applicants	Permits Issued	Draw Odds	Hunters	Hunter Days	Harvest					Hunt Success
											Bull	Spike	Cow	Calf	Total	
**ARCHERY (continued)**																
16A		2006	B	9/22-10/05	4	14	4	14.3	4	12	1	0	0	0	1	25
16A		2007	B	9/14-9/27	4	8	4	37.5	4	26	0	4	0	0	4	100
16A		2008	B	9/12-9/25	4	17	4	5.9	4	20	1	0	0	0	1	25
19A		2004	B	9/17-9/30	15	62	15	17.7	13	117	10	0	0	0	10	77
19A		2005	B	9/16-9/29	15	81	15	12.3	14	120	8	0	0	0	8	57
19A		2006	B	9/22-10/05	20	55	20	18.2	20	171	9	0	0	0	9	45
19A		2007	B	9/14-9/27	20	97	20	11.3	20	171	2	2	0	0	4	20
19A		2008	B	9/12-9/25	20	40	20	35.0	20	184	5	0	0	0	5	25
22		2004	B	11/12-11/25	160	238	160	39.5	158	1137	22	5	0	0	27	17
22		2008	B	9/12-9/25	25	205	25	7.8	25	137	17	0	0	0	17	68
22		2004	ALS	11/19-12/02	85	27	85	85.2	85	500	0	0	13	3	16	19
22		2005	ALS	11/18-12/01	85	26	85	80.8	85	651	0	0	21	4	25	29
22		2006	ALS	11/17-11/30	100	38	99	73.7	96	608	0	0	11	6	17	18
22		2007	ALS	11/16-11/29	50	14	50	78.6	50	340	0	0	16	0	16	32
22		2008	ALS	11/14-11/27	70	15	70	100.0	64	443	0	0	19	4	23	36
22N		2005	B	11/11-11/24	185	195	185	46.7	180	1326	49	13	0	0	62	34
22N		2006	B	11/10-11/23	200	238	200	44.5	197	1397	46	9	0	0	55	28
22N		2007	B	11/09-11/22	220	228	219	42.1	210	1609	34	11	0	0	45	21
22N		2008	B	11/14-11/27	300	172	300	79.7	281	2045	42	11	0	0	53	19
22S		2004	B	9/24-10/07	50	113	50	28.3	50	400	15	0	0	0	15	30
22S		2005	B	11/11-11/24	50	5	50	100.0	48	363	2	0	0	0	2	4
22S		2006	B	11/10-11/23	20	19	20	57.9	20	175	0	0	0	0	0	0
22S		2007	B	11/09-11/22	30	26	29	76.9	26	119	10	0	0	0	10	38
22S		2008	B	11/14-11/27	30	17	30	100.0	30	220	5	8	0	0	13	43
23		2004	B	11/12-11/25	125	122	125	52.5	125	915	21	0	0	0	21	17
23		2005	B	11/11-11/24	150	186	150	43.0	150	1160	12	5	0	0	17	11
23		2006	B	11/10-11/23	150	142	150	52.8	150	1133	22	10	0	0	32	21
23		2007	B	11/09-11/22	150	154	150	41.6	130	936	18	3	0	0	21	16
23		2008	B	11/07-11/20	150	118	150	62.7	144	1082	21	2	0	0	23	16
23		2004	ALS	11/19-12/02	100	15	100	100.0	95	481	0	0	11	3	14	15
23		2005	ALS	11/18-12/01	100	25	100	100.0	95	603	0	0	15	0	15	16
23		2006	ALS	11/17-11/30	100	24	100	100.0	97	621	0	0	24	0	24	25
23		2007	ALS	11/16-11/29	100	18	98	100.0	95	552	0	0	10	0	10	11
23		2008	ALS	11/14-11/27	100	14	100	100.0	97	605	0	0	26	3	29	30
23N		2008	B	9/12-9/25	15	594	15	2.2	15	135	8	0	0	0	8	53
23S		2005	B	9/23-10/06	25	212	25	9.0	24	165	13	0	0	0	13	54
23S		2007	B	9/14-9/27	35	438	35	4.1	35	223	28	0	0	0	28	80
23S		2008	B	9/12-9/25	15	102	15	8.8	14	112	7	0	0	0	7	50
27		2004	B	9/17-9/30	100	925	121	7.6	121	1064	47	0	0	0	47	39
27		2005	B	9/16-9/29	100	834	100	6.2	97	800	42	2	0	0	44	45
27		2006	B	9/22-10/05	100	850	100	7.4	100	824	43	2	0	0	45	45
27		2007	B	9/14-9/27	100	883	100	7.0	97	838	39	2	0	0	41	42
27		2008	B	9/12-9/25	150	960	150	9.9	146	1357	60	0	0	0	60	41
27		2008	B	11/14-11/27	25	40	25	30.0	19	183	0	0	0	0	0	0
27		2004	ALS	9/17-9/30	100	55	100	49.1	96	694	0	0	6	0	6	6
27		2005	ALS	9/16-9/29	175	31	175	100.0	164	1126	0	0	20	4	24	15
27		2006	ALS	9/22-10/05	145	54	145	66.7	136	805	0	0	21	2	23	17
27		2007	ALS	9/14-9/27	150	73	146	58.9	132	907	0	0	20	0	20	15
27		2008	ALS	9/12-9/25	75	52	75	46.2	65	498	0	0	8	0	8	12
28		2006	AE	9/22-10/05	5	12	5	25.0	5	50	0	0	0	0	0	0
28		2007	AE	9/14-9/27	5	13	5	30.8	5	38	3	0	0	0	3	60
28		2008	AE	9/12-9/25	5	4	5	50.0	5	30	5	0	0	0	5	100
28/31		2004	AE	9/17-9/30	5	17	5	23.5	5	15	3	0	0	0	3	60
28/31		2005	AE	9/16-9/29	5	8	5	37.5	5	23	0	0	0	0	0	0
31/32		2006	AE	9/22-10/05	5	3	5	66.7	3	25	0	0	0	0	0	0
31/32		2007	AE	9/14-9/27	5	3	5	100.0	5	20	0	0	0	0	0	0
31/32		2008	AE	9/12-9/25	5	8	5	37.5	5	30	0	0	0	0	0	0
CN		2004	ALS	8/30-9/10	17	0	17	–	17	123	0	0	4	0	4	24
CN		2004	ALS	8/30-9/10	3	2	3	100.0	3	9	0	0	0	0	0	0
CN		2004	ALS	9/17-9/23	17	0	17	–	17	79	0	0	0	3	3	18
CN		2004	ALS	9/17-9/23	3	0	3	–	0	0	0	0	0	0	0	–

BE = Early Bull **B** = Bull **ALS** = Antlerless **AE** = Any Elk **CN** = Camp Navajo **CH** = CHAMP Hunt **DV** = Disabled Veteran

Herd-Units:

CC = Canyon Creek
ES (Unit 1) = Escudilla
GV = Grapevine
HM = Hutch Mtn.
M = Martinez
ML = Marshall Lake
MM = Melatone Mesa
MZ = Mazatzal
RV = Round Valley
SM = East Sunset/West Sunset/Meteor Crater
TT = Twin Arrows/Two Guns/Grapevine
VV = Verde Valley
WI = Winslow
WS (17/20A/20C) = Williamson Valley/Skull Valley/Kirkland Junction

5-Year: 2004–2008 Harvest

Unit		Year	Hunt Type	Dates	Permits Authorized	1st Choice Applicants	Permits Issued	Draw Odds	Hunters	Hunter Days	Harvest					Hunt Success
											Bull	Spike	Cow	Calf	Total	
**ARCHERY (continued)**																
CN		2005	ALS	8/29-9/09	17	0	17	–	17	129	0	0	0	3	3	18
CN		2005	ALS	8/29-9/09	3	0	3	–	3	34	0	0	0	0	0	0
CN		2005	ALS	9/16-9/22	17	0	17	–	10	37	0	0	7	3	10	100
CN		2005	ALS	9/16-9/22	3	0	3	–	3	6	0	0	0	0	0	0
CN		2006	ALS	9/04-9/14	20	0	20	–	20	70	0	0	3	0	3	15
CN		2006	ALS	9/04-9/14	3	0	3	–	3	21	0	0	1	0	1	33
CN		2006	ALS	9/15-9/24	20	0	20	–	20	93	0	0	5	0	5	25
CN		2006	ALS	9/15-9/24	3	3	3	100.0	3	28	0	0	2	0	2	67
CN		2007	ALS	8/30-9/06	20	1	20	100.0	20	78	0	0	0	2	2	10
CN		2007	ALS	8/30-9/06	3	0	3	–	3	15	0	0	0	0	0	0
CN		2007	ALS	9/14-9/23	20	0	20	–	18	91	0	0	2	0	2	11
CN		2007	ALS	9/14-9/23	3	2	3	100.0	3	27	0	0	0	0	0	0
CN		2008	ALS	8/29-9/04	20	0	20	–	16	89	0	0	0	0	0	0
CN		2008	ALS	8/29-9/04	3	1	3	100.0	3	21	0	0	0	0	0	0
CN		2008	ALS	9/12-9/21	20	2	20	100.0	20	90	0	0	2	0	2	10
CN		2008	ALS	9/12-9/21	3	3	3	33.3	3	21	0	0	0	0	0	0
CN		2008	ALS	11/07-11/20	20	0	20	–	17	76	0	0	1	0	1	6
CN		2008	ALS	11/07-11/20	3	0	3	–	0	0	0	0	0	0	0	–
CN		2004	AE	8/30-9/10	10	8	10	87.5	10	65	0	0	0	0	0	0
CN		2004	AE	8/30-9/10	2	4	2	25.0	2	6	0	0	0	0	0	0
CN		2004	AE	9/17-9/23	20	20	20	85.0	20	83	5	0	0	0	5	25
CN		2004	AE	9/17-9/23	2	5	2	40.0	2	12	0	0	0	0	0	0
CN		2005	AE	8/29-9/09	10	25	12	48.0	12	67	2	2	0	0	4	33
CN		2005	AE	8/29-9/09	2	16	2	12.5	2	10	0	0	0	0	0	0
CN		2005	AE	9/16-9/22	20	26	20	57.7	18	95	5	0	3	0	8	44
CN		2005	AE	9/16-9/22	2	16	2	12.5	2	8	0	0	0	0	0	0
CN		2006	AE	9/04-9/14	17	10	17	80.0	17	95	3	0	0	0	3	18
CN		2006	AE	9/04-9/14	2	7	2	28.6	0	0	0	0	0	0	0	–
CN		2006	AE	9/15-9/24	17	26	17	57.7	17	95	3	3	0	0	6	35
CN		2006	AE	9/15-9/24	2	6	2	33.3	0	0	0	0	0	0	0	–
CN		2007	AE	8/30-9/06	17	4	16	75.0	16	92	2	2	2	0	6	38
CN		2007	AE	8/30-9/06	2	3	2	33.3	2	6	0	0	0	0	0	0
CN		2007	AE	9/14-9/23	17	32	17	40.6	13	77	4	0	4	0	8	62
CN		2007	AE	9/14-9/23	2	10	2	20.0	2	20	2	0	0	0	2	100
CN		2008	AE	8/29-9/04	17	12	17	91.7	15	64	0	0	0	0	0	0
CN		2008	AE	8/29-9/04	2	5	2	0.0	2	14	0	0	0	0	0	0
CN		2008	AE	9/12-9/21	17	32	17	40.6	14	68	3	0	0	0	3	21
CN		2008	AE	9/12-9/21	2	11	2	18.2	0	0	0	0	0	0	0	–
CN		2008	AE	11/07-11/20	17	1	17	100.0	17	116	0	0	0	0	0	0
CN		2008	AE	11/07-11/20	2	2	2	50.0	2	16	0	0	0	0	0	0

BE = Early Bull **B** = Bull **ALS** = Antlerless **AE** = Any Elk **CN** = Camp Navajo **CH** = CHAMP Hunt **DV** = Disabled Veteran

Turkey *(Meleagris gallopavo)*

Natural History

Arizona has two native subspecies of turkeys, Merriam's and Gould's. The Merriam's race of wild turkey (*M. g. merriami*) is found throughout the western United States, primarily in the ponderosa pine forests of Colorado, New Mexico, and northern Arizona. This turkey has also been transplanted into the pine forests of Utah, Idaho, Washington, Oregon, California, Montana, Wyoming, and South Dakota. The Gould's turkey (*M. g. mexicana*) is only found in Arizona and New Mexico. In Arizona, wild turkeys can be found not only in ponderosa pine forests but also in riparian deciduous forests and other vegetation types at elevations ranging from 3,500 to 10,000 feet. The best populations of Merriam's, however, occur in the ponderosa pine forests north of the Gila River. The Gould's occupy the sky island habitats in southeastern Arizona.

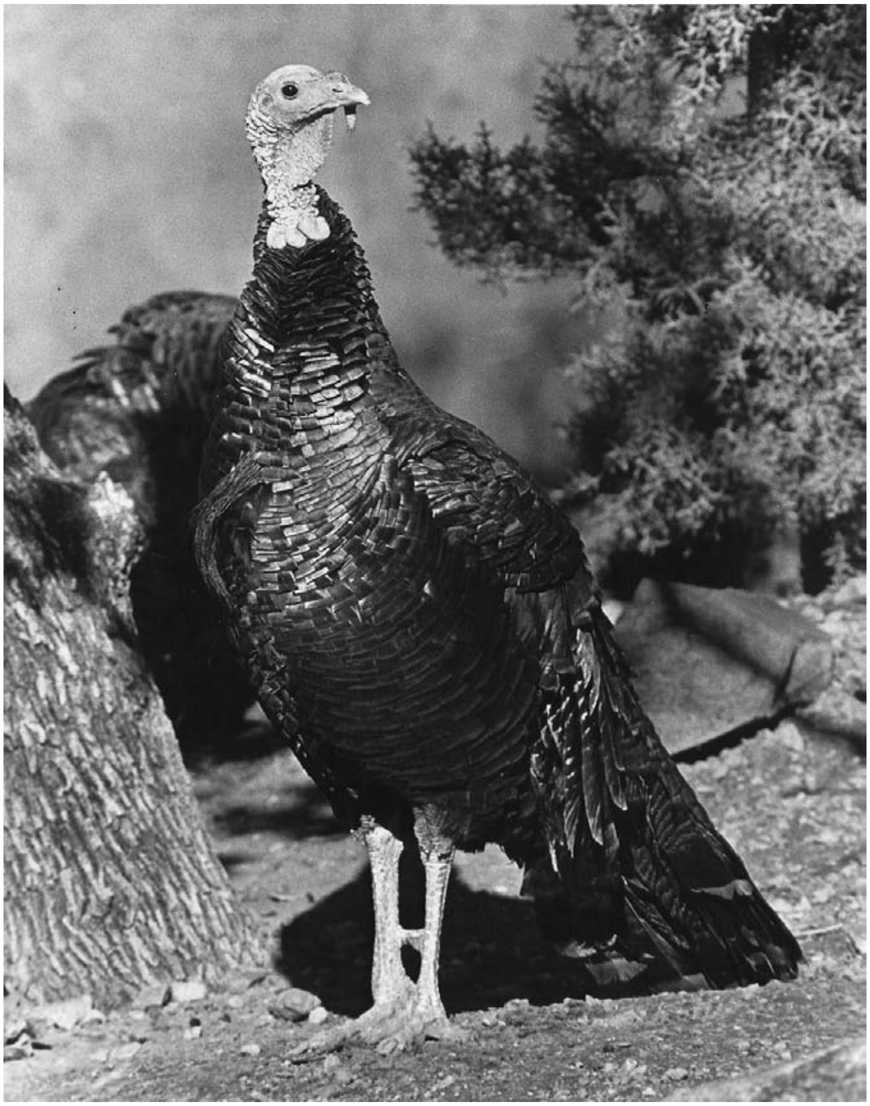
BOB MILES

In the spring, 2-year-old and older males weigh about 18 pounds on average, and yearling males or "jakes" weigh about 13 pounds. Hens more than a year old weigh between 8 and 12 pounds, depending partially on the contents of the crop, which may weigh up to a pound. As springtime temperatures warm, the onset of breeding is heralded by the commencement of gobbling. Gobbling may start as early as late February or early March, with a second peak of gobbling occurring in early May with some "toms" continuing to gobble into June. Hens mate once and lay between 8 and 12 eggs that take 28 days to incubate. The young are precocial and move from the nest soon after hatching.

Both hens and poults spend the rest of the summer eating, loafing, and gaining weight. As winter approaches, they begin to form flocks with other family groups. The flocks will usually spend the winter as high up on the mountain as

snow permits. The gobblers, too, have a defined wintering area in which they will flock together. During the winter, turkeys congregate in the pinyon pine-oak habitats just below the interface with the ponderosa pine forest. Following the snow line, both hen and tom turkey flocks work their way upslope to where gobbling toms attempt to accrue a harem of several hens. After mating, the hens often continue upslope into denser habitats to lay and incubate their eggs. Toms and hens are not usually seen together during the remainder of the year, although they may both frequent similar habitats.

During the summer months, the hens and poults spend much of their time searching for bugs and seeds in small meadows and forest openings. As winter approaches, the turkeys feed increasingly on acorns, pinyon nuts, and other mast crops. Later, with the onset of winter, the birds follow pine stringers downslope to snow-free areas where they feed on the seeds of ponderosa pine, junipers, pinyons, and other plants.

Hunt History

Wild turkeys have been classified as big game since 1913 when the first state legislature established a bag limit of three birds to be taken between October 1 and December 15. Turkey populations appeared to hold up fairly well, at least in northern Arizona, as the season was still a month long and the bag limit was only reduced to two in the new "game code" of 1929. After World War II, however, hunt pressure gradually increased, and hunt regulations became more stringent. Fall hunting was the only turkey hunting allowed, and by 1950 a hunter had to draw a permit to even hunt turkeys. Annual harvests ranged from a few hundred birds to more than 1,300.

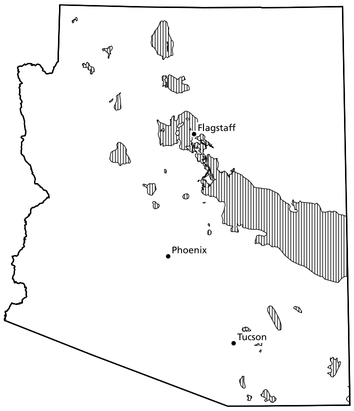

Arizona's turkey distribution

Turkey populations were fairly robust in the early 1960s, and the permit requirement was dropped in 1963; tag sales jumped from 8,050 in 1962 to 17,479 in 1963, but the turkey harvest only increased from 1,363 to 1,462. The first spring gobbler hunt was authorized in 1965 (100 permits), and by1969 the annual turkey harvest had climbed to 2,480 birds, with another 138 turkeys taken earlier that spring. That number remains an annual high.

Wild turkey populations have since been in a general decline. Current estimates number the population between 15,000 and 20,000 birds, depending on conditions. Fall hunting is again by permit-only, and in the spring the number of gobblers taken is equal to or greater than the fall harvest.

Historic Summary of Turkey Survey Data

Year	Tom	Hen	Poult	Unclassified	Total	Poults/Hen	Percent Young[1]
1960	343	267	544	31	1185	2.0	47
1961	297	260	634	64	1255	2.4	53
1962	248	293	847	28	1416	2.9	61
1963	273	374	1058	58	1763	2.8	62
1964	191	288	881	42	1402	3.1	65
1965	193	290	905	77	1465	3.1	65
1966	286	311	1034	34	1665	3.3	63
1967	337	413	809	111	1670	2.0	52
1968	299	295	978	188	1760	3.3	62
1969	236	304	1152	30	1722	3.8	68
1970	207	345	667	81	1300	1.9	55
1971	224	369	654	131	1378	1.8	52
1972	205	264	678	75	1222	2.6	59
1973	129	207	641	89	1066	3.1	66
1974	155	193	729	73	1150	3.8	68
1975	125	368	1406	351	2250	3.8	74
1976	98	262	1138	121	1619	4.3	76
1977	87	299	1391	74	1851	4.7	78
1978	179	307	1190	91	1767	3.9	71
1979	100	129	421	24	674	3.3	65
1980	42	111	401	81	635	3.6	72
1981	82	120	626	158	986	5.2	76
1982	105	157	586	17	865	3.7	69
1983	64	153	517	0	734	3.4	70
1984	156	202	664	159	1181	3.3	65
1985	88	332	1033	125	1578	3.1	71
1986	136	300	926	62	1424	3.1	68
1987	137	251	735	141	1264	2.9	65
1988	63	225	610	172	1070	2.7	68
1989	183	332	704	84	1303	2.1	58
1990	121	210	527	109	967	2.5	61
1991	117	176	389	162	844	2.2	57
1992	170	219	707	113	1209	3.2	65
1993	295	495	1148	120	2058	2.3	59
1994	251	381	559	24	1215	1.5	47
1995	130	306	527	12	975	1.7	55
1996	68	289	292	16	665	1.0	45
1997	37	270	708	15	1030	2.6	70
1998	122	228	497	4	851	2.2	59
1999	103	212	567	32	914	2.7	64
2000	144	198	303	50	695	1.5	47
2001	62	237	520	88	907	2.2	63
2002	86	44	25	85	240	0.6	16
2003	105	373	1156	50	1684	3.1	71
2004	124	144	202	37	507	1.4	43
2005	183	360	783	46	1372	2.2	59
2006	77	217	361	38	693	1.7	55
2007	102	192	298	25	617	1.6	50
2008	129	266	302	13	710	1.1	43

[1] Percent young is calculated from classified birds only.

5-Year: 2004–2008 Turkey Survey Data

Unit	Year	Tom	Hen	Poult	Unclassified	Total	Poults/Hen	Percent Young[1]
1	2004	2	25	27	0	54	1.1	50
1	2005	18	84	146	0	248	1.7	59
1	2006	38	81	102	0	221	1.3	46
1	2007	29	18	36	0	83	2.0	43
1	2008	43	99	91	13	246	0.9	39
3B	2005	0	4	0	0	4	0.0	0
3B	2007	19	0	0	0	19	–	0
3C	2005	17	37	26	1	81	0.7	33

[1] Percent young is calculated from classified birds only.

5-Year: 2004-2008 Turkey Survey Data

Unit	Year	Tom	Hen	Poult	Unclassified	Total	Poults/Hen	Percent Young[1]
3C	2006	4	14	41	0	59	2.9	69
3C	2007	1	19	47	19	86	2.5	70
3C	2008	11	15	6	0	32	0.4	19
4	2004	22	25	22	1	70	0.9	32
4	2005	42	29	67	0	138	2.3	49
4	2006	8	13	34	0	55	2.6	62
4	2007	2	8	31	0	41	3.9	76
4	2008	0	10	35	0	45	3.5	78
5A	2004	5	20	27	0	52	1.4	52
5A	2005	0	9	37	0	46	4.1	80
5A	2006	0	4	14	27	45	3.5	78
5A	2007	9	9	23	0	41	2.6	56
5A	2008	10	1	0	0	11	0.0	0
5B	2007	5	13	12	0	30	0.9	40
6A	2005	0	7	19	0	26	2.7	73
6A	2006	0	2	10	0	12	5.0	83
6B	2004	6	2	5	0	13	2.5	38
6B	2005	5	17	17	0	39	1.0	44
6B	2006	0	0	0	11	11	–	–
6B	2007	0	0	0	3	3	–	–
7	2005	4	3	12	0	19	4.0	63
7	2006	5	20	10	0	35	0.5	29
7	2007	10	0	0	0	10	–	0
8	2004	17	6	8	0	31	1.3	26
8	2005	18	31	76	22	147	2.5	61
8/10	2006	4	8	33	0	45	4.1	73
8/10	2007	8	42	19	0	69	0.5	28
9	2006	5	0	0	0	5	–	0
9	2008	2	14	18	0	34	1.3	53
12A	2004	13	14	45	0	72	3.2	63
12A	2005	4	12	55	0	71	4.6	77
12A	2006	7	12	34	0	53	2.8	64
12A	2007	3	11	31	0	45	2.8	69
12A	2008	0	22	56	0	78	2.6	72
13A	2004	8	11	3	0	22	0.3	14
13A	2005	19	14	28	3	64	2.0	46
13A	2006	2	2	3	0	7	1.5	43
13A	2007	7	36	0	0	43	0.0	0
13A	2008	10	7	3	0	20	0.4	15
13B	2004	2	14	0	0	16	0.0	0
13BS	2007	5	10	4	3	22	0.4	21
13B	2008	14	16	0	0	30	0.0	0
22	2004	8	5	12	0	25	2.4	48
22	2005	0	4	13	7	24	3.3	76
23	2004	7	17	40	32	96	2.4	63
23	2005	17	16	61	6	100	3.8	65
23	2006	4	42	31	0	77	0.7	40
27	2004	34	5	13	4	56	2.6	25
27	2005	39	93	226	7	365	2.4	63
27	2006	0	19	49	0	68	2.6	72
27	2007	4	26	95	0	125	3.7	76
27	2008	39	82	93	0	214	1.1	43

[1] Percent young is calculated from classified birds only.

Historic Summary of General Spring Turkey Hunts (juniors-only listed separately)

Year	Permits Authorized	1st Choice Applicants	Permits Issued	Hunters	Hunter Days	Total Harvest	Percent Success
1965	100	–	–	79	134	30	38.0
1966	500	–	–	417	716	58	13.9
1967	1100	–	–	878	–	151	17.2
1968	1600	–	–	1096	2440	98	8.9
1969	2200	–	–	1673	3719	138	8.2
1970	2600	–	–	1935	4579	215	11.1
1971	2650	–	–	2021	4702	260	12.9
1972	2800	–	–	1941	4674	153	7.9
1973	2550	–	–	1225	2705	71	5.8
1974	2550	–	2550	1747	4145	151	8.6
1975	3450	–	3450	2284	5582	205	9.0
1976	4001	–	4001	1869	4642	220	11.8
1977	4600	–	4600	2679	6848	326	12.2
1978	4865	–	4865	2952	7568	399	13.5
1979	4970	6275	3397	2853	7516	317	11.1
1980	4950	7894	4594	2692	7225	234	8.7
1981	4900	9143	4654	2542	8100	399	15.7
1982	4960	9444	4821	2648	8366	390	14.7
1983	4960	5106	4415	3073	10270	473	15.4
1984	4620	4725	4107	3455	11511	780	22.6
1985	4620	5863	4409	3382	11649	688	20.3
1986	4620	6663	4548	3581	12421	746	20.8
1987	4915	7132	4834	3734	13474	830	22.2
1988	4710	8216	4688	3736	13089	697	18.7
1989	4660	8171	4562	3691	12998	619	16.8
1990	4595	8553	4577	3684	13457	727	19.7
1991	4595	8044	4976	3994	15731	617	15.4
1992	4725	6413	4701	3757	14563	723	19.2
1993	4735	7260	4732	3820	15006	771	20.2
1994	4805	7730	4793	3795	14543	768	20.2
1995	4840	8591	4822	3806	14038	769	20.2
1996	5020	9258	5007	3820	13826	631	16.5
1997	5115	9312	5115	4021	15179	660	16.4
1998	4719	9460	4724	3722	13503	671	18.0
1999	4501	10260	4476	3497	12637	730	21
2000	4840	11120	4840	3833	13474	916	24
2001	5251	12815	5251	4232	15258	987	23
2002	5471	12643	5470	4301	16420	760	18
2003	5096	13819	5183	4234	16633	878	21
2004	5157	16020	5158	4055	15880	788	19
2005	5307	16355	5375	4264	16119	1155	27
2006	5593	14945	5599	4548	17705	1129	25
2007	6263	13583	6269	5092	19543	1269	25
2008	7007	13281	7001	5660	22725	1040	18

Historic Summary of Juniors-only Spring Turkey Hunts

Year	Permits Authorized	1st Choice Applicants	Permits Issued	Hunters	Hunter Days	Total Harvest	Percent Success
1999	175	120	163	150	666	30	20
2000	175	202	175	155	603	34	22
2001	180	307	180	156	523	30	19
2002	180	254	177	137	486	27	20
2003	150	290	153	125	443	23	18
2004	150	341	150	119	373	23	19
2005	150	327	153	122	450	24	20
2006	165	461	165	143	493	51	36
2007	225	563	225	202	636	105	52
2008	350	582	350	295	1094	82	28

Historic Summary of General Fall Turkey Hunts (juniors-only listed separately)

Year[1]	Permits Authorized	1st Choice Applicants	Permits Issued	Hunters	Hunter Days	Total Harvest	Percent Success
1946	9747	–	–	5406	–	526	9.7
1947	2147	–	–	1465	–	296	20.2
1948	2697	–	–	1990	–	403	20.3
1949	1243	–	–	945	–	307	32.5
1950	1657	–	–	1377	–	365	26.5
1951	3305	–	–	2780	–	549	19.7
1952	3454	–	–	2961	–	782	26.4
1953	4672	–	–	4096	–	1216	29.7
1954	5134	–	–	4448	–	971	21.8
1955	3012	–	–	2760	–	887	32.1
1956	4800	–	–	4218	–	1367	32.4
1957	2600	–	–	2138	–	647	30.3
1958	2800	–	–	2340	4308	569	24.3
1959	5700	–	–	4341	–	1050	24.2
1960	8150	–	–	6607	12058	1262	19.1
1961	–	–	–	7374	18216	1218	16.5
1962	–	–	–	9296	21543	1308	14.1
1963	–	–	17479	15847	35711	1434	9.0
1964	–	–	14803	13733	33614	1655	12.1
1965	–	–	15470	14367	34846	2001	13.9
1966	–	–	15681	14381	34353	1762	12.3
1967	–	–	17388	14626	37391	1601	10.9
1968	–	–	16782	15063	38754	1518	10.1
1969	–	–	18330	14768	37735	2392	16.2
1970	–	–	19222	15673	43147	2002	12.8
1971	–	–	17002	13176	34196	1200	9.1
1972	–	–	–	9584	26422	794	8.3
1973	–	–	–	13142	36597	2050	15.6
1974	–	–	–	12262	36634	1040	8.5
1975	–	–	–	9542	27676	1464	15.3
1976	–	–	–	8208	24754	508	6.2
1977	–	–	–	8652	28320	997	11.5
1978	–	–	–	9119	25395	1427	15.6
1979	–	–	–	8775	28646	856	9.8
1980	–	–	–	12578	34546	1192	9.5
1981	–	–	–	10640	36027	1390	13.1
1982	–	–	–	9923	34692	1496	15.1
1983	–	–	–	9286	31185	893	9.6
1984	–	–	7737	9302	30146	1236	13.3
1985	–	–	8271	9975	32701	1125	11.3
1986	–	–	7510	8740	29245	941	10.8
1987	–	–	8914	10912	37068	1935	17.7
1988	–	–	8259	10425	32224	1459	14.0
1989	–	–	9289	11156	32410	1927	17.3
1990	–	–	7836	9609	29003	982	10.2
1991	9280	3951	6332	5076	14330	955	18.8
1992	8730	5497	6731	5310	14563	1008	19.0
1993	8740	6123	7822	6310	17505	1048	16.6
1994	6965	6850	6921	5435	15051	1009	18.6
1995	6245	7322	6237	4857	13447	1034	21.3
1996	5350	7721	5350	4188	12203	486	11.6
1997	4050	7766	4050	3080	8492	511	16.6
1998	3700	7226	3700	2775	7648	508	18
1999	4160	8972	4160	3283	8935	872	27
2000	4760	9417	4760	3689	10660	793	21
2001	4635	9451	4635	3623	9723	1213	33
2002	5085	12240	5085	3933	11904	407	10
2003	4260	12774	4260	3199	8955	875	27
2004	4785	14455	4785	3676	11390	539	15
2005	4830	11563	4832	3811	10720	1117	29
2006	5310	14910	5302	3970	11224	640	16
2007	5870	9922	5868	4664	14317	1087	23
2008	6100	7820	5883	4659	14096	902	19

[1] Archery data are included in hunters, hunter days, and harvest from 1969-1990.

Turkey Harvest Data

Historic Summary of Juniors-only Fall Turkey Hunts

Year	Permits Authorized	1st Choice Applicants	Permits Issued	Hunters	Hunter Days	Total Harvest	Percent Success
1998	100	59	89	76	197	8	11
1999	100	105	100	86	236	21	24
2000	100	169	100	81	218	13	16
2001	125	164	125	96	264	33	34
2002	125	241	125	91	282	6	7
2003	125	240	125	103	231	18	17
2004	100	250	100	72	196	4	6
2005	100	137	100	71	191	16	23
2006	150	246	148	100	262	19	19
2007	150	179	150	114	304	12	11
2008	OTC	–	336	317	929	37	12

OTC = Over the counter nonpermit-tags.

Historic Summary of Archery Fall Turkey Hunts

Year	Permits Authorized	1st Choice Applicants	Permits Issued	Hunters	Hunter Days	Total Harvest	Percent Success
1991	–	–	1289	1072	4331	20	1.9
1992	–	–	1337	1245	4692	19	1.5
1993	–	–	1760	1465	6804	55	3.8
1994	–	–	1808	1533	7258	59	3.8
1995	–	–	1784	1426	7011	26	1.8
1996	–	–	1939	1479	7684	37	2.5
1997	–	–	1891	1390	7194	44	3.2
1998	–	–	2133	1739	8435	96	5.6
1999	–	–	2523	2082	10913	103	4.5
2000	–	–	3084	2539	13320	120	4.7
2001	–	–	3115	2722	13838	190	7.0
2002	–	–	3117	2583	12627	138	5.3
2003	–	–	2914	2485	12507	71	2.9
2004	–	–	3223	2630	12890	160	6.1
2005	–	–	3450	2586	12725	174	6.7
2006	–	–	3941	2820	13818	140	5.0
2007	–	–	4660	2719	14036	221	8.1
2008[1]	–	–	3831	2137	10403	103	4.8

[1] 2008 data is preliminary.

5-Year: 2004–2008 Harvest

Unit	Year	Dates	Permits Authorized	1st Choice Applicants	Permits Issued	Draw Odds	Hunters	Hunter Days	Harvest	Hunt Success
**SPRING GENERAL**										
1	2004	4/23-4/29	375	2076	376	17.5	303	1118	92	30
1	2004	4/30-5/20	400	674	400	32.9	286	1016	48	17
1	2005	4/22-4/28	400	2032	404	19.3	349	1191	136	39
1	2005	4/29-5/19	425	760	428	30.9	320	1120	63	20
1	2006	4/28-5/04	425	2037	425	20.2	354	1174	129	36
1	2006	5/05-5/25	450	331	450	43.5	363	1340	79	22
1	2007	4/27-5/03	450	1755	450	23.9	394	1309	163	41
1	2007	5/04-5/24	475	321	475	54.5	380	1383	91	24
1	2008	4/25-5/01	500	1912	500	24.9	435	1645	107	25
1	2008	5/02-5/22	525	253	525	61.3	409	1593	43	11
3B	2004	4/23-5/20	100	296	100	29.7	80	254	13	16
3B	2005	4/22-5/19	125	254	128	37.0	104	501	24	23
3B	2006	4/28-5/25	150	251	150	40.2	119	633	25	21
3B	2007	4/27-5/03	100	188	100	38.3	82	304	16	20
3B	2007	5/04-5/24	100	42	100	90.5	93	459	15	16
3B	2008	4/25-5/01	100	236	100	37.7	78	346	15	19
3B	2008	5/02-5/22	100	35	100	88.6	80	351	15	19
3C	2004	4/23-5/20	50	367	50	13.6	44	117	26	59
3C	2005	4/22-5/19	75	532	75	13.5	72	284	48	67

CN = Camp Navajo

5-Year: 2004-2008 Harvest

Unit	Year	Dates	Permits Authorized	1st Choice Applicants	Permits Issued	Draw Odds	Hunters	Hunter Days	Harvest	Hunt Success
**SPRING GENERAL (continued)**										
3C	2006	4/28-5/25	150	776	150	19.1	136	584	82	60
3C	2007	4/27-5/03	125	758	125	15.8	111	395	54	49
3C	2007	5/04-5/24	100	95	100	27.4	89	383	40	45
3C	2008	4/25-5/01	175	857	175	20.0	158	651	64	41
3C	2008	5/02-5/22	175	82	175	63.4	149	693	22	15
4	2004	4/23-5/20	275	1222	275	21.5	220	1042	35	16
4	2005	4/22-5/19	275	1128	277	22.3	230	868	66	29
4	2006	4/28-5/25	325	1224	325	24.4	243	1021	60	25
4	2007	4/27-5/03	200	719	200	26.0	159	675	43	27
4	2007	5/04-5/24	200	107	200	53.3	160	640	17	11
4	2008	4/25-5/01	250	700	250	31.6	189	755	47	25
4	2008	5/02-5/22	250	98	250	62.2	192	760	28	15
5A	2004	4/23-5/20	250	927	250	24.1	206	806	34	17
5A	2005	4/22-5/19	300	1100	300	23.1	260	1143	69	27
5A	2006	4/28-5/04	200	831	200	21.2	158	543	51	32
5A	2006	5/05-5/25	200	160	200	56.9	163	642	14	9
5A	2007	4/27-5/03	225	755	225	26.6	207	779	43	21
5A	2007	5/04-5/24	225	114	225	60.5	180	812	17	9
5A	2008	4/25-5/01	225	734	223	28.1	189	742	21	11
5A	2008	5/02-5/22	225	105	225	71.4	174	673	16	9
5B	2004	4/23-5/20	250	727	250	28.1	207	961	33	16
5B	2005	4/22-5/19	250	857	250	26.5	207	780	50	24
5B	2006	4/28-5/25	275	799	275	29.8	232	958	45	19
5B	2007	4/27-5/03	150	516	150	24.2	124	572	20	16
5B	2007	5/04-5/24	150	110	150	56.4	131	578	24	18
5B	2008	4/25-5/01	150	481	150	28.1	117	481	20	17
5B	2008	5/02-5/22	150	62	150	64.5	107	401	6	6
6A	2004	4/23-4/29	275	1970	275	13.8	246	863	43	17
6A	2004	4/30-5/20	225	558	225	21.3	157	563	29	18
6A	2005	4/22-4/28	275	1664	285	16.3	240	982	76	32
6A	2005	4/29-5/19	225	707	228	20.9	198	858	40	20
6A	2006	4/28-5/04	275	1784	275	15.4	230	914	45	20
6A	2006	5/05-5/25	275	250	275	27.6	228	925	52	23
6A	2007	4/27-5/03	300	1706	300	17.2	245	854	55	22
6A	2007	5/04-5/24	300	322	300	33.5	241	1012	42	17
6A	2008	4/25-5/01	350	1684	350	20.4	304	1343	46	15
6A	2008	5/02-5/22	350	226	350	38.5	296	1138	24	8
6B	2004	4/23-4/29	100	445	100	20.0	80	329	16	20
6B	2004	4/30-5/20	75	52	75	44.2	64	230	9	14
6B	2005	4/22-4/28	100	346	100	26.3	77	221	36	47
6B	2005	4/29-5/19	75	80	75	47.5	51	172	12	24
6B	2006	4/28-5/04	100	394	100	23.9	85	344	20	24
6B	2006	5/05-5/25	100	73	100	47.9	77	335	12	16
6B	2007	4/27-5/03	100	373	100	26.0	87	370	20	23
6B	2007	5/04-5/24	125	45	125	73.3	99	418	22	22
6B	2008	4/25-5/01	100	355	100	23.7	92	408	21	23
6B	2008	5/02-5/22	125	47	125	70.2	96	357	4	4
7	2004	4/23-5/20	275	397	275	41.8	199	928	31	16
7	2005	4/22-5/19	275	395	278	38.2	205	879	52	25
7	2006	4/28-5/25	275	433	275	43.9	220	906	37	17
7	2007	4/27-5/03	150	388	150	27.3	133	505	47	35
7	2007	5/04-5/24	150	68	150	80.9	115	481	25	22
7	2008	4/25-5/01	150	402	150	27.9	124	468	24	19
7	2008	5/02-5/22	175	58	175	67.2	137	695	17	12
8/10	2004	4/23-5/20	300	1600	300	17.5	253	1142	49	19
8/10	2005	4/22-5/19	300	1801	301	16.0	276	1185	67	24
8/10	2006	4/28-5/25	325	1507	325	20.4	290	1368	46	16
8/10	2007	4/27-5/03	200	1110	200	17.9	164	669	50	30
8/10	2007	5/04-5/24	125	160	125	33.8	98	369	13	13
8/10	2008	4/25-5/01	200	1002	200	18.8	177	774	66	37
8/10	2008	5/02-5/22	125	82	125	42.7	104	498	13	13
9	2004	4/23-5/20	40	60	40	33.3	27	109	7	26

CN = Camp Navajo

5-Year: 2004-2008 Harvest

Unit	Year	Dates	Permits Authorized	1st Choice Applicants	Permits Issued	Draw Odds	Hunters	Hunter Days	Harvest	Hunt Success
**SPRING GENERAL (continued)**										
9	2005	4/22-5/19	40	52	41	44.2	33	96	18	55
9	2006	4/28-5/25	40	71	40	31.0	32	97	17	53
9	2007	4/27-5/03	25	113	25	22.1	21	66	13	62
9	2007	5/04-5/24	25	13	25	30.8	22	117	3	14
9	2008	4/25-5/01	35	108	35	31.5	26	120	6	23
9	2008	5/02-5/22	35	20	35	85.0	30	118	5	17
12A	2004	4/23-4/29	350	551	350	51.2	239	986	14	6
12A	2004	4/30-5/20	350	269	350	71.7	287	1161	22	8
12A	2005	4/22-4/28	350	455	352	62.0	212	818	10	5
12A	2005	4/29-5/19	350	200	350	88.5	227	993	9	4
12A	2006	4/28-5/04	225	253	225	71.9	167	630	33	20
12A	2006	5/05-5/25	225	80	225	100.0	177	721	27	15
12A	2007	4/27-5/03	250	230	250	81.7	186	763	53	28
12A	2007	5/04-5/24	250	79	250	100.0	155	573	17	11
12A	2008	4/25-5/01	300	202	300	87.1	203	832	48	24
12A	2008	5/02-5/22	300	42	300	100.0	215	879	32	15
13A	2004	4/23-5/20	20	38	20	39.5	16	70	2	13
13A	2005	4/22-5/19	20	43	20	39.5	15	51	7	47
13A	2006	4/28-5/25	20	33	20	51.5	16	33	5	31
13A	2007	4/27-5/03	15	32	15	46.9	12	38	3	25
13A	2007	5/04-5/24	10	5	10	100.0	3	10	0	0
13A	2008	4/25-5/01	20	22	20	86.4	15	55	5	33
13A	2008	5/02-5/22	10	4	10	100.0	9	21	1	11
13BS	2004	4/23-4/29	15	91	15	15.4	12	37	10	83
13BS	2004	4/30-5/20	15	23	15	47.8	12	39	6	50
13BS	2005	4/22-4/28	15	73	17	19.2	12	32	5	42
13BS	2005	4/29-5/19	15	14	15	42.9	13	44	3	23
13BS	2006	4/28-5/04	15	48	15	31.3	15	51	5	33
13BS	2006	5/05-5/25	15	22	15	40.9	15	56	6	40
13BS	2007	4/27-5/03	15	35	15	40.0	7	22	5	71
13BS	2007	5/04-5/24	15	11	15	72.7	15	45	8	53
13BS	2008	4/25-5/01	20	42	20	40.5	17	74	1	6
13BS	2008	5/02-5/22	15	6	15	100.0	11	45	2	18
17/18B	2004	4/23-5/20	25	275	25	9.1	22	89	9	41
17/18B	2005	4/22-5/19	25	265	25	9.4	23	91	14	61
17/18B	2006	4/28-5/25	30	223	30	13.5	30	76	14	47
17/18B	2007	4/27-5/03	15	136	15	11.0	14	49	5	36
17/18B	2007	5/04-5/24	20	50	20	20.0	12	28	6	50
17/18B	2008	4/25-5/01	15	139	15	10.8	15	54	1	7
17/18B	2008	5/02-5/22	20	24	20	29.2	17	67	3	18
22	2004	4/23-5/20	125	452	125	25.9	94	281	29	31
22	2005	4/22-5/19	125	455	132	23.7	115	439	41	36
22	2006	4/28-5/25	125	387	125	28.7	91	359	7	8
22	2007	4/27-5/03	75	235	75	28.5	65	223	10	15
22	2007	5/04-5/24	75	34	75	61.8	61	197	14	23
22	2008	4/25-5/01	90	237	90	28.7	77	349	15	19
22	2008	5/02-5/22	90	37	90	67.6	80	321	16	20
23	2004	4/23-4/29	200	895	200	21.5	166	694	26	16
23	2004	4/30-5/20	200	229	200	33.2	165	610	33	20
23	2005	4/22-4/28	200	835	203	22.6	173	657	46	27
23	2005	4/29-5/19	200	381	208	33.3	161	527	15	9
23	2006	4/28-5/04	200	1057	200	18.1	160	569	33	21
23	2006	5/05-5/25	200	149	200	43.0	153	548	40	26
23	2007	4/27-5/03	200	1032	200	18.7	183	682	48	26
23	2007	5/04-5/24	225	175	225	42.3	180	690	32	18
23	2008	4/25-5/01	225	1135	225	19.4	201	783	46	23
23	2008	5/02-5/22	225	103	225	54.4	181	624	36	20
27	2004	4/23-4/29	425	1281	425	29.1	333	1202	105	32
27	2004	4/30-5/20	425	336	425	57.1	323	1169	65	20
27	2005	4/22-4/28	425	1270	428	30.2	364	1166	143	39
27	2005	4/29-5/19	425	413	432	50.4	305	937	95	31
27	2006	4/28-5/04	475	1249	475	30.4	401	1402	138	34

CN = Camp Navajo

5-Year: 2004–2008 Harvest

Unit	Year	Dates	Permits Authorized	1st Choice Applicants	Permits Issued	Draw Odds	Hunters	Hunter Days	Harvest	Hunt Success
**SPRING GENERAL (continued)**										
27	2006	5/05-5/25	475	190	475	78.9	376	1434	99	26
27	2007	4/27-5/03	550	1252	550	36.3	447	1543	149	33
27	2007	5/04-5/24	525	185	525	81.1	403	1490	79	20
27	2008	4/25-5/01	625	1271	625	37.5	503	1741	101	20
27	2008	5/02-5/22	550	90	550	100.0	436	1828	92	21
29	2008	4/25-5/01	1	61	1	1.6	1	4	1	100
29	2008	5/02-5/22	1	11	1	.0	1	2	1	100
35A	2004	4/23-5/20	2	194	2	1.0	2	24	0	0
35A	2005	4/22-5/19	2	227	2	.9	2	4	2	100
35A	2006	4/28-5/25	6	285	6	2.1	6	17	4	67
35A	2007	4/27-5/03	3	220	3	1.4	3	5	3	100
35A	2007	5/04-5/24	3	56	3	3.6	3	14	2	67
35A	2008	4/25-5/01	3	238	3	1.3	3	3	3	100
35A	2008	5/02-5/22	3	45	3	.0	3	9	3	100
CN	2004	4/23-5/20	10	10	10	100.0	8	27	2	25
CN	2004	4/23-5/20	5	5	5	100.0	4	13	0	0
CN	2005	4/22-5/19	10	3	15	100.0	15	69	8	53
CN	2005	4/22-5/19	5	13	6	46.2	5	11	0	0
CN	2006	4/28-5/25	10	16	15	93.8	4	8	0	0
CN	2006	4/28-5/25	5	18	6	27.8	5	11	2	40
CN	2007	4/27-5/24	10	10	15	100.0	6	19	0	0
CN	2007	4/27-5/24	5	18	6	33.3	0	0	0	–
CN	2008	4/25-5/22	15	4	12	100.0	5	15	2	40
CN	2008	4/25-5/22	6	3	5	100.0	3	5	0	0
FTHU	2006	4/28-5/25	2	14	2	14.3	2	6	2	100
FTHU	2007	4/27-5/24	1	2	1	50.0	1	1	1	100
FTHU	2007	4/27-5/24	1	8	1	12.5	1	1	1	100
FTHU	2008	4/25-5/22	3	26	3	11.5	1	4	1	100
**SPRING JUNIORS ONLY**										
1	2004	4/23-4/29	25	99	25	25.3	23	84	7	30
1	2005	4/22-5/19	25	95	25	25.3	23	65	3	13
1	2006	4/21-5/25	25	93	25	24.7	25	64	11	44
1	2007	4/20-5/24	35	86	35	40.7	33	70	15	45
1	2008	4/18-5/22	50	83	50	51.8	43	126	14	33
3C	2007	4/20-5/24	25	60	25	41.7	25	104	19	76
3C	2008	4/18-5/22	50	96	50	49.0	37	142	17	46
5B	2004	4/23-5/20	25	33	25	72.7	14	67	0	0
5B	2005	4/22-5/19	25	40	25	52.5	20	59	0	0
5B	2006	4/21-5/25	25	30	25	30.0	22	128	6	27
5B	2007	4/20-5/24	25	46	25	45.7	20	105	5	25
5B	2008	4/18-5/22	50	49	50	71.4	44	183	6	14
6A	2004	4/23-4/29	25	102	25	24.5	20	52	2	10
6A	2004	4/30-5/20	25	11	25	45.5	21	52	2	10
6A	2005	4/22-4/28	25	74	25	31.1	22	86	11	50
6A	2005	4/29-5/19	25	21	25	52.4	18	45	0	0
6A	2006	4/21-5/25	25	131	25	19.1	16	52	5	31
6A	2007	4/20-5/24	25	114	25	21.1	23	59	7	30
6A	2008	4/18-5/22	25	102	25	24.5	23	114	7	30
8/10	2004	4/23-5/20	25	57	25	42.1	19	54	4	21
8/10	2005	4/22-5/19	25	65	25	38.5	25	125	4	16
8/10	2006	4/21-5/25	40	79	40	44.3	34	107	11	32
8/10	2007	4/20-5/24	40	103	40	38.8	38	155	14	37
8/10	2008	4/18-5/22	50	97	50	48.5	41	196	7	17
23	2006	4/21-5/25	25	76	25	30.3	23	82	5	22
23	2007	4/20-5/24	25	76	25	32.9	23	50	18	78
23	2008	4/18-5/22	50	98	50	50.0	44	135	19	43
27	2004	4/23-5/20	25	39	25	48.7	22	64	8	36
27	2005	4/22-5/19	25	32	28	56.3	14	70	6	43
27	2006	4/21-5/25	25	52	25	44.2	23	60	13	57
27	2007	4/20-5/24	50	78	50	55.1	40	93	27	68
27	2008	4/18-5/22	75	57	75	89.5	63	198	12	19

CN = Camp Navajo

5-Year: 2004-2008 Harvest

Unit	Year	Dates	Permits Authorized	1st Choice Applicants	Permits Issued	Draw Odds	Hunters	Hunter Days	Harvest	Hunt Success
**FALL GENERAL**										
1	2004	10/08-10/14	625	2408	625	25.7	466	1483	57	12
1	2005	10/07-10/13	625	1847	625	33.5	491	1449	77	16
1	2006	10/13-10/19	625	2329	624	26.0	453	1279	65	14
1	2007	10/12-10/18	625	1358	625	45.2	487	1494	96	20
1	2008	10/03-10/09	625	1004	625	60.9	473	1411	84	18
4	2004	10/08-10/14	350	1313	350	24.5	257	797	28	11
4	2005	10/07-10/13	350	1059	350	31.4	283	792	58	20
4	2006	10/13-10/19	375	1307	375	27.9	297	826	52	18
4	2007	10/12-10/18	425	888	424	44.7	342	1052	84	25
4	2008	10/03-10/09	525	736	525	64.7	421	1289	74	18
5A	2004	10/08-10/14	300	916	300	26.1	246	763	32	13
5A	2005	10/07-10/13	300	784	300	32.0	238	652	76	32
5A	2006	10/13-10/19	400	1148	400	29.4	329	897	48	15
5A	2007	10/12-10/18	450	714	450	54.6	390	1208	40	10
5A	2008	10/03-10/09	450	523	450	68.6	353	1089	51	14
5B	2004	10/08-10/14	75	455	75	16.5	58	146	4	7
5B	2005	10/07-10/13	25	131	25	19.1	23	60	8	35
5BN	2006	10/13-10/19	35	255	35	13.7	19	39	2	11
5BN	2007	10/12-10/18	35	128	35	27.3	26	66	6	23
5BN	2008	10/03-10/09	35	88	35	38.6	27	81	5	19
5BS	2008	10/03-10/09	75	178	75	42.1	66	228	5	8
6A	2004	10/08-10/14	425	2584	425	16.1	353	1070	44	12
6A	2005	10/07-10/13	475	2055	475	22.8	393	1072	112	28
6A	2006	10/13-10/19	475	2420	475	18.8	380	1066	40	11
6A	2007	10/12-10/18	475	1519	475	30.9	407	1323	73	18
6A	2008	10/03-10/09	475	1114	475	41.2	381	1136	54	14
6B	2004	10/08-10/14	250	425	250	34.4	210	595	44	21
6B	2005	10/07-10/13	250	442	250	35.5	199	549	58	29
6B	2006	10/13-10/19	350	475	348	37.7	282	776	22	8
6B	2007	10/12-10/18	350	340	350	68.2	283	876	33	12
6B	2008	10/03-10/09	300	293	300	72.0	240	690	43	18
7	2006	10/13-10/19	75	352	75	20.2	57	168	13	23
7	2007	10/12-10/18	125	303	125	38.3	79	200	37	47
7	2008	10/03-10/09	200	242	200	69.0	171	533	52	30
8/10	2004	10/08-10/14	500	2132	500	22.2	410	1294	71	17
8/10	2005	10/07-10/13	550	1651	552	31.9	483	1439	225	47
8/10	2006	10/13-10/19	600	2341	600	24.2	470	1448	69	15
8/10	2007	10/12-10/18	700	1542	700	39.7	600	2082	43	7
8/10	2008	10/03-10/09	700	894	700	70.7	591	1971	79	13
9	2007	10/12-10/18	25	114	25	20.2	24	76	13	54
9	2008	10/03-10/09	25	110	25	22.7	18	48	11	61
12A	2004	10/08-10/14	750	1145	750	54.3	524	1758	94	18
12A	2005	10/07-10/13	750	787	750	78.0	532	1531	218	41
12A	2006	10/13-10/19	750	816	749	73.3	493	1416	138	28
12A	2007	10/12-10/18	1000	657	1000	100.0	725	2264	233	32
12A	2008	10/03-10/09	1000	427	783	100.0	536	1513	156	29
13A	2006	10/13-10/19	5	45	5	11.1	2	7	2	100
13A	2007	10/12-10/18	5	24	5	16.7	5	8	5	100
13A	2008	10/03-10/09	10	19	10	47.4	8	10	0	0
13B	2004	10/08-10/14	10	70	10	14.3	10	33	3	30
13B	2005	10/07-10/13	5	66	5	7.6	5	14	3	60
13B	2006	10/13-10/19	5	41	5	12.2	2	3	0	0
13B	2007	10/12-10/18	5	15	5	13.3	5	10	2	40
13B	2008	10/03-10/09	5	13	5	38.5	2	3	0	0
17/18B	2006	10/13-10/19	15	147	15	10.2	7	7	5	71
17/18B	2007	10/12-10/18	25	148	25	16.9	25	83	2	8
17/18B	2008	10/03-10/09	25	107	25	23.4	23	82	7	30
22	2004	10/08-10/14	75	351	75	21.4	52	127	5	10
22	2005	10/07-10/13	75	291	75	24.7	63	188	19	30
22	2006	10/13-10/19	100	406	100	23.4	67	187	13	19
22	2007	10/12-10/18	125	217	125	51.2	81	217	31	38

CN = Camp Navajo

5-Year: 2004-2008 Harvest

Unit	Year	Dates	Permits Authorized	1st Choice Applicants	Permits Issued	Draw Odds	Hunters	Hunter Days	Harvest	Hunt Success
**FALL GENERAL (continued)**										
22	2008	10/03-10/09	150	262	150	56.1	123	312	27	22
23	2004	10/08-10/14	700	1492	700	37.7	558	1699	83	15
23	2005	10/07-10/13	700	1415	700	42.0	550	1415	164	30
23	2006	10/13-10/19	700	1625	697	37.0	535	1435	107	20
23	2007	10/12-10/18	700	1225	699	50.9	579	1569	236	41
23	2008	10/03-10/09	700	1141	700	58.8	592	1815	116	20
27	2004	10/08-10/14	725	1164	725	43.3	532	1625	74	14
27	2005	10/07-10/13	725	1035	725	51.1	551	1573	102	19
27	2006	10/13-10/19	800	1203	799	46.7	577	1670	64	11
27	2007	10/12-10/18	800	730	800	71.8	606	1789	153	25
27	2008	10/03-10/09	800	669	800	91.5	634	1885	138	22
**FALL Juniors Only (all 2008 tags were issued over-the-counter)**										
1	2004	10/08-10/14	25	76	25	27.6	16	34	0	0
1	2005	10/07-10/13	25	53	25	47.2	20	61	2	10
1	2006	10/13-10/19	25	68	25	36.8	15	43	3	20
1	2007	10/12-10/18	25	40	25	62.5	20	42	2	10
1	2008	10/03-10/09	OTC	–	336	–	36	102	3	8
5B	2005	10/07-10/13	25	43	25	55.8	18	43	4	22
5BS	2006	10/13-10/19	50	85	50	52.9	43	102	14	33
5BS	2007	10/12-10/18	50	65	50	72.3	39	97	8	21
6A	2004	10/08-10/14	25	123	25	20.3	21	63	0	0
6A	2008	10/03-10/09	OTC	–	336	–	99	300	8	8
8	2008	10/03-10/09	OTC	–	336	–	44	124	0	0
8/10	2006	10/13-10/19	50	69	50	56.5	29	98	0	0
8/10	2007	10/12-10/18	50	49	50	79.6	40	125	2	5
10	2008	10/03-10/09	OTC	–	336	–	28	58	0	0
12A	2004	10/08-10/14	25	19	25	89.5	14	36	0	0
12A	2005	10/07-10/13	25	16	25	100.0	13	42	0	0
12A	2008	10/03-10/09	OTC	–	336	–	19	72	6	32
23	2008	10/03-10/09	OTC	–	336	–	69	212	17	25
27	2004	10/08-10/14	25	32	25	65.6	21	63	4	19
27	2005	10/07-10/13	25	25	25	92.0	20	45	10	50
27	2006	10/13-10/19	25	24	23	58.3	13	19	2	15
27	2007	10/12-10/18	25	25	25	84.0	15	40	0	0
27	2008	10/03-10/09	OTC	–	336	–	14	39	3	21

CN = Camp Navajo

Fall Archery-only Turkey 2004-2008 (2008 data is preliminary)

Unit	Year	Hunters	Hunter Days	Harvest	Hunt Success
1	2004	375	1277	30	8
1	2005	296	1136	6	2
1	2006	264	985	19	7
1	2007	313	1416	19	6
1	2008	157	555	14	9
3B	2007	88	432	13	15
3B	2008	69	315	5	7
3B/3C	2004	160	600	13	8
3B/3C	2005	151	634	18	12
3B/3C	2006	213	811	13	6
3C	2007	182	702	19	10
3C	2008	83	296	0	0
4	2004	160	570	0	0
4	2005	103	453	6	6
4	2006	129	457	6	5
4	2007	150	532	6	4
4	2008	116	490	0	0
5A	2004	147	738	4	3
5A	2005	181	773	18	10
5A	2006	155	586	0	0
5A	2007	257	952	0	0
5A	2008	153	555	5	3

Fall Archery-only Turkey 2004-2008 (2008 data is preliminary)

Unit	Year	Hunters	Hunter Days	Harvest	Hunt Success
5B	2004	173	544	9	5
5B	2005	109	501	6	6
5B	2006	167	599	0	0
5B	2007	188	733	0	0
5B	2008	106	500	9	8
6A	2004	337	1519	13	4
6A	2005	405	1674	12	3
6A	2006	386	1777	13	3
6A	2007	332	1334	13	4
6A	2008	282	1333	0	0
6B	2004	117	479	4	3
6B	2005	109	381	0	0
6B	2006	84	412	0	0
6B	2007	119	564	0	0
6B	2008	97	430	0	0
7	2004	147	652	9	6
7	2005	133	816	12	9
7	2006	225	1063	0	0
7	2007	119	695	0	0
7	2008	120	500	5	4
8	2004	198	768	13	7
8	2005	242	888	24	10
8	2006	270	1056	6	2
8	2007	113	507	6	5
8	2008	176	717	5	3
9	2004	26	95	0	0
9	2005	30	91	0	0
9	2006	26	109	0	0
9	2007	6	25	0	0
9	2008	28	69	5	18
10	2004	60	289	4	7
10	2005	97	369	6	6
10	2006	52	219	0	0
10	2007	25	144	0	0
10	2008	37	153	0	0
11M	2004	17	73	0	0
11M	2005	12	73	0	0
11M	2007	13	44	0	0
11M	2008	19	102	0	0
12A	2004	570	2861	30	5
12A	2005	550	2755	42	8
12A	2006	547	2827	19	3
12A	2007	382	2317	0	0
12A	2008	241	1356	9	4
17	2004	56	181	4	7
17	2005	48	290	0	0
17	2006	64	245	13	20
17	2007	6	25	0	0
17	2008	51	190	0	0
18B	2004	4	9	0	0
18B	2006	6	32	0	0
18B	2008	9	28	0	0
20A	2004	52	328	0	0
20A	2005	60	211	0	0
20A	2006	64	322	0	0
20A	2007	63	319	0	0
20A	2008	37	130	0	0
22	2004	91	293	9	10
22	2005	97	369	6	6
22	2006	109	373	6	6
22	2007	163	733	31	19
22	2008	125	444	5	4
23	2004	181	880	9	5
23	2005	193	689	12	6
23	2006	219	831	32	15

Fall Archery-only Turkey 2004-2008 (2008 data is preliminary)

Unit	Year	Hunters	Hunter Days	Harvest	Hunt Success
23	2007	357	1253	63	18
23	2008	291	1157	9	3
27	2004	186	626	9	5
27	2005	169	556	6	4
27	2006	219	792	13	6
27	2007	251	1071	38	15
27	2008	213	893	32	15
Unknown	2004	47	108	0	0
Unknown	2005	24	66	0	0
Unknown	2006	90	322	0	0
Unknown	2007	88	238	13	15
Unknown	2008	42	190	0	0

Javelina *(Tayassu tajacu)*

Natural History

The javelina, or collared peccary, is of tropical origin and only recently arrived in the Southwest. Peccary bones are not found in Arizona archaeological sites, and early settlers made infrequent references to the occurrence of javelina. Perhaps the javelina spread northward as scrub and cactus replaced Arizona's native grasslands. For whatever reason, the range of javelina is still expanding, primarily northwestward. The species occurs in the United States only in Arizona, Texas, and New Mexico, and currently occupies approximately 34 percent of Arizona with an estimated population of 38,000 post-hunt adults.

Adult javelina usually weigh between 35 and 60 pounds, the males being slightly heavier than the females. Newborn javelina only weigh about one pound. These "piglets" are tan or brown in color with a reddish dorsal stripe. They acquire the salt and pepper appearance of the adults in about three months. The whitish-banded black hairs are up to six inches long, with the hairs on the mane being the darkest and longest. In the winter, when the javelina's coat is dense and dark, a distinct, lighter-colored "collar" is visible. In summer, when the hair is shorter and lighter, this "collar" is less distinct.

Javelina continue to grow until they reach adult height in about 10 months. At this age both sexes are mature. Peccaries breed throughout the year, which, when combined with their early maturity and ability to have two litters per year, gives them the greatest reproductive potential of any North American big-game mammal. The gestation period is 145 days, with most births occurring in June, July, and August. A smaller birth peak occurs in spring, corresponding with Arizona's biseasonal rainfall seasons. Unlike other animals, javelina do not lick their offspring at birth, but roll or tumble their young. The usual litter size is two, and the precocial piglets closely follow their mothers from shortly after birth until they are weaned at about six weeks of age.

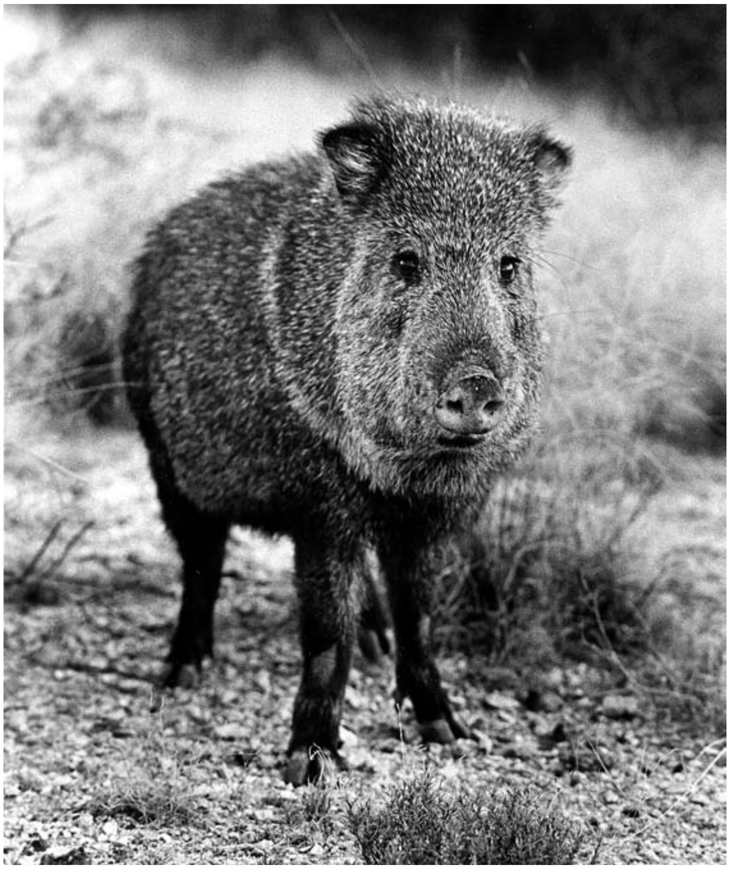

BOB MILES

Although javelina have lived as long as 24 years in captivity, the average life span in the wild is closer to seven or eight years. Coyotes and golden eagles are effective predators of juvenile javelina, and the adults are preyed upon by mountain lions, bobcats, and bears.

Javelina are opportunistic feeders, eating the flowers, fruits, nuts, and berries of a great variety of plants. Prickly pear cactus makes up the major portion of their diet, however, along with agaves, yucca roots, and other desert succulents.

Javelina are social animals with herd sizes averaging eight to nine animals. Territories are marked by droppings and by an

oily secretion produced by the animal's scent gland positioned on its back. Any intruding javelina will be met by an aggressive display, which will evolve into a fight unless the interloper withdraws. The size of a herd's territory varies with the productivity of the habitat, but averages about 750 acres.

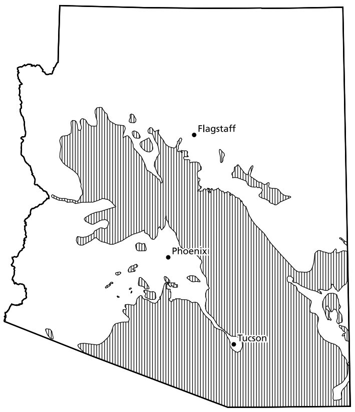

Javelina distribution

Hunt History

Javelina were not legally designated as big game until 1929, when a season from November 1 through January 31 was authorized and a bag limit of one javelina a year was imposed. Hunter interest gradually increased, particularly among non-residents, and the javelina became an important game animal in Arizona after World War II. By 1950, hunters were purchasing nearly 10,000 javelina tags and taking more than 1,000 animals a year. In 1959, an archery javelina season was initiated, and by 1971 more than 30,000 hunters were harvesting more than 6,000 javelina a year. This pressure was deemed excessive in some game management units, and permit-only firearm hunting was instituted in 1972. To further curtail hunt pressure and better distribute hunters, permit-only HAM (handgun, archery, and muzzleloader) hunts were initiated in 1974, and archery hunting was limited to permit-only hunting in 1992. In 1992, juniors only permits were authorized. Between 2003 and 2005, the average harvest for general firearms was 2,157, 131 for juniors-only, 873 for HAM, and 2,224 for archery.

Historic Summary of Javelina Survey Data

Year	No. of Herds Observed[1]	Total Javelina Observed	Average Herd Size[1]	Classification			Young/100 Adults
				Adult	Young	Unclassified	
1955	0	511	–	233	74	204	32
1956	0	324	–	201	75	48	37
1957	0	447	–	328	115	4	35
1958	35	346	9.9	258	88	0	34
1959	31	272	8.8	217	55	0	25
1960	87	677	7.8	391	120	166	31
1961	89	700	7.9	392	108	200	28
1962	109	1003	9.2	667	267	69	40
1963	123	1086	8.8	654	296	136	45
1964	114	768	6.7	560	207	1	37
1965	160	1033	6.5	709	219	105	31
1966	159	1197	7.5	703	362	132	51
1967	107	639	6.0	496	86	57	17
1968	123	872	7.1	613	221	38	36
1969	113	932	8.3	609	203	120	33
1970	216	1757	8.1	1266	414	77	33
1971	220	1666	7.6	1063	480	123	45
1972	143	1158	8.1	679	255	224	38
1973	184	1683	9.2	1121	372	190	33
1974	156	1408	9.0	1035	306	67	30
1975	234	1830	7.8	1418	332	80	23
1976	297	2435	8.2	1745	609	81	35
1977	213	1664	7.8	1119	362	183	32
1978	321	3051	9.5	2249	667	135	30
1979	326	3148	9.7	2385	688	75	29
1980	443	3688	8.3	2865	762	61	27
1981	384	3503	9.1	2635	807	61	31
1982	356	3266	9.2	2390	780	96	33
1983	328	3374	10.3	2502	796	76	32
1984	404	4074	10.1	3085	946	43	31
1985	561	5431	9.7	4043	1181	207	29
1986	536	5051	9.4	3903	1127	21	29
1987	719	6230	8.7	4923	1205	102	24
1988	656	5932	9.0	4606	1323	3	29
1989	663	5662	8.5	4645	1017	0	22
1990	559	4887	8.7	3839	1034	14	27
1991	596	5128	8.6	4008	1058	62	26
1992	571	5247	9.2	4142	1060	45	26
1993	591	5016	8.5	3969	1019	28	26
1994	767	6739	8.8	5485	1141	113	21
1995	682	5870	8.6	4763	1106	1	23
1996	674	5427	8.0	4582	817	28	18
1997	579	4684	8.1	3714	967	3	26
1998	538	4725	8.8	3666	1057	2	29
1999	553	4715	8.5	3831	807	77	21
2000	484	3907	8.0	3174	725	8	23
2001	562	4920	8.7	4007	904	9	23
2002	411	3058	7.4	2565	490	3	19
2003	468	3974	8.4	3128	831	15	27
2004	401	3435	8.5	2775	656	4	24
2005	450	3525	7.8	2843	675	7	24
2006	458	3867	8.4	3074	712	81	23
2007	448	3511	7.8	2913	584	14	20
2008	379	3237	8.5	2500	726	11	29

[1] Excluding single animals

Note: The year given represents the beginning of the survey period, which runs from December through March. Thus, surveys listed for 2007 were conducted from December 2007 through March 2008.

5-Year: 2004–2008 Javelina Survey Data

Unit	Year	No. of Herds Observed[1]	Total Javelina Observed	Average Herd Size[1]	Classification			Young/100 Adults
					Adult	Young	Unclassified	
1	2004	1	4	4.0	4	0	0	0
1	2008	3	20	6.7	13	4	3	31
3A/3C	2006	2	5	2.5	5	0	0	0
3A/3C	2007	2	4	2.0	4	0	0	0
4	2004	1	9	9.0	7	2	0	29
4	2005	1	6	6.0	4	2	0	50
4	2006	2	9	4.5	9	0	0	0
4	2007	2	10	5.0	8	2	0	25
5	2004	1	5	5.0	4	1	0	25
5	2005	1	1	0.0	1	0	0	0
5	2006	1	9	9.0	7	2	0	29
5	2007	2	10	5.0	9	1	0	11
5	2008	4	29	7.3	19	10	0	53
6A	2004	3	13	4.0	11	2	0	18
6A	2005	4	27	6.8	22	5	0	23
6A	2006	3	33	11.0	23	10	0	43
6A	2007	4	28	6.8	23	5	0	22
6A	2008	4	28	6.8	24	4	0	17
6B	2004	2	24	12.0	17	7	0	41
6B	2005	2	21	10.5	16	5	0	31
6B	2006	5	38	7.6	28	10	0	36
6B	2007	6	34	5.7	28	6	0	21
6B	2008	2	19	8.5	14	5	0	36
7	2004	2	4	2.0	4	0	0	0
7	2005	1	1	0.0	1	0	0	0
7	2007	1	2	2.0	2	0	0	0
7	2008	2	7	3.5	6	1	0	17
8	2005	1	5	5.0	4	1	0	25
8	2006	2	8	4.0	7	1	0	14
8	2007	3	19	6.3	13	6	0	46
9	2007	0	2	–	2	0	0	0
10	2004	6	51	8.5	35	16	0	46
10	2005	3	44	14.7	28	16	0	57
10	2006	3	33	10.7	27	6	0	22
10	2007	6	49	8.2	38	11	0	29
10	2008	2	26	12.0	15	11	0	73
15	2006	2	27	13.5	26	1	0	4
15A	2008	1	10	10.0	8	2	0	25
16A	2004	12	89	7.4	65	24	0	37
16A	2005	5	31	6.2	20	11	0	55
16A	2006	11	82	7.4	68	14	0	21
16A	2007	10	80	7.9	64	16	0	25
16A	2008	11	76	6.7	63	13	0	21
17	2004	7	77	10.9	63	14	0	22
17A	2005	1	10	10.0	6	4	0	67
17A	2006	3	24	7.7	17	7	0	41
17A	2007	6	77	12.8	64	13	0	20
17A	2008	4	36	9.0	26	10	0	38
17B	2005	5	53	10.6	32	21	0	66
17B	2006	8	86	10.8	62	24	0	39
17B	2007	10	125	12.5	107	18	0	17
17B	2008	6	72	11.8	53	19	0	36
18A	2004	6	40	6.7	24	16	0	67
18A	2005	5	18	3.6	13	5	0	38
18A	2006	1	10	10.0	7	3	0	43
18A	2007	7	56	8.0	53	3	0	6
18A	2008	5	29	5.8	18	11	0	61
18B	2004	4	61	15.0	49	12	0	24
18B	2005	29	232	8.0	204	28	0	14
18B	2006	13	133	10.2	95	23	15	24
18B	2007	10	116	11.5	99	17	0	17
18B	2008	12	77	6.3	65	12	0	18
19A	2005	11	115	10.5	88	27	0	31

5-Year: 2004-2008 Javelina Survey Data

Unit	Year	No. of Herds Observed[1]	Total Javelina Observed	Average Herd Size[1]	Classification			Young/100 Adults
					Adult	Young	Unclassified	
19A	2006	5	41	8.2	30	11	0	37
19A	2007	7	96	13.7	54	32	10	59
19A	2008	5	51	10.2	34	17	0	50
19B	2004	6	26	4.3	20	6	0	30
19B	2005	11	85	7.7	56	29	0	52
19B	2006	6	90	14.5	27	17	46	63
19B	2007	4	36	9.0	25	11	0	44
19B	2008	6	50	8.2	36	14	0	39
20A	2005	8	41	5.1	34	7	0	21
20A	2007	3	17	5.7	10	7	0	70
20A	2008	4	37	9.3	21	16	0	76
20B	2004	23	202	8.8	169	33	0	20
20B	2005	17	142	8.4	112	30	0	27
20B	2006	22	189	8.6	152	37	0	24
20B	2007	18	110	6.1	90	20	0	22
20B	2008	15	133	8.9	119	14	0	12
20C	2004	24	229	9.5	196	33	0	17
20C	2005	37	368	10.0	304	64	0	21
20C	2006	25	254	10.2	232	22	0	9
20C	2007	23	243	10.5	203	40	0	20
20C	2008	35	353	10.0	285	68	0	24
21	2004	16	143	8.9	117	26	0	22
21	2005	8	48	6.0	38	10	0	26
21	2006	11	79	7.2	67	12	0	18
21	2007	10	67	6.7	63	4	0	6
21	2008	9	122	13.6	94	28	0	30
22	2004	13	99	7.6	82	17	0	21
22	2005	11	67	6.1	48	19	0	40
22	2006	17	157	9.2	122	35	0	29
22	2007	12	75	6.2	62	13	0	21
22	2008	15	108	7.2	91	17	0	19
23	2004	16	177	10.9	131	46	0	35
23	2005	32	284	8.9	217	67	0	31
23	2006	27	231	8.6	196	35	0	18
23	2007	20	161	8.1	132	29	0	22
23	2008	12	113	9.4	87	26	0	30
24A	2004	20	185	9.2	151	34	0	23
24A	2005	22	182	8.3	148	33	1	22
24A	2006	22	214	9.7	176	38	0	22
24A	2007	9	61	6.8	50	11	0	22
24A	2008	10	85	8.4	51	34	0	67
24B	2004	9	83	9.2	64	19	0	30
24B	2005	9	94	10.4	82	12	0	15
24B	2006	8	66	8.3	52	14	0	27
24B	2007	11	75	6.8	65	10	0	15
24B	2008	6	47	7.8	36	11	0	31
25M	2006	4	31	7.8	20	11	0	55
26M	2006	5	30	6.0	24	6	0	25
27	2004	9	56	6.1	44	12	0	27
27	2005	4	29	7.3	27	2	0	7
27	2006	11	117	10.6	92	25	0	27
27	2007	3	23	7.7	16	7	0	44
28	2004	19	162	8.5	131	31	0	24
28	2005	27	158	5.9	141	17	0	12
28	2006	17	132	7.7	121	11	0	9
28	2007	21	148	6.9	129	19	0	15
28	2008	13	88	6.8	73	15	0	21
29	2004	10	53	5.3	44	9	0	20
29	2005	17	100	5.9	92	8	0	9
29	2006	10	69	6.9	61	8	0	13
29	2007	9	48	5.2	47	1	0	2
29	2008	4	21	5.0	18	3	0	17
30A	2004	8	40	5.0	33	7	0	21

5-Year: 2004-2008 Javelina Survey Data

Unit	Year	No. of Herds Observed[1]	Total Javelina Observed	Average Herd Size[1]	Classification			Young/100 Adults
					Adult	Young	Unclassified	
30A	2005	24	154	6.4	132	22	0	17
30A	2006	14	123	8.8	104	19	0	18
30A	2007	12	130	10.8	112	18	0	16
30A	2008	11	88	7.9	75	13	0	17
30B	2004	3	18	6.0	12	6	0	50
30B	2005	4	30	7.5	23	7	0	30
30B	2006	8	67	8.4	51	16	0	31
30B	2007	9	55	6.0	44	11	0	25
30B	2008	12	86	7.2	59	27	0	46
31	2004	12	82	6.8	63	19	0	30
31	2005	8	49	6.1	42	7	0	17
31	2006	16	99	6.1	80	19	0	24
31	2007	15	101	6.7	90	11	0	12
31	2008	14	113	8.1	86	27	0	31
32	2004	11	87	7.9	75	12	0	16
32	2005	17	140	8.2	101	35	4	35
32	2006	26	196	7.5	159	37	0	23
32	2007	18	101	5.6	86	15	0	17
32	2008	12	82	6.8	69	13	0	19
33	2004	13	153	11.8	132	21	0	16
33	2005	5	58	11.6	44	14	0	32
33	2006	8	70	8.8	53	17	0	32
33	2007	10	85	8.5	76	9	0	12
33	2008	11	133	12.1	97	36	0	37
34A	2004	16	115	7.1	90	25	0	28
34A	2005	21	159	7.6	132	27	0	20
34A	2006	14	115	8.1	99	16	0	16
34A	2007	23	190	8.3	149	37	4	25
34A	2008	12	108	9.0	88	20	0	23
34B	2004	11	100	9.1	72	28	0	39
34B	2005	6	55	9.2	47	8	0	17
34B	2006	11	79	7.2	61	18	0	30
34B	2007	12	71	5.8	53	18	0	34
34B	2008	5	58	11.4	41	17	0	41
35A	2004	16	97	6.0	83	14	0	17
35A	2005	10	75	7.5	54	21	0	39
35A	2006	9	61	6.8	40	21	0	53
35A	2007	15	112	7.4	82	30	0	37
35A	2008	11	74	6.7	52	22	0	42
35B	2004	8	79	9.6	59	20	0	34
35B	2005	3	23	7.7	19	4	0	21
35B	2006	6	64	10.7	45	19	0	42
35B	2007	9	106	11.8	85	21	0	25
35B	2008	7	70	10.0	53	17	0	32
36A	2004	18	140	7.8	120	20	0	17
36A	2005	21	161	7.7	141	20	0	14
36A	2006	18	110	6.1	87	23	0	26
36A	2007	16	106	6.6	87	19	0	22
36A	2008	16	147	9.2	107	40	0	37
36B	2004	16	146	9.1	128	18	0	14
36B	2005	18	149	8.3	119	30	0	25
36B	2006	16	127	7.9	101	26	0	26
36B	2007	14	123	8.6	98	25	0	26
36B	2008	9	77	8.6	56	21	0	38
36C	2004	9	50	5.4	45	5	0	11
36C	2005	12	88	7.3	68	18	2	26
36C	2006	13	94	7.2	78	16	0	21
36C	2007	17	126	7.4	105	21	0	20
36C	2008	17	166	9.8	114	47	5	41
37A	2004	13	130	9.9	100	27	3	27
37A	2005	7	56	8.0	41	15	0	37
37A	2006	16	128	8.0	104	24	0	23
37A	2007	27	194	7.2	172	22	0	13

5-Year: 2004-2008 Javelina Survey Data

Unit	Year	No. of Herds Observed[1]	Total Javelina Observed	Average Herd Size[1]	Classification			Young/100 Adults
					Adult	Young	Unclassified	
37A	2008	18	127	7.1	109	18	0	17
37B	2004	21	262	12.4	215	47	0	22
37B	2005	1	9	9.0	9	0	0	0
37B	2006	23	230	10.0	188	42	0	22
37B	2007	19	131	6.8	118	13	0	11
37B	2008	21	182	8.6	147	35	0	24
39	2004	1	4	4.0	3	1	0	33
39M	2005	1	7	7.0	7	0	0	0
40	2004	1	8	8.0	5	3	0	60
40A	2006	1	3	3.0	3	0	0	0
41	2006	1	12	12.0	0	0	12	–
41	2008	1	12	12.0	10	2	0	20
42	2004	7	60	8.3	46	13	1	28
42	2005	8	57	7.1	48	9	0	19
42	2006	6	50	8.3	33	9	8	27
42	2007	6	55	9.2	49	6	0	12
42	2008	7	41	5.7	35	3	3	9
44A	2004	7	72	10.0	62	10	0	16
44A	2005	12	93	7.8	78	15	0	19
44A	2006	6	42	7.0	35	7	0	20
44A	2007	7	53	7.6	47	6	0	13
44A	2008	5	36	7.2	33	3	0	9

Summary of Fall General Javelina Hunts

Year	Permits Authorized	1st Choice Applicants	Permits Issued	Hunters	Hunter Days	Total Harvest	Percent Success
2006	920	4437	917	620	1836	133	21
2007	920	3154	920	649	2113	122	19

Summary of Fall Juniors-Only Javelina Hunts

Year	Permits Authorized	1st Choice Applicants	Permits Issued	Hunters	Hunter Days	Total Harvest	Percent Success
2007	25	18	20	18	25	5	28
2008	2630	607	721	556	1513	134	24

Summary of Fall HAM Javelina Hunts

Year	Permits Authorized	1st Choice Applicants	Permits Issued	Hunters	Hunter Days	Total Harvest	Percent Success
2007	605	965	605	447	1365	50	11

Summary of Fall Archery Javelina Hunts

Year	Permits Authorized	1st Choice Applicants	Permits Issued	Hunters	Hunter Days	Total Harvest	Percent Success
2007	660	458	645	445	1443	21	5

Historic Summary of Spring General Javelina Hunts

Year[1]	Permits Authorized	1st Choice Applicants	Permits Issued	Hunters	Hunter Days	Total Harvest	Percent Success
1950	–	–	9294	7788	–	1344	17
1951	–	–	9995	8625	–	1851	22
1952	–	–	12581	10496	–	1762	17
1953	–	–	15095	13320	–	2510	19
1954	–	–	15299	14829	–	2661	18
1955	–	–	16832	14778	–	3142	21
1956	–	–	17644	14851	–	2930	20
1957	–	–	18724	16672	–	2236	13
1958	–	–	17156	12344	23716	2172	18
1959	–	–	14279	11900	23434	2725	23
1960	–	–	16070	13857	–	2759	20
1961	–	–	19817	17191	29735	3700	22
1962	–	–	22678	19138	41787	3845	20
1963	–	–	24940	21690	46093	4417	20
1964	–	–	24653	20985	46195	5247	25
1965	–	–	24393	20976	44818	4763	23
1966	–	–	25796	21838	46028	4849	22
1967	–	–	28386	23892	52780	4804	20
1968	–	–	29793	26551	62345	4794	18
1969	–	–	32400	28844	65775	5651	20
1970	–	–	33062	30603	66448	6278	21
1971	–	–	31208	27703	59943	5890	21
1972	25350	22855	25350	21450	44178	3819	18
1973	24275	26738	24275	20130	41189	4559	23
1974	22950	29708	22950	19222	39258	5007	26
1975	22300	30889	22300	19017	39409	4587	24
1976	20725	32657	20725	17435	35956	4172	24
1977	20525	33561	20525	17148	35890	4225	25
1978	19950	31685	19950	16075	32666	3449	22
1979	18560	28969	18560	15397	32551	3717	24
1980	17460	29690	17460	14354	33299	3672	26
1981	15785	32330	15785	12986	29477	3642	28
1982	15355	28007	15355	12627	30540	3075	24
1983	15170	21204	15170	13400	32250	3269	24
1984	16120	20052	16120	13975	35149	3638	26
1985	15145	20143	15145	13067	32970	3539	27
1986	15975	23247	15975	13725	33473	3743	27
1987	15890	21710	15890	13979	34330	4220	30
1988	15885	21737	15885	14129	35067	4432	31
1989	15310	20444	15310	13569	34861	3240	24
1990	14325	18859	14325	12565	31314	3468	28
1991	13225	16614	13900	12165	31618	2856	24
1992	13800	10394	13255	12360	32183	3158	26
1993	13880	10407	13787	11902	29035	3126	26
1994	13915	10867	13890	12382	31672	3536	29
1995	13440	11086	13433	11926	31928	2781	23
1996	13360	11151	13307	11938	31600	3444	29
1997	12620	11296	12622	11085	30147	2952	27
1998	12410	11835	12444	10493	27482	2520	24
1999	12200	12053	11937	10506	28005	2784	26
2000	12195	11603	12194	10793	27700	3182	29
2001	12105	12517	12110	10336	28124	2291	22
2002	11705	10941	11702	10256	27685	2823	28
2003	11900	11428	11920	10153	27419	2348	23
2004	11300	12879	11292	9747	26424	2393	25
2005	11090	13790	11207	8628	23772	1729	20
2006	11145	10972	11043	9538	26024	2544	27
2007	11500	9076	11170	9778	26632	2587	26
2008	11721	8106	11138	9536	26185	2008	21

[1] Ft. Huachuca hunt data was gathered using the hunter questionnaire program after 1995.

Javelina Harvest Data

Historic Summary of Spring Juniors-Only Javelina Hunts

Year	Permits Authorized	1st Choice Applicants	Permits Issued	Hunters	Hunter Days	Total Harvest	Percent Success
1992	150	75	120	103	225	20	19
1993	150	92	140	125	283	31	25
1994	150	88	148	138	289	49	36
1995	150	50	89	78	198	16	21
1996	250	131	197	191	396	65	34
1997	370	179	256	229	570	84	37
1998	420	244	324	300	738	74	25
1999	380	304	338	297	631	111	37
2000	480	353	444	397	824	159	40
2001	530	453	509	443	986	110	25
2002	460	942	460	385	812	149	39
2003	460	957	460	401	915	124	31
2004	485	609	485	430	1030	117	27
2005	535	611	550	447	983	151	34
2006	670	682	645	522	1241	172	33
2007	865	896	809	729	1841	314	43
2008	970	866	818	684	1614	273	40

Historic Summary of Spring HAM[1] Javelina Hunts

Year	Permits Authorized	1st Choice Applicants	Permits Issued	Hunters	Hunter Days	Total Harvest	Percent Success
1974	100	302	100	79	166	30	38
1975	100	246	100	82	127	29	35
1976	900	1046	900	711	1484	90	13
1977	925	1117	925	689	1385	104	15
1978	1700	1348	1700	1290	2623	145	11
1979	1850	1440	1850	1454	3128	212	15
1980	3000	2108	3000	2250	5178	367	16
1981	3750	2755	3750	2986	7545	544	18
1982	3850	3502	3850	3079	7771	482	16
1983	5990	3226	5990	4476	11313	824	18
1984	6375	3517	6375	4436	11775	878	20
1985	8180	4272	8116	5653	14835	1109	20
1986	7620	5446	7620	6316	16558	1180	19
1987	8200	5500	7719	6382	16289	1728	27
1988	6500	6208	6500	5655	15148	1133	20
1989	6075	6023	6075	5287	14271	991	19
1990	6980	6229	6980	5964	16286	1289	22
1991[2]	7340	6433	6991	6159	16796	929	15
1992	6740	4050	5786	5191	14667	951	18
1993	7665	4674	6839	5704	14961	973	17
1994	8150	5081	7875	7081	19553	1587	22
1995	8070	5553	8004	7034	19908	1186	17
1996	8210	5888	8012	7033	20053	1456	21
1997	8360	6088	8155	7229	20571	1387	19
1998	7685	5888	7531	6462	17451	1014	16
1999	7760	6184	7176	6287	17805	1281	20
2000	7260	6321	7262	6360	17621	1311	21
2001	6775	6034	6738	5812	17175	957	16
2002	6600	5377	6601	5705	16990	1148	20
2003	7050	5644	7059	5992	18169	860	14
2004	6550	5779	6550	5637	16683	1066	19
2005	6500	5342	6537	5018	14657	692	14
2006	6400	4868	6040	5226	15810	1141	22
2007	5465	4763	5205	4510	14278	878	19
2008	5440	4366	4838	4137	12768	692	17

[1] Prior to 1982, hunts were for handgun, handgun/archery, and/or archery/muzzleloader.
[2] Including special fall archery/shotgun hunts.

Historic Summary of Spring Archery Javelina Hunts

Year[1]	Permits Authorized	1st Choice Applicants	Permits Issued	Hunters	Hunter Days	Total Harvest	Percent Success
1963	–	–	–	1125	3519	111	10
1964	–	–	–	1226	3689	112	9
1965	–	–	–	1438	3574	118	8
1966	–	–	–	1441	3515	138	10
1967	–	–	–	1283	3855	120	9
1968	–	–	–	1608	5093	193	12
1969	–	–	–	2295	7720	206	9
1970	–	–	–	2455	8484	196	8
1971	–	–	–	2918	9663	354	12
1972	–	–	–	3795	12622	305	8
1973	–	–	–	4286	13613	469	11
1974	100	10	–	3680	13145	500	14
1975	100	37	–	4804	16129	650	14
1976	100	28	–	5478	18970	1044	19
1977	–	–	–	5472	20475	786	14
1978	–	–	–	6725	23940	824	12
1979	2400	510	–	4342	14722	786	18
1980	–	–	–	4902	19288	1222	25
1981	–	–	–	6643	36568	1527	23
1982	–	–	–	8735	39700	1543	18
1983	–	–	8987	7722	33638	1684	22
1984	–	–	9163	–	–	–	–
1985	–	–	9599	8883	32259	1946	22
1986	–	–	11088	10379	44358	2232	22
1987	–	–	12236	11200	50479	2870	26
1988	–	–	14625	13493	62771	3436	26
1989	–	–	14785	14011	62250	3605	26
1990	–	–	15104	14161	60256	3723	26
1991	–	–	13658	12504	54558	2263	18
1992	12926	6670	9490	8735	40906	2330	27
1993	11990	7239	9697	8657	38263	2439	28
1994	10205	7424	9944	9099	43001	2564	28
1995	10555	7639	10357	9430	45061	2764	29
1996	10125	7583	9908	8978	42000	2661	30
1997	9755	7809	9703	8725	40922	2672	31
1998	9450	8270	9444	8443	42692	2163	26
1999	9220	8972	9214	8242	41443	2187	27
2000	9650	8828	9646	8604	41072	2574	30
2001	9685	9736	9683	8438	41754	1862	22
2002	9685	9013	9673	8662	41735	2790	32
2003	9635	9756	9661	8545	43478	2236	26
2004	9435	10355	9434	8324	40575	2398	29
2005	9685	10351	9771	8506	42364	2038	24
2006	10000	9861	9930	8703	43174	2452	28
2007	9220	8311	8842	7675	34571	2305	30
2008	9661	8065	8939	7757	35110	2229	29

[1] Ft. Huachuca hunt data was gathered using the hunter questionnaire program after 1995.

5-Year: 2004-2008 Harvest

Unit	Year	Dates Authorized	Permits Authorized	1st Choice Applicants	Permits Issued	Draw Odds	Hunters	Hunter Days	Harvest	Hunt Success
**SPRING GENERAL**										
6A	2004	2/13-2/19	200	368	200	53.0	158	408	53	34
6A	2005	2/11-2/17	200	386	205	49.2	122	296	6	5
6A	2006	2/17-2/23	200	319	200	62.1	183	458	62	34
6A/6B/8	2007	2/23-3/01	150	258	150	54.3	112	275	42	38
6A/6B/8	2008	2/22-2/28	150	250	150	58.0	117	305	40	34
10/18A	2004	2/20-2/26	200	174	200	87.4	181	469	42	23
10/18A	2005	2/18-2/24	200	247	201	71.7	145	390	18	12
10/18A	2006	2/24-3/02	200	169	200	100.0	168	501	55	33
10/18A	2007	2/23-3/01	250	188	250	96.8	220	697	73	33
10/18A	2008	2/22-2/28	250	184	250	87.0	212	622	46	22
16A	2004	2/20-2/26	40	81	40	49.4	34	113	3	9
16A	2005	2/18-2/24	55	98	57	56.1	41	114	10	24
16A	2006	2/24-3/02	110	105	110	96.2	102	297	44	43
16A	2007	2/23-3/01	200	127	200	96.9	177	547	64	36
16A	2008	2/22-2/28	350	148	350	97.3	301	935	43	14
17	2004	2/13-2/19	400	547	400	69.3	339	976	109	32
17	2005	2/11-2/17	400	472	404	75.6	302	843	37	12
17	2006	2/17-2/23	400	455	400	82.0	355	1034	105	30
17	2007	2/23-3/01	400	305	400	98.7	359	1047	108	30
17	2008	2/22-2/28	450	289	446	97.9	385	1072	69	18
18B	2004	2/20-2/26	300	498	300	58.4	260	659	88	34
18B	2005	2/18-2/24	300	465	306	61.7	218	610	71	33
18B	2006	2/24-3/02	300	383	300	76.8	259	688	120	46
18B	2007	2/23-3/01	450	380	450	89.7	401	1066	189	47
18B	2008	2/22-2/28	500	361	500	87.8	412	1142	92	22
19A	2004	2/13-2/19	200	224	200	73.7	178	483	44	25
19A	2005	2/11-2/17	200	284	205	61.6	156	395	30	19
19A	2006	2/17-2/23	250	244	250	86.9	225	661	58	26
19A	2007	2/23-3/01	300	163	300	96.9	270	795	85	31
19A	2008	2/22-2/28	300	220	300	91.8	249	722	49	20
19B	2004	2/13-2/19	200	139	200	100.0	170	480	42	25
19B	2005	2/11-2/17	250	124	252	100.0	158	408	9	6
19B	2006	2/17-2/23	250	92	250	100.0	211	616	34	16
19B	2007	2/23-3/01	300	58	300	100.0	270	737	37	14
19B	2008	2/22-2/28	300	65	288	100.0	226	558	15	7
20A	2004	2/13-2/19	300	371	300	67.7	233	587	85	36
20A	2005	2/11-2/17	300	423	304	59.8	218	535	40	18
20A	2006	2/17-2/23	350	411	350	79.6	297	792	104	35
20A	2007	2/23-3/01	325	306	325	95.8	277	698	85	31
20A	2008	2/22-2/28	350	217	350	99.1	323	948	79	24
20B	2004	2/20-2/26	450	746	450	53.6	401	1046	129	32
20B	2005	2/18-2/24	550	1013	562	51.9	442	1137	96	22
20B	2006	2/24-3/02	550	767	550	66.4	485	1370	127	26
20B	2007	2/23-3/01	625	657	625	88.7	573	1430	157	27
20B	2008	2/22-2/28	625	539	625	98.0	535	1473	94	18
20C	2007	2/23-3/01	250	216	250	90.3	225	587	83	37
20C	2008	2/22-2/28	250	200	250	95.5	225	648	59	26
21	2004	2/20-2/26	700	1461	700	47.2	611	1467	116	19
21	2005	2/18-2/24	650	1525	652	42.0	443	1170	79	18
21	2006	2/24-3/02	600	935	600	61.1	548	1418	120	22
21	2007	2/23-3/01	550	697	550	74.8	473	1247	115	24
21	2008	2/22-2/28	500	672	500	72.0	437	1160	87	20
22	2004	2/20-2/26	775	1397	775	50.4	665	1728	70	11
22	2005	2/18-2/24	625	1244	630	46.7	425	1101	51	12
22	2006	2/24-3/02	625	922	625	59.8	541	1446	92	17
22	2007	2/23-3/01	700	870	700	72.4	597	1633	86	14
22	2008	2/22-2/28	675	627	675	85.3	534	1480	89	17
23	2004	2/20-2/26	225	575	225	39.1	193	495	41	21
23	2005	2/18-2/24	225	703	232	32.0	136	324	23	17
23	2006	2/24-3/02	225	708	225	31.5	185	450	62	34
23	2007	2/23-3/01	350	508	350	63.4	299	771	106	35
23	2008	2/22-2/28	300	480	300	59.0	238	680	54	23

FTHU = Fort Huachuca

5-Year: 2004-2008 Harvest

Unit	Year	Dates Authorized	Permits Authorized	1st Choice Applicants	Permits Issued	Draw Odds	Hunters	Hunter Days	Harvest	Hunt Success
**SPRING GENERAL (continued)**										
24A	2004	2/20-2/26	215	342	215	60.5	183	478	43	24
24A	2005	2/18-2/24	215	431	215	47.1	188	543	55	29
24A	2006	2/24-3/02	215	286	215	67.1	185	470	56	30
24A	2007	2/23-3/01	275	207	275	100.0	261	724	60	23
24A	2008	2/22-2/28	250	190	250	98.4	211	607	49	23
24B	2004	2/20-2/26	350	516	350	60.7	300	779	45	15
24B	2005	2/18-2/24	250	384	250	53.1	190	556	23	12
24B	2006	2/24-3/02	250	345	250	63.5	224	551	54	24
24B	2007	2/23-3/01	350	276	350	98.2	306	736	105	34
24B	2008	2/22-2/28	325	284	325	99.3	286	755	53	19
27	2004	2/13-2/19	250	116	250	100.0	216	583	56	26
27	2005	2/11-2/17	250	127	250	100.0	151	404	30	20
27	2006	2/17-2/23	250	85	250	100.0	186	546	54	29
27	2007	2/23-3/01	125	72	125	97.2	99	280	28	28
27	2008	2/22-2/28	125	72	114	95.8	91	228	31	34
28	2004	2/20-2/26	350	248	350	100.0	285	781	97	34
28	2005	2/18-2/24	350	279	357	100.0	285	786	117	41
28	2006	2/24-3/02	350	263	350	100.0	293	719	147	50
28	2007	2/23-3/01	250	182	250	96.7	225	626	86	38
28	2008	2/22-2/28	250	161	250	94.4	229	615	102	45
29	2004	2/20-2/26	250	35	250	100.0	208	550	42	20
29	2005	2/18-2/24	250	60	251	100.0	202	586	25	12
29	2006	2/24-3/02	250	43	153	100.0	119	340	26	22
29	2007	2/23-3/01	200	29	92	100.0	87	233	29	33
29	2008	2/22-2/28	200	19	78	100.0	76	165	30	39
30A	2004	2/20-2/26	350	221	349	100.0	297	762	82	28
30A	2005	2/18-2/24	350	312	352	100.0	302	866	85	28
30A	2006	2/24-3/02	350	170	347	100.0	287	792	103	36
30A	2007	2/23-3/01	300	145	274	96.6	228	659	71	31
30A	2008	2/22-2/28	300	146	250	94.5	217	522	85	39
30B	2004	2/20-2/26	200	106	200	100.0	175	543	42	24
30B	2005	2/18-2/24	200	80	201	100.0	163	504	9	6
30B	2006	2/24-3/02	150	69	150	100.0	135	398	34	25
30B	2007	2/23-3/01	125	57	125	100.0	84	240	19	23
30B	2008	2/22-2/28	125	41	106	100.0	104	303	20	19
31	2004	2/20-2/26	150	189	147	77.8	113	369	30	27
31	2005	2/18-2/24	150	169	151	82.3	118	371	33	28
31	2006	2/24-3/02	200	142	200	100.0	163	468	57	35
31	2007	2/23-3/01	175	97	175	97.9	146	414	18	12
31	2008	2/22-2/28	175	77	170	100.0	113	288	23	20
32	2004	2/20-2/26	500	545	500	85.7	425	1108	110	26
32	2005	2/18-2/24	450	520	454	80.6	369	1005	75	20
32	2006	2/24-3/02	500	479	500	99.4	402	1061	122	30
32	2007	2/23-3/01	450	316	450	97.5	392	1086	95	24
32	2008	2/22-2/28	475	290	475	97.6	405	1083	92	23
33	2004	2/20-2/26	700	563	697	100.0	605	1573	183	30
33	2005	2/18-2/24	700	644	710	95.3	583	1620	129	22
33	2006	2/24-3/02	700	619	700	100.0	601	1555	146	24
33	2007	2/23-3/01	600	521	600	93.5	508	1294	142	28
33	2008	2/22-2/28	600	458	600	95.0	511	1270	153	30
34A	2004	2/20-2/26	400	261	400	100.0	357	1028	92	26
34A	2005	2/18-2/24	400	339	401	100.0	336	945	90	27
34A	2006	2/24-3/02	400	271	400	100.0	341	951	91	27
34A	2007	2/23-3/01	625	188	509	100.0	446	1214	135	30
34A	2008	2/22-2/28	625	191	495	100.0	416	1150	84	20
34B	2004	2/20-2/26	100	68	100	100.0	87	266	17	20
34B	2005	2/18-2/24	100	79	101	100.0	88	292	15	17
34B	2006	2/24-3/02	100	62	100	100.0	78	198	22	28
34B	2007	2/23-3/01	100	47	100	100.0	83	231	12	14
34B	2008	2/22-2/28	100	32	77	96.9	66	165	9	14
35A	2004	2/20-2/26	100	69	100	100.0	80	205	25	31
35A	2005	2/18-2/24	100	74	100	100.0	86	293	14	16

FTHU = Fort Huachuca

5-Year: 2004-2008 Harvest

Unit	Year	Dates Authorized	Permits Authorized	1st Choice Applicants	Permits Issued	Draw Odds	Hunters	Hunter Days	Harvest	Hunt Success
**SPRING GENERAL (continued)**										
35A	2006	2/24-3/02	100	46	100	100.0	88	240	25	28
35A	2007	2/23-3/01	75	48	75	100.0	63	172	10	16
35A	2008	2/22-2/28	75	37	56	86.5	44	147	0	0
35B	2004	2/20-2/26	100	60	100	100.0	80	213	30	38
35B	2005	2/18-2/24	100	94	101	93.6	54	155	12	22
35B	2006	2/24-3/02	100	54	100	100.0	85	230	18	21
35B	2007	2/23-3/01	75	41	75	100.0	64	182	15	23
35B	2008	2/22-2/28	75	28	66	96.4	51	137	7	14
36A	2004	2/20-2/26	700	598	700	100.0	617	1763	162	26
36A	2005	2/18-2/24	700	584	711	100.0	600	1654	108	18
36A	2006	2/24-3/02	650	409	650	100.0	578	1635	129	22
36A	2007	2/23-3/01	375	269	375	94.1	330	968	66	20
36A	2008	2/22-2/28	375	233	375	94.0	327	907	87	27
36B	2004	2/20-2/26	400	369	400	99.7	360	977	97	27
36B	2005	2/18-2/24	400	388	403	94.6	335	1004	59	18
36B	2006	2/24-3/02	400	245	400	100.0	342	960	84	25
36B	2007	2/23-3/01	475	240	437	100.0	384	1049	83	22
36B	2008	2/22-2/28	475	181	347	95.0	313	871	69	22
36C	2004	2/20-2/26	475	299	475	100.0	418	1220	81	19
36C	2005	2/18-2/24	450	296	455	100.0	356	1009	63	18
36C	2006	2/24-3/02	350	179	350	100.0	306	921	88	29
36C	2007	2/23-3/01	275	137	237	100.0	223	606	49	22
36C	2008	2/22-2/28	275	112	228	100.0	206	652	34	17
37A	2004	2/20-2/26	800	629	800	100.0	707	2078	176	25
37A	2005	2/18-2/24	800	752	802	92.8	653	1800	149	23
37A	2006	2/24-3/02	800	649	800	100.0	725	2070	144	20
37A	2007	2/23-3/01	780	512	780	99.6	700	1936	143	20
37A	2008	2/22-2/28	780	415	780	96.6	702	1929	88	13
37B	2004	2/20-2/26	900	1049	901	77.9	794	2186	151	19
37B	2005	2/18-2/24	900	1179	914	71.3	745	2000	158	21
37B	2006	2/24-3/02	950	1025	950	88.6	823	2130	154	19
37B	2007	2/23-3/01	1000	932	1000	94.1	883	2424	187	21
37B	2008	2/22-2/28	1000	804	996	94.3	876	2368	159	18
42/44A	2008	2/22-2/28	100	60	100	93.3	82	239	8	10
FTHU	2004	2/13-2/19	20	15	18	100.0	17	51	10	59
FTHU	2005	2/11-2/17	20	15	18	100.0	18	56	10	56
FTHU	2006	2/17-2/23	20	21	18	85.7	18	58	7	39
FTHU	2007	2/16-2/22	20	27	16	59.3	13	28	4	31
FTHU	2008	2/15-2/21	16	23	16	65.2	16	39	9	56
**SPRING JUNIOR ONLY**										
6A/6B/8	2007	1/26-2/04	75	42	75	100.0	71	174	21	30
6A/6B/8	2008	1/25-2/03	75	54	75	96.3	63	150	21	33
10/17/18A/19/20A	2004	2/06-2/12	100	181	100	55.3	90	194	29	32
10/17/18A/19/20A	2005	2/04-2/10	100	156	105	63.5	87	169	33	38
10/17/18A/19/20A	2006	2/10-2/16	100	148	100	66.9	79	197	13	16
10/17/18A/19/20A	2007	1/26-2/04	100	96	100	85.4	89	223	35	39
10/17/18A/19/20A	2008	1/25-2/03	100	81	100	91.4	83	187	30	36
16A	2004	2/06-2/12	10	6	10	83.3	10	30	3	30
16A	2005	2/04-2/10	10	16	10	62.5	8	13	5	63
16A	2006	2/10-2/16	20	8	20	100.0	18	47	11	61
16A	2007	1/26-2/04	30	13	25	92.3	22	42	11	50
16A	2008	1/25-2/03	50	21	26	95.2	21	52	12	57
18B	2007	1/26-2/04	30	17	30	100.0	28	63	17	61
18B	2008	1/25-2/03	100	31	47	90.3	39	79	19	49
20B	2005	2/04-2/10	175	168	180	92.3	154	332	46	30
20B/21	2007	1/26-2/04	55	111	55	48.7	52	126	16	31
20B/21	2008	1/25-2/03	65	134	65	47.0	46	106	13	28
20C	2004	2/06-2/12	75	132	75	52.3	71	164	30	42
20C	2005	2/04-2/10	100	119	101	81.5	87	176	49	56
20C	2006	2/10-2/16	100	147	100	66.0	83	217	33	40
20C	2007	1/26-2/04	100	110	100	80.9	90	210	52	58
20C	2008	1/25-2/03	100	87	99	88.5	86	188	40	47

FTHU = Fort Huachuca

5-Year: 2004-2008 Harvest

Unit	Year	Dates Authorized	Permits Authorized	1st Choice Applicants	Permits Issued	Draw Odds	Hunters	Hunter Days	Harvest	Hunt Success
**SPRING JUNIOR ONLY (continued)**										
22/23	2007	1/26-2/04	50	146	50	34.3	43	119	21	49
22/23	2008	1/25-2/03	50	130	50	38.5	40	83	13	33
23	2006	2/10-2/16	200	188	200	85.1	169	377	48	28
24A/24B	2007	1/26-2/04	50	58	50	58.6	50	142	26	52
24A/24B	2008	1/25-2/03	45	58	45	56.9	36	76	17	47
24B	2004	2/06-2/12	150	123	150	89.4	126	316	29	23
27	2007	1/26-2/04	50	6	11	16.7	11	40	0	0
27	2008	1/25-2/03	50	6	13	83.3	13	36	5	38
28/28/30/31/32	2007	1/26-2/04	65	75	65	72.0	63	173	40	63
28/28/30/31/32	2008	1/25-2/03	75	75	75	78.7	60	134	35	58
33/37	2007	1/26-2/04	150	166	150	80.1	124	279	45	36
33/37	2008	1/25-2/03	150	140	150	90.7	128	347	38	30
33/37B	2006	2/10-2/16	150	140	150	85.7	115	279	47	41
34A/34B	2007	1/26-2/04	40	21	39	90.5	37	100	10	27
34A/34B	2008	1/25-2/03	40	14	20	71.4	18	51	7	39
35A/35B	2007	1/26-2/04	20	2	9	100.0	4	11	0	0
35A/35B	2008	1/25-2/03	20	3	5	100.0	5	11	1	20
36A/36C	2005	2/04-2/10	100	82	104	100.0	77	193	15	19
36A/36B	2006	2/10-2/16	100	51	75	86.3	58	124	20	34
36A/36B/36C	2007	1/26-2/04	50	33	50	93.9	45	139	20	44
36A/36B/36C	2008	1/25-2/03	50	32	48	90.6	46	114	22	48
37A	2005	2/04-2/10	50	70	50	71.4	34	100	3	9
37B	2004	2/06-2/12	150	167	150	85.6	133	326	26	20
**SPRING HAM**										
6A/6B/8	2007	2/09-2/18	50	117	50	41.9	46	141	13	28
6A/6B/8	2008	2/08-2/17	50	127	48	37.8	36	90	4	11
6B	2004	2/13-2/19	75	52	75	100.0	58	188	10	17
6B	2005	2/11-2/17	75	65	75	96.9	60	163	16	27
6B	2006	2/17-2/23	75	65	75	100.0	58	155	5	9
8	2004	2/13-2/19	50	38	50	100.0	47	117	12	26
8	2005	2/11-2/17	50	68	50	73.5	37	77	0	0
8	2006	2/17-2/23	75	39	75	100.0	52	196	10	19
10/18A	2007	2/09-2/18	100	29	100	96.6	88	265	20	23
10/18A	2008	2/08-2/17	100	52	100	100.0	78	280	15	19
16A	2007	2/09-2/18	100	34	100	100.0	84	235	33	39
16A	2008	2/08-2/17	100	43	100	100.0	88	265	15	17
17	2007	2/09-2/18	100	58	100	100.0	93	288	25	27
17	2008	2/08-2/17	100	84	100	92.9	91	300	27	30
18B	2004	2/13-2/19	200	236	200	78.0	170	514	46	27
18B	2005	2/11-2/17	200	266	200	72.6	176	616	48	27
18B	2006	2/17-2/23	200	199	200	91.5	175	590	55	31
18B	2007	2/09-2/18	200	180	200	78.3	182	550	59	32
18B	2008	2/08-2/17	200	226	200	85.8	163	488	45	28
19A	2007	2/09-2/18	100	40	100	100.0	91	253	15	16
19A	2008	2/08-2/17	100	57	100	100.0	90	273	12	13
19B	2007	2/09-2/18	100	16	100	100.0	78	227	4	5
19B	2008	2/08-2/17	100	28	91	100.0	69	170	17	25
20A	2007	2/09-2/18	100	49	100	100.0	91	309	16	18
20A	2008	2/08-2/17	100	75	100	100.0	91	277	15	16
20B	2004	2/13-2/19	450	450	450	86.0	391	1120	46	12
20B	2006	2/17-2/23	450	414	450	87.2	413	1155	61	15
20B	2007	2/09-2/18	325	327	325	81.7	296	948	57	19
20B	2008	2/08-2/17	325	329	325	88.8	289	889	21	7
20C	2004	2/13-2/19	250	552	250	45.3	210	719	62	30
20C	2004	2/20-2/26	250	99	250	90.9	229	632	45	20
20C	2005	2/11-2/17	375	615	376	57.7	285	785	49	17
20C	2005	2/18-2/24	275	69	280	100.0	214	562	52	24
20C	2006	2/17-2/23	375	553	375	66.6	343	1017	121	35
20C	2006	2/24-3/02	275	63	275	100.0	235	731	72	31
20C	2007	2/09-2/18	325	552	325	57.1	301	1080	49	16
20C	2008	2/08-2/17	325	367	325	84.7	270	872	57	21
21	2004	2/13-2/19	275	478	275	55.4	239	688	42	18

FTHU = Fort Huachuca

5-Year: 2004–2008 Harvest

Unit	Year	Dates Authorized	Permits Authorized	1st Choice Applicants	Permits Issued	Draw Odds	Hunters	Hunter Days	Harvest	Hunt Success
**SPRING HAM (continued)**										
21	2005	2/11-2/17	250	466	253	51.7	172	454	20	12
21	2006	2/17-2/23	225	373	225	60.3	190	500	38	20
21	2007	2/09-2/18	225	326	225	62.3	191	650	34	18
21	2008	2/08-2/17	200	280	200	69.3	168	488	21	13
22	2004	2/13-2/19	500	721	500	60.3	439	1273	74	17
22	2005	2/11-2/17	450	518	458	68.2	313	773	28	9
22	2006	2/17-2/23	450	573	450	66.7	361	1130	45	12
22	2007	2/09-2/18	325	385	325	72.2	294	911	66	22
22	2008	2/08-2/17	300	362	300	66.6	265	788	43	16
23	2004	2/13-2/19	300	583	300	49.7	272	813	62	23
23	2005	2/11-2/17	300	545	303	51.7	213	674	20	9
23	2007	2/09-2/18	150	374	150	40.1	128	405	37	29
23	2008	2/08-2/17	150	356	150	41.9	123	442	13	11
24A	2004	2/13-2/19	225	218	225	78.4	195	590	51	26
24A	2005	2/11-2/17	225	204	228	83.8	174	523	40	23
24A	2006	2/17-2/23	225	218	225	84.4	209	610	53	25
24A	2007	2/09-2/18	125	170	125	60.6	103	306	27	26
24A	2008	2/08-2/17	125	145	125	75.2	106	342	18	17
24B	2005	2/11-2/17	250	154	251	100.0	193	513	16	8
24B	2006	2/17-2/23	250	201	250	100.0	205	576	43	21
24B	2007	2/09-2/18	150	134	150	90.3	124	422	17	14
24B	2008	2/08-2/17	150	163	150	78.5	134	464	11	8
27	2007	2/09-2/18	65	7	58	100.0	39	106	5	13
27	2008	2/08-2/17	65	14	24	100.0	9	21	0	0
28	2004	2/13-2/19	100	68	100	100.0	79	219	33	42
28	2005	2/11-2/17	100	65	101	100.0	85	303	9	11
28	2006	2/17-2/23	100	62	100	100.0	94	308	38	40
28	2007	2/09-2/18	85	87	85	85.1	72	248	16	22
28	2008	2/08-2/17	85	50	80	96.0	67	242	17	25
29	2004	2/13-2/19	50	13	50	100.0	42	137	10	24
29	2005	2/11-2/17	50	23	51	100.0	35	91	5	14
29	2006	2/17-2/23	50	18	34	100.0	27	75	11	41
29	2007	2/09-2/18	40	14	27	100.0	25	74	13	52
29	2008	2/08-2/17	40	12	18	100.0	11	36	3	27
30A	2004	2/13-2/19	125	58	125	100.0	97	282	22	23
30A	2005	2/11-2/17	125	59	125	100.0	95	229	30	32
30A	2006	2/17-2/23	125	60	115	100.0	88	241	25	28
30A	2007	2/09-2/18	100	37	66	89.2	56	151	19	34
30A	2008	2/08-2/17	100	37	73	97.3	65	172	32	49
30B	2004	2/13-2/19	200	48	200	100.0	170	515	33	19
30B	2005	2/11-2/17	200	27	200	100.0	131	424	5	4
30B	2006	2/17-2/23	150	23	87	100.0	66	166	4	6
30B	2007	2/09-2/18	125	24	54	100.0	41	133	5	12
30B	2008	2/08-2/17	125	25	40	100.0	31	78	7	23
31	2004	2/13-2/19	250	95	250	100.0	222	681	50	23
31	2005	2/11-2/17	250	101	250	100.0	182	638	22	12
31	2006	2/17-2/23	150	86	150	100.0	132	427	18	14
31	2007	2/09-2/18	130	46	130	100.0	100	347	15	15
31	2008	2/08-2/17	130	63	118	92.1	104	333	21	20
32	2004	2/13-2/19	550	398	550	100.0	481	1397	90	19
32	2005	2/11-2/17	500	382	505	100.0	382	1180	44	12
32	2006	2/17-2/23	450	331	450	100.0	384	1186	108	28
32	2007	2/09-2/18	300	317	300	89.6	243	755	37	15
32	2008	2/08-2/17	325	224	304	97.3	265	815	22	8
33	2004	2/13-2/19	450	243	450	100.0	394	1087	60	15
33	2005	2/11-2/17	450	252	449	100.0	376	988	66	18
33	2006	2/17-2/23	450	295	446	100.0	375	1188	98	26
33	2007	2/09-2/18	380	263	380	97.0	309	938	76	25
33	2008	2/08-2/17	380	267	360	95.5	303	807	67	22
34A	2004	2/13-2/19	300	136	300	100.0	245	642	68	28
34A	2005	2/11-2/17	300	213	300	100.0	252	787	22	9

FTHU = Fort Huachuca

5-Year: 2004-2008 Harvest

Unit	Year	Dates Authorized	Permits Authorized	1st Choice Applicants	Permits Issued	Draw Odds	Hunters	Hunter Days	Harvest	Hunt Success
**SPRING HAM (continued)**										
34A	2006	2/17-2/23	300	149	300	100.0	278	771	62	22
34A	2007	2/09-2/18	285	119	253	97.5	222	656	38	17
34A	2008	2/08-2/17	285	121	195	98.4	173	510	24	14
34B	2004	2/13-2/19	75	46	75	100.0	66	182	9	14
34B	2005	2/11-2/17	75	39	75	100.0	58	184	7	12
34B	2006	2/17-2/23	75	29	75	100.0	69	200	6	9
34B	2007	2/09-2/18	30	20	30	80.0	28	79	0	0
34B	2008	2/08-2/17	30	22	22	86.4	19	85	6	32
35A	2004	2/13-2/19	100	39	100	100.0	72	221	14	19
35A	2005	2/11-2/17	100	49	98	100.0	80	252	10	13
35A	2006	2/17-2/23	100	33	82	100.0	68	292	14	21
35A	2007	2/09-2/18	80	47	62	89.4	42	193	2	5
35A	2008	2/08-2/17	80	10	37	100.0	35	99	7	20
35B	2004	2/13-2/19	100	37	100	100.0	86	262	11	13
35B	2005	2/11-2/17	100	49	100	100.0	74	233	8	11
35B	2006	2/17-2/23	100	44	79	100.0	61	198	14	23
35B	2007	2/09-2/18	80	17	57	100.0	50	154	4	8
35B	2008	2/08-2/17	80	15	32	100.0	28	113	9	32
36A	2007	2/09-2/18	165	39	111	100.0	91	294	26	29
36A	2008	2/08-2/17	165	52	106	100.0	92	258	16	17
36B	2004	2/13-2/19	600	289	600	100.0	507	1507	84	17
36B	2005	2/11-2/17	600	240	603	100.0	465	1297	52	11
36B	2006	2/17-2/23	600	207	391	100.0	340	1056	48	14
36B	2007	2/09-2/18	150	134	150	94.0	132	387	23	17
36B	2008	2/08-2/17	150	81	122	91.4	100	351	20	20
36C	2004	2/13-2/19	225	115	225	100.0	170	507	26	15
36C	2005	2/11-2/17	200	100	200	100.0	159	476	20	13
36C	2006	2/17-2/23	150	58	131	100.0	116	360	9	8
36C	2007	2/09-2/18	115	61	107	96.7	93	278	12	13
36C	2008	2/08-2/17	115	22	36	100.0	27	88	2	7
37A	2004	2/13-2/19	350	270	350	100.0	313	1003	41	13
37A	2005	2/11-2/17	350	269	350	100.0	298	934	43	14
37A	2006	2/17-2/23	350	261	350	100.0	305	918	55	18
37A	2007	2/09-2/18	360	209	360	94.7	323	1028	56	17
37A	2008	2/08-2/17	360	211	360	96.7	327	965	33	10
37B	2004	2/13-2/19	500	497	500	93.0	443	1387	65	15
37B	2005	2/11-2/17	650	504	656	100.0	509	1501	60	12
37B	2006	2/17-2/23	650	514	650	100.0	582	1764	128	22
37B	2007	2/09-2/18	500	531	500	89.3	454	1467	59	13
37B	2008	2/08-2/17	500	446	497	92.6	420	1367	72	17
**SPRING ARCHERY**										
Various Units	2004	1/01-1/31	1500	1727	1500	80.8	1313	6397	409	31
Various Units	2005	1/01-1/31	1800	1900	1819	89.0	1525	7887	366	24
Various Units	2006	1/01-1/31	2000	1759	2000	100.0	1699	8675	521	31
Various Units	2007	1/01-1/25	1500	1669	1500	85.3	1289	5779	486	38
Various Units	2008	1/01-1/24	1750	1630	1746	94.5	1465	6679	476	32
5/6/8/11M/19A	2008	1/01-1/24	800	639	800	98.9	676	2901	234	35
5/6/8/19A	2007	1/01-1/25	800	619	800	99.0	706	3039	223	32
5/6/19A	2004	1/01-1/31	800	754	799	92.7	705	3331	189	27
5/6/19A	2005	1/01-1/31	800	713	806	97.1	703	3353	151	21
5/6/19A	2006	1/01-1/31	800	776	800	94.7	725	3720	203	28
20A/20C	2004	1/01-1/31	500	742	500	65.6	448	2175	134	30
20A/20C	2005	1/01-1/31	500	767	504	63.4	446	1951	109	24
20A/20C	2006	1/01-1/31	500	720	500	68.3	426	2000	108	25
20A/20C	2007	1/01-1/25	400	580	400	67.1	363	1691	104	29
20A/20C	2008	1/01-1/24	400	537	400	71.9	366	1740	112	31
20B	2004	1/01-1/31	350	581	350	58.5	322	1451	79	25
20B	2005	1/01-1/31	475	534	478	71.7	440	1942	76	17
20B	2006	1/01-1/31	500	617	500	76.5	468	2080	112	24
20B	2007	1/01-1/25	475	474	475	89.5	433	1755	127	29
20B	2008	1/01-1/24	475	441	475	93.4	416	1795	97	23

FTHU = Fort Huachuca

5-Year: 2004-2008 Harvest

Unit	Year	Dates Authorized	Permits Authorized	1st Choice Applicants	Permits Issued	Draw Odds	Hunters	Hunter Days	Harvest	Hunt Success
**SPRING ARCHERY (continued)**										
21	2004	1/01-1/31	450	804	450	54.6	382	1593	102	27
21	2005	1/01-1/31	400	690	401	57.1	343	1380	73	21
21	2006	1/01-1/31	375	521	375	70.8	329	1377	63	19
21	2007	1/01-1/25	350	474	350	72.4	305	1140	83	27
21	2008	1/01-1/24	420	562	420	73.5	376	1434	95	25
22	2004	1/01-1/31	600	893	600	60.4	536	2372	92	17
22	2005	1/01-1/31	475	764	479	56.4	411	1858	76	18
22	2006	1/01-1/31	475	672	475	63.0	405	2019	126	31
22	2007	1/01-1/25	500	551	500	75.9	420	1540	97	23
22	2008	1/01-1/24	550	598	550	83.1	453	1836	111	25
23	2004	1/01-1/31	225	515	225	43.3	197	887	54	27
23	2005	1/01-1/31	225	552	232	40.8	194	957	33	17
23	2006	1/01-1/31	225	564	225	39.7	208	948	82	39
23	2007	1/01-1/25	225	543	225	41.4	192	771	78	41
23	2008	1/01-1/24	275	540	275	50.9	217	913	60	28
24A	2004	1/01-1/31	115	270	115	42.6	108	559	34	31
24A	2005	1/01-1/31	115	280	116	41.1	109	544	38	35
24A	2006	1/01-1/31	130	303	130	42.9	125	718	46	37
24A	2007	1/01-1/25	175	219	175	76.3	166	814	65	39
24A	2008	1/01-1/24	200	245	200	73.9	179	685	59	33
24B	2004	1/01-1/31	250	306	250	70.6	217	1043	35	16
24B	2005	1/01-1/31	250	226	252	79.2	231	997	34	15
24B	2006	1/01-1/31	250	251	250	76.9	216	983	50	23
24B	2007	1/01-1/25	225	213	225	90.1	196	769	42	21
24B	2008	1/01-1/24	250	174	250	98.9	218	933	52	24
25M/26M/47M	2007	1/01-1/25	175	111	175	99.1	143	791	54	38
25M/26M/47M	2008	1/01-1/24	175	135	175	100.0	138	674	37	27
27	2007	1/01-1/25	100	40	88	92.5	70	388	10	14
27	2008	1/01-1/24	100	31	71	93.6	62	277	14	23
27/28/29/30/31/32	2004	1/01-1/31	1500	1281	1498	100.0	1297	6309	487	38
27/28/29/30/31/32	2005	1/01-1/31	1500	1361	1522	100.0	1303	6651	379	29
27/28/29/30/31/32	2006	1/01-1/31	1500	1170	1500	100.0	1311	6226	387	30
28/29/30/31/32	2007	1/01-1/25	1200	922	1200	86.8	1017	4459	325	32
28/29/30/31/32	2008	1/01-1/24	1200	787	1042	87.9	914	4298	313	34
33/37B	2004	1/01-1/31	1150	900	1153	100.0	1049	5255	286	27
33/37B	2005	1/01-1/31	1150	1016	1155	100.0	1040	5188	249	24
33/37B	2006	1/01-1/31	1250	1108	1250	100.0	1109	5651	277	25
33/37B	2007	1/01-1/25	1250	899	1250	99.0	1066	4993	247	23
33/37B	2008	1/01-1/24	1250	823	1216	96.6	1115	4817	278	25
34A/37A/37M	2004	1/01-1/31	900	595	899	100.0	798	3983	219	27
34A/37A/37M	2005	1/01-1/31	900	579	898	100.0	804	4178	192	24
34A/37A/37M	2006	1/01-1/31	900	599	898	100.0	787	4068	210	27
34A/37A/38M	2007	1/01-1/25	700	485	700	98.4	625	2919	171	27
34A/37A/38M	2008	1/01-1/24	700	461	634	96.8	580	3063	133	23
34B/35A/35B	2004	1/01-1/31	300	193	300	100.0	243	1396	100	41
34B/35A/35B	2005	1/01-1/31	300	187	298	100.0	261	1398	75	29
34B/35A/35B	2006	1/01-1/31	300	155	234	100.0	213	1087	70	33
34B/35A/35B	2007	1/01-1/25	325	128	197	100.0	171	971	61	36
34B/35A/35B	2008	1/01-1/24	300	130	171	96.9	159	903	46	29
36A/36B/36C	2004	1/01-1/31	600	650	600	88.8	533	2979	120	23
36A/36B/36C	2005	1/01-1/31	600	650	616	89.1	541	3218	142	26
36A/36B/36C	2006	1/01-1/31	600	490	600	100.0	520	2767	130	25
36A/36B/36C	2007	1/01-1/25	800	373	566	99.5	497	2620	125	25
36A/36B/36C	2008	1/01-1/24	800	317	498	99.7	407	2060	105	26
39M/42M	2004	1/01-1/31	175	129	175	100.0	157	682	53	34
39M/42M	2005	1/01-1/31	175	116	177	100.0	137	733	34	25
39M/42M	2006	1/01-1/31	175	133	175	100.0	145	724	63	43
FTHU	2004	1/01-1/31	20	15	20	100.0	19	163	5	26
FTHU	2005	1/01-1/31	20	16	18	100.0	18	129	11	61
FTHU	2006	1/01-1/31	20	23	18	78.3	17	131	4	24
FTHU	2007	1/01-1/31	20	11	16	100.0	16	132	7	44
FTHU	2008	1/01-1/31	16	15	16	86.7	16	102	7	44

FTHU = Fort Huachuca

5-Year: 2004–2008 Harvest

Unit	Year	Dates Authorized	Permits Authorized	1st Choice Applicants	Permits Issued	Draw Odds	Hunters	Hunter Days	Harvest	Hunt Success
**FALL GENERAL**										
28	2006	11/10-11/19	50	262	50	17.2	25	82	7	28
28	2007	11/09-11/18	50	164	50	29.3	40	145	5	13
29	2006	11/10-11/19	40	61	38	34.4	10	35	0	0
29	2007	11/09-11/18	40	56	40	41.1	22	74	0	0
30A	2006	11/10-11/19	50	175	50	22.9	31	94	8	26
30A	2007	11/09-11/18	50	71	50	50.7	35	128	5	14
30B	2006	11/10-11/19	20	64	20	25.0	12	28	0	0
30B	2007	11/09-11/18	20	57	20	35.1	11	40	6	55
31	2006	11/10-11/19	30	208	30	14.4	19	74	0	0
31	2007	11/09-11/18	30	146	30	20.6	8	19	4	50
32	2006	11/10-11/19	75	313	75	18.9	49	107	12	24
32	2007	11/09-11/18	75	215	75	31.2	60	192	29	48
33	2006	11/10-11/19	105	653	104	13.5	63	137	4	6
33	2007	11/09-11/18	105	502	105	20.7	61	193	18	30
34A	2006	11/10-11/19	60	209	60	23.9	48	128	15	31
34A	2007	11/09-11/18	60	163	60	25.8	43	124	4	9
34B	2006	11/10-11/19	15	45	15	24.4	13	35	0	0
34B	2007	11/09-11/18	15	27	15	22.2	11	34	4	36
35A	2006	11/10-11/19	15	78	15	19.2	15	50	0	0
35A	2007	11/09-11/18	15	45	15	31.1	8	26	0	0
35B	2006	11/10-11/19	15	52	15	26.9	15	30	8	53
35B	2007	11/09-11/18	15	41	15	36.6	13	47	6	46
36A	2006	11/10-11/19	75	184	75	31.5	50	211	7	14
36A	2007	11/09-11/18	75	162	75	41.4	63	208	13	21
36B	2006	11/10-11/19	60	224	60	25.9	44	161	9	20
36B	2007	11/09-11/18	60	176	60	32.4	44	196	3	7
36C	2006	11/10-11/19	50	123	50	35.8	25	71	10	40
36C	2007	11/09-11/18	50	97	50	41.2	31	97	6	19
37A	2006	11/10-11/19	120	491	120	17.7	98	262	27	28
37A	2007	11/09-11/18	120	372	120	19.6	89	226	10	11
37B	2006	11/10-11/19	140	1295	140	10.7	103	331	26	25
37B	2007	11/09-11/18	140	860	140	16.3	110	364	9	8
**FALL JUNIOR ONLY**										
16A	2008	10/10-10/16	50	36	37	94.4	31	107	6	19
17B	2008	10/10-10/16	50	46	50	87.0	39	106	11	28
18B	2008	11/21-11/30	75	55	72	94.6	55	142	31	56
20A	2008	10/10-10/16	50	88	50	56.8	33	98	4	12
28	2008	10/10-10/16	50	28	37	96.4	33	90	8	24
28	2008	11/21-11/27	50	10	14	100.0	14	33	9	64
29	2008	10/10-10/16	50	1	3	100.0	3	5	1	33
29	2008	11/21-11/27	50	2	2	100.0	0	0	0	–
30A	2008	10/10-10/16	50	11	15	100.0	13	30	2	15
30A	2008	11/21-11/27	50	2	3	100.0	3	6	2	67
30B	2008	10/10-10/16	50	0	3	–	3	8	0	0
30B	2008	11/21-11/27	50	1	1	100.0	1	2	0	0
31	2008	10/10-10/16	50	23	23	73.9	17	50	4	24
31	2008	11/21-11/27	50	5	8	100.0	4	12	0	0
32	2008	10/10-10/16	100	12	22	91.7	11	24	2	18
32	2008	11/21-11/27	100	19	20	84.2	15	30	8	53
33	2008	10/10-10/16	135	73	73	86.3	57	149	7	12
33	2008	11/21-11/27	135	43	58	90.7	44	105	9	20
34A	2008	10/10-10/16	100	8	11	100.0	11	44	6	55
34A	2008	11/21-11/27	100	4	4	100.0	2	7	1	50
34B	2008	10/10-10/16	25	2	3	100.0	0	0	0	–
34B	2008	11/21-11/27	25	2	5	100.0	5	10	5	100
35A	2008	10/10-10/16	25	3	7	100.0	6	14	0	0
35A	2008	11/21-11/27	25	2	3	100.0	3	9	0	0
35B	2008	10/10-10/16	25	1	1	100.0	1	2	0	0
35B	2008	11/21-11/27	25	2	2	100.0	0	0	0	–
36A	2008	10/10-10/16	70	8	10	100.0	7	20	0	0
36A	2008	11/21-11/27	70	4	18	100.0	18	76	4	22

FTHU = Fort Huachuca

5-Year: 2004-2008 Harvest

Unit	Year	Dates Authorized	Permits Authorized	1st Choice Applicants	Permits Issued	Draw Odds	Hunters	Hunter Days	Harvest	Hunt Success
**FALL JUNIOR ONLY (continued)**										
36B	2008	10/10-10/16	100	7	8	100.0	4	8	0	0
36B	2008	11/21-11/27	100	7	12	100.0	10	31	5	50
36C	2008	10/10-10/16	75	1	2	100.0	0	0	0	–
36C	2008	11/21-11/27	75	4	2	25.0	0	0	0	–
37A	2008	10/10-10/16	125	18	27	100.0	20	52	2	10
37A	2008	11/21-11/27	125	8	13	100.0	13	35	4	31
37B	2008	10/10-10/16	170	29	46	100.0	39	96	0	0
37B	2008	11/21-11/27	165	38	51	100.0	38	102	3	8
39 (Archery)	2007	11/02-11/15	15	11	10	90.9	8	20	0	0
39 (Muzzleloader)	2007	11/16-11/25	10	7	10	85.7	10	15	5	50
39 (Archery)	2008	11/07-11/16	15	4	5	75.0	5	5	0	0
39 (Muzzleloader)	2008	11/21-11/27	10	4	5	75.0	3	10	0	0
**FALL HAM**										
28	2006	10/13-10/22	15	61	15	21.3	11	41	4	36
28	2007	10/12-10/21	15	29	15	41.4	15	35	0	0
29	2006	10/13-10/22	10	18	10	55.6	7	13	0	0
29	2007	10/12-10/21	10	10	10	90.0	8	28	3	38
30A	2006	10/13-10/22	20	21	20	71.4	15	45	0	0
30A	2007	10/12-10/21	20	29	20	69.0	4	8	0	0
30B	2006	10/13-10/22	25	29	25	65.5	13	25	0	0
30B	2007	10/12-10/21	25	16	25	81.3	8	28	0	0
31	2006	10/13-10/22	20	50	20	36.0	13	27	3	23
31	2007	10/12-10/21	20	35	20	25.7	15	45	3	20
32	2006	10/13-10/22	70	123	70	39.8	45	138	2	4
32	2007	10/12-10/21	70	89	70	61.8	51	154	0	0
33	2006	10/13-10/22	70	178	70	27.5	48	135	10	21
33	2007	10/12-10/21	70	149	70	37.6	60	145	13	22
34A	2006	10/13-10/22	45	112	45	33.9	39	119	0	0
34A	2007	10/12-10/21	45	66	45	56.1	39	139	6	15
34B	2006	10/13-10/22	10	24	10	33.3	10	23	0	0
34B	2007	10/12-10/21	10	15	10	53.3	8	24	0	0
35A	2006	10/13-10/22	15	15	15	66.7	13	35	0	0
35A	2007	10/12-10/21	15	22	15	59.1	12	23	0	0
35B	2006	10/13-10/22	15	12	15	50.0	15	49	0	0
35B	2007	10/12-10/21	15	18	15	61.1	8	28	0	0
36A	2006	10/13-10/22	25	38	25	57.9	22	78	3	14
36A	2007	10/12-10/21	25	17	25	82.4	19	71	0	0
36B	2006	10/13-10/22	90	96	89	63.5	61	161	8	13
36B	2007	10/12-10/21	90	42	90	97.6	57	163	7	12
36C	2006	10/13-10/22	25	38	25	44.7	18	42	2	11
36C	2007	10/12-10/21	25	22	25	90.9	21	69	2	10
37A	2006	10/13-10/22	50	159	50	22.0	48	140	6	13
37A	2007	10/12-10/21	50	107	50	29.9	40	118	3	8
37B	2006	10/13-10/22	100	407	100	17.0	77	214	5	6
37B	2007	10/12-10/21	100	299	100	26.8	82	287	13	16
**FALL ARCHERY**										
27/28/29/30A/31/32	2006	9/01-9/10	165	287	160	49.1	96	343	0	0
28/29/30A/31/32	2007	8/24-9/02	165	116	165	97.4	113	345	5	4
30B	2006	9/01-9/10	35	21	35	95.2	25	66	0	0
30B	2007	8/24-9/02	35	18	27	83.3	16	59	0	0
33/37B	2006	9/01-9/10	190	242	190	61.2	130	391	16	12
33/37B	2007	8/24-9/02	190	160	190	96.3	125	372	5	4
34A/37A/38M	2006	9/01-9/10	135	135	132	80.7	86	242	2	2
34A/37A/38M	2007	8/24-9/02	135	91	135	98.9	103	368	8	8
34B/35A/35B	2006	9/01-9/10	45	47	45	76.6	28	132	3	11
34B/35A/35B	2007	8/24-9/02	45	25	38	100.0	26	88	3	12
36A/36B/36C	2006	9/01-9/10	90	87	89	89.7	61	189	0	0
36A/36B/36C	2007	8/24-9/02	90	48	90	100.0	62	211	0	0

FTHU = Fort Huachuca

Bighorn Sheep *(Ovis canadensis)*

Natural History

Arizona's bighorn sheep population, consisting of both Rocky Mountain and desert races, is currently estimated at about 6,000 animals—a severe reduction from the numbers thought to once be present. The causes for this decline, which occurred primarily between 1860 and 1920, were exposure to livestock-borne parasites and diseases. Now, thanks to livestock-free refuges and an aggressive translocation program, bighorn sheep numbers are gradually edging upward.

Desert bighorn sheep weights vary considerably between the sexes. Adult rams weigh 160 and 200 pounds, with a maximum weight of 225 pounds. Adult ewes range from 75 to 130 pounds and average 110 pounds. The biggest visual difference between the two sexes is the horns. Ewe horns are generally 10 to 13 inches long with a circumference of three to six inches. An adult ram's horns may measure up to 40 inches along the outside curl with a basal circumference between 13 and 16 inches. The horn core is honeycombed with chambers, or sinuses, which reduce the weight of the skull.

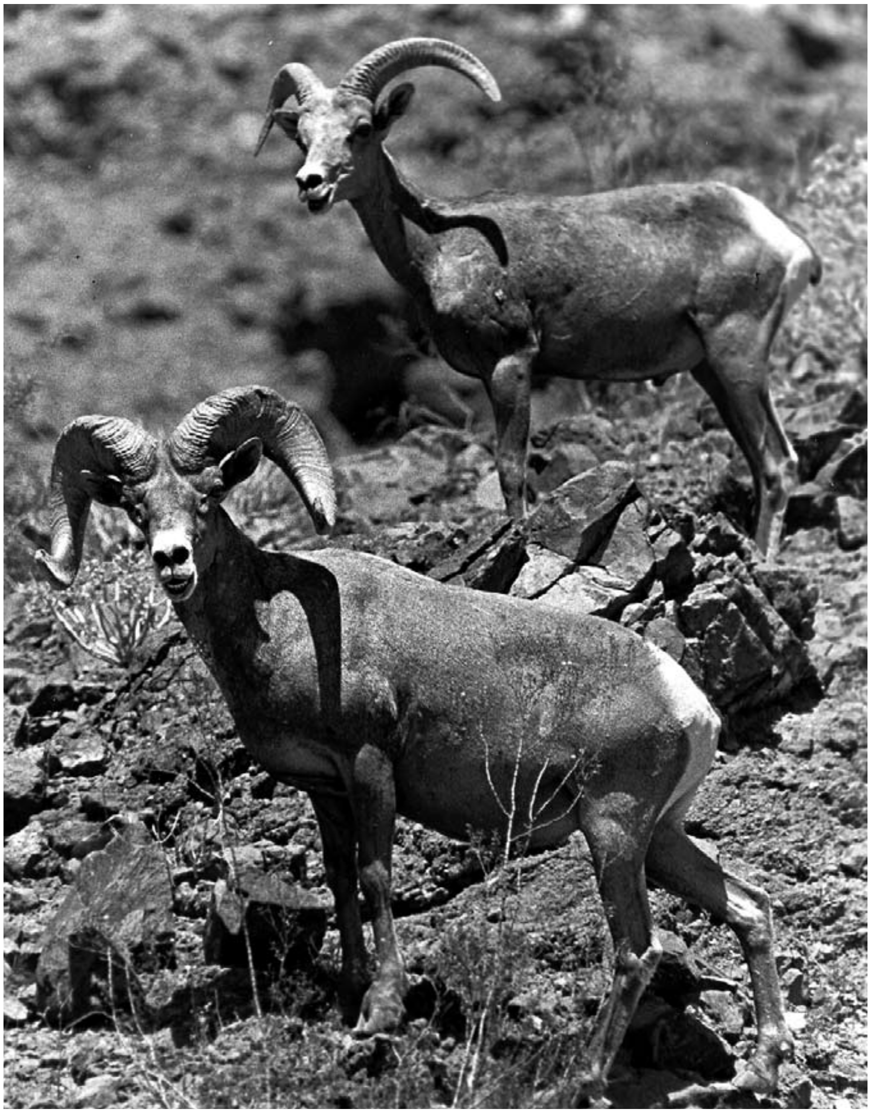

BOB MILES

Newborn bighorn lambs weigh from 8 to 10 pounds, have dark eyes and fuzzy, dark-gray hair, and are active within minutes of birth. As the lambs mature, their eyes take on the characteristic amber color of the adult's eyes. After several months, they also take on the adult's pale buff to dark, chocolate-brown coloration. This overall coat color is accentuated by a white muzzle, a white rump patch, light-colored eye rings, and a white edging on the rear legs. The tail is black, bordered in white.

Bighorn sheep have a life expectancy of 10 to 12 years, but may attain an age of 17 years or older. Usually one, rarely two, lambs are born. The youngsters typically stay with their moth-

ers until two years of age. The young rams then leave the nursery herds of ewes and lambs and join a bachelor herd. The adults usually remain segregated according to sex except during the summer breeding season, and sometimes during the spring with the sprouting of early vegetation.

Sexual maturity varies, both physiologically and behaviorally. Although rams as young as 6 months of age may be capable of breeding, they refrain due to the dominance of older rams. Ewes do not breed until they are about two-years old, and rams usually not until at least three years of age. The breeding season extends from early June through October, but the peak rutting activity takes place in August. The gestation period is about six months, and most lambs are born in late winter or early spring.

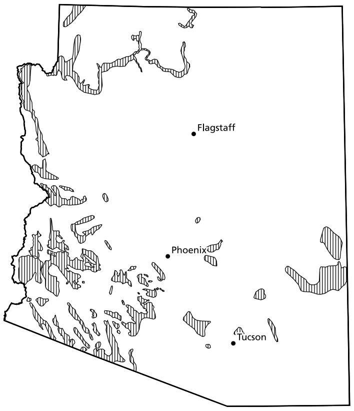

Bighorn distribution

Bighorn sheep are diurnal animals and are usually found in small groups, although herds of 50 or more are sometimes seen. Native grasses are important in the bighorn's diet, although the animals also feed heavily on jojoba and other woody plants. Pincushion, barrel, and saguaro cactuses provide needed moisture. Preferred plants vary with habitat quality, locality, and species availability. Mountain lions are the principal predator although golden eagles and bobcats have been observed taking lambs.

Hunt History

Totally protected by the territorial legislature in 1893, bighorn sheep were not legal game in Arizona until 1953, when it was determined that the limited hunting of trophy rams might be the only way to save these animals. Two limited hunts of 20 permits each were authorized, and 20 bighorn were taken. Since then, permit numbers, the number of units open to hunting, the number of rams taken, and hunt success have gradually increased. About 100 rams, mostly desert bighorns, are now being taken each year. This number will only increase, however, when the disease problem and other limiting factors are brought under control.

Historic Summary of Desert Bighorn Sheep Survey Data

Year	Ram	Ewe	Lamb	Yearling	Unclassified	Total	Number Per 100 Ewes		
							Rams	Lambs	Yearlings
1951	56	46	30	0	9	141	122	65	0
1952	48	36	15	0	8	107	133	42	0
1953	59	48	24	0	17	148	123	50	0
1955	159	129	29	0	41	358	123	22	0
1956	95	129	29	0	4	257	74	22	0
1957	43	48	0	0	0	91	90	0	0
1958	43	77	34	0	3	157	56	44	0
1959	15	46	10	26	5	102	33	22	57
1960	26	57	29	13	0	125	46	51	23
1961	47	106	46	1	0	200	44	43	1
1962	59	104	43	7	8	221	57	41	7
1963	47	109	48	6	8	218	43	44	6
1964	57	181	90	0	18	346	31	50	0
1965	75	134	69	0	41	319	56	51	0
1966	111	228	89	0	13	441	49	39	0
1967	109	341	145	0	46	641	32	43	0
1968	143	382	207	0	0	732	37	54	0
1969	142	407	152	0	2	703	35	37	0
1970	142	464	182	0	9	797	31	39	0
1971	131	264	138	0	20	553	50	52	0
1972	132	275	107	0	26	540	48	39	0
1973	95	214	76	0	7	392	44	36	0
1974	119	288	137	0	10	554	41	48	0
1975	213	418	170	0	12	813	51	41	0
1976	261	542	240	0	4	1047	48	44	0
1977	304	567	269	0	30	1170	54	47	0
1978	343	604	284	0	29	1260	57	47	0
1979	310	713	306	0	39	1368	43	43	0
1980	443	1073	459	0	3	1978	41	43	0
1981	374	775	272	0	1	1422	48	35	0
1982	478	892	301	0	9	1680	54	34	0
1983	554	934	278	0	4	1770	59	30	0
1984	527	819	212	173	0	1731	64	26	21
1985	590	1026	308	164	3	2091	58	30	16
1986	652	1137	383	220	2	2394	57	34	19
1987	648	1102	450	257	0	2457	59	41	23
1988	711	1306	470	259	0	2746	54	36	20
1989	571	1095	291	183	0	2140	52	27	17
1990	655	980	303	187	4	2129	67	31	19
1991	562	1008	301	190	9	2070	56	30	19
1992	696	1124	283	209	7	2319	62	25	19
1993	686	1051	264	167	5	2173	65	25	16
1994	789	1502	298	241	8	2838	53	20	16
1995	624	1224	299	107	6	2260	51	24	9
1996	474	870	134	96	4	1578	54	15	11
1997	742	1375	402	134	1	2654	54	29	10
1998	325	733	152	97	3	1310	44	21	13
1999	344	660	132	102	2	1240	52	20	15
2000	404	803	197	109	5	1518	50	25	14
2001	366	812	322	90	5	1595	45	40	11
2002	249	443	103	73	5	874	56	23	16
2003	288	739	224	84	3	1338	39	30	11
2004	197	443	179	43	1	863	44	40	10
2005	213	388	110	54	11	776	55	28	14
2006	381	635	154	71	8	1249	60	24	11
2007	396	690	215	93	9	1403	57	31	13
2008	433	764	260	99	7	1563	57	34	13

Bighorn Survey Data

Historic Summary of Rocky Mountain Bighorn Sheep Survey Data

Year	Ram	Ewe	Lamb	Yearling	Unclassified	Total	Number Per 100 Ewes		
							Rams	Lambs	Yearlings
1984	20	26	25	13	0	84	77	96	50
1985	16	22	9	3	0	50	73	41	14
1986	56	65	36	42	0	199	86	55	65
1987	54	54	14	40	76	238	100	26	74
1988	40	66	35	40	0	181	61	53	61
1989	50	69	41	50	0	210	72	59	72
1990	62	109	34	23	0	228	57	31	21
1991	43	68	27	18	0	156	63	40	26
1992	65	129	72	36	0	302	50	56	28
1993	75	157	82	18	0	332	48	52	11
1994	101	186	77	19	0	383	54	41	10
1995	68	171	82	24	0	345	40	48	14
1996	72	201	45	31	0	349	36	22	15
1997	71	150	69	20	12	322	47	46	13
1998	102	162	72	30	3	369	63	44	19
1999	65	188	71	25	0	349	35	38	13
2000	70	202	61	17	2	352	35	30	8
2001	75	190	60	11	0	336	39	32	6
2002	84	184	60	29	2	359	46	33	16
2003	11	25	5	2	0	43	44	20	8
2004	45	84	32	8	0	169	54	38	10
2005	89	155	49	17	38	348	57	32	11
2006	83	172	65	9	2	331	48	38	5
2007	59	115	62	17	0	253	51	54	15
2008	117	296	119	44	1	577	40	40	15

Historic Summary of Combined Bighorn Sheep Survey Data

Year	Ram	Ewe	Lamb	Yearling	Unclassified	Total	Number Per 100 Ewes		
							Rams	Lambs	Yearlings
1984	547	845	237	186	0	1815	65	28	22
1985	606	1048	317	167	3	2141	58	30	16
1986	708	1202	419	262	2	2593	59	35	22
1987	702	1156	464	297	76	2695	61	40	26
1988	751	1372	505	299	0	2927	55	37	22
1989	621	1164	332	233	0	2350	53	29	20
1990	717	1089	337	210	4	2357	66	31	19
1991	605	1076	328	208	9	2226	56	30	19
1992	761	1253	355	245	7	2621	61	28	20
1993	761	1208	346	185	5	2505	63	29	15
1994	890	1688	375	260	8	3221	53	22	15
1995	692	1395	381	131	6	2605	50	27	9
1996	546	1071	179	127	4	1927	51	17	12
1997	813	1525	471	154	13	2976	53	31	10
1998	427	895	224	127	6	1679	48	25	14
1999	409	848	203	127	2	1589	48	24	15
2000	474	1005	258	126	7	1870	47	26	13
2001	441	1002	382	101	5	1931	44	38	10
2002	333	627	163	102	8	1233	53	26	16
2003	299	764	229	86	3	1381	39	30	11
2004	242	527	211	51	1	1032	46	40	10
2005	302	543	159	71	49	1124	56	29	13
2006	464	807	219	80	10	1580	58	27	10
2007	455	805	277	110	9	1656	57	34	14
2008	550	1060	379	143	8	2140	52	36	13

5-Year: 2004-2008 Desert Bighorn Sheep Survey Data

Unit	Year	Ram	Ewe	Lamb	Yearling	Unclassified	Total	Number per 100 Ewes		
								Rams	Lambs	Yearlings
9/10	2006	9	20	5	0	0	34	45	25	0
12A/12B West	2005	5	11	3	2	0	21	45	27	18
12A/12B West	2006	9	8	2	0	0	19	113	25	0
12A/12B West	2008	4	11	0	3	0	18	36	0	27
12B East	2005	8	23	6	2	7	46	35	26	9
12B East	2008	28	44	7	11	0	90	64	16	25
13A	2005	18	19	7	0	0	44	95	37	0
13A	2006	25	38	4	5	0	72	66	11	13
13A	2008	3	5	1	0	0	9	60	20	0
13B North	2005	35	58	19	9	0	121	60	33	16
13B North	2006	32	90	18	13	0	153	36	20	14
13B North	2007	36	93	17	14	0	160	39	18	15
13B North	2008	34	44	8	0	0	86	77	18	0
13B South	2005	8	38	11	3	0	60	21	29	8
13B South	2008	13	22	6	1	0	42	59	27	5
15A/15B East	2004	7	20	11	0	0	38	35	55	0
15A/15B East	2007	3	13	1	1	0	18	23	8	8
15B West	2004	33	64	41	2	0	140	52	64	3
15B West	2007	31	64	33	3	0	131	48	52	5
15B West	2008	26	63	41	6	6	142	41	65	10
15C North	2004	18	58	43	1	0	120	31	74	2
15C North	2007	33	50	37	6	0	126	66	74	12
15C North	2008	41	85	63	9	0	198	48	74	11
15C South	2004	7	17	8	2	0	34	41	47	12
15C South	2005	8	16	6	0	0	30	50	38	0
15C South	2006	21	20	5	0	0	46	105	25	0
15C South	2007	6	7	4	0	0	17	86	57	0
15C South	2008	8	11	7	0	0	26	73	64	0
15D	2004	29	87	25	2	1	144	33	29	2
15D	2007	35	51	19	0	0	105	69	37	0
15D	2008	88	110	40	1	0	239	80	36	1
16A	2005	5	13	6	0	0	24	38	46	0
16A	2008	7	23	2	4	0	36	30	9	17
16B	2006	15	20	7	3	0	45	75	35	15
22	2006	13	22	8	5	6	54	59	36	23
23	2005	0	18	4	0	1	23	0	22	0
23	2006	11	26	9	1	0	47	42	35	4
24A	2005	2	1	1	0	0	4	200	100	0
24B	2006	18	22	6	3	0	49	82	27	14
28 (Peloncillos)	2007	15	23	4	1	1	44	65	17	4
31/32 (Aravaipa)	2004	6	18	1	4	0	29	33	6	22
31/32 (Aravaipa)	2006	25	47	11	0	0	83	53	23	0
32	2007	7	5	1	0	0	13	140	20	0
37A	2004	12	25	5	6	0	48	48	20	24
37A	2005	12	14	6	6	2	40	86	43	43
37A	2006	14	17	5	2	0	38	82	29	12
37A	2007	8	12	3	1	0	24	67	25	8
37A	2008	12	20	6	1	0	39	60	30	5
37B	2004	3	14	5	2	0	24	21	36	14
37B	2005	2	6	3	1	0	12	33	50	17
37B	2006	0	3	0	4	0	7	0	0	133
37B	2008	5	6	0	0	0	11	83	0	0
39 East	2006	8	11	4	0	0	23	73	36	0
39 West	2006	14	29	4	3	0	50	48	14	10
39 West	2008	12	24	3	1	0	40	50	13	4
40A North	2004	5	3	3	1	0	12	167	100	33
40A North	2007	0	1	1	0	0	2	0	100	0
40A South	2004	5	5	2	0	0	12	100	40	0
40A South	2007	4	7	1	2	0	14	57	14	29
40B East	2006	8	8	1	0	0	17	100	13	0
40B East	2008	1	4	4	2	0	11	25	100	50
40B West	2004	11	10	6	1	0	28	110	60	10
40B West	2006	40	47	17	5	2	111	85	36	11
40B West	2007	13	15	2	2	1	33	87	13	13

5-Year: 2004-2008 Desert Bighorn Sheep Survey Data (continued)

Unit	Year	Ram	Ewe	Lamb	Yearling	Unclassified	Total	Number per 100 Ewes		
								Rams	Lambs	Yearlings
41 East	2006	21	48	9	5	0	83	44	19	10
41 West	2006	21	32	6	4	0	63	66	19	13
42	2007	3	13	2	2	0	20	23	15	15
42	2008	8	20	4	4	0	36	40	20	20
43A	2004	9	12	3	2	0	26	75	25	17
43A	2007	7	14	7	6	0	34	50	50	43
43B	2004	42	86	19	15	0	162	49	22	17
43B	2007	54	114	30	17	2	217	47	26	15
44A East	2004	5	17	4	3	0	29	29	24	18
44A East	2005	9	16	2	1	0	28	56	13	6
44A East	2007	14	14	7	3	0	38	100	50	21
44A East	2008	5	16	3	0	0	24	31	19	0
44A West	2004	5	7	3	2	0	17	71	43	29
44A West	2007	5	8	1	0	0	14	63	13	0
44B North	2005	26	38	12	7	0	83	68	32	18
44B North	2007	31	33	3	10	0	77	94	9	30
44B South	2005	11	26	7	1	0	45	42	27	4
44B South	2007	8	15	3	0	0	26	53	20	0
45A	2006	33	51	14	4	0	102	65	27	8
45A	2007	28	55	12	8	0	103	51	22	15
45A	2008	20	50	14	8	0	92	40	28	16
45B	2006	15	41	8	7	0	71	37	20	17
45B	2007	20	37	13	7	0	77	54	35	19
45B	2008	19	39	10	4	0	72	49	26	10
45C	2006	29	35	11	7	0	82	83	31	20
45C	2007	35	46	14	10	5	110	76	30	22
45C	2008	27	43	15	11	0	96	63	35	26
46A	2005	21	30	7	8	0	66	70	23	27
46A	2008	15	25	11	5	0	56	60	44	20
46B	2005	43	61	10	14	1	129	70	16	23
46B	2008	57	99	15	28	1	200	58	15	28

5-Year: 2004-2008 Rocky Mountain Bighorn Sheep Survey Data

Unit	Year	Ram	Ewe	Lamb	Yearling	Unclassified	Total	Number per 100 Ewes		
								Rams	Lambs	Yearlings
1	2004	2	16	6	1	0	25	13	38	6
1	2005	3	21	4	5	0	33	14	19	24
1/27 North	2006	3	10	6	2	0	21	30	60	20
1/27 North	2007	5	0	0	0	0	5	–	–	–
1/27 North	2008	7	23	11	5	1	47	30	48	22
6A (West Clear Crk)	2008	9	22	12	0	0	43	41	55	0
23/24A	2008	3	16	5	0	0	24	19	31	0
27 (Bear Canyon)	2006	14	18	4	0	0	36	78	22	0
27 (Bear Canyon)	2007	6	16	5	2	0	29	38	31	13
27 (Bear Canyon)	2008	14	20	10	3	0	47	70	50	15
27 North	2004	34	59	22	7	0	122	58	37	12
27 North	2005	34	36	13	1	0	84	94	36	3
27 North	2006	13	28	9	0	2	52	46	32	0
27 North	2007	10	35	22	7	0	74	29	63	20
27 North	2008	25	61	24	10	0	120	41	39	16
27 South	2005	15	25	10	4	0	54	60	40	16
27 South	2008	14	15	3	7	0	39	93	20	47
27 South/28 North	2004	9	9	4	0	0	22	100	44	0
27 South/28 North	2005	37	73	22	7	38	177	51	30	10
27 South/28 North	2006	53	116	46	7	0	222	46	40	6
27 South/28 North	2007	38	64	35	8	0	145	59	55	13
27 South/28 North	2008	45	139	54	19	0	257	32	39	14

Historic Summary of Bighorn Sheep Hunts[1]

Year	Permits Authorized	1st Choice Applicants	Permits Issued	Hunters	Hunter Days	Total Harvest	Percent Success
1953	37	–	37	37	218	20	54.1
1954	20	–	20	19	103	12	63.2
1955	20	–	20	20	132	5	25.0
1956	20	–	20	19	112	6	31.6
1957	20	–	20	20	130	6	30.0
1958	40	–	40	37	–	18	48.6
1959	65	–	65	62	–	19	30.6
1960	80	–	80	80	–	24	30.0
1961	85	–	85	84	–	26	31.0
1962	90	–	90	89	–	27	30.3
1963	81	–	81	79	–	32	40.5
1964	78	–	78	76	–	25	32.9
1965	90	573	90	83	–	42	50.6
1966	84	601	84	84	–	35	41.7
1967	84	888	84	83	–	31	37.3
1968	81	1170	81	77	–	47	61.0
1969	86	1376	86	84	–	42	50.0
1970	79	1540	79	76	–	39	51.3
1971	82	1658	82	79	–	29	36.7
1972	71	1454	71	71	–	34	47.9
1973	65	1397	65	62	–	37	59.7
1974	57	1361	57	55	–	36	65.5
1975	54	1203	54	51	391	30	58.8
1976	55	1461	55	55	344	40	72.7
1977	51	1630	51	51	331	44	86.3
1978	52	1842	52	48	235	39	81.3
1979	52	1937	52	52	341	41	78.8
1980	50	2230	50	50	343	39	78.0
1981	45	2635	45	43	293	34	79.1
1982	42	2585	42	42	224	36	85.7
1983	48	2159	48	47	233	44	93.6
1984	55	2259	55	55	349	51	92.7
1985	56	2461	56	56	306	52	92.9
1986	65	2699	65	64	358	56	87.5
1987	72	3065	72	72	370	68	94.4
1988	78	3281	78	78	361	75	96.2
1989	82	3693	82	81	442	74	91.4
1990	78	3734	78	77	425	68	88.3
1991	85	4174	85	84	497	78	92.9
1992	82	4407	83	83	441	74	89.2
1993	99	4946	99	99	501	92	92.9
1994	112	5673	112	109	580	100	91.7
1995	113	6256	114	114	622	109	95.6
1996	108	6843	108	108	754	100	92.6
1997	99	7077	99	99	721	92	92.9
1998	109	7790	109	109	907	98	89.9
1999	111	8408	111	110	745	104	94.5
2000	105	8471	106	106	691	101	95.3
2001	105	8767	105	104	748	96	92.3
2002	104	13013	105	101	674	92	91.1
2003	99	16049	99	95	764	87	91.6
2004	84	18927	84	84	663	68	80.9
2005	82	11266	82	81	681	73	90.1
2006	96	16332	96	95	673	87	91.6
2007	99	10930	99	97	698	92	94.8
2008	93	9017	94	94	702	86	91.5

[1] Excluding Indian Reservation hunts; including raffle and auction tags.

5-Year: 2004-2008 Harvest

Unit	Year	Dates	Permits Authorized	1st Choice Applicants	2nd Choice Applicants	Permits Issued	Draw Odds	Hunters	Hunter Days	Harvest	Hunt Success
Auction	2004	–	1	0	0	0	–	1	9	1	100
Auction	2005	–	1	0	0	0	–	1	3	1	100
Auction (Desert)	2006	–	1	0	0	1	–	1	16	1	100
Auction (Rocky Mtn)	2006	–	1	0	0	1	–	1	14	1	100
Auction (Desert)	2007	–	1	0	0	1	–	1	12	1	100
Auction (Rocky Mtn)	2007	–	1	0	0	1	–	1	8	1	100
Auction (Desert)	2008	–	1	0	0	1	–	1	3	1	100
Auction (Rocky Mtn)	2008	–	1	0	0	1	–	1	30	1	100
Raffle	2004	–	1	0	0	0	–	1	4	1	100
Raffle	2005	–	1	0	0	0	–	1	21	1	100
Raffle	2006	–	1	0	0	1	–	1	7	1	100
Raffle	2007	–	1	0	0	1	–	1	7	1	100
Raffle	2008	–	1	0	0	1	–	1	6	1	100
1/27N Black River	2004	11/01-11/30	1	746	365	1	0.1	1	6	1	100
1/27N Black River	2005	11/01-11/30	1	404	262	1	0.2	1	4	1	100
1/27N Black River	2006	11/01-11/30	1	448	320	1	0.2	1	10	1	100
1/27N Black River	2007	11/01-11/30	1	240	154	1	0.0	1	16	1	100
1/27N Black River	2008	11/01-11/30	1	202	137	1	0.5	1	7	1	100
9/10	2005	10/01-12/31	1	71	23	1	1.4	0	0	0	–
9/10	2006	10/01-12/31	1	159	45	1	0.6	1	10	1	100
9/10	2007	10/01-12/31	1	54	25	1	0.0	1	14	0	0
9/10	2008	10/01-12/31	1	41	18	1	2.4	1	15	0	0
10	2004	10/01-12/31	1	331	113	1	0.3	1	14	0	0
12A/12B West	2005	12/01-12/31	2	142	276	2	0.7	2	34	2	100
12A/12B West	2006	12/01-12/31	2	177	163	2	1.1	2	38	1	50
12A/12B West	2007	12/01-12/31	3	134	235	3	0.7	3	29	1	33
12A/12B West	2008	12/01-12/31	2	53	49	2	3.8	2	4	2	100
12A/12BW/13A	2004	12/01-12/31	2	289	383	2	0.3	2	24	1	50
12B East	2004	12/01-12/31	2	509	371	2	0.4	2	6	2	100
12B East	2005	12/01-12/31	2	211	93	2	0.5	2	10	2	100
12B East	2006	12/01-12/31	1	126	97	1	0.0	1	7	1	100
12B East	2007	12/01-12/31	1	71	53	1	0.0	1	7	1	100
12B East	2008	12/01-12/31	1	44	30	1	2.3	1	19	1	100
13A	2004	12/01-12/31	2	402	352	2	0.2	2	28	2	100
13A	2005	12/01-12/31	2	290	376	2	0.0	2	60	2	100
13A	2006	12/01-12/31	3	678	502	3	0.3	3	9	3	100
13A	2007	12/01-12/31	5	506	450	5	0.6	5	33	5	100
13A	2008	12/01-12/31	3	256	248	3	0.4	3	21	3	100
13B North	2004	12/01-12/31	3	697	412	3	0.4	3	8	3	100
13B North	2005	12/01-12/31	4	602	515	4	0.5	4	78	3	75
13B North	2006	12/01-12/31	6	1907	1541	6	0.3	6	32	6	100
13B North	2007	12/01-12/31	5	1030	578	5	0.4	5	40	5	100
13B North	2008	12/01-12/31	4	722	340	4	0.6	4	12	4	100
13B South	2004	12/01-12/31	1	79	127	1	0.0	1	7	1	100
13B South	2005	12/01-12/31	1	24	49	1	0.0	1	17	1	100
13B South	2006	12/01-12/31	1	50	106	1	2.0	1	10	0	0
13B South	2007	12/01-12/31	1	24	56	1	4.2	1	7	0	0
13B South	2008	12/01-12/31	1	17	29	1	5.9	1	14	0	0
15A/15B East	2004	12/01-12/31	2	197	165	2	0.5	2	16	2	100
15A/15B East	2005	12/01-12/31	1	52	55	1	0.0	1	7	1	100
15A/15B East	2006	12/01-12/31	1	94	81	1	1.1	1	4	1	100
15A/15B East	2007	12/01-12/31	1	32	56	1	3.1	1	7	1	100
15A/15B East	2008	12/01-12/31	1	24	29	1	4.2	1	2	1	100
15B West	2004	12/01-12/31	4	994	853	4	0.2	4	26	3	75
15B West	2005	12/01-12/31	4	426	419	4	0.9	4	12	4	100
15B West	2006	12/01-12/31	3	385	532	3	0.8	3	21	3	100
15B West	2007	12/01-12/31	3	218	211	3	0.9	3	11	3	100
15B West	2008	12/01-12/31	3	190	178	3	1.6	3	19	3	100
15C North	2004	12/01-12/31	3	490	660	3	0.6	3	12	3	100
15C North	2005	12/01-12/31	2	213	267	2	0.9	2	19	2	100
15C North	2006	12/01-12/31	2	240	314	2	0.4	2	8	2	100
15C North	2007	12/01-12/31	2	100	146	2	1.0	2	9	2	100
15C North	2008	12/01-12/31	3	162	219	3	0.6	3	20	3	100
15C South	2004	12/01-12/31	1	115	246	1	0.0	1	11	1	100

5-Year: 2004-2008 Harvest (continued)

Unit	Year	Dates	Permits Authorized	1st Choice Applicants	2nd Choice Applicants	Permits Issued	Draw Odds	Hunters	Hunter Days	Harvest	Hunt Success
15C South	2005	12/01-12/31	1	60	152	1	0.0	1	1	1	100
15C South	2006	12/01-12/31	1	79	172	1	1.3	1	18	0	0
15C South	2007	12/01-12/31	1	21	84	1	0.0	1	3	1	100
15C South	2008	12/01-12/31	1	16	48	1	6.3	1	9	1	100
15D	2004	12/01-12/31	5	1591	860	5	0.3	5	22	5	100
15D	2005	12/01-12/31	5	775	641	5	0.6	5	24	5	100
15D	2006	12/01-12/31	7	1277	881	7	0.4	7	61	7	100
15D	2007	12/01-12/31	5	646	352	5	0.6	5	23	5	100
15D	2008	12/01-12/31	3	263	222	3	0.8	3	29	3	100
16A	2004	12/01-12/31	3	158	234	3	0.6	3	18	3	100
16A	2005	12/01-12/31	2	102	99	2	2.0	2	3	2	100
16A	2006	12/01-12/31	1	79	72	1	1.3	1	4	1	100
16A	2007	12/01-12/31	1	37	41	1	2.7	1	21	1	100
16A	2008	12/01-12/31	1	25	38	1	0.0	1	20	1	100
16B	2004	12/01-12/31	1	82	66	1	1.2	1	1	1	100
16B	2005	12/01-12/31	1	46	52	1	2.2	1	9	1	100
16B	2006	12/01-12/31	1	71	77	1	1.4	1	1	1	100
16B	2007	12/01-12/31	2	89	112	2	1.1	2	7	2	100
16B	2008	12/01-12/31	2	112	116	2	0.0	2	5	2	100
22	2004	12/01-12/31	1	674	345	1	0.1	1	30	1	100
22	2005	12/01-12/31	1	401	239	1	0.0	1	2	1	100
22	2006	12/01-12/31	1	489	306	1	0.0	1	3	1	100
22	2007	12/01-12/31	1	267	140	1	0.0	1	5	1	100
22	2008	12/01-12/31	1	261	130	1	0.4	1	3	1	100
23/24A	2006	12/01-12/31	1	388	197	1	0.3	1	21	1	100
23/24A	2007	12/01-12/31	1	181	104	1	0.6	1	28	0	0
23/24A	2008	12/01-12/31	1	143	68	1	0.7	1	29	0	0
24B	2004	12/01-12/31	1	406	339	1	0.2	1	6	1	100
24B	2005	12/01-12/31	1	304	245	1	0.3	1	11	1	100
24B	2006	12/01-12/31	1	372	286	1	0.3	1	7	1	100
24B North	2007	12/01-12/31	1	120	138	1	0.8	1	6	1	100
24B North	2008	12/01-12/31	1	138	169	1	0.0	1	2	1	100
24B South	2007	12/01-12/31	1	149	121	1	0.7	1	4	1	100
24B South	2008	12/01-12/31	1	136	128	1	0.7	1	8	1	100
27 Bear Canyon	2006	12/01-12/31	1	106	189	1	0.9	0	0	0	–
27 Bear Canyon	2007	12/01-12/31	1	83	106	1	0.0	1	6	1	100
27 Bear Canyon	2008	12/01-12/31	1	71	100	1	0.0	1	5	1	100
27N Foote Creek	2004	12/01-12/31	3	1383	634	3	0.2	3	6	3	100
27N Foote Creek	2005	12/01-12/31	4	1075	609	4	0.4	4	13	4	100
27N Foote Creek	2006	12/01-12/31	5	1308	673	5	0.3	5	31	3	60
27N Foote Creek	2007	12/01-12/31	5	788	468	5	0.4	5	20	5	100
27N Foote Creek	2008	12/01-12/31	3	618	379	3	0.5	3	14	3	100
27S/28N (early)	2004	12/01-12/15	2	838	1003	2	0.1	2	11	2	100
27S/28N (early)	2005	12/01-12/15	2	275	379	2	0.4	2	9	2	100
27S/28N (early)	2006	12/01-12/15	3	693	807	3	0.3	3	13	3	100
27S/28N (early)	2007	12/01-12/15	3	504	611	3	0.6	3	8	3	100
27S/28N (early)	2008	12/01-12/15	3	520	522	3	0.2	3	9	3	100
27S/28N (late)	2004	12/16-12/31	2	314	1050	2	0.3	2	23	2	100
27S/28N (late)	2005	12/16-12/31	2	334	791	2	0.3	2	10	2	100
27S/28N (late)	2006	12/16-12/31	3	197	965	3	0.0	3	5	3	100
27S/28N (late)	2007	12/16-12/31	3	226	749	3	0.4	3	3	3	100
27S/28N (late)	2008	12/16-12/31	3	189	677	3	0.0	3	16	3	100
28	2008	12/01-12/31	1	118	83	1	0.8	1	9	1	100
31/32	2004	12/01-12/31	1	958	238	1	0.1	1	2	1	100
31/32	2005	12/01-12/31	1	566	178	1	0.2	1	22	1	100
31/32	2006	12/01-12/31	1	694	283	1	0.1	1	3	1	100
31/32	2007	12/01-12/31	2	1169	312	2	0.2	2	26	2	100
31/32	2008	12/01-12/31	2	999	239	2	0.2	2	6	2	100
37A	2006	12/01-12/31	1	234	116	1	0.4	1	7	1	100
37A	2007	12/01-12/31	2	811	563	2	0.0	2	13	2	100
37A	2008	12/01-12/31	2	317	356	2	0.6	2	3	2	100
39 East	2004	12/01-12/31	1	120	116	1	0.0	1	21	0	0
39 East	2005	12/01-12/31	1	69	85	1	0.0	1	24	0	0
39 East	2006	12/01-12/31	1	78	77	1	0.0	1	9	1	100

5-Year: 2004-2008 Harvest (continued)

Unit	Year	Dates	Permits Authorized	1st Choice Applicants	2nd Choice Applicants	Permits Issued	Draw Odds	Hunters	Hunter Days	Harvest	Hunt Success
39 West	2004	12/01-12/31	2	322	317	2	0.3	2	8	2	100
39 West	2005	12/01-12/31	2	235	190	2	0.4	2	12	2	100
39 West	2006	12/01-12/31	2	293	774	2	0.0	2	6	2	100
39 West	2007	12/01-12/31	2	189	195	2	0.5	2	9	2	100
39 West	2008	12/01-12/31	2	184	215	2	0.0	2	24	2	100
40A North	2004	12/01-12/31	1	57	64	1	0.0	1	19	1	100
40A South	2004	12/01-12/31	1	71	53	1	1.4	1	7	1	100
40A	2005	12/01-12/31	1	68	49	1	1.5	1	17	0	0
40A	2006	12/01-12/31	1	53	44	1	1.9	1	3	1	100
40A	2007	12/01-12/31	1	68	53	1	1.5	1	19	1	100
40BW Gila Mts	2004	12/01-12/31	2	86	96	2	1.2	2	42	1	50
40BW Gila Mts	2005	12/01-12/31	2	43	125	2	2.3	2	36	1	50
40BW Gila Mts	2006	12/01-12/31	2	60	110	2	0.0	2	5	2	100
40BW Gila Mts	2007	12/01-12/31	3	66	178	3	1.5	3	27	3	100
40BW Gila Mts	2008	12/01-12/31	3	207	188	3	1.0	3	26	2	67
40BW Mohawk/Copper	2004	12/01-12/31	2	266	353	2	0.4	2	8	2	100
40BW Mohawk/Copper	2005	12/01-12/31	1	0	1	1	–	1	1	1	100
40BW Mohawk/Copper	2006	12/01-12/31	2	69	113	2	1.4	2	7	2	100
40BW Mohawk/Copper	2007	12/01-12/31	2	118	231	2	0.8	2	33	1	50
40BW Mohawk/Copper	2008	12/01-12/31	3	106	285	3	1.9	3	58	1	33
40BW Tinajas Altas	2004	12/01-12/31	1	95	83	1	1.1	1	11	0	0
40BW Tinajas Altas	2005	12/01-12/31	1	43	43	1	2.3	1	9	1	100
40BW Tinajas Altas	2006	12/01-12/31	1	41	40	1	0.0	1	4	1	100
40BW Tinajas Altas	2007	12/01-12/31	1	19	42	1	5.3	1	13	1	100
40BW Tinajas Altas	2008	12/01-12/31	1	26	24	1	3.8	1	2	1	100
41 East	2004	12/01-12/31	3	2272	456	3	0.1	3	27	3	100
41 East	2005	12/01-12/31	3	1277	340	3	0.2	3	37	3	100
41 East	2006	12/01-12/31	4	969	497	4	0.2	4	25	4	100
41 East	2007	12/01-12/31	2	328	426	2	0.6	2	34	2	100
41 East	2008	12/01-12/31	3	359	296	3	0.6	3	12	3	100
41 West	2004	12/01-12/31	2	199	350	2	1.0	2	4	2	100
41 West	2005	12/01-12/31	2	186	262	2	0.5	2	3	2	100
41 West	2006	12/01-12/31	2	325	432	2	0.3	2	11	2	100
41 West	2007	12/01-12/31	3	337	383	3	0.3	3	52	2	67
41 West	2008	12/01-12/31	3	287	301	3	0.3	3	19	3	100
43A	2004	12/01-12/31	1	127	76	1	0.8	1	1	0	0
43A	2005	12/01-12/31	1	55	34	1	1.8	1	4	1	100
43A	2006	12/01-12/31	1	62	68	1	0.0	1	5	1	100
43A	2007	12/01-12/31	1	28	31	1	0.0	1	2	1	100
43A	2008	12/01-12/31	1	27	44	1	0.0	1	3	1	100
43B	2004	12/01-12/31	3	330	317	3	0.9	3	36	3	100
43B	2005	12/01-12/31	4	377	321	4	0.8	4	11	4	100
43B	2006	12/01-12/31	5	486	610	5	0.6	5	38	5	100
43B	2007	12/01-12/31	5	536	384	5	0.9	5	16	5	100
43B	2008	12/01-12/31	5	335	343	5	1.5	5	32	5	100
44A West	2004	12/01-12/31	1	78	65	1	1.3	1	12	1	100
44A West	2005	12/01-12/31	1	93	65	1	0.0	1	3	1	100
44A West	2006	12/01-12/31	1	92	77	1	1.1	1	3	1	100
44A East	2007	12/01-12/31	1	63	72	1	0.0	1	9	1	100
44A East	2008	12/01-12/31	1	37	40	1	2.7	1	14	1	100
44A West	2007	12/01-12/31	1	52	45	1	1.9	1	5	1	100
44A West	2008	12/01-12/31	1	22	38	1	4.5	1	8	1	100
44B North	2004	12/01-12/31	2	353	975	2	0.3	2	2	1	50
44B North	2005	12/01-12/31	2	498	694	2	0.4	2	4	2	100
44B North	2006	12/01-12/31	2	385	316	2	0.5	2	3	2	100
44B North	2007	12/01-12/31	2	242	168	2	0.8	2	9	2	100
44B North	2008	12/01-12/31	3	548	430	3	0.5	3	15	3	100
44B South	2004	12/01-12/31	2	303	436	2	0.7	2	3	2	100
44B South	2005	12/01-12/31	2	213	480	2	0.5	2	14	2	100
44B South	2006	12/01-12/31	2	1234	543	2	0.2	2	28	1	50

5-Year: 2004-2008 Harvest (continued)

Unit	Year	Dates	Permits Authorized	1st Choice Applicants	2nd Choice Applicants	Permits Issued	Draw Odds	Hunters	Hunter Days	Harvest	Hunt Success
44B South	2007	12/01-12/31	2	101	177	2	1.0	2	7	2	100
44B South	2008	12/01-12/31	1	56	87	1	0.0	1	10	1	100
45A	2004	12/01-12/31	4	920	1119	4	0.3	4	23	2	50
45A	2005	12/01-12/31	4	417	459	4	1.0	4	26	3	75
45A	2006	12/01-12/31	4	678	571	4	0.4	4	29	4	100
45A	2007	12/01-12/31	5	580	485	5	0.7	5	34	5	100
45A	2008	12/01-12/31	3	174	253	3	1.7	3	26	3	100
45B	2004	12/01-12/31	2	196	403	2	0.5	2	39	0	0
45B	2005	12/01-12/31	2	89	315	2	0.0	2	5	2	100
45B	2006	12/01-12/31	2	110	297	2	0.9	2	2	2	100
45B	2007	12/01-12/31	1	34	99	1	2.9	1	3	1	100
45B	2008	12/01-12/31	2	110	281	2	0.0	2	11	2	100
45C	2004	12/01-12/31	5	1473	1282	5	0.3	5	47	4	80
45C	2005	12/01-12/31	5	938	705	5	0.5	5	61	5	100
45C	2006	12/01-12/31	5	693	635	5	0.6	5	64	4	80
45C	2007	12/01-12/31	4	291	505	4	0.3	4	46	4	100
45C	2008	12/01-12/31	4	527	495	4	0.8	4	23	4	100
46A	2004	12/01-12/31	2	113	176	2	0.0	2	22	1	50
46A	2005	12/01-12/31	2	62	70	2	1.6	2	14	2	100
46A	2006	12/01-12/31	2	113	185	2	1.8	2	25	1	50
46A	2007	12/01-12/31	2	55	107	2	1.8	2	6	2	100
46A	2008	12/01-12/31	2	146	179	2	1.4	3	33	2	67
46B	2004	12/01-12/31	4	293	376	4	1.0	4	41	2	50
46B	2005	12/01-12/31	4	230	285	4	1.3	4	31	2	50
46B	2006	12/01-12/31	6	340	426	6	1.5	6	46	6	100
46B	2007	12/01-12/31	6	323	274	6	1.5	6	29	6	100
46B	2008	12/01-12/31	6	229	299	6	1.3	6	47	5	83

Successful Hunters and the Measurements of their Bighorn Sheep – 2008 Season

		Curl (Inches)		Base (Inches)					
Hunt Number	Unit	Left	Right	Left	Right	Spread	Age	Arizona Score	Green Score
Special Tag	15D	32	32-6/8	14-1/8	14-4/8	23-5/8	5.0	93-3/8	152-6/8
Special Tag	27	36-7/8	34-4/8	13-3/8	13-3/8	20-1/8	8.0	98-1/8	161-5/8
Special Tag	32	32-4/8	37	15-1/8	15-1/8	23-7/8	6.0	99-6/8	155-2/8
6002	12A/12BW	33-3/8	33-2/8	14-5/8	14-4/8	21-3/8	7.0	95-6/8	162-1/8
6002	12A/12BW	28-6/8	28-3/8	15	15	22	7.0	87-1/8	155-1/8
6003	12BE	30-4/8	31-5/8	14-4/8	14	23-4/8	8.0	90-5/8	154-5/8
6004	13A	29-5/8	29	14-2/8	14-2/8	20	8.0	87-1/8	158-3/8
6004	13A	26-2/8	27-6/8	14-7/8	14-4/8	26	9.0	83-3/8	147-4/8
6004	13A	27-3/8	27-2/8	12	12	19-3/8	6.0	78-5/8	128-1/8
6005	13BN	32-7/8	29-3/8	14-6/8	14-6/8	20-5/8	7.0	91-6/8	150-6/8
6005	13BN	29	29-4/8	13-2/8	13-2/8	19	6.0	85	142-2/8
6005	13BN	33-2/8	33-2/8	13-2/8	13-6/8	19	10.0	93-4/8	158-4/8
6005	13BN	27	27-3/8	14-1/8	14-2/8	20-3/8	4.0	82-6/8	131-3/8
6007	15A/15B	26-6/8	29-3/8	14-1/8	14-1/8	18	9.0	84-3/8	141-3/8
6008	15BW	33	32	13-3/8	14-2/8	27-3/8	8.0	93-5/8	155-2/8
6008	15BW	26-3/8	25-1/8	12-6/8	12-4/8	17-2/8	5.0	76-6/8	117-4/8
6008	15BW	30	29	15	14-7/8	20-1/8	7.0	88-7/8	142-4/8
6009	15CN	29-1/8	28-2/8	15-1/8	15-2/8	22-2/8	5.0	87-6/8	145-7/8
6009	15CN	30-2/8	31	15-1/8	15-2/8	24-1/8	6.0	91-5/8	160-4/8
6009	15CN	33-6/8	29-5/8	14	13-7/8	26-5/8	6.0	91-2/8	148-3/8
6010	15CS	29	31-4/8	13-3/8	13-3/8	22-3/8	8.0	87-2/8	148-4/8
6011	15D	34-1/8	31-4/8	15-2/8	15-2/8	23-6/8	5.0	96-1/8	151-1/8
6011	15D	36	36-4/8	15-6/8	15-4/8	30	8.0	103-6/8	169-4/8
6011	15D	31-1/8	35-3/8	14-3/8	14-5/8	23-2/8	8.0	95-4/8	155-4/8
6012	16A	32	33	15-6/8	15-5/8	21-3/8	6.0	96-3/8	163-6/8
6013	16B	25-4/8	27-2/8	13-4/8	14	19-4/8	4.0	80-2/8	125-6/8
6013	16B	23	24-4/8	12	12	21	3.0	71-4/8	114
6014	22	33-5/8	34-2/8	14	13-4/8	23	11.0	95-3/8	163-5/8
6015	24BN	34-1/8	34-5/8	14-5/8	14-5/8	21-2/8	–	98	160
6016	24BS	33-4/8	34-1/8	14-7/8	15	20-4/8	7.0	97-4/8	167-5/8
6017	28S	31-4/8	31-4/8	16-5/8	15-6/8	21-4/8	6.0	95-3/8	165-6/8
6018	31/32	36	35-3/8	15	15	20-7/8	12.0	101-3/8	171-3/8
6018	31/32	34-5/8	32-5/8	13-3/8	13-2/8	22-1/8	11.0	93-7/8	155
6019	37A	32	33-4/8	15	14-7/8	20	7.0	95-3/8	164
6019	37A	35-4/8	33	15-1/8	15-1/8	20-4/8	8.5	98-6/8	168-6/8
6020	39W	35-6/8	36-4/8	13-7/8	14	20-5/8	8.0	100-1/8	169-6/8
6020	39W	33-4/8	33	14	13-2/8	20-2/8	11.0	93-6/8	153-6/8
6021	40BW	31	30	13-2/8	12-6/8	17-5/8	11.0	87	147-6/8
6021	40BW	31-7/8	31-5/8	14-6/8	14-7/8	21	10.0	93-1/8	159-4/8
6022	40BW	29-3/8	30-2/8	14-1/8	14-2/8	17-1/8	6.0	88	142-3/8
6023	40BW	32	32-6/8	14-3/8	14-6/8	20-3/8	8.0	93-7/8	158
6024	41E	33-6/8	34	14-6/8	14-5/8	21-2/8	10.0	97-1/8	168-2/8
6024	41E	34-4/8	34-5/8	15	15-2/8	23-4/8	5.0	99-3/8	157-3/8
6024	41E	32	35-3/8	14-7/8	14-7/8	22-7/8	7.0	97-1/8	163-1/8
6025	41W	31-4/8	30-2/8	14	14	18	8.5	89-6/8	152-2/8
6025	41W	30-2/8	32-6/8	14-1/8	14-4/8	23-6/8	6.0	91-5/8	149-2/8
6025	41W	27-4/8	29-6/8	14-1/8	14	20-6/8	6.0	85-3/8	144-6/8
6026	43A	26-4/8	31	14-6/8	14-6/8	24-4/8	7.0	87	145-2/8
6027	43B	33-1/8	32-5/8	14-4/8	14-3/8	21-5/8	8.0	94-5/8	160
6027	43B	30-7/8	32-3/8	15	15-1/8	21-4/8	6.0	93-3/8	155
6027	43B	34-2/8	34-2/8	14-6/8	14-6/8	22-4/8	9.0	98	165-6/8
6027	43B	33-1/8	29-6/8	14-4/8	14-4/8	19-5/8	7.0	91-7/8	154-5/8
6027	43B	30-6/8	30-6/8	13-3/8	13-5/8	19-6/8	8.0	88-4/8	151-6/8
6028	44AE	29-7/8	31	14	14	21	7.0	88-7/8	151-7/8
6029	44AW	33-7/8	34-5/8	16-2/8	15-7/8	23-7/8	7.0	100-5/8	169-2/8
6030	44BN	36	36	15-4/8	15-5/8	21-2/8	10.0	103-1/8	178
6030	44BN	30-2/8	32-5/8	13-6/8	14-2/8	21-5/8	8.0	90-7/8	156-7/8
6030	44BN	35-7/8	36-6/8	15	15	23-4/8	8.0	102-5/8	171-3/8
6031	44BS	34	32-1/8	15	15-1/8	20-4/8	9.0	96-2/8	169-5/8
6032	45A	29-6/8	32-7/8	14-1/8	14-3/8	21-4/8	7.0	91-1/8	156-1/8
6032	45A	18-1/8	18-2/8	11	11-2/8	18-2/8	2.0	58-5/8	94-3/8
6032	45A	31-2/8	30-6/8	14-1/8	14-2/8	20-4/8	4.0	90-3/8	150-6/8

Successful Hunters and the Measurements of their Bighorn Sheep – 2008 Season (continued)

		Curl (Inches)		Base (Inches)					
Hunt Number	Unit	Left	Right	Left	Right	Spread	Age	Arizona Score	Green Score
6033	45B	31-4/8	28-6/8	13	13	20-7/8	8.0	86-2/8	151
6033	45B	26-2/8	27-4/8	13-7/8	13-6/8	20-2/8	6.0	81-3/8	138
6034	45C	34-2/8	31-6/8	15-6/8	15-6/8	22-7/8	8.0	97-4/8	163
6034	45C	33-5/8	34	14-1/8	13-6/8	19	6.0	95-4/8	158-1/8
6034	45C	32	30	14-2/8	14-2/8	23	8.0	90-4/8	154
6034	45C	29-2/8	28-5/8	13-4/8	13-2/8	19-1/8	6.0	84-5/8	129-7/8
6035	46A	32-2/8	32-7/8	14-6/8	14-4/8	20-7/8	10.0	94-3/8	156-5/8
6035	46A	38-6/8	38-2/8	16-2/8	16-2/8	21-1/8	11.0	109-4/8	182-2/8
6036	46B	29-2/8	28-4/8	16	15-7/8	20-4/8	5.0	89-5/8	151-6/8
6036	46B	27-1/8	27	14-2/8	14-6/8	18-3/8	4.0	83-1/8	130-7/8
6036	46B	33-5/8	34	14-6/8	14-6/8	23-1/8	11.0	97-1/8	167-5/8
6036	46B	31-4/8	29-6/8	15	15	19-6/8	8.0	91-2/8	157-4/8
6036	46B	29-4/8	29-6/8	14	14	19-4/8	6.0	87-2/8	151-2/8
6051	01/27N	34	33-5/8	15-2/8	15-1/8	23-6/8	7.0	98	166-7/8
6053	27BC	35-7/8	35-7/8	15	15	20-7/8	5.0	101-6/8	171-6/8
6054	27/28	36	33-4/8	14-6/8	14-6/8	21-3/8	7.0	99	166-6/8
6054	27/28	35-2/8	35-5/8	15	15-1/8	21-2/8	8.0	101	172-3/8
6054	27/28	35-4/8	34	14-3/8	14-7/8	23-7/8	8.0	98-6/8	165-4/8
6055	27/28	37-3/8	37-6/8	14-6/8	14-7/8	23-7/8	8.0	104-6/8	174-3/8
6055	27/28	37-7/8	37-4/8	13-6/8	13-6/8	19-4/8	8.0	102-7/8	171-1/8
6055	27/28	39-2/8	35-6/8	16-2/8	16-1/8	21-4/8	8.0	107-3/8	183-2/8
6056	27N	34-4/8	36	16	16	25	8.0	102-4/8	174-4/8
6056	27N	34-6/8	31-7/8	16-1/8	15-5/8	21-4/8	7.0	98-3/8	167-3/8
6056	27N	34-7/8	39	15-4/8	15-4/8	21	8.0	104-7/8	179-1/8
Total	Minimum	18-1/8	18-2/8	11	11-2/8	17-1/8	2.0	58-5/8	94-3/8
Total	Maximum	39-2/8	39	16-5/8	16-2/8	30	12.0	109-4/8	183-2/8
Total	Average	32	32	14-4/8	14-4/8	21-4/8	7.4	92-7/8	155-6/8

Bighorn Sheep Horn Measurements

Year	Arizona Score[1]			Outside Curl	Average Basal Circumference	Average B&C Green Score
	Largest Head	Smallest Head	Average Head			
1953	102-1/8	56-2/8	85-2/8	29-1/8	13-4/8	–
1954	97	65-5/8	83-5/8	28-4/8	13-2/8	–
1955	93-6/8	66	84-6/8	28-2/8	14	–
1956	93-4/8	65-2/8	80	27-3/8	12-5/8	–
1957	82	60-2/8	73-5/8	24-6/8	12-1/8	–
1958	102-6/8	74	86-3/8	29-3/8	13-7/8	–
1959	100-2/8	63-4/8	84	28-4/8	13-4/8	–
1960	100-2/8	68-4/8	86-6/8	29-4/8	13-7/8	–
1961	110-5/8	63-2/8	84-1/8	28-5/8	13-3/8	–
1962	101-2/8	63-6/8	83-7/8	28-3/8	13-4/8	–
1963	105-4/8	60	82-2/8	27-6/8	13-3/8	–
1964	102-2/8	72-4/8	88-3/8	30-1/8	14-1/8	–
1965	113-1/8	71-4/8	89	30-1/8	14-3/8	–
1966	108-6/8	74	91-2/8	31	14-5/8	–
1967	104-5/8	76-2/8	91-4/8	31	14-6/8	–
1968	103-5/8	68-2/8	89-1/8	30	14-4/8	–
1969	106-2/8	71	89-2/8	30-2/8	14-3/8	–
1970	104-6/8	76-2/8	89-5/8	30-4/8	14-2/8	–
1971	103-6/8	70-2/8	87-3/8	29-3/8	14-2/8	–
1972	106-2/8	72-1/8	89	30	14-4/8	147-4/8
1973	103-5/8	72-7/8	89-5/8	30-5/8	14-2/8	150-3/8
1974	111-2/8	68-3/8	91	31-1/8	14-3/8	152-2/8
1975	106-6/8	74-4/8	89	30-4/8	14	148-7/8
1976	104-4/8	74-7/8	91-6/8	31-2/8	14-5/8	154-6/8
1977	104-1/8	75	91-5/8	31-3/8	14-3/8	153-6/8
1978	108	74-3/8	92-3/8	31-4/8	14-5/8	155-7/8
1979	108-4/8	71-3/8	91-3/8	31-2/8	14-4/8	153-1/8
1980	105	82-1/8	92-4/8	31-5/8	14-4/8	153-3/8
1981	110-3/8	82-6/8	94-3/8	32-2/8	15	160-2/8
1982	114-4/8	81-4/8	92-4/8	31-6/8	14-4/8	154-5/8
1983	112-3/8	71-5/8	93-3/8	32	14-5/8	156-7/8
1984	111-5/8	79-3/8	94	32-3/8	14-5/8	159-1/8
1985	107-6/8	74-4/8	92-6/8	31-7/8	14-4/8	156-2/8
1986	110-2/8	80-7/8	94-4/8	32-4/8	14-6/8	160-2/8
1987	110	77	94-3/8	32-5/8	14-5/8	159-4/8
1988	117-2/8	51-2/8	93-1/8	32	14-4/8	157-2/8
1989	103-4/8	78-6/8	92-6/8	31-7/8	14-3/8	157-4/8
1990	113-2/8	58-4/8	93-5/8	32-1/8	14-5/8	157-7/8
1991	107-2/8	67-1/8	92-7/8	31-6/8	14-5/8	157-5/8
1992	108-6/8	65	92-4/8	31-7/8	14-3/8	155-3/8
1993	112-3/8	69-2/8	92-4/8	31-7/8	14-3/8	156-3/8
1994	110-2/8	77-3/8	94-2/8	32-5/8	14-4/8	159-6/8
1995	110-3/8	77-2/8	92-5/8	31-7/8	14-3/8	156-3/8
1996	114-4/8	66-6/8	93	32-1/8	14-4/8	156-6/8
1997	108-3/8	69-2/8	92-1/8	31-6/8	14-4/8	156-5/8
1998	112	61-1/8	91-4/8	31-4/8	14-4/8	155-7/8
1999	109	63-6/8	91-6/8	31-5/8	14-4/8	156-7/8
2000	110-4/8	65-6/8	92-6/8	31-7/8	14-4/8	157-1/8
2001	107-6/8	73-6/8	91-6/8	31-5/8	14-3/8	155
2002	107-7/8	53-3/8	90-4/8	31-1/8	14-2/8	153-3/8
2003	108-6/8	66-7/8	89-3/8	30-5/8	14-2/8	151-6/8
2004	106-5/8	73-2/8	91-5/8	31-4/8	14-3/8	155-7/8
2005	104-3/8	69-2/8	90-6/8	31	14-2/8	152-3/8
2006	109-4/8	63	92-1/8	31-5/8	14-4/8	156-1/8
2007	113-5/8	74-3/8	93	32-1/8	14-3/8	156-5/8
2008	109-4/8	58-5/8	92-7/8	32	14-4/8	155-6/8

[1] Arizona score = sums of the 2 bases and the 2 curls.

2008 Bighorn Sheep Hunt Applications

Hunt No.	Unit	Permits Authorized	First Choice		Second Choice	
			Applicants	% Drawn	Applicants	% Drawn
6001	9/10	1	41	2.4	18	0.0
6002	12A/12B W	2	53	3.8	49	0.0
6003	12B E	1	44	2.3	30	0.0
6004	13A	3	256	0.4	248	0.8
6005	13B N	4	722	0.6	340	0.0
6006	13B S	1	17	5.9	29	0.0
6007	15A/15BE	1	24	4.2	29	0.0
6008	15B W	3	190	1.6	178	0.0
6009	15C N	3	162	0.6	219	0.9
6010	15C S	1	16	6.3	48	0.0
6011	15D	3	263	0.8	222	0.5
6012	16A	1	25	0.0	38	2.6
6013	16B	2	112	0.0	116	1.7
6014	22	1	261	0.4	130	0.0
6015	24B N	1	138	0.0	169	0.6
6016	24B S	1	136	0.7	128	0.0
6017	28	1	118	0.8	83	0.0
6018	31/32	2	999	0.2	239	0.0
6019	37A	2	317	0.6	356	0.0
6020	39 W	2	184	0.0	215	0.9
6021	40BW Gila Mtns	3	207	1.0	188	0.5
6022	40BW Mohawk/Copper	3	106	1.9	285	0.4
6023	40BW Tinajas Altas	1	26	3.8	24	0.0
6024	41 E	3	359	0.6	296	0.3
6025	41 W	3	287	0.3	301	0.7
6026	43A	1	27	0.0	44	2.3
6027	43B	5	335	1.5	343	0.0
6028	44A E	1	37	2.7	40	0.0
6029	44A W	1	22	4.5	38	0.0
6030	44B N	3	548	0.5	430	0.0
6031	44B S	1	56	0.0	87	1.1
6032	45A	3	174	1.7	253	0.0
6033	45B	2	110	0.0	281	0.7
6034	45C	4	527	0.8	495	0.0
6035	46A	2	146	1.4	179	0.0
6036	46B	6	229	1.3	299	1.0
6051	1/27N Black River	1	202	0.5	137	0.0
6052	23/24A	1	143	0.7	68	0.0
6053	27 Bear Canyon	1	71	0.0	100	1.0
6054	27S/28N E	3	520	0.2	522	0.4
6055	27S/28N L	3	189	0.0	677	0.4
6056	27N Foote Creek	3	618	0.5	379	0.0
6997	Auction	1	0	–	0	–
6998	Auction	1	0	–	0	–
6999	Raffle	1	0	–	0	–
TOTAL		93	9017	0.7	8350	0.3

One tag for hunt 6035 was issued by the Director after the draw. Total tags issued including the Auction and Raffle was 94.

Buffalo *(Bison bison)*

Natural History

Although these animals are not native to Arizona, American bison, more commonly known as buffalo, are found at two wildlife areas managed by the Arizona Game and Fish Department: Raymond Ranch Wildlife Area located east of Flagstaff, and House Rock Wildlife Area in House Rock Valley east of the North Kaibab National Forest. Approximately 250 buffalo inhabit the two areas, which are managed to provide both viewing and sport-hunting opportunities.

Buffalo are the largest living member of the cow family. Live adult weights range from 1,400 to 2,500 pounds for bulls and from 750 to 1,600 pounds for cows. Bulls have massive front quarters with a large hump above the shoulders covered with woolly hair up to 1.5 inches long that also covers the head and forelegs. This hair turns tan with age and is two to five times thicker than the hair on the hindquarters. The bull's head has a broad triangular appearance and possesses a beard or bell. Both bulls and cows possess horns, but the male's are much larger, attaining a length of up to 20 inches. Calves are reddish-tan at birth and change to brown or black in three months.

The senses of smell and hearing are acute, while the buffalo's eyesight is poor. Adult buffalo can run sprints

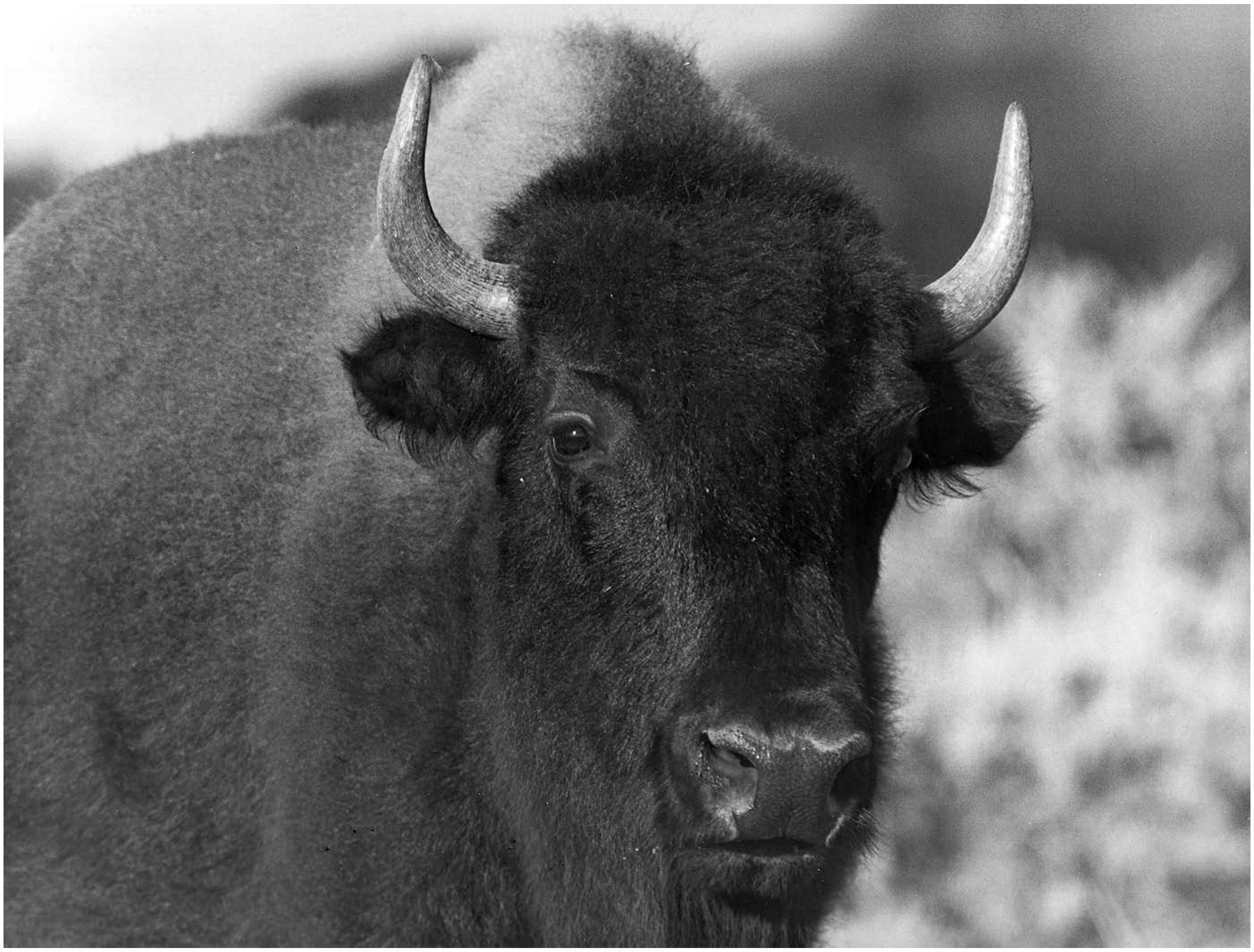

GEORGE ANDREJKO

of 35 mph for up to one-quarter mile and are capable of jumping over 6-foot-high fences. Buffalo are gregarious and often form large herds. Although the group composition of these herds changes constantly, the dominant animal is almost always a matriarchal cow. Adult buffalo eat approximately 35 pounds of forage per day, in general concentrating on the most abundant palatable forage, be it grasses, forbs, or browse. Buffalo may live as long as 28 years.

Breeding typically takes place from mid-July to early September. The bulls are polygamous, but do not maintain harems in the usual sense. Most of the breeding is done by mature bulls of five to eight years old. A bull can lose up to 300 pounds during the rut. Gestation ranges from 270 to 285 days, and typically a single calf is born in the spring from late April through May.

Numerous state and federal agencies, as well as private ranchers, have been trying to develop representative herds of free-ranging buffalo. Their goal is to maintain buffalo populations that provide recreational hunting, scientific research, and aesthetic uses with minimal management efforts. In these areas, hunting and live-animal sales are necessary to remove excess animals and keep the habitat within carrying capacity.

Hunt History

Public buffalo hunts have been held at House Rock Ranch since the 1920s. These buffalo, which were originally brought to Arizona by Charles Jesse "Buffalo" Jones, were sold to the state by Uncle Jimmie Owens after their "cattalo" experiment proved unsuccessful. When the number of buffalo was judged excessive for their Forest Service grazing lands in the mid-1940s, the Arizona Game and Fish Department moved some of them to the agency's newly acquired Raymond Ranch. Other buffalo were moved to Fort Huachuca, which the Department acquired after World War II. The tenure of these latter animals was short, however, as they had to be disposed of when the Fort was reactivated in the 1950s. Some were sold and sent to the state of Chihuahua, Mexico, and the remainder were removed through a public hunt.

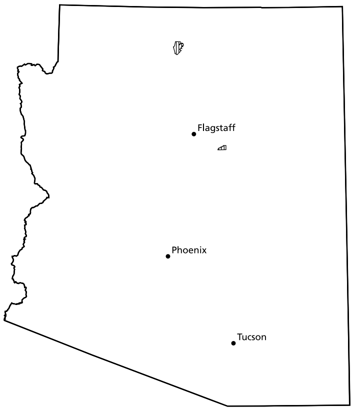

Buffalo distribution

The herds at House Rock and Raymond Ranch wildlife areas remained, however, and the Department set out to manage these herds on a sustained basis. A economic profit proved elusive, however, as it was impossible to sustain sufficient breeding stock without damaging the range. Moreover, the shooting of buffalo being driven out of a corral, while making economic sense, became increasingly difficult to justify from a sociological perspective. As a result, both herds were drastically reduced in the early 1970s by hunters who had to take their animals in the field. The management of the buffalo herds is now more in line with the carrying capacity of their respective ranges, with between 45 and 65 buffalo being harvested each year. A special permit has always been required for the taking of this species.

Buffalo Survey Data

Historic Summary of Buffalo Survey Data

Unit	Year	Bulls			Cows			Unclassified Calves	Total	Bulls/ 100 Cows[1]	Calves/ 100 Cows[1]
		Adults	Yearlings	Calves	Adults	Yearlings	Calves				
Statewide	1953[2]	66	111	–	168	–	–	–	345	39	–
	1954[2]	156	100	–	161	–	–	–	417	97	–
	1955[2]	–	–	–	–	–	–	–	–	–	–
	1956	37	86	53	103	–	–	–	279	34	51
	1957[3]	12	21	29	60	38	29	–	189	20	97
	1958[3]	38	28	25	69	27	25	–	212	55	85
	1959[3]	20	69	33	77	74	32	–	305	26	94
	1960[3]	36	18	25	85	19	25	–	208	42	59
	1961	75	24	30	131	25	30	–	315	57	46
	1962[3]	41	29	28	104	23	28	–	253	39	54
	1963[3]	52	25	28	107	25	28	–	265	49	52
	1964[3]	49	31	35	99	23	35	–	272	49	71
	1965[3]	51	32	35	115	31	35	–	299	44	61
	1966[3]	37	28	25	89	19	25	–	223	42	56
	1967[3]	43	22	28	97	22	26	–	238	44	56
	1968[3]	31	22	26	86	23	26	–	214	36	60
	1969[3]	36	27	30	91	27	30	–	241	40	66
	1970[3]	24	30	32	91	30	32	–	239	26	70
	1971[3]	37	21	22	80	21	22	–	203	46	55
	1972	47	30	30	108	30	30	–	275	44	56
	1973[3]	44	55	57	167	52	57	–	432	26	68
	1974	81	54	40	129	54	52	–	410	63	71
	1975	92	53	18	97	53	18	–	331	95	37
	1976	94	20	23	89	20	19	–	265	106	47
	1977	72	26	23	63	31	23	–	238	114	73
	1978	57	23	17	73	23	16	–	209	78	45
	1979	40	24	22	39	10	21	–	156	103	110
**From 1980 to present, data split by Wildlife Area**											
5B	1980	18	11	13	35	10	10	0	97	51	66
Raymond	1981	24	13	13	31	10	12	0	103	77	81
Wildlife Area	1982	20	13	10	29	12	7	0	91	69	59
	1983	26	10	8	29	7	9	0	89	90	59
	1984	16	8	15	34	10	10	0	93	47	74
	1985	15	14	17	39	10	12	0	107	38	74
	1986	12	16	15	37	12	15	0	107	32	81
	1987	16	15	16	34	15	13	0	109	47	85
	1988	16	16	14	37	13	17	0	113	43	84
	1989	15	14	9	40	17	21	0	116	38	75
	1990	10	9	14	42	21	17	0	113	24	74
	1991	10	14	12	43	17	15	0	111	23	63
	1992	14	12	14	43	15	16	0	114	33	70
	1993	14	14	17	41	16	17	0	119	34	83
	1994	12	17	17	39	17	14	0	116	31	79
	1995	10	15	12	40	18	18	0	113	25	75
	1996	11	0	18	43	0	13	0	85	26	72
	1997	4	0	11	41	3	12	0	71	10	56
	1998	3	8	14	43	12	12	0	92	7	60
	1999	7	13	14	41	14	14	0	103	17	68
	2000	5	14	9	37	14	13	0	92	14	59
	2001	13	10	0	39	8	0	20	90	33	51
	2002	18	12	0	40	9	0	21	100	45	53
	2003	20	11	0	38	11	0	24	104	53	63
	2004	10	10	0	32	9	0	14	75	31	44
	2005	6	3	0	27	–	0	14	50	22	52
	2006	5	8	0	24	–	0	14	51	21	58
	2007	4	4	0	27	7	0	14	56	24	41
	2008	4	0	–	19	4	–	12	39	21	63

[1] Based on adult animals only, yearlings excluded.
[2] Both sexes combined for yearlings and calves.
[3] Calf numbers are estimates.

Historic Summary of Buffalo Survey Data

Unit	Year	Bulls			Cows			Unclassified Calves	Total	Bulls/ 100 Cows[1]	Calves/ 100 Cows[1]
		Adults	Yearlings	Calves	Adults	Yearlings	Calves				
12	1980	14	11	10	45	11	15	0	106	31	56
House Rock	1981	18	10	10	34	15	10	0	97	53	59
Wildlife Area	1982	17	10	13	40	10	9	0	99	43	55
	1983	19	13	11	49	9	13	0	114	39	49
	1984	25	13	9	42	10	0	0	99	60	21
	1985	18	9	0	46	9	0	26	108	39	57
	1986	22	13	0	34	13	0	16	98	65	47
	1987	41	10	0	40	10	0	27	128	103	68
	1988	53	15	0	44	14	0	31	157	120	70
	1989	40	12	0	53	23	0	30	158	75	57
	1990	23	14	0	56	18	0	23	134	41	41
	1991	14	11	0	53	10	0	30	118	26	57
	1992	21	12	0	50	11	0	26	120	42	52
	1993	23	13	0	44	9	0	21	110	52	48
	1994	33	10	15	41	8	17	0	124	80	78
	1995	34	15	14	40	17	14	0	134	85	70
	1996	31	14	14	47	12	14	0	132	66	60
	1997	31	12	0	47	12	0	21	123	66	45
	1998	25	9	0	33	10	0	19	96	76	58
	1999	29	9	9	38	9	9	0	103	76	47
	2000	32	9	14	42	9	14	0	120	76	67
	2001	No Survey Conducted									
	2002	50	15	0	65	15	0	30	175	77	45
	2003	45	15	0	80	15	0	40	195	56	50
	2004	43	9	0	51	7	0	22	132	84	43
	2005	41	21	0	70	11	0	43	185	57	61
	2006	No Survey Conducted									
	2007	No Survey Conducted									
	2008	No Survey Conducted									

[1] Based on adult animals only, yearlings excluded.
[2] Both sexes combined for yearlings and calves.
[3] Calf numbers are estimates.

Buffalo Harvest Data

Historic Summary of Buffalo Hunts[1]

Year	1st Choice Applicants[2]	Permits Issued	Hunters	Hunter Days	Harvest				Calves	Total	Percent Success
					Bulls		Cows				
					Adults	Yearlings	Adults	Yearlings			
1950	–	–	–	–	–	–	–	–	–	92	–
1951	–	–	–	–	–	–	–	–	–	92	–
1953	–	–	–	–	–	–	–	–	–	25	–
1955	–	–	–	–	–	–	–	–	–	35	–
1956	–	–	–	–	–	–	–	–	–	30	–
1957	–	–	–	–	–	–	–	–	–	150	–
1958	–	–	–	–	28	19	18	20	0	85	–
1959	–	–	–	–	7	69	0	74	0	150	–
1960	–	–	–	–	26	8	18	8	0	60	–
1961	–	–	–	–	65	20	50	20	0	155	–
1962	–	–	–	–	29	20	32	15	0	96	–
1963	–	–	–	–	42	20	38	20	0	120	–
1964	–	–	–	–	39	28	42	21	0	130	–
1965	–	–	–	–	41	32	49	28	0	150	–
1966	–	–	–	–	28	28	30	14	0	100	–
1967	–	–	–	–	34	21	30	20	0	105	–
1968	–	–	–	–	21	20	14	20	0	75	–
1969	–	–	–	–	25	25	25	25	0	100	–
1970	–	–	–	–	12	25	18	25	0	80	–
1971	–	–	–	–	24	20	16	20	0	80	–
1972	–	–	–	–	32	30	33	30	0	125	–
1973	–	–	–	–	15	7	52	22	30	126	–
1974	–	–	–	–	9	35	52	34	0	130	–
1975	–	–	–	–	10	40	37	32	0	119	–
1976	–	–	–	–	7	18	34	16	0	75	–
1977	–	–	–	–	15	17	12	12	0	56	–
1978	–	–	–	–	26	18	5	9	0	58	–
1979	–	–	–	–	14	13	12	0	0	39	–
1980	545	57	57	–	23	6	21	5	0	55	96.5
1981	329	46	46	–	17	10	19	0	0	46	100.0
1982	198	38	38	51	28	0	9	0	0	37	97.4
1983	202	43	43	97	17	7	14	2	0	40	93.0
1984	209	40	40	76	24	5	9	2	0	40	100.0
1985	238	59	54	119	5	15	22	6	0	48	88.9
1986	225	47	43	108	18	5	12	4	0	39	90.7
1987	217	41	39	69	2	17	3	13	0	35	89.7
1988	366	61	58	154	11	19	15	5	0	50	86.2
1989	449	85	82	251	25	20	8	15	0	68	82.9
1990	417	91	89	369	13	11	14	17	0	55	61.8
1991	414	50	50	127	5	13	17	12	0	47	94.0
1992	551	65	64	210	9	9	15	16	0	49	76.6
1993	680	65	65	233	10	12	8	16	0	46	70.8
1994	742	64	60	176	8	16	7	16	0	47	78.3
1995	1075	95	90	352	10	20	8	23	0	61	67.8
1996	1175	71	71	273	14	10	8	13	0	45	63.4
1997	1193	61	61	152	11	12	20	15	0	58	95.1
1998	1431	64	64	216	11	9	8	15	0	41	64.1
1999	1380	49	45	131	3	15	6	12	0	36	80.0
2000	1325	54	52	164	3	12	7	10	1	33	63.5
2001	1360	72	70	432	4	8	11	6	0	29	41.4
2002	3316	50	48	198	20	14	1	3	0	38	79.2
2003	5154	53	52	203	10	1	27	1	0	39	75.0
2004	7788	97	84	380	9	20	7	5	0	41	48.8
2005	3043	26	24	37	4	4	12	2	0	22	91.7
2006	2640	21	21	70	3	1	9	5	0	18	85.7
2007	1232	28	28	151	5	10	8	0	0	23	82.1
2008	868	29	27	93	11	10	2	4	0	26	96.2

[1] Data from 1958 through 1979 are proposed harvest. Actual harvest may have varied slightly. Prior to 1980, hunters and hunter days are unknown but should have approximated harvest, with hunt success at or near 100%.
[2] Beginning in 1995, 1st Choice Applicants includes the spring and fall draws.

Buffalo Hunt Data

Population Management Season Results - Unit 12A

Year	Season[1]	Permits Issued	Hunters	Hunter Days	Harvest				Calves	Total	Percent Success
					Bulls		Cows				
					Adults	Yearlings	Adults	Yearlings			
2005	Companion	106	106	--	3	0	1	0	0	4	3.8
2005	Standard	20	19	39	5	5	0	2	0	12	63.2
2006	Companion	28	28	--	7	0	0	0	0	7	25.0
2006	Standard	25	24	52	4	1	1	3	0	9	37.5
2007	Companion	59	59	20	5	2	1	0	0	8	13.6
2007	Standard	8	7	10	0	0	3	1	0	4	57.1
2008	Companion	97	97	32	7	1	3	0	0	12	12.4
2008	Standard	16	12	16	0	3	7	0	0	10	83.3

[1] Designates the type of Population Management Season offered. "Companion" denotes tags issued to hunters with corresponding Kaibab deer hunts. "Standard" denotes seasons authorized through the typical Population Management Season process.

5-Year: 2004–2008 Harvest

Unit	Year	Season	Dates	Permits Authorized	1st Choice Applicants	2nd Choice Applicants	Draw Odds	Permits Issued	Hunters	Hunters Days	Bull Harvest	Cow Harvest	Total Harvest	Hunt Success
5B	2004	Bull	3/12-3/18	2	758	287	0.3	2	2	6	2	0	2	100
5B	2004	Bull	8/13-8/19	2	2294	606	0.1	2	2	2	2	0	2	100
5B	2004	Bull	3/19-3/25	2	157	647	0.0	2	2	4	2	0	2	100
5B	2005	Bull	5/27-6/05	2	950	249	0.2	2	2	6	2	0	2	100
5B	2006	Bull	5/26-6/04	2	253	54	0.8	2	2	3	2	0	2	100
5B	2007	Bull	9/22-9/23	1	444	11	0.2	1	1	1	1	0	1	100
5B	2004	Cow	12/03-12/06	2	153	305	1.3	2	2	2	0	2	2	100
5B	2004	Cow	11/19-11/23	4	201	261	1.5	4	3	3	0	3	3	100
5B	2005	Cow	9/23-9/26	3	287	98	1.1	3	3	3	0	3	3	100
5B	2005	Cow	11/18-11/21	3	185	398	1.6	4	4	4	0	4	4	100
5B	2005	Cow	12/02-12/05	3	320	148	0.9	3	3	3	0	3	3	100
5B	2006	Cow	9/09-9/11	2	842	100	0.2	2	2	2	0	2	2	100
5B	2006	Cow	9/23-9/25	2	198	771	1.0	2	2	2	0	2	2	100
5B	2006	Cow	9/30-10/02	2	271	531	0.4	2	2	2	0	2	2	100
5B	2006	Cow	10/21-10/23	2	686	177	0.3	2	2	2	0	2	2	100
5B	2007	Cow	9/22-9/23	1	54	122	0.0	1	1	1	0	1	1	100
5B	2007	Cow	9/29-9/30	2	95	117	2.1	2	2	2	0	2	2	100
5B	2007	Cow	10/13-10/14	2	80	156	0.0	2	2	2	0	2	2	100
5B	2007	Cow	10/27-10/28	2	106	89	0.9	2	2	2	0	2	2	100
5B	2008	Cow	9/27-9/28	2	146	8	1.4	2	2	3	0	1	1	50
5B	2004	Yrl	9/24-9/27	4	402	337	0.5	4	4	4	4	0	4	100
5B	2004	Yrl	10/15-10/18	4	245	430	0.8	4	4	4	4	0	4	100
5B	2004	Yrl	1/02-1/08	4	208	57	1.9	4	4	4	4	1	5	125
5B	2004	Yrl	1/09-1/15	4	49	206	0.0	4	4	4	3	1	4	100
5B	2004	Yrl	1/16-1/22	4	42	143	4.8	4	4	9	2	2	4	100
5B	2004	Yrl	1/23-1/29	4	89	66	2.3	4	4	4	3	1	4	100
5B	2005	Yrl	1/14-1/20	4	297	267	1.0	4	4	5	0	4	4	100
5B	2005	Yrl	1/21-1/27	4	110	364	0.9	5	4	4	0	4	4	100
5B	2006	Yrl	1/14-1/20	3	118	72	1.7	3	3	4	0	3	3	100
5B	2006	Yrl	2/03-2/10	3	59	135	1.7	3	3	3	0	3	3	100
5B	2007	Yrl	1/06-1/07	2	70	11	2.9	2	2	2	2	0	2	100
5B	2007	Yrl	1/13-1/14	2	19	76	0.0	2	2	2	2	0	2	100
5B	2007	Yrl	1/27-1/28	2	38	31	5.3	2	2	2	2	0	2	100
5B	2007	Yrl	2/03-2/04	2	45	74	4.4	2	2	2	2	0	2	100
5B	2008	Yrl	10/04-10/05	2	172	85	1.2	2	2	2	0	2	2	100
5B	2008	Yrl	10/11-10/12	2	81	195	1.2	2	2	2	2	0	2	100
5B	2008	Yrl	1/05-1/06	2	68	24	2.9	2	2	2	2	0	2	100
5B	2008	Yrl	1/12-1/13	2	20	67	5.0	2	2	2	2	0	2	100
5B	2008	Yrl	1/26-1/27	2	34	28	5.9	2	2	2	2	0	2	100
5B	2008	Yrl	2/02-2/03	2	22	49	4.6	2	2	2	0	2	2	100
5B	2008	Yrl	2/09-2/10	2	41	33	2.4	2	2	2	1	1	2	100
5B	2007	Any	2/10-2/11	2	55	29	3.6	2	2	2	2	0	2	100
12A	2004	AB	10/15-10/24	10	1962	718	0.5	10	9	64	0	0	0	0
12A	2004	CowYr	9/17-9/26	30	425	241	4.7	30	23	155	1	1	2	9
12A	2004	CowYr	10/01-10/10	15	52	375	5.8	15	12	64	0	0	0	0

Yrl = Yearling, **CY** = Cow or Yearling, **Any** = Any Buffalo, **SP** = Special raffle/auction permit.

Buffalo Hunt Data

5-Year: 2004-2008 Harvest

Unit	Year	Season	Dates	Permits Authorized	1st Choice Applicants	2nd Choice Applicants	Draw Odds	Permits Issued	Hunters	Hunters Days	Bull Harvest	Cow Harvest	Total Harvest	Hunt Success
12A	2004	Any	3/12-4/11	4	751	61	0.5	4	4	44	1	1	2	50
12A	2005	Any	–	0	0	0	–	106	106	0	3	1	4	4
12A	2005	Any	–	0	0	0	–	20	19	39	10	2	12	63
12A	2005	Any	3/11-4/10	5	894	238	0.6	5	4	12	2	0	2	50
12A	2006	Any	–	0	0	0	–	25	24	52	5	4	9	38
12A	2006	Any	–	0	0	0	–	28	28	0	7	0	7	25
12A	2006	Any	3/10-4/09	5	213	62	2.4	5	5	52	1	1	2	40
12A	2007	Any	–	8	0	0	–	8	7	10	0	4	4	57
12A	2007	Any	–	0	0	0	–	67	8	20	7	1	8	100
12A	2007	Any	3/09-4/08	5	167	43	3.0	5	5	92	0	0	0	0
12A	2007	Any	4/13-5/13	5	59	142	3.4	5	5	41	4	1	5	100
12A	2008	Any	1/01-5/31	10	284	1	3.5	10	8	64	8	0	8	100
12A	2004	SP	9/01-4/30	2	0	0	–	2	1	7	1	0	1	100
12A	2005	SP	–	2	0	0	–	2	1	2	1	0	1	100
12A	2006	SP	–	3	0	0	–	3	–	–	–	–	–	–
12A	2007	SP	–	3	0	0	–	3	–	–	–	–	–	–
12A	2008	SP	–	3	0	0	–	3	3	12	3	0	3	100
**TOTAL - SUMMARY FOR RAYMOND RANCH WILDLIFE AREA**														
05B	2004		–	36	4598	3345	0.5	36	35	46	26	10	36	103
05B	2005		–	19	2149	1524	0.7	21	20	25	2	18	20	100
05B	2006		–	16	2427	1840	0.5	16	16	18	2	14	16	100
05B	2007		–	18	1006	716	1.2	18	18	18	11	7	18	100
05B	2008		–	16	584	489	2.1	16	16	17	9	6	15	94
**TOTAL - SUMMARY FOR HOUSE ROCK WILDLIFE AREA (includes Population Management Season data)**														
12A	2004		–	61	3190	1395	1.2	61	49	334	3	2	5	10
12A	2005		–	7	894	238	0.6	133	130	53	16	3	19	15
12A	2006		–	8	213	62	2.4	58	57	104	13	5	18	32
12A	2007		–	21	226	185	3.1	77	25	163	11	6	17	68
12A	2008		–	29	284	1	3.5	126	35	124	22	10	32	91

Yrl = Yearling, **CY** = Cow or Yearling, **Any** = Any Buffalo, **SP** = Special raffle/auction permit.

Black Bear *(Ursus americanus)*

Natural History

Black bears in Arizona are found in a variety of habitats, including subalpine and montane conifer forests, riparian forests, evergreen woodlands, and chaparral. An interesting footnote to black bear distribution in the state is the absence of any sizeable population of black bears north of the Colorado River.

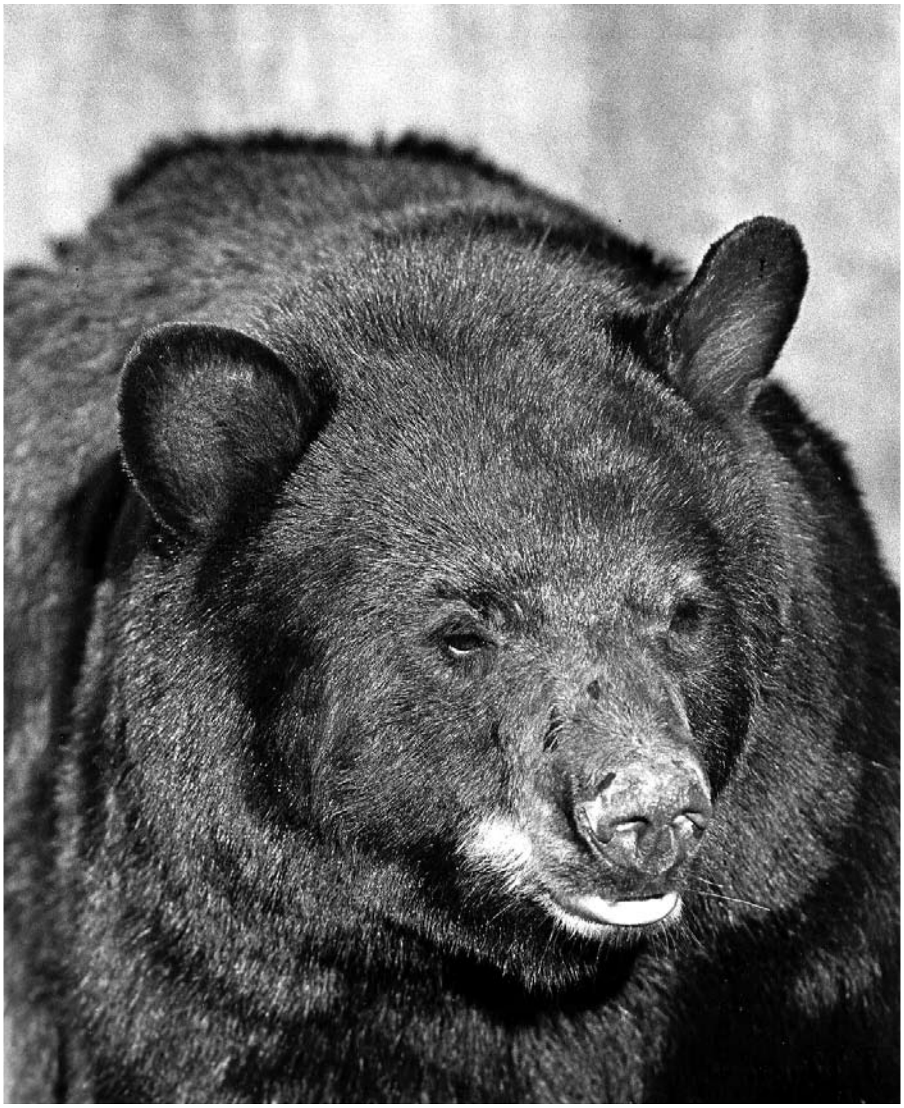

BOB MILES

Cubs are born in winter dens during January, usually in pairs, but larger litters are not uncommon. Cubs weigh only six to 12 ounces at birth and are helpless, but they grow and develop rapidly, emerging from the den with their mother in April. The mother stays with her cubs through the first summer and fall, and dens with them again the following winter. Female black bears in Arizona usually reach reproductive age in their fourth year, and generally breed every other year. Normal reproductive cycles in Arizona black bears may be adversely affected by drought, and/or poor physiological condition. Adult males weigh up to 350 pounds and adult females up to 250 pounds. Black bears are relatively long-lived animals, with some individuals exceeding 20 years of age. The statewide population is estimated at 2,500 bears.

Black bears are normally shy, secretive animals displaying high levels of intelligence and exploratory behavior. Although bears are generally most active in the early morning and late evening; they may alter their activity pattern to exploit sources of artificial food, becoming nocturnal at campgrounds and dumpsites. Nuisance activities are nearly always associated with artificial food sources (beehives, campgrounds, and livestock).

Bears are usually solitary animals; the exceptions are family groups (mother

and cubs), breeding pairs, and congregations at feeding sites. Both adults and sub-adults are known to move long distances (100 miles) to exploit isolated pockets of food. The mobility of black bears sometimes leads them to appear in uncharacteristic habitats and to return from long distances after being moved. Most Arizona black bears hibernate from November through March, during which time they reduce their body temperature, heart rate, and metabolic function, while still remaining somewhat conscious in the den.

Hunt History

Bear hunting has a long history in Arizona. As late as 1928, bears were classified as predatory animals and could be shot or trapped at any time. In 1929, however, a new "game code" classified bears of all kinds as big game, provided a month-long open season, and prescribed a bag limit of one. Bears could not be trapped, but they could be taken with dogs. Later years were even more restrictive; cubs were protected in 1934, and in 1936, the bear season was closed south of the Gila River.

The status of bears deteriorated drastically during World War II. In 1942 all of the state's refuges were open to bear hunting and the season was reopened in Cochise and Graham counties at the request of stockmen. In 1944, month-long fall and spring hunts were authorized. The following year, bears lost their designation as game animals, and in 1949 a year-long season was authorized for Apache, Greenlee, Graham, and eastern Coconino counties, except during the seasons for other big-game species. After reinstating spring and fall bear seasons in 1950, the Arizona Game and Fish Commission again opted for year-long seasons from 1951 to 1953.

After 1954, bear regulations became more restrictive, tags were required to take one, and in 1968 the black bear was again classified as big game. This designation was appropriate as hunter interest in the species was increasing. Hunt success varied with weather conditions and population vagaries, but annual bear harvests ranged from 131 to 313 for the years 1964 through 1980. Relatively few bears were taken under the stock-taking clause, most of them being taken by sport hunters. Concern about the bear's relatively low reproductive rate caused the Department to monitor the bear harvest more closely. Accordingly, mandatory check-out procedures were initiated in 1980. Other recent changes in regulations have included the authorization of a permit-only spring season in select units, the elimination of bear-baiting as a method of take, and unit harvest objectives in which the season is closed after a certain number of female bears are taken.

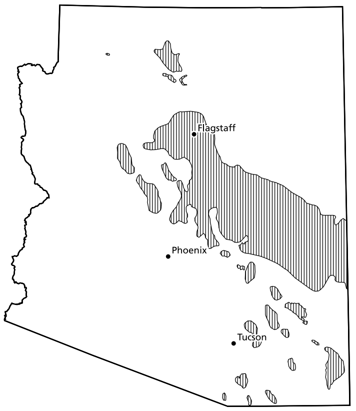

Black bear distribution

Historic Summary of Black Bear Harvest[1]

Year	Tags Issued	Sport[2]	Depredation	Other[3]	Total Harvest
1964	6638	178	0	0	178
1965	5974	131	0	0	131
1966	5798	134	0	0	134
1967	6344	219	0	0	219
1968	8264	242	0	0	242
1969	8978	268	0	0	268
1970	8454	236	0	0	236
1971	8042	241	33	0	274
1972	6009	187	17	0	204
1973	7162	225	2	0	227
1974	6839	202	12	0	214
1975	6746	224	9	0	233
1976	7055	265	10	0	275
1977	8707	309	4	0	313
1978	8985	264	6	0	270
1979	8833	251	2	0	253
1980	7820	255	2	0	257
1981	8494	287	5	0	292
1982	7178	260	8	0	268
1983	6183	273	1	0	274
1984	5258	246	5	0	251
1985	4917	251	6	0	257
1986	4816	182	7	0	189
1987	5117	302	9	0	311
1988	4272	146	7	2	155
1989	4714	271	18	3	292
1990	3711	149	11	1	161
1991	2843	96	4	1	101
1992	3217	121	1	0	122
1993	3329	117	1	3	121
1994	4376	236	2	14	252
1995	4586	197	1	0	198
1996	4462	254	5	19	278
1997	4093	224	2	6	232
1998	4461	142	0	13	155
1999	4163	181	0	5	186
2000	4413	320	2	46	368
2001	4293	178	6	6	184
2002	4535	230	1	16	252
2003	4525	214	5	34	249
2004	4521	160	5	11	176
2005	4850	158	0	2	160
2006	4840	197	1	40	238
2007	6110	217	2	19	238
2008	5950[4]	179	1	11	191

[1] Data from Indian Reservations are included through 1987 and excluded thereafter.
[2] Estimated from a mail questionnaire from 1964-1987 and from mandatory check-outs from 1988-present.
[3] Includes known kills other than sport or depredation (e.g., highway mortality, capture mortality, and illegal take).
[4] 2008 tags issued is preliminary pending a final audit.

5-Year: 2004–2008 Black Bear Harvest Data[1]

Unit	Year	Harvest			Sport Harv. Using Dogs	Sex of Sport Harv.		Month of Sport Harvest						
		Sport	Depredation	Other		Male	Female	Spring[2]	Aug.	Sept.	Oct.	Nov.	Dec.	Unknown
1	2004	7	1	1	5	4	3	0	2	4	0	1	0	0
1	2005	13	0	0	9	7	6	0	3	3	4	2	1	0
1	2006	23	0	1	14	15	8	0	9	3	7	4	0	0
1	2007	12	0	1	7	8	4	0	10	0	2	0	0	0
1	2008	19	0	1	13	16	3	0	17	0	1	1	0	0
3B	2004	8	0	1	7	2	6	0	5	2	1	0	0	0
3B	2005	9	0	0	7	8	1	0	6	1	1	1	0	0
3B	2006	13	0	0	9	10	3	0	4	6	3	0	0	0
3B	2007	3	0	4	1	2	1	1	2	0	0	0	0	0
3B	2008	3	0	1	1	2	1	1	2	0	0	0	0	0
3C	2004	5	0	2	0	3	2	0	0	4	1	0	0	0
3C	2005	4	0	0	0	2	2	1	1	2	0	0	0	0
3C	2006	3	0	3	0	2	1	2	0	0	1	0	0	0
3C	2007	2	0	3	1	1	1	1	0	0	0	0	1	0
3C	2008	3	0	3	0	3	0	0	0	0	2	1	0	0
4A	2004	4	0	1	2	3	1	0	0	0	4	0	0	0
4A	2005	1	0	0	0	0	1	0	0	0	1	0	0	0
4A	2006	8	0	1	7	6	2	0	0	0	8	0	0	0
4A	2007	4	0	1	1	2	2	0	0	0	4	0	0	0
4A	2008	3	0	0	1	2	1	0	0	0	3	0	0	0
4B	2004	2	0	0	2	1	1	0	2	0	0	0	0	0
4B	2005	5	0	0	2	4	1	0	3	2	0	0	0	0
4B	2006	1	0	0	0	0	1	0	0	1	0	0	0	0
4B	2007	2	0	0	0	2	0	0	0	0	2	0	0	0
4B	2008	4	0	0	1	2	2	0	3	1	0	0	0	0
5A	2004	8	0	0	6	5	3	0	0	0	5	3	0	0
5A	2005	10	0	0	5	8	2	0	0	0	8	2	0	0
5A	2006	1	0	0	0	1	0	0	0	0	1	0	0	0
5A	2007	5	0	0	1	1	3	0	0	0	4	0	0	0
5A	2008	8	0	0	5	4	4	0	0	0	8	0	0	0
5B	2004	5	0	0	3	3	2	0	0	0	1	4	0	0
5B	2005	2	0	1	2	2	0	0	0	0	0	2	0	0
5B	2006	3	0	0	2	2	1	0	0	0	3	0	0	0
5B	2007	4	0	0	2	3	1	0	0	0	3	1	0	0
5B	2008	7	0	0	3	6	1	0	0	0	7	0	0	0
6A	2004	5	0	0	2	3	2	0	3	2	0	0	0	0
6A	2005	6	0	0	0	5	1	0	5	0	1	0	0	0
6A	2006	11	0	0	6	8	3	0	0	0	8	3	0	0
6A	2007	10	0	0	2	8	2	0	0	0	8	2	0	0
6A	2008	12	0	0	1	9	3	0	6	0	6	0	0	0
6B	2004	6	0	0	0	4	2	0	3	2	1	0	0	0
6B	2005	3	0	0	0	3	0	1	1	1	0	0	0	0
6B	2006	8	0	0	2	5	3	0	8	0	0	0	0	0
6B	2007	10	0	0	0	6	4	0	9	1	0	0	0	0
6B	2008	4	0	0	1	3	1	0	3	1	0	0	0	0
7	2005	2	0	0	0	2	0	0	0	0	0	2	0	0
7	2006	1	0	0	0	1	0	0	0	0	1	0	0	0
7	2007	1	0	0	0	0	1	0	0	0	1	0	0	0
7	2008	3	0	0	0	1	2	0	0	0	3	0	0	0
8	2004	4	0	0	1	4	0	0	3	1	0	0	0	0
8	2005	3	0	0	1	2	1	0	3	0	0	0	0	0
8	2006	5	0	0	3	3	2	0	0	0	3	2	0	0
8	2007	9	0	0	1	5	4	0	0	0	9	0	0	0
8	2008	6	0	0	1	5	1	0	0	0	4	2	0	0
9	2008	1	0	0	0	0	1	0	0	0	1	0	0	0
11M	2004	0	0	1	–	–	–	–	–	–	–	–	–	–
11M	2006	1	0	0	0	0	1	0	0	1	0	0	0	0

[1] Excluding data from Indian Reservations.

[2] For Archery-Only Spring Bear hunts ending in August or September, bear harvest occurring in August or September will be reflected in the appropriate month of harvest column. All other spring harvest will be reflected in the Spring column.

5-Year: 2004–2008 Black Bear Harvest Data[1]

Unit	Year	Harvest			Sport Harv. Using Dogs	Sex of Sport Harv.		Month of Sport Harvest						
		Sport	Depredation	Other		Male	Female	Spring[2]	Aug.	Sept.	Oct.	Nov.	Dec.	Unknown
11M	2008	1	0	0	0	1	0	0	1	0	0	0	0	0
17A	2004	1	0	0	1	1	0	0	0	1	0	0	0	0
17A	2005	1	0	0	0	0	1	0	0	0	1	0	0	0
17B	2008	0	1	0	–	–	–	–	–	–	–	–	–	–
18A	2008	0	0	1	–	–	–	–	–	–	–	–	–	–
19A	2004	4	0	0	1	2	2	0	0	1	3	0	0	0
19A	2005	3	0	0	0	3	0	0	0	3	0	0	0	0
19A	2006	4	0	0	0	3	1	0	0	0	1	2	1	0
19A	2007	1	0	0	0	0	1	0	0	0	1	0	0	0
19A	2008	4	0	0	0	4	0	0	1	0	3	0	0	0
20B	2007	0	0	1	–	–	–	–	–	–	–	–	–	–
21	2004	7	0	0	0	5	2	0	0	5	2	0	0	0
21	2005	2	0	0	0	2	0	0	0	2	0	0	0	0
21	2006	2	0	0	0	0	2	0	0	0	2	0	0	0
21	2007	5	0	0	0	4	1	0	0	0	5	0	0	0
21	2008	6	0	0	0	4	2	0	0	0	6	0	0	0
22	2004	15	0	0	7	11	4	0	0	6	5	3	1	0
22	2005	12	0	0	2	10	2	1	0	8	0	2	1	0
22	2007	0	0	1	–	–	–	–	–	–	–	–	–	–
22N	2006	7	0	0	2	2	5	0	0	0	6	1	0	0
22N	2007	7	0	0	1	4	3	0	0	0	6	1	0	0
22N	2008	6	0	0	1	1	5	0	0	2	3	1	0	0
22S	2006	4	0	0	0	2	2	0	3	0	1	0	0	0
22S	2007	10	0	0	0	8	2	0	5	0	5	0	0	0
22S	2008	3	0	0	0	2	1	0	2	0	1	0	0	0
23	2004	1	0	0	1	1	0	0	0	0	1	0	0	0
23	2008	4	0	0	0	4	0	0	0	0	1	3	0	0
23N	2004	16	0	0	12	11	5	0	1	1	14	0	0	0
23N	2005	18	0	0	14	10	8	0	1	2	15	0	0	0
23N	2006	13	0	0	9	8	5	0	0	5	5	3	0	0
23N	2007	19	0	0	12	8	11	0	1	3	14	0	0	0
23N	2008	17	0	1	9	12	5	0	2	1	12	1	1	0
23S	2004	11	0	0	0	6	5	0	0	11	0	0	0	0
23S	2005	15	0	0	1	12	3	0	0	10	4	1	0	0
23S	2006	12	0	1	1	6	6	0	10	0	2	0	0	0
23S	2007	16	0	0	0	8	8	0	11	0	3	0	0	0
23S	2008	11	0	0	0	8	3	0	10	0	1	0	0	0
24A	2004	1	0	0	0	1	0	0	0	0	0	1	0	0
24A	2005	1	0	0	0	1	0	0	0	0	1	0	0	0
24A	2006	8	0	4	0	7	1	0	0	0	7	1	0	0
24A	2007	20	0	0	0	10	10	1	15	0	4	0	0	0
24A	2008	6	0	1	0	4	2	1	5	0	0	0	0	0
24B	2004	2	0	0	0	2	0	0	2	0	0	0	0	0
24B	2006	2	0	0	0	1	1	0	2	0	0	0	0	0
24B	2007	5	0	0	0	4	1	0	5	0	0	0	0	0
27	2004	24	2	2	5	17	7	0	10	7	2	4	1	0
27	2005	26	0	0	4	20	6	0	5	11	7	1	2	0
27	2006	30	0	1	9	16	14	0	0	10	13	7	0	0
27	2007	29	2	1	11	18	11	0	1	5	18	5	0	0
27	2008	26	0	0	12	13	13	0	4	4	14	3	1	0
28	2005	2	0	0	0	1	1	0	0	1	1	0	0	0
28	2006	3	0	1	0	3	0	0	0	0	2	1	0	0
28	2007	4	0	0	0	2	2	0	0	0	1	3	0	0
28	2008	1	0	1	0	0	1	0	0	0	1	0	0	0
29	2004	8	0	1	2	6	2	0	0	7	1	0	0	0
29	2005	6	0	0	0	1	5	0	0	6	0	0	0	0
29	2006	7	0	9	1	4	3	0	0	0	7	0	0	0
29	2007	10	0	2	0	3	7	0	0	0	9	0	0	0

[1] Excluding data from Indian Reservations.

[2] For Archery-Only Spring Bear hunts ending in August or September, bear harvest occurring in August or September will be reflected in the appropriate month of harvest column. All other spring harvest will be reflected in the Spring column.

5-Year: 2004–2008 Black Bear Harvest Data[1]

Unit	Year	Harvest			Sport Harv. Using Dogs	Sex of Sport Harv.		Month of Sport Harvest						
		Sport	Depredation	Other		Male	Female	Spring[2]	Aug.	Sept.	Oct.	Nov.	Dec.	Unknown
29	2008	6	0	0	1	5	1	0	0	0	4	2	0	0
30A	2004	4	0	0	0	4	0	0	0	1	3	0	0	0
30A	2005	0	0	1	–	–	–	–	–	–	–	–	–	–
30A	2006	3	0	3	2	0	3	0	0	0	3	0	0	0
30A	2007	5	0	0	0	3	2	0	0	0	4	0	0	0
31	2004	5	1	0	1	5	0	0	0	4	1	0	0	0
31	2005	7	0	0	0	5	2	1	0	5	1	0	0	0
31	2006	6	0	1	0	3	3	0	0	0	6	0	0	0
31	2007	8	0	0	0	3	5	0	0	0	8	0	0	0
31	2008	2	0	0	0	1	1	0	0	0	2	0	0	0
32	2004	4	1	0	0	2	2	0	0	4	0	0	0	0
32	2005	3	0	0	0	3	0	0	2	1	0	0	0	0
32	2006	7	1	1	1	4	3	0	0	4	3	0	0	0
32	2007	6	0	1	0	3	3	1	0	0	5	0	0	0
32	2008	4	0	0	0	2	2	0	0	2	2	0	0	0
33	2004	0	0	1	–	–	–	–	–	–	–	–	–	–
33	2005	1	0	0	1	0	1	0	0	0	0	0	1	0
33	2006	0	0	2	–	–	–	–	–	–	–	–	–	–
33	2007	0	0	1	–	–	–	–	–	–	–	–	–	–
33	2008	2	0	0	0	2	0	2	0	0	0	0	0	0
34A	2004	1	0	1	0	0	1	1	0	0	0	0	0	0
34A	2005	2	0	0	0	2	0	2	0	0	0	0	0	0
34A	2006	4	0	0	0	3	1	4	0	0	0	0	0	0
34A	2007	5	0	3	0	3	2	4	1	0	0	0	0	0
34A	2008	3	0	0	0	2	1	3	0	0	0	0	0	0
34B	2008	0	0	1	–	–	–	–	–	–	–	–	–	–
35A	2004	1	0	0	0	0	1	1	0	0	0	0	0	0
35A	2005	1	0	0	0	1	0	1	0	0	0	0	0	0
35A	2006	6	0	9	2	2	4	1	0	4	0	1	0	0
35A	2007	5	0	0	0	2	3	4	0	0	0	0	1	0
35A	2008	4	0	0	0	4	0	4	0	0	0	0	0	0
35B	2004	1	0	0	0	1	0	1	0	0	0	0	0	0
35B	2006	1	0	0	0	1	0	0	1	0	0	0	0	0
38M	2006	0	0	2	–	–	–	–	–	–	–	–	–	–
38M	2008	0	0	1	–	–	–	–	–	–	–	–	–	–
Unknown	2006	0	0	1	–	–	–	–	–	–	–	–	–	–
Total	2004	160	5	11	58	107	53	3	31	63	45	16	2	0
Total	2005	158	0	2	48	114	44	7	30	58	45	13	5	0
Total	2006	197	1	40	70	118	79	7	37	34	93	25	1	0
Total	2007	217	2	19	40	121	95	12	60	9	116	12	2	0
Total	2008	179	1	11	50	122	57	11	56	11	85	14	2	0
Percent	2004				36	67	33	2	19	39	28	10	1	
Percent	2005				30	72	28	4	19	37	28	8	3	
Percent	2006				36	60	40	4	19	17	47	13	1	
Percent	2007				18	56	44	6	28	4	53	6	1	
Percent	2008				28	68	32	6	31	6	47	8	1	

[1] Excluding data from Indian Reservations.

[2] For Archery-Only Spring Bear hunts ending in August or September, bear harvest occurring in August or September will be reflected in the appropriate month of harvest column. All other spring harvest will be reflected in the Spring column.

Mountain Lion *(Felis concolor)*

Natural History

In Arizona, mountain lions are absent only from the extremely arid southwest and those areas heavily impacted by human development. In general, the distribution of mountain lions in the state corresponds with the distribution of the animal's major prey species—mule and white-tailed deer.

Mountain lions may breed at any time of the year, and consequently litters may be born in any month. Summer is the peak period of kitten births, however, with litter sizes of two, three, and four being common. The kittens remain with their mother for 15 to 22 months learning the skills necessary for survival. Juvenile males tend to disperse long distances compared to the relatively short distances for young females. Mountain lions are essentially solitary animals. Adult females may be accompanied by kittens, but are normally not associated with other adult animals except for mating purposes. Mature males weigh up to 150 pounds and females 100 pounds. The statewide population is estimated at 2,500 mountain lions.

While deer are the principal mountain lion prey species in Arizona, javelina, elk, and/or livestock can be major components of the diet. Mountain lions will almost always attempt to cover the uneaten portion of a kill with leaves or other debris. An entire deer can be consumed in two nights. An experienced observer is usually able to detect the presence of a mountain lion in an area through the presence of tracks, scrapes, kills, or other sign.

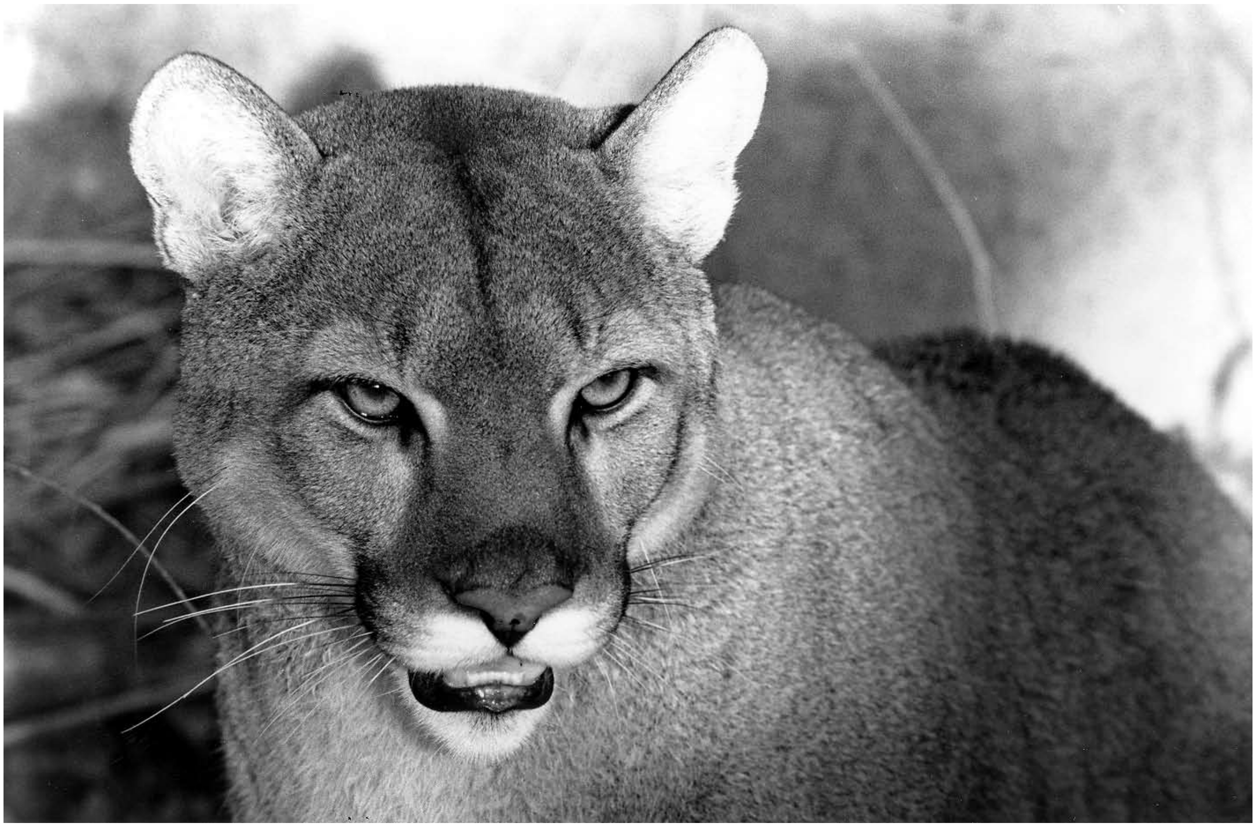

GEORGE ANDREJKO

Mountain lions are specialized predators and consequently do not normally exist in high concentrations. They maintain spatial separation between each other, thereby assuring that each individual has the resources necessary to survive. If these separations are not maintained, mountain lions will kill each other, which is the normal method of population regulation in undisturbed mountain lion populations. The cryptic system of boundary marking employed by resident mountain lions serves to provide for mutual avoidance and survival. Mountain lions in Arizona feed almost exclusively on large prey, usually killing one deer-sized animal every six to 12 days. Considerable skill in executing stalks and more importantly in consummating the kill is required to avoid debilitating injury.

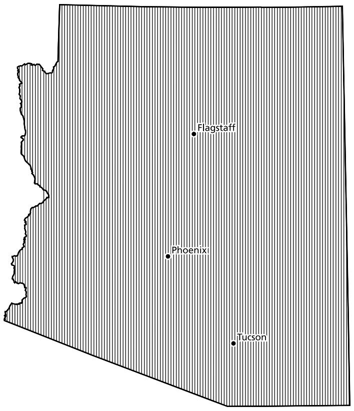

Mountain lion distribution

Hunt History

Lions were classified as a "predatory animal" by the territorial legislature and were subject to a statewide bounty of $50 dollars in 1919. This status continued until 1970 when the mountain lion was classified as a big-game animal and a tag was required to take one, even though ranchers and their agents could still take a depredating lion. A mandatory checkout procedure and other reporting requirements were instituted in 1982. Reporting information indicates that lion harvests have gradually increased over time. Recently, the annual kill has ranged between 250 and 350 animals, of which approximately 15 percent were taken by predator control agents.

Mountain Lion Harvest Data

Historic Summary of Mountain Lion Harvest[1]

Year	Tags Issued	Harvest				Sport Harvest Using Dogs	Sex of Sport Harvest		
		Sport[2]	Depredation[3]	Other[4]	Total		Male	Female	Unclassified
1951	–	–	181	0	181	–	–	–	–
1952	–	–	198	0	198	–	–	–	–
1953	–	–	200	0	200	–	–	–	–
1954	–	–	201	0	201	–	–	–	–
1955	–	–	230	0	230	–	–	–	–
1956	–	–	189	0	189	–	–	–	–
1957	–	–	266	0	266	–	–	–	–
1958	–	–	264	0	264	–	–	–	–
1959	–	–	243	0	243	–	–	–	–
1960	–	–	215	0	215	–	–	–	–
1961	–	–	242	0	242	–	–	–	–
1962	–	–	231	0	231	–	–	–	–
1963	–	–	197	0	197	–	–	–	–
1964	–	–	267	0	267	–	–	–	–
1965	–	–	286	0	286	–	–	–	–
1966	–	–	257	0	257	–	–	–	–
1967	–	–	257	0	257	–	–	–	–
1968	–	–	226	0	226	–	–	–	–
1969	–	–	217	0	217	–	–	–	–
1970	–	–	278	0	278	–	–	–	–
1971	3835	172	0	0	172	–	–	–	–
1972	4214	120	48	0	168	–	–	–	–
1973	4917	190	15	0	205	–	–	–	–
1974	4896	172	22	0	194	–	–	–	–
1975	5460	219	19	1	239	–	–	–	–
1976	6261	238	14	0	252	–	–	–	–
1977	7498	248	4	0	252	–	–	–	–
1978	7964	229	12	0	241	–	–	–	–
1979	7938	283	7	0	290	–	–	–	–
1980	7799	204	2	0	206	–	–	–	–
1981	7871	191	9	1	201	–	–	–	–
1982	8069	316	8	1	325	–	–	–	–
1983	7004	221	7	1	229	–	–	–	–
1984	6876	184	9	0	193	–	–	–	–
1985	7523	246	19	7	272	–	–	–	–
1986	7936	191	25	0	216	–	–	–	–
1987	8304	205	31	5	241	127	109	89	7
1988	8495	183	24	1	208	104	82	99	2
1989	3656	130	65	1	196	85	77	51	2
1990	3046	188	40	1	229	125	108	74	6
1991	3038	179	25	1	205	115	107	71	1
1992	3177	201	28	5	234	147	113	83	5
1993	3407	188	38	12	238	117	106	81	1
1994	4156	215	35	6	256	128	120	93	2
1995	4859	234	31	1	266	150	126	103	5
1996	5552	225	38	2	265	131	119	106	0
1997	5657	269	48	3	320	182	134	134	1
1998	6590	289	52	1	342	192	150	136	3
1999	6885	247	49	2	298	161	126	120	1
2000	7478	276	53	0	329	193	133	141	2
2001	8109	326	58	0	384	214	176	144	6
2002	8274	264	50	5	319	175	144	116	4
2003	8089	218	66	12	296	164	107	111	0
2004	8964	247	31	1	279	167	123	122	2
2005	10117	204	41	0	245	120	103	101	0
2006	10931	221	36	5	262	136	108	113	0
2007	10995	256	28	5	289	169	146	109	1
2008	10629[5]	264	42	5	311	168	142	120	2

[1]Data from Indian Reservations are included through 1987 and excluded thereafter.
[2]Estimated from a mail questionnaire from 1971-1987 and from mandatory check-outs from 1988-present.
[3]As reported by Arizona Livestock Sanitary Board through June 30, 1970, and reported stock-killers since 1971.
[4]Includes known kills other than sport or depredation (e.g., highway mortality, capture mortality, and illegal take).
[5]2008 tags sold is preliminary.

5-Year: 2004-2008 Mountain Lion Hunt Data

Unit	Year	Harvest			Sport Harvest Using Dogs	Sex of Sport Harvest		Month of Sport Harvest			
		Sport	Depredation	Other		Male	Female	Jan. to March	Apr. to June	July to Sept.	Oct. to Dec.
1	2004	2	0	0	2	1	1	0	0	0	2
1	2005	2	0	0	2	2	0	2	0	0	0
1	2006	3	0	0	2	3	0	0	0	0	3
1	2007	3	0	0	3	1	2	0	0	0	3
1	2008	5	0	0	3	1	4	0	1	1	3
2B	2004	3	0	0	3	1	2	1	1	0	1
2B	2006	1	0	0	0	0	1	0	0	0	1
3B	2006	1	0	0	0	0	1	0	0	0	1
3B	2007	2	0	0	2	2	0	1	0	0	1
3B	2008	1	0	0	1	1	0	0	0	0	1
3C	2004	2	0	0	1	1	1	1	0	0	1
3C	2005	3	0	0	1	2	1	2	0	0	1
3C	2006	3	0	0	3	2	1	1	1	0	1
3C	2007	2	0	0	2	2	0	2	0	0	0
3C	2008	1	0	0	1	1	0	0	0	0	1
4A	2004	2	0	0	1	1	1	0	0	0	2
4A	2005	1	0	0	1	1	0	1	0	0	0
4A	2006	3	0	0	2	3	0	2	0	1	0
4B	2004	3	0	0	3	2	1	3	0	0	0
4B	2005	1	0	0	0	0	1	0	1	0	0
4B	2006	2	0	0	1	1	1	1	0	1	0
4B	2007	3	0	0	3	2	1	2	0	0	1
4B	2008	1	0	0	1	1	0	1	0	0	0
5A	2005	1	0	0	0	1	0	0	1	0	0
5A	2006	1	0	0	1	0	1	0	0	0	1
5A	2008	1	0	0	1	1	0	0	0	1	0
5B	2004	2	0	0	2	2	0	2	0	0	0
5B	2005	2	0	0	0	0	2	0	0	1	1
5B	2006	4	0	0	2	4	0	3	0	0	1
5B	2007	4	0	0	4	2	2	3	0	0	1
5B	2008	1	0	1	1	0	1	1	0	0	0
6A	2004	6	0	0	4	4	2	5	0	0	1
6A	2005	11	0	0	9	4	7	8	1	1	1
6A	2006	6	0	1	6	4	2	4	0	0	2
6A	2007	6	0	0	4	3	3	3	1	1	1
6A	2008	2	0	0	0	0	2	0	0	0	2
6AS	2005	1	0	0	0	0	1	0	0	0	1
6AS	2006	4	0	1	1	1	3	1	1	0	2
6AS	2007	4	0	0	3	3	1	3	0	1	0
6AS	2008	6	0	0	2	4	2	1	1	3	1
6B	2004	4	0	0	4	1	3	4	0	0	0
6B	2006	4	0	0	2	0	4	1	0	1	2
6B	2007	3	0	0	2	1	2	2	0	1	0
7	2004	11	0	0	11	7	4	6	0	0	5
7	2005	1	0	0	1	1	0	1	0	0	0
7	2006	6	0	0	5	4	2	2	0	0	4
7	2007	8	0	0	7	5	3	5	0	0	3
7	2008	4	0	0	3	4	0	3	0	1	0
8	2004	5	0	0	3	1	4	3	0	1	1
8	2005	2	0	0	1	2	0	1	0	0	1
8	2006	4	0	0	3	2	2	3	0	0	1
8	2007	6	0	0	5	4	2	3	0	1	2
8	2008	7	0	0	6	6	1	3	0	1	3
9	2004	5	0	0	4	3	2	4	0	0	1
9	2005	2	0	0	1	1	1	1	0	0	1
9	2006	5	0	0	4	3	2	3	0	1	1
9	2007	5	0	0	2	1	4	2	0	0	3
9	2008	2	0	0	2	0	2	1	0	0	1
10	2004	4	0	0	3	2	2	3	1	0	0
10	2005	8	0	0	3	3	5	4	0	0	4
10	2006	3	0	0	1	0	3	2	0	0	1
10	2007	8	0	0	1	4	4	3	1	0	4

5-Year: 2004–2008 Mountain Lion Hunt Data

Unit	Year	Harvest			Sport Harvest Using Dogs	Sex of Sport Harvest		Month of Sport Harvest			
		Sport	Depredation	Other		Male	Female	Jan. to March	Apr. to June	July to Sept.	Oct. to Dec.
10	2008	11	0	0	8	6	5	9	0	0	2
11M	2006	1	0	0	1	0	1	0	0	0	1
11M	2007	2	0	0	2	2	0	1	1	0	0
11M	2008	1	0	0	1	1	0	0	0	0	1
12A	2004	8	0	0	8	4	4	4	0	0	4
12A	2005	5	0	0	4	3	2	0	0	0	5
12A	2006	8	0	0	7	4	4	4	0	1	3
12A	2007	11	0	0	11	6	5	3	0	0	8
12A	2008	9	0	0	5	5	4	3	0	0	5
12B	2004	1	0	0	1	1	0	0	0	0	1
12B	2006	1	0	0	0	1	0	0	0	0	1
13A	2005	2	0	0	2	0	2	0	0	1	1
13A	2008	1	0	0	0	1	0	1	0	0	0
13B	2004	3	0	0	3	2	1	3	0	0	0
13B	2005	1	0	0	1	1	0	1	0	0	0
13B	2006	1	0	0	1	1	0	1	0	0	0
13BS	2006	2	0	0	1	2	0	1	1	0	0
13BS	2007	1	0	0	0	0	1	0	0	0	1
13BS	2008	2	0	0	2	0	2	2	0	0	0
15A	2007	1	0	0	0	1	0	1	0	0	0
15A	2008	1	0	0	0	0	1	1	0	0	0
15B	2005	1	0	0	0	0	1	0	0	0	1
15B	2007	1	0	0	0	0	1	0	1	0	0
15B	2008	1	0	0	1	0	1	0	1	0	0
15BW	2005	1	0	0	1	0	1	1	0	0	0
15C	2004	1	0	0	1	1	0	0	1	0	0
15C	2006	0	0	1	–	–	–	–	–	–	–
15CS	2008	0	0	1	–	–	–	–	–	–	–
15D	2005	1	0	0	1	1	0	1	0	0	0
15D	2006	1	0	0	0	0	1	1	0	0	0
16A	2004	3	0	0	3	0	3	0	0	0	3
16A	2007	2	0	1	2	1	1	2	0	0	0
16A	2008	7	0	1	7	5	2	2	2	0	3
16AS	2007	1	0	0	1	1	0	0	1	0	0
17A	2004	10	1	0	8	2	8	8	0	0	2
17A	2005	8	0	0	5	4	4	5	0	0	3
17A	2006	1	1	0	1	0	1	0	0	0	1
17A	2007	3	0	0	2	3	0	2	0	0	1
17A	2008	8	0	0	6	6	2	4	1	0	3
17B	2004	7	1	0	5	2	5	1	1	0	5
17B	2005	5	4	0	5	3	2	3	0	0	2
17B	2006	7	0	0	6	5	2	4	2	1	0
17B	2007	7	1	0	6	6	1	6	0	0	1
17B	2008	4	3	0	2	3	1	2	0	0	2
18A	2004	2	0	0	2	1	1	2	0	0	0
18A	2005	2	0	0	1	1	1	1	0	1	0
18A	2006	4	0	0	2	1	3	2	0	0	2
18A	2008	1	1	0	1	0	1	1	0	0	0
18B	2004	6	2	0	3	3	3	2	1	0	3
18B	2005	4	2	0	2	1	3	1	1	0	2
18B	2006	8	3	0	5	3	5	2	2	0	4
18B	2007	8	3	0	6	4	4	2	0	3	3
18B	2008	5	1	0	2	1	4	3	0	1	1
18BS	2004	2	0	0	2	1	1	2	0	0	0
18BS	2007	1	0	0	0	1	0	0	0	0	1
18BS	2008	2	0	0	2	1	1	1	1	0	0
19A	2004	4	0	0	3	1	3	2	1	0	1
19A	2005	3	1	0	3	1	2	3	0	0	0
19A	2006	7	0	0	3	2	5	5	2	0	0
19A	2007	3	0	0	3	2	1	1	0	0	2
19A	2008	2	0	0	2	1	1	1	0	0	1
19B	2004	1	0	0	0	0	1	0	0	0	1

5-Year: 2004–2008 Mountain Lion Hunt Data

Unit	Year	Harvest			Sport Harvest Using Dogs	Sex of Sport Harvest		Month of Sport Harvest			
		Sport	Depredation	Other		Male	Female	Jan. to March	Apr. to June	July to Sept.	Oct. to Dec.
19B	2005	1	0	0	0	0	1	0	0	0	1
19B	2006	1	0	0	0	0	1	0	0	0	1
19B	2008	2	0	0	0	0	2	1	0	0	1
20A	2004	9	0	0	6	6	3	6	0	0	3
20A	2005	5	0	0	4	3	2	4	0	0	1
20A	2006	7	0	0	6	3	4	5	1	0	1
20A	2007	5	0	0	4	4	1	3	0	0	2
20A	2008	5	0	0	5	5	0	3	0	0	2
20B	2004	1	0	0	1	0	1	0	0	1	0
20B	2005	2	0	0	1	1	1	1	0	0	1
20B	2007	6	0	0	2	2	4	4	0	1	1
20B	2008	3	0	0	3	3	0	1	0	0	2
20C	2004	6	1	0	4	4	2	4	0	1	1
20C	2005	3	0	0	3	1	2	0	1	1	1
20C	2006	4	0	0	2	2	2	4	0	0	0
20C	2007	4	0	0	4	3	1	4	0	0	0
20C	2008	5	0	0	3	3	2	3	0	0	2
21	2004	1	0	0	1	1	0	1	0	0	0
21	2005	2	0	0	0	1	1	2	0	0	0
21	2006	1	0	0	1	1	0	1	0	0	0
21	2007	2	0	0	2	1	1	2	0	0	0
21	2008	6	0	0	3	1	5	2	1	0	3
21W	2004	4	0	0	3	1	3	3	1	0	0
21W	2005	6	0	0	5	3	3	2	1	0	3
21W	2006	3	0	0	3	0	3	3	0	0	0
21W	2007	1	0	0	1	0	1	0	1	0	0
22	2004	20	0	0	14	10	9	9	1	1	9
22	2005	4	0	0	4	3	1	3	0	0	1
22	2006	5	0	0	5	4	1	2	0	0	3
22	2007	8	0	0	7	5	3	4	1	0	3
22	2008	14	0	0	9	7	7	5	1	3	5
22S	2004	3	0	0	2	2	1	2	1	0	0
22S	2005	1	0	0	1	0	1	1	0	0	0
22S	2006	2	0	0	0	0	2	1	0	1	0
22S	2007	4	0	0	4	3	1	4	0	0	0
22S	2008	4	0	0	3	2	2	3	0	1	0
23	2004	11	0	0	8	6	5	6	1	1	3
23	2005	12	0	0	5	9	3	4	0	4	4
23	2006	16	0	0	10	6	10	5	4	1	6
23	2007	17	0	0	10	9	7	8	1	3	4
23	2008	12	0	0	10	6	6	4	2	0	6
24A	2004	7	0	0	3	5	2	3	1	0	3
24A	2005	10	0	0	4	5	5	1	1	3	5
24A	2006	13	2	0	10	10	3	5	3	3	2
24A	2007	11	1	1	7	8	3	2	1	0	7
24A	2008	9	0	1	7	4	5	2	1	0	6
24B	2004	5	0	0	3	2	3	2	0	1	2
24B	2005	3	0	0	1	1	2	0	1	0	2
24B	2006	3	0	0	3	3	0	3	0	0	0
24B	2007	2	0	0	1	1	1	2	0	0	0
24B	2008	7	0	0	4	3	4	4	0	0	1
27	2004	16	4	0	6	5	11	4	1	4	7
27	2005	5	2	0	2	4	1	3	0	1	1
27	2006	3	6	0	2	2	1	1	0	1	1
27	2007	5	1	1	3	1	4	2	0	0	3
27	2008	13	0	0	2	4	8	1	1	1	8
27BC	2005	1	0	0	0	1	0	0	0	0	1
27BC	2007	1	0	0	0	0	1	0	0	1	0
27BC	2008	1	0	0	0	1	0	0	0	1	0
27-P	2005	3	0	0	1	1	2	0	0	2	1
27-P	2008	1	0	0	1	1	0	0	1	0	0
28	2004	1	8	0	1	0	1	1	0	0	0

5-Year: 2004-2008 Mountain Lion Hunt Data

Unit	Year	Harvest			Sport Harvest Using Dogs	Sex of Sport Harvest		Month of Sport Harvest			
		Sport	Depredation	Other		Male	Female	Jan. to March	Apr. to June	July to Sept.	Oct. to Dec.
28	2005	2	16	0	2	2	0	0	1	0	1
28	2006	2	11	0	1	2	0	0	1	1	0
28	2007	2	10	0	1	1	1	2	0	0	0
28	2008	5	11	0	3	2	3	2	1	0	2
29	2004	3	0	0	2	1	2	2	0	0	1
29	2005	7	1	0	3	3	4	4	1	0	2
29	2006	8	0	0	6	3	5	4	1	0	3
29	2007	7	0	0	5	5	2	3	2	0	2
29	2008	4	0	0	4	3	1	2	0	0	2
30A	2004	9	0	0	7	7	2	3	1	0	5
30A	2005	11	0	0	9	4	7	4	1	1	5
30A	2006	7	0	0	6	4	3	4	1	0	2
30A	2007	5	0	0	3	2	3	3	1	0	1
30A	2008	6	0	0	5	4	2	5	0	0	1
30B	2005	3	0	0	1	2	1	1	0	0	2
30B	2008	0	0	1	–	–	–	–	–	–	–
31	2004	8	9	0	4	4	4	3	1	0	4
31	2005	8	11	0	4	3	5	2	1	0	5
31	2006	4	4	0	1	0	4	1	1	1	1
31	2007	4	7	0	2	2	2	3	1	0	0
31	2008	3	13	0	1	3	0	2	0	0	1
32	2004	6	5	0	1	5	1	2	0	0	4
32	2005	6	4	0	5	2	4	2	0	2	2
32	2006	12	9	1	4	6	6	1	2	0	9
32	2007	10	4	0	5	5	5	4	0	1	5
32	2008	13	13	0	9	6	7	7	0	1	5
33	2004	9	0	0	3	6	3	2	2	1	4
33	2005	11	0	0	5	7	4	4	2	0	5
33	2006	5	0	1	1	3	2	0	0	1	4
33	2007	16	0	0	13	12	4	9	4	1	2
33	2008	15	0	0	7	7	8	3	4	0	8
34A	2004	6	0	0	2	1	4	4	0	1	1
34A	2005	7	0	0	3	4	3	3	0	1	3
34A	2006	9	0	0	5	3	6	4	3	0	2
34A	2007	13	0	0	4	3	10	5	2	1	5
34A	2008	10	0	0	4	4	5	3	2	0	5
34B	2004	3	0	0	3	2	1	2	1	0	0
34B	2005	5	0	0	3	1	4	2	0	0	3
34B	2007	10	0	0	9	8	2	4	5	0	1
34B	2008	5	0	0	5	2	3	0	1	1	3
35A	2005	3	0	0	0	1	2	1	0	1	1
35A	2006	1	0	0	1	1	0	0	0	1	0
35A	2007	5	0	1	1	2	2	2	0	0	3
35A	2008	5	0	0	3	4	1	0	2	0	3
35B	2004	2	0	0	2	2	0	0	2	0	0
35B	2006	1	0	0	1	1	0	1	0	0	0
35B	2007	2	0	0	2	1	1	0	0	2	0
35B	2008	2	0	0	0	2	0	0	0	0	2
36A	2004	6	0	0	3	4	2	2	0	0	4
36A	2005	3	0	0	3	2	1	2	0	1	0
36A	2006	5	0	0	2	2	3	1	1	0	3
36A	2007	5	0	0	2	4	1	2	0	0	3
36A	2008	10	0	0	7	6	4	2	5	0	3
36B	2004	8	0	0	5	4	4	3	2	1	2
36B	2005	3	0	0	0	0	3	0	0	0	3
36B	2006	6	0	0	1	2	4	0	0	0	6
36B	2007	6	1	0	2	3	3	2	0	1	3
36B	2008	3	0	0	2	2	1	1	0	0	2
36C	2004	2	0	0	1	0	2	0	0	0	2
36C	2006	6	0	0	1	2	4	2	1	0	3
36C	2007	3	0	0	1	3	0	2	0	0	1
36C	2008	1	0	0	0	0	1	0	0	0	1

5-Year: 2004–2008 Mountain Lion Hunt Data

Unit	Year	Harvest			Sport Harvest Using Dogs	Sex of Sport Harvest		Month of Sport Harvest			
		Sport	Depredation	Other		Male	Female	Jan. to March	Apr. to June	July to Sept.	Oct. to Dec.
37A	2004	1	0	0	0	0	1	0	1	0	0
37A	2006	1	0	0	1	0	1	0	1	0	0
37A	2007	2	0	0	0	1	1	0	0	0	2
37B	2004	1	0	0	1	0	1	0	0	0	1
37B	2005	8	0	0	6	6	2	6	0	0	2
37B	2006	4	0	0	3	2	2	3	0	0	1
37B	2007	3	0	1	3	0	3	3	0	0	0
37B	2008	8	0	0	4	5	3	5	1	1	1
40A	2008	1	0	0	0	0	1	0	0	1	0
42	2004	2	0	0	2	1	1	0	0	2	0
42	2006	1	0	0	0	0	1	1	0	0	0
42	2007	1	0	0	0	0	1	1	0	0	0
42	2008	4	0	0	3	2	2	0	0	1	3
42M	2004	0	0	1	–	–	–	–	–	–	–
44A	2005	1	0	0	1	1	0	0	1	0	0
44A	2007	1	0	0	0	0	1	0	0	0	1
44A	2008	1	0	0	1	1	0	0	0	0	1
Total	2004	247	31	1	168	123	122	120	22	15	90
Total	2005	204	41	0	120	103	101	88	15	21	80
Total	2006	221	36	5	136	108	113	95	29	16	81
Total	2007	256	28	5	169	146	109	128	24	18	85
Total	2008	264	42	5	168	142	120	101	30	19	109
Percent	2004	89	11	0	68	50	50	49	9	6	36
Percent	2005	83	17	0	59	50	50	43	7	10	39
Percent	2006	84	14	2	62	49	51	43	13	7	37
Percent	2007	89	10	2	66	57	43	50	9	7	33
Percent	2008	85	14	2	64	54	46	38	11	7	41

Small Game

Quail

Arizonans have the privilege of hunting three species of quail—four, if the few California quail found along the Little Colorado River drainage in Apache County are included. These are the Gambel's quail, scaled quail, and Mearns' or Montezuma quail. Another quail, formerly found in Arizona, the masked bobwhite, is listed federally as an endangered species.

Of the above species, the Gambel's or desert quail is by far the best known. Found in most of the state's counties, these birds are often hunted in open desert country where they are more apt to run or flush than hold for a dog. The Gambel's jaunty, plumed topknot, carried by both sexes, makes for ready identification, along with the male's bright russet cap, black face and bib, and cream-colored belly marked with a black horseshoe. As with all species of quail, the young of the year can be distinguished through their first winter by their spotted secondary wing coverts. Adult males average only about 6 ounces; the slightly smaller females between 5.7 and 5.9 ounces.

The handsome—rather than gaudy—scaled quail is the second most commonly encountered quail in Arizona. A bird of the open country of eastern Arizona, this quail too is more likely to run than hold. Both sexes of this species display white, conical crests, hence the common name of "cottontop." The scaled appellation is appropriate, however, as the birds possess a distinctive scalloping on the breast, nape and belly. Otherwise, their overall color is tan above with a mixture of beige, grays, and whites below. A generally bigger bird than the Gambel's quail, adult male "scalies" average about 7.3 ounces, females 6.7 ounces.

Mearns' quail are the largest and most striking, yet also the most secretive of Arizona's quails. Male Mearns' quail have white and black harlequin-marked heads, capped by a russet shock of feathers that form an ill-fitting crest. These cock quail also possess handsome brown and black checkered backs interlaced with white darts, and white-spotted black flanks similar to a guinea fowl's. Their breasts and underparts are a rich mahogany that turns to black at the rump, which terminates in a stubby, almost non-existent tail. The hens are cinnamon colored with brown, black and buff markings. In winter, the males average about 6.9 ounces, the females about 6.2 ounces. Long, scythe-shaped claws that are used for digging show that these birds are ground-dwellers, and they hold so well to a dog that this species has come to be known as Arizona's greatest game bird.

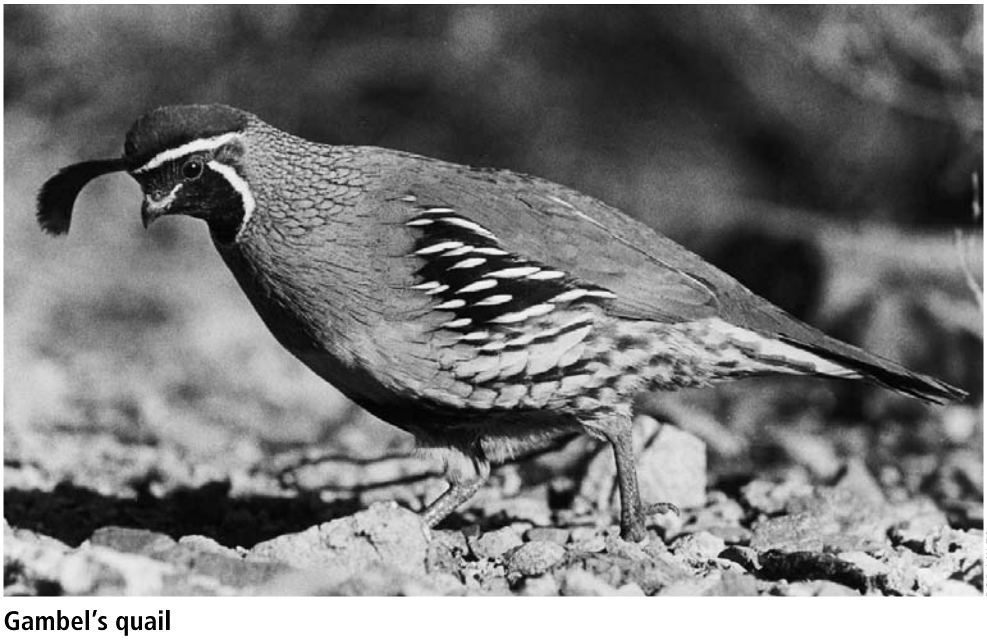
BOB MILES

Gambel's quail

Natural History

The sexes of all Arizona quails show some differences in plumage, and all of the species form seasonal pair bonds

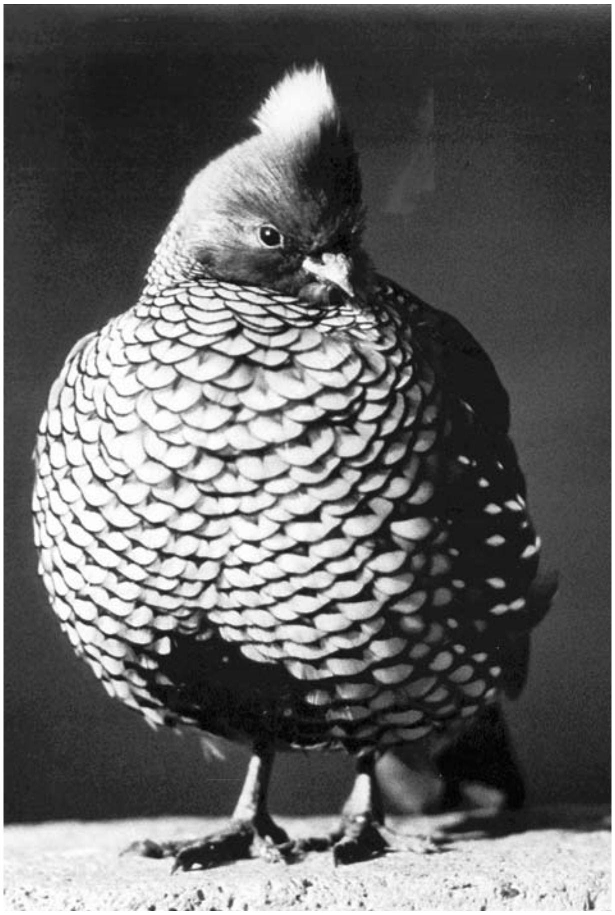
BOB MILES

Scaled quail

that last through incubation and brood-raising. Clutch and brood sizes are often large, ranging up to a dozen or more chicks, and both the cock and the hen care for the young. Individual birds have short life spans, however, and population sizes tend to fluctuate widely from year to year. All Arizona species form fall and winter coveys that are likely to remain in the same general area where they were raised.

Each species has its own habitat preferences. The Gambel's quail is found throughout the Sonoran and Mojave deserts upward in elevation through semi-desert grassland and chaparral to the edges of pinyon-juniper woodland and pine forest—wherever mesquites and other brushy cover occur. The scaled quail is a bird of semidesert grasslands and the Chihuahuan desert, preferring open plains and foothills; the Mearns' quail prefers oak woodlands and oak savannas in the southeastern portions of the state where grass cover is abundant enough to conceal its presence.

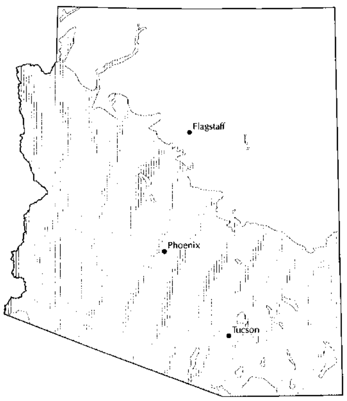

Gambel's quail distribution

Although all three major species of Arizona quail have formed pair bonds by March, they each have different breeding seasons. Gambel's quail breed only in spring and early summer, and breeding intensity and success are directly related to the amount of rainfall received during the previous October through March. The breeding season of scaled quail is more complex. They breed in spring after wet winters, but also during the summer months after the monsoons have started. Mearns' quail nest only after the summer monsoon season, and often postpone breeding until after the summer solstice when the days are getting shorter. The factors determining the population levels of the various species also differ. The numbers of Gambel's quail are related more to the success of the hatch than to carry-over from the previous year. Scaled quail numbers are determined by both the success of the hatch and the number of birds surviving from the year before. Mearns' quail generally have good hatching success, and their highly fluctuating numbers are determined largely by how many birds survive the winter. All of the birds experience relatively high winter mortality. The scaled and Mearns' quail are more dependent on grass cover for over-winter survival than is the Gambel's quail, and hence are more sensitive to livestock grazing pressures than the Gambel's.

Hunt History

By the turn of the century, quail hunting had become a popular pastime in Arizona, and a generous season and lack of a bag limit gave the state a reputation for harboring "game-hogs." Then, in 1909, the territorial legislature limited quail hunting to an open season of October 16 through January 31, an arrangement that was retained in the state game code of 1912 along with a bag limit of 25 quail. In 1929 quail numbers must have been thought to be in need of improvement, as the season was shortened to November 1 through December 31, and the following year the newly appointed Arizona Game and Fish Commission reduced the bag limit to 15 quail per day. There was no season on Mearns' or "fool quail" as this species was commonly known.

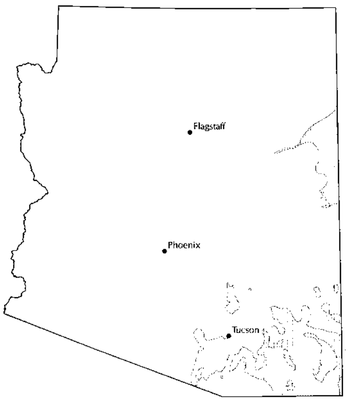

Scaled quail distribution

During the years that followed, quail seasons and bag limits varied in response to quail numbers and the success of the hatch, which in some years, such as 1946-48, was so poor that no season was authorized. It was believed that unless the ratio of young to adult quail observed on summer surveys was less than 2.1:1 a hunt could not be justified, and even when there was a season, it might be only two days long with a five-bird bag limit. Then, in the 1950s and early 1960s, research showed that hunting mortality was compensatory to natural mortality, and a standardized season from mid-October through the end of the month, followed by another season from November 1 through the end of January, gradually became the norm, along with a 15-bird bag limit. Later, the month of November was also opened to quail hunting and the closing date delayed until mid-February. This season, which applies to both Gambel's and scaled quail, has continued to the present day.

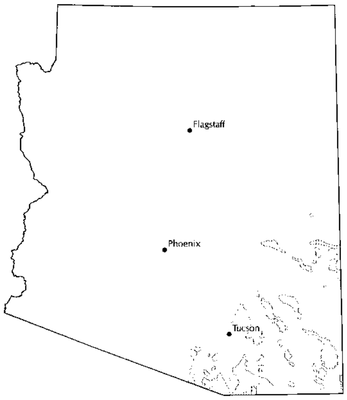

Mearns' quail distribution

In 1960 a two-day season on Mearns' quail was authorized for a limited area in the Santa Rita Mountains. Hunting was shown to have a negligible effect on this species also, and this season too was gradually expanded. Today, the season opens in mid-November in deference to the bird's late nesting habits, and continues to mid-February. This bird and season has become so popular with bird dog hunters that recent Commission meetings have often entertained proposals to lower the 15-bird bag limit to a lesser number in an attempt to "spread out the harvest."

Quail hunting in Arizona has always had its ups and downs. The top year in recent times was in 1979 when nearly 100,000 hunters reported harvesting more than 2.5 million quail. Since then, quail numbers and hunter interest have fallen off, with hunter numbers ranging from 44,000 to 75,000 each year between 1990 and 1999. The reported harvest of Gambel's quail during this same period has fluctuated from slightly more than 300,000 to just over 1.3 million, causing some hunters and wildlife managers to wonder if a long-term decline in quail numbers may have occurred.

White-winged Dove

This bird's hefty size and rounded off tail give the "whitewing" the appearance of being half dove and half pigeon, hence the older name of "Sonora pigeon." Whitewings differ from the more widespread mourning dove in having an overall grayer plumage, a white-tipped tail, and the white wing epaulets that give the bird its name. Unless pressed by gunners, the whitewing's flight also appears slower, less purposeful, and more pigeonlike than the mourning dove's. Adults can be distinguished by an unfeathered bright blue eye patch, red feet, and eyes that range from yellow-orange to orange-red. By way of contrast, birds of the year have dull purplish-brown feet and are marked mostly in grays, whites, and browns. Adult males are especially handsome birds, their brownish heads crowned in reddish purple with areas on the neck flecked with gold, green, and purple iridescence. The average weight of an adult male is about 5.5 ounces, although birds weighing up to 8 ounces have been recorded.

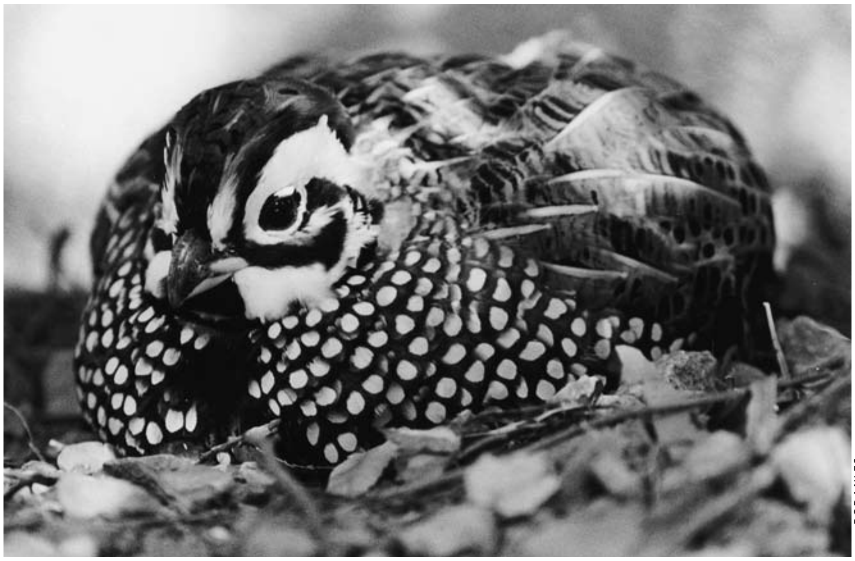
BOB MILES

Mearns' quail

Natural History

There are two types of white-winged dove populations in Arizona, a thinly scattered population found throughout the Sonoran Desert and the surrounding countryside (including towns and residential neighborhoods), and colonial populations that nest collectively along river bottoms adjacent to agricultural areas. Most of the desert and residential area whitewings nest only once and migrate out of the state prior to the opening of the dove season on September 1. The colonial whitewings, however, usually nest twice before departing for their wintering areas in

southwestern Mexico. These are the whitewings that are most often present after September 1, and which contribute most to the harvest.

Males of both populations begin courtship as soon as they arrive in Arizona in late April and early May. By late May, nesting is at its peak, both sexes sharing in the incubation of the eggs and the feeding and brooding of the two young squabs, most of which hatch toward the end of June. Fed a highly nutritious "pigeon-milk" by their parents, the squabs are usually fledged by late June or July. Should grains or other high-energy foods be available, the colonial-nesting birds will now attempt another nesting, while the "desert birds" begin migrating south. As the second nesting comes to a close in late July and August, both the juvenile birds and their parents form gregarious flocks in selected roost sites adjacent to favored feeding fields, which unlike those selected by mourning doves, are often composed of standing crops of barley, maize, and safflower. The stimuli for the mass migration from cultivated valleys that takes place about September 1 are not completely understood. Summer storms, a drop in nighttime temperatures, food shortages, and harassment by hunters have all been suggested as reasons for the movement. Nonetheless, there have been years when all of these events occurred with little or no influence on the onset of migration. Once migration is underway, the departure is often rapid, the adults usually leaving before the juveniles.

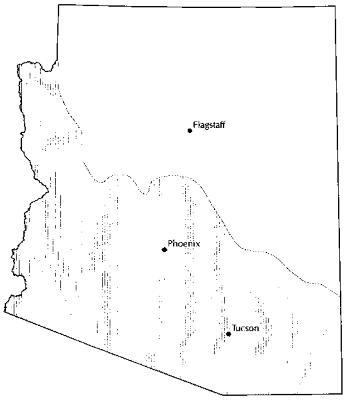

White-winged dove distribution

Hunt History

A favorable combination of nesting cover and grain crops resulted in two great heydays of white-winged dove hunting in Arizona. The first of these was in the years prior to World War I, and the second was during the years after World War II. So plentiful were these birds that the bag limit was 25 per day and 50 in possession. Numbers peaked in the 1960s when, in 1968, an all-time record harvest of more than 3/4 million was reached. Since then, declining nesting habitat and the virtual replacement of grain farming by cotton and alfalfa have greatly reduced whitewing hunting opportunities. But after reaching a low of 86,000 birds in 1980, whitewing harvests have again gradually increased. Although subject to half-day hunting and reduced bag limits, hunter numbers have stabilized during the past five years when an average of between 25,000 to 30,000 hunters have taken to the field, bagging from 122,000 to180,000 whitewings a year.

Mourning Dove

This is the most common and widely occurring game bird in Arizona, and the dove's trim, streamlined body, accentuated by its tiny head and sharply tapered tail is familiar to even the most casual observer of birds. This dove can also be differentiated from its white-winged cousin by its overall brown color, a lack of white on all but the outer tail feathers, the presence of black spots on the upper wing surfaces, and the distinctive rattling whistle that is emitted by the bird's wing feathers when it takes flight. The more richly colored adult males can usually be distinguished at all times of the year from the browner females by their pinkish rose breasts, flecks of metallic green and other iridescence on the napes of their necks, and their slate blue crowns. Adult males weigh about 4.3 ounces, females about 4 ounces, with an occasional male weighing up to 6 ounces. Juvenile birds can be identified up to 4 or 5 months of age by the white tipping on the margins of their wing feathers.

Natural History

Mourning doves occur from the lowest elevations along the Colorado River upward through forests of ponderosa pines to 8,500 feet. Their staple foods throughout the year are primarily small seeds and cultivated grains. Although some doves can be found nesting

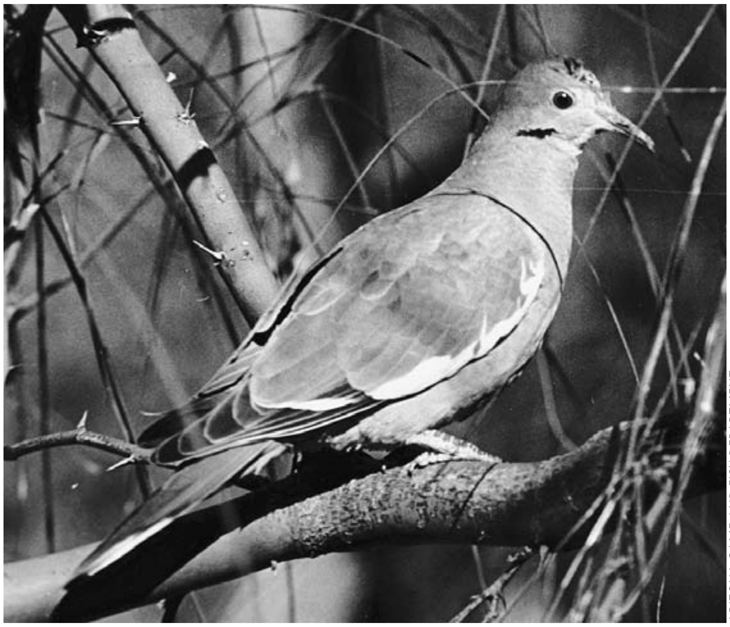

ARIZONA GAME AND FISH DEPARTMENT.

White-winged dove

on the ground in open prairies, the best nesting habitats are brushlands and woodlands within the Sonoran Desert. Here, the woeful call of breeding males can be heard as early as February, and pairs have been known to attempt as many as seven nestings in a single season. Productivity may therefore be high even though the usual clutch size is only two eggs. Incubation takes only about 15 days, and is accomplished by both parents, as is the brooding and feeding of the nearly naked squabs. The young doves are fed regurgitated "pigeon milk" by both parents, and they grow and develop rapidly. Fledglings leave the nest only 12 to 14 days after hatching. Even in southern Arizona, nesting is essentially over by mid-August, and some of the early-hatched juveniles have already migrated by late July. By the first week of September, the migration of most nesting populations is usually underway, the juveniles typically leaving before the adults.

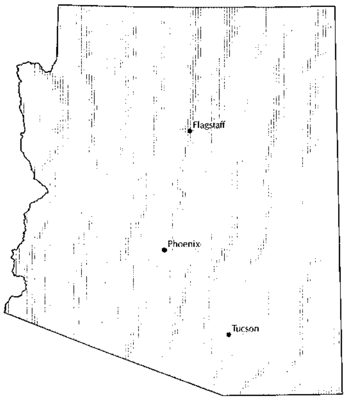

Mourning dove distribution

Hunt History

Prior to statehood this species was hunted primarily in conjunction with white-winged dove, and spring and summer shooting over grain fields was a common occurrence. In 1929, however, state and federal regulations curtailed the mourning dove season in Arizona to between September 1 and December 15, and established a 20-bird bag limit. As with the white-winged dove, the glory days of mourning dove shooting were in the 1960s and 1970s, when more than 100,000 hunters reported harvesting up to 2.5 million mourning doves a year. Although still ranked as one of Arizona's two most important game birds, mourning dove hunting has since fallen off due to urban expansion, changing farm practices, and more restrictive season arrangements. Questionnaire surveys indicate that during the past 10 years, an average of from 45,000 to 60,000 hunters bagged from 1 million to 1.3 million doves each year.

Cottontail Rabbit

Three species of cottontail occur in Arizona: the mountain cottontail, eastern cottontail, and desert cottontail. The smallest of these (22-30 ounces) is the relatively short-eared mountain cottontail, which is largely restricted to elevations above 7,500 feet from the Mogollon Rim northward. The generally larger eastern cottontail (28-52 ounces) is found in the mountains of southeastern and central Arizona where it occupies many of the same habitats as the Coues white-tailed deer. The most abundant and important rabbit by far, however, is the desert cottontail (26.5-44 ounces), which is found in every county in the state up to elevations exceeding 7,000 feet.

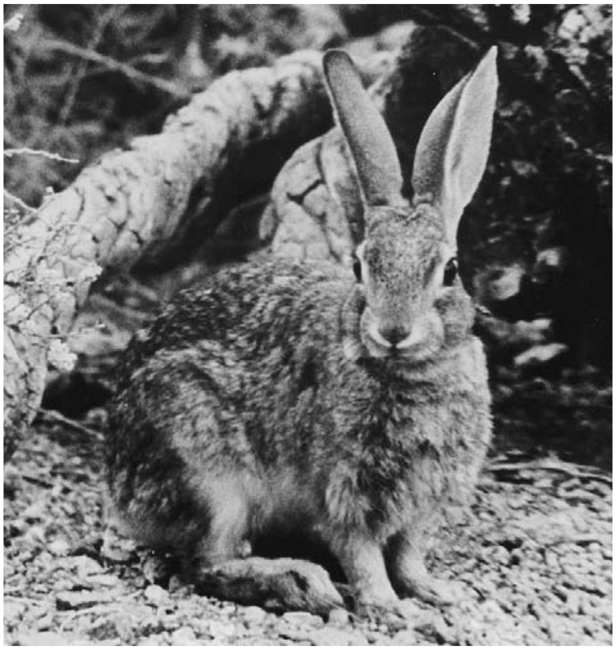
MARY IRELAND

Cottontail rabbit

Natural History

Despite, or perhaps because of, their relative abundance, little is known about the life histories of Arizona cottontails. Only one study has been conducted on desert cottontails, and none on eastern and mountain cottontails. Although we know that cottontail rabbits may vary from amazing abundance in one year to relative scarcity the next, we have little insight as to what factors other than winter rainfall control their numbers. Promiscuous and prolific, cottontails feeding on green growth may have up to five litters of two to four young a year. But, although the desert cottontail is able to breed throughout the year, most young rabbits are produced in spring

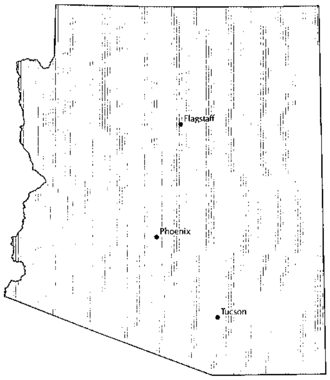

Cottontail rabbit distribution

when the new growth of plants is most available. At other times of the year, selected foods include twigs, newly emerging grasses, weeds, and even cacti. Cottontails rarely drink, and free water does not appear to be a requirement for either their survival or reproduction.

Hunt History

The cottontail hunting season has always been yearlong in Arizona, and the bag limit has been 10 rabbits per day for many years. Although some hunters consider cottontail hunting with a .22 rifle as their primary sport, cottontails traditionally have been taken in Arizona in conjunction with dove and quail hunting. As a consequence of the wide fluctuations in both cottontail and quail numbers, the annual take of cottontails is highly erratic, ranging from a reported high of about 850,000 rabbits in 1979 to less than 56,000 in 1998. The mean number of hunters reportedly hunting this animal during the past 10 years has been 18,662, and their average take has been 91,915 rabbits per year.

Tree Squirrels

No fewer than four species and eight subspecies of tree squirrels can be found in Arizona's forests. Of these, the Abert's or tassel-eared squirrel is the most widespread and contributes most to the annual squirrel harvest. This squirrel, with its easily discernible ear tufts, along with its close relatives, the black-bellied and white-tailed Kaibab squirrels, are exclusively inhabitants of ponderosa pine forests and the life cycles of the squirrels and the tree are remarkably intertwined. Less well known is the also white-bellied Arizona gray squirrel and its close relative, the rust-colored Chiricahua fox squirrel, both of which inhabit riparian deciduous forests and oak woodlands south of the Mogollon Rim. Another species is the chicaree or red squirrel (actually more olive or gray than red in Arizona), which is restricted to the higher forests of spruce and fir above 8,500 feet elevation. Both the tassel-eared and gray squirrels average a little under 1.5 pounds in weight, while the diminutive red squirrel averages just over 0.5 pounds.

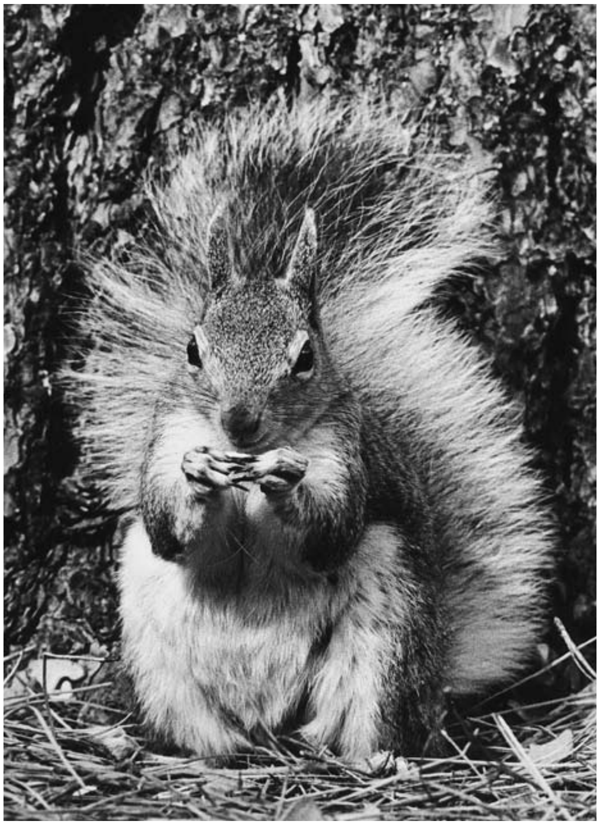
BOB MILES

Abert's Squirrels

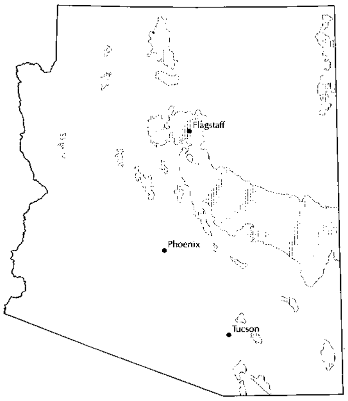

Tree squirrel distribution

Natural History

Tassel-eared squirrels have but one breeding season a year, which is closely correlated with the production of the staminate flowers of ponderosa pine in late April, May, or early June. After a lengthy chase, the female comes into estrus for only one day. She will later give birth to a single litter of from two to four young in a nest made of pine boughs. Throughout the summer, the squirrels feed on the seeds of developing cones as well as on underground fungi or truffles that grow under mature pine trees. These foods are the most nutritious for the squirrel, and only when they are exhausted does the animal resort to feeding on the inner bark of pine twigs—the discarded terminals of which are often seen littering the forest floor. These "clippings" of inner bark are only an emergency food, however, and if deep snow-cover or other factors force the squirrel to rely entirely on this food source, the animal will eventually go into shock and die. Only after years of research was it learned that the periods of tassel-eared squirrel scarcity and abundance were related to the amount of snow-cover and the availability of underground fungi. Most squirrel mortality is during the late winter, and when snow covers the ground for 80 or more days, the mortality rate exceeds the squirrel's rather modest recruitment rate. Hunting apparently has little effect on the animal's numbers as other research shows the lowest monthly mortality is during the October and November hunting season.

Hunt History

Tree squirrels have an uneven history as game in Arizona. Having gone from being totally ignored at the time of statehood, to having a limited season in conjunction with the deer and turkey seasons in the 1920s, the season was closed in 1935 due to a perceived lack of squirrels. Too many squirrels in the 1940s resulted in a re-opening of the season, and squirrel hunt regulations have since been liberalized gradually until every species and most subspecies are now subjected to limited hunting. Even the once sacrosanct Kaibab squirrel is now hunted, and the only totally protected squirrel is the federally endangered Graham Mountain spruce squirrel.

The tassel-eared or Abert's squirrel is the major game species, however, and the numbers of tree squirrel hunters and harvest depends largely on the vagaries of tassel-eared squirrel numbers. Questionnaire data collected since the early 1960s show that the peak number of hunters was in 1986 when 21,402 squirrel hunters took to the field and bagged nearly 165,000 squirrels for a hunter success of 2.5 squirrels per day. Since 1990 the number of hunters has generally averaged between 12,000 and 18,000 a year with the average annual harvest being between 50,000 and 100,000 tree squirrels.

Band-tailed Pigeon

About the size of a domestic pigeon, adult bandtails average just a little less than 8 ounces in weight, the females weighing about 0.8 ounces less than the males. Both sexes have an overall blue-gray appearance, and it is only after close inspection that one notices the male's rosier breast and more iridescence on the nape of the neck; otherwise, the sexes are similar. In autumn, adults can be differentiated from their young by the adult's chrome-yellow bills and feet, white crescent at the nape of the neck, and the dark gray band across the top of the tail that gives the bird its name.

Natural History

Bandtails are birds of the mountains and usually nest in mixed conifer forests, ponderosa pine forests, or in dense stands of evergreen oaks and pines between 4,500 and 9,100 feet elevation. As migratory birds, bandtails are usually only present in Arizona from late March thorough mid-October. Breeding generally takes place sometime in May and may continue through the summer, with some birds nesting twice and even three times in some years. The normal clutch is one glossy white egg, or occasionally two, so that the species' reproductive potential is low. After feeding on acorns and other fall mast crops, most Arizona bandtails migrate southward to the Sierra Madre Occidental in Mexico to spend the winter months.

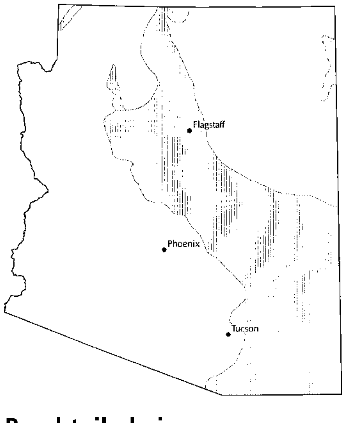

Band-tailed pigeon distribution

Hunt History

Bandtail hunting has an erratic history in Arizona. After the season was closed in 1951 for a perceived lack of birds, interest in band-tailed pigeons waned until a study was initiated in the "four-corner" states of Arizona, New Mexico, Colorado and Utah in the 1960s. These studies included an experimental season, which opened in 1968, and continued through 1972. Hunt information showed a limited but dedicated interest in the band-tailed pigeon as a game bird with the maximum number of hunters and birds harvested being 1,067 hunters and 3,545 pigeons in 1970. The numbers of both pigeons and pigeon hunters has since fallen off with only 146 bandtails reportedly taken in 1996. Now it appears that band-tailed pigeon numbers may again be inching upward.

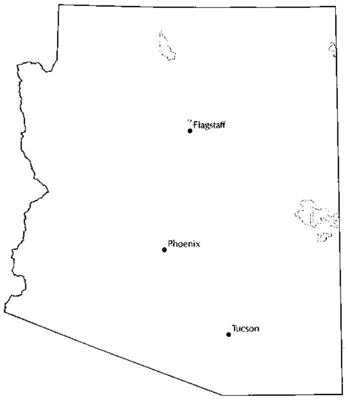

Blue grouse distribution

Blue Grouse

Blue grouse are bluish-gray, chickenlike birds restricted in Arizona to elevations above 8,500 feet in mixed conifer and aspen forests. As a consequence, these birds are only found in the White, Blue, Escudilla, Chuska, and Buckskin (North Kaibab) mountains, and on the San Francisco Peaks where they were introduced in the mid-1970s. Males are measurably larger than females, 2-year-old "cocks" weighing up to 3 pounds as opposed to the adult female's average weight of between 1.75 to 2 pounds. In comparison, first-year birds or poults typically weigh only 16 to 28 ounces during the early days of the September hunting season.

Natural History

Blue grouse in Arizona do not migrate downhill during the winter months as they do in the more northern

states. Instead, they spend the winter roosting in Douglas-fir trees, subsisting on needles until spring when the males form small "leks" or strutting grounds, which they occupy from April through June. Oftentimes these leks are located on a fallen log or in a small clearing in the forest, where the cock attempts to engage any hen that comes his way with soft "hooting" displays and "flutter flights." The peak of mating activity usually takes place during the last part of May or the first week of June, after which the male goes off to leave the hen to nest and raise the chicks on her own. Most broods are hatched between mid-June and mid-July during which time the hen and poults feed primarily on forbs and insects. Four to six is an average brood size, the young staying with the hen through the fall months. Fall usually finds the hens and poults at the edge of mountain meadows and in old burns feeding on forbs, while the now solitary males tend to favor aspen thickets and other dense cover.

Hunt History

The first legal season on blue grouse in Arizona did not take place until 1964 when 33 hunters spent 49 days to harvest 44 grouse. Since that time, a variety of grouse season dates have been authorized, but the number of grouse hunters has remained low due to the birds general scarcity and the steep terrain and high elevations of their habitat. Hunter numbers have never reached 800 in any given year, and the annual harvest since 1973 has been only 300 to 700 grouse.

Pheasant

Several attempts have been made to establish these natives of Asia as resident game birds in Arizona, the most recent being in the late 1960s and early 1970s when the small white-winged race of the ring-necked pheasant found in Afghanistan was released in farmlands along the Gila, San Pedro, and other river valleys. A handsome, unmistakable bird, both sexes of this pheasant have long pointed tails, but it is the cocks or roosters that are unrivaled in their plumage. Possessing iridescent green heads offset by ear-tufts and a crimson-wattled cheek patch, the rooster also has a purplish chest, a soot-colored belly, distinctively dotted golden flanks, white wing epaulets, and a handsomely barred tail. Cocks usually weigh more than 2.5 pounds, while the beige- and sand-colored hens average between 1.5 and 2 pounds. Both sexes, but especially the males, typically give a cackle on being flushed that once heard is always remembered.

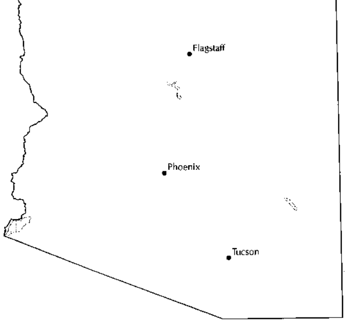

Ring-necked pheasant distribution

Natural History

Pheasant populations persisting in Arizona are largely confined to agricultural areas having a relatively high humidity (e.g., citrus orchards in the Yuma and Mesa areas) or high enough in elevation to escape the desiccating heat of Sonoran Desert summers (e.g., the Virgin River and Verde River valleys). In such locations, a rooster will acquire a harem of from one to three hens, with mating commencing in early April. By mid-May most of the hens are nesting and of no further interest to him, and he will abandon his territorial patrols by the end of the month. The peak of hatching is during the last week of May, the most arid time in Arizona, which is one of the reasons why pheasants have not become established here. The youngsters are covered with yellow and brown down, striped in brown and black, and are remarkably self-sufficient. After only about two weeks, they are capable of flight and remain with the hen for only another two months or so before making their own way in the world. Pheasants roost on the ground or the low branches of trees, and the typical hiding cover is a patch of rank weeds, a stand of cattails, or a dense jungle of salt-cedars. Primary foods are cultivated greens and grains—alfalfa, barley sprouts, and kernels of maize, barley, and corn.

Hunt History

Pheasants have always been a specialty game bird in Arizona, and are only taken by a small cadre of hunters, who either obtain one of the limited hunt permits periodically available, hunt with falcons, or hunt with a bow and arrows. With the cessation of the Department's experimental pheasant program in 1973, hunter numbers have never exceeded 100 in any given year and the annual harvest excluding birds taken in game farms has been less than 50 birds.

Summary of Small Game Harvest Information

Year	Hunters[1]	Hunter Days	Days/Hunter	Licensed Harvest	Junior Harvest	Total Harvest	Harvest/Day[2]
**MOURNING DOVE**							
1998	57,645	226,622	3.9	1,263.242	29,573	1,292,815	5.6
1999	56,157	229,623	4.1	1,278,193	44,487	1,322,680	5.6
2000	54,114	209,722	3.9	1,231,542	41,773	1,273,315	5.9
2001	49,305	229,943	4.7	1,419,539	61,934	1,481,473	6.2
2002	63,821	233,002	3.7	1,437,071	48,466	1,485,537	6.2
2003	55,672	253,989	4.6	1,332,458	46,400	1,378,858	5.3
2004	45,933	191,651	4.2	1,064,373	50,285	1,114,658	5.6
2005	62,745	270,826	4.3	1,635,491	67,648	1,703,139	6.0
The Small Game questionnaire was modified to collect unit specific data. Sample no longer weighted in analysis. The data is not comparable to historic data. In 2004 and 2005, the historic survey format and the new unit specific survey format were run simultaneously. Beginning in 2006, only the new unit specific survey format was used.							
2004	36,926	137,049	3.7	835,763	52,051	887,814	6.1
2005	33,244	131,795	4.0	825,550	75,464	901,014	6.3
2006	71,497	273,665	3.8	1,803,250	61,324	1,864,574	6.8
Dove were separated from the traditional Small Game questionnaire and surveyed using the new Dove and Band-tailed Pigeon questionnaire in 2007. The sample of hunters surveyed was derived for the list of Migratory Bird Stamp purchasers. The data is not comparable to historic data.							
2007	36,506	153,124	4.2	978,577	38,980	1,017,557	6.6

[1] Includes early and late hunters.
[2] Licensed hunters only; does not include junior harvest.

Year	Hunters	Hunter Days	Days/Hunter	Licensed Harvest	Junior Harvest	Total Harvest	Harvest/Day[1]
**WHITE-WINGED DOVE**							
1998	30,454	92,631	3.0	165,190	15,549	180,739	1.8
1999	26,689	89,709	3.4	135,226	7,903	143,129	1.5
2000	28,652	87,868	3.1	124,261	4,434	128,695	1.4
2001	21,180	77,462	3.7	97,026	5,915	102,941	1.3
2002	35,747	107,525	3.0	178,907	6,747	185,654	1.7
2003	26,598	86,120	3.2	142,269	5,442	147,711	1.7
2004	20,962	69,104	3.3	80,896	5,459	86,355	1.2
2005	29,057	98,411	3.4	134,519	5,465	139,984	1.4
The Small Game questionnaire was modified to collect unit specific data. Sample no longer weighted in analysis. The data is not comparable to historic data. In 2004 and 2005, the historic survey format and the new unit specific survey format were run simultaneously. Beginning in 2006, only the new unit specific survey format was used.							
2004	13,656	39,865	2.9	68,647	4,103	72,750	1.7
2005	12,636	36,196	2.9	64,717	7,322	72,039	1.8
2006	30,017	86,255	2.9	216,138	20,346	236,484	2.5
Dove were separated from the traditional Small Game questionnaire and surveyed using the new Dove and Band-tailed Pigeon questionnaire in 2007. The sample of hunters surveyed was derived for the list of Migratory Bird Stamp purchasers. The data is not comparable to historic data.							
2007	14,959	49,893	3.3	85,868	4,994	90,862	1.8

[1] Licensed hunters only; does not include junior harvest.

Year	Hunters	Hunter Days	Days/Hunter	Licensed Harvest	Junior Harvest	Total Harvest	Harvest/Day[1]
**COTTONTAIL RABBIT**							
1998	13,765	76,231	5.5	53,174	2,564	55,738	0.7
1999	14,366	62,033	4.3	59,661	2,593	62,254	1.0
2000	12,447	63,039	5.1	56,429	1,129	57,558	0.9
2001	12,959	62,005	4.8	50,403	3,242	53,645	0.8
2002	10,744	56,970	5.3	43,693	1,143	44,836	0.8
2003	13,614	65,741	4.8	39,092	11,968	51,060	0.7
2004	12,819	74,571	5.8	73,223	9,417	82,640	1.0
2005	18,696	93,314	5.0	77,011	3,416	80,427	0.8
The Small Game questionnaire was modified to collect unit specific data. Sample no longer weighted in analysis. The data is not comparable to historic data. In 2004 and 2005, the historic survey format and the new unit specific survey format were run simultaneously. Beginning in 2006, only the new unit specific survey format was used.							
2004	11,329	53,276	4.7	44,642	1,470	46,112	0.8
2005	13,404	75,581	5.6	59,638	3,543	63,181	0.8
2006	12,895	78,804	6.1	80,308	3,224	83,352	1.1
2007	7,015	163,222	9.6	109,781	10,398	120,179	0.7

[1] Licensed hunters only; does not include junior harvest.

Summary of Small Game Harvest Information (continued)

Year	Hunters	Hunter Days	Days/Hunter	Licensed Harvest			Junior Harvest	Total Harvest	Harvest/ Day[1]
				Gambel's	Scaled	Mearns'			
**QUAIL**									
1998	60,639	286,954	4.7	754,211	58,765	15,532	11,750	840,258	2.9
1999	60,104	311,586	5.2	708,764	44,595	29,200	11,671	794,230	2.5
2000	47,885	242,432	5.1	415,487	42,201	70,081	9,433	537,202	2.2
2001	52,432	287,878	5.5	712,215	52,204	38,556	11,584	814,559	2.8
2002	41,312	179,413	4.3	311,997	35,889	32,695	2,872	383,453	2.1
2003	51,511	274,155	5.3	674,652	28,795	44,250	12,192	759,889	2.7
2004	44,142	220,032	5.0	500,739	24,129	18,532	21,577	654,977	2.5
2005	74,991	394,749	5.3	1,455,007	55,516	33,917	23,409	1,566,849	3.9
The Small Game questionnaire was modified to collect unit specific data. Sample no longer weighted in analysis. The data is not comparable to historic data. In 2004 and 2005, the historic survey format and the new unit specific survey format were run simultaneously. Beginning in 2006, only the new unit specific survey format was used.									
**GAMBEL'S**									
2004	39,130	146,479	3.7	477,465			5,634	483,099	3.3
2005	45,644	192,910	4.2	861,392			17,478	878,870	4.6
2006	55,736	220,938	4.0	670,407			14,328	684,735	3.1
2007	37,623	239,350	6.4	481,410			7,562	488,972	2.0
**SCALED**									
2004	3,429	11,206	3.3		9,982		429	10,411	0.9
2005	3,956	13,640	3.4		23,678		531	26,209	1.8
2006	4,012	13,110	3.3		15,259		0	15,259	1.2
2007	6,302	41,404	6.6		47,265		567	47,832	1.2
**MEARNS'**									
2004	4,103	17,024	4.1			21,127	490	21,617	1.2
2005	3,366	10,274	3.1			18,069	709	18,778	1.8
2006	6,734	36,393	5.4			78,374	430	78,804	2.2
2007	6,743	34,850	5.2			80,918	1,260	82,178	2.4

[1] Licensed hunters only; does not include junior harvest.

Year	Hunters	Hunter Days	Days/Hunter	Licensed Harvest	Junior Harvest	Total Harvest	Harvest/Day[1]
**TREE SQUIRREL**							
1998	7,765	20,277	2.6	41,253	1,433	42,686	2.0
1999	15,039	47,201	3.1	83,574	3,413	86,987	1.8
2000	9,181	23,479	2.6	49,904	2,274	52,178	2.1
2001	8,972	23,241	2.6	39,751	5,129	44,880	1.7
2002	6,473	20,353	3.1	22,914	2,022	24,936	1.1
2003	7,274	24,935	3.4	26,171	3,950	30,121	1.1
2004	6,217	14,892	2.4	14,999	2,713	17,712	1.0
2005	10,755	27,201	2.5	38,442	2,410	40,852	1.4
The Small Game questionnaire was modified to collect unit specific data. Sample no longer weighted in analysis. The data is not comparable to historic data. In 2004 and 2005, the historic survey format and the new unit specific survey format were run simultaneously. Beginning in 2006, only the new unit specific survey format was used.							
2004	6,246	15,370	2.5	17,024	3,674	20,698	1.3
2005	7,263	29,642	4.1	31,591	3,307	34,898	1.2
2006	5,946	14,543	2.4	18,985	3,654	22,639	1.6
2007	9,138	29,430	3.2	40,018	2,458	42,476	1.4

[1] Licensed hunters only; does not include junior harvest.

Year	Hunters	Hunter Days	Days/Hunter	Licensed Harvest	Harvest/Day[1]
**BLUE GROUSE**					
The Small Game questionnaire was modified to collect unit specific data. The data is not comparable to historic data.					
2004	980	2,511	2.6	367	0.15
2005	1,004	3,336	3.4	295	0.09
2006	860	1,934	2.2	287	0.15
2007	945	2,899	3.1	630	0.22

Year	Hunters	Hunter Days	Days/Hunter	Licensed Harvest	Harvest/Day[1]
**CHUKAR PARTRIDGE**					
The Small Game questionnaire was modified to collect unit specific data. The data is not comparable to historic data.					
2007	252	819	3.3	189	0.23

Summary of Small Game Harvest Information (continued)

Year	Hunters	Hunter Days	Days/Hunter	Licensed Harvest	Junior Harvest	Total Harvest	Kill/Day[1]
BAND-TAILED PIGEON							
2004	612	1,531	2.5	919	0	919	0.6
2005	590	886	1.5	1,122	0	1,122	1.3
2006	501	1,791	3.6	2,006	0	2,006	1.1
Band-tailed pigeons were separated from the traditional Small Game questionnaire and surveyed using the new Dove and Band-tailed Pigeon questionnaire in 2007. The sample of hunters surveyed was derived for the list of Migratory Bird Stamp purchasers. The data is not comparable to historic data.							
2007	647	1,595	2.5	1,757	324	2,081	1.3

Summary of Willow Springs Quail Check Station Data

	2005-06	2006-07	2007-08	2008-09
No. of Hunter Days	214	323	62	133
No. of Quail Bagged	614	202	120	264
No. of Gambel's	609	181	114	222
No. of Scaled	5	3	6	0
Quail Per Day	2.9	0.6	1.8	2.0

	Gambel's	Scaled	Gambel's	Scaled	Gambel's	Scaled	Gambel's	Scaled
No. of Adult Quail Classified	129	1	108	1	66	2	25	0
No. of Young Quail Classified	240	1	29	1	40	3	96	0
Percent Young in the Bag	65	–	21	–	38	–	79	–

Summary of Freeman Road Quail Check Station Data

	2005-06	2006-07	2007-08	2008-09
No. of Hunter Days	256	248	161	131
No. of Quail Bagged	675	219	162	270
No. of Gambel's	675	219	162	234
No. of Scaled	0	0	0	0
Quail Per Day	2.6	0.9	1.0	2.1

	Gambel's	Gambel's	Gambel's	Gambel's
No. of Adult Quail Classified	73	50	61	26
No. of Young Quail Classified	272	21	32	138
Percent Young in the Bag	79	30	34	84

Summary of Punkin Center Quail Check Station Data

	2005-06	2006-07	2007-08	2008-09
No. of Hunter Days	218	94	–	102
No. of Gambel's	1436	184	69	285
Quail Per Day	6.6	2.0	–	2.8

	Gambel's	Gambel's	Gambel's	Gambel's
No. of Adult Quail Classified	228	64	11	30
No. of Young Quail Classified	1139	38	4	116
Percent Young in the Bag	83	37	–	79

Mearns' Quail Wing Barrel Data – Reported Data

Year	# of Birds Harvested	# of Hunter Days	Birds/Day	Hours Hunted	% Juvenile	Birds/Hour
1977						
1978		34		192	77.2	
1979	142	135	1.1	526	59.5	0.27
1980						
1981	101	113	0.9	488	84.9	0.21
1982	90	44	2.0		76.7	
1983		144	0.0	546	83.7	
1984	1047	277	3.8	1173.5	80.9	0.89
1985	1068	367	2.9	1513.5	68.4	0.71
1986	509	181	2.8		69.4	
1987	332	188	1.8	764.5	71.5	0.43
1988	644	305	2.1	1521.5	83.4	0.42
1989	244	213	1.1	810	55.9	0.30
1990	421	195	2.2	943	79.7	0.45
1991	750	319	2.4	1437.3	75.6	0.52
1992	703	256	2.7	1199	78.4	0.59
1993	275	172	1.6	814.5	72.9	0.34
1994	202	133	1.5	590	45.6	0.34
1995	115	150	0.8	606.5	75.3	0.19
1996	153	142	1.1	697	75.2	0.22
1997	166	128	1.3	494.5	71.1	0.34
1998	236	132	1.8	539	72.7	0.44
1999	642	226	2.8	1015	75.5	0.63
2000	1312	414	3.2	1710.25	73.8	0.77
2001	888	297	3.0	1199.5	79.7	0.74
2002	361	133	2.7	608	74.1	0.59
2003	606	218	2.8	937	77.6	0.65
2004	399	142	2.8	486	73.5	0.82
2005	591	186	3.2	735	69.4	0.80
2006	778	217	3.6	766	81.9	1.02
2007	2295	539	4.3	2044	72.7	1.12

Predators

Predatory mammals as defined by A.R.S. 17-101 are coyotes, bobcats, foxes, and skunks. Bobcats are the only predator also classified as a fur-bearer with an export tag required to ship a bobcat pelt out of state. There are no closed seasons or bag limits on any predator.

A word of caution: because of small sample sizes and vagaries in the sample frame of the hunt questionnaires, caution should be used in interpreting the annual harvests of both predators and furbearers. Most of these data are insufficient for making year-to-year comparisons, and are useful only in determining long-term harvest trends.

Coyotes

Arizona's premier predator is also an important fur resource. Found throughout Arizona, the coyote is probably the state's most familiar animal. Even where coyotes are not often seen, campers can hear their choruses of howls, yelps, and barks on almost any night. The animal's pointed ears, narrow nose, generally brown coat color, and black-tipped tail, which is usually held downward, help differentiate coyotes from dogs and wolves. The head and body length of coyotes is about 2 to 3 feet with the tail adding another foot or so. Adult males are larger than females, the two sexes averaging about 21 and 17 pounds, respectively. A very large male may attain a weight of 35 pounds. Contrary to popular belief, coyotes do not readily interbreed with either dogs or wolves.

Natural History

Coyotes are opportunists, feeding mainly on small mammals, but also on carrion, bird eggs, and vegetable matter such as manzanita and juniper berries. They also prey on pronghorn fawns, dead fish, and insects when such items are available (This is a peculiar choice of options). In urban areas, garbage, domestic cats, and small dogs are sometimes taken.

Coyotes form strong pair bonds, usually breeding between mid-January and March 15. After a two-month gestation period, from one to several young are born in a den or burrow; the average litter size being about five pups. The pups are fed regurgitated food by both parents. They leave the den when about 8 to 10 weeks old.

A coyote's home range may encompass up to 12 square miles during the spring and summer, with individual animals roaming up to 100 miles or more. Besides the ever-present threat of starvation, coyotes are also susceptible to diseases such as rabies and mange and human-caused mortality.

Hunting and Trapping History

The sport harvest of coyotes has been relatively stable during the past 10 years, about 13,000 hunters taking an average of between 30,000 and 40,000 coyotes a year. Most of these animals are taken while "varmint

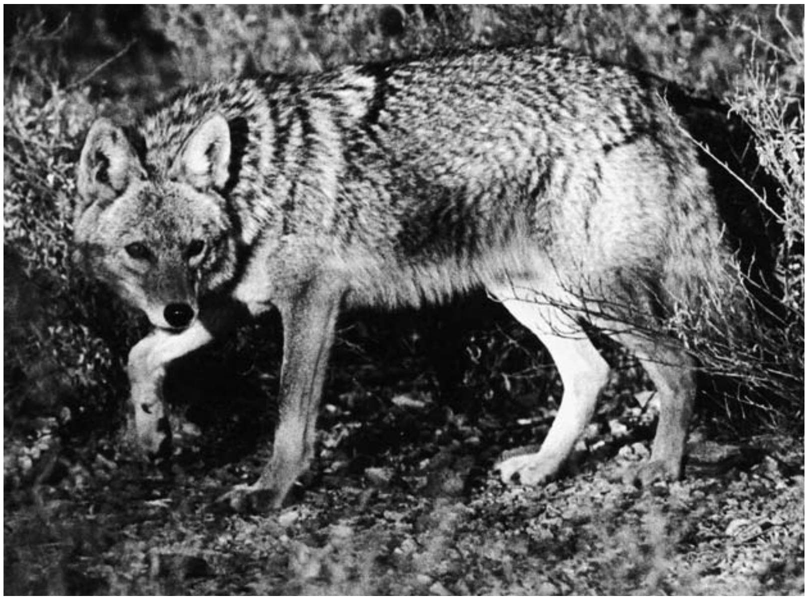

BOB MILES

Coyote

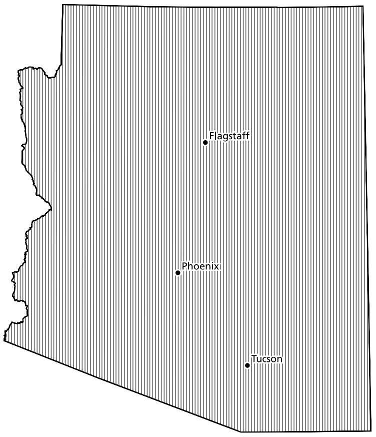

Coyote and bobcat distribution

calling," while hunting other game, or simply as opportunities arise. Formerly, trappers rivaled sport hunters in the number of coyotes taken, but the reported take of trapped coyotes during the past 10 years has averaged only a little more than 1,000 a year—a far cry from the yearly harvests of 10,000 or more coyotes reported in the late 1970s. Although some of this decline may be due to coyote population vagaries, the principal reason for this reduced take is undoubtedly a decline in trapping effort.

Bobcats

Found throughout the state in broken and brushy country, the bobcat, sometimes called wildcat, while rarely seen, is Arizona's most common wild feline. Usually an overall orange to gray in color with black markings, these medium-sized cats have a length of from 2 to 2 feet and weigh between 12 and 30 pounds. The underparts are whitish, and small ear tufts are usually present. The bobcat's most distinguishing characteristic, however, is its short, 5 inch tail, which is always less than ¼ of the length of its head and body. This feature, coupled with the animal's black spotting, can be used to distinguish bobcats from any other feline in Arizona, wild or domestic.

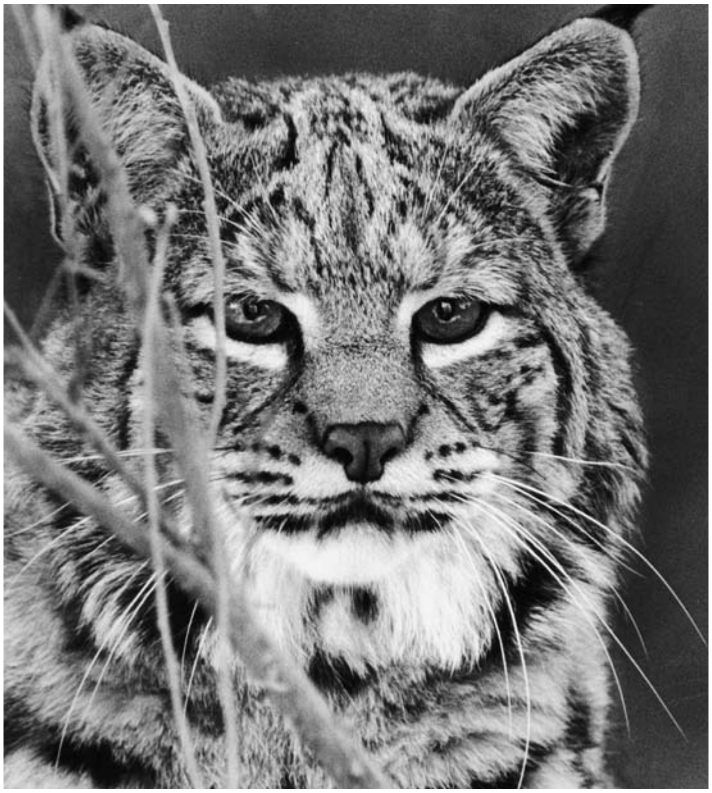
BOB MILES

Bobcat

Natural History

Little is known about Arizona's bobcats. Their principal prey are cottontail rabbits and jackrabbits, but they also take both smaller mammals such as pack rats and larger mammals including the young of some big game species. Snakes and lizards are also part of the bobcat's diet.

Bobcats require two years to mature and attain breeding age. The breeding season in Arizona is poorly documented, but appears to be mostly in late winter or early spring. The gestation period is from 50 to 60 days so that the one to three young are usually born in spring or early summer. As in most cats, the female raises the kittens alone, nursing them for two months before teaching them to hunt on their own.

Hunting and Trapping History

Sport hunters report taking between 1,200 and 1,300 bobcats a year. Most of these animals are taken while pursuing other game or by predator calling. This harvest appears relatively stable when compared to the numbers of bobcats trapped and tagged for export. Ten years ago the numbers of bobcats reportedly harvested and trapped were about equal, and 20 years ago the number of bobcats trapped was approximately seven times that taken by sport hunters. As recently as 1987, the number of bobcats trapped was reported to exceed 6,500, and more than 5,000 export tags were issued to trappers and fur dealers wanting to ship bobcat pelts out of state (Table 3). Fewer than 500 bobcats have reportedly been trapped each year since 1994.

Foxes

There are three species of foxes in Arizona–the red fox, kit fox, and gray fox. Of these, the 5- to 9-pound gray fox with its rust, black, and grizzled coloring and black longitudinally striped tail is by far the most common, occurring wherever there are mountains, wooded country, and broken terrain. The yellowish and paler red fox is of similar size (2-foot head and body with a 12

to 16 inch tail) but is uncommon in Arizona, occurring only in the northeast portions of the state. It can be differentiated from other foxes by its white-tipped tail and black ears. The 15 to 20 inch long kit fox has large, outsized ears, a 9- to 12-inch tail, and weighs less than 4 pounds. This diminutive fox is pale gray or buff in color, with a black-tipped tail. It is most often seen at night in valleys and on sandy plains in the southwestern deserts. For all three species, the sexes are similar in size and pelage.

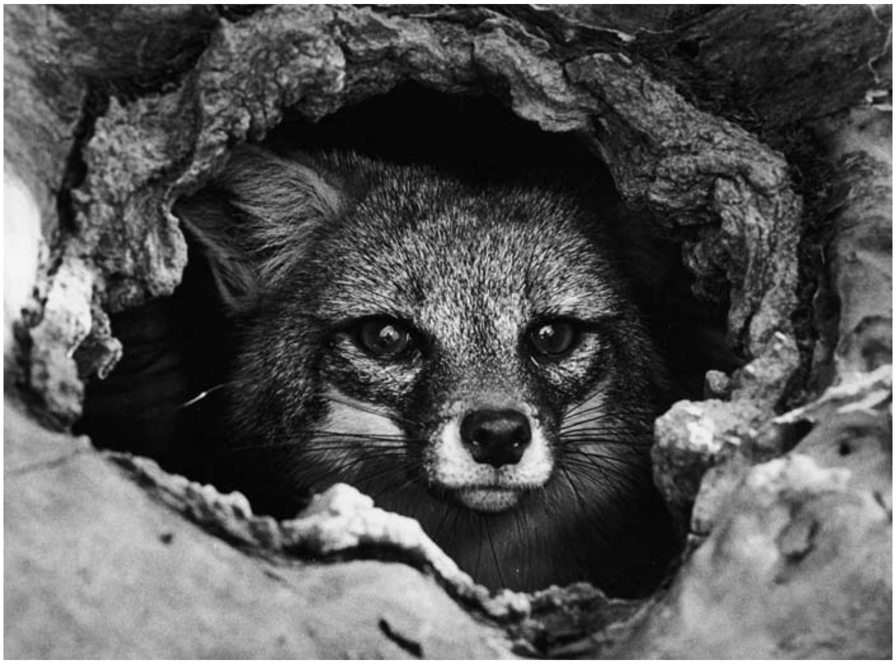

BOB MILES

Gray fox

Natural History

Gray foxes are the most often seen fox in that they are the most numerous species and are often active during daylight hours. And, although they favor brushy habitats, rock piles, and desert washes, they also climb trees and can be found in wooded areas. Kit foxes, on the other hand, prefer sandy areas, are almost exclusively nocturnal, and spend much of the day underground.

Hunting and Trapping History

More than 95 percent of the foxes taken and trapped in Arizona are undoubtedly the widely spread gray fox, the red fox occurring mostly on the Navajo Indian Reservation. Although kit foxes are remarkably easy to trap, their fur is of little value. Whatever the species, the annual take of about 3,500 foxes by predator callers and incidental hunters has been relatively stable in recent years despite any population changes due to rabies and other debilitating factors. Although the take by trappers once greatly exceeded the total taken by hunters, the average number of foxes trapped during the past 10 years was far less than the sport harvest.

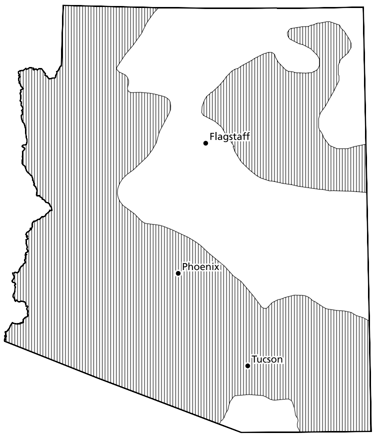

Kit fox distribution

Skunks

At least four species of skunks are found in Arizona. All of the species have scent glands on either side of their anal sphincter which secrete a secretion of musk that gives them their malodorous reputation. This defensive reaction and their striking white on black color patterns are usually enough to deter all but the most determined predator. Omnivorous, mostly nocturnal foragers, skunks are highly susceptible to the rabies virus. Indeed, early Arizonans so associated rabies with skunks that some species were termed “hydrophobia cats.”

The most common of the species by far is the cat-sized striped skunk that occurs throughout Arizona and constitutes the vast majority of the road-killed mammals seen on the state’s highways. The striped skunk is not only Arizona’s most frequently seen skunk, it is also the largest. Weights range from about 2 pounds for an adult female to an occasional 10 pounds or more for an obese male. The species always displays a thin white stripe on its face, even though the striping pattern may vary between individuals and populations. The usual markings, however, are two lateral stripes that form a chevron, merging toward the back of the head. The tail, which usually shows some white, is always shorter in length than the approximately foot-long body. Although “stripees” live almost everywhere but in the most extreme deserts, they are most often found near water. These skunks are active throughout the year and do not hibernate even in northern Arizona; the males instead form communal dens with several females.

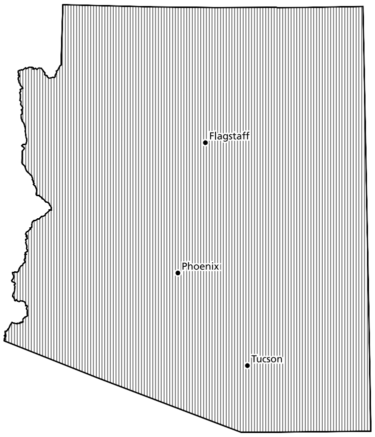

Gray fox, striped skunk, and spotted skunk distribution

The closely related hooded skunk is the striped skunk's Mexican counterpart. It is generally confined to southeastern Arizona, although specimens have reportedly been taken as far north as Flagstaff and the Mogollon Rim. Somewhat leaner than the striped skunk, hooded skunks weigh from 1 to 2 pounds and have a 12 to 16 inch long body. As for all species of skunks found in Arizona, the males are larger than the females. The white stripes on this animal are often solidly joined to form one large white streak down the center of the back, or in some individuals, are so totally separated that the skunk appears nearly solid black. The hooded skunk also differs from the striped skunk in that its foot-long tail is longer than its body. Both animals have the thin white stripe on the face and have the same general preferences for riparian habitats.

There is no problem distinguishing the western spotted skunk, also known as the civet. The average length of this diminutive fellow, including the tail, is only about 15 inches. Females average less than a pound; males are about a pound and a half. This skunk is also faster and more agile than its larger cousins. The spotted skunk's overall color is black with a white triangular patch on the forehead and a white spot under each ear. Five or six broken white stripes run down the neck, back, and sides, giving the impression of blotches or spots, and the animal its name. The animal's hair is finer than that of the other species, and the tail is tipped in white. Although reported from every county in Arizona, the spotted skunk appears to favor rocky, mountainous areas.

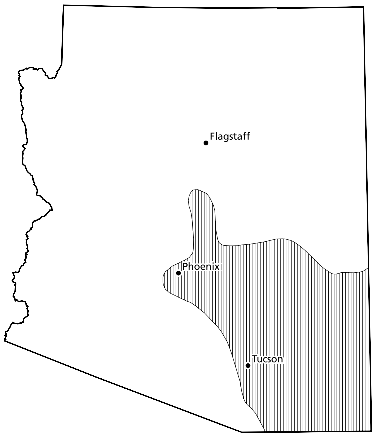

Hooded skunk distribution

The large, 2- to six-pound hog-nosed skunk is also easily identified by its entirely white back and tail and lack of any stripe on the forehead. Moreover, the elongated and slightly up-turned snout is largely naked, and the long claws on the feet are almost bear-like in appearance. This species occurs primarily in southeastern Arizona although specimens have been obtained from as far north as Flagstaff and the Hualapai Mountains.

Natural History

All of the skunks are more or less omnivores, feeding on grasshoppers and other insects, grubs, worms, mice, lizards, bulbs, carrion, and garbage. Some individuals even take to raiding hen houses, taking not only the eggs, but chickens as well. Even the hog-nosed skunk, which digs for most of its food, will eat fruits and carrion on occasion.

The striped, hooded, and hog-nosed skunks all mate in late winter and early spring, and produce from two to four young in April or May. The spotted skunk breeds in late September and early October, but the fertilized egg remains in a state of arrested development until March or April when implantation occurs with the two to four young being born about a month later. The young of all the skunk species are raised and on their own by early fall. Few skunks live more than a year or two.

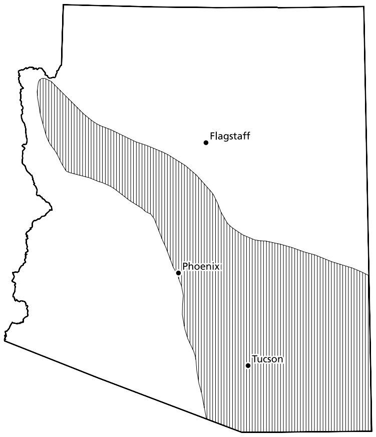

Hog-nosed skunk distribution

Trapping History

Formerly a major furbearer, striped skunks in Arizona have dropped in average take to fewer than 100 per year since 1995. This is in some ways unfortunate, as uncontrolled populations of these animals are prone to rabies and constitute a health hazard to other carnivores, as well as to humans. Although the amount is undoubtedly small, it would be interesting to know what percent of the number of skunks trapped constitutes spotted and hog-nosed skunks.

Furbearers

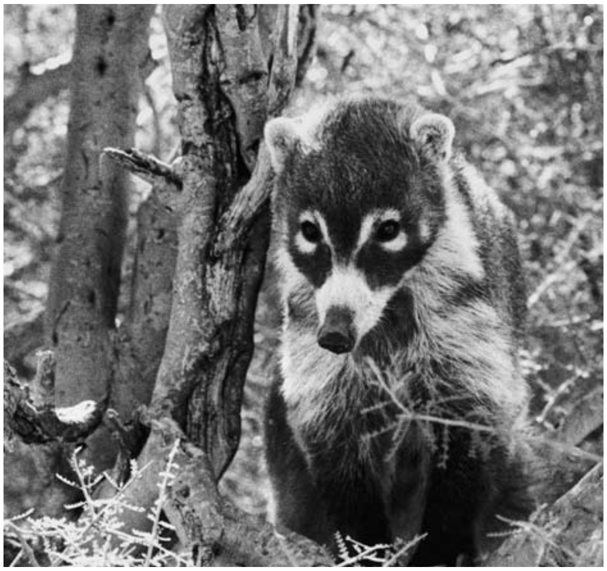

GEORGE ANDREJKO

Coati

Fur-bearing mammals are defined as muskrats, raccoons, otters, weasels, bobcats, beavers, badgers, and ringtails. Of these, only the bobcat is also considered a predatory animal. All mammals not classified as game mammals, predatory animals, or furbearers are considered "nongame mammals." These include opossums, coatis, black-footed ferrets, Gunnison's prairie dogs, black-tailed prairie dogs, wolves, jaguars, ocelots, and porcupines. Of these, only Gunnison's prairie dogs and coatis may be taken during an open season, with the bag limit on coatis being one per calendar year. No season for the taking of jaguars, ocelots, wolves, or porcupines exists.

Beaver

There is no mistaking a beaver–no other Arizona rodent even comes close to weighing between 30 and 60 pounds and exceeding two feet in length. Moreover, the beaver is uniquely adapted to an aquatic existence with a flattened, naked, nine to 10 inch long, oar-like tail, webbed hind feet, dense fur, and eyes positioned high on the head. Both sexes are similar in size and possess pungent scent glands called "castors" on either side of their anus. Arizona specimens are typically a light yellowish cinnamon color in contrast to the browner animals found in other states.

Beavers were at one time found nearly everywhere in Arizona that there was permanent water. With settlement, and the desiccation of the state's streams, beaver populations declined. This habitat loss, and in some cases, heavy trapping pressure, caused beavers to disappear from such former strongholds as the San Pedro and Santa Cruz rivers. Introductions and natural colonizations have since enabled the beaver to recover much of its former distribution, if not numbers, and these animals can now be found along several permanent streams, some of the larger river stretches, certain shallow lakes, and even a few dirt-lined canals.

Natural History

The beaver's diet is almost exclusively plant material with the bark of cottonwoods, aspen, and willow trees being especially important. Other reported foods include tamarisk or salt-cedar, mesquite, and the roots of such tuberous aquatic plants as cattail and bulrush. Even in those places where beavers are rarely seen, their activities are conspicuous–chiseled and felled trees, brush dams along small streams and backwaters, and stick houses or "lodges" constructed either as a separate residence or within the beaver dam itself. Even more common are "bank houses," dens excavated in river or canal banks. Whatever its construction, the den will be located above the water line, lined with cattails and grasses, and will provide a nursery area for the two to four "kits" or young beavers born in the spring.

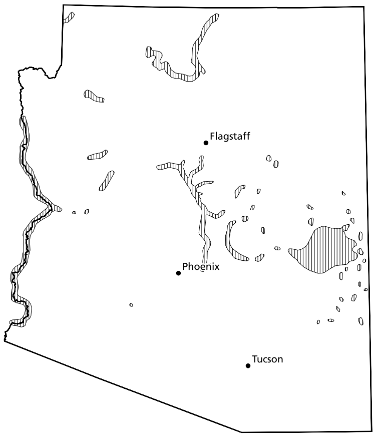

Beaver distribution

Trapping History

The average annual number of beavers trapped greatly declined since 1991 and is now virtually insignificant. Even if the fur market recovers, this species will probably never again be an important fur-bearer in Arizona due to the limitations on trapping and the limited areas of quality beaver habitat remaining.

Muskrat

A large water vole, this rodent is about a foot long with thick, silky fur and a naked, eight to 11 inch tail flattened on the side. The sexes are similar in size and weigh from 2 to 4 pounds. Most muskrats in Arizona are rusty reddish brown in color; young animals are darker than the adults, some being nearly black. Although the muskrat is highly adapted to an aquatic existence, its hind feet, while comparatively large, are not webbed like those of a beaver's.

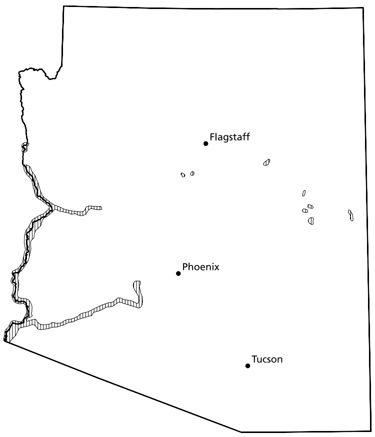

Muskrat distribution

Muskrats can be found along most of Arizona's perennial rivers and permanent marshes. Although they forsake most small streams, they can also be found along dirt-lined canals. Never particularly abundant other than locally (e.g., Montezuma Well and Peck's Lake off of the Verde River), muskrats have disappeared from some areas (e.g., the San Pedro River) and invaded others.

Natural History

Primarily a vegetarian, the muskrat feeds on aquatic grasses, pondweed, cattail roots, and the leaves of seep willows. Although many muskrats live in bank burrows, these animals also construct distinctive conical houses of shredded cattails and other marsh vegetation in quiet waters. These dens, which may serve as feeding areas, shelter areas, or nursery sites are all entered through submerged passageways. The nursery dens are the most elaborate, typically consisting of several chambers some of which are lined with grass and soft vegetation.

Muskrats in Arizona are reported to breed during every month of the year, but most of the young are born between March and October. The usual litter size is five or six.

Trapping History

Muskrats were never an important fur animal in Arizona, and the number trapped has been virtually nil since the late 1980s. Given the low state of the fur market and the limited distribution of this aquatic mammal, this status is likely to continue.

Raccoon

This medium sized carnivore is readily identified by its heavy-set body, grizzled brownish-gray appearance, black facial mask, and banded tail. The sexes are similar and measure from about 1 feet to 2 ⅓ feet in length with an eight to 12 inch tail that is alternately ringed in light and dark. Weights range from about 12 to 35 pounds.

A relatively common animal along Arizona's perennial streams, lakes, and reservoirs, raccoons can also be found near some of the larger stock tanks and in rural areas where permanent water is available. Although not often seen in the wild because of its nocturnal habits, the raccoon's distinctive five-toed tracks are commonly observed in mud around stock tanks and along river courses. These animals are adept climbers as well as swimmers.

Raccoons are omnivores, eating whatever food is available–aquatic insect larvae, beetle grubs, fish, frogs,

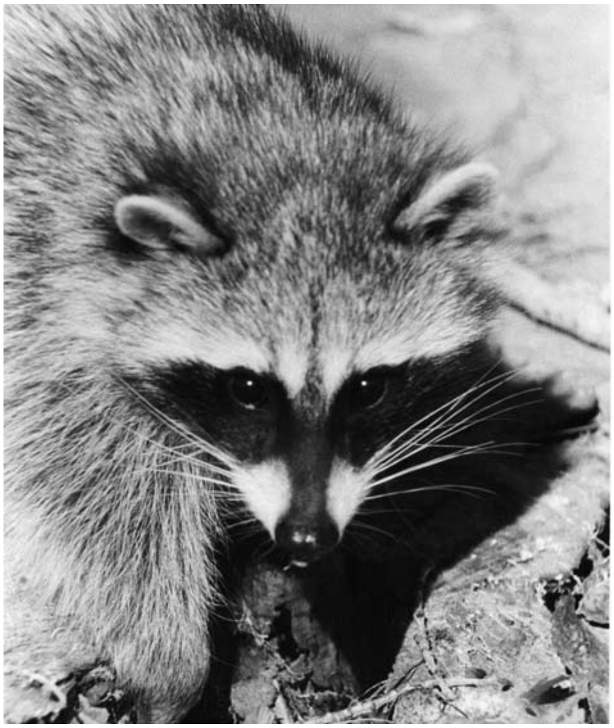

PAT O'BRIEN

Raccoon

crayfish, wild fruits, and even carrion. In certain areas, these animals can be a nuisance, not only raiding garbage cans, but also committing depredations on poultry houses, corn fields, and fruit trees. Nonetheless, raccoon meat is considered edible by some people, and the animal is considered more a game species than a furbearer.

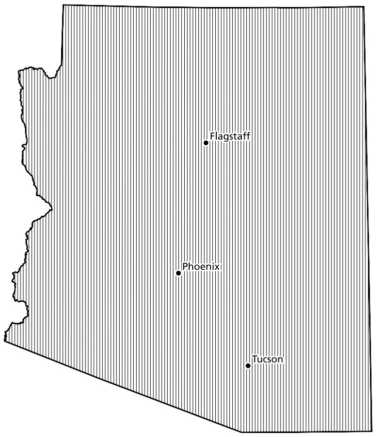

Raccoon and ringtail distribution

Natural History

Raccoons have been little studied in Arizona, and their life history here is not well documented. The two to five young are presumably born in spring in a den that may be located in a rocky crevice, brush-pile, or hollow tree. The young remain with the female until the fall when they are left to find their own way in the world.

Trapping and Hunt History

Both pursued with dogs as game, and trapped as a furbearer, the raccoon is somewhat unique in that it is the only animal in Arizona that can be legally taken with a firearm at night. Because of their limited distribution near water, "coons" have never been important furbearers, and annual harvests from trapping have rarely exceeded 1,000 pelts. With the decline in trapping activity over the past 10 years, this take has been reduced to only a few dozen raccoons a year. Although its nocturnal habits make for few incidental takings, the raccoon's status as a game animal appears more stable. Hunt questionnaire data from general license buyers indicate an annual harvest of another 1,200 animals a year. Most of this harvest is undoubtedly by hunters with hounds.

Ringtail

Ringtails have long, slender bodies from 14 to 16 inches in length with bushy, equally long black and white banded tails. The fur is a soft grayish brown with black-tipped hairs. Both the ears and eyes appear oversized, and the latter are outlined in white making them seem even larger. The legs are short, and the hind feet can be rotated 180 degrees like those of a tree squirrel, enabling the animal to descend vertical surfaces. Weights vary from 2 to 3 pounds, the males being slightly larger than the females. Primarily a night-time animal, ringtails can be extremely bold and unconcerned about the presence of humans. Calls consist of a repertoire of barks, chirps, growls, howls, and yips.

Ringtails are most common in the rocky regions of southern and western Arizona with the Grand Canyon being especially favored with the presence of these animals. About the only areas devoid of ringtails are flat, alluvial valleys in that the animal prefers rocky hillsides, canyons, rock-walled houses, and mine shafts.

Natural History

The ringtail's diet varies with the seasons but usually consists of small mammals, birds, lizards, and insects, as well as plant fruits, e.g., tomatillo berries. In farm areas, the ringtail may be an important predator on chickens and other poultry. Generally, four young are born in the spring.

Trapping History

Not having a particularly valuable pelt, the relatively easily trapped ringtail is most often trapped during times when fur prices and trapping activity are high. These animals can also be quite common, and in past years ringtails contributed substantially to the state's fur harvest. The take in ringtails has dropped off significantly in recent years, however, and now consists of only a couple of dozen animals.

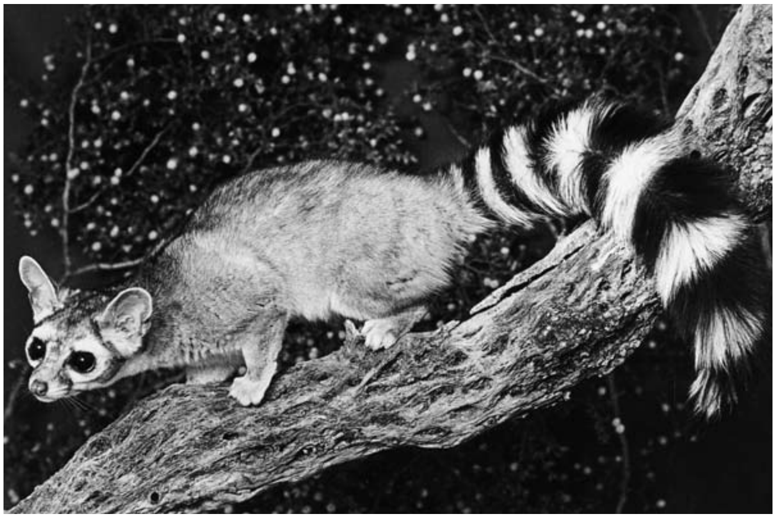
BOB MILES

Ringtail

Otter

Wonderfully adapted to an aquatic existence, the otter's elongated body terminates in a streamlined tail that tapers from a thick base to a pointed tip. Also contributing to the otter's fusiform shape is its flat-

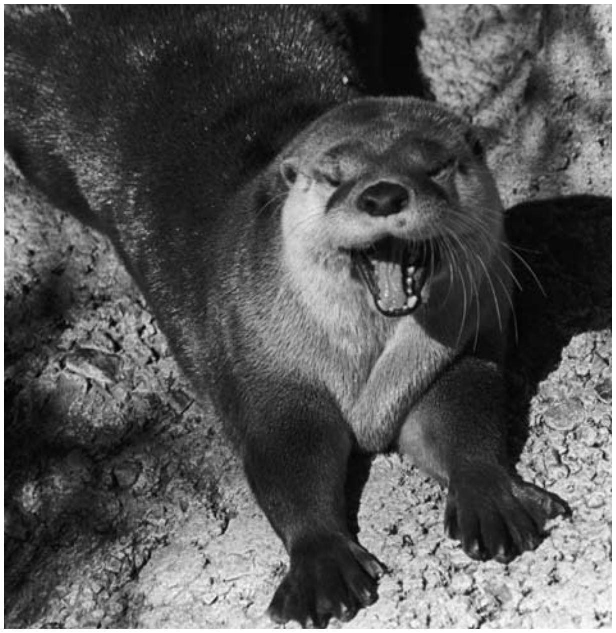
BOB MILES

Otter

tened head and small ears, the openings of which can be closed at will. The legs too are short, and the hind feet are webbed to the toes. The color of the densely furred coat is a rich chocolate brown with whitish underparts. Adults generally weigh from 12 to 20 pounds with lengths ranging from about 3 feet to just over 4 feet. The otter's webbed, rhomboid tracks are easily distinguishable from the also webbed, but elongated hind tracks of the beaver.

Once found throughout the Salt, Verde, Little Colorado, and probably also the Gila, and Colorado river systems, this species is now confined to the Verde River and its major tributaries where it was reintroduced in the early 1980s.

Natural History

Although most otter activity is at night, hunting is by sight as well as touch, and clear streams appear to be favorite haunts. The otter's usual fare is fish, waterbirds, turtles, eggs, and crawfish, the latter now being the most conspicuous food item in their droppings.

The breeding season in Arizona is uncertain, but otters elsewhere usually breed in late winter or early spring. Mating usually occurs in the water. Pregnancy lasts about two months, but because of delayed implantation gestation may take up to a year. Dens are located in natural shelters under rocks, logs, flood debris, or in river banks. Litter sizes vary, but usually consist of two or three pups. Weaning requires approximately three months, after which the young disperse.

Trapping and Hunt History

Otters were never numerous enough in Arizona to provide an important fur resource, although old photos show these animals being trapped and otherwise taken for their pelts prior to 1930. Secondhand reports indicate that some otters may also have been killed as fish predators. Whatever its past status, this species is now completely protected in Arizona and has been for many years.

Weasel

Only one species of weasel occurs in Arizona–the long-tailed weasel, which is readily identified by its dark brown coat and orangish underparts. Some white is often present on the head, and some animals may turn all white in winter. Male weasels are larger than the females, the animals ranging in length from 8 to 10 inches with the black-tipped tail adding another 4 to 6 inches. Weights range from 7 to 12 ounces for males and from 3 to 7 ounces for females. Voice is a high-pitched shriek.

Weasels in Arizona are largely restricted to high elevation wooded areas such as the Kaibab Plateau, Mogollon Rim, Chuska-Lukachukai mountains, and southern Arizona's sky-islands.

Natural History

Weasels are voracious predators, taking cottontail rabbits, hares, and rodents much larger than themselves. They also take birds, snakes, and lizards.

Weasels breed in midsummer, but, because of delayed implantation, the four to eight young are not born until the following spring. Usually nests in old burrows or under rock piles and other debris.

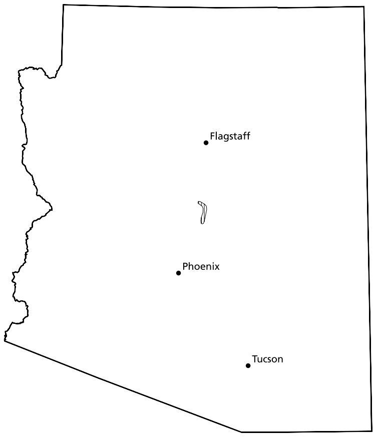

Otter distribution

Trapping History

No record is kept of the number of trappers who claim to take this animal. The number of

weasels trapped in Arizona is assumed to be very low, however, due to the animal's limited distribution and numbers, small pelt, and the current low number of trappers.

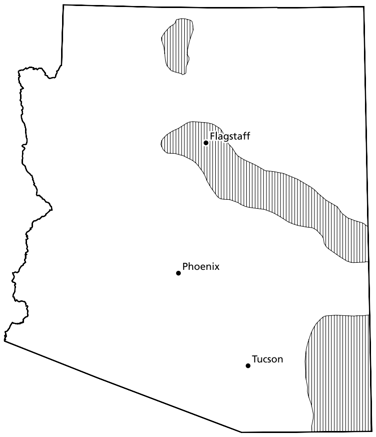

Long-tailed weasel distribution

Badger

A short, squat, medium-sized member of the weasel family, the badger is readily recognized by its grizzled gray, white, and black fur, cheek stripes, short legs, long claws, and the white stripe down its head and back. Adults may weigh from about 10 to 20 pounds and are approximately 20 inches long, with the tail adding another 4 to 6 inches in length. Widely distributed, the badger occurs almost anywhere in Arizona having ground suitable to dig in and excavate burrows.

Natural History

Badgers feed primarily on burrowing rodents such as prairie dogs and ground squirrels but also take snakes, lizards, and insects on occasion. Mating in these usually solitary animals takes place in the summer, the young being born the following spring due to delayed implantation. Primarily a nocturnal animal, badgers are sometimes encountered during the early morning hours.

Trapping History

Although the take of badger pelts averaged more than a 1,000 a year in the late 1970s and early 1980s, the number of these animals recently trapped in Arizona is virtually insignificant. A few badgers are undoubtedly also taken incidental to pursuing other game, but these numbers too must be very small. Probably less than 50 badgers a year are taken in the state.

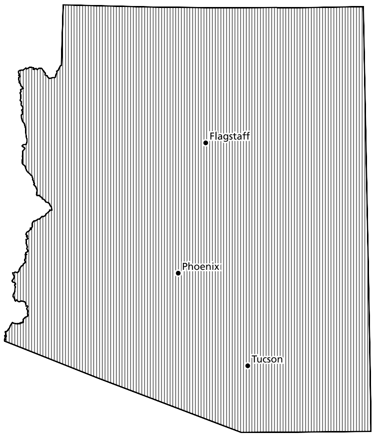

Badger distribution

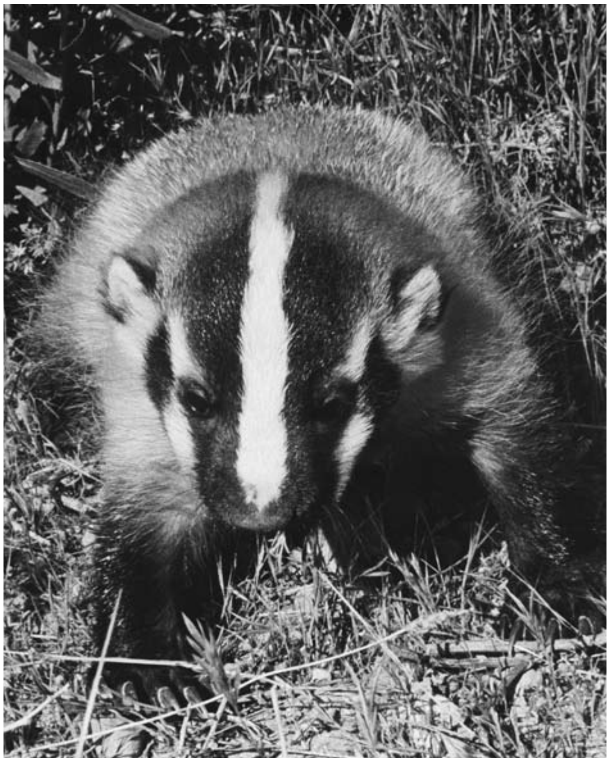
BOB MILES

Juvenile badger

Trapping

Trapping has had a long and interesting history in Arizona. Indeed, the first Anglo-American explorers to Arizona were trappers who worked the state's waterways for beaver in the 1820s and 1830s. Since that time, the popularity of trapping has fluctuated widely with the vagaries of the fur trade, the numbers of trappers and animals trapped increasing when fur prices were high, and decreasing when numbers were low. The popularity of beaver skin hats prior to 1850 fueled the early interest in trapping beaver in the Gila and Colorado river systems. Raccoon coats were popular in the 1920s as were a number of other furs. The most recent surge in trapping activity in Arizona was generated by prohibitions in the trade of spotted Neotropical cats during the 1970s. Spotted cat fur was then being highly used by foreign fashion houses as trim on ladies coats. This ban increased the demand and price for legal spotted cats, and the prices paid for bobcat pelts soared through the mid-1980s when they plummeted due to changes in fashion decorum.

Depredation activities have also greatly influenced the amount of trapping activity. Trapping was widely practiced around the turn of the 19th century due to generous bounties being paid on everything from coyotes to wolves. In addition to commercial trapping for furs and bounties, many ranchers and homesteaders also trapped, both to protect their livelihood and

to help make ends meet. Nor was all of the trapping carried out in the private sector; both the federal Predatory and Rodent Control branch of the U. S. Biological Survey and the state Arizona Game and Fish Commission employed professional trappers after 1915, and the federal government continues to do so. One of the oddest situations occurred in the late 1940s and early 1950s when the price of pelts was low. Plagued by complaints of beaver damaging irrigation canals, the Arizona Game and Fish Department hired crews of beaver trappers to reduce the number of depredation complaints.

Generally speaking, fur prices and trapping activity were high during the 1890s, and again during and shortly after World War I. After declining in the early 1920s, prices again rose in the mid-1920s before again falling in the 1930s. Prices picked up again during World War II, but collapsed shortly afterward before reaching another bottom in the 1950s. Prices gradually improved through the 1960s, and then accelerated in the early 1970s until the price of coyote and bobcat pelts peaked in the late 1970s and early 1980s. Since that time, competition from highly realistic faux fur and the declining use of fur in the highly volatile fashion industry have lowered fur prices even further. Another severe blow to the trapping industry was received in 1994 when a public initiative was passed in Arizona banning the use of leg-hold steel traps on public lands. Although trapping is still legal on private lands, all of these events served to depress the trapping industry until there are now fewer than 150 licensed trappers in the state of Arizona.

Summary of Predator and Furbearer Harvest

Year	Hunters	Hunter Days	HARVEST			
			Bobcats	Coyotes	Foxes	Raccoons
1981	13,004	96,598	1,212	24,877	3,231	0
1982	11,130	75,258	958	25,062	3,980	0
1983	11,342	71,954	817	19,780	1,361	0
1984	12,395	78,797	1,012	19,478	1,391	0
1985	13,835	85,793	655	26,933	1,555	0
1986	15,710	114,411	911	36,771	2,960	0
1987	11,442	82,558	1,011	24,527	1,896	0
1988	10,595	58,855	408	28,234	1,281	0
1989	10,558	99,284	676	27,876	1,664	0
1990	9,521	83,913	317	17,075	952	1,079
1991	10,128	76,131	1,274	23,275	1,140	805
1992	9,028	81,931	1,262	18,299	1,796	534
1993	13,083	86,968	907	30,455	3,156	1,101
1994	10,125	93,425q	880	22,378	1,395	240
1995	13,910	93,425	791	30,350	2,337	2,215
1996	13,997	119,052	547	37,929	3,516	2,977
1997	12,279	106,681	3,235	33,469	8,134	382
1998	11,134	68,727	630	19,231	2,306	948
1999	14,535	100,626	1,463	45,781	4,934	2,382
2000	15,385	101,679	1,539	42,526	7,028	932
2001	13,570	132,768	1,538	33,589	5,587	1,164
2002	10,489	68,404	1,484	22,054	2,239	123
2003	12,365	93,589	3,257	46,253	5,566	248
2004	13,346	104,243	4,076	35,354	4,272	114
2005	19,263	120,712	1,769	46,716	5,014	592
The Small Game questionnaire was modified to collect unit specific data. Sample no longer weighted in analysis. The data is not comparable to historic data. In 2004 and 2005, the historic survey format and the new unit specific survey format were run simultaneously. Beginning in 2006, only the new unit specific survey format was used.						
2004	12,615	114,146	2,388	22,107	3,368	245
2005	12,695	220,426	2,775	35,960	4,429	118
2006	13,970	182,180	2,006	45,133	2,426	215
2007	18,969	279,935	2,332	54,701	2,962	3,781

Summary of Trapping Numbers and Harvest Data For Predators and Furbearers [1]

Trapping Year	No. of Licensed Trappers	No. of Trappers	TRAPPING HARVEST							
			Coyote	Bobcat	Skunk	Muskrat	Ringtail	Badger	Raccoon	Beaver	Fox
1976-77	1,820	1,732	17,963	7,272	3,187	793	642	1,609	5,230	65	14,334
1977-78	1,621	1,070	13,732	4,695	554	301	356	595	520	57	12,648
1978-79	1,233	1,281	17,882	6,754	1,052	76	1,098	1,316	891	8	17,585
1979-80	2,098	1,888	16,605	6,648	4,119	593	2,055	1,065	894	268	21,780
1980-81	2,008	1,834	14,858	9,537	4,119	2,949	3,222	1,124	823	83	28,059
1981-82	2,219	1,964	25,379	8,036	4,115	14	4,027	1,384	1,127	117	29,124
1982-83	1,746	1,609	17,436	5,928	4,164	42	2,964	1,105	690	21	20,856
1983-84	1,129	1,006	11,763	4,827	3,275	0	2,371	874	518	0	15,857
1984-85	1,127	1,038	13,188	5,399	2,478	235	3,096	705	951	52	20,776
1985-86	1,129	1,022	11,263	4,942	3,082	111	2,649	697	735	40	18,065
1986-87	1,163	1,029	14,198	6,421	2,400	18	3,851	780	876	87	21,000
1987-88	1,315	1,165	13,335	6,609	2,537	23	4,475	748	834	127	22,009
1988-89	852	695	6,397	3,174	1,255	25	1,968	281	241	80	14,516
1989-90	444	348	3,140	1,253	590	0	1,091	89	190	202	5,210
1990-91	222	161	1,135	322	154	0	174	33	67	28	1,807
1991-92	265	189	2,214	878	336	0	403	151	84	52	2,864
1992-93	234	202	2,372	723	300	0	258	69	49	9	3,445
1993-94	194	181	2,683	1,362	271	12	372	44	74	0	5,312
1994-95	109	85	654	181	170	0	157	24	24	0	1,647
1995-96	34	24	178	55	46	0	12	8	0	0	144
1996-97	84	57	1,307	251	89	41	30	11	57	19	648
1997-98	86	46	1,437	286	61	3	15	21	49	52	685
1998-99	81	57	1,213	312	114	0	8	27	114	16	798
1999-00	75	58	1,096	144	144	0	29	17	37	0	470
2000-01	64	32	182	109	83	0	19	10	35	3	240
2001-02	66	29	305	97	25	0	3	7	7	9	143
2002-03	65	13	274	37	35	0	8	2	8	10	54
2003-04	122	58	635	267	97	0	31	25	23	3	312
2004-05	140	82	710	432	72	0	12	70	21	9	423
2005-06	122	76	820	742	119	0	17	33	25	13	484
2006-07	140	83	670	957	188	1	35	26	19	10	751
2007-08	133	94	806	944	123	0	49	41	169	22	1,008
2008-09[2]	192	113	707	1,124	268	0	33	35	14	5	1,173

[1] Not including Indian Reservations.
[2] 2008-09 data is preliminary.

Waterfowl

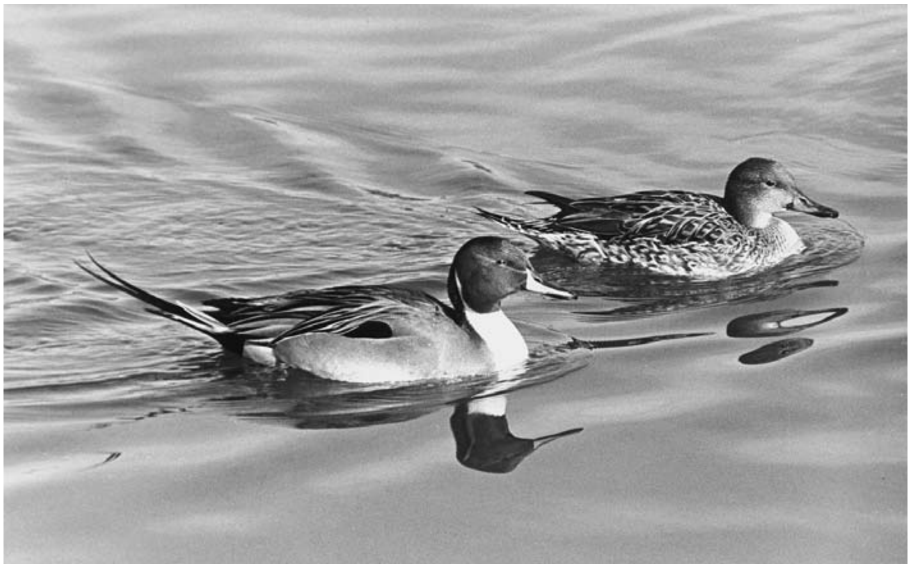
BOB MILES

Drakes

Natural History

Arizona's waterfowl can be grouped into two general classes—ducks, geese, and coots that nest in the state, and those that merely winter here or migrate through. The number of waterfowl raised in Arizona each summer, although few, is of great importance because these birds represent our state's breeding stock. The much more abundant migrants, though present only for limited periods of time between August and March, constitute most of Arizona's waterfowl harvest. Hunt regulations have been designed to accommodate both groups.

Arizona's principal waterfowl nesting grounds are the natural and modified marshes found above the Mogollon Rim and in the White Mountains. Most of these marshlands depend on winter precipitation and snow-melt rather than groundwater, are more or less seasonal, and are mostly located above 7,000 feet elevation. Examples include Mormon Lake and Marshall Lake on the Coconino Plateau, and Basin Lake and Nelson Reservoir in the White Mountains. Farm ponds and other small wetlands in the southeastern and southern parts of the state can also be expected to produce a few broods of Mexican ducks and black-bellied whistling ducks each year.

The principal duck species nesting in Arizona are mallards (especially in the White Mountains), pintails, cinnamon teal, redheads, and ruddy ducks. In addition to these "big five," smaller numbers of gadwall, green-winged teal, blue-winged teal, and ring-necked ducks are produced in northern Arizona marshes. Even less common are the occasional pair of canvasbacks, shovelers, and American widgeon. Most of the ducks that migrate through or winter in Arizona are from the Great Basin or "intermountain" states, with significant numbers of pintails and green-winged teal coming from the prairie states and provinces.

Arizona also hosts a few nesting Canada geese or honkers. These birds, which were introduced by the Arizona Game and Fish Department, are found primarily on shallow lakes east of the White Mountains between 6,000 and 7,500 feet elevation. Far more important to hunters are the more than 15,000 Canada geese that make their winter home in Arizona. The great majority of these birds are referred to as the Rocky Mountain Population of Canada goose, which nest in the intermountain states. A large goose, the males or ganders typically weigh about 9.75 pounds, the females about 8.25 pounds. The vast majority of these geese, along with several hundred snow geese, winter along the lower Colorado River on Cibola, Havasu, and Imperial National Wildlife Refuges, and in a few central Arizona locations such as Roosevelt Lake. A few white-fronted geese also pass through the state in September on their way to unknown wintering locales in Mexico.

The numbers of both nesting and wintering water-

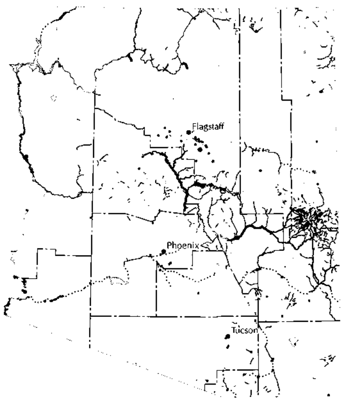

Watershed

fowl in Arizona vary sporadically from year to year depending on the vagaries of winter precipitation in the Great Basin region. Wet years generally see an increase in waterfowl production, while drought years result in fewer ducks being produced. A serious problem facing both nesting and migrating waterfowl is that our wetlands are increasingly difficult to manage for ducks and geese because of the limited occurrence of these habitats and the competing uses resulting from Arizona's human population boom. Nesting waterfowl require protection from disturbance, and many former nesting sites are no longer productive due to the introduction of predatory game fish and summer-long recreational use. One bright note of late has been the creation of wetlands using treated sewage effluent. These "municipal marshlands" are primarily managed as waterfowl nesting and resting areas. Working in conjunction with the Arizona Game and Fish Department and U.S. Forest Service, cities such as Pinetop-Lakeside, Show Low, and Sedona have developed a number of these nutrient-rich and highly productive wetlands that are heavily used by waterfowl, as well as a variety of other wetland dependent species.

Hunt History

When Anglo-Americans first arrived in Arizona, they found migrating and wintering waterfowl concentrated along the state's few major rivers. The lower Colorado and Gila rivers were especially noted as havens for waterfowl, with great clouds of the birds seen along the muddy banks by explorers, fur trappers, and steamboat passengers. Nor were nesting waterfowl in short supply; travelers across northern Arizona reported that they flushed a myriad of ducks in the shallow marshes on the San Francisco Plateau.

Unlike other states, early Arizona never experienced market hunting for waterfowl as a major enterprise. Prior to statehood, most duck shooting, when not for sport, was for personal subsistence. Settlers not only hunted waterfowl during spring, fall, and winter, they also gathered the ducks' eggs in spring. Gradually, with the development of the state's economies, this subsistence hunting gave way to sport-hunting, and irrigation ponds, canals and stock tanks became increasingly important waterfowl hunting locales. By the time that America entered World War I, waterfowling was one of the state's most popular outdoor pastimes—one that even attracted the attention of Arizona's often elected Governor George P. Hunt.

Being migratory birds, ducks and geese came under the protection of the federal government with the passage of the 1918 Migratory Bird Treaty Act. Arizona, unlike a number of other states, did not challenge the federal jurisdiction over migratory birds, and, prior to the Treaty's enactment, had even passed a number of protective measures for waterfowl. These included closing the hunting season during the spring months and prohibiting the gathering of eggs from nesting birds. All through the 1920s, and even into the drought years of the 1930s, waterfowl hunting was as popular a sport in Arizona as quail or dove hunting, if for no other reason than one got so much more game meat for the number of shells expended.

The drought years of the 1930s were hard on America's waterfowl populations, and it soon became apparent that nesting and other wetland habitats would have to be purchased and preserved if the public was to continue hunting ducks and geese. In 1934, a federal law was passed requiring persons 16 years of age and older to purchase a "duck stamp" if they wanted to hunt waterfowl. Soon after, a program was initiated to create a series of national wildlife refuges, many of which were primarily for waterfowl. From the 1940s through the 1950s Arizona saw the creation of two national waterfowl refuges on the Colorado River—Imperial and Havasu—as well as the acquisition of state wildlife areas such as Mittry Lake on the Colorado River, and Arlington and Robbins Butte on the middle Gila River. A number of waterfowl studies also started at this time, and banding investigations showed the value of managing waterfowl by flyways, a concept that was formalized in the hunt regulations in 1948. As a result, Arizona is included in the Pacific Flyway, which includes the Great Basin states as well as those on the Pacific Coast.

Major hunting restrictions incurred during the past 50 years have included limiting the take of such species as canvasbacks and redheads, closing certain portions of refuges and management areas to provide undisturbed resting and feeding places, and imposing the use of nontoxic steel shot rather than lead shot for the taking of waterfowl. Recently, favorable habitat conditions and resulting waterfowl production throughout the United States and Canadian breeding grounds has led to liberal season lengths and bag limits; although, long term declines of pintail and scaup have resulted in those species having bag limit restrictions.

The federal government, in conjunction with participating states, coordinates three major waterfowl surveys each year. The first of these, which does not include Arizona, is the "Breeding Ground Survey," which attempts to measure the coming year's productivity by

estimating the number of nesting ducks present on the continent's major nesting grounds in Alaska, Canada, and in the prairie states. The results of this survey are strongly linked to fall forecast flights of ducks and corresponding harvest frameworks. The "Winter Area Survey," which does include Arizona, is also conducted each year, and tallies the number of waterfowl using major wintering areas in the southern United States and Mexico. The number of birds counted on these surveys in Arizona has generally declined from the 1960s, when up to 42,000 ducks were observed in a given year, until the 1980s and '90s when counts often tallied less than 10,000. Conversely, the total number of Canada geese observed has increased from around 7,500 birds in 1960 to an average of 20,000 geese throughout the 1980s and '90s. The 1999 and 2000 survey revealed an increase in total ducks observed at about 35,000 with geese decreasing down to around 15,000 birds. The increase in ducks corresponds with the recent increase in the breeding ground surveys and the fall flight forecast.

The third survey is the annual hunt questionnaires sent to duck stamp purchasers requesting information on the number of ducks and geese bagged. Since 1979, to better evaluate the data obtained from this survey, Arizona has tried to maintain a standardized waterfowl season of approximately 100 days with a seven-bird bag limit (certain species excepted). As a result, Arizona's waterfowl regulations do not greatly vary from year-to-year, and bag-limit regulations do not provide for bonus (or penalty) points for taking certain species of waterfowl. The sample size of the state's hunt questionnaire survey greatly improved in 1988 when waterfowl hunters were required to purchase an Arizona waterfowl stamp in addition to a federal stamp.

The number of waterfowl hunters has fluctuated over the years, as much in response to duck stamp price increases as to any change in waterfowl numbers. Hunter numbers have been in a general downward trend since the mid-1980s, when more than 12,500 hunters took to the field, to the late 1990s when only about half that number participated. Recent estimates indicate that hunter numbers are again headed upward, and the long-term average of between 10,000 and 12,000 duck hunters a year may again be realized. Waterfowl hunting is nonetheless a resource-regulated sport, and Arizona's limited wetland areas will never accommodate high densities of hunters.

Annual waterfowl harvest figures are also sporadic. Estimates range from more than 150,000 ducks being harvested during the fall and winter of 1979-80, to less than 18,000 ducks being taken in 1990-91. The average annual take during the past three years has nonetheless been more than 50,000 birds. Goose harvests tend to be more predictable, with hunters usually claiming between 2,000 and 4,000 Canada geese and a few snows each year. Last year, however, survey estimates showed hunters taking 6,275 geese, the highest number since 1986-87.

Summary of January Waterfowl Survey[1]

Year	Ducks	Mergansers	Coots	Canada Geese	Snow Geese
1950	27,455	No survey	19,255	7,375	1,200
1951	10,965	1,350	4,780	5,155	1,150
1952	33,320	1,545	12,155	4,210	1,395
1953	25,050	1,335	22,060	3,050	1,400
1954	19,665	1,810	41,725	3,515	1,970
1955	27,115	965	8,570	2,860	900
1956	24,950	995	25,480	2,860	330
1957	44,455	610	31,840	3,640	215
1958	20,565	1,985	20,385	3,770	255
1959	34,700	1,795	24,055	5,865	335
1960	42,220	2,775	17,615	6,046	471
1961	27,100	4,395	19,055	5,526	583
1962	24,465	4,185	19,065	5,940	520
1963	22,260	4,145	40,625	6,650	805
1964	21,370	4,967	27,752	7,142	551
1965	21,304	3,298	15,900	4,431	229
1966	32,342	12,963	53,962	5,744	213
1967	19,425	3,980	12,278	3,602	192
1968	40,091	4,127	27,706	4,370	259
1969	11,020	4,854	9,839	3,052	500
1970	17,880	7,301	16,674	3,135	262
1971	19,212	3,552	15,649	3,502	221
1972	23,123	2,584	17,194	4,241	706
1973	19,684	4,682	12,935	4,745	503
1974	19,785	2,661	24,305	5,357	502
1975	9,828	1,775	17,831	2,534	228
1976	2,280	1,000	2,800	3,545	0
1977	4,680	700	1,900	3,511	4
1978	3,451	32	1,850	4,339	0
1979	18,326	220	3,160	4,962	7
1980	29,240	2,110	4,265	13,992	6
1981	10,550	281	3,033	9,170	2,500
1982	4,043	71	1,781	10,835	34
1983	5,176	202	1,026	13,373	2,527
1984	9,450	581	816	16,831	865
1985	7,306	830	162	17,619	1,443
1986	12,189	3,204	510	23,042	2,621
1987	9,623	2,321	1,337	14,131	1,103
1988	3,330	1,108	797	23,930	2,229
1989	6,317	298	1,409	22,594	1,303
1990	4,617	1,061	1,117	26,974	2,830
1991	7,114	1,894	1,135	31,897	4,434
1992	4,724	1,108	808	18,733	1,207
1993	7,961	826	143	22,596	1,265
1994	7,605	364	603	22,607	1,653
1995	11,933	881	1,051	21,078	2,941
1996	10,019	330	1,209	15,326	1,927
1997	9,776	220	2,356	18,598	1,325
1998	35,081[2]	1,749	757	14,164	2,965

[1] In 2001, this summary was revised to include Waterfowl from Cibola, Havasu and Imperial National Wildlife Refuges.Refuge data was collected by Refuge personnel.
[2] Resulting from excellent habitat condition.
[3] In 1999, the biologists conducting the survey changed; therefore, the observation rate may have changed.
[4] Resulting from poor habitat conditions (drought).
[5] Good late winter precipitation. Several lakes that had been nearly dry for years (specifically, San Carlos Reservoir) had water.

Summary of January Waterfowl Survey[1] (continued)

Year	Ducks	Mergansers	Coots	Canada Geese	Snow Geese
1999[3]	29,979	995	12,036	21,040	2,352
2000	29,376	450	12,924	9,169	446
2001	36,191	713	17,802	14,670	976
2002	20,498[4]	53	22,053	11,250	983
2003	22,489	220	9,517	13,351	261
2004	25,895	219	not counted	7,777	349
2005[5]	48,186	443	43,185	14,921	1,250
2006	16,974	633	12,727	13,849	911
2007	16,626	329	16,680	17,578	603
2008	18,360	292	30,973	7,695	750
2009	13,865	339	9,338	10,619	726

[1] In 2001, this summary was revised to include Waterfowl from Cibola, Havasu and Imperial National Wildlife Refuges.Refuge data was collected by Refuge personnel.
[2] Resulting from excellent habitat condition.
[3] In 1999, the biologists conducting the survey changed; therefore, the observation rate may have changed.
[4] Resulting from poor habitat conditions (drought).
[5] Good late winter precipitation. Several lakes that had been nearly dry for years (specifically, San Carlos Reservoir) had water.

Summary of Arizona Waterfowl Harvest

Year	Stamps Issued	Hunters	Hunter Days	HARVEST	
				Ducks	Geese
1981-82		10,904	57,184	81,091	5,169
1982-83		10,995	46,356	61,733	3,714
1983-84		8,438	39,470	46,820	3,357
1984-85		11,636	63,366	109,279	4,300
1985-86		12,508	64,508	79,653	4,994
1986-87		12,750	76,502	114,753	6,261
1987-88[1]	8,299	7,139	53,425	87,400	5,243
1988-89	7,104	5,101	33,683	34,662	4,054
1989-90	6,750	3,455	20,606	23,576	2,273
1990-91	6,292	2,513	16,324	17,683	2,219
1991-92	5,264	3,062	19,885	19,703	1,936
1992-93	5,383	3,389	22,464	23,241	3,631
1993-94	5,371	3,701	23,286	22,907	2,723
1994-95	5,107	4,138	30,041	35,971	3,009
1995-96	6,598	5,228	34,187	41,390	3,184
1996-97	6,908	5,513	35,784	41,603	3,247
1997-98	6,957	5,387	36,433	47,363	2,796
1998-99	7,951	5,964	42,853	61,685	2,911
1999-00	8,521	6,455	39,861	51,028	6,275
2000-01	9,019	5,677	44,431	48,788	4,504
2001-02	7,733	3,821	28,534	33,950	4,183
2002-03	6,775	4,885	35,146	35,128	2,859
2003-04	6,733	4,804	32,810	37,211	2,969
2004-05	6,334	4,459	31,373	35,421	3,051
2005-06	6,519	4,658	30,736	42,450	2,625
2006-07	6,776	4,001	28,107	42,771	1,996
2007-08	7,071	4,630	33,020	49,782	2,431
2008-09	5,580	3,775	30,305	37,494	1,666

[1] State waterfowl stamp implemented.

Sandhill Crane *(Grus canadensis)*

Natural History

Portions of three distinct populations of sandhill cranes winter in Arizona. Cranes from both the Rocky Mountain (RM) and Mid-Continent (M-C) populations winter in the Sulphur Springs and Gila River valleys in southeastern Arizona. Other sandhills from the Lower Colorado River Valley (LCRV) population winter along the lower Colorado River, primarily on the Colorado River Indian Reservation, Cibola National Wildlife Refuge, and below Gillespie Dam on the Gila River. RM cranes nest primarily in Idaho, Montana, Wyoming, and Utah, while cranes from the LCRV population mostly nest in northeastern Nevada. The

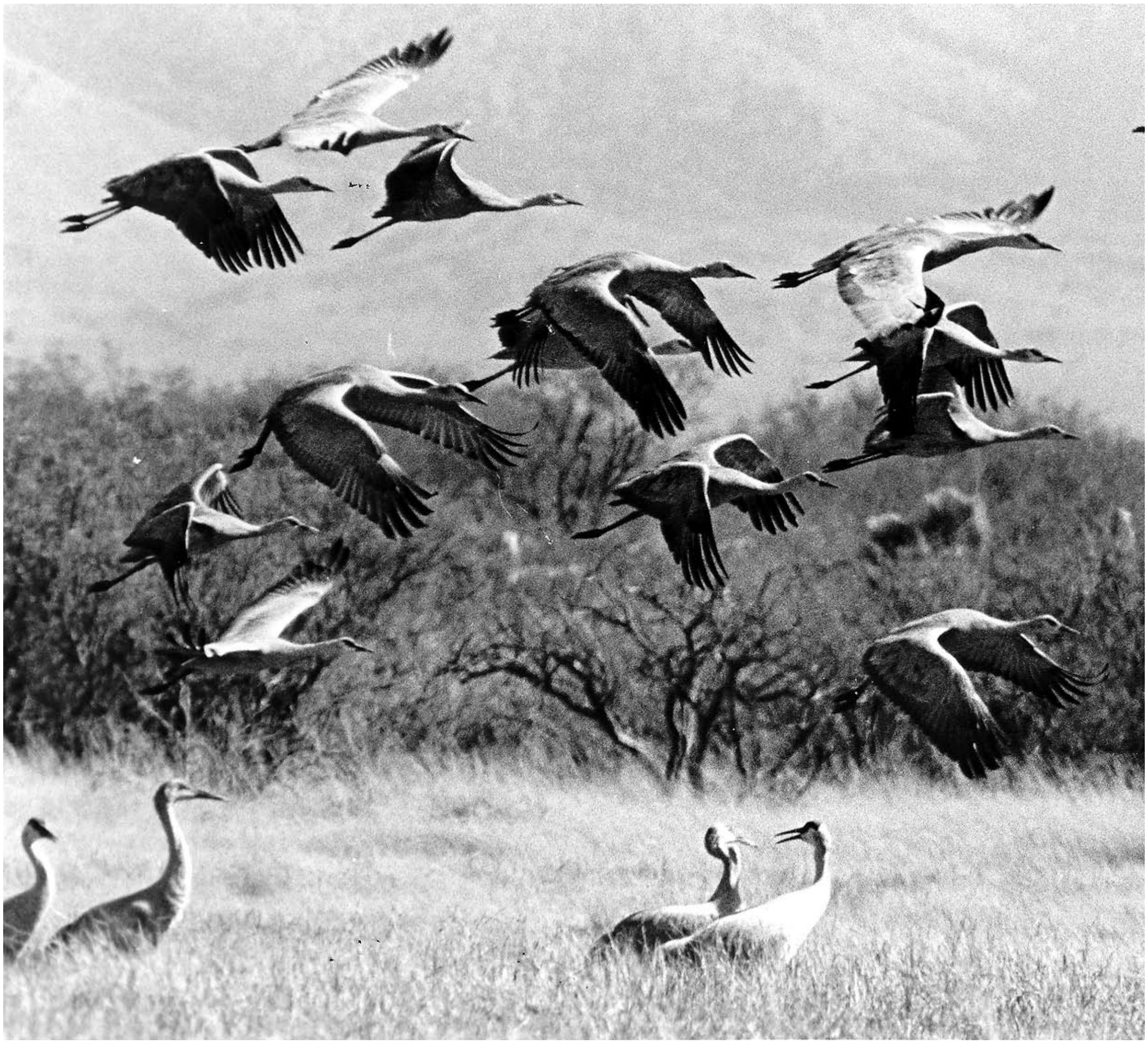

BOB MILES

nesting range of the M-C population includes much of Canada and Alaska. Birds from this latter population pass through the central plains before staging on the Platte River where they continue on to their wintering grounds in Texas, Oklahoma, New Mexico, Arizona, and Mexico.

Wintering areas selected by sandhill cranes feature shallow-water roosting sites with low or sparse vegetation including playa lakes and sandbars along shallow, braided river channels. Another requirement is the close proximity of harvested fields of grain, such as corn and milo. High-energy grains are needed to maintain the birds in sufficient condition to make their return migration in mid-to-late February.

Cranes leave their roosting areas in early morning, usually about sunrise, to fly to feeding areas where they typically spend from three to four hours eating. During midday the cranes return to the roost, or go to a nearby loafing area, which is commonly a grassland or wetland. In the late afternoon, cranes sometimes revisit their feeding area before returning to their nighttime roosts.

Sandhill cranes in the western United States nest in high elevation shallow marshes and wet meadows. Adult pairs do not nest until they are at least four or five years old, and typically have very poor success the first year or two. Cranes commonly lay two eggs, but only about one-third of the successful nesters are able to raise two young or "colts." In dry years, when wetlands shrink, predators, especially coyotes, take a heavy toll on the flightless young. During recent dry years the proportion of young-of-the-year birds in the fall population has been around four percent. Even in good production years, young-of-the-year birds rarely comprise more than 12 percent of the fall population.

Depending on habitat conditions, sandhills begin congregating in local agricultural areas, called pre-migration staging sites, in late August. Migration to wintering areas begins in September, the birds typically migrating in a few, high-altitude flights to traditional stopover areas. For cranes of the three populations that winter in Arizona, the major stopovers are the Platte River in Nebraska for the M-C, San Luis Valley in Colorado for the RM, and wetlands near Lund, Nevada, for the LCRV. Cranes begin arriving on their wintering areas between late September and mid-October.

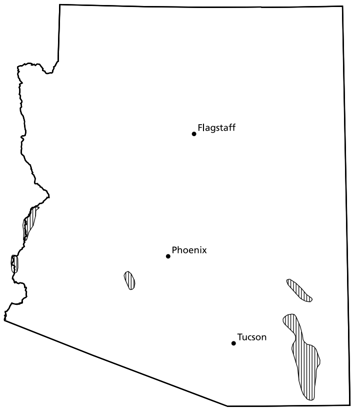

Sandhill crane distribution

Hunt History

A generally uncommon species in Arizona, sandhill cranes were protected by the Migratory Bird Treaty Act of 1918. In the early 1970s, however, counts of around 1,000 cranes wintering in Sulphur Springs Valley prompted concern that these birds might eventually cause crop damage. By 1980 more than 4,000 cranes were being tallied, and a limited hunt of 100 permits was authorized in 1981. This hunt was gradually expanded as crane numbers continued to increase and fears that the birds would winter elsewhere subsided. As of 1998, more than 300 permits were being authorized and census figures showed a wintering population of nearly 25,000 sandhill cranes in Sulphur Springs Valley.

Sandhill Crane Harvest Data

Summary of Sandhill Crane Harvest

Year	Mid-Winter Survey[1]	Permits Authorized	Total Applicants	Permits Issued[2]	Hunters Afield	Hunter Days	Percent Harvest	Hunter Success	Draw Odds[3]
1981	4,350	100	234	100	55	119	42	49	42.7
1982	5,640	100	279	100	55	95	73	78	35.8
1983	8,550	100	356	100	77	152	55	55	28.1
1984	8,350	100	239	104	72	110	69	74	41.8
1985	11,500	150	436	150	121	234	92	46	34.4
1986	11,450	150	239	150	124	217	138	69	62.8
1987	11,070	300	378	300	212	406	193	57	79.7
1988	6,670	300	505	300	228	446	207	58	59.4
1989	11,730	300	451	300	219	473	158	47	66.5
1990	11,990	165	512	165	139	275	123	53	32.3
1991	10,000	300	326	296	255	517	216	54	92.0
1992	2,4704	300	342	300	258	532	176	48	87.7
1993	12,740	300	381	300	217	401	174	50	78.7
1994	9,210	300	390	300	227	464	113	32	76.9
1995	24,190	270	390	270	211	423	157	48	69.2
1996	12,500	315	443	315	256	521	141	38	71.1
1997	21,050	315	389	315	235	430	193	47	81.0
1998[5]	24,616	310	440	321	232	450	151	40	72.9
1999	21,650	310	456	309	242	518	113	33	68.0
2000	21,131	310	383	305	218	389	203	57	80.9
2001	22,928	310	356	310	235	468	180	52	87.1
2002	21,327	310	349	310	253	489	239	58	88.8
2003	31,443	310	397	306	248	497	189	48	77.1
2004	29,208	325	367	311	263	319	192	59	84.7
2005	30,570	365	333	333	261	548	277	66	95.8
2006	28,156	365	353	353	222	559	180	55	97.4
2007	36,823	365	295	309	254	442	311	72	99.3
2008	29,103	375	368	318	261	485	162	48	84.5

[1] The Mid-Winter Survey occurs in December and January. The survey conducted in December 2008 and January 2009 is labeled 2008. The data listed is only for the Willcox Playa and surrounding areas.

[2] Permits Issued includes any tags via the draw and first-come, first-serve.

[3] Draw Odds is the number of permits issued through the draw divided by total applicants in the draw.

[4] Poor survey conditions.

[5] As of 1998, Sandhill crane check stations will be conducted every 3rd year (2008, 2011, 2014, etc.). Data will be based on the hunter questionnaire results unless a check station is conducted; then, harvest numbers will be taken from the check station results. Reminder questionnaires were sent if necessary.

Other Birds and Mammals

The Migratory Bird Treaty Act protects all birds except rock doves, European starlings, house sparrows, and all other non-native species. However, the federal government permits the states to open a season on certain birds and waterfowl. Mammals that are not classified as big or small game, predators, or furbearers are considered nongame and are managed by the Arizona Game and Fish Commission as "other mammals." Many of these mammals can be hunted by licensed individuals throughout the calendar year, with notable exceptions presented below. While there are no bag limits on most of these species, most nongame mammals are not hunted. As a result, harvest data for these species are not available.

Birds

Pigeon (Rock Dove)

Pigeons are closely associated with human developments including towns, parks, and agricultural landscapes. In their native settings, they nest along the seashore on airy cliffs and in rocky crevices or caves. In urban areas, they commonly nest on high-rise buildings, billboards, bridges and other structures. They average 12.5 inches in length. The coloration is highly variable, the most common being a dark gray head and neck with green and purplish iridescence on the neck, a back of lighter gray, and a whitish rump. The tail has a black band and the wings two black bars. The call is a soft coo familiar to most homeowners.

Natural History and Status

Pigeons nest year round in Arizona, building messy nests of sticks and roots. Nests are often placed under an overhang of some sort such as under eaves or bridges. The eggs are white. The species can raise four or five broods of one or two young in a single year. As with other pigeons, both sexes feed the young regurgitated "crop milk" exclusively for the first few days. After approximately five days, the young begin eating seeds and are soon eating the adult diet of grains and sometimes greens and insects. During the nonbreeding season, pigeons form large roosting and feeding flocks. Pigeons were introduced from Eurasia in the late 1800s and have become established throughout the United States.

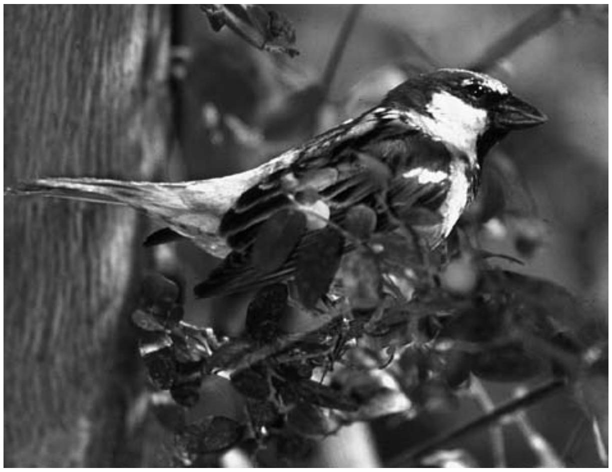

GEORGE ANDREJKO

House (English) Sparrow

House (English) Sparrow

House sparrows are common residents of cities and farms statewide. These brownish, conical-billed sparrows are approximately 5 inches in length. The males sport black bibs and beaks, white cheeks, blue-gray caps, chestnut napes, and black-streaked backs. Females are slightly smaller and less distinctive, with grayish, pale underparts, light-buff eye streaks, and striped backs. The house sparrow's lively calls and songs consist of chirps and cheeps that are familiar to almost every homeowner.

Natural History and Status

House sparrows nest from February through early summer, often having three broods per year. The nests, which may contain four to seven white to bluish colored eggs with gray or brown markings, are messy, woven affairs that may be located in eaves, palm fronds, bird houses, or most any other suitable site. House sparrows

will nest in cavities and aggressively compete with native species for nest sites. When they are not nesting, house sparrows commonly form flocks of up to a dozen or more birds. Highly adaptable, they feed on a wide variety of seeds, fruits, and insects. This resourceful bird greedily accepts almost any human handout and are commonly encountered foraging for morsels at fast-food restaurants.

House sparrows were introduced to Arizona from Europe via railroad cars from the East, and have been breeding residents since at least the early 1900s. They arrived in Tucson in 1903-04, had reached Winslow, Holbrook, and other railroad towns by 1909, and were widespread throughout the state by 1915. Despite its lack of protection, the species remains widely distributed, wherever humans and agricultural fields are found.

European Starling (Starling)

European Starlings are found in a wide variety of habitats, but are most numerous in or near human settlements that provide open, grassy areas for foraging and trees or structures for nesting. This dark, 8-inch, meadowlark-sized bird is a common resident of city parks, residential areas and agricultural lands below 7,500 feet elevation. Although usually found in urban, suburban and agricultural settings, starlings are also found in the desert, usually near small towns or dwellings. Starlings can be differentiated from other black birds by their short tails, robust build, narrow and light-colored bills, and short, pointed, brown wings. Both sexes are iridescent black in summer, and heavily speckled in winter. Starlings eat a varied diet including insects, fruits and seeds. When feeding, they walk, rather than hop, from site to site. Their principal call is a guttural squeak, although they also mimic other birdcalls.

Natural History and Status

European starlings reside in Arizona year-round and can initiate breeding activities as early as mid-January in warm areas of the state. Most breeding activity occurs from April to July, but nesting has been reported into early fall. Starlings take up residence in cavities such as woodpecker holes in saguaros or trees. Like the house sparrow, starlings are considered a pest species because they compete for nest sites with native species such as purple martins, woodpeckers and bluebirds. European Starlings will even evict nesting birds and destroy their eggs. Starlings typically lay four to six blue eggs and can raise two or three broods each year. When the species is not nesting, they form large communal roosts, which may contain hundreds of birds A more recent arrival than the house sparrow, European starlings were first recorded in Arizona in 1946 near Lupton, with the first nest reported near Glendale in 1954. The species is now both a breeding resident and a migrant in the vicinities of Phoenix, Tucson, Kingman, Yuma, and other Arizona cities and towns.

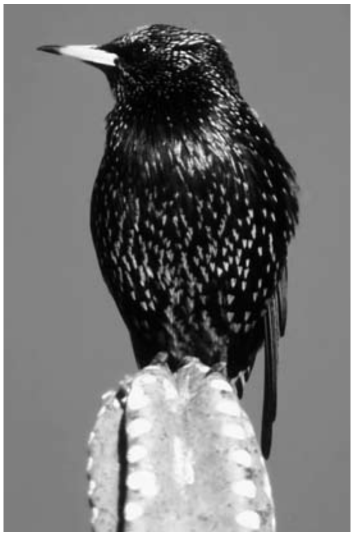
GEORGE ANDREJKO

European starling

Peach-faced Lovebird

In Africa peach-faced lovebirds prefer dry, open country including wooded savannas, palm groves, and arid mountain slopes. In Arizona they are primarily found among the ornamental plantings in desert urban and residential settings. Although locally established in and around the greater Phoenix metropolitan area, they do not venture into the surrounding desert lands. They are small, bright green, parrot-like birds with a pinkish face and light-colored bill. Regular visitors to many backyard water and feeding stations, they have also been observed feeding on cactus fruit, apples, palm fruit, and seed pods.

Natural History and Status

Like many other parrots, peach-faced lovebirds are cavity nesters and will take up residence in woodpecker holes in saguaros, under tile roof openings, and in untrimmed palm fronds. Lovebirds nest in groups and thus far there has been no evidence that they compete with native birds for nest sites. In Arizona, most nesting occurs from April through May. They will lay from three to eight eggs per clutch, possibly rearing two broods per year. The first free-ranging flock of peach-faced lovebirds in the Phoenix area was reported in 1987 near the border of Mesa and Apache Junction, and by the mid-1990s local flocks and colonies of lovebirds were discovered throughout the eastern half of the greater Phoenix metropolitan area.

American Crow (Crow)

In Arizona, American crows are far outnumbered by their larger and more heat-tolerant relative: the common raven. Crows occur as local breeding residents in the more open areas of the Mogollon Rim, along the South Rim of the Grand Canyon, in the higher portions of the Navajo Indian Reservation, and along the

San Francisco River. This shiny, all black 1.5-foot-long bird can be differentiated from the larger raven by its smaller beak and tail, smoother plumage, and distinctive "caw" call. Also unlike ravens, crows rarely soar, but instead flap their wings when flying directly from point to point. Because of crop depredations, an open season on this species is authorized from September 1 through December 31.

Natural History and Status

American crows are native to North America and reach their highest densities in the northeastern United States. They form large communal roosts during much of the year, sometimes in groups large enough to be problematic in towns or industrial areas. During the breeding season, however, the species is most often observed in smaller family units. They typically place their nests in well-hidden areas of their nest trees, generally close to the trunk. Nests are made of dead sticks, bark, corn stalks, twine, and cow dung, and lined with soft materials. Crows lay from three to nine bluish-green eggs marked with brown speckles. They feed on a variety of foods including insects, carrion, small mammals and birds, bird eggs and grains, including some agricultural crops. Numbers have probably increased significantly since European settlement because of agricultural developments and timber clearing. Human developments have also enabled breeding range expansions into portions of the West and Midwest.

Mammals

Coati

This relative of the raccoon is usually seen individually or in small bands called "troops." The lone males or "solos" may weigh up to 12 pounds, and greatly exceed the smaller 5.5- to 7-pound females in size. From 2.5 to just over 4 feet in length, coatis are approximately the size of a small dog. They range in color from ochre to cinnamon brown to nearly chocolate. Their most distinctive characteristics, however, are their clown-marked faces and faintly banded, tapered tails that commonly exceed 2 feet in length, giving coatis the superficial appearance of monkeys.

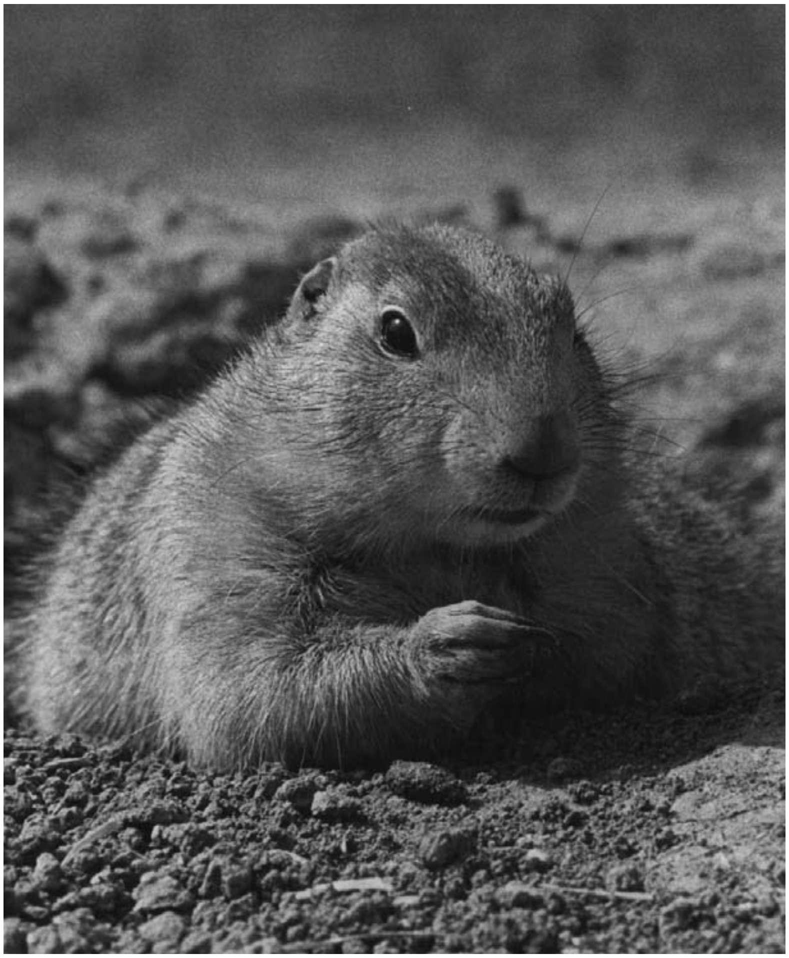
BOB MILES

Black-tailed prairie dog

Natural History and Status

Also known as chulos, coatis are semi-arboreal animals rarely found far from trees. Like tree squirrels, coatis have jointed hind feet, allowing the animals to descend the trunks of trees headfirst. These largely diurnal mammals are found primarily in mountains and canyons in the southeastern quarter of the state. Their principal habitats are Madrean oak-pine woodland and riparian deciduous forest. Highly omnivorous, their principal foods are lizards, insect larvae, bird eggs, acorns, fruits, and other mast.

Troops of coatis, which may range in size from one or two to up to 40 animals, are typically composed of females, sub-adults, and weaned young of the year. Males leave the troop when about 2 years old, after which they associate with the females only during the spring breeding season. Nursing females leave the troop for four to six weeks after giving birth. From one to six young are born in June or July. Born helpless in a den or hollow tree, the youngsters remain with their mother until old enough to forage with the troop in the fall.

Coati numbers fluctuate markedly, and at least two major population declines have been reported for Arizona. Recently, however, they appear to be expanding their range northward and are now common in such places as Aravaipa Canyon and the Sierra Ancha, where they were unheard of prior to 1970. Current hunt management authorizes a seven-month season, and a bag limit of one coati per calendar year.

Gunnison's Prairie Dog

Prairie dogs are robust, diurnal ground squirrels that live in underground colonies called "dogtowns." Their tails are relatively short, less than 25 percent of the body length. The animals get their name from their doglike barks, which warn the colony of intruders. Male Gunnison's prairie dogs are just over a foot long, with 2-inch, grayish to white-tipped tails. Adult males weigh about 1.75 pounds and females less than 1.5 pounds. Male and female are similar in appearance, both a pale buff in color. The species is now largely restricted to Great Basin grasslands above the Mogollon Rim, although colonies formerly extended south and east of Prescott to the Dewey and Dugas areas, as well as to the San Carlos Indian Reservation.

Natural History and Status

Gunnison's prairie dog colonies tend to be small, and usually contain fewer than 50 animals. Their burrow entrances are not typically built up into craters, unlike those of black-tailed prairie dogs. Gunnison's prairie dogs enter torpor below ground during winter months, and breed in February-March. The three to four pups typically appear in June. Grasses, forbs, and sedges are the usual dietary items.

Black-tailed Prairie Dog

Slightly larger than the Gunnison's prairie dog, this 15-inch-long rodent is yellowish tan in color with a usually dusky-tipped 3-inch tail. Male black-tailed prairie dogs average about 2 pounds; the females about 1.9 pounds. Unlike those of Gunnison's, the entrances to the burrows of black-tailed prairie dogs often have cratered mounds that can reach up to 3 feet in height. The underground burrow network may be extensive, and black-tailed prairie dog colonies were often large, especially those in the San Pedro and Sulphur Springs valleys

Natural History and Status

Black-tailed prairie dogs are active all year, and will come out on sunny days even in midwinter. The species breeds in late February; the young are born in March and appear in May. Dietary items include grass stems, grass roots, and shrubs.

Black-tailed prairie dogs formerly occurred in the semidesert grasslands of southeastern Arizona south of the Gila River, westward to the vicinity of Fort Huachuca. They have been extirpated in Arizona since 1959, although a small colony on the Day Ranch 15 miles southeast of Duncan on the Arizona-New Mexico border persisted until 1974. An attempt to reintroduce this animal to the Appleton Research Ranch (near Sonoita) in the summer of 1974 failed. The species has recently been protected in Arizona, in the hope that individuals from three colonies in Sonora within five miles of the United States-Mexico border might recolonize our state.

Specially protected mammals

The following mammals are protected at all times because they are endangered species, resemble endangered species, or are otherwise deemed in need of protection due to low numbers or vulnerability.

Bats

Arizona, with 28 species of bats belonging to four families (ghost-faced, leaf-nosed, vesper, and free-tailed), has one of the most diverse bat faunas of any state. Ranging in abundance from the American free-tailed bat, which numbers in the millions, to the seldom-seen ghost-faced bat, Arizona's bats are highly beneficial. They feed on insects and find their prey by emitting and receiving sonic waves—a process similar to sonar and known as echolocation. Each species has its own high-pitched call, some of which can be heard by human ears. Our largest bat species, the western mastiff bat, is about 7 inches long and has a wingspan of up to 18 inches. The western pipestrelle, at only 2.5-3 inches long is Arizona's smallest bat. Some species, such as the spotted bat with its death's-head markings and huge ears, are bizarre in appearance.

Natural History and Status

Although nearly all of Arizona's bats are insectivores, two, the lesser long-nosed bat and the Mexican long-tongued, feed on nectar and pollen. Some species, such as the red bat, are generally solitary, but most roost in colonies, selecting as their daytime retreat a particular cavern, rock fissure, or mine tunnel. Most bats are migratory, although a few over-winter by hibernating. To reduce competition, the various species use different habitats and feeding strategies. Pallid bats, for example, typically feed low to the ground; the western mastiff

bat tends to hunt high over water or in the tree canopy. It is also an unfortunate fact that bats transmit rabies, with the result that they expose dozens of people a year to this potentially deadly virus.

All bats are protected in Arizona due to their generally beneficial nature and the rarity of certain species. Colonial roost sites may also be protected, and certain caves have been declared “off-limits” because of their value to these intriguing flying mammals.

Black-footed Ferret

This uniquely North American mammal has always been extremely rare in Arizona, with only four specimens ever collected in the state. Until a reintroduction program began in 1996, the last ferret reported in Arizona was in 1931 when bubonic plague and rodent control programs killed off the ferret’s prairie dog prey. The black-footed ferret is a low slung, weasel-like animal less than 2 feet in length, with sooty black feet. The overall color is a yellow-buff, the face has a distinctive black mask, and the approximately 5-inch tail is tipped in black. Males are significantly larger than females, weighing about 2.25 pounds to the female’s 1.5 pounds.

Natural History and Status

Ferrets are almost exclusively restricted to prairie dog colonies, which provide most of the animal’s food. The ferret is primarily a nocturnal species. It breeds during mid-March or April, and after a gestation of 45 or so days give birth to from two to five young. The kits remain in a nest underground with their mother for 40 days or more and do not disperse to forage on their own until September, attaining breeding maturity at one year of age. The presence of ferrets can be detected by their tracks and diggings, which consist of 4-inch-deep trenches and lengthy piles of soil adjacent to prairie dog holes. Federally designated an endangered species in 1967, black-footed ferrets are the focus of an Arizona Game and Fish Department program to reintroduce captive-reared animals in Aubrey Valley. Although the project is still relatively young, some animals have already reproduced in the wild.

Hualapai Mexican Vole

Voles or meadow mice are dark brown, short-tailed (<1.5”) terrestrial rodents with short fur and small, rounded ears. The sexes are nearly identical in pelage and size. The Mexican vole, to which this race belongs, is widely distributed at higher elevations, with populations found in the White Mountains, the San Francisco Peaks, along the Mogollon Rim, and in such isolated ranges as the Sierra Ancha, Bradshaw Mountains, Navajo Mountain, and Hualapai Mountains. The latter population, and possibly those on the Hualapai Indian Reservation to the north, has been described as a separate subspecies due to its isolation. The identifying characters of this so-called Hualapai vole are not well defined, but are based on its having a smaller relative size, longer hind feet, and more cinnamon underparts than its closest neighbors.

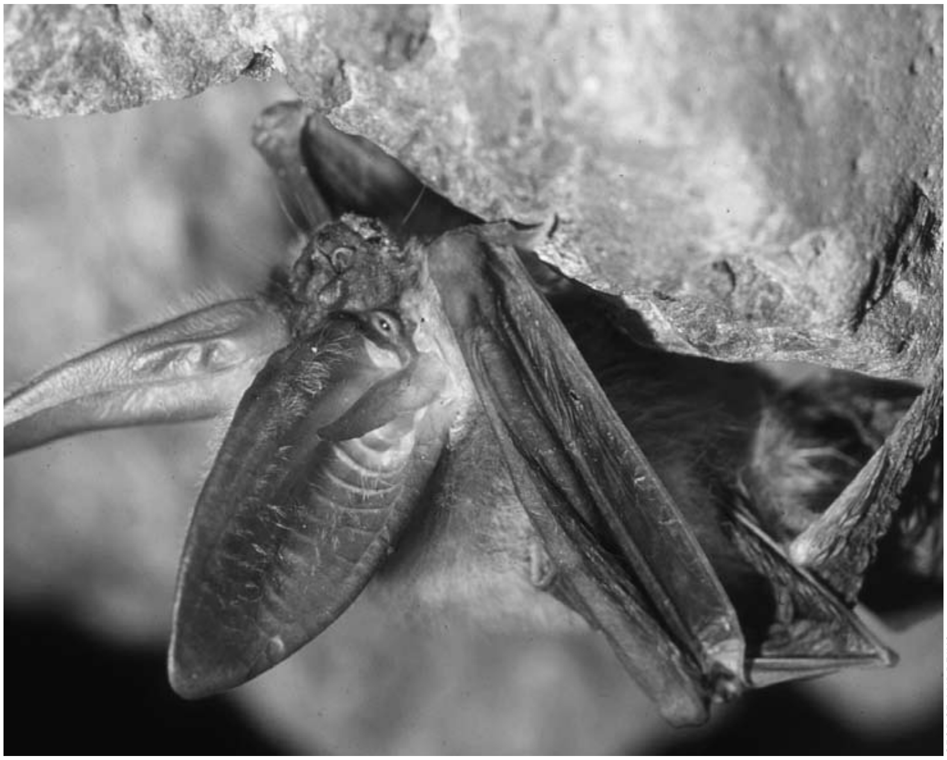
GEORGE ANDREJKO

Townsend’s big-eared bat

Natural History and Status

This 1.25-inch-long rodent prefers dry, grassy meadows and canyons in proximity to ponderosa pines, Gambel’s oaks, pinyon-juniper woodlands, and chaparral. As with most rodents, numbers may fluctuate from rare to abundant. Not as prolific as some other rodents, their litter

sizes vary from one to four and average about 2.5. This isolated population of Mexican voles is protected as a Federally endangered species

Jaguar

More than 50 of these large, spotted cats have been documented from Arizona since 1900. Although there are some early records of what appear to be young jaguars, almost all of the animals taken or photographed after 1950 have been wandering males from Sonora, Mexico. The last recorded female in Arizona was taken in 1962.

Borderland jaguars tend to be small when compared to those in South America. The males average from about 125 to 160 pounds; the females are smaller, averaging about 110 pounds—approximately the same as mountain lions. Adult jaguars stand about 2.25 feet to 2.5 feet tall at the shoulder. The males average about 7 feet in length and the females about 6 feet. The tail is relatively short, about 17 to 30 inches and less than half of the length of the head and body. Dorsal colors range from a pale yellow-buff to a golden orange. The black spots on the head and shoulders are relatively small, transforming to a complicated series of bars, splotches and broken rings or rosettes on the back, flanks, feet and tail. Underneath, the jaguar varies from a very pale gray to snow white with black markings. Although appearing garish in the open, jaguars are in fact wonderfully concealed in the dappled shadows of their wooded and scrubland habitats.

The only New World "roaring cat," jaguars call to each other by emitting a series of hoarse, rasping grunts.

Natural History and Status

Although jaguars have been recorded as far north as the Grand Canyon, most have been recovered or photographed in the borderland mountains in the southeastern quarter of the state. Found primarily in tropical thornscrub and deciduous forest in Mexico, most Arizona jaguars have been encountered in Madrean evergreen woodlands and scrub-invaded semidesert grassland. Several jaguars have been taken in proximity to water, and several have been taken in montane conifer forests, at least one above 9,000 feet elevation.

Jaguars hunt mostly at twilight and at night, seeking a wide variety of prey. Prey items in Arizona have ranged from frogs to elk, but white-tailed deer, javelina, and coatis appear to be the most important natural prey. Livestock is taken when available, especially calves.

Female jaguars reach sexual maturity at about 2.5 years. For biological and social reasons, most males do not breed until age 3 to 4. The breeding season in the Southwest borderlands appears to be in January or February, the young being born in spring after a 100-day gestation period. The one or two cubs are weaned at about 22 weeks, but female offspring may remain with the mother for more than a year. The average life span of Sonoran jaguars is thought to be less than 10 years due to the scarcity of game and their persecution as stock-killers. Jaguars have been protected in Arizona by state law since 1969, and U.S. populations were declared an endangered species in 1997. Prior to this time, jaguars have at various times been considered as furbearers, predators, or nongame mammals. The Department is engaged in a Conservation Team working to conserve jaguars of the Arizona-New Mexico-Mexico borderlands.

Jaguarundi

These low slung, 10- to 20-pound felines require dense tropical vegetation and are usually found near water. The animal's head and ears appear small for a cat, and the 1- to 2-foot tail is less than the body length. Two color phases of these uniformly colored cats occur—cinnamon and charcoal gray. Largely terrestrial, jaguarundis take to trees only when pursued by dogs, at which time they can display much arboreal agility. More diurnal than other wild felids, jaguarundis usually occur alone or in pairs. The species also emits a whistlelike call on occasion.

Natural History and Status

Jaguarundis feed on small mammals, such as cotton rats, as well as a variety of birds, lizards, and snakes. The breeding season varies with locality, but the gestation period is from 60 to 70 days after which from one to four kittens are born.

This species has never been documented as occurring in our state, or even southward in Sonora, Mexico. Jaguarundis have been protected here since 1972, on the basis of visual reports and the possibility that this animal might occur in Arizona. The species is included here only because it remains federally listed in Arizona.

Ocelot

These 18- to 22-pound felines are not residents of Arizona, but rare visitors from Sonora, Mexico, where they occur primarily in tropical thornscrub. Their background color is a grayish or brownish orange color with black stripes and dots. Solitary and terrestrial, the "gato galavis," as the species is known in Sonora, is largely nocturnal in its habits. Averaging about 22 pounds, male ocelots are slightly larger than the 19.5 pounds for the average female. The head and body length is approximately 3 to 3.5 feet, with the tail providing another 13 to 14 inches.

Natural History and Status

Only four specimens have been documented from Arizona since 1887. Two of these were male, one from the Huachuca Mountains and another from the Dragoon Mountains. The sex of the other two animals is unknown, as are the locations. The life history of the gato galavis in Sonora remains largely uninvestigated, but the litter size in other ocelot populations is one or two. The kittens' eyes are shut for 2.5 weeks and they remain with their mother for 18 to 20 months. Most ocelots do not reach breeding maturity until 2 years old or more. Ocelots feed mostly on terrestrial mammals, such as cottontails, but reptiles are also taken. Adults may have a home range of 3,000 acres or more.

Ocelots have been protected in Arizona since 1969. Any change in the species' status is therefore difficult to ascertain, as any ocelots taken by trappers and/or predator control agents are unlikely to be reported.

Otter (see Furbearers)

Porcupine

These large, bulky rodents are unmistakable. The large head, long spines intermixed with equally long or longer blackish, brownish, and yellowish hair, and heavy claws make for instant identification. The males are bigger than the females, but the females have longer tails. Overall, the animal's total length is about 2.5 feet, of which approximately 8 inches constitutes the tail. Weights range from 7.75 to 40 pounds depending on the porcupine's age and condition.

Natural History and Status

Possessed of poor vision but with a good sense of smell, porcupines are active mainly at night. Habitats occupied include forested mountains, riparian forests, meadows, semidesert grasslands and even deserts. During the winter months, porcupines may feed almost exclusively on the inner bark of pine trees, although the bark of cottonwoods, mesquites, and ocotillos is also taken. Porcupines lose weight when feeding only on inner-bark, however, and also eat mistletoe, acorns, fungi, cactus fruit, and other mast when available. During the summer months, the species feeds on the ground and is frequently seen in mountain meadows feeding on grasses and sedges. Porcupines are fond of salt and will gnaw ax handles and other objects having this mineral.

Solitary animals, porcupines den in hollow trees and burrows as well as in rocky outcrops and mine shafts, often using the same den site year after year.

Females mature in one year, males in 2.5. Mating takes place in September and October, often in a tree, and is usually accompanied by highly vocal grunts, squeals, and shrieks. The males are very aggressive at this time and will fight any other males they happen to come upon. Gestation is seven months and the single offspring is born in late April or early May. The youngster, weighing about a pound, is highly developed and well able to care for itself, staying with the mother only through its first summer. Probably because of their slow-paced life style, porcupines can live up to 9 years of age—a relatively long time for a rodent.

Although totally protected in Arizona, porcupines were unprotected for many years due to the damage inflicted on both mature ponderosa pines and pine seedlings, as evidenced by the trees' girdled trunks and white areas of peeled bark. As recently as the 1950s, hunters were encouraged to kill any porcupines encountered. Densities of porcupines appear to vary with time, however, and the species now appears much reduced in comparison to

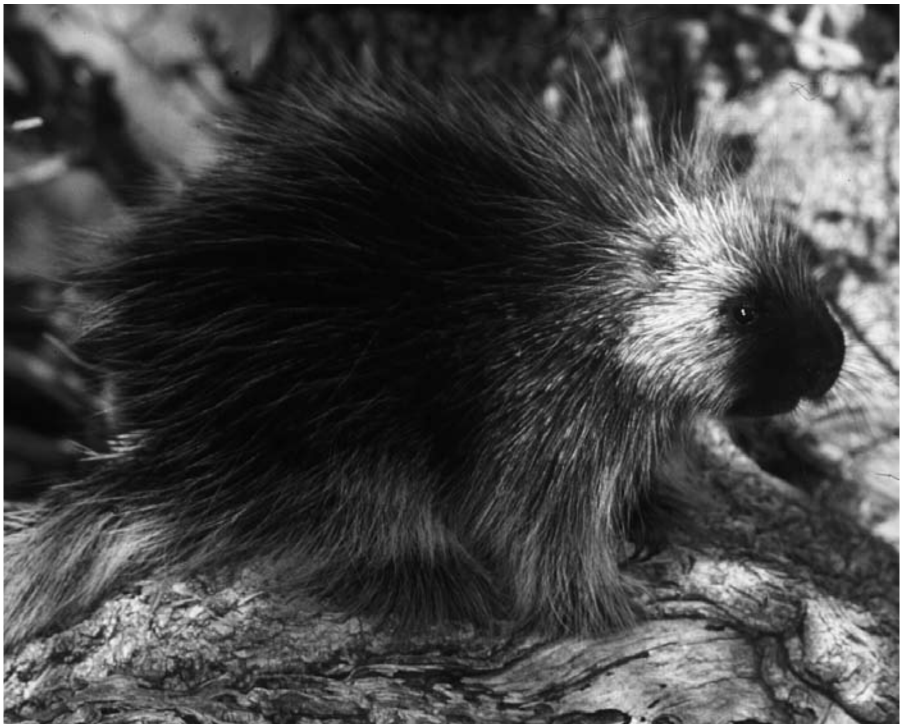

GEORGE ANDREJKO

Porcupine

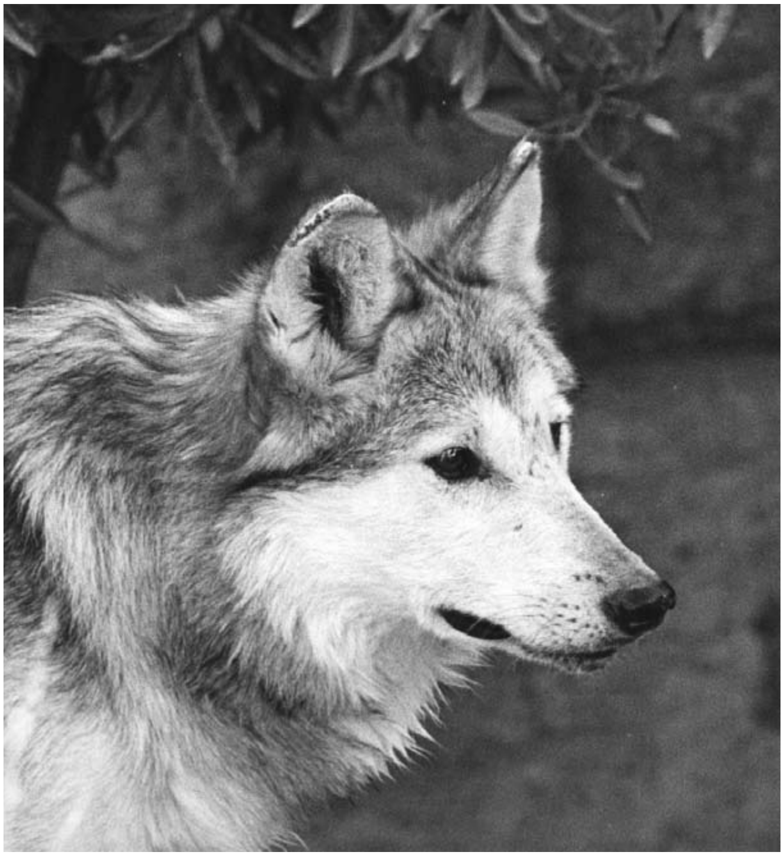
BOB MILES

Mexican gray wolf

numbers reported earlier. Nonetheless, porcupines may still cause problems locally and require relocation to other areas.

Gray Wolf

Now extirpated as a wild animal in Arizona, the gray wolf formerly occurred in small numbers throughout the eastern and northern portion of the state above 4,500 feet elevation. Southwestern wolves stand about 30 inches high at the shoulder, and differ from the much smaller (less than 35 pounds) coyotes by having heavier, deeper chests, impressive heads, shorter, thicker muzzles, larger nose pads, and a thicker neck that shows a ruff or mane when the animal's hackles are raised. Wolves also have long, slender forelegs and a dark-tipped tail. Coat color varies with season and individuals, some animals being so light as to be nearly white and others so dark as to appear almost black. The usual pelage, however, is a grizzled mixture of grays, browns, blacks, and whites on backs and flanks. Adults are about 4.5 to 5.5 feet long, with 14 to 17 inch tails. The males are about 10 pounds heavier than the females, weighing between 65 and 85 pounds, versus the female's 55 to 80 pounds.

Perhaps the wolf's most distinctive trademark is its mournful howl, which is usually given in late fall and early winter, and which once heard, is never forgotten.

Natural History and Status

Wolves are mostly active at night and hunt by trailing and running their prey to ground. Their preferred habitats are rolling woodlands, level forests, open meadows, and grasslands. Wolves historically fed on deer, elk, pronghorn, cottontails, and mice but readily adapted to taking sheep and cattle when livestock were introduced to Arizona.

For behavioral as well as biological reasons, wolves do not usually reach sexual maturity until they are about 2.5 years old. The breeding season in Arizona is between November and mid February, and the gestation period is 63 days. Den sites are selected by the female, and may consist of an enlarged burrow, hollow log, or a natural crevice. Four to eight sooty-brown pups are born in the spring and nursed for six to eight weeks. They are cared for by both parents. Although they are weaned in late fall, when they are 2.5 to 3 months old, the young wolves, especially the females, may remain with the parents for another year or so before dispersing.

Wolves are social animals, but packs in Arizona have historically been small, usually consisting of from one or two to seven animals. Wolves can have very large home ranges and travel long distances in search of food and mates.

Long persecuted as the state's premier livestock predator, the last record of wild wolves breeding in Arizona was in 1944. In an attempt to reintroduce the species, captive-raised wolves, descended from Mexican stock, have been released in and near the Blue Range Primitive Area beginning in 1996.

SUBSCRIBE TODAY!

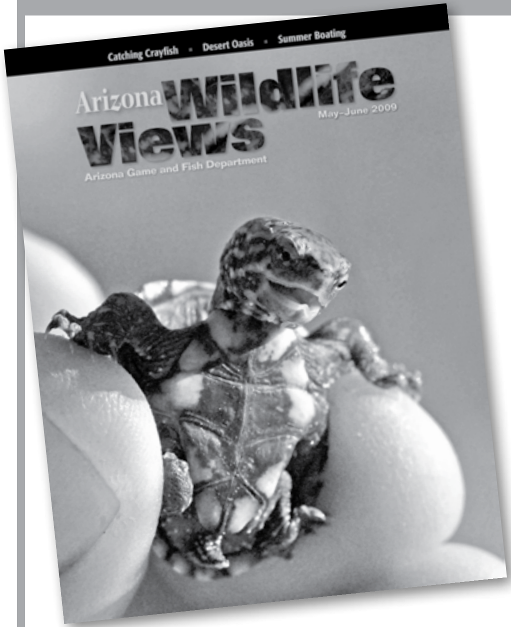

The official wildlife magazine of the Arizona Game and Fish Department published for outdoor enthusiasts!

Arizona Wildlife Views Magazine

Arizona Wildlife Views is filled with stunning photography and informative articles on hunting, fishing, boating and other outdoor recreation opportunities plus in-depth coverage of important wildlife issues. Subscribe today to Arizona's premier wildlife magazine and begin to enjoy the benefits of the best in outdoor information!

1 YEAR • 6 BIG ISSUES
40 FULL-COLOR PAGES
ONLY $8.50

Send no money now. We'll bill you later. CALL 1-800-777-0015

SIGN UP FOR FREE Arizona Game and Fish Department e-news

The Arizona Game and Fish Department wants to communicate with you! Our electronic information newsletters provide you with the latest news and information on wildlife issues and events, outdoor tips, education programs, regulations and more. Just provide us with your e-mail address and this information will come directly to you, **free of charge**.

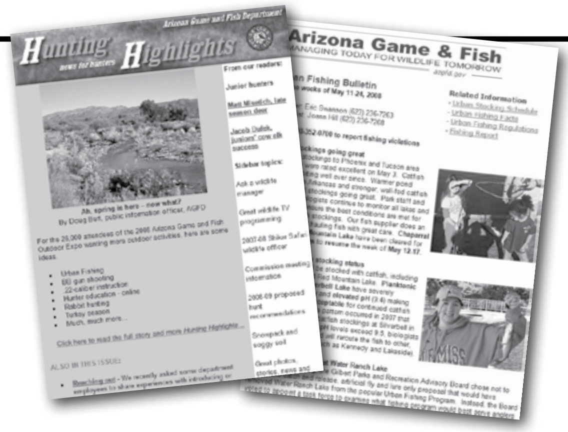

SIGN UP TODAY!
www.azgfd.gov/signup

To sign up for any of our products, visit www.azgfd.gov/signup and select the e-mail newsletters you would like to receive:

- **Wildlife News:** Biweekly news about conservation, recreation, department news, and upcoming activities for the public.
- **Fishing Report:** Weekly updates on fishing conditions around the state.
- **Hunting Highlights:** Bimonthly news about hunting techniques, outlooks, where to go, and youth/family participation.
- **Shooting Sports News:** Updates on pistol, rifle, shotgun and archery target shooting and shooting range activities.
- **Urban Fishing Bulletin:** Biweekly updates on fishing opportunities at urban lakes.
- **Endangered Species Updates:** News on issues pertaining to conservation of at-risk wildlife.
- **Working for Wildlife:** Updates to keep the public informed on efforts to secure funding to meet future wildlife conservation needs.
- **OHV:** Periodic updates for OHV enthusiasts about the Arizona Game and Fish Department's off-highway vehicle program.
- **EdNews:** Updates on wildlife education resources for educators, youth group leaders and students.

Game Management Unit Map

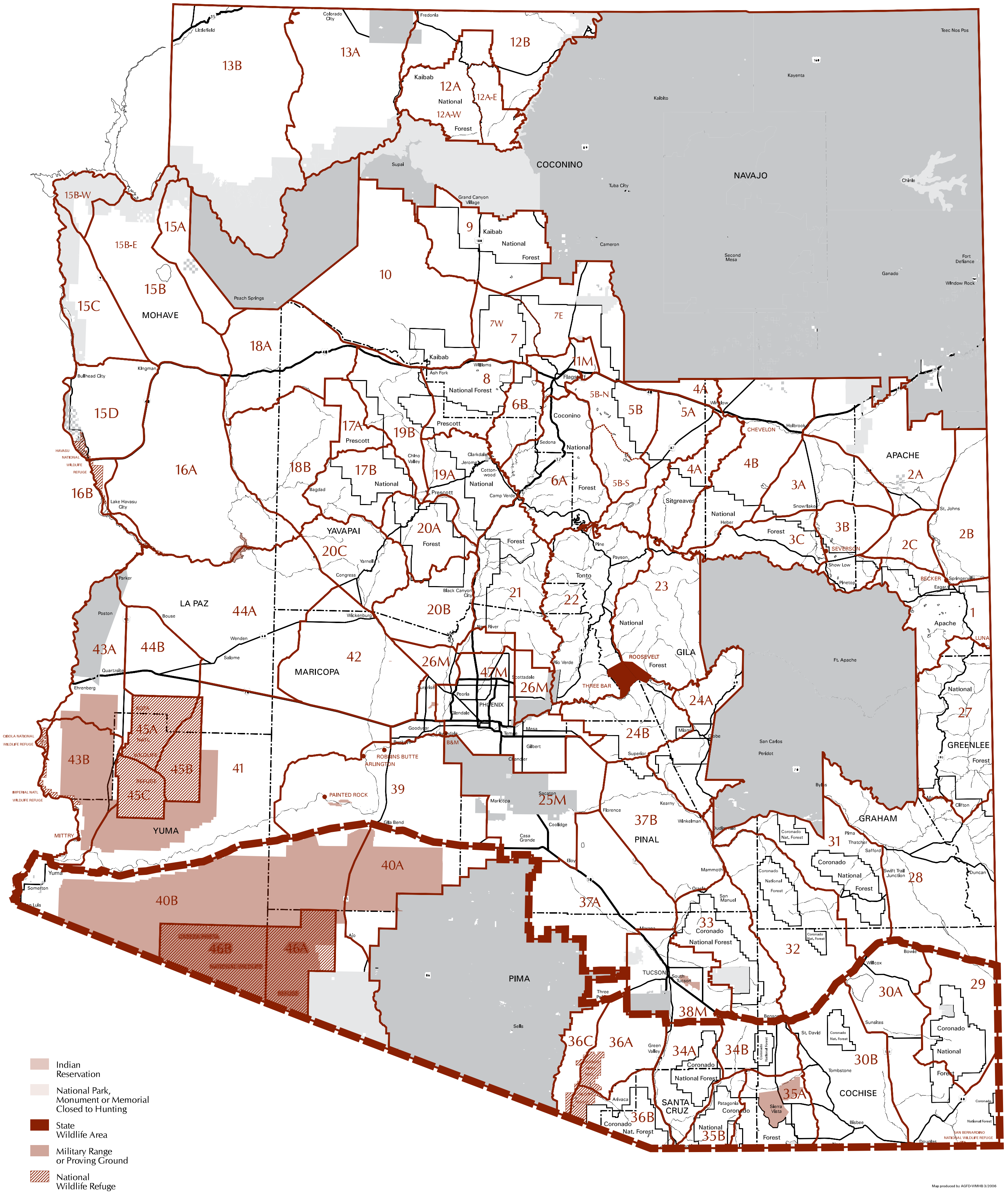

Homeland security issues along the international border may affect the quality of a person's hunt.

©2009

Arizona Game and Fish Department
5000 W. Carefree Highway • Phoenix, Arizona 85086 • (602) 942-3000

www.azgfd.gov
