# Supplementary material for: The Elephant in the room: What can we learn from California regarding the use of sport hunting of pumas (Puma concolor) as a management tool?
Source: PLoS One. 2020 Feb 13;15(2):e0224638. doi: 10.1371/journal.pone.0224638 (PMC7018503; doi:10.1371/journal.pone.0224638)
Supplement: S2 File — This is a zip file that contains sources of the data used in the analyses that are no longer available on the internet. (ZIP) [file pone.0224638.s003.zip › Montana deer kill 2004-2018.pdf]

Legend:

Deer Species: (md = mule deer, wt = white-tailed deer, all_deer = both species of deer combined)

Residency: Montana resdient or non-resident(SUM= both residents and non-residents).

Hunters: Estimate of how many people of this residency hunted in this area.

Days: Estimate of the number of days spent hunting in a particular area by hunters in a particular area.

Days per Hunter: Estimate of the number of days spent hunting per hunter in a particular area for a particular residency.

Total Harvest: Estimate of the number of animals harvested by a particular residency.

Deer Harvest Estimates - 2004 - 2018

License Year	Hunting District	Deer Species	Residency	Hunters	Days	Days per Hunter	Total Harvest	Bucks	Does	Fawns	Bow	Rifle	Less than 4 Points	4 or More Points
2004	State	all_deer	N	25780	146599	6	20375	14151	5543	579	611	19765	3397	10599
2004	State	all_deer	R	127467	872052	7	98891	60432	37174	1214	3787	95104	22141	36369
2004	State	all_deer	SUM	153247	1018651	7	119266	74583	42716	1793	4397	114869	25538	46968
2004	State	md	N	25780	146599	6	14409	10673	3389	278	322	14087	3038	7553
2004	State	md	R	127467	872052	7	50445	33488	16571	367	814	49631	14086	18479
2004	State	md	SUM	153247	1018651	7	64854	44160	19960	645	1136	63718	17123	26032
2004	State	wt	N	25780	146599	6	5569	3302	1962	288	271	5298	313	2919
2004	State	wt	R	127467	872052	7	48258	26858	20523	844	2960	45298	8026	17839
2004	State	wt	SUM	153247	1018651	7	53827	30160	22485	1132	3231	50595	8339	20758
2005	State	all_deer	N	25915	159116	6	18475	13208	5069	198	692	17710	2686	10389
2005	State	all_deer	R	124582	860345	7	96762	59292	36298	1132	4915	91074	20483	37406
2005	State	all_deer	SUM	150497	1019461	7	115238	72501	41367	1330	5607	108783	23168	47795
2005	State	md	N	25915	159116	6	12604	9836	2702	67	315	12241	2339	7415
2005	State	md	R	124582	860345	7	45508	31661	13529	313	925	44216	12358	18734
2005	State	md	SUM	150497	1019461	7	58112	41496	16231	380	1239	56457	14697	26149
2005	State	wt	N	25915	159116	6	5847	3358	2360	129	378	5446	346	2960
2005	State	wt	R	124582	860345	7	51076	27574	22691	798	3975	46734	8095	18651
2005	State	wt	SUM	150497	1019461	7	56922	30931	25050	927	4353	52179	8441	21611
2006	State	all_deer	N	26756	162497	6	20176	12949	6962	187	1093	18831	2559	10196
2006	State	all_deer	R	127014	925832	7	115878	65358	48709	1217	6584	107761	23114	40349
2006	State	all_deer	SUM	153770	1088328	7	136054	78307	55671	1404	7677	126593	25673	50544
2006	State	md	N	26756	162497	6	13203	9118	3979	71	471	12587	2041	6961
2006	State	md	R	127014	925832	7	51733	34268	16988	325	1448	49609	14019	19514
2006	State	md	SUM	153770	1088328	7	64936	43386	20966	395	1919	62195	16060	26475
2006	State	wt	N	26756	162497	6	6541	3524	2881	114	598	5863	441	3011
2006	State	wt	R	127014	925832	7	62450	30247	31092	874	5037	56714	8793	20336
2006	State	wt	SUM	153770	1088328	7	68991	33771	33973	987	5635	62577	9233	23347
2007	State	all_deer	N	28104	164330	6	21549	13497	7823	228	967	20582	2834	10544
2007	State	all_deer	R	128831	951148	7	104317	55307	47684	1326	5278	99039	18334	36127
2007	State	all_deer	SUM	156935	1115478	7	125866	68804	55508	1554	6245	119621	21168	46671

License Year	Hunting District	Deer Species	Residency	Hunters	Days	Days per Hunter	Total Harvest	Bucks	Does	Fawns	Bow	Rifle	Less than 4 Points	4 or More Points
2007	State	md	N	28104	164330	6	14725	10041	4587	97	416	14309	2461	7512
2007	State	md	R	128831	951148	7	48410	29648	18466	296	1141	47269	11841	17567
2007	State	md	SUM	156935	1115478	7	63135	39689	23053	393	1557	61578	14302	25079
2007	State	wt	N	28104	164330	6	6824	3457	3237	131	551	6273	373	3032
2007	State	wt	R	128831	951148	7	55907	25659	29218	1030	4137	51770	6493	18560
2007	State	wt	SUM	156935	1115478	7	62731	29115	32455	1161	4688	58043	6866	21592
2008	State	all_deer	N	27299	166395	6	20197	12448	7561	188	804	19393	2759	9603
2008	State	all_deer	R	133543	1031519	8	95545	51436	42839	1269	4580	90964	17055	33535
2008	State	all_deer	SUM	160842	1197914	7	115741	63884	50400	1457	5385	110357	19814	43138
2008	State	md	N	27299	166395	6	13721	8941	4686	94	348	13373	2403	6497
2008	State	md	R	133543	1031519	8	44717	27364	16988	365	1121	43596	10899	16187
2008	State	md	SUM	160842	1197914	7	58438	36306	21673	460	1469	56969	13302	22684
2008	State	wt	N	27299	166395	6	6476	3507	2875	94	456	6020	357	3105
2008	State	wt	R	133543	1031519	8	50827	24072	25852	904	3459	47368	6156	17349
2008	State	wt	SUM	160842	1197914	7	57303	27579	28727	997	3915	53388	6513	20454
2009	State	all_deer	N	25863	161216	6	19180	11901	7056	222	693	18488	2663	9159
2009	State	all_deer	R	132985	993099	8	85904	47640	37237	1027	3921	81982	15466	31256
2009	State	all_deer	SUM	158848	1154315	7	105084	59542	44293	1249	4614	100470	18130	40414
2009	State	md	N	25863	161216	6	12771	8657	4028	87	270	12501	2270	6330
2009	State	md	R	132985	993099	8	40909	25347	15271	291	843	40066	9659	15274
2009	State	md	SUM	158848	1154315	7	53680	34003	19299	378	1113	52567	11929	21604
2009	State	wt	N	25863	161216	6	6409	3245	3029	136	423	5986	393	2829
2009	State	wt	R	132985	993099	8	44995	22294	21965	736	3079	41916	5807	15982
2009	State	wt	SUM	158848	1154315	7	51404	25538	24994	872	3502	47902	6201	18811
2010	State	all_deer	N	26090	177228	7	18594	12679	5739	177	727	17867	2941	9730
2010	State	all_deer	R	126049	944804	8	76136	46141	29040	956	3353	72784	15617	30467
2010	State	all_deer	SUM	152139	1122032	7	94730	58819	34778	1133	4080	90650	18559	40197
2010	State	md	N	26090	177228	7	11583	8995	2529	59	266	11317	2484	6506
2010	State	md	R	126049	944804	8	32395	21978	10165	251	672	31723	8841	13115
2010	State	md	SUM	152139	1122032	7	43978	30973	12694	311	938	43040	11325	19620
2010	State	wt	N	26090	177228	7	7011	3684	3210	117	461	6550	458	3225
2010	State	wt	R	126049	944804	8	43742	24162	18875	705	2681	41060	6776	17352
2010	State	wt	SUM	152139	1122032	7	50753	27846	22085	822	3142	47610	7234	20577
2011	State	all_deer	N	25266	156612	6	15705	11789	3782	134	685	15019	2750	9033
2011	State	all_deer	R	122527	953993	8	66284	40287	25181	817	3377	62907	15533	24695
2011	State	all_deer	SUM	147793	1110605	8	81989	52076	28962	950	4063	77926	18283	33728
2011	State	md	N	25266	156612	6	10217	8839	1347	32	278	9939	2364	6474
2011	State	md	R	122527	953993	8	28289	20184	7920	185	733	27556	8891	11257
2011	State	md	SUM	147793	1110605	8	38506	29022	9267	217	1011	37496	11255	17730
2011	State	wt	N	25266	156612	6	5487	2951	2435	102	408	5080	386	2559
2011	State	wt	R	122527	953993	8	37995	20103	17260	632	2645	35350	6642	13439

License Year	Hunting District	Deer Species	Residency	Hunters	Days	Days per Hunter	Total Harvest	Bucks	Does	Fawns	Bow	Rifle	Less than 4 Points	4 or More Points
2011	State	wt	SUM	147793	1110605	8	43482	23054	19695	733	3052	40430	7029	15998
2012	State	all_deer	N	24904	0	0	16353	12690	3571	93	678	15675	3537	9140
2012	State	all_deer	R	122967	0	0	71285	44655	25972	658	4061	67224	17314	27280
2012	State	all_deer	SUM	147870	0	0	87638	57345	29542	751	4740	82898	20850	36420
2012	State	md	N	24904	0	0	10136	9171	943	22	280	9855	2974	6197
2012	State	md	R	122967	0	0	27658	21648	5890	120	715	26942	9828	11810
2012	State	md	SUM	147870	0	0	37793	30819	6832	142	996	36798	12803	18007
2012	State	wt	N	24904	0	0	6217	3519	2628	71	398	5819	562	2943
2012	State	wt	R	122967	0	0	43627	23007	20082	538	3346	40281	7485	15470
2012	State	wt	SUM	147870	0	0	49844	26526	22710	609	3744	46101	8048	18414
2013	State	all_deer	N	26346	164760	6	16292	13243	2959	91	518	15775	3662	9553
2013	State	all_deer	R	123764	972808	8	61945	42026	19373	546	3486	58460	17310	24645
2013	State	all_deer	SUM	150110	1137568	8	78238	55269	22331	637	4003	74234	20973	34198
2013	State	md	N	26346	164760	6	10706	9737	942	27	224	10482	3085	6649
2013	State	md	R	123764	972808	8	27088	21730	5217	141	655	26433	10165	11556
2013	State	md	SUM	150110	1137568	8	37794	31467	6158	168	879	36915	13251	18206
2013	State	wt	N	26346	164760	6	5586	3506	2017	63	294	5293	577	2904
2013	State	wt	R	123764	972808	8	34858	20296	14156	406	2831	32027	7145	13089
2013	State	wt	SUM	150110	1137568	8	40444	23802	16173	469	3124	37320	7722	15993
2014	State	all_deer	N	25133	0	0	15173	13562	1575	37	665	14509	3935	9622
2014	State	all_deer	R	119505	0	0	60507	48531	11538	438	3078	57429	19390	29108
2014	State	all_deer	SUM	144638	0	0	75680	62093	13112	475	3743	71938	23324	38730
2014	State	md	N	25133	0	0	9803	9694	108	1	309	9494	3237	6457
2014	State	md	R	119505	0	0	24678	23822	823	33	589	24089	10841	12975
2014	State	md	SUM	144638	0	0	34481	33515	931	35	898	33583	14078	19431
2014	State	wt	N	25133	0	0	5371	3868	1467	36	356	5015	698	3166
2014	State	wt	R	119505	0	0	35829	24710	10715	404	2489	33340	8549	16133
2014	State	wt	SUM	144638	0	0	41199	28578	12181	440	2845	38355	9246	19298
2015	State	all_deer	N	26225	0	0	17794	15282	2404	105	651	17101	4461	10816
2015	State	all_deer	R	128966	0	0	68788	52960	15206	600	3245	65417	20557	32370
2015	State	all_deer	SUM	155191	0	0	86582	68242	17610	705	3896	82517	25018	43186
2015	State	md	N	26225	0	0	11918	11168	722	28	329	11561	3762	7404
2015	State	md	R	128966	0	0	29814	27726	2018	70	634	29125	12362	15350
2015	State	md	SUM	155191	0	0	41733	38894	2740	98	963	40686	16123	22754
2015	State	wt	N	26225	0	0	5872	4114	1682	77	322	5539	699	3412
2015	State	wt	R	128966	0	0	38951	25234	13188	530	2611	36291	8196	17020
2015	State	wt	SUM	155191	0	0	44823	29347	14870	607	2932	41831	8895	20431
2016	State	all_deer	N	26486	167003	6	19556	15653	3791	112	848	18708	4530	11119
2016	State	all_deer	R	132410	1003970	8	85742	61387	23704	651	4437	81305	24801	36553
2016	State	all_deer	SUM	158896	1170973	7	105298	77040	27495	763	5285	100013	29331	47672
2016	State	md	N	26486	167003	6	14003	12093	1851	59	440	13563	3984	8106

License Year	Hunting District	Deer Species	Residency	Hunters	Days	Days per Hunter	Total Harvest	Bucks	Does	Fawns	Bow	Rifle	Less than 4 Points	4 or More Points
2016	State	md	R	132410	1003970	8	41273	33472	7600	201	1026	40247	15240	18229
2016	State	md	SUM	158896	1170973	7	55276	45564	9451	260	1465	53810	19224	26334
2016	State	wt	N	26486	167003	6	5553	3561	1940	53	408	5145	546	3014
2016	State	wt	R	132410	1003970	8	44469	27915	16104	451	3412	41057	9561	18324
2016	State	wt	SUM	158896	1170973	7	50023	31476	18044	503	3820	46203	10106	21338
2017	State	all_deer	N	26646	159741	6	20657	15404	5188	66	795	19862	3982	11419
2017	State	all_deer	R	125567	896044	7	83843	57290	26022	531	4292	79551	20811	36434
2017	State	all_deer	SUM	152213	1055785	7	104500	72694	31209	597	5087	99413	24793	47853
2017	State	md	N	26646	159741	6	14998	11907	3061	31	410	14589	3371	8535
2017	State	md	R	125567	896044	7	40546	30945	9407	195	1170	39376	12319	18615
2017	State	md	SUM	152213	1055785	7	55545	42851	12468	226	1580	53965	15690	27151
2017	State	wt	N	26646	159741	6	5659	3497	2127	35	386	5273	611	2884
2017	State	wt	R	125567	896044	7	43297	26346	16615	337	3122	40175	8493	17818
2017	State	wt	SUM	152213	1055785	7	48956	29843	18741	371	3508	45448	9103	20702
2018	State	all_deer	N	124736	0	0	20803	15130	5562	111	708	20096	3960	11148
2018	State	all_deer	R	28548	0	0	76352	50495	25389	469	3696	72653	18273	32171
2018	State	all_deer	SUM	153284	0	0	97156	65625	30951	580	4403	92749	22233	43320
2018	State	md	N	124736	0	0	15457	12034	3371	51	373	15084	3453	8577
2018	State	md	R	28548	0	0	36785	27267	9369	149	919	35866	11080	16174
2018	State	md	SUM	153284	0	0	52242	39302	12740	200	1292	50950	14533	24751
2018	State	wt	N	124736	0	0	5347	3096	2191	60	335	5012	507	2571
2018	State	wt	R	28548	0	0	39567	23228	16020	320	2777	36788	7193	15997
2018	State	wt	SUM	153284	0	0	44914	26323	18211	380	3111	41800	7700	18568
