# Supplementary material for: The Elephant in the room: What can we learn from California regarding the use of sport hunting of pumas (Puma concolor) as a management tool?
Source: PLoS One. 2020 Feb 13;15(2):e0224638. doi: 10.1371/journal.pone.0224638 (PMC7018503; doi:10.1371/journal.pone.0224638)
Supplement: S2 File — This is a zip file that contains sources of the data used in the analyses that are no longer available on the internet. (ZIP) [file pone.0224638.s003.zip › Montana Mountain Lion EIS 1996.pdf]

FINAL

ENVIRONMENTAL IMPACT STATEMENT

MANAGEMENT OF MOUNTAIN LIONS

IN MONTANA

January 1996

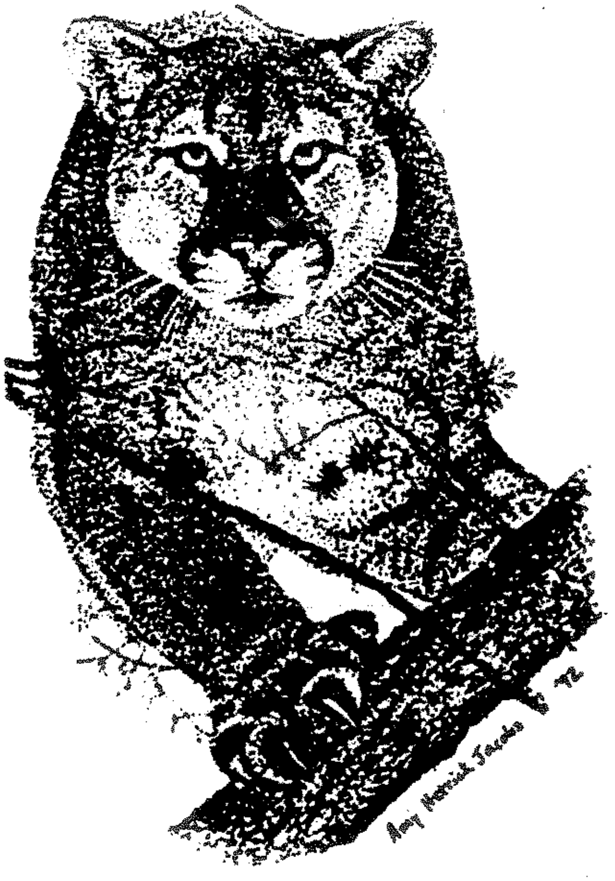

Montana Fish, Wildlife & Parks

COVER SHEET

Type of Statement

Final Environmental Impact Statement (FEIS)

Action

Management of Mountain Lion populations in Montana

Lead Agency

Montana Fish, Wildlife and Parks

Management Authority

Montana Fish, Wildlife and Parks

Abstract

The FEIS describes four Alternatives for the management of mountain lions in Montana; the process used to determine the Alternatives; Elements making up the Alternatives and the benefits, disadvantages, biological assessment and economic impact of implementing each Element. Current biological, social and economic values of mountain lions are described as is their current management in Montana. The four management Alternatives, including a no hunting Alternative, are described. Effects of implementing each on mountain lion habitat, prey populations, hunting and recreational opportunity are described and social conflicts and economic impacts are evaluated. Issues and concerns voiced by the public are addressed in the FEIS, as well as public comment on the draft EIS.

For further information, please contact:

Wildlife Division
Montana Fish, Wildlife and Parks
P.O. Box 200701
Helena, Mt 59620-0701
(406) 444-2612

PREPARERS, CONTRIBUTORS AND CONSULTATION

In addition to the public participation process, FWP consulted and coordinated with representatives of other public agencies that have responsibilities with respect to mountain lion management in Montana:

Larry Handegard, State Director, Animal Damage Control (ADC), U.S. Dept. of Agriculture, Billings

All National Forests in Montana

Montana Department of State Lands (DSL) Helena

Bureau of Land Management: Billings state office

Bud Solmonsson, Montana Department of Commerce, Executive Director Board of Outfitters and Guides, Helena

The following individuals participated in various phases of EIS preparation including research, compilation, writing, review, editing and consultation.

Name	Position
Montana Fish, Wildlife and Parks	
John J. McCarthy	Mtn. Lion EIS Coordinator
Shawn Riley	Cornell University
Glenn Erickson	Chief, Wildlife Management Bureau
Heidi Youmans	Small Game Bureau Chief
Don Childress	Administrator, Wildlife Division
Fay Moore	Administrative Assistant, Wildlife Division
Bill Phippen	Information Officer, Conservation Education
Jim Williams	Area Wildlife Biologist
Harvey Nyberg	Wildlife Manager, Region 1
John Firebaugh	Wildlife Manager, Region 2
Joel Peterson	Wildlife Manager, Region 3
Graham Taylor	Wildlife Manager, Region 4
Charles Eustace	Wildlife Manager, Region 5
Harold Wentland	Wildlife Manager, Region 6
Neil Martin	Wildlife Manager, Region 7
Keith Aune	Laboratory Supervisor, Research Bureau
Bridgitt Erickson	Legal Council
Beate Galda	Administrator, Enforcement Division

Tom Bivins	Program Manager, Enforcement Division
Bob Winfield	Chief Operations, Enforcement Division

Other Affiliation

John Beecham	Game & Research Manager, Idaho Dept. of Fish and Game

MONTANA MOUNTAIN LION MANAGEMENT PROGRAM
FINAL ENVIRONMENTAL IMPACT STATEMENT

SUMMARY

In keeping with the general management goals of Montana Fish, Wildlife & Parks (MFWP), the management of Montana's mountain lions (Felis concolor) was slated for review in 1992. Concerns raised both in the department and by the general public were used to shape the objectives for the program review. Because major changes in the management of mountain lions are being considered, an environmental impact statement (EIS) has been completed in compliance with the Montana Environmental Policy Act.

Montana's mountain lion populations occupy a wide variety of habitats, each with varying capabilities to support both mountain lions and their prey. Objectives of MFWP's mountain lion management program are to maintain both mountain lion and prey populations at levels that are compatible with outdoor recreational desires, and that minimize human-lion conflicts and livestock depredation. Montana's mountain lion management program will require strong administrative direction that will allow flexibility to address needs of diverse lion populations and habitat capabilities while being adaptable enough to allow regional control of mountain lion populations.

To achieve this level of management MFWP proposes to update and refine it's statewide management strategy for mountain lions. The objectives include: determining the carrying capacities of different habitats within the state for mountain lions and their prey; improving its ability to monitor populations and determine their status, composition and trend; improving the regulation of the annual harvest; improve the public understanding of mountain lion biology, habitat requirements and management; and developing policies and a proactive program to deal with human-lion confrontations and livestock depredation.

Preceded by press releases, a scoping document was released in September, 1994. After analysis of public input, a draft EIS was issued in April 1995 for further public review and input.

Four alternative management approaches encompassing a total of 36 elements are included in the EIS. Each element is a management strategy designed to resolve problems or meet objectives as determined by both MFWP and the public.

Each alternative and their major impacts are summarized. The alternatives differ in management objectives and public involvement in the mountain lion management program.

ALTERNATIVE 1 (Management with no hunting by the general public)
Under this alternative, mountain lion populations would continue to expand and be regulated by natural mortality, prey density, inherent social tolerances within populations, habitat

availability and human tolerances. Populations would be composed of older more productive animals. However recruitment would be dependent on the availability of territories and improving or expanding suitable habitat.

Predation rates on prey populations would increase and could contribute to extending low-density cycles of prey species. However, mountain lions by themselves would not significantly affect prey densities statewide, but could affect small, local populations.

The ability to track and monitor populations and collect biological information on populations would be significantly impeded under this alternative. It also could result in significant increases in the incidence of human-lion conflicts and livestock depredation.

Alternative 1 is not expected to impact air quality, water quality, soil or other geological features. Neither is this alternative expected to impact terrestrial or aquatic life, their habitats or vegetative cover. Hunting would be significantly impacted (eliminated), and while nonhunting recreation might increase, the extent of that impact is not known. Elimination of mountain lion hunting and chasing would have an economic impact on outfitters and guides as well as local communities that cater to participants in the mountain lion season. Elimination of license and trophy fees would significantly impact the funding of statewide management programs.

ALTERNATIVE 2 (Continue current management)

Alternative 2 would provide for mountain lion habitat protection as a result of protecting deer and elk habitats, but would not specifically attempt to preserve areas important to mountain lions. In the short term this would have little impact on populations. However, long-term impacts of habitat fragmentation could significantly affect some populations and eventually eradicate them. No attempts would be made to determine the capabilities of Montana's different ecosystems to support mountain lions under Alternative 2.

Alternative 2 would allow for continued expansion of mountain lion populations but make no adjustments to deal with current problems concerning habitat loss, timing of hunting seasons, harvests exceeding quotas, human-lion conflicts or livestock depredation issues. All of these are issues that, if left unaddressed, could have serious impacts on the future of hunting and mountain lion management in Montana.

Alternative 2 would not be expected to impact air quality, water quality, soil or other geological features. Neither is this alternative expected to impact terrestrial and aquatic life, their habitats or vegetation cover.

ALTERNATIVE 3 (Intensify harvest to decrease populations)

Alternative 3 allows the same protection of mountain lion habitats and evaluation of Montana's ecosystems to determine their capabilities of supporting mountain lions and the

prey as Alternative 2. Alternative 3 would direct FWP public education programs at improving awareness of the mountain lion's habitat requirements, encouraging thoughtful land use planning and providing information on living and recreating in mountain lion habitat. Alternative 3 also would require disclosure of the effects of new subdivisions on mountain lions and the prey species present to prospective subdivision homeowners.

Alternative 3 could result in significant reductions in mountain lion densities in some portions of the state. Such reductions would take place through institution of more liberal hunting seasons initiated for public safety, to reduce predation on prey species or to lower livestock depredation. Mountain lion populations would remain viable under Alternative 3, but would be maintained at significantly lower levels than currently present in Montana.

Alternative 3 would have significant long-terms effects on hunting opportunity, other recreation linked with mountain lion densities and the economies of individual outfitters, guides and local communities that cater to mountain lion hunters. It also would result in a loss of funds available to FWP by reducing license and trophy fee revenues.

Alternative 3 is not expected to impact air quality, water quality, soil or other geological features. Neither is this alternative expected to impact terrestrial or aquatic life and their habitats or vegetative cover.

PREFERRED ALTERNATIVE

ALTERNATIVE 4 (Regional management based on habitat capabilities)

Alternative 4 would entail evaluating Montana's ecosystems and their ability to sustain mountain lions and prey species by each FWP administrative region. Alternative 4 also provides for protection of mountain lion habitat and distribution of information that will provide the public with knowledge needed to make better land use decisions related to mountain lions and their ecological requirements. It would also require the development of a human/lion conflict policy and an evaluation of mountain lion hunting on WMAs.

Alternative 4 would give FWP regions the authority to recommend changes to the current season structure. This could result in significant changes (increases or decreases) in mountain lion densities and hunter numbers at the local level. Such changes could influence the numbers of human-lion conflicts and depredation situations, as well as result in economic impacts (positive or negative) on the local community.

Alternative 4 provides the flexibility in management needed to address changes in lion populations as they occur. Such changes would require approval of the FWP Commission.

TABLE OF CONTENTS

CHAPTER I - PURPOSE AND NEED FOR ACTION 1
Introduction 2
Need, Purpose & Benefits of the Proposed Action 2
Decisions to be Made 3
Other Agencies that have Jurisdiction or Responsibility 4
Results of Public Scoping 4
Issues and Impacts Evaluated in the DEIS 4
Issues and Impacts Not Evaluated in the DEIS 9
Analysis of the Elements Comprising the Issues/Impacts 11
Existing Activities or Programs that would Continue 11
Activities or Programs that would be Adjustments to Management 19
Activities or Programs added by Public Initiative 28

CHAPTER II - AFFECTED ENVIRONMENT 33
A Review of Mountain Lion Research & Biology 34
Evolution 34
Distribution 34
Habitats 34
Territories/home ranges 38
Reproduction and Natality 39
Emigration and Immigration 40
Mortality 41
Legal Status 42
Food habits and predator-prey relationships 42
Livestock depredation 43
Disease and Parasites 44
Mountain lion-human interactions 45
Translocations 45
Hunting 46
Values 55

CHAPTER III - ALTERNATIVES INCLUDING THE PROPOSED ACTION 57
Alternatives Selected for Analysis in this DEIS 58
Alternatives Not Considered by the DEIS 58
Description of the Alternatives Selected 60
Alternative No. 1 - No Hunting to Lions by the General Public 60
Alternative No. 2 - Continue the Current Management Program 61
Alternative No. 3 - Intensify the Harvest to Decrease the Population 61
Alternative No. 4 - Regional Management based on Habitat Capabilities 62

CHAPTER IV - ENVIRONMENTAL IMPACTS 64
Analysis of Environmental Consequences 65

Comparison of Alternatives 79
Comparative Summary of Environmental Consequences 82

LITERATURE CITED 101

APPENDIX 1 - MCA Statutes 108
APPENDIX 2 - MT 1994 Mountain Lion Regulations 112
APPENDIX 3 - Summary of Public Comments Received on the DEIS 114

LIST OF TABLES

1. Element description 12
2. Mountain lion mortalities by MFWP region, 1988-1993 88
3. Lion mortalities by mortality class in Montana, 1988-89 through 1992-93 89
4. Estimated value of livestock lost to mountain lions in Montana, 1984-93 90
5. Trichinella test results from Montana mountain lions, 1971-1989 90
6. Trichinella test results from mountain lions by MFWP Regions 1 - 5, 1971-1989 in order of declining prevalence 90
7. Resident, nonresident and total mountain lion hunting license sales in Montana . . 91
8, A list of mountain lion hunting season dates and structure in Montana, 1971-1993 92
9 A comparison of mountain lion hunting season dates, hunting season length, hunting license fees, hunter numbers and harvest during the 1992-93 or 1993-94 lion seasons in the western states and provinces 94
10. A summary by MFWP region, of recorded non-hunting mountain lion kills, 1971-1993. Years are from July 1 through June 30 95
11. A summary of MFWP region, of recorded mountain lion kill, 1981 -1993 96
12. Number of mountain lions killed in Montana by hunters having killed at least one mountain lion, 1971-1990 97
13. Percent of mountain lions harvested statewide by month in Montana 97
14. Montana's statewide outfitted mountain lion harvest, 1990-1993. 98
15. Chase information gathered for Montana from hunting season participants 1989 - 1993 99
16. Chase information gathered for Montana from chase season participants 1989 - 1993 99
17. Mountain lion hide and skull sale prices for 1990-1994 100
18. List of program elements by alternative and decision authority 59
19. Categories of impacts analyze by each alternative in the DEIS 100

LIST OF FIGURES

1. Distribution of mountain lions in Montana. 1994 35
2. Distribution of Montana counties that paid bounties for mountain lions. 1921-1922 36
3. Distribution of Montana hunting districts with a recorded mountain lion harvest. 1971-1972. 36
4. Distribution of Montana hunting districts with a recorded mountain lion harvest. 1981-1982 37
5. Distribution of Montana hunting districts with a recorded mountain lion harvest. 1991-1993 37
6. Hunting characteristics of Montana's mountain lion license holders. 47
7. Mountain lion licenses sold in Montana, 1971-1993 47
8. Nonhunting mountain lion mortality in Montana. 1971-1993 50
9. Percentage of total Montana mountain lion harvest taken in December. 1971-1993 51
10. Percent of Montana mountain lion harvest in December vs. licenses issued 51
11. Percent of Montana's mountain lion harvest that is female and taken by outfitted vs. nonoutfitted hunters. 52
12. Mountain lion distribution by Montana's ecosystems 54
13. Number of ADC justification reports filed and mountain lions killed in Montana. 1964-1992 54
14. All Montana cattle and calves, 1940-1994 71
15. All Montana sheep and lambs, 1940-1994 71

CHAPTER I

Purpose of and Need for Action

INTRODUCTION

The Environmental Impact Statement (EIS) describes four approaches to managing Montana's mountain lion populations, the process used to formulate the four alternative actions and the expected environmental consequences of implementing each. The potential impacts of each action on: mountain lion populations; hunting, chasing and viewing opportunities; regulation of harvest; assessment and monitoring of mountain lion population trends; illegal exploitation; economic impacts on local economies; program funding; and the incidence of human-lion conflicts and livestock depredation also are discussed. Three of the four alternatives include harvest by the hunting public as a management tool, one alternative does not. Issues and impacts voiced by the public at initial scoping meetings were considered and are addressed in the EIS.

NEED, PURPOSE AND BENEFITS OF THE PROPOSED ACTION

Montana Fish, Wildlife & Parks (FWP) is charged by law with protecting, preserving and perpetuating fish, game and furbearer populations as well as game and nongame bird populations within the state. FWP license revenues are used for that purpose (Section 87-1-201, MCA (Montana Codes Annotated). Management goals developed by FWP under the umbrella of its broad legal authority include:

> Manage with a focus on ecological systems to reflect the diversity of all wildlife and their habitats while maintaining our commitment to Montana's hunting and fishing heritage.
>
> Provide increased opportunities for public enjoyment of fish, wildlife and parks resources while maintaining our commitment to improve landowner/sportsperson relations.
>
> Elevate the importance of public education and participation in all program areas to afford citizens the opportunity to better understand, appreciate and make informed decisions about our natural and cultural resources.

Montana's mountain lion (<u>Felis concolor</u>) populations occupy a wide variety of habitats, each with varying capabilities to support both mountain lions and their prey. Objectives of FWP's mountain lion management program are to maintain mountain lion and prey populations, to maintain mountain lion populations at levels that are compatible with outdoor recreational desires, and to minimize human-lion conflicts and livestock depredation. Montana's mountain lion management program will require strong administrative direction that will allow flexibility to address needs of diverse lion populations and habitat capabilities while being adaptable enough to allow for FWP regional control of mountain lion populations.

To achieve this level of management, FWP proposes to update and refine its statewide management strategy for mountain lions. The objectives of the Proposed Action include: setting appropriate mountain lion management objectives for Montana's various mountain

lion habitats; improving our ability to monitor populations and determining their status, composition and trend; improving regulation of the annual harvest, improving the public understanding of mountain lion biology, habitat requirements and management; and developing policies and a proactive program to deal with human-lion confrontations and livestock depredation.

The ultimate benefit of the Proposed Action is long-term perpetuation of viable, healthy mountain lion populations that can provide aesthetic and recreational benefits to Montanans and visitors to our state.

The scope of this EIS is limited to elements of the mountain lion management program for which FWP, the FWP commission or the Montana Legislature has decision making authority. *The function of the EIS is to disclose potential consequences of each of four alternative actions to the public, as well as to involve the public in making changes to FWP's mountain lion management program.*

DECISIONS TO BE MADE

Each alternative action includes a number of individual program elements. Within each alternative, each proposed program element falls under the legal decision-making authority of one or more of the following decision-making bodies: 1) the FWP Commission; 2) FWP; and 3) the Montana Legislature.

FWP COMMISSION: Fifteen of the elements of the Proposed Action are subject to the authority of the FWP Commission and can, therefore, be considered recommendations made by FWP to the FWP Commission. The FWP Commission is the policy making body that oversees the state's wildlife management programs. Section 87-1-301(1), Montana Codes Annotated (MCA) requires the Commission to "set policies for the protection, preservation and propagation of the wildlife, fish, game, furbearers, waterfowl, nongame species and endangered species of the state for the fulfillment of all other responsibilities of the Department as provided by law." Recommendations that the FWP Commission chooses to approve would be in the form of hunting regulations, which would be adopted following public hearings during the season-setting process, and would become effective for the 1996 mountain lion hunting season. The FWP Commission adopts hunting seasons either annually or biennially under an exception to the Administrative Procedure Act for agency rules (Section 2-4-102(10)(d), MCA).

FWP: Elements subject to FWP authority can be implemented upon a change in department policy and/or a change in the Administrative Rules of Montana (ARM). ARM rules can be adopted by FWP following a formal rule-making process that includes public notification and an opportunity for public review and comment.

MONTANA LEGISLATURE: Decisions subject to legislative authority would require FWP to draft legislation for consideration by the Montana Legislature.

OTHER AGENCIES THAT HAVE JURISDICTION OR RESPONSIBILITY

The Animal Damage Control (ADC) division of the U.S. Department of Agriculture and the Animal and Plant Health Inspection Service (APHIS) also have responsibility for mountain lions in Montana. ADC has jurisdictional authority in cases of stock depredation.

Outfitters in Montana are under the jurisdiction of the Montana Department of Commerce, which is responsible for issuing outfitting licenses and the enforcement of laws regulating the outfitting industry.

RESULTS OF PUBLIC SCOPING FOR ISSUES AND IMPACTS

In November 1992, a draft mountain lion management plan was issued for public comment by FWP. Comments received included 127 letters from private individuals or organizations and 250 form letters. Issues raised by the draft plan were summarized and incorporated into a scoping document. This document, which outlined a draft of the Proposed Action and solicited public input on issues and alternatives was mailed to over 1,200 individuals and organizations in July 1994. It also was made available upon request (via press release) to the general public. Public meetings were held in Big Timber, Missoula and Kalispell to gather additional comments. Eighty-five written and 45 oral comments were received during the scoping process.

Comments received during the public scoping process addressed 18 issues or impacts. Of the 18, eleven were evaluated in this EIS. Seven other issues or impacts are discussed, but have not been included in the evaluation because they were not believed relevant to the process or were thought outside the scope of the process.

ISSUES AND IMPACTS EVALUATED IN THE EIS

Public comment indicated the following 11 issues and impacts needed to be evaluated in the EIS and addressed in the analysis.

1) Timing and structure of the mountain lion hunting season.

Montana's general mountain lion hunting season currently consists of two portions: a harvest season and a chase season. The general harvest season begins December 1 and runs until the harvest quota for a hunting district or districts is reached or February 15. The chase season begins either when district quotas have been reached or on February 16, and continues through April 30.

Three hunting districts that lie within the Bob Marshall and Scapegoat Wilderness complex open to mountain lion hunting on September 15, which is concurrent with the opening of the deer and elk season for these areas. Opening at this time allows hunters to access remote areas that, due to snow conditions, are not usually accessible when the remainder of the state opens to mountain lion hunting December 1.

The general mountain lion hunting season is set to coincide with the presence of snow cover in hunting areas, and periods of lower birth rates for Montana's mountain lion populations. Because birth rates peak in early summer in Montana, it is more likely at this time to observe the tracks of any dependent young that may be traveling with the female during winter months (Anderson 1983:32, Pall et al. 1988, Murphy et al. 1991). Snow cover gives hunters an opportunity to determine if the tracks they are following belong to a female accompanied by young prior to releasing dogs on the track. Snow cover also allows the opportunity to backtrack for kittens if a female is found to be lactating after being harvested. The mountain lion season also is timed to prevent the interference that hound hunting would cause during the general big game season.

A chase season has been established to provide an opportunity to train trailing hounds, provide additional public recreation and provide an opportunity for FWP to obtain mountain lion trend and distribution data from hound handlers.

Fifty-nine percent of the comments received during the public scoping process dealing with season structure indicated a desire to retain the current structure. Forty-one percent expressed the desire to begin the season with the opening of the general big game season. Of 86 letters and 47 oral comments received during the scoping process, one letter indicated FWP should abolish mountain lion hunting.

2) Regulation of nonresident hunters and hound handlers.

Montana currently does not limit the number of nonresidents that may hunt or chase mountain lions. Resident hunters are becoming increasingly aware of the impact of growing numbers of nonresident hunters on the overall mountain lion harvest. This is particularly evident in northwestern Montana, where quotas are being reached within 4 to 10 days of the season opening. Between 1990 and 1993, nonresidents accounted for 36% of the total mountain lion harvest in that area, as compared with 27% on a statewide level.

Hound hunting restrictions in other western states (Oregon abolished hound hunting in December 1994, California abolished mountain lion hunting in 1990, Idaho allows 55 nonresident hound hunters for mountain lions) would indicate Montana can expect a continued increase in nonresident interest in the sport. If it becomes necessary to regulate hunter numbers, by statute (87-3-303 MCA) nonresidents will be the first category to be restricted and it will be necessary to have a method to do this.

The state does not currently regulate or track numbers of resident or nonresident hound handlers that participate in the hunt and/or chase season. In 1994 regulations required that handlers in the field be accompanied by one person having a hunting license or chase permit. The handler is not required to have a license or permit to participate. In 1995 hound handlers were required to hold a current mountain lion license and a hound handler permit issued by FWP.

3) Length of the time period allowed for reporting kills, tagging hides and skulls and closing the seasons.

In 1992, 33% of the state's 42 mountain lion hunting districts exceeded the total harvest quota set for individual units. In 1994, that percentage rose to 42% of the 57 mountain lion hunting districts within the state. Factors leading to this situation include increasing mountain lion populations, increased hunting pressure, favorable snow conditions and a 48-hour time frame for closing the hunting seasons. Components of this problem that are under FWPs current authority include hunting pressure, procedures for closing the seasons, mountain lion densities and the abundance of their prey populations.

4) Levels of security afforded to females and young.

Hunting is recognized as being the principal cause of lion mortality in populations that are hunted (Ross and Jalkotzy, 1992, Logan et al. 1986, Currier et al. 1977). Population stability is most affected by the removal of reproductive adult females (Lindzey et al. 1992).

Females, because of their need to leave smaller kittens when hunting for long periods of time (Seidensticker et al. 1973), and their tendency to travel some distance apart from their dependent young (Barnhust and Lindzey, 1989), may become as vulnerable to harvest as lone animals.

To better assure population stability, and increase chances of young being raised to independence, it is important to afford females and dependent young a higher degree of security than adult males and transient young.

5) Recommendations regarding roadless and wilderness areas made by FWP to land management agencies.

Several comments received stated FWP should not recommend or support additions to Montana's wilderness or roadless areas.

FWP does not have constitutional or legislative authority to establish roadless or wilderness areas. It also has no authority to open or close roads on public lands. FWP provides technical assistance regarding wildlife to land management agencies, such as the U.S. Forest Service, Bureau of Land Management, Bureau of Reclamation, U. S. Fish and Wildlife Service, and Montana Department of State lands. One form this assistance can take is in comments and recommendations made to those agencies concerning proposed roadless and wilderness areas and their potential impacts on wildlife. Such recommendations are developed by FWP at the local level, and are presented to the land management agency to assure that wildlife values are considered in the decisions that agency is authorized to make. Because FWP makes recommendations only, impacts of the decision on the biological, social, cultural and economic environment of the area must be analyzed by the land management agency involved, not FWP.

In recent years the process of offering wildlife-related recommendations on proposed programs has become more important. Increasing public interest in participating in outdoor activities, wildlife management matters and the recreational opportunities wildlife affords, as well as in land management decisions, has resulted in expanding pressures on the state's wildlife resources and their habitats. Because FWP's recommendations are often the state's only opportunity to provide input on these land management decisions as they affect wildlife, it is vitally important that FWP continues to participate in this process.

6) Development of a policy dealing with human-lion and lion depredation conflicts.

Montana, like other western states, has seen an increase in the incidence of mountain lion attacks on people, pets and livestock in recent years. (Aune 1991, Beier 1991) This is a result of several factors, some of which are: increasing mountain lion populations; increasing numbers of people occupying or recreating in mountain lion habitat; subdivision development that create small refugia for prey species; and, a stable or increasing statewide prey base.

Numbers of incidents vary annually and differ with demographics and human land use and lion habitat patterns within the state (Aune and Schladweiler 1993). Incidents are currently monitored by FWP regional staffs using incident report forms, and statewide summaries are compiled annually. This level of monitoring would be continued under all the Alternatives considered in the EIS. In general, incidents that occur west of the Continental Divide involve people or pets, while those east of the divide involve livestock (Aune 1991). These situations are currently handled on an individual basis with no guidelines to direct what actions should be taken under the variety of circumstances that occur in the field. To assure consistency in handling these situations and to maximize the safety of humans, their pets, livestock and the individual lions involved, FWP needs to develop a human-lion conflict policy.

7) The effects of mountain lions on the prey base and on future big game hunting.

Mountain lions will kill and consume most vertebrates. However, mountain lions in the West are ubiquitously tied to deer as a staple food item. Among 16 food habit studies reviewed by Anderson (1983:50-51), 14 listed deer as the single major food item. Studies in Montana have recorded mountain lion consumption of elk and bighorn sheep where they are available. (Murphy et al. 1991, Williams 1992)

Effects on ungulate populations vary widely and depend on several factors including: age and sex of individual mountain lions; age and sex composition of the mountain lion population; reproductive status of individual female mountain lions; weather conditions; prey abundance; and habitat security for both the mountain lions and their prey species.

While the scope of an in-depth analysis of predator/prey relationships and prey abundance in Montana's various habitats is too complex for inclusion in this EIS, predation and prey abundance is partially addressed from the standpoint of the needs of individual mountain lions and the populations they comprise. It also is addressed through protection of threatened big game habitat and formulation of management objectives for deer and elk populations.

8) The role of public hunting in lion management

Hunting is perceived by various segments of the public as: 1) a necessary population management tool; and/or 2) an important form of recreation; or 3) an unnecessary, unethical exploitation of wildlife with no redeeming social value. Three of the four alternatives considered include general hunting harvest by the public as a primary management tool and valid recreational activity. (See the description of the alternatives beginning on Pp 58).

Application and regulation of hunting differs between alternatives. However, the direction of all alternatives is toward stricter regulation of mountain lion harvests, continued or increased protection of females and young, development of better population monitoring techniques and the development of management objectives. *It should be noted that stricter harvest regulations and additional protection do not necessarily equate to lower harvest objectives or no harvest.*

9) Use of nonlethal means to address depredating and nuisance mountain lion problems.

Some individuals and organizations that participated in the scoping process expressed the desire for nonlethal removal of mountain lions involved in the killing of livestock or close association with humans.

This will need to be addressed through research to evaluate the effectiveness of such removal and the level of response each individual situation demands. Three of the four alternatives require the development of a human-lion conflict policy that would establish guidelines to address such situations.

10) Prevention of illegal harvest and reporting activities.

Enforcement concerns such as reporting of harvests from the wrong areas, killing and abandonment of smaller mountain lions, and illegal outfitting were raised during the scoping process. These issues were discussed in relation to hunter ethics, privatization of wildlife, potential threat to sport hunting, the image projected by those who participate in Montana's mountain lion season and the concern over maintaining viable mountain lion populations.

These issues are addressed through proposals for closer control of the harvest, registration of all hound handlers and improving FWP's ability to collect and disseminate harvest information and regulate hunter numbers.

11) Application of Montana's game damage statutes to mountain lions.

Under Montana Statute 87-1-225 MCA, only those landowners that allow public hunting or do not significantly reduce public hunting are eligible for game damage assistance. The law also specifies that those lands where special circumstances have rendered public hunting inappropriate also are eligible for game damage assistance. Several participants in the

scoping process felt that landowners sustaining mountain lion depredation, who do not allow public hunting, should not be considered for assistance by FWP.

Mountain lion depredation and the rights of landowners in cases of depredation are addressed in three of the four alternatives.

ISSUES and IMPACTS NOT EVALUATED IN THE EIS

Seven issues or impacts raised during the scoping process were viewed by FWP as not relevant to decisions being made or were outside the scope of the EIS. They are described and the rationale for their exclusion is provided below.

1) Health concerns dealing with any requirement to use mountain lion meat

Due to the high incidence of trichinosis in Montana's mountain lion population, requiring use of the meat was dropped from consideration in the analysis.

No quantitative data is available documenting the number of successful hunters who utilize mountain lion meat. A survey of black bear hunters conducted by FWP indicated that 74% of residents and 57% of nonresidents utilized all or part of the meat of black bears they harvested (FWP 1993a). It is unknown if similar use rates may be assumed for mountain lions. However, it is known that some persons harvesting a mountain lion keep the meat.

Mountain lions have a much higher rate of trichinella infection than black bears. Tests carried out in Montana between 1971 and 1989 indicated 15% of black bears harvest were infected with trichinosis, while 54% of mountain lions were infected. Rates of infection varied by administrative region and ranged from 15% in Region 5 to 70% in Region one. (See Tables 5 and 6, pp 90). Because of these high infection rates for mountain lions, FWP did not feel the requirement to use mountain lion meat was in the public's best interest.

2) Orphaning of kittens

Each year FWP's wild animal shelter receives on average five mountain lion kittens. These animals have been orphaned as a result of hunting or accidents, or have been "found" by persons who assumed they had been abandoned. FWP attempts to place these animals in zoos. However, the captive population of the United States is currently increasing through natural reproduction and zoo placement has become more difficult with time. Annually two to three animals not placed in zoos have been raised to approximately 80 lbs. and reintroduced in remote areas of the state.

Current mountain lion densities, naturally expanding mountain lion populations, poor transplant success, rates of human encroachment into mountain lion habitat and liability concerns have made placement of animals in the wild much less feasible than in the past. As a result, FWP will be forced to euthanize animals that cannot be placed in zoos or

immediately returned to the wild. Because orphaned kittens would be handled similarly under all the Alternatives, this issue was not evaluated as part of the EIS.

3) Use of hunters to remove animals involved in depredation or human - lion conflicts.

FWP and ADC responsibilities are specified in statute. There are no provisions for state agencies to transfer responsibilities and public safety concerns, delegated to them by law to private parties. Due to the legal barriers surrounding this type of arrangement and public safety concerns, the gravity of these situations do not lend themselves to the time and inefficiency of sport hunting. For these reasons this issue was not evaluated further.

4) Regulating numbers of outfitters or limiting the harvest allowed by outfitted hunters.

Outfitters are regulated by the Montana Department of Commerce and are not under the jurisdictional authority of FWP. Outfitter use of public lands is regulated by land management agencies, and the level of use on those lands is managed by the controlling agency. Because of FWPs lack of authority in this matter, it was not considered further.

5) Retain and/or raise the trophy fee and earmark the funds collected for mountain lion management, research and trichinella testing.

Trophy licenses were first issued for grizzly bears in 1967. They were first required of mountain lion hunters in 1984. The concept behind issuance of trophy licenses was to require a fee on those animals that were not considered fit for human consumption and to direct the harvest towards the larger or "trophy' animals. The trophy license requirement was also an attempt to provide additional protection for the physically smaller females.

Over time the cost of the trophy license has not kept up with inflation and is no longer an economic barrier to hunters willing to take a female or smaller lion. License fees are established by the Legislature, as is the earmarking of funds for special programs. License fee increases were set by the 1993 Legislature. FWP projects those fees will be adequate until 1999. Because of these circumstances, this issue was not considered in the analysis.

6) The collecting of skulls for aging has resulted in damage to the skulls, lost skulls or long waiting periods before the skulls were returned.

Skulls are collected to ascertain the age of individual mountain lions harvested and the age composition of the total harvest. Age is determined by examination of the sutures in the skull and from rings laid down in the teeth. In the past, all skulls were cleaned to inspect the skull sutures. This process was time consuming and could result in damage to the skull. New methods of examining the skull sutures have been developed that do not require cleaning the skull and are not damaging. The turn around time for return of collected skulls has been greatly reduced.

Age information from harvested mountain lions is essential in determining the status and trend of mountain lion populations and is used in setting harvest regulations. Because the new method of examining of skulls, this issue was not included in the analysis.

7) Allowing trappers with current mountain lion licenses to keep a lion caught incidental to trapping operations.

A small number of mountain lions are taken each year by trappers incidental to their trapping operations (average three per year for the period 1990-93). Some trappers have expressed an interest in keeping mountain lions they incidently trap if the season is open and the trapper has a current mountain lion license.

This issue was not included in the analysis because it would be difficult to regulate this type of harvest and could increase the possibility of females with dependent young being taken. Incidental take would not stop when the quotas had closed and animals would still be trapped by some trappers who did not purchase a mountain lion license. Such a regulation also could encourage the snaring of mountain lions during open seasons, which would again increase the possibilities of taking a female with young.

ANALYSIS OF THE ELEMENTS COMPRISING THE ISSUES/IMPACTS

In order to facilitate analysis of the issues raised. each of the 11 issues or impacts analyzed was broken down into its fundamental elements. These elements then were grouped into activities and programs that are incorporated into the description of mountain lion management alternatives analyzed in the EIS. These activities and programs fell into three groups: 1) those currently used to manage mountain lions; 2) those that would constitute adjustments to management; and 3) additional activities or programs added to the analysis as a result of the scoping process.

The 11 issues and impacts that were to be analyzed comprised 26 elements that are listed in Table 1 (pp 13) and described below. As a result of comments received on the EIS changes have been made to Elements C, M, N, T and V. These changes are detailed and the reasons for the changes given in the Comments on the DEIS section.

EXISTING ACTIVITIES OR PROGRAMS THAT WOULD CONTINUE UNDER THE REFINED MANAGEMENT PROGRAM

Element A: Continue the use of the existing hunting season format.

Benefits:

The opening of mountain lion season occurs September 15 in portions of the Bob Marshall and Scapegoat Wilderness areas. These areas are usually inaccessible by December 1, when the remainder of the state opens to mountain lion hunting, and the early opening provides a limited amount of lion hunting opportunity (0.6% of the statewide harvest for the period 1990-93). The opening for the remainder of the state

Table 1. Element Description.

ELEMENTS	DESCRIPTION
	EXISTING
A	Continue the use of the existing hunting season format.
B	Continue the "chase season."
C	Continue to use quotas as one method of regulating harvest.
D	Assist landowners and ADC with cases of livestock depredation.
E	Continue public education projects on mountain lions.
F	Continue to assess road access on public lands.
G	Protect threatened habitats through the Habitat Montana Project.
H	Evaluate land management agencies proposed programs on their merits for wildlife.
I	Hides and skulls of mountain lions will continue to be tagged in the region harvested.
	ADJUSTMENTS
J	Reduce the minimum closure period for a season from 48 to 12 hours and allow FWP to announce closure prior to meeting quotas.
K	Reduce the reporting period for a lion kill to 24 hours.
L	Use season length to regulate harvest.
M	Reduce the inspection period for skull and hide to 24 hours after being reported.
N	Reword regulations to protect "Females and dependent young."
O	Re-evaluate the hunting of mountain lions on WMAs.
P	Provide for the establishment of intensive management zones around selected areas of human habitation.
Q	Amend Statute 76-3-608 MCA to require environmental assessments of subdivisions be provided to prospective buyers.
R	Clarify Statutes 87-3-127 (1) MCA and 87-3-130 MCA to allow landowners to use a hound handler to take depredating lions.
S	Determine and prioritize research needs to assess mountain lion management.
T	Estimated carrying capacities and biological and social constraints will be utilized to make season recommendations.
U	Develop human-lion conflict policy.
	PUBLIC INITIATIVES
V	Persons in control of dogs pursuing mountain lions must purchase a mountain lion hunting license and hold a hound handler permit. (Guides and outfitters excluded from license purchase).
W	Provide methods to regulate total hunters and nonresident hunters within the state or a portion of the state.
X	The department needs to improve its ability to obtain and disseminate harvest information.
Y	Increase the enforcement efforts to check kill sites, harvest information and hunter residency.
Z	Open mountain lion hunting with the general big game season.

is set to coincide with snow cover in mountain lion habitat. Snow cover aids hunters in determining whether the animal being tracked is a female with kittens or dependent young and, therefore, illegal to harvest. The season structure also allows for the closure of the season either based on the harvest or by a set date. That set date is currently February 15, but the closure date and the opening may be adjusted by the FWP Commission.

The season is of adequate length to allow most statewide harvest goals to be attained, and provides sufficient time for other recreational aspects of the hunt, such as photography, viewing and dog training, to take place. The structure also allows anyone interested in hunting a mountain lion the opportunity to purchase a license, as well as the time to participate.

Disadvantages:

The current season structure does not allow for the incidental harvest of mountain lions during the general big game season. While the current season structure was adopted to help protect females with young, increased mountain lion observations by deer and elk hunters have resulted in increased pressure to allow for this type of hunt.

The current season often opens on a weekday when many license holders are unable to hunt.

Biological assessment:

Mountain lion population dynamics and behavior allow for an annual sport take as long as annual recruitment is not exceeded, adult females are protected (Lindzey et al. 1992), or there are effective refuges from which transient young can move to vacant territories in hunted areas (Lindzey 1987:666). Current timing of the season is advantageous to identification and protection of females with young and does not conflict with the wildlife management objectives of the general big game season.

In some years, snow patterns can result in high concentrations of hunters and accelerated harvest rates in some areas of the state. At times, this has resulted in harvest quotas being exceeded.

Economic impact:

The current timing of the mountain lion season essentially increases the length of Montana's hunting season. Such an extension is economically important to local outfitters and other businesses catering to hunting and outdoor recreation. Excluding guide and outfitting fees, it is estimated that mountain lion hunting contributed a minimum of $900,000 to Montana's economy in 1993 (FWP, unpublished economic survey, 1994).

Decision Authority: FWP Commission

Element B: Continue the "chase season"

Benefits:

The chase season for each hunting unit currently begins either when the harvest quota is reached, or on February 16, and extends until April 30. This season provides additional recreational opportunity and allows for the training of trailing hounds. It provides FWP with winter mountain lion distribution and density information that is gathered from the participants by questionnaire. (See Tables 17, pp 100 and 18, pp 59).

Disadvantages:

The physiological effect of prolonged or repeated chases on an individual mountain lion are not well understood. It is known that mountain lions show a lowered plasma cortisol profile when subjected to simulated chase. This has been interpreted as either a negative condition brought on by stress or an adaption to stress that allows the mountain lion to adjust to higher stress levels. (Harlow et al. 1992)

The chasing of mountain lions with dogs is felt to be an unethical practice by some and leaves the participants, FWP and the sport itself open to criticism.

Biological assessment:

To effectively harvest mountain lions, use of trained hounds is needed. Mountain lion populations have increased in the state during the period of time this regulation has been in effect. Therefore, the chase season is not seen as a detriment to maintaining a viable mountain lion population. The physiological response of lions to the chase is open to examination and the impacts of the mountain lions' response to extended or repeated chases needs to be determined.

Economic impact:

Timing of the mountain lion season essentially increases the length of Montana's hunting season. Such an extension is economically important to outfitters and other businesses catering to hunting and outdoor recreation. Excluding guide and outfitter fees, participants in the chase season contributed an estimated $300,000 to Montana's economy in 1993 (FWP, unpublished economic survey, 1994).

Decision Authority: FWP Commission

Hunting as defined by statute (87-2-101(8)MCA) and as approved by the 1995 legislature includes the intent of taking or harvesting the animal being pursued or chased. This new definition clouds the authority of MFWP Commission to establish a "chase season" and needs to be clarified by the legislature.

Element C: Continue to use quotas as one method of regulating harvest

Benefits:

Quotas focus on, and allow a high level of control of, both total harvest and the sex of mountain lions harvested. Quotas also permit maximum levels of hunter participation when the harvest is limited and may be used with a variety of other regulations (permits, validation of hunting areas, etc.) to further control harvest or hunter numbers.

Disadvantages:

Quotas result in the creation of a scarcity model. That is, when people know there is a cap on the harvest, that when attained will close the season, they put more effort into hunting and into taking a lion than they would under less restrictive regulations. This has resulted in rapid harvests and quota overruns in some hunting areas.

Regulating statewide harvest with quotas does not allow a full range of management options or season structures to be implemented.

Quotas require strict control of season closures and put the impetus on FWP to have a procedure in place allowing the closure to take place in a timely fashion.

Biological assessment:

Quotas allow a maximum of hunter opportunity while providing for a strict control of the harvest.

Economic impact:

No significant economic impacts are anticipated.

Decision Authority: FWP Commission

Element D: Assist landowners and the ADC in livestock depredation cases.

Benefits:

Participation allows FWP to help direct control measures at particular animals. It also allows collection of biological information from animals taken as a result of livestock depredation and provides FWP with the opportunity to maintain contact with landowners and ADC. It also will provide more efficient service to landowners during times when ADC personnel are unavailable.

Disadvantages:

Requires time and personnel to be redirected from other projects in order to react in an efficient manner to depredation problems.

Biological assessment:

Additional protection may be provided to non-targeted animals by having more personnel available for depredation problems. It also allows for collection of biological data from mountain lions taken.

Economic impact:

Quick response will reduce depredation losses for individual stock growers. Estimated loss to mountain lion depredation in Montana was $12,875 in 1993. (Aune 1993)

Decision Authority: FWP

Element E: Continue public education projects on mountain lions.

Benefits:

The more local governments and the public know about the nature of mountain lions, their habitat requirements and mountain lion management, the greater the prospects are for maintaining healthy, viable mountain lion populations in Montana. Efforts directed at educating the public regarding safety measures and human-lion interactions also increase the security of both the human population in mountain lion habitat, their pets, and the mountain lion population in the state.

Disadvantages:

Education efforts require continuous commitments of personnel, time and expenditures and results are often difficult to measure.

Biological assessment:

Public education can directly affect our ability to influence the maintenance of mountain lion habitat, reduce depredation and human-lion conflicts, all of which ultimately improve the future outlook for Montana's mountain lion populations.

Economic impacts:

Some redirection of FWP's budget allocation may be needed.

Decision Authority: FWP

Element F: Continue to assess road access on public lands.

Benefits:

While FWP has no authority to open or close roads on public lands, participation in a land management agency's process of evaluating roads allows FWP input in determining security levels that will affect both mountain lions and their ungulate prey base.

Disadvantages:

Road closures are often perceived as FWP closing off public access for hunting, shutting down logging operations or other resource extraction activities rather than attempts to maintain or enhance big game security. As a result, FWP often is criticized and animosities develop between different user groups and the department.

Biological assessment:

The security of an area for mountain lions or their prey may be altered through increasing or reducing the density of open roads in that area and, subsequently, increasing or decreasing the ease of hunter access. FWPs ability to affect these situations relies on its ability to effectively evaluate and impact decisions of land management agencies regarding access and road density.

Economic impact:

FWP input, while affecting a decision to open or close roads, is in the form of recommendations. If the decision made has a significant impact on the local economy, the land management agency must analyze those impacts before a decision is finalized.

Decision Authority: FWP

Element G: Protect key ungulate habitats through the Habitat Montana Program.

Benefits:

The ability to purchase, hold easements for or lease key habitat for big game species affords long-term benefits for big game and other wildlife species in Montana through habitat protection. It provides tangible and intangible benefits to the people of Montana and their way of life.

Disadvantages:

Land management actions by FWP are not viewed as essential to the welfare of wildlife by some factions of the public. This can result in a confrontational situation that leaves the State of Montana and FWP open to public criticism and generates animosity towards hunters and other user groups served by FWP. This could weaken the Habitat Montana Program if it is perceived as a lack of public support by the Legislature.

Biological assessment:

In some areas of the state it is important to assure that habitats essential to the mountain lion and it's prey species are protected and perpetuated. The Habitat Montana Program allows FWP to accomplish this, as set forth in the program's policy through acquisition, easement or lease.

Economic impact:

Protection of lands through the Habitat Montana Program varies from fee title acquisition to the purchase of certain rights in perpetuity from a landowner. Each project varies in it's economic benefit to the landowner and may positively or negatively impact the local economy. In all projects, the economic impacts are analyzed prior to any action being taken by FWP and the FWP Commission.

Decision Authority: Mt. Legislature, FWP Commission, FWP

Element H: Evaluate land management agencies proposed programs on their merits for wildlife.

Benefits:

While FWP has no decision-making authority for programs carried out on public lands, its input into these processes assures that wildlife issues are considered in the decision process.

Disadvantages:

These actions are often perceived as FWP closing public lands for hunting, shutting down logging operations or other resource extraction activities or attempting to take over another agency's authority rather than attempts to maintain or enhance wildlife habitat. As a result, FWP is often criticized and animosities develop between different user groups, other agencies and the department.

Biological assessment:

Such evaluations allow FWP to have input regarding the effects of a land management agency's programs on wildlife, wildlife habitats and hunting opportunities in Montana. It also assures that the agency's decision has been made using the most current and complete wildlife information available for the area, and it becomes part of the public decision of record.

Economic impacts:

FWP input may affect the decision made on such projects. However, they are in the form of a recommendation. If the decision made has a significant economic impact, the land management agency must analyze the effects on the local economy before a decision is finalized.

Decision Authority: FWP

Element I: Hides and skulls of mountain lions will continue to be tagged in the FWP region where harvested.

Benefits:

This element assures FWP has contact with individual hunters and has the opportunity to collect biological and harvest information. It also reduces the incidence of lions

being taken in areas other than where reported, and gives FWP the opportunity to check if there are other discrepancies in harvest information.

Disadvantages:

This regulation poses some problems for resident and nonresident hunters who are unfamiliar with an area, and must locate local FWP personnel to tag a harvested mountain lion.

Biological assessment:

This regulation expedites the collection of biological and harvest information for the FWP regional staff and helps ensure harvest information provided by the hunter is accurate. It is also needed to allow quotas to be updated and seasons to be closed on a timely basis.

Economic Impact:

No significant impact is anticipated.

Decision Authority: FWP Commission

ACTIVITIES OR PROGRAMS THAT WOULD BE ADJUSTMENTS TO CURRENT MANAGEMENT

Element J: Reduce the minimum closure period for a season in hunting districts to 12 hours and allow FWP to announce closures prior to quotas being met.

Benefits:

When hunting pressure and snow conditions indicate an area quota could be met or exceeded, FWP will be able to anticipate and announce the closure as little as 12 hours before the closure is implemented. This change will allow improved control of the harvest and reduce the possibility of exceeding the quota for an area.

Disadvantages:

This change will require hunters to be more aware of the status of the harvest in the area they are hunting, which in turn will require FWP to develop and implement an information system which will accommodate this.

Under this system there will be times when the full quota will not be taken from an area.

Biological assessment:

Quotas are currently being exceeded in heavily hunted portions of the state under the current minimum of 48 hour notice of closure. While reducing the minimum notice of closure period to 12 hours will not eliminate this situation, it will help reduce the possibility of its occurrence. The authority to anticipate reaching quotas, and

beginning the closure procedure prior to a quota being met, also will further reduce the potential of exceeding that quota.

Exceeding a quota does not pose a threat to the long-term viability of mountain lion populations. Short-term effects will vary with mountain lion densities, harvest composition, numbers of reproductive females in the population and the availability of immigrants from other areas.

Exceeding quotas results in criticism from the public and can result in regulation changes or quota reductions that may unnecessarily restrict harvest.

Economic impacts:

Development and implementation of a policy that will allow season closures to efficiently take place will require some redirection of funds at FWPs state and regional level.

Decision Authority: FWP Commission

<u>Element K: Reduce the reporting period for a lion kill to 24 hours.</u>

Benefits:

This change will allow improved control of the harvest and reduce the possibility of exceeding the quota for an area.

Disadvantages:

This will reduce the time successful hunters have to report a kill which may be inconvenient in some circumstances. Because lions reported after a season is closed will be considered illegally taken, this element will require hunters to be more aware of the status of the harvest in the area they are hunting. Consequently, this will require FWP to develop and implement an information system that will accommodate hunter needs.

Biological assessment:

Quotas are being exceeded in heavily hunted portions of the state with the current 48-hour reporting period. While reducing the reporting period to 24 hours will not eliminate this situation, it will help reduce the possibility of its occurrence.

Economic impacts:

Development and implementation of a program that will allow season closure to efficiently take place under a 24-hour harvest reporting period will require some redirection of funds at FWPs state and regional level.

Decision Authority: FWP Commission

Element L: Use season length; rather than quotas, to regulate the harvest

Benefits:

Under some circumstances, such as when mountain lion densities are high, mountain lion populations are expanding in urban and urban-interface areas, or very secure habitat is available to lions, the size of the harvest could be controlled using season length rather than quotas. The stability of mountain lion populations is dependent on a core of reproductive females, their kittens and dependent young, established adult males and transient young attempting to become established. Harvest rates that remove established females potentially reduce the number of female kittens available to replace lost adults. This, in turn, effectively lowers reproductive potential. Over broad areas, such harvest rates may be used to reduce mountain lion densities. In areas with high lion populations, such a season could be used to reduce mountain lion densities to meet management objectives.

Disadvantages:

Such a season would result in a fluctuating harvest that would vary with weather, access and hunting pressure. Years of high harvest could be perceived by the public as being detrimental to the population and would require close monitoring of the future harvest and long-term population trends. Areas open to this type season would be expected to have high initial participation that would decline as the population density dropped and hunting became more difficult. As a consequence, human congestion, competition and conflicts would be high initially, but would decline with a drop in hunter participation.

Biological assessment:

Such a season could lower the reproductive rate of the population by increasing the adult female harvest during the period it takes to reduce densities to the desired level. Populations would regain reproductive stability at lower densities, and require continued hunting pressure to maintain such densities.

Economic impacts:

Some impact would be felt by the local area as hunting pressure initially increased and then declined over time.

Depredation complaints and human-lion conflicts would be expected to decrease with declining mountain lion densities. This will not occur if the density of the human population continues to increase and expand into mountain lion habitat.

Decision Authority: FWP, FWP Commission

Element M: Reduce the inspection period for the hide and skull to 24 hours after the harvest is reported. Tagging of the hide would take place at that time and skulls must be tagged within 5 days following inspection. EXCEPTION: Hunters with mountain lions harvested in those hunting districts with an early season (beginning Sept. 15) would have 24 hours after reaching the trailhead to present the hide and skull for inspection and the tagging of the hide, and five days after reaching the trailhead for the tagging of the skull.

Benefits:

This regulation will increase the potential for successfully backtracking a lactating female to her kittens, in the event one is mistakenly killed. It will help FWP monitor regional and statewide harvests and aid in season closures. It will help FWP assure that the reported harvest for an area is timely and accurate as to numbers and sex, and that seasons will not remain open after harvest quotas are met.

Disadvantages:

The regulation will require hunters to skin out at least the skull of a harvested mountain lion within 24 hours of harvest and present the carcass to FWP for tagging. It also requires FWP to have a program in place that will allow successful hunters to comply with this regulation.

Biological assessment:

Mandatory inspection and tagging of the hide and skull of harvested lions is a management tool that allows the skull to be collected for aging information and the hide to be inspected to determine sex and reproductive status (i.e. lactation) of the animal when harvested. This change will increase the likelihood that, if a lactating female has been taken, her kittens can be located and turned over to FWP.

Economic impact:

Development and implementation of a program that will provide for timely and efficient inspection of the hide and skull of harvested mountain lions will require some redirection of funds and work effort at the FWP state and regional level.

Decision Authority: FWP Commission

Element N: Reword regulations to read "Neither females with young nor young traveling with an adult female may be taken."

Benefits:

Current regulations state that it is illegal to take a "female with kittens." However, regulations make no provision for dependent young that may remain with the female for up to 24 months (Hemker et al. 1984, Logan et al. 1986, Ross and Jalkotzy 1992). A regulation change stating "females with young may not be taken" will afford lactating females, or females with young offspring that have not attained independence but are not considered kittens (still retaining spots), an added degree of security. This

additional security also will apply to the young, still-dependent animals in the population.

Disadvantages:

The change places additional responsibility on the hunter to determine if a female has dependent young prior to her being taken.

Biological assessment:

Young lions may become independent between one and two years of age (Robinette et al. 1961, Pall et al. 1988). During this period it is important to afford them a higher degree of security than needs to be given to transient or adult mountain lions.

Economic impact:

No significant impact is anticipated.

Decision Authority: FWP Commission

<u>Element O: Re-evaluate the hunting of mountain lions on Wildlife Management Areas (WMAs).</u>

Benefits:

All FWP's WMAs are closed to all hunting between December 1 and May 15. Opening of those WMAs where it did not interfere with other game management considerations would increase hunting opportunities and provide for a level of mountain lion management on those particular areas.

Disadvantages:

This would not be an acceptable management practice on all WMAs in Montana and would open FWP to criticism from some members of the public.

In some years elk, migrate early and such seasons could result in their displacement to private property.

In some instances, WMAs serve as reservoirs for producing mountain lions that will move to fill in vacant territories outside the WMA. Hunting would reduce the numbers of animals available from such a source.

Biological assessment:

Such an action would not affect mountain lion harvest levels as long as the harvest is controlled by a quota. It could serve to reduce levels of predation on a WMA by reducing the mountain lion population prior to arrival of high concentrations of wintering big game animals. In periods of low mountain lion densities, hunting on these WMAs might remove lions that would benefit adjacent populations where vacant territories exist.

Economic impact:

No significant impact is anticipated.

Decision Authority: FWP Commission

Element P: Provide for establishment of intensive management zones around selected areas of human habitation.

Benefits:

In many urban and urban interface areas of the state, mountain lions have either become or were previously established and now pose a threat to the human inhabitants and/or their pets or property. Human safety and losses due to depredation by mountain lions in these areas could be reduced by intensive removal of the mountain lion population.

Disadvantages:

The necessity of such population reductions, particularly with the use of hounds and hunters, is not well received by some residents of these areas and can become very controversial. To keep mountain lion population levels low, it would be necessary to reduce both the numbers of prey animals in these areas and the level of habitat security offered to mountain lions. Such programs can be expected to become controversial and, perhaps even, confrontational.

Population reductions and control actions taken in these areas also are perceived by hunters as reduced opportunities, especially if FWP or ADC personnel are responsible for carrying out the reduction.

In some urban and urban-interface areas, access for hunters and hound handlers may be difficult, resulting in little hunting pressure, and little control of mountain lion populations.

Methods of population reduction available for use in such areas, such as hunting by agency personnel, and trapping and transplanting, are inefficient, labor-intensive and costly.

Biological assessment:

Intensive removal of mountain lions from urban and urban-interface areas will have little effect if prey species or habitat alterations do not make the area less attractive to immigrating mountain lions. If adjacent areas have stable or expanding mountain lion populations, vacant territories within the control area will quickly become occupied with transient mountain lions from those populations. Urban and urban-interface areas may also act as refuges for mountain lions and, when adjacent populations are declining, would provide transient mountain lions to occupy territories left vacant in adjacent areas.

Effects of such reductions would be local and have a negligible effect on populations within a hunting district or region.

Economic impact:

Programs to reduce mountain lion populations in urban and urban-interface areas would have high initial costs that would decline with the necessity to remove lions. They would require a long-term commitment and redirection of FWP funds.

Decision Authority: FWP Commission

<u>Element Q: Require MFWPs wildlife evaluation of subdivisions be provided to prospective buyers.</u>

Benefits:

By statute (76-3-608 (3a) MCA) FWP is required to evaluate all proposed subdivisions for their wildlife values. Requiring this evaluation to be provided to prospective buyers would make them aware of potential wildlife conflicts, including those with mountain lions. This would allow them time to make decisions about architecture and landscape that would minimize the potential for such conflicts. It would also expand FWPs ability to help people learn to live with wildlife.

Disadvantages:

This will require legislative action and would be viewed by some as anti-development.

Biological assessment:

This requirement would not reduce the amount of mountain lion habitat lost to subdivisions. It would allow developers and clients to reduce future conflicts with pre-sales and pre-construction planning. This, in turn, would help reduce the growing numbers of human-lion conflicts.

Economic impact:

No significant impact is anticipated.

Decision Authority: Mt. Legislature

<u>Element R: Clarify Statutes 87-3-127(1) MCA and 87-3-130 MCA that allow stockgrowers to use hounds to take depredating mountain lions.</u>

Benefits:

Suggested changes would allow a stockgrower to appoint a hound handler as his agent to pursue and take depredating mountain lions and require FWP to be notified prior to such pursuit taking place. This would allow stock owners to react more quickly to depredation incidents and provide a better opportunity to target the specific mountain lion involved.

Disadvantages:

These changes place the burden of proof that a mountain lion was involved in a depredation situation on the stockgrower and will require FWP or ADC to investigate each incident. It also will be viewed by some as providing some stock growers an opportunity to abuse these rights by allowing them to take lions that may not be involved in depredation.

Biological assessment:

Such actions would have a negligible effect on mountain lion populations and would increase the probability that the animal involved in depredations is removed.

Economic assessment:

This legislative change would reduce the time needed to react to livestock depredation and help reduce livestock losses, which were estimated to be $12,875 in 1993 (Aune, Schladweiler 1993).

Decision Authority: Mt. Legislature

Element S: Determine and prioritize research needs to assess Montana's mountain lion management.

Benefits:

Research is needed to address current and future questions concerning Montana's mountain lion management. Assessment of FWP's research needs and the prioritization of those needs will result in a more efficient and economical research program for mountain lions.

Disadvantages:

Techniques and information applied, or misapplied, to Montana's mountain lion management from poorly designed or conducted studies results in lost time and money and can result in periods of mismanagement.

Biological assessment:

Research is needed to develop management techniques to monitor and assess Montana's mountain lion populations. It also is needed to answer other questions regarding the population dynamics of mountain lion populations and their ability to adjust to changes in harvest intensity. Information on the effects of long-term chasing of lions also is needed.

Economic impact:

Research on mountain lions would require a redirection or reallocation of FWP funds.

Decision Authority: Mt. Legislature, FWP

Element T: Estimated carrying capacities that reflect biological and social demands placed on both the mountain lion and prey populations in each FWP administrative region will be used when making Regional season recommendations.

Benefits:

Basing harvest objectives on biological and social parameters will allow adjustments to seasons and quotas that will follow fluctuations in prey densities, recreational demands for mountain lions and the prey base and shifting demographics within Montana.

Disadvantages:

Estimated carrying capacities will result in density ranges for mountain lion populations that will fluctuate with environmental conditions, urban expansion, hunting pressure, natural and human habitat modifications, predation and disease. During any significant fluctuation in populations prompted by these factors some segments of the public will want to retain densities at or near the highest estimated carrying capacity for each habitat type. Others will want to maintain lower population levels for reasons of public safety, lower levels of livestock depredation or to reduce predation on prey species.

Attitudes regarding the presence or absence of mountain lions around human population centers, numbers available for hunting and the effects of mountain lion predation on deer and elk vary considerably between people and locales. Harvest objectives that take into account social factors will always be met with disagreement from those portions of society that don't feel their point of view has been aptly considered.

Biological assessment:

This will help describe Montana's ecoregions and provide a reasonable estimate of the population levels of mountain lions game managers should attempt to maintain.

Economic impact:

No significant impact is anticipated.

Decision Authority: FWP

Element U: Develop a human-lion conflict policy.

Benefits:

The incidence of human-lion interactions will continue to increase as long as lion populations remain high and there is an expansion of human activities into lion habitat. In order to provide the highest level of security for both humans and lion population, guidelines for dealing with these incidents need to be formulated. These guidelines will describe the level of response that will be taken by FWP and what factors will dictate that response.

Disadvantages:

No policy will be able to cover all the situations that might arise, or guarantee that any individual, either a human or mountain lion, will never be killed or injured.

Biological assessment:

Such a policy will assure that mountain lions temporarily in proximity to humans, but posing no threat, will not be killed. It also will help to assure that any lion threatening human safety or that has been involved in livestock depredation is removed and destroyed. The policy also would establish guidelines that would help assure human-lion conflicts are monitored and handled similarly statewide.

Economic assessment:

Developing and implementing such a policy would require an initial outlay of FWP funds.

Decision Authority: FWP

ACTIVITIES OR PROGRAMS ADDED BY PUBLIC INITIATIVE

The following five initiatives were added to the list of elements sent out for discussion as a result of public input during the scoping process.

Element V: Require all hound handlers 12 years of age and older pursuing or hunting mountain lions with dogs to purchase a mountain lion hunting license and obtain a free hound handler permit at the time of license purchase.

> **Exception:** Guides and outfitters will be eligible to receive a free hound handler permit by presenting their Montana outfitters or guide license and their conservation license. Guides will also be required to provide the license number of the outfitter on whose license they are guiding.

Background:

Participants in the scoping process indicated they felt FWP needed more control over the numbers of people with dogs participating in the season, especially with increasing restrictions on hound handlers and hunters in other western states and the growing numbers of nonresident hound handlers and hunters in Montana. Montana law also requires persons "pursuing" or "chasing" game animals with the intention of harvesting that animal to possess a current hunting license during an open hunting season.

The exception for guides and outfitters to having to purchase a license brings this in line with current statutes that only require guides and outfitters to have a conservation license when guiding hunters for big game in Montana.

Benefits:

Requiring all dog handlers who participate in the hunt or chase of mountain lions to possess a current license or hound handler permit would allow these people to be contacted for information concerning their level of participation, locations of their activities and numbers of lions chased or taken while using their dogs. Such information would be included in questionnaire information obtained from licensed participants and provide a more complete picture of hunting and chase efforts.

This regulation would also provide FWP with a better idea of the numbers of nonresidents participating in the season, and allow a means of regulating those numbers if it became necessary. Such a regulation also could prove useful to the Board of Outfitters in detecting illegal outfitting for mountain lions.

Disadvantages:

This will add another regulation for participants to comply with and for FWP to enforce.

Economic impact:

Implementation will require some additional expenditure by FWP.

Decision Authority: FWP Commission

<u>Element W: Provide methods to regulate total hunters and nonresident hunters within the state or a portion of the state.</u>

Background:

High total hunter numbers, accelerated harvests and increasing interest by nonresident hunters in Montana's mountain lion season prompted several participants in the scoping process to ask that regulating total hunter numbers and/or the number of nonresident hunters be considered as part of the EIS process. Restrictions that would be considered include permits for nonresidents, permitting all hunting in an area, requiring hunters to pick an area that they will hunt (validation for an area) when licenses are purchased and restricting the number of people that can receive a validation for an area.

Benefits:

Limiting the numbers of hunters would reduce hunting pressure in areas that are currently experiencing rapid harvests and increasing conflicts between competing hunters. Fewer hunters also would enable those people participating in the hunt the time to be more selective in taking a mountain lion and improve the quality of the hunt. Unethical practices that result from people competing to take a mountain lion prior to the quota being reached and the season closed also would be expected to decline with fewer hunters.

Disadvantages:

Restricting hunter numbers can reduce the level of hunter opportunity currently available, and often means local residents are unable to hunt in the area where they reside. It would also reduce the number of hunters available to outfitters and those who are otherwise affecting the local economy. Restrictions also reduce the total numbers of participants in the sport, weakening support for mountain lion hunting and management programs.

Biological assessment:

Management goals would not be affected by the reduction of hunters in some areas of the state as long as interest in mountain lion hunting remains stable or continues to grow. Management goals may not be met if interest declines.

Economic impact:

Reduced hunter numbers would impact the outfitting industry in those areas of the state where nonresident or total hunters were restricted. There also would be an adverse affect on other businesses that cater to the outdoor recreation industry. If other areas of the state remained open to all hunters, outfitters and other businesses in those areas could be expected to profit from displaced hunters moving into those areas.

Restrictions could reduce the total number of hunters who purchase a mountain lion license, reducing revenues collected by FWP.

Decision Authority: FWP Commission

Element X: FWP needs to improve its ability to obtain and disseminate harvest information.

Background:

Through the scoping process it was determined that to better control harvest FWP will need to initiate a program that allows hunters access to the most current harvest and quota data. Better coordination between FWP's Helena and regional offices in updating and disseminating harvest information also will be required. Hunters need better access to FWP personnel to report harvested animals and comply with tagging regulations. Some suggested methods of gathering and disseminating information included 1-800 numbers, opening offices on weekends and establishing check stations in high harvest areas.

Benefits:

A system that would allow rapid accumulation and dissemination of information relating to mountain lion harvest and quota status would reduce the potential for exceeding harvest quotas. Such a system will be necessary if FWP reduce the closure period for seasons and the period for reporting and tagging harvested animals.

Disadvantages:

Such a system will need to be accessible from all areas of Montana, some of which do not have touch-tone phone capabilities.

Biological assessment:

Such a system is necessary if the mountain lion harvest is to continue to be managed with quotas.

Economic impact:

A one-time initial cost of implementing the system will require some redirection of funds by FWP. Costs will be lower is succeeding years.

Decision Authority: FWP

Element Y: Increase enforcement efforts to check kill sites and hunter residency.

Background:

Participants at scoping meetings indicated that increased enforcement efforts directed at checking kill sites, hunter activities and residency of hunters would result in a much more controlled harvest and collection of more accurate harvest information.

Benefits:

Accurate harvest information is necessary for proper management of mountain lions, and an increased law enforcement effort would result in better cooperation by hunters in providing that information.

Disadvantages:

Increasing law enforcement efforts during the mountain lion season will entail redirecting wardens from other activities.

Biological assessment:

More accurate harvest information will aid in the management of Montana's mountain lion population.

Economic impact:

FWP wardens are now restricted to 40-hour work weeks. Increased enforced efforts will require either a shift in work emphasis or an increase in personnel.

Decision Authority: FWP

Element Z: Open mountain lion hunting with the general big game season.

Background:

There are many people in Montana who do not have access to hounds but are interested in harvesting a mountain lion. This interest has grown in recent years as

hunters are seeing more mountain lions and mountain lion sign during the general big game season.

Benefits:

This alternative would provide those hunters who incidentally came across a mountain lion during the general season an opportunity to harvest that animal.

Disadvantages:

Drawbacks to this type of harvest include the hunter's lack of opportunity to identify the sex and age of mountain lions seen in the field. Hunting with hounds provides the opportunity to identify the animal being pursued either from its tracks or while it is in the tree. Trailing lions provides an opportunity for the hunter to check for the presence of dependent young traveling with the female before a chase is begun.

Incidental harvest in the field, under most circumstances, would not allow a lactating female or one with dependent young to be identified prior to harvest. If a lactating female were taken, the lack of snow during portions of the general season also would prevent backtracking to retrieve kittens.

Biological assessment:

This type of season would not have a long-term effect on the viability of Montana's mountain lion populations because the opportunity of taking a lion during the general season is very low. A similar type of season is conducted in Idaho, and it provides 10 - 15% of that state's mountain lion harvest (J. Beecham pers. comm March 1995).

This type of season would increase the potential of harvesting females with dependent young and decrease the possibility that lactating females could be backtracked to kittens.

Economic impacts:

While the number of licenses that would be sold is unknown, no significant impact is anticipated.

Decision Authority: FWP Commission

CHAPTER II

AFFECTED ENVIRONMENT

A REVIEW OF MOUNTAIN LION RESEARCH AND BIOLOGY:

Evolution:

Felids and canids diverged from a common ancestor nearly 50 million years ago (Simpson 1945). Felids with mountain lion-like characteristics occurred in the North American fossil record during the Pleistocene, approximately one million years ago. Physical specializations within the cat family, Felidae, are adaptations to each species' unique mode of attack (Vaughan 1978:223). In comparison with canids, such as wolves, mountain lion's have shorter legs, smaller lungs, fewer teeth, and shorter, flatter faces. All these morphological characteristics are expressions of the evolution towards a carnivore adapted for hunting in terrain with rugged topography or extensive cover, utilizing a stealthy stalk and a brief burst of speed (Gonyea 1976).

Distribution:

Mountain lions once were the most widely distributed terrestrial mammal in the western hemisphere. They were distributed from northern British Columbia to the southern tip of South America (Anderson 1983:8-14). The current distribution has become patchy with losses of populations in eastern North America and throughout most of Central America (Lindzey 1987:657). Within North America, 15 subspecies have been recognized from British Columbia to Florida and from the Baja of California to Nova Scotia (Hall 1981:1042). As late as 150 years ago, the mountain lion was common in the eastern deciduous forests of the United States, but with the exception of the Florida panther, it has recently been restricted to west of the Great Plains (Eaton 1973). Conversely, there are parts of the western United States that have abundant populations of mountain lions where they were historically rare or absent (Berger and Wehausen 1991), or where the increased distribution of an alternative prey species, such as white-tailed deer, has actually increased the mountain lion's range (Nero and Wrigley 1977).

The mountain lion has reclaimed much of its former habitats in Montana and is currently distributed in 46 of 56 counties (Figure 1). Much of that expansion has occurred during the past 24 years since mountain lions were classified as a game animal (Figures 2-5).

Habitats:

Historically in Montana, mountain lions were probably found in all habitats except the open plains and prairies where their stalking abilities were limited (Gonyea 1976) and the threat of attack from predators such as wolves (White and Boyd 1989) was great. Mountain lion habitat essentially is that of its primary prey species, deer and elk. It is limited by the vulnerability of prey (Seidensticker et al. 1973) and the energetics of females with kittens (Ackerman et al. 1982). Seidensticker et al. (1973), Murphy (1983) and Williams (1992) noted elevational changes between seasons, with higher elevations used in summer and lower elevation used in fall. These elevational shifts mimic observed movements of white-tailed deer (Odocoileus virginianus) (Dusek and Morgan 1990), mule deer (Odocoileus hemionus)

(Pac et al. 1991), and elk (Cervus elaphus) (Lemke 1975) in Montana's mountainous habitats. The mountain lion's physical characteristics, however, partially limit the animal to successfully stalking prey in dense vegetative cover or in steep terrain (Logan and Irwin 1985). These habitats allow mountain lions to stalk within a short attack range of their prey, and affinity for rugged terrain and dense cover can be great. Shaw (1980) reported mountain lions in Arizona starving to death in a severe winter rather than following migratory deer down into the lower, open valleys.

In many parts of the west, including Montana, there have been increasing numbers of mountain lion sightings at low elevations during summer. The increases in sightings probably have been associated with increased deer numbers and changes in ungulate distribution that have allowed for increased mountain lion abundance and distribution. Mule deer on the front range of the Colorado Rockies now occupy lowland habitats year round where once they only migrated to these sites during the winter (Halfpenny et al. 1991). This situation has corresponded with year-long occupancy of these same sites by mountain lions as well. This trend, as described by Halfpenny in Colorado, may also be occurring in parts of Montana.

Figure 1. Distribution of mountain lions in Montana, 1994.

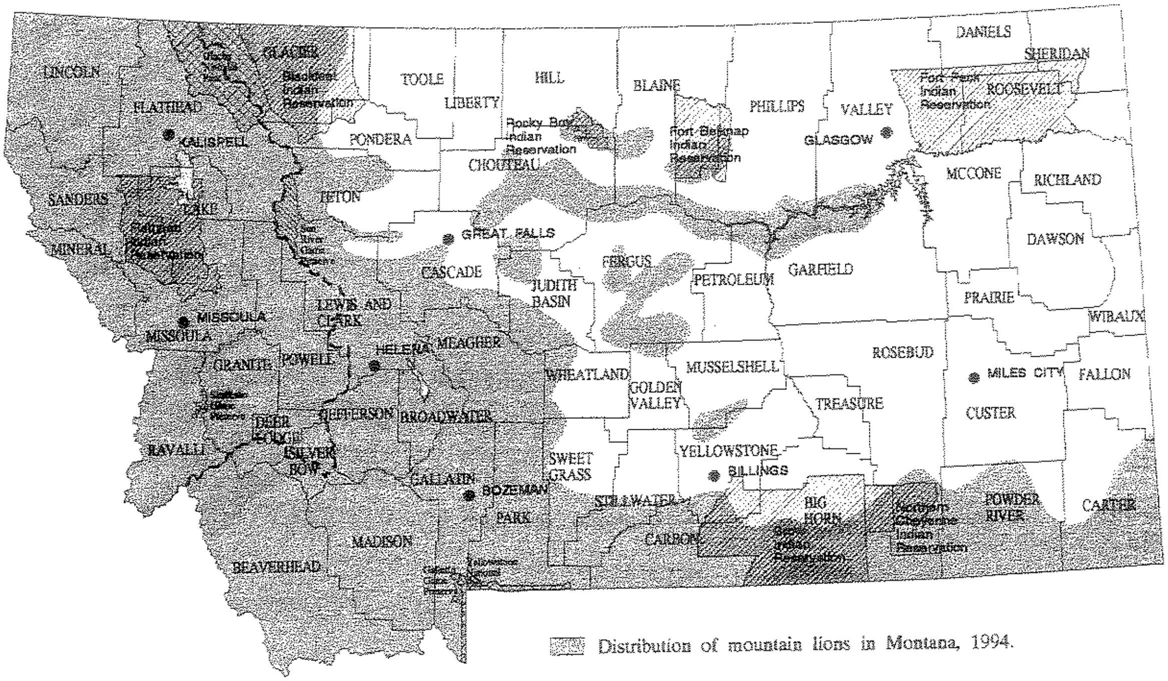

Distribution of mountain lions in Montana, 1994.

Figure 2. Distribution of Montana counties that paid bounties for mountain lions, 1921-1922.

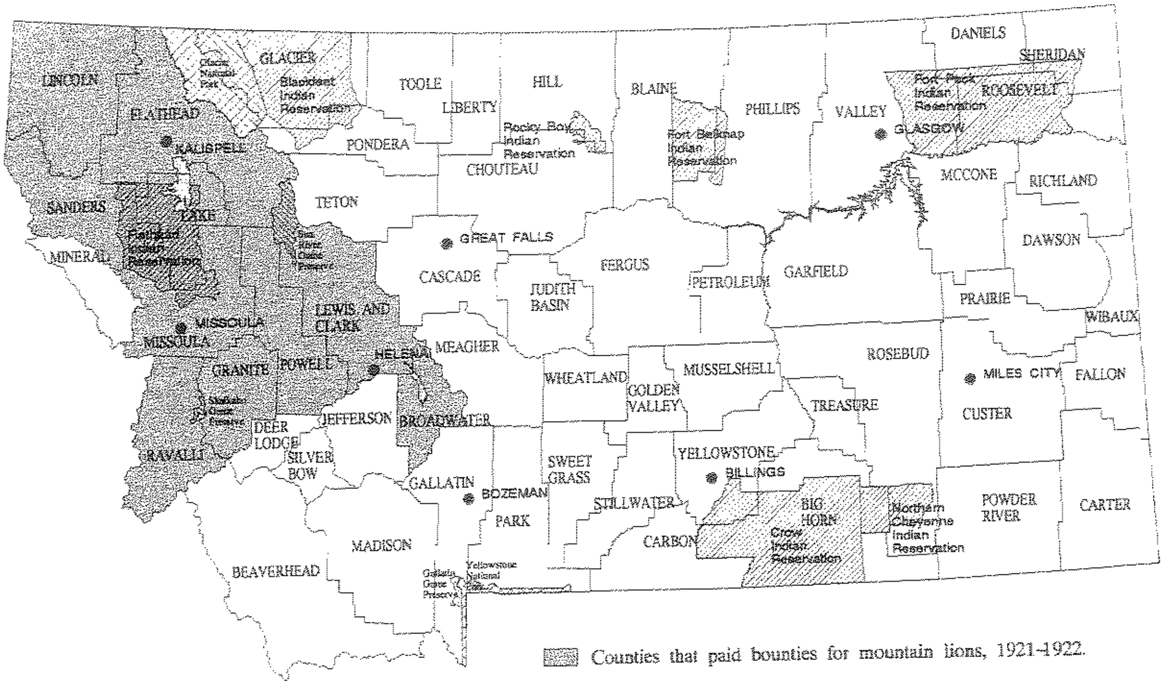

Figure 3. Lion hunting districts with recorded harvest, 1971-1972.

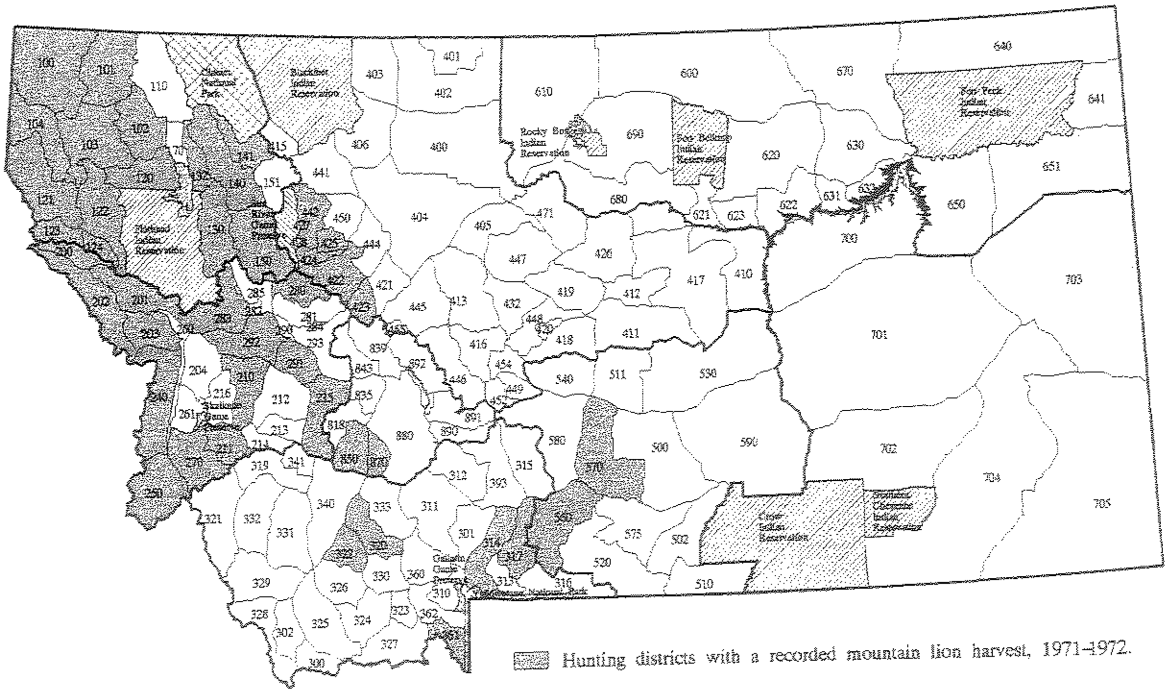

Figure 4. Lion hunting districts with recorded harvest, 1981-82.

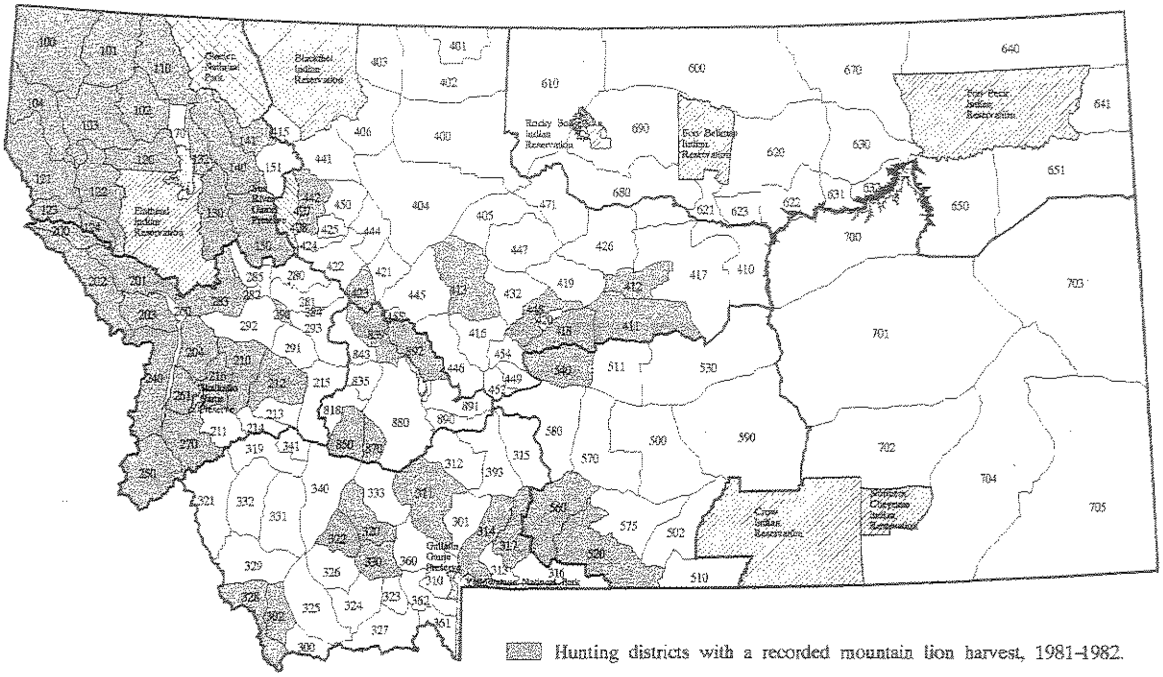

Figure 5. Lion hunting districts with recorded harvest, 1991-1993.

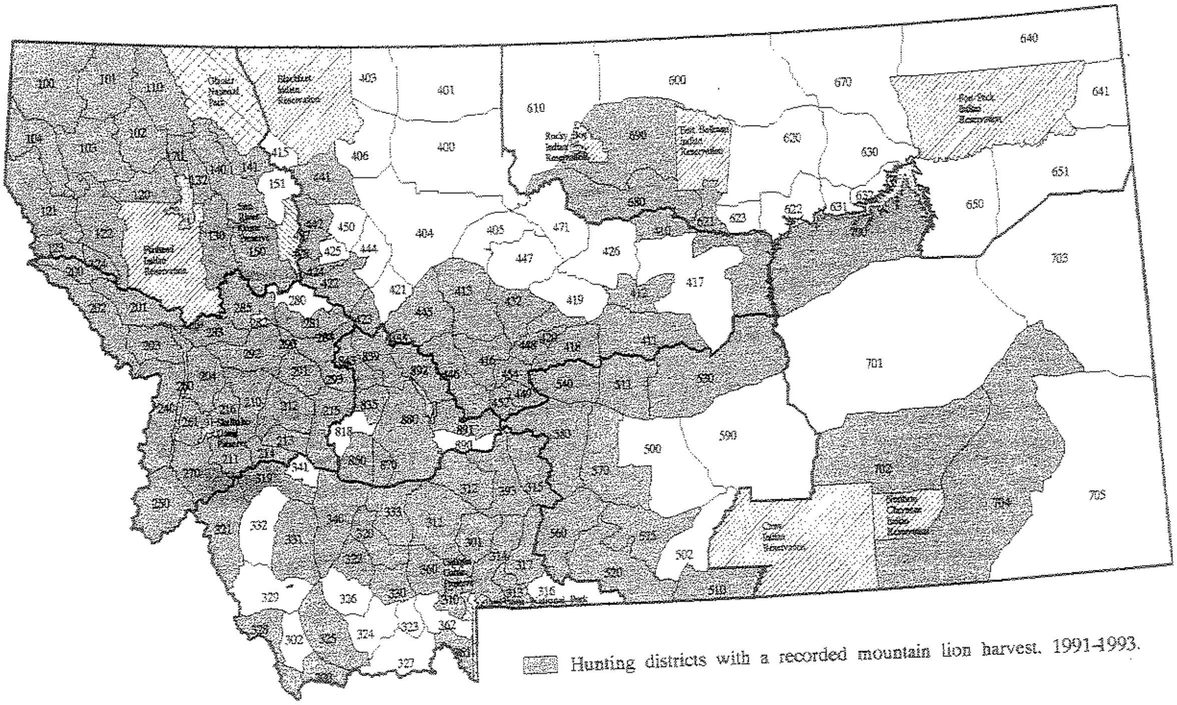

As the landscape of the western United States becomes more and more fragmented, primarily due to human development, secure mountain lion habitat becomes more and more restricted to remnant islands of continuous cover. Montana has not experienced the levels of habitat deterioration, linked to human development, observed in most other western states. We currently have sufficient refugia (wilderness, roadless areas, WMAs) to assure the mountain lion's survival in the immediate future. However, as the time to manage for conservation of non-fragmented mountain lion habitat is 30 to 50 years before fragmentation occurs (Beier 1991a), it is important to continually examine land use programs. Once depleted from fragmented habitats, mountain lion populations are slow to recover (Laundre' et al. 1991) or are subject to localized eradication (Beier 1991a).

Territories/home ranges:

Hornocker (1969) introduced, and Seidensticker et al. (1973) subsequently substantiated, the concept of "land tenure through mutual avoidance" in mountain lions. This concept entailed mountain lions confining their movements to specific areas during specific times of the year, but falling short of actively defending classic territories as described by Tinbergen (1957). The system of land tenure can best be described as dynamic and flexible. Home ranges overlap and a higher degree of overlap occurs between females, whose areas of use also are considerably smaller, than those of males. Females with kittens tend to have smaller territories than those with dependent juveniles or lone females (Ross and Jalkotzy, 1992). Male home ranges are the largest and encompass areas used by several females (Hornocker 1968, Seidensticker et al. 1973).

Because the home range of an adult male will overlap those of several adult females, the population structure in these situations then depends on the reproductive status of the females, numbers of kittens or dependent young and the number of transient young in the area. Adults (24+ months) constituted between 44% (Logan et al. 1986) and 75% (Anderson 1983) of populations in Wyoming, Nevada, New Mexico and Colorado. Neal et al. (1987) and Pall et al. (1988) found a high degree of home range overlap in males in both California and Alberta, but in such cases also observed mutual avoidance, thereby creating constantly shifting boundaries of home ranges.

Mountain lion home ranges have been reported to vary in size from 54 km^2 (20.8 mi^2) in Wyoming (Logan et al. 1986) to 1032 km^2 (397 mi^2) in Texas (McBride 1976). In western Montana, Murphy (1983) reported an average adult home range size of 79 km^2 (30 mi^2) in Fish Creek, a tributary to the Clark Fork, and average summer home ranges of 117 km^2 (45 mi^2) and 889 km^2 (342 mi^2) for females and males in the northern Yellowstone area (Murphy et al. 1991). Winter home ranges in the Yellowstone study area were significantly smaller, averaging 58 km^2 (22.3 mi^2) for females and 199 km^2 (77 mi^2) for males. Home ranges in the Sun River area of Montana averaged 137 km^2 (53 mi^2) for adult males and 65 km^2 (25 mi^2) for females (Williams, 1992).

Reproduction and Natality:

Mountain lions are promiscuous (Seidensticker et al. 1973), with a resident male breeding with numerous females whose home areas overlap with his own. Females are capable of reproducing at as early as 20 months of age (Lindzey et al, 1994), but usually do not breed until they have established a home area (Hornocker 1971). Murphy et al. (1991) reported the ages of first parturition in Montana among three females marked as kittens at 23, 31, and 36 months. The gestation period is approximately 92 days, and most females are believed to give birth on an average of 24 month intervals (Anderson 1983:33). Mountain lions are one of the few wild mammals that breed year-round. The peak time period of birth in northern latitudes is during May, June, and July (Anderson 1983:32, Pall et al. 1988, Murphy et al. 1991).

Tanner (1975) calculated intrinsic population growth rates for certain predators and their prey, and he suggested that the mountain lion's rate was nearly twice that of deer. However, estimates of annual recruitment typically have been less, averaging about 32% (Ashman et al. 1983, Robinette et al. 1977). Mountain lions produce litters of up to six young, with litter sizes of two or three most common (Robinette et al. 1961, Anderson 1983:33-34). Murphy et al. (1991) reported 10 litters averaging 2.7 kittens (range 2-4) in the northern Yellowstone region, similar to 2.9 kittens per litter found by Logan et al. (1986) in the Bighorn Mountains of Wyoming. Examination of five gravid females, killed in Montana from nonhunting sources, revealed a median litter size of 4 (range 3-4) (Aune and Schladweiler 1992).

Survival of kittens from first contact with researchers to the time of dispersal was 67% in a hunted population in Utah (Hemker et al. 1982) and 97% in a hunted population in Alberta (Ross and Jalkotzy 1992). Kitten survival in some hunted populations is probably less than in nonhunted populations because females with kittens can be inadvertently killed (Barnhurst and Lindzey 1989) and kittens have been captured and killed by trailing hounds (Lindzey 1987:659). Barnhurst and Lindzey (1989) found that females were with their kittens 67% of the time; however, kitten tracks were found with their mother's only 25% of the time. Mothers leave kittens while hunting (Seidensticker et al. 1973) for up to several days (Shaw 1989:14) before they are able to make a kill. This makes it difficult for hunters to determine with certainty that lions they are hunting do not have kittens.

Young lions become independent between one and two years of age (Robinette et al. 1961, Pall et al. 1988) with an average ranging between 15 and 19 months of age (Hemker et al. 1984, Logan et al. 1986, Ross and Jalkotzy 1992). Permanent family separation may take place at any time of the year (Pall et al. 1988), but most evidence (Hornocker 1970a, Seidensticker et al. 1973, Hemker et al. 1984, Murphy et al. 1991) suggests that the majority become independent between March and September.

Predatory-type play activities begin at as early as 10 days of age (Eaton and Velander 1977) and are an important component of learning to prey and survive on animals as large as elk (Hornocker 1970b). Hornocker (1970a) noted several instances where subadult lions were

unable to bring down young deer. Shaw (1980) and McBride (1976) documented mountain lions being independent between 6 and 10 months of age and having killed both deer and livestock.

Availability of small mammals such as rabbits and hares, ground squirrels, and voles may impact survival of subadult mountain lions and potentially influence rates of mountain lion-human interactions. The first few weeks after being displaced from the family group are the most critical. Beier (1991c) speculated that on Vancouver Island a lack of smaller prey species might be a contributing factor to the increasing numbers of attacks on humans. He also states that while yearlings make up approximately 25% of any population, they are responsible for nearly 40% of the attacks on humans in the United States and Canada (1991b). Both Beier (1991c) and Ackerman (1984) felt that small mammals must be present as an alternative food source for an individual lion to sustain itself.

Dispersal from maternal home ranges in spring has been attributed to the increase in availability of small prey species and less restricted travel conditions due to snow melt (Pall et al. 1988). Litter mates often are found traveling together within or near their maternal home range through their first winter after independence (Seidensticker et al. 1973). This fact probably accounts for most reports of several mountain lions traveling or hunting together.

Emigration and Immigration:

The processes of immigration and emigration play a vital role in the way mountain lion populations utilize the landscape (Laundre' et al. 1991) and regulate their numbers (Seidensticker et al. 1973). The primary mechanism for population growth is by recruitment of young, either with the offspring of females from the area (Lindzey et al. 1992, Laing and Lindzey 1993), or from adjacent and sometimes distant populations (Hemker et al. 1984, Logan and Irwin 1985). Immigration, is thus, a major factor in the speed of recovery of heavily hunted populations (Lindzey 1987:659). Isolated populations may recover more slowly from mortalities if immigration of transients is low. Seidensticker et al. (1973:51) articulated the problem with considering transients as strictly surplus animals: "... under the present environmental regime with its decimating factors, the transient represents a delayed addition to the resident population rather than a by-product of population regulation." Montana has several areas within its borders that function as important "reservoirs" for transient mountain lions (Lindzey 1987:659). Remote lightly hunted areas, WMAs and national parks all fit into this category.

Until the time an animal establishes a home area, it remains a transient with little social standing. While young females tend to establish themselves on or near the edge of their maternal home ranges (Lindzey et al. 1988, Ross and Jalkotzy 1992), males are more likely to disperse long distances, and have been found up to 480 km from their natal areas (Logan et al. 1986). Under most circumstances, females do not enter the breeding population until they have established a territory (Seidensticker et al. 1973, Lindzey 1987, Laing and Lindzey 1993).

Mortality:

There are inherent dangers for predators who attack prey several times their size. Occasionally, mountain lions are killed or injured during an attack on prey (Gashwiler and Robinette 1957, Barnes 1960, Hornocker 1970a), mountain lions are killed in accidents (McBride 1976), or struck by vehicles along highways (Sitton 1977). Recorded nonhunting mortalities for Montana increased from 5 in 1988-89 to 54 in 1993-94 (Table 2, pp 88). Self defense, animal damage control, vehicle collisions and nuisance lion control actions accounted for 60% of nonhunting mortalities from 1988 through 1993. (Aune and Schladweiler 1993). (Table 3, pp 89).

Intraspecific strife and cannibalism are additional sources of mortality, with reports of young males killing old males (Grinnell et al. 1937), males killing females (Robinette et al. 1959), adult females killing other adult females (Lindzey 1987:659), and cannibalism on kittens (Hornocker 1970a, Murphy et al. 1991). Stringham (1983) suggested adult males function as a population regulator through cannibalism in predator populations, but this hypothesis has yet to be adequately tested. Starvation can also be prevalent among very young and very old mountain lions or in extremely severe winters (Shaw 1980, Lindzey et al. 1988). Predation by wolves also has been documented in Montana (White and Boyd 1989, Boyd and Neale 1993).

The most substantial cause of mortality for adult mountain lions is human related. Hunting is the primary cause of mortality in hunted populations (Ross and Jalkotzy 1992, Logan et al. 1986, Currier et al. 1977). Where high density lion populations occupy habitat adjacent to livestock operations, control measures, as related to depredations, are a primary source of mortality in non-hunted populations (Weaver and Sitton 1978). Most mortality in lower density populations of unhunted lions occupying areas of low human habitation resulted from starvation, accidents and intraspecific strife (Lindzey et al. 1988, Hopkins et al. 1991)

Deaths caused by humans are probably additive to accidental mortalities in mountain lion populations, but may reduce the number of mountain lions killed by other mountain lions within the population (Lindzey 1987:659). In an unhunted population in southern Utah, annual mortality rates of adult females averaged 29% (range 0-53%), and for males and females combined averaged 28% (range 0-58%)(Lindzey et al. 1988). Total mortality in a hunted population in southwestern Alberta ranged between 3% and 14%. Over an eight-year period, hunting accounted for 77.8% of that mortality (Ross and Jalkotzy 1992).

Mountain lion population dynamics and behavior allow for an annual sport take as long as the sport harvest does not exceed annual recruitment and adult females are protected (Lindzey et al. 1992), or there are effective refuges adjacent to hunted areas (Lindzey 1987:666). Compensation for hunter mortality primarily will be through immigration into vacant, uncontested territories (Logan et al. 1986). To some degree, mountain lion-human interactions may be dependent upon the abundance of mountain lions (Aune 1991). Hunting

can lower the density of mountain lions, thereby potentially reducing conflicts with humans (Molini 1976).

Legal Status:

The mountain lion in Montana was a bountied animal from 1879 to 1962, an unclassified predator from 1963 to 1966, and a legislatively classified predator from 1966 to 1970 (Mitchell and Greer 1971:207-210). The 1971 Montana Legislature reclassified the mountain lion as a game animal for the first time in Montana and empowered the Fish and Game Commission with setting hunting seasons.

Status changes of mountain lions in Montana were mirrored throughout the western United States during the late 1960s and early 1970s. Colorado was the first state to reclassify the mountain lion to game animal status in 1965, and was soon followed by the rest of the western states with the exception of Texas. In recognition of its ecological importance, and the futility of most government-sponsored predator control programs, most states had declared the mountain lion a game animal by 1972 (Eaton 1973).

Food habits and predator-prey relationships:

Whereas they will kill and consume most vertebrates, mountain lions in the west are ubiquitously tied to deer as a staple food item. Among 16 food habits studies reviewed by Anderson (1983:50-51), 14 listed deer as the single major food item. Robinette et al. (1959) estimated deer comprised 77% and 64% of the mountain lion's winter and summer diets in Utah. Hornocker (1970a) also estimated that deer and elk comprised 70% of the mountain lion's winter diet in an Idaho wilderness area. A similar trend is also reflected in Montana's backcountry areas (Murphy 1983, Murphy et al. 1991, Williams 1992).

Many attempts have been made to estimate kill rate of prey by mountain lions (Anderson 1983:52). Those estimates have varied so greatly depending on habitat conditions, prey availability, type of prey, rate of kill decomposition, and time of year that they may have little practical value for generalizing about quantitative impacts of mountain lions on prey. The energy requirements of a mountain lion population also depend on the sex and age composition of the population. Consequently, changes in the composition of a lion population also alter its effects on the prey base (Ackerman et al. 1982).

Old deer appear to be most vulnerable to predation (Ackerman et al. 1984, Spalding and Lesowski 1971), and bucks are more prone to predation than does (Shaw 1977, Hornocker 1970a). Summer predation rates in Montana have been reported as one ungulate killed per 19, 21, and 15 days for adult males, lone females, and females with kittens, respectively (Murphy et al. 1991). Murphy (op sit.) also calculated winter rates as one ungulate per nine, six and eight days, respectively for those same classes of animals.

Anderson (1983:64) warned that difficult problems associated with enumerating both deer and mountain lions has resulted in ambiguous data and conclusions about mountain lion-prey

interactions. Tanner (1975) theorized that mountain lions have higher intrinsic rates of increase than deer and predicted that mountain lions can numerically control ungulate populations. This theory has not been substantiated by research to date.

Hornocker (1970a) hypothesized lion numbers could not ultimately control prey numbers and that lion numbers were determined by "... factors other than the food supply". Seidensticker et al. (1973) determined mountain lion populations were regulated more by territoriality than by the food supply. Research carried out in Utah (Lindzey et al, 1994) appears to support both these hypotheses when prey species are stable or increasing. However, low prey populations densities caused by other factors, such as habitat deterioration or excessive exploitation, may be further suppressed by mountain lion predation (Neal et al. 1987).

The presence of mountain lions may have beneficial effects on ungulate populations and their habitat. Hornocker (1970a:36) suggested that "the function of mountain lion predation is a more complex phenomenon than that shown on a purely numerical basis." Predation by lions dampens the highs and extends the lows of population cycles, removes the most vulnerable animals when prey populations exceed carrying capacities and redistributes prey species across a broader expanse of the ranges they occupy.

In an environment of human-induced habitat changes and/or increases of non-native ungulate species, an unchecked abundance of mountain lions potentially could narrow the niche of some prey species such as mule deer (R. J. Mackie pers. comm. 7/25/91). In turn, this phenomenon may limit the abundance of some local deer populations. Berger and Wehausen (1991) suggested that a predator-prey disequilibrium in the Great Basin desert has resulted from livestock grazing that favored mule deer, and hence mountain lions. They also feel an increase in the abundance of historically non-native mountain lions has impacted the community dynamics, including reducing the numbers of rare desert bighorn sheep. Nero and Wrigley (1977) suggested that a decrease in mule deer and elk distribution accounted for the temporary eradication of mountain lions in Manitoba during the early part of this century. However, a recent expansion of white-tailed deer distribution has lead to a net doubling of the historic mountain lion range in that province, a phenomenon that also may be occurring in Montana.

Predator-prey relationships are extremely complex and most authors have attempted to over-simplify the relationship, primarily focusing on the quantitative aspects or numerical impacts of predators on prey populations. Botkin (1990:75-89) concluded, after a review of the Kaibab deer-mountain lion-livestock interactions (Rasmussen 1941), that "What we learn from the mountain lion and the mule deer is about what we believed, not about what we know."

Livestock depredation:

Where mountain lion and livestock ranges overlap, livestock may become part of the prey base. Shaw (1977) reported that the species composition of 58 mountain lion kills on Arizona ranches was 64% mule deer, 32% cattle, and four percent other species. Greer (1976)

reported the stomach contents of 16 hunter-killed mountain lions. Deer were present in four stomachs, domestic sheep in one, and traces of grass, beaver, skunk, and porcupine in several others. Eight stomachs were completely empty. In other studies, where mountain lions and livestock occupied the same range, livestock occurred in only 0.4% of 239 scats collected during a 3-year feeding habits study (Ackerman et al. 1984).

Sheep have experienced the greatest amount of depredation both in Montana (Aune 1992) and other western states (Robinette et al. 1959, Weaver and Sitton 1978) and are often taken in multiple-killing episodes. Young (1946:142) reported 192 sheep having been killed in one night by one lion. While such incidents are rare, it does point out the propensity for individual mountain lions killing sheep.

Animal husbandry practices can greatly influence the likelihood of mountain lion depredation (Ackerman et al. 1984, Shaw 1977). Early turnout of cow-calf pairs into higher elevational pastures can lead to increased calf mortality. Likewise, species composition within a livestock operation can influence losses. In areas of higher mountain lion densities, cow-calf pair or sheep operations may not be economically feasible. However, livestock operations can only change to the degree that they remain a profitable business, and some level of depredation is likely to occur despite the best intended husbandry practices (Lindzey 1987:662).

In Montana the values placed on livestock lost to mountain lions by the U. S. Department of Agriculture's Animal Damage Control Division (ADC) between 1984 and 1993 ranged from $640 to $12,875 and has averaged $3,825.40 during the 10-year period (Table 4, pp 90). While livestock losses to mountain lions comprise a very small percentage of the total numbers of livestock raised in Montana and other western states, losses to individual operators can be devastating.

Management to prevent livestock depredation has been traditionally aimed at widespread predator control. This has proven to be ineffective both in terms of economics and in the extent to which depredation is reduced. Short of total eradication, livestock losses will still occur in occupied lion habitat depending on terrain, stocking rates, and availability of alternative prey. Evans (1983) suggested that the most effective means of controlling depredation are those aimed at eliminating individual depredating mountain lions.

Disease and Parasites:

Disease has seldom played a major role in the reduction of mountain lion populations. Despite popular belief, rabies is extremely rare (Beier 1991b). Fifty-four percent of 899 Montana mountain lions sampled between 1971 and 1989 tested positive for Trichinella. Infection rates varied from 15% in Region 5 to 70.3% in Region One (Tables 5 and 6, pp. 90). Dies and Gunson (1984) reported a similar incidence of Trichinella in mountain lions from Alberta. Trichinella has not had detrimental impacts to mountain lion populations, but infection rates should serve as a warning to those people who eat mountain lion meat that the

meat should be cooked thoroughly or until well done. Mountain lion remains should be disposed of in a manner that prevents spreading Trichinella to domestic pets or livestock.

Mountain lion-human interactions:

Mountain lion-human interactions have increased in Montana (Aune 1991) and elsewhere over the past 20 years. Reasons for the increase in incidents, both nationwide and in Montana, have included: a) habituation of mountain lions to people due to increases in both mountain lions and human housing in mountain lion habitat (Lay 1976); b) increased deer populations developing within human subdivisions; and c) increased use of recreational lands by an increasingly urban and urban-interface human population (Halfpenny et al. 1991).

More attacks on humans in North America have occurred over the past 20 years than during the previous 80 (Beier 1991c). Of 58 documented attack victims in North America over the past 100 years, 34 (64%) were less than 16 years of age, including nine of 10 fatalities (Beier 1991c). In Montana, a fatal attack on a child occurred within the Flathead Indian Reservation near Evaro in 1989. Another mountain lion attacked and injured a child within the Lake McDonald picnic area of Glacier National Park in 1990.

Yearlings and two-year old mountain lions accounted for 59% of the mountain lion-human and mountain lion-livestock incidents reported in Montana between 1989 and 1993 where age of nuisance mountain lion could be determined (Aune and Schladweiler 1993). VanDyke (1983) suggested that these animals, because of low social standing, are most apt to occur in human occupied habitats. Despite common belief, disease has not been determined to be a predisposing factor in any mountain lion attacks (Beier 1991c).

Translocations:

Various authors have had differing degrees of success with moving problem or orphaned lions. McBride (1976) moved eight lions in Texas, five of which remained within 8 km and 40 km (5 and 25 miles) of the release site. Seven lions released in Florida (Beldene et al. 1993) established territories until the opening of the hunting season, then moved to urban or agriculture areas where conflicts necessitated their removal. Ruth et al. (1993) reported on movements of 13 translocated lions in New Mexico. Two males returned to the capture area, an average distance of 478 km (296 mi.), and one female moved 285 km (177 mi.) in the direction of her capture site. Nine of the 13 lions translocated had died within 24 months of their release.

Approximately five orphaned lion kittens (range 3-10) are turned over annually to FWP. Most are found by hunters and turned in between October and March. While FWP attempts to place all these animals in zoos, this continues to become more difficult (Vince Yannone, FWP pers. comm. 11/10/94). Information obtained from the International Species Information System indicated that certified zoos in 1992 held 110 adults and subadults in captivity and had recorded 30 births and 17 deaths during the year.

Animals not placed in zoos are raised to approximately 80 lbs. and released in remote areas of the state. FWP has no long-term program in place to track these translocations. Results of some other translocations in Montana are available. Joslin and Brown (1978 FWP unpub. report) reported the successful release of an orphaned kitten after it had been pen-reared for approximately five months. This animal was tracked and is known to have reproduced and survived for at least six years (Joslin, FWP pers. comm. 12/20/94).

Williams (1992) describes a situation in which a pen-reared subadult was released into his Sun River study area, but had to be destroyed after showing aggressive behavior toward humans. Williams (pers. comm. 9/30/93) was involved with another release of a pen-reared female subadult that also had to be destroyed because of it's close association with humans. A young adult male mountain lion caught in a rabbit shed also was released into the study area, established a home range and was tracked for 12 months prior to being legally harvested (Williams 1992).

Griffith et al. (1989) in their analysis of translocations found several factors were generally associated with successful translocations. These included: (a) moving native species; (b) translocating them to high quality habitat; (c) relocating them to the core of historic range; and (d) releasing animals in areas that did not contain either the same or other species that would compete with the one being translocated. Ruth et al. (1993) also indicated younger lions ($\leq$2 years) appear to have a better chance of becoming successfully established than older animals that most likely had established territories prior to being moved.

Hunting:

Under the bounty system, mountain lion mortality fluctuated through the years and was probably related to mountain lion abundance, snow cover for tracking, and the level of livestock depredation. Total recorded bounties paid varied from a low of zero in 1931 to a high of 126 in 1894. Inconsistent record keeping precluded a detailed year to year analysis of bounty mortality, but did indicate the median number of mountain lions taken annually under the system was 38.

There are a number of ways to hunt mountain lions, but hunting with trailing hounds is the only effective means, and most mountain lions are harvested using hounds. Nearly 89% of the 1993 mountain lion hunters in Montana described themselves as either hunting with their hounds, a friend's hounds, or with an outfitter (Figure 6). Outfitters made up 3.5% of Montana's mountain lion hunters, while outfitted hunters made up an estimated 10% (FWP Tech. Serv. Div. 1993).

Montana's mountain lion license sales (Figure 7 and Table 7, pp 91) have increased 747% (31.1% annually) since the first licenses were issued in 1971. Nonresident license sales have averaged 9.5% of resident sales throughout the same period (Figure 7). Residents and nonresidents purchased 2,984 and 258 mountain lion licenses, respectively, in 1994. The first licenses issued in 1971 were free, while in 1994 license fees were $15.00 for residents and $320.00 for nonresidents.

Figure 6. Hunting characteristics of mountain lion license holders.

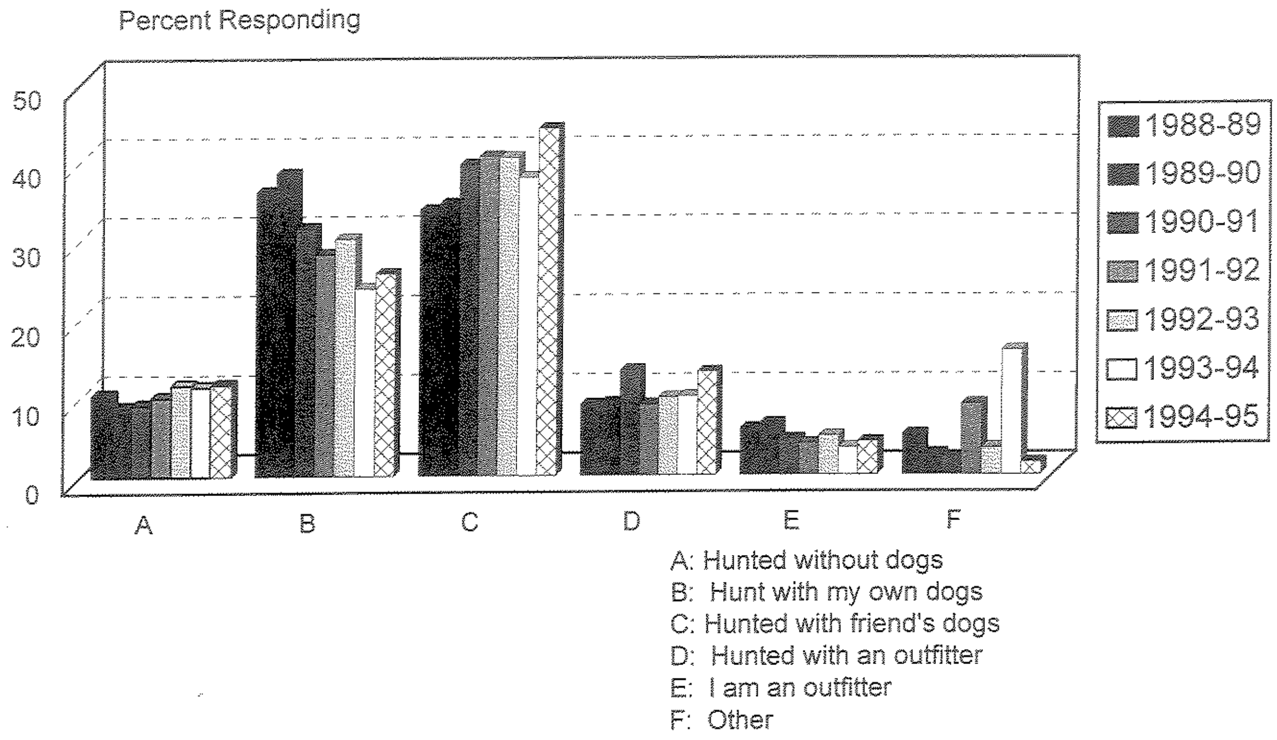

Figure 7. Mountain lion licenses sold, 1971 - 1993.

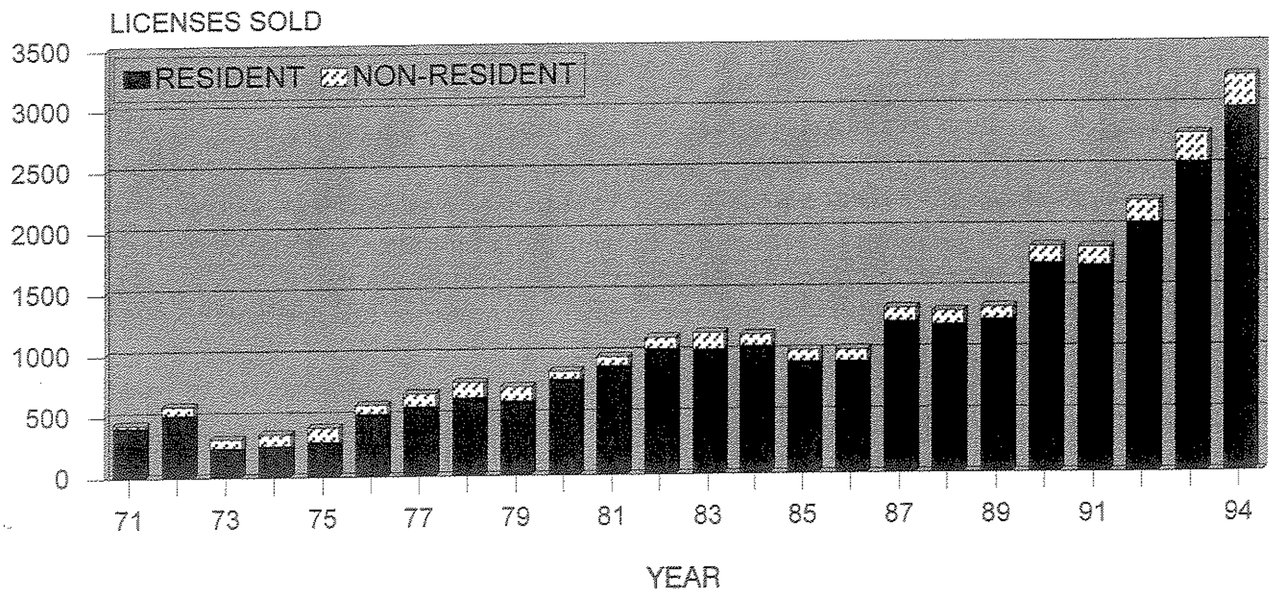

Between 1982 and 1993, Montana required the purchase of mountain lion licenses by August 31 each year, while in 1994 licenses were available until November 15. Historically, the cutoff date had been as early as June 15. The purpose for the cutoff date has been to prevent the purchase of a mountain lion license after a mountain lion has been treed or harvested.

In 1994, 55% of the mountain lion license holders said they did not go mountain lion hunting (Table 7, pp 91). Nonhunters comprised 41% of license purchases between 1988 and 1993. Early cutoff dates for purchase of mountain lion licenses may have caused some people to speculate about their chances of hunting three months into the future, and may have resulted in people purchasing licenses who never really intended to hunt. Increasing public concerns for mountain lions and mountain lion-human interactions in recent years also may have led more people to purchase licenses who never really intended to hunt. More than one-third of Montana's annual mountain lion hunters are first-time license buyers. Thirty-six percent of the 1991 license holders said that it was their first Montana mountain lion license, compared with 38% and 41% in 1992 and 1993 (FWP Tech. Serv. Div. 1991, 92, 93). These rates were more than twice as great as the overall rate of increase in license sales, suggesting a fairly high annual turnover of hunters. Causes for this phenomenon may include difficulties encountered in hunting mountain lions without owning hounds and the fact that few hunters desire to harvest a mountain lion annually.

Hunting regulations for mountain lions in Montana have evolved over the past 24 years from a six-month season to area quota systems (Table 8, pp 92). The winter timing of the hunting season, typical of most western states and provinces, was established because it is a time that hunters can effectively hunt with hounds over snow. Snow aids in determining the sex and reproductive status of the animal being tracked, and allows backtracking for kittens should a lactating female be mistakenly taken. The mountain lion season also is timed to prevent the interference that hound hunting would cause during the general big game season.

The initial 1971 mountain lion hunting season began the third weekend in October with the opening of the general big game season. The opening date was moved to a standard date of December 1 in 1976. The five-month season, running from December 1 to April 30, was subsequently shortened further to two-and-one-half months in 1978. A dog training, pursuit-only season, running from February 15 through April 30, was initiated in 1978 coincidental with the reduction in the length of the kill season. The purpose of the pursuit-only season was to allow for increased recreational opportunities, provide an opportunity for training hounds, and to allow for the collection of information on mountain lion abundance and distribution from hound handlers.

Harvest quotas were initiated in FWP regions one and three during 1986 for the purpose of regulating female harvest and controlling the total harvest under steadily increasing hunting pressure. Originally, when the female subquota was met, the kill season ended in that particular hunting unit. The remainder of the state was placed under the quota system in 1988. In 1989 the system was altered to allow the harvest of males to continue until an area quota was met or the season ended. A pursuit-only season automatically went into effect within a hunting unit once the harvest quotas were reached. Male and female quotas and

male subquotas were instituted in some areas of the state in 1994 to increase the female harvest in those hunting districts. Other regulations enforced during Montana's 1994 mountain lion season included: any area may be closed on 48 hours notice; successful hunters must report a kill within 48 hours; hides and skulls must be presented to FWP in the administrative region where the animal was taken within 10 days for inspection and tagging, (skulls are kept for aging purposes and returned to the hunter); and all successful hunters must pay a $50 trophy fee. (See Appendix 2)

Montana has one of the shortest general mountain lion hunting seasons when compared to other western states or provinces (Table 9, pp 94). The timing of the 1993-94 general harvest season was December 1 to February 15, followed by a pursuit-only season from February 16 to April 30. Two hunting areas within the Bob Marshall and Scapegoat wilderness areas open September 15 as they are generally inaccessible after December 1. A number of areas in Montana are either closed to the hunting of mountain lions or receive very light hunting pressure. Such areas may function as important "reservoirs" for repopulating vacated home ranges elsewhere (Lindzey 1987:659). These areas include most wildlife management areas managed by FWP, Indian Reservations, legislated wilderness areas, roadless areas, road closure areas, and national parks. Many other areas are effectively closed due to land ownership patterns that prevent access for hunters. Ruggedness and remoteness of terrain often preclude hound handlers from hunting localized areas. In all areas of Montana, the amount and timing of snowfall dictates hunting success, and in years of light snowfall many areas close before quotas are met.

Reported, nonhunting mountain lion mortality has been increasing (Figure 8). The median recorded annual mortality has been 10 mountain lions (range 4-64) since 1971 (Table 2, pp 88 and Table 10, pp 95). Nonhunting mortality was variable in location and degree from one year to the next, but consistently was greater west of the Continental Divide in FWP Regions One and Two. Since 1989 nonhunting mortality has steadily increased and now equals approximately 14% of the state's recorded harvest. More nonhunting mountain lion mortalities have been recorded between 1990 and 1994 than were recorded between 1971 and 1989 (213 vs. 190).

The legal take of mountain lions in Montana increased from 51 during the 1971-72 hunting season, to 566 during 1994-95 (Table 11, pp 96). The 1994 harvest was 38% female, compared to the twenty-three year average of 35%. The proportion of females in the harvest since the initiation of the quota system has averaged 34% compared with 38% in the years prior to quotas. Quotas were initially established based on an area's historical harvest. Adjustments have been made based on habitat considerations, the population dynamics of prey species, human-lion incidents and livestock depredation. One thousand, seven hundred and four hunters took at least one mountain lion between 1971 and 1991 (Table 12, pp 97). Of these, 1,419 (83.3%) took one, and 168 (9.9%) took two mountain lions. One person took 16.

Timing of snow cover and inclement weather, hunter access, and the abundance of mountain lions all play significant roles in determining the level and composition of mountain lion

harvest (Greer 1984). Regulations affecting season timing and length also have influenced the level and timing of the take (Table 13, pp 97). Whereas the harvest was initially spread out from October through April, the majority of the take currently occurs in December (Figure
9, Table 13, pp 97). The trend of increasing harvest has occurred since 1971 and is significantly correlated to the number of mountain lion licenses issued (Figure 10). The majority of the statewide mountain lion harvest has always occurred west of the Continental Divide in FWP Regions One and Two. Sixty percent of the 1993 statewide harvest and 63% of the 1994 harvest occurred in Regions One and Two, which was slightly below the 22-year (1971-92) average of 68%.

While outfitted hunters comprise 10% of hunters afield (Figure 7), they took 26% of the statewide harvest between 1990 and 1994. Between 1971 and 1990, outfitted hunters took 32% of the total harvest. While the percentage of lions taken by outfitted hunters has dropped, the total outfitter harvest has increased over the last several years as quotas have increased. Consequently, outfitted hunters took 26% or 61 of the 258 lions harvested in 1990 and 142 lions or 25% of the 1994 harvest of 566 lions (Table 14, pp 98).

Figure 8. Non-hunting mountain lion mortality 1971 - 1993.

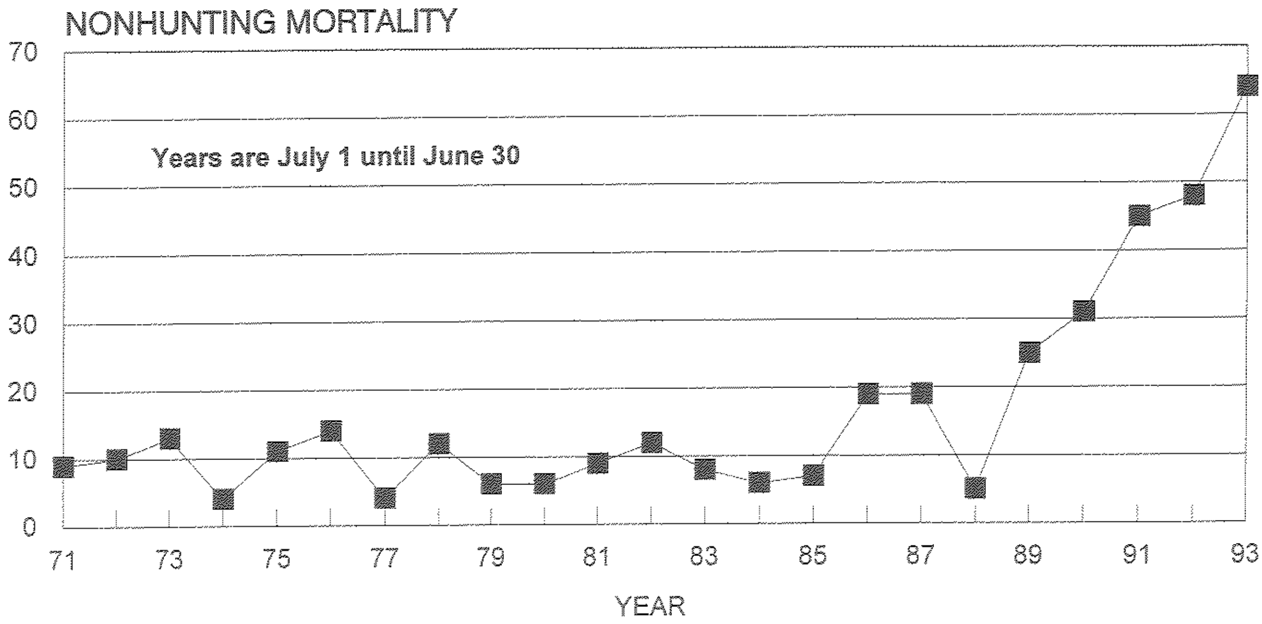

Figure 9. Percentage of total mountain lions harvested in December, 1971-1993.

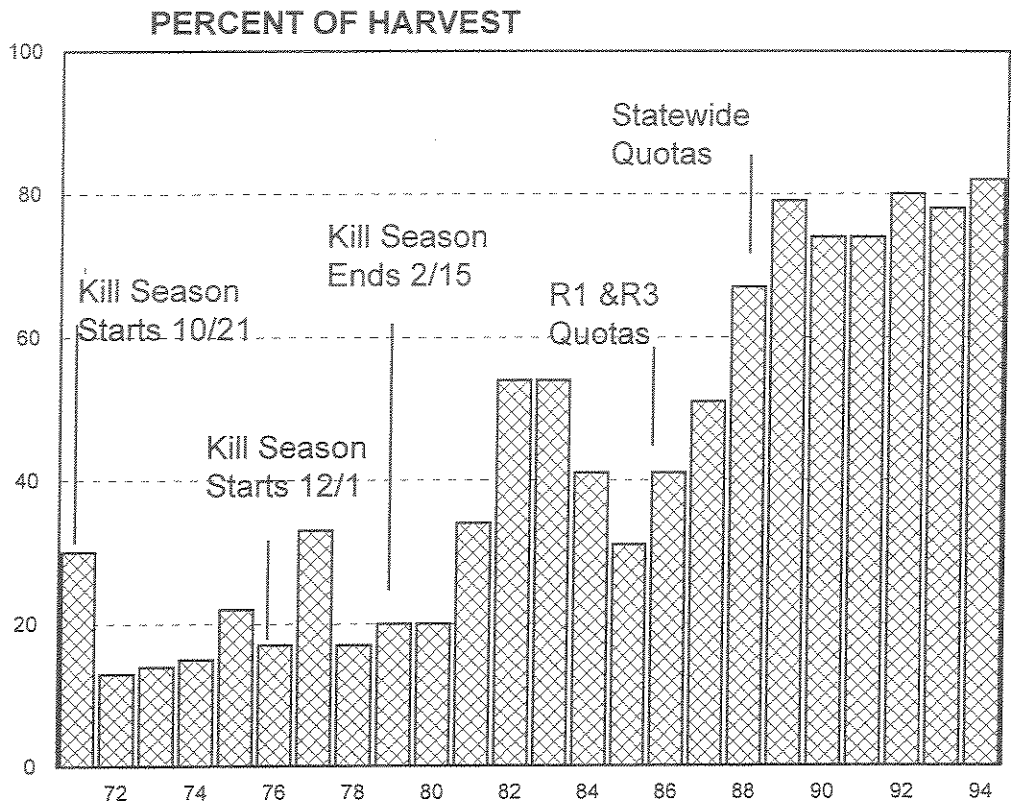

Figure 10. Percent of Montana mountain lions harvested in December vs. licenses issued.

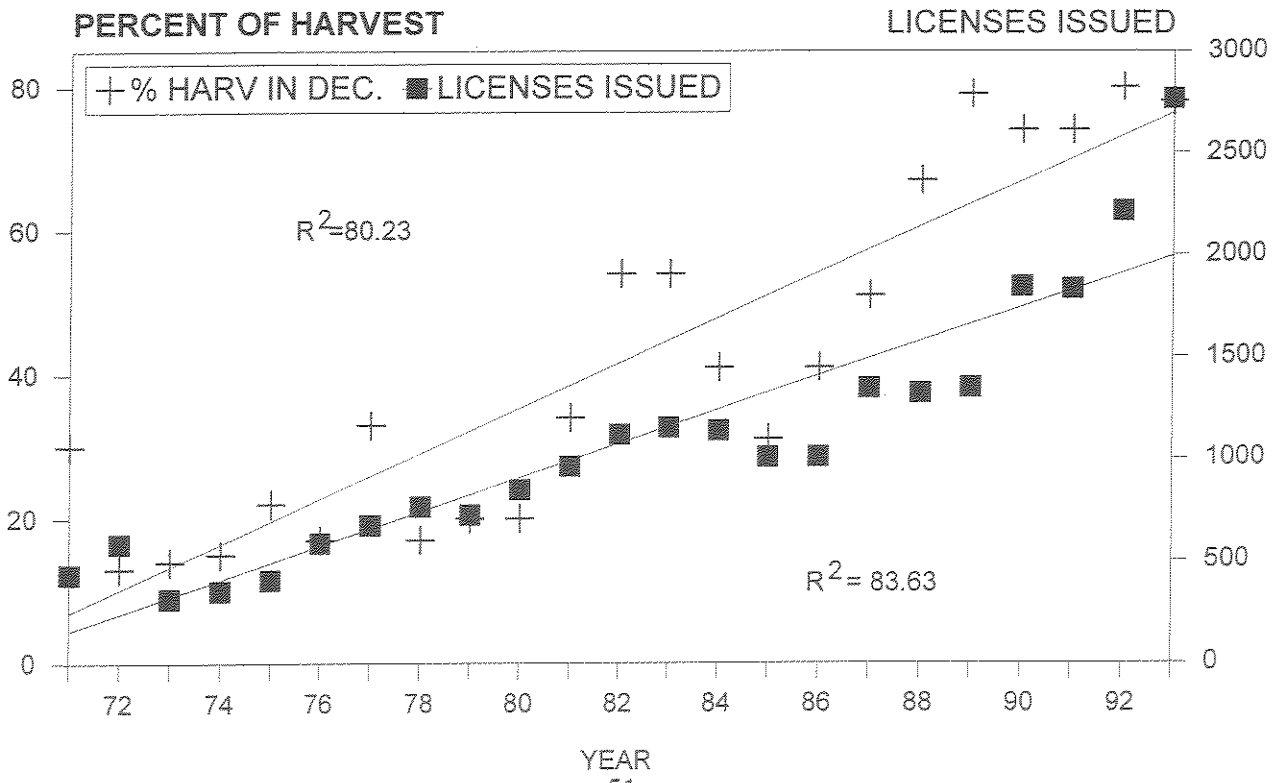

The outfitted hunter harvest has recently taken a slightly greater proportion of female lions (Figure 11) than the nonoutfitted hunter. The 1990 outfitted harvest was 34% females versus 30% for non-outfitted hunters. In the 1990 through 1993 period, outfitted hunters took 38% females as compared with 32% for nonoutfitted hunters. In 1994 outfitted and nonoutfitted hunters similarly took 36% females in the harvest. Outfitted hunters often are here for a short time and may be more inclined to harvest any adult lion than to leave without one.

A social conflict among lion hunters has arisen in some localities, such as the Clark Fork, Swan, and Bitterroot valleys. The most acute potential problem areas are in region one, where outfitted hunters accounted for 37% of the lion harvest between 1990 and 1993. Problems arise because guided hunters are hunting on a daily basis and have a strong influence on when quotas are reached and areas close. In some cases areas are closed before the "local weekend" hunters have an opportunity to get into the field.

The management of mountain lion has been chronically hindered by a paucity of information on population dynamics. Harvest objectives typically have been set based on the level of harvest a population sustained previously or in response to livestock depredations and mountain lion-human interactions. Nationally, a harvest level of 25 to 30% of the animals one year old or older has been employed where population information has been known (Ashman et al. 1983, Lindzey et al. 1992).

Figure 11. Percentage of the lion harvest comprising female lions taken by outfitted vs. non-outfitted hunters.

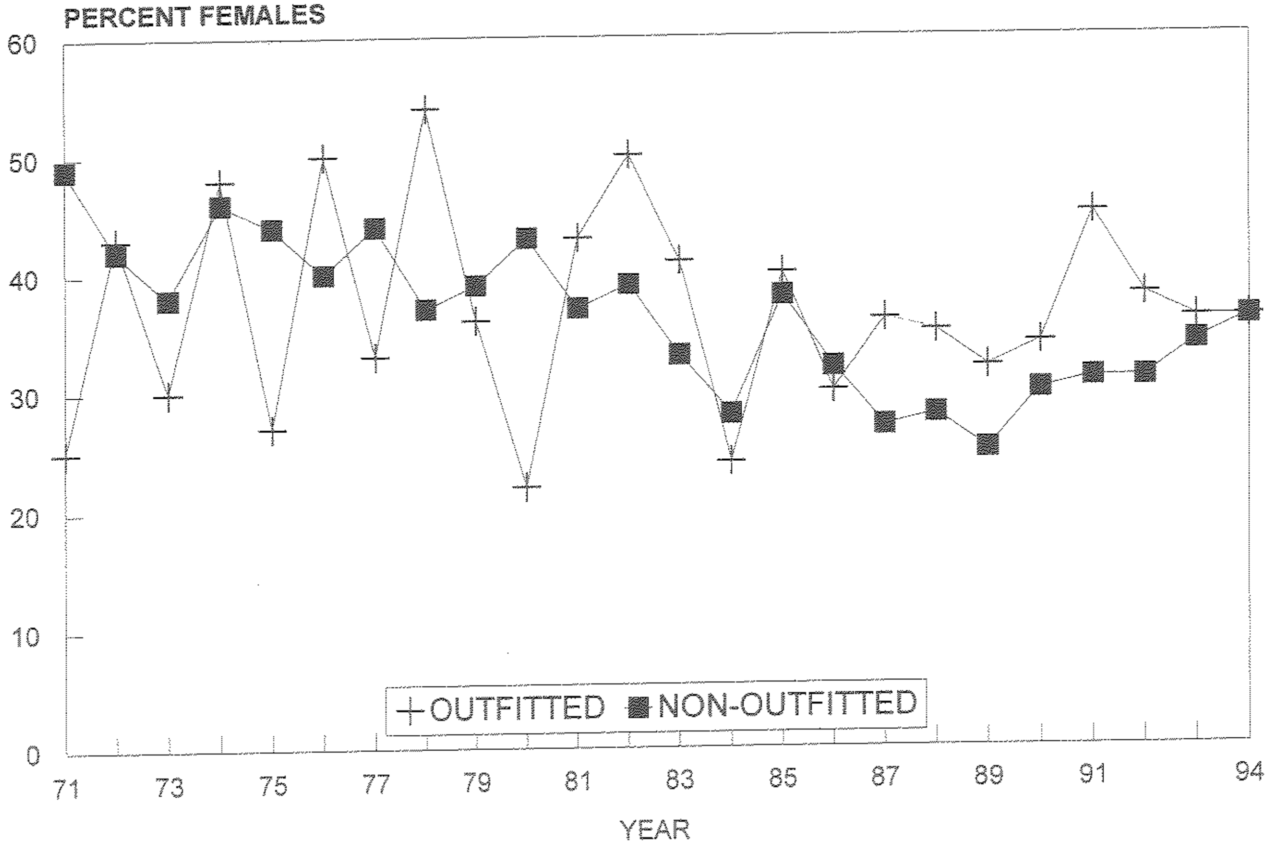

While population monitoring techniques have been studied and somewhat improved in recent years (Van Sickle 1991, Van Dyke et al. 1986, Ackerman et al. 1982, Fitzhugh 1987), they remain labor intensive, insensitive to subtle population changes, and questionable in their accuracy. Radio monitoring of populations continues to provide the most opportunity for collecting population information, but is cost prohibitive for large-scale monitoring programs. Because lions occupy all of Montana's ecosystems and ecoregions (Figure 12) with their various weather conditions, no one monitoring method is suitable for all areas. Long-term trends of harvest, numbers of sightings, human-lion conflicts, depredation (Figure 13) and numbers of lions pursued during the season remain the best indicators of lion population trends available to Montana's wildlife managers.

Lindzey et al. (1992) found that the effect of hunting on mountain lion populations depends not only on level of harvest (in his case a 27% harvest level was too high to maintain the population), but also on sex and age of animals removed. Populations are most sensitive to removal of adult females. Lindzey (1987:659) theorized that losses from hunting and control removal may be compensatory to deaths resulting from other lions, but are additive to accidental deaths in a population.

Whereas pursuit-only seasons have been regarded as nonconsumptive, the physiological effects of chasing mountain lions is poorly understood. Harlow et al. (1992) found a lowered plasma cortisol profile in mountain lions put through simulated pursuits, indicating an altered physiological response of the adrenals to the stress of repeated chases. Ramifications of this response remain unknown as they can be viewed as either a physiological adaptation to deal with stress or as a condition of by the body induced by stress.

In Montana, questionnaires are sent to all mountain lion license holders annually to obtain information on mountain lion harvest, distribution and density. This information is tracked by hunting district, region and on a statewide basis for use by wildlife managers. Information gathered through the questionnaire indicates that between 1989 and 1994 numbers of mountain lions treed during the hunting season increased from 1,259 to 3,169 and that while numbers of hunters almost doubled, chases per hunter remained stable. Chase season information for the same period indicates total chases increased from 1,003 in 1989 to 2,321 in 1994, a 131% increase, while participants show a 89% increase for those years (Tables 15 and 16, pp 99). The increased numbers of mountain lions being seen during the chase season would indicate mountain lion populations are continuing to increase despite increasing harvests during the same period.

Figure 12. Mountain lion distribution by ecosystem.

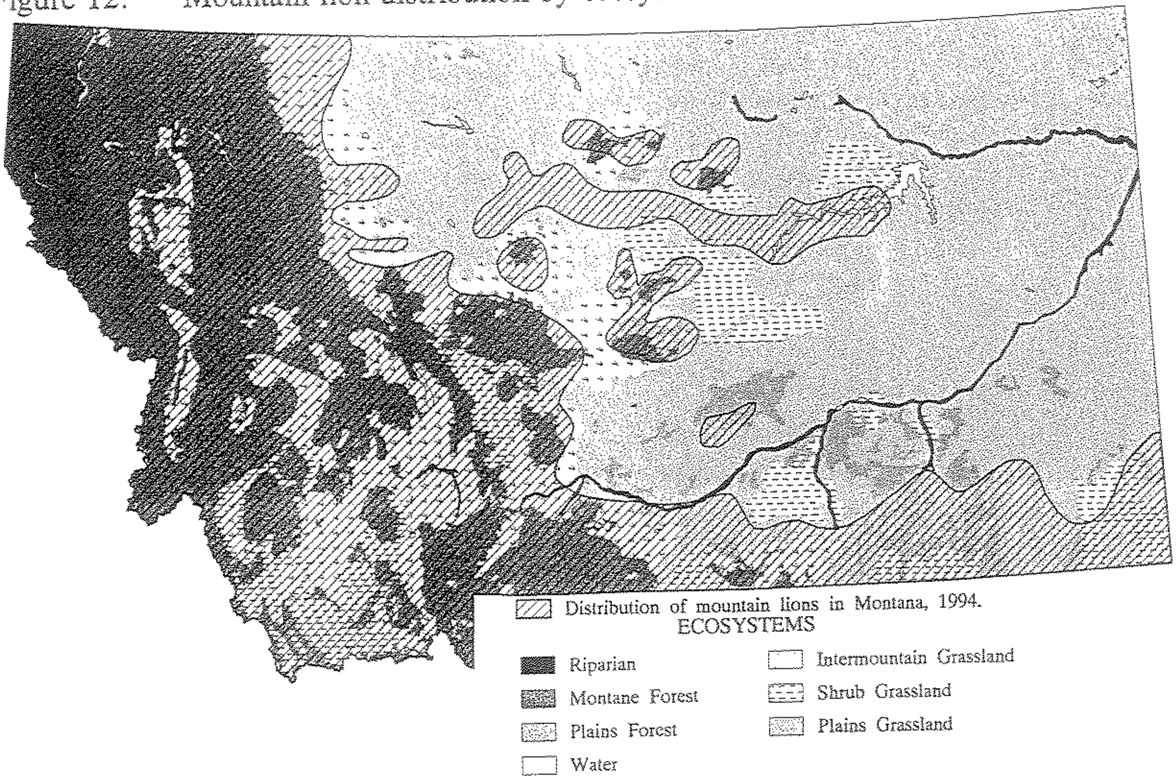

Figure 13. Number of ADC justification reports filed and mountain lions killed in Montana. 1964-1992.

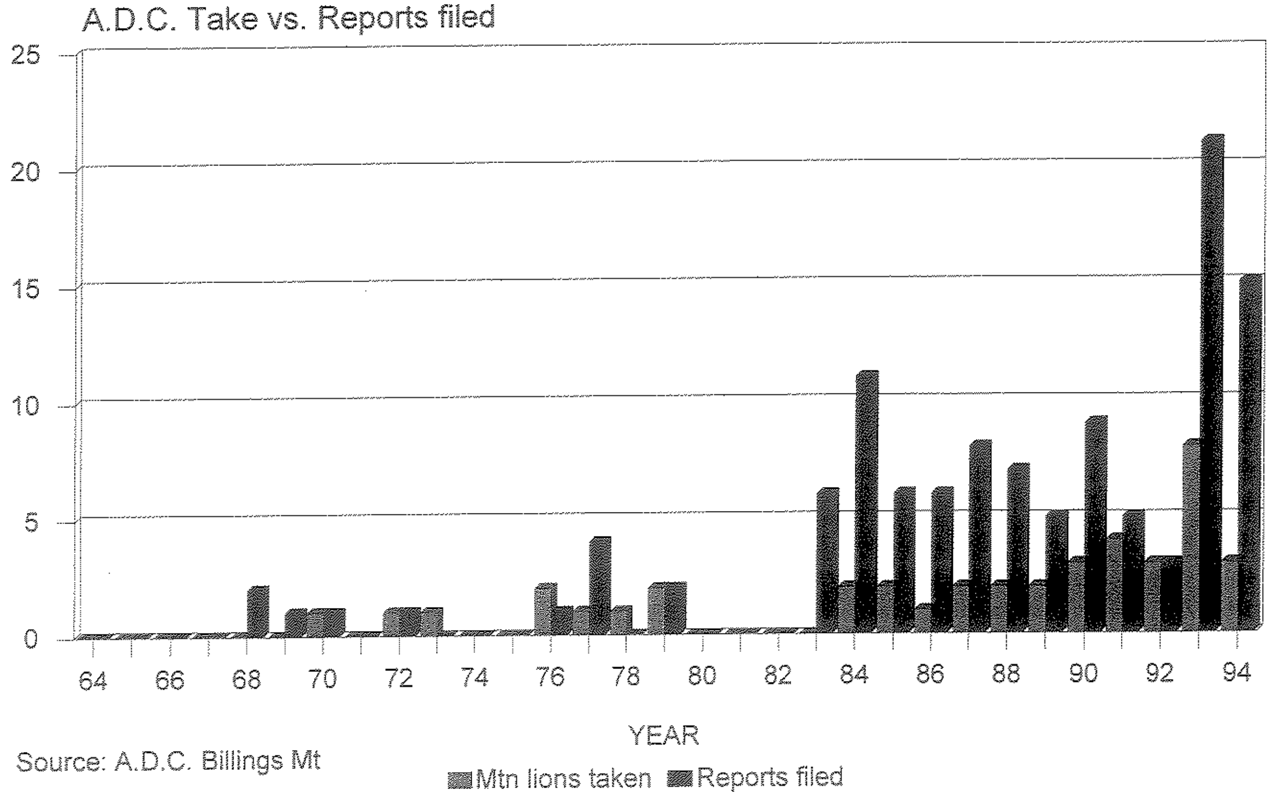

VALUES

A) Economic

In Montana the mountain lion has increased dramatically in both social and economic value over the last 30 years. In 1962, the last year the animal was bountied, the State of Montana paid $3,100 to have 62 mountain lions killed. FWP records show total revenue from the sale of mountain lion licenses has increased from $63,590 in 1990 to $127,320 in 1994. Travel and lodging by hunters, taxidermy fees, and other miscellaneous expenses make an unknown, but significant, contribution to the economy of Montana. Licensed participants in the hunt and chase season expended an estimated $1.2 million, in addition to guide and outfitter fees, during Montana's 1993 mountain lion season (FWP 1994). Idaho estimated that during their 1988 mountain lion hunting season, hunters contributed approximately $1,000,000 to the economy of that state (Harris 1991).

The mountain lion is not an economically important furbearer. There currently is little or no fur market in Montana for mountain lion hides (Pacific Hide, Steel and Recycling pers comm., 8/1/91). The amount paid for mountain lion hides at FWPs annual hide sale was $215.62 (n=9, range $65-325) in 1990 and $133.70 (n=58 range $45-275) in 1994. There is a limited market among taxidermists for large, premium hides used for life size mounted specimens (Table 17, pp 102).

B) Aesthetic

The intangible aesthetic values related to having large carnivores present in an ecosystem have not been adequately assessed. Mountain lions, as large predators, have an intrinsic value to the long-term health of ecosystems. Predation has been one of the strongest forces in the evolution of prey species. The mountain lion has been an influencing factor on big game species in Montana as well as North America as we know them. Errington (1967), in his classic text on predator-prey relationships, stated "... native predators belong in our natural outdoor scenes not so much because they have a postulated or demonstrated monetary value or utility in the so called Balance of Nature as because they are, it seems to me, a manifestation of Life's wholeness."

C) Social and Cultural

During the past decade, interest in Montana, its scenic and wildlife resources, and its recreational opportunities has elevated the stature of our state as a vacation destination. National and international interest has been reflected in an increased demand for Montana hunting licenses, increased recreational use of public lands and visitation to Glacier and Yellowstone National Parks. Montana also has become a destination for residents of other states wanting to participate in the quality of life Montana has to offer. Many of these newcomers are here as visitors, others as part-time residents, while still others are making Montana their home.

This influx of tourists and part-time and full-time residents has resulted in a diversification of public attitudes towards wildlife and wildlife-related recreational opportunities. Pursuit of a rural lifestyle by increasing numbers of people is fueling development of subdivision in Montana's urban and urban-interface areas. Many of these areas occur in occupied mountain lion habitat and others have resulted in the creation of small refuges that will maintain viable populations of mountain lions and their prey. These situations have resulted in increasing numbers of human-lion confrontations and conflicts.

Recently, a more stringent regulation of Montana's mountain lion harvest has coincided with an increasing prey base. As a result, mountain lion populations have increased and expanded into previously unoccupied habitats. These elevated mountain lion populations have resulted in outdoor recreationists observing more mountain lions, or their sign, and the general public has become more aware of the mountain lion's presence in Montana. Concerns have been raised about Montana's management of the species, increased livestock depredation, habitat losses, increased anti-hunting sentiment and the effects of such high populations on the prey base.

Many residents and nonresidents alike fear the expanding human population will result not only in the loss of habitat, but also in a lower tolerance for mountain lions or possibly the loss of our ability to use hunting as a management tool. Mountain lion hunters have expressed the desire for FWP's management program to be credible and able to withstand the scrutiny of those who do not hunt, or do not value the strong hunting tradition in Montana.

The alternatives considered in the EIS have the potential to impact mountain lion recreational opportunities available to the public, both hunting and nonhunting, as well as the incidence of human-lion conflicts. Changes in recreational opportunity and mountain lion densities statewide also may impact the economy of individuals and local communities.

CHAPTER III

ALTERNATIVES INCLUDING THE PROPOSED ACTION

ALTERNATIVES SELECTED FOR ANALYSIS

Four alternatives were selected for analysis, one other was considered and rejected. The choice of alternatives for analysis was based on:

1) FWP's responsibilities as laid down in the constitution of the State of Montana.

2) Requirements set forth by the Montana Environmental Protection Act (MEPA).

3) FWP's management goals as outlined in the "Needs, Purpose and Benefits of the Proposed Action" section.

4) Public input on the issues and impacts of implementing a statewide mountain lion management plan as discussed in Chapter I.

Mountain lion management programs that were selected to be analyzed in the EIS and described by the elements in Chapter I are:

1) No hunting of lions by the general public

2) Continue the current management program

3) Intensify the harvest to decrease mountain lion populations statewide

4) Regional management based on habitat capabilities (Preferred Alternative)

Elements that address each alternative are presented in Table 1, pp 12, & Table 18, pp 59.

ALTERNATIVES NOT CONSIDERED

One other alternative was suggested during the scoping process, but was not analyzed.

1) MANAGE USING BIOLOGICAL TARGETS TO MAINTAIN HIGH NUMBERS OF MATURE ADULTS IN MOUNTAIN LION POPULATIONS.

Due to the wide variety and distribution of occupied mountain lion habitat in Montana and the small harvest sample available from some populations to collect biological data, FWP does not believe this to be a viable management alternative.

Such an alternative would require the harvest from hunting districts or areas to meet certain biological parameters. Parameters set forth in the Black Bear EIS (FWP 1994) require the median age of the harvest to fall into categories, by sex, for each bear management unit. These parameters were identified based on black bear research. Equivalent research is not available for mountain lions, and, because of reproductive and social differences between

Table 18. List of program elements by alternative and decision authority.

Element	Alternative[1]				Decision Authority[2]			Changes needed to Implement	Additional FWP resources required to implement
	1	2	3	4	F	C	L		
**EXISTING**									
A - hunting season format		X	X	X		X		Hunting season format/regulations	None
B - chase season		X	X	X		X		Hunting season format/regulations	None
C - regulate harvest using quotas		X	X	X		X		Hunting season format/regulations	None
D - assist w/livestock depredation	X	X	X	X	X			Program procedures	None
E - public education projects	X	X	X	X	X			Program procedures	None
F - road access on public lands	X	X	X	X	X			Program procedures	None
G - protect threatened habitat	X	X	X	X	X	X	X	Montana law	None
H - evaluate land mgmt. agencies	X	X	X	X	X			Program procedures	None
I - hides/skulls tagged in region		X	X	X		X		Hunting season format/regulations	None
**ADJUSTMENTS**									
J - reduce closure period				X		X		Hunting season format/regulations	Redirection of funds
K - reduce reporting period				X		X		Hunting season format/regulations	Redirection of funds
L - change season length			X	X	X	X		Hunting season format/regulations	Redirection of funds
M - reduce inspection period				X		X		Hunting season format/regulations	Redirection of funds
N - protect dependent young			X	X		X		Hunting season format/regulations	None
O - re-evaluate hunting on WMAs			X	X		X		Hunting season format/regulations	None
P - intensive management zones	X		X	X		X		Hunting season format/regulations	None
Q - evaluation of subdivisions	X		X	X			X	Montana law	None
R - landowner use of houndsmen	X		X	X			X	Montana law	None
S - prioritize research needs	X	X	X	X	X			Program procedures	Redirection of funds
T - establish harvest objectives			X	X	X			Program procedures	Redirection of programs
U - human-lion conflict policy	X		X	X	X			Program procedures	Redirection of programs
**PUBLIC INITIATIVES**									
V - license and/or permit to "pursue"			X	X		X		Hunting season format/regulations	Redirection of policy
W - methods to regulate hunters				X		X		Hunting season format/regulations	Redirection of policy
X - improve harvest information		X		X	X			Program procedures	Redirection of programs/funds
Y - increase enforcement efforts	X		X	X	X			Program procedures	Redirection of priorities
Z - open with big game			X			X		Hunting season format/regulations	Redirection of policy

[1] ALTERNATIVES
Alternative 1 - No general hunting
Alternative 2 - Reactionary management
Alternative 3 - Intensive management
Alternative 4 - Population oriented management

[2] DECISION AUTHORITY
F - Montana Fish Wildlife & Parks
C - FWP Commission
L - Montana Legislature

black bears and mountain lions, may not be applicable. The sample size from many hunting units is too small for harvest information to be used in determining statistically significant biological parameters for those populations.

While it was not felt this method of management would be applicable statewide, if research information became available that would allow such parameters to be set, they would be utilized by regions having suitable harvests for setting management objectives.

DESCRIPTION OF THE ALTERNATIVES SELECTED

ALTERNATIVE NO. 1 - NO HUNTING OF LIONS BY THE GENERAL PUBLIC

Under this alternative, the general public would no longer be allowed to obtain hunting licenses or chase permits. Any harvest of lions would be either by FWP personnel or the ADC. Lions taken would be in association with threats to human life or livestock depredation.

The quota system would be dropped as would all regulations having to do with the harvest of mountain lions.No population objectives would be set for mountain lions.

The alternative would allow for continued increases in mountain lion populations in remote areas as well as in and around areas of human habitation. Zones of "no tolerance" would be established in selected areas of subdivision or development and attempts would be made to remove all lions from the area to protect human lives. The establishment of such areas would be through a public process and require input from those people occupying the area.

Implementation of this alternative, would be expected to have an increasingly negative impact on numbers of deer, elk and bighorn sheep available to hunters. Lion numbers would not control numbers of deer and elk (Hornocker 1970a), but would keep population numbers low over a longer period when their numbers declined as a result of a natural cycle or due to environmental or human-induced circumstances.(Neal et al. 1987)

Under this alternative, FWP would continue to: provide for public education about mountain lions, review programs proposed by land management agencies to determine their effects or merits for wildlife, and protect important habitat using the Habitat Montana Program. FWP would also develop a policy to deal with human-lion conflicts and livestock depredation and assess research needs for mountain lions in Montana. There would be legislation introduced that would: a) require FWP wildlife evaluation for subdivisions be made available to potential customers; and b) clarify a stockowners rights in dealing with depredating mountain lions.

This alternative would require an increase in FWP efforts aimed at dealing with mountain lions in nuisance situations, human-lion conflicts and stock depredation cases. Increased efforts to enforce the no hunting regulation also would be required.

ALTERNATIVE NO. 2 - CONTINUE THE CURRENT MANAGEMENT PROGRAM (NO ACTION ALTERNATIVE)

Under this alternative, the general hunting season would remain December 1 through February 15 and the chase season would open February 16 and continue through April 30. Early seasons would continue to be held in portions of the Bob Marshall and Scapegoat Wilderness areas beginning September 15. Harvest would be regulated with quotas for: a) total harvest; b) total harvest with a female subquota; c) male and female harvest; or d) total harvest with a male subquota. Quota type and level would be determined by FWP regional wildlife personnel and recommendations made to FWP Commission to finalize for the season. Public input on the seasons, harvest quotas and quota types would be addressed by the FWP Commission during the biennial season setting and annual quota setting processes.

Hunting regulations would require all harvested animals to be reported within 48 hours of being taken and to be presented to FWP for inspection and tagging within 10 days of being harvested. Seasons would be closed in hunting units 48 hours after the total quota was reached or to hunting for a specific sex of mountain lion upon 48 hours notice of a closure.

FWP would continue: education programs and projects on mountain lions; assisting the ADC and landowners with stock depredation problems; providing land management agencies with wildlife recommendations for roads, wilderness and development projects; using the Habitat Montana Program to protect threatened wildlife habitat; and, to inspect the hides and skulls of harvested mountain lions.

Research needs for mountain lions would be assessed and prioritized. Future budgets would include provisions to cover the expense of that level of research as is deemed necessary by FWP to meet mountain lion management goals.

Harvest quotas would be set based on the length of the previous season, sex and age data of mountain lions taken and the size of previous harvests, depredation complaints. and numbers of human-lion conflicts in an area. Quotas would also be based on the ability of an area to produce and sustain mountain lion, deer and elk populations.

ALTERNATIVE NO. 3 - INTENSIFY THE HARVEST TO DECREASE THE POPULATION

The objective of this alternative would be to reduce mountain lion populations for public safety or livestock protection purposes, while still maintaining a viable population, or to eradicate mountain lions on a small local scale. It could include the use of the current season structure with the quota system, or the harvest would be regulated by the length of the season. Under this scenario, season length could vary between FWP regions or hunting districts depending on harvest objectives. If season length were used to regulate harvest, there would be less need for a communications system to track harvest and area closures.

Under this alternative, FWP would continue to assist with mountain lion depredation cases, produce informational materials and programs on mountain lions for the public, make recommendations on roads, development projects and wilderness proposals to land management agencies, protect threatened habitats through the Habitat Montana Program, and inspect, tag and collect biological information on harvested animals.

Adjustments to the mountain lion management program would include changing regulations to make it illegal to take a female with dependent young and evaluating the opening of some WMAs to hunting. Management practices would include the establishment of low tolerance and no tolerance zones around selected sites of human habitation and the development of a human-lion conflict policy. Harvest objectives for lions would be set based on the ability of the habitat to sustain them and their prey base and would take into account local human tolerance and safety conditions. Legislation would be introduced to clarify a stockgrower's rights in dealing with depredating mountain lions and to require FWP's wildlife evaluation of any subdivision be provided to prospective buyers.

Adjustments would not include reducing the time periods for reporting harvest, having mountain lions tagged or closing of seasons. Because populations would be reduced, numbers of hunters participating also would decline and the length of time it takes to meet harvest goals would be extended. This also would negate the need to regulate hunter numbers. However, in order to track participation, all hound handlers participating in the hunt or chase season would be required to have a hound handler permit.

Enforcement efforts under this alternative would be directed at field checks for license possession, poaching, taking of lactating females and correct reporting of harvest area.

ALTERNATIVE No. 4 - REGIONAL MANAGEMENT BASED ON HABITAT CAPABILITIES (PREFERRED ALTERNATIVE)

This alternative bases mountain lion management on FWP regional population objectives for mountain lions as determined by the prey base density and the habitats ability to sustain both. Mountain lion densities would vary between habitat types, and population management goals would include considerations for habitat conditions, prey availability, other demands on the prey species, human encroachment and tolerance levels, depredation levels, and the biological health of the mountain lion population.

The current hunt and chase season formats would continue, as would the use of quotas to regulate the majority of the harvest. Season length could be substituted as a harvest strategy under this scenario and such recommendations would be made by the FWP regions for consideration by FWP Commission. Harvest intensity would vary depending on harvest objectives determined for habitat types, hunting areas or local urban and urban-interface management units.

As with the other alternatives, public education efforts would continue, assistance would be provided with animal depredation, recommendations would be made to land management agencies on projects affecting wildlife, the Habitat Montana Program would be used to protect habitat, and hides and skulls of harvested mountain lions would be inspected and tagged.

Adjustments would be made in season regulations that would:

a) allow seasons to close in as little as 12 hours (currently 48 hours) and closures to be announced prior to their being met.

b) require all harvest of lions to be reported within 24 hours

c) require all skulls and hides to be inspected within 24 hours of being reported harvested.

Such adjustments also would require FWP to have a system in place that would allow rapid dissemination of quota and harvest information as well as allow for timely reporting and tagging of animals. This will require some adjustments by regions in making this service available.

Additional adjustments to the management program would include making it illegal to take a female with dependent young or dependent young with an adult female, and evaluating the possibility of hunting lions on some WMAs. The alternative also would require all hound handlers participating in mountain lion seasons to possess a hound handler permit and provide methods for controlling hunter numbers.

Interactions between humans and mountain lions would be addressed through the development of a human-lion conflict policy and the establishment of intensive management or no tolerance zones around selected areas of human habitation or development. Legislation would be introduced to clarify the rights of stock- growers to handle mountain lion depredation and to require that potential buyers of property in subdivisions be provided a copy of the wildlife evaluation by FWP for the subdivision.

Research needs for mountain lions would be assessed and prioritized. Future budgets would include budget requests to the Legislature to cover research deemed necessary to achieve mountain lion management goals.

CHAPTER IV

ENVIRONMENTAL IMPACTS

ANALYSIS OF ENVIRONMENTAL CONSEQUENCES

This chapter describes the anticipated impacts of each of the alternatives on:

1) Habitats occupied by mountain lions
2) Mountain lion and prey populations
3) Recreational opportunity
4) Conflicts with management
5) Local, statewide and FWP economies

A comparative summary of the environmental consequences of each alternative begins on pp 82, and the components of each impact analyzed are shown in Table 19, pp 100.

ALTERNATIVE 1 - NO HUNTING OF MOUNTAIN LIONS BY THE GENERAL PUBLIC

IMPACTS ON HABITAT

The majority of Montana's mountain lion habitat lies within the montane forest ecosystem and is controlled by federal land management agencies or corporate interests. (See Figure 12, pp 54). The bulk of this land is expected to remain available and productive for wildlife into the future. Private lands within occupied habitat stand a much higher chance of being permanently converted to other uses and losing some, or all, of their value for mountain lions.

Under Alternative one the mountain lion's close ties with deer and elk would result in continued protection of mountain lion habitat as a secondary result of protecting deer and elk habitat. WMAs that currently harbor mountain lions would continue to do so, as would roadless and wilderness areas. The alternative still would require FWP to examine development projects proposed by land management agencies and the affects of those projects on Montana's wildlife resource. Authority granted FWP by the Legislature under the Habitat Montana Program would provide for some protection of threatened privately held habitat utilized by mountain lions and their prey. Under this alternative mountain lions would be given less consideration when evaluating federal programs or projects for the Habitat Montana Program than provided under Alternatives 2, 3 and 4.

Public education programs dealing with the needs of mountain lions, assisting landowners with depredation problems and providing information on living with wildlife in subdivisions are all programs that will continue, or be instituted, under this alternative. Each offers the opportunity to provide the public with information about land use changes and fosters more awareness of trade-offs that are made in connection with land use decisions.

IMPACTS ON POPULATIONS

Impacts on mountain lion populations:

In the absence of hunting by the general public, mountain lion populations would be regulated through natural mortality and social distribution within the populations. More individual mountain lions would be expected to be taken in human-lion conflicts and livestock depredation by FWP or the ADC.

Mountain lion populations would not be expected to expand dramatically in western Montana's montane forest habitats. These populations are believed to be near limits imposed by the mountain lion's social structure and available habitat. Populations in the eastern mountains, prairie foothills and prairie areas would be expected to continue to expand until limited by these same constraints.

Short-term population fluctuations would occur over time due to climatic variations and the cyclic nature of deer and elk populations. As prey populations declined, recruitment into mountain lion populations would also be expected to decline. The length and extent of such declines would vary with climatic conditions and availability of alternate prey species.

Populations would be comprised of a greater number of older mountain lions than occur in hunted populations and, while reproduction would be high, recruitment would decline. Older females would produce and successfully rear young to the transient juvenile stage when the young become independent. Such transients would not become established in a population unless additional habitat became available, unoccupied territories were found or habitat conditions improved to the point that they would support additional animals.

Absence of harvest information will result in the loss of age structure information currently obtained through the collection of skulls and teeth. Mountain lion density information currently obtained through the hunter and chase questionnaires will no longer be available. Some trend data gathered from depredation and human encounters will be maintained. However it will provide little information on local populations and will be more applicable over a broad area. This information will also be skewed toward areas with denser human populations.

Some monitoring of populations would occur during furbearer track surveys, but no priority would be put into long-term monitoring efforts unless in connection with research on other species.

Impacts on prey populations:

The impacts of this alternative would include an increase in density of mountain lions statewide and a subsequent increase in predation rates. Increasing mountain lion populations, by themselves, would have little effect on the deer and elk hunting

opportunities in Montana. Mountain lions do not control prey species, but do exert a dampening effect on prey populations, which is especially noticeable when prey densities are low. Such dampening can prolong the low density population cycles of prey species. The cumulative effects of mountain lion predation, hunting and predation by other species will extend such lows for longer periods of time.

IMPACTS ON RECREATION

Impacts on mountain lion hunting:

Under this alternative all hunting of mountain lions by the general public would cease.

Impacts on other recreational opportunities:

The closure to general hunting would also include closure of the chase season for mountain lions. Consequently, the secondary recreational opportunities currently offered by the chase, such as hound training, mountain lion viewing and photography, also would be lost.

Increased populations would slightly increase opportunities for outdoor recreationists to view and possibly photograph mountain lions. However, because of their secretive habits, such opportunities would remain sparse.

Impacts on the hunting of prey species:

High densities of mountain lions, by itself, would have little effect on statewide opportunities for hunting of deer and elk. Mountain lions could hold prey numbers below a local area's carrying capacity, which could result in local restrictions on hunting. With species such as bighorn sheep, heavy predation by mountain lions, especially when combined with other sources of predation, would result in FWP issuing fewer permits for areas exhibiting low or declining populations.

ANALYSIS OF CONFLICTS:

Human-lion conflicts:

Higher densities of mountain lions statewide would result in increasing numbers of confrontations with humans. This would require FWP to spend additional time dealing with such conflicts and to develop guidelines to equitably deal with them.

As available habitats became saturated with mountain lions, conflicts between expanding numbers of transient young, and older animals unable to defend their territories would be expected to increase. It would be necessary to destroy such animals when they became involved in conflicts, unless zoos or other states could be found to take them.

Livestock depredation:

Numbers of depredations would be expected to increase and the time FWP spends

handling, investigating or assisting ADC with such incidents also would increase. Legislation would be introduced to allow stockowners to appoint a hound handler as their agent to take depredating lions on their property.

Enforcement efforts:

Additional enforcement efforts would be required to enforce the ban on hunting. As mountain lion populations increased, more effort would be needed in dealing with human-lion conflicts, livestock depredation, illegal taking and accidental mortalities.

ECONOMIC IMPACTS

Mountain lion hunters spent approximately $1.2 million excluding guide and outfitter fees, in Montana in 1993 (FWP unpublished economic survey, 1995). Local economies would feel the brunt of this loss, as would outfitters, particularly in the northwestern portion of the state which supports the largest mountain lion harvests and the largest numbers taken by outfitters. FWP sold 2,984 resident and 258 nonresident mountain lion licenses in 1994 for $127,320. This source of revenue would be lost under this alternative, as would the $28,300 raised by trophy fees in 1994. Expenses of gathering and analyzing data and the collecting and aging of skulls would no longer be incurred. Additional funds would be needed to respond to human-lion conflicts and livestock depredation situations.

<u>ALTERNATIVE 2) CONTINUE THE CURRENT MANAGEMENT PROGRAM</u>

IMPACTS ON HABITAT

WMAs, wilderness areas and roadless areas will continue to harbor mountain lions and function as a source of transient young to fill vacant territories. More emphasis will be placed on the habitat needs of the mountain lion under this alternative than under Alternative one. This would be evident in the evaluation of federal projects and protection of land under the Habitat Montana Program. Additional effort would be put into the evaluation of roads in mountain lion habitat in order to provide security for the lions as well as access for hunters.

Ecosystems across the state would not be evaluated as to their potential to support mountain lions and the prey species on which they depend. No attempt would be made to educate purchasers of lands in subdivisions of the wildlife they might encounter or of methods to minimize impacts on species present.

IMPACTS ON POPULATIONS

Impacts on mountain lion populations:

Alternative 2 would allow the continued expansion, stabilization, or decrease of populations through the use of the quota system to regulate hunting and harvest.

Mountain lion populations would be expected to continue to expand in areas east of the Continental Divide and in those habitats outside the montane forest ecosystem. Populations within the montane forest ecosystem are currently believed to be at or near the habitat's capacity to support them and the limits their social structure will tolerate.

Population monitoring would continue to be based on harvest data, incidental data gathered during furbearer track surveys, questionnaire information from participants in the hunt or chase seasons, depredation complaints and numbers of human encounters. Priorities for research into the needs of mountain lion populations, or the density of mountain lions that can be supported by Montana's various habitats, would remain low and no funding requests would be made to the Legislature to support such studies or long-term monitoring projects.

Public education programs dealing with the needs of mountain lions, assisting landowners with depredation problems and providing information on living with lions and other wildlife would continue.

If populations expand, mountain lion mortality resulting from human encounters, livestock depredation, incidental trapping and car and train collisions is expected to increase. Mortality from these sources is not expected to reach levels that would threaten population stability or viability, either locally or statewide.

Impacts on prey populations:

Expanding mountain lion populations would result in increased rates of predation on their prey base. As with Alternative one, deer and elk populations would not be expected to be controlled by mountain lion predation alone. However, higher predation rates would dampen the rate of growth of prey species and could extend low density phases of population cycles.

IMPACTS ON RECREATION:

Impacts on mountain lion hunting:

Mountain lion hunting would continue under the current season structure. Numbers of hunters would be expected to continue to increase as neighboring states became more restrictive and interest by residents continued to grow. Harvest would be regulated by quotas. There would be no reduction in the notification period for season closures, reporting a harvested animal or the inspection and tagging of harvested mountain lions. Hunting females with dependent young would remain legal. However, taking females with kittens would continue to be illegal.

Conflicts between hunters in the heavily hunted areas of the state would be expected to escalate as hunter numbers increased and lion populations stabilized. Resident or

nonresident hunter numbers would not be limited, and hound handlers would not be required to be licensed or to hold hound handler permits to participate in the hunt.

Impacts on other recreational activities:

Numbers of mountain lions are expected to continue expanding statewide, increasing chances of incidental sightings by outdoor recreationists. Track observations and photographic opportunities also would increase slightly with expanding populations. A chase season would continue to offer those opportunities it does under the current season structure.

Impacts on the hunting of prey species:

Increased predation rates would result with expanding mountain lion populations. Mountain lion predation would not be expected to have long-term effects on prey species on a statewide basis. Some local populations may be reduced and, as a result, either hunting of that species would be restricted, or area quotas raised on mountain lions.

ANALYSIS OF CONFLICTS

Human-lion conflicts:

Confrontations between expanding human populations and expanding mountain lion populations would continue to increase. Such conflicts would continue to be resolved on a case-by-case basis under this Alternative. No policy would be developed to apply statewide guidelines to such incidents. FWP personnel would be expected to spend additional time and resources responding to such incidents.

"No tolerance" or "low tolerance" zones could be established in and around subdivisions and urban and urban-interface areas to reduce or eliminate mountain lions for reasons of public safety. No legislation would be introduced to require FWPs wildlife assessment of a subdivision be provided to prospective buyers.

Livestock depredation:

Numbers of depredation incidents would be expected to increase, but also would be expected to level out as numbers of cattle increase and numbers of sheep decline in Montana's agricultural community (Figures 14 and 15). Such incidents would continue to be handled through ADC with a low level of FWP assistance. No legislation would be introduced to clarify a stockowner's rights regarding the use of a hound handler to take a depredating mountain lion.

Enforcement efforts:

Enforcement efforts would continue at the current level during the hunting and chase seasons. Additional effort would be expended if mountain lion populations increase and conflicts with humans and livestock also increased.

Figure 14. All cattle and calves inventory: January 1, 1940-1994 (Source: MT Agriculture Reporting Service)

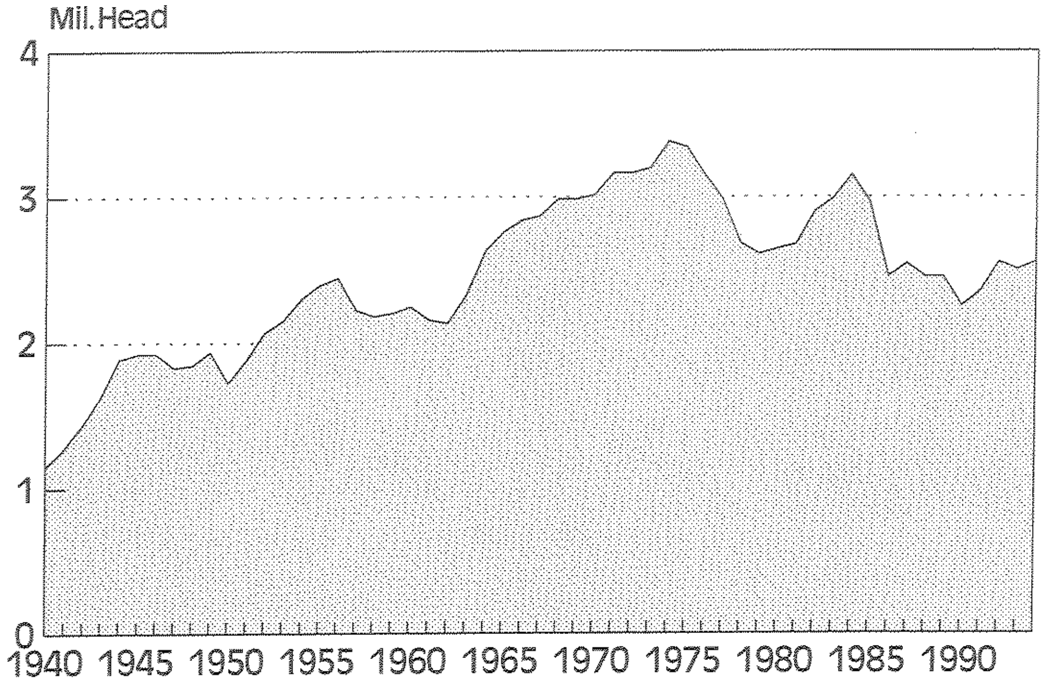

Figure 15. All sheep and lambs inventory: January 1, 1940-1994 (Source: MT Agriculture Reporting Service)

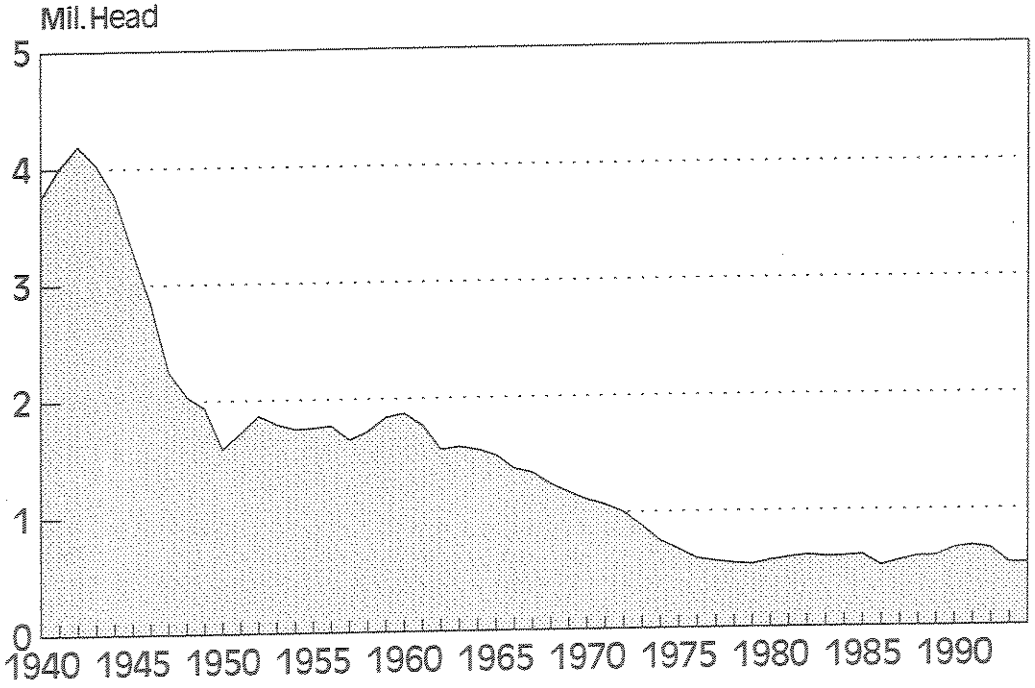

ECONOMIC IMPACTS

Expenditures by resident and nonresident lion hunters would be expected to increase above the $1.2 million spent in 1993. Those areas of the state currently receiving the heaviest hunting pressure are regions one and two. These areas would be expected to continue to be the largest beneficiaries of the economic activity generated by mountain lion hunters.

There is currently no method available to measure the economic impact of other recreational pursuits derived from the mountain lion. As part of the native fauna of Montana, their presence does account for some portion of the tourist dollars generated each year. Much of their value may lie in, the knowledge that mountain lions occupy some of the more secluded areas of the state and peoples willingness to pay to visit those areas.

Funds generated by the sale of hunting licenses is also expected to rise with increasing interest in mountain lion hunting in Montana. Additionally, the funds generated by successful hunters paying the $50 trophy fee are expected to increase gradually as mountain lion populations expand and harvest increases. In 1994, trophy fees paid on 566 harvested lions generated $28,300. Monies generated by neither the license nor trophy fee are earmarked for the mountain lion management program, but are deposited in the general license account. Authority to spend those funds is granted to FWP by the Mt. Legislature.

Increasing operating costs would be associated with an increased incidence of human-lion conflicts and mountain lion depredation.

ALTERNATIVE 3 - INTENSIFY HARVEST TO DECREASE POPULATIONS

IMPACTS ON HABITAT

Under Alternative 3, mountain lions would receive the same consideration in evaluation of federal land management projects, road proposals and protection of threatened lands under the Habitat Montana Program as under Alternative 2. Mountain lion habitats in Montana would be evaluated for their capacity to sustain both mountain lions and their prey base. Public education programs would be directed at improving awareness of the mountain lion's habitat requirements, informing the public about land use planning efforts that will improve mountain lion habitats, and providing information on living with mountain lions.

Legislation would be introduced that would make the wildlife evaluation prepared by FWP on new subdivisions available to prospective buyers.

IMPACTS ON POPULATIONS

Impacts on mountain lion populations:

As a result of more liberal hunting seasons, populations would be expected to stabilize statewide and be confined to those portions of the state currently occupied. Target densities would be determined for local areas by FWP regional wildlife management staff. These densities would be based on the habitat's capability to support both the mountain lion and its prey base and social tolerances. Harvest could be regulated by either the quota system or length of the season. Intensive management, or "low tolerance" zones, could be established in and around areas of human occupancy.

Mountain lion hunting would be evaluated on WMAs. FWP would consider the need to use such areas as a reservoir to supply transient young to adjacent areas before opening them to hunting. It also would be necessary to evaluate the effect such hunting would have on management objectives established for each WMA.

Populations in some areas would initially decline, then level out and stabilize at lower densities. Production of young also would decline, as would numbers of transient young. Increased hunting pressure would result in fewer juveniles becoming established as members of the breeding population.

Opening the mountain lion season to coincide with the opening of the general big game season would make some females more vulnerable to incidental harvest. The majority of animals taken during this season would be expected to be transient young, and rather than reproductive females. Opportunistic harvest of lactating females, or females with dependent young, would increase instances of orphaning and loss of kittens and dependent young.

A regulations change would make it illegal to take a female with "dependent young" rather than "kittens" in order to raise the security level of juvenile animals still dependent on the female. It would remain illegal to take any spotted animals, or dependent young traveling with adult females.

Population monitoring would include density information based on winter track surveys as well as harvest and observation data. Budget requests would be made to monitor population trends and to conduct research on the effects of increased hunting pressure on the social and biological functioning of mountain lion populations.

Numbers of accidental mortalities and mountain lions removed because of human-lion conflicts would be expected to decline under this alternative.

Impacts on prey populations:

Predation rates would decline with mountain lion densities. Effects on the population would depend on the numbers of other predatory species making use of the same prey

populations and the density of those populations. If prey population densities were high, little effect would be seen on prey numbers. If densities were low, and lions were the primary predator, some increase in prey numbers would be expected.

IMPACTS ON RECREATION

Impacts on mountain lion hunting:

Mountain lion hunting would continue with the season extended to begin with the general big game hunting season and hunting on WMAs will be evaluated. A recommendation would also be made to the FWP Commission to use season length to regulate harvest in some areas rather than a quota system.

Hides and skulls would continue to be tagged in the FWP region harvested, but no changes would be made in reporting or tagging requirements.

To track participation in both the hunt and chase seasons all persons over 12 years of age who are in control of dogs in the field would be required to purchase a license and/or possess a free hound handler permit.

Impacts on other recreational activities:

The chase season would continue and begin with the closure of the hunting season in an area or on a predetermined date set by the FWP Commission.

Lower population densities would reduce the opportunity for outdoor recreationists to observe or photograph a mountain lion. Numbers of tracks and other sign also would decrease, as would the potential of attacks on recreationists in mountain lion habitats.

Impacts on hunting prey species:

If prey populations were low, and mountain lions represented the main source of predation, such populations would be expected to increase. High prey populations would not be expected to show any dramatic increases. Local populations would be those most effected, while on a statewide level this alternative would have little effect on prey numbers.

ANALYSIS OF CONFLICTS

Human-lion conflicts:

Adoption of this alternative would reduce mountain lion densities, thereby lowering the probability of such conflicts. The alternative also provides for development of a conflict policy and the use of "no tolerance" or "low tolerance" zones around areas of human habitation. Educational efforts and programs would provide information to urban and urban-interface landowners to assist them in making wise land use decisions when building or living within mountain lion habitat. FWP activities associated with such conflicts also would decrease.

Livestock depredation:

Reduced mountain lion densities would lower the probability of depredation incidents. Legislation to permit landowners to use hound handlers to take depredating animals would be introduced. This could shorten the reaction time to depredation incidents and increase the probability of taking the animal responsible. While all such incidents would still have to be investigated, fewer incidents would result in fewer hours being expended by FWP for this type activity.

Enforcement efforts:

Enforcement efforts would be expected to decline as mountain lion densities declined.

ECONOMIC IMPACTS

Hunter numbers are expected to decline in some areas as a result of lower mountain lion densities and hunter success. The extent of this decline will be influenced by restrictions placed on hunting and chasing in other western states and the level of interest, both nationally and statewide, in mountain lion hunting.

Diminished numbers of hunters would have a greater impact on local economies in those areas currently receiving heavy hunting pressure. Most of these areas are located in northwestern Montana in FWP administrative regions one and two.

Funds collected from license sales by FWP would be expected to stabilize and could show a slight decline under this alternative. This would be influenced by restrictions in other western states and hunter interest in mountain lion hunting in Montana.

Additional funding authority would be requested from the Legislature to cover mountain lion research and monitoring needs. Funding needs would decline for activities associated with human-lion conflicts and stock depredation.

ALTERNATIVE 4 - REGIONAL MANAGEMENT BASED ON HABITAT CAPABILITIES.

IMPACTS ON HABITAT

Habitat and its capability to sustain viable populations of both mountain lions and prey would be closely examined under this alternative. Ecosystems within FWP regions would be analyzed and their estimated carrying capacity for mountain lions determined. Additionally, those factors that are affecting mountain lions and the prey base within habitats would be documented and utilized in determining harvest objectives.

Mountain lions would be considered in all FWP evaluations of development projects prepared by land management agencies, as well as in recommendations made on

proposals for roadless and wilderness areas. They also would be included in all evaluations of lands being considered under the Habitat Montana Program.

Legislation would be introduced to require that FWP's wildlife assessment of all new subdivisions be made available to prospective buyers in those subdivisions. FWP would develop programs to provide the public with information concerning: mountain lions habitat needs; the impacts of subdivisions on mountain lions; and enhancing public safety in mountain lion habitat, subdivisions and urban areas.

IMPACTS ON POPULATIONS

Impacts on mountain lion populations:

Alternative 4 would give broad management authority to FWP's administrative regions with a statewide goal of maintaining viable mountain lion populations. Each region would manage mountain lions based on: the capability of the habitat to support mountain lions and the prey base; prey density; other pressures influencing both mountain lion and prey populations; public safety and public tolerance for mountain lions. As a result, population parameters such as density, rates of expansion, age structure and reproductive status would vary across the state.

Populations within the western montane forest would not be expected to increase much beyond current densities, while those in the eastern mountains, prairie-foothills and prairie ecosystems could continue expanding, be stabilized or reduced, depending on regional management goals.

Regions would evaluate the possibilities of hunting on some WMAs as a means of decreasing predation on wintering big game or decreasing the numbers of transient young being supplied into adjacent populated areas. Such decisions would require evaluation of the disturbance to wintering ungulates and the management objectives of the WMA before such a hunt was held.

Harvest objectives would be based on the capabilities of habitats within the region to support both mountain lions and their prey and public safety concerns. Harvest objectives would be recommended by regional wildlife staff and subject to adoption by the Commission. The harvest could be regulated by either quotas or season length. Intensive management or low tolerance zones could be established around areas of human occupancy.

Statewide, the density of mountain lion populations would fluctuate over time depending on a variety of factors, including large-scale habitat alteration, drought, and the cyclic pattern of deer and elk populations. As prey populations declined, mountain lion populations would also decline. The length and extent of such declines would vary with a number of factors, including climatic conditions, habitat

improvement, competition from other predators and the availability of alternate prey species.

To increase the security of dependent young, regulations would be changed to prohibit the taking of a female with dependent young. No hunting of mountain lions would be recommended during the general big game season to protect lactating females and females with dependent young from incidental harvest.

Monitoring of population status and trends would require the establishment of trend routes within important habitats and hunting districts within regions. Statewide research goals would be established and prioritized by regional wildlife managers, with funding requested from the Legislature to carry out those projects. Population data would continue to be collected statewide from harvested mountain lions and participants in the hunt and chase seasons for use in regional evaluations of population management goals.

Impacts on prey populations:

Statewide impacts on prey populations would be negligible under this alternative. In local areas, populations may stabilize, increase, or decrease depending on regional mountain lion management strategies. The implementation of such strategies would be included as part of the season-setting process and would require the opportunity for public input before being adopted by the FWP Commission.

IMPACTS ON RECREATION:

Statewide regulation changes under this alternative would include reducing the reporting period for harvested animals from 48 to 24 hours and reducing the notification period for closing a season to 12 hours.

Regions would be given authority to anticipate when a quota would be reached and could begin closure of an area prior to quotas being reached. Hides and skulls would continue to be tagged and inspected in the region where an animal was harvested. However, the inspection period for the hide and skull would be reduced to 24 hours after a harvest is reported. Hides would be tagged at that time and the skull would be required to be turned in for aging and tagged within 5 days.

Harvest would be regulated by either the quota system or the length of the season. Season dates could be set outside current season dates to meet diverse regional management goals. They also would continue to be split into hunt and chase segments. Hunters could be required to choose what hunting district, area or region in which they wished to hunt, and limits could be placed on the numbers of hunters allowed in some areas.

Individuals 12 years of age and older hunting or pursuing mountain lions with dogs during the "hunt" would be required to possess a current mountain lion license and a free hound handler permit.

Owners and/or persons 12 years of age or older in control of dogs during the "chase" portion of the season would be required to possess the free hound handler permit.

Changes in regulations would require FWP to establish a statewide system for facilitating hunter compliance with these changes. Regional harvest and quota information would have to be readily available to hunters, as would the opportunities to report harvested animals and have animals inspected and tagged.

Impacts on other recreational activities:

Those recreational opportunities offered by the chase season would continue under this alternative. The opportunities to view or photograph mountain lions, observe tracks or other signs of the animals would vary with the density of populations across the state.

Impacts on the hunting of other prey species:

Statewide, this alternative would have little effect on the hunting of the mountain lion's prey species. In some local situations, prey populations may increase if mountain lion populations are having a dampening effect on them and the density of mountain lions declines. In those areas having both high numbers of prey and high numbers of mountain lions a decrease in the mountain lion population would not have a noticeable effect on prey density.

ANALYSIS OF CONFLICTS

Human-lion conflicts:

This alternative provides for development of a statewide conflict policy that would lay out guidelines for protection of the public and the responsible treatment of mountain lions involved in such situations. It also allows FWP regions to establish "no tolerance" or "low tolerance" zones for mountain lions in and around suburbs and populated urban and urban-interface areas.

Under this alternative legislation will be introduced to make FWP's wildlife evaluation of all new subdivisions available to prospective buyers. This would allow buyers time to take advantage of programs explaining ways to reduce the impacts on wildlife including mountain lions, and prevention of wildlife conflicts.

Livestock depredation:

FWP will continue to assist with depredation problems and will draft legislation that would allow stockowners to appoint hound handlers as their agents to take depredating mountain lions. Such incidents are expected to increase as mountain lions move into

previously unoccupied habitats in Montana. Levels of depredation are not expected to increase in currently occupied areas and may decline with declining domestic sheep numbers. The re-establishment of wolves in some areas may also reduce mountain lion depredation.

Enforcement efforts:

New regulations establishing shorter periods for notification of season closures and reporting and tagging periods will require an increase in enforcement effort. The requirement that all persons owning or controlling dogs in the hunt or chase season be licensed and/or hold a hound handler permit will also increase the need for additional enforcement patrols.

ECONOMIC IMPACTS

Expenditures by participants in the mountain lion seasons would be expected to increase with expansion of mountain lion populations, restrictions on hunting in other western states and a growing interest in mountain lion hunting by residents and nonresidents. Impacts will fluctuate regionally with variations in the density of mountain lions, hunting pressure and hunter success. Any regional or statewide restrictions put on hunter numbers will affect outfitters and guides as well as other businesses catering to the hunting and outdoor recreation industry.

Losses incurred by the livestock industry will fluctuate with mountain lion densities and may increase above the $12,875 loss incurred in 1993. Increased losses would result from both expansion of mountain lion populations and increased numbers of transient young from high density populations. Some of this increase may be offset by the current swing away from domestic sheep production to cattle by Montana's agricultural industry.

Funds generated from license sales and trophy fees are expected to increase with statewide expansion of occupied mountain lion habitat. Expenditures would increase under this alternative to fund research and monitoring programs, analyze harvest information, implement regulation changes and to react to increasing human confrontations and livestock depredation.

COMPARISON OF ALTERNATIVES

Alternative 1 (Management with no hunting by the general public)

Under this alternative, mountain lion populations would continue to expand and be regulated by natural mortality, prey density, inherent social tolerances within populations, habitat availability and human tolerances. Populations would be composed of older more productive animals. However, recruitment would be dependent on the availability of territories and improving or expanding suitable habitat.

Predation rates on prey populations would increase and could contribute to extending low density cycles of prey species. However, mountain lions by themselves would not significantly affect prey densities statewide, but could affect small, local populations.

The ability to track and monitor populations and collect biological information on populations would be significantly impeded under this alternative. It also could result in significant increases in the incidence of human-lion conflicts and livestock depredation.

Alternative 1 is not expected to impact air quality, water quality, soil or other geological features. Neither is this alternative expected to impact terrestrial or aquatic life, their habitats, or vegetative cover. Hunting would be significantly impacted (eliminated), and while nonhunting recreation might increase, the extent of that impact is not known. Elimination of mountain lion hunting and chasing would have an economic impact on outfitters and guides as well as local communities that cater to participants in the mountain lion season. Elimination of license and trophy fees would significantly impact the funding of statewide management programs.

ALTERNATIVE 2 (Continue current management)

Alternative 2 would provide for mountain lion habitat protection as a result of protecting deer and elk habitats, but would not specifically attempt to preserve areas important to mountain lions. In the short term this would have little impact on populations. However, long-term impacts of habitat fragmentation could significantly affect some populations and eventually eradicate them. No attempts would be made to determine the capabilities of Montana's different ecosystems to support mountain lions under Alternative 2.

Alternative 2 would allow for continued expansion of mountain lion populations but make no adjustments to deal with current problems concerning habitat loss, timing of hunting seasons, harvests exceeding quotas, human-lion conflicts or livestock depredation issues. All of these are issues that, if left unaddressed, could have serious impacts on the future of hunting and mountain lion management in Montana.

Alternative 2 would not be expected to impact air quality, water quality, soil or other geological features. Neither is this alternative expected to impact terrestrial and aquatic life, their habitats, or vegetation cover.

ALTERNATIVE 3 (Intensify harvest to decrease populations)

Alternative 3 allows the same protection of mountain lion habitats and evaluation of Montana's ecosystems to determine their capabilities of supporting mountain lions and the prey as Alternative 2. Alternative 3 would direct FWP public education programs at improving awareness of the mountain lion's habitat requirements, encouraging thoughtful land use planning and providing information on living and recreating in mountain lion

habitat. Alternative 3 also would require disclosure of the effects of new subdivisions on mountain lions and the prey species present to prospective subdivision homeowners.

Alternative 3 could result in significant reductions in mountain lion densities in some portions of the state. Such reductions would take place through institution of more liberal hunting seasons initiated for public safety, to reduce predation on prey species or to lower livestock depredation. Mountain lion populations would remain viable under Alternative 3, but would be maintained at significantly lower levels than currently present in Montana.

Alternative 3 would have significant long-terms effects on hunting opportunity, other recreation linked with mountain lion densities, and the economies of individual outfitters, guides and local communities that cater to mountain lion hunters. It also would result in a loss of funds available to FWP by reducing license and trophy fee revenues.

Alternative 3 is not expected to impact air quality, water quality, soil or other geological features. Neither is this alternative expected to impact terrestrial and aquatic life and their habitats, or vegetative cover.

ALTERNATIVE 4 (Regional management based on habitat capabilities)

Alternative 4 would entail evaluating Montana's ecosystems and their ability to sustain mountain lions and prey species by each FWP administrative region. Alternative 4 also provides for protection of mountain lion habitat and distribution of information that will provide the public with knowledge needed to make better land use decisions related to mountain lions and their ecological requirements. It would also require the development of a human/lion conflict policy and an evaluation of mountain lion hunting on WMAs.

Alternative 4 would give FWP regions the authority to recommend changes to the current season structure. This could result in significant changes (increases or decreases) in mountain lion densities and hunter numbers at the local level. Such changes could influence the numbers of human-lion conflicts and depredation situations, as well as result in economic impacts (positive or negative) on the local community.

Alternative 4 provides the flexibility in management to address changes in lion populations as they occur. Such changes would require approval of the FWP Commission.

Comparative Summary of Environmental Consequences

Impacts of Habitats

Alternative One	Alternative Two	Alternative Three	Alternative Four
No emphasis would be placed on the needs of mountain lions when evaluating Habitat Montana or public land projects.<br><br>Information on living with mountain lions and the effects of land use decisions on mountain lions would continue at the current level.	Mountain lion needs would be considered when evaluating Habitat Montana and public land projects.<br><br>Information on living with mountain lions and the effects of land use decisions on mountain lions would continue at the current level.	Mountain lion needs would be considered in the evaluation of Habitat Montana and public land projects.<br><br>Montana's habitats would be evaluated for their capability to support mountain lions and prey.<br><br>Information efforts on mountain lions and their requirements would be increased.<br><br>Legislation would be introduced to make FWP wildlife evaluation of subdivisions available to prospective buyers.	Mountain lion needs would be considered in the evaluation of Habitat Montana and public land projects.<br><br>Montana's habitats would be evaluated for their capability to support mountain lions and prey.<br><br>Information efforts on mountain lions and their requirements would be increased and programs developed on mountain lion habitat requirements, public safety and the effects of land management decisions.<br><br>Legislation would be introduced to make FWP evaluation of subdivisions available to prospective buyers.

Impacts on Mountain Lion Populations

ALTERNATIVE ONE	ALTERNATIVE TWO	ALTERNATIVE THREE	ALTERNATIVE FOUR
Expected to expand and be regulated by natural mortality, territoriality and prey density. Populations would have high reproduction but low recruitment.	Populations density would decrease, stabilize or expand depending on quotas.<br><br>Populations would continue to be monitored using harvest and questionnaire data.<br><br>Research would remain a low priority.<br><br>Expanding mountain lion populations would increase rates of predation and high densities could contribute to extending low density prey cycles.	Densities would decrease and stabilize.<br><br>Populations would be monitored using harvest data, track surveys, hunter questionnaire information and population monitoring studies.<br><br>Funding would be requested to meet research needs.	Density would vary with regional management objectives and habitat capabilities.<br><br>Populations would be monitored using harvest data, track surveys, hunter questionnaire information and population monitoring studies.<br><br>Funding would be requested to meet research needs.

Impacts on Prey Populations

ALTERNATIVE ONE	ALTERNATIVE TWO	ALTERNATIVE THREE	ALTERNATIVE FOUR
Mountain lions would exert a dampening effect on low density prey populations.	Expanding mountain lion populations would increase rates of predation and high densities could contribute to extending low density prey cycles.	Rates of predation on prey populations would decline.	Effects on prey populations would vary with habitat types, mountain lion densities and management objectives.

Impacts on Mountain Lion Hunting

Alternative One	Alternative Two	Alternative Three	Alternative Four
It would be illegal to hunt.	Continued under current season structure. Hunter conflicts would continue in areas of high hunting pressure.	Season would open with the general big game season and hunting on WMAs would be evaluated.<br><br>Harvest would be regulated with season lengths and quotas.<br><br>Hides and skulls would be tagged in region harvested.<br><br>Hound handlers would need a permit to pursue lions	Season dates could vary from current dates. Hunters could be required to validate for an area and numbers could be limited. Hunting on WMAs would be evaluated.<br><br>Harvest would be regulated with season lengths and quotas.<br><br>Hound handlers would need a permit to pursue lions.<br><br>Twenty-four hour reporting period, 24-hour inspection period, minimum of 12-hour season closure, regions could begin closure process before quota was reached, hides and skulls would be tagged in region harvested.

Impact on Other Recreational Opportunity

Alternative One	Alternative Two	Alternative Three	Alternative Four
Chase season would close, opportunity for viewing would increase with population density.	Chase opportunity would continue and opportunity for viewing would increase if population density increased.	Chase season would continue, viewing opportunities would decline with populations.	Chase season would continue, viewing would vary with population densities.

Impacts on Hunting Prey Species

Alternative One	Alternative Two	Alternative Three	Alternative Four
High densities of mountain lions could impact low density prey populations and would help hold such populations at low levels. May result in restrictive seasons on such prey populations.	High densities of mountain lions could impact low density prey populations and would help hold such populations at low levels. May result in restrictive seasons on such prey populations.	Local populations may increase as mountain lion densities decline.	Some local populations would respond inversely to increases or decreases in mountain lion densities.

Impacts on Human-lion Conflicts

Alternative One	Alternative Two	Alternative Three	Alternative Four
Expected to increase, guidelines and policy would be developed to manage such instances.<br><br>Amend MCA 76-3-608 requiring MFWPs wildlife evaluation be made available to prospective buyers in subdivisions.	Expected to increase, would be handled on a case-by-case basis.	Expected to decrease, guidelines and policy would be developed to manage such instances.<br><br>Amend MCA 76-3-608 requiring MFWPs wildlife evaluation be made available to prospective buyers in subdivisions.	Expected to increase, guidelines and policy would be developed to manage such instances.<br><br>Amend MCA 76-3-608 requiring MFWPs wildlife evaluation be made available to prospective buyers in subdivisions.

Impacts on Livestock Depredation

Alternative One	Alternative Two	Alternative Three	Alternative Four
Expected to increase, legislation would be introduced to allow stockowners to appoint hound handlers as their agents.	Expected to increase, would be handled on a case-by-case basis.	Expected to decrease, legislation would be introduced to allow stockowners to appoint hound handlers as their agents.	Expected to increase, legislation would be introduced to allow stockowners to appoint hound handlers as their agents.

Enforcement Activities

Alternative One	Alternative Two	Alternative Three	Alternative Four
Would require additional effort to enforce no hunting regulation and deal with conflicts and depredation.	Efforts would continue at current rate during seasons, additional time needed for conflicts and depredation as populations increased.	Reduced efforts needed as populations declined.	Additional effort required due to shorter closures and licensing requirements.

Economic impact

Alternative One	Alternative Two	Alternative Three	Alternative Four
$1.2 million in lost revenues plus guide/outfitter fees and licenses and trophy fees. Depredation costs would increase as would expense of responding to human/lion conflicts.	Positive impact as long as interest in lion hunting continued high.	Slight negative impact as mountain lion populations and numbers of hunters and hunter success declined.	Increased expenditures, license and trophy fees with expanding mountain lion populations. Some local fluctuations with increases and decreases in mountain lion densities statewide. Expanding human and mountain lion populations would result in increased numbers of conflicts and livestock losses which would increase costs for FWP.

Table 2. Mountain lion mortalities by FWP Region 1988-94[1]

Recorded Hunting Mortalities

REGION	1988-89	1989-90	1990-91	1991-92	1992-93	1993-94	1994-95	TOTAL
1	50	66	84	109	117	139	204	769
2	50	50	64	56	117	117	163	617
3	18	18	27	22	29	51	75	239
4	23	21	30	30	44	63	79	290
5	18	12	21	20	43	35	35	184
6	0	0	0	0	3	5	6	14
7	0	1	1	0	3	3	5	13
8					[15]	[12]	[37]	[52]
unk						1		1
TOTAL	159	168	227	237	356	414	566	2,127

RECORDED NONHUNTING MORTALITIES

REGION	1988-89	1989-90	1990-91	1991-92	1992-93	1993-94	1994-95	TOTAL
1	2	9	15	19	26	20	7	98
2	1	9	6	12	3	23	10	64
3	2	2	3	5	12	15	2	41
4	0	3	4	3	3	3	2	18
5	0	3	4	6	4	3	1	21
6	0	0	0	0	0	0	0	0
7	0	0	0	0	0	0	0	0
8					[3]		[3]	0
unk.							2	2
TOTAL	5	26	32	45	48	64	24	244
NATURAL[2]	0	2	5	6	5	2	2	22
HUM. CAUSED	5	24	27	39	43	62	22	222

[ ] Region 8 harvest from 1992-95 are integrated into Regions 3 and 4 to compare with administrative boundaries of previous years.

1 Years runs July 1 - June 30

2 Animals whose death was due to natural causes

Table 3. Lion Mortalities by mortality class, 1988-89 through 1992-93.

Mortality Class	No. of Lions*					
	1988-89	1989-90	1990-91	1991-92	1992-93	1993-94
Accidental Snare	2	3	1	2	4	2
Damage Control	0	4	7	7	8	11
Nuisance Control	0	1	6	4	9	4
Human Threat	0	2	2	0	1	0
Illegal	0	2	1	3	4	6
Dogs	2	0	0	0	1	1
Road Kill	1	4	2	10	7	16
Self Defense	0	5	5	4	7	4
Train	0	1	1	0	0	1
Natural	0	2	5	6	5	2
Unknown	0	1	1	8	2	15
Wounding	0	0	0	1	0	0
Trapping	0	0	0	0	0	2
Subtotal	5	25	31	45	48	64
Hunting						
Archery	30	38	44	49	66	74
Handgun	69	71	94	84	145	151
Rifle	60	57	87	100	142	185
Unknown	0	2	3	4	3	3
Subtotal	159	168	228	237	356	413
TOTAL	164	193	259	282	404	477

*This is an updated version of results for these four years. Some changes have been made in data from previous years.

Table 4. Estimated value of livestock lost to mountain lions in Montana 1984-93.

Year	Incidents	Lions Killed	Sheep	Cattle	Horses	Goats	Poultry	Estimated Dollar Value
1984	8.0	3.0	58.0		2.0		1.0	$4,170.00
1985	7.0	2.0	35.0		1.0			2,240.00
1986	6.0	1.0	66.0					4,379.00
1987	10.0	2.0	88.0					6,106.00
1988	8.0	2.0	31.0					2,200.00
1989	5.0	2.0	21.0					1,152.00
1990	9.0	3.0	15.0	4.0				3,010.00
1991	5.0	3.0	11.0					640.00
1992	3.0	3.0	24.0					1,482.00
1993	21.0	8.0	143.0	2.0		12.0		12,875.00
TOTAL	82.0	29.0	492.0	6.0	3.0	12.0	1.0	38,254.00
Ave.	8.2	2.9	49.2	.6	.3	1.2	.1	3825.40

Table 5. Trichinella test results from Montana mountain lions, 1971-1989.

Sex	Negative		Positive		Total	
	Number	Percent	Number	Percent	Number	Percent
Female	192	49.2	198	50.8	390	43.4
Male	218	42.8	291	57.2	509	56.6
**Total**	410	45.6	489	54.5	899	100

Table 6. Trichinella test results by FWP region 1971-1989 in order of declining prevalence.

Region	Negative		Positive	
	Number	Percent	Number	Percent
1	113	29.7	268	70.3
2	112	43.2	147	56.8
3	48	52.2	44	47.8
4	77	81.9	17	18.1
5	51	85.0	9	15.0

Table 7. Resident, nonresident and total mountain lion hunting license sales in Montana.

Year	Resident	Nonresident	Total	No. of Hunters Afield	% License Holders Not Hunting
1971	418	16	434		
1972	516	68	584		
1973	243	71	314		
1974	259	93	352		
1975	288	118	406		
1976	517	70	587		
1977	574	102	676		
1978	642	123	765		
1979	614	111	725		
1980	787	61	848		
1981	894	69	963		
1982	1027	91	1118		
1983	1021	132	1153		
1984	1045	92	1137		
1985	914	92	1006		
1986	916	92	1008		
1987	1237	108	1345		
1988	1210	109	1319	672	49
1989	1250	98	1348	768	43
1990	1708	136	1844	884	52
1991	1687	146	1833	843	54
1992	2038	177	2215	1211	45
1993	2535	230	2765	1353	51
1994	2984	258	3242	1794	55

95
96

Table 8. Mountain lion hunting season dates and fee structures in Montana, 1971 - 1994.

Season Structure	Season Dates	Resident License Fees	Nonresident License Fees	Trophy Fee**
One mountain lion/person	10/17/71 - 4/30/72	Free		
One mountain lion/person	10/22/72 - 4/30/73			
One mountain lion/person	10/21/73 - 4/30/74	$ 5.00	$ 25.00	
One mountain lion/person	*10/20/74 - 4/30/75	5.00	25.00	
One mountain lion/person	*10/19/75 - 4/30/76	5.00	25.00	
One mountain lion/person	*12/1/76 - 4/30/77	5.00	25.00	
One mountain lion/person	*12/1/77 - 4/30/78	5.00	25.00	
One mountain lion/person	*12/1/78 - 4/30/79	5.00	25.00	
One mountain lion/person	*12/1/79 - 2/15/80	5.00	25.00	
One mountain lion/person	*12/1/80 - 2/15/81	5.00	100.00	
One mountain lion/person	*12/1/81 - 2/15/82	5.00	100.00	
One mountain lion/person	*12/1/82 - 2/15/83	5.00	100.00	
One mountain lion/person	*12/1/83 - 2/15/84	5.00	100.00	
One mountain lion/person	*12/1/84 - 2/15/85	10.00	300.00	$ 50.00
One mountain lion/person	*12/2/85 - 2/15/86	10.00	300.00	50.00
One mountain lion/person; female & total quotas in Rg. 1 & 3	*12/1/86 - 2/15/87	10.00	300.00	50.00
One mountain lion/person; female & total quotas in Rg. 1 & 3	*12/1/87 - 2/15/88	10.00	300.00	50.00

One mountain lion/person; female & total quotas/hunting unit statewide	*12/1/88 - 2/15/89	10.00	320.00	50.00
One mountain lion/person; female & total quotas/hunting unit statewide	*12/1/89 - 2/15/90	10.00	320.00	50.00
One mountain lion/person; female & total quotas/hunting unit statewide	*12/1/90 - 2/15/91	10.00	320.00	50.00
One mountain lion/person; female & total quotas/hunting unit statewide	*12/1/91 - 2/15/92	10.00	320.00	50.00
One mountain lion/person; female & total quotas/hunting unit statewide	*12/1/92 - 2/15/93	13.00	320.00	50.00
One mountain lion/person; female & total quotas/hunting unit statewide	*12/1/93 - 2/15/94	10.00	320.00	50.00
One mountain lion/person; female, male & total quotas/hunting unit statewide	*12/1/94 - 2/15/95	15.00	320.00	50.00

* Some wilderness hunting districts open September 15 of each year.
**Trophy fee charged only when mountain lion was killed.

Table 9. A comparison of mountain lion hunting season dates, hunting season length, hunting license fees, hunter numbers and harvest during the 1992-93 or 1993-94 lion seasons in the western states and provinces.

State or province	Hunting Season Dates	Season Length (days)	License Fees ($)		Hunter Numbers		Harvest			Special Regulations[A]
			Res	N.Res	Res	N.Res	M	F	Total	
Arizona	7/1-6/30	365	11.00	150.00	2960	78	105	79	184	1;2
Alberta	12/1-2/28	90	41.00	221.00	132	10	41	21	62	
British Columbia										
Colorado	1/1-8/26									
	11/14-12/31	285	30.25	250.25						
Idaho	9/15-2/28	108-198	26.00	226.00			215	97	312	
Montana	9/15-2/15*	75-150	15.00	320.00	2535	230	274	134	415	3
Nevada	10/1-4/30	120	50.00	150.00	348	149	75	75	150	1;2
New Mexico	12/1-3/31	121	10.50	201.00	675	151	38	18	18	
Oregon	8/26-3/31	77-139	51.00	226.00	556	17	111	76	187	
Texas		365	10.00	200.00					134	
Utah	1/1-6/30	182	28.00	253.00	431	160	260	112	372	2
Washington	12/15-7/31	228	20.00	300.00						
Wyoming	11/22-1/15									
	9/1-3/31	210								

[A] Special Regulations
[1] Limited entry permits issued by area
[2] Season length varies by area
[3] Requires $50 Trophy fee
*Two areas open September 15. Fifty-seven areas open December 1.

Table 10. Recorded nonhunting mountain lion kills, FWP regions one - five 1971-1989. Years are from July 1 until June 30.

Year	One				Two				Three				Four				Five				State Total			
	M	F	Unk	Tot	M	F	Unk	Tot	M	F	Unk	Tot	M	F	Unk	Tot	M	F	Unk	Tot	M	F	Unk	Tot
1971	1	1	0	2	0	0	0	0	0	0	0	0	0	0	0	0	0	0	0	0	4	4	0	8
1972	0	1	1	2	0	1	1	2	0	0	1	1	0	0	0	0	0	2	1	3	0	4	4	8
1973	1	0	3	4	0	1	2	3	0	1	0	1	0	0	0	0	0	1	0	1	1	4	4	9
1974	0	0	1	1	1	0	0	1	0	0	2	2	0	0	0	0	0	0	0	0	1	0	3	4
1975	0	2	3	5	0	0	1	1	0	0	0	0	0	0	0	0	0	0	0	0	1	4	6	11
1976	1	3	1	5	0	0	1	1	0	1	0	1	1	3	0	4	0	1	1	2	2	8	3	13
1977	0	1	1	2	0	0	0	0	0	0	0	0	0	0	0	0	1	0	1	2	1	1	2	4
1978	0	5	0	5	0	0	1	1	0	2	0	2	0	0	2	2	0	2	0	2	0	10	4	14
1979	0	0	0	0	0	2	0	2	0	2	0	2	1	0	2	3	0	0	0	0	3	4	2	9
1980	0	0	0	0	1	0	0	1	0	2	0	2	0	1	2	3	0	0	0	0	1	3	2	6
1981	0	0	1	1	1	4	0	5	0	1	1	2	0	0	0	0	0	2	0	2	1	7	2	10
1982	0	0	0	0	1	0	0	1	0	0	0	0	0	0	0	0	0	0	0	0	1	0	0	1
1983	0	0	0	0	0	1	1	2	2	0	1	3	0	1	1	2	0	0	0	0	2	3	3	8
1984	0	0	0	0	0	1	0	1	0	2	1	3	0	0	0	0	0	0	2	2	0	4	3	7
1985	1	0	0	1	1	0	0	1	0	2	0	2	0	0	0	0	1	0	0	1	4	4	0	8
1986	2	0	0	2	0	0	0	0	2	1	2	5	0	1	0	1	0	1	1	2	12	4	3	19
1987	4	0	0	4	1	1	1	3	0	1	2	3	0	2	0	2	2	0	1	3	9	5	6	20
1988	0	1	1	2	0	1	0	1	0	1	1	2	0	0	0	0	0	0	0	0	0	3	2	5
1989	1	4	5	10	3	3	2	8	0	2	0	2	0	0	0	0	0	0	0	0	4	10	12	26

mltable.8

Table 11. A Summary, by MFWP Region, of the recorded Montana mountain lion harvest, male, female, unknown and total, 1971-1994.

Year	One				Two				Three				Four				Five				Seven				Unknown Region				State Total			
	Un	M	F	Tot	Un	M	F	Tot	Un	M	F	Tot	Un	M	F	Tot	Un	M	F	Tot	Un	M	F	Tot	Un	M	F	Tot	Un	M	F	Tot
1971		11	10	21		8	10	18		2	1	3		3	3	6		0	2	2						1	0	1		25	26	51
1972		13	9	22		10	10	20		2	2	4		4	2	6		1	1	2										30	24	54
1973		19	4	23	2	26	11	39		0	1	0		5	1	6		1	2	3									2	51	19	72
1974		23	23	46		19	16	35	1	2	2	5		4	2	6		0	0	0									1	48	43	92
1975		27	27	44		13	8	21		2	2	4		4	2	6		2	1	3										46	30	76
1976		20	18	38	1	12	7	20		0	2	2		5	1	6		1	3	4									1	42	31	72
1977		21	21	42		14	5	19		8	1	9		6	4	10		4	4	8										53	35	88
1978		14	12	26		16	8	24		6	7	13	1	2	2	5		0	3	3									1	38	32	71
1979		21	8	29		16	8	24		5	9	14		3	2	5		6	5	11										54	32	83
1980		6	9	15		14	6	20		6	1	7		7	5	12		4	4	8										37	25	62
1981		25	20	45		21	9	30		10	6	16		7	7	14		6	3	9										69	45	114
1982	1	26	18	45		17	13	30		11	7	18		5	4	9		2	3	5									1	61	45	107
1983		31	27	58	1	22	13	36	1	12	4	17		10	1	11		7	4	11						3	0	3	2	85	49	136
1984	1	29	13	43	1	34	14	49		21	5	26	1	18	7	26		12	2	14					1	4	2	7	4	118	43	165
1985	1	30	17	48		13	13	26	2	11	10	23	3	14	10	27		6	3	9						7	3	10	6	81	56	143
1986		32	16	48	1	22	9	32	1	13	4	18	1	7	4	12		11	4	15					1	8	7	15	4	93	44	141
1987		25	22	47	1	56	4	62		15	5	20		16	10	26		6	9	15					1	0	0	1	3	118	50	171
1988		33	17	50	1	33	16	50		17	1	18		17	6	23		11	7	18									1	111	47	159
1989		46	22	68		37	13	50		16	2	18		15	6	21		8	4	12		1	0	1						123	47	170
1990		55	29	84		45	19	64		22	5	27		20	10	30		13	8	21		0	1	1						155	72	227
1991		71	37	108	1	37	18	56		13	8	21		17	11	28		13	7	20					0	2	1	3	1	153	82	236
1992		68	50	118		87	30	117		19	9	28		30	15	45		21	10	31		3	0	3						239	118	357
1993		86	50	140		81	35	116		41	18	59	1	45	20	66		20	15	35		1	1	2		1	0	0	1	277	146	424
1994	1	119	85	204		101	62	163		48	26	74		51	28	79		20	15	35		4	2	6		5	0	5		348	218	566
TOTAL	3	851	554	1412	10	754	357	1121	5	302	138	445	7	315	163	485	0	175	119	294	0	9	4	13	3	31	13	46	28	2450	1359	3837

mltable.12

Table 12. Number of mountain lions killed by hunters that have killed at least one mountain lion, 1971 - 1990.

	Number of Lions Killed									
	1	2	3	4	5	6	7	8	10	16
No. of Hunters	1419	168	60	28	12	6	4	2	3	1

Table 13. Percentage of mountain lions taken by month statewide.

Year	Aug.	Sept.	Oct.	Nov.	Dec.	Jan.	Feb.	Mar.	Apr.
71-72			2	10	30	34	14	8	2
72-73	2		7	9	13	29	24	11	5
73-74				8	14	26	28	21	3
74-75		1	3	10	15	36	23	11	1
75-76			3	4	22	36	21	14	
76-77					17	39	13	23	6
77-78					33	27	26	12	2
78-79					17	42	21	16	4
79-80					20	49	31		
80-81					20	27	53		
81-82					34	43	23		
82-83					54	29	17		
83-84					54	39	6		
84-85		1		2	41	36	21		
85-86					31	47	22		
86-87					41	53	6		
87-88			.5	.5	51	36	12		
88-89				1	67	23	9		
89-90					79	16	5		
90-91				1	74	24	1		
91-92					73	26	1		
92-93	1		2	2	73	15	2	1	
93-94	1	3	1	2	72	16	3		
94-95				1	84	12	3		

Table 14. Statewide outfitter harvest, 1990 - 1994.

Year	Total Harvest			Outfitter Harvest			Percent of Total		
	Male	Fem.	Total	Male	Fem.	Total	Male	Fem.	Total
1990	155	72	227	40	21	61	0.26	0.29	0.27
1991	153	82	235	36	29	65	0.24	0.35	0.28
1992	239	118	357	58	35	93	0.24	0.30	0.26
1993	277	146	423	75	42	117	0.27	0.29	0.28
1994	362	204	566	92	52	144	0.25	0.25	0.25

Table 15. Chase information gathered for Montana[1] from hunting season participants 1989-1993.

Year	Participants	Total Chases	Total Chase Hours	Chases/ Hunter	Hours/ Chase	Lions Chased	Average Lions/ Chase	Chases Abandon	Chases where Treed	Lions Treed			
										♂	♀	Young	Unknown
1989	595	2085	6963	3.5	3.3	2652	1.3	814	1259	628	567	386	68
1990	691	2177	7023	3.2	3.2	2804	1.3	711	1403	654	720	503	78
1991	804	2380	7458	3.0	3.1	2925	1.2	962	1279	564	684	448	67
1992	919	3134	10545	3.4	3.4	4023	1.3	1017	2096	1002	931	708	123
1993	1134	3897	13040	3.4	3.3	4461	1.1	1371	2246	1065	1132	725	268
1994	1511	5064	16839	3.4	3.3	5819	1.1	1600	3186	1450	1582	857	286

[1] This information is collected by hunting district and compiled for each region and the state.

Table 16. Chase information gathered for Montana[1] from chase season participants 1989-1993.

Year	Participants	Total Chases	Total Chase Hours	Chases/ Hunter	Hours/ Chase	Lions Chased	Average Lions/ Chase	Chases Abandon	Chases where Treed	Lions Treed			
										♂	♀	Young	Unknown
1989	248	752	2165	3.0	2.9	1003	1.3	237	511	230	255	219	16
1990	327	927	2638	2.8	2.8	1254	1.4	239	680	274	397	293	63
1991	332	870	2855	2.6	3.3	1042	1.2	285	491	211	280	192	29
1992	411	1334	4295	3.2	3.2	1913	1.4	380	937	441	431	496	90
1993	382	1271	4356	3.3	3.4	1575	1.2	341	892	449	430	301	116
1994	467	1703	5772	3.6	3.4	2323	1.4	477	1217	615	687	508	138

[1] This information is collected by hunting district and compiled for each region and the state.

Table 17. Lion hide and skull sale prices for 1990-1994.

Year	N	Mean	Range
1990	9	$213.88	$65-325
1991	20	$164.00	$40-500
1992	27	$175.20	$55-400
1993	25	$174.00	$85-325
1994	58	$133.70	$45-275
All Years	139	$158.56	$40-500

Table 18. Table 18 is on page 59.

Table 19. Categories of impacts analyzed by each alternative in the DEIS

CATEGORY	IMPACT ANALYZED
1) Impacts on Habitat	a) impacts on mountain lion habitat
2) Impacts on Populations	a) impacts on mountain lion populations<br>b) impacts on prey populations
3) Impacts on Recreation	a) impacts on mountain lion hunting<br>b) impacts on other recreational opportunities<br>c) impacts on the hunting of prey species
4) Analysis of Conflicts	a) human-lion conflicts<br>b) livestock depredation<br>c) enforcement efforts
5) Economic Impacts	

LITERATURE CITED

Ackerman, B.B., F.G. Lindzey, and T.P. Hemker. 1982. Predictive energetics model for cougars. pp 333-352 in S.D. Miller and D.D. Everett, eds. Proc. Int. Cat. Symp., Kingsville, TX

____, ____, ____,. 1984. Cougar food habits in southern Utah. J. Wildl. Manage. 48:147-155.

Anderson, A.E. 1983. A critical review of literature on puma (Felis concolor). Colorado Division Wildlife Spec. Rep. 54. 91pp.

Ashman, D.L., G.L. Christensen, M.L. Hess, G.K. Tsukamoto, and M.S. Wickersham. 1983. The mountain lion in Nevada. P-R Project W-48-15, Nevada Dep. Wildl. 75pp.

Aune, K., and P. Schladweiler. 1992. Wildlife laboratory report (1989-1990). Job Prog. Rep. W-120-R-15 MT Fish, Wildlife and Parks, Helena. 36pp.

____, and ____. 1993. Wildlife laboratory report (C1989-1992). Job Prog. Rep. W-120-R-15. MT Fish, Wildlife and Parks, Helena. 68 pp.

____. 1991. Increasing mountain lion populations and human-lion interactions in Montana. in R. Tully ed. Proc. Mountain lion-human interaction symposium. Colo. Div. Wildl., Denver.

Barnes, C.T. 1960. The cougar or mountain lion. The Ralton Co., Salt Lake City, UT. 176 pp. Barnhurst, D. and F. Lindzey. 1989. Detecting female mountain lions with kittens. Northwest Sci 1:35-37.

Beecham, J. Pers. comm. March 1995. Idaho Fish and Game Dept. Boise, ID.

Beier, P. 1991a. Cougars, corridors, and conservation. in R. Tully, ed. Proc. Mountain lion -human interaction symp., Colo. Div. Wildl., Denver.

_______. 1991b. Cougar attacks on humans in the United States and Canada. in R. Tully ed. Proc. Mountain lion - human interaction symposium. Colo. Div. Wildl., Denver.

_______ 1991c. Cougar attacks on humans in the United States and Canada. Wildl Soc. Bull. 19:403-412.

Belden, R.C., B.W. Hagedorn, W.B. Frankenberger, 1993. Responses of translocated mountain lions to human disturbance. in R. Tully, ed. Proc. Mountain lion - human interactions symposium. Colorado Div. Wildl., Denver.

Berger, J. and J.D. Wehausen. 1991. Consequences of a mammalian predator-prey disequilibrium in the Great Basin Desert. Cons. Biol. 2:244-248.

Botkin, D.B. 1990. Discordant harmonies: a new ecology for the twenty-first century. Oxford University Press. N.Y. 241 pp.

Boyd, D.K. and G.K. Neale. 1992. An adult cougar Felis concolor killed by grey wolves, Canis lupus, in Glacier National Park, Montana. Can. Field Nat. 106 (4):524-525.

Currier, M.M.P., S.L. Sheriff and K.R. Russell. 1977. Mountain lion population near Canon City, CO, 1974-1977. Colo. Div. Wildl. Spec. Rep. 42. 12pp.

Dies, K.H. and J.R. Gunson. 1984. Prevalence and distribution of Trichinella sp. in cougars, Felis concolor L., and grizzly bears, Ursus arctos L., in Alberta. J. Wildl. Dis. 20:242-244.

Dusek, G. and J. Morgan. 1990. Population ecology of white-tailed deer in northwestern Montana. Job Prog. Rep., MT Fish, Wildlife and Parks, Helena. Fed Aid Job W-100-R-3. 29pp.

Eaton, R.L. 1973. The status, management and conservation of the cougar in the United States. World's Cats 1:68-89.

____, and K.A. Velander. 1977. Reproduction in the puma:biology, behavior, and ontogeny. World's Cats 3:45-70.

Errington, P.L. 1967. Of predation and life. Iowa Univ. Press. Ames. 277pp.

Evans, W. 1983. The cougar in New Mexico: biology, status, depredation of livestock, and management recommendations. New Mexico Dep. Game and Fish. 40pp.

Fitzhugh, E. L. and K. S. Smallwood. 1989. Techniques for monitoring mountain lion population levels in R. Smith ed. Proc. 3rd Mtn. Lion Workshop, Prescott, AZ.

Gashwiler, J.S., and W.L. Robinette. 1957. Accidental fatalities of the Utah cougar. J. Mammal. 38:123-126.

Gonyea, W.J. 1976. Adaptive differences in the body proportions of large felids. Acta Anat. 96:81-96.

Greer, K.G. 1976. Mountain lion studies (1975-1976). W-120-R-8 Study L-1.1, Job 3. MT Fish and Game Dep., Helena. 16pp.

____. 1984. Mountain lion studies (1983-84). MT Dept. Fish and Game Job Prog. Rept., Proj. W-120-R-8, No. L-1.

Griffith, B., J.M. Scott, J.W. Carpenter, C. Reed. 1989. Translocation as a species conservation tool:status and strategy. Science 245: 477-480.

Grinnell, J., J.S. Dixon, and J.M. Linsdale. 1937. Furbearing mammals of California. Univ. Calif. Press, Berkeley, 2 vols. 777pp.

Halfpenny, J.C., M.R. Sanders, K. Green, and K. Coughlon. 1991. Age and sex profiles for lions involved in human-lion encounters, Boulder County, CO. in R. Tully ed. Proc. Mountain lion - human interaction symposium. Colo. Div. Wildl., Denver.

Hall, E.R. 1981. The mammals of North America. 2nd ed. John Wiley and Sons, New York. 2 vols. 1081pp.

Harris, C.F. 1991. Draft Mountain lion management plan, 1991-95. Idaho Dept. Fish and Game, Boise. 32pp.

Harlow, H.J., F.G. Lindzey, W.D. VanSickle, and W.A.Gern. 1992. Stress response of cougars to a nonlethal pursuit by hunters. Can J. Zool.; 70(1):136-139.

Hemker, T.P., F.G. Lindzey, B.B. Ackerman, and A.J. Button. 1982. Survival of cougar cubs in a non-hunted population. pp 327-332 in S.D. Miller and D.D. Everett, eds. Proc. Int. Cat Symp. Kingsville, Tx.

____, ____, and ____. 1984. Population characteristics and movement patterns of cougars in southern Utah. J. Wildl. Manage. 48:1275-1284.

Hopkins, R. A. and R. W. Barrett 1991. Population characteristics of the mountain lions in the Diablo Range, Ca. Proc. Mountain lion human interaction symposium. Colo. Div. of Wildlife, Denver.

Hornocker, M.G. 1969. Winter territoriality in mountain lions. J. Wildl. Manage. 33:457-464.

____. 1970a. An analysis of mountain lion predation upon mule deer and elk in the Idaho Primitive Area. Wildl. Monogr. 21. 39pp.

____. 1970b. The American lion. Natural Hist. 79(9)40-49, 68-71.

____. 1971. Suggestions for the management of mountain lions as trophy species in the Intermountain region. Annu. Proc. Western Assoc. State Game and Fish Commissioners 51:399-402.

Joslin, G. and G. Brown. 1978. Capture & reintroduction of a mountain lion kitten. Unpub. rpt. MT Fish, Wildlife & Parks, Helena. 7pp.

Lang, S.P. and F.G. Lindzey, 1993. Patterns of replacement of resident cougars in southern Utah. J. Mamm. 74(4):1056-1058.

Laundre', J.W., M. Sellers, T. Clark, and D.P. Streubel. 1991. Behavior, ecology and conservation of mountain lions in fragmented habitat, a progress report. Idaho State University, Pocatello. 46pp.

Lay, J. 1976. Comments. pp 162-163 in G. C. Christensen and R. J. Fischer, chairmen, Trans. Mountain Lion Workshop. U.S. Fish Wildl. Serv., Portland, OR

Lemke, T.O. 1975. Movement and seasonal ranges of the Burdette Creek elk herd, and an investigation of sport hunting. M.S. Thesis., Univ. Mont., Missoula. 127pp.

Lindzey, F.G. 1987. Mountain Lion. pp 657-668 in Novak, M., J.A. Baker, M.E. Obbard and B. Malloch eds. Wild furbearer management and conservation in North America. Ontario Trappers Assoc., Toronto. 1150pp.

____, W.D. Van Sickle, B.B. Ackerman, D. Barnhurst, T.P. Hemker, S.P. Laing, 1994. Cougar population dynamics in southern Utah. J. Wildl. Manage 58(4):619-624.

____, B.B. Ackerman, D. Barnhurst, and T.P. Hemker. 1988. Survival of mountain lions in southern Utah. J.Wildl. Manage. 52:664-667.

____, W.D. Van Sickle, S.P. Laing and C. Mecham. 1992. Cougar population response to manipulation in southern Utah. Wildl. Soc. Bull. 20:224-227.

Logan, K.A., L.L. Irwin, and R. Skinner. 1986. Characteristics of a hunted mountain lion population in Wyoming. J. Wildl. Manage. 50:648-654.

____, and ____. 1985. Mountain lion habitats in the Big Horn Mountains, Wyoming, Wildl. Soc. Bull. 13:247-262.

McBride, R.T. 1976. The status and ecology of the mountain lion Felis concolor stanleyana of the Texas-Mexico border. M.S. Thesis, Sul Ross State Univ. Alpine, Texas. 160pp.

Mitchell, J.L. and K.R. Greer. 1971. Predators. pp 207-215 in T.W.Mussehl and F.W.Howell, eds. Game management in Montana. MT Fish and Game Dep., Helena. Fed Aid Proj. W-3-C. 238pp.

Molini, W. 1976. Comment pp 163-165 in G. C. Christensen and R. J. Fischer, Chairman, Trans. Mountain lion workshop. U. S. Fish & Wildl. Serv., Portland, OR

MT. Fish, Wildlife & Parks. 1990. Mountain lion hunting harvest and chase report. Technical Services Bureau, Bozeman.

____. 1991. Mountain lion hunting harvest and chase report. Technical Services Bureau, Bozeman.

____. 1992. Mountain lion hunting harvest and chase report. Technical Services Bureau, Bozeman.

____. 1993. Mountain lion hunting harvest and chase report. Technical Services Bureau, Bozeman.

____. 1993a. Montana bioeconomic study, A contingent valuation assessment of black bear hunting. 23 pp. Helena.

____. 1994. Unpublished mountain lion economic survey. 1994.

Murphy, K.A., G.S. Felzien, and M.G. Hornocker. 1991. Ecology of the mountain lion (Felis concolor missoulensis) in the northern Yellowstone ecosystem. Cumulative Prog. Rept No. 4. Wildlife Research Institute, Moscow, Idaho.

____. 1983. Characteristics of a hunted population of mountain lions in western Montana. M.S. Thesis, Univ. of Mt., Missoula. 48pp.

Neal, D.L., G. N.Steger, and R.C. Bertram. 1987. Mountain lions:preliminary findings on home range use and density in the central Sierra Nevada. Res. Note PSW - 392. Berkley, CA: Pacific S.W. Fur. Ser. U.S.D.A. 6pp.

Nero, R.W. and R.E. Wrigley. 1977. Status and habits of cougar in Manitoba. Can. Field Nat.91(1):28-40.

Pac, D., R.J. Mackie, and H.E. Jorgenson. 1991. Mule deer population organization, behavior and dynamics in a northern Rocky Mountain environment. MT Fish, Wild. and Parks Final Rept. Fed Aid Proj. W-120-R-7-18. 316pp.

Pall, O., M. Jalkotzy and I. Ross. 1988. The cougar in Alberta. Alberta For., Lands and Wildl. Calgary. 145pp.

Rasmussen, D.I. 1941. Biotic communities of the Kaibab Plateau, Arizona. Ecol. Monogr. 3:229-275.

Robinette, W.L., J.S. Gashwiler, and O.W. Morris. 1959. Food habits of the cougar in Utah and Nevada. J. Wildl. Manage. 23:261-273.

____, ____, and ____. 1961. Notes on cougar productivity and life history. J. Mammal. 42:204-217.

____, N.V. Hancock, and D.A. Jones. 1977. The Oak Creek mule deer herd in Utah. Utah Div. Wildl. Resour. Publ. 77915. 148pp.

Ross, I.P. and M.G. Jalkotzy. 1992. Characteristics of a hunted population of cougars in southwestern Alberta. J. Wildl. Manage. 56:417-426.

Ruth, T. K., L. A. Logan, L. L. Sweaner, J. F. Smith, L. J. Temple. 1993. Evaluating mountain lion translocation. Final report. USFWS grant agreement No. 14-16-0009-91-1216.

Seidensticker, J.C. IV., M.G. Hornocker, W.V. Wiles, and J. P. Messick. 1973. Mountain lion social organization in the Idaho Primitive Area. Wildl. Monogr. 35:1-60.

Shaw, H.G. 1977. Impact of mountain lions on mule deer and cattle in northwestern Arizona. pp 17-32 in R.L. Phillips and C. Jonkel, eds. Proc. 1975 Predator Symp. Univ. of Montana, Missoula. 268pp.

____. 1979. A mountain lion field guide. Arizona Game and Fish Dept. Spec. Rep. 9. 27pp.

____. 1980. Ecology of the mountain lion in Arizona. Final Report, P-R Proj. W-78-R, Work Plan 2, Job 13. Arizona Game and Fish Dep. 14pp.

____. 1989. Soul among lions: The cougar as peaceful adversary. Johnson Publ. Co, Boulder, Co. 140pp.

Simpson, G.G. 1945. The principles of classification and a classification of mammals. Bull. Am. Nat. Hist. 85:1-350.

Sitton, L.W. 1977. California mountain lion investigations with recommendations for management. Final Rep. to the State Legislature, P-R Project W-51-R, Sacramento: Calif. Fish and Game Dep. 35pp.

Smallwood, S.K. and E.L. Fitzhugh. 1991. Use of track counts for mountain lion population census. in R.Tully ed. Proc. Mountain lion-human interaction symposium. Colo. Div. Wildl., Denver.

Spalding, D.J. and J. Lesowski. 1971. Winter food of the cougar in south-central British Columbia. J. Wildl. Manage. 35:378-381.

Stringham, S. 1983. Role of adult males in grizzly bear population biology. Int. Conf. Bear Res. and manage. 5:140-151.

Tanner, J.T. 1975. The stability and the intrinsic growth rates of prey and predator populations. Ecology 56:855-867.

Tinbergen, N. 1957. The functions of territory. Bird Study 4:14-27.

VanDyke, F.G. 1983. A western study of cougar track surveys and environmental disturbances affecting cougars related to the status of the eastern cougar (Felis concolor cougar). Ph.D. Thesis, New York State Univ., Syracuse. 244pp.

____, R. H. Brocke, H. G. Shaw . 1986, Use of roadtrack counts as indicies of mountain lion presence, J. Wildl. Manage 50 (1):102-109.

Van Sickle, W.D. and F.G. Lindzey. 1991. Evaluation of a cougar population estimator based on probability sampling. J. Wildl. Manage. 55:738-743

Vaughan, T.A. 1978. Mammalogy. W. B. Saunders Co., Philadelphia. 522 pp.

Walters, C. 1986. Adaptive management of renewable resources. Macmillan Publ.Co., N.Y. 374 pp.

Weaver, R.A., and L.W. Sitton. 1978. Changing status of mountain lions in California and livestock depredation problems. Proc. Vertebr. Pest. Conf. 8:214-219.

White, P.A., and D.K. Boyd. 1989. A cougar, Felis concolor, kitten killed and eaten by gray wolves, Canis lupis in Glacier National Park, MT. Can. Field Nat. 103(3):408-409.

Williams, J. S. 1992. Ecology of mountain lions in the Sun River area of northern Montana. Ms Thesis. Montana State University, Bozeman. 109pp.

Young, S. P. 1946. History, life habits, economic studies and control, Part 1. pp 1- 173 in S. P. Young & E. A. Goldman. The puma, mysterious American Cat. The American Wildl. Inst., Washington, DC 258 pp.

lion.lit

Appendix 1. MCA Statutes regulating the hunting of mountain lions, use of dogs, stockowner rights and wildlife assessments for subdivisions.

Part 1
General Provisions

87-2-101. Definitions. As used in this chapter, chapter 3, and 87-1-102, unless the context clearly indicates otherwise, the following definitions apply:

(1) "Angling" or "fishing" means to take or the act of a person possessing any instrument, article, or substance for the purpose of taking fish in any location that a fish might inhabit.

(2) "Bait" means any animal matter, vegetable matter, or natural or artificial scent placed in an area inhabited by wildlife for the purpose of attracting game animals or game birds. The term does not include decoys, silhouettes, or other replicas of wildlife body forms; scents used only to mask human odor; or types of scents that are approved by the commission for attracting game animals or game birds.

(3) "Closed season" means the time during which game birds, fish, and game and fur-bearing animals may not be lawfully taken.

(4) "Commission" means the state fish, wildlife, and parks commission.

(5) "Fur-bearing animals" means marten or sable, otter, muskrat, fisher, mink, bobcat, lynx, wolverine, northern swift fox, and beaver.

(6) "Game animals" means deer, elk, moose, antelope, caribou, mountain sheep, mountain goat, mountain lion, bear, and wild buffalo.

(7) "Game fish" means all species of the family salmonidae (chars, trout, salmon, grayling, and whitefish); all species of the genus stizostedion (sandpike or sauger and walleyed pike or yellowpike perch); all species of the genus esox (northern pike, pickerel, and muskellunge); all species of the genus micropterus (bass); all species of the genus polyodon (paddlefish); all species of the family acipenseridae (sturgeon); all species of the genus lota (burbot or ling); and the species ictalurus punctatus (channel catfish).

(8) "Hunt" means to pursue, shoot, wound, kill, chase, lure, possess, or capture or the act of a person possessing a weapon, as defined in 45-2-101, or using a dog or a bird of prey for the purpose of shooting, wounding, killing, possessing, or capturing wildlife protected by the laws of this state in any location that wildlife may inhabit, whether or not the wildlife is then or subsequently taken. The term includes an attempt to take by any means, including but not limited to pursuing, shooting, wounding, killing, chasing, luring, possessing, or capturing.

(9) "Migratory game birds" means waterfowl, including wild ducks, wild geese, brant, and swans; cranes, including little brown and sandhill; rails, including coots; wilson's snipes or jacksnipes; and mourning doves; however, the open season on mourning doves is restricted to the open season on upland game birds as defined in subsection (15).

(10) "Nongame wildlife" means any wild mammal, bird, amphibian, reptile, fish, mollusk, crustacean, or other animal not otherwise legally classified by statute or regulation of this state.

(11) "Open season" means the time during which game birds, fish, and game and fur-bearing animals may be lawfully taken.

(12) "Person" means individuals, associations, partnerships, and corporations.

(13) "Predatory animals" means coyote, weasel, skunk, and civet cat.

(14) "Trap" means to take or participate in the taking of any wildlife protected by the laws of the state by setting or placing any mechanical device, snare, deadfall, pit, or device intended to take wildlife or to remove wildlife from any of these devices.

(15) "Upland game birds" means sharptailed grouse, blue grouse, spruce (Franklin) grouse, prairie chicken, sage hen or sage grouse, ruffed grouse, quail, pheasant, Hungarian partridge, ptarmigan, wild turkey, and chukar partridge.

(16) "Wild buffalo" means buffalo or bison that have not been reduced to captivity.

History: En. Sec. 1, Ch. 238, L. 1921; re-en. Sec. 3681, R.C.M. 1921; amd. Sec. 3, Ch. 77, L. 1923; amd. Sec. 12, Ch. 192, L. 1925; amd. Sec. 6, Ch. 59, L. 1927; re-en. Sec. 3681, R.C.M. 1935; amd. Sec. 1, Ch. 37, L. 1949; amd. Sec. 1, Ch. 36, L. 1951; amd. Sec. 1, Ch. 121, L. 1951; amd. Sec. 1, Ch. 19, L. 1953; amd. Sec. 1, Ch. 34, L. 1959; amd. Sec. 1, Ch. 11, L. 1965; amd. Sec. 1, Ch. 28, L. 1965; amd. Sec. 1, Ch. 46, L. 1971; amd. Sec. 1, Ch. 189, L. 1971; amd. Sec. 1, Ch. 167, L. 1973; amd. Sec. 1, Ch. 27, L. 1974; amd. Sec. 1, Ch. 67, L. 1975; amd. Sec. 1, Ch. 93, L. 1975; amd. Sec. 1, Ch. 113, L. 1975; amd. Sec. 1, Ch. 235, L. 1977; R.C.M. 1947, 26-201; amd. Sec. 6, Ch. 44, L. 1979; amd. Sec. 1, Ch. 46, L. 1979; amd. Sec. 1, Ch. 478, L. 1979; amd. Sec. 1, Ch. 420, L. 1983; amd. Sec. 1, Ch. 568, L. 1985; amd. Sec. 2, Ch. 28, L. 1991; amd. Sec. 5, Ch. 417, L. 1995.

Compiler's Comments

1995 Amendment: Chapter 417 in introductory clause inserted "and 87-1-102"; substituted definition of angling for former definition that read: ""Angling" or "fishing" means the taking of or attempting to take fish by hook and single line or single rod, in hand or within immediate control"; inserted definitions of bait, hunt, and trap; adjusted internal references; and made minor changes in style. Amendment effective July 1, 1995.

Cross-References

Fixing of seasons and limits on game, birds, fish, or fur-bearing animals, 87-1-304.

Unlawful for "person" to conduct contest based on size of game animals, 87-3-307.

Closed season on certain game birds, 87-3-402, 87-3-403.

87-2-103. License required. (1) Except as provided in subsection (2), it is unlawful for a person to:

(a) hunt or trap or attempt to hunt or trap any game animal, any game bird, or any fur-bearing animal or to fish for any fish within this state or possess within this state any game animal, game bird, fur-bearing animal, game fish, or parts of those animals or birds, except as provided by law or as provided by the department; or

(b) hunt or trap or attempt to hunt or trap any game animal, game bird, or fur-bearing animal or to fish for any fish, except at the places and during the periods and in the manner defined by law or as defined by the department; or

(c) hunt or trap or attempt to hunt or trap any game animal, game bird, or fur-bearing animal or fish for any fish within this state or possess, sell, purchase, ship, or reship any imported or other fur-bearing animal or parts of fur-bearing animals without first having obtained a proper license or permit from the department to do so; or

(d) trap or attempt to trap predatory animals or nongame wildlife without a license, as prescribed in 87-2-603, if that person is not a resident as defined in 87-2-102.

(2) The provisions of this section do not require a person who accompanies a licensed disabled hunter, as authorized under 87-2-803(4), to be licensed in order to kill or attempt to kill a game animal that has been wounded by a disabled hunter when the disabled hunter is unable to pursue and kill the wounded game animal. However, the person must meet the qualifications for a license in the person's state of residence.

History: En. Sec. 2, Ch. 238, L. 1921; re-en. Sec. 3682, R.C.M. 1921; amd. Sec. 13, Ch. 192, L. 1925; amd. Sec. 7, Ch. 59, L. 1927; amd. Sec. 3, Ch. 224, L. 1947; amd. Sec. 27, Ch. 9, L. 1977; amd. Sec. 13, Ch. 417, L. 1977; R.C.M. 1947, 26-202; amd. Sec. 1, Ch. 255, L. 1979; amd. Sec. 1, Ch. 49, L. 1993; amd. Sec. 8, Ch. 417, L. 1995.

Compiler's Comments

1995 Amendment: Chapter 417 at beginning of (1)(a), (1)(b), and (1)(c) substituted "hunt or trap or attempt to hunt or trap" for "pursue, hunt, trap, take, shoot, or kill or attempt to trap, take, shoot, or kill" and near middle of each subsection, after "fur-bearing animal", deleted "take, kill, trap"; in (1)(c), after "state or", deleted "have, keep"; in (1)(d), in two places after "trap", deleted "snare"; in (2), after "order to" and after "attempt to", deleted "pursue, hunt, take, shoot, or"; and made minor changes in style. Amendment effective July 1, 1995.

Cross-References

Rabies control — restrictions on possession of wild animals, Title 50, ch. 23, part 1.

Waste of fish or game or furbearers, 87-3-102, 87-3-506.

Limit on number of game animals, 87-3-103.

Unlawful to hunt or fish during closed season, 87-3-104, 87-3-402.

Unlawful to buy, sell, possess, or transport certain wildlife, 87-3-111, 87-3-112, 87-4-705, 87-5-201.

Taking of furbearers out of season, 87-3-501.

Sale of fish or spawn unlawful, 87-4-601.

1977; R.C.M. 1947, 26-301(3).

87-3-124. Restrictions on hunting with dogs. (1) (a) Except as provided in 87-3-127, no person may chase with dogs any of the game or fur-bearing animals as defined by the fish and game laws of this state.

(b) A person may take game birds during the appropriate open season with the aid of a dog or dogs. Any person or association organized for the protection of game may run field trials at any time upon obtaining written permission from the director.

(c) Any peace officer, game warden, or other person authorized to enforce the Montana fish and game laws who witnesses any dog attacking or killing hooved game animals may destroy that dog without criminal or civil liability.

(2) The department shall have authority to allow and regulate the use of dogs for hunting mountain lion and bobcat.

History: (1)En. Sec. 14, Ch. 238, L. 1921; re-en. Sec. 3694, R.C.M. 1921; amd. Sec. 5, Ch. 77, L. 1923; amd. Sec. 15, Ch. 192, L. 1925; amd. Sec. 12, Ch. 59, L. 1927; amd. Sec. 1, Ch. 162, L. 1931; amd. Sec. 1, Ch. 159, L. 1941; amd. Sec. 5, Ch. 224, L. 1947; amd. Sec. 1, Ch. 157, L. 1949; amd. Sec. 1, Ch. 126, L. 1951; amd. Sec. 1, Ch. 223, L. 1953; amd. Sec. 1, Ch. 193, L. 1955; amd. Sec. 1, Ch. 53, L. 1963; amd. Sec. 1, Ch. 34, L. 1967; amd. Sec. 1, Ch. 90, L. 1969; amd. Sec. 1, Ch. 201, L. 1969; amd. Sec. 1, Ch. 177, L. 1971; amd. Sec. 1, Ch. 124, L. 1973; amd. Sec. 1, Ch. 305, L. 1973; amd. Sec. 1, Ch. 108, L. 1975; amd. Sec. 1, Ch. 152, L. 1975; amd. Sec. 9, Ch. 9, L. 1977; amd. Sec. 1, Ch. 196, L. 1977; amd. Sec. 3, Ch. 235, L. 1977; amd. Sec. 1, Ch. 400, L. 1977; amd. Sec. 13, Ch. 417, L. 1977; amd. Sec. 1, Ch. 485, L. 1977; Sec. 26-301, R.C.M. 1947; (2)En. Sec. 1, Ch. 184, L. 1971; amd. Sec. 4, Ch. 235, L. 1977; amd. Sec. 13, Ch. 417, L. 1977; Sec. 26-303.5, R.C.M. 1947; R.C.M. 1947, 26-301(part (5)(a)), (5)(b), 26-303.5.

87-3-127. Taking of stock-killing animals. (1) Livestock owners or employees of the department or the federal fish and wildlife service may use dogs in pursuit of stock-killing bears, stock-killing mountain lions, and stock-killing bobcats. Other means of taking stock-killing bears, stock-killing mountain lions, and stock-killing bobcats may be used except the use of the deadfall.

(2) Traps used in capturing bears shall be inspected twice each day with the inspections 12 hours apart.

History: En. Sec. 14, Ch. 238, L. 1921; re-en. Sec. 3694, R.C.M. 1921; amd. Sec. 5, Ch. 77, L. 1923; amd. Sec. 15, Ch. 192, L. 1925; amd. Sec. 12, Ch. 59, L. 1927; amd. Sec. 1, Ch. 162, L. 1931; amd. Sec. 1, Ch. 159, L. 1941; amd. Sec. 5, Ch. 224, L. 1947; amd. Sec. 1, Ch. 157, L. 1949; amd. Sec. 1, Ch. 126, L. 1951; amd. Sec. 1, Ch. 223, L. 1953; amd. Sec. 1, Ch. 193, L. 1955; amd. Sec. 1, Ch. 53, L. 1963; amd. Sec. 1, Ch. 34, L. 1967; amd. Sec. 1, Ch. 90, L. 1969; amd. Sec. 1, Ch. 201, L. 1969; amd. Sec. 1, Ch. 177, L. 1971; amd. Sec. 1, Ch. 124, L. 1973; amd. Sec. 1, Ch. 305, L. 1973; amd. Sec. 1, Ch. 108, L. 1975; amd. Sec. 1, Ch. 152, L. 1975; amd. Sec. 9, Ch. 9, L. 1977; amd. Sec. 1, Ch. 196, L. 1977; amd. Sec. 3, Ch. 235, L. 1977; amd. Sec. 1, Ch. 400, L. 1977; amd. Sec. 13, Ch. 417, L. 1977; amd. Sec. 1, Ch. 485, L. 1977; R.C.M. 1947, 26-301(part (5)(a)).

87-3-128. Exceptions — department personnel. The provisions of this chapter relating to methods of herding, driving, capturing, taking, locating, or concentrating of fish, game animals, game birds, or fur-bearing animals do not apply to the department or to any employee thereof while acting within the scope and course of the powers and duties of the department.

History: En. Sec. 14, Ch. 238, L. 1921; re-en. Sec. 3694, R.C.M. 1921; amd. Sec. 5, Ch. 77, L. 1923; amd. Sec. 15, Ch. 192, L. 1925; amd. Sec. 12, Ch. 59, L. 1927; amd. Sec. 1, Ch. 162, L. 1931; amd. Sec. 1, Ch. 159, L. 1941; amd. Sec. 5, Ch. 224, L. 1947; amd. Sec. 1, Ch. 157, L. 1949; amd. Sec. 1, Ch. 126, L. 1951; amd. Sec. 1, Ch. 223, L. 1953; amd. Sec. 1, Ch. 193, L. 1955; amd. Sec. 1, Ch. 53, L. 1963; amd. Sec. 1, Ch. 34, L. 1967; amd. Sec. 1, Ch. 90, L. 1969; amd. Sec. 1, Ch. 201, L. 1969; amd. Sec. 1, Ch. 177, L. 1971; amd. Sec. 1, Ch. 124, [illegible] amd. Sec. 1, Ch. 305, L. 1973; amd. Sec. 1, Ch. 108, L. 1975; amd. Sec. 1, Ch. 152,

87-3-130. Taking of wildlife to protect persons or livestock. Nothing in this chapter may be construed to impose, by implication or otherwise, criminal liability for the taking of wildlife protected by this chapter if such wildlife is molesting, assaulting, killing, or threatening to kill any person or livestock. Any person who so takes any wildlife protected by this chapter shall notify the department within 72 hours.

History: En. Sec. 1, Ch. 306, L. 1981.

76-3-608. Criteria for local government review. (1) The basis for the governing body's decision to approve, conditionally approve, or disapprove a subdivision is whether the preliminary plat, applicable environmental assessment, public hearing, planning board recommendations, or additional information demonstrates that development of the subdivision meets the requirements of this chapter. A governing body may not deny approval of a subdivision based solely on the subdivision's impacts on educational services.

(2) The governing body shall issue written findings of fact that weigh the criteria in subsection (3), as applicable.

(3) A subdivision proposal must undergo review for the following primary criteria:

(a) the effect on agriculture, agricultural water user facilities, local services, the natural environment, wildlife and wildlife habitat, and public health and safety;

(b) compliance with:

(i) the survey requirements provided for in part 4 of this chapter;

(ii) the local subdivision regulations provided for in part 5 of this chapter; and

(iii) the local subdivision review procedure provided for in this part;

(c) the provision of easements for the location and installation of any planned utilities; and

(d) the provision of legal and physical access to each parcel within the subdivision and the required notation of that access on the applicable plat and any instrument of transfer concerning the parcel.

(4) The governing body may require the subdivider to design the subdivision to reasonably minimize potentially significant adverse impacts identified through the review required under subsection (3). The governing body shall issue written findings to justify the reasonable mitigation required under this subsection (4).

(5) (a) In reviewing a subdivision under subsection (3) and when requiring mitigation under subsection (4), a governing body may not unreasonably restrict a landowner's ability to develop land, but it is recognized that in some instances the unmitigated impacts of a proposed development may be unacceptable and will preclude approval of the plat.

(b) When requiring mitigation under subsection (4), a governing body shall consult with the subdivider and shall give due weight and consideration to the expressed preference of the subdivider.

(6) (a) When a minor subdivision is proposed in an area where a master plan has been adopted pursuant to chapter 1 and the proposed subdivision will comply with the plan, the subdivision is exempt from the review criteria contained in subsection (3)(a) but is subject to applicable zoning regulations.

(b) In order for a master plan to serve as the basis for the exemption provided by this subsection (6), the plan must, at a minimum, contain:

(i) housing, transportation, and land-use elements sufficient for the governing body to protect public health, safety, and welfare; and

(ii) a discussion of physical constraints on development that exist within the area encompassed by the proposed subdivision.

History: En. Sec. 8, Ch. 500, L. 1973; amd. Sec. 6, Ch. 334, L. 1974; amd. Sec. 3, Ch. 498, L. 1975; amd. Sec. 1, Ch. 555, L. 1977; R.C.M. 1947, 11-3866(4); amd. Sec. 5, Ch. 272, L. 1993; amd. Sec. 6, Ch. 468, L. 1995.

Compiler's Comments

1995 Amendment: Chapter 468 in (1) inserted second sentence concerning impacts on educational services; in (3)(a) inserted "agricultural water user facilities"; inserted (4) concerning design to minimize potentially significant adverse impacts; inserted (5) concerning not unreasonably restricting development and consultation with subdivider; and inserted (6) exempting certain minor subdivisions from review criteria.

Applicability: Section 13, Ch. 468, L. 1995, provided: "Funds in a park fund that exceed $10,000 as of [the effective date of this act] [October 1, 1995] must be used for park land acquisition and initial development. Funds in a park fund up to $10,000 as of [the effective date of this act] [October 1, 1995] may be used for park maintenance in accordance with a formally adopted park plan."

Appendix 2. Montana 1994 mountain lion regulations.

LICENSES & GENERAL INFORMATION

- A sportsperson may purchase only one mountain lion license per year.
- Mountain lion licenses are available only at Fish, Wildlife and Parks Headquarters.
- Applications must be postmarked by the U.S. Postal Service no later than November 15.
- A sportsperson may legally take one adult mountain lion per season.
- A sportsperson may legally begin the chase season any time after kill is made or when the quota is reached in a district and the hunting season is closed in its entirety.
- A sportsperson should obtain the chase permit at the time the lion license is purchased.
- When the total quota is reached in a hunting district or hunting district group, the mountain lion season for that area will close upon 48-hours notice, but no later than February 15.
- When the subquota is reached in a hunting district or hunting district group, the mountain lion subquota for that area will close upon 48 hour notice, but no later than February 15.
- Females with kittens may not be taken. If a lactating female is killed, she shall be backtracked and the kittens captured and turned over to the Department of Fish, Wildlife and Parks. (Kittens are young of the year). No juveniles (with body spots) shall be killed.
- The mountain lion license and tag must be attached to the animal immediately upon kill.
- Evidence of sex must remain naturally intact on the hide.
- Evidence of sex must be verified by department personnel before a trophy license is issued. It will also be mandatory that skulls be turned in and tagged at the time a trophy license is issued.
- All Wildlife Management Areas and National Wildlife Refuges are closed to lion hunting.
- Current harvest information may be obtained by calling the Fish, Wildlife and Parks Headquarters in the administrative region where the district is located during normal business hours. See Regional headquarters listing on page 5.

Use of dogs:

- Dogs may be used to chase or hunt mountain lions within prescribed seasons. Lion hunting and chase seasons are open for a portion of each day from one-half hour before sunrise to one-half hour after sunset for the dates listed by hunting district.
- It is prohibited for a dog owner or lion hunter to release dogs on a lion track, or allow dogs to chase a lion, or hold a lion at bay during any period of the day or year when the season is not open to hunting or chasing lions.
- Legitimate attempts to retrieve loose dogs after legal hunting hours does not constitute a violation of this rule.

Mountain Lion - Trophy

- A sportsperson harvesting a mountain lion must:
- Personally report their lion kill within 48 hours to an officer of the Department of Fish, Wildlife and Parks in the administrative region (see Regional Headquarters listing, page 5) where the kill was made, and must personally present the hide and skull within 10 days to an officer of the department residing in the administrative region in which the license was filled, for the purpose of inspection, tagging, recording of kill, and issuance of a trophy license.
- The hide tag must thereafter remain attached to the hide until tanned.
- The skull tag must remain attached to the skull for at least one year.
- The lion skull will be retained by the Department of Fish, Wildlife and Parks for processing and examination and then returned to the owner.
- It is unlawful for anyone to possess, ship or transport any mountain lion, or part thereof, unless they have been tagged as prescribed.

HUNTING OR CHASE SEASON

Hunting Districts (or groups)	Hunting Chase Season	FINAL Lion Harvest Quotas Total	Female	Male
**100 SERIES DISTRICTS**				
100	Dec. 1 - Feb. 15, 1995	14	8	6
101	Dec. 1 - Feb. 15, 1995	12	7	
102	Dec. 1 - Feb. 15, 1995	6	4	2
103	Dec. 1 - Feb. 15, 1995	10	6	4
104	Dec. 1 - Feb. 15, 1995	7	4	3
110	Dec. 1 - Feb. 15, 1995	10	5	
120	Dec. 1 - Feb. 15, 1995	5	3	2
121	Dec. 1 - Feb. 15, 1995	19 any lion		
122	Dec. 1 - Feb. 15, 1995	14 any lion		
123	Dec. 1 - Feb. 15, 1995	5 any lion		
124	Dec. 1 - Feb. 15, 1995	3 any lion		
130	Dec. 1 - Feb. 15, 1995	11	7	4
132, 170	Dec. 1 - Feb. 15, 1995	12	6	6
140	Dec. 1 - Feb. 15, 1995	8	4	4
141	Dec. 1 - Feb. 15, 1995	4	2	2
150,151	Sept. 15 - Feb. 15, 1995	5 any lion		
**200 SERIES DISTRICTS**				
*Except Missoula Special Management Area.				
200, 201*, 202, & 203*	Dec. 1 - Feb. 15, 1995	20	10	10
204*, 260 *& 261	Dec. 1 - Feb. 15, 1995	15	8	7
Missoula Special Management Area				
Portions of 201, 203, 204, 260, 283 & 292.	Dec. 1 - Feb. 15, 1995	25	15	10
(Legal description and map of this area available from Missoula FWP Headquarters, 542-5500).				
210, 211, & 216	Dec. 1 - Feb. 15, 1995	8	4	4
212 & 215	Dec. 1 - Feb. 15, 1995	6	3	3
213 & 214	Dec. 1 - Feb. 15, 1995	2	1	1

Hunting Districts (or groups)	Hunting Chase Season	FINAL Lion Harvest Quotas Total	Female	Male
240	Dec. 1 - Feb. 15, 1995	15	8	7
250 & 270	Dec. 1 - Feb. 15, 1995	15	5	10
280	Sept. 15 - Feb. 15, 1995	25	10	15
281, 284, 285 & 293	Dec. 1 - Feb. 15, 1995			
282	CLOSED			
283*, 290, 291 & 292*	Dec. 1 - Feb. 15, 1995	14	7	7
**300 SERIES DISTRICTS**				
300, 302, 328, & 329	Dec. 1 - Feb. 15, 1995	3	2	
301	Dec. 1 - Feb. 15, 1995	6	2	
310, 311, 360, 361 & 362	Dec. 1 - Feb. 15, 1995	6	2	
312, 315 & 393	Dec. 1 - Feb. 15, 1995	4	2	
313 & 316	Dec. 1 - Feb. 15, 1995	3	1	
Deckard Flats - Eagle Creek portion of hunting district 313 as posted, subject to closure to all hunting on 24 hour notice.				
314	Dec. 1 - Feb. 15, 1995	5	2	
317	Dec. 1 - Feb. 15, 1995	5	2	
319, 340 & 341	Dec. 1 - Feb. 15, 1995	3	2	
320 & 333	Dec. 1 - Feb. 15, 1995	3	2	
322, 326 & 330	Dec. 1 - Feb. 15, 1995	3	2	
323, 324, 325 & 327	Dec. 1 - Feb. 15, 1995	3	2	
321, 331 & 332	Dec. 1 - Feb. 15, 1995	3	2	
**400 SERIES DISTRICTS**				
400, 401, 403, 404 406, 415, 422, 424, 425, 441, 442, 444, 450, 427 & 428	Dec. 1 - Feb. 15, 1995	20	8	12
405, 418, 420, 432, 447, 448, 449, 452, 454 & 471	Dec. 1 - Feb. 15, 1995	14	7	7

Hunting Districts (or groups)	Hunting Chase Season	FINAL Lion Harvest Quotas Total	Female	Male
410, 411, 412, 417 419 & 426	Dec. 1 - Feb. 15, 1995	12	4	8
413	Dec. 1 - Feb. 15, 1995	7	3	4
416 & 446	Dec. 1 - Feb. 15, 1995	18	9	9
421, 423, 445 & 455	Dec. 1 - Feb. 15, 1995	9	3	6
**500 SERIES DISTRICTS**				
500, 511, 530 & 590	Dec. 1 - Feb. 15, 1995	6	3	
502 & 520	Dec. 1 - Feb. 15, 1995	8	4	
510	Dec. 1 - Feb. 15, 1995	2	1	
540	Dec. 1 - Feb. 15, 1995	6	3	
560	Dec. 1 - Feb. 15, 1995	12	6	
570 & 580	Dec. 1 - Feb. 15, 1995	6	3	
575	Dec. 1 - Feb. 15, 1995	4	2	
**600 SERIES DISTRICTS**				
	Dec. 1 - Feb. 15, 1995	5 any lion		
**700 SERIES DISTRICTS**				
	Dec. 1 - Feb. 15, 1995	5 any lion		
**800 SERIES DISTRICTS**				
818 & 850	Dec. 1 - Feb. 15, 1995	6	3	3
835, 839 & 843	Dec. 1 - Feb. 15, 1995	8	8	4
870 & 880	Dec. 1 - Feb. 15, 1995	8	4	4
890, 891 & 892	Dec. 1 - Feb. 15, 1995	12	6	6

FINAL MOUNTAIN LION QUOTAS

LICENSES & GENERAL INFORMATION

1994 - 1995

CHASE SEASON ONLY

- All hunting districts are open for a portion of each day, from one-half hour before sunrise to one-half hour after sunset, to the CHASE ONLY season if: 1) Sportsperson holding a chase permit has legally filled their mountain lion license, or 2) if the total district harvest quota has been met and the district is closed to hunting, or 3) from February 16 - April 30, 1995.
- Sportspersons wishing to participate in any chase season must:

  1) Hold a valid 1994 lion license (or show proof of purchasing a license in the event the license has been filled).

  2) Hold a free chase permit. Sportsperson should obtain the chase permit at the time the lion license is purchased.

  3) Free chase permits are available at all Fish, Wildlife and Parks Headquarters.
- Only one person in party must hold a chase permit to chase mountain lion.
- All Wildlife Management Areas, National Wildlife Refuges and Hunting District 282 are closed to the lion chase season.

All QUOTAS on this revised version of the mountain lion regulations are FINAL. Approved by Fish, Wildlife and Parks Commission on September 10, 1994. Please use this page with 1994 Big Game Regulations and Legal Descriptions.

Montana Fish, Wildlife & Parks

PUBLIC RESPONSES RECEIVED ON THE DRAFT MOUNTAIN LION EIS

The following summary was tallied from comments taken at 7 public hearings (119 attendants) and 128 written comments from individuals, groups or organizations, and governmental agencies. Comments have been categorized under those elements of the analysis it was felt they best addressed.

During the process many comments were received that were not substantive input to change or correct the content of the Draft EIS (DEIS) or the evaluation of the 4 Alternatives. Many of these were directed at proposed changes in season structures and harvest quotas rather than the content of the DEIS. In some cases it was felt these comments would be useful in analyzing public opinion of the elements used for analysis in the DEIS and they have been included in the summary.

Tallies of comments received are not meant to represent a vote but to give some representation of their significance in the process. Each written comment received has been tallied as 1 no matter how many signatures it contained or members of an organization it represented. As a result all comments represent the feelings of some segment of society, not their organizational ability.

All comments received on the DEIS, and transcripts and notes from public meetings are on file with the Wildlife Division of MFWP in the Helena headquarters. These are part of the public record of the FEIS and as such available for public examination.

COMMENTS RECEIVED AND RESPONSES

A. CONTINUE THE USE OF THE EXISTING HUNTING SEASON FORMAT

Maintain December 1 opening - 11
Open on a weekend: For - 2; Against - 7
Drop early (Sept 15) season - 1
Open for trappers - 1
No trapping - 1
No hunting - 1
Against using dogs - 2

COMMENT:

- "The timing and structure of the mountain lion season has to be one of the most important issues discussed in the EIS. The current season is just right beginning on December 1st, if it was made to start on a weekend it would be even more crowded than on the 1st."

- "Change the opening date to the weekend. This would allow not just the outfitters, but other hound hunter, lion hunters to get at least a chance to hunt, and would possibly cut down on some of the conflicts."

RESPONSE: Alternatives 2, 3 and 4 call for the continuance of the current season structure, including the early opening in the Bob Marshall Wilderness and the December 1 through February 15 regular season. Under those Alternatives that would be the recommendation MFWP would make to the MFWP Commission. However, it is within the Commissions authority to change the opening dates and the length of any season. Such changes do require public input prior to their adoption.In addition such a change would be adopted as an annual rule and may be changed the following year.

COMMENT:

- "I request the Dept to consider the placement of mountain lions with the bob cat, lynx wolverine, etc. and allow trappers with a valid general trapping license to harvest them. Limit the harvest per district as needed."

- "The trappers have expressed that they would like to purchase a lion tag in case they accidently trap a mountain lion. They already have the right to purchase a mountain lion permit, but can not trap one. We believe that they would be encouraging the accidents to happen by using larger traps, plus traps cannot determine sex or age. The size of a trap needed to hold a mountain lion is very injurious and larger than for most of the animals listed for taking by the trappers license. ... "

RESPONSE: The reason for excluding trapping as an element in the analysis is discussed in the text under Issues and Impacts not Evaluated in the DEIS.

COMMENT:

- "Obviously, we are so entrenched in management of wildlife, we simply can't pull out. But I'm still against hunting cougars for sport. Use of dogs is disgusting."

RESPONSE: Alternative 1 analyzes "no hunting" as a management alternative.

COMMENT:

- "Use of dogs in hunting lions must continue. I have never used dogs, and probably never will but consider it important that their use continue..."

- "I believe that hunting should only be allowed without using dogs. The hunting with

dogs is for ease of killing by treeing. If a person is a good hunter that person can track and tree the big cat without using dogs."

- "The best method of mountain lion harvesting and control is with hounds. Once the mountain lion is in a tree the sex of that lion can be determined, thus reducing the number of kittens or females with kittens from being harvested in large numbers and therefore fitting in with the quotas."

- "And what's the method of choice for the sport? Setting a bunch of hounds to run the cat up a tree; hunter arrives, relaxes, shoots as many times as it takes because where is the cat going? And tough luck for the orphaned kittens if the hounds and hunter bag a lactating female."

RESPONSE: MFWPs reasoning on continuing the use of trailing dogs for hunting mountain lions is discussed in the text in the Disadvantages and Biological assessment sections under the analysis of Element Z.

B. CONTINUE THE "CHASE SEASON"

For both hunt and chase seasons - 11
Against chase season - 1
Against using dogs - 1

- "The pursuit season is ridiculous for you to provide and even encourage. It has no wildlife value. Let the houndsmen exercise their dogs on their own, not within the auspices of State law."

- "As a resident of the state of Montana for my entire life, I would like to sustain the right to hunt and chase mountain lions for years to come."

- "The tradition and sport of pursuing the mountain lion is very important to me. I am very interested in preserving this tradition as well as the proper management of the lion in order to preserve the lion for ever in Montana."

- "We'll not argue that (page 15, draft EIS): "To effectively harvest mountain lion, use of trained hounds is needed ***" But we're not too fond of the chase only season, and agree with your statement (page 15, draft EIS, alluded to with reference to "Element B," and page 54, direct quote): "Whereas pursuit-only seasons have been regarded as non-consumptive, the physiological effects of chasing mountain lions is poorly understood." Well, there's got to be stress. Lactating females would, it would seem, have extra stress. Having said that, are you planning further work or research in an attempt to get an answer?"

RESPONSE: Alternatives 2, 3 and 4 would continue to utilize the "chase season" following the close of any hunting season. MFWP recognizes that the most effective method of harvesting mountain lions is with pursuing dogs, and that such animals need to be trained in order to become competent. As a big game animal it would be illegal to pursue mountain

lions outside the hunting season if the MFWP did not allow for the chase season as a training period.

Pursuit-only seasons have been recognized as an area that needs further examination from the standpoint of physiological stress on mountain lions. MFWP intends to include this in it's future research priorities and will take into consideration any work in this area done outside Montana.

Definition of Hunting: A change to the definition of "hunting" by the 1995 legislature resulted in the inclusion of the following statement under this element.

> Hunting as defined by statute (87-2-101(8)MCA) and as approved by the 1995 legislature includes the intent of taking for harvesting the animal being pursued or chased. This new definition clouds the authority of MFWP Commission to establish a "chase season" and needs to be clarified by the legislature.

C. CONTINUE TO REGULATE THE HARVEST USING QUOTAS

Drop the quota system - 4
Include depredation kills on quotas - 2
Put males on permits and females on quota - 1

REWORD TO READ: USE QUOTAS AS ONE METHOD OF REGULATING HARVEST.

The wording of this Element was changed in order to allow MFWP the flexibility to adopt to changes in mountain lion population densities and distribution across Montana. The change also allows for coordination between Element C and Element L which allows the use of season length to regulate harvest levels. MFWP recognizes that while quotas are effective in regulating the harvest they may not fit all situations or may be more restrictive than necessary under some circumstances.

COMMENT:

- "Several members feel that the recently adopted quota system has resulted in a net decrease in lion harvests. Although lion harvests have increased significantly in the past decade, populations have as well.

  Reinstatement of a general season determined by a specified time period, rather than harvest quotas, would more effectively control lion numbers, while also providing for increased hunter opportunity. Harvest numbers could still be monitored just as closely as under the quota system."

RESPONSE: Regulation of the harvest under the quota system has proven to be effective in obtaining the harvest and controlling the sex of animals harvested. It also provides the most opportunity for hunters while exerting a high degree of control over the harvest. With increased interest in mountain lion hunting in Montana, and the loss of hunting opportunity in neighboring states, MFWP feels it is in the best interest of Montana's mountain lion populations to retain the tight control of the harvest provided by the quota system.

Alternatives 3 and 4 would allow for the use of season length to control harvest in those hunting areas in which the Regions felt it would be justified. Such changes in management would require public input and approval of the Commission.

COMMENT:

- "Nonhunting fatalities should (must) be counted in the quotas"

RESPONSE: Harvest quotas are set by the Commission in August based on recommendations from the Regions. This allows the Regions to adjust quota recommendations, and to take into account any human caused nonhunting fatalities occurring between February and August. Previous years nonhunting human caused fatality data occurring between August and December are also factored into quota recommendations made to the Commission. If higher levels of human caused nonhunting mortalities occur than were expected in an area, the quotas for that area can be adjusted to compensate in coming years.

D. ASSIST LANDOWNERS AND ADC WITH DEPREDATION COMPLAINTS

For - 3

COMMENT:

Comments at public meetings supported providing assistance only to those landowners that are open to public hunting for all big game species.

RESPONSE: Montana Law (87-1-225 MCA) states that only those land owners that allow significant public hunting or do not impede public hunting will be afforded MFWP assistance with game damage.

COMMENT:

- "Allow livestock owners (or their employees) to kill an lion caught in the act of killing livestock, at that time or within a reasonable time after (perhaps 24 hours), without first contacting FWP and without having a license."

RESPONSE: Under Montana law (87-3-130 MCA) Landowners may now take any lion attacking or killing livestock and has 72 hours in which to report the killing of that animal.

E. CONTINUE PUBLIC EDUCATION PROJECTS ON MOUNTAIN LIONS

Increase current efforts - 3

F. CONTINUE TO ASSESS ROAD ACCESS ON PUBLIC LANDS

No comments on continuing with the program:

> Five comments were received indicating additional access to public lands was desired and no additional road closures were needed.

RESPONSE: Under each of the Alternatives analyzed these situations would be evaluated on a case by case basis and recommendations based on the effects of the roads on all wildlife would be made to the responsible land management agency.

Two comments were directed at prevention of damage to private lands by mountain lion hunters.

COMMENT:

- "Issue #11 addresses game damage statutes regarding the lion. Does this refer only to loss of livestock? how does a forest landowner collect for damage done to roads by rutting or collect for water quality violations: Who pays for damaged gates or trees cut for firewood?"

RESPONSE: MFWP, through it's Block Management Program, is willing to work with private landowners in developing programs that will continue to allow hunting while reducing damage to private lands in the program.

One comment was received indicating there is an economic impact in continuing this activity.

COMMENT:

- "In element F, page 17, you discuss open and closed roads and the FWP position of only recommending what happens. I am sure the department is aware that if they recommend a road be closed, the agency responsible for managing the land could not go against this recommendation. It is a cop out of responsibility to say, it is only a "recommendation". To say you have no effect on the economic impact, is incorrect."

RESPONSE: MFWP has no jurisdictional powers in regards to management decisions made on lands under another agencies control. MFWP can make only recommendations to land management agencies. Decisions made by those agencies may or may not be in accord with MFWPs recommendations.

G. PROTECT THREATENED HABITATS THROUGH "HABITAT MONTANA" PROGRAM

No comments received

H. EVALUATE LAND MANAGEMENT AGENCIES PROPOSED PROGRAMS BASED ON THEIR MERITS FOR WILDLIFE.

DSL indicated the preferred alternative (4) would not interfere with their mission of raising school trust funds.

I. HIDES AND SKULLS OF MOUNTAIN LIONS WILL CONTINUE TO BE TAGGED IN THE REGION HARVESTED

For - 2

Several general questions/comments on the need for available personnel to accommodate this regulation.

RESPONSE: Under Alternatives 2 and 4 MFWP would be required to provide for methods allowing hunters to comply with this regulation.

J. REDUCE THE MINIMUM CLOSURE PERIOD FOR A SEASON FROM 48 TO 12 HR. AND ALLOW FWP TO ANNOUNCE CLOSURE PRIOR TO MEETING QUOTAS.

For 12 hour closure - 6
Against 12 hour closure - 14 (12 of these suggested a 24 hour closure was more appropriate)

Keep current 48 hr. - 2
Allow regional control - 3
Against regional control - 1

COMMENT:

- "I support reducing the minimum closure period for a season in a specific hunting district to 12 hours and allowing the FWP to announce closures prior to quotas being met. This change,... will decrease the frequency that quotas are exceeded and thereby increase management precision."

- "Reducing the minimum closure period will alleviate overkill; the FWP should exercise careful judgement in specific areas to close areas prior to meeting quotas, only when an overkill seems imminent."

- "The 12 hour closure is too severe; a bad storm or vehicle breakdown could make people criminals in that short period of time."

RESPONSE: Extenuating circumstances would have to be allowed for if a 12 hour closure was put into effect.

COMMENT:

- "I feel the FWP should be given the authority to close the season with a 24 hour notice in order to avoid an overkill in certain areas."

- "We feel that allowing the season to be closed early upon one persons decision could cause much trouble in the future. The person with this power could close the season every year when half the quota had been met because he believed it was getting close.

RESPONSE: Any season closure requires a recommendation from the Region and notification and approval of the Commission. One person would not have the authority to close any season.

K. REDUCE THE REPORTING PERIOD FOR A LION KILL TO 24. HOURS

For - 25
Keep current regulation (48 hours) - 3
Reduce to 12 hours - 2

COMMENT:

- "The reporting period should be reduced; the FWP will need personnel available to implement these changes."

- "I do support a reduction to 24 hours in the lion kill reporting period and skull inspection. This is a reasonable but tight requirement and it may give enforcement officials a better opportunity to insure that lions are being harvested in the locations hunters claim."

RESPONSE: Under Alternatives 2 and 4 MFWP would be required to provide for methods to allow hunters to comply with this regulation.

COMMENT:

- "The 24 hour reporting period is longer than the 12 hour closure period; Element K, page 21 indicates that any lion reported in the second half of the 24 hour reporting period would be illegal if the 12 hour closure occurs in the first half."

RESPONSE: Any lion taken during an open season would be legal. An individual could report a kill up to 24 hours after a closure if the animal was harvested prior to the season closure.

COMMENT:

- "I support shortening the reporting period for a lion kill to 12 hours or less. There is no satisfactory explanation for leaving the reporting period longer than 12 hours."

RESPONSE: A 12 hour reporting period would aid in season closure. However, under many circumstances hunters are not able to comply with such a short reporting period, nor would is it be necessary to implement such restrictions on a statewide basis.

L. USE SEASON LENGTH TO REGULATE HARVEST

For - 2
Against - 3

COMMENT:

- "I don't agree with this. The seasons in place are tested and viable; a short season will result in indiscriminate harvest, and a longer season is not necessary as long as districting is used to regulate specific harvest."

- "Increase the harvest by: A. Longer season length; B. Opening hunting in currently closed areas near large cities(ex. Missoula) where human safety concerns exist.

- "Reinstatement of a general season determined by a specified time period, rather than harvest quotas, would more effectively control lion numbers, while also providing for increased hunter opportunity."

RESPONSE Alternatives 3 and 4 would allow for the use of season length to control harvest in those areas Regions felt it would be justified. Such justification would accompany any such recommendation made to the Commission which in turn would take public comment on that recommendation prior to adopting such a season.

M. REDUCE THE INSPECTION/TAGGING PERIOD FOR THE HIDE AND SKULL TO 24 HOURS AFTER BEING REPORTED

For - 11
For 72 hours - 3
Leave as is - 3

COMMENT:

- "Reduce inspection period for skull and hides to 24 hrs. after being reported, but have someone available after 5 pm to recorded and inspect the lion skull and hide."

- "Shorten the closure, reporting and turn-in time to 24 hours - with continuation and twice daily updating of the hotline information."

- "I feel the tagging period should be 3 days - not 24 hours."

- "I would like to see the recording period and season closures changed to 24 hours for better control, but I would like the check in period to be 3 days to allow for skinning + taxidermy preparations."

- "24 to 48 hours to present skull & hide - 5 days is ok but why not leave it at 10."

RESPONSE: **REWORD TO READ: REDUCE THE INSPECTION AND TAGGING PERIOD FOR THE HIDE TO 24 HOURS AFTER BEING REPORTED. TAGGING OF SKULLS WOULD BE ALLOWED UP TO 5 DAYS FOLLOWING HARVEST.** **EXCEPTION: Hunters with mountain lions harvested in those hunting districts with an early season (beginning Sept. 15) would have 24 hours after reaching the trailhead to**

present the animal for inspection and the tagging of the hide, and 5 days after reaching the trailhead for the tagging of the skull.

This suggestion was made at a public meeting in Columbus. It would allow timely inspection of the carcass to determine sex and reproductive status as well as allowing time for the hunter to have the skull skinned out to turn over to MFWP for aging.

N. REWORD REGULATIONS TO PROTECT "FEMALES WITH YOUNG"

For - 7
Against - 1

COMMENT:

- "Affording security to females with young is an excellent idea. This will maintain a viable population of lions in Montana and at the same time will add to a positive public perception. Young lions will not be left to fend for themselves at a time that nature intended them to spend learning with their mother."

- "Protection of maternal females should be increased beyond the changes discussed in the DEIS.... The regulation should read that any female in support of kittens <18 months old may not be taken regardless of her lactation status and regardless of whether kittens are present or not."

- "Dependents need to be defined. There is no way of knowing if they are dependent young (other than spots)."

RESPONSE: **SUGGESTED CHANGE:- REWORD THE REGULATIONS TO READ: "NEITHER FEMALES WITH YOUNG NOR YOUNG TRAVELING WITH AN ADULT FEMALE MAY BE TAKEN."**

Written comments, as well as those received during public meetings indicated the regulations should be changed in order to protect young, up to 24 months of age, that may still be accompanying their mother. This change was suggested as a method of preventing young that are still accompanying the female, but that have outgrown their spotted pelage from being taken.

O. RE-EVALUATE HUNTING ON WMA's

For - 8
Against - 1

COMMENT:

- "I encourage the opening of WMA's to lion hunting where this can be accomplished without jeopardizing the primary goals of the WMA."

RESPONSE: All evaluations of WMAs for hunting would require close evaluation of the primary goals for the acquisition. Mountain lion hunting would not be allowed on those areas where it would interfere with the primary use of the WMA.

COMMENT:

- "We do not support several elements listed in the EIS. For example element O, reevaluation of lion hunting on wildlife management areas, appears to have little justification. Given that there is no current shortage of lion hunting opportunity state-wide, and given the WMAs serve as refugia for lions, especially in portions of the state without large federal land holdings, we see no hunting might act to displace ungulates from critical winter range, thus negatively impacting some of the very resources for which WMAs are designated."

RESPONSE: Mountain lion hunting would be allowed only on those WMAs where it could be justified from the standpoint of managing both the mountain lion population and the designated purpose for the WMA.

P. PROVIDE FOR INTENSIVE MANAGEMENT ZONES

For - 2

COMMENT:

- "We need to establish criteria that will protect those animals that are "passing through" and not eliminate every lion that sets foot across the boundary."

RESPONSE: These types of situations would be covered under Alternatives 1, 3 and 4 by the development of a conflict policy as directed by Element U.

Q. REQUIRE WILDLIFE EVALUATIONS OF SUBDIVISIONS BE PROVIDED TO POTENTIAL BUYERS

For - 6
Against - 2

COMMENT:

- "Subdivision environmental assessments should be provided to buyers, but at the cost of the seller, not hunter funded."

RESPONSE: MFWP has been directed by the Legislature to develop wildlife evaluation on all new subdivisions addressing the effect of the subdivision on fisheries and wildlife. Assigning the cost of the evaluation to the developer would require Legislative action.

COMMENT:

- "In my mind I cannot fathom the loss of any small piece of mountain lion habitat to anything as insignificant as another new home."

- "Finally in discussing the preferred alternative I would rise in opposition to Adjustment Q. I know that it states that this is a recommend only situation. But I am reminded of the subdivision proposed last fall on Whitefish Stage, I believe, in which the Fish and Game sent in a letter opposing the subdivision believing it was in the Canyon rather than in the Whitefish Stage location. That was not a simple recommend -- it was outright opposition. It was not a statement of concern or a discussion with the developer --but dictatorial in its opposition. With this track record, and the apparent inability of Fish and Game to talk with the developer beforehand, thereby finding out that the proposed subdivision was in suburban Kalispell, I find little support for another assault on private property interests."

RESPONSE: By statute (76-3-608(3a)MCA) MFWP is required to evaluate all proposed subdivisions for their wildlife values. If the impacts of the subdivision are felt to be highly detrimental to wildlife the wildlife evaluation will reflect that loss of wildlife value.

COMMENT:

- "When you discuss the effects on subdivisions in element Q, I can not believe you can say "no significant impact is anticipated" from an economic point of view. Any burden you place on a developer has a cost!"

RESPONSE: Because MFWP is responsible for the development of the wildlife evaluation and assumes the cost of doing so, distribution of the final product is not felt to be a "significant economic impact" on the developer.

R. CLARIFY STATUTES ALLOWING LANDOWNERS TO USE DOGS FOR TAKING DEPREDATING LIONS

For - 2
Against - 1

COMMENT:

- "Clarify statutes 87-3-127(1) MCA and 87-3-130 MCA to allow landowners to use hound handlers to take depredating lions but they must be accompanied by a game warden.

RESPONSE: The intention of this element is to provide a fast response to depredation and to help assure the animal taken is the one involved in the incident. Requiring a warden to accompany the landowner would require additional time and reduce the possibilities of taking the depredating mountain lion.

COMMENT:

- "Allowing stockowners to appoint some one to remove what they consider a problem lion will cause more trouble then (sic) it will solve. ... Also these type of problems should only be assisted with for stockgrowers who allow public hunting during all hunting seasons."

RESPONSE: Montana law (87-3-130 MCA) currently allows for the taking of depredating lions and bears. The proposed change would require the landowner to provide proof of the depredation, and in most cases, would shorten the current notification time to MFWP.

S. DETERMINE AND PRIORITIZED RESEARCH NEEDS TO ASSESS MONTANA'S MOUNTAIN LION MANAGEMENT

We need additional research - 2
Test all animals for trichinella - 1

COMMENT:

- "I think all lions should be brought to FW&Parks offices for tagging and a meat specimen taken & tested for trichinella for a small mandatory fee regardless of meat usage for the exact percentages of infected animals, even if only for one season. I was unaware that cats carried trichinella!"

RESPONSE: Testing for trichinella would be considered as a possible study under this element.

T. ESTABLISH POPULATION OBJECTIVES FOR MOUNTAIN LIONS AND THEIR PRIMARY PREY

For - 1

- "Elements S and T are both essential for conservation of Montana's lion populations over the long term. Management-orientated research and research designed to understand broad lion/habitat interactions and relationships to human-caused environmental change should receive the highest priority; traditional radio-telemetry studies designed to gather site-specific ecologic data can then be conducted as funds become available."

- Regional game managers expressed concerns that as originally worded this element would require a number as the objective for the population.

RESPONSE: **REWORD TO READ: ESTIMATED CARRYING CAPACITIES THAT REFLECT BIOLOGICAL AND SOCIAL DEMANDS PLACED ON BOTH THE MOUNTAIN LION AND PREY POPULATIONS OF EACH FWP ADMINISTRATIVE REGION WILL BE USED WHEN MAKING SEASON RECOMMENDATION TO THE COMMISSION.**

MFWP is concerned the original wording would require the setting of a number as a population goal for mountain lions within Regions or habitats within the Regions. Because current survey methods will not allow an accurate census of mountain lions it would be impossible to ascertain if our population goals were being met. This would result in a situation similar to what we have with the grizzly bear.

Carrying capacity for some habitat types within Montana may be available from previously conducted studies, carried out either in Montana or neighboring states. Monitoring of these

habitats would determine mountain lion densities and provide a measure by which we could determine if goals were being met.

Additional base line studies would need to be carried out in other areas based on study priorities and funding in order to determine carrying capacities.

Landowner tolerance for hunters, the prey species and mountain lions, public safety concerns and the demands put upon the prey base by the hunting public will all need to be considered when harvest objectives are being set. Regional harvest recommendations will be required to undergo public review and adoption by the Commission before going into effect.

This change would not substantially change the analysis within the document and therefore does not require putting the document back out to the public for comments.

U. DEVELOP A HUMAN-LION CONFLICT POLICY

For - 13

COMMENT:

- "The draft document correctly points out that increased numbers of cats has had as a byproduct increased numbers of human/lion conflict. However, the draft document takes a particular point in saying that a human/lion conflict policy should be developed at a later date. In other words we ar no further ahead in addressing this admitted problem through the EIS process than when we started nearly two years ago. The general public has a right and the Fish and Game has an obligation to include this policy in plain English so that everyone can understand their individual rights and responsibilities under a human/lion conflict policy. It only makes good management sense, especially where the cat is concerned that a conflict policy be included in the draft EIS as that policy could have a major and direct bearing on harvest numbers."

RESPONSE: The EIS process is being used to determine in what direction the public wants MFWP to proceed with the management of mountain lions in Montana. Development of a conflict policy is an element of that management MFWP was seeking public opinion on. The inclusion of such a policy in the document would be premature as developing such a policy will require public input focused solely on that policy. It's inclusion in the EIS would only serve to cloud other issues involved in developing the EIS.

V. REQUIRE ALL HOUND HANDLERS PURSING OR HUNTING MOUNTAIN LIONS TO BE REGISTERED.

For - 2
Against - 23

COMMENT:

- "I am totally against any registration of dogs or hunters - I feel this is discrimination

of the worst kind; no other recreationists are required to register themselves <u>OR</u> their equipment."

- "I feel the registration of hound is only duplicating efforts, the chase permit should be sufficient, just use the information on it."

- "I don't think we need a hound handlers permit because we already have a chase permit, and if they want to get an idea on how many out of staters are running hounds in Montana then have the out-of-staters buy a out of state chase permit for Montana and charge them thus generating more revenue."

- "The ITEMS I oppose ARE:
  1) Hunter or dog handler registration"

- "V. I do not feel hounds or hound hunters should have to be registered."

- "#2) Houndsman permits. I feel this is a fine idea, but I also feel that it shouldn't mean that someone that is hunting with me shouldn't be able to handle by dogs if he doesn't have one (a houndsman permit)."

<u>RESPONSE:</u> SUGGESTED CHANGE: REWORD TO READ: REQUIRE ALL HOUND HANDLERS 12 YEARS OF AGE AND OLDER PURSUING OR HUNTING MOUNTAIN LIONS WITH DOGS TO PURCHASE A MOUNTAIN LION HUNTING LICENSE AND OBTAIN A FREE HOUND HANDLER PERMIT AT THE TIME OF LICENSE PURCHASE. EXCEPTION: Guides and outfitters will be eligible to receive a free hound handler permit by presenting their Montana outfitters or guide license and their conservation license. Guides will also be required to provide the license number of the outfitter on whose license they are guiding.

The word "registration" in this element was not well received by the public. As a consequence the intent of the change from the rules governing the chase permit to the requirement of having to purchase a license in order to release dogs on a mountain lion was lost.

This wording change would require anyone in charge of dogs in the field during the hunt or chase season, who is old enough to purchase a valid hunting license, to possess such a license. It also allows those same people a method to prove they have purchased a license if they harvest a lion during the hunting season and are participating in the chase season.

This will bring those people "pursuing" lions in compliance with the law and require non-resident houndsmen/outfitters to purchase a license in order to run their dogs in Montana. The change would also continue to allow outfitters and guides, licensed by Montana, to pursue lions with a client without having to purchase a mountain lion license.

W. PROVIDE METHODS TO REGULATE TOTAL HUNTERS AND NONRESIDENT HUNTERS WITHIN THE STATE OR A PORTION OF THE STATE

Nonresidents:

- For limiting non-residents to 10% of the harvest - 12
- For restricting the numbers of outfitters or outfitter harvest to 10% of the quota - 3
- For reducing or controlling nonresident numbers - 11
- For only selling nonresident licenses to reciprocating states - 2
- Increase nonresident fees - 1
- Charge for nonresident chase permit - 1

Validation: For - 7; Against - 6

No drawings or permits - 4
All drawings or permits - 1

RESPONSE: Comments received on this element indicated more of a willingness to regulate nonresident hunters (30 comments) than resident or local hunters (18 comments). The large number of comments do indicate a strong interest in such regulations by the public. They also enforce the need for MFWP and the Commission to maintain close ties with the public should any such regulation be considered.

X. MFWP NEEDS TO IMPROVE IT'S ABILITY TO OBTAIN AND DISSEMINATE HARVEST INFORMATION

COMMENT:
Several general comments were received at public meetings stating that reductions in closures, reporting and tagging periods would require an efficient method of gathering and disseminating information on the harvest to be developed by MFWP.

- "I agree with proposal (Alternative) #4 except I think a 12 hour reporting time for harvest of lions would be sufficient as long as there is a place to call and it is staffed to accept calls 24 hours a day, 7 days a week."

- "Something has to be done to get harvest information both into and out of FWP offices in a timely manner. All options have (to) be discussed."

RESPONSE: If the preferred alternative is adopted MFWP will be required to make those adjustments that are necessary to allow hunters comply with the regulations adopted by the Commission.

Y. INCREASE ENFORCEMENT EFFORTS TO CHECK KILL SITES AND HUNTER RESIDENCY

For checking kill sites - 19
Increased effort needed - 4

Z. OPEN WITH THE GENERAL SEASON

For - 10
Against - 17

- "The idea of opening mountain lion hunting with the general big game season is insane"

- "Currently, any person can buy a permit and hunt lions during the normal lion season. There are other ways of hunting lions, other than with hounds, however determining the sex and age will again be a problem."

- "I do support the extension of lion hunting into the general big game season. This is a unique and thrilling opportunity that should be returned to the hunter."

RESPONSE: MFWP does not think opening the mountain lion season with the general hunting season statewide would be in the best long term interests of either the mountain lion population or maintenance of the mountain lion season in Montana. A more detailed discussion addressing this element is given in the text.

OTHER COMMENTS RECEIVED:

COMMENT:
Support Alternative

1 - 0
2 - 1
3 - 11
4 - 58

COMMENT:
FEES: Increase trophy fee - 2
Eliminate trophy fee - 1
Keep all fees the same - 1
Charge for nonresident chase permit - 1

RESPONSE: License fees that MFWP may charge are set by the Legislature. Fees being charge currently are expected to carry MFWP through 1999. (See # 5 under the **ISSUES and IMPACTS NOT EVALUATED IN THE DEIS**)

COMMENT:
Hunting is not adequately addressed by the document - 1

- "The EIS in my opinion only address, and not very well, should we or should we not hunt lions."

RESPONSE: The EIS serves as a tool for MFWP and the MFWP Commission to use in making decisions about mountain lion management in Montana. The purpose of the document

is to: A) provide as much information on mountain lions and their management as possible; B) provide and analyze a reasonable array of management options and C) provide a method of documenting public interest and input in the project. An EIS document cannot analyze all aspects of mountain lion biology, ecology and management and their interworkings with the social and political demands placed on mountain lions and their prey by Montana's human population.

COMMENT:
USE OF DOG RADIOS - 2

- "I do not support the use of radio-transmitters/receivers for locating hounds or the use of citizen - ban radios for communication during lion or bobcat kill seasons."

- "I believe radio telemetry during the harvest season gives an unfair advantage and detracts from the sport. I support it's use during "chase season"."

RESPONSE: Neither the Commission or MFWP currently have the authority to regulate the use of radio equipment used to track trailing dogs or other communication equipment used during the hunting season.

COMMENT:
Reinstate the bounty - 2

- "A few years back there was a bounty on lions. That was a good deal. They stayed away from people (most of the time) and there was never a shortage of lions."

RESPONSE: Such an action would require the dropping of the mountain lion as a game animal and reinstating the bounty by the Legislature.

COMMENT:
Quality of the hunt - 2

- "The issue listed that is of greatest concern is the extreme competition between lion hunters prior to the time the quota is filled within lion hunting districts. This has substantially removed quality from hunting lions during this period. ... This concern however is not listed as an Issue in Chapter I. and therefore, specific strategies to resolve this concern have not been incorporated into the action alternatives."

RESPONSE: A combination of elements used to analyze the 4 Alternatives of the DEIS would allow each Region the latitude to address the competition for mountain lions by hunting district or area.

COMMENT:
Waiting period for successful hunters - 1

- "I support a waiting period for successful lion hunters similar to the system for sheep. Lions are a special animal and while the interest in hunting is so high we should have system to distribute the harvest and again encourage more folks to participate."

RESPONSE: As shown in Table 12. page 97 of the DEIS few hunters (<17%) take more than one mountain lion. Restricting all successful hunters from the opportunity to hunt on an annual basis would not serve to significantly reduce hunter numbers or significantly increase the opportunity of taking a mountain lion.

OFFICIAL STATEMENTS RECEIVED AT PUBLIC MEETINGS
(Number indicates the number of meetings where the issue was raised)

No weekend opener - 2
Reduce closure - 4
    for reduction to 24 hours - 4
24 hour reporting - For - 4
    Leave as is - 2
24 hour inspection - For - 2
    allow 5 days for skull tagging - 1
    not enough time - 3
Protect females and dependant young - 2
    do not allow dependent young (animals with females but without spots to be taken) - 3
Need additional base line research - 1
No registration - 6
    retain chase permit - 2
    hound handler permit takes care of this - 1
    change name i.e. drop "registration" - 1

Regulation of hunter numbers
    Limit nonresidents to 10% of the harvest - 2
    No drawings - 1
    Validation; For - 3; Against - 6
    Males on permits and females on quota - 1

Increase enforcement efforts - 7
    Check kill sites - 5

Open with the general season - 2
    Don't open with the general season - 5

Appendix 3, Table 1. Persons who provided oral comments on the DEIS at public hearings.

Havre
Greg Durward
Randy Rose
Dale Naber
Ron Cortese
Chris Faber
Laurina Burns

Eureka
Don Carvey
Nick Carvey
Clint Mills
Rupert Schreiver
Arlie Burk
Jack Burk
Steve Hawkins

Malta
Roger Licht
Louie Hould
Smucky Mann
Kevin Salsbery
Jerome Hould

Great Falls
Gary Knudson
Stan Meyer
Brad Lencioni
Larry Antonich
Don Davidson
Dan Lencioni
Nick Jung
Rick Cook
Gary Lankford
Kip Deboo
Buzz Martin
Mike Hofland
Scott Stroberg
Thomas Hardin
Ken Ouellette
Steve Tylish
Rocky & Lorell Heckman
Clyde Lankford
Gordon Lencioni
John Meyer
Tom Hutton

Missoula
William Schutter
Bob Wiesner
C. E. Bell
Bill Mitchell
Bobby Sutton
Scot Waletzko
John Simons
Dave Cousch
Dave Majors
Ken Schoening
Paul Rossignol
Greg Houska
Dave Boice
John Davidson
Steve Anderson
Larry Nelson
Jeff Freeman
Mike Fairclough
Mile Waletzko
Robert & Bev Tolker
Erick Lechleitner
Deirdre Anderson
Merle Bauer
Dave Barstad
Marianne Sytzforn
Steve Tenold
Mike Roy
Scott Muller
Mike Beeker
Don Earon
Bud Martin
Ben Hubbard
Scott Adder
Kathy Mauer
Robert Carroll
Jeff Hahn
Kent Anderson
Robert Anderson

Kalispell

Richelle Auger
Paul Grise
Steve Hawkins
Don Clark
Ray Bannon
Ruper Schneiver
Earl Wayne Weaver
Jared C. Wittake
Terry Suber
Dale Williams
Brenda Williams
Rob Tibbs
Randy Tolar
Charles Bogle
John Easton
Ronald Buentemeier
Rick Stevens
Will Collin
Arlan Kolodejduk
Jeff McCall
Ben Weidling
Roy Congdon
Steve D. Howe
Glen Gray
Clock Freeman
Todd Davis

Noxon

Robert Flansaas
Jerry C. Shively
Ben Mummert
Glenn Smith
Joel Smith
Dave Clay
Wayne Hill
Stan Arrants
Teri Burt
Bill Hagedorn
Kim Posselt
Jeff Smith
Speed Jessier
Rus Willis

Appendix 3. Table 2. Persons who provided written comments on the DEIS.

Ben Mummert,Trout Creek, MT 59874
Raymond J. Baemer, Libby, MT 59923
Teresa I. Mielke, Billings, MT 59106
Gerald L. Dwyer, Missoula, MT
Dan Rouse, Darby, MT 59829
Jack Atcheson, Butte, MT 59701
Emery O. Johnson, Great Falls, MT
Glen N. Gray, Swan Lake, MT 59911
Virgil W. Binkley, Townsend, MT 59644
Mike Beckel, Missoula, MT
L. A. Fatouros, Livingston, MT 59047
Kim Posselt, Trout Creek, MT 59874
Suzanna McDougal, Hamilton, MT 59840
Jeff Smith, Trout Creek, MT 59874
Marianne Spitzform, Missoula, MT 59802
John L. Sparks, Manhattan, MT 59741
Paul H. Martin, telephone comment
Shirley Kolodejchuk, Coram, MT 59913
Arlen Kolodejchuk, Coram, MT 59913
Dale W. Combs, Wise River, MT 59762
D. Brus,
Walter Fellows,Billings, MT 59101
Don Risland, Paradise, MT 59856
Kendall Creek Kennel, Clinton, MT 59825
Wayne Moore, Big Timber, MT 59011
Robert Nasheim,Glendive, MT 59330
David M. Skiera, Clancy, MT 59634
Richard O'Connor, Brusett, MT 59318
Lanny Perry, Utica, MT
John H. Voelker, Columbia Falls, MT
Mt Houndsmen Assn.,Emigrant, MT 59027
Will Collins, NW MT Houndsmen Assn.
Dave Majors, Stevensville, MT 59870
Larry Nelsen, Missoula, MT 59802
F. H. Stoltze Land and Lumber Co., Columbia Falls, MT 59912
D. W. Williams,Montanans for Multiple Use, Whitefish, MT
Don Clark, Libby, MT 59923
Rick Stevens, Whitefish, MT
Dale Naber, Butte, MT
Michael Roy, Natl. WL Federation, Missoula 59802
Toni K. Ruth, Hornocker WL Res., Columbia Falls, MT
Chris Marchion, Anaconda, MT 59711
Ben Hubbard, Missoula, MT 59801
Mick Waletzko, Missoula, MT 59802
Steve Antonioli, Skyline Sportsmen's Assn., Inc.,Butte, MT 59703
Dave Bryant, Kila, MT 59920

Tolker's, Alberton, MT 59820
Dan Hershman, Arlee, MT 59821
J. D. Lear, E. Missoula, MT 59802
Armand H. Johnson, Missoula, MT
Kelly A. Lloyd, no address given
Gary Creran, Missoula, MT
Bill Schutter, Lolo, MT 59847
Randy Strending, Missoula 59801
Joe A. Petersen, Alberton, MT 59820
Suzanne S. Bonner, Lolo, MT 59847
Monte and Mary Ellen Schnur, Townsend, MT 59644
Broadwater Outfitters & Guides Assn., Townsend, MT 59644
David Schneider, Helena, MT 59601
Richard Stavenow, Eureka, MT 59917
Timothy L. Ravndal, Townsend, MT 59644
Petition signed by 27 individuals - Broadwater, Meagher County
Scott Waletzko, Huson, MT 59846
Bob Wiesner, Missoula, MT 59802
Robert Flansaas, Noxon Rod and Gun Club, Noxon, MT 59853
Chuck Freeman, Columbia Falls, MT
David A. Pierce, Kalispell, MT 59901
Worth Nixon, Libby, MT 59923
Sabrina Ravndal, Townsend, MT 59644
Clyde Lankford, Great Falls, MT 59404
Deborah E. Boots, Noxon, MT 59853
Dr. & Mrs. Carlton W. Shaw, Bozeman, MT 59715
Ron and Marge Cheener, Townsend, MT 59644
Greg Houska, Missoula, MT 59801
Chester Wolter, Sheridan, MT 59749
Eric and Julie Weare, Noxon, MT 59853
Bill Reynolds, Kalispell, MT 59901
William J. Collins, Kalispell, MT 59901
Mike Shrah, Bitterroot Valley
James Phelps, Montana Audubon Council,Billings 59102
Dennis B. Harms, Livingston, MT 59047
James M. Clawson, Western MT F & G Assn., Missoula
Allen Jensen, no address
Robert L. Carroll, III, Clinton, MT 59825
Bob Heckel, Kalispell, MT 59801
John Easton, Kalispell, Mt 59801
Ben Weidling, Columbia Falls, MT 59912
Donald R. Page, Kalispell
Paul Petrusha, Colstrip, MT 59323
Scott Sallee, Emigrant, MT 59027
Merle Aus, Glendive, MT 59330
Wade and Jamie Johnson, Anaconda, MT 59711
Jefferson Valley Sportsmen's Assn. Inc., Whitehall, MT 59759
Keith Pietsch, Whitehall, MT 59759

Tom Madder, Biddle, MT
Roland Deane, Bozeman, MT 59715
Kerry Murphy
Chris Mehus, Montana Stockgrowers Association, Helena, MT 59624
Sandra Seaton, Montana Houndsmen Assn., Emigrant, MT
Jamie Sallee, Emigrant, MT 59027
Sandy Seaton, Emigrant, MT 59027
Susan Johnson, Gardiner, MT 59030
G. C. & Ida Sallee, Livingston, MT 59047
Gary W. Lankford, Great Falls, MT 59401
Harry Whitney, Bozeman, MT 59715
Grover L. Hedrick, Boulder, MT 59632
Scott L. Koelzer, Three Forks, MT 59752
Brian Koelzer, Three Forks, MT 59752
Nick Grenfell
Gary Grenfell
Richard Vetsch, Townsend, MT
Charles C. Bogle, Olney, MT 59927
Kevin Fox, Billings, MT 59101
Kenneth Schoening, Huson, MT 59846
Ronald W. Wilkerson, Missoula, MT 59802
Stephen V. Mayernik, Stockett, MT 59480
John Roseland, Lewistown, MT 59457
Mable L. Deane, Bozeman, MT 59715
Jerry L. Gillingham, no city listed
Shirley T. Seaton, Livingston, MT 59047
Floyd K. Bruce, no city listed
Don Davidson, Great Falls, MT 59404
Darrell Woodahl, Lincoln, MT 59639
P. J. Breithaupt, Troy, MT 59935
Rem Kohrt, Whitefish, MT 59937
Don Capp, Anaconda, MT 59711
Don Artley, Forestry Division, Missoula
Mike Hofland, Valier, MT
Jerry C. Shively, Thompson Falls, MT
Darrell L. Tessier Trout Creek MT
