# Supplementary material for: The Elephant in the room: What can we learn from California regarding the use of sport hunting of pumas (Puma concolor) as a management tool?
Source: PLoS One. 2020 Feb 13;15(2):e0224638. doi: 10.1371/journal.pone.0224638 (PMC7018503; doi:10.1371/journal.pone.0224638)
Supplement: S2 File — This is a zip file that contains sources of the data used in the analyses that are no longer available on the internet. (ZIP) [file pone.0224638.s003.zip › Mountain Lion Monitoring and Management Strategy_Final_Adopted.pdf]

MONTANA MOUNTAIN LION
MONITORING & MANAGEMENT STRATEGY

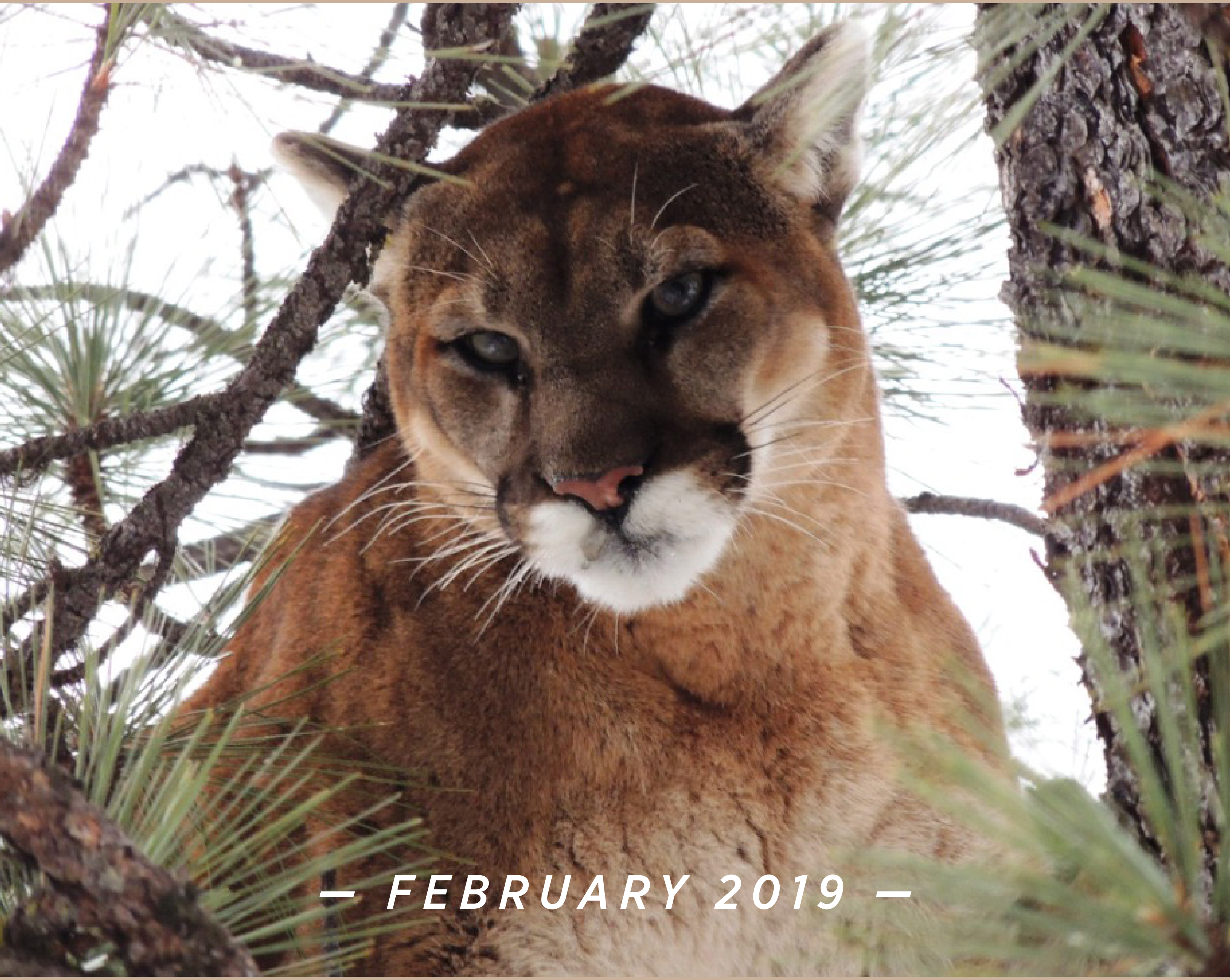

MONTANA FISH, WILDLIFE & PARKS

WILDLIFE RESTORATION

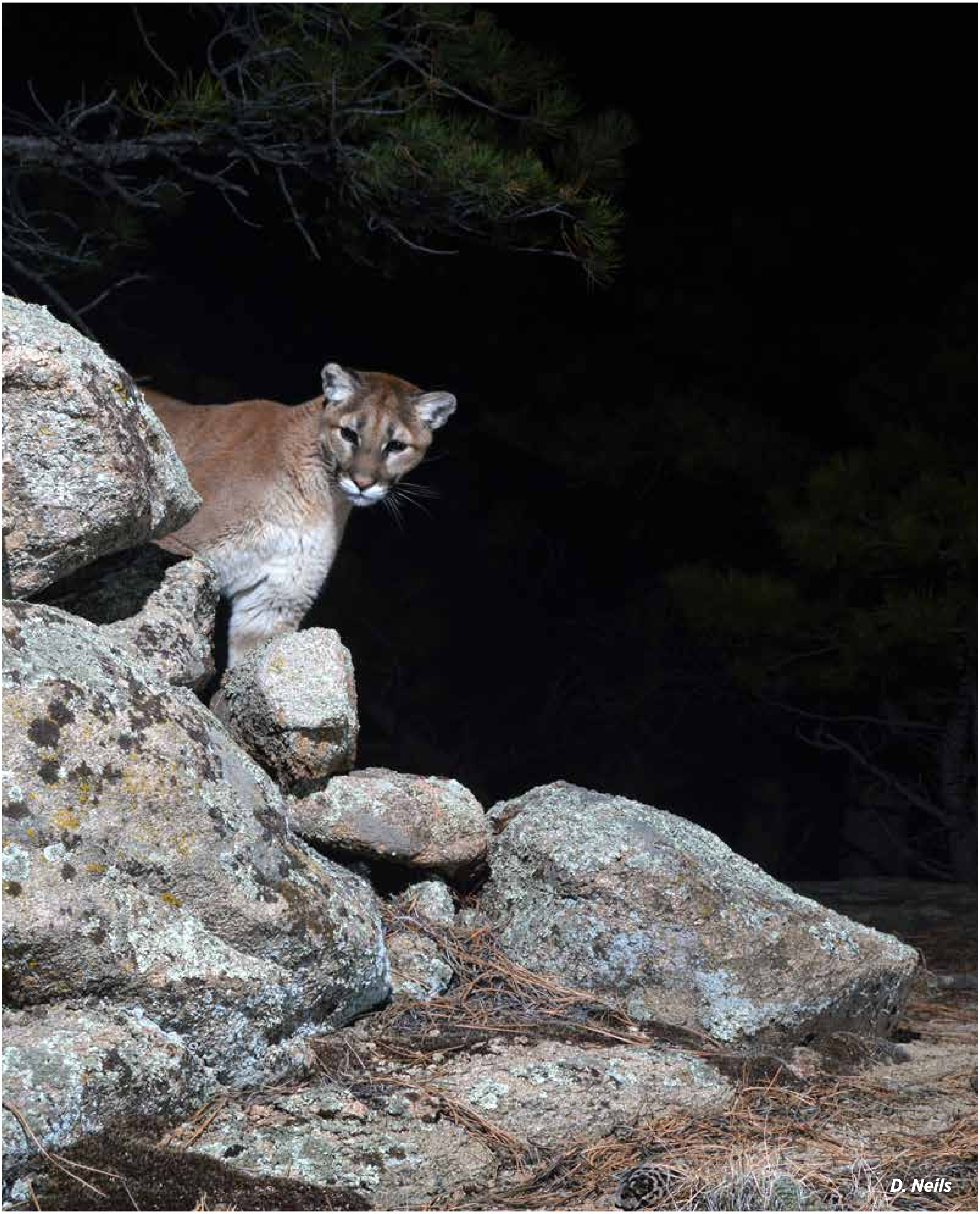
D. Neils

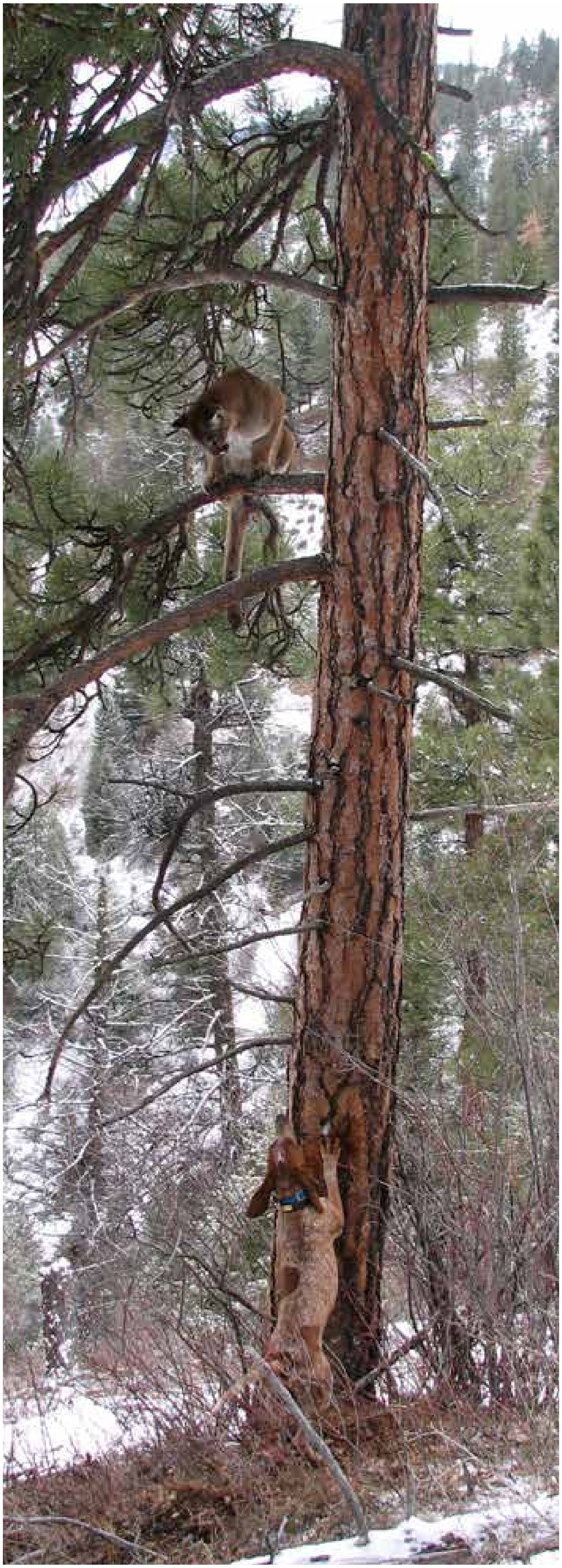

TABLE OF CONTENTS

MOUNTAIN LION CONSERVATION AND MANAGEMENT GUIDELINES 4

EXECUTIVE SUMMARY 6

ACKNOWLEDGMENTS 8

CHAPTER 1 MOUNTAIN LIONS IN MONTANA 10

CHAPTER 2 MOUNTAIN LION-HUMAN CONFLICT 19

CHAPTER 3 2016 MONTANA MOUNTAIN LION RESOURCE SELECTION FUNCTION 26

CHAPTER 4 MONTANA MOUNTAIN LION ECOREGIONS 31

CHAPTER 5 MONITORING MOUNTAIN LION ABUNDANCE 43

CHAPTER 6 THE MONTANA MOUNTAIN LION INTEGRATED POPULATION MODEL 50

CHAPTER 7 MOUNTAIN LION HARVEST REGULATION 56

CHAPTER 8 ADAPTIVE HARVEST MANAGEMENT 61

CHAPTER 9 REGIONAL MANAGEMENT CONSIDERATIONS 67

APPENDIX 1 POPULATION MONITORING, FIELD PROTOCOL 94
AND DATA ANALYSIS

APPENDIX 2 MOUNTAIN LION INTEGRATED POPULATION MODEL DEFINITION AND USER INPUTS 99

APPENDIX 3 MOUNTAIN LION DEPREDATION AND CONTROL GUIDELINES 103

APPENDIX 4 GUIDELINES FOR RESPONDING TO WILDLIFE ATTACKS THAT RESULT IN HUMAN INJURY OR DEATH: “WHART” GUIDELINES 106

APPENDIX 5 MONTANA MOUNTAIN LION LICENSE SALES, PRICE, AND REVENUE, 1973 - 2015 111

APPENDIX 6 APPLICABLE MONTANA STATUTE AND ADMINISTRATIVE RULES 113

APPENDIX 7 MONTANA MOUNTAIN LION IPM MODEL CODE 128

LITERATURE CITED 132

Cover photo: R. Wiesner

MOUNTAIN LION CONSERVATION AND MANAGEMENT GUIDELINES

With the publication of this document, Montana Fish, Wildlife & Parks (FWP) reaffirms its commitment, on behalf of the public, to the conservation and responsible management of mountain lion populations in Montana.

Many FWP wildlife biologists might find it redundant to first state that we are committed to conserving mountain lions. We tend to skip instead to describing specific strategies for mountain lion management, while taking our professional dedication to wildlife conservation for granted.

But we've learned over the years that an intensely interested and engaged public does not always accept FWP's commitment to mountain lion conservation as a given, and may not recognize FWP's management strategies as being consistent with conservation. Although our society has a long and evolving heritage of valuing wildlife, we acknowledge that Montana and other western states have risen relatively recently to the challenge of actively conserving mountain lions.

Many Montanans can still remember the bounty years when antagonistic public attitudes toward predatory wildlife were common. Since then, questions and concerns surrounding the management of mountain lions have increased as more people with a stake in mountain lion management come to the table.

One measure of Montana's commitment to wildlife conservation is the abundance, diversity, and distribution of our large predators. Wolves are now biologically and legally recovered, grizzly bear populations exceed restoration milestones, and the mountain lion has re-occupied its historic statewide habitat.

But with this success comes increased management complexity. Local declines in elk abundance and hunting opportunities, concerns about public safety, sharply responsive mountain lion hunting regulations, and uncertainties about management's effects on lion populations have sometimes strained a consensus about our values and management direction.

And conservation itself, we understand, is in the eye of the beholder. So, we strive to be clear. The following are the conservation and management guidelines that will direct FWP's decisions, and against which more specific management objectives will be measured.

FWP will conserve mountain lions as a functional and valued part of Montana's wildland ecosystems.

FWP will help manage suitable and connected habitat at a landscape scale for mountain lions and their prey.

FWP will responsibly manage mountain lions as a public trust resource and consistent with state law.

FWP will maintain and enhance public acceptance of mountain lions by helping landowners, homeowners, and the recreating public prevent conflicts with mountain lions. FWP will respond promptly and professionally when conflicts occur.

FWP will enhance public appreciation for mountain lions by providing information and insight about the role of mountain lions in the ecosystem and on practices for living and recreating in lion habitat.

FWP recognizes that mountain lion hunting is a highly valued recreational pursuit and that hunting plays a critical role in maintaining public advocacy and tolerance for the species. FWP will therefore recommend limited and sustainable mountain lion hunter-harvest opportunity on most lands within its jurisdiction. FWP will provide professional advice the Fish and Wildlife Commission to help ensure that hunting opportunities remain available to Montana resident, nonresident, and outfitted mountain lion hunters using simple and consistent regulations.

FWP will use an adaptive harvest management framework to develop and evaluate most mountain lion management decisions. Potential management objectives will be made explicit to all stakeholders throughout the decision-making process and the best available information will be used to evaluate whether those objectives are being met.

FWP will maintain a balance between mountain lion populations, their prey, and humans by helping direct local harvest of mountain lions, if and as needed, to manage prey survival and reduce human-lion conflicts. FWP specifically recognizes that mountain lion populations are most effectively conserved at the landscape scale, rather than within smaller individual Lion Management Units where prey survival or points of conflict may be concerns worthy of management.

FWP will develop informed public consent regarding the conservation status of mountain lions and the potential consequences of FWP management recommendations by instituting a credible, science-based system for estimating and monitoring Montana's lion populations.

FWP will consider, monitor, and conserve mountain lions at a landscape scale, consistent with the species' ecology. Specific management objectives will encourage sustainable and well-connected mountain lion populations within these landscapes.

EXECUTIVE SUMMARY

Despite historic persecution, mountain lions are thriving once again in Montana. Lions have reoccupied their historic statewide range and dispersing individuals now contribute to expanding populations across the western and midwestern U. S. This recovery is a testament to Montana's tradition of protecting habitat, conserving native wildlife populations, and investing in research that provides the scientific basis for sound wildlife management decisions.

The number of lion hunters and hound handlers has also increased during the last 40 years. These sportsmen and women became the state's most effective advocates for lion conservation and they have consistently encouraged FWP's efforts to improve lion management. Montanans, hunters and non-hunters alike, now expect assurances from FWP that lion populations remain healthy and that lion management decisions are informed by objective data instead of emotion.

Unfortunately, many past lion management decisions were controversial. Because it was impossible to precisely count lions or monitor population trends, Montanans who care deeply about lions and their prey often disagreed about the effects of lion harvest on both.

FWP clearly realized the need for better methods to track lion population changes and for a scientific framework upon which to base management recommendations. Over the last 25 years FWP made significant investments in field research that had improved our understanding of lion ecology and the way lions interact with their prey. FWP biologists and partners also developed new methods to monitor lion populations and built innovative population models that predict the effect of past and future harvest.

FWP intends to maintain sustainable lion populations across all suitable habitats within its jurisdiction. An important goal of this Strategy is to provide the public

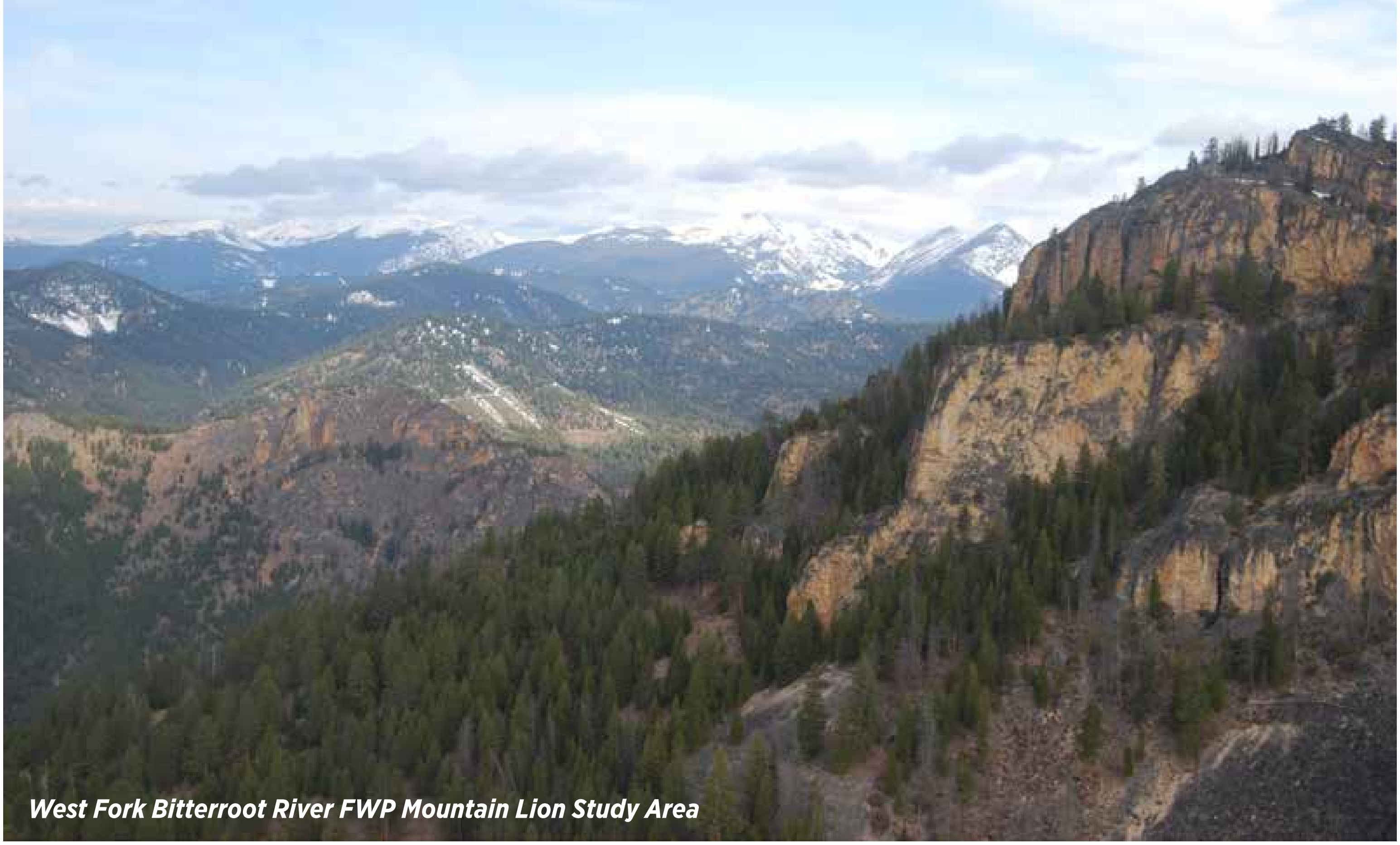

West Fork Bitterroot River FWP Mountain Lion Study Area

and the Department with accurate and timely information so that both populations and harvest are more stable over time. Accurate monitoring and modeling data will enable simpler harvest regulations, improve our ability to reduce conflicts, and allow FWP to better manage local lion densities while protecting regional populations.

Research in Montana and other states has revealed that lion ecology is remarkably similar across the species' western North American range. Populations in western North America are well connected and generally resilient to moderate harvest. However, hunter harvest is often additive to other forms of mortality and should be limited to prevent unwanted population declines. Critically, we now understand that lion populations are most effectively managed at large spatial scales.

For this management strategy FWP used a habitat model, built using Montana-based research and harvest data, to describe four biologically meaningful mountain lion "ecoregions" within the state. These ecoregions will be the spatial basis of FWP's lion management. FWP will periodically develop estimates of mountain lion abundance within most ecoregions using genetically-based field sampling.

Managers will then include these population estimates, our understanding of lion ecology, and lion harvest data to inform statistical models that predict the effects of lion harvest on statewide populations. Over time, this monitoring program will reduce uncertainty about the effects of lion harvest and will improve FWP's ability to meet lion management objectives.

An adaptive harvest management process will guide most of Montana's mountain lion harvest decisions. FWP will work with the public to develop clear and measurable population objectives at the ecoregion scale, as well as hunting seasons and harvest prescriptions that are most likely to meet those objectives. The effects of lion harvest will be regularly monitored so that harvest can be adjusted based on current information.

Although overall management objectives and harvest prescriptions will be developed at a large (ecoregional)

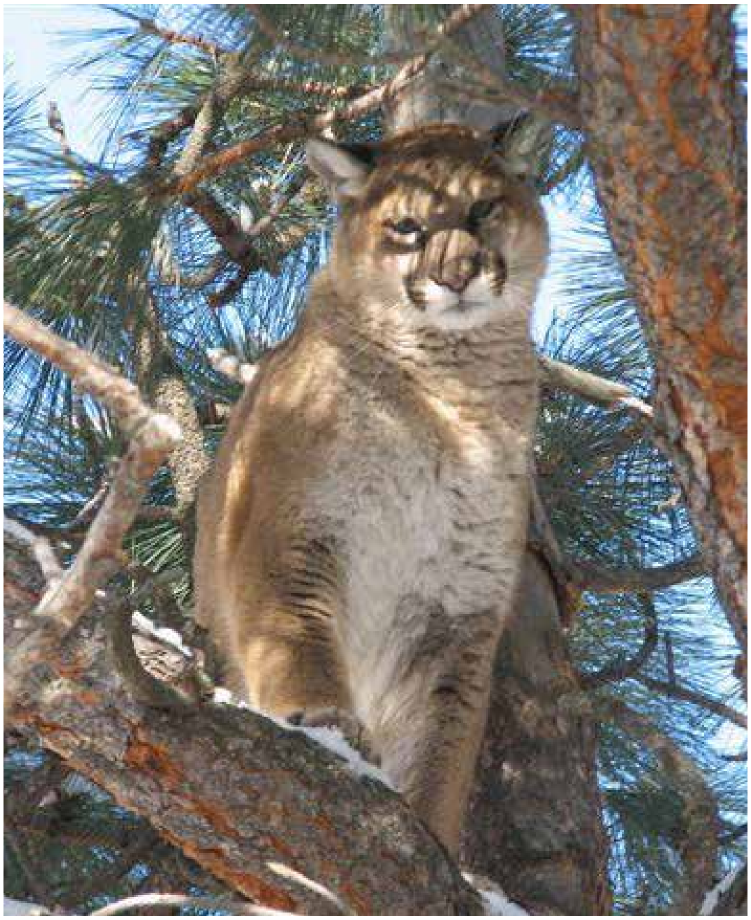

scale, harvest limits will generally be distributed across an ecoregion's lion management units to address social concerns, reduce hunter crowding, and focus or limit harvest where needed.

The following chapters describe FWP's mountain lion monitoring program and methods to produce periodic estimates of mountain lion abundance across the state. This Strategy includes a population model that will allow managers to effectively use those field-based estimates and other information to make predictions about the effect of future mountain lion harvest. We present policies detailing how FWP will reduce and respond to human-lion conflicts. Finally, we describe an adaptive harvest management process that will help FWP and the public build realistic lion management objectives and how to evaluate whether those objectives are being met.

This Management Strategy represents FWP's long term commitment to use the best available scientific information to ensure that mountain lion management decisions are as objective, transparent, and adaptive as possible.

ACKNOWLEDGMENTS

This document is a synthesis, and practical application, of fundamental mountain lion field research conducted over decades in western North America. We sincerely thank the many wildlife biologists, technicians, and managers whose efforts have contributed to our understanding of lion ecology. Their body of work specifically informed this effort and will help ensure the continued conservation of mountain lions in Montana.

Several biologists made specific and fundamental contributions to this strategy. Dr. Hugh Robinson of Panthera guided important Montana lion field research to publication and built lion habitat models that became critical components of this strategy.

Dr. Josh Nowak and Dr. Paul Lukacs, both with the University of Montana, worked with FWP to construct an interactive model that describes how harvest affects mountain lion populations. This model, and the web-based interface they built, will allow FWP to make better lion management decisions going forward.

FWP research scientist Dr. Kelly Proffitt developed innovative field and statistical methods to estimate local lion abundance and to extrapolate those estimates more broadly. Dr. Proffitt's work, and good advice, made this strategy possible.

FWP Game Management Bureau Chief John Vore patiently guided this strategy from its inception. His council and critical reviews vastly improved this document.

Justin Gude, FWP's Wildlife Research Chief, effectively advocated for and helped implement many of the projects that developed core components of this strategy. It would not have been possible without his vision and support.

FWP's Mike Thompson helped make clear that this strategy is intended to conserve Montana's mountain lions, not simply manage them. We sincerely appreciate both his perspective and eloquence.

Many FWP biologists and managers reviewed earlier drafts of this strategy and it was much improved by those efforts. Julie Cunningham, Adam Grove, Jessy Coltrane, Heather Harris, Elizabeth Bradley, Howard Burt, Ben Jimenez, James Jonkel, Jay Newell, Scott Eggeman, Justin Gude, Kelly Proffitt, Nick DeCesare, and Brent Lonner contributed and/or compiled particularly thorough and valuable comment.

Members of the Montana State Houndsmen Association, Northwest Houndsmen Association, Ravalli Co. Fish and Wildlife Association, Montana Outfitters and Guides Association, unaffiliated hound handlers, and others with a stake in lion management provided important input during the development of this Strategy. Their continued engagement as the strategy is finalized and implemented will be critical.

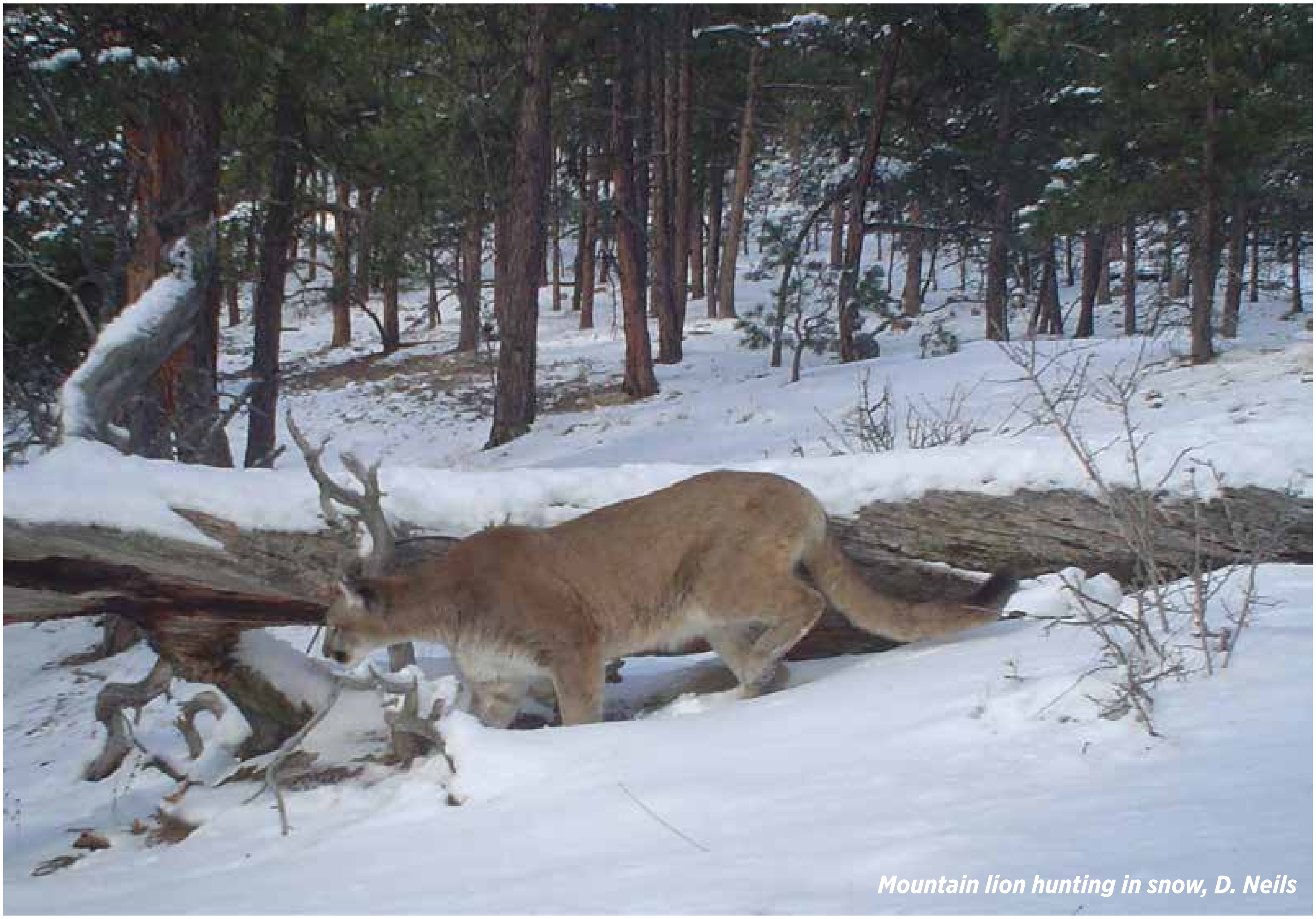

Mountain lion hunting in snow, D. Neils

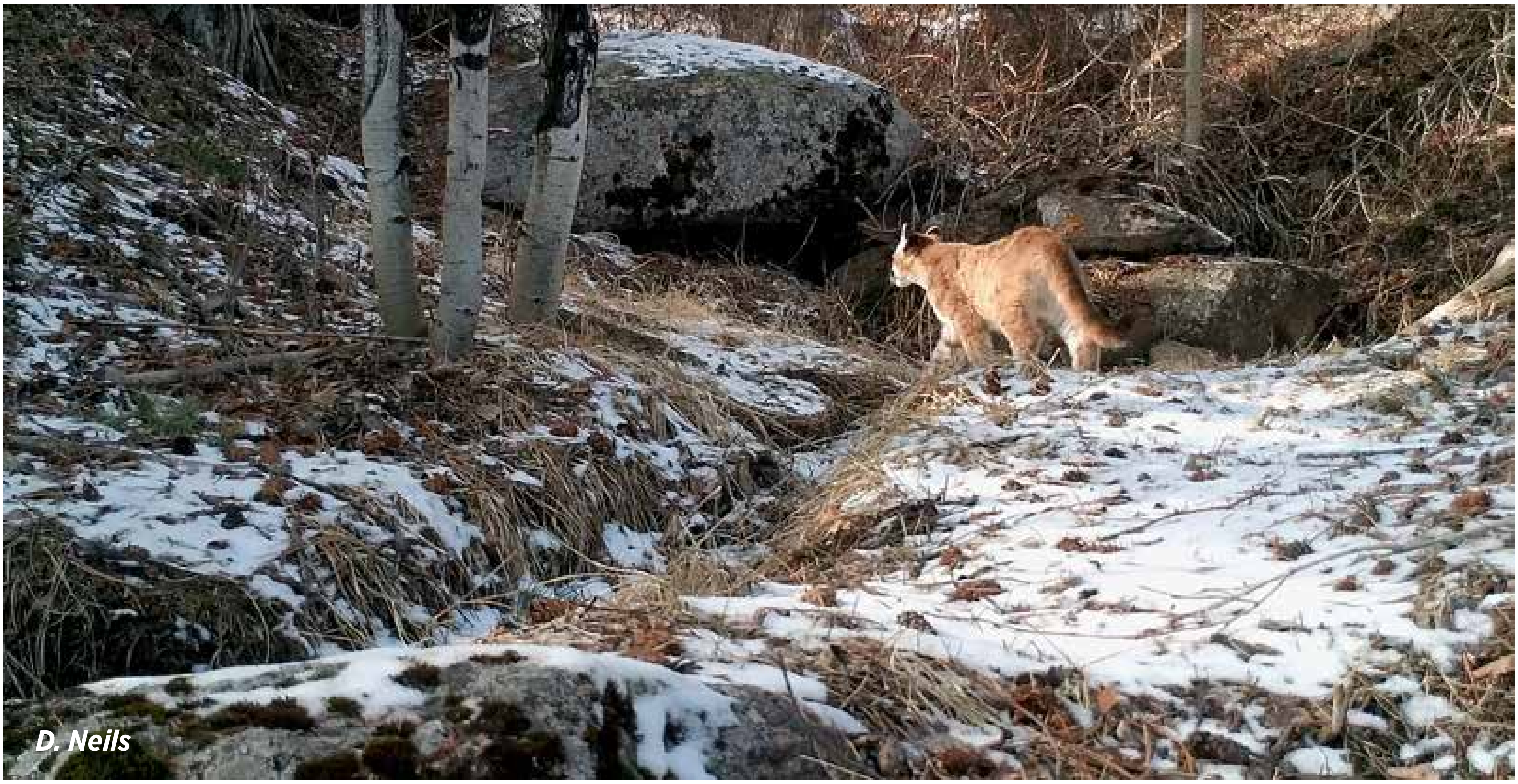

D. Neils

CHAPTER 1

MOUNTAIN LIONS IN MONTANA

Mountain lions were historically found in most of Montana except on its open plains and prairies (Young & Goldman 1946). Like other predators, Montana mountain lions had a bounty placed on them from 1879 to 1962. The number of bounties paid declined from a high of 177 in 1908 (at $8) to fewer than 5 per year by 1925 (at $25; $350 in 2016 dollars). At least 1,562 lion bounties were paid between 1900 and 1930 (Riley 1998). Mountain lions were nearly extirpated from the state by 1930 due to widespread persecution and the severe depletion of their ungulate prey.

Mountain lions began to recover in core Montana habitats during the 1950s as deer and elk numbers increased. Lions were designated as a predator from 1963 until 1971 when the state legislature reclassified the species as a game animal and transferred their management to the Fish and Game Commission.

Martin Bright and Ed Lord, Bitterroot Valley, 1890.

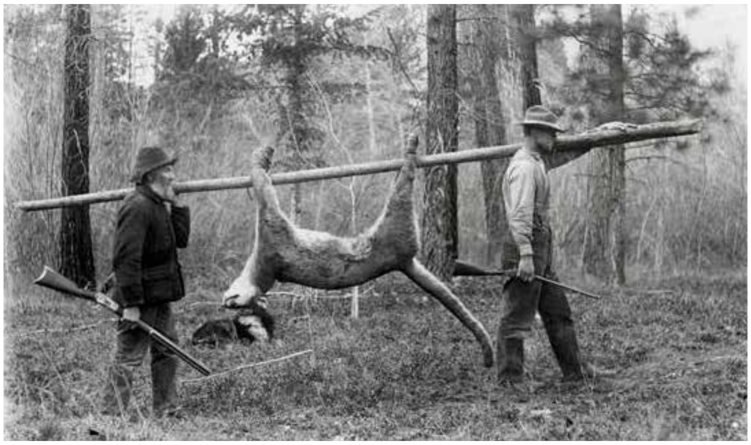

Lions expanded their range, and legal harvest increased, over the next 20 years (Figure 1, Table 1). In western Montana during the mid- to late-1990s the number of public lion sightings grew, human-lion conflicts became increasingly common, and harvest quotas filled quickly.

After the severe winter of 1996-97 caused white-tailed deer herds in west-central and northwest Montana to decline by as much as 50% (Montana Fish, Wildlife and Parks 2006), human-lion conflicts (including several nonfatal attacks)

Figure 1. Montana statewide mountain lion harvest, 1971 – 2016.

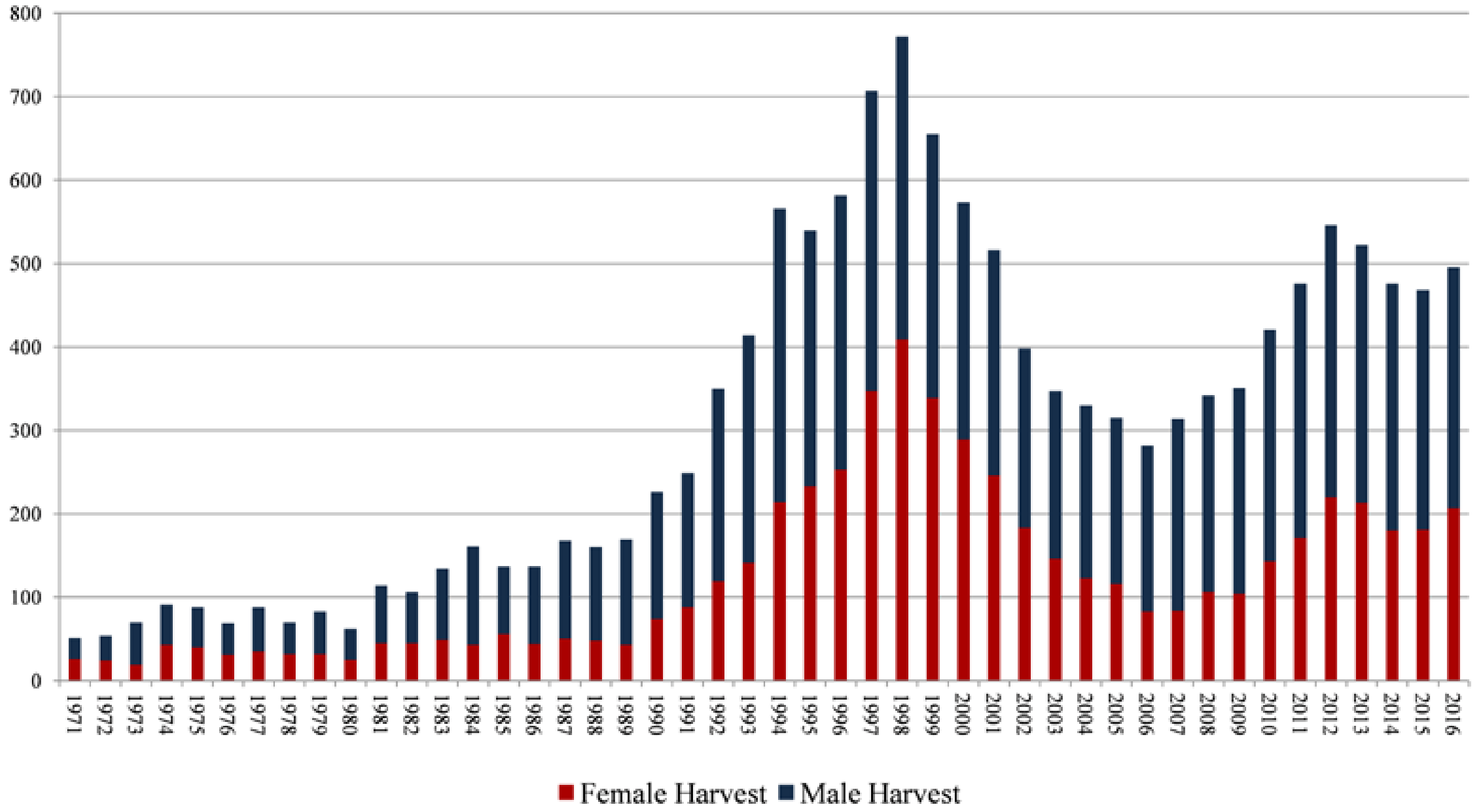

Figure. 2. Distribution of Montana mountain lion harvest, 1988 – 2015 (unshaded counties have had no harvests).

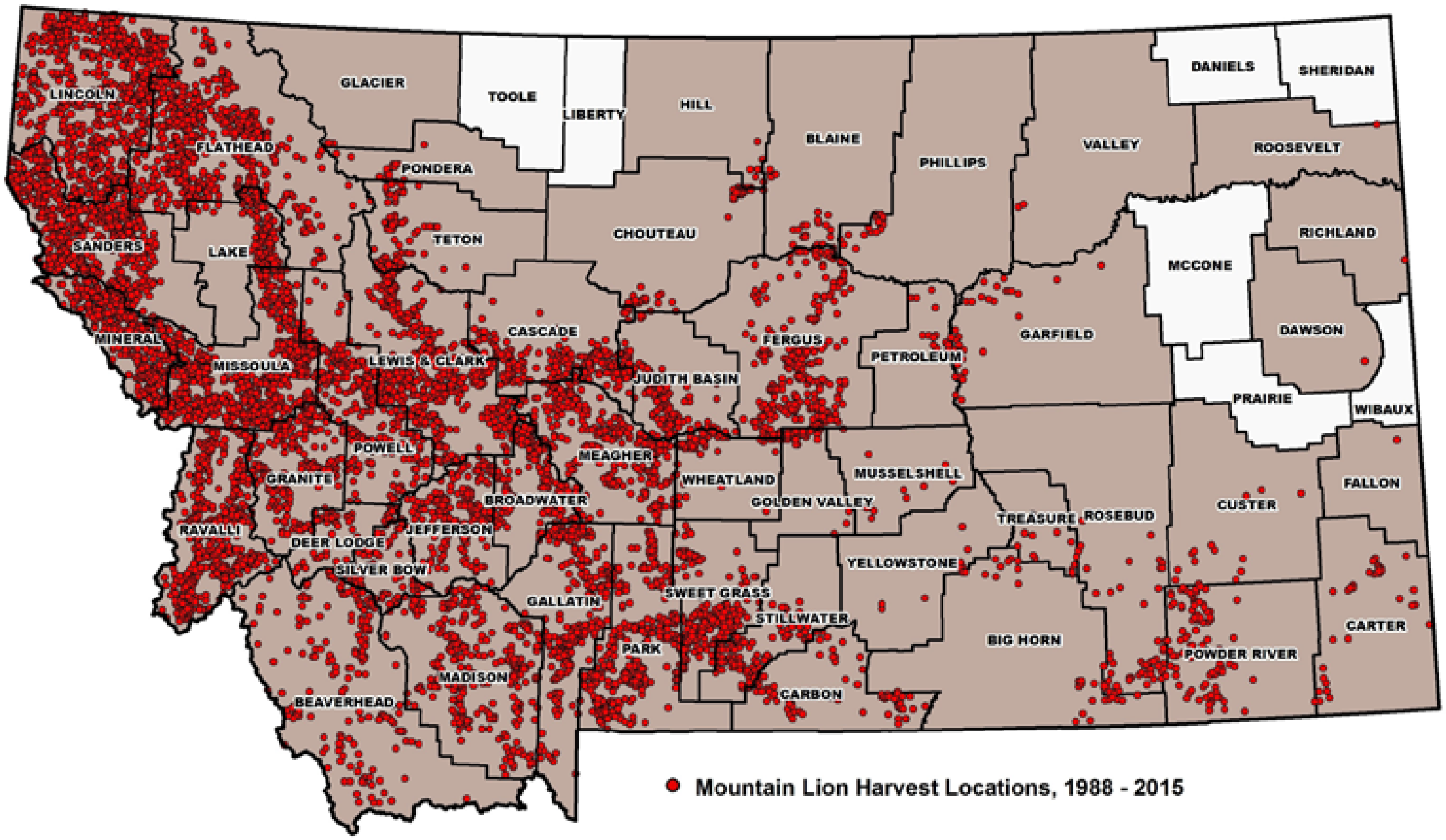

Table 1. Montana statewide mountain lion harvest, 1971 – 2016.

License	Statewide			
Year	F	M	Unk	Tot.
1971	26	25	0	51
1972	24	30	0	54
1973	19	51	2	72
1974	43	48	1	92
1975	40	48	0	88
1976	31	38	1	70
1977	35	53	0	88
1978	32	38	1	71
1979	32	51	0	83
1980	25	37	0	62
1981	45	69	0	114
1982	45	61	1	107
1983	49	85	2	134[1]
1984	43	118	4	161[1]
1985	56	81	6	137[1]
1986	44	93	4	137[1]
1987	50	118	2	168[1]
1988	48	112	1	160
1989	43	126	0	169
1990	74	152	0	226
1991	88	161	0	249[1]
1992	119	231	1	350
1993	141	273	0	414[1]
1994	214	352	0	566[1]
1995	233	307	0	540
1996	253	329	0	582
1997	347	360	0	707
1998	409	363	3	772
1999	339	316	0	655
2000	289	284	1	573
2001	246	270	0	516
2002	183	215	0	398
2003	146	201	0	347
2004	123	207	0	330
2005	116	199	3	315
2006	83	199	0	282
2007	84	230	0	314
2008	106	236	0	342
2009	104	247	0	351
2010	143	278	0	421
2011	171	305	0	476
2012	220	326	0	546
2013	213	309	0	522
2014	180	296	0	476
2015	181	287	0	468
2016	207	288	0	495

[1] Statewide totals differ from the Regions' sum because some harvest was reported as "unknown Region"

spiked. Managers were pressed to maintain historically high lion quotas in FWP Regions 1 and 2 because of concerns about public safety and to aid struggling prey populations. Lion harvest also reached record high levels during the late 1990s in Fish, Wildlife & Parks (FWP) Regions 3, 4, and 5.

By the early 2000s, many hound handlers believed that lion densities had significantly declined—an observation supported by ongoing FWP research in the Garnet Mountains. In response, the Fish and Wildlife Commission restricted the harvest of female lions during that decade in much of the state. By 2006, the Garnet Mountains research population had recovered to near 1990s densities (Robinson et al. 2014). Lions became increasingly common in eastern Montana FWP Regions 6 and 7 during the same period.

Mountain lions are now present in all suitable Montana habitats and continue to reoccupy neighboring states to the east. Between 1990 – 2016, an average of 450 lions were taken by licensed Montana hunters each year. Lions have been legally harvested in 49 of the state's 56 counties (Figure 2).

Harvest can be the most important factor affecting population size and growth where harvest occurs

Montana likely includes some of the most productive mountain lion habitat in North America. Although directly comparing lion densities across research projects and study areas is complicated (because of differences in field methods, inclusion of different sex-age classes in estimates, and the use of different areas over which density is calculated), reported North American lion densities generally range from 1 to 4 lions per 100 km^2 (37 $mile^2$; Hornocker & Negri 2009). In western Montana, researchers using DNA based detection methods have recently documented mountain lion densities exceeding 5 lions per 100 km^2 (Russell et al. 2012, Robinson et al. 2014, Proffitt et al. 2015).

GENETIC CONNECTIVITY

Mountain lion populations across the central Rocky Mountain west are genetically well connected. When wildlife populations are small and isolated, individuals can become more genetically similar over time. Although male lions are more frequent long-range dispersers (Logan & Sweanor 2001), Biek et al. (2006a) found that in Montana and Wyoming, neither male nor female resident lions shared more genes than expected by chance. Thus, frequent introduction of new genes by immigrating males is likely sufficient to maintain genetic diversity in females despite their lower dispersal rates and distances (Goudet et al. 2002).

Similarly, Anderson et al. (2004) found that there is ample gene flow between mountain lion populations in Wyoming and Colorado despite their being separated by large areas of relatively poor habitat. Even small and geographically isolated lion populations in North and South Dakota have maintained genetic diversity over time (Juarez et al. 2016).

In Montana, researchers genetically analyzed the fast-evolving feline immunodeficiency virus that commonly infects wild mountain lions. Although the study's 352 samples were collected as far as 1,000 km apart, there was no evidence of genetic sub-structuring, genetic drift, or barriers to gene flow within Montana populations (Biek et al. 2006b).

MOUNTAIN LION DISEASE, PARASITES, AND HUMAN HEALTH RISK

Mountain lions carry few communicable diseases that potentially threaten humans but certain precautions should still be taken when handling both live animals and carcasses. Fifty-four percent of lions sampled in Montana between 1971 and 1989 tested positive for the Trichinella roundworm. All harvested lions should be treated as if they are infected because a negative lab test does not mean Trichinella is not present. This parasite is transmissible to humans and pets if they consume undercooked infected mountain lion meat. Although mountain lion hunters are not required by Montana law to retain a harvested lion's meat (MCA 87-6-205), many hunters do. Trichinella infected lion meat that has been cooked to at least 165 degrees Fahrenheit is safe for human consumption (Western Wildlife Disease Workshop 2009).

Precautions protecting against the ingestion of other rare, but potentially fatal, air or blood-borne pathogens (i.e. pneumonic plague) should also be taken when handling a harvested lion carcass or one encountered in the field (Wong 2009). Pathogen infections or disease epizootics are not known to limit wild mountain lion populations in Montana.

EFFECTS OF HUNTER HARVEST

Mountain lion reproduction (age at first parturition, maternity, interbirth interval, litter size) and annual non-harvest mortality rates are remarkably consistent across western North American populations. Reproduction and non-harvest survival are also generally unaffected by hunter harvest. However, harvest can be additive to other forms of mortality and is often the most important factor affecting population size and growth in areas where harvest occurs. Lion populations are particularly sensitive to changes in adult female harvest rate (Anderson &

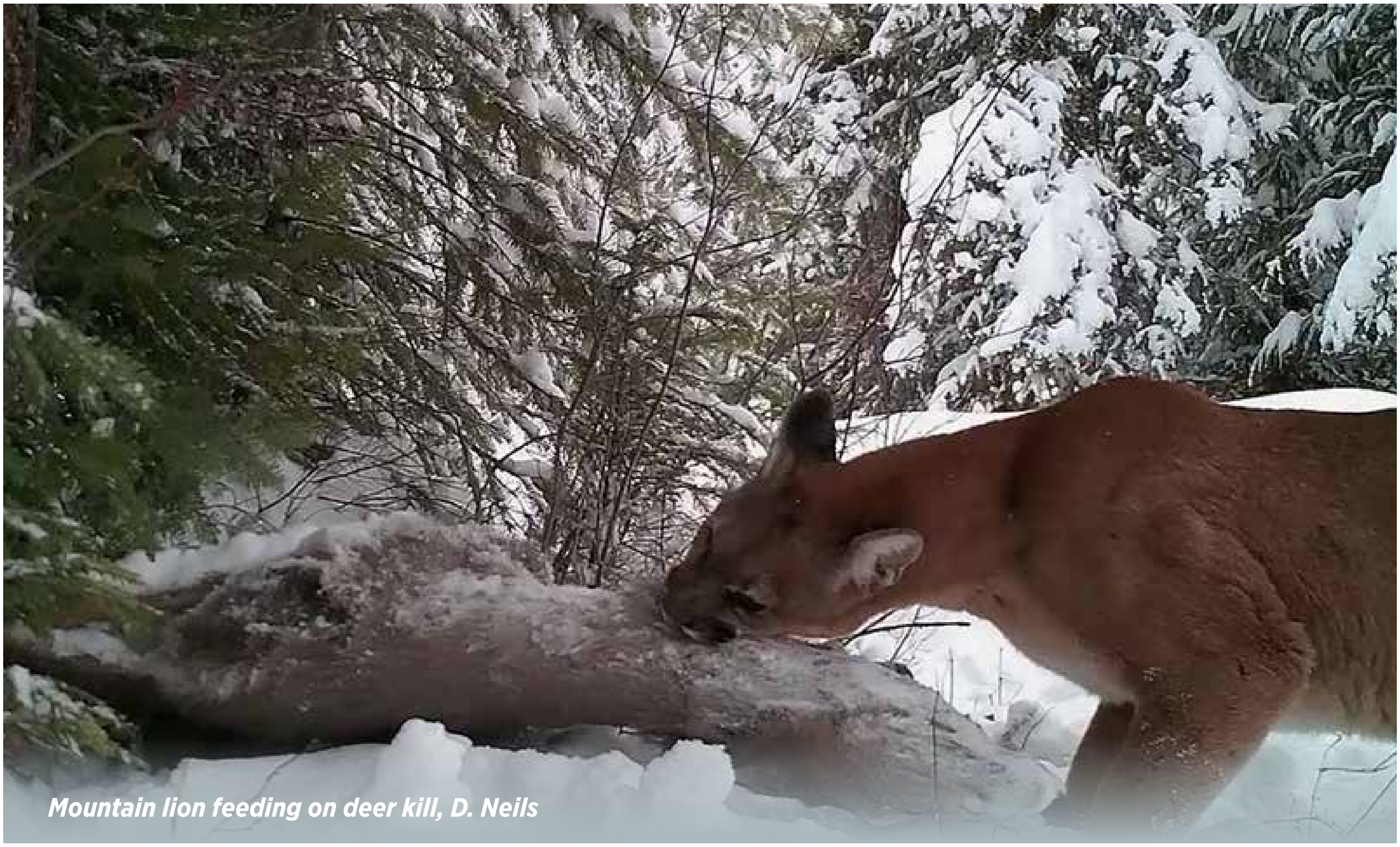

Mountain lion feeding on deer kill, D. Neils

Lindzey 2005, Stoner et al. 2006, Robinson et al. 2008, Cooley et al. 2009, Robinson et al. 2014).

Local mountain lion populations that are reduced by harvest can recover rapidly. Populations that are below prey limited densities can increase up to 30% annually when harvest (especially of females) declines and lions from other areas are able to immigrate (Ross & Jalkotzy 1992, Sweanor et al. 2000, Jenks 2011, Clark et al. 2014a). For example, in Utah, mountain lion densities that were reduced >60% over a 6-year period recovered to pretreatment levels after 5 years of reduced hunter harvest (Stoner et al. 2006). In New Mexico, an adult population that was experimentally reduced by >50% fully recovered in 31 months (Logan & Sweanor 2001), and in Wyoming a population that was lowered >40% by heavy harvest recovered in 3 years after harvest was reduced (Anderson & Lindzey 2005).

Montana lion populations are similarly resilient. Lion numbers in the Garnet Mountains declined nearly 50% during a period of heavy harvest but fully recovered within 5 years after the harvest rate was reduced there and in surrounding areas (Robinson et al. 2014).

The influence of dispersal and immigration on mountain lion population growth cannot be overemphasized. Even heavily hunted local populations may fail to decline if immigrants readily replace harvested lions (Cooley et al. 2009). On the other hand, a population (such as the one within the Garnet Mountains study area) may recover more slowly where high harvest rates are applied across a broader landscape.

Harvest can also alter a population's age structure. However, the interpretation of trends in the age of harvested mountain lions may be confounded by immigration, hunter selectivity, harvest regulations, and other factors. Monitoring changes in harvest-age composition can be a useful indication of a population's status in some cases. In general, the proportion of older age-class mountain lions in harvest—especially females—is higher within growing populations (Anderson & Lindzey 2005, Stoner et al. 2006, Wolfe et al. 2015). This index

should only be used when monitored over a period of 3 or more years (Anderson 2003), and after considering other factors (i.e. immigration and harvest) that may be influencing age-at-harvest.

Within a lightly hunted lion population in western Montana's Bitterroot Mountains, 60% of independent aged lions were female (Proffitt et al. 2015). This is similar to the proportion of juvenile (13-24 month) females documented during a 10-year study of a lion population in west-central Montana, although the proportion of adult males to females varied widely during the study period depending on the level of hunter harvest (Robinson et al. 2014). Male:female ratios of 1:2 to 1:3 were commonly reported in other hunted populations (Hornocker & Negri 2009).

MOUNTAIN LION-PREY INTERACTIONS

The relationship between mountain lion predation and their prey populations is complex. This is especially true in Montana where lions often occupy multi-predator/ multi-prey species systems. Mountain lions are the most influential ungulate carnivore across much of the state, especially where grizzly bears and wolves are absent or recovering. Therefore, wildlife managers must carefully consider the potential effects of mountain lion predation on prey populations when developing management prescriptions for both.

Mountain lions are opportunistic and adaptable foragers that prey or scavenge on a variety of species (Bauer et al. 2005, Murphy & Ruth 2011). In Montana, lions are obligate ungulate predators primarily preying on deer and elk. Mountain lion diet varies across the state depending on available prey, and lions may switch preferred prey seasonally as ungulate newborns become available or ungulate distribution changes (Williams 1992, Murphy 1998, Kunkel et al. 1999, Ruth & Buotte 2007). Mountain lions may also increasingly prey on pets, livestock (Torres et al. 1996), or other wildlife species (Logan & Sweanor 2001) following a significant decline in wild ungulate populations. Where hunter harvest is not an overriding factor, mountain lion densities are ultimately regulated by prey availability (Pierce et al. 2000a, Logan & Sweanor 2001, Stoner et al. 2006).

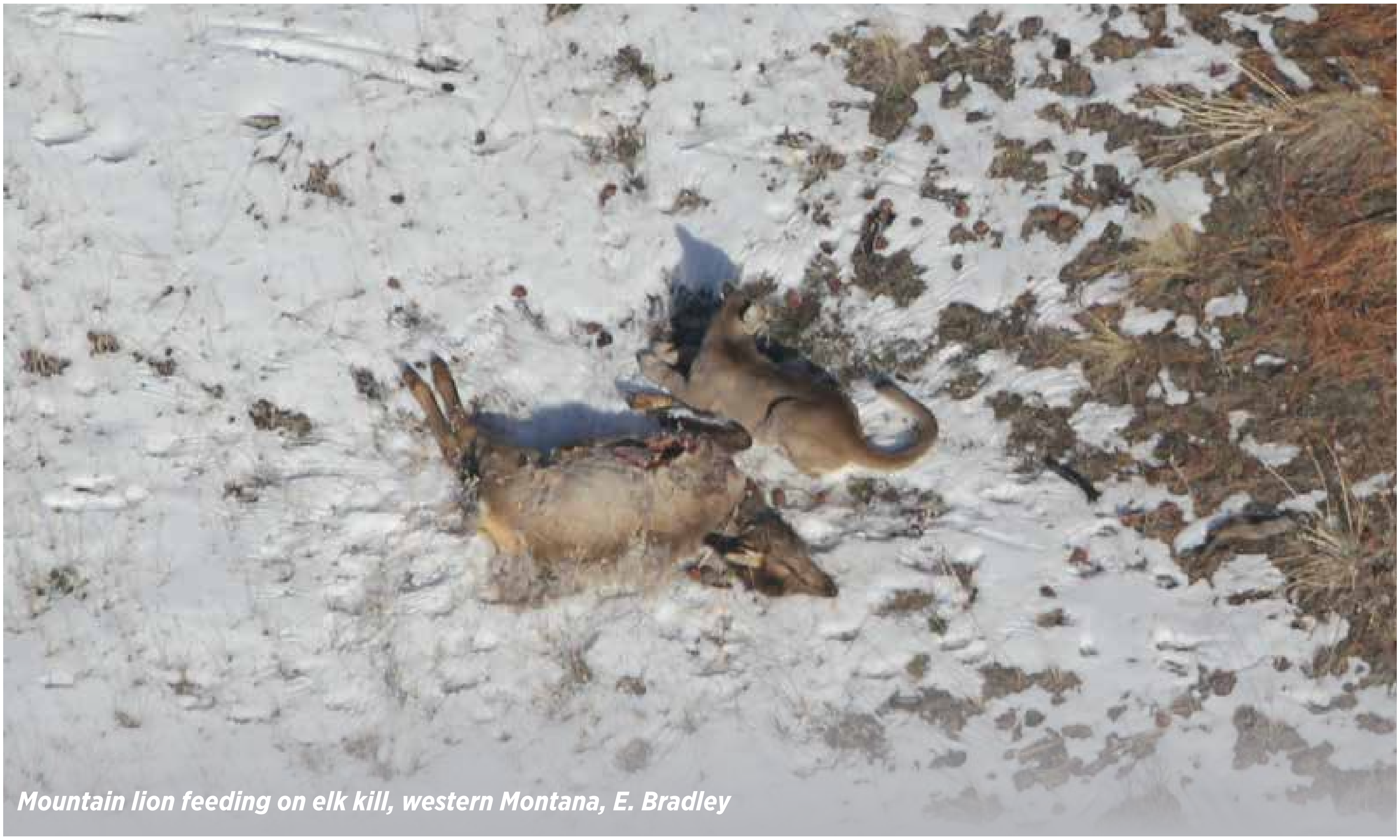

Mountain lion feeding on elk kill, western Montana, E. Bradley

GENERAL PREDATOR-PREY RELATIONSHIPS

In theory, compensatory predation removes a number of prey animals from a population that would have died anyway from another cause. Additive predation removes prey that would have otherwise survived. Predators regulate prey populations when the rate at which they remove prey changes along with prey population levels. Predation can limit prey population growth if the predation rate is independent of changes to a prey species' abundance—in these cases, predation can depress, rather than stabilize, prey populations.

Predation is more likely to limit a prey population when 1) an alternative and abundant prey species supports high predator densities, 2) prey is below carrying capacity despite weather and habitat that allow adequate survival and recruitment, and 3) there is a high predation rate relative to recruitment.

Predators can limit prey populations when predation is additive to other sources of mortality (i.e. severe weather or starvation). For example, in Idaho, when experimental mountain lion removals immediately increased mule deer fawn and adult survival, the effect of mountain lion predation initially appeared to be additive. However, reducing lion densities did not significantly affect overall deer population growth. In this case, weather and annual changes in forage quality ultimately regulated mule deer numbers — mountain lion predation was, in fact, compensatory over the long term (Bishop et al. 2009, Hurley et al. 2011).

In systems where most prey biomass is composed of a single, fecund, species (e.g. white-tailed or mule deer), predation itself is unlikely to depress prey populations for extended periods. However, when severe weather or other factors decrease populations significantly below habitat carrying capacity, mountain lion predation can delay the prey species' recovery (Ballard et al. 2001, Logan & Sweanor 2001, Pierce et al. 2012).

Where predator populations are sustained at high densities by an abundant prey species, populations of other relatively vulnerable or scarce prey species might decline or remain depressed (Messier 1994, Mills 2007). This apparent competition (Holt 1977) has been implicated in declines of mule deer (Robinson et al. 2002, Cooley et al. 2008), bighorn sheep (Logan & Sweanor 2001), mountain caribou (Kinley & Apps 2001) and other species (Sweitzer et al. 1997) due to lion predation.

Montana includes some of the most highly productive mountain lion habitat in North America

Winter severity explained most variation in annual white-tailed deer recruitment in northwest Montana. There, when harsh winter weather depressed reproduction and survival of hunted deer, predation (primarily by lions) became additive to other forms of mortality and exacerbated population declines (Montana Fish, Wildlife & Parks 2006).

Mountain lion kill rates vary by location and ecological system, but are generally reported as 1 kill per 7 days in deer dominated systems and 1 kill per 10 days in systems where elk are also available (Murphy 1998, Anderson & Lindzey 2003, Cooley et al. 2009). Lions tend to kill more frequently in warmer months, when ungulate newborns are available, and when competition with or rates of displacement by other predators is high.

Predation rates also vary depending on a mountain lion's age, sex, and reproductive status. Adults kill prey more frequently than younger lions. While adult females with dependent kittens exhibit the highest kill rate of any lion age/sex class, adult males kill a greater prey biomass on an annual basis (Nowak 1999, Buotte et al. 2008, Clark et al. 2014b). In Alberta, the annual live weight biomass of prey killed by mountain lions averaged 3,180 lbs. for subadult females, 4,520 lbs. for subadult males, 10,380 lbs. for adult males, 5,340 lbs. for adult females, 6,160 lbs. for females with kittens < 6 months, and 9,440 lbs. for females with kittens > 6 months (Knopff et al. 2010).

Deer are the most common mountain lion prey species in Montana. In northwest Montana's Salish Mountains, lions were the most common predator of radio marked white-tailed deer (Montana Fish, Wildlife & Parks 2006). Similarly, 87% of lion kills documented in Montana's North Fork of the Flathead River drainage were white-tailed deer, where elk, mule deer, and moose were also present in lower numbers (Kunkel 1999).

However, in northeast Washington mountain lions disproportionately selected for mule deer even though white-tailed deer were more abundant (Cooley et al. 2008). The same was true in south-central British Columbia where mountain lion predation was implicated in mule deer declines (Robinson et al. 2002). Where both elk and mule deer were present, female mountain lions were more likely to kill mule deer, whereas male mountain lions killed elk more frequently (Anderson & Lindzey 2003). Female lions may also select for calf elk and younger or older mule deer (Nowak 1999, Pierce et al. 2000b).

Although most researchers found that mountain lions selected for male elk and deer (Hornocker 1970, Kunkel et al. 1999, Anderson & Lindsey 2003, Atwood et al. 2007, Blake & Gese 2016), others did not (Clark et al. 2014b). Adult male elk and deer are more often killed by mountain lions during and after the rut while most adult female elk and deer are killed before giving birth in late spring (Knopff et al. 2010, Clark et al. 2014b).

The annual risk of mountain lion predation to adult female elk across the western U. S. (Brodie et al. 2013) and in Montana (Hamlin & Cunningham 2009, Eacker et al. 2016) is low compared to other sources of mortality, including hunting. This is important because, in certain situations, adult female survival explains more of the variation in overall elk population growth rate than elk calf survival (Eacker et al. 2017).

Lions are often one of the primary predators of elk during their first year of life. The rate of calf predation by mountain lions increases with overall lion density, decreases when other predators (especially wolves and grizzly bears) are abundant, and increases when herds are nutritionally limited and concentrated during winter (Kortello et al. 2007, White et al. 2010, Griffin et al. 2011, Johnson et al. 2013, Eacker et al. 2016).

Elk calf survival and recruitment can influence a herd's growth and, subsequently, the number of elk available for hunter harvest (Raithel et al. 2007). Although calf survival does not appear to be strongly influenced by the physical (nutritional) condition of cow elk, poor forage on summer range can reduce a herd's pregnancy rate (Reardon 2005, Proffitt et al. 2016). Depressed calf production may then predispose that herd to the effects of mountain lion predation and exacerbate population declines (Clark et al. 2014b, Eacker et al. 2016).

Unlike bears, which primarily kill elk calves during the first 30 days of life, mountain lions prey on them throughout the year. Mountain lions were responsible for 70% of elk calf mortalities in northeastern Oregon where there are black bears but no wolves or grizzly bears (Reardon 2005). On a study site in western Montana where there were wolves

Wildlife managers must carefully consider the potential effects of mountain lion predation on prey populations when developing management prescriptions for both

and black bears (but no grizzlies), Eacker et al. (2016) found that 60% of known cause calf mortality was by mountain lions and male calves were 50% more likely to die than females.

Elk migration to areas of greater or lesser exposure to predation can also affect calf survival (Hebblewhite & Merrill 2007). For example, in Montana, seasonal migration of elk to ranges dominated by agriculture (where predators were rare) lowered predation risk while concentration on winter ranges increased it (Eacker et al. 2016).

The density of mountain lions in an area may itself be enough to explain predation's influence on elk calf recruitment. Where mountain lion densities are high they are capable of limiting elk recruitment enough that annual variation in lion densities explains most of the variation in annual calf survival (Johnson et al. 2013). In Montana's Bitterroot Range, where lion densities were relatively high, grizzlies absent, and wolves were present, lion predation accounted for most calf elk mortality (Eacker et al. 2016). In contrast, on Yellowstone's Northern Range and in Montana's Garnet Mountains where mountain lion density was relatively low, the rate of lion predation of elk calves was also low (Raithel 2005, Barber-Meyer et al. 2008).

The effect of mountain lion predation on bighorn sheep populations varies, but is most likely to limit population growth where herds are small and isolated (Ruth & Murphy 2011). The rate of predation can simply be a function of the overall mountain lion density within a sheep herd's range. However, in some cases bighorn sheep predation is a specialized behavior adopted by individual lions (Logan & Sweanor 2001).

Lion predation of bighorn sheep can increase where lion densities are buoyed by an abundant primary prey species or when a decline in the primary prey causes lions to switch to bighorn sheep (Kamler et al. 2002). Targeted removals of individual lions that specialize on sheep, or sustained efforts to suppress lion density in core bighorn sheep habitat, can effectively reduce the impact of lion predation on small, isolated herds (Ernest et al. 2002, McKinney et al. 2006).

MANAGEMENT CONSIDERATIONS

- Weather and forage availability are more likely than predation to explain chronically low ungulate populations. The influence of these potentially limiting factors should be evaluated before predation is implicated.

- Mountain lion predation is more likely to limit a prey population's growth if that population is below habitat carrying capacity and the lion predation rate is high. For instance, if a severe winter causes a significant deer die off but overall forage availability remains unchanged, mountain lion predation may slow the herd's recovery. In this case, preemptively and temporarily reducing mountain lion density through hunting could increase the deer population's growth rate while potentially reducing human-mountain lion conflicts.

- Mountain lion predation can limit a prey population where lions are the most abundant predator, lion density is supported by another abundant prey species, and the prey population is below its habitat's carrying capacity. In this case, managers should consider whether apparent competition is the ultimate cause of a secondary prey species' (e.g. mule deer or bighorn sheep) decline. Where abundant primary prey support dense mountain lion populations, sympatric populations of more vulnerable secondary prey may be disproportionately affected.

- The effect of predation on elk survival increases with the diversity of the predator community – the addition of grizzlies and wolves to a system with established mountain lions and black bears can change the influence of predation on ungulate prey.

- Mountain lion predation is unlikely to limit adult elk survival but can significantly reduce elk calf recruitment where lions are the predominant predator, lions occur at high densities, and where weather and/or habitat quality has reduced elk pregnancy rates.

- Targeted removal of individual lions that specialize on bighorn sheep, or sustained efforts to suppress lion density in core bighorn sheep habitat, may reduce the influence of mountain lion predation on the growth of small and isolated sheep herds.

-Attempts to locally reduce mountain lion populations will likely be confounded by the effect of immigration. Harvest treatments intended to reduce lion density should be sustained, broad scale, or both.

- Any proposal to reduce mountain lion density to benefit prey should be explicitly developed in an adaptive management framework. Managers should make measurable predictions about the outcome of a mountain lion harvest prescription (on lion and prey populations), monitor and evaluate the treatment's effects after a predetermined period, and be prepared to modify management based on that evaluation.

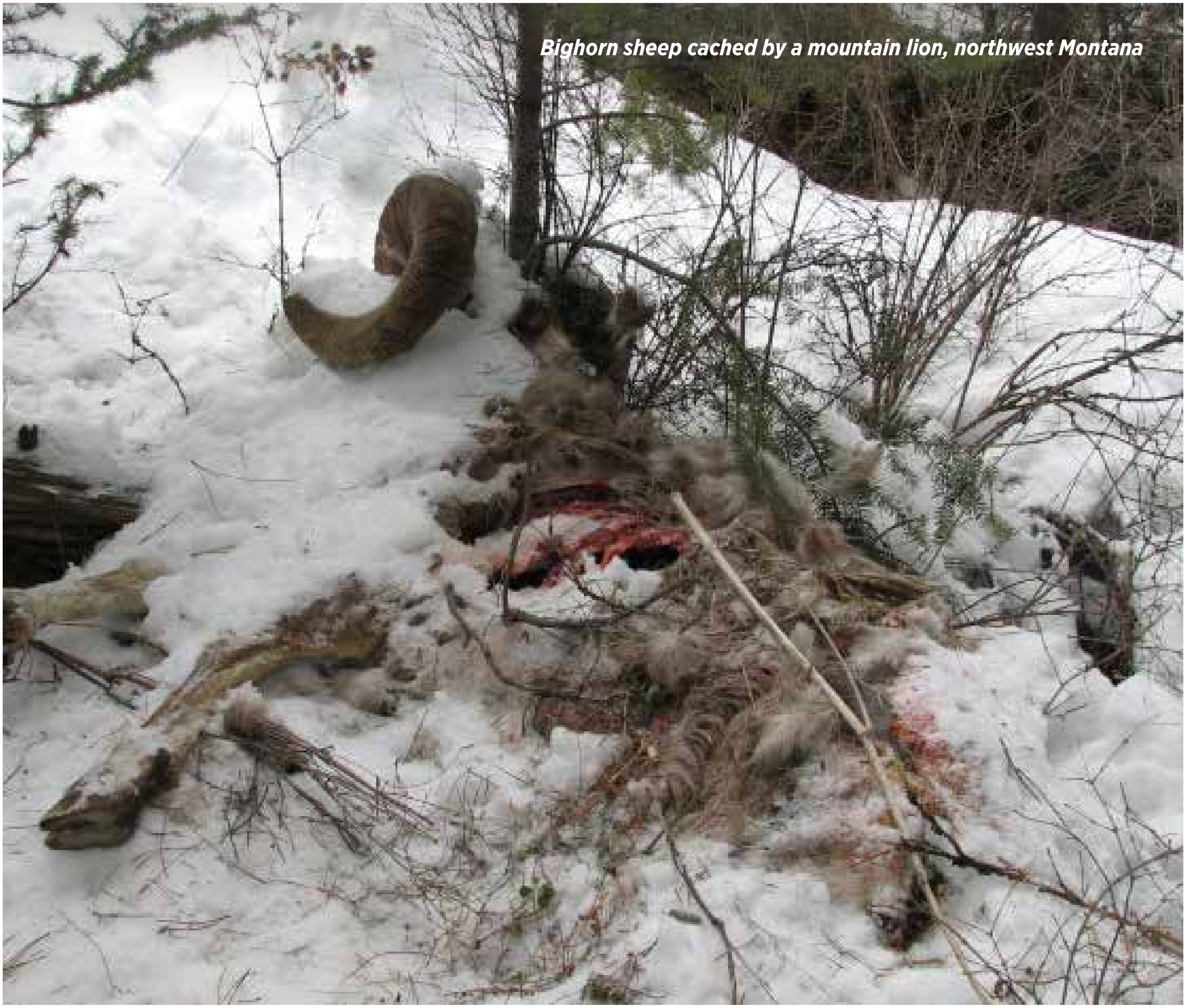
Bighorn sheep cached by a mountain lion, northwest Montana

CHAPTER 2

MOUNTAIN LION-HUMAN CONFLICT

Montana law grants FWP and the Fish and Wildlife Commission broad authority and discretion to manage wildlife. However, the legislature provided specific direction to the Department regarding the management of large predators, including mountain lions, that clearly emphasizes the protection of people and property over sport hunting of either mountain lions or their prey:

> 87-1-217. Policy For Management Of Large Predators - Legislative Intent
>
> (1) In managing large predators, the primary goals of the department, in the order of listed priority, are to:
>
> (a) protect humans, livestock, and pets;
> (b) preserve and enhance the safety of the public during outdoor recreational and livelihood activities; and
> (c) preserve citizens' opportunities to hunt large game species.

A mountain lion becomes a public safety concern when it appears habituated to human activity or development, attacks livestock or pets, or in any way behaves aggressively toward humans. FWP has developed specific Mountain Lion Depredation and Control Guidelines (Appendix 3) which describe and direct the Department's actions following a reported conflict between a human and a mountain lion.

The types and rate of conflicts between mountain lions, humans, and livestock are affected by mountain lion abundance, location, presence of attractants, and individual lion behavior. FWP will rely on the expertise and judgment of its field staff and agents (i.e. USDA Wildlife Services personnel) to investigate reported conflicts and determine the most appropriate response to a given situation. FWP's principal consideration when making these decisions will be reducing future risk of harm to people and/or property.

FWP will respond to human-lion conflicts in a manner that protects public safety, reduces property loss, and increases public tolerance for mountain lions. FWP will enforce state law (MCA 87-6-216) and local ordinances that prohibit certain wildlife attractants and will work to remove or contain attractants when a lion localizes in a problematic location. FWP will use hunter harvest when and where appropriate to manage lion density in high conflict areas. Finally, FWP may use targeted hazing or removal of individual offending mountain lions to mitigate ongoing or potential risk to people, pets, or livestock.

FWP will implement and facilitate programs that help livestock and pet owners protect their animals such as those currently offered by FWP, the Montana Livestock Loss Board, and nongovernmental organizations. FWP will continue to emphasize the importance of preventative efforts intended to reduce the risk of livestock loss in memoranda of understanding entered into with USDA Wildlife Services.

FWP does not maintain facilities to rear, hold, or rehabilitate mountain lions. Mountain lions that are injured so severely that they could pose a risk to humans or those that are unlikely to survive without intervention will be euthanized.

Montana hunting regulations prohibit the taking of a female lion accompanied by spotted kittens. However, in the unfortunate circumstance that a lactating female lion is mistakenly taken by a hunter or is otherwise killed, FWP staff may attempt to find the kittens and humanely euthanize them, unless an approved zoo or other facility is prepared to permanently assume responsibility for their care.

Capturing and relocating habituated, aggressive, or depredating mountain lions is not an effective conflict management response (Hornocker & Negri 2009). Mountain lions that are captured and translocated are

Table 2. Recorded non-harvest human-caused lion mortality, 1989 – 2015.

Year [2]	89	90	91	92	93	94	95	96	97	98	99	00	01	02	03	04	05	06	07	08	09	10	11	12	13	14	15	16	17
**FWP, Private Party, & Other Removals [1,3]**	10	14	17	20	23	15	9	23	53	19	21		3			5		8	22	32	24	23	39	27	35	21	16	41	9
Public Safety																			4	10	9	9	15	9	10	6	1	16	2
Depredation or Protection of Pets			1	2						3	2								5	4	5	2	6	5	2	6	5	10	2
Depredation or Protection of Livestock	1			1						6	1								5	8	4	6	15	6	14	8	4	2	3
Self Defense																			4	10	4	2	2	3	7	1	5	10	1
Other/Unknown	9	14	16	17	23	15	9	23	53	10	18		3			5		8	4		2	4	1	4	2	0	1	3	1
% Female (of those known)																			50%	50%	55%	41%	47%	36%	64%	42%	44%	32%	67%
**Livestock Depredation USDAWS/APHIS [4]**	2	3	3	4	7	3	9	8	13	21	16	18	9	12	7	3	7	5	8	13	12	14	17	19	15	12	12	3	4
**Illegal**	2	2	2	4	9	5	11	6	18	7	5	0	2	0	1	0	0	2	4	2	5	6	7	5	9	7	3	3	7
**Incidental Trapping [5]**	2	2	2	4	5	9	7	4	6	4	2	0	1	3	3	1	1	1	10	9	9	8	8	16	16	12	13	6	4
Snare																			7	2	7	4	6	7	2	6	8	5	3
Foothold																			2	7	2	4	2	9	13	6	5	1	1
Conibear																									1				
Unknown	2	2	2	4	5	9	7	4	6	4	2	0	1	3	3	1	1	1	1										
% Female (of those known)																			60%	78%	63%	50%	88%	75%	50%	89%	73%	67%	50%
**Total**	16	21	24	32	44	32	36	41	90	51	44	18	15	15	11	9	8	16	44	56	50	51	71	67	75	52	44	53	24

[1] Roadkill incidents are inconsistently reported in MT and are not included in this table

[2] FWP License Year, 8/1 - 7-31, unless otherwise noted

[3] Data from License Year 2000 to 2006 are incomplete and should be considered minimums

[4] Source: USDAWS/APHIS. Data recorded by Federal Fiscal Year, 10/1 - 9/30

[5] Data prior to 2007 are incomplete and should be considered minimums

unlikely to survive, often return (or attempt to return) to their capture location (Ross & Jalkotzy 1995, Ruth et al. 1998), and can cause future conflicts (Belden et al. 1991, Williams 1992). For these reasons, mountain lions shall not be captured and translocated under any circumstances. Mountain lions involved in any form of conflict will be dealt with per the Mountain Lion Depredation and Control Guidelines (Appendix 3).

Statewide records of reported mountain lion-human conflicts are historically incomplete (Table 2). In 2007, FWP created a centralized database to track harvest and most reported human caused non-harvest lion mortality. The same database has since been updated to also archive records of animals, including mountain lions, that are incidentally caught by recreational trappers and successfully released. This system will also be used to record all reported human-mountain lion conflict incidents, and their resolution. These more complete records will allow FWP to identify sources of and trends in mountain lion conflicts so that they can be more effectively addressed.

FWP actively educates the public about safely living with mountain lions, avoiding human-lion conflicts, and reducing the risk of property loss. The agency will continue to employ biologists and technicians who specialize in educating the public about, and responding to, human-predator conflicts. FWP will also maintain and periodically update educational materials and programs that teach the public about lion biology and behavior, ways to avoid and diffuse conflicts, strategies and methods to protect pets and livestock, and how to responsibly live and recreate in mountain lion habitat.

LIVESTOCK DEPREDATION

Mountain lions were confirmed to have killed an average of 136 head of livestock in Montana annually between 2006 and 2015 (USDA Wildlife Services, Table 3). However, only a fraction of actual livestock losses to mountain lions are found and formally documented (Jenks 2011). In Montana, male mountain lions were more likely than females to be removed in response to livestock depredation and most depredating lions were younger adults (1-4 years old) in good physical condition. The peak time period for both

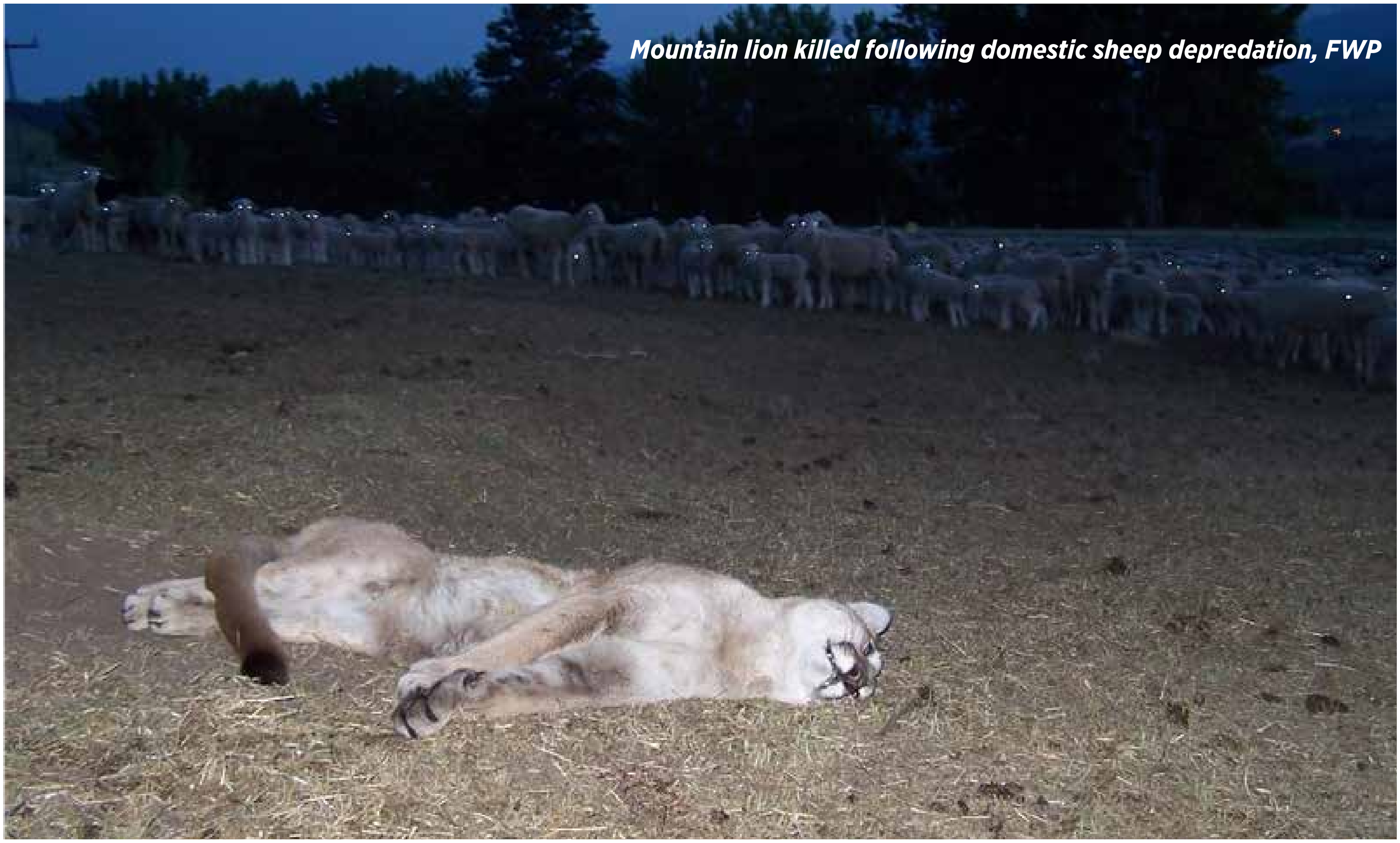

Mountain lion killed following domestic sheep depredation, FWP

The rate of livestock loss may be partly a function of an area's mountain lion density

livestock and human conflict incidents was between June and November (Riley & Aune 1997).

Mountain lions most commonly kill livestock that weigh less than 300 pounds. Although full grown cattle and horses are occasionally killed, mountain lions mainly kill calves/foals and yearlings. Losses are highest where calves or foals are born in lion habitat (Cougar Management Guidelines Working Group 2005). Small livestock (sheep, goats, and fowl) are the domestic species most vulnerable to mountain lion predation in Montana (Figure 3). Livestock depredation predominately occurred in central Montana where sheep production is more common and in western valleys where there is a greater number of hobby livestock.

Montana law (MCA 87-6-106) allows private citizens to legally kill any mountain lion that is attacking, killing, or threatening to kill a person or livestock. Private citizens may also legally kill a mountain lion that is in the act of attacking or killing a domestic dog. A person who kills a mountain lion under this statute must notify a FWP employee within 72 hours and surrender the carcass. FWP may issue a permit to kill a mountain lion to a landowner which allows them to take a mountain lion, within a specific area and time period, that is threatening to or suspected of killing livestock.

FWP annually contracts USDA Wildlife Services to respond to reported depredation of commercial livestock. When a loss is reported, a Wildlife Services agent conducts a field investigation to determine whether the loss is a "probable" or "confirmed" depredation and what predator species is responsible. Based on that investigation, and whether predation is determined to be the likely cause, the agent decides what response is most likely to prevent further livestock losses. This may, but does not always, include attempting to lethally remove the offending individual predator. The annual FWP contract requires Wildlife Services to provide records of all reported incidents (including lethal removals) to FWP at the end of the federal fiscal year (October 1).

Montana's Livestock Loss Board may reimburse stock growers for up to fair market value of probable or confirmed livestock losses due to mountain lion predation. The Board may also issue grants supporting efforts to reduce or mitigate the risk of mountain lion depredation of livestock (MCA 2-15-3110 through 3113).

Table 3. Domestic livestock reported to and/or verified by USDA APHIS Wildlife Services as injured or killed by mountain lions, federal fiscal years 2006 – 2015.

	2006		2007		2008		2009		2010	
	Injured	Killed	Injured	Killed	Injured	Killed	Injured	Killed	Injured	Killed
Cattle		2		10		2		18	2	8
Horses	6	2	8	8	1	2	5	2		3
Goat		2		16	2	20		23	1	22
Llama		1				3		2		4
Sheep		23	1	26	4	115	2	157	2	128
Swine										
Fowl				7		8		49		25
Total	6	30	9	67	7	150	7	251	5	190

The rate of livestock loss may be partly a function of an area's mountain lion density. In Oregon, Hiller et al. (2015) found that as mountain lion population density increased, so did the number of mountain lions killed as a result of livestock predation. This relationship was especially strong at higher mountain lion densities. Livestock conflicts either decreased when mountain lion hunter harvest increased or remained constant where mountain lion densities were relatively low.

There is evidence that a similar relationship between lion abundance and livestock conflict may exist in Montana. There is a correlation ($r^2 = 0.66$) between the number of mountain lions that Wildlife Services agents annually killed in response to livestock depredations and the statewide mountain lion population estimated by FWP's Integrated Population Model (1990 – 2013; Chapter 6; Figure 4). Hunter harvest that maintains mountain lions at moderate densities may be a useful tool in managing livestock predation in some circumstances (Hiller et al. 2015).

Figure 3. Proportion of livestock killed by mountain lions by species, 2006 – 2015.

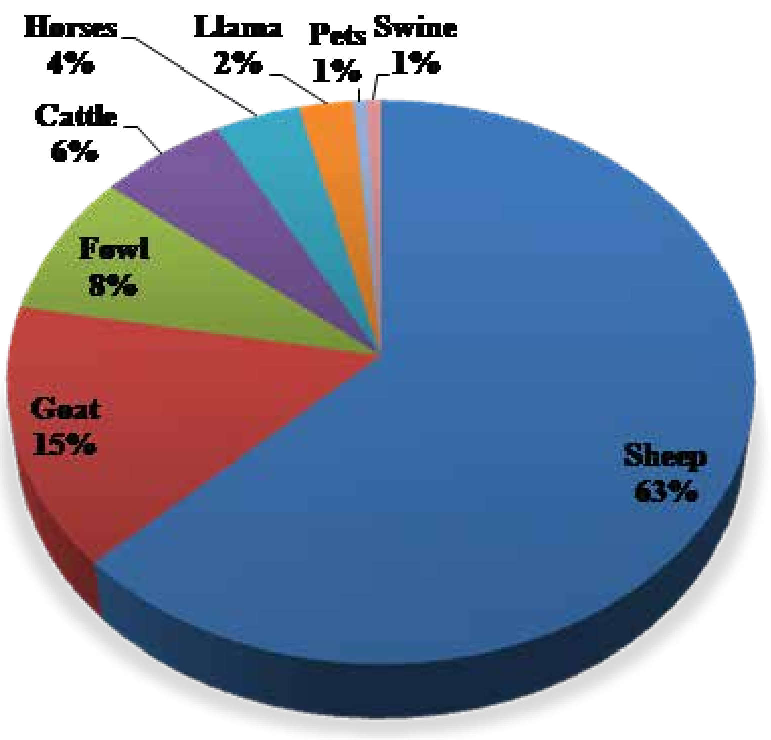

Otherwise, there are few practical measures that can completely prevent the loss of commercial livestock to mountain lions. Delaying turnout of cow-calf pairs into remote lion occupied pastures may reduce calf loss. Although guard dogs can reduce livestock losses to canine predators, guard dogs do not effectively protect against mountain lion depredation (Jenks 2011). If economically feasible, switching from raising small livestock (i.e. sheep) to less vulnerable species where mountain lions are common may also reduce depredation losses (Lindzey 1987). Owners of hobby livestock can effectively use practices unavailable to commercial producers such as night penning, lights, and clearing brush around paddocks to reduce depredation risk.

2011		2012		2013		2014		2015	
Injured	Killed	Injured	Killed	Injured	Killed	Injured	Killed	Injured	Killed
	3		14		14		2		10
2	2	2	4	2	1	3	1	1	2
	17	3	44		6		11		45
	10		10		5		1		
	67	1	79		162		64		55
					2		2		
	3						24		
2	102	6	151	2	190	3	105	1	112

MOUNTAIN LION-HUMAN INTERACTIONS

Mountain lion attacks on humans in Montana are extremely rare. The only fatal mountain lion attack in modern times was that of a 5-year old boy killed near Evaro, on the Flathead Indian Reservation, in September of 1989. Several nonfatal attacks have also occurred in the state and, like elsewhere, overwhelmingly involved children (Beier 1991). Juvenile and subadult mountain lions are responsible for most human-lion conflicts across the western U. S. (Mattson 2007), including Montana.

Subadult lions of both sexes are also more likely than adults to use urban and exurban residential areas (Kertson et al. 2013). Although in Montana males were more likely than females to take livestock, sex ratios of lions involved in human incidents were not significantly different from 50:50. Human incidents mostly occurred near western intermountain valley communities.

Mountain lions commonly live adjacent to, or travel through, developed areas but most lions travel at night and are rarely seen (Kertson et al. 2013). Individuals that are routinely sighted during daylight hours near homes and people, or those that appear accustomed to human activity and development, have become habituated and are a public safety concern. Individual lion behavior often escalates from natural to habituated to nuisance to dangerous, at which point the lion may begin to kill pets in populated areas and/or to display aggression toward humans (Cougar Management Guidelines Working Group 2005).

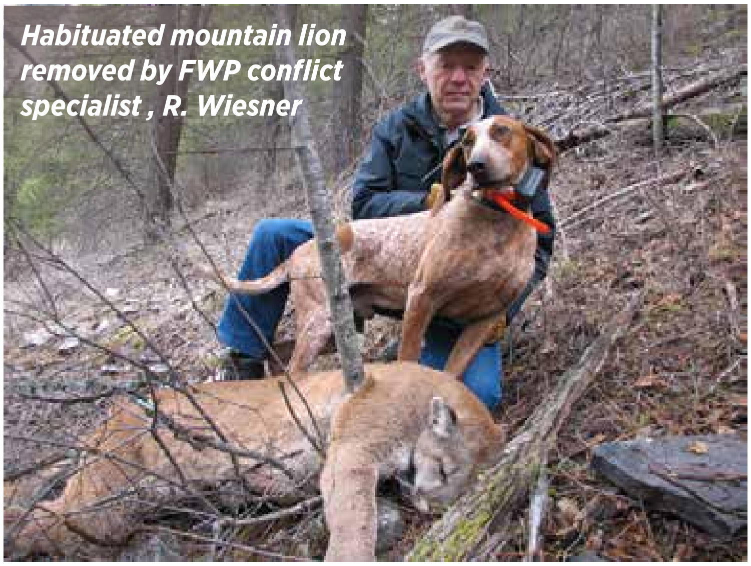
Habituated mountain lion removed by FWP conflict specialist , R. Wiesner

Mountain lions commonly live adjacent to, or travel through, developed areas but most lions travel at night and are rarely seen

If an investigation reveals that a habituated mountain lion has become a nuisance or aggressive, FWP staff should document the behavior, notify area residents of the situation (especially those with children and/or outdoor pets), and immediately attempt to either aversively haze or lethally remove the offending individual.

Field staff should closely follow the approved protocols for responding to human-lion conflicts in the Mountain Lion Depredation and Control Guidelines (Appendix 3).

SPECIAL MANAGEMENT AREAS

Montana has designed certain Lion Management Units (LMUs) specifically to encompass urban, suburban, or agricultural areas where the tolerance for mountain lion presence is low and the potential for human-mountain lion conflict is high. The Commission may designate these LMUs “Special Management Areas” (described by Logan & Sweanor 2001) and either elect to assign an “unlimited” harvest quota (e.g. LMU 170, immediately surrounding Kalispell) or a high annual quota that it is rarely, if ever, met.

If a Special Management Area contains suitable mountain lion habitat, the management approach may not significantly reduce mountain lion densities because of

rapid immigration into vacated home ranges (Robinson et al. 2008, Cooley et al. 2009). However, specifically designating Special Management Areas can ease social and political concerns (Jenks 2011) and, importantly, ensure that legal hunter harvest remains a management tool throughout the fall and winter hunting seasons.

For example, the Missoula Special Management Area (MSMA), a LMU surrounding the highly developed Missoula Valley, was established in 1994. Relatively high quotas in this LMU are rarely met even though the area contains high-quality lion habitat and General License hunting was allowed for nearly 7 months each year.

The average age of a mountain lion harvested within the MSMA between 2000 and 2015 (3.09 years; n = 421) was slightly lower than that of lions harvested during the same period in the remainder of Region 2 (3.58 years; n = 2319). However, this small difference does not indicate that higher hunter harvest opportunity meaningfully increased the proportion of more conflict prone juveniles in the LMU. Although FWP staff lethally removed several nuisance mountain lions from the MSMA each year, FWP hunting regulations were not publicly perceived as limiting legal hunter harvest during established seasons in this high conflict area.

Figure 4. The relationship between Montana's modeled mountain lion population trend and annual mountain lion removals by Wildlife Services in response to livestock depredation, 1989 - 2013.

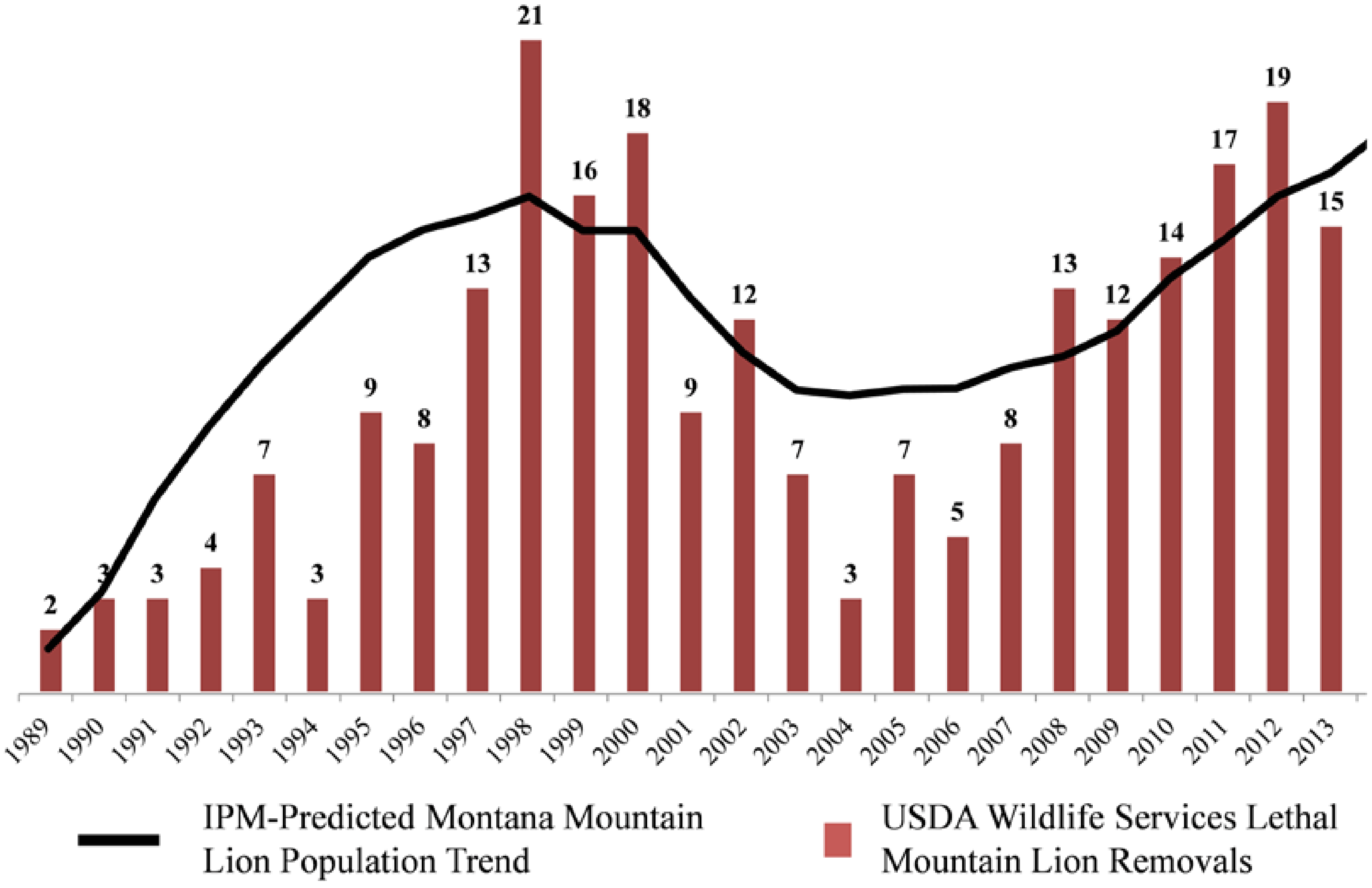

CHAPTER 3

2016 MONTANA MOUNTAIN LION RESOURCE SELECTION FUNCTION

INTRODUCTION

To produce accurate estimates of mountain lion abundance, managers first need to understand what habitat features are important to lions and how that habitat is distributed across the state. Accurate spatial models that describe mountain lion habitat use can also be used to monitor lion populations over time. While producing reliable maps of relative mountain lion habitat quality and landscape linkages is critically important (Cougar Management Guidelines Working Group 2005, Jenks 2011) they have previously been difficult to produce and validate.

Managers need accurate spatial data that depict mountain lions' use of their habitat in order to predict lion abundance and to monitor their populations over time

Montana FWP will use a "resource selection function" (RSF) model to depict and analyze the state's mountain lion habitat. A RSF is a statistical model that represents the relative probability that an animal will select a particular place or resource (Manly et al. 2002). A RSF is simply a spatial surface of pixels or cells that are each assigned a statistical value based on what we know about a species' habitat selection. This surface can then be used to mathematically analyze and describe the species' habitat use at larger scales.

A RSF is often displayed as a map showing the relative likelihood a species will use a particular resource or available habitat. Biologists construct RSFs from field data that describe an animal's spatial use (such as telemetry relocations collected using radio or GPS collars) and the habitat variables that likely cause the animal to select (or avoid) certain resources or areas. Habitat variables may include vegetation type, canopy closure, elevation, terrain, or other features that affect an animal's habitat selection.

It's impossible to quantify all the habitat variables that cause an animal to select a certain location. However, we can often identify a combination of measurable factors that accurately predict the relative likelihood that a species is present in a certain habitat type. If we also have information about a population's vital rates and population density, we can also estimate how many individuals a larger area likely supports.

A well designed RSF can help biologists better manage wildlife in many important ways. RSFs can describe the kind of habitat where we'd expect to find a certain species, map corridors that are potentially important connections between larger habitat patches, and identify isolated areas of suitable habitat that may support a species, even if the species is not currently there. RSFs help managers identify resources that are important for the conservation of a species or that may be limiting its use of an area. Finally, a RSF allows biologists to make inferences about an animal's abundance across broad landscapes using monitoring data that provides information on the population's current density.

FWP will use a statewide mountain lion RSF to:

1. Define distinct mountain lion ecoregions.
   The RSF surface consists of many small cells, or "pixels", that are each assigned a value based on the habitat features present within them. The average RSF value of all the pixels within a hunting district or lion management unit generally describes the overall quality of that unit's lion habitat. FWP used these average values to define large, biologically meaningful, ecoregions within the state where lion habitat is similar in type and

distribution. These ecoregions will be the primary spatial basis of its mountain lion population monitoring program (Chapter 4).

2. Improve population monitoring.
   The RSF helped FWP identify representative population Trend Monitoring Areas within the Northwest, West-central, and Southwest ecoregions. The RSF will also be used to guide periodic field sampling within these Monitoring Areas (Chapter 4).

3. Enable FWP to estimate mountain lion abundance.
   When the relationship between observed lion abundance and the RSF is known, we can estimate lion abundance within both Trend Monitoring Area(s) and the larger ecoregion. Integrating the RSF with field sampling such as spatial capture-recapture (Chapter 5) makes these monitoring methods more effective. Including a RSF as a covariate in the density estimation model—that is, formally assuming that an animal's activity center is more likely to fall in higher quality habitat—significantly improves the population estimate's biological realism and precision.

MONTANA MOUNTAIN LION RESOURCE SELECTION FUNCTION

Robinson et al. (2015) produced the first comprehensive winter mountain lion resource selection function for the state of Montana. The authors used mountain lion telemetry relocations (both VHF and GPS) from 10 individual mountain lion field research projects conducted throughout Montana and Yellowstone National Park between 1979 and 2012 to train and validate the RSF (Table 4). A significant number of telemetry locations were withheld from the training data for internal model validation. Mountain lion harvest locations (1988 – 2011; generalized to the center of the 640-acre section of harvest) were also used to validate the model. The original manuscript contains a detailed description of how this original RSF was constructed, was tested, and performed.

The most important measure of a RSF's utility is its ability to predict a species' use of available habitat (Boyce et al. 2002). The 2015 RSF model predicted both out-of-sample lion telemetry locations and hunter harvest locations quite well across Montana. Although there was generally excellent agreement between the location of harvested animals and predicted areas of lion habitat use, the 2015 model was most predictive in FWP Regions 1, 2, 4 and 6. In Regions 3, 5, and 7, a higher proportion of animals were harvested in areas that the RSF predicted to be lower quality habitat, compared to other FWP Regions.

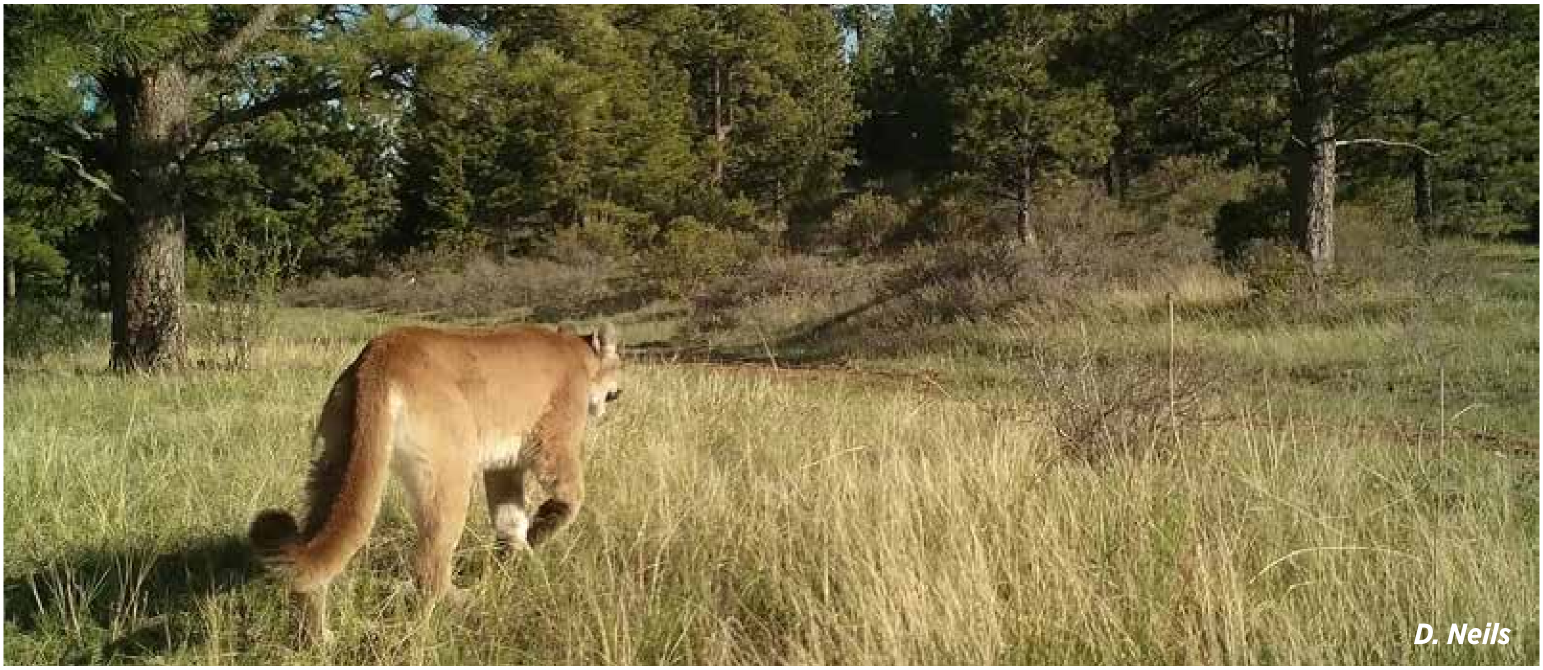
D. Neils

Table 4. Field studies and sampling data used to develop the Robinson et al. (2015) and 2016 MT Mountain Lion Resource Selection Function.

Study	Location	Years	N	Telemetry Method	2016 Model Training Locations
Murphy (1983)	Fish Creek	1979–1982	9 (6F, 3M)	VHF	127
Williams (1992)	Sun River	1991–1992	24 (15F, 9M)	VHF	104
Murphy (1998)	Yellowstone National Park	1987–1995	41 (29F, 12M)	VHF	1335
Ruth (2004)	North Fork Flathead	1993–1997	38 (28F, 8M)	VHF	692
Ruth & Buotte (2007)	Yellowstone National Park	1986–2006	39 (21F, 18M)	VHF and GPS	2782
Choate (2009)	National Bison Range	2000–2003	8 (7F, 1M)	VHF	576
Robinson & DeSimone (2011)	Garnet Range	1998–2006	39 (31F, 8M)	VHF and GPS	14,127
Kunkel et al. (2012)	Rocky Boys Reservation	2006–2009	6 (2F, 4M)	GPS	1786
Kunkel et al. (2012)	Fort Belknap Reservation	2008–2010	3 (2F, 1M)	GPS	281
Matchett (2012)	Missouri Breaks	2011–2012	2 (2M)	GPS	785

Table 5. Montana mountain lion winter Resource Selection Functions developed as part of Robinson et al. (2015) and the revised 2016 model.

Covariate	Robinson et al. 2015<br>Coefficient (SE)	2016 (revised) RSF<br>Coefficient (SE)
South Aspect	0.3181 (0.0274)	0.3716 (0.0249)
High Montane	-1.3883 (0.3093)	-0.4619 (0.2116)
Agriculture	-1.9151 (0.1512)	-1.5664 (0.1115)
Developed	-0.6110 (0.1706)	-1.0656 (0.1642)
Transitional Vegetation	-0.7200 (0.0453)	-1.3047 (0.0417)
Elevation	0.0191 (0.0002)	0.0084 (0.0002)
Elevation^2	-0.000006 (8.67E-08)	-0.000003 (7.13 E-08)
Percent Slope	0.0264 (0.0017)	0.0229 (0.0014)
Percent Slope^2	-0.00015 (1.96E-05)	-0.0001 (1.3E-05)
Distance from forest	-0.0078 (0.0002)	N/A
Canopy	N/A	0.1688 (0.0029)
Canopy^2	N/A	-0.0022 (0.00004)
Constant	-14.9483 (0.2250)	-6.4305 (0.1551)

Figure 5. The 2016 Montana Mountain Lion Resource Selection Function map. Higher values indicate an area is more likely to be used by mountain lions.

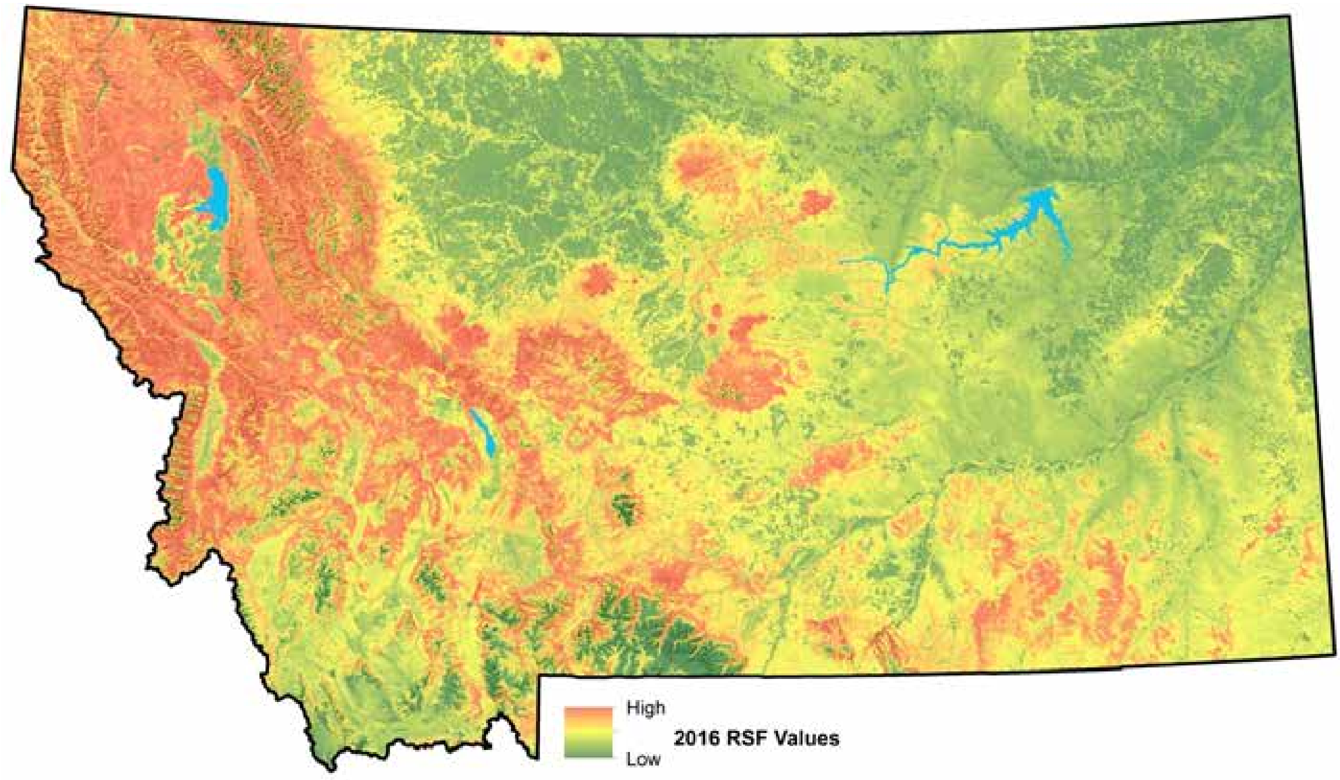

Figure 6. The 2016 Montana Mountain Lion Resource Selection Function map with 22,595 mountain lion telemetry model training points (1979 – 2012) and 10,503 harvest location validation points (1988 – 2015).

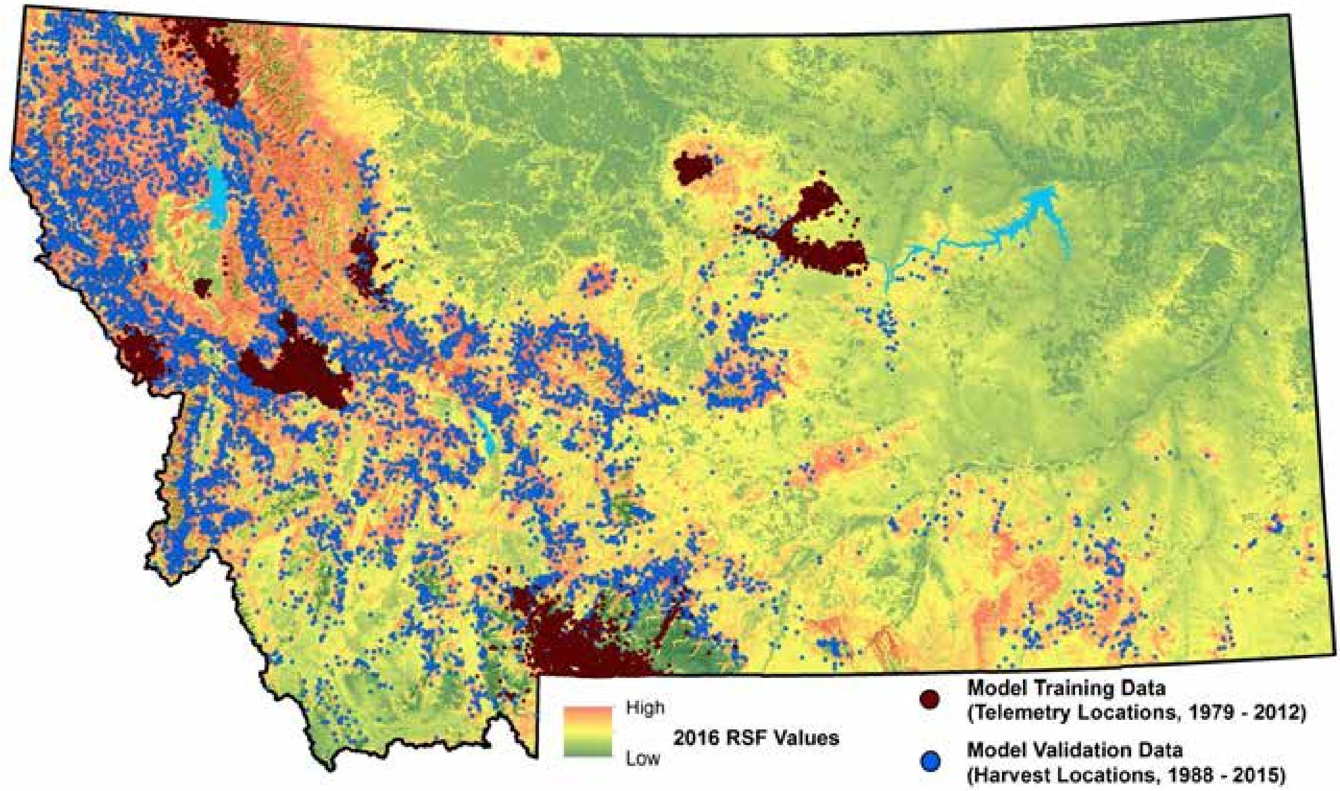

Figure 7. 2016 Montana Mountain Lion Resource Selection Function values and proportion of lion harvest locations per equal-sized bin (bin 1 = lowest quality predicted habitat; bin10 = highest quality habitat) by FWP administrative Region.

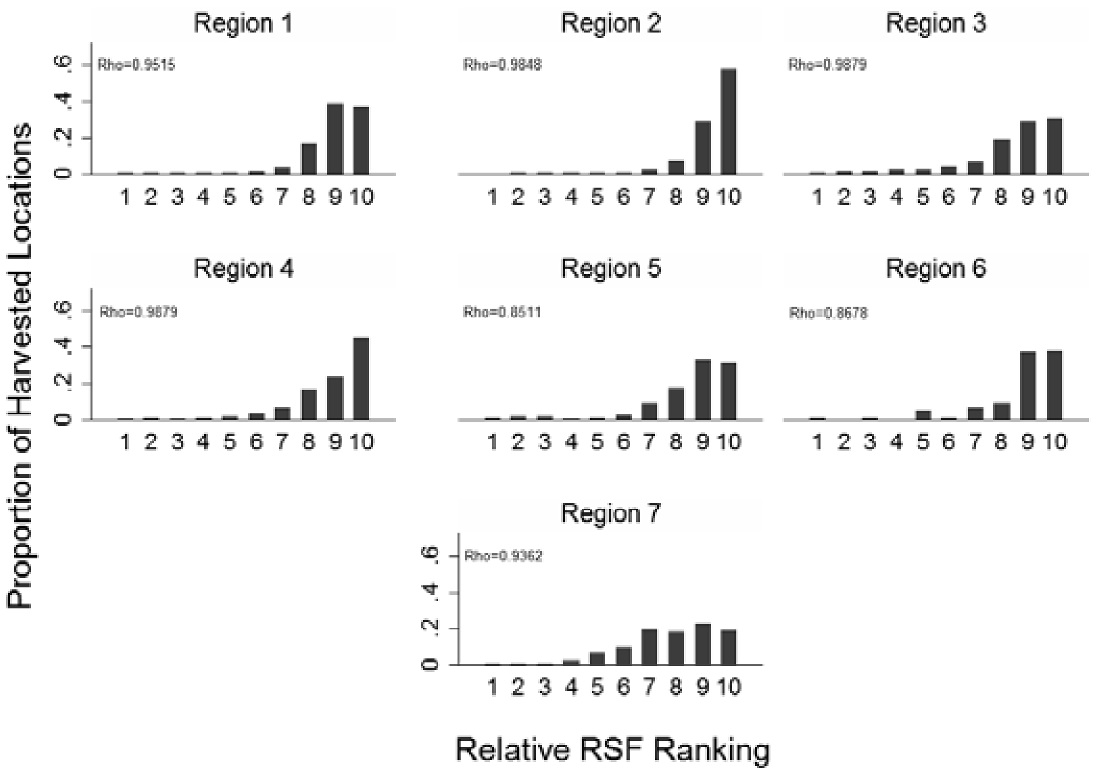

2016 MONTANA MOUNTAIN LION RSF

In 2016, FWP and Dr. Robinson worked together to improve the mountain lion RSF's ability to predict lion habitat selection statewide — specifically, in southern and eastern Montana. The same methods used by Robinson et al. (2015) were used to develop a revised version of the RSF, with three important refinements:

1. All available mountain lion telemetry relocations (n = 22,595) from the 10 Montana and Yellowstone National Park studies were used to train the revised model. "Study Area" was then used in the Generalized Linear Model as a random effect to account for varying levels of sampling intensity amongst studies.

2. FWP reexamined approximately 3,800 individual harvest locations reported between 2007 and 2015 - hundreds of location errors were found and corrected. The more accurate and complete 1988 – 2015 harvest data set (totaling 10,503 mountain lion harvest locations) was then used for external validation of the refined winter RSF model.

3. The revised winter RSF contained the same variables as described by Robinson et al. (2015) except that the variable "distance to forest" was replaced by a quadratic of "canopy closure" (Table 5). The revised model included a random intercept for each study area/data set.

We refer to this refined model (Figures 5 and 6) as the 2016 MT Mountain Lion RSF and it is the model used throughout this Strategy. The 2016 RSF performed similarly to Robinson et al.'s original 2015 model in FWP Regions 1, 2, 4, and 6 while the agreement between harvest locations and predicted high-quality habitat in Regions 3, 5 and 7 was significantly improved (Figure 7).

It is important to note that the RSF does not describe all the variables that affect mountain lion distribution or abundance. There are factors such as prey density, habitat disturbance (i.e. wildfire), or harvest history that are important to mountain lions and that vary over time. Therefore, it will be necessary to periodically reassess the relationship between the RSF and actual mountain lion density in an area (as described in Chapter 5).

CHAPTER 4

MONTANA MOUNTAIN LION ECOREGIONS

Mountain lions currently occupy nearly all of their suitable habitat in Montana. However, the quality, quantity, and arrangement of that habitat— thus the number of lions an area can support—varies significantly across the state. Mountain lion habitat in northwest Montana is nearly continuous, but habitat quality generally declines and becomes patchily distributed in more southern and eastern portions of the state (Figure 5).

The average RSF values of individual Lion Management Units reflects this pattern (Figure 8). This gradient in lion habitat quality across Montana allowed FWP to partition the state into distinct mountain lion "ecoregions". These ecoregions are large, contiguous areas of the state within which lion habitat is broadly similar. Mountain lion ecoregions are the spatial basis of FWP's lion population monitoring program.

Mountain lion harvest management is most effective when it's done at a large and biologically meaningful scale (Cougar Management Guidelines Working Group 2005, Jenks 2011). In lightly hunted populations, virtually all males and a significant proportion of females disperse from their natal area. Lion populations are best thought of as many connected sub-populations linked by dispersing animals. Local areas generally depend on immigration to recruit breeding males and, often, a large portion of breeding females.

These local sub-populations (i.e. within a LMU) can be resilient to harvest because lions are able to readily emigrate from adjacent areas and refill available habitat. Dispersal can also cause local populations to exhibit lower growth rates than expected, given their intrinsic vital rates (Sweanor et al. 2000, Logan & Sweanor 2001, Stoner et al. 2006, Cooley et al. 2009, Robinson et al. 2008 & 2011, Newby et al. 2011). Therefore, even if a LMU's harvest rate appears sustainable (when supported by immigration), the same harvest level could cause the unit's population to decline if harvest in adjacent areas increases. Similarly, specific attempts to reduce local lion populations can fail over the long term because of increased immigration from outside the treatment unit (Clark 2014a).

Monitoring and management programs are most effective when implemented across large landscapes. The effects of immigration and emigration on local population dynamics are less pronounced when considering large scale trends (Robinson et al. 2015). Importantly, large-landscape (i.e. > 35,000 km^2, an area ~ 115 x 115 miles) lion populations can be considered statistically "closed" (that is, the influence of immigration/emigration is eliminated) for most analyses (Robinson et al. 2008). Harvest treatments and abundance estimates are therefore less likely to be confounded by metapopulation dynamics if they are conducted across broad landscapes.

Montana includes a diverse range of habitat types, prey communities, weather patterns and other factors that affect mountain lion abundance. The relationship between an area's lion abundance and the range of RSF values within that area is unlikely to be the same across the state. Therefore, conducting field population monitoring and modeling efforts within large but discrete ecoregions (containing similar lion habitat) helps take this habitat variability into account.

FWP can more accurately estimate broad scale (ecoregion) lion abundance when using monitoring data collected from within that same ecoregion because mountain lion habitat and harvest history is more similar within ecoregions than across them (Boyce & McDonald 1999). FWP will produce periodic estimates of lion abundance and forecast the effects of harvest based only on monitoring data collected within those respective ecoregions (Chapters 5 and 6).

Mountain lion harvest management is most effective when it's done at a large and biologically meaningful scale

Figure 8. Avg. Mountain Lion Resource Selection Function values (0 = lowest quality habitat, 1 = highest quality habitat) for Montana's 2016 Lion Management Units.

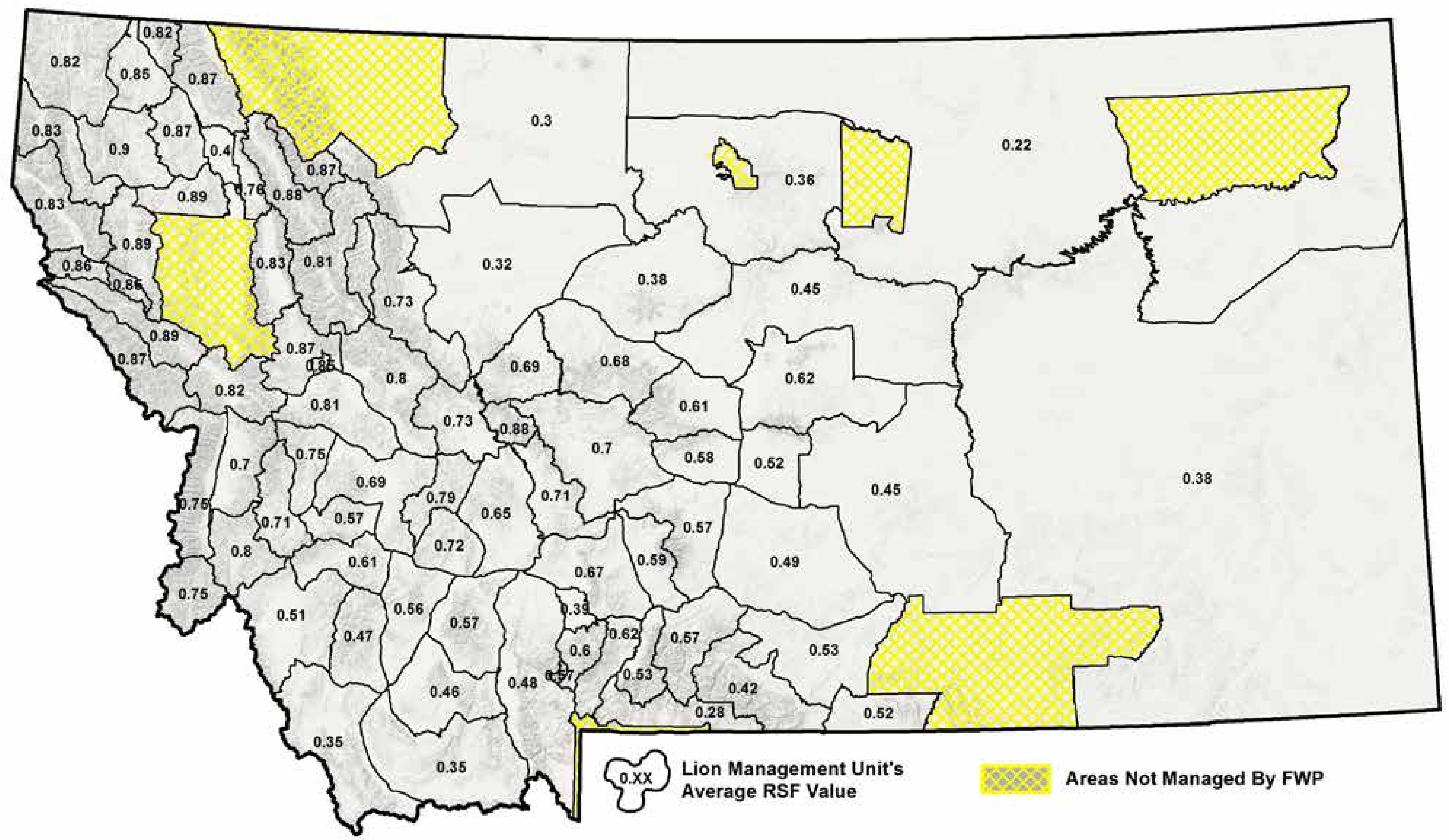

Figure 9. Montana's four mountain lion ecoregions and seven FWP administrative regions.

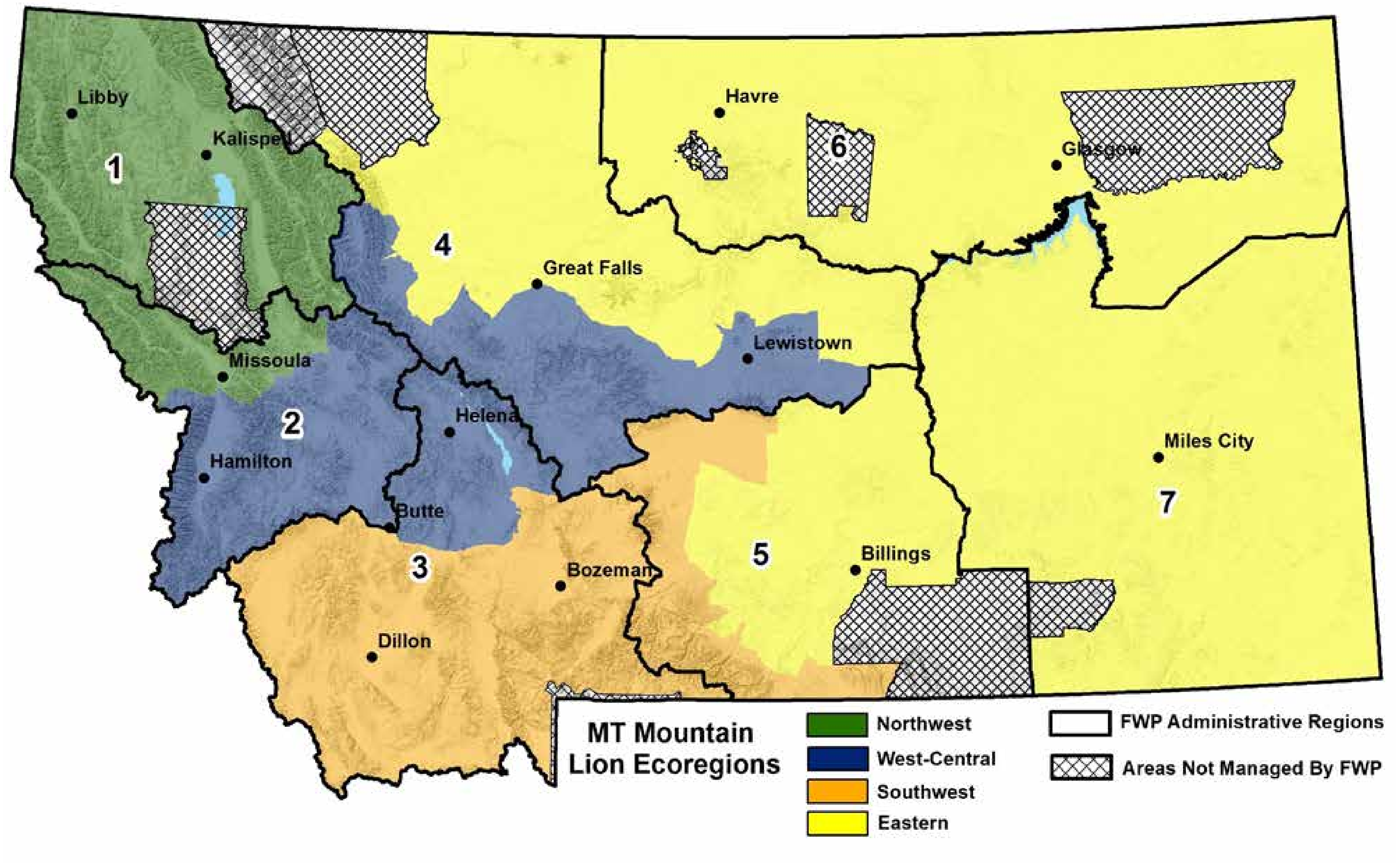

For the same reason, it is also only statistically and logistically practical to estimate lion population trend at a large scale. Mountain lion ecoregions should contain enough lions that populations can be modeled assuming that those populations are statistically closed. Population models then consider vital rates (from research on marked animals), harvest records, and periodic abundance estimates to allow managers to better understand past and future population trends (Chapter 6). This ability to describe the effects of past harvest and to predict the effect of future harvest prescriptions is a cornerstone of an adaptive harvest management program (Chapter 8).

FWP considered four factors when identifying individual mountain lion ecoregions:

1. They include contiguous LMUs with broadly similar habitat quality (RSF values).

2. They are large enough to allow management prescriptions to be effective despite internal lion metapopulation dynamics.

3. They are well distributed and represent the range of Montana lion habitat types.

4. The total number of ecoregions is limited so that monitoring can occur frequently enough to provide meaningful and timely data to managers. There is a tradeoff between the number of statewide ecoregions and how often each of them can be monitored. Budgets and available personnel limit the amount of effort FWP can expend field sampling lion populations.

FWP grouped 2016 LMUs using a k-Nearest Neighbor algorithm (ESRI ArcGIS 10.1) based on their RSF values and proximity. Local biologists then helped identify four contiguous mountain lion ecoregions that met the above criteria and that could be reasonably managed as distinct units (Figure 9). FWP will periodically collect field data to produce abundance estimates for each of the three western MT ecoregions (where approximately 90% of harvest annually occurs). Estimates of future lion abundance and trend will also be modeled for these ecoregions.

Each Montana mountain lion ecoregion includes all or portions of two or more FWP administrative Regions. FWP managers and the public from different administrative Regions will collectively evaluate an ecoregion's monitoring data, develop management objectives, and decide on an overall management prescription (harvest) for the ecoregion. Managers will then recommend individual LMU harvest limits that implement the prescription, distribute hunter effort, and address local concerns.

FWP also identified a permanent population Trend Monitoring Area in each of the state's three western ecoregions. These Trend Monitoring Areas will be periodically sampled to produce estimates of lion abundance within them, and in their respective ecoregions. The criteria used to select Trend Monitoring Areas are described in Appendix 1.

To be clear, the following ecoregions will be the basis of Montana's mountain lion population monitoring program. Information about the status and trend of lion populations within these ecoregions will inform adaptive management proposals that affect lion populations at the ecoregion scale. FWP and the public in two or more FWP administrative regions will periodically collaborate to develop certain population objectives for each ecoregion. For example, biologists and the public in FWP Regions 1 and 2 may agree to an objective of a moderately positive, negative, or stable population growth rate over the following 6 years in the Northwest ecoregion.

However, biologists and the public in each of the seven FWP administrative regions have local expertise, experience, and relationships. FWP public meetings and many wildlife advocacy groups are also organized by FWP administrative region. Therefore, specific management recommendations about harvest prescriptions and season structure for individual LMUs will be developed by FWP staff and the public in each of the seven administrative regions. The cumulative effect of these individual LMU prescriptions (i.e. the overall harvest within an ecoregion) will be considered, and periodically assessed, at the ecoregion scale.

Northwest Ecoregion

The Northwest mountain lion ecoregion encompasses all of FWP Region 1 (except for the Flathead Indian Reservation) and Region 2's northern Blackfoot and middle Clark Fork River drainages (Figure 10). It is Montana's smallest ecoregion at 36,893 km^2 but it contains the state's most continuous and highest quality lion habitat (average LMU RSF value = 0.83). Forests cover more than 90% of the Northwest ecoregion due to its Pacific maritime climate and moderate elevations.

Most of this ecoregion's lion habitat is either public land or publicly accessible private land. Hunter access during winter is extensive outside of designated wilderness areas. Tracking snow is generally present throughout the Winter Season.

The 2,550 km^2 Northwest mountain lion ecoregion Trend Monitoring Area includes the Libby Cr., Thompson River, and Fisher River drainages southeast of Libby. (Figure 11).

Figure 10. The Northwest mountain lion ecoregion, trend monitoring area, and 2016 FWP hunting districts.

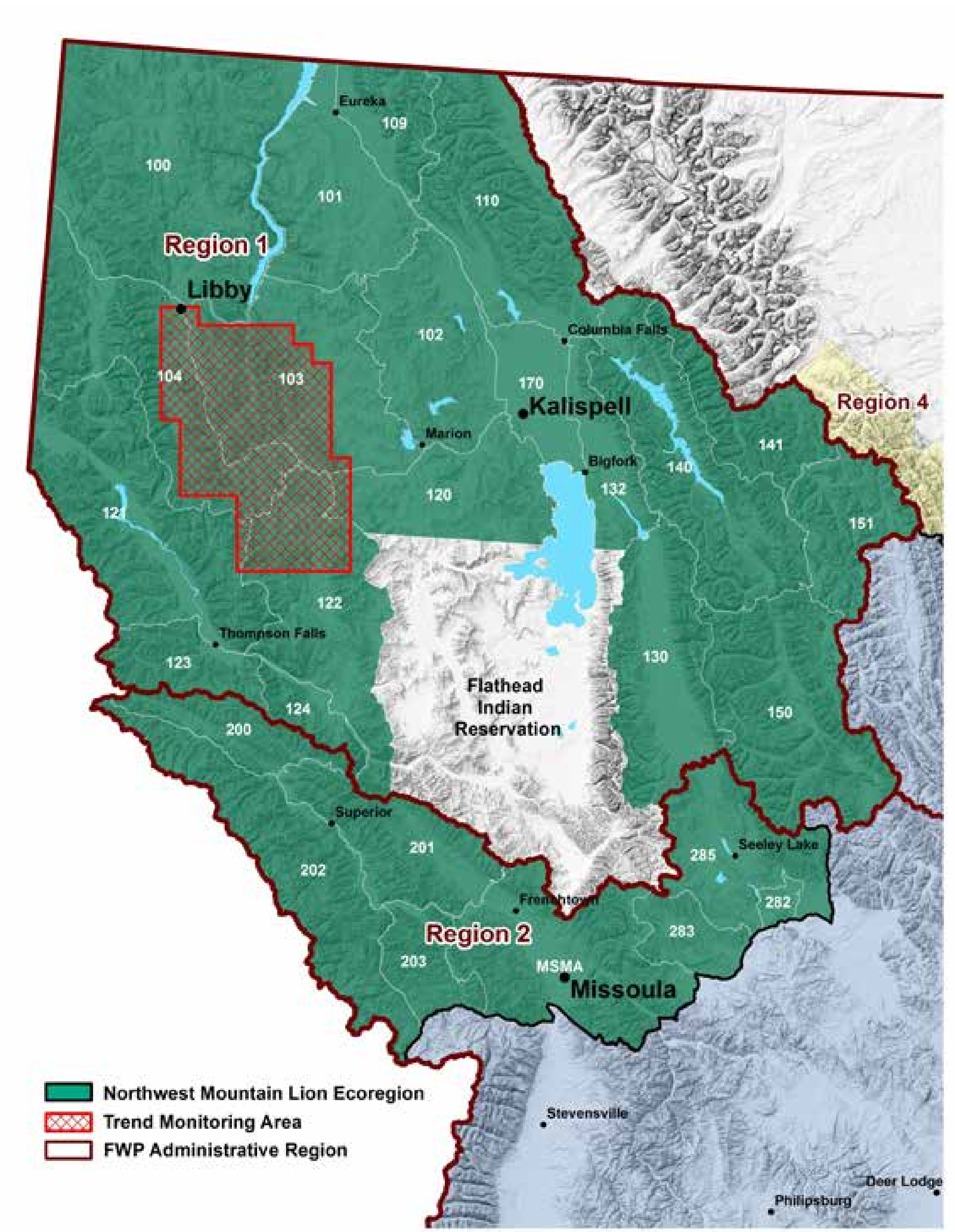

Mountain lion harvest in the Northwest ecoregion steadily increased during the 1990s, reaching a historic high of 344 (57% females) in 1998 (Fig 12). White-tailed deer make up as much as 90% of mountain lion prey in northwest Montana (Kunkel 1999, Montana Fish, Wildlife & Parks 2006). The ecoregion's white-tailed deer numbers were high in the mid-1990s before the severe 1996-97 winter significantly reduced this prey base. Lion harvest density, especially of females, was low during the 2000s but increased through the mid-2010s to approximately 4.6 lions per 1,000 km^2 (42% female), less than half the harvest density observed in the late 1990s.

Figure 11. The Northwest mountain lion ecoregion trend monitoring area divided into a grid of 102 5x5 km sampling cells.

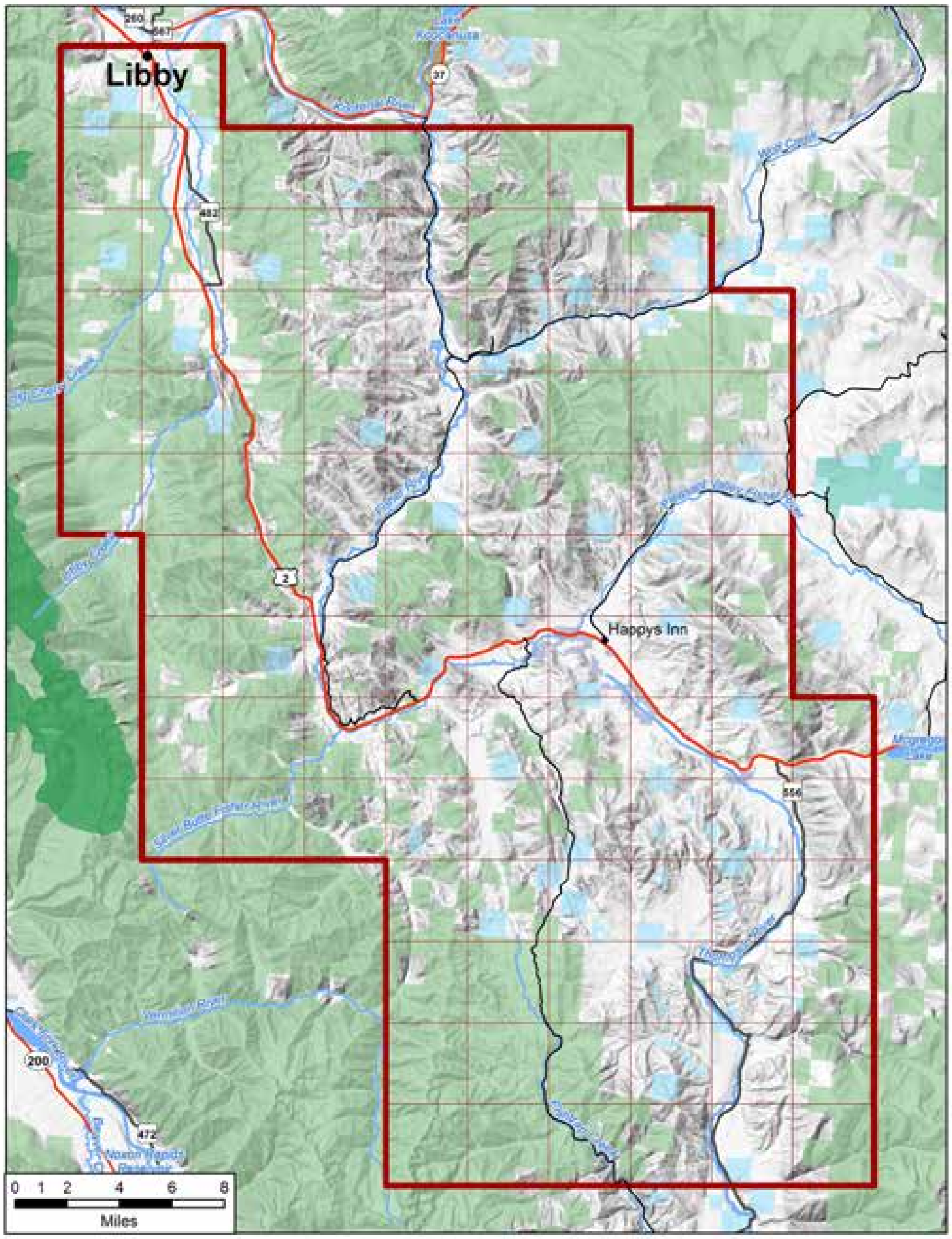

Figure 12. Northwest ecoregion mountain lion harvest, 1990 – 2015.

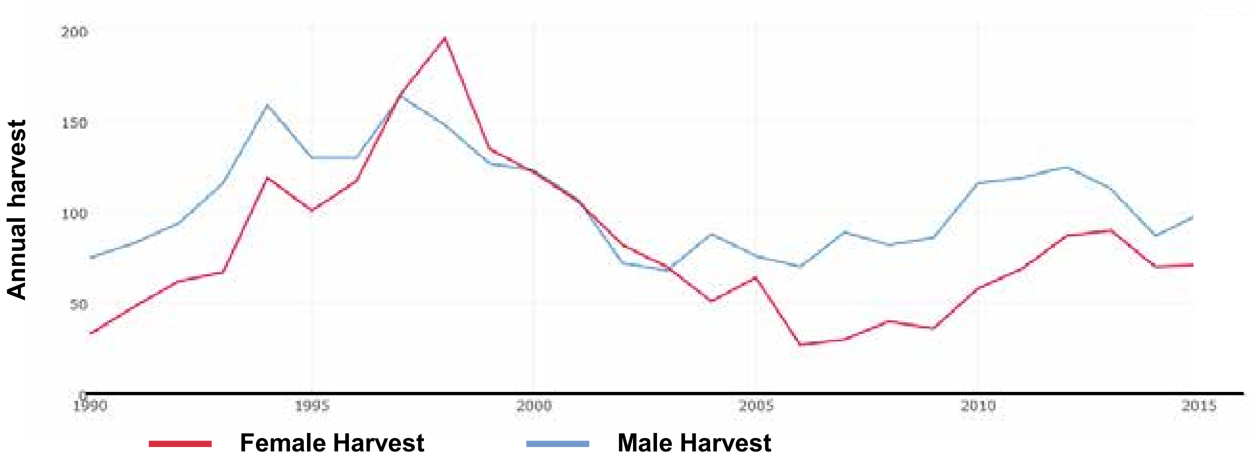

West-central Ecoregion

The 51,665 km^2 West-central ecoregion includes the forested mountain ranges and intermountain valleys of the Bitterroot, southern Blackfoot, and upper Clark Fork watersheds west of the Continental Divide and the Rocky Mountain Front, Helena/Boulder valleys, Belt and Snowy Mountains to the east (Figure 13). The ecoregion includes portions of FWP Regions 2, 3 and 4.

Forests across the ecoregion are diverse and often separated by broad intermountain valleys. The average mountain lion habitat quality (average LMU RSF value = 0.72) is generally lower than in northwest Montana because high-quality lion habitat is more intermittent. There is extensive and well distributed public recreational access to winter lion habitat, although some local private land refuges exist. Snow conditions annually vary within and between watersheds—a lack of adequate tracking snow occasionally limits winter lion harvest in some areas.

The ungulate prey base and density varies across the ecoregion. Although white-tailed deer are generally common, mule deer and elk make up a greater proportion of available ungulates than in northwest Montana.

The 2,200 km^2 West-central ecoregion's Trend Monitoring Area includes the upper Blackfoot and east Nevada Cr. Valleys west of the Continental Divide (Region 2) and the Canyon Creek/Little Prickly Pear drainages east of the Divide in Region 3 (Figure 14).

Mountain lion harvest in the West-central ecoregion climbed to a high of 294 lions (53% female) in 1998 (Figure 15). Hunter harvest, particularly of females, was significantly reduced in the 2000s following perceived population declines. By 2015, overall harvest density increased to 3.1 per 1,000 km^2, well below the nearly 6.0 per 1,000 km^2 in the late 1990s—specifically, the 2015 female harvest was one third of the 1998 peak.

Figure 13. The West-central mountain lion ecoregion, trend monitoring area, and 2016 FWP hunting districts.

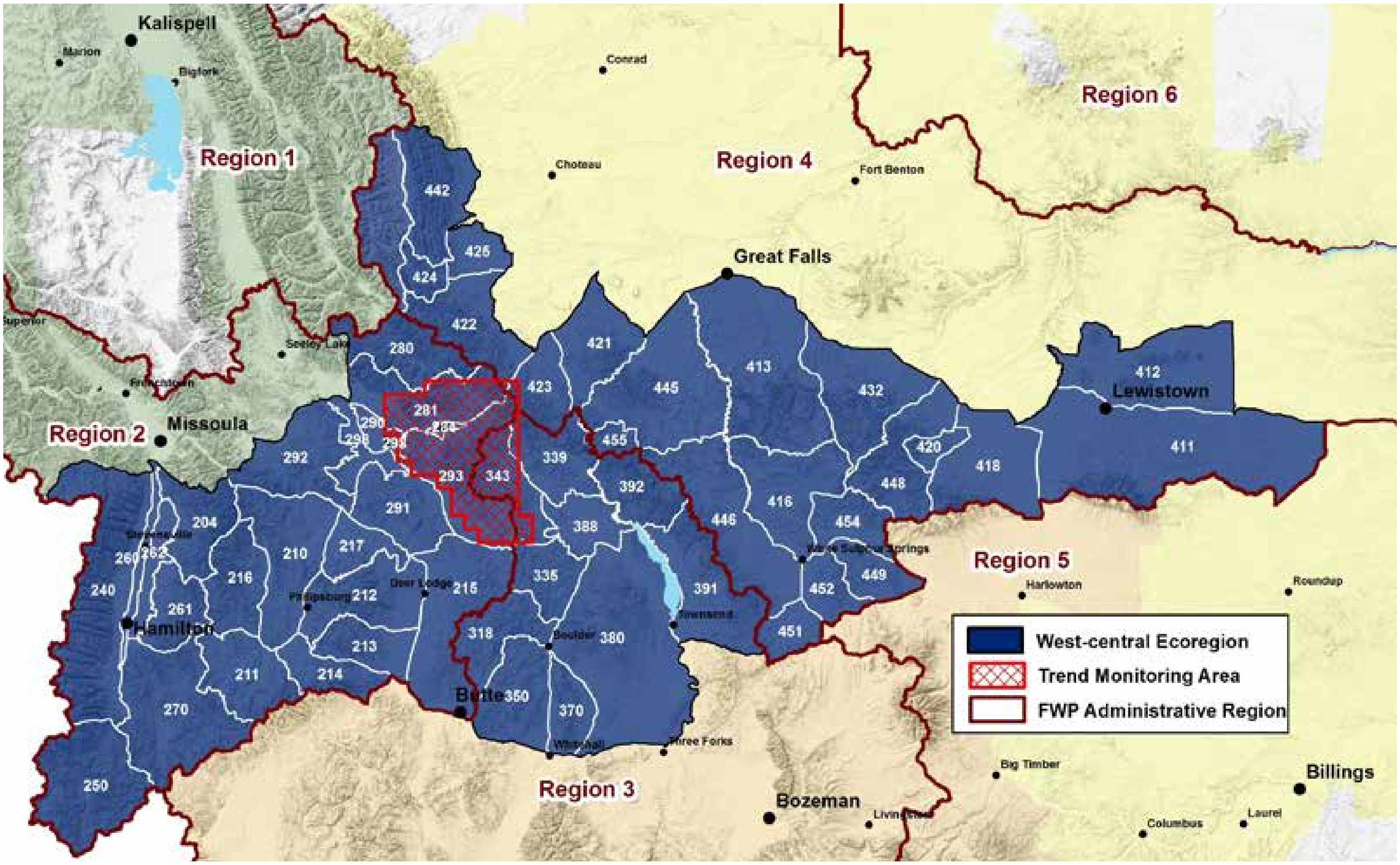

Figure 14. The West-central mountain lion ecoregion trend monitoring area divided into a grid of 101 5x5 km sampling cells.

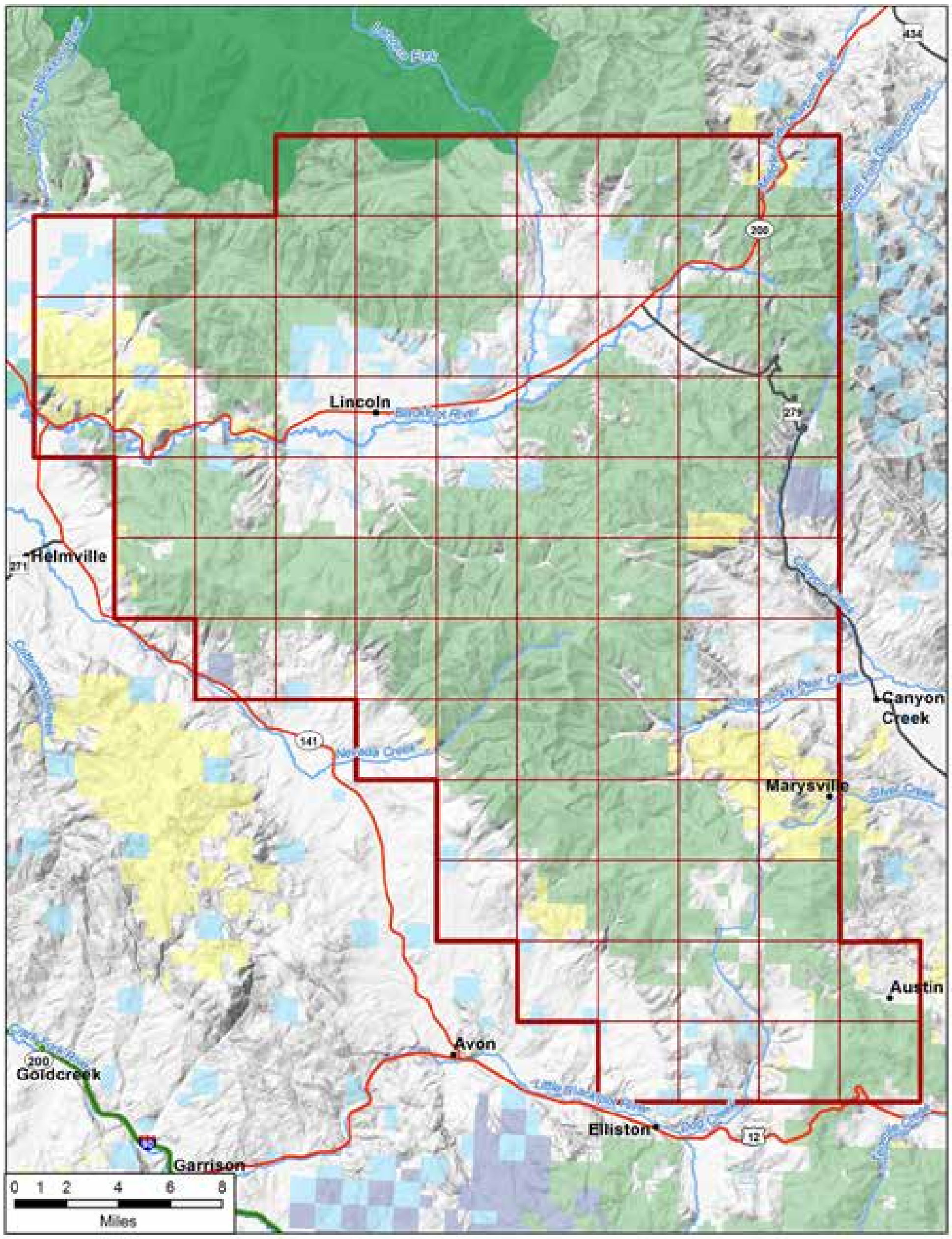

Fig. 15. West-central ecoregion mountain lion harvest, 1990 – 2015.

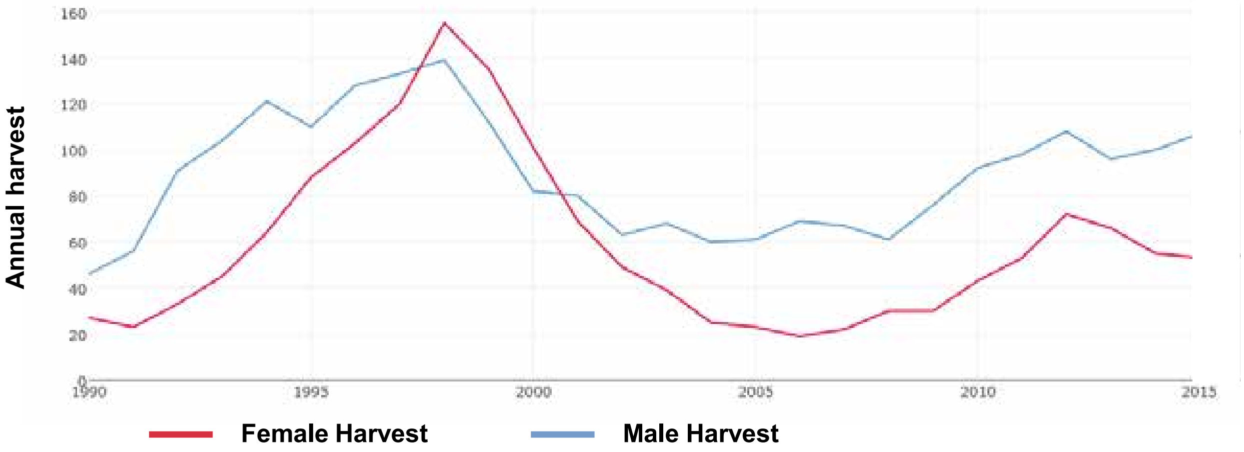

Southwest Ecoregion

Mountain lion habitat is relatively patchy and linearly distributed in much of the 52,487 km^2 Southwest ecoregion. This area extends from the Continental Divide and southwest Montana's island ranges, across the Greater Yellowstone Ecosystem to the Beartooths, Crazy Mountains, southeastern Little Belts, and southern Big Snowy Mountains. The ecoregion includes much of FWP Region 3 and western Region 5 (Figure 16). Although many portions of the ecoregion include high-quality lion habitat, only about a third of the total area is forested—the average LMU's RSF value in this ecoregion is 0.51.

Public access to winter mountain lion habitat is mixed; approximately 75% of lions harvested between 2007 and 2015 were taken on public land. Winter snow tracking conditions vary and can, at times, limit effective harvest.

The 2,525 km^2 Southwest ecoregion mountain lion Trend Monitoring Area is located in the Gallatin Range between Bozeman and Yellowstone National Park (Figure 17).

Total mountain lion harvest in this ecoregion peaked in the late 1990s, declined in the 2000s, then returned to near the 25-year average level by 2015. Much of this variation, however, was due to fluctuations in female lion harvest; male harvest has remained relatively constant since the mid-1990s (Fig. 18). Overall Southwest ecoregion harvest density was 1.75 lions per 1,000 km^2 in 2015.

Figure 16. The Southwest mountain lion ecoregion, trend monitoring area, and 2016 FWP hunting districts.

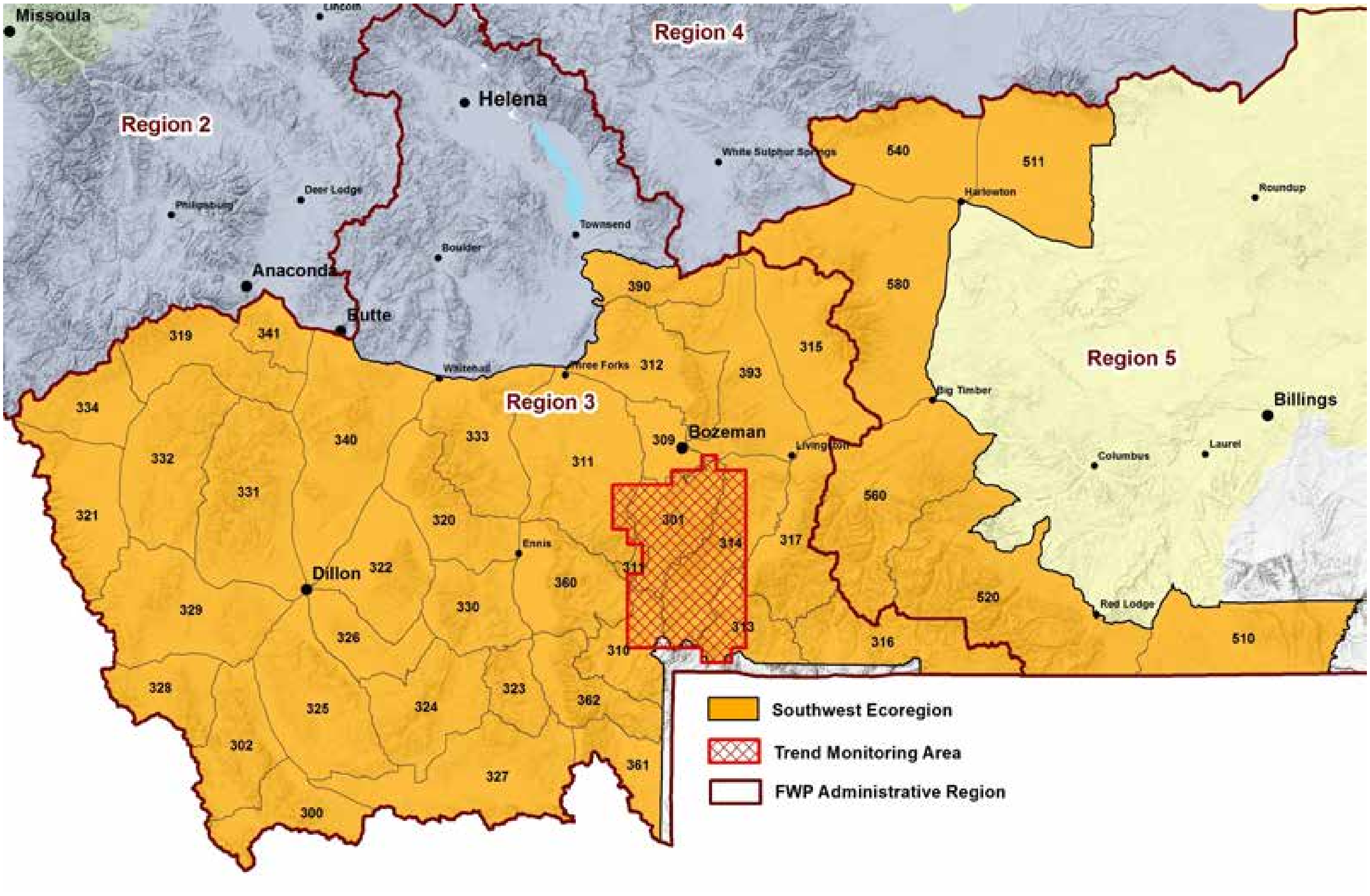

Figure 17. The Southwest mountain lion ecoregion trend monitoring area divided into a grid of 101 5x5 km sampling cells.

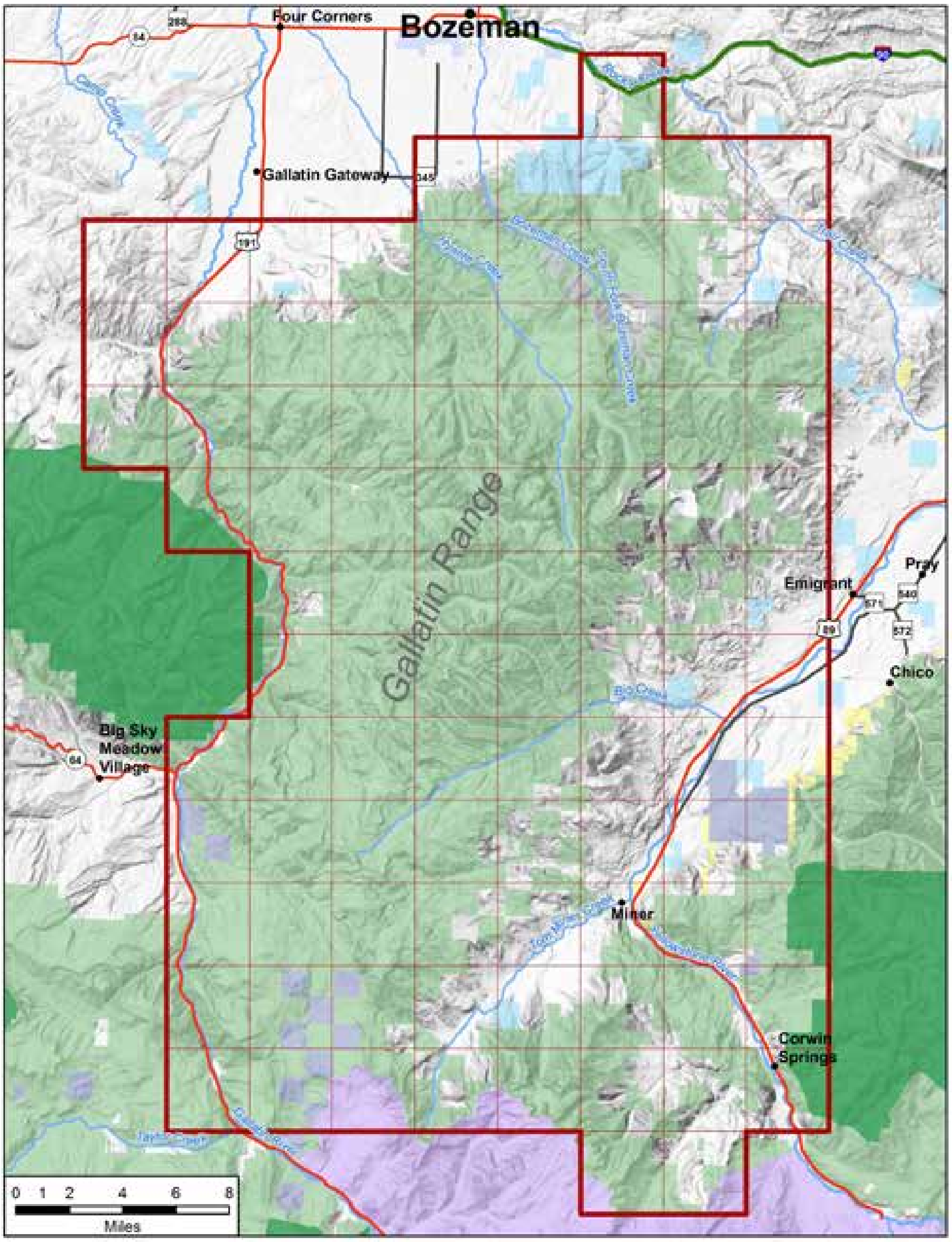

Fig. 18. Southwest ecoregion mountain lion harvest, 1990 – 2015.

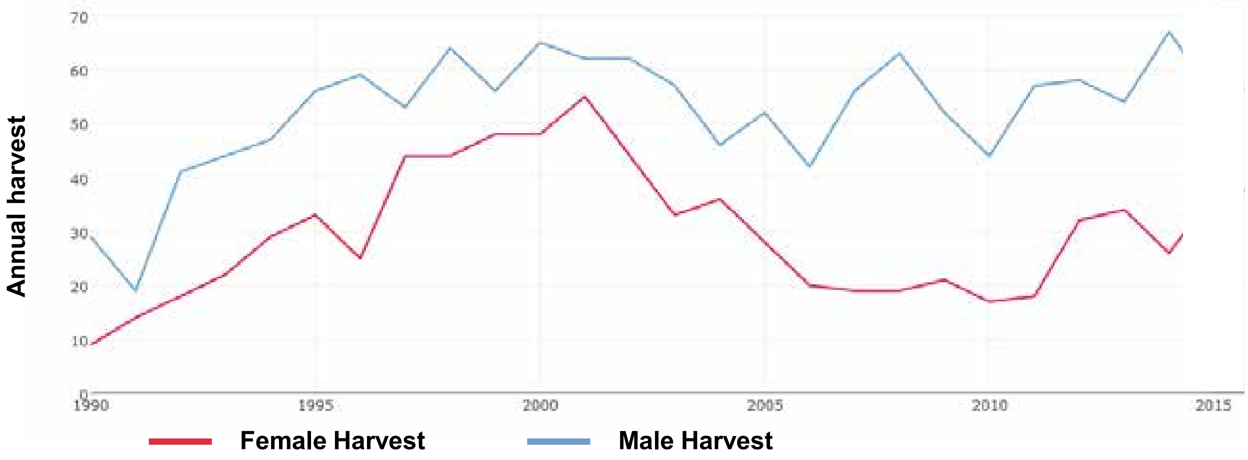

Eastern Ecoregion

The 198,175 km^2 Eastern ecoregion is, by far, the largest in the state and includes all or portions of FWP Regions 4, 5, 6 and 7 (Fig 19). Much of the highest quality mountain lion habitat in eastern Montana lies within Indian reservations—FWP does not have routine mountain lion management jurisdiction on these reservations and they are excluded from the ecoregion for analysis and planning purposes. Less than 10% of the remaining ecoregion supports ponderosa pine or juniper-dominated forest. In general, patches of high-quality lion habitat are relatively small and widely distributed (average LMU RSF value = 0.38).

Genetic field monitoring data will not be routinely collected in the Eastern ecoregion and, therefore, no permanent Trend Monitoring Area has been designated. Lions in this ecoregion occur at an overall low density and sub-populations occur in discontinuous patches of suitable habitat. Inferences drawn from field sampling in one area would be of limited use for broad scale management of this ecoregion.

Mountain lion distribution and abundance has significantly increased in eastern Montana since the 1980s and recovery likely continued through the 2010s. Harvest has steadily increased since the 1990s (Fig. 20). Intermittent snow cover in eastern Montana can significantly reduce hound hunting's effectiveness. Therefore, in this ecoregion, quotas are more likely to serve as limits on harvest during years when snow conditions are favorable than as reliable annual harvest prescriptions.

Lion harvest in the Eastern ecoregion generally occurs in areas that the RSF describes as high-quality habitat on or near the Custer National Forest, Bureau of Land Management lands surrounding the Charles M. Russell National Wildlife Refuge, private land in the Bears Paw Mountains, in the Highwood Mountains, and along the northern Rocky Mountain Front.

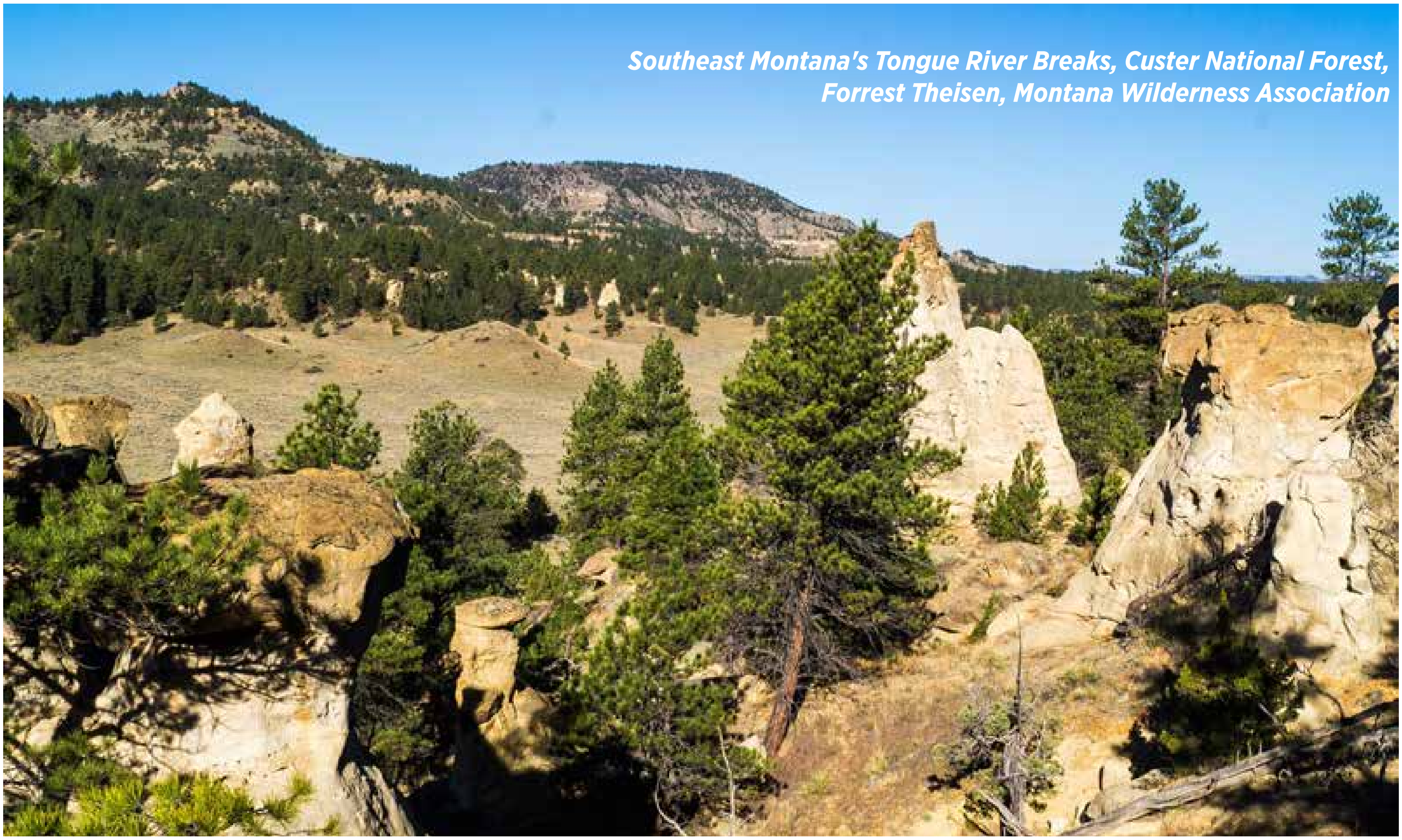

Southeast Montana's Tongue River Breaks, Custer National Forest, Forrest Theisen, Montana Wilderness Association

Figure 19. The Eastern mountain lion ecoregion and 2016 FWP hunting districts.

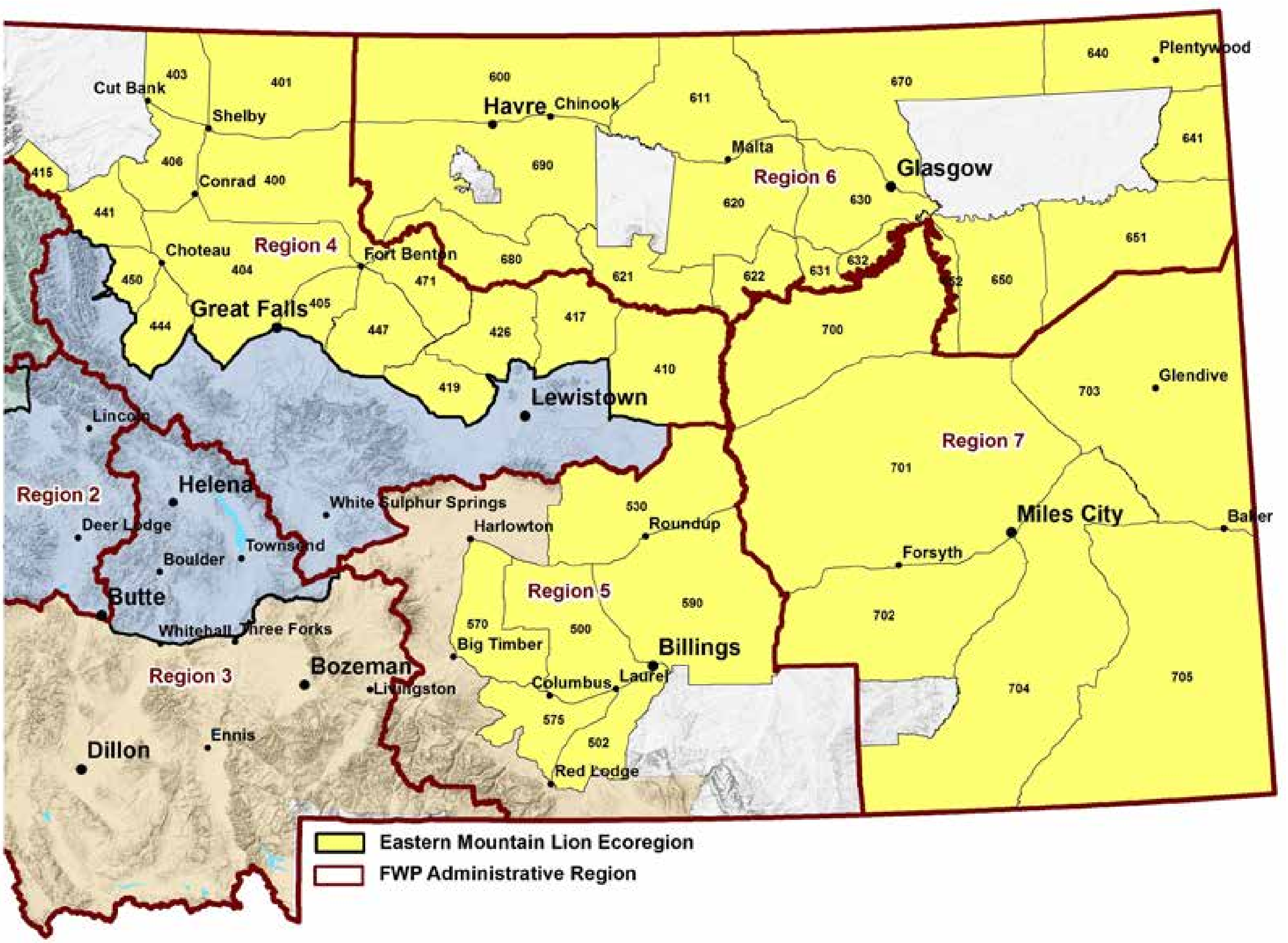

Fig. 20. Eastern ecoregion mountain lion harvest, 1990 – 2015.

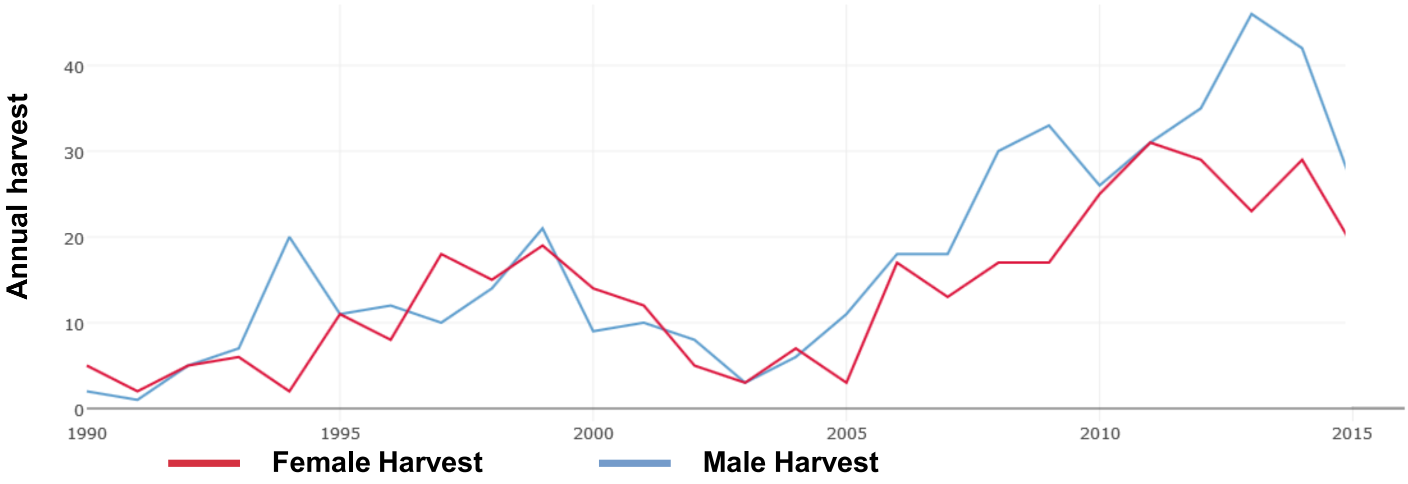

CHAPTER 5

MONITORING MOUNTAIN LION ABUNDANCE

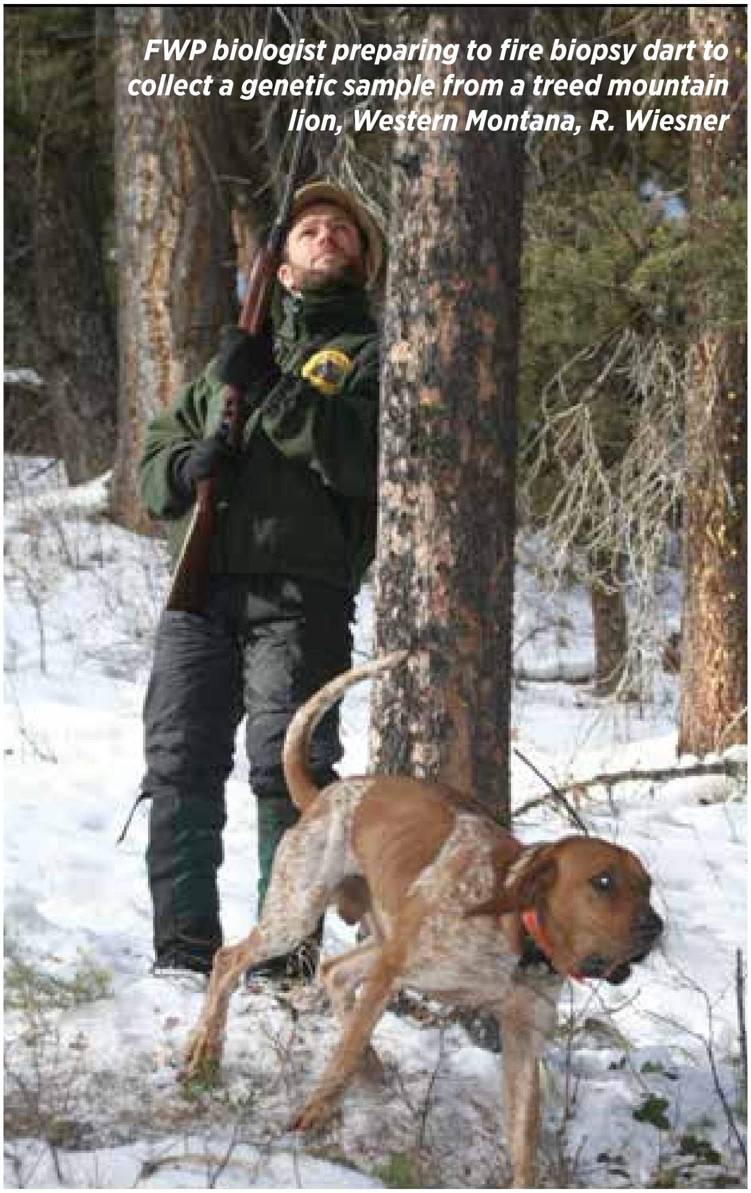
FWP biologist preparing to fire biopsy dart to collect a genetic sample from a treed mountain lion, Western Montana, R. Wiesner

" The Holy Grail of cougar management has always been the question of 'How many are there?'"

**Managing Cougars in North America—
J. A. Jenks, editor (2011)**

INTRODUCTION

To conserve mountain lions while ensuring sustainable recreational hunting opportunities, FWP needs accurate and up-to-date information about mountain lion population size and trend. In the past, managers used indirect measures of lion abundance, inferences drawn from long term field research projects, or anecdotal information about population status to inform decisions. Unfortunately, these sources of information often fail to accurately describe the effects of previous management actions and don't allow us to precisely predict the effects of future harvest (Beausoleil et al. 2013).

Developing a method to obtain regular, accurate, extensive, and affordable estimates of the size of lion populations has been one of the highest priority mountain lion management needs (Beausoleil et al. 2008, Jenks 2011). Until recently, there was no cost effective and relatively quick way to produce reliable lion population estimates at a large enough scale to be meaningful for management (Choate et al. 2006, Beausoleil et al. 2016).

Many agencies that are charged with managing mountain lions rely on indirect measures, or indices, of lion abundance to make inferences about population changes because these indirect data are already available or relatively easy to collect. However, the actual relationship (if one exists) between a population index and true population size is rarely known and may be inconsistent over time (Anderson 2003).

When potential indices of abundance were formally compared to known populations, the indices often proved too insensitive to be useful management triggers. For example, Wolfe et al. (2015) found that although the number of lions treed-per-day, permit fill rate, and the proportion of females in harvest were correlated with abundance, those relationships were weak. These indices are also not generally relevant in Montana where most harvest is regulated by sex-specific quotas.

Although the sex and age of harvested lions can eventually indicate significant changes in a lion population's size or

growth rate, these harvest indices are only able to detect relatively large and long term increases or declines (Stoner 2004, Anderson & Lindzey 2005, Robinson & DeSimone 2011).

In Montana, changes in harvest-age structure appear to broadly correspond to observed, long term, changes in lion abundance. When populations were thought to be high and growing during the early 1990s, a greater proportion of the harvest consisted of older lions (Table 6). Lion populations apparently declined during the early 2000s before recovering; both the average ages of harvested lions and the proportion of older lions in the harvest reflected this trend. A similar relationship was documented in western Montana's Garnet Mountains between 1997 and 2006 (Figure 21).

Statewide lion density declined and recovered dramatically between the mid-1990s and late 2000s. This pattern was, in part, driven by dramatic changes in statewide harvest rates that are unlikely to be applied in the future. The current magnitude of variation in statewide age-at-harvest is relatively small and annually variable. During periods when the amplitude of population change is moderate, trends in harvest-age are less informative.

Tracking changes in the ages of harvested animals may be somewhat useful where more direct measures of population trend are not available (such as eastern Montana), but the index is too insensitive to detect moderate, short term changes in an area's lion density. The proportion of older adult animals in harvest (especially females)

Table 6. Montana mountain lion age-in-harvest, 1988 – 2015.

	Female, Average Age	Male, Average Age	Combined, Average Age	% of Harvest > 5 Y.O.
**1988**	3.45	2.94	3.10	10.3
**1989**	3.21	3.94	3.75	16.7
**1990**	4.38	4.38	4.38	26.1
**1991**	5.04	4.94	4.97	37.6
**1992**	6.59	6.40	6.46	58.8
**1993**	4.99	5.39	5.25	39.0
**1994**	3.67	3.98	3.86	24.0
**1995**	4.29	4.30	4.29	27.0
**1996**	3.81	3.07	3.39	13.3
**1997**	3.70	3.18	3.44	15.4
**1998**	3.34	2.71	3.04	11.3
**1999**	3.21	2.60	2.91	12.5
**2000**	3.42	3.18	3.30	13.2
**2001**	3.33	3.28	3.31	12.6
**2002**	3.17	3.07	3.11	4.3
**2003**	2.93	2.73	2.81	5.5
**2004**	3.24	2.53	2.79	9.7
**2005**	3.22	2.95	3.05	10.6
**2006**	2.76	2.89	2.85	4.7
**2007**	3.46	3.43	3.44	11.8
**2008**	3.14	3.53	3.41	14.3
**2009**	3.34	3.13	3.19	10.8
**2010**	4.02	3.45	3.65	17.2
**2011**	3.42	3.00	3.14	10.1
**2012**	3.76	3.45	3.57	16.7
**2013**	3.62	3.13	3.33	13.1
**2014**	3.34	3.35	3.34	11.0
**2015**	3.09	2.89	2.97	6.8

Figure 21. Minimum mountain lion population estimate, and mean adult (> 24 months) age of harvested lions, Garnet Mountains, MT (Robinson & DeSimone 2011)

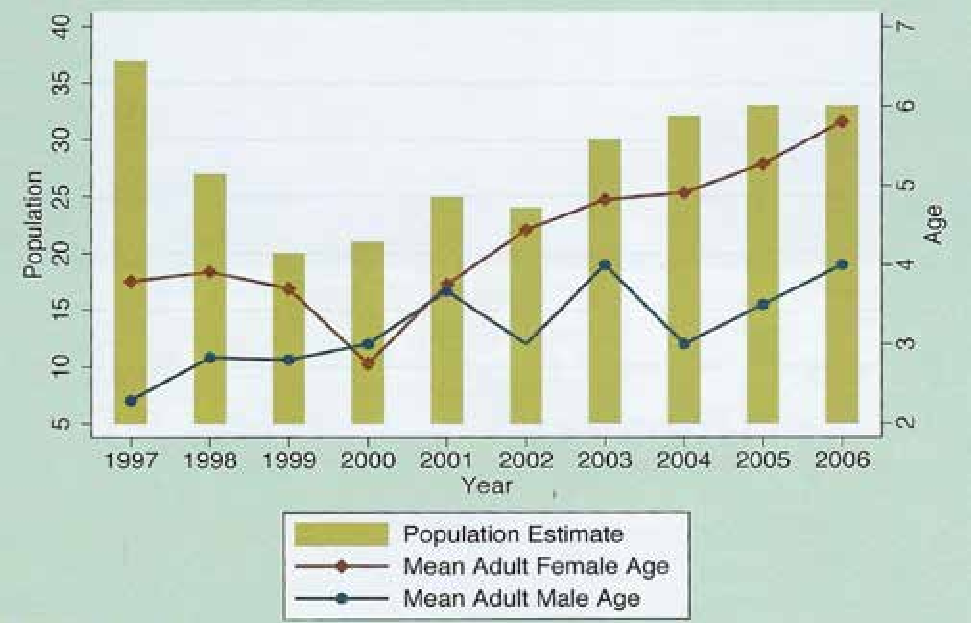

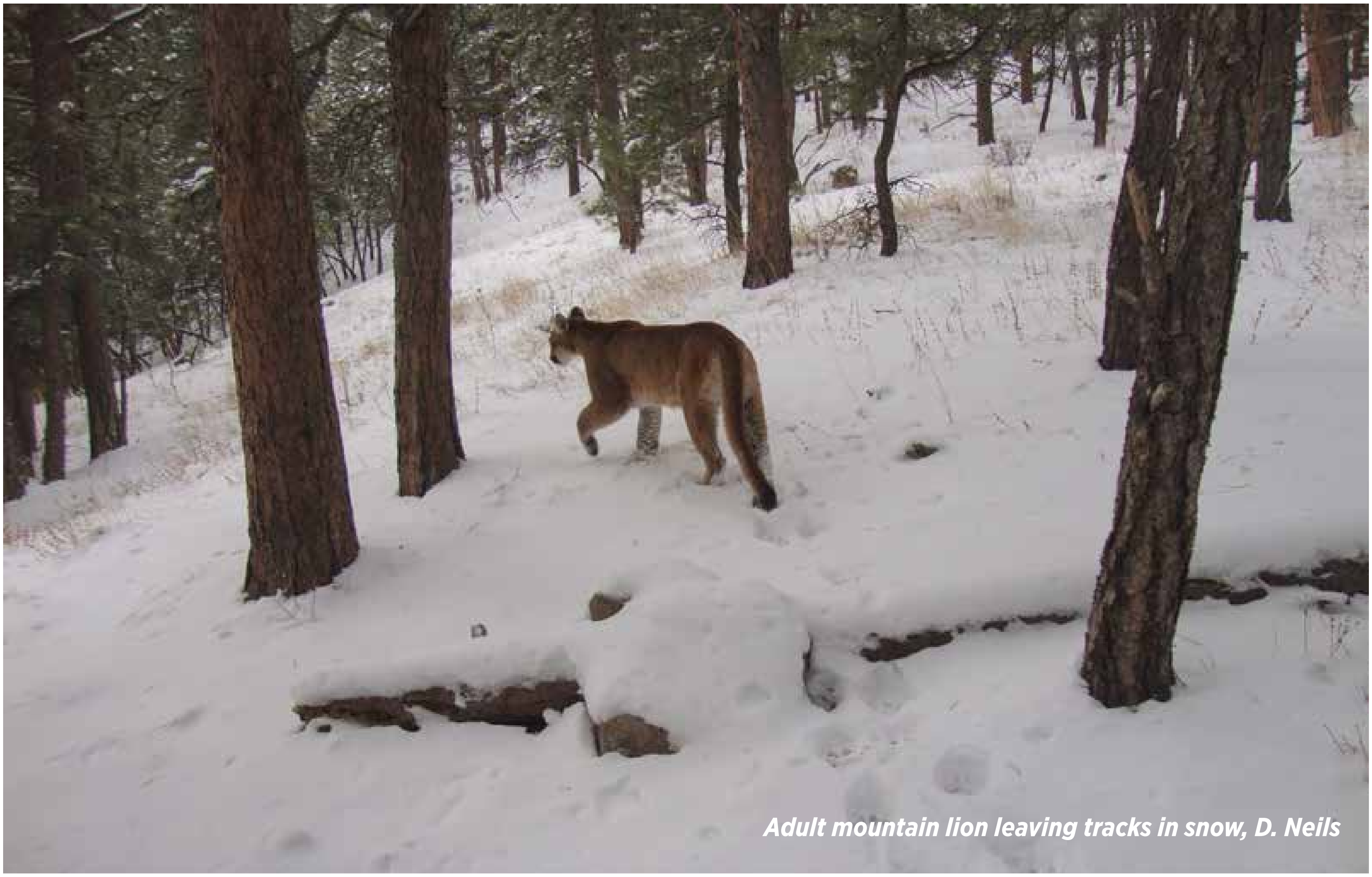

Adult mountain lion leaving tracks in snow, D. Neils

is more strongly correlated with annual adult survival than is the overall mean or median age-in-harvest (Wolfe et al. 2015).

Relying on past years' harvest to inform future quotas is also problematic. This "sledgehammer approach" (Logan & Sweanor 2001) uses previous seasons' hunter success rates to determine future harvest quotas. Even if managers reduce harvest quotas as hunter success decreases, these incremental reductions may not match existing population levels and can lead to further declines. Harvest indices are also much less informative in jurisdictions, like Montana, where most harvest is limited by sex-specific quotas.

Patterns in total annual harvest or days required to fill an area's quota can be misleading when factors that are independent of mountain lion population trend most strongly predict year-to-year harvest. For example, in much of the Eastern ecoregion adequate tracking snow is present only sporadically— during winters when there is snow cover, harvest increases. In these cases, quotas effectively prevent excessive harvest during years with favorable tracking conditions even though they will not be routinely met in other years.

Intensive winter track surveys, surveys of public lion observations, and hunter effort generally failed to detect known lion population changes quickly or before large changes in population size had already occurred (Beier & Cunningham 1996, Jenks 2011, Robinson & DeSimone 2011).

Long term capture and radio-telemetry studies were traditionally considered to be the most reliable way to estimate local lion populations (Cougar Management Guidelines Working Group 2005, Jenks 2011). This method requires researchers to attempt to capture and mark all resident individuals within a study area, account for additional unmarked animals, and then extrapolate observed and suspected home ranges across a study area to produce an estimate of abundance (Lambert et al. 2006, Cooley et al. 2009, Robinson et al. 2008 & 2014).

However, capturing, marking, and counting individual lions is impractical for routine lion population monitoring. Intensive capture and radio-tracking projects can take many years to complete, require significant field resources, and are prohibitively expensive (Hornocker & Negri 2009). The uncertainty around estimates developed using this field method is also often difficult, or impossible, to assess. Finally, this technique usually produces only minimum counts because all individuals in a study area are rarely captured and nonresident (transient) individuals are often either missed or discounted (Robinson et al. 2015).

Because it was so difficult to directly monitor mountain lion population size and trend at a large scale, some researchers suggested implementing "zone management" (Logan & Sweanor 2001) or a similar "metapopulation model" (Laundre & Clark 2003) instead. These strategies advise maintaining large and well-distributed lightly hunted refuge areas (sources) that sustain more heavily-hunted areas (sinks) through emigration. Although metapopulation management doesn't rely on accurate population estimates, it does require knowledge of immigration rates between heavily and lightly-hunted areas. Few studies have rigorously estimated these immigration rates and the metapopulation management model's effectiveness remains largely untested.

Although several large patches of un- or lightly-hunted lion habitat (including national parks, wilderness areas, and Indian reservations) undoubtedly act as sources of

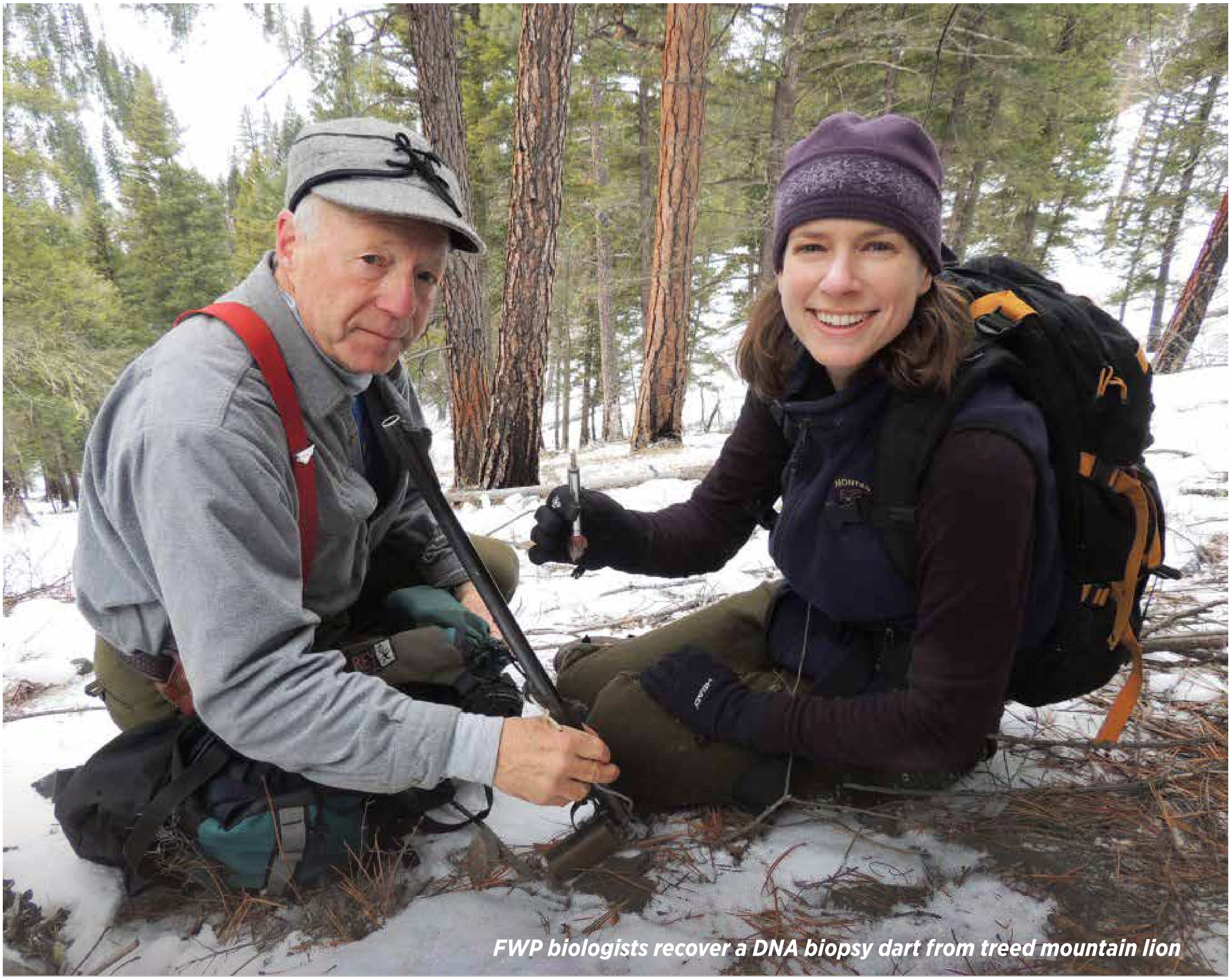

FWP biologists recover a DNA biopsy dart from treed mountain lion

lions that disperse to other areas in Montana (Robinson et al. 2015), these refuges are neither extensive or well distributed enough to subsidize unlimited harvest in the remainder of the state.

FWP will not further restrict lion harvest across broad areas of the state in order to create additional specific "source" areas and, therefore, does not intend to use the metapopulation model as the basis for its mountain lion Management Strategy.

Instead, FWP will manage for limited and sustainable mountain lion hunter-harvest opportunity on most lands within its jurisdiction. To enable this approach, FWP will periodically monitor the size and trend of lion populations in the Northwest, West-central and Southwest ecoregions. We will use rigorous, field-based techniques to estimate population size and trend, and we will remain open to incorporating new monitoring methods as they are developed and validated. Distributing this monitoring effort across these three biologically distinct ecoregions will reduce the uncertainty of the estimates developed using local monitoring data (Walters & Holling 1990, Conroy et al. 2012).

Subsequent Trend Monitoring Area abundance estimates can be directly compared to past estimates from the same area. Abundance estimates for the Trend and Supplemental Monitoring Areas (see Montana Mountain Lion Monitoring section, Chapter 5) can also be used to develop abundance estimates (presented as a range of likely values) for their respective ecoregions. These periodic ecoregional estimates will allow managers to track changes in mountain lion abundance over time and will be included in the Integrated Population Model (Chapter 6) to predict the effect of future harvest prescriptions.

The same regular field monitoring will not be conducted in the Eastern ecoregion. There, lion subpopulations are patchily distributed and the ecoregion annually produces <15% of the state's annual harvest. Other population indices and harvest management strategies will be used in this ecoregion to conserve hunted populations. However, Eastern ecoregion managers may choose to sample lion abundance in specific areas of interest to better understand local populations.

ESTIMATING MOUNTAIN LION POPULATIONS

Capture-recapture (CR) sampling has been a standard method used to estimate a population's abundance for many years (Seber 1982). To produce a traditional CR estimate, some animals in a population are captured, marked, and released. Later, there is another capture effort and the number of marked animals within the second sample is counted. The proportion of the first sample detected in the subsequent sample is then used to calculate a population estimate.

Conventional CR sampling assumes that the effective sampling area's size is known, that animals don't enter or leave the study area, and that all animals have a similar probability of detection (Royle et al. 2013). Species like mountain lion that are wide-ranging, occur at low densities, and are difficult to detect violate these assumptions and may cause CR methods to produce misleading results.

SPATIAL CAPTURE-RECAPTURE

A newer spatial capture-recapture (SCR) method specifically addresses the shortcomings of traditional CR techniques when working with wide ranging, low-density species. SCR has been successfully used to estimate carnivore populations (Royle et al. 2011, Blanc et al. 2013) including mountain lions in Montana (Russell et al. 2012, Proffitt et al. 2015). SCR also works well with less invasive data collection techniques such as acquiring genetic samples from biopsy darts, hair, or scat.

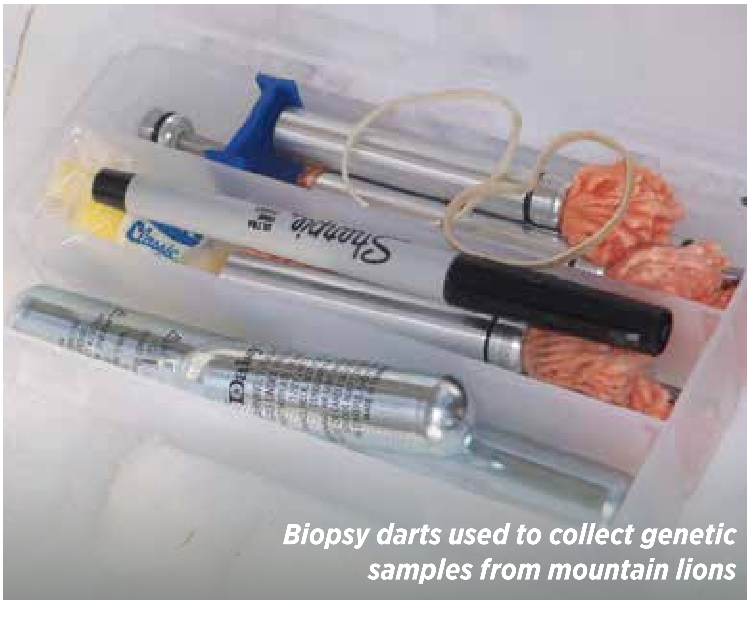

Biopsy darts used to collect genetic samples from mountain lions

The SCR approach allows biologists to estimate population abundance within a defined area while also accounting for animals whose ranges partially or occasionally overlap the area surveyed. SCR methods consider the spatial organization of individual animals and the fact that the probability of an individual being recaptured decreases the farther that animal is from where it was originally detected or is known to reside. SCR methods also allow for sampling effort to vary across a study area when sampling wide ranging species (such as mountain lion) that use heterogeneous habitat.

Mountain lions in Montana prefer areas with habitat features such as forest cover, moderate slopes, forest edges, and intermediate elevations (Newby 2011, Robinson et al. 2015). Consequently, lions are not evenly distributed across different habitat types within an area. SCR methods use information about lion habitat preferences (specifically, the 2016 Montana mountain lion RSF) to inform estimates of population abundance.

Because estimated abundances are spatially explicit, population abundances associated with habitat of a certain quality within a sampling area can be extrapolated across broad landscapes as a function of that landscape's habitat quality. This allows information about lion abundance within Monitoring Areas to be used to estimate lion populations at the ecoregion scale.

SCR methods can also include information from harvested animals in population estimation models, thus allowing sampling to occur where hunter harvest is expected on and around the study area during the period the sampling is taking place (Efford 2014).

ABUNDANCE ESTIMATES

Monitoring an area's mountain lion abundance over time is essential to understanding the effect of hunter harvest on lion populations. However, variation in the ways researchers have defined their study areas, inconsistent reporting of age-classes included in population estimates, and the differences in estimation methodology make directly comparing lion densities reported in the literature nearly impossible (Hornocker & Negri 2009).

FWP will monitor and report the estimated winter density of all non-dependent individual lions—that is, lions that are legal to harvest—within an area

For example, researchers have variously reported densities of all mountain lions (including dependent kittens), the minimum number of resident adults, and the density of lions estimated across both seasonal and annual ranges. FWP will monitor and report the estimated winter density of all non-dependent individual lions—that is, lions that are legal to harvest—within an area.

In Montana, the average age that a young lion becomes independent of its mother is approximately 15 months (Robinson & DeSimone 2011). Montana law prohibits the harvest of young lions with body spots; these spots are nearly gone by 15 months of age (Currier 1983, Lindzey 1987).

Young lions make up a significant proportion of legal harvest. Of the known age lions legally harvested in Montana between 1988 and 2014, 42% were <3 years old and 15% were <2 years old. Many of these juveniles and subadults are transient, having yet to establish a fixed home range. The number of transient mountain lions in a population is difficult to quantify using traditional field sampling methods and this age class is often underrepresented in population estimates reported in the literature (Logan & Sweanor 2001).

Thus, an advantage of the SCR monitoring approach is that abundance estimates will include resident and transient animals, both of which are legal to harvest. The SCR method that FWP will initially use estimates the abundance of all independent aged lions within Trend Monitoring Areas and ecoregions during the winter monitoring period. Because all independent aged lions (including transients) are included, genetically based SCR abundance estimates may well be higher than estimates previously developed using other methods.

MONTANA MOUNTAIN LION MONITORING

FWP will use scientifically sound techniques to monitor Montana lion populations and produce periodic estimates of their size and trend. However, currently available monitoring techniques are both expensive and labor intensive. As field-based monitoring and analytical techniques improve and become more practical, FWP will remain open to incorporating them.

Initially, FWP will use the SCR sampling and analysis methods described by Proffitt et al. (2015) to periodically estimate independent aged mountain lion populations in the Northwest, West-central, and Southwest ecoregions. FWP has identified permanent Trend Monitoring Areas within each of these three western ecoregions which will be sampled on a rotating basis.

An additional Supplemental Monitoring Area within each ecoregion may also be sampled the year after the Trend Monitoring Area is sampled. Unlike the Trend Monitoring Areas, the location of Supplemental Monitoring Areas can change over time. These additional Monitoring Areas will allow FWP to sample a broader range of habitats within the ecoregions. Estimates of abundance calculated by sampling Supplemental Trend Monitoring Areas can also be compared to estimates for the same area calculated by extrapolating the previous year's Trend Monitoring Area sample data across the broader ecoregion. Thus, the subsequent Supplemental Area estimate can help validate the extrapolated ecoregional estimate. Methods for selecting the permanent Trend and Supplemental Monitoring Areas, the field protocol for collecting data, and a description of the data analysis are included in Appendix 1.

Each new estimate of a Trend Monitoring Area's lion population can be directly compared to past estimates for that same area. In addition, the relationship between lion density and the 2016 RSF within an ecoregion's Trend Monitoring Area (sampled Year 1) and Supplemental Monitoring Area (sampled in subsequent years) can be combined to develop an estimate of population abundance for the larger ecoregion. If, over time, pooling the two Monitoring Areas' data produces ecoregional estimates that are functionally similar to estimates calculated from using the Trend Monitoring Area data alone, continued sampling of Supplemental Monitoring Areas may not be necessary.

Finally, an ecoregion's population estimate (with error distribution) will be input into the Mountain Lion Integrated Population Model (Chapter 6) to increase our understanding of past and predicted mountain lion population trend and to evaluate alternative harvest prescriptions. Uncertainty about mountain lion abundance impedes effective harvest management. More accurate abundance estimates will be used in an adaptive management framework to make management more predictable over time. The frequency of monitoring will affect the rate at which this uncertainty is reduced, but monitoring frequency will also depend on the availability of funding and other priorities.

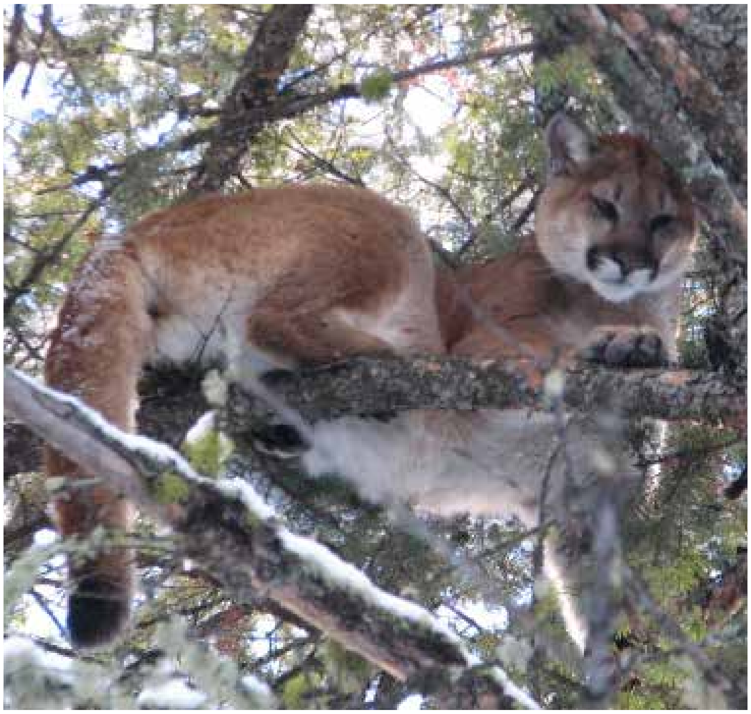

CHAPTER 6

THE MONTANA MOUNTAIN LION INTEGRATED POPULATION MODEL

INTRODUCTION

Wildlife biologists use mathematical models to approximate the real ecological systems they manage. These models allow them to better understand how populations work and to make more accurate predictions about how they're likely to change in the future. The most useful models are built using rigorously collected field research data and have a clearly defined purpose. These data (such as the age a male lion will most likely disperse or an adult female's annual survival rate) describe what's most likely to occur as well as the range of probable outcomes we should expect. By combining the best information available about a species or system we can better understand them.

Dr. Paul M. Lukacs and Dr. Joshua Nowak of the University of Montana collaborated with FWP to develop the Montana Mountain Lion Integrated Population Model (IPM; Nowak et al. 2018). The IPM is a tool that combines available information about a mountain lion population (i.e. harvest, abundance, survival, and reproduction) into a single analysis of that population's demography. Managers can use the IPM to describe the effects of past management and make predictions about future population trends.

PREDICTING LION POPULATIONS USING THE IPM

The primary purpose of the IPM is to help wildlife managers, decision makers, and the public understand the effect of past and future harvest on mountain lion populations. The IPM is directly linked to the FWP lion harvest database, and a web interface allows users to input future possible harvest prescriptions (by sex and age class).

Using this information, the model forecasts the future population trend that would likely result from an ecoregion's proposed harvest prescription. The output clearly shows the range and magnitude of the predictions' uncertainty for each year of the analysis; this uncertainty increases the further into the future the model is asked to make predictions.

Periodic abundance estimates that are developed from field-based monitoring (described in Chapter 5) can also be input into the model. These estimates (also input as a range of likely values) make the IPM's predictions more precise. The IPM outputs the results of model runs as graphs (by population and by age and sex-class) as well as in a tabular format.

Montana's mountain lion IPM was built using the software program PopR which was developed in collaboration with Idaho Fish and Game, South Dakota Game, Fish and Parks and The University of Montana in 2014 (Nowak et al. 2018). PopR is a web based application linked directly to agency harvest databases through an interactive graphic user interface. It allows non-expert users to easily update data and change model parameters (such as assumed survival rates or reproduction) to evaluate the potential effects of future harvest levels. The IPM and web application were specifically designed to be repeatable, transparent, and easy for biologists to use.

The Montana mountain lion IPM can analyze populations within the three western Montana mountain lion ecoregions. Harvest data are input into and analyses are output by the IPM at the ecoregion scale.

The Integrated Population Model is a tool that combines all available information into a single analysis of mountain lion population demographics

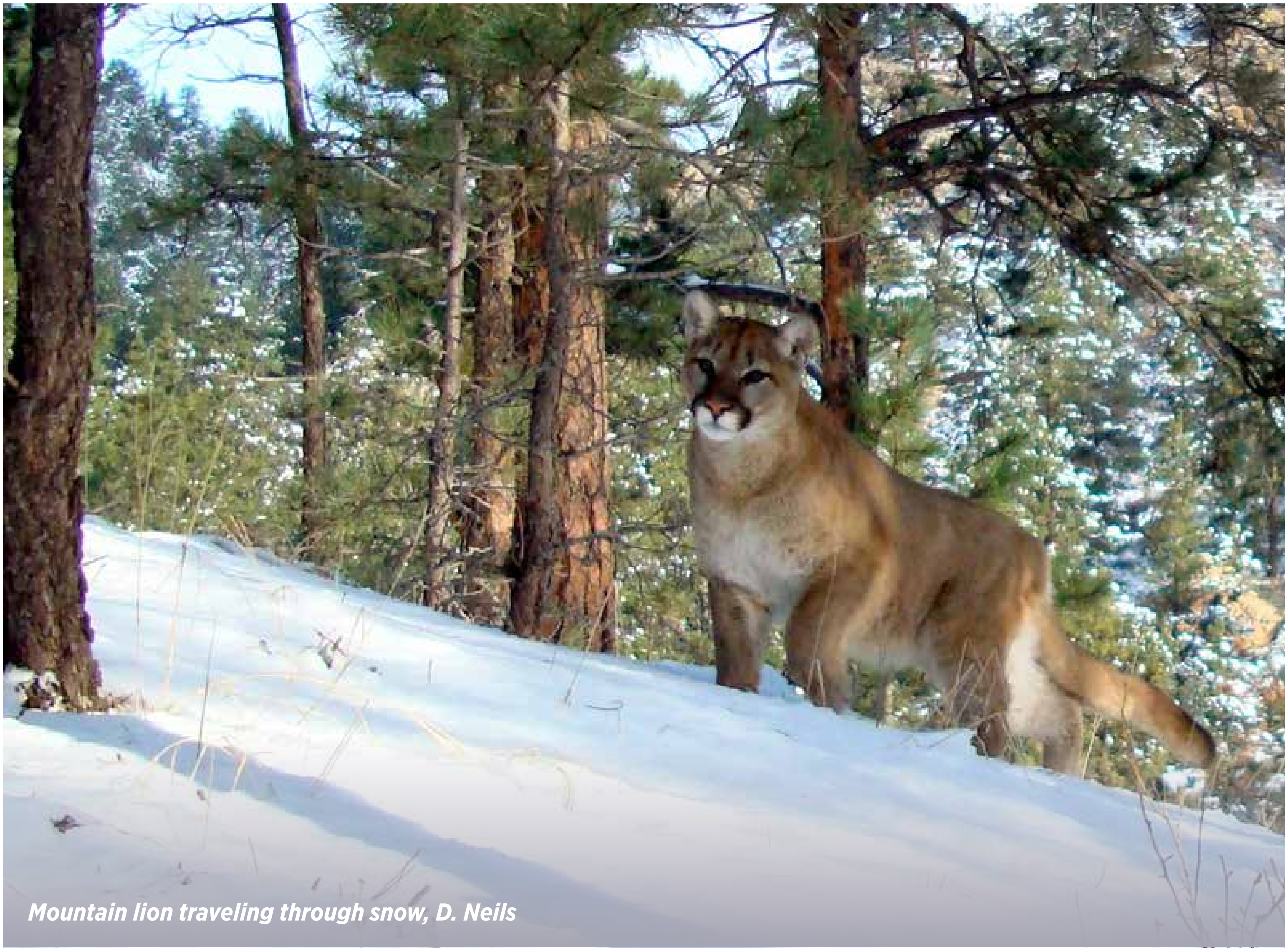
Mountain lion traveling through snow, D. Neils

The IPM contains two underlying model components: a biological process model and an observation model (Schaub & Abadi 2011). The biological process model describes what we know about lion population dynamics and vital rates (Caswell 2001). It uses parameters including age-class and sex-specific survival probabilities, fecundity by age-class, and estimates of overall population size (when those field estimates are periodically available). The observation model describes the data collection process and the link between field data, harvest records, and biological parameters.

Field-based estimates of population vital rates have some statistical uncertainty and fluctuate over time. That is, field data (i.e. litter size) occur as a distribution of observed values that produce both a point estimate and a range of likely values. The IPM combines and considers all sources of uncertainty when predicting mountain lion population size and trend.

Field research has shown us that although many lion population vital rates (including reproduction and non-hunting survival) are remarkably consistent across the species' range, variability around average rates can significantly influence populations (Robinson et al. 2014). This variability is explicitly incorporated into the model and carried forward into predictions. The IPM allows users to estimate sex and age-specific population size and growth, as well as the precision of those predictions.

It's difficult to directly measure mountain lion vital rates and population trend frequently or extensively. Fortunately, lion ecology has been studied for decades in Montana and throughout the western U.S. The lion IPM allows for

a straightforward application of expert knowledge even when specific information about local or contemporary populations is sparse. The model generates reasonable estimates of those parameters managers cannot directly measure based on the range of values researchers have previously collected in the field.

The IPM uses Bayesian statistics that allow a range of possible but uncertain values to be substituted in lieu of new field data. The range of values can be ‘uninformative’ (allowing a wide range of values to be equally likely) or ‘informative’ (where values known to be more likely are given a higher probability). For example, the annual survival probability for mountain lions can take any value from 0 (certain to die) to 1 (certain to live). Field research suggests that annual adult female mountain lion survival is near 0.85 in the absence of harvest. Therefore, an uninformative range of values could be a uniform (0,1) while a more useful informative range of values would have a mean of 0.85 with a standard deviation based on the range of values reported in the research literature. Montana’s lion IPM uses informative values based on previous field research to improve model performance because it’s impossible to directly measure vital rates every place or every year.

MOUNTAIN LION IPM MULTI-STATE SURVIVAL MODEL

Long-lived species with moderate reproductive rates (like lions) are particularly sensitive to changes in survival rates (Gaillard et al. 1998). The chances of a lion surviving each year also changes as it grows older. Kitten survival is the lowest of any age-class. Field estimates of kitten survival are often biased high because dens are usually located sometime after birth occurs (eg. Robinson et al. 2014) and kitten deaths between birth and when researchers discover the den may not be accounted for. Juveniles and subadults typically experience higher mortality during transient and dispersal movements (Sweaner et al. 2000, Robinson et al. 2008). Once a lion establishes a home range, nonhunting mortality risk decreases until the lion reaches old age. Adult lions typically die from intraspecific strife and human caused sources like road kills, management removals, and sport hunting (Hornocker 1970, Logan et al. 1986, Cooley et al. 2009, Robinson et al. 2014).

The model generates reasonable estimates of parameters managers cannot directly measure based on the range of values researchers have previously collected in the field

Reported rates of lion survival vary and are plagued by small sample sizes (Hornocker & Negri 2009). The lion IPM default parameters are based on telemetry data from marked lions in Wyoming’s Teton Mountains (n = 100, 2001-2012), Washington’s Kettle Range (n = 36, 2002-2006) and studies in Montana’s Garnet Mountains and National Bison Range (combined n = 127, 1998-2006). These field data describe age and sex-class annual survival probabilities and error distributions used in the model (Appendix 2). Biologists can easily adjust input values if they have reason to believe that vital rates in their area are different from those observed during these field studies.

The IPM uses a known-fate multi-state survival model (Lebreton et al. 1992, Schaub et al. 2010, Servanty et al. 2010, Kery & Schaub 2011). The known fate assumption was necessary because the data included summaries of collar deployments but not true encounter histories. The IPM assumes that at the end of each month an animal could be in one of four states: a lion could be alive, dead by harvest, dead by other causes, or already dead at the beginning of that month. Animals whose fate was unknown because they left the area or whose collar failed are only included in the analysis up until the time they were last observed. Similarly, animals harvested outside Montana were only included up until they left the state so they did not contribute to Montana’s estimated harvest rates. A description of these specific biological inputs and assumptions is included in Appendix 2.

POPULATION RECONSTRUCTION

With the exception of kittens, Montana mountain lion reproduction and nonhunting mortality is not significantly affected by typical changes in harvest levels. That is, harvest doesn't reduce the probability of animals otherwise dying and changes in a population's harvest rates don't significantly affect the surviving individuals' fecundity. In much of Montana hunter harvest is the most likely cause of lion mortality. Research on hunted populations in Montana's Garnet Mountains showed harvest to be largely additive to more consistent background nonhunting mortality risk (Robinson et al. 2014), and FWP is not aware of research results demonstrating that harvest of independent aged mountain lions is compensatory with other mortality sources. Because nonhunting mortality occurs at a relatively constant rate, the overall number of animals that die from nonhunting causes will vary with increases or decreases of the overall population.

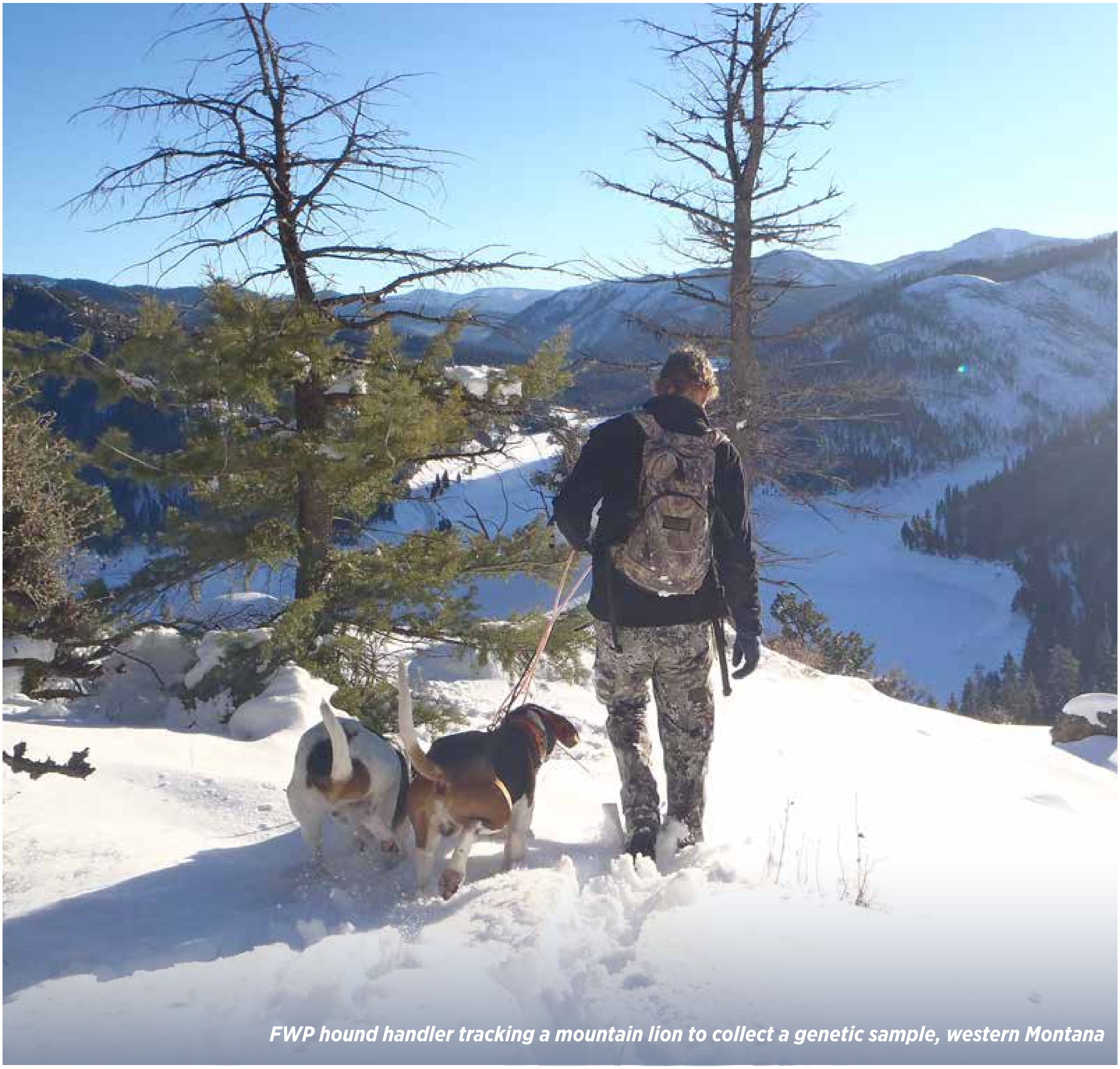

FWP hound handler tracking a mountain lion to collect a genetic sample, western Montana

Hunter harvest can, and often does, affect lion population growth (Cooley et al. 2009, Hornocker & Negri 2009). Harvest data also gives managers information about past population numbers and sex/age composition in an area. When managers have reliable estimates of past and current population levels, they are better able to predict the effect of future harvest prescriptions on the lion populations they manage. The IPM uses survival estimates along with the annual harvest records to reconstruct past mountain lion populations (Gove et al. 2002, Conn et al. 2008). A description of these specific biological inputs and assumptions is included in Appendix 2.

If we have an estimate of the harvest mortality rate (from telemetry data) and know the number of lions harvested, dividing the number harvested by the harvest mortality rate gives us an estimate of the pre-hunt population size. This is then corrected for an "other mortality" rate, which is relatively constant.

“Population reconstruction” methods have been successfully used to estimate the size and trend of harvested fish and wildlife populations for over 70 years. The technique uses age-at-harvest, total harvest, harvest rate, and the rate of non-harvest mortality to “rebuild” the past population that must have existed in order to have produced the known type and level of harvest.

The IPM uses these age and sex-specific survival estimates (from field research studies) along with the annual harvest rate to reconstruct past mountain lion populations. Current hunter harvest by sex, age, and location (data that, in Montana, are collected during the mandatory lion harvest inspection) is input to the model after the close of the harvest season each year. By combining survival models with observed harvest data, the IPM estimates annual population size as well as a confidence interval around these estimates.

Direct, field-based estimates of population abundance may be input into the model when they are available. These periodic field estimates can significantly improve past and future population estimates for individual lion ecoregions.

MOUNTAIN LION REPRODUCTION INTEGRATED POPULATION MODEL INPUTS

Lions can begin reproducing as early as 17 months of age or as late as 3 years old (López-González & González-Romero 1998). Studies focused on modeling cougar population dynamics often assume females reproduce for the first time at 24 months (Robinson et al. 2008, 2014; Cooley et al. 2009); the IPM uses this same convention.

Lions are induced ovulators, they can conceive during any month of the year (Bonney et al. 1981, Robinson et al. 2014), and gestation lasts about 92 days (Logan & Sweanor 2001). Despite their ability to give birth year round, most researchers working in northern latitudes report a birth pulse in mid or late summer (Laundre & Hernandez 2007, Robinson et al. 2014). The IPM assumes a default birth date of July 1.

Montana mountain lion reproduction and non-hunting mortality is not significantly affected by typical changes in harvest levels

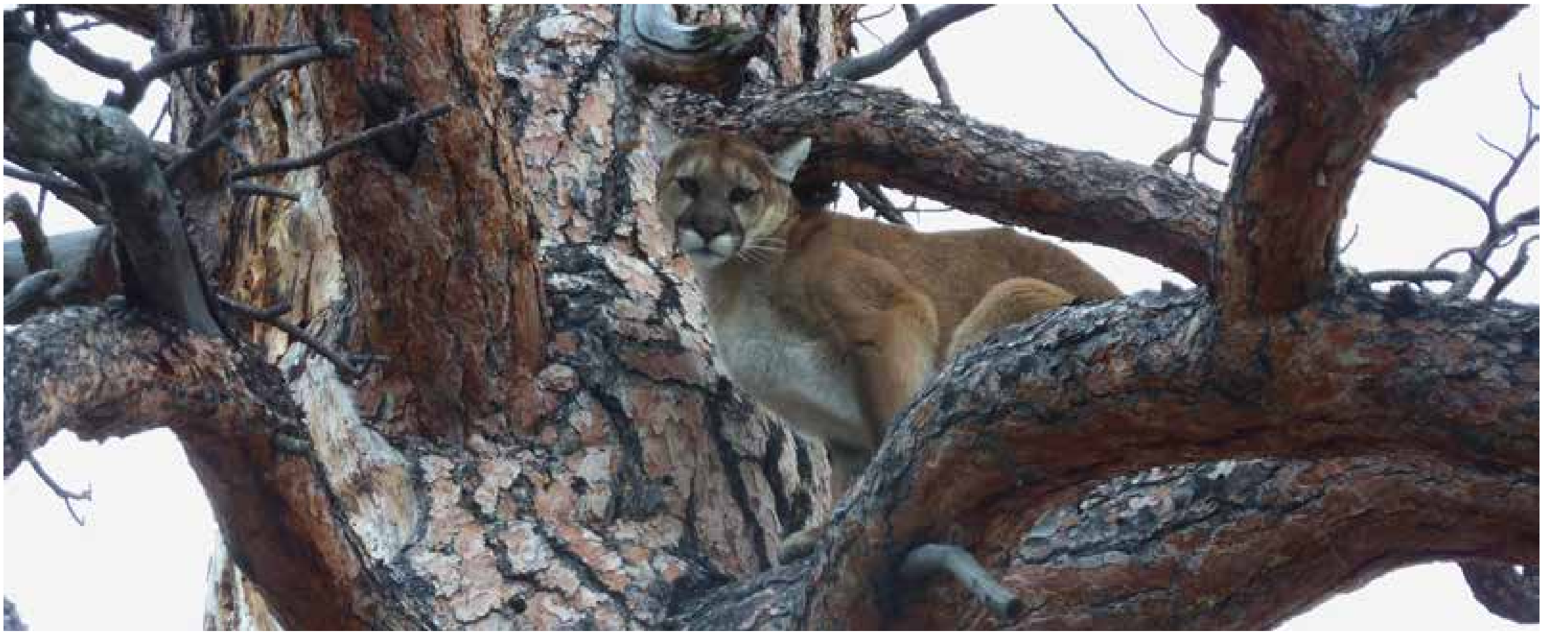

Intervals between subsequent births are a function of gestation length, kitten time to independence, and any lag that may exist between rearing and breeding. Previous population models have assumed a 24-month interbirth period (Robinson et al. 2008 & 2014, Cooley et al. 2009). Field researchers measuring interbirth intervals in the wild report a range of about 17 to 24-months between litters (Lindzey et al. 1994, Logan & Sweanor 2001, Hornocker & Negri 2009). Newborn kittens trail their mothers for 1 to 2 years before dispersing or achieving independence (Hornocker & Negri 2009). In the Garnet Mountains of Montana, Robinson et al. (2014) observed an average dispersal age of 15 months (n = 33, range: 11-23 months), similar to that observed by others (Sweanor et al. 2000; Logan & Sweanor 2001). The IPM uses an interbirth interval of 24 months as the model default.

Mountain lion litter sizes are remarkably similar across a wide range of locations and conditions. A common estimate of litter size is 3 kittens (Spreadbury et al. 1996, Logan & Sweanor 2001, Robinson et al. 2014). Litter size does not appear to vary with harvest intensity, but may fluctuate with prey density (Wilson et al. 2004, Stoner et al. 2006, Robinson et al. 2014). The IPM uses the estimate of an average of 2.92 kittens per litter derived from recent research in Montana's Garnet Mountains (Robinson et al. 2014; n = 24 litters) and it assumes that half of the kittens are female. Throughout the model, the average and range of litter sizes observed in the Garnet study is used to describe a normal distribution of litter sizes truncated between 0 and 3. The model also assumes that litter size remains constant through time and does not fluctuate with population size, prey density, or the female's age. A description of the specific biological inputs and assumptions used is included in Appendix 2.

USER CONTROLS

Biologists can adjust most model inputs such as biological assumptions, future harvest prescriptions, and model controls. The default biological assumptions are based on field research data and should only be changed if users believe that future or local circumstances have changed lion reproduction or non-harvest survival.

Users can easily use sliding scales provided on the user interface to change future harvest prescriptions by sex and to allow the model to estimate the effects of those changes. Users only need to input total anticipated hunting mortality by sex—the model will assign future harvest mortality to age classes that are consistent with the distribution of previously observed harvest ages. If the user believes that the harvest-age distribution will be different than past years, a different distribution can be manually assigned.

For more information on the model controls and settings, including the IPM model's computer code in programming language R, see Appendices 2 and 7.

CHAPTER 7

MOUNTAIN LION HARVEST REGULATION

REGULATION HISTORY

Montana's mountain lion hunting regulations became increasingly complex, and inconsistent, during the 45 years since lions were designated as a big game species (Chapter 9). New and modified regulations were adopted in an ad hoc fashion as various Fish and Wildlife Commissions struggled to address public concerns about harvest levels, prey populations, harvest distribution, parity between hound handlers and hunters without dogs, nonresident and outfitter participation, human-lion conflicts, and scores of other issues.

In FWP regions where hunting was allowed, mountain lion harvest was not restricted by quotas or limited licenses until the mid-1980s. Hunters were simply required to purchase a license and allow FWP personnel to inspect lions following harvest. By 1988, most FWP regions had established Lion Management Units with individual harvest quotas (and/or female subquotas) to limit harvest. The Department began to require harvested lions to be reported to a hotline within 48 hours and presented for physical inspection within 10 days. The reporting period was reduced to 12 hours in subsequent seasons.

Until 1997, most Winter lion hunting seasons ran from 12/1 to 2/15, after which hound handlers could continue to pursue lions with dogs during dedicated "chase" or "training seasons" that extended into April. More recently, hound training seasons open 12/2 and run concurrent with established harvest seasons.

Montana lion populations appeared to significantly expand and grow after 1980, as did the popularity of recreational hound hunting. Both resident and nonresident hunter participation increased to historically high levels by the mid-1990s (Figure 22) and the number of nonresident hunters was not limited. During that period, conflicts among lion hunters were common in some areas.

In 2000, FWP's Region 1 began to issue resident mountain lion hunting permits which, in effect, limited nonresident hunters' opportunity. Beginning in 2005, most Region 1 LMUs were managed using limited Special Mountain Lion Licenses that restricted nonresidents to no more than 10% of the licenses offered in a drawing.

Similarly, in 2006, the Fish and Wildlife Commission began to require that nonresidents draw a Special Mountain Lion License to harvest a lion in most of Region 2. Resident lion harvest was managed using a quota and nonresident Special License numbers could not exceed 10% of an LMU's total quota. The Commission required that both resident and nonresident hunters draw a Special Mountain Lion License in most Region 2 LMUs beginning in 2008.

Montana's mountain lion hunting regulations became increasingly complex, and inconsistent, during the 45 years since lions were designated as a big game species

In Region 2, harvest was less predictable using only these Special Mountain Lion Licenses compared to quotas as license fill rates varied widely from year-to-year and across LMUs. Female lion harvest was also virtually eliminated despite increasing populations. In 2012, Region 2 introduced an additional Late Winter Season (opening 2/1) during which hunters with a General Lion License could hunt until any quotas previously unfilled by Special Mountain License holders were met (this became known as a "hybrid" season). Nonresident participation was unlimited during the Late Winter Season and nonresident harvest rates more than doubled after the Late Winter Season was adopted.

Figure 22. Montana mountain lion license sales, 1973 – 2015.

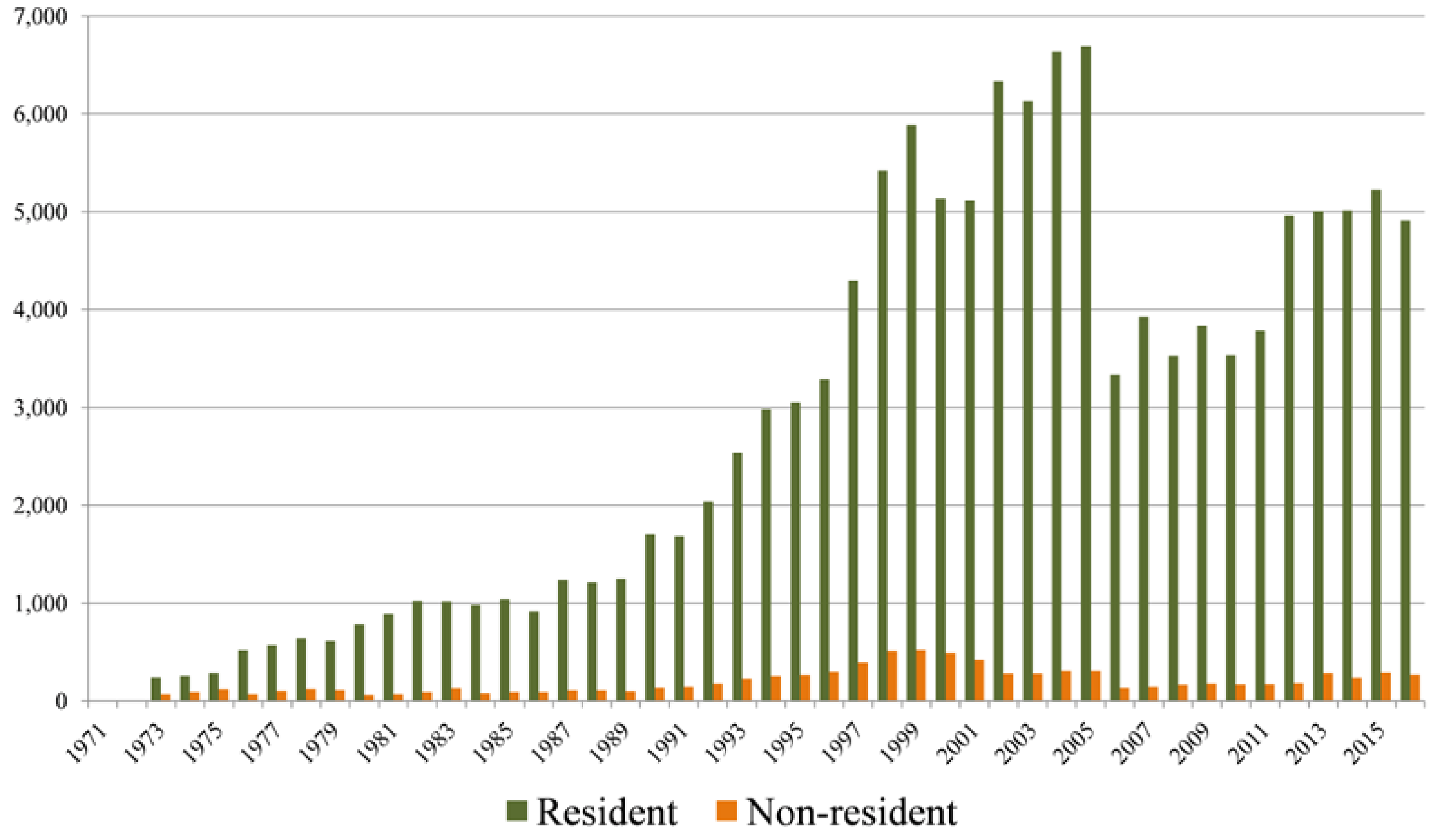

Regions 3-7 continued to limit harvest during this period using sex-specific quotas and subquotas. The Fish and Wildlife Commission did not adopt regulations that limited nonresident harvest opportunity in these Regions.

Prior to 1997, all legal harvest occurred during the Winter Season (that immediately followed the 5-week fall General Deer/Elk season) during which hunting with the aid of dogs was allowed. Beginning that year, portions of the state began to also allow lion harvest during the fall General Deer/Elk Season but without the use of dogs—fall seasons were adopted statewide in 1999. In 2010, the Commission added a statewide Archery Only Season that corresponded with the Archery Only Deer/Elk Season.

The Commission responded to concerns that Fall Season harvest could significantly reduce winter hound harvest opportunity by adopting separate LMU harvest quotas for the combined Archery Only and Fall Seasons. In most cases, if harvest prior to the Winter Season(s) exceeded 20% of a lion management unit's total quota or number of Special Lion Licenses, that LMU's Fall Season would be closed.

The separate quota for Archery Only and Fall Season harvest did not appear to meaningfully affect the seasonal distribution of lion harvest. Between 2007 and 2016, 95% of all hunter harvested lions in Montana were taken during the Winter Seasons with the aid of dogs. During that same period 11% of the state's LMUs were closed during any given Archery Only or Fall Season due to the 20% quota being met, and 85% of those LMUs had an Archery Only/Fall quota equal to only one lion. Harvest that met fall quotas in these LMUs occurred a median of 16 days from the end of the 85-day Archery Only/Fall Season. The Archery Only/Fall Season quota was unlikely to reduce overall harvest in LMUs because that harvest was deducted from the LMU's quota and subquota.

However, harvest during the fall seasons is additive to prescribed Winter Season harvest in LMUs where the number of Special Mountain Lion Licenses issued serves

as the effective harvest limit. Because of this difference, maintaining a separate Archery Only/Fall Season harvest quota may be necessary in LMUs where harvest is managed using Special Mountain Lion Licenses, instead of quotas.

HARVEST SEASON SETTING

This Strategy identifies four mountain lion ecoregions within the state that will be the basis for both monitoring populations and establishing broad harvest objectives. Within an ecoregion, FWP managers will work with the public and the Fish and Wildlife Commission to:

1. Develop clear and measurable population, harvest, and hunter opportunity objectives for the ecoregion.

2. Determine an overall harvest prescription that is likely to achieve the ecoregion's explicit population objectives.

3. Distribute harvest opportunity across the ecoregions' LMUs to address local concerns, reduce hunter crowding, and to focus or limit harvest where necessary.

4. Actively monitor the effect of the harvest prescription over time.

5. Adjust management objectives and harvest prescriptions, as necessary.

This process is described, in detail, in Chapter 8.

The amount of harvest that occurs in any one LMU matters much less to an ecoregion's mountain lion population than the overall harvest within that LMU's ecoregion. That is, whether an individual LMU's harvest limit (or quota) is reached or exceeded during a given year (due to weather, hunter participation, or other factors) is less important that the total annual ecoregional harvest.

Managers may intentionally recommend a relatively high harvest rate in certain LMUs (e.g. those including urban areas) or relatively low harvest rate in others (where access is challenging or tolerance for lions is high). As long as harvest is generally distributed across an ecoregion, the sum total of harvest is what will affect the ecoregion's population status and trend.

Therefore, in an LMU where harvest is limited by a quota, that quota will simply serve as "trigger" to initiate the closing of the LMU to further harvest. A quota is not necessarily a harvest objective for the LMU. When setting LMU quotas, biologists will anticipate how much additional harvest (if any) is likely to occur between the time the LMU's Season closure is publicly noticed and when the closure is effective. Subsequent ecoregional harvest decisions will consider the actual harvest that occurred in previous years' Seasons. Individual LMU quota "over runs" or "under runs" will be fully accounted for in future management decisions.

In LMUs managed using Special Mountain Lion Licenses, an area's average Special License fill rate (by sex) can be used to determine the overall number of licenses that should be offered to meet the ecoregion's harvest objective. Any differences between projected and observed Special License fill rates will be considered when determining future license levels. As with General License areas, decisions about future harvest prescriptions will be based on the modeled and measured effect the actual past harvest had on ecoregional populations.

From a population standpoint, harvest that occurs in any one LMU matters much less than the overall level of harvest within that LMU's ecoregion

There is little biological justification to frequently adjust mountain lion harvest prescriptions. Large scale mountain lion populations are very resilient to moderate changes in harvest and updated population estimates (both within trend areas and for the western ecoregions) will be available only periodically. Therefore, although FWP will routinely consider changes to mountain lion hunting season structure and quota levels, actual adjustments could be made less frequently.

LEGAL AUTHORITY

The Montana Fish and Wildlife Commission has statutory authority to regulate the management of wildlife (87-1-201), specifically "Large Predators" (87-1-217), including mountain lions. The Commission may determine seasons, bag limits, possession limits, and means of take for mountain lions as it deems appropriate (87-1-304). Montana statute describes specific resident and nonresident licenses required to hunt mountain lions (87-2-507, 508) and the license necessary for residents to pursue lions with dogs during the Training Season (87-2-521). Montana law limits hunters to taking no more than one mountain lion per license year (87-2-702) and allows the use of dogs to hunt or capture mountain lions during designated seasons (87-6-404). It is legal to kill a mountain lion at any time that is attacking, killing, or threatening to kill a person or livestock (87-6-106), using dogs if necessary (87-3-127).

Consistent with Montana law and Administrative Rules when harvest opportunity within a LMU is limited (ie. a limited number of Special Licenses is available) nonresident licenses will be limited to numbers not exceeding 10% of the total licenses or quotas assigned to a given hunting area (87-2-506, 12.3.105). LMUs with a number of licenses of less than 10 will be combined with similar Regional LMUs and a number of nonresident licenses, not exceeding 10% of the combined total quota(s), will be allocated among those districts on a rotating basis (as described in ARM 12.3.116)

Montana law specifically allows the Commission broad discretion to regulate the allocation of hunting opportunity among resident and nonresident hunters:

87-1-301. Powers Of Commission

(6) (a) The commission may adopt rules to:

(i) limit the number of nonresident mountain lion hunters in designated hunting districts; and

(ii) determine the conditions under which nonresidents may hunt mountain lion in designated hunting districts

(b) The commission shall consider, but is not limited to consideration of, the following factors:

(i) harvest of lions by resident and nonresident hunters;

(ii) history of quota overruns;

(iii) composition, including age and sex, of the lion harvest;

(iv) historical outfitter use;

(v) conflicts among hunter groups;

(vi) availability of public and private lands; and;

(vii) whether restrictions on nonresident hunters are more appropriate than restrictions on all hunters.

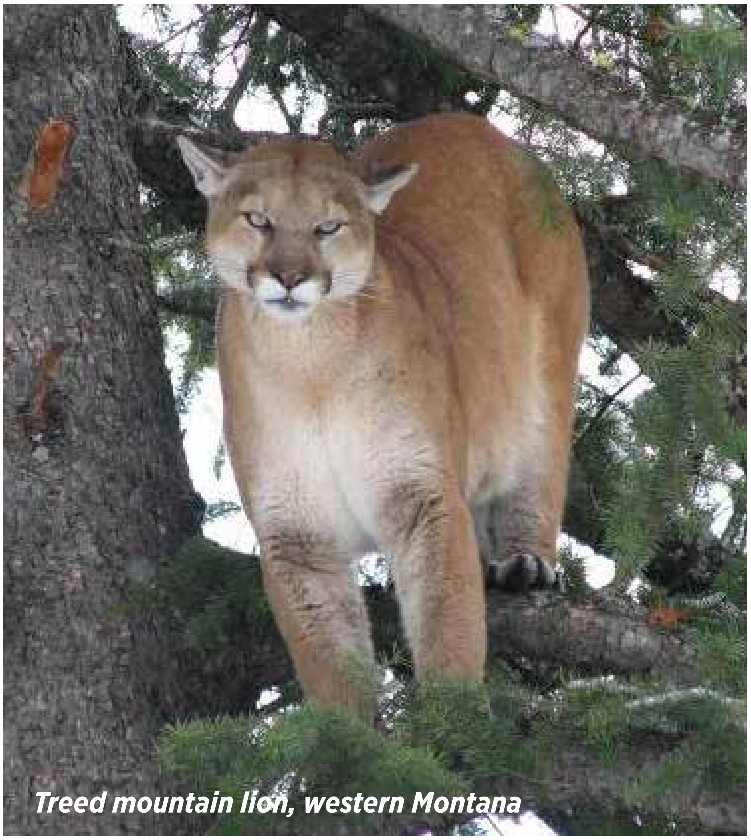

Treed mountain lion, western Montana

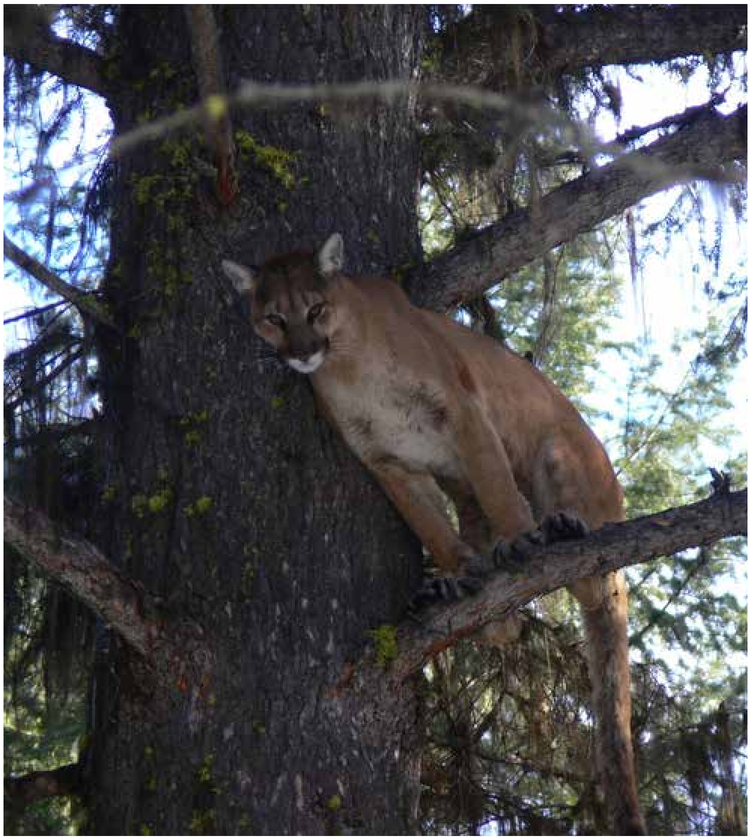

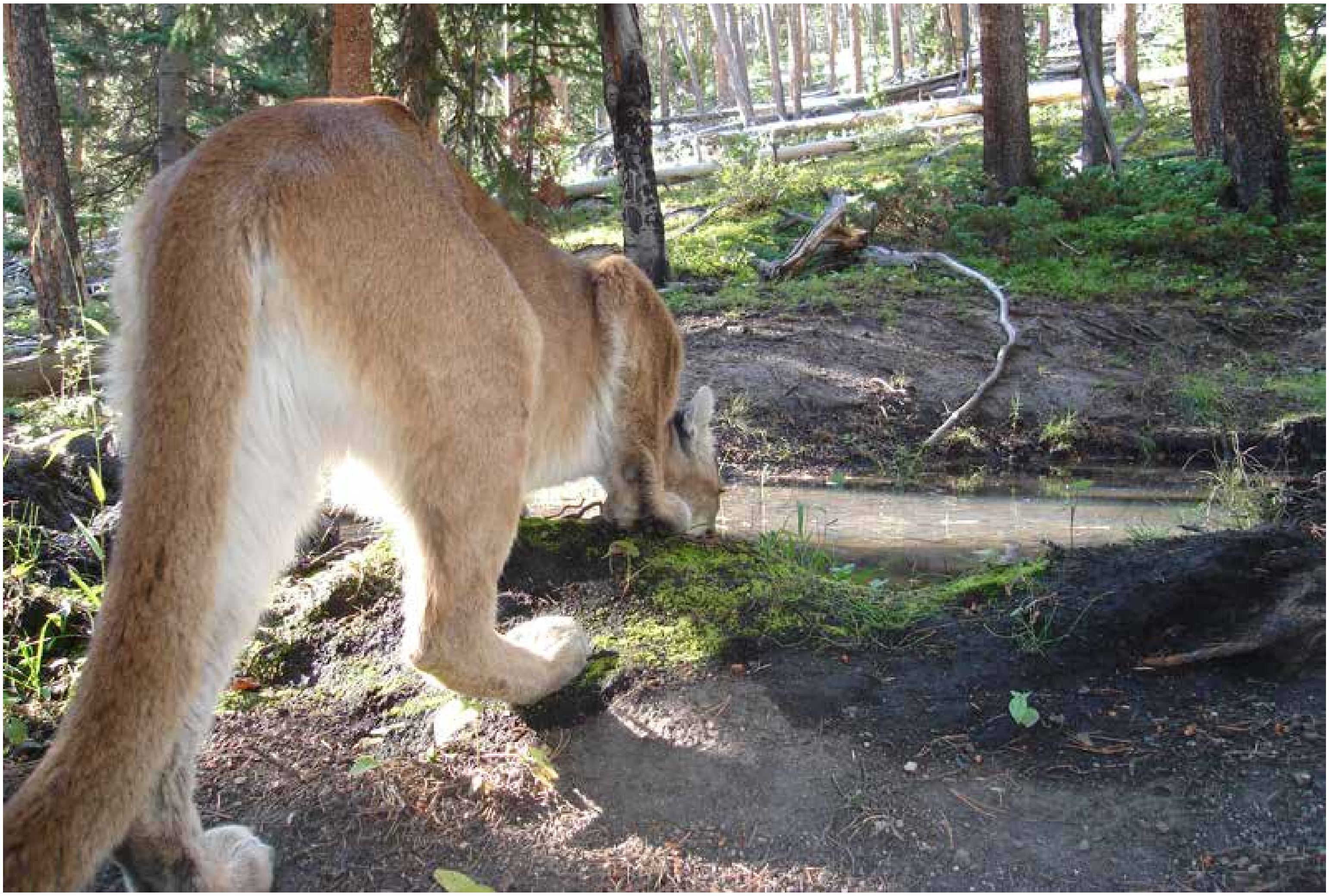

CHAPTER 8

ADAPTIVE HARVEST MANAGEMENT

This Strategy will provide FWP and the public with more accurate information about Montana's current, and likely future, mountain lion populations. However, there will always be some uncertainty about the precise effects of our management actions on lion populations. Although the overriding Conservation and Management Guidelines that direct Montana's mountain lion management decisions will not change, specific local management objectives may well need to be refined over time as more information becomes available and conditions change.

In this chapter, we describe the adaptive harvest management process FWP will use to develop, evaluate, and adjust specific mountain lion management actions. This process relies on field monitoring and population modeling data (described earlier in this Strategy) to measure the results of management actions against explicit objectives that the public, FWP, and the Fish and Wildlife Commission collaboratively develop.

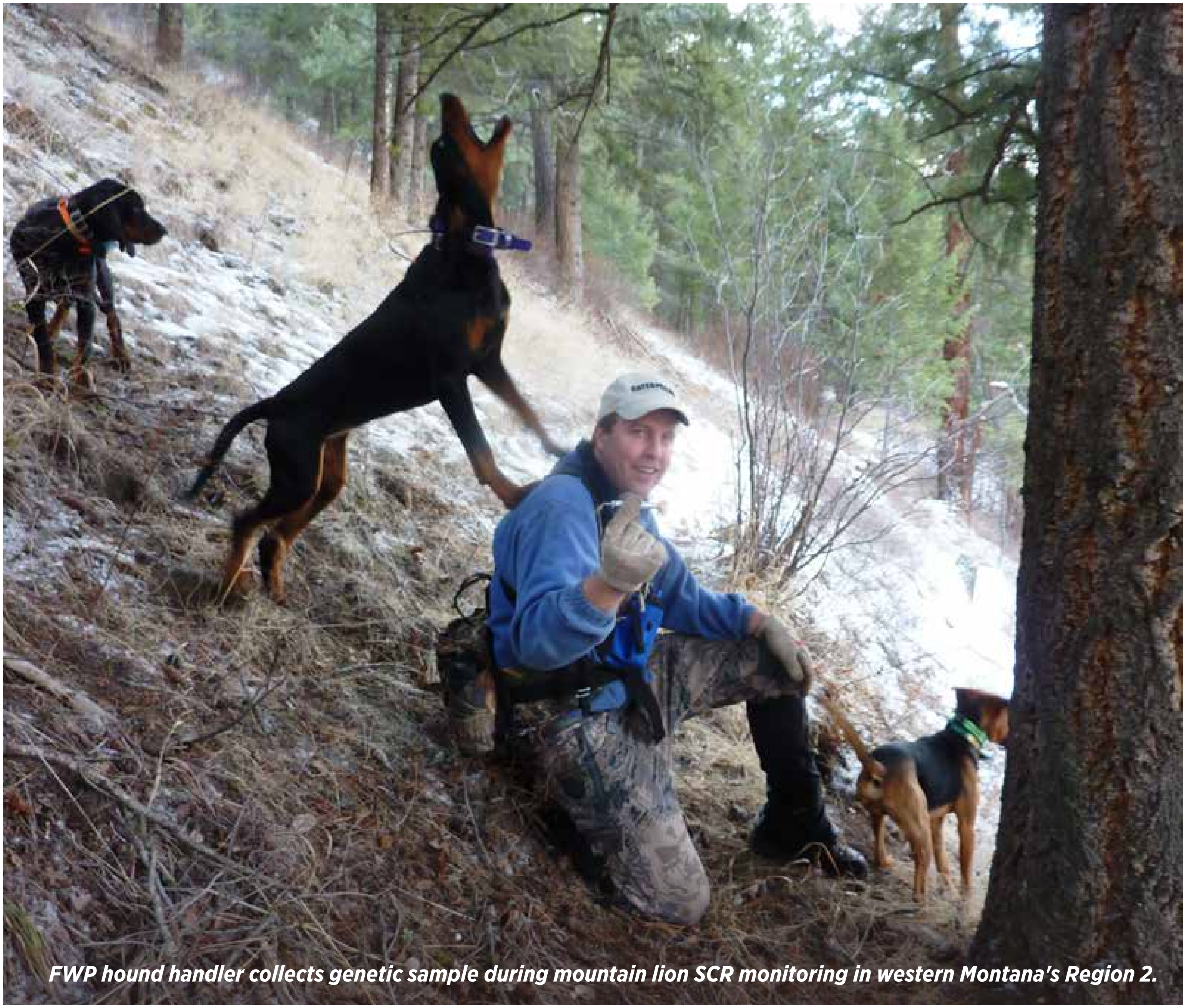

FWP hound handler collects genetic sample during mountain lion SCR monitoring in western Montana's Region 2.

Adaptive management can help reduce decision-making gridlock by making it clear that decisions are provisional, their effects will be carefully monitored, and that modifications are expected

Adaptive management is a science based approach to decision making that's useful when there is uncertainty about a decision's outcome. It is a cycle of planning for an action, doing the action, measuring what happened, and then modifying the next action (if needed) based on what you learned. The basic principles of adaptive management have been used for centuries (Falaruw 1984) and are increasingly employed by natural resource management agencies, including FWP (Montana Fish, Wildlife and Parks 2001).

The process works to continually improve our understanding of a system by comparing the resource's actual versus predicted response to management treatments (Nichols & Williams 2006, Williams et al. 2007). Adaptive management emphasizes 'learning while doing' and then adjusting management based on what was learned (Walters & Holling 1990). It is specifically not 'trial and error'— instead, managers explore alternative ways to meet management objectives, predict the outcomes of those alternatives based on the current state of knowledge, implement one or more alternatives, monitor the impacts of the management actions, and then use the results to adjust management actions as needed to more effectively meet objectives. Over time, resource management improves while uncertainty is reduced.

An adaptive management system requires the following conditions (Williams & Brown 2012):

- Resources are responsive to management but actual outcomes are uncertain;
- Management objectives are clear and measurable;
- There is both a range of management alternatives and the flexibility to change prescriptions as understanding improves over time;
- Monitoring can effectively describe the effect of the management action;
- There is a sustained commitment to the process by both stakeholders and decision makers.

Resource models are a critical component of the adaptive management approach. Models allow managers to use the most current information to predict the effect of possible treatments. They also represent what we don't yet know about how the system works—these uncertainties are explicitly incorporated into the model. The credibility of predictive models can improve through time as new information becomes available and uncertainty is reduced.

The effects of management actions must also be monitored so that the actual response can be compared to what was initially predicted. A successful monitoring program provides data that specifically describes the effects of the management action. Monitoring efforts must be designed from the start with that goal in mind (Szaro et al. 1999, Nichols and Williams 2006).

Disagreement about the past, and potential, effects of management decisions often leads to conflict among stakeholders. Adaptive management can help reduce decision making gridlock by making it clear that decisions are provisional, that their effects will be carefully monitored, and that modifications are expected. Management itself allows us to learn about, and therefore better manage, the resource through time.

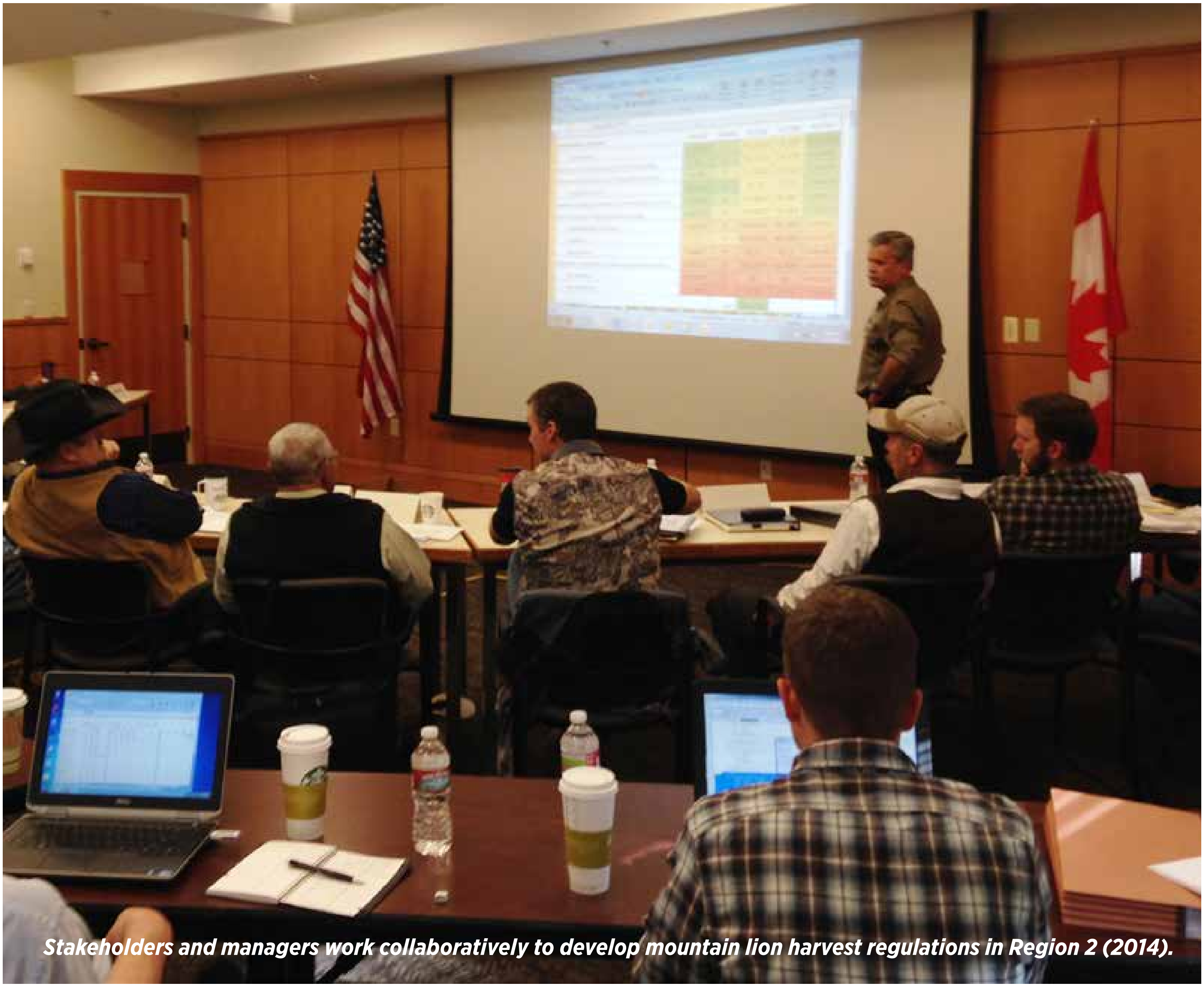

Stakeholders and managers work collaboratively to develop mountain lion harvest regulations in Region 2 (2014).

MONTANA'S ADAPTIVE MOUNTAIN LION HARVEST MANAGEMENT PROGRAM

An adaptive harvest management process will guide most of Montana's mountain lion harvest decisions. FWP will use the best available science to develop the modeling and monitoring methods necessary to fully implement this Strategy. The modeling and monitoring techniques described in this document will be periodically reviewed and updated to ensure that we continue to use the most rigorous and up-to-date scientific methods practically available.

FWP used a habitat model (Chapter 3) to describe four distinct and biologically meaningful mountain lion "ecoregions" within the state (Chapter 4). These ecoregions will be the spatial basis of FWP's lion monitoring program. FWP will work with stakeholders to periodically develop measurable mountain lion management objectives for each of these ecoregions. These objectives will be periodically reviewed, and potentially refined, by FWP and the public.

The likely effects of alternative harvest prescriptions will be evaluated using an Integrated Population Model (Chapter 6). These predictions will help stakeholders and FWP recommend an alternative to the Fish and Wildlife Commission that is most likely to meet that ecoregion's objectives.

In most cases, management alternatives will include an overall harvest prescription for each ecoregion. Harvest opportunity will then be allocated among the ecoregions' individual lion management units to distribute hunter effort and address local issues.

FWP will use field data to periodically estimate mountain lion population size, composition, and trend within the Northwest, West-central, and Southwest ecoregions (Chapter 5). These periodic population estimates will be used to improve the IPM's predictions, to assess how well management objectives are being met, and to inform decisions about future harvest prescriptions.

Other monitoring data including hunter effort and success, location and age of harvested animals, conflict rates, and prey status will be collected annually throughout the state. These additional data will be considered when evaluating management alternatives. Harvest data, weather, patterns of conflict, harvest success and other metrics will be the primary data used to guide management in the Eastern ecoregion.

The adaptive management approach includes the following basic steps (Figure 23):

Step 1 – Involve stakeholders

Stakeholders (including the public, managers, and decision makers) help design an adaptive management program, set management objectives, and develop management actions. Stakeholders must be committed to the adaptive management process for the long term.

FWP biologists and managers routinely meet with hound handlers, other hunters, and mountain lion advocates to share data and solicit public input concerning ongoing mountain lion management. The Fish and Wildlife Commission will generally consider proposals to adjust harvest season structure and/or harvest quotas every two years during the biennial season setting process.

Step 2 – Set objectives

Objectives must be clear and measurable. These objectives are benchmarks against which to compare the potential effects of management alternatives. They also serve as means to evaluate how effective management actions were, once implemented.

There may be discrete objectives for population composition and trend, hunter experience, harvest distribution, rates of reported conflict, etc. It's important that an objective identifies a clear time by which it should be met and clearly describes how progress toward that objective will be measured.

An example of clear and measurable objectives would be:

> ***"The 2023 Northwest ecoregion estimated population of independent age mountain lions will be between 1,100 and 1,300 animals", and***
>
> ***"The proportion of >5-year-old male mountain lions harvested in the Northwest ecoregion will exceed 12% during 4 of the next 6 hunting seasons"***

Step 3 – Develop management alternatives

Identify a set of potential management actions that, based on the best information available, are likely to help meet the objectives.

For example, competing harvest alternatives could be:

> ***Alternative 1: "Offer a total of 160 Special Licenses with a male subquota of 70 in LMUs 100 – 130; maintain a total "any legal" mountain lion quota of 30 in LMUs 132 – 170; and maintain a quota of 30 females and 50 males distributed across LMUs 200 – 203, the MSMA, and 283/285 during the 2018 – 2019 hunting seasons in order to harvest an average of 130 male and 90 female lions annually", or***
>
> ***Alternative 2: "Offer a total of 200 Special Licenses with a male subquota of 80 in LMUs 100 – 130 and maintain a total any legal mountain lion quota of 30 in LMUs 132 – 170; and maintain a quota of 45 females and 70 males distributed across LMUs 200 – 203, the MSMA, and 283/285 during the 2018 – 2019 hunting seasons in order to harvest an average of 150 male and 110 female lions annually"***

Step 4 – Use models to predict the alternatives' effects

Models can describe our current understanding about how a system works and explicitly represent our uncertainties. Models are used to predict likely responses of a resource to management actions.

In our example, biologists would use the Integrated Population Model to evaluate which of the previous alternatives is most likely to move the overall Northwest Ecoregion's independent aged mountain lion population toward the 1,100-1,300-objective range in 6 years and recruit sufficient older age-class toms each year to also meet the harvest-age composition objective. If neither alternative is likely to meet both objectives, new alternatives will be developed and evaluated.

Step 5 – Develop monitoring plans

Design a monitoring plan that effectively tracks the resource's status relative to the objectives. Monitoring must produce data relevant to the management situation that motivated the monitoring in the first place.

For our example, there would be three monitoring plans in place:

> ***1. Teeth will be extracted from all harvested lions upon mandatory inspection resulting in a >90% age assignment rate using cementum annuli analysis, and***
>
> ***2. Actual 2018 and 2019 Northwest ecoregion harvest, by sex, will be input into the Integrated Population Model following the 2019 season to reassess population trend relative to the population objective, and***
>
> ***3. A Spatial Capture-Recapture field estimate of lion abundance will be developed for the Northwest ecoregion Trend Monitoring Area in 2023 and Supplemental Monitoring Area in 2024. Biologists will directly compare the 2018 and 2023 Trend Monitoring Area population estimates. The relationship between observed mountain lion abundance and the RSF for both monitoring areas will be combined to produce an estimate of independent age mountain lions in 2024, which will be input into the IPM.***

Step 6 – Make management decisions

Select management actions that are likely to move the resource toward the objectives.

For our example:

> ***Managers will recommend a preferred alternative or alternatives to the Fish and Wildlife Commission who will make a management decision for the upcoming hunting seasons.***

Step 7 – Monitor the resource

Measure the resources' response to management actions.

FWP will implement the monitoring plans described in Step 5.

Step 8 – Assess management success

Compare the predicted vs. observed changes in the resource's status to improve our understanding of the system and allow better decisions to be made in the future.

For our example:

> ***Monitoring data indicate that the overall population objective has been (or is likely to be) achieved but the harvest-age composition objective has not.***

Step 9 – Repeat the process

Cycle back to Step 6 and, less frequently, to Step 1. Predictive models will improve based on new information. Objectives can change over time.

For our example:

> ***Managers propose a revised harvest prescription that maintains female harvest at a similar level while reducing male harvest.***

Figure 23. Adaptive mountain lion harvest management process.

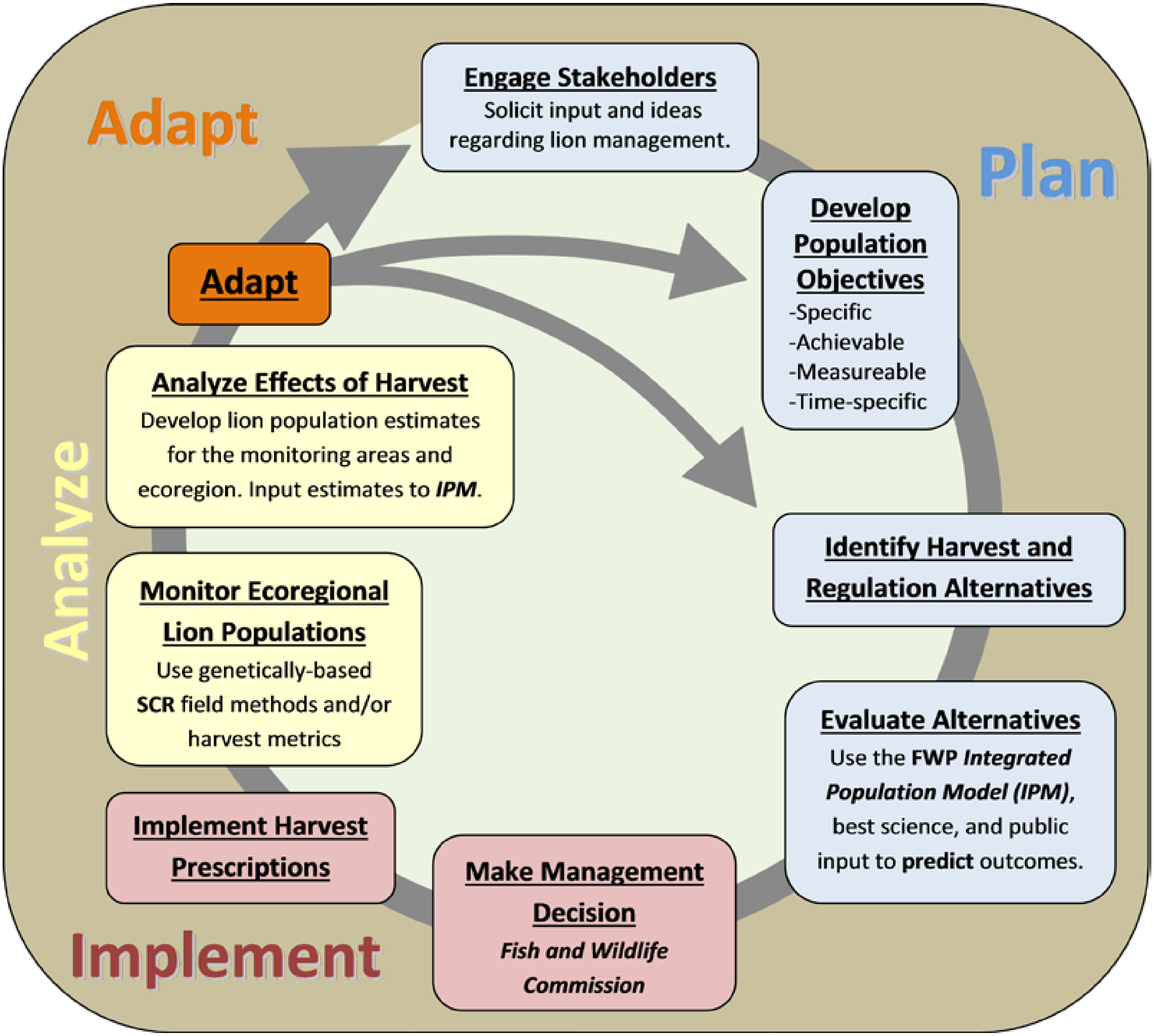

CHAPTER 9

REGIONAL MANAGEMENT CONSIDERATIONS

Mountain lion populations will be monitored, modeled, and managed at the ecoregion scale. However, it is important to recognize the social and biological issues that are unique to each FWP administrative Region. FWP wildlife managers are experts in their regional landscapes and communities, opportunities to gather public input are organized regionally, and regional managers develop and submit individual hunting season proposals for Fish and Wildlife Commission consideration. Responses to human-lion conflicts are also coordinated by Regional managers and field staff.

This Strategy will require that FWP and the public work across FWP regional boundaries to develop management objectives and alternatives for each of the 4 broader mountain lion ecoregions. They will also need to collaboratively work to distribute an ecoregion's harvest prescription because the ecoregion's constituent LMUs lie within more than one FWP administrative region.

This chapter presents each FWP administrative region's mountain lion management history and some local factors that will need to be considered as ecoregional management proposals are developed and evaluated.

This Strategy will require that FWP and the public work across FWP regional boundaries to develop management objectives and alternatives for each of the 4 broader mountain lion ecoregions

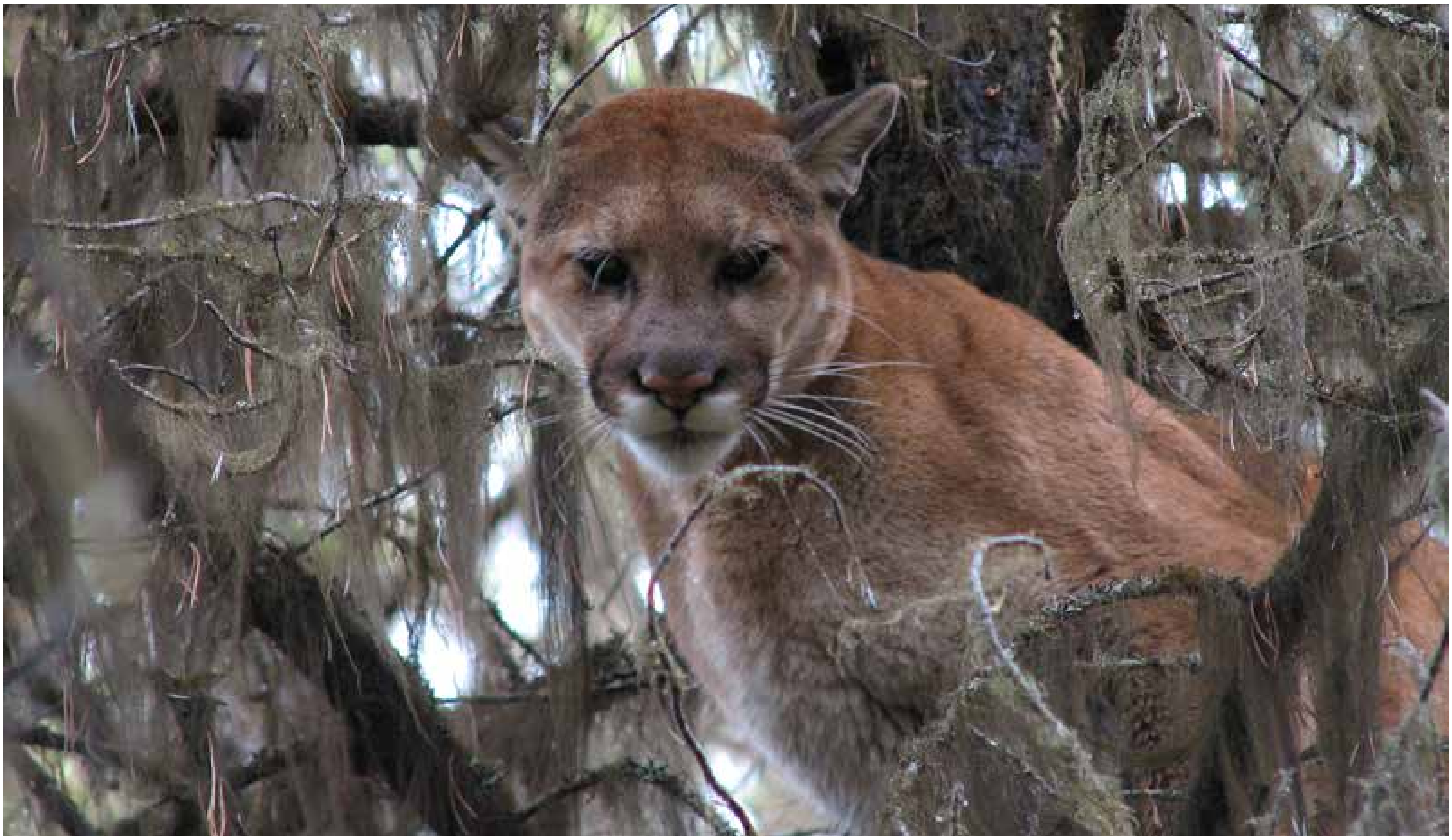

REGION 1

Approximately 80% of FWP Region 1's area is high-quality mountain lion habitat (Chapter 3), the most of any of the state's 7 administrative Regions (Figure 24). Because of this, and the Region's abundant white-tailed deer, it may support the highest overall mountain lion density in the state. Mountain lion habitat occurs almost entirely on either public or publicly accessible private land and tracking snow is generally present throughout the Winter Season.

Region 1 lion harvest was unlimited until specific LMU quotas were adopted in 1986. Harvest was managed using a system of total quotas and female subquotas through 1994, followed by a total quota system until 1999 (Table 8).

Regional harvest steadily increased throughout the 1990s (Table 7) and the average age of harvested lions also increased during this same period. In the late 1980s, only 38% of the harvest was made up of older (≥ 3 years) lions. That proportion increased to 66% older individuals as the harvest steadily increased from 1990 to 1996.

Mountain lion harvest increased during the 1990's such that even historically-high quotas were exceeded in 1995 and 1997. Harvest then began to decline in 1999 following a decline in harvest-age structure that began in 1997. The effect of high harvest levels (especially of females) was likely exacerbated by a severe winter in 1996-1997 that significantly reduced both the Region's deer populations and subsequent recruitment (Montana Fish, Wildlife and Parks 2006).

Quota-based, General License harvest regulations did not limit nonresident hunter participation during the 1990s and conflicts among lion hunters became increasingly common during that decade.

Between 1997 and 2004, only 39% of harvested lions were 3 years old or older. In 2000, concerned that lion populations were declining and in an attempt to quell ongoing conflicts among hound handlers, the Fish and Wildlife Commission changed the Region's management approach. The Commission restricted both resident and nonresident harvest by requiring a Special Lion License, obtained through a drawing, across much of the Region that year.

Table 7. Region 1 mountain lion harvest, 1971 – 2016.

License Year	R1			
	F	M	Unk	Tot.
1971	10	11	0	21
1972	9	13	0	22
1973	4	19	0	23
1974	23	23	0	46
1975	27	27	0	54
1976	18	20	0	38
1977	21	21	0	42
1978	12	14	0	26
1979	8	21	0	29
1980	9	6	0	15
1981	20	25	0	45
1982	18	26	1	45
1983	27	31	0	58
1984	13	29	1	43
1985	17	30	1	48
1986	16	32	0	48
1987	22	25	0	47
1988	18	34	0	52
1989	20	46	0	66
1990	30	55	0	85
1991	40	69	0	109
1992	50	67	1	118
1993	53	86	0	139
1994	81	122	0	203
1995	80	100	0	180
1996	87	94	0	181
1997	119	112	0	231
1998	139	105	1	245
1999	92	86	0	178
2000	103	93	0	196
2001	80	83	0	163
2002	67	61	0	128
2003	57	47	0	104
2004	42	69	0	111
2005	52	59	2	113
2006	20	50	0	70
2007	20	64	0	84
2008	32	62	0	94
2009	29	63	0	92
2010	42	83	0	125
2011	53	89	0	142
2012	46	78	0	124
2013	50	79	0	129
2014	43	57	0	100
2015	41	68	0	109
2016	49	56	0	105

In 2005, a combination of limited entry (Special Licenses) and quota systems were adopted in Region 1. At the time, the stated goals of this harvest management strategy were to 1) maintain a high-quality hunting experience, 2) limit nonresident hunter harvest in some LMUs, 3) prevent the overharvest of adult females while recruiting more mature males into the population, and 4) prevent FWP regulations from limiting effective harvest in LMUs where tolerance for lion presence was low. Region 1 documented a higher percentage (55%) of older individuals (≥ 3 years) in the harvest during the years following the change (2005 –

Figure 24. FWP Region 1 2016 mountain lion winter RSF and hunting districts.

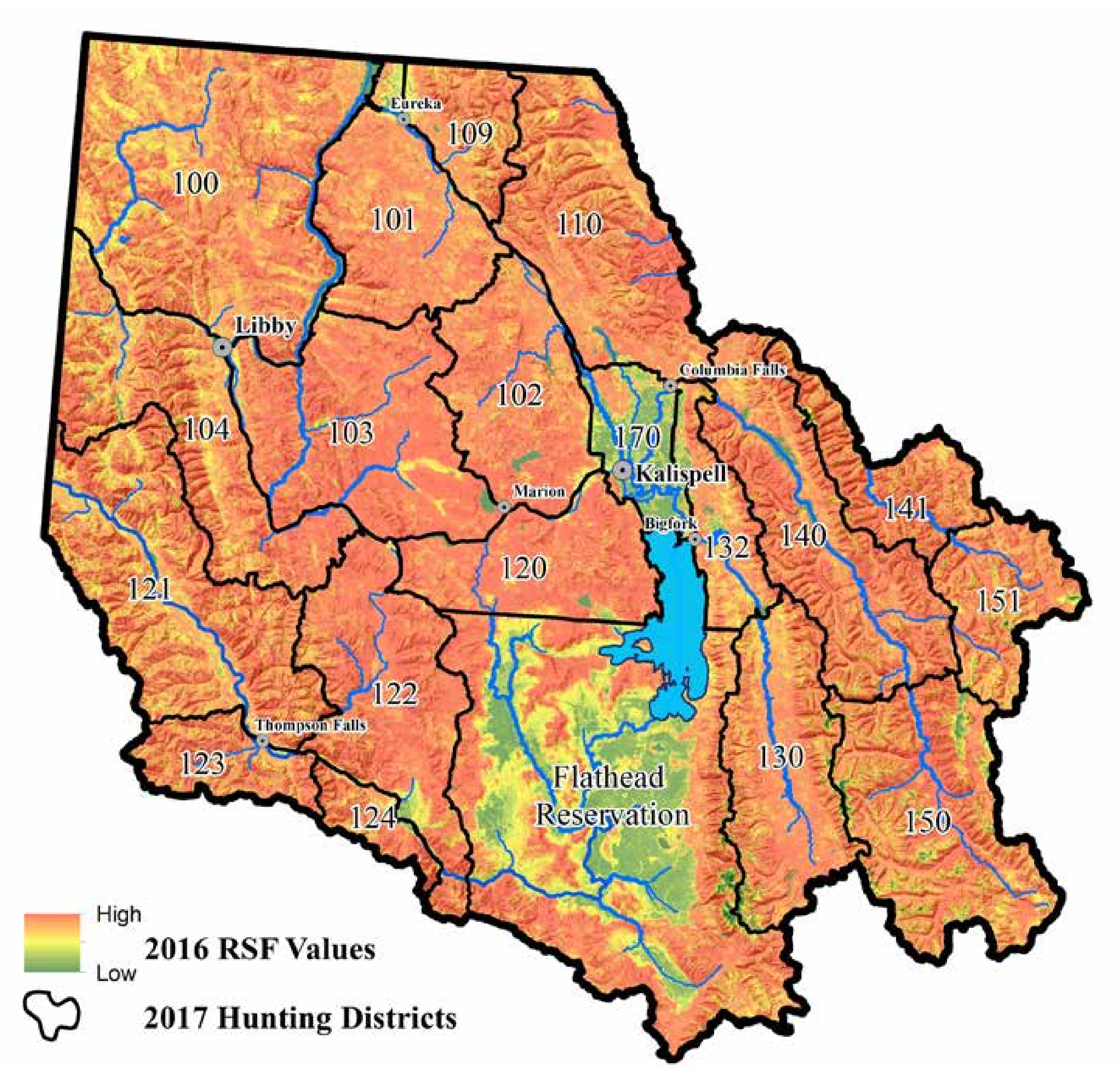

2013). In 2014, the Commission adopted a male subquota, limited entry hunting season type for most Region 1 LMUs.

In 2017, 13 of the Region's 18 LMUs were issued a limited number of Special Licenses, available through a drawing, with nonresidents limited to 10% of the total number of Licenses offered. The Region's remaining 5 LMUs were managed using General Lion Licenses; harvest in these Units is generally limited by overall quotas and male subquotas. LMU 170 (the Flathead Valley) is the single exception. An unlimited number of lions could be taken each season in this highly developed, urban, LMU. In practice, however, lions are rarely harvested in LMU 170—only 4 lions were taken by hunters in that Unit between 2007 and 2016.

The predominant use of limited Special Licenses in Region 1 has effectively emphasized resident hunter harvest—between 2007 and 2016 an average of only 13% of harvested lions were taken by nonresidents there.

Figure 25. FWP Region 1 hunting districts and mountain lion ecoregion.

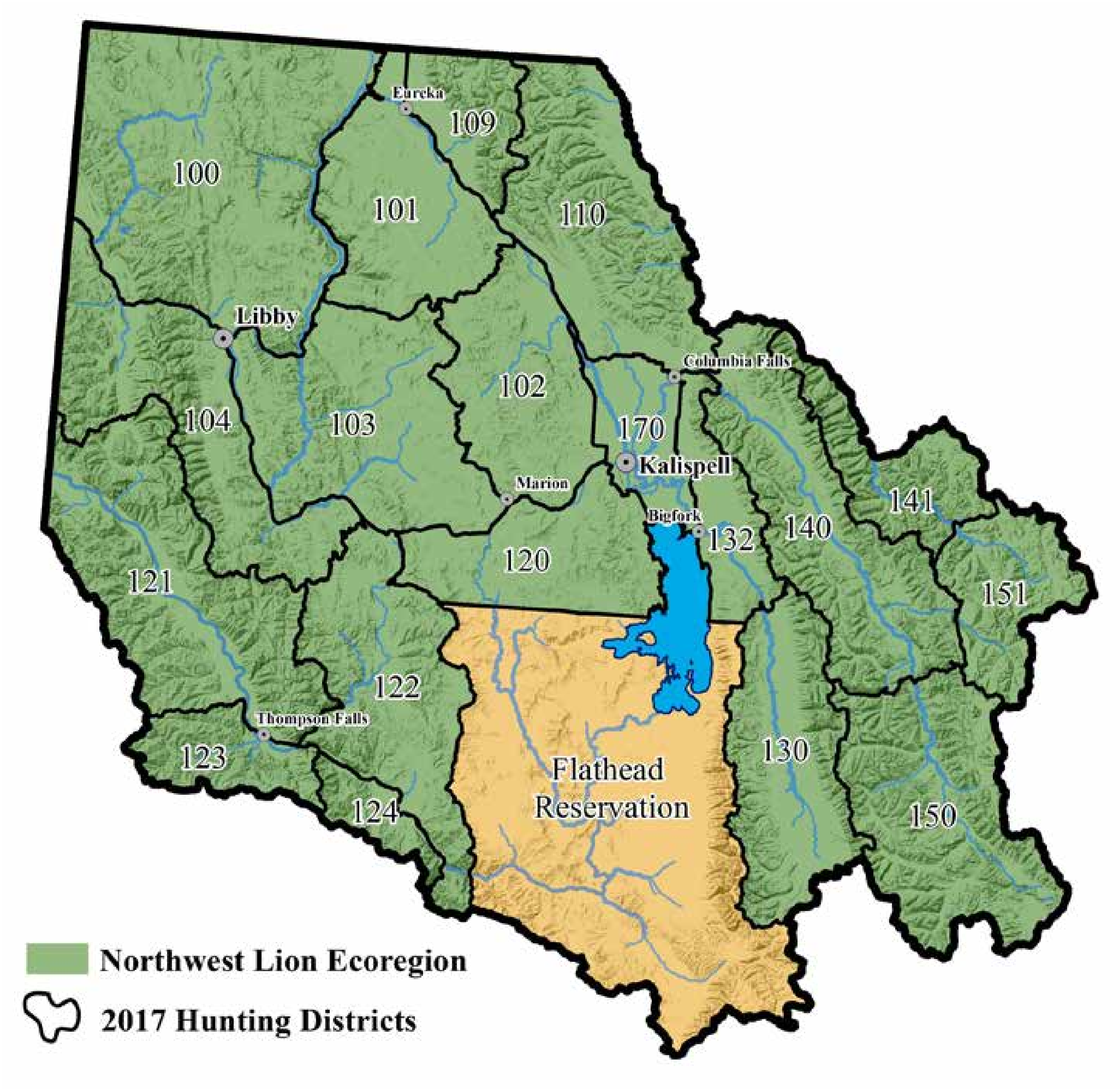

Region 1 lies entirely within the Northwest mountain lion ecoregion (Figure 25). The Region's biologists and public will work with their counterparts in Region 2 (that includes the remainder of the Northwest ecoregion) to adaptively manage the ecoregion's mountain lion population.

Specific harvest and population objectives will be identified and evaluated through the adaptive harvest management process (Chapter 8).

Region 1 will also ensure that hunting regulations do not limit hunter harvest in densely populated areas of the Region (such as LMU 170) where human-lion conflicts are likely. Human-lion conflicts will be mitigated using both hunter harvest and effective responses to individual incidents that are consistent with the Depredation and Control Guidelines (Appendix 3).

Table 8. Summary of Region 1 mountain lion harvest regulations, 1971 – 2017.

License Year	1971	1972	1973	1974	1975	1976	1977	1978	1979	1980	1981	1982	1983	1984	1985	1986
Mandatory Inspection	None		10 Day Inspection					4 Day Inspection				48 Hr. Inspection		10 Day Inspection	72 Hr. Inspection	48 Hr. Inspection
Hunting season	Opening of General D/E - 4/30			Opening of General D/E - 4/30; HD 150 9/15 - 11/24			12/1 - 4/30; HD 150 9/15 - 11/27	12/1 - 4/30; HD 150 9/15 - 4/30	12/1 - 2/15; HD 150 9/15 - 2/15	12/1 - 2/15; HD 150 & 151 9/15 - 2/15						
Chase/Hound Training	None								2/16 - 4/30							
Regional Quotas	UNLIMITED; One ES Adult Lion per Hunter															Total = 52; FSQ = 26

License Year	1987	1988	1989	1990	1991	1992	1993	1994	1995	1996	1997	1998	1999	2000	2001	2002
Mandatory Inspection	48 Hr. Report; 10 Day Inspection								24 Hr. Report; 5 Day Inspection			24 Hr. Report; 10 Day Inspection	12 Hr. Report; 10 Day Inspection			
Hunting season	12/1 - 2/15; HD 150 & 151 9/15 - 2/15										Fall Season w/o dogs; 12/1 - 2/15; HDs 150 & 151, 9/15 - 2/15	Fall Season w/o dogs; Winter Season, 12/1 - 4/14; HDs 150 & 151, 9/15 - 4/14				
Chase/Hound Training Season	2/16 - 4/30											No dedicated Chase Season, Hound Training allowed during Winter Hunting Season				
Regional Quotas	Total = 52; FSQ = 26	Total = 55; FSQ = 28	Total = 68; FSQ = 32	Total = 77; FSQ = 38	Total = 98; FSQ = 51	Total = 95; FSQ = 53	Total = 97; FSQ = 51	Total = 119; FSQ = 90	Total = 145, Any Legal Lion	Total = 175, Any Legal Lion	Total = 204, Any Legal Lion	Total = 229, Any Legal Lion	Total = 216, Any Legal Lion	Total = 203, Any Legal Lion	Total = 199, Any Legal Lion	Total = 164, Any Legal Lion

License Year	2003	2004	2005	2006	2007	2008	2009	2010	2011	2012	2013	2014	2015	2016	2017
Mandatory Inspection	12 Hr. Report; 10 Day Inspection														
Hunting season	Fall Season w/o dogs; Winter Season, 12/1 - 4/14; HDs 150 & 151, 9/15 - 4/14							Archery-only Season w/o dogs; Fall Season w/o dogs; Winter Season, 12/1 - 4/14; HDs 150 & 151, 9/15 - 4/14							
Chase/Hound Training Season	Hound Training Season 12/2 - 4/14														
Regional Quotas	Total = 154, Any Legal Lion	Total = 141, Any Legal Lion	Total = 141, Any Legal Lion	Total = 136; FSQ (some LMUs) = 41	Total = 148; FSQ (some LMUs) = 41	Total = 158; FSQ (some LMUs) = 51	Total = 172; FSQ (some LMUs) = 54	Total = 191; FSQ (some LMUs) = 55	Total = 191; FSQ (some LMUs) = 55	Total = 223; FSQ (some LMUs) = 69	Total = 223; FSQ (some LMUs) = 69	Total = 190; MSQ (some LMUs) = 71	Total = 190; MSQ (some LMUs) = 71	Total = 190; MSQ (some LMUs) = 71	Total = 190; MSQ (some LMUs) = 71

REGION 2

High-quality mountain lion habitat is distributed throughout FWP Region 2, especially in the lower Clark Fork, Blackfoot, and portions of the Bitterroot Valleys (Figure 26). The Region has a diverse and abundant ungulate prey base. Recent field estimates of mountain lion abundance (using SCR) in portions of the Blackfoot and Bitterroot Valleys were high compared to the range of densities previously reported for western North America.

Important field research into mountain lion ecology, the effects of harvest, and new population monitoring techniques has been conducted in Region 2 and the results of this work were used to develop this Strategy (Hornocker & Negri 2009, Robinson & DeSimone 2011, Russell et al. 2012, Proffitt et al. 2015).

Region 2 mountain lion harvest increased dramatically during the 1990s, reaching a peak of 267 lions taken (more than half of them females) during the 1998 seasons (Table 9). Historically high harvest continued through the late 1990s, even after the severe winter of 1996-97 reduced deer and elk herds in several portions of the Region.

By the early 2000s, the average age of harvested lions had fallen. FWP significantly reduced harvest quotas during the 2000s after both ongoing research and hound handlers' field observations indicated that lion numbers had declined (Table 10). Research in the Garnet Mountains (Robinson & DeSimone 2011), public observations, and rates of human-lion conflict all suggested that Region 2 lion populations had recovered to near 1990s levels by the late 2000s.

In 1994, Region 2 established a new LMU—the Missoula Special Management Area—surrounding the densely populated Missoula Valley. FWP prescribed high quotas (that were rarely met) in this LMU to ensure that hunting regulations were not publicly perceived as limiting legal hunter harvest in this high conflict area.

By 2005, nonresident hunters harvested nearly 50% of the Region's lions. In 2006, The Fish and Wildlife Commission began to require that nonresident hunters draw a limited Special Lion License to harvest a lion in most Region 2 LMUs—the number of these nonresident Special Licenses were equal to 10% of the total harvest quota.

Table 9. Region 2 mountain lion harvest, 1971 – 2016.

License Year	R2			
	F	M	Unk	Tot.
1971	10	8	0	18
1972	10	10	0	20
1973	11	26	2	39
1974	16	19	0	35
1975	8	13	0	21
1976	7	12	1	20
1977	5	14	0	19
1978	8	16	0	24
1979	8	16	0	24
1980	6	14	0	20
1981	9	21	0	30
1982	13	17	0	30
1983	13	22	1	36
1984	14	34	1	49
1985	13	13	0	26
1986	9	22	1	32
1987	4	56	1	61
1988	16	34	1	51
1989	12	39	0	51
1990	19	44	0	63
1991	18	42	0	60
1992	30	84	0	114
1993	36	82	0	118
1994	62	99	0	161
1995	64	88	0	152
1996	84	103	0	187
1997	112	127	0	239
1998	143	123	1	267
1999	107	101	0	208
2000	60	70	0	130
2001	43	56	0	99
2002	26	36	0	62
2003	26	47	0	73
2004	14	37	0	51
2005	12	41	0	53
2006	8	43	0	51
2007	10	48	0	58
2008	10	36	0	46
2009	10	52	0	62
2010	31	73	0	104
2011	34	74	0	108
2012	76	97	0	173
2013	68	72	0	140
2014	45	71	0	116
2015	47	78	0	125
2016	47	69	0	116

In 2008, the Commission required both resident and nonresident hunters draw a Special Lion License to harvest a lion in most of the Region's LMUs. This season type resulted in less predictable harvest rates and female harvest objectives were rarely met using Special Lion Licenses alone. In 2012 the Commission adopted a Late Winter Season (beginning 2/1) in most Region 2 LMUs. During the late Winter Season, hunters with a General Lion License could harvest lions until any quotas previously unfilled by Special Lion License holders were met (this became known as a "hybrid" season). Nonresident

Figure 26. FWP Region 2 2016 mountain lion winter RSF and hunting districts.

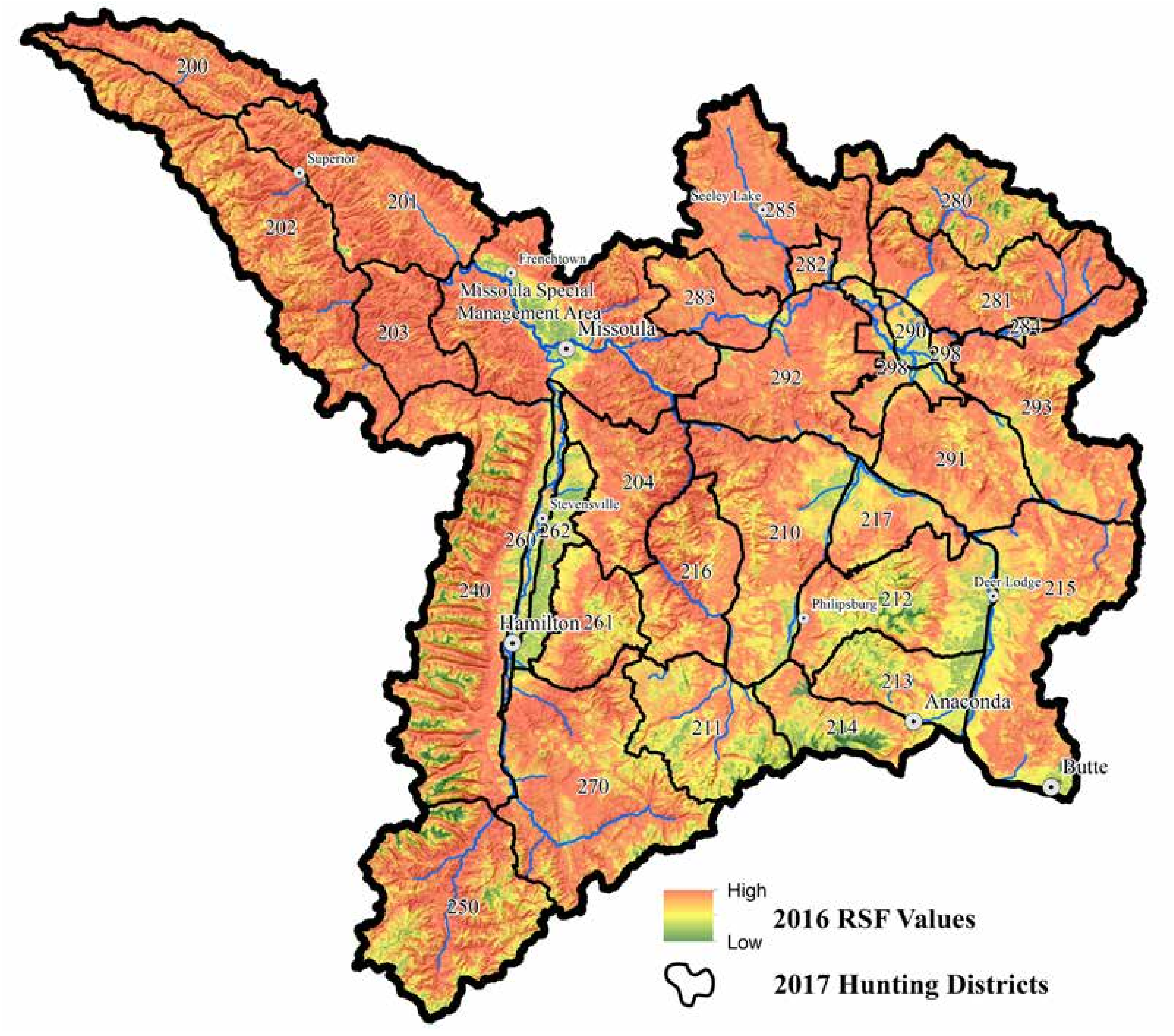

participation was unlimited during the Late Winter Season and Region 2 nonresident harvest rates more than doubled after the Late Winter Season was adopted.

Most Region 2 lion habitat is on public or publicly accessible private land. Tracking snow is generally present during the Winter Season, although snow conditions are more likely to limit effective harvest in the upper Clark Fork and Bitterroot drainages.

FWP Region 2 includes portions of both the Northwest and West-central mountain lion ecoregions (Figure 27). Region 2's biologists and public will work with their counterparts in Regions 1, 3 and 4 to set specific

Figure 27. FWP Region 2 hunting districts and mountain lion ecoregions.

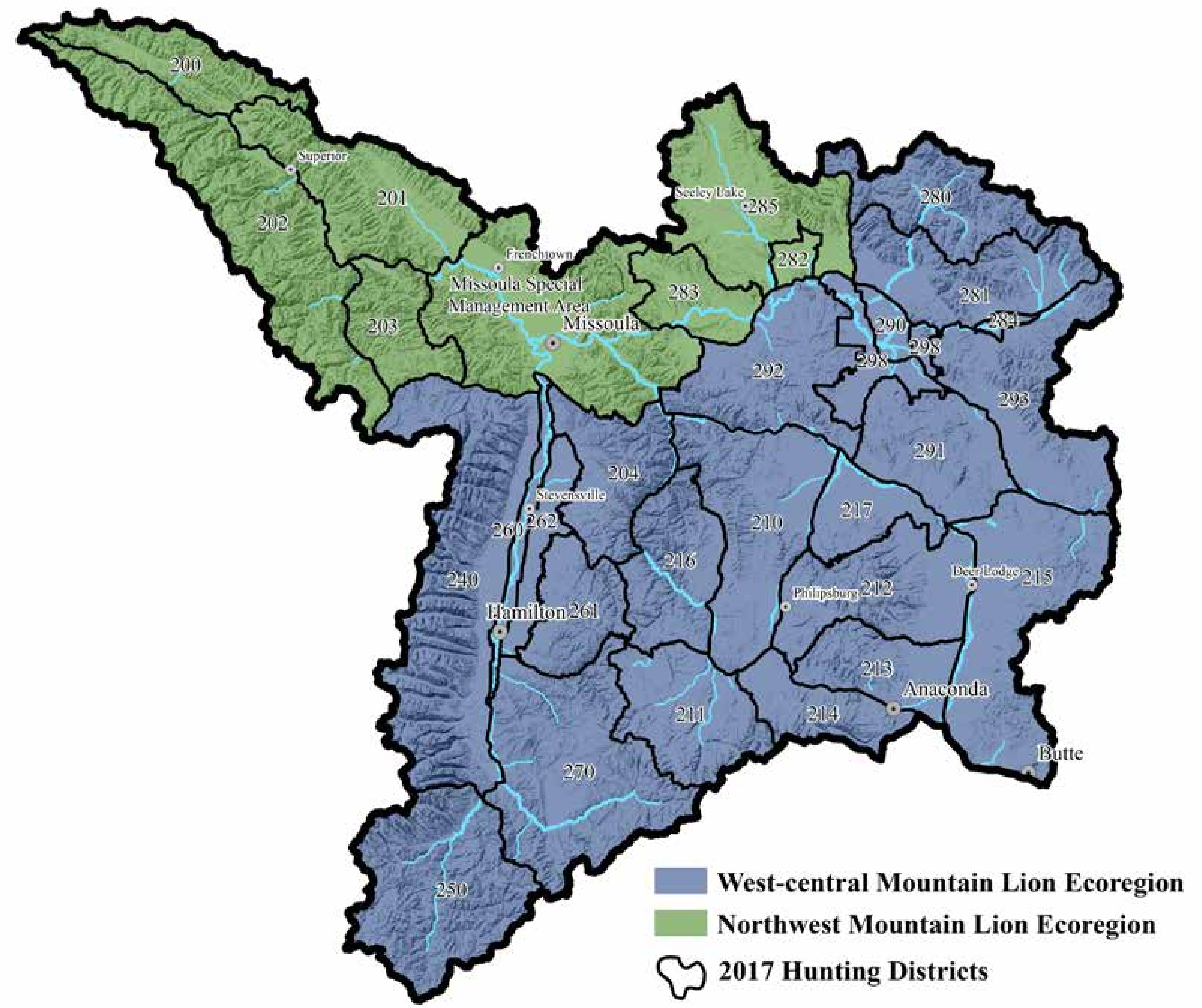

objectives for, and adaptively manage, these ecoregions' mountain lion populations.

Specific harvest and population objectives will be identified and evaluated through the adaptive harvest management process (Chapter 8).

Region 2 will minimize human-lion conflicts using both hunter harvest and effective responses to individual incidents that are consistent with the Depredation and Control Guidelines. Hunting regulations and harvest quotas for the Missoula Special Management Area will not significantly limit hunter harvest opportunity there during open seasons.

Figure 28. Region 2's four major watersheds and the Missoula Special Management Area.

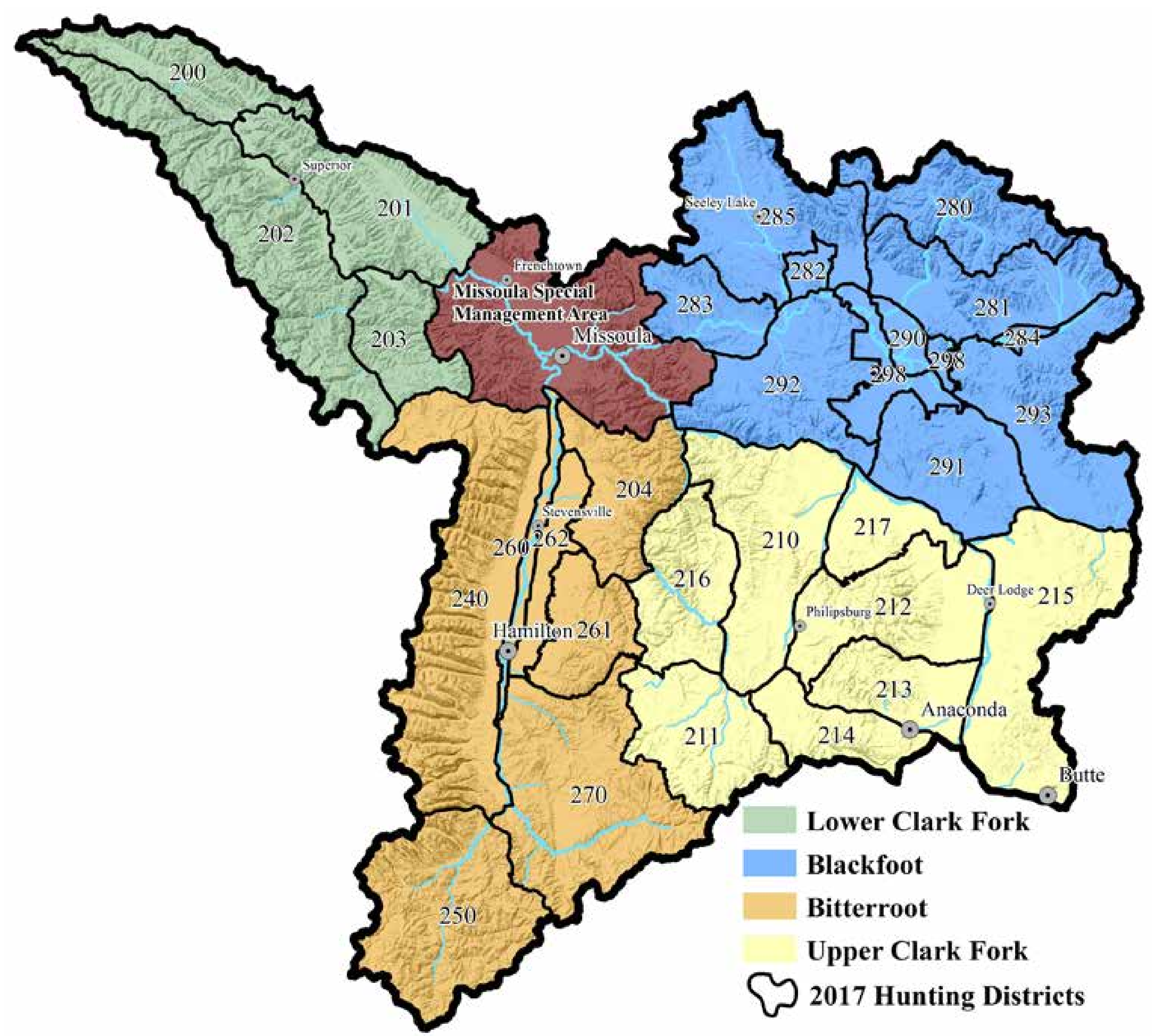

Table 10. Summary of Region 2 mountain lion harvest regulations, 1971 – 2017.

License Year	1971	1972	1973	1974	1975	1976	1977	1978	1979	1980	1981	1982	1983	1984	1985	1986
Mandatory Inspection	None		10 Day Inspection					4 Day Inspection				48 Hr. Inspection		10 Day Inspection	72 Hr. Inspection	48 Hr. Inspection
Hunting season	Opening of General D/E - 4/30			Opening of General D/E - 4/30; HD 280, 9/15 - 11/24	Opening of General D/E - 4/30; HD 280 9/15 - 4/30	12/1 - 4/30; HD 280 9/15 - 4/30	12/1 - 4/30; HD 280 9/15 - 11/27; HD 282, CLOSED	12/1 - 4/30; HD 280 9/15 - 4/30; HD 282, CLOSED	12/1 - 2/15; HD 280 9/15 - 2/15; HD 282, CLOSED							
Chase/Hound Training Season	None								2/16 - 4/30; HD282, CLOSED							
Regional Quotas	UNLIMITED; One ES Adult Lion per Hunter															

License Year	1987	1988	1989	1990	1991	1992	1993	1994	1995	1996	1997	1998	1999	2000	2001	2002
Mandatory Inspection	48 Hr. Report; 10 Day Inspection								24 Hr. Report; 5 Day Inspection			24 Hr. Report; 10 Day Inspection	12 Hr. Report; 10 Day Inspection			
Hunting season	12/1 - 2/15; HD 280, 9/15 - 2/15; HD282, CLOSED											12/1 - 4/14; HD 280, 9/15 - 4/14; HD282, CLOSED	Fall Season w/o dogs; 12/1 - 4/14; HD282, CLOSED			
Chase/Hound Training Season	2/16 - 4/30; HD282, CLOSED											No dedicated Chase Season, Hound Training allowed during Winter Hunting Season; HD 282 Closed				
Regional Quotas	UNLIMITED One ES Adult Lion per Hunter	Total = 46; FSQ = 21	Total = 52; FSQ = 21	Total = 55; FSQ = 22	Total = 74; FSQ = 28	Total = 104; FSQ = 46	Total = 106; FSQ = 47	Total = 133; FSQ = 78	Total = 172; Male = 94; Female = 78	Total = 212; Male = 109; Female = 103	Total = 299; Male = 135; Female = 164	Total = 305; Male = 127; Female = 178	Total = 232; Male = 101; Female = 131	Total = 167; Male = 96; Female = 71	Total = 111; Male = 61; Female = 50	Total = 93; Male = 57; Female = 36

License Year	2003	2004	2005	2006	2007	2008	2009	2010	2011	2012	2013	2014	2015	2016	2017
Mandatory Inspection	12 Hr. Report; 10 Day Inspection														
Hunting season	Fall Season w/o dogs; 12/1 - 4/14; HD282, CLOSED							Archery-only Season; Fall Season; 12/1 - 4/14; HD282, CLOSED		Archery-only Season; Fall Season w/o dogs; 12/1 - 4/14; Late Winter Season, 2/1 - 4/14; HD282, CLOSED					
Chase/Hound Training Season	Hound Training Season 12/2 - 4/14														
Regional Quotas	Total = 85; Male = 48; Female = 36	Total = 72; Male = 44; Female = 28	Total = 70; Male = 47; Female = 23	Total = 71; Male = 52; Female = 12	Total = 64; Male = 48; Female = 12	Total = 88; FSQ (some LMUs) = 16	Total = 126; FSQ (some LMUs) = 19	Total = 192; FSQ (some LMUs) = 38	Total = 219; FSQ (some LMUs) = 54	Total = 202; Male = 119; Female = 81	Total = 197; Male = 110; Female = 85	Total = 163; Male = 96; Female = 65	Total = 160; Male = 102; Female = 56	Total = 158; Male = 100; Female = 56	Total = 158; Male = 100; Female = 56

REGION 3

Mountain lions occur throughout their suitable habitat in southwest Montana's Region 3 (Figure 29). The Region has a diverse and abundant ungulate prey base that inhabits a mix of publicly accessible and privately-owned land.

Lion abundance increased in Region 3 during the 1980s and 1990s but, unlike other areas of the state, did not appear to fall as sharply during the 2000s. Instead, anecdotal evidence and harvest records suggest that mountain lion distribution and abundance have remained relatively stable in the Region since the mid-1990s. Variation in the total annual harvest (Table 11) is almost entirely due to changes in female harvest quotas. Sustained harvest in the late 2010s was similar to harvest levels in both Regions' 1 and 2 during the same period.

Figure 29. FWP Region 3 2016 mountain lion winter RSF and hunting districts.

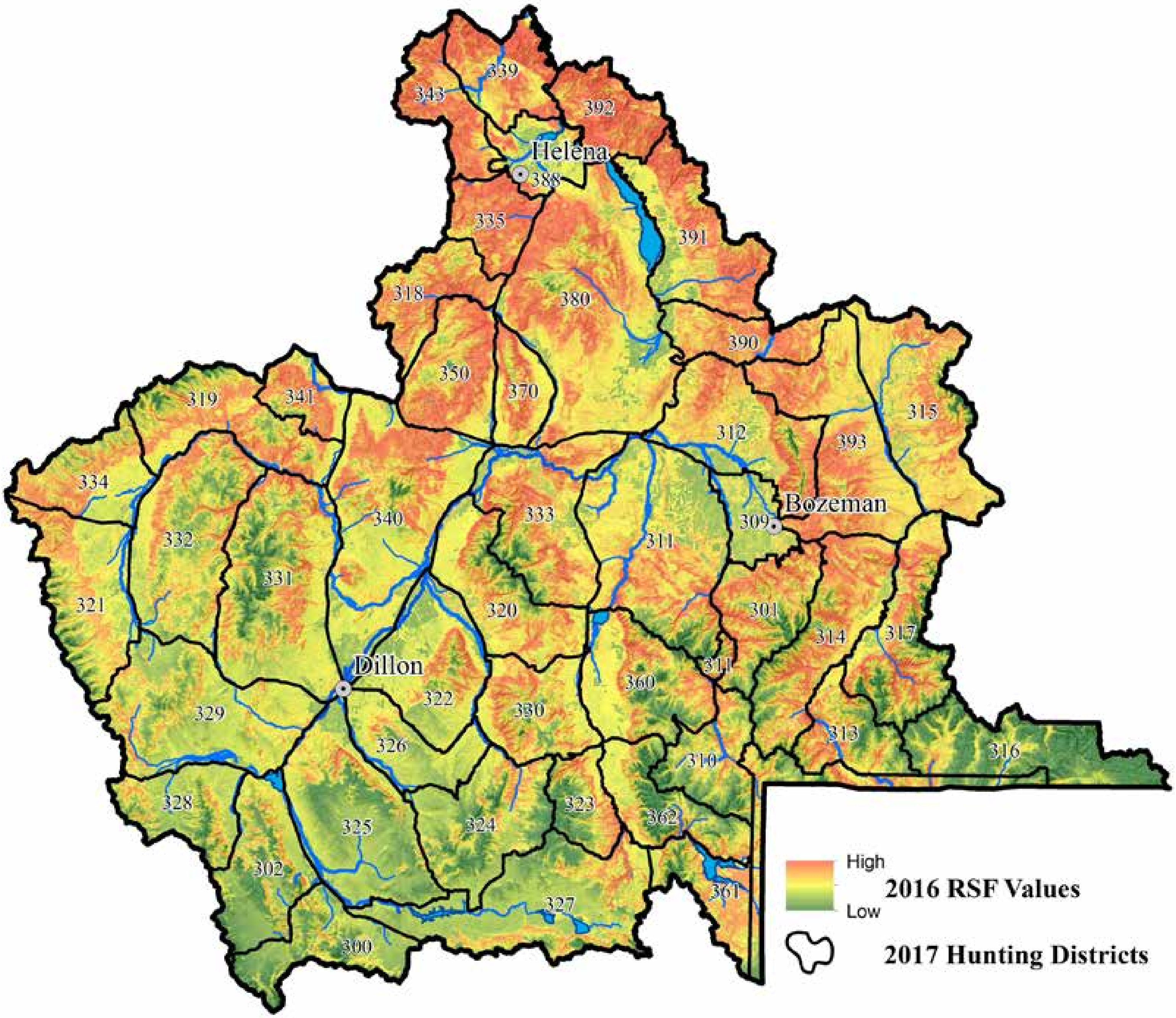

Region 3 generally managed harvest using simple harvest quotas and female subquotas (Table 12). However, the Region historically designated a relatively large number of LMUS (23 in 2017)—the number of these individual LMUs may be reduced during future season setting processes. In Region 3, quotas serve as harvest limits in all LMUs.

Public access to winter mountain lion habitat is mixed, although most harvest occurs on public land. Winter snow tracking conditions vary annually and can, at times, limit effective harvest. Nonresidents accounted for 15% of all successful hunters in the Region between 2007 and 2016 even though there was no regulatory limit on nonresident hunter harvest during that period.

Region 3 manages LMU 309, (the Gallatin Valley around Bozeman) as a Special Management Area. Lions are rarely harvested in this LMU (2 between 2007 and 2016), but the quota is high enough to ensure that FWP regulations do not limit legal harvest. Similarly, the Fall Season Without Dogs in LMU 309 opened with the beginning of the Deer/Elk Archery Only Season and remained open through the General Deer/Elk Season. The Region also designated a specific quota for the Spanish Peaks portion of LMU 311 to reduce lion predation on the resident bighorn sheep herd.

FWP Region 3 contains portions of both the Southwest and West-central Mountain Lion Ecoregions (Figure 30). Region 3's biologists and public will work with their counterparts in Regions 2, 4 and 5 to set objectives for, and adaptively manage, these ecoregions' mountain lion populations.

FWP and public stakeholders will determine and evaluate specific lion population objectives using the Adaptive Harvest Management process (Chapter 8).

Hunting regulations will not limit hunter harvest in highly developed areas where human-lion conflicts are likely (such as LMU 309) or where suppression of local lion density is desired (such as the Spanish Peaks portion of LMU 311).

FWP will minimize human-lion conflicts using both hunter harvest and effective responses to individual incidents that are consistent with the Depredation and Control Guidelines.

Table 11. Region 3 mountain lion harvest, 1971 – 2016.

License Year	R3			
	F	M	Unk	Tot.
1971	1	2	0	3
1972	2	2	0	4
1973	1	0	0	1
1974	2	2	1	5
1975	2	2	0	4
1976	2	0	0	2
1977	1	8	0	9
1978	7	6	0	13
1979	9	5	0	14
1980	1	6	0	7
1981	6	10	0	16
1982	7	11	0	18
1983	4	12	1	17
1984	5	21	0	26
1985	10	11	2	23
1986	4	13	1	18
1987	5	15	0	20
1988	1	17	0	18
1989	2	16	0	18
1990	6	23	0	29
1991	11	19	0	30
1992	11	33	0	44
1993	18	41	0	59
1994	32	52	0	84
1995	33	53	0	86
1996	29	60	0	89
1997	43	56	0	99
1998	51	66	0	117
1999	54	63	0	117
2000	55	55	1	111
2001	52	57	0	109
2002	46	64	0	110
2003	32	57	0	89
2004	34	44	0	78
2005	23	51	1	75
2006	16	45	0	61
2007	12	57	0	69
2008	13	61	0	74
2009	14	53	0	67
2010	17	50	0	67
2011	17	57	0	74
2012	33	68	0	101
2013	33	61	0	94
2014	33	70	0	103
2015	44	72	0	116
2016	44	69	0	113

Figure 30. FWP Region 3 hunting districts and mountain lion ecoregions.

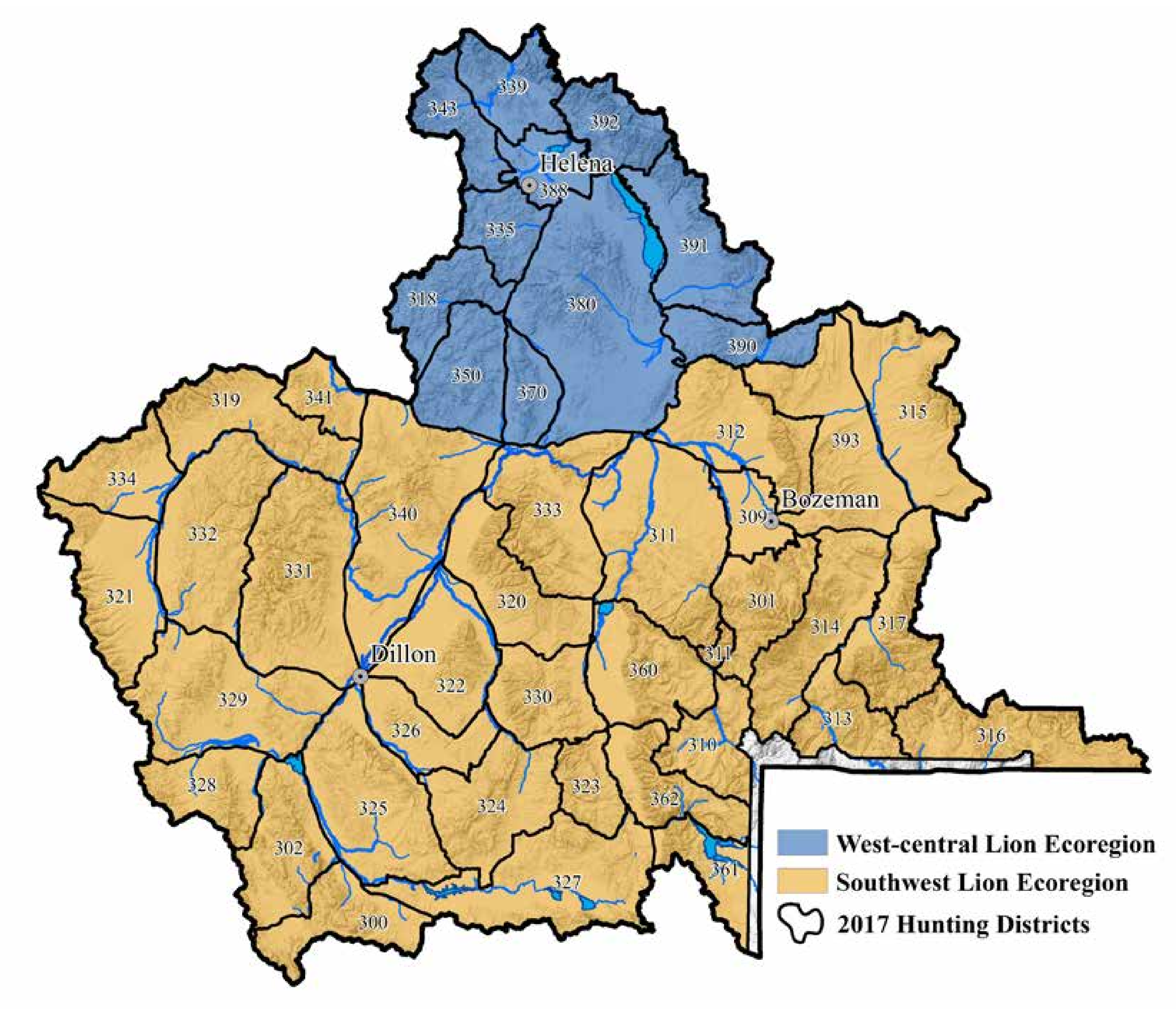

Table 12. Summary of Region 3 mountain lion harvest regulations, 1971 – 2017.

License Year	1971	1972	1973	1974	1975	1976	1977	1978	1979	1980	1981	1982	1983	1984	1985	1986
Mandatory Inspection	None		10 Day Inspection					4 Day Inspection				48 Hr. Inspection		10 Day Inspection	72 Hr. Inspection	48 Hr. Inspection
Hunting season	Opening of General D/E - 4/30					12/1 - 4/30			12/1 - 2/15							
Chase/Hound Training Season	None								2/16 - 4/30;							
Regional Quotas	UNLIMITED; One ES Adult Lion per Hunter															Total = 32; FSQ = 16 [1]

License Year	1987	1988	1989	1990	1991	1992	1993	1994	1995	1996	1997	1998	1999	2000	2001	2002
Mandatory Inspection	48 Hr. Report; 10 Day Inspection								24 Hr. Report; 5 Day Inspection			24 Hr. Report; 10 Day Inspection	12 Hr. Report; 10 Day Inspection			
Hunting season	12/1 - 2/15											12/1 - 4/14	Fall Season w/o dogs; 12/1 - 4/14			
Chase/Hound Training Season	2/16 - 4/30											No dedicated Chase Season, Hound Training allowed during Winter Hunting Season				
Regional Quotas	Total = 32; FSQ = 20	Total = 34; FSQ = 21	Total = 34; FSQ = 21	Total = 34; FSQ = 21	Total = 39; FSQ = 21	Total = 37; FSQ = 21	Total = 45; FSQ = 23	Total = 69; FSQ = 33	Total = 86; FSQ = 39; MSQ (R8) = 19	Total = 89; FSQ = 41; MSQ (some LMUs) = 19	Total = 104; FSQ = 49; MSQ (some LMUs) = 21	Total = 123; FSQ = 47; MSQ (some LMUs) = 22	Total = 123; FSQ = 60; MSQ (some LMUs) = 22	Total = 134; FSQ = 67; MSQ (some LMUs) = 25	Total = 132; FSQ = 60; MSQ (some LMUs) = 19	Total = 136; FSQ = 63; MSQ (some LMUs) = 23

License Year	2003	2004	2005	2006	2007	2008	2009	2010	2011	2012	2013	2014	2015	2016	2017
Mandatory Inspection	12 Hr. Report; 10 Day Inspection														
Hunting season	Fall Season w/o dogs; 12/1 - 4/14							Archery-only Season; Fall Season w/o dogs; 12/1 - 4/14							
Chase/Hound Training Season	Hound Training Season 12/2 - 4/14														
Regional Quotas	Total = 117; FSQ (some LMUs) = 41	Total = 105; FSQ (some LMUs) = 40	Total = 101; FSQ (some LMUs) = 30	Total = 76; FSQ (some LMUs) = 16	Total = 66; FSQ (some LMUs) = 12	Total = 73; FSQ (some LMUs) = 12	Total = 72; FSQ (some LMUs) = 12	Total = 80; FSQ (some LMUs) = 16	Total = 77; FSQ (some LMUs) = 16	Total = 109; FSQ (some LMUs) = 34	Total = 109; FSQ (some LMUs) = 34	Total = 126; FSQ (some LMUs) = 41	Total = 135; FSQ (some LMUs) = 46	Total = 140; FSQ (some LMUs) = 48	Total = 138; FSQ (some LMUs) = 48

REGION 4

Mountain lion abundance and distribution generally increased in Region 4 from the 1980s to mid-2010s — only toward the end of that period was all suitable habitat (including the Missouri River Breaks and Sweet Grass Hills) fully reoccupied (Figure 31).

Region 4 includes portions of both the West-central and Eastern Mountain Lion Ecoregions (Figure 32). Most of the Region's high-quality lion habitat lies within the West-central ecoregion, although quality habitat exists in portions of the Eastern ecoregion along the northern Rocky Mountain front, the Highwoods, the Sweet Grass Hills and Missouri River Breaks. Most lion harvest within Region 4 occurs on public land.

Region 4's annual harvest peaked in the late 1990s and stabilized somewhat below those historic high levels in the mid-2010s (Table 13). The Region traditionally managed harvest by prescribing male and female quotas to individual LMUs. Nonresident hunters accounted for 19% of all lions harvested between 2007 and 2016; less than 20% of those successful nonresident hunters used the services of an outfitter.

Reducing and mitigating conflicts between lions and agricultural interests is a high Regional priority. Region 4 staff will actively respond to potential and ongoing mountain lion conflicts, consistent with the Depredation and Control Guidelines, in order to maintain landowner tolerance for lions.

Region 4 will generally support management objectives that maintain stable lion abundance, distribution, and harvest across the Region's suitable habitat. Region 4's biologists and public will work with their counterparts in other Regions to set objectives for, and adaptively manage, the West-central and Eastern ecoregions' mountain lion populations.

Region 4 will generally support management objectives that maintain stable lion abundance, distribution, and harvest across the Region's suitable habitat. Region 4's biologists and public will work with their counterparts in other Regions to set objectives for, and adaptively manage, the West-central and Eastern ecoregions' mountain lion populations.

Table 13. Region 4 mountain lion harvest, 1971 – 2016.

License Year	R4			
	F	M	Unk	Tot.
1971	3	3	0	6
1972	2	4	0	6
1973	1	5	0	6
1974	2	4	0	6
1975	2	4	0	6
1976	1	5	0	6
1977	4	6	0	10
1978	2	2	1	5
1979	2	3	0	5
1980	5	7	0	12
1981	7	7	0	14
1982	4	5	0	9
1983	1	10	0	11
1984	7	18	1	26
1985	10	14	3	27
1986	4	7	1	12
1987	10	16	0	26
1988	6	16	0	22
1989	5	16	0	21
1990	10	17	0	27
1991	10	17	0	27
1992	15	22	0	37
1993	16	39	0	55
1994	24	46	0	70
1995	32	39	0	71
1996	37	47	0	84
1997	44	41	0	85
1998	54	39	0	93
1999	56	37	0	93
2000	45	36	0	81
2001	39	36	0	75
2002	24	26	0	50
2003	21	27	0	48
2004	17	27	0	44
2005	17	26	0	43
2006	18	35	0	53
2007	25	30	0	55
2008	32	37	0	69
2009	30	35	0	65
2010	32	43	0	75
2011	32	46	0	78
2012	35	44	0	79
2013	34	48	0	82
2014	31	47	0	78
2015	28	37	0	65
2016	38	42	0	80

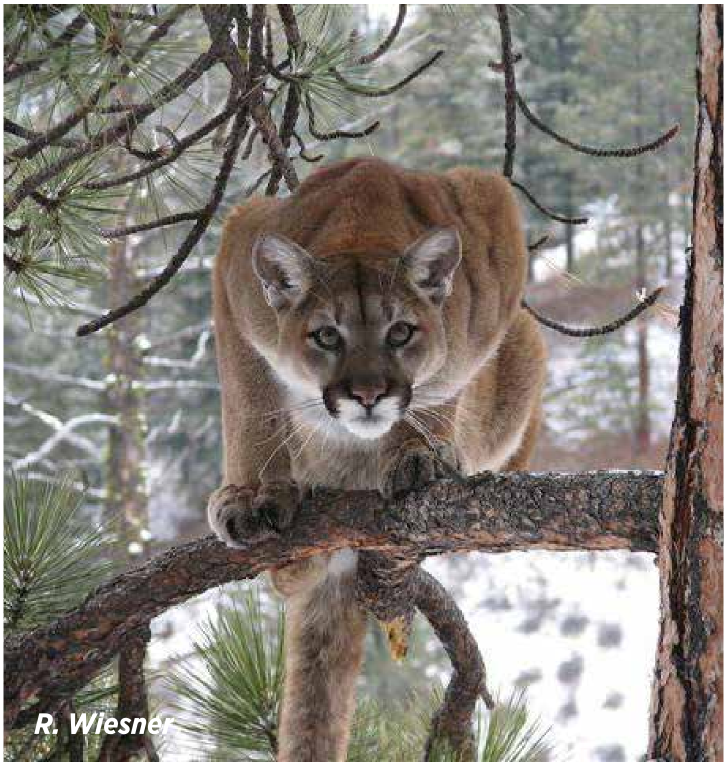

Figure 31. FWP Region 4 2016 mountain lion winter RSF and hunting districts.

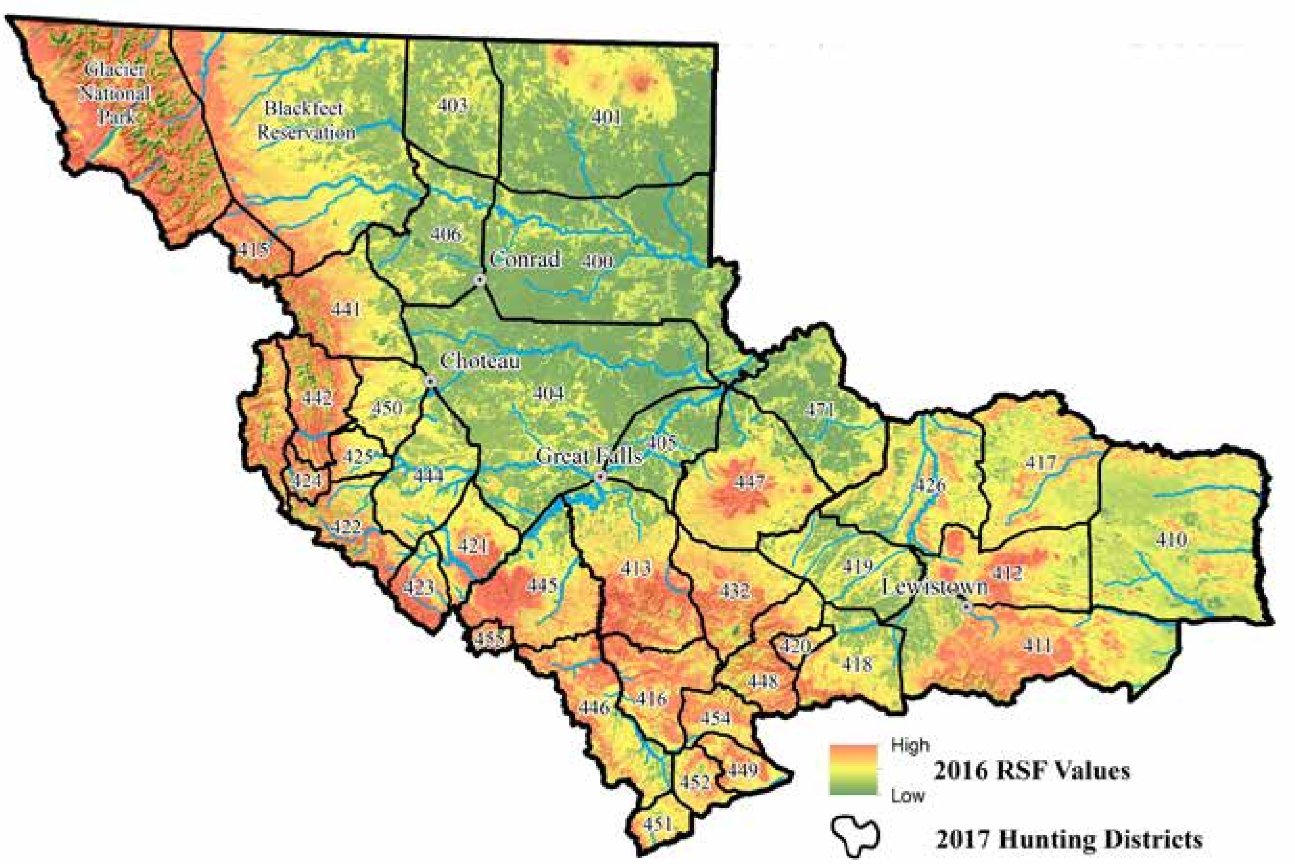

Figure 32. FWP Region 4 hunting districts and mountain lion ecoregions.

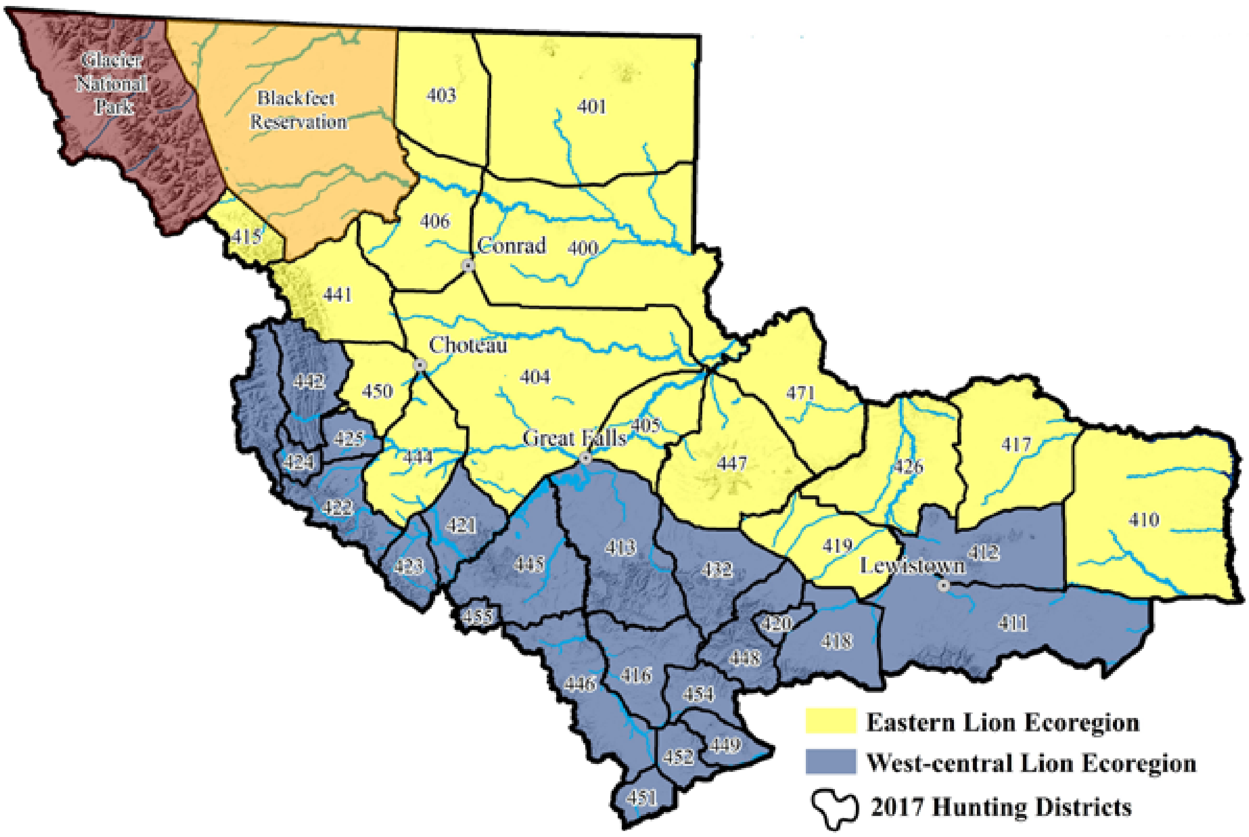

Table 14. Summary of Region 4 mountain lion harvest regulations, 1971 – 2017.

License Year	1971	1972	1973	1974	1975	1976	1977	1978	1979	1980	1981	1982	1983	1984	1985	1986
Mandatory Inspection	None		10 Day Inspection					4 Day Inspection				48 Hr. Inspection		10 Day Inspection	72 Hr. Inspection	48 Hr. Inspection
Hunting season	Opening of General D/E - 4/30					12/1 - 4/30			12/1 - 2/15			12/1 - 2/15; HDs 427 & 428, 1/1 - 2/15				
Chase/Hound Training Season	None								2/16 - 4/30							
Regional Quotas	UNLIMITED; One ES Adult Lion per Hunter															

License Year	1987	1988	1989	1990	1991	1992	1993	1994	1995	1996	1997	1998	1999	2000	2001	2002
Mandatory Inspection	48 Hr. Report; 10 Day Inspection								24 Hr. Report; 5 Day Inspection			24 Hr. Report; 10 Day Inspection	12 Hr. Report; 10 Day Inspection			
Hunting season	12/1 - 2/15; HDs 427 & 428, 1/1 - 2/15							12/1 - 2/15				12/1 - 4/14	Fall Season w/o dogs; 12/1 - 4/14			
Chase/Hound Training Season	2/16 - 4/30											No dedicated Chase Season, Hound Training allowed during Winter Hunting Season				
Regional Quotas	UNLIMITED One ES Adult Lion per Hunter	Total = 30; FSQ = 10	Total = 35; FSQ = 10	Total = 35; FSQ = 10	Total = 40; FSQ = 12	Total = 46; FSQ = 14	Total = 46; FSQ = 14	Total = 65; FSQ = 26	Total = 80; Male = 46; F = 34	Total = 110; Male = 57; F = 53	Total = 108; Male = 49; Female = 59	Total = 133; Male = 52; Female = 81	Total = 126; Male = 48; Female = 78	Total = 124; Male = 48; Female = 76	Total = 110; Male = 47; Female = 63	Total = 106; Male = 48; Female = 58

License Year	2003	2004	2005	2006	2007	2008	2009	2010	2011	2012	2013	2014	2015	2016	2017
Mandatory Inspection	12 Hr. Report; 10 Day Inspection														
Hunting season	Fall Season w/o dogs; 12/1 - 4/14							Archery-only Season; Fall Season w/o dogs; 12/1 - 4/14							
Chase/Hound Training Season	Hound Training Season 12/2 - 4/14														
Regional Quotas	Total = 103; Male = 48; Female = 55	Total = 91; Male = 45; Female = 46	Total = 91; Male = 45; Female = 46	Total = 83; Male = 43; Female = 40	Total = 85; Male = 45; Female = 40	Total = 80; Male = 40; Female = 40	Total = 80; Male = 40; Female = 40	Total = 88; Male = 44; Female = 44	Total = 91; Male = 46; Female = 45	Total = 93; Male = 46; Female = 47	Total = 93; Male = 46; Female = 47	Total = 98; Male = 48; Female = 50	Total = 98; Male = 48; Female = 50	Total = 98; Male = 48; Female = 50	Total = 100; Male = 48; Female = 52

REGION 5

Mountain lion hunter harvest opportunity was generally stable in Region 5 from the 1990s to late 2010s. However, annual harvest success varied year-to-year depending on winter snow-tracking conditions. Most of the Region's publicly accessible, high-quality, lion habitat lies in its peripheral mountain foothills (Figure 33). While the Region includes portions of both the Southwest and Eastern Mountain Lion ecoregions, most lions are harvested in the Southwest ecoregion (Figure 34). Nonresidents took 18% of all lions harvested in Region 5 between 2007 and 2016, most without the aid of an outfitter.

Although Region 5 harvest is well distributed across suitable lion habitat, individual LMU quotas may not be consistently reached because annual harvest is dependent on the presence of adequate tracking snow. Region 5 may consider eliminating or grouping a number of Regional LMUs to simplify harvest management.

Biologists and the public will work with their counterparts in other Regions to set objectives for, and adaptively manage, the Southwest and Eastern Ecoregions' mountain lion populations. Region 5 historically used overall LMU quotas (with female subquotas) to manage harvest (Table 16).

Figure 33. FWP Region 5 2016 mountain lion winter RSF and hunting districts.

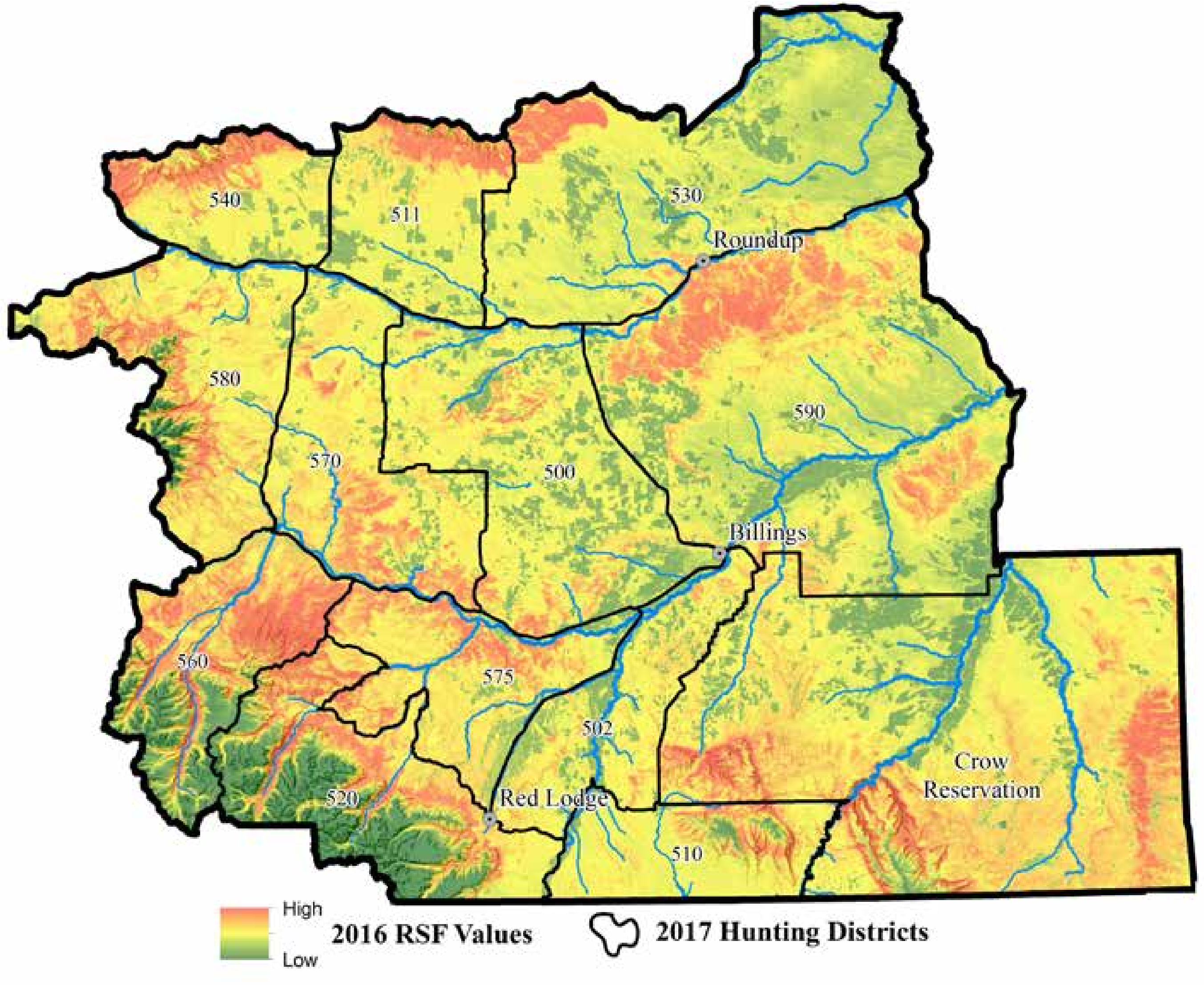

Minimizing human-lion conflicts and livestock depredation is a high Regional priority. Region 5 will use both hunter harvest and effective responses to individual incidents that are consistent with the Depredation and Control Guidelines to reduce potential conflicts.

Table 15. Region 5 mountain lion harvest, 1971 – 2016.

License Year	R5 F	R5 M	R5 Unk	R5 Tot.
1971	2	0	0	2
1972	1	1	0	2
1973	2	1	0	3
1974	0	0	0	0
1975	1	2	0	3
1976	3	1	0	4
1977	4	4	0	8
1978	3	0	0	3
1979	5	6	0	11
1980	4	4	0	8
1981	3	6	0	9
1982	3	2	0	5
1983	4	7	0	11
1984	2	12	0	14
1985	3	6	0	9
1986	4	11	0	15
1987	9	6	0	15
1988	7	11	0	18
1989	4	9	0	13
1990	8	13	0	21
1991	8	12	0	20
1992	10	21	0	31
1993	15	20	0	35
1994	13	19	0	32
1995	19	23	0	42
1996	13	22	0	35
1997	23	21	0	44
1998	17	23	1	41
1999	23	21	0	44
2000	19	24	0	43
2001	25	25	0	50
2002	16	17	0	33
2003	9	18	0	27
2004	12	22	0	34
2005	12	15	0	27
2006	12	13	0	25
2007	10	18	0	28
2008	10	21	0	31
2009	12	24	0	36
2010	8	10	0	18
2011	13	21	0	34
2012	11	20	0	31
2013	16	20	0	36
2014	8	28	0	36
2015	11	12	0	23
2016	13	26	0	39

Figure 34. FWP Region 5 hunting districts and mountain lion ecoregions.

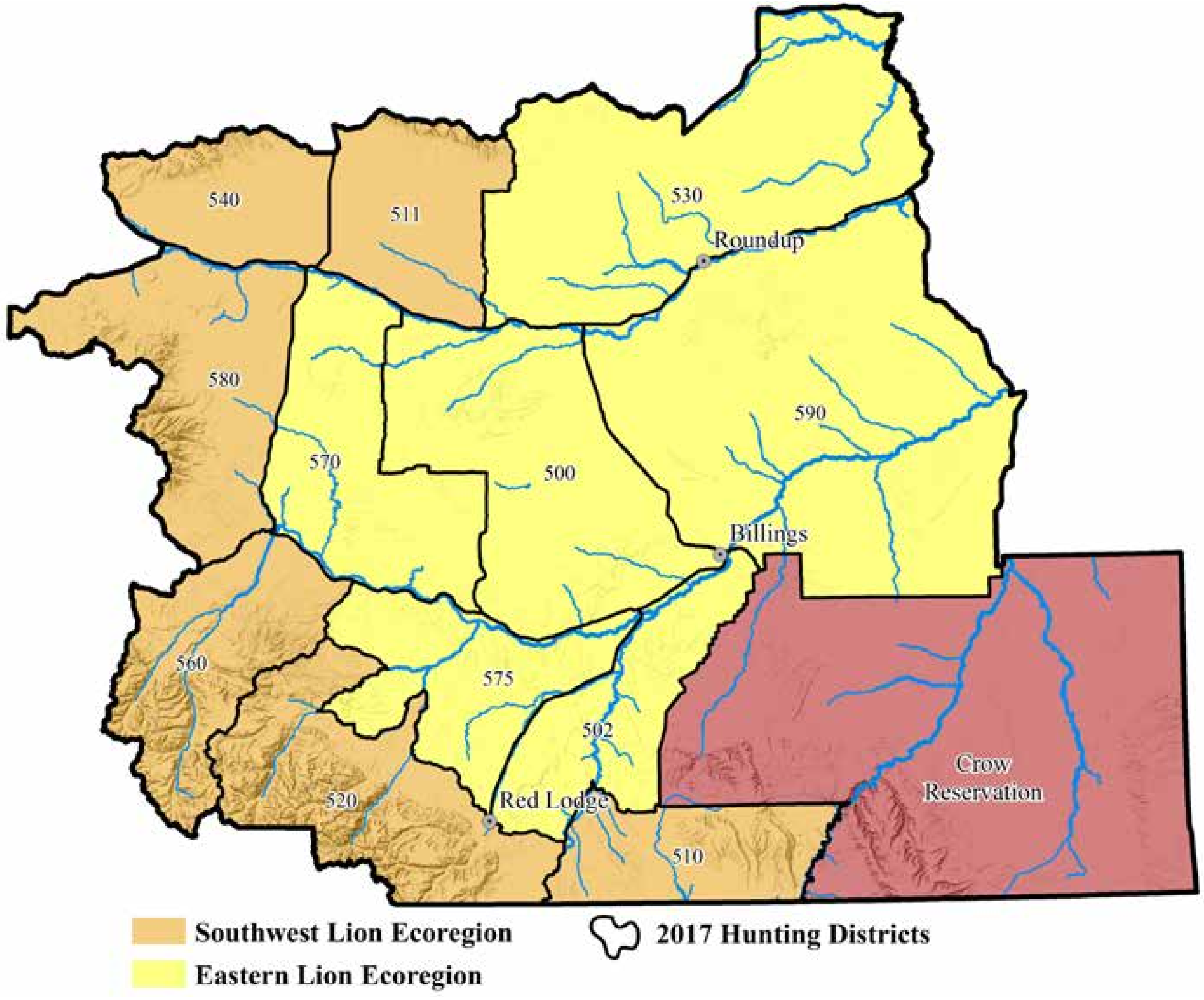

Table 16. Summary of Region 5 mountain lion harvest regulations, 1971 – 2017.

License Year	1971	1972	1973	1974	1975	1976	1977	1978	1979	1980	1981	1982	1983	1984	1985	1986
Mandatory Inspection	None		10 Day Inspection					4 Day Inspection				48 Hr. Inspection		10 Day Inspection	72 Hr. Inspection	48 Hr. Inspection
Hunting season	Opening of General D/E - 4/30					12/1 - 4/30			12/1 - 2/15							
Chase/Hound Training Season	None								2/16 - 4/30							
Regional Quotas	UNLIMITED; One ES Adult Lion per Hunter															

License Year	1987	1988	1989	1990	1991	1992	1993	1994	1995	1996	1997	1998	1999	2000	2001	2002
Mandatory Inspection	48 Hr. Report; 10 Day Inspection								24 Hr. Report; 5 Day Inspection			24 Hr. Report; 10 Day Inspection	12 Hr. Report; 10 Day Inspection			
Hunting season	12/1 - 2/15											12/1 - 4/14	Fall Season w/o dogs; 12/1 - 4/14			
Chase/Hound Training Season	2/16 - 4/30											No dedicated Chase Season, Hound Training allowed during Winter Hunting Season				
Regional Quotas	UNLIMITED One ES Adult Lion per Hunter	Total = 22; FSQ = 10	Total = 25; FSQ = 11	Total = 30; FSQ = 13	Total = 33; FSQ = 13	Total = 37; FSQ = 15	Total = 37; FSQ = 15	Total = 44; FSQ = 22	Total = 44; FSQ = 22	Total = 50; FSQ (some LMUs) = 20	Total = 52; FSQ (some LMUs) = 21	Total = 58; FSQ (some LMUs) = 23	Total = 56; FSQ (some LMUs) = 22	Total = 56; FSQ (some LMUs) = 22	Total = 57; FSQ (some LMUs) = 22	Total = 57; FSQ (some LMUs) = 22

License Year	2003	2004	2005	2006	2007	2008	2009	2010	2011	2012	2013	2014	2015	2016	2017
Mandatory Inspection	12 Hr. Report; 10 Day Inspection														
Hunting season	Fall Season w/o dogs; 12/1 - 4/14							Archery-only Season; Fall Season w/o dogs; 12/1 - 4/14							
Chase/Hound Training Season	Hound Training Season 12/2 - 4/14														
Regional Quotas	Total = 57; FSQ (some LMUs) = 22	Total = 49; FSQ (some LMUs) = 18	Total = 49; FSQ (some LMUs) = 18	Total = 49; FSQ (some LMUs) = 18	Total = 49; FSQ (some LMUs) = 18	Total = 49; FSQ (some LMUs) = 18	Total = 49; FSQ (some LMUs) = 21	Total = 44; FSQ (some LMUs) = 15	Total = 44; FSQ (some LMUs) = 15	Total = 44; FSQ (some LMUs) = 15	Total = 44; FSQ (some LMUs) = 15	Total = 47; FSQ (some LMUs) = 15	Total = 47; FSQ (some LMUs) = 15	Total = 47; FSQ (some LMUs) = 15	Total = 47; FSQ (some LMUs) = 15

REGION 6

Most suitable mountain lion habitat in Region 6 lies in the Bears Paw and Little Rockies ranges, as well as along the Missouri River (Figure 35). A significant portion of the Region's lion habitat is included within the Rocky Boy's and Fort Belknap Reservations—FWP does not have wildlife management authority within these jurisdictions.

There was no open mountain lion hunting season between 1976 and 1992 in Region 6 (Table 18); mountain lions became increasingly common in the Region 6 during this period. Harvest quotas have remained relatively stable since hunting seasons were re-opened in 1993 but the annual FWP-managed harvest varies annually depending on winter tracking conditions, hunter access, and individual hunters' participation in the harvest season (Table 17).

Mountain lion harvest that occurs on the Rocky Boy's and Fort Belknap reservations may not be reported to FWP, and thus, regional harvest totals should be viewed as minimums. Kunkel et al. (2012) documented a relatively high annual hunter harvest rate and low adult survival for Region 6 lions during their study. The authors suggested that Region 6 lion populations may be sustained by immigration rather than local recruitment. If so, continuing to protect adult females from harvest may allow local reproduction to supplement lions that disperse into the Region.

Lions are only likely to be consistently resident in hunting districts 680, 690, 621, 622, 631 and 632. The remainder of the Region may be considered a Special Management Area where tolerance for lions is low. In this area, liberal quotas may be recommended so that hunter harvest is available when needed to minimize conflict while still allowing for lion movement between resident populations.

All of Region 6 lies within the Eastern Mountain Lion ecoregion (Figure 36). Routine lion abundance estimates and population modeling will not be available in this ecoregion. Because of annual variations in tracking snow cover, annual harvest varies independent of population trend. Regional managers will therefore rely on indirect indications of lion abundance and public input to monitor lion populations. Region 6 may also choose to produce a baseline Regional abundance estimate (either alone or in collaboration with Tribal or NGO partners) following SCR or other field methods (Chapter 5) if funding is available.

Table 17. Region 6 mountain lion harvest, 1971 – 2016.

License Year	R6		
	F	M	Tot.
1971	0	0	0
1972	0	0	0
1973	0	0	0
1974	0	0	0
1975	0	0	0
1976	0	0	0
1977	0	0	0
1978	0	0	0
1979	0	0	0
1980	0	0	0
1981	0	0	0
1982	0	0	0
1983	0	0	0
1984	0	0	0
1985	0	0	0
1986	0	0	0
1987	0	0	0
1988	0	0	0
1989	0	0	0
1990	0	0	0
1991	0	0	0
1992	2	2	4
1993	2	2	4
1994	2	4	6
1995	3	3	6
1996	1	2	3
1997	5	2	7
1998	4	3	7
1999	4	4	8
2000	2	1	3
2001	3	2	5
2002	1	1	2
2003	0	0	0
2004	0	1	1
2005	0	0	0
2006	0	1	1
2007	1	2	3
2008	0	7	7
2009	1	3	4
2010	2	4	6
2011	5	4	9
2012	4	3	7
2013	2	3	5
2014	2	3	5
2015	2	4	6
2016	4	9	13

Figure 35. FWP Region 6 2016 mountain lion winter RSF and hunting districts.

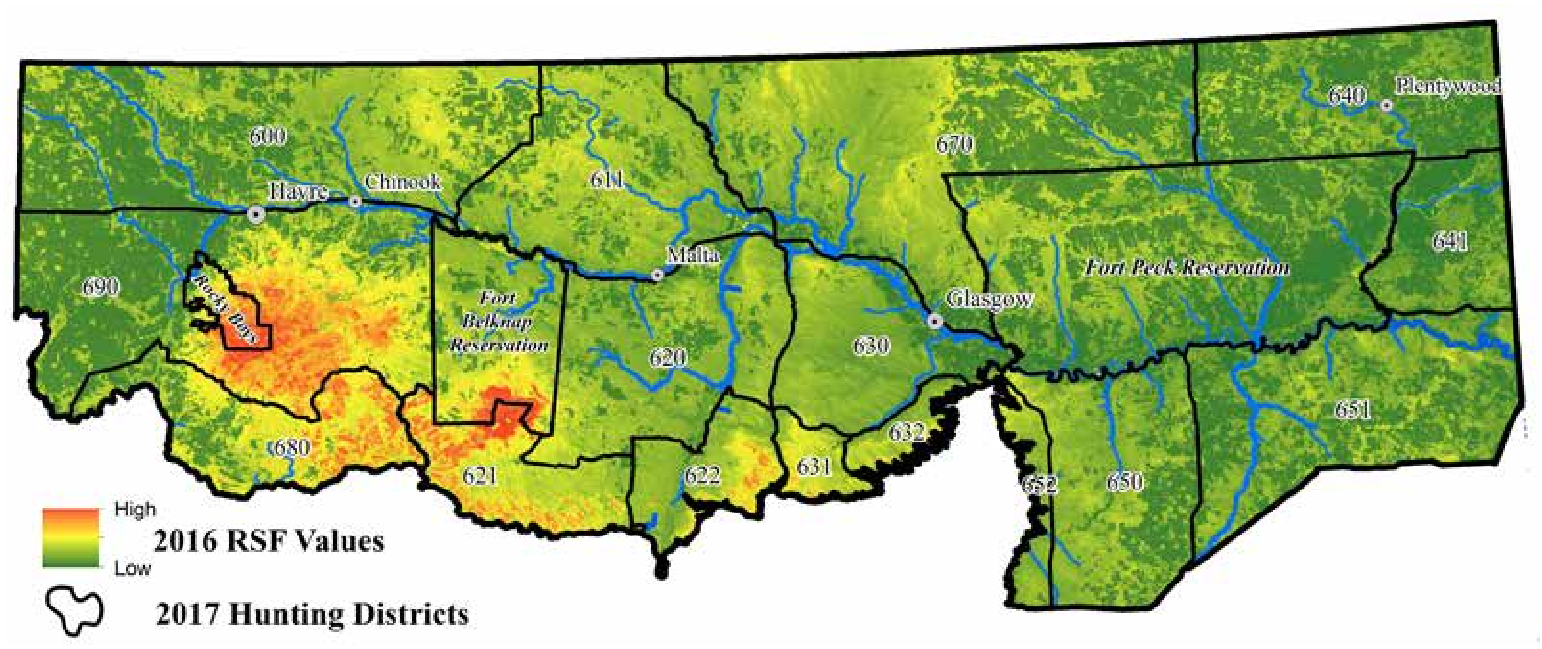

Figure 36. FWP Region 6 hunting districts and mountain lion ecoregion.

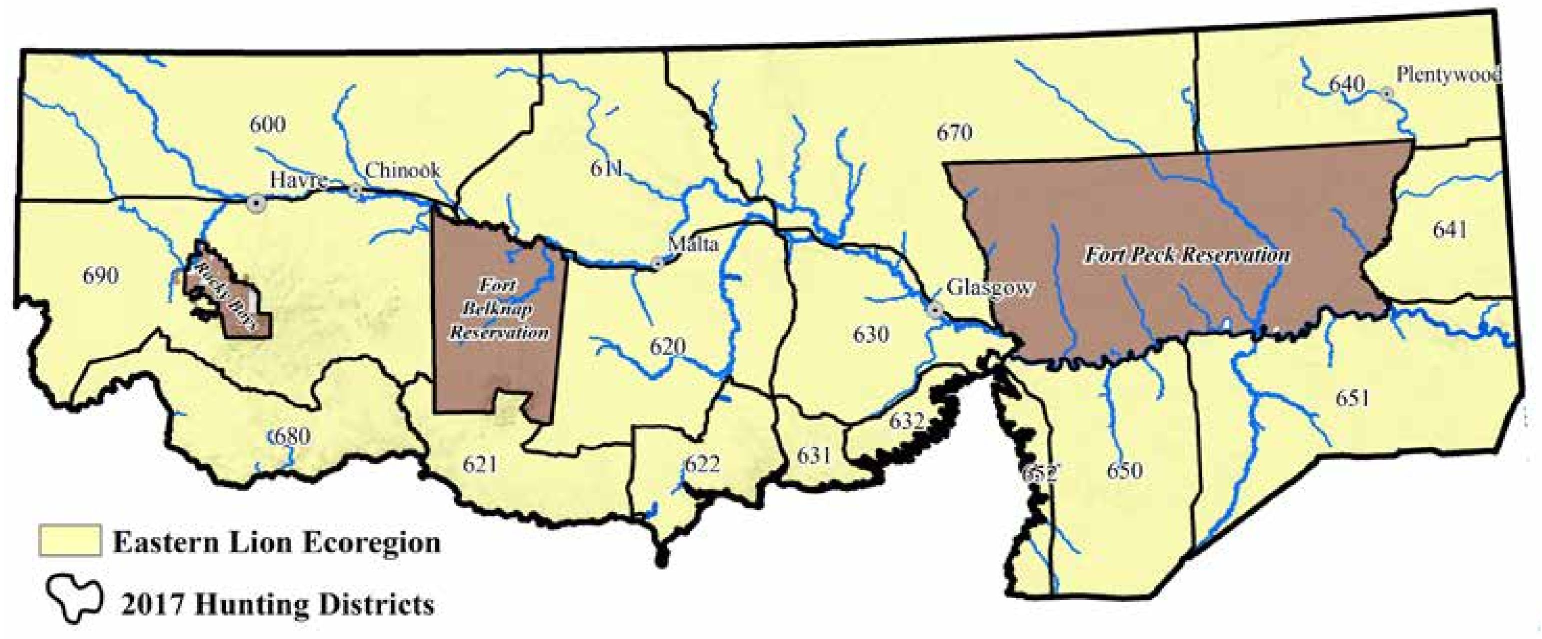

Table 18. Summary of Region 6 mountain lion harvest regulations, 1971 – 2017.

License Year	1971	1972	1973	1974	1975	1976	1977	1978	1979	1980	1981	1982	1983	1984	1985	1986
Mandatory Inspection	NONE		10 Day Inspection													
Hunting season	Opening of General D/E - 4/30					CLOSED										
Chase/Hound Training Season	NONE								CLOSED						2/16 - 4/30	
Regional Quotas	UNLIMITED; One ES Adult Lion per Hunter					CLOSED										

License Year	1987	1988	1989	1990	1991	1992	1993	1994	1995	1996	1997	1998	1999	2000	2001	2002
Mandatory Inspection						48 Hr. Report; 10 Day Inspection			24 Hr. Report; 5 Day Inspection			24 Hr. Report; 10 Day Inspection	12 Hr. Report; 10 Day Inspection			
Hunting season	CLOSED					12/1 - 2/15						12/1 - 4/14	Fall Season w/o dogs; 12/1 - 4/14			
Chase/Hound Training Season	12/1 - 4/30					2/16 - 4/30						No dedicated Chase Season, Hound Training allowed during Winter Hunting Season				
Regional Quotas	CLOSED					3 Any Legal Lion	3 Any Legal Lion	5 Any Legal Lion	Total = 10; FSQ = 3	Total = 10; FSQ = 3	Total = 13; FSQ (some LMUs) = 5; MSQ (some LMUs) = 3	Total = 13; FSQ (some LMUs) = 6	Total = 13; FSQ (one LMUs) = 6	Total = 16; FSQ (one LMUs) = 5	Total = 11; FSQ (one LMUs) = 3	Total = 11; FSQ (one LMU) = 3

License Year	2003	2004	2005	2006	2007	2008	2009	2010	2011	2012	2013	2014	2015	2016	2017
Mandatory Inspection	12 Hr. Report; 10 Day Inspection														
Hunting season	Fall Season w/o dogs; 12/1 - 4/14							Archery-only Season; Fall Season w/o dogs; 12/1 - 4/14							
Chase/Hound Training Season	Hound Training Season 12/2 - 4/14														
Regional Quotas	Total = 11; FSQ (one LMU) = 3	Total = 11; FSQ (one LMU) = 3	Total = 11; FSQ (one LMU) = 3	Total = 11; FSQ (one LMU) = 3	Total = 11; FSQ (one LMU) = 3	Total = 11; FSQ (one LMU) = 3	Total = 12; FSQ = 3	Total = 11; FSQ (one LMU) = 2	Total = 11; FSQ (one LMU) = 2	Total = 11; FSQ (one LMU) = 2	Total = 12; FSQ = 4	Total = 12; FSQ = 4	Total = 12; FSQ = 4	Total = 12; FSQ = 4	Total = 12; FSQ = 4

REGION 7

Mountain lions have expanded their range into eastern Montana since the 1980s and are now found in all suitable Region 7 habitats (Figure 37). The first mountain lion hunting season in Region 7 occurred in 1985 but no harvest was recorded until 1990. The Fish and Wildlife Commission incrementally raised quotas as the Region's lion abundance and distribution increased. Mountain lion age-in-harvest, harvest sex ratios, and hunter effort remained stable through the late 2010s.

Table 19. Region 7 mountain lion harvest, 1971 – 2016.

License	R7		
Year	F	M	Tot.
1971	0	1	1
1972	0	0	0
1973	0	0	0
1974	0	0	0
1975	0	0	0
1976	0	0	0
1977	0	0	0
1978	0	0	0
1979	0	0	0
1980	0	0	0
1981	0	0	0
1982	0	0	0
1983	0	0	0
1984	0	0	0
1985	0	0	0
1986	0	0	0
1987	0	0	0
1988	0	0	0
1989	0	0	0
1990	1	0	1
1991	0	0	0
1992	1	2	3
1993	1	2	3
1994	0	5	5
1995	2	1	3
1996	2	1	3
1997	1	1	2
1998	1	4	5
1999	3	4	7
2000	5	5	10
2001	4	11	15
2002	3	10	13
2003	1	5	6
2004	4	7	11
2005	0	7	7
2006	9	12	21
2007	6	11	17
2008	9	12	21
2009	8	17	25
2010	11	15	26
2011	17	14	31
2012	15	16	31
2013	10	26	36
2014	18	20	38
2015	8	16	24
2016	12	17	29

Because lions only recently recovered in Region 7, neither biological nor social carrying capacities are well known. Incidents of human-lion conflict and livestock depredation remained low through the mid-2010s and landowners were generally tolerant of mountain lion presence.

Region 7 lies entirely within the Eastern mountain lion ecoregion (Figure 38). Estimates of lion abundance will not be routinely produced using SCR or other field methods for this ecoregion. Managers will need to instead rely in indirect indices of abundance, harvest success, and public input to help guide management decisions.

Intermittent winter snow cover in the Region limits hound hunting's effectiveness. Annual lion harvest is correlated with the number of days the Region has snow cover (FWP data). Therefore, Region 7 quotas are more likely to serve as limits on harvest during years when snow conditions are favorable than as reliable annual harvest prescriptions. If quotas are met despite annually variable environmental conditions, managers may consider whether an increase is appropriate. Overharvest in Region 7 is unlikely because these favorable tracking conditions are rare and hunters have limited access to occupied habitat.

Region 7 traditionally prescribed a single, Region-wide, harvest quota. This approach was intended to both maximize hunter opportunity and regulation simplicity. It also allowed flexibility to direct harvest to areas with higher lion densities, more conflicts, or better tracking conditions.

FWP biologists will carefully monitor harvest distribution within the Region. Region 7 contains three lion management areas: 1) the Ashland Ranger District of the Custer National Forest (where the majority of Region 7 mountain lion harvests occurs) and adjacent lands, 2) the Sioux Ranger District (Chalk Butte, Ekalaka Hills and Long Pines units) of the Custer National Forest, plus several adjacent large tracts of BLM and private land and, 3) lands on and adjacent to the Charles M. Russell Wildlife Refuge.

Patterns in harvest among these units will be tracked over time. If there is a significant reduction in the distribution of harvest that cannot be attributed to tracking conditions or changes in hunter access, the Region may recommend management alternatives. Regional managers will also

Figure 37. FWP Region 7 2016 mountain lion winter RSF and hunting districts.

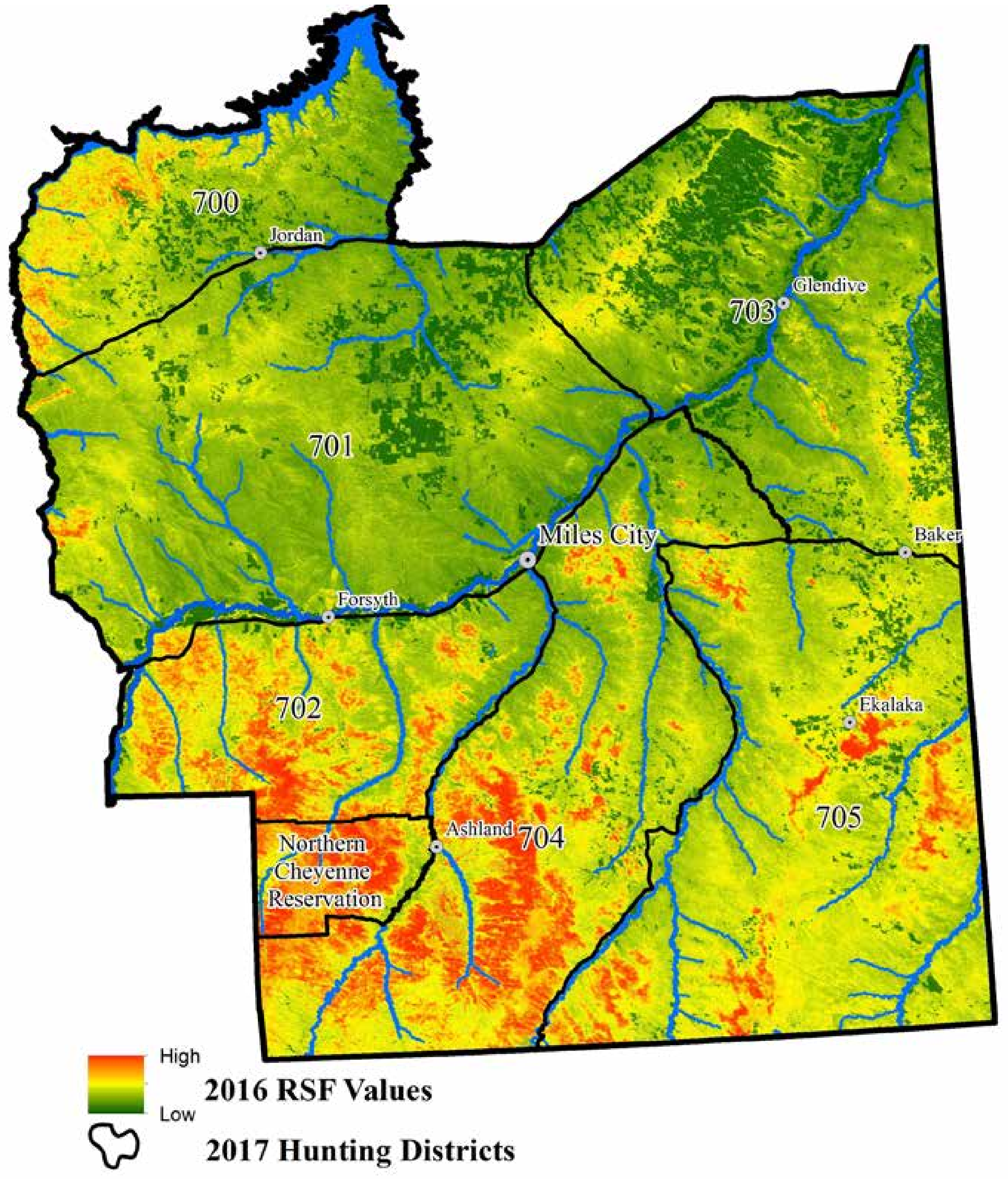

consider the pattern and rate of Regional human-lion conflicts and landowner input when evaluating these alternatives.

Nonresident hunters take an average of 15% of the lions harvested in Region 7 each year.

Minimizing human-lion conflicts and livestock depredation is a high priority in Region 7. The Region will use both hunter harvest and effective responses to individual incidents that are consistent with the Depredation and Control Guidelines to minimize potential conflicts.

Figure 38. FWP Region 7 hunting districts and mountain lion ecoregion.

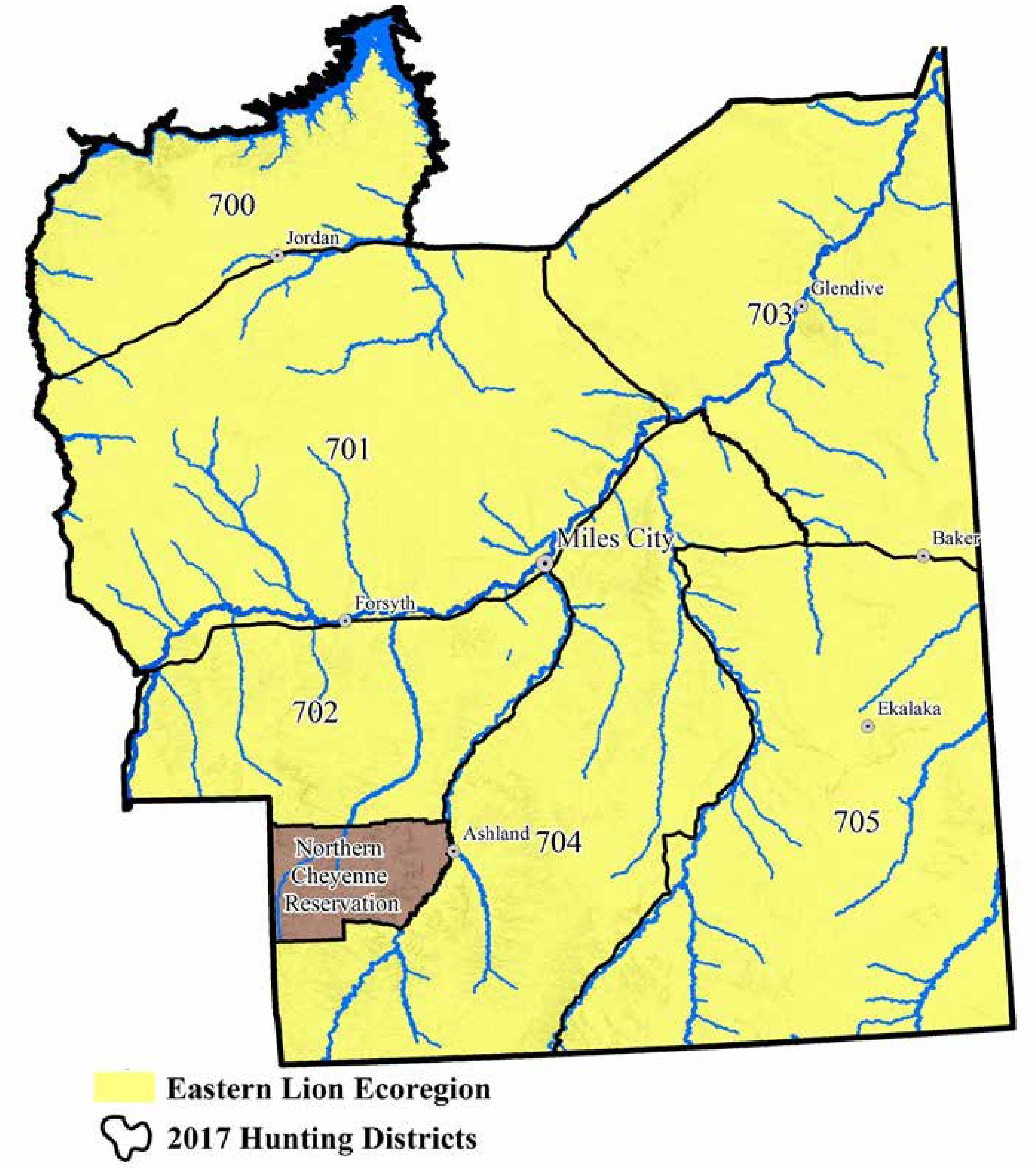

Table 20. Summary of Region 7 mountain lion harvest regulations, 1971 – 2017.

License Year	1971	1972	1973	1974	1975	1976	1977	1978	1979	1980	1981	1982	1983	1984	1985	1986
Mandatory Inspection	None		10 Day Inspection					4 Day Inspection				48 Hr. Inspection		10 Day Inspection	72 Hr. Inspection	48 Hr. Inspection
Hunting season	Opening of General D/E - 4/30					CLOSED									12/2 - 2/15	
Chase/Hound Training Season	NONE								CLOSED							
Regional Quotas	UNLIMITED; One ES Adult Lion per Hunter					CLOSED									3 Any Legal Lion	

License Year	1987	1988	1989	1990	1991	1992	1993	1994	1995	1996	1997	1998	1999	2000	2001	2002
Mandatory Inspection	48 Hr. Report; 10 Day Inspection								24 Hr. Report; 5 Day Inspection			24 Hr. Report; 10 Day Inspection	12 Hr. Report; 10 Day Inspection			
Hunting season	12/1 - 2/15											12/1 - 4/14	Fall Season w/o dogs; 12/1 - 4/14			
Chase/Hound Training Season	CLOSED		2/16 - 4/30									No dedicated Chase Season, Hound Training allowed during Winter Hunting Season				
Regional Quotas	3 Any Legal Lion	3 Any Legal Lion	3 Any Legal Lion	3 Any Legal Lion	3 Any Legal Lion	3 Any Legal Lion	3 Any Legal Lion	5 Any Legal Lion	7 Any Legal Lion	7 Any Legal Lion	7 Any Legal Lion	7 Any Legal Lion	7 Any Legal Lion	10 Any Legal Lion	15 Any Legal Lion	20 Any Legal Lion

License Year	2003	2004	2005	2006	2007	2008	2009	2010	2011	2012	2013	2014	2015	2016	2017
Mandatory Inspection	12 Hr. Report; 10 Day Inspection														
Hunting season	Fall Season w/o dogs; 12/1 - 4/14							Archery-only Season; Fall Season w/o dogs; 12/1 - 4/14							
Chase/Hound Training Season	Hound Training Season 12/2 - 4/14														
Regional Quotas	20 Any Legal Lion	20 Any Legal Lion	20 Any Legal Lion	20 Any Legal Lion	25 Any Legal Lion	25 Any Legal Lion	25 Any Legal Lion	25 Any Legal Lion	30 Any Legal Lion	30 Any Legal Lion	35 Any Legal Lion	45 Any Legal Lion	45 Any Legal Lion	45 Any Legal Lion	45 Any Legal Lion

APPENDIX 1

POPULATION MONITORING, FIELD PROTOCOL, AND DATA ANALYSIS

Trend Monitoring Area Selection

FWP identified permanent trend monitoring areas within the Northwest, West-central, and Southwest ecoregions based on the following criteria:

- The area is approximately 2,600 km^2 (1,000 mi^2) in size, and
- The habitat quality (assessed both qualitatively and as predicted by the 2016 RSF) within the trend area is representative of the lion habitat type and quality present in the remainder of the ecoregion, and
- There is current and long term physical and legal access to the majority of the trend monitoring area during winter, and
- Regional wildlife managers and the public are committed to prescribing annual mountain lion harvest rates for the trend monitoring area's LMUs that are representative of the annual harvest rate in the larger ecoregion.

Locations of the Northwest, West-central, and Southwest trend monitoring areas are shown in Chapter 4.

Supplemental Monitoring Area Selection

Supplemental monitoring areas in each of the Northwest, West-central, and Southwest ecoregions may be sampled the year after each ecoregion's trend monitoring area is sampled. The supplemental monitoring areas will be selected using the following criteria:

- The area is approximately 2,600 km^2 (1,000 mi.2) in size, and
- There is sufficient physical and legal access (i.e. public land or prior permission from private landowners) to allow sampling of most of the predicted mountain lion habitat in the monitoring area during winter, and
- Harvest rates for the proposed supplemental monitoring area's LMUs have been representative of the annual harvest rate in the larger ecoregion for at least the last 6 years.

Figure 39. An example of a sampling grid overlaid on a 3,400 km^2 monitoring area and the underlying 2016 RSF for the area (Proffitt et al. 2014; Upper Clark Fork River, MT).

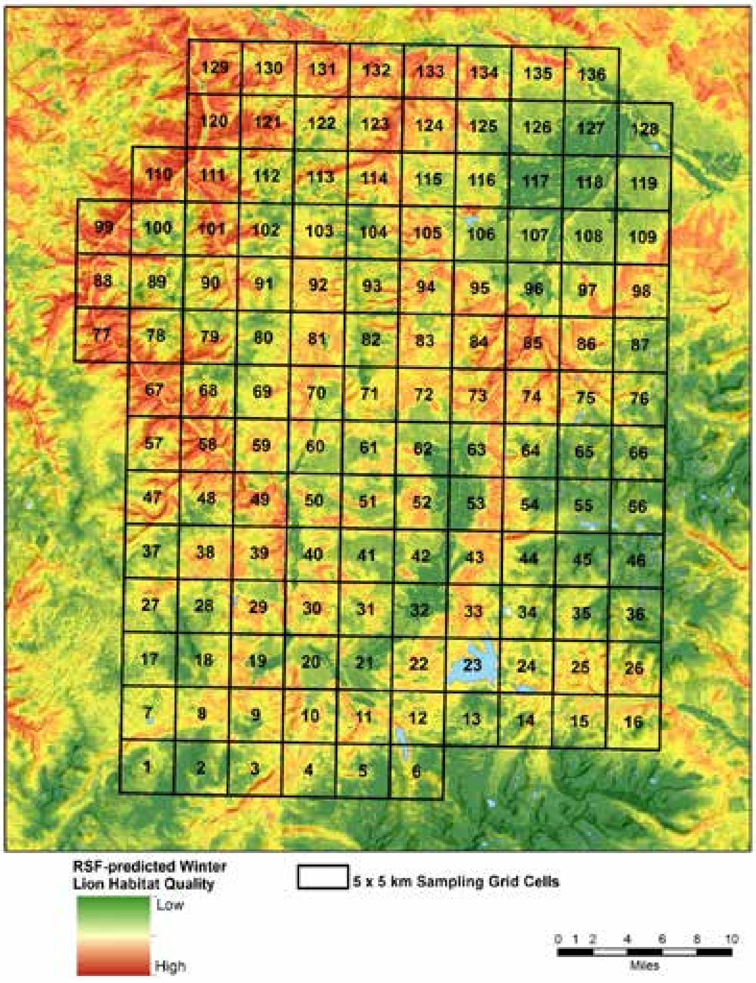

Initial Field Protocol

Collection and analysis of field data will initially follow methods described in detail by Proffitt et al. (2015). Population monitoring and field sampling techniques may change as improved methods are developed and validated in the future.

Monitoring areas will be sampled between 12/1 and 4/15. Field staff will overlay a 5x5 km grid across the study area and assign each cell a number. Cells will then be stratified into classes according to their habitat quality (RSF value) and a random search order will be assigned to cells in each class. Although each day's search effort will begin in a randomly assigned grid cell, more overall search effort will be dedicated to cells with higher quality habitat (Figure 39).

Trackers and hound handlers will search their assigned cell(s) to collect genetic samples from mountain lion hair,

scat, and muscle. The location where each sample is collected will be recorded, as will the search route trackers used to survey the cells (Figure 40).

When a fresh track of a suspected independent-aged mountain lion is located, the hound handlers will attempt to tree the lion and collect a muscle sample using a biopsy dart fired from a pneumatic gun. The tracks will then be backtracked and inspected to determine if the lion was independent or associated with a family group—if it was traveling with other animals, the group size will be recorded. Sex of the treed lion will be determined based on genetic analysis.

When older mountain lion tracks are located, a tracker or hound handler will backtrack and collect any hair or scat samples present along the track. All field crews will use a Global Positioning System to record the length and location of their search effort (Figure 40).

Figure 40. An example of the distribution of search effort within a SCR sampling area. In total, 12,785 km of trails within 127 grid cells were sampled over 121 days (Proffitt et al. 2014; Upper Clark Fork River, MT).

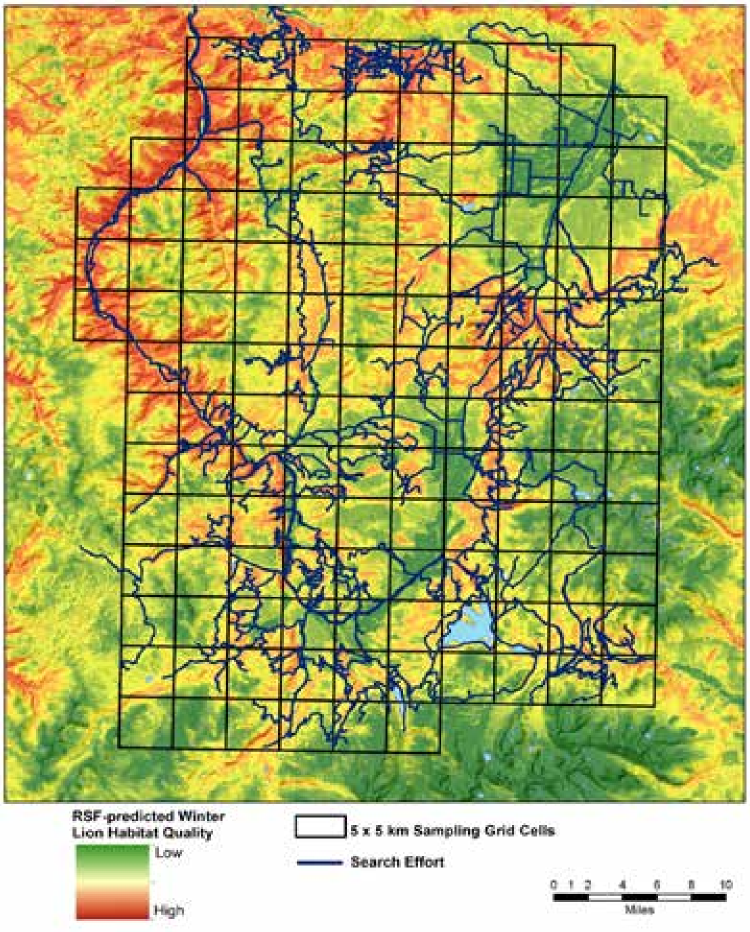

Figure 41. An example of a SCR sampling area and the locations of 132 mountain lion tissue samples (from both field sampling and harvest) that had DNA successfully extracted and analyzed to determine individual ID (Proffitt et al. 2014).

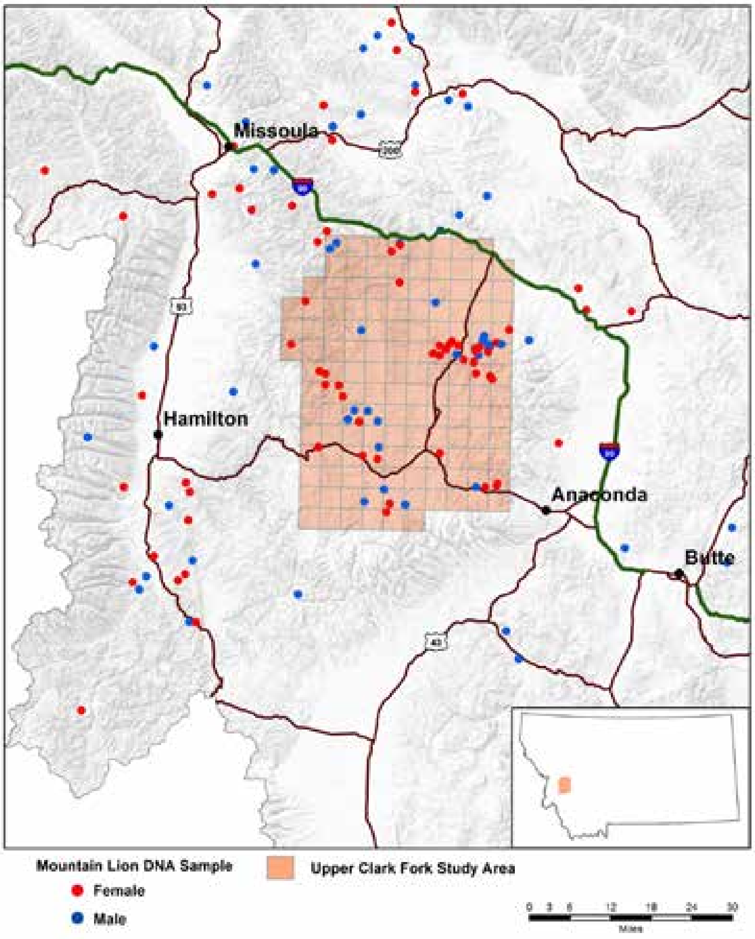

In Montana, the hide and skull of all harvested mountain lions must be presented to a FWP employee within 10 days. FWP will collect genetic samples from all know lion mortalities that occur in or adjacent to the monitoring area. Hair and muscle samples from these lions will be genetically analyzed to determine sex and the individual lions' identities (Figure 41).

Field Sampling Recommendations

A "sample" is a successfully extracted and identified individual mountain lion DNA sequence. Because not all non-invasive DNA samples will generate amplifiable DNA, not all material collected in the field will provide a useful DNA sample. Even after a single sample is collected in a cell, field staff are generally encouraged to continue to expend effort in that cell to obtain either additional lower quality samples (scat, hairs) or a high-quality sample (muscle biopsy). For hound handlers, this means collecting

a biopsy dart sample, and a backup high-quality hair sample. For snow backtrackers, multiple scat samples from different scats, and/or hair samples are ideal.

Field staff will collect tissue from biopsy darts, scats from backtracking, hairs from both biopsy darting (as a backup sample) and hairs from snow tracking, and harvested lion muscle samples. During previous studies (Russell et al. 2012, Proffitt et al. 2015) DNA extraction success was highest for muscle/biopsy samples and lowest for hair and scat. Because not all biopsy samples generate successful DNA sequences, a second set of high-quality hair samples (with follicles attached) should also be collected. Hound handlers should collect these samples opportunistically while tracking the animal to the tree, then search for hair and/or scat around the tree and while back tracking from the tree.

There is a critical difference between when a survey cell has been searched versus when a cell has been successfully sampled. Survey effort was an important predictor of detection in previous SCR studies of lions (Russell et al. 2012). Therefore, field staff must carefully collect a GPS track log of all daily search effort. If a cell is searched and lion sign is present but a sample is not obtained, then the cell was not successfully sampled.

Search effort should be spatially distributed by randomly assigning cells to be searched each day. These random grid cells are the starting point for the day's search. However, if new tracks are encountered while traveling to the days starting grid cell, the tracker should follow those tracks if that grid cell has not been successfully sampled yet. If tracks of a lion previously captured in that grid cell are detected, however, the tracker should proceed to the day's assigned starting location.

The hound handler/tracker should confine search activity to the assigned focal cell or its 8 adjacent grid cells on any particular day. Field crews may choose to skip a randomly assigned cell if multiple teams are working nearby and the randomly assigned cell could lead to survey overlap. Likewise, assigned cells may be skipped if that cell has been surveyed within the previous month and a high-quality sample already obtained. Field crews may choose to skip assigned cells if conditions in the assigned cell will not allow snow tracking.

Once a hound handler is assigned a starting grid cell, subsequent sampling effort may proceed in one of several ways. If the assigned cell and adjacent cells are searched, no sign is detected, and the hound handler believes the area is likely void of lions at that time (e.g. too high of an elevation, too much snow, etc.), the hound handler will receive a new randomly assigned starting cell the next day. The cell will remain on the sampling list for that period.

If after the assigned cell and adjacent cells are searched, all tracks are followed, and the hound handler believes that all lions currently detected within the area have been sampled, the cell(s) from which samples were collected will be removed from the sample list for that period. The hound handler will then get a new starting cell from the sampling list the next day.

If the assigned cell and adjacent cells are searched, multiple tracks are found, and the hound handler believes that NOT all lions currently within the area have been sampled, only the cell(s) from which samples were collected will be removed from the list. The hound handler will then return to the area and continue to work there until their shift is over, or they believe they have sampled all of the lions thought to be in the area. A new starting cell from the sampling list will be assigned the next day.

All samples will be carefully stored in desiccant and labeled with a unique sample ID. Hound handlers and trackers will record their daily search effort using GPS tracks from GPS units.

Estimating Ecoregional Lion Abundance

Montana FWP will monitor and manage mountain lions within large (>35,000 km^2) ecoregions. To do so, managers will need to periodically estimate lion population size within these ecoregions and make predictions about the effect of future harvest at this scale. Once an overall harvest prescription has been developed for an ecoregion, individual harvest limits will be assigned to the ecoregions' LMUs to distribute harvest and address local management objectives.

Spatially explicit abundance estimates from representative sampling areas can be extrapolated across a broader area of inference to estimate that landscape's population size (Boyce & McDonald 1999). This method of extrapolating animal abundance as a function of RSF-predicted habitat quality has been used to estimate populations of many species (Boyce et al. 2016), including mountain lions in Montana (Robinson et al. 2015).

Several important factors must be considered when using data collected from sampling areas to estimate a species' population size across a larger area (Wiens et al. 2008, Boyce et al. 2016):

- The relationship between the observed number of animals and available habitat (ie. the 2016 RSF) within a sampling area should be similar to that same relationship across the larger landscape, and
- Harvest management within sampling areas should be representative of the broader area of inference (Reynolds et al. 2016). Specifically, it's important that the long-term mountain lion hunter-harvest rate within an ecoregion's monitoring areas is similar to the harvest rate within the larger landscape for which the estimate is being made, and
- Because a species' abundance can vary over time for reasons unrelated to habitat quality (ie. hunting or changes in prey density), representative sampling area(s) must be periodically re-sampled. This helps ensure that up-to-date relationships between abundance and RSF values are used to estimate current populations.

Producing Ecoregion Population Estimates

The relationship between mountain lion density and habitat within an ecoregion's monitoring area(s) will be most similar to other areas within that same ecoregion. Therefore, the mountain lion abundance data collected on monitoring areas will only be used to estimate the population size of the ecoregion where that monitoring area is located—they will not be used to develop population estimates for other ecoregions.

Even within ecoregions, the relationship between mountain lion abundance and habitat quality varies. To improve the accuracy of an ecoregion's population estimate, FWP may initially collect data from both a fixed Trend Monitoring Area (sampled Year 1) and a Supplemental Monitoring Area (sampled Year 2). The locations of Supplemental Monitoring Areas may vary over time, Trend Monitoring Area locations will not.

Abundance estimates from the Supplemental Monitoring Area will be compared to the estimate for the same area that was extrapolated prom the previous year's Trend Monitoring Area estimate. Therefore, the Supplemental Monitoring Area sample will help FWP compare and validate the accuracy of the extrapolated ecoregional population estimate.

Combining the data collected from both the trend and supplemental monitoring areas may generate a more representative ecoregional estimate of the relationship between lion abundance and the RSF as compared to using data from the trend monitoring area alone (Howe et al. 2013). Therefore, the results of the two subsequent samples will be pooled to describe the current relationship between lion abundance and the RSF within an ecoregion. This pooled relationship will be used to estimate the population of independent-aged mountain lions within that ecoregion.

Ecoregion population estimates will also be produced using monitoring data from the fixed trend monitoring area alone. FWP will compare the estimate derived using the pooled areas' data and the estimate using only the trend monitoring area data. If the two methods consistently produce similar estimates, supplemental monitoring areas will not continue to be sampled.

The initial FWP SCR model predicts the abundance of independent-aged mountain lions at a 4 km^2 resolution (Proffitt et al. 2015). The following regression equation is an example of one way to estimate the effect of RSF on abundance across the ecoregion:

$$\text{Abundance} = \beta 0 + \beta 1 * \text{RSF} + e$$

FWP continues to test and validate extrapolation methods.

FWP will estimate the mean RSF value over the same spatial extent (4 km^2) for both the trend and supplemental monitoring areas, and use these mean RSF values in the regression model. The above regression equation represents the effect of the mean 4 km^2 RSF on predicted spatial abundances within the pooled trend and supplemental monitoring areas. Using this relationship, FWP will predict mountain lion abundance for the entire ecoregion by extrapolating the observed relationship between RSF values and mountain lion abundance (Boyce & McDonald 1999). FWP will use the 95% confidence interval around β RSF to estimate the 95% upper and lower confidence intervals around the predicted mean abundance for the ecoregion.

FWP will periodically sample mountain lion populations and produce estimates for the Northwest, West-central, and Southwest ecoregions. An estimate of the overall abundance of mountain lions within these ecoregions will then be developed based on the sampling data. These estimates will be input into the IPM (Chapter 6) as additional data. The IPM then considers the field-based abundance estimates along with harvest prescriptions and lion vital rates when generating more complete predictions of past and future ecoregional population trends.

Data Analysis

Genotyping errors (genetic misidentification of DNA field samples) can have a significant impact on capture-mark-recapture and spatial-capture-recapture abundance estimates (McKelvey and Schwartz 2004). FWP will ensure that contracted genetic analysis laboratories employ rigorous testing so that genotyping errors are minimized and quantified.

To estimate the abundance of independent lions in the sampling area, FWP will initially fit the SCR model to a dataset that includes only samples from independent animals or the adult female of a family group. This eliminates multiple samples from within family groups as well as all groups where only a subadult animal was sampled.

The monitoring period will be divided into sampling periods within the winter season (December, January, February, and March-April). An encounter history will be developed for each detected individual during each sampling period and the detection probability for harvested animals will be adjusted to '0' for the sampling periods following their death.

FWP will initially use a Bayesian SCR model to estimate the number of mountain lions present within the sampling area. This method explicitly incorporates the spatial organization of individuals through the estimation of specific capture probabilities (Efford 2004, Efford et al. 2009, Gardner et al. 2010, Royle et al. 2013).

To account for individuals that had a home range only partially within the sampling area, FWP will buffer the study area by 10 km and estimate spatial densities within the larger area. We will then evaluate potential models that include all possible combinations of the covariates for search effort and sex, RSF-driven densities, and sex-specific activity center distributions (Russell et al. 2012). We will conduct model selection using a combination of Bayesian Information Criteria (BIC), examination of the posterior significance of the parameters in each model, and two goodness of fit statistics (as described in Proffitt et al. 2015). All of these factors will be weighted by our prior knowledge of mountain lion biology.

We will then estimate the independent-aged lion abundance, with confidence intervals, for the trend and supplemental monitoring areas. Because these abundances are spatially explicit functions of the areas' underlying habitat quality, we will then extrapolate the monitoring areas' relationship between abundance and the RSF to produce an estimate of lion abundance across the larger ecoregion.

Cost

Field monitoring will occur at a significant periodic cost to Fish, Wildlife and Parks. The Department will need to hire one staff biologist who will work half-time (6 months) to plan and organize logistics, contract field staff, coordinate day-to-day field operations, and prepare data for analysis. Enough hound handlers will be contracted to successfully sample approximately 60% of grid cells within the Monitoring Area during the four sampling periods. The number of contractors may vary depending on each contractor's seasonal availability. Genetic analysis of the collected samples will also be contracted through an independent laboratory.

Table 21. Approximate costs (2016) to collect and analyze mountain lion monitoring area data.

Contracted Hound Handlers	$65,000
Genetic Analysis	$9,500
Fuel and housing	$6,500
FWP Biologist (1/2 FTE)	$32,500
Misc. Supplies	$2,000
Total	$115,500

APPENDIX 2

MOUNTAIN LION INTEGRATED POPULATION MODEL DEFINITION AND USER INPUTS

The Montana mountain lion integrated population model is generally described in Chapter 6 and in Nowak et al. 2018. Following are more complete descriptions of the several internal models, the data and prior assumptions that the IPM includes, and an explanation of the controls that users can manipulate to improve the IPM's outputs.

Reproduction Model Definition

The equation describing the number of kittens in year y is as follows:

$$N_{kit,f,y} = \left(N_{sa,f,y} * P_s a * LS_s a * 0.5 + N_{a,f,y} * P_a * LS_a * 0.5 * 0.5\right) * Survival_{kit,y-1}$$

Thus, we calculate the number of female kittens ***f*** in year ***y*** as a function of the number of subadult ***SA*** and adult ***A*** females ***f*** in year ***y***. For the subadult contribution we take the product of the number of subadults, the age specific pregnancy rate ***P***, and litter size ***LS***.

Only a fraction of the resulting kittens will be female and so the final term in the product simply assumes that half of the kittens born are female. The adult contribution to the kitten population is calculated as the product of the number of adults, the age specific pregnancy rate, litter size, and 0.25 (0.5 * 0.5). Because we assume the adult inter-birth interval is 24 months, only half of the adult females are available to reproduce in any given year. We therefore multiply the reproductive term by 0.5. Said another way, the first 0.5 represents the assumption that half of the kittens born are females and the second 0.5 reflects our assumption that the birth interval is 24 months, which results in half of the adult female population giving birth each year.

Multi-state Survival Model Definition

The mountain lion IPM in ***PopR*** is built around a 4-age class and 2-sex population model. The 4 age classes are ***kittens*** (0-6 months), ***juveniles*** (6-18 months), ***subadults*** (18-30 months) and ***adults*** (30+ months). We assume a 50:50 sex ratio at birth but, starting with the juvenile age class, each sex is modeled separately. The process model describing lion ecology is represented by a series of equations that describe transitions from one age class to the next each year.

$$N_{kit,f,y} = \left(N_{sa,f,y} * P_s a * LS_s a * 0.5 + N_{a,f,y} * P_a * LS_a * 0.5 * 0.5\right) * Survival_{kit,y-1}$$

$$N_{juv,f,y} = N_{kit,f,y-1} * Survival_{juv,f,y-1} - harvest_{juv,f,y-1} + \epsilon_{juv,f,y}$$

$$N_{sa,f,y} = N_{juv,f,y-1} * Survival_{sa,f,y-1} - harvest_{sa,f,y-1} + \epsilon_{sa,f,y}$$

$$N_{ad,f,y} = \left(N_{sa,f,y-1} + N_{ad,f,y-1}\right) * Survival_{ad,f,y-1} - harvest_{ad,f,y-1} + \epsilon_{ad,f,y}$$

where,

$$N_{age,sex,y}$$
is the abundance of age class age, sex sex in year y

$$Survival_{age,sex,y}$$
is the survival of age class age, sex sex in year y

$$P_{age}$$
is the age-specific pregnancy rate

$$LS_{age}$$
LS is the age-specific litter size

$$\epsilon_{age,sex,y}$$
is the age, sex and year-specific residual variation

Kittens born to subadults and adults the previous year are recruited as juveniles on December 1st each year. The number of subadults and adults is indexed to year ***y*** based on the number of reproductive females in the population on December 1. The model then takes into account the probability these females will survive until they give birth (assumed to be July 1). We also assume that kittens whose mothers die within the first six months after giving birth will not survive.

The model does not make kittens available for harvest because it assumes they become juveniles on December 1 at 6 months old but would not be independent (and legally harvestable) until after the winter hunting season ends. Although some subadults may reproduce, they do so at a lower rate than adults. Subadults transition to adults on December 1st of the following year. Any mountain lion older than 30 months is considered either an adult male or female. As adults, the model assumes that each sex survives (except for harvest) and reproduces at the same respective rate for the remainder of their lives.

The lion IPM primarily uses estimates and variability of documented vital rates (from the research literature) rather than raw field data itself (Table 22). This model structure provides several advantages. First, it allows lion research data collected using a wide variety of field sampling protocols to fit into the IPM framework—once the parameter and its error distribution is described it can be entered into the IPM. Because we also include a measure of the field estimate's precision, all sources of uncertainty remain in the IPM.

The general form of the observation model in ***PopR*** is:

$$\hat{\theta} \sim Normal\left(\bar{\theta}, \widehat{SE}(\hat{\theta})\right)$$

where,

$\theta =$ field estimate

$\widehat{SE}(\hat{\theta}) =$ estimated standard error of

$\bar{\theta} =$ IPM parameter.

The observation model is like a multi-dimension regression model. The model fitting process seeks to minimize the distance between the IPM parameter (ie. Adult Female Survival) and the associated field estimate simultaneously across all IPM parameters.

Population Reconstruction Model Definition
The IPM uses survival estimates along with the annual harvest rate to reconstruct past mountain lion populations. It is based on examples of live recapture/dead recovery models from the literature that consider sex, age and year specific abundance estimates from records of harvested animals (Brownie et al. 1985, Link et al. 2003, Conn et al. 2008, Buderman et al. 2014). Current hunter harvest by sex, age, and location is input to the model after the close of the harvest season each year. By combining the multi-state survival model with observed harvest data, we can intuitively estimate population size by assuming a simple binomial distribution whose expectation is equivalent to:

$$N_{age,sex,y} = \frac{harvest_{age,sex,y}}{harvestMortality_{age,sex,y}}$$

where,

$$harvest_{age,sex,y}$$

is the number of age a, sex s, animals harvested in year y

$$N_{age,sex,y}$$

is the age, sex and year specific abundance

$$harvestMorality_{age,sex,y}$$

describes the relationship between abundance and harvest.

In practice, we implement harvest reconstruction as a binomial distribution:

$$harvest_{age,sex,y} \sim Binomial\left(harvestMortality_{age,sex,y}, N_{age,sex,y}\right)$$

Because the model requires that annual harvest data are input annually by both sex and age, FWP determines the age of harvested lions using cementum age analysis (Trainer & Matson 1988). In cases where teeth cannot be successfully extracted or an age confidently determined, the model randomly samples the distribution of known-age animals by sex and assigns an age to that animal for the purpose of the population reconstruction.

Direct estimates of population abundance (Proffitt et al. 2015) will be input into the model when they are available. These periodic field estimates can significantly improve past and future population estimates for individual lion ecoregions. Direct population estimates will be periodically developed for most lion ecoregions following the methods described in Chapter 5.

PopR uses Markov Chain Monte Carlo (MCMC) methods to “fit” IPM population estimates to the available data. MCMC methods estimate parameters in complex models by systematically updating informed prior distributions with information gleaned from field data (e.g. observed harvest). Therefore, they allow us to describe each parameter in terms of a distribution and that distribution’s shape. Parameters described by a narrow and peaked distribution are more precisely estimated than those that are flatter and less peaked.

PopR provides generally acceptable default MCMC settings but also allows users to easily adjust them in the web-based user interface. Typically, 25,000-100,000 MCMC iterations will be required to fit an IPM. ***PopR*** provides convergence diagnostics in the output report.

IPM USER CONTROLS

Demographic Variation

These settings allow users to decide whether to allow estimates of population vital rates to be drawn from a single distribution (“Constant”) or from a range of all possible distributions that differs every year (“Time Varying”). Biologists should only choose “Time Varying” if they have reason to believe that non-harvest factors (such as weather or prey density) introduce additional volatility in these vital rates that would not have been present during the field research projects from which the “Constant” rate distribution was developed. Research has demonstrated that mountain lion non-harvest survival and reproductive rates are remarkably stable and the “Constant” setting should be considered the default.

Burn-in Length

“Burn-in” is a colloquial term for an initial process that gives the Markov Chain time to approach the solution to the problem by throwing away some less reasonable starting points at the beginning of a Markov Chain Monte Carlo run. Allowing the Burn-in process to establish an equilibrium distribution reduces the number of subsequent MCMC sampling iterations needed to provide an estimate with reasonable certainty. In PopR, managers should simply use the default Burn-in Length setting when developing an estimate through the standard user interface.

Markov Chain Monte Carlo (MCMC) Iterations

If the number of MCMC iterations is set too low the uncertainty about an estimate is likely to be misrepresented. In ***PopR***, we use the Brooks-Gelman-Rubin (BGR) statistic as an initial assessment and this is the statistic used when automating convergence. The BGR statistic suggests convergence when estimates of Rhat are below 1.1 or more generally close to 1. This statistic is reported under the “Table” tab and highlighted in red when Rhat estimates are above 1.1. The default settings will produce results that are unlikely to change even if run longer, but users should increase the number of MCMC iterations to 15,000 or greater if either Rhat estimates are above 1.1 and/or computing time allows.

Thinning Rate

Thinning tells the sampler to only retain every nth value from the chains. This technique is sometimes used to reduce autocorrelation in the chains, but comes at the cost of reduced efficiency of the sampler. A more reasonable use of thinning is when hardware limitations are being reached, which typically comes in the form of running out of memory. This will not be an issue in ***PopR*** and, therefore, the recommended setting for the Thinning slider is ***1.***

Automate Convergence

Users may choose to simply check the “Automate Convergence” box below the MCMC sliders menu in the ***PopR*** interface. Although this option will increase the time necessary to produce an estimate, it will assure that an adequate Burn-in Length and number of MCMC Iterations have been used to produce a statistically sound estimate and error distribution.

Table 22. Default mountain lion vital rates used in Montana’s 2016 Integrated Population Model. Rates are based on field data collected from 263 radio-monitored lions from Montana, Wyoming and Washington.

Parameter	Age	Sex	Mean	SE
Survival	YOY	F	0.5	0.1
Survival	Juvenile	F	0.75	0.1
Survival	SubAdult	F	0.57	0.1
Survival	Adult	F	0.8	0.05
Survival	YOY	M	0.5	0.1
Survival	Juvenile	M	0.75	0.1
Survival	SubAdult	M	0.49	0.1
Survival	Adult	M	0.65	0.05
HarvMort	Juvenile	F	0.01	0.01
HarvMort	SubAdult	F	0.25	0.1
HarvMort	Adult	F	0.1	0.1
HarvMort	Juvenile	M	0.01	0.1
HarvMort	SubAdult	M	0.35	0.1
HarvMort	Adult	M	0.2	0.1
OtherMort	Juvenile	F	0.24	0.1
OtherMort	SubAdult	F	0.18	0.1
OtherMort	Adult	F	0.05	0.1
OtherMort	Juvenile	M	0.24	0.1
OtherMort	SubAdult	M	0.16	0.1
OtherMort	Adult	M	0.15	0.1
Fetus Count	SubAdult	F	3	0.1
Fetus Count	Adult	F	3	0.1
Pregnancy	SubAdult	F	0.5	0.01
Pregnancy	Adult	F	1	0.01

APPENDIX 3

MOUNTAIN LION DEPREDATION AND CONTROL GUIDELINES

In accordance with Montana Code Annotated 87-1-201, 87-1-217, 87-1-225, 87-1-301, 87-1-304, 87-3-127, 87-3-128, 87-5-713, 87-5-725, and 87-6-106, Montana Fish, Wildlife and Parks (FWP) and the Fish and Wildlife Commission are both authorized and charged with the duties of protecting persons and personal property from damage and depredation resulting from ingress or attack by wildlife. The goal of the **Mountain Lion Depredation and Control Guidelines** is to minimize damage to property and to prevent public safety problems. For the purpose of these Guidelines, a Public Safety Problem is defined as: Any situation where a FWP employee (or their agent) reasonably determines that a human has been physically injured or killed as a result of contact with a mountain lion, that an attack by a mountain lion has resulted in the loss of livestock or pets, or that the continued presence of a mountain lion poses a threat to human safety.

Any mountain lion that is lethally removed by FWP or its agents must be retained and transferred to the Montana Livestock Loss Board for sale or auction pursuant to MCA 2-15-3110 to 3113 and 87-1-217.

I. DEFINITIONS

The following are definitions designed to standardize the vocabulary used in the investigation and reporting of human/lion conflicts. It is important that the same terms be used to describe the different types of encounters that occur between humans and mountain lions. The definitions presented here are similar to those used in other western states.

Sighting: A visual observation of a mountain lion.

Encounter: An unexpected direct meeting between a human and a mountain lion without incident or the recurrent sighting in close proximity to human development or habitation.

Incident: A conflict between a human and mountain lion that may have serious results (i.e. a mountain lion killing or attempting to kill a pet that must be forced to back down).

Attack: When a human is bodily injured or killed by physical contact by a mountain lion.

Nuisance Lion: A mountain lion involved in encounters and incidents (i.e. pet attacks, continual presence around humans or areas of high human activity, presence near where children are or will be shortly) but is showing no aggression and/or flees when encountered by a human.

Depredation Lion: A mountain lion involved in the killing of livestock.

Aggressive Lion: An individual mountain lion exhibiting aggressive behavior towards humans including a mountain lion that attacks a person without provocation, intentionally approaches humans or fails to retreat when a human takes aggressive actions, or forces a human to take evasive action to avoid attack.

Livestock Depredation: Livestock attacked or killed by a mountain lion.

Conflict: When a human and mountain lion are involved in an encounter, incident or attack, or a mountain lion is determined to be aggressive, a nuisance, or involved in livestock depredation.

II. DOCUMENTATION OF HUMAN-MOUNTAIN LION CONFLICTS

1. Each FWP Region is responsible for responding to reports of mountain lion damage to property and human-mountain lion encounters, incidents, or attacks. Regional Supervisors shall ensure the following procedures are used upon FWP employees' receiving such reports.

    a. Obtain the name, address, and telephone number of the person making the report, the person receiving the call, and the time and date of the call.

    b. Record if the conflict involves an Encounter, an Incident, an Attack, or a Livestock Depredation.

    c. If a Livestock Depredation is reported or suspected, record the number and type of livestock involved and immediately contact the USDA APHIS Wildlife Services agent with responsibility for the area where the incident occurred.

    d. Record the number of mountain lions involved, its/their age class (if known), and the date and time of the conflict.

    e. If the conflict was a human Attack, record the name, sex, and age of the victim, location, and the extent of any injuries. IMMEDIATELY notify both 911 (if that had not already occurred) AND FWP Enforcement Division staff, who will determine whether a Wildlife Human Attack Response Team (WHART) should be convened to initiate a response following WHART Guidelines (Appendix 4).

    f. Record the location of Encounters, Incidents, and Attacks as specifically as possible, including physical address and/or geospatial coordinates.

    g. For Encounters, Incidents, or Attacks, record the behavior of the mountain lion and what, if any, action was taken on the part of the person involved.

    h. Record which FWP personnel responded to investigate, the time and date of the response, and what action(s) was taken.

2. A description of all reported conflict incidents, including the above information, will be entered into the designated FWP wildlife conflict database as soon as possible following receipt of the report. This record should be updated when the situation is resolved.

III. FWP ACTIONS TO BE TAKEN WHEN HUMAN-MOUNTAIN LION CONFLICTS ARE REPORTED

A FWP employee shall promptly investigate the validity, severity, and details of any reported human-mountain lion conflict. The following guidelines are the minimum actions required of FWP when conflicts are reported. Additional investigation into a conflict, or higher levels of response, will occur at the discretion of the Regional Supervisor and the investigating FWP employee. All interviews and investigations will begin no more than 48 hours after the conflict is reported in accordance with MCA 87-1-225.

CONFLICT	ACTIONS THAT WILL BE TAKEN
Encounter	The reporting party will be contacted and the details of the Encounter (Section II. (1)) will be documented. If the mountain lion involved in the conflict is determined to be a Nuisance Lion, the responding FWP employee and Regional Supervisor may choose to either haze (i.e. using less-than-lethal ammunition or pursued with trained dogs) or lethally remove the mountain lion(s). This decision will depend on the severity of the conflict, location, pattern of habituation, escalation of behavior, or other relevant factors. FWP may also issue a kill permit to the affected landowner. Mountain lions shall not be captured and translocated under any circumstances. Information about the Encounter and FWP's response will be

	recorded and entered into the FWP wildlife conflict database.
Incident Depredation	A FWP employee will conduct an on-site investigation to determine if the mountain lion involved in the conflict is Aggressive. All Aggressive mountain lions will be lethally removed as soon as is practical. If the mountain lion involved in the conflict is determined to be a Nuisance Lion, the responding FWP employee and Regional Supervisor may choose to either haze (i.e. using less-than-lethal ammunition or pursued with trained dogs) or lethally remove the mountain lion(s) depending on the severity of the conflict, location, pattern of habituation, escalation of behavior, or other relevant factors. FWP may also issue a kill permit to the affected landowner. Mountain lions shall not be captured and translocated under any circumstances. Information about the Encounter and FWP's response will be recorded and entered into the FWP wildlife conflict database.
Attack	The FWP employee receiving a report of an Attack will record the name, sex, and age of the victim, location, and the extent of any injuries. The employee will IMMEDIATELY notify both 911 (if that had not already occurred) AND FWP Enforcement Division staff, who will determine whether a Wildlife Human Attack Response Team should be convened and to initiate a response following WHART Guidelines. Measures to lethally remove the offending mountain lion(s) will be immediately initiated.<br><br>Montana law (MCA 87-6-106) gives private citizens the right to kill, without fear of penalty, any mountain lion attacking, killing, or threatening to kill a person or livestock. Private citizens may also kill a mountain lion that is in the act of attacking or killing a domestic dog. A person who kills a mountain lion under this statute must notify a FWP employee within 72 hours and surrender the carcass to FWP.
Livestock	If a Livestock Depredation is reported or suspected, the FWP employee will record the number and type of livestock involved, location, livestock owner's contact information, and number of mountain lions involved. The FWP employee will then immediately contact the USDA APHIS Wildlife Services agent with responsibility for the area where the incident occurred and convey that information. That Wildlife Services agent will be responsible for investigating the reported Livestock Depredation and determining the appropriate response.<br><br>Montana law (MCA 87-6-106) gives private citizens the right to kill, without fear of penalty, any mountain lion attacking, killing, or threatening to kill a person or livestock. Private citizens may also kill a mountain lion that is in the act of attacking or killing a domestic dog. A person who kills a mountain lion under this statute must notify a FWP employee within 72 hours and surrender the carcass to FWP.

These **Mountain Lion Depredation and Control Guideline**s are effective upon Fish and Wildlife Commission's adoption of this Strategy and supersede any previously-adopted versions.

APPENDIX 4

GUIDELINES FOR RESPONDING TO WILDLIFE ATTACKS THAT RESULT IN HUMAN INJURY OR DEATH: "WHART" GUIDELINES

(Note: attachments and appendices referenced in this section are available from FWP Enforcement Division, upon request)

INTRODUCTION:
This document will provide guidance in the process for handling responses to a wildlife attack that causes human injury or death. In order to provide guidance and standardize the response of FWP personnel, the following guidelines will direct their actions in dealing with wildlife attacks on humans that result in injury and/or death to human victims. It may not be possible to follow these guidelines in every situation.

FIRST RESPONDERS:
An immediate field response is required for any wildlife-caused human injury or death.

In the event of an attack, the responding department employee may take any action necessary that is in the scope of the employee's authority to protect public safety. The following steps should be taken:

1. Secure the safety of the public (ensure proper medical aid for the victim, aid with evacuation of injured or other members of a group, and assist other agencies in removal of the body or victim. Identify the victim's name, address and phone number).

2. Report the incident to 911.

3. Immediately notify the Regional FWP Enforcement Personnel and/or WHART Team personnel.

4. FWP Enforcement personnel confirm as wildlife attack and identify species if possible; if the offending animal is identified the wild animal may be humanely killed, if possible and depending on the circumstances. Always consult with WHART Team leader and Warden Captain if unsure of actions to be taken with offending animal.

5. If medical, rescue and/or sheriff department personnel arrive on scene before the FWP Incident Commander, advise them about the Wildlife Attack-Victim Kit (Attachment 1 (follow guidelines in Appendix B)) for collecting possible animal saliva stains or hair that might be on the victim prior to cleaning the victim's wounds.

INITIATE THE INCIDENT COMMAND SYSTEM:

- **If a human death or injury has occurred, the Region Warden Captain or other Enforcement designee shall:**

    - Respond to the scene and assume the lead role for FWP.

    - The County Sheriff's Office/Coroner has the initial lead in the investigation of a human death and at first FWP's role is that of assistance.

    - The Warden Captain or Enforcement designee holds FWP Incident Commander responsibility and authority over the scene, locating the animal, its resultant carcass, and any other physical evidence from the attack.

    - The Warden Captain or Enforcement designee will ensure proper collection, transfer, and disposition of all physical evidence and reports.

    - Contact the appropriate landownership, enforcement, and wildlife governing agencies. (refer to Inter Agency Jurisdiction Section)

- **The first warden on the scene shall secure the area in order:**

    1. To protect as much of the immediate attack scene as possible, establishing a perimeter as large as possible to avoid contamination or destruction of any evidence.

    2. To determine the offending animal and preserve as much on-scene evidence as possible.

    3. The area should be excluded from public access by using flagging tape and/or signing stating "Do Not Enter".

    4. To preserve the scene, one entry and exit port should be established; only essential personnel should be permitted in the area.

- **If a warden is the first Law Enforcement person on the scene of an attack:**

    1. Their first notification should be the County Sheriff's Office.

    2. If it appears the incident is an attack only and not a death then FWP will be the lead agency in the in the incident investigation.

    3. If it appears there is a human death the warden should advise the Sheriff's Office that a Coroner will be needed.

    4. In the case of a death it should be clear that FWP would at first be in an assisting role to the Sheriff's Office and the Coroner, but FWP's guidelines should be followed as closely as possible.

    5. In a human fatality FWP is the lead agency in processing and handling of the offending wildlife, if possible in coordination with County Sheriff/Coroner.

    6. Before the victim's body is removed and with the Coroners assistance it is important to use a Wildlife Attack -Victim Kit (Appendix B and Attachment 1) to collect any forensic evidence possible.

    7. The lead investigator must complete Attachment 5 and the investigator will need to work with the Coroner, in the case of a fatality, or the attending physician/medical personnel, in the case of an attack incident victim(s).

- **Once the Warden Captain or the Enforcement designee has been notified of an attack that resulted in human injury or death, he/she must:**

    1. Notify the FWP Regional Supervisor (who will notify the Directors Office), FWP Regional Wildlife Management Specialist, and Regional Wildlife Manager.

    2. Notify the Regional Information Officer to give him/her initial information; and once notified the Regional Information Officer will become the only contact with the media for FWP in regards to this incident.

- **Upon arrival on scene the Warden Captain or Enforcement designee will set up an area outside the initial crime scene as the Command Post.**

- **The Warden Captain or Enforcement designee will formulate a plan for the systematic investigation of the scene using available manpower and resources.**

- **If applicable, (not all FWP regions utilize this option) activate the Wildlife Human Attack Response Team (WHART).**

- **If applicable, the Enforcement designee, shall assume the role of WHART leader, and shall coordinate and delegate duties before attending the attack site and are responsible for the management of the attack scene from the FWP purview.**

- **WHART Team members will wear fluorescent vests with the Team leader wearing a different color fluorescent vest. These vests will designate the team to other individuals and aid in the safety of the team members while at the scene.**

At this time, with the information available, options should be discussed with the Regional Supervisor and Regional Wildlife Manager on what actions to take regarding the offending animal.

- The suggested approach to a systematic investigation would include:

    1. The Warden Captain, Enforcement designee, or WHART leader will appoint a lead investigator. The lead investigator will conduct the investigation and write a final report of their investigation findings. The lead investigator will be responsible for the investigation at the attack site. The lead investigator should have a team of at least three individuals to assist in evidence collection, securing the scene and photographing and logging of all evidence. One of those members should be the Wildlife Management Specialist or another person that is very experienced in wildlife behavior. The lead investigator shall refer to the "Forensic Guidelines/Wildlife attack Scene Investigation/ Management" (Appendix A) as a possible baseline to conduct their investigation and should have attended at least one Wildlife Human Attack Response Training Course. If necessary, the Warden Captain, Enforcement Designee, or WHART leader will appoint a lead person for the potential capture or kill of the offending animal. This person will have to rely on their experience/training and the resources available to locate the offending animal as quickly as possible. If necessary, the animal may be tranquilized, captured, held for DNA testing, or removed from the system. The animal should be shot in the body, to preserve the head. After capture, use the Wildlife Carcass Collection Kit (Appendix C & Attachment 4) and the Wildlife Attack Kit for Sampling the Animal and Evidence at the Scene (Appendix D & Attachment 4); and the listed Appendices are only suggested guides. The animal should be handled with rubber gloves. The animal must be treated as evidence and be handled to protect the animal's external body from loss of bloodstains or other such physical evidence originating from the victim. Tape paper or cloth bags over the head and paws. Plug wounds with tight gauze to minimize contamination of the animal with its own blood. Place the carcass inside a protective durable body bag. Avoid dragging the carcass, if possible.

    2. The Warden Captain, Enforcement Designee, or WHART leader will designate the task of notifying surrounding residences or persons of the event and safety concerns (usually wildlife biologists will be assigned this task). Land/ area closures will have to involve the agencies or owner of the property involved, but it is necessary to restrict public access to the area until the attack scene has been processed and the offending animal captured.

    3. The Warden Captain, Enforcement Designee, or WHART leader will notify the FWP Wildlife Lab of the attack and inform them that a potential offending animal will be transported as quickly as possible to the FWP Lab directly for forensic examination/necropsy. A completed Wildlife Attack Response Form and Animal Necropsy form (Appendix E & F) must accompany the animal to the lab.

    4. In a fatal incident, the Warden Captain and the Enforcement Designee or WHART leader will meet with the County Coroner/Sheriff, the Regional Supervisor, and the Regional Information Officer to decide how and who will approach the victim's family to gather information and to provide the family with investigation information.

5. In an attack incident, the Warden Captain, Enforcement Designee, or WHART leader will determine who will meet with the victim and family members in order to obtain investigative information and disseminate investigation information to the victim and family. All interviews will follow Attachment 2 and should be recorded when possible.

6. All media questions should be directed to the Regional Information Officer and the media will not be allowed on scene or at the Command Post.

7. Once evidence has been collected, photographed and logged (Attachment 3) it shall be placed into the custody of the Regional Investigator or designee, who will maintain the evidence and the chain of custody.

8. The Warden Captain, Enforcement designee, or WHART leader will keep a log of the events (Attachment 6) as they occurred at the Command Post and this will be included in the final report.

INFORMATION/MEDIA:

In conjunction with the wildlife attack response guidelines listed above, the following provides direction and guidance in handling the media in the event of an attack on a human by wildlife.

1. The Regional Information Officer (RIO) will be notified immediately in the event of an attack resulting in human injury from big game animals or any wildlife species. Complete and accurate information should be provided to the RIO and inquiries regarding the incident should be handled by the RIO or Regional Supervisor. Media consultation regarding human injuries resulting from federally listed grizzly bears will be coordinated with the USFWS.

   Incidents that result from interaction with other species of wildlife will be managed by personnel within the region where the incident occurred.

   County Sheriff/Coroner's offices will coordinate all media regarding status of human deaths. In the event of taking of federally listed species by a public citizen, the USFWS will coordinate all media responses.

2. Department personnel should be helpful and open with the media, but specific questions relating to the incident should be directed to the RIO. It is imperative that appropriate personnel with the region be kept current on developments and all involved receive the same information.

3. A fact sheet and/or statewide press release may be developed with information about the situation and provided upon request to media outlets.

4. If deemed necessary by the RIO, Regional Supervisor, Regional Wildlife Manager, and Warden Captain or Enforcement designee a press conference may be initiated.

5. Appropriate information will be made available to citizens in the vicinity of the incident upon request.

GUIDELINE TRAINING:

The Warden Captain or Enforcement designee is responsible for the distribution of the guidelines and annual training of employees that may be involved in wildlife attack incidents, including first responders.

The Warden Captain or Enforcement designee will assign employees to contact County Sheriff and Search and Rescue teams, and Land Management agencies and offer a review of the guidelines and training.

Employees' responding to attacks incidences, as investigators on the incident shall participate in at least one formal Wildlife Attack Response training each year. The FWP Law Enforcement Program Training Officer will approve these annual Wildlife Human Attack Response training sessions.

INTER-AGENCY JURISDICTION ISSUES:

U.S. Fish and Wildlife Service, Grizzly Bear Recovery Coordinator

U.S. Fish and Wildlife Service Special Agent – based upon their administrative region.

Land Management Agencies, Companies and Emergency Response Teams

The Warden Captain or Enforcement designee will delegate FWP personnel to work in advance with the US Forest Service, BLM, DNRC, Plum Creek Timber, and Search & Rescue Teams to arrange for FWP to enact temporary closures or post warnings to protect the public at a moment's notice as needed. This advanced contact will include an offer to review the guidelines with all contacts. As soon as possible thereafter, FWP would follow up with the agencies to keep them informed and address any issues or concerns. Search and Rescue Teams and other emergency response units should be kept abreast of special risks on recreational lands in the event that these teams are deployed while the risk of a dangerous bear encounter is elevated.

County Sheriff and Coroner

If an FWP employee is the first on the scene of an attack their first notification should be the County Sheriff's Office and if it appears there is a human death the employee should advise the Sheriff's Office that a Coroner will be needed. In the event of a human death, FWP will, at first, be in an assisting role to the Sheriff's Office and the Coroner, but FWP's guidelines should be followed as closely as possible. Before the victim's body is removed and with the Coroners assistance it is important to use a Wildlife Attack -Victim Kit (Attachment 1 & Attachment 5) to collect any forensic evidence possible.

FINAL REPORT:

The Warden Captain, Enforcement designee, or WHART leader is responsible for producing a final report. The report will include a detailed Investigative Summary of the events, how it was resolved, evidence and lab reports, and conclusions. The completed report will be reviewed and released in a timely manner by the Regional Supervisor.

Attachments and WHART Appendices (available from FWP Enforcement Division, upon request)

Attachment 1 – First Responder Kit Wildlife Attack Human Victim Kit

Attachment 2 – Interview with Victim and/or witness

Attachment 3 – Wildlife Attack Scene Evidence Log

Attachment 4 – Wildlife Attack Animal Evidence Collection Information

Attachment 5 – Wildlife Attack Victim Evidence Collection Information

Attachment 6 – Events/Contacts Log

Appendix A – Wildlife Attack Scene Investigations/ Management

Appendix B – Carnivore Attack Victim Sampling Kit

Appendix C – Carnivore Carcass Collection Kit

Appendix D – Carnivore Attack Animal Sampling Kit

Appendix E – Wildlife Attack Response Form

Appendix F – Wildlife Attack Animal Necropsy Form

APPENDIX 5

MONTANA MOUNTAIN LION LICENSE SALES, PRICE, AND REVENUE, 1973 - 2015

	1973	1974	1975	1976	1977	1978	1979	1980	1981	1982	1983
**License Type**											
Res. Mountain Lion	**241**	**259**	**286**	**517**	**574**	**639**	**614**	**787**	**893**	**1,027**	**1,021**
Nonres. Mountain Lion	**70**	**92**	**120**	**70**	**102**	**123**	**111**	**61**	**69**	**91**	**132**
**Total**	**311**	**351**	**406**	**587**	**676**	**762**	**725**	**848**	**962**	**1,118**	**1,153**
**Fees**											
Res. Mountain Lion	$5	$5	$5	$5	$5	$5	$5	$5	$5	$5	$5
Nonres. Mountain Lion	$25	$25	$25	$25	$25	$25	$25	$100	$100	$100	$100
**License Revenue**											
Res. Mountain Lion	$1,205	$1,295	$1,430	$2,585	$2,870	$3,195	$3,070	$3,935	$4,465	$5,135	$5,105
Nonres. Mountain Lion	$1,750	$2,300	$3,000	$1,750	$2,550	$3,075	$2,775	$6,100	$6,900	$9,100	$13,200
**Total**	$2,955	$3,595	$4,430	$4,335	$5,420	$6,270	$5,845	$10,035	$11,365	$14,235	$18,305

	1984	1985	1986	1987	1988	1989	1990	1991	1992	1993	1994
**License Type**											
Res. Mountain Lion	**984**	**1,045**	**916**	**1,237**	**1,210**	**1,250**	**1,708**	**1,687**	**2,038**	**2,535**	**2,984**
Nonres. Mountain Lion	**80**	**92**	**92**	**108**	**109**	**98**	**136**	**146**	**177**	**230**	**258**
**Total**	**1,064**	**1,137**	**1,008**	**1,345**	**1,319**	**1,348**	**1,844**	**1,833**	**2,215**	**2,765**	**3,242**
**Fees**											
Res. Mountain Lion	$10	$10	$10	$10	$10	$10	$10	$10	$13	$13	$15
Nonres. Mountain Lion	$300	$300	$300	$300	$320	$320	$320	$320	$320	$320	$320
**License Revenue**											
Res. Mountain Lion	$9,840	$10,450	$9,160	$12,370	$12,100	$12,500	$17,080	$16,870	$26,494	$32,955	$44,760
Nonres. Mountain Lion	$24,000	$27,600	$27,600	$32,400	$34,880	$31,360	$43,520	$46,720	$56,640	$73,600	$82,560
**Total**	$33,840	$38,050	$36,760	$44,770	$46,980	$43,860	$60,600	$63,590	$83,134	$106,555	$127,320

	1995	1996	1997	1998	1999	2000	2001	2002	2003	2004	2005
**License Type**											
Res. Mountain Lion	**3,056**	**3,287**	**4,297**	**5,421**	**5,886**	**5,138**	**5,116**	**6,337**	**6,130**	**6,635**	**6,688**
Nonres. Mountain Lion	**270**	**301**	**394**	**510**	**519**	**493**	**421**	**281**	**282**	**312**	**311**
Res. Hound Training									**207**	**289**	**340**
**Total**	**3,326**	**3,588**	**4,691**	**5,931**	**6,405**	**5,631**	**5,537**	**6,618**	**6,619**	**7,236**	**7,339**
**Fees**											
Res. Mountain Lion	$15	$15	$15	$15	$15	$15	$15	$15	$15	$15	$15
Nonres. Mountain Lion	$320	$320	$320	$320	$320	$320	$320	$320	$320	$320	$320
Res. Hound Training									$5	$5	$5
**License Revenue**											
Res. Mountain Lion	$45,840	$49,305	$64,455	$81,315	$88,290	$77,070	$76,740	$95,055	$91,950	$99,525	$100,320
Nonres. Mountain Lion	$86,400	$96,320	$126,080	$163,200	$166,080	$157,760	$134,720	$89,920	$90,240	$99,840	$99,520
Res. Hound Training									$1,035	$1,445	$1,700
**Total**	$132,240	$145,625	$190,535	$244,515	$254,370	$234,830	$211,460	$184,975	$183,225	$200,810	$201,540

	2006	2007	2008	2009	2010	2011	2012	2013	2014	2015	2016
**License Type**											
Res. Mountain Lion	**3,331**	**3,922**	**3,529**	**3,832**	**3,535**	**3,788**	**4,964**	**5,007**	**5,016**	**5,221**	**4,912**
Nonres. Mountain Lion	**133**	**145**	**167**	**179**	**170**	**172**	**182**	**286**	**240**	**292**	**271**
Res. Hound Training	**488**	**423**	**471**	**424**	**441**	**405**	**352**	**364**	**389**	**239**	**216**
**Total**	**3,952**	**4,490**	**4,167**	**4,435**	**4,146**	**4,365**	**5,498**	**5,657**	**5,645**	**5,752**	**5,399**
**Fees**											
Res. Mountain Lion	$19	$19	$19	$19	$19	$19	$19	$19	$19	$19	$19
Nonres. Mountain Lion	$320	$320	$320	$320	$320	$320	$320	$320	$320	$320	$320
Res. Hound Training	$5	$5	$5	$5	$5	$5	$5	$5	$5	$5	$5
**License Revenue**											
Res. Mountain Lion	$63,289	$74,518	$67,051	$72,808	$67,165	$71,972	$94,316	$95,133	$95,304	$99,199	$93,328
Nonres. Mountain Lion	$42,560	$46,400	$53,440	$57,280	$54,400	$55,040	$58,240	$91,520	$76,800	$93,440	$86,720
Res. Hound Training	$2,440	$2,115	$2,355	$2,120	$2,205	$2,025	$1,760	$1,820	$1,945	$1,195	$1,080
**Total**	$108,289	$123,033	$122,846	$132,208	$123,770	$129,037	$154,316	$188,473	$174,049	$193,834	$181,128

APPENDIX 6

APPLICABLE MONTANA STATUTE AND ADMINISTRATIVE RULES

Montana Code Annotated statutes and Administrative Rules of Montana describing FWP and the Fish & Wildlife Commission's authorities and responsibilities, regulation of the licensed hunting of mountain lions, enumeration of stock grower and personal protection rights, and disclosure of information.

2-15-3110. (Temporary) Livestock loss board - purpose, membership, and qualifications

(1) There is a livestock loss board. The purpose of the board is to administer the programs called for in the Montana gray wolf conservation and management plan, the Montana mountain lion management plan, and the Montana grizzly bear management plan and established in 2-15-3111 through 2-15-3113, with funds provided through the accounts established in 81-1-110, in order to minimize losses caused by wolves, mountain lions, and grizzly bears to livestock producers and to reimburse livestock producers for livestock losses from wolf, mountain lion, and grizzly bear predation.
(2) The board consists of five members, appointed by the governor, as follows:
(a) three members who are actively involved in the livestock industry and who have knowledge and experience with regard to wildlife impacts or management; and
(b) two members of the general public who are or have been actively involved in wildlife conservation or wildlife management and who have knowledge and experience with regard to livestock production or management.
(3) The board is designated as a quasi-judicial board for the purposes of 2-15-124. Notwithstanding the provisions of 2-15-124(1), the governor is not required to appoint an attorney to serve as a member of the board.
(4) The board is allocated to the department of livestock for administrative purposes only as provided in 2-15-121.
(5) The board shall adopt rules to implement the provisions of 2-15-3110 through 2-15-3114 and
(6) The board shall prioritize grants for prevention of wolf and grizzly bear predation over those for mountain lion predation.

2-15-3111. Livestock loss reduction program

The livestock loss board shall establish and administer a program to cost-share with individuals or incorporated entities in implementing measures to prevent wolf, mountain lion, and grizzly bear predation on livestock, including:
(1) eligibility requirements for program participation;
(2) application procedures for program participation and procedures for awarding grants for wolf, mountain lion, and grizzly bear predation prevention measures, subject to grant priorities and the availability of funds;
(3) criteria for the selection of projects and program participants, which may include establishment of grant priorities based on factors such as chronic depredation, multiple depredation incidents, single depredation incidents, and potential high-risk geographical or habitat location;
(4) grant guidelines for prevention measures on public and private lands, including:
(a) grant terms that clearly set out the obligations of the livestock producer and that provide for a term of up to 12 months subject to renewal based on availability of funds, satisfaction of program requirements, and prioritization of the project;
(b) cost-share for prevention measures, which may be a combination of grant and livestock producer responsibility, payable in cash or in appropriate services, such as labor to install or implement preventive measures, unless the board adjusts the cost-share because of extenuating circumstances related to chronic or multiple depredation; and
(c) proactive preventive measures, including but not limited to fencing, fladry, night penning, increased human presence in the form of livestock herders and riders, guard animals, providing hay and dog food, rental of private land or alternative pasture allotments, delayed turnouts, and other preventive measures as information on new or different successful prevent ion measures becomes available; and
(5) reporting requirements for program participants to assist in determining the effectiveness of loss reduction relative to each grant."

2-15-3112. Livestock loss mitigation program - definitions

The livestock loss board shall establish and administer a

program to reimburse livestock producers for livestock losses caused by wolves, mountain lions, and grizzly bears, subject to the following provisions:
(1) The board shall establish eligibility requirement s for reimbursement, which must provide that all Montana livestock producers are eligible for coverage for losses by wolves, mountain lions, and grizzly bears to cattle, swine, horses, mules, sheep, goats, llamas, and livestock guard animals on state, federal, and private land and on tribal land that is eligible through agreement pursuant to 2-15-3113(2).
(2) Confirmed and probable livestock losses must be reimbursed at an amount not to exceed fair market value as determined by the board.
(3) Other losses may be reimbursed at rates determined by the board.
(4) A claim process must be established to be used when a livestock producer suffers a livestock loss for which wolves, mountain lions, or grizzly bears may be responsible. The claim process must set out a clear and concise method for documenting and processing claims for reimbursement for livestock losses.
(5) A process must be established to allow livestock producers to appeal reimbursement decisions. A producer may appeal a staff adjuster's decision by notifying the staff adjuster and the board in writing, stating the reasons for the appeal and providing documentation supporting the appeal. If the documentation is incomplete, the board or a producer may consult with the U.S. department of agriculture wildlife services to complete the documentation. The board may not accept any appeal on the question of whether the loss was or was not a confirmed or probable loss because that final determination lies solely with the U.S. department of agriculture wildlife services and may not be changed by the board. The board shall hold a hearing on the appeal within 90 days of receipt of the written appeal, allowing the staff adjuster and the producer to present their positions. A decision must be rendered by the board within 30 days after the hearing. The producer must be notified in writing of the board's decision.
(6) As used in this section, the following definitions apply:
(a) "Confirmed" means reasonable physical evidence that livestock was actually attacked or killed by a wolf, mountain lion, or grizzly bear, including but not limited to the presence of bite marks indicative of the spacing of tooth punctures of wolves, mountain lions, or grizzly bears and associated subcutaneous hemorrhaging and tissue damage indicating that the attack occurred while the animal was alive, feeding patterns on the carcass, fresh tracks, scat, hair rubbed off on fences or brush, eyewitness accounts, or other physical evidence that allows a reasonable inference of wolf, mountain lion, or grizzly bear predation on an animal that has been largely consumed.
(b) "Fair market value" means:
(i) for commercial sheep more than 1 year old, the average price of sheep of similar age and sex paid at the most recent Billings livestock sale ring or other ring as determined by the board;
(ii) for commercial lambs, the average market weaning value;
(iii) for registered sheep, the average price paid to the specific breeder for sheep of similar age and sex during the past year at public or private sales for that registered breed;
(iv) for commercial cattle more than 1 year old, the average price of cattle of similar age and sex paid at the most recent Billings livestock sale ring or other ring as determined by the board;
(v) for commercial calves, the average market weaning value;
(vi) for registered cattle, the average price paid to the owner for cattle of similar age and sex during the past year at public or private sales for that registered breed;
(vii) for other registered livestock, the average price paid to the producer at public or private sales for animals of similar age and sex. A producer may provide documentation that a registered animal has a fair market value in excess of the average price, in which case the board shall seek additional verification of the value of the animal from independent sources. If the board determines that the value of that animal is greater than the average price, then the increased value must be accepted as the fair market value for that animal.
(viii) for other livestock, the average price paid at the most recent public auction for the type of animal lost or the replacement price as determined by the board.
(c) "Probable" means the presence of some evidence to suggest possible predation but a lack of sufficient evidence to clearly confirm predation by a particular species. A

kill may be classified as probable depending on factors including but not limited to recent confirmed predation by the suspected depredating species in the same or a nearby area, recent observation of the livestock by the owner or the owner's employees, and telemetry monitoring data, sightings, howling, or fresh tracks suggesting that the suspected depredating species may have been in the area when the depredation occurred."

2-15-3113. Additional powers and duties of livestock loss board

(1) The livestock loss board shall:
(a) process claims;
(b) seek information necessary to ensure that claim documentation is complete;
(c) provide payments authorized by the board for confirmed and probable livestock losses, along with a written explanation of payment;
(d) submit monthly and annual reports to the board of livestock summarizing claims and expenditures and the results of action taken on claims and maintain files of all claims received, including supporting documentation;
(e) provide information to the board of livestock regarding appealed claims and implement any decision by the board;
(f) prepare the annual budget for the board; and
(g) provide proper documentation of staff time and expenditures.
(2) The livestock loss board may enter into an agreement with any Montana tribe, if the tribe has adopted a wolf, mountain lion, or grizzly bear management plan for reservation lands that is consistent with the state wolf, mountain lion, or grizzly bear management plan, to provide that tribal lands within reservation boundaries are eligible for mitigation grants pursuant to 2-15-3111 and that livestock losses on tribal lands within reservation boundaries are eligible for reimbursement payments pursuant to 2-15-3112.
(3) The livestock loss board shall:
(a) coordinate and share information with state, federal, and tribal officials, livestock producers, nongovernmental organizations, and the general public in an effort to reduce livestock losses caused by wolves, mountain lions, and grizzly bears;
(b) establish an annual budget for the prevention, mitigation, and reimbursement of livestock losses caused by wolves, mountain lions, and grizzly bears;
(c) perform or contract for the performance of periodic program audits and reviews of program expenditures, including payments to Individuals, incorporated entities, and producers who receive loss reduction grants and reimbursement payments;
(d) adjudicate appeals of claims;
(e) investigate alternative or enhanced funding sources, including possible agreements with public entities and private wildlife or livestock organizations that have active livestock loss reimbursement programs in place;
(f) meet as necessary to conduct business; and
(g) report annually to the governor, the legislature, members of the Montana congressional delegation, the board of livestock, the fish and wildlife commission, and the public regarding results of the programs established in 2-15-3111 through 2-15-3113.
(4) The livestock loss board may sell or auction any carcasses or parts of carcasses from wolves or mountain lions received pursuant to 87-1-217. The proceeds, minus the costs of the sale including the preparation of the carcass or part of the carcass for sale, must be deposited into the livestock loss reduction and mitigation special revenue account established in 81-1-110 and used for the purposes of 215-3111 through 2-15-3114."

81-1-110. Livestock loss reduction and mitigation accounts

(1) There are livestock loss reduction and mitigation special revenue accounts administered by the department within the state special revenue fund and the federal special revenue fund established in 17-2-102.
(2)(a) All state proceeds allocated or budgeted for the purposes of 2-15-3110 through 2-15-3114, 81-1-110, and 81-1-111, except those transferred to the account provided for in 81-1-112 [or 81-1-113] or appropriated to the department of livestock, must be deposited in the state special revenue account provided for in subsection (1) of this section.
(b) Money received by the state in the form of gifts, grants, reimbursements, or allocations from any source intended to be used for the purposes of 2-15-3111 through 2-15-3113 must be deposited in the appropriate account provided for in subsection (1) of this section.
(c) All federal funds awarded to the state for compensation for wolf, mountain lion, or grizzly bear depredations on livestock must be deposited in the federal special revenue

account provided for in subsection (1) for the purposes of 2-15-3112.
(3) The livestock loss board may spend funds in the accounts only to carry out the provisions of 2-15-3111 through 2-15-3113.

<u>87-1-201. Powers And Duties</u>

(1) Except as provided in subsection (11), the department shall supervise all the wildlife, fish, game, game and nongame birds, waterfowl, and the game and fur-bearing animals of the state and may implement voluntary programs that encourage hunting access on private lands and that promote harmonious relations between landowners and the hunting public. The department possesses all powers necessary to fulfill the duties prescribed by law and to bring actions in the proper courts of this state for the enforcement of the fish and game laws and the rules adopted by the department.
(2) Except as provided in subsection (11), the department shall enforce all the laws of the state regarding the protection, preservation, management, and propagation of fish, game, fur-bearing animals, and game and nongame birds within the state.
(3) The department has the exclusive power to spend for the protection, preservation, management, and propagation of fish, game, fur-bearing animals, and game and nongame birds all state funds collected or acquired for that purpose, whether arising from state appropriation, licenses, fines, gifts, or otherwise. Money collected or received from the sale of hunting and fishing licenses or permits, from the sale of seized game or hides, from fines or damages collected for violations of the fish and game laws, or from appropriations or received by the department from any other sources is under the control of the department and is available for appropriation to the department.
(4) The department may discharge any appointee or employee of the department for cause at any time.
(5) The department may dispose of all property owned by the state used for the protection, preservation, management, and propagation of fish, game, fur-bearing animals, and game and nongame birds that is of no further value or use to the state and shall turn over the proceeds from the sale to the state treasurer to be credited to the fish and game account in the state special revenue fund.
(6) The department may not issue permits to carry firearms within this state to anyone except regularly appointed officers or wardens.
(7) Except as provided in subsection (11), the department is authorized to make, promulgate, and enforce reasonable rules and regulations not inconsistent with the provisions of Title 87, chapter 2, that in its judgment will accomplish the purpose of chapter 2.
(8) The department is authorized to promulgate rules relative to tagging, possession, or transportation of bear within or outside of the state.
(9) (a) The department shall implement programs that:
(i) manage wildlife, fish, game, and nongame animals in a manner that prevents the need for listing under 87-5-107 or under the federal Endangered Species Act, 16 U.S.C. 1531, et seq.;
(ii) manage listed species, sensitive species, or a species that is a potential candidate for listing under 87-5-107 or under the federal Endangered Species Act, 16 U.S.C. 1531, et seq., in a manner that assists in the maintenance or recovery of those species;
(iii) manage elk, deer, and antelope populations based on habitat estimates determined as provided in 87-1-322 and maintain elk, deer, and antelope population numbers at or below population estimates as provided in 87-1-323. In implementing an elk management plan, the department shall, as necessary to achieve harvest and population objectives, request that land management agencies open public lands and public roads to public access during the big game hunting season.
(iv) in accordance with the forest management plan required by 87-1-622, address fire mitigation, pine beetle infestation, and wildlife habitat enhancement giving priority to forested lands in excess of 50 contiguous acres in any state park, fishing access site, or wildlife management area under the department's jurisdiction.
(b) In maintaining or recovering a listed species, a sensitive species, or a species that is a potential candidate for listing, the department shall seek, to the fullest extent possible, to balance maintenance or recovery of those species with the social and economic impacts of species maintenance or recovery.
(c) Any management plan developed by the department pursuant to this subsection (9) is subject to the requirements of Title 75, chapter 1, part 1.

(d) This subsection (9) does not affect the ownership or possession, as authorized under law, of a privately held listed species, a sensitive species, or a species that is a potential candidate for listing.
(10) The department shall publish an annual game count, estimating to the department's best ability the numbers of each species of game animal, as defined in 87-2-101, in the hunting districts and administrative regions of the state. In preparing the publication, the department may incorporate field observations, hunter reporting statistics, or any other suitable method of determining game numbers. The publication must include an explanation of the basis used in determining the game count.
(11) The department may not regulate the use or possession of firearms, firearm accessories, or ammunition, including the chemical elements of ammunition used for hunting. This does not prevent:
(a) the restriction of certain hunting seasons to the use of specified hunting arms, such as the establishment of special archery seasons;
(b) for human safety, the restriction of certain areas to the use of only specified hunting arms, including bows and arrows, traditional handguns, and muzzle loading rifles;
(c) the restriction of the use of shotguns for the hunting of deer and elk pursuant to 87-6-401(1)(f);
(d) the regulation of migratory game bird hunting pursuant to 87-3-403; or
(e) the restriction of the use of rifles for bird hunting pursuant to 87-6-401(1)(g) or (1)(h).

87-1-214. Disclosure Of Information - Legislative Finding - Large Predators

(1) Except for information that is required by law to be reported to state or federal officials, the department may not disclose any information that identifies any person who has lawfully taken a large predator as defined in 87-1-217 during a hunt without the written consent of the person affected. Information that may not be disclosed includes but is not limited to a person's name, address, phone number, date of birth, social security number, and driver's license number.
(2) The legislature finds that the prohibition on disclosure of information pursuant to subsection (1) is necessary to protect an individual's privacy, safety, and welfare.

87-1-217. Policy For Management Of Large Predators - Legislative Intent
(1) In managing large predators, the primary goals of the department, in the order of listed priority, are to:
(a) protect humans, livestock, and pets;
(b) preserve and enhance the safety of the public during outdoor recreational and livelihood activities; and
(c) preserve citizens' opportunities to hunt large game species.
(2) With regard to large predators, it is the intent of the legislature that the specific provisions of this section concerning the management of large predators will control the general supervisory authority of the department regarding the management of all wildlife.
(3) For the management of wolves in accordance with the priorities established in subsection (1), the department may use lethal action to take problem wolves that attack livestock if the state objective for breeding pairs has been met. For the purposes of this subsection, "problem wolves" means any individual wolf or pack of wolves with a history of livestock predation.
(4) The department shall work with the livestock loss board and the United States department of agriculture wildlife services to establish the conditions under which carcasses or parts of carcasses from wolves or mountain lions are retrieved during management activities and when those carcasses or parts of carcasses are made available to the livestock loss board for sale or auction pursuant to 2-15-3113.
(5) The department shall ensure that county commissioners and tribal governments in areas that have identifiable populations of large predators have the opportunity for consultation and coordination with state and federal agencies prior to state and federal policy decisions involving large predators and large game species.
(6) As used in this section:
(a) "consultation" means to actively provide information to a county or tribal government regarding proposed policy decisions on matters that may have a harmful effect on agricultural production or livestock operations or that may pose a risk to human health or safety in that county or on those tribal lands and to seek information and advice from counties or tribal governments on these matters;
(b) "large game species" means deer, elk, mountain sheep, moose, antelope, and mountain goats; and

(c) “large predators” means bears, mountain lions, and wolves.

87-1-225. Regulation of Wild Animals Damaging Property - Public Hunting Requirements

(1) Subject to the provisions of subsection (2), a landowner is eligible for game damage assistance under subsection (3) if the landowner:
(a) allows public hunting during established hunting seasons; or
(b) does not significantly reduce public hunting through imposed restrictions.
(2) The department may provide game damage assistance when public hunting on a landowner’s property has been denied because of unique or special circumstances that have rendered public hunting inappropriate.
(3) Within 48 hours after receiving a request or complaint from any landholder or person in possession and having charge of any land in the state that wild animals of the state, protected by the fish and game laws and regulations, are doing damage to the property or crops on the property, the department shall investigate and arrange to study the situation with respect to damage and depredation. The department may then decide to open a special season on the game or, if the special season method is not feasible, the department may destroy the animals causing the damage. The department may authorize and grant the holders of the property permission to kill or destroy a specified number of the animals causing the damage. A wild, ferocious animal damaging property or endangering life is not covered by this section.

87-1-271. Annual Lottery Of Hunting Licenses - Proceeds Dedicated To Hunting Access Enhancement

(1) The commission may issue through a lottery one license each year for each of the following:
(a) deer;
(b) elk;
(c) shiras moose;
(d) mountain sheep;
(e) mountain goat;
(f) wild buffalo or bison;
(g) antelope; and
(h) mountain lion.
(2) The restriction in 87-2-702(4) that a person who receives a moose, mountain goat, or mountain sheep special license is not eligible to receive another license for that species for the next 7 years does not apply to a person who receives a license through a lottery conducted pursuant to this section.
(3) The commission shall establish rules regarding:
(a) the conduct of the lottery authorized in this section;
(b) the use of licenses issued through the lottery; and
(c) the price of lottery tickets.
(4) Except as provided in 87-2-903, all proceeds from a lottery conducted pursuant to this section must be used by the department for hunting access enhancement programs and law enforcement.

87-1-301. Powers Of Commission

(1) Except as provided in subsections (7) and (8), the commission:
(a) shall set the policies for the protection, preservation, management, and propagation of the wildlife, fish, game, furbearers, waterfowl, nongame species, and endangered species of the state and for the fulfillment of all other responsibilities of the department related to fish and wildlife as provided by law;
(b) shall establish the hunting, fishing, and trapping rules of the department;
(c) except as provided in 23-1-111 and 87-1-303(3), shall establish the rules of the department governing the use of lands owned or controlled by the department and waters under the jurisdiction of the department;
(d) must have the power within the department to establish wildlife refuges and bird and game preserves;
(e) shall approve all acquisitions or transfers by the department of interests in land or water, except as provided in 23-1-111 and 87-1-209(2) and (4);
(f) except as provided in 23-1-111, shall review and approve the budget of the department prior to its transmittal to the office of budget and program planning;
(g) except as provided in 23-1-111, shall review and approve construction projects that have an estimated cost of more than $1,000 but less than $5,000;
(h) shall manage elk, deer, and antelope populations based on habitat estimates determined as provided in 87-1-322 and maintain elk, deer, and antelope population numbers at or below population estimates as provided in 87-1-323. In developing or implementing an elk management plan,

the commission shall consider landowner tolerance when deciding whether to restrict elk hunting on surrounding public land in a particular hunting district. As used in this subsection (1)(h), "landowner tolerance" means the written or documented verbal opinion of an affected landowner regarding the impact upon the landowner's property within the particular hunting district where a restriction on elk hunting on public property is proposed.
(i) shall set the policies for the salvage of antelope, deer, elk, or moose pursuant to 87-3-145; and
(j) shall comply with, adopt policies that comply with, and ensure the department implements in each region the provisions of state wildlife management plans adopted following an environmental review conducted pursuant to Title 75, chapter 1, parts 1 through 3.
(2) The commission may adopt rules regarding the use and type of archery equipment that may be employed for hunting and fishing purposes, taking into account applicable standards as technical innovations in archery equipment change.
(3) The commission may adopt rules regarding the establishment of special licenses or permits, seasons, conditions, programs, or other provisions that the commission considers appropriate to promote or enhance hunting by Montana's youth and persons with disabilities.
(4) (a) The commission may adopt rules regarding nonresident big game combination licenses to:
(i) separate deer licenses from nonresident elk combination licenses;
(ii) set the fees for the separated deer combination licenses and the elk combination licenses without the deer tag;
(iii) condition the use of the deer licenses; and
(iv) limit the number of licenses sold.
(b) The commission may exercise the rulemaking authority in subsection (4)(a) when it is necessary and appropriate to regulate the harvest by nonresident big game combination license holders:
(i) for the biologically sound management of big game populations of elk, deer, and antelope;
(ii) to control the impacts of those elk, deer, and antelope populations on uses of private property; and
(iii) to ensure that elk, deer, and antelope populations are at a sustainable level as provided in 87-1-321 through 87-1-325.
(5) (a) Subject to the provisions of 87-2-115, the commission may adopt rules establishing license preference systems to distribute hunting licenses and permits:
(i) giving an applicant who has been unsuccessful for a longer period of time priority over an applicant who has been unsuccessful for a shorter period of time; and
(ii) giving a qualifying landowner a preference in drawings. As used in this subsection (5)(a), "qualifying landowner" means the owner of land that provides some significant habitat benefit for wildlife, as determined by the commission.
(b) The commission shall square the number of points purchased by an applicant per species when conducting drawings for licenses and permits.
(6) (a) The commission may adopt rules to:
(i) limit the number of nonresident mountain lion hunters in designated hunting districts; and
(ii) determine the conditions under which nonresidents may hunt mountain lion in designated hunting districts.
(b) The commission shall consider, but is not limited to consideration of, the following factors:
(i) harvest of lions by resident and nonresident hunters;
(ii) history of quota overruns;
(iii) composition, including age and sex, of the lion harvest;
(iv) historical outfitter use;
(v) conflicts among hunter groups;
(vi) availability of public and private lands; and
(vii) whether restrictions on nonresident hunters are more appropriate than restrictions on all hunters.
(7) The commission may not regulate the use or possession of firearms, firearm accessories, or ammunition, including the chemical elements of ammunition used for hunting. This does not prevent:
(a) the restriction of certain hunting seasons to the use of specified hunting arms, such as the establishment of special archery seasons;
(b) for human safety, the restriction of certain areas to the use of only specified hunting arms, including bows and arrows, traditional handguns, and muzzle loading rifles;
(c) the restriction of the use of shotguns for the hunting of deer and elk pursuant to 87-6-401(1)(f);
(d) the regulation of migratory game bird hunting pursuant to 87-3-403; or
(e) the restriction of the use of rifles for bird hunting pursuant to 87-6-401(1)(g) or (1)(h).

(8) Pursuant to 23-1-111, the commission does not oversee department activities related to the administration of state parks, primitive parks, state recreational areas, public camping grounds, state historic sites, state monuments, and other heritage and recreational resources, land, and water administered pursuant to Title 23, chapter 1, and Title 23, chapter 2, parts 1, 4, and 9.

87-1-304. Fixing Of Seasons And Bag And Possession Limits

(1) Subject to the provisions of 87-5-302 and subsection (7) of this section, the commission may:
(a) fix seasons, bag limits, possession limits, and season limits;
(b) open or close or shorten or lengthen seasons on any species of game, bird, fish, or fur-bearing animal as defined by 87-2-101;
(c) declare areas open to the hunting of deer, antelope, elk, moose, sheep, goat, mountain lion, bear, wild buffalo or bison, and wolf by persons holding an archery stamp and the required license, permit, or tag and designate times when only bows and arrows may be used to hunt deer, antelope, elk, moose, sheep, goat, mountain lion, bear, wild buffalo or bison, and wolf in those areas;
(d) subject to the provisions of 87-1-301(7), restrict areas and species to hunting with only specified hunting arms, including bow and arrow, for the reasons of safety or of providing diverse hunting opportunities and experiences; and
(e) declare areas open to special license holders only and issue special licenses in a limited number when the commission determines, after proper investigation, that a special season is necessary to ensure the maintenance of an adequate supply of game birds, fish, or animals or fur-bearing animals. The commission may declare a special season and issue special licenses when game birds, animals, or fur-bearing animals are causing damage to private property or when a written complaint of damage has been filed with the commission by the owner of that property. In determining to whom special licenses must be issued, the commission may, when more applications are received than the number of animals to be killed, award permits to those chosen under a drawing system. The procedures used for awarding the permits from the drawing system must be determined by the commission.
(2) The commission may adopt rules governing the use of livestock and vehicles by archers during special archery seasons.
(3) Subject to the provisions of 87-5-302 and subsection (7) of this section, the commission may divide the state into fish and game districts and create fish, game, or fur-bearing animal districts throughout the state. The commission may declare a closed season for hunting, fishing, or trapping in any of those districts and later may open those districts to hunting, fishing, or trapping.
(4) The commission may declare a closed season on any species of game, fish, game birds, or fur-bearing animals threatened with undue depletion from any cause. The commission may close any area or district of any stream, public lake, or public water or portions thereof to hunting, trapping, or fishing for limited periods of time when necessary to protect a recently stocked area, district, water, spawning waters, spawn-taking waters, or spawn-taking stations or to prevent the undue depletion of fish, game, fur-bearing animals, game birds, and nongame birds. The commission may open the area or district upon consent of a majority of the property owners affected.
(5) The commission may authorize the director to open or close any special season upon 12 hours' notice to the public.
(6) The commission may declare certain fishing waters closed to fishing except by persons under 15 years of age. The purpose of this subsection is to provide suitable fishing waters for the exclusive use and enjoyment of juveniles under 15 years of age, at times and in areas the commission in its discretion considers advisable and consistent with its policies relating to fishing.
(7) In an area immediately adjacent to a national park, the commission may not:
(a) prohibit the hunting or trapping of wolves; or
(b) close the area to wolf hunting or trapping unless a wolf harvest quota established by the commission for that area has been met.

87-2-101. Definitions

As used in Title 87, chapter 3, and this chapter, unless the context clearly indicates otherwise, the following definitions apply:
(1) "Angling" or "fishing" means to take or the act of a person possessing any instrument, article, or substance for

the purpose of taking fish in any location that a fish might inhabit.
(2) (a) “Bait” means any animal matter, vegetable matter, or natural or artificial scent placed in an area inhabited by wildlife for the purpose of attracting game animals or game birds.
(b) The term does not include:
(i) decoys, silhouettes, or other replicas of wildlife body forms;
(ii) scents used only to mask human odor; or
(iii) types of scents that are approved by the commission for attracting game animals or game birds.
(3) “Fur-bearing animals” means marten or sable, otter, muskrat, fisher, mink, bobcat, lynx, wolverine, northern swift fox, and beaver.
(4) “Game animals” means deer, elk, moose, antelope, caribou, mountain sheep, mountain goat, mountain lion, bear, and wild buffalo.
(5) “Game fish” means all species of the family Salmonidae (chars, trout, salmon, grayling, and whitefish); all species of the genus Sander (sandpike or sauger and walleyed pike or yellowpike perch); all species of the genus Esox (northern pike, pickerel, and muskellunge); all species of the genus Micropterus (bass); all species of the genus Polyodon (paddlefish); all species of the family Acipenseridae (sturgeon); all species of the genus Lota (burbot or ling); the species Perca flavescens (yellow perch); all species of the genus Pomoxis (crappie); and the species Ictalurus punctatus (channel catfish).
(6) “Hunt” means to pursue, shoot, wound, kill, chase, lure, possess, or capture or the act of a person possessing a weapon, as defined in 45-2-101, or using a dog or a bird of prey for the purpose of shooting, wounding, killing, possessing, or capturing wildlife protected by the laws of this state in any location that wildlife may inhabit, whether or not the wildlife is then or subsequently taken. The term includes an attempt to take by any means, including but not limited to pursuing, shooting, wounding, killing, chasing, luring, possessing, or capturing.
(7) “Migratory game birds” means waterfowl, including wild ducks, wild geese, brant, and swans; cranes, including little brown and sandhill; rails, including coots; Wilson’s snipes or jacksnipes; and mourning doves.
(8) “Nongame wildlife” means any wild mammal, bird, amphibian, reptile, fish, mollusk, crustacean, or other animal not otherwise legally classified by statute or regulation of this state.
(9) “Open season” means the time during which game birds, game fish, game animals, and fur-bearing animals may be lawfully taken.
(10) “Person” means an individual, association, partnership, or corporation.
(11) “Predatory animals” means coyote, weasel, skunk, and civet cat.
(12) “Trap” means to take or participate in the taking of any wildlife protected by the laws of the state by setting or placing any mechanical device, snare, deadfall, pit, or device intended to take wildlife or to remove wildlife from any of these devices.
(13) “Upland game birds” means sharp-tailed grouse, blue grouse, spruce (Franklin) grouse, prairie chicken, sage hen or sage grouse, ruffed grouse, ring-necked pheasant, Hungarian partridge, ptarmigan, wild turkey, and chukar partridge.
(14) “Wild buffalo” means buffalo or bison that have not been reduced to captivity.

87-2-506. Restrictions On Hunting Licenses

Restrictions on hunting licenses. (1) The department may prescribe by rule the number of hunting licenses to be issued. Any license sold may be restricted to a specific administrative region, hunting district, or other designated area and may specify the species, age, and sex to be taken and the time period for which the license is valid.
(2) When the number of valid resident applications for big game licenses or permits of a single class or type exceeds the number of licenses or permits the department desires to issue in an administrative region, hunting district, or other designated area, then the number of big game licenses or permits issued to nonresident license or permit holders in the region, district, or area may not exceed 10% of the total issued.
(3) Disabled veterans who meet the qualifying criteria provided in 87-2-817(1) must be provided a total of 50 Class A-3 deer A tags, 50 Class A-4 deer B tags, 50 Class B-7 deer A tags, 50 Class B-8 deer B tags, and 50 special antelope licenses annually, which may be used within the administrative region, hunting district, or other designated area of the disabled veteran’s choice, except in a region, district, or area where the number of licenses are less than

the number of applicants, in which case qualifying disabled veterans are eligible for no more than 10% of the total licenses for that region, district, or area.

87-2-507. Class D-1-Nonresident Mountain Lion License

Except as otherwise provided in this chapter, a person who is not a resident, as defined in 87-2-102, but who is 12 years of age or older or who will turn 12 years old before or during the season for which the license is issued may, upon payment of a fee of $320, receive a Class D-1 license that entitles a holder who is 12 years of age or older to hunt mountain lion and possess the carcass of the mountain lion as authorized by department rules.

87-2-508. Class D-2-Resident Mountain Lion License

Except as otherwise provided in this chapter, a person who is a resident, as defined in 87-2-102, and who is 12 years of age or older or who will turn 12 years old before or during the season for which the license is issued may, upon payment of a fee of $19, receive a Class D-2 license that entitles a holder who is 12 years of age or older to hunt mountain lion and possess the carcass of the mountain lion as authorized by department rules.

87-2-521. Class D-3-Resident Hound Training License

A person who is a resident, as defined in 87-2-102, and who is 12 years of age or older or who will turn 12 years old before or during the season for which the license is issued, upon payment of a fee of $5, may receive a Class D-3 hound training license that entitles the holder to use a dog or dogs to aid in pursuing mountain lions or bobcats during the training season established in 87-6-404(4).

87-2-702. Restrictions On Special Licenses - Availability Of Bear And Mountain Lion Licenses

(1) A person who has killed or taken any game animal, except a deer, an elk, or an antelope, during the current license year is not permitted to receive a special license under this chapter to hunt or kill a second game animal of the same species.
(2) The commission may require applicants for special permits authorized by this chapter to obtain a valid big game license for that species for the current year prior to applying for a special permit.
(3) Except as provided in 87-2-815, a person may take only one grizzly bear in Montana with a license authorized by 87-2-701.
(4) (a) Except as provided in 87-1-271(2) and 87-2-815, a person who receives a moose, mountain goat, or limited mountain sheep license, as authorized by 87-2-701, with the exception of an antlerless moose or an adult ewe game management license issued under 87-2-104, is not eligible to receive another special license for that species for the next 7 years. For the purposes of this subsection (4)(a), “limited mountain sheep license” means a license that is valid for an area in which the number of licenses issued is restricted.
(b) Except as provided in 87-1-271(2) and 87-2-815, a person who takes a mountain sheep using an unlimited mountain sheep license, with the exception of a mountain sheep taken pursuant to an adult ewe license, as authorized by 87-2-701, is not eligible to receive another special license for that species for the next 7 years. For the purposes of this subsection (4)(b), “unlimited mountain sheep license” means a license that is valid for an area in which the number of licenses issued is not restricted.
(5) An application for a wild buffalo or bison license must be made on the same form and is subject to the same license application deadline as the special license for moose, mountain goat, and mountain sheep.
(6) (a) Licenses for spring bear hunts must be available for purchase at department offices after April 15 of any license year. However, a person who purchases a license for a spring bear hunt after April 15 of any license year may not use the license until 24 hours after the license is issued.
(b) Licenses for fall bear hunts must be available for purchase at department offices after August 31 of any license year. However, a person who purchases a license for a fall bear hunt after August 31 of any license year may not use the license until 24 hours after the license is issued.
(7) Licenses for mountain lion hunts must be available for purchase at department offices after August 31 of any license year. However, a person who purchases a license for a mountain lion hunt after August 31 of any license year may not use the license until 5 days after the license is issued.

87-2-806. Taking Fish Or Game For Scientific Purposes

(1) An accredited representative of an accredited school, college, university, or other institution of learning or

of any governmental agency or an individual who is investigating a scientific subject for which collection is necessary, may take, kill, capture, and possess for that purpose any birds, fish, or animals protected by Montana law or department or commission rule if a permit to collect is authorized by the department. Under the provisions of this section, a permittee may take, kill, and capture protected or unprotected birds, fish, or animals in any way that is approved by the department, except by the use of explosives. A permittee may not take, kill, or capture more birds, fish, or animals than are necessary for the investigation. A collection permit may not be given for a species for which a taking is prohibited by statute or rule.
(2) A person who desires to engage in the scientific investigation shall apply to the department for a permit. The department may require the applicant to submit a plan of operations that includes the purpose for the collection, collection methodology to be employed, and the qualifications of the person who will be doing the collecting. The department may set qualifications for persons to whom permits are issued and may place special authorizations or special requirements and limitations on any permit. If the department is satisfied of the good faith and qualifications of the applicant and that the collecting is necessary for a valid purpose, the department:
(a) may issue a permit that must place a time limit on the collections and may place a restriction on the number of birds, fish, or animals to be taken; and
(b) shall require a report of the numbers and species of animals taken by collection areas.
(3) The department may deny a permit if:
(a) the applicant is not qualified to make the scientific investigation;
(b) the proposed collecting is not necessary for the proposed scientific investigation;
(c) the method of collecting is not appropriate;
(d) the proposed collecting may threaten the viability of the species; or
(e) there is no valid reason or need for the proposed scientific investigation.
(4) By December 31 of each year, a permittee shall submit a report to the department that lists the species and numbers of individuals of the species taken and locations from which collections were taken. A permittee who fails to file a required report may not be issued another permit.
(5) The permittee shall pay $50 for the permit, except that a permittee who is a representative of an accredited school, college, university, or other institution of learning or of any governmental agency is exempt from payment of the fee.
(6) The permittee may not take, have, or capture any other or greater number of birds, fish, or animals than are allowed in the permit.
(7) A representative of an accredited school, college, university, or other institution of learning or an individual permittee who may have various students or associates assisting throughout the year may apply to have a permit issued that includes the individual and the students or associates. The department shall approve the qualifications of a student or an associate and the level of supervision required by the primary permittee. The students or associates, when carrying a copy of the permit, have the same authorizations and restrictions as the primary applicant. The primary applicant shall keep a record of all students or associates listed on the permit and of the dates when each student or associate conducts a collection under the permit. The primary applicant is responsible for the students' or associates' use of the permit or copies of the permit.

87-3-127. Taking Of Stock-killing Animals

(1) Livestock owners, their agents, or employees of the department or a federal agency may use dogs in pursuit of stock-killing black bears, stock-killing mountain lions, and stock-killing bobcats. Other means of taking stock-killing black bears, stock-killing mountain lions, and stock-killing bobcats may be used, except the deadfall.
(2) Traps used in capturing bears must be inspected twice each day with the inspections 12 hours apart.

87-3-128. Exceptions - Department Personnel

The provisions of this chapter relating to methods of herding, driving, capturing, taking, locating, or concentrating of fish, game animals, game birds, or fur-bearing animals do not apply to the department or to any employee thereof while acting within the scope and course of the powers and duties of the department.

87-5-713. Control Of Wildlife Species Permitted To Be Transplanted Or Introduced

Any wildlife species listed in 87-5-714 or approved by the commission for introduction or transplantation may be introduced or transplanted only subject to a plan developed by the department to assure that the population can be controlled if any unforeseen harm should occur.

87-5-725. Notification Of Transplantation Or Introduction Of Wildlife

Notification of transplantation or introduction of wildlife.
(1) When the decision to introduce or transplant a wolf, bear, or mountain lion is made pursuant to this part, the department shall:
(a) provide public notice on its website and, when practical, by personal contact in the general area where the animal is released; and
(b) notify the public through print and broadcast media of the availability of release information on the department's website.
(2) Prior permission from the landowner is required before any animal may be transplanted onto private property.

87-6-106. Lawful Taking To Protect Livestock Or Person

(1) This chapter may not be construed to impose, by implication or otherwise, criminal liability for the taking of wildlife protected by this title if the wildlife is attacking, killing, or threatening to kill a person or livestock. However, for purposes of protecting livestock, a person may not kill or attempt to kill a grizzly bear unless the grizzly bear is in the act of attacking or killing livestock.
(2) A person may kill or attempt to kill a wolf or mountain lion that is in the act of attacking or killing a domestic dog.
(3) A person who, under this section, takes wildlife protected by this title shall notify the department within 72 hours and shall surrender or arrange to surrender the wildlife to the department.

87-6-404. Unlawful Use Of Dog While Hunting

(1) Except as provided in subsections (3) through (6), a person may not:
(a) chase any game animal or fur-bearing animal with a dog; or
(b) purposely, knowingly, or negligently permit a dog to chase, stalk, pursue, attack, or kill a hooved game animal. If the dog is not under the control of an adult at the time of the violation, the owner of the dog is personally responsible. A defense that the dog was allowed to run at large by another person is not allowable unless it is shown that at the time of the violation, the dog was running at large without the consent of the owner and that the owner took reasonable precautions to prevent the dog from running at large.
(2) Except as provided in subsection (3)(d), a peace officer, game warden, or other person authorized to enforce the Montana fish and game laws who witnesses a dog chasing, stalking, pursuing, attacking, or killing a hooved game animal may destroy that dog on public land or on private land at the request of the landowner without criminal or civil liability.
(3) A person may:
(a) take game birds during the appropriate open season with the aid of a dog;
(b) hunt mountain lions during the winter open season, as established by the commission, with the aid of a dog or dogs;
(c) hunt bobcats during the trapping season, as established by the commission, with the aid of a dog or dogs; and
(d) use trained or controlled dogs to chase or herd away game animals or fur-bearing animals to protect humans, lawns, gardens, livestock, or agricultural products, including growing crops and stored hay and grain. The dog may not be destroyed pursuant to subsection (2).
(4) A resident who possesses a Class D-3 resident hound training license may pursue mountain lions and bobcats with a dog or dogs during a training season from December 2 of each year to April 14 of the following year.
(5) (a) A person with a valid hunting license issued pursuant to Title 87, chapter 2, may use a dog to track a wounded game animal during an appropriate open season. Any person using a dog in this manner:
(i) shall maintain physical control of the dog at all times by means of a maximum 50-foot lead attached to the dog's collar or harness;
(ii) during the general season, whether handling or accompanying the dog, shall wear hunter orange material pursuant to 87-6-414;
(iii) may carry any weapon allowed by law;
(iv) may dispose of the wounded game animal using any weapon allowed by the valid hunting license; and
(v) shall tag an animal that has been reduced to possession in accordance with 87-6-411.

(b) Dog handlers tracking a wounded game animal with a dog are exempt from licensing requirements under Title 87, chapter 2, as long as they are accompanied by the licensed hunter who wounded the game animal.
(6) Any person or association organized for the protection of game may run field trials at any time upon obtaining written permission from the director.
(7) A person who is convicted of or who forfeits bond or bail after being charged with a violation of this section shall be fined not less than $50 or more than $1,000 or be imprisoned in the county detention center for not more than 6 months, or both. In addition, the person, upon conviction or forfeiture of bond or bail, may be subject to forfeiture of any current hunting, fishing, or trapping license issued by this state and the privilege to hunt, fish, and trap in this state or to use state lands, as defined in 77-1-101, for recreational purposes for a period of time set by the court.
(8) A violation of this section may also result in an order to pay restitution pursuant to 87-6-905 through 87-6-907.

87-6-701. Failure To Report Or Tattoo

Failure to report or tattoo. (1) Any bear, wolf, tiger, mountain lion, or coyote that is captured alive to be released later or that is held in captivity for any purpose must be reported to the department within 3 days of the capture or commencement of captivity.
(2) Each animal reported as required in subsection (1) must be permanently tattooed or otherwise permanently identified in a manner that will provide positive individual identification of the animal. No tattoo is required if the animal is subject to a permanent, individual identification process by another state or federal agency.
(3) Any person holding a bear, wolf, tiger, mountain lion, or coyote in captivity shall immediately report to the department any death, escape, release, transfer of custody, or other disposition of the animal.
(4) A person convicted of a violation of this section shall be fined not less than $50 or more than $1,000 or be imprisoned in the county detention center for not more than 6 months, or both. In addition, the person, upon conviction or forfeiture of bond or bail, may be subject to forfeiture of any current hunting, fishing, or trapping license issued by this state and the privilege to hunt, fish, or trap in this state or to use state lands, as defined in 77-1-101, for recreational purposes for a period of time set by the court.

ADMINISTRATIVE RULES OF MONTANA

12.3.105 Limitation On Number Of Hunting Licenses

(1) When the department sets a limitation or quota for the number of hunting licenses to be issued in any hunting district or other designated area, resident applicants shall receive at least 90% of the total hunting licenses to be issued for that game species in that district. When the number of resident applicants totals less than 90% of the quota for that district, all resident applicants shall receive a hunting license for that game species.
(2) The remaining licenses will be issued to the nonresident applicants for that district by drawing.
(3) Any thereafter remaining licenses for that district shall be issued in such manner as the director determines.

12.3.111. License/Permit Prerequisites

(1) Deer. All valid resident conservation license holders and all valid nonresident big game (class B-10) and deer combination (class B-11) license holders may apply for deer permits. However, a holder of a B-11 license obtained through a landowner sponsor can only apply for a deer permit where the permitted area includes the landowner sponsor's property and can only use the permit for hunting on the landowner sponsor's property. All valid conservation license holders may apply for deer B licenses. All nonresident conservation license holders who do not possess a B-10 or B-11 license may apply for a nonresident deer A (B-7) license, if available.
(2) Elk. Only persons who possess a valid resident A-5 elk license or a valid nonresident class B-10 license may apply for a special elk permit or A-7 license.
(3) All valid conservation license holders may apply for moose, sheep, goat, deer B, antelope, black bear, grizzly bear, buffalo, swan, and mountain lion licenses, and turkey permits/licenses. Resident sportsman and nonresident big game combination license holders may not apply for a black bear license if the black bear license is included as part of the combination license.
(4) A nonresident who uses a class B-11 landowner sponsored license in conjunction with a deer permit or a wild turkey license may hunt only on the landowner

sponsor's property. A nonresident who possesses a class B-1 landowner sponsored license and who hunts turkey off the landowner sponsor's property must also hold a class B-1, nonresident bird license valid statewide which is different than the restrictive B-1 license contained in the B-11 license. A nonresident holding both the class B-11 license and the class B-1 license valid statewide may purchase only the number of wild turkey licenses specified on the annual regulations for that season.

12.3.116 Moose, Sheep, And Goat Licenses

(1) The department shall issue moose, sheep, and goat licenses as described in sections 87-2-701 and 87-2-506 , MCA according to the following policy and procedures:
(a) Applicants for moose and goat must specify one choice for a hunting district. However, for bighorn sheep, an applicant may specify a second choice.
(b) Application for unlimited sheep must be postmarked no later than May 1. The deadline may be extended by the department if necessary to provide adequate time for the applicants to apply.
(2) The following procedure will be used when allocating 10% license opportunities for nonresidents in moose, sheep and goat drawings:
(a) The total regional license quota, by species and region, will be used to determine 10% nonresident quota.
(b) Nonresident license allocations will be applied to those hunting districts and season types with a quota of ten or more in the tentative regulations.
(c) Any remaining license allocation will be put, on a rotating basis, in those districts and season types with a quota of less than ten of the tentative regulations.
(d) If no district in a region has a quota of ten or more licenses on the tentative regulations, all of the nonresident license authority will be allocated as described in (c).
(e) If a region has a total quota of less than ten, no nonresident license allocations will be made for that region.

12.3.140 Application For Drawings

(1) The deadline date for the moose, sheep, and goat special drawings is on or before May 1. The deadline date for elk, deer and antelope special drawings is on or before June 1. All applications for participation in any special permit/license drawing, except drawings under ARM 12.9.801 (damage hunts) provided for by these regulations must be postmarked by the U.S. Postal Service on or before the deadline date of the current license year, or delivered by private mail service on or before the deadline date; or if personally delivered, received in the Helena Fish, Wildlife and Parks office by 5:00 p.m., on the deadline date of the current license year. If the deadline date for application for any license or drawings, as set by the department, falls on a Sunday or state holiday, that date shall be automatically extended to 5:00 p.m. of the next full work day. The deadline may be extended by the department if necessary to provide adequate time for the applicants to apply.
(2) The department shall reject an application for any permit/license drawing or for surplus, mountain lion, black bear, trapping, buffalo, or grizzly bear licenses if:
(a) application is not made on the current year's form provided by the department;
(b) applicant fails to provide mandatory information on the form;
(c) applicant fails to sign the application; or
(d) applicant fails to submit the proper fee. The department will not accept personal checks from nonresidents for nonresident license applications and drawing fees.
(3) Submittal of more than one application for any one drawing by an individual will disqualify that individual's applications from the drawing for which the multiple applications were submitted.
(4) No corrections or changes may be made after the department has received the drawing application, except those types that can be made without contacting the applicant. These include:
(a) adding hunter safety numbers;
(b) moving valid district choices up to replace invalid choices;
(c) eliminating species choices on those applications that are short money when the shortfall is the amount for that species; and
(d) adjusting party applications to insure party consistency.
(5) Any category of correction made by the department must be applied to all applications. In addition, the department will accept corrections on the applications of those seeking landowner preference. Unless otherwise provided by these rules, all drawings will take place in Helena.
(6) All applications for participation in buffalo, spring grizzly bear, swan and turkey drawings must be

postmarked by the U.S. Postal Service by the advertised deadline date, or delivered by private mail service on or before the date to the address indicated for the particular drawing which is being applied for.
(7) If an application for any species is rejected by the department pursuant to this rule:
(a) the application must not be included in the procedure for awarding the permits/licenses applied for;
(b) the applicant must not be awarded a bonus point for that drawing for that species; and
(c) the drawing fee, and any bonus point fee, once the application is entered into the drawing, will be retained by the department. Applications not processed in the drawing because of errors will be returned to the applicant with all fees.

12.3.185. Super-tag Hunting Licenses
(1) The department will issue one deer, one elk, one shiras moose, one mountain sheep, one mountain goat, one wild buffalo or bison, one antelope, and one mountain lion hunting license each year through a lottery. These hunting licenses are known as "super-tags."
(2) For each species, an unlimited number of chances to draw a super-tag will be sold at $5 per chance. Chances will be sold by license agents as defined in ARM 12.3.201A or through the department authorized web site on the internet. License agents will receive a commission of $0.50 for each super-tag transaction for a species. A transaction in this case means the purchase of one or more super-tag chances of the same species at one time. Individuals purchasing a ticket through the internet shall pay a convenience fee in accordance with the current internet provider contract.
(3) After the completion of the special license drawing for a species, the department will conduct a computerized drawing selecting randomly the super-tag winner for that species. The department shall issue the appropriate super-tag to the lottery winner.
(4) Only a person legally able to be licensed under current Montana statutes may purchase chances to draw a super-tag or use a super-tag. A person must possess a valid conservation license to be eligible to purchase a chance to draw a super-tag.
(5) The super-tag is valid for the taking of one animal of the species for which it is issued and is valid only for the current license year. A super-tag may be used in any legally described hunting district open for hunting of that species. A super-tag may be used only during the legal hunting season for the species for which it is issued. The person using the super-tag may use it only during a hunting district's open season and is subject to all hunting regulations, including special weapons regulations, that apply to a hunting district. However, if a hunting district requires a permit to hunt that species in that district, a super-tag can be used without the special permit.
(6) In the event that a person who drew a license or purchased a license is also drawn for the super-tag for the same species, the person must surrender the license to the department before receiving the super-tag. The department will refund the license fee paid by the winner of the super-tag. The person winning the super-tag shall retain any accumulated bonus points for that species.
(7) The super-tag is a nontransferable license.

APPENDIX 7

MONTANA MOUNTAIN LION IPM MODEL CODE

The Montana Mountain Lion Integrated Population Model was constructed using the statistical programming language R (R Development Core Team 2013).

```
model{
     #  Naming
     #  Parameter names begin with a capitalized letter
     #  Data are all lower case
     #  Indexing always follows - DAU, Year, Age, Sex
     #  If fewer indices are needed they follow the same order despite
     #   omissions

     #  Priors
     #  Pregnancy rates - [age, sex, mean:tau]
     Preg[1] ~ dnorm(preg[3,1,1], preg[3,1,2])T(0,1)
     Preg[2] ~ dnorm(preg[4,1,1], preg[4,1,2])T(0,1)

     #  Fetus Counts - [age, sex, mean:tau]
     FC[1] ~ dnorm(fc[3,1,1], fc[3,1,2])T(0,3)
     FC[2] ~ dnorm(fc[4,1,1], fc[4,1,2])T(0,3)

     #  Survival
     #  Priors on survival - First age class, not available for harvest, so
     #   survival is the only parameter
     #  Informative prior stored as probability
     yS_mu ~ dnorm(means[1,1,1], means[1,1,2])T(0,1)

     #  Transform probability back to real scale and use as the intercept
     for(u in 1:ndau){
       for(yr in 1:nyr){
         for(s in 1:2){
           logit(S[u,yr, 1, s]) <- log(yS_mu/(1 - yS_mu))
           H[u,yr,1,s] <- 0
           O[u,yr,1,s] <- 0
         }
       }
     }

     #  Priors on survival - Juveniles - two sexes, cause specific mortality
     for(s in 1:2){
       #  Informative priors are stored as probabilities
       jS_tmp[1,s] ~ dnorm(means[2,s,1], means[2,s,2])T(0, 1)
       jS_tmp[2,s] ~ dnorm(meanh[2,s,1], meanh[2,s,2])T(0, 1)
       jS_tmp[3,s] ~ dnorm(meano[2,s,1], meano[2,s,2])T(0, 1)

       #  Transform probability to real scale
       for(i in 1:3){
         jS_mu[i,s] <- log(jS_tmp[i,s]/jS_tmp[3,s])
       }

       #  Describe rate as function of linear predictor and define link
       #   function
       for(u in 1:ndau){
         for(yr in 1:nyr){
           log(jS_log[u,yr,s]) <- jS_mu[1,s]
           log(jH_log[u,yr,s]) <- jS_mu[2,s]
```

```
      log(jO_log[u,yr,s]) <- 0
      jSums[u,yr,s] <- jS_log[u,yr,s] + jH_log[u,yr,s] + jO_log[u,yr,s]
      S[u,yr,2,s] <- jS_log[u,yr,s]/jSums[u,yr,s]
      H[u,yr,2,s] <- jH_log[u,yr,s]/jSums[u,yr,s]
      O[u,yr,2,s] <- jO_log[u,yr,s]/jSums[u,yr,s]
    }
  }
}

#  Priors on survival - SubAdults - two sexes, cause specific mortality
for(s in 1:2){
  #  Informative priors are stored as probabilities
  sS_tmp[1,s] ~ dnorm(means[3,s,1], means[3,s,2])T(0, 1)
  sS_tmp[2,s] ~ dnorm(meanh[3,s,1], meanh[3,s,2])T(0, 1)
  sS_tmp[3,s] ~ dnorm(meano[3,s,1], meano[3,s,2])T(0, 1)

  #  Transform probability to real scale
  for(i in 1:3){
    sS_mu[i,s] <- log(sS_tmp[i,s]/sS_tmp[3,s])
  }

  #  Describe rate as function of linear predictor and define link
  #   function
  for(u in 1:ndau){
    for(yr in 1:nyr){
      log(sS_log[u,yr,s]) <- sS_mu[1,s]
      log(sH_log[u,yr,s]) <- sS_mu[2,s]
      log(sO_log[u,yr,s]) <- 0
      sSums[u,yr,s] <- sS_log[u,yr,s] + sH_log[u,yr,s] + sO_log[u,yr,s]
      S[u,yr,3,s] <- sS_log[u,yr,s]/sSums[u,yr,s]
      H[u,yr,3,s] <- sH_log[u,yr,s]/sSums[u,yr,s]
      O[u,yr,3,s] <- sO_log[u,yr,s]/sSums[u,yr,s]
    }
  }
}

#  Priors on survival - Adults, two sexes, cause specific mortality
for(s in 1:2){
  #  Informative priors are stored as probabilities
  aS_tmp[1,s] ~ dnorm(means[4,s,1], means[4,s,2])T(0, 1)
  aS_tmp[2,s] ~ dnorm(meanh[4,s,1], meanh[4,s,2])T(0, 1)
  aS_tmp[3,s] ~ dnorm(meano[4,s,1], meano[4,s,2])T(0, 1)

  #  Transform probability to real scale
  for(i in 1:3){
    aS_mu[i,s] <- log(aS_tmp[i,s]/aS_tmp[3,s])
  }


  #  Describe rate as function of linear predictor and define link
  #   function
  for(u in 1:ndau){
    for(yr in 1:nyr){
      log(aS_log[u,yr,s]) <- aS_mu[1,s]
      log(aH_log[u,yr,s]) <- aS_mu[2,s]
      log(aO_log[u,yr,s]) <- 0
      aSums[u,yr,s] <- aS_log[u,yr,s] + aH_log[u,yr,s] + aO_log[u,yr,s]
      S[u,yr,4,s] <- aS_log[u,yr,s]/aSums[u,yr,s]
      H[u,yr,4,s] <- aH_log[u,yr,s]/aSums[u,yr,s]
      O[u,yr,4,s] <- aO_log[u,yr,s]/aSums[u,yr,s]
    }
  }
}

###  Prior on first year population size
#  Indexing - Year, Age, Sex
for(u in 1:ndau){
  N[u,1,1,1] ~ dnorm(n1[1,2], 1/n1[1,2])T(0,)
```

```
  N[u,1,1,2] <- N[u,1,1,1]

  for(a in 2:nage){
    for(s in 1:2){
      N[u,1,a,s] ~ dnorm(n1[a,s+1], 1/n1[a,s+1])T(0,)
    }
  }

  yN[u,1] <- N[u,1,1,1] + N[u,1,1,2]
  fN[u,1] <- N[u,1,2,1] + N[u,1,3,1] + N[u,1,4,1]
  mN[u,1] <- N[u,1,2,2] + N[u,1,3,2] + N[u,1,4,2]
  totN[u,1] <- yN[u,1] + fN[u,1] + mN[u,1]
}

###  Process model - 4 ages, 2 sex
#  Using normal approximation because it is fast and mixes well
#  Sex = 1 is a female
#  Indexing follows - DAU, Year, Age, Sex
for(u in 1:ndau){
  for(yr in 2:nyr){
    #  Kittens
    #  Normal approximation of Poisson
    nMu[u,yr,1,1] <-
      ((N[u,yr,3,1] * 0.5 * FC[1] * Preg[1]) +
        (N[u,yr,4,1] * 0.5 * FC[2] * Preg[2])) *
        S[u,yr-1,1,1]
    nMu[u,yr,1,2] <- nMu[u,yr,1,1]

    N[u,yr,1,1] ~ dnorm(nMu[u,yr,1,1], 1/(nMu[u,yr,1,1]))
    N[u,yr,1,2] <- N[u,yr,1,1]

    for(s in 1:2){
      #  Juveniles
      #  Normal approximation of Binomial
      nMu[u,yr,2,s] <-
        (1 - O[u,yr-1,2,s]) * (N[u,yr-1,1,s] - harv[u,yr-1,2,s])

      nTau[u,yr,2,s] <- 1/((N[u,yr-1,1,s] - harv[u,yr-1,2,s]) *
        (O[u,yr-1,2,s]) * (1 - O[u,yr-1,2,s]))

      N[u,yr,2,s] ~ dnorm(nMu[u,yr,2,s], nTau[u,yr,2,s])

      #  SubAdults
      #  Normal approximation of Binomial
      nMu[u,yr,3,s] <-
        (1 - O[u,yr-1,3,s]) * (N[u,yr-1,2,s] - harv[u,yr-1,3,s])

      nTau[u,yr,3,s] <- 1/((N[u,yr-1,2,s] - harv[u,yr-1,3,s]) *
        (O[u,yr-1,3,s]) * (1 - O[u,yr-1,3,s]))

      N[u,yr,3,s] ~ dnorm(nMu[u,yr,3,s], nTau[u,yr,3,s])


      #  Adults
      #  Normal approximation of Binomial
      #  Female Other Mortality shared between the sexes
      nMu[u,yr,4,s] <-

        (N[u,yr-1,3,s] + N[u,yr-1,4,s] - harv[u,yr-1,4,s]) *
          (1 - O[u,yr-1,4,s])

      nTau[u,yr,4,s] <-
        1/((N[u,yr-1,3,s] + N[u,yr-1,4,s] - harv[u,yr-1,4,s]) *
        (O[u,yr-1,4,s]) * (1 - O[u,yr-1,4,s]))


      N[u,yr,4,s] ~ dnorm(nMu[u,yr,4,s], nTau[u,yr,4,s])
```

```
      }

    #  Totals in each year
    yN[u,yr] <- N[u,yr,1,1] + N[u,yr,1,2]
    fN[u,yr] <- N[u,yr,2,1] + N[u,yr,3,1] + N[u,yr,4,1]
    mN[u,yr] <- N[u,yr,2,2] + N[u,yr,3,2] + N[u,yr,4,2]
    totN[u,yr] <- yN[u,yr] + fN[u,yr] + mN[u,yr]
    }
  }

  ####################  Observation Models
  #  Indexing/columns always follows
  #    1   2     3    4    5    6
  #  DAU, Year, Age, Sex, Mean, Tau

  #  Abundance Observation - [dau, yr]
  for(i in 1:nn){
    ndat[i,5] ~ dnorm(totN[1,ndat[i,2]], ndat[i,6])T(0,)
  }

  #  Harvest Observations - [dau,yr,a,s]
  for(u in 1:ndau){
    for(yr in 1:nobs_yr){
      for(a in 1:nage){
        for(s in 1:2){
          harv[u,yr,a,s] ~ dbinom(H[u,yr,a,s], round(N[u,yr,a,s]))
        }
      }
    }
  }

  #  Survival Observations
  for(i in 1:ns){
    sdat[i,5] ~ dnorm(S[1, sdat[i,2], sdat[i,3], sdat[i,4]], sdat[i,6])T(0, 1)
  }
  #  Harvest Mortality Rate Observations
  for(i in 1:nhm){
    hmdat[i,5] ~ dnorm(H[1, hmdat[i,2], hmdat[i,3], hmdat[i,4]], hmdat[i,6])T(0, 1)
  }
  #  Other (Non-Harvest) Mortality Rate Observations
  for(i in 1:nom){
    omdat[i,5] ~ dnorm(O[1, omdat[i,2], omdat[i,3], omdat[i,4]], omdat[i,6])T(0, 1)
  }

  #  Derived - the constant is added to avoid division by 0
  for(u in 1:ndau){
    for(yr in 1:nyr){

      mf[u,yr] <- (mN[u,yr] + 0.001)/(fN[u,yr] + 0.001)
    }
  }

  #  Incomplete vectors cannot be monitored, so aribitrary value is given
  #  to the first year
  #  Same constant trick is used here for the division
  #  Using the log and exp handles 0 gracefully, recall that
  #  log(x) + log(y) = log(xy), so the geometric mean is calculated using
  #  an algebraic rearrangment that is more robust to 0's
  for(u in 1:ndau){
    lambda[u,1] <- 1
    for(yr in 2:nyr){
      lambda[u,yr] <- (totN[u,yr] + 0.001)/(totN[u,yr-1] + 0.001)
      logla[u,yr] <- log(lambda[u,yr])
    }
    geoLambda[u] <- exp((1/(nyr-1))*sum(logla[u,2:(nyr)]))
  }
}
```

LITERATURE CITED

Anderson, D. R. 2003. Response to Engeman: index values rarely constitute reliable information. Wildlife Society Bulletin 31:288-291.

Anderson, C. R. Jr., and F. G. Lindzey. 2003. Estimating cougar predation rates from GPS location clusters. Journal of Wildlife Management 67:307-316.

Anderson, C. R. Jr., F. G. Lindzey, and D. B. McDonald. 2004. Genetic structure of cougar populations across the Wyoming Basin: metapopulation or megapopulation. Journal of Mammalogy 85:1207-1214.

Anderson, C. R., and F. G. Lindzey. 2005. Experimental evaluation of population trend and harvest composition in a Wyoming cougar population. Wildlife Society Bulletin 33:179-188.

Atwood, T. C., E. M. Gese, and K. Kunkel. 2007. Comparing patterns of predation by cougars and recolonizing wolves in Montana's Madison Range. Journal of Wildlife Management 71(4):1098-1106.

Ballard, W. B., D. Lutz, T. W. Keegan, L. H. Carpenter, and J. C. DeVos. 2001. Deer-predator relationships: A review of recent North American studies with emphasis on mule deer and black-tailed deer. Wildlife Society Bulletin 29:99-115.

Bauer, J. W., K. A. Logan, L. Sweanor, and W. M. Boyce. 2005. Scavenging behavior in puma. Southwestern Naturalist 50:466-471.

Barber-Meyer, S. M., L. D. Mech, and P. J. White. 2008. Elk calf survival and mortality following wolf restoration to Yellowstone National Park. Wildlife Monographs 169:1-30.

Beausoleil, R. A., D. Dawn, D. A. Martorello, and C. P. Morgan. 2008. Cougar management protocols: a survey of wildlife agencies in North America in D. Toweill, S.

Nadeau, and D. Smith, editors, Proceedings of the ninth mountain lion workshop. Idaho Game and Fish Dept, Boise, ID.

Beausoleil, R. A., G. M. Koehler, B. T. Maletzke, B. N. Kertson, and R. B. Wielgus. 2013. Research to regulation: Cougar social behavior as a guide for management. Wildlife Society Bulletin 37:680-688.

Beausoleil, R. A., J. D. Clark, and B. T. Maletzke. 2016. A long-term evaluation of biopsy darts and DNA to estimate cougar density: An agency-citizen science collaboration. Wildlife Society Bulletin 40(3):583-592.

Beier, P. 1991. Cougar attacks on humans in the United States and Canada. Wildlife Society Bulletin 19:403-412.

Beier, P., and S. C. Cunningham. 1996. Power of track surveys to detect changes in cougar populations. Wildlife Society Bulletin 24:540-546.

Belden, R. C., B. W. Hagedorn, and W. B. Frankenberger. 1991. Responses of translocated mountain lions to human disturbance in C. Braun, editor, Proceedings Mountain lion-human interactions symposium. Colorado Division of Wildlife.

Biek, R., N. Akamine, M. Schwartz, T. Ruth, K. Murphy, and M. Poss. 2006a. Genetic consequences of sex-biased dispersal in a solitary carnivore: Yellowstone cougars. Biological Letters 2:312-315.

Biek, R., A. J. Drummond, and M. Poss. 2006b. A virus reveals population structure and recent demographic history of its carnivore host. Science 311:538-541.

Bishop, C. J., G. C. White, D. J. Freddy, B. E. Watkins, and T. R. Stephenson. 2009. Effect of enhanced nutrition on mule deer population rate of change. Wildlife Monographs 172:1–28.

Blake, L. W., and E. M. Gese. 2016. Cougar predation rates and prey composition in the Pryor Mountains of Wyoming and Montana. Northwest Science 90(4):394-410.

Blanc, L., E. Marboutin, S. Gatti, and O. Gimenez. 2013. Abundance of rare and elusive species: Empirical investigation of closed versus spatially explicit capture-recapture models with lynx as a case study. Journal of Wildlife Management 77:372-378.

Bonney, R. C., H. D. M. Moore, and D. M. Jones. 1981. Plasma concentrations of oestradiol-17 and progesterone, and laparoscopic observations of the ovary in the puma (Felis concolor) during oestrus, pseudopregnancy and pregnancy. Journal of Reproduction and Fertility 63: 523-531.

Boyce, M. S., and L. L. McDonald. 1999. Relating populations to habitats using resource selection functions. Trends Ecology and Evolution 14:268-272.

Boyce, M. S., P. R. Vernier, S. E. Nielsen, and F. K. A. Schmiegelow. 2002. Evaluating resource selection functions. Ecological Modelling 157:281-300.

Boyce, M. S., C. J. Johnson, E. H. Merrill, S. E. Nielsen, E. J. Solberg, and B. VanMoorter. 2016. Can habitat selection predict abundance? Journal of Animal Ecology 85:11-20.

Brodie, J., H. Johnson, M. Mitchell, P. Zager, K. Proffitt, M. Hebblewhite, M. Kauffman, B. Johnson, J. Bissonette, C. Bishop, J. Gude, J. Herbert, K. Hersey, M. Hurley, P. M. Lukacs, S. McCorquodale, E. McIntire, J. Nowak, H. Sawyer, D. Smith and P. J. White. 2013. Relative influence of human harvest, carnivores, and weather on adult female elk survival across western North America. Journal of Applied Ecology 50:295-305.

Brownie, C., D. R. Anderson, K. P. Burnham, and D. S. Robson. 1985. Statistical Inference from Band Recovery Data: A Handbook. United States Geological Survey.

Buderman, F. E., D. R. Diefenbach, M. J. Casalena, C. S. Rosenberry, and B. D. Wallingford. 2014. Accounting for tagging-to-harvest mortality in a Brownie tag-recovery model by incorporating radio-telemetry data. Ecology and Evolution 4:1439-1450.

Buotte, P. C., T. K. Ruth, and M. G. Hornocker. 2008. Wolf and bear detection of cougar killed ungulates on the Northern Range of Yellowstone National Park in D. Toweill, S. Nadeau, and D. Smith, editors, Proceedings of the ninth mountain lion workshop. Idaho Game and Fish Department, Boise, ID.

Caswell, H. 2006. Matrix population models. Encyclopedia of Environmetrics. John Wiley & Sons, Ltd.

Choate, D. M., M. L.Wolfe, and D. C. Stoner. 2006. Evaluation of cougar population estimators in Utah. Wildlife Society Bulletin 34:782-799.

Choate, D. M. 2009. Cougar-induced behavioral plasticity: Ungulate behavior under the risk of predation on the National Bison Range. Biological Sciences. Univeristy of Notre Dame, Notre Dame, IN, pp. 281.

Clark, D. A. 2014a. Population growth rates and simulated responses of cougar populations to density reduction under variable immigration and emigration. in D. A. Clark, author. Implications of cougar prey selection and demography on population dynamics of elk in northeast Oregon. Oregon State University, Corvallis, OR, pp. 96-137.

Clark, D. A., G. A. Davidson, B. K. Johnson, and R. G Anthony. 2014b. Cougar kill rates and prey selection in a multiple-prey system in northeast Oregon. Journal of Wildlife Management 78(7):1161-1176.

Conn, P. B., D. R. Diefenbach, J. L. Laake, M. A. Ternent and G. C. White. 2008. Bayesian analysis of wildlife age-at-harvest data. Biometrics 64:1170-1177.

Conroy, M. J., K. W. Stodola, and R. J. Cooper. 2012. Effective use of data from monitoring programs and field studies for conservation decision making: Predictions, designs and models working together. Journal of Ornithology 152:325-338.

Cooley, H. S., H. S. Robinson, R. B. Wieglus, and C. S. Lambert. 2008. Cougar prey selection in a white-tailed deer and mule deer community. Journal of Wildlife Management 72(1):99-106.

Cooley, H. S., R. B. Wielgus, G. M. Koehler, H. S. Robinson, and B. T. Maletzke. 2009. Does hunting regulate cougar populations? A test of the compensatory mortality hypothesis. Ecology 90:2913-2921.

Cougar Management Guidelines Working Group. 2005. Cougar Management Guidelines, First Edition. WildFutures. Bainbridge Island, WA.

Currier, M. J. P. 1983. Felis concolor. Mammalian Species No. 200. American Society of Mammalogists.

Eacker, D. R., M. Hebblewhite, K. M. Proffitt, B. S. Jimenez, M. S. Mitchell, and H. S. Robinson. 2016. Annual elk calf survival in a multiple carnivore system. Journal of Wildlife management 80(8):1345-1359.

Eacker, D. R., P. M. Lukacs, K. M. Proffitt, and M. Hebblewhite. 2017. Assessing the importance of demographic parameters for population dynamics using Bayesian integrated population modeling. Ecological Applications 27(4):1280-1293.

Efford, M. G., D. K. Dawson, and D. L. Borchers. 2009. Population density estimated from locations of individuals on a passive detector array. Ecology 90:2676-2682.

Efford, M. G. 2014. SECR: Spatially explicit capture recapture models. R package version 2.8.2. https://cran.r project.org/web/packages/secr/secr.pdf

Ernest, H. B., E. S. Rubin, and W. M. Boyce. 2002. Fecal DNA analysis and risk assessment of mountain lion predation of bighorn sheep. Journal of Wildlife Management 66:75-85.

Falaruw, M. V. C. 1984. People pressure and management of limited resources on Yap in J.A.

McNeeley and K.R. Miller, editors, National Parks, conservation and development: The role of protected areas in sustaining people. Smithsonian Institution Press, Washington, D.C.

Gaillard, J. M., M. Festa-Bianchet, and N. G.Yoccoz. 1998. Population dynamics of large herbivores: Variable recruitment with constant adult survival. Trends in Ecology and Evolution 13:58-63.

Gardner, B., J. Reppucci, M. Lucherini, and J. A. Royle. 2010. Spatially explicit inference for open populations: Estimating demographic parameters from camera-trap studies. Ecology 91:3376-3383.

Goudet, J., N. Perrin, and P. Waser. 2002. Tests for sex-biased dispersal using bi-parentally inherited genetic markers. Molecular Ecolology 11:1103-1114.

Gove, N. E., J. R. Skalski, P. Zager, and R. L. Townsend. 2002. Statistical models for population reconstruction using age-at-harvest data. Journal of Wildlife Management 66:310-320.

Griffin, D. A., M. Hebblewhite, H. S. Robinson, P. Zager, S. M. Barber-Meyer, D. Christianson, S. Creel, N. C. Harris, M. A. Hurley, D. H. Jackson, B. K. Johnson, W. L. Myers, J. D. Raithel, M. Schelgel, B. L. Smith, C. White, and P. J. White. 2011. Neonatal mortality of elk driven by climate, predator phenology and predator community composition. Journal of Animal Ecology 80:1246-1257.

Hamlin, K. L., and J. A. Cunningham. 2009. Monitoring and assessment of wolf-ungulate interactions and population trends within the greater Yellowstone area, southwestern Montana, and Montana statewide. Montana Department of Fish, Wildlife, and Parks, Wildlife Division, Helena, MT, USA.

Hebblewhite, M., and E. H. Merrill. 2007. Multiscale wolf predation risk for elk: Does migration reduce risk? Oecologia 152(2):377-387.

Hiller, T. L., J. E. McFadden-Hiller, S. R. Jenkins, J. L. Belant, and A. J. Tyre. 2015. Demography, prey abundance, and management affect number of cougar mortalities associated with livestock conflicts. Journal of Wildlife Management 79(6):978-988.

Holt, R. D. 1977. Predation, apparent competition, and the structure of prey communities. Theoretical Population Biology 12:197-229.

Hornocker, M. G. 1970. An analysis of mountain lion predation upon mule deer and elk in the Idaho Primitive Area. Wildlife Monographs 21:3-39.

Hornocker, M. G., and S. Negri. 2009. Cougar Ecology and Conservation. University of Chicago Press, Illinois, USA.

Howe, E. J., M. E. Obbard, and C. J. Kyle. 2013. Combining data from 43 standardized surveys to estimate densities of female American black bears by spatially explicit capture recapture. Population Ecology 55:595-607.

Hurley, M. A., J. W. Unsworth, P. Zager, M. Hebblewhite, E. O. Garton, D. M. Montgomery, J. R. Skalski, and C. L. Maycock. 2011. Demographic response of mule deer to experimental reduction of coyotes and mountain lions in southeastern Idaho. Wildlife Monographs 178:1-33.

Jenks., J. A., editor. 2011. Managing Cougars in North America. Jack H. Berryman Institute, Utah State University, Logan, Utah, USA.

Johnson, B. K., P. K. Coe, and R. L. Green. 2013. Abiotic, bottom-up, and top-down influences on recruitment of Rocky Mountain elk in Oregon: A retrospective analysis. Journal of Wildlife Management 77:102-116.

Juarez, R. L., M. K. Schwartz, K. L. Pilgrim, D. J. Thompson, S. A. Tucker, J. B. Smith, and J. A. Jenks. 2016. Assessing temporal genetic variation in a cougar population: Influence of harvest and neighboring populations. Natural Resource Management Faculty Publications. Paper 59. http://openprairie.sdstate.edu/nrm_pubs/59

Kamler, J. F., R. M. Lee, J. C. deVos Jr., W. B. Ballard, and H. A. Whitlaw. 2002. Survival and cougar predation of translocated bighorn sheep in Arizona. Journal of Wildlife Management 66:1267-72.

Karanth, K. U., and J. D. Nichols. 1998. Estimation of tiger densities in India using photographic captures and recaptures. Ecology 79:2852-2862.

Kertson, B. N., R. D. Spencer, and C. E. Grue. 2013. Demographic influences on cougar residential use and interactions with people in western Washington. Journal of Mammalogy 94(2):269–281.

Kery, M., and M. Schaub. 2011. Bayesian population analysis using WinBUGS: A hierarchical perspective, First edition. Academic Press, Boston, MA.
Kinley, T. A., and C. D. Apps. 2001. Mortality patterns in a subpopulation of endangered mountain caribou. Wildlife Society Bulletin 29:158-164.

Knopff, K. H., A. A. Knopff, A. Kortello, and M. S. Boyce. 2010. Cougar kill rate and prey composition in a multiprey system. Journal of Wildlife Management 74:1435–1447.

Kortello, A. D., T. E. Hurd, and D. L. Murray. 2007. Interactions between cougars (Puma concolor) and gray wolves (Canis lupus) in Banff National Park, Alberta. Ecoscience 14:214-222.

Kunkel, K., T. K. Ruth, D. H. Pletscher, and M. G. Hornocker. 1999. Winter prey selection by wolves and cougars in and near Glacier National Park Montana. Journal of Wildlife Management 63(3):901-910.

Kunkel, K., T. Vosburgh, and H. Robinson. 2012. Ecology of cougars (Puma concolor) in north-central Montana. Final report to the US fish and wildlife service. W.W.F.N.G.P., Bozeman, MT.

Lambert, M. S., R. B. Wielgus, H. S. Robinson, D. D. Katnik, and H. S. Cruickshank. 2006. Cougar population dynamics in the Pacific Northwest. Journal of Wildlife Management 70:246-254.

Laundre, J. W., and T. W. Clark. 2003. Managing puma hunting in the western United States through a metapopulation approach. Animal Conservation 6:159–170.

Laundre, J. W., and L. Hernández. 2007. Do female pumas (Puma concolor) exhibit a birth pulse? Journal of Mammalogy 88:1300-1304.

Lebreton, J. D., K. P. Burnham, J. Clobert, and D. R. Anderson. 1992. Modeling survival and testing biological hypotheses using marked animals: A unified approach with case studies. Ecological Monographs 62:67-118.

Lindzey, F. 1987. Mountain lion in M. Novak, J. A. Baker, M. E. Obbard, and B. Malloch, editors, Wild furbearer management and conservation in North America. Ministry of Natural Resources, Ontario, Canada.

Lindzey, F. G., W. D. V. Sickle, B. B. Ackerman, D. Barnhurst, T. P. Hemker, and S. P. Laing. 1994. Cougar population dynamics in southern Utah. Journal of Wildlife Management 58:619-624.

Link, W. A., J. A. Royle, and J. S. Hatfield. 2003. Demographic analysis from summaries of an age-structured population. Biometrics 59:778-785.

Logan, K. A., L. L. Irwin, and R. Skinner. 1986. Characteristics of a hunted mountain lion population in Wyoming. Journal of Wildlife Management 50:648-654.

Logan, K. A., and L. L. Sweanor. 2001. Desert puma: Evolutionary ecology and conservation of an enduring carnivore. Island Press, Washington, D. C.

López -González, C. A., and A. González-Romero. 1998. A synthesis of current literature and knowledge about the ecology of the puma (Puma concolor Linnaeus). Acta Zoologica Mexicana Nueva Serie 0(75):171-190.

Manly, B. F. J., L. L. McDonald, D. L. Thomas, T. L. McDonald, and W. P. Erickson. 2002. Resource selection by animals: Statistical design and analysis for field studies, 2nd ed. Kluwer Academic Publishers, Dordrecht/Boston.

Matchett, R. 2012. Mountain lion research in the Missouri River Breaks. U.S. Fish and Wildlife Service, Charles M. Russell National Wildlife Refuge, Lewistown, MT.

Mattson, D. J. 2007. Managing for human safety in mountain lion range. in Mountain lions of the Flagstaff uplands. U. S. Department of the Interior, U. S. Geological Survey.

McKelvey, K S. and M. K. Schwartz. 2004. Providing reliable and accurate genetic capture-mark-recapture estimates in a cost-effective way. Journal of Wildlife Management 68(3)

McKinney, T., T. W. Smith, and J. C. deVos, Jr. 2006. Evaluation of factors potentially influencing a desert bighorn sheep population. Wildlife Monographs 164:1-36.

Messier, F. 1994. Ungulate population models with predation: A case study with the North American moose. Ecology 75:478-488.

Mills, L. S. 2007. Conservation of wildlife populations: demography, genetics, and management. Blackwell Publishing, Malden, MA.

Montana Fish, Wildlife & Parks. 2001. Adaptive Harvest Management. Montana Fish, Wildlife and Parks, Helena, Montana.

Montana Fish, Wildlife & Parks. 2006. White-tailed deer studies in the Salish Mountains, Northwest Montana. Fed. Aid in Wildlife Restoration Project W-120-R: pp. 263.

Murphy, K. 1983. Relationships between a mountain lion population and hunting pressure in western Montana. Thesis. University of Montana, Missoula, MT.

Murphy, K. 1998. The ecology of the cougar (Puma concolor) in the northern Yellowstone ecosystem: Interactions with prey, bears, and humans. Thesis. Forestry, Wildlife, and Range Sciences, University of Idaho, Moscow, ID, pp. 147.

Murphy, K., and T. Ruth. 2011. Diet and Prey Selection of a Perfect Predator. in M. Hornocker and S. Negri, editors, Cougar: Ecology and Conservation. University of Chicago Press, Chicago, Illinois.

Newby, J. R. 2011. Puma dispersal ecology in the central Rocky Mountains. Thesis. University of Montana, Missoula, MT.

Nichols, J. D., and B. K. Williams. 2006. Monitoring for conservation. Trends in Ecology & Evolution 21:668-673.

Nowak, M. C. 1999. Predation rates and foraging ecology of adult female mountain lions in northeastern Oregon. Thesis. Washington State University, Pullman, WA.

Nowak, J. J., P. M. Lukacs, M. A. Hurley, A. J. Lindbloom, K. A. Robling, C. Krause, J. A. Gude, and H. Robinson. 2018. Customized software to streamline routine analyses for wildlife management. Wildlife Society Bulletin 42(1):144-149.

Pierce, B. M., V. C. Bleich, and R. T. Bowyer. 2000a. Social organization in mountain lions: Does a land-tenure system regulate population size? Ecology 81(6):1533-1543.

Pierce, B. M., V. C. Bleich, and R. T. Bowyer. 2000b. Selection of mule deer by mountain lions and coyotes: Effects of hunting style, body size, and reproductive status. Journal of Mammalogy 81:462-472.

Pierce, B. M, V. C. Bleich, K. L. Monteith, and R. T. Bowyer. 2012. Top-down versus bottom-up forcing: Evidence from mountain lions and mule deer. Journal of Mammalogy 93(4):977-988.

Proffitt, K., M. Hebblewhite, J. Goldberg, B. Jimenez, R. Vinkey, and M. Thompson. 2014. Philipsburg area mountain lion population estimate. Montana Fish, Wildlife & Parks, Helena, MT.

Proffitt, K. M., J. F. Goldberg, M. Hebblewhite, R. Russell, B. S. Jimenez, H. S. Robinson, K. Pilgrim, and M. K. Schwartz. 2015. Integrating resource selection into spatial capture-recapture models for large carnivores. Ecosphere 6(11): Article 239.

Proffitt, K. M., M. Hebblewhite, W. Peters, N. Hupp, and J. Shamhart. 2016. Linking landscape-scale differences in forage to ungulate nutritional ecology. Ecological Applications 26(7):2156-2174.

R Development Core Team. 2013. R: A language and environment for statistical computing. R Foundation for Statistical Computing, Vienna, Austria.

Raithel, J. D. 2005. Impact of calf survival on elk population dynamics in west-central Montana. Thesis. University of Montana, Missoula, MT.

Raithel, J., M. Kauffman, and D. Pletscher. 2007. Impact of spatial and temporal variation in calf survival on the growth of elk populations. Journal of Wildlife Management 71:795–803.

Rearden, S. N. 2005. Juvenile survival and birth site selection of Rocky Mountain elk in northeastern Oregon. Thesis. Oregon State University, Corvallis, OR.

Reynolds, J. H., M. G. Knutson, K. B. Newman, E. D. Silverman, and W. L. Thompson. 2016. A road map for designing and implementing a biological monitoring program. Environmental Monitoring and Assessment 188(7):1-25.

Riley, S. J., and K. E. Aune. 1997. Mountain lion–human and mountain lion–livestock incidents in Montana. in Proceedings of the Fifth Mountain Lion Workshop, W. D. Padley, editor, California Department of Fish and Game, San Diego, California.

Riley, S. J. 1998. Integration of environmental, biological, and human dimensions for management of mountain lions (Puma concolor) in Montana. Thesis. Cornell University, New York, NY.

Robinson, H. S., R. B. Wielgus, and J. C. Gwilliam. 2002. Cougar population and growth of sympatric mule deer and white-tailed deer. Canadian Journal of Zoology 80:556-568.

Robinson, H. S., R. B. Wielgus, H. S. Cooley, and S. W. Cooley. 2008. Sink populations in carnivore management: Cougar demography and immigration in a hunted population. Ecological Applications 18:1028-1037.

Robinson, H. S., and R. M. DeSimone. 2011. The Garnet Range mountain lion study: Characteristics of a hunted population in west-central Montana. Federal Aid in Wildlife Restoration Project W-154-R, Helena, MT.

Robinson, H. S., R. Desimone, C. Hartway, J. A. Gude, M. J. Thompson, M. S. Mitchell, and M. Hebblewhite. 2014. A test of the compensatory mortality hypothesis in mountain lions: A management experiment in West-Central Montana. Journal of Wildlife Management 78(5):791-807.

Robinson, H. S., T. K. Ruth, J. A. Gude, D. Choate, R. DeSimone, M. Hebblewhite, M. R. Matchett, M. S. Mitchell, K. Murphy, and J. Williams. 2015. Linking resource selection and mortality modeling for population estimation of mountain lions in Montana. Ecological Modeling 312:11-25.

Ross, P. I., and M. G. Jalkotzy. 1992. Characteristics of a hunted population of cougars in southwestern Alberta. Journal of Wildlife Management 56:417-426.

Ross, P. I., and M. G. Jalkotzy. 1995. Fates of trans-located cougars, Felis concolor, in Alberta. Canadian Field-Naturalist 109:475-476.

Royle, J. A., A. J. Magoun, B. Gardner, P. Valkenburg, and R. E. Lowell. 2011. Density estimation in a wolverine population using spatial capture-recapture models. Journal of Wildlife Management 75:604-611.

Royle, J. A., R. B. Chandler, R. Sollmann, and B. Gardner. 2013. Spatial Capture-Recapture. Academic Press/Elsevier, Waltham, MA.

Russell, R. E., J. A. Royle, R. Desimone, M. K. Schwartz, V. L. Edwards, K. P. Pilgrim, and K. S. Mckelvey. 2012. Estimating abundance of mountain lions from unstructured spatial sampling. Journal of Wildlife Management 76:1551-1561.

Ruth, T. K., K. A. Logan, L. L. Sweanor, M. G. Hornocker, and L. J. Temple. 1998. Evaluating cougar translocation in New Mexico. Journal of Wildlife Management 62:1264-1275.

Ruth, T. K. 2004. Patterns of resource use among cougars and wolves in north-western Montana and southeastern British Columbia. Thesis. University of Idaho, Moscow, ID.

Ruth, T. K., and P. C. Buotte. 2007. Cougar ecology and cougar–carnivore interactions in Yellowstone National Park. in Final Technical Report, Hornocker Wildlife Institute-Wildlife Conservation Society, Bozeman, MT.

Ruth, T. K., and K. Murphy. 2011. Cougar-Prey Relationships. in M. Hornocker and S. Negri, editors, Cougar Ecology and Conservation. University of Chicago Press, Chicago, Illinois.

Schaub, M., and F. Abadi. 2011. Integrated population models: A novel analysis framework for deeper insights into population dynamics. Journal of Ornithology 152:227-237.

Schaub, M., A. Aebischer, O. Gimenez, S. Berger, and R. Arlettaz. 2010. Massive immigration balances high anthropogenic mortality in a stable eagle owl population: Lessons for conservation. Biological Conservation 143:1911-1918.

Seber, G. A. 1982. The estimation of animal abundance and related parameters. Griffin, London.

Servanty, S., R. Choquet, É Baubet, S. Brandt, J.-M. Gaillard, M. Schaub, C. Toïgo, J.-D. Lebreton, M. Buoro, and O. Gimenez. 2010. Assessing whether mortality is additive using marked animals: A Bayesian state-space modeling approach. Ecology 91:1916-1923.

Spreadbury, B. R., R. R. K. Musil, J. Musil, C. Kaisner, and J. Kovak. 1996. Cougar population characteristics in southeastern British Columbia. Journal of Wildlife Management 60:962-969.

Stoner, D. C. 2004. Cougar exploitation levels in Utah: Implications for demographic structure, metapopulation dynamics, and population recovery. Thesis. Utah State University, Logan, UT.

Stoner, D. C., M. L. Wolfe, and D. L. Choate. 2006. Cougar exploitation levels in Utah: Implications for demographic structure, population recovery, and metapopulation dynamics. Journal of Wildlife Management 70:1588-1600.

Sweanor, L. L., K. A. Logan, and M. G. Hornocker. 2000. Cougar dispersal patterns, metapopulation dynamics, and conservation. Conservation Biology 14:798-808.

Sweitzer, R. A., S. H. Jenkins, and J. Berger. 1997. Near-extinction of porcupines by mountain lions and consequences for ecosystem changes in the Great Basin Desert. Conservation Biology 11:1407-1417.

Szaro, R. C., D. Maddox, T. Tolle, and M. McBurney. 1999. Monitoring and evaluation. in Johnson, N., A. Malk, R. C. Szaro and W. T. Sexton, editors, Ecological stewardship: A common reference for ecosystem management, Volume 1: Key findings. Elsevier Science, Oxford, UK.

Torres, S. G., T. M. Mansfield, J. E. Foley, T. Lupo, and A. Brinkhaus. 1996. Mountain lion and human activity in California: Testing speculations. Wildlife Society Bulletin 24:451-460.

Trainer, C. E, and G. Matson. 1988. Age determination in cougar from cementum annuli counts of tooth sections. Proceedings of the Mountain Lion Workshop 3:71.

Walters, C. J., and C. S. Holling. 1990. Large-scale management experiments and learning by doing. Ecology 71:2060-2068.

Western Wildlife Disease Workshop Notebook. 2009. Southeastern Cooperative Wildlife Disease Study. College of Veterinary Medicine, University of Georgia, GA.

White, C. G., P. Zager, and M. W. Gratson. 2010. Influence of predator harvest, biological factors, and landscape on elk calf survival in Idaho. Journal of Wildlife Management 74:355-369.

Wiens T. S., B. C. Dale, M. S. Boyce, and G. P. Kershaw. 2008. Three-way k-fold cross validation of resource selection functions. Ecological Modelling 212: 244-55.

Williams, J. 1992. Ecology of mountain lions in the Sun River area of northern Montana. Thesis. Montana State University, Bozeman, MT.

Williams, B. K., R. C. Szaro, C. D. Shapiro. 2007. Adaptive management: The U. S. Department of the Interior Technical Guide. U. S. Department of the Interior, Washington, DC.

Williams, B. K., and E. D. Brown. 2012. Adaptive Management: The U.S. Department of the Interior Applications Guide. Adaptive Management Working Group, U.S. Department of the Interior, Washington, DC.

Wilson, S. F., A. Hahn, A. Gladders, K. M. L. Goh, and D. M. Shackleton. 2004. Morphology and population characteristics of Vancouver Island cougars, Puma concolor vancouverensis. Canadian Field-Naturalist 118:159-163.

Wolfe, M. T., E. M. Gese, P. Terletzky, D. C. Stoner, and L. M. Aubry. 2015. Evaluation of harvest indices for monitoring cougar survival and abundance. Journal of Wildlife Management 80(1):27-36.

Wong, D., M. A. Wild, M. A. Walburger, C. L. Higgins, M. Callahan, M. L. A. Czarnecki, E. W. Lawaczeck, C. E. Levy, J. G. Patterson, R. Sunenshine, P. Adem, C. D. Paddock, S. R. Zaki, J. M. Peterson, M. E. Schriefer, R. J. Eisen, K. L. Gage, K. S. Griffith, I. B. Weber, T. R. Spraker, and P. S. Mead. 2009. Primary pneumonic plague contracted from a mountain lion carcass. Clinical Infectious Diseases 49(3):33-38.

Young, S. P., and E. A. Goldman. 1946. The puma: Mysterious American cat. American Wildlife Institute, Washington, DC.

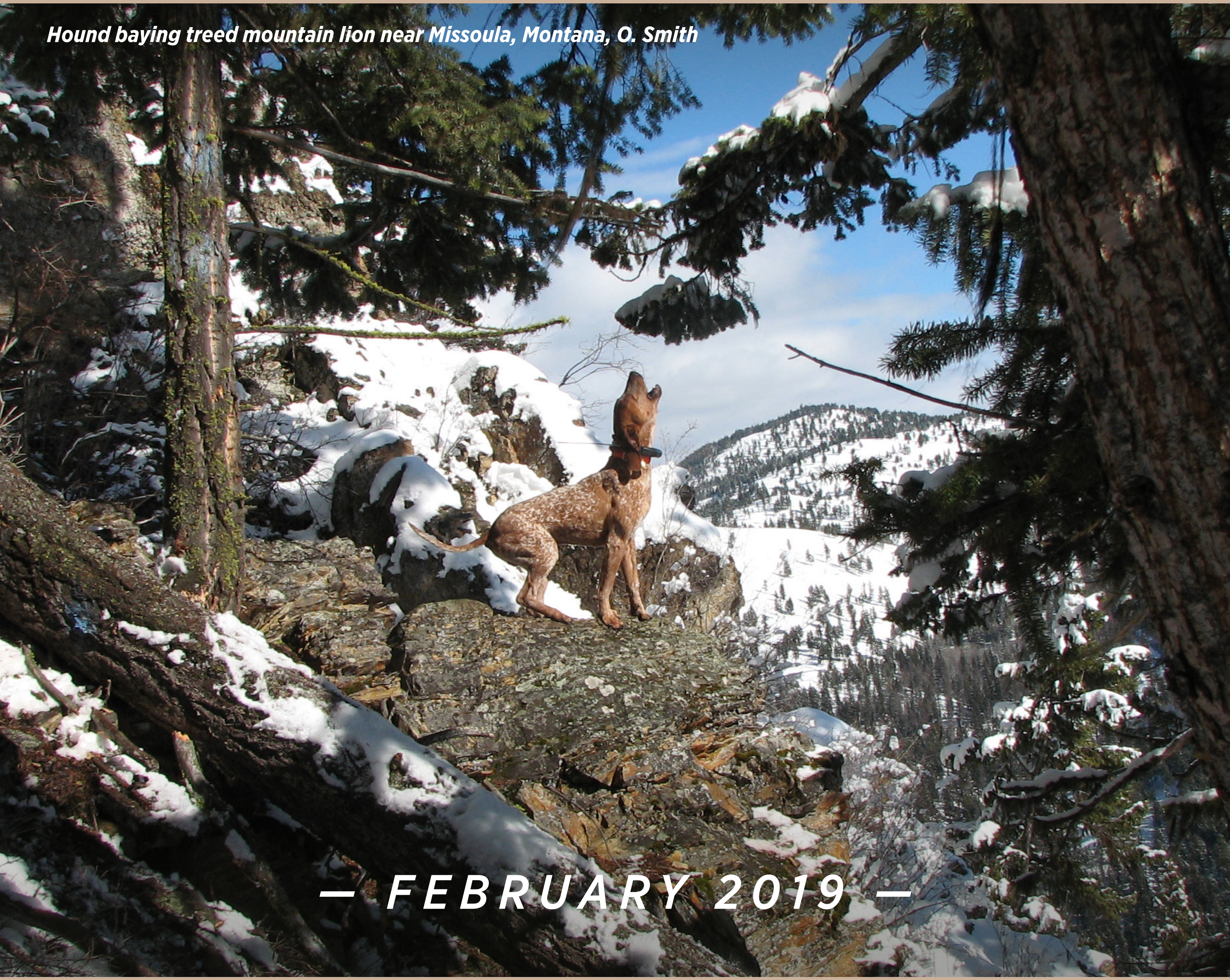

Hound baying treed mountain lion near Missoula, Montana, O. Smith

This publication produced in part with Pittman-Roberts funds, under grant #W-154-M.
