# Supplementary material for: The Elephant in the room: What can we learn from California regarding the use of sport hunting of pumas (Puma concolor) as a management tool?
Source: PLoS One. 2020 Feb 13;15(2):e0224638. doi: 10.1371/journal.pone.0224638 (PMC7018503; doi:10.1371/journal.pone.0224638)
Supplement: S2 File — This is a zip file that contains sources of the data used in the analyses that are no longer available on the internet. (ZIP) [file pone.0224638.s003.zip › OR-A-ODFW-Broman-2018-Lion-Mortality.pdf]

Oregon
Kate Brown., Governor

Department of Fish and Wildlife
Office of the Director
4034 Fairview Industrial Drive SE
Salem, OR 97302
(503) 947-6044
FAX (503) 947-6042
odfw.com

Date: February 21, 2019

To: The Honorable Brad Witt, Chair
House Committee on Natural Resources

House Bill 2795
Derek Broman, Carnivore-Furbearer Coordinator
Dr. Doug Cottam, Wildlife Division Administrator
Oregon Department of Fish and Wildlife

The Oregon Department of Fish and Wildlife (Department) appreciates the opportunity to provide information regarding cougar management in Oregon.

SUMMARY

- Cougar management in Oregon has been guided by a detailed, standalone cougar management plan for over 30 years (1987). Plan updates in 1993, 2006, and 2017 have produced a 200+ page document that guides all aspects of cougar management now and into the future.
- The Department maintains numerous, highly detailed databases for use in monitoring and managing cougars. This large amount of information is possible through mandatory check in and sample collection of all known cougar mortalities, mandatory reporting for hunters, and Department staff use of the Wildlife Damage Database where detailed records of complaints are recorded.
- The extensive amounts of information collected by the Department allow for the construction and maintenance of a peer-reviewed population model to monitor populations. Data also allows for examining various indexes on population trends based on research findings.
- Cougar research has been conducted in Oregon for over 40 years. Research findings have providing invaluable insight on many areas such as cougar behavior, ecology, habitat, predator-prey relationships, management responses, and population size and status. These efforts have produced over 40 scientific publications, dissertations, and theses on Oregon cougars. Two research projects are currently underway.

Oregon Cougar Management History

The status and management of cougars in Oregon has a long and varied history. Three time periods best represent that history: (1) Unprotected Predator, (2) Game Animal Classification, and (3) Current Management.

1800s – 1967 - Unprotected Predator -- Cougar population decline

Bounties were placed on cougars and other "predators" as early as 1843. During this time there were no restrictions on how cougars could be killed. By statute cougars were defined as

"predators" and were not protected by any laws or regulations. Similar to wolves, many large carnivores were greatly impacted by poison used for predator removals. The 1961 statewide cougar population was estimated at approximately 200. In 1967, the Oregon Legislature, at the urging of houndsmen and other concerned sportsmen, classified the cougar as a game animal which gave the Oregon State Game Commission (now the Department) management responsibility.

1967- 1994 - Game Animal Classification -- Cougar population recovery

Since 1967, the statewide cougar population has made a remarkable recovery. A 1980 statewide wildlife planning update delineated approximately 50 percent of the state as cougar habitat and estimated the population at 1,800 animals. By 1993, the Department estimated the statewide population at about 3,000 animals occupying approximately 80 percent of the state. The controlled hunt system was considered appropriate for addressing cougar damage complaints while meeting goals to maintain healthy cougar populations and provide hunting opportunity. The system allowed the Department to change harvest rates annually in response to changing conditions, as well as concentrate hunting efforts in areas with excessive damage problems.

Game animal status allowed the Department to manage cougars by setting hunting seasons and controlling harvest rates. Primarily in response to livestock damage complaints in northeast Oregon, the Department authorized the first controlled cougar season in 1970 (25 tags available). The number of controlled hunt areas and tags available gradually increased through 1994 (588 tags available). The number of controlled cougar seasons, tag numbers, and extent of hunting areas were based on a combination of previous cougar mortality patterns in the area, age and sex composition of the known mortalities, and trend in the number and type of complaints received.

Post-1994 - Management Changes -- Current cougar populations

Cougar management changed dramatically in 1994 when Measure 18, a citizen Ballot Initiative, passed making it unlawful for cougar hunters to use dogs (ORS 498.164). With expectations of a marked decline in hunter success rates, the Department changed cougar hunting from controlled hunts with a limited number of highly successful hunters (hunter success of 27–64%, typically ~40%) to an unlimited general statewide season with hunter success ~1%.

In 1999, the Oregon Legislature adopted a new statute allowing persons to legally take cougars posing a threat to human safety without a permit (ORS 498.166). In 2003, ORS 498.012 was modified to expand allowable take of wildlife causing damage, including cougars, to also allow take of animals posing a public health risk, or causing a public nuisance. Although the absolute number of cougars harvested has returned to the pre-1994 level, the impacts of harvest before and after 1994 are not directly comparable. Changes in hunting techniques, distribution of the harvest, and age structure of the harvest post-1994 resulted in additional cougar population growth. Associated with these changes in management goals and cougar populations, there have been changes in public attitudes regarding cougar management in Oregon. Based on what the Department heard, the public comments can be broken into two general groups. Those concerned about the impacts of an increasing cougar population on big game herds, damage to livestock and threats to human safety, and those concerned that cougars should be protected from hunting or all lethal take.

The Department has evaluated all these changes and incorporated them into the development of the 2017 Cougar Management Plan.

Oregon Cougar Management Plan

The Department's first cougar management plan was adopted by the Oregon Fish and Wildlife Commission (Commission) in 1987. An updated and revised plan was adopted by the Commission in 1993 and 2006. In 2016, the Department undertook an update of the 2006 plan which resulted in the 2017 Oregon Cougar Management Plan. The draft plan chapters include information on Oregon cougars; cougar management objectives; and cougar management goals incorporated into an adaptive management approach for the future. These chapters contain a significant amount of information and data on cougar biology, population trends, research findings, damage and conflict, and management activities. The Plan can be found on the Department website: www.odfw.com/wildlife/cougar/docs/2017_Oregon_Cougar_Management_Plan.pdf

Similar to the 2006 Plan, the 2017 plan establishes objectives that seek to maintain viable and healthy cougar populations in Oregon, reduce conflicts with cougars, and manage cougars in a manner compatible with other game mammal species.

> **Objective 1** seeks to manage the state's cougar population at a level well above that required for long term sustainability. Achieving and monitoring this objective is complicated but empirical data and numerous indices are used to assess population status. Zone management with mortality quotas are used to ensure harvest does not reduce the population below objective levels. Population models and proportions of adult females in the harvest are used to monitor cougar populations and data will continue to be collected in intensive, small scale research studies.
>
> **Objectives 2 – 3** address solving conflict. The primary strategy to solve conflict since 1995 has been to give advice and, when necessary, remove the problem animal.
>
> **Objective 4** seeks to achieve established management objectives for other game mammal species.

All management activities are carried out in an adaptive management approach, as suggested in the Cougar Management Guidelines, which allows for monitoring, evaluation, and changes in management based on results. Numerous indicators will be used to monitor success. Total mortality, hunter harvest success rates, and biological data will continue to be collected. These data will contribute to population modeling for each Cougar Management Zone. Cougar-human conflict will continue to be monitored using non-hunting mortalities and complaints concerning human safety, pets, and livestock. Research projects will collect information on movements, density, predation rates, and will be able to better detect other factors such as disease.

The Department's mission is to protect and enhance Oregon's fish and wildlife and their habitats for use and enjoyment by present and future generations. Cougar management is complicated by the dichotomy of sentiment toward cougars among Oregon residents. This plan presents the Department's strategy to meet its mission and incorporate public attitudes and desires. It is a plan that will be updated and rewritten as agency policies, new biological data, and human and/or cougar populations change.

Zone-based Quota Management

The Department established a zone-management system with mortality quotas starting in 1995 which is used to ensure harvest does not reduce cougar populations below minimum population levels. During 1995-2005, cougars counted towards quotas were only those harvested by hunters. Since adoption of the 2006 Cougar Plan, all known mortalities (e.g., hunter-harvest, damage take, human-safety take, administrative removal, road-killed) count toward zone quotas. Oregon is

divided into 6 cougar management zones that were delineated to include similar habitats, human demographics, land use patterns, prey base, and cougar density (Figure 1). The six cougar zones average 16,195 mi^2 (range 8,465 mi^2 in Zone D to 28,003 mi^2 Zone F) and consist of multiple Wildlife Management Units (WMU). If a zone quota is met, that zone is closed to hunting and target area administrative removals for the remainder of the year, but the zone does not close to take related to livestock damage and human safety. Because hunting seasons for cougar are January 1 to December 31 each year, any closed zone reopens for the next season on January 1 of the year following the closure.

Based on the April 2017 cougar model update, from 2006-2016, annual zone quotas represented on average 13% of the modeled state population of all age classes, but over that time an average of 4% mortality from hunting and 8% mortality from all sources occurred. The most recent zone closure occurred in Zone A (Coast/North Cascades) in 2018 when the quota of 180 cougars was reached for the first time and the zone was closed to hunter-harvest of cougars. This is the seventh time since implementation of the quota system in 1995 that a zone quota has been met (Table 1).

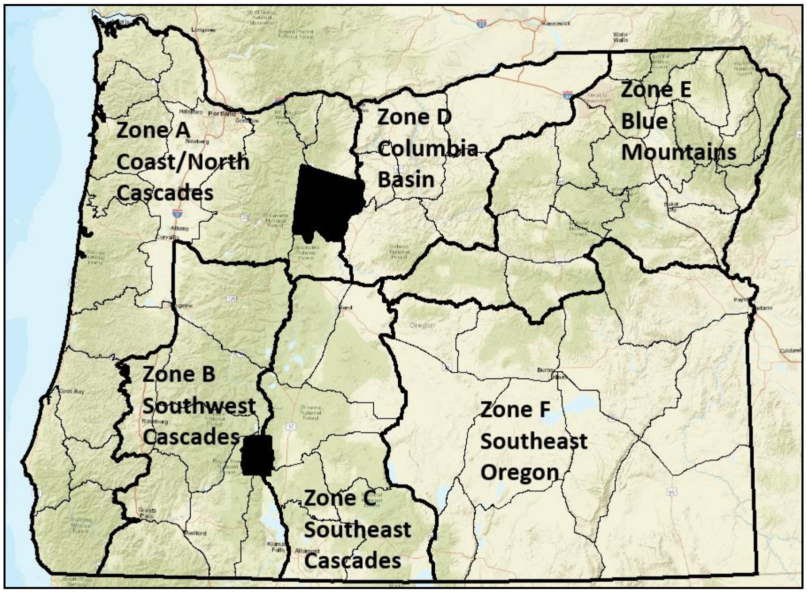

Figure 1. Location and name of the six Oregon cougar management zones. Wildlife Management Units (WMUs) delineated by thinner black lines.

Table 1. Total cougar mortality by cougar management zone in Oregon 2008 – 2018. Years where Zone Quota was met are highlighted in **bold**. Quotas have been in place since 1995 and other Zone closures include Zone D (2002) and Zone E (2001, 2002).

Zone	*Zone Quota*	Total Mortality 2008	2009	2010	2011	2012	2013	2014	*Zone Quota*	Total Mortality 2015	2016	2017	2018
A Coast/N Casc.	*120*	95	99	103	**120**	**121**	**130**	101	*180*	117	177	157	**194**
B SW Cascades	*165*	106	90	96	109	106	143	100	*200*	98	131	140	155
C SE Cascades	*65*	25	*25*	20	15	24	21	17	*80*	25	42	43	40
D Col. Basin	*62*	35	38	31	36	38	50	26	*100*	41	27	38	39
E Blue Mtns.	*245*	174	158	163	169	164	135	93	*270*	106	114	142	115
F SE Oregon	*120*	57	63	69	57	77	52	45	*140*	42	55	51	45
Total	*777*	492	473	482	506	530	531	383	*970*	427	546	571	588

Population Monitoring

The Department monitors cougar abundance using two criteria: a deterministic, density-dependent model is used to estimate population abundance at zone and state levels, and the proportion of adult female cougars in the harvest is used at the zone level. For example, given sufficient sample sizes (i.e., annual harvest of >25% of total population), there is scientific evidence that cougar populations do not begin to decline until adult (2:3 yr old) females comprise at least 25% of the harvest (Anderson and Lindzey 2005). At the zone level, the Department has an objective of maintaining a 3-year average proportion of adult females in the total mortality at no more than 25-35%. No zones currently meet this objective.

Deterministic Model

Since 1995, the Department has used a published, deterministic, density-dependent population model to monitor cougar populations (Keister and Van Dyke 2002). The cougar model utilizes extensive, long-term data collected from cougars in Oregon. The model incorporates measured productivity and observed mortality (all sources including illegal take) to calculate changes in the cougar population.

During development of the 2006 Plan, the Keister and Van Dyke (2002) model was updated with new data collected in Oregon. In addition, models were created for each of six cougar zones and each model incorporates zone-specific parameters such as habitat and density. The statewide cougar population estimate is the sum of the six zone estimates. Each zone model is updated annually using cougar mortality data and any new biological information if available. These zone and statewide estimates provide insight on population trends and determining potential impacts of management activities and mortality sources, but less attention is given to exact population estimates.

The model has indicated growing cougar populations across the Oregon for many years. When considering all the research and biological information available such as mortality rates, proportions of adult females in total mortalities, age structure, survival rates, growth rates, distribution, densities, reproductive rates, and other factors, the model's indication of growing cougar populations is thoroughly supported. Published literature also supports the model's suggested growth, particularly those identifying population responses to varying mortality rates.

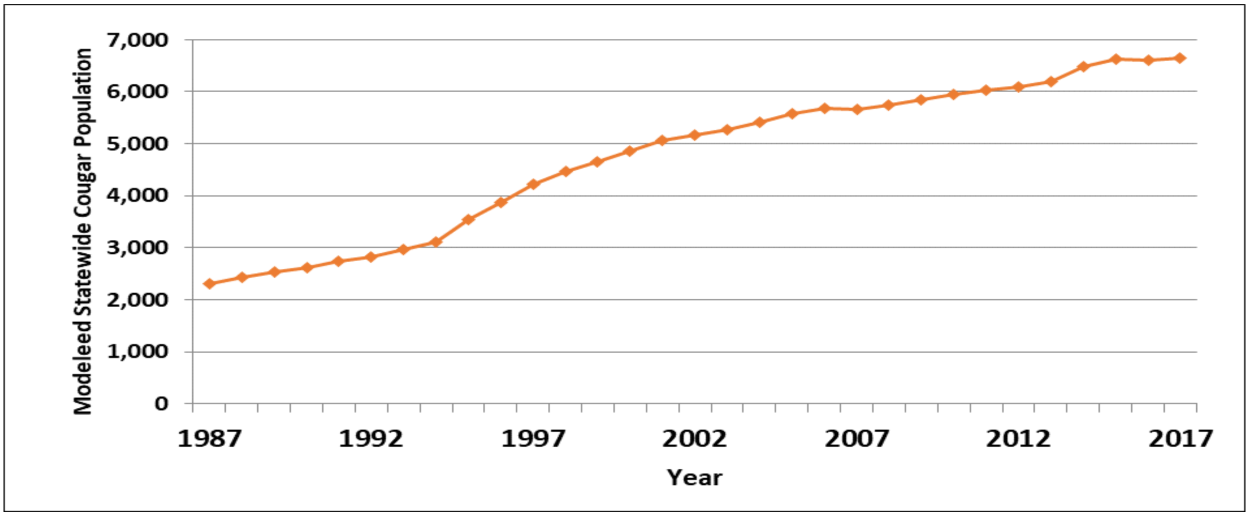

Figure 2. Modeled statewide population abundance of all age classes of cougars in Oregon during 1987-2017, based on results from the deterministic, density-dependent population model (updated in 2018).

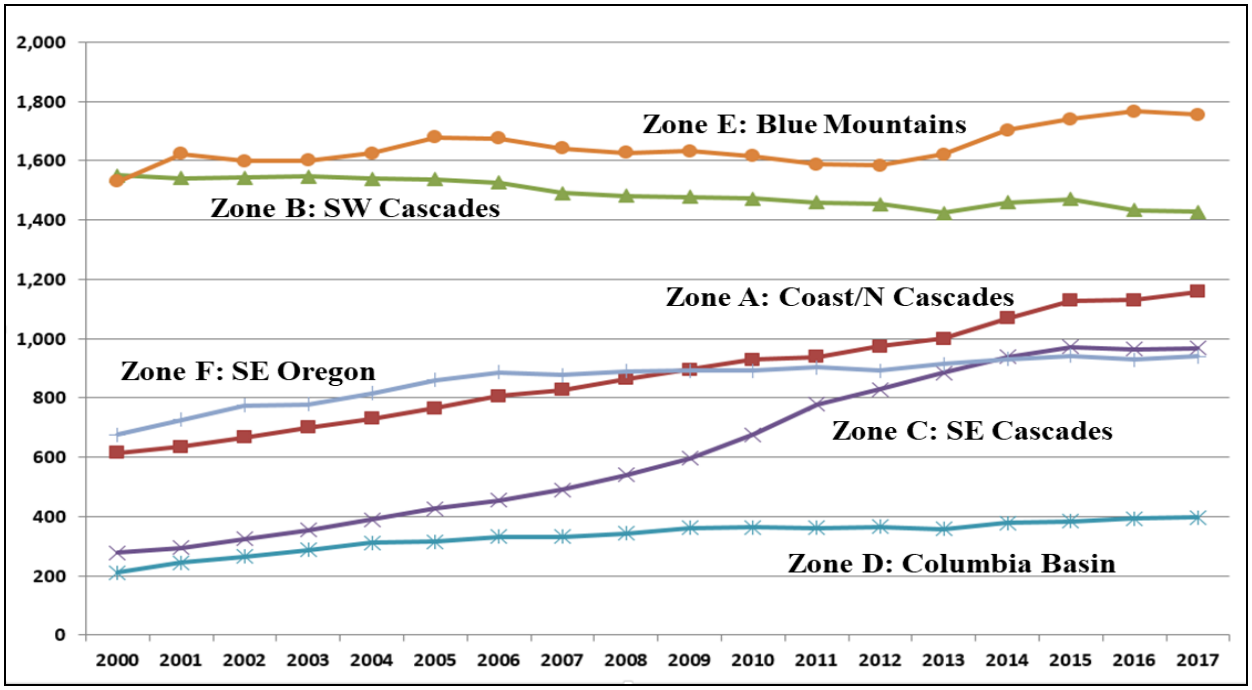

Figure 3. Estimated zone-level population abundance of all age classes of cougars in Oregon during 2000–2017, based on results from deterministic, density-dependent population model (updated 2018).

Hunting Season Structure

During 1994, the passage of Measure 18 resulted in the prohibition of the use of dogs to hunt or pursue cougars, with certain exemptions such as for agents appointed by and acting on behalf of the Department to implement management actions, or for landowners to address damage or human safety concerns. Over the next several years, the Department implemented several regulatory changes in an effort to address the expected dramatic decline in hunter success rates. During 1995, the Department changed cougar hunting from a controlled hunt system to a statewide, unlimited general season using a quota-based system (see below) and increased season length from 2½--4 months to 7 months; the season length was increased to 10 months in 2001 and to the current year-long statewide season based on the calendar year (Jan 1-Dec 31) in 2010.

During 1997, the Oregon State Legislature decreased the cost of a cougar tag from $51.00 to $10.00 and created the Sports Pac license option for residents, which automatically issued a cougar tag with purchase of this license package. During 2010, cougar tags were set at the current $14.50 for both resident and non-resident hunters. If a hunter purchases their general season cougar tag prior to the established tag sales deadline, they may also purchase an additional general season cougar tag.

Successful hunters must present the pelt with skull and proof of sex attached at a Department office within 10 days of harvest. The Department collects harvest data during this mandatory check-in process, including a tooth to age individual cougars, and tags each pelt; the reproductive tract of female cougars is also required for collection of reproductive data. This process is required for all known cougar mortalities including damage, human safety, or road-killed animals.

Currently, most cougars are harvested by hunters that randomly encounter a cougar while hunting for other species, but are in possession of a cougar tag. In 2015, of the reporting cougar tag holders that harvested a cougar, 66% did so while pursuing other game. Some hunters continue to hunt specifically for cougar outside of the deer and elk seasons when snow conditions allow animals to be tracked or by using a predator call. Hunter harvest has remained relatively stable for over a decade and average annual statewide harvest was 261 (range 209-309) from 2007-2018. Hunter success rates are low with current harvest techniques and range from 1-2%.

Table 2. Trends in cougar complaints, damage, harvest, and other mortality in Oregon during 2007–2018. Complaint and mortality data current through 1/9/19. Numbers may change as late data are added.

Year	Number of Complaints[a]	Tags Sold[b]	Hunter-Harvest	Damage[c]	Human Safety[d]	Administrative Removals[e]	Roadkill	Other[f]	Total
2007	453	41,813	309	114	21	52	19	22	537
2008	515	43,211	272	109	23	34	19	35	492
2009	432	45,375	274	110	31	21	15	22	473
2010	465	48,776	240	99	25	79	14	25	482
2011	500	50,889	241	139	23	71	12	20	506
2012	419	53,698	253	130	46	56	17	28	530
2013	359	55,072	292	148	24	36	9	22	531
2014	404	56,114	209	124	27	0	16	9	385
2015	444	57,387	233	134	22	0	24	20	433
2016	421	58,091	268	152	18	71	19	18	544
2017	462	57,950	292	151	27	60	16	25	571
2018*	661	61,022	271	177	37	67	22	14	588

[a]Number of complaints received during the calendar year. Sightings not associated with damage or public safety concerns are not included.
[b]Includes general and additional tags (including Sports Pac licenses).
[c]Number of animals killed as a result of damage during a calendar year.
[d]Animals killed as a result of real or perceived threat to humans or pets.
[e]Administrative removals on cougar target areas (2007–present only).
[f]Includes roadkill, accidental, found dead, and illegal kill.
[g]Hunting season changed to calendar year.
*as of 1/9/19

Cougar Complaints

Cougar complaints consist of the contacts received by the Department and USDA Wildlife Services (WS) regarding conflict with cougar. The Department has been recording complaints for over 30 years, although a standardized reporting system was implemented in 2001. The Department currently manages complaints in the Wildlife Damage Database and there have been a few updates to the database with the most recent occurring in 2017. The Wildlife Damage Database has 18 primary complaint types to describe the particular complaint but are primarily grouped into three main categories: Safety, Damage and Other. Sightings reported to the Department with no discernable concern expressed by the reporting person are not counted as a complaint. Numerous other details are included in the database including if the complaint was verified, the complaint occurred inside city limits, the complaint was a repeat occurrence, and the estimated cost of pet/livestock loss. All of these additional details aid in quantifying the situation at hand and help determine the appropriate response.

Department staff evaluates each complaint and respond within established legal and policy frameworks and Appendix G of the Plan provides a summary of how the Department responds to complaints. At every opportunity, the Department provides advice and education to the public to reduce current and future conflict.

With the exception of Zone A (Coast/N Cascades), cougar complaints are stable or declining across much of Oregon. Department staff speculate that declining cougar complaints may be due to the local public being familiar with how to live with cougars, they know how to resolve their issue, or they are familiar with their legal options (ability to lethally remove via ORS 498.012). On the other hand, cougar complaints have been increasing in areas of cougar population growth, where human-cougar interactions are a relatively new occurrence, or the local public is unexperienced with how to live with cougars (e.g. Zone A).

Damage and Safety Mortalities

The number of cougars killed in Oregon due to livestock damage or human safety/pet conflict has been stable statewide and in eastern Oregon, but has been increasing in western Oregon. The average number of cougars taken annually on damage/safety statewide has increased from 23 cougars per year in 1987-1994, to 121 per year in 1995-2005, and 150 per year in 2006-2016. Over the same time, cougar populations have been increasing and expanding into new areas, some highly susceptible to conflict (e.g. urban, agricultural landscapes). Such is the case in western Oregon where the majority of Oregon's human population resides and there are significant numbers of small- and medium-sized livestock (e.g. goats, sheep). From 2006-2016, damage and safety mortalities comprise 31% of annual known cougar mortalities and the majority (80%) are the result of cougars killed as a result of causing damage to livestock.

Oregon Cougar Research

Cougar research has been conducted in Oregon for many decades and the Department has conducted four research projects, and has fifth underway (Table 3). Research has provided information for many biological parameters needed to model cougar populations, establishing population density in two management zones, and greater understandings of the relationship between cougars and their prey. This rich body of information includes over 40 scientific publications, dissertations, and theses, in addition to numerous project reports.

Table 3. Time span of primary field-based cougar research projects conducted in Oregon. The Mt. Emily and Alsea studies are underway at time of writing.

	1985	1986	1987	1988	1989	1990	1991	1992	1993	1994	1995	1996	1997	1998	1999	2000	2001	2002	2003	2004	2005	2006	2007	2008	2009	2010	2011	2012	2013	2014	2015	2016	2017	2018	2019
**Gagliuso (1991) Toketee**	■	■	■																																
**Catherine Creek**					■	■	■	■	■	■	■	■																							
**Jackson Creek**									■	■	■	■	■	■	■	■	■	■																	
**Nowak (1999) Catherine Creek**												■	■	■																					
**Wenaha-Sled Springs-Mt Emily**																	■	■	■	■	■	■	■	■											
**Sled Springs**																	■	■	■	■	■	■	■	■											
**Toketee/ Steamboat**																		■	■	■	■	■													
**Mt Emily**																									■	■	■	■			■	■	■	■	■
**Alsea**																																	■	■	■

Catherine Creek Study

The Department initiated a study in the Catherine Creek WMU in 1988 to determine cougar population density. Additional objectives included documenting cougar diet, productivity, survival, dispersal, and effects of hunting on the population. Hunting regulations at that time included controlled hunting and use of dogs.

Between January 1989 and April 1995, 72 cougars were captured and 58 individuals were radio-collared during seven capture seasons. Twenty-seven mortalities of radio-collared cougars were documented. Five different mortality causes were determined, with hunting accounting for 67% of all cougar deaths. Documented deaths did not all occur within the study area, but included radio-collared cougars that dispersed or were otherwise outside the study area boundary. Hunting accounted for 18 cougar deaths within the study boundary and included 11 radio-collared and seven unmarked cougars.

Jackson Creek Study

In December 1993, the Department initiated the Jackson Creek study to determine cougar population parameters in the south Cascades. A total of 113 cougars (58 male, 55 female) were captured and radio-collared during 11 capture periods between December 1992 and May 2003. When the study ended in 2004, 33 cougars were being monitored, 65 had died (37 males, 28 females) and 15 were unaccounted for (transmitter failure was suspected in some cases). Regulated hunting during the study varied greatly. During the first 2 years, hunting was regulated via controlled hunt drawing with limited tags and the use of dogs was legal. After passage of Measure 18 in 1994, use of dogs was prohibited statewide and cougar hunting opportunity has gradually shifted to longer, general cougar seasons and increasing numbers of cougar tags.

Percentage of mortalities due to a single cause varied annually. Prior to Measure 18 in 1994, legal harvest was the highest mortality cause. After 1997, natural mortality (particularly disease/parasites) was the highest mortality cause for adult and sub-adult cougars. Between May 2000 and June 2002, 14 natural-caused mortalities were documented. During the early study years (1993- 1997) the percentage of radio-collared cougars that died annually was variable, and reached nearly 70% during 1996 when eight of 12 radio-collared cougars died. During 1998-2002, more than 30 cougars were marked, and fluctuations in annual mortality were less erratic. Additional data analyses on capture, home range size and overlap, reproduction, sub-adult dispersal, population density estimates, age-specific survival rates, and causes of mortality are currently being conducted.

Toketee, Steamboat, and Sled Springs-Wenaha Project

This research was designed to examine how two factors, carnivore (black bear, cougar) density and elk nutritional condition may act independently or interact to affect calf recruitment. Elk nutritional condition and landscape carrying capacity was used as a surrogate to habitat quality. This research approach was conducted in both northeast and southwest Oregon to provide a broad geographic and physiographic contrast.

The cougar component of this research had two primary objectives: (1) to estimate movements, survival, and densities of cougar on the study sites in southwest and northeast Oregon, and (2) to test whether predation by cougars is an additive or compensatory source of mortality for elk calves in southwest and northeast Oregon. Methods included capturing and radio-collared cougars within the study sites and estimating densities based on home range size, movements, and capture effort. Survival of radio-collared juvenile elk increased as cougar density decreased. The highest survival rates of elk calves were in the Toketee study area where cougar density was the lowest of the four study sites.

Mt. Emily Project

The most recent cougar research project completed by the Department is the Mt. Emily project conducted during 2009–2012 in northeast Oregon. This project was developed with five primary objectives: 1) to investigate the diet, kill rates, and prey selection of cougars; 2) to develop methods to estimate cougar populations; 3) to compare survival and mortality patterns of cougars from 3 studies conducted from 1989 to 2011; 4) to develop a population model for cougars that can be used to evaluate management scenarios that incorporates hunting, immigration, and emigration; and 5) to develop a population model for elk incorporating cougar predation rates and nutritional components for elk.

The Department captured and radio-collared 25 adult cougars with GPS collars to identify potential kill sites through field investigation of clustered locations of individual cougars. The results of this study indicated cougars killed more frequently during summer when their diets were dominated by juvenile ungulates, females killed more frequently than males, and females with kittens killed more frequently than those without kittens. Female cougars had a larger percentage of deer in their diets (~80%) than males (~50%). While deer comprised about 70% of the prey items, cougars did not show selection for any age or sex class of deer during summer, but selectively preyed upon fawns during winter. Cougars did show a strong selection for elk calves during summer, but did not show patterns of selection for any age class or sex of elk during the remainder of the year.

A second manuscript was also published in the Journal of Wildlife Management on a method to estimate cougar populations using DNA samples from cougars. In this work, innovative methods were developed that relied on using dogs trained to locate cougar scat from which DNA could be isolated to identify individuals. Density estimates were among the highest reported in western North America (*see* Density section). Using recent statistical advances in estimating populations, this method may provide a useful tool to estimate cougar populations. Rather than relying on multiple-year capture-recapture efforts, cougar population estimates can be obtained in less than 1 year.

The Mt. Emily project was restarted in late 2014, with data collection continuing into 2019. The current research is occurring following the establishment of wolves in the Mt. Emily WMU. The objectives of the current research are to determine the effects of wolves on cougar diets, kill rates, habitat use, survival, and densities. Any potential changes that are observed will guide cougar management in the presence of wolves and identify potential effects on deer and elk populations.

Alsea Cougar Study

A cougar study was initiated in 2017 in the Alsea Wildlife Management Unit in the central coast range. To date, 12 cougars have been GPS-collared and 19 genetic samples have been collected. This study is examining habitat and area use in the coast range among other things, and information from this study can be used to update the Zone A cougar model.

Other Analyses

A third and fourth manuscript were published in the Journal of Wildlife Management and Northwest Science, from the Mt. Emily study and other studies. These manuscripts summarize survival rates of cougars under different management scenarios (pre- and post-Measure 18) by sex and age class. Survival rates of male cougars varied across three study areas (Catherine Creek 1989–1997; Jackson Creek 1993–2001, Wenaha-Sled Springs-Mt. Emily 2001–2012); with male survival

lowest during periods it was legal to hunt cougars with dogs. Survival rates of female cougars and cougar kittens were similar among study areas. Human-caused mortality was the primary cause of mortality in northeast Oregon (~70%), regardless of whether it was legal to hunt cougars with dogs, however, disease and natural mortality were the primary causes of cougar mortality (~70%) in southwest Oregon. Survival rates in both areas were similar, suggesting cougar harvest is at least partially compensatory for natural mortality. Further, survival rates of radio-collared cougars in the post-Measure 18 era in Oregon are high and only slightly below survival rates reported for cougar populations that were lightly hunted in and adjacent to Yellowstone National Park and in the San Andres Mountains, New Mexico. The high survival rates of cougars in Oregon provide evidence that cougar populations are sustainable and not threatened by hunting.

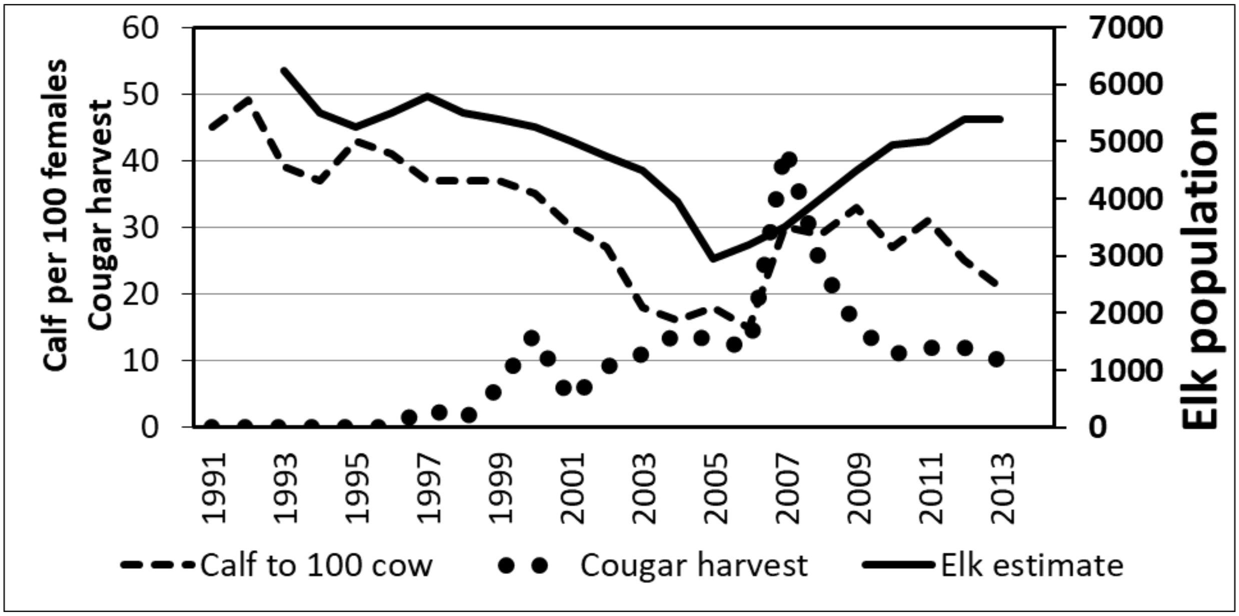

Figure 4. Trend in elk population and cougar harvest for the Heppner Cougar Target Area.

A fourth analysis estimates cougar population growth rates under a variety of management scenarios and how quickly a local cougar population can recover from heavy exploitation such as demonstrated in the Heppner Target area (Appendix J of 2017 Plan). Evaluation of data from the Heppner target area indicated that with removal of 50% of cougars, the elk population responded rather quickly (Figure 4). Observed calf ratios increased from the teens to the low 30's. The elk population increased from about 3,000 to over 5,000. Modeling incorporated information from the body of cougar research conducted in Oregon and adjacent states. Results indicate that even in the absence of immigration, cougar populations can recover to pre-reduction numbers within five years. With moderate rates of immigration, cougar populations can recover within about two to three years.
