# Supplementary material for: The Elephant in the room: What can we learn from California regarding the use of sport hunting of pumas (Puma concolor) as a management tool?
Source: PLoS One. 2020 Feb 13;15(2):e0224638. doi: 10.1371/journal.pone.0224638 (PMC7018503; doi:10.1371/journal.pone.0224638)
Supplement: S2 File — This is a zip file that contains sources of the data used in the analyses that are no longer available on the internet. (ZIP) [file pone.0224638.s003.zip › sheep predator loss 1990.pdf]

Sheep and Goat Predator Loss

National Agricultural Statistics Service

United States Department of Agriculture

Agricultural Statistics Board

Washington, D.C.

RELEASED: April 24, 1991
3:00 P. M. ET

In this issue:

* Losses of Sheep and Lambs from Coyotes, Dogs and Total Predators by State
* Losses from All Predators by Region
* Value of All Predator losses for Sheep and Lambs by State
* Number and Value of Goat Losses for 5 States

A total of 490 thousand sheep and lambs were lost from animal predators during 1990, in the United States, according to the Agricultural Statistics Board. This resulted in a loss of $21.7 million for farmers and ranchers. This is 36.9 percent (%) of the total losses from all causes.

Coyotes were the largest cause of sheep and lamb losses to predators, with 63.7% of the total losses from all animal predators. Most losses were in the West, but substantial losses from coyotes were prevalent throughout the United States. The value of losses for both sheep and lambs caused by coyotes was $13.6 million. **Dogs** were the second largest cause, accounting for 13.6% of the sheep and lamb losses due to predators.

Other common predators were mountain lions, bears, foxes, eagles, and bobcats. Wild pigs were a major threat in Hawaii.

Losses of Sheep and Lambs from Predators,
Number of Head, and Total Value, United States, 1990

Predator	Sheep and Lambs	Total Predators	Total Value
	**Head**	**Percent**	**Dollars**
Coyotes	311,900	63.7	13,555,500
Dogs	66,400	13.6	3,424,875
Mountain Lions	16,800	3.4	814,875
Bears	8,000	1.6	454,475
Foxes	12,800	2.6	451,550
Eagles	17,700	3.6	622,500
Bobcats	13,600	2.8	493,750
All Other Animals	42,300	8.7	1,878,175
US	489,500	100.0	21,695,700

 For Information Call Linda Simpson 447-3578

Coyotes were also the largest predator of **Goats** in the five major producing States, accounting for over half of the total losses caused by predators. The value lost was $5.66 million.

Survey Procedures: Primary data for sheep and lamb loss estimates were from a sample of agricultural producers across the **US** using probability surveys in all States **(except Alaska which was not in the survey)** and an additional non-probability survey in a few Western States. The five major goat producing States (see **Terms and Definitions**) also collected losses for Angora, Spanish, and other goats. The probability survey included information from a list of about 57,300 agricultural producers plus additional information from operators of about 7,500 small land area tracts. Information from the probability survey was collected during the first half of January by mail, telephone, and personal interview. Data for the non-probability sample was obtained later. No similar Nationwide survey on predator losses for sheep, lambs, or goats is currently planned for any future date.

Revision Policy: Estimates for inventory numbers and losses to all causes are subject to revision in the following year's report and also after data for the five year Census of Agriculture, conducted by the Department of Commerce, are available. No revision to predator loss estimates is planned.

Estimation Procedures: The estimates were prepared by the Livestock, Dairy, and Poultry Branch, National Agricultural Statistics Service **(NASS)**, and reviewed by all 49 states. Total death losses from all causes for sheep and lambs were as reported in **Meat Animals, Production, Disposition, and Income - 1990 (released April 12, 1991)**. In setting the predator loss estimates, first total predator losses were estimated as a percent of total losses then specific predator losses were generally estimated and rounded to the nearest 100 head using the percent of total predator losses as indicated by the survey data. States were combined into regions (see **Terms and Definitions**) when sampling variability did not permit individual State data to be released.

Reliability: Since all operations with sheep were not included in the sample, survey estimates are subject to sampling variability. This variation, as measured by the relative standard error, was 4.1 percent at the **US** level for stock sheep inventory and 4.3 percent for sheep on feed inventory. This means that chances are approximately 95 out of 100 that the survey estimate will be within plus or minus 8.2 percent for stock sheep inventory and 8.6 percent for sheep on feed inventory of the value that would result from averaging all possible samples selected from the same population and surveyed using the same procedures. The relative standard error for the specific losses caused by predators was higher.

Survey estimates are subject to non-sampling errors such as omissions, duplications, and mistakes in reporting, recording, and processing the data. These errors cannot be measured directly, but they are minimized through rigid quality controls in the data collection process and a careful review of all reported data for consistency and reasonableness.

Terms and Definitions:

Docked: Lambs with tails removed.

Goats: Any of various hollow-horned ruminant animals (especially of Genus Capra) related to sheep but of lighter build and with backwardly arching horns. For this report included Angora (raised for mohair production) and other goats (for example, Spanish goats raised for meat and milk goats). Major goat producing States are Arizona, Michigan, New Mexico, Oklahoma, and Texas.

Lamb: Sheep less than one year old. In the Western States lamb losses are after docking. For this report, lambs on feed are included.

Regions: Regions were defined to allow comparison with earlier USDA research on sheep and lamb losses. States in each region are:

Mountain and Western:

Arizona	California	Colorado	Hawaii
Idaho	Kansas	Montana	Nebraska
Nevada	New Mexico	North Dakota	Oklahoma
Oregon	South Dakota	Texas	Utah
Washington	Wyoming		

Midwest:

Arkansas	Illinois	Indiana	Iowa
Michigan	Minnesota	Missouri	Ohio
Wisconsin			

Southern and Eastern:

Alabama	Connecticut	Delaware	Florida
Georgia	Kentucky	Louisiana	Maine
Maryland	Massachusetts	Mississippi	New Hampshire
New Jersey	New York	North Carolina	Pennsylvania
Rhode Island	South Carolina	Tennessee	Vermont
Virginia	West Virginia		

Sheep: Mature animals, one year old and older. For this report, includes stock sheep and sheep on feed.

Sheep and Lambs on Feed: Sheep and lambs intended for slaughter market that are being fed grain or other concentrates, or are being pastured on succulent grasses (for example, alfalfa) or crop residues (corn stalks, beet tops) are expected to produce a carcass that will grade select or better.

Stock Sheep: Sheep in the breeding flock, including ewes and rams used for breeding, wethers one year old and older, ewe lambs, and ram lambs.

Value: Sheep - A straight average of the value per head of ewes one year old and older from the January 1, 1990, NASS probability agricultural survey and the January 1, 1991, NASS probability agricultural survey (see **Survey Procedures**) for each State. Lambs - Each State's 1990 market year average price for lambs applied to an average weight of 60 pounds per lamb. This reflects the opportunity losses of lambs.

If a State was not part of the NASS price estimating program in 1990 (Alabama, Arkansas, Delaware, Florida, Georgia, Mississippi, Rhode Island, and South Carolina), a value for sheep and a price for lambs were estimated based on surrounding States. Hawaii submitted a value and price estimate although they were not part of the program.

Losses of Sheep and Lambs by Coyotes and Dogs, by States, and United States, 1990

State	Coyotes: Sheep	Coyotes: Sheep Total Pred.	Coyotes: Lambs	Coyotes: Lambs Total Pred.	Dogs: Sheep	Dogs: Sheep Total Pred.	Dogs: Lambs	Dogs: Lambs Total Pred.
	Head	Percent	Head	Percent	Head	Percent	Head	Percent
AL	100	33.3	200	50.0	100	33.3	100	25.0
AZ	2,600	65.0	5,100	68.0	1,000	25.0	1,800	24.0
AR	100	50.0	400	50.0	*	---	300	37.5
CA	5,300	53.5	12,800	72.3	3,300	33.3	2,800	15.8
CO	5,900	65.6	26,300	86.2	400	4.4	300	1.0
CT	*	---	*	---	300	75.0	*	---
DE	0	---	0	---	*	---	0	---
FL	*	---	200	66.7	100	50.0	*	---
GA	*	---	*	---	100	50.0	200	66.7
HI	0	---	0	---	100	5.6	*	---
ID	2,600	72.2	6,200	81.6	400	11.1	500	6.6
IL	700	36.8	1,200	42.9	1,100	57.9	800	28.6
IN	100	5.0	200	8.7	1,800	90.0	2,000	87.0
IA	5,800	61.7	2,900	56.9	3,100	33.0	2,100	41.2
KS	1,300	65.0	1,300	72.2	600	30.0	400	22.2
KY	100	20.0	800	66.7	300	60.0	300	25.0
LA	200	50.0	200	66.7	*	---	*	---
ME	*	---	300	75.0	*	---	0	---
MD	0	---	*	---	100	50.0	100	33.3
MA	*	---	0	---	*	---	*	---
MI	100	25.0	200	25.0	200	50.0	500	62.5
MN	300	18.8	1,700	53.1	400	25.0	500	15.6
MS	100	33.3	300	75.0	100	33.3	*	---
MO	500	50.0	1,500	62.5	400	40.0	800	33.3
MT	6,100	80.3	19,100	83.0	600	7.9	400	1.7
NE	1,500	88.2	4,200	91.3	100	5.9	300	6.5
NV	3,200	71.1	6,300	68.5	300	6.7	400	4.4
NH	*	---	100	50.0	200	66.7	*	---
NJ	0	---	0	---	*	---	*	---
NM	4,300	43.0	10,600	39.3	300	3.0	1,100	4.1
NY	200	20.0	200	28.6	700	70.0	300	42.9
NC	0	---	0	---	500	83.3	1,400	93.3
ND	1,100	64.7	4,500	84.9	500	29.4	600	11.3
OH	200	13.3	1,700	54.8	1,200	80.0	1,300	41.9
OK	2,800	93.3	4,400	89.8	100	3.3	400	8.2
OR	3,200	62.8	9,900	52.7	1,200	23.5	7,100	37.8
PA	*	---	*	---	500	83.3	400	66.7
RI	0	---	0	---	*	---	*	---
SC	0	---	0	---	*	---	*	---
SD	8,200	94.3	20,700	91.2	300	3.5	300	1.3
TN	*	---	100	25.0	200	66.7	200	50.0
TX	16,000	59.2	40,000	50.0	4,000	14.8	4,000	5.0
UT	6,500	69.9	15,000	67.9	700	7.5	1,400	6.3
VT	*	---	300	100.0	*	---	0	---
VA	200	8.0	3,900	70.9	2,100	84.0	1,200	21.8
WA	200	50.0	1,100	78.6	100	25.0	200	14.3
WV	*	---	500	27.8	600	66.7	1,200	66.7
WI	300	60.0	900	69.2	100	20.0	200	15.4
WY	4,300	75.4	21,900	82.3	700	12.3	700	2.6
US	84,500	59.2	227,400	65.4	29,400	20.6	37,000	10.7

* Less than 100 head for the State, but number is included in US total.

Losses of Sheep and Lambs by Mountain Lions, Bears, Foxes and Eagles, by Selected States, and United States, 1990

State	Mountain Lions				Bears			
	Sheep	Total Pred.	Lambs	Total Pred.	Sheep	Total Pred.	Lambs	Total Pred.
	**Head**	**Percent**	**Head**	**Percent**	**Head**	**Percent**	**Head**	**Percent**
AZ	200	5.0	200	2.7	1/	---	1/	---
CA	900	9.1	500	2.8	100	1.0	1/	---
CO	1,000	11.1	1,500	4.9	1,500	16.7	1,100	3.6
ID	100	2.8	200	2.6	500	13.9	500	6.6
MT	100	1.3	500	2.2	400	5.3	300	1.3
NV	700	15.6	1,800	19.6	1/	---	100	1.1
NM	200	2.0	400	1.5	1/	---	100	0.4
OR	400	7.8	300	1.6	200	3.9	200	1.1
TX	1,500	5.5	1,000	1.2	0	---	0	---
UT	1,200	12.9	3,100	14.0	500	5.4	1,200	5.4
WA	1/	---	1/	---	0	---	0	---
WY	200	3.5	700	2.6	200	3.5	200	0.8
Oth	0	---	0	---	2/400	9.5	3/300	3.6
US	6,500	4.6	10,300	3.0	3,900	2.7	4,100	1.2

State	Foxes				Eagles			
	Sheep	Total Pred.	Lambs	Total Pred.	Sheep	Total Pred.	Lambs	Total Pred.
	**Head**	**Percent**	**Head**	**Percent**	**Head**	**Percent**	**Head**	**Percent**
AZ	0	---	0	---	0	---	100	1.3
CA	1/	---	1/	---	1/	---	700	4.0
CO	0	---	100	0.3	0	---	600	2.0
ID	0	---	100	1.3	0	---	0	---
MT	100	1.3	1,700	7.4	100	1.3	900	3.9
NV	0	---	0	---	100	2.2	300	3.3
NM	0	---	0	---	100	1.0	2,700	10.0
OR	0	---	300	1.6	0	---	700	3.7
TX	500	1.9	6,000	7.5	500	1.9	8,000	10.0
UT	0	---	100	0.5	0	---	400	1.8
WA	0	---	0	---	1/	---	1/	---
WY	100	1.8	1,500	5.6	200	3.5	1,500	5.6
Oth	4/100	1.1	5/2,000	5.2	6/100	0.9	7/500	1.6
US	900	0.6	11,900	3.4	1,200	0.8	16,500	4.8

1/ Less than 100 head for the State, but number is included in US total.
2/ Includes ME, MI, NH, VA and WV. 3/ Includes MI, NH, VA and WV.
4/ Includes MD and SD. 5/ Includes MD, NY, NC, ND, PA, SD, TN, VA and WI.
6/ Includes ME, SD and VA. 7/ Includes FL, GA, ME, SC, SD, TN, VA and WI.

Losses of Sheep and Lambs by Bobcats,
by Selected States, and United States, 1990

State	Bobcats			
	Sheep	Total Predators	Lambs	Total Predators
	**Head**	**Percent**	**Head**	**Percent**
AZ	1/	---	100	1.3
CA	1/	---	400	2.3
CO	0	---	100	0.3
ID	0	---	100	1.3
MT	0	---	0	---
NV	100	2.2	200	2.2
NM	200	2.0	2,800	10.4
OR	0	---	100	0.5
TX	500	1.9	8,000	10.0
UT	100	1.1	600	2.7
WA	0	---	1/	---
WY	0	---	0	---
Oth	1/ 2/	---	3/100	1.2
US	1,100	0.8	12,500	3.6

1/ Less than 100 head for the State, but number is included in **US** total.
2/ Includes FL and OK.
3/ Includes NC, OK and WV.

Losses of Sheep and Lambs by Other Animals, by States, and United States, 1990

State	All Other Animals			
	Sheep	Total Predators	Lambs	Total Predators
	Head	Percent	Head	Percent
AL	*	---	*	---
AZ	100	2.5	*	---
AR	*	---	100	12.5
CA	200	2.0	400	2.2
CO	200	2.2	400	1.3
CT	*	---	*	---
DE	0	---	*	---
FL	0	---	*	---
GA	*	---	*	---
HI	1,700	94.4	100	50.0
ID	0	---	0	---
IL	100	5.3	800	28.5
IN	100	5.0	100	4.3
IA	500	5.3	100	1.9
KS	100	5.0	100	5.6
KY	*	---	*	---
LA	100	25.0	*	---
ME	0	---	*	---
MD	*	---	*	---
MA	0	---	0	---
MI	*	---	*	---
MN	900	56.2	1,000	31.3
MS	*	---	*	---
MO	*	---	100	4.2
MT	200	2.6	100	0.5
NE	100	5.9	100	2.2
NV	*	---	100	0.9
NH	*	---	0	---
NJ	0	---	0	---
NM	4,900	49.0	9,300	34.3
NY	*	---	0	---
NC	*	---	*	---
ND	100	5.9	100	1.9
OH	100	6.7	100	3.3
OK	*	---	100	2.0
OR	100	2.0	200	1.0
PA	*	---	*	---
RI	0	---	0	---
SC	0	---	0	---
SD	100	1.0	100	0.5
TN	*	---	0	---
TX	4,000	14.8	13,000	16.3
UT	300	3.2	300	1.4
VT	0	---	0	---
VA	*	---	100	1.9
WA	*	---	*	---
WV	*	---	*	---
WI	*	---	*	---
WY	0	---	100	0.5
US	14,700	10.3	27,600	7.9

* Less than 100 head for the State, but number is included in US total.

Losses of Sheep by All Causes and by Predators,
Number and Total Value, by States, and United States, 1990

State	All Causes	Losses by Predators		
	Sheep	Sheep	Per Head	Total Value
	------- Head ----------		------- Dollars ----------	
AL	1/1,000	300	120	36,000
AZ	8,000	4,000	92	368,000
AR	1/1,200	200	78	15,600
CA	32,000	9,900	85	841,500
CO	25,000	9,000	93	837,000
CT	800	400	115	46,000
DE	1/ 2/	---	---	---
FL	1/400	200	120	24,000
GA	1/600	200	120	24,000
HI	1/3,600	1,800	47	84,600
ID	13,000	3,600	81	291,600
IL	7,000	1,900	84	159,600
IN	5,000	2,000	86	172,000
IA	36,000	9,400	71	667,400
KS	9,000	2,000	62	124,000
KY	2,000	500	77	38,500
LA	800	400	78	31,200
ME	700	200	95	19,000
MD	1,000	200	101	20,200
MA	500	100	120	12,000
MI	3,000	400	77	30,800
MN	12,000	1,600	72	115,200
MS	1/500	300	78	23,400
MO	4,500	1,000	67	67,000
MT	40,000	7,600	76	577,600
NE	11,000	1,700	71	120,700
NV	9,000	4,500	82	369,000
NH	700	300	115	34,500
NJ	600	2/	---	---
NM	35,000	10,000	69	690,000
NY	6,000	1,000	93	93,000
NC	1,000	600	76	45,600
ND	9,000	1,700	77	130,900
OH	10,000	1,500	83	124,500
OK	5,000	3,000	65	195,000
OR	19,000	5,100	58	295,800
PA	5,000	600	85	51,000
RI	2/	2/	---	---
SC	2/	2/	---	---
SD	30,000	8,700	75	652,500
TN	1,000	300	88	26,400
TX	92,000	27,000	59	1,593,000
UT	25,000	9,300	80	744,000
VT	600	100	95	9,500
VA	10,000	2,500	65	162,500
WA	3,000	400	79	31,600
WV	4,000	900	64	57,600
WI	5,000	500	82	41,000
WY	29,000	5,700	75	427,500
US	518,500	3/142,200		3/10,534,850

1/ State data not published separately in **Production, Disposition, and Income - 1990**. 2/ Less than 100 head for the State, but number is included in US total. 3/ Sum of individual State data is 500 head more than US total shown due to rounding at the State level. Sum of individual State data is $53,175 less than **US** total shown due to rounding of head at the State level.

Losses of Lambs by All Causes, All Predators, Number and Total Value, by States, and United States, 1990

State	All Causes	Losses by Predators		
	Lambs	Lambs	Per Head	Total Value
	Head		Dollars	
AL	1/1,500	400	34	13,600
AZ	11,000	7,500	37	277,500
AR	1/2,500	800	34	27,200
CA	30,000	17,700	36	637,200
CO	50,000	30,500	33	1,006,500
CT	800	100	60	6,000
DE	1/ 2/	---	---	---
FL	1/600	300	34	10,200
GA	1/1,500	300	34	10,200
HI	1/600	200	31	6,200
ID	16,000	7,600	29	220,400
IL	14,000	2,800	32	89,600
IN	10,000	2,300	31	71,300
IA	53,000	5,100	32	163,200
KS	15,000	1,800	33	59,400
KY	5,000	1,200	30	36,000
LA	3,000	300	34	10,200
ME	1,100	400	60	24,000
MD	3,000	300	34	10,200
MA	1,200	2/	---	---
MI	10,000	800	33	26,400
MN	23,000	3,200	32	102,400
MS	1/900	400	34	13,600
MO	16,100	2,400	30	72,000
MT	40,000	23,000	29	667,000
NE	21,000	4,600	32	147,200
NV	15,000	9,200	29	266,800
NH	1,200	200	60	12,000
NJ	1,000	2/	---	---
NM	35,000	27,000	30	810,000
NY	7,000	700	35	24,500
NC	2,500	1,500	30	45,000
ND	21,000	5,300	31	164,300
OH	28,000	3,100	33	102,300
OK	10,000	4,900	31	151,900
OR	39,000	18,800	31	582,800
PA	12,000	600	35	21,000
RI	1/200	2/	---	---
SC	1/400	100	30	3,000
SD	55,000	22,700	33	749,100
TN	1,200	400	34	13,600
TX	120,000	80,000	35	2,800,000
UT	34,000	22,100	29	640,900
VT	1,500	300	59	17,700
VA	20,000	5,500	32	176,000
WA	7,000	1,400	32	44,800
WV	9,000	1,800	31	55,800
WI	14,000	1,300	33	42,900
WY	45,000	26,600	30	798,000
US	809,900	3/347,300		3/11,235,625

1/ State data not published individually in **Meat Animals, Production, Disposition and Income - 1990.** 2/ Less than 100 head for the State, but number is included in **US** total. 3/ Sum of individual State data is 200 head more than US total shown due to rounding at the State level. Sum of individual State data is $21,600 less than **US** total shown due to rounding of head data at the State level.

Losses of Sheep and Lambs by Specific Predators and Total Value, by Regions, and United States, 1990

Region	Total Predator Losses: Sheep	Total Predator Losses: Lambs	Total Predator Losses: Total Sheep and Lambs Value	Coyotes: Sheep	Coyotes: Lambs	Coyotes: Total Sheep and Lambs Value
	Head	Head	Dollars	Head	Head	Dollars
Mtn and West	115,000	310,700	18,405,825	75,100	209,400	12,250,600
Midwest	18,500	21,900	2,083,775	8,100	10,700	933,800
South and East	8,700	14,700	1,206,100	1,300	7,300	371,100
US	142,200	347,300	21,695,700	84,500	227,400	13,555,500

Region	Dogs: Sheep	Dogs: Lambs	Dogs: Total Sheep and Lambs Value	Mountain Lions: Sheep	Mountain Lions: Lambs	Mountain Lions: Total Sheep and Lambs Value
	Head	Head	Dollars	Head	Head	Dollars
Mtn and West	14,700	22,700	1,812,075	6,500	10,300	814,875
Midwest	8,400	8,500	922,950	0	0	0
South and East	6,300	5,800	689,850	0	0	0
US	29,400	37,000	3,424,875	6,500	10,300	814,875

Losses of Sheep and Lambs by Specific Predators and Total Value, by Regions, and United States, 1990

Region	Bears: Sheep	Bears: Lambs	Bears: Total Sheep and Lambs Value	Foxes: Sheep	Foxes: Lambs	Foxes: Total Sheep and Lambs Value
	----- Head ------		Dollars	----- Head ------		Dollars
Mtn and West	3,500	3,800	411,400	800	11,400	429,700
Midwest	*	100	9,075	0	*	1,650
South and East	300	200	34,000	*	400	20,200
US	3,900	4,100	454,475	900	11,900	451,550

Region	Eagles: Sheep	Eagles: Lambs	Eagles: Total Sheep and Lambs Value	Bobcats: Sheep	Bobcats: Lambs	Bobcats: Total Sheep and Lambs Value
	----- Head -------		Dollars	----- Head ------		Dollars
Mtn and West	1,100	16,100	604,075	1,000	12,400	489,225
Midwest	0	100	3,300	0	0	0
South and East	*	300	15,125	*	*	4,525
US	1,200	16,500	622,500	1,100	12,500	493,750

* Less than 100 head for the region, but the number is included in the US total.

Losses of Sheep and Lambs by Specific Predators and Total Value, by Regions, and United States, 1990

Region	All Other Animals		
	Sheep	Lambs	Sheep and Lambs Value
	Head	Head	Dollars
Mountain and Western	12,300	24,600	1,593,875
Midwest	1,800	2,400	213,000
Southern and Eastern	600	600	71,300
US	14,700	27,600	1,878,175

Losses of All Goats (Angora and Other) by Specific Predators and Total Value, 5-State Total, 1990

Predator	Goats	Predator	Goats	Total Value
	Head		Head	Dollars
Coyotes	64,900	Eagles	16,300	
Dogs	9,700	Bobcats	20,600	
Mountain Lions	2,900	All Other Animals	10,900	
Bears	0			
Foxes	4,100	5-State Total	129,400	5,661,250
