# Supplementary material for: The Elephant in the room: What can we learn from California regarding the use of sport hunting of pumas (Puma concolor) as a management tool?
Source: PLoS One. 2020 Feb 13;15(2):e0224638. doi: 10.1371/journal.pone.0224638 (PMC7018503; doi:10.1371/journal.pone.0224638)
Supplement: S2 File — This is a zip file that contains sources of the data used in the analyses that are no longer available on the internet. (ZIP) [file pone.0224638.s003.zip › sheep predator loss 1994.pdf]

Sheep and Goat Predator Loss

National Agricultural Statistics Service

United States Department of Agriculture

Washington, D.C.

Released April 27, 1995, by the Agricultural Statistics Board. Estimates refer to calendar year 1994.

A total of 368,050 sheep and lambs were lost from animal predators during 1994 *in the United States. This represented 38.9 percent of the total losses from* all causes and resulted in a loss of $17.7 million due to predators to farmers and ranchers. Other non-predator causes include digestive and respiratory problems, weather-related causes and theft.

Coyotes were the largest cause of sheep and lamb losses to predators, with 66.2 percent of the total losses in 1994 from all animal predators. The West and Mountain states sustained the most losses with 91.8 percent of the total coyote losses. Nationally, losses from dogs were more widespread with Virginia losing 3,800 head of sheep and lambs, 400 head more than second place Texas.

Coyotes were also the largest predator of **Goats** *in the five major producing* states (AZ, MI, NM, OK, and TX) accounting for 29.3 percent. The value of goats lost from all predators was $5.48 million.

This report is released as a cooperative effort between the National Agricultural Statistics Service and Animal and Plant Health Inspection Service - Animal Damage Control and National Animal Health Monitoring System.

Losses of Sheep and Lambs from Predators: Number of Head and Total Value, United States, 1994 1/

Predator	Number of Head	% of Total Predators	Value
	Number	Percent	Dollars
Coyotes	243,800	66.2	11,504,900
Dogs	40,325	11.0	2,206,975
Mountain Lions, Cougars, or Pumas	28,500	7.7	1,460,600
Bears	12,250	3.3	640,150
Foxes	12,350	3.4	507,250
Eagles	15,000	4.1	641,150
Bobcats	9,200	2.5	418,425
All Other Animals	6,625	1.8	337,450
US	368,050	100.0	17,716,900

1/ Lambs are after docking.

For information call Linda Simpson at (202) 720-3578. Office hours are 8:00 a.m. to 4:30 p.m. ET.

Lv Gn 1 (4-95)

Losses of Sheep and Lambs: Number and Percent by Coyotes and Dogs by State and United States, 1994 2/

State	Coyotes				Dogs			
	Sheep	% Total Pred.	Lambs	% Total Pred.	Sheep	% Total Pred.	Lambs	% Total Pred.
	Head	Pct	Head	Pct	Head	Pct	Head	Pct
AL	800	94.1	1,500	92.3	1/		100	6.2
AZ	1,500	85.7	2,100	79.2	150	8.6	450	17.0
AR	1/		1/		1/		125	62.5
CA	5,750	61.5	10,800	71.6	925	9.9	1,625	10.8
CO	2,700	41.5	12,500	62.5	1,000	15.4	1,300	6.5
CT	375	88.2	300	100.0	0		0	
DE	0		0		0		0	
FL	1/		125	62.5	0		1/	
GA	1/		200	88.9	0		1/	
HI	0		0		1/		1/	
ID	2,900	76.3	8,600	86.9	300	7.9	400	4.0
IL	450	43.9	1,100	49.4	575	56.1	825	37.1
IN	300	32.4	175	41.2	625	67.6	250	58.8
IA	300	24.0	925	36.6	900	72.0	1,575	62.4
KS	575	51.1	600	58.5	550	48.9	125	12.2
KY	450	85.7	425	51.5	1/		400	48.5
LA	175	77.8	700	100.0	1/		0	
ME	1/		1/		0		0	
MD	0		0		100	100.0	275	91.7
MA	1/		150	75.0	1/		1/	
MI	125	15.6	700	68.3	650	81.3	325	31.7
MN	1/		1,000	47.1	750	69.8	800	37.6
MS	0		150	75.0	100	100.0	1/	
MO	100	19.0	425	41.5	425	81.0	600	58.5
MT	5,000	75.8	19,100	70.2	500	7.6	400	1.5
NE	800	88.9	1,350	84.4	1/		200	12.5
NV	3,075	81.5	10,375	83.8	1/		1/	
NH	1/		1/		0		0	
NJ	0		0		1/		1/	
NM	3,450	49.5	6,100	50.8	1,425	20.4	450	3.8
NY	175	25.0	300	42.9	525	75.0	350	50.0
NC	0		0		300	100.0	500	95.2
ND	675	81.8	2,675	89.2	150	18.2	250	8.3
OH	350	34.1	1,025	71.9	675	65.9	350	24.6
OK	500	48.8	800	56.1	500	48.8	575	40.4
OR	2,750	64.3	9,100	69.5	750	17.5	1,200	9.2
PA	600	85.7	675	81.8	100	14.3	150	18.2
RI	1/		1/		1/		0	
SC	1/		1/		0		1/	
SD	1,925	79.4	8,775	68.8	425	17.5	700	5.5
TN	1/		1/		175	77.8	175	77.8
TX	7,400	45.1	19,700	63.5	2,300	14.0	1,100	3.5
UT	5,950	60.3	15,925	65.4	550	5.6	525	2.2
VT	225	69.2	200	100.0	1/		0	
VA	100	4.8	1,025	33.6	2,000	95.2	1,800	59.0
WA	575	76.7	800	80.0	125	16.7	200	20.0
WV	300	22.2	2,000	47.3	1,000	74.1	2,000	47.3
WI	100	50.0	1,300	94.5	1/		1/	
WY	11,500	76.7	37,500	81.5	400	2.7	200	.4
US	62,325	58.5	181,475	69.4	19,600	18.4	20,725	7.9

1/ Less than 100 head for the State but data are included in the US totals.
2/ Lambs are after docking.

1994 Sheep and Lamb Losses from Coyotes
10 Largest States

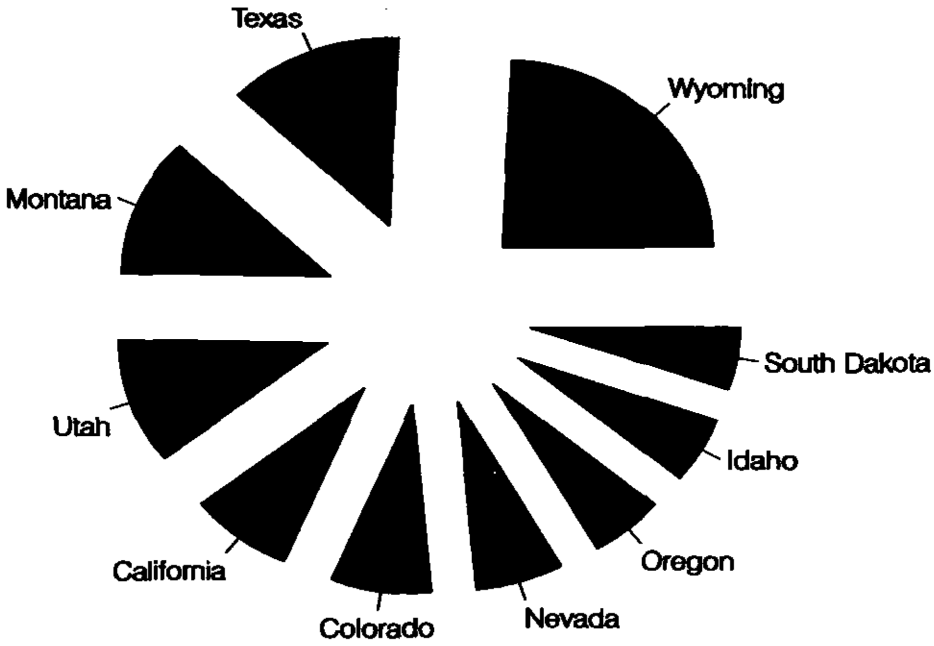

1994 Sheep and Lamb Losses from Dogs
10 Largest States

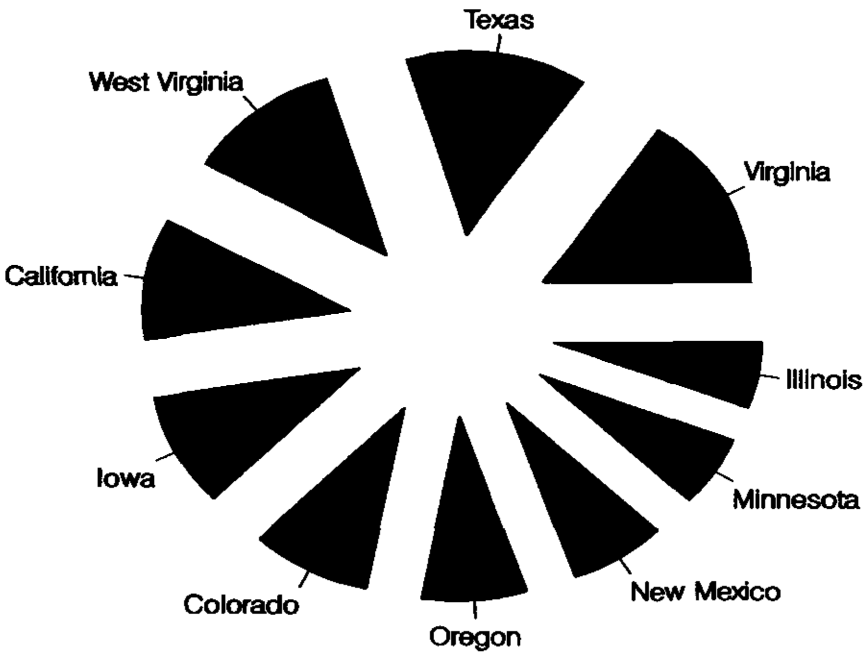

Losses of Sheep and Lambs: Number and Percent by Mountain Lions, Cougars, or Pumas, Bears, Foxes, and Eagles by State and United States, 1994 5/

State	Mountain Lions, Cougars, or Pumas				Bears			
	Sheep	% Total Pred.	Lambs	% Total Pred.	Sheep	% Total Pred.	Lambs	% Total Pred.
	Head	Pct	Head	Pct	Head	Pct	Head	Pct
AZ	100	5.7	100	3.8	0		0	
CA	2,275	24.3	1,850	12.3	275	2.9	325	2.2
CO	1,200	18.5	2,900	14.5	1,300	20.0	2,000	10.0
ID	100	2.6	200	2.0	300	7.9	500	5.1
MT	400	6.1	500	1.8	200	3.0	400	1.5
NV	475	12.6	1,050	8.5	1/		225	1.8
NM	450	6.5	700	5.8	325	4.7	300	2.5
OR	450	10.5	1,625	12.4	225	5.3	400	3.1
TX	3,300	20.1	1,600	5.2	0		100	.3
UT	2,200	22.3	5,350	22.0	875	8.9	1,900	7.8
WA	0		0		0		0	
WY	500	3.3	1,100	2.4	1,000	6.7	1,400	3.0
Oth Sts	0		1/	2/	1/	2/	1/	2/
US	11,450	10.7	17,050	6.5	4,625	4.3	7,625	2.9
	Foxes				Eagles			
AZ	0		0		0		0	
CA	0		0		0		125	.8
CO	0		100	.5	0		600	3.0
ID	0		100	1.0	0		0	
MT	100	1.5	3,600	13.2	100	1.5	3,200	11.8
NV	0		0		0		0	
NM	0		325	2.7	600	8.6	1,325	11.0
OR	0		150	1.1	0		375	2.9
TX	500	3.0	2,000	6.5	200	1.2	3,200	10.3
UT	125	1.3	450	1.8	0		125	.5
WA	0		0		0		0	
WY	100	.7	2,400	5.2	1,000	6.7	2,900	6.3
Oth Sts	0		3/2,400	2/	0		4/1,250	2/
US	825	.8	11,525	4.4	1,900	1.8	13,100	5.0

1/ Less than 100 head for the State(s) but data are included in US totals.
2/ Less than 0.1 percent.
3/ Includes MD, ND, NY, SD, VA, and WV.
4/ Includes IA, SC, SD and VA.
5/ Lambs are after docking.

Losses of Sheep and Lambs: Number and Percent by Bobcats by State and United States, 1994 3/

State	Bobcats			
	Sheep	% Total Pred.	Lambs	% Total Pred.
	Head	Pct	Head	Pct
AZ	0		0	
CA	0		175	1.2
CO	0		100	.5
ID	0		0	
MT	200	3.0	0	
NV	1/	2.0	225	1.8
NM	700	10.0	2,800	23.3
OR	1/	1.8	175	1.3
TX	1,400	8.5	2,300	7.4
UT	125	1.3	1/	.2
WA	0		0	
WY	200	1.3	200	.4
Oth Sts	100	2/	300	2/
US	2,875	2.7	6,325	2.4

1/ Less than 100 head for the State but data are included in the US totals.
2/ Less than 0.1 percent. 3/ Lambs are after docking.

Sheep and Lambs Lost from Mountain Lions, Bears, Foxes, Eagles, Bobcats and Other Animals, United States 1994

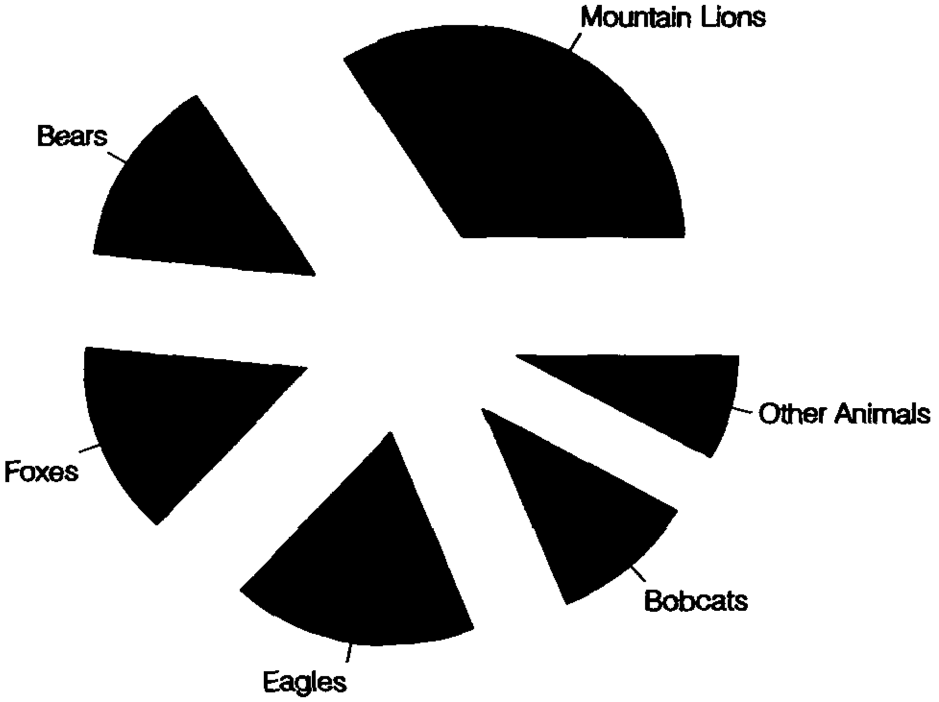

Losses of Sheep and Lambs: Number and Percent by All Other Animals by State and United States, 1994 2/

State	All Other Animals 3/			
	Sheep	% Total Pred.	Lambs	% Total Pred.
	Head	Percent	Head	Percent
AL	0		1/	
AZ	0		0	
AR	0		0	
CA	125	1.3	175	1.2
CO	300	4.6	500	2.5
CT	1/		0	
DE	0		0	
FL	0		0	
GA	0		0	
HI	0		0	
ID	200	5.3	100	1.0
IL	0		300	13.5
IN	0		0	
IA	1/		0	
KS	0		1/	
KY	0		0	
LA	0		0	
ME	0		0	
MD	0		0	
MA	0		0	
MI	1/		0	
MN	250	23.3	325	15.3
MS	0		0	
MO	0		0	
MT	100	1.5	0	
NE	0		1/	
NV	0		475	3.8
NH	0		0	
NJ	0		0	
NM	1/		0	
NY	0		0	
NC	0		1/	
ND	0		0	
OH	0		1/	
OK	0		1/	
OR	1/		1/	
PA	0		0	
RI	0		0	
SC	0		1/	
SD	0		1/	
TN	0		0	
TX	1,300	7.9	1,000	3.2
UT	1/		1/	
VT	1/		0	
VA	0		1/	
WA	1/		0	
WV	1/		1/	
WI	1/		0	
WY	300	2.0	300	.7
US	2,975	2.8	3,650	1.4

1/ Less than 100 head for the State, but data are included in US totals.
2/ Lambs are after docking.
3/ Includes wolves, ravens, crows, pigs, etc.

Number of Sheep and Lambs Lost from Predators United States 1994

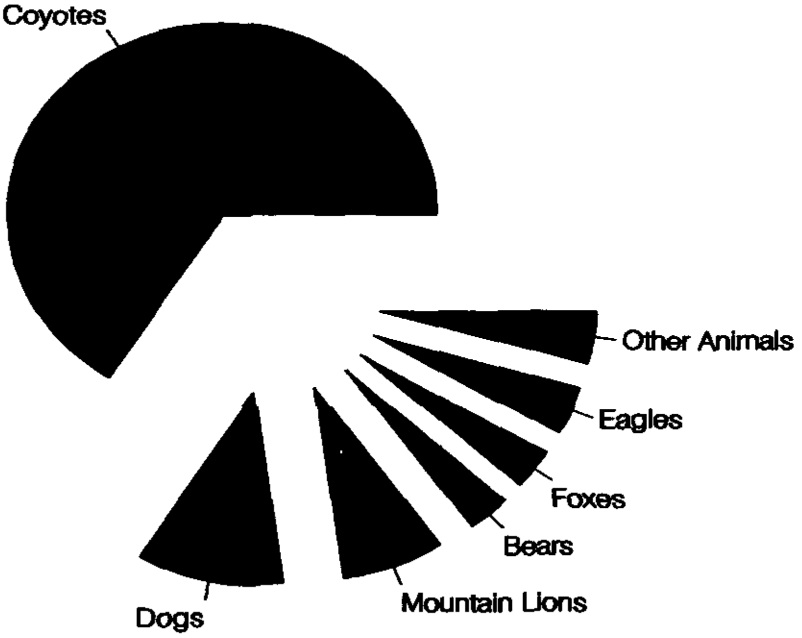

Value of Sheep and Lambs Lost from Predators United States 1994

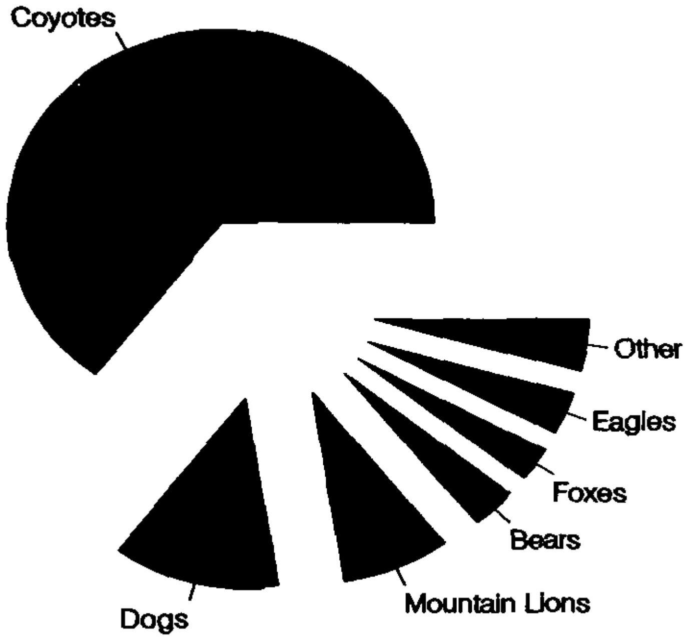

Losses of Lambs: By All Causes and Predators, Number
and Total Value by State and United States, 1994 3/

State	All Causes Lambs	Predators Lambs	Predators per Head 1/	Predators Value
	Head	Head	Dollars	Dollars
AL	2,500	1,625	30.00	48,750
AZ	5,000	2,650	38.00	100,700
AR	400	200	39.00	7,800
CA	24,000	15,075	39.00	587,925
CO	55,000	20,000	39.00	780,000
CT	1,000	300	60.00	18,000
DE	50	2/		
FL	400	200	38.00	7,600
GA	400	225	38.00	8,550
HI	2,000	2/		
ID	18,000	9,900	38.00	376,200
IL	14,000	2,225	37.00	82,325
IN	4,000	425	39.00	16,575
IA	30,000	2,525	41.00	103,525
KS	11,000	1,025	40.00	41,000
KY	4,500	825	38.00	31,350
LA	1,500	700	39.00	27,300
ME	500	2/		
MD	2,000	300	44.00	13,200
MA	800	200	66.00	13,200
MI	10,000	1,025	42.00	43,050
MN	21,000	2,125	39.00	82,875
MS	300	200	39.00	7,800
MO	4,700	1,025	39.00	39,975
MT	40,000	27,200	40.00	1,088,000
NE	13,000	1,600	39.00	62,400
NV	13,000	12,375	31.00	383,625
NH	900	2/		
NJ	1,700	2/		
NM	21,000	12,000	38.00	456,000
NY	6,000	700	41.00	28,700
NC	2,100	525	37.00	19,425
ND	14,000	3,000	37.00	111,000
OH	12,500	1,425	40.00	57,000
OK	8,000	1,425	36.00	51,300
OR	22,000	13,100	36.00	471,600
PA	10,000	825	43.00	35,475
RI	50	2/		
SC	200	150	37.00	5,550
SD	45,000	12,750	40.00	510,000
TN	1,600	225	41.00	9,225
TX	50,000	31,000	39.00	1,209,000
UT	32,000	24,350	38.00	925,300
VT	2,300	200	57.00	11,400
VA	15,000	3,050	41.00	125,050
WA	2,000	1,000	34.00	34,000
WV	12,000	4,225	41.00	173,225
WI	12,000	1,375	40.00	55,000
WY	60,000	46,000	40.00	1,840,000
US	609,400	261,475		10,110,350

1/ Lamb value per head is based on the USDA annual average price received by farmers and ranchers in each State for a 60 pound lamb.
2/ Less than 100 head for the State but data are included in US totals.
3/ Lambs are after docking.

Losses of Sheep: By All Causes and Predators, Number and Total Value by State and United States, 1994

State	All Causes Sheep	Predators Sheep	Predators per Head 1/	Predators Value
	Head	Head	Dollars	Dollars
AL	1,000	850	62.00	52,700
AZ	3,000	1,750	92.00	161,000
AR	50	2/		
CA	24,000	9,350	85.00	794,750
CO	15,000	6,500	78.00	507,000
CT	700	425	115.00	48,875
DE	50	0		
FL	50	2/		
GA	50	2/		
HI	1,700	2/		
ID	12,000	3,800	83.00	315,400
IL	4,000	1,025	93.00	95,325
IN	2,000	925	88.00	81,400
IA	8,000	1,250	76.00	95,000
KS	7,000	1,125	68.00	76,500
KY	1,300	525	77.00	40,425
LA	600	225	85.00	19,125
ME	200	2/		
MD	1,000	100	110.00	11,000
MA	500	100	115.00	11,500
MI	4,000	800	80.00	64,000
MN	9,000	1,075	71.00	76,325
MS	100	100	70.00	7,000
MO	1,900	525	66.00	34,650
MT	29,000	6,600	65.00	429,000
NE	5,000	900	69.00	62,100
NV	8,000	3,775	77.00	290,675
NH	400	2/		
NJ	700	2/		
NM	21,000	6,975	65.00	453,375
NY	5,000	700	100.00	70,000
NC	1,300	300	78.00	23,400
ND	4,000	825	72.00	59,400
OH	4,500	1,025	83.00	85,075
OK	4,000	1,025	66.00	67,650
OR	14,000	4,275	66.00	282,150
PA	4,000	700	90.00	63,000
RI	50	2/		
SC	50	2/		
SD	20,000	2,425	67.00	162,475
TN	1,000	225	84.00	18,900
TX	46,000	16,400	55.00	902,000
UT	18,000	9,875	78.00	770,250
VT	800	325	110.00	35,750
VA	8,000	2,100	55.00	115,500
WA	2,000	750	78.00	58,500
WV	4,600	1,350	58.00	78,300
WI	3,000	200	86.00	17,200
WY	35,000	15,000	69.00	1,035,000
US	336,600	106,575		7,606,550

1/ Sheep value per head is based on a two-year straight average of the value of ewes one year old and older from the January 1, 1994 and the January 1, 1995 NASS Agricultural Surveys.
2/ Less than 100 head for the State but data are included in US totals.

1994 Sheep Losses from Predators
10 Largest States

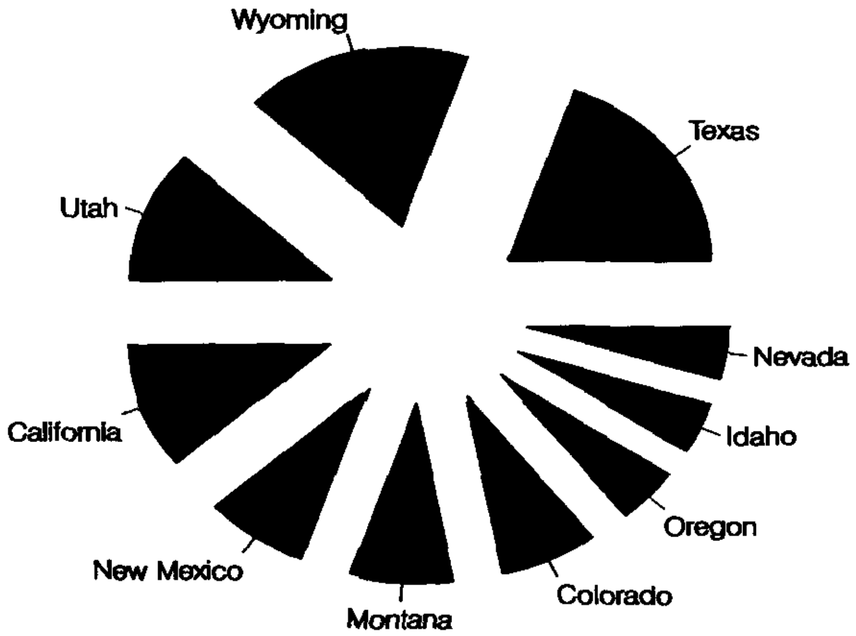

1994 Lamb Losses After Docking from Predators
10 Largest States

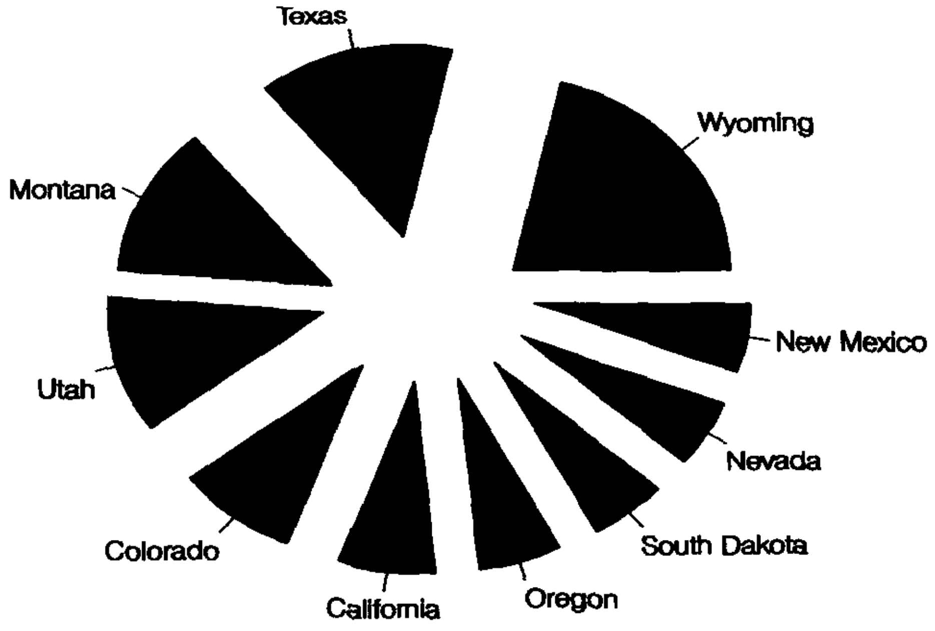

Losses of Sheep and Lambs: Number by Specific Predators
and Total Value by Region and United States, 1994 1/

Region	Predator Losses: Sheep	Predator Losses: Lambs	Predator Losses: Sheep and Lambs	Coyotes: Sheep	Coyotes: Lambs	Coyotes: Sheep and Lambs
	Head	Head	Dollars	Head	Head	Dollars
Mtn and West	91,400	234,475	15,459,150	57,025	166,800	10,476,300
Mid-west	6,875	12,350	1,040,600	1,825	6,725	418,725
South and East	8,300	14,650	1,217,150	3,475	7,950	609,875
US	106,575	261,475	17,716,900	62,325	181,475	11,504,900

Region	Dogs: Sheep	Dogs: Lambs	Dogs: Sheep and Lambs	Mountain Lions, Cougars, or Pumas: Sheep	Mountain Lions, Cougars, or Pumas: Lambs	Mountain Lions, Cougars, or Pumas: Sheep and Lambs
Mtn and West	10,250	9,750	1,073,050	11,450	17,050	1,460,600
Mid-west	4,675	4,925	567,225	0	0	0
South and East	4,675	6,050	566,700	0	0	0
US	19,600	20,725	2,206,975	11,450	17,050	1,460,600

Region	Bears: Sheep	Bears: Lambs	Bears: Sheep and Lambs	Foxes: Sheep	Foxes: Lambs	Foxes: Sheep and Lambs
Mtn and West	4,600	7,550	633,950	825	11,325	498,975
Mid-west	0	0	0	0	0	0
South and East	2/	2/	6,200	0	200	8,275
US	4,625	7,625	640,150	825	11,525	507,250

-- continued

Losses of Sheep and Lambs: Number by Specific Predators and Total Value by Region and United States, 1994 (continued) 1/

Region	Eagles			Bobcats		
	Sheep	Lambs	Sheep and Lambs	Sheep	Lambs	Sheep and Lambs
	Head	Head	Dollars	Head	Head	Dollars
Mtn and West	1,900	12,925	634,175	2,875	6,250	415,350
Mid-west	0	2/	1,025	0	0	0
South and East	0	150	5,950	0	2/	3,075
US	1,900	13,100	641,150	2,875	6,325	418,425
	All Other Animals 3/					
Mtn and West	2,475	2,825	266,750			
Mid-west	375	675	53,625			
South and East	125	150	17,075			
US	2,975	3,650	337,450			

1/ Lambs are after docking.
2/ Less than 100 head for the region but data are included in US totals.
3/ Includes wolves, ravens, crows, pigs, etc.

Losses of All Goats (Angora and Other): Number by Specific Predators and Total Value, 5-States, 1994 2/

Predator	Losses	Value 1/
	Head	Dollars
Coyotes	41,000	
Dogs	15,000	
Mountain Lions, Cougars, or Pumas	5,000	
Bears	1,000	
Foxes	7,000	
Eagles	25,000	
Bobcats	21,000	
All Other Animals 3/	25,000	
5-State	140,000	5,481,000

1/ Goat value is based on a straight average of the value per head of all goats from the January 1, 1994 and January 1, 1995 NASS Agricultural Surveys.
2/ Includes AZ, MI, NM, OK, and TX.
3/ Includes wolves, ravens, crows, pigs, etc.

Number of Goats Lost from Predators United States 1994

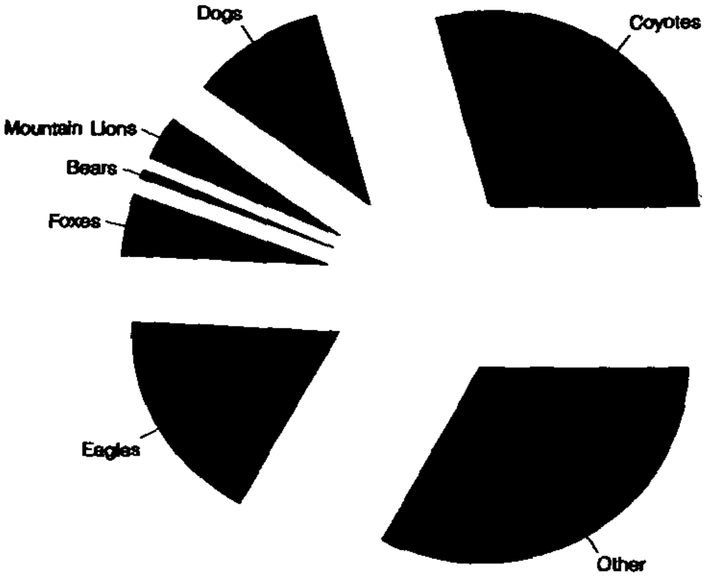

Reliability of Sheep and Goat Predator Loss Estimates

Survey Procedures: A random sample of U. S. producers was surveyed to provide data for these estimates. Survey procedures ensured that all sheep and goat producers, regardless of size, had a chance to be included in the survey. (Alaska was not included.) Large producers were sampled more heavily than small operations. About 75,000 operators were contacted during the first half of January by mail, telephone, and face-to-face personal interviews. An additional non-probability survey was also conducted in a few western States.

Revision Policy: Revisions to previous estimates are made to improve the current estimate. Previous year estimates are subject to revision when current estimates are made. Estimates for losses from all causes are subject to revision in NASS' following year's **"Meat Animals, Production, Disposition and Income Report"**. No revision to predator losses is planned.

Estimation Procedures: Total death losses from all causes for sheep and lambs were as reported in **"Meat Animals, Production, Disposition, and Income - 1994"** (released April 13, 1995).

In setting the predator loss estimates, first total predator losses were estimated as a percent of total losses, then specific predator losses were estimated as a percent of total predator losses. States were combined into regions, when sampling variability did not permit individual State data to be released.

Terms and Definitions

Lamb: Sheep less than one year old. In the Western States, lamb losses are after docking.

Regions: Regions were defined to allow comparison with earlier USDA research on sheep and lamb losses and a similar study done by NASS released April 21, 1991.

States in each region were:

Mountain and Western:

Arizona (AZ)	California (CA)	Colorado (CO)	Hawaii (HI)
Idaho (ID)	Kansas (KS)	Montana (MT)	Nebraska (NE)
Nevada (NV)	New Mexico (NM)	North Dakota (ND)	Oklahoma (OK)
Oregon (OR)	South Dakota (SD)	Texas (TX)	Utah (UT)
Washington (WA)	Wyoming (WY)		

Midwest:

Arkansas (AR)	Illinois (IL)	Indiana (IN)	Iowa (IA)
Michigan (MI)	Minnesota (MN)	Missouri (MO)	Ohio (OH)
Wisconsin (WI)			

Southern and Eastern:

Alabama (AL)	Connecticut (CT)	Delaware (DE)	Florida (FL)
Georgia (GA)	Kentucky (KY)	Louisiana (LA)	Maine (ME)
Maryland (MD)	Massachusetts (MA)	Mississippi (MS)	New Hampshire (NH)
New Jersey (NJ)	New York (NY)	North Carolina (NC)	Pennsylvania (PA)
Rhode Island (RI)	South Carolina (SC)	Tennessee (TN)	Vermont (VT)
Virginia (VA)	West Virginia (WV)		

The next report on sheep and lamb losses from all causes, including predator losses by size of flock, will be released in May 1995.

The United States Department of Agriculture (USDA) prohibits discrimination in its programs on the basis of race, color, national origin, sex, religion, age, disability, political beliefs, and marital or familial status. Persons with disabilities who require alternative means for communication of program information (braille, large print, audiotape, etc.) should contact the USDA Office of Communications at (202) 720-5881 (voice) or (202) 720-7808 (TDD).

To file a complaint, write the Secretary of Agriculture, USDA, Washington, D.C., 20250, or call (202) 720-7327 (voice) or (202) 720-1127 (TDD). USDA is an equal employment opportunity employer.

Subscription Information

To subscribe to **NASS** reports or to order single copies, call toll free, 1-800-999-6779 (weekdays 8:30 a.m.-5:00 p.m. ET). You may write: **ERS/NASS, 341 Victory Drive, Herndon VA 22070.**
