# Supplementary material for: The Elephant in the room: What can we learn from California regarding the use of sport hunting of pumas (Puma concolor) as a management tool?
Source: PLoS One. 2020 Feb 13;15(2):e0224638. doi: 10.1371/journal.pone.0224638 (PMC7018503; doi:10.1371/journal.pone.0224638)
Supplement: S2 File — This is a zip file that contains sources of the data used in the analyses that are no longer available on the internet. (ZIP) [file pone.0224638.s003.zip › sheep predator loss 1999.pdf]

NASS
FACT FINDERS FOR AGRICULTURE
UNITED STATES DEPARTMENT OF AGRICULTURE
Washington, D.C.

Sheep and Goats Predator Loss

Released May 5, 2000, by the National Agricultural Statistics Service (NASS), Agricultural Statistics Board, U.S. Department of Agriculture. For information on "Sheep and Goats Predator Loss" call Steve Anderson at 202-690-3236, office hours 7:30 a.m. to 4:30 p.m. ET.

U.S. Sheep and Lamb Predator Loss

Sheep and lamb losses from animal predators in the United States totaled 273,000 during 1999. This represented 36.7 percent of the total losses from all causes and resulted in a loss of $16.5 million due to predators to farmers and ranchers.

Coyotes caused the majority of sheep and lamb losses to predators. They accounted for 60.7 percent of the total.

Coyotes were also the largest predator of **goats** in the three major states (AZ, NM, and TX) accounting for 35.6 percent of predator death losses. The value of goats lost from all predators was $3.4 million.

Use of non-lethal predator controls is also highlighted in this publication. Farmers and ranchers throughout the United States spent $8.8 million on non-lethal methods to prevent predator loss of sheep and lambs. Another $1.0 million was spent on non-lethal predator controls for goats and kids.

This report is released as a cooperative effort between the National Agricultural Statistics Service and Animal and Plant Health Inspection Service - Wildlife Services and National Animal Health Monitoring System.

Losses of Sheep and Lambs from Predators: Number of Head and Total Value, United States, 1999

Predator	Number of Head	% of Total Predators	Total Value
	Number	*Percent*	*1,000 Dollars*
Coyotes	165,800	60.7	9,637
Dogs	41,300	15.1	2,982
Mountain Lions, Cougars, or Pumas	15,600	5.7	998
Bears	7,800	2.9	555
Foxes	8,100	3.0	400
Eagles	10,700	3.9	522
Bobcats	12,700	4.7	650
All Other Animals	11,000	4.0	758
US	273,000	100.0	16,502

Losses of Sheep and Lambs: Number and Percent by Coyotes and Dogs by State and United States, 1999

State	Coyotes				Dogs [1]			
	Sheep	% Total Pred.	Lambs	% Total Pred.	Sheep	% Total Pred.	Lambs	% Total Pred.
	Head	*Pct*	*Head*	*Pct*	*Head*	*Pct*	*Head*	*Pct*
AZ	400	80.0	900	60.0			400	26.7
CA	5,000	55.6	6,500	75.6	3,000	33.3	1,000	11.6
CO	1,000	33.3	6,400	71.1	400	13.3	1,100	12.2
ID	1,700	60.7	6,100	82.4	500	17.9	400	5.4
IL	300	50.0	800	53.3	300	50.0	600	40.0
IN	100	10.0	300	50.0	900	90.0	300	50.0
IA	600	42.9	1,400	56.0	800	57.1	1,100	44.0
KS	300	60.0	500	71.4	200	40.0	200	28.6
MI	100	50.0	800	61.5	100	50.0	400	30.8
MN	100	5.9	400	17.4	1,000	58.8	1,000	43.5
MO	400	44.4	1,600	64.0	400	44.4	700	28.0
MT	2,800	73.7	10,000	79.4	300	7.9	200	1.6
NE	800	66.7	1,700	73.9	300	25.0	500	21.7
NV	2,700	67.5	8,000	80.0				
N ENG [2]	200	100.0	300	100.0				
NM	900	32.1	3,800	50.7	1,200	42.9	300	4.0
NY	400	50.0	500	71.4	300	37.5	100	14.3
ND	800	88.9	3,100	88.6			300	8.6
OH	500	71.4	800	66.7	200	28.6	300	25.0
OK	200	40.0	1,100	52.4	300	60.0	900	42.9
OR	1,800	54.5	5,500	73.3	700	21.2	800	10.7
PA	100	25.0	400	44.4	300	75.0	200	22.2
SD	2,300	82.1	7,500	68.2	400	14.3	600	5.5
TX	6,000	42.9	23,000	51.1	4,200	30.0	4,000	8.9
UT	3,800	57.6	12,000	64.2	500	7.6	1,200	6.4
VA	400	44.4	1,500	60.0	400	44.4	500	20.0
WA	200	33.3	500	38.5			300	23.1
WV	300	37.5	1,800	64.3	400	50.0	700	25.0
WI	200	50.0	300	60.0				
WY	4,000	66.7	17,000	77.3	400	6.7	400	1.8
Oth Sts [3]	1,400	29.8	1,500	26.3	2,000	42.6	2,800	49.1
Unpub					300		200	
US	39,800	51.7	126,000	64.3	19,800	25.7	21,500	11.0

[1] Unpublished States include NV and WI for sheep and lambs; AZ, ND, and WA for sheep only.
[2] N ENG includes CT, ME, MA, NH, RI, and VT.
[3] Other States include AL, AK, AR, DE, FL, GA, HI, KY, LA, MD, MS, NJ, NC, SC, and TN.

Losses of Sheep and Lambs: Number and Percent by Mountain Lions, Cougars, or Pumas, Bears, Foxes, and Eagles by State and United States, 1999

State	Mountain Lions, Cougars, or Pumas [1]				Bears [2]			
	Sheep	% Total Pred.	Lambs	% Total Pred.	Sheep	% Total Pred.	Lambs	% Total Pred.
	Head	*Pct*	*Head*	*Pct*	*Head*	*Pct*	*Head*	*Pct*
CA	600	6.7	700	8.1				
CO	400	13.3	300	3.3	1,000	33.3	700	7.8
ID	200	7.1	400	5.4	400	14.3	300	4.1
MT	200	5.3	200	1.6	200	5.3	200	1.6
NV	1,100	27.5	1,300	13.0			400	4.0
NM	400	14.3	400	5.3				
OR	500	15.2	800	10.7	200	6.1		
SD			400	3.6				
TX	300	2.1	800	1.8				
UT	1,200	18.2	2,900	15.5	1,000	15.2	1,500	8.0
WA	200	33.3	400	30.8			100	7.7
WV					100	12.5	200	7.1
WY	700	11.7	900	4.1	300	5.0	500	2.3
Unpub	100		200		400		300	
US	5,900	7.6	9,700	4.9	3,600	4.6	4,200	2.1
	Foxes [3]				Eagles [4]			
CO			200	2.2				
MT			600	4.8	100	2.6	900	7.1
NM							700	9.3
OR							200	2.7
SD			1,700	15.5			700	6.4
TX	600	4.3	3,100	6.9	300	2.1	4,600	10.2
UT			200	1.1			300	1.6
WY	100	1.7	1,000	4.5	400	6.7	2,200	10.0
Unpub			600				300	
US	700	0.9	7,400	3.8	800	1.0	9,900	5.1

[1] Unpublished States includes AZ for sheep and lambs; SD for sheep only; MO and NE for lambs only.
[2] Unpublished States includes CA for sheep and lambs; NV, NM, VA, and WA for sheep only; OR, TX, and WI for lambs only.
[3] Unpublished States include ID, MN, NM, NY, ND, OK, OR, VA, and WV for lambs only.
[4] Unpublished States include CA, CO, MN, NV, and ND for lambs only.

Losses of Sheep and Lambs: Number and Percent by Bobcats and All Other Animals by State and United States, 1999

State	Bobcat [1]				All Other Animals [2 3]			
	Sheep	% Total Pred.	Lambs	% Total Pred.	Sheep	% Total Pred.	Lambs	% Total Pred.
	Head	*Pct*	*Head*	*Pct*	*Head*	*Pct*	*Head*	*Pct*
CA			200	2.3	200	2.2		
IL							100	6.7
MI							100	7.7
MN					600	35.3	800	34.8
MO					100	11.1		
MT					200	5.3	400	3.2
NM	200	7.1	2,100	28.0				
OH							100	8.3
PA							300	33.3
TX	1,800	12.9	7,000	15.6	800	5.7	2,400	5.3
UT	100	1.5	500	2.7			100	0.5
VA			300	12.0				
WY					100	1.7		
Oth Sts [4]					1,300	27.7	1,400	24.6
Unpub	200		300		800		1,200	
US	2,300	3.0	10,400	5.3	4,100	5.3	6,900	3.5

[1] Unpublished States include CA and NE for sheep only; CO, MT, NV, OK, and OR for lambs only.
[2] Unpublished States include CO, NM, NY, OR, SD, VA, and WI for sheep and lambs; ND and WA for sheep only; AZ, CA, ID, MO, NE, NV, and WV for lambs only.
[3] Other predators include ravens, vultures, wolves, pigs, and other animals.
[4] Other States include AL, AK, AR, DE, FL, GA, HI, KY, LA, MD, MS, NJ, NC, SC, and TN.

Losses of Lambs: By All Causes and Predators, Number by State and United States, 1999

State	All Causes		Predators	
	Sheep	Lambs	Sheep	Lambs
	Head	*Head*	*Head*	*Head*
AZ	2,000	2,000	500	1,500
CA	23,000	12,000	9,000	8,600
CO	8,000	40,000	3,000	9,000
ID	10,000	16,000	2,800	7,400
IL	4,000	8,000	600	1,500
IN	3,000	6,000	1,000	600
IA	10,000	28,000	1,400	2,500
KS	4,500	7,000	500	700
MI	2,500	7,000	200	1,300
MN	9,000	20,000	1,700	2,300
MO	2,500	8,000	900	2,500
MT	18,000	23,000	3,800	12,600
NE	5,000	11,000	1,200	2,300
NV	7,000	13,000	4,000	10,000
N ENG [1]	1,900	3,500	200	300
NM	10,000	11,000	2,800	7,500
NY	3,000	6,000	800	700
ND	5,000	15,000	900	3,500
OH	6,000	14,000	700	1,200
OK	3,000	6,000	500	2,100
OR	8,000	13,000	3,300	7,500
PA	3,000	8,000	400	900
SD	16,000	39,000	2,800	11,000
TX	40,000	65,000	14,000	45,000
UT	18,000	26,000	6,600	18,700
VA	3,000	7,000	900	2,500
WA	2,000	3,000	600	1,300
WV	2,000	6,000	800	2,800
WI	2,500	8,500	400	500
WY	18,000	35,000	6,000	22,000
Oth Sts [2]	11,000	15,000	4,700	5,700
US	260,900	482,000	77,000	196,000

[1] N ENG includes CT, ME, MA, NH, RI, and VT.
[2] Other States include AL, AK, AR, DE, FL, GA, HI, KY, LA, MD, MS, NJ, NC, SC, and TN.

Losses of Sheep and Lambs from Predators: Total Value per Head and Total Value by State and United States, 1999

State	Value per Head		Total Value	
	Sheep [1]	Lambs [2]	Sheep	Lambs
	Dollars	*Dollars*	*1,000 Dollars*	*1,000 Dollars*
AZ	100.00	45.00	50	68
CA	104.00	45.00	936	387
CO	99.00	67.00	297	603
ID	101.00	42.00	283	311
IL	136.00	41.00	82	62
IN	145.00	43.00	145	26
IA	115.00	42.00	161	105
KS	96.00	45.00	48	32
MI	124.00	41.00	25	53
MN	93.00	44.00	158	101
MO	94.00	45.00	85	113
MT	88.00	47.00	334	592
NE	97.00	44.00	116	101
NV	102.00	42.00	408	420
N ENG [3]	171.00	66.00	34	20
NM	81.00	45.00	227	338
NY	118.00	50.00	94	35
ND	85.00	43.00	77	151
OH	141.00	44.00	99	53
OK	128.00	44.00	64	92
OR	80.00	40.00	264	300
PA	115.00	49.00	46	44
SD	93.00	48.00	260	528
TX	71.00	46.00	994	2,070
UT	103.00	46.00	680	860
VA	111.00	45.00	100	113
WA	93.00	40.00	56	52
WV	89.00	44.00	71	123
WI	101.00	42.00	40	21
WY	88.00	46.00	528	1,012
Oth Sts [4]	146.00	47.00	686	268
US [5]	97.00	46.00	7,448	9,054

[1] Sheep value per head is based on a two-year straight average of the value of ewes reported in the January 1 Sheep Survey from 1999 to 2000.
[2] Lamb value per head is based on the market year average lamb price. An average lamb weight of 60 pounds was used in all States except Colorado, where a 90 pound average was used.
[3] N ENG includes CT, ME, MA, NH, RI, and VT.
[4] Other States include AL, AK, AR, DE, FL, GA, HI, KY, LA, MD, MS, NJ, NC, SC, and TN.
[5] US value per head rounded to the nearest dollar.

Losses of All Goats (Angora and Other): Number by Specific Predators and Total Value, 3-State Total, 1999 [1]

Predator	Losses	Total Value
	Head	*1,000 Dollars*
Coyotes	21,700	
Dogs	10,700	
Mountain Lions, Cougars, or Pumas	800	
Foxes	3,800	
Eagles	5,300	
Bobcats	11,700	
All Other Animals [2]	7,000	
3-State Total [3]	61,000	3,355

[1] Goat value per head is based on a two-year straight average of the value of all goats reported in the January 1 Sheep Survey from 1999 to 2000.
[2] Other predators include bears, wolves, ravens, vultures, and other animals.
[3] AZ, NM, and TX.

Non-Lethal Methods Used to Prevent Losses of Sheep and Lambs to Predators, by State and United States, 1999

State	Fencing	Guard Dogs	Llamas	Donkeys	Lamb Shed
	Percent	*Percent*	*Percent*	*Percent*	*Percent*
AZ	21.7	23.2	60.9	6.0	23.8
CA	69.1	21.6	12.4	5.2	56.1
CO	31.3	23.0	9.1	3.4	66.6
ID	46.4	55.2	9.9	2.5	45.5
IL	38.0	18.8	6.7	5.6	68.3
IN	29.9	27.8	5.2	3.9	57.4
IA	25.9	38.4	20.9	8.1	28.1
KS	64.5	27.2	3.2	7.5	82.8
MI	67.6	37.1	18.6	5.2	45.2
MN	66.8	41.4	4.4	5.5	53.7
MO	44.3	56.3	5.2	9.1	26.0
MT	36.0	27.5	22.7	15.1	65.6
NE	50.0	24.2	10.9	0.5	68.5
NV	47.3	50.7	8.3	2.8	57.0
N ENG [1]	78.3	16.3	3.9	7.3	19.6
NM	83.9	8.4	70.4	1.9	78.6
NY	77.6	16.2	15.2	14.7	45.3
ND	45.6	42.8	8.6	19.7	45.5
OH	73.9	15.6	5.1	6.5	40.5
OK	46.2	49.3	9.0	10.2	17.6
OR	61.9	27.2	37.4	2.1	49.9
PA	72.0	14.9	3.4	2.8	49.5
SD	10.4	22.7	5.9	21.6	41.6
TX	46.7	34.6	6.1	24.4	29.8
UT	53.6	28.5	7.4	2.3	46.5
VA	69.1	32.2	15.7	25.1	43.5
WA	82.6	39.3	14.3	1.3	60.6
WV	58.7	13.9	5.3	16.8	35.5
WI	73.2	12.1	6.4	3.5	49.5
WY	27.0	36.0	20.0	7.9	55.7
Oth Sts [2]	67.0	32.5	14.4	15.4	32.5
US	57.0	28.2	13.2	9.0	46.0

[1] N ENG includes CT, ME, MA, NH, RI, and VT.
[2] Other States include AL, AK, AR, DE, FL, GA, HI, KY, LA, MD, MS, NJ, NC, SC, and TN.

Non-Lethal Methods Used to Prevent Losses of Sheep and Lambs to Predators, by State and United States, 1999

State	Herding	Night Penning	Fright Tactics	Other
	Percent	*Percent*	*Percent*	*Percent*
AZ	8.7	20.4	6.3	0.3
CA	8.4	54.0	7.3	11.5
CO	7.1	79.4	5.6	3.6
ID	11.3	50.2	7.3	0.8
IL	3.2	58.1	1.5	4.1
IN	7.2	36.4	0.6	15.9
IA	3.2	43.8	2.8	2.5
KS	1.6	80.0	10.9	18.0
MI	1.1	36.6	3.3	2.3
MN	3.4	37.0	15.3	0.6
MO	2.6	50.2	1.1	2.9
MT	12.9	44.4	3.3	1.2
NE	14.8	43.4	5.0	7.0
NV	7.5	52.1	1.4	1.4
N ENG [1]	2.9	24.2	3.1	7.8
NM	5.0	86.0	3.6	2.2
NY	11.1	49.2	5.6	13.6
ND	9.2	49.5	14.6	6.8
OH	0.6	13.9	4.0	8.8
OK	0.2	59.7	3.3	2.9
OR	4.3	28.1	5.4	2.0
PA	0.6	33.9	1.2	2.0
SD	1.7	50.6	1.3	5.3
TX	9.3	35.0	9.2	7.3
UT	11.9	34.4	5.8	0.9
VA	21.4	36.5	8.6	9.2
WA	14.8	60.9	4.7	0.0
WV	7.0	40.2	2.9	8.6
WI	3.3	29.3	0.5	1.1
WY	13.4	53.5	9.2	4.0
Oth Sts [2]	10.9	34.6	6.0	14.0
US	6.6	42.6	5.1	6.2

[1] N ENG includes CT, ME, MA, NH, RI, and VT.
[2] Other States include AL, AK, AR, DE, FL, GA, HI, KY, LA, MD, MS, NJ, NC, SC, and TN.

Reliability of Sheep and Goat Predator Loss Estimates

Survey Procedures: A random sample of U.S. producers was surveyed to provide data for these estimates. Survey procedures ensured that all sheep producers, regardless of size, had a chance to be included in the survey. Large producers were sampled more heavily than small operations. About 13,800 operators were contacted during the first half of January by mail, telephone and face-to-face personal interview.

Estimation Procedures: Total death losses from all causes for sheep and lambs were as reported in "*Meat Animals, Production, Disposition, and Income - 2000*" (released April 27, 2000).

In setting the predator loss estimates, first total predator losses were estimated as a percent of total losses, then specific predator losses were estimated as a percent of total predator losses.

Revision Policy: Revisions to previous estimates are made to improve the current estimate. Previous year estimates are subject to revision when current estimates are made. Estimates for losses from all causes are subject to revision in next year's "*Meat Animals, Production, Disposition, and Income*". No revision to predator losses is planned.

The United States Department of Agriculture (USDA) prohibits discrimination in all its programs on the basis of race, color, national origin, gender, religion, age, disability, political beliefs, sexual orientation, and marital or family status. (Not all prohibited bases apply to all programs.) Persons with disabilities who require alternative means for communication of program information (braille, large print, audiotape, etc.) should contact the USDA's TARGET Center at 202-720-2600 (voice and TDD).

To file a complaint of discrimination, write USDA, Director, Office of Civil Rights, Room 326-W, Whitten Building, 14th and Independence Avenue, SW, Washington, D.C., 20250-9410, or call 202-720-5964 (voice or TDD). USDA is an equal opportunity provider and employer.

ACCESS TO REPORTS!!

For your convenience, there are several ways to obtain NASS reports, data products, and services:

INTERNET ACCESS

All NASS reports are available free of charge on the worldwide Internet. For access, connect to the Internet and go to the NASS Home Page at: **http://www.usda.gov/nass/**. Select "Today's Reports" or Publications and then Reports by Calendar or Publications and then Search, by Title or Subject.

E-MAIL SUBSCRIPTION

There are two options for subscribing via e-mail. All NASS reports are available by subscription free of charge direct to your e-mail address. 1) Starting with the NASS Home Page at **http://www.usda.gov/nass/**, click on **Publications**, then click on the **Subscribe by E-mail** button which takes you to the page describing e-mail delivery of reports. Finally, click on **Go to the Subscription Page** and follow the instructions. 2) If you do NOT have Internet access, send an e-mail message to: **usda-reports@usda.mannlib.cornell.edu**. In the body of the message type the word: **list**.

AUTOFAX ACCESS

NASSFax service is available for some reports from your fax machine. Please call 202-720-2000, using the handset attached to your fax. Respond to the voice prompts. Document 0411 is a list of available reports.

- -

PRINTED REPORTS OR DATA PRODUCTS

CALL OUR TOLL-FREE ORDER DESK: 800-999-6779 (U.S. and Canada)
Other areas, please call 703-834-0125 FAX: 703-834-0110
(Visa, MasterCard, check, or money order acceptable for payment.)

- -

ASSISTANCE

For **assistance** with general agricultural statistics or further information about NASS or its products or services, contact the **Agricultural Statistics Hotline** at **800-727-9540**, 7:30 a.m. to 4:00 p.m. ET, or e-mail: **nass@nass.usda.gov.**
