# Supplementary material for: The Elephant in the room: What can we learn from California regarding the use of sport hunting of pumas (Puma concolor) as a management tool?
Source: PLoS One. 2020 Feb 13;15(2):e0224638. doi: 10.1371/journal.pone.0224638 (PMC7018503; doi:10.1371/journal.pone.0224638)
Supplement: S2 File — This is a zip file that contains sources of the data used in the analyses that are no longer available on the internet. (ZIP) [file pone.0224638.s003.zip › Sheep predator loss 2004.pdf]

NASS
FACT FINDERS FOR AGRICULTURE
UNITED STATES DEPARTMENT OF AGRICULTURE
Washington, D.C.

Sheep and Goats Death Loss

Released May 6, 2005, by the National Agricultural Statistics Service (NASS), Agricultural Statistics Board, U.S. Department of Agriculture. For information on *Sheep and Goats Death Loss* call Scott Hollis at 202-720-4751, office hours 7:30 a.m. to 4:30 p.m. ET.

Sheep and Lamb Death Losses

Sheep and lamb losses from animal predators and non-predator causes in the United States totaled 600,300. This report provides detailed breakouts of sheep and lamb losses after docking by predators and non-predator causes, as well as data on non-lethal predator control measures.

Sheep and lamb losses from animal predators totaled 224,200. This represented 37.3 percent of the total losses from all causes and resulted in a loss of $18.3 million to farmers and ranchers. Coyotes and dogs caused the majority of sheep and lamb losses accounting for 60.5 percent and 13.3 percent respectively.

Sheep and lamb losses from non-predator causes totaled 376,100 or 62.7 percent of the total losses. Respiratory problems was the leading cause of non-predator deaths accounting for 17.5 percent, followed by digestive problems at 17.1 percent.

Farmers and ranchers throughout the United States spent 9.8 million dollars on non-lethal methods to control predators. Fencing was the most frequently used method at 52.5 percent. Night penning, guard dogs, and lamb sheds were the next most commonly used methods of preventing sheep and lamb losses at 32.9 percent, 31.8 percent, and 30.8 percent respectively.

A total of 415,200 **goats and kids** were lost in 2004. Diseases and other known causes accounted for 180,000 deaths or 43.4 percent of the total deaths. Predators accounted for 155,000 deaths or 37.3 percent of the total while the remainder of goat and kid losses at 80,200 or 19.3 percent resulted from unknown causes.

This report is released as a cooperative effort between the National Agricultural Statistics Service and Animal and Plant Health Inspection Service - Wildlife Services and Veterinary Services.

Contents

Page

Number and Value of Losses of Sheep and Lambs 1

Losses of Sheep and Lambs by Predators 4

Losses of Sheep and Lambs from Non-Predator Causes 8

Non-Lethal Prevention Methods 14

Losses of Goats and Kids 17

Reliability of Sheep and Goat Predator and Non-Predator Loss Estimates 18

Losses of Sheep and Lambs from Predators: Number of Head and Total Value, United States, 2004

Predator	Number of Head	% of Total Predators	Total Value
	Number	*Percent*	*1,000 Dollars*
Coyotes	135,600	60.5	10,707
Dogs	29,800	13.3	2,807
Mountain Lions, Cougars, or Pumas	12,700	5.7	1,101
Bears	8,500	3.8	769
Foxes	4,200	1.9	285
Eagles	6,300	2.8	438
Bobcats	11,100	4.9	814
All Other Predators	16,000	7.1	1,376
US	224,200	100.0	18,297

Losses of Sheep and Lambs from Non-Predators: Number of Head and Total Value, United States, 2004

Non-Predator	Number of Head	% of Total Non-Predators	Total Value
	Number	*Percent*	*1,000 Dollars*
Digestive Problems	64,250	17.1	5,216
Respiratory Problems	65,800	17.5	5,064
Metabolic Problems	11,800	3.1	1,099
Other Diseases	17,500	4.7	1,701
Weather Related	39,450	10.5	2,840
Lambing Problems	53,400	14.2	4,715
Old Age	39,900	10.6	5,181
On Their Back	3,800	1.0	436
Poisioning	10,300	2.7	1,010
Theft	2,400	0.6	199
Other Non-Predator	19,400	5.2	1,659
Unknown Non-Predator	48,100	12.8	4,164
US	376,100	100.0	33,284

Losses of Sheep and Lambs: By All Causes, Predators and Non-Predators, Number by State and United States, 2004

State	All Causes		Predators		Non-Predators	
	Sheep	Lambs	Sheep	Lambs	Sheep	Lambs
	Head	*Head*	*Head*	*Head*	*Head*	*Head*
AZ	2,000	2,000	1,500	500	500	1,500
CA	15,000	11,000	4,000	7,000	11,000	4,000
CO	5,000	19,000	2,200	8,000	2,800	11,000
ID	10,000	12,000	4,100	6,800	5,900	5,200
IL	3,000	6,000	900	800	2,100	5,200
IN	2,500	7,000	600	700	1,900	6,300
IA	6,000	25,000	500	1,700	5,500	23,300
KS	4,000	8,000	900	2,000	3,100	6,000
KY	1,400	3,200	600	1,300	800	1,900
MD	600	1,900	200	700	400	1,200
MI	3,000	6,000	400	800	2,600	5,200
MN	8,000	17,000	1,200	1,900	6,800	15,100
MO	2,500	7,500	200	900	2,300	6,600
MT	13,000	14,000	2,900	7,900	10,100	6,100
NE	4,000	10,000	500	1,500	3,500	8,500
NV	6,000	12,000	3,400	9,000	2,600	3,000
N ENG [1]	1,000	2,600	300	400	700	2,200
NM	7,000	6,000	2,700	4,000	4,300	2,000
NY	5,000	6,000	1,300	1,100	3,700	4,900
NC	1,300	2,300	300	500	1,000	1,800
ND	4,000	8,000	700	3,000	3,300	5,000
OH	5,000	15,000	500	1,500	4,500	13,500
OK	3,000	8,000	1,000	2,700	2,000	5,300
OR	8,000	8,000	2,800	5,800	5,200	2,200
PA	4,000	9,000	600	1,200	3,400	7,800
SD	10,000	27,000	2,000	5,000	8,000	22,000
TN	1,000	2,000	600	1,100	400	900
TX	41,000	58,000	16,000	41,000	25,000	17,000
UT	11,000	18,000	5,700	14,800	5,300	3,200
VA	2,500	7,500	600	2,400	1,900	5,100
WA	3,000	2,000	900	1,400	2,100	600
WV	2,500	5,000	1,100	2,200	1,400	2,800
WI	3,000	10,000	400	1,200	2,600	8,800
WY	11,000	18,000	3,000	14,000	8,000	4,000
Oth Sts [2]	6,000	11,000	1,800	3,000	4,200	8,000
US	215,300	385,000	66,400	157,800	148,900	227,200

[1] N ENG includes CT, ME, MA, NH, RI, and VT.

[2] Other States include AL, AK, AR, DE, FL, GA, HI, LA, MS, NJ, and SC. These states appear in the January Sheep and Goat report as Other States and all losses for this category in this report will pertain to these same states.

Losses of Sheep and Lambs from Predators and Non-Predators:
Total Value per Head and Total Value, by State and United States, 2004

State	Value per Head		Total Value Predator Losses		Total Value Non-Predator Losses	
	Sheep [1]	Lambs [2]	Sheep	Lambs	Sheep	Lambs
	Dollars	*Dollars*	*1,000 Dollars*	*1,000 Dollars*	*1,000 Dollars*	*1,000 Dollars*
AZ	145.00	57.00	218	29	73	86
CA	129.00	54.00	516	378	1,419	216
CO	136.00	91.00	299	728	381	1,001
ID	129.00	57.00	529	388	761	296
IL	165.00	60.00	149	48	347	312
IN	170.00	61.00	102	43	323	384
IA	141.00	57.00	71	97	776	1,328
KS	127.00	57.00	114	114	394	342
KY	160.00	61.00	96	79	128	116
MD	142.00	62.00	28	43	57	74
MI	143.00	56.00	57	45	372	291
MN	129.00	57.00	155	108	877	861
MO	133.00	61.00	27	55	306	403
MT	124.00	67.00	360	529	1,252	409
NE	142.00	59.00	71	89	497	502
NV	129.00	59.00	439	531	335	177
N ENG [3]	222.00	75.00	67	30	155	165
NM	111.00	60.00	300	240	477	120
NY	150.00	68.00	195	75	555	333
NC	140.00	63.00	42	32	140	113
ND	125.00	62.00	88	186	413	310
OH	150.00	59.00	75	89	675	797
OK	145.00	58.00	145	157	290	307
OR	116.00	57.00	325	331	603	125
PA	146.00	69.00	88	83	496	538
SD	129.00	69.00	258	345	1,032	1,518
TN	137.00	61.00	82	67	55	55
TX	100.00	66.00	1,600	2,706	2,500	1,122
UT	128.00	58.00	730	858	678	186
VA	139.00	61.00	83	146	264	311
WA	123.00	58.00	111	81	258	35
WV	120.00	61.00	132	134	168	171
WI	162.00	56.00	65	67	421	493
WY	120.00	68.00	360	952	960	272
Oth Sts [4]	146.00	58.00	263	174	613	464
US [5]	127.00	63.00	8,240	10,057	19,051	14,233

[1] Sheep value per head is based on a two-year straight average of the value of ewes reported in the January 1 Sheep Survey from 2004 to 2005.
[2] Lamb value per head is based on the market year average lamb price. An average lamb weight of 60 pounds was used in all States except Colorado, where a 90 pound average was used.
[3] N ENG includes CT, ME, MA, NH, RI, and VT.
[4] Other States include AL, AK, AR, DE, FL, GA, HI, LA, MS, NJ, and SC.
[5] US value per head is weighted between predator and non-predator losses for sheep and lambs. US value of sheep and lamb losses is the sum of state sheep and lamb losses.

Losses of Sheep and Lambs: Number and Percent by Coyotes and Dogs by State and United States, 2004

State	Coyotes				Dogs			
	Sheep	% Total Predator	Lambs	% Total Predator	Sheep	% Total Predator	Lambs	% Total Predator
	Head	*Pct*	*Head*	*Pct*	*Head*	*Pct*	*Head*	*Pct*
AZ	700	46.7	400	80.0	500	33.3	100	20.0
CA	2,000	50.0	5,200	74.3	800	20.0	900	12.9
CO	1,100	50.0	5,000	62.5	200	9.1	700	8.8
ID	2,600	63.4	4,500	66.2	400	9.8	1,000	14.7
IL	300	33.3	500	62.5	300	33.3		
IN	100	16.7	400	57.1	500	83.3	300	42.9
IA	200	40.0	1,000	58.8	200	40.0	700	41.2
KS	300	33.3	1,000	50.0	500	55.6	800	40.0
KY	200	33.3	600	46.2	100	16.7	600	46.2
MD			100	14.3	100	50.0	100	14.3
MI	200	50.0	500	62.5	100	25.0	100	12.5
MN	200	16.7	400	21.1	700	58.3	1,000	52.6
MO	100	50.0	600	66.7	100	50.0	300	33.3
MT	1,500	51.7	6,200	78.5	400	13.8	100	1.3
NE	300	60.0	1,200	80.0	100	20.0	100	6.7
NV	2,300	67.6	7,400	82.2	100	2.9		
N ENG [1]	300	100.0	400	100.0				
NM	1,100	40.7	2,000	50.0	900	33.3		
NY	1,000	76.9	700	63.6	200	15.4	200	18.2
NC	100	33.3	200	40.0	100	33.3	100	20.0
ND	700	100.0	2,800	93.3			200	6.7
OH	100	20.0	700	46.7	300	60.0	300	20.0
OK	600	60.0	1,800	66.7	400	40.0	600	22.2
OR	1,400	50.0	4,300	74.1	400	14.3	300	5.2
PA	200	33.3	900	75.0	400	66.7	200	16.7
SD	1,700	85.0	3,700	74.0	100	5.0	200	4.0
TN	300	50.0	700	63.6	300	50.0	300	27.3
TX	7,500	46.9	22,000	53.7	4,500	28.1	3,000	7.3
UT	3,200	56.1	9,500	64.2	300	5.3	300	2.0
VA	400	66.7	1,700	70.8	200	33.3	300	12.5
WA	500	55.6	1,000	71.4	100	11.1	300	21.4
WV	700	63.6	1,400	63.6	300	27.3	300	13.6
WI	200	50.0	900	75.0	200	50.0	200	16.7
WY	2,100	70.0	10,500	75.0	100	3.3	300	2.1
Oth Sts [2]	100	5.6	1,100	36.7	1,200	66.7	800	26.7
US	34,300	51.7	101,300	64.2	15,100	22.7	14,700	9.3

[1] N ENG includes CT, ME, MA, NH, RI, and VT.
[2] Other States include AL, AK, AR, DE, FL, GA, HI, LA, MS, NJ, and SC.

Losses of Sheep and Lambs: Number and Percent by Mountain Lions, Cougars, or Pumas, and Bears by State and United States, 2004

State	Mountain Lions, Cougars, or Pumas [1]				Bears [2]			
	Sheep	% Total Predator	Lambs	% Total Predator	Sheep	% Total Predator	Lambs	% Total Predator
	Head	*Pct*	*Head*	*Pct*	*Head*	*Pct*	*Head*	*Pct*
AZ	200	13.3						
CA	500	12.5	500	7.1			100	1.4
CO	200	9.1	100	1.3	600	27.3	1,700	21.3
ID	200	4.9	200	2.9	500	12.2	500	7.4
IA	100	20.0						
MN							100	5.3
MT			400	5.1	500	17.2	300	3.8
NV	900	26.5	1,200	13.3			200	2.2
NM	100	3.7	400	10.0	100	3.7	200	5.0
NC	100	33.3	100	20.0				
OR	500	17.9	700	12.1	100	3.6		
SD			100	2.0				
TX	300	1.9	600	1.5				
UT	1,300	22.8	2,600	17.6	700	12.3	1,500	10.1
WA	200	22.2					100	7.1
WV					100	9.1	100	4.5
WY	400	13.3	600	4.3	200	6.7	700	5.0
Oth Sts [3]			100	3.3	100	5.6		
Unpub [4]	100				100			
US	5,100	7.7	7,600	4.8	3,000	4.5	5,500	3.5

[1] Unpublished States include KS, NE, and SD for sheep.
[2] Unpublished States include CA, NV and N ENG for sheep.
[3] Other States include AL, AK, AR, DE, FL, GA, HI, LA, MS, NJ and SC.
[4] States with values that did not meet publication requirements were combined into an Unpublished States category.

Losses of Sheep and Lambs: Number and Percent by Foxes and Eagles by State and United States, 2004

State	Foxes				Eagles			
	Sheep	% Total Predator	Lambs	% Total Predator	Sheep	% Total Predator	Lambs	% Total Predator
	Head	*Pct*	*Head*	*Pct*	*Head*	*Pct*	*Head*	*Pct*
MD			100	14.3				
MI							100	12.5
MT			300	3.8			200	2.5
NE							200	13.3
NM					200	7.4	400	10.0
OR							200	3.4
SD			200	4.0			400	8.0
TX	300	1.9	2,000	4.9	400	2.5	3,000	7.3
UT			400	2.7			100	0.7
VA							200	8.3
WV			100	4.5				
WY			800	5.7	100	3.3	800	5.7
US	300	0.5	3,900	2.5	700	1.1	5,600	3.5

Losses of Sheep and Lambs: Number and Percent by Bobcats and All Other Animals by State and United States, 2004

State	Bobcats [1]				All Other Predators [2]			
	Sheep	% Total Predator	Lambs	% Total Predator	Sheep	% Total Predator	Lambs	% Total Predator
	Head	*Pct*	*Head*	*Pct*	*Head*	*Pct*	*Head*	*Pct*
AZ					100	6.7		
CA			100	1.4	700	17.5	200	2.9
CO					100	4.5	500	6.3
ID					400	9.8	600	8.8
IL	200	22.2	200	25.0	100	11.1	100	12.5
KS					100	11.1	200	10.0
KY					300	50.0	100	7.7
MD					100	50.0	400	57.1
MI					100	25.0	100	12.5
MN					300	25.0	400	21.1
MT					500	17.2	400	5.1
NE					100	20.0		
NV					100	2.9	200	2.2
NM	200	7.4	1,000	25.0	100	3.7		
NY					100	7.7	200	18.2
NC							100	20.0
OH					100	20.0	500	33.3
OK			100	3.7			200	7.4
OR					400	14.3	300	5.2
PA							100	8.3
SD					200	10.0	400	8.0
TN							100	9.1
TX	1,700	10.6	7,000	17.1	1,300	8.1	3,400	8.3
UT	100	1.8	100	0.7	100	1.8	300	2.0
VA			100	4.2			100	4.2
WA					100	11.1		
WV							300	13.6
WI							100	8.3
WY			100	0.7	100	3.3	200	1.4
Oth Sts [3]			100	3.3	200	11.1	800	26.7
Unpub [4]			100					
US	2,200	3.3	8,900	5.6	5,700	8.6	10,300	6.5

[1] Unpublished States include AZ, KS, NV, and SD for lambs.
[2] Other predators include wolves, ravens, vultures, and other animals.
[3] Other States include AL, AK, AR, DE, FL, GA, HI, LA, MS, NJ, and SC.
[4] States with values that did not meet publication requirements were combined into an Unpublished States category.

Losses of Sheep and Lambs: Number and Percent from Digestive and Respiratory Problems by State and United States, 2004

State	Digestive Problems [1]				Respiratory Problems [2]			
	Sheep	% Total Non-Pred	Lambs	% Total Non-Pred	Sheep	% Total Non-Pred	Lambs	% Total Non-Pred
	Head	*Pct*	*Head*	*Pct*	*Head*	*Pct*	*Head*	*Pct*
AZ	50	10.0	100	6.7	100	20.0	1,200	80.0
CA	1,300	11.8	800	20.0	1,800	16.4	900	22.5
CO	200	7.1	3,600	32.7	100	3.6	4,100	37.3
ID	200	3.4	1,400	26.9	500	8.5	800	15.4
IL	200	9.5	1,000	19.2	500	23.8	1,200	23.1
IN	300	15.8	800	12.7	200	10.5	2,200	34.9
IA	600	10.9	3,400	14.6	700	12.7	8,600	36.9
KS	300	9.7	900	15.0	500	16.1	2,200	36.7
KY	100	12.5	200	10.5	100	12.5	200	10.5
MD	100	25.0	100	8.3			100	8.3
MI	500	19.2	1,400	26.9	200	7.7	1,300	25.0
MN	600	8.8	3,000	19.9	800	11.8	4,000	26.5
MO	400	17.4	1,600	24.2	100	4.3	1,000	15.2
MT	500	5.0	1,200	19.7	800	7.9	1,000	16.4
NE	300	8.6	1,300	15.3	400	11.4	1,100	12.9
NV	100	3.8	100	3.3	800	30.8	1,500	50.0
N ENG [3]	100	14.3	300	13.6			100	4.5
NM	600	14.0	400	20.0	500	11.6	200	10.0
NY	100	2.7	200	4.1	400	10.8	600	12.2
NC	100	10.0	800	44.4			100	5.6
ND	300	9.1	700	14.0	300	9.1	1,200	24.0
OH	400	8.9	2,800	20.7	200	4.4	3,000	22.2
OK	400	20.0	500	9.4	400	20.0	800	15.1
OR	500	9.6	400	18.2	300	5.8	900	40.9
PA	400	11.8	900	11.5	100	2.9	900	11.5
SD	900	11.3	3,200	14.5	1,000	12.5	4,800	21.8
TN	100	25.0	100	11.1			200	22.2
TX	6,800	27.2	5,500	32.4	1,700	6.8	2,700	15.9
UT	400	7.5	500	15.6	200	3.8	300	9.4
VA	200	10.5	800	15.7	200	10.5	700	13.7
WA	200	9.5	100	16.7	200	9.5	200	33.3
WV	300	21.4	400	14.3	200	14.3	500	17.9
WI	200	7.7	1,400	15.9	200	7.7	1,700	19.3
WY	500	6.3	1,000	25.0	300	3.8	700	17.5
Oth Sts [4]	1,000	23.8	4,100	51.3	200	4.8	800	10.0
US	19,250	12.9	45,000	19.8	14,000	9.4	51,800	22.8

[1] Includes bloat, scours, parasites, enterotoxemia, acidosis, etc.
[2] Includes pneumonia, shipping fever, etc.
[3] N ENG includes CT, ME, MA, NH, RI, and VT.
[4] Other States include AL, AK, AR, DE, FL, GA, HI, LA, MS, NJ, and SC.

Losses of Sheep and Lambs: Number and Percent from Metabolic Problems and Other Diseases by State and United States, 2004

State	Metabolic Problems [1]				Other Diseases [2]			
	Sheep	% Total Non-Pred	Lambs	% Total Non-Pred	Sheep	% Total Non-Pred	Lambs	% Total Non-Pred
	Head	*Pct*	*Head*	*Pct*	*Head*	*Pct*	*Head*	*Pct*
AZ								
CA	200	1.8	100	2.5	1,300	11.8	200	5.0
CO					300	10.7	100	0.9
ID	900	15.3	100	1.9	500	8.5	400	7.7
IL	100	4.8	100	1.9	100	4.8		
IN			100	1.6			300	4.8
IA	100	1.8	500	2.1	100	1.8	800	3.4
KS	100	3.2			100	3.2	100	1.7
KY							100	5.3
MD			100	8.3				
MI	100	3.8	100	1.9	100	3.8	200	3.8
MN	600	8.8	500	3.3	300	4.4	400	2.6
MO	100	4.3	400	6.1	100	4.3	500	7.6
MT	300	3.0	200	3.3	900	8.9	200	3.3
NE	100	2.9	100	1.2	200	5.7	700	8.2
NV	100	3.8					100	3.3
N ENG [3]	100	14.3					100	4.5
NM	100	2.3			200	4.7	200	10.0
NY	100	2.7	100	2.0	600	16.2		
NC			300	16.7				
ND	100	3.0	200	4.0	200	6.1	100	2.0
OH	200	4.4	500	3.7	200	4.4	100	0.7
OK			100	1.9			100	1.9
OR	300	5.8	100	4.5	200	3.8	100	4.5
PA	100	2.9	700	9.0	300	8.8	300	3.8
SD	200	2.5	500	2.3	400	5.0	600	2.7
TN							100	11.1
TX	800	3.2	700	4.1	2,500	10.0	800	4.7
UT	100	1.9	100	3.1	500	9.4	200	6.3
VA	100	5.3	100	2.0				
WA	100	4.8	100	16.7				
WV								
WI	100	3.8	200	2.3	200	7.7	300	3.4
WY	300	3.8	300	7.5	400	5.0	300	7.5
Oth Sts [4]					100	2.4	100	1.3
Unpub [5]	100				100		100	
US	5,500	3.7	6,300	2.8	9,900	6.6	7,600	3.3

[1] Unpublished States include IN, KY, MD, and OK for sheep. Includes milk fever, twin lamb disease, pregnancy toxemia, etc.

[2] Unpublished States inlcude AZ, KY and N Eng for sheep only; IL and NY for lambs only; MD, NC, VA, WA, and WV for sheep and lambs. Includes mastitis, foot rot, boils, etc.

[3] New England includes CT, ME, MA, NH, RI, and VT.

[4] Other States include AL, AK, AR, DE, FL, GA, HI, LA, MS, NJ, and SC.

[5] States with values that did not meet publication requirements were combined into an Unpublished States category.

Losses of Sheep and Lambs: Number and Percent from Weather and Lambing Problems by State and United States, 2004

State	Weather Related [1]				Lambing Problems [2]			
	Sheep	% Total Non-Pred	Lambs	% Total Non-Pred	Sheep	% Total Non-Pred	Lambs	% Total Non-Pred
	Head	*Pct*	*Head*	*Pct*	*Head*	*Pct*	*Head*	*Pct*
AZ	50	10.0						
CA	200	1.8	600	15.0	600	5.5		
CO	100	3.6	800	7.3	500	17.9		
ID	100	1.7	500	9.6	1,100	18.6		
IL			1,100	21.2	200	9.5	1,300	25.0
IN			1,300	20.6	500	26.3	1,000	15.9
IA	100	1.8	1,000	4.3	1,000	18.2	5,700	24.5
KS	200	6.5	1,600	26.7	400	12.9	600	10.0
KY			200	10.5	400	50.0	1,000	52.6
MD			400	33.3	100	25.0	300	25.0
MI			900	17.3	300	11.5	400	7.7
MN	200	2.9	1,400	9.3	1,000	14.7	2,400	15.9
MO	100	4.3	900	13.6	500	21.7	1,600	24.2
MT	500	5.0	1,400	23.0	1,300	12.9		
NE	800	22.9	1,200	14.1	300	8.6	2,400	28.2
NV			500	16.7	100	3.8		
N ENG [3]			300	13.6	100	14.3	1,000	45.5
NM	300	7.0	200	10.0	500	11.6		
NY			2,000	40.8	800	21.6	1,800	36.7
NC			100	5.6	100	10.0	200	11.1
ND	300	9.1	600	12.0	300	9.1	1,500	30.0
OH	200	4.4	2,600	19.3	1,100	24.4	1,000	7.4
OK			800	15.1	500	25.0	1,100	20.8
OR	300	5.8	200	9.1	500	9.6		
PA	200	5.9	2,000	25.6	600	17.6	1,600	20.5
SD	300	3.8	3,500	15.9	900	11.3	5,500	25.0
TN			300	33.3	100	25.0	100	11.1
TX	1,000	4.0	3,000	17.6	2,400	9.6		
UT	100	1.9	300	9.4	600	11.3		
VA			800	15.7	200	10.5	1,000	19.6
WA					200	9.5		
WV			200	7.1	100	7.1	600	21.4
WI	100	3.8	1,100	12.5	700	26.9	1,000	11.4
WY	400	5.0	800	20.0	1,800	22.5		
Oth Sts [4]	100	2.4	1,100	13.8	200	4.8	300	3.8
Unpub [5]	100							
US	5,750	3.9	33,700	14.8	20,000	13.4	33,400	14.7

[1] Unpublished States include KY, N Eng, NY, TN, and VA for sheep. Includes chilling, drowning, lightning, etc.
[2] Lambs lost due to lambing problems are not applicable for AZ, CA, ID, MT, NV, NM, OR, TX, UT, WA, and WY. These States ask lambs lost before and after docking while all other States only ask for lambs lost. Estimates shown here are after docking losses only. Before docking loss estimates are available in state level publications for CO, ID, MT, UT, and WY.
[3] New England includes CT, ME, MA, NH, RI, and VT.
[4] Other States include AL, AK, AR, DE, FL, GA, HI, LA, MS, NJ, and SC.
[5] States with values that did not meet publication requirements were combined into an Unpublished States category.

Losses of Sheep and Lambs: Number and Percent from Old Age and Being on Their Back by State and United States, 2004

State	Old Age [1]				On Their Back [2]			
	Sheep	% Total Non-Pred	Lambs	% Total Non-Pred	Sheep	% Total Non-Pred	Lambs	% Total Non-Pred
	Head	*Pct*	*Head*	*Pct*	*Head*	*Pct*	*Head*	*Pct*
AZ	100	20.0						
CA	3,900	35.5			100	0.9		
CO	600	21.4			100	3.6	100	0.9
ID	1,100	18.6			200	3.4	100	1.9
IL	600	28.6						
IN	400	21.1						
IA	2,200	40.0					100	0.4
KS	1,000	32.3						
KY	100	12.5						
MD	200	50.0					100	8.3
MI	900	34.6			100	3.8		
MN	1,700	25.0			100	1.5		
MO	800	34.8						
MT	3,400	33.7			400	4.0		
NE	700	20.0						
NV	1,000	38.5						
N ENG [3]	300	42.9						
NM	700	16.3			200	4.7		
NY	900	24.3			200	5.4		
NC	200	20.0						
ND	1,200	36.4			100	3.0		
OH	1,100	24.4			100	2.2	100	0.7
OK	300	15.0						
OR	1,100	21.2			200	3.8		
PA	1,100	32.4			100	2.9		
SD	2,800	35.0			200	2.5	100	0.5
TN	100	25.0						
TX	4,600	18.4			100	0.4		
UT	1,200	22.6			100	1.9		
VA	700	36.8			100	5.3		
WA	800	38.1						
WV	600	42.9						
WI	800	30.8						
WY	1,900	23.8			600	7.5	100	2.5
Oth Sts [4]	800	19.0						
Unpub [5]							100	
US	39,900	26.8			3,000	2.0	800	0.4

[1] Not applicable for Lambs.
[2] Unpublished States include IL, MI, MO, and OR for lambs.
[3] New England includes CT, ME, MA, NH, RI, and VT.
[4] Other States include AL, AK, AR, DE, FL, GA, HI, LA, MS, NJ, and SC.
[5] States with values that did not meet publication requirements were combined into an Unpublished States category.

Losses of Sheep and Lambs: Number and Percent from Poisioning and Theft by State and United States, 2004

State	Poisioning [1]				Theft			
	Sheep	% Total Non-Pred	Lambs	% Total Non-Pred	Sheep	% Total Non-Pred	Lambs	% Total Non-Pred
	Head	*Pct*	*Head*	*Pct*	*Head*	*Pct*	*Head*	*Pct*
AZ							100	6.7
CA	500	4.5	200	5.0			100	2.5
CO	500	17.9	400	3.6			200	1.8
ID	200	3.4	600	11.5				
IL	100	4.8						
IN	100	5.3						
IA	100	1.8	100	0.4				
MI			100	1.9			100	1.9
MN	100	1.5	200	1.3				
MT	500	5.0	300	4.9				
NE			100	1.2				
NV	100	3.8						
NM	400	9.3	100	5.0	100	2.3	400	20.0
NY							100	2.0
ND	100	3.0						
OH	300	6.7	200	1.5			100	0.7
OK			300	5.7	100	5.0		
OR	100	1.9	100	4.5				
SD	100	1.3	200	0.9				
TX	800	3.2	600	3.5	100	0.4	600	3.5
UT	500	9.4	200	6.3				
VA			100	2.0				
WV			100	3.6				
WY	1,200	15.0	600	15.0	300	3.8		
Oth Sts [3]					100	2.4		
Unpub [4]	100							
US	5,800	3.9	4,500	2.0	700	0.5	1,700	0.7

[1] Unpublished States include KS, MO, NE, and WV for sheep. Includes nitrate poisioning, noxious feeds, noxious weeds, etc.
[2] New England includes CT, ME, MA, NH, RI, and VT.
[3] Other States include AL, AK, AR, DE, FL, GA, HI, LA, MS, NJ, and SC.
[4] States with values that did not meet publication requirements were combined into an Unpublished States category.

Losses of Sheep and Lambs: Number and Percent from Other Non-Predators and Unknown Non-Predators by State and United States, 2004

State	Other Non-Predator [1]				Unknown Non-Predator [2]			
	Sheep	% Total Non-Pred	Lambs	% Total Non-Pred	Sheep	% Total Non-Pred	Lambs	% Total Non-Pred
	Head	*Pct*	*Head*	*Pct*	*Head*	*Pct*	*Head*	*Pct*
AZ	100	20.0			100	20.0	100	6.7
CA	600	5.5	200	5.0	500	4.5	900	22.5
CO			600	5.5	400	14.3	1,100	10.0
ID	400	6.8	200	3.8	700	11.9	1,100	21.2
IL	100	4.8	200	3.8	200	9.5	300	5.8
IN	100	5.3	300	4.8	300	15.8	300	4.8
IA	300	5.5	1,700	7.3	300	5.5	1,400	6.0
KS	100	3.2	300	5.0	400	12.9	300	5.0
KY			100	5.3	100	12.5	100	5.3
MD							100	8.3
MI	100	3.8	400	7.7	300	11.5	300	5.8
MN	600	8.8	1,300	8.6	800	11.8	1,900	12.6
MO			300	4.5	200	8.7	300	4.5
MT	300	3.0	400	6.6	1,200	11.9	1,400	23.0
NE	100	2.9	200	2.4	600	17.1	1,400	16.5
NV					400	15.4	800	26.7
N ENG [3]			300	13.6	100	14.3	100	4.5
NM	200	4.7	200	10.0	500	11.6	300	15.0
NY	200	5.4			400	10.8	100	2.0
NC			200	11.1	600	60.0	100	5.6
ND	100	3.0	600	12.0	300	9.1	100	2.0
OH	200	4.4	900	6.7	500	11.1	2,200	16.3
OK	100	5.0	300	5.7	200	10.0	1,300	24.5
OR	1,000	19.2	100	4.5	700	13.5	300	13.6
PA	100	2.9	400	5.1	400	11.8	1,000	12.8
SD	200	2.5	700	3.2	1,000	12.5	2,900	13.2
TN					100	25.0	100	11.1
TX	1,300	5.2	600	3.5	2,900	11.6	2,500	14.7
UT					1,600	30.2	1,600	50.0
VA	100	5.3	400	7.8	300	15.8	1,200	23.5
WA	400	19.0	100	16.7	200	9.5	100	16.7
WV			300	10.7	200	14.3	700	25.0
WI	100	3.8	400	4.5	200	7.7	2,700	30.7
WY					300	3.8	200	5.0
Oth Sts [4]	300	7.1	600	7.5	1,000	23.8	800	10.0
US	7,100	4.8	12,300	5.4	18,000	12.1	30,100	13.2

[1] Includes accidents, fire, starvation, dehydration, etc.
[2] Exact cause of death is unidentifiable.
[3] New England includes CT, ME, MA, NH, RI, and VT.
[4] Other States include AL, AK, AR, DE, FL, GA, HI, LA, MS, NJ, and SC.

Non-Lethal Methods Used to Prevent Losses of Sheep and Lambs to Predators, by State and United States, 2004

State	Fencing	Guard Dogs	Llamas	Donkeys	Lamb Shed
	Percent	*Percent*	*Percent*	*Percent*	*Percent*
AZ	40.1	86.5	1.2	2.4	32.2
CA	71.9	39.1	8.6	3.4	33.8
CO	50.7	28.8	39.0	2.4	43.7
ID	55.7	57.8	12.6	2.3	40.8
IL	52.6	25.8	7.2	7.8	52.6
IN	58.6	23.4	19.3	7.6	52.7
IA	27.9	48.5	16.3	3.3	29.5
KS	26.9	23.6	13.8	9.3	37.3
KY	59.7	54.4	8.4	10.9	13.7
MD	64.9	9.1	1.3	17.5	32.3
MI	85.0	5.2	3.4	0.8	9.5
MN	58.7	29.2	6.5	3.5	57.8
MO	20.9	40.8	22.5	7.2	9.9
MT	23.4	25.3	27.7	7.0	44.4
NE	35.9	44.0	10.9	7.7	45.1
NV	77.5	19.7	20.1	2.6	30.4
N ENG [1]	60.6	53.6	18.3	3.1	18.4
NM	42.7	34.2	13.1	4.5	32.7
NY	81.1	10.1	5.6	2.1	1.2
NC	50.1	24.8	24.2	47.9	24.6
ND	21.0	30.8	22.7	6.7	4.5
OH	48.2	26.5	20.4	10.2	23.2
OK	67.6	35.2	19.2	16.5	13.4
OR	53.6	17.0	19.4	9.7	47.9
PA	58.0	41.7	6.7	10.2	57.6
SD	19.4	23.1	20.8	12.1	41.4
TN	27.3	28.5	4.3	21.7	16.0
TX	32.0	28.5	10.5	23.5	16.3
UT	48.4	31.4	23.4	7.9	49.4
VA	57.3	31.5	11.6	9.4	22.8
WA	68.5	25.0	16.4	6.7	35.4
WV	67.3	15.5	0.4	12.9	22.8
WI	35.7	15.5	11.8	6.2	36.5
WY	48.3	56.9	50.3	3.4	18.0
Oth Sts [2]	69.0	44.9	1.7	11.7	33.5
US	52.5	31.8	14.0	9.1	30.8

[1] N ENG includes CT, ME, MA, NH, RI, and VT.
[2] Other States include AL, AK, AR, DE, FL, GA, HI, LA, MS, NJ, and SC.

Non-Lethal Methods Used to Prevent Losses of Sheep and Lambs to Predators, by State and United States, 2004

State	Herding	Night Penning	Fright Tactics	Removing Carrion
	Percent	*Percent*	*Percent*	*Percent*
AZ	11.6	27.8	2.4	4.2
CA	10.0	35.6	5.9	12.5
CO	4.7	69.8	2.7	31.6
ID	5.7	14.8	4.2	5.3
IL	8.3	53.9	5.5	23.5
IN	6.4	39.3	3.4	27.1
IA	1.7	14.9	0.8	1.4
KS	2.1	39.7	1.8	9.6
KY	1.4	45.0	3.8	1.5
MD	0.6	3.3	7.5	1.8
MI	1.9	10.2	0.4	1.5
MN	7.1	37.8	1.6	28.4
MO	5.7	34.2		0.4
MT	18.7	33.7	7.1	28.6
NE	5.5	56.7	2.5	8.8
NV	18.5	51.0	0.7	15.0
N ENG [1]	1.7	34.5	0.1	1.0
NM	5.4	53.5	11.3	26.0
NY	0.3	31.8	0.3	2.0
NC	0.7	10.5	0.3	12.0
ND	0.5	34.8	2.9	1.6
OH	0.8	38.3	1.8	6.0
OK	2.2	31.2		2.6
OR	4.6	39.8	2.3	37.4
PA	22.4	41.0	3.2	7.3
SD	6.0	39.3	2.4	16.4
TN	8.2	41.8	4.0	4.3
TX	5.2	23.7	0.8	4.7
UT	14.4	27.7	5.5	33.3
VA	5.8	21.5	3.2	12.0
WA	2.4	36.6	2.0	1.0
WV	14.2	12.0	1.3	10.6
WI	3.8	27.9	3.1	18.4
WY	6.5	55.7	1.6	2.7
Oth Sts [2]	4.5	19.3	0.2	16.1
US	5.7	32.9	2.2	11.7

[1] N ENG includes CT, ME, MA, NH, RI, and VT.
[2] Other States include AL, AK, AR, DE, FL, GA, HI, LA, MS, NJ, and SC.

Non-Lethal Methods Used to Prevent Losses of Sheep and Lambs to Predators, by State and United States, 2004

State	Culling	Change Bedding	Frequent Checks	Other
	Percent	*Percent*	*Percent*	*Percent*
AZ	15.0	22.9	27.1	1.2
CA	16.2	12.3	11.8	2.4
CO	32.4	3.4	9.5	2.1
ID	6.9	5.9	15.6	1.0
IL	23.6	7.9	22.4	7.3
IN	42.6	34.5	36.5	3.4
IA	1.4	0.3	1.5	2.0
KS	8.0	1.7	3.9	7.8
KY	4.7	8.7	5.3	13.3
MD	9.9	0.9	16.2	0.6
MI	4.7	3.2	8.4	1.6
MN	32.9	7.6	17.3	3.2
MO	0.8	0.2	5.9	1.0
MT	32.4	19.2	31.1	1.5
NE	16.9	9.0	9.2	6.8
NV	4.0	18.3	16.4	1.4
N ENG [1]	1.1	14.5	2.0	4.7
NM	14.5	11.0	33.5	6.4
NY	0.2	2.0		2.2
NC	5.8	9.9	13.1	0.7
ND	2.9	2.0	6.2	10.7
OH	11.7	6.6	11.4	5.3
OK	2.9	0.7	3.6	6.5
OR	28.7	10.3	32.2	1.9
PA	21.7	11.6	19.2	0.8
SD	25.1	14.2	18.9	5.2
TN	4.7	3.6	20.8	0.4
TX	11.2	6.5	16.7	4.2
UT	42.1	27.5	24.1	4.6
VA	18.5	11.8	18.6	4.4
WA	4.0	0.1	2.5	2.0
WV	10.9	13.6	65.1	2.8
WI	14.1	10.0	3.9	9.1
WY	6.6	3.3	11.7	7.2
Oth Sts [2]	3.4	6.8	3.1	4.3
US	13.8	8.9	14.0	3.8

[1] N ENG includes CT, ME, MA, NH, RI, and VT.
[2] Other States include AL, AK, AR, DE, FL, GA, HI, LA, MS, NJ, and SC.

Losses of All Goats: Number by Predators,
Diseases or Other Known Causes, Unknown Causes, and Total Value, 2004

Item	Losses		Total Value
	Goats	Kids	All Goat Losses
	Head	*Head*	*1,000 Dollars*
Predator	40,000	115,000	15,965
Diseases and Other Known Causes	60,000	120,000	18,540
Unknown Causes	29,200	51,000	8,261
Total	129,200	286,000	42,766

[1] Goat value per head is based on a two-year straight average, using Texas data, of the value of all goats reported in the January 1 Sheep and Goat Survey from 2004 to 2005.

Reliability of Sheep and Goat Predator and Non-Predator Loss Estimates

Survey Procedures: A random sample of U.S. producers was surveyed to provide data for these estimates. Survey procedures ensured that all sheep producers, regardless of size, had a chance to be included in the survey. Large producers were sampled more heavily than small operations. About 22,000 operators were contacted during the first half of January by mail, telephone and face-to-face personal interview.

Estimation Procedures: Total death losses from all causes for sheep and lambs were as reported in "*Meat Animals, Production, Disposition, and Income - 2004*" (released April 28, 2005).

In setting the predator and non-predator loss estimates, first total predator and non-predator losses were estimated as a percent of total losses, then specific predator and non-predator losses were estimated as a percent of total predator and non-predator losses.

Revision Policy: Revisions to previous estimates are made to improve the current estimate. Previous year estimates are subject to revision when current estimates are made. Estimates for losses from all causes are subject to revision in next year's "*Meat Animals, Production, Disposition, and Income*". No revision to predator or non-predator losses is planned.

ACCESS TO REPORTS!!

For your convenience, there are several ways to obtain NASS reports, data products, and services:

INTERNET ACCESS

All NASS reports are available free of charge on the worldwide Internet. For access, connect to the Internet and go to the NASS Home Page at: **www.usda.gov/nass/**. Select "Today's Reports" or Publications and then Reports Calendar or Publications and then Search, by Title or Subject.

E-MAIL SUBSCRIPTION

All NASS reports are available by subscription free of charge direct to your e-mail address. Starting with the NASS Home Page at **www.usda.gov/nass/**, click on **Publications**, then click on the **Subscribe by E-mail** button which takes you to the page describing e-mail delivery of reports. Finally, click on **Go to the Subscription Page** and follow the instructions.

\- -

PRINTED REPORTS OR DATA PRODUCTS

CALL OUR TOLL-FREE ORDER DESK: 800-999-6779 (U.S. and Canada)
Other areas, please call 703-605-6220 FAX: 703-605-6900
(Visa, MasterCard, check, or money order acceptable for payment.)

\- -

ASSISTANCE

For **assistance** with general agricultural statistics or further information about NASS or its products or services, contact the **Agricultural Statistics Hotline** at **800-727-9540**, 7:30 a.m. to 4:00 p.m. ET, or e-mail: **nass@nass.usda.gov.**

The U.S. Department of Agriculture (USDA) prohibits discrimination in all its programs and activities on the basis of race, color, national origin, sex, religion, age, disability, political beliefs, sexual orientation, and marital or family status. (Not all prohibited bases apply to all programs.) Persons with disabilities who require alternative means for communication of program information (Braille, large print, audiotape, etc.) should contact USDA's TARGET Center at 202-720-2600 (voice and TDD).

To file a complaint of discrimination, write USDA, Director, Office of Civil Rights, Room 326-W, Whitten Building, 14th and Independence Avenue, SW, Washington, DC 20250-9410 or call 202-720-5964 (voice and TDD). USDA is an equal opportunity provider and employer.
