# Supplementary material for: The Elephant in the room: What can we learn from California regarding the use of sport hunting of pumas (Puma concolor) as a management tool?
Source: PLoS One. 2020 Feb 13;15(2):e0224638. doi: 10.1371/journal.pone.0224638 (PMC7018503; doi:10.1371/journal.pone.0224638)
Supplement: S2 File — This is a zip file that contains sources of the data used in the analyses that are no longer available on the internet. (ZIP) [file pone.0224638.s003.zip › WY-2006-Mountain-lion-management-plan.pdf]

WYOMING GAME & FISH DEPARTMENT

Mountain Lion Management Plan

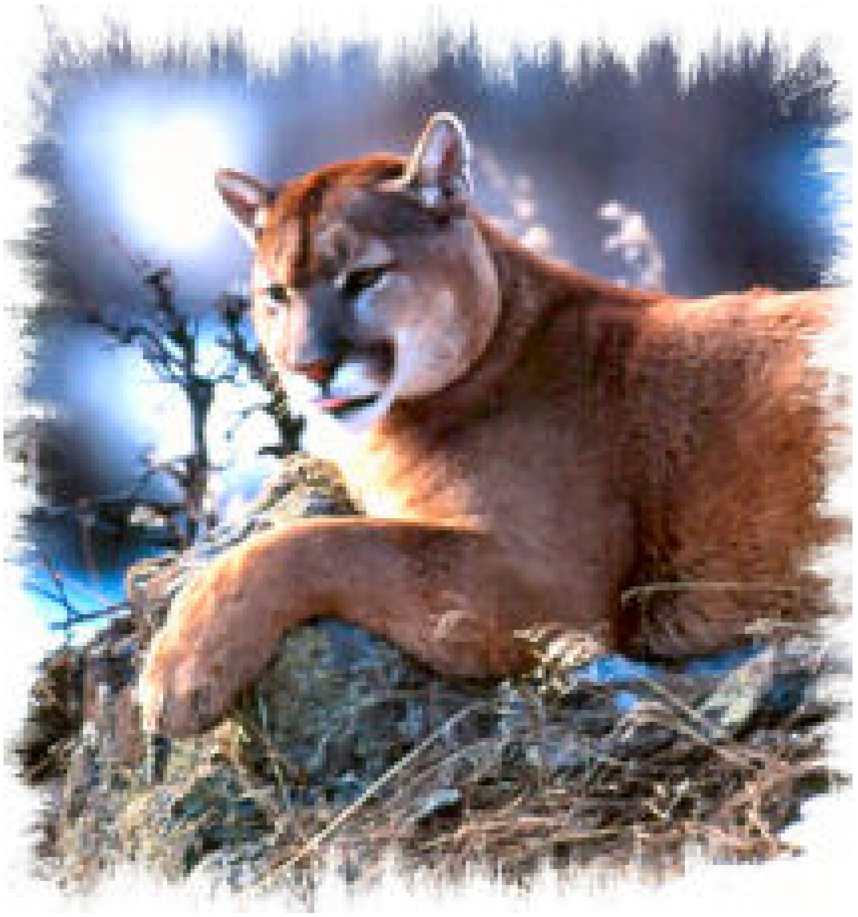

Prepared By: Trophy Game Section (Management/Research Branch), Wyoming Game & Fish Department, Lander, Wyoming

Sept. 7, 2006

TABLE OF CONTENTS

PAGE

TABLE OF CONTENTS i

EXECUTIVE SUMMARY iii

MOUNTAIN LION LIFE HISTORY AND ECOLOGY 1
- Distribution 1
- Habitat Use 1
- Mountain lion Social Structure and Reproduction 2
- Food Habits and Prey Relationships 3

TRADITIONAL MOUNTAIN LION MANAGEMENT IN WYOMING 4
- Local and Regional Mountain lion Management and Annual Data Collection 5
- Mountain Lion Hunting Season Structure 7
- Methods of Mountain Lion Hunting 7
- Potential for Orphaning Young 8
- Mountain Lion Habitat Management 11
- Mountain Lion Population Monitoring 11
  - Monitoring mountain lion population trend 11
  - Population estimation methods 15

ADAPTIVE MOUNTAIN LION MANAGEMENT APPROACH FOR WYOMING 16
  - Hunting season structure, hunting methods, and hunter effort indices 16
  - Mountain lion habitat management 17
  - Management criteria for establishing mountain lion management objectives.. 18

NUISANCE MOUNTAIN LION MANAGEMENT 26
- Livestock Depredations 26
- Mountain lion/Human Interactions 28

PUBLIC INFORMATION AND EDUCATION EFFORTS 29

FUTURE RESEARCH AND MANAGEMENT NEEDS 31

LITERATURE CITED 32

APPENDIX I – History of mountain lion management regulations in Wyoming 37

APPENDIX II – Mountain lion harvest, hunter effort, and human caused mortalities 40

APPENDIX III – Wyoming mountain lion management units and hunt areas 42

APPENDIX IV – Wyoming mountain lion mortality form 43

APPENDIX V – Interpretation of mountain lion behaviors 44

APPENDIX VI – Preventative measures for mountain lion encounters 45

EXECUTIVE SUMMARY

- The goal of mountain lion management in Wyoming is to sustain mountain lion populations throughout core habitat at varying densities depending on management objectives to provide for recreational/hunting opportunity, maintain ungulate populations at established objectives or in line with current habitat conditions, and minimize mountain lion depredation to pets and livestock and reduce the potential for human injury.

- The intent of this document is to provide guidelines to direct future management efforts for mountain lion populations in Wyoming and not to specifically address local management issues throughout the state; a process that occurs during the 3 year season setting process, when hunt area specific data are presented in the annual mountain lion mortality summaries. The management approach addressed in this document favors an adaptive management process where management objectives are established based on local biological and social conditions and modified/adapted over time relative to management criteria suggesting whether or not objectives have been met, to achieve balance between predator and prey populations, and address changing social factors related to depredation incidents and human-mountain lion interactions.

- Core occupied habitats for adult mountain lions during the winter will be delineated statewide to evaluate impacts from the density of human-caused mountain lion mortalities and to evaluate potential impacts from future development projects. Local (by hunt area) and regional (by Mountain Lion Management Unit-LMU) management objectives will be developed and evaluated based on harvest data. A source-stable-sink adaptive management approach will be applied evaluating (1) density of human-caused mortalities, (2) sex-age composition of mountain lion harvest focusing on relative proportion of adult female harvest, and (3) the relative age of harvested adult females.

- Hunt area management objectives will be based on Regional desires to meet localized situations relative to maintaining low population densities (sink), stable population densities, or to maintain areas with low mountain lion mortality to serve as source areas for mountain lion dispersal into areas experiencing negative population growth (sink areas). Sink management will be applied to maintain low mountain lion densities in areas experiencing high nuisance incidents (livestock depredation, human-lion interactions) and areas where ungulate populations are believed to be depressed primarily due to mountain lion predation; stable management objectives will be implemented to sustain long term hunting opportunity; and source management objectives will be applied to areas where nuisance incidents and predation impacts to prey populations are not an issue. Management objectives at the LMU level will strive for a combination of source, stable, and sink management that will allow for the department to sustain mountain lion populations throughout core habitat at varying densities depending on management objectives.

- Status of representative source areas will be periodically evaluated to verify that these areas are functioning as source areas for mountain lion dispersal using monitoring

techniques that can be reasonably applied relative to Department budget constraints. Success of sink management to address nuisance incidents or predation pressures on ungulate populations will be evaluated over time following the adaptive management process outlined in this plan. Similarly, mountain lion population monitoring criteria will be evaluated and modified as information becomes available addressing the utility of the proposed criteria in defining source, stable, or sink mountain lion habitats.

- Hunting season structure will be based on mountain lion mortality quotas. Mortality quotas will be established for each hunt area, and the hunting season will be closed when the quota has been met. Most of the hunting seasons will run from September 1 through March 31, with the exception of a few hunt areas with chronic livestock depredations. Hunting with hounds will continue to be allowed. Hunters shall present the pelt and skull of harvested mountain lions to Department personnel within 72 hours of harvest so specific data can be recorded. These data will be used to determine the management status, age and sex structure of harvested mountain lions, distribution of mortalities, hunter effort, hunter success, and to account for and set future mortality quotas. Mortality quotas will be established every 3 years to allow sufficient time to reach management objectives and to permit adequate analysis of potential impacts of specific harvest quotas. The process by which these 3-year mortality quotas are set includes annual data analyses and summary by the Trophy Game Section, internal review and recommendations at the regional level, public review of the recommendations, and final approval by the Commission.

- The Department will continue to use a variety of options ranging from no action to lethal removal, which will be assessed on a case-by-case basis, to address mountain lion depredation on domestic livestock and pets and mountain lion/human interactions. All management actions and responses will be documented for future evaluation.

- Adaptive management will be implemented to address short and long-term management needs where appropriate, and additional research efforts will be conducted to address other management priorities as funds become available relative to other Department priorities.

- A previous draft of this management plan was revised based on comments received from 4 peer reviewers and 73 separate public comments. We thank Brad Compton, Idaho Department of Fish and Game, Fred Lindzey, Wyoming Cooperative Fish & Wildlife Research Unit-retired, Ken Logan, Colorado Division of Wildlife, Dale Strickland, Western Ecosystems Technology, Inc, Cheyenne, WY, and members of the public submitting comments for suggestions on improving this management plan. Comments from peer reviewers were evaluated and most have been addressed throughout the revised document. Comments concerning various aspects of the proposed plan (e.g. surveying all mountain lion license holders for hunter effort data, educating hunters about sexing lions in the field, including all human-caused mortality towards quotas, oppose sink management every 3 years, balance source-sink management and reducing the reporting period for harvested lions to 48 hours) were addressed and included in the plan for consideration by the Commission.

- The Department will continue to update and expand, where feasible, information and education efforts across the state including development of a website to educate hunters on sexing mountain lions in the field, and periodically conducting public attitude surveys of Wyoming residents.

- The Department will begin to survey all mountain license holders to enhance the management database.

- All human caused mountain lion mortalities will be counted towards quotas.

MOUNTAIN LION LIFE HISTORY AND ECOLOGY

Distribution

The historic range of the mountain lion was the largest of any terrestrial mammal in the western Hemisphere, with the exception of humans (Logan and Sweanor 2001). The mountain lion continues to range from the southern tip of South America to northern British Columbia (Logan and Sweanor 2001), but were apparently extirpated from the eastern US and Canada, with the exception of southern Florida, by the late 1800s to early 1900s. Between the mid 1960s and the early 1990s, mountain lion populations increased in many western states and they expanded their distribution into some of the mid-western states including Nebraska, South Dakota, and North Dakota likely due to reclassifying mountain lions from unregulated predator status to game animals and the restricted use of predicides since the early 1970s. Similarly, mountain lions in Wyoming have increased in abundance and distribution and currently occupy most timbered and tall-shrub covered regions statewide. In the early part of the 20th century, efforts to remove mountain lions from many areas of Wyoming caused local extirpations. However, robust populations are currently found in the Black Hills of northeastern Wyoming, the pinyon-juniper country of southwestern Wyoming, and all major mountain ranges throughout the state. This reestablishment of mountain lions throughout Wyoming (and likely throughout much of their former range) is likely due to a shift in management practices and policies that favored increases in numbers and distribution (see Appendix I for mountain lion management history in Wyoming) and habitat conditions favoring increases in some prey abundance (e.g., elk, *Cervus elaphus*, white-tailed deer, *Odocoileus virginianus*).

Dispersal patterns and genetic evidence suggest mountain lion populations throughout most of the western US are well connected (Culver et al. 2000, Sinclair et al. 2001, Anderson et al. 2004). Movements of male mountain lions in excess of 1,000 km have been documented (Thompson and Jenks 2005). These long-range movements provide a very effective means of genetic transfer and population maintenance to mountain lion populations in distant regions. In addition, much of Wyoming's mountain lion habitats are extensions of mountain ranges in other states. This provides excellent connectivity to other habitats, and hence, other mountain lion populations. Overall, gene flow among mountain lion populations in the Central Rocky Mountains suggests this region exists as one large mountain lion population with rapid genetic exchange among suitable habitat patches throughout the region (Anderson et al. 2004).

Habitat Use

The broad geographic distribution of the mountain lion in North America attests to its ability to persist anywhere that provides adequate prey and cover [Cougar Management Guidelines Working Group (CMGWG) 2005]. Previous mountain lion habitat studies in the western US suggest mountain lions select conifer, deciduous timber, riparian, and tall shrub habitat types at mid-high elevations in steep or rugged terrain (Logan and Irwin 1985, Laing 1988, Koehler and Hornocker 1991, Williams et al. 1995, Dickson and Beier 2002). Tall vegetation or rugged terrain sufficient for concealment provides the necessary hiding and stalking cover for securing prey and raising young (CMGWG 2005). Mountain lions may be found in climates ranging from arid regions of desert environments to temperate rainforests of the Pacific Coast. Besides prey

availability, the only biophysical limitations for mountain lions are vast, open areas with little hiding cover and severely cold winter temperatures of northern climates (Pierce and Bleich 2003).

Despite the mountain lions broad distribution and adaptability, human impacts from development and habitat fragmentation can negatively impact mountain lion populations (Beier 1993). Increased construction of roads and homes in mountain lion habitat not only reduces the amount and quality of habitat available to mountain lions and their prey [e.g., deer (*Odocoileus* spp.) and elk (*Cervus* spp.)], but also increases human presence in these areas. Increased human activity ultimately leads to increases in mountain lion/human interactions and mountain lion deaths (CMGWG 2005). Even in sparsely human populated states like Wyoming, where most mountain lion range is still relatively contiguous, subdivisions, new road construction, and oil and gas development may negatively impact mountain lion habitats.

Mountain Lion Social Structure and Reproduction

Social behavior of mountain lions likely evolved to maximize individual survival and reproductive success (Logan and Sweanor 2001). Mountain lions are solitary carnivores exhibiting a polygynous breeding strategy where dominant males typically breed with females that reside within their home range (Murphy 1998). Resident males aggressively defend their territories against male intruders, whereas females allow more overlap, but express mutual avoidance (Lindzey et al. 1989, Ross and Jalkotzy 1992, Logan and Sweanor 2001). Size of female home ranges tend to be large enough to provide sufficient prey for themselves and their young (~50-100 km^2, 20-40 mi^2), while male home ranges tend to be larger (~150-300 km^2, 60-120 mi^2), overlapping several females, apparently to maximize their reproductive success (Murphy 1998). Young females commonly express philopatric behavior (remain in their natal range) upon independence, but males typically disperse from their natal range (Anderson et al. 1992, Ross and Jalkotzy 1992, Lindzey et al. 1994, Logan and Sweanor 2001). Partially due to their solitary and territorial nature and ultimately limited by prey abundance, mountain lion densities are low relative to other large mammals ranging from about 10 independent (>1 year old and self sufficient) mountain lions/1,000 km^2 (386 mi^2) in arid climates (e.g., southern Utah, Lindzey et al. 1989) to about 35 independent mountain lions/1,000 km^2 in more mesic areas (e.g., the Diablo Range, California, Hopkins 1989, southwest Alberta, Ross and Jalkotzy 1992).

Female mountain lions typically produce their first litter at 2-3 years old (Anderson 1983, Ashman et al. 1983, Logan and Sweanor 2001) and may breed at any time of the year, but exhibit seasonal birth pulses. Data from 7 mountain lion studies in western North America indicate May through October are the peak months for mountain lion parturition (CMGWG 2005). Gestation lasts 82-96 days and mountain lions typically produce 2 to 4 young. The average size of 53 nursling litters documented in New Mexico was 3.0, with 13 (26%) 2-kitten litters, 26 (49%) 3-kitten litters, and 14 (26%) 4-kitten litters (Logan and Sweanor 2001). Other studies reported average litter sizes <6 months old, ranging from 2.2 in Alberta (Ross and Jalkotzy 1992) to 2.9 in Wyoming (Logan et al. 1986). Kittens are usually weaned at 2–3 months and typically remain with the female for 12–18 months before becoming independent (Pierce and Bleich 2003).

Food Habits and Prey Relationships

Mountain lion diets consist primarily of large vertebrate prey species. In much of North America, deer comprise the majority of mountain lion diets (Pierce and Bleich 2003), but other large ungulates such as elk, bighorn sheep (*Ovis canadensis*), moose (*Alces alces*), and pronghorn (*Antilocapra americana*) may also be consumed (Ross and Jalkotzy 1996, Ross et al. 1997, Murphy 1998, Anderson and Lindzey 2003). Although mountain lions primarily subsist on large ungulates, small mammals including porcupines (*Erethizon dorsatum*), lagomorphs (hares and rabbits), ground squirrels (*Spermophilus* spp.), and beavers (*Castor canadensis*) may also supplement mountain lion diets. Mountain lions also occasionally prey on domestic livestock and pets. Sheep and goats are the most commonly killed domestic livestock, but mountain lions also kill cattle, horses, and pets including dogs, and cats (CMGWG 2005).

The mountain lion can be an influential predator on some ungulate populations. Mountain lions were an important source of predation on a bighorn sheep population in Alberta (Ross et al. 1997), and were implicated in the decline of another bighorn population by causing avoidance of high quality forage (Wehausan 1996). Logan and Sweanor (2001) reported that mountain lion predation was the strongest proximate cause limiting a New Mexico mule deer (*O. hemionus*) population by slowing the rate of growth during a population increase phase, and hastening the decline of the population during drought conditions that degraded forage quantity and quality. Mountain lions have annually removed an estimated 15-20% of a mule deer population on the Kaibab Plateau, Arizona (Shaw 1980), 8-12% of a mule deer population on the Uncompahgre Plateau, Colorado (Anderson et al. 1992), and 2-3% of elk and 3-5% of mule deer in the northern Yellowstone Ecosystem (Murphy 1998). Mountain lion predation, however, does not necessarily indicate suppression or regulation of the prey population. Regulation is more likely in systems with multiple prey and multiple predator species. In these situations, predator populations that would normally decrease as their prey populations are reduced, are supported by other, more numerous prey populations (Pierce and Bleich 2003).

The potential impacts of mountain lions on prey populations are largely dependent on the condition of the prey and their habitat. In areas where prey habitat is in good condition, prey body condition will also be greater. Thus, most individuals in the prey population are likely to survive in the absence of predation. In prey populations where individuals are in poor condition due to poor forage quality, however, those individuals are more likely to die regardless of predation. Therefore, mountain lion predation on ungulates in good physical condition is more likely to be *additive* to other causes of mortality. Conversely, mountain lion predation on ungulates in poor physical condition is more likely to be *compensatory* (Logan and Sweanor 2001). In addition, healthy prey populations likely exhibit higher reproductive rates and are more likely to offset predatory regulation by producing more young than are consumed by predators. Ungulate populations exhibiting the characteristics of limitation by predation (Table 1) may benefit from increased mountain lion harvest. Populations limited mainly by habitat conditions will not likely benefit from increases in local mountain lion harvest except during the initial phases of habitat recovery allowing more rapid response of the prey population to improved forage conditions. Additionally, in situations where alternative prey species are lacking, a decline in mountain lion numbers will naturally follow the decrease in the ungulate population regardless of mountain lion harvest levels (CMGWG 2005).

Table 1. Characteristics of ungulate-prey populations regulated by predation and populations regulated by forage conditions (from the Cougar Management Guidelines 2005, page 15).

Life history characteristic	Population size mainly affected by predation[b]	Population size mainly affected by forage
Physical condition of adult females	better	poorer
Pregnancy rate of adult females	higher	lower
Pause in annual production by adult females	less likely	more likely
Yearlings pregnant[a]	usually	seldom
Corpora lutea counts of adult females[a]	higher	lower
Litter size[a]	higher	lower
Age at first reproduction for females	younger	older
Weight of neonates	heavier	lighter
Mortality of young	additive	compensatory
Age at extensive tooth wear	older	younger
Diet quality	higher	lower

[a]Some species of ungulates may show limited variability in these characteristics.
[b]These traits will be evident in *any* population far below carrying capacity, even if it experiences *no* predation. The manager should have evidence that predation is a limiting factor before concluding that reducing predation would increase ungulate recruitment.

TRADITIONAL MOUNTAIN LION MANAGEMENT IN WYOMING

Mountain lion management in Wyoming (and throughout its range) has traditionally consisted of more art than science largely due to the secretive nature and naturally low densities typical of this solitary large carnivore and the rugged terrain it typically inhabits. Agencies charged with mountain lion management attempt to address the public's desires, where values vary and sometimes compete between maintaining abundant populations, providing hunting opportunity, and minimizing human conflicts by addressing depredation incidents and potential for mountain lion-human interactions. The goal of mountain lion management in Wyoming is to sustain mountain lion populations throughout suitable mountain lion habitat at varying densities depending on management objectives, and to provide for recreation/hunting opportunity, maintain ungulate populations at established objectives or in line with current habitat conditions, and minimize mountain lion depredation and potential for human injury resulting from mountain lion-human encounters.

Although population estimates have traditionally been lacking, evidence based on professional experience and opinion (i.e., local wildlife biologists, game wardens), increasing mountain lion harvest levels (Appendix II, Fig. II-1), hunter observations, sightings, and nonharvest-human caused mortalities (Appendix II, Fig. II-3) indicate mountain lion populations have increased in Wyoming over the past 30 years. In response to perceived increases in mountain lion numbers, harvest quotas were increased annually during the mid to late 1990s (Appendix II, Fig. II-1). Approaches to how we manage mountain lion populations have changed gradually since 1974 when regulated hunting was first established in Wyoming, including establishment of fall-winter hunting seasons, developing management units and hunt areas to address local management issues, requiring mandatory inspection of harvested mountain lions for annual data collection, and developing total and female harvest quotas to address hunt area management objectives (Appendix I). Traditionally, mountain lion harvest quotas were set based on perceived densities and the history of or potential for human conflicts (e.g., mountain lion-human interactions, depredation incidents, potential impacts to big game species) and adjusted based on perceived mountain population trends relative to annual harvest data, and how quickly quotas were filled each year loosely reflecting hunter effort. Although mountain lion populations in Wyoming increased under this management scheme, this general approach to mountain lion management provided managers with limited ability to determine whether or not management objectives were achieved. The previous Draft Wyoming Mountain Lion Management Plan (1997) identified the lack of data necessary to identify whether or not management objectives have been met and supported research investigating potential methods to adequately monitor mountain lion population responses to varying management prescriptions. Subsequently, mountain lion research was conducted from 1997-2003 (Anderson 2003) to investigate potential approaches for evaluating mountain lion management.

Local and Regional Mountain Lion Management and Annual Data Collection

Wyoming is currently divided into 5 Mountain Lion Management Units (LMU), which are further divided into 29 mountain lion hunt areas (Appendix III). Due to the large size of the West LMU, covering several connected mountain ranges and associated foothill winter mountain lion habitats, the West LMU is divided into 3 separate Data Analysis Units (DAUs) called the Absaroka (hunt areas 19 and 20), Wyoming Range (hunt areas 2, 14, 17, 26, and 29) and Wind River (hunt areas 3, 4, 18 and 28) DAUs (Appendix III). This subdivision provides managers improved capability to monitor the effects of harvest strategies designed to meet potentially different management objectives among these 3 regions.

Mountain lion management units primarily represent connected regions of contiguous mountain lion habitat (i.e., geographic populations), and the smaller hunt areas allow managers to address local management issues while maintaining the overall management objective for the regional population (i.e., within the LMU). The Cougar Management Guidelines Working Group (2005) recently suggested managing mountain lion populations with respect to source-sink dynamics, where source areas would be managed for positive growth and sustain sink areas where management objectives call for reducing mountain lion densities. The current hunt area and management unit structure in Wyoming lends itself well to this concept, where hunt areas within management units can be managed as source and sink subpopulations, depending on local

management issues, and can continue to support desired mountain lion population densities at landscape levels.

Mountain lion management objectives shall be based on ecological data and social conditions to ensure management strategies benefit both the species of concern and the people who are impacted by mountain lion conflicts. Mountain lion mortality data in Wyoming include information obtained annually from harvest or other documented forms of mortality [e.g., natural causes, damage removals, road kills; Appendix II]. Since 1974, hunters have been required to present the pelt and skull of harvested mountain lions to a district game warden, biologist, or a Wyoming Game and Fish Department regional office for registration. Information collected include: harvest date, location (legal description, Universal Transverse Mercator location, and hunt area), sex, lactation history (whether or not females have ever produced young from nipple characteristics; Anderson and Lindzey 2000), estimated age from tooth wear and degree of staining, and collection of teeth for cementum annuli aging, number of days spent hunting, hunting method, and number of mountain lions and mountain lion tracks observed while hunting (Appendix IV). Trainer and Golly (1992) reported 76% agreement ≤1 year of annuli ages compared using blind tests of 2 premolars from the same mountain lion (n = 426; 92% agreement for lions <4 years old), and annuli age comparisons of known age mountain lions were 95% accurate (within 1 year; Trainer and Golly 1992:14/15, Anderson 2003:6/6). In addition to mortality data, the Wyoming Game & Fish Department compiles data on mountain lion observations, sign, depredations, human interactions and gauges social concerns through public meetings, hunter surveys, public attitude surveys, and contacts with the public.

Mountain lion mortality data are used to assess: (1) population status, (2) age and sex structure of harvested mountain lions, (3) distribution of mountain lion mortalities, (4) effort expended per mountain lion harvested (Appendix II, Fig. II-2), and (5) to account for and set mortality quotas. Sex and age composition of mountain lion harvests are useful to assess mountain lion population trends (Anderson and Lindzey 2005), and the age of reproductive females can be useful to examine the reproductive potential of mountain lion populations (Stoner 2004, Anderson and Lindzey 2005); populations maintaining older-age females have higher reproductive potential, and thus resiliency, than populations where female survival is reduced. Recording distribution of mountain lion harvest and other human-caused mortalities allows assessment of potential source areas where little or no mountain lion mortality occurs, and sink areas where mountain lion mortalities may be relatively high. Changes in hunter effort may indicate changes in mountain lion densities, assuming the time required to harvest a mountain lion is related to the number of mountain lions in an area. This information is used to establish total and/or female mortality quotas by hunt area every 3 years. Setting mountain lion seasons every 3 years allows sufficient time for management reductions in areas with sufficient hunter access (Anderson and Lindzey 2005) and recovery for previously suppressed populations (Logan and Sweanor 2001, Anderson and Lindzey 2005). The process by which these 3-year mortality quotas are set include (1) annual data analyses and summary by the Trophy Game Section, (2) internal regional review and recommendations provided by each of the 7 Wyoming Game and Fish regions, (3) a public input process, and (4) final hunting season regulations submitted from the regions for action to the Wyoming Game and Fish Commission.

Mountain Lion Hunting Season Structure

Regulation of sport hunting for mountain lions in the western states typically follows 1 of 3 harvest strategies including general seasons, limited entry, and harvest quota systems (CMGWG 2005). General seasons allow unlimited hunting of mountain lions of either sex, and the only restrictions include the number of licenses issued per hunter (typically 1 per season) and timing and length of the hunting season. General seasons provide the highest hunting opportunity, but likely result in uneven hunting pressure (i.e., accessible areas are heavily hunted and inaccessible areas are not) limiting control over harvest level, composition of the harvest, and distribution of the harvest. Limited entry programs limit the number of hunters per hunt area through limited license allocation, using either first come first serve or lottery license sales. This approach is most limiting in terms of hunter opportunity, but can be useful to disperse hunting pressure, control harvest levels, and may increase the opportunity for hunters to be selective (increasing male harvest) in areas where hunting pressure is low. Harvest quota management requires setting a limit on the total harvest and/or number of female mountain lions harvested from an area. The hunting season is closed in an area once the harvest quota has been met. Hunters are required to monitor status of the hunting season by calling a harvest quota hotline. Advantages to the quota management approach are that hunting opportunity remains high and harvest distribution and level can be regulated. Female sub quotas can be used to support a management objective of sustaining harvest levels with reduced impact on the mountain lion population. Potential disadvantages of harvest quota management include the number of hunters per hunt area is unlimited until quotas are filled and harvest quotas may be exceeded if more than 1 mountain lion is harvested the same day the quotas is filled. Harvest quota management has traditionally been used in Wyoming for mountain lion management.

Methods of Mountain Lion Hunting

Mountain lion hunting in Wyoming is accomplished using various hunting methods including opportunistic harvest (spot and stalk) during big game (e.g., elk and deer) seasons, calling mountain lions using predator calls, and tracking and baying mountain lions using trained hunting dogs (i.e., hunting with hounds). The majority of mountain lions harvested annually in Wyoming are taken by hunting with hounds (typically >90%).

Some groups and individuals, both nationally and locally (Gasson and Moody 1995), are concerned about the use of dogs as a hunting method for mountain lions, and some states have recently banned hunting with hounds (e.g., Oregon, Washington). In states where hunting with hounds is not allowed, opportunistic mountain lion hunting (during big game seasons, predator calling) appears comparably successful based on harvest levels observed in Washington and South Dakota. Results from Washington (Martorello and Beausoleil 2003) suggest opportunistic mountain lion hunting is less selective than hunting with hounds and/or female mountain lions are more vulnerable to opportunistic hunting; relative female harvest levels increased from 42% to 59% when hunting with hounds was banned in Washington (mean annual harvest before hound hunting ban = 157 and after hound hunting ban = 199, but harvest rates were not significantly different due to annual harvest variability).

Mountain lion harvest data from Wyoming the past 5 years suggest an average of 32% of successful hound hunters (range = 25-44%; mean total lion harvest from hunting with hounds = 176/year) report being selective while mountain lion hunting and averaged 1.8 days longer in the field than unselective hunters (4.8 days versus 3.0 days). Harvest comparisons indicate on average 49% of unselective and 32% of selective hunters harvest females each year (mean total female harvest = 44%), averaging 9 fewer females and 9 additional males harvested by selective hound hunters in Wyoming annually. Although selectivity reduces female mountain lion harvest, it does not completely explain differences observed between Washington and Wyoming. These differences likely also relate to differences in mountain lion vulnerability between hunting methods.

Anderson (2003) observed that nightly movement distances from Global Positioning System (GPS) data averaged over 3 times longer for male mountain lions than for females (mean end-point distance = 4.6 km versus 1.5 km, 2.9 mi versus 0.9 mi). These longer distance movements expose males more than females to hunting methods where tracking is involved (i.e., hunting with hounds). Opportunistic hunters who do not track mountain lions while hunting are also more likely to harvest the less mobile and more abundant sex (typically females, CMGWG 2005:40) because relative abundance rather than movement patterns drive harvest vulnerability when mountain lions are hunted opportunistically. In addition, hunters with hounds have an increased ability to avoid family groups by detecting young while tracking mountain lions, whereas opportunistic hunters have limited opportunity to determine if young are present.

Potential for Orphaning Young

Because mountain lions can breed and reproduce any time of the year, orphaning of young can result from the harvest of female mountain lions with young. This issue draws emotionally negative responses from some segments of the public and deserves formal appraisal of the potential biological consequences of orphaning young from the harvest of adult female mountain lions. Wyoming law prohibits the harvest of mountain lions accompanied by young, but females may not be accompanied by young while searching for prey (Barnhurst and Lindzey 1989), and therefore may mistakenly be harvested by mountain lion hunters.

Number of mountain lion litters orphaned from hunting can be estimated if data are collected addressing the number of adult females harvested annually. All mountain lions harvested in Wyoming are subjected to mandatory inspection where sex, age, and lactation history data (from nipple characteristics; Anderson and Lindzey 2000) are collected to determine the number of subadult (estimated age <4 years old and have never nursed young) and adult females (nipple characteristics suggest previous lactation and/or estimated age >3 years old) harvested each year. Logan and Sweanor (2001) reported that on average 50% of adult females reproduce and 75% were with dependent young each year. Thus, about 25% of adult females are without young and 25% are with yearlings. Because young may become independent as early as 12 months old or earlier and average dispersal age is about 14-15 months (Anderson et al. 1992, Sweanor et al. 2000), it is unlikely yearling survival is influenced by death of their mother, but survival of young ≤12 months old is likely reduced. Applying these assumptions, timing of female mountain lion harvest, and estimates of monthly birthing rates we can estimate the number of litters orphaned each year due to hunting. Two Wyoming mountain lion studies identified birth

month for 31 litters in north central (n = 10, Logan 1983) and southeast Wyoming (n = 21, Anderson 2003) and provide estimates of monthly birth rates for Wyoming mountain lions (Table 2). Female harvest of both age classes (non-reproducing subadults, reproductive adults) averaged 88 the past 5 years (fall 2000-spring 2005) and averaged 32 adult females (Table 3). Assuming 50% of reproductive females produce young each year, we estimated about 16 litters ≤12 months old may be orphaned in Wyoming annually due to harvest of adult female mountain lions (Table 3).

Table 2. Monthly birth rate from 2 Wyoming mountain lion studies.

	Number of litters			
Birth month	North-central, Wyo.[a]	Southeast, Wyo.[b]	Total	Monthly birth rate
January	0	1	1	0.032
February	0	1	1	0.032
March	0	0	0	0
April	0	1	1	0.032
May	2	1	3	0.097
June	0	4	4	0.129
July	0	3	3	0.097
August	2	5	7	0.226
September	2	1	3	0.097
October	0	1	1	0.032
November	3	2	5	0.161
December	1	1	2	0.065

[a]From Logan 1983.
[b]From data collected by Anderson 2003.

This annual estimate of the number of mountain lion litters orphaned in Wyoming may be high (i.e., assumes 50% of adult females are with young when harvested) because our approach ignores the possibility of hunters detecting and passing females with young while hunting, therefore shifting the harvest toward barren females, which likely occurs at some level when mountain lion tracks are followed in the snow while hunting with hounds. To investigate the estimate, we compared the average number of lactating females harvested the past 5 years (mean = 2.6, range 1-3/year) to that expected when compared to data from Tables 2 and 3. Assuming juvenile mountain lions quit nursing at 2-3 months of age (Pierce and Bleich 2003), we would expect annual harvest of lactating females to range somewhere between 2.8 and 4.7. Whether the lower than expected harvest of lactating females is due more to hunter selectivity or reduced

vulnerability resulting from the more sedentary nature of young family groups is unknown but further indicates that some degree of harvest selectivity is occurring.

Based on the estimate of orphaned litters from average adult female mountain lion harvest in Wyoming the past 5 years, 8.7 litters <6 months old and 7.5 litters 6-12 months old (Table 3) would be orphaned in a given year. Survival of orphaned young <6 months old is unlikely, but survival of orphaned young 6-12 months has been documented during at least 3 mountain lion studies (Lindzey et al. 1989, Logan and Sweanor 2001, Anderson 2003) suggesting about 71% survival for this age group; total sample size from the 3 studies was small, resulting in 5 of 7 young orphaned at 6-10 months old surviving. If we assume on average 2 kittens/litter survive to independence (Logan and Sweanor 2001), orphaned young <6 months do not survive, and about 71% of orphaned young 6-12 months old survive, the estimated biological impact to Wyoming mountain lion populations would be an average loss of about 22 juvenile mountain lions annually [$2 \times 8.7 = 17.4$ young <6 months old, $(2 \times 7.5) \times 0.29 = 4.4$ young 6-12 months old]. Based on mountain lion occupancy throughout most timbered and shrub-covered habitats statewide, this level of loss is biologically insignificant, but is still a concern to some segments of the public. If opportunistic hunting increased and hunting with hounds were reduced, we would expect the actual number of young being orphaned to increase because of the apparent increased vulnerability and the higher proportion of females harvested when compared to hunting with hounds (Martorello and Beausoleil 2003).

Table 3. Monthly female mountain lion harvest in Wyoming (recent 5 year average), and estimated number of litters orphaned (<6 months old, 6-12 months old) from adult female harvest.

Month	Mean total female harvest	Mean adult female harvest	Est. mean No. of females w/young[a]	Est. mean No. orphaned litters <6 moths old[b]	Est. mean No. orphaned litters 6-12 months old[c]
Sept.	1.4	0.4	0.2	0.12	0.08
Oct.	6.0	2.4	1.2	0.77	0.43
Nov.	17.2	6.0	3.0	1.74	1.26
Dec.	26.4	8.6	4.3	2.64	1.66
Jan.	15.6	6.2	3.1	1.80	1.30
Feb.	15.8	5.8	2.9	1.12	1.78
Mar.	6.0	3.0	1.5	0.48	1.02
Total	88.4	32.4	16.2	8.67	7.53

[a]Assumes 50% of adult females reproduce annually (Logan and Sweanor 2001).
[b]Estimated number of females w/young × sum of previous 5-month birth rate from Table 2.
[c]Estimated number of females w/young – estimated number of litters <6 months old.

Mountain Lion Habitat Management

Mountain lions are habitat generalists evident in their broad geographic distribution ranging throughout a variety of habitat types in much of the western hemisphere. The primary habitat component necessary for mountain lion survival includes some form of hiding cover for securing large prey (e.g., ungulates) and raising young. Although open vegetative communities are rarely used, mountain lions are found in virtually all other vegetation types including coniferous and deciduous forests, woodlands, swamps, savannahs, chaparral, riparian forests, desert canyons and mountains, and semi-arid shrub lands (Hansen 1992). In Wyoming, Logan and Irwin (1985) reported that mountain lions preferred mixed conifer-curlleaf mountain mahogany (*Cercocarpus ledifolius*) habitats in rugged terrain, and Anderson et al. (in review) reported mountain lion use of timbered and tall-shrub covered regions occurring near the base of mountain ranges during winter.

Mountain lions, depend on healthy prey populations (e.g., deer, elk), therefore, habitats supporting abundant prey are also important to mountain lion populations. Habitat protection and improvement projects are currently in place for ungulate populations in Wyoming (Wyoming Game & Fish Department 2001), which will undoubtedly benefit mountain lion populations. In addition, Anderson et al. (in review) recently developed a mountain lion habitat model and efforts are currently in place to delineate core winter mountain lion habitat statewide (Fig. 1). Current habitat projects for mountain lion prey species and application of the mountain lion habitat model allow evaluation of potential impacts of proposed development projects to habitats supporting mountain lions and their prey.

Mountain Lion Population Monitoring

Monitoring Mountain Lion Population Trend: Although mountain lion populations have previously been monitored with intensive capture efforts over relatively small areas, reliable and affordable techniques to monitor mountain lion populations for large-scale management programs are lacking. Mountain lion management has traditionally employed harvest strategies with little understanding of the quantitative effect differing harvest levels have on mountain lion population demographics. Sex and age classes of mountain lions exhibit different and relatively predictable movement patterns, where males move longer distances than females and subadults (1-2.5 years old) generally move longer distances than adults (Barnhurst 1986, Anderson 2003). Conceptually, the likelihood of a specific sex or age class of mountain lion being harvested would reflect its relative abundance in the population and its relative vulnerability based on daily movement patterns. In areas where dogs are used to track mountain lions, those mountain lions that typically move longer distances would most likely be detected first (males/subadults). The least vulnerable individuals (adult females) should become prominent in the harvest only after the population has been reduced in size by removal of more vulnerable/available mountain lions. Anderson and Lindzey (2005) tested these predictions applying varying levels of hunter harvest and found harvest composition to be predominantly subadults for a high-density population with low harvest levels, shift to adult males as harvest levels increased, and then a shift from adult males to adult females with continued high harvest as the population declined. When harvest levels were reduced, composition of the harvest returned to primarily subadults. The male segment of the reduced population recovered within 2 years primarily due to male immigration

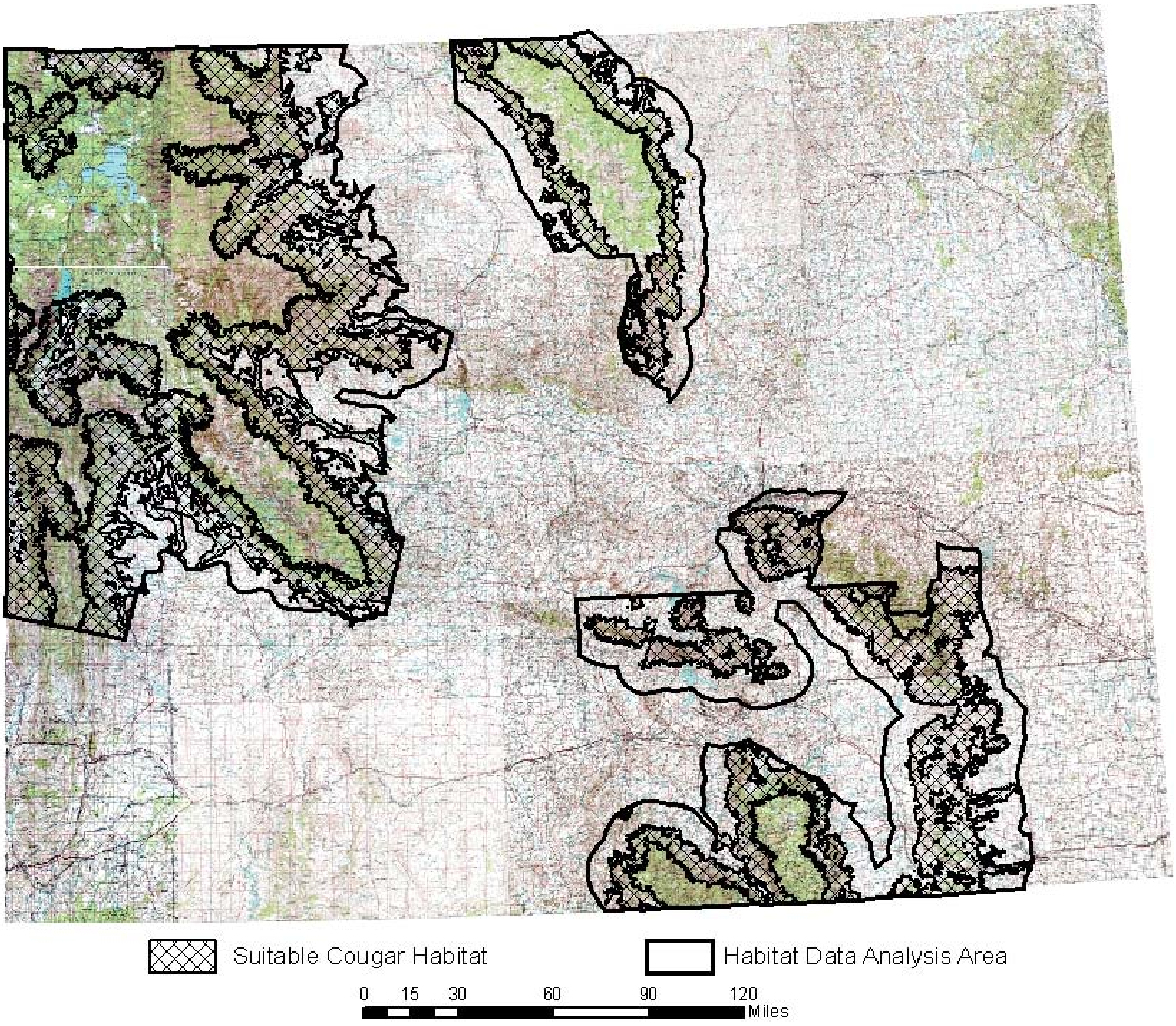

Figure 1. Wyoming mountain lion winter habitat based on model predictions for those portions of Wyoming with suitable vegetation data available for analyses (Anderson et al. in review). Winter mountain lion habitat represents areas suitable for resident adult mountain lions and not necessarily transient subadults (i.e., core mountain lion habitat). Background represents USGS 1:250,000 scale maps. Mountain lion habitat analyses will be completed for areas outside the habitat data analysis area (e.g., northeast and southwest Wyoming) when sufficient vegetation data layers are developed for those regions of the state.

from other populations and the female segment within 3 years from an increased number of females producing young within the population (Anderson and Lindzey 2005).

We compared harvest composition and age of harvested adult females from the Snowy Range (Fig. 2; Anderson and Lindzey 2005) to 2 other areas in Wyoming (Fig. 3; Star Valley and the Laramie Range) where management objectives called for increasing harvest levels to reduce mountain lion populations (i.e., where comparable data were available). We then applied the

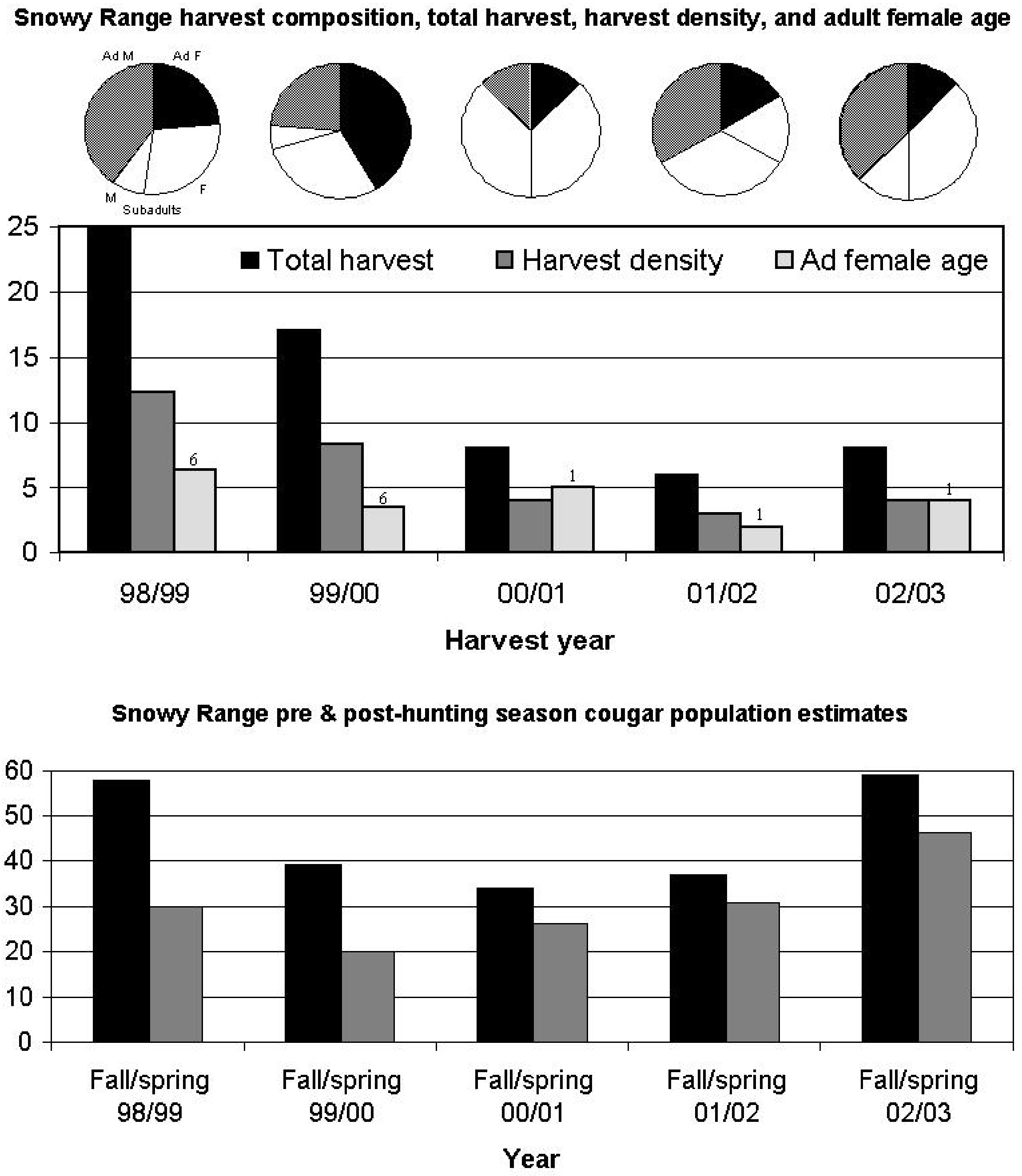

Figure 2. Sex/age composition of mountain lion harvest (pie charts), total harvest, harvest density (mountain lions/1,000 km^2), and mean annuli age of adult females (top bar graph) and pre and post-hunting season mountain lion population estimates (bottom bar graph; Anderson and Lindzey 2005) from the Snowy Range, Wyoming, 1998-2003. Numbers above adult female age represent sample size. Note initial high harvest density (>12 mountain lions/1,000 km^2), decline in adult male harvest, increase in adult female harvest, and decline in age of harvested adult females as the population decreased in size. Also note low harvest densities (<5 mountain lions/1,000 km^2) and low adult female harvest levels during population increase.

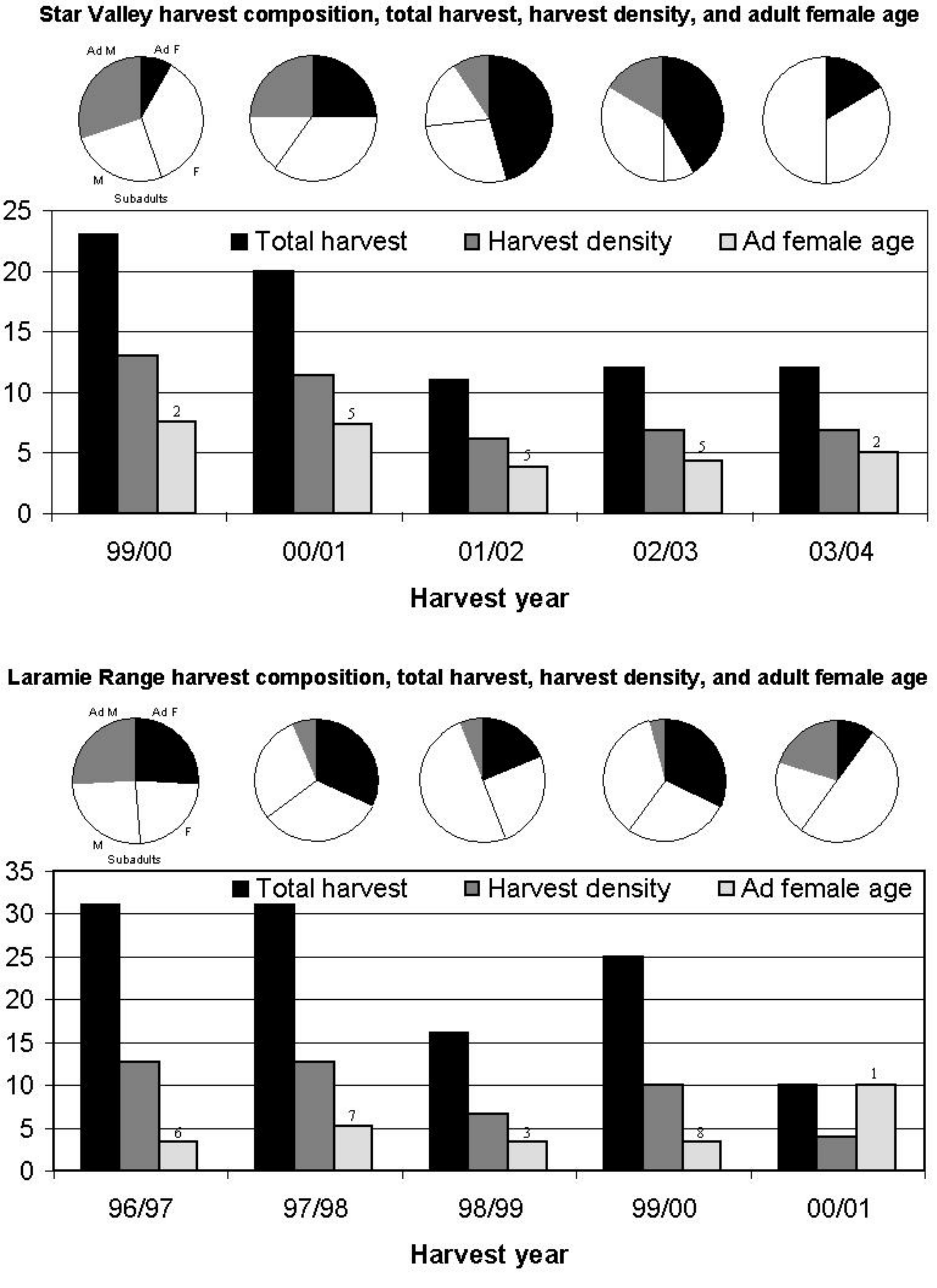

Figure 3. Sex/age composition of mountain lion harvest (pie charts), total harvest, harvest density (mountain lions/1,000 km^2), and mean age of adult females harvested from Star Valley (hunt area 26), Wyoming, 1999-2004 (top bar graph) and from the Laramie Range (hunt areas 6 and 27), Wyoming, 1996-2001 (bottom bar graph). Numbers above adult female age represent sample size. Mountain lion harvest was increased >40% during the first harvest year in each area to achieve the management objective of reducing mountain lion populations.

Wyoming mountain lion habitat model (Anderson et al. in review; Fig. 1) to evaluate harvest densities among areas. The Snowy Range mountain lion population declined about 33% (fall population estimates) following a harvest density of 12.3 mountain lions/1,000 km^2 (386 mi^2; 1998/99 harvest year) and continued to decline another 13% following a harvest density of 8.4 mountain lions/1,000 km^2 (386 mi^2; 1999/00 harvest year). Harvest composition shifted from primarily adult males to adult females and mean annuli age of harvested adult females declined from 6.3 to 3.6 years old as the population declined (Fig. 2). The Snowy Range mountain lion population recovered to previous levels following a 3-year period where harvest densities were between 3.0-4.0 mountain lions/1,000 km^2 (386 mi^2) and harvest composition consisted primarily of subadults, buffering the adult female segment of the population during recovery (2000/01-2002/03 harvest years; Fig. 2). We noted similar progressions in harvest density, harvest composition, and mean age of harvested adult females for Star Valley and the Laramie Range (Fig. 3), except that harvest composition shifting from adult males to adult females was more gradual in Star Valley. Harvest densities remained moderate (typically between 6-7 mountain lions/1,000 km^2) following initial high harvest densities (>10/1,000 km^2) in both areas, and older age females (>5 years old) were not evident in the harvest until the second year of high harvest density in the Laramie Range. The more gradual increase in adult female harvest for Star Valley is likely due to this area being more connected to adjacent mountain lion habitat than the Snowy or Laramie ranges (i.e., more resilient to mountain lion harvest allowing animals from adjacent areas to replace harvested animals). Based on relatively high adult female harvest and intermediate harvest densities (Fig. 3), Star Valley and Laramie Range mountain lion populations were likely maintained at low-moderate densities during the periods examined.

Population Estimation Methods: Obtaining accurate and precise estimates of mountain lion population size for each managed population can be logistically and financially challenging, limiting application of estimation methods to relatively small areas every several years. Methods that have been evaluated or hold promise for estimating mountain lion populations for large-scale management programs include ground-based track surveys, sampling mountain lion tracks during helicopter surveys (i.e., helicopter probability sampling; Van Sickle and Lindzey 1991), and DNA or camera-based mark-recapture efforts. Application of DNA or camera-based mark-recapture methods to estimate mountain lion populations is currently limited because there does not appear to be a reliable attractant for luring mountain lions into hair collection or photo detection sites and individual identification of mountain lions from photos appears unreliable for the camera approach. Until these methods are further developed for mountain lions, track surveys and helicopter probability sampling mountain lion tracks appear most promising in estimating mountain lion populations for management application.

Track surveys have been used to monitor mountain lion populations in California (Smallwood 1994, Smallwood and Fitzhugh 1995) and Arizona (Cunningham et al. 1995). This method requires transect sampling areas where mountain lion tracks are detectable and provides presence-absence data with confidence interval estimates. Beier and Cunningham (1996) reported that sampling 140 and 110 8-km-long transects would be required to detect 30% and 50% population declines, respectively (80% power, $\alpha = 0.05$). The difficulty in implementing track surveys is ensuring transects are well distributed throughout the population in areas where access may be limited and the unpredictability of favorable tracking conditions. The level of

effort required to detect useful population changes likely limits application of this method to once every few to several years.

Becker (1991) and Becker et al. (1998) addressed helicopter probability sampling of snow tracks to estimate lynx and wolf population size in Alaska. This method requires sampling animal tracks during helicopter surveys and then following tracks from beginning to end to estimate the probability of detection for each track observed during surveys, and therefore requires consistent snow conditions for the duration of the survey. Helicopter probability sampling provides population and confidence interval estimates derived from the inverse of the detection probabilities for tracks in the sample. Van Sickle and Lindzey (1991) applied this method to a low-density Utah mountain lion population of known size and obtained an accurate but imprecise (high variance) population estimate. Anderson et al. (2003) investigated this method further using computer simulations of mountain lion GPS data (≤6 locations/night) to simulate mountain lion tracks and reported that mountain lion population changes of 15-30% could be detected (90% probability) for medium-high density mountain lion populations (23-35 independent mountain lions/1,000 km^2 or 386 mi^2) depending on sampling effort (transects spaced 2 to 3 km apart). Both Becker (1991) and Anderson et al. (2003) noted the logistical difficulty and added expense of completely following tracks during surveys and suggested using telemetry data from radiocollared animals in the population or GPS movement data from similar habitat types during similar seasons to estimate track lengths. Anderson et al. (2003) noted that an area of about 2,000 km^2 (771 mi^2) could be surveyed in 2 helicopter days for about $8,000-$10,000. Thus, helicopter probability sampling mountain lion populations would be limited to relatively small areas and likely only affordable to management agencies every few to several years.

ADAPTIVE MOUNTAIN LION MANAGEMENT APROACH FOR WYOMING

Mountain Lion Hunting Season Structure, Hunting Methods, and Hunter Effort Indices: Since 1980, mountain lion harvest in Wyoming has been controlled using harvest quota management. Harvest quota management maximizes management flexibility by maintaining high hunting opportunity and controlling harvest by assigning total and sometimes female subquotas by hunt area depending on local management objectives. Rarely are harvest quotas exceeded in Wyoming, but heavily roaded areas are more prone to multiple hunters harvesting mountain lions at the end of the season thereby exceeding harvest quotas. If exceeding harvest quotas becomes a recurring problem, limited entry seasons could be established in those areas or quotas could be adjusted anticipating additional harvest similar to past seasons.

Mountain lion hunting seasons in Wyoming typically occur from September 1 through March 31 lasting 212 days. Year round seasons are established in 2 areas with high depredation incidents to provide opportunity for licensed hunters to take depredating mountain lions as a substitute for removal by agency personnel. Most mountain lion harvest (>90% annually) occurs during the winter months (November-March) when snow cover provides optimal tracking conditions. Although few mountain lions are harvested during September and October, this period provides hunting opportunity for hunters opportunistically during big game seasons or using predator calls.

Although some individuals and groups criticize the use of hounds for hunting mountain lions, this hunting method is an efficient management tool, which allows optimal dispersal of hunting pressure and minimizes harvest of adult females primarily due to vulnerability differences between hunting methods. Tracking mountain lions while hunting with hounds also increases the opportunity for hunters to detect and avoid family groups.

Currently, hunting information is only recorded from successful hunters when registering harvested mountain lions during the mandatory inspection process. Catch-per-unit-effort indices can be useful to monitor impacts to hunted populations assuming there is an identifiable relationship between hunter effort and the number of animals in the area hunted. Hunter effort data from only successful hunters has changed little the past 20 years has not proved useful in assessing mountain lion population trends (Appendix II, Fig. II-2). Additional information from unsuccessful hunters may prove more useful in evaluating these indices and knowledge about the number of unsuccessful and successful hunters hunting an area may explain changes in harvest level in cases where other information does not (i.e., due to changes in the number of hunters hunting an area). Regardless, data from unsuccessful hunters will enhance the management database and likely contribute to other harvest data currently collected.

Mountain Lion Habitat Management: Anderson et al. (in review) developed a winter mountain lion habitat model from GPS data collected in the Snowy Range, Wyoming, and validated model predictions using historic harvest locations 1996-2005 from the Bighorn, Sierra Madre, and Snowy Mountain Ranges. Habitat modeling efforts by Anderson et al. (in review) focused on the winter period (November-May) because this is the period when mountain lion activity is most limited due to deep snow at higher elevations resulting in ungulate concentrations on low elevation winter ranges, human development projects are vastly more common on low elevation winter ranges than on higher elevation summer ranges, and the vast majority of human-caused mountain lion mortality occurs during this period (>90% annually). The winter mountain lion habitat model is currently being used to delineate core winter mountain lion habitat statewide (Figs. 1 and 5). Thus far, most contiguous core mountain lion habitat in Wyoming has been delineated with the exception of the Southwest LMU, Northeast LMU, and hunt areas 14, 22, 25 and the Converse County portion of hunt area 6 (refer to Appendix III). Habitat maps for the other areas will be completed when detailed vegetation data layers are mapped and ground verified (e.g., Landsat Enhanced Thematic Mapper data at 30 m resolution); efforts are currently in place to complete vegetation data layers statewide.

Our intent for the mountain lion habitat model is to delineate suitable winter mountain lion habitat for resident adults (i.e., core mountain lion habitat) and exclude marginal habitats used as transition areas by transient subadults. Delineating core mountain lion habitat allows assessment of potential impacts from proposed development projects and application of mountain lion mortality densities to be used in development and assessment of management objectives (see next section below). Based on evaluations using historic harvest distribution (Fig. 4), the model appears to work well in most regions of Wyoming. Final acceptance of mountain lion habitat model predictions is pending regional review based on local knowledge of mountain lion habitat use during winter.

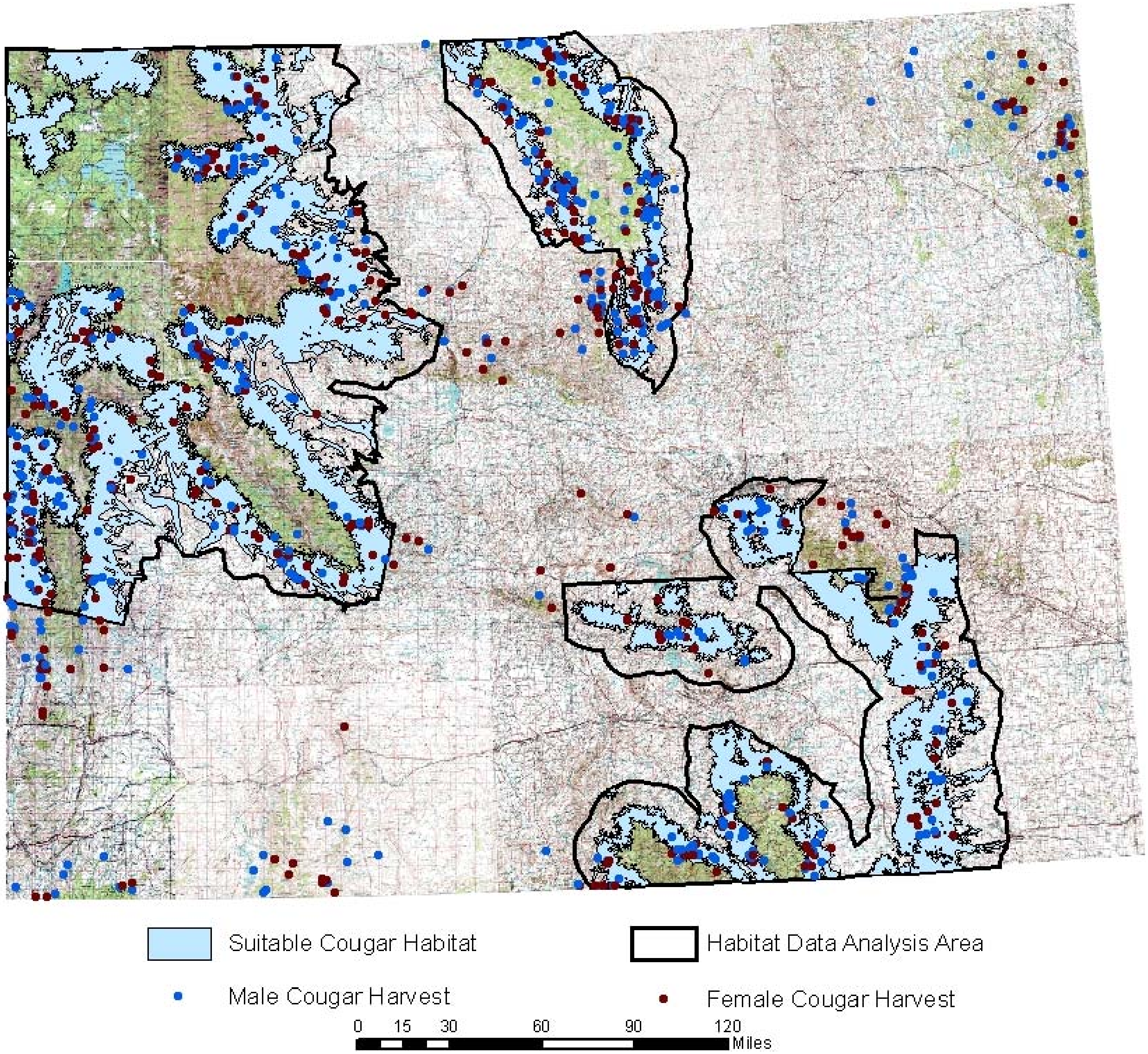

Figure 4. Winter mountain lion habitat model predictions relative to mountain lion harvest locations by sex, fall 2000-spring 2005. Winter mountain lion habitat represents core habitat of resident adult mountain lions and excludes marginal habitats occasionally used as transition areas by transient subadult mountain lions.

Habitat management efforts should include conserving large tracts of connected habitats that have the characteristics preferred by mountain lions and their prey. The Department's efforts to maintain high quality ungulate habitat should benefit mountain lion populations, and application of the mountain lion habitat model will provide opportunity to evaluate potential impacts from proposed development projects.

Management Criteria for Establishing Mountain Lion Management Objectives: The Cougar Management Guidelines Working Group (2005) suggested managing mountain lion populations

by managing source and sink subpopulations. As stated previously, the hunt area and management unit approach currently used in Wyoming lends itself well to this concept and has likely, by default, maintained source-sink mountain lion population dynamics since the early 1970s by maintaining relatively high lion densities in some portions of the state (i.e., source areas) which support recruitment of young lions into other areas managed at low population densities (i.e., sink areas); maintaining source mountain lion habitats allow persistence of mountain lions in other habitats experiencing high mortality rates. The CMGWG did not provide specific guidelines on how to delineate source and sink mountain lion habitats other than to establish large-unhunted refuge areas to offset population sinks that experience high human-caused mortality. However, refining this approach by applying sex-age composition of harvest and annuli age of harvested adult females addressed by Anderson and Lindzey (2005) and applying the Wyoming mountain lion habitat model (Anderson et al. in review) to evaluate density of human-caused mortality provides criteria to establish source and sink mountain lion management. Based on Anderson and Lindzey (2005) and evaluation of harvest densities presented here for mountain lion population decline (Figs. 2 and 3) and increase (Fig. 2), the following criteria appear appropriate for establishing source-stable-sink mountain lion management:

Hunt area management objectives:

1. Sink management: reduce mountain lion densities
   a) Maintain density of human-caused mortality >8 mountain lions/1,000 km^2 (386 mi^2).
   b) Achieve adult female harvest >25% of total harvest for 2 of 3 seasons.
   c) Progression in mean age of harvested adult females should decline to <5 years old.

2. Source management: maintain human-caused mortality levels that allow mountain lion population growth or maintenance of relatively high mountain lion densities.

   a) Maintain density of human-caused mortality <5 mountain lions/1,000 km^2 (386 mi^2)
   b) Maintain adult female harvest <20% of total harvest.
   c) Maintain older-age adult females in the population (>5 years old). This will be difficult to identify without additional sampling due to low sample size from harvest, but would be expected for lightly hunted populations.

3. Manage for stable mountain lion populations: maximize long-term hunting opportunity.

   a) Maintain human-caused mortality density between 5-8 mountain lions/1,000 km^2 (386 mi^2)
   b) Adult female harvest should not exceed 20% of total harvest for more than 1 season.

c) Maintain intermediate aged adult females (mean ≅ 4-6 years old) in the harvest. Adequate age evaluation may require averaging age data over time to achieve meaningful sample sizes.

LMU management objectives:

- The LMU management objective should attempt to achieve the criteria above for source, stable, or sink mountain lion management at the LMU level. The objectives chosen by managers will be based on the adjacent management priorities, size of the LMU, maintaining recreational opportunity, maintaining source mountain lion populations, as well as depredations and other factors to achieve the overall management goal of sustaining mountain lion populations throughout core habitat at varying densities depending on management objectives.

- Coordinating management efforts with adjacent states would be most desirable for the smaller LMUs (i.e., Northeast and Southwest LMUs) where the majority of connected mountain lion habitat extends beyond Wyoming. Source or stable management could be maintained without interagency coordination, but sink management could also be implemented when sufficient source habitat has been identified in adjacent areas.

Acknowledging managers rarely, if ever, have precise information to measure success of management objectives, that mountain lion densities vary regionally, and the criteria proposed here are general guidelines, these guidelines should be compared to one another and applied adaptively to assess success of management prescriptions. For example, an area managed with the objective of stability and receiving a mountain lion removal density of 7 mountain lions/1,000 km^2 (386 mi^2), but relative adult female harvest exceeds 25% and harvested adult female annuli ages have declined below 5 years old likely suggests mountain lion population decline rather than stability. Conversely, an area managed with the objective of sink and receiving harvest densities of 10 mountain lions/1,000 km^2 (386 mi^2), but relative adult female harvest remains below 20% and older-age females (>5 years old) are consistently harvested suggests population stability (e.g., hunt area 23 in Table 4). Applying management objectives in an adaptive management framework, where density of human-caused mortality, harvest composition, and age of harvested adult females are monitored relative to expectations (criteria above) allows assessment of whether or not management objectives are being achieved and if management strategies should be modified to produce the desired outcome. Based on mountain lion management criteria averaged over the past 5 years for single or combined hunt areas of at least 1,000 km^2 of core mountain lion habitat (Table 4), 9 regions (1 to 3 hunt areas each) currently qualify as source areas, 7 as stable areas, and 1 as a sink area; 2 regions appear intermediate between source and stable and 2 regions intermediate between stable and sink (Fig. 5).

In implementing and evaluating mountain lion management objectives based on human-caused mortality density, proportion of total harvest comprised of adult females, and mean age of harvested adult females, it may be necessary to maintain consistent harvest objectives and combine data spatially or temporally to obtain meaningful information. Examples include hunt

Table 4. Annual 5-year average (fall 2001-spring 2006) of human-caused mountain lion mortality density (mountain lions/1,000 km^2), proportion of adult females in the total harvest, adult female annuli age (*n* = sample size), management status (source, stable, or sink), and area of core winter mountain lion habitat for Wyoming mountain lion hunt areas[a] and management units (LMU).

LMU<br>Hunt area	Density of human caused mortalities	Proportion of total harvest including adult females	*n*/Annuli age[b]	Management status[c]	Core habitat (km^2)
Northeast					
1 & 24[d]	a	0.13	5/4.4	source/stable[e]	Undetermined
Southeast					
5 & 25[d]	1.9	0.26	3/7.0	Source/stable[e]	2,889[f]
7	6.2	0.20	8/4.1	Stable to stable/sink[e]	2,185
8 & 16[d]	2.9	0.08	3/5.3	Source	1,475[f]
9 & 10[d]	6.3	0.12	3/5.0	Stable	1,138
6 & 27[d]	5.6	0.13	6/4.2	Stable	2,480[f]
Southwest					
11, 12 & 13[d]	a	0.06	2/4.0	Source	Undetermined
North central					
15	15.4	0.11	8/4.4	Sink	1,221
21	9.6	0.14	6/4.8	Sink to stable[e]	1,295
22	a	0.19	8/3.4	stable to stable/sink	Undetermined
23	11.2	0.12	7/6.6	Stable	1,377
West					
Absoraka DAU					
19	4.6	0.13	8/6.8	Source	3,905
20	2.8	0.15	4/6.3	Stable to source[e]	3,045
Wind River DAU					
18	6.8	0.16	5/6.4	Stable	1,235
28	0.5	0.00	0/-	Source	1,720
4	4.5	0.16	3/4.3	Source	1,023
3	3.4	0.14	3/7.0	Source	2,151

Continued

Table 4. Continued.

LMU<br>Hunt area	Density of human caused mortalities	Proportion of total harvest including adult females	*n*/Annuli age[b]	Management status[c]	Core habitat (km^2)
West (cont.)					
Wyoming Range DAU					
2 & 29[d]	3.2	0.23	12/6.4	Source	3,372
26	6.2	0.27	13/4.3	Sink to stable[e]	1,762
17	2.0	0.09	1/2.0	Source	1,838
14	a	0.22	10/5.5	Stable	Undetermined

[a]Insufficient vegetative data for hunt areas 1, 11-14, 16, 22, and 24-25 to calculate core mountain lion habitat and mortality density.

[b]Annuli age estimated from the number of rings evident after cross sectioning of the first premolar. Mean annuli ages from small sample sizes ($n < 5$) should be interpreted with caution.

[c]Status assigned based on the majority of the 3 criteria examined. Status criteria: source = mortality density <5 mountain lions/1,000 km^2, <20% of total harvest includes adult females, mean adult female annuli age >5 years old; stable = mortality density of 5-8 mountain lions/1,000 km^2, proportion of harvested adult females should not exceed 25% of total harvest for more than 1 year, mean annuli age of adult females should be intermediate to source and sink areas (e.g., 4-6 years old); sink = mortality density >8 mountain lions/1,000 km^2, >25% of total harvest includes adult females for 2 years, mean adult female annuli age declines to <5 years old.

[d]Hunt areas with <1,000 km^2 of core mountain lion habitat were combined with adjacent hunt areas within the same mountain range.

[e]Criteria separated with " / " indicate intermediate management status. Management criteria separated with "to" indicate a transition in management status over the 5-year period based on trends in annual data.

[f]Amount of core mountain lion habitat subject to change in hunt areas 5 and 6 following completion of improved habitat data layers and Regional review. Lack of vegetative data for hunt areas 16 and 25 precludes core habitat delineation and mortality density calculations for these hunt areas.

areas receiving low harvest levels or hunt areas of small geographic size. Small hunt areas can be combined with adjacent hunt areas and information from lightly hunted areas can be averaged over time to improve sample sizes (e.g., Table 4). Evaluating annual changes in management criteria are also important to determine if the population may be changing due to annual shifts in mortality density, harvest sex/age composition, and/or age of adult females, especially in areas experiencing moderate to high harvest levels; averaging management criteria over time may mask shifts in management status that are otherwise evident from annual changes in management criteria (e.g., hunt areas 7, 21, 22, 20, 2 & 29, and 26; Table 4). For example, mountain lion population reduction can be achieved in a short time period (>50% reduction; Logan and Sweanor 2001, Anderson and Lindzey 2005) in areas that are accessible to hunters where high harvest densities, increase in adult female harvest, and decline in age of adult females occurs within 2-3 years and subsequent management criteria suggest stability following the initial reduction (Fig. 3).

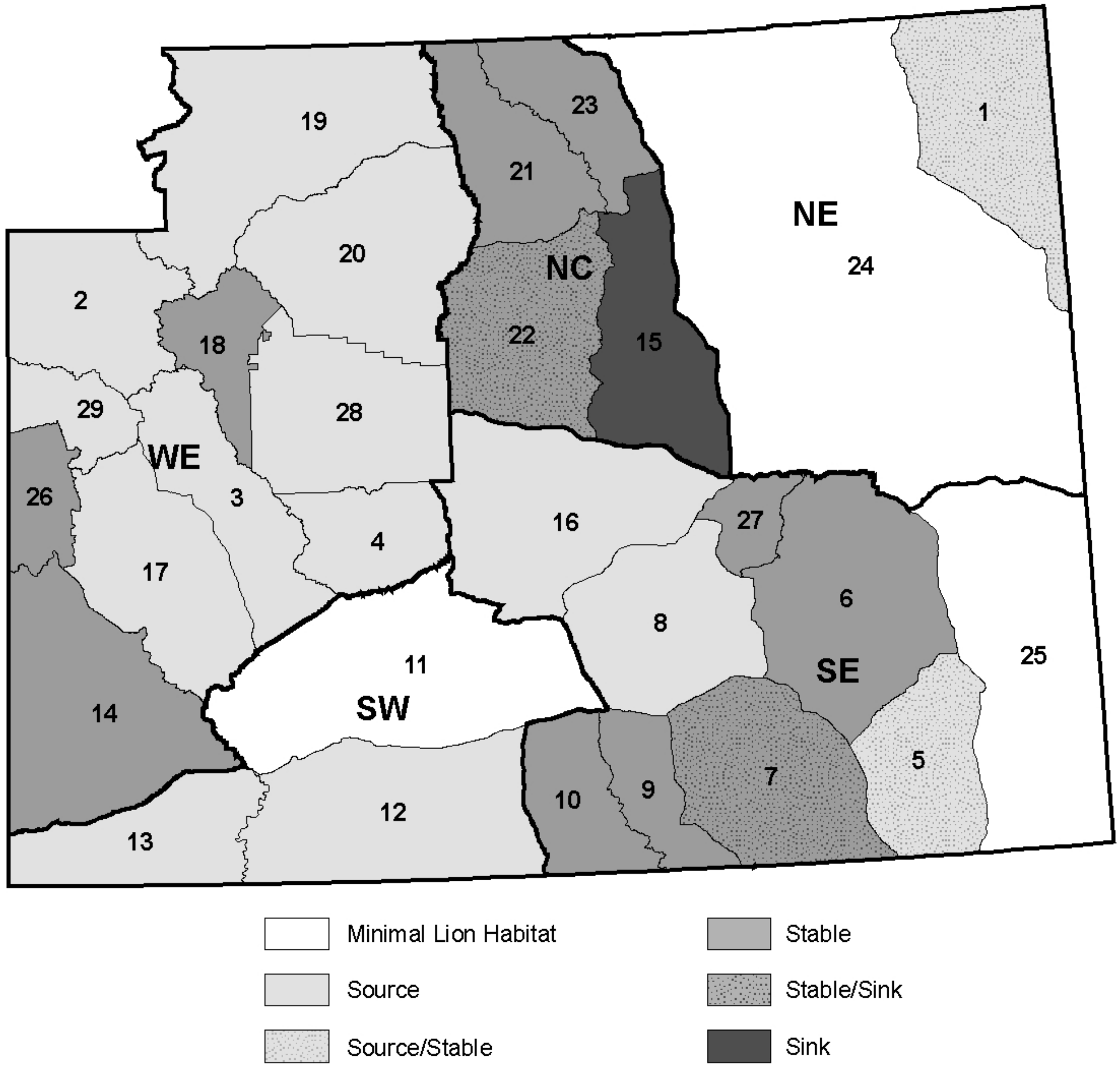

Figure 5. Current Wyoming mountain lion management status by hunt areas (numbered) within mountain lion management units (WE = west, NC = north central, NE = northeast, SE = southeast, SW = southwest). Status assigned based on the majority of the 3 criteria examined: source = human caused mortality density <5 mountain lions/1,000 km^2, <20% of total harvest includes adult females, mean adult female annuli age >5 years old; stable = human caused mortality density of 5-8 mountain lions/1,000 km^2, proportion of harvested adult females should not exceed 25% of total harvest for more than 1 year, mean annuli age of adult females should be intermediate to source and sink areas (e.g., 4-6 years old); sink = human caused mortality density >8 mountain lions/1,000 km^2, >25% of total harvest includes adult females for 2 years, mean adult female annuli age declines to <5 years old (Table 4). Unable to calculate mortality density for hunt areas 1, 12, 13, 14, 16, and 22 due to incomplete habitat data. White areas represent primarily open vegetative types and contain low-density mountain lion habitats.

Other factors to consider are the similarity in harvest composition for high and low-density populations and the duration for establishing source management areas. Anderson and Lindzey (2005) observed that harvest composition progressed from primarily subadults, to adult males, and finally to adult females with mountain lion population decline, but observed similar harvest composition to a high-density population, composed primarily of subadults, when the population was at low density. Harvest composition composed primarily of subadults may suggest a high density population where the less vulnerable adults have not yet been greatly exposed to harvest or conversely that the population is actually at low density where the majority of the adult segment of the population has previously been removed (via disease, past harvest levels, etc.) and most of the individuals in the population are immigrants from other populations. Approaches to determining whether high subadult harvest/low adult harvest suggests high or low mountain lion densities include comparing other harvest criteria, evaluating changes in harvest data over time (e.g., Table 4), and evaluating relative harvest of subadult females. Based on the current season setting structure in Wyoming where management objectives are established every 3 years, we suggest monitoring management criteria for the previous 2 management cycles (6 years) to adequately determine whether populations may be increasing, decreasing, or remaining stable. Low density of human-caused mortalities (<5/1,000 km^2) for a 6-year period would indicate a high-density population, as would a majority of females in the subadult harvest suggesting numerous adult females producing young within the population. Ideally, source management areas should be maintained over time. If changes in social or biological conditions warrant shifting from source to sink management, 3 years should be sufficient to reduce mountain lion densities assuming sufficient access, but returning to source status will likely take longer. Numerical recovery can occur within 3 years (Logan and Sweanor 2001, Anderson and Lindzey 2005), but returning to the older age structure consistent with a functioning source population will benefit from source management for 2 management cycles (i.e., 6 years).

Another issue relative to source-stable-sink mountain lion management that should be addressed is the size at which an area may serve as a source subpopulation and the relative area and juxtaposition of source-sink mountain lion habitat necessary to sustain mountain lion populations at landscape levels. This issue has not been well addressed at this time, but work by Beier (1993) may offer some guidance. Beier (1993) suggested areas as small as 600-1,600 km^2 (231-617 mi^2) would likely sustain viable mountain lion populations assuming 4 immigrants every 10 years, and higher levels of immigration would allow even smaller areas to support mountain lions. Genetic evidence suggests Wyoming mountain lion populations are well connected, with the estimated number of migrants per generation ranging from 6-30 among geographically distinct regions (i.e., LMUs; Anderson et al. 2004). Thus, areas of at least 1,000 km^2 (386 mi^2) would appear sufficient to serve as source areas in Wyoming. The amount and juxtaposition of source mountain lion habitat relative to sink habitat necessary to sustain mountain lion populations at landscape levels, however, is still unresolved. Past mountain lion management and recent management status (Table 4, Fig. 5) suggests the current amount of source mountain lion habitat has been sufficient to sustain mountain lion populations statewide. In addition, maintaining source or stable management objectives at the LMU level should support large-scale mountain lion population persistence and this approach may preclude the need to specifically delineate the ratio of source:sink mountain lion habitat relative to hunt area management objectives.

In addition to assessing mountain lion population trends for stable or sink management areas, periodic mountain lion population monitoring will also be useful to confirm the status of source populations. Harvest data may be sufficient to reasonably evaluate trends for areas managed as stable or sink populations, but likely insufficient to adequately evaluate status of source populations. Confirming the status of areas intended to support mountain lions at landscape scales will be a useful component in source-stable-sink management of mountain lion populations in Wyoming. Population estimation methods (e.g., track surveys, helicopter probability sampling, mark-recapture methods if they become applicable for estimating mountain lion populations) should be applied every 3-5 years (e.g., 1 hunt area/LMU) to confirm mountain lion densities are consistent with populations that are at or near carrying capacity. Ability to formally survey source areas, however, will be dependent on Department budget constraints. If budget constraints do not allow formal surveys of source areas, other approaches should be investigated to confirm the status of source populations (e.g., less intensive track surveys, hunter interviews, etc.).

Mountain lion management objectives should be based on local and regional biological and social considerations. Management objectives to reduce mountain lion densities should be proposed when the expected outcome will result in (1) reduced human conflicts (e.g., human-mountain lion encounters, mountain lion incidents near human development), (2) reduced depredation incidents, or (3) to alleviate predation pressures on ungulate populations that are below the ungulate population management objective primarily due to mountain lion predation rather than habitat conditions. Success of management actions should be monitored to determine if reducing mountain lion densities achieve the desired outcome by recording changes in human conflict levels, depredation incidents, or ungulate population parameters (e.g., changes in female:young ratios). In the case of predation impacts to ungulate populations, additional data collection may be necessary to determine if reducing mountain lion numbers has resulted in increased ungulate numbers, and will depend on the availability of additional funding to monitor the ungulate population response. Changing management strategies over time, while monitoring the effects will provide an adaptive management approach to evaluate the success of mountain lion management prescriptions.

In areas where human conflicts and depredation incidents are not an issue and ungulate populations do not appear to be strongly influenced by predation, stable or source management objectives should be implemented. Managing areas for stable mountain lion populations should maximize long-term hunting opportunity, and source population management should offset reduction in other areas managed as sink populations. In areas of Wyoming where hunter access is limited (National Parks, refuges, ungulate winter range closures, private lands), sink (e.g., hunt area 2) or even stable management at lower densities (e.g., hunt area 28) may not be possible. These areas have served and will continue to serve as source mountain lion populations as long as access remains limited.

NUISANCE MOUNTAIN LION MANAGEMENT

Livestock Depredations

Mountain lions will kill most species of domestic livestock, although sheep and cattle tend to dominate depredation records (Lindzey 1987). In Arizona, Shaw (1983) reported that 93% of mountain lion-killed cattle examined were calves (typically <300 lbs.), and although all age classes of sheep were killed, lambs were preferred. Cattle losses to mountain lions are rare in Wyoming (Fig. 6) primarily due to calves being born away from mountain lion habitat compared to other areas of the southwestern U.S. where calves are born in mountain lion habitat (e.g., the desert southwest; Shaw 1977, Cunningham et al. 1995). Mountain lion depredations of horses, llamas, goats, poultry, pigs, and other types of livestock have also been documented (Tully 1991). Data from Wyoming, 2000-2005, indicate approximately 97% of the damage claims submitted for reimbursement were for sheep, primarily lambs and ewes (Fig. 6; Wyoming Game & Fish Department 2005). Other livestock occasionally killed include horses, cattle, goats, and pigs. The loss of domestic pets near residential areas is also on the increase in urban areas, primarily due to human development into occupied mountain lion habitat (Davies 1991).

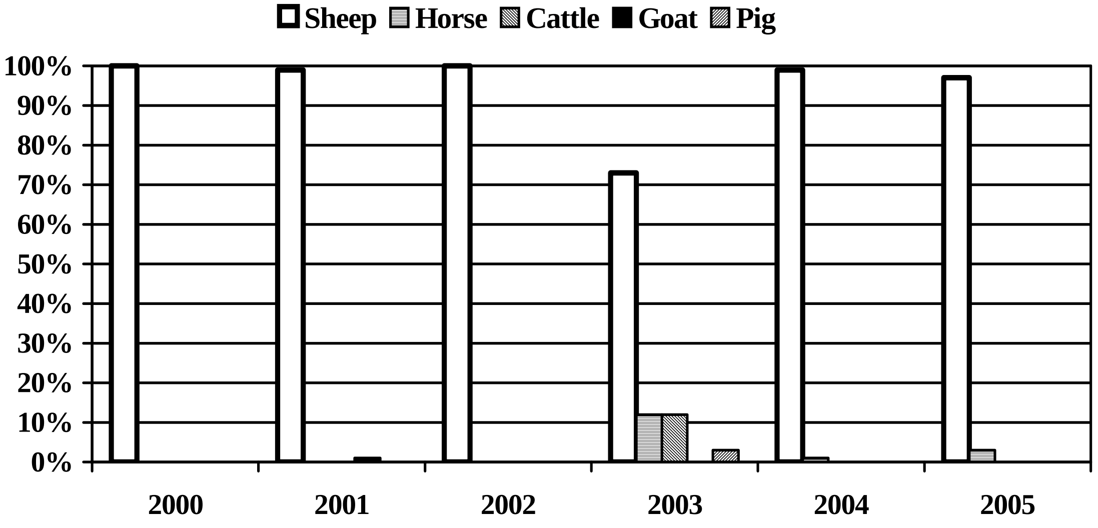

Figure 6. Percentage of mountain lion damage compensation in Wyoming by type, fiscal year 2000-2005.

Wyoming Statute §23-1-901 provides for monetary compensation of damage to livestock caused by mountain lions, and W.S. §§23-3-115 allows property owners or their employees and lessees to kill mountain lions damaging private property, given they immediately notify the nearest game warden of the incident. They may keep the pelt and skull if they purchase a Wyoming game tag. Because of this statute, Wyoming obtains annual information on the number of reported conflicts between mountain lions and domestic livestock and provides compensation for those losses. The number of damage claims submitted to the Department has varied between 1980 and 2005, ranging from under 5 to over 40 (Fig. 7). During that same time period, compensation paid to

livestock producers ranged from just over $7,400 to just under $110,000 (Fig. 8). Compensation does not correspond to the number of claims submitted in all years. For example, in fiscal year 2003, 21 damage claims were submitted for payment and only $10,131 was paid to producers compared to 2005 when only 10 claims were submitted that resulted in $39,000 in compensation. This is due primarily to the loss of expensive livestock, primarily horses, in some years.

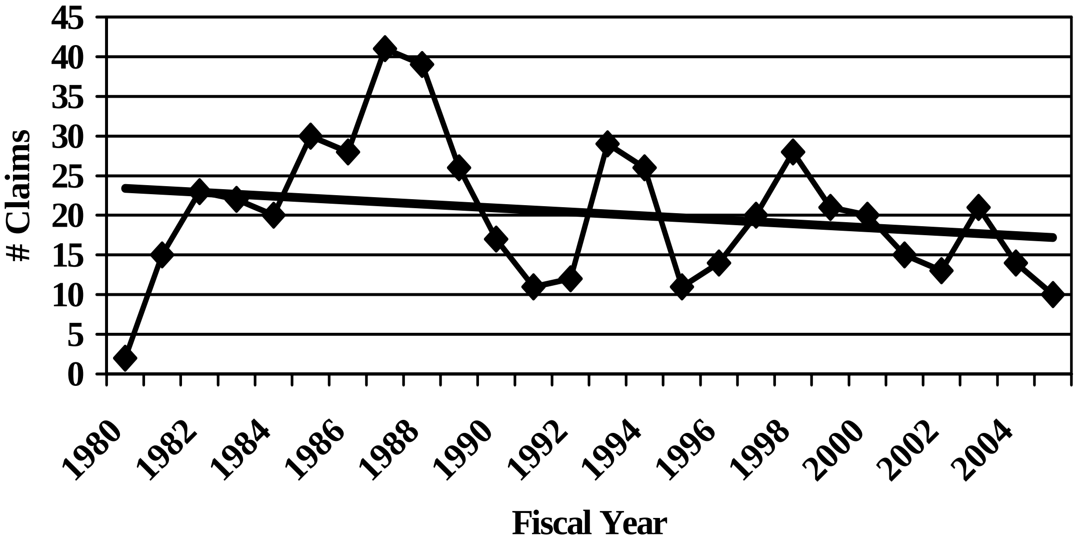

Figure 7. Trend in the number of damage claims submitted for Wyoming mountain lion depredations, fiscal year 1980-2005.

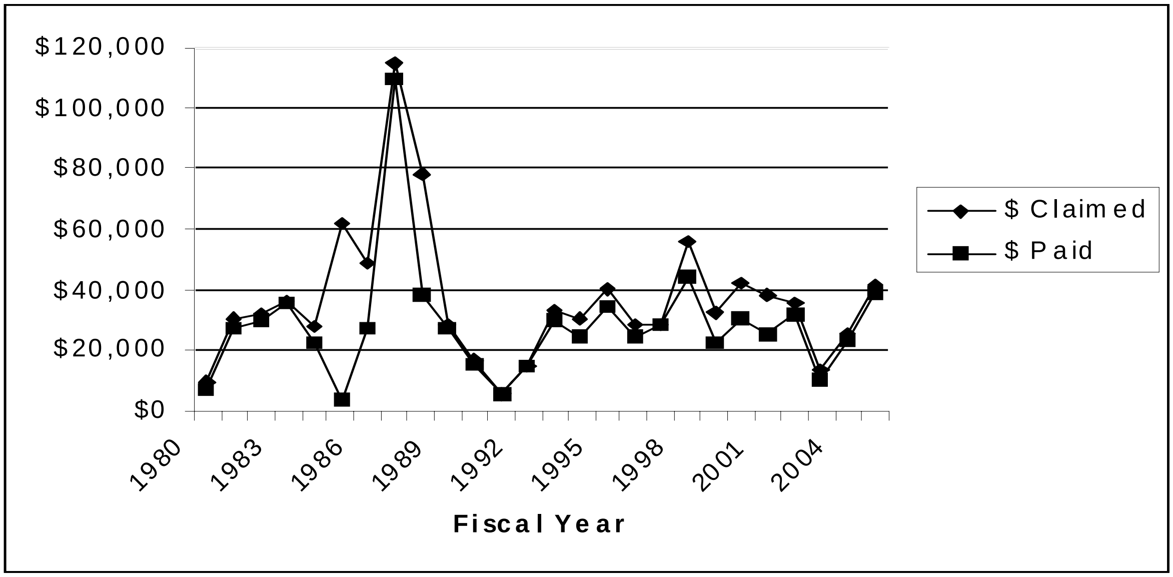

Figure 8. Mountain lion damage claims versus payments to livestock producers in Wyoming, fiscal year 1980-2005.

Although Wyoming Statute allows for the take of mountain lions depredating livestock, mountain lions also have aesthetic value, trophy value, and removal costs that should be considered when making removal decisions (Lindzey 1987). In Wyoming, there are currently 2 approaches to reduce mountain lion damage including (1) remove the offending mountain lion and (2) increase take through sport hunting. Removal of individuals appears to be more accepted by the public than overall population reductions (Gasson and Moody 1995). Killing the offending mountain lion has been successful as a short-term solution, but livestock losses may eventually continue in the future where livestock remain in mountain lion habitat. Conversely, attempting to reduce mountain lion populations also does not appear to entirely resolve the depredation issue because it is usually very difficult to maintain a reduction program that is sufficient to reduce a population to the level required to reduce depredations. Public acceptance of such a program may or may not be maintained over a sustained period of time. We currently do not know the harvest level or length of time required to reduce lion populations to the point that livestock reductions would be reduced, but the adaptive management approach outlined in this plan will allow evaluation of this issue in the future. Therefore the Department will continue to consider all issues, including livestock depredation, to establish harvest quotas. Mountain lion populations have the ability to rebound from this level of reduction fairly quickly. Lindzey et al. (1992) documented that a population of mountain lions in Utah recovered from a reduction of approximately 42% in only 9 months. Similarly, mountain lion populations recovered from comparable reductions in New Mexico and Wyoming in 31 and 36 months, respectively (Logan and Sweanor 2001, Anderson and Lindzey 2005). Licensed hunters are occasionally directed to areas with damage in hopes of removing problem individuals, but agency personnel, either the Department of Agriculture's APHIS-Wildlife Services or the Wyoming Game & Fish Department, do most individual removals.

Management actions that target mountain lions that are a potential threat to human safety or cause livestock damage normally result in the lethal removal of the offending mountain lion. Current protocols provide agency personnel with a variety of options to address conflicts ranging from no action to relocation of the offending animal to lethal removal. Agency personnel respond and resolve incidents based on site-specific conditions. The Department will continue to document incident circumstances and outcomes.

Reducing non-harvest mortality should allow for increased hunter opportunity through season/quota regulations. Nevertheless, in most instances agency removal of specific individuals will be necessary to resolve specific depredation incidents. Striving for removal of only responsible individuals should help minimize losses, increase public acceptance, and maintain hunter opportunity.

Mountain Lion - Human Interactions

Interactions between humans and mountain lions have increased during the last 2 decades throughout most of the western United States and Canada (Beier 1991). Although mountain lion attacks are extremely rare, there were 9 fatal and at least 44 non-fatal attacks reported in North America between 1890 and 1990 (Beier 1991). The majority (66%) of the humans attacked were either unsupervised children or lone adults. Approximately 30% of the attacks occurred within sight of some type of developed area. Fitzhugh et. al. (2003) updated this information through

2003, and determined an additional 7 fatal and 38 non-fatal attacks had occurred since Beier (1991) published his data. The first recorded physical injury resulting from a human-mountain lion encounter in Wyoming occurred in 2006 near Laramie; fortunately, the injuries were minor. It appears younger-aged males, primarily yearlings, accounted for 42% of the attacks on humans (Beier 1991). Increased mountain lion numbers along with increased recreational use and urbanization of mountain lion habitat has created greater opportunity for mountain lion-human encounters. For example, new homes have been built on traditional mule deer winter range in Boulder County, Colorado, resulting in increased mountain lion sightings along with a dramatic increase in mountain lion predation on domestic pets (Sanders and Halfpenny 1991). Typically, when a mountain lion interacts with another animal, including a human, it determines whether the other animal is either prey or non-prey. If the animal is determined to be non-prey, it might become the target of aggressive behavior as the mountain lion may think the animal is a threat. Humans should attempt to maintain eye contact with an aggressive mountain lion and attempt to increase one's potential size by standing erect. It appears that attacks can be reduced if the mountain lion is aware that you are not a typical prey species. If an attack does occur, humans should fight back as aggressively as possible. Several attacks have been broken off due to this type of response (Fitzhugh et al. 2003). If humans have the ability to observe a mountain lion prior to an attack, they can interpret specific mountain lion behavior to assess the level of threat from the mountain lion (Appendix IV).

Not all mountain lion-human interactions can be avoided and, in some cases, humans do have the opportunity to modify their behavior to reduce the chance of an attack. It is much more effective for humans to modify their behavior than it is for people to modify mountain lion behavior. Guidelines that can reduce the chance of an attack are presented in Appendix V.

The Wyoming Game and Fish Department strives to minimize human conflicts with mountain lions while maintaining sustainable mountain lion populations for ecological, recreational, scientific, and aesthetic purposes. Coordination with county planning boards to minimize conflicts in suitable mountain lion habitats (Anderson et al. in review) should help reduce conflicts.

A "Protocol for Managing Aggressive Wildlife/Human Interactions", which includes mountain lions, was completed in 1999 (Moody et al. 1999). Major components of this protocol include procedures for reporting, documenting, and investigating incidents. This document is designed to aid Wyoming Game and Fish Department personnel in conducting investigations and assure appropriate coordination with other State and/or Federal agencies. Accurate reporting and periodic analysis of this information will improve our understanding of the factors that promote conflicts and how to better address them.

PUBLIC INFORMATION AND EDUCATION EFFORTS

As with all large predators, some aspects of mountain lion management are increasingly controversial. The public is much more cognizant of issues associated with mountain lion management compared to the early 1990s. The Department traditionally relied on public contacts, open houses, and public meetings held in conjunction with season setting meetings to gauge constituent attitudes and values about managed species. This process does not appear to

provide a forum that all interest groups are comfortable participating in. The Department will consider alternative methods to engage these segments of the public, such as increased involvement in establishing population management objectives.

The Wyoming Game & Fish Department completed an attitude survey of Wyoming residents to assess public values and attitudes that might influence mountain lion management (Gasson and Moody 1995). No attempt was made to calculate confidence intervals around the survey results. As a result, these data are qualitative indicators of public attitudes. The distribution of the sample by county roughly approximated the distribution of Wyoming's population. Approximately 67% of the respondents reported they hunted at some point in their lives, and over 54% presently engaged in some form of hunting. Less than 9% of the respondents hunted mountain lions, and 65% of mountain lion hunters used dogs to pursue mountain lions. Over 71% of the respondents felt that mountain lions were a benefit to Wyoming. Only 11% felt that mountain lions were not a benefit to the state. Approximately 50% agreed or strongly agreed that mountain lion hunting should continue, while 29% of respondents believed mountain lion hunting should be discontinued, and 57% felt hunting with dogs should be eliminated. However, only 51% of the people surveyed were aware mountain lion hunting was legal in Wyoming, suggesting the Wyoming public may be uninformed about the issues surrounding mountain lion management in the state. Sixty percent of the respondents indicated they would benefit from additional information and education about this species.

Based on the results of this survey it was apparent the Wyoming Game and Fish Department should expand its efforts to educate the public on mountain lion management and provide those interested with the information necessary to aid the Wyoming Game and Fish Commission/Department in future management strategies. The Wyoming Game and Fish Commission/Department recognize the importance of keeping the public informed.

To address these concerns, the Department provided additional information to the public about mountain lion biology, management, and how to avoid conflicts with lions beginning in 1996. One specific publication entitled "Living in Lion Country" was developed and distributed to WGFD Regional offices throughout the state. The Department has worked closely with The Center for Wildlife Information to integrate this material into existing programs that have traditionally focused on grizzly bears. Mountain lion information has been included in the Department's "Living in Lion and Bear Country" workshops that are presented every spring around the state. These workshops include information on grizzly bear, black bear, and mountain lion biology and how to reduce conflicts. An updated public attitude survey would be useful to assess the success of additional information and education efforts implemented since the previous survey in 1995.

Although a species management plan provides direction for the responsible agency, it also provides a concise, complete overview of important issues surrounding the species, which can easily be circulated to the public. Thus, wide circulation of this plan will help inform and educate the public about current mountain lion management topics. Issues can change, as well as attitudes, so periodically surveying public opinion will be necessary, along with education updates following completion of surveys. Collectively, adequate ongoing education and

information efforts coupled with periodic public surveys will help the Commission optimally manage mountain lions to address the public trust.

The Department will institute new programs. Additional information will be put on the Game and Fish web site to assist hunters in being able to differentiate sex of individuals. Additional and continued training of Department employees will be implemented to assure personnel who field check harvested lions are adequately trained to determine sex and age.

FUTURE RESEARCH AND MANAGEMENT NEEDS

The adaptive management approach outlined in this plan will provide opportunity to evaluate many of the management needs listed below, while other management needs will likely require additional research efforts. Addressing mountain lion management needs that require additional research efforts will be implemented when and if additional funding becomes available with respect to other management priorities for the Wyoming Game & Fish Department.

Short Term Needs:

- ➢ Develop or cooperate with other agencies in the development of vegetation data layers sufficient for application of the mountain lion habitat model in regions of the state where data are currently lacking.
- ➢ Further evaluation and refinement of population monitoring techniques.
  - Explore the potential for new approaches that are cost effective and logistically feasible for management application.
  - Evaluate track surveys and helicopter probability sampling for periodically monitoring mountain lion subpopulations the size of hunt areas.
  - Investigate the utility of DNA and camera based mark-recapture methods for estimating mountain lion populations. Explore reliability of different attractants for enticing mountain lions into hair collection or photo detection sites, and evaluate ability of photographic technology to differentiate individual mountain lions from digital photographs.
  - Include hunter effort data from unsuccessful hunters to that collected from successful hunters to better evaluate catch-per-unit-effort indices in evaluating mountain lion population trends.
- ➢ Test mountain lion habitat model predictions using independent data sets (e.g., GPS locations) as they become available.
- ➢ Monitor success of sink management objectives in reducing human conflicts and depredation incidents.
- ➢ Conduct placental analyses from harvested females to confirm accuracy of female age class determination.

Long-Term Needs:

- ➢ Identify juxtaposition and amount of source mountain lion habitat necessary to sustain mountain lion populations at landscape scales.

- Evaluate the level at which sink management successfully reduces human conflicts, depredation incidents, and predation impacts to prey populations.
- Develop and evaluate application of simulation models to examine vital rates relative to source-sink mountain lion management.
- Improve knowledge of mountain lion-prey relationships.
- Investigate population dynamics of multi predator-prey systems.
- Investigate potential influences of exploitation on mountain lion population dynamics.

LITERATURE CITED

Anderson, A. E. 1983. A critical review of literature on puma (Felis concolor). Special Report No. 54. Colorado Division of Wildlife, Fort Collins, USA.

Anderson, A. E., D. C. Bowden, and D. M. Kattner. 1992. The puma on Uncompahgre Plateau, Colorado. Colorado Division of Wildlife Technical Publication No. 40.

Anderson, C. R., Jr. 2003. Cougar ecology, management, and population genetics in Wyoming. Dissertation, University of Wyoming, Laramie, USA.

Anderson, C. R., Jr., and F. G. Lindzey. 2000. A photographic guide to estimating mountain lion age classes. Wyoming Cooperative Fish & Wildlife Research Unit, Laramie, USA.

Anderson, C. R., Jr., and F. G. Lindzey. 2003. Estimating cougar predation rates from GPS location clusters. Journal of Wildlife Management 67:307-316.

Anderson, C. R., Jr., and F. G. Lindzey. 2005. Experimental evaluation of population trend and harvest composition in a Wyoming cougar population. Wildlife Society Bulletin 33:179-188.

Anderson, C. R., Jr., F. G. Lindzey, and N. P. Nibbelink. 2003. Estimating cougar abundance using probability sampling: an evaluation of transect versus block design. Pages 31-64 *in* C. R. Anderson. Cougar management, ecology, and population genetics in Wyoming. Dissertation, University of Wyoming, Laramie, USA.

Anderson, C. R., Jr., F. G. Lindzey, and D. B. McDonald. 2004. Genetic structure of cougar populaions across the Wyoming Basin: metapopulation or megapopulation. Journal of Mammalogy 85:1207-1214.

Anderson, C. R., Jr., F. G. Lindzey, D. D. Bjornlie, H. Sawyer, R. M. Nielson, and D. S. Moody. In review. Development and evaluation of winter resource selection functions of cougars in the northern Rocky Mountains. Journal of Applied Ecology.

Ashman, D., G. C. Christensen, M. L. Hess, G. K. Tsukamoto, and M. S. Wickersham. 1983. The mountain lion in Nevada. Nevada Game and Fish Department, Federal Aid in Wildlife Restoration Project W-14-15, Final Report.

Barnhurst, D. 1986. Vulnerability of cougars to hunting. Thesis, Utah State University, Logan, USA.

Barnhurst, D., and F. G. Lindzey. 1989. Detecting female mountain lions with kittens. Northwest Science 63:35-37.

Beier, P. 1991. Cougar attacks on humans in the United States and Canada. Wildlife Society Bulletin 19:403-412.

Beier, P. 1993. Determining minimum habitat areas and habitat corridors for cougars. Conservation Biology 7:94-108.

Beier, P., and S. C. Cunningham. 1996. Power of track surveys to detect changes in cougar populations. Wildlife Society Bulletin 24:540-546.

Beier, P., D. Choate, and R. H. Barrett. 1995. Movement patterns of mountain lions during different behaviors. Journal of Mammalogy 76:1056-1070.

Becker, E. F. 1991. A terrestrial furbearer estimator based on probability sampling. Journal of Wildlife Management 55:730-737.

Becker, E. F., M. A. Spindler, and T. O. Osborne. 1998. A population estimator based on network sampling of tracks in the snow. Journal of Wildlife Management 62:968-977.

Cougar Management Guidelines Working Group. 2005. Cougar management guidelines—first edition. WildFutures, Bainbridge Island, Washington, USA.

Culver, M., W. E. Johnson, J. Pecon-Slattery, and S. J. O'Brien. 2000. Genomic ancestry of the puma (puma concolor). Journal of Heredity 91:186-197.

Cunningham, S. C., L. A. Haynes, C. Gustavson, and D. D. Haywood. 1995. Evaluation of the interaction between mountain lions and cattle in the Aravaipa-Klondyke region of southeast Arizona. Arizona Game and Fish Department Technical Report No. 17.

Davies, R. B. 1991. Lion damage to pets in urban Colorado Springs, Colorado. Pages 79-80 *in* C.E. Braun, editor. Proceedings of the 4th Mountain Lion Workshop. Colorado Division of Wildlife, Denver, USA.

Dickson, B. G., and P. Beier. 2002. Home-range and habitat selection by adult cougars in southern California. Journal of Wildlife Management 66:1235-1245.

Fitzhugh, E. L., S. Schmid-Holmes, M. W. Kenyon, and K. Etling. 2003. Lessening the impact of a puma attack on a human. Pages 89-103 *in* S. A. Becker, D. D. Bjornlie, F. G. Lindzey, and D. S. Moody, editors. Proceedings of the 7th Mountain Lion Workshop. Wyoming Game & Fish Department, Jackson, USA.

Gasson, W., and D. Moody. 1995. Attitudes of Wyoming residents on mountain lion management. Wyoming Game & Fish Department, Planning Report No. 40.

Hansen, K. 1992. Cougar, the American lion. Northland Publishing, Flagstaff, Arizona, USA.

Hopkins, R. A. 1989. Ecology of the puma in the Diablo Range. Dissertation, University of California, Berkeley, USA.

Koehler, G. M., and M. G. Horncker. 1991. Seasonal resource use among mountain lions, bobcats, and coyotes. Journal of Mammalogy 72:391-396.

Laing, S. P. 1988. Cougar habitat selection and spatial use patterns in southern Utah. Thesis, University of Wyoming, Laramie, USA.

Lindzey, F. G. 1987. Mountain Lion. Pages 657-668 *in* M. Novak, J.A. Baker, M.E. Obbard, and B. Malloch, editors. Wild Furbearer Management and Conservation in North America. Ontario Ministry of Natural Resources and Ontario Trappers Association, Toronto, Canada.

Lindzey, F. G., B. B. Ackerman, D. Barnhurst, T. Becker, T. P. Hemker, S. P. Laing, C. Mecham, and W. D. Van Sickle. 1989. Boulder-Escalante cougar project. Final Report. Utah Division of Wildlife Research, Salt Lake City, USA.

Lindzey, F. G., W. D. Van Sickle, S. P. Laing, and C. S. Mecham. 1992. Cougar population response to manipulation in southern Utah. Wildlife Society Bulletin 20:224-227.

Lindzey, F. G., W. D. Van Sickle, B. B. Ackerman, D. Barnhurst, T. P. Hemker, and S. P. Laing. 1994. Cougar population dynamics in southern Utah. Journal of Wildlife Management 58:619-624.

Logan, K. A. 1983. Mountain lion population and habitat characteristics in the Big Horn Mountains of Wyoming. Thesis, University of Wyoming, Laramie, USA.

Logan, K. A., and L. L. Irwin. 1985. Mountain lion habitats in the Big Horn Mountains, Wyoming. Wildlife Society Bulletin 13:257-262.

Logan, K. A., L. L. Irwin, and R. Skinner. 1986. Characteristics of a hunted mountain lion population in Wyoming. Journal of Wildlife Management 50:648–654.

Logan, K. A., and L. L. Sweanor. 2001. Desert puma: evolutionary ecology and conservation of an enduring carnivore. Island Press, Washington, D. C., USA.

Martorello, D. A., and R. A. Beausoliel. 2003. Cougar harvest characteristics with and without the use of hounds. Pages 129-135 *in* S. A. Becker, D. D. Bjornlie, F. G. Lindzey, and D. S. Moody, editors. Proceedings of the 7th Mountain Lion Workshop. Wyoming Game and Fish Department, Lander, USA.

Moody, D., C. Anderson, G. Shorma, T. Moore, E. Williams, T. Kreeger, A. Langston, and S. Mays. 1999. Protocol For Managing Aggressive Wildlife/Human Interactions. Wyoming Game & Fish Department, Cheyenne, USA.

Murphy, K. M. 1998. The ecology of the cougar (*Puma concolor*) in the northern Yellowstone ecosystem: Interactions with prey, bears, and humans. Dissertation, University of Idaho, Moscow, USA.

Pierce, B .M. and V. C. Bleich. 2003. Mountain Lion. Pages 744–757 *in* G.A. Feldhamer, B.C. Thompson, and J.A. Chapman, editors. Wild Mammals of North America: Biolgy, Management, and Conservation. 2nd edition. The Johns Hopkins University Press, Baltimore, Maryland, USA.

Ross, I. P., and M. G. Jalkotzy. 1992. Characteristics of a hunted population of cougars in southwestern Alberta. Journal of Wildlife Management 56:417–426.

Ross, I. P., and M. G. Jalkotzy. 1996. Cougar predation on moose in southwestern Alberta. Alces 32:1-8.

Ross, P.I. M.G. Jalkotzy, and M. Festa-Bianchet. 1997. Cougar predation on bighorn sheep in southwestern Alberta during winter. Canadian Journal of Zoology 74:771–775.

Sanders, M. R. and J. C. Halfpenny. 1991. Human-lion interactions in Boulder County, Colorado: behavior patterns. Page 17 *in* C.E. Braun, editor. Proceedings of the 4th Mountain Lion Workshop. Colorado Division of Wildlife, Denver, USA.

Shaw, H. G. 1977. Impacts of mountain lion on mule deer and cattle in northwestern Arizona. Pages 17-32 *in* R. L. Phillips and C. J. Jonkel, editors. Proceedings of the 1975 predator Symposium. Montana Forest and Conservation Experiment Station, University of Montana, Missoula, USA.

Shaw, H. G. 1980. Ecology of the mountain lion in Arizona. Final Report, Federal Aid in Wildlife Restoration Project W-78-R, Work Plan 2, Job 13. Arizona Game and Fish Department, Phoenix, USA.

Shaw, H. G. 1983. Mountain lion field guide. Arizona Game and Fish Department Special Report No. 9. Arizona Game and Fish Department, Phoenix, USA.

Sinclair, E. A., E. L. Swenson, M. L. Wolfe, D. C. Choate, B. Gates, and K. A. Crandall. 2001. Gene flow estimates in Utah cougars imply management beyond Utah. Animal Conservation 4:257-264.

Smallwood, K. S. 1994. Trends in California mountain lion populations. Southwest Naturalist 39:67-72.

Smallwood, K. S., and E. L. Fitzhugh. 1995. A track count for estimating mountain lion, *Felis concolor californica*, population trend. Biological Conservation 71:251-259.

Stoner, D. C. 2004. Cougar exploitation levels in Utah: implications for demographic structure, metapopulation dynamics, and population recovery. Thesis, University of Utah, Logan, USA.

Sweanor, L. L., K. A. Logan, and M. G. Hornocker. 2000. Cougar dispersal patterns, metapopulation dynamics, and conservation. Conservation Biology 14:789-808.

Thompson, D. J., and J. Jenks. 2005. Long-distance dispersal by a sub-adult male cougar from the Black Hills, South Dakota. Journal of Wildlife Management 69:818-820

Trainer, C. E., and N. E. Golly. 1992. Cougar age and reproduction. Oregon Department of Fish and Wildlife, Corvallis, USA.

Tully, R. J. 1991. Results, 1991 questionnaire on damage to livestock by mountain lion. Pages 68-74, *in* C.E. Braun, editor. Proceedings of the 4th Mountain Lion Workshop. Colorado Division of Wildlife, Denver, USA.

Van Sickle, W. D., and F. G. Lindzey. 1991. Evaluation of a cougar population estimator based on probability sampling. Journal of Wildlife Management 55:738-743.

Wehausan, J. D. 1996. Effects of mountain lion predation on bighorn sheep in the Sierra Nevada and Granite Mountains of California. Wildlife Society Bulletin 24:471–479.

Williams, J. S., J. J. McCarthy, and H. D. Picton. 1995. Mountain lion habitat use and food habits on the Montana Rocky Mountain front. Intermountain Journal of Sciences 1:16-28.

Wyoming Game & Fish Department. 1997. Mountain lion management plan. Lander, Wyoming, USA.

Wyoming Game & Fish Department. 2001. Strategic habitat plan. Cheyenne, Wyoming, USA.

Wyoming Game & Fish Department. 2000-2005. Annual Wildlife Damage Claim Summaries. Cheyenne, Wyoming, USA.

APPENDIX I. History of mountain lion management regulations in Wyoming.

As in other western states, management in Wyoming became increasingly conservative during the mid 1970s through the early 1990s, primarily to control the number and sex of lions harvested. Emphasis was placed on controlling the take of females until sufficient information was available to warrant increased harvest. Harvest quotas have been increased since that time in an effort to limit population increase in specific portions of the state.

From territorial days to 1973, mountain lions received no legal protection. The earliest statutory reference to mountain lions was in 1882 when the Council and House of Representatives of the Territory of Wyoming enacted Chapter 108, Section 1. This legislation authorized county commissioners to encourage the destruction of wolves (*Canis lupus*), wild cats (i.e., bobcats; *Lynx rufus*), lynx (*Lynx canadensis*), bears (*Ursus* spp.), and mountain lions by offering bounty payments. Although property owners, employees, and lessees are still allowed to kill any mountain lion causing damage to private property, bounty payments are no longer authorized. In 1973, the mountain lion was reclassified from a predator to a trophy game animal. Since then, regulations governing the take of mountain lions have become more restrictive with the establishment of shorter seasons, total mortality quotas, and female sub-quotas.

CHRONOLOGICAL SUMMARY OF MOUNTAIN LION MANAGEMENT REGULATIONS IN WYOMING

1882 The Wyoming Territorial Legislature passed a law authorizing County Commissioners to encourage the destruction of wolves, bobcats, lynx, bears, and mountain lions. The County Fund paid $2.50 for each mountain lion killed. This was the first law authorizing bounty payments for mountain lions.

1884 The bounty payment for mountain lions was raised to $5.00.

1890 The bounty payment was raised to $6.00. The Territorial Legislature passed a law prohibiting the killing of mountain lions outside of the Wyoming Territory. Violation of the law resulted in a penalty ranging from $25.00 to $50.00.

1907 Applications for bounty payments had to be accompanied by an affidavit stating that the person presenting the skin, in said county, and within Wyoming, killed the animal. The animal had to be taken after March 1st. Persons could take predators (mountain lions) within State Game Preserves with the permission of the State Game Warden.

1910-1911 It was unlawful to enter the forest reserves of Wyoming for the purpose of chasing or coursing predators with dogs, unless the dogs were licensed. The license was $1.00 per dog, per calendar year. It was permissible to take mountain lions during closed big game seasons on State Game Preserves with a permit from the State Game Warden.

1913-1914 It was lawful to use dogs on predatory species and on State Game Preserves with permit from State Game Warden.

1915-1916 Game animals could not be used as bait for the purpose of trapping predatory animals within Wyoming.

1917-1972 No changes in mountain lion regulations.

1973 The mountain lion was reclassified from a predator to a trophy game animal.

1974 The first mountain lion hunting season established. The hunt area was considered the entire state. The season ran for the entire calendar year, with a bag limit of 1 mountain lion per season. A license and fee was required, and hunters had to present the pelt and skull to the nearest Wyoming Game and Fish District Office within 10 days of harvest. Hunting with dogs was allowed and females with kittens at side and kittens were protected from harvest. The owner, employees, or lessee of said property could take mountain lions damaging private property.

1978 Mountain lion season ran from September 1—December 31 and January 1—March 31.

1980 Wyoming was divided into 22 hunt areas and 5 LMUs. Mortality quotas (total mountain lions) by hunt area were established. The season ran from September 1 - March 31.

1983 Hunt area 15 was divided into hunt areas 15 and 23.

1985 Hunters must report mountain lion kills within 72 hours to nearest Wyoming Game and Fish District Office or game warden.

1993 The pelt and skull were required to be presented in an unfrozen condition to allow extraction of two premolar teeth for aging, and to allow examination of the pelt to determine sex. Female mortality quotas established in some hunt areas.

1994 Hunt area boundaries revised to more closely correspond with known distribution. A total of 27 hunt areas existed.

1999 Hunt area 26 was eliminated from the Southeast LMU. Hunt area 6 was expanded in its place. Regulations revised to allow for the take of 2 mountain lions per person per year in hunt areas 7 and 21 to assist the Snowy Range mountain lion study. Hunters must purchase an additional license ($15 for resident and $75 for non-resident). Hunt Area 25 added to the southeast LMU.

2000 Hunt area 17 split with hunt area 26 being created in the West LMU to separate the Wyoming Range from the Salt River Range in the Jackson Region. Hunt area 27 added to the areas where two mountain lions can be taken in a calendar year. Biological year for analysis of harvest information changed to September 1–August 31. Hunt area 28 created to address potential harvest and damage on fee title lands within the Wind River Reservation. Hunt area 7 was eliminated from those where 2 mountain lions can be harvested annually.

2001 Hunt area 21 eliminated from those where 2 mountain lions can be harvested annually.

2003 Hunt area 2 in the Jackson region split to address hunter pressure issues. Hunt area 29 established in the southern portion of hunt area 2. Quotas set for three-year cycle to address data assessment issues.

Appendix II. Wyoming mountain lion harvest and harvest quotas, hunter effort for successful mountain lion hunters, and nonharvest-human caused mountain lion mortalities.

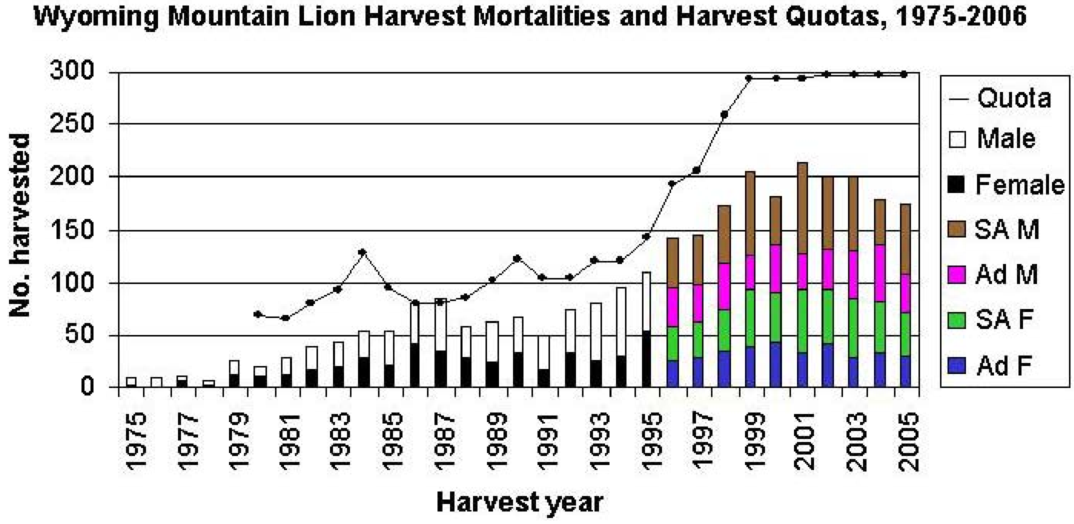

Figure II-1. Wyoming mountain lion harvest mortalities by sex (1975-1995) and age class (subadult = SA, adult = Ad; 1996-2006) and annual harvest quotas (1980-2006). Harvest year represents September of the given year through March of the following year; quotas reported from 1980-1984 were based on calendar year (Jan.-Mar. and Sept.-Dec. of the year reported). No harvest quotas were in place 1975-1979 and for hunt areas 15 and 22 (i.e., the southern Bighorn Mtns.) from 1986-1989.

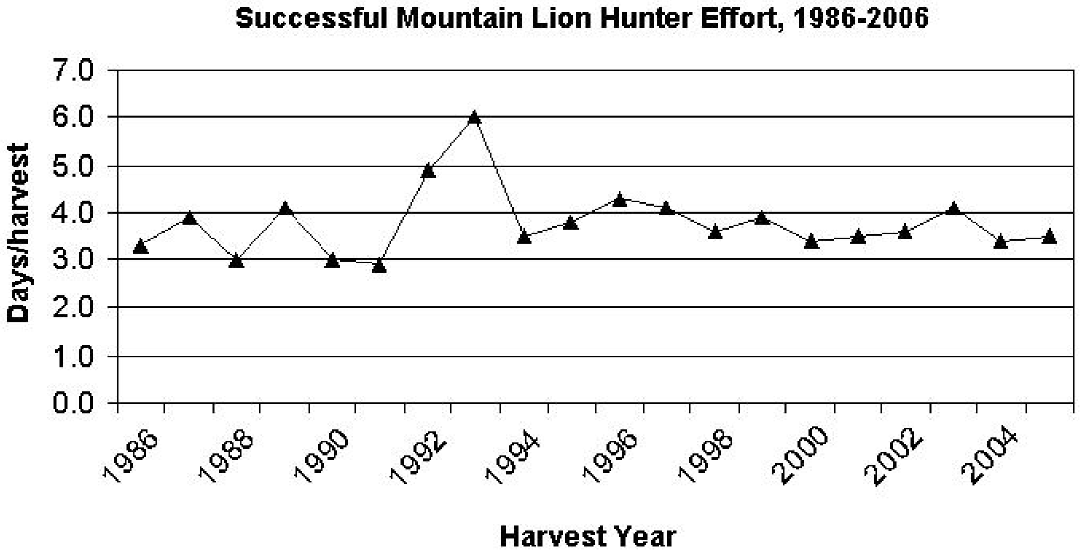

Figure II-2. Hunter effort (average days hunted per harvest) for hunters successfully harvesting a mountain lion, 1986-2006. Harvest year represents September of the given year through March of the following year. Harvest years exceeding 4 days per harvest were primarily due to a single hunter hunting for unusually long periods during the hunting season (e.g., a hunter reported hunting for 90 days in 1993).

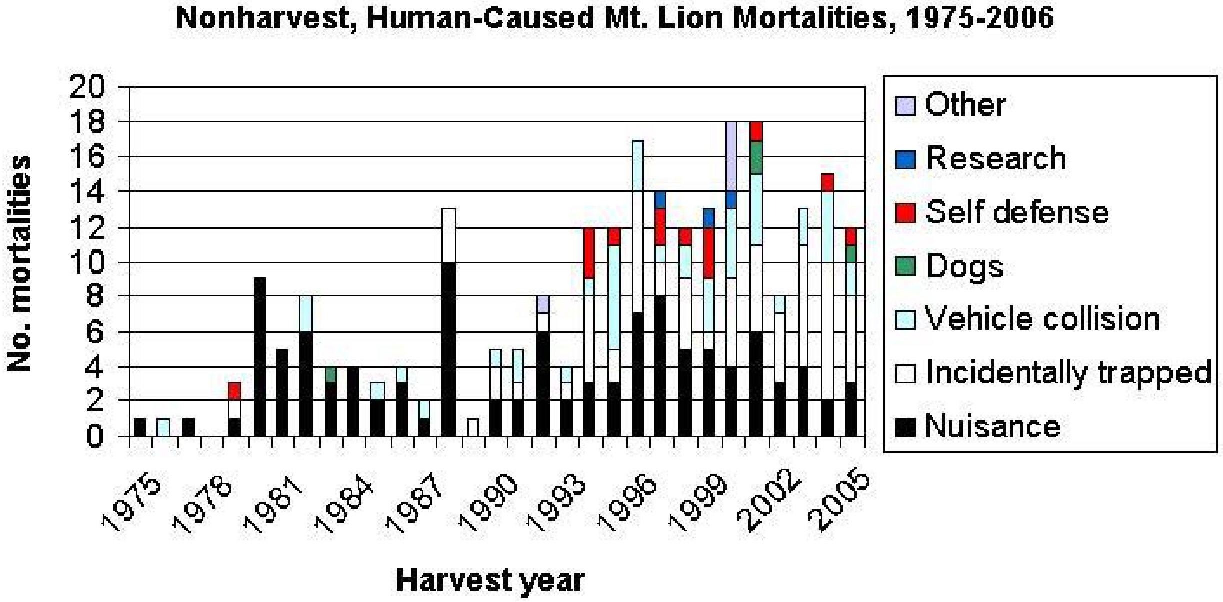

Figure II-3. Nonharvest, human caused mountain lion mortalities by cause reported in Wyoming, 1975-2006. Harvest year represents September of the given year through March of the following year. Other represents an electrocution in 1992 and a family group (1 female with 3 young) illegally poisoned in 2000. Nuisance mortalities include mountain lions depredating livestock or coming into close contact with human residence.

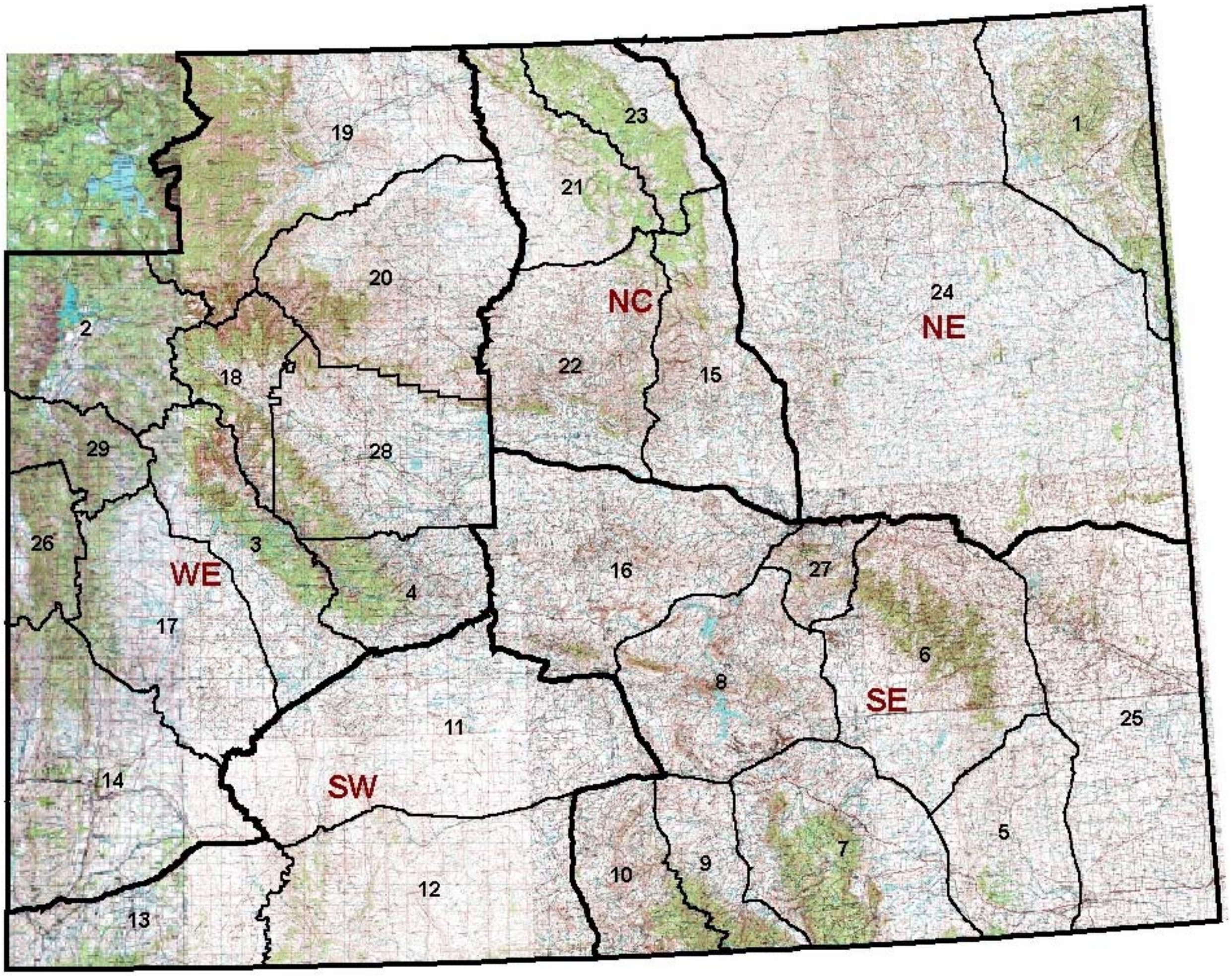

APPENDIX III. Wyoming mountain lion management units and hunt areas (numbered). Mountain lion management units: WE = West, SW = Southwest, SE = Southeast, NE = Northeast, and NC = North central.

APPENDIX IV. Wyoming mountain lion mortality form.

MOUNTAIN LION MORTALITY FORM Hunt Area _____ Region _____

Date of kill: _______________ TYPE: Legal_____; Illegal_____; Damage Control_____; Other_____; Unknown_____

If "Other" or "Unknown", probable cause of mortality ______________________________

PERSON WHO HARVESTED LION: Name: ______________________________

Address: ______________________________ City: ______________

State: ______ Zip: _______________ Phone: ______________________ Resident: _____ Nonresident: _____

METHODS/EFFORT: Days hunted: _____ Were dogs used? (Y/N) _____ If not, how was lion harvested? ______________

Was a guide/outfitter used? (Y/N): _____ Name: ____________________ Dog owner: ______________

Number of lions observed: _____ Were you selective while hunting? (Y/N): _____ Number of lions treed and released: _____

Number of lions that were marked: ______ (Ear tag / tattoo / radio collar frequency : ______________________)

Number of fresh tracks not pursued: _____ (How many were single adults?: _____ How many were adults with kittens?: _____)

LOCATION/DRAINAGE: Where was lion harvested? ______________________________

Sec: _____ Twnshp: _____ Rng: _____ UTM Zone: _____ UTM Easting: __________ UTM Northing: ____________

SEX AND AGE: Sex: _____ Est. Age: _____

If female, presently lactating? (Y[≥2] / N) _____

Appear to have lactated in past? (Y / N) _____

Canine ridge below gumline? (Y[≥2.5] / N) _____

Any visible spotting on rear legs? (Y[≤3] / N / ?) _____

Visible bars on inside of front legs? (Y[<4] / N / ?) _____

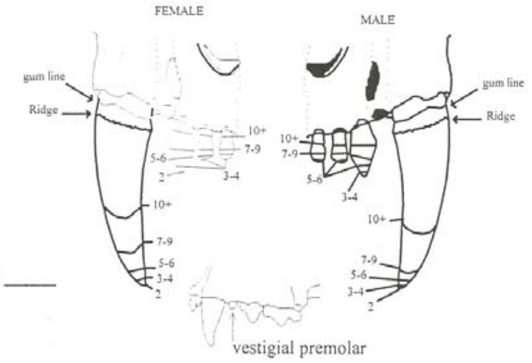

REQUIRED SAMPLES:

Number of teeth collected: 0 1 2 Pictures of teeth (Y/N): _____

Hair/Hide sample (1/2" X 1/2") taken (Y/N): _____

Remarks: ______________________________

____

____

Date record was WOFed: ____________________ Date Biological Services Called: ______________________

I, ______________________________ of ______________________________

being duly sworn, depose and say that I am the holder of Wyoming Mountain Lion license # _______________,

and lawfully took the above lion on __________ - ____________, 20_____ in Hunt Area # __________.

______________________ ______________ ______________________
Inspected by Date Hunter's Signature

Any person who makes a false statement on the registration form regarding the date the mountain lion was taken or the hunt area in which it was taken shall be in violation of this regulation and, such violation shall be punishable as provided by Title 23, Wyoming statutes for violation of Commission regulations.

Note: The person that checked the lion should forward the completed form and all tooth & hair samples to the Regional Office of registration and call Biological Services to update the harvest database. The Regional Office of registration will keep a copy of the completed form and send the original, along with the tooth and hair samples to the Trophy Game Section. Revised 01/04.

Appendix V. Interpretation of mountain lion behaviors arranged in order of increasing risk to a human interacting with the mountain lion. Do not rely solely on these behaviors to assess risk, because mountain lions are ambush predators whose behavior usually is not observed before an attack on a human (from the Cougar Management Guidelines 2005, page 89).

Observation	Interpretation	Human Risk
Opportunistically viewed at distance	Secretive	Low
Flight, hiding	Avoidance	Low
Lack of attention, various movements not directed toward person	Indifference, or actively avoiding inducing aggression	Low
Various body positions, ears up, may be shifting positions, intent attention, following behavior.	Curiosity	Low-provided human response is appropriate
Intense staring, following and hiding behavior	Assessing success of attack	Moderate
Hissing, snarling, vocalization	Defensive behaviors, attack may be imminent	Moderate, depending on distance to animal
Crouching, tail twitching, intense staring, ears flattened like wings, body low to ground, head may be up	Pre-attack	High
Ears flat, fur out, tail twitching, body and head low to ground, rear legs "pumping"	Imminent attack	Very high and immediate

Appendix VI. Some measures, with supporting information, that humans can take during an encounter to prevent injury (from the Cougar Management Guidelines 2005, page 93).

Recommendations	Supporting Information
Keep children under close control, and in view. Pick up small children immediately if you Encounter a mountain lion. Do not hike alone.	60% of victims have been unsupervised children or lone adults.
Do not run.	Running and quick movements may Stimulate chasing and catching response.
Stand. Wave your arms. Raise jacket over your Head. Appear as large as possible. Move to higher ground if nearby. Throw sticks, rocks, or other objects if within reach and accessible without bending to low.	Prey size vulnerability, and "positioning" influences mountain lion response.
Avoid dead animals and never approach kittens. Talk calmly. Back away.	Non-prey may be attacked if viewed as a threat.
Maintain eye contact. Do not look away. But if mountain lion appears agitated use peripheral vision to keep track if its location.	Eye-to-eye contact often restrains large cats. Direct eye contact from prey may inhibit predatory action.
Be alert to your surroundings.	Cats exploit all vantage points/cover when investigating prey.
If attacked, fight back. Humans have successfully deterred attacks by becoming aggressive.	A cat grasps with its teeth only if it meets with no resistance. Violently struggling Prey may be released.
Secure pets and hobby animals in predator proof enclosures between dusk and dawn. Keep pets on leashes and off trails in the backcountry.	Domestic prey animals may sustain mountain lion populations at unnaturally high levels.
Keep garbage under control to avoid attracting raccoons, skunks, etc. Do not feed pets outside and remove extra feed from domestic animal pens. Do not feed wildlife.	Mountain lions may be attracted to concentrations of potential prey.
A mountain lion that treats humans as prey is a public safety threat.	Once a learned behavior develops it may not be possible to modify this behavior.
Mountain lions that enter yards or campsites to kill pets may be candidates for removal. Keep pets under control.	Once a learned behavior develops it may not be modifiable.
