# Supplementary material for: The Elephant in the room: What can we learn from California regarding the use of sport hunting of pumas (Puma concolor) as a management tool?
Source: PLoS One. 2020 Feb 13;15(2):e0224638. doi: 10.1371/journal.pone.0224638 (PMC7018503; doi:10.1371/journal.pone.0224638)
Supplement: S2 File — This is a zip file that contains sources of the data used in the analyses that are no longer available on the internet. (ZIP) [file pone.0224638.s003.zip › Wyoming Big Game Report 2007.pdf]

ANNUAL
REPORT

OF

BIG
&
TROPHY
GAME
HARVEST
2007

ANNUAL REPORT

OF

BIG & TROPHY

GAME HARVEST

2007

For the Biological Year
June 1, 2007 to May 31, 2008

Wyoming Game and Fish Department **July 15, 2008**
Prepared by
Christine Leonard, Harvest Survey Coordinator
and Gail Sheridan, Administrative Secretary
Biological Services

Table of Contents

Big Game Survey

Project Statement: Big Game Harvest Survey 1
Summary of Big and Trophy Game Harvest Statewide 2
Summary of Big Game Hunters 3
Resident Hunter Satisfaction Results 4
Nonresident Hunter Satisfaction Results 5
Antelope Survey Form 6
Deer Survey Form 10
Elk Survey Form 14
Black Bear Survey Form 18
Mountain Lion Survey Form 22
Moose Survey Form 26
Bighorn Sheep Survey Form 27

Pronghorn Antelope Harvest

Antelope Harvest Statewide by Total Hunters: Table I 29
Antelope Ten-Year Comparison of Antelope Harvest: Table II 29
Antelope Harvest by Harvest Tool 29
Antelope Harvest, Hunting Pressure and Hunter Success by Hunt Area: Table 1-A 30
Antelope Harvest by Herd Unit: Table I-H 43
Confidence Intervals for Total Antelope Harvest by Herd Unit: Table III 47
Antelope Hunters Hunting on Opening Day by Hunt Area and Residency: Table IV 48
Antelope License Data Summary: Table VII 50
Antelope Hunting Seasons: Chapter 5 54
Antelope Hunt Area Map 77

Deer Harvest

Mule Deer Harvest Statewide by Total Hunters: Table I 79
Mule Deer Harvest by Harvest Tool 79
White-tailed Deer Harvest Statewide by Total Hunters: Table II 80
White-tailed Deer Harvest by Harvest Tool 80
Total Deer Harvest Statewide by Total Hunters: Table III 81
Statewide Deer Herd Ten-Year Comparison: Table IV 81
Mule Deer Harvest, Hunting Pressure and Hunter Success by Hunt Area: Table I-A.... 82
Mule Deer Harvest by Herd Unit: Table I-H 98
White-tailed Deer Harvest, Hunting Pressure, Hunter Success
by Hunt Area: Table II-A 103
White-tailed Deer Harvest by Herd Unit: Table II-H 120
Confidence Intervals for Total Deer Harvest by Herd Unit: Table V 121
Deer Hunters Hunting on Opening Day by Hunt Area and Residency: Table VI 122
Deer License Data Summary: Table VII 125
Deer Hunting Seasons: Chapter 6 128
Deer Hunt Area Map 156

Table of Contents

Elk Harvest

Elk Harvest Statewide by Total Hunters: Table I 157
Elk Ten-Year Comparison of Harvest by Total Hunters: Table II 157
Elk Harvest by Harvest Tool 157
Elk Harvest, Hunting Pressure, Hunter Success by Hunt Area: Table I-A 158
Elk Harvest by Herd Unit: Table I-H 172
Confidence Intervals for Total Elk Harvest by Herd Unit: Table III 176
Elk Hunters Hunting on Opening Day by Hunt Area and Residency: Table IV 177
Elk License Data Summary: Table V 179
Elk Hunting Seasons: Chapter 7 183
Elk Hunt Area Map 209

Moose Harvest

Moose Harvest Statewide by Total Hunters 211
Moose Ten-Year Comparison of Harvest 211
Moose Harvest by Harvest Tool 211
Moose Harvest by Hunt Area: Table I 212
Moose Harvest by Herd Unit: Table II 215
Moose Hunting Seasons: Chapter 8 216
Moose Hunt Area Map 224

Bighorn Sheep Harvest

Bighorn Sheep Harvest Statewide by Total Hunters 225
Bighorn Sheep Ten-Year Comparison of Harvest 225
Bighorn Sheep Harvest by Harvest Tool 225
Bighorn Sheep Harvest by Hunt Area 226
Bighorn Sheep Hunting Seasons: Chapter 9 228
Bighorn Sheep Hunt Area Map 233

Mountain Goat Harvest

Mountain Goat Harvest Statewide 235
Mountain Goat Ten-Year Comparison of Harvest 235
Mountain Goat Harvest Statewide by Hunt Area 236
Mountain Goat Hunting Seasons: Chapter 24 237
Mountain Goat Hunt Area Map 239

Bison Harvest

Bull Bison Harvest Statewide by Total Hunters 241
Bull Bison Nine-Year Comparison of Harvest 241
Cow Bison Harvest Statewide by Total Hunter 242
Cow Bison Nine-Year Comparison of Harvest 242
Wild Bison Recreational Hunting Seasons: Chapter 15 243
Wild Bison Recreational Hunting Seasons: Chapter 15 Emergency Rule 247
Bison Hunt Area Map 251

Table of Contents

Black Bear Harvest

Black Bear Hunts, Recreation Days, Hunter Days Per Bear: Table I 253
Black Bear Statewide Totals: Table II 256
Black Bear License Data Summary: Table III 256
Black Bear Harvest by Harvest Tool 257
Black Bear Hunting Seasons: Chapter 3 258
Black Bear Hunt Area Map 267

Mountain Lion Harvest

Mountain Lion Hunters, Hunter Days, Harvest, Days Per Harvest, and Hunter Success Rate: Table I 269
Mountain Lion Statewide Totals:Table II 272
Mountain Lion License Data Summary: Table III 272
Mountain Lion Harvest by Harvest Tool: Table IV 272
Mountain Lion Hunting Seasons: Chapter 42 273
Mountain Lion Hunting Seasons: Chapter 42 Emergency Rule.................................... 283
Mountain Lion Hunt Area Map 293

Archery

Antelope Archery Hunting - Harvest by Hunters Holding an Archery Tag 295
Antelope Archery Hunting - Total Days by Hunters Holding an Archery Tag 295
Antelope Archery Hunting - Equipment Used 295
Bear Archery Hunting - Harvest by Hunters Holding an Archery Tag 296
Bear Archery Hunting - Total Days by Hunters Holding an Archery Tag 296
Bear Archery Hunting - Equipment Used 296
Deer Archery Hunting - Harvest by Hunters Holding an Archery Tag 297
Deer Archery Hunting - Total Days by Hunters Holding an Archery Tag 297
Deer Archery Hunting - Equipment Used 297
Elk Archery Hunting - Harvest by Hunters Holding an Archery Tag 298
Elk Archery Hunting - Total Days by Hunters Holding an Archery Tag 298
Elk Archery Hunting - Equipment Used 298

JOB COMPLETION REPORT
INVESTIGATIONS PROJECT

STATE OF Wyoming **NAME: Big and Trophy Game Harvest Surveys**

PROJECT N. W-27-R-43 **PREPARED BY: Biological Services Section**
Reg. Rothwell, Supervisor

PROJECT OBJECTIVE NO. 1 JOB NO. 1

PERIOD COVERED: June 1, 2007 to May 31, 2008

ABSTRACT:
This report contains the results of harvest surveys completed following the 2007 big and trophy game hunting seasons. Harvest and hunting activity estimates for all big game, black bear and mountain lion were obtained by sampling licensed hunters with Internet and mailed questionnaires. Big and trophy game archery harvest for each species was determined as part of these surveys.

OBJECTIVES:
1. To determine the 2007 big and trophy game harvest.
2. To determine the number of active hunters, days spent hunting big and trophy game, the number of days required to harvest an animal, and the percent hunter success during 2007 hunting season.

PROCEDURES:
The 2007 pronghorn, deer, elk, black bear, and mountain lion harvest surveys were conducted by PA Consulting, Inc. (PA), the Department's harvest survey contractor for these species. At the close of the 2007 fall hunting season, license holders selected for the harvest survey were sent a postcard, asking them to complete the 2007 harvest survey on the internet site PA established for this purpose. Mountain lion hunters were contacted after the close of the majority of hunting seasons closed on March 31, 2008 (Three hunt areas remain open year round). After a period of time, each non-respondent was a sent harvest questionnaire by mail.

The procedure used in making the harvest and hunting estimates was similar for pronghorn, deer and elk. In general, a stratified systematic random sample was drawn from holders of pronghorn antelope, deer and elk licenses. License holders were stratified by residency, license type, hunt area(s), and/or region in which their licenses were valid. Resident general deer and elk license holders were also stratified by the county in which their licenses were purchased. For black bear and mountain lion, only information on hunter effort and success was requested since registration of all harvested black bears and mountain lions is mandatory. Black bear and mountain lion surveys are censuses. Because 3 mountain lion hunt areas are open from September 1 through August 31 (essentially year round), hunters were asked to estimate how much they would hunt in those hunt areas from April 1 through August 31, and PA then estimated effort and success for them.

Big game license holder samples adequate to meet survey objectives for both resident and nonresident hunters were drawn from license data, and they included both general and limited quota license types. Big game harvest estimates were made for each hunt area and for each herd unit within the state. Herd units consist of one or more hunt areas. Harvest for a herd unit was determined by combining harvest estimates from the appropriate hunt areas.

Hunters with general licenses are allowed to hunt many areas. Even hunters with limited quota licenses are allowed to hunt more than one area in some herd units. As a result, two steps are required to eliminate double or triple counting of hunters and provide accurate estimates of individual hunter numbers. When estimates of the number of hunters per herd unit are made, we count those who hunted more than one area within a herd unit as one hunter. Likewise, for statewide hunter estimates, we considered those who hunted more than one herd unit only one hunter. Thus, adding hunter numbers for hunt areas, herd units and the entire state would produce three different estimates of hunter numbers. All are accurate, but with a different frame of reference.

Additional questions were asked of hunters in an attempt to determine hunter satisfaction and hunter use of the lands enrolled in the Department's Private Lands Public Wildlife program.

2007
Summary of Big and Trophy Game Harvest
Statewide

Species	Number of Hunters	Days Hunted	Harvest	Hunter Success
Pronghorn	52,774	169,419	51,883	98.4%
Mule Deer	65,503	328,020	41,106	62.8%
White-tailed Deer	25,008	113,668	13,955	55.8%
Elk	52,151	387,973	22,523	43.2%
Moose	750	4,674	669	89.2%
Bighorn Sheep	237	2,225	201	84.8%
Mountain Goat	20	113	19	95.0%
Bison	277	824	267	96.3%
Black Bear	1,981	19,379	213	*
Mountain Lion	1,068	10,944	198	*

*** success not applicable, harvest is regulated by area quotas**

2007
Summary of Big Game Hunters*

License Combinations	Resident	Nonresident
Antelope	3445	12301
Deer	14193	18085
Elk	10355	7073
Bear	108	184
Mt Lion**	165	99
Multiple Antelope	1224	16488
Multiple Deer	2985	6087
Multiple Elk	2597	869
Antelope and Deer	5063	10066
Antelope and Elk	3076	668
Deer and Elk	19083	1242
Antelope Deer Elk	10654	446
Antelope Bear	22	19
Antelope Bear Elk	60	1
Antelope Bear Deer	71	13
Bear Elk	130	23
Bear Deer	134	25
Bear Elk Deer	1046	8
Antelope Bear Elk Deer	869	4
Mt Lion Antelope	21	6
Mt Lion Bear	16	0
Mt Lion Deer	193	7
Mt Lion Elk	118	3
Mt Lion Antelope Bear	1	0
Mt Lion Antelope Deer	83	3
Mt Lion Antelope Elk	24	0
Mt Lion Bear Deer	25	0
Mt Lion Bear Elk	10	0
Mt Lion Deer Elk	450	2
Mt Lion Antelope Bear Deer	7	1
Mt Lion Antelope Bear Elk	7	0
Mt Lion Antelope Deer Elk	479	2
Mt Lion Bear Deer Elk	189	3
Mt Lion Antelope Bear Deer Elk	271	2
Totals	77173	73728

*License combinations for all Big Game license holders (regardless of whether or not they hunted).
** Mountain lion hunt season: September 1, 2007 - March 31, 2008

2007 Hunter Satisfaction Questions*
Resident Response, by Species

Antelope:

Question #	Very Satisfied	Satisfied	Neutral	Dissatisfied	Very Dissatisfied
1	45.7%	42.6%	7.8%	2.8%	1.1%
2	45.6%	34.4%	17.9%	1.3%	0.7%

Deer:

Question #	Very Satisfied	Satisfied	Neutral	Dissatisfied	Very Dissatisfied
1	26.6%	45.8%	15.6%	7.9%	4.1%
2	35.2%	41.1%	19.0%	2.2%	2.3%

Elk:

Question #	Very Satisfied	Satisfied	Neutral	Dissatisfied	Very Dissatisfied
1	25.8%	40.6%	16.7%	10.7%	6.2%
2	36.5%	42.2%	16.3%	2.3%	2.6%

Black Bear:

Question #	Very Satisfied	Satisfied	Neutral	Dissatisfied	Very Dissatisfied
1	18.2%	40.4%	26.2%	10.3%	4.9%
2	30.2%	38.3%	25.8%	3.6%	2.0%

*unweighted numbers

Questions:

1. The overall quality of your hunt?
2. If you were contacted by WY Game and Fish personnel while you were in the field, rate your experience?

2007/2008 Hunter Satisfaction Questions*
Resident Response

Mountain Lion:

Question #	Very Satisfied	Satisfied	Neutral	Dissatisfied	Very Dissatisfied
1	21.8%	40.4%	29.0%	6.3%	2.6%
2	33.5%	38.6%	20.3%	3.2%	4.4%

*unweighted numbers

Questions:

1. The overall quality of your hunt?
2. If you were contacted by WY Game and Fish personnel in the field or if you registered a harvested Mountain Lion, rate your experience…

2007 Hunter Satisfaction Questions*
Nonresident Response, by Species

Antelope:

Question #	Very Satisfied	Satisfied	Neutral	Dissatisfied	Very Dissatisfied
1	64.3%	29.8%	3.9%	1.4%	0.7%
2	61.7%	26.8%	8.5%	1.6%	1.3%

Deer:

Question #	Very Satisfied	Satisfied	Neutral	Dissatisfied	Very Dissatisfied
1	42.5%	39.1%	9.8%	5.7%	2.9%
2	51.2%	34.7%	10.1%	2.4%	1.6%

Elk:

Question #	Very Satisfied	Satisfied	Neutral	Dissatisfied	Very Dissatisfied
1	41.1%	36.4%	10.2%	7.6%	4.7%
2	54.8%	31.9%	11.2%	1.1%	1.0%

Black Bear:

Question #	Very Satisfied	Satisfied	Neutral	Dissatisfied	Very Dissatisfied
1	31.2%	37.7%	20.1%	7.1%	3.9%
2	50.0%	23.3%	26.7%	0.0%	0.0%

*unweighted numbers

Questions:

1. The overall quality of your hunt?
2. If you were contacted by WY Game and Fish personnel while you were in the field, rate your experience?

2007/2008 Hunter Satisfaction Questions*
Nonresident Response

Mountain Lion:

Question #	Very Satisfied	Satisfied	Neutral	Dissatisfied	Very Dissatisfied
1	54.3%	30.4%	10.9%	4.3%	0.0%
2	62.9%	28.6%	8.6%	0.0%	0.0%

*unweighted numbers

Questions:

1. The overall quality of your hunt?
2. If you were contacted by WY Game and Fish personnel in the field or if you registered a harvested Mountain Lion, rate your experience…

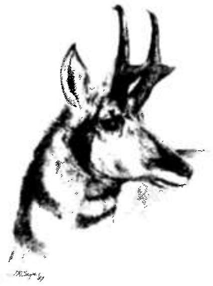

2007 WYOMING PRONGHORN HUNTER SURVEY

If you prefer to fill out the survey on the Internet (www.wyhunter.org/pronghorn), please use the 8-digit password on the back of this survey.

If you have questions about the survey or prefer to complete it over the phone, please contact Chris Mayhew at 1-800-216-0477 or e-mail: chris.mayhew@paconsulting.com

Please complete this survey after you have finished hunting or decided not to hunt Pronghorn for the season.

Q1. Did you hunt Pronghorn in Wyoming during the 2007 season? *(Check one box)*

☐ *Yes* ☐ *No* ***(IF YOU DID NOT HUNT PRONGHORN DURING THE 2007 SEASON, SKIP TO Q15)***

Q2. How many **different** days did you hunt Pronghorn in Wyoming during the 2007 season? *(**NOTE:** Count each partial day of hunting as 1 day of Pronghorn hunting) (Enter number in space below)*

☐☐ *Number of different days*

Q3. Please indicate your satisfaction when hunting Pronghorn in Wyoming during the 2007 season. *(Check one box for each question below)*

	Very Satisfied	*Satisfied*	*Neutral*	*Dissatisfied*	*Very Dissatisfied*	*Not Applicable*
How satisfied were you with the overall quality of your hunt..................	☐	☐	☐	☐	☐	☐
If you were contacted by WY Game and Fish personnel while you were in the field, rate your experience.............	☐	☐	☐	☐	☐	☐

Q4. What was the PRIMARY weapon you used to hunt Pronghorn in Wyoming during the 2007 season? *(This would be the weapon you used the most amount of time to hunt Pronghorn and may or may not be the weapon you actually used to harvest a Pronghorn) (Check one box only)*

☐ *Rifle or Pistol* ☐ *Muzzleloader* ☐ *Archery* ***(Skip to Q6)***

Q5. Did you hunt Pronghorn with archery equipment in Wyoming during the 2007 season? *(Check one box)*

☐ *Yes* ☐ *No* ***(Skip to Q8)***

Q6. What type of archery equipment did you use during the 2007 Pronghorn season? *(Check all that apply)*

☐ *Compound bow* ☐ *Recurve bow* ☐ *Long bow* ☐ *Crossbow*

Q7. How many **different** days did you hunt Pronghorn with archery equipment in Wyoming during the 2007 archery and gun season? *(**NOTE:** Count each partial day of hunting as 1 day of Pronghorn hunting) (Enter number in space below)*

☐☐ *Number of different days hunted with archery equipment during the archery-only season*

☐☐ *Number of different days hunted with archery equipment during the gun season*

2007 Pronghorn Hunt Information

Q8. How many **FULL PRICE** Pronghorn licenses did you hold? *(Reminder: Full Price means General or Limited Quota regular, youth, pioneer or landowner Pronghorn license other than Type 6 or 7)*

☐ *None* ***(Skip to Q9)*** ☐ *One* ☐ *Two*

Q8a. For each **FULL PRICE** license please write the Hunt Area, License Type, and indicate whether you hunted with this license. *(Hunt Area and License Type are on your license documents)*

Type of Full Price License(s) Held	***Did you hunt with this license?***
1st FULL PRICE license Hunt Area: ☐☐☐ License Type: ☐ (Type 1, 2, 8, or 9)	☐ *Yes* ☐ *No*
2nd FULL PRICE license Hunt Area: ☐☐☐ License Type: ☐ (Type 1, 2, 8, or 9)	☐ *Yes* ☐ *No*

Q8b. Please fill in the table below for up to 2 Hunt Areas you mainly hunted with each **FULL PRICE** Pronghorn license you held, even if you did not harvest a Pronghorn with one or more of these licenses.

	Number of the Hunt Area(s) where you hunted* (Hunt Area numbers range from 1 to 117)	Did you hunt opening day of the gun season in this Hunt Area? (Check one box)	Number of different days hunted in this Hunt Area** (Enter number below)	Did you harvest a Pronghorn in this Hunt Area? (Check one box)	Age/sex of Pronghorn harvested in this Hunt Area (Check one box)	Weapon used to harvest this Pronghorn (Check one box)
**1st Full Price License**	**Main Hunt Area hunted:** ☐☐☐	☐ *Yes* ☐ *No*	☐☐ *days*	☐ *Yes* ☐ *No* ***(Skip to 2nd Hunt Area)***	☐ *Adult Buck* ☐ *Adult Doe* ☐ *Fawn (either sex)*	☐ *Rifle or Pistol* ☐ *Muzzleloader* ☐ *Archery*
	**2nd Hunt Area hunted:** ☐☐☐	☐ *Yes* ☐ *No*	☐☐ *days*	☐ *Yes* ☐ *No* ***(Skip to 2nd license)***	☐ *Adult Buck* ☐ *Adult Doe* ☐ *Fawn (either sex)*	☐ *Rifle or Pistol* ☐ *Muzzleloader* ☐ *Archery*
**2nd Full Price License**	**Main Hunt Area hunted:** ☐☐☐	☐ *Yes* ☐ *No*	☐☐ *days*	☐ *Yes* ☐ *No* ***(Skip to 2nd Hunt Area)***	☐ *Adult Buck* ☐ *Adult Doe* ☐ *Fawn (either sex)*	☐ *Rifle or Pistol* ☐ *Muzzleloader* ☐ *Archery*
	**2nd Hunt Area hunted:** ☐☐☐	☐ *Yes* ☐ *No*	☐☐ *days*	☐ *Yes* ☐ *No* ***(Skip to Q9)***	☐ *Adult Buck* ☐ *Adult Doe* ☐ *Fawn (either sex)*	☐ *Rifle or Pistol* ☐ *Muzzleloader* ☐ *Archery*

* **NOTE:** Hunt Area map can be found on the back of the survey cover letter
** **NOTE:** Please count each partial day of hunting as 1 day of Pronghorn hunting

Q9. How many **Type 6 or 7 Reduced Price DOE/FAWN** Pronghorn licenses did you hold? *(Reminder: all Reduced Price Pronghorn licenses, including Reduced Price youth licenses and Type 6 or 7 pioneer licenses, have a Type 6 or 7 designation)*

☐ *None* ***(Skip to Q10)*** ☐ *One* ☐ *Two* ☐ *Three* ☐ *Four*

5693516993

Q9a. For each **Type 6 or 7 Reduced Price DOE/FAWN** Pronghorn license please write the Hunt Area and the License Type, and indicate whether you hunted with this license. *(Hunt Area and License Type are on your license documents)*

Type of Reduced Price Doe/Fawn License(s) Held			***Did you hunt with this license?***
1st DOE/FAWN license	Hunt Area: ☐☐☐	License Type: ☐ (Type 6 or 7)	☐ *Yes* ☐ *No*
2nd DOE/FAWN license	Hunt Area: ☐☐☐	License Type: ☐ (Type 6 or 7)	☐ *Yes* ☐ *No*
3rd DOE/FAWN license	Hunt Area: ☐☐☐	License Type: ☐ (Type 6 or 7)	☐ *Yes* ☐ *No*
4th DOE/FAWN license	Hunt Area: ☐☐☐	License Type: ☐ (Type 6 or 7)	☐ *Yes* ☐ *No*

Q9b. Please fill in the table below for up to 2 Hunt Areas you mainly hunted with each **Type 6 or 7 Reduced Price DOE/FAWN** Pronghorn license you held, even if you did not harvest a Pronghorn with one or more of these licenses.

	Number of the Hunt Area(s) where you hunted* (Hunt Area numbers range from 1 to 117)	Did you hunt opening day of the gun season in this Hunt Area? (Check one box)	Number of different days hunted in this Hunt Area** (Enter number below)	Did you harvest a Pronghorn in this Hunt Area? (Check one box)	Age/sex of Pronghorn harvested in this Hunt Area (Check one box)	Weapon used to harvest this Pronghorn (Check one box)
**1st Doe/Fawn License**	**Main Hunt Area hunted:** ☐☐☐	☐ *Yes* ☐ *No*	☐☐ *days*	☐ *Yes* ☐ *No* ***(Skip to 2nd Hunt Area)***	☐ *Adult Doe* ☐ *Fawn (either sex)*	☐ *Rifle or Pistol* ☐ *Muzzleloader* ☐ *Archery*
	**2nd Hunt Area hunted:** ☐☐☐	☐ *Yes* ☐ *No*	☐☐ *days*	☐ *Yes* ☐ *No* ***(Skip to 2nd license)***	☐ *Adult Doe* ☐ *Fawn (either sex)*	☐ *Rifle or Pistol* ☐ *Muzzleloader* ☐ *Archery*
**2nd Doe/Fawn License**	**Main Hunt Area hunted:** ☐☐☐	☐ *Yes* ☐ *No*	☐☐ *days*	☐ *Yes* ☐ *No* ***(Skip to 2nd Hunt Area)***	☐ *Adult Doe* ☐ *Fawn (either sex)*	☐ *Rifle or Pistol* ☐ *Muzzleloader* ☐ *Archery*
	**2nd Hunt Area hunted:** ☐☐☐	☐ *Yes* ☐ *No*	☐☐ *days*	☐ *Yes* ☐ *No* ***(Skip to 3rd license)***	☐ *Adult Doe* ☐ *Fawn (either sex)*	☐ *Rifle or Pistol* ☐ *Muzzleloader* ☐ *Archery*
**3rd Doe/Fawn License**	**Main Hunt Area hunted:** ☐☐☐	☐ *Yes* ☐ *No*	☐☐ *days*	☐ *Yes* ☐ *No* ***(Skip to 2nd Hunt Area)***	☐ *Adult Doe* ☐ *Fawn (either sex)*	☐ *Rifle or Pistol* ☐ *Muzzleloader* ☐ *Archery*
	**2nd Hunt Area hunted:** ☐☐☐	☐ *Yes* ☐ *No*	☐☐ *days*	☐ *Yes* ☐ *No* ***(Skip to 4th license)***	☐ *Adult Doe* ☐ *Fawn (either sex)*	☐ *Rifle or Pistol* ☐ *Muzzleloader* ☐ *Archery*
**4th Doe/Fawn License**	**Main Hunt Area hunted:** ☐☐☐	☐ *Yes* ☐ *No*	☐☐ *days*	☐ *Yes* ☐ *No* ***(Skip to 2nd Hunt Area)***	☐ *Adult Doe* ☐ *Fawn (either sex)*	☐ *Rifle or Pistol* ☐ *Muzzleloader* ☐ *Archery*
	**2nd Hunt Area hunted:** ☐☐☐	☐ *Yes* ☐ *No*	☐☐ *days*	☐ *Yes* ☐ *No* ***(Skip to Q10)***	☐ *Adult Doe* ☐ *Fawn (either sex)*	☐ *Rifle or Pistol* ☐ *Muzzleloader* ☐ *Archery*

* **NOTE:** Hunt Area map can be found on the back of the survey cover letter
** **NOTE:** Please count each partial day of hunting as 1 day of Pronghorn hunting

OVER→

Private Lands Public Wildlife Access Program

In 1997, the Wyoming Game and Fish Department began the Private Lands Public Wildlife Access Program (PLPW) to improve public hunting and fishing access on private and land-locked public lands through the **Walk-in Area** and **Hunter Management Area** programs. Your response to the following questions will help the Department improve this program to better serve hunters.

Q10. Did you hunt Pronghorn on any of the PLPW Walk-in Areas or Hunter Management Areas during the 2007 season? *(**NOTE:** Walk-in Areas are open to hunting without a permission slip, but a permission slip is needed for Hunter Management Areas)*

☐ *Yes* ☐ *No **(Skip to Q15)*** ☐ *Don't know **(Skip to Q15)***

Q11. How many different days did you hunt Pronghorn on PLPW Walk-in Areas or Hunter Management Areas during the 2007 season? *(**NOTE:** Count each partial day of hunting as 1 day of Pronghorn hunting) (Enter number in space below; if no days, write 0)*

☐☐ *Days hunted in **Walk-in Areas*** ☐☐ *Days hunted in **Hunter Management Areas***

Q12. On how many different PLPW Walk-in Areas or Hunter Management Areas did you hunt Pronghorn during the 2007 season? *(If no areas, write 0)*

☐☐ *Different **Walk-in Areas*** ☐☐ *Different **Hunter Management Areas***

Q13. Overall, how satisfied were you with the opportunity Wyoming Game and Fish provided through the PLPW Walk-in Area and Hunter Management Area programs? *(check one box below)*

Very Satisfied	*Satisfied*	*Neutral*	*Dissatisfied*	*Very Dissatisfied*
☐	☐	☐	☐	☐

Q14. If you were dissatisfied, why?

____

Other 2007 Big Game Licenses

Q15. What other 2007 Wyoming big game licenses did you hold? *(Check all that apply)*

☐ *Black Bear* ☐ *Deer* ☐ *Elk* ☐ *Mountain Lion* ☐ *No other big game license*

Thank you for your help with this important study!

COMMENTS: Feel free to attach a sheet of paper with any comments you would like to share with the Wyoming Game and Fish Department. **Please include the hunt area as well as the species hunted with your comments.** These comments along with your contact information will be sent directly to the Wyoming Game and Fish Department.

*(**NOTE:** individual responses to survey questions are confidential and will not be shared with the Wyoming Game and Fish Department)*

Please return your completed survey in the enclosed, postage-paid envelope to:
Wyoming Big Game Hunter Survey, c/o PA Consulting Group, 6410 Enterprise Lane, Suite 300, Madison WI 53719

password:

9943516995 | 94 1 95 7 95 7 | 11999980

12156-1

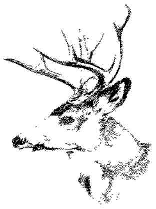

2007 WYOMING DEER HUNTER SURVEY

If you prefer to fill out the survey on the Internet (www.wyhunter.org/deer), please use the 8-digit password on the back of this survey.

If you have questions about the survey or prefer to complete it over the phone, please contact Chris Mayhew at 1-800-216-0477 or e-mail: chris.mayhew@paconsulting.com

Please complete this survey after you have finished hunting or decided not to hunt Deer for the season.

Q1. Did you hunt Deer in Wyoming during the 2007 season? *(Check one box)*

☐ *Yes* ☐ *No* ***(IF YOU DID NOT HUNT DEER DURING THE 2007 SEASON, SKIP TO Q15)***

Q2. How many **different** days did you hunt Deer in Wyoming during the 2007 season? (***NOTE:*** *Count each partial day of hunting as 1 day of Deer hunting) (Enter number in space below)*

☐☐ *Number of different days*

Q3. Please indicate your satisfaction when hunting Deer in Wyoming during the 2007 season. *(Check one box for each question below)*

	Very Satisfied	*Satisfied*	*Neutral*	*Dissatisfied*	*Very Dissatisfied*	*Not Applicable*
How satisfied were you with the overall quality of your hunt.................	☐	☐	☐	☐	☐	☐
If you were contacted by WY Game and Fish personnel while you were in the field, rate your experience...........	☐	☐	☐	☐	☐	☐

Q4. What was the PRIMARY weapon you used to hunt Deer in Wyoming during the 2007 season? *(This would be the weapon you spent the <u>most amount of time hunting Deer</u> and may or may not be the weapon you actually used to harvest a Deer) (Check <u>one</u> box only)*

☐ *Rifle or Pistol* ☐ *Muzzleloader* ☐ *Archery* ***(Skip to Q5a)***

Q5. Did you hunt Deer with archery equipment in Wyoming during the 2007 season? *(Check one box)*

☐ *Yes* ☐ *No* ***(Skip to Q8)***

Q5a. What percent of time did you spend hunting Mule Deer vs. White-tailed Deer with archery equipment during the 2007 Deer season? *(Enter percents below so that the total equals 100%)*

Mule Deer: ☐☐☐ % + *White-tailed Deer:* ☐☐☐ % = ***100% TOTAL***

Q6. What type of archery equipment did you use during the 2007 Deer season? *(Check all that apply)*

☐ *Compound bow* ☐ *Recurve bow* ☐ *Long bow* ☐ *Crossbow*

Q7. How many **different** days did you hunt Deer with archery equipment in Wyoming during the 2007 archery and gun seasons? (***NOTE:*** *Count each partial day of hunting as 1 day of Deer hunting) (Enter number in space below)*

☐☐ *Number of different days hunted with archery equipment during the archery-only season*

☐☐ *Number of different days hunted with archery equipment during the gun season*

2007 Deer Hunt Information

Q8. Did you hold a **FULL PRICE** Deer license? *(Reminder: Full Price means General or Limited Quota regular, youth, pioneer or landowner Deer license other than Type 6 or 7)*

☐ *Yes* ☐ *No* ***(Skip to Q9)***

Q8a. Did you hunt with this **FULL PRICE** Deer license?

☐ *Yes* ☐ *No* ***(Skip to Q9)***

Q8b. Please fill in the table below for up to 3 Hunt Areas you mainly hunted with this **FULL PRICE** Deer license, even if you did not harvest with this license.

Number of the Hunt Area(s) where you hunted* ***(Hunt Area numbers range from 1 to 171)***	Did you hunt opening day of the gun season in this Hunt Area? *(Check one box)*	Number of different days hunted in this Hunt Area** *(Enter number below)*	Percent of time hunted Mule Deer vs. White-tailed Deer in this Hunt Area *(Enter percent below)*	Did you harvest a Deer in this Hunt Area? *(Check one box)*	Age/sex of Deer harvested in this Hunt Area *(Check one box)*	Weapon used to harvest this Deer *(Check one box)*
**Main Hunt Area hunted:** (do <u>not</u> list region letter) ☐☐☐	☐ Yes<br>☐ No	☐☐ *days*	*Mule Deer:* ☐☐☐ %<br>*White-tailed Deer:* ☐☐☐ %<br>***100% TOTAL***	☐ *Yes, a Mule deer*<br>☐ *Yes, a White-tailed deer*<br>☐ *No* ***(Skip to 2nd Hunt Area)***	☐ *Adult Buck*<br>☐ *Adult Doe*<br>☐ *Fawn (either sex)*	☐ *Rifle or Pistol*<br>☐ *Muzzleloader*<br>☐ *Archery*
**2nd Hunt Area hunted:** (do <u>not</u> list region letter) ☐☐☐	☐ Yes<br>☐ No	☐☐ *days*	*Mule Deer:* ☐☐☐ %<br>*White-tailed Deer:* ☐☐☐ %<br>***100% TOTAL***	☐ *Yes, a Mule deer*<br>☐ *Yes, a White-tailed deer*<br>☐ *No* ***(Skip to 3rd Hunt Area)***	☐ *Adult Buck*<br>☐ *Adult Doe*<br>☐ *Fawn (either sex)*	☐ *Rifle or Pistol*<br>☐ *Muzzleloader*<br>☐ *Archery*
**3rd Hunt Area hunted:** (do <u>not</u> list region letter) ☐☐☐	☐ Yes<br>☐ No	☐☐ *days*	*Mule Deer:* ☐☐☐ %<br>*White-tailed Deer:* ☐☐☐ %<br>***100% TOTAL***	☐ *Yes, a Mule deer*<br>☐ *Yes, a White-tailed deer*<br>☐ *No* ***(Skip to Q9)***	☐ *Adult Buck*<br>☐ *Adult Doe*<br>☐ *Fawn (either sex)*	☐ *Rifle or Pistol*<br>☐ *Muzzleloader*<br>☐ *Archery*

****Note:*** *Hunt Area map can be found on the back of the survey cover letter*

*****Note:*** *Please count each partial day of hunting as 1 day of Deer hunting*

Q9. How many **Type 6 or 7 Reduced Price DOE/FAWN** Deer licenses did you hold? *(Reminder: <u>all</u> Reduced Price Deer licenses, including Reduced Price youth licenses and Type 6 or 7 pioneer licenses, have a Type 6 or 7 designation)*

☐ *None* ***(Skip to Q10)*** ☐ *One* ☐ *Two* ☐ *Three* ☐ *Four*

Q9a. For each **Type 6 or 7 Reduced Price DOE/FAWN** Deer license, please write the Hunt Area, License Type, and indicate whether you hunted with this license. *(Hunt Area and License Type are on your license documents)*

Type of Reduced Price DOE/FAWN License(s) Held			***Did you hunt with this license?***	
1st DOE/FAWN license	Hunt Area: ☐☐☐	License Type: ☐ (Type 6 or 7)	☐ *Yes*	☐ *No*
2nd DOE/FAWN license	Hunt Area: ☐☐☐	License Type: ☐ (Type 6 or 7)	☐ *Yes*	☐ *No*
3rd DOE/FAWN license	Hunt Area: ☐☐☐	License Type: ☐ (Type 6 or 7)	☐ *Yes*	☐ *No*
4th DOE/FAWN license	Hunt Area: ☐☐☐	License Type: ☐ (Type 6 or 7)	☐ *Yes*	☐ *No*

2888506636

Q9b. Please fill in the table below for up to 2 Hunt Areas you mainly hunted with each **Type 6 or 7 Reduced Price DOE/FAWN** Deer license you held, even if you did not harvest a Deer with one or more of these licenses.

		Number of the Hunt Area(s) where you hunted* ***(Hunt Area numbers range from 1 to 171)***	Did you hunt opening day of the gun season in this Hunt Area? *(Check one box)*	Number of different days hunted in this Hunt Area** *(Enter number below)*	Percent of time hunted Mule Deer vs. White-tailed Deer in this Hunt Area *(Enter percent below)*	Did you harvest a Deer in this Hunt Area? *(Check one box)*	Age/sex of Deer harvested in this Hunt Area *(Check one box)*	Weapon used to harvest this Deer *(Check one box)*
**1st Doe/Fawn License**	**Main Hunt Area hunted:**	☐☐☐	☐ Yes<br>☐ No	☐☐ days	*Mule Deer:* ☐☐☐ %<br>*White-tailed Deer:* ☐☐☐ %<br>**100% TOTAL**	☐ *Yes, a Mule deer*<br>☐ *Yes, a White-tailed deer*<br>☐ *No* ***(Skip to 2nd Hunt Area)***	☐ *Adult Doe*<br>☐ *Fawn (either sex)*	☐ *Rifle or Pistol*<br>☐ *Muzzleloader*<br>☐ *Archery*
	**2nd Hunt Area hunted:**	☐☐☐	☐ Yes<br>☐ No	☐☐ days	*Mule Deer:* ☐☐☐ %<br>*White-tailed Deer:* ☐☐☐ %<br>**100% TOTAL**	☐ *Yes, a Mule deer*<br>☐ *Yes, a White-tailed deer*<br>☐ *No* ***(Skip to 2nd license)***	☐ *Adult Doe*<br>☐ *Fawn (either sex)*	☐ *Rifle or Pistol*<br>☐ *Muzzleloader*<br>☐ *Archery*
**2nd Doe/Fawn License**	**Main Hunt Area hunted:**	☐☐☐	☐ Yes<br>☐ No	☐☐ days	*Mule Deer:* ☐☐☐ %<br>*White-tailed Deer:* ☐☐☐ %<br>**100% TOTAL**	☐ *Yes, a Mule deer*<br>☐ *Yes, a White-tailed deer*<br>☐ *No* ***(Skip to 2nd Hunt Area)***	☐ *Adult Doe*<br>☐ *Fawn (either sex)*	☐ *Rifle or Pistol*<br>☐ *Muzzleloader*<br>☐ *Archery*
	**2nd Hunt Area hunted:**	☐☐☐	☐ Yes<br>☐ No	☐☐ days	*Mule Deer:* ☐☐☐ %<br>*White-tailed Deer:* ☐☐☐ %<br>**100% TOTAL**	☐ *Yes, a Mule deer*<br>☐ *Yes, a White-tailed deer*<br>☐ *No* ***(Skip to 3rd license)***	☐ *Adult Doe*<br>☐ *Fawn (either sex)*	☐ *Rifle or Pistol*<br>☐ *Muzzleloader*<br>☐ *Archery*
**3rd Doe/Fawn License**	**Main Hunt Area hunted:**	☐☐☐	☐ Yes<br>☐ No	☐☐ days	*Mule Deer:* ☐☐☐ %<br>*White-tailed Deer:* ☐☐☐ %<br>**100% TOTAL**	☐ *Yes, a Mule deer*<br>☐ *Yes, a White-tailed deer*<br>☐ *No* ***(Skip to 2nd Hunt Area)***	☐ *Adult Doe*<br>☐ *Fawn (either sex)*	☐ *Rifle or Pistol*<br>☐ *Muzzleloader*<br>☐ *Archery*
	**2nd Hunt Area hunted:**	☐☐☐	☐ Yes<br>☐ No	☐☐ days	*Mule Deer:* ☐☐☐ %<br>*White-tailed Deer:* ☐☐☐ %<br>**100% TOTAL**	☐ *Yes, a Mule deer*<br>☐ *Yes, a White-tailed deer*<br>☐ *No* ***(Skip to 4th license)***	☐ *Adult Doe*<br>☐ *Fawn (either sex)*	☐ *Rifle or Pistol*<br>☐ *Muzzleloader*<br>☐ *Archery*
**4th Doe/Fawn License**	**Main Hunt Area hunted:**	☐☐☐	☐ Yes<br>☐ No	☐☐ days	*Mule Deer:* ☐☐☐ %<br>*White-tailed Deer:* ☐☐☐ %<br>**100% TOTAL**	☐ *Yes, a Mule deer*<br>☐ *Yes, a White-tailed deer*<br>☐ *No* ***(Skip to 2nd Hunt Area)***	☐ *Adult Doe*<br>☐ *Fawn (either sex)*	☐ *Rifle or Pistol*<br>☐ *Muzzleloader*<br>☐ *Archery*
	**2nd Hunt Area hunted:**	☐☐☐	☐ Yes<br>☐ No	☐☐ days	*Mule Deer:* ☐☐☐ %<br>*White-tailed Deer:* ☐☐☐ %<br>**100% TOTAL**	☐ *Yes, a Mule deer*<br>☐ *Yes, a White-tailed deer*<br>☐ *No* ***(Skip to Q10)***	☐ *Adult Doe*<br>☐ *Fawn (either sex)*	☐ *Rifle or Pistol*<br>☐ *Muzzleloader*<br>☐ *Archery*

****Note:*** *Hunt Area map can be found on the back of the survey cover letter*
*****Note:*** *Please count each partial day of hunting as 1 day of Deer hunting*

OVER →

Private Lands Public Wildlife Access Program

In 1997, the Wyoming Game and Fish Department began the Private Lands Public Wildlife Access Program (PLPW) to improve public hunting and fishing access on private and land-locked public lands through the **Walk-in Area** and **Hunter Management Area** programs. Your response to the following questions will help the Department improve this program to better serve hunters.

Q10. Did you hunt Deer on any of the PLPW Walk-in Areas or Hunter Management Areas during the 2007 season? *(**NOTE:** Walk-in Areas are open to hunting without a permission slip, but a permission slip is needed for Hunter Management Areas)*

☐ *Yes* ☐ *No **(Skip to Q15)*** ☐ *Don't know **(Skip to Q15)***

Q11. How many different days did you hunt Deer on PLPW Walk-in Areas or Hunter Management Areas during the 2007 season? *(**NOTE:** Count each partial day of hunting as 1 day of Deer hunting)* *(Enter number in space below; if no days, write 0)*

☐☐ *Days hunted in **Walk-in Areas*** ☐☐ *Days hunted in **Hunter Management Areas***

Q12. On how many different PLPW Walk-in Areas or Hunter Management Areas did you hunt Deer during the 2007 season? *(If no areas, write 0)*

☐☐ *Different **Walk-in Areas*** ☐☐ *Different **Hunter Management Areas***

Q13. Overall, how satisfied were you with the opportunity Wyoming Game and Fish provided through the PLPW Walk-in Area and Hunter Management Area programs? *(check one box below)*

Very Satisfied	*Satisfied*	*Neutral*	*Dissatisfied*	*Very Dissatisfied*
☐	☐	☐	☐	☐

Q14. If you were dissatisfied, why?

Other 2007 Big Game Licenses

Q15. What other 2007 Wyoming big game licenses did you hold? *(Check all that apply)*

☐ *Pronghorn* ☐ *Black Bear* ☐ *Elk* ☐ *Mountain Lion* ☐ *No other big game license*

Thank you for your help with this important study!

COMMENTS: Feel free to attach a sheet of paper with any comments you would like to share with the Wyoming Game and Fish Department. **Please include the hunt area as well as the species hunted with your comments.** These comments along with your contact information will be sent directly to the Wyoming Game and Fish Department.

*(**NOTE:** individual responses to survey questions are confidential and will not be shared with the Wyoming Game and Fish Department)*

Please return your completed survey in the enclosed, postage-paid envelope to:
Wyoming Big Game Hunter Survey, c/o PA Consulting Group, 6410 Enterprise Lane, Suite 300, Madison WI 53719

password:

6 6

22023784

9095506633

16266-2

2007 WYOMING ELK HUNTER SURVEY

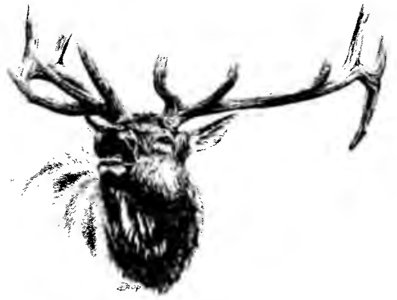

If you prefer to fill out the survey on the Internet (www.wyhunter.org/elk), please use the 8-digit password on the back of this survey.

If you have questions about the survey or prefer to complete it over the phone, please contact Chris Mayhew at 1-800-216-0477 or e-mail: chris.mayhew@paconsulting.com

---

Please complete this survey after you have finished hunting or decided not to hunt Elk for the season.

Q1. Did you hunt Elk in Wyoming during the 2007 season? *(Check one box)*

☐ *Yes* ☐ *No* ***(IF YOU DID NOT HUNT ELK DURING THE 2007 SEASON, SKIP TO Q15)***

Q2. How many **different** days did you hunt Elk in Wyoming during the 2007 season? *(**NOTE:** Count each partial day of hunting as 1 day of Elk hunting) (Enter number in space below)*

☐☐ *Number of different days*

Q3. Please indicate your satisfaction when hunting Elk in Wyoming during the 2007 season. *(Check one box for each question below)*

	Very Satisfied	*Satisfied*	*Neutral*	*Dissatisfied*	*Very Dissatisfied*	*Not Applicable*
How satisfied were you with the overall quality of your hunt................	☐	☐	☐	☐	☐	☐
If you were contacted by WY Game and Fish personnel while you were in the field, rate your experience..........	☐	☐	☐	☐	☐	☐

Q4. What was the PRIMARY weapon you used to hunt Elk in Wyoming during the 2007 season? *(This would be the weapon you used the most amount of time to hunt Elk and may or may not be the weapon you actually used to harvest an Elk) (Check one box only)*

☐ *Rifle or Pistol* ☐ *Muzzleloader* ☐ *Archery* ***(Skip to Q6)***

Q5. Did you hunt Elk with archery equipment in Wyoming during the 2007 season? *(Check one box)*

☐ *Yes* ☐ *No* ***(Skip to Q8)***

Q6. What type of <u>archery equipment</u> did you use during the 2007 Elk season? *(Check all that apply)*

☐ *Compound bow* ☐ *Recurve bow* ☐ *Long bow* ☐ *Crossbow*

Q7. How many **different** days did you hunt Elk with <u>archery equipment</u> in Wyoming during the 2007 archery and gun seasons? ***(NOTE:** Count each partial day of hunting as 1 day of Elk hunting) (Enter number in space below)*

☐☐ *Number of different days hunted with archery equipment during the archery-only season*

☐☐ *Number of different days hunted with archery equipment during the gun season*

2007 Elk Hunt Information

Q8. Did you hold a **FULL PRICE** Elk license? *(Reminder: Full Price means General or Limited Quota regular, youth, pioneer or landowner Elk license other than Type 5, 6 or 7)*

☐ *Yes* ☐ *No* ***(Skip to Q9)***

Q8a. Did you hunt with this **FULL PRICE** Elk license?

☐ *Yes* ☐ *No* ***(Skip to Q9)***

Q8b. Please fill in the table below for up to 3 Hunt Areas where you hunted with this **FULL PRICE** Elk license, even if you did not harvest with this license.

Number of the Hunt Area(s) where you hunted* ***(Hunt Area numbers range from 1 to 130)***	Did you hunt opening day of the gun season in this Hunt Area? *(Check one box)*	Number of different days hunted in this Hunt Area** *(Enter number below)*	Did you harvest an Elk in this Hunt Area? *(Check one box)*	Age/sex of Elk harvested in this Hunt Area *(A mature bull is any branch-antlered bull; a calf includes both male and female calf) (Check one box)*	Weapon used to harvest this Elk *(Check one box)*
**Main Hunt Area hunted:** ☐☐☐	☐ Yes ☐ No	☐☐ days	☐ Yes ☐ No ***(Skip to 2nd Hunt Area)***	☐ Mature Bull ☐ Spike Bull ☐ Adult Cow ☐ Calf	☐ Rifle or Pistol ☐ Muzzleloader ☐ Archery
**2nd Hunt Area hunted:** ☐☐☐	☐ Yes ☐ No	☐☐ days	☐ Yes ☐ No ***(Skip to 3rd Hunt Area)***	☐ Mature Bull ☐ Spike Bull ☐ Adult Cow ☐ Calf	☐ Rifle or Pistol ☐ Muzzleloader ☐ Archery
**3rd Hunt Area hunted:** ☐☐☐	☐ Yes ☐ No	☐☐ days	☐ Yes ☐ No ***(Skip to Q9)***	☐ Mature Bull ☐ Spike Bull ☐ Adult Cow ☐ Calf	☐ Rifle or Pistol ☐ Muzzleloader ☐ Archery

****Note:*** *Hunt Area map can be found on the back of the survey cover letter*
*****Note:*** *Please count each partial day of hunting as 1 day of Elk hunting*

2687155936

Q9. Did you hold a **Type 5, 6 or 7 Reduced Price COW/CALF** Elk license? *(Reminder: all Reduced Price Elk licenses, including Reduced Price youth licenses and Type 5, 6 or 7 pioneer licenses, have a Type 5, 6 or 7 designation)*

☐ *Yes* ☐ *No* ***(Skip to Q10)***

Q9a. Did you hunt with this **Type 5, 6 or 7 Reduced Price COW/CALF** Elk license?

☐ *Yes* ☐ *No* ***(Skip to Q10)***

Q9b. Please fill in the table below for up to 3 Hunt Areas where you hunted with this **Type 5, 6 or 7 Reduced Price COW/CALF** Elk license, even if you did not harvest with this license.

Number of the Hunt Area(s) where you hunted* ***(Hunt Area numbers range from 1 to 130)***	Did you hunt opening day of the gun season in this Hunt Area? *(Check one box)*	Number of different days hunted in this Hunt Area** *(Enter number below)*	Did you harvest an Elk in this Hunt Area? *(Check one box)*	Age/sex of Elk harvested in this Hunt Area *(A calf includes both a male and female calf) (Check one box)*	Weapon used to harvest this Elk *(Check one box)*
**Main Hunt Area hunted:** ☐☐☐	☐ *Yes* ☐ *No*	☐☐ *days*	☐ *Yes* ☐ *No* ***(Skip to 2nd Hunt Area)***	☐ *Adult Cow* ☐ *Calf*	☐ *Rifle or Pistol* ☐ *Muzzleloader* ☐ *Archery*
**2nd Hunt Area hunted:** ☐☐☐	☐ *Yes* ☐ *No*	☐☐ *days*	☐ *Yes* ☐ *No* ***(Skip to 3rd Hunt Area)***	☐ *Adult Cow* ☐ *Calf*	☐ *Rifle or Pistol* ☐ *Muzzleloader* ☐ *Archery*
**3rd Hunt Area hunted:** ☐☐☐	☐ *Yes* ☐ *No*	☐☐ *days*	☐ *Yes* ☐ *No* ***(Skip to Q10)***	☐ *Adult Cow* ☐ *Calf*	☐ *Rifle or Pistol* ☐ *Muzzleloader* ☐ *Archery*

****Note:*** *Hunt Area map can be found on the back of the survey cover letter*
*****Note:*** *Please count each partial day of hunting as 1 day of Elk hunting*

OVER→

Private Lands Public Wildlife Access Program

In 1997, the Wyoming Game and Fish Department began the Private Lands Public Wildlife Access Program (PLPW) to improve public hunting and fishing access on private and land-locked public lands through the **Walk-in Area** and **Hunter Management Area** programs. Your response to the following questions will help the Department improve this program to better serve hunters.

Q10. Did you hunt Elk on any of the PLPW Walk-in Areas or Hunter Management Areas during the 2007 season? *(**NOTE:** Walk-in Areas are open to hunting without a permission slip, but a permission slip is needed for Hunter Management Areas)*

☐ *Yes* ☐ *No **(Skip to Q15)*** ☐ *Don't know **(Skip to Q15)***

Q11. How many different days did you hunt Elk on PLPW Walk-in Areas or Hunter Management Areas during the 2007 season? *(**NOTE:** Count each partial day of hunting as 1 day of Elk hunting)* *(Enter number in space below; if no days, write 0)*

☐☐ *Days hunted in **Walk-in Areas*** ☐☐ *Days hunted in **Hunter Management Areas***

Q12. On how many different PLPW Walk-in Areas or Hunter Management Areas did you hunt Elk during the 2007 season? *(If no areas, write 0)*

☐☐ *Different **Walk-in Areas*** ☐☐ *Different **Hunter Management Areas***

Q13. Overall, how satisfied were you with the opportunity Wyoming Game and Fish provided through the PLPW Walk-in Area and Hunter Management Area programs? *(check one box below)*

Very Satisfied	*Satisfied*	*Neutral*	*Dissatisfied*	*Very Dissatisfied*
☐	☐	☐	☐	☐

Q14. If you were dissatisfied, why?

____

Other 2007 Big Game Licenses

Q15. What other 2007 Wyoming big game licenses did you hold? *(Check all that apply)*

☐ *Pronghorn* ☐ *Black Bear* ☐ *Deer* ☐ *Mountain Lion* ☐ *No other big game license*

Thank you for your help with this important study!

COMMENTS: Feel free to attach a sheet of paper with any comments you would like to share with the Wyoming Game and Fish Department. **Please include the hunt area as well as the species hunted with your comments.** These comments along with your contact information will be sent directly to the Wyoming Game and Fish Department.

*(**NOTE:** individual responses to survey questions are confidential and will not be shared with the Wyoming Game and Fish Department)*

Please return your completed survey in the enclosed, postage-paid envelope to:
Wyoming Big Game Hunter Survey, c/o PA Consulting Group, 6410 Enterprise Lane, Suite 300, Madison WI 53719

password:

2195155930 94 1 95 7 31999980

15180-1

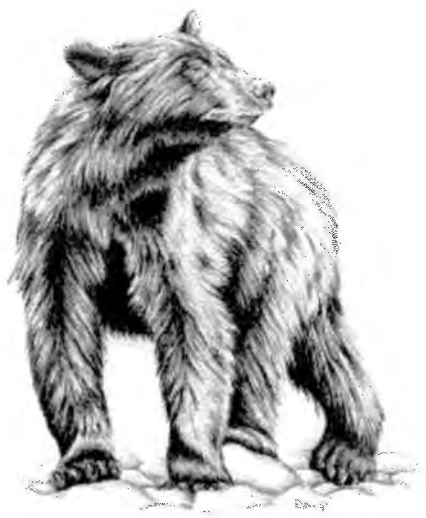

2007 WYOMING BLACK BEAR HUNTER SURVEY

If you prefer to fill out the survey on the Internet (www.wyhunter.org/blackbear), please use the 8-digit password on the back of this survey.

If you have questions about the survey or prefer to complete it over the phone, please contact Chris Mayhew at 1-800-216-0477 or e-mail: chris.mayhew@paconsulting.com

---

Please complete this survey after the fall 2007 Black Bear season or after you decided not to hunt Black Bear for the season.

Q1. Did you hunt Black Bear in Wyoming at any time during 2007? *(Check one box)*

☐ *Yes* ☐ *No* ***(IF YOU DID NOT HUNT BLACK BEAR DURING 2007, SKIP TO Q12)***

Q2. Please indicate your satisfaction when hunting Black Bear in Wyoming during 2007. *(Check one box for each question below)*

	Very Satisfied	*Satisfied*	*Neutral*	*Dissatisfied*	*Very Dissatisfied*	*Not Applicable*
How satisfied were you with the overall quality of your hunt..................	☐	☐	☐	☐	☐	☐
If you were contacted by WY Game and Fish personnel while you were in the field, rate your experience............	☐	☐	☐	☐	☐	☐

Q3. Did you hunt Black Bear with archery equipment in Wyoming during 2007? *(Check one box)*

☐ *Yes* ☐ *No* ***(Skip to Q6)***

Q4. What type of archery equipment did you use to hunt Black Bear during 2007? *(Check all that apply)*

☐ *Compound bow* ☐ *Recurve bow* ☐ *Long bow* ☐ *Crossbow*

Q5. How many **different** days did you hunt Black Bear with archery equipment in Wyoming during the 2007 archery and gun seasons? *(**NOTE:** Count each partial day of hunting as 1 day of Black Bear hunting) (Enter number in space below)*

☐☐ *Number of different days hunted with archery equipment during the archery-only season*

☐☐ *Number of different days hunted with archery equipment during the gun season*

Spring 2007 Black Bear Hunting Experiences

Q6. Did you hunt Black Bear in Wyoming during the spring of 2007? *(Check one box)*

☐ *Yes*

☐ *No* ***(Skip to Q9)***

Q7. How many **different** days did you hunt Black Bear during the spring of 2007? *(**NOTE:** Count each partial day of hunting as 1 day of Black Bear hunting) (Enter number in space below)*

☐☐ *Number of different days you hunted Black Bear in the spring of 2007*

Q8. Please tell us about your Black Bear hunting effort during the spring of 2007. *(Fill in the table below for each Hunt Area hunted)*

Number of the Hunt Area(s) where you hunted Black Bear during the spring of 2007* *(Hunt Area numbers range from 1 to 32)*	Number of different days you hunted Black Bear in this Hunt Area during the spring of 2007** *(Enter number below)*	Did you harvest a Black Bear during the spring of 2007 in this Hunt Area? *(Check one box)*	Weapon used to harvest this Bear *(Check one box)*
Main Hunt Area hunted: ☐☐☐	☐☐ *days*	☐ *Yes* ☐ *No* ***(Skip to 2nd Hunt Area)***	☐ *Rifle or Pistol* ☐ *Muzzleloader* ☐ *Archery*
2nd Hunt Area hunted: ☐☐☐	☐☐ *days*	☐ *Yes* ☐ *No* ***(Skip to 3rd Hunt Area)***	☐ *Rifle or Pistol* ☐ *Muzzleloader* ☐ *Archery*
3rd Hunt Area hunted: ☐☐☐	☐☐ *days*	☐ *Yes* ☐ *No* ***(Skip to 4th Hunt Area)***	☐ *Rifle or Pistol* ☐ *Muzzleloader* ☐ *Archery*
4th Hunt Area hunted: ☐☐☐	☐☐ *days*	☐ *Yes* ☐ *No* ***(Skip to 5th Hunt Area)***	☐ *Rifle or Pistol* ☐ *Muzzleloader* ☐ *Archery*
5th Hunt Area hunted: ☐☐☐	☐☐ *days*	☐ *Yes* ☐ *No* ***(Skip to Q9)***	☐ *Rifle or Pistol* ☐ *Muzzleloader* ☐ *Archery*

NOTE: *Hunt Area map can be found on the back of the survey cover letter*
***NOTE:** *Please count each partial day of hunting as 1 day of Black Bear hunting*

3698065186

Fall 2007 Black Bear Hunting Experiences

Q9. Did you hunt Black Bear in Wyoming during the fall of 2007? *(Check one box)*

☐ *Yes*

☐ *No* ***(Skip to Q12)***

Q10. How many **different** days did you hunt Black Bear during the fall of 2007? ***(NOTE:** Count each partial day of hunting as 1 day of Black Bear hunting) (Enter number in space below)*

☐☐ *Number of different days you hunted Black Bear during the fall of 2007*

Q11. Please tell us about your Black Bear hunting effort during the fall of 2007. *(Fill in the table below for each Hunt Area hunted)*

Number of the Hunt Area(s) where you hunted Black Bear during the fall of 2007* *(Hunt Area numbers range from 1 to 32)*	Number of different days you hunted Black Bear in this Hunt Area during the fall of 2007** *(Enter number below)*	Did you harvest a Black Bear during the fall of 2007 in this Hunt Area? *(Check one box)*	Weapon used to harvest this Bear *(Check one box)*
Main Hunt Area hunted: ☐☐☐	☐☐ *days*	☐ *Yes*<br>☐ *No* ***(Skip to 2nd Hunt Area)***	☐ *Rifle or Pistol*<br>☐ *Muzzleloader*<br>☐ *Archery*
2nd Hunt Area hunted: ☐☐☐	☐☐ *days*	☐ *Yes*<br>☐ *No* ***(Skip to 3rd Hunt Area)***	☐ *Rifle or Pistol*<br>☐ *Muzzleloader*<br>☐ *Archery*
3rd Hunt Area hunted: ☐☐☐	☐☐ *days*	☐ *Yes*<br>☐ *No* ***(Skip to 4th Hunt Area)***	☐ *Rifle or Pistol*<br>☐ *Muzzleloader*<br>☐ *Archery*
4th Hunt Area hunted: ☐☐☐	☐☐ *days*	☐ *Yes*<br>☐ *No* ***(Skip to 5th Hunt Area)***	☐ *Rifle or Pistol*<br>☐ *Muzzleloader*<br>☐ *Archery*
5th Hunt Area hunted: ☐☐☐	☐☐ *days*	☐ *Yes*<br>☐ *No* ***(Skip to Q12)***	☐ *Rifle or Pistol*<br>☐ *Muzzleloader*<br>☐ *Archery*

****NOTE:*** *Hunt Area map can be found on the back of the survey cover letter*
*****NOTE:*** *Please count each partial day of hunting as 1 day of Black Bear hunting*

Other 2007 Big Game Licenses

Q12. What other 2007 Wyoming big game licenses did you hold? *(Check all that apply)*

☐ *Pronghorn* ☐ *Deer* ☐ *Elk* ☐ *Mountain Lion* ☐ *No other big game license*

OVER →

Thank you for your help with this important study!

Feel free to attach a sheet of paper with any comments you would like to share with the Wyoming Game and Fish Department. **Please include the hunt area as well as the species hunted with your comments.** These comments along with your contact information will be sent directly to the Wyoming Game and Fish Department.

*(**NOTE:** individual responses to survey questions are confidential and will not be shared with the Wyoming Game and Fish Department)*

Please return your completed survey in the enclosed, postage-paid envelope to:
Wyoming Big Game Hunter Survey, c/o PA Consulting Group, 6410 Enterprise Lane, Suite 300, Madison WI 53719

password:

41999980

1079065187

1551-1

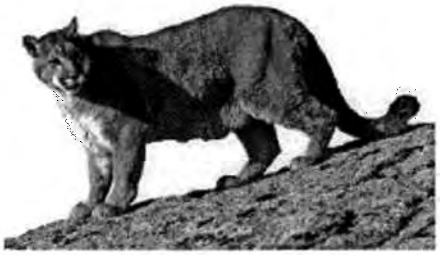

2007/2008 WYOMING MOUNTAIN LION HUNTER SURVEY

If you prefer to fill out the survey on the Internet (www.wyhunter.org/mtlion), please use the 8-digit password on the back of this survey.

If you have questions about the survey or prefer to complete it over the phone, please contact Chris Mayhew at 1-800-216-0477 or e-mail: chris.mayhew@paconsulting.com

---

Q1. This survey is about hunting effort for Mountain Lion in Wyoming between September 1, 2007 and March 31, 2008. Did you hunt Mountain Lion during this time period? *(Check one box)*

☐ *Yes* ☐ *No* ***(IF YOU DID NOT HUNT MOUNTAIN LION BETWEEN SEPTEMBER 1, 2007 AND MARCH 31, 2008, SKIP TO Q17)***

Q2. Please indicate your satisfaction when hunting Mountain Lion in Wyoming between September 1, 2007 and March 31, 2008. *(Check one box for each question below)*

	Very Satisfied	*Satisfied*	*Neutral*	*Dissatisfied*	*Very Dissatisfied*	*Not Applicable*
How satisfied were you with the overall quality of your hunt..................	☐	☐	☐	☐	☐	☐
If you were contacted by WY Game and Fish personnel in the field or if you registered a harvested Mountain Lion, please rate your experience.......	☐	☐	☐	☐	☐	☐

Q3. Did you hunt Mountain Lion with archery equipment in Wyoming between September 1, 2007 and March 31, 2008? *(Check one box)*

☐ *Yes* ☐ *No* ***(Skip to Q5)***

Q4. What type of archery equipment did you use to hunt Mountain Lion between September 1, 2007 and March 31, 2008? *(Check all that apply)*

☐ *Compound bow* ☐ *Recurve bow* ☐ *Long bow* ☐ *Crossbow*

Q5. Did you hunt Mountain Lion with dogs between September 1, 2007 and March 31, 2008? *(Check one box)*

☐ *Yes* ☐ *No*

Q6. Did you use a guide or an outfitter to hunt Mountain Lion between September 1, 2007 and March 31, 2008? *(Check one box)*

☐ *Yes* ☐ *No*

Q7. How many Mountain Lion did you see while hunting between September 1, 2007 and March 31, 2008? *(Enter number in space below)*

☐☐ *Number of Mountain Lion seen*

Q8. How many separate sets of Mountain Lion tracks did you see while hunting Mountain Lion between September 1, 2007 and March 31, 2008? *(Enter number in space below)*

☐☐ *Separate sets of tracks*

2007 MOUNTAIN LION LICENSE HOLDERS

Q9. Did you purchase a 2007 Mountain Lion license? *(Check one box)*

☐ *Yes* ☐ *No* ***(Skip to Q13)***

Q10. Did you hunt Mountain Lion in Wyoming between September 1, 2007 and December 31, 2007? *(Check one box)*

☐ *Yes* ☐ *No* ***(IF YOU DID NOT HUNT MOUNTAIN LION DURING THIS TIME, SKIP TO Q13)***

Q11. How many **different** days did you hunt Mountain Lion between September 1, 2007 and December 31, 2007? ***(NOTE:** Count each partial day of hunting as 1 day of Mountain Lion hunting)* *(Enter number in space below)*

☐☐☐ *Number of different days spent hunting Mountain Lion*

Q12. Please tell us about your Mountain Lion hunting effort between September 1, 2007 and December 31, 2007. (Fill in table below for each Hunt Area hunted.)

*Number of the Hunt Area(s) where you hunted Mountain Lion during this period (hunt area numbers range from 1 to 31)**	*Number of different days you hunted Mountain Lion in this Hunt Area during this period*** *(Enter number below)*	*Did you harvest a Mountain Lion during this period in this Hunt Area? (Check one box)*	*Sex of Mountain Lion harvested in this Hunt Area (Check one box)*	Weapon used to harvest this Mountain Lion *(Check one box)*
Main Hunt Area hunted: ☐☐☐	☐☐☐ *days*	☐ *Yes* ☐ *No* ***(Skip to 2nd Hunt Area)***	☐ *Male* ☐ *Female*	☐ *Rifle or Pistol* ☐ *Muzzleloader* ☐ *Archery*
2nd Hunt Area hunted: ☐☐☐	☐☐☐ *days*	☐ *Yes* ☐ *No* ***(Skip to 3rd Hunt Area)***	☐ *Male* ☐ *Female*	☐ *Rifle or Pistol* ☐ *Muzzleloader* ☐ *Archery*
3rd Hunt Area hunted: ☐☐☐	☐☐☐ *days*	☐ *Yes* ☐ *No* ***(Skip to 4th Hunt Area)***	☐ *Male* ☐ *Female*	☐ *Rifle or Pistol* ☐ *Muzzleloader* ☐ *Archery*
4th Hunt Area hunted: ☐☐☐	☐☐☐ *days*	☐ *Yes* ☐ *No* ***(Skip to 5th Hunt Area)***	☐ *Male* ☐ *Female*	☐ *Rifle or Pistol* ☐ *Muzzleloader* ☐ *Archery*
5th Hunt Area hunted: ☐☐☐	☐☐☐ *days*	☐ *Yes* ☐ *No* ***(Skip to Q13)***	☐ *Male* ☐ *Female*	☐ *Rifle or Pistol* ☐ *Muzzleloader* ☐ *Archery*

****NOTE:** Hunt Area map can be found on the back of the survey cover letter*
*****NOTE:** Please count each partial day of hunting as 1 day of Mountain Lion hunting*

2093478227

2008 MOUNTAIN LION LICENSE HOLDERS

Q13. Did you purchase a 2008 Mountain Lion license? *(Check one box)*

☐ *Yes* ☐ *No* ***(Skip to Q19)***

Q14. Did you hunt Mountain Lion in Wyoming between January 1, 2008 and March 31, 2008? *(Check one box)*

☐ *Yes* ☐ *No* ***(IF YOU DID NOT HUNT MOUNTAIN LION DURING THIS TIME, SKIP TO Q17)***

Q15. How many different days did you hunt Mountain Lion between January 1, 2008 and March 31, 2008? ***(NOTE:** Count each partial day of hunting as 1 day of Mountain Lion hunting)* *(Enter number in space below)*

☐☐☐ *Number of different days spent hunting Mountain Lion*

Q16. Please tell us about your Mountain Lion hunting effort between January 1, 2008 and March 31, 2008. (Fill in table below for each Hunt Area hunted.)

*Number of the Hunt Area(s) where you hunted Mountain Lion during this period (hunt area numbers range from 1 to 31)**	*Number of different days you hunted Mountain Lion in this Hunt Area during this period** (Enter number below)*	*Did you harvest a Mountain Lion during this period in this Hunt Area? (Check one box)*	*Sex of Mountain Lion harvested in this Hunt Area (Check one box)*	Weapon used to harvest this Mountain Lion *(Check one box)*
Main Hunt Area hunted: ☐☐☐	☐☐☐ *days*	☐ *Yes* ☐ *No* ***(Skip to 2nd Hunt Area)***	☐ *Male* ☐ *Female*	☐ *Rifle or Pistol* ☐ *Muzzleloader* ☐ *Archery*
2nd Hunt Area hunted: ☐☐☐	☐☐☐ *days*	☐ *Yes* ☐ *No* ***(Skip to 3rd Hunt Area)***	☐ *Male* ☐ *Female*	☐ *Rifle or Pistol* ☐ *Muzzleloader* ☐ *Archery*
3rd Hunt Area hunted: ☐☐☐	☐☐☐ *days*	☐ *Yes* ☐ *No* ***(Skip to 4th Hunt Area)***	☐ *Male* ☐ *Female*	☐ *Rifle or Pistol* ☐ *Muzzleloader* ☐ *Archery*
4th Hunt Area hunted: ☐☐☐	☐☐☐ *days*	☐ *Yes* ☐ *No* ***(Skip to 5th Hunt Area)***	☐ *Male* ☐ *Female*	☐ *Rifle or Pistol* ☐ *Muzzleloader* ☐ *Archery*
5th Hunt Area hunted: ☐☐☐	☐☐☐ *days*	☐ *Yes* ☐ *No* ***(Skip to Q17)***	☐ *Male* ☐ *Female*	☐ *Rifle or Pistol* ☐ *Muzzleloader* ☐ *Archery*

***NOTE:** Hunt Area map can be found on the back of the survey cover letter*
*****NOTE:** Please count each partial day of hunting as 1 day of Mountain Lion hunting*

OVER →

Q17. If you have not yet hunted or harvested, do you still plan to hunt Mountain Lion between April 1, 2008 and August 31, 2008? *(Check one box)*

☐ *Yes* ☐ *No* ***(Skip to Q19)***

Q18. Approximately how many days do you plan to hunt Mountain Lion between April 1, 2008 and August 31, 2008?

☐☐☐ *Number of different days spent hunting Mountain Lion*

OTHER BIG GAME LICENSES

Q19. What other 2007 Wyoming big game licenses did you hold? *(Check all that apply)*

☐ *Pronghorn* ☐ *Deer* ☐ *Elk* ☐ *Black Bear* ☐ *No other big game license*

Thank you for your help with this important study!

Feel free to attach a sheet of paper with any comments you would like to share with the Wyoming Game and Fish Department. **Please include the hunt area as well as the species hunted with your comments.** These comments along with your contact information will be sent directly to the Wyoming Game and Fish Department. *(**NOTE:** individual responses to survey questions are confidential and will not be shared with the Wyoming Game and Fish Department)*

Please return your completed survey in the enclosed, postage-paid envelope to:
Wyoming Big Game Hunter Survey, c/o PA Consulting Group, 6410 Enterprise Lane, Suite 300, Madison WI 53719

Did you know.....

(1) The Wyoming Game and Fish Department offers mountain lion education and identification information and an exam to aid hunters, guides, outfitters, houndsmen, and all others in correctly identifying male and female mountain lions in the field. The information and exam can be found at: http://gf.state.wy.us/mountainlion/index.asp

(2) The Wyoming Game and Fish Department is managing Mountain Lion according to Mountain Lion Management plan. The Final Mountain Lion Management Plan released by Wyoming Game and Fish can be found at: http://gf.state.wy.us/wildlife/MoutainLionPlan/index.asp

2701478223

2007

password: 54000074

WGFD MOOSE HARVEST SURVEY

Dear Hunter, We are asking you to participate in the 2007 Wyoming Game and Fish Department Moose Harvest Survey. Our biologists use the results of this survey to monitor populations and recommend hunting seasons. Your responses are confidential and are very important **even if you did not hunt or were unsuccessful.**

You may complete this survey in one of two ways: via the internet go to **http://gf.state.wy.us/HarvestSurvey/** and use the **Hunter Key** as it appears on the top of the attached survey postcard OR complete the survey postcard, detach it and mail it back to us, the postage is paid.

All surveys will be destroyed after the harvest survey has been completed. We appreciate your assistance with the survey and thank you for your help.

MOOSE HUNT AREA MAP

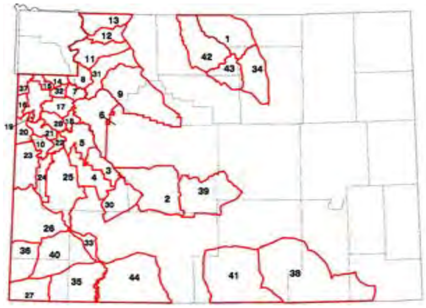

NO POSTAGE NECESSARY IF MAILED IN THE UNITED STATES

BUSINESS REPLY MAIL
FIRST CLASS PERMIT NO. 661 CHEYENNE, WY

POSTAGE WILL BE PAID BY ADDRESSEE

WYOMING GAME & FISH DEPARTMENT
BIOLOGICAL SERVICES
5400 BISHOP BLVD
CHEYENNE WY 82010-0023

Wyoming Game and Fish Department
Biological Services
5400 Bishop Blvd
Cheyenne, WY 82006-0001

First Class
US Postage Paid
Cheyenne, WY
Permit #196

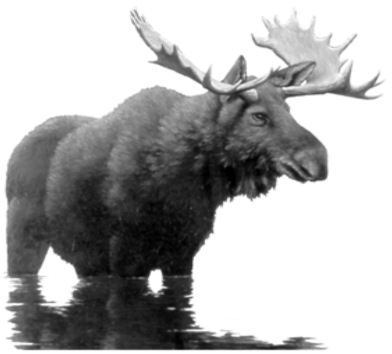

HUNTER KEY

Did you hunt moose? ☐yes ☐no (if "no" - skip to comments section below)

hunt area you hunted (see map)	total # of days hunting moose	Did you harvest a moose?	harvested	weapon used to harvest	date of harvest
______	______	☐yes ☐no	☐bull ☐cow ☐calf	☐firearm ☐archery	______
Enter # of days hunted with each weapon type	firearm	compound bow	crossbow	recurved bow	long bow

Approximately, how many moose did you see during your hunt? ☐

COMMENTS: ______

WGFD BIGHORN SHEEP HARVEST SURVEY

Dear Hunter, We are asking you to participate in the 2007 Wyoming Game and Fish Department BighornSheep Harvest Survey. Our biologists use the results of this survey to monitor populations and recommend hunting seasons. Your responses are confidential and are very important **even if you registered your sheep, did not hunt or were unsuccessful.**

You may complete this survey in one of two ways: via the internet go to http://gf.state.wy.us/HarvestSurvey/ and use the **Hunter Key** as it appears on the top of the attached survey postcard OR complete the survey postcard, detach it and mail it back to us, the postage is paid.

All surveys will be destroyed after the harvest survey has been completed. We appreciate your assistance with the survey and thank you for your help.

BIGHORN SHEEP HUNT AREA MAP

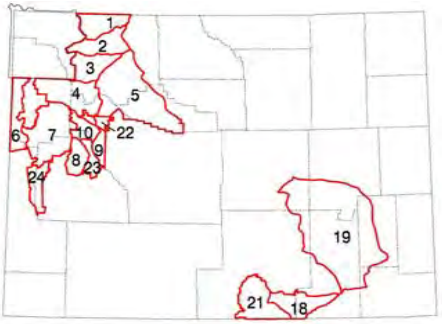

Wyoming Game and Fish Department
Biological Services
5400 Bishop Blvd
Cheyenne, WY 82006-0001

First Class
US Postage Paid
Cheyenne, WY
Permit #196

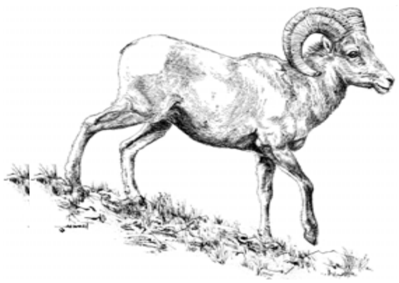

HUNTER KEY

Did you hunt bighorn sheep? ☐yes ☐no (if "no" - skip to comments section below)

hunt area you hunted (see map)	total # of days hunting sheep	Did you harvest a sheep?	harvested	weapon used to harvest	date of harvest
____	____	☐yes ☐no	☐ram ☐ewe ☐lamb	☐firearm ☐archery	____
Enter # of days hunted with each weapon type	firearm	compound bow	crossbow	recurved bow	long bow

Approximately, how many sheep did you see during your hunt? ☐

COMMENTS: ____

NO POSTAGE NECESSARY IF MAILED IN THE UNITED STATES

BUSINESS REPLY MAIL
FIRST CLASS PERMIT NO. 661 CHEYENNE, WY

POSTAGE WILL BE PAID BY ADDRESSEE

WYOMING GAME & FISH DEPARTMENT
BIOLOGICAL SERVICES
5400 BISHOP BLVD
CHEYENNE WY 82010-0023

TABLE I

ANTELOPE

HARVEST STATEWIDE

BY TOTAL HUNTERS

2007

	LICENSES SOLD	NUMBER ACTIVE HUNTERS	HARVEST BUCK	DOE	FAWN	TOTAL	TOTAL RECREATION DAYS	HUNTER SUCCESS	DAYS/ HARVEST
STATE TOTAL	65,397	52,744	30,345	19,076	2,462	51,883	169,419	98.4%	3.3
RESIDENT	25,377	20,269	13,101	5,058	609	18,768	66,858	92.6%	3.6
NONRESIDENT	40,020	32,475	17,244	14,018	1,853	33,115	102,561	102.0%	3.1

TABLE II

ANTELOPE

TEN-YEAR COMPARISON OF HARVEST BY TOTAL HUNTERS

1998–2007

YEAR	1998	1999	2000	2001	2002	2003	2004	2005	2006	2007
HARVEST	28,138	30,694	33,977	26,864	30,260	34,393	36,383	39,526	45,615	51,883

ANTELOPE

HARVEST BY HARVEST TOOL

2007

ARCHERY	FIREARM	MUZZLE LOADER
4.81%	94.44%	0.75%

TABLE I-A

ANTELOPE

HARVEST, HUNTING PRESSURE, HUNTER SUCCESS

BY HUNT AREA

2007

AREA (cross hunt areas)	TYPE	ACTIVE LICS/HTRS	HARVEST BUCK	DOE	FAWN	TOTAL	HUNTER SUCCESS	DAYS/ HARVEST	HUNTER DAYS	LICENSES SOLD (cross hunt incl)
1 Crook										
	Limited 1	258	207	9	0	216	83.7%	3.5	750	289
	D/F 6	113	0	65	9	74	65.5%	4.3	317	119
Pooled Total		328	207	74	9	290	88.4%	3.7	1067	
Pooled Resident		39	12	8	0	20	51.3%	6.7	133	
Pooled Nonresident		289	195	66	9	270	93.4%	3.5	934	
2 Bearlodge										
	Limited 1	337	282	9	5	296	87.8%	3.9	1147	351
	D/F 6	214	0	137	28	165	77.1%	4.2	698	239
Pooled Total		488	282	146	33	461	94.5%	4.0	1845	
Pooled Resident		83	54	20	4	78	94.0%	7.3	570	
Pooled Nonresident		405	228	126	29	383	94.6%	3.3	1275	
3 Keyhole										
	Limited 1	215	196	11	0	207	96.3%	2.8	589	237
	D/F 6	152	0	131	12	143	94.1%	3.3	472	167
Pooled Total		295	196	142	12	350	118.6%	3.0	1061	
Pooled Resident		86	43	46	4	93	108.1%	4.6	425	
Pooled Nonresident		209	153	96	8	257	123.0%	2.5	636	
4 Arch Creek										
	Limited 1	221	201	4	0	205	92.8%	2.2	459	229
	D/F 6	124	0	84	11	95	76.6%	3.3	315	135
Pooled Total		273	201	88	11	300	109.9%	2.6	774	
Pooled Resident		70	48	22	3	73	104.3%	2.0	146	
Pooled Nonresident		203	153	66	8	227	111.8%	2.8	628	
5 Inyan Kara										
	Limited 1	270	238	0	0	238	88.1%	3.6	854	302
	D/F 6	201	0	134	26	160	79.6%	4.4	698	213
Pooled Total		418	238	134	26	398	95.2%	3.9	1552	
Pooled Resident		171	111	20	0	131	76.6%	6.5	846	
Pooled Nonresident		247	127	114	26	267	108.1%	2.6	706	
6 South Newcastle										
	Limited 1	326	295	5	0	300	92.0%	2.6	784	339
	D/F 6	126	0	106	7	113	89.7%	2.2	245	134
Pooled Total		412	295	111	7	413	100.2%	2.5	1029	
Pooled Resident		79	37	35	0	72	91.1%	2.3	167	
Pooled Nonresident		333	258	76	7	341	102.4%	2.5	862	
7 South Upton										
	Limited 1	859	785	0	0	785	91.4%	3.1	2417	859
	D/F 6	513	0	416	28	444	86.5%	3.1	1370	559
Pooled Total		1228	785	416	28	1229	100.1%	3.1	3787	
Pooled Resident		263	166	64	0	230	87.5%	2.9	664	
Pooled Nonresident		965	619	352	28	999	103.5%	3.1	3123	
8 Niobrara										
	Limited 1	279	269	0	0	269	96.4%	3.3	877	301
	D/F 6	47	0	34	9	43	91.5%	3.8	163	50
Pooled Total		308	269	34	9	312	101.3%	3.3	1040	
Pooled Resident		19	13	6	0	19	100.0%	2.7	51	
Pooled Nonresident		289	256	28	9	293	101.4%	3.4	989	
9 Lance Creek										
	Limited 1	552	468	32	0	500	90.6%	3.1	1566	552
	D/F 6	400	0	315	26	341	85.3%	3.6	1242	400
Pooled Total		857	468	347	26	841	98.1%	3.3	2808	
Pooled Resident		104	57	38	0	95	91.3%	2.6	245	
Pooled Nonresident		753	411	309	26	746	99.1%	3.4	2563	

TABLE I-A (Continued)

ANTELOPE HUNT AREA REPORT

AREA (cross hunt areas)	TYPE	ACTIVE LICS/HTRS	HARVEST BUCK	DOE	FAWN	TOTAL	HUNTER SUCCESS	DAYS/ HARVEST	HUNTER DAYS	LICENSES SOLD (cross hunt incl)
10 Lake De Smet										
	Limited 1	161	136	0	0	136	84.5%	3.8	520	185
	D/F 6	152	0	130	10	140	92.1%	4.0	562	179
Pooled Total		219	136	130	10	276	126.0%	3.9	1082	
Pooled Resident		66	40	38	0	78	118.2%	6.6	511	
Pooled Nonresident		153	96	92	10	198	129.4%	2.9	571	
11 Rawhide Butte										
	Limited 1	235	214	9	0	223	94.9%	3.8	854	252
	D/F 6	262	0	208	5	213	81.3%	4.6	970	300
Pooled Total		443	214	217	5	436	98.4%	4.2	1824	
Pooled Resident		129	81	63	5	149	115.5%	5.6	827	
Pooled Nonresident		314	133	154	0	287	91.4%	3.5	997	
12 Meadowdale										
	Limited 1	188	180	0	0	180	95.7%	2.5	453	201
	D/F 6	142	0	121	13	134	94.4%	3.0	396	153
Pooled Total		280	180	121	13	314	112.1%	2.7	849	
Pooled Resident		42	27	10	0	37	88.1%	3.9	143	
Pooled Nonresident		238	153	111	13	277	116.4%	2.5	706	
15 Clearmont										
	Limited 1	523	409	10	0	419	80.1%	5.0	2083	589
	D/F 6	365	0	225	35	260	71.2%	4.8	1241	423
Pooled Total		786	409	235	35	679	86.4%	4.9	3324	
Pooled Resident		217	144	39	15	198	91.2%	5.4	1062	
Pooled Nonresident		569	265	196	20	481	84.5%	4.7	2262	
16 Ucross										
	Limited 1	372	324	0	0	324	87.1%	2.9	933	382
	D/F 6	119	0	95	6	101	84.9%	3.4	341	128
Pooled Total		419	324	95	6	425	101.4%	3.0	1274	
Pooled Resident		96	53	32	0	85	88.5%	2.3	197	
Pooled Nonresident		323	271	63	6	340	105.3%	3.2	1077	
17 Gillette										
	Limited 1	976	948	0	0	948	97.1%	3.2	3071	1049
**(18, 19)**	D/F 6	585	0	533	0	533	91.1%	2.9	1556	902
Pooled Total		1410	948	533	0	1481	105.0%	3.1	4627	
Pooled Resident		456	279	190	0	469	102.9%	3.1	1447	
Pooled Nonresident		954	669	343	0	1012	106.1%	3.1	3180	
18 Rockypoint										
	Limited 1	316	240	8	0	248	78.5%	3.3	806	330
**(17, 19)**	D/F 6	82	0	71	0	71	86.6%	2.5	175	902
Pooled Total		398	240	79	0	319	80.2%	3.1	981	
Pooled Resident		80	28	14	0	42	52.5%	4.3	179	
Pooled Nonresident		318	212	65	0	277	87.1%	2.9	802	
19 Rozet										
	Limited 1	365	326	6	0	332	91.0%	3.0	1009	391
**(17, 18)**	D/F 6	178	0	156	11	167	93.8%	2.0	331	902
Pooled Total		512	326	162	11	499	97.5%	2.7	1340	
Pooled Resident		137	96	41	0	137	100.0%	2.5	336	
Pooled Nonresident		375	230	121	11	362	96.5%	2.8	1004	
20 Upper Powder River										
	Limited 1	491	375	8	8	391	79.6%	3.4	1334	538
	D/F 6	347	0	242	33	275	79.3%	4.2	1152	380
Pooled Total		758	375	250	41	666	87.9%	3.7	2486	
Pooled Resident		115	62	19	11	92	80.0%	3.9	363	
Pooled Nonresident		643	313	231	30	574	89.3%	3.7	2123	

TABLE I-A (Continued)

ANTELOPE HUNT AREA REPORT

AREA (cross hunt areas)	TYPE	ACTIVE LICS/HTRS	HARVEST BUCK	HARVEST DOE	HARVEST FAWN	HARVEST TOTAL	HUNTER SUCCESS	DAYS/ HARVEST	HUNTER DAYS	LICENSES SOLD (cross hunt incl)
21 Middle Fork										
	Limited 1	438	363	32	6	401	91.6%	2.7	1069	457
	D/F 6	330	0	231	29	260	78.8%	3.4	878	361
Pooled Total		725	363	263	35	661	91.2%	2.9	1947	
Pooled Resident		66	56	5	5	66	100.0%	1.8	118	
Pooled Nonresident		659	307	258	30	595	90.3%	3.1	1829	
22 Crazy Woman										
	Limited 1	477	462	0	0	462	96.9%	2.2	1025	528
	D/F 6	356	0	299	26	325	91.3%	2.4	780	371
Pooled Total		737	462	299	26	787	106.8%	2.3	1805	
Pooled Resident		113	92	38	4	134	118.6%	1.6	220	
Pooled Nonresident		624	370	261	22	653	104.6%	2.4	1585	
23 Pumpkin Buttes										
	Limited 1	1606	1452	0	0	1452	90.4%	2.9	4159	1648
	D/F 6	965	0	870	52	922	95.5%	3.1	2836	993
Pooled Total		2424	1452	870	52	2374	97.9%	2.9	6995	
Pooled Resident		378	255	119	24	398	105.3%	2.6	1022	
Pooled Nonresident		2046	1197	751	28	1976	96.6%	3.0	5973	
24 Thunder Basin										
	Limited 1	624	565	10	0	575	92.1%	3.3	1900	707
	D/F 6	489	0	472	9	481	98.4%	2.2	1064	581
Pooled Total		1027	565	482	9	1056	102.8%	2.8	2964	
Pooled Resident		245	150	79	0	229	93.5%	4.7	1067	
Pooled Nonresident		782	415	403	9	827	105.8%	2.3	1897	
25 Ormsby										
	Limited 1	543	444	34	0	478	88.0%	3.5	1651	581
	D/F 6	231	0	119	44	163	70.6%	4.6	757	256
Pooled Total		693	444	153	44	641	92.5%	3.8	2408	
Pooled Resident		266	146	76	19	241	90.6%	4.7	1140	
Pooled Nonresident		427	298	77	25	400	93.7%	3.2	1268	
26 Bear Creek										
**(also valid 28)**	Limited 1	845	820	12	0	832	98.5%	2.4	1989	926
**(also valid 28)**	D/F 6	436	0	381	35	416	95.4%	2.9	1202	481
Pooled Total		1167	820	393	35	1248	106.9%	2.6	3191	
Pooled Resident		106	93	12	0	105	99.1%	2.6	274	
Pooled Nonresident		1061	727	381	35	1143	107.7%	2.6	2917	
27 Bill										
	Limited 1	422	357	0	0	357	84.6%	3.1	1122	453
	D/F 6	216	0	174	29	203	94.0%	2.5	504	256
Pooled Total		600	357	174	29	560	93.3%	2.9	1626	
Pooled Resident		327	242	24	5	271	82.9%	3.1	840	
Pooled Nonresident		273	115	150	24	289	105.9%	2.7	786	
28 Sage Creek										
**(also valid 26)**	Limited 1	237	222	0	0	222	93.7%	2.6	583	241
**(also valid 26)**	D/F 6	180	0	162	5	167	92.8%	3.0	509	205
Pooled Total		335	222	162	5	389	116.1%	2.8	1092	
Pooled Resident		33	26	0	0	26	78.8%	2.8	74	
Pooled Nonresident		302	196	162	5	363	120.2%	2.8	1018	
29 Shawnee										
	Limited 1	1174	1078	17	0	1095	93.3%	3.1	3393	1204
	D/F 6	761	0	614	86	700	92.0%	4.1	2889	786
	D/F 7	77	0	64	5	69	89.6%	3.7	252	77
Pooled Total		1916	1078	695	91	1864	97.3%	3.5	6534	
Pooled Resident		255	175	80	0	255	100.0%	5.2	1325	
Pooled Nonresident		1661	903	615	91	1609	96.9%	3.2	5209	

TABLE I-A (Continued)

ANTELOPE HUNT AREA REPORT

AREA (cross hunt areas)	TYPE	ACTIVE LICS/HTRS	HARVEST BUCK	DOE	FAWN	TOTAL	HUNTER SUCCESS	DAYS/ HARVEST	HUNTER DAYS	LICENSES SOLD (cross hunt incl)
30 La Prele										
	Limited 1	567	455	9	0	464	81.8%	3.3	1531	603
	D/F 6	559	0	406	57	463	82.8%	3.6	1659	596
Pooled Total		1017	455	415	57	927	91.2%	3.4	3190	
Pooled Resident		240	126	61	20	207	86.3%	3.8	794	
Pooled Nonresident		777	329	354	37	720	92.7%	3.3	2396	
31 Hat Six										
	Limited 1	364	336	0	6	342	94.0%	3.0	1043	402
	D/F 6	387	0	265	89	354	91.5%	3.8	1357	448
Pooled Total		607	336	265	95	696	114.7%	3.4	2400	
Pooled Resident		137	83	54	17	154	112.4%	3.8	588	
Pooled Nonresident		470	253	211	78	542	115.3%	3.3	1812	
32 Bates Creek										
	Limited 1	405	303	15	0	318	78.5%	3.1	980	454
	Limited 2	235	191	21	0	212	90.2%	3.8	815	252
	D/F 6	592	0	420	116	536	90.5%	3.4	1809	650
Pooled Total		1160	494	456	116	1066	91.9%	3.4	3604	
Pooled Resident		216	104	46	0	150	69.4%	3.9	592	
Pooled Nonresident		944	390	410	116	916	97.0%	3.3	3012	
34 Rock Eagle										
	Limited 1	219	149	19	0	168	76.7%	4.4	737	226
	D/F 6	157	0	115	12	127	80.9%	3.8	477	200
Pooled Total		348	149	134	12	295	84.8%	4.1	1214	
Pooled Resident		210	120	64	0	184	87.6%	4.6	850	
Pooled Nonresident		138	29	70	12	111	80.4%	3.3	364	
35 Horse Creek										
	Limited 1	279	199	13	6	218	78.1%	3.4	747	301
	D/F 6	179	0	105	32	137	76.5%	3.9	537	207
Pooled Total		401	199	118	38	355	88.5%	3.6	1284	
Pooled Resident		151	58	34	20	112	74.2%	3.7	409	
Pooled Nonresident		250	141	84	18	243	97.2%	3.6	875	
36 Burns										
	Limited 1	186	116	16	0	132	71.0%	4.8	629	201
	D/F 6	167	0	91	31	122	73.1%	4.2	507	199
Pooled Total		317	116	107	31	254	80.1%	4.5	1136	
Pooled Resident		197	89	58	16	163	82.7%	4.3	707	
Pooled Nonresident		120	27	49	15	91	75.8%	4.7	429	
37 Harriman										
	Limited 1	261	210	10	5	225	86.2%	2.5	569	276
	D/F 6	65	0	45	3	48	73.8%	4.1	195	75
Pooled Total		278	210	55	8	273	98.2%	2.8	764	
Pooled Resident		170	117	20	6	143	84.1%	2.9	418	
Pooled Nonresident		108	93	35	2	130	120.4%	2.7	346	
38 Little Bear Creek										
	Limited 1	491	371	11	10	392	79.8%	4.2	1641	551
	D/F 6	410	0	335	0	335	81.7%	3.7	1236	446
Pooled Total		784	371	346	10	727	92.7%	4.0	2877	
Pooled Resident		270	128	92	0	220	81.5%	5.1	1124	
Pooled Nonresident		514	243	254	10	507	98.6%	3.5	1753	
39 Bluegrass										
	Limited 1	287	249	6	0	255	88.9%	3.1	780	300
	D/F 6	87	0	60	2	62	71.3%	4.2	263	100
Pooled Total		349	249	66	2	317	90.8%	3.3	1043	
Pooled Resident		141	98	22	0	120	85.1%	3.7	442	
Pooled Nonresident		208	151	44	2	197	94.7%	3.1	601	

TABLE I-A (Continued)

ANTELOPE HUNT AREA REPORT

AREA (cross hunt areas)	TYPE	ACTIVE LICS/HTRS	HARVEST BUCK	HARVEST DOE	HARVEST FAWN	HARVEST TOTAL	HUNTER SUCCESS	DAYS/ HARVEST	HUNTER DAYS	LICENSES SOLD (cross hunt incl)
40 East Laramie										
	Limited 1	194	168	6	0	174	89.7%	2.8	483	207
	D/F 6	196	0	144	33	177	90.3%	2.1	373	203
Pooled Total		300	168	150	33	351	117.0%	2.4	856	
Pooled Resident		87	60	25	0	85	97.7%	3.1	261	
Pooled Nonresident		213	108	125	33	266	124.9%	2.2	595	
41 Laramie Peak										
	Limited 1	50	44	0	0	44	88.0%	2.3	101	58
	D/F 6	46	0	30	2	32	69.6%	3.7	117	50
Pooled Total		75	44	30	2	76	101.3%	2.9	218	
Pooled Resident		28	23	0	0	23	82.1%	2.5	58	
Pooled Nonresident		47	21	30	2	53	112.8%	3.0	160	
42 Laramie Plains										
	Limited 1	605	529	0	0	529	87.4%	3.3	1747	650
	D/F 6	262	0	169	38	207	79.0%	4.0	818	300
	D/F 7	175	0	126	14	140	80.0%	4.8	674	200
Pooled Total		907	529	295	52	876	96.6%	3.7	3239	
Pooled Resident		141	100	25	0	125	88.7%	3.2	398	
Pooled Nonresident		766	429	270	52	751	98.0%	3.8	2841	
43 Cooper Lake										
	Limited 1	382	325	0	0	325	85.1%	3.5	1124	402
	D/F 6	338	0	206	48	254	75.1%	3.4	875	400
Pooled Total		672	325	206	48	579	86.2%	3.5	1999	
Pooled Resident		121	54	18	5	77	63.6%	5.5	425	
Pooled Nonresident		551	271	188	43	502	91.1%	3.1	1574	
44 South Laramie										
	Limited 1	244	205	12	0	217	88.9%	3.3	719	258
	D/F 6	262	0	147	37	184	70.2%	5.6	1034	298
Pooled Total		420	205	159	37	401	95.5%	4.4	1753	
Pooled Resident		125	73	23	11	107	85.6%	5.2	558	
Pooled Nonresident		295	132	136	26	294	99.7%	4.1	1195	
45 West Laramie										
	Limited 1	567	467	0	10	477	84.1%	3.1	1494	657
	D/F 6	510	0	362	25	387	75.9%	4.7	1833	600
Pooled Total		999	467	362	35	864	86.5%	3.9	3327	
Pooled Resident		327	150	74	0	224	68.5%	5.1	1135	
Pooled Nonresident		672	317	288	35	640	95.2%	3.4	2192	
46 Kyle										
	Limited 1	455	363	7	0	370	81.3%	3.0	1118	504
	D/F 6	269	0	188	34	222	82.5%	2.9	633	311
	D/F 7	172	0	112	19	131	76.2%	3.8	500	200
Pooled Total		789	363	307	53	723	91.6%	3.1	2251	
Pooled Resident		219	123	48	0	171	78.1%	3.4	577	
Pooled Nonresident		570	240	259	53	552	96.8%	3.0	1674	
47 Shirley Basin										
	Limited 1	941	714	15	15	744	79.1%	3.1	2282	1006
	Limited 2	336	310	9	0	319	94.9%	2.7	851	350
	D/F 6	607	0	449	82	531	87.5%	3.1	1670	706
	D/F 7	359	0	249	60	309	86.1%	3.2	997	400
Pooled Total		2144	1024	722	157	1903	88.8%	3.0	5800	
Pooled Resident		980	604	200	19	823	84.0%	3.2	2622	
Pooled Nonresident		1164	420	522	138	1080	92.8%	2.9	3178	

TABLE I-A (Continued)
ANTELOPE HUNT AREA REPORT

AREA (cross hunt areas)	TYPE	ACTIVE LICS/HTRS	HARVEST BUCK	HARVEST DOE	HARVEST FAWN	HARVEST TOTAL	HUNTER SUCCESS	DAYS/ HARVEST	HUNTER DAYS	LICENSES SOLD (cross hunt incl)
48 Leo-Hanna										
	Limited 1	307	246	17	0	263	85.7%	2.6	685	356
	Limited 2	141	127	0	2	129	91.5%	2.9	376	156
	D/F 6	273	0	217	16	233	85.3%	3.2	746	375
	D/F 7	399	0	271	30	301	75.4%	3.4	1015	476
Pooled Total		997	373	505	48	926	92.9%	3.0	2822	
Pooled Resident		303	209	50	0	259	85.5%	2.7	695	
Pooled Nonresident		694	164	455	48	667	96.1%	3.2	2127	
50 Elk Mountain										
	Limited 1	470	412	0	0	412	87.7%	3.6	1480	506
	D/F 6	466	0	363	41	404	86.7%	3.3	1342	500
	Limited 8	48	39	0	0	39	81.3%	3.7	144	51
Pooled Total		908	451	363	41	855	94.2%	3.5	2966	
Pooled Resident		162	90	47	0	137	84.6%	4.0	551	
Pooled Nonresident		746	361	316	41	718	96.2%	3.4	2415	
51 Big Creek										
	Limited 1	96	77	4	0	81	84.4%	4.1	329	104
	D/F 6	125	0	101	8	109	87.2%	3.5	382	150
Pooled Total		185	77	105	8	190	102.7%	3.7	711	
Pooled Resident		99	58	33	0	91	91.9%	4.2	378	
Pooled Nonresident		86	19	72	8	99	115.1%	3.4	333	
52 Spring Creek										
	Limited 1	235	206	0	0	206	87.7%	2.6	544	254
	Limited 2	239	203	0	0	203	84.9%	3.5	709	250
	D/F 6	152	0	131	14	145	95.4%	2.4	341	202
	D/F 7	85	0	60	14	74	87.1%	4.3	316	101
Pooled Total		636	409	191	28	628	98.7%	3.0	1910	
Pooled Resident		263	209	36	0	245	93.2%	2.9	722	
Pooled Nonresident		373	200	155	28	383	102.7%	3.1	1188	
53 Baggs										
	Limited 1	531	444	0	0	444	83.6%	4.0	1767	556
	D/F 6	523	0	376	58	434	83.0%	3.1	1328	751
	D/F 7	20	0	16	2	18	90.0%	5.7	102	25
Pooled Total		981	444	392	60	896	91.3%	3.6	3197	
Pooled Resident		487	360	55	2	417	85.6%	4.0	1651	
Pooled Nonresident		494	84	337	58	479	97.0%	3.2	1546	
55 Red Rim										
	Limited 1	131	106	0	0	106	80.9%	3.6	383	152
	D/F 6	78	0	71	2	73	93.6%	2.2	161	100
Pooled Total		181	106	71	2	179	98.9%	3.0	544	
Pooled Resident		116	80	14	0	94	81.0%	4.1	384	
Pooled Nonresident		65	26	57	2	85	130.8%	1.9	160	
56 Iron Springs										
	Limited 1	177	164	0	0	164	92.7%	2.8	456	201
Pooled Total		177	164	0	0	164	92.7%	2.8	456	
Pooled Resident		25	25	0	0	25	100.0%	1.4	35	
Pooled Nonresident		152	139	0	0	139	91.4%	3.0	421	
57 South Wamsutter										
	Limited 1	283	229	0	0	229	80.9%	3.6	833	302
	D/F 6	28	0	21	7	28	100.0%	4.7	132	28
Pooled Total		301	229	21	7	257	85.4%	3.8	965	
Pooled Resident		241	175	13	7	195	80.9%	4.2	811	
Pooled Nonresident		60	54	8	0	62	103.3%	2.5	154	
58 Black Butte-Kenny Rim										
	Limited 1	140	118	0	0	118	84.3%	2.5	296	152
Pooled Total		140	118	0	0	118	84.3%	2.5	296	
Pooled Resident		110	88	0	0	88	80.0%	2.7	234	
Pooled Nonresident		30	30	0	0	30	100.0%	2.1	62	

TABLE I-A (Continued)

ANTELOPE HUNT AREA REPORT

AREA (cross hunt areas)	TYPE	ACTIVE LICS/HTRS	HARVEST BUCK	HARVEST DOE	HARVEST FAWN	HARVEST TOTAL	HUNTER SUCCESS	DAYS/ HARVEST	HUNTER DAYS	LICENSES SOLD (cross hunt incl)
59 Aspen Mountain										
	Limited 1	173	148	0	0	148	85.5%	3.0	451	177
	D/F 6	114	0	103	7	110	96.5%	1.5	170	128
Pooled Total		255	148	103	7	258	101.2%	2.4	621	
Pooled Resident		191	116	61	3	180	94.2%	2.5	454	
Pooled Nonresident		64	32	42	4	78	121.9%	2.1	167	
60 Table Rock										
	Limited 1	99	89	0	0	89	89.9%	4.3	386	100
Pooled Total		99	89	0	0	89	89.9%	4.3	386	
Pooled Resident		80	70	0	0	70	87.5%	4.4	307	
Pooled Nonresident		19	19	0	0	19	100.0%	4.2	79	
61 Chain Lakes										
	Limited 1	135	115	0	0	115	85.2%	3.0	344	154
Pooled Total		135	115	0	0	115	85.2%	3.0	344	
Pooled Resident		104	84	0	0	84	80.8%	3.2	269	
Pooled Nonresident		31	31	0	0	31	100.0%	2.4	75	
62 South Ferris										
	Limited 1	359	305	0	0	305	85.0%	3.6	1088	407
Pooled Total		359	305	0	0	305	85.0%	3.6	1088	
Pooled Resident		274	226	0	0	226	82.5%	3.8	850	
Pooled Nonresident		85	79	0	0	79	92.9%	3.0	238	
63 North Ferris										
	Limited 1	385	310	6	0	316	82.1%	2.6	808	404
	D/F 6	120	0	102	5	107	89.2%	3.0	324	150
Pooled Total		471	310	108	5	423	89.8%	2.7	1132	
Pooled Resident		329	226	42	0	268	81.5%	2.4	646	
Pooled Nonresident		142	84	66	5	155	109.2%	3.1	486	
64 Bison Basin										
	Limited 1	236	196	0	0	196	83.1%	3.7	721	251
Pooled Total		236	196	0	0	196	83.1%	3.7	721	
Pooled Resident		185	145	0	0	145	78.4%	4.2	607	
Pooled Nonresident		51	51	0	0	51	100.0%	2.2	114	
65 South Pass										
	Limited 1	259	238	0	0	238	91.9%	4.5	1076	273
	D/F 6	222	0	176	9	185	83.3%	4.0	744	252
Pooled Total		440	238	176	9	423	96.1%	4.3	1820	
Pooled Resident		288	172	90	5	267	92.7%	4.2	1111	
Pooled Nonresident		152	66	86	4	156	102.6%	4.5	709	
66 Lander										
	Limited 1	81	66	0	0	66	81.5%	4.6	302	83
	D/F 6	61	0	49	0	49	80.3%	5.6	272	74
Pooled Total		118	66	49	0	115	97.5%	5.0	574	
Pooled Resident		86	49	39	0	88	102.3%	5.6	489	
Pooled Nonresident		32	17	10	0	27	84.4%	3.1	85	
67 Sand Draw										
	Limited 1	208	193	0	0	193	92.8%	2.3	450	222
	D/F 6	98	0	74	6	80	81.6%	3.1	245	125
Pooled Total		272	193	74	6	273	100.4%	2.5	695	
Pooled Resident		198	145	44	3	192	97.0%	2.6	490	
Pooled Nonresident		74	48	30	3	81	109.5%	2.5	205	

TABLE I-A (Continued)

ANTELOPE HUNT AREA REPORT

AREA (cross hunt areas)	TYPE	ACTIVE LICS/HTRS	HARVEST BUCK	HARVEST DOE	HARVEST FAWN	HARVEST TOTAL	HUNTER SUCCESS	DAYS/ HARVEST	HUNTER DAYS	LICENSES SOLD (cross hunt incl)
68 Split Rock										
	Limited 1	306	256	0	0	256	83.7%	3.5	887	326
	D/F 6	181	0	148	16	164	90.6%	2.6	421	243
Pooled Total		438	256	148	16	420	95.9%	3.1	1308	
Pooled Resident		254	187	33	0	220	86.6%	3.2	704	
Pooled Nonresident		184	69	115	16	200	108.7%	3.0	604	
69 South Rattlesnake										
	Limited 1	89	62	1	6	69	77.5%	3.4	234	101
	D/F 6	56	0	37	9	46	82.1%	2.7	123	56
Pooled Total		124	62	38	15	115	92.7%	3.1	357	
Pooled Resident		88	50	17	12	79	89.8%	2.8	218	
Pooled Nonresident		36	12	21	3	36	100.0%	3.9	139	
70 Kendrick										
	Limited 1	184	174	0	0	174	94.6%	2.6	444	201
	D/F 6	270	0	183	50	233	86.3%	3.6	835	300
Pooled Total		360	174	183	50	407	113.1%	3.1	1279	
Pooled Resident		97	54	40	10	104	107.2%	3.1	318	
Pooled Nonresident		263	120	143	40	303	115.2%	3.2	961	
71 East Poison Spider										
	Limited 1	277	267	0	0	267	96.4%	1.8	484	303
	D/F 6	196	0	157	31	188	95.9%	3.4	630	199
Pooled Total		449	267	157	31	455	101.3%	2.4	1114	
Pooled Resident		229	156	52	15	223	97.4%	2.0	445	
Pooled Nonresident		220	111	105	16	232	105.5%	2.9	669	
72 West Poison Spider										
	Limited 1	834	766	0	0	766	91.8%	2.9	2234	906
	D/F 6	404	0	336	50	386	95.5%	2.6	985	452
Pooled Total		1121	766	336	50	1152	102.8%	2.8	3219	
Pooled Resident		590	422	129	28	579	98.1%	3.2	1873	
Pooled Nonresident		531	344	207	22	573	107.9%	2.3	1346	
73 North Natrona										
	Limited 1	451	403	28	0	431	95.6%	2.2	940	504
	Limited 2	247	212	0	0	212	85.8%	3.2	672	305
	D/F 6	312	0	244	29	273	87.5%	3.0	825	399
	D/F 7	35	0	25	4	29	82.9%	4.1	120	52
Pooled Total		1008	615	297	33	945	93.8%	2.7	2557	
Pooled Resident		664	460	140	11	611	92.0%	2.5	1535	
Pooled Nonresident		344	155	157	22	334	97.1%	3.1	1022	
74 Deer Creek										
	Limited 1	189	181	5	0	186	98.4%	2.8	523	202
	D/F 6	157	0	113	14	127	80.9%	3.6	452	173
Pooled Total		285	181	118	14	313	109.8%	3.1	975	
Pooled Resident		223	145	90	14	249	111.7%	3.2	807	
Pooled Nonresident		62	36	28	0	64	103.2%	2.6	168	
75 Badwater										
	Limited 1	382	331	0	0	331	86.6%	3.2	1049	404
	D/F 6	251	0	208	14	222	88.4%	3.2	714	273
Pooled Total		578	331	208	14	553	95.7%	3.2	1763	
Pooled Resident		437	261	137	14	412	94.3%	3.1	1283	
Pooled Nonresident		141	70	71	0	141	100.0%	3.4	480	
76 Copper Mountain										
	Limited 1	89	72	0	0	72	80.9%	5.3	383	100
	D/F 6	33	0	24	5	29	87.9%	3.5	101	36
Pooled Total		102	72	24	5	101	99.0%	4.8	484	
Pooled Resident		78	56	18	3	77	98.7%	5.6	430	
Pooled Nonresident		24	16	6	2	24	100.0%	2.3	54	

ANTELOPE HUNT AREA REPORT

AREA (cross hunt areas)	TYPE	ACTIVE LICS/HTRS	HARVEST BUCK	HARVEST DOE	HARVEST FAWN	HARVEST TOTAL	HUNTER SUCCESS	DAYS/ HARVEST	HUNTER DAYS	LICENSES SOLD (cross hunt incl)
77 Fifteen Mile Creek										
	Limited 1	52	46	0	0	46	88.5%	4.7	218	54
	D/F 6	28	0	24	1	25	89.3%	3.3	83	31
	D/F 7	25	0	9	12	21	84.0%	2.7	56	25
Pooled Total		92	46	33	13	92	100.0%	3.9	357	
Pooled Resident		70	32	25	10	67	95.7%	4.3	287	
Pooled Nonresident		22	14	8	3	25	113.6%	2.8	70	
78 McCullough Peaks										
	Limited 1	41	37	1	0	38	92.7%	4.2	158	50
	D/F 6	49	0	34	4	38	77.6%	5.0	190	51
	D/F 7	24	0	23	0	23	95.8%	4.7	109	35
Pooled Total		90	37	58	4	99	110.0%	4.6	457	
Pooled Resident		71	29	39	4	72	101.4%	4.7	341	
Pooled Nonresident		19	8	19	0	27	142.1%	4.3	116	
79 Crystal Creek										
**(116)**	Limited 9	17	10	0	0	10	58.8%	15.2	152	30
Pooled Total		17	10	0	0	10	58.8%	15.2	152	
Pooled Resident		14	7	0	0	7	50.0%	19.6	137	
Pooled Nonresident		3	3	0	0	3	100.0%	5.0	15	
80 Badger Basin										
	Limited 1	101	95	0	0	95	94.1%	3.8	362	106
	D/F 6	92	0	75	6	81	88.0%	3.2	256	101
Pooled Total		168	95	75	6	176	104.8%	3.5	618	
Pooled Resident		133	73	60	4	137	103.0%	3.9	533	
Pooled Nonresident		35	22	15	2	39	111.4%	2.2	85	
81 South Fork										
	Limited 1	25	23	0	0	23	92.0%	4.9	112	25
	Limited 2	47	37	2	0	39	83.0%	2.4	95	51
Pooled Total		72	60	2	0	62	86.1%	3.3	207	
Pooled Resident		56	46	2	0	48	85.7%	3.7	178	
Pooled Nonresident		16	14	0	0	14	87.5%	2.1	29	
82 North Greybull River										
	Limited 1	71	68	2	0	70	98.6%	2.7	190	80
	D/F 6	35	0	33	0	33	94.3%	3.1	102	35
Pooled Total		92	68	35	0	103	112.0%	2.8	292	
Pooled Resident		72	52	31	0	83	115.3%	2.9	242	
Pooled Nonresident		20	16	4	0	20	100.0%	2.5	50	
83 Owl Creek										
	Limited 1	72	61	5	0	66	91.7%	2.7	176	78
	D/F 6	47	0	40	5	45	95.7%	7.9	354	50
Pooled Total		102	61	45	5	111	108.8%	4.8	530	
Pooled Resident		79	46	39	5	90	113.9%	5.4	482	
Pooled Nonresident		23	15	6	0	21	91.3%	2.3	48	
84 Wind River										
	Limited 1	32	23	3	0	26	81.3%	3.9	101	36
	D/F 6	23	0	18	1	19	82.6%	4.8	91	25
Pooled Total		38	23	21	1	45	118.4%	4.3	192	
Pooled Resident		29	16	17	0	33	113.8%	4.9	163	
Pooled Nonresident		9	7	4	1	12	133.3%	2.4	29	
85 Gros Ventre										
	Limited 1	15	11	2	0	13	86.7%	6.1	79	16
Pooled Total		15	11	2	0	13	86.7%	6.1	79	
Pooled Resident		12	8	2	0	10	83.3%	6.6	66	
Pooled Nonresident		3	3	0	0	3	100.0%	4.3	13	

ANTELOPE HUNT AREA REPORT

AREA (cross hunt areas)	TYPE	ACTIVE LICS/HTRS	HARVEST BUCK	HARVEST DOE	HARVEST FAWN	HARVEST TOTAL	HUNTER SUCCESS	DAYS/ HARVEST	HUNTER DAYS	LICENSES SOLD (cross hunt incl)
86 Hoback										
	Limited 1	32	24	0	0	24	75.0%	5.4	130	34
	D/F 6	20	0	7	1	8	40.0%	12.6	101	25
Pooled Total		41	24	7	1	32	78.0%	7.2	231	
Pooled Resident		28	19	3	0	22	78.6%	9.5	210	
Pooled Nonresident		13	5	4	1	10	76.9%	2.1	21	
87 Pinedale										
	Limited 1	186	177	0	0	177	95.2%	3.1	542	203
	Limited 2	132	102	4	0	106	80.3%	4.4	471	152
	D/F 6	230	0	176	35	211	91.7%	3.0	632	250
	D/F 7	232	0	180	26	206	88.8%	4.6	951	250
Pooled Total		673	279	360	61	700	104.0%	3.7	2596	
Pooled Resident		389	207	133	43	383	98.5%	3.9	1478	
Pooled Nonresident		284	72	227	18	317	111.6%	3.5	1118	
88 Beaver										
	Limited 1	236	173	17	9	199	84.3%	3.1	610	254
	D/F 6	239	0	174	24	198	82.8%	3.3	663	275
Pooled Total		417	173	191	33	397	95.2%	3.2	1273	
Pooled Resident		249	140	64	14	218	87.6%	3.4	738	
Pooled Nonresident		168	33	127	19	179	106.5%	3.0	535	
89 Piney										
	Limited 1	249	206	0	0	206	82.7%	3.4	701	252
	D/F 6	149	0	126	6	132	88.6%	2.1	274	176
	D/F 7	77	0	57	8	65	84.4%	2.5	165	79
Pooled Total		414	206	183	14	403	97.3%	2.8	1140	
Pooled Resident		325	163	133	10	306	94.2%	2.8	872	
Pooled Nonresident		89	43	50	4	97	109.0%	2.8	268	
90 Yellow Point										
	Limited 1	185	174	0	0	174	94.1%	2.2	380	207
	Limited 2	177	156	10	0	166	93.8%	2.3	390	202
	D/F 6	144	0	115	17	132	91.7%	3.7	492	175
	D/F 7	130	0	112	2	114	87.7%	2.7	309	175
Pooled Total		557	330	237	19	586	105.2%	2.7	1571	
Pooled Resident		328	244	73	9	326	99.4%	2.5	820	
Pooled Nonresident		229	86	164	10	260	113.5%	2.9	751	
91 Dry Sandy										
	Limited 1	373	348	0	0	348	93.3%	2.3	783	403
	D/F 6	93	0	67	13	80	86.0%	1.9	148	127
	D/F 7	25	0	17	3	20	80.0%	3.7	74	25
Pooled Total		465	348	84	16	448	96.3%	2.2	1005	
Pooled Resident		356	266	47	11	324	91.0%	2.2	701	
Pooled Nonresident		109	82	37	5	124	113.8%	2.5	304	
92 Steamboat										
	Limited 1	294	261	0	0	261	88.8%	3.6	943	303
	D/F 6	82	0	65	2	67	81.7%	4.3	290	101
	D/F 7	23	0	17	3	20	87.0%	2.5	50	25
Pooled Total		354	261	82	5	348	98.3%	3.7	1283	
Pooled Resident		273	202	58	0	260	95.2%	4.0	1040	
Pooled Nonresident		81	59	24	5	88	108.6%	2.8	243	
93 West Green River										
	Limited 1	649	601	0	0	601	92.6%	2.9	1730	703
	D/F 6	534	0	440	48	488	91.4%	2.7	1327	600
	D/F 7	86	0	68	12	80	93.0%	3.7	292	100
Pooled Total		1170	601	508	60	1169	99.9%	2.9	3349	
Pooled Resident		730	463	235	34	732	100.3%	2.8	2070	
Pooled Nonresident		440	138	273	26	437	99.3%	2.9	1279	

TABLE I-A (Continued)

ANTELOPE HUNT AREA REPORT

AREA (cross hunt areas)	TYPE	ACTIVE LICS/HTRS	HARVEST BUCK	HARVEST DOE	HARVEST FAWN	HARVEST TOTAL	HUNTER SUCCESS	DAYS/ HARVEST	HUNTER DAYS	LICENSES SOLD (cross hunt incl)
94 Carter Lease										
	Limited 1	464	409	0	0	409	88.1%	3.4	1379	501
	D/F 6	158	0	110	19	129	81.6%	3.5	452	202
	D/F 7	264	0	217	21	238	90.2%	3.6	849	301
Pooled Total		805	409	327	40	776	96.4%	3.5	2680	
Pooled Resident		514	314	132	14	460	89.5%	4.1	1890	
Pooled Nonresident		291	95	195	26	316	108.6%	2.5	790	
95 Cedar Mountain										
	Limited 1	315	290	5	0	295	93.7%	3.5	1043	331
	D/F 7	43	0	30	6	36	83.7%	3.1	112	51
Pooled Total		336	290	35	6	331	98.5%	3.5	1155	
Pooled Resident		259	222	31	0	253	97.7%	3.8	949	
Pooled Nonresident		77	68	4	6	78	101.3%	2.6	206	
96 West Farson										
	Limited 1	261	213	0	0	213	81.6%	4.4	934	279
	D/F 6	114	0	96	5	101	88.6%	3.2	320	125
	D/F 7	22	0	21	0	21	95.5%	4.2	88	25
Pooled Total		341	213	117	5	335	98.2%	4.0	1342	
Pooled Resident		237	161	60	0	221	93.2%	4.5	987	
Pooled Nonresident		104	52	57	5	114	109.6%	3.1	355	
97 Boysen										
**(117)**	Limited 1	110	102	0	0	102	92.7%	2.8	282	126
**(117)**	D/F 6	105	0	94	5	99	94.3%	2.4	236	111
**(117)**	D/F 7	13	0	11	0	11	84.6%	4.7	52	20
Pooled Total		165	102	105	5	212	128.5%	2.7	570	
Pooled Resident		127	82	82	2	166	130.7%	2.7	444	
Pooled Nonresident		38	20	23	3	46	121.1%	2.7	126	
98 Sage										
	Limited 1	157	148	0	0	148	94.3%	3.8	569	157
	D/F 6	249	0	181	38	219	88.0%	3.0	650	300
Pooled Total		365	148	181	38	367	100.5%	3.3	1219	
Pooled Resident		148	113	16	10	139	93.9%	3.7	517	
Pooled Nonresident		217	35	165	28	228	105.1%	3.1	702	
99 Uinta										
	Limited 1	116	96	0	0	96	82.8%	4.5	432	128
	D/F 6	83	0	62	5	67	80.7%	4.4	294	100
	D/F 7	181	0	119	40	159	87.8%	3.2	505	199
	Limited 8	48	38	0	0	38	79.2%	5.2	199	51
Pooled Total		348	134	181	45	360	103.4%	4.0	1430	
Pooled Resident		214	99	67	25	191	89.3%	4.7	899	
Pooled Nonresident		134	35	114	20	169	126.1%	3.1	531	
100 Bear River Divide										
	Limited 1	93	86	1	3	90	96.8%	3.0	267	100
	D/F 6	227	0	159	26	185	81.5%	3.6	665	300
Pooled Total		284	86	160	29	275	96.8%	3.4	932	
Pooled Resident		109	67	33	12	112	102.8%	3.0	340	
Pooled Nonresident		175	19	127	17	163	93.1%	3.6	592	
102 Buffalo										
	Limited 1	381	289	28	0	317	83.2%	3.3	1057	449
	D/F 6	316	0	208	73	281	88.9%	3.2	894	350
Pooled Total		649	289	236	73	598	92.1%	3.3	1951	
Pooled Resident		175	53	71	10	134	76.6%	3.2	424	
Pooled Nonresident		474	236	165	63	464	97.9%	3.3	1527	
103 Dwyer										
	Limited 1	343	284	15	0	299	87.2%	3.0	901	363
	D/F 6	48	0	42	0	42	87.5%	2.6	110	50
	D/F 7	168	0	126	21	147	87.5%	3.7	541	200
Pooled Total		486	284	183	21	488	100.4%	3.2	1552	
Pooled Resident		242	152	99	7	258	106.6%	3.4	867	
Pooled Nonresident		244	132	84	14	230	94.3%	3.0	685	

TABLE I-A (Continued)

ANTELOPE HUNT AREA REPORT

AREA (cross hunt areas)	TYPE	ACTIVE LICS/HTRS	HARVEST BUCK	DOE	FAWN	TOTAL	HUNTER SUCCESS	DAYS/ HARVEST	HUNTER DAYS	LICENSES SOLD (cross hunt incl)
104 Crow Creek										
	Limited 1	189	134	12	0	146	77.2%	4.9	713	204
	D/F 6	161	0	101	21	122	75.8%	3.5	423	175
Pooled Total		277	134	113	21	268	96.8%	4.2	1136	
Pooled Resident		151	61	50	14	125	82.8%	5.4	672	
Pooled Nonresident		126	73	63	7	143	113.5%	3.2	464	
106 Long Creek										
	Limited 1	270	216	4	0	220	81.5%	3.1	674	274
	D/F 6	170	0	127	7	134	78.8%	3.2	429	200
Pooled Total		386	216	131	7	354	91.7%	3.1	1103	
Pooled Resident		249	148	55	4	207	83.1%	3.2	664	
Pooled Nonresident		137	68	76	3	147	107.3%	3.0	439	
107 Upper Sweetwater										
	Limited 1	102	80	4	2	86	84.3%	2.7	235	106
	D/F 6	118	0	103	11	114	96.6%	2.7	304	150
	Limited 8	124	58	11	0	69	55.6%	5.6	389	134
Pooled Total		307	138	118	13	269	87.6%	3.4	928	
Pooled Resident		184	92	35	2	129	70.1%	3.9	507	
Pooled Nonresident		123	46	83	11	140	113.8%	3.0	421	
108 Bridger Pass										
	Limited 1	92	80	0	0	80	87.0%	2.8	222	106
Pooled Total		92	80	0	0	80	87.0%	2.8	222	
Pooled Resident		68	56	0	0	56	82.4%	2.9	165	
Pooled Nonresident		24	24	0	0	24	100.0%	2.4	57	
109 Beckton										
	Limited 1	185	135	0	0	135	73.0%	4.6	616	203
	D/F 6	151	0	107	6	113	74.8%	4.1	458	173
Pooled Total		283	135	107	6	248	87.6%	4.3	1074	
Pooled Resident		97	48	16	0	64	66.0%	5.5	350	
Pooled Nonresident		186	87	91	6	184	98.9%	3.9	724	
110 South Greybull River										
	Limited 1	59	58	0	0	58	98.3%	2.3	136	63
	D/F 6	22	0	19	0	19	86.4%	3.4	64	25
Pooled Total		77	58	19	0	77	100.0%	2.6	200	
Pooled Resident		61	48	15	0	63	103.3%	2.4	152	
Pooled Nonresident		16	10	4	0	14	87.5%	3.4	48	
111 Chalk Bluffs										
	Limited 1	146	118	0	7	125	85.6%	3.9	492	150
	D/F 6	168	0	88	25	113	67.3%	3.5	390	200
Pooled Total		276	118	88	32	238	86.2%	3.7	882	
Pooled Resident		170	85	31	16	132	77.6%	4.3	570	
Pooled Nonresident		106	33	57	16	106	100.0%	2.9	312	
112 Pine Mountain										
	Limited 1	163	150	0	0	150	92.0%	3.3	488	177
	D/F 6	61	0	41	10	51	83.6%	4.4	224	76
Pooled Total		178	150	41	10	201	112.9%	3.5	712	
Pooled Resident		136	121	25	5	151	111.0%	3.8	573	
Pooled Nonresident		42	29	16	5	50	119.0%	2.8	139	
113 Salt Creek										
	Limited 1	170	149	5	0	154	90.6%	2.8	438	177
	Limited 2	135	127	2	4	133	98.5%	2.5	338	154
	D/F 6	243	0	217	13	230	94.7%	3.0	688	247
Pooled Total		439	276	224	17	517	117.8%	2.8	1464	
Pooled Resident		101	58	54	0	112	110.9%	2.9	328	
Pooled Nonresident		338	218	170	17	405	119.8%	2.8	1136	

ANTELOPE HUNT AREA REPORT

AREA (cross hunt areas)	TYPE	ACTIVE LICS/HTRS	HARVEST BUCK	DOE	FAWN	TOTAL	HUNTER SUCCESS	DAYS/ HARVEST	HUNTER DAYS	LICENSES SOLD (cross hunt incl)
114 Nowater										
	Limited 1	55	44	0	0	44	80.0%	4.7	205	57
	D/F 6	25	0	23	0	23	92.0%	2.8	64	26
Pooled Total		71	44	23	0	67	94.4%	4.0	269	
Pooled Resident		50	29	13	0	42	84.0%	5.2	217	
Pooled Nonresident		21	15	10	0	25	119.0%	2.1	52	
115 Upper Nowood										
	Limited 1	92	84	0	0	84	91.3%	2.7	227	102
	D/F 6	112	0	89	11	100	89.3%	3.2	322	125
Pooled Total		188	84	89	11	184	97.9%	3.0	549	
Pooled Resident		90	62	23	4	89	98.9%	2.7	237	
Pooled Nonresident		98	22	66	7	95	96.9%	3.3	312	
116 Manderson										
**(79)**	Limited 9	13	6	0	0	6	46.2%	11.8	71	30
Pooled Total		13	6	0	0	6	46.2%	11.8	71	
Pooled Resident		10	3	0	0	3	30.0%	17.7	53	
Pooled Nonresident		3	3	0	0	3	100.0%	6.0	18	
117 Crowheart Butte										
**(97)**	Limited 1	10	3	2	0	5	50.0%	8.4	42	126
**(97)**	D/F 6	3	0	3	0	3	100.0%	3.3	10	111
**(97)**	D/F 7	7	0	5	2	7	100.0%	3.3	23	20
Pooled Total		17	3	10	2	15	88.2%	5.0	75	
Pooled Resident		9	0	7	0	7	77.8%	6.9	48	
Pooled Nonresident		8	3	3	2	8	100.0%	3.4	27	

TABLE I-H

ANTELOPE HARVEST

BY HERD UNIT

2007

HERD	TYPE	ACTIVE LICS/HTRS	HARVEST BUCK	DOE	FAWN	TOTAL	HUNTER SUCCESS	DAYS/ HARVEST	HUNTER DAYS	LICENSES SOLD (cross hunt incl)
202 Bighorn	Limited	28	16	0	0	16	57.1%	13.9	223	30
Total Hunters		28	16	0	0	16	57.1%	13.9	223	
Resident		22	10	0	0	10	45.5%	19.0	190	
Nonresident		6	6	0	0	6	100.0%	5.5	33	
203 Copper Mountain	D/F	170	0	136	16	152	89.4%	3.2	487	187
	Limited	236	200	0	0	200	84.7%	4.1	815	259
Total Hunters		360	200	136	16	352	97.8%	3.7	1302	
Resident		217	147	54	7	208	95.9%	4.3	884	
Nonresident		143	53	82	9	144	100.7%	2.9	418	
204 Fifteenmile	D/F	122	0	92	18	110	90.2%	5.1	557	131
	Limited	183	165	5	0	170	92.9%	3.1	530	195
Total Hunters		270	165	97	18	280	103.7%	3.9	1087	
Resident		210	126	79	15	220	104.8%	4.2	921	
Nonresident		60	39	18	3	60	100.0%	2.8	166	
205 Carter Mountain	D/F	108	0	90	4	94	87.0%	4.3	401	121
	Limited	184	165	5	0	170	92.4%	3.3	555	206
Total Hunters		254	165	95	4	264	103.9%	3.6	956	
Resident		199	127	72	4	203	102.0%	3.7	761	
Nonresident		55	38	23	0	61	110.9%	3.2	195	
207 Badger Basin	D/F	92	0	75	6	81	88.0%	3.2	256	101
	Limited	101	95	0	0	95	94.1%	3.8	362	106
Total Hunters		168	95	75	6	176	104.8%	3.5	618	
Resident		133	73	60	4	137	103.0%	3.9	533	
Nonresident		35	22	15	2	39	111.4%	2.2	85	
308 Clearmont	D/F	365	0	225	35	260	71.2%	4.8	1241	423
	Limited	523	409	10	0	419	80.1%	5.0	2083	589
Total Hunters		786	409	235	35	679	86.4%	4.9	3324	
Resident		217	144	39	15	198	91.2%	5.4	1062	
Nonresident		569	265	196	20	481	84.5%	4.7	2262	
309 Pumpkin Buttes	D/F	965	0	870	52	922	95.5%	3.1	2836	993
	Limited	1606	1452	0	0	1452	90.4%	2.9	4159	1648
Total Hunters		2424	1452	870	52	2374	97.9%	2.9	6995	
Resident		378	255	119	24	398	105.3%	2.6	1022	
Nonresident		2046	1197	751	28	1976	96.6%	3.0	5973	
310 Upper Powder River	D/F	347	0	242	33	275	79.3%	4.2	1152	380
	Limited	491	375	8	8	391	79.6%	3.4	1334	538
Total Hunters		758	375	250	41	666	87.9%	3.7	2486	
Resident		115	62	19	11	92	80.0%	3.9	363	
Nonresident		643	313	231	30	574	89.3%	3.7	2123	
316 Highlight	D/F	489	0	472	9	481	98.4%	2.2	1064	581
	Limited	624	565	10	0	575	92.1%	3.3	1900	707
Total Hunters		1027	565	482	9	1056	102.8%	2.8	2964	
Resident		245	150	79	0	229	93.5%	4.7	1067	
Nonresident		782	415	403	9	827	105.8%	2.3	1897	
318 Crazy Woman	D/F	599	0	516	39	555	92.7%	2.6	1468	618
	Limited	782	738	7	4	749	95.8%	2.4	1801	859
Total Hunters		1177	738	523	43	1304	110.8%	2.5	3269	
Resident		214	150	92	4	246	115.0%	2.2	548	
Nonresident		963	588	431	39	1058	109.9%	2.6	2721	
339 North Black Hills	D/F	739	0	560	60	620	83.9%	3.2	1993	1427
	Limited	1491	1251	43	5	1299	87.1%	3.3	4301	1598
Total Hunters		1991	1251	603	65	1919	96.4%	3.3	6294	
Resident		420	233	129	8	370	88.1%	4.4	1643	
Nonresident		1571	1018	474	57	1549	98.6%	3.0	4651	

TABLE I-H (Continued)
ANTELOPE HERD UNIT REPORT

HERD	TYPE	ACTIVE LICS/HTRS	HARVEST BUCK	DOE	FAWN	TOTAL	HUNTER SUCCESS	DAYS/ HARVEST	HUNTER DAYS	LICENSES SOLD (cross hunt incl)
351 Gillette	D/F	585	0	533	0	533	91.1%	2.9	1556	902
	Limited	976	948	0	0	948	97.1%	3.2	3071	1049
Total Hunters		1410	948	533	0	1481	105.0%	3.1	4627	
Resident		456	279	190	0	469	102.9%	3.1	1447	
Nonresident		954	669	343	0	1012	106.1%	3.1	3180	
352 Middle Fork	D/F	330	0	231	29	260	78.8%	3.4	878	361
	Limited	438	363	32	6	401	91.6%	2.7	1069	457
Total Hunters		725	363	263	35	661	91.2%	2.9	1947	
Resident		66	56	5	5	66	100.0%	1.8	118	
Nonresident		659	307	258	30	595	90.3%	3.1	1829	
353 Ucross	D/F	271	0	225	16	241	88.9%	3.7	903	307
	Limited	533	460	0	0	460	86.3%	3.2	1453	567
Total Hunters		634	460	225	16	701	110.6%	3.4	2356	
Resident		158	93	70	0	163	103.2%	4.3	708	
Nonresident		476	367	155	16	538	113.0%	3.1	1648	
354 Buffalo	D/F	316	0	208	73	281	88.9%	3.2	894	350
	Limited	381	289	28	0	317	83.2%	3.3	1057	449
Total Hunters		649	289	236	73	598	92.1%	3.3	1951	
Resident		175	53	71	10	134	76.6%	3.2	424	
Nonresident		474	236	165	63	464	97.9%	3.3	1527	
355 Beckton	D/F	151	0	107	6	113	74.8%	4.1	458	173
	Limited	185	135	0	0	135	73.0%	4.6	616	203
Total Hunters		283	135	107	6	248	87.6%	4.3	1074	
Resident		97	48	16	0	64	66.0%	5.5	350	
Nonresident		186	87	91	6	184	98.9%	3.9	724	
401 Sublette	D/F	2318	0	1841	216	2057	88.7%	3.2	6480	2683
	Limited	3015	2584	48	11	2643	87.7%	3.1	8317	3248
Total Hunters		4728	2584	1889	227	4700	99.4%	3.1	14797	
Resident		3095	1965	843	123	2931	94.7%	3.2	9489	
Nonresident		1633	619	1046	104	1769	108.3%	3.0	5308	
411 Uinta-Cedar Mountain	D/F	307	0	211	51	262	85.3%	3.5	911	350
	Limited	479	424	5	0	429	89.6%	3.9	1674	510
Total Hunters		684	424	216	51	691	101.0%	3.7	2585	
Resident		473	321	98	25	444	93.9%	4.2	1848	
Nonresident		211	103	118	26	247	117.1%	3.0	737	
412 South Rock Springs	D/F	175	0	144	17	161	92.0%	2.4	394	204
	Limited	336	298	0	0	298	88.7%	3.2	939	354
Total Hunters		434	298	144	17	459	105.8%	2.9	1333	
Resident		327	237	86	8	331	101.2%	3.1	1027	
Nonresident		107	61	58	9	128	119.6%	2.4	306	
414 Bitter Creek	D/F	28	0	21	7	28	100.0%	4.7	132	28
	Limited	423	347	0	0	347	82.0%	3.3	1129	454
Total Hunters		440	347	21	7	375	85.2%	3.4	1261	
Resident		351	263	13	7	283	80.6%	3.7	1045	
Nonresident		89	84	8	0	92	103.4%	2.3	216	
419 Carter Lease	D/F	898	0	667	104	771	85.9%	3.4	2616	1103
	Limited	714	643	1	3	647	90.6%	3.4	2215	758
Total Hunters		1454	643	668	107	1418	97.5%	3.4	4831	
Resident		771	494	181	36	711	92.2%	3.9	2747	
Nonresident		683	149	487	71	707	103.5%	2.9	2084	
438 Baggs	D/F	621	0	463	62	525	84.5%	3.0	1591	876
	Limited	662	550	0	0	550	83.1%	3.9	2150	708
Total Hunters		1162	550	463	62	1075	92.5%	3.5	3741	
Resident		603	440	69	2	511	84.7%	4.0	2035	
Nonresident		559	110	394	60	564	100.9%	3.0	1706	

TABLE I-H (Continued)

ANTELOPE HERD UNIT REPORT

HERD	TYPE	ACTIVE LICS/HTRS	HARVEST BUCK	DOE	FAWN	TOTAL	HUNTER SUCCESS	DAYS/ HARVEST	HUNTER DAYS	LICENSES SOLD (cross hunt incl)
520 Chalk Bluffs	D/F	168	0	88	25	113	67.3%	3.5	390	200
	Limited	146	118	0	7	125	85.6%	3.9	492	150
Total Hunters		276	118	88	32	238	86.2%	3.7	882	
Resident		170	85	31	16	132	77.6%	4.3	570	
Nonresident		106	33	57	16	106	100.0%	2.9	312	
521 Hawk Springs	D/F	503	0	311	75	386	76.7%	3.9	1521	606
	Limited	684	464	48	6	518	75.7%	4.1	2113	728
Total Hunters		1053	464	359	81	904	85.8%	4.0	3634	
Resident		557	267	156	36	459	82.4%	4.3	1966	
Nonresident		496	197	203	45	445	89.7%	3.7	1668	
522 Meadowdale	D/F	404	0	329	18	347	85.9%	3.9	1366	453
	Limited	423	394	9	0	403	95.3%	3.2	1307	453
Total Hunters		718	394	338	18	750	104.5%	3.6	2673	
Resident		171	108	73	5	186	108.8%	5.2	970	
Nonresident		547	286	265	13	564	103.1%	3.0	1703	
523 Iron Mountain	D/F	854	0	640	56	696	81.5%	3.3	2295	924
	Limited	1161	922	35	10	967	83.3%	3.7	3617	1262
Total Hunters		1698	922	675	66	1663	97.9%	3.6	5912	
Resident		649	347	189	14	550	84.7%	4.5	2499	
Nonresident		1049	575	486	52	1113	106.1%	3.1	3413	
524 Dwyer	D/F	216	0	168	21	189	87.5%	3.4	651	250
	Limited	343	284	15	0	299	87.2%	3.0	901	363
Total Hunters		486	284	183	21	488	100.4%	3.2	1552	
Resident		242	152	99	7	258	106.6%	3.4	867	
Nonresident		244	132	84	14	230	94.3%	3.0	685	
525 Medicine Bow	D/F	4100	0	2902	557	3459	84.4%	3.5	11995	4712
	Limited	4406	3618	93	23	3734	84.7%	3.1	11529	4791
Total Hunters		7655	3618	2995	580	7193	94.0%	3.3	23524	
Resident		2264	1372	484	56	1912	84.5%	3.3	6324	
Nonresident		5391	2246	2511	524	5281	98.0%	3.3	17200	
526 Cooper Lake	D/F	338	0	206	48	254	75.1%	3.4	875	400
	Limited	382	325	0	0	325	85.1%	3.5	1124	402
Total Hunters		672	325	206	48	579	86.2%	3.5	1999	
Resident		121	54	18	5	77	63.6%	5.5	425	
Nonresident		551	271	188	43	502	91.1%	3.1	1574	
527 Centennial	D/F	837	0	554	65	619	74.0%	4.9	3062	973
	Limited	1072	882	22	15	919	85.7%	3.0	2782	1191
Total Hunters		1697	882	576	80	1538	90.6%	3.8	5844	
Resident		622	340	117	17	474	76.2%	4.5	2111	
Nonresident		1075	542	459	63	1064	99.0%	3.5	3733	
528 Elk Mountain	D/F	466	0	363	41	404	86.7%	3.3	1342	500
	Limited	518	451	0	0	451	87.1%	3.6	1624	557
Total Hunters		908	451	363	41	855	94.2%	3.5	2966	
Resident		162	90	47	0	137	84.6%	4.0	551	
Nonresident		746	361	316	41	718	96.2%	3.4	2415	
529 Big Creek	D/F	125	0	101	8	109	87.2%	3.5	382	150
	Limited	96	77	4	0	81	84.4%	4.1	329	104
Total Hunters		185	77	105	8	190	102.7%	3.7	711	
Resident		99	58	33	0	91	91.9%	4.2	378	
Nonresident		86	19	72	8	99	115.1%	3.4	333	
615 Red Desert	Limited	470	400	0	0	400	85.1%	3.6	1451	505
Total Hunters		470	400	0	0	400	85.1%	3.6	1451	
Resident		369	299	0	0	299	81.0%	4.0	1183	
Nonresident		101	101	0	0	101	100.0%	2.7	268	

TABLE I-H (Continued)

ANTELOPE HERD UNIT REPORT

HERD	TYPE	ACTIVE LICS/HTRS	HARVEST BUCK	HARVEST DOE	HARVEST FAWN	HARVEST TOTAL	HUNTER SUCCESS	DAYS/ HARVEST	HUNTER DAYS	LICENSES SOLD (cross hunt incl)
630 Iron Springs	D/F	237	0	191	28	219	92.4%	3.0	657	303
	Limited	743	653	0	0	653	87.9%	3.0	1931	811
Total Hunters		906	653	191	28	872	96.2%	3.0	2588	
Resident		356	290	36	0	326	91.6%	2.8	922	
Nonresident		550	363	155	28	546	99.3%	3.1	1666	
631 Wind River	D/F	23	0	18	1	19	82.6%	4.8	91	25
	Limited	32	23	3	0	26	81.3%	3.9	101	36
Total Hunters		38	23	21	1	45	118.4%	4.3	192	
Resident		29	16	17	0	33	113.8%	4.9	163	
Nonresident		9	7	4	1	12	133.3%	2.4	29	
632 Beaver Rim	D/F	945	0	724	61	785	83.1%	3.4	2686	1123
	Limited	1402	1212	10	6	1228	87.6%	3.4	4146	1481
Total Hunters		2051	1212	734	67	2013	98.1%	3.4	6832	
Resident		1387	896	368	38	1302	93.9%	3.4	4483	
Nonresident		664	316	366	29	711	107.1%	3.3	2349	
634 Badwater	D/F	251	0	208	14	222	88.4%	3.2	714	273
	Limited	382	331	0	0	331	86.6%	3.2	1049	404
Total Hunters		578	331	208	14	553	95.7%	3.2	1763	
Resident		437	261	137	14	412	94.3%	3.1	1283	
Nonresident		141	70	71	0	141	100.0%	3.4	480	
635 Project	D/F	128	0	113	7	120	93.8%	2.7	321	131
	Limited	117	105	2	0	107	91.5%	3.0	324	126
Total Hunters		179	105	115	7	227	126.8%	2.8	645	
Resident		134	82	89	2	173	129.1%	2.8	492	
Nonresident		45	23	26	5	54	120.0%	2.8	153	
636 North Ferris	D/F	120	0	102	5	107	89.2%	3.0	324	150
	Limited	385	310	6	0	316	82.1%	2.6	808	404
Total Hunters		471	310	108	5	423	89.8%	2.7	1132	
Resident		329	226	42	0	268	81.5%	2.4	646	
Nonresident		142	84	66	5	155	109.2%	3.1	486	
637 South Ferris	Limited	359	305	0	0	305	85.0%	3.6	1088	407
Total Hunters		359	305	0	0	305	85.0%	3.6	1088	
Resident		274	226	0	0	226	82.5%	3.8	850	
Nonresident		85	79	0	0	79	92.9%	3.0	238	
740 Cheyenne River	D/F	2465	0	1941	227	2168	88.0%	3.5	7678	2610
	Limited	4103	3691	58	0	3749	91.4%	3.1	11472	4239
Total Hunters		5915	3691	1999	227	5917	100.0%	3.2	19150	
Resident		1246	849	289	8	1146	92.0%	3.7	4284	
Nonresident		4669	2842	1710	219	4771	102.2%	3.1	14866	
745 Rattlesnake	D/F	870	0	676	131	807	92.8%	3.0	2450	951
	Limited	1295	1207	0	0	1207	93.2%	2.6	3162	1410
Total Hunters		1923	1207	676	131	2014	104.7%	2.8	5612	
Resident		916	632	221	53	906	98.9%	2.9	2636	
Nonresident		1007	575	455	78	1108	110.0%	2.7	2976	
746 North Natrona	D/F	347	0	269	33	302	87.0%	3.1	945	451
	Limited	698	615	28	0	643	92.1%	2.5	1612	809
Total Hunters		1008	615	297	33	945	93.8%	2.7	2557	
Resident		664	460	140	11	611	92.0%	2.5	1535	
Nonresident		344	155	157	22	334	97.1%	3.1	1022	
748 North Converse	D/F	827	0	662	84	746	90.2%	3.3	2468	942
	Limited	1625	1486	46	0	1532	94.3%	2.8	4223	1748
Total Hunters		2183	1486	708	84	2278	104.4%	2.9	6691	
Resident		405	265	88	19	372	91.9%	4.0	1488	
Nonresident		1778	1221	620	65	1906	107.2%	2.7	5203	

TABLE III

CONFIDENCE INTERVALS FOR TOTAL ANTELOPE HARVEST

BY HERD UNIT

2007

HERD		TOTAL HARVEST	90% CONFIDENCE LIMITS		
202	Bighorn	16	11	-	21
203	Copper Mountain	352	341	-	363
204	Fifteenmile	280	272	-	288
205	Carter Mountain	264	255	-	273
207	Badger Basin	176	170	-	182
308	Clearmont	679	658	-	700
309	Pumpkin Buttes	2374	2352	-	2396
310	Upper Powder River	666	647	-	685
316	Highlight	1056	1044	-	1068
318	Crazy Woman	1304	1290	-	1318
339	North Black Hills	1919	1892	-	1946
351	Gillette	1481	1467	-	1495
352	Middle Fork	661	645	-	677
353	Ucross	701	686	-	716
354	Buffalo	598	583	-	613
355	Beckton	248	235	-	261
401	Sublette	4700	4661	-	4739
411	Uinta-Cedar Mountain	691	676	-	706
412	South Rock Springs	459	448	-	470
414	Bitter Creek	375	362	-	388
419	Carter Lease	1418	1396	-	1440
438	Baggs	1075	1053	-	1097
520	Chalk Bluffs	238	225	-	251
521	Hawk Springs	904	880	-	928
522	Meadowdale	750	736	-	764
523	Iron Mountain	1663	1635	-	1691
524	Dwyer	488	475	-	501
525	Medicine Bow	7193	7138	-	7248
526	Cooper Lake	579	561	-	597
527	Centennial	1538	1510	-	1566
528	Elk Mountain	855	838	-	872
529	Big Creek	190	181	-	199
615	Red Desert	400	387	-	413
630	Iron Springs	872	856	-	888
631	Wind River	45	40	-	50
632	Beaver Rim	2013	1985	-	2041
634	Badwater	553	539	-	567
635	Project	227	220	-	234
636	North Ferris	423	409	-	437
637	South Ferris	305	294	-	316
740	Cheyenne River	5917	5877	-	5957
745	Rattlesnake	2014	1995	-	2033
746	North Natrona	945	929	-	961
748	North Converse	2278	2256	-	2300

TABLE IV

ANTELOPE HUNTERS HUNTING ON OPENING DAY

BY HUNT AREA AND RESIDENCY

2007

HUNT AREA	RESIDENT HUNTERS HUNTING ON OPENING DAY	PERCENT WHO HUNTED ON OPENING DAY	NONRESIDENT HUNTERS HUNTING ON OPENING DAY	PERCENT WHO HUNTED ON OPENING DAY
1	8	19.2%	73	25.3%
2	9	10.8%	92	22.8%
3	22	25.9%	78	37.5%
4	52	73.2%	83	40.7%
5	56	32.5%	82	33.2%
6	43	54.7%	130	39.0%
7	143	54.1%	251	26.1%
8	12	61.8%	40	14.0%
9	38	36.4%	158	21.0%
10	17	25.5%	66	43.0%
11	45	34.7%	49	15.7%
12	14	32.7%	66	27.7%
15	61	28.2%	128	22.5%
16	33	34.5%	77	23.8%
17	201	44.1%	186	19.5%
18	0	0.0%	90	28.1%
19	62	45.0%	108	28.9%
20	46	40.4%	113	17.5%
21	28	42.4%	165	25.0%
22	70	62.0%	240	38.4%
23	156	41.3%	506	24.7%
24	73	29.6%	291	37.3%
25	112	42.2%	119	27.9%
26	0	0.0%	287	27.1%
27	161	49.3%	79	29.0%
28	22	66.7%	102	33.8%
29	103	40.5%	383	23.1%
30	101	41.8%	188	24.1%
31	58	42.5%	103	21.8%
32	59	27.5%	239	25.3%
34	65	31.0%	47	33.9%
35	72	47.8%	67	26.6%
36	85	43.1%	60	49.6%
37	84	49.5%	53	49.3%
38	74	27.4%	94	18.3%
39	60	42.6%	57	27.2%
40	41	46.9%	84	39.6%
41	13	46.7%	20	43.0%
42	52	36.5%	152	19.8%
43	29	24.2%	125	22.6%
44	55	43.6%	71	24.2%
45	91	27.8%	198	29.5%
46	105	47.8%	180	31.6%
47	407	41.6%	307	26.4%
48	148	48.9%	157	22.6%
50	36	22.2%	131	17.5%
51	40	40.2%	20	23.6%
52	138	52.4%	102	27.4%
53	168	34.4%	116	23.6%
55	29	25.0%	19	28.6%
56	20	80.0%	38	25.0%
57	82	34.1%	20	33.1%
58	62	55.9%	11	35.7%
59	93	48.7%	28	42.9%
60	21	25.8%	5	28.6%
61	44	41.9%	12	38.9%
62	89	32.5%	23	26.7%
63	197	59.8%	19	13.6%
64	47	25.5%	16	30.8%
65	80	27.9%	27	18.0%

TABLE IV (Continued)

ANTELOPE OPENING DAY HUNTERS REPORT

HUNT AREA	RESIDENT HUNTERS	PERCENT WHO HUNTED ON OPENING DAY	NONRESIDENT HUNTERS	PERCENT WHO HUNTED ON OPENING DAY
66	48	55.3%	10	30.4%
67	89	44.8%	28	37.7%
68	115	45.3%	64	34.7%
69	47	53.7%	5	13.6%
70	71	72.6%	77	29.3%
71	130	56.8%	103	46.7%
72	221	37.5%	129	24.3%
73	334	50.2%	84	24.5%
74	88	39.6%	18	28.3%
75	201	46.1%	36	25.7%
76	22	28.4%	14	60.0%
77	18	26.3%	8	36.4%
78	18	25.0%	7	39.3%
79	0	0.0%	0	0.0%
80	62	46.5%	13	38.2%
81	19	34.4%	10	59.5%
82	34	46.9%	12	58.0%
83	34	42.4%	14	62.2%
84	7	25.1%	3	28.2%
85	3	25.0%	0	0.0%
86	9	31.1%	12	89.6%
87	155	39.7%	96	33.7%
88	123	49.4%	71	42.3%
89	170	52.3%	26	28.9%
90	189	57.5%	70	30.7%
91	185	51.8%	31	28.2%
92	78	28.6%	15	18.1%
93	287	39.3%	130	29.6%
94	176	34.2%	68	23.3%
95	110	42.4%	24	30.7%
96	83	34.9%	22	21.6%
97	77	60.7%	19	51.0%
98	27	18.6%	74	34.0%
99	68	31.6%	47	35.0%
100	45	41.3%	46	26.2%
102	51	29.3%	104	22.0%
103	88	36.3%	45	18.3%
104	37	24.8%	43	33.8%
106	111	44.6%	42	30.5%
107	54	29.4%	24	19.5%
108	22	32.1%	4	14.3%
109	18	18.8%	34	18.2%
110	36	59.8%	8	49.2%
111	59	34.7%	31	28.9%
112	59	43.3%	9	21.9%
113	45	44.6%	74	22.0%
114	15	29.9%	14	67.0%
115	54	59.3%	34	34.5%
116	0	0.0%	0	0.0%
117	4	39.3%	3	34.8%

TABLE VII

ANTELOPE LICENSE DATA SUMMARY
2007

HUNT AREA	LICENSE TYPE	NUMBER SOLD	NUMBER SURVEYED	NUMBER USABLE SURVEYS (both hunters and nonhunters)	% USABLE RESPONSE
1	1	289	116	71	61.2%
1	6	119	106	40	37.7%
2	1	351	116	68	58.6%
2	6	239	116	68	58.6%
3	1	237	118	68	57.6%
3	6	167	116	56	48.3%
4	1	229	114	56	49.1%
4	6	135	110	48	43.6%
5	1	302	110	59	53.6%
5	6	213	116	71	61.2%
6	1	339	114	79	69.3%
6	6	134	94	53	56.4%
7	1	859	126	74	58.7%
7	6	559	124	73	58.9%
8	1	301	110	67	60.9%
8	6	50	50	22	44.0%
9	1	552	120	72	60.0%
9	6	400	116	74	63.8%
10	1	185	114	67	58.8%
10	6	179	120	63	52.5%
11	1	252	106	62	58.5%
11	6	300	110	56	50.9%
12	1	201	102	60	58.8%
12	6	153	106	54	50.9%
15	1	589	124	67	54.0%
15	6	423	124	46	37.1%
16	1	382	120	74	61.7%
16	6	128	116	42	36.2%
17	1	1049	128	73	57.0%
17, 18, 19	6	902	192	75	39.1%
18	1	330	120	69	57.5%
19	1	391	124	60	48.4%
20	1	538	124	69	55.7%
20	6	380	122	51	41.8%
21	1	457	122	68	55.7%
21	6	361	122	47	38.5%
22	1	528	124	68	54.8%
22	6	371	122	55	45.1%
23	1	1648	130	75	57.7%
23	6	993	128	66	51.6%
24	1	707	124	62	50.0%
24	6	581	124	63	50.8%
25	1	581	122	71	58.2%
25	6	256	116	41	35.3%
26	1	926	128	69	53.9%
26	6	481	120	68	56.7%
27	1	453	118	66	55.9%
27	6	256	118	62	52.5%
28	1	241	116	54	46.6%
28	6	205	75	38	50.7%
29	1	1204	128	76	59.4%
29	6	786	124	60	48.4%
29	7	77	69	25	36.2%
30	1	603	122	65	53.3%
30	6	596	124	58	46.8%
31	1	402	116	74	63.8%
31	6	448	118	74	62.7%
32	1	454	118	74	62.7%
32	2	252	106	74	69.8%
32	6	650	122	81	66.4%
34	1	226	104	59	56.7%
34	6	200	102	51	50.0%
35	1	301	110	58	52.7%
35	6	207	105	51	48.6%

ANTELOPE LICENSE DATA SUMMARY

HUNT AREA	LICENSE TYPE	NUMBER SOLD	NUMBER SURVEYED	NUMBER USABLE SURVEYS (both hunters and nonhunters)	% USABLE RESPONSE
36	1	201	102	49	48.0%
36	6	199	102	46	45.1%
37	1	276	108	59	54.6%
37	6	75	72	47	65.3%
38	1	551	120	55	45.8%
38	6	446	120	60	50.0%
39	1	300	110	55	50.0%
39	6	100	80	40	50.0%
40	1	207	102	56	54.9%
40	6	203	102	55	53.9%
41	1	58	58	28	48.3%
41	6	50	51 *	23	45.1%
42	1	650	122	75	61.5%
42	6	300	110	79	71.8%
42	7	200	102	54	52.9%
43	1	402	116	63	54.3%
43	6	400	116	58	50.0%
44	1	258	108	57	52.8%
44	6	298	110	68	61.8%
45	1	657	122	66	54.1%
45	6	600	122	73	59.8%
46	1	504	120	73	60.8%
46	6	311	112	69	61.6%
46	7	200	102	48	47.1%
47	1	1006	126	74	58.7%
47	2	350	114	78	68.4%
47	6	706	124	64	51.6%
47	7	400	116	74	63.8%
48	1	356	114	65	57.0%
48	2	156	94	66	70.2%
48	6	375	114	67	58.8%
48	7	476	118	63	53.4%
50	1	506	120	72	60.0%
50	6	500	120	73	60.8%
50	8	51	51	37	72.6%
51	1	104	82	50	61.0%
51	6	150	94	55	58.5%
52	1	254	106	64	60.4%
52	2	250	106	69	65.1%
52	6	202	102	71	69.6%
52	7	101	82	50	61.0%
53	1	556	120	73	60.8%
53	6	751	124	86	69.4%
53	7	25	24	12	50.0%
55	1	152	94	43	45.7%
55	6	100	80	46	57.5%
56	1	201	102	60	58.8%
57	1	302	110	66	60.0%
57	6	28	28	18	64.3%
58	1	152	94	52	55.3%
59	1	177	98	45	45.9%
59	6	128	89	47	52.8%
60	1	100	82	46	56.1%
61	1	154	94	55	58.5%
62	1	407	116	62	53.5%
63	1	404	116	68	58.6%
63	6	150	94	61	64.9%
64	1	251	106	68	64.2%
65	1	273	108	55	50.9%
65	6	252	106	58	54.7%
66	1	83	72	40	55.6%
66	6	74	72	35	48.6%
67	1	222	102	69	67.7%
67	6	125	88	41	46.6%
68	1	326	110	65	59.1%
68	6	243	106	66	62.3%

TABLE VII (Continued)

ANTELOPE LICENSE DATA SUMMARY

HUNT AREA	LICENSE TYPE	NUMBER SOLD	NUMBER SURVEYED	NUMBER USABLE SURVEYS (both hunters and nonhunters)	% USABLE RESPONSE
69	1	101	82	38	46.3%
69	6	56	54	24	44.4%
70	1	201	102	65	63.7%
70	6	300	110	65	59.1%
71	1	303	110	61	55.5%
71	6	199	102	50	49.0%
72	1	906	126	77	61.1%
72	6	452	118	45	38.1%
73	1	504	120	58	48.3%
73	2	305	110	54	49.1%
73	6	399	117	65	55.6%
73	7	52	52	26	50.0%
74	1	202	102	46	45.1%
74	6	173	98	46	46.9%
75	1	404	116	73	62.9%
75	6	273	108	46	42.6%
76	1	100	80	45	56.3%
76	6	36	36	26	72.2%
77	1	54	54	35	64.8%
77	6	31	32 *	15	46.9%
77	7	25	25	9	36.0%
78	1	50	50	38	76.0%
78	6	51	51	24	47.1%
78	7	35	35	15	42.9%
79, 116	9	30	30	18	60.0%
80	1	106	82	39	47.6%
80	6	101	82	46	56.1%
81	1	25	25	15	60.0%
81	2	51	51	29	56.9%
82	1	80	74	45	60.8%
82	6	35	35	16	45.7%
83	1	78	72	38	52.8%
83	6	50	49	20	40.8%
84	1	36	36	25	69.4%
84	6	25	25	15	60.0%
85	1	16	16	12	75.0%
86	1	34	34	13	38.2%
86	6	25	25	13	52.0%
87	1	203	102	57	55.9%
87	2	152	94	52	55.3%
87	6	250	106	64	60.4%
87	7	250	106	68	64.2%
88	1	254	106	56	52.8%
88	6	275	108	69	63.9%
89	1	252	106	61	57.6%
89	6	176	98	58	59.2%
89	7	79	75	27	36.0%
90	1	207	102	57	55.9%
90	2	202	102	60	58.8%
90	6	175	98	62	63.3%
90	7	175	98	75	76.5%
91	1	403	116	72	62.1%
91	6	127	88	48	54.6%
91	7	25	25	17	68.0%
92	1	303	110	65	59.1%
92	6	101	82	49	59.8%
92	7	25	25	16	64.0%
93	1	703	124	78	62.9%
93	6	600	122	67	54.9%
93	7	100	80	49	61.3%
94	1	501	120	69	57.5%
94	6	202	102	51	50.0%
94	7	301	110	45	40.9%
95	1	331	112	71	63.4%
95	7	51	51	25	49.0%

TABLE VII (Continued)

ANTELOPE LICENSE DATA SUMMARY

HUNT AREA	LICENSE TYPE	NUMBER SOLD	NUMBER SURVEYED	NUMBER USABLE SURVEYS (both hunters and nonhunters)	% USABLE RESPONSE
96	1	279	108	53	49.1%
96	6	125	88	47	53.4%
96	7	25	25	15	60.0%
97, 117	1	126	126	68	54.0%
97, 117	6	111	106	58	54.7%
97, 117	7	20	25 *	12	48.0%
98	1	157	94	56	59.6%
98	6	300	110	73	66.4%
99	1	128	88	48	54.6%
99	6	100	80	45	56.3%
99	7	199	102	35	34.3%
99	8	51	51	39	76.5%
100	1	100	80	52	65.0%
100	6	300	110	70	63.6%
102	1	449	120	61	50.8%
102	6	350	120	42	35.0%
103	1	363	114	72	63.2%
103	6	50	52 *	26	50.0%
103	7	200	102	46	45.1%
104	1	204	102	52	51.0%
104	6	175	98	35	35.7%
106	1	274	106	70	66.0%
106	6	200	106	53	50.0%
107	1	106	82	54	65.9%
107	6	150	94	70	74.5%
107	8	134	90	60	66.7%
108	1	106	82	48	58.5%
109	1	203	110	47	42.7%
109	6	173	110	55	50.0%
110	1	63	63	37	58.7%
110	6	25	25	10	40.0%
111	1	150	94	43	45.7%
111	6	200	103	39	37.9%
112	1	177	98	53	54.1%
112	6	76	72	44	61.1%
113	1	177	99	58	58.6%
113	2	154	98	68	69.4%
113	6	247	110	50	45.5%
114	1	57	57	37	64.9%
114	6	26	26	18	69.2%
115	1	102	82	42	51.2%
115	6	125	88	55	62.5%
TOTAL		65397	22918	12639	55.1%

* Actual downloads exceed WG&F sales data.

WYOMING GAME AND FISH COMMISSION

CHAPTER 5

ANTELOPE HUNTING SEASONS

Section 1. Authority. This regulation is promulgated by authority of W.S. §23-1-302, §23-1-703 and §23-2-104.

Section 2. Hunting Seasons Established. There shall be open seasons during 2007 for the hunting of antelope as set forth in this Chapter and in accordance with Chapter 2, General Hunting Regulation.

Section 3. Regular Hunting Seasons.

(a) Hunt areas, season dates and limitations.

		Date of Seasons		
Hunt Area	Type	Opens	Closes	Limitations
1	1	Oct. 1	Nov. 20	Limited quota; 400 licenses any antelope
	6	Oct. 1	Nov. 20	Limited quota; 250 licenses doe or fawn valid on private land
2	1	Oct. 1	Nov. 20	Limited quota; 400 licenses any antelope
	6	Oct. 1	Nov. 20	Limited quota; 400 licenses doe or fawn
3	1	Oct. 1	Nov. 20	Limited quota; 450 licenses any antelope
	6	Oct. 1	Nov. 20	Limited quota; 400 licenses doe or fawn valid on private land
4	1	Oct. 1	Nov. 20	Limited quota; 350 licenses any antelope
	6	Oct. 1	Nov. 20	Limited quota; 300 licenses doe or fawn
5	1	Oct. 1	Nov. 20	Limited quota; 300 licenses any antelope
	6	Oct. 1	Nov. 20	Limited quota; 400 licenses doe or fawn valid on private land
6	1	Oct. 1	Oct. 15	Limited quota; 350 licenses any antelope
	6	Oct. 1	Oct. 15	Limited quota; 150 licenses doe or fawn
7	1	Oct. 1	Oct. 15	Limited quota; 1,000 licenses any antelope
	6	Oct. 1	Oct. 15	Limited quota; 800 licenses doe or fawn valid in that portion of Area 7 north of Lodgepole Creek
8	1	Oct. 1	Oct. 15	Limited quota; 300 licenses any antelope
	6	Oct. 1	Oct. 15	Limited quota; 50 licenses doe or fawn
9	1	Oct. 1	Oct. 15	Limited quota; 550 licenses any antelope
	6	Oct. 1	Oct. 31	Limited quota; 400 licenses doe or fawn
10	1	Oct. 1	Oct. 14	Limited quota; 350 licenses any antelope
	6	Oct. 1	Oct. 31	Limited quota; 500 licenses doe or fawn
11	1	Oct. 1	Oct. 15	Limited quota; 250 licenses any antelope
	6	Oct. 1	Oct. 15	Limited quota; 300 licenses doe or fawn
		Oct. 16	Oct. 31	Unused Area 11 Type 6 licenses valid in that portion of Area 11 in Niobrara County.
12	1	Oct. 1	Oct. 15	Limited quota; 200 licenses any antelope
	6	Oct. 1	Oct. 15	Limited quota; 250 licenses doe or fawn
		Oct. 16	Oct. 31	Unused Area 12 Type 6 licenses valid in that portion of Area 12 in Converse and Niobrara Counties

Hunt Area	Type	Date of Seasons Opens	Closes	Limitations
15	1	Oct. 1	Oct. 14	Limited quota; 800 licenses any antelope
	6	Oct. 1	Oct. 31	Limited quota; 800 licenses doe or fawn
16	1	Oct. 1	Oct. 14	Limited quota; 500 licenses any antelope
	6	Oct. 1	Oct. 31	Limited quota; 400 licenses doe or fawn
17	1	Oct. 1	Oct. 31	Limited quota; 1,200 licenses any antelope
18	1	Oct. 1	Oct. 20	Limited quota; 500 licenses any antelope
19	1	Oct. 1	Oct. 20	Limited quota; 700 licenses any antelope
17, 18, 19	6	Oct. 1	Oct. 31	Limited quota; 1,200 licenses doe or fawn valid on private land
20	1	Oct. 15	Nov. 15	Limited quota; 800 licenses any antelope
	6	Oct. 15	Nov. 15	Limited quota; 600 licenses doe or fawn
21	1	Oct. 15	Oct. 31	Limited quota; 600 licenses any antelope
	6	Oct. 15	Oct. 31	Limited quota; 600 licenses doe or fawn valid on private land
22	1	Oct. 1	Oct. 31	Limited quota; 800 licenses any antelope
	6	Oct. 1	Oct. 31	Limited quota; 600 licenses doe or fawn
23	1	Oct. 1	Oct. 31	Limited quota; 2,000 licenses any antelope
	6	Oct. 1	Oct. 31	Limited quota; 1,300 licenses doe or fawn valid on private land
24	1	Oct. 1	Oct. 31	Limited quota; 800 licenses any antelope
	6	Oct. 1	Oct. 31	Limited quota; 700 licenses doe or fawn valid on private land
25	1	Oct. 1	Oct. 14	Limited quota; 650 licenses any antelope
	6	Oct. 1	Oct. 14	Limited quota; 400 licenses doe or fawn valid in that portion of Area 25 south of the Ormsby Road (Natrona County Road 705)
26	1	Sept. 24	Oct. 14	Limited quota; 1,100 licenses any antelope, also valid in Area 28
	6	Sept. 24	Oct. 14	Limited quota; 500 licenses doe or fawn, also valid in Area 28
27	1	Oct. 1	Oct. 15	Limited quota; 450 licenses any antelope
	6	Oct. 1	Oct. 15	Limited quota; 450 licenses doe or fawn
28	1	Sept. 24	Oct. 14	Limited quota; 400 licenses any antelope, also valid in Area 26
	6	Sept. 24	Oct. 14	Limited quota; 300 licenses doe or fawn, also valid in Area 26
29	1	Oct. 1	Oct. 15	Limited quota; 1,200 licenses any antelope
	6	Oct. 1	Oct. 15	Limited quota; 800 licenses doe or fawn
	7	Oct. 1	Oct. 31	Limited quota; 200 licenses doe or fawn valid in that portion of Area 29 west of Interstate Highway 25
30	1	Oct. 10	Oct. 31	Limited quota; 600 licenses any antelope
	6	Oct. 10	Oct. 31	Limited quota; 700 licenses doe or fawn
31	1	Sept. 25	Oct. 25	Limited quota; 400 licenses any antelope
	6	Sept. 25	Oct. 25	Limited quota; 450 licenses doe or fawn

Hunt Area	Type	Date of Seasons Opens	Closes	Limitations
32	1	Sept. 25	Oct. 25	Limited quota; 450 licenses any antelope
	2	Oct. 5	Oct. 25	Limited quota; 250 licenses any antelope
	6	Sept. 25	Oct. 25	Limited quota; 650 licenses doe or fawn
34	1	Oct. 1	Oct. 14	Limited quota; 225 licenses any antelope
	6	Oct. 1	Oct. 14	Limited quota; 200 licenses doe or fawn
		Nov. 15	Dec. 31	Unused Area 34 Type 6 licenses
35	1	Oct. 1	Oct. 14	Limited quota; 300 licenses any antelope
	6	Oct. 1	Oct. 14	Limited quota; 200 licenses doe or fawn
		Nov. 15	Dec. 31	Unused Area 35 Type 6 licenses
36	1	Oct. 1	Oct. 14	Limited quota; 200 licenses any antelope
	6	Oct. 1	Oct. 14	Limited quota; 200 licenses doe or fawn
		Nov. 15	Dec. 31	Unused Area 36 Type 6 licenses
37	1	Sept. 15	Oct. 14	Limited quota; 275 licenses any antelope
	6	Sept. 15	Oct. 14	Limited quota; 75 licenses doe or fawn valid in that portion of Area 37 east of the Harriman Road (Wyoming Highway 218, Laramie County Road 102)
38	1	Oct. 5	Oct. 31	Limited quota; 550 licenses any antelope
	6	Oct. 5	Oct. 31	Limited quota; 500 licenses doe or fawn
39	1	Oct. 5	Oct. 31	Limited quota; 300 licenses any antelope
	6	Oct. 5	Oct. 31	Limited quota; 100 licenses doe or fawn
40	1	Oct. 5	Oct. 31	Limited quota; 200 licenses any antelope
	6	Oct. 5	Oct. 31	Limited quota; 200 licenses doe or fawn
41	1	Sept. 25	Oct. 31	Limited quota; 50 licenses any antelope
	6	Sept. 25	Oct. 31	Limited quota; 50 licenses doe or fawn
42	1	Sept. 25	Oct. 31	Limited quota; 650 licenses any antelope
	6	Sept. 25	Oct. 31	Limited quota; 300 licenses doe or fawn
	7	Oct. 5	Oct. 31	Limited quota; 200 licenses doe or fawn
43	1	Sept. 15	Oct. 14	Limited quota; 400 licenses any antelope
	6	Sept. 15	Oct. 14	Limited quota; 400 licenses doe or fawn
44	1	Sept. 15	Oct. 14	Limited quota; 250 licenses any antelope
	6	Sept. 15	Oct. 14	Limited quota; 300 licenses doe or fawn
45	1	Sept. 15	Oct. 14	Limited quota; 650 licenses any antelope
	6	Sept. 15	Oct. 14	Limited quota; 600 licenses doe or fawn
46	1	Sept. 25	Oct. 25	Limited quota; 500 licenses any antelope
	6	Sept. 25	Oct. 25	Limited quota; 300 licenses doe or fawn
	7	Oct. 5	Oct. 25	Limited quota; 200 licenses doe or fawn

Hunt Area	Type	Date of Seasons Opens	Closes	Limitations
47	1	Sept. 25	Oct. 31	Limited quota; 1,000 licenses any antelope
	2	Oct. 5	Oct. 31	Limited quota; 350 licenses any antelope
	6	Sept. 25	Oct. 31	Limited quota; 700 licenses doe or fawn
	7	Oct. 5	Oct. 31	Limited quota; 400 licenses doe or fawn
48	1	Sept. 25	Oct. 31	Limited quota; 350 licenses any antelope
	2	Oct. 5	Oct. 31	Limited quota; 150 licenses any antelope
	6	Sept. 25	Oct. 31	Limited quota; 375 licenses doe or fawn
	7	Oct. 5	Oct. 31	Limited quota; 475 licenses doe or fawn valid in that portion of Area 48 north of the Medicine Bow River
		Nov. 1	Nov. 30	Unused Area 48 Type 6 and Type 7 licenses valid in that portion of Area 48 north of the Lost Creek-Medicine Bow River hydrographic divide and west of Carbon County Road 291; or north of Carbon County Road 102 and west of Carbon County Road 103
50	1	Sept. 15	Oct. 31	Limited quota; 500 licenses any antelope
	6	Sept. 15	Oct. 31	Limited quota; 500 licenses doe or fawn
	8	Sept. 1	Sept. 14	Limited quota; 50 licenses any antelope, muzzle-loading firearms only
51	1	Sept. 15	Nov. 14	Limited quota; 100 licenses any antelope
	6	Sept. 15	Nov. 14	Limited quota; 150 licenses doe or fawn
52	1	Sept. 25	Oct. 31	Limited quota; 250 licenses any antelope
	2	Sept. 25	Oct. 31	Limited quota; 250 licenses any antelope valid in that portion of Area 52 south of North Spring Creek
	6	Sept. 25	Oct. 31	Limited quota; 200 licenses doe or fawn
	7	Sept. 25	Nov. 14	Limited quota; 100 licenses doe or fawn valid in that portion of Area 52 south of North Spring Creek
53	1	Sept. 20	Oct. 14	Limited quota; 550 licenses any antelope
	6	Sept. 20	Oct. 14	Limited quota; 750 licenses doe or fawn
	7	Sept. 1	Oct. 14	Limited quota; 25 licenses doe or fawn valid on private land
55	1	Sept. 20	Oct. 14	Limited quota; 150 licenses any antelope
	6	Sept. 20	Oct. 14	Limited quota; 100 licenses doe or fawn
56	1	Sept. 20	Oct. 14	Limited quota; 200 licenses any antelope
57	1	Sept. 20	Oct. 14	Limited quota; 300 licenses any antelope
	6	Sept. 1	Oct. 14	Limited quota; 25 licenses doe or fawn
58	1	Sept. 20	Oct. 14	Limited quota; 150 licenses any antelope
59	1	Sept. 20	Oct. 14	Limited quota; 175 licenses any antelope
	6	Sept. 20	Oct. 14	Limited quota; 125 licenses doe or fawn
60	1	Sept. 15	Oct. 14	Limited quota; 100 licenses any antelope
61	1	Sept. 1	Sept. 30	Limited quota; 150 licenses any antelope
62	1	Sept. 8	Oct. 14	Limited quota; 400 licenses any antelope
63	1	Sept. 11	Oct. 14	Limited quota; 400 licenses any antelope
	6	Sept. 11	Oct. 14	Limited quota; 150 licenses doe or fawn

Hunt Area	Type	Date of Seasons Opens	Closes	Limitations
64	1	Sept. 15	Oct. 14	Limited quota; 250 licenses any antelope
65	1	Sept. 15	Oct. 22	Limited quota; 250 licenses any antelope
	6	Sept. 15	Oct. 22	Limited quota; 250 licenses doe or fawn
66	1	Sept. 15	Oct. 22	Limited quota; 75 licenses any antelope
	6	Sept. 15	Oct. 22	Limited quota; 75 licenses doe or fawn
67	1	Sept. 15	Oct. 22	Limited quota; 200 licenses any antelope
	6	Sept. 15	Oct. 22	Limited quota; 125 licenses doe or fawn
68	1	Sept. 15	Oct. 22	Limited quota; 300 licenses any antelope
	6	Sept. 15	Oct. 22	Limited quota; 250 licenses doe or fawn
69	1	Sept. 15	Oct. 22	Limited quota; 100 licenses any antelope
	6	Sept. 15	Oct. 22	Limited quota; 50 licenses doe or fawn
70	1	Sept. 15	Oct. 22	Limited quota; 200 licenses any antelope
	6	Sept. 15	Nov. 30	Limited quota; 300 licenses doe or fawn
71	1	Sept. 15	Oct. 22	Limited quota; 300 licenses any antelope
	6	Sept. 15	Oct. 22	Limited quota; 200 licenses doe or fawn
72	1	Sept. 15	Oct. 22	Limited quota; 900 licenses any antelope
	6	Sept. 15	Oct. 22	Limited quota; 450 licenses doe or fawn
73	1	Sept. 15	Sept. 30	Limited quota; 500 licenses any antelope
	2	Oct. 12	Oct. 31	Limited quota; 300 licenses any antelope
	6	Sept. 15	Oct. 31	Limited quota; 400 licenses doe or fawn
	7	Nov. 1	Nov. 20	Limited quota; 50 licenses doe or fawn valid in that portion of Area 73 east of the Bucknum Road (Natrona County Road 125) in the Casper Creek drainage
74	1	Sept. 15	Oct. 22	Limited quota; 200 licenses any antelope
	6	Sept. 15	Oct. 22	Limited quota; 175 licenses doe or fawn
75	1	Sept. 15	Oct. 22	Limited quota; 400 licenses any antelope
	6	Sept. 15	Oct. 22	Limited quota; 275 licenses doe or fawn
76	1	Oct. 9	Oct. 31	Limited quota; 100 licenses any antelope
	6	Sept. 1	Oct. 31	Limited quota; 35 licenses doe or fawn
77	1	Sept. 20	Oct. 14	Limited quota; 50 licenses any antelope
	6	Sept. 1	Oct. 14	Limited quota; 30 licenses doe or fawn valid on private land in Big Horn County or in that portion of Area 77 north of Wyoming Highway 30 or east of U.S. Highway 16-20
	7	Sept. 1	Oct. 14	Limited quota; 25 licenses doe or fawn valid on private land in that portion of Area 77 in Washakie County
78	1	Sept. 1	Sept. 15	Limited quota; 50 licenses any antelope
	6	Sept. 1	Oct. 31	Limited quota; 50 licenses doe or fawn valid in that portion of Area 78 south of U.S. Highway 14-16-20 and that portion of Area 78 south of Little Dry Creek east of Wyoming Highway 789
	7	Sept. 1	Oct. 31	Limited quota; 35 licenses doe or fawn valid in that portion of Area 78 west of the Oregon Basin/Garland power line

Hunt Area	Type	Date of Seasons Opens	Closes	Limitations
79, 116	9	Aug. 15	Sept. 30	Limited quota; 30 licenses any antelope, archery only
80	1	Sept. 1	Sept. 30	Limited quota; 100 licenses any antelope
	6	Sept. 1	Sept. 30	Limited quota; 100 licenses doe or fawn valid on private land
81	1	Oct. 1	Nov. 15	Limited quota; 25 licenses any antelope valid in that portion of Area 81 east of Wyoming Highway 120
	2	Nov. 1	Nov. 15	Limited quota; 50 licenses any antelope
82	1	Sept. 20	Oct. 14	Limited quota; 75 licenses any antelope
	6	Sept. 1	Sept. 19	Limited quota; 35 licenses doe or fawn valid in that portion of Area 82 south of the Lower Greybull River Road (Park County Road 3LE) and east of Coyote Canyon Road (Park County Road 3SL) to the Park-Big Horn County line
		Sept. 20	Oct. 14	Unused Area 82 Type 6 doe or fawn valid in that portion of Area 82 west of Wyoming Highway 120
83	1	Oct. 9	Oct. 31	Limited quota; 75 licenses any antelope
	6	Sept. 1	Oct. 31	Limited quota; 50 licenses doe or fawn valid on private land
84	1	Sept. 15	Oct. 22	Limited quota; 35 licenses any antelope
	6	Sept. 15	Oct. 22	Limited quota; 25 licenses doe or fawn
85	1	Sept. 10	Oct. 31	Limited quota; 15 licenses any antelope
86	1	Sept. 10	Oct. 31	Limited quota; 30 licenses any antelope
	6	Sept. 10	Oct. 31	Limited quota; 25 licenses doe or fawn
87	1	Sept. 10	Oct. 31	Limited quota; 200 licenses any antelope
	2	Sept. 25	Oct. 31	Limited quota; 150 licenses any antelope
	6	Sept. 10	Oct. 31	Limited quota; 250 licenses doe or fawn
	7	Sept. 25	Oct. 31	Limited quota; 250 licenses doe or fawn
88	1	Sept. 10	Oct. 31	Limited quota; 250 licenses any antelope
	6	Oct. 1	Oct. 31	Limited quota; 275 licenses doe or fawn
89	1	Sept. 10	Oct. 31	Limited quota; 250 licenses any antelope
	6	Oct. 1	Oct. 31	Limited quota; 175 licenses doe or fawn
	7	Oct. 1	Oct. 31	Limited quota; 75 licenses doe or fawn valid in that portion of Area 89 south of Wyoming Highway 351 and east of U.S. Highway 189
90	1	Sept. 10	Oct. 31	Limited quota; 200 licenses any antelope valid in that portion of Area 90 east of U.S. Highway 191
	2	Sept. 10	Oct. 31	Limited quota; 200 licenses any antelope valid in that portion of Area 90 west of U.S. Highway 191
	6	Sept. 1	Sept. 9	Limited quota; 175 licenses doe or fawn valid in that portion of Area 90 east of U.S. Highway 191 on private land
		Sept. 10	Oct. 31	Unused Area 90 Type 6 licenses doe or fawn valid in that portion of Area 90 east of U.S. Highway 191
	7	Sept. 10	Oct. 31	Limited quota; 175 licenses doe or fawn valid in that portion of Area 90 west of U.S. Highway 191

Hunt Area	Type	Date of Seasons Opens	Closes	Limitations
91	1	Sept. 10	Oct. 14	Limited quota; 400 licenses any antelope
	6	Sept. 10	Oct. 14	Limited quota; 125 licenses doe or fawn
	7	Sept. 10	Oct. 14	Limited quota; 25 licenses doe or fawn valid in that portion of Area 91 within the Farson-Eden Irrigation Project
92	1	Sept. 10	Oct. 14	Limited quota; 300 licenses any antelope
	6	Sept. 10	Oct. 14	Limited quota; 100 licenses doe or fawn
	7	Sept. 10	Oct. 14	Limited quota; 25 licenses doe or fawn valid in that portion of Area 92 within the Farson-Eden Irrigation Project
93	1	Sept. 10	Oct. 14	Limited quota; 700 licenses any antelope
	6	Sept. 10	Oct. 14	Limited quota; 600 licenses doe or fawn
	7	Sept. 10	Oct. 14	Limited quota; 100 licenses doe or fawn valid in that portion of Area 93 north and west of Wyoming Highway 189 on private land
94	1	Sept. 10	Oct. 14	Limited quota; 500 licenses any antelope
	6	Sept. 10	Oct. 14	Limited quota; 200 licenses doe or fawn
	7	Sept. 10	Oct. 14	Limited quota; 300 licenses doe or fawn valid in that portion of Area 94 on irrigated lands
95	1	Sept. 10	Oct. 14	Limited quota; 325 licenses any antelope
	7	Sept. 10	Oct. 14	Limited quota; 50 licenses doe or fawn valid in that portion of Area 95 west of Cottonwood Creek or the Smith’s Fork River
96	1	Sept. 10	Oct. 14	Limited quota; 275 licenses any antelope
	6	Sept. 10	Oct. 14	Limited quota; 125 licenses doe or fawn
	7	Sept. 10	Oct. 14	Limited quota; 25 licenses doe or fawn valid in that portion of Area 96 within the Farson-Eden Irrigation Project
97, 117	1	Sept. 15	Oct. 22	Limited quota; 125 licenses any antelope
	6	Sept. 15	Oct. 22	Limited quota; 100 licenses doe or fawn
	7	Sept. 15	Oct. 22	Limited quota; 25 licenses doe or fawn valid in that portion of Area 97 south of U.S. Highway 26 and in all of Area 117
98	1	Sept. 10	Oct. 14	Limited quota; 150 licenses any antelope
	6	Sept. 10	Oct. 14	Limited quota; 300 licenses doe or fawn
99	1	Sept. 10	Oct. 14	Limited quota; 125 licenses any antelope
	6	Sept. 10	Oct. 14	Limited quota; 100 licenses doe or fawn valid in that portion of Area 99 west of Wyoming Highway 410 and Uinta County Road 283 (U.S. Forest Service Road 075)
	7	Sept. 10	Oct. 14	Limited quota; 200 licenses doe or fawn valid in that portion of Area 99 east of Wyoming Highway 410 and Uinta County Road 283 (U.S. Forest Service Road 075)
	8	Sept. 1	Sept. 30	Limited quota; 50 licenses any antelope, muzzle-loading firearms only
100	1	Sept. 10	Oct. 14	Limited quota; 100 licenses any antelope
	6	Sept. 10	Oct. 14	Limited quota; 300 licenses doe or fawn
102	1	Oct. 15	Nov. 15	Limited quota; 500 licenses any antelope
	6	Oct. 15	Nov. 15	Limited quota; 500 licenses doe or fawn

Hunt Area	Type	Date of Seasons Opens	Closes	Limitations
103	1	Oct. 5	Oct. 31	Limited quota; 350 licenses any antelope
	6	Oct. 5	Oct. 31	Limited quota; 50 licenses doe or fawn
	7	Oct. 5	Oct. 31	Limited quota; 200 licenses doe or fawn valid in that portion of Area 103 south of Cottonwood Creek
104	1	Oct. 20	Nov. 11	Limited quota; 200 licenses any antelope
	6	Oct. 20	Nov. 11	Limited quota; 175 licenses doe or fawn
		Nov. 12	Dec. 31	Unused Area 104 Type 6 licenses valid in that portion of Area 104 off national forest
106	1	Sept. 15	Oct. 22	Limited quota; 250 licenses any antelope
	6	Sept. 15	Oct. 22	Limited quota; 250 licenses doe or fawn
107	1	Sept. 10	Sept. 30	Limited quota; 100 licenses any antelope
	6	Sept. 10	Sept. 30	Limited quota; 150 licenses doe or fawn
	8	Aug. 20	Sept. 9	Limited quota; 125 licenses any antelope, muzzle-loading firearms and handguns only
108	1	Sept. 20	Oct. 14	Limited quota; 100 licenses any antelope
109	1	Sept. 15	Nov. 30	Limited quota; 300 licenses any antelope
	6	Sept. 15	Nov. 30	Limited quota; 300 licenses doe or fawn
110	1	Sept. 20	Oct. 14	Limited quota; 60 licenses any antelope
	6	Sept. 20	Oct. 14	Limited quota; 25 licenses doe or fawn valid on private land
111	1	Sept. 15	Oct. 14	Limited quota; 150 licenses any antelope
	6	Sept. 15	Oct. 14	Limited quota; 200 licenses doe or fawn
		Nov. 15	Dec. 31	Unused Area 111 Type 6 licenses
112	1	Sept. 20	Oct. 14	Limited quota; 175 licenses any antelope
	6	Sept. 20	Oct. 14	Limited quota; 75 licenses doe or fawn
113	1	Oct. 1	Oct. 31	Limited quota; 175 licenses any antelope
	2	Oct. 11	Oct. 31	Limited quota; 175 licenses any antelope
	6	Oct. 1	Oct. 31	Limited quota; 300 licenses doe or fawn
114	1	Oct. 1	Oct. 31	Limited quota; 50 licenses any antelope
	6	Sept. 1	Oct. 31	Limited quota; 25 licenses doe or fawn valid on private land
115	1	Oct. 9	Oct. 31	Limited quota; 100 licenses any antelope
	6	Oct. 9	Oct. 31	Limited quota; 125 licenses doe or fawn valid in that portion of Area 115 east of the Upper Nowood Road or south and west of Nowater Stock Trail (BLM Road 1411)

Section 4. Special Archery Seasons. Hunt Areas listed in this section shall have special archery hunting seasons starting on the dates as specified in this section. Special archery seasons close when the earliest any antelope season for the Hunt Area opens as listed in Section 3.

Archers shall possess a limited quota antelope license in addition to an archery license in order to hunt antelope during any special archery season. Archers shall hunt only in the Hunt Area(s) and for the sex of antelope set forth by the limitations of their license as specified in Section 3 of this Chapter.

Hunt Area	Opening Date	Limitations
1-10, 15-24, 27, 29, 30, 102, 113	Sept. 1	Refer to Section 3 of this Chapter
11,12, 25, 26, 28, 31, 32, 34-48, 50-53, 55-78, 80-100, 103, 104, 106-112, 114, 115, 117	Aug. 15	Refer to Section 3 of this Chapter

Section 5. Hunt Area Descriptions.

(a) Area and Number.

Area 1. Crook. Beginning where Wyoming Highway 112 crosses the Wyoming-Montana state line; southwesterly along said highway to Wyoming Highway 24 in the town of Hulett; southerly along said highway to U.S. Highway 14; westerly along said highway to the Cabin Creek Road; northwesterly along said road to Oshoto and the Oshoto Road connection; westerly along said road to the "D" Road; northerly along said road to the Rocky Point Road; northerly along said road to the Wyoming-Montana state line.

Area 2. Bearlodge. Beginning where Wyoming Highway 112 crosses the Wyoming-Montana state line; easterly along said line to the Wyoming-South Dakota state line; southerly along said line to Interstate Highway 90; southwesterly along said highway to the town of Sundance and U.S. Highway 14; westerly and northerly along said highway to Wyoming Highway 24; northerly along said highway to Wyoming Highway 112 in the town of Hulett; northeasterly along said highway to the Wyoming-Montana state line.

Area 3. Keyhole. Beginning where Interstate Highway 90 intersects U.S. Highway 14 in the town of Sundance; southerly and westerly along Interstate Highway 90 to its intersection with the "D" Road; northerly along said road to the Oshoto Road connection; easterly along said road to Oshoto and the Cabin Creek Road; southerly and easterly along the Cabin Creek Road to U.S. Highway 14; easterly and southerly along said highway to Interstate Highway 90 in the town of Sundance.

Area 4. Arch Creek. Beginning in the town of Upton where U.S. Highway 16 intersects Wyoming Highway 116; westerly and northerly along U.S. Highway 16 to Interstate Highway 90 in the town of Moorcroft; easterly and northerly along said highway to U.S. Highway 14; easterly along said highway to Wyoming Highway 116; southerly and westerly along said highway to its junction with U.S. Highway 16 in the town of Upton.

Area 5. Inyan Kara. Beginning where Interstate Highway 90 intersects the Wyoming-South Dakota state line; southerly along said line to U.S. Highway 16; northerly and westerly along said highway to Wyoming Highway 116 in the town of Upton; northerly and easterly along said highway to U.S. Highway 14; westerly along said highway to Interstate Highway 90; northerly and easterly along said highway to the Wyoming-South Dakota state line.

Area 6. South Newcastle. Beginning where U.S. Highway 16 intersects the Wyoming-South Dakota state line; southerly along said line to the Dewey Road; northwesterly along said road to U.S. Highway 85; southerly along said highway to Cheyenne River Road N. (Weston County); westerly along said road to the Lynch Road; northerly along said road to Wyoming Highway 450; northeasterly along said highway to U.S. Highway 16; southeasterly along said highway to the Wyoming-South Dakota state line.

Area 7. South Upton. Beginning where Wyoming Highway 450 intersects U.S. Highway 16; southwesterly along Wyoming Highway 450 to the Keeline Road (U.S.F.S. Road 930); northwesterly along said road to Black Thunder Creek; easterly down said creek to Bacon Creek; northerly up said creek to Newel Prong; northerly up said prong to the divide between the Cheyenne River and Belle Fourche River; northeasterly along said divide to the divide between Four Horse Creek and Raven Creek; northwesterly along said divide to the Belle Fourche River; northeasterly down said river to U.S. Highway 16; southeasterly along said highway to Wyoming Highway 450.

Area 8. Niobrara. Beginning where the Boner Road crosses the Wyoming-South Dakota state line; westerly along said road to U.S. Highway 85; southerly along said highway to Wyoming Highway 270; westerly

along said highway to Wyoming Highway 272; northerly along said highway to the North Lance Creek Road; northerly and westerly along said road to the Niobrara-Converse county line and the Dull Center Road (Converse County Road 38); westerly along said road to the Clareton Road (Converse County Road 39); northeasterly along said road to the Niobrara-Weston county line and the Lynch Road; northerly along said road to the Cheyenne River Road N. (Weston County); easterly along said road to U.S. Highway 85; northerly along said highway to the Dewey Road; southeasterly along said road to the Wyoming-South Dakota state line; southerly along said line to the Boner Road.

Area 9. Lance Creek. Beginning where U.S. Highway 20 crosses the Wyoming-Nebraska state line; westerly along said highway to the Twenty Mile Creek Road (Converse County Road 46) at Lost Springs; northerly along said road to the Manning Road (Converse County Road 53); easterly along said road to the Twenty Mile Road (Niobrara County) at the Converse-Niobrara county line; easterly along said road to Wyoming Highway 271; easterly along said highway to the town of Lance Creek and Wyoming Highway 270; northerly and easterly along said highway to U.S. Highway 85; northerly along said highway to the Boner Road; easterly along said road to the Wyoming-South Dakota state line; southerly along said line to the Wyoming-Nebraska state line; southerly along said line to U.S. Highway 20.

Area 10. Lake De Smet. Beginning at the town of Ucross and U.S. Highway 16; southerly along said highway to Interstate Highway 90; northwesterly along said highway to the Meade Creek Road (Sheridan County Road 131); northeasterly along said road to U.S. Highway 14; southeasterly along said highway to the town of Ucross and U.S. Highway 16.

Area 11. Rawhide Butte. Beginning where U.S. Highway 20 intersects the Wyoming-Nebraska state line; southerly along said line to the North Platte River; northwesterly up said river to U.S. Highway 26 at the town of Guernsey; easterly along said highway to Wyoming Highway 270; northerly along said highway to U.S. Highway 20; easterly along said highway to the Wyoming-Nebraska state line.

Area 12. Meadowdale. Beginning where U.S. Highway 20 intersects Wyoming Highway 270; southerly along Wyoming Highway 270 to U.S. Highway 26; westerly along said highway to the North Platte River; northwesterly up said river to Glendo Dam; northerly along the eastern shore of Glendo Reservoir to the North Platte River; northwesterly up said river to Interstate Highway 25; northerly along said highway to U.S. Highway 20 at Orin Junction; easterly along said highway to Wyoming Highway 270.

Area 15. Clearmont. Beginning where the Powder River crosses the Wyoming-Montana state line; southerly up said river to U.S. Highway 14; westerly along said highway to the Meade Creek Road (Sheridan County Road 131); southwesterly along said road to Interstate Highway 90; northwesterly along said highway to the Wyoming-Montana state line; easterly along said line to the Powder River.

Area 16. Ucross. Beginning where U.S. Highway 14 crosses the Powder River; southerly up said river to Interstate Highway 90; northwesterly along said highway to U.S. Highway 16; northeasterly along said highway to U.S. Highway 14; easterly along said highway to the Powder River.

Area 17. Gillette. Beginning where Wyoming Highway 59 crosses the Wyoming-Montana state line; southerly along said highway to U.S. Highway 14-16; southerly along said highway to Interstate Highway 90 west of the town of Gillette; westerly along said highway to the Powder River; northerly down said river to the Wyoming-Montana state line; easterly along said line to Wyoming Highway 59.

Area 18. Rockypoint. Beginning where the Rocky Point Road crosses the Wyoming-Montana state line; southerly along said road to the “D” Road; southerly along said road to Cabin Creek; westerly up said creek to the Cabin-Mitchell Creek hydrographic divide; westerly along said divide to Mitchell Creek; westerly down said creek to the Spring Creek Road north of Adon; southerly and westerly along said road to the Cow Creek Road; westerly along said road to Wyoming Highway 59; northerly along said highway to the Wyoming-Montana state line; easterly along said line to the Rocky Point Road.

Area 19. Rozet. Beginning where the "D" Road crosses Cabin Creek; southerly along said road to Interstate Highway 90; westerly along said highway to U.S. Highway 14-16 west of the city of Gillette; northerly

along said highway to Wyoming Highway 59; northerly along said highway to the Cow Creek Road; easterly along said road to the Spring Creek Road north of Adon; northeasterly along said road to Mitchell Creek; easterly up said creek to the Mitchell Creek-Cabin Creek hydrographic divide; easterly along said divide to Cabin Creek; easterly down said creek to the "D" Road.

Area 20. Upper Powder River. Beginning at the junction of the Trabing Road (Johnson County Road 13) and Interstate Highway 25; southerly along said highway to Wyoming Highway 191; westerly along said highway to Wyoming Highway 190; southerly along said highway to the town of Barnum and the Barnum Mountain Road; westerly along said road to the Hazelton Road (Washakie County Road 81-Johnson County Road 3); northerly along said road to the Gold Mine Road (U.S.F.S. Road 452); northerly along said road to the Canyon Creek Road (U.S.F.S. Road 25); northerly along said road to U.S. Highway 16; easterly along said highway to the Crazy Woman Canyon Road (Johnson County Road 14); easterly along said road to Wyoming Highway 196; northerly along said highway to the Trabing Road (Johnson County Road 13); easterly along said road to Interstate Highway 25.

Area 21. Middle Fork. Beginning at the town of Kaycee and Interstate Highway 25; southeasterly along said highway to the Midwest interchange and the Smoky Gap Road (Natrona County Road 115); westerly along said road to the Dead Horse Road (Natrona County Road 114); southwesterly along said road to the Wild Horse Trail (Natrona County Road 113); westerly along said road to the 33 Mile Road (Natrona County Road 110); northwesterly along said road to the Hazelton Road (Washakie County Road 81); northerly along said road to the Barnum Mountain Road; easterly along said road to the town of Barnum and Wyoming Highway 190; northeasterly along said highway to Wyoming Highway 191; easterly along said highway to Interstate Highway 25.

Area 22. Crazy Woman. Beginning where Interstate Highway 90 crosses the Powder River; southerly up said river to Wyoming Highway 192; westerly along said highway to Interstate Highway 25; northerly along said highway to Interstate Highway 90; southeasterly along said highway to the Powder River.

Area 23. Pumpkin Buttes. Beginning at the junction of U.S. Highway 14-16 and Wyoming Highway 59 at the city of Gillette; southerly along Wyoming Highway 59 to Wyoming Highway 387 at Reno Junction; southwesterly along Wyoming Highway 387 to Wyoming Highway 192; northwesterly along said highway to the Powder River at Sussex; northerly down said river to Interstate Highway 90; easterly along said highway to U.S. Highway 14-16 at the city of Gillette; easterly along said highway to Wyoming Highway 59.

Area 24. Thunder Basin. Beginning where Interstate Highway 90 crosses the Belle Fourche River; southwesterly up said river to the divide between Four Horse Creek and Raven Creek; southeasterly along said divide to the divide between the Cheyenne River and Belle Fourche River Divide; southwesterly along said divide to Newel Prong; southerly down said prong to Bacon Creek; southerly down said creek to Black Thunder Creek; westerly up said creek to the Keeline Road (U.S.F.S Road 930); southeasterly along said road to Wyoming Highway 450; westerly along said highway to Wyoming Highway 59; northerly along said highway to Interstate Highway 90; easterly along said highway to the Belle Fourche River.

Area 25. Ormsby. Beginning where Wyoming Highway 259 intersects Wyoming Highway 387 at the town of Midwest; northeasterly along Wyoming Highway 387 to the top of Pine Ridge; southerly along Pine Ridge to the head of Sand Creek; southeasterly down said creek to the 55 Ranch Road (Converse County Road 23); southerly along said road to Wyoming Highway 95; southwesterly along said highway to the North Platte River; westerly up said river to Interstate Highway 25 in the city of Casper; northerly along said highway to Wyoming Highway 259; easterly along said highway to Wyoming Highway 387 at the town of Midwest.

Area 26. Bear Creek. Beginning where Wyoming Highway 387 intersects Wyoming Highway 59 at Reno Junction; southerly along Wyoming Highway 59 to the Highland Loop Road (Converse County Road 32); westerly along said road to its junction with Wyoming Highway 93 and the Ross-Highland Loop Cutoff Road; westerly along said cutoff road to its junction with the Ross Road (Converse County Road 31) and the Blizzard Heights-Sage Creek Divide Road; westerly along said divide road to the 55 Ranch Road (Converse County Road 23); southerly along said road to Sand Creek; northwesterly up said creek to the top of Pine Ridge; northerly along the top of Pine Ridge to Wyoming Highway 387; northeasterly along said highway to Wyoming Highway 59 at Reno Junction.

Area 27. Bill. Beginning at the junction of Wyoming Highways 59 and 450; easterly along Wyoming Highway 450 to the Lynch Road (Weston County Road 7A); southerly along said road to the Weston-Converse county line and the Clareton Road (Converse County Road 39); southerly along said road to the Dull Center Road (Converse County Road 38); westerly along said road to the Rochelle Hills Road (U.S.F.S. Road 933); northerly along said road to the Cheyenne River; westerly up said river to its confluence with Antelope Creek and the Dry Fork of the Cheyenne River; southwesterly up the Dry Fork of the Cheyenne River to Wyoming Highway 59; northerly along said highway to the junction of Wyoming Highways 59 and 450.

Area 28. Sage Creek. Beginning at the junction of the North Platte River and Wyoming Highway 95 at the town of Glenrock; northeasterly along said highway to the 55 Ranch Road (Converse County Road 23); northerly along said road to the Blizzard Heights-Sage Creek Divide Road; easterly along said road to the Ross Road (Converse County Road 31) and the Ross-Highland Loop Cutoff Road; easterly along said cutoff road to Wyoming Highway 93 and Highland Loop Road (Converse County Road 32); easterly along said road to Wyoming Highway 59; southerly along said highway to the North Platte River at the town of Douglas; westerly up said river to Wyoming Highway 95.

Area 29. Shawnee. Beginning where Interstate Highway 25 crosses the North Platte River immediately south of Orin Junction; westerly and northerly up said river to Wyoming Highway 59 at the town of Douglas; northerly along said highway to the Dry Fork of the Cheyenne River; northeasterly down said fork to its confluence with Antelope Creek and the Cheyenne River; easterly down the Cheyenne River to the Rochelle Hills Road (U.S.F.S. Road 933); southerly along said road to the Dull Center Road (Converse County Road 38); easterly along said road to the Converse-Niobrara county line and the North Lance Creek Road (Niobrara County); easterly and southerly along said road to Wyoming Highway 272; southerly along said highway to Wyoming Highway 270; southwesterly along said highway to the town of Lance Creek and Wyoming Highway 271; westerly along said highway to the Twenty Mile Creek Road (Niobrara County); westerly along said road to the Converse-Niobrara County line and the Manning Road (Converse County Road 53); westerly along said road to the Twenty Mile Creek Road (Converse County Road 46); southerly along said road to U.S. Highway 18-20 at the town of Lost Springs; westerly along said highway to Interstate Highway 25 at Orin Junction; southerly along said highway to the North Platte River.

Area 30. La Prele. Beginning where Wyoming Highway 95 crosses the North Platte River at the town of Glenrock; easterly and southerly down said river to the Jackalope Warm Springs and the Esterbrook Highway (Wyoming Highway 94); southerly along said highway to the Esterbrook Road (Converse County Road 5); southerly along said road to the town of Esterbrook and U.S.F.S. Road 633; easterly and southerly along said road to the divide between Soldier Creek and Horseshoe Creek; southerly along said divide to the divide between Horseshoe Creek and the North Laramie River; westerly and northerly along said divide to Blacktail Peak and the head of South Fork Creek tributary to LaBonte Creek; northerly down said creek to U.S.F.S. Road 658; westerly along said road to the Old Fort Fetterman Road (Converse County Road 16); northerly along said road to the divide between Corduroy Creek and Rocky Ford Creek; westerly along said divide to the divide between La Prele Creek and the Little Medicine Bow River; westerly along said divide to the Converse-Albany county line; westerly along said line to the Little Medicine Road (Albany County Road 62); southwesterly along said road to U.S.F.S. Road 660; northwesterly along said road to Curry Creek; northwesterly down said creek to Deer Creek; northerly down said creek to the Stephens Road (Converse County Road 20); northerly along said road to the Deer Creek Road (Converse County Road 19); northerly along said road to Wyoming Highway 95; northerly along said highway to the North Platte River.

Area 31. Hat Six. Beginning where the Casper city limits intersect Wyoming Highway 251 (Casper Mountain Road); easterly and northerly along said city limits to the North Platte River; easterly down said river to Wyoming Highway 95 at the town of Glenrock; southerly along said highway to the Deer Creek Road (Converse County Road 19); southerly along said road to the Stephens Road (Converse County Road 20); southerly along said road to Deer Creek; southerly up said creek to Sawmill Creek; southerly and westerly up said creek to the top of Reno Hill and the divide between Soldier Creek and the West Fork of Deer Creek; westerly along said divide to the divide between the East Fork of Bates Creek and the West Fork of Deer Creek; westerly along said divide to the divide between Kerfoot Creek and the West Fork of Deer Creek; westerly along said divide to the divide between the West Fork of Deer Creek, Kerfoot Creek and Smith Creek; northerly and westerly along said divide to the head of Smith Creek; northerly across Muddy Mountain along the divide of Corral Creek and Otter Creek to the divide

between Red Creek and the Clear Fork of Muddy Creek; northerly along said divide to the Circle Drive Road (Natrona County Road 505); northerly along said road to Wyoming Highway 251; northerly along said highway to the Casper city limits.

Area 32. Bates Creek. Beginning where the Pathfinder Dam crosses the North Platte River; easterly and northerly down said river to the Casper city limits; southerly and easterly along said limits to Wyoming Highway 251 (Casper Mountain Road); southerly along said road to the Circle Drive Road (Natrona County Road 505); southerly along said road to the divide between Red Creek and the Clear Fork of Muddy Creek; southerly along said divide to the divide between Corral Creek and Otter Creek; southerly across Muddy Mountain along said divide to the head of Smith Creek; southerly and easterly to the divide between Smith Creek, Kerfoot Creek and the West Fork of Deer Creek; easterly along said divide to the divide between Kerfoot Creek and the West Fork of Deer Creek; easterly along said divide to the divide between Spruce Creek and the West Fork of Deer Creek; easterly along said divide to the divide between the East Fork of Bates Creek and West Fork of Deer Creek; easterly along said divide to the divide between Soldier Creek and the West Fork of Deer Creek; easterly along said divide to Reno Hill and the head of Sawmill Creek; easterly and northerly down said creek to Deer Creek; southerly up said creek to Curry Creek; southeasterly up said creek to U.S.F.S. Road 660; westerly along said road to the Squaw Spring Trail Road; southwesterly along said road to the divide between the Little Medicine Bow River and Bates Creek; southwesterly along said divide to Wyoming Highway 77; southerly along said highway to the Chalk Mountain Road (BLM Road 3129); westerly and northerly along said road to the Dry Creek Road (Carbon County Road 103); northwesterly along said road to the south fork of Canyon Creek; westerly and northerly down said fork to Canyon Creek; westerly down said creek to the east shore of Pathfinder Reservoir; northeasterly along said east shore to the Pathfinder Dam and the North Platte River.

Area 34. Rock Eagle. Beginning where Wyoming Highway 151 crosses the Wyoming-Nebraska state line; westerly along said highway to U.S. Highway 85 at Bear Mountain Station; northerly along said highway to Wyoming Highway 313; westerly along said highway to Interstate Highway 25; northerly along said highway to the Laramie River; northeasterly down said river to the North Platte River; southeasterly down said river to the Wyoming-Nebraska state line; south along said line to Wyoming Highway 151.

Area 35. Horse Creek. Beginning at the town of Chugwater and Wyoming Highway 313; easterly along said highway to U.S. Highway 85; southwesterly along said highway to Interstate Highway 25; north along said highway to Wyoming Highway 313.

Area 36. Burns. Beginning at the junction of Wyoming Highway 151 and the Wyoming-Nebraska state line; southerly along said line to Interstate Highway 80; westerly along said highway to Interstate Highway 25; northeasterly along said highway to U.S. Highway 85; easterly and northerly along said highway to Wyoming Highway 151; easterly along said highway to the Wyoming-Nebraska state line.

Area 37. Harriman. Beginning at the city of Laramie and Interstate Highway 80; southeasterly along said highway to Interstate Highway 25 at the city of Cheyenne; southerly along said highway to the Wyoming-Colorado state line; west along said line to U.S. Highway 287; northerly along said highway to Interstate Highway 80.

Area 38. Little Bear Creek. Beginning at the junction of Wyoming Highway 34 and Interstate Highway 25, near the city of Wheatland; southerly along said Interstate to Wyoming Highway 211, near the city of Cheyenne; northwesterly along said highway to the McLees Road (Laramie County Road 103-1); northwesterly along said road to the Bell Ranch Road; northwesterly along said road to the Sybille Road (Albany County Road 12); northeasterly along said road to Wyoming Highway 34; northeasterly along said highway to Interstate Highway 25.

Area 39. Bluegrass. Beginning where U.S. Highway 30 crosses the Laramie River; south of the town of Bosler; northeasterly down said river to Interstate Highway 25; north of the city of Wheatland; southerly along said highway to Wyoming Highway 34; southwesterly along said highway to the Sybille Road (Albany County Road 12); southerly and westerly along said road to Wyoming Highway 34; southwesterly along said highway to U.S. Highway 30; northerly along said highway to the Laramie River.

Area 40. East Laramie. Beginning in the city of Laramie and U.S. Highway 30; northerly along said highway to Wyoming Highway 34; northeasterly along said highway to the Sybille Road (Albany County Road 12);

easterly along said road to the Bell Ranch Road; easterly along said road to the McLees Road (Laramie County Road 103); southeasterly along said road to Wyoming Highway 211; southerly along said highway to the Fisher Canyon-Rogers Canyon Road (Laramie County Road 228-Albany County Road 17); westerly along said road to U.S. Highway 30.

Area 41. Laramie Peak. Beginning where the Big Laramie River crosses the Dodge Ranch Road; northerly along said road to the Garrett Road (Albany County Road 713); northerly and westerly along said road to the Fetterman Road (Albany County Road 61); southerly along said road to the Mule Creek Road (Albany County Road 64); northwesterly along said road to Albany County Road 610; northerly along said road to Sheep Creek; northerly up said creek to the southern boundary of the Laramie Peak Division of the Medicine Bow National Forest; northwesterly along said boundary to the Albany-Converse county line; east along said line to LaPrele Creek; southerly up said creek to the divide between LaPrele Creek and the Little Medicine Bow River; easterly along said divide to the divide between Corduroy Creek and Rocky Ford Creek; easterly along said divide to the Fetterman Road (Albany County Road 61); southerly along said road to U.S.F.S. Road 658; easterly along said road to South Fork Creek, tributary to LaBonte Creek; southerly up said creek to the divide between Horseshoe Creek and the North Laramie River; southeasterly along said divide to the head of Friend Creek; southerly down said creek to Bear Creek; easterly down said creek to the North Laramie River; easterly down said river to the Cottonwood Park Road (Albany County Road 71); southerly down said road to its junction with the Palmer Canyon Road (Albany County Road 721); easterly along said road to Ashley Creek; southerly down said creek to Duck Creek; easterly down said creek to the Big Laramie River; southerly and westerly up said river to the Dodge Ranch Road.

Area 42. Laramie Plains. Beginning at the junction of the Marshall Road (Albany County Road 610) and U.S. Highway 30 north of the town of Rock River; northeasterly along said road to the Mule Creek Road (Albany County Road 64); southerly along said road to the Fetterman Road (Albany County Road 61); northerly along said road to the Garrett Road (Albany County Road 713); southeasterly along said road to the Dodge Ranch Road; southerly along said road to the Big Laramie River; southwesterly up said river to U.S. Highway 30; northwesterly along said highway to the Marshall Road (Albany County Road 610).

Area 43. Cooper Lake. Beginning at the junction of Wyoming Highway 13 and U.S. Highway 30 north of the town of Rock River; southeasterly along U.S. Highway 30 to Interstate Highway 80; northwesterly along said highway to Rock Creek; northeasterly down said creek to Wyoming Highway 13 west of the town of McFadden; northeasterly along said highway to U.S. Highway 30.

Area 44. South Laramie. Beginning at the city of Laramie and U.S. Highway 287; southerly along said highway to the Wyoming-Colorado state line; west along said line to Wyoming Highway 230; northeasterly along said highway to U.S. Highway 287.

Area 45. West Laramie. Beginning at the city of Laramie and Wyoming Highway 230; southwesterly along said highway to the divide between the Laramie River and the North Platte River; northerly along said divide to the divide between the Medicine Bow River and the Laramie River at Medicine Bow Peak; northeasterly along said divide to Deep Creek; northeasterly down said creek to Rock Creek; northeasterly down said creek to Interstate Highway 80 at Arlington; southeasterly along said highway to Wyoming Highway 230.

Area 46. Kyle. Beginning where U. S. Highway 30 intersects Wyoming Highway 72; easterly along U.S. Highway 30 to Wyoming Highway 13; southwesterly along said highway to Rock Creek west of the town of McFadden; southwesterly up said creek to Interstate Highway 80; northwesterly along said highway to Wyoming Highway 72; northerly along said highway to U.S. Highway 30.

Area 47. Shirley Basin. Beginning at U.S. Highway 30 in the town of Medicine Bow; westerly along said highway to Wyoming Highway 72; northerly along said highway to the Hanna-Leo Road (Carbon County Road 291); northerly along said road to Troublesome Creek; northerly up said creek to the Troublesome Spring Road on Shirley Mountain; northerly along said road to the BLM Fire Camp Road (BLM Road 3115); northwesterly along said road to the Leo-Shirley Basin Road (Carbon County Road 102) at Pryor Flat; northeasterly along said road to the Dry Creek Road (Carbon County Road 103); northerly along said road to the Chalk Mountain Road (BLM Road 3129); easterly along said road to Wyoming Highway 77; northerly along said highway to the divide between Bates Creek and the Little Medicine Bow River; northeasterly along said divide to the Old Medicine Bow Road (Carbon

County Road 97); northerly along said road to the Squaw Springs Trail Road; northerly along said road to U.S.F.S. Road 660; southeasterly along said road to the Little Medicine Road (Albany County Road 62); northeasterly along said road to the south boundary of the Laramie Peak Division of the Medicine Bow Forest; southerly and easterly along said boundary to Sheep Creek; southerly along said creek to its second crossing with the Marshall Road (Albany County Road 610); southerly along said road to U.S. Highway 30; westerly along said highway to the town of Medicine Bow.

Area 48. Leo-Hanna. Beginning at the junction of Wyoming Highway 72 and Interstate Highway 80; westerly along Interstate Highway 80 to the North Platte River; northerly down said river to Canyon Creek; easterly up said creek to the south fork of Canyon Creek; southerly and easterly up said fork to the Dry Creek Road (Carbon County Road 103); southerly along said road to the Leo-Shirley Basin Road (Carbon County Road 102); southwesterly along said road to Pryor Flat and the BLM Fire Camp Road (BLM Road 3115); southeasterly along said road to the Troublesome Spring Road; southerly along said road to Troublesome Creek; southerly along said creek to the Hanna-Leo Road (Carbon County Road 291); southerly along said road to the town of Hanna and Wyoming Highway 72; southerly along said highway to Interstate Highway 80.

Area 50. Elk Mountain. Beginning where Interstate Highway 80 crosses the North Platte River; easterly along said highway to Rock Creek at Arlington; southerly up said creek to Deep Creek; southwesterly up said creek to the divide between the Laramie River and the North Platte River; southerly along said divide to Wyoming Highway 230; southwesterly along said highway to the Wyoming-Colorado state line; west along said line to the North Platte River; northerly down said river to Interstate Highway 80.

Area 51. Big Creek. Beginning where the North Platte River crosses the Wyoming-Colorado state line; west along said line to the Encampment River; northeasterly down said river to the North Platte River; southerly up said river to the Wyoming-Colorado state line.

Area 52. Spring Creek. Beginning where the Continental Divide crosses the Wyoming-Colorado state line; northerly along said divide to the Sage Creek Road (Carbon County Road 401); northerly along said road to Sage Creek; northeasterly down said creek to the North Platte River; southerly up said river to the Encampment River; southwesterly up said river to the Wyoming-Colorado state line; westerly along said line to the Continental Divide.

Area 53. Baggs. Beginning at the junction of Wyoming Highway 789 and the Wyoming-Colorado state line; northerly along said highway to Muddy Creek north of the town of Dad; easterly up said creek to the Muddy Creek Road (BLM Road 3306); easterly along said road to the Miller Hill Road (BLM Road 3328); southeasterly along said road to the McCarty Canyon Road (Carbon County Road 503); northeasterly along said road to the Rawlins City Water Works Road; northerly along said road to Sage Creek below Sage Creek Reservoir; northeasterly down said creek to the Sage Creek Road (Carbon County Road 401); southerly along said road to the Continental Divide at Middlewood Hill; southerly along said divide to the Wyoming-Colorado state line; west along said line to Wyoming Highway 789.

Area 55. Red Rim. Beginning at the city of Rawlins; southerly along Wyoming Highway 71 to the divide along Five Mile Ridge; southwesterly along the divide on Five Mile Ridge and Atlantic Rim to the crossing of the Sulphur Springs Ranch Road over Muddy Creek; westerly down said creek to Wyoming Highway 789; northerly along said highway to Interstate Highway 80; easterly along said highway to the city of Rawlins.

Area 56. Iron Springs. Beginning at the city of Rawlins; easterly along Interstate Highway 80 to the North Platte River; southerly up said river to Sage Creek; southwesterly up said creek to the Sage Creek Road (Carbon County Road 401); northerly along said road to Wyoming Highway 71; northerly along said highway to the city of Rawlins.

Area 57. South Wamsutter. Beginning at the junction of Wyoming Highway 789 and the Wyoming-Colorado state line; westerly along said line to the Cow Creek-Powder Wash Road north of Powder Wash, Colorado; northwesterly along said road to the Bitter Creek Road (Sweetwater County Road 19); northwesterly along said road to Interstate Highway 80; easterly along said highway to Creston Junction and Wyoming Highway 789; southerly along said highway to the Wyoming-Colorado state line.

Area 58. Black Butte-Kenny Rim. Beginning at the junction of Wyoming Highway 430 and the Wyoming-Colorado state line; northwesterly along said highway to Interstate Highway 80 in the town of Rock Springs; easterly along said highway to the Bitter Creek Road (Sweetwater County Road 19); southeasterly along said road to the Carson Springs Junction and the Cow Creek-Powder Wash Road; southeasterly along said road to the Wyoming-Colorado state line; westerly along said line to Wyoming Highway 430.

Area 59. Aspen Mountain. Beginning where Interstate Highway 80 crosses the Green River; easterly along said highway to the town of Rock Springs and Wyoming Highway 430; southeasterly along said highway to the Mud Springs Road (Sweetwater County Road 32); westerly along said road to the Aspen Mountain Road (Sweetwater County Road 27); southerly along said road to the Clay Basin Pipeline Road (Sweetwater County Road 71); southwesterly along said road to the Ramsey Ranch Road (Sweetwater County Road 34); westerly along said road to U.S. Highway 191; westerly along said highway to the Ramsey Ranch Road (Sweetwater County Road 34); northerly along said road to the Sage Creek Road (Sweetwater County Road 36); westerly along said road to Sage Creek; westerly down said creek to Flaming Gorge Reservoir; northerly along the east shore of said reservoir to the Green River; northeasterly then northwesterly up said river to Interstate Highway 80.

Area 60. Table Rock. Beginning at the junction of Wamsutter-Crooks Gap Road (Sweetwater County Road 23) and Interstate Highway 80 at the town of Wamsutter; westerly along said highway to Wyoming Highway 377 at Point of Rocks; northerly along said highway to the Nine Mile Road (Sweetwater County Road 15); northerly along said road to the Bar X Road (Sweetwater County Road 21) at the Bar X Ranch; northwesterly along said road to the Oregon Buttes Road (Sweetwater County Road 74); northerly along said road to the Continental Divide Road; northeasterly along said road to the Sand Creek Road; easterly along said road to the Red Creek Road (BLM Road 3219); southeasterly along said road to the Cyclone Ridge Road (BLM Road 3216); easterly along said road to the Bison Basin Road (BLM Road 3221); southerly along said road to the Hadsell Road (BLM Road 3217); easterly along said road to the Osborne Road (BLM Road 3212); easterly along said road to the Wamsutter-Crooks Gap Road (Sweetwater County Road 23); southerly along said road to Interstate Highway 80.

Area 61. Chain Lakes. Beginning at the city of Rawlins; westerly along Interstate Highway 80 to the Wamsutter-Crooks Gap Road (Sweetwater County Road 23); northerly along said road to Crooks Gap and the divide along Green Mountain; southeasterly along said divide to Whiskey Peak and the head of Coal Creek; northeasterly down said creek to U.S. Highway 287; southerly along said highway to the city of Rawlins.

Area 62. South Ferris. Beginning at the city of Rawlins; northerly along U.S. Highway 287 to Muddy Gap Junction and the divide along the Ferris Mountains; southeasterly along said divide to Ferris Peak and the northeast branch of the Continental Divide; southeasterly along said branch to Bradley Peak and the divide along the Seminoe Mountains; easterly along said divide to Seminoe Dam and the North Platte River; southerly up said river to Interstate Highway 80; westerly along said highway to the city of Rawlins.

Area 63. North Ferris. Beginning at Muddy Gap Junction; northeasterly along Wyoming Highway 220 to the Kortes Road (Natrona County Road 407); southerly along said road to the North Platte River; southerly up said river to Seminoe Dam and the divide along the Seminoe Mountains; westerly along said divide to Bradley Peak and the northeast branch of the Continental Divide; northwesterly along said divide to Ferris Peak and the divide along the Ferris Mountains; northwesterly along said divide to Muddy Gap Junction.

Area 64. Bison Basin. Beginning at the confluence of the Sweetwater River and Alkali Creek southwest of Sweetwater Station; southeasterly along the divide between Alkali Creek and the Sweetwater River to the northeast branch of the Continental Divide; northeasterly along said divide to the Happy Springs Oil Field Road; southeasterly along said road to the Wamsutter-Crooks Gap Road (Fremont County Road and Sweetwater County Road 23); southerly along said road to the Osborne Road (BLM Road 3212); westerly along said road to the Hadsell Road (BLM Road 3217); westerly along said road to the Bison Basin Road (BLM Road 3221); northerly along said road to the Cyclone Ridge Road (BLM Road 3216); westerly along said road to the Red Creek Road (BLM Road 3219); northwesterly along said road to the Sand Creek Road; westerly along said road to the Continental Divide Road; northeasterly along said road to the Harris Slough Road; northerly along said road to the Three Forks-Atlantic City Road (BLM Road 2317); northwesterly along said road to the Sweetwater River at Phelps-Dodge Bridge; northeasterly down said river to Alkali Creek.

Area 65. South Pass. Beginning at the southwest corner of the Wind River Indian Reservation boundary; easterly along said boundary to U.S. Highway 287; southerly and southeasterly along said highway to the Sweetwater River at Sweetwater Station; westerly up said river to Rock Creek; northwesterly up said creek to Granite Peak and the divide between the Sweetwater River and Wind River drainages; northwesterly along said divide to the Continental Divide; northwesterly along said divide to the southwest corner of the Wind River Indian Reservation boundary.

Area 66. Lander. Beginning where U.S. Highway 287 crosses the south boundary of the Wind River Indian Reservation at the North Popo Agie River; easterly along said boundary to Wyoming Highway 135 (Sand Draw Road); southerly along said highway to U.S. Highway 287; northwesterly and northerly along said highway to the south boundary of the Wind River Indian Reservation.

Area 67. Sand Draw. Beginning where U.S. Highway 20-26 crosses the east boundary of the Wind River Indian Reservation; easterly along said highway to the Castle Gardens Road; southerly along said road to Wyoming Highway 136 (Gas Hills Road); southeasterly along said road to the Ore Road (Fremont County Road); southerly along said road to Beaver Rim; westerly and southerly along said rim to Wyoming Highway 135 (Sand Draw Road); northerly along said highway to the Wind River Indian Reservation boundary; easterly and northerly along said boundary to U.S. Highway 20-26.

Area 68. Split Rock. Beginning at the town of Jeffrey City; northerly along the Ore Road to Beaver Rim; northeasterly along said rim to the divide between Deer Creek and Dry Creek; northeasterly along said divide to the Dry Creek Road (Natrona County Road 321); southeasterly along said road to Wyoming Highway 220; southwesterly along said highway to U.S. Highway 287; southerly along said highway to Coal Creek; southwesterly up said creek to Whiskey Peak and the divide along Green Mountain; northwesterly along said divide to Crooks Gap and the Wamsutter-Crooks Gap Road (Sweetwater County Road 23); northerly along said road to the town of Jeffrey City.

Area 69. South Rattlesnake. Beginning where the Dry Creek Road (Natrona County Road 321) intersects Wyoming Highway 220; northwesterly along said road to the Gas Hills Road (Natrona County Road 212); northeasterly along said road to the divide along the Rattlesnake Mountain range; easterly and southerly along said divide to Wyoming Highway 220 at the top of Alcova Hill; westerly along said highway to the Dry Creek Road (Natrona County Road 321).

Area 70. Kendrick. Beginning at the crossing of the North Platte River by U.S. Highway 20-26 in the city of Casper; southwesterly up said river to the Kortes Road (Natrona County Road 407) below Alcova Dam; northerly along said road to Wyoming Highway 220; westerly along said highway to the divide along the Rattlesnake Mountain range at the top of Alcova Hill; northerly along said divide to the Oregon Trail (Natrona County Road 319) at the top of Ryan Hill; northeasterly along said trail to the Kendrick Canal at the Iron Creek Oil Field; northerly along said canal to U.S. Highway 20-26; easterly along said highway to the North Platte River.

Area 71. East Poison Spider. Beginning where U.S. Highway 20-26 crosses the Kendrick Canal; southerly along said canal to the Poison Spider Road (Natrona County Road 201); westerly along said road to the Oil Camp Road (Natrona County Road 210); northwesterly along said road to U.S. Highway 20-26 at the town of Powder River; southeasterly along said highway to the Kendrick Canal.

Area 72. West Poison Spider. Beginning at the intersection of the Gas Hills Road (Natrona County Road 212) and U.S. Highway 20-26 at the town of Waltman; easterly along said highway to the Oil Camp Road (Natrona County Road 210) at the town of Powder River; southeasterly along said road to the Poison Spider Road (Natrona County Road 201); easterly along said road to the Kendrick Canal; southerly along said canal to the Iron Creek Oil Field and the Oregon Trail (Natrona County Road 319); southwesterly along said trail to the divide along the Rattlesnake Mountain range at the top of Ryan Hill; northerly and westerly along said divide to the Gas Hills Road (Natrona County Road 212); northeasterly along said road to the town of Waltman and U.S. Highway 20-26.

Area 73. North Natrona. Beginning where Interstate Highway 25 intersects U.S. Highway 20-26 in the city of Casper; westerly along U.S. Highway 20-26 to the town of Waltman and the Waltman-Arminto Road

(Natrona County Road 104); northerly along said road to the town of Arminto and the Buffalo Creek Road (Natrona County Road 105); northerly along said road to the E-K Trail; northerly along said trail to the Bighorn Trail; northerly along said trail to the divide between the Middle Fork of the Powder River and North Buffalo Creek; easterly and northerly along said divide to the Big Horn Mountain Road (Natrona County Road 109); northwesterly along said road to the 33 Mile Road (Natrona County Road 110); southeasterly along said road to the Wild Horse Trail (Natrona County Road 113); easterly along said trail to the Dead Horse Road (Natrona County Road 114); northeasterly along said road to the Smoky Gap Road (Natrona County Road 115); easterly along said road to U.S. Highway 387; easterly along said highway to Wyoming Highway 259 at the town of Midwest; southerly along said highway to Interstate Highway 25; southerly along said highway to U.S. Highway 20-26 in the city of Casper.

Area 74. Deer Creek. Beginning where the Castle Gardens Road intersects U.S. Highway 20-26; easterly along said highway to the town of Waltman and the Gas Hills Road (Natrona County Road 212); southwesterly along said road to the Dry Creek Road (Natrona County Road 321); southeasterly along said road to the divide between Deer Creek and Dry Creek; southwesterly along said divide to Beaver Rim; southwesterly along said rim to the Ore Road; northerly along said road to Wyoming Highway 136 (Gas Hills Road); westerly along said road to the Castle Gardens Road; northerly along said road to U.S. Highway 20-26.

Area 75. Badwater. Beginning at the town of Shoshoni and U.S. Highway 20-26; westerly along said highway to the east shoreline of Boysen Reservoir; northerly along said shoreline to Boysen Dam and the Wind River; northerly down said river to Gold Creek (excluding Indian tribal trust lands immediately north of U.S. Highway 20-26 near Boysen Reservoir and northeast of Boysen Dam); easterly up Gold Creek to the divide between Buffalo Creek and Badwater Creek on Copper Mountain; easterly along said divide to the divide between Badwater Creek and the Kirby-Nowater-Nowood Creek drainages; northeasterly then southeasterly along said divide to Cottonwood Pass and the divide between Nowood Creek and Badwater Creek; easterly along said divide to the Devil's Slide Road; easterly along said road to Sioux Pass and the Oakie Trail (Natrona County Road 101); easterly along said trail to the Big Horn Trail; easterly along said trail to the E-K Trail; southerly along said trail to the Buffalo Creek Road (Natrona County Road 105); southerly along said road to the town of Arminto and the Arminto Road (Natrona County Road 104); southerly along said road to the town of Waltman and U.S. Highway 20-26; westerly along said highway to the town of Shoshoni.

Area 76. Copper Mountain. Beginning where Gold Creek intersects the east boundary of the Wind River Indian Reservation; northerly and westerly along said boundary to the Big Horn River; northerly down said river to Walters Draw; easterly up said draw to the divide between Nowater Creek and Kirby Creek; easterly along said divide to the Murphy Dome-Mud Creek Road (BLM Road 1409); northeasterly along said road to the Nowater Stock Drive Road (BLM Road 1404); easterly along said road to the divide between the Nowood River and Nowater Creek; southerly along said divide to Hawks Butte and the divide between the Nowood River/Nowater Creek/Kirby Creek and Badwater Creek; westerly along said divide to the divide between Badwater Creek and Buffalo Creek on Copper Mountain; westerly along said divide to the head of Gold Creek; westerly down said creek to the east boundary of the Wind River Indian Reservation.

Area 77. Fifteen Mile Creek. Beginning at the town of Meeteetse; easterly down the Greybull River to the Big Horn River; southerly up said river to Wyoming Highway 432; westerly along said highway to its junction with Wyoming Highway 431 and U.S. Highway 20; westerly along Wyoming Highway 431 to U.S. Highway 120; northwesterly along U.S. Highway 120 to the town of Meeteetse.

Area 78. McCullough Peaks. Beginning at the junction of U.S. Highway 120 and U.S. Highway 14-16-20 in the town of Cody; northerly along U.S. Highway 120 to the Shoshone River; easterly down said river to the Bighorn River; southerly up said river to the Greybull River; westerly up said river to Bighorn County Road 8; northerly up said road to Wyoming Highway 30; northerly up said highway to U.S. Highway 14-16-20; westerly along said highway to U.S. Highway 120.

Area 79. Crystal Creek. Beginning where U.S. Highway 14 crosses the Big Horn River at the town of Greybull; northerly down said river to the Wyoming-Montana state line; easterly along said line to the Bighorn National Forest boundary; southerly along said boundary to U.S. Highway 14; westerly along said highway to the Big Horn River.

Area 80. Badger Basin. Beginning where the Big Horn River crosses the Wyoming-Montana state line; southerly up said river to the Shoshone River; westerly up said river to the North Fork of the Shoshone River; westerly up said river to the Shoshone National Forest boundary; easterly then northerly along said boundary to Wyoming Highway 296 (Sunlight Road); northerly along said highway to U.S. Highway 212; easterly along said highway to the Wyoming-Montana state line; east along said line to the Big Horn River.

Area 81. South Fork. Beginning where the South Fork of Dry Creek crosses Wyoming Highway 120 at the Halfway House historical marker; southwesterly up said creek to the divide between Meeteetse Creek and Sage Creek; westerly along said divide to the divide between the Greybull River and the South Fork of the Shoshone River; westerly then southwesterly along said divide to the divide between Boulder Creek and Needle Creek; westerly along said divide to the divide between Boulder Creek and the South Fork of the Shoshone River; northerly along said divide to the head of Aspen Creek; northwesterly down said creek to the South Fork of the Shoshone River; northeasterly down said river to the Shoshone National Forest boundary; northerly along said boundary to the North Fork of the Shoshone River; easterly down said river to the Shoshone River; easterly along said river to Wyoming Highway 120; southerly along said highway to U.S. Highway 14-16-20; easterly along said highway to Dry Creek; southwesterly up said creek to the South Fork of Dry Creek; southwesterly up said creek to Wyoming Highway 120 and the Halfway House historical marker.

Area 82. North Greybull River. Beginning where the South Fork of Dry Creek crosses Wyoming Highway 120 at the Halfway House historical marker; northeasterly down said creek to Dry Creek; northeasterly down said creek to U.S. Highway 14-16-20; easterly along said highway to the Wyoming Highway 30 (Burlington Road); southerly along said road to Big Horn County Road 8; southerly along said road to the Greybull River; westerly then southerly along said river to the divide between the Greybull River and the Wind River; northwesterly along said divide to the divide between the Greybull River and the South Fork of the Shoshone River; northerly then easterly along said divide to the divide between Meeteetse Creek and Sage Creek; easterly along said divide to the South Fork of Dry Creek; northeasterly down said creek to Wyoming Highway 120 at the Halfway House historical marker.

Area 83. Owl Creek. Beginning where the Big Horn River leaves the Wind River Indian Reservation and the reservation boundary; westerly then northerly along said boundary to Owl Creek and the north boundary of the Wind River Indian Reservation; westerly along said boundary to the divide between Owl Creek/Cottonwood Creek/Grass Creek and Wood River/Gooseberry Creek; northeasterly along said divide to Wyoming Highway 120; southeasterly down said highway to Wyoming Highway 431; easterly along said highway to the junction with Wyoming Highway 432; easterly along said highway to the Big Horn River; southerly along said river to the Wind River Indian Reservation boundary, in addition, all non-Indian fee title lands in that portion of Hot Springs County within the exterior boundaries of the Wind River Indian Reservation.

Area 84. Wind River. All of the drainage of the Wind River west of the western boundary of the Wind River Indian Reservation including areas of the Spence and Moriarity Wildlife Management Area east of the East Fork of the Wind River.

Area 85. Gros Ventre. Beginning where Flat Creek crosses the National Elk Refuge boundary; northerly along said boundary to the Grand Teton National Park boundary; northerly along said boundary to U.S. Highway 26-287; easterly along said highway to the Continental Divide at Togwotee Pass; southerly along said divide to the Union Pass Road (USFS Road 600); southwesterly along said road to the Darwin Ranch Road (USFS Road 620); westerly along said road to the divide between the Green River and the Gros Ventre River; southwesterly along said divide to the divide between the Gros Ventre River and the Hoback River at Hodges Peak; westerly along said divide to the divide between Flat Creek and the Hoback River; westerly along said divide to Flat Creek; northerly down said creek to the National Elk Refuge boundary.

Area 86. Hoback. All of the drainage of the Hoback River.

Area 87. Pinedale. Beginning where the East Green River Road (Sublette County Road 23-110) intersects Wyoming Highway 351; northerly along said road to U.S. Highway 191 at Cora Junction; westerly then northerly along said highway to the divide between the Hoback River and the Green River (Hoback Rim); northerly along said divide to the divide between the Hoback River and the Gros Ventre River at Hodges Peak; northeasterly along said

divide to the Darwin Ranch Road (USFS Road 620); easterly along said road to the Union Pass Road (USFS Road 600); northeasterly along said road to the Continental Divide at Union Pass; southerly along said divide to the Middle Fork of Boulder Creek; southwesterly down said creek to Boulder Creek; westerly down said creek to the New Fork River; southwesterly down said river to Wyoming Highway 351; westerly along said highway to the East Green River Road (Sublette County Road 23-110).

Area 88. Beaver. Beginning where the East Green River Road (Sublette County Road 23-110) intersects Wyoming Highway 351; westerly along said highway to the Green River; northerly up said river to Cottonwood Creek; westerly up said creek to South Cottonwood Creek; westerly up said creek to the South Fork of South Cottonwood Creek; westerly up said creek to the divide between the Green River and the Greys River; northerly along said divide to the Hoback Rim; easterly along said rim to U.S. Highway 191; southerly along said highway to the East Green River Road (Sublette County Road 23-110) at the Cora Junction; southerly along said road to Wyoming Highway 351.

Area 89. Piney. Beginning where LaBarge Creek enters the Green River; westerly up said creek to the Greys River Road; northerly up said road to the divide between the Green River and the Greys River; northerly along said divide to the South Fork of South Cottonwood Creek; easterly down said creek to the South Fork of Cottonwood Creek; easterly down said creek to Cottonwood Creek; easterly down said creek to the Green River; southerly down said river to LaBarge Creek.

Area 90. Yellow Point. Beginning where the County Line Road (Sweetwater County Road 52) crosses the Green River; northerly up said river to Wyoming Highway 351; easterly along said highway to the New Fork River; northeasterly up said river to Boulder Creek; easterly up said creek to the Middle Fork of Boulder Creek; northeasterly up said creek to the Continental Divide; southerly along said divide to the Big Sandy River; southerly down said river to the Big Sandy Reservoir Road (Sweetwater County Road 28); westerly along said road to U.S. Highway 191; southerly along said highway to the Eighteen Mile Road (Sweetwater County Road 49); westerly then southerly along said road to the County Line Road (Sweetwater County Road 52); southerly along said road to the Green River.

Area 91. Dry Sandy. Beginning at the town of Farson and U.S. Highway 191; northerly along said highway to the Big Sandy Reservoir Road (Sweetwater County Road 28); easterly along said road to the Big Sandy River at the Big Sandy Reservoir Dam; northerly up said river to the Continental Divide; southeasterly along said divide to Mt. Nystrom; easterly from Mt. Nystrom to Sweetwater Gap and the headwaters of the Sweetwater River; southerly down said river to Wyoming Highway 28; southwesterly along said highway to the town of Farson.

Area 92. Steamboat. Beginning at the junction of Interstate Highway 80 and U.S. Highway 191 in the city of Rock Springs; northerly along U.S. Highway 191 to U.S. Highway 28; northeasterly along U.S. Highway 28 to the Oregon Buttes Road (Sweetwater County Road 74); southerly along said road to the Bar X Road (Sweetwater County Road 21); easterly along said road to the Nine Mile Road (Sweetwater County Road 15) at the Bar X Ranch; southerly along said road to Wyoming Highway 377; southerly along said highway to Point of Rocks and Interstate Highway 80; westerly along said highway to U.S. Highway 191.

Area 93. West Green River. Beginning where the Green River crosses Interstate Highway 80; westerly along said highway to U.S. Highway 30; northwesterly along said highway to the town of Kemmerer and Ham's Fork Creek; northerly up said creek to Commissary Ridge; northerly along said ridge to the head of LaBarge Creek; southeasterly down said creek to the Green River; southeasterly down said river to Interstate Highway 80.

Area 94. Carter Lease. Beginning at the junction of Interstate Highway 80 and U.S. Highway 189; northerly along U.S. Highway 189 to U.S. Highway 30; easterly then southerly along said highway to Interstate Highway 80; westerly along said highway to U.S. Highway 189.

Area 95. Cedar Mountain. Beginning where the Flaming Gorge Reservoir crosses the Wyoming-Utah state line; west along said line to the Hoop Lake-Hole-in-the-Rock Road (Uinta County Road 295); northerly along said road to Wyoming Highway 414 at the town of Lone Tree; northerly along said highway to Interstate Highway 80; easterly along said highway to the Green River; southerly down said river to Flaming Gorge Reservoir; southerly along the east shore of Flaming Gorge Reservoir to the Wyoming-Utah state line.

Area 96. West Farson. Beginning where the Green River crosses Interstate Highway 80; northerly up said river to the CCC Bridge and the County Line Road (Sweetwater County Road 52); northerly along said road to the Eighteen Mile Road (Sweetwater County Road 49); northerly then easterly along said road to U.S. Highway 191; southerly along said highway to Interstate Highway 80; westerly along said highway to the Green River.

Area 97. Boysen. All lands within the Bureau of Reclamation's Riverton and Boysen Unit boundaries and those lands within Boysen State Park south of Cottonwood Creek and west of Boysen Reservoir and those lands within Boysen State Park south of U.S. Highway 20-26.

Area 98. Sage. Beginning at the town of Kemmerer and U.S. Highway 30; westerly along said highway to Wyoming Highway 89 at Sage Junction; southwesterly along said highway to the Wyoming-Utah state line; northerly along said line to the Wyoming-Idaho state line; northerly along said line to the divide between the Salt River and Bear River; easterly along said divide to the divide between the Salt River and Smith's Fork; northeasterly along said divide to the divide between the Smith's Fork and Greys River; southeasterly along said divide to Commissary Ridge; southerly along said ridge to the head of the Ham's Fork Creek; southerly down said creek to U.S. Highway 30.

Area 99. Uinta. Beginning where Interstate Highway 80 crosses the Wyoming-Utah state line; easterly along said highway to Wyoming Highway 414; southerly along said highway through the town of Lone Tree to the Hoop Lake-Hole-in-the-Rock Road (Uinta County Road 295); southerly along said road to the Wyoming-Utah state line; westerly then northerly along said line to Interstate Highway 80.

Area 100. Bear River Divide. Beginning where Interstate Highway 80 crosses the Wyoming-Utah state line; northerly along said line to Wyoming Highway 89 west of Sage Junction; easterly along said highway to U.S. Highway 30; easterly along said highway to U.S. Highway 189; southerly along said highway to Interstate Highway 80; westerly along said highway to the Wyoming-Utah state line.

Area 102. Buffalo. Beginning where Interstate Highway 90 crosses Piney Creek; southerly along said highway to Interstate Highway 25; southerly along said highway to the Trabing Road (Johnson County Road 13); westerly along said road to Wyoming Highway 196; southerly along said highway to the Crazy Woman Canyon Road (Johnson County Road 14); westerly along said road to U.S. Highway 16; westerly along said highway to the divide along the Big Horn Mountain range at Powder River Pass; northerly along said divide to South Piney Creek; northeasterly down said creek to Piney Creek; southeasterly down said creek to Interstate Highway 90.

Area 103. Dwyer. Beginning at the confluence of the North Platte River and the Laramie River southwest of Ft. Laramie; westerly up the Laramie River to Duck Creek; westerly up said creek to Ashley Creek; northerly up said creek to the Palmer Canyon Road (Albany County Road 721); westerly along said road to its junction with the Cottonwood Park Road (Albany County Road 71); northerly along said road to the North Laramie River; westerly up said river to Bear Creek; northerly up said creek to Friend Creek; northerly up said creek to the divide between Horseshoe Creek and the North Laramie River; northerly along said divide to the divide between Horseshoe Creek and Soldier Creek; northerly along said divide to U.S.F.S. Road 633; northerly and westerly along said road to the town of Esterbrook and the Esterbrook Road (Converse County Road 5); northerly along said road to the Esterbrook Highway (Wyoming Highway 94); northerly along said highway to the North Platte River at Jackalope Warm Springs; easterly and southerly down said river to the Laramie River.

Area 104. Crow Creek. Beginning in the city of Laramie and the Rogers Canyon-Fisher Canyon Road (Albany County Road 17-Laramie County Road 228); northeasterly along said road to Wyoming Highway 211 (Horse Creek-Chugwater Road); southeasterly along said highway to Interstate Highway 25; southerly along said highway to Interstate Highway 80; westerly along said highway to the city of Laramie.

Area 106. Long Creek. Beginning at the town of Jeffrey City; southerly along the Crooks Gap Road to the Happy Springs Oil Field Road; northwesterly along said road to the northeast branch of the Continental Divide; southwesterly along said divide to the divide between Alkali Creek and the Sweetwater River; northwesterly along said divide to the confluence of Alkali Creek and the Sweetwater River; northeasterly down said river to U.S. Highway 287; northwesterly along said highway to Wyoming Highway 135 (Sand Draw Road); northerly along said

highway to Beaver Rim; easterly along said rim to the Ore Road; southerly along said road to the town of Jeffrey City.

Area 107. Upper Sweetwater. Beginning at the intersection of Wyoming Highway 28 and the Sweetwater River; northwesterly up said river to the divide between the Sweetwater River and the Wind River; southeasterly along said divide to Granite Peak and the head of Rock Creek; southeasterly down said creek to the Sweetwater River; westerly up said river to the Phelps-Dodge Bridge and the Three Forks-Atlantic City Road (BLM Road 2317); southerly along said road to the Harris Slough Road; southerly along said road to the Continental Divide Road northeast of Continental Peak; westerly along said road to the Oregon Buttes Road; northerly along said road to Wyoming Highway 28; northeasterly along said highway to the Sweetwater River.

Area 108. Bridger Pass. Beginning where Wyoming Highway 71 crosses Five Mile Ridge south of the city of Rawlins; southerly along said highway to the Sage Creek Road (Carbon County Road 401); southerly along said road to Sage Creek; southwesterly up said creek to the Rawlins City Water Works Road below Sage Creek Reservoir; southerly along said road to the McCarty Canyon Road (Carbon County Road 503); southwesterly along said road to the Miller Hill Road (BLM Road 3328); northwesterly along said road to the Muddy Creek Road (BLM Road 3306); westerly along said road to Muddy Creek; westerly down said creek to the Sulphur Springs Ranch Road crossing; northeasterly from said crossing along the divide on Atlantic Rim and Five Mile Ridge to Wyoming Highway 71.

Area 109. Beckton. Beginning where Interstate Highway 90 crosses the Wyoming-Montana state line; southerly along said highway to Piney Creek; northwesterly up said creek to South Piney Creek; westerly up said creek to the Bighorn National Forest boundary; northwesterly along said boundary to the Wyoming-Montana state line; easterly along said line to Interstate Highway 90.

Area 110. South Greybull River. All of the drainage of the Greybull River south of the Greybull River and west of Wyoming Highway 120; all of the drainage of Gooseberry Creek west of Wyoming Highway 120.

Area 111. Chalk Bluffs. Beginning at the junction of Interstate Highway 80 and the Wyoming-Nebraska state line; southerly along said line to where the Wyoming-Nebraska-Colorado state lines intersect; westerly along the Wyoming-Colorado state line to Interstate Highway 25; northerly along said highway to Interstate Highway 80; easterly along said highway to the Wyoming-Nebraska state line.

Area 112. Pine Mountain. Beginning where Wyoming Highway 430 crosses the Wyoming-Colorado state line; west along said line to Flaming Gorge Reservoir; northerly along the east shore of said reservoir to Sage Creek; easterly up said creek to Sage Creek Road (Sweetwater County Road 36); easterly along said road to Ramsey Ranch Road (Sweetwater County Road 34); southerly along said road to U.S. Highway 191; easterly along said highway to Ramsey Ranch Road (Sweetwater County Road 34); easterly along said road to Clay Basin Pipeline Road (Sweetwater County Road 71); northeasterly along said road to Aspen Mountain Road (Sweetwater County Road 27); northerly along said road to Mud Springs Road (Sweetwater County Road 32); northeasterly along said road to Wyoming Highway 430; southerly along said highway to the Wyoming-Colorado state line.

Area 113. Salt Creek. Beginning at the junction of Wyoming Highway 192 and Wyoming Highway 387; southwesterly along Wyoming Highway 387 to Interstate Highway 25; northwesterly along said highway to Wyoming Highway 192; easterly and southerly along said highway to Wyoming Highway 387.

Area 114. Nowater. Beginning at the confluence of Walters Draw and the Bighorn River; northerly down said river to the Nowood River; southeasterly up said river to U.S. Highway 16; westerly along said highway to the Blue Bank Road (BLM Road 1411); southerly along said road to the Nowater Stock Drive Road (BLM Road 1404); westerly along said road to the Murphy Dome-Mud Creek Road (BLM Road 1409); southwesterly along said road to the divide between Nowater Creek and Kirby Creek; westerly along said divide to the head of Walters Draw; westerly down said draw to the Bighorn River.

Area 115. Upper Nowood. Beginning at the junction of the Blue Bank Road and U.S. Highway 16; easterly along said highway to the Canyon Creek Road (U.S.F.S. Road 25); southerly along said road to the Gold Mine Road (U.S.F.S. Road 452); southerly along said road to the Hazelton Road (Johnson County Road 3);

southerly along said road to the Big Horn Mountain Road (Natrona County Road 109); southerly along said road to the Big Horn Trail; westerly along said trail to the Oakie Road (Natrona County Road 101); westerly along said road to Sioux Pass and the divide between the Nowood River and Badwater Creek; westerly then northerly along said divide to Hawks Butte and the divide between the Nowood River and Nowater Creek; northerly along said divide to the Nowater Stock Drive Road (BLM Road 1404); northeasterly along said road to the Blue Bank Road (BLM Road 1411); northerly along said road to U.S. Highway 16.

Area 116. Manderson. Beginning at the junction of the Nowood River and the Big Horn River; northerly down said river to U.S. Highway 14 at the town of Greybull; easterly along said highway to the Bighorn National Forest boundary; easterly and southerly along said boundary to U.S. Highway 16; southwesterly along said highway to the Nowood River; northwesterly down said river to the Big Horn River at the town of Manderson.

Area 117. Crowheart Butte. All non-Indian owned fee title lands within the exterior boundaries of the Wind River Indian Reservation; excluding Antelope Hunt Area 97, those areas of the Spence and Moriarity Wildlife Management Area that lie east of the East Fork of the Wind River, and those lands within Hot Springs County.

WYOMING GAME AND FISH COMMISSION

By:

Bill Williams, DVM, President

Dated: April 24, 2007

2007 ANTELOPE HUNT AREAS

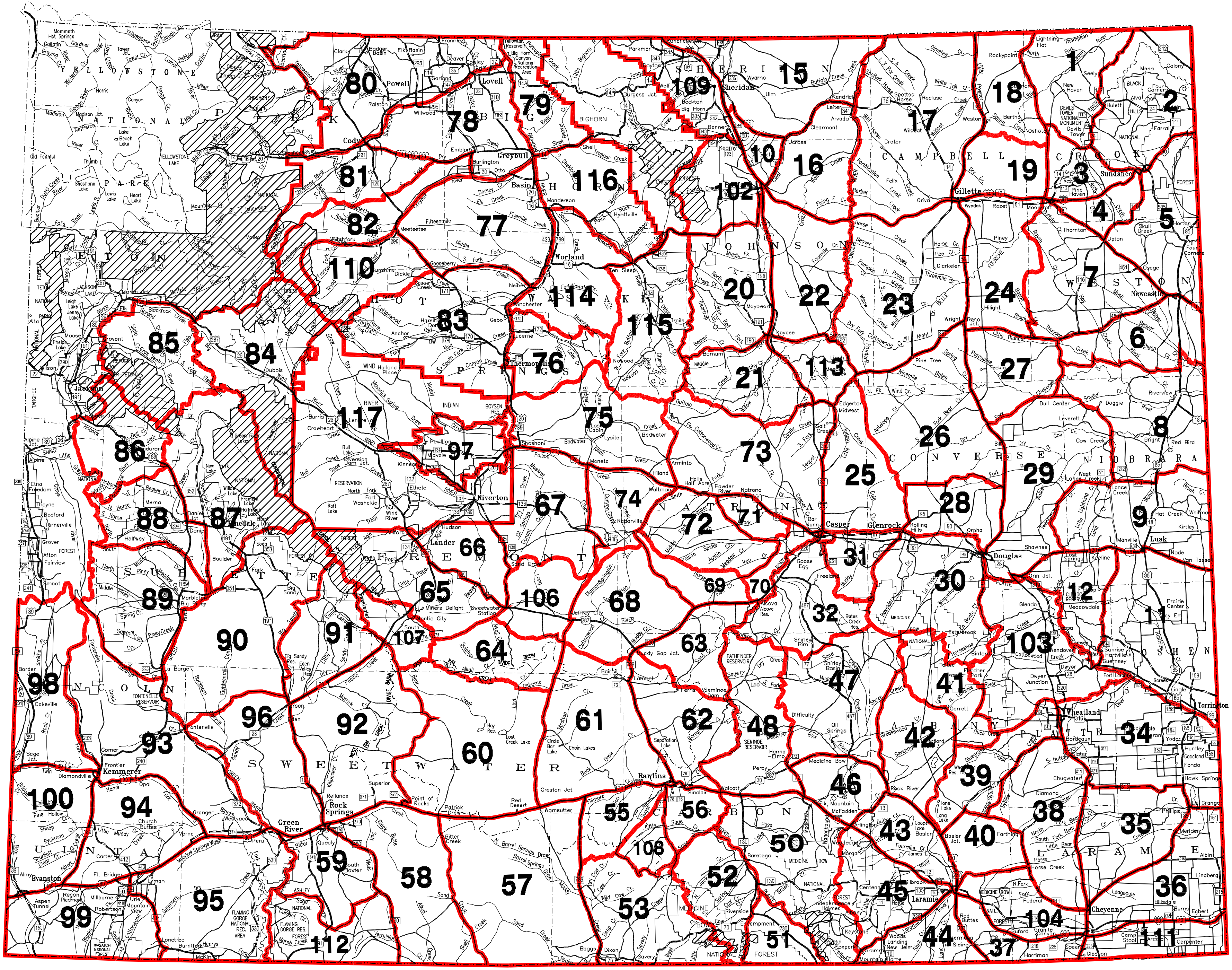

THIS MAP IS FOR GENERAL REFERENCE ONLY. Please use the written boundary descriptions in this regulation for detailed boundary information.

Note: Wilderness area, nonresidents must have guides.

TABLE I

MULE DEER

HARVEST STATEWIDE

BY TOTAL HUNTERS

2007

	LICENSES SOLD	NUMBER ACTIVE HUNTERS	HARVEST BUCK	DOE	FAWN	TOTAL	TOTAL RECREATION DAYS	HUNTER SUCCESS	DAYS/ HARVEST
STATE TOTAL	91,789	65,503	31,480	8,986	640	41,106	328,020	62.8%	8.0
RESIDENT	55,795	38,389	16,748	4,712	356	21,816	207,169	56.8%	9.5
NONRESIDENT	35,994	27,114	14,732	4,274	284	19,290	120,851	71.1%	6.3

MULE DEER

HARVEST BY HARVEST TOOL

2007

ARCHERY	FIREARM	MUZZLE LOADER
3.82%	95.68%	0.50%

TABLE II

WHITE-TAILED DEER

HARVEST STATEWIDE

BY TOTAL HUNTERS

2007

	LICENSES SOLD	NUMBER ACTIVE HUNTERS	HARVEST BUCK	DOE	FAWN	TOTAL	TOTAL RECREATION DAYS	HUNTER SUCCESS	DAYS/ HARVEST
STATE TOTAL	91,789	25,008	7,975	5,109	871	13,955	113,668	55.8%	8.1
RESIDENT	55,795	15,705	4,688	3,079	553	8,320	81,280	53.0%	9.8
NONRESIDENT	35,994	9,303	3,287	2,030	318	5,635	32,388	60.6%	5.7

WHITE-TAILED DEER

HARVEST BY HARVEST TOOL

2007

ARCHERY	FIREARM	MUZZLE LOADER
7.49%	91.51%	1.00%

TABLE III

TOTAL DEER

HARVEST STATEWIDE

BY TOTAL HUNTERS

2007

	LICENSES SOLD	NUMBER ACTIVE HUNTERS	HARVEST				TOTAL RECREATION DAYS	HUNTER SUCCESS	DAYS/ HARVEST
			BUCK	DOE	FAWN	TOTAL			
STATE TOTAL	91,789	77,307	39,455	14,095	1,511	55,061	403,857	71.2%	7.3
RESIDENT	55,795	46,383	21,436	7,791	909	30,136	266,227	65.0%	8.8
NONRESIDENT	35,994	30,924	18,019	6,304	602	24,925	137,630	80.6%	5.5

TABLE IV

STATEWIDE DEER HERD

TEN-YEAR COMPARISON OF HARVEST

1998–2007

YEAR	1998	1999	2000	2001	2002	2003	2004	2005	2006	2007
HARVEST	37,026	45,570	54,377	47,943	46,796	45,710	47,466	47,599	53,925	55,061

TABLE I-A

MULE DEER

HARVEST, HUNTING PRESSURE, HUNTER SUCCESS

BY HUNT AREA

2007

AREA (cross hunt areas)	TYPE	ACTIVE LICS/HTRS	HARVEST BUCK	DOE	FAWN	TOTAL	HUNTER SUCCESS	DAYS/ HARVEST	HUNTER DAYS	LICENSES SOLD (cross hunt incl.)
1 Crook										
**(2, 3)**	D/F Type 6	438	0	204	37	241	55.0%	4.5	1073	2722
	General	1488	614	54	0	668	44.9%	5.8	3874	
Pooled Total		1847	614	258	37	909	49.2%	5.4	4947	
Pooled Resident		519	197	16	0	213	41.0%	5.7	1218	
Pooled Nonresident		1328	417	242	37	696	52.4%	5.4	3729	
2 Bearlodge										
**(1, 3)**	D/F Type 6	738	0	338	0	338	45.8%	6.8	2303	2722
	General	2274	686	46	0	732	32.2%	8.3	6054	
Pooled Total		2832	686	384	0	1070	37.8%	7.8	8357	
Pooled Resident		840	142	89	0	231	27.5%	13.4	3097	
Pooled Nonresident		1992	544	295	0	839	42.1%	6.3	5260	
3 Keyhole										
**(1, 2)**	D/F Type 6	283	0	246	0	246	86.9%	4.9	1211	2722
	General	582	250	16	3	269	46.2%	7.8	2098	
Pooled Total		799	250	262	3	515	64.5%	6.4	3309	
Pooled Resident		293	96	70	0	166	56.7%	7.6	1262	
Pooled Nonresident		506	154	192	3	349	69.0%	5.9	2047	
4 Sand Creek										
	D/F Type 6	90	0	54	0	54	60.0%	3.9	213	324
	General	603	120	3	3	126	20.9%	13.1	1646	
Pooled Total		651	120	57	3	180	27.6%	10.3	1859	
Pooled Resident		301	62	34	0	96	31.9%	9.1	878	
Pooled Nonresident		350	58	23	3	84	24.0%	11.7	981	
5 Inyan Kara										
	D/F Type 6	84	0	60	5	65	77.4%	3.5	230	112
	General	456	201	17	2	220	48.2%	7.7	1685	
Pooled Total		506	201	77	7	285	56.3%	6.7	1915	
Pooled Resident		268	95	34	5	134	50.0%	8.9	1189	
Pooled Nonresident		238	106	43	2	151	63.4%	4.8	726	
6 Skull Creek										
**(9)**	D/F Type 6	139	0	69	7	76	54.7%	5.4	407	233
	General	640	304	29	4	337	52.7%	7.0	2354	
Pooled Total		710	304	98	11	413	58.2%	6.7	2761	
Pooled Resident		392	177	44	3	224	57.1%	6.7	1490	
Pooled Nonresident		318	127	54	8	189	59.4%	6.7	1271	
7 South Osage										
	General	312	166	28	0	194	62.2%	5.0	976	
Pooled Total		312	166	28	0	194	62.2%	5.0	976	
Pooled Resident		103	24	14	0	38	36.9%	6.8	258	
Pooled Nonresident		209	142	14	0	156	74.6%	4.6	718	
8 Upton-Four Horse										
	D/F Type 6	64	0	55	0	55	85.9%	6.0	328	96
	General	264	143	15	0	158	59.8%	6.8	1068	
Pooled Total		298	143	70	0	213	71.5%	6.6	1396	
Pooled Resident		94	44	0	0	44	46.8%	8.5	374	
Pooled Nonresident		204	99	70	0	169	82.8%	6.0	1022	
9 Blacktail Creek										
**(6)**	D/F Type 6	28	0	14	0	14	50.0%	10.6	149	233
	General	245	143	15	0	158	64.5%	5.8	923	
Pooled Total		261	143	29	0	172	65.9%	6.2	1072	
Pooled Resident		87	37	11	0	48	55.2%	9.4	453	
Pooled Nonresident		174	106	18	0	124	71.3%	5.0	619	

TABLE I-A (Continued)

MULE DEER HUNT AREA REPORT

AREA (cross hunt areas)	TYPE	ACTIVE LICS/HTRS	HARVEST BUCK	DOE	FAWN	TOTAL	HUNTER SUCCESS	DAYS/ HARVEST	HUNTER DAYS	LICENSES SOLD (cross hunt incl.)
10 Teckla										
	D/F Type 6	29	0	16	5	21	72.4%	4.1	86	32
	General	250	104	0	0	104	41.6%	8.6	898	
Pooled Total		261	104	16	5	125	47.9%	7.9	984	
Pooled Resident		143	37	0	0	37	25.9%	14.6	541	
Pooled Nonresident		118	67	16	5	88	74.6%	5.0	443	
11 Mule Creek										
	D/F Type 6	100	0	85	0	85	85.0%	3.9	332	134
	General	277	167	7	4	178	64.3%	5.3	939	
Pooled Total		342	167	92	4	263	76.9%	4.8	1271	
Pooled Resident		51	22	9	0	31	60.8%	3.7	115	
Pooled Nonresident		291	145	83	4	232	79.7%	5.0	1156	
12 Hat Creek										
**(13, 14)**	D/F Type 6	181	0	130	4	134	74.0%	5.1	682	500
	General	337	163	33	0	196	58.2%	6.5	1270	
Pooled Total		468	163	163	4	330	70.5%	5.9	1952	
Pooled Resident		101	25	58	0	83	82.2%	5.7	477	
Pooled Nonresident		367	138	105	4	247	67.3%	6.0	1475	
13 Lusk										
**(12, 14)**	D/F Type 6	153	0	106	13	119	77.8%	3.8	448	500
	General	648	412	44	0	456	70.4%	5.3	2404	
Pooled Total		771	412	150	13	575	74.6%	5.0	2852	
Pooled Resident		192	90	51	0	141	73.4%	5.0	700	
Pooled Nonresident		579	322	99	13	434	75.0%	5.0	2152	
14 Twenty Mile										
**(12, 13)**	D/F Type 6	119	0	69	0	69	58.0%	6.0	412	500
	General	467	291	24	4	319	68.3%	7.2	2288	
Pooled Total		560	291	93	4	388	69.3%	7.0	2700	
Pooled Resident		119	47	17	4	68	57.1%	12.7	861	
Pooled Nonresident		441	244	76	0	320	72.6%	5.7	1839	
15 South Keeline										
	D/F Type 6	14	0	11	0	11	78.6%	5.0	55	18
	General	167	92	11	0	103	61.7%	5.5	564	
Pooled Total		174	92	22	0	114	65.5%	5.4	619	
Pooled Resident		85	35	11	0	46	54.1%	6.8	311	
Pooled Nonresident		89	57	11	0	68	76.4%	4.5	308	
16 Meadowdale/Jay Em										
	Limited Type 2	21	9	4	0	13	61.9%	7.1	92	28
	General	422	212	0	0	212	50.2%	6.8	1447	
Pooled Total		443	221	4	0	225	50.8%	6.8	1539	
Pooled Resident		293	123	4	0	127	43.3%	7.6	967	
Pooled Nonresident		150	98	0	0	98	65.3%	5.8	572	
17 Northwest Gillette										
**(18, 19, 20, 21)**	D/F Type 6	286	0	238	11	249	87.1%	4.1	1026	928
	General	1077	647	31	0	678	63.0%	6.9	4690	
Pooled Total		1310	647	269	11	927	70.8%	6.2	5716	
Pooled Resident		607	252	154	11	417	68.7%	6.4	2666	
Pooled Nonresident		703	395	115	0	510	72.5%	6.0	3050	
18 Campbell										
**(17, 19, 20, 21)**	D/F Type 6	242	0	208	0	208	86.0%	3.4	701	928
	General	1370	657	47	0	704	51.4%	7.2	5046	
Pooled Total		1553	657	255	0	912	58.7%	6.3	5747	
Pooled Resident		705	187	127	0	314	44.5%	8.4	2629	
Pooled Nonresident		848	470	128	0	598	70.5%	5.2	3118	

TABLE I-A (Continued)

MULE DEER HUNT AREA REPORT

AREA (cross hunt areas)	TYPE	ACTIVE LICS/HTRS	HARVEST BUCK	HARVEST DOE	HARVEST FAWN	HARVEST TOTAL	HUNTER SUCCESS	DAYS/ HARVEST	HUNTER DAYS	LICENSES SOLD (cross hunt incl.)
19 Pumpkin Buttes										
**(17, 18, 20, 21)**	D/F Type 6	90	0	83	0	83	92.2%	2.9	241	928
	General	390	257	8	0	265	67.9%	5.0	1335	
Pooled Total		459	257	91	0	348	75.8%	4.5	1576	
Pooled Resident		131	53	40	0	93	71.0%	5.1	471	
Pooled Nonresident		328	204	51	0	255	77.7%	4.3	1105	
20 Clarkelen										
**(17, 18, 19, 21)**	D/F Type 6	30	0	17	0	17	56.7%	4.6	79	928
	General	121	64	2	0	66	54.5%	6.4	423	
Pooled Total		151	64	19	0	83	55.0%	6.0	502	
Pooled Resident		53	17	13	0	30	56.6%	4.9	147	
Pooled Nonresident		98	47	6	0	53	54.1%	6.7	355	
21 Thunder Basin										
**(17, 18, 19, 20)**	D/F Type 6	163	0	83	13	96	58.9%	7.2	689	928
	General	489	243	49	0	292	59.7%	6.6	1913	
Pooled Total		614	243	132	13	388	63.2%	6.7	2602	
Pooled Resident		351	112	74	0	186	53.0%	9.9	1840	
Pooled Nonresident		263	131	58	13	202	76.8%	3.8	762	
22 Douglas										
	Limited Type 1	293	254	15	0	269	91.8%	2.9	788	338
**(158)**	D/F Type 6	149	0	102	0	102	68.5%	3.7	379	286
Pooled Total		391	254	117	0	371	94.9%	3.1	1167	
Pooled Resident		40	7	24	0	31	77.5%	4.5	139	
Pooled Nonresident		351	247	93	0	340	96.9%	3.0	1028	
23 Clearmont										
**(26)**	D/F Type 6	520	0	427	24	451	86.7%	4.4	1984	783
	General	1392	857	61	4	922	66.2%	6.3	5779	
Pooled Total		1748	857	488	28	1373	78.5%	5.7	7763	
Pooled Resident		693	219	203	18	440	63.5%	7.2	3185	
Pooled Nonresident		1055	638	285	10	933	88.4%	4.9	4578	
24 Sheridan										
	D/F Type 6	335	0	293	12	305	91.0%	5.1	1549	998
	General	1148	406	69	4	479	41.7%	11.7	5602	
Pooled Total		1421	406	362	16	784	55.2%	9.1	7151	
Pooled Resident		843	184	179	16	379	45.0%	12.7	4813	
Pooled Nonresident		578	222	183	0	405	70.1%	5.8	2338	
25 Tongue-Goose										
	General	818	177	0	0	177	21.6%	24.4	4314	
Pooled Total		818	177	0	0	177	21.6%	24.4	4314	
Pooled Resident		484	80	0	0	80	16.5%	34.1	2730	
Pooled Nonresident		334	97	0	0	97	29.0%	16.3	1584	
26 Ucross										
**(23)**	D/F Type 6	75	0	56	0	56	74.7%	3.2	181	783
	General	719	429	8	5	442	61.5%	5.0	2196	
Pooled Total		784	429	64	5	498	63.5%	4.8	2377	
Pooled Resident		291	99	34	5	138	47.4%	5.6	768	
Pooled Nonresident		493	330	30	0	360	73.0%	4.5	1609	
27 Buffalo										
	D/F Type 6	106	0	64	0	64	60.4%	7.1	452	200
	General	650	212	34	4	250	38.5%	9.2	2308	
Pooled Total		712	212	98	4	314	44.1%	8.8	2760	
Pooled Resident		329	43	50	4	97	29.5%	15.1	1460	
Pooled Nonresident		383	169	48	0	217	56.7%	6.0	1300	

TABLE I-A (Continued)

MULE DEER HUNT AREA REPORT

AREA (cross hunt areas)	TYPE	ACTIVE LICS/HTRS	HARVEST BUCK	HARVEST DOE	HARVEST FAWN	HARVEST TOTAL	HUNTER SUCCESS	DAYS/ HARVEST	HUNTER DAYS	LICENSES SOLD (cross hunt incl.)
28 Hunter Mesa										
	General	497	69	0	0	69	13.9%	37.2	2569	
Pooled Total		497	69	0	0	69	13.9%	37.2	2569	
Pooled Resident		310	26	0	0	26	8.4%	75.0	1951	
Pooled Nonresident		187	43	0	0	43	23.0%	14.4	618	
29 Johnson										
	D/F Type 6	163	0	102	14	116	71.2%	4.6	536	259
	General	413	249	8	0	257	62.2%	5.4	1386	
Pooled Total		537	249	110	14	373	69.5%	5.2	1922	
Pooled Resident		157	37	22	5	64	40.8%	9.5	605	
Pooled Nonresident		380	212	88	9	309	81.3%	4.3	1317	
30 Upper Powder River										
	D/F Type 6	118	0	98	0	98	83.1%	5.3	524	219
	General	394	176	35	0	211	53.6%	8.2	1732	
Pooled Total		457	176	133	0	309	67.6%	7.3	2256	
Pooled Resident		147	36	42	0	78	53.1%	14.2	1110	
Pooled Nonresident		310	140	91	0	231	74.5%	5.0	1146	
31 Salt Creek										
	General	72	43	0	0	43	59.7%	9.0	385	
Pooled Total		72	43	0	0	43	59.7%	9.0	385	
Pooled Resident		33	11	0	0	11	33.3%	22.9	252	
Pooled Nonresident		39	32	0	0	32	82.1%	4.2	133	
32 Beartrap Creek										
	General	97	54	13	0	67	69.1%	4.8	319	
Pooled Total		97	54	13	0	67	69.1%	4.8	319	
Pooled Resident		50	15	13	0	28	56.0%	5.0	141	
Pooled Nonresident		47	39	0	0	39	83.0%	4.6	178	
33 Red Fork										
	D/F Type 6	146	0	76	9	85	58.2%	7.9	674	199
	General	793	468	39	4	511	64.4%	6.9	3511	
Pooled Total		883	468	115	13	596	67.5%	7.0	4185	
Pooled Resident		168	63	26	0	89	53.0%	8.2	733	
Pooled Nonresident		715	405	89	13	507	70.9%	6.8	3452	
34 Natrona										
	Limited Type 1	294	226	4	0	230	78.2%	5.5	1271	351
	D/F Type 6	134	0	99	7	106	79.1%	4.4	470	151
Pooled Total		381	226	103	7	336	88.2%	5.2	1741	
Pooled Resident		274	166	70	7	243	88.7%	5.1	1241	
Pooled Nonresident		107	60	33	0	93	86.9%	5.4	500	
35 Upper Nowood										
	General	175	87	9	0	96	54.9%	5.7	549	
Pooled Total		175	87	9	0	96	54.9%	5.7	549	
Pooled Resident		75	19	9	0	28	37.3%	8.5	237	
Pooled Nonresident		100	68	0	0	68	68.0%	4.6	312	
36 Badwater										
	General	447	245	0	0	245	54.8%	6.2	1514	
Pooled Total		447	245	0	0	245	54.8%	6.2	1514	
Pooled Resident		300	152	0	0	152	50.7%	7.2	1090	
Pooled Nonresident		147	93	0	0	93	63.3%	4.6	424	
37 Copper Mountain										
	Limited Type 1	168	126	12	0	138	82.1%	5.1	708	179
	D/F Type 6	108	0	92	4	96	88.9%	3.3	312	148
Pooled Total		236	126	104	4	234	99.2%	4.4	1020	
Pooled Resident		177	94	80	2	176	99.4%	3.9	694	
Pooled Nonresident		59	32	24	2	58	98.3%	5.6	326	

MULE DEER HUNT AREA REPORT

AREA (cross hunt areas)		TYPE	ACTIVE LICS/HTRS	HARVEST BUCK	DOE	FAWN	TOTAL	HUNTER SUCCESS	DAYS/ HARVEST	HUNTER DAYS	LICENSES SOLD (cross hunt incl.)
38 Black Mountain											
		General	167	71	0	0	71	42.5%	8.1	577	
Pooled Total			167	71	0	0	71	42.5%	8.1	577	
Pooled Resident			92	35	0	0	35	38.0%	9.0	315	
Pooled Nonresident			75	36	0	0	36	48.0%	7.3	262	
39 Bates Creek											
		D/F Type 6	40	0	24	0	24	60.0%	5.0	121	50
		General	165	73	0	0	73	44.2%	6.4	469	
Pooled Total			193	73	24	0	97	50.3%	6.1	590	
Pooled Resident			57	23	0	0	23	40.4%	5.8	133	
Pooled Nonresident			136	50	24	0	74	54.4%	6.2	457	
40 Buffalo Creek											
		D/F Type 6	87	0	37	8	45	51.7%	7.4	335	121
		General	258	147	10	0	157	60.9%	5.3	835	
Pooled Total			306	147	47	8	202	66.0%	5.8	1170	
Pooled Resident			80	36	6	0	42	52.5%	5.8	243	
Pooled Nonresident			226	111	41	8	160	70.8%	5.8	927	
41 Sand Creek											
	**(45)**	D/F Type 6	121	0	80	12	92	76.0%	7.7	708	197
		General	322	102	35	0	137	42.5%	9.9	1357	
Pooled Total			397	102	115	12	229	57.7%	9.0	2065	
Pooled Resident			212	41	45	0	86	40.6%	9.9	848	
Pooled Nonresident			185	61	70	12	143	77.3%	8.5	1217	
42 Otter Creek											
		D/F Type 6	147	0	105	11	116	78.9%	5.7	657	175
		General	983	434	89	4	527	53.6%	7.8	4124	
Pooled Total			1050	434	194	15	643	61.2%	7.4	4781	
Pooled Resident			335	126	38	8	172	51.3%	9.3	1591	
Pooled Nonresident			715	308	156	7	471	65.9%	6.8	3190	
43 Leigh Creek											
		General	120	26	0	0	26	21.7%	22.7	591	
Pooled Total			120	26	0	0	26	21.7%	22.7	591	
Pooled Resident			41	12	0	0	12	29.3%	17.7	212	
Pooled Nonresident			79	14	0	0	14	17.7%	27.1	379	
44 Brokenback Creek											
		General	146	50	9	0	59	40.4%	9.4	556	
Pooled Total			146	50	9	0	59	40.4%	9.4	556	
Pooled Resident			82	10	6	0	16	19.5%	19.6	313	
Pooled Nonresident			64	40	3	0	43	67.2%	5.7	243	
45 Paint Rock Creek											
	**(41)**	D/F Type 6	24	0	19	2	21	87.5%	4.9	103	197
		General	244	90	17	0	107	43.9%	9.5	1021	
Pooled Total			258	90	36	2	128	49.6%	8.8	1124	
Pooled Resident			123	33	7	2	42	34.1%	14.4	605	
Pooled Nonresident			135	57	29	0	86	63.7%	6.0	519	
46 Cloud Peak											
		General	342	77	29	0	106	31.0%	13.7	1449	
Pooled Total			342	77	29	0	106	31.0%	13.7	1449	
Pooled Resident			231	33	12	0	45	19.5%	23.0	1033	
. Pooled Nonresident			111	44	17	0	61	55.0%	6.8	416	
47 Manderson											
	**(49)**	D/F Type 6	68	0	47	12	59	86.8%	3.8	227	149
		General	373	142	33	4	179	48.0%	7.6	1362	
Pooled Total			405	142	80	16	238	58.8%	6.7	1589	
Pooled Resident			250	81	61	9	151	60.4%	6.7	1006	
Pooled Nonresident			155	61	19	7	87	56.1%	6.7	583	

TABLE I-A (Continued)

MULE DEER HUNT AREA REPORT

AREA (cross hunt areas)	TYPE	ACTIVE LICS/HTRS	HARVEST BUCK	DOE	FAWN	TOTAL	HUNTER SUCCESS	DAYS/ HARVEST	HUNTER DAYS	LICENSES SOLD (cross hunt incl.)
49 Trapper Creek										
**(47)**	D/F Type 6	53	0	41	3	44	83.0%	3.9	170	149
	General	287	124	19	0	143	49.8%	8.2	1170	
Pooled Total		308	124	60	3	187	60.7%	7.2	1340	
Pooled Resident		182	46	38	0	84	46.2%	9.8	822	
Pooled Nonresident		126	78	22	3	103	81.7%	5.0	518	
50 Upper Shell-Beaver Creek										
	General	245	113	9	0	122	49.8%	6.8	830	
Pooled Total		245	113	9	0	122	49.8%	6.8	830	
Pooled Resident		151	49	9	0	58	38.4%	9.4	547	
Pooled Nonresident		94	64	0	0	64	68.1%	4.4	283	
51 Beaver Creek										
	D/F Type 6	162	0	134	7	141	87.0%	3.8	538	260
	General	375	179	54	0	233	62.1%	5.9	1382	
Pooled Total		472	179	188	7	374	79.2%	5.1	1920	
Pooled Resident		236	64	86	0	150	63.6%	6.7	998	
Pooled Nonresident		236	115	102	7	224	94.9%	4.1	922	
52 Crystal Creek										
	General	148	55	0	0	55	37.2%	8.0	442	
Pooled Total		148	55	0	0	55	37.2%	8.0	442	
Pooled Resident		111	35	0	0	35	31.5%	9.5	331	
Pooled Nonresident		37	20	0	0	20	54.1%	5.6	111	
53 Devils Canyon										
	General	425	178	4	0	182	42.8%	9.2	1676	
Pooled Total		425	178	4	0	182	42.8%	9.2	1676	
Pooled Resident		331	111	4	0	115	34.7%	12.0	1376	
Pooled Nonresident		94	67	0	0	67	71.3%	4.5	300	
55 Rock Eagle										
	D/F Type 6	122	0	77	3	80	65.6%	5.8	466	150
	General	621	263	0	0	263	42.4%	8.6	2263	
Pooled Total		692	263	77	3	343	49.6%	8.0	2729	
Pooled Resident		512	175	49	0	224	43.8%	9.3	2089	
Pooled Nonresident		180	88	28	3	119	66.1%	5.4	640	
57 Southeast										
	General	429	150	46	15	211	49.2%	7.7	1634	
Pooled Total		429	150	46	15	211	49.2%	7.7	1634	
Pooled Resident		379	131	43	15	189	49.9%	7.6	1431	
Pooled Nonresident		50	19	3	0	22	44.0%	9.2	203	
59 Little Bear										
	D/F Type 6	38	0	32	0	32	84.2%	4.3	138	51
	General	128	79	0	0	79	61.7%	6.6	518	
Pooled Total		150	79	32	0	111	74.0%	5.9	656	
Pooled Resident		109	59	18	0	77	70.6%	6.8	523	
Pooled Nonresident		41	20	14	0	34	82.9%	3.9	133	
60 Pole Mountain										
	Limited Type 1	93	69	0	0	69	74.2%	8.4	581	105
	Limited Type 2	137	83	27	0	110	80.3%	5.6	616	157
	D/F Type 6	43	0	26	0	26	60.5%	6.4	166	50
Pooled Total		259	152	53	0	205	79.2%	6.6	1363	
Pooled Resident		211	110	51	0	161	76.3%	6.9	1108	
Pooled Nonresident		48	42	2	0	44	91.7%	5.8	255	
61 Harriman										
	General	190	52	7	4	63	33.2%	14.5	914	
Pooled Total		190	52	7	4	63	33.2%	14.5	914	
Pooled Resident		158	32	7	4	43	27.2%	18.8	808	
Pooled Nonresident		32	20	0	0	20	62.5%	5.3	106	

MULE DEER HUNT AREA REPORT

AREA (cross hunt areas)	TYPE	ACTIVE LICS/HTRS	HARVEST BUCK	HARVEST DOE	HARVEST FAWN	HARVEST TOTAL	HUNTER SUCCESS	DAYS/ HARVEST	HUNTER DAYS	LICENSES SOLD (cross hunt incl.)
62 Iron Mountain										
**(63, 64)**	D/F Type 6	20	0	9	0	9	45.0%	10.2	92	177
	General	114	93	0	0	93	81.6%	5.8	539	
Pooled Total		134	93	9	0	102	76.1%	6.2	631	
Pooled Resident		80	58	4	0	62	77.5%	6.6	411	
Pooled Nonresident		54	35	5	0	40	74.1%	5.5	220	
63 Richeau										
**(62, 64)**	D/F Type 6	15	0	2	4	6	40.0%	6.3	38	177
	General	262	171	0	0	171	65.3%	5.4	921	
Pooled Total		273	171	2	4	177	64.8%	5.4	959	
Pooled Resident		122	67	2	2	71	58.2%	4.9	350	
Pooled Nonresident		151	104	0	2	106	70.2%	5.7	609	
64 Laramie Peak										
	Limited Type 2	122	75	0	0	75	61.5%	7.7	576	149
**(62, 63)**	D/F Type 6	107	0	63	15	78	72.9%	4.8	377	177
	General	1252	712	0	0	712	56.9%	7.3	5200	
Pooled Total		1439	787	63	15	865	60.1%	7.1	6153	
Pooled Resident		958	467	32	6	505	52.7%	8.4	4243	
Pooled Nonresident		481	320	31	9	360	74.8%	5.3	1910	
65 South Converse										
	D/F Type 6	157	0	106	7	113	72.0%	5.2	583	196
	General	1108	616	0	0	616	55.6%	6.9	4224	
Pooled Total		1185	616	106	7	729	61.5%	6.6	4807	
Pooled Resident		539	265	21	4	290	53.8%	7.2	2088	
Pooled Nonresident		646	351	85	3	439	68.0%	6.2	2719	
66 Bates Hole-Hat Six										
	D/F Type 6	167	0	121	25	146	87.4%	4.1	594	200
	General	1050	512	0	0	512	48.8%	8.2	4191	
Pooled Total		1163	512	121	25	658	56.6%	7.3	4785	
Pooled Resident		785	310	47	3	360	45.9%	8.6	3112	
Pooled Nonresident		378	202	74	22	298	78.8%	5.6	1673	
70 Shirley Mountain										
	D/F Type 6	71	0	54	4	58	81.7%	3.9	225	100
	General	879	511	0	0	511	58.1%	6.7	3406	
Pooled Total		923	511	54	4	569	61.6%	6.4	3631	
Pooled Resident		478	210	20	2	232	48.5%	5.8	1340	
Pooled Nonresident		445	301	34	2	337	75.7%	6.8	2291	
73 Boswell										
	General	289	120	0	0	120	41.5%	9.6	1155	
Pooled Total		289	120	0	0	120	41.5%	9.6	1155	
Pooled Resident		174	60	0	0	60	34.5%	12.1	725	
Pooled Nonresident		115	60	0	0	60	52.2%	7.2	430	
74 Kyle										
**(75)**	D/F Type 6	3	0	2	0	2	66.7%	2.0	4	25
	General	242	105	0	0	105	43.4%	8.7	913	
Pooled Total		245	105	2	0	107	43.7%	8.6	917	
Pooled Resident		162	72	0	0	72	44.4%	8.3	599	
Pooled Nonresident		83	33	2	0	35	42.2%	9.1	318	
75 Four Mile										
**(74)**	D/F Type 6	2	0	0	0	0	0.0%	0.0	19	25
	General	717	176	0	0	176	24.5%	16.7	2940	
Pooled Total		719	176	0	0	176	24.5%	16.8	2959	
Pooled Resident		591	114	0	0	114	19.3%	21.4	2435	
Pooled Nonresident		128	62	0	0	62	48.4%	8.5	524	

TABLE I-A (Continued)

MULE DEER HUNT AREA REPORT

AREA (cross hunt areas)	TYPE	ACTIVE LICS/HTRS	HARVEST BUCK	DOE	FAWN	TOTAL	HUNTER SUCCESS	DAYS/ HARVEST	HUNTER DAYS	LICENSES SOLD (cross hunt incl.)
76 Sheep Mountain										
	General	916	140	0	0	140	15.3%	27.3	3818	
Pooled Total		916	140	0	0	140	15.3%	27.3	3818	
Pooled Resident		747	96	0	0	96	12.9%	32.8	3151	
Pooled Nonresident		169	44	0	0	44	26.0%	15.2	667	
77 Boulder Ridge										
	General	256	44	0	0	44	17.2%	21.8	961	
Pooled Total		256	44	0	0	44	17.2%	21.8	961	
Pooled Resident		201	37	0	0	37	18.4%	19.9	737	
Pooled Nonresident		55	7	0	0	7	12.7%	32.0	224	
78 French Creek										
**(79, 80, 81)**	Limited Type 2	18	16	0	0	16	88.9%	4.6	74	27
**(79, 80, 81)**	D/F Type 6	80	0	31	8	39	48.8%	7.4	287	203
	General	1680	306	164	15	485	28.9%	16.0	7771	
Pooled Total		1731	322	195	23	540	31.2%	15.1	8132	
Pooled Resident		1311	201	151	19	371	28.3%	16.8	6216	
Pooled Nonresident		420	121	44	4	169	40.2%	11.3	1916	
79 Kennaday Peak										
**(78, 80, 81)**	Limited Type 2	11	6	0	0	6	54.5%	4.2	25	27
**(78, 80, 81)**	D/F Type 6	47	0	29	4	33	70.2%	6.7	221	203
	General	996	255	99	3	357	35.8%	12.0	4298	
Pooled Total		1026	261	128	7	396	38.6%	11.5	4544	
Pooled Resident		651	129	70	0	199	30.6%	15.1	3000	
Pooled Nonresident		375	132	58	7	197	52.5%	7.8	1544	
80 Spring Creek										
**(78, 79, 81)**	Limited Type 2	10	3	0	0	3	30.0%	11.3	34	27
**(78, 79, 81)**	D/F Type 6	23	0	9	2	11	47.8%	10.7	118	203
	General	963	296	79	9	384	39.9%	11.6	4442	
Pooled Total		982	299	88	11	398	40.5%	11.5	4594	
Pooled Resident		561	147	48	9	204	36.4%	13.4	2737	
Pooled Nonresident		421	152	40	2	194	46.1%	9.6	1857	
81 Blackhall										
**(78, 79, 80)**	Limited Type 2	2	2	0	0	2	100.0%	1.5	3	27
**(78, 79, 80)**	D/F Type 6	29	0	18	0	18	62.1%	7.2	130	203
	General	967	259	125	12	396	41.0%	11.0	4342	
Pooled Total		968	261	143	12	416	43.0%	10.8	4475	
Pooled Resident		608	175	92	12	279	45.9%	9.6	2685	
Pooled Nonresident		360	86	51	0	137	38.1%	13.1	1790	
82 Baggs										
**(also valid 84)**	Limited Type 1	60	42	3	0	45	75.0%	7.6	344	33
**(also valid 84)**	D/F Type 6	490	0	399	8	407	83.1%	6.0	2442	600
	General	2845	1649	206	24	1879	66.0%	6.9	12982	
Pooled Total		3216	1691	608	32	2331	72.5%	6.8	15768	
Pooled Resident		2050	959	375	23	1357	66.2%	7.3	9898	
Pooled Nonresident		1166	732	233	9	974	83.5%	6.0	5870	
83 Bolten Rim										
	General	84	31	0	0	31	36.9%	11.9	370	
Pooled Total		84	31	0	0	31	36.9%	11.9	370	
Pooled Resident		40	13	0	0	13	32.5%	17.2	223	
Pooled Nonresident		44	18	0	0	18	40.9%	8.2	147	
84 Atlantic Rim										
	Limited Type 1	133	81	3	0	84	63.2%	6.7	565	150
	D/F Type 6	29	0	19	5	24	82.8%	1.9	46	59
Pooled Total		149	81	22	5	108	72.5%	5.7	611	
Pooled Resident		107	60	4	0	64	59.8%	7.5	477	
Pooled Nonresident		42	21	18	5	44	104.8%	3.0	134	

MULE DEER HUNT AREA REPORT

AREA (cross hunt areas)	TYPE	ACTIVE LICS/HTRS	HARVEST BUCK	HARVEST DOE	HARVEST FAWN	HARVEST TOTAL	HUNTER SUCCESS	DAYS/ HARVEST	HUNTER DAYS	LICENSES SOLD (cross hunt incl.)
87 Ferris										
	Limited Type 1	275	226	0	0	226	82.2%	6.3	1427	301
Pooled Total		275	226	0	0	226	82.2%	6.3	1427	
Pooled Resident		222	184	0	0	184	82.9%	5.1	942	
Pooled Nonresident		53	42	0	0	42	79.2%	11.5	485	
88 Kendrick										
	D/F Type 6	199	0	132	8	140	70.4%	7.2	1006	256
	General	279	144	0	0	144	51.6%	6.3	909	
Pooled Total		413	144	132	8	284	68.8%	6.7	1915	
Pooled Resident		365	134	110	4	248	67.9%	7.0	1741	
Pooled Nonresident		48	10	22	4	36	75.0%	4.8	174	
89 Rattlesnake										
	Limited Type 1	278	234	0	0	234	84.2%	4.3	998	278
	D/F Type 6	83	0	71	5	76	91.6%	5.5	420	94
Pooled Total		338	234	71	5	310	91.7%	4.6	1418	
Pooled Resident		272	183	65	5	253	93.0%	4.9	1239	
Pooled Nonresident		66	51	6	0	57	86.4%	3.1	179	
90 Muskrat										
	Limited Type 1	73	64	0	0	64	87.7%	5.8	368	75
Pooled Total		73	64	0	0	64	87.7%	5.8	368	
Pooled Resident		58	51	0	0	51	87.9%	5.7	291	
Pooled Nonresident		15	13	0	0	13	86.7%	5.9	77	
91 Popo Agie										
	General	403	129	0	0	129	32.0%	12.8	1654	
Pooled Total		403	129	0	0	129	32.0%	12.8	1654	
Pooled Resident		343	99	0	0	99	28.9%	14.1	1394	
Pooled Nonresident		60	30	0	0	30	50.0%	8.7	260	
92 Table Mountain										
	D/F Type 6	44	0	44	0	44	100.0%	5.7	250	50
	General	506	306	0	0	306	60.5%	4.7	1448	
Pooled Total		533	306	44	0	350	65.7%	4.9	1698	
Pooled Resident		460	269	41	0	310	67.4%	4.6	1423	
Pooled Nonresident		73	37	3	0	40	54.8%	6.9	275	
93 Upper Sweetwater										
	General	85	22	0	0	22	25.9%	16.6	365	
Pooled Total		85	22	0	0	22	25.9%	16.6	365	
Pooled Resident		75	19	0	0	19	25.3%	17.5	332	
Pooled Nonresident		10	3	0	0	3	30.0%	11.0	33	
94 Twin Creek										
**(160)**	D/F Type 6	28	0	20	0	20	71.4%	3.7	74	54
	General	422	167	0	0	167	39.6%	7.2	1210	
Pooled Total		440	167	20	0	187	42.5%	6.9	1284	
Pooled Resident		377	130	14	0	144	38.2%	8.1	1161	
Pooled Nonresident		63	37	6	0	43	68.3%	2.9	123	
95 South Pass										
	General	243	47	0	0	47	19.3%	15.7	737	
Pooled Total		243	47	0	0	47	19.3%	15.7	737	
Pooled Resident		180	47	0	0	47	26.1%	12.1	570	
Pooled Nonresident		63	0	0	0	0	0.0%	0.0	167	
96 Green Mountain										
	D/F Type 6	46	0	37	4	41	89.1%	2.3	93	60
	General	643	301	0	0	301	46.8%	6.4	1937	
Pooled Total		677	301	37	4	342	50.5%	5.9	2030	
Pooled Resident		480	168	28	4	200	41.7%	6.3	1268	
Pooled Nonresident		197	133	9	0	142	72.1%	5.4	762	

TABLE I-A (Continued)

MULE DEER HUNT AREA REPORT

AREA (cross hunt areas)	TYPE	ACTIVE LICS/HTRS	HARVEST BUCK	DOE	FAWN	TOTAL	HUNTER SUCCESS	DAYS/ HARVEST	HUNTER DAYS	LICENSES SOLD (cross hunt incl.)
97 Sweetwater Rocks										
	D/F Type 6	86	0	64	11	75	87.2%	1.5	112	102
	General	401	195	0	0	195	48.6%	7.3	1414	
Pooled Total		448	195	64	11	270	60.3%	5.7	1526	
Pooled Resident		368	158	54	4	216	58.7%	5.6	1204	
Pooled Nonresident		80	37	10	7	54	67.5%	6.0	322	
98 Chain Lakes										
	General	108	60	0	0	60	55.6%	5.6	338	
Pooled Total		108	60	0	0	60	55.6%	5.6	338	
Pooled Resident		68	33	0	0	33	48.5%	5.7	188	
Pooled Nonresident		40	27	0	0	27	67.5%	5.6	150	
100 South Wamsutter										
	General	375	193	0	0	193	51.5%	7.0	1351	
Pooled Total		375	193	0	0	193	51.5%	7.0	1351	
Pooled Resident		245	120	0	0	120	49.0%	6.9	832	
Pooled Nonresident		130	73	0	0	73	56.2%	7.1	519	
101 Black Butte										
	Limited Type 1	75	55	0	0	55	73.3%	7.0	385	79
Pooled Total		75	55	0	0	55	73.3%	7.0	385	
Pooled Resident		57	41	0	0	41	71.9%	6.8	277	
Pooled Nonresident		18	14	0	0	14	77.8%	7.7	108	
102 Aspen Mountain										
	Limited Type 1	374	296	0	0	296	79.1%	6.9	2039	402
Pooled Total		374	296	0	0	296	79.1%	6.9	2039	
Pooled Resident		300	245	0	0	245	81.7%	6.5	1590	
Pooled Nonresident		74	51	0	0	51	68.9%	8.8	449	
105 Beartooth										
	General	365	173	38	0	211	57.8%	5.6	1188	
Pooled Total		365	173	38	0	211	57.8%	5.6	1188	
Pooled Resident		254	107	35	0	142	55.9%	5.3	746	
Pooled Nonresident		111	66	3	0	69	62.2%	6.4	442	
106 Crandall-Sunlight										
	General	622	299	0	0	299	48.1%	9.0	2691	
Pooled Total		622	299	0	0	299	48.1%	9.0	2691	
Pooled Resident		397	161	0	0	161	40.6%	10.6	1713	
Pooled Nonresident		225	138	0	0	138	61.3%	7.1	978	
109 Bald Ridge										
	Limited Type 1	46	39	0	0	39	84.8%	4.9	190	50
Pooled Total		46	39	0	0	39	84.8%	4.9	190	
Pooled Resident		40	33	0	0	33	82.5%	5.1	169	
Pooled Nonresident		6	6	0	0	6	100.0%	3.5	21	
110 Grinnell										
	General	219	64	0	0	64	29.2%	15.6	998	
Pooled Total		219	64	0	0	64	29.2%	15.6	998	
Pooled Resident		119	16	0	0	16	13.4%	36.3	581	
Pooled Nonresident		100	48	0	0	48	48.0%	8.7	417	
111 Elk Fork										
	D/F Type 6	54	0	41	0	41	75.9%	4.8	196	76
	General	692	352	18	4	374	54.0%	7.1	2645	
Pooled Total		723	352	59	4	415	57.4%	6.8	2841	
Pooled Resident		444	183	51	4	238	53.6%	7.5	1775	
Pooled Nonresident		279	169	8	0	177	63.4%	6.0	1066	

TABLE I-A (Continued)

MULE DEER HUNT AREA REPORT

AREA (cross hunt areas)	TYPE	ACTIVE LICS/HTRS	HARVEST BUCK	HARVEST DOE	HARVEST FAWN	HARVEST TOTAL	HUNTER SUCCESS	DAYS/ HARVEST	HUNTER DAYS	LICENSES SOLD (cross hunt incl.)
112 Ishawooa										
**(113)**	D/F Type 6	97	0	69	15	84	86.6%	3.5	296	252
	General	608	300	34	4	338	55.6%	6.7	2276	
Pooled Total		656	300	103	19	422	64.3%	6.1	2572	
Pooled Resident		385	138	73	4	215	55.8%	7.3	1568	
Pooled Nonresident		271	162	30	15	207	76.4%	4.9	1004	
113 Boulder										
**(112)**	D/F Type 6	101	0	86	0	86	85.1%	4.9	424	252
	General	640	325	21	4	350	54.7%	7.1	2484	
Pooled Total		691	325	107	4	436	63.1%	6.7	2908	
Pooled Resident		398	149	56	4	209	52.5%	8.6	1789	
Pooled Nonresident		293	176	51	0	227	77.5%	4.9	1119	
114 Wall Mountain										
	General	10	4	0	0	4	40.0%	10.8	43	
Pooled Total		10	4	0	0	4	40.0%	10.8	43	
Pooled Resident		7	4	0	0	4	57.1%	7.3	29	
Pooled Nonresident		3	0	0	0	0	0.0%	0.0	14	
115 Thorofare										
	General	110	47	0	0	47	42.7%	10.9	512	
Pooled Total		110	47	0	0	47	42.7%	10.9	512	
Pooled Resident		44	12	0	0	12	27.3%	20.8	249	
Pooled Nonresident		66	35	0	0	35	53.0%	7.5	263	
116 Meeteetse										
	Limited Type 1	123	68	2	0	70	56.9%	7.6	532	153
	D/F Type 6	70	0	39	7	46	65.7%	4.5	205	104
Pooled Total		162	68	41	7	116	71.6%	6.4	737	
Pooled Resident		125	47	22	7	76	60.8%	7.8	589	
Pooled Nonresident		37	21	19	0	40	108.1%	3.7	148	
117 Franc's Peak										
	Limited Type 1	47	16	0	0	16	34.0%	18.3	292	53
Pooled Total		47	16	0	0	16	34.0%	18.3	292	
Pooled Resident		35	9	0	0	9	25.7%	23.2	209	
Pooled Nonresident		12	7	0	0	7	58.3%	11.9	83	
118 Upper Gooseberry Creek										
	Limited Type 1	71	43	2	0	45	63.4%	7.2	322	78
	D/F Type 6	28	0	24	4	28	100.0%	3.1	86	35
Pooled Total		81	43	26	4	73	90.1%	5.6	408	
Pooled Resident		61	32	20	4	56	91.8%	5.2	291	
Pooled Nonresident		20	11	6	0	17	85.0%	6.9	117	
119 Anchor										
	Limited Type 1	98	84	0	0	84	85.7%	4.9	412	104
	D/F Type 6	39	0	31	2	33	84.6%	3.3	108	50
Pooled Total		123	84	31	2	117	95.1%	4.4	520	
Pooled Resident		94	64	23	2	89	94.7%	4.3	379	
Pooled Nonresident		29	20	8	0	28	96.6%	5.0	141	
120 Hamilton Dome										
	Limited Type 1	98	61	0	0	61	62.2%	7.0	430	128
	D/F Type 6	68	0	45	0	45	66.2%	6.2	279	100
Pooled Total		129	61	45	0	106	82.2%	6.7	709	
Pooled Resident		89	42	22	0	64	71.9%	7.4	474	
Pooled Nonresident		40	19	23	0	42	105.0%	5.6	235	
121 Heart Mountain										
	General	610	322	37	2	361	59.2%	4.7	1708	
Pooled Total		610	322	37	2	361	59.2%	4.7	1708	
Pooled Resident		548	284	37	2	323	58.9%	4.7	1534	
Pooled Nonresident		62	38	0	0	38	61.3%	4.6	174	

TABLE I-A (Continued)

MULE DEER HUNT AREA REPORT

AREA (cross hunt areas)	TYPE	ACTIVE LICS/HTRS	HARVEST BUCK	DOE	FAWN	TOTAL	HUNTER SUCCESS	DAYS/ HARVEST	HUNTER DAYS	LICENSES SOLD (cross hunt incl.)
122 Shoshone River										
	General	773	277	58	6	341	44.1%	7.1	2405	
Pooled Total		773	277	58	6	341	44.1%	7.1	2405	
Pooled Resident		711	260	51	6	317	44.6%	6.0	1908	
Pooled Nonresident		62	17	7	0	24	38.7%	20.7	497	
123 Yellowtail										
	General	110	13	0	0	13	11.8%	22.3	290	
Pooled Total		110	13	0	0	13	11.8%	22.3	290	
Pooled Resident		89	6	0	0	6	6.7%	43.2	259	
Pooled Nonresident		21	7	0	0	7	33.3%	4.4	31	
124 Emblem										
	D/F Type 6	156	0	103	17	120	76.9%	4.0	477	204
	D/F Type 7	86	0	61	6	67	77.9%	3.8	257	125
	General	658	251	80	8	339	51.5%	6.8	2316	
Pooled Total		816	251	244	31	526	64.5%	5.8	3050	
Pooled Resident		698	178	201	19	398	57.0%	6.5	2580	
Pooled Nonresident		118	73	43	12	128	108.5%	3.7	470	
125 Fifteen Mile										
	Limited Type 1	129	82	0	0	82	63.6%	10.0	822	135
	D/F Type 6	24	0	19	0	19	79.2%	8.1	154	24
Pooled Total		150	82	19	0	101	67.3%	9.7	976	
Pooled Resident		109	60	10	0	70	64.2%	9.7	678	
Pooled Nonresident		41	22	9	0	31	75.6%	9.6	298	
127 Cottonwood										
	D/F Type 6	29	0	22	2	24	82.8%	3.7	88	87
	General	208	73	14	0	87	41.8%	9.9	857	
Pooled Total		220	73	36	2	111	50.5%	8.5	945	
Pooled Resident		185	56	26	0	82	44.3%	9.7	798	
Pooled Nonresident		35	17	10	2	29	82.9%	5.1	147	
128 Wind River										
	Limited Type 1	101	80	2	0	82	81.2%	8.2	674	107
	D/F Type 6	59	0	55	3	58	98.3%	3.3	194	100
	D/F Type 7	115	0	95	10	105	91.3%	3.2	332	150
	General	1073	411	0	0	411	38.3%	14.7	6042	
Pooled Total		1249	491	152	13	656	52.5%	11.0	7242	
Pooled Resident		1025	387	125	13	525	51.2%	11.5	6042	
Pooled Nonresident		224	104	27	0	131	58.5%	9.2	1200	
130 Big Sandy										
	Limited Type 1	24	24	0	0	24	100.0%	4.6	111	26
	D/F Type 6	8	0	2	0	2	25.0%	5.5	11	11
	General	274	96	0	0	96	35.0%	10.0	960	
Pooled Total		306	120	2	0	122	39.9%	8.9	1082	
Pooled Resident		291	107	0	0	107	36.8%	9.6	1023	
Pooled Nonresident		15	13	2	0	15	100.0%	3.9	59	
131 Steamboat										
	Limited Type 2	36	0	25	2	27	75.0%	2.9	78	55
	General	1034	357	0	0	357	34.5%	10.3	3678	
Pooled Total		1070	357	25	2	384	35.9%	9.8	3756	
Pooled Resident		987	310	25	2	337	34.1%	10.1	3417	
Pooled Nonresident		83	47	0	0	47	56.6%	7.2	339	

TABLE I-A (Continued)

MULE DEER HUNT AREA REPORT

AREA (cross hunt areas)	TYPE	ACTIVE LICS/HTRS	HARVEST BUCK	HARVEST DOE	HARVEST FAWN	HARVEST TOTAL	HUNTER SUCCESS	DAYS/ HARVEST	HUNTER DAYS	LICENSES SOLD (cross hunt incl.)
132 Cedar Mountain										
	General	499	137	0	0	137	27.5%	14.5	1986	
Pooled Total		499	137	0	0	137	27.5%	14.5	1986	
Pooled Resident		443	99	0	0	99	22.3%	17.9	1772	
Pooled Nonresident		56	38	0	0	38	67.9%	5.6	214	
133 Uinta										
	General	964	421	0	0	421	43.7%	9.4	3938	
Pooled Total		964	421	0	0	421	43.7%	9.4	3938	
Pooled Resident		835	351	0	0	351	42.0%	9.5	3327	
Pooled Nonresident		129	70	0	0	70	54.3%	8.7	611	
134 Bear River Divide										
	General	732	389	0	0	389	53.1%	6.2	2397	
Pooled Total		732	389	0	0	389	53.1%	6.2	2397	
Pooled Resident		571	293	0	0	293	51.3%	5.9	1724	
Pooled Nonresident		161	96	0	0	96	59.6%	7.0	673	
135 West Green River										
	General	2141	982	0	0	982	45.9%	10.3	10070	
Pooled Total		2141	982	0	0	982	45.9%	10.3	10070	
Pooled Resident		1891	845	0	0	845	44.7%	10.6	8987	
Pooled Nonresident		250	137	0	0	137	54.8%	7.9	1083	
138 Boulder										
	General	523	128	34	8	170	32.5%	13.3	2266	
Pooled Total		523	128	34	8	170	32.5%	13.3	2266	
Pooled Resident		481	121	34	8	163	33.9%	12.3	2001	
Pooled Nonresident		42	7	0	0	7	16.7%	37.9	265	
139 Pinedale										
	General	466	147	83	9	239	51.3%	8.2	1950	
Pooled Total		466	147	83	9	239	51.3%	8.2	1950	
Pooled Resident		427	133	76	9	218	51.1%	8.2	1781	
Pooled Nonresident		39	14	7	0	21	53.8%	8.0	169	
140 New Fork										
	General	370	88	70	0	158	42.7%	9.7	1530	
Pooled Total		370	88	70	0	158	42.7%	9.7	1530	
Pooled Resident		331	77	70	0	147	44.4%	9.2	1354	
Pooled Nonresident		39	11	0	0	11	28.2%	16.0	176	
141 Green River										
**(162)**	Limited Type 1	78	36	2	0	38	48.7%	13.1	499	125
Pooled Total		78	36	2	0	38	48.7%	13.1	499	
Pooled Resident		61	30	2	0	32	52.5%	13.2	421	
Pooled Nonresident		17	6	0	0	6	35.3%	13.0	78	
142 Piney-Horse Creek										
	General	716	206	0	0	206	28.8%	18.5	3805	
Pooled Total		716	206	0	0	206	28.8%	18.5	3805	
Pooled Resident		557	150	0	0	150	26.9%	18.8	2822	
Pooled Nonresident		159	56	0	0	56	35.2%	17.6	983	
143 South Piney										
	General	888	259	0	0	259	29.2%	16.6	4295	
Pooled Total		888	259	0	0	259	29.2%	16.6	4295	
Pooled Resident		813	234	0	0	234	28.8%	16.9	3964	
Pooled Nonresident		75	25	0	0	25	33.3%	13.2	331	

MULE DEER HUNT AREA REPORT

AREA (cross hunt areas)	TYPE	ACTIVE LICS/HTRS	HARVEST BUCK	HARVEST DOE	HARVEST FAWN	HARVEST TOTAL	HUNTER SUCCESS	DAYS/ HARVEST	HUNTER DAYS	LICENSES SOLD (cross hunt incl.)
144 Greys River										
	General	1092	344	0	0	344	31.5%	14.4	4938	
Pooled Total		1092	344	0	0	344	31.5%	14.4	4938	
Pooled Resident		742	179	0	0	179	24.1%	17.7	3175	
Pooled Nonresident		350	165	0	0	165	47.1%	10.7	1763	
145 Salt River										
	General	909	297	0	0	297	32.7%	14.4	4286	
Pooled Total		909	297	0	0	297	32.7%	14.4	4286	
Pooled Resident		750	213	0	0	213	28.4%	16.2	3450	
Pooled Nonresident		159	84	0	0	84	52.8%	10.0	836	
146 Fish Creek										
	General	243	80	15	0	95	39.1%	11.5	1097	
Pooled Total		243	80	15	0	95	39.1%	11.5	1097	
Pooled Resident		222	73	15	0	88	39.6%	11.3	991	
Pooled Nonresident		21	7	0	0	7	33.3%	15.1	106	
148 Teton										
	General	232	72	0	0	72	31.0%	25.0	1800	
Pooled Total		232	72	0	0	72	31.0%	25.0	1800	
Pooled Resident		202	59	0	0	59	29.2%	27.9	1647	
Pooled Nonresident		30	13	0	0	13	43.3%	11.8	153	
149 Targhee										
	General	179	38	0	0	38	21.2%	30.4	1154	
Pooled Total		179	38	0	0	38	21.2%	30.4	1154	
Pooled Resident		115	6	0	0	6	5.2%	128.7	772	
Pooled Nonresident		64	32	0	0	32	50.0%	11.9	382	
150 Wilson										
	General	57	14	0	4	18	31.6%	18.6	334	
Pooled Total		57	14	0	4	18	31.6%	18.6	334	
Pooled Resident		39	0	0	4	4	10.3%	60.5	242	
Pooled Nonresident		18	14	0	0	14	77.8%	6.6	92	
151 Fall Creek										
	General	529	117	18	4	139	26.3%	20.1	2791	
Pooled Total		529	117	18	4	139	26.3%	20.1	2791	
Pooled Resident		420	75	18	4	97	23.1%	22.2	2149	
Pooled Nonresident		109	42	0	0	42	38.5%	15.3	642	
152 Lower Hoback										
	General	1057	389	33	0	422	39.9%	11.7	4938	
Pooled Total		1057	389	33	0	422	39.9%	11.7	4938	
Pooled Resident		736	227	33	0	260	35.3%	13.5	3498	
Pooled Nonresident		321	162	0	0	162	50.5%	8.9	1440	
153 Monument Ridge										
	General	357	100	0	0	100	28.0%	18.7	1869	
Pooled Total		357	100	0	0	100	28.0%	18.7	1869	
Pooled Resident		212	40	0	0	40	18.9%	29.7	1188	
Pooled Nonresident		145	60	0	0	60	41.4%	11.4	681	
154 Raspberry Ridge										
	General	603	206	7	0	213	35.3%	16.6	3530	
Pooled Total		603	206	7	0	213	35.3%	16.6	3530	
Pooled Resident		437	132	7	0	139	31.8%	17.7	2463	
Pooled Nonresident		166	74	0	0	74	44.6%	14.4	1067	

TABLE I-A (Continued)

MULE DEER HUNT AREA REPORT

AREA (cross hunt areas)		TYPE	ACTIVE LICS/HTRS	HARVEST BUCK	DOE	FAWN	TOTAL	HUNTER SUCCESS	DAYS/ HARVEST	HUNTER DAYS	LICENSES SOLD (cross hunt incl.)
155 Crystal Peak											
		General	286	90	21	0	111	38.8%	12.5	1391	
Pooled Total			286	90	21	0	111	38.8%	12.5	1391	
Pooled Resident			258	83	21	0	104	40.3%	12.1	1260	
Pooled Nonresident			28	7	0	0	7	25.0%	18.7	131	
156 Spread Creek											
		General	476	123	37	0	160	33.6%	15.2	2436	
Pooled Total			476	123	37	0	160	33.6%	15.2	2436	
Pooled Resident			384	91	37	0	128	33.3%	14.9	1909	
Pooled Nonresident			92	32	0	0	32	34.8%	16.5	527	
157 Boysen											
	**(170)**	Limited Type 1	213	168	14	0	182	85.4%	4.9	889	255
	**(170)**	D/F Type 6	267	0	185	30	215	80.5%	4.1	892	330
Pooled Total			385	168	199	30	397	103.1%	4.5	1781	
Pooled Resident			323	129	176	24	329	101.9%	4.4	1446	
Pooled Nonresident			62	39	23	6	68	109.7%	4.9	335	
158 Ormsby											
	**(also valid 22)**	Limited Type 1	336	252	13	9	274	81.5%	3.6	992	411
	**(22)**	D/F Type 6	88	0	56	4	60	68.2%	5.0	298	286
Pooled Total			413	252	69	13	334	80.9%	3.9	1290	
Pooled Resident			132	42	39	13	94	71.2%	4.1	390	
Pooled Nonresident			281	210	30	0	240	85.4%	3.8	900	
160 Beaver Creek											
	**(94)**	D/F Type 6	22	0	10	2	12	54.5%	5.7	68	54
		General	332	137	0	0	137	41.3%	8.9	1225	
Pooled Total			343	137	10	2	149	43.4%	8.7	1293	
Pooled Resident			276	100	10	2	112	40.6%	9.4	1050	
Pooled Nonresident			67	37	0	0	37	55.2%	6.6	243	
161 St. Mary's Creek											
		General	86	36	0	0	36	41.9%	7.6	275	
Pooled Total			86	36	0	0	36	41.9%	7.6	275	
Pooled Resident			71	29	0	0	29	40.8%	8.3	242	
Pooled Nonresident			15	7	0	0	7	46.7%	4.7	33	
162 Water Dog Lakes											
	**(141)**	Limited Type 1	51	21	0	0	21	41.2%	14.8	310	125
Pooled Total			51	21	0	0	21	41.2%	14.8	310	
Pooled Resident			35	16	0	0	16	45.7%	13.5	216	
Pooled Nonresident			16	5	0	0	5	31.3%	18.8	94	
163 Middle Fork											
		General	269	153	0	0	153	56.9%	7.7	1180	
Pooled Total			269	153	0	0	153	56.9%	7.7	1180	
Pooled Resident			50	17	0	0	17	34.0%	14.0	238	
Pooled Nonresident			219	136	0	0	136	62.1%	6.9	942	
164 Nowater											
		D/F Type 6	49	0	30	2	32	65.3%	8.4	270	75
		General	339	95	22	0	117	34.5%	11.1	1293	
Pooled Total			360	95	52	2	149	41.4%	10.5	1563	
Pooled Resident			238	45	38	2	85	35.7%	12.2	1041	
Pooled Nonresident			122	50	14	0	64	52.5%	8.2	522	

TABLE I-A (Continued)

MULE DEER HUNT AREA REPORT

AREA (cross hunt areas)		TYPE	ACTIVE LICS/HTRS	HARVEST BUCK	HARVEST DOE	HARVEST FAWN	HARVEST TOTAL	HUNTER SUCCESS	DAYS/ HARVEST	HUNTER DAYS	LICENSES SOLD (cross hunt incl.)
165 YU Bench											
		Limited Type 1	99	69	2	0	71	71.7%	4.8	343	107
		D/F Type 6	101	0	85	3	88	87.1%	5.5	485	167
Pooled Total			158	69	87	3	159	100.6%	5.2	828	
Pooled Resident			117	49	61	3	113	96.6%	5.1	571	
Pooled Nonresident			41	20	26	0	46	112.2%	5.6	257	
167 Dull Center											
		General	221	117	0	0	117	52.9%	6.0	707	
Pooled Total			221	117	0	0	117	52.9%	6.0	707	
Pooled Resident			65	25	0	0	25	38.5%	7.2	180	
Pooled Nonresident			156	92	0	0	92	59.0%	5.7	527	
168 Medicine Butte											
		General	1005	460	0	0	460	45.8%	8.1	3724	
Pooled Total			1005	460	0	0	460	45.8%	8.1	3724	
Pooled Resident			838	355	0	0	355	42.4%	8.8	3133	
Pooled Nonresident			167	105	0	0	105	62.9%	5.6	591	
169 Tisdale Mountain											
		General	204	152	0	0	152	74.5%	3.9	591	
Pooled Total			204	152	0	0	152	74.5%	3.9	591	
Pooled Resident			57	44	0	0	44	77.2%	2.0	90	
Pooled Nonresident			147	108	0	0	108	73.5%	4.6	501	
170 Riverview											
	**(157)**	Limited Type 1	20	2	0	0	2	10.0%	27.0	54	255
	**(157)**	D/F Type 6	19	0	10	3	13	68.4%	4.1	53	330
Pooled Total			36	2	10	3	15	41.7%	7.1	107	
Pooled Resident			32	2	6	3	11	34.4%	8.0	88	
Pooled Nonresident			4	0	4	0	4	100.0%	4.8	19	
171 Crowheart Butte											
		General	172	54	12	2	68	39.5%	8.3	563	
Pooled Total			172	54	12	2	68	39.5%	8.3	563	
Pooled Resident			152	51	12	2	65	42.8%	7.4	483	
Pooled Nonresident			20	3	0	0	3	15.0%	26.7	80	

TABLE I-H

MULE DEER HARVEST

BY HERD UNIT

2007

HERD	TYPE	ACTIVE LICS/HTRS	HARVEST BUCK	DOE	FAWN	TOTAL	HUNTER SUCCESS	DAYS/ HARVEST	HUNTER DAYS	LICENSES SOLD (cross hunt incl.)
101 Targhee	General	179	38	0	0	38	21.2%	30.4	1154	
Total Hunters		179	38	0	0	38	21.2%	30.4	1154	
Resident		115	6	0	0	6	5.2%	128.7	772	
Nonresident		64	32	0	0	32	50.0%	11.9	382	
104 Sublette	D/F	8	0	2	0	2	25.0%	5.5	11	11
	Limited	141	81	2	0	83	58.9%	11.1	920	151
	General	5115	1784	318	25	2127	41.6%	13.6	28897	
Total Hunters		5262	1865	322	25	2212	42.0%	13.5	29828	
Resident		4215	1355	313	25	1693	40.2%	13.8	23318	
Nonresident		1047	510	9	0	519	49.6%	12.5	6510	
131 Wyoming Range	General	5027	2271	0	0	2271	45.2%	11.4	25986	
Total Hunters		5027	2271	0	0	2271	45.2%	11.4	25986	
Resident		4120	1764	0	0	1764	42.8%	12.1	21300	
Nonresident		907	507	0	0	507	55.9%	9.2	4686	
207 Paintrock	D/F	264	0	187	29	216	81.8%	5.6	1208	346
	General	1551	585	142	4	731	47.1%	9.5	6915	
Total Hunters		1684	585	329	33	947	56.2%	8.6	8123	
Resident		999	244	169	11	424	42.4%	10.9	4627	
Nonresident		685	341	160	22	523	76.4%	6.7	3496	
208 Southwest Bighorns	D/F	431	0	288	25	313	72.6%	5.4	1695	569
	Limited	168	126	12	0	138	82.1%	5.1	708	179
	General	2367	1178	130	4	1312	55.4%	7.6	9952	
Total Hunters		2760	1304	430	29	1763	63.9%	7.0	12355	
Resident		1299	542	171	12	725	55.8%	7.7	5556	
Nonresident		1461	762	259	17	1038	71.0%	6.6	6799	
209 Basin	D/F	53	0	41	2	43	81.1%	5.6	242	111
	Limited	129	82	0	0	82	63.6%	10.0	822	135
	General	208	73	14	0	87	41.8%	9.9	857	
Total Hunters		370	155	55	2	212	57.3%	9.1	1921	
Resident		295	116	36	0	152	51.5%	9.7	1476	
Nonresident		75	39	19	2	60	80.0%	7.4	445	
210 Greybull River	D/F	343	0	249	26	275	80.2%	4.4	1219	496
	Limited	99	69	2	0	71	71.7%	4.8	343	107
	General	658	251	80	8	339	51.5%	6.8	2316	
Total Hunters		971	320	331	34	685	70.5%	5.7	3878	
Resident		813	227	262	22	511	62.9%	6.2	3151	
Nonresident		158	93	69	12	174	110.1%	4.2	727	
211 Shoshone River	General	828	290	58	6	354	42.8%	7.6	2695	
Total Hunters		828	290	58	6	354	42.8%	7.6	2695	
Resident		745	266	51	6	323	43.4%	6.7	2167	
Nonresident		83	24	7	0	31	37.3%	17.0	528	
212 Owl Creek/Meeteetse	D/F	205	0	139	13	152	74.1%	4.5	678	289
	Limited	437	272	4	0	276	63.2%	7.2	1988	516
Total Hunters		543	272	143	13	428	78.8%	6.2	2666	
Resident		404	194	87	13	294	72.8%	6.6	1942	
Nonresident		139	78	56	0	134	96.4%	5.4	724	

TABLE I-H (Continued)

MULE DEER HERD UNIT REPORT

HERD	TYPE	ACTIVE LICS/HTRS	HARVEST BUCK	HARVEST DOE	HARVEST FAWN	HARVEST TOTAL	HUNTER SUCCESS	DAYS/ HARVEST	HUNTER DAYS	LICENSES SOLD (cross hunt incl.)
215 Upper Shoshone	D/F	247	0	196	15	211	85.4%	4.3	916	328
	General	1964	1092	73	12	1177	59.9%	7.6	8958	
Total Hunters		2063	1092	269	27	1388	67.3%	7.1	9874	
Resident		1191	502	180	12	694	58.3%	8.6	5991	
Nonresident		872	590	89	15	694	79.6%	5.6	3883	
216 Clark's Fork	Limited	46	39	0	0	39	84.8%	4.9	190	50
	General	1481	794	75	2	871	58.8%	6.4	5587	
Total Hunters		1527	833	75	2	910	59.6%	6.3	5777	
Resident		1172	585	72	2	659	56.2%	6.3	4162	
Nonresident		355	248	3	0	251	70.7%	6.4	1615	
319 Powder River	D/F	1100	0	929	35	964	87.6%	4.0	3892	1711
	General	4349	2590	147	9	2746	63.1%	6.4	17711	
Total Hunters		5167	2590	1076	44	3710	71.8%	5.8	21603	
Resident		2185	757	518	34	1309	59.9%	7.1	9248	
Nonresident		2982	1833	558	10	2401	80.5%	5.1	12355	
320 Pumpkin Buttes	D/F	283	0	202	14	216	76.3%	4.0	856	1187
	General	983	613	18	0	631	64.2%	5.6	3529	
Total Hunters		1209	613	220	14	847	70.1%	5.2	4385	
Resident		377	118	75	5	198	52.5%	7.4	1475	
Nonresident		832	495	145	9	649	78.0%	4.5	2910	
321 North Bighorn	D/F	603	0	491	19	510	84.6%	5.0	2539	1458
	General	3916	1389	170	8	1567	40.0%	12.2	19123	
Total Hunters		4348	1389	661	27	2077	47.8%	10.4	21662	
Resident		2557	592	328	20	940	36.8%	15.1	14206	
Nonresident		1791	797	333	7	1137	63.5%	6.6	7456	
322 Upper Powder River	D/F	264	0	174	9	183	69.3%	6.5	1198	418
	General	1650	1003	87	4	1094	66.3%	6.7	7333	
Total Hunters		1797	1003	261	13	1277	71.1%	6.7	8531	
Resident		454	175	81	0	256	56.4%	9.0	2312	
Nonresident		1343	828	180	13	1021	76.0%	6.1	6219	
423 Uinta	General	2176	1018	0	0	1018	46.8%	9.5	9648	
Total Hunters		2176	1018	0	0	1018	46.8%	9.5	9648	
Resident		1849	805	0	0	805	43.5%	10.2	8232	
Nonresident		327	213	0	0	213	65.1%	6.6	1416	
424 South Rock Springs	Limited	449	351	0	0	351	78.2%	6.9	2424	481
Total Hunters		449	351	0	0	351	78.2%	6.9	2424	
Resident		357	286	0	0	286	80.1%	6.5	1867	
Nonresident		92	65	0	0	65	70.7%	8.6	557	
427 Baggs	D/F	519	0	418	13	431	83.0%	5.8	2488	659
	Limited	176	123	6	0	129	73.3%	7.0	909	183
	General	3153	1842	206	24	2072	65.7%	6.9	14333	
Total Hunters		3670	1965	630	37	2632	71.7%	6.7	17730	
Resident		2375	1139	379	23	1541	64.9%	7.3	11207	
Nonresident		1295	826	251	14	1091	84.2%	6.0	6523	
430 Steamboat	Limited	36	0	25	2	27	75.0%	2.9	78	55
	General	1034	357	0	0	357	34.5%	10.3	3678	
Total Hunters		1070	357	25	2	384	35.9%	9.8	3756	
Resident		987	310	25	2	337	34.1%	10.1	3417	
Nonresident		83	47	0	0	47	56.6%	7.2	339	

TABLE I-H (Continued)

MULE DEER HERD UNIT REPORT

HERD	TYPE	ACTIVE LICS/HTRS	HARVEST BUCK	HARVEST DOE	HARVEST FAWN	HARVEST TOTAL	HUNTER SUCCESS	DAYS/ HARVEST	HUNTER DAYS	LICENSES SOLD (cross hunt incl.)
534 Goshen Rim	D/F	136	0	88	3	91	66.9%	5.7	521	168
	Limited	21	9	4	0	13	61.9%	7.1	92	28
	General	1526	717	57	15	789	51.7%	7.5	5908	
Total Hunters		1622	726	149	18	893	55.1%	7.3	6521	
Resident		1187	464	107	15	586	49.4%	8.2	4798	
Nonresident		435	262	42	3	307	70.6%	5.6	1723	
537 Laramie Mountains	D/F	219	0	132	19	151	68.9%	5.4	811	278
	Limited	352	227	27	0	254	72.2%	7.0	1773	411
	General	1981	1175	0	0	1175	59.3%	7.1	8333	
Total Hunters		2474	1402	159	19	1580	63.9%	6.9	10917	
Resident		1619	821	107	8	936	57.8%	7.9	7360	
Nonresident		855	581	52	11	644	75.3%	5.5	3557	
539 Sheep Mountain	D/F	5	0	2	0	2	40.0%	11.5	23	25
	General	2077	517	7	4	528	25.4%	18.1	9546	
Total Hunters		2082	517	9	4	530	25.5%	18.1	9569	
Resident		1656	351	7	4	362	21.9%	21.4	7730	
Nonresident		426	166	2	0	168	39.4%	10.9	1839	
540 Shirley Mountain	D/F	71	0	54	4	58	81.7%	3.9	225	100
	General	879	511	0	0	511	58.1%	6.7	3406	
Total Hunters		924	511	54	4	569	61.6%	6.4	3631	
Resident		478	210	20	2	232	48.5%	5.8	1340	
Nonresident		446	301	34	2	337	75.6%	6.8	2291	
541 Platte Valley	D/F	158	0	87	14	101	63.9%	7.5	756	203
	Limited	27	27	0	0	27	100.0%	5.0	136	27
	General	4277	1183	467	39	1689	39.5%	12.7	21498	
Total Hunters		4357	1210	554	53	1817	41.7%	12.3	22390	
Resident		2977	694	361	40	1095	36.8%	13.8	15103	
Nonresident		1380	516	193	13	722	52.3%	10.1	7287	
642 Dubois	D/F	174	0	150	13	163	93.7%	3.2	526	250
	Limited	101	80	2	0	82	81.2%	8.2	674	107
	General	1287	483	0	0	483	37.5%	16.2	7842	
Total Hunters		1466	563	152	13	728	49.7%	12.4	9042	
Resident		1212	446	125	13	584	48.2%	13.2	7689	
Nonresident		254	117	27	0	144	56.7%	9.4	1353	
643 Project	D/F	278	0	195	33	228	82.0%	4.1	945	330
	Limited	225	170	14	0	184	81.8%	5.1	943	255
	General	172	54	12	2	68	39.5%	8.3	563	
Total Hunters		572	224	221	35	480	83.9%	5.1	2451	
Resident		488	182	194	29	405	83.0%	5.0	2017	
Nonresident		84	42	27	6	75	89.3%	5.8	434	
644 South Wind River	D/F	92	0	74	2	76	82.6%	5.2	392	104
	General	1680	808	0	0	808	48.1%	8.2	6639	
Total Hunters		1732	808	74	2	884	51.0%	8.0	7031	
Resident		1470	664	65	2	731	49.7%	8.1	5930	
Nonresident		262	144	9	0	153	58.4%	7.2	1101	
646 Sweetwater	D/F	132	0	101	15	116	87.9%	1.8	205	162
	General	996	496	0	0	496	49.8%	6.8	3351	
Total Hunters		1076	496	101	15	612	56.9%	5.8	3556	
Resident		805	326	82	8	416	51.7%	5.9	2472	
Nonresident		271	170	19	7	196	72.3%	5.5	1084	

TABLE I-H (Continued)

MULE DEER HERD UNIT REPORT

HERD	TYPE	ACTIVE LICS/HTRS	HARVEST BUCK	HARVEST DOE	HARVEST FAWN	HARVEST TOTAL	HUNTER SUCCESS	DAYS/ HARVEST	HUNTER DAYS	LICENSES SOLD (cross hunt incl.)
647 Ferris	Limited	275	226	0	0	226	82.2%	6.3	1427	301
Total Hunters		275	226	0	0	226	82.2%	6.3	1427	
Resident		222	184	0	0	184	82.9%	5.1	942	
Nonresident		53	42	0	0	42	79.2%	11.5	485	
648 Beaver Rim	Limited	73	64	0	0	64	87.7%	5.8	368	75
Total Hunters		73	64	0	0	64	87.7%	5.8	368	
Resident		58	51	0	0	51	87.9%	5.7	291	
Nonresident		15	13	0	0	13	86.7%	5.9	77	
650 Chain Lakes	General	108	60	0	0	60	55.6%	5.6	338	
Total Hunters		108	60	0	0	60	55.6%	5.6	338	
Resident		68	33	0	0	33	48.5%	5.7	188	
Nonresident		40	27	0	0	27	67.5%	5.6	150	
751 Black Hills	D/F	1755	0	971	49	1020	58.1%	5.3	5437	3391
	General	5523	2175	165	12	2352	42.6%	7.5	17711	
Total Hunters		6917	2175	1136	61	3372	48.7%	6.9	23148	
Resident		2437	769	287	8	1064	43.7%	8.6	9134	
Nonresident		4480	1406	849	53	2308	51.5%	6.1	14014	
752 Thunder Basin	D/F	384	0	253	18	271	70.6%	5.8	1584	495
	General	1689	966	114	4	1084	64.2%	6.2	6717	
Total Hunters		1942	966	367	22	1355	69.8%	6.1	8301	
Resident		768	276	108	0	384	50.0%	9.3	3581	
Nonresident		1174	690	259	22	971	82.7%	4.9	4720	
753 Lance Creek	D/F	420	0	305	17	322	76.7%	4.8	1542	500
	General	1605	983	101	4	1088	67.8%	6.1	6669	
Total Hunters		1914	983	406	21	1410	73.7%	5.8	8211	
Resident		454	187	126	4	317	69.8%	7.0	2218	
Nonresident		1460	796	280	17	1093	74.9%	5.5	5993	
755 North Converse	D/F	230	0	158	4	162	70.4%	4.2	677	286
	Limited	626	506	28	9	543	86.7%	3.3	1780	749
Total Hunters		794	506	186	13	705	88.8%	3.5	2457	
Resident		168	49	63	13	125	74.4%	4.2	529	
Nonresident		626	457	123	0	580	92.7%	3.3	1928	
756 South Converse	D/F	157	0	106	7	113	72.0%	5.2	583	196
	General	1108	616	0	0	616	55.6%	6.9	4224	
Total Hunters		1185	616	106	7	729	61.5%	6.6	4807	
Resident		539	265	21	4	290	53.8%	7.2	2088	
Nonresident		646	351	85	3	439	68.0%	6.2	2719	
757 Bates Hole/Hat Six	D/F	167	0	121	25	146	87.4%	4.1	594	200
	General	1050	512	0	0	512	48.8%	8.2	4191	
Total Hunters		1164	512	121	25	658	56.5%	7.3	4785	
Resident		785	310	47	3	360	45.9%	8.6	3112	
Nonresident		379	202	74	22	298	78.6%	5.6	1673	
758 Rattlesnake	D/F	282	0	203	13	216	76.6%	6.6	1426	350
	Limited	278	234	0	0	234	84.2%	4.3	998	278
	General	279	144	0	0	144	51.6%	6.3	909	
Total Hunters		743	378	203	13	594	79.9%	5.6	3333	
Resident		632	317	175	9	501	79.3%	5.9	2980	
Nonresident		111	61	28	4	93	83.8%	3.8	353	

TABLE I-H (Continued)

MULE DEER HERD UNIT REPORT

HERD	TYPE	ACTIVE LICS/HTRS	HARVEST BUCK	DOE	FAWN	TOTAL	HUNTER SUCCESS	DAYS/ HARVEST	HUNTER DAYS	LICENSES SOLD (cross hunt incl.)
759 North Natrona	D/F	134	0	99	7	106	79.1%	4.4	470	151
	Limited	294	226	4	0	230	78.2%	5.5	1271	351
Total Hunters		381	226	103	7	336	88.2%	5.2	1741	
Resident		274	166	70	7	243	88.7%	5.1	1241	
Nonresident		107	60	33	0	93	86.9%	5.4	500	

TABLE II-A

WHITE-TAILED DEER

HARVEST, HUNTING PRESSURE, HUNTER SUCCESS

BY HUNT AREA

2007

AREA (cross hunt areas)	TYPE	ACTIVE LICS/HTRS	HARVEST BUCK	DOE	FAWN	TOTAL	HUNTER SUCCESS	DAYS/ HARVEST	HUNTER DAYS	LICENSES SOLD (cross hunt incl.)
1 Crook										
**(2, 3)**	D/F Type 6	639	0	460	37	497	77.8%	5.9	2955	2722
	General	1685	853	57	5	915	54.3%	6.3	5754	
Pooled Total		2170	853	517	42	1412	65.1%	6.2	8709	
Pooled Resident		922	319	201	5	525	56.9%	9.0	4700	
Pooled Nonresident		1248	534	316	37	887	71.1%	4.5	4009	
2 Bearlodge										
**(1, 3)**	D/F Type 6	1264	0	846	49	895	70.8%	5.4	4836	2722
	General	4456	2251	122	47	2420	54.3%	6.8	16520	
Pooled Total		5476	2251	968	96	3315	60.5%	6.4	21356	
Pooled Resident		2644	975	458	60	1493	56.5%	8.0	11948	
Pooled Nonresident		2832	1276	510	36	1822	64.3%	5.2	9408	
3 Keyhole										
**(1, 2)**	D/F Type 6	179	0	74	0	74	41.3%	5.8	430	2722
	General	575	221	28	16	265	46.1%	7.4	1954	
Pooled Total		743	221	102	16	339	45.6%	7.0	2384	
Pooled Resident		352	94	21	13	128	36.4%	8.4	1079	
Pooled Nonresident		391	127	81	3	211	54.0%	6.2	1305	
4 Sand Creek										
	D/F Type 6	251	0	171	15	186	74.1%	4.8	887	324
	General	1364	532	15	0	547	40.1%	11.0	6006	
Pooled Total		1500	532	186	15	733	48.9%	9.4	6893	
Pooled Resident		925	275	35	0	310	33.5%	13.6	4214	
Pooled Nonresident		575	257	151	15	423	73.6%	6.3	2679	
5 Inyan Kara										
	D/F Type 6	49	0	16	3	19	38.8%	5.2	98	112
	General	330	103	14	0	117	35.5%	10.3	1210	
Pooled Total		362	103	30	3	136	37.6%	9.6	1308	
Pooled Resident		221	62	13	3	78	35.3%	11.9	932	
Pooled Nonresident		141	41	17	0	58	41.1%	6.5	376	
6 Skull Creek										
**(9)**	D/F Type 6	109	0	53	5	58	53.2%	7.7	445	233
	General	368	122	10	0	132	35.9%	8.7	1152	
Pooled Total		428	122	63	5	190	44.4%	8.4	1597	
Pooled Resident		262	64	30	3	97	37.0%	9.1	883	
Pooled Nonresident		166	58	33	2	93	56.0%	7.7	714	
7 South Osage										
	General	62	9	0	0	9	14.5%	9.7	87	
Pooled Total		62	9	0	0	9	14.5%	9.7	87	
Pooled Resident		30	2	0	0	2	6.7%	19.5	39	
Pooled Nonresident		32	7	0	0	7	21.9%	6.9	48	
8 Upton-Four Horse										
	D/F Type 6	14	0	6	0	6	42.9%	6.3	38	96
	General	46	4	0	0	4	8.7%	22.5	90	
Pooled Total		57	4	6	0	10	17.5%	12.8	128	
Pooled Resident		25	0	0	0	0	0.0%	0.0	51	
Pooled Nonresident		32	4	6	0	10	31.3%	7.7	77	
9 Blacktail Creek										
**(6)**	D/F Type 6	33	0	8	3	11	33.3%	9.5	104	233
	General	57	11	0	0	11	19.3%	11.8	130	
Pooled Total		79	11	8	3	22	27.8%	10.6	234	
Pooled Resident		33	4	6	3	13	39.4%	13.1	170	
Pooled Nonresident		46	7	2	0	9	19.6%	7.1	64	

TABLE II-A (Continued)

WHITE-TAILED DEER HUNT AREA REPORT

AREA (cross hunt areas)	TYPE	ACTIVE LICS/HTRS	HARVEST BUCK	DOE	FAWN	TOTAL	HUNTER SUCCESS	DAYS/ HARVEST	HUNTER DAYS	LICENSES SOLD (cross hunt incl.)
10 Teckla										
	D/F Type 6	8	0	6	0	6	75.0%	1.3	8	32
	General	16	0	0	0	0	0.0%	0.0	20	
Pooled Total		20	0	6	0	6	30.0%	4.7	28	
Pooled Resident		11	0	6	0	6	54.5%	2.3	14	
Pooled Nonresident		9	0	0	0	0	0.0%	0.0	14	
11 Mule Creek										
**(12, 13, 14, 15, 167)**	Limited Type 1	84	47	8	0	55	65.5%	6.5	356	300
	D/F Type 6	33	0	17	4	21	63.6%	2.4	50	134
**(12, 13, 14, 15, 167)**	D/F Type 7	59	0	35	12	47	79.7%	4.8	225	158
	General	90	25	4	4	33	36.7%	3.8	124	
Pooled Total		233	72	64	20	156	67.0%	4.8	755	
Pooled Resident		75	29	22	9	60	80.0%	6.9	416	
Pooled Nonresident		158	43	42	11	96	60.8%	3.5	339	
12 Hat Creek										
**(11, 13, 14, 15, 167)**	Limited Type 1	34	10	2	0	12	35.3%	9.7	116	300
**(13, 14)**	D/F Type 6	24	0	0	0	0	0.0%	0.0	36	500
**(11, 13, 14, 15, 167)**	D/F Type 7	15	0	10	2	12	80.0%	5.7	68	158
	General	81	25	0	0	25	30.9%	7.6	191	
Pooled Total		140	35	12	2	49	35.0%	8.4	411	
Pooled Resident		45	4	2	2	8	17.8%	13.9	111	
Pooled Nonresident		95	31	10	0	41	43.2%	7.3	300	
13 Lusk										
**(11, 12, 14, 15, 167)**	Limited Type 1	110	56	13	7	76	69.1%	5.8	444	300
**(12, 14)**	D/F Type 6	51	0	23	9	32	62.7%	3.8	121	500
**(11, 12, 14, 15, 167)**	D/F Type 7	53	0	24	16	40	75.5%	5.8	232	158
	General	153	26	3	4	33	21.6%	6.8	224	
Pooled Total		320	82	63	36	181	56.6%	5.6	1021	
Pooled Resident		159	44	44	25	113	71.1%	5.8	661	
Pooled Nonresident		161	38	19	11	68	42.2%	5.3	360	
14 Twenty Mile										
**(11, 12, 13, 15, 167)**	Limited Type 1	24	13	5	0	18	75.0%	7.7	139	300
**(12, 13)**	D/F Type 6	9	0	4	0	4	44.4%	3.5	14	500
**(11, 12, 13, 15, 167)**	D/F Type 7	10	0	5	0	5	50.0%	8.8	44	158
	General	45	4	0	0	4	8.9%	41.8	167	
Pooled Total		80	17	14	0	31	38.8%	11.7	364	
Pooled Resident		45	11	4	0	15	33.3%	17.3	260	
Pooled Nonresident		35	6	10	0	16	45.7%	6.5	104	
15 South Keeline										
**(11, 12, 13, 14, 167)**	Limited Type 1	36	25	0	0	25	69.4%	9.8	246	300
	D/F Type 6	0	0	0	0	0	0.0%	0.0	0	18
**(11, 12, 13, 14, 167)**	D/F Type 7	10	0	10	0	10	100.0%	6.2	62	158
	General	26	6	0	0	6	23.1%	8.2	49	
Pooled Total		63	31	10	0	41	65.1%	8.7	357	
Pooled Resident		48	25	5	0	30	62.5%	11.0	329	
Pooled Nonresident		15	6	5	0	11	73.3%	2.5	28	
16 Meadowdale/Jay Em										
**(55)**	Limited Type 1	38	12	4	0	16	42.1%	14.9	238	125
	Limited Type 2	9	2	4	0	6	66.7%	4.5	27	28
	General	81	0	0	0	0	0.0%	0.0	170	
Pooled Total		128	14	8	0	22	17.2%	19.8	435	
Pooled Resident		92	10	8	0	18	19.6%	19.8	356	
Pooled Nonresident		36	4	0	0	4	11.1%	19.8	79	

TABLE II-A (Continued)

WHITE-TAILED DEER HUNT AREA REPORT

AREA (cross hunt areas)	TYPE	ACTIVE LICS/HTRS	HARVEST BUCK	DOE	FAWN	TOTAL	HUNTER SUCCESS	DAYS/ HARVEST	HUNTER DAYS	LICENSES SOLD (cross hunt incl.)
17 Northwest Gillette										
**(18, 19, 20, 21)**	D/F Type 6	54	0	0	6	6	11.1%	16.3	98	928
	General	231	74	10	0	84	36.4%	7.3	609	
Pooled Total		285	74	10	6	90	31.6%	7.9	707	
Pooled Resident		181	45	10	0	55	30.4%	8.6	473	
Pooled Nonresident		104	29	0	6	35	33.7%	6.7	234	
18 Campbell										
**(17, 19, 20, 21)**	D/F Type 6	64	0	17	13	30	46.9%	5.3	158	928
	General	301	43	0	0	43	14.3%	11.1	476	
Pooled Total		359	43	17	13	73	20.3%	8.7	634	
Pooled Resident		219	14	11	0	25	11.4%	13.0	326	
Pooled Nonresident		140	29	6	13	48	34.3%	6.4	308	
19 Pumpkin Buttes										
**(17, 18, 20, 21)**	D/F Type 6	7	0	0	0	0	0.0%	0.0	19	928
	General	34	9	4	0	13	38.2%	7.1	92	
Pooled Total		41	9	4	0	13	31.7%	8.5	111	
Pooled Resident		24	5	0	0	5	20.8%	15.6	78	
Pooled Nonresident		17	4	4	0	8	47.1%	4.1	33	
20 Clarkelen										
**(17, 18, 19, 21)**	D/F Type 6	6	0	0	0	0	0.0%	0.0	13	928
	General	29	4	0	0	4	13.8%	17.5	70	
Pooled Total		35	4	0	0	4	11.4%	20.8	83	
Pooled Resident		11	0	0	0	0	0.0%	0.0	32	
Pooled Nonresident		24	4	0	0	4	16.7%	12.8	51	
21 Thunder Basin										
**(17, 18, 19, 20)**	D/F Type 6	13	0	6	0	6	46.2%	2.3	14	928
	General	52	0	4	0	4	7.7%	25.3	101	
Pooled Total		65	0	10	0	10	15.4%	11.5	115	
Pooled Resident		42	0	0	0	0	0.0%	0.0	83	
Pooled Nonresident		23	0	10	0	10	43.5%	3.2	32	
22 Douglas										
	Limited Type 1	29	0	4	0	4	13.8%	13.5	54	338
**(158)**	D/F Type 6	25	0	8	0	8	32.0%	5.8	46	286
	General	62	20	10	4	34	54.8%	4.5	152	
Pooled Total		109	20	22	4	46	42.2%	5.5	252	
Pooled Resident		51	13	10	4	27	52.9%	4.7	127	
Pooled Nonresident		58	7	12	0	19	32.8%	6.6	125	
23 Clearmont										
**(26)**	Limited Type 3	20	11	2	0	13	65.0%	5.8	75	43
**(26)**	D/F Type 6	159	0	48	9	57	35.8%	6.5	372	783
**(26)**	D/F Type 7	122	0	77	13	90	73.8%	6.1	549	240
	General	476	191	30	16	237	49.8%	7.5	1774	
Pooled Total		659	202	157	38	397	60.2%	7.0	2770	
Pooled Resident		441	155	104	30	289	65.5%	7.5	2163	
Pooled Nonresident		218	47	53	8	108	49.5%	5.6	607	
24 Sheridan										
**(27)**	Limited Type 3	56	44	5	2	51	91.1%	3.5	178	102
	D/F Type 6	511	0	299	18	317	62.0%	6.4	2024	998
	D/F Type 7	949	0	650	104	754	79.5%	8.7	6563	1142
	General	1511	771	139	12	922	61.0%	9.2	8479	
Pooled Total		2661	815	1093	136	2044	76.8%	8.4	17244	
Pooled Resident		1814	613	705	80	1398	77.1%	9.7	13536	
Pooled Nonresident		847	202	388	56	646	76.3%	5.7	3708	

TABLE II-A (Continued)

WHITE-TAILED DEER HUNT AREA REPORT

AREA (cross hunt areas)		TYPE	ACTIVE LICS/HTRS	HARVEST BUCK	DOE	FAWN	TOTAL	HUNTER SUCCESS	DAYS/ HARVEST	HUNTER DAYS	LICENSES SOLD (cross hunt incl.)
25 Tongue-Goose											
		General	131	37	0	0	37	28.2%	8.1	299	
Pooled Total			131	37	0	0	37	28.2%	8.1	299	
Pooled Resident			109	23	0	0	23	21.1%	9.8	225	
Pooled Nonresident			22	14	0	0	14	63.6%	5.3	74	
26 Ucross											
	**(23)**	Limited Type 3	9	9	0	0	9	100.0%	3.4	31	43
	**(23)**	D/F Type 6	90	0	57	9	66	73.3%	7.3	479	783
	**(23)**	D/F Type 7	83	0	63	5	68	81.9%	5.9	399	240
		General	403	190	24	0	214	53.1%	8.3	1780	
Pooled Total			510	199	144	14	357	70.0%	7.5	2689	
Pooled Resident			429	161	132	14	307	71.6%	8.1	2481	
Pooled Nonresident			81	38	12	0	50	61.7%	4.2	208	
27 Buffalo											
	**(24)**	Limited Type 3	40	26	0	0	26	65.0%	6.3	163	102
		D/F Type 6	116	0	63	8	71	61.2%	7.8	557	200
		D/F Type 7	440	0	279	73	352	80.0%	11.1	3924	519
		General	743	345	66	29	440	59.2%	7.3	3193	
Pooled Total			1182	371	408	110	889	75.2%	8.8	7837	
Pooled Resident			857	261	329	60	650	75.8%	10.4	6756	
Pooled Nonresident			325	110	79	50	239	73.5%	4.5	1081	
28 Hunter Mesa											
		General	201	59	0	0	59	29.4%	9.7	575	
Pooled Total			201	59	0	0	59	29.4%	9.7	575	
Pooled Resident			133	45	0	0	45	33.8%	9.9	446	
Pooled Nonresident			68	14	0	0	14	20.6%	9.2	129	
29 Johnson											
		D/F Type 6	130	0	57	9	66	50.8%	5.2	343	259
		D/F Type 7	30	0	25	0	25	83.3%	2.2	54	30
		General	213	73	21	0	94	44.1%	5.1	475	
Pooled Total			310	73	103	9	185	59.7%	4.7	872	
Pooled Resident			195	51	61	5	117	60.0%	5.4	628	
Pooled Nonresident			115	22	42	4	68	59.1%	3.6	244	
30 Upper Powder River											
		D/F Type 6	149	0	80	8	88	59.1%	3.5	304	219
		General	263	91	20	9	120	45.6%	7.7	921	
Pooled Total			335	91	100	17	208	62.1%	5.9	1225	
Pooled Resident			180	66	60	9	135	75.0%	5.5	740	
Pooled Nonresident			155	25	40	8	73	47.1%	6.6	485	
31 Salt Creek											
		General	0	0	0	0	0	0.0%	0.0	0	
Pooled Total			0	0	0	0	0	0.0%	0.0	0	
Pooled Resident			0	0	0	0	0	0.0%	0.0	0	
Pooled Nonresident			0	0	0	0	0	0.0%	0.0	0	
32 Beartrap Creek											
	**(33)**	D/F Type 7	5	0	5	0	5	100.0%	27.2	136	42
		General	16	4	4	0	8	50.0%	5.9	47	
Pooled Total			21	4	9	0	13	61.9%	14.1	183	
Pooled Resident			15	4	7	0	11	73.3%	15.5	170	
Pooled Nonresident			6	0	2	0	2	33.3%	6.5	13	

TABLE II-A (Continued)

WHITE-TAILED DEER HUNT AREA REPORT

AREA (cross hunt areas)		TYPE	ACTIVE LICS/HTRS	HARVEST BUCK	DOE	FAWN	TOTAL	HUNTER SUCCESS	DAYS/ HARVEST	HUNTER DAYS	LICENSES SOLD (cross hunt incl.)
33 Red Fork											
		D/F Type 6	45	0	7	10	17	37.8%	17.2	293	199
	**(32)**	D/F Type 7	26	0	20	3	23	88.5%	2.8	64	42
		General	286	64	21	9	94	32.9%	7.3	690	
Pooled Total			317	64	48	22	134	42.3%	7.8	1047	
Pooled Resident			162	39	23	20	82	50.6%	7.2	594	
Pooled Nonresident			155	25	25	2	52	33.5%	8.7	453	
34 Natrona											
		Limited Type 1	30	13	0	0	13	43.3%	12.4	161	351
		D/F Type 6	18	0	0	4	4	22.2%	5.8	23	151
Pooled Total			40	13	0	4	17	42.5%	10.8	184	
Pooled Resident			33	13	0	4	17	51.5%	10.1	172	
Pooled Nonresident			7	0	0	0	0	0.0%	0.0	12	
35 Upper Nowood											
		General	26	0	0	0	0	0.0%	0.0	99	
Pooled Total			26	0	0	0	0	0.0%	0.0	99	
Pooled Resident			22	0	0	0	0	0.0%	0.0	95	
Pooled Nonresident			4	0	0	0	0	0.0%	0.0	4	
36 Badwater											
		D/F Type 7	10	0	0	0	0	0.0%	0.0	27	12
		General	55	0	0	0	0	0.0%	0.0	101	
Pooled Total			59	0	0	0	0	0.0%	0.0	128	
Pooled Resident			46	0	0	0	0	0.0%	0.0	110	
Pooled Nonresident			13	0	0	0	0	0.0%	0.0	18	
37 Copper Mountain											
		Limited Type 1	30	6	0	0	6	20.0%	30.7	184	179
	**(127)**	Limited Type 2	17	13	0	0	13	76.5%	10.8	141	25
		D/F Type 6	31	0	6	6	12	38.7%	9.7	116	148
	**(127)**	D/F Type 7	14	0	7	2	9	64.3%	4.3	39	35
Pooled Total			66	19	13	8	40	60.6%	12.0	480	
Pooled Resident			65	18	13	8	39	60.0%	12.2	475	
Pooled Nonresident			1	1	0	0	1	100.0%	5.0	5	
38 Black Mountain											
		General	6	0	0	0	0	0.0%	0.0	18	
Pooled Total			6	0	0	0	0	0.0%	0.0	18	
Pooled Resident			6	0	0	0	0	0.0%	0.0	18	
Pooled Nonresident			0	0	0	0	0	0.0%	0.0	0	
39 Bates Creek											
		D/F Type 6	12	0	0	0	0	0.0%	0.0	19	50
		General	16	2	0	0	2	12.5%	8.0	16	
Pooled Total			24	2	0	0	2	8.3%	17.5	35	
Pooled Resident			5	2	0	0	2	40.0%	2.5	5	
Pooled Nonresident			19	0	0	0	0	0.0%	0.0	30	
40 Buffalo Creek											
		D/F Type 6	16	0	11	0	11	68.8%	3.0	33	121
		General	64	8	8	0	16	25.0%	6.2	99	
Pooled Total			80	8	19	0	27	33.8%	4.9	132	
Pooled Resident			28	4	7	0	11	39.3%	5.2	57	
Pooled Nonresident			52	4	12	0	16	30.8%	4.7	75	
41 Sand Creek											
	**(44, 45)**	Limited Type 1	47	27	2	0	29	61.7%	11.8	343	54
	**(45)**	D/F Type 6	68	0	19	10	29	42.6%	5.8	168	197
	**(45)**	D/F Type 7	18	0	0	12	12	66.7%	9.0	108	29
		General	120	25	0	0	25	20.8%	12.2	305	
Pooled Total			203	52	21	22	95	46.8%	9.7	924	
Pooled Resident			154	45	14	17	76	49.4%	10.2	776	
Pooled Nonresident			49	7	7	5	19	38.8%	7.8	148	

TABLE II-A (Continued)

WHITE-TAILED DEER HUNT AREA REPORT

AREA (cross hunt areas)	TYPE	ACTIVE LICS/HTRS	HARVEST BUCK	DOE	FAWN	TOTAL	HUNTER SUCCESS	DAYS/ HARVEST	HUNTER DAYS	LICENSES SOLD (cross hunt incl.)
42 Otter Creek										
	D/F Type 6	28	0	15	5	20	71.4%	6.6	131	175
	General	224	18	8	0	26	11.6%	21.2	552	
Pooled Total		238	18	23	5	46	19.3%	14.8	683	
Pooled Resident		104	4	14	0	18	17.3%	25.7	463	
Pooled Nonresident		134	14	9	5	28	20.9%	7.9	220	
43 Leigh Creek										
	General	22	7	0	0	7	31.8%	6.6	46	
Pooled Total		22	7	0	0	7	31.8%	6.6	46	
Pooled Resident		8	0	0	0	0	0.0%	0.0	17	
Pooled Nonresident		14	7	0	0	7	50.0%	4.1	29	
44 Brokenback Creek										
**(41, 45)**	Limited Type 1	6	2	0	0	2	33.3%	9.5	19	54
	General	29	4	0	0	4	13.8%	15.3	61	
Pooled Total		35	6	0	0	6	17.1%	13.3	80	
Pooled Resident		32	6	0	0	6	18.8%	12.2	73	
Pooled Nonresident		3	0	0	0	0	0.0%	0.0	7	
45 Paint Rock Creek										
**(41, 44)**	Limited Type 1	6	6	0	0	6	100.0%	1.8	11	54
**(41)**	D/F Type 6	12	0	5	2	7	58.3%	4.1	29	197
**(41)**	D/F Type 7	0	0	0	0	0	0.0%	0.0	0	29
	General	48	14	0	0	14	29.2%	20.3	284	
Pooled Total		64	20	5	2	27	42.2%	12.0	324	
Pooled Resident		42	17	0	2	19	45.2%	15.2	288	
Pooled Nonresident		22	3	5	0	8	36.4%	4.5	36	
46 Cloud Peak										
	General	37	0	0	0	0	0.0%	0.0	61	
Pooled Total		37	0	0	0	0	0.0%	0.0	61	
Pooled Resident		34	0	0	0	0	0.0%	0.0	56	
. Pooled Nonresident		3	0	0	0	0	0.0%	0.0	5	
47 Manderson										
**(also valid 51)**	Limited Type 1	7	1	1	0	2	28.6%	43.5	87	
**(49)**	D/F Type 6	15	0	5	0	5	33.3%	4.6	23	149
	General	95	16	0	0	16	16.8%	16.4	262	
Pooled Total		106	17	6	0	23	21.7%	16.2	372	
Pooled Resident		71	16	3	0	19	26.8%	14.8	281	
Pooled Nonresident		35	1	3	0	4	11.4%	22.8	91	
49 Trapper Creek										
**(also valid 51)**	Limited Type 1	1	0	0	0	0	0.0%	0.0	6	
**(47)**	D/F Type 6	11	0	6	0	6	54.5%	1.8	11	149
	General	18	0	0	0	0	0.0%	0.0	37	
Pooled Total		26	0	6	0	6	23.1%	9.0	54	
Pooled Resident		21	0	6	0	6	28.6%	7.2	43	
Pooled Nonresident		5	0	0	0	0	0.0%	0.0	11	
50 Upper Shell-Beaver Creek										
	General	26	7	0	0	7	26.9%	6.1	43	
Pooled Total		26	7	0	0	7	26.9%	6.1	43	
Pooled Resident		19	4	0	0	4	21.1%	8.3	33	
Pooled Nonresident		7	3	0	0	3	42.9%	3.3	10	
51 Beaver Creek										
	Limited Type 1	48	30	0	0	30	62.5%	10.1	302	75
	D/F Type 6	57	0	19	0	19	33.3%	6.2	118	260
	General	92	10	3	0	13	14.1%	15.8	205	
Pooled Total		182	40	22	0	62	34.1%	10.1	625	
Pooled Resident		118	21	19	0	40	33.9%	11.3	452	
Pooled Nonresident		64	19	3	0	22	34.4%	7.9	173	

TABLE II-A (Continued)

WHITE-TAILED DEER HUNT AREA REPORT

AREA (cross hunt areas)		TYPE	ACTIVE LICS/HTRS	HARVEST BUCK	HARVEST DOE	HARVEST FAWN	HARVEST TOTAL	HUNTER SUCCESS	DAYS/ HARVEST	HUNTER DAYS	LICENSES SOLD (cross hunt incl.)
52 Crystal Creek											
		General	27	2	0	0	2	7.4%	18.5	37	
Pooled Total			27	2	0	0	2	7.4%	18.5	37	
Pooled Resident			24	2	0	0	2	8.3%	17.0	34	
Pooled Nonresident			3	0	0	0	0	0.0%	0.0	3	
53 Devils Canyon											
		General	23	4	0	0	4	17.4%	8.3	33	
Pooled Total			23	4	0	0	4	17.4%	8.3	33	
Pooled Resident			20	4	0	0	4	20.0%	6.5	26	
Pooled Nonresident			3	0	0	0	0	0.0%	0.0	7	
55 Rock Eagle											
	**(16)**	Limited Type 1	79	54	4	2	60	75.9%	8.9	536	125
		D/F Type 6	50	0	13	2	15	30.0%	12.0	180	150
		General	137	0	0	0	0	0.0%	0.0	320	
Pooled Total			244	54	17	4	75	30.7%	13.8	1036	
Pooled Resident			196	42	10	4	56	28.6%	15.9	892	
Pooled Nonresident			48	12	7	0	19	39.6%	7.6	144	
57 Southeast											
		Limited Type 1	33	20	2	0	22	66.7%	10.1	223	50
		General	145	25	4	3	32	22.1%	13.9	445	
Pooled Total			178	45	6	3	54	30.3%	12.4	668	
Pooled Resident			156	36	4	3	43	27.6%	13.9	598	
Pooled Nonresident			22	9	2	0	11	50.0%	6.4	70	
59 Little Bear											
	**(62, 63, 64)**	Limited Type 1	31	20	2	0	22	71.0%	8.5	187	126
		D/F Type 6	11	0	8	0	8	72.7%	2.6	21	51
		General	29	0	0	0	0	0.0%	0.0	85	
Pooled Total			67	20	10	0	30	44.8%	9.8	293	
Pooled Resident			50	14	8	0	22	44.0%	11.1	244	
Pooled Nonresident			17	6	2	0	8	47.1%	6.1	49	
60 Pole Mountain											
	**(also valid 59, 62, 63, 64)**	Limited Type 1	23	8	0	0	8	34.8%	5.1	41	105
		Limited Type 2	20	6	0	0	6	30.0%	7.2	43	157
		D/F Type 6	22	0	11	0	11	50.0%	8.1	89	50
Pooled Total			53	14	11	0	25	47.2%	6.9	173	
Pooled Resident			45	12	7	0	19	42.2%	7.2	137	
Pooled Nonresident			8	2	4	0	6	75.0%	6.0	36	
61 Harriman											
		General	17	0	0	0	0	0.0%	0.0	45	
Pooled Total			17	0	0	0	0	0.0%	0.0	45	
Pooled Resident			17	0	0	0	0	0.0%	0.0	45	
Pooled Nonresident			0	0	0	0	0	0.0%	0.0	0	
62 Iron Mountain											
	**(59, 63, 64)**	Limited Type 1	6	2	2	2	6	100.0%	1.3	8	126
	**(63, 64)**	D/F Type 6	6	0	0	0	0	0.0%	0.0	17	177
		General	22	3	0	0	3	13.6%	10.3	31	
Pooled Total			34	5	2	2	9	26.5%	6.2	56	
Pooled Resident			27	2	2	2	6	22.2%	6.5	39	
Pooled Nonresident			7	3	0	0	3	42.9%	5.7	17	
63 Richeau											
	**(59, 62, 64)**	Limited Type 1	6	2	0	0	2	33.3%	21.0	42	126
	**(62, 64)**	D/F Type 6	9	0	4	0	4	44.4%	3.8	15	177
		General	29	7	0	0	7	24.1%	5.4	38	
Pooled Total			40	9	4	0	13	32.5%	7.3	95	
Pooled Resident			31	6	4	0	10	32.3%	7.9	79	
Pooled Nonresident			9	3	0	0	3	33.3%	5.3	16	

WHITE-TAILED DEER HUNT AREA REPORT

AREA (cross hunt areas)	TYPE	ACTIVE LICS/HTRS	HARVEST BUCK	DOE	FAWN	TOTAL	HUNTER SUCCESS	DAYS/ HARVEST	HUNTER DAYS	LICENSES SOLD (cross hunt incl.)
64 Laramie Peak										
**(59, 62, 63)**	Limited Type 1	60	46	0	2	48	80.0%	7.0	336	126
	Limited Type 2	13	9	0	0	9	69.2%	5.0	45	149
**(62, 63)**	D/F Type 6	37	0	15	2	17	45.9%	5.8	99	177
	General	216	44	0	0	44	20.4%	12.5	551	
Pooled Total		306	99	15	4	118	38.6%	8.7	1031	
Pooled Resident		234	68	10	4	82	35.0%	10.1	826	
Pooled Nonresident		72	31	5	0	36	50.0%	5.7	205	
65 South Converse										
**(66, 88)**	Limited Type 1	214	133	10	0	143	66.8%	6.3	896	400
	D/F Type 6	70	0	12	6	18	25.7%	6.4	116	196
**(66, 88)**	D/F Type 7	157	0	97	48	145	92.4%	3.8	545	389
	General	287	52	0	0	52	18.1%	11.1	577	
Pooled Total		623	185	119	54	358	57.5%	6.0	2134	
Pooled Resident		394	143	74	35	252	64.0%	6.0	1503	
Pooled Nonresident		229	42	45	19	106	46.3%	6.0	631	
66 Bates Hole-Hat Six										
**(65, 88)**	Limited Type 1	75	46	8	0	54	72.0%	8.6	465	400
	D/F Type 6	6	0	0	0	0	0.0%	0.0	8	200
**(65, 88)**	D/F Type 7	108	0	62	20	82	75.9%	5.6	461	389
	General	155	30	0	0	30	19.4%	12.7	380	
Pooled Total		302	76	70	20	166	55.0%	7.9	1314	
Pooled Resident		263	68	61	13	142	54.0%	8.6	1227	
Pooled Nonresident		39	8	9	7	24	61.5%	3.6	87	
70 Shirley Mountain										
	Limited Type 1	7	4	0	0	4	57.1%	13.5	54	11
	D/F Type 6	17	0	6	2	8	47.1%	3.6	29	100
	General	64	0	0	0	0	0.0%	0.0	97	
Pooled Total		80	4	6	2	12	15.0%	15.0	180	
Pooled Resident		45	4	4	0	8	17.8%	12.3	98	
Pooled Nonresident		35	0	2	2	4	11.4%	20.5	82	
73 Boswell										
	General	23	0	0	0	0	0.0%	0.0	29	
Pooled Total		23	0	0	0	0	0.0%	0.0	29	
Pooled Resident		20	0	0	0	0	0.0%	0.0	25	
Pooled Nonresident		3	0	0	0	0	0.0%	0.0	4	
74 Kyle										
**(75)**	Limited Type 1	16	9	0	0	9	56.3%	11.9	107	26
**(75)**	D/F Type 6	2	0	2	0	2	100.0%	2.0	4	25
	General	43	0	0	0	0	0.0%	0.0	96	
Pooled Total		61	9	2	0	11	18.0%	18.8	207	
Pooled Resident		44	6	2	0	8	18.2%	21.9	175	
Pooled Nonresident		17	3	0	0	3	17.6%	10.7	32	
75 Four Mile										
**(74)**	Limited Type 1	8	3	0	0	3	37.5%	4.7	14	26
**(74)**	D/F Type 6	2	0	2	0	2	100.0%	4.0	8	25
	General	70	0	0	0	0	0.0%	0.0	167	
Pooled Total		80	3	2	0	5	6.3%	37.8	189	
Pooled Resident		76	3	2	0	5	6.6%	37.0	185	
Pooled Nonresident		4	0	0	0	0	0.0%	0.0	4	

TABLE II-A (Continued)

WHITE-TAILED DEER HUNT AREA REPORT

AREA (cross hunt areas)	TYPE	ACTIVE LICS/HTRS	HARVEST BUCK	DOE	FAWN	TOTAL	HUNTER SUCCESS	DAYS/ HARVEST	HUNTER DAYS	LICENSES SOLD (cross hunt incl.)
76 Sheep Mountain										
	General	83	0	0	0	0	0.0%	0.0	269	
Pooled Total		83	0	0	0	0	0.0%	0.0	269	
Pooled Resident		79	0	0	0	0	0.0%	0.0	265	
Pooled Nonresident		4	0	0	0	0	0.0%	0.0	4	
77 Boulder Ridge										
	General	44	0	0	0	0	0.0%	0.0	116	
Pooled Total		44	0	0	0	0	0.0%	0.0	116	
Pooled Resident		37	0	0	0	0	0.0%	0.0	89	
Pooled Nonresident		7	0	0	0	0	0.0%	0.0	27	
78 French Creek										
**(79, 80, 81)**	Limited Type 1	5	5	0	0	5	100.0%	3.4	17	28
**(79, 80, 81)**	Limited Type 2	0	0	0	0	0	0.0%	0.0	0	27
**(79, 80, 81)**	D/F Type 6	4	0	2	0	2	50.0%	5.5	11	203
	General	78	0	0	4	4	5.1%	35.0	140	
Pooled Total		87	5	2	4	11	12.6%	15.3	168	
Pooled Resident		61	5	2	0	7	11.5%	20.1	141	
Pooled Nonresident		26	0	0	4	4	15.4%	6.8	27	
79 Kennaday Peak										
**(78, 80, 81)**	Limited Type 1	7	5	0	0	5	71.4%	6.8	34	28
**(78, 80, 81)**	Limited Type 2	0	0	0	0	0	0.0%	0.0	0	27
**(78, 80, 81)**	D/F Type 6	13	0	8	0	8	61.5%	3.1	25	203
	General	56	4	13	0	17	30.4%	12.2	208	
Pooled Total		70	9	21	0	30	42.9%	8.9	267	
Pooled Resident		61	5	19	0	24	39.3%	9.7	233	
Pooled Nonresident		9	4	2	0	6	66.7%	5.7	34	
80 Spring Creek										
**(78, 79, 81)**	Limited Type 1	0	0	0	0	0	0.0%	0.0	0	28
**(78, 79, 81)**	Limited Type 2	0	0	0	0	0	0.0%	0.0	0	27
**(78, 79, 81)**	D/F Type 6	6	0	2	0	2	33.3%	3.0	6	203
	General	72	0	0	0	0	0.0%	0.0	189	
Pooled Total		76	0	2	0	2	2.6%	97.5	195	
Pooled Resident		54	0	2	0	2	3.7%	73.5	147	
Pooled Nonresident		22	0	0	0	0	0.0%	0.0	48	
81 Blackhall										
**(78, 79, 80)**	Limited Type 1	2	2	0	0	2	100.0%	1.0	2	28
**(78, 79, 80)**	Limited Type 2	0	0	0	0	0	0.0%	0.0	0	27
**(78, 79, 80)**	D/F Type 6	0	0	0	0	0	0.0%	0.0	0	203
	General	28	4	4	0	8	28.6%	7.9	63	
Pooled Total		30	6	4	0	10	33.3%	6.5	65	
Pooled Resident		19	6	4	0	10	52.6%	3.6	36	
Pooled Nonresident		11	0	0	0	0	0.0%	0.0	29	
82 Baggs										
	Limited Type 1	3	0	0	0	0	0.0%	0.0	5	33
	D/F Type 6	11	0	0	6	6	54.5%	10.8	65	600
	General	119	15	0	0	15	12.6%	16.5	248	
Pooled Total		129	15	0	6	21	16.3%	15.1	318	
Pooled Resident		102	8	0	0	8	7.8%	24.4	195	
Pooled Nonresident		27	7	0	6	13	48.1%	9.5	123	
83 Bolten Rim										
	General	5	0	0	0	0	0.0%	0.0	7	
Pooled Total		5	0	0	0	0	0.0%	0.0	7	
Pooled Resident		5	0	0	0	0	0.0%	0.0	7	
Pooled Nonresident		0	0	0	0	0	0.0%	0.0	0	

TABLE II-A (Continued)

WHITE-TAILED DEER HUNT AREA REPORT

AREA (cross hunt areas)		TYPE	ACTIVE LICS/HTRS	HARVEST BUCK	HARVEST DOE	HARVEST FAWN	HARVEST TOTAL	HUNTER SUCCESS	DAYS/ HARVEST	HUNTER DAYS	LICENSES SOLD (cross hunt incl.)
84 Atlantic Rim											
		Limited Type 1	0	0	0	0	0	0.0%	0.0	0	150
		D/F Type 6	0	0	0	0	0	0.0%	0.0	0	59
Pooled Total			0	0	0	0	0	0.0%	0.0	0	
Pooled Resident			0	0	0	0	0	0.0%	0.0	0	
Pooled Nonresident			0	0	0	0	0	0.0%	0.0	0	
87 Ferris											
		Limited Type 1	0	0	0	0	0	0.0%	0.0	0	301
Pooled Total			0	0	0	0	0	0.0%	0.0	0	
Pooled Resident			0	0	0	0	0	0.0%	0.0	0	
Pooled Nonresident			0	0	0	0	0	0.0%	0.0	0	
88 Kendrick											
	**(65, 66)**	Limited Type 1	42	11	6	0	17	40.5%	7.6	130	400
		D/F Type 6	62	0	22	0	22	35.5%	7.0	155	256
	**(65, 66)**	D/F Type 7	61	0	43	4	47	77.0%	6.0	283	389
		General	98	13	0	0	13	13.3%	15.5	202	
Pooled Total			228	24	71	4	99	43.4%	7.8	770	
Pooled Resident			217	24	71	4	99	45.6%	7.6	753	
Pooled Nonresident			11	0	0	0	0	0.0%	0.0	17	
89 Rattlesnake											
		Limited Type 1	5	0	0	0	0	0.0%	0.0	5	278
		D/F Type 6	7	0	0	0	0	0.0%	0.0	11	94
Pooled Total			12	0	0	0	0	0.0%	0.0	16	
Pooled Resident			10	0	0	0	0	0.0%	0.0	12	
Pooled Nonresident			2	0	0	0	0	0.0%	0.0	4	
90 Muskrat											
		Limited Type 1	0	0	0	0	0	0.0%	0.0	0	75
Pooled Total			0	0	0	0	0	0.0%	0.0	0	
Pooled Resident			0	0	0	0	0	0.0%	0.0	0	
Pooled Nonresident			0	0	0	0	0	0.0%	0.0	0	
91 Popo Agie											
		General	54	0	0	0	0	0.0%	0.0	69	
Pooled Total			54	0	0	0	0	0.0%	0.0	69	
Pooled Resident			51	0	0	0	0	0.0%	0.0	66	
Pooled Nonresident			3	0	0	0	0	0.0%	0.0	3	
92 Table Mountain											
		D/F Type 6	0	0	0	0	0	0.0%	0.0	0	50
		General	67	0	0	0	0	0.0%	0.0	126	
Pooled Total			67	0	0	0	0	0.0%	0.0	126	
Pooled Resident			64	0	0	0	0	0.0%	0.0	119	
Pooled Nonresident			3	0	0	0	0	0.0%	0.0	7	
93 Upper Sweetwater											
		General	17	0	0	0	0	0.0%	0.0	81	
Pooled Total			17	0	0	0	0	0.0%	0.0	81	
Pooled Resident			17	0	0	0	0	0.0%	0.0	81	
Pooled Nonresident			0	0	0	0	0	0.0%	0.0	0	
94 Twin Creek											
	**(160)**	D/F Type 6	6	0	2	0	2	33.3%	12.0	24	54
		General	41	0	0	0	0	0.0%	0.0	48	
Pooled Total			47	0	2	0	2	4.3%	36.0	72	
Pooled Resident			47	0	2	0	2	4.3%	36.0	72	
Pooled Nonresident			0	0	0	0	0	0.0%	0.0	0	
95 South Pass											
		General	11	0	0	0	0	0.0%	0.0	15	
Pooled Total			11	0	0	0	0	0.0%	0.0	15	
Pooled Resident			8	0	0	0	0	0.0%	0.0	12	
Pooled Nonresident			3	0	0	0	0	0.0%	0.0	3	

TABLE II-A (Continued)

WHITE-TAILED DEER HUNT AREA REPORT

AREA (cross hunt areas)	TYPE	ACTIVE LICS/HTRS	HARVEST BUCK	DOE	FAWN	TOTAL	HUNTER SUCCESS	DAYS/ HARVEST	HUNTER DAYS	LICENSES SOLD (cross hunt incl.)
96 Green Mountain										
	D/F Type 6	11	0	2	0	2	18.2%	9.5	19	60
	General	11	0	0	0	0	0.0%	0.0	12	
Pooled Total		19	0	2	0	2	10.5%	15.5	31	
Pooled Resident		12	0	0	0	0	0.0%	0.0	12	
Pooled Nonresident		7	0	2	0	2	28.6%	9.5	19	
97 Sweetwater Rocks										
	Limited Type 1	23	6	0	0	6	26.1%	17.7	106	27
	D/F Type 6	8	0	1	0	1	12.5%	8.0	8	102
	General	41	0	0	0	0	0.0%	0.0	244	
Pooled Total		70	6	1	0	7	10.0%	51.1	358	
Pooled Resident		57	5	0	0	5	8.8%	63.6	318	
Pooled Nonresident		13	1	1	0	2	15.4%	20.0	40	
98 Chain Lakes										
	General	9	0	0	0	0	0.0%	0.0	14	
Pooled Total		9	0	0	0	0	0.0%	0.0	14	
Pooled Resident		9	0	0	0	0	0.0%	0.0	14	
Pooled Nonresident		0	0	0	0	0	0.0%	0.0	0	
100 South Wamsutter										
	General	9	0	0	0	0	0.0%	0.0	9	
Pooled Total		9	0	0	0	0	0.0%	0.0	9	
Pooled Resident		9	0	0	0	0	0.0%	0.0	9	
Pooled Nonresident		0	0	0	0	0	0.0%	0.0	0	
101 Black Butte										
	Limited Type 1	0	0	0	0	0	0.0%	0.0	0	79
Pooled Total		0	0	0	0	0	0.0%	0.0	0	
Pooled Resident		0	0	0	0	0	0.0%	0.0	0	
Pooled Nonresident		0	0	0	0	0	0.0%	0.0	0	
102 Aspen Mountain										
	Limited Type 1	0	0	0	0	0	0.0%	0.0	0	402
Pooled Total		0	0	0	0	0	0.0%	0.0	0	
Pooled Resident		0	0	0	0	0	0.0%	0.0	0	
Pooled Nonresident		0	0	0	0	0	0.0%	0.0	0	
105 Beartooth										
	General	93	17	7	4	28	30.1%	6.3	176	
Pooled Total		93	17	7	4	28	30.1%	6.3	176	
Pooled Resident		86	14	7	4	25	29.1%	6.2	155	
Pooled Nonresident		7	3	0	0	3	42.9%	7.0	21	
106 Crandall-Sunlight										
	General	61	0	0	0	0	0.0%	0.0	173	
Pooled Total		61	0	0	0	0	0.0%	0.0	173	
Pooled Resident		47	0	0	0	0	0.0%	0.0	151	
Pooled Nonresident		14	0	0	0	0	0.0%	0.0	22	
109 Bald Ridge										
	Limited Type 1	26	0	0	0	0	0.0%	0.0	38	50
	D/F Type 6	25	0	22	3	25	100.0%	1.0	25	25
Pooled Total		42	0	22	3	25	59.5%	2.5	63	
Pooled Resident		40	0	22	3	25	62.5%	2.4	60	
Pooled Nonresident		2	0	0	0	0	0.0%	0.0	3	
110 Grinnell										
	General	22	7	0	0	7	31.8%	8.6	60	
Pooled Total		22	7	0	0	7	31.8%	8.6	60	
Pooled Resident		15	7	0	0	7	46.7%	7.6	53	
Pooled Nonresident		7	0	0	0	0	0.0%	0.0	7	

WHITE-TAILED DEER HUNT AREA REPORT

AREA (cross hunt areas)		TYPE	ACTIVE LICS/HTRS	HARVEST BUCK	DOE	FAWN	TOTAL	HUNTER SUCCESS	DAYS/ HARVEST	HUNTER DAYS	LICENSES SOLD (cross hunt incl.)
111 Elk Fork											
		D/F Type 6	12	0	8	4	12	100.0%	1.0	12	76
		General	82	7	0	0	7	8.5%	22.9	160	
Pooled Total			91	7	8	4	19	20.9%	9.1	172	
Pooled Resident			77	4	8	4	16	20.8%	9.4	151	
Pooled Nonresident			14	3	0	0	3	21.4%	7.0	21	
112 Ishawooa											
	**(113)**	D/F Type 6	24	0	6	0	6	25.0%	10.7	64	252
		General	105	18	3	0	21	20.0%	9.8	206	
Pooled Total			118	18	9	0	27	22.9%	10.0	270	
Pooled Resident			88	15	6	0	21	23.9%	8.6	180	
Pooled Nonresident			30	3	3	0	6	20.0%	15.0	90	
113 Boulder											
	**(112)**	D/F Type 6	37	0	2	2	4	10.8%	26.0	104	252
		General	154	20	4	0	24	15.6%	23.5	565	
Pooled Total			174	20	6	2	28	16.1%	23.9	669	
Pooled Resident			129	17	6	2	25	19.4%	23.6	591	
Pooled Nonresident			45	3	0	0	3	6.7%	26.0	78	
114 Wall Mountain											
		General	4	0	0	0	0	0.0%	0.0	11	
Pooled Total			4	0	0	0	0	0.0%	0.0	11	
Pooled Resident			4	0	0	0	0	0.0%	0.0	11	
Pooled Nonresident			0	0	0	0	0	0.0%	0.0	0	
115 Thorofare											
		General	4	0	0	0	0	0.0%	0.0	20	
Pooled Total			4	0	0	0	0	0.0%	0.0	20	
Pooled Resident			4	0	0	0	0	0.0%	0.0	20	
Pooled Nonresident			0	0	0	0	0	0.0%	0.0	0	
116 Meeteetse											
		Limited Type 1	76	20	0	0	20	26.3%	12.6	252	153
		Limited Type 2	36	30	0	0	30	83.3%	6.1	184	40
		D/F Type 6	46	0	20	0	20	43.5%	6.3	126	104
Pooled Total			137	50	20	0	70	51.1%	8.0	562	
Pooled Resident			115	39	16	0	55	47.8%	8.6	475	
Pooled Nonresident			22	11	4	0	15	68.2%	5.8	87	
117 Franc's Peak											
		Limited Type 1	6	0	0	0	0	0.0%	0.0	6	53
Pooled Total			6	0	0	0	0	0.0%	0.0	6	
Pooled Resident			6	0	0	0	0	0.0%	0.0	6	
Pooled Nonresident			0	0	0	0	0	0.0%	0.0	0	
118 Upper Gooseberry Creek											
		Limited Type 1	12	0	0	0	0	0.0%	0.0	27	78
	**(also valid 116)**	Limited Type 2	0	0	0	0	0	0.0%	0.0	0	
		D/F Type 6	12	0	2	0	2	16.7%	7.5	15	35
Pooled Total			20	0	2	0	2	10.0%	21.0	42	
Pooled Resident			14	0	2	0	2	14.3%	17.5	35	
Pooled Nonresident			6	0	0	0	0	0.0%	0.0	7	
119 Anchor											
		Limited Type 1	10	0	0	0	0	0.0%	0.0	11	104
	**(also valid 120)**	Limited Type 2	2	0	0	0	0	0.0%	0.0	9	
		D/F Type 6	15	0	8	0	8	53.3%	2.5	20	50
Pooled Total			22	0	8	0	8	36.4%	5.0	40	
Pooled Resident			19	0	8	0	8	42.1%	4.5	36	
Pooled Nonresident			3	0	0	0	0	0.0%	0.0	4	

WHITE-TAILED DEER HUNT AREA REPORT

AREA (cross hunt areas)		TYPE	ACTIVE LICS/HTRS	HARVEST BUCK	DOE	FAWN	TOTAL	HUNTER SUCCESS	DAYS/ HARVEST	HUNTER DAYS	LICENSES SOLD (cross hunt incl.)
120 Hamilton Dome											
		Limited Type 1	55	14	4	0	18	32.7%	14.4	260	128
		Limited Type 2	24	20	0	0	20	83.3%	4.5	90	26
		D/F Type 6	50	0	28	0	28	56.0%	4.1	114	100
		D/F Type 7	27	0	14	5	19	70.4%	11.9	226	35
Pooled Total			121	34	46	5	85	70.2%	8.1	690	
Pooled Resident			90	27	34	3	64	71.1%	7.3	464	
Pooled Nonresident			31	7	12	2	21	67.7%	10.8	226	
121 Heart Mountain											
	**(122)**	Limited Type 1	13	11	0	0	11	84.6%	8.6	95	28
		General	312	62	22	4	88	28.2%	10.2	894	
Pooled Total			325	73	22	4	99	30.5%	10.0	989	
Pooled Resident			315	73	22	4	99	31.4%	9.9	977	
Pooled Nonresident			10	0	0	0	0	0.0%	0.0	12	
122 Shoshone River											
	**(121)**	Limited Type 1	11	11	0	0	11	100.0%	9.8	108	28
		D/F Type 6	26	0	5	6	11	42.3%	22.2	244	50
		General	560	117	33	7	157	28.0%	13.1	2051	
Pooled Total			581	128	38	13	179	30.8%	13.4	2403	
Pooled Resident			547	114	38	12	164	30.0%	12.0	1966	
Pooled Nonresident			34	14	0	1	15	44.1%	29.1	437	
123 Yellowtail											
		General	167	18	4	0	22	13.2%	23.6	520	
Pooled Total			167	18	4	0	22	13.2%	23.6	520	
Pooled Resident			150	18	4	0	22	14.7%	22.6	497	
Pooled Nonresident			17	0	0	0	0	0.0%	0.0	23	
124 Emblem											
		Limited Type 1	54	28	2	0	30	55.6%	10.7	320	58
		D/F Type 6	116	0	15	13	28	24.1%	18.4	515	204
		D/F Type 7	49	0	24	5	29	59.2%	10.2	295	125
		General	346	59	22	0	81	23.4%	12.9	1043	
Pooled Total			506	87	63	18	168	33.2%	12.9	2173	
Pooled Resident			434	76	54	16	146	33.6%	13.7	1996	
Pooled Nonresident			72	11	9	2	22	30.6%	8.0	177	
125 Fifteen Mile											
		Limited Type 1	7	0	0	0	0	0.0%	0.0	7	135
		D/F Type 6	0	0	0	0	0	0.0%	0.0	0	24
Pooled Total			7	0	0	0	0	0.0%	0.0	7	
Pooled Resident			7	0	0	0	0	0.0%	0.0	7	
Pooled Nonresident			0	0	0	0	0	0.0%	0.0	0	
127 Cottonwood											
	**(37)**	Limited Type 2	9	6	0	0	6	66.7%	9.5	57	25
		D/F Type 6	41	0	20	9	29	70.7%	3.8	110	87
	**(37)**	D/F Type 7	16	0	7	2	9	56.3%	18.3	165	35
		General	67	7	4	0	11	16.4%	28.8	317	
Pooled Total			113	13	31	11	55	48.7%	11.8	649	
Pooled Resident			111	11	31	11	53	47.7%	12.0	636	
Pooled Nonresident			2	2	0	0	2	100.0%	6.5	13	
128 Wind River											
		Limited Type 1	7	0	0	0	0	0.0%	0.0	15	107
		Limited Type 2	41	7	9	2	18	43.9%	16.6	299	50
		D/F Type 6	9	0	4	0	4	44.4%	17.8	71	100
		D/F Type 7	5	0	0	0	0	0.0%	0.0	20	150
		General	118	9	0	0	9	7.6%	26.7	240	
Pooled Total			174	16	13	2	31	17.8%	20.8	645	
Pooled Resident			163	16	13	2	31	19.0%	18.6	576	
Pooled Nonresident			11	0	0	0	0	0.0%	0.0	69	

TABLE II-A (Continued)

WHITE-TAILED DEER HUNT AREA REPORT

AREA (cross hunt areas)	TYPE	ACTIVE LICS/HTRS	HARVEST BUCK	DOE	FAWN	TOTAL	HUNTER SUCCESS	DAYS/ HARVEST	HUNTER DAYS	LICENSES SOLD (cross hunt incl.)
130 Big Sandy										
	Limited Type 1	0	0	0	0	0	0.0%	0.0	0	26
	D/F Type 6	0	0	0	0	0	0.0%	0.0	0	11
	General	12	0	0	0	0	0.0%	0.0	12	
Pooled Total		12	0	0	0	0	0.0%	0.0	12	
Pooled Resident		12	0	0	0	0	0.0%	0.0	12	
Pooled Nonresident		0	0	0	0	0	0.0%	0.0	0	
131 Steamboat										
	Limited Type 2	0	0	0	0	0	0.0%	0.0	0	55
	General	24	4	0	0	4	16.7%	8.5	34	
Pooled Total		24	4	0	0	4	16.7%	8.5	34	
Pooled Resident		24	4	0	0	4	16.7%	8.5	34	
Pooled Nonresident		0	0	0	0	0	0.0%	0.0	0	
132 Cedar Mountain										
	General	27	3	0	0	3	11.1%	22.3	67	
Pooled Total		27	3	0	0	3	11.1%	22.3	67	
Pooled Resident		24	0	0	0	0	0.0%	0.0	58	
Pooled Nonresident		3	3	0	0	3	100.0%	3.0	9	
133 Uinta										
	General	33	0	0	0	0	0.0%	0.0	65	
Pooled Total		33	0	0	0	0	0.0%	0.0	65	
Pooled Resident		33	0	0	0	0	0.0%	0.0	65	
Pooled Nonresident		0	0	0	0	0	0.0%	0.0	0	
134 Bear River Divide										
	General	20	8	0	0	8	40.0%	5.9	47	
Pooled Total		20	8	0	0	8	40.0%	5.9	47	
Pooled Resident		20	8	0	0	8	40.0%	5.9	47	
Pooled Nonresident		0	0	0	0	0	0.0%	0.0	0	
135 West Green River										
	General	58	0	0	0	0	0.0%	0.0	119	
Pooled Total		58	0	0	0	0	0.0%	0.0	119	
Pooled Resident		58	0	0	0	0	0.0%	0.0	119	
Pooled Nonresident		0	0	0	0	0	0.0%	0.0	0	
138 Boulder										
	General	56	0	0	0	0	0.0%	0.0	196	
Pooled Total		56	0	0	0	0	0.0%	0.0	196	
Pooled Resident		56	0	0	0	0	0.0%	0.0	196	
Pooled Nonresident		0	0	0	0	0	0.0%	0.0	0	
139 Pinedale										
	General	93	5	4	0	9	9.7%	21.7	195	
Pooled Total		93	5	4	0	9	9.7%	21.7	195	
Pooled Resident		89	5	4	0	9	10.1%	18.6	167	
Pooled Nonresident		4	0	0	0	0	0.0%	0.0	28	
140 New Fork										
	General	29	0	0	0	0	0.0%	0.0	74	
Pooled Total		29	0	0	0	0	0.0%	0.0	74	
Pooled Resident		25	0	0	0	0	0.0%	0.0	67	
Pooled Nonresident		4	0	0	0	0	0.0%	0.0	7	
141 Green River										
**(162)**	Limited Type 1	2	0	0	0	0	0.0%	0.0	3	125
Pooled Total		2	0	0	0	0	0.0%	0.0	3	
Pooled Resident		0	0	0	0	0	0.0%	0.0	0	
Pooled Nonresident		2	0	0	0	0	0.0%	0.0	3	

TABLE II-A (Continued)

WHITE-TAILED DEER HUNT AREA REPORT

AREA (cross hunt areas)	TYPE	ACTIVE LICS/HTRS	HARVEST BUCK	DOE	FAWN	TOTAL	HUNTER SUCCESS	DAYS/ HARVEST	HUNTER DAYS	LICENSES SOLD (cross hunt incl.)
142 Piney-Horse Creek										
	General	30	5	0	0	5	16.7%	43.4	217	
Pooled Total		30	5	0	0	5	16.7%	43.4	217	
Pooled Resident		26	5	0	0	5	19.2%	8.4	42	
Pooled Nonresident		4	0	0	0	0	0.0%	0.0	175	
143 South Piney										
	General	25	0	0	0	0	0.0%	0.0	61	
Pooled Total		25	0	0	0	0	0.0%	0.0	61	
Pooled Resident		25	0	0	0	0	0.0%	0.0	61	
Pooled Nonresident		0	0	0	0	0	0.0%	0.0	0	
144 Greys River										
	General	20	0	0	0	0	0.0%	0.0	47	
Pooled Total		20	0	0	0	0	0.0%	0.0	47	
Pooled Resident		20	0	0	0	0	0.0%	0.0	47	
Pooled Nonresident		0	0	0	0	0	0.0%	0.0	0	
145 Salt River										
	General	52	15	0	0	15	28.8%	13.5	203	
Pooled Total		52	15	0	0	15	28.8%	13.5	203	
Pooled Resident		49	15	0	0	15	30.6%	12.5	187	
Pooled Nonresident		3	0	0	0	0	0.0%	0.0	16	
146 Fish Creek										
	General	4	4	0	0	4	100.0%	1.0	4	
Pooled Total		4	4	0	0	4	100.0%	1.0	4	
Pooled Resident		0	0	0	0	0	0.0%	0.0	0	
Pooled Nonresident		4	4	0	0	4	100.0%	1.0	4	
148 Teton										
	General	4	0	0	0	0	0.0%	0.0	8	
Pooled Total		4	0	0	0	0	0.0%	0.0	8	
Pooled Resident		4	0	0	0	0	0.0%	0.0	8	
Pooled Nonresident		0	0	0	0	0	0.0%	0.0	0	
149 Targhee										
	General	15	0	0	0	0	0.0%	0.0	29	
Pooled Total		15	0	0	0	0	0.0%	0.0	29	
Pooled Resident		11	0	0	0	0	0.0%	0.0	16	
Pooled Nonresident		4	0	0	0	0	0.0%	0.0	13	
150 Wilson										
	General	0	0	0	0	0	0.0%	0.0	0	
Pooled Total		0	0	0	0	0	0.0%	0.0	0	
Pooled Resident		0	0	0	0	0	0.0%	0.0	0	
Pooled Nonresident		0	0	0	0	0	0.0%	0.0	0	
151 Fall Creek										
	General	27	4	0	0	4	14.8%	8.3	33	
Pooled Total		27	4	0	0	4	14.8%	8.3	33	
Pooled Resident		27	4	0	0	4	14.8%	8.3	33	
Pooled Nonresident		0	0	0	0	0	0.0%	0.0	0	
152 Lower Hoback										
	General	29	4	4	0	8	27.6%	5.8	46	
Pooled Total		29	4	4	0	8	27.6%	5.8	46	
Pooled Resident		29	4	4	0	8	27.6%	5.8	46	
Pooled Nonresident		0	0	0	0	0	0.0%	0.0	0	
153 Monument Ridge										
	General	4	0	0	0	0	0.0%	0.0	5	
Pooled Total		4	0	0	0	0	0.0%	0.0	5	
Pooled Resident		4	0	0	0	0	0.0%	0.0	5	
Pooled Nonresident		0	0	0	0	0	0.0%	0.0	0	

TABLE II-A (Continued)

WHITE-TAILED DEER HUNT AREA REPORT

AREA (cross hunt areas)		TYPE	ACTIVE LICS/HTRS	HARVEST BUCK	DOE	FAWN	TOTAL	HUNTER SUCCESS	DAYS/ HARVEST	HUNTER DAYS	LICENSES SOLD (cross hunt incl.)
154 Raspberry Ridge											
		General	15	0	0	0	0	0.0%	0.0	238	
Pooled Total			15	0	0	0	0	0.0%	0.0	238	
Pooled Resident			11	0	0	0	0	0.0%	0.0	63	
Pooled Nonresident			4	0	0	0	0	0.0%	0.0	175	
155 Crystal Peak											
		General	12	0	4	0	4	33.3%	12.0	48	
Pooled Total			12	0	4	0	4	33.3%	12.0	48	
Pooled Resident			12	0	4	0	4	33.3%	12.0	48	
Pooled Nonresident			0	0	0	0	0	0.0%	0.0	0	
156 Spread Creek											
		General	19	0	0	0	0	0.0%	0.0	50	
Pooled Total			19	0	0	0	0	0.0%	0.0	50	
Pooled Resident			15	0	0	0	0	0.0%	0.0	41	
Pooled Nonresident			4	0	0	0	0	0.0%	0.0	9	
157 Boysen											
	**(170)**	Limited Type 1	49	8	0	0	8	16.3%	17.9	143	255
	**(170)**	Limited Type 2	65	36	6	0	42	64.6%	12.0	506	77
	**(170)**	D/F Type 6	87	0	8	15	23	26.4%	7.9	181	330
	**(170)**	D/F Type 7	82	0	40	8	48	58.5%	8.5	407	97
Pooled Total			214	44	54	23	121	56.5%	10.2	1237	
Pooled Resident			189	37	49	22	108	57.1%	10.8	1165	
Pooled Nonresident			25	7	5	1	13	52.0%	5.5	72	
158 Ormsby											
	**(also valid 22)**	Limited Type 1	18	0	0	4	4	22.2%	6.0	24	411
	**(22)**	D/F Type 6	33	0	13	5	18	54.5%	4.4	80	286
		General	52	4	4	4	12	23.1%	17.8	214	
Pooled Total			98	4	17	13	34	34.7%	9.4	318	
Pooled Resident			87	4	17	13	34	39.1%	8.9	304	
Pooled Nonresident			11	0	0	0	0	0.0%	0.0	14	
160 Beaver Creek											
	**(94)**	D/F Type 6	6	0	0	2	2	33.3%	4.0	8	54
		General	37	0	0	0	0	0.0%	0.0	289	
Pooled Total			43	0	0	2	2	4.7%	148.5	297	
Pooled Resident			43	0	0	2	2	4.7%	148.5	297	
Pooled Nonresident			0	0	0	0	0	0.0%	0.0	0	
161 St. Mary's Creek											
		General	12	0	0	0	0	0.0%	0.0	20	
Pooled Total			12	0	0	0	0	0.0%	0.0	20	
Pooled Resident			12	0	0	0	0	0.0%	0.0	20	
Pooled Nonresident			0	0	0	0	0	0.0%	0.0	0	
162 Water Dog Lakes											
	**(141)**	Limited Type 1	0	0	0	0	0	0.0%	0.0	0	125
Pooled Total			0	0	0	0	0	0.0%	0.0	0	
Pooled Resident			0	0	0	0	0	0.0%	0.0	0	
Pooled Nonresident			0	0	0	0	0	0.0%	0.0	0	

WHITE-TAILED DEER HUNT AREA REPORT

AREA (cross hunt areas)		TYPE	ACTIVE LICS/HTRS	HARVEST BUCK	DOE	FAWN	TOTAL	HUNTER SUCCESS	DAYS/ HARVEST	HUNTER DAYS	LICENSES SOLD (cross hunt incl.)
163 Middle Fork											
		General	7	0	0	0	0	0.0%	0.0	8	
Pooled Total			7	0	0	0	0	0.0%	0.0	8	
Pooled Resident			0	0	0	0	0	0.0%	0.0	0	
Pooled Nonresident			7	0	0	0	0	0.0%	0.0	8	
164 Nowater											
		Limited Type 1	25	11	5	0	16	64.0%	11.5	184	27
		D/F Type 6	12	0	2	2	4	33.3%	12.0	48	75
		General	131	43	0	4	47	35.9%	8.8	412	
Pooled Total			165	54	7	6	67	40.6%	9.6	644	
Pooled Resident			139	47	7	6	60	43.2%	9.4	566	
Pooled Nonresident			26	7	0	0	7	26.9%	11.1	78	
165 YU Bench											
		Limited Type 1	41	7	2	0	9	22.0%	8.0	72	107
		Limited Type 2	47	23	7	4	34	72.3%	11.7	397	54
		D/F Type 6	62	0	24	6	30	48.4%	9.3	280	167
Pooled Total			114	30	33	10	73	64.0%	10.3	749	
Pooled Resident			95	23	31	8	62	65.3%	10.5	648	
Pooled Nonresident			19	7	2	2	11	57.9%	9.2	101	
167 Dull Center											
	**(11, 12, 13, 14, 15)**	Limited Type 1	20	6	4	0	10	50.0%	7.6	76	300
	**(11, 12, 13, 14, 15)**	D/F Type 7	3	0	0	0	0	0.0%	0.0	3	158
		General	27	4	0	0	4	14.8%	16.3	65	
Pooled Total			50	10	4	0	14	28.0%	10.3	144	
Pooled Resident			38	8	4	0	12	31.6%	10.8	130	
Pooled Nonresident			12	2	0	0	2	16.7%	7.0	14	
168 Medicine Butte											
		General	15	0	0	0	0	0.0%	0.0	33	
Pooled Total			15	0	0	0	0	0.0%	0.0	33	
Pooled Resident			15	0	0	0	0	0.0%	0.0	33	
Pooled Nonresident			0	0	0	0	0	0.0%	0.0	0	
169 Tisdale Mountain											
		General	4	0	0	0	0	0.0%	0.0	4	
Pooled Total			4	0	0	0	0	0.0%	0.0	4	
Pooled Resident			0	0	0	0	0	0.0%	0.0	0	
Pooled Nonresident			4	0	0	0	0	0.0%	0.0	4	
170 Riverview											
	**(157)**	Limited Type 1	14	2	3	0	5	35.7%	5.2	26	255
	**(157)**	Limited Type 2	17	8	2	0	10	58.8%	11.4	114	77
	**(157)**	D/F Type 6	9	0	6	0	6	66.7%	1.5	9	330
	**(157)**	D/F Type 7	3	0	2	0	2	66.7%	1.5	3	97
Pooled Total			43	10	13	0	23	53.5%	6.6	152	
Pooled Resident			39	9	13	0	22	56.4%	5.7	126	
Pooled Nonresident			4	1	0	0	1	25.0%	26.0	26	
171 Crowheart Butte											
		Limited 1	20	18	0	0	18	90.0%	5.9	106	35
		General	113	40	4	0	44	38.9%	7.5	332	
Pooled Total			133	58	4	0	62	46.6%	7.1	438	
Pooled Resident			133	58	4	0	62	46.6%	7.1	438	
Pooled Nonresident			0	0	0	0	0	0.0%	0.0	0	

TABLE II-H

WHITE-TAILED DEER HARVEST

BY HERD UNIT

2007

HERD	TYPE	ACTIVE LICS/HTRS	HARVEST BUCK	DOE	FAWN	TOTAL	HUNTER SUCCESS	DAYS/ HARVEST	HUNTER DAYS	LICENSES SOLD (cross hunt incl.)
201 Bighorn Basin	D/F	847	0	300	94	394	46.5%	8.0	3158	2573
	Limited	595	266	23	4	293	49.2%	11.0	3210	1374
	General	2600	492	118	19	629	24.2%	13.9	8766	
Total Hunters		3619	758	441	117	1316	36.4%	11.5	15134	
Resident		2906	628	372	100	1100	37.9%	11.7	12873	
Nonresident		713	130	69	17	216	30.3%	10.5	2261	
303 Powder River	D/F	2943	0	1747	288	2035	69.1%	8.0	16336	5559
	Limited	120	90	7	2	99	82.5%	4.5	447	145
	General	4391	1951	339	75	2365	53.9%	8.2	19422	
Total Hunters		6635	2041	2093	365	4499	67.8%	8.0	36205	
Resident		4438	1482	1442	218	3142	70.8%	9.1	28616	
Nonresident		2197	559	651	147	1357	61.8%	5.6	7589	
504 Southeast Wyoming	D/F	174	0	73	6	79	45.4%	6.4	504	756
	Limited	352	209	18	6	233	66.2%	8.4	1954	832
	General	1134	87	21	7	115	10.1%	26.8	3086	
Total Hunters		1589	296	112	19	427	26.9%	13.0	5544	
Resident		1238	219	88	13	320	25.8%	14.5	4637	
Nonresident		351	77	24	6	107	30.5%	8.5	907	
706 Black Hills	D/F	2453	0	1620	109	1729	70.5%	5.6	9651	3391
	General	8059	4082	246	68	4396	54.5%	7.4	32596	
Total Hunters		10081	4082	1866	177	6125	60.8%	6.9	42247	
Resident		4965	1789	758	84	2631	53.0%	9.0	23756	
Nonresident		5116	2293	1108	93	3494	68.3%	5.3	18491	
707 Central	D/F	859	0	411	133	544	63.3%	5.0	2747	2743
	Limited	669	360	60	11	431	64.4%	7.2	3112	2078
	General	1255	233	25	16	274	21.8%	10.1	2773	
Total Hunters		2421	593	496	160	1249	51.6%	6.9	8632	
Resident		1492	392	326	112	830	55.6%	7.7	6362	
Nonresident		929	201	170	48	419	45.1%	5.4	2270	
999 Rest of State	D/F	248	0	65	31	96	38.7%	8.9	855	2650
	Limited	228	85	20	2	107	46.9%	12.4	1323	1797
	General	1208	120	16	0	136	11.3%	27.4	3729	
Total Hunters		1590	205	101	33	339	21.3%	17.4	5907	
Resident		1432	178	93	26	297	20.7%	17.0	5037	
Nonresident		158	27	8	7	42	26.6%	20.7	870	

TABLE V

CONFIDENCE INTERVALS FOR TOTAL DEER HARVEST

BY HERD UNIT

2007

HERD (W = White Tail)		TOTAL HARVEST	90% CONFIDENCE LIMITS		
101	Targhee	38	29	-	47
104	Sublette	2212	2150	-	2274
131	Wyoming Range	2271	2210	-	2332
201 (W)	Bighorn Basin	1316	1266	-	1366
207	Paintrock	947	910	-	984
208	Southwest Bighorns	1763	1716	-	1810
209	Basin	212	196	-	228
210	Greybull River	685	659	-	711
211	Shoshone River	354	330	-	378
212	Owl Creek/Meeteetse	428	408	-	448
215	Upper Shoshone	1388	1347	-	1429
216	Clark's Fork	910	877	-	943
303 (W)	Powder River	4499	4426	-	4572
319	Powder River	3710	3651	-	3769
320	Pumpkin Buttes	847	819	-	875
321	North Bighorn	2077	2020	-	2134
322	Upper Powder River	1277	1241	-	1313
423	Uinta	1018	978	-	1058
424	South Rock Springs	351	337	-	365
427	Baggs	2632	2583	-	2681
430	Steamboat	384	358	-	410
504 (W)	Southeast Wyoming	427	397	-	457
534	Goeshen Rim	893	858	-	928
537	Laramie Mountains	1580	1539	-	1621
539	Sheep Mountain	530	497	-	563
540	Shirley Mountain	569	544	-	594
541	Platte Valley	1817	1761	-	1873
642	Dubois	728	695	-	761
643	Project	480	460	-	500
644	South Wind River	884	847	-	921
646	Sweetwater	612	584	-	640
647	Ferris	226	215	-	237
648	Beaver Rim	64	59	-	69
650	Chain Lakes	60	51	-	69
706 (W)	Black Hills	6125	6038	-	6212
707 (W)	Central	1249	1205	-	1293
751	Black Hills	3372	3300	-	3444
752	Thunder Basin	1355	1317	-	1393
753	Lance Creek	1410	1374	-	1446
755	North Converse	705	686	-	724
756	South Converse	729	700	-	758
757	Bates Hole-Hat Six	658	629	-	687
758	Rattlesnake	594	572	-	616
759	North Natrona	336	322	-	350
999 (W)	Rest of State	339	312	-	366

TABLE VI

DEER HUNTERS HUNTING ON OPENING DAY

BY HUNT AREA AND RESIDENCY

2007

AREA	RESIDENT HUNTERS HUNTING ON OPENING DAY	PERCENT WHO HUNTED ON OPENING DAY	NONRESIDENT HUNTERS HUNTING ON OPENING DAY	PERCENT WHO HUNTED ON OPENING DAY
1	184	15.3%	368	20.4%
2	441	16.0%	661	20.8%
3	75	15.4%	244	40.9%
4	220	22.1%	174	27.4%
5	66	19.7%	82	29.6%
6	135	27.8%	130	37.6%
7	34	29.7%	110	50.8%
8	34	32.9%	122	57.4%
9	19	20.9%	101	56.6%
10	61	41.5%	90	71.9%
11	22	17.0%	176	49.0%
12	38	28.5%	230	56.5%
13	56	18.3%	387	60.7%
14	39	26.2%	311	67.6%
15	31	25.5%	60	62.5%
16	167	51.2%	77	47.4%
17	147	21.7%	333	45.6%
18	214	28.5%	409	46.5%
19	16	11.1%	168	50.7%
20	7	12.0%	42	41.6%
21	105	29.8%	122	46.2%
22	17	16.6%	183	47.2%
23	220	22.2%	543	46.1%
24	489	21.2%	337	28.1%
25	215	39.9%	212	60.8%
26	126	20.3%	263	48.3%
27	132	13.1%	262	44.6%
28	138	37.2%	97	48.2%
29	81	29.5%	185	43.8%
30	58	19.8%	133	39.3%
31	9	26.4%	32	81.8%
32	15	23.4%	18	36.6%
33	47	15.7%	402	54.3%
34	72	23.4%	57	53.3%
35	9	10.4%	61	60.7%
36	125	41.4%	111	74.3%
37	77	35.5%	30	48.5%
38	31	32.8%	54	71.4%
39	11	17.3%	69	50.9%
40	34	33.7%	143	59.0%
41	106	34.2%	144	73.1%
42	118	31.2%	511	69.9%
43	12	25.7%	64	78.3%
44	39	41.2%	51	75.0%
45	66	46.7%	94	67.4%
46	130	52.0%	91	81.8%
47	126	44.6%	109	64.7%
49	91	48.8%	105	82.3%
50	96	58.9%	74	75.9%
51	111	38.8%	151	59.1%
52	43	36.5%	27	66.7%
53	153	44.1%	57	56.7%
55	231	40.1%	85	43.8%
57	213	49.2%	49	72.3%
59	43	28.5%	23	40.5%
60	125	54.1%	25	52.4%
61	79	50.1%	20	63.6%
62	30	32.1%	20	32.8%
63	24	18.6%	100	64.5%
64	433	40.2%	315	60.8%

TABLE VI (Continued)

DEER OPENING DAY HUNTERS REPORT

AREA	RESIDENT HUNTERS HUNTING ON OPENING DAY	PERCENT WHO HUNTED ON OPENING DAY	NONRESIDENT HUNTERS HUNTING ON OPENING DAY	PERCENT WHO HUNTED ON OPENING DAY
65	231	26.9%	431	59.3%
66	310	32.0%	302	75.3%
70	156	32.2%	324	72.3%
73	62	34.9%	63	55.0%
74	58	32.7%	37	43.0%
75	177	29.5%	77	58.2%
76	288	38.5%	73	43.5%
77	71	35.5%	40	73.3%
78	503	37.0%	187	42.3%
79	254	36.0%	188	48.6%
80	214	36.5%	259	60.1%
81	229	34.6%	191	51.6%
82	866	40.5%	786	66.4%
83	17	42.3%	22	50.0%
84	38	35.4%	26	63.0%
87	88	39.7%	31	55.0%
88	191	37.6%	28	57.5%
89	110	40.5%	34	50.7%
90	42	73.1%	0	0.0%
91	120	34.9%	50	83.3%
92	208	45.3%	50	68.5%
93	18	24.4%	3	33.3%
94	151	39.9%	53	84.0%
95	96	53.2%	30	47.4%
96	238	49.5%	182	91.6%
97	196	50.7%	60	69.4%
98	18	25.2%	23	53.8%
100	111	44.4%	66	51.3%
101	41	67.9%	10	57.1%
102	200	63.5%	31	40.0%
105	73	26.0%	35	30.3%
106	99	24.1%	38	16.7%
109	27	48.4%	0	0.0%
110	17	13.6%	17	17.2%
111	120	24.0%	57	20.1%
112	121	30.9%	64	22.2%
113	134	30.8%	69	23.3%
114	0	0.0%	0	0.0%
115	16	37.0%	21	31.6%
116	80	46.9%	13	27.0%
117	28	68.2%	12	100.0%
118	21	31.4%	17	82.5%
119	35	34.9%	6	20.6%
120	44	32.3%	15	24.9%
121	307	46.3%	14	21.1%
122	432	48.2%	36	47.1%
123	78	42.1%	7	33.3%
124	337	39.5%	84	58.0%
125	65	59.9%	11	26.9%
127	71	29.4%	22	58.1%
128	360	32.6%	52	22.8%
130	137	46.6%	4	23.4%
131	566	55.3%	63	76.0%
132	233	47.1%	44	71.4%
133	411	47.2%	91	68.9%
134	333	54.4%	132	81.8%
135	905	47.0%	178	71.3%
138	208	39.9%	25	53.8%
139	133	28.2%	18	38.5%
140	78	23.0%	11	25.0%

TABLE VI (Continued)

DEER OPENING DAY HUNTERS REPORT

AREA	RESIDENT HUNTERS HUNTING ON OPENING DAY	PERCENT WHO HUNTED ON OPENING DAY	NONRESIDENT HUNTERS HUNTING ON OPENING DAY	PERCENT WHO HUNTED ON OPENING DAY
141	33	51.4%	6	36.4%
142	247	42.5%	81	51.1%
143	332	38.9%	37	50.0%
144	400	51.3%	197	55.8%
145	438	55.8%	112	66.7%
146	74	33.1%	14	50.0%
148	23	10.7%	3	10.0%
149	37	28.9%	35	55.6%
150	14	28.7%	18	100.0%
151	147	33.7%	64	56.3%
152	295	38.8%	180	56.0%
153	89	41.2%	81	53.5%
154	219	49.0%	78	45.8%
155	92	33.7%	21	66.7%
156	139	34.1%	35	38.5%
157	175	39.6%	57	75.2%
158	60	29.7%	119	41.3%
160	112	39.9%	57	85.0%
161	28	39.8%	4	25.0%
162	23	65.0%	6	40.0%
163	13	25.7%	172	78.7%
164	115	38.4%	82	63.2%
165	77	46.2%	28	51.7%
167	32	36.1%	144	86.9%
168	397	45.7%	111	65.5%
169	35	62.0%	100	68.3%
170	20	36.4%	0	0.0%
171	74	31.9%	7	33.3%
171	53	34.8%	7	40.0%

TABLE VII

DEER LICENSE DATA SUMMARY
2007

HUNT AREA	LICENSE TYPE	NUMBER SOLD	NUMBER SURVEYED	NUMBER USABLE SURVEYS (both hunters and nonhunters)	% USABLE RESPONSE
GENERAL	RES	43200	31747	11209	35.3%
GENERAL	NONRES	25949	12787	7554	59.1%
1, 2, 3	6	2722	259	129	49.8%
4	6	324	149	61	40.9%
5	6	112	117*	43	36.8%
6, 9	6	233	210	93	44.3%
8	6	96	96	31	32.3%
10	6	32	32	18	56.3%
11, 12, 13, 15, 15, 167	1	300	216	103	47.7%
11	6	134	123	62	50.4%
11, 12, 13, 15, 15, 167	7	158	151	64	42.4%
12, 13, 14	6	500	235	108	46.0%
15	6	18	18	10	55.6%
16, 55	1	125	125	66	52.8%
16	2	28	28	15	53.6%
17, 18, 19, 20, 21	6	928	247	119	48.2%
22	1	338	154	85	55.2%
22, 158	6	286	217	90	41.5%
23, 26	3	43	42	18	42.9%
23, 26	6	783	247	107	43.3%
23, 26	7	240	227	52	22.9%
24, 27	3	102	99	51	51.5%
24	6	998	165	71	43.0%
24	7	1142	170	65	38.2%
27	6	200	135	58	43.0%
27	7	519	162	53	32.7%
29	6	259	144	55	38.2%
29	7	30	29	9	31.0%
30	6	219	144	51	35.4%
32, 33	7	42	40	16	40.0%
33	6	199	133	73	54.9%
34	1	351	149	83	55.7%
34	6	151	123	43	35.0%
36	7	12	11	3	27.3%
37	1	179	128	67	52.3%
37, 127	2	25	25	16	64.0%
37	6	148	123	73	59.4%
37, 127	7	35	35	15	42.9%
39	6	50	50	12	24.0%
40	6	121	72	30	41.7%
41, 44, 45	1	54	54	25	46.3%
41, 45	6	197	197	81	41.1%
41, 45	7	29	29	5	17.2%
42	6	175	128	79	61.7%
47, 49	6	149	149	67	45.0%
51	1	75	75	40	53.3%
51	6	260	139	64	46.0%
55	6	150	123	68	55.3%
57	1	50	50	20	40.0%
59, 62, 63, 64	1	126	126	55	43.7%
59	6	51	51	24	47.1%
60	1	105	105	49	46.7%
60	2	157	123	62	50.4%
60	6	50	50	22	44.0%
62, 63, 64	6	177	177	95	53.7%
64	2	149	123	69	56.1%
65, 66, 88	1	400	227	85	37.4%
65	6	196	133	65	48.9%
65, 66, 88	7	389	235	96	40.9%
66	6	200	133	73	54.9%
70	1	11	11	4	36.4%

DEER LICENSE DATA SUMMARY

HUNT AREA	LICENSE TYPE	NUMBER SOLD	NUMBER SURVEYED	NUMBER USABLE SURVEYS (both hunters and nonhunters)	% USABLE RESPONSE
70	6	100	99	42	42.4%
74, 75	1	26	26	11	42.3%
74, 75	6	25	25	13	52.0%
78, 79, 80, 81	1	28	28	17	60.7%
78, 79, 80, 81	2	27	27	17	63.0%
78, 79, 80, 81	6	203	200	112	56.0%
82	1	33	22	12	54.6%
82	6	600	159	87	54.7%
84	1	150	123	63	51.2%
84	6	59	59	36	61.0%
87	1	301	144	90	62.5%
88	6	256	139	58	41.7%
89	1	278	141	72	51.1%
89	6	94	94	38	40.4%
90	1	75	74	34	46.0%
92	6	50	50	19	38.0%
94, 160	6	54	49	25	51.0%
96	6	60	59	23	39.0%
97	1	27	27	13	48.2%
97	6	102	102	52	51.0%
101	1	79	78	42	53.9%
102	1	402	152	85	55.9%
109	1	50	50	22	44.0%
109	6	25	23	8	34.8%
111	6	76	64	23	35.9%
112, 113	6	252	209	117	56.0%
116	1	153	123	71	57.7%
116	2	40	40	22	55.0%
116	6	104	99	50	50.5%
117	1	53	53	31	58.5%
118	1	78	78	47	60.3%
118	6	35	35	20	57.1%
119	1	104	104	67	64.4%
119	6	50	50	27	54.0%
120	1	128	115	64	55.7%
120	2	26	26	13	50.0%
120	6	100	100	44	44.0%
120	7	35	36*	19	52.8%
121, 122	1	28	28	13	46.4%
122	6	50	50	28	56.0%
124	1	58	58	24	41.4%
124	6	204	135	65	48.2%
124	7	125	118	31	26.3%
125	1	135	118	70	59.3%
125	6	24	23	7	30.4%
127	6	87	87	32	36.8%
128	1	107	105	63	60.0%
128	2	50	50	30	60.0%
128	6	100	100	57	57.0%
128	7	150	123	59	48.0%
130	1	26	26	15	57.7%
130	6	11	11	7	63.6%
131	2	55	55	29	52.7%
141, 162	1	125	125	73	58.4%
157, 170	1	255	208	129	62.0%
157, 170	2	77	77	38	49.4%
157, 170	6	330	220	115	52.3%
157	7	97	99*	44	44.4%
158	1	411	159	90	56.6%
164	1	27	27	12	44.4%
164	6	75	74	31	41.9%

TABLE VII (Continued)

DEER LICENSE DATA SUMMARY

HUNT AREA		LICENSE TYPE	NUMBER SOLD	NUMBER SURVEYED	NUMBER USABLE SURVEYS (both hunters and nonhunters)	% USABLE RESPONSE
165		1	107	107	59	55.1%
165		2	54	54	32	59.3%
165		6	167	128	61	47.7%
171		1	35	35	17	48.6%
	TOTAL		91789	57284	24866	43.4%

* Actual downloads exceed WG&F sales data.

WYOMING GAME AND FISH COMMISSION

CHAPTER 6

DEER HUNTING SEASONS

Section 1. Authority. This regulation is promulgated by authority of W.S. §23-1-302, §23-1-703 and §23-2-104.

Section 2. Hunting Seasons Established. There shall be open seasons during 2007 for the hunting of deer as set forth in this Chapter and in accordance with Chapter 2, General Hunting Regulation.

Section 3. Regular Hunting Seasons.

(a) Hunt areas, season dates and limitations.

Hunt Area	Type	Date of Seasons Opens	Closes	Limitations
1, 2, 3		Nov. 1	Nov. 30	General license; antlered deer off private land; any deer on private land
1, 2, 3	6	Nov. 1	Nov. 30	Limited quota; 3,000 licenses doe or fawn valid on private land
*4, 5, *6		Nov. 1	Nov. 20	General license; antlered deer off private land; any deer on private land except the lands of the State of Wyoming's Ranch A property shall be closed
*4	6	Nov. 1	Nov. 30	Limited quota; 350 licenses doe or fawn valid on private land
5	6	Nov. 1	Nov. 30	Limited quota; 250 licenses doe or fawn
*6, *9	6	Nov. 1	Nov. 30	Limited quota; 300 licenses doe or fawn valid in that portion of Area 6 and Area 9 east of U.S. Highway 85
7, *8, *9		Oct. 1	Oct. 15	General license; any deer
*8	6	Oct. 1	Oct. 15	Limited quota; 300 licenses doe or fawn valid in that portion of Area 8 north of Lodgepole Creek
*10		Oct. 1	Oct. 5	General license; antlered deer
	6	Oct. 1	Oct. 15	Limited quota; 50 licenses doe or fawn valid on private land
11		Oct. 1	Oct. 15	General license; any deer
	6	Oct. 1	Nov. 30	Limited quota; 150 licenses doe or fawn
12,*13,*14		Oct. 1	Oct. 15	General license; any deer
	6	Oct. 1	Oct. 31	Limited quota; 500 licenses doe or fawn
11, 12, *13, *14, *15, 167	1	Oct. 1	Nov. 30	Limited quota; 300 licenses any white-tailed deer
	7	Oct. 1	Nov. 30	Limited quota; 300 licenses doe or fawn white-tailed deer
*15		Oct. 1	Oct. 15	General license; any deer
	6	Oct. 1	Oct. 15	Limited quota; 25 licenses doe or fawn
*16		Oct. 1	Oct. 10	General license; antlered mule deer or any white-tailed deer
	2	Nov. 1	Nov. 30	Limited quota; 25 licenses any deer valid in that portion of Area 16 east of U.S. Highway 85 and south of the Tea Kettle Road and Goshen County Road 94
*16, *55	1	Nov. 1	Nov. 30	Limited quota; 125 licenses any white-tailed deer
17, 18, 19, 20, 21		Oct. 1	Oct. 20	General license; antlered deer off private land, any deer on private land

Hunt Area	Type	Date of Seasons Opens	Closes	Limitations
17		Nov. 1	Nov. 30	General license; any white-tailed deer
17, 18, 19, 20, 21	6	Oct. 1	Oct. 31	Limited quota; 1,000 licenses doe or fawn valid on private land
*22	1	Oct. 1	Oct. 14	Limited quota; 450 licenses any deer, also valid in Area 158
*22, *158		Nov. 1	Nov. 30	General license; any white-tailed deer
	6	Oct. 1	Oct. 14	Limited quota; 800 licenses doe or fawn
		Nov. 1	Nov. 30	Unused Area 22, 158 Type 6 licenses valid for doe or fawn white-tailed deer
23, 26		Oct. 1	Oct. 14	General license; antlered deer off private land, any deer on private land
		Nov. 1	Nov. 30	General license; any white-tailed deer
	3	Nov. 1	Nov. 30	Limited quota; 50 licenses any white-tailed deer
	6	Oct. 1	Dec. 16	Limited quota; 1,000 licenses doe or fawn valid on private land
	7	Oct. 1	Dec. 16	Limited quota; 750 licenses doe or fawn white-tailed deer
24		Oct. 15	Oct. 31	General license; antlered deer off private land, any deer on private land
		Nov. 1	Nov. 30	General license; any white-tailed deer
		Dec. 1	Dec. 16	General license; antlerless white-tailed deer
	6	Sept. 1	Dec. 16	Limited quota; 1,000 licenses doe or fawn valid on private land
	7	Sept. 1	Dec. 16	Limited quota; 2,000 licenses doe or fawn white-tailed deer
24, 27	3	Nov. 1	Nov. 30	Limited quota; 100 licenses any white-tailed deer
25		Oct. 15	Oct. 31	General license; antlered deer
27		Oct. 15	Oct. 31	General license; any deer
		Nov. 1	Nov. 30	General license; any white-tailed deer
		Dec. 1	Dec. 16	General license; antlerless white-tailed deer
	6	Oct. 15	Dec. 16	Limited quota; 200 licenses doe or fawn
	7	Oct. 15	Dec. 16	Limited quota; 700 licenses doe or fawn white-tailed deer
28		Oct. 15	Oct. 31	General license; antlered deer
29		Oct. 1	Oct. 14	General license; antlered deer off private land, any deer on private land
		Nov. 1	Nov. 15	General license; any white-tailed deer
	6	Oct. 1	Nov. 30	Limited quota; 300 licenses doe or fawn valid on private land
	7	Oct. 1	Nov. 30	Limited quota; 300 licenses doe or fawn white-tailed deer
*30		Oct. 15	Oct. 31	General license; any deer
		Nov. 1	Nov. 30	General license; any white-tailed deer
	6	Oct. 15	Nov. 30	Limited quota; 300 licenses doe or fawn valid on private land
31		Oct. 1	Oct. 10	General license; antlered deer
32, *33		Oct. 15	Oct. 31	General license; any deer
		Nov. 1	Nov. 15	General license; any white-tailed deer
	7	Oct. 15	Nov. 15	Limited quota; 150 licenses doe or fawn white-tailed deer
*33	6	Oct. 15	Nov. 15	Limited quota; 200 licenses doe or fawn

Hunt Area	Type	Date of Seasons Opens	Closes	Limitations
*34	1	Oct. 15	Oct. 31	Limited quota; 350 licenses antlered deer; any deer valid in that portion of Area 34 east of the Bucknum Road (Natrona County Road 125) in the Casper Creek drainage
		Nov. 1	Nov. 30	Unused Area 34 Type 1 licenses valid for any white-tailed deer
	6	Oct. 15	Oct. 31	Limited quota; 150 licenses doe or fawn
35		Oct. 15	Oct. 31	General license; any deer
36		Oct. 15	Oct. 22	General license; antlered mule deer or any white-tailed deer
	7	Oct. 15	Oct. 22	Limited quota; 25 licenses doe or fawn white-tailed deer
*37	1	Oct. 15	Oct. 31	Limited quota; 175 licenses any deer
	6	Sept. 15	Nov. 15	Limited quota; 150 licenses doe or fawn
*37, *127	2	Nov. 1	Nov. 30	Limited quota; 25 licenses any white-tailed deer
	7	Nov. 1	Nov. 30	Limited quota; 35 licenses doe or fawn white-tailed deer
38, 39		Oct. 15	Oct. 31	General license; antlered deer
39	6	Oct. 15	Oct. 31	Limited quota; 50 licenses doe or fawn valid in that portion of Area 39 within one (1) mile of the Upper Nowood Road
40		Oct. 15	Nov. 4	General license; any deer
	6	Oct. 15	Nov. 4	Limited quota; 125 licenses doe or fawn
*41, 45, 47, 49		Oct. 15	Oct. 24	General license; any deer
		Oct. 25	Oct. 31	General license; antlerless deer on private land
*41, 44, 45	1	Nov. 1	Nov. 30	Limited quota; 50 licenses any white-tailed deer
*41, 45	6	Oct. 1	Nov. 30	Limited quota; 200 licenses doe or fawn valid on private land
*41, 45	7	Oct. 1	Nov. 30	Limited quota; 50 licenses doe or fawn white-tailed deer
42		Oct. 15	Nov. 4	General license; any deer
	6	Oct. 15	Nov. 4	Limited quota; 175 licenses doe or fawn
43		Oct. 15	Oct. 31	General license; antlered deer
44, 46		Oct. 15	Oct. 24	General license; any deer
47, 49	6	Oct. 1	Nov. 30	Limited quota; 150 licenses doe or fawn valid on private land
50, 52		Oct. 15	Oct. 24	General license; any deer
51		Oct. 15	Oct. 24	General license; any deer
		Oct. 25	Oct. 31	General license; antlerless deer on private land
	1	Oct. 15	Nov. 30	Limited quota; 75 licenses any white-tailed deer; also valid in Hunt Areas 47 and 49
	6	Oct. 1	Nov. 30	Limited quota; 250 licenses doe or fawn valid on private land
53		Oct. 15	Oct. 24	General license; any deer
		Oct. 25	Nov. 4	General license; antlered deer
*55		Oct. 1	Oct. 14	General license; antlered mule deer or any white-tailed deer
	6	Oct. 1	Oct. 14	Limited quota; 150 licenses doe or fawn
		Nov. 1	Nov. 30	Unused Area 55 Type 6 licenses valid in that portion of Area 55 in Goshen County and north of the Laramie River in Platte County
*57		Oct. 1	Oct. 14	General license; any deer
	1	Nov. 1	Nov. 30	Limited quota; 50 licenses any white-tailed deer
*59,*62,*63		Oct. 15	Oct. 31	General license; antlered deer, except the Wyoming Game

Hunt Area	Type	Date of Seasons Opens	Closes	Limitations
				and Fish Commission's Tom Thorne/Beth Williams Wildlife Research Center at Sybille shall be closed
*59, *62, *63, *64	1	Nov. 1	Nov. 30	Limited quota; 125 licenses any white-tailed deer, also valid in Area 60, except the Wyoming Game and Fish Commission's Tom Thorne/Beth Williams Wildlife Research Center at Sybille south of Wyoming Highway 34 shall be closed
		Dec. 1	Dec. 15	Unused Area 59, 62, 63, 64 Type 1 licenses valid for doe or fawn white-tailed deer in Area 64, also valid in Area 60
*59	6	Oct. 15	Nov. 30	Limited quota; 50 licenses doe or fawn
*60	1	Oct. 20	Nov. 4	Limited quota; 100 licenses antlered deer on national forest, any deer off national forest; all lands within Curt Gowdy State Park, archery only
	2	Oct. 20	Nov. 4	Limited quota; 150 licenses any deer off national forest; all lands within Curt Gowdy State Park, archery only
		Nov. 15	Nov. 30	Unused Area 60 Type 1 and Type 2 licenses valid for doe or fawn white-tailed deer off national forest; all lands within Curt Gowdy State Park, archery only
	6	Oct. 20	Nov. 4	Limited quota; 50 licenses doe or fawn valid in that portion of Area 60 in Laramie County
*61		Oct. 1	Oct. 14	General license; any deer
*62, *63, *64	6	Oct. 15	Oct. 31	Limited quota; 200 licenses doe or fawn, except the Wyoming Game and Fish Commission's Tom Thorne/Beth Williams Wildlife Habitat Management Area and the Laramie Peak Wildlife Habitat Management Area north of the Tunnel Road (Albany County Road 727), archery only. The Wyoming Game and Fish Commission's Tom Thorne/Beth Williams Wildlife Research Center at Sybille south of Wyoming Highway 34 shall be closed.
*64		Oct. 15	Oct. 31	General license; antlered deer, except the Wyoming Game and Fish Commission's Tom Thorne/Beth Williams Wildlife Habitat Management Area and the Laramie Peak Wildlife Habitat Management Area north of the Tunnel Road (Albany County Road 727), archery only
	2	Oct. 15	Oct. 31	Limited quota; 150 licenses antlered deer
*65		Oct. 15	Oct. 25	General license; antlered deer
	6	Oct. 15	Oct. 25	Limited quota; 200 licenses doe or fawn valid on private land
*65, *66, *88	1	Nov. 1	Nov. 30	Limited quota; 400 licenses any white-tailed deer
	7	Nov. 1	Nov. 30	Limited quota; 500 licenses doe or fawn white-tailed deer
*66		Oct. 15	Oct. 25	General license; antlered deer
	6	Oct. 15	Oct. 25	Limited quota; 200 licenses doe or fawn
*67				CLOSED
*70		Oct. 15	Oct. 25	General license; antlered mule deer or any white-tailed deer
	1	Nov. 1	Nov. 30	Limited quota; 25 licenses any white-tailed deer
	6	Oct. 15	Nov. 30	Limited quota; 100 licenses doe or fawn valid in that portion of Area 70 north of the Lost Creek-Medicine Bow River hydrographic divide and west of Carbon County Road 291; or north of Carbon County Road 102 and west of Carbon County Road 103

Hunt Area	Type	Date of Seasons Opens	Closes	Limitations
*73		Oct. 15	Oct. 31	General license; antlered deer
*74,*75,*76, *77		Oct. 1	Oct. 14	General license; antlered mule deer or any white-tailed deer
*74, *75	1	Nov. 1	Nov. 30	Limited quota; 25 licenses any white-tailed deer valid in all of Area 74 and that portion of Area 75 west of Rock Creek
	6	Nov. 1	Nov. 30	Limited quota; 25 licenses doe or fawn valid in all of Area 74 and that portion of Area 75 west of Rock Creek
*78, *79, *80, *81		Oct. 1	Oct. 12	General license; any deer
		Oct. 13	Oct. 14	General license; antlered deer
	1	Nov. 1	Nov. 30	Limited quota; 25 licenses any white-tailed deer
	2	Oct. 15	Oct. 31	Limited quota; 25 licenses any deer
*78, *79, *80, *81	6	Oct. 1	Oct. 12	Limited quota; 200 licenses doe or fawn
*82		Oct. 1	Oct. 14	General license; any deer
	1	Oct. 15	Oct. 31	Limited quota; 20 licenses any deer
	6	Oct. 1	Oct. 14	Limited quota; 600 licenses doe or fawn
83		Oct. 1	Oct. 14	General license; antlered mule deer or any white-tailed deer
84	1	Oct. 1	Oct. 14	Limited quota; 150 licenses any deer; also valid in Area 82
	6	Oct. 1	Oct. 14	Limited quota; 50 licenses doe or fawn; also valid in Area 82
87	1	Oct. 15	Oct. 31	Limited quota; 300 licenses antlered deer
*88		Oct. 15	Oct. 25	General license; antlered deer
	6	Oct. 15	Nov. 30	Limited quota; 250 licenses doe or fawn
*89	1	Oct. 15	Oct. 31	Limited quota; 275 licenses antlered deer
	6	Oct. 15	Oct. 31	Limited quota; 100 licenses doe or fawn
90	1	Oct. 15	Oct. 31	Limited quota; 75 licenses any deer
91, 92, 93, 94, 95, 96, 97		Oct. 15	Oct. 22	General license; antlered mule deer or any white-tailed deer
92	6	Oct. 15	Oct. 22	Limited quota; 50 license doe or fawn
94, 160	6	Oct. 15	Oct. 22	Limited quota; 50 licenses doe or fawn
96	6	Oct. 15	Oct. 22	Limited quota; 75 licenses doe or fawn
97	1	Nov. 1	Nov. 30	Limited quota; 25 licenses any white-tailed deer
	6	Nov. 1	Nov. 30	Limited quota; 100 licenses doe or fawn
98		Oct. 15	Oct. 22	General license; any deer, archery or muzzle-loading firearms only
100		Oct. 1	Oct. 8	General license; antlered deer
101	1	Oct. 15	Oct. 31	Limited quota; 75 licenses antlered deer
102	1	Oct. 15	Oct. 31	Limited quota; 400 licenses antlered deer
105		Oct. 15	Oct. 31	General license; antlered deer on national forest
		Nov. 1	Nov. 5	General license; antlered deer
		Nov. 1	Nov. 10	General license; antlerless deer off national forest
106		Oct. 15	Nov. 5	General license; antlered deer
109	1	Nov. 1	Nov. 15	Limited quota; 50 licenses antlered deer
	6	Nov. 15	Nov. 30	Limited quota; 25 licenses doe or fawn white-tailed deer

Hunt Area	Type	Date of Seasons Opens	Closes	Limitations
110		Oct. 15	Nov. 10	General license; antlered deer
111, 112, 113		Oct. 15	Nov. 10	General license; antlered deer on national forest
		Nov. 1	Nov. 10	General license; any deer off national forest
111	6	Oct. 15	Nov. 10	Limited quota; 150 licenses doe or fawn off national forest
112, 113	6	Oct. 15	Nov. 10	Limited quota; 250 licenses doe or fawn off national forest
114		Oct. 15	Nov. 10	General license; antlered deer
115		Sept. 10	Oct. 21	General license; antlered deer
116	1	Oct. 15	Oct. 31	Limited quota; 150 licenses any deer
	2	Oct. 15	Nov. 7	Limited quota; 40 licenses any white-tailed deer; also valid in Area 118
		Nov. 8	Nov. 15	Unused Area 116 Type 2 licenses valid for antlerless white-tailed deer; also valid in Area 118
	6	Oct. 15	Nov. 15	Limited quota; 100 licenses doe or fawn valid on private land
117	1	Oct. 1	Oct. 15	Limited quota; 50 licenses antlered deer
118	1	Oct. 15	Oct. 31	Limited quota; 75 licenses any deer
	6	Oct. 15	Oct. 31	Limited quota; 35 licenses doe or fawn
119	1	Nov. 1	Nov. 15	Limited quota; 100 licenses any deer
	6	Sept. 15	Nov. 15	Limited quota; 50 licenses doe or fawn
*120	1	Nov. 1	Nov. 15	Limited quota; 125 licenses any deer
	2	Nov. 15	Nov. 30	Limited quota; 25 licenses any white-tailed deer; also valid in Area 119
	6	Sept. 15	Nov. 15	Limited quota; 100 licenses doe or fawn valid on private land
	7	Nov. 1	Nov. 30	Limited quota; 35 licenses doe or fawn white-tailed deer
121, 122	1	Nov. 1	Nov. 30	Limited quota; 25 licenses any white-tailed deer
121		Nov. 1	Nov. 7	General license; antlered deer in that portion of Area 121 west of the Heart Mountain Canal and west of Wyoming Highway 294
		Nov. 1	Nov. 11	General license; any deer in that portion of Area 121 east of Heart Mountain Canal and east of Wyoming Highway 294
		Nov. 12	Nov. 18	General license; antlerless white-tailed deer in that portion of Area 121 east of Heart Mountain Canal and east of Wyoming Highway 294
122		Nov. 1	Nov. 11	General license; antlered deer off private land; any deer on private land
		Nov. 12	Nov. 18	General license; antlerless white-tailed deer
	6	Nov. 1	Nov. 30	Limited quota; 50 licenses doe or fawn white-tailed deer
123		Oct. 15	Nov. 2	General license; any deer
124		Nov. 1	Nov. 7	General license; antlered deer off private land, any deer on private land
		Nov. 8	Nov. 15	General license; antlerless deer on private land
	1	Nov. 1	Nov. 30	Limited quota; 50 licenses any white-tailed deer
	6	Oct. 1	Nov. 30	Limited quota; 200 licenses doe or fawn valid on private land
	7	Sept. 15	Nov. 30	Limited quota; 150 licenses doe or fawn valid on private land in that portion of Area 124 in Park County

Hunt Area	Type	Date of Seasons Opens	Closes	Limitations
125	1	Nov. 1	Nov. 15	Limited quota; 125 licenses any deer
	6	Sept. 1	Sept. 30	Limited quota; 25 licenses doe or fawn valid on private land in that portion of Area 125 in Washakie County
*127		Oct. 15	Oct. 31	General license; antlered deer off private land, any deer on private land
	6	Sept. 15	Nov. 15	Limited quota; 125 licenses doe or fawn valid on private land
128		Oct. 1	Oct. 22	General license; antlered deer
	1	Nov. 1	Nov. 20	Limited quota; 100 licenses any deer
	2	Nov. 1	Nov. 20	Limited quota; 50 licenses any white-tailed deer
	6	Nov. 1	Nov. 20	Limited quota; 100 licenses doe or fawn
	7	Nov. 1	Nov. 20	Limited quota; 150 licenses doe or fawn valid in Area 128, excluding the Wiggins Fork River, East Fork River and Torrey Creek drainages
130		Oct. 1	Oct. 8	General license; antlered deer
	1	Oct. 15	Oct. 31	Limited quota; 25 licenses antlered deer
	6	Nov. 1	Dec. 15	Limited quota; 10 licenses doe or fawn valid in that portion of Area 130 within Sweetwater County
131		Oct. 1	Oct. 8	General license; antlered deer
	2	Nov. 1	Nov. 11	Limited quota; 50 licenses antlerless deer valid in that portion of Area 131 west of the Lower Farson Cut Off Road (Sweetwater County Road 8) and the Blue Rim Road (Sweetwater County Road 5)
132		Oct. 1	Oct. 10	General license; antlered deer 4 points or more on either antler
133, 134, 135		Oct. 1	Oct. 10	General license; antlered deer
138, 139, 140		Sept. 15	Sept. 30	General license; antlered deer
		Oct. 1	Oct. 8	General license; any deer
		Oct. 9	Nov. 15	General license; any white-tailed deer
141, 162	1	Oct. 15	Oct. 31	Limited quota; 125 licenses any deer
142, 143, 144, 153		Sept. 15	Oct. 5	General license; antlered deer
145		Sept. 15	Sept. 30	General license; antlered deer
146, 149, 151, 152, 154, 155, 156		Sept. 15	Sept. 30	General license; any deer
		Oct. 1	Oct. 10	General license; antlered deer
148		Sept. 15	Oct. 25	General license; antlered deer
150		Sept. 15	Oct. 10	General license; any deer valid in that portion of Area 150 west of Wyoming Highway 390
		Oct. 1	Oct. 10	General license; archery only, any deer valid in that portion of Area 150 east of Wyoming Highway 390
157, 170	1	Oct. 15	Oct. 31	Limited quota; 250 licenses any deer
	2	Nov. 1	Nov. 30	Limited quota; 75 licenses any white-tailed deer
	6	Oct. 15	Oct. 19	Limited quota; 325 licenses doe or fawn
		Oct. 20	Oct. 31	Unused Areas 157, 170 Type 6 licenses valid on private land
	7	Oct. 15	Nov. 30	Limited quota; 100 licenses doe or fawn white-tailed deer

Hunt Area	Type	Date of Seasons Opens	Closes	Limitations
*158	1	Oct. 1	Oct. 14	Limited quota; 600 licenses any deer, also valid in Area 22
160		Oct. 15	Oct. 22	General license; antlered mule deer or any white-tailed deer
161		Oct. 15	Oct. 25	General license; antlered mule deer or any white-tailed deer
163		Oct. 15	Oct. 21	General license; antlered mule deer or any white-tailed deer
		Nov. 1	Nov. 15	General license; any white-tailed deer
*164		Oct. 1	Oct. 10	General license; any deer
	1	Nov. 1	Nov. 30	Limited quota; 25 licenses any white-tailed deer
	6	Oct. 1	Nov. 30	Limited quota; 75 licenses doe or fawn valid on private land
165	1	Oct. 15	Oct. 31	Limited quota; 100 licenses any deer
	2	Oct. 15	Nov. 7	Limited quota; 50 licenses any white-tailed deer
		Nov. 8	Nov. 15	Unused Area 165 Type 2 licenses valid for antlerless white-tailed deer
	6	Oct. 15	Nov. 15	Limited quota; 175 licenses doe or fawn valid on private land
167		Oct. 1	Oct. 5	General license; antlered deer
168		Oct. 1	Oct. 10	General license; antlered deer
169		Oct. 15	Oct. 21	General license; antlered mule deer or any white-tailed deer
		Nov. 1	Nov. 15	General license; any white-tailed deer
171		Oct. 15	Oct. 31	General license; any deer
	1	Nov. 1	Nov. 20	Limited quota; 35 licenses any white-tailed deer

*ASTERISK DENOTES A CWD HUNT AREA. PLEASE SEE IMPORTANT INFORMATION SECTION OF THE DEER REGULATION BROCHURE REGARDING CHRONIC WASTING DISEASE.

Section 4. Special Archery Seasons. Hunt Areas listed in this section shall have special archery hunting seasons during the dates specified in this section. Archers shall possess a general deer license or a limited quota deer license in addition to an archery license in order to hunt deer during any special archery season.

Archers with general deer licenses shall only hunt in those Hunt Areas specified in Section 3 as open to hunting with a general license and may take any deer.

Archers with limited quota deer licenses shall only hunt in the hunt area(s) and for the sex and species of deer set forth by the limitations of their license as specified in Section 3 of this Chapter.

Archers who possess resident general deer licenses or nonresident region B general deer licenses may archery hunt any deer during the special archery season in limited quota Hunt Areas 22 and 158. Archers who possess resident general deer licenses or nonresident region E licenses may archery hunt any deer during the special archery season in limited quota Hunt Area 34.

**Hunt Area**	**Date of Seasons Opens**	**Closes**
1-47, 49-53, 55, 57, 59-66, 70, 73-84, 87-98, 100-102, 105, 106, 109-114, 116-120, 124, 125, 127, 128, 130-135, 141, 157, 158, 160-165, 167-171. Curt Gowdy State Park in Area 60 closed.	Sept. 1	Sept. 30
138-140, 142-146, 148-156	Sept. 1	Sept. 14
115	Sept. 1	Sept. 9
121, 122, 123	Sept. 15	Oct. 14

Section 5. Nonresident Region General Deer License Quotas.

Nonresident region general deer licenses shall only be valid within the specific region for which they are issued and for only those Hunt Areas in the Region as listed in this section.
Quotas of nonresident region general deer licenses are established for the regions and shall not exceed the numbers specified in this section.

Region	Deer Hunt Areas	Quotas
A	1-6	5,000
B	7-14, 21, 167	2,300
C	17-20, 23-33, 163, 169	6,000
D	66, 70, 74-81, 83, 161	2,500
E	88, 91-98, 128, 148, 160, 171	800
F	105, 106, 110-115, 121-124, 127	1,450
G	135, 143-145	800
H	130, 138-140, 142, 146, 149-156	1,200
J	59, 61-65, 73	1,800
K	132-134, 168	500
M	35, 36, 38-40, 42, 43, 164	1,500
R	41, 44-47, 49-53	1,000
T	15, 16, 55, 57	500
W	82, 100, 131	1,200

Section 6. Hunt Area Descriptions.

(a) Area and Number.

Area 1. Crook. Beginning where Wyoming Highway 112 crosses the Wyoming-Montana state line; southwesterly along said highway to Wyoming Highway 24 in the town of Hulett; southerly along said highway to U.S. Highway 14; westerly along said highway to the Cabin Creek Road; northwesterly along said road to Oshoto and the Oshoto Road connection; westerly along said road to the "D" Road; northerly along said road to the Rocky Point Road; northerly along said road to the Wyoming-Montana state line.

Area 2. Bearlodge. Beginning where Wyoming Highway 112 crosses the Wyoming-Montana state line; easterly along said line to the Wyoming-South Dakota state line; southerly along said line to Interstate Highway 90; southwesterly along said highway to the town of Sundance and the interchange with U.S. Highway 14; westerly and northerly along said highway to Wyoming Highway 24; northerly along said highway to Wyoming Highway 112 at the town of Hulett; northeasterly along said highway to the Wyoming-Montana state line.

Area 3. Keyhole. Beginning at the intersection of Interstate Highway 90 and U.S. Highway 14 in the town of Sundance; southerly and westerly along Interstate Highway 90 to its intersection with the "D" Road; northerly along said road to the Oshoto Road Connection; easterly along said road to Oshoto and the Cabin Creek Road; southerly and easterly along the Cabin Creek Road to U.S. Highway 14; easterly and southerly along said highway to Interstate Highway 90 in the town of Sundance.

Area 4. Sand Creek. Beginning where Interstate Highway 90 intersects the Wyoming-South Dakota state line; southerly along said line to U.S. Highway 85; southerly along said highway to Wyoming Highway 585 at Four Corners; northerly and westerly along said highway to the town of Sundance and Interstate Highway 90; northerly and easterly along said highway to the Wyoming-South Dakota state line.

Area 5. Inyan Kara. Beginning where Interstate Highway 90 intersects Wyoming Highway 585 in the town of Sundance; southeasterly along Wyoming Highway 585 to the Skull Creek Road; westerly along said road to the Green Mountain Road; northerly along said road to the Dry Creek Road; westerly and northerly along said road

to Wyoming Highway 116; westerly and southerly along said highway to U.S. Highway 16 in the town of Upton; northwesterly along said highway to Interstate Highway 90 at the town of Moorcroft; easterly and northerly along said highway to Wyoming Highway 585 at the town of Sundance.

Area 6. Skull Creek. Beginning where U.S. Highway 85 intersects the Wyoming-South Dakota state line; southerly along said line to U.S. Highway 16; northwesterly along said highway to Wyoming Highway 116 in the town of Upton; northerly and easterly along said highway to the Dry Creek Road; southerly and easterly along said road to the Green Mountain Road; southerly along said road to the Skull Creek Road; easterly along said road to Wyoming Highway 585; southerly and easterly along said highway to U.S. Highway 85; northerly along said highway to the Wyoming-South Dakota state line.

Area 7. South Osage. Beginning at the junction of U.S. Highway 16 and Wyoming Highway 450; southwesterly along Wyoming Highway 450 to Wyoming Highway 116; northerly along said highway to U.S. Highway 16 in the town of Upton; southeasterly along said highway to Wyoming Highway 450.

Area 8. Upton-Four Horse. Beginning at the junction of Wyoming Highway 116 and Wyoming Highway 450; westerly along Wyoming Highway 450 to the Keeline Road (U.S.F.S. Road 930); northwesterly along said road to Black Thunder Creek; easterly down said creek to Bacon Creek; northerly up said creek to Newel Prong; northerly up said prong to the Cheyenne River/Belle Fourche River Divide; northeasterly along said divide to the divide between Four Horse Creek and Raven Creek; northwesterly along said divide to the Belle Fourche River; northeasterly down said river to U.S. Highway 16; southeasterly along said highway to Wyoming Highway 116 at the town of Upton; southerly along said highway to Wyoming Highway 450.

Area 9. Blacktail Creek. Beginning where U.S. Highway 16 crosses the Wyoming-South Dakota state line; southerly along said line to the Dewey Road; northwesterly along said road to U.S. Highway 85; southerly along said highway to the Cheyenne River Road N. (Weston County); westerly along said road to the Lynch Road; northerly along said road to Wyoming Highway 450; northeasterly along said highway to U.S. Highway 16; southeasterly along said highway to the Wyoming-South Dakota state line.

Area 10. Teckla. Beginning at the junction of Wyoming Highway 450 and Wyoming Highway 59; easterly along Wyoming Highway 450 to the Lynch Road; southerly along said road to the Campbell-Converse county line and the Clareton Road (Converse County Road 39); southwesterly along said road to the Dull Center Road (Converse County Road 38); westerly along said road to the Rochelle Hills Road (U.S.F.S. Road 933); northerly and westerly along said road to the North Antelope Road (U.S.F.S. Road 949); northerly and westerly along said road to the Steckley Road (U.S.F.S. Road 942); westerly along said road to Wyoming Highway 59; northerly along said highway to Wyoming Highway 450.

Area 11. Mule Creek. Beginning where the Dewey Road crosses the Wyoming-South Dakota state line; southerly along said line to U.S. Highway 18; westerly along said highway to the Old Highway 85 Road; southwesterly along said road to U.S. Highway 85; southerly along said highway to the Wasserburger Road; westerly along said road to the North Lance Creek Road; northwesterly along said road to the Niobrara-Converse county line and the Dull Center Road (Converse County Road 38); westerly along said road to the Clareton Road (Converse County Road 39); northeasterly along said road to the Converse-Weston county line and the Lynch Road; northerly along said road to the Cheyenne River Road N. (Weston County); easterly along said road to U.S. Highway 85; northerly along said highway to the Dewey Road; southeasterly along said road to the Wyoming-South Dakota state line.

Area 12. Hat Creek. Beginning where U.S. Highway 18 intersects the Wyoming-South Dakota state line; southerly along said line to the Wyoming-Nebraska state line; southerly along said line to the Hat Creek Road; westerly along said road to U.S. Highway 85; northerly along said highway to Wyoming Highway 270; westerly along said highway to Wyoming Highway 272; northerly along said highway to the North Lance Creek Road; northerly along said road to the Wasserburger Road; easterly along said road to U.S. Highway 85; northerly along said highway to the Old Highway 85 Road; northeasterly along said road to U.S. Highway 18; easterly along said highway to the Wyoming-South Dakota state line.

Area 13. Lusk. Beginning where the Hat Creek Road intersects the Wyoming-Nebraska state line; southerly along said line to U.S. Highway 20; westerly along said highway to the Twenty Mile Creek Road (Converse County Road 46) at the town of Lost Springs; northerly along said road to the Manning Road (Converse County Road 53); easterly along said road to the Twenty Mile Road (Niobrara County) at the Converse-Niobrara county line; easterly along said road to Wyoming Highway 271; easterly along said highway to Wyoming Highway 270 at the town of Lance Creek; northerly and easterly along said highway to U.S. Highway 85; southerly along said highway to the Hat Creek Road; easterly along said road to the Wyoming-Nebraska state line.

Area 14. Twenty Mile. Beginning at the junction of Wyoming Highway 59 and the Dull Center Road (Converse County Road 38); easterly along said road to the East Bill Road (U.S.F.S. Road 958); easterly along said road to U.S.F.S. Road 958C; southeasterly along said road to the Iberlin Ranch Road; southerly along said road to Dry Creek; easterly down said creek to the Walker Creek Road (Converse County Road 43); easterly along said road to the Converse-Niobrara county line and the Walker Creek-Cow Creek Interconnect Road; northerly along said road to the Converse-Niobrara county line and the Cow Creek Road (Converse County Road 45); northerly along said road to the Tillard Ranch Road; easterly along said road to the Niobrara-Converse county line and the Tillard Ranch-Leverett Road; easterly along said road to the Cow Creek Road (Niobrara County); northerly along said road to the North Lance Creek Road; southerly along said road to Wyoming Highway 272; southerly along said highway to Wyoming Highway 270; southwesterly along said highway to the town of Lance Creek and Wyoming Highway 271; westerly along said highway to the Twenty Mile Road; westerly along said road to the Niobrara-Converse county line and the Manning Road (Converse County Road 53); southwesterly along said road to the Twenty Mile Creek Road (Converse County Road 46); southerly along said road to U.S. Highway 20 at the town of Lost Springs; southwesterly along said highway to Interstate Highway 25; southerly along said highway to the North Platte River; westerly and northerly up said river to Wyoming Highway 59 at the town of Douglas; northerly along said highway to the Dull Center Road.

Area 15. South Keeline. Beginning where U.S. Highway 20 intersects the Wyoming-Nebraska state line; southerly along said line to the Niobrara-Goshen county line trail and road (Goshen County Road 197); westerly along said trail and road to the Cundall Ranch Road (Goshen County Road 235); southwesterly along said road to U.S. Highway 85; northerly along said highway to the Niobrara-Goshen county line; westerly along said line to the radar tower and the Radar Tower Road (Goshen County Road 238); westerly along said road to Wyoming Highway 270; southerly along said highway to the Jireh Road (Platte County Road 201); westerly along said road to the Flat Top Road (Platte County Road 121); westerly along said road to the Cedar Top Road (Platte County Road 175); northerly along said road to Spanish Creek; westerly down said creek to Muddy Creek; southerly down said creek to Glendo Reservoir and the North Platte River; northwesterly up said river to Interstate Highway 25; northerly along said highway to U.S. Highway 20 at Orin Junction; easterly along said highway to the Wyoming-Nebraska state line.

Area 16. Meadowdale/Jay Em. Beginning where U.S. Highway 26 crosses the Wyoming-Nebraska state line; westerly along said highway to the North Platte River; up said river to Muddy Creek; northerly up said creek to Spanish Creek; easterly up said creek to the Cedar Top Road (Platte County Road 175); southerly along said road to the Flat Top Road (Platte County Road 121); easterly along said road to the Jireh Road (Platte County Road 201); easterly along said road to Wyoming Highway 270; northerly along said highway to the Radar Tower Road (Goshen County Road 238); easterly along said road to the radar tower and the Niobrara-Goshen county line; easterly along said line to U.S. Highway 85; southerly along said highway to the Cundall Ranch Road (Goshen County Road 235); northeasterly along said road to the Niobrara-Goshen county line trail and road (Goshen County Road 197); east along said trail and road to the Wyoming-Nebraska state line; south along said line to U.S. Highway 26.

Area 17. Northwest Gillette. Beginning where the Buffalo Creek Road intersects the Wyoming-Montana state line; southerly along said road to the Beason Road; southerly along said road to the Recluse Road; southerly along said road to U.S. Highway 14-16; southeasterly along said highway to Interstate Highway 90; westerly along said highway to the Powder River; northerly down said river to the Wyoming-Montana state line; easterly along said line to the Buffalo Creek Road.

Area 18. Campbell. Beginning where the Rocky Point Road crosses the Wyoming-Montana state line; southerly along said road to the “D” Road; southerly along said road to Interstate Highway 90; westerly along said highway to U.S. Highway 14-16; northerly along said highway to the Recluse Road; northerly along said road to the

Beason Road; northerly along said road to the Buffalo Creek Road; northerly along said road to the Wyoming-Montana state line; easterly along said line to the Rocky Point Road.

Area 19. Pumpkin Buttes. Beginning at the intersection of Interstate Highway 90 and the 4-J Road at the city of Gillette; southerly along said road to Wyoming Highway 50; southwesterly along said highway to Wyoming Highway 387; southwesterly along said highway to Wyoming Highway 192; northwesterly along said highway to the Streeter Road (Johnson County Road 135); northerly along said road to the Powder River; northerly down said river to Interstate Highway 90; easterly along said highway to the intersection of Interstate Highway 90 and the 4-J Road at the city of Gillette.

Area 20. Clarkelen. Beginning at the intersection of Interstate Highway 90 and Wyoming Highway 59 at the city of Gillette; southerly along said highway to Wyoming Highway 387; southwesterly along said highway to Wyoming Highway 50; northeasterly along said highway to the 4-J Road; northeasterly along said road to Interstate Highway 90; easterly along said highway to Wyoming Highway 59.

Area 21. Thunder Basin. Beginning where Interstate Highway 90 crosses the Belle Fourche River; southwesterly up said river to the divide between Four Horse Creek and Raven Creek; southerly along said divide to the divide between the Cheyenne River and Belle Fourche River; southwesterly along said divide to Newel Prong; southerly down said prong to Bacon Creek; southerly down said creek to Black Thunder Creek; westerly up said creek to the Keeline Road (U.S.F.S Road 930); southeasterly along said road to Wyoming Highway 450; westerly along said highway to Wyoming Highway 59; northerly along said highway to Interstate Highway 90 at the city of Gillette; easterly along said highway to the Belle Fourche River.

Area 22. Douglas. Beginning at the intersection of the North Platte River and Wyoming Highway 95 at the town of Glenrock; northeasterly along said highway to the 55 Ranch Road (Converse County Road 23); northerly along said road to the Sage Creek Divide-Blizzard Heights Road; easterly along said road to the Ross Road (Converse County Road 31); northwesterly along said road into Campbell County and to Wyoming Highway 387; northeasterly along said highway to Wyoming Highway 59 at Reno Junction; southerly along said highway to the North Platte River at the town of Douglas; westerly up said river to Wyoming Highway 95.

Area 23. Clearmont. Beginning where the Powder River crosses the Wyoming-Montana state line; southerly up said river to U.S. Highway 14; westerly along said highway to the Meade Creek Road (Sheridan County Road 131); southwesterly along said road to Interstate Highway 90; northerly along said highway to Wyoming Highway 338; northeasterly along said highway to the Wyoming-Montana state line; easterly along said line to the Powder River.

Area 24. Sheridan. Beginning where Wyoming Highway 338 crosses the Wyoming-Montana state line; southerly along said highway to Interstate Highway 90; southerly along said highway to the Piney Creek interchange and U.S. Highway 87; northwesterly along said highway to Wyoming Highway 193; northwesterly along said highway to South Piney Creek; westerly up said creek to the Bighorn National Forest boundary; northwesterly along said boundary to the Wyoming-Montana state line; easterly along said line to Wyoming Highway 338.

Area 25. Tongue-Goose. Beginning where the Wyoming-Montana state line and the east boundary of the Bighorn National Forest intersect; southeasterly along said boundary to South Piney Creek; southwesterly up said creek to the divide along the Big Horn Mountain range; northwesterly along said divide to U.S. Highway 14 at Granite Pass; northerly along said highway to the Hunt Mountain Road (U.S.F.S. Road 10); northwesterly along said road to U.S. Highway 14A; northwesterly along said highway to the Devils Canyon Road (U.S.F.S. Road 14); northerly along said road to the Sheep Mountain Road (U.S.F.S. Road 11); northerly along said road to the Wyoming-Montana state line; easterly along said line to the east boundary of the Bighorn National Forest.

Area 26. Ucross. Beginning where U.S. Highway 14 crosses the Powder River; southerly up said river to Interstate Highway 90; northwesterly along said highway to the Meade Creek Road (Sheridan County Road 131); northeasterly along said road to U.S. Highway 14; easterly along said highway to the Powder River.

Area 27. Buffalo. Beginning where Wyoming Highway 193 crosses South Piney Creek; southeasterly along said highway to U.S. Highway 87; southeasterly along said highway to Interstate Highway 90; southerly along

said highway to Interstate Highway 25; southerly along said highway to the Trabing Road (Johnson County Road 13); westerly along said road to Wyoming Highway 196; southerly along said highway to the Crazy Woman Canyon Road (Johnson County Road 14); westerly along said road to the Bighorn National Forest boundary; northerly along said boundary to South Piney Creek; easterly down said creek to Wyoming Highway 193.

Area 28. Hunter Mesa. Beginning where South Piney Creek crosses the east boundary of the Bighorn National Forest; southerly and westerly along said boundary to the Gold Mine Road (U.S.F.S. Road 452); northerly along said road to the Canyon Creek Road (U.S.F.S. Road 25); northerly along said road to U.S. Highway 16 and the divide along the Big Horn Mountain range at Powder River Pass; northerly along said divide to the head of South Piney Creek; northeasterly down said creek to the east boundary of the Bighorn National Forest.

Area 29. Johnson. Beginning where Interstate Highway 90 crosses the Powder River; southerly up said river to the Streeter Road (Johnson County Road 135); southerly along said road to Wyoming Highway 192; southeasterly along said highway to the West Sussex Oil Field Road at the town of Linch; westerly along said road to old U.S. Highway 87; west from the junction of the West Sussex Oil Field Road and old U.S. Highway 87 to Interstate Highway 25; northerly along said highway to Interstate Highway 90; southeasterly along said highway to the Powder River.

Area 30. Upper Powder River. Beginning at the junction of the Trabing Road (Johnson County Road 13) and Interstate Highway 25; southerly along Interstate Highway 25 to the point east of the U.S. Highway 196-Mesa Road (Johnson County Road 115) junction; west to the U.S. Highway 196-Mesa Road junction; southwesterly along the Mesa Road to the Mayoworth Road (Johnson County Road 266) at the town of Mayoworth; westerly along said road to the Slip Road (Johnson County Road 67); westerly along said road to the Hazelton Road (Johnson County Road 3); northerly along said road to the Gold Mine Road (U.S.F.S. Road 452); northwesterly along said road to the Bighorn National Forest boundary; easterly and northerly along said boundary to the Crazy Woman Canyon Road (Johnson County Road 14); easterly along said road to Wyoming Highway 196; northerly along said highway to the Trabing Road; easterly along said road to Interstate Highway 25.

Area 31. Salt Creek. Beginning at the junction of the West Sussex Oil Field Road and Wyoming Highway 192 at the town of Linch; southeasterly along said highway to Wyoming Highway 387; southwesterly along said highway to Interstate Highway 25; northwesterly along said highway to the point on Interstate Highway 25 due west of the junction of old U.S. Highway 87 and the West Sussex Oil Field Road; east from Interstate Highway 25 to the junction of old U.S. Highway 87 and the West Sussex Oil Field Road; easterly along said road to Wyoming Highway 192 at the town of Linch.

Area 32. Beartrap Creek. Beginning where the Slip Road (Johnson County Road 67) crosses Arch Creek west of the town of Mayoworth; southerly down said creek to Beartrap Creek; southerly down said creek to the North Fork of the Red Fork of the Powder River; southeasterly down said river to the Ken Graves Ranch Road; southerly down said road to the Barnum Road (Johnson County Road 78); southerly along said road to the Barnum Mountain Road; westerly along said road to the Hazelton Road (Washakie County Road 81-Johnson County Road 3); northerly along said road to the Slip Road; southeasterly along said road to Arch Creek.

Area 33. Red Fork. Beginning at the town of Kaycee and Interstate Highway 25; southeasterly along said highway to the South Fork of the Powder River; southerly up said river to Willow Creek; southwesterly up said creek to the Lone Bear Road (Natrona County Road 112); northerly along said road to the Willow Creek Road (Natrona County Road 111); southwesterly along said road to the 33 Mile Road (Natrona County Road 110); northwesterly along said road to Buffalo Creek; northerly down said creek to the Middle Fork of the Powder River; westerly up said river to the Outlaw Cave Access Road; northerly along said road to the Barnum Road (Johnson County Road 78) at the town of Barnum; northerly along said road to the Ken Graves Ranch Road; northerly along said road to the North Fork of the Red Fork of the Powder River at the Ken Graves Ranch; northwesterly up said river to Beartrap Creek; northwesterly up said creek to Arch Creek; northerly up said creek to the Slip Road (Johnson County Road 67); easterly along said road to the Mayoworth Road (Johnson County Road 266); easterly along said road to the Mesa Road (Johnson County Road 115) at Mayoworth; northeasterly along said road to Wyoming Highway 196; due east from the junction of the Mesa Road and Wyoming Highway 196 to the Interstate Highway 25; southerly along said highway to the town of Kaycee.

Area 34. Natrona. Beginning at the intersection of Interstate Highway 25 and U.S. Highway 20-26 in the city of Casper; westerly along U.S. Highway 20-26 to the Waltman-Arminto Road (Natrona County Road 104); northerly along said road to Natrona County Road 105; northerly along said road to the E-K Stock Trail; northerly along said trail to the Bighorn Trail Road; northerly along said road to the divide between the Middle Fork of the Powder River and North Buffalo Creek; easterly and northerly along said divide to the Big Horn Mountain Road (Natrona County Road 109); northeasterly along said road to the 33 Mile Road (Natrona County Road 110); southeasterly along said road to the Wild Horse Trail Road (Natrona County Road 113); easterly along said road to the Dead Horse Road (Natrona County Road 114); northeasterly along said road to the Smoky Gap Road (Natrona County Road 115); easterly along said road to U.S. Highway 387; easterly along said highway to Wyoming Highway 259 at the town of Midwest; southerly along said highway to Interstate Highway 25; southerly along said highway to U.S. Highway 20-26 in the city of Casper.

Area 35. Upper Nowood. Beginning at Cottonwood Pass and the Nowood Road; northeasterly along said road to Deep Creek; up said creek to Lost Creek; up said creek to the Cherry Creek Hill Stock Drive Road; northeasterly along said road to the Hazelton Road (Big Horn Mountain Road); southerly along said road to the divide between the Middle Fork of the Powder River and the north fork of Buffalo Creek; southwesterly along said divide to the Big Horn Trail; southerly and westerly along said trail to the Oakie Road (Natrona County Road 101); westerly along said road to Sioux Pass and the Devil's Slide Road; westerly along said road to the divide between Nowood Creek and Badwater Creek; southwesterly then northwesterly along said divide to the Nowood Road at Cottonwood Pass.

Area 36. Badwater. Beginning at the town of Shoshoni and U.S. Highway 20-26; westerly along said highway to the east shoreline of Boysen Reservoir; northerly along said shoreline to Boysen Dam and the Wind River; northerly down said river to Gold Creek (excluding Indian tribal trust lands immediately north of U.S. Highway 20-26 near Boysen Reservoir and northeast of Boysen Dam); easterly up Gold Creek to the divide between Buffalo Creek and Badwater Creek on Copper Mountain; easterly along said divide to West Bridger Creek; easterly down said creek to the Kirby Creek Road (Hot Springs County Road 7); northerly along said road to Little V-H Draw; northeasterly up said draw to the Packsaddle Road; southerly along said road to the Bridger Creek Road; northeasterly along said road to the Pine Creek Road at the Gardner Summer Ranch; easterly up said road to the Seaman Ranch Road; easterly, southerly, then easterly along said road to the divide between Nowood Creek and Badwater Creek at Cottonwood Pass; southeasterly then northeasterly along said divide to the Devil's Slide Road; easterly along said road to Sioux Pass and the Oakie Trail (Natrona County Road 101); easterly along said trail to the Big Horn Trail; easterly along said trail to the E-K Stock Trail; southerly along said trail to the Buffalo Creek Road (Natrona County Road 105); southerly along said road to the Arminto Road (Natrona County Road 104); southerly along said road to Waltman and U.S. Highway 20-26; westerly along said highway to the town of Shoshoni.

Area 37. Copper Mountain. Beginning at the confluence of the Big Horn River and Walter's Draw; easterly up said draw to the divide between Nowater Creek and Kirby Creek; easterly along said divide to the Murphy Dome-Mud Creek Road (BLM Road 1409); southerly along said road to Wyoming Highway 172 (Black Mountain Road); westerly along said highway to Kirby Creek Road (Hot Springs County Road 7); southeasterly along said road to West Bridger Creek; westerly up said creek to the divide between Buffalo Creek and Badwater Creek; westerly along said divide to the head of Gold Creek; westerly down said creek to the east boundary of the Wind River Indian Reservation; north and west along said boundary to the Big Horn River; northerly down said river to Walter's Draw.

Area 38. Black Mountain. Beginning at the intersection of the Kirby Creek Road (Hot Springs County Road 7) and Wyoming Highway 172 (Black Mountain Road); easterly along said highway to Murphy Dome-Mud Creek Road (BLM Road 1409); northeasterly along said road to the Nowater Stock Drive Road (BLM Road 1404); easterly along said road to the Seaman Ranch Road (Lysite Mountain Road); southerly along said road to the Pine Creek Road; westerly along said road to the Bridger Creek Road at the Gardner Summer Ranch; southwesterly along said road to the Packsaddle Road; northerly along said road to the head of Little V-H Draw; southwesterly down said draw to the Kirby Creek Road (Hot Springs County Road 7); northerly along said road to Wyoming Highway 172.

Area 39. Bates Creek. Beginning at the junction of the Upper Nowood Road and the Seaman Ranch Road (Lysite Mountain Road) at Cottonwood Pass; westerly then northerly along the Seaman Ranch Road (Lysite Mountain Road) to the Nowater Stock Drive Road (BLM Road 1404); southeasterly along said road to the Hampton Stock Drive Road; easterly along said road to the Hampton Ranch and the Upper Nowood Road; southerly along said road to the Seaman Ranch Road (Lysite Mountain Road).

Area 40. Buffalo Creek. Beginning at the junction of the Upper Nowood Road and the Dry Farm Road (Big Trails Stock Drive Road); easterly along said road to the Hazelton Road (Big Horn Divide Road); southerly along said road to the Cherry Creek Hill Stock Drive Road; westerly along said road to Lost Creek; northwesterly down said creek to Deep Creek; northerly down said creek to the Upper Nowood Road; northeasterly along said road to the Hampton Stock Drive Road (SV Draw); westerly along said road to the Nowater Stock Drive Road (BLM Road 1404); northwesterly along said road to Buffalo Creek; northeasterly down said creek to the Nowood River; southerly up said river to Crooked Creek; southeasterly up said creek to the Upper Nowood Road; southerly along said road to Dry Farm Road (Big Trails Stock Drive Road).

Area 41. Sand Creek. Beginning at the town of Hyattville; southerly along the Hyattville-Ten Sleep Road (Washakie County Road 54) to U.S. Highway 16; westerly along said highway to U.S. Highway 20-Wyoming Highway 789; southerly along said highway to its junction with the Big Horn Canal three quarter mile south of the town of Worland; northerly along said canal to U.S. Highway 16-20; easterly along said highway to Wyoming Highway 31; easterly along said highway to the town of Hyattville.

Area 42. Otter Creek. Beginning at the intersection of U.S. Highway 16 and the Bighorn National Forest western boundary; southerly then easterly along said boundary to the Hazelton Road (Big Horn Mountain Divide Road); southerly along said road to the Dry Farm Road (Big Trails Road Stock Drive Road); westerly along said road to the Upper Nowood Road; northerly along said road to Crooked Creek; northwesterly down said creek to the Nowood River; northerly down said river to Buffalo Creek; southwesterly up said creek to the Nowater Stock Drive Road (BLM Road 1404); northwesterly along said road to the Bluebank Road (BLM Road 1411); northerly along said road to U.S. Highway 16; easterly along said highway to the Bighorn National Forest boundary.

Area 43. Leigh Creek. Beginning at the intersection of U.S. Highway 16 and the Gold Mine Road (U.S.F.S. Road 25 and 452); southerly along said road to the Bighorn National Forest boundary; westerly along said boundary to U.S. Highway 16; northeasterly then easterly along said highway to the Gold Mine Road (U.S.F.S. Road 25 and 452).

Area 44. Brokenback Creek. Beginning at Washakie County Road 54 in the town of Ten Sleep; northerly along said road to the Hyattville Logging Road (BLM Road 1117); southeasterly along said road to the Bighorn National Forest boundary; southerly along said boundary to U.S. Highway 16; southwesterly along said highway to the town of Ten Sleep.

Area 45. Paint Rock Creek. Beginning at the intersection of Wyoming Highway 31 and the Cold Springs Road (becomes BLM/U.S.F.S. Road 344 at forest boundary) near the town of Hyattville; northeasterly along said road to the Bighorn National Forest boundary; southeasterly along said boundary to the Hyattville Logging Road (becomes U.S.F.S. Road 408 at forest boundary) (BLM Road 1117); westerly along said road to Washakie County Road 54; northerly along said road to the town of Hyattville.

Area 46. Cloud Peak. Beginning at Granite Pass on U.S. Highway 14 and the divide along the Bighorn Mountain range; southeasterly along said divide to the U.S. Highway 16 at Powder River Pass; westerly along said highway to the Bighorn National Forest Boundary; northerly along said boundary to the Alkali Road (U.S.F.S. Road 338); easterly along said road to the Shell Ranger Station-Paintrock Lakes Road (U.S.F.S. Road 17); northwesterly along said road to U.S. Highway 14; northerly along said highway to Granite Pass.

Area 47. Manderson. Beginning at the junction of U.S. Highway 16-20 and U.S. Highway 14 in the town of Greybull; easterly along U.S. Highway 14 to the Red Gulch Road (BLM Road 1109); southeasterly along said road to the Alkali Road (BLM Road 1111); northeasterly along said road to the Bighorn National Forest boundary; southeasterly along said boundary to the Cold Springs Road (BLM/U.S.F.S. Road 344); southwesterly along said

road to Wyoming Highway 31; westerly along said highway to U.S. Highway 16-20; northerly along said highway to U.S. Highway 14 in the town of Greybull.

Area 49. Trapper Creek. Beginning at the town of Shell on U.S. Highway 14; easterly along said highway to the Shell Ranger Station-Paint Rock Lakes Road (U.S.F.S. Road 17); southeasterly along said road to the Alkali Road (U.S.F.S. Road 338); southwesterly along said road to the Red Gulch Road (BLM Road 1109); westerly then northwesterly along said road to U.S. Highway 14; easterly along said highway to the town of Shell.

Area 50. Upper Shell-Beaver Creek. Beginning at the Bighorn National Forest boundary and U.S. Highway 14A; easterly along said highway to the Hunt Mountain Road (U.S.F.S. Road 10); southwesterly along said road to U.S. Highway 14; southerly then westerly along said highway to the Bighorn National Forest boundary; northwesterly along said boundary to U.S. Highway 14A.

Area 51. Beaver Creek. Beginning at the Big Horn River in the town of Greybull; northerly down said river to Bear Creek; northeasterly up said creek to the Bighorn National Forest boundary; southeasterly along said boundary to U.S. Highway 14; westerly along said highway to the Big Horn River.

Area 52. Crystal Creek. Beginning at the Big Horn River and U.S. Highway 14A nine (9) miles east of the town of Lovell; easterly along said highway to the Bighorn National Forest boundary; southeasterly along said boundary to Bear Creek; southwesterly down said creek to the Big Horn River; northerly down said river to U.S. Highway 14A.

Area 53. Devils Canyon. Beginning where U.S. Highway 14A crosses the Big Horn River; northerly down said river to the Wyoming-Montana state line; easterly along said line to the Sheep Mountain Road (U.S.F.S. Road 11); southerly along said road to the Devils Canyon Road (U.S.F.S. Road 14); southerly along said road to U.S. Highway 14A; westerly along said highway to the Big Horn River.

Area 55. Rock Eagle. Beginning where U.S. Highway 26 crosses the Wyoming-Nebraska state line; south along said line to Wyoming Highway 151; westerly along said highway to U.S. Highway 85; northerly along said highway to Wyoming Highway 313; westerly along said highway to the town of Chugwater and Interstate Highway 25; northerly along said highway to U. S. Highway 26; easterly along said highway to the Wyoming-Nebraska state line.

Area 57. Southeast. Beginning at the junction of Wyoming Highway 151 and the Wyoming-Nebraska state line; south along said line to the Wyoming-Colorado state line; west along said line to Interstate Highway 25; northerly along said highway to the town of Chugwater and Wyoming Highway 313; easterly along said highway to U.S. Highway 85; southeasterly along said highway to Wyoming Highway 151; easterly along said highway to the Wyoming-Nebraska state line.

Area 59. Little Bear. Beginning at the junction of Wyoming Highway 211 and Interstate Highway 25 near the town of Chugwater; southerly along Interstate Highway 25 to Wyoming Highway 211 near the city of Cheyenne; northwesterly along said highway to the Horse Creek-Iron Mountain Road (Laramie County Road 106-2-Platte County Road 10); northeasterly along said road to Wyoming Highway 211; northeasterly along said road to Interstate Highway 25.

Area 60. Pole Mountain. Beginning in the city of Laramie and the Rogers Canyon-Ninth Street Road (Albany County Road 17-Laramie County Road 228); northeasterly along said road to Wyoming Highway 211 (Horse Creek-Chugwater Road); southeasterly along said highway to Interstate Highway 25; southerly along said highway to Interstate Highway 80; westerly along said highway to the city of Laramie.

Area 61. Harriman. Beginning at the city of Laramie and Interstate Highway 80; southeasterly along said highway to Interstate Highway 25 near the city of Cheyenne; southerly along said highway to the Wyoming-Colorado state line; west along said line to U.S. Highway 287; northerly along said highway to Interstate Highway 80.

Area 62. Iron Mountain. Beginning at the junction of U.S. Highway 30 and Wyoming Highway 34; northeasterly along Wyoming Highway 34 to the Sybille Canyon Road (Albany County Road 12); easterly along said road to the Bell Ranch Road; southeasterly along said road to the McLees Road (Laramie County Road 103-1); southeasterly along said road to Wyoming Highway 211; southerly along said highway to the Fisher Canyon-Rogers Canyon Road (Laramie County Road 228-1-Albany County Road 17) at Horse Creek Station; westerly along said road to U.S. Highway 30; northerly along said highway to Wyoming Highway 34.

Area 63. Richeau. Beginning at the junction of Wyoming Highway 34 and the Sybille Canyon Road (Albany County Road 12); northeasterly along said highway to Interstate Highway 25; southerly along said highway to Wyoming Highway 211; southwesterly along said highway to Platte County Road 210-Laramie County Road 103; southwesterly along said road to the McLees Road (Laramie County Road 103); westerly along said road to the Bell Ranch Road; northwesterly along said road to the Sybille Canyon Road (Albany County Road 12); southerly and westerly along said road to Wyoming Highway 34.

Area 64. Laramie Peak. Beginning at the junction of Interstate Highway 25 and Wyoming Highway 34; southwesterly along Highway 34 to the Flat Top-Round Top Road west of Morton Pass; northwesterly along said road to the Big Laramie River (at the steel bridge); northerly down said river to the Dodge Ranch Road; northerly along said road to the Garrett Road (Albany County Road 713); northwesterly along said road to the Bear Creek Road (Albany County Road 77); northerly along said road to U.S.F.S. Road 671; northerly and westerly along said road to the Esterbrook Road (Albany County Road 710-Converse County Road 5 and 7-U.S.F.S. Road 653); northeasterly along said road to the Glendo-Esterbrook Road (Ridge Road; Converse County Road 6-Platte County Road 135); easterly along said road to the Glendo Park Road (Platte County Road 17) at the town of Glendo; easterly along said road to the North Platte River; southeasterly down said river to U.S. Highway 26; westerly along said highway to Interstate Highway 25; southerly along said highway to Wyoming Highway 34.

Area 65. South Converse. Beginning where Wyoming Highway 95 crosses the North Platte River at the town of Glenrock; easterly and southerly down said river to the Glendo Reservoir Dam and the Glendo Park Road (Platte County Road 17); westerly along said road to the town of Glendo and the Glendo-Esterbrook Road (Ridge Road; Platte County Road 135-Converse County Road 6); westerly along said road to the Esterbrook Road (Converse County Road 5 and 7-U.S.F.S. Road 653-Albany County Road 710); southwesterly along said road to the divide between Horseshoe Creek and the North Laramie River; westerly and northerly along said divide to the head of South Fork Creek, a tributary of LaBonte Creek; northeasterly along said creek to U.S.F.S. Road 658; westerly along said road to the Fetterman Road (Albany County Road 61); northerly along said road to the divide between Corduroy Creek and Rocky Ford Creek; westerly along said divide to the divide between LaPrele Creek and the Little Medicine Bow River; westerly along said divide to the Cold Springs Road (Converse County Road 24); southwesterly along said road to U.S.F.S. Road 660; northwesterly along said road to Curry Creek; northwesterly down said creek to Deer Creek; northerly down said creek to the Stephens Road (Converse County Road 20); northerly along said road to the Deer Creek Road (Converse County Road 19); northerly along said road to Wyoming Highway 95; northerly along said highway to the North Platte River at the town of Glenrock.

Area 66. Bates Hole-Hat Six. Beginning where Wyoming Highway 95 crosses the North Platte River at the town of Glenrock; southerly along said highway to the Deer Creek Road (Converse County Road 19); southerly along said road to the Stephens Road (Converse County Road 20); southerly along said road to Deer Creek; southerly up said creek to Curry Creek; southeasterly up said creek to U.S.F.S. Road 660; westerly along said road to the Squaw Spring Trail Road; southwesterly along said road to the divide between Spring Creek and the Bates Creek drainage; westerly along said divide to the divide between the Bates Creek drainage and Sand Creek; westerly along said divide to Wyoming Highway 487; southerly along said highway to the Shirley Ridge Road (Carbon County Road 2); southwesterly along said road to Wyoming Highway 77; southerly along said highway to the Chalk Mountain Road (BLM Road 3129); westerly and northerly along said road to the Dry Creek Road (Carbon County Road 103); northwesterly along said road to the south fork of Canyon Creek; westerly and northerly down said fork to Canyon Creek; westerly down said creek to Pathfinder Reservoir and the North Platte River; northerly and easterly down said river to Wolf Creek; southerly up said creek to its head; southerly one and one-eighth (1 1/8) miles to the Casper Mountain area boundary marker; due east two and one-quarter (2 1/4) miles to the west boundary of Star Wallow; southerly along said boundary one and one-half (1 1/2) miles to the switchback of the Circle Drive Road (Natrona County Road 505); southerly along said road to the head of the Clear Fork of Muddy Creek; easterly down said fork to the Casper Mountain area boundary marker; north two (2) miles to the southeast

corner of Crimson Dawn Park; northerly and westerly along the east and north boundary of said park to the north entrance road; northerly along said road to the East End Road (Natrona County Road 506); northeasterly along said road to the head of the East Fork of Elkhorn Creek; northerly down said creek to the lower timberline on Casper Mountain; westerly along said timberline to the Casper Mountain Road (Wyoming Highway 251); northerly along said road to the Casper city limits; easterly and northerly along said city limits to Interstate Highway 25; easterly along said highway to the Cole Creek Road (Wyoming Highway 256); northerly along said highway to the North Platte River; easterly down said river to Wyoming Highway 95 at the town of Glenrock.

Area 67. Casper Mountain. Beginning where Wolf Creek enters the North Platte River; easterly down said river to Cole Creek Road (Wyoming Highway 256); southerly along said highway to Interstate Highway 25; westerly along said highway to the Casper city limits; southerly and westerly along said city limits to Casper Mountain Road (Wyoming Highway 251); southerly along said road to the lower timberline on Casper Mountain; easterly along said timberline to the East Fork of Elkhorn Creek; southerly up said creek to the East End Road (Natrona County Road 506); southwesterly along said road to the north entrance road to Crimson Dawn Park; southerly along said road to the Crimson Dawn Park boundary; easterly and southerly along said boundary to the southeast corner of the park; southerly to the Clear Fork of Muddy Creek and the Casper Mountain area boundary marker; southwesterly up said creek to the Circle Drive Road (Natrona County Road 505); northerly along said road to the switchback; northerly one and one-half (1 ½) miles along the west boundary of Star Wallow; west two and one-fourth (2 ¼) miles to the Casper Mountain area boundary marker; northerly to the head of Wolf Creek; northerly down said creek to the North Platte River.

Area 70. Shirley Mountain. Beginning where Wyoming Highway 487 intersects U.S. Highway 30 at the town of Medicine Bow; westerly along U.S. Highway 30 to Wyoming Highway 72; northerly along said highway to the town of Hanna and the Hanna-Leo Road (Carbon County Road 291); northerly along said road to the Medicine Bow River; westerly down said river to Seminoe Reservoir and the North Platte River; northerly down said river to where Canyon Creek enters Pathfinder Reservoir; easterly up said creek to the south fork of Canyon Creek; easterly and southerly up said fork to the Dry Creek Road (Carbon County Road 103); southeasterly along said road to the Chalk Mountain Road (BLM Road 3129); easterly and southerly along said road to Wyoming Highway 77; northerly along said highway to the Shirley Ridge Road (Carbon County Road 2); northeasterly along said road to Wyoming Highway 487; southerly along said highway to the intersection with U.S. Highway 30 at the town of Medicine Bow.

Area 73. Boswell. Beginning at the junction of the Marshall Road (Albany County Road 610) and U.S. Highway 30 north of the town of Rock River; northwesterly along said highway to the junction with Wyoming Highway 487 at the town of Medicine Bow; northerly along said highway to the divide between Hunt Creek and Sand Creek; easterly along said divide to the divide between Lone Tree Creek and Spring Creek; easterly along said divide to the Old Medicine Bow Road (Carbon County Road 97); northerly along said road to the Squaw Springs Trail Road; northerly along said road to U.S.F.S. Road 660; southeasterly along said road to the Cold Springs Road (Converse County Road 24); easterly along said road to the divide between the Little Medicine Bow River and LaPrele Creek; easterly along said divide to the divide between Corduroy Creek and Rocky Ford Creek; easterly along said divide to the Fetterman Road (Albany County Road 61); southerly along said road to U.S.F.S. Road 658; easterly along said road to South Fork Creek, a tributary to LaBonte Creek; southerly up said creek to the divide between Horseshoe Creek and the North Laramie River; southerly and easterly along said divide to the Esterbrook Road (Albany County Road 710); northeasterly along said road to U.S.F.S. Road 671; easterly and southerly along said road to the Bear Creek Road (Albany County Road 77); southwesterly along said road to the Garrett Road (Albany County Road 713); southeasterly along said road to the Dodge Ranch Road; southerly along said road to the Big Laramie River; southerly up said river to the Round Top-Flat Top Road at the steel bridge; southeasterly along said road to Wyoming Highway 34; southwesterly along said highway to U.S. Highway 30; northwesterly along said highway to the Marshall Road (Albany County Road 610).

Area 74. Kyle. Beginning where U.S. Highway 30 intersects Wyoming Highway 72; southeasterly along said highway to the city of Laramie and Interstate Highway 80; northwesterly along said highway to Wyoming Highway 72; northerly along said highway to U.S. Highway 30.

Area 75. Four Mile. Beginning at the city of Laramie and Wyoming Highway 130; westerly along said highway to the divide between the Laramie River and the North Platte River; northerly along said divide to

Medicine Bow Peak and the divide between the Medicine Bow River and Brush Creek; northeasterly along said divide to the North Brush Creek Road (U.S.F.S Road 100); northerly along said road to the Sand Lake Road (U.S.F.S. Road 101); northwesterly along said road to the Cedar Pass Road (U.S.F.S. Road 261); southwesterly along said road to U.S.F.S. Road 115; northerly along said road to the Pass Creek Basin Road; northerly along said road to the Pass Creek Road (Carbon County Road 404); northerly along said road to Wyoming Highway 72; northwesterly along said highway to Interstate Highway 80 to the west Elk Mountain interchange; southeasterly along said highway to Wyoming Highway 130.

Area 76. Sheep Mountain. Beginning at the city of Laramie and Wyoming Highway 230; southwesterly along said highway to Wyoming Highway 10 at Woods Landing; southerly along said highway to the Wyoming-Colorado state line; west along said line to Wyoming Highway 230; northeasterly along said highway to U.S.F.S. Road 512; northwesterly along said road to U.S.F.S. Road 543; northerly along said road to Douglas Creek at Keystone; northerly up said creek to the divide between the Laramie River and the North Platte River; northerly along said divide to Wyoming Highway 130; easterly along said highway to Wyoming Highway 230.

Area 77. Boulder Ridge. Beginning at the city of Laramie and U.S. Highway 287; southerly along said highway to the Wyoming-Colorado state line; westerly along said line to Wyoming Highway 10; northerly along said highway to Wyoming Highway 230 at Woods Landing; northeasterly along said highway to U.S. Highway 287.

Area 78. French Creek. Beginning where Wyoming Highway 130 crosses the divide between the Laramie River and the North Platte River; southerly along said divide to Douglas Creek; southerly down said creek to U.S.F.S. Road 543 at Keystone; southerly along said road to U.S.F.S. Road 512; southeasterly along said road to Wyoming Highway 230; southwesterly along said highway to the Wyoming-Colorado state line; westerly along said line to Wyoming Highway 230; northwesterly along said highway to Wyoming Highway 130; easterly along said highway to the divide between the Laramie River and the North Platte River.

Area 79. Kennaday Peak. Beginning where Interstate Highway 80 crosses the North Platte River; easterly along said highway to U.S. Highway 30; easterly along said highway to Wyoming Highway 72; southerly along said highway to the Pass Creek Road (Carbon County Road 404); southerly along said road to the Pass Creek Basin Road; southerly along said road to U.S.F.S. Road 115; southerly along said road to the Cedar Pass Road (U.S.F.S. Road 261); northeasterly along said road to the Sand Lake Road (U.S.F.S. Road 101); southeasterly along said road to the North Brush Creek Road (U.S.F.S. Road 100); southerly along said road to the divide between Brush Creek and the Medicine Bow River; southeasterly along said divide to Medicine Bow Peak and the divide between the Laramie River and the North Platte River; southerly along said divide to Wyoming Highway 130; westerly and northerly along said highway to the North Platte River in the town of Saratoga; northerly down said river to Interstate Highway 80.

Area 80. Spring Creek. Beginning at the Continental Divide on Wyoming Highway 70; northerly along said divide to the Rawlins-Sandstone Road; northerly along said road to Sage Creek; northeasterly down said creek to the North Platte River; southerly up said river to the town of Saratoga and Wyoming Highway 130; southerly along said highway to Wyoming Highway 230; southerly along said highway to Wyoming Highway 70 at the town of Riverside; southwesterly along said highway to the Continental Divide.

Area 81. Blackhall. Beginning at the Wyoming-Colorado state line and Wyoming Highway 230; westerly along said line to the Continental Divide; northerly along said divide to Wyoming Highway 70; northeasterly along said highway to Wyoming Highway 230 at the town of Riverside; easterly and southeasterly along said highway to the Wyoming-Colorado state line.

Area 82. Baggs. Beginning at the junction of Wyoming Highway 789 and the Wyoming-Colorado state line; northerly along said highway to Muddy Creek north of Dad; easterly up said creek to the Muddy Creek Road (BLM Road 3306); easterly along said road to Miller Hill Road (BLM Road 3328); southeasterly along said road to McCarty Canyon Road (Carbon County Road 503); northeasterly along said road to Rawlins City Water Works Road; northerly along said road to Sage Creek below Sage Creek Reservoir; northeasterly down said creek to Sage Creek Road (Carbon County Road 401); southerly along said road to the Continental Divide at Middlewood Hill; southerly along said divide to the Wyoming-Colorado state line; westerly along said line to Wyoming Highway 789.

Area 83. Bolten Rim. Beginning at the city of Rawlins; easterly along Interstate Highway 80 to the North Platte River; southerly up said river to Sage Creek; southwesterly up said creek to the Sage Creek Road (Carbon County Road 401); northerly along said road to Wyoming Highway 71; northerly along said highway to the city of Rawlins.

Area 84. Atlantic Rim. Beginning at the city of Rawlins; southerly along Wyoming Highway 71 to the Sage Creek Road (Carbon County Road 401); southerly along said road to Sage Creek; southwesterly up said creek to the Rawlins City Water Works Road below Sage Creek Reservoir; southerly along said road to the McCarty Canyon Road (Carbon County Road 503); southwesterly along said road to the Miller Hill Road (BLM Road 3328); northwesterly along said road to the Muddy Creek Road (BLM Road 3306); westerly along said road to Muddy Creek; westerly down said creek to Wyoming Highway 789; northerly along said highway to Interstate Highway 80; easterly along said highway to the city of Rawlins.

Area 87. Ferris. Beginning at the city of Rawlins; northerly along U.S. Highway 287 to Muddy Gap Junction and Wyoming Highway 220; northeasterly along said highway to the Pathfinder Road (Natrona County Road 409); southerly along said road to the Pathfinder Dam service road; southerly along said road to the North Platte River at Pathfinder Dam; southerly up said river to Interstate Highway 80; westerly along said highway to the city of Rawlins.

Area 88. Kendrick. Beginning where U.S. Highway 20-26 crosses the North Platte River in the city of Casper; southwesterly up said river to the Pathfinder Dam; northerly along the Pathfinder Dam service road to Wyoming Highway 220; westerly along said highway to the Rattlesnake Divide between Eagle Creek and Fish Creek; northerly along said divide to the Oregon Trail (Natrona County Road 319) at the top of Ryan Hill; northeasterly along said trail to the Kendrick Canal at the Iron Creek Oil Field; northerly along said canal to U.S. Highway 20-26; easterly along said highway to the North Platte River.

Area 89. Rattlesnake. Beginning where the Gas Hills Road (Natrona County Road 212) intersects U.S. Highway 20-26 at the town of Waltman; easterly along said highway to the Kendrick Canal; southerly along said canal to the Oregon Trail (Natrona County Road 319) at the Iron Creek Oil Field; southwesterly along said road to the Rattlesnake Divide at Ryan Hill; southerly along said divide between Eagle Creek and Fish Creek to Wyoming Highway 220; westerly along said highway to the Dry Creek Road (Natrona County Road 321); northwesterly along said road to the Gas Hills Road (Natrona County Road 212); northeasterly along said road to U.S. Highway 20-26 at the town of Waltman.

Area 90. Muskrat. Beginning where U.S. Highway 26 intersects the Wind River Indian Reservation boundary; easterly along said highway to the town of Waltman and the Gas Hills Road (Natrona County Road 212); southwesterly along said road to the Dry Creek Road (Natrona County Road 321); southeasterly along said road to the divide between Deer Creek and Dry Creek; southwesterly along said divide to Beaver Rim; southwesterly along said rim to the Ore Road; southerly along said road to the Beaver Rim Road (BLM Road 2401); westerly along said road to the Cedar Rim Draw Road (BLM Road 2301); southwesterly along said road to Wyoming Highway 135 (Sand Draw Road); northerly along said highway to the south boundary of the Wind River Indian Reservation; easterly and northerly along said boundary to its intersection with U.S. Highway 26.

Area 91. Popo Agie. Beginning where Wyoming Highway 28 crosses Willow Creek; northwesterly up said creek to the divide between the Sweetwater River and the Wind River at Granite Peak; northwesterly along said divide to the Continental Divide; northwesterly along said divide to the Wind River Indian Reservation boundary; easterly along said boundary to the Shoshone National Forest boundary; southeasterly along said boundary to Wyoming Highway 28; southwesterly along said highway to Willow Creek.

Area 92. Table Mountain. Beginning where U.S. Highway 287 intersects the Wind River Indian Reservation boundary; southeasterly along said highway to Wyoming Highway 28; southerly along said highway to the Shoshone National Forest boundary; northwesterly along said boundary to the Wind River Indian Reservation boundary; easterly along said boundary to U.S. Highway 287.

Area 93. Upper Sweetwater. Beginning where Wyoming Highway 28 crosses the Sweetwater River; northwesterly and northerly up said river to the divide between the Sweetwater River and the Wind River at Sweetwater Gap; southeasterly along said divide to Granite Peak and the head of Willow Creek; southeasterly down said creek to Wyoming Highway 28; southwesterly along said highway to the Sweetwater River.

Area 94. Twin Creek. Beginning where Wyoming Highway 28 intersects U.S. Highway 287 south of the city of Lander; southeasterly along U.S. Highway 287 to the Hudson-Atlantic City Road (BLM Road 2302); westerly along said road to the Fort Stambaugh Road (BLM Road 2324); westerly along said road to the Atlantic City Road (Fremont County Road 237); northerly along said road to Wyoming Highway 28; northerly along said highway to U.S. Highway 287.

Area 95. South Pass. Beginning where the Bison Basin Road (Fremont County Road and BLM Road 3221) intersects U.S. Highway 287; southerly along said road to the Cyclone Ridge Road (BLM Road 3216); westerly along said road to the Red Creek Road (BLM Road 3219); northwesterly along said road to the Sand Creek Road; westerly along said road to the Continental Divide Road north of Continental Peak; westerly along said road to the Oregon Buttes Road; northerly along said road to Wyoming Highway 28; northeasterly along said highway to the Atlantic City Road (Fremont County Road 237) at the Wyoming Department of Transportation South Pass Maintenance Shop; southerly along said road to the Fort Stambaugh Road (BLM Road 2324) at Atlantic City; easterly along said road to the Hudson-Atlantic City Road (BLM Road 2302); easterly along said road to U.S. Highway 287; southeasterly along said highway to the Bison Basin Road (Fremont County Road and BLM Road 3221).

Area 96. Green Mountain. Beginning at the town of Lamont; westerly along Wyoming Highway 73 to the Bairoil Road (Sweetwater County Road 22); westerly along said road to the Wamsutter-Crooks Gap Road (Sweetwater County Road 23); southerly along said road to the Osborne Road (BLM Road 3212); westerly along said road to the Hadsell Road (BLM Road 3217); westerly along said road to the Bison Basin Road (Fremont County Road and BLM Road 3221); northerly along said road to U.S. Highway 287; southeasterly along said highway to the town of Lamont.

Area 97. Sweetwater Rocks. Beginning at Muddy Gap Junction; northwesterly along U.S. Highway 287 to Wyoming Highway 135 (Sand Draw Road); northerly along said highway to the Cedar Rim Draw Road (BLM Road 2301); northeasterly along said road to the Beaver Rim Road (BLM Road 2401); easterly along said road to the Ore Road; northerly along said road to Beaver Rim; northeasterly along said rim to the divide between Deer Creek and Dry Creek; northeasterly along said divide to the Dry Creek Road (Natrona County Road 321); southeasterly along said road to Wyoming Highway 220; southwesterly along said highway to Muddy Gap Junction.

Area 98. Chain Lakes. Beginning at the city of Rawlins; westerly along Interstate Highway 80 to the Wamsutter-Crooks Gap Road (Sweetwater County Road 23); northerly along said road to the Bairoil Road (Sweetwater County Road 22); easterly along said road to Wyoming Highway 73; easterly along said highway to U.S. Highway 287; southerly along said highway to the city of Rawlins.

Area 100. South Wamsutter. Beginning where Wyoming Highway 789 crosses the Wyoming-Colorado state line; west along said line to the Cow Creek-Powder Wash Road north of Powder Wash, Colorado; northwesterly along said road to the Bitter Creek Road (Sweetwater County Road 19); northwesterly along said road to Interstate Highway 80; easterly along said highway to Wyoming Highway 789 at Creston Junction; southerly along said highway to the Wyoming-Colorado state line.

Area 101. Black Butte. Beginning at the junction of Wyoming Highway 430 and the Wyoming-Colorado state line; northwesterly along said highway to Interstate Highway 80 in the town of Rock Springs; easterly along said interstate to the Bitter Creek Road (Sweetwater County Road 19); southeasterly along said road to Carson Springs Junction and the Cow Creek-Powder Wash Road; southeasterly along said road to the Wyoming-Colorado state line; westerly along said line to Wyoming Highway 430.

Area 102. Aspen Mountain. Beginning where Interstate Highway 80 crosses the Green River; easterly along said highway to the town of Rock Springs and Wyoming Highway 430; southeasterly along said highway to

the Wyoming-Colorado state line; westerly along said line to the Flaming Gorge Reservoir; northerly along the east shore of said reservoir to the Green River; up the east bank of said river to Interstate Highway 80.

Area 105. Beartooth. All of the area within Wyoming north of Clark's Fork River and west of Wyoming Highway 120.

Area 106. Crandall-Sunlight. All of the drainage of Soda Butte Creek outside of Yellowstone National Park; all of the south drainage of Clark's Fork River down to and including Dead Indian Creek drainage.

Area 109. Bald Ridge. All of the drainage of Pat O'Hara Creek, Paint Creek, Trail Creek, Cottonwood Creek, Dry Creek, Newmeyer Creek and Dead Indian Gulch north of the North Fork of the Shoshone River and west of Wyoming Highway 120.

Area 110. Grinnell. All of the north and west drainage of the North Fork of the Shoshone River down to and including the drainage of Clearwater Creek; all of the south drainage of the North Fork of the Shoshone River down to the drainage of Elk Fork Creek.

Area 111. Elk Fork. All of the north drainage of the North Fork of the Shoshone River below the drainage of Clearwater Creek; all of the south drainage of the North Fork of the Shoshone River below and including Elk Fork Creek.

Area 112. Ishawooa. All of the north and west drainage of the South Fork of the Shoshone River up to the drainage of Fall Creek.

Area 113. Boulder. All of the east and south drainage of the South Fork of the Shoshone River up to the drainage of Needle Creek; all of the drainage of Sage Creek and Dry Creek west of Wyoming Highway 120; all of the drainage of Sulphur Creek.

Area 114. Wall Mountain. All of the drainage of the South Fork of the Shoshone River above and including the drainage of Fall Creek on the west side of the South Fork of the Shoshone River, and above and including the drainage of Needle Creek on the east side of the South Fork of the Shoshone River.

Area 115. Thorofare. All of the drainage of the Yellowstone River upstream from Yellowstone Lake and outside of Yellowstone National Park.

Area 116. Meeteetse. All of the Greybull and Wood River drainages off national forest west of Wyoming Highway 120.

Area 117. Franc's Peak. All of the Greybull and Wood River drainages on national forest.

Area 118. Upper Gooseberry Creek. All of the drainages of Gooseberry Creek and Little Buffalo Creek located west of Wyoming Highway 120.

Area 119. Anchor. Beginning where the divide between Gooseberry Creek and Grass Creek crosses Wyoming Highway 120; southerly along said highway to the Upper Cottonwood Creek Road (Hot Springs County Road 10); westerly then southerly along said road to the Lake Creek Road; southerly along said road to the Anchor Dam Road at Anchor Reservoir (Owl Creek Road, Hot Springs County Road 1); southerly along said road to the Wind River Indian Reservation boundary; westerly along said boundary to the divide between Owl Creek and the South Fork of the Wood River; easterly along said divide to the divide between Cottonwood Creek and Gooseberry Creek; northeasterly along said divide to the divide between Gooseberry Creek and Grass Creek; easterly along said divide to Wyoming Highway 120.

Area 120. Hamilton Dome. Beginning at the junction of the Upper Cottonwood Creek Road (Hot Springs County Road 10) and Wyoming Highway 120; southerly along said highway to the town of Thermopolis and the Broadway Street bridge over the Bighorn River; southerly up said river to the Wind River Indian Reservation boundary; westerly, northerly, then westerly along said boundary to the Anchor Dam Road at Anchor Reservoir

(Owl Creek Road-Hot Springs County Road 1); northerly and easterly along said road to the Lake Creek Road; northeasterly along said road to the Upper Cottonwood Creek Road (Hot Springs County Road 10); easterly along said road to Wyoming Highway 120; in addition, all non-Indian fee title lands in that portion of Hot Springs County within the exterior boundaries of the Wind River Indian Reservation.

Area 121. Heart Mountain. All of the north drainage of the Shoshone River east of Wyoming Highway 120 and west of Wyoming Highway 295; all of the drainage of the Clark's Fork River east of Wyoming Highway 120 and west of Wyoming Highway 295 and Elk Basin Road.

Area 122. Shoshone River. Beginning where the Elk Basin Road (Wyoming Highway 295) crosses the Wyoming-Montana state line; easterly along said line to the Bighorn River; southerly up said river to Horseshoe Bend boat ramp; westerly along the Horseshoe Bend Road to Wyoming Highway 37; southerly along said highway to U.S. Highway 14A; easterly along said highway to the Bighorn River; southerly up said river to Stucco (WyoBen Bentonite Plant); from Stucco south to BLM Road 1138; northwesterly along said road to U.S. Highway 310; northerly along said highway to the divide between the Shoshone River and Dry Creek; southwesterly along said divide to the Park-Big Horn county line; southerly along said line to U.S. Highway 14-16-20; westerly along said highway to the town of Cody and the Shoshone River; northeasterly down said river to the Willwood Road (Wyoming Highway 295); northerly along said highway to the Wyoming-Montana state line.

Area 123. Yellowtail. Beginning at the Horseshoe Bend boat ramp on the Bighorn River; southerly up said river to U.S. Highway 14A; westerly along said highway to Wyoming Highway 37; northerly along said highway to the Horseshoe Bend Road; easterly along said road to the Horseshoe Bend boat ramp.

Area 124. Emblem. Beginning in the town of Greybull; southerly along U.S. Highway 16-20 to the divide between Antelope Creek and Elk Creek; westerly along said divide to the divide between Elk Creek and Dorsey Creek; westerly along said divide to the divide between Fifteen Mile Creek and the Greybull River; westerly along said divide to Fenton Pass and the Fenton Pass Road (BLM Road 1222); northerly along said road to Bighorn County Lane 40 1/2 (becomes Park County Road 3XQ); southwesterly along said road to the Roach Gulch Road; southwesterly along said road to Roach Gulch Reservoir; southerly along the west shore of said reservoir to the Roach Gulch intake canal; westerly along said canal to Renner Ranch Lane; northerly along said lane to the Coyote Canyon Road (Park County Road 3SL); northwesterly along said road to the Greybull River Road (Park County Road 3LE); northeasterly along said road to the Park-Bighorn county line; northerly along said line to the divide between Dry Creek and the Shoshone River; northeasterly along said divide to U.S. Highway 310 (Wyoming Highway 789); southerly along said highway to BLM Road 1138; southeasterly along said road to Stucco (WyoBen Bentonite Plant) and the Big Horn River; southerly up said river to the town of Greybull.

Area 125. Fifteen Mile. Beginning where Wyoming Highway 120 crosses the divide between the Greybull River and Fifteen Mile Creek; northerly and easterly along said divide to the divide between Dorsey Creek and Elk Creek; easterly along said divide to the divide between Elk Creek and Antelope Creek; easterly along said divide to U.S. Highway 16-20; southerly then easterly along said highway to its junction with the Big Horn Canal three (3) miles west of Manderson; southerly along said canal to its junction with Wyoming Highway 20-U.S. Highway 789 three quarters of a mile south of Worland; southwesterly along said highway to Tie Down Gulch; westerly along said gulch to the divide between Cottonwood Creek and Gooseberry Creek; westerly along said divide to the divide between Gooseberry Creek and Grass Creek; westerly along said divide to Wyoming Highway 120; northwesterly along said highway to the divide between the Greybull River and Fifteen Mile Creek.

Area 127. Cottonwood. Beginning where Wyoming Highway 120 crosses the divide between Gooseberry Creek and Grass Creek; easterly along said divide to the divide between Gooseberry Creek and Cottonwood Creek; easterly along said divide to Tie Down Gulch; easterly down said gulch to the Big Horn River; southerly up said river to the Broadway Street bridge at the town of Thermopolis; westerly along said street to Wyoming Highway 120; northwesterly along said highway to the divide between Gooseberry Creek and Cottonwood Creek.

Area 128. Wind River. All of the drainage of the Wind River west of the west boundary of the Wind River Indian Reservation and those areas of the Spence and Moriarty Wildlife Habitat Management Area that lie east of the East Fork of the Wind River.

Area 130. Big Sandy. Beginning at the town of Farson and U.S. Highway 191; northerly along said highway to the Big Sandy Reservoir Road (Sweetwater County Road 28); easterly along said road to the Big Sandy River at the Big Sandy Reservoir dam; northerly up said river to the Continental Divide; southeasterly along said divide to Mt. Nystrom; easterly from Mt. Nystrom to Sweetwater Gap and the headwaters of the Sweetwater River; southerly down said river to Wyoming Highway 28; southwesterly along said highway to U.S. Highway 191 at the town of Farson.

Area 131. Steamboat. Beginning at the junction of Interstate Highway 80 and Wyoming Highway 372; northwesterly along Wyoming Highway 372 to the Fontenelle Dam Road (Lincoln County Road 313); northerly then easterly along said road to the County Line Road (Sweetwater County Road 52); southerly along said road to the Eighteen Mile Road (Sweetwater County Road 49); northeasterly along said road to U.S. Highway 191; southerly along said highway to U.S. Highway 28; northeasterly along said highway to the Oregon Buttes Road (Sweetwater County Road 74); southerly along said road to the Continental Divide Road; northerly along said road to the Sand Creek Road; easterly along said road to the Red Creek Road (BLM Road 3219); southeasterly along said road to the Cyclone Ridge Road (BLM Road 3216); easterly along said road to the Bison Basin Road (BLM Road 3221); southerly along said road to the Hadsell Road (BLM Road 3217); easterly along said road to the Osborne Road (BLM Road 3212); easterly along said road to the Wamsutter-Crooks Gap Road (Sweetwater County Road 23); southerly along said road to Interstate Highway 80; westerly along said highway to Wyoming Highway 372.

Area 132. Cedar Mountain. Beginning where the Flaming Gorge Reservoir crosses the Wyoming-Utah state line; west along said line to the Stateline Dam Road (U.S.F.S. Road 072, Uinta County Road 283); northerly along said road to Wyoming Highway 410; northerly along said road to Interstate Highway 80; easterly along said highway to the Green River; southerly down the east bank of said river to Flaming Gorge Reservoir; southerly along the east shore of said reservoir to the Wyoming-Utah state line.

Area 133. Uinta. Beginning where Interstate Highway 80 crosses the Wyoming-Utah state line; easterly along said highway to Wyoming Highway 414; southerly along said highway to Wyoming Highway 410 at the town of Mountain View; southerly along said highway to the Stateline Dam Road (U.S.F.S. Road 072, Uinta County Road 283); southerly along said road to the Wyoming-Utah state line; westerly then northerly along said line to Interstate Highway 80.

Area 134. Bear River Divide. Beginning where Wyoming Highway 89 crosses the Wyoming-Utah state line west of Sage Junction; northeasterly along said highway to Wyoming U.S. Highway 30; easterly then southerly along said highway to Interstate Highway 80; westerly along said highway to Wyoming Highway 412; northwesterly along said highway to U.S. Highway 189; southerly along said highway to Muddy Creek; westerly along said creek to the Amoco Sulfur Haul Road; southerly then westerly along said road to the Whitney Canyon Road; westerly along said road to Uinta County Road 103; southerly along said road to Wyoming Highway 89; northerly along said highway to the Wyoming-Utah state line; northerly along said line to Wyoming Highway 89.

Area 135. West Green River. Beginning at the junction of Wyoming Highway 372 and Interstate Highway 80; westerly along said interstate highway to U.S. Highway 30; northwesterly along said highway to Sage Junction and Wyoming Highway 89; southwesterly along said highway to the Wyoming-Utah state line; northerly along said line to the Wyoming-Idaho state line; northerly along said line to the divide between the Salt River and Bear River; easterly along said divide to the divide between the Salt River and Smith's Fork; northeasterly along said divide to Commissary Ridge; southeasterly along said ridge to the head of LaBarge Creek; southeasterly down said creek to the Green River; southeasterly along said river to Fontenelle Dam and the Fontenelle Dam Road (Lincoln County Road 313); westerly along said road to Lincoln County Road 316; southerly along said road to Wyoming Highway 372; southeasterly along said highway to Interstate Highway 80.

Area 138. Boulder. Beginning at the Fontenelle Dam on the Green River; northerly up said river to Wyoming Highway 351; easterly along said highway to the New Fork River; northerly up said river to Boulder Creek; easterly up said creek to the middle fork of Boulder Creek; easterly up said creek to the Continental Divide; southerly along said divide to the Big Sandy River; southerly down said river to the Big Sandy Reservoir Road (Sweetwater County Road 28); westerly along said road to U.S. Highway 191; southerly along said highway to the 18 Mile Road (Sweetwater County Road 49); southwesterly along said road to the County Line Road (Sweetwater

County Road 52); northerly along said road to the Fontenelle Dam Road (Lincoln County Road 313); westerly along said road to Fontenelle Dam and the Green River.

Area 139. Pinedale. Beginning where the East Green River Road (Sublette County Road 23-110) intersects Wyoming Highway 351; northerly along said highway to Cora Junction and U.S. Highway 191; easterly along said highway to Pine Creek; northerly up said creek to Summit Lake; easterly to Mt. Helen and the Continental Divide; southerly along said divide to the Middle Fork of Boulder Creek; westerly down said creek to Boulder Creek; westerly down said creek to the New Fork River; southerly down said river to Wyoming Highway 351; westerly along said highway to the East Green River Road (Sublette County Road 23-110).

Area 140. New Fork. Beginning where U.S. Highway 191 crosses Pine Creek in the town of Pinedale; westerly and northerly along said highway to the Green River at Warren Bridge; northerly up said river to Jim Creek; easterly up said creek to the divide between the New Fork River and the Green River; southerly along said divide to the divide between the Green River and Pine Creek; southeasterly along said divide to Summit Lake and the head of Pine Creek; southerly down said creek to U.S. Highway 191.

Area 141. Green River. Beginning where Tosi Creek enters the Green River; westerly up said creek to the Union Pass Road (U.S.F.S. Road 600); northerly and easterly along said road to the Continental Divide; southerly along said divide to Mt. Helen; westerly to Summit Lake and the divide between the Green River and Pine Creek; northwesterly along said divide to the divide between the Green River and the New Fork River; northerly along said divide to the head of Jim Creek; westerly down said creek to the Green River; northerly up said river to Tosi Creek.

Area 142. Piney-Horse Creek. Beginning where the East Green River Road (Sublette County Road 23-110) intersects Wyoming Highway 351; westerly along said road to the Green River; northerly up said river to Cottonwood Creek; westerly up said creek to South Cottonwood Creek; westerly up said creek to the South Fork of South Cottonwood Creek; westerly up said creek to the divide between the Green River and the Greys River; northerly along said divide to the divide between the Hoback River and the Green River; easterly along said divide to U.S. Highway 191 at Hoback Rim; southerly along said highway to the East Green River Road (Sublette County Road 23-110) at Cora Junction; southerly along said road to Wyoming Highway 351.

Area 143. South Piney. Beginning where LaBarge Creek enters the Green River; westerly up said creek to the divide between the Green River and Greys River; northerly along said divide to the head of the South Fork of South Cottonwood Creek; easterly down said creek to South Cottonwood Creek; easterly down said creek to Cottonwood Creek; easterly down said creek to the Green River; southerly down said river to LaBarge Creek.

Area 144. Greys River. Beginning at the confluence of Greys River and the Snake River; easterly up the Snake River to Bailey Creek; southerly up said creek to Dry Wash Draw; easterly up said draw to the top of the Greyback Ridge; southerly along said ridge to the divide between the Greys River and the Green River; southerly, westerly and northerly along said divide to the divide between the Greys River and Smith Fork Creek; northerly and westerly along said divide to the divide between the Greys River and Salt River; northerly along said divide to the ridge between Squaw Creek and the Greys River; northeasterly along said ridge to the confluence of Squaw Creek and the Greys River; westerly down said river to the Snake River.

Area 145. Salt River. Beginning at the Wyoming-Idaho state line on the Snake River; easterly up said river to the Greys River; easterly up said river to the confluence of the Greys River and Squaw Creek; southwesterly from said confluence to the ridge between Squaw Creek and the Greys River; southerly up said ridge to the divide between the Greys River and the Salt River; southerly along said divide to the divide between the Bear River and the Salt River; westerly along said divide to the Wyoming-Idaho state line; northerly along said line to the Snake River.

Area 146. Fish Creek. Beginning where Kinky Creek enters the Gros Ventre River; northerly down said river to Fish Creek; northerly up said creek to the North Fork of Fish Creek; northerly up said creek to the Moccasin Basin Road (U.S.F.S. Road 30750) at Calf Creek; northerly up said road to the Continental Divide; southerly along said divide to the Union Pass Road (U.S.F.S. Road 600); southerly along said road to the Darwin Ranch Road (U.S.F.S. Road 620); westerly along said road to Kinky Creek; westerly down said creek to the Gros Ventre River.

Area 148. Teton. Beginning where U.S. Highway 26-287 crosses the Grand Teton National Park boundary east of Moran; northerly along said boundary to the Targhee National Forest boundary at the northwest corner of the park; northerly along said boundary to the Yellowstone National Park boundary; easterly along said boundary to the Continental Divide; southerly and easterly along said divide to Togwotee Pass and U.S. Highway 26-287; westerly along said highway to the Grand Teton National Park boundary.

Area 149. Targhee. Beginning where Wyoming Highway 22 crosses the Wyoming-Idaho state line; northerly along said line to the southern boundary of Yellowstone National Park; easterly along said boundary to the Targhee National Forest boundary; southerly along said boundary to Teton Pass and Wyoming Highway 22; westerly along said highway to the Wyoming-Idaho state line.

Area 150. Wilson. Beginning where the Targhee National Forest boundary crosses Wyoming Highway 22 at Teton Pass; northerly along said boundary to the Grand Teton National Park boundary; northerly and southerly along said boundary to U.S. Highway 191; southerly along said highway to its junction with Wyoming Highway 22; westerly along said highway to Teton Pass.

Area 151. Fall Creek. Beginning where Wyoming Highway 22 crosses the Snake River; southerly and westerly down said river to the Wyoming-Idaho state line; northerly along said line to Wyoming Highway 22; easterly along said highway to the Snake River.

Area 152. Lower Hoback. Beginning where U.S. Highway 191 crosses Flat Creek at the north edge of the town of Jackson; due east to the National Elk Refuge boundary; easterly along said boundary to the Bridger-Teton National Forest boundary and the ridge between Twin Creeks and Cache Creek; easterly along said ridge to Jackson Peak; southerly along the divide between Flat Creek and Cache Creek to Cache Peak; easterly along the divide between Flat Creek and Granite Creek to Pyramid Peak; southerly along the divide between the Gros Ventre River and the Hoback River to Steamboat Peak; southerly and westerly along the divide between Shoal Creek and Dell Creek to the Riling Draw road; southerly along said road to the Dell Creek Road; westerly along said road to the Hoback River; westerly down said river to Cliff Creek; southerly up Cliff Creek to the divide between the Greys River and the Hoback River; northwesterly along said divide to the divide between the Greys River and Willow Creek; northerly along said divide to the divide between Willow Creek and Bailey Creek (Greyback Ridge); northerly along said divide to Dry Wash Draw; westerly down said draw to Bailey Creek; northwesterly down said creek to the Snake River; northerly up said river to Wyoming Highway 22; easterly along said highway to U.S. Highway 191; easterly along said highway to Flat Creek at the north edge of the town of Jackson.

Area 153. Monument Ridge. Beginning at the head of Cliff Creek; northerly down said creek to the Hoback River; southerly up said river to the South Fork of the Hoback River; easterly to the Hoback Rim and the divide between the Hoback River and the Green River; southwesterly along said divide to the divide between the Hoback River and the Greys River; northerly along said divide to the head of Cliff Creek.

Area 154. Raspberry Ridge. Beginning where U.S. Highway 191 crosses the divide between the Green River and the Hoback River on the Hoback Rim; southwesterly along said divide to a point due east of the confluence of the South Fork of the Hoback River and the Hoback River; due west to the Hoback River; northerly down said river to the Dell Creek road; easterly along said road to the Riling Draw Road; northerly along said road to the divide between Shoal Creek and Dell Creek; northeasterly along said divide to the divide between the Gros Ventre River and the Hoback River at Steamboat Peak; southeasterly along said divide to the divide between the Green River and the Hoback River at Hodges Peak; southerly along said divide to U.S. Highway 191.

Area 155. Crystal Peak. Beginning at the National Elk Refuge boundary at the north edge of the town of Jackson; northeasterly along said boundary to the Gros Ventre River; easterly up said river to Kinky Creek; easterly up said creek to the Darwin Ranch Road (U.S.F.S. Road 620); easterly along said road to the divide between the Gros Ventre River and the Green River; southwesterly along said divide to the divide between the Gros Ventre River and the Hoback River at Hodges Peak; westerly along said divide to the divide between Granite Creek and Flat Creek at Pyramid Peak; westerly along said divide to the divide between Flat Creek and Cache Creek at Cache Peak; northerly along said divide to the divide between Twin Creeks and Cache Creek at Jackson Peak; westerly along said divide to the National Elk Refuge boundary; westerly along said boundary to the town of Jackson.

Area 156. Spread Creek. Beginning where the Grand Teton National Park boundary crosses the Gros Ventre River; northerly along said boundary to U.S. Highway 26-287; easterly along said highway to Togwotee Pass and the Continental Divide; southerly along said divide to the Moccasin Basin Road (U.S.F.S. Road 30750); southerly down said road to the North Fork of Fish Creek at Calf Creek; southerly down said creek to Fish Creek; southwesterly down said creek to the Gros Ventre River; westerly down said river to the Grand Teton National Park boundary.

Area 157. Boysen. All lands within the Bureau of Reclamation's Riverton and Boysen Unit boundaries and those lands within Boysen State Park south of Cottonwood Creek and west of Boysen Reservoir and those lands within Boysen State Park south of U.S. Highway 20-26.

Area 158. Ormsby. Beginning where Interstate Highway 25 crosses the North Platte River in the city of Casper; northerly along said highway to Wyoming Highway 259; northerly along said highway to the town of Midwest and Wyoming Highway 387; northeasterly along said highway to the Ross Road (in Converse County, County Road 31); southeasterly along said road to the Blizzard Heights-Sage Creek Divide Road; westerly along said road to 55 Ranch Road (Converse County Road 23); southerly along said road to Wyoming Highway 95; southwesterly along said highway to the North Platte River; westerly up said river to Interstate Highway 25 in the city of Casper.

Area 160. Beaver Creek. Beginning where U.S. Highway 287 intersects the south boundary of the Wind River Indian Reservation; easterly along said boundary to Wyoming Highway 135 (Sand Draw Road); southerly along said highway to U.S. Highway 287; northwesterly along said highway to the south boundary of the Wind River Indian Reservation.

Area 161. St. Mary's Creek. Beginning on Wyoming Highway 72 at the town of Hanna on Wyoming Highway 72; southerly along said highway to U.S. Highway 30; westerly along said highway to Interstate Highway 80 at Walcott; westerly along said highway to the North Platte River; northerly down said river to the Medicine Bow River; easterly up said river to the Hanna-Leo Road (Carbon County Road 291); southerly along said road to Wyoming Highway 72 at the town of Hanna.

Area 162. Water Dog Lakes. Beginning where U.S. Highway 191 crosses the Green River at Warren Bridge; northwesterly along said highway to the divide between the Hoback River and the Green River (Hoback Rim); northerly along said divide to the divide between the Green River and the Gros Ventre River at Hodges Peak; northeasterly along said divide to the Darwin Ranch Road (U.S.F.S. Road 620); easterly along said road to the Union Pass Road (U.S.F.S. Road 600); southerly and easterly along said road to Tosi Creek; easterly down said creek to the Green River; southerly down said river to U.S. Highway 191.

Area 163. Middle Fork. Beginning at the junction of the Barnum Mountain Road and the Outlaw Cave Access Road west of the town of Barnum; southerly along the Outlaw Cave Access Road to the Middle Fork of the Powder River; easterly down said river to Buffalo Creek; southerly down said creek to the 33 Mile Road (Natrona County Road 110); northwesterly along said road to the Hazelton Road (Washakie County Road 81); northerly along said road to the Barnum Mountain Road; easterly along said road to the Outlaw Cave Access Road.

Area 164. Nowater. Beginning at the junction of U.S. Highway 16 and Bluebank Road (BLM Road 1411); southerly along said road to the Nowater Stock Drive Road; westerly along said road to the Mud Creek Road; southerly along said road to the divide between Nowater Creek and Kirby Creek; westerly along said divide to the head of Walter's Draw; westerly down said draw to the Big Horn River; northerly down said river to Tie Down Gulch; northwesterly up said gulch to U.S. Highway 20; northeasterly along said highway to U.S. Highway 16; easterly along said highway to the Bluebank Road.

Area 165. YU Bench. Beginning at the junction of Wyoming Highway 120 and U.S. Highway 14-16-20; easterly along said highway to the Park-Bighorn County line; southerly along said line to the Greybull River Road (Park County Road 3LE); southwesterly along said road to the Coyote Canyon Road (Park County Road 3SL); southeasterly along said road to Renner Ranch Lane; southerly along said lane to the Roach Gulch intake canal; easterly along said canal to Roach Gulch Reservoir; northerly along the west shore of said reservoir to the Roach Gulch Road; northeasterly along said road to Park County Road 3XQ (becomes Big Horn County Lane 40 ½);

northeasterly along said road to the Fenton Pass Road (BLM Road 1222); southerly along said road to the divide between the Greybull River and Fifteen Mile Creek; southwesterly along said divide to Wyoming Highway 120; northwesterly along said highway to U. S. Highway 14-16-20.

Area 167. Dull Center. Beginning at the intersection of Wyoming Highway 59 and the Steckley Road (U.S.F.S. Road 942); easterly along said road to the North Antelope Road (U.S.F.S. Road 949); easterly and southerly along said road to the Rochelle Hills Road (U.S.F.S. Road 933); easterly and southerly along said road to the Dull Center Road (Converse County Road 38); easterly along said road to the Converse-Niobrara county line and the North Lance Creek Road; easterly and southerly along said road to the Cow Creek Road; southerly along said road to the Tillard Ranch-Cow Creek Road; westerly along said road to the Converse-Niobrara county line and the Tillard Ranch Road; westerly along said road to the Cow Creek Road (Converse County Road 45); southerly along said road to the Converse-Niobrara county line and the Cow Creek-Walker Creek Interconnect Road; southerly along said road to its junction with the Walker Creek Road (Converse County Road 43) at the Converse-Niobrara county line; westerly along said road to Dry Creek; westerly up said creek to the Iberlin Ranch Road; northerly along said road to the Thunder Basin National Grassland and U.S.F.S. Road 958C; northwesterly along said road to the East Bill Road (U.S.F.S. Road 958); westerly along said road to the Dull Center Road (Converse County Road 38); westerly along said road to Wyoming Highway 59; northerly along said highway to the Steckley Road.

Area 168. Medicine Butte. Beginning where Interstate Highway 80 crosses the Wyoming-Utah state line; northerly along said line to Wyoming Highway 89; southeasterly along said highway to its junction with Uinta County Road 103; northerly along said road to the Whitney Canyon Road; easterly along said road to the Amoco Sulfur Haul Road; easterly then northerly along said road to Muddy Creek; easterly down said creek to U.S. Highway 189; northerly along said highway to Wyoming Highway 412; southeasterly along said highway to Interstate Highway 80; westerly along said highway to the Wyoming-Utah state line.

Area 169. Tisdale Mountain. Beginning where Interstate Highway 25 crosses the South Fork of the Powder River; southeasterly along said highway to the Midwest interchange and the Smoky Gap Road (Natrona County Road 115); westerly along said road to the Dead Horse Road (Natrona County Road 114); southwesterly along said road to the Wild Horse Trail Road (Natrona County Road 113); westerly along said road to the 33 Mile Road (Natrona County Road 110); northwesterly along said road to the Willow Creek Road (Natrona County Road 111); northeasterly along said road to the Lone Bear Road (Natrona County Road 112); southerly along said road to Willow Creek; northwesterly down said creek to the South Fork of the Powder River; northerly down said river to Interstate 25.

Area 170. Riverview. All non-Indian owned fee title lands within the following described area: Beginning where Wyoming Highway 789 crosses the Big Wind River near the City of Riverton; northwesterly up said river to Diversion Dam and the southern Riverton Reclamation Unit boundary; easterly, southerly, and northeasterly along said boundary to the Boysen State Park boundary south of Wyoming Highway 789; southerly, easterly, then northerly along said boundary to the eastern boundary of the Wind River Indian Reservation; southerly, then westerly along said boundary to Wyoming Highway 135; northwesterly along said highway to Wyoming Highway 136; northwesterly along said highway to Wyoming Highway 789; northwesterly along said highway to the Big Wind River near the City of Riverton.

Area 171. Crowheart Butte. All non-Indian owned fee title lands within the exterior boundaries of the Wind River Indian Reservation; excluding Deer Hunt Areas 157 and 170, those areas of the Spence and Moriarity Wildlife Management Area that lie east of the East Fork of the Wind River, and those lands within Hot Springs County.

WYOMING GAME AND FISH COMMISSION

By:

Bill Williams, DVM, President

Dated: April 24, 2007

2007 DEER HUNT AREAS

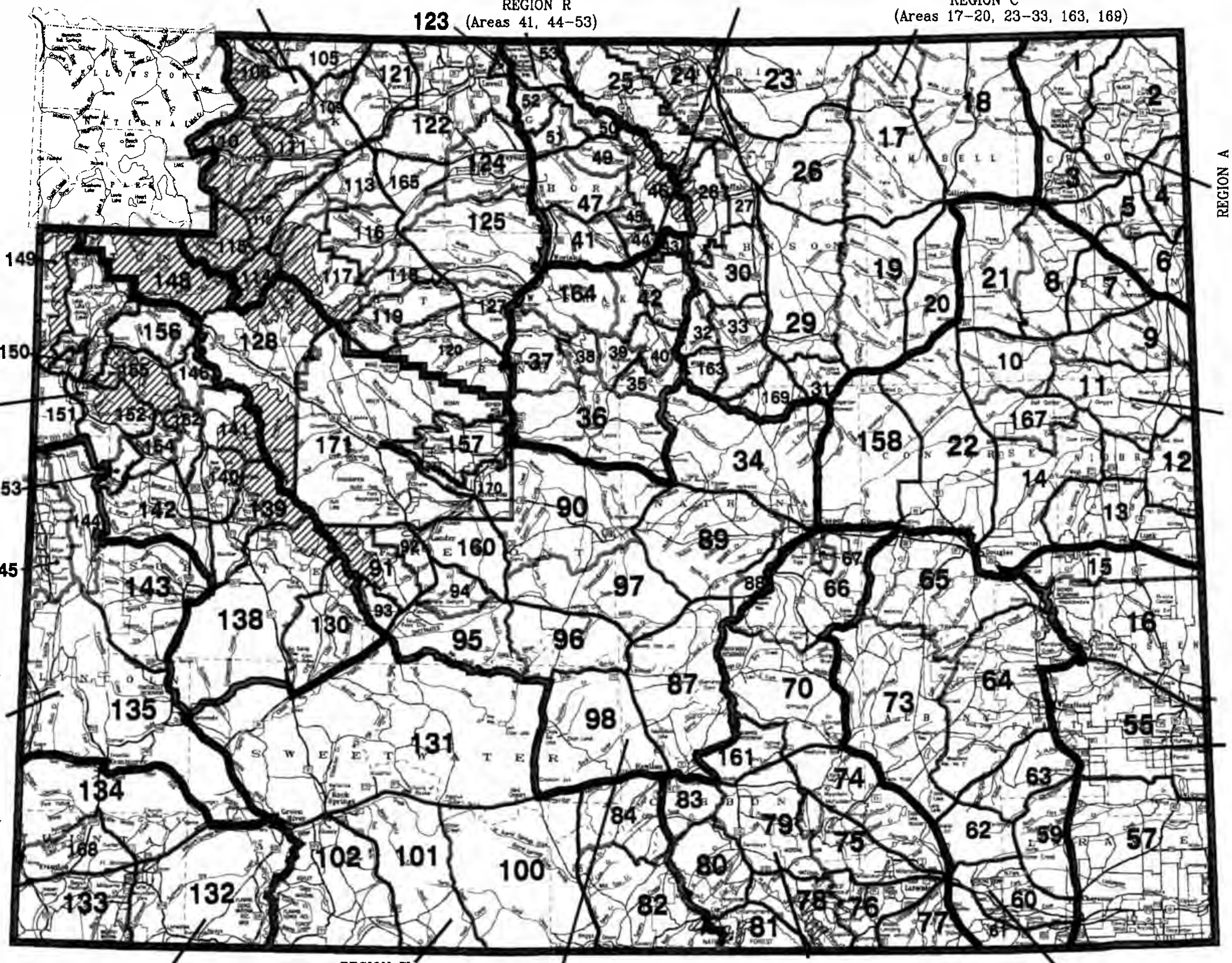

* LIMITED QUOTA AREAS – Region General Licenses not valid for limited quota areas.

THIS MAP IS FOR GENERAL REFERENCE ONLY. Please use the written boundary descriptions in this regulation for detailed boundary information.

Note: Wilderness area, nonresidents must have guides.

TABLE I

ELK

HARVEST STATEWIDE

BY TOTAL HUNTERS

2007

	LICENSES SOLD	NUMBER ACTIVE HUNTERS	HARVEST BULL	SPIKE	COW	CALF	TOTAL	TOTAL RECREATION DAYS	HUNTER SUCCESS	DAYS/ HARVEST
STATE TOTAL	59,763	52,151	9,597	1,943	9,123	1,860	22,523	387,973	43.2%	17.2
RESIDENT	49,419	42,575	6,826	1,693	7,564	1,598	17,681	330,126	41.5%	18.7
NONRESIDENT	10,344	9,576	2,771	250	1,559	262	4,842	57,847	50.6%	11.9

TABLE II

ELK

TEN-YEAR COMPARISON OF HARVEST BY TOTAL HUNTERS

1998–2007

YEAR	1998	1999	2000	2001	2002	2003	2004	2005	2006	2007
HARVEST	22,586	21,830	23,727	22,772	21,462	21,365	21,252	19,708	21,680	22,523

ELK

HARVEST BY HARVEST TOOL

2007

ARCHERY	FIREARM	MUZZLE LOADER
8.20%	91.41%	0.38%

TABLE I-A

ELK

HARVEST, HUNTING PRESSURE, HUNTER SUCCESS

BY HUNT AREA

2007

AREA (cross hunt areas)	TYPE	ACTIVE LICS/HTRS	HARVEST BULL	SPIKE	COW	CALF	TOTAL	HUNTER SUCCESS	DAYS/ HARVEST	HUNTER DAYS	LICENSES SOLD (cross hunt incl.)
1 Black Hills											
	Limited 1	149	46	12	16	3	77	51.7%	20.4	1570	152
	Limited 2	103	0	0	45	10	55	53.4%	16.5	906	105
Pooled Total		252	46	12	61	13	132	52.4%	18.8	2476	
Pooled Resident		205	34	12	47	9	102	49.8%	19.7	2007	
Pooled Nonresident		47	12	0	14	4	30	63.8%	15.6	469	
2 Fortification											
	Limited 1	36	13	2	0	0	15	41.7%	11.8	177	38
	Limited 2	33	0	0	9	0	9	27.3%	16.3	147	35
	Limited 6	38	0	0	17	1	18	47.4%	6.4	115	40
Pooled Total		104	13	2	26	1	42	40.4%	10.5	439	
Pooled Resident		93	10	2	26	0	38	40.9%	10.2	388	
Pooled Nonresident		11	3	0	0	1	4	36.4%	12.8	51	
3 Rawhide											
	Limited 1	50	27	3	5	0	35	70.0%	11.4	398	51
	Limited 2	36	0	0	4	4	8	22.2%	77.8	622	50
	Limited 6	41	0	0	15	7	22	53.7%	24.3	535	50
Pooled Total		114	27	3	24	11	65	57.0%	23.9	1555	
Pooled Resident		103	21	3	23	11	58	56.3%	25.9	1505	
Pooled Nonresident		11	6	0	1	0	7	63.6%	7.1	50	
5 Iron Mountain											
	Limited 1	224	93	11	23	0	127	56.7%	9.1	1159	255
	Limited 6	172	0	0	82	4	86	50.0%	12.0	1031	226
Pooled Total		393	93	11	105	4	213	54.2%	10.3	2190	
Pooled Resident		308	57	11	82	4	154	50.0%	12.0	1853	
Pooled Nonresident		85	36	0	23	0	59	69.4%	5.7	337	
6 Pole Mountain											
	Limited 1	159	56	3	13	0	72	45.3%	19.9	1435	163
	Limited 2	78	0	0	44	3	47	60.3%	13.9	654	100
	Limited 6	81	0	0	35	6	41	50.6%	17.6	720	100
Pooled Total		298	56	3	92	9	160	53.7%	17.6	2809	
Pooled Resident		251	35	3	75	9	122	48.6%	20.1	2447	
Pooled Nonresident		47	21	0	17	0	38	80.9%	9.5	362	
7 Laramie Peak											
	Limited 1	1331	637	83	98	0	818	61.5%	11.5	9445	1509
	Limited 2	984	0	0	390	170	560	56.9%	14.2	7927	1002
	Limited 3	387	0	0	180	34	214	55.3%	11.1	2371	473
	Limited 5	45	0	0	23	6	29	64.4%	8.7	252	100
	Limited 6	400	0	0	161	62	223	55.8%	14.2	3160	500
	Limited 7	80	0	0	37	10	47	58.8%	11.1	521	102
Pooled Total		3082	637	83	889	282	1891	61.4%	12.5	23676	
Pooled Resident		2637	538	83	764	246	1631	61.9%	13.0	21249	
Pooled Nonresident		445	99	0	125	36	260	58.4%	9.3	2427	
8 Boulder Ridge											
	Limited 1	107	15	7	30	4	56	52.3%	12.2	684	126
Pooled Total		107	15	7	30	4	56	52.3%	12.2	684	
Pooled Resident		93	12	4	27	4	47	50.5%	13.2	619	
Pooled Nonresident		14	3	3	3	0	9	64.3%	7.2	65	
9 South Snowy Range											
	General	848	100	14	26	6	146	17.2%	33.7	4927	
Pooled Total		848	100	14	26	6	146	17.2%	33.7	4927	
Pooled Resident		796	80	14	23	6	123	15.5%	37.6	4622	
Pooled Nonresident		52	20	0	3	0	23	44.2%	13.3	305	

TABLE I-A (Continued)

ELK HUNT AREA REPORT

AREA (cross hunt areas)	TYPE	ACTIVE LICS/HTRS	HARVEST BULL	SPIKE	COW	CALF	TOTAL	HUNTER SUCCESS	DAYS/ HARVEST	HUNTER DAYS	LICENSES SOLD (cross hunt incl.)
10 Rock Creek											
	Limited 2	92	0	0	24	0	24	26.1%	23.6	567	105
	Limited 6	128	0	0	28	6	34	26.6%	29.1	989	151
	General	900	141	24	34	0	199	22.1%	26.8	5342	
Pooled Total		1079	141	24	86	6	257	23.8%	26.8	6898	
Pooled Resident		926	111	21	74	6	212	22.9%	28.3	6002	
Pooled Nonresident		153	30	3	12	0	45	29.4%	19.9	896	
11 Medicine Bow River											
	Limited 1	147	49	9	8	6	72	49.0%	15.6	1125	153
	Limited 2	273	0	0	45	22	67	24.5%	29.4	1967	301
Pooled Total		420	49	9	53	28	139	33.1%	22.2	3092	
Pooled Resident		364	32	9	33	28	102	28.0%	27.3	2781	
Pooled Nonresident		56	17	0	20	0	37	66.1%	8.4	311	
12 Kennaday Peak											
	Limited 2	105	0	0	21	13	34	32.4%	19.1	648	114
**(13, 15, 110)**	Limited 6	14	0	0	8	2	10	71.4%	3.7	37	75
	General	1129	143	54	128	27	352	31.2%	19.2	6773	
Pooled Total		1248	143	54	157	42	396	31.7%	18.8	7458	
Pooled Resident		1118	117	54	140	34	345	30.9%	19.6	6771	
Pooled Nonresident		130	26	0	17	8	51	39.2%	13.5	687	
13 Big Creek											
	Limited 2	128	0	0	28	1	29	22.7%	29.9	866	140
**(12, 15, 110)**	Limited 6	8	0	0	4	2	6	75.0%	7.0	42	75
	General	1112	183	52	69	29	333	29.9%	20.6	6847	
Pooled Total		1245	183	52	101	32	368	29.6%	21.1	7755	
Pooled Resident		979	114	36	68	21	239	24.4%	25.7	6136	
Pooled Nonresident		266	69	16	33	11	129	48.5%	12.6	1619	
15 Spring Creek											
	Limited 2	81	0	0	27	6	33	40.7%	16.9	558	85
**(12, 13, 110)**	Limited 6	7	0	0	1	0	1	14.3%	13.0	13	75
	General	885	79	61	44	7	191	21.6%	27.0	5150	
Pooled Total		973	79	61	72	13	225	23.1%	25.4	5721	
Pooled Resident		810	36	55	58	4	153	18.9%	30.9	4723	
Pooled Nonresident		163	43	6	14	9	72	44.2%	13.9	998	
16 Shirley Mountain											
	Limited 1	187	95	10	3	3	111	59.4%	10.6	1173	204
	Limited 2	200	0	0	98	21	119	59.5%	8.5	1010	227
	Limited 6	125	0	0	47	15	62	49.6%	12.1	751	153
	Limited 7	30	0	0	12	8	20	66.7%	4.4	88	35
Pooled Total		528	95	10	160	47	312	59.1%	9.7	3022	
Pooled Resident		452	65	10	135	43	253	56.0%	10.5	2667	
Pooled Nonresident		76	30	0	25	4	59	77.6%	6.0	355	
19 Muddy Mountain											
	Limited 1	140	62	3	0	3	68	48.6%	16.9	1148	154
	Limited 2	138	58	0	0	0	58	42.0%	21.9	1268	155
	Limited 3	102	0	0	19	13	32	31.4%	22.3	714	121
	Limited 4	52	0	0	8	2	10	19.2%	29.8	298	66
	Limited 6	119	0	0	9	9	18	15.1%	59.9	1079	150
Pooled Total		514	120	3	36	27	186	36.2%	24.2	4507	
Pooled Resident		460	91	3	36	27	157	34.1%	26.2	4107	
Pooled Nonresident		54	29	0	0	0	29	53.7%	13.8	400	
21 Baggs											
	Limited 2	474	0	0	182	48	230	48.5%	12.3	2824	542
	Limited 6	16	0	0	5	4	9	56.3%	11.7	105	25
	General	2553	368	279	180	15	842	33.0%	19.0	15972	
Pooled Total		3026	368	279	367	67	1081	35.7%	17.5	18901	
Pooled Resident		2672	312	240	308	36	896	33.5%	19.0	16995	
Pooled Nonresident		354	56	39	59	31	185	52.3%	10.3	1906	

TABLE I-A (Continued)

ELK HUNT AREA REPORT

AREA (cross hunt areas)	TYPE	ACTIVE LICS/HTRS	HARVEST BULL	SPIKE	COW	CALF	TOTAL	HUNTER SUCCESS	DAYS/ HARVEST	HUNTER DAYS	LICENSES SOLD (cross hunt incl.)
22 Ferris											
	Limited 1	51	30	2	0	0	32	62.7%	13.9	445	52
	Limited 2	66	0	0	13	4	17	25.8%	18.1	307	79
Pooled Total		117	30	2	13	4	49	41.9%	15.3	752	
Pooled Resident		99	26	2	9	4	41	41.4%	15.9	652	
Pooled Nonresident		18	4	0	4	0	8	44.4%	12.5	100	
23 Rattlesnake											
	Limited 1	105	62	0	5	0	67	63.8%	9.1	612	105
	Limited 2	80	0	0	36	3	39	48.8%	17.7	689	100
	Limited 6	137	0	0	30	9	39	28.5%	24.8	968	150
Pooled Total		295	62	0	71	12	145	49.2%	15.6	2269	
Pooled Resident		254	53	0	62	10	125	49.2%	16.8	2103	
Pooled Nonresident		41	9	0	9	2	20	48.8%	8.3	166	
24 Green Mountain											
	Limited 1	127	64	7	0	0	71	55.9%	11.1	785	131
	Limited 2	71	0	0	43	8	51	71.8%	5.3	270	75
	Limited 3	117	0	0	48	3	51	43.6%	11.5	584	128
Pooled Total		315	64	7	91	11	173	54.9%	9.5	1639	
Pooled Resident		278	48	7	81	10	146	52.5%	10.0	1455	
Pooled Nonresident		37	16	0	10	1	27	73.0%	6.8	184	
25 South Pass											
**(27)**	Limited 1	123	43	6	8	1	58	47.2%	13.8	798	155
**(27)**	Limited 2	72	0	0	26	2	28	38.9%	19.9	557	100
	Limited 6	137	0	0	50	16	66	48.2%	10.5	695	152
Pooled Total		310	43	6	84	19	152	49.0%	13.5	2050	
Pooled Resident		265	36	6	65	7	114	43.0%	15.5	1769	
Pooled Nonresident		45	7	0	19	12	38	84.4%	7.4	281	
27 Upper Sweetwater											
**(25)**	Limited 1	52	10	2	4	0	16	30.8%	17.0	272	155
**(25)**	Limited 2	38	0	0	10	0	10	26.3%	19.3	193	100
Pooled Total		90	10	2	14	0	26	28.9%	17.9	465	
Pooled Resident		69	6	2	14	0	22	31.9%	14.7	323	
Pooled Nonresident		21	4	0	0	0	4	19.0%	35.5	142	
28 Cony Mountain											
	Limited 2	174	0	0	40	7	47	27.0%	24.1	1132	201
	Limited 6	141	0	0	33	7	40	28.4%	19.2	768	152
	General	1047	175	104	0	0	279	26.6%	26.2	7304	
Pooled Total		1346	175	104	73	14	366	27.2%	25.1	9204	
Pooled Resident		1252	159	91	53	14	317	25.3%	27.0	8564	
Pooled Nonresident		94	16	13	20	0	49	52.1%	13.1	640	
30 Aspen Mountain											
	Limited 1	52	39	0	5	0	44	84.6%	10.2	447	54
	Limited 2	130	0	0	68	18	86	66.2%	9.5	815	130
Pooled Total		182	39	0	73	18	130	71.4%	9.7	1262	
Pooled Resident		157	28	0	66	18	112	71.3%	10.1	1136	
Pooled Nonresident		25	11	0	7	0	18	72.0%	7.0	126	
31 Little Mountain											
	Limited 1	106	88	3	0	2	93	87.7%	9.1	845	106
	Limited 2	172	0	0	116	14	130	75.6%	5.3	689	179
Pooled Total		278	88	3	116	16	223	80.2%	6.9	1534	
Pooled Resident		240	71	3	103	16	193	80.4%	6.8	1303	
Pooled Nonresident		38	17	0	13	0	30	78.9%	7.7	231	
32 Pine Mountain											
	Limited 1	95	41	3	2	0	46	48.4%	15.9	732	99
	Limited 2	51	0	0	21	0	21	41.2%	19.4	408	55
Pooled Total		146	41	3	23	0	67	45.9%	17.0	1140	
Pooled Resident		115	32	0	19	0	51	44.3%	17.3	880	
Pooled Nonresident		31	9	3	4	0	16	51.6%	16.3	260	

AREA (cross hunt areas)	TYPE	ACTIVE LICS/HTRS	HARVEST BULL	SPIKE	COW	CALF	TOTAL	HUNTER SUCCESS	DAYS/ HARVEST	HUNTER DAYS	LICENSES SOLD (cross hunt incl.)
33 Middle Fork											
	Limited 1	91	32	3	12	2	49	53.8%	12.6	619	101
	Limited 2	51	0	0	17	0	17	33.3%	14.1	240	55
Pooled Total		142	32	3	29	2	66	46.5%	13.0	859	
Pooled Resident		114	20	3	21	0	44	38.6%	16.3	718	
Pooled Nonresident		28	12	0	8	2	22	78.6%	6.4	141	
34 Upper Powder River											
	Limited 1	698	173	30	66	13	282	40.4%	17.1	4832	807
	Limited 6	283	0	0	44	28	72	25.4%	21.7	1563	344
Pooled Total		970	173	30	110	41	354	36.5%	18.1	6395	
Pooled Resident		761	106	13	84	41	244	32.1%	21.4	5231	
Pooled Nonresident		209	67	17	26	0	110	52.6%	10.6	1164	
35 Hunter Mesa											
	Limited 1	148	46	14	0	0	60	40.5%	18.6	1116	151
	Limited 2	72	0	0	31	4	35	48.6%	18.0	631	76
	Limited 6	28	0	0	16	3	19	67.9%	5.4	102	50
	Limited 9	49	15	3	0	0	18	36.7%	27.3	491	53
Pooled Total		293	61	17	47	7	132	45.1%	17.7	2340	
Pooled Resident		247	47	14	41	6	108	43.7%	18.9	2045	
Pooled Nonresident		46	14	3	6	1	24	52.2%	12.3	295	
36 Rock Creek											
	Limited 2	149	0	0	32	12	44	29.5%	22.4	984	156
	Limited 9	50	0	0	0	0	0	0.0%	0.0	317	57
	General	584	52	22	0	0	74	12.7%	44.9	3324	
Pooled Total		783	52	22	32	12	118	15.1%	39.2	4625	
Pooled Resident		689	49	19	27	12	107	15.5%	38.4	4112	
Pooled Nonresident		94	3	3	5	0	11	11.7%	46.6	513	
37 Goose											
	Limited 6	321	0	0	71	8	79	24.6%	26.1	2060	409
	Limited 9	138	13	3	0	0	16	11.6%	79.8	1277	154
	General	928	100	22	43	4	169	18.2%	36.1	6096	
Pooled Total		1356	113	25	114	12	264	19.5%	35.7	9433	
Pooled Resident		1186	97	25	100	12	234	19.7%	35.5	8303	
Pooled Nonresident		170	16	0	14	0	30	17.6%	37.7	1130	
38 Tongue											
	Limited 1	378	92	19	11	12	134	35.4%	18.7	2505	403
	Limited 2	425	0	0	16	16	32	7.5%	78.9	2526	451
	Limited 9	248	51	0	10	0	61	24.6%	43.6	2661	253
Pooled Total		1051	143	19	37	28	227	21.6%	33.9	7692	
Pooled Resident		868	117	19	19	21	176	20.3%	36.8	6479	
Pooled Nonresident		183	26	0	18	7	51	27.9%	23.8	1213	
39 Deer Creek											
	Limited 1	88	14	9	28	6	57	64.8%	7.3	417	93
	Limited 2	76	23	7	0	0	30	39.5%	14.9	448	80
	Limited 9	71	14	6	4	0	24	33.8%	33.8	812	71
Pooled Total		235	51	22	32	6	111	47.2%	15.1	1677	
Pooled Resident		197	35	18	32	2	87	44.2%	16.1	1404	
Pooled Nonresident		38	16	4	0	4	24	63.2%	11.4	273	
40 Horse Creek											
	Limited 1	155	44	7	20	0	71	45.8%	9.0	637	177
	Limited 2	93	0	0	30	4	34	36.6%	13.9	471	103
	Limited 9	47	5	2	3	0	10	21.3%	46.4	464	54
Pooled Total		295	49	9	53	4	115	39.0%	13.7	1572	
Pooled Resident		242	35	9	41	4	89	36.8%	15.0	1334	
Pooled Nonresident		53	14	0	12	0	26	49.1%	9.2	238	

TABLE I-A (Continued)

ELK HUNT AREA REPORT

AREA (cross hunt areas)	TYPE	ACTIVE LICS/HTRS	HARVEST BULL	SPIKE	COW	CALF	TOTAL	HUNTER SUCCESS	DAYS/ HARVEST	HUNTER DAYS	LICENSES SOLD (cross hunt incl.)
41 North Medicine Lodge											
**(42)**	Limited 1	286	55	21	29	0	105	36.7%	17.7	1859	355
**(42)**	Limited 2	228	0	0	49	3	52	22.8%	30.0	1560	327
**(42)**	Limited 9	92	11	0	0	0	11	12.0%	75.6	832	106
Pooled Total		606	66	21	78	3	168	27.7%	25.3	4251	
Pooled Resident		511	40	16	69	3	128	25.0%	28.7	3679	
Pooled Nonresident		95	26	5	9	0	40	42.1%	14.3	572	
42 Medicine Lodge											
**(41)**	Limited 1	103	22	0	8	0	30	29.1%	23.7	711	355
**(41)**	Limited 2	146	0	0	24	6	30	20.5%	32.9	987	327
**(41)**	Limited 9	16	1	1	0	0	2	12.5%	51.0	102	106
Pooled Total		265	23	1	32	6	62	23.4%	29.0	1800	
Pooled Resident		220	12	0	18	6	36	16.4%	42.1	1515	
Pooled Nonresident		45	11	1	14	0	26	57.8%	11.0	285	
45 Paint Rock Creek											
**(46)**	Limited 1	286	128	15	11	0	154	53.8%	10.2	1576	351
	Limited 2	172	0	0	84	19	103	59.9%	11.6	1194	179
**(46)**	Limited 3	131	0	0	52	8	60	45.8%	11.6	696	155
**(46)**	Limited 9	120	32	0	4	1	37	30.8%	37.5	1388	142
Pooled Total		709	160	15	151	28	354	49.9%	13.7	4854	
Pooled Resident		606	131	15	113	27	286	47.2%	14.3	4104	
Pooled Nonresident		103	29	0	38	1	68	66.0%	11.0	750	
46 Leigh Creek											
**(45)**	Limited 1	111	38	4	11	0	53	47.7%	10.5	557	351
**(45)**	Limited 3	34	0	0	13	0	13	38.2%	10.2	133	155
**(45)**	Limited 9	21	6	0	0	0	6	28.6%	27.5	165	142
Pooled Total		166	44	4	24	0	72	43.4%	11.9	855	
Pooled Resident		147	34	4	24	0	62	42.2%	11.6	720	
Pooled Nonresident		19	10	0	0	0	10	52.6%	13.5	135	
47 Copper Mountain											
	Limited 1	97	37	2	5	5	49	50.5%	9.0	441	102
	Limited 3	24	13	3	1	0	17	70.8%	4.9	84	25
	Limited 6	43	0	0	12	2	14	32.6%	18.4	258	50
Pooled Total		142	50	5	18	7	80	56.3%	9.8	783	
Pooled Resident		107	42	3	11	7	63	58.9%	8.7	545	
Pooled Nonresident		35	8	2	7	0	17	48.6%	14.0	238	
48 Upper Nowood											
	Limited 1	278	127	0	29	6	162	58.3%	12.8	2069	302
	Limited 2	60	0	0	17	5	22	36.7%	13.2	290	68
	Limited 6	367	0	0	164	11	175	47.7%	11.0	1923	428
Pooled Total		663	127	0	210	22	359	54.1%	11.9	4282	
Pooled Resident		484	100	0	150	18	268	55.4%	13.4	3598	
Pooled Nonresident		179	27	0	60	4	91	50.8%	7.5	684	
49 Otter Creek											
	Limited 1	267	66	6	39	0	111	41.6%	16.8	1862	277
	Limited 2	65	0	0	23	3	26	40.0%	16.3	423	72
	Limited 6	222	0	0	83	17	100	45.0%	12.4	1239	251
Pooled Total		545	66	6	145	20	237	43.5%	14.9	3524	
Pooled Resident		435	45	6	122	20	193	44.4%	15.2	2940	
Pooled Nonresident		110	21	0	23	0	44	40.0%	13.3	584	
50 Beartooth											
	Limited 1	24	0	7	8	0	15	62.5%	10.4	156	27
	Limited 2	39	0	0	36	0	36	92.3%	6.6	237	41
	Limited 9	12	2	0	0	0	2	16.7%	46.0	92	12
	General	157	34	15	0	0	49	31.2%	18.9	928	
Pooled Total		232	36	22	44	0	102	44.0%	13.9	1413	
Pooled Resident		205	34	15	39	0	88	42.9%	14.4	1263	
Pooled Nonresident		27	2	7	5	0	14	51.9%	10.7	150	

TABLE I-A (Continued)

ELK HUNT AREA REPORT

AREA (cross hunt areas)	TYPE	ACTIVE LICS/HTRS	HARVEST BULL	SPIKE	COW	CALF	TOTAL	HUNTER SUCCESS	DAYS/ HARVEST	HUNTER DAYS	LICENSES SOLD (cross hunt incl.)
51 Crandall											
	Limited 1	40	14	4	4	2	24	60.0%	9.3	223	42
**(52)**	Limited 2	67	0	0	48	5	53	79.1%	6.0	317	153
	Limited 9	28	8	0	0	0	8	28.6%	23.6	189	30
	General	276	93	7	0	0	100	36.2%	16.8	1683	
Pooled Total		411	115	11	52	7	185	45.0%	13.0	2412	
Pooled Resident		279	54	8	44	6	112	40.1%	15.8	1769	
Pooled Nonresident		132	61	3	8	1	73	55.3%	8.8	643	
52 Sunlight											
	Limited 1	28	9	3	4	0	16	57.1%	11.8	189	28
**(51)**	Limited 2	89	0	0	75	0	75	84.3%	4.9	364	153
	Limited 9	6	0	0	0	0	0	0.0%	0.0	38	6
	General	196	31	0	0	0	31	15.8%	40.5	1256	
Pooled Total		319	40	3	79	0	122	38.2%	15.1	1847	
Pooled Resident		284	30	3	75	0	108	38.0%	15.5	1669	
Pooled Nonresident		35	10	0	4	0	14	40.0%	12.7	178	
53 Dead Indian											
**(54, 65)**	Limited 1	29	7	2	0	0	9	31.0%	22.7	204	51
**(54, 65)**	Limited 2	68	0	0	26	2	28	41.2%	18.1	506	150
**(54, 65)**	Limited 6	58	0	0	28	2	30	51.7%	8.5	256	150
**(54, 65)**	Limited 9	11	2	0	0	0	2	18.2%	46.0	92	26
	General	57	6	0	0	0	6	10.5%	44.5	267	
Pooled Total		215	15	2	54	4	75	34.9%	17.7	1325	
Pooled Resident		202	15	0	52	4	71	35.1%	17.7	1259	
Pooled Nonresident		13	0	2	2	0	4	30.8%	16.5	66	
54 Bald Ridge											
**(53, 65)**	Limited 1	31	5	0	2	0	7	22.6%	30.0	210	51
**(53, 65, also valid 121)**	Limited 2	91	0	0	36	0	36	39.6%	15.6	560	150
	Limited 3	25	0	0	23	0	23	92.0%	2.8	65	25
**(53, 65, also valid 121)**	Limited 6	63	0	0	26	2	28	44.4%	9.4	262	150
**(53, 65)**	Limited 9	22	5	0	0	0	5	22.7%	44.8	224	26
Pooled Total		224	10	0	87	2	99	44.2%	13.3	1321	
Pooled Resident		198	6	0	82	2	90	45.5%	13.3	1196	
Pooled Nonresident		26	4	0	5	0	9	34.6%	13.9	125	
55 Grinnell											
	Limited 1	25	5	0	6	0	11	44.0%	16.6	183	25
	Limited 2	65	0	0	44	4	48	73.8%	6.6	318	75
	Limited 9	18	1	0	0	0	1	5.6%	93.0	93	19
	General	343	114	0	0	0	114	33.2%	14.4	1642	
Pooled Total		451	120	0	50	4	174	38.6%	12.9	2236	
Pooled Resident		334	60	0	47	4	111	33.2%	15.2	1692	
Pooled Nonresident		117	60	0	3	0	63	53.8%	8.6	544	
56 Wapiti Ridge											
	Limited 1	47	20	4	0	0	24	51.1%	12.3	294	47
	Limited 2	158	0	0	76	12	88	55.7%	11.1	977	168
	Limited 3	37	0	0	21	0	21	56.8%	10.2	215	50
	Limited 9	22	6	0	0	0	6	27.3%	22.8	137	23
	General	366	96	21	0	0	117	32.0%	15.6	1827	
Pooled Total		630	122	25	97	12	256	40.6%	13.5	3450	
Pooled Resident		494	84	23	75	9	191	38.7%	14.9	2844	
Pooled Nonresident		136	38	2	22	3	65	47.8%	9.3	606	
58 Sage Creek											
	Limited 1	64	47	0	0	0	47	73.4%	13.1	616	70
	Limited 2	134	0	0	75	8	83	61.9%	14.2	1176	151
Pooled Total		198	47	0	75	8	130	65.7%	13.8	1792	
Pooled Resident		163	36	0	72	8	116	71.2%	13.4	1558	
Pooled Nonresident		35	11	0	3	0	14	40.0%	16.7	234	

AREA (cross hunt areas)	TYPE	ACTIVE LICS/HTRS	HARVEST BULL	SPIKE	COW	CALF	TOTAL	HUNTER SUCCESS	DAYS/ HARVEST	HUNTER DAYS	LICENSES SOLD (cross hunt incl.)
59 Boulder Basin											
	Limited 1	11	10	0	0	0	10	90.9%	10.4	104	13
	Limited 2	151	0	0	92	10	102	67.5%	8.5	863	154
	Limited 6	84	0	0	43	12	55	65.5%	8.1	445	101
	Limited 9	25	0	0	0	0	0	0.0%	0.0	155	33
	General	362	115	20	0	0	135	37.3%	13.2	1784	
Pooled Total		607	125	20	135	22	302	49.8%	11.1	3351	
Pooled Resident		457	74	20	105	22	221	48.4%	11.9	2626	
Pooled Nonresident		150	51	0	30	0	81	54.0%	9.0	725	
60 Thorofare											
	Limited 9	19	4	0	0	0	4	21.1%	24.8	99	19
	General	379	200	3	7	0	210	55.4%	10.4	2193	
Pooled Total		398	204	3	7	0	214	53.8%	10.7	2292	
Pooled Resident		136	59	0	0	0	59	43.4%	14.3	846	
Pooled Nonresident		262	145	3	7	0	155	59.2%	9.3	1446	
61 North Greybull River											
	Limited 1	135	81	9	3	0	93	68.9%	6.9	639	157
	Limited 2	28	17	0	3	0	20	71.4%	9.7	194	28
	Limited 3	71	0	0	39	2	41	57.7%	6.1	252	82
	General	65	0	0	28	4	32	49.2%	8.1	260	
Pooled Total		299	98	9	73	6	186	62.2%	7.2	1345	
Pooled Resident		223	60	9	57	4	130	58.3%	7.7	1001	
Pooled Nonresident		76	38	0	16	2	56	73.7%	6.1	344	
62 South Greybull River											
	Limited 1	149	64	0	6	0	70	47.0%	13.5	945	158
	Limited 2	44	0	0	15	3	18	40.9%	14.4	259	54
	Limited 3	147	0	0	32	6	38	25.9%	25.7	978	155
Pooled Total		340	64	0	53	9	126	37.1%	17.3	2182	
Pooled Resident		278	44	0	49	5	98	35.3%	18.1	1771	
Pooled Nonresident		62	20	0	4	4	28	45.2%	14.7	411	
63 Wood River-Gooseberry Creek											
**(64)**	Limited 1	151	91	8	0	3	102	67.5%	7.9	805	210
	Limited 2	141	0	0	58	5	63	44.7%	13.5	851	159
	Limited 6	90	0	0	35	9	44	48.9%	10.1	445	100
Pooled Total		349	91	8	93	17	209	59.9%	10.1	2101	
Pooled Resident		294	66	8	76	16	166	56.5%	11.0	1832	
Pooled Nonresident		55	25	0	17	1	43	78.2%	6.3	269	
64 Owl Creek											
**(63)**	Limited 1	92	48	3	3	0	54	58.7%	9.7	525	210
	Limited 2	95	33	10	3	0	46	48.4%	10.6	489	103
	Limited 6	106	0	0	48	13	61	57.5%	8.7	530	125
Pooled Total		268	81	13	54	13	161	60.1%	9.6	1544	
Pooled Resident		216	57	13	41	10	121	56.0%	10.6	1282	
Pooled Nonresident		52	24	0	13	3	40	76.9%	6.6	262	
65 McCullough Peaks											
**(53, 54, also valid 121)**	Limited 1	42	2	0	3	2	7	16.7%	27.0	189	51
**(53, 54, also valid 121)**	Limited 2	91	0	0	16	7	23	25.3%	28.3	652	150
	Limited 5	45	0	0	13	0	13	28.9%	22.4	291	95
**(53, 54, also valid 121)**	Limited 6	89	0	0	29	5	34	38.2%	12.9	438	150
	Limited 7	93	0	0	16	8	24	25.8%	15.2	365	103
**(53, 54)**	Limited 9	2	0	0	0	0	0	0.0%	0.0	4	26
Pooled Total		350	2	0	77	22	101	28.9%	19.2	1939	
Pooled Resident		320	2	0	65	21	88	27.5%	20.5	1801	
Pooled Nonresident		30	0	0	12	1	13	43.3%	10.6	138	
66 Basin											
**(also valid 61)**	Limited 2	2	0	0	0	0	0	0.0%	0.0	2	
	General	105	0	0	7	0	7	6.7%	71.7	502	
Pooled Total		107	0	0	7	0	7	6.5%	72.0	504	
Pooled Resident		107	0	0	7	0	7	6.5%	72.0	504	
Pooled Nonresident		0	0	0	0	0	0	0.0%	0.0	0	

TABLE I-A (Continued)

ELK HUNT AREA REPORT

AREA (cross hunt areas)	TYPE	ACTIVE LICS/HTRS	HARVEST BULL	SPIKE	COW	CALF	TOTAL	HUNTER SUCCESS	DAYS/ HARVEST	HUNTER DAYS	LICENSES SOLD (cross hunt incl.)
67 Wiggins Fork											
**(68)**	Limited 2	213	0	0	103	9	112	52.6%	11.6	1304	255
**(68, 69)**	Limited 9	74	8	0	0	0	8	10.8%	68.3	546	129
	General	1032	238	90	0	0	328	31.8%	18.8	6157	
Pooled Total		1319	246	90	103	9	448	34.0%	17.9	8007	
Pooled Resident		1099	186	80	84	9	359	32.7%	18.7	6727	
Pooled Nonresident		220	60	10	19	0	89	40.5%	14.4	1280	
68 Warm Springs											
**(67)**	Limited 2	49	0	0	12	0	12	24.5%	14.3	171	255
**(67, 69)**	Limited 9	55	6	0	2	1	9	16.4%	47.9	431	129
	General	541	80	26	0	0	106	19.6%	31.1	3298	
Pooled Total		645	86	26	14	1	127	19.7%	30.7	3900	
Pooled Resident		537	80	26	9	0	115	21.4%	27.9	3214	
Pooled Nonresident		108	6	0	5	1	12	11.1%	57.2	686	
69 Whiskey Mountain											
	Limited 6	36	0	0	11	2	13	36.1%	13.6	177.0	50
**(67, 68)**	Limited 9	15	0	0	0	0	0	0.0%	0.0	72	129
	General	279	58	0	32	8	98	35.1%	14.1	1380	
Pooled Total		319	58	0	43	10	111	34.8%	14.7	1629	
Pooled Resident		245	32	0	41	10	83	33.9%	14.3	1189	
Pooled Nonresident		74	26	0	2	0	28	37.8%	15.7	440	
70 Buffalo Fork											
	General	554	160	4	0	0	164	29.6%	20.7	3401	
Pooled Total		554	160	4	0	0	164	29.6%	20.7	3401	
Pooled Resident		410	85	4	0	0	89	21.7%	29.9	2660	
Pooled Nonresident		144	75	0	0	0	75	52.1%	9.9	741	
71 Pacific Creek											
	General	340	82	7	0	0	89	26.2%	25.3	2248	
Pooled Total		340	82	7	0	0	89	26.2%	25.3	2248	
Pooled Resident		176	23	7	0	0	30	17.0%	41.1	1232	
Pooled Nonresident		164	59	0	0	0	59	36.0%	17.2	1016	
73 Targhee-Alta											
	Limited 2	7	0	0	0	0	0	0.0%	0.0	35	9
	Limited 6	32	0	0	3	3	6	18.8%	37.0	222	49
	General	281	74	7	7	0	88	31.3%	21.1	1854	
Pooled Total		315	74	7	10	3	94	29.8%	22.5	2111	
Pooled Resident		179	22	7	10	0	39	21.8%	33.8	1318	
Pooled Nonresident		136	52	0	0	3	55	40.4%	14.4	793	
74 Ditch Creek											
	General	143	14	0	8	8	30	21.0%	31.9	956	
Pooled Total		143	14	0	8	8	30	21.0%	31.9	956	
Pooled Resident		127	11	0	8	8	27	21.3%	32.7	884	
Pooled Nonresident		16	3	0	0	0	3	18.8%	24.0	72	
75 Snake River											
	Limited 1	102	44	6	7	2	59	57.8%	14.6	861	106
**(also valid 79)**	Limited 2	240	0	0	57	15	72	30.0%	21.3	1532	240
**(also valid 79)**	Limited 6	132	0	0	64	4	68	51.5%	10.2	696	
Pooled Total		474	44	6	128	21	199	42.0%	15.5	3089	
Pooled Resident		292	29	6	60	6	101	34.6%	19.9	2009	
Pooled Nonresident		182	15	0	68	15	98	53.8%	11.0	1080	

TABLE I-A (Continued)

ELK HUNT AREA REPORT

AREA (cross hunt areas)	TYPE	ACTIVE LICS/HTRS	HARVEST BULL	SPIKE	COW	CALF	TOTAL	HUNTER SUCCESS	DAYS/ HARVEST	HUNTER DAYS	LICENSES SOLD (cross hunt incl.)
77 National Elk Refuge											
**(also valid limited)**	Limited 1	33	5	0	2	3	10	30.3%	8.5	85	
**(also valid limited)**	Limited 2	46	0	0	22	0	22	47.8%	7.0	154	
**(also valid limited)**	Limited 3	0	0	0	0	0	0	0.0%	0.0	0	
**(also valid limited)**	Limited 4	0	0	0	0	0	0	0.0%	0.0	0	
**(also valid limited)**	Limited 5	0	0	0	0	0	0	0.0%	0.0	0	
**(also valid limited)**	Limited 6	94	0	0	31	6	37	39.4%	12.8	473	
**(also valid limited)**	Limited 7	0	0	0	0	0	0	0.0%	0.0	0	
**(also valid limited)**	Limited 8	0	0	0	0	0	0	0.0%	0.0	0	
	General	263	23	12	61	21	117	44.5%	9.5	1117	
Pooled Total		422	28	12	116	30	186	44.1%	9.8	1829	
Pooled Resident		355	20	12	98	30	160	45.1%	10.2	1637	
Pooled Nonresident		67	8	0	18	0	26	38.8%	7.4	192	
78 Wilson											
	Limited 1	27	24	0	0	0	24	88.9%	9.2	221	27
	Limited 2	14	0	0	7	2	9	64.3%	7.4	67	25
	Limited 6	40	0	0	18	2	20	50.0%	11.0	220	75
Pooled Total		78	24	0	25	4	53	67.9%	9.6	508	
Pooled Resident		63	20	0	22	4	46	73.0%	10.3	473	
Pooled Nonresident		15	4	0	3	0	7	46.7%	5.0	35	
79 Teton Park											
	Limited 1	178	44	9	12	3	68	38.2%	16.4	1115	200
	Limited 2	48	0	0	20	0	20	41.7%	13.5	269	58
	Limited 6	154	0	0	21	0	21	13.6%	33.6	705	300
Pooled Total		371	44	9	53	3	109	29.4%	19.2	2089	
Pooled Resident		233	34	9	18	3	64	27.5%	21.4	1370	
Pooled Nonresident		138	10	0	35	0	45	32.6%	16.0	719	
80 Sheep Creek											
	Limited 2	6	0	0	2	2	4	66.7%	8.8	35	7
	General	498	40	7	162	16	225	45.2%	11.2	2526	
Pooled Total		504	40	7	164	18	229	45.4%	11.2	2561	
Pooled Resident		475	34	7	159	16	216	45.5%	11.2	2412	
Pooled Nonresident		29	6	0	5	2	13	44.8%	11.5	149	
81 Spread Creek											
	Limited 2	28	0	0	5	0	5	17.9%	48.2	241	28
	General	951	153	0	131	24	308	32.4%	17.5	5389	
Pooled Total		979	153	0	136	24	313	32.0%	18.0	5630	
Pooled Resident		741	88	0	111	24	223	30.1%	19.0	4245	
Pooled Nonresident		238	65	0	25	0	90	37.8%	15.4	1385	
82 Crystal Peak											
	Limited 2	25	0	0	8	3	11	44.0%	12.3	135	25
	General	476	103	0	61	6	170	35.7%	14.2	2419	
Pooled Total		501	103	0	69	9	181	36.1%	14.1	2554	
Pooled Resident		394	61	0	59	6	126	32.0%	15.7	1977	
Pooled Nonresident		107	42	0	10	3	55	51.4%	10.5	577	
83 Fish Creek											
	Limited 2	30	0	0	11	6	17	56.7%	9.7	165	31
	General	588	86	0	83	16	185	31.5%	18.5	3415	
Pooled Total		618	86	0	94	22	202	32.7%	17.7	3580	
Pooled Resident		540	70	0	74	16	160	29.6%	19.4	3101	
Pooled Nonresident		78	16	0	20	6	42	53.8%	11.4	479	
84 Lower Hoback											
	Limited 1	8	3	1	0	0	4	50.0%	9.0	36	22
	Limited 2	42	0	0	9	2	11	26.2%	25.5	281	49
	Limited 6	221	0	0	49	17	66	29.9%	24.4	1611	275
	General	1159	194	68	123	36	421	36.3%	16.5	6955	
Pooled Total		1391	197	69	181	55	502	36.1%	17.7	8883	
Pooled Resident		1020	112	48	132	47	339	33.2%	19.2	6510	
Pooled Nonresident		371	85	21	49	8	163	43.9%	14.6	2373	

TABLE I-A (Continued)

ELK HUNT AREA REPORT

AREA (cross hunt areas)	TYPE	ACTIVE LICS/HTRS	HARVEST BULL	SPIKE	COW	CALF	TOTAL	HUNTER SUCCESS	DAYS/ HARVEST	HUNTER DAYS	LICENSES SOLD (cross hunt incl.)
85 Fall Creek											
	Limited 1	12	2	0	0	0	2	16.7%	83.0	166	20
	Limited 2	44	0	0	14	3	17	38.6%	19.9	338	50
	Limited 6	233	0	0	85	18	103	44.2%	16.3	1683	277
	General	976	162	33	97	18	310	31.8%	19.2	5950	
Pooled Total		1228	164	33	196	39	432	35.2%	18.8	8137	
Pooled Resident		886	89	26	137	28	280	31.6%	21.6	6054	
Pooled Nonresident		342	75	7	59	11	152	44.4%	13.7	2083	
86 Monument Ridge											
	General	325	59	12	28	9	108	33.2%	16.0	1730	
Pooled Total		325	59	12	28	9	108	33.2%	16.0	1730	
Pooled Resident		250	23	12	22	9	66	26.4%	20.3	1343	
Pooled Nonresident		75	36	0	6	0	42	56.0%	9.2	387	
87 Raspberry Ridge											
	Limited 6	21	0	0	5	4	9	42.9%	13.3	120	56
	General	590	66	48	40	4	158	26.8%	24.4	3853	
Pooled Total		607	66	48	45	8	167	27.5%	23.8	3973	
Pooled Resident		551	56	41	42	4	143	26.0%	24.9	3565	
Pooled Nonresident		56	10	7	3	4	24	42.9%	17.0	408	
88 Greys Feedground											
	Limited 1	51	5	2	8	2	17	33.3%	24.4	415	60
Pooled Total		51	5	2	8	2	17	33.3%	24.4	415	
Pooled Resident		43	2	2	8	2	14	32.6%	24.3	340	
Pooled Nonresident		8	3	0	0	0	3	37.5%	25.0	75	
89 Lower Greys River											
	General	978	146	15	78	15	254	26.0%	18.0	4576	
Pooled Total		978	146	15	78	15	254	26.0%	18.0	4576	
Pooled Resident		749	84	12	78	15	189	25.2%	18.5	3503	
Pooled Nonresident		229	62	3	0	0	65	28.4%	16.5	1073	
90 Upper Greys River											
	Limited 6	75	0	0	11	4	15	20.0%	46.1	692	101
	General	805	85	22	61	16	184	22.9%	23.7	4367	
Pooled Total		867	85	22	72	20	199	23.0%	25.4	5059	
Pooled Resident		687	33	16	62	16	127	18.5%	30.4	3863	
Pooled Nonresident		180	52	6	10	4	72	40.0%	16.6	1196	
91 Salt River											
	Limited 1	296	34	6	96	12	148	50.0%	16.8	2486	355
	Limited 6	67	0	0	24	4	28	41.8%	14.1	394	100
	General	654	91	11	43	3	148	22.6%	23.5	3480	
Pooled Total		984	125	17	163	19	324	32.9%	19.6	6360	
Pooled Resident		770	75	11	159	17	262	34.0%	19.6	5144	
Pooled Nonresident		214	50	6	4	2	62	29.0%	19.6	1216	
92 Piney-Horse Creek											
**(also valid 94)**	Limited 1	3	0	0	0	0	0	0.0%	0.0	16	
**(also valid 94)**	Limited 6	121	0	0	55	17	72	59.5%	11.2	805	203
	General	1053	160	35	139	33	367	34.9%	18.4	6747	
Pooled Total		1145	160	35	194	50	439	38.3%	17.2	7568	
Pooled Resident		966	114	25	152	45	336	34.8%	19.7	6616	
Pooled Nonresident		179	46	10	42	5	103	57.5%	9.2	952	
93 Waterdog Lakes											
	Limited 1	142	20	7	14	7	48	33.8%	21.5	1034	155
	Limited 2	24	0	0	8	2	10	41.7%	15.5	155	26
	Limited 6	118	0	0	44	3	47	39.8%	17.1	806	140
Pooled Total		269	20	7	66	12	105	39.0%	19.0	1995	
Pooled Resident		186	18	7	47	10	82	44.1%	17.5	1439	
Pooled Nonresident		83	2	0	19	2	23	27.7%	24.2	556	

TABLE I-A (Continued)

ELK HUNT AREA REPORT

AREA (cross hunt areas)	TYPE	ACTIVE LICS/HTRS	HARVEST BULL	SPIKE	COW	CALF	TOTAL	HUNTER SUCCESS	DAYS/ HARVEST	HUNTER DAYS	LICENSES SOLD (cross hunt incl.)
94 South Piney											
	Limited 1	47	6	0	7	0	13	27.7%	29.5	384	62
	Limited 6	221	0	0	49	26	75	33.9%	20.1	1505	276
	General	1422	163	76	132	45	416	29.3%	20.9	8705	
Pooled Total		1625	169	76	188	71	504	31.0%	21.0	10594	
Pooled Resident		1427	137	69	157	71	434	30.4%	21.8	9476	
Pooled Nonresident		198	32	7	31	0	70	35.4%	16.0	1118	
95 Green River											
	Limited 1	152	49	8	16	0	73	48.0%	17.2	1258	155
	Limited 2	240	0	0	79	22	101	42.1%	15.3	1548	248
	Limited 3	26	12	0	2	0	14	53.8%	15.9	222	32
	Limited 4	10	0	0	4	0	4	40.0%	7.3	29	11
	Limited 6	22	0	0	5	0	5	22.7%	30.0	150	25
Pooled Total		434	61	8	106	22	197	45.4%	16.3	3207	
Pooled Resident		348	44	6	73	22	145	41.7%	18.0	2610	
Pooled Nonresident		86	17	2	33	0	52	60.5%	11.5	597	
96 New Fork											
**(also valid 93)**	Limited 1	189	39	12	11	4	66	34.9%	23.8	1571	206
**(also valid 93)**	Limited 2	9	0	0	2	0	2	22.2%	40.0	80	9
**(also valid 93)**	Limited 6	63	0	0	15	2	17	27.0%	40.4	687	75
	General	217	15	4	8	9	36	16.6%	33.2	1195	
Pooled Total		457	54	16	36	15	121	26.5%	29.2	3533	
Pooled Resident		360	34	16	33	11	94	26.1%	31.7	2984	
Pooled Nonresident		97	20	0	3	4	27	27.8%	20.3	549	
97 Pinedale											
	Limited 1	265	82	12	25	0	119	44.9%	13.4	1597	301
	Limited 2	15	0	0	0	0	0	0.0%	0.0	79	16
	Limited 6	58	0	0	12	0	12	20.7%	28.7	344	75
	General	230	34	4	18	4	60	26.1%	18.5	1110	
Pooled Total		547	116	16	55	4	191	34.9%	16.4	3130	
Pooled Resident		325	55	4	46	4	109	33.5%	16.6	1811	
Pooled Nonresident		222	61	12	9	0	82	36.9%	16.1	1319	
98 Boulder											
	Limited 1	334	75	10	28	10	123	36.8%	20.7	2546	353
	Limited 2	59	0	0	6	5	11	18.6%	31.0	341	86
	Limited 6	106	0	0	23	8	31	29.2%	24.9	772	151
	General	345	42	4	9	0	55	15.9%	31.3	1722	
Pooled Total		811	117	14	66	23	220	27.1%	24.5	5381	
Pooled Resident		669	79	10	56	17	162	24.2%	28.5	4621	
Pooled Nonresident		142	38	4	10	6	58	40.8%	13.1	760	
99 Big Sandy											
	Limited 1	157	47	12	0	0	59	37.6%	20.5	1208	179
	Limited 2	216	0	0	23	0	23	10.6%	74.6	1716	228
Pooled Total		373	47	12	23	0	82	22.0%	35.7	2924	
Pooled Resident		317	28	12	23	0	63	19.9%	41.8	2631	
Pooled Nonresident		56	19	0	0	0	19	33.9%	15.4	293	
100 Steamboat											
	Limited 1	233	192	0	0	0	192	82.4%	7.3	1396	233
	Limited 2	194	0	0	126	27	153	78.9%	5.0	759	202
Pooled Total		427	192	0	126	27	345	80.8%	6.2	2155	
Pooled Resident		369	157	0	114	23	294	79.7%	6.7	1969	
Pooled Nonresident		58	35	0	12	4	51	87.9%	3.6	186	
102 West Green River											
	General	1548	193	86	222	44	545	35.2%	15.4	8391	
Pooled Total		1548	193	86	222	44	545	35.2%	15.4	8391	
Pooled Resident		1446	174	76	199	44	493	34.1%	15.9	7833	
Pooled Nonresident		102	19	10	23	0	52	51.0%	10.7	558	

ELK HUNT AREA REPORT

AREA (cross hunt areas)	TYPE	ACTIVE LICS/HTRS	HARVEST BULL	SPIKE	COW	CALF	TOTAL	HUNTER SUCCESS	DAYS/ HARVEST	HUNTER DAYS	LICENSES SOLD (cross hunt incl.)
103 Raymond Mountain											
	Limited 6	29	0	0	12	1	13	44.8%	6.5	84	53
	General	381	37	18	67	14	136	35.7%	11.7	1591	
Pooled Total		405	37	18	79	15	149	36.8%	11.2	1675	
Pooled Resident		340	24	15	71	14	124	36.5%	10.6	1313	
Pooled Nonresident		65	13	3	8	1	25	38.5%	14.5	362	
104 Lincoln											
	Limited 6	125	0	0	39	29	68	54.4%	10.3	699	151
	General	1136	178	48	120	45	391	34.4%	14.7	5749	
Pooled Total		1228	178	48	159	74	459	37.4%	14.0	6448	
Pooled Resident		1099	145	45	143	61	394	35.9%	14.7	5777	
Pooled Nonresident		129	33	3	16	13	65	50.4%	10.3	671	
105 Carter Lease											
	General	422	43	15	31	11	100	23.7%	19.3	1932	
Pooled Total		422	43	15	31	11	100	23.7%	19.3	1932	
Pooled Resident		419	43	15	31	11	100	23.9%	19.1	1906	
Pooled Nonresident		3	0	0	0	0	0	0.0%	0.0	26	
106 Uinta											
	Limited 1	30	17	5	3	0	25	83.3%	10.0	250	34
	Limited 2	98	0	0	21	2	23	23.5%	22.6	520	149
	Limited 6	24	0	0	8	0	8	33.3%	25.8	206	25
	General	697	133	25	44	7	209	30.0%	17.9	3751	
Pooled Total		849	150	30	76	9	265	31.2%	17.8	4727	
Pooled Resident		783	123	30	66	7	226	28.9%	19.7	4441	
Pooled Nonresident		66	27	0	10	2	39	59.1%	7.3	286	
107 Hickey Mountain											
	Limited 2	71	0	0	25	0	25	35.2%	21.5	538	101
	General	524	47	25	72	4	148	28.2%	14.8	2183	
Pooled Total		595	47	25	97	4	173	29.1%	15.7	2721	
Pooled Resident		583	47	25	85	4	161	27.6%	16.6	2674	
Pooled Nonresident		12	0	0	12	0	12	100.0%	3.9	47	
108 South Rawlins											
	Limited 1	60	34	0	2	0	36	60.0%	8.4	302	61
	Limited 2	76	0	0	43	8	51	67.1%	8.1	411	84
	Limited 3	12	0	0	7	0	7	58.3%	4.9	34	13
Pooled Total		148	34	0	52	8	94	63.5%	7.9	747	
Pooled Resident		97	18	0	26	7	51	52.6%	11.5	587	
Pooled Nonresident		51	16	0	26	1	43	84.3%	3.7	160	
110 Upper Platte River											
	Limited 2	68	0	0	7	0	7	10.3%	53.9	377	76
**(12, 13, 15)**	Limited 6	32	0	0	23	2	25	78.1%	5.5	138	75
	General	1738	156	41	136	33	366	21.1%	28.9	10576	
Pooled Total		1833	156	41	166	35	398	21.7%	27.9	11091	
Pooled Resident		1724	140	41	159	35	375	21.8%	27.7	10390	
Pooled Nonresident		109	16	0	7	0	23	21.1%	30.5	701	
111 Seminoe											
	Limited 1	43	34	2	0	0	36	83.7%	7.1	257	47
	Limited 2	87	0	0	27	4	31	35.6%	16.2	503	95
	Limited 3	38	0	0	2	2	4	10.5%	47.0	188	53
Pooled Total		168	34	2	29	6	71	42.3%	13.4	948	
Pooled Resident		137	24	2	20	6	52	38.0%	16.1	839	
Pooled Nonresident		31	10	0	9	0	19	61.3%	5.7	109	
113 Rochelle Hills											
	Limited 1	47	37	2	3	0	42	89.4%	4.7	199	47
	Limited 2	56	0	0	42	6	48	85.7%	2.9	138	61
Pooled Total		103	37	2	45	6	90	87.4%	3.7	337	
Pooled Resident		84	28	2	41	6	77	91.7%	3.0	232	
Pooled Nonresident		19	9	0	4	0	13	68.4%	8.1	105	

TABLE I-A (Continued)

ELK HUNT AREA REPORT

AREA (cross hunt areas)	TYPE	ACTIVE LICS/HTRS	HARVEST BULL	SPIKE	COW	CALF	TOTAL	HUNTER SUCCESS	DAYS/ HARVEST	HUNTER DAYS	LICENSES SOLD (cross hunt incl.)
114 Kyle											
	Limited 1	49	22	5	1	0	28	57.1%	12.3	344	51
	Limited 2	36	0	0	17	4	21	58.3%	8.8	184	41
	Limited 6	38	0	0	14	7	21	55.3%	9.4	197	50
Pooled Total		109	22	5	32	11	70	64.2%	10.4	725	
Pooled Resident		80	17	5	23	9	54	67.5%	10.3	556	
Pooled Nonresident		29	5	0	9	2	16	55.2%	10.6	169	
116 Bear Lodge											
	Limited 1	164	67	4	0	0	71	43.3%	31.4	2229	178
	Limited 2	127	0	0	38	3	41	32.3%	40.5	1662	150
	Limited 6	86	0	0	27	0	27	31.4%	42.2	1140	100
Pooled Total		353	67	4	65	3	139	39.4%	36.2	5031	
Pooled Resident		294	57	0	57	0	114	38.8%	40.5	4617	
Pooled Nonresident		59	10	4	8	3	25	42.4%	16.6	414	
117 South Black Hills											
	Limited 1	220	145	14	19	0	178	80.9%	11.5	2050	232
	Limited 2	160	0	0	57	3	60	37.5%	24.9	1491	175
	Limited 6	55	0	0	23	4	27	49.1%	18.3	495	62
Pooled Total		421	145	14	99	7	265	62.9%	15.2	4036	
Pooled Resident		356	110	14	82	6	212	59.6%	17.1	3629	
Pooled Nonresident		65	35	0	17	1	53	81.5%	7.7	407	
118 Shamrock Hills											
	Limited 1	36	27	0	0	0	27	75.0%	5.8	156	38
	Limited 2	36	0	0	9	2	11	30.6%	18.7	206	38
	Limited 6	26	0	0	12	2	14	53.8%	12.9	181	36
Pooled Total		90	27	0	21	4	52	57.8%	10.4	543	
Pooled Resident		72	16	0	17	2	35	48.6%	11.9	415	
Pooled Nonresident		18	11	0	4	2	17	94.4%	7.5	128	
120 Buffalo Creek											
	Limited 1	103	42	3	8	0	53	51.5%	9.1	482	109
	Limited 2	99	0	0	40	11	51	51.5%	14.0	716	102
	Limited 3	72	0	0	24	0	24	33.3%	20.6	495	74
Pooled Total		274	42	3	72	11	128	46.7%	13.2	1693	
Pooled Resident		233	24	3	64	11	102	43.8%	15.3	1562	
Pooled Nonresident		41	18	0	8	0	26	63.4%	5.0	131	
121 Trout Creek-Rattlesnake Mountain											
	Limited 1	97	40	0	0	0	40	41.2%	21.0	840	105
**(also valid 53, 54, 65)**	Limited 2	99	0	0	39	3	42	42.4%	10.0	419	125
	Limited 3	27	0	0	18	2	20	74.1%	3.6	72	36
**(also valid 53, 54, 65)**	Limited 6	77	0	0	19	2	21	27.3%	20.7	434	100
Pooled Total		293	40	0	76	7	123	42.0%	14.3	1765	
Pooled Resident		236	32	0	50	7	89	37.7%	17.1	1519	
Pooled Nonresident		57	8	0	26	0	34	59.6%	7.2	246	
122 Pine Ridge											
	Limited 1	48	22	0	10	3	35	72.9%	10.0	350	51
	Limited 2	0	0	0	0	0	0	0.0%	0.0	0	5
	Limited 6	33	0	0	13	2	15	45.5%	8.7	131	33
Pooled Total		77	22	0	23	5	50	64.9%	9.6	481	
Pooled Resident		71	19	0	23	3	45	63.4%	9.9	447	
Pooled Nonresident		6	3	0	0	2	5	83.3%	6.8	34	
123 Black Thunder											
	Limited 2	83	0	0	53	6	59	71.1%	5.7	338	100
Pooled Total		83	0	0	53	6	59	71.1%	5.7	338	
Pooled Resident		81	0	0	53	6	59	72.8%	5.5	322	
Pooled Nonresident		2	0	0	0	0	0	0.0%	0.0	16	

TABLE I-A (Continued)

ELK HUNT AREA REPORT

AREA (cross hunt areas)	TYPE	ACTIVE LICS/HTRS	HARVEST BULL	SPIKE	COW	CALF	TOTAL	HUNTER SUCCESS	DAYS/ HARVEST	HUNTER DAYS	LICENSES SOLD (cross hunt incl.)
124 Powder Rim											
	Limited 1	18	10	0	0	0	10	55.6%	9.2	92	18
	Limited 2	58	0	0	28	4	32	55.2%	12.2	390	62
Pooled Total		76	10	0	28	4	42	55.3%	11.5	482	
Pooled Resident		60	6	0	19	4	29	48.3%	13.9	404	
Pooled Nonresident		16	4	0	9	0	13	81.3%	6.0	78	
125 Elk Mountain											
	Limited 1	116	57	3	12	0	72	62.1%	6.7	483	138
**(also valid 114)**	Limited 2	13	0	0	2	0	2	15.4%	21.0	42	13
**(also valid 114)**	Limited 6	46	0	0	19	2	21	45.7%	5.8	121	76
Pooled Total		173	57	3	33	2	95	54.9%	6.8	646	
Pooled Resident		89	10	3	21	2	36	40.4%	10.0	361	
Pooled Nonresident		84	47	0	12	0	59	70.2%	4.8	285	
126 Flat Top											
	Limited 1	27	16	0	0	0	16	59.3%	7.7	123	27
	Limited 2	33	0	0	13	6	19	57.6%	7.9	151	35
Pooled Total		60	16	0	13	6	35	58.3%	7.8	274	
Pooled Resident		49	14	0	10	4	28	57.1%	6.6	186	
Pooled Nonresident		11	2	0	3	2	7	63.6%	12.6	88	
127 Crowheart Butte											
	General	54	4	0	0	0	4	7.4%	93.3	373	
Pooled Total		54	4	0	0	0	4	7.4%	93.3	373	
Pooled Resident		44	4	0	0	0	4	9.1%	70.3	281	
Pooled Nonresident		10	0	0	0	0	0	0.0%	0.0	92	
128 Muskrat											
	Limited 6	11	0	0	2	0	2	18.2%	17.5	35	20
	General	221	11	0	8	4	23	10.4%	41.4	953	
Pooled Total		232	11	0	10	4	25	10.8%	39.5	988	
Pooled Resident		227	11	0	10	4	25	11.0%	38.6	966	
Pooled Nonresident		5	0	0	0	0	0	0.0%	0.0	22	
129 Rest of State											
	Limited 6	60	0	0	12	10	22	36.7%	24.0	529	67
	General	635	103	8	44	5	160	25.2%	30.1	4812	
Pooled Total		670	103	8	56	15	182	27.2%	29.3	5341	
Pooled Resident		634	90	8	51	15	164	25.9%	31.5	5158	
Pooled Nonresident		36	13	0	5	0	18	50.0%	10.2	183	
130 Bolten Rim											
	General	22	0	0	0	0	0	0.0%	0.0	107	
Pooled Total		22	0	0	0	0	0	0.0%	0.0	107	
Pooled Resident		19	0	0	0	0	0	0.0%	0.0	87	
Pooled Nonresident		3	0	0	0	0	0	0.0%	0.0	20	

TABLE I-H

ELK HARVEST

BY HERD UNIT

2007

HERD	TYPE	ACTIVE LICS/HTRS	HARVEST BULL	SPIKE	COW	CALF	TOTAL	HUNTER SUCCESS	DAYS/ HARVEST	HUNTER DAYS	LICENSES SOLD (cross hunt incl.)
101 Targhee	Cow/Calf	32	0	0	3	3	6	18.8%	37.0	222	49
	Limited	7	0	0	0	0	0	0.0%	0.0	35	9
	General	281	74	7	7	0	88	31.3%	21.1	1854	
Total Hunters		315	74	7	10	3	94	29.8%	22.5	2111	
Resident		179	22	7	10	0	39	21.8%	33.8	1318	
Nonresident		136	52	0	0	3	55	40.4%	14.4	793	
102 Jackson	Cow/Calf	318	0	0	134	12	146	45.9%	14.3	2094	375
	Limited	698	117	15	153	36	321	46.0%	15.2	4880	747
	General	3357	661	30	506	91	1288	38.4%	16.7	21471	
Total Hunters		4317	778	45	793	139	1755	40.7%	16.2	28445	
Resident		3274	475	45	609	113	1242	37.9%	17.7	22000	
Nonresident		1043	303	0	184	26	513	49.2%	12.6	6445	
103 Fall Creek	Cow/Calf	454	0	0	134	35	169	37.2%	19.5	3294	552
	Limited	106	5	1	23	5	34	32.1%	24.1	821	141
	General	1984	356	101	220	54	731	36.8%	17.7	12905	
Total Hunters		2472	361	102	377	94	934	37.8%	18.2	17020	
Resident		1777	201	74	269	75	619	34.8%	20.3	12564	
Nonresident		695	160	28	108	19	315	45.3%	14.1	4456	
104 Hoback	Cow/Calf	21	0	0	5	4	9	42.9%	13.3	120	56
	General	873	125	60	68	13	266	30.5%	21.0	5583	
Total Hunters		890	125	60	73	17	275	30.9%	20.7	5703	
Resident		765	79	53	64	13	209	27.3%	23.5	4908	
Nonresident		125	46	7	9	4	66	52.8%	12.0	795	
105 Afton	Cow/Calf	142	0	0	35	8	43	30.3%	25.3	1086	201
	Limited	347	39	8	104	14	165	47.6%	17.6	2901	415
	General	2046	322	48	182	34	586	28.6%	21.2	12423	
Total Hunters		2483	361	56	321	56	794	32.0%	20.7	16410	
Resident		1913	194	41	307	50	592	30.9%	21.7	12850	
Nonresident		570	167	15	14	6	202	35.4%	17.6	3560	
106 Piney	Cow/Calf	342	0	0	104	43	147	43.0%	15.7	2310	479
	Limited	50	6	0	7	0	13	26.0%	30.8	400	62
	General	2324	323	111	271	78	783	33.7%	19.7	15452	
Total Hunters		2630	329	111	382	121	943	35.9%	19.3	18162	
Resident		2256	251	94	309	116	770	34.1%	20.9	16092	
Nonresident		374	78	17	73	5	173	46.3%	12.0	2070	
107 Green River	Cow/Calf	203	0	0	64	5	69	34.0%	23.8	1643	240
	Limited	790	120	27	136	35	318	40.3%	18.5	5897	842
	General	217	15	4	8	9	36	16.6%	33.2	1195	
Total Hunters		1153	135	31	208	49	423	36.7%	20.7	8735	
Resident		890	96	29	153	43	321	36.1%	21.9	7033	
Nonresident		263	39	2	55	6	102	38.8%	16.7	1702	
108 Pinedale	Cow/Calf	164	0	0	35	8	43	26.2%	26.0	1116	226
	Limited	673	157	22	59	15	253	37.6%	18.0	4563	756
	General	556	76	8	27	4	115	20.7%	24.6	2832	
Total Hunters		1343	233	30	121	27	411	30.6%	20.7	8511	
Resident		978	134	14	102	21	271	27.7%	23.7	6432	
Nonresident		365	99	16	19	6	140	38.4%	14.9	2079	
211 Medicine Lodge	Limited	1536	293	41	285	37	656	42.7%	17.9	11760	1615
Total Hunters		1536	293	41	285	37	656	42.7%	17.9	11760	
Resident		1293	217	35	224	36	512	39.6%	19.6	10018	
Nonresident		243	76	6	61	1	144	59.3%	12.1	1742	
214 Gooseberry	Cow/Calf	196	0	0	83	22	105	53.6%	9.3	975	225
	Limited	783	236	21	117	17	391	49.9%	12.4	4852	839
Total Hunters		914	236	21	200	39	496	54.3%	11.7	5827	
Resident		749	167	21	166	31	385	51.4%	12.7	4885	
Nonresident		165	69	0	34	8	111	67.3%	8.5	942	

ELK HERD UNIT REPORT

HERD	TYPE	ACTIVE LICS/HTRS	HARVEST BULL	SPIKE	COW	CALF	TOTAL	HUNTER SUCCESS	DAYS/ HARVEST	HUNTER DAYS	LICENSES SOLD (cross hunt incl.)
216 Cody	Cow/Calf	84	0	0	43	12	55	65.5%	8.1	445	101
	Limited	1010	191	13	359	36	599	59.3%	10.5	6317	1114
	General	1433	525	44	42	4	615	42.9%	13.3	8208	
Total Hunters		2493	716	57	444	52	1269	50.9%	11.8	14970	
Resident		1723	373	52	363	47	835	48.5%	13.3	11071	
Nonresident		770	343	5	81	5	434	56.4%	9.0	3899	
217 Clark's Fork	Cow/Calf	303	0	0	102	11	113	37.3%	12.3	1390	353
	Limited	830	94	16	367	31	508	61.2%	12.8	6498	952
	General	595	164	22	0	0	186	31.3%	22.2	4134	
Total Hunters		1676	258	38	469	42	807	48.2%	14.9	12022	
Resident		1386	173	26	407	40	646	46.6%	16.2	10476	
Nonresident		290	85	12	62	2	161	55.5%	9.6	1546	
320 Fortification	Cow/Calf	38	0	0	17	1	18	47.4%	6.4	115	40
	Limited	69	13	2	9	0	24	34.8%	13.5	324	73
Total Hunters		104	13	2	26	1	42	40.4%	10.5	439	
Resident		93	10	2	26	0	38	40.9%	10.2	388	
Nonresident		11	3	0	0	1	4	36.4%	12.8	51	
321 North Bighorn	Cow/Calf	349	0	0	87	11	98	28.1%	22.1	2162	459
	Limited	2187	317	70	185	54	626	28.6%	25.2	15757	2332
	General	1398	152	44	43	4	243	17.4%	38.8	9420	
Total Hunters		3883	469	114	315	69	967	24.9%	28.3	27339	
Resident		3308	380	104	260	57	801	24.2%	29.6	23677	
Nonresident		575	89	10	55	12	166	28.9%	22.1	3662	
322 South Bighorn	Cow/Calf	915	0	0	303	58	361	39.5%	13.8	4983	1073
	Limited	1905	490	47	281	45	863	45.3%	14.5	12553	2094
Total Hunters		2736	490	47	584	103	1224	44.7%	14.3	17536	
Resident		2135	337	28	452	97	914	42.8%	16.0	14594	
Nonresident		601	153	19	132	6	310	51.6%	9.5	2942	
344 Rochelle Hills	Limited	246	53	2	111	18	184	74.8%	5.2	949	270
Total Hunters		246	53	2	111	18	184	74.8%	5.2	949	
Resident		214	42	2	104	16	164	76.6%	4.5	740	
Nonresident		32	11	0	7	2	20	62.5%	10.5	209	
423 Uinta	Cow/Calf	24	0	0	8	0	8	33.3%	25.8	206	25
	Limited	199	17	5	49	2	73	36.7%	17.9	1308	284
	General	1129	180	50	116	11	357	31.6%	16.6	5934	
Total Hunters		1347	197	55	173	13	438	32.5%	17.0	7448	
Resident		1270	170	55	151	11	387	30.5%	18.4	7115	
Nonresident		77	27	0	22	2	51	66.2%	6.5	333	
424 South Rock Springs	Limited	606	168	6	212	34	420	69.3%	9.4	3936	623
Total Hunters		606	168	6	212	34	420	69.3%	9.4	3936	
Resident		512	131	3	188	34	356	69.5%	9.3	3319	
Nonresident		94	37	3	24	0	64	68.1%	9.6	617	
425 Sierra Madre	Cow/Calf	31	0	0	10	6	16	51.6%	10.0	160	100
	Limited	831	34	0	289	63	386	46.5%	12.9	4995	925
	General	4213	630	392	293	51	1366	32.4%	20.6	28076	
Total Hunters		5059	664	392	592	120	1768	34.9%	18.8	33231	
Resident		4270	480	331	460	68	1339	31.4%	21.3	28528	
Nonresident		789	184	61	132	52	429	54.4%	11.0	4703	
426 Steamboat	Limited	427	192	0	126	27	345	80.8%	6.2	2155	435
Total Hunters		427	192	0	126	27	345	80.8%	6.2	2155	
Resident		369	157	0	114	23	294	79.7%	6.7	1969	
Nonresident		58	35	0	12	4	51	87.9%	3.6	186	
428 West Green River	Cow/Calf	154	0	0	51	30	81	52.6%	9.7	783	204
	General	3101	451	167	440	114	1172	37.8%	15.1	17663	
Total Hunters		3213	451	167	491	144	1253	39.0%	14.7	18446	
Resident		2931	386	151	444	130	1111	37.9%	15.1	16829	
Nonresident		282	65	16	47	14	142	50.4%	11.4	1617	

TABLE I-H (Continued)

ELK HERD UNIT REPORT

HERD	TYPE	ACTIVE LICS/HTRS	HARVEST BULL	SPIKE	COW	CALF	TOTAL	HUNTER SUCCESS	DAYS/ HARVEST	HUNTER DAYS	LICENSES SOLD (cross hunt incl.)
430 Petition	Limited	76	10	0	28	4	42	55.3%	11.5	482	80
Total Hunters		76	10	0	28	4	42	55.3%	11.5	482	
Resident		60	6	0	19	4	29	48.3%	13.9	404	
Nonresident		16	4	0	9	0	13	81.3%	6.0	78	
531 Iron Mountain	Cow/Calf	253	0	0	117	10	127	50.2%	13.8	1751	326
	Limited	461	149	14	80	3	246	53.4%	13.2	3248	518
Total Hunters		692	149	14	197	13	373	53.9%	13.4	4999	
Resident		560	92	14	157	13	276	49.3%	15.6	4300	
Nonresident		132	57	0	40	0	97	73.5%	7.2	699	
533 Snowy Range	Cow/Calf	258	0	0	92	19	111	43.0%	13.4	1482	352
	Limited	1004	143	24	167	49	383	38.1%	16.8	6421	1118
	General	4017	540	133	324	66	1063	26.5%	26.0	27618	
Total Hunters		5174	683	157	583	134	1557	30.1%	22.8	35521	
Resident		4564	519	151	500	124	1294	28.4%	24.8	32102	
Nonresident		610	164	6	83	10	263	43.1%	13.0	3419	
534 Shirley Mountain	Cow/Calf	155	0	0	47	15	62	40.0%	12.1	751	188
	Limited	387	95	10	113	32	250	64.6%	9.1	2271	431
Total Hunters		529	95	10	160	47	312	59.0%	9.7	3022	
Resident		453	65	10	135	43	253	55.8%	10.5	2667	
Nonresident		76	30	0	25	4	59	77.6%	6.0	355	
635 Wiggins Fork	Cow/Calf	36	0	0	11	2	13	36.1%	13.6	177	50
	Limited	345	14	0	117	10	141	40.9%	17.9	2524	384
	General	1667	380	116	32	8	536	32.2%	20.9	11208	
Total Hunters		2034	394	116	160	20	690	33.9%	20.2	13909	
Resident		1660	302	106	134	19	561	33.8%	20.3	11411	
Nonresident		374	92	10	26	1	129	34.5%	19.4	2498	
637 South Wind River	Cow/Calf	278	0	0	83	23	106	38.1%	13.8	1463	304
	Limited	782	100	20	111	10	241	30.8%	24.4	5876	863
	General	1047	175	104	0	0	279	26.6%	26.2	7304	
Total Hunters		2054	275	124	194	33	626	30.5%	23.4	14643	
Resident		1844	229	111	155	21	516	28.0%	25.8	13287	
Nonresident		210	46	13	39	12	110	52.4%	12.3	1356	
638 Green Mountain	Cow/Calf	11	0	0	2	0	2	18.2%	17.5	35	20
	Limited	315	64	7	91	11	173	54.9%	9.5	1639	334
	General	221	11	0	8	4	23	10.4%	41.4	953	
Total Hunters		545	75	7	101	15	198	36.3%	13.3	2627	
Resident		503	59	7	91	14	171	34.0%	14.2	2421	
Nonresident		42	16	0	10	1	27	64.3%	7.6	206	
639 Ferris	Limited	285	64	4	42	10	120	42.1%	14.2	1700	326
Total Hunters		285	64	4	42	10	120	42.1%	14.2	1700	
Resident		236	50	4	29	10	93	39.4%	16.0	1491	
Nonresident		49	14	0	13	0	27	55.1%	7.7	209	
643 Shamrock	Cow/Calf	26	0	0	12	2	14	53.8%	12.9	181	36
	Limited	72	27	0	9	2	38	52.8%	9.5	362	76
Total Hunters		90	27	0	21	4	52	57.8%	10.4	543	
Resident		72	16	0	17	2	35	48.6%	11.9	415	
Nonresident		18	11	0	4	2	17	94.4%	7.5	128	
730 Rawhide	Cow/Calf	41	0	0	15	7	22	53.7%	24.3	535	50
	Limited	86	27	3	9	4	43	50.0%	23.7	1020	101
Total Hunters		114	27	3	24	11	65	57.0%	23.9	1555	
Resident		103	21	3	23	11	58	56.3%	25.9	1505	
Nonresident		11	6	0	1	0	7	63.6%	7.1	50	
740 Black Hills	Cow/Calf	141	0	0	50	4	54	38.3%	30.3	1635	162
	Limited	923	258	30	175	19	482	52.2%	20.6	9908	992
Total Hunters		1028	258	30	225	23	536	52.1%	21.5	11543	
Resident		857	201	26	186	15	428	49.9%	24.0	10253	
Nonresident		171	57	4	39	8	108	63.2%	11.9	1290	

TABLE I-H (Continued)

ELK HERD UNIT REPORT

HERD	TYPE	ACTIVE LICS/HTRS	HARVEST BULL	SPIKE	COW	CALF	TOTAL	HUNTER SUCCESS	DAYS/ HARVEST	HUNTER DAYS	LICENSES SOLD (cross hunt incl.)
741 Laramie Peak-	Cow/Calf	599	0	0	170	71	241	40.2%	17.6	4239	752
Muddy Mountain	Limited	3179	757	86	755	238	1836	57.8%	13.0	23944	3580
Total Hunters		3648	757	86	925	309	2077	56.9%	13.6	28183	
Resident		3146	629	86	800	273	1788	56.8%	14.2	25356	
Nonresident		502	128	0	125	36	289	57.6%	9.8	2827	
742 Rattlesnake	Cow/Calf	137	0	0	30	9	39	28.5%	24.8	968	150
	Limited	185	62	0	41	3	106	57.3%	12.3	1301	205
Total Hunters		296	62	0	71	12	145	49.0%	15.6	2269	
Resident		255	53	0	62	10	125	49.0%	16.8	2103	
Nonresident		41	9	0	9	2	20	48.8%	8.3	166	
743 Pine Ridge	Cow/Calf	33	0	0	13	2	15	45.5%	8.7	131	33
	Limited	48	22	0	10	3	35	72.9%	10.0	350	56
Total Hunters		77	22	0	23	5	50	64.9%	9.6	481	
Resident		71	19	0	23	3	45	63.4%	9.9	447	
Nonresident		6	3	0	0	2	5	83.3%	6.8	34	
999 Rest of State (hunt area 129)											
	Cow/Calf	60	0	0	12	10	22	36.7%	24.0	529	67
	General	635	103	8	44	5	160	25.2%	30.1	4812	
Total Hunters		672	103	8	56	15	182	27.1%	29.3	5341	
Resident		636	90	8	51	15	164	25.8%	31.5	5158	
Nonresident		36	13	0	5	0	18	50.0%	10.2	183	

TABLE III

CONFIDENCE INTERVALS FOR TOTAL ELK HARVEST

BY HERD UNIT

2007

HERD		TOTAL HARVEST	90% CONFIDENCE LIMITS		
101	Targhee	94	81	-	107
102	Jackson	1755	1699	-	1811
103	Fall Creek	934	893	-	975
104	Hoback	275	252	-	298
105	Afton	794	754	-	834
106	Piney	943	902	-	984
107	Green River	423	396	-	450
108	Pinedale	411	383	-	439
211	Medicine Lodge	656	623	-	689
214	Gooseberry	496	470	-	522
216	Cody	1269	1226	-	1312
217	Clark's Fork	807	770	-	844
320	Fortification	42	34	-	50
321	North Bighorn	967	922	-	1012
322	South Bighorn	1224	1181	-	1267
344	Rochelle Hills	184	173	-	195
423	Uinta	438	409	-	467
424	South Rock Springs	420	401	-	439
425	Sierra Madre	1768	1711	-	1825
426	Steamboat	345	332	-	358
428	West Green River	1253	1206	-	1300
430	Petition	42	35	-	49
531	Iron Mountain	373	351	-	395
533	Snowy Range	1557	1501	-	1613
534	Shirley Mountain	312	293	-	331
635	Wiggins Fork	690	654	-	726
637	South Wind River	626	591	-	661
638	Green Mountain	198	179	-	217
639	Ferris	120	106	-	134
643	Shamrock	52	44	-	60
730	Rawhide	65	56	-	74
740	Black Hills	536	509	-	563
741	Laramie Peak-Muddy Mountain	2077	2027	-	2127
742	Rattlesnake	145	130	-	160
743	Pine Ridge	50	43	-	57
999	Rest of State	182	163	-	201

TABLE IV

ELK HUNTERS HUNTING ON OPENING DAY

BY HUNT AREA AND RESIDENCY

2007

HUNT AREA	RESIDENT HUNTERS HUNTING ON OPENING DAY	PERCENT WHO HUNTED ON OPENING DAY	NONRESIDENT HUNTERS HUNTING ON OPENING DAY	PERCENT WHO HUNTED ON OPENING DAY
1	98	48.0%	27	56.6%
2	64	68.6%	5	40.4%
3	47	45.7%	5	49.4%
5	112	36.3%	18	21.3%
6	151	60.0%	25	52.1%
7	982	37.2%	176	39.5%
8	35	37.5%	7	50.0%
9	283	35.6%	23	43.8%
10	323	34.9%	60	39.1%
11	210	57.7%	46	81.5%
12	462	41.3%	61	46.8%
13	460	46.9%	123	46.2%
15	351	43.3%	100	61.5%
16	261	57.9%	52	68.2%
19	278	60.5%	34	62.3%
21	1302	48.7%	155	43.9%
22	48	48.5%	11	59.5%
23	137	53.9%	25	61.3%
24	175	62.8%	31	83.8%
25	116	43.8%	18	40.5%
27	24	35.3%	8	38.2%
28	469	37.5%	65	68.7%
30	69	44.1%	14	57.5%
31	120	49.8%	16	43.3%
32	73	63.6%	15	49.0%
33	44	38.8%	23	81.8%
34	301	39.6%	76	36.6%
35	125	50.5%	23	49.8%
36	237	34.4%	45	47.2%
37	392	33.1%	57	33.7%
38	460	53.0%	94	51.3%
39	113	57.3%	21	55.6%
40	159	65.6%	31	58.2%
41	215	42.1%	43	44.8%
42	101	45.8%	23	50.6%
45	265	43.8%	60	58.2%
46	67	45.7%	12	67.1%
47	61	57.1%	28	80.7%
48	194	40.0%	57	31.9%
49	163	37.4%	38	34.5%
50	108	52.6%	5	18.6%
51	134	48.2%	44	33.6%
52	130	45.8%	8	23.4%
53	52	26.0%	6	41.1%
54	52	26.5%	5	20.8%
55	107	32.0%	14	11.7%
56	128	25.9%	36	26.7%
58	42	25.6%	12	32.5%
59	169	37.1%	45	29.8%
60	56	40.8%	81	30.9%
61	102	45.9%	37	49.0%
62	118	42.6%	39	63.0%
63	136	46.2%	31	57.1%
64	99	45.7%	26	48.9%
65	127	39.6%	7	24.8%
66	43	40.2%	0	0.0%
67	391	35.6%	55	25.2%
68	187	34.9%	13	12.2%
69	43	17.7%	33	44.2%
70	121	29.6%	59	40.9%
71	57	32.7%	46	28.0%
73	74	41.3%	61	45.2%
74	39	30.5%	10	60.0%
75	80	27.4%	36	19.6%
77	55	15.6%	14	21.3%

TABLE IV (Continued)

ELK OPENING DAY HUNTERS REPORT

HUNT AREA	RESIDENT HUNTERS	PERCENT WHO HUNTED ON OPENING DAY	NONRESIDENT HUNTERS	PERCENT WHO HUNTED ON OPENING DAY
78	10	15.9%	0	0.0%
79	106	45.4%	31	22.3%
80	41	8.6%	5	18.2%
81	246	33.2%	121	51.0%
82	102	25.8%	44	41.2%
83	198	36.7%	34	43.1%
84	302	29.6%	138	37.0%
85	282	31.8%	140	40.9%
86	101	40.3%	36	47.8%
87	319	57.9%	23	40.9%
88	25	59.3%	5	60.0%
89	466	62.2%	102	44.3%
90	252	36.8%	97	54.1%
91	306	39.8%	103	48.1%
92	415	42.9%	93	51.8%
93	78	41.7%	43	51.2%
94	677	47.4%	118	59.7%
95	201	57.7%	54	62.1%
96	124	34.3%	35	35.9%
97	108	33.3%	83	37.4%
98	253	37.8%	58	41.1%
99	161	50.7%	39	70.7%
100	253	68.4%	43	74.4%
102	728	50.3%	43	41.9%
103	141	41.6%	46	71.1%
104	492	44.8%	59	45.6%
105	209	49.8%	0	0.0%
106	311	39.7%	39	59.5%
107	281	48.2%	3	27.4%
108	48	49.3%	22	43.0%
110	727	42.1%	64	58.8%
111	64	46.5%	25	80.9%
113	41	48.6%	6	33.6%
114	40	49.5%	7	25.9%
116	142	48.2%	22	37.7%
117	175	49.0%	35	53.5%
118	45	61.7%	15	80.0%
120	132	56.5%	31	75.6%
121	45	19.0%	12	21.3%
122	17	23.9%	0	0.0%
123	42	51.7%	2	100.0%
124	21	35.0%	12	76.0%
125	6	7.2%	8	9.8%
126	27	55.1%	5	40.9%
127	26	57.9%	7	66.7%
128	108	47.7%	0	0.0%
129	185	29.2%	12	34.0%
130	12	65.6%	0	0.0%

TABLE V
ELK LICENSE DATA SUMMARY
2007

HUNT AREA	LICENSE TYPE	NUMBER SOLD	NUMBER SURVEYED	NUMBER USABLE SURVEYS (both hunters and nonhunters)	% USABLE RESPONSE
GENERAL	RES	24940	17386	6660	38.3%
GENERAL	NONRES	4058	1887	1238	65.6%
1	1	152	89	54	60.7%
1	2	105	77	51	66.2%
2	1	38	38	20	52.6%
2	2	35	35	16	45.7%
2	6	40	40	17	42.5%
3	1	51	51	24	47.1%
3	2	50	50	23	46.0%
3	6	50	50	30	60.0%
5	1	255	100	52	52.0%
5	6	226	100	57	57.0%
6	1	163	91	49	53.9%
6	2	100	75	31	41.3%
6	6	100	75	31	41.3%
7	1	1509	140	76	54.3%
7	2	1002	136	58	42.7%
7	3	473	129	46	35.7%
7	5	100	75	18	24.0%
7	6	500	129	80	62.0%
7	7	102	86	51	59.3%
8	1	126	83	33	39.8%
10	2	105	77	41	53.3%
10	6	151	89	53	59.6%
11	1	153	89	57	64.0%
11	2	301	104	54	51.9%
12	2	114	89	61	68.5%
12, 13, 15, 110	6	75	75	43	57.3%
13	2	140	89	57	64.0%
15	2	85	82	48	58.5%
16	1	204	96	61	63.5%
16	2	227	98	44	44.9%
16	6	153	89	53	59.6%
16	7	35	35	23	65.7%
19	1	154	89	45	50.6%
19	2	155	89	46	51.7%
19	3	121	83	39	47.0%
19	4	66	66	33	50.0%
19	6	150	89	46	51.7%
21	2	542	115	58	50.4%
21	6	25	25	12	48.0%
22	1	52	52	30	57.7%
22	2	79	68	40	58.8%
23	1	105	77	28	36.4%
23	2	100	75	38	50.7%
23	6	150	89	44	49.4%
24	1	131	85	56	65.9%
24	2	75	68	46	67.7%
24	3	128	83	49	59.0%
25, 27	1	155	133	76	57.1%
25, 27	2	100	100	61	61.0%
25	6	152	89	43	48.3%
28	2	201	96	59	61.5%
28	6	152	89	46	51.7%
30	1	54	54	27	50.0%
30	2	130	83	53	63.9%
31	1	106	77	48	62.3%
31	2	179	92	57	62.0%
32	1	99	75	45	60.0%
32	2	55	54	31	57.4%
33	1	101	77	35	45.5%
33	2	55	55	34	61.8%
34	1	807	117	59	50.4%
34	6	344	115	42	36.5%

TABLE V (Continued)

ELK LICENSE DATA SUMMARY

HUNT AREA	LICENSE TYPE	NUMBER SOLD	NUMBER SURVEYED	NUMBER USABLE SURVEYS (both hunters and nonhunters)	% USABLE RESPONSE
35	1	151	89	52	58.4%
35	2	76	68	41	60.3%
35	6	50	50	25	50.0%
35	9	53	53	28	52.8%
36	2	156	96	63	65.6%
36	9	57	57	30	52.6%
37	6	409	110	60	54.6%
37	9	154	89	50	56.2%
38	1	403	109	66	60.6%
38	2	451	115	54	47.0%
38	9	253	100	54	54.0%
39	1	93	74	39	52.7%
39	2	80	70	43	61.4%
39	9	71	66	36	54.6%
40	1	177	92	51	55.4%
40	2	103	77	47	61.0%
40	9	54	54	33	61.1%
41, 42	1	355	161	86	53.4%
41, 42	2	327	158	98	62.0%
41, 42	9	106	106	58	54.7%
45, 46	1	351	161	99	61.5%
45	2	179	92	45	48.9%
45, 46	3	155	133	82	61.7%
45, 46	9	142	130	68	52.3%
47	1	102	77	42	54.6%
47	3	25	25	19	76.0%
47	6	50	50	24	48.0%
48	1	302	104	58	55.8%
48	2	68	68	40	58.8%
48	6	428	111	46	41.4%
49	1	277	102	52	51.0%
49	2	72	69	31	44.9%
49	6	251	100	61	61.0%
50	1	27	27	12	44.4%
50	2	41	41	16	39.0%
50	9	12	11	6	54.6%
51	1	42	37	19	51.4%
51, 52	2	153	133	86	64.7%
51	9	30	30	23	76.7%
52	1	28	26	18	69.2%
52	9	6	6	3	50.0%
53, 54, 65	1	51	51	28	54.9%
53, 54, 65	2	150	133	74	55.6%
53, 54, 65	6	150	133	71	53.4%
53, 54, 65	9	26	26	12	46.2%
54	3	25	25	16	64.0%
55	1	25	23	15	65.2%
55	2	75	68	40	58.8%
55	9	19	19	12	63.2%
56	1	47	40	26	65.0%
56	2	168	100	53	53.0%
56	3	50	50	21	42.0%
56	9	23	23	15	65.2%
58	1	70	56	39	69.6%
58	2	151	89	37	41.6%
59	1	13	12	9	75.0%
59	2	154	90	47	52.2%
59	6	101	77	36	46.8%
59	9	33	33	11	33.3%
60	9	19	19	9	47.4%
61	1	157	89	52	58.4%
61	2	28	27	17	63.0%
61	3	82	82	38	46.3%
62	1	158	89	56	62.9%
62	2	54	54	33	61.1%
62	3	155	89	51	57.3%

HUNT AREA	LICENSE TYPE	NUMBER SOLD	NUMBER SURVEYED	NUMBER USABLE SURVEYS (both hunters and nonhunters)	% USABLE RESPONSE
63, 64	1	210	144	82	56.9%
63	2	159	92	53	57.6%
63	6	100	75	44	58.7%
64	2	103	77	47	61.0%
64	6	125	83	43	51.8%
65	5	95	75	13	17.3%
65	7	103	75	42	56.0%
67, 68	2	255	150	84	56.0%
67, 68, 69	9	129	125	76	60.8%
69	6	50	50	28	56.0%
73	2	9	9	3	33.3%
73	6	49	46	18	39.1%
75	1	106	77	50	64.9%
75	2	240	109	67	61.5%
78	1	27	27	17	63.0%
78	2	25	25	13	52.0%
78	6	75	68	33	48.5%
79	1	200	96	64	66.7%
79	2	58	58	33	56.9%
79	6	300	104	63	60.6%
80	2	7	6	3	50.0%
81	2	28	28	14	50.0%
82	2	25	26 *	18	69.2%
83	2	31	31	15	48.4%
84	1	22	22	8	36.4%
84	2	49	49	28	57.1%
84	6	275	105	48	45.7%
85	1	20	20	10	50.0%
85	2	50	50	34	68.0%
85	6	277	102	59	57.8%
87	6	56	50	19	38.0%
88	1	60	60	38	63.3%
90	6	101	64	27	42.2%
91	1	355	108	42	38.9%
91	6	100	75	42	56.0%
92	6	203	96	46	47.9%
93	1	155	89	48	53.9%
93	2	26	26	13	50.0%
93	6	140	98	51	52.0%
94	1	62	60	32	53.3%
94	6	276	104	45	43.3%
95	1	155	89	57	64.0%
95	2	248	100	58	58.0%
95	3	32	32	18	56.3%
95	4	11	11	8	72.7%
95	6	25	25	16	64.0%
96	1	206	96	52	54.2%
96	2	9	8	4	50.0%
96	6	75	68	36	52.9%
97	1	301	104	56	53.9%
97	2	16	16	11	68.8%
97	6	75	68	43	63.2%
98	1	353	108	61	56.5%
98	2	86	75	35	46.7%
98	6	151	89	43	48.3%
99	1	179	92	47	51.1%
99	2	228	98	56	57.1%
100	1	233	98	60	61.2%
100	2	202	96	53	55.2%
103	6	53	53	33	62.3%
104	6	151	82	37	45.1%
106	1	34	34	15	44.1%
106	2	149	89	33	37.1%
106	6	25	25	12	48.0%
107	2	101	77	40	52.0%
108	1	61	60	32	53.3%
108	2	84	75	55	73.3%
108	3	13	13	10	76.9%
110	2	76	68	45	66.2%

TABLE V (Continued)

ELK LICENSE DATA SUMMARY

HUNT AREA	LICENSE TYPE	NUMBER SOLD	NUMBER SURVEYED	NUMBER USABLE SURVEYS (both hunters and nonhunters)	% USABLE RESPONSE
111	1	47	47	25	53.2%
111	2	95	75	47	62.7%
111	3	53	53	29	54.7%
113	1	47	43	24	55.8%
113	2	61	60	33	55.0%
114	1	51	51	32	62.8%
114	2	41	41	19	46.3%
114	6	50	50	25	50.0%
116	1	178	92	45	48.9%
116	2	150	89	40	44.9%
116	6	100	75	37	49.3%
117	1	232	98	54	55.1%
117	2	175	92	47	51.1%
117	6	62	60	29	48.3%
118	1	38	38	21	55.3%
118	2	38	38	20	52.6%
118	6	36	36	19	52.8%
120	1	109	79	40	50.6%
120	2	102	77	42	54.6%
120	3	74	68	30	44.1%
121	1	105	77	40	52.0%
121	2	125	83	47	56.6%
121	3	36	35	16	45.7%
121	6	100	75	40	53.3%
122	1	51	51	19	37.3%
122	2	5	5	1	20.0%
122	6	33	31	8	25.8%
123	2	100	75	36	48.0%
124	1	18	18	11	61.1%
124	2	62	60	33	55.0%
125	1	138	89	47	52.8%
125	2	13	13	6	46.2%
125	6	76	68	38	55.9%
126	1	27	27	13	48.2%
126	2	35	35	18	51.4%
128	6	20	20	9	45.0%
129	6	67	65	29	44.6%
TOTAL		59763	35989	16926	47.0%

* Actual downloads exceed WG&F sales data.

WYOMING GAME AND FISH COMMISSION

CHAPTER 7

ELK HUNTING SEASONS

Section 1. Authority. This regulation is promulgated by authority of W.S. §23-1-302, §23-1-703 and §23-2-104.

Section 2. Hunting Seasons Established. There shall be open seasons during 2007 and January, 2008 for the hunting of elk as set forth in this Chapter and in accordance with Chapter 2, General Hunting Regulation.

Section 3. Regular Hunting Seasons.

(a) Hunt areas, season dates and limitations.

Hunt Area	Type	Date of Seasons Opens	Closes	Limitations
*1	1	Oct. 15	Nov. 30	Limited quota; 150 licenses any elk
	2	Oct. 15	Nov. 30	Limited quota; 100 licenses antlerless elk
2	1	Oct. 22	Oct. 31	Limited quota; 35 licenses any elk
	2	Oct. 22	Oct. 31	Limited quota; 35 licenses antlerless elk
	6	Sept. 12	Sept. 18	Limited quota; 40 licenses cow or calf
3	1	Sept. 15	Oct. 14	Limited quota; 50 licenses any elk
	2	Sept. 15	Oct. 14	Limited quota; 50 licenses antlerless elk
	6	Sept. 15	Oct. 14	Limited quota; 50 licenses cow or calf
		Nov. 1	Dec. 15	Unused Area 3 Type 1, Type 2, and Type 6 licenses valid for antlerless elk
*5	1	Oct. 1	Dec. 15	Limited quota; 250 licenses any elk
	6	Oct. 1	Dec. 15	Limited quota; 250 licenses cow or calf
*6	1	Oct. 20	Nov. 11	Limited quota; 150 licenses any elk
		Nov. 12	Jan. 31	Unused Area 6 Type 1 licenses valid for antlerless elk off national forest
	2	Oct. 20	Jan. 31	Limited quota; 100 licenses antlerless elk off national forest
	6	Oct. 1	Jan. 31	Limited quota; 100 licenses cow or calf off national forest
*7	1	Oct. 15	Nov. 25	Limited quota; 1,500 licenses any elk
	2	Oct. 15	Nov. 25	Limited quota; 1,000 licenses antlerless elk
	3	Nov. 1	Nov. 25	Limited quota; 500 licenses antlerless elk
	5	Aug. 25	Sept. 9	Limited quota; 100 licenses cow or calf valid in that portion of Area 7 in Converse County off national forest
	6	Oct. 15	Nov. 25	Limited quota; 500 licenses cow or calf
		Dec. 1	Dec. 16	Unused Area 7 Type 1, Type 2, Type 3, Type 5 and Type 6 licenses valid for antlerless elk, except that the Wyoming Game and Fish Commission Laramie Peak Wildlife Habitat Management Area will be closed.
		Dec. 17	Jan. 4	Unused Area 7 Type 1, Type 2, Type 3, Type 5 and Type 6 licenses valid for antlerless elk, valid in that portion of Area 7 in Converse County
		Jan. 5	Jan. 20	Unused Area 7 Type 1, Type 2, Type 3, Type 5 and Type 6 licenses valid for antlerless elk, except that the Wyoming Game and Fish Commission Laramie Peak Wildlife Habitat Management Area will be closed.

Hunt Area	Type	Date of Seasons Opens	Closes	Limitations
		Jan. 21	Jan. 31	Unused Area 7 Type 1, Type 2, Type 3, Type 5 and Type 6 licenses valid for antlerless elk, valid in that portion of Area 7 in Converse County
	7	Sept. 1	Oct. 14	Limited quota; 100 licenses cow or calf valid in that portion of Area 7 south and east of the Fetterman Road (Albany County Road 61), south and west of the Garrett Road (Albany County Road 713) and south of the Tunnel Road (Albany County Road 727 - Platte County Road 266) or in that portion of Area 7 in Platte County north of Cottonwood Creek
*8	1	Oct. 1	Oct. 31	Limited quota; 125 licenses any elk
		Nov. 1	Dec. 31	Unused Area 8 Type 1 licenses; valid for antlerless elk
9		Oct. 15	Oct. 21	General license; any elk
		Oct. 22	Oct. 31	General license; antlered elk
10		Oct. 15	Oct. 31	General license; any elk
	2	Oct. 1	Nov. 30	Limited quota; 100 licenses antlerless elk
	6	Oct. 1	Nov. 30	Limited quota; 150 licenses cow or calf
11	1	Oct. 9	Oct. 31	Limited quota; 150 licenses any elk
	2	Oct. 9	Oct. 31	Limited quota; 300 licenses antlerless elk
		Nov. 1	Nov. 14	Unused Area 11 Type 2 licenses valid in that portion of Area 11 off national forest and off the Wyoming Game and Fish Commission's Wick Brothers Wildlife Habitat Management Area
12		Oct. 15	Oct. 31	General license; any elk
	2	Oct. 15	Nov. 5	Limited quota; 150 licenses antlerless elk
12, *13, 15, 110	6	Sept. 1	Jan. 31	Limited quota; 75 licenses cow or calf valid on private land
*13		Oct. 15	Oct. 31	General license; any elk
	2	Oct. 15	Nov. 14	Limited quota; 150 licenses antlerless elk
15, *21		Oct. 15	Oct. 24	General license; antlered elk
		Oct. 25	Nov. 4	General license; antlerless elk
15	2	Oct. 15	Nov. 14	Limited quota; 150 licenses antlerless elk
*16	1	Oct. 1	Oct. 14	Limited quota; 200 licenses any elk
		Nov. 1	Nov. 20	Unused Area 16 Type 1 licenses valid on the Beer Mug Mountain Hunter Management Area
	2	Oct. 1	Oct. 14	Limited quota; 225 licenses antlerless elk
	6	Oct. 1	Oct. 14	Limited quota; 150 licenses cow or calf
		Nov. 1	Nov. 20	Unused Area 16 Type 2 and Type 6 licenses valid for antlerless elk
	7	Oct. 1	Oct. 14	Limited quota; 35 licenses cow or calf
		Oct. 26	Nov. 30	Unused Area 16 Type 7 licenses
		Dec. 1	Dec. 14	Unused Area 16 Type 2, Type 6 and Type 7 licenses valid in that portion of Area 16 north of Canyon Creek and west of the Bolton Creek Road (Natrona County Road 404 – Carbon County Road 103) and in that portion of Area 23 between the North Platte River and U.S. Highway 220.

Hunt Area	Type	Date of Seasons Opens	Closes	Limitations
19	1	Oct. 1	Oct. 14	Limited quota; 150 licenses any elk
	2	Nov. 1	Nov. 25	Limited quota; 150 licenses any elk
	3	Oct. 1	Oct. 14	Limited quota; 125 licenses antlerless elk
	4	Nov. 1	Nov. 25	Limited quota; 125 licenses antlerless elk
	6	Oct. 1	Oct. 14	Limited quota; 150 licenses cow or calf
		Nov. 1	Nov. 25	Unused Area 19 Type 6 licenses
		Dec. 1	Dec. 16	Unused Area 19 Type 1, Type 2, Type 3, Type 4 and Type 6 licenses valid for antlerless elk
21	2	Oct. 25	Nov. 30	Limited quota; 650 licenses antlerless elk
	6	Aug. 15	Aug. 30	Limited quota; 25 licenses cow or calf valid in that portion of Area 21 off national forest south of Wyoming Highway 70 and the Baggs-Dixon Road (Carbon County Road 702)
		Sept. 1	Dec. 15	Unused Area 21 Type 6 licenses valid in that portion of Area 21 on national forest on Battle Mountain and all lands off national forest south of Wyoming Highway 70 and the Baggs-Dixon Road (Carbon County Road 702)
*22	1	Oct. 8	Oct. 21	Limited quota; 50 licenses antlered elk
	2	Oct. 8	Nov. 20	Limited quota; 75 licenses antlerless elk
23	1	Oct. 1	Oct. 31	Limited quota; 100 licenses any elk
	2	Oct. 1	Oct. 31	Limited quota; 100 licenses antlerless elk
	6	Oct. 1	Oct. 31	Limited quota; 150 licenses cow or calf
		Nov. 6	Nov. 30	Unused Area 23 Type 1, Type 2 and Type 6 licenses valid for antlerless elk
24	1	Oct. 1	Oct. 14	Limited quota; 125 licenses antlered elk
	2	Oct. 1	Oct. 14	Limited quota; 75 licenses antlerless elk
	3	Nov. 1	Nov. 15	Limited quota; 125 licenses antlerless elk
25, 27	1	Oct. 1	Oct. 31	Limited quota; 150 licenses any elk
	2	Oct. 1	Oct. 31	Limited quota; 100 licenses antlerless elk
25	6	Oct. 1	Oct. 31	Limited quota; 150 licenses cow or calf
28		Oct. 1	Oct. 22	General license; antlered elk
	2	Oct. 23	Nov. 15	Limited quota; 200 licenses antlerless elk
	6	Oct. 23	Nov. 15	Limited quota; 150 licenses cow or calf
30	1	Oct. 1	Oct. 31	Limited quota; 50 licenses any elk
	2	Oct. 6	Nov. 30	Limited quota; 125 licenses antlerless elk
31	1	Oct. 1	Oct. 31	Limited quota; 100 licenses any elk
	2	Oct. 6	Nov. 30	Limited quota; 175 licenses antlerless elk
32	1	Oct. 1	Oct. 31	Limited quota; 90 licenses any elk
	2	Oct. 6	Nov. 30	Limited quota; 50 licenses antlerless elk
33	1	Oct. 9	Oct. 31	Limited quota; 100 licenses any elk
		Nov. 1	Nov. 30	Unused Area 33 Type 1 licenses valid for antlerless elk
	2	Oct. 9	Nov. 30	Limited quota; 50 licenses antlerless elk
34	1	Oct. 15	Nov. 15	Limited quota; 800 licenses any elk
		Nov. 16	Nov. 30	Unused Area 34 Type 1 licenses valid for antlerless elk
	6	Oct. 15	Nov. 30	Limited quota; 600 licenses cow or calf valid in that portion of Area 34 off national forest

Hunt Area	Type	Date of Seasons<br>Opens	Closes	Limitations
35	1	Oct. 15	Nov. 5	Limited quota; 150 licenses antlered elk
	2	Oct. 15	Nov. 30	Limited quota; 75 licenses antlerless elk
	6	Oct. 15	Nov. 30	Limited quota; 50 licenses cow or calf valid in that portion of Area 35 off national forest
	9	Sept. 1	Sept. 30	Limited quota; 50 licenses any elk, archery only
36		Oct. 15	Nov. 5	General license; antlered elk
	2	Oct. 15	Nov. 30	Limited quota; 200 licenses antlerless elk
	9	Sept. 1	Sept. 30	Limited quota; 50 licenses any elk, archery only
37		Oct. 15	Nov. 5	General license; any elk
	6	Oct. 1	Nov. 30	Limited quota; 400 licenses cow or calf
		Dec. 1	Dec. 16	Unused Area 37 Type 6 licenses valid in that portion of Area 37 south and east of Little Goose Creek
	9	Sept. 1	Sept. 30	Limited quota; 150 licenses any elk, archery only
38	1	Oct. 15	Nov. 5	Limited quota; 400 licenses any elk
		Nov. 6	Nov. 15	Unused Area 38 Type 1 licenses valid for antlerless elk
	2	Oct. 15	Nov. 15	Limited quota; 600 licenses antlerless elk valid in that portion of Area 38 north of Columbus Creek, the Fools Creek Road (U.S.F.S. Road 168), the Burgess Road (U.S.F.S. Road 15) to Burgess Junction and U.S. Highway 14A
	9	Sept. 1	Sept. 30	Limited quota; 250 licenses any elk, archery only
39	1	Oct. 15	Nov. 4	Limited quota; 90 licenses any elk
	2	Oct. 15	Nov. 4	Limited quota; 75 licenses antlered elk
	9	Sept. 1	Sept. 30	Limited quota; 70 licenses any elk, archery only
40	1	Oct. 15	Nov. 4	Limited quota; 175 licenses any elk
	2	Oct. 15	Nov. 15	Limited quota; 100 licenses antlerless elk
	9	Sept. 1	Sept. 30	Limited quota; 50 licenses any elk, archery only
41, 42	1	Oct. 15	Nov. 4	Limited quota; 350 licenses any elk
	2	Oct. 15	Nov. 15	Limited quota; 325 licenses antlerless elk
		Nov. 16	Nov. 25	Unused Area 41, 42 Type 2 licenses antlerless elk valid in Area 41
	9	Sept. 1	Sept. 30	Limited quota; 100 licenses any elk, archery only
45, 46	1	Oct. 15	Nov. 4	Limited quota; 350 licenses any elk
	3	Oct. 15	Nov. 15	Limited quota; 150 licenses antlerless elk
	9	Sept. 1	Sept. 30	Limited quota; 140 licenses any elk, archery only
45	2	Oct. 1	Oct. 9	Limited quota; 175 licenses antlerless elk valid in that portion of Area 45 off national forest
		Oct. 10	Nov. 15	Unused Area 45 Type 2 licenses valid for antlerless elk in the entire area
47	1	Oct. 9	Oct. 31	Limited quota; 100 licenses any elk
	3	Oct. 9	Oct. 31	Limited quota; 25 licenses any elk valid in that portion of Area 47 in Fremont County
	6	Oct. 9	Oct. 31	Limited quota; 50 licenses cow or calf
48	1	Oct. 9	Oct. 31	Limited quota; 300 licenses any elk
	2	Oct. 9	Oct. 31	Limited quota; 100 licenses antlerless elk
	6	Oct. 9	Oct. 31	Limited quota; 450 licenses cow or calf
		Nov. 10	Nov. 30	Unused Area 48 Type 1, Type 2 and Type 6 licenses valid for antlerless elk

Hunt Area	Type	Date of Seasons Opens	Closes	Limitations
49	1	Oct. 9	Oct. 31	Limited quota; 275 licenses any elk
		Nov. 1	Nov. 30	Unused Area 49 Type 1 licenses valid for antlerless elk
	2	Oct. 9	Nov. 30	Limited quota; 100 licenses antlerless elk
	6	Sept. 1	Nov. 30	Limited quota; 250 licenses cow or calf
50		Oct. 1	Oct. 21	General license; antlered elk
	1	Nov. 18	Dec. 7	Limited quota; 20 licenses any elk
	2	Nov. 16	Dec. 21	Limited quota; 40 licenses antlerless elk
	9	Sept. 1	Sept. 30	Limited quota; 15 licenses any elk, archery only
51		Oct. 1	Oct. 21	General license; antlered elk
	1	Nov. 18	Dec. 7	Limited quota; 35 licenses any elk
	9	Sept. 1	Sept. 30	Limited quota; 25 licenses any elk, archery only
51, 52	2	Nov. 16	Dec. 21	Limited quota; 150 licenses antlerless elk
52		Oct. 1	Oct. 21	General license; antlered elk
	1	Nov. 18	Dec. 7	Limited quota; 25 licenses any elk
	9	Sept. 1	Sept. 30	Limited quota; 15 licenses any elk, archery only
53		Oct. 1	Oct. 21	General license; antlered elk
53,54,65	1	Nov. 1	Nov. 30	Limited quota; 50 licenses any elk
	2	Nov. 1	Dec. 31	Limited quota; 150 licenses antlerless elk; also valid in Area 121
	6	Nov. 1	Dec. 31	Limited quota; 150 licenses cow or calf; also valid in Area 121
	9	Sept. 1	Sept. 30	Limited quota; 25 licenses any elk, archery only
54	3	Sept. 1	Sept. 30	Limited quota; 25 licenses antlerless elk valid in that portion of Area 54 off national forest
55		Oct. 1	Oct. 21	General license; antlered elk
	1	Nov. 18	Dec. 7	Limited quota; 20 licenses any elk
	2	Nov. 16	Dec. 21	Limited quota; 75 licenses antlerless elk
	9	Sept. 1	Sept. 30	Limited quota; 20 licenses any elk, archery only
56		Oct. 1	Oct. 21	General license; antlered elk
	1	Nov. 18	Dec. 7	Limited quota; 35 licenses any elk
	2	Nov. 1	Nov. 15	Limited quota; 250 licenses antlerless elk valid in that portion of Area 56 in the South Fork of the Shoshone River drainage
		Nov. 16	Dec. 21	Unused Area 56 Type 2 licenses valid in the entire area
	3	Nov. 1	Dec. 31	Limited quota; 50 licenses antlerless elk valid in that portion of Area 56 off national forest
	9	Sept. 1	Sept. 30	Limited quota; 30 licenses any elk, archery only
58	1	Oct. 1	Nov. 30	Limited quota; 50 licenses any elk
	2	Oct. 1	Dec. 21	Limited quota; 150 licenses antlerless elk
59		Oct. 1	Oct. 21	General license; antlered elk
	1	Nov. 1	Nov. 15	Limited quota; 10 licenses any elk valid in that portion of Area 59 on national forest
	2	Nov. 1	Dec. 21	Limited quota; 150 licenses antlerless elk
	6	Nov. 1	Dec. 21	Limited quota; 100 licenses cow or calf
	9	Sept. 1	Sept. 30	Limited quota; 25 licenses any elk, archery only
60		Sept. 10	Oct. 21	General license; any elk
	9	Sept. 1	Sept. 30	Limited quota; 20 licenses any elk, archery only

Hunt Area	Type	Date of Seasons Opens	Closes	Limitations
61		Nov. 1	Nov. 30	General license; antlerless elk valid in that portion of Area 61 north of and including the Rawhide Creek drainage
	1	Oct. 1	Oct. 21	Limited quota; 150 licenses any elk valid in that portion of Area 61 within the Washakie Wilderness
	2	Nov. 1	Nov. 15	Limited quota; 25 licenses any elk valid in that portion of Area 61 outside the Washakie Wilderness; also valid in Area 66 from Nov. 1 to Nov. 15 for any elk
	3	Dec. 1	Dec. 21	Limited quota; 150 licenses antlerless elk
62	1	Oct. 1	Oct. 21	Limited quota; 150 licenses any elk
	2	Oct. 1	Oct. 21	Limited quota; 50 licenses antlerless elk
	3	Oct. 22	Nov. 15	Limited quota; 150 licenses antlerless elk
63, 64	1	Oct. 1	Oct. 21	Limited quota; 200 licenses any elk
63	2	Oct. 1	Nov. 25	Limited quota; 175 licenses antlerless elk
	6	Sept. 1	Sept. 30	Limited quota; 100 licenses cow or calf valid in that portion of Area 63 north of Gooseberry Creek off national forest
		Oct. 1	Nov. 25	Unused Area 63 Type 6 licenses valid for cow or calf elk in Area 63
64	2	Nov. 1	Nov. 15	Limited quota; 100 licenses any elk
	6	Nov. 1	Nov. 30	Limited quota; 125 licenses cow or calf
		Dec. 1	Dec. 15	Unused Area 64 Type 6 licenses valid in that portion of Area 64 south of and including the Cottonwood Creek drainage
65	7	Sept. 1	Sept. 30	Limited quota; 100 licenses cow or calf
	5	Oct. 1	Oct. 31	Limited quota; 100 licenses cow or calf
66		Sept. 10	Nov. 15	General license; antlerless elk
67, 68		Oct. 1	Oct. 31	General license; antlered elk
	2	Nov. 1	Nov. 20	Limited quota; 250 licenses antlerless elk
67,68,69	9	Sept. 1	Sept. 30	Limited quota; 125 licenses any elk, archery only
69		Oct. 1	Oct. 31	General license; any elk
		Nov. 1	Nov. 20	General license; antlerless elk
	6	Oct. 1	Nov. 20	Limited quota; 50 licenses cow or calf
70		Sept. 20	Oct. 31	General license; antlered elk. SEE SECTION 5
		Nov. 1	Nov. 15	General license; antlered elk valid in that portion of Area 70 north and west of Pacific Creek and Mink Creek. SEE SECTION 5
71		Sept. 20	Nov. 15	General license; antlered elk. SEE SECTION 5
72				CLOSED
73		Sept. 20	Oct. 31	General license; any elk
	2	Sept. 10	Oct. 31	Limited quota; 25 licenses antlerless elk
	6	Sept. 10	Oct. 31	Limited quota; 50 licenses cow or calf valid in that portion of Area 73 south of Teton Creek
		Nov. 1	Jan. 31	Unused Area 73 Type 6 licenses valid for cow or calf on private land
74		Sept. 26	Oct. 31	General license; any elk. SEE SECTION 5
		Nov. 1	Nov. 25	General license; antlerless elk. SEE SECTION 5
75	1	Oct. 20	Nov. 25	Limited quota; 100 licenses any elk, antlerless elk on hayfields and Antelope Flats portion of Area 75. SEE SECTIONS 5 and 6
	2	Oct. 20	Nov. 25	Limited quota; 400 licenses antlerless elk. SEE SECTIONS 5 and 6

Hunt Area	Type	Date of Seasons Opens	Closes	Limitations
77		Oct. 13	Oct. 14	General license and unused limited quota licenses, excluding limited quota cow or calf licenses and limited quota archery only licenses, valid for any elk; 70 National Elk Refuge permits may be issued only for youths 12 through 17 years of age. SEE SECTIONS 5 and 6
		Oct. 15	Oct. 19	General license and unused limited quota licenses, excluding limited quota cow or calf licenses and limited quota archery only licenses, valid for any elk. SEE SECTIONS 5 and 6
		Oct. 20	Nov. 30	General license and unused limited quota licenses; excluding limited quota archery only licenses, antlerless elk only. SEE SECTIONS 5 and 6
78	1	Sept. 26	Dec. 15	Limited quota; 25 licenses any elk. SEE SECTION 5
	2	Sept. 10	Jan. 31	Limited quota; 25 licenses antlerless elk. SEE SECTION 5
	6	Sept. 10	Jan. 31	Limited quota; 75 licenses cow or calf. SEE SECTION 5
79	1	Oct. 20	Nov. 15	Limited quota; 200 licenses any elk. SEE SECTIONS 5 and 6
	2	Oct. 20	Nov. 15	Limited quota; 150 licenses antlerless elk; also valid in Area 75 for antlerless elk. SEE SECTIONS 5 and 6
	6	Oct. 20	Nov. 15	Limited quota; 300 licenses cow or calf; also valid in Area 75 for cow or calf. SEE SECTIONS 5 and 6
		Oct. 20	Nov. 25	Unused Area 79 Type 2 and Type 6 licenses valid in Area 75. SEE SECTIONS 5 and 6
80		Sept. 26	Oct. 31	General license; any elk. SEE SECTION 5
	2	Sept. 10	Oct. 31	Limited quota; 75 licenses antlerless elk. SEE SECTION 5
		Nov. 1	Nov. 15	General license and Unused Area 80 Type 2 licenses; valid for antlerless elk. SEE SECTION 5
		Nov. 16	Nov. 30	General licenses and Unused Area 80 Type 2 licenses valid for antlerless elk in that portion of Area 80 south of Flat Creek. SEE SECTION 5
81		Sept. 26	Oct. 25	General license; any elk, spikes excluded. SEE SECTION 5
		Oct. 26	Oct. 31	General license; antlerless elk. SEE SECTION 5
	2	Sept. 10	Oct. 31	Limited quota; 25 licenses antlerless elk. SEE SECTION 5
82		Sept. 26	Oct. 25	General license; any elk, spikes excluded. SEE SECTION 5
		Oct. 26	Oct. 31	General license; antlerless elk. SEE SECTION 5
	2	Sept. 10	Oct. 31	Limited quota; 25 licenses antlerless elk. SEE SECTION 5
83		Oct. 1	Oct. 25	General license; any elk, spikes excluded. SEE SECTION 5
		Oct. 26	Oct. 31	General license; antlerless elk. SEE SECTION 5
	2	Oct. 1	Oct. 31	Limited quota; 25 licenses antlerless elk. SEE SECTION 5
84		Sept. 26	Oct. 31	General license; any elk. SEE SECTION 5
		Nov. 1	Nov. 11	General license; antlerless elk. SEE SECTION 5
		Nov. 12	Nov. 30	General license; antlerless elk valid in that portion of Area 84 in the Cache Creek drainage. SEE SECTION 5
	1	Nov. 1	Jan. 31	Limited quota; 20 licenses any elk valid only in that portion of Area 84 on private land west of U.S. Highway 191 and north and east of the Snake River starting at the South Park Bridge; access to private land is limited. SEE SECTION 5
	2	Sept. 10	Nov. 18	Limited quota; 50 licenses antlerless elk. SEE SECTION 5
	6	Sept. 10	Nov. 18	Limited quota; 250 licenses cow or calf. SEE SECTION 5

Hunt Area	Type	Date of Seasons Opens	Closes	Limitations
		Nov. 19	Jan. 31	Unused Area 84 Type 6 licenses valid for cow or calf elk valid in that portion of Area 84 on private land west of U.S. Highway 191 and north and east of the Snake River starting at the South Park Bridge; access to private land is limited. SEE SECTION 5
85		Sept. 26	Oct. 31	General license; any elk. SEE SECTION 5
		Nov. 1	Nov. 11	General license; antlerless elk valid in that portion of Area 85 upstream from Wolf Creek within the Snake River drainage. SEE SECTION 5
	1	Nov. 1	Jan. 31	Limited quota; 20 licenses any elk valid only in that portion of Area 85 on private land north of Butler Creek; access to private land is limited. SEE SECTION 5
	2	Sept. 10	Nov. 18	Limited quota; 50 licenses antlerless elk. SEE SECTION 5
	6	Sept. 10	Nov. 18	Limited quota; 250 licenses cow or calf. SEE SECTION 5
		Nov. 19	Jan. 31	Unused Area 85 Type 6 licenses valid for cow or calf elk on private land in that portion of Area 85 north of Butler Creek; access to private land is limited. SEE SECTION 5
86		Sept. 26	Oct. 31	General license; any elk. SEE SECTION 5
87		Oct. 15	Oct. 31	General license; any elk. SEE SECTION 5
	6	Nov. 19	Jan. 31	Limited quota; 50 licenses cow or calf valid in that portion of Area 87 south and east of Dell Creek, north of U.S. Highway 191, and west of the North Fork of Fisherman Creek. SEE SECTION 5
88	1	Oct. 1	Oct. 31	Limited quota; 60 licenses any elk. SEE SECTION 5
89		Oct. 15	Oct. 17	General license; any elk. SEE SECTION 5
		Oct. 18	Oct. 31	General license; antlered elk. SEE SECTION 5
90		Oct. 15	Oct. 31	General license; any elk. SEE SECTION 5
		Nov. 1	Nov. 11	General license; antlerless elk. SEE SECTION 5
	6	Oct. 15	Nov. 11	Limited quota; 100 licenses cow or calf. SEE SECTION 5
91		Oct. 1	Oct. 31	General license; any elk. SEE SECTION 5
	1	Oct. 1	Oct. 31	Limited quota; 350 licenses any elk. SEE SECTION 5
		Nov. 1	Jan. 31	Unused Area 91 Type 1 licenses valid for antlerless elk. SEE SECTION 5
	6	Oct. 1	Jan. 31	Limited quota; 100 licenses cow or calf. SEE SECTION 5
92		Oct. 15	Oct. 31	General license; any elk. SEE SECTION 5
		Nov. 1	Nov. 4	General license; antlerless elk. SEE SECTION 5
	6	Oct. 15	Nov. 4	Limited quota; 200 licenses cow or calf. SEE SECTION 5
		Nov. 16	Jan. 31	Unused Area 92 Type 6 licenses valid only in that portion of Area 92 east of Sublette County Roads 115, 116 and 117 and south of the North Beaver Road, off national forest lands. SEE SECTION 5
93	1	Oct. 1	Oct. 31	Limited quota; 150 licenses any elk; also valid in that portion of Area 96 west of Wyoming Highway 352. SEE SECTION 5
		Nov. 1	Nov. 15	Unused Area 93 Type 1 licenses valid for antlerless elk; also valid in that portion of Area 96 west of Wyoming Highway 352. SEE SECTION 5
	2	Oct. 1	Nov. 15	Limited quota; 75 licenses antlerless elk; also valid in that portion of Area 96 west of Wyoming Highway 352. SEE SECTION 5
	6	Oct. 1	Nov. 15	Limited quota; 225 licenses cow or calf; also valid in that portion of Area 96 west of Wyoming Highway 352. SEE SECTION 5

Hunt Area	Type	Date of Seasons Opens	Closes	Limitations
94		Oct. 15	Oct. 31	General license; any elk. SEE SECTION 5
		Nov. 1	Nov. 4	General license; antlerless elk. SEE SECTION 5
	1	Oct. 15	Oct. 21	Limited quota; 50 licenses any elk. SEE SECTION 5
		Oct. 22	Nov. 4	Unused Area 94 Type 1 licenses valid for antlerless elk. SEE SECTION 5
	6	Oct. 15	Nov. 4	Limited quota; 300 licenses cow or calf. SEE SECTION 5
		Nov. 16	Jan. 31	Unused Area 94 Type 1 and Type 6 licenses; 100 access permits will be issued for antlerless elk only on those lands enrolled in the Big Piney Hunter Management Area in Hunt Areas 92 and 94. Access permits will be issued at the Pinedale Regional Office on Nov. 10. SEE SECTION 5
95	1	Oct. 15	Nov. 5	Limited quota; 150 licenses any elk. SEE SECTION 5
	2	Oct. 15	Nov. 5	Limited quota; 250 licenses antlerless elk. SEE SECTION 5
	3	Oct. 1	Nov. 5	Limited quota; 25 licenses any elk valid in that portion of Area 95 that is within the Green River Drainage upstream from the outlet of Lower Green River Lake, including that portion east and south of Mill Creek. SEE SECTION 5
	4	Oct. 1	Nov. 5	Limited quota; 25 licenses antlerless elk valid in that portion of Area 95 that is within the Green River Drainage upstream from the outlet of Lower Green River Lake, including that portion east and south of Mill Creek. SEE SECTION 5
	6	Oct. 15	Nov. 5	Limited quota; 25 licenses cow or calf. SEE SECTION 5
96		Oct. 15	Oct. 31	General license; any elk. SEE SECTION 5
	1	Oct. 1	Oct. 31	Limited quota; 200 licenses any elk. SEE SECTION 5
	2	Oct. 1	Oct. 31	Limited quota; 50 licenses antlerless elk. SEE SECTION 5
	6	Oct. 1	Oct. 31	Limited quota; 75 licenses cow or calf. SEE SECTION 5
		Nov. 1	Nov. 15	Unused Area 96 Type 1, 2, and 6 licenses antlerless elk. SEE SECTION 5
97		Oct. 1	Oct. 31	General license; any elk. SEE SECTION 5
	1	Sept. 20	Oct. 31	Limited quota; 300 licenses any elk. SEE SECTION 5
		Nov. 1	Nov. 6	Unused Area 97 Type 1 licenses valid for antlerless elk. SEE SECTION 5
	2	Sept. 20	Nov. 6	Limited quota; 50 licenses antlerless elk. SEE SECTION 5
	6	Sept. 20	Nov. 6	Limited quota; 75 licenses cow or calf. SEE SECTION 5
98		Oct. 1	Oct. 31	General license; any elk. SEE SECTION 5
	1	Sept. 20	Oct. 31	Limited quota; 350 licenses any elk. SEE SECTION 5
		Nov. 1	Nov. 15	Unused Area 98 Type 1 licenses valid for antlerless elk. SEE SECTION 5
	2	Sept. 20	Nov. 15	Limited quota; 100 licenses antlerless elk. SEE SECTION 5
	6	Sept. 20	Nov. 15	Limited quota; 150 licenses cow or calf. SEE SECTION 5
		Nov. 16	Dec. 31	Unused Area 98 Type 1, Type 2 and Type 6 licenses valid for antlerless elk in that portion of Area 98 between Scab Creek and the East Fork River drainage, excluding Irish Canyon Creek and Muddy Creek. SEE SECTION 5
99	1	Oct. 1	Oct. 31	Limited quota; 175 licenses antlered elk
	2	Oct. 1	Nov. 15	Limited quota; 225 licenses antlerless elk
100	1	Oct. 15	Oct. 31	Limited quota; 225 licenses antlered elk
	2	Oct. 20	Nov. 11	Limited quota; 200 licenses antlerless elk
102		Oct. 15	Oct. 24	General license; any elk
		Oct. 25	Nov. 4	General license; antlerless elk

Hunt Area	Type	Date of Seasons Opens	Closes	Limitations
103		Oct. 15	Oct. 24	General license; any elk
		Oct. 25	Nov. 4	General license; antlerless elk
	6	Dec. 15	Jan. 31	Limited quota; 50 licenses cow or calf
104		Oct. 15	Oct. 24	General license; any elk
		Oct. 25	Nov. 4	General license; antlerless elk
	6	Oct. 10	Oct. 14	Limited quota; 150 licenses cow or calf valid in that portion of Area 104 west of U. S. Highway 30
		Oct. 15	Nov. 4	Unused Area 104 Type 6 licenses cow or calf valid in the entire area
105		Oct. 15	Oct. 31	General license; any elk
106		Oct. 15	Oct. 31	General license; any elk
	1	Nov. 15	Dec. 31	Limited quota; 30 licenses any elk valid in that portion of Area 106 west of Muddy Creek, or north of Wyoming Highway 410 and Uinta County Road 204
	2	Nov. 15	Dec. 31	Limited quota; 150 licenses antlerless elk
	6	Sept. 10	Oct. 31	Limited quota; 25 licenses cow or calf valid east of Wyoming Highway 150 and north and west of Uinta County Road 173
		Nov. 15	Dec. 31	Unused Area 106 Type 6 licenses cow or calf valid in the entire area
107		Oct. 15	Oct. 31	General license; any elk
	2	Nov. 15	Dec. 31	Limited quota; 100 licenses antlerless elk
		Jan. 1	Jan. 31	Unused Area 107 Type 2 licenses antlerless elk off national forest
108	1	Oct. 11	Oct. 31	Limited quota; 50 licenses any elk
	2	Oct. 11	Nov. 20	Limited quota; 100 licenses antlerless elk
	3	Nov. 1	Nov. 20	Limited quota; 50 licenses antlerless elk
110		Oct. 15	Oct. 31	General license; any elk
	2	Oct. 15	Nov. 5	Limited quota; 75 licenses antlerless elk
111	1	Oct. 10	Oct. 31	Limited quota; 40 licenses antlered elk
	2	Oct. 10	Nov. 20	Limited quota; 90 licenses antlerless elk
	3	Oct. 22	Nov. 20	Limited quota; 60 licenses antlerless elk
113	1	Nov. 5	Nov. 30	Limited quota; 40 licenses any elk
	2	Nov. 5	Nov. 30	Limited quota; 60 licenses antlerless elk
114	1	Oct. 1	Oct. 31	Limited quota; 50 licenses any elk
	2	Oct. 1	Nov. 14	Limited quota; 50 licenses antlerless elk; also valid in Area 125 north of Interstate Highway 80
	6	Oct. 1	Nov. 14	Limited quota; 50 licenses cow or calf; also valid in Area 125 north of Interstate Highway 80
116	1	Oct. 15	Dec. 15	Limited quota; 175 licenses any elk
	2	Oct. 15	Dec. 15	Limited quota; 150 licenses antlerless elk
	6	Oct. 15	Dec. 15	Limited quota; 100 licenses cow or calf
117	1	Oct. 15	Nov. 30	Limited quota; 225 licenses any elk
	2	Oct. 15	Nov. 30	Limited quota; 175 licenses antlerless elk
	6	Oct. 15	Nov. 30	Limited quota; 60 licenses cow or calf
		Dec. 1	Dec. 15	Unused Area 117 Type 1, Type 2 and Type 6 licenses valid for antlerless elk on private land
118	1	Oct. 23	Nov. 4	Limited quota; 25 licenses antlered elk
	2	Oct. 23	Nov. 4	Limited quota; 35 licenses antlerless elk
	6	Oct. 1	Nov. 30	Limited quota; 35 licenses cow or calf valid in that portion of Area 118 south and east of Separation Creek

Hunt Area	Type	Date of Seasons Opens	Closes	Limitations
120	1	Oct. 9	Oct. 31	Limited quota; 100 licenses any elk
		Nov. 1	Nov. 30	Unused Area 120 Type 1 licenses valid for antlerless elk
	2	Oct. 9	Nov. 30	Limited quota; 100 licenses antlerless elk
	3	Nov. 1	Nov. 30	Limited quota; 75 licenses antlerless elk
121	1	Nov. 1	Nov. 30	Limited quota; 100 licenses any elk; also valid in Area 65
	2	Nov. 1	Dec. 31	Limited quota; 125 licenses antlerless elk; also valid in Areas 54 and 65
	3	Oct. 1	Oct. 21	Limited quota; 50 licenses antlerless elk
	6	Nov. 1	Dec. 31	Limited quota; 100 licenses cow or calf; also valid in Areas 54 and 65
122	1	Oct. 20	Nov. 19	Limited quota; 50 licenses any elk
		Nov. 20	Nov. 30	Unused Area 122 Type 1 licenses valid for antlerless elk
	2	Oct. 20	Nov. 30	Limited quota; 25 licenses antlerless elk
	6	Oct. 20	Nov. 30	Limited quota; 75 licenses cow or calf
123	2	Sept. 8	Sept. 16	Limited quota; 100 licenses antlerless elk
		Nov. 5	Nov. 14	Unused Area 123 Type 2 licenses antlerless elk
124	1	Oct. 15	Nov. 30	Limited quota; 15 licenses any elk
	2	Oct. 15	Nov. 30	Limited quota; 60 licenses antlerless elk
*125	1	Oct. 1	Nov. 6	Limited quota; 150 licenses any elk
		Nov. 7	Dec. 31	Unused Area 125 Type 1 licenses valid for antlerless elk
	2	Oct. 1	Dec. 31	Limited quota; 50 licenses antlerless elk
	6	Oct. 1	Dec. 31	Limited quota; 75 licenses cow or calf
126	1	Nov. 5	Dec. 10	Limited quota; 25 licenses any elk
	2	Nov. 5	Dec. 10	Limited quota; 35 licenses antlerless elk
127		Oct. 15	Oct. 31	General license; any elk
128		Oct. 1	Oct. 14	General license; any elk
	6	Nov. 1	Nov. 30	Limited quota; 25 licenses cow or calf
129		Sept. 1	Nov. 30	General license; any elk
	6	Sept. 1	Nov. 30	Limited quota; 100 licenses cow or calf
130		Oct. 1	Oct. 22	General license, any elk

*ASTERISK DENOTES A CWD HUNT AREA. PLEASE SEE IMPORTANT INFORMATION SECTION OF THE ELK REGULATION BROCHURE REGARDING CHRONIC WASTING DISEASE.

Section 4. Special Archery Seasons. Hunt Areas listed in this section shall have special archery hunting seasons during the dates specified in this section. Archers shall possess a general elk license or a limited quota elk license in addition to an archery license in order to hunt elk during any special archery season.

Archers with general elk licenses shall only hunt in those Hunt Areas specified in Section 3 as open to hunting with a general license and may take any elk, unless specified as antlerless elk in Section 3.

Archers with limited quota elk licenses may only hunt in the Hunt Area specified in Section 3 for which their license is valid. They shall only hunt the sex of elk allowed to be taken within the Hunt Area on the earliest opening date for which their license type is valid as specified in Section 3. Archers with limited quota Type 9 elk licenses are not required to obtain an archery license in addition to the Type 9 license.

Hunt Area	Date of Seasons Opens	Closes
1, 5-16, 19, 21-25, 27, 28, 30-34, 47-56, 58, 59, 61-65, 83, 87-96, 99, 100, 102-111, 113, 114, 116-118, 120-122, 124-128, 130	Sept. 1	Sept. 30
3	Sept. 1	Sept. 14
35-37, 41, 42, 45, 46, 67-69	Sept. 15	Sept. 30
60, 66	Sept. 1	Sept. 9
70, 71, 73, 97, 98	Sept. 1	Sept. 19
74, 78, 80-82, 84-86	Sept. 1	Sept. 25

Section 5. Elk Feedground Special Management Permit (Fee $10.50). The Department's elk feedground program is a special management program that requires additional expenditures for the feeding of wildlife. An elk feedground special management permit shall be required of any person who hunts elk in elk hunt areas listed in subsection (a) of this section. The elk feedground special management permit shall be in possession of any person while hunting elk in those hunt areas and shall be immediately produced upon request from an authorized Department representative. Any person who purchases an elk feedground special management permit for the purpose set forth in this section shall sign his or her name in ink across the face of the permit. The permit shall cost ten dollars and fifty cents ($10.50) and shall be available at Cheyenne Headquarters, Department Regional Offices, and at designated license selling agents.

(a) Elk Hunt Areas 70, 71, 74, 75, 77, 78, 79, 80, 81, 82, 83, 84, 85, 86, 87, 88, 89, 90, 91, 92, 93, 94, 95, 96, 97, and 98.

Section 6. Regulations Governing Elk Hunting In Grand Teton National Park (Hunt Areas 75 and 79) and the National Elk Refuge (Hunt Area 77).

(a) NO HUNTER MAY HARVEST MORE THAN ONE (1) ELK IN GRAND TETON NATIONAL PARK.

(b) Successful completion of a hunter safety course is required, regardless of a hunter's age, prior to hunting in Grand Teton National Park or the National Elk Refuge and a hunter safety certificate shall be in possession when hunting elk.

(c) A Park or National Elk Refuge Permit is required prior to hunting elk and shall be in possession while hunting elk in Grand Teton National Park or the National Elk Refuge.

(d) That portion of Hunt Area 75 beginning at the Lost Creek Road (U.S.F.S. Road 30340) at U.S. Highway 26-89-191; easterly along said road to the Grand Teton National Park boundary; southerly along said boundary to the intersection with the Shadow Mountain-Kelly Road (U.S.F.S. Road 30340) at Carpenter Draw; southwesterly along said road to the junction of the Mormon Row Road; northerly along said road to the Antelope Flats Road; westerly along said road to U.S. Highway 26-89-191; northeasterly along said highway to the Lost Creek Road (U.S.F.S. Road 30340), described as the Antelope Flats and the hayfields areas shall be open only to antlerless elk hunting.

(e) An area one-quarter (1/4) mile wide along either side of U.S. Highway 26-89-191 shall be closed to all elk hunting within Grand Teton National Park.

(f) An area within one-half (1/2) mile of any building shall be closed to elk hunting within Grand Teton National Park.

(g) A portion of Hunt Area 75 in the SW 1/4 of Section 22, T43N, R115W, S1/2, S1/2 of Sections 3 and 4, T42N, R115W shall be closed to elk hunting, as posted in the field.

(h) Legal firearms include rifles with a barrel bore diameter of at least .23 caliber that fire a cartridge that is two (2) inches or more in length. Handguns and archery hunting are prohibited in Grand Teton National Park and the National Elk Refuge.

Section 7. Hunt Area Descriptions.

(a) Area and Number.

Area 1. Black Hills. Beginning where the north boundary of the Black Hills National Forest crosses the Wyoming-South Dakota state line; southerly along said line to U.S.F.S. Road 807; northwesterly along said road to Moskee; northerly along the Moskee Road to its junction with U.S.F.S. Road 863; northerly along said road to the Black Hills National Forest boundary marker and one-quarter mile south of Ranch A; easterly along said boundary to the Wyoming-South Dakota state line.

Area 2. Fortification. Beginning where the Wild Horse Road (Sheridan County Road 38) crosses the Powder River at Arvada; easterly along said road to the Echeta Road (Sheridan County Road 293); southeasterly along said road to the Montgomery Road; westerly along said road to the Kingsbury Road; southerly along said road to Interstate Highway 90; westerly along said highway to the Powder River; northerly down said river to the Wild Horse Road (Sheridan County Road 38).

Area 3. Rawhide. Beginning at the intersection of U.S. Highway 20 and U.S. Highway 85 in the town of Lusk; easterly along U.S. Highway 20 to the Wyoming-Nebraska state line; southerly along said line to Wyoming State Highway 151; westerly along said highway to U.S. Highway 85; northerly along said highway to Wyoming State Highway 313; westerly along said highway to the town of Chugwater and Interstate Highway 25; northerly along said highway to U.S. Highway 26; easterly along said highway to the North Platte River; northerly and westerly up said river to Interstate Highway 25; northerly along said Interstate to U.S. Highway 20; easterly along said highway to the intersection of U.S. Highway 20 and U.S. Highway 85 in the town of Lusk.

Area 5. Iron Mountain. Beginning at the city of Laramie and U.S. Highway 30-287; northerly along said highway to Wyoming Highway 34; northerly and easterly along said highway to Interstate Highway 25; southerly along said highway to Wyoming Highway 211; southwesterly along said highway to the Fisher Canyon-Rogers Canyon Road (Laramie County Road 228-Albany County Road 17); westerly and southerly along said road to U.S. Highway 30-287.

Area 6. Pole Mountain. Beginning where Interstate Highway 25 crosses the Wyoming-Colorado state line; westerly along said line to U.S. Highway 287; northerly along said highway to the Rogers Canyon-Fisher Canyon Road (Albany County Road 17-Laramie County Road 228); northeasterly along said road to Wyoming Highway 211; southeasterly along said highway to Interstate Highway 25; southerly along said highway to the Wyoming-Colorado state line.

Area 7. Laramie Peak. Beginning at the confluence of Deer Creek and the North Platte River in the town of Glenrock; easterly and southerly down said river to the town of Guernsey and U.S. Highway 26; westerly along said highway to Interstate Highway 25; southerly along said highway to Wyoming Highway 34; southwesterly along said highway to U.S. Highway 30-287; northerly along said highway to Wyoming Highway 487 at the town of Medicine Bow; northerly along said highway to the Little Medicine Bow River; northerly up said river to the Little Medicine Road (Carbon County Road 99); northeasterly along said road to the Shirley Ridge Road (Carbon County Road 2); northerly and easterly along said road to Wyoming Highway 487; northerly and westerly along said highway to the divide between Spring Creek and Bates Creek; easterly along said divide to the Bates Creek Road (Natrona County Road 402); easterly along said road to the Squaw Springs Trail Road; northeasterly along said road to U.S.F.S. Road 660; easterly along said road to Curry Creek; northwesterly down said creek to Deer Creek; northerly down said creek to the Stephens Road (Converse County Road 20); northerly along said road to the Deer Creek Road (Converse County Road 19); northerly along said road to Wyoming Highway 20-26 in the town of Glenrock; easterly along said highway to Deer Creek; northerly down said creek to the North Platte River.

Area 8. Boulder Ridge. Beginning at the city of Laramie and U.S. Highway 287; southerly along said highway to the Wyoming-Colorado state line; westerly along said line to Wyoming Highway 10; northerly along

said highway to Wyoming Highway 230 at Woods Landing; northeasterly along said highway to U.S. Highway 287 at the city of Laramie.

Area 9. South Snowy Range. Beginning at the city of Laramie and Wyoming Highway 230; southwesterly along said highway to Woods Landing and Wyoming Highway 10; southerly along said highway to the Wyoming-Colorado state line; westerly along said line to Wyoming Highway 230; northeasterly along said highway to U.S.F.S. Road 512; northwesterly along said road to U.S.F.S. Road 543; northerly along said road to Douglas Creek at Keystone; northerly up said creek to the divide between the Laramie River and the North Platte River; northerly along said divide to Wyoming Highway 130; easterly along said highway to Wyoming Highway 230.

Area 10. Rock Creek. Beginning at the city of Laramie and Wyoming Highway 130; westerly along said highway to the divide between the Laramie River and the North Platte River; northerly along said divide to the divide between the Medicine Bow River and the Laramie River at Medicine Bow Peak; northeasterly along said divide to Sheep Lake Trail 389; northwesterly along said trail to Deep Creek; northeasterly down said creek to Rock Creek; northeasterly down said creek to Interstate Highway 80 at Arlington; southeasterly along said highway to Wyoming Highway 130.

Area 11. Medicine Bow River. Beginning where Interstate Highway 80 crosses Rock Creek at Arlington; southwesterly up said creek to Deep Creek; southwesterly up said creek to Sheep Lake Trail 389; southeasterly along said trail to the divide between the Laramie River and the Medicine Bow River; southwesterly along said divide to Medicine Bow Peak and the divide between the Medicine Bow River and Brush Creek; northwesterly along said divide to U.S.F.S. Road 103; northwesterly along said road to the North Brush Creek Road (U.S.F.S. Road 100); northerly along said road to the Sand Lake Road (U.S.F.S. Road 101); northwesterly along said road to the Cedar Pass Road (U.S.F.S. Road 261); southwesterly along said road to U.S.F.S. Road 115; northerly along said road to the Pass Creek Basin Road; northerly along said road to the Pass Creek Road (Carbon County Road 404); northerly along said road to Wyoming Highway 72; northwesterly along said highway to Interstate Highway 80 at the west Elk Mountain interchange; southeasterly along said highway to Rock Creek.

Area 12. Kennaday Peak. Beginning at the junction of the North Platte River and Interstate Highway 80 at Fort Steele Bridge; easterly along said highway to Wyoming Highway 130 at the Saratoga interchange; southerly along said highway to the Pass Creek Road (Carbon County Road 404); easterly along said road to the Pass Creek Basin Road; southerly along said road to U.S.F.S. Road 115; southerly along said road to the Cedar Pass Road (U.S.F.S. Road 261); northeasterly along said road to the Sand Lake Road (U.S.F.S. Road 101); southeasterly along said road to the North Brush Creek Road (U.S.F.S. Road 100); southerly along said road to U.S.F.S. Road 103; southeasterly along said road to the divide between Brush Creek and the Medicine Bow River; southeasterly along said divide to Medicine Bow Peak and the Laramie River-North Platte River divide; southerly along said divide to Wyoming Highway 130; westerly and northerly along said highway to the North Platte River at Saratoga; northwesterly down said river to Interstate Highway 80.

Area 13. Big Creek. Beginning where the Continental Divide crosses the Wyoming-Colorado state line; northerly along said divide to the Encampment-Savery Road (Wyoming Highway 70); easterly along said road to Wyoming Highway 230; easterly along said highway to the Blackhall Mountain Road (U.S.F.S. Road 409); southerly along said road to the Blackhall Lookout Station; southerly along the divide between Big Creek and the Encampment River to the Wyoming-Colorado state line; westerly along said line to the Continental Divide.

Area 15. Spring Creek. Beginning where Wyoming Highway 71 (Rawlins-Sandstone Road) crosses the Continental Divide; northerly along said highway to Sage Creek; easterly down said creek to the North Platte River; southerly up said river to Wyoming Highway 130; southerly along said highway to Wyoming Highway 230; southeasterly along said highway to Wyoming Highway 70; westerly along said highway to the Continental Divide; northerly along said divide to Wyoming Highway 71.

Area 16. Shirley Mountain. Beginning at the junction of Interstate Highway 80 and the North Platte River; northeasterly down said river to Wyoming Highway 220 at Government Bridge; northeasterly along said highway to the Bolton Creek Road (Natrona County Road 404); southeasterly along said road to the Bates Hole Stock Trail (Natrona County Road 403); southeasterly along said road to Wyoming Highway 487; southerly along

said highway to the Shirley Ridge Road (Carbon County Road 2); easterly and southerly along said road to the Little Medicine Road (Carbon County Road 99); southwesterly along said road to the Little Medicine Bow River; southerly along said river to Wyoming Highway 487; southerly along said highway to U.S. Highway 30; southwesterly along said highway to Interstate Highway 80; westerly along said interstate to the North Platte River.

Area 19. Muddy Mountain. Beginning where Wyoming Highway 95 crosses the North Platte River; easterly down said river to Deer Creek; southerly up said creek to Wyoming Highway 20-26 in the town of Glenrock; westerly along said highway to the Deer Creek road (Converse County Road 19); southerly along said road to the Stephens Road (Converse County Road 20); southerly along said road to Deer Creek; southerly up said creek to Curry Creek; southeasterly up said creek to U.S.F.S. Road 660; westerly along said road to the Squaw Springs Trail Road; southwesterly along said road to the Bates Creek Road (Natrona County Road 402); southwesterly along said road to the divide between Spring Creek and the Bates Creek drainage; westerly along said divide to Wyoming Highway 487; northwesterly along said highway to the Bates Hole Stock Trail (Natrona County Road 403); northwesterly along said road to the Bolton Creek Road (Natrona County Road 404); northwesterly along said road to Wyoming Highway 220; southwesterly along said highway to the North Platte River at Government Bridge; easterly down said river to Wyoming Highway 95.

Area 21. Baggs. Beginning at the junction of Wyoming Highway 789 and the Wyoming-Colorado state line; northerly along said highway to Muddy Creek north of Dad; easterly up said creek to the Muddy Creek Road (BLM Road 3306); easterly along said road to Miller Hill Road (BLM Road 3328); southeasterly along said road to the McCarty Canyon Road (Carbon County Road 503); northeasterly along said road to the Rawlins City Water Works Road; northerly along said road to Sage Creek below Sage Creek Reservoir; northeasterly down said creek to the Sage Creek Road (Carbon County Road 401); southerly along said road to the Continental Divide at Middlewood Hill; southerly along said divide to the Wyoming-Colorado state line; westerly along said line to Wyoming Highway 789.

Area 22. Ferris. Beginning where Wyoming Highway 287 intersects Wyoming Highway 220 at Muddy Gap Junction; northeasterly along Wyoming Highway 220 to the Buzzard Road (Natrona County Road 410-Carbon County Road 497); southerly along said road to the South Ferris Road (Carbon County Road 100); westerly along said road to U.S. Highway 287; northerly along said highway to Muddy Gap Junction.

Area 23. Rattlesnake. Beginning where Wyoming Highway 220 intersects the Dry Creek Road (Natrona County Road 321); northwesterly along said road to the Gas Hills Road (Natrona County Road 212); northeasterly along said road to U.S. Highway 20-26 at the town of Waltman; easterly along said highway to the North Platte River at the city of Casper; southwesterly up said river to the Kortes Road (Natrona County Road 407); northerly along said road to Wyoming Highway 220; westerly along said highway to the Dry Creek Road.

Area 24. Green Mountain. Beginning where Wyoming Highway 287 intersects Wyoming Highway 220 at Muddy Gap Junction; southerly along U.S. Highway 287 to the town of Lamont; westerly along Wyoming Highway 73 to the Bairoil Road (Sweetwater County Road 22); westerly along said road to the Wamsutter-Crooks Gap Road (Sweetwater County Road 23); northerly along said road to the Three Forks-Atlantic City Road (BLM Road 2317); westerly along said road to the Bison Basin Road (BLM Road 3221); northerly along said road to U.S. Highway 287; easterly along said highway to Muddy Gap Junction.

Area 25. South Pass. Beginning where U.S. Highway 287 crosses the south boundary of the Wind River Indian Reservation at the North Popo Agie River; easterly along said boundary to Wyoming Highway 135 (Sand Draw Road); southerly along said highway to U.S. Highway 287 at Sweetwater Station; easterly along said highway to the Bison Basin Road (Fremont County and BLM Road 3221); southerly along said road to the Atlantic City/Three Forks Road (BLM Road 2317; westerly along said road to the Sweetwater River at Phelps-Dodge Bridge; southwesterly up said river to Wyoming Highway 28; northeasterly up said highway to its junction with U.S. Highway 287; northerly along said highway to the south boundary of the Wind River Indian Reservation.

Area 27. Upper Sweetwater. Beginning where Wyoming Highway 28 crosses the Sweetwater River; northwesterly and northerly up said river to the divide between the Sweetwater River and the Wind River at Sweetwater Gap; southeasterly along said divide to Granite Peak and the head of Willow Creek; southeasterly down said creek to Wyoming Highway 28; southwesterly along said highway to the Sweetwater River.

Area 28. Cony Mountain. Beginning where U.S. Highway 287 intersects the southern Wind River Indian Reservation boundary; southeasterly along said highway to Wyoming Highway 28; southwesterly along said highway to Willow Creek; northwesterly up said creek to the divide between the Sweetwater River and Wind River drainages at Granite Peak; northwesterly along said divide to the Continental Divide; northwesterly along said divide to the southern boundary of the Wind River Indian Reservation; easterly along said boundary to U.S. Highway 287.

Area 30. Aspen Mountain. Beginning where Interstate Highway 80 crosses the Green River; easterly along said highway to the town of Rock Springs and Wyoming Highway 430; southeasterly along said highway to the Mud Springs Road (Sweetwater County Road 32); southwesterly along said road to Aspen Mountain Road (Sweetwater County Road 27); southerly along said road to Clay Basin Pipeline Road (Sweetwater County Road 71); southwesterly along said road to the Ramsey Ranch Road (Sweetwater County Road 34); westerly along said road to U.S. Highway 191; westerly along said highway to the Ramsey Ranch Road (Sweetwater County Road 34); northerly along said road to Sage Creek Road (Sweetwater County Road 36); westerly along said road to Sage Creek; westerly down said creek to Flaming Gorge Reservoir; northerly along the east shore of said reservoir to the Green River; northeasterly then northwesterly up said river to Interstate Highway 80.

Area 31. Little Mountain. Beginning where the east shore of Flaming Gorge Reservoir crosses the Wyoming-Utah state line; northerly along the east shore of Flaming Gorge Reservoir to its confluence with Sage Creek; easterly up said creek to the Sage Creek Road (Sweetwater County Road 36); easterly along said road to the Ramsey Ranch Road (Sweetwater County Road 34); southerly along said road to U.S. Highway 191; southerly along said highway to the Wyoming-Utah state line; westerly along said line to the east shore of Flaming Gorge Reservoir.

Area 32. Pine Mountain. Beginning where Wyoming Highway 430 crosses the Wyoming-Colorado state line; westerly along said line to the Wyoming-Utah state line; westerly along said line to U.S. Highway 191; northerly along said highway to the Ramsey Ranch Road (Sweetwater County Road 34); easterly along said road to the Clay Basin Pipeline Road (Sweetwater County Road 71); northeasterly along said road to the Aspen Mountain Road (Sweetwater County Road 27); northerly along said road to the Mud Springs Road (Sweetwater County Road 32); northeasterly long said road to Wyoming Highway 430; southerly along said highway to the Wyoming-Colorado state line.

Area 33. Middle Fork. Beginning at the Kaycee interchange on Interstate Highway 25; southeasterly along said highway to the Midwest interchange and the Smoky Gap Road (Natrona County Road 115); westerly along said road to the Dead Horse Road (Natrona County Road 114); southwesterly along said road to the Wild Horse Trail Road (Natrona County Road 113); westerly along said road to the 33 Mile Road (Natrona County Road 110); northwesterly along said road to the Hazelton Road (Washakie County Road 81); northerly along said road to the Barnum Mountain Road; easterly along said road to the Barnum Road (Johnson County Road 78) at the town of Barnum; easterly along said road to Wyoming Highway 190; northeasterly along said road to Wyoming Highway 191; easterly along said highway to Interstate Highway 25.

Area 34. Upper Powder River. Beginning at the junction of Wyoming Highway 196 and the Crazy Woman Canyon Road (Johnson County Road 14); southerly along said highway to Wyoming Highway 191 at the town of Kaycee; westerly along said highway to Wyoming Highway 190; westerly along said highway to the town of Barnum and the Barnum Mountain Road; westerly along said road to the Hazelton Road (Washakie County Road 81-Johnson County Road 3); northerly along said road to the Gold Mine Road (U.S.F.S. Road 452); northerly along said road to the Canyon Creek Road (U.S.F.S. Road 25); northerly along said road to U.S. Highway 16; easterly along said highway to the Crazy Woman Canyon Road; easterly along said road to Wyoming Highway 196.

Area 35. Hunter Mesa. Beginning where U.S. Highway 87 business loop crosses French Creek; southerly along said highway to Wyoming Highway 196; southerly along said highway to the Crazy Woman Canyon Road (Johnson County Road 14); westerly along said road to U.S. Highway 16; westerly along said highway to the divide along the Big Horn Mountain range at Powder River Pass; northwesterly along said divide to North Clear Creek; easterly down said creek to the North Fork Ditch; easterly down said ditch to French Creek; easterly down said creek to the U.S. Highway 87 business loop.

Area 36. Rock Creek. Beginning where Interstate Highway 90 crosses Piney Creek; southeasterly along said highway to Interstate Highway 25; southerly along said highway to French Creek east of the town of Buffalo; westerly up said creek to the North Fork Ditch; westerly up said ditch to the North Fork of Clear Creek; westerly up said creek to the divide along the Big Horn Mountain range; northerly along said divide to South Piney Creek; northeasterly down said creek to Piney Creek; easterly down said creek to Interstate Highway 90.

Area 37. Goose. Beginning at the junction of U.S. Highway 14 and Interstate Highway 90 east of the town of Ranchester; southerly along said highway to Piney Creek; westerly up said creek to South Piney Creek; southwesterly up said creek to the divide along the Big Horn Mountain range; northwesterly along said divide to the Woodchuck Pass Road (U.S.F.S. Road 226); northerly along said road to the Goose (Red Grade) Road (U.S.F.S. Road 26); northwesterly along said road to the Black Mountain Road (U.S.F.S. Road 16); northeasterly along said road to U.S. Highway 14; easterly along said road to Interstate Highway 90.

Area 38. Tongue. Beginning where Interstate Highway 90 crosses the Wyoming-Montana state line; southeasterly along said highway to U.S. Highway 14; westerly along said highway to the Black Mountain Road (U.S.F.S. Road 16); southerly along said road to the Goose (Red Grade) Road (U.S.F.S. Road 26); southerly along said road to the Woodchuck Pass Road (U.S.F.S. Road 226); southerly along said road to Woodchuck Pass and the divide along the Big Horn Mountain range; northwesterly along said divide to U. S. Highway 14 at Granite Pass; northerly along said highway to the Hunt Mountain Road (U.S.F.S. Road 10); northwesterly along said road to U. S. Highway 14A; westerly along said highway to the Devils Canyon Road (U.S.F.S. Road 14); northerly along said road to the Sheep Mountain Road (U.S.F.S. Road 11); northerly along said road to the Wyoming-Montana state line; easterly along said line to Interstate Highway 90.

Area 39. Deer Creek. Beginning where U.S. Highway 14A crosses the Big Horn River; northerly down said river to the Wyoming-Montana state line; easterly along said line to the Sheep Mountain Road (U.S.F.S. Road 11); southerly along said road to the Devils Canyon Road (U.S.F.S. Road 14); southerly along said road to U.S. Highway 14A; westerly along said highway to the Big Horn River.

Area 40. Horse Creek. Beginning on the Big Horn River in the town of Greybull; northerly down river to U.S. Highway 14A; easterly along said highway to the Hunt Mountain Road (U.S.F.S. Road 10); southeasterly along said road to U.S. Highway 14 at Granite Pass; southwesterly along said highway to the Big Horn River at the town of Greybull.

Area 41. North Medicine Lodge. Beginning where U.S. Highway 14 crosses the Bighorn River in the town of Greybull; easterly along said highway to Granite Pass and the divide along the Bighorn Mountain range; southeasterly along said divide to the divide between Shell Creek and Medicine Lodge Creek; westerly along said divide to U.S.F.S. Road 341; westerly along said road to the Shell Ranger Station-Paintrock Lakes Road (U.S.F.S. road 17); southeasterly along said road to the Alkali Road (U.S.F.S. Road 338-BLM Road 1111); southwesterly along said road to Wyoming Highway 31; westerly along said highway to the Bighorn River at the town of Manderson; northerly down said river to U.S. Highway 14 in the town of Greybull.

Area 42. Medicine Lodge. Beginning at the junction of the Cold Springs Road and the Alkali Road near the town of Hyattville; northerly along the Alkali Road (becomes U.S.F.S. Road 338) to the Paint Rock Lakes Road (U.S.F.S. Road 17); northerly along said road to U.S.F.S. Road 341; easterly along said road to the divide between Shell Creek and Medicine Lodge Creek; easterly along said divide to the divide between Medicine Lodge Creek and Paint Rock Creek; southwesterly along said divide to the Cold Springs Road (U.S.F.S. Road 344) at Upper Medicine Lodge Lake; southwesterly along said road to its junction with the Alkali Road.

Area 45. Paint Rock Creek. Beginning where U.S. Highway 16 crosses the Big Horn River in the town of Worland; northerly down said river to Wyoming Highway 31 at the town of Manderson; easterly along said highway to the Cold Springs Road (BLM/U.S.F.S. Road 344); northeasterly along said road to the divide between Medicine Lodge Creek and Paint Rock Creek at Upper Medicine Lodge Lake; easterly along said divide to the divide along the Big Horn Mountain range; southeasterly along said divide to U.S. Highway 16 at Powder River Pass; westerly along said highway to the Big Horn River.

Area 46. Leigh Creek. Beginning at the junction of Wyoming Highway 436 (Rome Hill Road) and U.S. Highway 16 approximately four (4) miles east of the town of Ten Sleep; northeasterly along U.S. Highway 16 to the Canyon Creek Road (U.S.F.S. Road 25); southerly along said road to the Gold Mine Road (U.S.F.S. Road 452); southerly along said road to the Hazelton Road (Big Horn Divide Road-Johnson County Road 3); southerly along said road to the Rome Hill Road (Wyoming Highway 436); northwesterly along said road to U.S. Highway 16.

Area 47. Copper Mountain. Beginning where the Big Horn River intersects Wyoming Highway 172 (Black Mountain Road); easterly along said highway to the Kirby Creek Road (Hot Springs County Road 7); southerly along said road to the Bridger Creek Road (Fremont County Road 259); southerly along said road to the Badwater Road (Fremont County Road 117); southeasterly along said road to the Lysite-Moneta Road (Fremont County Road 176); southerly along said road to U.S. Highway 20-26; westerly along said highway to the town of Shoshoni; northerly along U.S. Highway 20 to the Wind River Indian Reservation boundary; north and west along said boundary to the Big Horn River; northerly down said river to Wyoming Highway 172.

Area 48. Upper Nowood. Beginning at the town of Moneta and U.S. Highway 20-26; northerly along the Lysite-Moneta Road (Fremont County Road 176) to the Badwater Road (Fremont County Road 117) at the town of Lysite; westerly along said road to the Bridger Creek Road (Fremont County Road 259); northerly along the Bridger Creek Road to the Kirby Creek Road (Hot Springs County Road 7); northerly along said road to Wyoming Highway 172 (Black Mountain Road); easterly along said road to the Murphy Dome-Mud Creek Road (BLM Road 1409); northeasterly along said road to the Nowater Stock Drive Road (BLM Road 1404); easterly along said road to the Upper Nowood Road; northeasterly along said road to Deep Creek; southerly up said creek to Lost Creek; southerly up said creek to the Cherry Creek Hill Stock Drive Road; northeasterly along said road to the Hazelton Road (Big Horn Divide Road); southerly along said road to the divide between the Middle Fork of the Powder River and Buffalo Creek; westerly along said divide to the divide between Sawmill Creek and Little Lost Creek; westerly along said divide to Deep Creek; southerly up said creek to the divide with Badwater Creek; southerly down said creek to the Badwater Road (Natrona County Road 102); southerly along said road to the Lost Cabin-Arminto Road (Natrona County Road 103); southeasterly along said road to the Buffalo Creek Road (Natrona County Road 105); southerly along said road to the Arminto-Waltman Road (Natrona County Road 104); southerly along said road to U.S. Highway 20-26; westerly along said highway to the town of Moneta.

Area 49. Otter Creek. Beginning at the junction of U.S. Highway 16 and Wyoming Highway 436 (Rome Hill Road) approximately four (4) miles east of the town of Ten Sleep; easterly along Wyoming Highway 436 to the Hazelton Road (Big Horn Divide Road-Johnson County Road 3); southerly along said road to the Cherry Creek Hill Stock Drive Road; southwesterly down said road to Lost Creek; northwesterly down said creek to Deep Creek; northwesterly down said creek to the Upper Nowood Road; southwesterly along said road to the Nowater Stock Drive (BLM Road 1404); westerly along said road to the Blue Bank Road (BLM Road 1411); northerly along said road to U.S. Highway 16; easterly along said highway to Wyoming Highway 436 (Rome Hill Road).

Area 50. Beartooth. All of the area north of the Clark's Fork River and west of Wyoming Highway 120.

Area 51. Crandall. All of the drainage of Soda Butte Creek outside of Yellowstone National Park; all of the south drainage of the Clark's Fork River down to and excluding the drainage of Russell Creek.

Area 52. Sunlight. All of the drainage of Sunlight Creek and Russell Creek.

Area 53. Dead Indian. All of the drainage of the Dead Indian Creek.

Area 54. Bald Ridge. All of the drainage of Pat O'Hara Creek, Paint Creek, Newmeyer Creek and Dead Indian Gulch west of Wyoming Highway 120.

Area 55. Grinnell. All of the north and west drainage of the North Fork of the Shoshone River down to but excluding the drainage of Sweetwater Creek; all of the south drainage of the North Fork of the Shoshone River down to but excluding the drainage of Elk Fork Creek.

Area 56. Wapiti Ridge. All of the drainage of the North Fork of the Shoshone River below and including the drainage of Sweetwater Creek and above the drainage of Jim Creek; all of the south drainage of the North Fork

of the Shoshone River below and including the drainage of Elk Fork Creek; all of the north and west drainage of the South Fork of the Shoshone River below and including the drainage of Ishawooa Creek.

Area 58. Sage Creek. Beginning where Wyoming Highway 120 leaves the town of Cody; southerly along said highway to Meeteetse Rim; westerly along said rim to the divide between the Greybull River and the South Fork of the Shoshone River; westerly along said divide to the west fork of Belknap Creek; northwesterly down said creek to the South Fork of the Shoshone River; northeasterly down said river to the town of Cody.

Area 59. Boulder Basin. All of the south and east drainage of the South Fork of the Shoshone River, west of the west fork and mainstream of Belknap Creek; down to the South Fork of the Shoshone River; all of the north and west drainage of the South Fork of the Shoshone River, above the drainage of Ishawooa Creek.

Area 60. Thorofare. All of the drainage of the Yellowstone River upstream from Yellowstone Lake outside of Yellowstone National Park.

Area 61. North Greybull River. All of the north and west drainage of the Greybull River west of Wyoming Highway 120.

Area 62. South Greybull River. All of the south and east drainage of the Greybull River located west of the mouth of the Wood River; all of the north and west drainage of the Wood River.

Area 63. Wood River-Gooseberry Creek. All of the south and east drainage of the Wood River; all of the south and east drainage of the Greybull River located east of the mouth of the Wood River; all of the drainage of Gooseberry Creek located west of Wyoming Highway 120.

Area 64. Owl Creek. Beginning where the divide between Gooseberry Creek and Grass Creek crosses Wyoming Highway 120; southeasterly along said highway to the Big Horn River; southerly up said river to the Wind River Indian Reservation boundary; westerly, then northerly, then westerly along said boundary to the divide between Owl Creek and the South Fork of the Wood River; northeasterly along said divide to the divide between Cottonwood Creek and Gooseberry Creek; northeasterly along said divide to the divide between Gooseberry Creek and Grass Creek; easterly along said divide to Wyoming Highway 120; in addition, all non-Indian fee title lands in that portion of Hot Springs County within the exterior boundaries of the Wind River Indian Reservation.

Area 65. McCullough Peaks. All of the area west of the Big Horn River that lies north of U.S. Highway 14-16-20 and east of Wyoming Highway 120.

Area 66. Basin. All of the area west of the Big Horn River; south of U.S. Highway 14-16-20 and U.S. Highway 14, east of Wyoming Highway 120 and north of Broadway Street in the town of Thermopolis.

Area 67. Wiggins Fork. All of the drainage of the Wind River that lies north of U.S. Highway 287 and west of the Wind River Indian Reservation and those areas of the Spence and Moriarity Wildlife Management Area east of the East Fork of the Wind River.

Area 68. Warm Springs. Beginning where U.S. Highway 287 crosses Jakey's Fork Creek; southwesterly up said creek to the Continental Divide; northwesterly along said divide to U.S. Highway 287 at Togwotee Pass; southeasterly along said highway to Jakey's Fork Creek.

Area 69. Whiskey Mountain. Beginning where U.S. Highway 287 crosses the west boundary of the Wind River Indian Reservation; southerly along said boundary to the Continental Divide; northwesterly along said divide to Jakey's Fork Creek; northeasterly down said creek to U.S. Highway 287; southeasterly along said highway to the Wind River Indian Reservation boundary.

Area 70. Buffalo Fork. Beginning where U.S. Highway 26-287 intersects the Grand Teton National Park boundary; northerly along said boundary to Pacific Creek; northeasterly up said creek to Gravel Creek; northerly up said creek to the Snake River Trail; northerly along said trail to the south boundary of Yellowstone National Park;

easterly along said boundary to the Continental Divide; southerly and easterly along said divide to U.S. Highway 26-287 at Togwotee Pass; westerly along said highway to the east boundary of Grand Teton National Park.

Area 71. Pacific Creek. Beginning where Pacific Creek crosses the Grand Teton National Park boundary; northerly along said boundary to the Targhee National Forest boundary; northerly along said boundary to the southern boundary of Yellowstone National Park; easterly along said boundary to the Snake River Trail at Fox Park; southwesterly along said trail over Big Game Ridge to Gravel Creek; southerly down said creek to Pacific Creek; southerly down said creek to the Grand Teton National Park boundary.

Area 72. Berry Creek. All of the drainages of Berry Creek, Owl Creek and Webb Canyon Creek and that area north of Berry Creek to the Grand Teton National Park boundary.

Area 73. Targhee-Alta. Beginning where Wyoming Highway 22 crosses the Wyoming-Idaho state line; northerly along said line to the south boundary of Yellowstone National Park; easterly along said boundary to the Targhee National Forest boundary; southerly along said boundary to the Grand Teton National Park boundary; southerly along said boundary to Wyoming Highway 390; southerly along said highway to Wyoming Highway 22; westerly along said highway to the Wyoming-Idaho state line.

Area 74. Ditch Creek. Beginning where Ditch Creek crosses the Grand Teton National Park boundary; northeasterly along said boundary to Brush Creek; southerly up said creek to the divide between Brush Creek and Ditch Creek; southerly along a posted boundary to Ditch Creek; southerly down said creek to the Grand Teton National Park boundary.

Area 75. Snake River. Beginning at the junction of the Gros Ventre-Kelly Road and U.S. Highway 26-89-191; northerly along said highway to Ditch Creek; westerly along said creek to the Snake River proper; northerly along the easternmost bank of the main channel of the Snake river to the northernmost channel of Spread Creek; easterly along said creek to U.S. Highway 26-89-191; southwesterly along said highway to U.S.F.S. Road 30310 at the Cunningham Cabin; southerly along said road to the Grand Teton National Park boundary; southerly along said boundary to the southernmost intersection with the Shadow Mountain-Kelly Road (U.S.F.S. Road 30340); southwesterly along said road to the junction of the Teton Science School road; northeasterly along said road one and one-quarter (1¼) miles to a marked boundary; easterly one (1) mile to the Grand Teton National Park boundary; southerly along the Grand Teton National Park boundary to the Gros Ventre-Kelly Road; southwesterly along said road to U.S. Highway 26-89-191.

Area 77. National Elk Refuge. Beginning where U.S. Highway 26 crosses the Gros Ventre River; easterly up said river to the Bridger-Teton National Forest boundary; southerly along said boundary to the town of Jackson and the National Elk Refuge boundary; westerly along said boundary to U.S. Highway 26; northerly along said highway to the Gros Ventre River.

Area 78. Wilson. Beginning at the junction of Wyoming Highway 22 and Wyoming Highway 390; northerly along Wyoming Highway 390 to the Grand Teton National Park boundary; northerly, easterly and southerly along said boundary to U.S. Highway 26-89-191; southerly along said highway to its junction with Wyoming Highway 22; westerly along said highway to its junction with Wyoming Highway 390.

Area 79. Teton Park. Beginning where the U.S. Forest Service access road (U.S.F.S. Road 30310) near Cunningham Cabin intersects U.S. Highway 26-89-191; northerly along said highway to the junction of U.S. Highway 89-287 at Moran; northwesterly along said highway to the Grand Teton National Park boundary easterly and southerly along said boundary to the U.S. Forest Service access road (U.S. F.S. Road 30310) near Cunningham Cabin; westerly along said road to U.S. Highway 26-89-191.

Area 80. Sheep Creek. All of the drainages of Flat Creek, Sheep Creek and Nowlin Creek east of the National Elk Refuge.

Area 81. Spread Creek. Beginning where the Grand Teton National Park boundary crosses the Gros Ventre River east of the town of Kelly; northerly along said boundary to Ditch Creek; northeasterly up said creek to a posted boundary; straight north along the posted boundary to the divide between Brush Creek and Ditch Creek; northerly down Brush Creek to the Grand Teton National Park boundary; northerly along said boundary to U.S.

Highway 26-287; easterly along said highway to Togwotee Pass and the Continental Divide; southerly along said divide to the Moccasin Basin Road (U.S.F.S. Road 30750); southerly down said road to the North Fork of Fish Creek at Calf Creek; southerly down said creek to Fish Creek; southeasterly down said creek to the Gros Ventre River; westerly down said river to the Grand Teton National Park boundary.

Area 82. Crystal Peak. Beginning where the Gros Ventre River crosses the National Elk Refuge boundary near the town of Kelly; easterly up said river to Kinky Creek; easterly up said creek to the Darwin Ranch Road (U.S.F.S. Road 620); easterly along said road to the divide between the Green River and Gros Ventre River; southwesterly along said divide to the divide between the Green River and Hoback River at Hodges Peak; westerly along the divide between Flat Creek and the Gros Ventre River; northerly along said divide to the National Elk Refuge boundary; northerly along said boundary to the Gros Ventre River.

Area 83. Fish Creek. Beginning where Kinky Creek enters the Gros Ventre River; northerly down said river to Fish Creek; northeasterly up said creek to the North Fork of Fish Creek; northerly up said creek to the Moccasin Basin Road (U.S.F.S. Road 30750) at Calf Creek; northerly up said road to the Continental Divide; southerly along said divide to the Union Pass Road (U.S.F.S. Road 600); southwesterly along said road to the Darwin Ranch Road (U.S.F.S. Road 620); westerly along said road to Kinky Creek; westerly down said creek to the Gros Ventre River.

Area 84. Lower Hoback. Beginning where U.S. Highway 191 crosses Flat Creek at the north edge of the town of Jackson; due east to the National Elk Refuge boundary; easterly along said boundary to the Bridger-Teton National Forest boundary and the ridge between Twin Creeks and Cache Creek; easterly along said ridge to Jackson Peak; southerly along the divide between Flat Creek and Cache Creek to Cache Peak; easterly along the divide between Flat Creek and Granite Creek to Pyramid Peak; southerly along the divide between the Gros Ventre River and the Hoback River to Steamboat Peak; southerly and westerly along the divide between Shoal Creek and Dell Creek to the Riling Draw Road; southerly along said road to the Dell Creek Road; westerly along said road to the Hoback River; westerly down said river to Cliff Creek; southerly up Cliff Creek to the divide between the Greys River and the Hoback River; northwesterly along said divide to the divide between the Greys River and Willow Creek; northerly along said divide to the divide between the Bailey Creek and Willow Creek (Greyback Ridge); northerly along said divide to Dry Wash Draw; westerly down said draw to Baily Creek; northwesterly down said creek to the Snake River; northerly up said river to Wyoming Highway 22; easterly along said highway to U.S. Highway 191; easterly along said highway to Flat Creek at the north edge of the town of Jackson.

Area 85. Fall Creek. Beginning where Wyoming Highway 22 crosses the Snake River; southerly and westerly down the Snake River to the Wyoming-Idaho state line; northerly along said line to Wyoming Highway 22; easterly along said highway to the Snake River.

Area 86. Monument Ridge. Beginning at the head of Cliff Creek; northerly down said creek to the Hoback River; southerly up said river to the South Fork of the Hoback River; due east to the Hoback Rim and the divide between the Hoback River and the Green River; southwesterly along said divide to the divide between the Greys River and the Hoback River; northerly along said divide to the head of Cliff Creek.

Area 87. Raspberry Ridge. Beginning where U.S. Highway 191 crosses the divide between the Green River and the Hoback River on the Hoback Rim; southwesterly along said divide to a point east of the confluence of the South Fork of the Hoback River and the Hoback River; due west to the Hoback River; northerly down said river to the Dell Creek Road; easterly along said road to the Riling Draw Road; northerly along said road to the divide between Shoal Creek and Dell Creek; northeasterly along said divide to the divide between the Gros Ventre River and the Hoback River at Steamboat Peak; southeasterly along said divide to the divide between the Green River and the Hoback River at Hodges Peak; southerly along said divide to U.S. Highway 191.

Area 88. Greys Feedground. Beginning at the Wyoming-Idaho state line on the Snake River; easterly up said river to the Greys River; easterly up said river to the confluence of the Greys River and Squaw Creek; southwesterly from said confluence to the ridge between the Squaw Creek drainage and the Greys River; southerly up said ridge to the divide between the Greys River and the Salt River; southerly along said divide to the Stewart Sheep Trail; westerly along said trail to the Stewart Creek Road; westerly and southerly along said road to U.S.

Highway 89; northerly along said highway to the McCoy Creek Road; westerly and northerly along said road to the Wyoming-Idaho state line; north along said line to the Snake River.

Area 89. Lower Greys River. Beginning at the confluence of Greys River and the Snake River near Alpine; easterly up the Snake River to Bailey Creek; southeasterly up said creek to Dry Wash Draw; easterly up said draw to the divide between Bailey Creek and Willow Creek (Greyback Ridge); southerly along said divide to the divide between Willow Creek and the Greys River; southerly along said divide to the divide between the Greys River and the Hoback River; southerly and easterly along said divide between the Greys River and the Green River; southerly along said divide to the head of Blind Bull Creek; westerly down said creek to Greys River; southerly up said river to Meadow Creek; westerly up said creek to the divide between the Greys River and the Salt River; northerly along said divide to the ridge between the Squaw Creek drainage and the Greys River; northeasterly along said ridge to the confluence of Squaw Creek and the Greys River; westerly down said river to the Snake River.

Area 90. Upper Greys River. Beginning at the point where Blind Bull Creek runs into the Greys River; easterly up said creek to the divide between the Greys River and the Green River; southerly and westerly along said divide to the divide between the Greys River and the Smith Fork Creek; northerly along said divide to the divide between the Greys River and Salt River; northerly along said divide to the head of Meadow Creek; easterly down said creek to Greys River; northerly down said river to Blind Bull Creek.

Area 91. Salt River. Beginning at the point where the McCoy Creek Road crosses the Wyoming-Idaho state line; easterly and southerly along said road to U.S. Highway 89; southerly along said highway to the Stewart Creek Road; northerly and easterly along said road to the Stewart Driveway Trail; easterly along said trail to the divide between the Salt River and the Greys River; southerly along said divide to the divide between the Bear River and the Salt River; westerly along said divide to the Wyoming-Idaho state line; north along said line to the McCoy Creek Road.

Area 92. Piney-Horse Creek. Beginning where the East Green River Road (Sublette County Road 23-110) intersects Wyoming Highway 351; westerly along said highway to the Green River; northerly up said river to Cottonwood Creek; westerly up said creek to the South Cottonwood Creek; westerly up said creek to the South Fork of the South Fork of Cottonwood Creek; westerly up said creek to the divide between the Greys River and the Green River; northerly along said divide to the divide between the Green River and the Hoback River; northwesterly along said divide (Hoback Rim) to U.S. Highway 191; southerly along said highway to the East Green River Road (Sublette County Road 23-110) at Cora Junction; southerly along said road to Wyoming Highway 351.

Area 93. Waterdog Lakes. Beginning where U.S. Highway 191 crosses the Green River at Warren Bridge; northwesterly along said highway to the divide between the Hoback River and the Green River (Hoback Rim); northerly along said divide to the divide between the Green River and the Gros Ventre River at Hodges Peak; northeasterly along said divide to the Darwin Ranch Road (U.S.F.S. Road 620); easterly along said road to the Union Pass Road (U.S.F.S. Road 600); southerly and easterly along said road to Tosi Creek; easterly down said creek to the Green River; southerly down said river to U.S. Highway 191.

Area 94. South Piney. Beginning where LaBarge Creek enters the Green River; westerly up said river to the divide between the Green River and the Greys River; northerly along said divide to the head of the South Fork of South Cottonwood Creek; easterly down said creek to South Cottonwood Creek; easterly down said creek to Cottonwood Creek; easterly down said creek to the Green River; southerly down said river to LaBarge Creek.

Area 95. Green River. Beginning where Tosi Creek enters the Green River; westerly up said creek to the Union Pass Road (U.S.F.S. Road 600); northerly and easterly along said road to the Continental Divide; southerly along said divide to Mt. Helen; westerly to Summit Lake and the divide between the Green River and Pine Creek; northwesterly along said divide to the divide between the Green River and the New Fork River; northerly along said divide to the head of Jim Creek; westerly down said creek to the Green River; northerly up said river to Tosi Creek.

Area 96. New Fork. Beginning where U.S. Highway 191 crosses Pine Creek in the town of Pinedale; westerly and northerly along said highway to the Green River at Warren Bridge; northerly up said river to Jim Creek; easterly up said creek to the divide between the New Fork River and the Green River; southerly along said

divide to the divide between the Green River and Pine Creek; southeasterly along said divide to Summit Lake and Pine Creek; southerly down said creek to U.S. Highway 191.

Area 97. Pinedale. Beginning where the East Green River Road (Sublette County Road 23-110) intersects Wyoming Highway 351; northerly along said highway to Cora Junction and U.S. Highway 191; easterly along said highway to Pine Creek; northerly up said creek to Summit Lake; easterly to Mt. Helen and the Continental Divide; southerly along said divide to the Middle Fork of Boulder Creek; westerly down said creek to Boulder Creek; westerly down said creek to the New Fork River; southerly down said river to Wyoming Highway 351; westerly along said highway to the East Green River Road (Sublette County Road 23-110).

Area 98. Boulder. Beginning where the Big Sandy River enters the Green River; northerly up the Green River to Wyoming Highway 351; easterly along said highway to the New Fork River; northerly up said river to Boulder Creek; easterly up said creek to the Middle Fork of Boulder Creek; easterly up said creek to the Continental Divide; southerly along said divide to the Big Sandy River; southerly along said river to the Big Sandy Reservoir Road (Sweetwater County Road 28); westerly along said road to U.S. Highway 191; southerly along said highway to the Big Sandy River; southwesterly down said river to the Green River.

Area 99. Big Sandy. Beginning at the town of Farson and U.S. Highway 191; northerly along said highway to the Big Sandy Reservoir Road (Sweetwater County Road 28); easterly along said road to the Big Sandy River at the dam; northerly up said river to the Continental Divide; southeasterly along said divide to Mt. Nystrom; easterly from Mt. Nystrom to Sweetwater Gap and the headwaters of the Sweetwater River; southerly down said river to Wyoming Highway 28; southwesterly along said highway to U.S. Highway 191 at the town of Farson.

Area 100. Steamboat. Beginning where Interstate Highway 80 crosses the Green River; northerly up said river to the Big Sandy River; northeasterly along said river to U.S. Highway 28; northeasterly along said highway to the Sweetwater River; easterly down said river to the Three Forks-Atlantic City Road (BLM Road 2317) at Phelps-Dodge Bridge; easterly along said road to the Wamsutter-Crooks Gap Road (Sweetwater County Road 23); southerly along said road to Interstate Highway 80; westerly along said highway to the Green River.

Area 102. West Green River. Beginning where Interstate Highway 80 crosses the Green River; westerly along said highway to U.S. Highway 30; northwesterly along said highway to the town of Kemmerer and Ham's Fork Creek; northerly up said creek to the Commissary Ridge; northerly along said ridge to the head of LaBarge Creek; southeasterly down said creek to the Green River; southeasterly down said river to Interstate Highway 80.

Area 103. Raymond Mountain. Beginning where the Bear River crosses the Wyoming-Idaho state line; northerly along said line to the divide between the Salt River and Bear River; easterly along said divide to the divide between the Salt River and Smiths Fork; northeasterly along said divide to the head of Smiths Fork Creek; southerly down said creek to the Bear River at the town of Cokeville; northwesterly down said river to the Wyoming-Idaho state line.

Area 104. Lincoln. Beginning at the town of Kemmerer and U.S. Highway 30; westerly along said highway to Sage Junction and Wyoming Highway 89; southwesterly along said highway to the Wyoming-Utah state line; northerly along said line to the Wyoming-Idaho state line; northerly along said line to the Bear River; southeasterly up said river to the town of Cokeville and Smith's Fork Creek; northerly up said creek to the divide between the Smith's Fork and Greys River; southeasterly along said divide to Commissary Ridge; southerly along said ridge to the head of Ham's Fork Creek; southerly down said creek to U.S. Highway 30.

Area 105. Carter Lease. Beginning where Interstate Highway 80 crosses the Wyoming-Utah state line; northerly along said line to Wyoming Highway 89 west of Sage Junction; easterly along said highway to U.S. Highway 30; easterly then southerly along said highway to Interstate Highway 80; westerly along said highway to the Wyoming-Utah state line.

Area 106. Uinta. Beginning where Interstate Highway 80 crosses the Wyoming-Utah state line; easterly along said highway to Wyoming Highway 414; southerly along said highway to Wyoming Highway 410 at the town of Mountain View; southerly along said highway to the Stateline Dam Road (U.S.F.S. Road 072, Uinta County

Road 283); southerly along said road to the Wyoming-Utah state line; west then north along said line to Interstate Highway 80.

Area 107. Hickey Mountain. Beginning where the Flaming Gorge Reservoir crosses the Wyoming-Utah state line; west along said line to the Stateline Dam Road (U.S.F.S. Road 072, Uinta County Road 283); northerly along said road to Wyoming Highway 410; northerly along said highway to Interstate Highway 80; easterly along said highway to the Green River; southerly down said river to Flaming Gorge Reservoir; southerly along the east shore of said reservoir to the Wyoming-Utah state line.

Area 108. South Rawlins. Beginning at the city of Rawlins; southerly along Wyoming Highway 71 to the Sage Creek Road (Carbon County Road 401); southerly along said road to Sage Creek; southwesterly up said creek to the Rawlins City Water Works Road below Sage Creek Reservoir; southerly along said road to the McCarty Canyon Road (Carbon County Road 503); southwesterly along said road to the Miller Hill Road (BLM Road 3328); northwesterly along said road to the Muddy Creek Road (BLM Road 3306); westerly along said road to Muddy Creek; westerly down said creek to Wyoming Highway 789; northerly along said highway to Interstate Highway 80; easterly along said highway to the city of Rawlins.

Area 110. Upper Platte River. Beginning where Wyoming Highway 130 crosses the Laramie River-North Platte River Divide; southerly along said divide to Douglas Creek; southerly down said creek to U.S.F.S. Road 543 at Keystone; southerly along said road to U.S.F.S. Road 512; southeasterly along said road to Wyoming Highway 230; southwesterly along said highway to the Wyoming-Colorado state line; westerly along said line to Wyoming Highway 230; northwesterly along said highway to Wyoming Highway 130; easterly along said highway to the divide between the Laramie River and the North Platte River.

Area 111. Seminoe. Beginning at the city of Rawlins; northerly along U.S. Highway 287 to the South Ferris Road (Carbon County Road 100); easterly along said road to the Buzzard Road (Carbon County Road 497-Natrona County Road 410); northerly along said road to Wyoming Highway 220; northeasterly along said highway to the Kortes Road (Natrona County Road 407); southerly along said road to the North Platte River; southerly up said river to Interstate Highway 80; westerly along said highway to the city of Rawlins.

Area 113. Rochelle Hills. Beginning at the junction of Wyoming Highway 59 and Wyoming Highway 450; easterly along Wyoming Highway 450 to the Lynch Road; southerly along said road to the Clareton Road (Converse County Road 39) at the Weston-Converse County line; southwesterly along said road to the Dull Center Road (Converse County Road 38); easterly along said road to the North Lance Creek Road at the Converse-Niobrara County Line; easterly along said road to the Cow Creek Road (Niobrara County); southerly along said road to the Burke Road; westerly along said road to the Tilliard Ranch Road at the Niobrara-Converse County line; westerly along said Road to the Cow Creek Road (Converse County Road 45); westerly along said road to the Dull Center Road (Converse County Road 38); southerly and westerly along said road to Wyoming Highway 59; northerly along said highway to its junction with Wyoming Highway 450.

Area 114. Kyle. Beginning at the city of Laramie at the junction of Interstate Highway 80 and U.S. Highway 30-287; westerly along Interstate Highway 80 to Wyoming Highway 72; northerly along said highway to U.S. Highway 30-287; southeasterly along said highway to Interstate Highway 80.

Area 116. Bear Lodge. Beginning where the Belle Fourche River crosses the Wyoming-South Dakota state line; southerly along said line to Interstate Highway 90; southwesterly along said highway to U. S. Highway 14 at the town of Moorcroft; northeasterly along said highway to Wyoming Highway 24; northerly along said highway to the Belle Fourche River in the town of Hulett; northeasterly and southeasterly down said river to the Wyoming-South Dakota state line.

Area 117. South Black Hills. Beginning where Interstate Highway 90 crosses the Wyoming-South Dakota state line; southerly along said line to the Black Hills National Forest boundary; westerly along said boundary to the Sand Creek Road (U.S.F.S. Road 863) at the State of Wyoming’s Ranch A property; southerly along said road to the Moskee Road; southeasterly along said road to the Deer Creek Road (U.S.F.S. Road 807); southeasterly along said road to the Wyoming-South Dakota state line; southerly along said line to U.S. Highway

16; northwesterly along said highway to Interstate Highway 90; northeasterly along said highway to the Wyoming-South Dakota state line.

Area 118. Shamrock Hills. Beginning at the city of Rawlins; westerly along Interstate Highway 80 to the Wamsutter-Crooks Gap Road (Sweetwater County Road 23); northerly along said road to the Bairoil Road (Sweetwater County Road 22); easterly along said road to Wyoming Highway 73; easterly along said highway to U.S. Highway 287; southerly along said highway to Interstate Highway 80.

Area 120. Buffalo Creek. Beginning at the intersection of the 33 Mile Road (Natrona County Road 110) and the Buffalo Creek Road (Natrona County Road 105); northwesterly along the 33 Mile Road (Natrona County Road 110 in Natrona County) to the Big Horn Mountain Road; southwesterly along said road (Natrona County Road 109 in Natrona County) to the divide between the Middle Fork of the Powder River and Buffalo Creek; westerly along said divide to the divide between Sawmill Creek and Little Lost Creek; westerly along said divide to Deep Creek; southerly up said creek to the divide between Deep Creek and Badwater Creek; southerly down Badwater Creek to the Badwater Road (Natrona County Road 102); southeasterly along said road to the Arminto-Lost Cabin Road (Natrona County Road 103); southeasterly along said road to the Waltman-Arminto Road (Natrona County Road 104); southerly along said road to Wyoming Highway 20-26 at Waltman; easterly along said highway to Interstate Highway 25 in the city of Casper; northerly along said highway to Wyoming Highway 259; northerly along said highway to U.S. Highway 387 at the town of Midwest; westerly along said highway to the Smoky Gap Road (Natrona County Road 115); westerly along said road to the Dead Horse Road (Natrona County Road 114); southwesterly along said road to the Wild Horse Trail Road (Natrona County Road 113); westerly along said road to the 33 Mile Road (Natrona County Road 110); northwesterly along said road to the Buffalo Creek Road intersection (Natrona County Road 105).

Area 121. Trout Creek-Rattlesnake Mountain. All of the drainage of Jim Creek, Trout Creek and Rattlesnake Creek north of the North Fork of the Shoshone River; all of the drainage of Cottonwood Creek, Dry Creek and Trail Creek west of Wyoming Highway 120.

Area 122. Pine Ridge. Beginning at the intersection of Interstate Highway 25 and Wyoming Highway 259; northerly along Wyoming Highway 259 to U.S. Highway 387 at the town of Midwest; northeasterly along U.S. Highway 387 to the Ross Road (in Converse County, County Road 31); southeasterly along said road to Wyoming Highway 95; southwesterly along said highway to the North Platte River at the town of Glenrock; westerly up said river to Interstate Highway 25 in the city of Casper; northerly along said highway to Wyoming Highway 259.

Area 123. Black Thunder. Beginning where Wyoming Highway 59 crosses the Belle Fourche River; northeasterly down said river to the mouth of Raven Creek; southeasterly up said creek to the Raven Creek Road; southerly along said road to Wyoming Highway 116; southerly along said highway to Wyoming Highway 450; westerly along said highway to Wyoming Highway 59; northerly along said highway to the Belle Fourche River.

Area 124. Powder Rim. Beginning at the junction of Wyoming Highway 789 and the Wyoming-Colorado state line; westerly along said line to Wyoming Highway 430; northerly along said highway to Interstate Highway 80; easterly along said highway to Wyoming Highway 789; southerly along said highway to the Wyoming-Colorado state line.

Area 125. Elk Mountain. Beginning at the junction of Interstate Highway 80 and U.S. Highway 30 at the Saratoga interchange; northeasterly along U.S. Highway 30 to Wyoming Highway 72 at the Hanna interchange; southeasterly along Wyoming Highway 72 to the Pass Creek Road (Carbon County Road 404) west of the town of Elk Mountain; southerly and westerly along said road to Wyoming Highway 130; northerly along said highway to Interstate Highway 80.

Area 126. Flat Top. Beginning at the town of Douglas and Wyoming Highway 59, northerly along said highway to the Dull Center Road (Converse County Road 38) at the town of Bill; easterly and northerly along said road to the Cow Creek Road (Converse County Road 45); easterly along said road to the Tilliard Ranch Road; easterly along said road to the Burke Road at the Converse-Niobrara County Line; easterly along said road to the Cow Creek Road; southeasterly along said road to the North Lance Creek Road; southerly along said road to Wyoming Highway 272; southerly along said highway to Wyoming Highway 270; westerly and southerly along said

highway to U.S. Highway 18-20 at the town of Manville; westerly along said highway to Interstate Highway 25 at Orin Junction; southerly along said highway to the North Platte River; westerly and northerly up said river to Wyoming Highway 59 at the town of Douglas.

Area 127. Crowheart Butte. All non-Indian owned fee title lands within the exterior boundaries of the Wind River Indian Reservation, and all lands within the Bureau of Reclamation's Riverton and Boysen Unit boundaries, and those lands within Boysen State Park south of Cottonwood Creek, west of Boysen Reservoir, and south of U.S. Highway 20-26; excluding those areas of the Spence and Moriarity Wildlife Management Area that lie east of the East Fork of the Wind River, and excluding those lands within Hot Springs County.

Area 128. Muskrat. Beginning where U.S. Highway 20-26 intersects the Wind River Indian Reservation boundary; easterly along said highway to the town of Waltman and the Gas Hills Road (Natrona County Road 212); southwesterly along said road to the Dry Creek Road (Natrona County Road 321); southeasterly along said road to Wyoming Highway 220; southwesterly along said highway to U.S. Highway 287; westerly along said highway to Wyoming Highway 135 (Sand Draw Road); northerly along said highway to the south boundary of the Wind River Indian Reservation; easterly and northerly along said boundary to U.S. Highway 20-26.

Area 129. Non-Herd Unit. All of Campbell, Converse, Crook, Johnson, Natrona, Niobrara, Sheridan, and Weston Counties, excluding lands within other described and numbered elk hunt areas.

Area 130. Bolten Rim. Beginning at the City of Rawlins; easterly along Interstate Highway 80 to the North Platte River; southerly up said river to Sage Creek; southwesterly up said creek to the Sage Creek Road (Carbon County Road 401); northerly along said road to Wyoming Highway 71; northerly along said highway to the City of Rawlins.

WYOMING GAME AND FISH COMMISSION

By:

Bill Williams, DVM, President

Dated: April 24, 2007

2007 ELK HUNT AREAS

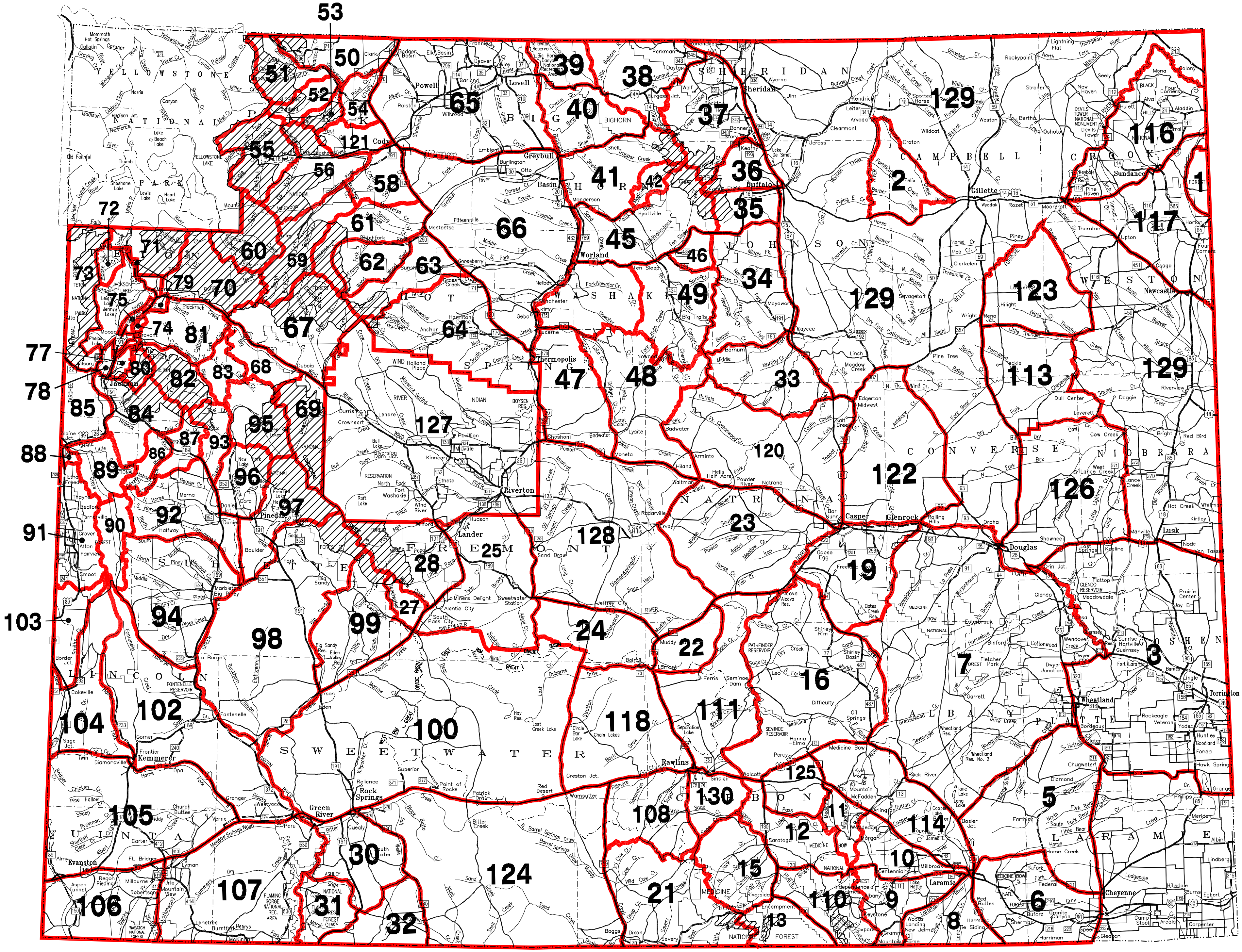

THIS MAP IS FOR GENERAL REFERENCE ONLY. Please use the written boundary descriptions in this regulation for detailed boundary information.

Note: Wilderness area, nonresidents must have guides.

MOOSE
HARVEST STATEWIDE
BY TOTAL HUNTERS
2007

	LICENSES SOLD	NUMBER ACTIVE HUNTERS	HARVEST BULL	HARVEST COW	HARVEST CALF	HARVEST TOTAL	TOTAL RECREATION DAYS	HUNTER SUCCESS	DAYS/ HARVEST
STATE TOTAL	759	750	503	146	20	669	4674	89.2%	6.99
RESIDENT	630	624	402	135	19	556	3983	89.1%	7.16
NONRESIDENT	129	126	101	11	1	113	691	89.68%	6.11

TEN-YEAR COMPARISON OF HARVEST TABLE

YEAR	1998	1999	2000	2001	2002	2003	2004	2005	2006	2007
HARVEST	1061	1101	1209	1215	1160	999	770	682	636	669

HARVEST BY HARVEST TOOL

ARCHERY	FIREARM	TOTAL
13.2	86.8	100%

TABLE I

MOOSE

HARVEST BY HUNT AREA

2007

AREA (cross hunt areas)	TYPE	ACTIVE LICS/HTRS	HARVEST BULL	COW	CALF	TOTAL	HUNTER SUCCESS	DAYS/ HARVEST	HUNTER DAYS	(cross hunt incl) LICENSES SOLD
1 Goose Creek	Limited 1									
	Resident	17	12	1	0	13	76.47%	14.10	183	17
	Nonresident	9	9	0	0	9	100.00%	4.33	39	9
	Limited 2									
	Resident	19	1	18	0	19	100.00%	2.75	52	19
	Area Total	45	22	19	0	41	91.11%	6.70	275	45
2 Lander Area	Limited 1									
	Resident	4	3	0	0	3	75.00%	10.33	31	4
	Nonresident	1	1	0	0	1	100.00%	1.00	1	1
	Area Total	5	4	0	0	4	80.00%	8.00	32	5
3 Pinedale-Sandy	Limited 1									
	Resident	5	4	0	0	4	80.00%	3.75	15	5
	Nonresident	1	1	0	0	1	100.00%	3.00	3	1
	Area Total	6	5	0	0	5	83.33%	3.60	18	6
4 Pinedale-Off NTL	Limited 1									
	Resident	13	13	0	0	13	100.00%	6.60	86	13
	Nonresident	3	2	0	0	2	66.67%	6.00	12	3
	Limited 2									
	Resident	5	0	3	1	4	80.00%	3.75	15	5
	Area Total	21	15	3	1	19	90.48%	5.94	113	21
5 Upper Green River	Limited 1									
	Resident	25	22	0	0	22	88.00%	9.45	208	25
	Nonresident	6	5	0	0	5	83.33%	5.80	29	6
	Limited 2									
	Resident	12	0	5	2	7	58.33%	8.10	57	12
	Nonresident	2	0	2	0	2	100.00%	10.00	20	3
	Area Total	45	27	7	2	36	80.00%	8.71	314	46
6 Wind River	Limited 1									
	Resident	8	7	0	0	7	87.50%	9.33	65	8
	Nonresident	2	0	0	0	0	0.00%	-	14	2
	Limited 3									
	Resident	4	4	0	0	4	100.00%	6.50	26	4
	Nonresident	0	0	0	0	0	0.00%	-	0	1
	Area Total	14	11	0	0	11	78.57%	9.58	105	15
7 Buffalo River	Limited 1									
	Resident	4	3	0	0	3	75.00%	12.67	38	4
	Nonresident	1	1	0	0	1	100.00%	1.00	1	1
	Area Total	5	4	0	0	4	80.00%	9.75	39	5
8 Thorofare/Yellowstone	CLOSED									
9 Greybull-Owl Creek	Limited 1									
	Resident	4	4	0	0	4	100.00%	7.00	28	4
	Nonresident	1	1	0	0	1	100.00%	4.00	4	1
	Area Total	5	5	0	0	5	100.00%	6.40	32	5
10 South Hoback	Limited 1									
	Resident	12	12	0	0	12	100.00%	6.00	72	12
	Nonresident	3	3	0	0	3	100.00%	6.00	18	3
	Limited 2									
	Resident	5	0	3	0	3	60.00%	10.56	32	5
	Area Total	20	15	3	0	18	90.00%	6.76	122	20
11 North Fork	Limited 1									
(12, 13, 31)	Resident	3	3	0	0	3	100.00%	9.00	27	3
	Area Total	3	3	0	0	3	100.00%	9.00	27	3
12 Sunlight	ALL LICENSES EXERCISED IN CROSS HUNT AREAS									
(11, 13, 31)										
13 Crandall - Beartooth	Limited 1									
(11, 12, 31)	Resident	1	1	0	0	1	100.00%	1.00	1	1
	Area Total	1	1	0	0	1	100.00%	1.00	1	1
14 Fox Park	Limited 1									
	Resident	1	1	0	0	1	100.00%	12.00	12	1
	Nonresident	1	0	0	0	0	0.00%	-	9	1
	Area Total	2	1	0	0	1	50.00%	21.00	21	2
15 Glade Creek-Pilgrim Creek	Limited 1									
	Resident	5	5	0	0	5	100.00%	10.50	53	5
	Area Total	5	5	0	0	5	100.00%	10.50	53	5

TABLE I

MOOSE

HARVEST BY HUNT AREA

2007

AREA (cross hunt areas)	TYPE	ACTIVE LICS/HTRS	HARVEST BULL	COW	CALF	TOTAL	HUNTER SUCCESS	DAYS/ HARVEST	HUNTER DAYS	(cross hunt incl) LICENSES SOLD
16 Alta	Limited 1									
	Resident	16	15	0	0	15	93.75%	8.08	121	16
	Nonresident	5	5	0	0	5	100.00%	4.80	24	5
	Area Total	21	20	0	0	20	95.24%	7.26	145	21
17 Lower Gros Ventre-Spreak Creek	Limited 1									
	Resident	20	19	0	0	19	95.00%	9.08	173	20
	Nonresident	5	5	0	0	5	100.00%	7.20	36	5
	Area Total	25	24	0	0	24	96.00%	8.69	209	25
18 Fish Creek	Limited 1									
	Resident	22	20	0	0	20	90.91%	6.81	136	22
	Nonresident	5	4	0	0	4	80.00%	10.00	40	5
	Area Total	27	24	0	0	24	88.89%	7.34	176	27
19 Wilson	CLOSED									
20 Fall Creek-Targee	Limited 1									
	Resident	23	21	0	0	21	91.30%	6.05	127	23
	Nonresident	6	6	0	0	6	100.00%	3.67	22	6
	Limited 2									
	Resident	4	0	3	1	4	100.00%	3.00	12	5
	Area Total	33	27	3	1	31	93.94%	5.19	161	34
21 North Hoback	Limited 1									
	Resident	8	7	0	0	7	87.50%	11.43	80	8
	Nonresident	2	2	0	0	2	100.00%	4.00	8	2
	Limited 2									
	Resident	3	0	3	0	3	100.00%	4.89	15	4
	Nonresident	1	0	1	0	1	100.00%	1.00	1	1
	Area Total	14	9	4	0	13	92.80%	8.00	104	15
22 Raspberry Ridge	Limited 1									
	Resident	12	11	0	0	11	91.67%	10.77	119	12
	Nonresident	3	2	0	0	2	66.67%	11.50	23	3
	Limited 2									
	Resident	5	0	3	1	4	80.00%	10.75	43	5
	Area Total	20	13	3	1	17	85.00%	10.85	185	20
23 Grey's River - Salt River	Limited 1									
	Resident	27	24	0	0	24	88.89%	7.40	178	27
	Nonresident	6	6	0	0	6	100.00%	1.83	11	7
	Limited 2									
	Resident	4	0	4	0	4	100.00%	4.33	17	4
	Nonresident	1	0	1	0	1	100.00%	4.00	4	1
	Area Total	38	30	5	0	35	92.10%	6.00	210	39
24 Big Piney - on NTL Forest	Limited 1									
	Resident	24	22	0	0	22	91.67%	8.48	187	24
	Nonresident	6	6	0	0	6	100.00%	4.83	29	6
	Limited 2									
	Resident	4	0	4	0	4	100.00%	1.00	4	4
	Area Total	34	28	4	0	32	94.12%	6.86	220	34
25 Big Piney - off NTL Forest	Limited 1									
	Resident	34	31	0	0	31	91.18%	6.25	194	35
	Nonresident	9	9	0	0	9	100.00%	2.67	24	9
	Limited 2									
	Resident	21	0	13	3	16	76.19%	3.92	63	22
	Nonresident	3	0	3	0	3	100.00%	3.50	11	3
	Area Total	69	40	16	3	59	85.55%	4.95	292	69
26 Lincoln	Limited 1									
	Resident	48	44	0	0	44	91.67%	7.24	318	48
	Nonresident	12	11	0	0	11	91.67%	7.34	81	12
	Limited 2									
	Resident	20	0	17	1	18	90.00%	3.33	60	20
	Nonresident	4	0	3	1	4	100.00%	2.33	9	4
	Area Total	84	55	20	2	77	91.67%	6.08	468	84

TABLE I
MOOSE
HARVEST BY HUNT AREA
2007

AREA (cross hunt areas)	TYPE	ACTIVE LICS/HTRS	HARVEST BULL	COW	CALF	TOTAL	HUNTER SUCCESS	DAYS/ HARVEST	HUNTER DAYS	(cross hunt incl) LICENSES SOLD
27 Uinta	Limited 1									
	Resident	24	23	0	0	23	95.83%	9.80	225	24
	Nonresident	6	4	0	0	4	66.67%	17.50	70	6
	Limited 2									
	Resident	30	0	20	6	26	86.67%	7.27	189	30
	Area Total	60	27	20	6	53	88.33%	9.14	484	60
28 Crystal Creek - Flat Creek	Limited 1									
	Resident	4	4	0	0	4	100.00%	4.75	19	4
	Nonresident	1	1	0	0	1	100.00%	6.00	6	1
	Area Total	5	5	0	0	5	100.00%	5.00	25	5
30 Sweetwater	Limited 1									
	Resident	4	4	0	0	4	100.00%	1.00	4	4
	Nonresident	1	1	0	0	1	100.00%	9.00	9	1
	Area Total	5	5	0	0	5	100.00%	2.60	13	5
31 South Fork	Limited 1									
(11, 12, 13)	Nonresident	1	1	0	0	1	100.00%	21.00	21	1
	Area Total	1	1	0	0	1	100.00%	21.00	21	1
32 Enos Lake	Limited 1									
	Resident	3	3	0	0	3	100.00%	12.00	36	3
	Area Total	3	3	0	0	3	100.00%	12.00	36	3
33 Lower Green River	CLOSED									
34 Hunter Mesa	Limited 1									
	Resident	12	11	0	1	12	100.00%	5.42	65	12
	Nonresident	3	3	0	0	3	100.00%	1.67	5	3
	Limited 2									
	Resident	20	1	14	1	16	80.00%	3.88	62	20
	Area Total	35	15	14	2	31	88.57%	4.26	132	35
35 Henry's Fork	Limited 1									
	Resident	8	7	0	0	7	87.50%	10.12	71	8
	Nonresident	2	2	0	0	2	100.00%	3.50	7	2
	Limited 2									
	Resident	8	0	3	0	3	37.50%	16.00	48	8
	Nonresident	1	0	0	0	0	0.00%	-	4	1
	Area Total	19	9	3	0	12	63.16%	10.82	130	19
36 Bear River Divide	Limited 1									
	Resident	8	8	0	0	8	100.00%	6.43	51	8
	Nonresident	2	2	0	0	2	100.00%	2.00	4	2
	Area Total	10	10	0	0	10	100.00%	5.54	55	10
37 Grassy Lake	Limited 1									
	Resident	11	8	0	0	8	72.73%	12.90	103	12
	Nonresident	3	3	0	0	3	100.00%	14.33	43	3
	Area Total	14	11	0	0	11	78.57%	13.29	146	15
38 Snowy Range	Limited 1									
	Resident	12	12	0	0	12	100.00%	4.20	50	12
	Nonresident	3	3	0	0	3	100.00%	3.67	11	3
	Limited 2									
	Resident	24	0	21	2	23	95.83%	3.37	77	24
	Nonresident	1	0	1	0	1	100.00%	12.00	12	1
	Area Total	40	15	22	2	39	97.50%	3.87	151	40
39 Jeffrey City	CLOSED									
40 Lower Hams Fork	Limited 1									
	Resident	3	2	0	0	2	66.67%	6.75	14	3
	Nonresident	1	1	0	0	1	100.00%	3.00	3	1
	Area Total	4	3	0	0	3	75.00%	5.50	17	4
42 Shell	Limited 1									
	Resident	4	4	0	0	4	100.00%	14.50	58	4
	Nonresident	1	0	0	0	0	0.00%	-	15	1
	Area Total	5	4	0	0	4	80.00%	18.25	73	5
43 Ten Sleep	Limited 1									
	Resident	4	4	0	0	4	100.00%	6.00	24	4
	Nonresident	1	0	0	0	0	0.00%	0.00	5	1
	Area Total	5	4	0	0	4	80.00%	7.25	29	5
44 East Flaming Gorge	Limited 1									
	Resident	3	2	0	0	2	66.67%	20.00	40	4
	Nonresident	1	1	0	0	1	100.00%	3.00	3	1
	Area Total	4	3	0	0	3	75.00%	14.33	43	5

TABLE II

MOOSE

HARVEST BY HERD UNIT

2007

HERD UNIT	TYPE	ACTIVE LICS/HTRS	HARVEST BULL	COW	CALF	TOTAL	HUNTER SUCCESS	DAYS/ HARVEST	HUNTER DAYS	LICENSES SOLD
101 Targhee										
	Resident	27	23	0	0	23	85.19%	9.75	224	28
	Nonresident	8	8	0	0	8	100.00%	8.38	67	8
	Total	35	31	0	0	31	88.57%	9.40	291	36
103 Jackson	Resident	59	55	0	0	55	93.22%	8.48	466	59
	Nonresident	13	11	0	0	11	84.62%	8.36	92	13
	Total	72	66	0	0	66	91.67%	8.46	558	72
105 Sublette	Resident	246	167	41	8	216	87.80%	7.04	1521	250
	Nonresident	52	42	7	0	49	94.23%	4.38	215	54
	Total	298	209	48	8	265	88.93%	6.55	1736	304
207 Clark's Fork	Resident	1	1	0	0	1	100.00%	1.00	1	1
	Total	1	1	0	0	1	100.00%	1.00	1	1
210 North Fork/Shoshone	Resident	3	3	0	0	3	100.00%	9.00	27	3
	Nonresident	1	1	0	0	1	100.00%	21.00	21	1
	Total	4	4	0	0	4	100	12	48	4
212 Gooseberry	Resident	4	4	0	0	4	100.00%	7.00	28	4
	Nonresident	1	1	0	0	1	100.00%	4.00	4	1
	Total	5	5	0	0	5	100	6	32	5
313 Gooseberry	Resident	76	33	33	2	68	89.47%	6.54	445	76
	Nonresident	14	12	0	0	12	85.71%	5.33	64	14
	Total	90	45	33	2	80	88.89%	6.36	509	90
415 Uinta	Resident	73	32	23	6	61	83.56%	9.40	573	74
	Nonresident	10	7	0	0	7	70.00%	2.00	84	10
	Total	83	39	23	6	68	81.93%	9.66	657	84
417 Lincoln	Resident	71	46	17	1	64	90.14%	6.12	392	71
	Nonresident	17	12	3	1	16	94.12%	5.82	93	17
	Total	88	58	20	2	80	90.91%	6.06	485	88
419 Bear River Divide	Resident	8	8	0	0	8	100.00%	6.43	51	8
	Nonresident	2	2	0	0	2	100.00%	2.00	4	2
	Total	10	10	0	0	10	100.00%	5.54	55	10
545 Snowy Range	Resident	36	12	21	2	35	97.22%	3.65	128	36
	Nonresident	4	3	1	0	4	100.00%	5.75	23	4
	Total	40	15	22	2	39	97.50%	3.87	151	40
620 Lander	Resident	8	7	0	0	7	87.50%	5.00	35	8
	Nonresident	2	2	0	0	2	100.00%	5.00	10	2
	Total	10	9	0	0	9	90.00%	5.00	45	10
621 Dubois	Resident	12	11	0	0	11	91.67%	8.30	91	12
	Nonresident	2	0	0	0	0	0.00%	0.00	14	3
	Total	14	11	0	0	11	78.57%	9.58	105	15

WYOMING GAME AND FISH COMMISSION

CHAPTER 8

MOOSE HUNTING SEASONS

Section 1. Authority. This regulation is promulgated by authority of W.S. §23-1-302, §23-1-703 and §23-2-104.

Section 2. Hunting Seasons Established. There shall be open seasons during 2007 for the hunting of moose as set forth in this Chapter and in accordance with Chapter 2, General Hunting Regulation.

Section 3. Regular Hunting Seasons.

(a) Hunt areas, season dates and limitations.

Hunt Area	Type	Date of Season Opens	Closes	Limitations
1	1	Oct. 1	Oct. 14	Limited quota; 20 licenses any moose, except cow moose with calf at side
	2	Oct. 1	Oct. 14	Limited quota; 20 licenses antlerless moose, except cow moose with calf at side
2	1	Oct. 1	Nov. 15	Limited quota; 5 licenses antlered moose
3	1	Sept. 20	Oct. 31	Limited quota; 5 licenses antlered moose
4	1	Sept. 20	Oct. 31	Limited quota; 15 licenses antlered moose
	2	Sept. 20	Oct. 31	Limited quota; 5 licenses antlerless moose, except cow moose with calf at side
5	1	Oct. 1	Oct. 31	Limited quota; 30 licenses antlered moose
	2	Oct. 1	Oct. 31	Limited quota; 15 licenses antlerless moose, except cow moose with calf at side
6	1	Oct. 1	Nov. 20	Limited quota; 10 licenses antlered moose
	3	Sept. 15	Oct. 31	Limited quota; 5 licenses antlered moose, valid in that portion of Area 6 within the Fitzpatrick Wilderness
7, 14, 32	1	Sept. 10	Oct. 31	Limited quota; 10 licenses antlered moose
8				CLOSED
9	1	Oct. 1	Oct. 31	Limited quota; 5 licenses antlered moose
10	1	Sept. 10	Oct. 31	Limited quota; 15 licenses antlered moose
	2	Sept. 10	Oct. 31	Limited quota; 5 licenses antlerless moose, except cow moose with calf at side
11, 12, 13, 31	1	Sept. 10	Nov. 10	Limited quota; 5 licenses antlered moose
15	1	Sept. 10	Oct. 31	Limited quota; 5 licenses antlered moose
16	1	Sept. 10	Nov. 15	Limited quota; 20 licenses antlered moose
17	1	Sept. 10	Oct. 31	Limited quota; 25 licenses antlered moose
18	1	Oct. 1	Oct. 31	Limited quota; 25 licenses antlered moose
19				CLOSED
20	1	Sept. 10	Oct. 31	Limited quota; 30 licenses antlered moose
	2	Sept. 10	Oct. 31	Limited quota; 5 licenses antlerless moose, except cow moose with calf at side
21	1	Sept. 10	Oct. 31	Limited quota; 10 licenses antlered moose

Hunt Area	Type	Date of Season Opens	Closes	Limitations
	2	Sept. 10	Oct. 31	Limited quota; 5 licenses antlerless moose, except cow moose with calf at side
22	1	Oct. 1	Oct. 31	Limited quota; 15 licenses antlered moose
	2	Oct. 1	Oct. 31	Limited quota; 5 licenses antlerless moose, except cow moose with calf at side
23	1	Sept. 15	Oct.31	Limited quota; 35 licenses antlered moose
	2	Sept. 15	Oct. 31	Limited quota; 5 licenses antlerless moose, except cow moose with calf at side
24	1	Oct. 1	Oct. 31	Limited quota; 30 licenses antlered moose
	2	Oct. 1	Oct. 31	Limited quota; 5 licenses antlerless moose, except cow moose with calf at side
25	1	Oct. 1	Oct. 31	Limited quota; 45 licenses antlered moose
	2	Oct. 1	Oct. 31	Limited quota; 25 licenses antlerless moose, except cow moose with calf at side
26	1	Oct. 1	Oct. 31	Limited quota; 60 licenses antlered moose
	2	Oct. 1	Oct. 31	Limited quota; 25 licenses antlerless moose, except cow moose with calf at side
27	1	Oct. 1	Nov. 15	Limited quota; 30 licenses antlered moose
	2	Oct. 1	Oct. 31	Limited quota; 30 licenses antlerless moose, except cow moose with calf at side
28	1	Sept. 10	Oct. 31	Limited quota; 5 licenses antlered moose
30	1	Oct. 1	Nov. 15	Limited quota; 5 licenses antlered moose
33				CLOSED
34	1	Oct. 1	Oct. 14	Limited quota; 15 licenses any moose, except cow moose with calf at side
	2	Oct. 1	Oct. 14	Limited quota; 20 licenses antlerless moose, except cow moose with calf at side
35	1	Oct. 1	Nov. 15	Limited quota; 10 licenses any moose, except cow moose with calf at side
	2	Oct. 1	Oct. 31	Limited quota; 10 licenses antlerless moose, except cow moose with calf at side
36	1	Oct. 1	Oct. 31	Limited quota; 10 licenses any moose, except cow moose with calf at side
37	1	Sept. 10	Nov. 15	Limited quota; 15 licenses antlered moose
38, 41	1	Oct. 1	Oct. 31	Limited quota; 15 licenses any moose, except cow moose with calf at side
	2	Oct. 1	Oct. 31	Limited quota; 25 licenses antlerless moose, except cow moose with calf at side
39				CLOSED
40	1	Oct. 1	Oct. 31	Limited quota; 5 licenses any moose, except cow moose with calf at side
42	1	Oct. 1	Oct. 14	Limited quota; 5 licenses any moose, except cow moose with calf at side
43	1	Oct. 1	Oct. 14	Limited quota; 5 licenses any moose, except cow moose with calf at side
44	1	Oct. 1	Nov. 30	Limited quota; 5 licenses any moose, except cow moose with calf at side

Section 4. Special Archery Seasons. Hunt areas listed in this section shall have special archery hunting seasons during the dates and with limitations specified in this section. Archers shall possess a limited quota moose license in addition to an archery license in order to hunt moose during any special archery season. Archers shall hunt only in the hunt area and for the type of moose set forth by the limitations of their license as specified in Section 3 of this Chapter.

	Dates of Seasons		
**Hunt Area(s)**	**Opens**	**Closes**	**Limitations**
1, 6, 34, 42, 43	Sept. 15	Sept. 30	Refer to Section 3 of this Chapter
2, 5, 9, 18, 22, 24-27, 30, 35, 36, 38, 40, 41, 44	Sept. 1	Sept. 30	Refer to Section 3 of this Chapter
3, 4	Sept. 1	Sept. 19	Refer to Section 3 of this Chapter
7, 10-17, 20, 21, 28, 31, 32, 37	Sept. 1	Sept. 9	Refer to Section 3 of this Chapter
23	Sept. 1	Sept. 14	Refer to Section 3 of this Chapter

Section 5. Hunt Area Descriptions.

(a) Area and Number

Area 1. Goose Creek. Beginning where Interstate Highway 90 crosses the Wyoming-Montana state line; southeasterly along said highway to Piney Creek; northwesterly up said creek to South Piney Creek; southwesterly up said creek to the divide along the Big Horn Mountain range; northwesterly along said divide to the Wyoming-Montana state line; easterly along said line to Interstate Highway 90.

Area 2. Lander Area. Beginning where U.S. Highway 287 intersects the south boundary of the Wind River Indian Reservation; easterly along said boundary to Wyoming Highway 135 (Sand Draw Road); southerly along said highway to U.S. Highway 287; easterly along said highway to the Bison Basin Road (BLM Road 3221); southerly along said road to the Cyclone Ridge Road (BLM Road 3216); westerly along said road to the Red Creek Road (BLM Road 3219); northwesterly along said road to the Sand Creek Road; westerly along said road to the Continental Divide Road north of Continental Peak; westerly along said road to the Oregon Buttes Road; northerly along said road to Wyoming Highway 28; northeasterly along said highway to Willow Creek; northwesterly up said creek to the divide between the Sweetwater River and the Wind River at Granite Peak; northwesterly along said divide to the Continental Divide; northwesterly along said divide to the Wind River Indian Reservation boundary; easterly along said boundary to U.S. Highway 287.

Area 3. Pinedale-Sandy. Beginning where Pine Creek crosses the Bridger-Teton National Forest boundary below Fremont Lake; northerly up said creek to Summit Lake; easterly to Mt. Helen and the Continental Divide; southerly along said divide to the head of the Big Sandy River; southwesterly down said river to the Bridger-Teton National Forest boundary; northerly along said boundary to Pine Creek.

Area 4. Pinedale-Off National Forest. Beginning where the Big Sandy River crosses the Bridger-Teton National Forest boundary; southwesterly down said river to the Erramouspe Ranch Road; westerly along said road to U.S. Highway 191; northerly along said highway to Wyoming Highway 351; westerly along said highway to the East Green River Road (Sublette County Road 23-110); northerly along said road to U.S. Highway 191; northwesterly along said highway to the Green River; northeasterly up said river to Jim Creek; easterly up said creek to the Bridger-Teton National Forest boundary; southeasterly along said boundary to the Big Sandy River.

Area 5. Upper Green River. Beginning where U.S. Highway 191 crosses the Green River at Warren Bridge; northwesterly along said highway to the divide between the Hoback River and the Green River (Hoback Rim); northerly along said divide to the divide between the Green River and the Gro Ventre River at Hodges Peak; northeasterly along said divide to the Darwin Ranch Road (USFS Road 620); easterly along said road to the Union Pass Road (USFS Road 600); northeasterly along said road to the Continental Divide; southerly along said divide to Mt. Helen; west to Summit Lake and Pine Creek; southerly down said creek to the Bridger-Teton National Forest

boundary; northwesterly along said boundary to Jim Creek; westerly down said creek to the Green River; southerly down said river to U.S. Highway 191.

Area 6. Wind River. All of the drainage of the Wind River west of the west boundary of the Wind River Indian Reservation and those areas of the Spence and Moriarity Wildlife Management Area east of the East Fork of the Wind River.

Area 7. Buffalo River. Beginning at the confluence of the South Fork and the North Fork of the Buffalo River; northerly up the North Fork to the Trail Creek Trail; northerly along said trail to Pacific Creek; easterly along said creek to the Continental Divide; easterly and southerly along said divide to the Teton Wilderness boundary and the divide between Blackrock Creek and the South Fork of the Buffalo River; westerly along said divide to Togwotee Lodge; northerly along the South Fork of the Buffalo River-Togwotee Lodge Trail to the South Fork of the Buffalo River; westerly down said river to the North Fork of the Buffalo River.

Area 8. Thorofare-Yellowstone. All of the drainages of Mountain Creek, the Thorofare River and the Yellowstone River upstream from the Yellowstone National Park boundary.

Area 9. Greybull-Owl Creek. Beginning where Wyoming Highway 120 crosses the divide between Dry Creek and the Greybull River drainage (Meeteetse Rim); southeasterly along said highway to the Bighorn River; southerly up said river to the Wind River Indian Reservation boundary; westerly along said boundary to the divide between the Wood River and the Wind River; northwesterly along said divide to the divide between the Greybull River and the Wind River; northwesterly along said divide to the divide between the Greybull River and the South Fork of the Shoshone River; northeasterly along said divide to the divide between Meeteetse Creek and Sage Creek; easterly along said divide to the divide between Dry Creek and the Greybull River; easterly along said divide to Wyoming Highway 120; in addition, all non-Indian fee title lands in that portion of Hot Springs County within the exterior boundaries of the Wind River Indian Reservation.

Area 10. South Hoback. Beginning at the confluence of the Hoback and Snake rivers; southeasterly up the Hoback River to the South Fork of the Hoback River; east to Hoback Rim and the divide between the Green River and Hoback River; southwesterly along said divide to the divide between the Greys River and the Hoback River; northerly along said divide to the divide between the Greys River and Willow Creek; northerly along said divide to the divide between Bailey Creek and Willow Creek; northerly along said divide to Drywash Draw; westerly down said draw to Bailey Creek; northwesterly down said creek to the Snake River; northerly up said river to the confluence of the Hoback and Snake rivers.

Area 11. North Fork. All of the drainage of the North Fork of the Shoshone River; all of the north drainage of the Shoshone River located west of Wyoming Highway 120.

Area 12. Sunlight. All of the south drainage of the Clark's Fork River west of Wyoming Highway 120 up to and including the drainage of Russell Creek.

Area 13. Crandall-Beartooth. All of the north drainage of the Clark's Fork River; all of the south drainage of the Clark's Fork River above the drainage of Russell Creek; all of the drainage of Soda Butte Creek outside of Yellowstone National Park.

Area 14. Fox Park. Beginning at the head of the Pacific Creek at the Continental Divide; westerly down Pacific Creek to the Whetstone Creek-Wildcat Peak Trail (#3034); northerly and westerly along said trail to Wildcat Ridge and the West Pilgrim Creek Trail; northerly along said trail to Brown Meadows and the Arizona Creek-Sheffield Creek Trail (#3026); northerly along said trail to Huckleberry Ridge and the divide between Sheffield Creek and Rodent Creek; northerly along said divide to the Yellowstone National Park boundary; easterly along said boundary to the Continental Divide; southerly along said divide to the head of Pacific Creek.

Area 15. Glade Creek-Pilgrim Creek. Beginning where Pacific Creek crosses the Grand Teton National Park boundary; northerly and westerly along said boundary to the Targhee National Forest boundary; northerly along said boundary to the Yellowstone National Park boundary; easterly along said boundary to Huckleberry Ridge; southerly along said ridge to the Sheffield Creek-Arizona Creek Trail (#3026); southerly along said trail to

Brown Meadows and the West Pilgrim Creek Trail; southerly along said trail to Wildcat Ridge and the Wildcat Peak-Whetstone Creek Trail (#3034); southerly and easterly along said trail to Pacific Creek; southwesterly down said creek to the Grand Teton National Park boundary.

Area 16. Alta. Beginning at the intersection of Wyoming Highway 22 and the Wyoming-Idaho state line; northerly along said line to the South Fork of Badger Creek; easterly up said creek to the Targhee National Forest-Grand Teton National Park boundary; southerly along said boundary to the Targhee/Bridger-Teton interforest boundary; southerly along said boundary to Wyoming Highway 22; westerly along said highway to the Wyoming-Idaho state line.

Area 17. Lower Gros Ventre-Spread Creek. Beginning where the Gros Ventre River crosses the Grand Teton National Park boundary; northerly along said boundary to U.S. Highway 26-287; easterly along said highway to Togwotee Lodge and the divide between the Buffalo River and Blackrock Creek; easterly along said divide and the Teton Wilderness boundary to the Continental Divide; southerly along said divide to the Moccasin Basin Road (U.S.F.S. Road 30750); southerly along said road to the North Fork of Fish Creek at Calf Creek; southerly down said creek to Fish Creek; westerly down said creek to the Gros Ventre River; westerly down said river to the Grand Teton National Park boundary.

Area 18. Fish Creek. Beginning at the confluence of the Gros Ventre River and Fish Creek; northeasterly up said creek to North Fork of Fish Creek; northerly up said creek to the Moccasin Basin Road (U.S.F.S. Road 30750) at Calf Creek; northerly up said road to the Continental Divide; southeasterly along said divide to the Union Pass Road (USFS Road 600); southwesterly along said road to the Darwin Ranch Road (USFS Road 620); westerly along said road to Kinky Creek; northwesterly down said creek to the Gros Ventre River; northwesterly down said river to Fish Creek.

Area 19. Wilson. Beginning at the junction of Wyoming Highway 22 and Wyoming Highway 390 west of the Snake River; northerly along Wyoming Highway 390 to the Grand Teton National Park boundary; northerly, easterly and southerly along said boundary to U.S. Highway 191; southerly along said highway to Wyoming Highway 22; westerly along said highway to Wyoming Highway 390.

Area 20. Fall Creek-Targhee. Beginning at the junction of Wyoming Highway 22 and Wyoming Highway 390 west of the Snake River; easterly along Wyoming Highway 22 to the Snake River; southerly and westerly down said river to the Wyoming-Idaho state line; northerly along said line to Wyoming Highway 22; easterly along said highway to the Targhee/Bridger-Teton interforest boundary at Teton Pass; northerly along said boundary to the Grand Teton National Park boundary; northeasterly along said boundary to Wyoming Highway 390; southerly along said highway to Wyoming Highway 22.

Area 21. North Hoback. Beginning where U.S. Highway 191 crosses Flat Creek at the north edge of the town of Jackson; due east to the National Elk Refuge boundary; easterly and northerly along said boundary to Flat Creek; easterly and southerly up Flat Creek to Cache Peak; southeasterly along the divide between Flat Creek and Granite Creek to Pyramid Peak; southeasterly along the divide between the Gros Ventre River and the Hoback River to Steamboat Peak; southerly and westerly along the divide between Shoal Creek and Dell Creek to the Riling Draw Road; southerly along said road to the Dell Creek Road; westerly along said road to the Hoback River; westerly down said river to the Snake River; northerly up said river to Wyoming Highway 22; easterly along said highway to U.S. Highway 191; easterly and northerly along said highway to Flat Creek at the north edge of the town of Jackson.

Area 22. Raspberry Ridge. Beginning where U.S. Highway 191 crosses the divide between the Green River and the Hoback River on Hoback Rim; southwesterly along said divide to a point east of the confluence of the South Fork of the Hoback River and the Hoback River; due west to the Hoback River; northerly along said river to the Dell Creek Road; easterly along said road to the Riling Draw Road; northerly along said road to the divide between Shoal Creek and Dell Creek; easterly and northerly along said divide to the divide between the Gros Ventre River and the Hoback River at Steamboat Peak; southeasterly along the divide between the Gros Ventre River and the Hoback River to the divide between the Green River and the Hoback River at Hodges Peak; southerly along the divide between the Green River and the Hoback River to U.S. Highway 191.

Area 23. Greys River-Salt River. Beginning at the confluence of the Greys River and the Snake River; easterly up the Snake River to Bailey Creek; southerly up said creek to Dry Wash Draw; easterly up said draw to Greyback Ridge; southerly along said ridge to the divide between the Little Grays River and the Hoback River; southerly and easterly along said divide to the divide between the Greys River and the Green River; southerly, westerly, and northerly along said divide to the divide between the Greys River and the Smiths Fork River; northerly and westerly along said divide to the divide between the Bear River and the Salt River; westerly along said divide to the Wyoming-Idaho state line; northerly along said line to the Snake River; easterly up said river to the Greys River.

Area 24. Big Piney-On National Forest. Beginning at Deadman Peak and the divide between the Green River and the Hoback River; northeasterly along said divide to the Bridger-Teton National Forest boundary; southerly along said boundary to LaBarge Creek Road (U.S.F.S. Road 10-138); northwesterly along said road to the Smiths Fork Road (U.S.F.S. Road 10-072); northerly along said road to the divide between the Green River and the Greys River; northerly along said divide to Deadman Peak.

Area 25. Big Piney-Off National Forest. Beginning where U.S. Highway 191 crosses the divide between the Green River and the Hoback River (Hoback Rim); southeasterly along said highway to the East Green River Road (Sublette County Road 23-110) at Cora Junction; southerly along said road to Wyoming Highway 351; easterly along said highway to the Burma Road (BLM Road 5406); southerly along said road to the Yellow Point Road (BLM Road 5409); westerly along said road to Colorado Point and the CCC Road (BLM Road 5402); southerly along said road to the Fontenelle Reservoir Dam Road; westerly along said road to the Fontenelle Reservoir Dam (Lincoln County Road 313) on the Green River; northerly up said river to LaBarge Creek; westerly up said creek to U.S. Highway 189; northerly along said highway to the LaBarge Creek Road (Lincoln County Road 315) northwesterly along said road to the forest boundary; northerly up said boundary to the divide between the Green River and the Hoback River; northeasterly along said divide to U.S. Highway 191.

Area 26. Lincoln. Beginning at the town of Kemmerer and U.S. Highway 30; westerly along said highway to Wyoming Highway 89 at Sage Junction; southwesterly along said highway to the Wyoming-Utah state line; northerly along said line to the Wyoming-Idaho state line; northerly along said line to the divide between the Salt River and the Bear River; easterly along said divide to the divide between the Salt River and the Smiths Fork; northeasterly along said divide to the divide between the Greys River and the Smiths Fork; southeasterly along said divide to the divide between the Greys River and LaBarge Creek; southeasterly along said divide to the LaBarge Creek Road (U.S.F.S. Road 10-138 and Lincoln County Road 315); southeasterly along said road to U.S. Highway 189; southerly along said highway to LaBarge Creek; easterly down said creek to the Green River; southeasterly down said river to the Fontenelle Reservoir Dam Road (Lincoln County Road 313); westerly along said road to Wyoming Highway 189; southwesterly along said road to the town of Kemmerer.

Area 27. Uinta. Beginning where Interstate Highway 80 crosses the Wyoming-Utah state line; easterly along said highway to Wyoming Highway 410; southerly along said highway to the Stateline Dam Road (U.S.F.S. Road 072); southerly along said road to the Wyoming-Utah state line; west then north along said line to Interstate Highway 80.

Area 28. Crystal Creek-Flat Creek. Beginning where the Gros Ventre River crosses the Bridger-Teton National Forest boundary; easterly and southerly up said river to Kinky Creek; southeasterly up said creek to the Darwin Ranch Road (USFS Road 620); easterly along said road to the divide between the Green River and the Gros Ventre River; southwesterly along said divide to the divide between the Green River and the Hoback River at Hodges Peak; westerly along said divide to the divide between the Granite Creek-Cache Creek-Flat Creek and the head of Flat Creek; northwesterly down Flat Creek to the Bridger-Teton National Forest boundary; northerly along said boundary to the Gros Ventre River.

Area 30. Sweetwater. Beginning where Wyoming Highway 28 crosses Willow Creek; southwesterly along said highway to U.S. Highway 191 at the town of Farson; northerly along said highway to the Big Sandy Reservoir Road (Sweetwater County Road 28); easterly along said road to the Big Sandy River at the dam; northerly up said river to the Continental Divide; southeasterly along said divide to Mt. Nystrom and the divide between the Sweetwater River and the Wind River; southeasterly along said divide to Granite Peak and the headwaters of Willow Creek; southeasterly down said creek to Wyoming Highway 28.

Area 31. South Fork. All of the drainage of the South Fork of the Shoshone River; all of the south drainage of the Shoshone River located west of Wyoming Highway 120; and all of the Dry Creek drainage west of Wyoming Highway 120.

Area 32. Enos Lake. Beginning where Pacific Creek crosses the Grand Teton National Park boundary; easterly and northerly up said creek to Trail Creek and the Trail Creek trail; southerly along said trail to the North Fork of the Buffalo River; southerly down said river to the South Fork of the Buffalo River; easterly up the South Fork to the South Fork of the Buffalo-Togwotee Lodge Trail; southerly along said trail to Togwotee Lodge and U.S. Highway 26-287; westerly along said highway to the Grand Teton National Park boundary; westerly and northerly along said boundary to Pacific Creek.

Area 33. Lower Green River. Beginning at the junction of Interstate Highway 80 and Wyoming Highway 372; northwesterly along Wyoming Highway 372 to Lincoln County Road 316 at the Fontenelle Junction; northerly along said road to the Fontenelle Reservoir Dam Road (Lincoln County Road 313); easterly along said road to the County Line Road (Sweetwater County Road 52); southerly along said road to the Lower Farson Cutoff Road (Sweetwater County Road 8); easterly along said road to Wyoming Highway 28; easterly along said highway to the continuation of the Lower Farson Cutoff Road (Sweetwater County Road 8); southerly along said road to the Blue Rim Road (Sweetwater County Road 5); southerly along said road to Interstate Highway 80; westerly along said highway to Wyoming Highway 372.

Area 34. Hunter Mesa. Beginning at the junction of Piney Creek and Interstate Highway 90; southerly along said highway to Interstate Highway 25; southerly along said highway to the North Fork of the Powder River; northwesterly up said river to Pass Creek; westerly up said creek to the Hazelton Road (Johnson County Road 3); northerly along said road to the Gold Mine Road (U.S.F.S. Road 452); northerly along said road to the Canyon Creek Road (U.S.F.S. Road 25); northerly along said road to U.S. Highway 16; easterly along said highway to the divide along the Big Horn Mountain range at Powder River Pass; northerly along said divide to South Piney Creek; northeasterly down said creek to Piney Creek; southeasterly down said creek to Interstate Highway 90.

Area 35. Henry's Fork. Beginning where Interstate Highway 80 crosses the Green River; southerly along the east bank of said river to Flaming Gorge Reservoir; southerly along the east shore of said reservoir to the Wyoming-Utah state line; westerly along said line to the Stateline Dam Road (U.S.F. S. Road 072); northerly along said road to Wyoming Highway 410; northerly along said highway to Interstate Highway 80; easterly along said highway to the Green River.

Area 36. Bear River Divide. Beginning where Interstate Highway 80 crosses the Wyoming-Utah state line; northerly along said line to Wyoming Highway 89 west of Sage Junction; easterly along said highway to U.S. Highway 189; southerly along said highway to Interstate Highway 80; westerly along said highway to the Wyoming-Utah state line.

Area 37. Grassy Lake. Beginning where the South Fork of Badger Creek crosses the Wyoming-Idaho state line; northerly along said line to the south boundary of Yellowstone National Park; easterly along said boundary to the Targhee National Forest boundary; southerly along said boundary to the Targhee National Forest-Grand Teton National Park boundary; southerly along said boundary to the South Fork of Badger Creek; westerly down said creek to the Wyoming-Idaho state line.

Area 38. Snowy Range. Beginning where Interstate Highway 25 crosses the Wyoming-Colorado state line; westerly along said line to Wyoming Highway 230 west of the North Platte River; northwesterly along said highway to Wyoming Highway 130; northerly along said highway to U.S. Highway 30 at Walcott Junction; easterly and southerly along said highway to the town of Laramie and the Rogers Canyon-Fisher Canyon Road (Albany County Road 17-Laramie County Road 228); easterly along said road to Wyoming Highway 211; southeasterly along said highway to Interstate Highway 25; southerly along said highway to the Wyoming-Colorado state line.

Area 39. Jeffrey City. Beginning at the junction of U.S. Highway 287 and Wyoming Highway 135 (Sand Draw Road); northerly along Wyoming Highway 135 to Beaver Rim; easterly along said rim to the divide between Deer Creek and Dry Creek; northeasterly along said divide to the Dry Creek Road (Natrona County Road 321); southeasterly along said road to Wyoming Highway 220; southwesterly along said highway to U.S. Highway 287;

southerly along said highway to Lamont and Wyoming Highway 73; westerly along said highway to the Bairoil Road (Sweetwater County Road 22); westerly along said road to the Wamsutter-Crooks Gap Road (Sweetwater County Road 23); southerly along said road to the Osborne Road (BLM Road 3212); westerly along said road to the Hadsell Road (BLM Road 3217); westerly along said road to the Bison Basin Road (BLM Road 3221); northerly along said road to U.S. Highway 287; westerly along said highway to Wyoming Highway 135 (Sand Draw Road).

Area 40. Lower Hams Fork. Beginning at the town of Kemmerer and U.S. Highway 189; northerly then northeasterly along said highway to Wyoming Highway 372; easterly then southeasterly along said highway to Interstate Highway 80; westerly along said highway to U.S. Highway 189; northerly along said highway to the town of Kemmerer.

Area 41. Sierra Madres. Beginning where Wyoming Highway 230 west of the North Platte River crosses the Wyoming-Colorado state line; westerly along said line to Wyoming Highway 789; northerly along said highway to Interstate Highway 80; easterly along said highway to Wyoming Highway 130; southerly along said highway to Wyoming Highway 230; southeasterly along said highway to the Wyoming-Colorado state line.

Area 42. Shell. Beginning where the Big Horn River crosses the Wyoming-Montana state line; easterly along said line to the divide along the Big Horn Mountain range; southeasterly along said divide to the divide between Medicine Lodge Creek and Paint Rock Creek; southwesterly along said divide to the Cold Springs Road (U.S.F.S. Road 344, becomes BLM Road 268A); southwesterly along said road to the Alkali Road (BLM Road 1111); southerly along said road to Wyoming Highway 31; westerly along said highway to U.S. Highway 16-20; westerly along said highway to the Big Horn River; northerly down said river to the Wyoming-Montana state line.

Area 43. Ten Sleep. Beginning at the intersection of Wyoming Highway 31 and the Alkali Road (BLM Road 1111) just north of Hyattville; northerly along said road to the Cold Springs Road (BLM Road 268A-U.S.F.S. Road 344); northeasterly along said road to the divide between Medicine Lodge Creek and Paint Rock Creek; northeasterly along said divide to the divide along the Bighorn Mountain range; southerly along said divide to U.S. Highway 16 at the Powder River Pass; westerly along highway to the Canyon Creek Road (U.S.F.S. Road 25); southerly along said road to the Gold Mine Road (U.S.F.S. Road 452); southerly along said road to the Hazelton Road (Big Horn Divide Road-Johnson County Road 3); southerly along said road to the Rome Hill Road (Wyoming Highway 436); northwesterly along said road to U.S. Highway 16; westerly along said highway to the Nowood River; northwesterly down said river to Wyoming Highway 31; northeasterly along said highway to the Alkali Road (BLM Road 1111).

Area 44. East Flaming Gorge. Beginning where Interstate Highway 80 crosses the Green River; easterly along said highway to the Bitter Creek Road (Sweetwater County Road 19); southerly along said road to Carson Springs Junction and the Cow Creek-Powder Wash Road; southerly along said road to the Wyoming-Colorado state line; westerly along said line to the Wyoming-Utah state line; westerly along said line to Flaming Gorge Reservoir; northerly along the east shore of said reservoir to the Green River; northerly up the east bank of said river to Interstate Highway 80.

WYOMING GAME AND FISH COMMISSION

By:

Bill Williams, DVM, President

Dated: April 24, 2007

2007 MOOSE HUNT AREAS

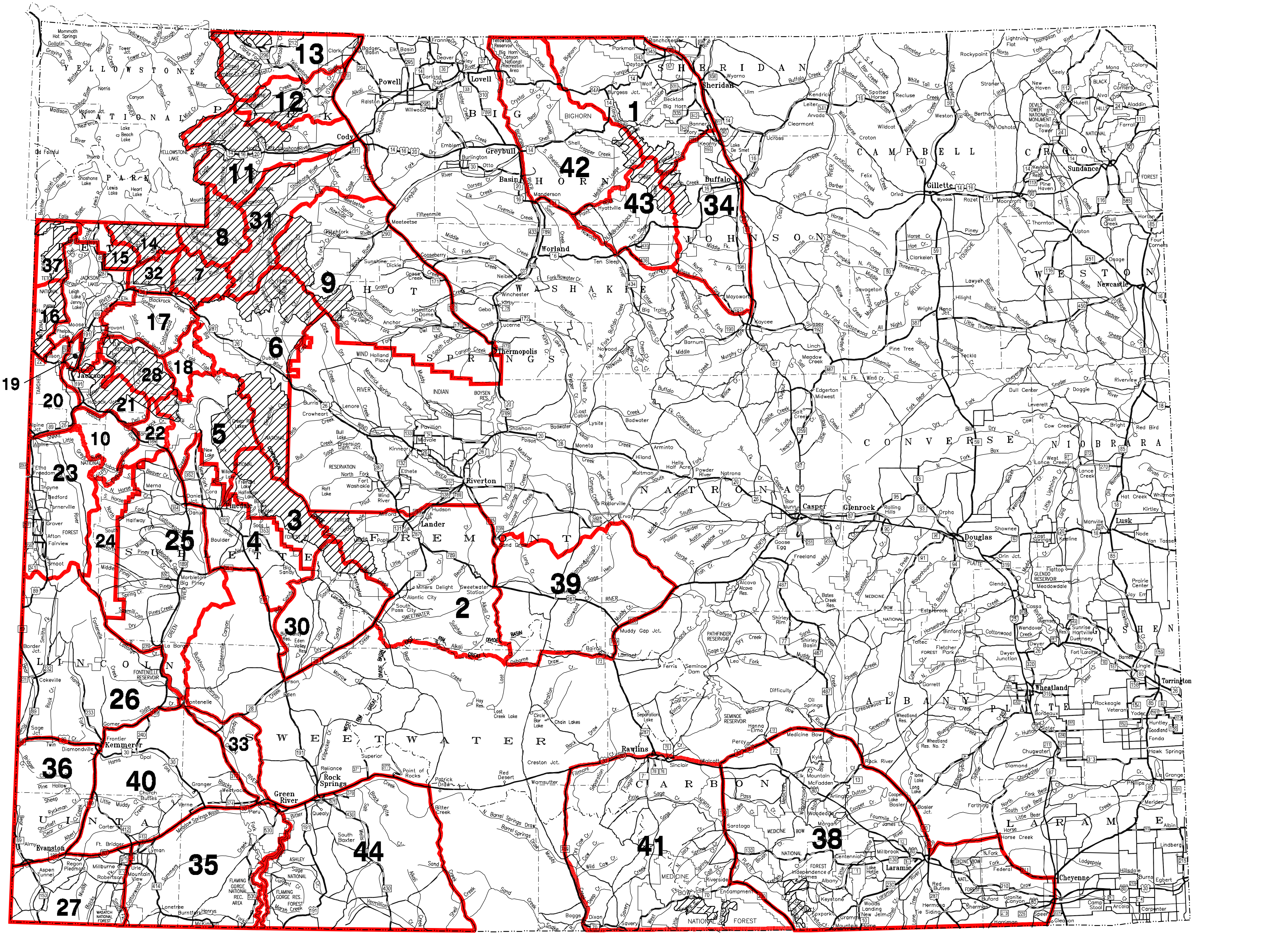

THIS MAP IS FOR GENERAL REFERENCE ONLY. Please use the written boundary descriptions in this regulation for detailed boundary information.

Note: Wilderness area, nonresidents must have guides.

BIGHORN SHEEP
HARVEST STATEWIDE
BY TOTAL HUNTERS
2007

	LICENSES SOLD	NUMBER ACTIVE HUNTERS	HARVEST RAM	HARVEST EWE	HARVEST LAMB	HARVEST TOTAL	TOTAL RECREATION DAYS	HUNTER SUCCESS	DAYS/ HARVEST
STATE TOTAL	238	237	200	1	0	201	2225	84.81	11.07
RESIDENT	177	176	150	1	0	151	1739	85.80	11.52
NONRESIDENT	61	61	50	0	0	50	486	81.97	9.72

TEN-YEAR COMPARISON OF HARVEST TABLE

YEAR	1998	1999	2000	2001	2002	2003	2004	2005	2006	2007
HARVEST	183	173	185	206	183	183	205	172	186	201

HARVEST BY HARVEST TOOL

ARCHERY	FIREARM	TOTAL
1%	99%	100%

BIGHORN SHEEP

HARVEST BY HUNT AREA

2007

AREA (cross hunt areas)	TYPE	ACTIVE LICS/HTRS	HARVEST RAM	EWE	LAMB	TOTAL	HUNTER SUCCESS	DAYS/ HARVEST	HUNTER DAYS	(cross hunt incl) LICENSES SOLD
1 Clark's Fork	Limited 1									
	Resident	15	12	0	0	12	80.00%	26.25	315	15
	Nonresident	5	4	0	0	4	80.00%	17.25	69	5
	Area Total	20	16	0	0	16	80.00%	24.00	384	20
2 Trout Peak	Limited 1									
	Resident	19	14	0	0	14	73.68%	12.57	176	19
	Nonresident	6	3	0	0	3	50.00%	12.67	38	6
	Area Total	25	17	0	0	17	68.00%	12.59	214	25
3 Wapiti Ridge	Limited 1									
	Resident	31	29	0	0	29	93.55%	8.76	254	31
	Nonresident	11	8	0	0	8	72.73%	15.13	121	11
	Area Total	42	37	0	0	37	88.10%	10.14	375	42
4 Yount's Peak	Limited 1									
	Resident	28	24	0	0	24	85.71%	9.04	217	28
	Nonresident	10	7	0	0	7	70.00%	13.57	95	10
	Area Total	38	31	0	0	31	81.58%	10.06	312	38
5 Franc's Peak	Limited 1									
(22)	Resident	52	46	0	0	46	88.46%	7.67	353	52
	Nonresident	18	18	0	0	18	100.00%	3.06	55	18
	Area Total	70	64	0	0	64	91.43%	6.38	408	70
6 Targhee	Limited 1									
	Resident*	2	1	0	0	1	50.00%	25.00	25	3
	Nonresident	1	0	0	0	0	0.00%	0.00	9	1
	Area Total	3	1	0	0	1	33.33%	34.00	34	4
*1 resident did not hunt										
7 Jackson	Limited 1									
	Resident	7	4	1	0	5	71.43%	41.20	206	7
	Nonresident	2	2	0	0	2	100.00%	12.50	25	2
	Area Total	9	6	1	0	7	77.78%	33.00	231	9
8 Sheep Mountain	Limited 1									
(23)	Nonresident	1	1	0	0	1	100.00%	9.00	9	1
	Area Total	1	1	0	0	1	100.00%	9.00	9	1
9 Dinwoody	Limited 1									
	Resident	3	2	0	0	2	66.67%	10.00	20	3
	Nonresident	1	1	0	0	1	100.00%	11.00	11	1
	Area Total	4	3	0	0	3	75.00%	10.33	31	4
10 Jakey's Fork	Limited 1									
	Resident	6	6	0	0	6	100.00%	11.00	66	6
	Nonresident	2	2	0	0	2	100.00%	8.00	16	2
	Area Total	8	8	0	0	8	100.00%	10.25	82	8
18 Douglas Creek	CLOSED									
19 Laramie Peak	Limited 1									
	Resident	3	3	0	0	3	100.00%	6.67	20	3
	Nonresident	1	1	0	0	1	100.00%	24.00	24	1
	Area Total	4	4	0	0	4	100.00%	11.00	44	4
21 Encampment River	CLOSED									
22 Dubois Badlands	Limited 1									
(5)	Resident	3	3	0	0	3	100.00%	6.33	19	3
	Nonresident	1	1	0	0	1	100.00%	4.00	4	1

BIGHORN SHEEP

HARVEST BY HUNT AREA

2007

AREA (cross hunt areas)	TYPE	ACTIVE LICS/HTRS	HARVEST RAM	EWE	LAMB	TOTAL	HUNTER SUCCESS	DAYS/ HARVEST	HUNTER DAYS	(cross hunt incl) LICENSES SOLD
	Area Total	4	4	0	0	4	100.00%	5.75	23	4
23 Desolation Peak	Limited 1									
(8)	Resident	7	6	0	0	6	85.71%	11.33	68	7
	Nonresident	2	2	0	0	2	100.00%	5.00	10	2
	Area Total	9	8	0	0	8	88.89%	9.75	78	9

WYOMING GAME AND FISH COMMISSION

CHAPTER 9

BIGHORN SHEEP HUNTING SEASONS

Section 1. Authority. This regulation is promulgated by authority of W.S. §23-1-302, §23-1-703, §23-2-104 and §23-3-117.

Section 2. Hunting Seasons Established. There shall be open seasons during 2007 for the hunting of bighorn sheep as set forth in this Chapter and in accordance with Chapter 2, General Hunting Regulation.

Section 3. Regular Hunting Seasons.

(a) Hunt areas, season dates and limitations.

		Date of Seasons		
**Hunt Area**	**Type**	**Opens**	**Closes**	**Limitations**
1	1	Sept. 1	Oct. 31	Limited quota; 20 licenses any ram
2	1	Sept. 1	Oct. 31	Limited quota; 24 licenses any ram
3	1	Sept. 1	Oct. 20	Limited quota; 44 licenses any ram
		Oct. 21	Oct. 31	Unused Area 3 Type 1 licenses valid only in that portion of Area 3 in the North Fork of the Shoshone River drainage and the South Fork of the Shoshone River drainage northeast of Ishawooa Creek
4	1	Sept. 1	Oct. 20	Limited quota; 40 licenses any ram
5	1	Sept. 1	Oct. 20	Limited quota; 48 licenses any ram
6	1	Aug. 15	Oct. 31	Limited quota; 4 licenses any ram
7	1	Sept. 1	Oct. 31	Limited quota; 8 licenses any bighorn sheep
8, 23	1	Sept. 1	Oct. 15	Limited quota; 12 licenses any ram
9	1	Aug. 15	Oct. 15	Limited quota; 4 licenses any ram
10	1	Aug. 15	Oct. 15	Limited quota; 8 licenses any ram
18, 21				CLOSED
19	1	Sept. 1	Oct. 31	Limited quota; 4 licenses any ram
22	1	Sept. 1	Oct. 20	Limited quota; 4 licenses any ram; also valid in Area 5 after September 30 for any ram
24				CLOSED

Section 4. Special Archery Seasons.

(a) Hunt areas listed in this section shall have special archery hunting seasons during the dates and with limitations specified in this section. Archers shall possess a limited quota bighorn sheep license in addition to an archery license in order to hunt bighorn sheep during any special archery season. Archers shall hunt only in the hunt area(s) and for the type of bighorn sheep set forth by the limitations of their license as specified in Section 3 of this Chapter.

	Dates of Seasons		
**Hunt Area**	**Opens**	**Closes**	**Limitations**
1-5, 7, 8, 19, 22, 23	Aug. 15	Aug. 31	Refer to Section 3 of this Chapter
9, 10	Aug. 1	Aug. 14	Refer to Section 3 of this Chapter

Section 5. Definitions.

(a) “Bighorn sheep horns” mean the hollow horn sheaths of male bighorn sheep, either attached to the skull or separated.

(b) “Plugging” means placement of a permanent metal plug provided and attached by the Wyoming Game and Fish Department.

(c) “Ram” means a male bighorn sheep.

Section 6. Registration and Plugging of Bighorn Sheep Horns. Any person harvesting a bighorn sheep in Wyoming shall within fifteen (15) consecutive days following the harvest present the horns attached to the skull plate for registration and plugging. Any person picking up any bighorn sheep horns in Wyoming shall within fifteen (15) consecutive days following the pickup of the horns present the horn or horns for registration, plugging and interstate game tagging. Plugging may be omitted if the Game and Fish Department determines that the size or condition of the horn prevents proper plugging. Any person taking bighorn sheep horns shall present them in person at one of the following Game and Fish Department offices, Monday through Friday, excluding legal holidays, 8:00 a.m. - 12:00 p.m. and 1:00 p.m. - 5:00 p.m. Bighorn sheep horns not originating in Wyoming shall not be plugged.

Game and Fish Department Headquarters	Cheyenne	1(800) 842-1934
Casper Region	Casper	1(800) 233-8544
Cody Region	Cody	1(800) 654-1178
Green River Region	Green River	1(800) 843-8096
Lander Region	Lander	1(800) 654-7862
Laramie Region	Laramie	1(800) 843-2352
Pinedale/Jackson Region	Jackson	1(800) 423-4113
	Pinedale	1(800) 452-9107
Sheridan Region	Sheridan	1(800) 331-9834

(a) The Wyoming Game and Fish Department may require substantive proof from individuals that do not possess a valid bighorn sheep license and take bighorn sheep horns that the horns were legally acquired. Failure to provide such proof may result in seizure of the horns. Before plugging any bighorn sheep horns, the person may be required to take a Wyoming Game and Fish Department enforcement officer to the location where the bighorn sheep horns were acquired. Failure to comply with this request shall be a violation of this regulation and the bighorn sheep horns may be seized.

(b) No person shall alter, deface or remove a plug placed in a bighorn sheep horn by the Wyoming Game and Fish Department.

(c) No person shall possess the horn(s) of a bighorn sheep that bears an altered, defaced or counterfeit Wyoming plug or from which the plug has been removed.

(d) The insertion of a Wyoming plug does not in itself certify that the animal was legally taken or possessed.

(e) Any person who makes a false statement on the registration form regarding the date the bighorn sheep was taken or the hunt area in which it was taken shall be in violation of this regulation. Such violation shall be punishable as provided by Title 23, Wyoming Statutes for violation of Commission regulations.

Section 7. Hunt Area Descriptions.

(a) Area and Number

Area 1. Clark's Fork. All of the drainage of the Clark's Fork River north of Sunlight Creek and north of the Clark's Fork River below its confluence with Sunlight Creek; and the drainage of Soda Butte Creek outside of Yellowstone National Park.

Area 2. Trout Peak. All of the north drainage of the North Fork of the Shoshone River and north of U.S. Highway 14-16-20 between Pahaska Teepee and the east boundary of Yellowstone National Park; all of the south drainage of the Clark's Fork River south and east of Sunlight Creek and west of Wyoming Highway 120; all of the north drainage of the Shoshone River west of Wyoming Highway 120.

Area 3. Wapiti Ridge. Beginning where U.S. Highway 14-16-20 intersects the east boundary of Yellowstone National Park; easterly down said highway to the North Fork of the Shoshone River; easterly down said river to the South Fork of the Shoshone River at the Buffalo Bill Reservoir; southwesterly up said river to Deer Creek; westerly up said creek to the head of Butte Creek; westerly down said creek to the Thorofare River; westerly down said river to the Yellowstone National Park boundary; easterly then northerly along said boundary to U.S. Highway 14-16-20.

Area 4. Yount's Peak. Beginning where Deer Creek enters the South Fork of the Shoshone River; southerly up said river to Needle Creek; southerly up said creek to the head of Emerald Creek; southerly down said creek to Wiggins Fork Creek; southerly down said creek to the Wiggins Fork Road (Shoshone National Forest Road 285); southerly down said road to the Shoshone National Forest boundary; westerly then southerly along said boundary to U.S. Highway 287; northwesterly along said highway to the east boundary of Grand Teton National Park; northerly and westerly along said boundary to U.S. Highway 287; northerly along said highway to the south boundary of Yellowstone National Park; easterly along said boundary to the Thorofare River; easterly up said river to Butte Creek; northeasterly up said creek to the head of Deer Creek; easterly down said creek to the South Fork of the Shoshone River.

Area 5. Franc's Peak. Beginning where Needle Creek enters the South Fork of the Shoshone River; northeasterly down said river to the Shoshone River; easterly down said river to Wyoming Highway 120; southeasterly along said highway to the Bighorn River; southerly up said river to the Wind River Indian Reservation boundary; westerly along said boundary to the Shoshone National Forest boundary; northwesterly, southwesterly, then westerly along said boundary to the Wiggins Fork Road (Shoshone National Forest Road 285); northerly along said road to Wiggins Fork Creek; northerly up said creek to Emerald Creek; northerly up said creek to Needle Creek; northerly down said creek to the South Fork of the Shoshone River; in addition, all non-Indian fee title lands in that portion of Hot Springs County within the exterior boundaries of the Wind River Indian Reservation.

Area 6. Targhee. Beginning where the Snake River crosses the Wyoming-Idaho state line; northerly along said line to the Yellowstone National Park boundary; easterly along said boundary to the Targhee-John D. Rockefeller Jr. Memorial Parkway boundary; southerly along said boundary to the Targhee Forest-Grand Teton National Park boundary; southerly along said boundary to the Bridger-Teton National Forest-Grand Teton National Park boundary; northerly, easterly and southerly along said boundary to Wyoming Highway 390 west of the Snake River; southerly along said highway to Wyoming Highway 22; easterly along said highway to the Snake River; southerly along said river to the Wyoming-Idaho state line.

Area 7. Jackson. Beginning at Togwotee Pass on the Continental Divide; southerly along said divide to the Union Pass Road (USFS Road 600); westerly along said road to Wagon Creek; southerly down said creek to the Green River; southerly down said river to the Bridger-Teton National Forest boundary; westerly along said boundary to the divide between the Green River and the Hoback River at the Hoback Rim where U.S. Highway 191 crosses the Rim; westerly along said divide to the divide between the Greys River and the Hoback River; northerly along said divide to the divide between the Greys River

and Willow Creek; northerly along said divide to the divide between Bailey Creek and Willow Creek (Greyback Ridge); northerly along said divide to the Dry Wash Draw; westerly down said draw to Bailey Creek; northwesterly down said creek to the Snake River; northerly along said river to Wyoming Highway 22; easterly along said highway to U.S. Highway 191; easterly along said highway to the National Elk Refuge boundary at the north end of the town of Jackson; southerly, easterly and northerly along said boundary to the Grand Teton National Park boundary; northerly along said boundary to U.S. Highway 26-287; easterly along said highway to Togwotee Pass.

Area 8. Sheep Mountain. Beginning where Pine Creek crosses U.S. Highway 191 in the town of Pinedale; northwesterly along said highway to the Green River at Warren Bridge; northerly, easterly and southerly up said river to Trail Creek; southerly up said creek to Summit Lake and the head of Pine Creek; southerly down said creek to U.S. Highway 191.

Area 9. Dinwoody. Beginning where U.S. Highway 287 crosses Torrey Creek; southerly along said highway to the Wind River Indian Reservation boundary; southerly along said boundary to the Continental Divide; northwesterly along said divide to the head of Bomber Creek (East Fork of Torrey Creek); northeasterly down said creek to Torrey Creek; northerly down said creek to U.S. Highway 287.

Area 10. Jakey's Fork. Beginning where Torrey Creek crosses U.S. Highway 287; southwesterly up said creek to Bomber Creek (East Fork of Torrey Creek); southwesterly up said creek to the Continental Divide; northerly along said divide to the head of Roaring Fork Creek; southerly down said creek to the Green River; westerly and southerly down said river to Wagon Creek; northwesterly up said creek to the Union Pass Road (USFS Road 600); northerly along said road to the Continental Divide; northwesterly along said divide to U.S. Highway 287; southeasterly along said highway to Torrey Creek.

Area 18. Douglas Creek. Beginning where Wyoming Highway 230 crosses the Wyoming-Colorado state line near Mountain Home; westerly along said line to Wyoming Highway 230; northwesterly along said highway to Wyoming Highway 130; easterly along said highway to Wyoming Highway 230; southwesterly along said highway to the Wyoming-Colorado state line.

Area 19. Laramie Peak. Beginning at the junction of Interstate Highway 25 and Wyoming Highway 211 at the town of Chugwater; southwesterly along Wyoming Highway 211 to the Iron Mountain-Jordan Road (Platte County Road 10-Laramie County Road 106-2), southwesterly along said road to Wyoming Highway 211; southerly along said road to the Fisher Canyon-Rogers Canyon Road (Laramie County Road 228-1 - Albany County Road 17) at Horse Creek Station; southwesterly along said road to U.S. Highway 30 at the town of Laramie; northwesterly along said highway to Wyoming Highway 487; northerly along said highway to Wyoming Highway 220; northeasterly along said highway to Interstate Highway 25; southeasterly along said interstate to Wyoming Highway 211.

Area 21. Encampment River. Beginning where Wyoming Highway 130 crosses the North Platte River in the town of Saratoga; southerly along said highway to Wyoming Highway 230; southeasterly along said highway to the Wyoming-Colorado state line; westerly along said line to the Medicine Bow National Forest boundary near the confluence of Spring Creek and the Little Snake River; westerly and northerly along said boundary to Wyoming Highway 70; northeasterly along said highway to the Sage Creek Road (U.S.F.S. Road 801-Carbon County Road 401); northerly along said road to Sage Creek; easterly down said creek to the North Platte River; southerly up said river to Wyoming Highway 130 in the town of Saratoga.

Area 22. Dubois Badlands. Beginning where U.S. Highway 287 crosses the Wind River Indian Reservation boundary; westerly along said highway to the Wiggins Fork Road (Fremont County Road 285); northerly along said road to the Shoshone National Forest boundary; easterly along said boundary to the Wind River Indian Reservation boundary; southerly along said boundary to U.S. Highway 287. This includes those areas of the Spence-Moriarity Wildlife Habitat Area east of the East Fork of the Wind River.

Area 23. Desolation Peak. Beginning where Roaring Fork Creek enters the Green River; easterly up said creek to the Continental Divide; southwesterly along said divide to Hay Pass and the head of the

North Fork of Boulder Creek; southwesterly down said creek to the Bridger-Teton National Forest boundary; northwesterly along said boundary to Pine Creek; northerly up said creek to Summit Lake and the head of Trail Creek; northerly down said creek to the Green River; northerly down said river to Roaring Fork Creek.

Area 24. Big Piney. Beginning at a point where the Bridger-Teton National Forest boundary crosses LaBarge Creek; westerly up said creek to the head of the Greys River; northerly down said river to Deadman Creek; easterly up said creek to the divide between the Greys River and the Hoback River, southerly along said divide to the divide between the Hoback River and the Green River; easterly along said divide to the Bridger-Teton National Forest boundary on Hoback Rim; southerly along said boundary to LaBarge Creek.

WYOMING GAME AND FISH COMMISSION

By:

Bill Williams, DVM, President

Dated: April 24, 2007

2007 BIGHORN SHEEP HUNT AREAS

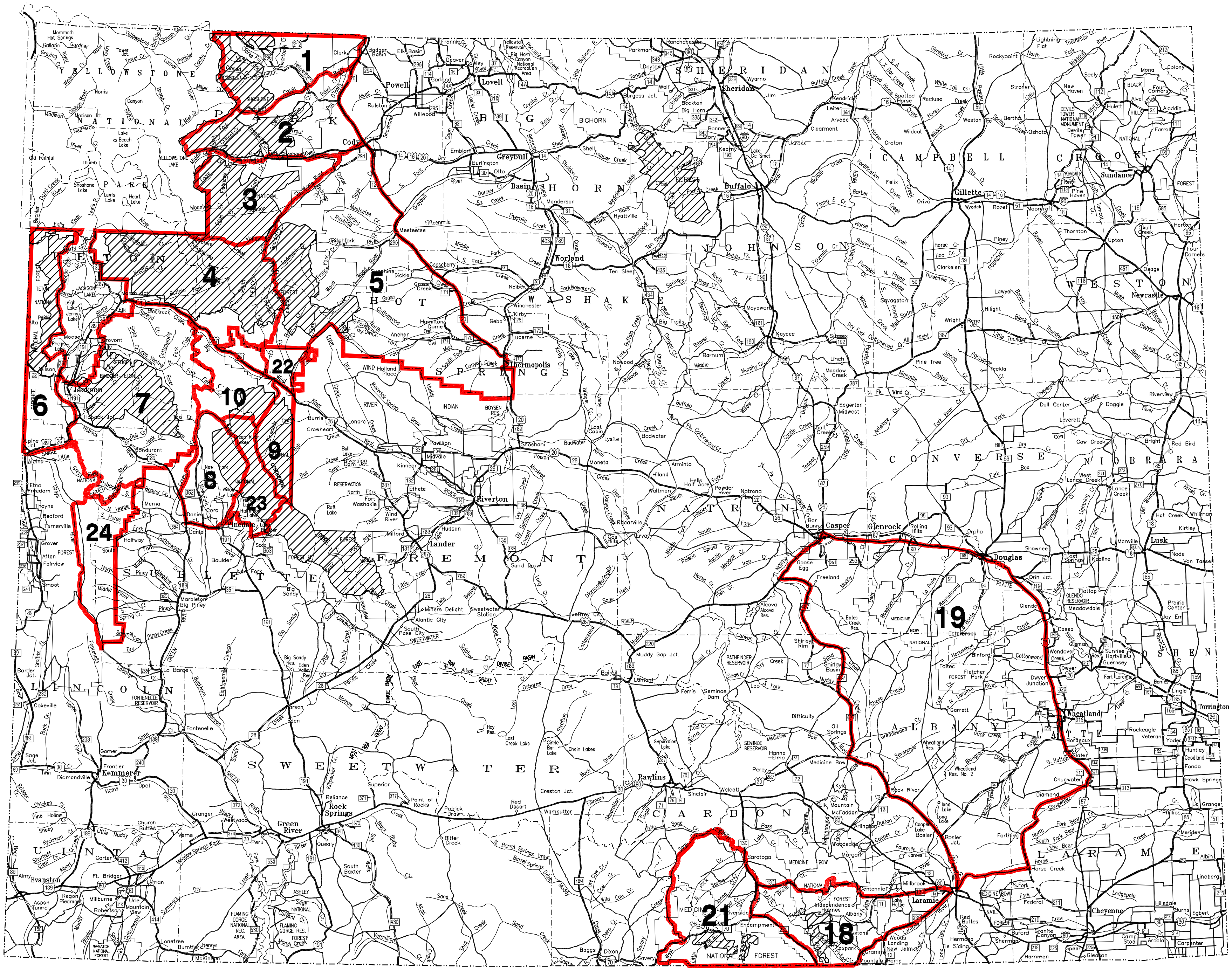

THIS MAP IS FOR GENERAL REFERENCE ONLY. Please use the written boundary descriptions in this regulation for detailed boundary information.

Note: Wilderness area, nonresidents must have guides.

MOUNTAIN GOAT
HARVEST STATEWIDE
BY TOTAL HUNTERS
2007

	LICENSES SOLD	NUMBER ACTIVE HUNTERS	HARVEST TOTAL	TOTAL RECREATION DAYS	HUNTER SUCCESS	DAYS/ HARVEST
STATE TOTAL	20	20	19	113	95%	5.947
RESIDENT	15	15	14	94	100%	6.714
NONRESIDENT	5	5	5	19	100%	3.80

TEN YEAR COMPARISON OF HARVEST

YEAR	1998	1999	2000	2001	2002	2003	2004	2005	2006	2007
HARVEST	12	16	13	17	15	15	15	19	20	19

MOUNTAIN GOAT
HARVEST STATEWIDE
BY HUNT AREA
2007

HUNT AREA #1	LICENSES SOLD	NUMBER ACTIVE HUNTERS	HARVEST BILLY	HARVEST NANNY	HARVEST TOTAL	TOTAL RECREATION DAYS	HUNTER SUCCESS	DAYS/ HARVEST
RESIDENT	9	9	5	3	8	72	88.89%	9.00
NONRESIDENT	3	3	3	0	3	13	100%	4.33
AREA TOTAL	12	12	8	3	11	85	91.67%	7.73

HUNT AREA #2	LICENSES SOLD	NUMBER ACTIVE HUNTERS	HARVEST BILLY	HARVEST NANNY	HARVEST TOTAL	TOTAL RECREATION DAYS	HUNTER SUCCESS	DAYS/ HARVEST
RESIDENT	6	6	4	2	6	22	100%	3.67
NONRESIDENT	2	2	2	0	2	6	100%	3.00
AREA TOTAL	8	8	6	2	8	28	100%	3.50

WYOMING GAME AND FISH COMMISSION

CHAPTER 24

MOUNTAIN GOAT HUNTING SEASONS

Section 1. Authority. This regulation is promulgated by authority of W.S. §23-1-302, §23-1-703 and §23-2-104.

Section 2. Hunting Seasons Established. There shall be open seasons during 2007 for the hunting of mountain goat as set forth in this Chapter and in accordance with Chapter 2, General Hunting Regulation.

Section 3. Regular Hunting Seasons.

(a) Hunt areas, season dates and limitations.

Hunt Area	Type	Date of Seasons Opens	Closes	Limitations
1	1	Sept. 1	Oct. 31	Limited quota; 12 licenses any mountain goat
2	1	Sept. 1	Oct. 31	Limited quota; 8 licenses any mountain goat

Section 4. Special Archery Seasons. Hunt areas listed in this section shall have special archery hunting seasons during the dates and with limitations as specified in this section. Archers shall possess a limited quota mountain goat license in addition to an archery license in order to hunt mountain goat during any special archery season. Archers shall hunt only in the hunt area(s) and for the type of mountain goat as set forth by the limitations for their license as specified in Section 3 of this Chapter.

Hunt Area	Date of Seasons Opens	Closes	Limitations
1, 2	Aug. 15	Aug. 31	Refer to Section 3 of this Chapter

Section 5. Registration of Mountain Goat.

(a) Any person harvesting a mountain goat in Wyoming shall within fifteen (15) consecutive days following the harvest present the skull plate and horns for registration. Any person harvesting a mountain goat shall register the harvested mountain goat in person at one of the following Game and Fish Department Offices Monday through Friday, excluding legal holidays, 8:00 a.m. - 12:00 p.m. and 1:00 p.m. - 5:00 p.m.

Game and Fish Department Headquarters	Cheyenne	1(800) 842-1934
	(307)777-4583	
Casper Region	Casper	1(800) 233-8544
Cody Region	Cody	1(800) 654-1178
Green River Region	Green River	1(800) 843-8096
Lander Region	Lander	1(800) 654-7862
Laramie Region	Laramie	1(800) 843-2352
Pinedale/Jackson Region	Jackson	1(800) 423-4113
	Pinedale	1(800) 452-9107
Sheridan Region	Sheridan	1(800) 331-9834

(b) Any person who makes a false statement on the registration form regarding the date the mountain goat was taken or the hunt area in which it was taken shall be in violation of this regulation. Such violation shall be punishable as provided by Title 23, Wyoming Statutes, for violation of Commission regulations.

Section 6. Hunt Area Descriptions.

(a) Area and Number.

Area 1. Beartooth. All of the drainage of the Clark's Fork River west of Wyoming Highway 120; all of the drainage of Soda Butte Creek outside of Yellowstone National Park.

Area 2. Palisades. Beginning where Wyoming Highway 22 crosses the Snake River; southerly and westerly down said river to the Wyoming-Idaho state line; northerly along said line to Wyoming Highway 22; easterly along said highway to the Snake River.

WYOMING GAME AND FISH COMMISSION

By:

Bill Williams, DVM, President

Dated: April 24, 2007

MOUNTAIN GOAT HUNT AREAS

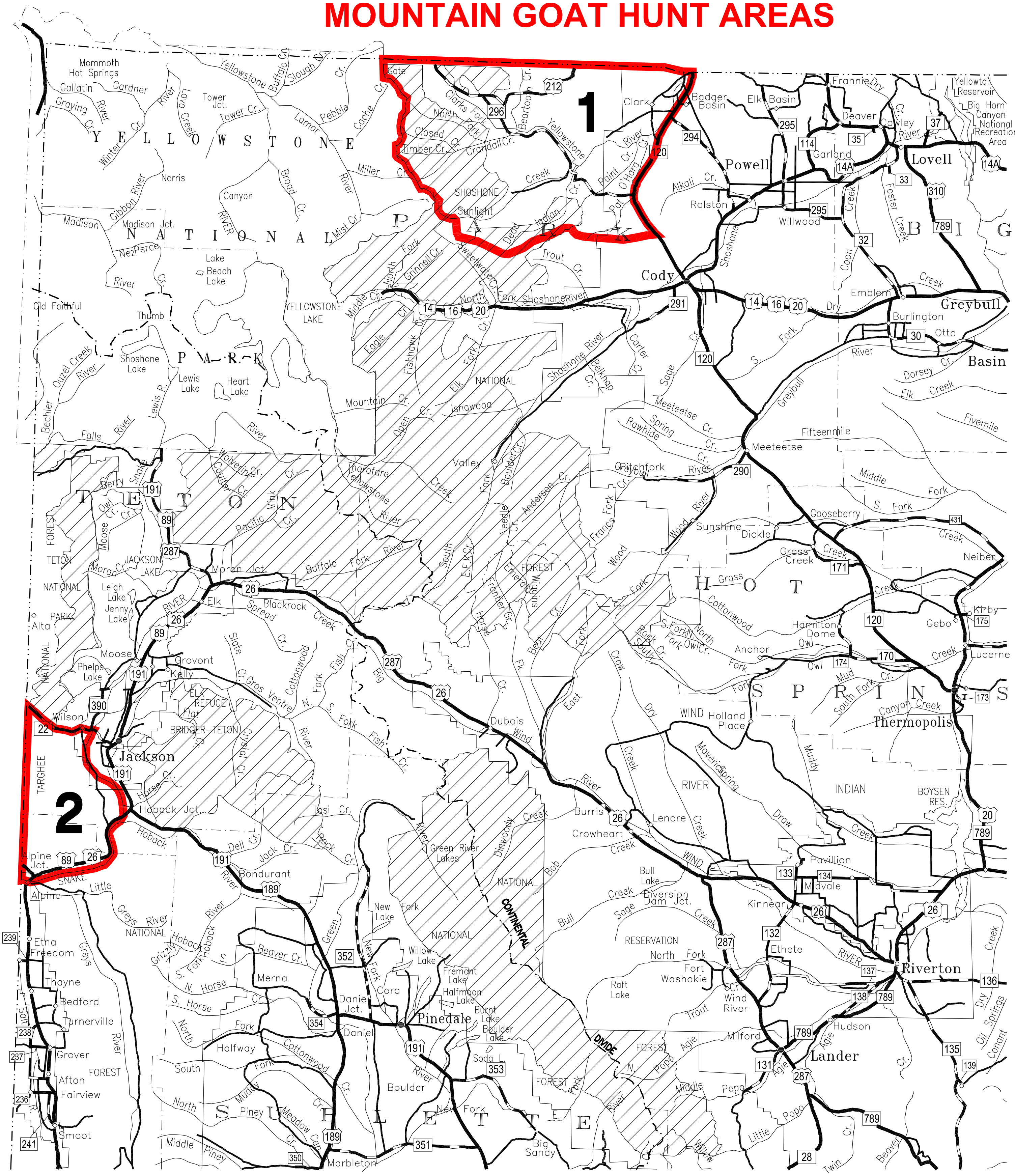

THIS MAP IS FOR GENERAL REFERENCE ONLY. Please use the written boundary descriptions in this regulation for detailed boundary information.

Note: Wilderness area, nonresidents must have guides.

BULL BISON
HARVEST STATEWIDE
BY TOTAL HUNTERS
2007

	LICENSES SOLD	NUMBER ACTIVE HUNTERS	HARVEST TOTAL	TOTAL RECREATION DAYS	HUNTER SUCCESS	DAYS/ HARVEST
STATE TOTAL	113	113	113	185	100%	1.637
RESIDENT	96	96	96	159	100%	1.656
NONRESIDENT	17	17	17	26	100%	1.529

NINE-YEAR COMPARISON OF HARVEST

YEAR	1999	2000	2001	2002	2003	2004	2005	2006	2007
HARVEST	4	13	19	25	27	21	23	23	*113

*The area availble for bison hunting expanded in 2007, resulting in an increase in licenses available for sale.

COW BISON
HARVEST STATEWIDE
BY TOTAL HUNTERS
2007

	LICENSES SOLD	NUMBER ACTIVE HUNTERS	HARVEST TOTAL	TOTAL RECREATION DAYS	HUNTER SUCCESS	DAYS/ HARVEST
STATE TOTAL	164	164	154	639	93.90%	4.149
RESIDENT	161	161	151	629	93.79%	4.166
NONRESIDENT	3	3	3	10	100.00%	3.333

NINE YEAR COMPARISON OF HARVEST

YEAR	1999	2000	2001	2002	2003	2004	2005	2006	2007
HARVEST	5	6	22	22	13	10	13	25	*154

*The area availble for bison hunting expanded in 2007, resulting in an increase in licenses available for sale.

WYOMING GAME AND FISH COMMISSION

CHAPTER 15

WILD BISON RECREATIONAL HUNTING SEASON

Section 1. Authority. This regulation is promulgated by authority of W.S. §23-1-101, §23-1-302 and §23-2-107.

Section 2. Regulation. The Wyoming Game and Fish Commission hereby adopts the following regulation governing wild bison recreational hunting seasons. This regulation shall remain in effect until modified or repealed by the Commission.

Section 3. Definitions. For the purpose of this regulation, definitions shall be as set forth in Title 23, Wyoming Statutes, and the Commission also adopts the following definitions:

(a) "Edible portions" means the meat of the front quarters as far down as the knees, the meat of the hindquarters as far down as the hocks, and the meat along the backbone between the neck and hindquarters, excluding meat on the ribs and neck.

(b) "Harvest year" means seven (7) consecutive months beginning July 1 of each year through January 31 of the next succeeding year.

Section 4. Recreational Hunting Season Established And Effective Date. When the Department determines a wild bison recreational hunting opportunity exists, there shall be a wild bison recreational hunting season as established in accordance with Section 5 through Section 18 of this regulation.

Section 5. Supervision Of Licensees. Wild bison recreational hunting may be done under the supervision and/or direction of Department personnel.

Section 6. Notification.

(a) Applicants shall be notified of their placement on the wild bison priority list by the Department. Applicants on the wild bison priority list shall also be notified by mail or by telephone, in order of their priority ranking, when the Department determines a wild bison recreational hunting opportunity may exist. If telephone calls are made, the calls shall be done by the Department during the evening hours between 4:00 p.m. and 10:00 p.m. If telephone calls are made, a minimum of three (3) attempts by telephone shall be made that evening and the subsequent evening to contact and notify any individual applicant. Notification attempts shall be spaced at a minimum of one-half (½) hour intervals. Department personnel must speak directly to the applicant during the notification call. If an individual cannot be contacted, the next individual on the list shall be notified and those who could not be contacted shall retain their positions on the wild bison priority list. An applicant who declines participation shall have his/her name removed from the wild bison priority list.

(b) At the Department's discretion, notification may be made by mail. The Department shall send notification by first-class mail to the address listed on the

applicant's application for placement on the wild bison priority list. The notification shall be mailed a minimum of ten (10) days immediately prior to the scheduled wild bison hunting season.

(c) Applicants shall be advised during the notification call or by mail of: the date, time, and location of their scheduled wild bison recreational hunt.

Section 7. Selection Of Bison To Be Harvested.

(a) Hunters may be directed by Department personnel to take a specific age class of wild bison in a specific geographic portion of a hunt area and during a specific time period in order to meet the Department's management objectives. Numbers and age classes of wild bison available when the applicant arrives may differ from those available at the time of the notification call or letter. Issuance of a wild bison license provides the licensee an opportunity to hunt a wild bison; it does not guarantee that a licensee shall harvest a wild bison.

Section 8. Wild Bison Hunting Orientation Information.

(a) After notification, individuals interested in participating in the wild bison recreational hunting season shall be provided wild bison hunting orientation information in a manner determined by the Department.

Section 9. Firearms. Wild bison shall only be taken with a firearm which has a barrel bore diameter of at least twenty-seven hundredths (27/100) of an inch and is chambered to fire a center-fire cartridge not less than two (2) inches in overall length, including a soft or expanding point bullet seated to a normal depth. The cartridge shall have a minimum of a one hundred fifty (150) grain bullet.

Section 10. Licensing. Licenses shall be issued upon payment by the applicant of the proper license fee at the Department Regional Office administering the wild bison recreational hunt. A license must be purchased prior to participating in the wild bison recreational hunt. A conservation stamp shall be in possession of the licensee while exercising the privileges under the wild bison license.

Section 11. Tagging Requirements. When any wild bison is killed under a license, the licensee shall detach, sign and date the carcass coupon and attach the coupon to the carcass before leaving the site of the kill. The coupon shall remain on the wild bison carcass at all times until the meat undergoes processing, except that during transportation of the carcass the coupon may be removed to prevent its loss. If the coupon is removed for transportation of the carcass, it shall be in the possession of the person accompanying the carcass at all times.

Section 12. Retrieval of Edible Portions. No person shall take and leave, abandon, or allow any wild bison, or edible portion to intentionally or needlessly go to waste. The failure of any person to properly dress and care for any wild bison killed by that person and if the carcass is reasonably accessible, within forty-eight (48) hours to take or transport the carcass to the camp of that person, and to properly care for the carcass is prima facie evidence of a violation of this section.

Section 13. Wearing of Fluorescent Orange Clothing. No person shall hunt any wild bison without wearing in a visible manner one (1) or more exterior garments, which shall include a hat, shirt, jacket, coat, vest or sweater of a fluorescent orange color.

Section 14. Transportation And Exportation.

(a) No person shall ship, transport, or receive for shipment or transportation within Wyoming, any wild bison or part thereof, unless tagged with a Wyoming interstate game tag, or unless:

(i) The transportation is by a person accompanying the carcass of a wild bison who is in possession of a proper coupon.

(b) No wild bison or any part thereof shall be shipped or transported from the State unless accompanied by the licensee who harvested the animal, in possession of a proper coupon, or unless:

(i) The part to be exported from the state is of a nonedible trophy or hidelike nature and properly tagged with a Wyoming interstate game tag.

(ii) The wild bison was legally harvested by a nonresident and is tagged with a Wyoming interstate game tag.

Section 15. Hunting Regulations.

(a) Shooting Hours. Shooting hours for the hunting of wild bison shall be from one-half (1/2) hour before sunrise to one-half (1/2) hour after sunset.

(b) Within three (3) consecutive days (seventy-two (72) hours) after taking a wild bison, the licensee shall contact the Department Regional Office where the licensee obtained his/her wild bison license during business hours to report the harvest. The licensee shall report the date of the harvest, the sex, age class (adult, yearling, calf) of the bison harvested, supply biological samples as required by the Department and provide the legal description of where the bison was harvested to include section, range and township or UTM coordinates.

(c) Bag and Possession Limits. The bag and possession limit for wild bison shall be one (1) wild bison during any one (1) harvest year. No person who is issued a wild bison hunting license and who harvests a wild bison after January 31, 2001, shall be eligible to apply for or receive a hunting license for wild bison in any future year.

(d) Open Areas, Season Dates and Limitations.

Hunt Area	Type	Date of Seasons Opens	Closes	Limitations
1, 2	1	July 1	Jan. 31	Any male Wild Bison; other limitations specified during notification
	2	July 1	Jan. 31	Any female/calf Wild Bison; other limitations specified during notification
2	3	Sept. 1	Nov. 30	Any male Wild Bison; other limitations specified during notification
2	4	Sept. 1	Nov. 30	Any female/calf Wild Bison; other limitations specified during notification

Section 16. Hunt Area Descriptions.

(a) Area and Number.

Area 1. Absaroka. All lands within Park, Hot Springs, Fremont, Sublette and Teton Counties east of the Continental Divide, excluding lands administered by the Wind River Indian Reservation and Yellowstone National Park.

Area 2. Jackson. All lands within Lincoln, Sublette, Fremont and Teton Counties west of the Continental Divide, excluding Grand Teton National Park, John D. Rockefeller Jr. Memorial Parkway, Yellowstone National Park and the National Elk Refuge.

Section 17. Violation of Commission Regulations. Failure to abide by the provisions of this regulation shall be punishable as provided by Wyoming statutes for violation of Commission regulations.

Section 18. Savings Clause. If any provision of this regulation shall be held to be illegal or unconstitutional, such a ruling shall not affect other provisions of this regulation which can be given effect without the illegal or unconstitutional provision; and, to this end, the provisions of this regulation shall be severable.

WYOMING GAME AND FISH COMMISSION

By:
M. Hale Kreycik, President

Date: July 15, 2004

WYOMING GAME AND FISH COMMISSION

CHAPTER 15

WILD BISON RECREATIONAL HUNTING SEASON

EMERGENCY RULE

(For the 120 days the emergency rules are in effect, the following rules shall apply. Emergency rules are no longer in effect 120 days after filing with the Secretary of State.)

Section 1. Authority. This regulation is promulgated by authority of W.S. §23-1-101, §23-1-302 and §23-2-107.

Section 2. Regulation. The Wyoming Game and Fish Commission hereby adopts the following regulation governing wild bison recreational hunting seasons. This regulation shall remain in effect until modified or repealed by the Commission.

Section 3. Definitions. For the purpose of this regulation, definitions shall be as set forth in Title 23, Wyoming Statutes, and the Commission also adopts the following definitions:

(a) "Edible portions" means the meat of the front quarters as far down as the knees, the meat of the hindquarters as far down as the hocks, and the meat along the backbone between the neck and hindquarters, excluding meat on the ribs and neck.

(b) "Harvest year" means seven (7) consecutive months beginning July 1 of each year through January 31 of the next succeeding year.

Section 4. Recreational Hunting Season Established And Effective Date. When the Department determines a wild bison recreational hunting opportunity exists, there shall be a wild bison recreational hunting season as established in accordance with Section 5 through Section 19 of this regulation.

Section 5. Supervision Of Licensees. Wild bison recreational hunting may be done under the supervision and/or direction of Department personnel.

Section 6. Notification.

(a) Applicants shall be notified of their placement on the wild bison priority list by the Department. Applicants on the wild bison priority list shall also be notified by mail or by telephone, in order of their priority ranking, when the Department determines a wild bison recreational hunting opportunity may exist. If telephone calls are made, the calls shall be done by the Department during the evening hours between 4:00 p.m. and 10:00 p.m. If telephone calls are made, a minimum of three (3) attempts by telephone shall be made that evening and the subsequent evening to contact and notify any individual applicant. Notification attempts shall be spaced at a minimum of one-half (½) hour intervals. Department personnel must speak directly to the applicant during the notification call. If an individual cannot be contacted, the next individual on the list shall be notified and those who could not be contacted shall retain their positions on the wild bison priority list. An applicant who declines participation shall have his/her name removed from the wild bison priority list.

(b) At the Department's discretion, notification may be made by mail. The Department shall send notification by first-class mail to the address listed on the applicant's application for placement on the wild bison

priority list. The notification shall be mailed a minimum of ten (10) days immediately prior to the scheduled wild bison hunting season.

(c) Applicants shall be advised during the notification call or by mail of: the date, time, and location of their scheduled wild bison recreational hunt. Applicants contacted may have an opportunity to receive a permit valid for hunting wild bison on the National Elk Refuge.

Section 7. Selection Of Bison To Be Harvested.

(a) Hunters may be directed by Department personnel to take a specific age class of wild bison in a specific geographic portion of a hunt area and during a specific time period in order to meet the Department's management objectives. Numbers and age classes of wild bison available when the applicant arrives may differ from those available at the time of the notification call or letter. Issuance of a wild bison license provides the licensee an opportunity to hunt a wild bison; it does not guarantee that a licensee shall harvest a wild bison.

Section 8. Wild Bison Hunting Orientation Information.

(a) After notification, individuals interested in participating in the wild bison recreational hunting season shall be provided wild bison hunting orientation information in a manner determined by the Department.

Section 9. Firearms. Wild bison shall only be taken with a firearm which has a barrel bore diameter of at least twenty-seven hundredths (27/100) of an inch and is chambered to fire a center-fire cartridge not less than two (2) inches in overall length, including a soft or expanding point bullet seated to a normal depth. The cartridge shall have a minimum of a one hundred fifty (150) grain bullet.

Section 10. Licensing. Licenses shall be issued upon payment by the applicant of the proper license fee at the Department Regional Office administering the wild bison recreational hunt. A license must be purchased prior to participating in the wild bison recreational hunt. A conservation stamp shall be in possession of the licensee while exercising the privileges under the wild bison license.

Section 11. National Elk Refuge Permits. A National Elk Refuge permit is required prior to hunting wild bison on the National Elk Refuge and shall be in possession of the hunter while hunting wild bison on the National Elk Refuge. A limited number of permits shall be available to hunt wild bison on the National Elk Refuge. Permits shall be issued by the Department to licensed wild bison hunters in the order of their placement on the wild bison priority list, and shall be valid only for specified dates.

Section 12. Tagging Requirements. When any wild bison is killed under a license, the licensee shall detach, sign and date the carcass coupon and attach the coupon to the carcass before leaving the site of the kill. The coupon shall remain on the wild bison carcass at all times until the meat undergoes processing, except that during transportation of the carcass the coupon may be removed to prevent its loss. If the coupon is removed for transportation of the carcass, it shall be in the possession of the person accompanying the carcass at all times.

Section 13. Retrieval of Edible Portions. No person shall take and leave, abandon, or allow any wild bison, or edible portion to intentionally or needlessly go to waste. The failure of any person to properly dress and care for any wild bison killed by that person and if the carcass is reasonably accessible, within forty-eight (48) hours to take or transport the carcass to the camp of that person, and to properly care for the carcass is prima facie evidence of a violation of this section.

Section 14. Wearing of Fluorescent Orange Clothing. No person shall hunt any wild bison without wearing in a visible manner one (1) or more exterior garments, which shall include a hat, shirt, jacket, coat, vest or sweater of a fluorescent orange color.

Section 15. Transportation And Exportation.

(a) No person shall ship, transport, or receive for shipment or transportation within Wyoming, any wild bison or part thereof, unless tagged with a Wyoming interstate game tag, or unless:

(i) The transportation is by a person accompanying the carcass of a wild bison who is in possession of a proper coupon.

(b) No wild bison or any part thereof shall be shipped or transported from the State unless accompanied by the licensee who harvested the animal, in possession of a proper coupon, or unless:

(i) The part to be exported from the state is of a nonedible trophy or hidelike nature and properly tagged with a Wyoming interstate game tag.

(ii) The wild bison was legally harvested by a nonresident and is tagged with a Wyoming interstate game tag.

Section 16. Hunting Regulations.

(a) Shooting Hours. Shooting hours for the hunting of wild bison shall be from one-half (1/2) hour before sunrise to one-half (1/2) hour after sunset.

(b) Within three (3) consecutive days (seventy-two (72) hours) after taking a wild bison, the licensee shall contact the Department Regional Office where the licensee obtained his/her wild bison license during business hours to report the harvest. The licensee shall report the date of the harvest, the sex, age class (adult, yearling, calf) of the bison harvested, supply biological samples as required by the Department and provide the legal description of where the bison was harvested to include section, range and township or UTM coordinates.

(c) Bag and Possession Limits. The bag and possession limit for wild bison shall be one (1) wild bison during any one (1) harvest year. No person who is issued a wild bison hunting license and who harvests a wild bison after January 31, 2001, shall be eligible to apply for or receive a hunting license for wild bison in any future year.

(d) Open Areas, Season Dates and Limitations.

**Hunt Area**	**Type**	**Date of Seasons**		**Limitations**
		**Opens**	**Closes**	
1, 2	1	July 1	Jan. 31	Any male Wild Bison; other limitations specified during notification
	2	July 1	Jan. 31	Any female/calf Wild Bison; other limitations specified during notification
2	3	Sept. 1	Nov. 30	Any male Wild Bison; other limitations specified during notification
2	4	Sept. 1	Nov. 30	Any female/calf Wild Bison; other limitations specified during notification

Section 17. Hunt Area Descriptions.

(a) Area and Number.

Area 1. Absaroka. All lands within Park, Hot Springs, Fremont, Sublette and Teton Counties east of the Continental Divide, excluding lands administered by the Wind River Indian Reservation and Yellowstone National Park.

Area 2. Jackson. All lands within Lincoln, Sublette, Fremont and Teton Counties west of the Continental Divide, excluding Grand Teton National Park, John D. Rockefeller Jr. Memorial Parkway and Yellowstone National Park.

Section 18. Violation of Commission Regulations. Failure to abide by the provisions of this regulation shall be punishable as provided by Wyoming statutes for violation of Commission regulations.

Section 19. Savings Clause. If any provision of this regulation shall be held to be illegal or unconstitutional, such a ruling shall not affect other provisions of this regulation which can be given effect without the illegal or unconstitutional provision; and, to this end, the provisions of this regulation shall be severable.

WYOMING GAME AND FISH COMMISSION

By:

Bill Williams, DVM, President

Date: August 14, 2007

BISON 2007 HUNT AREAS

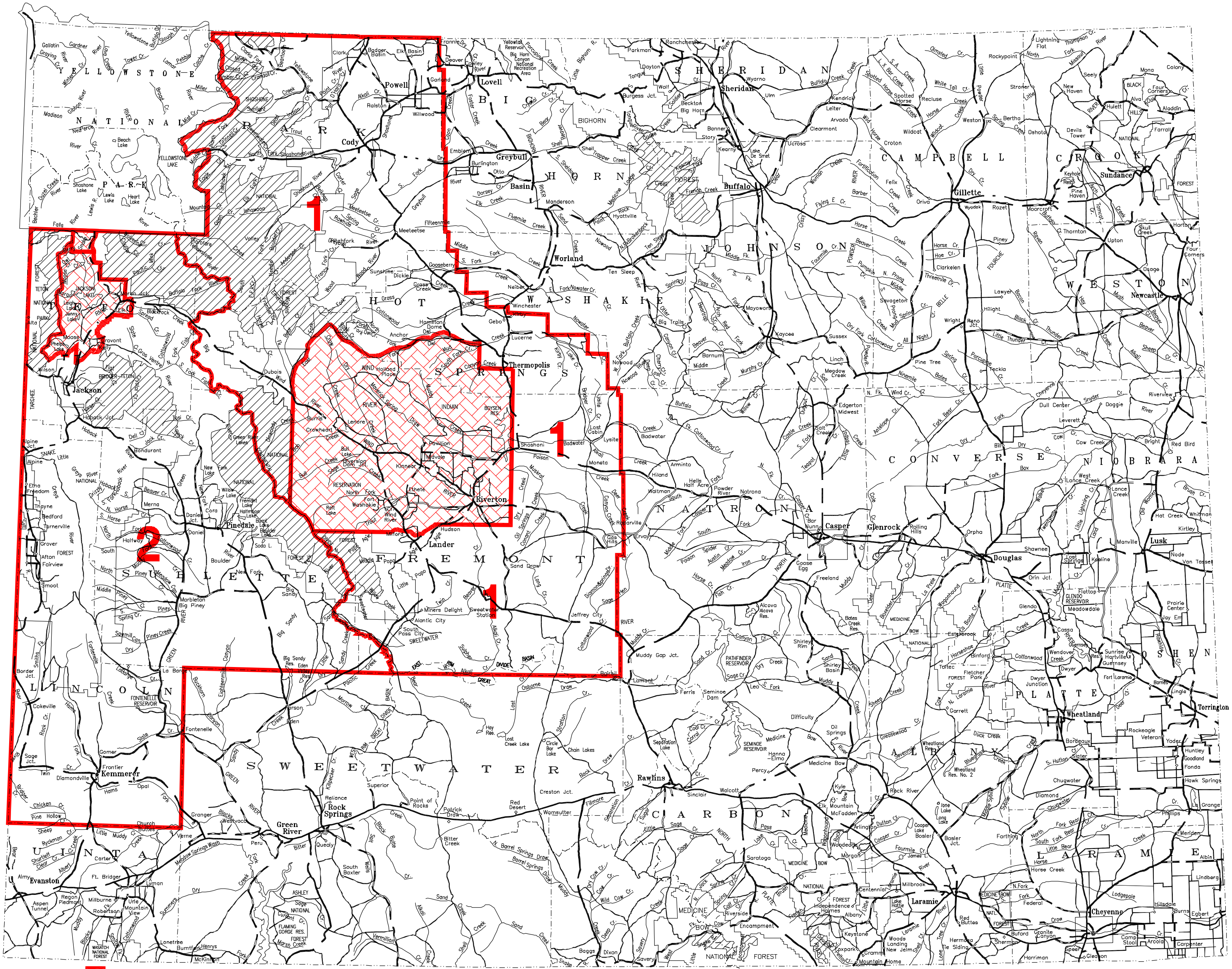

These areas closed to hunting.

THIS MAP IS FOR GENERAL REFERENCE ONLY. Please use the written boundary descriptions in this regulation for detailed boundary information.

Note: Wilderness area, nonresidents must have guides.

TABLE I

BLACK BEAR

HUNTS, RECREATION DAYS, HUNTER DAYS PER BEAR

2007

AREA	SPRING			FALL			TOTAL				Hunter Days/ Bear
	Hunters	Days	Harvest	Hunters	Days	Harvest	Hunters	Hunts	Days	Harvest	
**1. Northwest Bighorn**											
Resident	78	590	7	25	178	1	88	103	768	8	96.0
Nonresident	3	5	1	2	18	0	5	5	23	1	23.0
Total	81	595	8	27	196	1	93	108	791	9	87.9
**2. Burgess Junction**											
Resident	57	328	5	31	235	2	76	88	563	7	80.4
Nonresident *	8	33	3	2	8	3	10	10	41	6	6.8
Total	65	361	8	33	243	5	86	98	604	13	46.5
**3. Paint Rock**											
Resident	22	112	2	12	102	0	29	34	214	2	107.0
Nonresident *	2	8	0	0	0	1	2	2	8	1	8.0
Total	24	120	2	12	102	1	31	36	222	3	74.0
**4. Story**											
Resident	57	269	6	29	269	0	76	86	538	6	89.7
Nonresident	10	44	1	2	8	0	10	12	52	1	52.0
Total	67	313	7	31	277	0	86	98	590	7	84.3
**5. Upper Nowood**											
Resident	12	257	0	2	4	0	12	14	261	0	0.0
Nonresident	0	0	0	0	0	0	0	0	0	0	0.0
Total	12	257	0	2	4	0	12	14	261	0	0.0
**6. Mayoworth**											
Resident	12	251	2	16	75	0	22	28	326	2	163.0
Nonresident *	0	0	2	2	7	0	2	2	7	2	3.5
Total	12	251	4	18	82	0	24	30	333	4	83.3
**7. Laramie Peak**											
Resident	41	228	7	37	314	7	69	78	542	14	38.7
Nonresident	3	7	0	5	33	1	8	8	40	1	40.0
Total	44	235	7	42	347	8	77	86	582	15	38.8
**8. Snowy Range**											
Resident	106	600	10	59	208	4	139	165	808	14	57.7
Nonresident	18	91	5	8	33	3	24	26	124	8	15.5
Total	124	691	15	67	241	7	163	191	932	22	42.4
**9. Sierra Madre**											
Resident	75	677	14	102	800	6	149	177	1477	20	73.9
Nonresident	11	51	5	8	31	1	16	19	82	6	13.7
Total	86	728	19	110	831	7	165	196	1559	26	60.0

*NOTE: Number of hunters estimated from survey data is lower than actual harvest data provided by WY Game and Fish.

TABLE I (Continued)

BLACK BEAR SPRING FALL REPORT

AREA	SPRING Hunters	SPRING Days	SPRING Harvest	FALL Hunters	FALL Days	FALL Harvest	TOTAL Hunters	TOTAL Hunts	TOTAL Days	TOTAL Harvest	Hunter Days/ Bear
**10. Uinta**											
Resident	10	106	0	0	0	0	10	10	106	0	0.0
Nonresident	2	5	0	0	0	0	2	2	5	0	0.0
Total	12	111	0	0	0	0	12	12	111	0	0.0
**11. Smith's Fork**											
Resident	71	637	8	84	663	2	131	155	1300	10	130.0
Nonresident	7	29	0	8	54	1	13	15	83	1	83.0
Total	78	666	8	92	717	3	144	170	1383	11	125.7
**13. Lander**											
Resident	82	830	8	41	406	0	102	123	1236	8	154.5
Nonresident	0	0	0	0	0	0	0	0	0	0	0.0
Total	82	830	8	41	406	0	102	123	1236	8	154.5
**14. South Piney**											
Resident	73	426	6	51	318	4	110	124	744	10	74.4
Nonresident	0	0	0	0	0	0	0	0	0	0	0.0
Total	73	426	6	51	318	4	110	124	744	10	74.4
**15. Piney-Horse Creek**											
Resident	24	177	2	41	324	1	61	65	501	3	167.0
Nonresident	2	7	0	7	42	0	8	9	49	0	0.0
Total	26	184	2	48	366	1	69	74	550	3	183.3
**16. Greys River**											
Resident	31	163	2	65	331	10	82	96	494	12	41.2
Nonresident	15	52	7	5	31	1	20	20	83	8	10.4
Total	46	215	9	70	362	11	102	116	577	20	28.9
**17. Hoback**											
Resident	35	206	5	61	335	5	88	96	541	10	54.1
Nonresident	7	24	1	16	98	2	21	23	122	3	40.7
Total	42	230	6	77	433	7	109	119	663	13	51.0
**18. Fall Creek**											
Resident	29	218	2	22	196	0	45	51	414	2	207.0
Nonresident	20	73	6	7	21	0	23	27	94	6	15.7
Total	49	291	8	29	217	0	68	78	508	8	63.5
**19. Green River**											
Resident	102	641	8	100	796	4	177	202	1437	12	119.8
Nonresident	8	34	5	8	34	0	15	16	68	5	13.6
Total	110	675	13	108	830	4	192	218	1505	17	88.5

TABLE I (Continued)

BLACK BEAR SPRING FALL REPORT

AREA	SPRING Hunters	SPRING Days	SPRING Harvest	FALL Hunters	FALL Days	FALL Harvest	TOTAL Hunters	TOTAL Hunts	TOTAL Days	TOTAL Harvest	Hunter Days/ Bear
**20. Gros Ventre**											
Resident	22	186	9	90	588	4	106	112	774	13	59.5
Nonresident	23	78	4	7	49	3	29	30	127	7	18.1
Total	45	264	13	97	637	7	135	142	901	20	45.1
**21. Conant Basin**											
Resident	25	196	7	22	178	1	41	47	374	8	46.8
Nonresident *	2	3	4	3	18	3	5	5	21	7	3.0
Total	27	199	11	25	196	4	46	52	395	15	26.3
**22. Pacific Creek**											
Resident	12	75	1	33	182	2	39	45	257	3	85.7
Nonresident	0	0	0	2	8	0	2	2	8	0	0.0
Total	12	75	1	35	190	2	41	47	265	3	88.3
**23. Ramshorn**											
Resident	14	104	0	35	218	3	47	49	322	3	107.3
Nonresident	2	10	0	2	8	0	3	4	18	0	0.0
Total	16	114	0	37	226	3	50	53	340	3	113.3
**24. Darby Creek**											
Resident	12	135	0	16	108	1	24	28	243	1	243.0
Nonresident	5	8	1	8	21	4	13	13	29	5	5.8
Total	17	143	1	24	129	5	37	41	272	6	45.3
**25. North Absaroka**											
Resident	104	630	7	98	792	1	153	202	1422	8	177.8
Nonresident	7	44	0	2	7	1	7	9	51	1	51.0
Total	111	674	7	100	799	2	160	211	1473	9	163.7
**26. South Absaroka**											
Resident	45	249	3	75	526	7	100	120	775	10	77.5
Nonresident	8	55	0	5	39	0	13	13	94	0	0.0
Total	53	304	3	80	565	7	113	133	869	10	86.9
**27. Grass Creek**											
Resident	37	210	3	45	330	2	67	82	540	5	108.0
Nonresident	2	2	0	0	0	0	2	2	2	0	0.0
Total	39	212	3	45	330	2	69	84	542	5	108.4
**28. Wind River**											
Resident	22	151	4	24	218	3	37	46	369	7	52.7
Nonresident	0	0	0	7	57	2	7	7	57	2	28.5
Total	22	151	4	31	275	5	44	53	426	9	47.3

*NOTE: Number of hunters estimated from survey data is lower than actual harvest data provided by WY Game and Fish.

TABLE I (Continued)

BLACK BEAR SPRING FALL REPORT

AREA	SPRING Hunters	SPRING Days	SPRING Harvest	FALL Hunters	FALL Days	FALL Harvest	TOTAL Hunters	TOTAL Hunts	TOTAL Days	TOTAL Harvest	Hunter Days/ Bear
**29. Spread Creek**											
Resident	22	265	1	22	106	0	39	44	371	1	371.0
Nonresident	3	26	0	5	23	0	8	8	49	0	0.0
Total	25	291	1	27	129	0	47	52	420	1	420.0
**30. Star Valley**											
Resident	69	551	4	61	412	3	104	130	963	7	137.6
Nonresident	8	23	1	2	8	0	10	10	31	1	31.0
Total	77	574	5	63	420	3	114	140	994	8	124.3
**31. Crowheart Butte**											
Resident	2	20	0	4	35	0	6	6	55	0	0.0
Nonresident	0	0	0	2	3	0	2	2	3	0	0.0
Total	2	20	0	6	38	0	8	8	58	0	0.0
**32. Cody**											
Resident	35	269	3	55	375	4	75	90	644	7	92.0
Nonresident	5	11	0	2	7	0	7	7	18	0	0.0
Total	40	280	3	57	382	4	82	97	662	7	94.6

TABLE II

BLACK BEAR
STATEWIDE TOTALS
2007

	LICENSES SOLD	SPRING Hunters	SPRING Days	SPRING Harvest	FALL Hunters	FALL Days	FALL Harvest	TOTAL Hunters	TOTAL Hunts	TOTAL Days	TOTAL Harvest	Hunter Days/ Bear
Resident	2965	1224	9757	136	1210	9622	77	1981	2434	19379	213	91.0
Nonresident	282	161	723	46	114	666	26	254	275	1389	72	19.3
Total	3247	1385	10480	182	1324	10288	103	2235	2709	20768	285	72.9

TABLE III

BLACK BEAR LICENSE DATA SUMMARY
2007

	LICENSES SOLD	NUMBER SURVEYED	NUMBER USABLE (hunters and nonhunters)	RESPONSE RATE (# USABLE/# SURVEYED)
Resident	2965	2901	1507	51.9%
Nonresident	282	277	173	62.5%
Total	3247	3178	1680	52.9%

BLACK BEAR
HARVEST BY HARVEST TOOL
2007

ARCHERY	FIREARM	MUZZLE LOADER
17.32%	79.78%	2.90%

WYOMING GAME AND FISH COMMISSION

CHAPTER 3

BLACK BEAR HUNTING SEASONS

Section 1. Authority. This regulation is promulgated by authority of W.S. §23-1-302 and W.S. §23-2-104.

Section 2. Definitions. For the purpose of this regulation, definitions shall be as set forth in Title 23, Wyoming Statutes and the Commission also adopts the following definitions:

(a) “Baiting” means placing or utilizing a processed bait or an unprocessed bait as a lure or attractant for the purpose of taking black bear.

(b) “Cub” means any black bear less than one (1) year old.

(c) “Immediately” means following the observation of a grizzly bear using a bait, the person registering the bait, the person placing the bait and/or the person hunting over the bait shall report, without delay, said use to the Wyoming Game and Fish Department and/or the U.S. Fish and Wildlife Service; immediately also means any person taking a grizzly bear shall report, without delay, to the Wyoming Game and Fish Department and/or to the U.S. Fish and Wildlife Service in accordance with Section 12 of this regulation.

(d) “Mortality” means any legal or illegal human caused female black bear death, excluding female black bears taken by the Department, known female natural mortalities, and accidental female black bear deaths.

(e) “Processed bait” means a nontoxic biodegradable substance. A chemical used as an attractant or mask rather than for consumption shall not be considered bait.

(f) “Prohibited bait” means big game animals, small game animals, game birds, game fish, protected animals, protected birds, or parts thereof.

(g) “Unprocessed bait” means livestock or livestock parts that have not been processed for human consumption, or wildlife or wildlife parts that are not otherwise prohibited in Section 2(f). A chemical used as an attractant or mask rather than for consumption shall not be considered bait.

(h) “Water” means permanent rivers, streams, lakes or ponds.

Section 3. Hunting Seasons Established. There shall be open seasons for the hunting of black bear as set forth in the Hunting Regulations (Section 4); Registration, Number, Placement, Removal and Identification of Baits on Federal and State Lands (Section 5); Areas Opened and Areas Closed to Black Bear Baiting; Restrictions on use of Processed and Unprocessed Baits (Section 6); Reporting Use of Bait by a Grizzly Bear (Section 7); Prohibited Bait (Section 8); Area Mortality Quota Limitation (Section 9); Open areas, general and special archery season dates and limitations (Section 10); Special Archery Seasons (Section 11); Reporting Grizzly Bear Mortalities (Section 12); and Hunt Area Descriptions (Section 13) of this Chapter. This regulation shall remain in effect until modified or repealed by the Commission.

Section 4. Hunting Regulations.

(a) Open Hours for the Taking of Black Bears. Black bears shall only be taken from one-half (1/2) hour before sunrise to one-half (1/2) hour after sunset.

(b) Bag and Possession Limits. Bag and possession limits for black bear shall be one (1) black bear during any one (1) calendar year (January 1 - December 31). Hunters may take any black bear except cubs and female black bears with cubs at side.

(c) Registering Kills. Hunters taking a black bear shall retain the pelt and skull from each black bear taken for registration purposes. Even if the skull is damaged, it shall accompany the pelt. Visible external evidence of sex shall remain naturally attached to the pelt. Within three (3) days, (seventy-two (72) hours), after taking a black bear, the licensee shall present the pelt and skull to a district game warden, district wildlife biologist, or Department personnel at a Game and Fish Department Regional Office during business hours for registration. The pelt and skull shall be presented in an unfrozen condition in order to allow collection of two (2) premolar teeth to be utilized to determine the age of the black bear and to allow examination of the pelt to determine the sex of the black bear. At the time of registration, the licensee shall furnish the Department the location of the site of kill to include section, township and range, or UTM coordinates.

(i) Any person who makes a false statement on the registration form regarding the date the black bear was taken or the hunt area in which it was taken shall be in violation of this regulation. Such violation shall be punishable as provided by Title 23, Wyoming statutes for violation of Commission regulations.

Section 5. Registration, Number, Placement, Removal and Identification of Baits on Federal and State Lands. The provisions in this section shall only apply to the registration, number, placement, removal and identification of baits on federal lands and state lands, including lands owned and/or administered by the Wyoming Game and Fish Commission.

(a) Registration of bait site(s).

(i) A person may register the same bait site(s) the person registered in the previous calendar year by telephone, mail, facsimile, in person, or by a representative. A person who registers a bait site(s) for the first time shall only register in person or by a representative. A bait site shall not be transferred to another person in the same calendar year in which the bait site was registered. Bait sites that are not renewed during the preference period shall be available for registration beginning April 1.

(ii) Prior to placement of bait(s), the bait site(s) registration shall be approved by the Department.

(A) To register a bait site(s), the person or representative registering the bait site(s) shall provide all information that is required on the bait site registration form. Any person who makes a false statement on the bait site registration form shall be in violation of this regulation. Such violation shall be punishable as provided by Title 23, Wyoming statutes for violation of Commission regulations.

(B) The Department shall review the information provided on the bait site(s) registration form. If the Department approves the bait site registration, the applicant shall be furnished a copy indicating the site has been approved. The registration form shall include the name, address, telephone number and the number of the valid Wyoming Black Bear Hunting License of the person desiring to register the bait; and, the name of the person registering the bait site(s) if the registration is by a representative other than a licensed outfitter. In the case of a licensed outfitter who desires to utilize the bait for outfitting black bear hunters, the outfitter's license number shall be furnished in lieu of the number of the valid black bear hunting license. The registration form shall include the legal description of the bait site location to include the range, township, section and quarter section, or UTM coordinates. A registration form which has not been completed with all required information shall not be considered a valid registration and shall be denied by the Department.

(iii) Registrations for bait site(s) in black bear hunt areas shall be presented to the Regional Wildlife Supervisor or his designee as follows: See Section 6 for areas closed to black bear baiting and restrictions on the use of bait.

Regional Offices	Black Bear Hunt Areas
Casper	7
Cody	1, 3, 5, 27
Green River	9, 10, 11, 19
Jackson	14, 15, 16, 17, 18, 19, 20, 21, 24, 29, 30
Lander	13, 23, 28, 31
Laramie	7, 8, 9
Pinedale	14, 15, 16, 17, 18, 19, 20, 21, 24, 29, 30
Sheridan	2, 4, 6
	Areas closed to baiting, See Section 6

(iv) Persons who had a bait site(s) registered the previous calendar year shall have a preference period during March 1 through March 20 to register the same bait site(s). Applications to register a bait site(s) shall not be accepted by the Department prior to 8:00 a.m. March 1, or 8:00 a.m. of the first business day following March 1 if March 1 occurs on a day when the Regional Game and Fish Department Office is closed. After the preference period ending March 20, applications will be accepted only if the bait site(s) location is still available beginning April 1. Following the preference period, first time registration of a bait site(s) shall be accepted in person on a first come, first served basis. A person cannot change the bait site(s) location in the specific hunt area or any other hunt area during the calendar year for which the bait site(s) was approved by the Department.

(b) Number of black bear baits and bait density. No hunter and/or licensed outfitter shall register and/or place more than two (2) baits at any one time during the calendar year except as provided in this subsection. A person licensed in Wyoming in the current year as an outfitter shall place no more than two (2) baits per black bear hunting client at any one time during the calendar year. Bait density shall be no more than one (1) bait per section as illustrated on U.S. Forest Service or Bureau of Land Management maps.

(c) Placement and removal of baits. No person shall place a bait or hunt over a bait that is not enclosed in a rigid container(s) of wood, metal or plastic not to exceed a combined total of eight (8) cubic feet, the approximate size of a fifty-five (55) gallon drum. Holes in containers shall be smooth and not constructed to function as a trap. No person shall place a bait or hunt over a bait that has not been registered with the Department in accordance with this regulation. No person shall place a bait or hunt over a bait that is less than two-hundred (200) yards from water; less than two-hundred (200) yards from roads and/or pack trails; nor less than one-half (1/2) mile from a developed campground, picnic ground, and/or building. Upon request, persons placing bait(s) shall be required to escort the Regional Wildlife Supervisor or his designee to the bait site(s). No person shall place a bait within a hunt area more than seven (7) days prior to the opening day of the black bear special archery season as listed in Section 10 of this regulation. No person who has registered and/or placed a bait shall fail to remove all containers and bait materials within seven (7) days after the last date of the black bear hunting season within the hunt area in which the bait has been placed, as listed in Section 10 of this regulation. Baits placed in the spring season, including all containers and bait materials, shall be removed within seven (7) days after the close of the spring season. Baits placed in the fall season, including all containers and bait materials, shall be removed within seven (7) days after the close of the fall season. Burial of the bait shall be specifically prohibited and shall not constitute removal of the bait.

(d) Identification of bait site(s). The name and address of the hunter and/or the licensed outfitter registering the bait site(s) shall be legible and permanently affixed to or painted on the outside of the container(s) enclosing the bait.

Section 6. Areas Opened and Areas Closed to Black Bear Baiting; Restrictions on Use of Processed and Unprocessed Baits.

(a) All black bear hunt areas shall be open to the baiting of black bear except those hunt areas and those portions of hunt areas listed in this section.

(i) Black Bear Hunt Areas 22, 25, 26, 32 and that portion of Black Bear Hunt Area 23 on U.S. Forest Service, Bureau of Land Management, and State lands, including lands administered by the Wyoming Game and Fish Commission, shall be closed.

(ii) That portion of Black Bear Hunt Area 27 north and west of the Wood River on U.S. Forest Service, Bureau of Land Management, and State lands including lands administered by the Wyoming Game and Fish Commission, shall be closed.

(iii) That portion of Black Bear Hunt Area 28 on the Shoshone National Forest north of Pelham Lake, Pelham Lake Creek and Lava Mountain shall be closed.

(iv) That portion of Black Bear Hunt Area 7 on the Pole Mountain Unit of the Medicine Bow National Forest shall be closed.

(v) All designated Wilderness Areas in National Forests shall be closed. Hunters should check maps of designated Wilderness Areas at the Wyoming Game and Fish Department's Headquarters and/or Regional Offices.

(vi) The designated recovery area for grizzly bears shall be closed. Hunters should check maps of the recovery area for grizzly bears at the Wyoming Game and Fish Department's Headquarters and/or Regional Offices, or refer to the Wyoming Game and Fish Department's website at http://gf.state.wy.us.

(vii) Areas administered by the U.S. Forest Service, Bureau of Land Management and/or Wyoming Game and Fish Commission in which human access has been restricted to protect wintering wildlife shall be closed. Such areas, if not already closed by this section, shall be closed to black bear baiting during the time period for which the areas have been closed to human access.

(b) Only unprocessed baits shall be utilized for the baiting of black bears in the following black bear hunt areas and in accordance with the provisions of this regulation.

(i) Black Bear Hunt Area 18 and those portions of Hunt Areas 19, 20, 21, 23, 24, 27, 28 and 29 in which bear baiting is not excluded by Section 6 (a).

(c) Processed or unprocessed baits shall be utilized for the baiting of black bears in the following black bear hunt areas, in accordance with the provisions of this regulation.

(i) Black Bear Hunt Areas 1, 2, 3, 4, 5, 6, 7, 8, 9, 10, 11, 13, 14, 15, 16, 17, 30, 31.

Section 7. Reporting Use of Bait by a Grizzly Bear. Any person registering a bait site, placing a bait, and/or hunting at a bait site shall immediately report use of the bait by a grizzly bear to the Wyoming Game and Fish Department (1-800-442-4331) and/or the U. S. Fish and Wildlife Service. Regardless of the land status albeit federal, State, or private, no person shall hunt black bear over such a bait site for the remainder of the calendar year. The bait site(s) shall be closed for the remainder of the calendar year and the bait shall be removed as soon as possible by personnel of the Wyoming Game and Fish Department and/or U.S. Fish and Wildlife Service.

Section 8. Prohibited Bait. No person shall place any prohibited bait for the purpose of baiting black bears. Baiting for black bear with a substance other than defined as a bait in Section 2 or in a manner other than as specified in Section 5 shall be prohibited on Federal or State lands, including lands administered by the Wyoming Game and Fish Commission. Chemicals used as an attractant or mask rather than for consumption, shall not be considered bait and shall not be prohibited.

Section 9. Area Mortality Quota Limitation. The black bear hunting season shall close in each black bear hunt area or management unit when the female mortality quota specified for the respective hunt area or management unit has been reached. If the female mortality quota is not reached, the season shall close upon the date specified in Section 10. If the female mortality quotas for the spring hunting season are exceeded, the additional mortalities

shall be subtracted from the fall mortality quotas. Conversely, if female mortality quotas in the spring have not been reached, the portion of the quota remaining shall be added to the fall mortality quota. It is the hunter's responsibility to confirm that the hunt area the person intends to hunt is open. The status of hunt area closures shall be available twenty-four (24) hours a day by calling toll free **1-800-264-1280.**

Section 10. Open Areas, General and Special Archery Season Dates and Limitations.

Black Bear Management Unit	Hunt Area	Season Dates Archery	General	Annual Female Mortality Quota
Bighorns	1-5	May 1-14<br>Sept. 1-14	May 15-June 15<br>Sept. 15-Oct. 31	10 Spring<br>5 Fall
	6		May 1-June 15<br>Sept. 1-Oct. 31	1 Spring<br>1 Fall
Laramie Peak	7	April 15-30<br>Aug. 15-31	May 1-June 7<br>Sept. 1-Oct. 31	4 Spring<br>3 Fall
Snowy Range	8	April 15-30<br>Sept. 1-30	May 1-June 7<br>Oct. 1-31	4 Spring<br>3 Fall
Sierra Madres	9	April 15-30<br>Sept. 1-30	May 1-June 15<br>Oct. 1-31	4 Spring<br>3 Fall
Uinta	10	April 15-30<br>Sept. 1-30	May 1-June 15<br>Oct. 1-31	1 Spring<br>1 Fall
Greys River	11	April 1-30<br>Aug. 15-31	May 1-June 15<br>Sept. 1-Nov. 15	3 Spring<br>3 Fall
	14,15,16,17,30	April 15-30<br>Aug. 15-31	May 1-June 15<br>Sept. 1-Nov. 15	14 Spring<br>10 Fall
Wind River	13	April 15-30<br>Aug. 15-31	May 1-June 15<br>Aug. 1-31, valid only in that portion of Area 13 within the Popo Agie Wilderness<br>Sept. 1-Oct. 31	3 Spring<br>2 Fall
	19	April 15-30<br>Aug. 15-31	May 1-June 15<br>Sept. 1-Nov. 15	6 Spring<br>4 Fall
	28	April 15-30<br>Aug. 1-14	May 1-June 15<br>Aug. 15-Oct. 31	3 Spring<br>3 Fall
	31	April 15-30<br>Aug. 1-14	May 1-June 15<br>Aug. 15-Oct. 31	2 Spring<br>2 Fall
Jackson	18,20,21,22,<br>24,29	April 15-30<br>Aug. 15-31	May 1-June 15<br>Sept. 1-Nov. 15	11 Spring<br>9 Fall
Absaroka	23	April 15-30<br>Aug. 1-14	May 1-June 15<br>Aug. 15-Oct. 31	8 Spring*<br>10 Fall*
	25,26,27	April 15-30<br>Aug. 15-31	May 1-June 15<br>Sept. 1-Oct. 31	
	32	April 15-30	May 1-June 15<br>Aug. 1-Oct. 31	2 Spring<br>3 Fall

***inclusive to areas 23, 25, 26, and 27**

Section 11. Special Archery Seasons.

(a) There shall be special archery seasons for the hunting of black bear by the use of legal archery equipment in areas as set forth in Section 10 of this Chapter.

Section 12. Reporting Grizzly Bear Mortalities. Any taking of a grizzly bear shall be reported immediately to the Wyoming Game and Fish Department, 1-800-442-4331, and/or to the U.S. Fish and Wildlife Service, Division of Law Enforcement, (307) 261-6365, or (307) 332-7607, or (307) 587-5349.

Section 13. Hunt Area Descriptions.

(a) Area and Number.

Area 1. Northwest Bighorn. Beginning where U.S. Highway 310 crosses the Wyoming-Montana state line; east along said line to the Bighorn Mountain divide; southeasterly along said divide to U.S. Highway 14 at Granite Pass; southerly then westerly along said highway to U. S. Highway 14-16-20; westerly along said highway to U.S. Highway 310; northwesterly along said highway to the Wyoming-Montana state line.

Area 2. Burgess Junction. Beginning where Interstate Highway 90 crosses the Wyoming-Montana state line; southeasterly along said highway to U.S. Highway 14 at the town of Ranchester; southwesterly along said highway to the Big Horn Mountain Divide at Granite Pass; northwesterly along said divide to the Wyoming-Montana state line; easterly along said line to Interstate Highway 90.

Area 3. Paint Rock. Beginning at the town of Greybull; easterly and northerly along U.S. Highway 14 to the Bighorn Mountain Divide; southerly along said divide to Powder River Pass and U.S. Highway 16; westerly along said highway to the Canyon Creek Road (U.S.F.S. Road 25); southerly along said road to the Gold Mine Road (U.S.F.S. Road 452); southerly along said road to the Big Horn Mountain Divide Road/Hazelton Road (Johnson County Road 3); southerly along said road to the Rome Hill Road (Johnson County Road 3A-Washakie County Road 56); westerly along said road to U.S. Highway 16; westerly along said highway to U.S. Highway 20; westerly along said highway to Wyoming Highway 433; northerly along said highway to U.S. Highway 16-20; northerly along said highway to the town of Greybull.

Area 4. Story. Beginning at Interstate Highway 90 at the town of Ranchester; southeasterly along said highway to Interstate Highway 25; southerly along said highway to the Trabing Road (Johnson County Road 13); westerly along said road to Wyoming Highway 196; southerly along said highway to the Crazy Woman Canyon Road (Johnson County Road 14); westerly along said road to U.S. Highway 16; westerly along said highway to the Bighorn Mountain Divide at Powder River Pass; northwesterly along said divide to U.S. Highway 14 at Granite Pass; northeasterly along said highway to Interstate Highway 90 at the town of Ranchester.

Area 5. Upper Nowood. Beginning at the town of Ten Sleep; easterly along U.S. Highway 16 to the Rome Hill Road (Washakie County Road 56-Johnson County Road 3A); easterly along said road to the Big Horn Mountain Divide Road/Hazelton Road (Johnson County Road 3-Washakie County Road 81-Natrona County Road 109); southerly along said road to the Buffalo Creek Road (Natrona County Road 105); southwesterly along said road to the Arminto Road (Natrona County Road 104); southerly along said road to Waltman and U.S. Highway 20-26; westerly along said highway to the town of Shoshoni; northerly along U.S. Highway 20 to the eastern boundary of the Wind River Indian Reservation; northerly, then westerly along said boundary to U.S. Highway 20; northerly along said highway to the town of Worland and U.S. Highway 16; easterly along said highway to the town of Ten Sleep.

Area 6. Mayoworth. Beginning at the junction of Interstate Highway 25 and the Trabing Road (Johnson County Road 13); southerly along said highway to the Midwest interchange and the Smoky Gap Road (Natrona County Road 115); westerly along said road to the Dead Horse Road (Natrona County Road 114); southwesterly along said road to the Wild Horse Trail Road (Natrona County Road 113); westerly along said road to the 33 Mile Road (Natrona County Road 110); northwesterly along said road to the Buffalo Creek Road (Natrona County Road 105); southwesterly along said road to the Bighorn Mountain Divide Road/Hazelton Road (Natrona County Road

109-Washakie County Road 81-Johnson County Road 3); northerly along said road to the Gold Mine Road (U.S.F.S. Road 452); northerly along said road to the Canyon Creek Road (U.S.F.S. Road 25); northerly along said road to U.S. Highway 16; easterly along said highway to the Crazy Woman Canyon Road (Johnson County Road 14); easterly along said road to Wyoming Highway 196; northerly along said highway to the Trabing Road (Johnson County Road 13); easterly along said road to Interstate Highway 25.

Area 7. Laramie Peak. Beginning at the junction of U.S. Highway 30/287 and Wyoming Highway 487 in the town of Medicine Bow; northerly and westerly along Wyoming Highway 487 to Wyoming Highway 220; southwesterly along said highway to the North Platte River; easterly and southerly down said river to Interstate Highway 25 at the City of Douglas; southerly along said highway to the Wyoming-Colorado state line; westerly along said line to U.S. Highway 287; northerly along said highway to U.S. Highway 30/287; northerly and westerly along said highway to Wyoming Highway 487 in the town of Medicine Bow.

Area 8. Snowy Range. Beginning where U.S. Highway 287 crosses the Wyoming-Colorado state line; west along said line to Wyoming Highway 230 west of the North Platte River; northerly along said highway to Wyoming Highway 130; northerly along said highway to U.S. Highway 30; easterly and southerly along said highway to U.S. Highway 287; southerly along said highway to the Wyoming-Colorado state line.

Area 9. Sierra Madre. Beginning where Wyoming Highway 789 crosses the Wyoming-Colorado state line; northerly along said highway to Interstate Highway 80; easterly along said highway to Wyoming Highway 130; southerly along said highway to Wyoming Highway 230; southeasterly along said highway to the Wyoming-Colorado state line; westerly along said line to Wyoming Highway 789.

Area 10. Uinta. Beginning where Interstate Highway 80 crosses the Wyoming-Utah state line; easterly along said highway to Wyoming Highway 414; southerly along said highway through the town of Lonetree to the Hoop Lake-Hole-in-the-Rock Road (Uinta County Road 295); southerly along said road to the Wyoming-Utah state line; westerly then northerly along said line to Interstate Highway 80.

Area 11. Smith's Fork. Beginning where Wyoming Highway 89 crosses the Wyoming-Utah state line west of Sage Junction; northerly along said line to the Wyoming-Idaho state line; northerly along said line to the divide between the Bear River and Salt River; easterly along said divide to the divide between the Salt River and Smith's Fork River; northeasterly along said divide to the divide between the Smith's Fork River and Greys River; southeasterly along said divide to LaBarge Creek; southeasterly down said creek to the Green River; southeasterly down said river to the CCC Bridge and the Lincoln-Sweetwater Road (Lincoln County Road 311); westerly along said road to Wyoming Highway 372; westerly along said highway to U.S. Highway 189; westerly then southerly along said highway to U.S. Highway 30; westerly along said highway to Wyoming Highway 89 at Sage Junction; westerly along said highway to the Wyoming-Utah state line.

Area 13. Lander. Beginning where U.S. Highway 287 crosses the south boundary of the Wind River Indian Reservation; southerly and easterly along said highway to the Sweetwater River; westerly up said river to Wyoming Highway 28; southwesterly along said highway to the Continental Divide; northwesterly along said divide to the south boundary of the Wind River Indian Reservation; easterly along said boundary to U.S. Highway 287.

Area 14. South Piney. Beginning where LaBarge Creek enters the Green River; westerly up said creek to the divide between the Green River and Greys River; northerly along said divide to the South Fork of South Cottonwood Creek; easterly down said creek to South Cottonwood Creek; easterly down said creek to Cottonwood Creek; easterly down said creek to the Green River; southerly down said river to LaBarge Creek.

Area 15. Piney-Horse Creek. Beginning where the Mesa Road (Sublette County Road 23-110) joins Wyoming Highway 351; westerly along said highway to the Green River; northerly up said river to Cottonwood Creek; westerly up said creek to South Cottonwood Creek; westerly up said creek to the South Fork of South Cottonwood Creek; westerly up said creek to the divide between the Green River and Greys River; northerly along said divide to the divide between the Green River and the Hoback River (Hoback Rim); easterly along said divide to U.S. Highway 191; southerly along said highway to the Mesa Road (Sublette County Road 23-110) at Cora Junction; southerly along said road to Wyoming Highway 351.

Area 16. Greys River. All of the drainages of Greys River, Little Greys River and the drainages south of the Snake River from Alpine east to and including Bailey Creek.

Area 17. Hoback. Beginning where U.S. Highway 191 crosses the divide between the Green River and the Hoback River (Hoback Rim); westerly along said divide to the divide between the Greys River and the Hoback River; northerly along said divide to the divide between the Greys River and Willow Creek; northerly along said divide to the divide between Bailey Creek and Willow Creek (Greyback Ridge); northerly along said divide to the divide between Bailey Creek and the Snake River; westerly along said divide to the Snake River; northerly up said river to the Hoback River; southeasterly up said river to Highway 191 at Bondurant; southerly and easterly along said highway to the divide between the Green River and the Hoback River (Hoback Rim).

Area 18. Fall Creek. Beginning where Wyoming Highway 22 crosses the Snake River; southerly and westerly down said river to the Wyoming-Idaho state line; northerly along said line to Wyoming Highway 22; easterly along said highway to the Snake River.

Area 19. Green River. Beginning at the town of Farson; northerly along U.S. Highway 191 to the divide between the Green River and the Hoback River (Hoback Rim); northerly along said divide to the divide between the Green River and the Gros Ventre River at Hodges Peak; northeasterly along said divide to the Darwin Ranch road (U.S.F.S. Road 620); easterly along said road to the Union Pass Road (U.S.F.S. Road 600); northeasterly along said road to the Continental Divide; southerly along said divide to U.S. Highway 28; southwesterly along said highway to the town of Farson.

Area 20. Gros Ventre. Beginning at the southeast corner boundary of the National Elk Refuge near the town of Jackson; northerly along the refuge boundary to the Gros Ventre River; easterly up said river to Kinky Creek; easterly up said creek to the Darwin Ranch Road (U.S.F.S. Road 620); easterly along said road to the divide between the Green River and the Gros Ventre River; southwesterly along said divide to the divide between the Green River and the Hoback River at Hodges Peak; southerly along said divide to Highway 191 at Hoback Rim; northerly and westerly along said highway to the Hoback River at Bondurant; westerly down said river to the Snake River; northerly along said river to Wyoming Highway 22; easterly along said highway to Highway 26; northerly along said highway to the National Elk Refuge boundary; easterly along said boundary to the southeast corner of the National Elk Refuge.

Area 21. Conant Basin. Beginning where U.S. Highway 26-287 intersects the south boundary of John D. Rockefeller Memorial Parkway; westerly along said boundary to the Targhee National Forest boundary; southerly along said boundary to the head of the north fork of Teton Creek; westerly down said creek to the Wyoming-Idaho state line; northerly along said line to the Yellowstone National Park boundary; easterly along said boundary to U.S. Highway 26-287; southerly along said highway to the south boundary of John D. Rockefeller Memorial Parkway.

Area 22. Pacific Creek. Beginning where U.S. Highway 26-287 intersects the Grand Teton National Park boundary; northwesterly along said boundary to the John D. Rockefeller Memorial Parkway boundary; westerly along said boundary to U.S. Highway 26-287; northerly along said highway to the Yellowstone National Park boundary; easterly along said boundary to the Continental Divide; southeasterly along said divide to U.S. Highway 26-287; westerly along said highway to the Grand Teton National Park boundary.

Area 23. Ramshorn. All of the drainage of the Wind River that lies north of U.S. Highway 287 and west of the Wind River Indian Reservation and those areas of the Spence and Moriarity Wildlife Management Area east of the East Fork of the Wind River.

Area 24. Darby Creek. Beginning at the town of Jackson; westerly along Wyoming Highway 22 to the Wyoming-Idaho state line; northerly along said line to Teton Creek; easterly along said creek to the north fork to Teton Creek; northeasterly along said creek to the Grand Teton National Park boundary; southerly and easterly along said boundary to the Snake River; northerly and southerly along said park boundary to U.S. Highway 189; southerly along said highway to Wyoming Highway 22.

Area 25. North Absaroka. Beginning where the east boundary of Yellowstone National Park crosses the Wyoming-Montana state line; easterly along said line to the Shoshone National Forest boundary; southerly along

said boundary to U.S. Highway 14-16-20; westerly along said highway to the east boundary of Yellowstone National Park; northerly along said boundary to the Wyoming-Montana state line.

Area 26. South Absaroka. Beginning where the east boundary of Yellowstone National Park crosses U.S. Highway 14-16-20; easterly along said highway to the Shoshone National Forest boundary; southerly along said boundary to the Greybull River; westerly then southerly up said river to the divide between the Greybull River and the Wind River; westerly along said divide to the divide between the South Fork of the Shoshone River and the Wind River; westerly along said divide to the Continental Divide; northwesterly along said divide to the south boundary of Yellowstone National Park; easterly then northerly along said boundary to U.S. Highway 14-16-20.

Area 27. Grass Creek. Beginning where U.S. Highway 120 crosses the Greybull River; southeasterly along said highway to U. S. Highway 20; southerly along said highway to the northern boundary of the Wind River Indian Reservation; westerly along said boundary to the divide between the Wood River and the Wind River; northwesterly along said divide to the divide between the Greybull River and the Wind River; northwesterly along said divide to the Greybull River; northerly then easterly down said river to U.S. Highway 120; and all non-Indian fee title lands in that portion of Hot Springs County within the exterior boundaries of the Wind River Indian Reservation.

Area 28. Wind River. Beginning where U.S. Highway 287 crosses the west boundary of the Wind River Indian Reservation; southerly along said boundary to the Continental Divide; northwesterly along said divide to U.S. Highway 287 at Togwotee Pass; southeasterly along said highway to the Wind River Indian Reservation boundary.

Area 29. Spread Creek. Beginning where the Grand Teton National Park boundary crosses the Gros Ventre River; northerly along said boundary to U.S. Highway 26-287; easterly along said highway to Togwotee Pass and the Continental Divide; southerly along said divide to the Union Pass Road (U.S.F.S. Road 600); southwesterly along said road to the Darwin Ranch Road (U.S.F.S. Road 620); westerly along said road to Kinky Creek; westerly down said creek to the Gros Ventre River; westerly down said river to the Grand Teton National Park boundary.

Area 30. Star Valley. All of the drainage of the Salt River east of the Wyoming-Idaho state line.

Area 31. Crowheart Butte. All non-Indian owned fee title lands within the exterior boundaries of the Wind River Indian Reservation, and all lands within the Bureau of Reclamation's Riverton and Boysen Unit boundaries, and those lands within Boysen State Park south of Cottonwood Creek, west of Boysen Reservoir, and south of U.S. Highway 20-26; excluding those areas of the Spence and Moriarity Wildlife Management Area that lie east of the East Fork of the Wind River, and those lands within Hot Springs County.

Area 32. Cody. Beginning where the Shoshone National Forest boundary meets the Wyoming-Montana state line; easterly along said line to U.S. Highway 310; southeasterly along said highway to U.S. Highway 14-16-20; easterly along said highway to U.S. Highway 16-20; southerly along said highway to Wyoming Highway 433; southerly along said highway to U.S. Highway 20; southerly along said highway to U.S. Highway 120; northwesterly along said highway to the Greybull River; southwesterly up said river to the Shoshone National Forest Boundary; northerly along said boundary to the Wyoming-Montana state line.

WYOMING GAME AND FISH COMMISSION

BY: ____________________________________

Ron Lovercheck, President

Dated: January 26, 2007

BLACK BEAR HUNTING AREAS

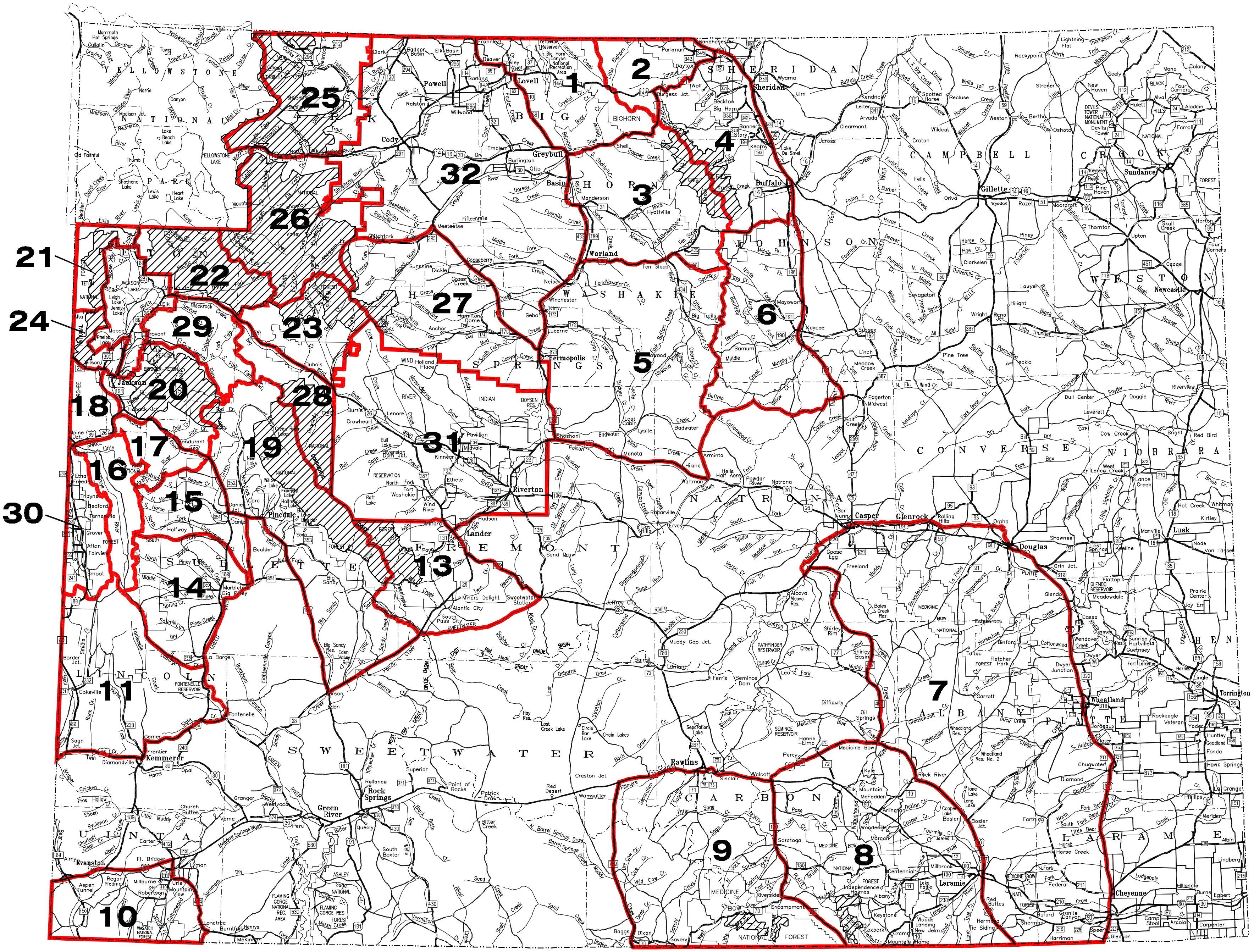

This map is for general reference only.

Note: [hatched box] Wilderness area, nonresidents must have guides.

TABLE I

MOUNTAIN LION

HUNTERS, HUNTER DAYS, HARVEST, DAYS PER HARVEST, AND HUNTER SUCCESS RATE

HUNT SEASON: SEPTEMBER 1, 2007–MARCH 31, 2008

AREA	Hunters	Hunter Days	Harvest	Days/ Harvest	Hunter Success
**1. Black Hills**					
Resident	123	828	9	92.0	7.3%
Nonresident	6	18	3	6.0	50.0%
Total	129	846	12	70.5	9.3%
**2. Teton**					
Resident	23	262	1	262.0	4.3%
Nonresident	1	5	1	5.0	100.0%
Total	24	267	2	133.5	8.3%
**3. Bridger**					
Resident	40	268	3	89.3	7.5%
Nonresident	15	35	6	5.8	40.0%
Total	55	303	9	33.7	16.4%
**4. Popo Agie**					
Resident	19	122	4	30.5	21.1%
Nonresident*	1	9	3	3.0	—
Total	20	131	7	18.7	35.0%
**5. Iron Mountain**					
Resident	35	322	4	80.5	11.4%
Nonresident	1	7	0	0.0	0.0%
Total	36	329	4	82.3	11.1%
**6. Laramie Peak**					
Resident	91	585	10	58.5	11.0%
Nonresident	1	5	0	0.0	0.0%
Total	92	590	10	59.0	10.9%
**7. South Snowy Range**					
Resident	61	333	4	83.3	6.6%
Nonresident	6	15	3	5.0	50.0%
Total	67	348	7	49.7	10.4%
**8. Seminoe**					
Resident	68	308	6	51.3	8.8%
Nonresident	0	0	0	0.0	0.0%
Total	68	308	6	51.3	8.8%
**9. Sierra Madre**					
Resident	19	222	2	111.0	10.5%
Nonresident*	2	7	4	1.8	—
Total	21	229	6	38.2	28.6%

*NOTE: Number of hunters estimated from survey data is lower than actual harvest data provided by WY Game and Fish.

MOUNTAIN LION HUNT SEASON: SEPTEMBER 1, 2007–MARCH 31, 2008

AREA	Hunters	Hunter Days	Harvest	Days/ Harvest	Hunter Success
**10. Haystacks**					
Resident	17	137	5	27.4	29.4%
Nonresident	0	0	0	0.0	0.0%
Total	17	137	5	27.4	29.4%
**11. Red Desert**					
Resident	1	10	0	0.0	0.0%
Nonresident	0	0	0	0.0	0.0%
Total	1	10	0	0.0	0.0%
**12. Flaming Gorge**					
Resident	47	455	3	151.7	6.4%
Nonresident	0	0	0	0.0	0.0%
Total	47	455	3	151.7	6.4%
**13. Wasatch**					
Resident	21	156	2	78.0	9.5%
Nonresident	0	0	0	0.0	0.0%
Total	21	156	2	78.0	9.5%
**14. Lincoln**					
Resident	42	507	7	72.4	16.7%
Nonresident*	4	5	8	0.6	0.0%
Total	46	512	15	34.1	32.6%
**15. Kaycee**					
Resident	106	505	22	23.0	20.8%
Nonresident*	1	1	3	0.3	—
Total	107	506	25	20.2	23.4%
**16. Gas Hills**					
Resident	23	167	1	167.0	4.3%
Nonresident	0	0	0	0.0	0.0%
Total	23	167	1	167.0	4.3%
**17. Piney**					
Resident	47	334	2	167.0	4.3%
Nonresident	1	7	0	0.0	0.0%
Total	48	341	2	170.5	4.2%
**18. Wind River**					
Resident	17	120	2	60.0	11.8%
Nonresident	0	0	0	0.0	0.0%
Total	17	120	2	60.0	11.8%
**19. Northwest**					
Resident	75	806	8	100.8	10.7%
Nonresident	29	125	2	62.5	6.9%
Total	104	931	10	93.1	9.6%

*NOTE: Number of hunters estimated from survey data is lower than actual harvest data provided by WY Game and Fish.

AREA	Hunters	Hunter Days	Harvest	Days/ Harvest	Hunter Success
**20. Grass Creek**					
Resident	25	173	2	86.5	8.0%
Nonresident*	0	0	1	0.0	—
Total	25	173	3	57.7	12.0%
**21. Shell**					
Resident	54	609	6	101.5	11.1%
Nonresident	7	29	5	5.8	71.4%
Total	61	638	11	58.0	18.0%
**22. Ten Sleep**					
Resident	48	624	5	124.8	10.4%
Nonresident*	1	10	3	3.3	—
Total	49	634	8	79.3	16.3%
**23. Sheridan**					
Resident	77	830	9	92.2	11.7%
Nonresident*	4	16	6	2.7	—
Total	81	846	15	56.4	18.5%
**24. Northeast**					
Resident	36	323	4	80.8	11.1%
Nonresident	0	0	0	0.0	0.0%
Total	36	323	4	80.8	11.1%
**25. Hartville**					
Resident	28	369	1	369.0	3.6%
Nonresident	1	4	0	0.0	0.0%
Total	29	373	1	373.0	3.4%
**26. Greys River**					
Resident	32	389	4	97.3	12.5%
Nonresident	4	12	4	3.0	100.0%
Total	36	401	8	50.1	22.2%
**27. Casper Mountain**					
Resident	25	95	6	15.8	24.0%
Nonresident	1	2	0	0.0	0.0%
Total	26	97	6	16.2	23.1%
**28. Crowheart Butte**					
Resident	2	15	1	15.0	50.0%
Nonresident	0	0	0	0.0	0.0%
Total	2	15	1	15.0	50.0%
**29. Hoback**					
Resident	33	157	2	78.5	6.1%
Nonresident	4	11	0	0.0	0.0%
Total	37	168	2	84.0	5.4%

*NOTE: Number of hunters estimated from survey data is lower than actual harvest data provided by WY Game and Fish.

TABLE I (Continued)

MOUNTAIN LION HUNT SEASON: SEPTEMBER 1, 2007–MARCH 31, 2008

AREA	Hunters	Hunter Days	Harvest	Days/ Harvest	Hunter Success
**30. Sundance**					
Resident	57	456	6	76.0	10.5%
Nonresident	1	5	0	0.0	0.0%
Total	58	461	6	76.8	10.3%
**31. Elk Mountain**					
Resident	15	112	3	37.3	20.0%
Nonresident	4	17	2	8.5	50.0%
Total	19	129	5	25.8	26.3%

*NOTE: Number of hunters estimated from survey data is lower than actual harvest data provided by WY Game and Fish.

TABLE II

MOUNTAIN LION

STATEWIDE TOTALS

HUNT SEASON: SEPTEMBER 1, 2007–MARCH 31, 2008

	LICENSES SOLD	Hunters	Days	Harvest	Hunter Days/ Mt. Lion
Resident	2057	979	10599	144	73.6
Nonresident	126	89	345	54	6.4
Total	2183	1068	10944	198	55.3

TABLE III

MOUNTAIN LION LICENSE DATA SUMMARY

HUNT SEASON: SEPTEMBER 1, 2007–MARCH 31, 2008

	LICENSES SOLD	NUMBER SURVEYED	NUMBER USABLE (hunters and nonhunters)	RESPONSE RATE (# USABLE/# SURVEYED)
Resident	2057	1670	807	48.3%
Nonresident	126	115	73	63.5%
Total	2183	1785	880	49.3%

TABLE IV

MOUNTAIN LION

HARVEST BY HARVEST TOOL

HUNT SEASON: SEPTEMBER 1, 2007–MARCH 31, 2008

ARCHERY	FIREARM	MUZZLE LOADER
15.29%	83.78%	0.94%

WYOMING GAME AND FISH COMMISSION

CHAPTER 42

MOUNTAIN LION HUNTING SEASONS

Section 1. Authority. This regulation is promulgated by authority of W.S. §23-1-302 and W.S. §23-2-101(d).

Section 2. Definitions. For the purpose of this regulation, definitions shall be as set forth in Title 23, Wyoming Statutes and the Commission also adopts the following definitions:

(a) "Biological year" means twelve (12) consecutive months from September 1 to August 31.

(b) "Kitten" means any mountain lion less than one (1) year of age.

(c) "Mortality" means any legal or illegal human caused mountain lion death, excluding mountain lions taken by the Department, known natural mortalities and accidental mountain lion deaths.

Section 3. Hunting Seasons Established. There shall be open seasons for the hunting of mountain lion as set forth in this regulation. This regulation shall remain in effect until modified or repealed by the Commission.

Section 4. Hunting Regulations.

(a) Mountain lions shall only be taken during open seasons, except as otherwise provided by State statute and Commission regulations.

(b) Open Hours for Taking of Mountain Lions. Mountain lions shall only be taken one-half (1/2) hour before sunrise to one-half (1/2) hour after sunset.

(c) Registering Kills. Hunters taking mountain lions shall retain the pelt and skull from each mountain lion taken for registration purposes. Even if the skull is damaged, it shall accompany the pelt. Visible external evidence of sex shall remain naturally attached to the pelt. Within three (3) days (seventy-two (72) hours) after taking a mountain lion, the licensee shall present the pelt and skull to a district game warden, district wildlife biologist, or Department personnel at a Department Regional Office during business hours for registration. The pelt and skull shall be presented in an unfrozen condition in order to allow collection of two (2) premolar teeth to be utilized to determine the age of the mountain lion and to allow examination of the pelt to determine the sex of the mountain lion. At the time of registration, the licensee shall furnish the Department the location of the site of kill to include section, township and range, or UTM coordinates.

(d) Any person who makes a false statement on the registration form regarding the date the mountain lion was taken or the hunt area in which it was taken shall be in violation of this regulation and, such violation shall be punishable as provided by Title 23, Wyoming statutes for violation of Commission regulations.

(e) After a mountain lion has been pursued, treed, cornered or held at bay, a properly licensed person shall immediately harvest or release the mountain lion. No person shall in any manner restrict or hinder the mountain lion's ability to escape for the purpose of allowing a person who was not present at the time the mountain lion was treed to arrive and take the mountain lion.

(f) Use of Dogs. Dogs may be used to take mountain lions during the open season.

(g) Bag and Possession Limits. The bag and possession limit for any person with a proper license shall be one (1) mountain lion during any one (1) calendar year (January 1-December 31). Hunters may take any mountain lion, except kittens and female mountain lions with kittens at side shall not be taken.

(h) Open Areas, Season Dates and Limitations.

**Mountain Lion Management Unit**	**Hunt Area**	**Dates of Seasons**	**Annual Mortality Quota**	**Annual Female Mortality Quota**
Northeast	1	Sept. 1-Mar. 31	16	
	24	Sept. 1-Mar. 31	4	
	30	Sept. 1-Mar. 31	8	
Southeast	5	Sept. 1-Mar. 31	12	
	6	Sept. 1-Mar. 31	12	
	7	Sept. 1-Mar. 31	14	
	8	Sept. 1-Mar. 31	10	
	9	Sept. 1-Mar. 31	7	
	10	Sept. 1-Mar. 31	7	
	16	Sept. 1-Mar. 31	6	
	25	Sept. 1-Mar. 31	3	
	27	Sept. 1-Aug. 31	10	
	31	Sept. 1-Mar. 31	6	
Southwest	11	Sept. 1-Mar. 31	2	
	12	Sept. 1-Mar. 31	6	3
	13	Sept. 1-Mar. 31	3	
North-Central	15	Sept. 1-Aug. 31	25	
	21	Sept. 1-Mar. 31	20	
	22	Sept. 1-Aug. 31	15	
	23	Sept. 1-Mar. 31	18	
West	2	Sept. 1-Mar. 31	7	3
	3	Sept. 1-Mar. 31	12	
	4	Sept. 1-Mar. 31	8	
	14	Sept. 1-Mar. 31	15	
	17	Sept. 1-Mar. 31	9	
	18	Sept. 1-Mar. 31	12	
	19	Sept. 1-Mar. 31	20	
	20	Sept. 1-Mar. 31	4	
	26	Sept. 1-Mar. 31	15	
	28	Sept. 1-Mar. 31	3	
	29	Sept. 1-Mar. 31	9	4

(i) Area Mortality Quota Limitation. The mountain lion season in a hunt area shall close when the mortality quota is reached. If the female mortality quota is not reached, the season shall close upon the date specified in subsection (h) of this section. In areas for which a female quota is listed, the hunt area shall close if the female mortality quota is reached prior to the annual mortality quota being reached. Mortality quotas shall be for fall through spring. It is the hunter's responsibility to confirm that the hunt area he/she intends to hunt is open. The status of hunt area closures is available by calling toll free **1-800-637-0809** twenty-four (24) hours a

day. Any mountain lion mortality documented by Department personnel after the close of the season shall be subtracted from the mortality quota for the next biological year.

Section 5. Archery Regulations. Mountain lions may be taken with archery equipment in all areas as set forth in Section 4 of this Chapter.

Section 6. Hunt Area Descriptions.

(a) Area and Number

Area 1. Black Hills. Beginning where the Wyoming-Montana-South Dakota state lines meet; southerly along the Wyoming-South Dakota state line to U.S. Highway 85; southwesterly along said highway to Wyoming Highway 585; northwesterly along said highway to Interstate Highway 90; westerly along said highway to U.S. Highway 14 at the town of Sundance; northwesterly along said highway to Wyoming Highway 24; northerly along said highway to Wyoming Highway 112 in the town of Hulett; northerly along said highway to the Wyoming-Montana state line; easterly along said line to the Wyoming-South Dakota state line.

Area 2. Teton. Beginning where the Continental Divide crosses the southern boundary of Yellowstone National Park; southerly along said divide to the Union Pass Road (U.S.F.S. Road 600); westerly and southerly along said road to the Darwin Ranch Road (U.S.F.S. Road 620); westerly along said road to the divide between the Green River and the Gros Ventre River; southwesterly along said divide to the divide between the Gros Ventre River and the Hoback River at Hodges Peak; westerly along said divide to the divide between Flat Creek and the Granite Creek; southwesterly along said divide to Cache Peak; northerly along the divide between Flat Creek and Cache Creek at Jackson Peak; westerly along the ridge between Cache Creek and Twin Creeks to the Bridger-Teton National Forest/National Elk Refuge Boundary; southwesterly along the National Elk Refuge boundary to where Flat Creek crosses U. S. Highway 26-89-191 at the north edge of the town of Jackson; southwesterly along said highway to Wyoming Highway 22; westerly along said highway to the Wyoming-Idaho state line; northerly along said line to the south boundary of Yellowstone National Park; easterly along said boundary to the Continental Divide.

Area 3. Bridger. Beginning where the Union Pass Road (U.S.F.S. Road 600) crosses the Continental Divide; southeasterly along said divide to U.S. Highway 28; southwesterly along said highway to U.S. Highway 191; northerly along said highway to the divide between the Green River and the Hoback River (Hoback Rim); northerly along said divide to the divide between the Green River and the Gros Ventre River at Hodges Peak; northeasterly along said divide to the Darwin Ranch Road (U.S.F.S. Road 620); easterly along said road to the Union Pass Road (U.S.F.S. Road 600); northerly and easterly along said road to the Continental Divide.

Area 4. Popo Agie. Beginning where Wyoming Highway 789 crosses the southern boundary of the Wind River Indian Reservation; westerly and southerly along said highway to Wyoming Highway 28; southwesterly along said highway to the Continental Divide; northwesterly along said divide to the south boundary of the Wind River Indian Reservation; easterly along said boundary to Wyoming Highway 789.

Area 5. Iron Mountain. Beginning where Interstate Highway 25 crosses the Wyoming-Colorado state line; westerly along said line to U.S. Highway 287; northerly along said highway to Wyoming Highway 34; northerly and easterly along said highway to Interstate Highway 25; southerly along said highway to the Wyoming-Colorado state line.

Area 6. Laramie Peak. Beginning where Interstate Highway 25 crosses the North Platte River at the city of Douglas; southerly along said highway to Wyoming Highway 34; westerly along said highway to U.S. Highway 287; northerly and westerly along said highway to Wyoming Highway 487; northerly and westerly along said highway to the divide between Spring Creek and Lone Tree Creek at Shirley Rim; northerly and easterly along said divide to the divide between Spring Creek and Bates Creek; northeasterly along said divide to the Bates Creek Road (Natrona County Road 402); easterly along said road to the Squaw Springs Trail; northerly along said road to U.S.F.S. Road 660; easterly along said road to Curry Creek; northwesterly down said creek to Deer Creek; northeasterly down said creek to the confluence of Deer Creek and the North Platte River; easterly down said river to Interstate Highway 25.

Area 7. South Snowy Range. Beginning at the city of Laramie and U.S. Highway 287; southerly along said highway to the Wyoming-Colorado state line; westerly along said line to Wyoming Highway 230 (west of the North Platte River); northerly along said highway to Wyoming Highway 130; easterly along said highway to the city of Laramie.

Area 8. Seminoe. Beginning at the junction of Wyoming Highway 220 and Wyoming Highway 487; southerly along Wyoming Highway 487 to U.S. Highway 287; westerly along said highway to Interstate Highway 80; westerly along said highway to U.S. Highway 287 in the city of Rawlins; northerly along said highway to Wyoming Highway 220; northeasterly along said highway to Wyoming Highway 487.

Area 9. Sierra Madre. Beginning at the junction of Interstate Highway 80 and Wyoming Highway 130; southerly along Wyoming Highway 130 to Wyoming Highway 230; southeasterly along said highway to the Wyoming-Colorado state line; westerly along said line to the Continental Divide; northerly along said divide to the Sage Creek Road (Carbon County Road 401); northerly along said road to Wyoming Highway 71; northerly along said highway to Interstate Highway 80; easterly along said highway to Wyoming Highway 130.

Area 10. Haystacks. Beginning at the junction of Interstate Highway 80 and the Bitter Creek Road (Sweetwater County Road 19); easterly along Interstate Highway 80 to the junction of Wyoming Highway 71; southerly along said highway to the Sage Creek Road (Carbon County Road 401); southerly along said road to the Continental Divide at Middlewood Hill; southerly along said divide to the Wyoming-Colorado state line; westerly along said line to the Cow Creek-Powder Wash Road north of Powder Wash, Colorado; northwesterly along said road to the Bitter Creek Road; northerly along said road to Interstate Highway 80.

Area 11. Red Desert. Beginning at the junction of Wyoming Highway 73 and U.S. Highway 287; southerly along U.S. Highway 287 to Interstate Highway 80; westerly along said highway to the Green River; northerly up said river to Big Sandy River; northeasterly up said

river to U.S. Highway 191; southerly along said highway to Wyoming Highway 28; northeasterly along said highway to the Sweetwater River; northeasterly down said river to U.S. Highway 287; easterly along said highway to the Bison Basin Road; southerly along said road to the Alkali Creek Road (BLM 2317); easterly along said road to the Wamsutter-Crooks Gap Road; southerly along said road to the Bairoil Road (Sweetwater County Road 22); easterly along said road to Wyoming Highway 73; easterly along said highway to U.S. Highway 287.

Area 12. Flaming Gorge. Beginning where Interstate Highway 80 intersects the Bitter Creek Road (Sweetwater County Road 19); southerly along the Bitter Creek Road to the Cow Creek-Powder Wash Road north of Powder Wash, Colorado; southeasterly along said road to the Wyoming-Colorado state line; westerly along said line to the Wyoming-Utah state line; westerly along said line to Flaming Gorge Reservoir and the Green River; northerly up said river to Interstate Highway 80; easterly along said highway to the junction of the Bitter Creek Road.

Area 13. Wasatch. Beginning where Interstate Highway 80 crosses the Green River; southerly down said river to the Wyoming-Utah state line; westerly and northerly along said line to Interstate Highway 80; easterly along said highway to the Green River.

Area 14. Lincoln. Beginning where the Green River crosses Interstate Highway 80; westerly along said highway to the Wyoming-Utah state line; northerly along said line to the Wyoming-Idaho state line; northerly along said line to the divide between the Salt River and Bear River; easterly along said divide to the divide between the Salt River and the Smiths Fork River; northeasterly along said divide to Commissary Ridge; southeasterly along said ridge to the head of LaBarge Creek; southeasterly down said creek to the Green River; southeasterly down said river to Interstate Highway 80.

Area 15. Kaycee. Beginning at the junction of U.S. Highway 16 and Interstate Highway 25 at the town of Buffalo; southerly along Interstate Highway 25 to U.S. Highway 20-26 at the city of Casper; westerly along said highway to the town of Waltman and the Waltman-Arminto Road (Natrona County Road 104); northerly along said road to the Buffalo Creek Road (Natrona County Road 105); northeasterly along said road to the Big Horn Mountain Divide Road/Hazelton Road (Natrona County Road 109-Washakie County Road 81-Johnson County Road 3); northerly along said road to the Gold Mine Road (U.S.F.S. Road 452); northerly along said road to the Canyon Creek Road (U.S.F.S. Road 25); northerly along said road to U.S. Highway 16; northeasterly along said highway to Interstate Highway 25.

Area 16. Gas Hills. Beginning at the city of Casper and Wyoming Highway 220; southwesterly along said highway to U.S. Highway 287; southerly along said highway to Wyoming Highway 73 at Lamont; westerly along said highway to the Bairoil Road (Sweetwater County Road 22); westerly along said road to the Wamsutter Crooks Gap Road; northerly along said road to the Alkali Creek Road (BLM 2317); westerly along said road to the Bison Basin Road; northerly along said road to U.S. Highway 287; westerly along said highway to the Sweetwater River; southwesterly up said river to Wyoming Highway 28; northerly along said highway to Wyoming Highway 789; northerly and easterly along said highway to the boundary of the Wind River Indian Reservation; easterly and northerly along said boundary to U.S. Highway 20-26; easterly along said highway to Wyoming Highway 220 in the city of Casper.

Area 17. Piney. Beginning where U.S. Highway 191 crosses the Green River; southeasterly along said highway to the Big Sandy River; southwesterly down said river to the Green River; northerly up said river to LaBarge Creek; northwesterly up said creek to the Greys River Road at Tri Basin Divide; northerly along said road to the divide between the Green River and the Greys River; easterly and northerly along said divide to the divide between the Green River and the Hoback River (Hoback Rim); easterly along said divide to U.S. Highway 191; southerly along said highway to the Green River.

Area 18. Wind River. All of the drainage of the Wind River, which lies west of the west boundary of the Wind River Indian Reservation.

Area 19. Northwest. Beginning where the Big Horn River crosses the Wyoming-Montana state line; southerly up said river to Dry Creek; southwesterly up said creek to the South Fork of Dry Creek; southwesterly up said creek, past the Halfway House historical marker on Wyoming Highway 120, to the divide between Meeteetse Creek and Sage Creek; westerly along said divide to the divide between the Greybull River and the South Fork of the Shoshone River; southwesterly along said divide to the divide between the South Fork of the Shoshone River and the Wind River; westerly along said divide to the Continental Divide; northwesterly along said divide to the south boundary of Yellowstone National Park; easterly then northerly along said boundary to the Wyoming-Montana state line; easterly along said line to the Big Horn River.

Area 20. Grass Creek. Beginning where Dry Creek flows into the Big Horn River; southerly up said river to the north boundary of the Wind River Indian Reservation; westerly along said boundary to the divide between the Wood River and Wind River; northwesterly along said divide to the divide between the Greybull River and the Wind River; northwesterly along said divide to the divide between the Greybull River and the South Fork of the Shoshone River; northeasterly along said divide to the divide between Meeteetse Creek and Sage Creek; easterly along said divide to the head of the South Fork of Dry Creek; northeasterly down said creek, past the Halfway House historical maker on Wyoming Highway 120, to Dry Creek; northeasterly down said creek to the Big Horn River; and all non-Indian fee title lands in that portion of Hot Springs County within the exterior boundaries of the Wind River Indian Reservation.

Area 21. Shell. Beginning where the Big Horn Mountain Divide crosses the Wyoming-Montana state line; southerly along said divide to U.S. Highway 16; westerly along said highway to the Big Horn River; northerly down said river to the Wyoming-Montana state line; easterly along said line to the Big Horn Mountain Divide.

Area 22. Ten Sleep. Beginning at the junction of U.S. Highway 16 and the Canyon Creek Road (U.S.F.S. Road 25); southerly along said road to the Gold Mine Road (U.S.F.S. Road 452); southerly along said road to the Big Horn Mountain Divide Road-Hazelton Road (Johnson County Road 3-Washakie County Road 81-Natrona County Road 109); southerly along said road to the Buffalo Creek Road (Natrona county Road 105); southwesterly along said road to the Waltman-Arminto Road (Natrona County Road 104); southerly along said road to the town of Waltman and U.S. Highway 20-26; westerly along said highway to the east boundary of

the Wind River Indian Reservation; northerly then westerly along said boundary to the Big Horn River; northerly down said river to U.S. Highway 16; easterly along said highway to the Canyon Creek Road (U.S.F.S. Road 25). Also included are all lands of the Wind River Indian Reservation within the Bureau of Reclamation Riverton and Boysen Unit boundaries and Boysen State Park, excluding Indian sole occupancy lands north of Cottonwood Creek.

Area 23. Sheridan. Beginning where Interstate Highway 90 crosses the Wyoming-Montana state line; southerly along said highway to Interstate Highway 25; southerly along said highway to U.S. Highway 16; southwesterly along said highway to the Big Horn Mountain Divide at Powder River Pass; northerly along said divide to the Wyoming-Montana state line; easterly along said line to Interstate Highway 90.

Area 24. Northeast. Beginning where the Rocky Point Road crosses the Wyoming-Montana state line; southerly along said road to the “D” Road; southerly along said road to Interstate Highway 90; easterly along said highway to U.S. Highway 16; southeasterly along said highway to the Wyoming-South Dakota state line; southerly along said line to the Wyoming-Nebraska state line; southerly along said line to U.S. Highway 20; westerly along said highway to Interstate Highway 25; northwesterly along said highway to the North Platte River at the town of Douglas; westerly up said river to the city of Casper and Interstate Highway 25; northerly along said highway to Interstate Highway 90; northerly along said highway to the Wyoming-Montana state line; easterly along said line to the Rocky Point Road.

Area 25. Hartville. Beginning where U.S. Highway 20 crosses the Wyoming-Nebraska state line; southerly along said line to the Wyoming-Colorado state line; westerly along said line to Interstate Highway 25; northerly along said highway to U.S. Highway 20; easterly along said highway to the Wyoming-Nebraska state line.

Area 26. Greys River. Beginning at the Wyoming-Idaho state line on the Snake River; easterly up said river to Bailey Creek; southeasterly up said creek to Dry Wash Draw; easterly up said draw to the divide between Bailey Creek and Willow Creek (Greyback Ridge); southerly along said divide to the divide between the Greys River and Willow Creek; southerly along said divide to the divide between the Greys River and the Hoback River; southerly along said divide to the divide between the Greys River and the Green River; southerly and westerly along said divide to the Greys River Road at the Tri Basin Divide; southerly along said road to La Barge Creek; northwesterly up said creek to Commissary Ridge; northwesterly along said ridge to the divide between the Salt River and the Smiths Fork River; southwesterly along said divide to the divide between the Salt river and the Bear River; westerly along said divide to the Wyoming-Idaho state line; northerly along said line to the Snake River.

Area 27. Casper Mountain. Beginning at the confluence of the North Platte River and Deer Creek at the town of Glenrock; southerly up said creek to the confluence of Curry Creek and Deer Creek; southeasterly up Curry Creek to U.S.F.S. Road 660; westerly along said road to the Squaw Spring Trail Road; southerly along said road to the Bates Creek Road (Natrona County Road 402); westerly along said road to the divide between Bates Creek and Spring Creek; westerly and southerly along said divide to the divide between Spring Creek and Lone Tree Creek; westerly and southerly along said divide to Wyoming Highway 487; northerly and

westerly along said highway to Wyoming Highway 220; northerly and easterly along said highway to the Casper city limits; northerly along said city limits to the North Platte River; easterly along said river to Deer Creek.

Area 28. Crowheart Butte. All non-Indian owned fee title land within the exterior boundaries of the Wind River Indian Reservation excluding Mountain Lion Hunt Area 22, those areas of the Spence and Moriarity Wildlife Management Area that lie east of the East Fork of the Wind River, and those lands within Hot Springs County.

Area 29. Hoback. Beginning where U.S. Highway 191 crosses Flat Creek at the north edge of the town of Jackson; due east to the National Elk Refuge boundary; easterly along said boundary to the Bridger-Teton National Forest boundary and the ridge between Twin Creeks and Cache Creek; easterly along said ridge to Jackson Peak; southerly along the divide between Flat Creek and Cache Creek to Cache Peak; easterly along the divide between Flat Creek and Granite Creek to Pyramid Peak; southerly along the divide between the Gros Ventre River and the Hoback River to Steamboat Peak; southeasterly along said divide to the divide between the Green River and the Hoback River at Hodges Peak; southeasterly along the divide between the Hoback River and Green River; southwesterly along said divide to the divide between the Greys River and Hoback River; northeasterly and westerly along said divide to the divide between the Greys River and Willow Creek; northerly along said divide to the divide between the Bailey Creek and Willow Creek (Greyback Ridge); northerly along said divide to Dry Wash Draw; westerly down said draw to Baily Creek; northwesterly down said creek to the Snake River; southerly and westerly down the Snake River to the Wyoming-Idaho state line; northerly along said line to Wyoming Highway 22; easterly along said highway to U.S. Highway 191; northeasterly along said highway to Flat Creek.

Area 30. Sundance. Beginning where the Wyoming-Montana state line crosses Wyoming Highway 112; southerly along said highway to the town of Hulett and Wyoming Highway 24; southerly along said highway to U.S. Highway 14; southeasterly along said highway to Interstate Highway 90 at the town of Sundance; easterly along said highway to Wyoming Highway 585; southeasterly along said highway to U.S. Highway 85; northeasterly along said highway to Wyoming-South Dakota state line; southerly along said line to U.S. Highway 16; northwesterly along said highway to Interstate Highway 90 at the town of Moorcroft; westerly along said highway to the “D” Road; northerly along said road to the Rocky Point Road; northerly along said road to the Wyoming-Montana state line; easterly along said line to Wyoming Highway 112.

Area 31. Elk Mountain. Beginning at the city of Laramie and Wyoming Highway 130; westerly then northerly along said highway to U.S. Highway 287; easterly and southerly along said highway to the city of Laramie.

Section 7. Violation of Commission Regulations. Failure to abide by the provisions of this regulation shall be punishable as provided by Wyoming statutes for violation of Commission regulations.

Section 8. Savings Clause. If any provision of this regulation is held to be illegal or unconstitutional, such a ruling shall not affect other provisions of this regulation which can be given effect without the illegal or unconstitutional provision; and, to this end, the provisions of the regulation are severable.

WYOMING GAME AND FISH COMMISSION

By:

Bill Williams, DVM, President

Dated: July 20, 2007

WYOMING GAME AND FISH COMMISSION

CHAPTER 42

MOUNTAIN LION HUNTING SEASONS

EMERGENCY RULE

(For the 120 days the emergency rules are in effect, the following rules shall apply. Emergency rules are no longer in effect 120 days after filing with the Secretary of State.)

Section 1. Authority. This regulation is promulgated by authority of W.S. §23-1-302 and W.S. §23-2-101(d).

Section 2. Definitions. For the purpose of this regulation, definitions shall be as set forth in Title 23, Wyoming Statutes and the Commission also adopts the following definitions:

(a) "Biological year" means twelve (12) consecutive months from September 1 to August 31.

(b) "Kitten" means any mountain lion less than one (1) year of age.

(c) "Mortality" means any human caused mountain lion death documented by the Department.

Section 3. Hunting Seasons Established. There shall be open seasons for the hunting of mountain lion as set forth in this regulation. This regulation shall remain in effect until modified or repealed by the Commission.

Section 4. Hunting Regulations.

(a) Mountain lions shall only be taken during open seasons, except as otherwise provided by State statute and Commission regulations.

(b) Open Hours for Taking of Mountain Lions. Mountain lions shall only be taken one-half (1/2) hour before sunrise to one-half (1/2) hour after sunset.

(c) Registering Kills. Hunters taking mountain lions shall retain the pelt and skull from each mountain lion taken for registration purposes. Even if the skull is damaged, it shall accompany the pelt. Visible external evidence of sex shall remain naturally attached to the pelt. Within three (3) days (seventy-two (72) hours) after taking a mountain lion, the licensee shall present the pelt and skull to a district game warden, district wildlife biologist, or Department personnel at a Department Regional Office

during business hours for registration. The pelt and skull shall be presented in an unfrozen condition in order to allow collection of two (2) premolar teeth to be utilized to determine the age of the mountain lion and to allow examination of the pelt to determine the sex of the mountain lion. At the time of registration, the licensee shall furnish the Department the location of the site of kill to include section, township and range, or UTM coordinates.

(d) Any person who makes a false statement on the registration form regarding the date the mountain lion was taken or the hunt area in which it was taken shall be in violation of this regulation and, such violation shall be punishable as provided by Title 23, Wyoming statutes for violation of Commission regulations.

(e) After a mountain lion has been pursued, treed, cornered or held at bay, a properly licensed person shall immediately harvest or release the mountain lion. No person shall in any manner restrict or hinder the mountain lion’s ability to escape for the purpose of allowing a person who was not present at the time the mountain lion was treed to arrive and take the mountain lion.

(f) Use of Dogs. Dogs may be used to take mountain lions during the open season.

(g) Bag and Possession Limits. The bag and possession limit for any person with a proper license shall be one (1) mountain lion during any one (1) calendar year (January 1-December 31). Hunters may take any mountain lion, except kittens and female mountain lions with kittens at side shall not be taken.

(h) Open Areas, Season Dates and Limitations.

Mountain Lion Management Unit	Hunt Area	Dates of Seasons	Annual Mortality Quota	Annual Female Mortality Quota
Northeast	1	Sept. 1-Mar. 31	16	
	24	Sept. 1-Mar. 31	4	
	30	Sept. 1-Mar. 31	8	
Southeast	5	Sept. 1-Mar. 31	12	
	6	Sept. 1-Mar. 31	12	
	7	Sept. 1-Mar. 31	14	
	8	Sept. 1-Mar. 31	10	
	9	Sept. 1-Mar. 31	7	
	10	Sept. 1-Mar. 31	7	
	16	Sept. 1-Mar. 31	6	
	25	Sept. 1-Mar. 31	3	
	27	Sept. 1-Aug. 31	10	
	31	Sept. 1-Mar. 31	6	
Southwest	11	Sept. 1-Mar. 31	2	
	12	Sept. 1-Mar. 31	6	3
	13	Sept. 1-Mar. 31	3	
North-Central	15	Sept. 1-Aug. 31	25	
	21	Sept. 1-Mar. 31	20	
	22	Sept. 1-Aug. 31	15	
	23	Sept. 1-Mar. 31	18	
West	2	Sept. 1-Mar. 31	7	3
	3	Sept. 1-Mar. 31	12	
	4	Sept. 1-Mar. 31	8	
	14	Sept. 1-Mar. 31	15	
	17	Sept. 1-Mar. 31	9	
	18	Sept. 1-Mar. 31	12	
	19	Sept. 1-Mar. 31	20	
	20	Sept. 1-Mar. 31	4	
	26	Sept. 1-Mar. 31	15	
	28	Sept. 1-Mar. 31	3	
	29	Sept. 1-Mar. 31	9	4

(i) Area Mortality Quota Limitation. The mountain lion season in a hunt area shall close when the mortality quota is reached. If the female mortality quota is not reached, the season shall close upon the date specified in subsection (h) of this section. In areas for which a female quota is listed, the hunt area shall close if the female mortality quota is reached prior to the annual mortality quota being reached. Mortality quotas shall be for fall through spring. It is the hunter's responsibility to confirm that the

hunt area he/she intends to hunt is open. The status of hunt area closures is available by calling toll free **1-800-637-0809** twenty-four (24) hours a day. Any mountain lion mortality documented by Department personnel after the close of the season shall be subtracted from the mortality quota for the next biological year.

Section 5. Archery Regulations. Mountain lions may be taken with archery equipment in all areas as set forth in Section 4 of this Chapter.

Section 6. Hunt Area Descriptions.

(a) Area and Number

Area 1. Black Hills. Beginning where the Wyoming-Montana-South Dakota state lines meet; southerly along the Wyoming-South Dakota state line to U.S. Highway 85; southwesterly along said highway to Wyoming Highway 585; northwesterly along said highway to Interstate Highway 90; westerly along said highway to U.S. Highway 14 at the town of Sundance; northwesterly along said highway to Wyoming Highway 24; northerly along said highway to Wyoming Highway 112 in the town of Hulett; northerly along said highway to the Wyoming-Montana state line; easterly along said line to the Wyoming-South Dakota state line.

Area 2. Teton. Beginning where the Continental Divide crosses the southern boundary of Yellowstone National Park; southerly along said divide to the Union Pass Road (U.S.F.S. Road 600); westerly and southerly along said road to the Darwin Ranch Road (U.S.F.S. Road 620); westerly along said road to the divide between the Green River and the Gros Ventre River; southwesterly along said divide to the divide between the Gros Ventre River and the Hoback River at Hodges Peak; westerly along said divide to the divide between Flat Creek and the Granite Creek; southwesterly along said divide to Cache Peak; northerly along the divide between Flat Creek and Cache Creek at Jackson Peak; westerly along the ridge between Cache Creek and Twin Creeks to the Bridger-Teton National Forest/National Elk Refuge Boundary; southwesterly along the National Elk Refuge boundary to where Flat Creek crosses U. S. Highway 26-89-191 at the north edge of the town of Jackson; southwesterly along said highway to Wyoming Highway 22; westerly along said highway to the Wyoming-Idaho state line; northerly along said line to the south boundary of Yellowstone National Park; easterly along said boundary to the Continental Divide.

Area 3. Bridger. Beginning where the Union Pass Road (U.S.F.S. Road 600) crosses the Continental Divide; southeasterly along said divide to U.S. Highway 28; southwesterly along said highway to U.S. Highway 191; northerly along said highway to the divide between the Green River and the Hoback River (Hoback Rim); northerly along said divide to the divide between the Green River and the Gros Ventre River at Hodges Peak; northeasterly along said divide to the Darwin Ranch Road (U.S.F.S. Road 620);

easterly along said road to the Union Pass Road (U.S.F.S. Road 600); northerly and easterly along said road to the Continental Divide.

Area 4. Popo Agie. Beginning where Wyoming Highway 789 crosses the southern boundary of the Wind River Indian Reservation; westerly and southerly along said highway to Wyoming Highway 28; southwesterly along said highway to the Continental Divide; northwesterly along said divide to the south boundary of the Wind River Indian Reservation; easterly along said boundary to Wyoming Highway 789.

Area 5. Iron Mountain. Beginning where Interstate Highway 25 crosses the Wyoming-Colorado state line; westerly along said line to U.S. Highway 287; northerly along said highway to Wyoming Highway 34; northerly and easterly along said highway to Interstate Highway 25; southerly along said highway to the Wyoming-Colorado state line.

Area 6. Laramie Peak. Beginning where Interstate Highway 25 crosses the North Platte River at the city of Douglas; southerly along said highway to Wyoming Highway 34; westerly along said highway to U.S. Highway 287; northerly and westerly along said highway to Wyoming Highway 487; northerly and westerly along said highway to the divide between Spring Creek and Lone Tree Creek at Shirley Rim; northerly and easterly along said divide to the divide between Spring Creek and Bates Creek; northeasterly along said divide to the Bates Creek Road (Natrona County Road 402); easterly along said road to the Squaw Springs Trail; northerly along said road to U.S.F.S. Road 660; easterly along said road to Curry Creek; northwesterly down said creek to Deer Creek; northeasterly down said creek to the confluence of Deer Creek and the North Platte River; easterly down said river to Interstate Highway 25.

Area 7. South Snowy Range. Beginning at the city of Laramie and U.S. Highway 287; southerly along said highway to the Wyoming-Colorado state line; westerly along said line to Wyoming Highway 230 (west of the North Platte River); northerly along said highway to Wyoming Highway 130; easterly along said highway to the city of Laramie.

Area 8. Seminoe. Beginning at the junction of Wyoming Highway 220 and Wyoming Highway 487; southerly along Wyoming Highway 487 to U.S. Highway 287; westerly along said highway to Interstate Highway 80; westerly along said highway to U.S. Highway 287 in the city of Rawlins; northerly along said highway to Wyoming Highway 220; northeasterly along said highway to Wyoming Highway 487.

Area 9. Sierra Madre. Beginning at the junction of Interstate Highway 80 and Wyoming Highway 130; southerly along Wyoming Highway 130 to Wyoming Highway 230; southeasterly along said highway to the Wyoming-Colorado state line; westerly along said line to the Continental Divide; northerly along said divide to the Sage Creek Road (Carbon County Road 401); northerly along said road to Wyoming Highway 71;

northerly along said highway to Interstate Highway 80; easterly along said highway to Wyoming Highway 130.

Area 10. Haystacks. Beginning at the junction of Interstate Highway 80 and the Bitter Creek Road (Sweetwater County Road 19); easterly along Interstate Highway 80 to the junction of Wyoming Highway 71; southerly along said highway to the Sage Creek Road (Carbon County Road 401); southerly along said road to the Continental Divide at Middlewood Hill; southerly along said divide to the Wyoming-Colorado state line; westerly along said line to the Cow Creek-Powder Wash Road north of Powder Wash, Colorado; northwesterly along said road to the Bitter Creek Road; northerly along said road to Interstate Highway 80.

Area 11. Red Desert. Beginning at the junction of Wyoming Highway 73 and U.S. Highway 287; southerly along U.S. Highway 287 to Interstate Highway 80; westerly along said highway to the Green River; northerly up said river to Big Sandy River; northeasterly up said river to U.S. Highway 191; southerly along said highway to Wyoming Highway 28; northeasterly along said highway to the Sweetwater River; northeasterly down said river to U.S. Highway 287; easterly along said highway to the Bison Basin Road; southerly along said road to the Alkali Creek Road (BLM 2317); easterly along said road to the Wamsutter-Crooks Gap Road; southerly along said road to the Bairoil Road (Sweetwater County Road 22); easterly along said road to Wyoming Highway 73; easterly along said highway to U.S. Highway 287.

Area 12. Flaming Gorge. Beginning where Interstate Highway 80 intersects the Bitter Creek Road (Sweetwater County Road 19); southerly along the Bitter Creek Road to the Cow Creek-Powder Wash Road north of Powder Wash, Colorado; southeasterly along said road to the Wyoming-Colorado state line; westerly along said line to the Wyoming-Utah state line; westerly along said line to Flaming Gorge Reservoir and the Green River; northerly up said river to Interstate Highway 80; easterly along said highway to the junction of the Bitter Creek Road.

Area 13. Wasatch. Beginning where Interstate Highway 80 crosses the Green River; southerly down said river to the Wyoming-Utah state line; westerly and northerly along said line to Interstate Highway 80; easterly along said highway to the Green River.

Area 14. Lincoln. Beginning where the Green River crosses Interstate Highway 80; westerly along said highway to the Wyoming-Utah state line; northerly along said line to the Wyoming-Idaho state line; northerly along said line to the divide between the Salt River and Bear River; easterly along said divide to the divide between the Salt River and the Smiths Fork River; northeasterly along said divide to Commissary Ridge; southeasterly along said ridge to the head of LaBarge Creek; southeasterly down said creek to the Green River; southeasterly down said river to Interstate Highway 80.

Area 15. Kaycee. Beginning at the junction of U.S. Highway 16 and Interstate Highway 25 at the town of Buffalo; southerly along Interstate Highway 25 to U.S.

Highway 20-26 at the city of Casper; westerly along said highway to the town of Waltman and the Waltman-Arminto Road (Natrona County Road 104); northerly along said road to the Buffalo Creek Road (Natrona County Road 105); northeasterly along said road to the Big Horn Mountain Divide Road/Hazelton Road (Natrona County Road 109-Washakie County Road 81-Johnson County Road 3); northerly along said road to the Gold Mine Road (U.S.F.S. Road 452); northerly along said road to the Canyon Creek Road (U.S.F.S. Road 25); northerly along said road to U.S. Highway 16; northeasterly along said highway to Interstate Highway 25.

Area 16. Gas Hills. Beginning at the city of Casper and Wyoming Highway 220; southwesterly along said highway to U.S. Highway 287; southerly along said highway to Wyoming Highway 73 at Lamont; westerly along said highway to the Bairoil Road (Sweetwater County Road 22); westerly along said road to the Wamsutter Crooks Gap Road; northerly along said road to the Alkali Creek Road (BLM 2317); westerly along said road to the Bison Basin Road; northerly along said road to U.S. Highway 287; westerly along said highway to the Sweetwater River; southwesterly up said river to Wyoming Highway 28; northerly along said highway to Wyoming Highway 789; northerly and easterly along said highway to the boundary of the Wind River Indian Reservation; easterly and northerly along said boundary to U.S. Highway 20-26; easterly along said highway to Wyoming Highway 220 in the city of Casper.

Area 17. Piney. Beginning where U.S. Highway 191 crosses the Green River; southeasterly along said highway to the Big Sandy River; southwesterly down said river to the Green River; northerly up said river to LaBarge Creek; northwesterly up said creek to the Greys River Road at Tri Basin Divide; northerly along said road to the divide between the Green River and the Greys River; easterly and northerly along said divide to the divide between the Green River and the Hoback River (Hoback Rim); easterly along said divide to U.S. Highway 191; southerly along said highway to the Green River.

Area 18. Wind River. All of the drainage of the Wind River, which lies west of the west boundary of the Wind River Indian Reservation.

Area 19. Northwest. Beginning where the Big Horn River crosses the Wyoming-Montana state line; southerly up said river to Dry Creek; southwesterly up said creek to the South Fork of Dry Creek; southwesterly up said creek, past the Halfway House historical marker on Wyoming Highway 120, to the divide between Meeteetse Creek and Sage Creek; westerly along said divide to the divide between the Greybull River and the South Fork of the Shoshone River; southwesterly along said divide to the divide between the South Fork of the Shoshone River and the Wind River; westerly along said divide to the Continental Divide; northwesterly along said divide to the south boundary of Yellowstone National Park; easterly then northerly along said boundary to the Wyoming-Montana state line; easterly along said line to the Big Horn River.

Area 20. Grass Creek. Beginning where Dry Creek flows into the Big Horn River; southerly up said river to the north boundary of the Wind River Indian

Reservation; westerly along said boundary to the divide between the Wood River and Wind River; northwesterly along said divide to the divide between the Greybull River and the Wind River; northwesterly along said divide to the divide between the Greybull River and the South Fork of the Shoshone River; northeasterly along said divide to the divide between Meeteetse Creek and Sage Creek; easterly along said divide to the head of the South Fork of Dry Creek; northeasterly down said creek, past the Halfway House historical maker on Wyoming Highway 120, to Dry Creek; northeasterly down said creek to the Big Horn River; and all non-Indian fee title lands in that portion of Hot Springs County within the exterior boundaries of the Wind River Indian Reservation.

Area 21. Shell. Beginning where the Big Horn Mountain Divide crosses the Wyoming-Montana state line; southerly along said divide to U.S. Highway 16; westerly along said highway to the Big Horn River; northerly down said river to the Wyoming-Montana state line; easterly along said line to the Big Horn Mountain Divide.

Area 22. Ten Sleep. Beginning at the junction of U.S. Highway 16 and the Canyon Creek Road (U.S.F.S. Road 25); southerly along said road to the Gold Mine Road (U.S.F.S. Road 452); southerly along said road to the Big Horn Mountain Divide Road-Hazelton Road (Johnson County Road 3-Washakie County Road 81-Natrona County Road 109); southerly along said road to the Buffalo Creek Road (Natrona county Road 105); southwesterly along said road to the Waltman-Arminto Road (Natrona County Road 104); southerly along said road to the town of Waltman and U.S. Highway 20-26; westerly along said highway to the east boundary of the Wind River Indian Reservation; northerly then westerly along said boundary to the Big Horn River; northerly down said river to U.S. Highway 16; easterly along said highway to the Canyon Creek Road (U.S.F.S. Road 25). Also included are all lands of the Wind River Indian Reservation within the Bureau of Reclamation Riverton and Boysen Unit boundaries and Boysen State Park, excluding Indian sole occupancy lands north of Cottonwood Creek.

Area 23. Sheridan. Beginning where Interstate Highway 90 crosses the Wyoming-Montana state line; southerly along said highway to Interstate Highway 25; southerly along said highway to U.S. Highway 16; southwesterly along said highway to the Big Horn Mountain Divide at Powder River Pass; northerly along said divide to the Wyoming-Montana state line; easterly along said line to Interstate Highway 90.

Area 24. Northeast. Beginning where the Rocky Point Road crosses the Wyoming-Montana state line; southerly along said road to the "D" Road; southerly along said road to Interstate Highway 90; easterly along said highway to U.S. Highway 16; southeasterly along said highway to the Wyoming-South Dakota state line; southerly along said line to the Wyoming-Nebraska state line; southerly along said line to U.S. Highway 20; westerly along said highway to Interstate Highway 25; northwesterly along said highway to the North Platte River at the town of Douglas; westerly up said river to the city of Casper and Interstate Highway 25; northerly along said highway to Interstate Highway 90; northerly along said highway to the Wyoming-Montana state line; easterly along said line to the Rocky Point Road.

Area 25. Hartville. Beginning where U.S. Highway 20 crosses the Wyoming-Nebraska state line; southerly along said line to the Wyoming-Colorado state line; westerly along said line to Interstate Highway 25; northerly along said highway to U.S. Highway 20; easterly along said highway to the Wyoming-Nebraska state line.

Area 26. Greys River. Beginning at the Wyoming-Idaho state line on the Snake River; easterly up said river to Bailey Creek; southeasterly up said creek to Dry Wash Draw; easterly up said draw to the divide between Bailey Creek and Willow Creek (Greyback Ridge); southerly along said divide to the divide between the Greys River and Willow Creek; southerly along said divide to the divide between the Greys River and the Hoback River; southerly along said divide to the divide between the Greys River and the Green River; southerly and westerly along said divide to the Greys River Road at the Tri Basin Divide; southerly along said road to La Barge Creek; northwesterly up said creek to Commissary Ridge; northwesterly along said ridge to the divide between the Salt River and the Smiths Fork River; southwesterly along said divide to the divide between the Salt river and the Bear River; westerly along said divide to the Wyoming-Idaho state line; northerly along said line to the Snake River.

Area 27. Casper Mountain. Beginning at the confluence of the North Platte River and Deer Creek at the town of Glenrock; southerly up said creek to the confluence of Curry Creek and Deer Creek; southeasterly up Curry Creek to U.S.F.S. Road 660; westerly along said road to the Squaw Spring Trail Road; southerly along said road to the Bates Creek Road (Natrona County Road 402); westerly along said road to the divide between Bates Creek and Spring Creek; westerly and southerly along said divide to the divide between Spring Creek and Lone Tree Creek; westerly and southerly along said divide to Wyoming Highway 487; northerly and westerly along said highway to Wyoming Highway 220; northerly and easterly along said highway to the Casper city limits; northerly along said city limits to the North Platte River; easterly along said river to Deer Creek.

Area 28. Crowheart Butte. All non-Indian owned fee title land within the exterior boundaries of the Wind River Indian Reservation excluding Mountain Lion Hunt Area 22, those areas of the Spence and Moriarity Wildlife Management Area that lie east of the East Fork of the Wind River, and those lands within Hot Springs County.

Area 29. Hoback. Beginning where U.S. Highway 191 crosses Flat Creek at the north edge of the town of Jackson; due east to the National Elk Refuge boundary; easterly along said boundary to the Bridger-Teton National Forest boundary and the ridge between Twin Creeks and Cache Creek; easterly along said ridge to Jackson Peak; southerly along the divide between Flat Creek and Cache Creek to Cache Peak; easterly along the divide between Flat Creek and Granite Creek to Pyramid Peak; southerly along the divide between the Gros Ventre River and the Hoback River to Steamboat Peak; southeasterly along said divide to the divide between the Green River and the Hoback River at Hodges Peak; southeasterly along the divide between the Hoback River and

Green River; southwesterly along said divide to the divide between the Greys River and Hoback River; northeasterly and westerly along said divide to the divide between the Greys River and Willow Creek; northerly along said divide to the divide between the Bailey Creek and Willow Creek (Greyback Ridge); northerly along said divide to Dry Wash Draw; westerly down said draw to Baily Creek; northwesterly down said creek to the Snake River; southerly and westerly down the Snake River to the Wyoming-Idaho state line; northerly along said line to Wyoming Highway 22; easterly along said highway to U.S. Highway 191; northeasterly along said highway to Flat Creek.

Area 30. Sundance. Beginning where the Wyoming-Montana state line crosses Wyoming Highway 112; southerly along said highway to the town of Hulett and Wyoming Highway 24; southerly along said highway to U.S. Highway 14; southeasterly along said highway to Interstate Highway 90 at the town of Sundance; easterly along said highway to Wyoming Highway 585; southeasterly along said highway to U.S. Highway 85; northeasterly along said highway to Wyoming-South Dakota state line; southerly along said line to U.S. Highway 16; northwesterly along said highway to Interstate Highway 90 at the town of Moorcroft; westerly along said highway to the “D” Road; northerly along said road to the Rocky Point Road; northerly along said road to the Wyoming-Montana state line; easterly along said line to Wyoming Highway 112.

Area 31. Elk Mountain. Beginning at the city of Laramie and Wyoming Highway 130; westerly then northerly along said highway to U.S. Highway 287; easterly and southerly along said highway to the city of Laramie.

Section 7. Violation of Commission Regulations. Failure to abide by the provisions of this regulation shall be punishable as provided by Wyoming statutes for violation of Commission regulations.

Section 8. Savings Clause. If any provision of this regulation is held to be illegal or unconstitutional, such a ruling shall not affect other provisions of this regulation which can be given effect without the illegal or unconstitutional provision; and, to this end, the provisions of the regulation are severable.

WYOMING GAME AND FISH COMMISSION

By:

Bill Williams, DVM, President

Dated: August 14, 2007

2007 MOUNTAIN LION HUNT AREAS

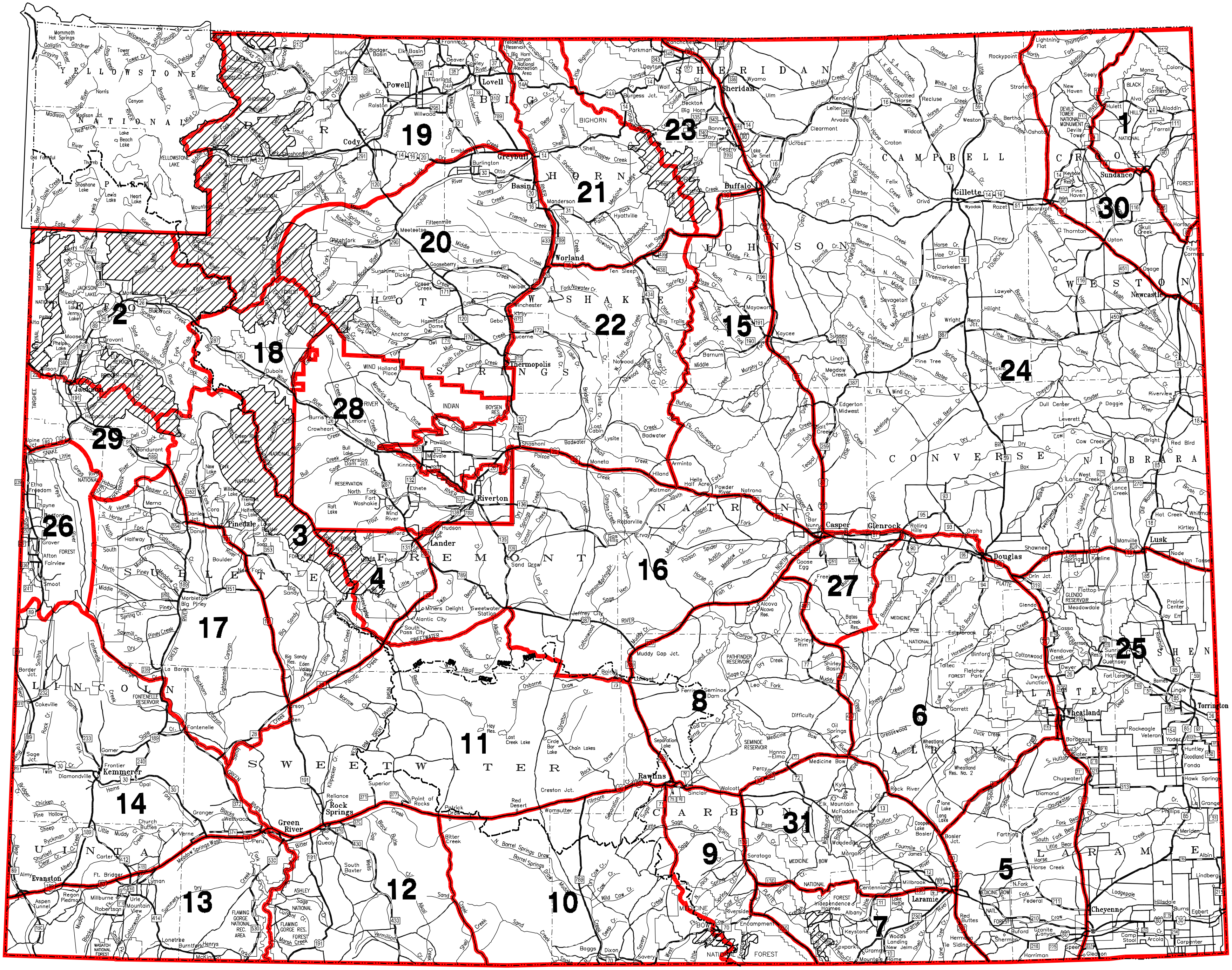

THIS MAP IS FOR GENERAL REFERENCE ONLY. Please use the written boundary descriptions in this regulation for detailed boundary information.

Note: Wilderness area, nonresidents must have guides.

ANTELOPE
ARCHERY HUNTING - Harvest
BY HUNTERS HOLDING AN ARCHERY TAG
2007

	NUMBER ACTIVE ARCHERY HUNTERS	HARVEST				HUNTER SUCCESS	DAYS/ HARVEST
		BUCK	DOE	FAWN	TOTAL		
STATE TOTAL	3,899	1,511	771	65	2,347	60.2%	8.3
RESIDENT	2,456	766	357	18	1,141	46.5%	11.9
NONRESIDENT	1,443	745	414	47	1,206	83.6%	5.0

ANTELOPE
ARCHERY HUNTING - Total Days
BY HUNTERS HOLDING AN ARCHERY TAG
2007

	TOTAL		ARCHERY ONLY SEASON		GUN SEASON	
	HUNTERS	DAYS	HUNTERS	DAYS	HUNTERS	DAYS
STATE TOTAL	3,899	19,556	3,831	17,240	765	2,316
RESIDENT	2,456	13,522	2,415	11,516	597	2,006
NONRESIDENT	1,443	6,034	1,416	5,724	168	310

ANTELOPE
ARCHERY HUNTING - EQUIPMENT USED*
2007

COMPOUND BOW	RECURVE BOW	LONG BOW	CROSSBOW
84.4%	4.0%	2.6%	10.2%

*may total more than 100% as hunters could report using multiple types of equipment

BEAR
ARCHERY HUNTING - Harvest
BY HUNTERS HOLDING AN ARCHERY TAG
2007

	NUMBER ACTIVE ARCHERY HUNTERS	HARVEST			HUNTER	DAYS/
		SPRING	FALL	TOTAL	SUCCESS	HARVEST
STATE TOTAL	619	14	24	38	6.1%	135.3
RESIDENT	568	14	15	29	5.1%	166.0
NONRESIDENT	51	0	9	9	17.6%	36.4

BEAR
ARCHERY HUNTING - Total Days
BY HUNTERS HOLDING AN ARCHERY TAG
2007

	TOTAL		ARCHERY ONLY SEASON		GUN SEASON	
	HUNTERS	DAYS	HUNTERS	DAYS	HUNTERS	DAYS
STATE TOTAL	619	5,141	526	3,361	286	1,780
RESIDENT	568	4,813	490	3,159	268	1,654
NONRESIDENT	51	328	36	202	18	126

BEAR
ARCHERY HUNTING - EQUIPMENT USED*
2007

COMPOUND BOW	RECURVE BOW	LONG BOW	CROSSBOW
78.4%	5.0%	6.9%	10.3%

*may total more than 100% as hunters could report using multiple types of equipment

DEER
ARCHERY HUNTING - Harvest
BY HUNTERS HOLDING AN ARCHERY TAG
2007

	NUMBER ACTIVE ARCHERY HUNTERS	HARVEST				HUNTER SUCCESS	DAYS/ HARVEST
		BUCK	DOE	FAWN	TOTAL		
STATE TOTAL	8,237	1,515	1,153	111	2,779	33.7%	26.8
RESIDENT	6,493	902	752	62	1,716	26.4%	36.0
Mule Deer	5,993	666	384	23	1,073	17.9%	41.1
White Tail	2,507	236	368	39	643	25.6%	27.6
NONRESIDENT	1,744	613	401	49	1,063	61.0%	11.9
Mule Deer	1,527	372	229	29	630	41.3%	13.7
White Tail	805	241	172	20	433	53.8%	9.3

DEER
ARCHERY HUNTING - Total Days
BY HUNTERS HOLDING AN ARCHERY TAG
2007

	TOTAL		ARCHERY ONLY SEASON		GUN SEASON	
	HUNTERS	DAYS	HUNTERS	DAYS	HUNTERS	DAYS
STATE TOTAL	8,237	74,471	7,972	60,973	1,935	13,498
RESIDENT	6,493	61,855	6,261	49,382	1,743	12,473
Mule Deer	5,993	44,118	5,873	38,058	1,503	6,060
White Tail	2,507	17,737	2,259	11,324	1,108	6,413
NONRESIDENT	1,744	12,616	1,711	11,591	192	1,025
Mule Deer	1,527	8,601	1,507	8,092	147	509
White Tail	805	4,015	784	3,499	139	516

DEER
ARCHERY HUNTING - EQUIPMENT USED*
2007

	COMPOUND BOW	RECURVE BOW	LONG BOW	CROSSBOW
Mule Deer	79.3%	4.2%	2.4%	14.8%
White Tail	81.3%	5.5%	3.2%	11.1%

*may total more than 100% as hunters could report using multiple types of equipment

ELK
ARCHERY HUNTING - Harvest
BY HUNTERS HOLDING AN ARCHERY TAG
2007

	NUMBER ACTIVE ARCHERY HUNTERS	HARVEST					HUNTER SUCCESS	DAYS/ HARVEST
		BULL	SPIKE	COW	CALF	TOTAL		
STATE TOTAL	9,756	1,195	171	155	36	1,557	16.0%	52.7
RESIDENT	8,149	842	155	135	28	1,160	14.2%	60.2
NONRESIDENT	1,607	353	16	20	8	397	24.7%	30.9

ELK
ARCHERY HUNTING - Total Days
BY HUNTERS HOLDING AN ARCHERY TAG
2007

	TOTAL		ARCHERY ONLY SEASON		GUN SEASON	
	HUNTERS	DAYS	HUNTERS	DAYS	HUNTERS	DAYS
STATE TOTAL	9,756	82,058	9,709	74,290	1,572	7,768
RESIDENT	8,149	69,808	8,102	62,560	1,441	7,248
NONRESIDENT	1,607	12,250	1,607	11,730	131	520

ELK
ARCHERY HUNTING - EQUIPMENT USED*
2007

COMPOUND BOW	RECURVE BOW	LONG BOW	CROSSBOW
77.3%	4.0%	2.4%	16.5%

*may total more than 100% as hunters could report using multiple types of equipment
