# Supplementary material for: The Elephant in the room: What can we learn from California regarding the use of sport hunting of pumas (Puma concolor) as a management tool?
Source: PLoS One. 2020 Feb 13;15(2):e0224638. doi: 10.1371/journal.pone.0224638 (PMC7018503; doi:10.1371/journal.pone.0224638)
Supplement: S2 File — This is a zip file that contains sources of the data used in the analyses that are no longer available on the internet. (ZIP) [file pone.0224638.s003.zip › Wyoming Mtn-Lion-Mortality-Report-HY2014.pdf]

WYOMING MOUNTAIN LION MORTALITY REPORT

HARVEST YEAR – 2014

September 1, 2014 – August 31, 2015

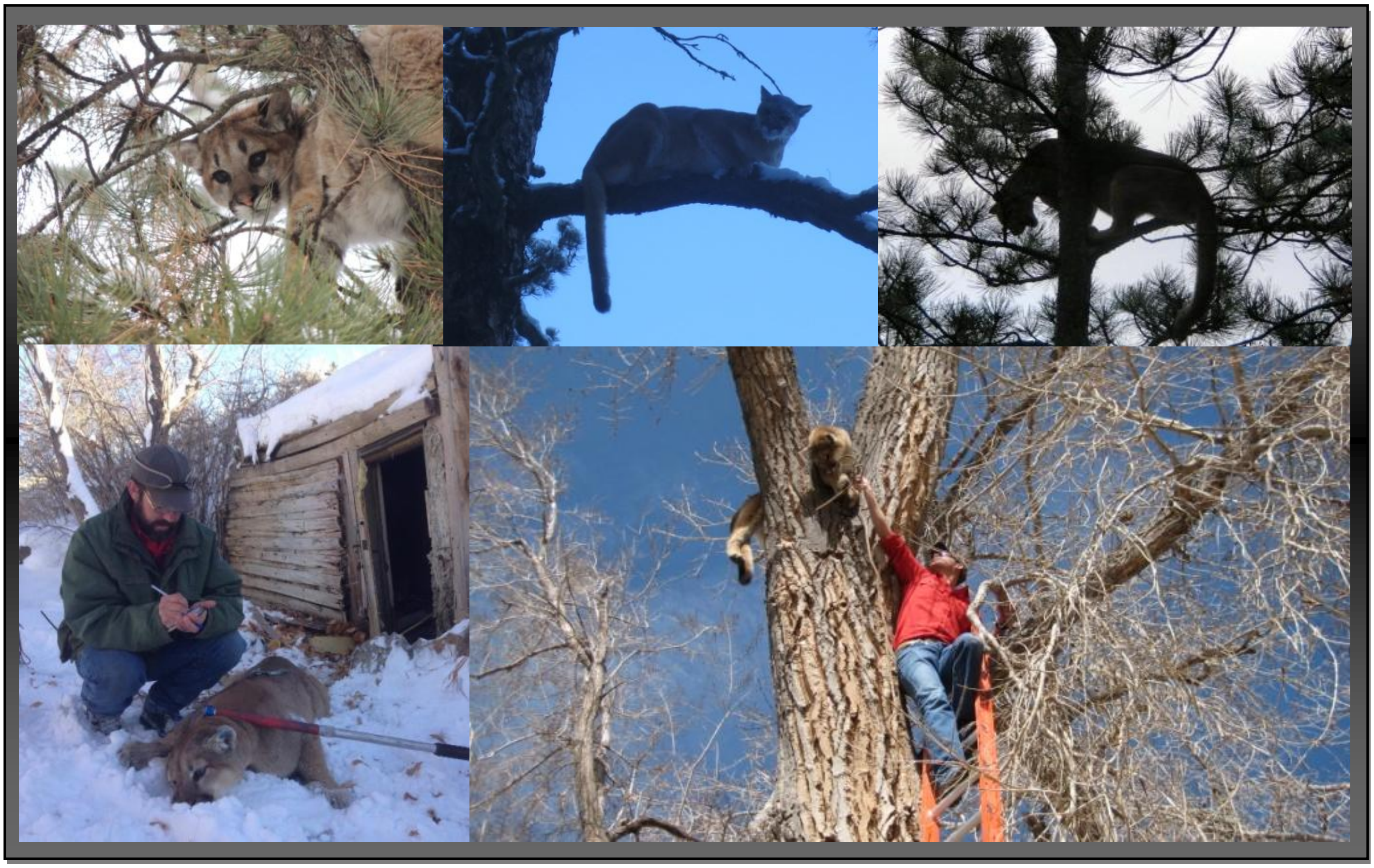

Prepared by:
Justin Clapp/Large Carnivore Section
Wyoming Game and Fish Department
Lander, Wyoming

September 2015

INTRODUCTION

The following report contains mountain lion mortality data and harvest composition for Wyoming's 33 Hunt Areas (HAs) and 5 Mountain Lion Management Units (MLMUs; Figure 1) for Harvest Year (HY) 2014 beginning 1 September 2014 through 31 August 2015. HY 2014 marks the midpoint of the third 3-year management cycle for mountain lions used by the Wyoming Game and Fish Department (WGFD). Because harvest limits are evaluated and revised every three years, the next revision is not scheduled until the conclusion of HY 2015. This report summarizes statewide mountain lion mortality, but does not propose any recommendations for future management. For an in-depth explanation of data analysis, harvest criteria, and discussions on statewide mountain lion management, the Mountain Lion Management Plan (WGFD 2006) or the Wyoming Mountain Lion Harvest/Mortality Report: Harvest Years 2007-2009 or 2010-2012 (Thompson et al. 2010; 2013) are available through the WGFD Large Carnivore Section or the WGFD website: http://gf.state.wy.us/wildlife/MountainLionPlan/.

Management of mountain lions in Wyoming is an adaptive management process, evaluating harvest and management as it relates to mountain lion population status and trend. The overall objective of mountain lion management in Wyoming is to sustain mountain lion populations in core habitat at varying densities depending on local management objectives (WGFD 2006). The data presented in this report supersedes previous reports, as information about previous harvest data has been updated due to various season lengths, subsequent reporting of harvested lions, and biological data acquired from laboratory results.

Cover photos: Mountain lions captured or bayed for monitoring and conflict resolution:
Bottom left: Luke Ellsbury takes information on a mountain lion captured and relocated from Cody.
Bottom right: Justin Clapp safely extricates an immobilized animal from a residence in Fremont County.

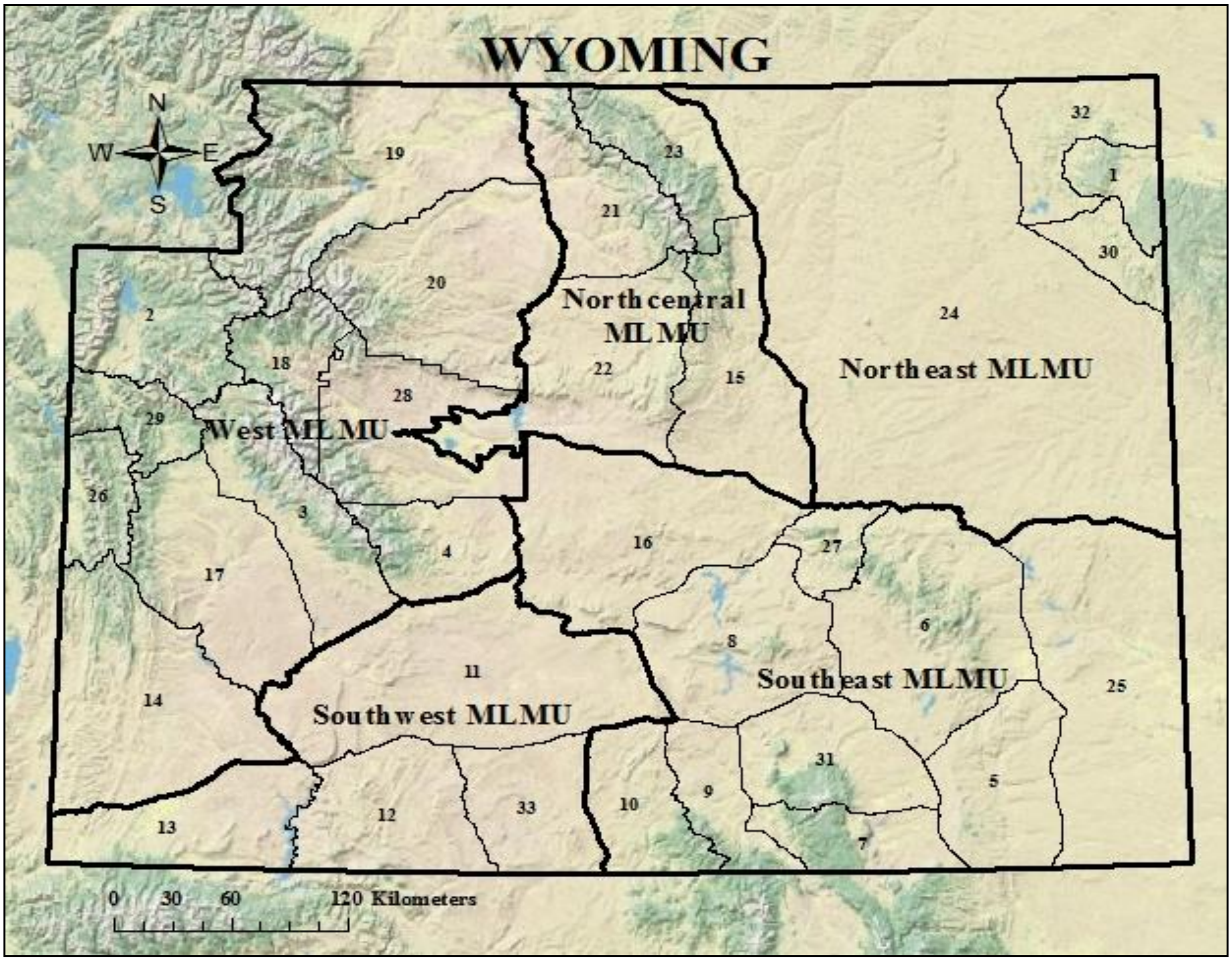

FIGURE 1. WYOMING MOUNTAIN LION MANAGEMENT UNIT AND HUNT AREA MAP FOR HY 2014.

STATEWIDE MOUNTAIN LION MORTALITY

Statewide mountain lion mortalities were reduced in Wyoming for HY 2014 compared to recent years. Total harvest for HY 2014 (*n* = 268) resulted in 38 fewer harvests (12% less) than the highest harvest recorded in HY 2013 (*n* = 306; Figure 2). Total documented mortality for HY 2014 was 286, resulting in 18 mountain lion mortalities not related to harvest; an 18% reduction in statewide non-harvest mortality from the previous season. Non-legal mortalities were maintained or reduced in most categories in comparison to previous seasons (Figure 3). Overall,

statewide harvest comprised 63% of the total allowable mountain lion mortality across the state for HY 2014.

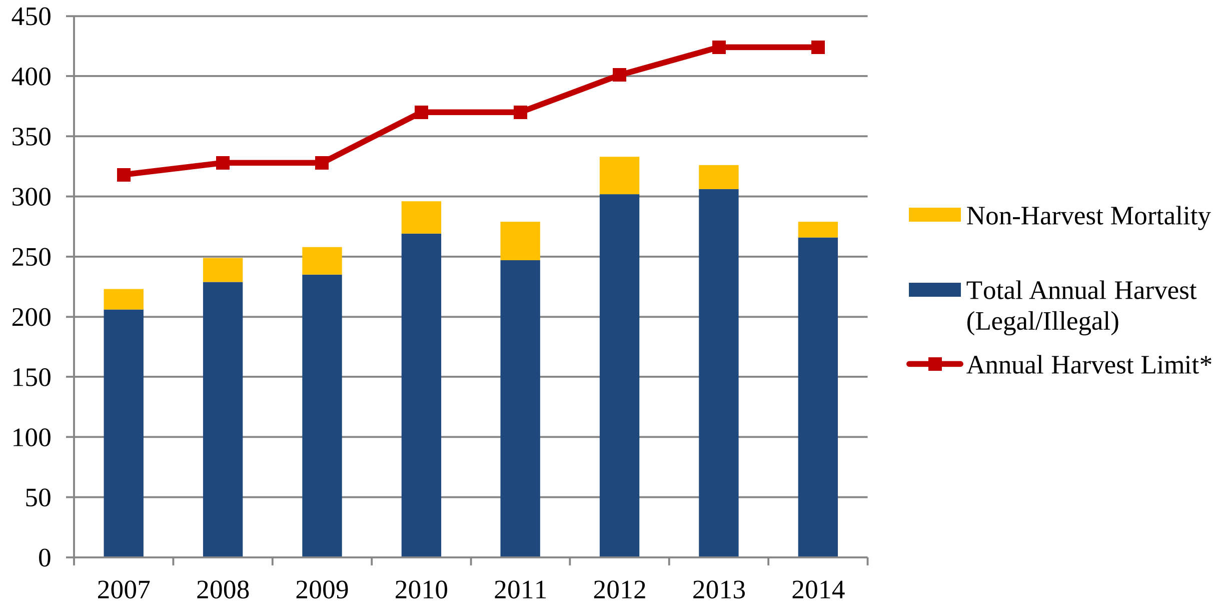

* Beginning in 2010, statewide harvest limits include hunt areas allowing unlimited harvest (i.e. HAs 15, 24, and 27), and are therefore represented by the last applicable and numerical harvest limit.

FIGURE 2. ANNUAL MOUNTAIN LION HARVEST AND MORTALITY DATA FOR WYOMING, HYS 2007-2014.

Only 5 hunt areas (HAs 1, 8, 12, 23, and 32), roughly 15%, closed due to mortality limits being reached or exceeded in HY 2014. Nineteen hunt areas closed per season regulations on 31 March 2015, with an additional 3 areas with extended seasons to accommodate late-season tracking conditions for hunters (e.g. spring snow) closing on 30 April (HAs 5 & 6) and 31 May (HA 24). Five hunt areas in the southeast MLMU (HAs 7, 8, 9, 27, and 31) and two in the north-central MLMU (HAs 15 & 22) remain open all year, and hunt areas 15, 24, and 27 allow unlimited harvest to address concerns with potential domestic livestock depredation and/or proximity to residential areas or in areas with minimal mountain lion habitat.

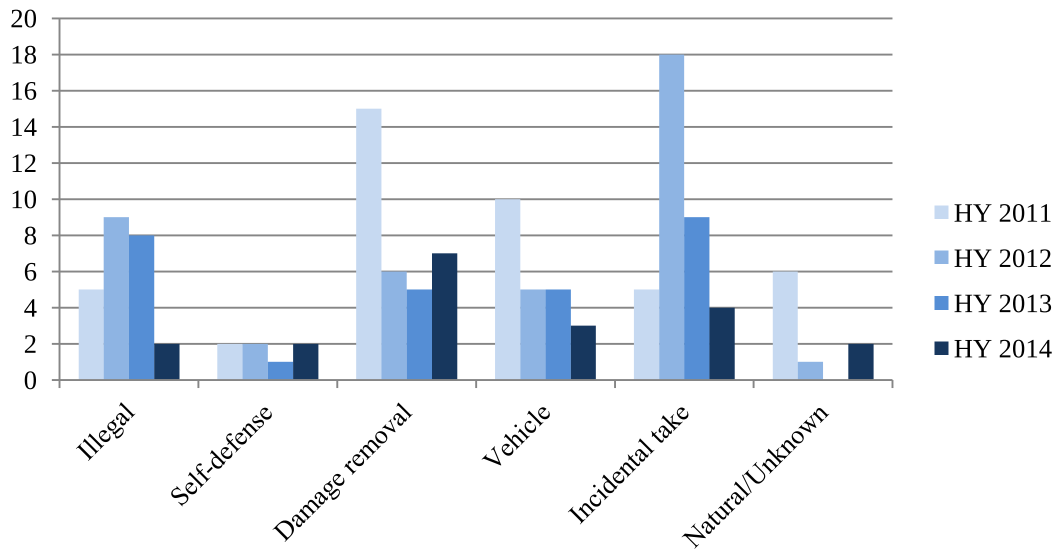

FIGURE 3. NON-LEGAL MORTALITY OF MOUNTAIN LIONS IN WYOMING, HYS 2011-2014.

The decrease in overall statewide harvest can be attributed to reduced harvests within the northeast, southeast, and west MLMUs. From the previous year, the northeast MLMU saw a reduction from 66 to 62 harvests, with HA 30 not reaching the set mortality limit for the first time since HY 2009. This was to be expected as recent management objectives are to reduce mountain lion densities in the Black Hills. The southeast MLMU reduced harvests from 74 to 62 mountain lions, with hunt area 6 only harvesting 15 animals. The west MLMU showed the largest decrease in harvest, reduced from 98 to 64 mountain lions taken, with no hunt areas showing increased harvest. Hunt areas 14, 18, and 19 within the west MLMU all resulted in 50% or more reductions from HY 2013.

Similar to previous seasons, mountain lion hunters spent an average of 3.9 days to successfully harvest a mountain lion in HY 2014 (Range: 1−45 days; Median: 2 days), although a majority of hunters spent only one day in the field for a successful hunt (44.4%). The primary method used to successfully harvest a mountain lion was with trained dogs (92.4%). Other methods of harvest included spot and stalk or tracking techniques (n = 6 harvests; 2.2%),

opportunistic harvests during big game hunting (n = 5 harvests; 1.9%), and using predator calls (n = 2 harvests; 0.7%). Non-resident hunters accounted for 38.3% of all successful mountain lion hunters in HY 2014. Overall, 27% of hunters used an outfitter or guide when hunting, with 53% of non-residents using outfitters or guides for successful hunting, a slight reduction from the previous harvest year. Hunters that successfully harvested mountain lions primarily used modern firearms (88.4%), but archery equipment was used in 11.6% of harvests, and no hunters reported the use of muzzleloaders. Mountain lion harvest occurred on a variety of land status, including: Bureau of Land Management (21.3%), Bureau of Reclamation (<0.1%), private property (33.8%), U.S. Forest Service (35.0%), and State Lands (9.5%), resulting in approximately 66% of harvested mountain lions taken on public land and 34% on private land.

Statewide harvest and non-harvest mortalities, as well as male and female harvest locations are illustrated in Figure 4. The majority of the mountain lion harvest occurs during the winter, and most harvest is distributed in areas where ungulate prey densities are highest (typically ungulate winter ranges). Areas within the north-central and northeast MLMUs showed the highest density of harvest compared to management units in other areas of the state.

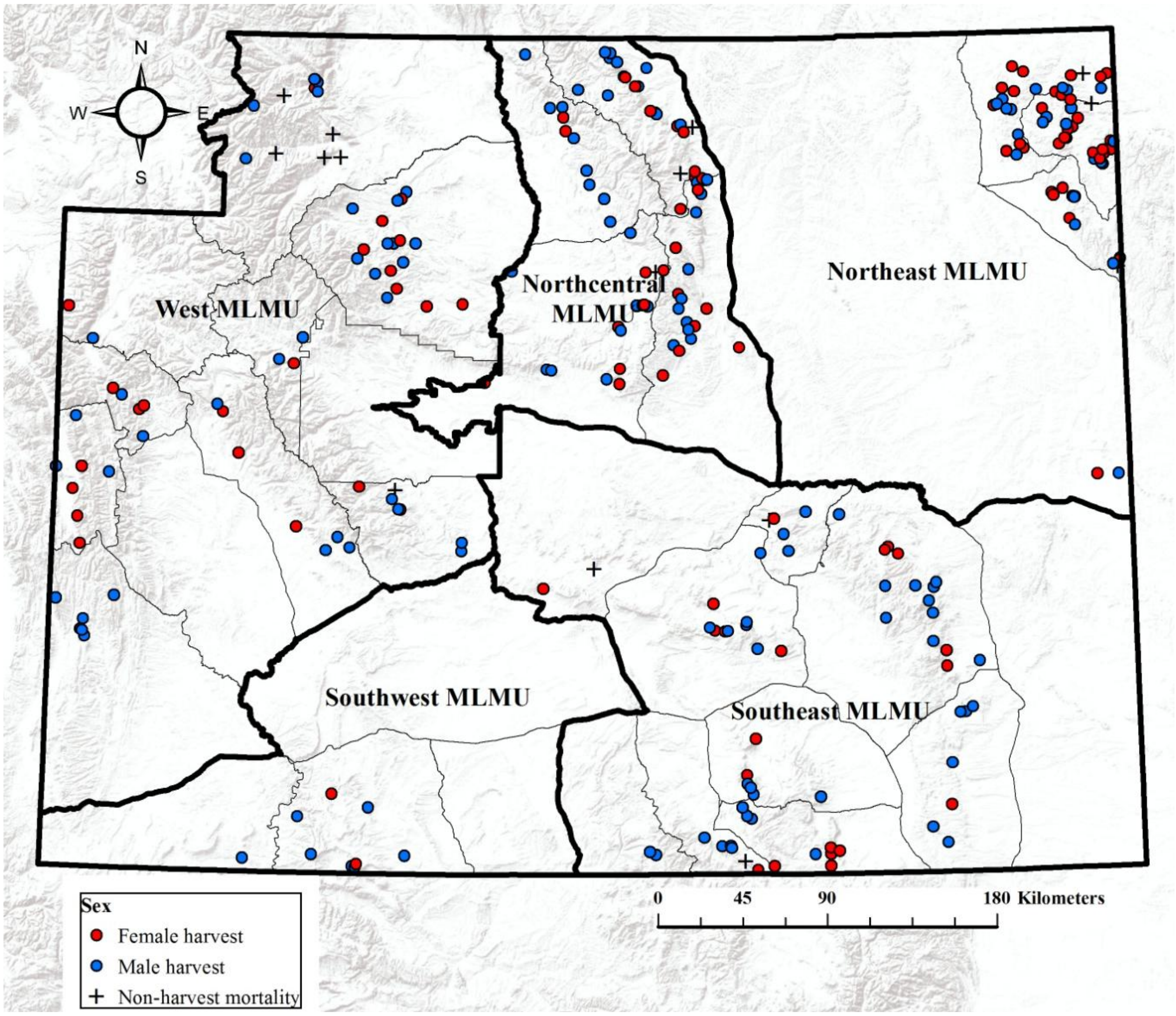

FIGURE 4. LOCATIONS OF MOUNTAIN LION HARVEST AND OTHER DOCUMENTED MOUNTAIN LION MORTALITIES IN WYOMING, HY 2014.

Hunters have the option to release any treed mountain lion during a hunt in preference for a different animal (e.g., trophy quality, males). In HY 2014, 41% of hunters reported being "selective" while hunting, but 18% of total hunters being selective harvested the first mountain lion treed. The remaining 23% of successful hunters opted to release lions in search of a different animal to harvest. In addition, hunter surveys for the HY 2014 season indicated that 58% of residents and 74% of non-residents were satisfied or very satisfied with the quality of mountain lion hunting in the state (Figure 5).

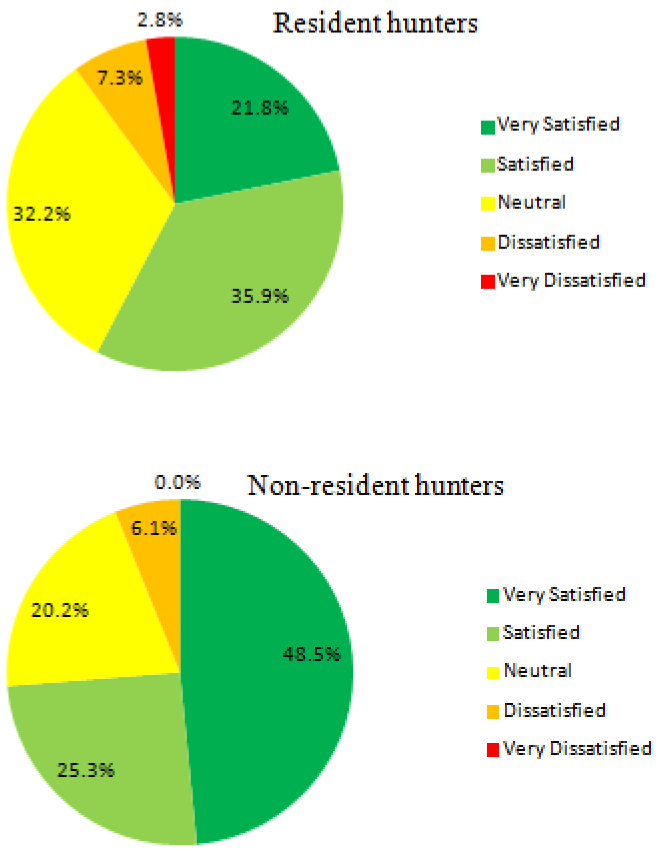

FIGURE 5. SURVEY RESULTS FOR RESIDENT AND NON-RESIDENT MOUNTAIN LION HUNTER SATISFACTION IN WYOMING, HY 2014.

MOUNTAIN LION BIOLOGICAL SAMPLING

The Large Carnivore Section of the WGFD acquires biological samples and harvest data from reported mountain lion mortalities on an annual basis (Appendix B). A tooth is typically extracted from each animal, and sent to the WGFD laboratory where annuli aging techniques are applied to estimate the age of sampled animals. Starting in HY 2014, unless reporting is necessary to inform management and season setting recommendations at the conclusion of 3-year management cycles, mortality reports will be prepared after lab ages are acquired upon closure of most hunt areas. In previous years, information included in annual reports used estimated ages from personnel registering and reporting mountain lion harvests, and were later

updated when lab results were returned to the Large Carnivore Section. However, estimated ages are typically a close approximation to lab results. For example, in HY 2014 the average difference in field estimated age to annuli age was 0.64 years (Range = 0–4 years; Median = 0.5 years), with 86% of field estimates within 1 year of estimated lab ages. While these discrepancies are minimal, potential inaccuracies applied to hunt areas with few animals harvested has the potential to influence age classifications and population trend analyses. Excellent response from personnel registering harvested lions resulted in tooth samples from 94% of harvested animals, and annuli ages estimated for 88% of harvests in HY 2014.

In addition to implementation of annuli ages via tooth samples, in HY 2014 the WGFD began collecting tissue samples for DNA extraction and various analyses. Tissue samples were collected from 73% of harvested mountain lions across Wyoming, which can be used to construct genetic profiles to identify genetically distinct segments of a population, investigate genetic susceptibility to disease, inform mark-recapture studies, and identify populations from which animals disperse. Tissue samples from harvested animals near the northeast MLMU were recently used from 2010-2012 in mark-recapture efforts, and the Large Carnivore Section plans to continue to collect samples and expand efforts by collecting samples from all statewide mountain lion mortalities.

HARVEST COMPOSITION AND MORTALITY DENSITY

Three primary monitoring criteria are used to evaluate management objectives and assess mountain lion population status:

1. *Density of human-caused mountain lion mortalities/1,000 km*^{2}
2. *Percent (%) of adult females in the harvest by HA*
3. *Average age of adult female harvest by HA.*

These criteria are based on research conducted in Wyoming (Anderson and Lindzey 2005) and are used as the foundation of the state management plan. When the density of mountain lion mortalities increased above 8.0 lions/1,000km^2 of winter lion habitat, the resident mountain lion population decreased. To reach this level of mortality it was also noted that an increase in the proportion of adult female mortality occurred. If harvest densities were maintained to reduce populations, it was postulated that managers would see a reduction in the age of adult females harvested (WGFD 2006). Appendix C provides data relative to these monitoring criteria separated by Hunt Area. These data are quantified into trends and assessed at the end of each 3-year management cycle prior to any alterations made to current management strategies. We will further evaluate the efficacy of these mortality densities depending on habitat differences and topography in relation to population status in upcoming harvest years.

TABLE 1. KNOWN SEX AND AGE COMPOSITION OF HARVESTED MOUNTAIN LIONS BY MOUNTAIN LION MANAGEMENT UNIT FOR HY 2014.

Management Unit	Adult Female	Subadult Female	Adult Male	Subadult Male	Totals
Northeast	16	23	5	19	63
Northcentral	12	17	18	24	71
Southeast	5	15	21	21	62
Southwest	1	1	3	4	9
Absaroka DAU	6	4	6	11	27
Wind River DAU	4	2	5	5	16
WY Range DAU	2	7	2	9	20
Totals	46	69	60	93	268

Table 1 provides sex and age class data for harvested mountain lions, separated by Mountain Lion Management Units. Also, harvest and sex/age class composition of harvest separated at the Hunt Area level are provided (Appendix D); note that as sample sizes decrease, interpretation of data is more difficult. Age class for females is determined by lactation status,

with any female currently or previously lactating considered an adult. Male mountain lions > 3 years of age are considered adults.

Examination of the age/sex composition of harvested animals allows an assessment of the proportion of each cohort available for harvest, and may be used as a sample that represents the local population. Criteria 2 of the WGFD management plan states the proportion of adult females harvested can inform current population trend, with consecutive years of adult female harvest above 25% indicating population reduction. However, at the hunt area or management unit level, low sample sizes can cause dramatic fluctuations in proportions, and estimates of adult female average age in the harvest (monitoring criteria 3). In addition, higher proportions of adult females harvested in response to increased mortality limits can erratically shift to a relatively low proportion due to assumed suppression in overall abundance of mature mountain lions. Under high levels of harvest (resulting in high mortality densities), the elevated proportion of adult females harvested cannot be sustained for prolonged periods, and scenarios such as these convey the importance of the Department's assessment of harvest data through time to identify trends and determine the status of the population.

While hunt area and MLMU harvest composition data is available in Appendix D, statewide trends in harvest from the implementation of the WGFD management plan in HY 2007 are shown in Figure 6. Statewide composition shows that the overall proportion of adult females harvested was slightly higher in earlier years than currently. Of note, the proportion of adult mountain lions harvested has steadily declined in recent years, from almost 60% adults harvested in HY 2007 to below 40% in HY 2014.

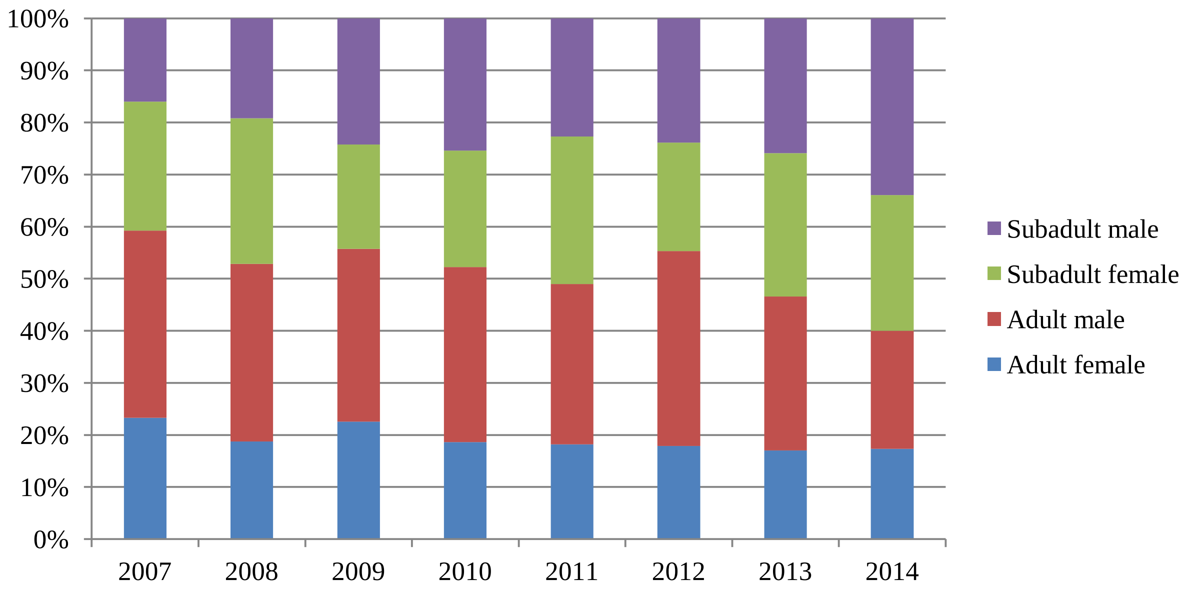

FIGURE 6. STATEWIDE WYOMING MOUNTAIN LION HARVEST COMPOSITION, HYS 2007-2014.

Overall, we documented a decrease in the average statewide mortality density (5.9 mortalities/1,000 km^2). We also identified adult female harvest of 17.3%, and an average estimated age of adult females at 5.1 years. This monitoring criteria indicates that on a statewide level, mountain lion populations are likely stable in Wyoming. Steady increases in harvests up to this year have roughly doubled reported mortality occurring during the mid-1990's, likely a response to increasing and expanding populations during that time. Recent liberalization of mortlaity limits has resulted in many hunt areas with increased harvest pressure. Many of these hunt areas demonstrate high harvest initially when mortality limit increases occur, followed by a decrease in the number of animals taken in subsequent seasons (likely in response to a decrease in local densities and/or hunter participation). Data indicate that several areas are achieving desired local reductions, but increasing harvest pressure has also resulted in a decline in the proportion of mature mountain lions available for harvest on a statewide level. Based on harvest data and supported by public surveys, juvenile mountain lions comprise the majority of what is currently available for harvest in certain locales where population reduction is the objective.

SUPPLEMENTAL POPULATION ASSESSMENTS

A variety of methods can be applied to gain insight into mountain lion population dynamics, and we discuss some of the ongoing management efforts to improve our knowledge of the species and apply best management practices accordingly.

Population reconstruction is a method of recreating a population based from mortality information, primarily from harvest. It involves quantifying mortalities for a given year, and using estimated ages to back-calculate the age of each animal in previous years back to birth. These animals are tallied for each retrospective year, resulting in a “minimum known abundance” estimate for a given population. These data can then be classified by sex and age. Certain conditions and assumptions must be met to insure accuracy of this methodology. Population reconstruction assumes a closed population, meaning no immigration into or emmigration from the geographic area. Also, this method works best when most all animals eventually result in a known mortality (typically harvested) to be included in the sample, as unknown or natural mortalities can bias recontruction estimates. High selectivity may also skew results if hunters intentionally avoid certain cohorts, and mortality limits that restrict harvest to the point that undocumented natural mortality increases can hinder analysis; but consistent hunter effort improves model reliability. As previously described, population reconstruction is a retrospective methodology, meaning that information is used to reconstruct past populations. Therefore, the most accurate estimates occur only after currently harvested animals are not old enough to contribute back to previous years’ tallied amounts. Because of this, population reconstruction is not typically used for current population estimates, but can be used to validate other population monitoring techniques and to evaluate management objectives in an adaptive manner. Overall, population reconstruction can be most informative when animals are assumed

not to move in or out of the population, have a high “turnover rate” or are short lived, and have a high tendency to eventually be harvested and captured in the sample.

We applied this model the to the Black Hills region of northeast Wyoming and western South Dakota. Because these animals readily occupy habitat across state lines we used mortality data from both states to reconstruct the relatively isolated mountain lion population in that geographic range. It is certain that the reestablishment of mountain lions in the Black Hills was ignited via dispersal from other areas of Wyoming and Montana, but the population is considered “semi-isolated” in that it is > 180 km of current breeding populations of extant mountain lions in surrounding regions (Thompson and Jenks 2010). In a conservative effort not to overestimate, we knew that not all natural mortalities were documented, and we assumed that immigration into the Black Hills was no greater than emmigration/dispersal out of the area once the Black Hills population was established and documented as having high mountain lion densities. Also, little hunter selectivity has been documented in this area, and mortaltiy limits have been increased toward a management objective for a reduction in mountain lion densities.

We populated the model back to 1988, and although the median age of harvests in 2014 was 2 years, we used mean age (3 years) to censor uninformative (most recent) years from the results (Figure 7). From 1988 to 2003, male mountain lions comprised the majority of the harvested population. This may be because male mountain lions likely dispersed/immigrated farther and at higher rates than females while reestablighing breeding populations in the Black Hills. Afterward, females may have comprised a higher proportion of the harvest due to moderate selectivity of hunters for male mountain lions and more available females on the landscape. All known animals alive during each year are included in Figure 7, so although the highest estimate was recorded in 2010 (n = 515), the actual number of independent (> 1 year)

mountain lions was lower during that time (combined adults and juvenile estimate = 422; Figure 8). Kittens that survived to independence and whose mortalities were documented are therefore included, while high natural mortality rate for mountain lion kittens less than 1 year in age would not be identified in estimates.

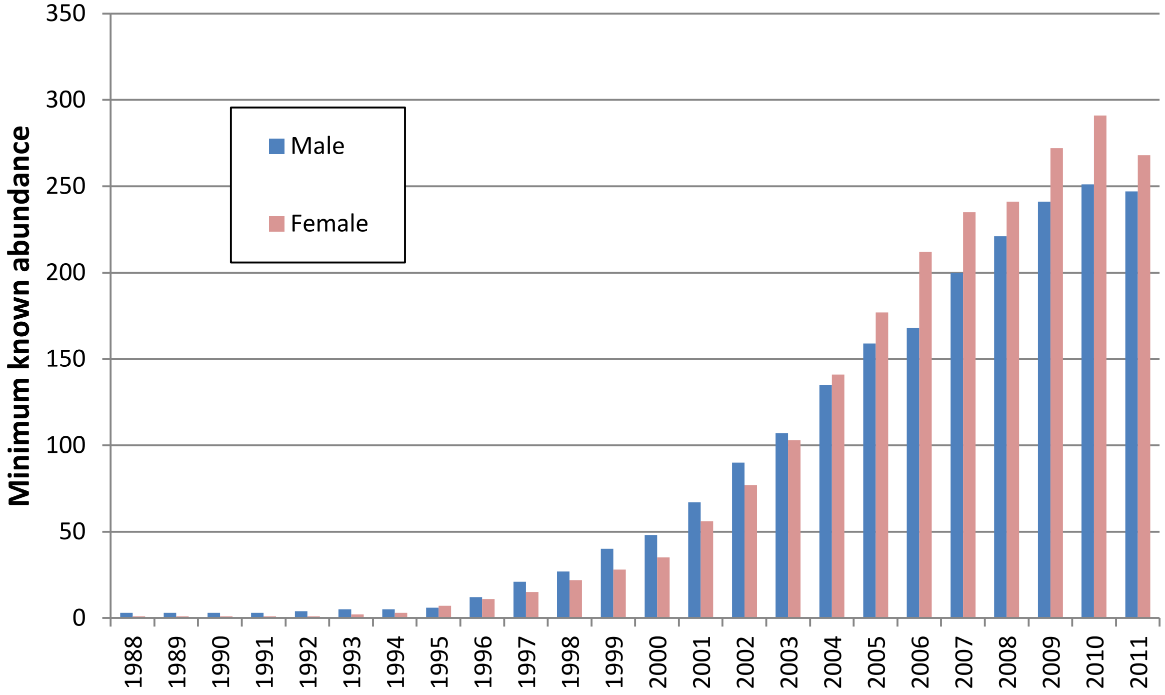

FIGURE 7. POPULATION RECONSTRUCTION FOR MALE AND FEMALE MOUNTAIN LIONS IN THE BLACK HILLS FROM 1988-2011.

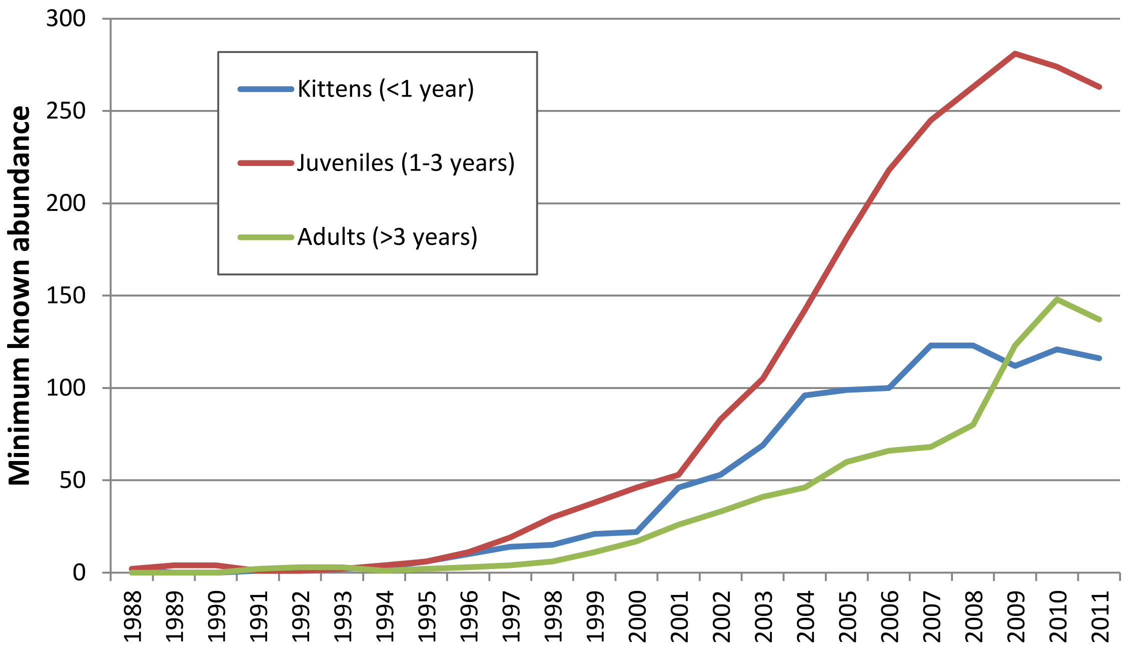

FIGURE 8. POPULATION RECONSTRUCTION FOR DIFFERENT AGE CLASSIFICATIONS OF MOUNTAIN LIONS IN THE BLACK HILLS FROM 1988-2011.

While this methodology may be far from a perfect representation of the true population dynamic, it gives managers the avantage of applying a variety of monitoring techniques to study populations, and may be used in concert with harvest criteria used in the management plan to identify trends in mountain lion populations as well as comparisons with other methods used to estimate mountain lion densities. We used the Black Hills as a specific example as an area where population reconstruction could be reasonably applied, and will further compare these results with estimates from DNA-derived mark-recapture efforts in the Black Hills.

Aerial Infra Red (AIR) technology is increasing in popularity to conduct wildlife research, particularly in identifying animals from fixed-wing aircraft. While current methods that dominate density estimation tecniques include a variety of mark-recapture studies (biopsy sampling, camera traps, capture-recapture, hair snares, scat sampling), little is known about the effectiveness of estimating mountain lion densities with the use of infrared camera technology.

The WGFD Large Carnivore Section is initiating testing on the efficacy of such technology, and plans to conduct preliminary AIR flights to assess detection probabilities under differing landscape characteristics. Generally, this would include capturing a sample of mountain lions in a distinct area and collaring animals with satellite GPS collars. GPS collars would transmit location data at high fix-rates (e.g., 5 min intervals) while survey flights were conducted over the area. Detection probabilities could then be estimated using the number of known marked animals in the area, the number of marked animals detected, and the total number of animals identified to estimate mountain lion densities for that area.

Whether using minimum known abundances from population reconstruction, various mark-recapture methods via DNA sampling, or developing new technology to survey animals, these techniques are valuable options available to estimate abundance and density of populations. Accurately estimating densities of mountain lions has always been a challenge for researchers and managers, but the ability to do so can greatly increase our ability to gather data to improve management of the species.

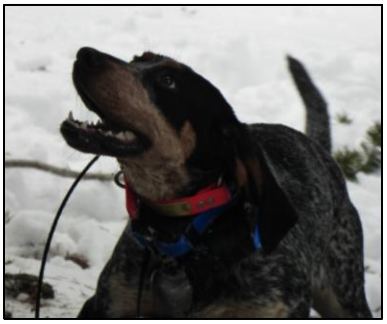
"Lily" assisting with biological sampling - Luke Ellsbury, WGFD

STATEWIDE MOUNTAIN LION HABITAT EVAUATION

According to WGFD's Mountain Lion Management Plan (2006), the key criteria used to determine population trend is derived from quantifying the number of human-caused mountain lion mortalities per unit area of suitable winter habitat. Therefore, much of the current methodology is dependent on estimates of suitable mountain lion habitat using the most current and best available data. Suitable habitat for much of the state has been derived from resource selection models that use model parameters such as distances to ecotones or habitat edges, slope,

elevation, and aspect to predict high-quality mountain lion habitats (Anderson 2003). These models likely apply well to areas of Wyoming similar to the study areas used in model construction, but a variety of habitats across Wyoming likely do not align well with model predictions. Therefore, a combination of habitat modeling and biologist perspectives are used in concert to predict high quality habitats across the state, and are verified using historic winter harvest locations and animal location data. Large carnivore biologists are tasked with continuing to update, improve, and refine techniques and estimates as data becomes available, and winter habitat estimates have been recently reconstructed (Figure 9).

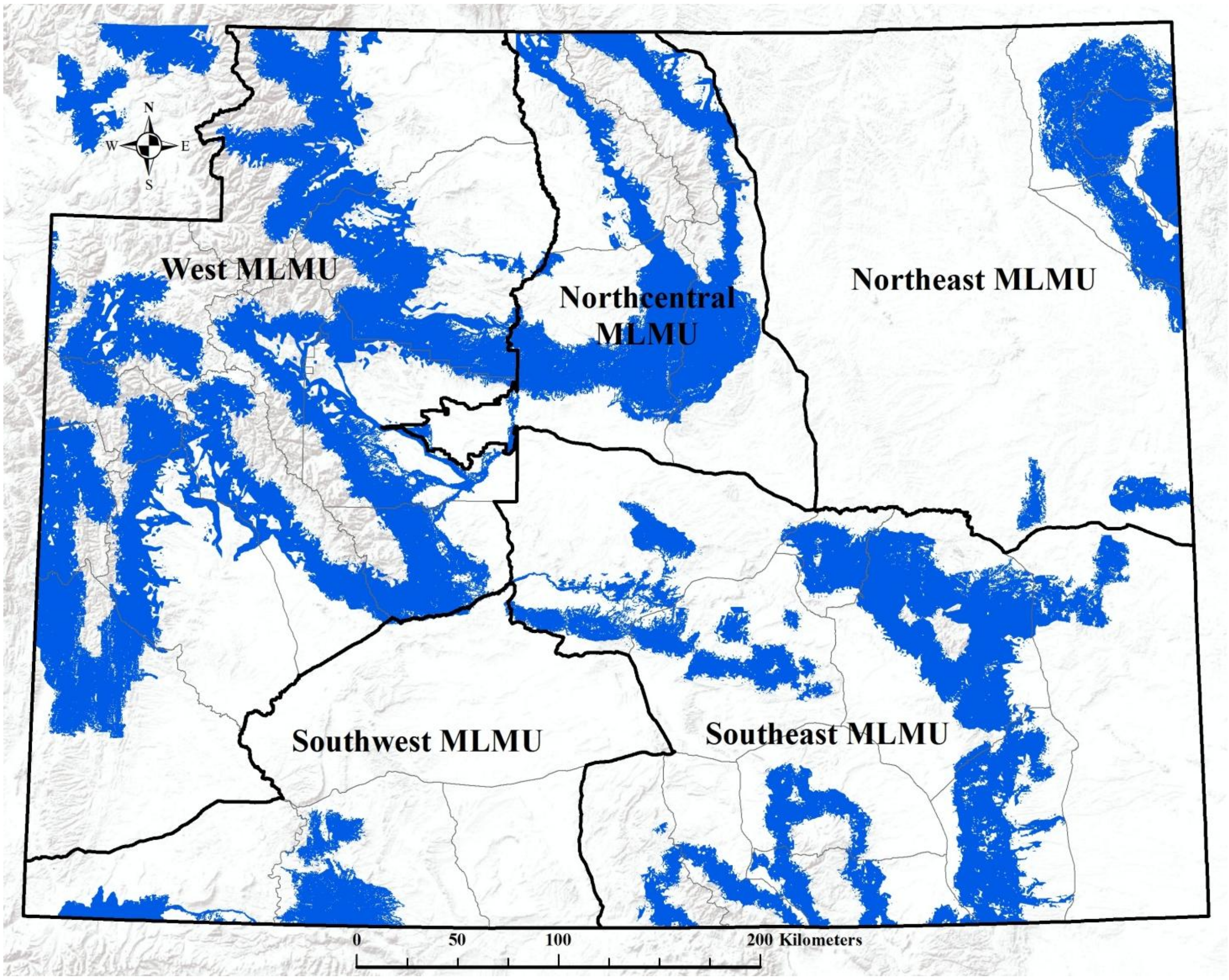

FIGURE 9. UPDATED ESTIMATES OF SUITABLE MOUNTAIN LION WINTER HABITAT ACROSS WYOMING.

These improvements bolster our ability to accurately estimate mortality densities and population trends. Although not drastically different from previous estimates, changes in the area of suitable habitat has the potential to shift estimated mountain lion mortality densites, particularly within specific regions and at the hunt area level. Because HY2014 marks the midpoint in the current 3-year management cycle, the WGFD's Large Carnivore Section plans to implement new habitat estimates for the next management cycle beginning in HY2016.

WYOMING HOUNDSMEN ASSOCIATION

Over the past several years the WGFD has met with local houndsmen across the state to provide information regarding Wyoming's management of mountain lion populations. In the fall of 2014, many of these houndsmen initiated a Wyoming Houndsmen Association to organize sportsmen in favor of preserving wildlife and perpetuating the use of coursing dogs for hunting opportunities. This organization seeks to keep members updated and informed on current mountain lion management, and to provide sportsmen-based recommendations during adaptive mountain lion management strategies implemented across the state. The newly-formed Wyoming Houndsmen Association's mission statement follows:

> "The Wyoming Houndsmen Association is comprised of a multifaceted group of sportsmen across Wyoming aimed at promoting the merits and ethics of hunting with the use of hounds and other coursing dogs. We support sound data-based management of all wildlife and provide a unified voice for the conservation and management of animals such as mountain lions and bobcats. The Association strives to educate others on the ethics and sportsmanship of hound hunting through public awareness and education as well as supporting the long-term perpetuity of all wildlife (big game, trophy game, and furbearer) for the future generations."

This year, members of this association have made introductions and began to familiarize themselves with commission meetings and WGFD proceedings, and plan to be more active at upcoming meetings as mountain lion season settings and various management issues arise. The Large Carnivore Section and the WGFD encourage the public to provide comments and recommendations and look forward to positive interactions with all groups holding interest in large carnivores.

CONCLUSION

The WGFD continues to collect mountain lion mortality data on an annual basis to monitor population trends across the state. Mortality data collected in HY 2014 provide the second year of information required to monitor population trends and the effectiveness of adaptations made for the current management cycle (HY 2013−HY 2015), but data from previous management cycles are also used to study long-term population dynamics. This is a valuable resource, especially applied toward areas where management strategies typically do not change. This information, coupled with ongoing research and improved monitoring techniques, increases our knowledge and understanding of the species, resulting in better mountain lion management for the state.

LITERATURE CITED

ANDERSON, C. R. JR. 2003. Cougar ecology, management, and population genetics in Wyoming, Ph.D., Department of Zoology and Physiology, University of Wyoming.

ANDERSON, C.R., JR., AND F.G. LINDZEY. 2005. Experimental evaluation of population trend and harvest composition in a Wyoming cougar population. Wildlife Society Bulletin 33(1):179-188.

THOMPSON, D.J., AND J.A. JENKS. 2010. Dispersal movements of subadult cougars from the Black Hills: the notions of range expansion and recolonization. Ecosphere 1(4):art8. doi:10.1890/ES10-00028.1

THOMPSON, D.J., AND LARGE CARNIVORE SECTION. 2013. Wyoming mountain lion harvest/mortality report: Harvest years 2010-2012 (1 September 2010 – 15 April 2012). Wyoming Game and Fish Department, Lander, Wyoming, USA.

THOMPSON, D.J., AND TROPHY GAME MANAGEMENT/RESEARCH BRANCH. 2010. Wyoming mountain lion harvest/mortality report: Harvest years 2007-2009 (1 September 2007 – 23 April 2010). Wyoming Game and Fish Department, Lander, Wyoming, USA.

WYOMING GAME AND FISH DEPARTMENT. 2006. Mountain Lion Management Plan. Wyoming Game and Fish Department, Cheyenne, Wyoming, USA.

APPENDIX A. Statewide Hunt Areas, season dates, and limitations for HY 2014.

Hunt Area	Dates of Seasons	Mortality Limit	Limitations
1	Sep. 1 - Mar. 31	24	
2	Sep. 1 - Mar. 31	5	
3	Sep. 1 - Mar. 31	12	
4	Sep. 1 - Mar. 31	10	
5	Sep. 1 - Mar. 31	12	Additional license valid
	Apr. 1 - Apr. 30*		Valid off national forest*
6	Sep. 1 - Apr. 30	21	Additional license valid
7	Sep. 1 - Aug. 31	14	Additional license valid
8	Sep. 1 - Aug. 31	10	Additional license valid
9	Sep. 1 - Aug. 31	12	Additional license valid
10	Sep. 1 - Mar. 31	7	
11	Sep. 1 - Mar. 31	2	
12	Sep. 1 - Mar. 31	8	
13	Sep. 1 - Mar. 31	5	
14	Sep. 1 - Mar. 31	15	
15	Sep. 1 - Aug. 31	Unlimited	Additional license valid
16	Sep. 1 - Mar. 31	6	Additional license valid
17	Sep. 1 - Mar. 31	9	
18	Sep. 1 - Mar. 31	12	
19	Sep. 1 - Mar. 31	20	Additional license valid
20	Sep. 1 - Mar. 31	20	
21	Sep. 1 - Mar. 31	20	
22	Sep. 1 - Aug. 31	25	
23	Sep. 1 - Mar. 31	20	
24	Sep. 1 - May 31	Unlimited	Additional license valid
25	Sep. 1 - Mar. 31	12	Additional license valid
26	Sep. 1 - Mar. 31	15	
27	Sep. 1 - Aug. 31	Unlimited	Additional license valid
28	Sep. 1 - Mar. 31	3	
29	Sep. 1 - Mar. 31	6	
30	Sep. 1 - Mar. 31	12	
31	Sep. 1 - Aug. 31	11	Additional license valid
32	Sep. 1 - Mar. 31	25	
33	Sep. 1 - Mar. 31	2	

***Brown = year-round harvest *Orange = extended season dates**

APPENDIX B. WGFD form for documenting mountain lion mortality data in Wyoming in 2014.

MOUNTAIN LION MORTALITY FORM

Hunt Area _____ Region _____

Date of kill: _______________ TYPE: Legal_____; Illegal_____; Damage Control_____; Other_____; Unknown_____

If "Other" or "Unknown", probable cause of mortality ____

PERSON WHO HARVESTED LION: Name: ____

Address: ____ City: _______________________

State: ______ Zip: _______________ Phone: _______________________________ Resident: _____ Nonresident: _____

METHODS/EFFORT: Days hunted: _____ Were dogs used? (Y/N) _____ If not, how was lion harvested? ______________

Was a guide/outfitter used? (Y/N): _____ Name: ___________________________ Dog owner: _______________________

Number of lions observed including harvest: __________ Weapon used: ___________________________

Were you selective while hunting? (Y/N): __________ Number treed and released: _________

Number of lions that were marked: ______ (Ear tag / tattoo / radio collar frequency : ___________________________________)

Number of fresh tracks not pursued: _____ (How many were single adults?: _____ How many were adults with kittens?: _____)

LOCATION/DRAINAGE: Where was lion harvested? ____

Sec: _____ Twnshp: _____ Rng: _____ **UTM Zone: _____ UTM Easting: ___________ UTM Northing**: ____________

SEX AND AGE: Sex: _____ Est. Age: _____

If female, presently lactating? (Y[≥2] / N) _____

Appear to have lactated in past? (Y / N) _____

Canine ridge below gumline? (Y[≥2.5] / N) _____

Any visible spotting on rear legs? (Y[≤3] / N / ?) _____

Visible bars on inside of front legs? (Y[<4] / N / ?) _____

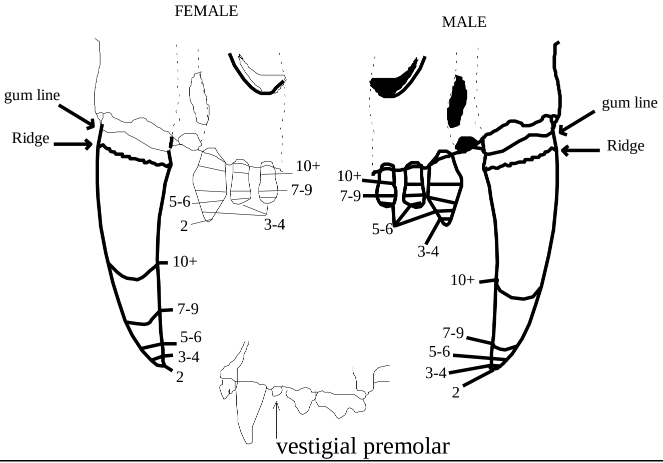

REQUIRED SAMPLES:

Teeth collected (Y/N):_____ Pictures of teeth (Y/N): _____

Tissue sample (Y/N):_____

Remarks: ____

____

____

Date record was WOFed: ____________________ Date Biological Services Called: _______________________

I, ________________________________ of ____

being duly sworn, depose and say that I am the holder of Wyoming Mountain Lion license # _______________,

and lawfully took the above lion on __________ - _____________, 20_____ in Hunt Area # __________.

________________________________ ________________ ________________________________

Inspected by / GF Number   Date   Hunter's Signature

Any person who makes a false statement on the registration form regarding the date the mountain lion was taken or the hunt area in which it was taken shall be in violation of this regulation and, such violation shall be punishable as provided by Title 23, Wyoming statutes for violation of Commission regulations.

Note: The person that checked the lion should forward the completed form and all samples to the Regional Office of registration and call Biological Services to update the harvest database. The Regional Office of registration will keep a copy of the completed form and send the original, along with the tooth and hair samples to the Large Carnivore Section. Revised 6/14.

APPENDIX C. Table of HY 2014 mountain lion data relative to WGFD mountain lion management plan monitoring criteria.

MLMU	HA	**Mortality Density** Mortalities/1,000 km^2	**Adult Female Take** % of Harvest	**Mean Age of Adult Females** Years (sample size in parentheses)
Northeast	1	15.84	25.0	4.0 (6)
	30	12.44	36.4	5.0 (4)
	32	18.56	20.0	4.8 (5)
	24	2.06	50.0	4.0 (1)
	Total	13.25	25.8	4.5 (16)
Northcentral	15	16.34	10.0	6.5 (2)
	21	9.26	8.3	4.0 (1)
	22	9.22	26.3	5.5 (5)
	23	15.95	20.0	4.8 (4)
	Total	12.19	16.9	5.3 (12)
Southeast	5	2.41	0.0	NA
	6	5.45	13.3	4.5 (2)
	7	8.13	0.0	NA
	8	6.77	20.0	5.5 (2)
	9	9.43	0.0	NA
	10	5.96	0.0	NA
	16	2.44	0.0	NA
	25	*	NA	NA
	27	5.09	0.0	NA
	31	5.52	16.7	6.0 (1)
	Total	5.14	8.3	5.2 (5)
Southwest	11	*	NA	NA
	12	9.41	12.5	5.0 (1)
	13	1.54	0.0	NA
	33	*	NA	NA
	Total	6.0	11.1	5.0 (1)
Absaroka DAU	19	3.00	12.5	5.0 (1)
	20	6.22	26.3	4.6 (5)
	Total	4.46	22.2	4.7 (6)
Wind River DAU	3	3.14	28.6	7.5 (2)
	4	5.84	16.7	12.0 (1)
	18	2.38	33.3	4.0 (1)
	28	0.00	NA	NA
	Total	2.63	25.0	7.8 (4)
WY Range DAU	2	0.91	0.0	NA
	14	3.22	0.0	NA
	17	0.00	NA	NA
	26	3.87	0.0	NA
	29	3.94	40.0	4.5 (2)
	Total	2.25	9.5	4.5 (2)
STATEWIDE		5.90	17.3	5.1 (46)

*****Represents a Hunt Area with minimal mountain lion habitat and not managed by WGFD for long term population viability.

APPENDIX D. Table of known sex and age class composition by Hunt Area and MLMU, HY 2014. Table excludes 1 harvest of unknown classification in HA14.

MLMU	HA	Adult Female	Subadult Female	Adult Male	Subadult Male	Other Mortalities	Total
Northeast	1	6	10	2	6	0	24
	30	4	3	0	4	1	12
	32	5	10	3	7	2	27
	24	1	0	0	1	1	3
	Total	16	23	5	18	4	66
Northcentral	15	2	7	3	8	1	21
	21	1	3	5	3	0	12
	22	5	3	5	6	1	20
	23	4	4	5	7	2	22
	Total	12	17	18	24	4	75
Southeast	5	0	1	3	3	0	7
	6	2	3	7	3	0	15
	7	0	5	2	2	0	9
	8	2	2	2	4	0	10
	9	0	1	1	4	1	7
	10	0	0	1	2	0	3
	16	0	1	0	0	1	2
	25	0	0	0	0	1	1
	27	0	1	2	1	1	5
	31	1	1	3	2	0	7
	Total	5	15	21	21	4	66
Southwest	11	0	0	0	0	0	0
	12	1	1	2	4	0	8
	13	0	0	1	0	0	1
	33	0	0	0	0	0	0
	Total	1	1	3	4	0	9
Absaroka DAU	19	1	1	2	4	5	13
	20	5	3	4	7	0	19
	Total	6	4	6	11	5	32
Wind River DAU	3	2	1	3	1	0	7
	4	1	1	0	4	1	7
	18	1	0	2	0	0	3
	28	0	0	0	0	0	0
	Total	4	2	5	5	1	17
Wyoming Range DAU	2	0	1	0	1	0	2
	14	0	1	2	3	0	7
	17	0	0	0	0	0	0
	26	0	4	0	3	0	7
	29	2	1	0	2	0	5
	Total	2	7	2	9	0	21
STATEWIDE		46	69	60	90	18	286

APPENDIX E. Table showing mean age of harvested male and female mountain lions separated by Hunt Area and MLMU, HY 2014.

MLMU	Hunt Area	Mean Age (years) Male	Female
Northeast	1	3.6	2.5
	30	2.3	3.7
	32	2.4	3.2
	24	3.0	4.0
	**NE MLMU**	**2.8**	**3.0**
Northcentral	15	3.2	3.8
	21	3.7	2.5
	22	3.3	4.1
	23	3.5	3.5
	**NC MLMU**	**3.4**	**3.6**
Southeast	5	4.6	2.0
	6	4.3	3.2
	7	4.4	2.8
	8	3.1	4.1
	9	2.8	3.0
	10	3.3	NA
	16	NA	2.0
	25	NA	NA
	27	4.2	2.0
	31	4.8	4.0
	**SE MLMU**	**4.0**	**3.2**
Southwest	11	NA	NA
	12	3.6	4.0
	13	4.0	NA
	33	NA	NA
	**SW MLMU**	**3.6**	**4.0**
Absaroka DAU	19	4.0	4.5
	20	3.3	3.5
	**ABS DAU**	**3.5**	**3.7**
Wind River DAU	3	3.3	6.0
	4	2.8	7.5
	18	5.3	4.0
	28	NA	NA
	**WR DAU**	**3.5**	**6.2**
Wyoming Range DAU	2	2.0	3.0
	14	3.6	2.0
	17	NA	NA
	26	2.7	2.3
	29	2.5	3.7
	**WY Range DAU**	**3.0**	**2.8**
	**STATEWIDE**	**3.5**	**3.4**
